# Protein remote homology detection based on bidirectional long short-term memory

**DOI:** 10.1186/s12859-017-1842-2

**Published:** 2017-10-10

**Authors:** Shumin Li, Junjie Chen, Bin Liu

**Affiliations:** School of Computer Science and Technology, Harbin Institute of Technology Shenzhen Graduate School, HIT Campus Shenzhen University Town, Xili, Shenzhen, 518055 China

**Keywords:** Protein sequence analysis, Protein remote homology detection, Neural network, Bidirectional Long Short-Term Memory

## Abstract

**Background:**

Protein remote homology detection plays a vital role in studies of protein structures and functions. Almost all of the traditional machine leaning methods require fixed length features to represent the protein sequences. However, it is never an easy task to extract the discriminative features with limited knowledge of proteins. On the other hand, deep learning technique has demonstrated its advantage in automatically learning representations. It is worthwhile to explore the applications of deep learning techniques to the protein remote homology detection.

**Results:**

In this study, we employ the Bidirectional Long Short-Term Memory (BLSTM) to learn effective features from pseudo proteins, also propose a predictor called **ProDec-BLSTM**: it includes input layer, bidirectional LSTM, time distributed dense layer and output layer. This neural network can automatically extract the discriminative features by using bidirectional LSTM and the time distributed dense layer.

**Conclusion:**

Experimental results on a widely-used benchmark dataset show that **ProDec-BLSTM** outperforms other related methods in terms of both the mean ROC and mean ROC50 scores. This promising result shows that **ProDec-BLSTM** is a useful tool for protein remote homology detection. Furthermore, the hidden patterns learnt by **ProDec-BLSTM** can be interpreted and visualized, and therefore, additional useful information can be obtained.

**Electronic supplementary material:**

The online version of this article (10.1186/s12859-017-1842-2) contains supplementary material, which is available to authorized users.

## Background

Protein remote protein homology detection plays a vital role in the field of bioinformatics since remote homologous proteins share similar structures and functions, which is critical for the studies of protein 3D structure and function [1, 2]. Unfortunately, because of their low protein sequence similarities, the performance of predictors is still too low to be applied to real world applications [3]. During the past decades, some powerful computational methods have been proposed to deal with this problem. The earliest and most widely used methods are alignment-based approaches, including sequence alignment [4–8], profile alignment [9–14] and HMM alignment [15–17]. Later, discriminative methods have been proposed, which treat protein remote homology protein detection as a superfamily level classification task. These methods take the advantages of machine learning algorithms by using both positive and negative samples to train a classifier [18, 19]. A key of these methods is to find an effective representation of proteins. In this regard, several feature extraction methods have been proposed, for example, Top-n-gram extracted the evolutionary information from the profiles [20], Thomas Lingner proposed an approach to incorporate the distances between short oligomers [21], and some methods incorporated physicochemical properties of amino acids into the feature vector representation, such as SVM-RQA [22], SVM-PCD [23], SVM-PDT [24], disPseAAC [25]. Kernel tricks are also employed in discriminative methods, which are used to measure the similarity between protein pairs [26]. Several kernels have been proposed to calculate the similarity between protein samples, such as mismatch kernel [27], motif kernel [28], LA kernel [29], SW-PSSM [30], SVM-Pairwise [31], etc. For more information of these methods, please refer to a recent review paper [1].

The aforementioned methods have obviously facilitated the development of this important field. However, further studies are still required. Almost all the machine learning methods require fixed length vectors as inputs. Nevertheless, the lengths of protein sequences vary significantly. During the vectorization process, the sequence-order information and the position dependency effects are lost, and this information is critical for protein sequence analysis and nucleic acid analysis [32–34]. Although some studies attempted to incorporate this information into the predictors [21, 24, 35, 36], it is never an easy task due to the limited knowledge of proteins.

Recently, deep learning techniques have demonstrated their ability for improving the discriminative power compared with other machine learning methods [37, 38], and have been widely applied to the field of bioinformatics [39], such as the estimation of protein model quality [40], protein structure prediction [41–43], protein disorder prediction [44], etc. Recurrent Neural Network (RNN) is one of the most successful deep learning techniques, which is designed to utilize sequential information of input data with cyclic connections among building blocks, such as Long Short-Term Memory (LSTM) [45, 46], and gated recurrent units (GRUs) [47]. LSTM can automatically detect the long-terms and short-terms dependency relationships in protein sequences, and decides how to process a current subsequence according to the information extracted from the prior subsequences [48]. LSTM has also been applied to protein remote homology detection to automatically to generate the representation of proteins [48]. Compared with other methods, it is able to identify effective patterns of protein sequences. Although this approach has achieved state-of-the-art performance, it has several shortcomings: 1) Hochreiter’s neural network [48] only has two layers: LSTM and output layer. Its capacity is too limited to capture sequence-order effects, especially for the long proteins; 2) Features are generated only based on the last output of LSTM. However, as protein sequences contains hundreds of amino acids, it is hard to detect the dependency relationships of all the subsequences by only considering information contained in the last output of LSTM; 3) The last output generated from LSTM contains complex dependencies, which cannot be traced to any specific subsequence for further analysis.

Here, we are to propose a computational predictor for protein remote homology detection based on Bidirectional Long Short-Term Memory [45, 46, 49], called **ProDec-BLSTM**, to address the aforementioned disadvantages of the existing methods in this field. **ProDec-BLSTM** consisted of input layer, bidirectional LSTM layer, time distributed dense layer and output layer. With this neural network, both the long and short dependency information of pseudo proteins can be captured by tapping the information from every mediate hidden value of bidirectional LSTM. Experimental results on a widely used benchmark dataset and an updated independent dataset show that **ProDec-BLSTM** outperforms other existing methods. Furthermore, the patterns learnt by **ProDec-BLSTM** can be interpreted and visualized, providing additional information for further analysis.

## Methods

### SCOP benchmark dataset

A widely used benchmark dataset has been used to evaluate the performance of various methods [28], which was constructed based on the SCOP database [50] by Hochreiter [48]. This dataset can be accessed from http://www.bioinf.jku.at/software/LSTM_protein/.

The SCOP database [50] classifies the protein sequences into a hierarchy structure, whose levels from top to bottom are class, fold, superfamily, and family. 4019 proteins sequences are extracted from SCOP database, whose identities are lower than 95%, and they are divided into 102 families and 52 superfamilies. For each family, there are at least 10 positive samples. For the 102 families in the database, the training and testing datasets are defined as:

1 $$ \left\{\begin{array}{c}{\mathbb{S}}_{\mathrm{train}}(k)={\mathbb{S}}_{\mathrm{train}}^{+}(k)\cup {\mathrm{E}}_{\mathrm{train}}^{+}(k)\cup {\mathbb{S}}_{\mathrm{train}}^{-}(k)\\ {}{\mathbb{S}}_{\mathrm{test}}(k)={\mathbb{S}}_{\mathrm{test}}^{+}(k)\cup {\mathbb{S}}_{\mathrm{test}}^{-}(k)\ \end{array}\right.\left(k=1,2,...,102\right) $$

where ${\mathbb{S}}_{\mathrm{test}}^{+}(k)$ represents the *k* ^th^ positive testing dataset with proteins in *k* ^th^ family, and ${\mathbb{S}}_{\mathrm{train}}^{+}(k)$ represents the *k* ^th^ positive training dataset containing proteins in the same superfamily and not in the *k* ^th^ family. ${\mathrm{E}}_{\mathrm{train}}^{+}(k)$ denotes the extended positive training dataset for *k* ^th^ training dataset. The added training samples are extracted from Uniref50 [51] by using PSI-BLAST [9] with default parameters except that the e-value was set as 10.0. For all of the superfamilies except which *k* ^th^ family belongs to, select one family in each of the superfamilies respectively, to form the *k* ^th^ negative testing dataset ${\mathbb{S}}_{\mathrm{test}}^{-}(k)$ and the rest of proteins in these superfamilies are included in the negative training dataset ${\mathbb{S}}_{\mathrm{train}}^{-}(k).$ The average number of samples of all the 102 training datasets is 9077.

### Neural network architectures based on bidirectional LSTM

**Fig. 1:**
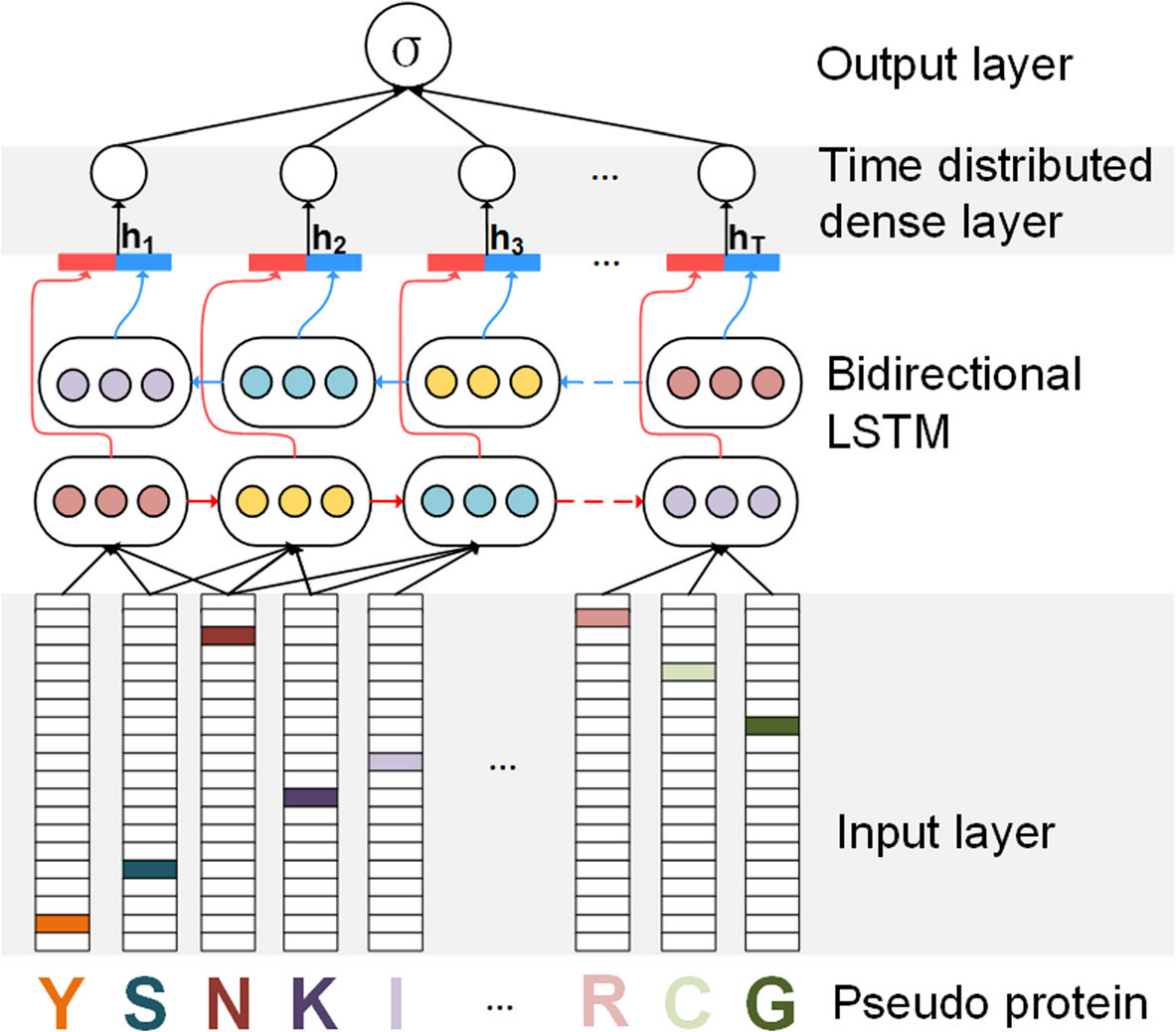
The structure of ProDec-BLSTM. The input layer converts the pseudo proteins into feature vectors by one-hot encoding. Next, the subsequences within the sliding window are fed into the bidirectional LSTM layer for extracting the sequence patterns. Then, the time distributed dense layer weights the extracted patterns. Finally, the extracted feature vectors are fed into output layer for prediction

In this section, we will introduce the network architecture of **ProDec-BLSTM**, as shown in Fig. 1. This network has four layers: input layer, bidirectional LSTM layer, time distributed dense layer, and output layer. The input layer is designed to encode the pseudo protein by one-hot encoding [52].Bidirectional LSTM extracts the dependency relationships between subsequences. We take the advantages of every intermediate hidden value from bidirectional LSTM to better handling the long length of protein sequences. More comprehensive dependency information can be included into the hidden values by using bidirectional LSTM. Then, those intermediate hidden values are connected to the time distributed dense layer. Because memory cells in one block extract different levels of dependency information, the time distributed dense layer is designed to weight the dependency relationships extracted from different cells. The outputs of time distributed dense layer are concatenated into one feature vector and be fed into the output layer for prediction. Next, we will introduce the four layers in more details.

### Input layer

The input layer transfers the protein sequence into a representing matrix, and fed it into the bidirectional LSTM layer.

Given a protein sequence **P**:

2 $$ \mathbf{P}={\mathrm{R}}_1,{\mathrm{R}}_2,\dots, {\mathrm{R}}_l $$

where R_1_ denotes the 1st residue, R_2_ denotes the 2nd residue and so forth, *l* represents the length of **P**. Then the **P** is converted into pseudo protein **P′’** based on PSSM [26, 53] generated by PSI-BLAST with command line “-evalue 0.001 -num_iterations 3″.

The input matrix at the *t* ^th^ time step can be obtained by one-hot encoding of **P′’** [52], shown as:

3 $$ {\mathbf{M}}_t=\left({\mathbf{v}}_i,{\mathbf{v}}_{i+1},,\dots, {\mathbf{v}}_{i+w-1}\right) $$

4 $$ {\mathbf{v}}_i={\left({e}_{i1},{e}_{i2},,\dots, {e}_{i20}\right)}^T,{e}_{ij}=\left\{\begin{array}{c}1,{\mathrm{R}}_i=\mathrm{A}{\mathrm{A}}_j\\ {}0,\mathrm{otherwise}\end{array}\right. $$

where **v**_*i*_ is the representing vector for R_*i*_, *w* denotes the size of the sliding window, *i* represents the start position of the subsequence, AA_*j*_ denotes the *j* ^th^ standard amino acid.

### Bidirectional LSTM Layer

**Fig. 2:**
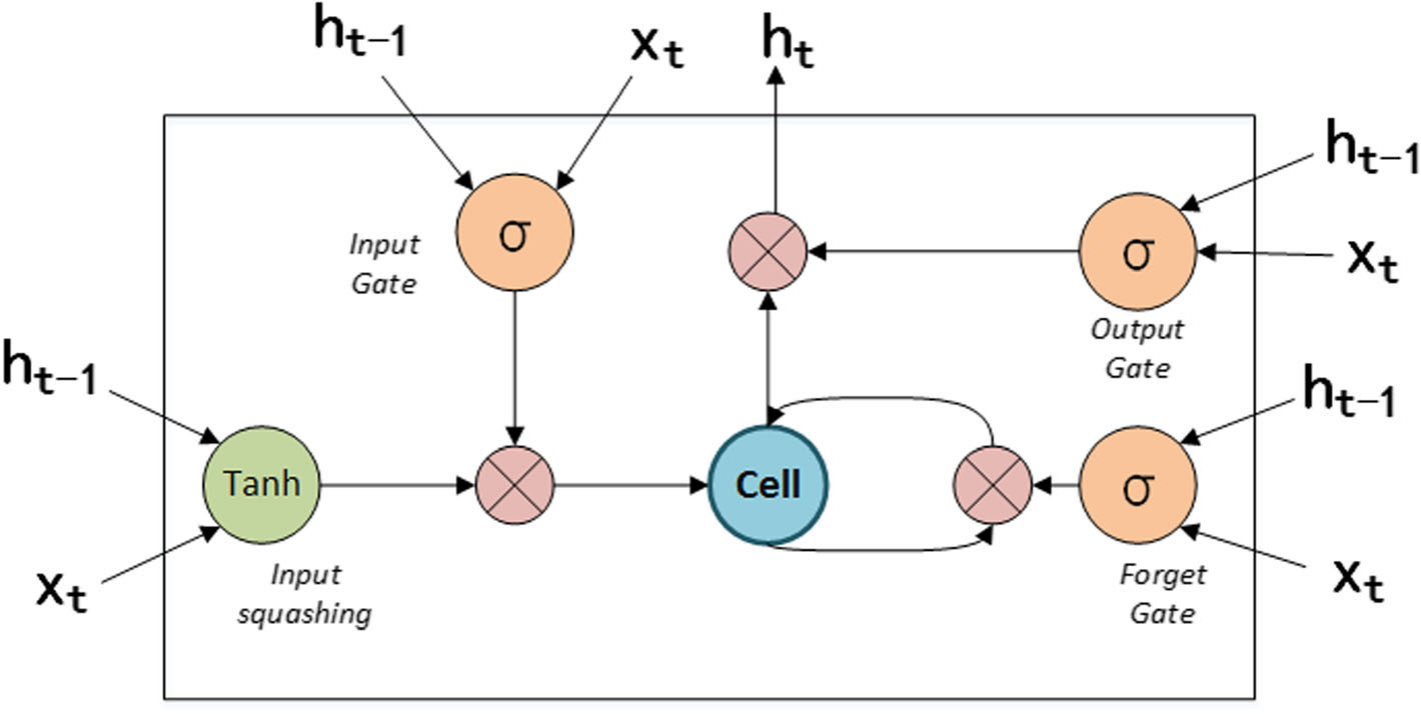
The structure of LSTM memory cell. There are three gates, including input gate (marked as *i*), forget gate (marked as *f*), output gate (marked as *o*), to control the information stream flowing in and out the block. *σ* denotes the sigmoid function, which produces a value bounded by 0 and 1. The internal cell state is maintained and updated by the coordination of input gate and forget gate. The output gate controls outputting information stored in the cell. h is the output of the memory cell, x is representing matrix of the input subsequence and *t* mean the *t* ^th^ time step

Bidirectional LSTM layer is the most important part in **ProDec-BLSTM**, aiming to extract the sequence patterns from pseudo proteins. The basic unit of LSTM is the memory cell. In this study, we adopted the memory cell described in [46], whose structure is shown in Fig. 2. The memory cell receives two input streams: the subsequence within the sliding window, and the output of LSTM from the last time step. Based on the two information streams, the three gates coordinate with each other to update and output the cell state. The input gate controls how much of new information can flow into the cell; The forget gate decides how much stored information in the cell will be kept. By coordination of input gate and forget gate, the cell state is updated. The output gate controls outputting the information stored in the cell, which is hidden value (denoted as **h**_*t*_ in Fig. 2).

The bidirectional LSTM is made up of two reversed unidirectional LSTM. To handle the long pseudo protein sequences, and better capture the dependency information of subsequences, we tap into all of the intermediate hidden values generated by bidirectional LSTM. The hidden values generated by the forward LSTM and backward LSTM for the same input subsequence are concatenated into a vector, which is shown in Eq. (5).

5 $$ {\mathbf{h}}_t=\left({\mathbf{h}}_t^{\mathrm{f}},{\mathbf{h}}_t^{\mathrm{b}}\right) $$

where **h** is hidden value, f represents the forward LSTM, b represents the backward LSTM, *t* means the *t* ^th^ time step.

In the bidirectional LSTM layer, the pseudo protein is processed N-terminus to C-terminus and C-terminus to N-terminus simultaneously. Therefore, ${\mathbf{h}}_t^{\mathrm{f}}$ contains dependencies between the target subsequence and its left neighbouring subsequence. ${\mathbf{h}}_t^{\mathrm{b}}$ contains dependencies between the target subsequence and its right neighbouring subsequence. These two dependency relationships are concatenated into one vector **h**_*t*_, which can be interpreted as the feature of the target subsequence. Therefore, more comprehensive dependencies can be included into the intermediate hidden values by using bidirectional LSTM.

### Time distributed dense layer

Different memory cells in one block extracts different levels of dependency relationships. Thus, we add the time distributed dense layer after the bidirectional LSTM layer to give weights to the hidden values generated from different memory cells. The time distributed dense receives the hidden value generated from memory block, and outputs a single value for one subsequence. The outputs of time distributed dense layer at every position are then concatenated into one vector, which is fed into the output layer for prediction.

### Output layer

The output layer is a fully connected network with one node and it performs the binary prediction based on the representing vectors generated by the time distributed dense layer. Therefore, for each protein, its probability of belonging to a specific superfamily is produced.

### Implementation details

This network was implemented by using Keras 2.0.6 (https://github.com/fchollet/keras) with the backend of Theano (0.9.0) [54].

The size of the sliding window was set as 3, and the protein sequence length was fixed as 400. The bidirectional LSTM has 50 memory cells in one block. The time distributed fully dense layer was a fully connected layer with the one output node, using ReLu activation function [55]. All the initializations of weights and bias were set as the default in Keras. The model was optimized by the algorithm of RMSprop [56] with the loss function of binary crossentropy at learning rate 0.01. The batch size was 32. Dropout [57] was included in bidirectional LSTM layer and the proportion of disconnection was 0.2. Each model was optimized by training for 150 epochs.

### Performance measure

In this study, ROC score and ROC50 score are used to evaluate the performance of various methods. Receiver operating characteristics (ROC) curve is plotted by using the true positive rate as the *x* axis and the false positive rate as the *y* axis, which are calculated based on different classification threshold [58]. ROC score refers to the normalized area under ROC curve. ROC50 is the normalized area when the first 50 false positive samples occur. For a perfect classification, ROC score and ROC50 are equal to 1.

## Results and discussion

### Comparison with various methods

We compared **ProDec-BLSTM** with various related methods, including GPkernel [28], GPextended [28], GPboost [28], SVM-Pairwise [31], Mismatch [27], eMOTIF [59], LA-kernel [29], PSI-BLAST [9] and LSTM [48]. The results are shown in Table 1, from which we can see that **ProDec-BLSTM** outperforms all of other methods. Both **ProDec-BLSTM** and LSTM [48] are based on deep learning techniques with smart representation of proteins, and all the other approaches are based on Support Vector Machines (SVMs). These results indicate that the LSTM method is a suitable approach for protein remote homology detection. As discussed above, the SVM-based methods rely on the quality of hand-made features and kernel tricks. However, due to the imited knowledges of proteins, their discriminative power is still low. In contrast, the deep learning algorithms, especially LSTM are able to automatically extract the features from proteins sequences, and capture the sequence-order effects. The *t*-test is employed to measure the differences between **ProDec-BLSTM** and LSTM [48]. The results show that **ProDec-BLSTM** significantly outperforms LSTM [48] in terms of ROC scores (*P*-value = 0.05) and ROC50 scores (*P*-value = 3.04e-09). There are four main reasons for **ProDec-BLSTM** outperforms LSTM: 1) **ProDec-BLSTM** taps into all of the intermediate hidden values generated by bidirectional LSTM to better handle the long proteins and pay attention to local as well as global dependencies; 2) **ProDec-BLSTM** used bidirectional LSTM layer which is able to include the dependency information from both N-terminal to C-terminal and from C-terminal to N-terminal into the intermediate hidden values; 3) the time distributed dense layer gives weights to different levels of dependency information to fuse information. 4) Evolutionary information extracted from PSSMs is incorporated into the predictor by using pseudo proteins.

**Table 1:** Mean ROC and ROC50 scores of various methods on the SCOP benchmark dataset (Eq. 1)

Methods	Mean ROC	Mean ROC50	classifier
GPkernel	0.902	0.591	SVM
GPextended	0.869	0.542	SVM
GPboost	0.797	0.375	SVM
SVM-Pairwise	0.849	0.555	SVM
Mismatch	0.878	0.543	SVM
eMOTIF	0.857	0.551	SVM
LA-kernel	0.919	0.686	SVM
PSI-BLAST	0.575	0.175	NA
LSTM	0.943	0.735	LSTM
ProDec-BLSTM	0.969	0.849	LSTM

### Visualizations

The hidden patterns learnt by **ProDec-BLSTM** can be interpreted and visualized. We explore the reason why the proposed **ProDec-BLSTM** showed higher discriminative power based on the visualization of hidden patterns.

Given a pseudo protein **P′**, it can be converted into a feature vector:

6 $$ \mathbf{V}=\left[{\upalpha}_1,{\upalpha}_2,,\dots, {\upalpha}_t\right] $$

where α_*t*_ indicates the output of time distributed dense layer at the *t* ^th^ time step. The feature vector **V** is generated by concatenating all the outputs of time distributed dense layer and each value of **V** represents the fused dependency relationships of a subsequence. Thus, **V** contains global sequence characteristics.

**Fig. 3:**
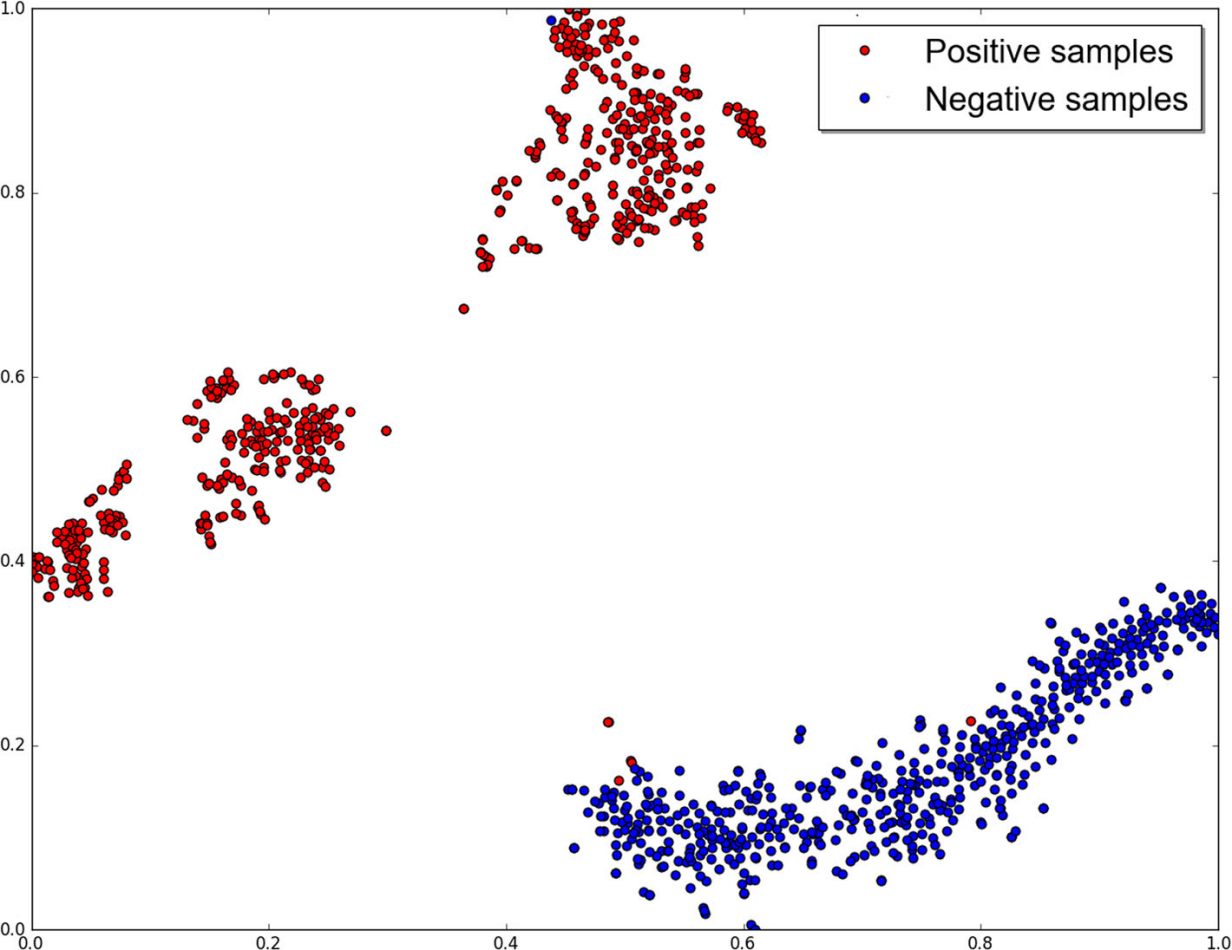
Feature visualization of ProDec-BLSTM for the protein family *b.1.1.1*. The positive samples and negative samples are shown in red color and blue color, respectively

Here, we demonstrate the testing set of the family *b.1.1.1* in SCOP benchmark dataset (**Eq.** **1**), which has 538 positive samples and 543 negative samples, as an example: the representing vector of each sample are generated by the trained **ProDec-BLSTM** model, and then t-SNE [60] is employed to reduce the their dimensions into two in order to visualize their distributions (shown in Fig. 3). The ranges of x and y axis are both normalized. From Fig. 3, we can see that most of the positive and negative samples are clustered and clearly apart from each other, indicating that the feature vectors automatically generated by **ProDec-BLSTM** are effective for protein remote homology detection.

### Independent test on SCOPe dataset

Moreover, as a demonstration, we also extend the comparison with other methods via an updated independent dataset set constructed based on SCOPe (latest version: 2.06) [61]. To avoid the homology bias, the CD-HIT [62] is used to remove those proteins from SCOPe that have more than 95% sequence identity to any protein in the SCOP benchmark dataset (**Eq.** **1**). Finally, 4679 proteins in SCOPe are obtained using as the independent dataset (see Additional file 1). Trained with SCOP benchmark dataset, **ProDec-BLSTM** predictor is used to identify the proteins in the SCOPe independent dataset set. Four related methods are compared with **ProDec-BLSTM**, including HHblits [16], Hmmer [15], PSI-BLAST [9] and ProDec-LTR [3, 63]. HHblits and PSI-BLAST are employed in the top-performing methods in CASP [64] and ProDec-LTR [3] is a recent method that combines different alignment-based methods. The results thus obtained are given in Table 2 **,** and their implementations are listed below. It can be clearly seen from there that the new predictor outperforms all the existing approaches for protein remote homology detection.

**Table 2:** Mean ROC and ROC50 scores of related methods on the SCOPe independent dataset

Method	Mean ROC	Mean ROC50
HHblits^a^	0.725	0.443
Hmmer^b^	0.556	0.145
PSI-BLAST^c^	0.668	0.096
ProtDec-LTR^d^	0.742	0.445
ProDec-BLSTM	0.970	0.714

^a^the command line of HHblits is ‘-e 1 -p 0 -E inf -Z 10000 -B 10000 -b 10000’

^b^The parameters of Hmmer are set as default

^c^The paramters of PSI-BLAST are set as default

^d^The above three alignment-based methods are combined by ProDec-LTR. The model is trained with SCOP benchmark dataset (Eq. 1)

## Conclusion

In this study, we propose a predictor **ProDec-BLSTM** based on bidirectional LSTM for protein remote homology detection, which can automatically extract the discriminative features and capture sequence-order effects. Experimental results showed that **ProDec-BLSTM** achieved the top performance comparing with other existing methods on an SCOP benchmark dataset and a SCOPe independent dataset. Comparing with hand-made protein features used by traditional machine learning methods, the features learnt by **ProDec-BLSTM** have more discriminative power.

Such high performance of **ProDec-BLSTM** benefits from bidirectional LSTM, and time distributed dense layer, by which it is able to extract the global and local sequence order effects. Every intermediate hidden values of bidirectional LSTM are also incorporated into the proposed predictor so as to capture context dependency information of subsequences. The time distributed dense layer gives weights to different level of dependency relationships, and fuses the dependency information.

In the future, we will focus on exploring new features to further improve the performance of **ProDec-BLSTM**, such as directly learning from PSSM [65].

## Additional files

Additional file 1: The SCOP ID of the independent SCOPe testing dataset. (PDF 7601 kb)

Additional file 2: The source code and its document of ProDec-BLSTM. (ZIP 316 kb)

## Abbreviations

GRU: Recurrent gated unit
HMM: Hidden Markov model
LSTM: Long-Short Term Memory
ReLu: Rectified Linear Units
RMSProp: Root Mean Square Propagation
RNN: Recurrent neural network
ROC: Receiving operating characteristics
SVM: Support vector machine
