# Supplementary material for: Protein remote homology detection based on bidirectional long short-term memory
Source: BMC Bioinformatics. 2017 Oct 10;18:443. doi: 10.1186/s12859-017-1842-2 (PMC5634958; doi:10.1186/s12859-017-1842-2)
Supplement: Supplementary file 1 — The SCOP ID of the independent SCOPe testing dataset. (PDF 7601 kb) [file 12859_2017_1842_MOESM1_ESM.pdf]

**Additional file S1**. This independent testing dataset contains 4,679 protein sequences extracted from SCOPe (version 2.06), and none of these proteins share ≥95% sequence identity to any protein in the SCOP benchmark dataset (**Eq. 1**).

---

**Family-1 (a.1.1.2)**

**Positive dataset contains 67 protein sequences.**

d2r80b_ d3boma_ d2h8fb_ d2raoa_ d3bj1a_ d4hrrb_ d2r50a_ d3d1kb_
d2aa1b1 d3zhwa_ d3hf4b_ d3gdja_ d3bj1b_ d1x9fb_ d1wmub_ d3bcqb_
d3d4xb_ d2dc3a_ d3hf4a_ d4yu3a_ d3k8ba_ d2r80a_ d3bcqa_ d1v4wa_
d3wtgb_ d4esaa_ d3d1ab_ d4h2la_ d1v4wb_ d1q1fa_ d1tu9a1 d3d1aa_
d3hyub_ d2zfbb_ d3fs4a_ d1shrb_ d4h2lb_ d4yu3b_ d3hrwa_ d3qqra_
d3at5a_ d4wchd_ d3hyua_ d2raob_ d4esab_ d3gdjb_ d3qqqa_ d1x9fc_
d3vrfa_ d3mkba_ d3d4xa_ d3mjpa_ d3fh9a_ d2dc3b_ d3eoka_ d3g46a_
d3vrfb_ d1x9fd_ d2zfba_ d3mkbb_ d3wtga_ d3fh9b_ d3bomb_ d3cy5a_
d3at5b_ d1x9fa_ d2oifa_

**Negative dataset contains 3908 protein sequences.**

d1xfda2 d1c7ga_ d2v1pa1 d2ez1a_ d1u08a_ d1lc5a_ d1b5pa_ d2ay1a_
d1bw0a_ d3pd6a_ d7aata_ d2csta_ d1j32a_ d1fg7a_ d4wlha_ d1o4sa_
d1v2da_ d1gdea_ d1m6sa_ d1v72a1 d1svva_ d1ajsa_ d1wsta1 d2f8ja2
d1lk9a_ d1vp4a1 d1xi9a1 d2gb3a1 d1yaaa_ d2bnma2 d2cfba1 d1o69a_
d1bj4a_ d3k28a_ d2bwpa_ d3lg0a_ d2fnua_ d3ou5a_ d1z7da1 d1b9ha_
d1iaya_ d4msoa_ d1bs0a_ d1s0aa_ d1sffa_ d3tqxa_ d1mdoa_ d3qboa_
d3pgya_ d1bjna_ d2dkja_ d1ejia_ d1vefa1 d4wxga_ d3bs8a_ d3gbxa1
d1w23a_ d2tbvc_ d1c8nc_ d1x36a_ d1smvc_ d1ng0c_ d4sbvc_ d1opoa_
d1f2nc_ d1g7ea_ d1qnia1 d1fwxa1 d2wo3b_ d1shxa_ d1f56a_ d1bypa_
d1azca_ d1ws8a_ d3u25a_ d1cuoa_ d1id2a_ d1rkra_ d2iaac_ d4dp1x_
d1nwpa_ d2gima_ d2i7sa_ d1qhqa_ d3ehbb1 d1fftb1 d1gyca3 d1hfua2
d1kbva1 d1kbva2 d1sdda1 d1sdda2 d1gyca1 d1gyca2 d1mzya1 d2bw4a1
d1aoza1 d1aoza3 d1aoza2 d1kyaa2 d1kyaa3 d1kyaa1 d1mzya2 d4e9sa1
d1sddb1 d1sddb2 d1hfua1 d1hfua3 d1oe1a2 d1oe1a1 d1v10a2 d1v10a3
d1v10a1 d1novc_ d2bbvc_ g1gk9.1 g1or0.1 g1e3a.1 d2bema_ d1pama1
d1ea9c1 d1qhoa1 d2fhfa2 d2fhfa1 d1k3ia1 d1j0ha1 d1cxla1 d2bhua1
d1edqa1 d1f1sa2 d1ut9a2 d1gvia1 d1txka1 d1zx5a1 d2phla2 d2phla1
d1fxza2 d1fxza1 d1lrha_ d1uija1 g1dgw.1 d2v09a_ d1uika2 d1uika1
d1dgwa_ d1ogpa1 d1ktja_ d1ds6b_ d1fstb_ d1ex0a1 d1g0da1 d1p8ba_
d4alac_ d1pjwa_ d1rera1 d1s6na_ d3uzqb_ d1tafa_ d1bh9b_ d1tafb_
d1jfib_ d1q9ca_ d1h3ob_ d2byka1 d1n1ja_ d4g92b_ d1bh9a_ d1jfia_
d1h3oa_ d2bykb1 d1eixa_ d3exra_ d2ffca1 d2fdsa1 d3n3ma1 d2czda_
d1q6oa_ d2yyua_ d1vqta1 d1dbta_ d3ldva1 d2bjca1 d2hsga1 d1qpza1
d1efaa1 d1mqta_ d1d4m3_ d1fmd3_ d2mev1_ g1aym.1 d1ev13_ d4q4w1_
d1r1a3_ d2x5ia_ d1rhi1_ g1r1a.1 g1d4m.1 g1rhi.1 d1b35b_ d1oopa_
d1tme1_ d4iv1a_ g1pvc.1 g1fpn.1 d1fpn1_ d4gb31_ d1d4m1_ d1fmd1_
d1ev11_ d2wzr3_ g1cov.1 d1qqp3_ g1fmd.1 d2x5ic_ d1rhi3_ g1oop.1
d1hxs1_ d1fpn3_ d1bev1_ g2mev.1 d1eah3_ d1aym1_ d1pvc3_ d1qqp1_

d1zba3_ d1h8ta_ d3vbhc_ g1b35.1 g1bev.1 d1hxs3_ g1ev1.1 d1bev3_
g1tmf.1 d1tmf1_ d1b35a_ d2mev3_ g1eah.1 g1h8t.1 d1eah1_ d4q4w3_
d1r1a1_ d1aym3_ d1pvc1_ d1zba1_ d1h8tc_ d1oopc_ d1tme3_ g1qqp.1
d4iv1c_ g1tme.1 d1r0ka3 d1j5pa3 d1vkoa2 d1vm6a2 d3qvsa2 d1gr0a2
d1p1ja2 d2ovga_ d1sq8a_ d1rzsa_ d3kz3a_ d3orca_ d3gpdg2 d2q49a2
d1hdgo2 d2g17a2 d2cvoa2 d2czca1 d3pwka2 d1obfo2 d1i32a2 d1zyao2
d2pkqo2 d1b7go2 d2hjsa2 d1cf2o2 d1vkna2 d1nvmb2 d2msya_ d2djoa1
d2r5za_ d1f43a_ d2lmda1 d1akhb_ d1s7ea1 d3nara_ d1zq3p1 d2craa1
d1mija_ d4uuta_ d3a01a_ d1x58a1 d3rkqa1 d3cmya1 d2cqxa1 d2da6a1
d1e3oc1 d1fjla_ d1mh3a1 d1ig7a_ d1x41a1 d1wh7a1 d1gt0c1 d2mw8a_
d1lfup_ d1x2ma1 d1b72a_ d1wi3a1 d1jgga_ d1wjha1 d1x2na1 d1mnmc_
d2cuea1 d3k2aa_ d2hosa_ d2jwta2 d1pufa_ d1octc1 d2hi3a_ d1b8ia_
d4j19a_ d3lnqa_ d1wh5a1 d1b8ib_ d1fexa_ d2cqra1 d1ug2a1 d2cqqa1
d2ajea1 d4a69c_ d1h8ac1 d2crga1 d2iw5b1 d1xc5a1 d1ofcx1 d2cu7a1
d1wgxa1 d2cjja1 d2ckxa1 d1gvda_ d1hcra_ d1tc3c_ d1gdta1 d1u78a2
d1igna2 d1bl0a1 d1bl0a2 d1d5ya1 d3lv8a_ d1s3ga1 d1zaka1 d1s4qa_
d3tvta2 d1uf9a_ d1khta_ d1ckea_ d1osna_ d1p5zb_ d4tmka_ d3cm0a_
d1tmka_ d1jaga_ d3h86a_ d1t0hb_ d1nn5a_ d2h92a_ d3kb2a1 d2c95a1
d1yj5a2 d1kgda_ d1ly1a_ d2ak3a1 d1vyva2 d1deka_ d2grja1 d1p3ja1
d1y63a_ d1e4ya1 d1s96a_ d3iija1 d1lvga_ d1nksa_ d1vyua2 d4e22a_
d1qf9a_ d1jjva_ d1q3ta_ d1ki9a_ d1lw7a2 d1teva_ d1kjwa2 d2iyva2
d1kaga_ d1viaa_ d1e6ca_ d1g3ma_ d1t8ta1 d1nsta_ d1fmja_ d1zrha_
d1ls6a_ d2a3ra_ d1aqua_ d2zptx_ d3ckla_ d1texa_ d1q44a_ d2gwha_
d1odfa_ d1uj2a_ d4gi7a_ d2jeoa1 d1rz3a_ d1z0ja1 d3umra2 d3gj0a1
d2gaoa1 d1yzga1 d1x3sa1 d1g7sa4 d1puja_ d1udxa2 d1kk1a3 d2o52a_
d1jwyb_ d1zcaa2 d1zcba2 d2fv8a1 d2cjwa_ d3dz8a1 d1xzpa2 d1vg8a_
d4qxaa_ d2ew1a1 d1x1ra1 d2bcgy_ d3doea1 d3cona_ d3rega_ d3wyga_
d1upta_ d2b6ha1 d1zd9a1 d1r5ba3 d1n0ua2 d1r2qa_ d2f9la1 d2rhda_
d3q3jb_ d3q85a_ d1efma1 d1r8qa_ d2gf9a1 d2akab1 d1ek0a_ d1z08a1
d2gcoa1 d2dy1a2 d4g01b_ d2cxxa1 d1svia_ d3q72a_ d1ni3a1 d4ac9a4
d2erxa1 d2bmea_ d1wxqa1 d2gf0a1 d4ldja1 d1zunb3 d1d5ca_ d4lv5b_
d1efca3 d1kmqa_ d2g6ba1 d3lrpa_ d1tada2 d2y8ea_ d4rd4a1 d1z06a1
d2heia_ d3a58b_ d1u0la2 d1d2ea3 d1zj6a1 d2ocba_ d3rwoa_ d1mr3f_
d2e9sa1 d4u5xa1 d2iwra_ d2fn4a1 d3bwdd_ d2nzja1 d2atva1 d2ksqa_
d3l43a_ d1mkya2 d1mkya1 d1wmsa_ d1jnya3 d2rmka_ d1f6ba_ d1f60a3
d1gota2 d1jala1 d2fh5b1 d1g16a_ d2wblc_ d1m7ba_ d1wf3a1 d1f5na2
d1t9ha2 d1z6xa1 d1z0aa1 d2fg5a1 d2a5ja1 d2bcjq2 d1s0ua3 d1ky3a_
d4y0va_ d4lhwa_ d2qmea1 d2atxa1 d1egaa1 d1ryha_ d1moza_ d1nrjb_
d2erya1 d3bfka_ d1puia_ d1fzqa_ d3j3az1 d3t5ga_ d1shza2 d3defa_
d2f7sa1 d3raba_ d3tsoa_ d3aq4a_ d2al7a1 d3sfva1 d3bc1a_ d2ncda_
d1sdma_ d3h4sa1 d2heha_ d2grya_ g3kin.1 d1kk8a2 d1br2a2 d1goja_
d1f9va_ d2mysa2 d1lkxa_ d3t0qa_ d4db1a1 d3gbja_ d1ry6a_ d1v8ka_
d1k8ka2 d1k8ka1 d3uleb1 d3i33a2 d3i33a1 d1bupa1 d1dkgd2 d1dkgd1
d4ci6a2 d1jcfa1 d4a2ba2 d2fxua1 d1yaga2 d1yaga1 d4cbua2 d1bupa2

d1g99a2 d2e1za2 d2e1za1 d1saza1 d1saza2 d1iqca2 d1iqca1 d1eb7a1
d1nmla2 d1nmla1 d3h1jd1 d1hroa_ d4gedb_ d3zooa_ d1mz4a_ d1gu2a_
d1f1fa_ d1dvva_ d1c6ra_ d1c75a_ d1e29a_ d1gdva_ d1cnoa_ d1h32b_
d1co6a_ d2bh4x_ d2aiua_ d5cytr_ d1jdla_ d1i8oa_ d2zboa_ d3dmia_
d1ls9a_ d1wvec_ d2yk3a_ d3ph2b_ d1dw0a_ d5aura_ d1ql3a_ d1qn2a_
d1f1ca_ d2nqya_ d1h4ga_ d1hixa_ d1oa2a_ d1olra_ d3b7ma_ d4h7ma1
d3wp3a_ d2vgda_ d1igoa_ d3zsea_ d2nlra_ d3wx5a_ d1oa3a_ d1pvxa_
d1m4wa_ d1f5ja_ d1oa4a_ d1ks5a_ d4ixla1 d1g66a_ d5ajha1 d1evsa_
d1cnt1_ d1pvhb_ d1z7ca_ d1huwa_ d1bgea_ d1rhga_ d1f6fa_ d1f45b_
d1i1rb_ d1axia_ d1etea_ d3tgxb_ d1scfa_ d2z3qa1 d3qb1a_ d1hula_
d1m48a_ d4nkqb_ d1eera_ d1v7mv_ d5e4ea_ d2o27a_ d3b5ka_ d2psma1
d4zf7a_ d3uf5a_ d4x51a_ d4ypgc1 d3oq3a_ d1lqsl_ d1n1fa_ d1au1a_
d1wu3i_ d3besl_ d3ux9a_ d1v8fa_ d2ejca_ d3muea1 d1ihoa_ d3q12a1
d4dnxa2 d1m8pa2 d1g8fa2 d1jhda2 d1x6va2 d3rbaa_ d2qtra_ d1nupa_
d3l92a_ d1f9aa_ d1qjca_ d2h29a_ d1vlha_ d1od6a_ d1lw7a1 d1mrza2
d4ruka_ d2el7a_ d1iq0a2 d1irxa2 d1r6ta2 d1ivsa4 d2cyba_ d1rqga2
d1j09a2 d2yxna_ d1f7ua2 d1h3fa1 d1ffya3 d2g36a1 d1jila_ d1n3la_
d1i6la_ d1pfva2 d1h3na3 d3n9ia_ d3sz3a1 d3sw2a_ d1moxc_ d1dx5i3
d1edmb_ d1autl2 d1autl1 d1nqlb_ d1dval1 d1szba2 d1kigl_ d2y5fl_
d1ijqa2 d1gk5a_ d1g1sa2 d1nt0a3 d1xdtr_ d1lmja2 d1lmja1 d1nzia2
d2wphe_ d1klil_ d1pfxl2 d1pfxl1 d1p9ja_ d1g1ta2 d1f7ea_ d4yzub_
d2mgpa1 d1b9wa1 d1b9wa2 d2mgpa2 d1ob1c2 d1n1ia2 d1n1ia1 d2npra2
d2npra1 d1m1xb5 d1qx2a_ d2y5ia_ d1psra_ d3n22a_ d2egda_ d4ggfc_
d2wcea_ d2luca_ d4aqia_ d3nsia_ d2lp3a_ d1j55a_ d1a4pa_ d1k8ua_
d1nsha_ d1pvaa_ d3fs7a_ d2pvba_ d1ttxa_ d1rwya_ d2kyfa_ d1rk9a_
d1a75a_ d1exra_ d1tiza1 d1nyaa_ d1g8ia_ d1ggwa_ d1wdcc_ d3fwba_
d1ggza_ d2o5ga_ d1ctaa_ d3i5gc_ d1sl8a_ d1fpwa_ d2scpa_ d2roaa_
d4aqra_ d3pn7b_ d1br1b_ d2mysb_ d1s6ca_ d1bjfa_ d2obha_ d1jfka_
d1jf0a_ d1qv0a_ d1lkja_ d2mlfc_ d2m97a_ d2d8na_ d4by5a_ d1jbaa_
d4nqga_ d1wdcb_ d2mysc_ d1j7qa_ d1s6ia_ d1uhka2 d1r2ua_ d1dtla_
d3kf9a_ d3pn7c_ d1qjta_ d1fi6a_ d1gjya_ d1y1xa_ d1hqva_ d1dvia_
d1df0a1 d1qxpa2 d4usla_ d1k94a_ d1wkya2 d1uuqa_ d3wh9a_ d4m1ra_
d1xyza_ d4xuya_ d2je8a5 d4bf7a1 d3cuia_ d1ur4a_ d1odza_ d2vzsa5
d1bqca_ d2x2ya1 d1hjqa_ d1qnra_ d4y7ea1 d1vjza_ d4k68a_ d2v3ga_
d4hpga_ d1nofa2 d1nq6a_ d1h4pa_ d1aq0a_ d3ziza_ d4bs0a_ d1ecea_
d1h1na_ d1ta3b_ d1w91a2 d1i1wa_ d1uhva2 d1us3a2 d1egza_ d7a3ha_
d1tvna1 d3nyda1 d1ghsa_ d2cyga1 d4xv0a_ d2c8na2 d1w32a_ d3emca_
d1v6ya1 d1foba_ d2uwfa1 d2fgla_ d3niya1 d2c0ha1 d1rh9a1 d1yq2a5
d1szna2 d1fa2a_ d1pama4 d5do8a1 d1ea9c3 d1hx0a2 d2fhfa5 d1qhoa4
d1avaa2 d1m53a2 d4m8ua1 d2gjpa2 d1lwha2 d4howa1 d1ktba2 d1j0ha3
d3vm5a1 d2x1ia_ d1wzaa2 d1uasa2 d2diea1 d1mxga2 d1ht6a2 d1eswa_
d5cpsa_ d1ud2a2 d1zjaa1 d3gbea1 d1kwga2 d1wdpa_ d1gcya2 d1g5aa2
d1hvxa2 d3dhpa2 d2bhua3 d4do4a1 d3qgva1 d1e43a2 d1g94a2 d2d3na2
d1gjwa2 d1gvia3 d1x1na1 d1r7aa2 d1tz7a1 d4c7ga2 d2gjxa1 d1jaka1

d1yhta1 d4toqa_ d1wb0a1 d1w9pa1 d1eoka_ d1itxa1 d1kfwa1 d1goia2
d2gsja_ d1hjxa1 d1jnda1 d1vf8a1 d3wdpp1 d3wq5a_ d1gnxa_ d4gxpa_
d4hz8a_ d1e4mm_ d3aiua_ d1wcga1 d1qvba_ d3ahxa_ d1v02a_ d2dgaa_
d1pbga_ d1vffa1 d1qoxa_ d4ha4a_ d1mixa2 d1e5wa2 d1isna2 d2al6b2
d3syxa1 d1mkea1 d2p8va_ d1xoda1 d1i7aa_ d1evha_ d1qc6a_ d1egxa_
d1xl4a1 d1n9pa_ d3vsqa_ d2gixa1 d2v76a_ d1wj1a1 d1aqca_ d1qqga2
d1qqga1 d1n3ha_ d4dbba_ d3f0wa1 d1p3ra_ d1wgua1 d2cy4a1 d3hqca_
d1wvha1 d1xr0b1 d3nvva1 d1krha3 d2h88b1 d1n62a2 d1t3qa2 d1kf6b2
d1zoyb1 d1ffva2 d1dgja2 d1rm6c2 d1h6ua1 d9ldta1 d2ewda3 d1up7a1
d2dfda1 d1s6ya1 d1vjta1 d7mdha1 d1hyea1 d1t2da1 d1ojsa3 d1b8pa1
d1ldna1 d1hyha1 d1civa1 d2a92a1 d1ez4a1 d1i0za1 d1y6ja1 d1gv0a1
d1ur5a1 d1llda1 d1v8ba1 d1gdha1 d1l7da1 d2dlda1 d1mx3a1 d1ygya1
d1qp8a1 d1j4aa1 d2naca1 d1npya1 d3t4ea2 d1a4ia1 d1o0sa1 d2hk9a2
d2egga2 d1b26a1 d1bvua1 d1gq2a1 d1c1da1 d1leha1 d1gtma1 d1euza1
d3dona2 d1edza1 d1gpja2 d1vi2a1 d1p77a1 d1nyta1 d1b0aa1 d1vl6a1
d1v9la1 d1txga2 d3f47a1 d1mv8a2 d2pv7a2 d1xc2a3 d2ahra2 d4xiya1
d2f1ka2 d1pgja2 d1i36a2 d1np3a2 d1lj8a4 d1teaa3 d1qmga2 d2i76a2
d2g5ca2 d1wdka3 d1dlja2 d3cuma3 d1kola2 d1f8fa2 d1qora2 d1jqba2
d1vj0a2 d1cdoa2 d1gu7a2 d1d1ta2 d1xa0a2 d2dm6a2 d1jvba2 d1rjwa2
d1llua2 d1h2ba2 d1tt7a2 d1piwa2 d1o89a2 d1iz0a2 d1e3ja2 d1yb5a2
d1uufa2 d1e3ia2 d3gpdg1 d1vc2a1 d2nvwa1 d1u8fo1 d1ydwa1 d1hdgo1
d2g17a1 d1cf2o1 d2czca2 d1r0ka2 d1obfo1 d2cvoa1 d1ebfa1 d1h6da1
d1i32a1 d1zh8a1 d2q49a1 d1qkia1 d3pzra1 d1xeaa1 d1vkoa1 d1vm6a3
d3qvsa1 d2pkqo1 d1b7go1 d1gr0a1 d1p1ja1 d1lc0a1 d1j0xo1 d3pwka1
d2hjsa1 d1zyao1 d1vkna1 d1tlta1 d1nvmb1 d3sjua_ d3ojfa1 d1xu9a1
d2jjya1 d1n7ha_ d1y1pa1 d1h5qa_ d1jtva_ d4fs3a_ d2bkaa1 d1gy8a_
d2c5aa1 d1ulsa_ d1ooea_ d2b69a1 d1y5ma1 d1spxa_ d1ae1a_ d1bxka_
d1ydea1 d1gega_ d1hdoa_ d1o5ia_ d1e6ua_ d1snya1 d2rhca_ d3zv4a_
d5ceja_ d2d1ya1 d1fjha_ d3w8ea_ d2fmua1 d1x1ta_ d1rpna_ d3ay7a1
d1k2wa_ d1eq2a_ d2hrba1 d3ruaa_ d2ew8a1 d4hwka_ d1xgka_ d2bd0a1
d1yo6a1 d2a35a1 d2ntva_ d1vl8a_ d1hdca_ d4bkua_ d2ag5a1 d2q45a_
d1nffa_ d4iy1a_ d1zbqa1 d1kewa_ d1ek6a_ d1ja9a_ d2q46a2 d1iy8a_
d1cyda_ d2ptga_ d1hxha_ d1uzma1 d3lyla_ d4m89a_ d3wyea_ d2blla1
d1w6ua_ d1sb8a_ d2q2va_ d4nr0a_ d1e6wa_ d2gasa_ d3oiga1 d2bgka1
d1mxha_ d1vl0a1 d1rkxa_ d1r6da_ d1wmaa1 d1e7wa_ d2c07a1 d1xkqa_
d3tzha_ d1g0oa_ d3lz6a_ d1qyca_ d1edoa_ d1gz6a_ d1oc2a_ d1t2aa_
d1zema1 d2xoxa_ d1z45a2 d1geea_ d3lt0a_ d4e6pa_ d2wyua_ d1db3a_
d4q9na_ d1xhla_ d4o1ma_ d1fmca_ d2uvda_ d2gdza1 d1uaya_ d2z1ma_
d2o23a2 d2fr1a2 d2fr1a1 d1qsga_ d1qyda_ d4zjua_ d2a4ka1 d1yb1a_
d1wvga1 d1yxma1 d3f4ba_ d1mg5a_ d1xg5a1 d1r55a1 d3gboa_ d1atla_
d1kufa_ d4q1la_ d1quaa_ d1yp1a_ d1wnia_ d3nqyb_ d4gera_ d1mdwa_
d1ziva1 d1zcma1 d3ioqa_ d1fh0a_ d1o0ea_ d1khqa_ d1m6da_ d2cb5a_
d1cs8a_ g8pch.1 d1s4va_ d2pnsa_ d1vsna1 d1mira_ d1iwda_ d2oula_
d7pcka_ d3bwka_ d3bcna_ d2p86a_ d1pvja_ d1pcia_ d1gece_ d1deua_

d4bs6a_ d1xkga1 d1cqda_ d1jqpa2 d2c0ya_ d2bdza_ d3p5ua_ d2o36a_
d1k9xa_ d1ex4a2 d1c6va_ d2jk6a2 d2jk6a1 d1feca1 d1feca2 d1lpfa1
d1lpfa2 d1xhca1 d1xhca2 d1dxla2 d1dxla1 d1gera2 d1m6ia2 d1onfa1
d1onfa2 d3lada2 d3lada1 d1w4xa1 d1w4xa2 d1gesa2 d1mo9a1 d1xdia1
d1fl2a2 d1fl2a1 d1fcda1 d1fcda2 d1h6va2 d1h6va1 d1mo9a2 d1gv4a2
d1gv4a1 d2gv8a2 d2gv8a1 d1aoga1 d1aoga2 d1hyua1 d1ebda1 d1ebda2
d3dk9a1 d1q1ra2 d1q1ra1 d1gesa1 d1zbfa1 d2z1ha_ d2etja1 d3qioa1
d1jl1a_ d2e4la_ d2lsna1 d1io2a_ d1uaxa_ d2yv0x_ d1mu2a1 d1ekea_
d1i39a_ d1pn0a1 d1reoa1 d1ju2a1 d2f5va1 d3c96a1 d2gmha1 d1gpea1
d1o5wa1 d1n4wa1 d1k0ia1 d2dw4a2 d2ivda1 d1seza1 d2voua1 d1kdga1
d1s5ja1 d2hbja2 d2igia_ d1wlja_ d1qhta1 d4dlea1 d1tgoa1 d2f96a1
d3tr8a1 d3mxma_ d1q8ia1 d1y97a1 d1w0ha_ d1kfsa1 d2py5a1 d1noya_
d1d5aa1 d1yt3a3 d2guia_ d3mwpa1 d4kb1a_ d1js9c_ d1f15b_ d1za7a_
d1laja_ d2csua1 d3q9ub_ d2yv1a1 d3q9na_ d2d59a_ d1oi7a1 d2yv2a1
d1euca1 d1y81a1 d4iuaa3 d4bvva_ d5hpga_ d3k65a_ d1ki0a3 d1ki0a2
d1ki0a1 d1a0ha1 d1pmla_ d3sp8a3 d2feba1 d1wgqa1 d1v89a1 d2coda1
d1zc3b_ d1wg7a1 d1u5ea1 d1v61a1 d2coca1 d2ys1a1 d1v5pa1 d1ntya2
d1wjma1 d1wi1a1 d1u29a_ d1xx0a1 d1unqa_ d2zkmx3 d1fhoa_ d1v88a1
d2adza1 d1x1fa1 d1p6sa_ d2coaa1 d1btka_ d1v5ma1 d1faoa_ d2x18a_
d1dyna_ d1x1ga1 d4kaxb_ d2j59m1 d1xcga2 d1txda2 d1u5da1 d1dbha2
d1w1ha_ d1upqa_ d1v5ua1 d1foea2 d2fjla1 d1eaza_ d2dfka2 d2cofa1
d1kz7a2 d1r7ha_ d1faaa_ d1wika1 d1xfla_ d1ttza1 d2f51a1 d1ti3a_
d1nhoa_ d1sena_ d2i4aa_ d1ep7a_ d1zmaa1 d1fo5a_ d3lefa1 d3diea1
d2yana1 d1z6na1 d1oaza_ d3zywa1 d4aj8a_ d1nm3a1 d4fiwa_ d1wmja_
d4hs1a1 d1iloa_ d1xwaa1 d1wjka1 d2vm1a_ d1fb6a_ d1gh2a_ d1h75a_
d1nw2a_ d1kx5b_ d3a6na_ d4m6ba2 d2nqbd_ d3wkjd_ d2yfwe_ d4h9na_
d5bnxa_ d1kx5c_ d3r45a_ d1eqzb_ d4jjnb_ d1f66c_ d1eqza_ d4wnnb_
d1kx5a_ d1u35c1 d1id3a_ d2nqbc_ d1kx5d_ d3us3a3 d3us3a1 d1um8a_
d1ixza_ d1qvra2 d1qvra3 d1qzma_ d1sxjc2 d1jr3d2 d2p65a_ d1l8qa2
d1g8pa_ d3hu3a3 d1sxjd2 d1njfa_ d1in4a2 d1sxja2 d1tuea1 d2ce7a2
d1w5sa2 d1sxjb2 d1d2na_ d1ny5a2 d1ofha_ d2gnoa2 d2a5yb3 d1fnna2
d1u0ja1 d1lv7a_ d1iqpa2 d2fnaa2 d1ixsb2 d4gita_ d1sxje2 d1g41a_
d1pd212 d1nhya2 d2a2ra2 d1k0dd2 d1f2ea2 d1gsua2 d1f3ba2 d2fhea2
d2pvqa1 d1tw9a2 d1oyja2 d1e6ba2 d1k0ma2 d3ik7a1 d4is0a1 d1r5aa2
d1jlva2 d1m0ua2 d3gtub2 d1z9ha2 d1okta2 d1axda2 d2gsra2 d1tu7a2
d1k3ya2 d1zgma1 d1b48a2 d1gnwa2 d1gwca2 d2gsta2 d1glqa2 d1oe8a2
d1pn9a2 d1v2aa2 d1fw1a2 d1duga2 d3vrda1 d3vrda2 d1fcdc2 d1fcdc1
d1h1oa2 d1h1oa1 d1m70a2 d3zxha_ d1cgla_ d1rm8a_ d1eaka2 d1hy7a_
d2jsda_ d1cxva_ d1hv5a_ d1i76a_ d1ig8a1 d1czan2 d1czan3 d1czan1
d1ig8a2 d1czan4 d1bg3a4 d1bg3a3 d1bg3a1 d1m32a_ d1ibja_ d1c7na_
d1iuga_ d4q75a_ d2bkwa1 d1t3ia_ d1n8pa_ d1qz9a_ d1jf9a_ d1eg5a_
d1p3wa_ d3kgwa_ d1cl1a_ d2ctza1 d1vjoa_ d2rfva_ d1gc0a_ d1cs1a_
d1d2fa_ d2ch1a1 d1qgna_ d1pffa_ d1elua_ d3wyha_ d4g9sa1 d3gxra_
d1qqya_ d1f6ra_ d1ivma_ d2gv0a_ d1iiza_ d2z2fa_ d1j8xa_ d1ghla_

d1b9oa_ d174la_ d169la_ d2o4wa_ d1p37a_ d1k28a3 d1xjta1 d1jtma_
d2f2qa_ d4e97a1 d1g4ma1 d3w3ea_ d4tx7a_ d3cqla_ d4msta_ d3iwra_
d1dxja_ d2z37a_ d4dyga_ d2pjsa1 d1klla_ d2i7ra1 d1ecsa_ d1xqaa1
d5cj3a1 d1npba_ d1jifa_ d2p7oa_ d1r9ca_ d1nkia_ d1mpya2 d1kw3b2
d1kw3b1 d4ghga1 d1tfza1 d3isqa1 d3isqa2 d1sqia1 d1sqia2 d1lkda2
d1lkda1 d1mpya1 d1cjxa2 d1sp9b2 d1t47a1 d1t47a2 d1f1ua2 d1sp8a1
d1sp8a2 d1f1ua1 d1etob_ d1umqa_ d1g2ha_ d1ro5a1 d1w5qa_ d2c1ha_
d1ohla_ d1l6sa_ d1qsta_ d2beia1 d4m3sa_ d1u6ma1 d1ygha_ d1yk3a1
d3dr6a_ d1xmta_ d1z4ea1 d2ge3a1 d1bo4a_ d2fiaa1 d2jdca1 d1y9wa1
d3to7a_ d1wwza1 d2fl4a1 d2ozua1 d2b5ga_ d2cy2a1 d2p0wa_ d4pswa_
d1yrea1 d1yr0a1 d1m4ia_ d2gana1 d1yvka1 d1ghea_ d2g3aa1 d1y7ra1
d2euia1 d1q2ya_ d2fsra1 d1xeba_ d2ae6a1 d1cjwa_ d1qsma_ d2aj6a1
d1y9ka1 d1i12a_ d1vkca_ d2atra1 d1n71a_ d2d4pa1 d2fiwa1 d2fe7a1
d1tiqa1 d2ou2a_ d1yx0a1 d2fcka1 d1p0ha_ d2j8ma_ d2pq8a_ d1z4ra1
d1s3za1 d1ufha_ d1xvqa1 d1pn0a2 d3hy2a_ d2cvba1 d1o8xa_ d1lu4a_
d1we0a1 d2bmxa1 d2a4va1 d1psqa_ d1xiya1 d1q98a_ d1n8ja_ d1zofa1
d2ggta_ d3tjja_ d4je1a1 d1o73a_ d3s9fa_ d2zcta_ d1qmva_ d1jfua_
d2b1ka_ d5dvba1 d2b5xa1 d2v2ga_ d1st9a_ d3tb2a_ d4llra_ d4fh8a1
d1e2ya_ d1knga_ d2h30a_ d1i5ga_ d2z9sa_ d2f8aa1 d1tp9a1 d2b7ka_
d1nm3a2 d3hvva_ d2wfca1 d4kcea_ d1xvwa1 d4gqca1 d3qpma_ d4k1fa_
d1zzoa1 d1gp1a_ d1prxa_ d2m5ta_ d1cqqa_ d1wqsa_ d5bpea_ d2ipha_
d1lvoa_ d2bhga1 d3tnta_ d3sjia_ d3d23a1 d2fyqa_ d3tloa_ d3zvea1
d2hrva_ d3q3ya1 d4asha1 d1e88a1 d1l6ja5 d1l6ja4 d1eaka4 d1eaka3
d1l6ja3 d1h8pa2 d1h8pa1 d1etha2 d1w52x1 d2ppla1 d1lpbb2 d2oxea1
d1bu8a2 d1rp1a2 d1ys1x_ d4hs9a_ d1ex9a_ d1ku0a_ d3qmma_ d1lgya_
d4l3wa_ d1gz7a_ d1ukca_ d2ftba_ d1pmpa_ d2rcqa1 d1fdqa_ d4lkpa_
d1sa8a_ d1vyfa_ d3vg7a1 d4azqa1 d1o8va_ d5bvqa_ d1p6pa_ d2qo4a_
d1opba_ d1yiva1 d1o1va_ d4bvma1 d4tkba_ d4i3ba_ d1bwya_ d1ftpa_
d1ggla_ d4gkca_ d1cbia_ d3p6da1 d1kqwa_ d2a0aa1 d1xwdc1 d3kj4a_
d1w8aa_ d2ifga3 d1p9ag_ d1xkua_ d3p0ya1 d1igra1 d1igra2 d1n8yc2
d1n8yc1 d1m6ba2 d1m4la_ d2bk7a_ d3fjua_ d1z5ra1 d1kwma1 d1uwya2
d1jqga1 d2c1ca1 d1dtda_ d1h8la2 d1i26a_ d1cixa_ d1y29a_ d1v7fa_
d2jtba_ d1d1ha_ d1g9pa_ d1lupa_ d1qk7a_ d1mb6a_ d2a2va_ d1niya_
d1i25a_ d2mpqa_ d1ie6a_ d1kqha_ d1la4a_ d2mxoa1 d1dl0a_ d1koza_
d1qk6a_ d1emxa_ d1lqra_ d1c6wa_ d1rmka_ d1ixta_ d1eyoa_ d1fu3a_
d1g1pa_ d1f3ka_ d1fyga_ d1rypd_ d1irua_ d1rypc_ d3unfk_ d1rypa_
d1j2pa_ d1irui_ d1j2qh_ d3unfh_ d1m4ya_ d1rypb_ d1irul_ d1irug_
d1iru2_ d1q5rh_ d1rypg_ d1g3ka_ d1iruf_ d1q5qa_ d1e94a_ d1irub_
d1ryph_ d3ty6a_ d1iruj_ d1iruk_ d1rype_ d1irud_ d1irue_ d3mi01_
d1rypf_ d1iruh_ d2z3ba_ d1iruc_ d3mka2_ d1iru1_ d1ny721 d1a6ca3
d1a6ca2 d1a6ca1 d1pgl22 d1pgl21 d1pgl11 d1ny711 d1ofda3 d1ea0a3
d1ecfa2 d1q15a2 d1ct9a2 d1ao0a2 d1te5a_ d3ubub_ d1egia_ d1qo3c_
d1v7pa_ d1tdqb_ d3ubua_ d1c3ab_ d1h8ua_ d3bx4b_ d1t8ca1 d1sb2a1
d1qdda_ d2ziba1 d3cada_ d1byfa_ d1j34b_ d1jwia_ d1jzna_ d1c3aa_

d1wmza_ d3bdwb_ d1hq8a_ d1fifa1 d1dv8a_ d3hupa_ d1wk1a1 d1umra_
d2afpa_ d3bx4a_ d1gz2a_ d1sl6a_ d1v7pb_ d3g8ka_ d1ixxa_ d1j34a_
d1jwib_ d1g1sa1 d1y17a1 d1ja3a_ d1fvub_ d3bdwa_ d1sb2b1 d1fvua_
d1umrc_ d1oz7a_ d4m17a_ d3cfwa1 d1ypqa1 d1g1ta1 d3fd4a_ d3l9jc_
d4dn8a1 d1v4lb_ d1rdl1_ d2msba_ d4w9ta_ d1jvna1 d4x2ra1 d1ka9f_
d4axka1 d1thfd_ d1h5ya_ d4u28a_ d2y88a_ d4wd0a_ d1vzwa1 d1ix2a_
d4lt9a1 d3tc7a1 d3hoja_ d1i4na_ d3vnda_ d3nl8a1 d1geqa_ d3nava1
d1v5xa_ d4a2ra1 d3tc6a1 d1ujpa_ d4ijba_ d1xcfa_ d1vc4a_ d1rd5a_
d1qopa_ d3b5va_ d1jx4a2 d3gv5b1 d1t94a2 d3bq0a2 d1kyqa1 g1gxs.1
g1wht.1 d1azwa_ d1mtza_ d2xqfa_ d1f6wa_ d1dx4a_ d1qe3a_ d1k4ya_
d4qwwa_ d2ha2a_ d3c8da2 d2pbla1 d1jkma_ d1jjia_ d1lzla_ d1gl9b1
d1gkub2 d2qvba_ d2v9za_ d4kafa1 d1jmxa4 d1jmxa3 d1pbya4 d1b9ra_
d1offa_ d1frra_ d3wcqa_ d1e9ma_ d1wria_ d1i7ha_ d3av8a_ d1czpa_
d1rfka_ d3p1ma1 d3b2ga_ d2bt6a2 d1xm5a_ d1xaxa_ d1tvia_ d1s5ja2
d1qhta2 d4dlea2 d1tgoa2 d3py8a2 d1kfsa2 d2py5a2 d1d5aa2 d1xova2
d1f74a_ d3l21a_ d2e1da_ d1to3a1 d1w3ia_ d1sfla_ d1n7ka_ d1vcva1
d4imea_ d1wa3a_ d2ocza1 d1f2ja_ d3m16a_ d2yxga_ d1ub3a_ d3a5fa_
d1fbaa_ d1l6wa_ d1xfba1 d1wx0a1 d1vpxa1 d1mxsa_ d2a4aa1 d1xkya1
d3cq0a1 d3s1xa_ d1f05a_ d1fdja_ d3cpra1 d4d2ja_ d1yloa2 d1y7ea2
d1y0ya3 d1lfwa1 d1vixa1 d1fnoa4 d1vhoa2 d1ysja1 d1vhea2 d1z2la1
d2fvga2 d1vgya1 d1r3na1 d2grea2 d1xmba1 g1q7l.1 d1rtqa_ d3rp2a_
g1ucy.1 d1fuja_ d2zchp_ d3w94a_ d1si5h_ d1lo6a_ g2pka.1 d1cgha_
d1kigh_ d1a5ia_ d3s9ba_ d1hj8a_ d1ao5a_ d2anya_ d1q3xa1 d1gvza_
d1azza_ d4gsoa_ d2f91a1 d1iaua_ d1gdna_ d1eufa_ g1fiw.1 d1eq9a_
d4nfea_ d2rdla_ d1spja_ d1elva1 d1a7sa_ d1ekbb_ d1pfxc_ d1yc0a_
d2aiqa_ g1fiz.1 d1fi8a_ d4k60a_ d4dgja_ d1mzaa_ d1op0a_ d3tjva_
d1pytd_ d1sgfg_ g1gg6.1 d2eeka_ g1rtf.1 d1a0ja_ d3g01a_ d1pytc_
d4e7na_ d1ddja_ d1z8ga1 d2hlca_ d1npma_ d1brup_ g1jou.1 d1sgfa_
d1autc_ g2hnt.1 g1kdq.1 d1orfa_ d3s69a_ d4crga_ d1fxya_ d1bqya_
d1svpa_ d1mbma_ d3su6a1 d1cu1a1 d4agka_ d1vcpa_ d1q31a_ d3e90b_
d1ep5a_ d3u1jb_ d1dzka_ d4tlja_ d2a2ua_ d4wfva_ d3t1da_ d4iaxa_
d3zq3a_ d2nnda_ d1i4ua_ d1kxoa_ d1bbpa_ d1kt7a_ d1exsa_ d3s26a1
d1lf7a_ d1qfta_ d4gh7a_ d1z24a1 d1qabe_ d1xkia_ d1ew3a_ d3bx7a_
d4iawa1 d1n0sa_ d1gkab_ d3kzaa_ d2k23a_ d2l9ca1 d2joza1 d1gm6a_
d3kffa_ d3saoa1 d1e5pa_ d1yupa1 d1iiua_ d3lgia_ d3i77a_ d2z9ia2
d4nsva_ d1lcya2 d4jcna_ d1agja_ d1os8a_ d1hpga_ d1p3ca_ d1l1ja_
d1e69a_ d1mv5a_ d3g60a4 g1ii8.1 d1ewqa2 d1ji0a_ d1qhla_ d1v43a3
d2wmfa1 d1vpla_ d4a82a2 g1f2t.1 d2d62a1 d3tuic1 d2bbsa1 d3g60a3
d2ixea_ d1g6ha_ d1sgwa1 d2qi9c_ d3rlfa1 d3b60a1 d3d31a2 d2pzea1
g1xew.1 d1jj7a_ d1oxxk2 d2onka1 d1w1wa_ d1iwea_ d1qf5a_ d1vmaa2
d1nija1 d1np6a_ d2p67a1 d3ihla_ d1okkd2 d1ls1a2 d1s1ma2 d1iona_
d2v40a_ d1dj3a_ d3hida_ d2vo1a1 d2g0ta1 d1vcoa2 d1j8mf2 d1g3qa_
d1xjca_ d1p9ba_ d1qzxa3 d1ihua2 d1ihua1 d2qm8a_ d1dj2a_ d4ioja_
d1cp2a_ d1yrba2 d1hyqa_ d3gqba2 d1skye3 d1xp8a1 d1tf7a2 d1tf7a1

d1ye8a1 d1skyb3 d2qe7d2 d4blta_ d4huta_ d3ldaa2 d4hyya_ d2i3ba1
d1cr1a_ d3j9tb2 d3ntua2 d1vdza1 d2z43a_ d1e9ra_ d1nlfa_ d3ziaa2
d1n0wa_ d1g6oa_ d3ziad2 d3j9ta2 d1pzna2 d3gqbb2 d2i49a_ d1nnfa_
d1xvxa_ d1ursa_ d1h3da1 d1iuda_ d4m1va1 d1twya_ d3e13x_ d2nxoa1
d4pfua_ d3ttma_ d1elja_ d2qmwa1 d1us5a_ d1y3na1 d1jeta_ d1uqwa_
d4f19a1 d2esna2 d3thia_ d3fasa1 d2uyeb_ d2xwva1 d1r9la_ d2fyia1
d1pb7a_ d2ozza1 d1sw5a_ d1nh8a1 d1vr5a1 d2g29a_ d1xs5a_ d3lswa_
d2z23a_ d3w9va_ d3tcha_ d1q35a_ d1z7me1 d3q27a_ d1p99a_ d1ve4a1
d2i6ea1 d4mcoa1 d4n8ya_ d1eu8a_ d1zbma1 d1hsla_ d1ixca2 d1j1na_
d1xvya_ d1y9ua_ d2z22a1 d1xoca1 d4oera_ d1wdna_ d5f1qa1 d1xt8a1
d1i6aa_ d4gwoa_ d4igta1 d1pc3a_ d4mmpa1 d1o63a_ d2czla1 d2onsa2
d4mija1 d4ok5a1 d2bmfa1 d2bmfa2 d1yksa1 d1yksa2 d2qeqa1 d2jlqa1
d1a1va2 d1m8pa3 d1c4oa1 d2p6ra3 d2p6ra4 d1oywa2 d1oywa3 d1wrba1
d1w36b2 d1w36b1 d1c4oa2 d3bora_ d1t6na_ d2eyqa5 d2eyqa3 d2eyqa2
d2zu6a2 d1veca_ d1uaaa1 d1w36d1 d2g9na1 d1z3ix2 d2rb4a_ d1hv8a1
d1hv8a2 d1t5ia_ d1w36c2 d2j0sa2 d2j0sa1 d1fuka_ d3hjha1 d1z5za1
d1qdea_ d1z63a1 d1s2ma2 d1s2ma1 d1wp9a1 d1wp9a2 d1gm5a4 d3dina4
d3dina3 d1q0ua_ d1w36c1 d1z3ix1 d1nkta3 d1nkta4 d1uaaa2 d1w36d2
g1qhh.1 d2fwra2 d2fwra1 d3q3ma_ d1za0a1 d2inca_ d1jk0a_ d2incb_
d1mhyb_ d3hf1b_ d1mtyb_ d1r2fa_ d2uw1a_ d1mhyd_ d3n37a_ d1afra_
d1oquc_ d1syya_ d1mtyd_ d2o1za_ d1uzra_ d1smsa_ d2fkza_ d1zuja1
d1s3qa1 d2fjca1 d1z6om1 d1veia_ d4dyya_ d1jiga_ d3gvya_ d2pyba_
d4cy9a1 d3bvfa1 d1r03a_ d2cf7a_ d1nf4a_ d1zs3a1 d4iwka_ d3ka8a_
d3np0x_ d1n1qa_ d1lb3a_ d4e6ka_ d1z6oa1 d2fzfa1 d4dyua_ d3ge4a_
d1jgca_ d4am4a_ d2z6ma_ d1ji5a_ d1vlga_ d1lkoa1 d1krqa_ d3qz3a_
d1o9ra_ d2ffxj_ d1euma_ d1yv1a1 d2v2pa_ d1j30a_ d4di0a_ d1dpsa_
d1oewa_ d2qzxa_ d2asia_ d1psoe_ d1miqa_ d1smra_ d2h6ta_ d1pfza_
d1izda_ d3hd8a_ d1j71a_ d1dpja_ d4aa9a_ d1t6ex_ g1b5f.1 g1lya.1
g1htr.1 d1qdma2 d1bxoa_ d1wkra_ d1tzsa_ d1ibqa_ d2qzwa_ d1bdqa_
d3ggta_ d3ixoa_ d1mrxa_ d2haha_ d1tw7a_ d4njsa_ d1sgua_ d2p3da_
d1nsoa_ d3d3ta_ d2rkfa_ d1c6ya_ d2rspa_ d3ogpa_ d3ekwa_ d2o4na_
d2j9ja_ d1baia_ d3ggua_ d3lzua_ d4npua_ d4j55a_ d2r8na_ d2fxda_
d2p3ca_ d4dqea_ d3ttpa_ d3t3ca_ d2p3aa_ d2hc0a_ d2fdda_ d3mwsa_
d1eh3a_ d3skpa_ d1dtza1 d1dtza2 d1ieja_ d1jnfa2 d1ce2a1 d1jnfa1
d1b1xa1 d1jw1a1 d1jw1a2 d1ryoa_ d1h76a1 d1h76a2 d1k8rb_ d1wgra1
d1ef5a_ d1wfya1 d1e8ya3 d1wgya1 d1lfda_ d2cs4a1 d1wxma1 d2byea1
d3kh0a1 d2byfa1 d1wxaa1 d1e7ua3 d1e57b_ d1auyb_ d3h33a_ d1rwja_
d19hca_ d1os6a_ d3bxua_ d3h4na_ d3caoa_ d1j0pa_ d2ctha_ d1duwa_
d1ofwa_ d2e84a_ d1hh5a_ d1gyoa_ d3h34a_ d1dszb_ d1a6ya_ d3cbba_
d1ynwa1 d1r4ia_ d1hcqa_ d2hana_ d2hanb_ d2enva1 d1kb4b_ d2nllb_
d1lo1a_ d1cita_ d2dara2 d2dara1 d1wyha2 d2dloa2 d1rutx4 d1rutx3
d2cuqa2 d1x4la2 d1x4la1 d1x3ha2 d1x3ha1 d2d8za1 d1x61a2 d1x6aa1
d1x6aa2 d2cora2 d2cora1 d1v6ga1 d1v6ga2 d2d8za2 d1x64a1 d1x64a2
d1b8ta1 d2co8a1 d1x63a2 d2dj7a2 d2dj7a1 d2cu8a2 d2cu8a1 d1g47a2

d1g47a1 d1j2oa1 d1wyha1 d2dloa1 d1wiga1 d1wiga2 d2cupa2 d2cupa3
d1x68a1 d2d8ya2 d2d8ya1 d1m3va2 d1x62a1 d1x68a2 d1x61a1 d1b8ta3
d1b8ta2 d2d8xa2 d2d8xa1 d1x4ka1 d1x4ka2 d2cuqa1 d1m3va1 d2co8a2
d1j2oa2 d1x63a1 d2cura1 d2cura2 d1x62a2 d2lj7a_ d1n4na_ d2m8bb1
d2kyqa_ d3psma_ d1jxca_ d2gl1a_ d1ugla_ d4uj0a_ d2k4ua_ d1hp2a_
d2lu9a_ d1baha_ d2meoa1 d1wm7a_ d1hlya_ d1n8ma_ d1jlza_ d1wpda_
d1c55a_ d1pvza_ d1wt7a_ d1wz5a_ d1m2sa_ d1pjva_ d1c49a_ d1qkya_
d2ktca1 d1wmta_ d1du9a_ d1j5ja_ d1r1ga_ d1t0za_ d2i61a_ d1t1ta_
d1kv0a_ d2lkba_ d1npia_ d1wwna_ d1pe4a_ d2kbka_ d2li7a_ d1djta_
d2kbha_ d1dq7a_ d1sega_ d1omya_ d1zywa1 d1jzaa_ d1t61c1 d1iyjb4
d1iyjb5 d1iyjb3 d1o7ia_ d4ipca1 d1x3ea_ d4apva_ d1jb7a1 d1jb7a2
d1jb7a3 d3kdfa_ d1ue1b_ d1wjja1 d3eiva_ d1xjva2 d1xjva1 d2ccza2
d1se8a_ d1l1oc_ d1jb7b_ d1qvca_ d3ulla_ d1miua4 d1miua5 d1miua3
d3g48a1 d1gd7a_ d1mkha_ d1jjcb3 d1pyba_ d1y14b1 d2nn6i1 d1i94l_
d2zjra2 d1smxa_ d2qamc2 d1hh2p1 d2r7da1 d1rl2a2 d1wfqa1 d3bzka4
d1x6oa2 d3a0ja_ d2j01d2 d2ja9a1 d4ql5a_ d1hr0w_ d1luza_ d2n3sa_
d2vnud1 d1h95a_ d1go3e1 d2nn6h1 d1ne3a_ d2mo1a_ d1iz6a2 d1jjga_
d1e3pa2 d2ix0a3 d2ix0a2 d2lxja_ d1ueba3 d1ueba2 d2z0sa1 d1u0la1
d2ix0a1 d2eifa2 d2je6i1 d3i2za1 d2c35b1 d2ahob2 d1t9ha1 d3i4oa_
d2qall1 d1jt8a_ d2nn6g1 d1g6pa_ d3cama_ d1i94q_ d1c9oa_ d1d7qa_
d2ba0a1 d2vnud2 d2vnud3 d1khia2 d2qalq1 d1wi5a1 d1xtda2 d1dgsa2
d1ckma1 d1p16a1 d1x9na2 d1pfsa_ d2a1ka_ d1je5a_ d2shpa1 d1yfoa_
d1l8ka_ d2ooqa1 d1g4us2 d1p15a_ d1lara2 d1lara1 d1wcha_ d2fh7a2
d2fh7a1 d1ohea1 d1ohea2 d2f4da_ d1rxda_ d2hxpa_ d1xria_ d1d5ra2
d1fpza_ d3ezza_ d4y2ea_ d1i9sa_ d3azya_ d3wvja_ d1upsa1 d1ajoa_
d2hyka_ d1ajka_ d2vy0a_ d1o4ya_ d3o5sa_ d3d6ea_ d3atga_ d1dypa_
d1umza_ d3i4ia_ g1qmo.1 g1len.1 d3zyra_ d1n47a_ g2ltn.1 d1avba_
d3usua_ d1dhkb_ d1f9ka_ d1ukga_ d1g8wa_ d1g9fa_ d1wbfa_ d3wcsa_
d1g7ya_ g1loe.1 d1mvqa_ d1hqla_ d1v6ia_ d1dbna_ d1qnwa_ d1fx5a_
d4l8qa_ d1ioaa_ d3n35a_ d3ubra_ d1qo8a1 d1d4ca1 d2k3va_ d1ft5a_
d1qdba_ d1m1qa_ d2j7aa_ d1h21a_ d1ogyb_ d4fasa_ d1jnia_ d1sp3a_
d2h0ba_ d2erfa_ d1h30a2 d1h30a1 d3mw4a1 d1pz7a_ d2r1ba_ d3v64a1
d1d2sa_ d3bopa1 d3mw3a1 d1okqa1 d1okqa2 d1wioa4 d1vcaa1 d1dr9a2
d1ic1a1 d2d9qb1 d2nxyb2 d1adqa1 d1fnga1 d1uvqb1 d1zs8a1 d2vola1
d2vola2 d1mfbl2 d2jelh2 d1uwel2 d3qq4b1 d1kjvb_ d1k5na1 d4odhl2
d1ow0a1 d1ow0a2 d1bfob2 d3l9ra2 d3jtth1 d2fath2 d4elkb2 d1fn4b2
d2xfxb_ d1pg7x2 d1cqka_ d4k71b2 d1lk2a1 d1eapb2 d4lfhg2 d1i4fa1
d2nw2a2 d1kfah2 d1mhca1 d1h3ua1 d1wejh2 d1h0db2 d1a5fh2 d1kcvh2
d1mjuh2 d1qqda1 d1kcrh2 d1k8ia1 d3huja2 d2nnab1 d1nfdf2 d3mffb2
d1s9va1 d1lk3h2 d1q0xl2 d1fv1b1 d1neza1 d4rfeb2 d1r3ha1 d1a6tb2
d1kjva1 d1pz5b2 d1onqa1 d2bc4a2 d1de4a1 d1k8da1 d1tcra2 d1dn0b2
d1nfde2 d2xwtb2 d1bfoa2 d1mexh2 d1iakb1 d1ypzf2 d4f7ea2 d1q72l2
d2fbjh2 d1fe8h2 d1clzh2 d1fngb1 d1uvqa1 d1vgka1 d1c16a1 d4j12a2
d4x99b2 d1igtb3 d1je6a1 d1q72h2 d1nldh2 d1teth2 d2hmic2 d1i1ca2

d1i1ca1 d1k8ib1 d1q0yh2 d1kfal2 d3frua1 d1pg7w2 d1u58a1 d1igyb4
d1igyb3 d1hyrc1 d1klub1 d1deeb2 d1c12b2 d2bc4b2 d2ig2h2 d1kcvl2
d3wn5b1 d1lk2b_ d1lk3l2 d1mjul2 d1sm3h2 d1q0xh2 d1zvsa1 d1ydpa1
d1u8ib2 d1ypze2 d1kcuh2 d1op9a_ d1y0la1 d1etzb1 d1egjl1 d2gsib1
d1nj9a1 d4jfhd1 d1aqkl1 d1t2ja_ d1lo4h1 d1zvya1 d3v4vl1 d4enec1
d1kxtb_ d5e8ea1 d2pxya_ d4weud_ d1xiwc_ d2esvd1 d1qnzh_ d1ogae1
d1f4xh1 d4n1hb_ d3ezjb1 d3mlwl1 d1kfah1 d1etza1 d1b4jh1 d3cfic_
d4w68a_ d2arja1 d1ek3a_ d2ok0l1 d1vera_ d1uwgh1 d1blnb1 d1fpth1
d1rzga1 d2giya1 d4m3kb1 d1nc2a1 d2nyyd1 d1ncna_ d1fbih1 d5dxwa_
d4fqqa1 d1dqdh1 d1jfql1 d1kelh1 d1faih1 d1frgl1 d1figl1 d4m6nl1
d1fo0a_ d4hxbl1 d3b5ga_ d3quyd1 d2h9gb1 d1i8kb_ d2dd8h1 d2ck0h1
d1eaja_ d4w6wb1 d2a6ib3 d1t66d1 d2e27h_ d1ob1b1 d1f11b1 d2v7na1
d1ad0a1 d2xt1b1 d1pskh1 d4i13b1 d2op4l1 d1a0ql1 d1uwel1 d1za3a1
d1ncbl1 d4uu9a_ d1bm3h1 d1cloh1 d1nqba2 d1cf8h1 d4y5va_ d1mhph1
d5anma1 d4cdgc1 d4ldeb_ d1y0lb1 d2pcpb1 d1z3gl1 d1dfbl1 d1fh5h1
d3g5xa1 d1tzib1 d4dn3l1 d3zhka1 d2b1hh1 d2uyla1 d1f3dh1 d1nj9b1
d2agjh1 d2h1pl1 d1q72h1 d8fabb1 d1ndmb1 d1rjca1 d4u6hb1 d3upca_
d2fbjl1 d1osph1 d1tvda_ d2cjvh_ d4fhbd1 d1bd2d1 d1rz7h1 d3nn8a_
d1blna1 d1knoa1 d1nfda1 d2bmkb1 d1dqll_ d1igyb1 d1igfl1 d12e8h1
d3utza1 d1nc2b1 d1rihh1 d4mwfb1 d4jvpa1 d1mieh1 d1rhfa1 d1fgnl1
d1sbsh1 d1lp9e1 d1dn0a1 d4lqfl1 d1iaih1 d1kgcd1 d1mh5b1 d1gpol1
d2g60h3 d1f8tl1 d2jixd1 d4grwh_ d1fo0b_ d1bfob1 d1axsa1 d1u9ka_
d1nmbh_ d1h8na1 d1h8na2 d1jguh1 d4eura1 d3sdyl1 d35c8h1 d1w72l1
d1mvub_ d1i9rh1 d1ob1a1 d4wenb_ d1yeeh1 d3ietc1 d1f58h1 d1ad0b1
d1mqkl_ d1nbvh1 d4b50a_ d3r8bb_ d1bafl1 d1mamh1 d4rgne1 d2gsgb_
d35c8l1 d1bafh1 d1ai1h1 d2pcpa1 d2fath1 d2x1pa_ d1orsb1 d1ibgl1
d1shma_ d1a2yb_ d1u0qa_ d2coqa_ d1hilb1 d1qfwi_ d1jv5b_ d1fn4b1
d5e0qa_ d1nsnh1 d1plgl1 d4ocxl1 d1kb5h1 d1rzfh1 d1pg7x1 d2fx7h1
d2oi9c_ d1l7il1 d3eyva1 d1d5il1 d4ffyl_ d1a6wh_ d1ac6a_ d3dvfa_
d1nfde1 d1mimh1 d1ri8a1 d1jhlh_ d3dura_ d1fskb1 d1f97a1 d3so3b1
d2ak4d1 d1lp9f1 d4z7wf1 d1l7th1 d4y5ya_ d4x7ec1 d1emth1 d1ikfl1
d1nlbh1 d4utal1 d1bjma1 d1eapb1 d1u6ah1 d2dtgd1 d1kj2b_ d1xeda1
d1c1eh1 d1fgvh_ d2bnub1 d2fb4h1 d2dtgb1 d1ktrh_ d1jpth1 d1qkzl1
d4hepg_ d2atpb1 d1flrh1 d2hrpl1 d1mvfa_ d1iaim1 d3k80a1 d2jb5h1
d3zkxc_ d4grwe_ d1it9h1 d1rhhb1 d3w9ec_ d1e4wh1 d1sy6h1 d2j6eh1
d3qxta_ d1jglh1 d1p7ka1 d1tzhb1 d4n8pa1 d1gafl1 d2jelh1 d1wioa2
d4i0cc1 d1cicb1 d1lmka2 d1lmka1 d1iqwh1 d1orqb1 d1ejoh1 d1jnlh1
d4zg1a_ d43c9b_ d1ggbl1 d1wejh1 d3q6fa1 d1f3dj1 d3c6la1 d1bqlh1
d3p9wb_ d1a3rl1 d1h0db1 d2uzil_ d1eo8l1 d1qfwl_ d1n7ml1 d1igja1
d1c5ch1 d2a9mh_ d1rmfh1 d1fj1b1 d4heme_ d1ccza1 d1a5fh1 d1ogad1
d1dlfh_ d1qa9b_ d1vgel1 d3qskb_ d1sjxa_ d4ozfg1 d1sq2n_ d1qd0a_
d1kcvh1 d1c5db1 d3qcul1 d8faba1 d3bn9d1 d1mjuh1 d4y8dc_ d7fabh1
d3dvgb1 d1lo0l1 d1a6tb1 d1gigh1 d1kcrh1 d2hwzl1 d1yjdc1 d3clfl1
d4krna_ d1dl7l_ d1e6ol1 d1mexh1 d3r0ma_ d4kkna_ d1fnsl1 d1oaqh_

d1rzia1 d1ucbh1 d1ncwl1 d4b41a_ d1nfdf1 d2mpah1 d2d7tl1 d4grwf_
d4b5ea_ d1lk3h1 d3u79a_ d4u3xa_ d1ghfl1 d1bvkb_ d1mcww1 d1b88a_
d1jrhh1 d32c2b1 d4wemb_ d1dsfh_ d1op3h1 d1ce1l1 d1iqda1 d1qoka1
d1qoka2 d1qfuh1 d2crya1 d1ay1h1 d1oaql_ d3ab0b_ d1rurl1 d1t4kb1
d1bz7b1 d1n0xh1 d1forl1 d1ucbl1 d4w6yb_ d43c9a_ d2aabl1 d1tzia1
d4n1ca_ d1k6qh1 d3g9ab_ d4tvsa1 d1igmh_ d1pz5b1 d3dx9a1 d3wxwh_
d1nakh1 d1kcul1 d3eaka_ d4qlra1 d1cr9l1 d1d9ka_ d1f4xl1 d1deea1
d2i27n2 d1c12a1 d2orbl1 d1j8hd1 d1mcph1 d1c5da1 d1kenh1 d1bd2e1
d1h5ba_ d4x7fc1 d3upaa_ d1t3fa1 d1indh1 d2dtgc1 d4hc1l1 d1zv5a1
d4wgvb_ d1uwgl1 d4f37k1 d1aqkh1 d1vesa_ d3zkqd_ d1fptl1 d1lo4l1
d1knob1 d3rrqa_ d1ct8b1 d4orzc_ d1mlbb1 d4hgkc1 d1dqdl1 d1bogb1
d4ozhe1 d1dr9a1 d1i7za1 d1dn0b1 d1kell1 d1rzib1 d1ypzf1 d1wtla_
d1hkfa_ d1fail1 d1figh1 d2vyre_ d1sjva_ d1ohqa_ d1bfoa1 d1pkqb1
d1mjul1 d1b4jl1 d1nsnl1 d2arjb1 d1p4bh_ d4aj0a_ d3nn8b_ d2rcja1
d1f2xk_ d4v1dd_ d1adqh1 d2eksa_ d4k3ga_ d1jfqh1 d1uweh1 d1frgh1
d4gftb_ d1uz8b1 d4n1ea_ d1bm3l1 d1clol1 d3mcka1 d2vq1a1 d1ae6h1
d4aq1b1 d1uj3b1 d1nbqa1 d1fh5l1 d2ck0l1 d2ywza_ d1q72l1 d4nbxb_
d1hila1 d4hzlb1 d2j88l1 d1zmya1 d1cd0a_ d1a4ka1 d1pskl1 d1a0qh1
d4krmb_ d1ncbh1 d2fbjh1 d1j8he1 d1i3ua_ d1cf8l1 d1fe8h1 d2p1ya1
d1eeqa_ d2x89a_ d1ol0a_ d2r0lh1 d1ub5a1 d1dfbh1 d3eyvb1 d4jdvb1
d4p2cg_ d2gsia1 d2agjl1 d1u3ha1 d2h1ph1 d1rihl1 d1fskc1 d1boga1
d2a77l1 d1ospl1 d1qfoa_ d3dwta_ d2fx7l1 d1rz7l1 d3stba1 d1pkqa1
d1b6da1 d1dzba2 d1dzba1 d1kj2a_ d4l1ha_ d1rzgb1 d1dqlh_ d12e8l1
d1miel1 d1jmaa_ d1sbsl1 d1fgnh1 d1f58l1 d1mqkh_ d1ndgb1 d3qegl1
d1iail1 d2aw2a1 d1tqbc1 d3fo2a1 d4dkaa_ d1f8th1 d2g60l1 d2ywya_
d1uj3a1 d1yc7a_ d3bd3a_ d4w6xb1 d1nmbl_ d1i8ka_ d1rjlb1 d1ypze1
d1p7kb1 d1kxqe_ d1gpoh1 d2z4qa1 d1w72h1 d1i9rl1 d4qgya1 d4xt1c_
d2qqnh1 d1cica1 d3c08h1 d2cd0a_ d2bdnh1 d1hnga1 d1maml1 d1nldh1
d1cr9h1 d4lcih1 d3bkyh1 d3qg7l1 d1ai1l1 d1kxvc_ d4iosd_ d1l7ih1
d2x1qa_ d1ub6b1 d3k74b_ d1ibgh1 d1teth1 d1igya1 d1hxma1 d2x6ma_
d2e7la_ d5e03a_ d1qfwm_ d2r0kh1 d1igjb1 d1rurh1 d1plgh1 d1mj8h1
d1yuhb1 d3fmgl1 d1rzfl1 d1emtl1 d1ad9b1 d1igtb1 d1nlbl1 d1d5ih1
d3k7ua_ d4ffyh_ d1bwwa_ d3dvga1 d1a6ta1 d1fgvl_ d4qxgl1 d1miml1
d1jhll_ d2hffb1 d2hh0l1 d1qkzh1 d1l7tl1 d1afvh1 d1iaii1 d2cjuh_
d1um5h1 d2ghwb2 d2ghwb1 d1kgce1 d2fgwh1 d1ikfh1 d1dvfd_ d2i24n1
d2xtjb_ d1za3b1 d2yc1a_ d2hh0h1 d2x1oa_ d1bvka_ d1rjla1 d5e7ba1
d1igta1 d1il1b1 d2fb4l1 d1mvua_ d1jptl1 d32c2a1 d1fvca_ d1iqdb1
d2hrph1 d2p4ab_ d4qo1a_ d1p2cb1 d1wt5c_ d1t4ka1 d5c1mb_ d1it9l1
d1bz7a1 d1b2wh1 d1e4wl1 d1jnll1 d1yedb1 d1za6b1 d3u7aa_ d1igfh1
d3gnml1 d1orsa1 d1wejl1 d1a2ya_ d1hxmb1 d1gafh1 d2j4wh1 d1pz5a1
d1j05a_ d1a3rh1 d4fqll1 d1qfwh_ d1eo8h1 d1n7mh1 d4s11a_ d1kegh1
d5da4a_ d1iqwl1 d3whxb1 d1vgeh1 d1deeb1 d1c12b1 d1t2ql1 d1ad9a1
d3gsnb1 d3b2uc1 d1ggbh1 d1t3fb1 d1nakl1 d4ocmc_ d1lila1 d2v7hb3
d2esve1 d1mcoh1 d2qr0b1 d2q3aa_ d1ct8a1 d1kcrl1 d2pxyb_ d2uzih_

d5c3ld_ d3durb_ d1c5cl1 d2ak4e1 d1dl7h_ d1xiwd_ d1mlba1 d1rmfl1 d1i7zb1 d1dlfl_ d2xa3a_ d5bq7b1 d1dqqb1 d3h42l1 d1egjh1 d1kcvl1 d1fe8l1 d2j6el1 d1f3rb2 d1lk3l1 d1sm3h1 d1q0xh1 d1ghfh1 d7fabl1 d1igch1 d1lo0h1 d4idla_ d5c2bl_ d1jrhl1 d1il1a1 d3zhda1 d4kfzc_ d1b0wa_ d1e6oh1 d1mexl1 d4krpb_ d1fnsh1 d1ncwh1 d4hgma_ d4eigb1 d4rzca_ d2mpal1 d4y5xa_ d5en2b1 d3lrga1 d4ej1c1 d1rhha1 d1j05b_ d1forh1 d1q9wb1 d2wzpd_ d2aq2a1 d1h0da1 d3revb1 d2ij0c1 d1tzha1 d4m7jl1 d1k6ql1 d3b9kc1 d1igml_ d3qeua1 d1dsfl_ d1ce1h1 d1kcuh1 d1zvha1 d1zvoa1 d1tqbb1 d1ay1l1 d2qscl1 d2cgrh1 d1nl0h1 d1kenl1 d3eo0a1 d4n9ob1 d1n0xl1 d3k1kc_ d1c4qa_ d1prtc1 d2wv6d_ d1prtf_ d1ltrd_ d4l63b1 d1r4pb_ d1prtd_ d1tl0f_ d2bosa_ d1prtb1 d3chbd_ d2aq2b1 d1ck1a1 d1enfa1 d1i4pa1 d1v1oa1 d1et9a1 d1esfa1 d2g9hd1 d4ohja1 d1klud1 d1bxta1 d1eu3a1 d1ty0a1 d1fnua1 d2cqva1 d1fhga_ d2k1ma_ d2dl2a1 d1biha3 d1biha1 d1biha4 d3qp3a1 d1g1ca_ d1gxea_ d1cvsc2 d1cvsc1 d1xmwa2 d2otpa2 d1biha2 d1rhfa2 d2fcba2 d2fcba1 d1wwbx_ d1gsma2 d1epfa1 d1epfa2 d2dava1 d1waaa_ d1f97a2 d1x44a1 d1vcaa2 d1fnla2 d1fnla1 d3b5ha2 d3h8na2 d3h8na1 d1jbja1 d1jbja2 d1pd6a_ d1f2qa2 d1f2qa1 d3cx2a_ d1nbqa2 d3b5ha1 d1n26a1 d1xiwb_ d1xaua_ d1fltx_ d2ifga1 d1olla1 d1olla2 d1cs6a4 d1cs6a3 d1cs6a2 d1cs6a1 d2gw5a2 d1sy6a1 d1xiwa_ d1ucta2 d1ucta1 d1ry7b2 d1gl4b_ d1qz1a3 d1olza1 d2c9aa1 d1wwca_

**Family-2 (a.1.1.3)**

**Positive dataset contains 28 protein sequences.**

d4lmxb_ d3v57a_ d4f0ta_ d2vjha_ d4po5b_ d4f0tb_ d4h0mb_ d2vjhb_ d4lm6b_ d2vmlb_ d2vjta_ d3dbja_ d2vmla_ d2vjtb_ d4f0ub_ d4rmpa1 d2uulb_ d4lmsb_ d4f0ua_ d2bv8a_ d2bv8b_ d2c7lb_ d3dbjb_ d4rmpb1 d4h0ma_ d3v57b_ d2uula_ d2j96a_

**Negative dataset contains 3969 protein sequences.**

d1m32a_ d1ibja_ d1c7na_ d1iuga_ d4q75a_ d2bkwa1 d1t3ia_ d1n8pa_ d1qz9a_ d1jf9a_ d1eg5a_ d1p3wa_ d3kgwa_ d1cl1a_ d2ctza1 d1vjoa_ d2rfva_ d1gc0a_ d1cs1a_ d1d2fa_ d2ch1a1 d1qgna_ d1pffa_ d1elua_ d1tq5a1 d1u08a_ d1lc5a_ d1b5pa_ d2ay1a_ d1bw0a_ d3pd6a_ d7aata_ d2csta_ d1j32a_ d1fg7a_ d4wlha_ d1o4sa_ d1v2da_ d1gdea_ d1m6sa_ d1v72a1 d1svva_ d1ajsa_ d1wsta1 d2f8ja2 d1lk9a_ d1vp4a1 d1xi9a1 d2gb3a1 d3rcha_ d3k40a_ d1pmma_ d2cfba1 d1dfoa_ d1o69a_ d1bj4a_ d3k28a_ d2bwpa_ d3lg0a_ d2fnua_ d3ou5a_ d1z7da1 d1b9ha_ d1iaya_ d4msoa_ d1bs0a_ d1s0aa_ d1sffa_ d3tqxa_ d3qboa_ d3pgya_ d1bjna_ d2dkja_ d1ejia_ d1vefa1 d4wxga_ d3bs8a_ d3gbxa1 d1w23a_ d1i26a_ d1ddla_ d1e57b_ d2wo3b_ d1shxa_ d1f56a_ d1bypa_ d1azca_ d1ws8a_ d3u25a_ d1cuoa_ d1id2a_ d1rkra_ d2iaac_ d4dp1x_ d1nwpa_ d2gima_ d2i7sa_ d1qhqa_ d3ehbb1 d1fftb1 d1gyca3 d1hfua2 d1kbva1 d1kbva2 d1sdda1 d1sdda2 d1gyca2 d1mzya1 d2bw4a1 d1aoza1 d1aoza3 d1aoza2 d1kyaa2 d1kyaa3 d1kyaa1 d1mzya2 d4e9sa1 d1sddb1 d1sddb2 d1hfua1 d1hfua3 d1oe1a2 d1oe1a1 d1v10a2 d1v10a3 d1v10a1 d2dara2 d2dara1

d1wyha2 d2dloa2 d1rutx4 d1rutx3 d2cuqa2 d1x4la2 d1x4la1 d1x3ha2
d1x3ha1 d2d8za1 d1x61a2 d1x6aa1 d1x6aa2 d2cora2 d2cora1 d1v6ga1
d1v6ga2 d2d8za2 d1x64a1 d1x64a2 d1b8ta1 d2co8a1 d1x63a2 d2dj7a2
d2dj7a1 d2cu8a2 d2cu8a1 d1g47a2 d1g47a1 d1j2oa1 d1wyha1 d2dloa1
d1wiga1 d1wiga2 d2cupa2 d2cupa3 d1x68a1 d2d8ya2 d2d8ya1 d1m3va2
d1x62a1 d1x68a2 d1x61a1 d1b8ta3 d1b8ta2 d2d8xa2 d2d8xa1 d1x4ka1
d1x4ka2 d2cuqa1 d1m3va1 d2co8a2 d1j2oa2 d1x63a1 d2cura1 d2cura2
d1x62a2 d2y5ia_ d1psra_ d3n22a_ d2egda_ d4ggfc_ d2wcea_ d2luca_
d4aqia_ d3nsia_ d2lp3a_ d1j55a_ d1a4pa_ d1k8ua_ d1nsha_ d3kasa1
d3bi1a3 d4qgna_ d1vr3a1 d2bema_ d1pama1 d1ea9c1 d1qhoa1 d2fhfa2
d2fhfa1 d1k3ia1 d1j0ha1 d1cxla1 d2bhua1 d1edqa1 d1f1sa2 d1ut9a2
d1gvia1 d1txka1 d4alac_ d1pjwa_ d1rera1 d1s6na_ d3uzqb_ d1jgta2
d1ea0a3 d1ecfa2 d1q15a2 d1ct9a2 d1ao0a2 d1te5a_ d2pa7a1 d1wlta1
d1nxma_ d2c0za1 d1fstb_ d1g0da1 d1v70a_ d2f4pa1 d1yhfa1 d4mh8a_
d4i7ga1 d1tafa_ d1bh9b_ d1tafb_ d1jfib_ d1h3ob_ d2byka1 d1n1ja_
d4g92b_ d1bh9a_ d1jfia_ d1h3oa_ d2bykb1 d1n1jb_ d2bjca1 d2hsga1
d1qpza1 d1efaa1 d1r0ka3 d1j5pa3 d1vkoa2 d1vm6a2 d3qvsa2 d1p1ja2
d2ovga_ d1sq8a_ d1rzsa_ d3kz3a_ d3orca_ d3gpdg2 d2q49a2 d1hdgo2
d2g17a2 d2cvoa2 d2czca1 d3pwka2 d1obfo2 d1i32a2 d1zyao2 d2pkqo2
d1b7go2 d2hjsa2 d1cf2o2 d1vkna2 d1nvmb2 d2msya_ d2djoa1 d2r5za_
d1au7a1 d1f43a_ d2lmda1 d1akhb_ d1s7ea1 d3nara_ d1zq3p1 d2craa1
d1mija_ d4uuta_ d3a01a_ d1x58a1 d3rkqa1 d3cmya1 d2cqxa1 d2da6a1
d1e3oc1 d1fjla_ d1mh3a1 d1x41a1 d1wh7a1 d1gt0c1 d2mw8a_ d1lfup_
d1x2ma1 d1b72a_ d1wi3a1 d1jgga_ d1wjha1 d1x2na1 d1mnmc_ d2cuea1
d3k2aa_ d2hosa_ d2jwta2 d1pufa_ d1octc1 d2hi3a_ d1b8ia_ d4j19a_
d3lnqa_ d1wh5a1 d1b8ib_ d1fexa_ d2cqra1 d1guua_ d1ug2a1 d2cqqa1
d2ajea1 d4a69c_ d1h8ac1 d2crga1 d2iw5b1 d1xc5a1 d2cu7a1 d1wgxa1
d2cjja1 d2ckxa1 d1gvda_ d6paxa1 d1k78a1 d1k78a2 d1hlva1 d1hlva2
d1iufa2 d1iufa1 d2iu5a1 d1zk8a1 d2g7sa1 d2fd5a1 d2np5a1 d1z0xa1
d2id6a1 d2d6ya1 d2i10a1 d1vi0a1 d2oi8a1 d3c07a1 d2ns7a1 d2fq4a1
d2yvea1 d1t33a1 d2o7ta1 d2np3b1 d2g7ga1 d2gena1 d2fbqa1 d2g7la1
d2gfna1 d2g3ba1 d2jj7a1 d2hyja1 d1sgma1 d1ui5a1 d2hkua1 d2id3a1
d4jyka1 d3lv8a_ d1s3ga1 d1zaka1 d1s4qa_ d3tvta2 d1uf9a_ d1khta_
d1ckea_ d1osna_ d1p5zb_ d4tmka_ d3cm0a_ d1tmka_ d1jaga_ d3h86a_
d1t0hb_ d1nn5a_ d2h92a_ d3kb2a1 d2c95a1 d1yj5a2 d1kgda_ d1ly1a_
d2ak3a1 d1vyva2 d1deka_ d2grja1 d1p3ja1 d1y63a_ d1e4ya1 d1s96a_
d3iija1 d1lvga_ d1nksa_ d1vyua2 d4e22a_ d1qf9a_ d1jjva_ d1q3ta_
d1ki9a_ d1lw7a2 d1teva_ d1kjwa2 d2iyva2 d1kaga_ d1viaa_ d1m7ga_
d1x6va3 d1g3ma_ d1j99a_ d1t8ta1 d1fmja_ d1zrha_ d1ls6a_ d2a3ra_
d1aqua_ d2zptx_ d3ckla_ d1texa_ d1q44a_ d2gwha_ d1odfa_ d1uj2a_
d4gi7a_ d2jeoa1 d1rz3a_ d1z0ja1 d3umra2 d3gj0a1 d2gaoa1 d1yzga1
d1x3sa1 d1g7sa4 d1puja_ d1udxa2 d1kk1a3 d2o52a_ d1jwyb_ d1zcaa2
d1zcba2 d2fv8a1 d2cjwa_ d3dz8a1 d1xzpa2 d1vg8a_ d4qxaa_ d2ew1a1
d1x1ra1 d2bcgy_ d3doea1 d3cona_ d3rega_ d3wyga_ d1upta_ d2b6ha1

d1zd9a1 d1lnza2 d1r5ba3 d1n0ua2 d1r2qa_ d2f9la1 d2rhda_ d3q3jb_
d3q85a_ d1efma1 d1r8qa_ d2gf9a1 d2akab1 d1ek0a_ d1z08a1 d2gcoa1
d2dy1a2 d4g01b_ d2cxxa1 d1svia_ d3q72a_ d1ni3a1 d4ac9a4 d2erxa1
d2bmea_ d1wxqa1 d2gf0a1 d4ldja1 d1zunb3 d1d5ca_ d4lv5b_ d1efca3
d1kmqa_ d2g6ba1 d3lrpa_ d1tada2 d2y8ea_ d4rd4a1 d1z06a1 d2heia_
d3a58b_ d1u0la2 d1d2ea3 d1zj6a1 d2ocba_ d3rwoa_ d1mr3f_ d2e9sa1
d4u5xa1 d2iwra_ d2fn4a1 d3bwdd_ d2nzja1 d2atva1 d2ksqa_ d3l43a_
d1mkya2 d1mkya1 d1wmsa_ d1jnya3 d2rmka_ d1f6ba_ d1f60a3 d1gota2
d1jala1 d2fh5b1 d1g16a_ d2wblc_ d1m7ba_ d1wf3a1 d1f5na2 d1t9ha2
d1z6xa1 d1z0aa1 d2fg5a1 d2a5ja1 d2bcjq2 d1ky3a_ d4y0va_ d4lhwa_
d2qmea1 d2atxa1 d1egaa1 d1ryha_ d1moza_ d1nrjb_ d2erya1 d3bfka_
d1puia_ d1fzqa_ d3j3az1 d3t5ga_ d1shza2 d3defa_ d2f7sa1 d3raba_
d3tsoa_ d3aq4a_ d2al7a1 d3sfva1 d3bc1a_ d1vava_ d1uaia_ d1jmxa2
d1jmxa1 d1pbya1 d1ig8a1 d1czan2 d1czan3 d1czan1 d1ig8a2 d1czan4
d1bg3a4 d1bg3a2 d1bg3a1 d3h3no1 d3h3no2 d3ezwa2 d1zc6a1 d1zbsa1
d1zbsa2 d1zc6a2 d2ch5a2 d2ch5a1 d1zxoa2 d1zxoa1 d2d0oa3 d2d0oa2
d1nbwa3 d1hroa_ d4gedb_ d3zooa_ d1mz4a_ d1gu2a_ d1f1fa_ d1dvva_
d1c6ra_ d1c75a_ d1e29a_ d1gdva_ d1cnoa_ d1h32b_ d1co6a_ d2bh4x_
d2aiua_ d5cytr_ d1jdla_ d1i8oa_ d2zboa_ d3dmia_ d1ls9a_ d1wvec_
d2yk3a_ d3ph2b_ d1dw0a_ d5aura_ d1ql3a_ d1qn2a_ d1f1ca_ d2nqya_
d1h4ga_ d1hixa_ d1oa2a_ d1olra_ d3b7ma_ d4h7ma1 d3wp3a_ d2vgda_
d1igoa_ d3zsea_ d2nlra_ d3wx5a_ d1oa3a_ d1pvxa_ d1m4wa_ d1f5ja_
d1oa4a_ d1ks5a_ d4ixla1 d2ah2a1 d1n1ta1 d3i2ka1 d1mpxa2 d1evsa_
d1cnt1_ d1pvhb_ d1z7ca_ d1huwa_ d1bgea_ d1rhga_ d1f6fa_ d1f45b_
d1i1rb_ d1axia_ d4x51a_ d1d9ca_ d4ypgc1 d3oq3a_ d1n1fa_ d1au1a_
d1wu3i_ d3besl_ d3ux9a_ d1v8fa_ d2ejca_ d3muea1 d3q12a1 d3rbaa_
d2qtra_ d1nupa_ d3l92a_ d1qjca_ d2h29a_ d1vlha_ d1od6a_ d1lw7a1
d1mrza2 d4ruka_ d2el7a_ d1iq0a2 d1irxa2 d1r6ta2 d1ivsa4 d2cyba_
d1rqga2 d1j09a2 d2yxna_ d1f7ua2 d1h3fa1 d1ffya3 d2g36a1 d1jila_
d1n3la_ d1i6la_ d1pfva2 d1h3na3 d3n9ia_ d3sz3a1 d1m4la_ d2bk7a_
d3fjua_ d1z5ra1 d1kwma1 d1uwya2 d1jqga1 d2c1ca1 d1dtda_ d3sw2a_
d1moxc_ d1dx5i3 d1edmb_ d1autl2 d1autl1 d1nqlb_ d1dval1 d1szba2
d1kigl_ d2y5fl_ d1ijqa2 d1gk5a_ d1g1sa2 d1nt0a3 d1xdtr_ d1lmja2
d1lmja1 d1nzia2 d2wphe_ d1klil_ d1pfxl2 d1pfxl1 d1p9ja_ d1g1ta2
d1f7ea_ d4yzub_ d2mgpa1 d1b9wa1 d1b9wa2 d2mgpa2 d1ob1c1 d1ob1c2
d1n1ia2 d1n1ia1 d2npra2 d2npra1 d1s5ja2 d1qhta2 d4dlea2 d1tgoa2
d3py8a2 d1q8ia2 d2py5a2 d1d5aa2 d1pvaa_ d3fs7a_ d2pvba_ d1ttxa_
d1rwya_ d2kyfa_ d1rk9a_ d1a75a_ d1exra_ d1tiza1 d1nyaa_ d1g8ia_
d1ggwa_ d1wdcc_ d3fwba_ d1ggza_ d2o5ga_ d1ctaa_ d1m45a_ d3i5gc_
d1sl8a_ d1fpwa_ d2scpa_ d2roaa_ d4aqra_ d3pn7b_ d1br1b_ d2mysb_
d1s6ca_ d1bjfa_ d2obha_ d1jfka_ d1jf0a_ d1qv0a_ d1lkja_ d2mlfc_
d2m97a_ d2d8na_ d4by5a_ d1jbaa_ d4nqga_ d1wdcb_ d2mysc_ d1j7qa_
d1s6ia_ d1uhka2 d1r2ua_ d1dtla_ d3kf9a_ d3pn7c_ d1qjta_ d1c07a_
d1gjya_ d1y1xa_ d1hqva_ d1dvia_ d1df0a1 d1qxpa2 d4usla_ d1k94a_

d1wkya2 d1uuqa_ d3wh9a_ d4m1ra_ d4xuya_ d2je8a5 d4bf7a1 d3cuia_
d1ur4a_ d1qw9a2 d1odza_ d2vzsa5 d1bqca_ d2x2ya1 d1hjqa_ d1qnra_
d4y7ea1 d1vjza_ d4k68a_ d2v3ga_ d4hpga_ d1nofa2 d1nq6a_ d1h4pa_
d1aq0a_ d3ziza_ d4bs0a_ d1ecea_ d1h1na_ d1ta3b_ d1w91a2 d1i1wa_
d1uhva2 d1us3a2 d1egza_ d7a3ha_ d1tvna1 d3nyda1 d1ghsa_ d2cyga1
d4xv0a_ d2c8na2 d1w32a_ d3emca_ d1v6ya1 d1foba_ d2uwfa1 d2fgla_
d3niya1 d2c0ha1 d1rh9a1 d1yq2a5 d1szna2 d1fa2a_ d1pama4 d5do8a1
d1ea9c3 d2fhfa5 d1qhoa4 d1avaa2 d1m53a2 d4m8ua1 d2gjpa2 d1lwha2
d4howa1 d1ktba2 d1j0ha3 d3vm5a1 d2x1ia_ d1wzaa2 d1uasa2 d2diea1
d1mxga2 d1ht6a2 d1eswa_ d1iv8a2 d5cpsa_ d1ud2a2 d1zjaa1 d3gbea1
d1kwga2 d1wdpa_ d1gcya2 d1g5aa2 d1hvxa2 d3dhpa2 d2bhua3 d4do4a1
d3qgva1 d1e43a2 d1g94a2 d2d3na2 d1gjwa2 d1gvia3 d1x1na1 d1r7aa2
d1tz7a1 d4toqa_ d1wb0a1 d1w9pa1 d1eoka_ d1itxa1 d1kfwa1 d1goia2
d1edqa2 d2gsja_ d1hjxa1 d1jnda1 d1vf8a1 d1etob_ d1umqa_ d1g2ha_
d2w0pa_ d2diaa1 d2e9ia2 d2e9ja1 d2dj4a1 d2eeba1 d2diba1 d2dmba1
d2dmca1 d2d7oa1 d2ee6a1 d2d7pa1 d2eeaa1 d2di8a1 d1wlha1 d2d7na1
d2nqca_ d2d7ma1 d2dica1 d3cnka_ d2k3ta1 d2eeca1 d1qfha1 d1qfha2
d2bp3a1 d1v05a_ d2ee9a1 d2eeda1 d2di9a1 d3syxa1 d1mkea1 d2p8va_
d1xoda1 d1evha_ d1qc6a_ d1egxa_ d1id1a_ d2hmta_ d2v76a_ d1wj1a1
d1qqga2 d1qqga1 d1ntva_ d1n3ha_ d4dbba_ d3f0wa1 d1p3ra_ d1wgua1
d2cy4a1 d3hqca_ d1wvha1 d1xr0b1 d1wgqa1 d1v89a1 d2coda1 d1zc3b_
d1wg7a1 d1u5ea1 d1v61a1 d2coca1 d2ys1a1 d1v5pa1 d1ntya2 d1wjma1
d1wi1a1 d1u29a_ d1xx0a1 d1unqa_ d2zkmx3 d1fhoa_ d1v88a1 d2adza1
d1x1fa1 d1p6sa_ d2coaa1 d1v5ma1 d1faoa_ d2x18a_ d1dyna_ d1x1ga1
d4kaxb_ d2j59m1 d1ki1b2 d1xcga2 d1txda2 d1u5da1 d1dbha2 d1w1ha_
d1upqa_ d1v5ua1 d1foea2 d2fjla1 d1eaza_ d2dfka2 d2cofa1 d1kz7a2
d1h6ta1 d1h6ua1 d9ldta1 d2ewda3 d1up7a1 d2dfda1 d1s6ya1 d1vjta1
d7mdha1 d1hyea1 d1t2da1 d1ojsa3 d1b8pa1 d1ldna1 d1hyha1 d1civa1
d2a92a1 d1ez4a1 d1i0za1 d1y6ja1 d1gv0a1 d1ur5a1 d1llda1 d1obba1
d1v8ba1 d1l7da1 d2dlda1 d1mx3a1 d1ygya1 d1qp8a1 d1li4a1 d1j4aa1
d2naca1 d1npya1 d3t4ea2 d1a4ia1 d1o0sa1 d2hk9a2 d2egga2 d1b26a1
d1bvua1 d1gq2a1 d1c1da1 d1leha1 d1gtma1 d1euza1 d3dona2 d1edza1
d1gpja2 d1vi2a1 d1p77a1 d1nyta1 d1b0aa1 d1vl6a1 d1v9la1 d3wdpp1
d3wq5a_ d1gnxa_ d4gxpa_ d4hz8a_ d1e4mm_ d3aiua_ d1wcga1 d1qvba_
d3ahxa_ d1v02a_ d2dgaa_ d1pbga_ d1vffa1 d4ha4a_ d1kola2 d1f8fa2
d1qora2 d1jqba2 d1vj0a2 d1cdoa2 d1gu7a2 d1d1ta2 d1xa0a2 d2dm6a2
d1jvba2 d1rjwa2 d1llua2 d1h2ba2 d1tt7a2 d1piwa2 d1o89a2 d1iz0a2
d1e3ja2 d1yb5a2 d1uufa2 d1vj1a2 d1e3ia2 d3gpdg1 d1vc2a1 d2nvwa1
d1u8fo1 d1ydwa1 d1hdgo1 d2g17a1 d1cf2o1 d2czca2 d1r0ka2 d1obfo1
d2cvoa1 d1ebfa1 d1h6da1 d1i32a1 d1zh8a1 d2q49a1 d1qkia1 d1j5pa4
d3pzra1 d1xeaa1 d1vkoa1 d1vm6a3 d3qvsa1 d2pkqo1 d1b7go1 d1gr0a1
d1p1ja1 d1lc0a1 d1j0xo1 d3pwka1 d2hjsa1 d1zyao1 d1vkna1 d1tlta1
d1nvmb1 d3sjua_ d3ojfa1 d1xu9a1 d2jjya1 d1n7ha_ d1y1pa1 d1h5qa_
d1jtva_ d4fs3a_ d2bkaa1 d1gy8a_ d2c5aa1 d1ulsa_ d1ooea_ d2b69a1

d1y5ma1 d1spxa_ d1ae1a_ d1bxka_ d1ydea1 d1gega_ d1hdoa_ d1o5ia_
d1e6ua_ d1snya1 d2rhca_ d3zv4a_ d5ceja_ d2d1ya1 d3w8ea_ d2fmua1
d1x1ta_ d1rpna_ d3ay7a1 d1k2wa_ d1eq2a_ d2hrba1 d3ruaa_ d2ew8a1
d4hwka_ d1xgka_ d2bd0a1 d1yo6a1 d2a35a1 d2ntva_ d1vl8a_ d1hdca_
d4bkua_ d2ag5a1 d2q45a_ d1nffa_ d4iy1a_ d1zbqa1 d1kewa_ d1ek6a_
d1ja9a_ d2q46a2 d1iy8a_ d1cyda_ d2ptga_ d1hxha_ d1uzma1 d3lyla_
d4m89a_ d3wyea_ d2blla1 d1w6ua_ d1sb8a_ d2q2va_ d4nr0a_ d1e6wa_
d2gasa_ d3oiga1 d2bgka1 d1mxha_ d1vl0a1 d1rkxa_ d1r6da_ d1wmaa1
d1e7wa_ d2c07a1 d1xkqa_ d3tzha_ d1g0oa_ d3lz6a_ d1qyca_ d1edoa_
d1gz6a_ d1oc2a_ d1t2aa_ d1zema1 d2xoxa_ d1z45a2 d1geea_ d1p33a_
d3lt0a_ d4e6pa_ d2wyua_ d1db3a_ d4q9na_ d1xhla_ d4o1ma_ d1fmca_
d2uvda_ d2gdza1 d1uaya_ d2z1ma_ d2o23a2 d2fr1a2 d2fr1a1 d1qsga_
d1qyda_ d4zjua_ d2a4ka1 d1yb1a_ d1wvga1 d1yxma1 d3f4ba_ d1mg5a_
d1xg5a1 d1ex0a4 d1g0da4 d2f4ma1 d1x3za_ d3u1ra1 d1g9ka2 d1kapp2
d1k7ia2 d1t3ca_ d1epwa3 d2a97a_ d3fiea_ g1e1h.1 d3ioqa_ d1fh0a_
d1o0ea_ d1khqa_ d1m6da_ d2cb5a_ d1cs8a_ g8pch.1 d1s4va_ d2pnsa_
d1vsna1 d1mira_ d1iwda_ d2oula_ d7pcka_ d3bwka_ d3bcna_ d2p86a_
d1pvja_ d1pcia_ d1gece_ d1deua_ d4bs6a_ d1xkga1 d1cqda_ d1jqpa2
d2c0ya_ d2bdza_ d3p5ua_ d1zoya1 d1qo8a2 d1d4ca2 d1chua2 d1jnra2
d2jk6a2 d2jk6a1 d1feca1 d1feca2 d1lpfa1 d1lpfa2 d1xhca1 d1xhca2
d1dxla2 d1dxla1 d1gera2 d1m6ia2 d1onfa1 d3lada2 d3lada1 d1w4xa1
d1w4xa2 d1gesa2 d1mo9a1 d1xdia1 d1fl2a2 d1fl2a1 d1fcda1 d1fcda2
d1h6va2 d1h6va1 d1mo9a2 d1gv4a2 d1gv4a1 d2gv8a2 d2gv8a1 d1aoga1
d1aoga2 d1hyua1 d1ebda1 d1ebda2 d3dk9a1 d1q1ra2 d1q1ra1 d1gesa1
d1zbfa1 d2z1ha_ d2etja1 d3qioa1 d2e4la_ d1jl2a_ d2lsna1 d1io2a_
d1uaxa_ d2yv0x_ d1mu2a1 d1ekea_ d1i39a_ d1s5ja1 d2hbja2 d2igia_
d1wlja_ d1qhta1 d4dlea1 d1tgoa1 d2f96a1 d3tr8a1 d3mxma_ d1q8ia1
d1y97a1 d1w0ha_ d1kfsa1 d2py5a1 d1noya_ d1d5aa1 d1yt3a3 d2guia_
d3mwpa1 d4kb1a_ d1iuka_ d2csua1 d3q9ub_ d2yv1a1 d3q9na_ d2d59a_
d1oi7a1 d2yv2a1 d1euca1 d1y81a1 d3nvva1 d1krha3 d2h88b1 d1n62a2
d1t3qa2 d1zoyb1 d1jq4a_ d1ffva2 d1dgja2 d1rm6c2 d1k8ka2 d1k8ka1
d3uleb1 d3i33a2 d3i33a1 d1bupa1 d1dkgd2 d1dkgd1 d4ci6a2 d1jcfa1
d4a2ba2 d2fxua1 d1yaga2 d4cbua2 d1bupa2 d1r7ha_ d1faaa_ d1wika1
d1xfla_ d1ttza1 d2f51a1 d1ti3a_ d1nhoa_ d1sena_ d2i4aa_ d1zmaa1
d1fo5a_ d3lefa1 d3diea1 d2yana1 d1z6na1 d1oaza_ d3zywa1 d4aj8a_
d1nm3a1 d4fiwa_ d1wmja_ d4hs1a1 d1iloa_ d1xwaa1 d1wjka1 d2vm1a_
d1fb6a_ d1gh2a_ d1h75a_ d1nw2a_ d1kx5b_ d3a6na_ d4m6ba2 d2nqbd_
d3wkjd_ d2yfwe_ d4h9na_ d5bnxa_ d1kx5c_ d3r45a_ d1eqzb_ d4jjnb_
d1f66c_ d1eqza_ d4wnnb_ d1kx5a_ d1u35c1 d1id3a_ d2nqbc_ d1kx5d_
d2b5ea2 d2b5ea3 d2b5ea1 d2b5ea4 d2kp1a1 d2kp2a1 d1hyua3 d1hyua4
d1j08a1 d1j08a2 d1pd212 d1nhya2 d2a2ra2 d1k0dd2 d1f2ea2 d1gsua2
d2fhea2 d2pvqa1 d1tw9a2 d1oyja2 d1e6ba2 d1k0ma2 d3ik7a1 d4is0a1
d1r5aa2 d1jlva2 d1m0ua2 d3gtub2 d1z9ha2 d1okta2 d1axda2 d2gsra2
d1tu7a2 d1k3ya2 d1zgma1 d1b48a2 d1gnwa2 d1gwca2 d2gsta2 d1glqa2

d1oe8a2 d1pn9a2 d1v2aa2 d1fw1a2 d1duga2 d1ovna2 d1nira1 d1qksa1
d4ilya_ d1chka_ d4olta_ d1txga2 d3f47a1 d1mv8a2 d2pv7a2 d1xc2a3
d2ahra2 d4xiya1 d2f1ka2 d1pgja2 d1i36a2 d1np3a2 d1lj8a4 d1teaa3
d1qmga2 d2i76a2 d2g5ca2 d1wdka3 d1dlja2 d3cuma3 d1qqya_ d1gd6a_
d1f6ra_ d1ivma_ d2gv0a_ d1iiza_ d2z2fa_ d1j8xa_ d1ghla_ d1b9oa_
d174la_ d169la_ d2o4wa_ d1p37a_ d1k28a3 d1xjta1 d1jtma_ d2f2qa_
d4e97a1 d1a3qa1 d1iknc_ d3brda1 d1oy3c_ d1imhc1 d1gjia1 d1zk9a1
d2cxka1 d2k4ua_ d1hp2a_ d2lu9a_ d1baha_ d2meoa1 d1wm7a_ d1hlya_
d1n8ma_ d1jlza_ d1wpda_ d1c55a_ d1pvza_ d1wt7a_ d1rjia_ d1wz5a_
d1m2sa_ d1pjva_ d1c49a_ d1qkya_ d2ktca1 d1wmta_ d1du9a_ d1j5ja_
d1r1ga_ d2pjsa1 d1klla_ d2i7ra1 d1ecsa_ d1xqaa1 d5cj3a1 d1npba_
d1jifa_ d2p7oa_ d1r9ca_ d1mpya2 d1kw3b2 d1kw3b1 d4ghga1 d1tfza1
d3isqa1 d3isqa2 d1sqia1 d1sqia2 d1lkda1 d1mpya1 d1cjxa2 d1cjxa1
d1sp9b2 d1t47a1 d1t47a2 d1f1ua2 d1sp8a1 d1sp8a2 d1f1ua1 d1w5qa_
d2c1ha_ d1ohla_ d1l6sa_ d1qsta_ d2beia1 d4m3sa_ d1u6ma1 d1ygha_
d1yk3a1 d3dr6a_ d1xmta_ d1z4ea1 d2ge3a1 d1bo4a_ d2fiaa1 d2jdca1
d1y9wa1 d3to7a_ d1wwza1 d2fl4a1 d2ozua1 d2b5ga_ d2cy2a1 d2p0wa_
d1cm0a_ d4pswa_ d1yrea1 d1yr0a1 d1m4ia_ d2gana1 d1yvka1 d1ghea_
d2g3aa1 d1y7ra1 d2euia1 d1q2ya_ d2fsra1 d1xeba_ d2ae6a1 d1cjwa_
d1qsma_ d2aj6a1 d1y9ka1 d1i12a_ d1vkca_ d2atra1 d1n71a_ d2d4pa1
d2fiwa1 d2fe7a1 d1tiqa1 d2ou2a_ d1yx0a1 d2fcka1 d1p0ha_ d2j8ma_
d2pq8a_ d1z4ra1 d1s3za1 d1ufha_ d1dosa_ d1gvfa_ d3qm3a1 d2fjka_
d1xvqa1 d1pn0a2 d3hy2a_ d2cvba1 d1o8xa_ d1we0a1 d2bmxa1 d2a4va1
d1psqa_ d1xiya1 d1q98a_ d1n8ja_ d1zofa1 d2ggta_ d3tjja_ d4je1a1
d1o73a_ d3s9fa_ d2zcta_ d1qmva_ d1jfua_ d2b1ka_ d5dvba1 d2b5xa1
d2v2ga_ d1st9a_ d3tb2a_ d4llra_ d4fh8a1 d1e2ya_ d1knga_ d2h30a_
d1i5ga_ d2z9sa_ d2f8aa1 d1tp9a1 d2b7ka_ d1nm3a2 d3hvva_ d2wfca1
d4kcea_ d1xvwa1 d4gqca1 d3qpma_ d4k1fa_ d1zzoa1 d1gp1a_ d1prxa_
d4od7a1 d1z6ma1 d4mcua1 d4dvca1 d1r4wa_ d3l9sa_ d1un2a_ d3rppa_
d2m5ta_ d1cqqa_ d1wqsa_ d5bpea_ d2ipha_ d1lvoa_ d2bhga1 d3tnta_
d3sjia_ d3d23a1 d2fyqa_ d3tloa_ d3zvea1 d2hrva_ d3q3ya1 d4asha1
d2phla2 d2phla1 d1fxza2 d1fxza1 d1lrha_ d1uija1 g1dgw.1 d2v09a_
d1uika2 d1uika1 d1dgwa_ d1e88a1 d1l6ja5 d1l6ja4 d1eaka4 d1eaka3
d1l6ja3 d1h8pa1 d1etha2 d1w52x1 d2ppla1 d1hpla2 d1lpbb2 d2oxea1
d1bu8a2 d1rp1a2 d1ra6a_ d1muka_ d3gsza_ d1te9a_ d2xwha_ d3uqsa_
d3hkwa_ d3n6ma_ d2cjqa_ d2xyma_ d1teba_ d1te8a_ d1s48a_ d1u09a1
d4wfxa_ d3tyqa_ d2pgga1 d1gyta2 d1lgya_ d4l3wa_ d1llfa_ d1gz7a_
d1ukca_ d2ftba_ d1pmpa_ d2rcqa1 d1fdqa_ d4lkpa_ d1sa8a_ d1vyfa_
d1lpja_ d3vg7a1 d4azqa1 d5bvqa_ d1p6pa_ d2qo4a_ d1opba_ d1yiva1
d1o1va_ d4bvma1 d4tkba_ d4i3ba_ d1bwya_ d1ftpa_ d1ggla_ d4gkca_
d1cbia_ d3p6da1 d1kqwa_ d2a0aa1 d3zxha_ d1cgla_ d1rm8a_ d1eaka2
d1hy7a_ d2jsda_ d1cxva_ d1hv5a_ d1i76a_ d1eb6a_ d3p0ya1 d1igra1
d1igra2 d1n8yc2 d1n8yc1 d1m6ba2 d4dv8a2 d1j7na1 d1jx4a2 d3gv5b1
d1t94a2 d3bq0a2 d2tbvc_ d1c8nc_ d1x36a_ d1smvc_ d1ng0c_ d4sbvc_

d1opoa_ d1f2nc_ d1cixa_ d1y29a_ d1v7fa_ d2jtba_ d1d1ha_ d1g9pa_
d1lupa_ d1qk7a_ d1mb6a_ d2a2va_ d1niya_ d1i25a_ d1hvwa_ d2mpqa_
d1ie6a_ d1la4a_ d2mxoa1 d1dl0a_ d1koza_ d1qk6a_ d1emxa_ d1lqra_
d1c6wa_ d1rmka_ d1ixta_ d1eyoa_ d1cnna_ d1g1pa_ d1f3ka_ d1fyga_
d1rypd_ d1irua_ d1rypc_ d3unfk_ d1rypa_ d1j2pa_ d1irui_ d1j2qh_
d3unfh_ d1m4ya_ d1rypb_ d1irul_ d1irug_ d1iru2_ d1q5rh_ d1rypg_
d1iruf_ d1q5qa_ d1e94a_ d1irub_ d1ryph_ d3ty6a_ d1iruj_ d1iruk_
d1rype_ d1irud_ d1irue_ d3mi01_ d1rypf_ d1iruh_ d2z3ba_ d1iruc_
d3mka2_ d1iru1_ d1ny721 d1ny722 d1a6ca3 d1a6ca2 d1a6ca1 d1pgl22
d1pgl21 d1pgl11 d1mqta_ d1d4m3_ d1fmd3_ d2mev1_ g1aym.1 d1ev13_
d4q4w1_ d1r1a3_ d2x5ia_ d1rhi1_ g1r1a.1 g1d4m.1 g1rhi.1 d1b35b_
d1oopa_ d1tme1_ d4iv1a_ g1pvc.1 g1fpn.1 d1fpn1_ d4gb31_ d1d4m1_
d1fmd1_ d1ev11_ d2wzr3_ g1cov.1 d1qqp3_ g1fmd.1 d2x5ic_ d1rhi3_
g1oop.1 d1hxs1_ g1hxs.1 d1fpn3_ d1bev1_ g2mev.1 d1eah3_ d1aym1_
d1pvc3_ d1zba3_ d1h8ta_ d3vbhc_ g1b35.1 g1bev.1 d1hxs3_ g1ev1.1
d1bev3_ g1tmf.1 d1tmf1_ d1b35a_ d2mev3_ g1eah.1 g1h8t.1 d1eah1_
d4q4w3_ d1r1a1_ d1aym3_ d1pvc1_ d1zba1_ d1h8tc_ d1oopc_ d1tme3_
g1qqp.1 d4iv1c_ g1tme.1 d1y14b1 d2nn6i1 d1i94l_ d2zjra2 d1smxa_
d2qamc2 d1hh2p1 d2r7da1 d1rl2a2 d1wfqa1 d3bzka4 d1x6oa2 d3a0ja_
d2j01d2 d2ja9a1 d4ql5a_ d1hr0w_ d1luza_ d2n3sa_ d2vnud1 d1h95a_
d1go3e1 d2nn6h1 d1ne3a_ d2mo1a_ d1iz6a2 d1jjga_ d1e3pa2 d2ix0a3
d2ix0a2 d2lxja_ d1ueba3 d1ueba2 d2z0sa1 d1u0la1 d2ix0a1 d2eifa2
d2je6i1 d3i2za1 d2c35b1 d2ahob2 d1t9ha1 d3i4oa_ d2qall1 d1jt8a_
d2nn6g1 d1g6pa_ d3cama_ d1i94q_ d1c9oa_ d1d7qa_ d2ba0a1 d2vnud2
d2vnud3 d1khia2 d2qalq1 d1wi5a1 d1xtda2 d4w9ta_ d4x2ra1 d1ka9f_
d1qo2a_ d4axka1 d1thfd_ d1h5ya_ d4u28a_ d2y88a_ d4wd0a_ d1vzwa1
d1eixa_ d3exra_ d2ffca1 d2fdsa1 d3n3ma1 d2czda_ d1q6oa_ d2yyua_
d1vqta1 d1dbta_ d3ldva1 d4lt9a1 d3tc7a1 d3hoja_ d1i4na_ d3vnda_
d3nl8a1 d1geqa_ d3nava1 d1v5xa_ d4a2ra1 d3tc6a1 d1ujpa_ d4ijba_
d1xcfa_ d1vc4a_ d1rd5a_ d1qopa_ d3b5va_ d1azwa_ d1mtza_ d2xqfa_
d1f6wa_ d1dx4a_ d1qe3a_ d1k4ya_ d4qwwa_ d2ha2a_ d1sfra_ d1f0na_
d4qeka_ d3c8da2 d2pbla1 d1jkma_ d1jjia_ d1lzla_ d1jjfa_ d1jmxa4
d1pbya3 d1pbya4 d4iuaa3 d4bvva_ d5hpga_ d3k65a_ d1ki0a3 d1ki0a2
d1ki0a1 d1a0ha1 d1pmla_ d3sp8a3 d2feba1 d1xova2 d1f74a_ d3l21a_
d2e1da_ d1to3a1 d1w3ia_ d1sfla_ d1n7ka_ d1vcva1 d4imea_ d1wa3a_
d2ocza1 d1f2ja_ d3m16a_ d2yxga_ d1ub3a_ d3a5fa_ d1fbaa_ d1l6wa_
d1xfba1 d1wx0a1 d1vpxa1 d1ojxa_ d2a4aa1 d1xkya1 d3cq0a1 d3s1xa_
d1f05a_ d1fdja_ d3cpra1 d4d2ja_ d1cg2a1 d1yloa2 d1y7ea2 d1y0ya3
d1lfwa1 d1vixa1 d1fnoa4 d1vhoa2 d1ysja1 d1vhea2 d1z2la1 d2fvga2
d1vgya1 d1r3na1 d2grea2 d1xmba1 g1q7l.1 d3rp2a_ g1ucy.1 d1fuja_
d2zchp_ d3w94a_ d1si5h_ d1lo6a_ g2pka.1 d1cgha_ d1kigh_ d1a5ia_
d3s9ba_ d1hj8a_ d1ao5a_ d2anya_ d1q3xa1 d1gvza_ d1azza_ d4gsoa_
d2f91a1 d1iaua_ d1gdna_ d1eufa_ g1fiw.1 d1eq9a_ d4nfea_ d2rdla_
d1spja_ d1elva1 d1a7sa_ d1ekbb_ d1pfxc_ d1yc0a_ d2aiqa_ g1fiz.1

| | | | | | | | |
|---|---|---|---|---|---|---|---|
| d1fi8a_ | d4k60a_ | d4dgja_ | d1mzaa_ | d1op0a_ | d3tjva_ | d1pytd_ | d1sgfg_ |
| g1gg6.1 | d2eeka_ | g1rtf.1 | d1a0ja_ | d3g01a_ | d1pytc_ | d4e7na_ | d1ddja_ |
| d1z8ga1 | d2hlca_ | d1npma_ | g1jou.1 | d1sgfa_ | d1autc_ | g2hnt.1 | g1kdq.1 |
| d1orfa_ | d3s69a_ | d4crga_ | d1fxya_ | d1bqya_ | d1svpa_ | d1mbma_ | d3su6a1 |
| d1cu1a1 | d4agka_ | d1vcpa_ | d1q31a_ | g1n1l.1 | d3e90b_ | d1ep5a_ | d3u1jb_ |
| d1dzka_ | d4tlja_ | d2a2ua_ | d4wfva_ | d3t1da_ | d4iaxa_ | d3zq3a_ | d2nnda_ |
| d1i4ua_ | d1kxoa_ | d1bbpa_ | d1kt7a_ | d1exsa_ | d3s26a1 | d1qfta_ | d4gh7a_ |
| d1z24a1 | d1qabe_ | d1xkia_ | d1ew3a_ | d3bx7a_ | d4iawa1 | d1n0sa_ | d1gkab_ |
| d3kzaa_ | d2k23a_ | d2l9ca1 | d2joza1 | d1gm6a_ | d3kffa_ | d3saoa1 | d1e5pa_ |
| d1yupa1 | d1iiua_ | d3lgia_ | d3i77a_ | d2z9ia2 | d4nsva_ | d1lcya2 | d4jcna_ |
| d1agja_ | d1os8a_ | d1hpga_ | d1p3ca_ | d1l1ja_ | d1qtfa_ | d1e69a_ | d1mv5a_ |
| d3g60a4 | g1ii8.1 | d1ewqa2 | d1ji0a_ | d1qhla_ | d1v43a3 | d2wmfa1 | d1vpla_ |
| d1g2912 | d4a82a2 | g1f2t.1 | d2d62a1 | d3tuic1 | d2bbsa1 | d3g60a3 | d2ixea_ |
| d1g6ha_ | d1sgwa1 | d2qi9c_ | d3rlfa1 | d3b60a1 | d3d31a2 | d2pzea1 | g1xew.1 |
| d1jj7a_ | d1oxxk2 | d2onka1 | d1w1wa_ | d1iwea_ | d1qf5a_ | d1vmaa2 | d1nija1 |
| d1np6a_ | d2p67a1 | d3ihla_ | d1okkd2 | d1ls1a2 | d1s1ma2 | d1iona_ | d2v40a_ |
| d1dj3a_ | d3hida_ | d2vo1a1 | d2g0ta1 | d1vcoa2 | d1j8mf2 | d1g3qa_ | d1xjca_ |
| d1p9ba_ | d1qzxa3 | d1ihua2 | d1ihua1 | d2qm8a_ | d1dj2a_ | d4ioja_ | d1cp2a_ |
| d1yrba2 | d1hyqa_ | d3gqba2 | d1skye3 | d1xp8a1 | d1tf7a2 | d1tf7a1 | d1ye8a1 |
| d1skyb3 | d2qe7d2 | d4blta_ | d4huta_ | d3ldaa2 | d4hyya_ | d2i3ba1 | d1cr1a_ |
| d3j9tb2 | d3ntua2 | d1vdza1 | d2z43a_ | d1e9ra_ | d1nlfa_ | d3ziaa2 | d1n0wa_ |
| d1g6oa_ | d3ziad2 | d3j9ta2 | d1pzna2 | d3gqbb2 | d2i49a_ | d1nnfa_ | d1xvxa_ |
| d1ursa_ | d1h3da1 | d1iuda_ | d4m1va1 | d1twya_ | d3e13x_ | d2nxoa1 | d4pfua_ |
| d3ttma_ | d1elja_ | d2qmwa1 | d1us5a_ | d1y3na1 | d1jeta_ | d1uqwa_ | d4f19a1 |
| d2esna2 | d3thia_ | d3fasa1 | d2uyeb_ | d2xwva1 | d1r9la_ | d2fyia1 | d1pb7a_ |
| d2ozza1 | d1sw5a_ | d1vr5a1 | d2g29a_ | d1xs5a_ | d3lswa_ | d2z23a_ | d3w9va_ |
| d3tcha_ | d1q35a_ | d1z7me1 | d3q27a_ | d1p99a_ | d1ve4a1 | d2i6ea1 | d4mcoa1 |
| d4n8ya_ | d1eu8a_ | d1zbma1 | d1hsla_ | d1ixca2 | d1j1na_ | d1xvya_ | d1y9ua_ |
| d2z22a1 | d1xoca1 | d4oera_ | d1wdna_ | d5f1qa1 | d1xt8a1 | d1i6aa_ | d4gwoa_ |
| d4igta1 | d1pc3a_ | d4mmpa1 | d1o63a_ | d2czla1 | d2onsa2 | d4mija1 | d4ok5a1 |
| d2bmfa1 | d2bmfa2 | d1yksa1 | d1yksa2 | d2qeqa1 | d2jlqa1 | d1a1va2 | d1eh3a_ |
| d3skpa_ | d1dtza2 | d1ieja_ | d1jnfa2 | d1ce2a1 | d1jnfa1 | d1b1xa2 | d1b1xa1 |
| d1jw1a1 | d1jw1a2 | d1ryoa_ | d1h76a1 | d1h76a2 | d1c4oa1 | d2p6ra3 | d2p6ra4 |
| d1oywa2 | d1oywa3 | d1wrba1 | d1w36b2 | d1w36b1 | d1c4oa2 | d3bora_ | d1t6na_ |
| d2eyqa5 | d2eyqa3 | d2eyqa2 | d2zu6a2 | d1veca_ | d1uaaa1 | d1w36d1 | d2g9na1 |
| d1z3ix2 | d2rb4a_ | d1hv8a1 | d1hv8a2 | d1t5ia_ | d1w36c2 | d2j0sa2 | d2j0sa1 |
| d1fuka_ | d3hjha1 | d1z5za1 | d1qdea_ | d1z63a1 | d1s2ma2 | d1s2ma1 | d1wp9a1 |
| d1wp9a2 | d1gm5a3 | d1gm5a4 | d3dina4 | d3dina3 | d1q0ua_ | d1w36c1 | d1z3ix1 |
| d1nkta3 | d1nkta4 | d1uaaa2 | d1w36d2 | g1qhh.1 | d2fwra2 | d2fwra1 | d3q3ma_ |
| d1za0a1 | d1mxra_ | d2inca_ | d1jk0a_ | d2incb_ | d1mhyb_ | d3hf1b_ | d1mtyb_ |
| d1r2fa_ | d2uw1a_ | d1mhyd_ | d3n37a_ | d1afra_ | d1oquc_ | d1syya_ | d1mtyd_ |
| d2o1za_ | d1uzra_ | d1smsa_ | d2fkza_ | d1zuja1 | d1s3qa1 | d2fjca1 | d1z6om1 |
| d1veia_ | d4dyya_ | d1jiga_ | d3gvya_ | d2pyba_ | d4cy9a1 | d3bvfa1 | d1r03a_ |
| d2cf7a_ | d1nf4a_ | d1zs3a1 | d4iwka_ | d3ka8a_ | d3np0x_ | d1n1qa_ | d1lb3a_ |

d4e6ka_ d1z6oa1 d2fzfa1 d4dyua_ d3ge4a_ d1jgca_ d4am4a_ d2z6ma_
d1ji5a_ d1vlga_ d1lkoa1 d3qz3a_ d1o9ra_ d2ffxj_ d1euma_ d1yv1a1
d2v2pa_ d1j30a_ d4di0a_ d1dpsa_ d1oewa_ d2qzxa_ d2asia_ d1psoe_
d1miqa_ d1smra_ d2h6ta_ d1pfza_ d1izda_ d3hd8a_ d1j71a_ d1dpja_
d4aa9a_ d1t6ex_ g1b5f.1 g1lya.1 g1htr.1 d1qdma2 d1bxoa_ d1wkra_
d1tzsa_ d1ibqa_ d2qzwa_ d1bdqa_ d3ggta_ d3ixoa_ d1mrxa_ d2haha_
d1tw7a_ d4njsa_ d1sgua_ d2p3da_ d1nsoa_ d3d3ta_ d2rkfa_ d1c6ya_
d3ogpa_ d3ekwa_ d2o4na_ d2j9ja_ d1baia_ d3ggua_ d3lzua_ d4npua_
d4j55a_ d2r8na_ d2fxda_ d2p3ca_ d4dqea_ d3ttpa_ d3t3ca_ d2p3aa_
d2hc0a_ d2fdda_ d3mwsa_ d2za0a_ d2c21a1 d1f9za_ d1dgsa2 d1fvia1
d1ckma1 d1x9na2 d1h4ra3 d4yl8a1 d2al6a3 d1wgda1 d1wgga1 d4q5eb_
d1wjna1 d1se9a_ d3n3kb_ d1wz0a1 d1we6a1 d2m0xa_ d2gbka_ d3rt3b1
d1wh3a1 d1sifa_ d2ekec2 d4ii2b1 d4hk2a1 d1c3ta_ d1m94a_ d1wgha1
d1ttna1 d1j8ca_ d1wx8a1 d3v6cb1 d2ma9b_ d4i6lb1 d1bt0a_ d2jvca1
d1iyfa_ d2k1fa_ d1wxva1 d1wf9a1 d1t0ya_ d2io0b2 d1x1ma1 d2gbma_
d1zw7a1 d2m17a_ d1ud7a_ d1we7a1 d2lwpa_ d2gbja_ d1wjua1 d1v6ea1
d1v86a1 d4eewa_ d1wiaa1 d4icva_ d4gu2a_ d1wx7a1 d1wy8a1 d4hjka1
d2faza1 d1v5oa1 d2al3a1 d2cr5a1 d1wj4a1 d1s3sg_ d1g66a_ d1qoza_
d5ajha1 d4lu0a_ d1of8a_ d3unda1 d3e9aa1 d1vr6a2 d1n8fa_ d2qkfa_
d1d9ea_ d3h33a_ d1rwja_ d19hca_ d1os6a_ d3bxua_ d3h4na_ d1i77a_
d3caoa_ d1j0pa_ d2ctha_ d1ofwa_ d2e84a_ d1hh5a_ d1gyoa_ d3h34a_
d1mixa2 d1e5wa2 d1j19a2 d1isna2 d2al6b2 d1dszb_ d1a6ya_ d3cbba_
d1ynwa1 d1r4ia_ d1hcqa_ d2hana_ d2hanb_ d2enva1 d1kb4b_ d2nllb_
d1lo1a_ d1cita_ d1um8a_ d1ixza_ d1qvra2 d1qvra3 d1qzma_ d1sxjc2
d1jr3d2 d2p65a_ d1l8qa2 d3hu3a3 d1sxjd2 d1njfa_ d1in4a2 d1sxja2
d1tuea1 d2ce7a2 d1w5sa2 d1sxjb2 d1d2na_ d1ny5a2 d1ofha_ d2gnoa2
d2a5yb3 d1fnna2 d1u0ja1 d1lv7a_ d1iqpa2 d2fnaa2 d1ixsb2 d4gita_
d1sxje2 d1g41a_ d3ia2a_ d1a88a_ d4dgqa1 d1zoia_ d1f15b_ d1za7a_
d1laja_ d1t0za_ d2i61a_ d1t1ta_ d1sn4a_ d1kv0a_ d2lkba_ d1npia_
d1wwna_ d1pe4a_ d2kbka_ d2li7a_ d1djta_ d2kbha_ d1dq7a_ d1sega_
d1zywa1 d1jzaa_ d1n9wa1 d1bbua1 d1e1oa1 d1b8aa1 d1l0wa1 d3a74a1
d1eova1 d1iyjb4 d1iyjb5 d1iyjb3 d1o7ia_ d4ipca1 d1x3ea_ d4apva_
d1jb7a1 d1jb7a2 d1jb7a3 d3kdfa_ d1ue1b_ d1wjja1 d3eiva_ d1xjva2
d1xjva1 d2ccza2 d1se8a_ d1l1oc_ d1jb7b_ d1qvca_ d1qzga_ d1miua4
d1miua5 d1miua3 d3g48a1 d1gd7a_ d1mkha_ d1jjcb3 d3ubub_ d1egia_
d1qo3c_ d1v7pa_ d1tdqb_ d3ubua_ d1c3ab_ d1h8ua_ d3bx4b_ d1t8ca1
d1sb2a1 d1qdda_ d2ziba1 d3cada_ d1byfa_ d1j34b_ d1jwia_ d1jzna_
d1c3aa_ d1oz7b_ d1wmza_ d3bdwb_ d1hq8a_ d1fifa1 d1dv8a_ d3hupa_
d1wk1a1 d1umra_ d2afpa_ d3bx4a_ d1gz2a_ d1sl6a_ d1v7pb_ d3g8ka_
d1ixxa_ d1j34a_ d1jwib_ d1g1sa1 d1y17a1 d1ja3a_ d1fvub_ d3bdwa_
d1sb2b1 d1fvua_ d1umrc_ d1oz7a_ d4m17a_ d3cfwa1 d1ypqa1 d1g1ta1
d3fd4a_ d3l9jc_ d4dn8a1 d1v4lb_ d1rdl1_ d2msba_ d1prtc2 d1prtb2
d1xwdc1 d3kj4a_ d1w8aa_ d2ifga3 d1p9ag_ d1xkua_ d1ohea1 d1ohea2
d2f4da_ d1rxda_ d2hxpa_ d1xria_ d1d5ra2 d1fpza_ d3ezza_ d4y2ea_

d3azya_ d3wvja_ d1upsa1 d1ajoa_ d2hyka_ d1ajka_ d2vy0a_ d1o4ya_
d3o5sa_ d3d6ea_ d3atga_ d1dypa_ d1umza_ d3i4ia_ g1qmo.1 g1len.1
d3zyra_ d1fnya_ d1n47a_ g2ltn.1 d1avba_ d3usua_ d1dhkb_ d1f9ka_
d1ukga_ d1g8wa_ d1g9fa_ d1wbfa_ d3wcsa_ d1g7ya_ g1loe.1 d1mvqa_
d1hqla_ d1v6ia_ d1dbna_ d1qnwa_ d1fx5a_ d4l8qa_ d1ioaa_ d3n35a_
d3ubra_ d1qo8a1 d1d4ca1 d2k3va_ d1ft5a_ d1qdba_ d1m1qa_ d2j7aa_
d1h21a_ d1ogyb_ d4fasa_ d1jnia_ d1oaha_ d1sp3a_ d2h0ba_ d2erfa_
d1h30a2 d1h30a1 d3mw4a1 d1pz7a_ d2r1ba_ d3v64a1 d1d2sa_ d3bopa1
d3mw3a1 d1okqa1 d1okqa2 d1ccza2 d1vcaa1 d1dr9a2 d1ic1a1 d2d9qb1
d2nxyb2 d1adqa1 d1fnga1 d1uvqb1 d1zs8a1 d2vola1 d2vola2 d1mfbl2
d2jelh2 d1uwel2 d3qq4b1 d1kjvb_ d1k5na1 d1osph2 d4odhl2 d1ow0a1
d1ow0a2 d1bfob2 d3l9ra2 d3jtth1 d2fath2 d4elkb2 d1fn4b2 d2xfxb_
d1pg7x2 d1cqka_ d4k71b2 d1lk2a1 d1eapb2 d4lfhg2 d1i4fa1 d2nw2a2
d1kfah2 d1mhca1 d1h3ua1 d1wejh2 d1h0db2 d1a5fh2 d1kcvh2 d1mjuh2
d1qqda1 d1kcrh2 d1k8ia1 d3huja2 d2nnab1 d1nfdf2 d3mffb2 d1s9va1
d1lk3h2 d1q0xl2 d1fv1b1 d1neza1 d4rfeb2 d1r3ha1 d1a6tb2 d1kjva1
d1pz5b2 d1onqa1 d2bc4a2 d1de4a1 d1k8da1 d1tcra2 d1dn0b2 d1nfde2
d2xwtb2 d1bfoa2 d1mexh2 d1iakb1 d1ypzf2 d4f7ea2 d1q72l2 d2fbjh2
d1fe8h2 d1clzh2 d1fngb1 d1uvqa1 d1vgka1 d1c16a1 d4j12a2 d4x99b2
d1igtb3 d1je6a1 d1q72h2 d1nldh2 d1teth2 d2hmic2 d1i1ca2 d1i1ca1
d1k8ib1 d1q0yh2 d1kfal2 d3frua1 d1pg7w2 d1u58a1 d1igyb4 d1igyb3
d1hyrc1 d1klub1 d1deeb2 d1c12b2 d2bc4b2 d2ig2h2 d1kcvl2 d3wn5b1
d1lk2b_ d1lk3l2 d1mjul2 d1sm3h2 d1q0xh2 d1zvsa1 d1ydpa1 d1u8ib2
d1ypze2 d1kcuh2 d1op9a_ d1y0la1 d1etzb1 d1egjl1 d2gsib1 d1nj9a1
d4jfhd1 d1aqkl1 d1t2ja_ d1lo4h1 d1zvya1 d3v4vl1 d4enec1 d1kxtb_
d5e8ea1 d2pxya_ d4weud_ d1xiwc_ d2esvd1 d1qnzh_ d1ogae1 d1f4xh1
d4n1hb_ d3ezjb1 d3mlwl1 d1kfah1 d1etza1 d1b4jh1 d3cfic_ d4w68a_
d2arja1 d1ek3a_ d2ok0l1 d1vera_ d1uwgh1 d1blnb1 d1fpth1 d1rzga1
d2giya1 d4m3kb1 d1nc2a1 d2nyyd1 d1ncna_ d1fbih1 d5dxwa_ d4fqqa1
d1dqdh1 d1jfql1 d1kelh1 d1faih1 d1frgl1 d1figl1 d4m6nl1 d1fo0a_
d4hxbl1 d3b5ga_ d3quyd1 d2h9gb1 d1i8kb_ d2dd8h1 d2ck0h1 d1eaja_
d4w6wb1 d2a6ib3 d1t66d1 d2e27h_ d1ob1b1 d1f11b1 d2v7na1 d1ad0a1
d2xt1b1 d1pskh1 d4i13b1 d2op4l1 d1a0ql1 d1uwel1 d1za3a1 d1ncbl1
d4uu9a_ d1bm3h1 d1cloh1 d1nqba2 d1cf8h1 d4y5va_ d1mhph1 d5anma1
d4cdgc1 d4ldeb_ d1y0lb1 d2pcpb1 d1z3gl1 d1dfbl1 d1fh5h1 d3g5xa1
d1tzib1 d4dn3l1 d3zhka1 d2b1hh1 d2uyla1 d1f3dh1 d1nj9b1 d2agjh1
d2h1pl1 d1q72h1 d8fabb1 d1ndmb1 d1rjca1 d4u6hb1 d3upca_ d2fbjl1
d1osph1 d1tvda_ d2cjvh_ d4fhbd1 d1bd2d1 d1rz7h1 d3nn8a_ d1blna1
d1knoa1 d1nfda1 d2bmkb1 d1dqll_ d1igyb1 d1igfl1 d12e8h1 d3utza1
d1nc2b1 d1rihh1 d4mwfb1 d4jvpa1 d1mieh1 d1rhfa1 d1fgnl1 d1sbsh1
d1lp9e1 d1dn0a1 d4lqfl1 d1iaih1 d1kgcd1 d1mh5b1 d1gpol1 d2g60h3
d1f8tl1 d2jixd1 d4grwh_ d1fo0b_ d1bfob1 d1axsa1 d1u9ka_ d1nmbh_
d1h8na1 d1h8na2 d1jguh1 d4eura1 d3sdyl1 d35c8h1 d1w72l1 d1mvub_
d1i9rh1 d1ob1a1 d4wenb_ d1yeeh1 d3ietc1 d1f58h1 d1ad0b1 d1mqkl_

d1nbvh1 d4b50a_ d3r8bb_ d1bafl1 d1mamh1 d4rgne1 d2gsgb_ d35c8l1
d1bafh1 d1ai1h1 d2pcpa1 d2fath1 d2x1pa_ d1orsb1 d1ibgl1 d1shma_
d1a2yb_ d1u0qa_ d2coqa_ d1hilb1 d1qfwi_ d1jv5b_ d1fn4b1 d5e0qa_
d1nsnh1 d1plgl1 d4ocxl1 d1kb5h1 d1rzfh1 d1pg7x1 d2fx7h1 d2oi9c_
d1l7il1 d3eyva1 d1d5il1 d4ffyl_ d1tetl1 d1a6wh_ d1ac6a_ d3dvfa_
d1nfde1 d1mimh1 d1ri8a1 d1jhlh_ d3dura_ d1fskb1 d1f97a1 d3so3b1
d2ak4d1 d1lp9f1 d4z7wf1 d1l7th1 d4y5ya_ d4x7ec1 d1emth1 d1ikfl1
d1nlbh1 d4utal1 d1bjma1 d1eapb1 d1u6ah1 d2dtgd1 d1kj2b_ d1xeda1
d1c1eh1 d1fgvh_ d2bnub1 d2fb4h1 d2dtgb1 d1ktrh_ d1jpth1 d1qkzl1
d4hepg_ d2atpb1 d1flrh1 d2hrpl1 d1mvfa_ d1iaim1 d3k80a1 d2jb5h1
d3zkxc_ d4grwe_ d1it9h1 d1rhhb1 d3w9ec_ d1e4wh1 d1sy6h1 d2j6eh1
d3qxta_ d1jglh1 d1p7ka1 d1tzhb1 d4n8pa1 d1gafl1 d2jelh1 d1wioa2
d4i0cc1 d1cicb1 d1lmka2 d1lmka1 d1iqwh1 d1orqb1 d1ejoh1 d1jnlh1
d4zg1a_ d43c9b_ d1ggbl1 d1wejh1 d3q6fa1 d1f3dj1 d3c6la1 d1bqlh1
d3p9wb_ d1a3rl1 d1h0db1 d2uzil_ d1eo8l1 d1qfwl_ d1n7ml1 d1igja1
d1c5ch1 d2a9mh_ d1rmfh1 d1fj1b1 d4heme_ d1ccza1 d1a5fh1 d1ogad1
d1dlfh_ d1qa9b_ d1vgel1 d3qskb_ d1sjxa_ d4ozfg1 d1sq2n_ d1qd0a_
d1kcvh1 d1c5db1 d3qcul1 d8faba1 d3bn9d1 d1mjuh1 d4y8dc_ d7fabh1
d3dvgb1 d1lo0l1 d1a6tb1 d1gigh1 d1kcrh1 d2hwzl1 d1yjdc1 d3clfl1
d4krna_ d1dl7l_ d1e6ol1 d1mexh1 d3r0ma_ d4kkna_ d1fnsl1 d1oaqh_
d1rzia1 d1ucbh1 d1ncwl1 d4b41a_ d1nfdf1 d2mpah1 d2d7tl1 d4grwf_
d4b5ea_ d1lk3h1 d3u79a_ d4u3xa_ d1ghfl1 d1bvkb_ d1mcww1 d1b88a_
d1jrhh1 d32c2b1 d4wemb_ d1dsfh_ d1op3h1 d1ce1l1 d1iqda1 d1qoka1
d1qoka2 d1qfuh1 d2crya1 d1ay1h1 d1oaql_ d3ab0b_ d1rurl1 d1t4kb1
d1bz7b1 d1n0xh1 d1forl1 d1ucbl1 d4w6yb_ d43c9a_ d2aabl1 d1tzia1
d4n1ca_ d1k6qh1 d3g9ab_ d4tvsa1 d1igmh_ d1pz5b1 d3dx9a1 d3wxwh_
d1kcul1 d3eaka_ d4qlra1 d1cr9l1 d1d9ka_ d1f4xl1 d1deea1 d2i27n2
d1c12a1 d2orbl1 d1j8hd1 d1mcph1 d1c5da1 d1kenh1 d1bd2e1 d1h5ba_
d4x7fc1 d3upaa_ d1t3fa1 d1indh1 d2dtgc1 d4hc1l1 d1zv5a1 d4wgvb_
d1uwgl1 d4f37k1 d1aqkh1 d1vesa_ d3zkqd_ d1fptl1 d1lo4l1 d1knob1
d3rrqa_ d1ct8b1 d4orzc_ d1mlbb1 d4hgkc1 d1dqdl1 d1bogb1 d4ozhe1
d1dr9a1 d1i7za1 d1dn0b1 d1kell1 d1rzib1 d1ypzf1 d1wtla_ d1hkfa_
d1fail1 d1figh1 d2vyre_ d1sjva_ d1ohqa_ d1bfoa1 d1pkqb1 d1mjul1
d1b4jl1 d1nsnl1 d2arjb1 d1p4bh_ d4aj0a_ d3nn8b_ d2rcja1 d1f2xk_
d4v1dd_ d1adqh1 d2eksa_ d4k3ga_ d1jfqh1 d1uweh1 d1frgh1 d4gftb_
d1uz8b1 d4n1ea_ d1bm3l1 d1clol1 d3mcka1 d2vq1a1 d1ae6h1 d4aq1b1
d1uj3b1 d1nbqa1 d1fh5l1 d2ck0l1 d2ywza_ d1q72l1 d4nbxb_ d1hila1
d4hzlb1 d2j88l1 d1zmya1 d1cd0a_ d1a4ka1 d1pskl1 d1a0qh1 d4krmb_
d1ncbh1 d2fbjh1 d1j8he1 d1i3ua_ d1cf8l1 d1fe8h1 d2p1ya1 d1eeqa_
d2x89a_ d1ol0a_ d2r0lh1 d1ub5a1 d1dfbh1 d3eyvb1 d4jdvb1 d4p2cg_
d2gsia1 d2agjl1 d1u3ha1 d2h1ph1 d1rihl1 d1fskc1 d1boga1 d2a77l1
d1ospl1 d1qfoa_ d3dwta_ d2fx7l1 d1rz7l1 d3stba1 d1pkqa1 d1b6da1
d1dzba2 d1dzba1 d1kj2a_ d4l1ha_ d1rzgb1 d1dqlh_ d12e8l1 d1miel1
d1jmaa_ d1sbsl1 d1fgnh1 d1f58l1 d1mqkh_ d1ndgb1 d3qegl1 d1iail1

d2aw2a1 d1tqbc1 d3fo2a1 d4dkaa_ d1f8th1 d2g60l1 d2ywya_ d1uj3a1 d1yc7a_ d3bd3a_ d4w6xb1 d1nmbl_ d1i8ka_ d1rjlb1 d1ypze1 d1p7kb1 d1kxqe_ d1gpoh1 d2z4qa1 d1w72h1 d1i9rl1 d4qgya1 d4xt1c_ d2qqnh1 d1cica1 d3c08h1 d2cd0a_ d2bdnh1 d1hnga1 d1maml1 d1nldh1 d1cr9h1 d4lcih1 d3bkyh1 d3qg7l1 d1ai1l1 d1kxvc_ d4iosd_ d1l7ih1 d2x1qa_ d1ub6b1 d3k74b_ d1ibgh1 d1teth1 d1igya1 d1hxma1 d2x6ma_ d2e7la_ d5e03a_ d1qfwm_ d2r0kh1 d1igjb1 d1rurh1 d1plgh1 d1mj8h1 d1yuhb1 d3fmgl1 d1rzfl1 d1emtl1 d1ad9b1 d1igtb1 d1nlbl1 d1d5ih1 d3k7ua_ d4ffyh_ d1bwwa_ d3dvga1 d1a6ta1 d1fgvl_ d4qxgl1 d1miml1 d1jhll_ d2hffb1 d2hh0l1 d1qkzh1 d1l7tl1 d1afvh1 d1iaii1 d2cjuh_ d1um5h1 d2ghwb2 d2ghwb1 d1kgce1 d2fgwh1 d1ikfh1 d1dvfd_ d2i24n1 d2xtjb_ d1za3b1 d2yc1a_ d2hh0h1 d2x1oa_ d1bvka_ d1rjla1 d5e7ba1 d1igta1 d1il1b1 d2fb4l1 d1mvua_ d1jptl1 d32c2a1 d1fvca_ d1iqdb1 d2hrph1 d2p4ab_ d4qo1a_ d1p2cb1 d1wt5c_ d1t4ka1 d5c1mb_ d1it9l1 d1bz7a1 d1b2wh1 d1e4wl1 d1jnll1 d1yedb1 d1za6b1 d3u7aa_ d1igfh1 d3gnml1 d1orsa1 d1wejl1 d1a2ya_ d1hxmb1 d1gafh1 d2j4wh1 d1pz5a1 d1j05a_ d1a3rh1 d4fqll1 d1qfwh_ d1eo8h1 d1n7mh1 d4s11a_ d1kegh1 d5da4a_ d1iqwl1 d3whxb1 d1vgeh1 d1deeb1 d1c12b1 d1t2ql1 d1ad9a1 d3gsnb1 d3b2uc1 d1ggbh1 d1t3fb1 d1nakl1 d4ocmc_ d1lila1 d2v7hb3 d2esve1 d1mcoh1 d2qr0b1 d2q3aa_ d1ct8a1 d1kcrl1 d2pxyb_ d2uzih_ d5c3ld_ d3durb_ d1c5cl1 d2ak4e1 d1dl7h_ d1xiwd_ d1mlba1 d1rmfl1 d1i7zb1 d1dlfl_ d2xa3a_ d5bq7b1 d1dqqb1 d3h42l1 d1egjh1 d1kcvl1 d1fe8l1 d2j6el1 d1f3rb2 d1lk3l1 d1sm3h1 d1q0xh1 d1ghfh1 d7fabl1 d1igch1 d1lo0h1 d4idla_ d5c2bl_ d1jrhl1 d1il1a1 d3zhda1 d4kfzc_ d1b0wa_ d1e6oh1 d1mexl1 d4krpb_ d1fnsh1 d1ncwh1 d4hgma_ d4eigb1 d4rzca_ d2mpal1 d4y5xa_ d5en2b1 d3lrga1 d4ej1c1 d1rhha1 d1j05b_ d1forh1 d1q9wb1 d2wzpd_ d2aq2a1 d1h0da1 d3revb1 d2ij0c1 d1tzha1 d4m7jl1 d1k6ql1 d3b9kc1 d1igml_ d3qeua1 d1dsfl_ d1ce1h1 d1kcuh1 d1zvha1 d1zvoa1 d1tqbb1 d1ay1l1 d2qscl1 d2cgrh1 d1nl0h1 d1kenl1 d3eo0a1 d4n9ob1 d1n0xl1 d3k1kc_ d1c4qa_ d1prtc1 d2wv6d_ d1prtf_ d1ltrd_ d4l63b1 d1r4pb_ d1prtd_ d1tl0f_ d1prtb1 d3chbd_ d2aq2b1 d1ck1a1 d1enfa1 d1i4pa1 d1v1oa1 d1et9a1 d1esfa1 d2g9hd1 d4ohja1 d1klud1 d1bxta1 d1ty0a1 d1fnua1 d2cqva1 d1fhga_ d2k1ma_ d2dl2a1 d1biha3 d1biha1 d1biha4 d3qp3a1 d1g1ca_ d1gxea_ d1cvsc2 d1cvsc1 d1xmwa2 d2otpa2 d1biha2 d1rhfa2 d2fcba2 d2fcba1 d1wwbx_ d1gsma2 d1epfa1 d1epfa2 d2dava1 d1waaa_ d1f97a2 d1x44a1 d1vcaa2 d1fnla2 d1fnla1 d3b5ha2 d3h8na2 d3h8na1 d1jbja1 d1jbja2 d1pd6a_ d1f2qa2 d1f2qa1 d3cx2a_ d1nbqa2 d3b5ha1 d1n26a1 d1xiwb_ d1xaua_ d1fltx_ d2ifga1 d1olla1 d1olla2 d1cs6a4 d1cs6a3 d1cs6a2 d1cs6a1 d2gw5a2 d1sy6a1 d1xiwa_ d1ucta2 d1ucta1 d1ry7b2 d1gl4b_ d1qz1a3 d1olza1 d2c9aa1 d1wwca_

**Family-3 (a.138.1.1)**

**Positive dataset contains 6 protein sequences.**

d3h33a_ d1rwja_ d3bxua_ d3h4na_ d2e84a_ d3h34a_

**Negative dataset contains 4019 protein sequences.**

d1m32a_ d1ibja_ d1c7na_ d1iuga_ d4q75a_ d2bkwa1 d1t3ia_ d1n8pa_
d1qz9a_ d1jf9a_ d1eg5a_ d1p3wa_ d3kgwa_ d1cl1a_ d2ctza1 d1vjoa_
d2rfva_ d1gc0a_ d1cs1a_ d1d2fa_ d2ch1a1 d1qgna_ d1pffa_ d1elua_
d1ax4a_ d2v1pa1 d2ez1a_ d1u08a_ d1lc5a_ d1b5pa_ d2ay1a_ d1bw0a_
d3pd6a_ d7aata_ d2csta_ d1j32a_ d1fg7a_ d4wlha_ d1o4sa_ d1v2da_
d1gdea_ d1m6sa_ d1v72a1 d1svva_ d1ajsa_ d1wsta1 d2f8ja2 d1lk9a_
d1vp4a1 d1xi9a1 d2gb3a1 d1xkta1 d2hfka_ d5d3ka_ d2cfba1 d1dfoa_
d1o69a_ d1bj4a_ d3k28a_ d2bwpa_ d3lg0a_ d2fnua_ d3ou5a_ d1z7da1
d1b9ha_ d1iaya_ d4msoa_ d1bs0a_ d1s0aa_ d1sffa_ d3tqxa_ d3qboa_
d3pgya_ d1bjna_ d2dkja_ d1ejia_ d1vefa1 d4wxga_ d3bs8a_ d3gbxa1
d1w23a_ d2tbvc_ d1c8nc_ d1x36a_ d1smvc_ d1ng0c_ d4sbvc_ d1opoa_
d1f2nc_ d3lgia_ d3i77a_ d2z9ia2 d4nsva_ d1lcya2 d4jcna_ d1agja_
d1os8a_ d1hpga_ d1l1ja_ d1qtfa_ d1ddla_ d1e57b_ d1auyb_ d1qnia1
d1ibya_ d1f56a_ d1bypa_ d1azca_ d1ws8a_ d3u25a_ d1cuoa_ d1id2a_
d1rkra_ d2iaac_ d4dp1x_ d1nwpa_ d2gima_ d2i7sa_ d1qhqa_ d3ehbb1
d1fftb1 d1gyca3 d1kbva1 d1kbva2 d1sdda1 d1sdda2 d1gyca1 d1gyca2
d1mzya1 d2bw4a1 d1aoza1 d1aoza3 d1aoza2 d1kyaa2 d1kyaa3 d1kyaa1
d1mzya2 d4e9sa1 d1sddb1 d1sddb2 d1hfua1 d1hfua3 d1oe1a2 d1oe1a1
d1v10a2 d1v10a3 d1v10a1 d1iknc_ d3brda1 d1p7hl1 d1oy3c_ d1imhc1
d1gjia1 d1zk9a1 d2cxka1 d2bema_ d1pama1 d1ea9c1 d1qhoa1 d2fhfa2
d2fhfa1 d1k3ia1 d1j0ha1 d1cxla1 d2bhua1 d1edqa1 d1f1sa2 d1ut9a2
d1gvia1 d1txka1 d4alac_ d1rera1 d1s6na_ d3uzqb_ d2phla2 d2phla1
d1fxza2 d1lrha_ d1uija1 g1dgw.1 d2v09a_ d1uika2 d1uika1 d1dgwa_
d1ep0a_ d2pa7a1 d1wlta1 d2c0za1 d1ds6b_ d1fstb_ d1ex0a1 d1g0da1
d1cg2a1 d1yloa2 d1y7ea2 d1y0ya3 d1lfwa1 d1vixa1 d1fnoa4 d1vhoa2
d1ysja1 d1vhea2 d1z2la1 d2fvga2 d1vgya1 d1r3na1 d2grea2 d1xmba1
d1rtqa_ d2ovga_ d1sq8a_ d1rzsa_ d3kz3a_ d3orca_ d3gpdg2 d2q49a2
d1hdgo2 d2g17a2 d2cvoa2 d2czca1 d3pwka2 d1obfo2 d1i32a2 d1zyao2
d2pkqo2 d1b7go2 d2hjsa2 d1cf2o2 d1vkna2 d1nvmb2 d1ux8a_ d4i0va_
d2bkma_ d1dlwa_ d2bmma_ d2msya_ d2djoa1 d2r5za_ d1au7a1 d1f43a_
d2lmda1 d1akhb_ d1s7ea1 d3nara_ d1zq3p1 d2craa1 d1mija_ d4uuta_
d3a01a_ d1x58a1 d3rkqa1 d3cmya1 d2cqxa1 d2da6a1 d1e3oc1 d1fjla_
d1mh3a1 d1ig7a_ d1x41a1 d1wh7a1 d1gt0c1 d2mw8a_ d1lfup_ d1x2ma1
d1b72a_ d1wi3a1 d1jgga_ d1wjha1 d1x2na1 d1mnmc_ d2cuea1 d3k2aa_
d2hosa_ d2jwta2 d1pufa_ d1octc1 d2hi3a_ d1b8ia_ d4j19a_ d3lnqa_
d1wh5a1 d1fexa_ d2cqra1 d1guua_ d1ug2a1 d2cqqa1 d2ajea1 d4a69c_
d1h8ac1 d2crga1 d2iw5b1 d1xc5a1 d1ofcx1 d2cu7a1 d1wgxa1 d2cjja1
d2ckxa1 d1gvda_ d1hcra_ d1tc3c_ d1gdta1 d1u78a2 d6paxa1 d6paxa2
d1k78a1 d1hlva1 d1hlva2 d1iufa2 d1iufa1 d2iu5a1 d1zk8a1 d2g7sa1
d2fd5a1 d2np5a1 d1z0xa1 d2id6a1 d2d6ya1 d2i10a1 d1vi0a1 d2oi8a1
d3c07a1 d2ns7a1 d2fq4a1 d2yvea1 d1t33a1 d2o7ta1 d1rkta1 d2np3b1
d2g7ga1 d2gena1 d2fbqa1 d2g7la1 d2gfna1 d2g3ba1 d2jj7a1 d2hyja1
d1sgma1 d1ui5a1 d2hkua1 d2id3a1 d4jyka1 d1bl0a2 d1d5ya1 d1d5ya2

d3ubub_ d1egia_ d1qo3c_ d1v7pa_ d1tdqb_ d3ubua_ d1c3ab_ d1h8ua_
d3bx4b_ d1t8ca1 d1sb2a1 d1qdda_ d2ziba1 d3cada_ d1byfa_ d1j34b_
d1jwia_ d1jzna_ d1c3aa_ d1wmza_ d3bdwb_ d1hq8a_ d1fifa1 d1dv8a_
d3hupa_ d1wk1a1 d1umra_ d2afpa_ d3bx4a_ d1gz2a_ d1sl6a_ d1v7pb_
d3g8ka_ d1ixxa_ d1j34a_ d1jwib_ d1g1sa1 d1y17a1 d1ja3a_ d1fvub_
d3bdwa_ d1sb2b1 d1fvua_ d1umrc_ d1oz7a_ d4m17a_ d3cfwa1 d1ypqa1
d1g1ta1 d3fd4a_ d3l9jc_ d4dn8a1 d1v4lb_ d1rdl1_ d2msba_ d3lv8a_
d1s3ga1 d1zaka1 d1s4qa_ d3tvta2 d1uf9a_ d1khta_ d1ckea_ d1osna_
d1p5zb_ d3cm0a_ d1tmka_ d1jaga_ d3h86a_ d1t0hb_ d1nn5a_ d2h92a_
d3kb2a1 d2c95a1 d1yj5a2 d1kgda_ d1ly1a_ d2ak3a1 d1vyva2 d1deka_
d2grja1 d1p3ja1 d1y63a_ d1e4ya1 d1s96a_ d3iija1 d1lvga_ d1nksa_
d1vyua2 d4e22a_ d1qf9a_ d1jjva_ d1q3ta_ d1ki9a_ d1lw7a2 d1teva_
d1kjwa2 d1gsia_ d2iyva2 d1kaga_ d1viaa_ d1e6ca_ d2xlba_ d3m83a1
d1g3ma_ d1j99a_ d1t8ta1 d1nsta_ d1fmja_ d1zrha_ d1ls6a_ d2a3ra_
d2zptx_ d3ckla_ d1texa_ d1q44a_ d2gwha_ d1uj2a_ d4gi7a_ d2jeoa1
d1rz3a_ d1z0ja1 d3umra2 d3gj0a1 d2gaoa1 d1yzga1 d1x3sa1 d1g7sa4
d1puja_ d1udxa2 d1kk1a3 d2o52a_ d1jwyb_ d1zcaa2 d1zcba2 d2fv8a1
d2cjwa_ d3dz8a1 d1xzpa2 d1vg8a_ d4qxaa_ d2ew1a1 d1x1ra1 d2bcgy_
d3doea1 d3cona_ d3rega_ d3wyga_ d1upta_ d2b6ha1 d1zd9a1 d1lnza2
d1r5ba3 d1n0ua2 d1r2qa_ d2f9la1 d2rhda_ d3q3jb_ d3q85a_ d1efma1
d1r8qa_ d2gf9a1 d2akab1 d1ek0a_ d1z08a1 d2gcoa1 d2dy1a2 d4g01b_
d2cxxa1 d1svia_ d3q72a_ d1ni3a1 d4ac9a4 d2erxa1 d2bmea_ d1wxqa1
d2gf0a1 d4ldja1 d1zunb3 d4lv5b_ d1efca3 d1kmqa_ d2g6ba1 d3lrpa_
d1tada2 d2y8ea_ d4rd4a1 d1z06a1 d2heia_ d3a58b_ d1u0la2 d1d2ea3
d1zj6a1 d2ocba_ d3rwoa_ d1mr3f_ d2e9sa1 d4u5xa1 d2iwra_ d2fn4a1
d3bwdd_ d2nzja1 d2atva1 d2ksqa_ d3l43a_ d1mkya2 d1mkya1 d1wmsa_
d1jnya3 d2rmka_ d1f6ba_ d1f60a3 d1gota2 d1jala1 d2fh5b1 d1g16a_
d2wblc_ d1m7ba_ d1wf3a1 d1f5na2 d1t9ha2 d1z6xa1 d1z0aa1 d2fg5a1
d2a5ja1 d2bcjq2 d1s0ua3 d1ky3a_ d4y0va_ d4lhwa_ d2qmea1 d2atxa1
d1egaa1 d1ryha_ d1moza_ d1nrjb_ d2erya1 d3bfka_ d1puia_ d1fzqa_
d3j3az1 d3t5ga_ d1shza2 d3defa_ d2f7sa1 d3raba_ d3tsoa_ d3aq4a_
d2al7a1 d3sfva1 d3bc1a_ d2ncda_ d1sdma_ d3h4sa1 d2heha_ d2grya_
g3kin.1 d1kk8a2 d1br2a2 d1goja_ d2mysa2 d1lkxa_ d3t0qa_ d4db1a1
d3gbja_ d1ry6a_ d1v8ka_ d1s5ja2 d1qhta2 d4dlea2 d1tgoa2 d3py8a2
d1q8ia2 d1kfsa2 d2py5a2 d1d5aa2 d1k8ka2 d1k8ka1 d3uleb1 d3i33a2
d3i33a1 d1bupa1 d1dkgd2 d1dkgd1 d4ci6a2 d1jcfa1 d4a2ba2 d2fxua1
d1yaga2 d1yaga1 d4cbua2 d1bupa2 d1iqca2 d1iqca1 d1eb7a2 d1eb7a1
d1nmla1 d1nira1 d1hroa_ d4gedb_ d3zooa_ d1mz4a_ d1gu2a_ d1f1fa_
d1dvva_ d1c6ra_ d1c75a_ d1e29a_ d1gdva_ d1cnoa_ d1h32b_ d1co6a_
d2bh4x_ d2aiua_ d5cytr_ d1jdla_ d1i8oa_ d2zboa_ d3dmia_ d1ls9a_
d1wvec_ d2yk3a_ d3ph2b_ d1dw0a_ d5aura_ d1ql3a_ d1qn2a_ d1f1ca_
d2nqya_ d1h4ga_ d1oa2a_ d1olra_ d3b7ma_ d4h7ma1 d3wp3a_ d2vgda_
d1igoa_ d3zsea_ d2nlra_ d3wx5a_ d1oa3a_ d1pvxa_ d1m4wa_ d1f5ja_
d1oa4a_ d1ks5a_ d4ixla1 d1evsa_ d1cnt1_ d1pvhb_ d1z7ca_ d1huwa_

d1bgea_ d1rhga_ d1f6fa_ d1f45b_ d1i1rb_ d1axia_ d1etea_ d3tgxb_
d2z3qa1 d3qb1a_ d1hula_ d1m48a_ d4nkqb_ d1eera_ d1v7mv_ d5e4ea_
d2o27a_ d3b5ka_ d2psma1 d4zf7a_ d3uf5a_ d4x51a_ d1d9ca_ d4ypgc1
d3oq3a_ d1lqsl_ d1n1fa_ d1au1a_ d1wu3i_ d3besl_ d3ux9a_ d1v8fa_
d2ejca_ d3muea1 d1ihoa_ d3q12a1 d2b7la_ d3rbaa_ d2qtra_ d1nupa_
d3l92a_ d1f9aa_ d1qjca_ d2h29a_ d1vlha_ d1od6a_ d1lw7a1 d1mrza2
d4ruka_ d2el7a_ d1iq0a2 d1irxa2 d1r6ta2 d1ivsa4 d2cyba_ d1rqga2
d1j09a2 d2yxna_ d1f7ua2 d1h3fa1 d1ffya3 d2g36a1 d1nzja_ d1jila_
d1n3la_ d1i6la_ d1pfva2 d1h3na3 d3n9ia_ d3sz3a1 d1m4la_ d2bk7a_
d3fjua_ d1z5ra1 d1kwma1 d1jqga1 d2c1ca1 d1dtda_ d1h8la2 d3sw2a_
d1moxc_ d1dx5i3 d1edmb_ d1autl2 d1autl1 d1nqlb_ d1dval1 d1szba2
d1kigl_ d2y5fl_ d1ijqa2 d1gk5a_ d1g1sa2 d1xdtr_ d1lmja2 d1lmja1
d1nzia2 d2wphe_ d1klil_ d1pfxl2 d1pfxl1 d1p9ja_ d1g1ta2 d1f7ea_
d4yzub_ d2mgpa1 d1b9wa1 d1b9wa2 d2mgpa2 d1ob1c1 d1ob1c2 d1n1ia1
d2npra2 d2npra1 d2y5ia_ d1qlsa_ d1psra_ d3n22a_ d2egda_ d4ggfc_
d2wcea_ d2luca_ d4aqia_ d3nsia_ d2lp3a_ d1j55a_ d1a4pa_ d1nsha_
d1pvaa_ d3fs7a_ d2pvba_ d1ttxa_ d1rwya_ d2kyfa_ d1rk9a_ d1a75a_
d1exra_ d1tiza1 d1nyaa_ d1g8ia_ d1wdcc_ d3fwba_ d1ggza_ d2o5ga_
d1ctaa_ d1m45a_ d3i5gc_ d1sl8a_ d1fpwa_ d2scpa_ d2roaa_ d4aqra_
d3pn7b_ d1br1b_ d2mysb_ d1s6ca_ d1bjfa_ d2obha_ d1jfka_ d1jf0a_
d1qv0a_ d1lkja_ d2mlfc_ d2m97a_ d2d8na_ d4by5a_ d1jbaa_ d4nqga_
d1wdcb_ d2mysc_ d1j7qa_ d1s6ia_ d1uhka2 d1r2ua_ d1dtla_ d3kf9a_
d3pn7c_ d5d67d_ d1tuza_ d1h8ba_ d1eg3a1 d1eg3a2 d2zkmx1 d1snla_
d1gjya_ d1y1xa_ d1hqva_ d1df0a1 d1qxpa2 d4usla_ d1k94a_ d1wkya2
d1uuqa_ d3wh9a_ d4m1ra_ d1xyza_ d4xuya_ d2je8a5 d4bf7a1 d3cuia_
d1ur4a_ d1odza_ d2vzsa5 d1bqca_ d2x2ya1 d1hjqa_ d1qnra_ d4y7ea1
d1vjza_ d4k68a_ d2v3ga_ d4hpga_ d1nofa2 d1nq6a_ d1h4pa_ d1aq0a_
d3ziza_ d4bs0a_ d1ecea_ d1h1na_ d1ta3b_ d1w91a2 d1i1wa_ d1uhva2
d1us3a2 d1egza_ d7a3ha_ d1tvna1 d3nyda1 d1ghsa_ d2cyga1 d4xv0a_
d2c8na2 d1w32a_ d3emca_ d1v6ya1 d1foba_ d2uwfa1 d2fgla_ d3niya1
d2c0ha1 d1rh9a1 d1yq2a5 d1szna2 d1fa2a_ d1pama4 d5do8a1 d1ea9c3
d1hx0a2 d2fhfa5 d1qhoa4 d1avaa2 d1m53a2 d4m8ua1 d2gjpa2 d1lwha2
d4howa1 d1ktba2 d1j0ha3 d3vm5a1 d2x1ia_ d1wzaa2 d1uasa2 d2diea1
d1mxga2 d1ht6a2 d1eswa_ d5cpsa_ d1ud2a2 d1zjaa1 d3gbea1 d1kwga2
d1wdpa_ d1gcya2 d1g5aa2 d1hvxa2 d3dhpa2 d2bhua3 d4do4a1 d3qgva1
d1e43a2 d1g94a2 d2d3na2 d1gjwa2 d1gvia3 d1x1na1 d1r7aa2 d1tz7a1
d4toqa_ d1wb0a1 d1w9pa1 d1itxa1 d1kfwa1 d1goia2 d1edqa2 d2gsja_
d1hjxa1 d1jnda1 d1vf8a1 d3wdpp1 d3wq5a_ d1gnxa_ d4gxpa_ d4hz8a_
d1e4mm_ d3aiua_ d1wcga1 d1qvba_ d3ahxa_ d1v02a_ d2dgaa_ d1pbga_
d1vffa1 d1qoxa_ d4ha4a_ d1mixa2 d1e5wa2 d1j19a2 d1isna2 d2al6b2
d1g4ma1 d1g4ma2 d1eh3a_ d3skpa_ d1dtza1 d1dtza2 d1jnfa2 d1ce2a1
d1jnfa1 d1b1xa2 d1b1xa1 d1jw1a1 d1jw1a2 d1ryoa_ d1h76a1 d1h76a2
d1iuka_ d2csua1 d3q9ub_ d2yv1a1 d3q9na_ d2d59a_ d1oi7a1 d2yv2a1
d1y81a1 d1wgqa1 d1v89a1 d2coda1 d1zc3b_ d1wg7a1 d1u5ea1 d1v61a1

d2coca1 d2ys1a1 d1v5pa1 d1ntya2 d1wjma1 d1wi1a1 d1u29a_ d1xx0a1
d1unqa_ d2zkmx3 d1fhoa_ d1v88a1 d2adza1 d1x1fa1 d1p6sa_ d2coaa1
d1btka_ d1v5ma1 d1faoa_ d2x18a_ d1dyna_ d1x1ga1 d4kaxb_ d2j59m1
d1ki1b2 d1xcga2 d1txda2 d1u5da1 d1dbha2 d1w1ha_ d1upqa_ d1v5ua1
d1foea2 d2fjla1 d1eaza_ d2dfka2 d2cofa1 d1kz7a2 d1h6ua1 d9ldta1
d2ewda3 d1up7a1 d2dfda1 d1s6ya1 d1vjta1 d7mdha1 d1hyea1 d1t2da1
d1ojsa3 d1b8pa1 d1ldna1 d1hyha1 d1civa1 d2a92a1 d1ez4a1 d1i0za1
d1y6ja1 d1gv0a1 d1ur5a1 d1llda1 d1obba1 d1v8ba1 d1gdha1 d2dlda1
d1mx3a1 d1ygya1 d1qp8a1 d1li4a1 d1j4aa1 d2naca1 d1npya1 d3t4ea2
d1a4ia1 d1o0sa1 d2hk9a2 d2egga2 d1b26a1 d1bvua1 d1gq2a1 d1c1da1
d1leha1 d1gtma1 d1nvta1 d3dona2 d1edza1 d1gpja2 d1vi2a1 d1p77a1
d1nyta1 d1b0aa1 d1vl6a1 d1v9la1 d1txga2 d3f47a1 d1mv8a2 d2pv7a2
d1xc2a3 d2ahra2 d4xiya1 d2f1ka2 d1pgja2 d1np3a2 d1lj8a4 d1teaa3
d1qmga2 d2i76a2 d2g5ca2 d1wdka3 d1dlja2 d3cuma3 d1kola2 d1qora2
d1jqba2 d1vj0a2 d1cdoa2 d1gu7a2 d1d1ta2 d1xa0a2 d2dm6a2 d1jvba2
d1rjwa2 d1llua2 d1h2ba2 d1tt7a2 d1piwa2 d1o89a2 d1iz0a2 d1e3ja2
d1yb5a2 d1uufa2 d1vj1a2 d1e3ia2 d3gpdg1 d1vc2a1 d2nvwa1 d1u8fo1
d1ydwa1 d1hdgo1 d2g17a1 d1cf2o1 d2czca2 d1r0ka2 d1obfo1 d2cvoa1
d1ebfa1 d1h6da1 d1i32a1 d1zh8a1 d2q49a1 d1qkia1 d1j5pa4 d3pzra1
d1xeaa1 d1vkoa1 d1vm6a3 d3qvsa1 d2pkqo1 d1b7go1 d1gr0a1 d1p1ja1
d1lc0a1 d3pwka1 d2hjsa1 d1zyao1 d1vkna1 d1tlta1 d1nvmb1 d3sjua_
d3ojfa1 d1xu9a1 d2jjya1 d1n7ha_ d1y1pa1 d1h5qa_ d1jtva_ d4fs3a_
d2bkaa1 d1gy8a_ d2c5aa1 d1ulsa_ d1ooea_ d2b69a1 d1y5ma1 d1spxa_
d1bxka_ d1ydea1 d1gega_ d1hdoa_ d1o5ia_ d1e6ua_ d1snya1 d2rhca_
d3zv4a_ d5ceja_ d2d1ya1 d1fjha_ d3w8ea_ d2fmua1 d1x1ta_ d1rpna_
d3ay7a1 d1k2wa_ d1eq2a_ d2hrba1 d3ruaa_ d2ew8a1 d4hwka_ d1xgka_
d2bd0a1 d1yo6a1 d2a35a1 d2ntva_ d1vl8a_ d1hdca_ d4bkua_ d2ag5a1
d2q45a_ d1nffa_ d4iy1a_ d1zbqa1 d1kewa_ d1ek6a_ d1ja9a_ d2q46a2
d1iy8a_ d1cyda_ d2ptga_ d1hxha_ d1uzma1 d3lyla_ d4m89a_ d3wyea_
d2blla1 d1w6ua_ d1sb8a_ d2q2va_ d4nr0a_ d1e6wa_ d2gasa_ d3oiga1
d2bgka1 d1mxha_ d1vl0a1 d1rkxa_ d1r6da_ d1wmaa1 d1e7wa_ d2c07a1
d1xkqa_ d3tzha_ d1g0oa_ d3lz6a_ d1qyca_ d1edoa_ d1gz6a_ d1oc2a_
d1t2aa_ d1zema1 d2xoxa_ d1z45a2 d1geea_ d1p33a_ d3lt0a_ d4e6pa_
d2wyua_ d1db3a_ d4q9na_ d1xhla_ d4o1ma_ d1fmca_ d2uvda_ d2gdza1
d1uaya_ d2z1ma_ d2o23a2 d2fr1a2 d2fr1a1 d1qsga_ d1qyda_ d4zjua_
d2a4ka1 d1yb1a_ d1wvga1 d1yxma1 d3f4ba_ d1mg5a_ d1xg5a1 d1r55a1
d3gboa_ d1atla_ d1kufa_ d1buda_ d4q1la_ d1quaa_ d1yp1a_ d1wnia_
d3nqyb_ d4gera_ d1bqba_ d1ex0a4 d2f4ma1 d1x3za_ d2w0pa_ d2diaa1
d2e9ia2 d2e9ja1 d2dj4a1 d2eeba1 d2diba1 d2dmba1 d2dmca1 d2d7oa1
d2ee6a1 d2d7pa1 d2eeaa1 d2di8a1 d1wlha1 d2d7na1 d2nqca_ d2d7ma1
d2dica1 d3cnka_ d2k3ta1 d2eeca1 d1qfha1 d2bp3a1 d1v05a_ d2ee9a1
d2eeda1 d2di9a1 d1t3ca_ d1epwa3 d2a97a_ d3fiea_ g1e1h.1 d3ioqa_
d1fh0a_ d1o0ea_ d1khqa_ d1m6da_ d2cb5a_ g8pch.1 d1s4va_ d2pnsa_
d1vsna1 d1mira_ d1iwda_ d2oula_ d7pcka_ d3bwka_ d3bcna_ d2p86a_

d1pvja_ d1pcia_ d1gece_ d1deua_ d4bs6a_ d1xkga1 d1cqda_ d1jqpa2
d2c0ya_ d2bdza_ d3p5ua_ d2jk6a2 d2jk6a1 d1feca1 d1feca2 d1lpfa1
d1lpfa2 d1xhca1 d1xhca2 d1dxla2 d1dxla1 d1gera2 d1m6ia2 d1onfa1
d3lada2 d3lada1 d1w4xa1 d1w4xa2 d1gesa2 d1mo9a1 d1xdia1 d1fl2a2
d1fl2a1 d1fcda1 d1fcda2 d1h6va2 d1h6va1 d1mo9a2 d1gv4a2 d1gv4a1
d2gv8a2 d2gv8a1 d1aoga1 d1aoga2 d1hyua1 d1ebda1 d1ebda2 d3dk9a1
d1q1ra2 d1q1ra1 d1gesa1 d1zbfa1 d2z1ha_ d2etja1 d3qioa1 d1jl1a_
d2e4la_ d1jl2a_ d2lsna1 d1io2a_ d1uaxa_ d2yv0x_ d1mu2a1 d1ekea_
d1i39a_ d1pn0a1 d1reoa1 d1ju2a1 d2f5va1 d3c96a1 d2gmha1 d1pj5a2
d1gpea1 d1o5wa1 d1n4wa1 d1k0ia1 d2dw4a2 d2ivda1 d2voua1 d1kdga1
d1s5ja1 d2hbja2 d2igia_ d1wlja_ d1qhta1 d4dlea1 d1tgoa1 d2f96a1
d3tr8a1 d3mxma_ d1q8ia1 d1y97a1 d1w0ha_ d1kfsa1 d2py5a1 d1noya_
d1d5aa1 d1yt3a3 d2guia_ d3mwpa1 d4kb1a_ d2y8ga_ d1xkea1 d2y8fa_
d2v76a_ d1wj1a1 d1aqca_ d1qqga1 d1ntva_ d1n3ha_ d4dbba_ d3f0wa1
d1p3ra_ d1wgua1 d2cy4a1 d3hqca_ d1wvha1 d1xr0b1 d2av4a1 d3nvva1
d1krha3 d2h88b1 d1n62a2 d1t3qa2 d1kf6b2 d1zoyb1 d1jq4a_ d1dgja2
d1rm6c2 d1r7ha_ d1faaa_ d1wika1 d1xfla_ d1ttza1 d2f51a1 d1ti3a_
d1nhoa_ d1sena_ d2i4aa_ d1ep7a_ d1zmaa1 d1fo5a_ d3lefa1 d3diea1
d2yana1 d1z6na1 d1oaza_ d3zywa1 d4aj8a_ d1nm3a1 d4fiwa_ d1wmja_
d4hs1a1 d1iloa_ d1xwaa1 d1wjka1 d2vm1a_ d1fb6a_ d1gh2a_ d1h75a_
d1nw2a_ d1kx5b_ d3a6na_ d4m6ba2 d2nqbd_ d3wkjd_ d2yfwe_ d4h9na_
d5bnxa_ d1kx5c_ d3r45a_ d1eqzb_ d4jjnb_ d1f66c_ d1eqza_ d4wnnb_
d1kx5a_ d1u35c1 d1id3a_ d2nqbc_ d1kx5d_ d1tafa_ d1bh9b_ d1jfib_
d1q9ca_ d1h3ob_ d2byka1 d1n1ja_ d4g92b_ d1bh9a_ d1jfia_ d1h3oa_
d2bykb1 d1n1jb_ d1pd212 d1nhya2 d2a2ra2 d1k0dd2 d1f2ea2 d1gsua2
d1f3ba2 d2fhea2 d2pvqa1 d1tw9a2 d1oyja2 d1k0ma2 d3ik7a1 d4is0a1
d1r5aa2 d1jlva2 d1m0ua2 d3gtub2 d1z9ha2 d1okta2 d1axda2 d2gsra2
d1tu7a2 d1k3ya2 d1zgma1 d1b48a2 d1gnwa2 d1gwca2 d2gsta2 d1glqa2
d1oe8a2 d1pn9a2 d1v2aa2 d1fw1a2 d1duga2 d2m9wa1 d3dfxa_ d1ig8a1
d1czan2 d1czan3 d1czan1 d1ig8a2 d1czan4 d1bg3a3 d1bg3a2 d1bg3a1
d1qqya_ d1gd6a_ d1f6ra_ d1ivma_ d2gv0a_ d1iiza_ d2z2fa_ d1j8xa_
d1ghla_ d1b9oa_ d174la_ d169la_ d2o4wa_ d1p37a_ d1k28a3 d1xjta1
d1jtma_ d2f2qa_ d4e97a1 d3w3ea_ d4tx7a_ d3cqla_ d4msta_ d3iwra_
d1dxja_ d2z37a_ d4dyga_ d2pjsa1 d2i7ra1 d1ecsa_ d1xqaa1 d5cj3a1
d1npba_ d1jifa_ d2p7oa_ d1r9ca_ d1nkia_ d1mpya2 d1kw3b2 d1kw3b1
d4ghga1 d1tfza1 d3isqa1 d3isqa2 d1sqia1 d1sqia2 d1lkda2 d1lkda1
d1mpya1 d1cjxa1 d1sp9b2 d1t47a1 d1t47a2 d1f1ua2 d1sp8a1 d1sp8a2
d1f1ua1 d1etob_ d1umqa_ d1g2ha_ d1pv8a_ d1w5qa_ d2c1ha_ d1l6sa_
d1qsta_ d2beia1 d4m3sa_ d1u6ma1 d1ygha_ d1yk3a1 d3dr6a_ d1xmta_
d1z4ea1 d2ge3a1 d1bo4a_ d2fiaa1 d2jdca1 d1y9wa1 d3to7a_ d1wwza1
d2fl4a1 d2ozua1 d2b5ga_ d2cy2a1 d2p0wa_ d1cm0a_ d4pswa_ d1yrea1
d1yr0a1 d1m4ia_ d2gana1 d1yvka1 d1ghea_ d2g3aa1 d1y7ra1 d2euia1
d1q2ya_ d2fsra1 d1xeba_ d2ae6a1 d1cjwa_ d1qsma_ d2aj6a1 d1y9ka1
d1i12a_ d1vkca_ d2atra1 d1n71a_ d2d4pa1 d2fiwa1 d2fe7a1 d1tiqa1

d2ou2a_ d1yx0a1 d2fcka1 d1p0ha_ d2j8ma_ d2pq8a_ d1z4ra1 d1s3za1
d1ufha_ d1xvqa1 d1pn0a2 d3hy2a_ d2cvba1 d1o8xa_ d1lu4a_ d1we0a1
d2bmxa1 d2a4va1 d1psqa_ d1xiya1 d1q98a_ d1n8ja_ d1zofa1 d2ggta_
d3tjja_ d4je1a1 d1o73a_ d3s9fa_ d2zcta_ d1qmva_ d1jfua_ d2b1ka_
d5dvba1 d2b5xa1 d2v2ga_ d1st9a_ d3tb2a_ d4llra_ d4fh8a1 d1e2ya_
d1knga_ d2h30a_ d1i5ga_ d2z9sa_ d2f8aa1 d1tp9a1 d2b7ka_ d1nm3a2
d3hvva_ d2wfca1 d4kcea_ d1xvwa1 d4gqca1 d3qpma_ d4k1fa_ d1zzoa1
d1gp1a_ d1prxa_ d4od7a1 d1z6ma1 d4mcua1 d4dvca1 d3l9sa_ d1un2a_
d3rppa_ d3kasa1 d3bi1a3 d1e88a1 d1l6ja5 d1l6ja4 d1eaka4 d1eaka3
d1h8pa2 d1h8pa1 d1jgta2 d1ea0a3 d1ecfa2 d1q15a2 d1ct9a2 d1ao0a2
d1te5a_ d1ys1x_ d4hs9a_ d1ispa_ d1ex9a_ d1ku0a_ d3qmma_ d1ra6a_
d3gsza_ d1khva_ d1te9a_ d2xwha_ d3uqsa_ d3hkwa_ d3n6ma_ d2cjqa_
d2xyma_ d1teba_ d1te8a_ d1s48a_ d1u09a1 d4wfxa_ d3tyqa_ d2pgga1
d1dgsa2 d1fvia1 d1p16a1 d1x9na2 d1lgya_ d4l3wa_ d1llfa_ d1gz7a_
d1ukca_ d2ftba_ d1pmpa_ d2rcqa1 d1fdqa_ d4lkpa_ d1sa8a_ d1vyfa_
d1lpja_ d3vg7a1 d4azqa1 d1o8va_ d5bvqa_ d1p6pa_ d2qo4a_ d1opba_
d1yiva1 d1o1va_ d4bvma1 d4tkba_ d4i3ba_ d1bwya_ d1ftpa_ d1ggla_
d4gkca_ d1cbia_ d3p6da1 d1kqwa_ d2a0aa1 d3zxha_ d1cgla_ d1rm8a_
d1eaka2 d1hy7a_ d2jsda_ d1cxva_ d1hv5a_ d1i76a_ d3p0ya1 d1igra1
d1igra2 d1n8yc2 d1n8yc1 d1m6ba2 d1m6ba1 d1sr9a2 d1rqba2 d1h6ta2
d1lmra_ d1cixa_ d1y29a_ d1v7fa_ d2jtba_ d1d1ha_ d1g9pa_ d1lupa_
d1qk7a_ d1mb6a_ d2a2va_ d1niya_ d1i25a_ d1hvwa_ d2mpqa_ d1ie6a_
d1kqha_ d1la4a_ d2mxoa1 d1dl0a_ d1koza_ d1qk6a_ d1emxa_ d1lqra_
d1c6wa_ d1rypd_ d1irua_ d1rypc_ d3unfk_ d1rypa_ d1j2pa_ d1irui_
d1j2qh_ d3unfh_ d1m4ya_ d1rypb_ d1irul_ d1irug_ d1iru2_ d1rypg_
d1g3ka_ d1iruf_ d1q5qa_ d1e94a_ d1irub_ d1ryph_ d3ty6a_ d1iruj_
d1iruk_ d1rype_ d1irud_ d1irue_ d3mi01_ d1rypf_ d1iruh_ d2z3ba_
d1iruc_ d3mka2_ d1iru1_ d1ny721 d1ny722 d1a6ca3 d1a6ca2 d1pgl22
d1pgl21 d1pgl11 d1ny711 d1mqta_ d1d4m3_ d1fmd3_ d2mev1_ g1aym.1
d1ev13_ d4q4w1_ d1r1a3_ d2x5ia_ d1rhi1_ g1r1a.1 g1d4m.1 g1rhi.1
d1b35b_ d1oopa_ d1tme1_ d4iv1a_ g1pvc.1 g1fpn.1 d1fpn1_ d4gb31_
d1d4m1_ d1fmd1_ d1ev11_ d2wzr3_ g1cov.1 d1qqp3_ g1fmd.1 d2x5ic_
d1rhi3_ g1oop.1 d1hxs1_ g1hxs.1 d1fpn3_ g2mev.1 d1eah3_ d1aym1_
d1pvc3_ d1qqp1_ d1zba3_ d1h8ta_ d3vbhc_ g1b35.1 g1bev.1 d1hxs3_
g1ev1.1 d1bev3_ g1tmf.1 d1tmf1_ d1b35a_ d2mev3_ g1eah.1 g1h8t.1
d1eah1_ d4q4w3_ d1r1a1_ d1aym3_ d1pvc1_ d1zba1_ d1h8tc_ d1oopc_
d1tme3_ g1qqp.1 d4iv1c_ g1tme.1 d1ju8a_ d4lt9a1 d3tc7a1 d3hoja_
d1i4na_ d3vnda_ d3nl8a1 d1geqa_ d3nava1 d1v5xa_ d4a2ra1 d3tc6a1
d1ujpa_ d4ijba_ d1xcfa_ d1vc4a_ d1rd5a_ d1qopa_ d3b5va_ d2r80b_
d1cqxa1 d1cg5a_ d1gcvb_ d3boma_ d2h8fb_ d2raoa_ d3bj1a_ d4hrrb_
d2r50a_ d3d1kb_ d2aa1b1 d3zhwa_ d3hf4b_ d3gdja_ d1jebb_ d3bj1b_
d1x9fb_ d1wmub_ d1gcva_ d3bcqb_ d3d4xb_ d1gvha1 d2dc3a_ d3hf4a_
d4yu3a_ d3k8ba_ d1qpwb_ d2r80a_ d3bcqa_ d1v4wa_ d3wtgb_ d1jl7a_
d4esaa_ d1a9we_ d3d1ab_ d1fsla_ d4h2la_ d1v4wb_ d1mwca_ d1vhba_

d1jeba_ d1spgb_ d1h97a_ d1q1fa_ d1tu9a1 d3d1aa_ d1hdsb_ d3hyub_
d2zfbb_ d3fs4a_ d1oj6a_ d1shrb_ d4h2lb_ d4yu3b_ d1spga_ d3hrwa_
d3qqra_ d3at5a_ d4wchd_ d3hyua_ d1cg5b_ d1uc3a_ d1hbra_ d2raob_
d1outb_ d4esab_ d3gdjb_ d3qqqa_ d1x9fc_ d3vrfa_ d1itha_ d3mkba_
d3d4xa_ d3mjpa_ d1jl6a_ d3fh9a_ d1outa_ d2dc3b_ d1it2a_ d3eoka_
d3g46a_ d1qpwa_ d3vrfb_ d1ch4a_ d1x9fd_ d1hdsa_ d2zfba_ d3mkbb_
d1or4a_ d3wtga_ d1a4fa_ d3fh9b_ d3bomb_ d3cy5a_ d3at5b_ d1x9fa_
d2oifa_ d4lmxb_ d1cpcb_ d3v57a_ d1kn1a_ d1cpca_ d4f0ta_ d2vjha_
d4po5b_ d1eyxa_ d1alla_ d4f0tb_ d1b33b_ d1b8db_ d4h0mb_ d1b33a_
d2vjhb_ d4lm6b_ d2vmlb_ d1allb_ d2vjta_ d3dbja_ d2vmla_ d2vjtb_
d4f0ub_ d1f99b_ d4rmpa1 d1liaa_ d1eyxb_ d2uulb_ d4lmsb_ d4f0ua_
d2bv8a_ d1f99a_ d2bv8b_ d2c7lb_ d3dbjb_ d4rmpb1 d1b8da_ d4h0ma_
d3v57b_ d1kn1b_ d2uula_ d2j96a_ d1kyqa1 g1gxs.1 d1azwa_ d1mtza_
d2xqfa_ d1ea5a_ d1f6wa_ d1dx4a_ d1qe3a_ d1k4ya_ d4qwwa_ d2ha2a_
d1r88a_ d1sfra_ d4qeka_ d3c8da2 d2pbla1 d1jkma_ d1jjia_ d1lzla_
d2m5ta_ d1wqsa_ d5bpea_ d2ipha_ d1lvoa_ d2bhga1 d3tnta_ d3sjia_
d3d23a1 d2fyqa_ d3tloa_ d3zvea1 d2hrva_ d3q3ya1 d4asha1 d1jmxa3
d1pbya3 d1pbya4 d1b9ra_ d1offa_ d1frra_ d3wcqa_ d1e9ma_ d1l5pa_
d1wria_ d1i7ha_ d3av8a_ d1rfka_ d3p1ma1 d3b2ga_ d2bt6a2 d4iuaa3
d4bvva_ d5hpga_ d3k65a_ d1ki0a3 d1ki0a2 d1ki0a1 d1a0ha1 d1pmla_
d3sp8a3 d2feba1 d1f74a_ d3l21a_ d2e1da_ d1to3a1 d1w3ia_ d1sfla_
d1n7ka_ d1vcva1 d4imea_ d1wa3a_ d2ocza1 d1f2ja_ d3m16a_ d2yxga_
d1ub3a_ d3a5fa_ d1fbaa_ d1l6wa_ d1xfba1 d1wx0a1 d1vpxa1 d1ojxa_
d1mxsa_ d2a4aa1 d1xkya1 d3cq0a1 d3s1xa_ d1f05a_ d1fdja_ d3cpra1
d4d2ja_ d4mh8a_ d1mu2a2 d4i7ga1 d3rp2a_ g1ucy.1 d1fuja_ d2zchp_
d3w94a_ d1si5h_ d1lo6a_ g2pka.1 d1cgha_ d1kigh_ d1a5ia_ d3s9ba_
d1hj8a_ d1ao5a_ d2anya_ d1q3xa1 d1gvza_ d1azza_ d4gsoa_ d2f91a1
d1iaua_ d1gdna_ d1eufa_ g1fiw.1 d1eq9a_ d4nfea_ d2rdla_ d1spja_
d1elva1 d1a7sa_ d1ekbb_ d1pfxc_ d1yc0a_ d2aiqa_ g1fiz.1 d1fi8a_
d4k60a_ d4dgja_ d1mzaa_ d1op0a_ d3tjva_ d1pytd_ d1sgfg_ g1gg6.1
d2eeka_ g1rtf.1 d1a0ja_ d3g01a_ d1pytc_ d4e7na_ d1ddja_ d1z8ga1
d2hlca_ d1npma_ d1brup_ g1jou.1 d1sgfa_ d1autc_ g2hnt.1 g1kdq.1
d1orfa_ d3s69a_ d4crga_ d1fxya_ d1bqya_ d1svpa_ d1mbma_ d3su6a1
d1cu1a1 d4agka_ d1vcpa_ d1q31a_ g1n1l.1 d3e90b_ d1ep5a_ d3u1jb_
d1dzka_ d4tlja_ d2a2ua_ d4wfva_ d3t1da_ d4iaxa_ d3zq3a_ d2nnda_
d1i4ua_ d1kxoa_ d1bbpa_ d1kt7a_ d1exsa_ d3s26a1 d1lf7a_ d1qfta_
d4gh7a_ d1z24a1 d1qabe_ d1xkia_ d1ew3a_ d3bx7a_ d4iawa1 d1n0sa_
d3kzaa_ d2k23a_ d2l9ca1 d2joza1 d1gm6a_ d3kffa_ d3saoa1 d1e5pa_
d1yupa1 d1iiua_ d1e69a_ d1mv5a_ d3g60a4 g1ii8.1 d1ewqa2 d1ji0a_
d1qhla_ d1v43a3 d2wmfa1 d1vpla_ d1g2912 d4a82a2 g1f2t.1 d2d62a1
d3tuic1 d2bbsa1 d3g60a3 d2ixea_ d1g6ha_ d1sgwa1 d2qi9c_ d3rlfa1
d3b60a1 d3d31a2 d2pzea1 g1xew.1 d1jj7a_ d1oxxk2 d2onka1 d1w1wa_
d1iwea_ d1qf5a_ d1vmaa2 d1nija1 d1np6a_ d2p67a1 d3ihla_ d1okkd2
d1s1ma2 d1iona_ d2v40a_ d1dj3a_ d3hida_ d2vo1a1 d2g0ta1 d1vcoa2

d1j8mf2 d1g3qa_ d1xjca_ d1p9ba_ d1qzxa3 d1ihua2 d1ihua1 d2qm8a_
d1dj2a_ d4ioja_ d1cp2a_ d1yrba2 d1hyqa_ d3gqba2 d1skye3 d1xp8a1
d1tf7a2 d1tf7a1 d1ye8a1 d1skyb3 d2qe7d2 d4blta_ d4huta_ d3ldaa2
d4hyya_ d2i3ba1 d1cr1a_ d3j9tb2 d3ntua2 d1vdza1 d2z43a_ d1e9ra_
d1nlfa_ d3ziaa2 d1n0wa_ d1g6oa_ d3ziad2 d3j9ta2 d1pzna2 d3gqbb2
d2i49a_ d1nnfa_ d1xvxa_ d1ursa_ d1h3da1 d1iuda_ d4m1va1 d1twya_
d3e13x_ d2nxoa1 d4pfua_ d3ttma_ d1elja_ d2qmwa1 d1us5a_ d1y3na1
d1jeta_ d1uqwa_ d4f19a1 d2esna2 d3thia_ d3fasa1 d2uyeb_ d2xwva1
d1r9la_ d2fyia1 d2ozza1 d1sw5a_ d1nh8a1 d1vr5a1 d2g29a_ d1xs5a_
d3lswa_ d2z23a_ d3w9va_ d3tcha_ d1q35a_ d1z7me1 d3q27a_ d1p99a_
d1ve4a1 d2i6ea1 d4mcoa1 d4n8ya_ d1eu8a_ d1zbma1 d1hsla_ d1ixca2
d1j1na_ d1xvya_ d1y9ua_ d2z22a1 d1xoca1 d4oera_ d1wdna_ d5f1qa1
d1xt8a1 d1i6aa_ d4gwoa_ d4igta1 d1pc3a_ d4mmpa1 d1o63a_ d2czla1
d2onsa2 d4mija1 d1g8fa3 d1c4oa1 d2p6ra3 d2p6ra4 d1oywa2 d1oywa3
d1wrba1 d1w36b2 d1w36b1 d1c4oa2 d3bora_ d1t6na_ d2eyqa5 d2eyqa3
d2eyqa2 d2zu6a2 d1veca_ d1uaaa1 d1w36d1 d2g9na1 d1z3ix2 d2rb4a_
d1hv8a1 d1hv8a2 d1t5ia_ d1w36c2 d2j0sa2 d2j0sa1 d1fuka_ d3hjha1
d1z5za1 d1qdea_ d1z63a1 d1s2ma2 d1s2ma1 d1wp9a1 d1wp9a2 d1gm5a3
d1gm5a4 d3dina4 d3dina3 d1q0ua_ d1w36c1 d1z3ix1 d1nkta3 d1nkta4
d1uaaa2 d1w36d2 g1qhh.1 d2fwra2 d2fwra1 d3q3ma_ d1za0a1 d1mxra_
d2inca_ d1jk0a_ d2incb_ d1mhyb_ d3hf1b_ d1mtyb_ d1r2fa_ d2uw1a_
d1mhyd_ d3n37a_ d1afra_ d1syya_ d1mtyd_ d2o1za_ d1uzra_ d1smsa_
d2fkza_ d1zuja1 d1s3qa1 d2fjca1 d1z6om1 d1veia_ d4dyya_ d1jiga_
d3gvya_ d2pyba_ d4cy9a1 d3bvfa1 d1r03a_ d2cf7a_ d1nf4a_ d1zs3a1
d4iwka_ d3ka8a_ d3np0x_ d1n1qa_ d1lb3a_ d4e6ka_ d1z6oa1 d2fzfa1
d4dyua_ d3ge4a_ d1jgca_ d4am4a_ d2z6ma_ d1ji5a_ d1vlga_ d1lkoa1
d1krqa_ d3qz3a_ d1o9ra_ d2ffxj_ d1euma_ d1yv1a1 d2v2pa_ d1j30a_
d4di0a_ d1dpsa_ d2cbia2 d1gqia1 d2choa2 d1oewa_ d2qzxa_ d2asia_
d1psoe_ d1smra_ d2h6ta_ d1pfza_ d1izda_ d3hd8a_ d1j71a_ d1dpja_
d4aa9a_ d1t6ex_ g1b5f.1 g1lya.1 g1htr.1 d1qdma2 d1bxoa_ d1wkra_
d1tzsa_ d1ibqa_ d2qzwa_ d1bdqa_ d3ggta_ d3ixoa_ d1mrxa_ d2haha_
d1tw7a_ d4njsa_ d1sgua_ d2p3da_ d1nsoa_ d3d3ta_ d2rkfa_ d1c6ya_
d2rspa_ d3ogpa_ d3ekwa_ d2o4na_ d2j9ja_ d1baia_ d3ggua_ d3lzua_
d4npua_ d4j55a_ d2r8na_ d2fxda_ d2p3ca_ d4dqea_ d3ttpa_ d3t3ca_
d2p3aa_ d2hc0a_ d2fdda_ d3mwsa_ d1h4ra3 d4yl8a1 d2al6a3 d1wgda1
d1wgga1 d4q5eb_ d1wjna1 d1se9a_ d3n3kb_ d1wz0a1 d1we6a1 d2m0xa_
d2gbka_ d3rt3b1 d1wh3a1 d1sifa_ d2ekec2 d4ii2b1 d4hk2a1 d1c3ta_
d1m94a_ d1wgha1 d1ttna1 d1j8ca_ d1wx8a1 d3v6cb1 d2ma9b_ d4i6lb1
d1bt0a_ d2jvca1 d1iyfa_ d2k1fa_ d1wxva1 d1wf9a1 d1t0ya_ d2io0b2
d1x1ma1 d2gbma_ d1zw7a1 d2m17a_ d1ud7a_ d1we7a1 d2lwpa_ d2gbja_
d1wjua1 d1v6ea1 d1v86a1 d4eewa_ d1wiaa1 d4icva_ d4gu2a_ d1wx7a1
d1wy8a1 d4hjka1 d2faza1 d1v5oa1 d2al3a1 d2cr5a1 d1wj4a1 d1s3sg_
d1yvca1 d1yeza1 d1dszb_ d1a6ya_ d3cbba_ d1ynwa1 d1r4ia_ d1hcqa_
d2hana_ d2hanb_ d2enva1 d1kb4b_ d2nllb_ d1lo1a_ d1cita_ d1um8a_

d1qvra2 d1qvra3 d1qzma_ d1sxjc2 d1jr3d2 d2p65a_ d1l8qa2 d1g8pa_
d3hu3a3 d1sxjd2 d1njfa_ d1in4a2 d1sxja2 d1tuea1 d2ce7a2 d1w5sa2
d1sxjb2 d1d2na_ d1ny5a2 d1ofha_ d2gnoa2 d2a5yb3 d1fnna2 d1u0ja1
d1lv7a_ d1iqpa2 d2fnaa2 d1ixsb2 d4gita_ d1sxje2 d1g41a_ d2e3fa_
d1mm0a_ d2e3ea_ d2llda_ d2ny8x_ d2e3ga_ d2ny9x_ d2nz3a_ d2k4ua_
d1hp2a_ d2lu9a_ d1baha_ d2meoa1 d1wm7a_ d1hlya_ d1n8ma_ d1jlza_
d1wpda_ d1c55a_ d1pvza_ d1wt7a_ d1wz5a_ d1m2sa_ d1pjva_ d1c49a_
d1qkya_ d2ktca1 d1wmta_ d1du9a_ d1j5ja_ d1r1ga_ d1t0za_ d2i61a_
d1t1ta_ d1sn4a_ d1kv0a_ d2lkba_ d1npia_ d1wwna_ d1pe4a_ d2kbka_
d2li7a_ d1djta_ d2kbha_ d1dq7a_ d1sega_ d1omya_ d1zywa1 d1jzaa_
d1n9wa1 d1bbua1 d1c0aa1 d1b8aa1 d1l0wa1 d3a74a1 d1eova1 d1iyjb4
d1iyjb3 d1o7ia_ d4ipca1 d1x3ea_ d4apva_ d1jb7a1 d1jb7a2 d1jb7a3
d3kdfa_ d1ue1b_ d1wjja1 d3eiva_ d1xjva2 d1xjva1 d2ccza2 d1se8a_
d1l1oc_ d1jb7b_ d1qvca_ d3ulla_ d1qzga_ d1miua4 d1miua5 d1miua3
d1y14b1 d2nn6i1 d1i94l_ d2zjra2 d1smxa_ d2qamc2 d1hh2p1 d2r7da1
d1rl2a2 d1wfqa1 d3bzka4 d1x6oa2 d3a0ja_ d2j01d2 d2ja9a1 d4ql5a_
d1hr0w_ d1luza_ d2n3sa_ d2vnud1 d1h95a_ d1go3e1 d2nn6h1 d1ne3a_
d2mo1a_ d1iz6a2 d1jjga_ d1e3pa2 d2ix0a3 d2ix0a2 d2lxja_ d1ueba3
d1ueba2 d2z0sa1 d1u0la1 d2ix0a1 d2eifa2 d2je6i1 d3i2za1 d2c35b1
d2ahob2 d1t9ha1 d3i4oa_ d2qall1 d1jt8a_ d2nn6g1 d1g6pa_ d3cama_
d1i94q_ d1c9oa_ d1d7qa_ d2ba0a1 d2vnud2 d2vnud3 d1khia2 d2qalq1
d1wi5a1 d1xtda2 d1prtc2 d1pfsa_ d2a1ka_ d1ohea1 d1ohea2 d2f4da_
d1rxda_ d2hxpa_ d1xria_ d1d5ra2 d1fpza_ d3ezza_ d4y2ea_ d1i9sa_
d1k9ua_ d2opoa_ d3azya_ d3wvja_ d1upsa1 d1ajoa_ d2hyka_ d1ajka_
d2vy0a_ d1o4ya_ d3o5sa_ d3d6ea_ d3atga_ d1dypa_ d1umza_ d3i4ia_
d1c1la_ d4lbqa_ d1a78a_ d1slta_ d4no4a_ d3zsja_ d3duia_ d1g86a_
d1qmja_ d1hlca_ d1tdha3 d1q3ba3 d1k82a3 g1qmo.1 g1len.1 d3zyra_
d1fnya_ d1n47a_ g2ltn.1 d1avba_ d3usua_ d1dhkb_ d1f9ka_ d1ukga_
d1g8wa_ d1g9fa_ d1wbfa_ d3wcsa_ d1g7ya_ g1loe.1 d1mvqa_ d1hqla_
d1v6ia_ d1dbna_ d1qnwa_ d1fx5a_ d4l8qa_ d3n35a_ d2h0ba_ d2erfa_
d1h30a2 d1h30a1 d3mw4a1 d1pz7a_ d2r1ba_ d3v64a1 d1d2sa_ d3bopa1
d3mw3a1 d1okqa1 d1okqa2 d1wioa4 d1ccza2 d1vcaa1 d1dr9a2 d1ic1a1
d2d9qb1 d2nxyb2 d1adqa1 d1fnga1 d1uvqb1 d1zs8a1 d2vola1 d2vola2
d1mfbl2 d2jelh2 d1uwel2 d3qq4b1 d1kjvb_ d1k5na1 d1osph2 d4odhl2
d1ow0a1 d1ow0a2 d1bfob2 d3l9ra2 d3jtth1 d2fath2 d4elkb2 d1fn4b2
d2xfxb_ d1pg7x2 d1cqka_ d4k71b2 d1lk2a1 d1eapb2 d4lfhg2 d1i4fa1
d2nw2a2 d1kfah2 d1mhca1 d1h3ua1 d1wejh2 d1h0db2 d1a5fh2 d1kcvh2
d1mjuh2 d1qqda1 d1kcrh2 d1k8ia1 d3huja2 d2nnab1 d1nfdf2 d3mffb2
d1s9va1 d1lk3h2 d1q0xl2 d1fv1b1 d1neza1 d4rfeb2 d1r3ha1 d1a6tb2
d1kjva1 d1pz5b2 d1onqa1 d2bc4a2 d1de4a1 d1k8da1 d1tcra2 d1dn0b2
d1nfde2 d2xwtb2 d1bfoa2 d1mexh2 d1iakb1 d1ypzf2 d4f7ea2 d1q72l2
d2fbjh2 d1fe8h2 d1clzh2 d1fngb1 d1uvqa1 d1vgka1 d1c16a1 d4j12a2
d4x99b2 d1igtb3 d1je6a1 d1q72h2 d1nldh2 d1teth2 d2hmic2 d1i1ca2
d1i1ca1 d1k8ib1 d1q0yh2 d1kfal2 d3frua1 d1pg7w2 d1u58a1 d1igyb4

d1igyb3 d1hyrc1 d1klub1 d1deeb2 d1c12b2 d2bc4b2 d2ig2h2 d1kcvl2
d3wn5b1 d1lk2b_ d1lk3l2 d1mjul2 d1sm3h2 d1q0xh2 d1zvsa1 d1ydpa1
d1u8ib2 d1ypze2 d1kcuh2 d1op9a_ d1y0la1 d1etzb1 d1egjl1 d2gsib1
d1nj9a1 d4jfhd1 d1aqkl1 d1t2ja_ d1lo4h1 d1zvya1 d3v4vl1 d4enec1
d1kxtb_ d5e8ea1 d2pxya_ d4weud_ d1xiwc_ d2esvd1 d1qnzh_ d1ogae1
d1f4xh1 d4n1hb_ d3ezjb1 d3mlwl1 d1kfah1 d1etza1 d1b4jh1 d3cfic_
d4w68a_ d2arja1 d1ek3a_ d2ok0l1 d1vera_ d1uwgh1 d1blnb1 d1fpth1
d1rzga1 d2giya1 d4m3kb1 d1nc2a1 d2nyyd1 d1ncna_ d1fbih1 d5dxwa_
d4fqqa1 d1dqdh1 d1jfql1 d1kelh1 d1faih1 d1frgl1 d1figl1 d4m6nl1
d1fo0a_ d4hxbl1 d3b5ga_ d3quyd1 d2h9gb1 d1i8kb_ d2dd8h1 d2ck0h1
d1eaja_ d4w6wb1 d2a6ib3 d1t66d1 d2e27h_ d1ob1b1 d1f11b1 d2v7na1
d1ad0a1 d2xt1b1 d1pskh1 d4i13b1 d2op4l1 d1a0ql1 d1uwel1 d1za3a1
d1ncbl1 d4uu9a_ d1bm3h1 d1cloh1 d1nqba2 d1cf8h1 d4y5va_ d1mhph1
d5anma1 d4cdgc1 d4ldeb_ d1y0lb1 d2pcpb1 d1z3gl1 d1dfbl1 d1fh5h1
d3g5xa1 d1tzib1 d4dn3l1 d3zhka1 d2b1hh1 d2uyla1 d1f3dh1 d1nj9b1
d2agjh1 d2h1pl1 d1q72h1 d8fabb1 d1ndmb1 d1rjca1 d4u6hb1 d3upca_
d2fbjl1 d1osph1 d1tvda_ d2cjvh_ d4fhbd1 d1bd2d1 d1rz7h1 d3nn8a_
d1blna1 d1knoa1 d1nfda1 d2bmkb1 d1dqll_ d1igyb1 d1igfl1 d12e8h1
d3utza1 d1nc2b1 d1rihh1 d4mwfb1 d4jvpa1 d1mieh1 d1rhfa1 d1fgnl1
d1sbsh1 d1lp9e1 d1dn0a1 d4lqfl1 d1iaih1 d1kgcd1 d1mh5b1 d1gpol1
d2g60h3 d1f8tl1 d2jixd1 d4grwh_ d1fo0b_ d1bfob1 d1axsa1 d1u9ka_
d1nmbh_ d1h8na1 d1h8na2 d1jguh1 d4eura1 d3sdyl1 d35c8h1 d1w72l1
d1mvub_ d1i9rh1 d1ob1a1 d4wenb_ d1yeeh1 d3ietc1 d1f58h1 d1ad0b1
d1mqkl_ d1nbvh1 d4b50a_ d3r8bb_ d1bafl1 d1mamh1 d4rgne1 d2gsgb_
d35c8l1 d1bafh1 d1ai1h1 d2pcpa1 d2fath1 d2x1pa_ d1orsb1 d1ibgl1
d1shma_ d1a2yb_ d1u0qa_ d2coqa_ d1hilb1 d1qfwi_ d1jv5b_ d1fn4b1
d5e0qa_ d1nsnh1 d1plgl1 d4ocxl1 d1kb5h1 d1rzfh1 d1pg7x1 d2fx7h1
d2oi9c_ d1l7il1 d3eyva1 d1d5il1 d4ffyl_ d1tetl1 d1a6wh_ d1ac6a_
d3dvfa_ d1nfde1 d1mimh1 d1ri8a1 d1jhlh_ d3dura_ d1fskb1 d1f97a1
d3so3b1 d2ak4d1 d1lp9f1 d4z7wf1 d1l7th1 d4y5ya_ d4x7ec1 d1emth1
d1ikfl1 d1nlbh1 d4utal1 d1bjma1 d1eapb1 d1u6ah1 d2dtgd1 d1kj2b_
d1xeda1 d1c1eh1 d1fgvh_ d2bnub1 d2fb4h1 d2dtgb1 d1ktrh_ d1jpth1
d1qkzl1 d4hepg_ d2atpb1 d1flrh1 d2hrpl1 d1mvfa_ d1iaim1 d3k80a1
d2jb5h1 d3zkxc_ d4grwe_ d1it9h1 d1rhhb1 d3w9ec_ d1e4wh1 d1sy6h1
d2j6eh1 d3qxta_ d1jglh1 d1p7ka1 d1tzhb1 d4n8pa1 d1gafl1 d2jelh1
d1wioa2 d4i0cc1 d1cicb1 d1lmka2 d1lmka1 d1iqwh1 d1orqb1 d1ejoh1
d1jnlh1 d4zg1a_ d43c9b_ d1ggbl1 d1wejh1 d3q6fa1 d1f3dj1 d3c6la1
d1bqlh1 d3p9wb_ d1a3rl1 d1h0db1 d2uzil_ d1eo8l1 d1qfwl_ d1n7ml1
d1igja1 d1c5ch1 d2a9mh_ d1rmfh1 d1fj1b1 d4heme_ d1ccza1 d1a5fh1
d1ogad1 d1dlfh_ d1qa9b_ d1vgel1 d3qskb_ d1sjxa_ d4ozfg1 d1sq2n_
d1qd0a_ d1kcvh1 d1c5db1 d3qcul1 d8faba1 d3bn9d1 d1mjuh1 d4y8dc_
d7fabh1 d3dvgb1 d1lo0l1 d1a6tb1 d1gigh1 d1kcrh1 d2hwzl1 d1yjdc1
d3clfl1 d4krna_ d1dl7l_ d1e6ol1 d1mexh1 d3r0ma_ d4kkna_ d1fnsl1
d1oaqh_ d1rzia1 d1ucbh1 d1ncwl1 d4b41a_ d1nfdf1 d2mpah1 d2d7tl1

d4grwf_ d4b5ea_ d1lk3h1 d3u79a_ d4u3xa_ d1ghfl1 d1bvkb_ d1mcww1
d1jrhh1 d32c2b1 d4wemb_ d1dsfh_ d1op3h1 d1ce1l1 d1iqda1 d1qoka1
d1qoka2 d1qfuh1 d2crya1 d1ay1h1 d1oaql_ d3ab0b_ d1rurl1 d1t4kb1
d1bz7b1 d1n0xh1 d1forl1 d1ucbl1 d4w6yb_ d43c9a_ d2aabl1 d1tzia1
d4n1ca_ d1k6qh1 d3g9ab_ d4tvsa1 d1igmh_ d1pz5b1 d3dx9a1 d3wxwh_
d1nakh1 d1kcul1 d3eaka_ d4qlra1 d1cr9l1 d1d9ka_ d1f4xl1 d1deea1
d2i27n2 d1c12a1 d2orbl1 d1j8hd1 d1mcph1 d1c5da1 d1kenh1 d1bd2e1
d1h5ba_ d4x7fc1 d3upaa_ d1t3fa1 d1indh1 d2dtgc1 d4hc1l1 d1zv5a1
d4wgvb_ d1uwgl1 d4f37k1 d1aqkh1 d1vesa_ d3zkqd_ d1fptl1 d1lo4l1
d1knob1 d3rrqa_ d1ct8b1 d4orzc_ d1mlbb1 d4hgkc1 d1dqdl1 d1bogb1
d4ozhe1 d1dr9a1 d1i7za1 d1dn0b1 d1kell1 d1rzib1 d1ypzf1 d1wtla_
d1hkfa_ d1fail1 d1figh1 d2vyre_ d1sjva_ d1ohqa_ d1bfoa1 d1pkqb1
d1mjul1 d1b4jl1 d1nsnl1 d2arjb1 d1p4bh_ d4aj0a_ d3nn8b_ d2rcja1
d1f2xk_ d4v1dd_ d1adqh1 d2eksa_ d4k3ga_ d1jfqh1 d1uweh1 d1frgh1
d4gftb_ d1uz8b1 d4n1ea_ d1bm3l1 d1clol1 d3mcka1 d2vq1a1 d1ae6h1
d4aq1b1 d1uj3b1 d1nbqa1 d1fh5l1 d2ck0l1 d2ywza_ d1q72l1 d4nbxb_
d1hila1 d4hzlb1 d2j88l1 d1zmya1 d1cd0a_ d1a4ka1 d1pskl1 d1a0qh1
d4krmb_ d1ncbh1 d2fbjh1 d1j8he1 d1i3ua_ d1cf8l1 d1fe8h1 d2p1ya1
d1eeqa_ d2x89a_ d1ol0a_ d2r0lh1 d1ub5a1 d1dfbh1 d3eyvb1 d4jdvb1
d4p2cg_ d2gsia1 d2agjl1 d1u3ha1 d2h1ph1 d1rihl1 d1fskc1 d1boga1
d2a77l1 d1ospl1 d1qfoa_ d3dwta_ d2fx7l1 d1rz7l1 d3stba1 d1pkqa1
d1b6da1 d1dzba2 d1dzba1 d1kj2a_ d4l1ha_ d1rzgb1 d1dqlh_ d12e8l1
d1miel1 d1jmaa_ d1sbsl1 d1fgnh1 d1f58l1 d1mqkh_ d1ndgb1 d3qegl1
d1iail1 d2aw2a1 d1tqbc1 d3fo2a1 d4dkaa_ d1f8th1 d2g60l1 d2ywya_
d1uj3a1 d1yc7a_ d3bd3a_ d4w6xb1 d1nmbl_ d1i8ka_ d1rjlb1 d1ypze1
d1p7kb1 d1kxqe_ d1gpoh1 d2z4qa1 d1w72h1 d1i9rl1 d4qgya1 d4xt1c_
d2qqnh1 d1cica1 d3c08h1 d2cd0a_ d2bdnh1 d1hnga1 d1maml1 d1nldh1
d1cr9h1 d4lcih1 d3bkyh1 d3qg7l1 d1ai1l1 d1kxvc_ d4iosd_ d1l7ih1
d2x1qa_ d1ub6b1 d3k74b_ d1ibgh1 d1teth1 d1igya1 d1hxma1 d2x6ma_
d2e7la_ d5e03a_ d1qfwm_ d2r0kh1 d1igjb1 d1rurh1 d1plgh1 d1mj8h1
d1yuhb1 d3fmgl1 d1rzfl1 d1emtl1 d1ad9b1 d1igtb1 d1nlbl1 d1d5ih1
d3k7ua_ d4ffyh_ d1bwwa_ d3dvga1 d1a6ta1 d1fgvl_ d4qxgl1 d1miml1
d1jhll_ d2hffb1 d2hh0l1 d1qkzh1 d1l7tl1 d1afvh1 d1iaii1 d2cjuh_
d1um5h1 d2ghwb2 d2ghwb1 d1kgce1 d2fgwh1 d1ikfh1 d1dvfd_ d2i24n1
d2xtjb_ d1za3b1 d2yc1a_ d2hh0h1 d2x1oa_ d1bvka_ d1rjla1 d5e7ba1
d1igta1 d1il1b1 d2fb4l1 d1mvua_ d1jptl1 d32c2a1 d1fvca_ d1iqdb1
d2hrph1 d2p4ab_ d4qo1a_ d1p2cb1 d1wt5c_ d1t4ka1 d5c1mb_ d1it9l1
d1bz7a1 d1b2wh1 d1e4wl1 d1jnll1 d1yedb1 d1za6b1 d3u7aa_ d1igfh1
d3gnml1 d1orsa1 d1wejl1 d1a2ya_ d1hxmb1 d1gafh1 d2j4wh1 d1pz5a1
d1j05a_ d1a3rh1 d4fqll1 d1qfwh_ d1eo8h1 d1n7mh1 d4s11a_ d1kegh1
d5da4a_ d1iqwl1 d3whxb1 d1vgeh1 d1deeb1 d1c12b1 d1t2ql1 d1ad9a1
d3gsnb1 d3b2uc1 d1ggbh1 d1t3fb1 d1nakl1 d4ocmc_ d1lila1 d2v7hb3
d2esve1 d1mcoh1 d2qr0b1 d2q3aa_ d1ct8a1 d1kcrl1 d2pxyb_ d2uzih_
d5c3ld_ d3durb_ d1c5cl1 d2ak4e1 d1dl7h_ d1xiwd_ d1mlba1 d1rmfl1

d1i7zb1 d1dlfl_ d2xa3a_ d5bq7b1 d1dqqb1 d3h42l1 d1egjh1 d1kcvl1
d1fe8l1 d2j6el1 d1f3rb2 d1lk3l1 d1sm3h1 d1q0xh1 d1ghfh1 d7fabl1
d1igch1 d1lo0h1 d4idla_ d5c2bl_ d1jrhl1 d1il1a1 d3zhda1 d4kfzc_
d1b0wa_ d1e6oh1 d1mexl1 d4krpb_ d1fnsh1 d1ncwh1 d4hgma_ d4eigb1
d4rzca_ d2mpal1 d4y5xa_ d5en2b1 d3lrga1 d4ej1c1 d1rhha1 d1j05b_
d1forh1 d1q9wb1 d2wzpd_ d2aq2a1 d1h0da1 d3revb1 d2ij0c1 d1tzha1
d4m7jl1 d1k6ql1 d3b9kc1 d1igml_ d3qeua1 d1dsfl_ d1ce1h1 d1kcuh1
d1zvha1 d1zvoa1 d1tqbb1 d1ay1l1 d2qscl1 d2cgrh1 d1nl0h1 d1kenl1
d3eo0a1 d4n9ob1 d1n0xl1 d3k1kc_ d2cqva1 d1fhga_ d2k1ma_ d2dl2a1
d1biha3 d1biha1 d1biha4 d3qp3a1 d1g1ca_ d1gxea_ d1cvsc2 d1cvsc1
d1xmwa2 d1m4ka2 d2otpa2 d1biha2 d1rhfa2 d2fcba2 d2fcba1 d1wwbx_
d1gsma2 d1epfa1 d1epfa2 d2dava1 d1waaa_ d1f97a2 d1x44a1 d1vcaa2
d1fnla2 d1fnla1 d3b5ha2 d3h8na2 d3h8na1 d1jbja1 d1jbja2 d1pd6a_
d1f2qa2 d1f2qa1 d3cx2a_ d1nbqa2 d3b5ha1 d1n26a1 d1xiwb_ d1xaua_
d1fltx_ d2ifga1 d1olla1 d1olla2 d1cs6a4 d1cs6a3 d1cs6a2 d1cs6a1
d2gw5a2 d1sy6a1 d1xiwa_ d1ucta2 d1ucta1 d1ry7b2 d1gl4b_ d1qz1a3
d1olza1 d2c9aa1 d1wwca_ d2aq2b1 d1ck1a1 d1enfa1 d1i4pa1 d1v1oa1
d1et9a1 d1esfa1 d2g9hd1 d4ohja1 d1bxta1 d1eu3a1 d1ty0a1 d1fnua1
d1c4qa_ d1prtc1 d2wv6d_ d1prtf_ d1ltrd_ d4l63b1 d1r4pb_ d1prtd_
d1tl0f_ d2bosa_ d3chbd_

**Family-4 (a.138.1.3)**

**Positive dataset contains 6 protein sequences.**

d3ubra_ d1d4ca1 d2k3va_ d2j7aa_ d4fasa_ d1sp3a_

**Negative dataset contains 3985 protein sequences.**

d1m32a_ d1ibja_ d1c7na_ d4q75a_ d2bkwa1 d1t3ia_ d1n8pa_ d1qz9a_
d1jf9a_ d1eg5a_ d1p3wa_ d3kgwa_ d1cl1a_ d2ctza1 d1vjoa_ d2rfva_
d1gc0a_ d1cs1a_ d1d2fa_ d2ch1a1 d1qgna_ d1pffa_ d1elua_ d2xlba_
d3m83a1 d1u08a_ d1lc5a_ d1b5pa_ d2ay1a_ d1bw0a_ d3pd6a_ d7aata_
d2csta_ d1j32a_ d4wlha_ d1o4sa_ d1v2da_ d1gdea_ d1m6sa_ d1v72a1
d1svva_ d1ajsa_ d1wsta1 d2f8ja2 d1lk9a_ d1vp4a1 d1xi9a1 d2gb3a1
d1yaaa_ d1jx4a2 d3gv5b1 d1t94a2 d3bq0a2 d3i2ka1 d1lnsa3 d1mpxa2
d1xkta1 d2hfka_ d1jmkc_ d5d3ka_ d2cfba1 d1o69a_ d1bj4a_ d3k28a_
d2bwpa_ d3lg0a_ d2fnua_ d3ou5a_ d1z7da1 d1b9ha_ d1iaya_ d4msoa_
d1bs0a_ d1s0aa_ d1sffa_ d3tqxa_ d1mdoa_ d3qboa_ d3pgya_ d1bjna_
d2dkja_ d1ejia_ d1vefa1 d4wxga_ d3bs8a_ d3gbxa1 d1w23a_ d1r0ka3
d1vkoa2 d1vm6a2 d3qvsa2 d1gr0a2 d1p1ja2 d1t61c1 d1g8fa3 d1f56a_
d1bypa_ d1azca_ d1ws8a_ d3u25a_ d1cuoa_ d1id2a_ d1rkra_ d2iaac_
d4dp1x_ d1nwpa_ d2gima_ d2i7sa_ d1qhqa_ d3ehbb1 d1fftb1 d1gyca3
d1hfua2 d1kbva1 d1kbva2 d1sdda1 d1sdda2 d1gyca1 d1gyca2 d1mzya1
d2bw4a1 d1aoza1 d1aoza3 d1aoza2 d1kyaa2 d1kyaa3 d1kyaa1 d1mzya2
d4e9sa1 d1sddb1 d1sddb2 d1hfua1 d1hfua3 d1oe1a2 d1oe1a1 d1v10a2
d1v10a3 d1v10a1 d1rypd_ d1irua_ d1rypc_ d3unfk_ d1rypa_ d1j2pa_
d1irui_ d1j2qh_ d3unfh_ d1m4ya_ d1rypb_ d1irul_ d1irug_ d1iru2_
d1q5rh_ d1rypg_ d1g3ka_ d1iruf_ d1q5qa_ d1e94a_ d1irub_ d1ryph_

d3ty6a_ d1iruj_ d1iruk_ d1rype_ d1irud_ d1irue_ d3mi01_ d1rypf_
d1iruh_ d2z3ba_ d1iruc_ d3mka2_ d1iru1_ d1a3qa1 d1iknc_ d3brda1
d1p7hl1 d1oy3c_ d1imhc1 d1gjia1 d1zk9a1 d2cxka1 d2bema_ d1pama1
d1ea9c1 d1qhoa1 d2fhfa2 d2fhfa1 d1k3ia1 d1j0ha1 d1cxla1 d2bhua1
d1edqa1 d1f1sa2 d1ut9a2 d1gvia1 d1txka1 d4alac_ d1pjwa_ d1rera1
d1s6na_ d3uzqb_ d2phla1 d1fxza2 d1fxza1 d1lrha_ d1uija1 g1dgw.1
d2v09a_ d1uika2 d1uika1 d1dgwa_ d1ds6b_ d1fstb_ d3us3a3 d3us3a1
d4mh8a_ d4i7ga1 d2b5ea2 d2b5ea3 d2b5ea1 d2b5ea4 d2kp1a1 d2kp2a1
d1hyua3 d1hyua4 d1j08a1 d2bjca1 d2hsga1 d1qpza1 d1ydwa2 d2nvwa2
d1zh8a2 d1qkia2 d1xeaa2 d1tlta2 d1mqta_ d1d4m3_ d1fmd3_ d2mev1_
g1aym.1 d1ev13_ d4q4w1_ d1r1a3_ d2x5ia_ d1rhi1_ g1r1a.1 g1d4m.1
g1rhi.1 d1b35b_ d1oopa_ d1tme1_ d4iv1a_ g1pvc.1 g1fpn.1 d1fpn1_
d4gb31_ d1d4m1_ d1fmd1_ d1ev11_ d2wzr3_ g1cov.1 d1qqp3_ g1fmd.1
d2x5ic_ d1rhi3_ g1oop.1 d1hxs1_ g1hxs.1 d1fpn3_ d1bev1_ g2mev.1
d1eah3_ d1aym1_ d1pvc3_ d1qqp1_ d1zba3_ d1h8ta_ d3vbhc_ g1b35.1
g1bev.1 d1hxs3_ g1ev1.1 d1bev3_ g1tmf.1 d1tmf1_ d1b35a_ d2mev3_
g1eah.1 g1h8t.1 d1eah1_ d4q4w3_ d1r1a1_ d1aym3_ d1pvc1_ d1zba1_
d1h8tc_ d1oopc_ d1tme3_ g1qqp.1 d4iv1c_ g1tme.1 d1au7a2 d1e3oc2
d2ovga_ d1sq8a_ d1rzsa_ d3kz3a_ d3orca_ d3gpdg2 d2q49a2 d1hdgo2
d2g17a2 d2cvoa2 d2czca1 d3pwka2 d1obfo2 d1i32a2 d1zyao2 d2pkqo2
d1b7go2 d2hjsa2 d1cf2o2 d1vkna2 d1nvmb2 d2msya_ d2djoa1 d2r5za_
d1au7a1 d1f43a_ d2lmda1 d1akhb_ d1s7ea1 d3nara_ d1zq3p1 d2craa1
d1mija_ d4uuta_ d3a01a_ d1x58a1 d3rkqa1 d3cmya1 d2cqxa1 d2da6a1
d1e3oc1 d1fjla_ d1mh3a1 d1ig7a_ d1x41a1 d1wh7a1 d1gt0c1 d2mw8a_
d1lfup_ d1x2ma1 d1b72a_ d1wi3a1 d1jgga_ d1wjha1 d1x2na1 d1mnmc_
d2cuea1 d3k2aa_ d2hosa_ d2jwta2 d1pufa_ d1octc1 d2hi3a_ d1b8ia_
d4j19a_ d3lnqa_ d1wh5a1 d1b8ib_ d1fexa_ d2cqra1 d1ug2a1 d2cqqa1
d2ajea1 d4a69c_ d1h8ac1 d2crga1 d2iw5b1 d1xc5a1 d1ofcx1 d2cu7a1
d1wgxa1 d2cjja1 d2ckxa1 d1gvda_ d1hcra_ d1tc3c_ d1gdta1 d1u78a2
d1igna2 d2iu5a1 d1zk8a1 d2g7sa1 d2fd5a1 d2np5a1 d1z0xa1 d2id6a1
d2d6ya1 d2i10a1 d1vi0a1 d2oi8a1 d3c07a1 d2ns7a1 d2fq4a1 d2yvea1
d1t33a1 d2o7ta1 d1rkta1 d2np3b1 d2g7ga1 d2gena1 d2fbqa1 d2g7la1
d2gfna1 d2g3ba1 d2jj7a1 d2hyja1 d1sgma1 d1ui5a1 d2hkua1 d2id3a1
d4jyka1 d3lv8a_ d1s3ga1 d1zaka1 d1s4qa_ d3tvta2 d1uf9a_ d1khta_
d1ckea_ d1osna_ d1p5zb_ d4tmka_ d3cm0a_ d1tmka_ d1jaga_ d3h86a_
d1t0hb_ d1nn5a_ d2h92a_ d3kb2a1 d2c95a1 d1yj5a2 d1kgda_ d2ak3a1
d1vyva2 d1deka_ d2grja1 d1p3ja1 d1y63a_ d1e4ya1 d1s96a_ d3iija1
d1lvga_ d1nksa_ d1vyua2 d4e22a_ d1qf9a_ d1jjva_ d1q3ta_ d1ki9a_
d1lw7a2 d1teva_ d1kjwa2 d1gsia_ d1g3ma_ d1j99a_ d1t8ta1 d1nsta_
d1fmja_ d1zrha_ d1ls6a_ d2a3ra_ d1aqua_ d2zptx_ d3ckla_ d1texa_
d1q44a_ d2gwha_ d1k6ma1 d1z0ja1 d3umra2 d3gj0a1 d2gaoa1 d1yzga1
d1x3sa1 d1g7sa4 d1puja_ d1udxa2 d1kk1a3 d2o52a_ d1jwyb_ d1zcaa2
d1zcba2 d2fv8a1 d2cjwa_ d3dz8a1 d1xzpa2 d1vg8a_ d4qxaa_ d2ew1a1
d1x1ra1 d2bcgy_ d3doea1 d3cona_ d3rega_ d3wyga_ d1upta_ d2b6ha1

d1zd9a1 d1lnza2 d1r5ba3 d1n0ua2 d1r2qa_ d2f9la1 d2rhda_ d3q3jb_
d3q85a_ d1efma1 d1r8qa_ d2gf9a1 d2akab1 d1ek0a_ d1z08a1 d2gcoa1
d2dy1a2 d4g01b_ d2cxxa1 d1svia_ d3q72a_ d1ni3a1 d4ac9a4 d2erxa1
d2bmea_ d1wxqa1 d2gf0a1 d4ldja1 d1zunb3 d4lv5b_ d1efca3 d1kmqa_
d2g6ba1 d3lrpa_ d1tada2 d2y8ea_ d4rd4a1 d1z06a1 d2heia_ d3a58b_
d1u0la2 d1d2ea3 d1zj6a1 d2ocba_ d3rwoa_ d1mr3f_ d2e9sa1 d4u5xa1
d2iwra_ d2fn4a1 d3bwdd_ d2nzja1 d2atva1 d2ksqa_ d3l43a_ d1mkya2
d1mkya1 d1wmsa_ d1jnya3 d2rmka_ d1f6ba_ d1f60a3 d1gota2 d1jala1
d2fh5b1 d1g16a_ d2wblc_ d1m7ba_ d1wf3a1 d1f5na2 d1t9ha2 d1z6xa1
d1z0aa1 d2fg5a1 d2a5ja1 d2bcjq2 d1s0ua3 d1ky3a_ d4y0va_ d4lhwa_
d2qmea1 d2atxa1 d1egaa1 d1ryha_ d1moza_ d1nrjb_ d2erya1 d3bfka_
d1puia_ d1fzqa_ d3j3az1 d3t5ga_ d1shza2 d3defa_ d2f7sa1 d3raba_
d3tsoa_ d3aq4a_ d2al7a1 d3sfva1 d3bc1a_ d2ncda_ d1sdma_ d3h4sa1
d2heha_ d2grya_ g3kin.1 d1kk8a2 d1br2a2 d1goja_ d1f9va_ d2mysa2
d1lkxa_ d3t0qa_ d4db1a1 d3gbja_ d1ry6a_ d1v8ka_ d1k8ka2 d1k8ka1
d3uleb1 d3i33a2 d3i33a1 d1bupa1 d1dkgd2 d1dkgd1 d4ci6a2 d1jcfa1
d4a2ba2 d2fxua1 d1yaga2 d1yaga1 d4cbua2 d1bupa2 d1iqca2 d1iqca1
d1eb7a2 d1eb7a1 d1nmla1 d1nira1 d1qksa1 d1zc6a1 d1zbsa1 d1zbsa2
d1zc6a2 d2ch5a2 d2ch5a1 d1zxoa2 d1zxoa1 d1hroa_ d4gedb_ d3zooa_
d1mz4a_ d1gu2a_ d1f1fa_ d1dvva_ d1c6ra_ d1c75a_ d1e29a_ d1gdva_
d1cnoa_ d1h32b_ d1co6a_ d2bh4x_ d2aiua_ d5cytr_ d1jdla_ d1i8oa_
d2zboa_ d3dmia_ d1ls9a_ d1wvec_ d2yk3a_ d3ph2b_ d1dw0a_ d5aura_
d1ql3a_ d1qn2a_ d1f1ca_ d4zzva_ d2xspa_ d1eg1a_ d4v20a_ d1ojja_
d4ipma1 d4csia_ d3pfza_ d3ovwa_ d2yoka_ d1evsa_ d1cnt1_ d1pvhb_
d1z7ca_ d1huwa_ d1bgea_ d1rhga_ d1f6fa_ d1f45b_ d1i1rb_ d1axia_
d1etea_ d3tgxb_ d2z3qa1 d3qb1a_ d1hula_ d1m48a_ d4nkqb_ d1eera_
d1v7mv_ d5e4ea_ d2o27a_ d3b5ka_ d2psma1 d4zf7a_ d3uf5a_ d4x51a_
d4ypgc1 d3oq3a_ d1lqsl_ d1n1fa_ d1au1a_ d1wu3i_ d3besl_ d3ux9a_
d1v8fa_ d2ejca_ d3muea1 d3q12a1 d4dnxa2 d1m8pa2 d1v47a2 d1g8fa2
d1x6va2 d3rbaa_ d2qtra_ d3l92a_ d1f9aa_ d1qjca_ d2h29a_ d1vlha_
d1od6a_ d1lw7a1 d1mrza2 d4ruka_ d2el7a_ d1iq0a2 d1irxa2 d1r6ta2
d1ivsa4 d2cyba_ d1rqga2 d1j09a2 d2yxna_ d1f7ua2 d1h3fa1 d1ffya3
d2g36a1 d1nzja_ d1jila_ d1n3la_ d1pfva2 d1h3na3 d3n9ia_ d3sz3a1
d3sw2a_ d1moxc_ d1dx5i3 d1edmb_ d1autl2 d1autl1 d1nqlb_ d1dval1
d1szba2 d1kigl_ d2y5fl_ d1ijqa2 d1gk5a_ d1g1sa2 d1nt0a3 d1xdtr_
d1lmja2 d1lmja1 d1nzia2 d2wphe_ d1klil_ d1pfxl2 d1pfxl1 d1p9ja_
d1g1ta2 d1f7ea_ d4yzub_ d2mgpa1 d1b9wa1 d1b9wa2 d2mgpa2 d1ob1c1
d1n1ia2 d1n1ia1 d2npra2 d2npra1 d2y5ia_ d1qlsa_ d1psra_ d3n22a_
d2egda_ d4ggfc_ d2wcea_ d2luca_ d4aqia_ d3nsia_ d2lp3a_ d1j55a_
d1a4pa_ d1k8ua_ d3fs7a_ d2pvba_ d1ttxa_ d1rwya_ d2kyfa_ d1rk9a_
d1a75a_ d1exra_ d1tiza1 d1nyaa_ d1g8ia_ d1ggwa_ d1wdcc_ d3fwba_
d1ggza_ d2o5ga_ d1ctaa_ d1m45a_ d3i5gc_ d1sl8a_ d1fpwa_ d2scpa_
d2roaa_ d4aqra_ d3pn7b_ d1br1b_ d2mysb_ d1s6ca_ d1bjfa_ d2obha_
d1jfka_ d1jf0a_ d1qv0a_ d1lkja_ d2mlfc_ d2m97a_ d2d8na_ d4by5a_

d1jbaa_ d4nqga_ d1wdcb_ d2mysc_ d1j7qa_ d1s6ia_ d1uhka2 d1r2ua_

d1dtla_ d3kf9a_ d3pn7c_ d1qjta_ d1fi6a_ d1c07a_ d5d67d_ d1tuza_

d1h8ba_ d1eg3a1 d1eg3a2 d2zkmx1 d1snla_ d1wkya2 d1uuqa_ d3wh9a_

d4m1ra_ d1xyza_ d4xuya_ d2je8a5 d4bf7a1 d3cuia_ d1ur4a_ d1qw9a2

d1odza_ d2vzsa5 d1bqca_ d2x2ya1 d1hjqa_ d1qnra_ d4y7ea1 d4k68a_

d2v3ga_ d4hpga_ d1nofa2 d1nq6a_ d1h4pa_ d1aq0a_ d3ziza_ d4bs0a_

d1ecea_ d1h1na_ d1ta3b_ d1w91a2 d1i1wa_ d1uhva2 d1us3a2 d1egza_

d7a3ha_ d1tvna1 d3nyda1 d1ghsa_ d2cyga1 d4xv0a_ d2c8na2 d1w32a_

d3emca_ d1v6ya1 d1foba_ d2uwfa1 d2fgla_ d3niya1 d2c0ha1 d1rh9a1

d1yq2a5 d1szna2 d1fa2a_ d1pama4 d5do8a1 d1ea9c3 d1hx0a2 d2fhfa5

d1qhoa4 d1avaa2 d1m53a2 d4m8ua1 d2gjpa2 d1lwha2 d4howa1 d1ktba2

d1j0ha3 d3vm5a1 d2x1ia_ d1wzaa2 d1uasa2 d2diea1 d1mxga2 d1ht6a2

d1eswa_ d1iv8a2 d5cpsa_ d1ud2a2 d1zjaa1 d3gbea1 d1kwga2 d1wdpa_

d1gcya2 d1g5aa2 d1hvxa2 d3dhpa2 d2bhua3 d4do4a1 d3qgva1 d1e43a2

d1g94a2 d2d3na2 d1gjwa2 d1gvia3 d1x1na1 d1r7aa2 d1tz7a1 d2x42a1

d1tr9a1 d3abza1 d4c7ga2 d2gjxa1 d1nowa1 d1yhta1 d4toqa_ d1wb0a1

d1w9pa1 d1eoka_ d1itxa1 d1kfwa1 d1goia2 d1edqa2 d2gsja_ d1hjxa1

d1jnda1 d1vf8a1 d3wdpp1 d3wq5a_ d1gnxa_ d4gxpa_ d4hz8a_ d1e4mm_

d3aiua_ d1wcga1 d3ahxa_ d1v02a_ d2dgaa_ d1pbga_ d1vffa1 d1qoxa_

d4ha4a_ d1g4ma1 d1g4ma2 d1offa_ d1frra_ d3wcqa_ d1e9ma_ d1l5pa_

d1wria_ d1i7ha_ d3av8a_ d1czpa_ d1rfka_ d3p1ma1 d3b2ga_ d2bt6a2

d3nvva1 d1krha3 d2h88b1 d1n62a2 d1t3qa2 d1kf6b2 d1zoyb1 d1jq4a_

d1ffva2 d1dgja2 d1rm6c2 d1h6ta1 d1h6ua1 d9ldta1 d2ewda3 d1up7a1

d2dfda1 d1s6ya1 d1vjta1 d7mdha1 d1hyea1 d1t2da1 d1ojsa3 d1ldna1

d1hyha1 d1civa1 d2a92a1 d1ez4a1 d1i0za1 d1y6ja1 d1gv0a1 d1ur5a1

d1llda1 d1obba1 d1v8ba1 d1gdha1 d1l7da1 d2dlda1 d1mx3a1 d1ygya1

d1qp8a1 d1li4a1 d1j4aa1 d1npya1 d3t4ea2 d1a4ia1 d1o0sa1 d2hk9a2

d2egga2 d1b26a1 d1bvua1 d1gq2a1 d1c1da1 d1leha1 d1gtma1 d1euza1

d1nvta1 d3dona2 d1edza1 d1vi2a1 d1p77a1 d1nyta1 d1b0aa1 d1vl6a1

d1v9la1 d1txga2 d3f47a1 d1mv8a2 d2pv7a2 d1xc2a3 d2ahra2 d4xiya1

d2f1ka2 d1pgja2 d1i36a2 d1np3a2 d1lj8a4 d1teaa3 d1qmga2 d2i76a2

d2g5ca2 d1wdka3 d1dlja2 d3cuma3 d1kola2 d1f8fa2 d1qora2 d1jqba2

d1vj0a2 d1cdoa2 d1gu7a2 d1d1ta2 d1xa0a2 d2dm6a2 d1jvba2 d1rjwa2

d1llua2 d1tt7a2 d1piwa2 d1o89a2 d1iz0a2 d1e3ja2 d1yb5a2 d1uufa2

d1vj1a2 d1e3ia2 d3gpdg1 d1vc2a1 d2nvwa1 d1u8fo1 d1ydwa1 d1hdgo1

d2g17a1 d1cf2o1 d2czca2 d1r0ka2 d1obfo1 d2cvoa1 d1ebfa1 d1h6da1

d1i32a1 d1zh8a1 d2q49a1 d1qkia1 d3pzra1 d1xeaa1 d1vkoa1 d1vm6a3

d3qvsa1 d2pkqo1 d1b7go1 d1gr0a1 d1p1ja1 d1lc0a1 d1j0xo1 d3pwka1

d2hjsa1 d1zyao1 d1vkna1 d1tlta1 d1nvmb1 d3sjua_ d3ojfa1 d1xu9a1

d2jjya1 d1n7ha_ d1y1pa1 d1h5qa_ d1jtva_ d4fs3a_ d2bkaa1 d1gy8a_

d2c5aa1 d1ulsa_ d1ooea_ d2b69a1 d1y5ma1 d1spxa_ d1ae1a_ d1bxka_

d1ydea1 d1gega_ d1hdoa_ d1o5ia_ d1e6ua_ d1snya1 d2rhca_ d3zv4a_

d5ceja_ d2d1ya1 d1fjha_ d3w8ea_ d2fmua1 d1x1ta_ d1rpna_ d3ay7a1

d1k2wa_ d1eq2a_ d2hrba1 d3ruaa_ d2ew8a1 d4hwka_ d1xgka_ d2bd0a1

d1yo6a1 d2a35a1 d2ntva_ d1vl8a_ d1hdca_ d4bkua_ d2ag5a1 d2q45a_
d1nffa_ d4iy1a_ d1zbqa1 d1kewa_ d1ek6a_ d1ja9a_ d2q46a2 d1iy8a_
d1cyda_ d2ptga_ d1hxha_ d1uzma1 d3lyla_ d4m89a_ d3wyea_ d2blla1
d1w6ua_ d1sb8a_ d2q2va_ d4nr0a_ d1e6wa_ d2gasa_ d3oiga1 d2bgka1
d1mxha_ d1vl0a1 d1rkxa_ d1r6da_ d1wmaa1 d1e7wa_ d2c07a1 d1xkqa_
d3tzha_ d1g0oa_ d3lz6a_ d1qyca_ d1edoa_ d1gz6a_ d1oc2a_ d1t2aa_
d1zema1 d2xoxa_ d1z45a2 d1geea_ d1p33a_ d3lt0a_ d4e6pa_ d2wyua_
d1db3a_ d4q9na_ d1xhla_ d4o1ma_ d1fmca_ d2uvda_ d2gdza1 d1uaya_
d2z1ma_ d2o23a2 d2fr1a2 d2fr1a1 d1qsga_ d1qyda_ d4zjua_ d2a4ka1
d1yb1a_ d1wvga1 d1yxma1 d3f4ba_ d1mg5a_ d1xg5a1 d4ekfa_ d2ckga_
d1th0a_ d2bkra_ d1g0da4 d2f4ma1 d1x3za_ d1mdwa_ d1qxpa4 d1ziva1
d1zcma1 d3ioqa_ d1fh0a_ d1o0ea_ d1khqa_ d1m6da_ d2cb5a_ d1cs8a_
g8pch.1 d1s4va_ d2pnsa_ d1vsna1 d1mira_ d1iwda_ d2oula_ d7pcka_
d3bwka_ d3bcna_ d2p86a_ d1pvja_ d1pcia_ d1gece_ d1deua_ d4bs6a_
d1xkga1 d1cqda_ d1jqpa2 d2c0ya_ d2bdza_ d3p5ua_ d1ex4a2 d1c6va_
d2jk6a2 d2jk6a1 d1feca1 d1feca2 d1lpfa1 d1lpfa2 d1xhca1 d1xhca2
d1dxla2 d1dxla1 d1gera2 d1m6ia2 d1onfa2 d3lada2 d3lada1 d1w4xa1
d1w4xa2 d1gesa2 d1mo9a1 d1xdia1 d1fl2a2 d1fl2a1 d1fcda1 d1fcda2
d1h6va2 d1h6va1 d1mo9a2 d1gv4a2 d1gv4a1 d2gv8a2 d2gv8a1 d1aoga1
d1aoga2 d1hyua1 d1ebda1 d1ebda2 d3dk9a1 d1q1ra2 d1q1ra1 d1gesa1
d1w5qa_ d2c1ha_ d1ohla_ d1l6sa_ d1zbfa1 d2z1ha_ d2etja1 d3qioa1
d1jl1a_ d2e4la_ d1jl2a_ d2lsna1 d1uaxa_ d2yv0x_ d1mu2a1 d1ekea_
d1i39a_ d1pn0a1 d1reoa1 d1ju2a1 d2f5va1 d3c96a1 d2gmha1 d1gpea1
d1o5wa1 d1n4wa1 d1k0ia1 d2dw4a2 d2ivda1 d1seza1 d2voua1 d1kdga1
d1s5ja1 d2hbja2 d2igia_ d1wlja_ d1qhta1 d4dlea1 d1tgoa1 d2f96a1
d3tr8a1 d3mxma_ d1q8ia1 d1y97a1 d1w0ha_ d1kfsa1 d2py5a1 d1noya_
d1d5aa1 d1yt3a3 d2guia_ d3mwpa1 d4kb1a_ d1js9c_ d1za7a_ d1laja_
d1wgqa1 d1v89a1 d2coda1 d1zc3b_ d1wg7a1 d1u5ea1 d1v61a1 d2coca1
d2ys1a1 d1v5pa1 d1ntya2 d1wjma1 d1wi1a1 d1u29a_ d1xx0a1 d1unqa_
d2zkmx3 d1fhoa_ d1v88a1 d2adza1 d1x1fa1 d1p6sa_ d2coaa1 d1btka_
d1v5ma1 d1faoa_ d2x18a_ d1dyna_ d1x1ga1 d4kaxb_ d2j59m1 d1ki1b2
d1xcga2 d1txda2 d1u5da1 d1dbha2 d1w1ha_ d1upqa_ d1v5ua1 d1foea2
d2fjla1 d1eaza_ d2dfka2 d2cofa1 d1kz7a2 d1jmxa2 d1jmxa1 d1pbya2
d1r7ha_ d1faaa_ d1wika1 d1xfla_ d1ttza1 d2f51a1 d1ti3a_ d1nhoa_
d1sena_ d2i4aa_ d1ep7a_ d1zmaa1 d1fo5a_ d3lefa1 d3diea1 d2yana1
d1z6na1 d1oaza_ d3zywa1 d4aj8a_ d1nm3a1 d4fiwa_ d1wmja_ d4hs1a1
d1iloa_ d1xwaa1 d1wjka1 d2vm1a_ d1fb6a_ d1gh2a_ d1h75a_ d1nw2a_
d1kx5b_ d3a6na_ d4m6ba2 d2nqbd_ d3wkjd_ d2yfwe_ d4h9na_ d5bnxa_
d1kx5c_ d3r45a_ d1eqzb_ d4jjnb_ d1f66c_ d1eqza_ d4wnnb_ d1kx5a_
d1u35c1 d1id3a_ d2nqbc_ d1kx5d_ d1ku5a_ d1f1ea_ d1b67a_ d1tafa_
d1bh9b_ d1jfib_ d1q9ca_ d1h3ob_ d2byka1 d1n1ja_ d4g92b_ d1bh9a_
d1jfia_ d1h3oa_ d2bykb1 d1n1jb_ d1pd212 d1nhya2 d2a2ra2 d1k0dd2
d1f2ea2 d1gsua2 d1f3ba2 d2fhea2 d2pvqa1 d1tw9a2 d1oyja2 d1e6ba2
d3ik7a1 d4is0a1 d1r5aa2 d1jlva2 d1m0ua2 d3gtub2 d1z9ha2 d1okta2

d1axda2 d2gsra2 d1tu7a2 d1k3ya2 d1zgma1 d1b48a2 d1gnwa2 d1gwca2
d2gsta2 d1glqa2 d1oe8a2 d1pn9a2 d1v2aa2 d1fw1a2 d1duga2 d1ovna2
d2trcp_ d1ig8a1 d1czan2 d1czan3 d1czan1 d1ig8a2 d1czan4 d1bg3a4
d1bg3a3 d1bg3a2 d1bg3a1 d1novc_ d2bbvc_ d3h1jd1 d1qusa_ d4ilya_
d4olta_ d1qgia_ d2lj7a_ d2m8bb1 d2kyqa_ d3psma_ d1jkza_ d1jxca_
d2gl1a_ d1ugla_ d4uj0a_ d1qqya_ d1gd6a_ d1f6ra_ d1ivma_ d2gv0a_
d1iiza_ d2z2fa_ d1j8xa_ d1ghla_ d1b9oa_ d174la_ d169la_ d2o4wa_
d1p37a_ d1k28a3 d1xjta1 d1jtma_ d2f2qa_ d4e97a1 d1iyka2 d1rxta1
d1iica2 d1iica1 d2za0a_ d2c21a1 d1f9za_ d1mpya2 d1kw3b2 d1kw3b1
d4ghga1 d1tfza1 d3isqa1 d3isqa2 d1sqia1 d1sqia2 d1lkda2 d1lkda1
d1mpya1 d1cjxa2 d1cjxa1 d1sp9b2 d1t47a1 d1t47a2 d1f1ua2 d1sp8a1
d1sp8a2 d1etob_ d1umqa_ d1s5ja2 d1qhta2 d4dlea2 d1tgoa2 d3py8a2
d1q8ia2 d1kfsa2 d2py5a2 d1d5aa2 d1qsta_ d2beia1 d4m3sa_ d1u6ma1
d1ygha_ d1yk3a1 d3dr6a_ d1xmta_ d1z4ea1 d2ge3a1 d1bo4a_ d2fiaa1
d2jdca1 d1y9wa1 d3to7a_ d1wwza1 d2fl4a1 d2ozua1 d2b5ga_ d2cy2a1
d2p0wa_ d1cm0a_ d4pswa_ d1yrea1 d1yr0a1 d1m4ia_ d2gana1 d1yvka1
d1ghea_ d2g3aa1 d1y7ra1 d2euia1 d1q2ya_ d2fsra1 d1xeba_ d2ae6a1
d1cjwa_ d1qsma_ d2aj6a1 d1y9ka1 d1i12a_ d1vkca_ d2atra1 d1n71a_
d2d4pa1 d2fiwa1 d2fe7a1 d1tiqa1 d2ou2a_ d1yx0a1 d2fcka1 d1p0ha_
d2j8ma_ d2pq8a_ d1z4ra1 d1s3za1 d1ufha_ d2hqya1 d2hqya2 d1ne9a1
d1lrza3 d1ne9a2 d1xvqa1 d1pn0a2 d3hy2a_ d2cvba1 d1o8xa_ d1lu4a_
d1we0a1 d2bmxa1 d2a4va1 d1psqa_ d1xiya1 d1q98a_ d1n8ja_ d1zofa1
d2ggta_ d3tjja_ d4je1a1 d1o73a_ d3s9fa_ d2zcta_ d1qmva_ d2b1ka_
d5dvba1 d2b5xa1 d2v2ga_ d1st9a_ d3tb2a_ d4llra_ d4fh8a1 d1e2ya_
d1knga_ d2h30a_ d1i5ga_ d2z9sa_ d2f8aa1 d1tp9a1 d2b7ka_ d1nm3a2
d3hvva_ d2wfca1 d4kcea_ d1xvwa1 d4gqca1 d3qpma_ d4k1fa_ d1zzoa1
d1gp1a_ d1prxa_ d1e88a1 d1l6ja4 d1eaka4 d1eaka3 d1l6ja3 d1h8pa2
d1h8pa1 d1ys1x_ d4hs9a_ d1ispa_ d1ex9a_ d1ku0a_ d3qmma_ d2h1ia1
d2r8ba1 d3b5ea1 d3cn9a_ d1lgya_ d4l3wa_ d1llfa_ d1gz7a_ d1ukca_
d1c4xa_ d1j1ia_ d2rhwa_ d3groa_ d1thta_ d1ei9a_ d2ftba_ d1pmpa_
d2rcqa1 d1fdqa_ d4lkpa_ d1sa8a_ d1vyfa_ d1lpja_ d3vg7a1 d4azqa1
d1o8va_ d5bvqa_ d1p6pa_ d2qo4a_ d1opba_ d1yiva1 d1o1va_ d4bvma1
d4tkba_ d4i3ba_ d1bwya_ d1ftpa_ d1ggla_ d4gkca_ d3p6da1 d1kqwa_
d2a0aa1 d3zxha_ d1cgla_ d1rm8a_ d1eaka2 d1hy7a_ d2jsda_ d1cxva_
d1hv5a_ d1i76a_ d3p0ya1 d1igra1 d1n8yc2 d1n8yc1 d1m6ba2 d1m6ba1
d1m4la_ d2bk7a_ d3fjua_ d1z5ra1 d1kwma1 d1uwya2 d1jqga1 d2c1ca1
d1dtda_ d1h8la2 d2tbvc_ d1c8nc_ d1x36a_ d1smvc_ d1ng0c_ d4sbvc_
d1opoa_ d1f2nc_ d1cixa_ d1y29a_ d1v7fa_ d2jtba_ d1d1ha_ d1g9pa_
d1qk7a_ d1mb6a_ d2a2va_ d1niya_ d1i25a_ d1hvwa_ d2mpqa_ d1ie6a_
d1kqha_ d1la4a_ d2mxoa1 d1dl0a_ d1koza_ d1qk6a_ d1emxa_ d1lqra_
d1c6wa_ d1rmka_ d1ixta_ d1eyoa_ d1cnna_ d1g1pa_ d1f3ka_ d1fyga_
d4lu0a_ d1of8a_ d3unda1 d3e9aa1 d1vr6a2 d1n8fa_ d2qkfa_ d1d9ea_
d1o60a_ d1ny721 d1ny722 d1a6ca3 d1a6ca2 d1a6ca1 d1pgl22 d1pgl21
d1pgl11 d1ny711 d1jgta2 d1ofda3 d1ea0a3 d1q15a2 d1ct9a2 d1ao0a2

| | | | | | | | |
|---|---|---|---|---|---|---|---|
| d1te5a_ | d1y14b1 | d2nn6i1 | d1i94l_ | d2zjra2 | d1smxa_ | d2qamc2 | d1hh2p1 |
| d2r7da1 | d1rl2a2 | d1wfqa1 | d3bzka4 | d1x6oa2 | d3a0ja_ | d2j01d2 | d2ja9a1 |
| d4ql5a_ | d1hr0w_ | d1luza_ | d2n3sa_ | d2vnud1 | d1h95a_ | d1go3e1 | d2nn6h1 |
| d1ne3a_ | d2mo1a_ | d1iz6a2 | d1jjga_ | d1e3pa2 | d2ix0a3 | d2ix0a2 | d2lxja_ |
| d1ueba3 | d1ueba2 | d2z0sa1 | d1u0la1 | d2ix0a1 | d2eifa2 | d2je6i1 | d3i2za1 |
| d2c35b1 | d2ahob2 | d1t9ha1 | d3i4oa_ | d2qall1 | d1jt8a_ | d2nn6g1 | d1g6pa_ |
| d3cama_ | d1c9oa_ | d1d7qa_ | d2ba0a1 | d2vnud2 | d2vnud3 | d1khia2 | d2qalq1 |
| d1wi5a1 | d1xtda2 | d1ny4a_ | d1eixa_ | d3exra_ | d2ffca1 | d2fdsa1 | d3n3ma1 |
| d2czda_ | d1q6oa_ | d2yyua_ | d1vqta1 | d1dbta_ | d3ldva1 | d2flia1 | d1rpxa_ |
| d1tqja_ | d1tqxa_ | d4lt9a1 | d3tc7a1 | d3hoja_ | d1i4na_ | d3vnda_ | d3nl8a1 |
| d1geqa_ | d3nava1 | d1v5xa_ | d4a2ra1 | d3tc6a1 | d1ujpa_ | d4ijba_ | d1xcfa_ |
| d1vc4a_ | d1rd5a_ | d1qopa_ | d3b5va_ | d2r80b_ | d1cqxa1 | d1cg5a_ | d1gcvb_ |
| d3boma_ | d2h8fb_ | d2raoa_ | d3bj1a_ | d4hrrb_ | d2r50a_ | d3d1kb_ | d2aa1b1 |
| d3zhwa_ | d3hf4b_ | d3gdja_ | d1jebb_ | d3bj1b_ | d1x9fb_ | d1wmub_ | d1gcva_ |
| d3bcqb_ | d3d4xb_ | d1gvha1 | d2dc3a_ | d3hf4a_ | d4yu3a_ | d3k8ba_ | d1qpwb_ |
| d2r80a_ | d3bcqa_ | d1v4wa_ | d3wtgb_ | d1jl7a_ | d4esaa_ | d1a9we_ | d3d1ab_ |
| d1fsla_ | d4h2la_ | d1v4wb_ | d1mwca_ | d1vhba_ | d1jeba_ | d1spgb_ | d1h97a_ |
| d1q1fa_ | d1tu9a1 | d3d1aa_ | d1hdsb_ | d3hyub_ | d2zfbb_ | d3fs4a_ | d1oj6a_ |
| d1shrb_ | d4h2lb_ | d4yu3b_ | d1spga_ | d3hrwa_ | d3qqra_ | d3at5a_ | d4wchd_ |
| d3hyua_ | d1cg5b_ | d1uc3a_ | d1hbra_ | d2raob_ | d1outb_ | d4esab_ | d3gdjb_ |
| d3qqqa_ | d1x9fc_ | d3vrfa_ | d1itha_ | d3mkba_ | d3d4xa_ | d3mjpa_ | d1jl6a_ |
| d3fh9a_ | d1outa_ | d2dc3b_ | d1it2a_ | d3eoka_ | d3g46a_ | d1qpwa_ | d3vrfb_ |
| d1ch4a_ | d1x9fd_ | d1hdsa_ | d2zfba_ | d3mkbb_ | d1or4a_ | d3wtga_ | d1a4fa_ |
| d3fh9b_ | d3bomb_ | d3cy5a_ | d3at5b_ | d1x9fa_ | d2oifa_ | d4lmxb_ | d1cpcb_ |
| d3v57a_ | d1kn1a_ | d1cpca_ | d4f0ta_ | d2vjha_ | d4po5b_ | d1eyxa_ | d1alla_ |
| d4f0tb_ | d1b33b_ | d4h0mb_ | d1b33a_ | d2vjhb_ | d4lm6b_ | d2vmlb_ | d1allb_ |
| d2vjta_ | d3dbja_ | d2vmla_ | d2vjtb_ | d4f0ub_ | d1f99b_ | d4rmpa1 | d1liaa_ |
| d1eyxb_ | d2uulb_ | d4lmsb_ | d4f0ua_ | d2bv8a_ | d1f99a_ | d2bv8b_ | d2c7lb_ |
| d3dbjb_ | d4rmpb1 | d1b8da_ | d4h0ma_ | d3v57b_ | d1kn1b_ | d1liab_ | d2uula_ |
| d2j96a_ | g1wht.1 | d2xqfa_ | d1ea5a_ | d1f6wa_ | d1dx4a_ | d1qe3a_ | d4qwwa_ |
| d2ha2a_ | d3c8da2 | d2pbla1 | d1jkma_ | d1jjia_ | d1jjfa_ | d2i49a_ | d1nnfa_ |
| d1xvxa_ | d1ursa_ | d1h3da1 | d1iuda_ | d4m1va1 | d1twya_ | d3e13x_ | d2nxoa1 |
| d4pfua_ | d3ttma_ | d1elja_ | d2qmwa1 | d1us5a_ | d1y3na1 | d1jeta_ | d1uqwa_ |
| d4f19a1 | d2esna2 | d3thia_ | d3fasa1 | d2uyeb_ | d2xwva1 | d1r9la_ | d2fyia1 |
| d1pb7a_ | d2ozza1 | d1sw5a_ | d1nh8a1 | d1vr5a1 | d2g29a_ | d1xs5a_ | d3lswa_ |
| d2z23a_ | d3w9va_ | d3tcha_ | d1q35a_ | d1z7me1 | d3q27a_ | d1p99a_ | d1ve4a1 |
| d2i6ea1 | d4mcoa1 | d4n8ya_ | d1eu8a_ | d1zbma1 | d1ixca2 | d1j1na_ | d1xvya_ |
| d1y9ua_ | d2z22a1 | d1xoca1 | d4oera_ | d1wdna_ | d5f1qa1 | d1xt8a1 | d1i6aa_ |
| d4gwoa_ | d4igta1 | d1pc3a_ | d4mmpa1 | d1o63a_ | d2czla1 | d2onsa2 | d4mija1 |
| d4iuaa3 | d4bvva_ | d5hpga_ | d3k65a_ | d1ki0a3 | d1ki0a2 | d1ki0a1 | d1a0ha1 |
| d1pmla_ | d3sp8a3 | d2feba1 | d1xova2 | d1f74a_ | d3l21a_ | d2e1da_ | d1to3a1 |
| d1w3ia_ | d1sfla_ | d1vcva1 | d4imea_ | d1wa3a_ | d2ocza1 | d1f2ja_ | d3m16a_ |
| d2yxga_ | d1ub3a_ | d3a5fa_ | d1fbaa_ | d1l6wa_ | d1xfba1 | d1wx0a1 | d1vpxa1 |
| d1ojxa_ | d1mxsa_ | d2a4aa1 | d1xkya1 | d3cq0a1 | d3s1xa_ | d1f05a_ | d1fdja_ |

d3cpra1 d4d2ja_ d1cg2a1 d1yloa2 d1y7ea2 d1y0ya3 d1lfwa1 d1vixa1
d1fnoa4 d1vhoa2 d1ysja1 d1vhea2 d1z2la1 d2fvga2 d1r3na1 d2grea2
d1xmba1 g1q7l.1 d1rtqa_ d3rp2a_ g1ucy.1 d1fuja_ d2zchp_ d3w94a_
d1si5h_ d1lo6a_ g2pka.1 d1cgha_ d1kigh_ d1a5ia_ d3s9ba_ d1hj8a_
d1ao5a_ d2anya_ d1q3xa1 d1gvza_ d4gsoa_ d2f91a1 d1iaua_ d1gdna_
d1eufa_ g1fiw.1 d1eq9a_ d4nfea_ d2rdla_ d1spja_ d1elva1 d1a7sa_
d1ekbb_ d1pfxc_ d1yc0a_ d2aiqa_ g1fiz.1 d1fi8a_ d4k60a_ d4dgja_
d1mzaa_ d1op0a_ d3tjva_ d1pytd_ d1sgfg_ g1gg6.1 d2eeka_ g1rtf.1
d1a0ja_ d3g01a_ d1pytc_ d4e7na_ d1ddja_ d1z8ga1 d2hlca_ d1npma_
d1brup_ g1jou.1 d1sgfa_ d1autc_ g2hnt.1 g1kdq.1 d1orfa_ d3s69a_
d4crga_ d1fxya_ d1bqya_ d1svpa_ d1mbma_ d3su6a1 d1cu1a1 d4agka_
d1q31a_ g1n1l.1 d3e90b_ d1ep5a_ d3u1jb_ d1dzka_ d4tlja_ d2a2ua_
d4wfva_ d3t1da_ d4iaxa_ d3zq3a_ d2nnda_ d1i4ua_ d1kxoa_ d1bbpa_
d1kt7a_ d1exsa_ d3s26a1 d1lf7a_ d4gh7a_ d1z24a1 d1qabe_ d1xkia_
d1ew3a_ d3bx7a_ d4iawa1 d1n0sa_ d1gkab_ d3kzaa_ d2k23a_ d2l9ca1
d2joza1 d1gm6a_ d3kffa_ d3saoa1 d1e5pa_ d1yupa1 d1iiua_ d3lgia_
d3i77a_ d2z9ia2 d4nsva_ d1lcya2 d4jcna_ d1agja_ d1os8a_ d1hpga_
d1p3ca_ d1l1ja_ d1qtfa_ d1e69a_ d1mv5a_ d3g60a4 g1ii8.1 d1ewqa2
d1ji0a_ d1qhla_ d1v43a3 d2wmfa1 d1vpla_ d1g2912 d4a82a2 g1f2t.1
d2d62a1 d3tuic1 d2bbsa1 d3g60a3 d2ixea_ d1g6ha_ d1sgwa1 d2qi9c_
d3rlfa1 d3b60a1 d3d31a2 d2pzea1 g1xew.1 d1jj7a_ d1oxxk2 d2onka1
d1w1wa_ d1iwea_ d1qf5a_ d1vmaa2 d1nija1 d1np6a_ d2p67a1 d3ihla_
d1okkd2 d1ls1a2 d1s1ma2 d1iona_ d2v40a_ d1dj3a_ d3hida_ d2vo1a1
d2g0ta1 d1vcoa2 d1j8mf2 d1g3qa_ d1xjca_ d1p9ba_ d1qzxa3 d1ihua2
d1ihua1 d2qm8a_ d1dj2a_ d4ioja_ d1cp2a_ d1yrba2 d3gqba2 d1skye3
d1xp8a1 d1tf7a2 d1tf7a1 d1ye8a1 d1skyb3 d2qe7d2 d4blta_ d4huta_
d3ldaa2 d4hyya_ d2i3ba1 d1cr1a_ d3j9tb2 d3ntua2 d1vdza1 d2z43a_
d1e9ra_ d1nlfa_ d3ziaa2 d1n0wa_ d3ziad2 d3j9ta2 d1pzna2 d3gqbb2
d1gl9b1 d1gkub1 d4ok5a1 d2bmfa1 d2bmfa2 d1yksa1 d1yksa2 d1jr6a_
d2qeqa1 d2jlqa1 d1a1va2 d1eh3a_ d3skpa_ d1dtza2 d1ieja_ d1jnfa2
d1ce2a1 d1jnfa1 d1b1xa2 d1b1xa1 d1jw1a1 d1jw1a2 d1ryoa_ d1h76a1
d1h76a2 d1c4oa1 d2p6ra3 d2p6ra4 d1oywa2 d1oywa3 d1wrba1 d1w36b2
d1w36b1 d1c4oa2 d3bora_ d1t6na_ d2eyqa5 d2eyqa3 d2eyqa2 d2zu6a2
d1veca_ d1uaaa1 d1w36d1 d2g9na1 d1z3ix2 d2rb4a_ d1hv8a1 d1t5ia_
d1w36c2 d2j0sa2 d2j0sa1 d1fuka_ d3hjha1 d1z5za1 d1qdea_ d1z63a1
d1s2ma2 d1s2ma1 d1wp9a1 d1wp9a2 d1gm5a3 d1gm5a4 d3dina4 d3dina3
d1q0ua_ d1w36c1 d1z3ix1 d1nkta3 d1nkta4 d1uaaa2 d1w36d2 g1qhh.1
d2fwra2 d2fwra1 d2fkza_ d1zuja1 d1s3qa1 d2fjca1 d1z6om1 d1veia_
d4dyya_ d1jiga_ d3gvya_ d2pyba_ d4cy9a1 d3bvfa1 d1r03a_ d2cf7a_
d1nf4a_ d1zs3a1 d4iwka_ d3ka8a_ d3np0x_ d1n1qa_ d1lb3a_ d4e6ka_
d1z6oa1 d2fzfa1 d4dyua_ d3ge4a_ d1jgca_ d4am4a_ d2z6ma_ d1ji5a_
d1vlga_ d1lkoa1 d1krqa_ d3qz3a_ d1o9ra_ d2ffxj_ d1euma_ d1yv1a1
d2v2pa_ d1j30a_ d4di0a_ d1dpsa_ d1oewa_ d2qzxa_ d2asia_ d1psoe_
d1smra_ d2h6ta_ d1pfza_ d1izda_ d3hd8a_ d1j71a_ d1dpja_ d4aa9a_

| | | | | | | | |
|---|---|---|---|---|---|---|---|
| d1t6ex_ | g1b5f.1 | g1lya.1 | g1htr.1 | d1qdma2 | d1bxoa_ | d1wkra_ | d1tzsa_ |
| d1ibqa_ | d2qzwa_ | d1zoya1 | d1qo8a2 | d1d4ca2 | d1chua2 | d1jnra2 | d1sr9a2 |
| d1rqba2 | d1k8rb_ | d1wgra1 | d1ef5a_ | d1wfya1 | d1e8ya3 | d1wgya1 | d1lfda_ |
| d2cs4a1 | d1wxma1 | d2byea1 | d3kh0a1 | d2byfa1 | d1wxaa1 | d1e7ua3 | d1wgda1 |
| d1wgga1 | d4q5eb_ | d1wjna1 | d1se9a_ | d3n3kb_ | d1wz0a1 | d1we6a1 | d2m0xa_ |
| d2gbka_ | d3rt3b1 | d1wh3a1 | d1sifa_ | d2ekec2 | d4ii2b1 | d4hk2a1 | d1c3ta_ |
| d1wgha1 | d1ttna1 | d1j8ca_ | d1wx8a1 | d3v6cb1 | d2ma9b_ | d4i6lb1 | d1bt0a_ |
| d2jvca1 | d1iyfa_ | d2k1fa_ | d1wxva1 | d1wf9a1 | d1t0ya_ | d2io0b2 | d1x1ma1 |
| d2gbma_ | d1zw7a1 | d2m17a_ | d1ud7a_ | d1we7a1 | d2lwpa_ | d2gbja_ | d1wjua1 |
| d1v6ea1 | d1v86a1 | d4eewa_ | d1wiaa1 | d4icva_ | d4gu2a_ | d1wx7a1 | d1wy8a1 |
| d4hjka1 | d2faza1 | d1v5oa1 | d3ruib_ | d1eo6a_ | d3waob_ | d2r2qa1 | d3m95a_ |
| d3wana_ | d2al3a1 | d2cr5a1 | d1wj4a1 | d1s3sg_ | d1ps9a2 | d1o94a2 | d1lqta1 |
| d1cjca1 | d2dara2 | d2dara1 | d1wyha2 | d2dloa2 | d1rutx4 | d1rutx3 | d2cuqa2 |
| d1x4la2 | d1x4la1 | d1x3ha2 | d1x3ha1 | d2d8za1 | d1x61a2 | d1x6aa1 | d1x6aa2 |
| d2cora2 | d2cora1 | d1v6ga1 | d1v6ga2 | d2d8za2 | d1x64a1 | d1x64a2 | d1b8ta1 |
| d2co8a1 | d1x63a2 | d2dj7a2 | d2dj7a1 | d2cu8a2 | d2cu8a1 | d1g47a1 | d1j2oa1 |
| d1wyha1 | d2dloa1 | d1wiga1 | d1wiga2 | d2cupa2 | d2cupa3 | d1x68a1 | d2d8ya2 |
| d2d8ya1 | d1m3va2 | d1x62a1 | d1x68a2 | d1x61a1 | d1b8ta3 | d1b8ta2 | d2d8xa2 |
| d2d8xa1 | d1x4ka1 | d1x4ka2 | d2cuqa1 | d1m3va1 | d2co8a2 | d1j2oa2 | d1x63a1 |
| d2cura1 | d2cura2 | d1x62a2 | d1wioa4 | d1ccza2 | d1vcaa1 | d1dr9a2 | d1ic1a1 |
| d2d9qb1 | d2nxyb2 | d1um8a_ | d1ixza_ | d1qvra2 | d1qvra3 | d1qzma_ | d1sxjc2 |
| d1jr3d2 | d2p65a_ | d1l8qa2 | d3hu3a3 | d1sxjd2 | d1njfa_ | d1in4a2 | d1sxja2 |
| d1tuea1 | d2ce7a2 | d1w5sa2 | d1sxjb2 | d1d2na_ | d1ny5a2 | d1ofha_ | d2gnoa2 |
| d2a5yb3 | d1fnna2 | d1u0ja1 | d1lv7a_ | d1iqpa2 | d2fnaa2 | d1ixsb2 | d4gita_ |
| d1sxje2 | d1g41a_ | d3ia2a_ | d1a88a_ | d4dgqa1 | d1zoia_ | d2k4ua_ | d1hp2a_ |
| d2lu9a_ | d1baha_ | d2meoa1 | d1wm7a_ | d1hlya_ | d1jlza_ | d1wpda_ | d1c55a_ |
| d1pvza_ | d1wt7a_ | d1rjia_ | d1wz5a_ | d1m2sa_ | d1pjva_ | d1c49a_ | d1qkya_ |
| d2ktca1 | d1wmta_ | d1du9a_ | d1j5ja_ | d1r1ga_ | d1t0za_ | d2i61a_ | d1t1ta_ |
| d1sn4a_ | d1kv0a_ | d2lkba_ | d1npia_ | d1wwna_ | d1pe4a_ | d2kbka_ | d2li7a_ |
| d2kbha_ | d1dq7a_ | d1sega_ | d1omya_ | d1zywa1 | d1jzaa_ | d1bvsa3 | d1iyjb4 |
| d1iyjb5 | d1iyjb3 | d1o7ia_ | d4ipca1 | d1x3ea_ | d4apva_ | d1jb7a1 | d1jb7a2 |
| d1jb7a3 | d3kdfa_ | d1ue1b_ | d1wjja1 | d3eiva_ | d1xjva2 | d1xjva1 | d2ccza2 |
| d1se8a_ | d1l1oc_ | d1jb7b_ | d1qvca_ | d3ulla_ | d1qzga_ | d1miua4 | d1miua5 |
| d1miua3 | d3g48a1 | d1gd7a_ | d1mkha_ | d1jjcb3 | d3ubub_ | d1egia_ | d1qo3c_ |
| d1v7pa_ | d1tdqb_ | d3ubua_ | d1c3ab_ | d1h8ua_ | d3bx4b_ | d1t8ca1 | d1sb2a1 |
| d1qdda_ | d2ziba1 | d3cada_ | d1byfa_ | d1j34b_ | d1jwia_ | d1jzna_ | d1c3aa_ |
| d1oz7b_ | d1wmza_ | d3bdwb_ | d1hq8a_ | d1fifa1 | d3hupa_ | d1wk1a1 | d1umra_ |
| d2afpa_ | d3bx4a_ | d1gz2a_ | d1sl6a_ | d1v7pb_ | d3g8ka_ | d1ixxa_ | d1j34a_ |
| d1jwib_ | d1g1sa1 | d1y17a1 | d1ja3a_ | d1fvub_ | d3bdwa_ | d1sb2b1 | d1fvua_ |
| d1umrc_ | d1oz7a_ | d4m17a_ | d3cfwa1 | d1ypqa1 | d1g1ta1 | d3fd4a_ | d3l9jc_ |
| d4dn8a1 | d1v4lb_ | d1rdl1_ | d2msba_ | d1prtc2 | d1prtb2 | d1xwdc1 | d3kj4a_ |
| d1ozna_ | d1w8aa_ | d2ifga3 | d1xkua_ | d1rpma_ | d2shpa1 | d1yfoa_ | d1l8ka_ |
| d2ooqa1 | d1g4us2 | d1p15a_ | d1lara2 | d1lara1 | d1wcha_ | d2fh7a2 | d2fh7a1 |
| d3azya_ | d3wvja_ | d1upsa1 | d1ajoa_ | d2hyka_ | d1ajka_ | d2vy0a_ | d1o4ya_ |

d3o5sa_ d3d6ea_ d3atga_ d1dypa_ d1umza_ d3i4ia_ d4lbqa_ d1a78a_
d1slta_ d4no4a_ d3zsja_ d3duia_ d1g86a_ d1is3a_ d1qmja_ d1hlca_
g1qmo.1 g1len.1 d3zyra_ d1fnya_ d1n47a_ g2ltn.1 d1avba_ d3usua_
d1dhkb_ d1f9ka_ d1ukga_ d1g8wa_ d1g9fa_ d1wbfa_ d3wcsa_ d1g7ya_
g1loe.1 d1mvqa_ d1hqla_ d1v6ia_ d1dbna_ d1qnwa_ d4l8qa_ d1ioaa_
d3n35a_ d2h0ba_ d2erfa_ d1h30a2 d1h30a1 d3mw4a1 d1pz7a_ d2r1ba_
d3v64a1 d1d2sa_ d3bopa1 d3mw3a1 d1okqa1 d1dszb_ d1a6ya_ d3cbba_
d1ynwa1 d1r4ia_ d1hcqa_ d2hana_ d2hanb_ d2enva1 d1kb4b_ d2nllb_
d1cita_ d1adqa1 d1fnga1 d1uvqb1 d1zs8a1 d2vola1 d2vola2 d1mfbl2
d2jelh2 d1uwel2 d3qq4b1 d1kjvb_ d1k5na1 d1osph2 d4odhl2 d1ow0a1
d1ow0a2 d1bfob2 d3l9ra2 d3jtth1 d2fath2 d4elkb2 d1fn4b2 d2xfxb_
d1pg7x2 d1cqka_ d4k71b2 d1lk2a1 d1eapb2 d4lfhg2 d1i4fa1 d2nw2a2
d1kfah2 d1mhca1 d1h3ua1 d1wejh2 d1h0db2 d1a5fh2 d1kcvh2 d1mjuh2
d1qqda1 d1kcrh2 d1k8ia1 d3huja2 d2nnab1 d1nfdf2 d3mffb2 d1s9va1
d1lk3h2 d1q0xl2 d1fv1b1 d1neza1 d4rfeb2 d1r3ha1 d1a6tb2 d1kjva1
d1pz5b2 d1onqa1 d2bc4a2 d1de4a1 d1k8da1 d1tcra2 d1dn0b2 d1nfde2
d2xwtb2 d1bfoa2 d1mexh2 d1iakb1 d1ypzf2 d4f7ea2 d1q72l2 d2fbjh2
d1fe8h2 d1clzh2 d1fngb1 d1uvqa1 d1vgka1 d1c16a1 d4j12a2 d4x99b2
d1igtb3 d1je6a1 d1q72h2 d1nldh2 d1teth2 d2hmic2 d1i1ca2 d1i1ca1
d1k8ib1 d1q0yh2 d1kfal2 d3frua1 d1pg7w2 d1u58a1 d1igyb4 d1igyb3
d1hyrc1 d1klub1 d1deeb2 d1c12b2 d2bc4b2 d2ig2h2 d1kcvl2 d3wn5b1
d1lk2b_ d1lk3l2 d1mjul2 d1sm3h2 d1q0xh2 d1zvsa1 d1ydpa1 d1u8ib2
d1ypze2 d1kcuh2 d1op9a_ d1y0la1 d1etzb1 d1egjl1 d2gsib1 d1nj9a1
d4jfhd1 d1aqkl1 d1t2ja_ d1lo4h1 d1zvya1 d3v4vl1 d4enec1 d1kxtb_
d5e8ea1 d2pxya_ d4weud_ d1xiwc_ d2esvd1 d1qnzh_ d1ogae1 d1f4xh1
d4n1hb_ d3ezjb1 d3mlwl1 d1kfah1 d1etza1 d1b4jh1 d3cfic_ d4w68a_
d2arja1 d1ek3a_ d2ok0l1 d1vera_ d1uwgh1 d1blnb1 d1fpth1 d1rzga1
d2giya1 d4m3kb1 d1nc2a1 d2nyyd1 d1ncna_ d1fbih1 d5dxwa_ d4fqqa1
d1dqdh1 d1jfql1 d1kelh1 d1faih1 d1frgl1 d1figl1 d4m6nl1 d1fo0a_
d4hxbl1 d3b5ga_ d3quyd1 d2h9gb1 d1i8kb_ d2dd8h1 d2ck0h1 d1eaja_
d4w6wb1 d2a6ib3 d1t66d1 d2e27h_ d1ob1b1 d1f11b1 d2v7na1 d2xt1b1
d1pskh1 d4i13b1 d2op4l1 d1a0ql1 d1uwel1 d1za3a1 d1ncbl1 d4uu9a_
d1bm3h1 d1cloh1 d1nqba2 d1cf8h1 d4y5va_ d1mhph1 d5anma1 d4cdgc1
d4ldeb_ d1y0lb1 d2pcpb1 d1z3gl1 d1dfbl1 d1fh5h1 d3g5xa1 d1tzib1
d4dn3l1 d3zhka1 d2b1hh1 d2uyla1 d1f3dh1 d1nj9b1 d2agjh1 d2h1pl1
d1q72h1 d8fabb1 d1ndmb1 d1rjca1 d4u6hb1 d3upca_ d2fbjl1 d1osph1
d1tvda_ d2cjvh_ d4fhbd1 d1bd2d1 d1rz7h1 d3nn8a_ d1blna1 d1knoa1
d1nfda1 d2bmkb1 d1dqll_ d1igyb1 d1igfl1 d12e8h1 d3utza1 d1nc2b1
d1rihh1 d4mwfb1 d4jvpa1 d1mieh1 d1rhfa1 d1fgnl1 d1sbsh1 d1lp9e1
d1dn0a1 d4lqfl1 d1iaih1 d1kgcd1 d1mh5b1 d1gpol1 d2g60h3 d1f8tl1
d2jixd1 d4grwh_ d1fo0b_ d1bfob1 d1axsa1 d1u9ka_ d1nmbh_ d1h8na1
d1h8na2 d1jguh1 d4eura1 d3sdyl1 d35c8h1 d1w72l1 d1mvub_ d1i9rh1
d1ob1a1 d4wenb_ d1yeeh1 d3ietc1 d1f58h1 d1ad0b1 d1mqkl_ d1nbvh1
d4b50a_ d3r8bb_ d1bafl1 d1mamh1 d4rgne1 d2gsgb_ d35c8l1 d1bafh1

d1ai1h1 d2pcpa1 d2fath1 d2x1pa_ d1orsb1 d1ibgl1 d1shma_ d1a2yb_
d1u0qa_ d2coqa_ d1hilb1 d1qfwi_ d1jv5b_ d1fn4b1 d5e0qa_ d1nsnh1
d1plgl1 d4ocxl1 d1kb5h1 d1rzfh1 d1pg7x1 d2fx7h1 d2oi9c_ d1l7il1
d3eyva1 d1d5il1 d4ffyl_ d1tetl1 d1a6wh_ d1ac6a_ d3dvfa_ d1nfde1
d1mimh1 d1ri8a1 d1jhlh_ d3dura_ d1fskb1 d1f97a1 d3so3b1 d2ak4d1
d1lp9f1 d4z7wf1 d1l7th1 d4y5ya_ d4x7ec1 d1emth1 d1ikfl1 d1nlbh1
d4utal1 d1bjma1 d1eapb1 d1u6ah1 d2dtgd1 d1kj2b_ d1xeda1 d1c1eh1
d1fgvh_ d2bnub1 d2fb4h1 d2dtgb1 d1ktrh_ d1jpth1 d1qkzl1 d4hepg_
d2atpb1 d1flrh1 d2hrpl1 d1mvfa_ d1iaim1 d3k80a1 d2jb5h1 d3zkxc_
d4grwe_ d1it9h1 d1rhhb1 d3w9ec_ d1e4wh1 d1sy6h1 d2j6eh1 d3qxta_
d1jglh1 d1p7ka1 d1tzhb1 d4n8pa1 d1gafl1 d2jelh1 d1wioa2 d4i0cc1
d1cicb1 d1lmka2 d1lmka1 d1iqwh1 d1orqb1 d1ejoh1 d1jnlh1 d4zg1a_
d43c9b_ d1ggbl1 d1wejh1 d3q6fa1 d1f3dj1 d3c6la1 d1bqlh1 d3p9wb_
d1a3rl1 d1h0db1 d2uzil_ d1eo8l1 d1qfwl_ d1n7ml1 d1igja1 d1c5ch1
d2a9mh_ d1rmfh1 d1fj1b1 d4heme_ d1ccza1 d1a5fh1 d1ogad1 d1dlfh_
d1qa9b_ d1vgel1 d3qskb_ d1sjxa_ d4ozfg1 d1sq2n_ d1qd0a_ d1kcvh1
d1c5db1 d3qcul1 d8faba1 d3bn9d1 d1mjuh1 d4y8dc_ d7fabh1 d3dvgb1
d1lo0l1 d1a6tb1 d1gigh1 d1kcrh1 d2hwzl1 d1yjdc1 d3clfl1 d4krna_
d1dl7l_ d1e6ol1 d1mexh1 d3r0ma_ d4kkna_ d1fnsl1 d1oaqh_ d1rzia1
d1ucbh1 d1ncwl1 d4b41a_ d1nfdf1 d2mpah1 d2d7tl1 d4grwf_ d4b5ea_
d1lk3h1 d3u79a_ d4u3xa_ d1ghfl1 d1bvkb_ d1mcww1 d1b88a_ d1jrhh1
d32c2b1 d4wemb_ d1dsfh_ d1op3h1 d1cell1 d1iqda1 d1qoka1 d1qoka2
d1qfuh1 d2crya1 d1ay1h1 d1oaql_ d3ab0b_ d1rurl1 d1t4kb1 d1bz7b1
d1n0xh1 d1forl1 d1ucbl1 d4w6yb_ d43c9a_ d2aabl1 d1tzia1 d4n1ca_
d1k6qh1 d3g9ab_ d4tvsa1 d1igmh_ d1pz5b1 d3dx9a1 d3wxwh_ d1nakh1
d1kcul1 d3eaka_ d4qlra1 d1cr9l1 d1d9ka_ d1f4xl1 d1deea1 d2i27n2
d1c12a1 d2orbl1 d1j8hd1 d1mcph1 d1c5da1 d1kenh1 d1bd2e1 d1h5ba_
d4x7fc1 d3upaa_ d1t3fa1 d1indh1 d2dtgc1 d4hc1l1 d1zv5a1 d4wgvb_
d1uwgl1 d4f37k1 d1aqkh1 d1vesa_ d3zkqd_ d1fptl1 d1lo4l1 d1knob1
d3rrqa_ d1ct8b1 d4orzc_ d1mlbb1 d4hgkc1 d1dqdl1 d1bogb1 d4ozhe1
d1dr9a1 d1i7za1 d1dn0b1 d1kell1 d1rzib1 d1ypzf1 d1wtla_ d1hkfa_
d1fail1 d1figh1 d2vyre_ d1sjva_ d1ohqa_ d1bfoa1 d1pkqb1 d1mjul1
d1b4jl1 d1nsnl1 d2arjb1 d1p4bh_ d4aj0a_ d3nn8b_ d2rcja1 d1f2xk_
d4v1dd_ d1adqh1 d2eksa_ d4k3ga_ d1jfqh1 d1uweh1 d1frgh1 d4gftb_
d1uz8b1 d4n1ea_ d1bm3l1 d1clol1 d3mcka1 d2vq1a1 d1ae6h1 d4aq1b1
d1uj3b1 d1nbqa1 d1fh5l1 d2ck0l1 d2ywza_ d1q72l1 d4nbxb_ d1hila1
d4hzlb1 d2j88l1 d1zmya1 d1cd0a_ d1a4ka1 d1pskl1 d1a0qh1 d4krmb_
d1ncbh1 d2fbjh1 d1j8he1 d1i3ua_ d1cf8l1 d1fe8h1 d2p1ya1 d1eeqa_
d2x89a_ d1ol0a_ d2r0lh1 d1ub5a1 d1dfbh1 d3eyvb1 d4jdvb1 d4p2cg_
d2gsia1 d2agjl1 d1u3ha1 d2h1ph1 d1rihl1 d1fskc1 d1boga1 d2a77l1
d1ospl1 d1qfoa_ d3dwta_ d2fx7l1 d1rz7l1 d3stba1 d1pkqa1 d1b6da1
d1dzba2 d1dzba1 d1kj2a_ d4l1ha_ d1rzgb1 d1dqlh_ d12e8l1 d1miel1
d1jmaa_ d1sbsl1 d1fgnh1 d1f58l1 d1mqkh_ d1ndgb1 d3qegl1 d1iail1
d2aw2a1 d1tqbc1 d3fo2a1 d4dkaa_ d1f8th1 d2g60l1 d2ywya_ d1uj3a1

d1yc7a_ d3bd3a_ d4w6xb1 d1nmbl_ d1i8ka_ d1rjlb1 d1ypze1 d1p7kb1 d1kxqe_ d1gpoh1 d2z4qa1 d1w72h1 d1i9rl1 d4qgya1 d4xt1c_ d2qqnh1 d1cica1 d3c08h1 d2cd0a_ d2bdnh1 d1hnga1 d1maml1 d1nldh1 d1cr9h1 d4lcih1 d3bkyh1 d3qg7l1 d1ai1l1 d1kxvc_ d4iosd_ d1l7ih1 d2x1qa_ d1ub6b1 d3k74b_ d1ibgh1 d1teth1 d1igya1 d1hxma1 d2x6ma_ d2e7la_ d5e03a_ d1qfwm_ d2r0kh1 d1igjb1 d1rurh1 d1plgh1 d1mj8h1 d1yuhb1 d3fmgl1 d1rzfl1 d1emtl1 d1ad9b1 d1igtb1 d1nlbl1 d1d5ih1 d3k7ua_ d4ffyh_ d1bwwa_ d3dvga1 d1a6ta1 d1fgvl_ d4qxgl1 d1miml1 d1jhll_ d2hffb1 d2hh0l1 d1qkzh1 d1l7tl1 d1afvh1 d1iaii1 d2cjuh_ d1um5h1 d2ghwb2 d2ghwb1 d1kgce1 d2fgwh1 d1ikfh1 d1dvfd_ d2i24n1 d2xtjb_ d1za3b1 d2yc1a_ d2hh0h1 d2x1oa_ d1bvka_ d1rjla1 d5e7ba1 d1igta1 d1il1b1 d2fb4l1 d1mvua_ d1jptl1 d32c2a1 d1fvca_ d1iqdb1 d2hrph1 d2p4ab_ d4qo1a_ d1p2cb1 d1wt5c_ d1t4ka1 d5c1mb_ d1it9l1 d1bz7a1 d1b2wh1 d1e4wl1 d1jnll1 d1yedb1 d1za6b1 d3u7aa_ d1igfh1 d3gnml1 d1orsa1 d1wejl1 d1a2ya_ d1hxmb1 d1gafh1 d2j4wh1 d1pz5a1 d1j05a_ d1a3rh1 d4fqll1 d1qfwh_ d1eo8h1 d1n7mh1 d4s11a_ d1kegh1 d5da4a_ d1iqwl1 d3whxb1 d1vgeh1 d1deeb1 d1c12b1 d1t2ql1 d1ad9a1 d3gsnb1 d3b2uc1 d1ggbh1 d1t3fb1 d1nakl1 d4ocmc_ d1lila1 d2v7hb3 d2esve1 d1mcoh1 d2qr0b1 d2q3aa_ d1ct8a1 d1kcrl1 d2pxyb_ d2uzih_ d5c3ld_ d3durb_ d1c5cl1 d2ak4e1 d1dl7h_ d1xiwd_ d1mlba1 d1rmfl1 d1i7zb1 d1dlfl_ d2xa3a_ d5bq7b1 d1dqqb1 d3h42l1 d1egjh1 d1kcvl1 d1fe8l1 d2j6el1 d1f3rb2 d1lk3l1 d1sm3h1 d1q0xh1 d1ghfh1 d7fabl1 d1igch1 d1lo0h1 d4idla_ d5c2bl_ d1jrhl1 d1il1a1 d3zhda1 d4kfzc_ d1b0wa_ d1e6oh1 d1mexl1 d4krpb_ d1fnsh1 d1ncwh1 d4hgma_ d4eigb1 d4rzca_ d2mpal1 d4y5xa_ d5en2b1 d3lrga1 d4ej1c1 d1rhha1 d1j05b_ d1forh1 d1q9wb1 d2wzpd_ d2aq2a1 d1h0da1 d3revb1 d2ij0c1 d1tzha1 d4m7jl1 d1k6ql1 d3b9kc1 d1igml_ d3qeua1 d1dsfl_ d1ce1h1 d1kcuh1 d1zvha1 d1zvoa1 d1tqbb1 d1ay1l1 d2qscl1 d2cgrh1 d1nl0h1 d1kenl1 d3eo0a1 d4n9ob1 d1n0xl1 d3k1kc_ d1d4ua2 d2aq2b1 d1ck1a1 d1enfa1 d1i4pa1 d1v1oa1 d1et9a1 d1esfa1 d2g9hd1 d4ohja1 d1klud1 d1bxta1 d1eu3a1 d1ty0a1 d1fnua1 d2cqva1 d1fhga_ d2k1ma_ d2dl2a1 d1biha3 d1biha1 d1biha4 d3qp3a1 d1g1ca_ d1gxea_ d1cvsc2 d1cvsc1 d1xmwa2 d1m4ka2 d2otpa2 d1biha2 d1rhfa2 d2fcba2 d2fcba1 d1wwbx_ d1gsma2 d1epfa1 d1epfa2 d2dava1 d1waaa_ d1f97a2 d1x44a1 d1vcaa2 d1fnla2 d1fnla1 d3b5ha2 d3h8na2 d3h8na1 d1jbja1 d1jbja2 d1pd6a_ d1f2qa2 d1f2qa1 d3cx2a_ d1nbqa2 d3b5ha1 d1n26a1 d1xiwb_ d1xaua_ d1fltx_ d2ifga1 d1olla1 d1olla2 d1cs6a4 d1cs6a3 d1cs6a2 d1cs6a1 d2gw5a2 d1sy6a1 d1xiwa_ d1ucta2 d1ucta1 d1ry7b2 d1gl4b_ d1qz1a3 d1olza1 d2c9aa1 d1wwca_

**Family-5 (a.22.1.1)**

**Positive dataset contains 12 protein sequences.**

d3a6na_ d4m6ba2 d2nqbd_ d3wkjd_ d2yfwe_ d4h9na_ d5bnxa_ d3r45a_ d4jjnb_ d4wnnb_ d1u35c1 d2nqbc_

**Negative dataset contains 4031 protein sequences.**

| | | | | | | | |
|---|---|---|---|---|---|---|---|
| d1m32a_ | d1ibja_ | d1c7na_ | d1iuga_ | d4q75a_ | d2bkwa1 | d1t3ia_ | d1qz9a_ |
| d1jf9a_ | d1eg5a_ | d1p3wa_ | d3kgwa_ | d1cl1a_ | d2ctza1 | d1vjoa_ | d2rfva_ |
| d1gc0a_ | d1cs1a_ | d1d2fa_ | d2ch1a1 | d1qgna_ | d1pffa_ | d1elua_ | d1u08a_ |
| d1lc5a_ | d1b5pa_ | d2ay1a_ | d1bw0a_ | d3pd6a_ | d2csta_ | d1j32a_ | d1fg7a_ |
| d4wlha_ | d1o4sa_ | d1v2da_ | d1gdea_ | d1m6sa_ | d1v72a1 | d1svva_ | d1ajsa_ |
| d1wsta1 | d2f8ja2 | d1lk9a_ | d1vp4a1 | d1xi9a1 | d2gb3a1 | d1yaaa_ | d2bnma2 |
| d3rcha_ | d3k40a_ | d1pmma_ | d2cfba1 | d1dfoa_ | d1o69a_ | d1bj4a_ | d3k28a_ |
| d2bwpa_ | d3lg0a_ | d2fnua_ | d3ou5a_ | d1z7da1 | d1b9ha_ | d1iaya_ | d4msoa_ |
| d1bs0a_ | d1s0aa_ | d1sffa_ | d3tqxa_ | d1mdoa_ | d3qboa_ | d3pgya_ | d1bjna_ |
| d2dkja_ | d1ejia_ | d1vefa1 | d4wxga_ | d3bs8a_ | d3gbxa1 | d1w23a_ | d2b5aa1 |
| d2ofya1 | d4mcxa_ | d2xi8a_ | d2icta_ | d2bnma1 | d2ppxa1 | d1y7ya1 | d1r0ka3 |
| d1j5pa3 | d1vkoa2 | d1vm6a2 | d3qvsa2 | d1p1ja2 | d1f56a_ | d1bypa_ | d1azca_ |
| d1ws8a_ | d3u25a_ | d1cuoa_ | d1rkra_ | d2iaac_ | d4dp1x_ | d1nwpa_ | d2gima_ |
| d2i7sa_ | d1qhqa_ | d1gyca3 | d1hfua2 | d1kbva1 | d1kbva2 | d1sdda1 | d1sdda2 |
| d1gyca1 | d1gyca2 | d1mzya1 | d2bw4a1 | d1aoza1 | d1aoza2 | d1kyaa2 | d1kyaa3 |
| d1kyaa1 | d1mzya2 | d4e9sa1 | d1sddb1 | d1sddb2 | d1hfua1 | d1hfua3 | d1oe1a2 |
| d1oe1a1 | d1v10a2 | d1v10a3 | d1v10a1 | d2dara2 | d2dara1 | d1wyha2 | d2dloa2 |
| d1rutx4 | d1rutx3 | d2cuqa2 | d1x4la2 | d1x4la1 | d1x3ha2 | d1x3ha1 | d2d8za1 |
| d1x61a2 | d1x6aa1 | d1x6aa2 | d2cora2 | d2cora1 | d1v6ga1 | d1v6ga2 | d2d8za2 |
| d1x64a1 | d1x64a2 | d1b8ta1 | d2co8a1 | d1x63a2 | d2dj7a2 | d2dj7a1 | d2cu8a2 |
| d2cu8a1 | d1g47a2 | d1g47a1 | d1j2oa1 | d1wyha1 | d2dloa1 | d1wiga1 | d1wiga2 |
| d2cupa2 | d2cupa3 | d1x68a1 | d2d8ya2 | d2d8ya1 | d1m3va2 | d1x62a1 | d1x68a2 |
| d1x61a1 | d1b8ta3 | d1b8ta2 | d2d8xa2 | d2d8xa1 | d1x4ka1 | d1x4ka2 | d2cuqa1 |
| d1m3va1 | d2co8a2 | d1j2oa2 | d1x63a1 | d2cura1 | d2cura2 | d1x62a2 | d1iknc_ |
| d3brda1 | d1p7hl1 | d1oy3c_ | d1imhc1 | d1gjia1 | d1zk9a1 | d2cxka1 | d3sxwa1 |
| d4luka_ | d4qgna_ | d1vr3a1 | d2bema_ | d1pama1 | d1ea9c1 | d1qhoa1 | d2fhfa2 |
| d2fhfa1 | d1k3ia1 | d1cxla1 | d2bhua1 | d1edqa1 | d1f1sa2 | d1ut9a2 | d1gvia1 |
| d1txka1 | d1zx5a1 | d1qwra_ | d2phla2 | d2phla1 | d1fxza2 | d1fxza1 | d1lrha_ |
| d1uija1 | g1dgw.1 | d2v09a_ | d1uika2 | d1uika1 | d1dgwa_ | d1ep0a_ | d2pa7a1 |
| d1wlta1 | d2c0za1 | d1ds6b_ | d1fstb_ | d1ju8a_ | d4alac_ | d1rera1 | d1s6na_ |
| d3uzqb_ | d2bjca1 | d2hsga1 | d1qpza1 | d1efaa1 | d1au7a2 | d1ic8a2 | d2ovga_ |
| d1sq8a_ | d1rzsa_ | d3kz3a_ | d3orca_ | d3gpdg2 | d2q49a2 | d1hdgo2 | d2g17a2 |
| d2cvoa2 | d2czca1 | d3pwka2 | d1obfo2 | d1i32a2 | d1zyao2 | d2pkqo2 | d1b7go2 |
| d2hjsa2 | d1cf2o2 | d1vkna2 | d1nvmb2 | d2m9wa1 | d3dfxa_ | d2msya_ | d2djoa1 |
| d2r5za_ | d1au7a1 | d1f43a_ | d2lmda1 | d1akhb_ | d1s7ea1 | d3nara_ | d1zq3p1 |
| d2craa1 | d1mija_ | d4uuta_ | d3a01a_ | d1x58a1 | d3rkqa1 | d3cmya1 | d2cqxa1 |
| d2da6a1 | d1e3oc1 | d1fjla_ | d1mh3a1 | d1ig7a_ | d1x41a1 | d1wh7a1 | d1gt0c1 |
| d2mw8a_ | d1lfup_ | d1x2ma1 | d1b72a_ | d1wi3a1 | d1jgga_ | d1wjha1 | d1x2na1 |
| d1mnmc_ | d2cuea1 | d3k2aa_ | d2hosa_ | d2jwta2 | d1pufa_ | d1octc1 | d2hi3a_ |
| d1b8ia_ | d4j19a_ | d3lnqa_ | d1wh5a1 | d1fexa_ | d2cqra1 | d1guua_ | d1ug2a1 |
| d2cqqa1 | d2ajea1 | d4a69c_ | d2crga1 | d2iw5b1 | d1xc5a1 | d1ofcx1 | d2cu7a1 |
| d1wgxa1 | d2cjja1 | d2ckxa1 | d1gvda_ | d1hcra_ | d1tc3c_ | d1gdta1 | d1u78a2 |
| d6paxa1 | d6paxa2 | d1k78a2 | d1hlva1 | d1iufa2 | d1iufa1 | d1bl0a1 | d1bl0a2 |
| d1d5ya2 | d3lv8a_ | d1s3ga1 | d1zaka1 | d1s4qa_ | d3tvta2 | d1uf9a_ | d1khta_ |

| | | | | | | | |
|---|---|---|---|---|---|---|---|
| d1ckea_ | d1osna_ | d1p5zb_ | d3cm0a_ | d1tmka_ | d1jaga_ | d3h86a_ | d1t0hb_ |
| d1nn5a_ | d2h92a_ | d3kb2a1 | d2c95a1 | d1yj5a2 | d1kgda_ | d1ly1a_ | d2ak3a1 |
| d1vyva2 | d1deka_ | d2grja1 | d1p3ja1 | d1y63a_ | d1e4ya1 | d1s96a_ | d3iija1 |
| d1lvga_ | d1nksa_ | d1vyua2 | d4e22a_ | d1qf9a_ | d1jjva_ | d1q3ta_ | d1ki9a_ |
| d1lw7a2 | d1teva_ | d1kjwa2 | d1gsia_ | d2iyva2 | d1kaga_ | d1e6ca_ | d1g3ma_ |
| d1j99a_ | d1t8ta1 | d1nsta_ | d1fmja_ | d1zrha_ | d2a3ra_ | d1aqua_ | d2zptx_ |
| d3ckla_ | d1texa_ | d1q44a_ | d2gwha_ | d1z0ja1 | d3umra2 | d3gj0a1 | d2gaoa1 |
| d1yzga1 | d1x3sa1 | d1g7sa4 | d1puja_ | d1udxa2 | d1kk1a3 | d2o52a_ | d1jwyb_ |
| d1zcaa2 | d1zcba2 | d2fv8a1 | d2cjwa_ | d3dz8a1 | d1xzpa2 | d1vg8a_ | d4qxaa_ |
| d2ew1a1 | d1x1ra1 | d2bcgy_ | d3doea1 | d3cona_ | d3rega_ | d3wyga_ | d1upta_ |
| d2b6ha1 | d1zd9a1 | d1lnza2 | d1r5ba3 | d1n0ua2 | d1r2qa_ | d2f9la1 | d2rhda_ |
| d3q3jb_ | d3q85a_ | d1efma1 | d1r8qa_ | d2gf9a1 | d2akab1 | d1ek0a_ | d1z08a1 |
| d2gcoa1 | d2dy1a2 | d4g01b_ | d2cxxa1 | d1svia_ | d3q72a_ | d1ni3a1 | d4ac9a4 |
| d2erxa1 | d2bmea_ | d1wxqa1 | d2gf0a1 | d4ldja1 | d1zunb3 | d4lv5b_ | d1efca3 |
| d1kmqa_ | d2g6ba1 | d3lrpa_ | d1tada2 | d2y8ea_ | d4rd4a1 | d1z06a1 | d2heia_ |
| d3a58b_ | d1u0la2 | d1d2ea3 | d1zj6a1 | d2ocba_ | d3rwoa_ | d1mr3f_ | d2e9sa1 |
| d4u5xa1 | d2iwra_ | d2fn4a1 | d3bwdd_ | d2nzja1 | d2atva1 | d2ksqa_ | d3l43a_ |
| d1mkya2 | d1mkya1 | d1wmsa_ | d1jnya3 | d2rmka_ | d1f6ba_ | d1f60a3 | d1gota2 |
| d1jala1 | d2fh5b1 | d1g16a_ | d2wblc_ | d1m7ba_ | d1wf3a1 | d1f5na2 | d1t9ha2 |
| d1z6xa1 | d1z0aa1 | d2fg5a1 | d2a5ja1 | d2bcjq2 | d1s0ua3 | d1ky3a_ | d4y0va_ |
| d4lhwa_ | d2qmea1 | d2atxa1 | d1egaa1 | d1ryha_ | d1moza_ | d1nrjb_ | d2erya1 |
| d3bfka_ | d1puia_ | d1fzqa_ | d3j3az1 | d3t5ga_ | d1shza2 | d3defa_ | d2f7sa1 |
| d3raba_ | d3tsoa_ | d3aq4a_ | d2al7a1 | d3sfva1 | d3bc1a_ | d2ncda_ | d1sdma_ |
| d3h4sa1 | d2heha_ | d2grya_ | g3kin.1 | d1kk8a2 | d1br2a2 | d1goja_ | d1f9va_ |
| d2mysa2 | d3t0qa_ | d4db1a1 | d3gbja_ | d1ry6a_ | d1v8ka_ | d1k8ka2 | d1k8ka1 |
| d3uleb1 | d3i33a2 | d3i33a1 | d1bupa1 | d1dkgd2 | d1dkgd1 | d4ci6a2 | d1jcfa1 |
| d4a2ba2 | d2fxua1 | d1yaga2 | d1yaga1 | d4cbua2 | d1bupa2 | d1g99a1 | d2e1za2 |
| d2e1za1 | d1saza1 | d1saza2 | d1iqca2 | d1iqca1 | d1eb7a2 | d1eb7a1 | d1nmla2 |
| d3h1jd1 | d1hroa_ | d4gedb_ | d3zooa_ | d1mz4a_ | d1gu2a_ | d1f1fa_ | d1dvva_ |
| d1c6ra_ | d1c75a_ | d1e29a_ | d1gdva_ | d1cnoa_ | d1h32b_ | d1co6a_ | d2bh4x_ |
| d2aiua_ | d5cytr_ | d1jdla_ | d1i8oa_ | d2zboa_ | d3dmia_ | d1ls9a_ | d1wvec_ |
| d2yk3a_ | d3ph2b_ | d1dw0a_ | d5aura_ | d1ql3a_ | d1qn2a_ | d1f1ca_ | d2nqya_ |
| d1h4ga_ | d1hixa_ | d1oa2a_ | d1olra_ | d3b7ma_ | d4h7ma1 | d3wp3a_ | d2vgda_ |
| d1igoa_ | d3zsea_ | d3wx5a_ | d1oa3a_ | d1pvxa_ | d1m4wa_ | d1f5ja_ | d1oa4a_ |
| d1ks5a_ | d4ixla1 | d3i2ka1 | d1lnsa3 | d1mpxa2 | d1etea_ | d3tgxb_ | d2z3qa1 |
| d3qb1a_ | d1hula_ | d1m48a_ | d4nkqb_ | d1eera_ | d1v7mv_ | d5e4ea_ | d2o27a_ |
| d3b5ka_ | d2psma1 | d4zf7a_ | d3uf5a_ | d4x51a_ | d1d9ca_ | d4ypgc1 | d3oq3a_ |
| d1lqsl_ | d1n1fa_ | d1au1a_ | d1wu3i_ | d3besl_ | d3ux9a_ | d2ejca_ | d3muea1 |
| d1ihoa_ | d3q12a1 | d4dnxa2 | d1m8pa2 | d1g8fa2 | d1jhda2 | d1x6va2 | d2b7la_ |
| d3rbaa_ | d2qtra_ | d1nupa_ | d3l92a_ | d1f9aa_ | d1qjca_ | d2h29a_ | d1vlha_ |
| d1od6a_ | d1lw7a1 | d1mrza2 | d4ruka_ | d2el7a_ | d1iq0a2 | d1irxa2 | d1r6ta2 |
| d1ivsa4 | d2cyba_ | d1rqga2 | d1j09a2 | d2yxna_ | d1f7ua2 | d1h3fa1 | d1ffya3 |
| d2g36a1 | d1jila_ | d1n3la_ | d1i6la_ | d1pfva2 | d1h3na3 | d3n9ia_ | d3sz3a1 |
| d3sw2a_ | d1moxc_ | d1dx5i3 | d1edmb_ | d1autl2 | d1autl1 | d1nqlb_ | d1dval1 |

d1szba2 d1kigl_ d2y5fl_ d1ijqa2 d1gk5a_ d1g1sa2 d1nt0a3 d1xdtr_
d1lmja2 d1lmja1 d1nzia2 d2wphe_ d1klil_ d1pfxl2 d1pfxl1 d1p9ja_
d1g1ta2 d1f7ea_ d4yzub_ d2mgpa1 d1b9wa1 d1b9wa2 d2mgpa2 d1ob1c1
d1n1ia2 d1n1ia1 d2npra2 d2npra1 d1m1xb4 d1pvaa_ d3fs7a_ d1ttxa_
d1rwya_ d2kyfa_ d1rk9a_ d1a75a_ d1exra_ d1tiza1 d1nyaa_ d1g8ia_
d1wdcc_ d3fwba_ d1ggza_ d2o5ga_ d1ctaa_ d1m45a_ d3i5gc_ d1sl8a_
d1fpwa_ d2scpa_ d2roaa_ d4aqra_ d3pn7b_ d1br1b_ d2mysb_ d1s6ca_
d1bjfa_ d2obha_ d1jfka_ d1jf0a_ d1qv0a_ d1lkja_ d2mlfc_ d2m97a_
d2d8na_ d4by5a_ d1jbaa_ d4nqga_ d1wdcb_ d2mysc_ d1j7qa_ d1s6ia_
d1uhka2 d1r2ua_ d1dtla_ d3kf9a_ d3pn7c_ d5d67d_ d1tuza_ d1h8ba_
d1eg3a1 d1eg3a2 d2zkmx1 d1snla_ d1gjya_ d1y1xa_ d1hqva_ d1dvia_
d1df0a1 d1qxpa2 d4usla_ d1k94a_ d1wkya2 d1uuqa_ d3wh9a_ d4m1ra_
d1xyza_ d4xuya_ d2je8a5 d4bf7a1 d3cuia_ d1ur4a_ d1qw9a2 d1odza_
d2vzsa5 d1bqca_ d2x2ya1 d1hjqa_ d1qnra_ d4y7ea1 d1vjza_ d4k68a_
d2v3ga_ d4hpga_ d1nofa2 d1nq6a_ d1h4pa_ d1aq0a_ d3ziza_ d4bs0a_
d1ecea_ d1h1na_ d1ta3b_ d1w91a2 d1i1wa_ d1uhva2 d1us3a2 d1egza_
d7a3ha_ d1tvna1 d3nyda1 d1ghsa_ d2cyga1 d4xv0a_ d2c8na2 d1w32a_
d3emca_ d1v6ya1 d1foba_ d2uwfa1 d2fgla_ d3niya1 d2c0ha1 d1rh9a1
d1yq2a5 d1szna2 d1fa2a_ d1pama4 d5do8a1 d1ea9c3 d1hx0a2 d2fhfa5
d1qhoa4 d1avaa2 d1m53a2 d4m8ua1 d2gjpa2 d1lwha2 d4howa1 d1ktba2
d1j0ha3 d3vm5a1 d2x1ia_ d1wzaa2 d1uasa2 d2diea1 d1mxga2 d1ht6a2
d1eswa_ d1iv8a2 d5cpsa_ d1ud2a2 d1zjaa1 d3gbea1 d1kwga2 d1wdpa_
d1gcya2 d1g5aa2 d1hvxa2 d3dhpa2 d2bhua3 d4do4a1 d3qgva1 d1e43a2
d1g94a2 d2d3na2 d1gjwa2 d1gvia3 d1x1na1 d1r7aa2 d1tz7a1 d2x42a1
d1tr9a1 d3abza1 d4toqa_ d1wb0a1 d1w9pa1 d1eoka_ d1itxa1 d1kfwa1
d1goia2 d1edqa2 d2gsja_ d1hjxa1 d1jnda1 d1vf8a1 d1etob_ d1umqa_
d1g2ha_ d2w0pa_ d2diaa1 d2e9ia2 d2e9ja1 d2dj4a1 d2eeba1 d2diba1
d2dmba1 d2dmca1 d2d7oa1 d2ee6a1 d2d7pa1 d2eeaa1 d2di8a1 d1wlha1
d2d7na1 d2nqca_ d2d7ma1 d2dica1 d3cnka_ d2k3ta1 d2eeca1 d1qfha2
d2bp3a1 d1v05a_ d2ee9a1 d2eeda1 d2di9a1 d3syxa1 d1mkea1 d2p8va_
d1xoda1 d1i7aa_ d1evha_ d1qc6a_ d2v76a_ d1wj1a1 d1aqca_ d1qqga2
d1qqga1 d1n3ha_ d4dbba_ d3f0wa1 d1p3ra_ d1wgua1 d2cy4a1 d3hqca_
d1wvha1 d1xr0b1 d1wgqa1 d1v89a1 d2coda1 d1zc3b_ d1wg7a1 d1u5ea1
d1v61a1 d2coca1 d2ys1a1 d1v5pa1 d1ntya2 d1wjma1 d1wi1a1 d1u29a_
d1xx0a1 d1unqa_ d2zkmx3 d1fhoa_ d1v88a1 d2adza1 d1x1fa1 d1p6sa_
d2coaa1 d1btka_ d1v5ma1 d1faoa_ d2x18a_ d1dyna_ d1x1ga1 d4kaxb_
d2j59m1 d1ki1b2 d1xcga2 d1txda2 d1u5da1 d1dbha2 d1w1ha_ d1upqa_
d1v5ua1 d1foea2 d2fjla1 d1eaza_ d2dfka2 d2cofa1 d1kz7a2 d9ldta1
d2ewda3 d1up7a1 d2dfda1 d1s6ya1 d1vjta1 d7mdha1 d1hyea1 d1t2da1
d1ojsa3 d1b8pa1 d1ldna1 d1hyha1 d1civa1 d2a92a1 d1ez4a1 d1i0za1
d1y6ja1 d1gv0a1 d1llda1 d1obba1 d3t4ea2 d1a4ia1 d1o0sa1 d2hk9a2
d2egga2 d1b26a1 d1bvua1 d1gq2a1 d1c1da1 d1leha1 d1gtma1 d1euza1
d1nvta1 d3dona2 d1edza1 d1gpja2 d1vi2a1 d1p77a1 d1nyta1 d1b0aa1
d1vl6a1 d1v9la1 d3wdpp1 d3wq5a_ d1gnxa_ d4gxpa_ d4hz8a_ d1e4mm_

d3aiua_ d1wcga1 d1qvba_ d3ahxa_ d1v02a_ d2dgaa_ d1pbga_ d1vffa1
d1qoxa_ d4ha4a_ d1kola2 d1f8fa2 d1qora2 d1jqba2 d1vj0a2 d1cdoa2
d1gu7a2 d1d1ta2 d1xa0a2 d2dm6a2 d1jvba2 d1rjwa2 d1llua2 d1h2ba2
d1tt7a2 d1piwa2 d1o89a2 d1iz0a2 d1e3ja2 d1yb5a2 d1uufa2 d1e3ia2
d3gpdg1 d1vc2a1 d2nvwa1 d1u8fo1 d1ydwa1 d1hdgo1 d2g17a1 d1cf2o1
d2czca2 d1r0ka2 d1obfo1 d2cvoa1 d1ebfa1 d1h6da1 d1i32a1 d1zh8a1
d2q49a1 d1qkia1 d1j5pa4 d3pzra1 d1xeaa1 d1vkoa1 d1vm6a3 d3qvsa1
d2pkqo1 d1b7go1 d1gr0a1 d1p1ja1 d1lc0a1 d1j0xo1 d3pwka1 d2hjsa1
d1zyao1 d1vkna1 d1tlta1 d1nvmb1 d3sjua_ d3ojfa1 d1xu9a1 d2jjya1
d1n7ha_ d1y1pa1 d1h5qa_ d1jtva_ d4fs3a_ d2bkaa1 d1gy8a_ d2c5aa1
d1ulsa_ d1ooea_ d2b69a1 d1y5ma1 d1spxa_ d1ae1a_ d1bxka_ d1ydea1
d1gega_ d1hdoa_ d1o5ia_ d1e6ua_ d1snya1 d2rhca_ d3zv4a_ d5ceja_
d2d1ya1 d1fjha_ d3w8ea_ d2fmua1 d1x1ta_ d1rpna_ d3ay7a1 d1k2wa_
d1eq2a_ d2hrba1 d3ruaa_ d2ew8a1 d4hwka_ d1xgka_ d2bd0a1 d1yo6a1
d2a35a1 d2ntva_ d1vl8a_ d1hdca_ d4bkua_ d2ag5a1 d2q45a_ d1nffa_
d4iy1a_ d1zbqa1 d1kewa_ d1ek6a_ d1ja9a_ d2q46a2 d1iy8a_ d1cyda_
d2ptga_ d1hxha_ d1uzma1 d3lyla_ d4m89a_ d3wyea_ d2blla1 d1w6ua_
d1sb8a_ d2q2va_ d4nr0a_ d1e6wa_ d2gasa_ d3oiga1 d2bgka1 d1mxha_
d1vl0a1 d1rkxa_ d1r6da_ d1wmaa1 d1e7wa_ d2c07a1 d1xkqa_ d3tzha_
d1g0oa_ d3lz6a_ d1qyca_ d1edoa_ d1gz6a_ d1oc2a_ d1t2aa_ d1zema1
d2xoxa_ d1z45a2 d1geea_ d1p33a_ d3lt0a_ d4e6pa_ d2wyua_ d1db3a_
d4q9na_ d1xhla_ d4o1ma_ d1fmca_ d2uvda_ d2gdza1 d1uaya_ d2z1ma_
d2o23a2 d2fr1a2 d2fr1a1 d1qsga_ d1qyda_ d4zjua_ d2a4ka1 d1yb1a_
d1wvga1 d1yxma1 d3f4ba_ d1mg5a_ d1xg5a1 d1r55a1 d3gboa_ d1atla_
d1buda_ d4q1la_ d1quaa_ d1yp1a_ d1wnia_ d3nqyb_ d4gera_ d1bqba_
d1g0da4 d2f4ma1 d1x3za_ d3u1ra1 d1kapp2 d1k7ia2 d3ioqa_ d1fh0a_
d1o0ea_ d1khqa_ d1m6da_ d2cb5a_ d1cs8a_ g8pch.1 d1s4va_ d2pnsa_
d1vsna1 d1mira_ d1iwda_ d2oula_ d7pcka_ d3bwka_ d3bcna_ d2p86a_
d1pvja_ d1pcia_ d1gece_ d1deua_ d4bs6a_ d1xkga1 d1cqda_ d1jqpa2
d2c0ya_ d2bdza_ d3p5ua_ d2o36a_ d1k9xa_ d1zoya1 d1d4ca2 d1chua2
d1jnra2 d1kf6a2 d2jk6a2 d2jk6a1 d1feca1 d1feca2 d1lpfa1 d1lpfa2
d1xhca1 d1xhca2 d1dxla2 d1dxla1 d1gera2 d1m6ia2 d1onfa1 d1onfa2
d3lada2 d3lada1 d1w4xa1 d1w4xa2 d1gesa2 d1xdia1 d1fl2a2 d1fl2a1
d1fcda1 d1fcda2 d1h6va2 d1h6va1 d1mo9a2 d1gv4a2 d1gv4a1 d2gv8a2
d2gv8a1 d1aoga1 d1aoga2 d1hyua1 d1ebda1 d1ebda2 d3dk9a1 d1q1ra2
d1q1ra1 d1gesa1 d1zbfa1 d2z1ha_ d2etja1 d3qioa1 d1jl1a_ d2e4la_
d1jl2a_ d2lsna1 d1io2a_ d1uaxa_ d2yv0x_ d1mu2a1 d1ekea_ d1i39a_
d1pn0a1 d1reoa1 d1ju2a1 d2f5va1 d3c96a1 d2gmha1 d1pj5a2 d1gpea1
d1o5wa1 d1n4wa1 d2dw4a2 d2ivda1 d1seza1 d2voua1 d1kdga1 d1s5ja1
d2hbja2 d2igia_ d1wlja_ d1qhta1 d4dlea1 d1tgoa1 d2f96a1 d3tr8a1
d3mxma_ d1q8ia1 d1y97a1 d1w0ha_ d1kfsa1 d2py5a1 d1noya_ d1d5aa1
d1yt3a3 d2guia_ d3mwpa1 d4kb1a_ g1apy.1 d3ljqa_ g1k2x.1 d1iuka_
d2csua1 d3q9ub_ d2yv1a1 d3q9na_ d2d59a_ d1oi7a1 d2yv2a1 d1euca1
d1y81a1 d2av4a1 d3nvva1 d1krha3 d2h88b1 d1n62a2 d1t3qa2 d1kf6b2

d1zoyb1 d1jq4a_ d1dgja2 d1rm6c2 d1r7ha_ d1faaa_ d1wika1 d1xfla_
d1ttza1 d2f51a1 d1ti3a_ d1nhoa_ d1sena_ d2i4aa_ d1ep7a_ d1zmaa1
d1fo5a_ d3lefa1 d3diea1 d2yana1 d1z6na1 d1oaza_ d3zywa1 d4aj8a_
d1nm3a1 d4fiwa_ d1wmja_ d4hs1a1 d1iloa_ d1xwaa1 d1wjka1 d2vm1a_
d1fb6a_ d1gh2a_ d1h75a_ d1nw2a_ d3us3a3 d3us3a1 d1pd212 d1nhya2
d2a2ra2 d1k0dd2 d1f2ea2 d1gsua2 d1f3ba2 d2fhea2 d2pvqa1 d1tw9a2
d1oyja2 d1e6ba2 d1k0ma2 d3ik7a1 d4is0a1 d1r5aa2 d1jlva2 d1m0ua2
d3gtub2 d1z9ha2 d1okta2 d1axda2 d2gsra2 d1tu7a2 d1k3ya2 d1zgma1
d1b48a2 d1gnwa2 d2gsta2 d1glqa2 d1oe8a2 d1pn9a2 d1v2aa2 d1fw1a2
d1duga2 d1g7ea_ d1a0rp_ d1ig8a1 d1czan2 d1czan3 d1czan1 d1ig8a2
d1czan4 d1bg3a4 d1bg3a3 d1bg3a1 d1qusa_ d1txga2 d3f47a1 d1mv8a2
d2pv7a2 d1xc2a3 d2ahra2 d4xiya1 d2f1ka2 d1pgja2 d1i36a2 d1np3a2
d1lj8a4 d1teaa3 d1qmga2 d2i76a2 d2g5ca2 d1wdka3 d1dlja2 d3cuma3
d1qqya_ d1gd6a_ d1f6ra_ d1ivma_ d2gv0a_ d1iiza_ d2z2fa_ d1j8xa_
d1ghla_ d1b9oa_ d169la_ d2o4wa_ d1p37a_ d1k28a3 d1xjta1 d1jtma_
d2f2qa_ d4e97a1 d1g4ma1 d1g4ma2 d3w3ea_ d4tx7a_ d3cqla_ d4msta_
d3iwra_ d1dxja_ d2z37a_ d4dyga_ d2pjsa1 d1klla_ d2i7ra1 d1ecsa_
d1xqaa1 d5cj3a1 d1npba_ d2p7oa_ d1r9ca_ d1nkia_ d1mpya2 d1kw3b2
d1kw3b1 d4ghga1 d1tfza1 d3isqa1 d3isqa2 d1sqia1 d1sqia2 d1lkda2
d1lkda1 d1mpya1 d1cjxa2 d1sp9b2 d1t47a1 d1t47a2 d1f1ua2 d1sp8a1
d1sp8a2 d1f1ua1 d1pv8a_ d1w5qa_ d2c1ha_ d1l6sa_ d1qsta_ d2beia1
d4m3sa_ d1u6ma1 d1ygha_ d1yk3a1 d3dr6a_ d1xmta_ d1z4ea1 d2ge3a1
d1bo4a_ d2fiaa1 d2jdca1 d1y9wa1 d3to7a_ d1wwza1 d2fl4a1 d2ozua1
d2b5ga_ d2cy2a1 d2p0wa_ d1cm0a_ d4pswa_ d1yrea1 d1yr0a1 d1m4ia_
d2gana1 d1yvka1 d1ghea_ d2g3aa1 d1y7ra1 d2euia1 d1q2ya_ d2fsra1
d1xeba_ d2ae6a1 d1qsma_ d2aj6a1 d1y9ka1 d1i12a_ d1vkca_ d2atra1
d1n71a_ d2d4pa1 d2fiwa1 d2fe7a1 d1tiqa1 d2ou2a_ d1yx0a1 d2fcka1
d1p0ha_ d2j8ma_ d2pq8a_ d1z4ra1 d1s3za1 d1ufha_ d1dosa_ d1gvfa_
d3qm3a1 d2fjka_ d1xvqa1 d1pn0a2 d3hy2a_ d2cvba1 d1o8xa_ d1lu4a_
d1we0a1 d2bmxa1 d2a4va1 d1psqa_ d1xiya1 d1q98a_ d1n8ja_ d1zofa1
d2ggta_ d3tjja_ d4je1a1 d3s9fa_ d2zcta_ d1qmva_ d1jfua_ d2b1ka_
d5dvba1 d2b5xa1 d2v2ga_ d1st9a_ d3tb2a_ d4llra_ d4fh8a1 d1e2ya_
d1knga_ d2h30a_ d1i5ga_ d2z9sa_ d2f8aa1 d1tp9a1 d2b7ka_ d1nm3a2
d3hvva_ d2wfca1 d4kcea_ d1xvwa1 d4gqca1 d3qpma_ d4k1fa_ d1zzoa1
d1gp1a_ d1prxa_ d1f74a_ d3l21a_ d2e1da_ d1to3a1 d1w3ia_ d1sfla_
d1n7ka_ d1vcva1 d4imea_ d1wa3a_ d2ocza1 d1f2ja_ d3m16a_ d2yxga_
d1ub3a_ d3a5fa_ d1fbaa_ d1l6wa_ d1xfba1 d1wx0a1 d1vpxa1 d1mxsa_
d2a4aa1 d1xkya1 d3cq0a1 d3s1xa_ d1f05a_ d1fdja_ d3cpra1 d4d2ja_
d1cg2a1 d1yloa2 d1y7ea2 d1y0ya3 d1lfwa1 d1vixa1 d1fnoa4 d1vhoa2
d1ysja1 d1vhea2 d1z2la1 d2fvga2 d1vgya1 d1r3na1 d2grea2 d1xmba1
d1rtqa_ d1etha2 d1w52x1 d2ppla1 d1hpla2 d1lpbb2 d2oxea1 d1rp1a2
d1ys1x_ d4hs9a_ d1ispa_ d1ex9a_ d1ku0a_ d3qmma_ d3rp2a_ g1ucy.1
d1fuja_ d2zchp_ d3w94a_ d1si5h_ d1lo6a_ g2pka.1 d1cgha_ d1kigh_
d1a5ia_ d3s9ba_ d1hj8a_ d1ao5a_ d2anya_ d1q3xa1 d1gvza_ d1azza_

d4gsoa_ d2f91a1 d1iaua_ d1gdna_ d1eufa_ g1fiw.1 d1eq9a_ d4nfea_
d2rdla_ d1spja_ d1elva1 d1a7sa_ d1ekbb_ d1pfxc_ d1yc0a_ d2aiqa_
g1fiz.1 d1fi8a_ d4k60a_ d4dgja_ d1mzaa_ d1op0a_ d3tjva_ d1pytd_
d1sgfg_ g1gg6.1 d2eeka_ g1rtf.1 d1a0ja_ d3g01a_ d1pytc_ d4e7na_
d1ddja_ d1z8ga1 d2hlca_ d1npma_ g1jou.1 d1sgfa_ d1autc_ g2hnt.1
g1kdq.1 d1orfa_ d3s69a_ d4crga_ d1fxya_ d1bqya_ d1lgya_ d4l3wa_
d1llfa_ d1gz7a_ d1ukca_ d2hqya1 d2hqya2 d1lrza2 d1ne9a1 d1lrza3
d3groa_ d1pjaa_ d1thta_ d1ra6a_ d1muka_ d3gsza_ d1khva_ d1te9a_
d2xwha_ d3uqsa_ d3hkwa_ d3n6ma_ d2cjqa_ d2xyma_ d1teba_ d1te8a_
d1s48a_ d1u09a1 d4wfxa_ d3tyqa_ d2pgga1 d3zxha_ d1cgla_ d1rm8a_
d1eaka2 d1hy7a_ d2jsda_ d1cxva_ d1hv5a_ d3p0ya1 d1igra1 d1igra2
d1n8yc2 d1n8yc1 d1m6ba1 d1dzka_ d4tlja_ d2a2ua_ d4wfva_ d3t1da_
d4iaxa_ d3zq3a_ d2nnda_ d1i4ua_ d1kxoa_ d1bbpa_ d1kt7a_ d1exsa_
d3s26a1 d1lf7a_ d1qfta_ d4gh7a_ d1z24a1 d1qabe_ d1xkia_ d1ew3a_
d3bx7a_ d4iawa1 d1n0sa_ d1gkab_ d3kzaa_ d2k23a_ d2l9ca1 d2joza1
d1gm6a_ d3kffa_ d3saoa1 d1e5pa_ d1yupa1 d1iiua_ d2tbvc_ d1c8nc_
d1x36a_ d1smvc_ d1ng0c_ d1opoa_ d1f2nc_ d1cixa_ d1y29a_ d1v7fa_
d2jtba_ d1d1ha_ d1g9pa_ d1lupa_ d1qk7a_ d1mb6a_ d2a2va_ d1niya_
d1i25a_ d1hvwa_ d2mpqa_ d1ie6a_ d1kqha_ d1la4a_ d2mxoa1 d1koza_
d1qk6a_ d1emxa_ d1lqra_ d1c6wa_ d1rmka_ d1eyoa_ d1cnna_ d1fu3a_
d1g1pa_ d1f3ka_ d1fyga_ d1rypd_ d1irua_ d1rypc_ d3unfk_ d1rypa_
d1j2pa_ d1irui_ d1j2qh_ d3unfh_ d1m4ya_ d1rypb_ d1irul_ d1irug_
d1iru2_ d1q5rh_ d1rypg_ d1g3ka_ d1iruf_ d1q5qa_ d1e94a_ d1irub_
d1ryph_ d3ty6a_ d1iruj_ d1iruk_ d1rype_ d1irud_ d1irue_ d3mi01_
d1rypf_ d1iruh_ d2z3ba_ d1iruc_ d3mka2_ d1iru1_ g1gk9.1 g1or0.1
g1e3a.1 d1mqta_ d1d4m3_ d1fmd3_ d2mev1_ g1aym.1 d1ev13_ d4q4w1_
d1r1a3_ d2x5ia_ d1rhi1_ g1r1a.1 g1d4m.1 g1rhi.1 d1b35b_ d1oopa_
d1tme1_ d4iv1a_ g1pvc.1 g1fpn.1 d1fpn1_ d4gb31_ d1d4m1_ d1fmd1_
d1ev11_ d2wzr3_ g1cov.1 d1qqp3_ g1fmd.1 d2x5ic_ d1rhi3_ g1oop.1
d1hxs1_ g1hxs.1 d1fpn3_ d1bev1_ g2mev.1 d1eah3_ d1aym1_ d1pvc3_
d1qqp1_ d1zba3_ d1h8ta_ d3vbhc_ g1b35.1 g1bev.1 d1hxs3_ g1ev1.1
d1bev3_ g1tmf.1 d1b35a_ d2mev3_ g1eah.1 g1h8t.1 d1eah1_ d4q4w3_
d1r1a1_ d1aym3_ d1pvc1_ d1zba1_ d1h8tc_ d1oopc_ d1tme3_ g1qqp.1
d4iv1c_ g1tme.1 d1y14b1 d2nn6i1 d1i94l_ d2zjra2 d1smxa_ d2qamc2
d1hh2p1 d2r7da1 d1rl2a2 d1wfqa1 d3bzka4 d1x6oa2 d3a0ja_ d2j01d2
d2ja9a1 d4ql5a_ d1hr0w_ d1luza_ d2n3sa_ d2vnud1 d1h95a_ d1go3e1
d2nn6h1 d1ne3a_ d2mo1a_ d1iz6a2 d1jjga_ d1e3pa2 d2ix0a3 d2ix0a2
d2lxja_ d1ueba3 d1ueba2 d2z0sa1 d1u0la1 d2ix0a1 d2eifa2 d2je6i1
d3i2za1 d2c35b1 d2ahob2 d1t9ha1 d3i4oa_ d2qall1 d1jt8a_ d2nn6g1
d1g6pa_ d3cama_ d1i94q_ d1c9oa_ d1d7qa_ d2ba0a1 d2vnud2 d2vnud3
d1khia2 d2qalq1 d1wi5a1 d1xtda2 d1ny4a_ d4w9ta_ d4x2ra1 d1ka9f_
d1qo2a_ d4axka1 d1thfd_ d1h5ya_ d4u28a_ d2y88a_ d4wd0a_ d1vzwa1
d1ux8a_ d1dlya_ d4i0va_ d2bkma_ d2bmma_ d2r80b_ d1cqxa1 d1cg5a_
d1gcvb_ d3boma_ d2h8fb_ d2raoa_ d3bj1a_ d4hrrb_ d2r50a_ d3d1kb_

d2aa1b1 d3zhwa_ d3hf4b_ d3gdja_ d1jebb_ d3bj1b_ d1x9fb_ d1wmub_
d1gcva_ d3bcqb_ d3d4xb_ d1gvha1 d2dc3a_ d3hf4a_ d4yu3a_ d3k8ba_
d1qpwb_ d2r80a_ d3bcqa_ d1v4wa_ d3wtgb_ d1jl7a_ d4esaa_ d1a9we_
d3d1ab_ d1fsla_ d4h2la_ d1v4wb_ d1mwca_ d1vhba_ d1jeba_ d1spgb_
d1h97a_ d1q1fa_ d1tu9a1 d3d1aa_ d1hdsb_ d3hyub_ d2zfbb_ d3fs4a_
d1oj6a_ d1shrb_ d4h2lb_ d4yu3b_ d1spga_ d3hrwa_ d3qqra_ d3at5a_
d4wchd_ d3hyua_ d1cg5b_ d1uc3a_ d1hbra_ d2raob_ d1outb_ d4esab_
d3gdjb_ d3qqqa_ d1x9fc_ d3vrfa_ d1itha_ d3mkba_ d3d4xa_ d3mjpa_
d1jl6a_ d3fh9a_ d1outa_ d2dc3b_ d1it2a_ d3eoka_ d3g46a_ d1qpwa_
d3vrfb_ d1ch4a_ d1x9fd_ d1hdsa_ d2zfba_ d3mkbb_ d1or4a_ d3wtga_
d1a4fa_ d3fh9b_ d3bomb_ d3cy5a_ d3at5b_ d1x9fa_ d2oifa_ d4lmxb_
d1cpcb_ d3v57a_ d1kn1a_ d1cpca_ d4f0ta_ d2vjha_ d4po5b_ d1eyxa_
d1alla_ d4f0tb_ d1b33b_ d1b8db_ d4h0mb_ d1b33a_ d2vjhb_ d4lm6b_
d2vmlb_ d1allb_ d2vjta_ d3dbja_ d2vmla_ d2vjtb_ d4f0ub_ d1f99b_
d4rmpa1 d1liaa_ d1eyxb_ d2uulb_ d4lmsb_ d4f0ua_ d2bv8a_ d1f99a_
d2bv8b_ d2c7lb_ d3dbjb_ d4rmpb1 d1b8da_ d4h0ma_ d3v57b_ d1kn1b_
d2uula_ d2j96a_ g1gxs.1 d1azwa_ d2xqfa_ d1ea5a_ d1f6wa_ d1dx4a_
d1qe3a_ d1k4ya_ d4qwwa_ d2ha2a_ d1sfra_ d1f0na_ d4qeka_ d3c8da2
d2pbla1 d1jkma_ d1jjia_ d1jjfa_ d1jmxa4 d1pbya3 d1pbya4 d1b9ra_
d1offa_ d1frra_ d3wcqa_ d1e9ma_ d1l5pa_ d1wria_ d1i7ha_ d3av8a_
d1czpa_ d1rfka_ d3p1ma1 d3b2ga_ d2bt6a2 d4iuaa3 d4bvva_ d5hpga_
d3k65a_ d1ki0a3 d1ki0a2 d1ki0a1 d1a0ha1 d3sp8a3 d2feba1 d1mdwa_
d1ziva1 d1zcma1 d2m5ta_ d1cqqa_ d1wqsa_ d5bpea_ d2ipha_ d1lvoa_
d2bhga1 d3tnta_ d3sjia_ d3d23a1 d2fyqa_ d3tloa_ d3zvea1 d2hrva_
d3q3ya1 d4asha1 d1e88a1 d1l6ja5 d1l6ja4 d1eaka4 d1eaka3 d1l6ja3
d1h8pa2 d1h8pa1 d2ftba_ d1pmpa_ d2rcqa1 d1fdqa_ d4lkpa_ d1sa8a_
d1vyfa_ d1lpja_ d3vg7a1 d4azqa1 d1o8va_ d5bvqa_ d1p6pa_ d2qo4a_
d1opba_ d1yiva1 d1o1va_ d4bvma1 d4tkba_ d4i3ba_ d1bwya_ d1ftpa_
d4gkca_ d1cbia_ d3p6da1 d1kqwa_ d2a0aa1 d3gv5b1 d1t94a2 d3bq0a2
d3lgia_ d3i77a_ d2z9ia2 d4nsva_ d1lcya2 d4jcna_ d1agja_ d1os8a_
d1hpga_ d1p3ca_ d1l1ja_ d1qtfa_ d1e69a_ d1mv5a_ d3g60a4 d1ewqa2
d1ji0a_ d1qhla_ d1v43a3 d2wmfa1 d1vpla_ d1g2912 d4a82a2 g1f2t.1
d2d62a1 d3tuic1 d2bbsa1 d3g60a3 d2ixea_ d1g6ha_ d1sgwa1 d2qi9c_
d3rlfa1 d3b60a1 d3d31a2 d2pzea1 g1xew.1 d1jj7a_ d1oxxk2 d2onka1
d1w1wa_ d1iwea_ d1qf5a_ d1vmaa2 d1nija1 d1np6a_ d2p67a1 d3ihla_
d1okkd2 d1ls1a2 d1s1ma2 d1iona_ d2v40a_ d3hida_ d2vo1a1 d2g0ta1
d1vcoa2 d1j8mf2 d1g3qa_ d1xjca_ d1p9ba_ d1qzxa3 d1ihua2 d1ihua1
d2qm8a_ d1dj2a_ d4ioja_ d1cp2a_ d1yrba2 d1hyqa_ d3gqba2 d1skye3
d1xp8a1 d1tf7a2 d1tf7a1 d1ye8a1 d1skyb3 d2qe7d2 d4blta_ d4huta_
d3ldaa2 d4hyya_ d2i3ba1 d1cr1a_ d3j9tb2 d3ntua2 d1vdza1 d2z43a_
d1e9ra_ d1nlfa_ d3ziaa2 d1n0wa_ d1g6oa_ d3ziad2 d3j9ta2 d1pzna2
d3gqbb2 d2i49a_ d1nnfa_ d1xvxa_ d1ursa_ d1h3da1 d1iuda_ d4m1va1
d1twya_ d3e13x_ d2nxoa1 d4pfua_ d3ttma_ d1elja_ d2qmwa1 d1us5a_
d1y3na1 d1jeta_ d1uqwa_ d4f19a1 d2esna2 d3thia_ d3fasa1 d2uyeb_

d2xwva1 d1r9la_ d2fyia1 d1pb7a_ d2ozza1 d1sw5a_ d1nh8a1 d1vr5a1
d2g29a_ d1xs5a_ d3lswa_ d2z23a_ d3w9va_ d3tcha_ d1q35a_ d1z7me1
d3q27a_ d1p99a_ d1ve4a1 d2i6ea1 d4mcoa1 d4n8ya_ d1eu8a_ d1zbma1
d1hsla_ d1ixca2 d1j1na_ d1xvya_ d1y9ua_ d2z22a1 d1xoca1 d4oera_
d1wdna_ d5f1qa1 d1xt8a1 d1i6aa_ d4gwoa_ d4igta1 d1pc3a_ d4mmpa1
d1o63a_ d2czla1 d2onsa2 d4mija1 d4ok5a1 d2bmfa1 d2bmfa2 d1yksa1
d1yksa2 d2qeqa1 d2jlqa1 d1a1va2 d1eh3a_ d3skpa_ d1dtza1 d1dtza2
d1ieja_ d1jnfa2 d1ce2a1 d1jnfa1 d1b1xa2 d1b1xa1 d1jw1a1 d1jw1a2
d1ryoa_ d1h76a1 d1h76a2 d1c4oa1 d2p6ra3 d2p6ra4 d1oywa2 d1oywa3
d1wrba1 d1w36b2 d1w36b1 d1c4oa2 d3bora_ d1t6na_ d2eyqa5 d2eyqa3
d2eyqa2 d2zu6a2 d1veca_ d1uaaa1 d1w36d1 d2g9na1 d1z3ix2 d2rb4a_
d1hv8a1 d1hv8a2 d1t5ia_ d1w36c2 d2j0sa2 d2j0sa1 d1fuka_ d3hjha1
d1z5za1 d1qdea_ d1z63a1 d1s2ma2 d1s2ma1 d1wp9a1 d1wp9a2 d1gm5a3
d1gm5a4 d3dina4 d3dina3 d1q0ua_ d1w36c1 d1z3ix1 d1nkta3 d1uaaa2
d1w36d2 g1qhh.1 d2fwra2 d2fwra1 d3q3ma_ d1za0a1 d1mxra_ d2inca_
d1jk0a_ d2incb_ d1mhyb_ d3hf1b_ d1mtyb_ d1r2fa_ d2uw1a_ d1mhyd_
d3n37a_ d1afra_ d1syya_ d1mtyd_ d2o1za_ d1uzra_ d1smsa_ d2fkza_
d1zuja1 d1s3qa1 d2fjca1 d1z6om1 d1veia_ d4dyya_ d1jiga_ d3gvya_
d2pyba_ d4cy9a1 d3bvfa1 d1r03a_ d2cf7a_ d1nf4a_ d1zs3a1 d4iwka_
d3ka8a_ d3np0x_ d1n1qa_ d1lb3a_ d4e6ka_ d1z6oa1 d2fzfa1 d4dyua_
d3ge4a_ d1jgca_ d4am4a_ d2z6ma_ d1ji5a_ d1vlga_ d1lkoa1 d1krqa_
d3qz3a_ d1o9ra_ d2ffxj_ d1euma_ d1yv1a1 d2v2pa_ d1j30a_ d4di0a_
d1dpsa_ d2cbia2 d2choa2 d1l8na1 d1oewa_ d2qzxa_ d2asia_ d1psoe_
d1smra_ d2h6ta_ d1pfza_ d1izda_ d3hd8a_ d1j71a_ d1dpja_ d4aa9a_
d1t6ex_ g1b5f.1 g1lya.1 g1htr.1 d1qdma2 d1bxoa_ d1wkra_ d1tzsa_
d1ibqa_ d2qzwa_ d1ex4a2 d1c6va_ d1bdqa_ d3ggta_ d3ixoa_ d1mrxa_
d2haha_ d1tw7a_ d4njsa_ d1sgua_ d2p3da_ d1nsoa_ d3d3ta_ d2rkfa_
d1c6ya_ d3ogpa_ d3ekwa_ d2o4na_ d2j9ja_ d1baia_ d3ggua_ d3lzua_
d4npua_ d4j55a_ d2r8na_ d2fxda_ d2p3ca_ d4dqea_ d3ttpa_ d3t3ca_
d2p3aa_ d2hc0a_ d2fdda_ d3mwsa_ d1k8rb_ d1wgra1 d1ef5a_ d1wfya1
d1e8ya3 d1wgya1 d2cs4a1 d1wxma1 d2byea1 d3kh0a1 d2byfa1 d1wxaa1
d1e7ua3 d1wgda1 d1wgga1 d4q5eb_ d1wjna1 d1se9a_ d3n3kb_ d1wz0a1
d1we6a1 d2m0xa_ d2gbka_ d3rt3b1 d1wh3a1 d1sifa_ d2ekec2 d4ii2b1
d4hk2a1 d1c3ta_ d1m94a_ d1wgha1 d1ttna1 d1j8ca_ d1wx8a1 d3v6cb1
d2ma9b_ d4i6lb1 d1bt0a_ d2jvca1 d1iyfa_ d2k1fa_ d1wxva1 d1wf9a1
d1t0ya_ d2io0b2 d1x1ma1 d2gbma_ d1zw7a1 d2m17a_ d1ud7a_ d1we7a1
d2lwpa_ d2gbja_ d1wjua1 d1v6ea1 d1v86a1 d4eewa_ d1wiaa1 d4icva_
d4gu2a_ d1wx7a1 d1wy8a1 d4hjka1 d2faza1 d1v5oa1 d2al3a1 d2cr5a1
d1wj4a1 d3h33a_ d1rwja_ d19hca_ d1os6a_ d3bxua_ d3h4na_ d1i77a_
d3caoa_ d1j0pa_ d2ctha_ d1duwa_ d1ofwa_ d2e84a_ d1hh5a_ d1gyoa_
d3h34a_ d2bcgg1 d2bcgg2 d1d5ta1 d1wioa4 d1ccza2 d1vcaa1 d1dr9a2
d1ic1a1 d2d9qb1 d2nxyb2 d1um8a_ d1ixza_ d1qvra2 d1qvra3 d1qzma_
d1sxjc2 d1jr3d2 d2p65a_ d1g8pa_ d3hu3a3 d1sxjd2 d1njfa_ d1in4a2
d1sxja2 d1tuea1 d2ce7a2 d1w5sa2 d1sxjb2 d1d2na_ d1ny5a2 d1ofha_

d2gnoa2 d2a5yb3 d1fnna2 d1u0ja1 d1lv7a_ d1iqpa2 d2fnaa2 d1ixsb2
d4gita_ d1sxje2 d1g41a_ d2k4ua_ d1hp2a_ d2lu9a_ d1baha_ d2meoa1
d1wm7a_ d1hlya_ d1n8ma_ d1jlza_ d1wpda_ d1c55a_ d1pvza_ d1wt7a_
d1rjia_ d1wz5a_ d1m2sa_ d1pjva_ d1c49a_ d1qkya_ d2ktca1 d1wmta_
d1du9a_ d1j5ja_ d1r1ga_ d1t0za_ d2i61a_ d1t1ta_ d1sn4a_ d1kv0a_
d2lkba_ d1npia_ d1wwna_ d1pe4a_ d2kbka_ d2li7a_ d1djta_ d2kbha_
d1dq7a_ d1sega_ d1omya_ d1zywa1 d1jzaa_ d1bbua1 d1e1oa1 d1c0aa1
d1b8aa1 d1l0wa1 d3a74a1 d1eova1 d1t61c1 d1iyjb4 d1iyjb5 d1iyjb3
d1o7ia_ d4ipca1 d1x3ea_ d4apva_ d1jb7a1 d1jb7a2 d1jb7a3 d3kdfa_
d1ue1b_ d1wjja1 d3eiva_ d1xjva2 d1xjva1 d2ccza2 d1se8a_ d1l1oc_
d1jb7b_ d3ulla_ d1qzga_ d1miua4 d1miua5 d1miua3 d3g48a1 d1gd7a_
d1mkha_ d1pyba_ d3ubub_ d1egia_ d1qo3c_ d1v7pa_ d1tdqb_ d3ubua_
d1c3ab_ d1h8ua_ d3bx4b_ d1t8ca1 d1sb2a1 d1qdda_ d2ziba1 d3cada_
d1byfa_ d1j34b_ d1jwia_ d1jzna_ d1oz7b_ d1wmza_ d3bdwb_ d1hq8a_
d1fifa1 d1dv8a_ d3hupa_ d1wk1a1 d1umra_ d2afpa_ d3bx4a_ d1gz2a_
d1sl6a_ d1v7pb_ d3g8ka_ d1ixxa_ d1j34a_ d1jwib_ d1g1sa1 d1y17a1
d1ja3a_ d1fvub_ d3bdwa_ d1sb2b1 d1fvua_ d1umrc_ d1oz7a_ d4m17a_
d3cfwa1 d1ypqa1 d1g1ta1 d3fd4a_ d3l9jc_ d4dn8a1 d1v4lb_ d1rdl1_
d2msba_ d1dgsa2 d1fvia1 d1ckma1 d1p16a1 d1x9na2 d1rpma_ d2shpa1
d1yfoa_ d1l8ka_ d2ooqa1 d1g4us2 d1p15a_ d1lara2 d1lara1 d1wcha_
d2fh7a2 d2fh7a1 d1ohea2 d2f4da_ d1rxda_ d2hxpa_ d1xria_ d1d5ra2
d1fpza_ d3ezza_ d4y2ea_ d1i9sa_ d3azya_ d3wvja_ d1upsa1 d1ajoa_
d2hyka_ d2vy0a_ d1o4ya_ d3o5sa_ d3d6ea_ d3atga_ d1dypa_ d1umza_
d3i4ia_ d1c1la_ d4lbqa_ d1a78a_ d1slta_ d4no4a_ d3zsja_ d3duia_
d1g86a_ d1is3a_ d1qmja_ d1hlca_ d1tdha3 d1q3ba3 d1ee8a3 d1k82a3
g1qmo.1 g1len.1 d3zyra_ d1fnya_ d1n47a_ g2ltn.1 d1avba_ d3usua_
d1dhkb_ d1f9ka_ d1ukga_ d1g8wa_ d1g9fa_ d1wbfa_ d3wcsa_ g1loe.1
d1mvqa_ d1hqla_ d1v6ia_ d1dbna_ d1qnwa_ d1fx5a_ d4l8qa_ d1ioaa_
d3n35a_ d3ubra_ d1qo8a1 d1d4ca1 d2k3va_ d1ft5a_ d1qdba_ d1m1qa_
d2j7aa_ d1h21a_ d1ogyb_ d4fasa_ d1jnia_ d1oaha_ d1sp3a_ d2h0ba_
d2erfa_ d1h30a2 d3mw4a1 d1pz7a_ d2r1ba_ d3v64a1 d1d2sa_ d3bopa1
d3mw3a1 d1okqa1 d1okqa2 d1dszb_ d1a6ya_ d3cbba_ d1ynwa1 d1r4ia_
d1hcqa_ d2hana_ d2hanb_ d2enva1 d1kb4b_ d2nllb_ d1cita_ d1adqa1
d1fnga1 d1uvqb1 d1zs8a1 d2vola1 d2vola2 d1mfbl2 d2jelh2 d1uwel2
d3qq4b1 d1kjvb_ d1k5na1 d1osph2 d4odhl2 d1ow0a1 d1ow0a2 d1bfob2
d3l9ra2 d3jtth1 d2fath2 d4elkb2 d1fn4b2 d2xfxb_ d1pg7x2 d1cqka_
d4k71b2 d1lk2a1 d1eapb2 d4lfhg2 d1i4fa1 d2nw2a2 d1kfah2 d1mhca1
d1h3ua1 d1wejh2 d1h0db2 d1a5fh2 d1kcvh2 d1mjuh2 d1qqda1 d1kcrh2
d1k8ia1 d3huja2 d2nnab1 d1nfdf2 d3mffb2 d1s9va1 d1lk3h2 d1q0xl2
d1fv1b1 d1neza1 d4rfeb2 d1r3ha1 d1a6tb2 d1kjva1 d1pz5b2 d1onqa1
d2bc4a2 d1de4a1 d1k8da1 d1tcra2 d1dn0b2 d1nfde2 d2xwtb2 d1bfoa2
d1mexh2 d1iakb1 d1ypzf2 d4f7ea2 d1q72l2 d2fbjh2 d1fe8h2 d1clzh2
d1fngb1 d1uvqa1 d1vgka1 d1c16a1 d4j12a2 d4x99b2 d1igtb3 d1je6a1
d1q72h2 d1nldh2 d1teth2 d2hmic2 d1i1ca2 d1i1ca1 d1k8ib1 d1q0yh2

d1kfal2 d3frua1 d1pg7w2 d1u58a1 d1igyb4 d1igyb3 d1hyrc1 d1deeb2
d1c12b2 d2bc4b2 d2ig2h2 d1kcvl2 d3wn5b1 d1lk2b_ d1lk3l2 d1mjul2
d1sm3h2 d1q0xh2 d1zvsa1 d1ydpa1 d1u8ib2 d1ypze2 d1kcuh2 d1op9a_
d1y0la1 d1etzb1 d1egjl1 d2gsib1 d1nj9a1 d4jfhd1 d1aqkl1 d1t2ja_
d1lo4h1 d1zvya1 d3v4vl1 d4enec1 d1kxtb_ d5e8ea1 d2pxya_ d4weud_
d1xiwc_ d2esvd1 d1qnzh_ d1ogae1 d1f4xh1 d4n1hb_ d3ezjb1 d3mlwl1
d1kfah1 d1etza1 d1b4jh1 d3cfic_ d4w68a_ d2arja1 d1ek3a_ d2ok0l1
d1vera_ d1uwgh1 d1blnb1 d1fpth1 d1rzga1 d2giya1 d4m3kb1 d1nc2a1
d2nyyd1 d1ncna_ d1fbih1 d5dxwa_ d4fqqa1 d1dqdh1 d1jfql1 d1kelh1
d1faih1 d1frgl1 d1figl1 d4m6nl1 d1fo0a_ d4hxbl1 d3b5ga_ d3quyd1
d2h9gb1 d1i8kb_ d2dd8h1 d2ck0h1 d1eaja_ d4w6wb1 d2a6ib3 d1t66d1
d2e27h_ d1ob1b1 d1f11b1 d2v7na1 d1ad0a1 d2xt1b1 d1pskh1 d4i13b1
d2op4l1 d1a0ql1 d1uwel1 d1za3a1 d1ncbl1 d4uu9a_ d1bm3h1 d1cloh1
d1nqba2 d1cf8h1 d4y5va_ d1mhph1 d5anma1 d4cdgc1 d4ldeb_ d1y0lb1
d2pcpb1 d1z3gl1 d1dfbl1 d1fh5h1 d3g5xa1 d1tzib1 d4dn3l1 d3zhka1
d2b1hh1 d2uyla1 d1f3dh1 d1nj9b1 d2agjh1 d2h1pl1 d1q72h1 d8fabb1
d1ndmb1 d1rjca1 d4u6hb1 d3upca_ d2fbjl1 d1osph1 d1tvda_ d2cjvh_
d4fhbd1 d1bd2d1 d1rz7h1 d3nn8a_ d1blna1 d1knoa1 d1nfda1 d2bmkb1
d1dqll_ d1igyb1 d1igfl1 d12e8h1 d3utza1 d1nc2b1 d1rihh1 d4mwfb1
d4jvpa1 d1mieh1 d1rhfa1 d1fgnl1 d1sbsh1 d1lp9e1 d1dn0a1 d4lqfl1
d1iaih1 d1kgcd1 d1mh5b1 d1gpol1 d2g60h3 d1f8tl1 d2jixd1 d4grwh_
d1fo0b_ d1bfob1 d1axsa1 d1u9ka_ d1nmbh_ d1h8na1 d1h8na2 d1jguh1
d4eura1 d3sdyl1 d35c8h1 d1w72l1 d1mvub_ d1i9rh1 d1ob1a1 d4wenb_
d1yeeh1 d3ietc1 d1f58h1 d1ad0b1 d1mqkl_ d1nbvh1 d4b50a_ d3r8bb_
d1bafl1 d1mamh1 d4rgne1 d2gsgb_ d35c8l1 d1bafh1 d1ai1h1 d2pcpa1
d2fath1 d2x1pa_ d1orsb1 d1ibgl1 d1shma_ d1a2yb_ d1u0qa_ d2coqa_
d1hilb1 d1qfwi_ d1jv5b_ d1fn4b1 d5e0qa_ d1nsnh1 d1plgl1 d4ocxl1
d1kb5h1 d1rzfh1 d1pg7x1 d2fx7h1 d2oi9c_ d1l7il1 d3eyva1 d1d5il1
d4ffyl_ d1tetl1 d1a6wh_ d1ac6a_ d3dvfa_ d1nfde1 d1mimh1 d1ri8a1
d1jhlh_ d3dura_ d1fskb1 d1f97a1 d3so3b1 d2ak4d1 d1lp9f1 d4z7wf1
d1l7th1 d4y5ya_ d4x7ec1 d1emth1 d1ikfl1 d1nlbh1 d4utal1 d1bjma1
d1eapb1 d1u6ah1 d2dtgd1 d1kj2b_ d1xeda1 d1c1eh1 d1fgvh_ d2bnub1
d2fb4h1 d2dtgb1 d1ktrh_ d1jpth1 d1qkzl1 d4hepg_ d2atpb1 d1flrh1
d2hrpl1 d1mvfa_ d1iaim1 d3k80a1 d2jb5h1 d3zkxc_ d4grwe_ d1it9h1
d1rhhb1 d3w9ec_ d1e4wh1 d1sy6h1 d2j6eh1 d3qxta_ d1jglh1 d1p7ka1
d1tzhb1 d4n8pa1 d1gafl1 d2jelh1 d1wioa2 d4i0cc1 d1cicb1 d1lmka2
d1lmka1 d1iqwh1 d1orqb1 d1ejoh1 d1jnlh1 d4zg1a_ d43c9b_ d1ggbl1
d1wejh1 d3q6fa1 d1f3dj1 d3c6la1 d1bqlh1 d3p9wb_ d1a3rl1 d1h0db1
d2uzil_ d1eo8l1 d1qfwl_ d1n7ml1 d1igja1 d1c5ch1 d2a9mh_ d1rmfh1
d1fj1b1 d4heme_ d1ccza1 d1a5fh1 d1ogad1 d1dlfh_ d1qa9b_ d1vgel1
d3qskb_ d1sjxa_ d4ozfg1 d1sq2n_ d1qd0a_ d1kcvh1 d1c5db1 d3qcul1
d8faba1 d3bn9d1 d1mjuh1 d4y8dc_ d7fabh1 d3dvgb1 d1lo0l1 d1a6tb1
d1gigh1 d1kcrh1 d2hwzl1 d1yjdc1 d3clfl1 d4krna_ d1dl7l_ d1e6ol1
d1mexh1 d3r0ma_ d4kkna_ d1fnsl1 d1oaqh_ d1rzia1 d1ucbh1 d1ncwl1

| | | | | | | | |
|---|---|---|---|---|---|---|---|
| d4b41a_ | d1nfdf1 | d2mpah1 | d2d7tl1 | d4grwf_ | d4b5ea_ | d1lk3h1 | d3u79a_ |
| d4u3xa_ | d1ghfl1 | d1bvkb_ | d1mcww1 | d1b88a_ | d1jrhh1 | d32c2b1 | d4wemb_ |
| d1dsfh_ | d1op3h1 | d1ce1l1 | d1iqda1 | d1qoka1 | d1qoka2 | d1qfuh1 | d2crya1 |
| d1ay1h1 | d1oaql_ | d3ab0b_ | d1rurl1 | d1t4kb1 | d1bz7b1 | d1n0xh1 | d1forl1 |
| d1ucbl1 | d4w6yb_ | d43c9a_ | d2aabl1 | d1tzia1 | d4n1ca_ | d1k6qh1 | d3g9ab_ |
| d4tvsa1 | d1igmh_ | d1pz5b1 | d3dx9a1 | d3wxwh_ | d1nakh1 | d1kcul1 | d3eaka_ |
| d4qlra1 | d1cr9l1 | d1d9ka_ | d1f4xl1 | d1deea1 | d2i27n2 | d1c12a1 | d2orbl1 |
| d1j8hd1 | d1mcph1 | d1c5da1 | d1kenh1 | d1bd2e1 | d1h5ba_ | d4x7fc1 | d3upaa_ |
| d1t3fa1 | d1indh1 | d2dtgc1 | d4hc1l1 | d1zv5a1 | d4wgvb_ | d1uwgl1 | d4f37k1 |
| d1aqkh1 | d1vesa_ | d3zkqd_ | d1fptl1 | d1lo4l1 | d1knob1 | d3rrqa_ | d1ct8b1 |
| d4orzc_ | d1mlbb1 | d4hgkc1 | d1dqdl1 | d1bogb1 | d4ozhe1 | d1dr9a1 | d1i7za1 |
| d1dn0b1 | d1kell1 | d1rzib1 | d1ypzf1 | d1wtla_ | d1hkfa_ | d1fail1 | d1figh1 |
| d2vyre_ | d1sjva_ | d1ohqa_ | d1bfoa1 | d1pkqb1 | d1mjul1 | d1b4jl1 | d1nsnl1 |
| d2arjb1 | d1p4bh_ | d4aj0a_ | d3nn8b_ | d2rcja1 | d1f2xk_ | d4v1dd_ | d1adqh1 |
| d2eksa_ | d4k3ga_ | d1jfqh1 | d1uweh1 | d1frgh1 | d4gftb_ | d1uz8b1 | d4n1ea_ |
| d1bm3l1 | d1clol1 | d3mcka1 | d2vq1a1 | d1ae6h1 | d4aq1b1 | d1uj3b1 | d1nbqa1 |
| d1fh5l1 | d2ck0l1 | d2ywza_ | d1q72l1 | d4nbxb_ | d1hila1 | d4hzlb1 | d2j88l1 |
| d1zmya1 | d1cd0a_ | d1a4ka1 | d1pskl1 | d1a0qh1 | d4krmb_ | d1ncbh1 | d2fbjh1 |
| d1j8he1 | d1i3ua_ | d1cf8l1 | d1fe8h1 | d2p1ya1 | d1eeqa_ | d2x89a_ | d1ol0a_ |
| d2r0lh1 | d1ub5a1 | d1dfbh1 | d3eyvb1 | d4jdvb1 | d4p2cg_ | d2gsia1 | d2agjl1 |
| d1u3ha1 | d2h1ph1 | d1rihl1 | d1fskc1 | d1boga1 | d2a77l1 | d1ospl1 | d1qfoa_ |
| d3dwta_ | d2fx7l1 | d1rz7l1 | d3stba1 | d1pkqa1 | d1b6da1 | d1dzba2 | d1dzba1 |
| d1kj2a_ | d4l1ha_ | d1rzgb1 | d1dqlh_ | d12e8l1 | d1miel1 | d1jmaa_ | d1sbsl1 |
| d1fgnh1 | d1f58l1 | d1mqkh_ | d1ndgb1 | d3qegl1 | d1iail1 | d2aw2a1 | d1tqbc1 |
| d3fo2a1 | d4dkaa_ | d1f8th1 | d2g60l1 | d2ywya_ | d1uj3a1 | d1yc7a_ | d3bd3a_ |
| d4w6xb1 | d1nmbl_ | d1i8ka_ | d1rjlb1 | d1ypze1 | d1p7kb1 | d1kxqe_ | d1gpoh1 |
| d2z4qa1 | d1w72h1 | d1i9rl1 | d4qgya1 | d4xt1c_ | d2qqnh1 | d1cica1 | d3c08h1 |
| d2cd0a_ | d2bdnh1 | d1hnga1 | d1maml1 | d1nldh1 | d1cr9h1 | d4lcih1 | d3bkyh1 |
| d3qg7l1 | d1ai1l1 | d1kxvc_ | d4iosd_ | d1l7ih1 | d2x1qa_ | d1ub6b1 | d3k74b_ |
| d1ibgh1 | d1teth1 | d1igya1 | d1hxma1 | d2x6ma_ | d2e7la_ | d5e03a_ | d1qfwm_ |
| d2r0kh1 | d1igjb1 | d1rurh1 | d1plgh1 | d1mj8h1 | d1yuhb1 | d3fmgl1 | d1rzfl1 |
| d1emtl1 | d1ad9b1 | d1igtb1 | d1nlbl1 | d1d5ih1 | d3k7ua_ | d4ffyh_ | d1bwwa_ |
| d3dvga1 | d1a6ta1 | d1fgvl_ | d4qxgl1 | d1miml1 | d1jhll_ | d2hffb1 | d2hh0l1 |
| d1qkzh1 | d1l7tl1 | d1afvh1 | d1iaii1 | d2cjuh_ | d1um5h1 | d2ghwb2 | d2ghwb1 |
| d1kgce1 | d2fgwh1 | d1ikfh1 | d1dvfd_ | d2i24n1 | d2xtjb_ | d1za3b1 | d2yc1a_ |
| d2hh0h1 | d2x1oa_ | d1bvka_ | d1rjla1 | d5e7ba1 | d1igta1 | d1il1b1 | d2fb4l1 |
| d1mvua_ | d1jptl1 | d32c2a1 | d1fvca_ | d1iqdb1 | d2hrph1 | d2p4ab_ | d4qo1a_ |
| d1p2cb1 | d1wt5c_ | d1t4ka1 | d5c1mb_ | d1it9l1 | d1bz7a1 | d1b2wh1 | d1e4wl1 |
| d1jnll1 | d1yedb1 | d1za6b1 | d3u7aa_ | d1igfh1 | d3gnml1 | d1orsa1 | d1wejl1 |
| d1a2ya_ | d1hxmb1 | d1gafh1 | d2j4wh1 | d1pz5a1 | d1j05a_ | d1a3rh1 | d4fqll1 |
| d1qfwh_ | d1eo8h1 | d1n7mh1 | d4s11a_ | d1kegh1 | d5da4a_ | d1iqwl1 | d3whxb1 |
| d1vgeh1 | d1deeb1 | d1c12b1 | d1t2ql1 | d1ad9a1 | d3gsnb1 | d3b2uc1 | d1ggbh1 |
| d1t3fb1 | d1nakl1 | d4ocmc_ | d1lila1 | d2v7hb3 | d2esve1 | d1mcoh1 | d2qr0b1 |
| d2q3aa_ | d1ct8a1 | d1kcrl1 | d2pxyb_ | d2uzih_ | d5c3ld_ | d3durb_ | d2ak4e1 |

d1dl7h_ d1xiwd_ d1mlba1 d1rmfl1 d1i7zb1 d1dlfl_ d2xa3a_ d5bq7b1
d1dqqb1 d3h42l1 d1egjh1 d1kcvl1 d1fe8l1 d2j6el1 d1f3rb2 d1lk3l1
d1sm3h1 d1q0xh1 d1ghfh1 d7fabl1 d1igch1 d1lo0h1 d4idla_ d5c2bl_
d1jrhl1 d1il1a1 d3zhda1 d4kfzc_ d1b0wa_ d1e6oh1 d1mexl1 d4krpb_
d1fnsh1 d1ncwh1 d4hgma_ d4eigb1 d4rzca_ d2mpal1 d4y5xa_ d5en2b1
d3lrga1 d4ej1c1 d1rhha1 d1j05b_ d1forh1 d1q9wb1 d2wzpd_ d2aq2a1
d1h0da1 d3revb1 d2ij0c1 d1tzha1 d4m7jl1 d1k6ql1 d3b9kc1 d1igml_
d3qeua1 d1dsfl_ d1ce1h1 d1kcuh1 d1zvha1 d1zvoa1 d1tqbb1 d1ay1l1
d2qscl1 d2cgrh1 d1nl0h1 d1kenl1 d3eo0a1 d4n9ob1 d1n0xl1 d3k1kc_
d1c4qa_ d1prtc1 d2wv6d_ d1prtf_ d1ltrd_ d4l63b1 d1r4pb_ d1prtd_
d1tl0f_ d1prtb1 d3chbd_ d2aq2b1 d1ck1a1 d1enfa1 d1i4pa1 d1v1oa1
d1et9a1 d1esfa1 d2g9hd1 d4ohja1 d1klud1 d1bxta1 d1eu3a1 d1ty0a1
d2cqva1 d1fhga_ d2k1ma_ d2dl2a1 d1biha3 d1biha1 d1biha4 d3qp3a1
d1g1ca_ d1gxea_ d1cvsc2 d1cvsc1 d1xmwa2 d1m4ka2 d2otpa2 d1biha2
d1rhfa2 d2fcba2 d2fcba1 d1wwbx_ d1gsma2 d1epfa1 d1epfa2 d2dava1
d1waaa_ d1f97a2 d1x44a1 d1vcaa2 d1fnla2 d1fnla1 d3b5ha2 d3h8na2
d3h8na1 d1jbja1 d1jbja2 d1pd6a_ d1f2qa2 d1f2qa1 d3cx2a_ d1nbqa2
d3b5ha1 d1n26a1 d1xiwb_ d1xaua_ d1fltx_ d2ifga1 d1olla1 d1olla2
d1cs6a4 d1cs6a3 d1cs6a2 d1cs6a1 d2gw5a2 d1sy6a1 d1xiwa_ d1ucta2
d1ucta1 d1ry7b2 d1gl4b_ d1qz1a3 d1olza1 d2c9aa1 d1wwca_

**Family-6 (a.22.1.3)**

**Positive dataset contains 3 protein sequences.**

d2byka1 d4g92b_ d2bykb1

**Negative dataset contains 4008 protein sequences.**

d1m32a_ d1ibja_ d1c7na_ d1iuga_ d4q75a_ d2bkwa1 d1t3ia_ d1n8pa_
d1qz9a_ d1jf9a_ d1p3wa_ d3kgwa_ d1cl1a_ d2ctza1 d1vjoa_ d2rfva_
d1gc0a_ d1cs1a_ d1d2fa_ d2ch1a1 d1qgna_ d1pffa_ d1elua_ d1u08a_
d1lc5a_ d1b5pa_ d2ay1a_ d1bw0a_ d3pd6a_ d7aata_ d2csta_ d1j32a_
d1fg7a_ d4wlha_ d1o4sa_ d1v2da_ d1gdea_ d1m6sa_ d1v72a1 d1svva_
d1ajsa_ d1wsta1 d2f8ja2 d1lk9a_ d1vp4a1 d1xi9a1 d2gb3a1 d1js3a_
d3rcha_ d3k40a_ d1xkta1 d2hfka_ d1jmkc_ d5d3ka_ d2cfba1 d1dfoa_
d1bj4a_ d3k28a_ d2bwpa_ d3lg0a_ d2fnua_ d3ou5a_ d1z7da1 d1b9ha_
d1iaya_ d4msoa_ d1bs0a_ d1s0aa_ d1sffa_ d3tqxa_ d1mdoa_ d3qboa_
d3pgya_ d1bjna_ d2dkja_ d1ejia_ d1vefa1 d4wxga_ d3bs8a_ d3gbxa1
d1w23a_ d2b5aa1 d2ofya1 d4mcxa_ d2xi8a_ d2icta_ d2bnma1 d2ppxa1
d1y7ya1 d1sefa_ d1sfna_ d1sq4a_ d1g8fa3 d1qnia1 d1ibya_ d1js9c_
d1f15b_ d1za7a_ d1gyca3 d1hfua2 d1kbva1 d1kbva2 d1sdda1 d1sdda2
d1gyca1 d1gyca2 d1mzya1 d2bw4a1 d1aoza1 d1aoza2 d1kyaa2 d1kyaa3
d1kyaa1 d1mzya2 d4e9sa1 d1sddb1 d1sddb2 d1hfua1 d1hfua3 d1oe1a2
d1oe1a1 d1v10a2 d1v10a3 d1v10a1 d2dara2 d2dara1 d1wyha2 d2dloa2
d1rutx4 d1rutx3 d2cuqa2 d1x4la2 d1x4la1 d1x3ha2 d1x3ha1 d2d8za1
d1x61a2 d1x6aa1 d1x6aa2 d2cora2 d2cora1 d1v6ga1 d1v6ga2 d2d8za2
d1x64a1 d1x64a2 d1b8ta1 d2co8a1 d1x63a2 d2dj7a2 d2dj7a1 d2cu8a2
d2cu8a1 d1g47a2 d1g47a1 d1j2oa1 d1wyha1 d2dloa1 d1wiga1 d1wiga2

d2cupa2 d2cupa3 d1x68a1 d2d8ya2 d2d8ya1 d1m3va2 d1x62a1 d1x68a2
d1x61a1 d1b8ta3 d1b8ta2 d2d8xa2 d2d8xa1 d1x4ka1 d1x4ka2 d2cuqa1
d1m3va1 d2co8a2 d1x63a1 d2cura1 d2cura2 d1x62a2 d3sxwa1 d1j3qa_
d4luka_ d1a3qa1 d1iknc_ d3brda1 d1p7hl1 d1oy3c_ d1gjia1 d1zk9a1
d2cxka1 d2bema_ d1pama1 d1ea9c1 d1qhoa1 d2fhfa2 d2fhfa1 d1k3ia1
d1j0ha1 d1cxla1 d2bhua1 d1edqa1 d1f1sa2 d1ut9a2 d1gvia1 d1txka1
d1zx5a1 d2phla2 d1fxza2 d1fxza1 d1lrha_ d1uija1 g1dgw.1 d2v09a_
d1uika2 d1uika1 d1dgwa_ d2pa7a1 d1wlta1 d1nxma_ d2c0za1 d1nepa_
d1ds6b_ d1fstb_ d1g0da1 d1v70a_ d2f4pa1 d1yhfa1 d2bjca1 d2hsga1
d1qpza1 d1ebfa2 d1e3oc2 d1ic8a2 d2ovga_ d1sq8a_ d1rzsa_ d3kz3a_
d3orca_ d3gpdg2 d2q49a2 d1hdgo2 d2g17a2 d2cvoa2 d2czca1 d3pwka2
d1obfo2 d1i32a2 d1zyao2 d2pkqo2 d1b7go2 d2hjsa2 d1cf2o2 d1vkna2
d1nvmb2 d2msya_ d2djoa1 d2r5za_ d1au7a1 d1f43a_ d2lmda1 d1akhb_
d1s7ea1 d3nara_ d1zq3p1 d2craa1 d1mija_ d4uuta_ d3a01a_ d1x58a1
d3rkqa1 d3cmya1 d2cqxa1 d2da6a1 d1e3oc1 d1fjla_ d1mh3a1 d1ig7a_
d1x41a1 d1wh7a1 d1gt0c1 d2mw8a_ d1lfup_ d1x2ma1 d1b72a_ d1wi3a1
d1wjha1 d1x2na1 d1mnmc_ d2cuea1 d3k2aa_ d2hosa_ d2jwta2 d1pufa_
d1octc1 d2hi3a_ d1b8ia_ d4j19a_ d3lnqa_ d1wh5a1 d1b8ib_ d1hcra_
d1gdta1 d1u78a2 d6paxa1 d1k78a1 d1k78a2 d1hlva1 d1hlva2 d1iufa2
d1iufa1 d1bl0a2 d1d5ya1 d1d5ya2 d1prtb2 d3lv8a_ d1s3ga1 d1zaka1
d1s4qa_ d3tvta2 d1uf9a_ d1khta_ d1ckea_ d1osna_ d1p5zb_ d4tmka_
d3cm0a_ d1tmka_ d1jaga_ d3h86a_ d1t0hb_ d1nn5a_ d2h92a_ d3kb2a1
d2c95a1 d1yj5a2 d1kgda_ d2ak3a1 d1vyva2 d1deka_ d2grja1 d1p3ja1
d1y63a_ d1e4ya1 d1s96a_ d3iija1 d1lvga_ d1nksa_ d1vyua2 d4e22a_
d1qf9a_ d1jjva_ d1q3ta_ d1ki9a_ d1lw7a2 d1teva_ d1kjwa2 d1gsia_
d1z0ja1 d3umra2 d3gj0a1 d2gaoa1 d1yzga1 d1x3sa1 d1g7sa4 d1puja_
d1udxa2 d1kk1a3 d2o52a_ d1jwyb_ d1zcaa2 d1zcba2 d2fv8a1 d2cjwa_
d3dz8a1 d1xzpa2 d1vg8a_ d4qxaa_ d2ew1a1 d1x1ra1 d2bcgy_ d3doea1
d3cona_ d3rega_ d3wyga_ d1upta_ d2b6ha1 d1zd9a1 d1lnza2 d1r5ba3
d1n0ua2 d1r2qa_ d2f9la1 d2rhda_ d3q3jb_ d3q85a_ d1efma1 d1r8qa_
d2gf9a1 d2akab1 d1ek0a_ d1z08a1 d2gcoa1 d2dy1a2 d4g01b_ d2cxxa1
d1svia_ d3q72a_ d1ni3a1 d4ac9a4 d2erxa1 d2bmea_ d1wxqa1 d2gf0a1
d4ldja1 d1zunb3 d4lv5b_ d1efca3 d1kmqa_ d2g6ba1 d3lrpa_ d1tada2
d2y8ea_ d4rd4a1 d1z06a1 d2heia_ d3a58b_ d1u0la2 d1d2ea3 d1zj6a1
d2ocba_ d3rwoa_ d1mr3f_ d2e9sa1 d4u5xa1 d2iwra_ d2fn4a1 d3bwdd_
d2nzja1 d2atva1 d2ksqa_ d3l43a_ d1mkya2 d1mkya1 d1wmsa_ d1jnya3
d2rmka_ d1f6ba_ d1f60a3 d1gota2 d1jala1 d2fh5b1 d1g16a_ d2wblc_
d1m7ba_ d1wf3a1 d1f5na2 d1t9ha2 d1z6xa1 d1z0aa1 d2fg5a1 d2a5ja1
d2bcjq2 d1s0ua3 d1ky3a_ d4y0va_ d4lhwa_ d2qmea1 d2atxa1 d1egaa1
d1ryha_ d1moza_ d1nrjb_ d2erya1 d3bfka_ d1puia_ d1fzqa_ d3j3az1
d3t5ga_ d1shza2 d3defa_ d2f7sa1 d3raba_ d3tsoa_ d3aq4a_ d2al7a1
d3sfva1 d3bc1a_ d2ncda_ d1sdma_ d3h4sa1 d2heha_ d2grya_ g3kin.1
d1kk8a2 d1br2a2 d1goja_ d1f9va_ d2mysa2 d1lkxa_ d3t0qa_ d4db1a1
d3gbja_ d1ry6a_ d1kv9a1 d1k8ka2 d1k8ka1 d3uleb1 d3i33a2 d3i33a1

d1bupa1 d1dkgd2 d1dkgd1 d4ci6a2 d1jcfa1 d4a2ba2 d2fxua1 d1yaga2
d1yaga1 d4cbua2 d1bupa2 d3vrda1 d3vrda2 d1fcdc2 d1h1oa2 d1h1oa1
d1m70a2 d1m70a1 d1iqca2 d1iqca1 d1eb7a1 d1nmla2 d1nmla1 d1nira1
d1hroa_ d4gedb_ d3zooa_ d1mz4a_ d1gu2a_ d1f1fa_ d1dvva_ d1c6ra_
d1c75a_ d1e29a_ d1gdva_ d1cnoa_ d1h32b_ d1co6a_ d2bh4x_ d2aiua_
d5cytr_ d1jdla_ d1i8oa_ d2zboa_ d3dmia_ d1ls9a_ d1wvec_ d2yk3a_
d3ph2b_ d1dw0a_ d5aura_ d1ql3a_ d1qn2a_ d1f1ca_ d2nqya_ d1h4ga_
d1hixa_ d1oa2a_ d1olra_ d3b7ma_ d4h7ma1 d3wp3a_ d2vgda_ d1igoa_
d3zsea_ d2nlra_ d3wx5a_ d1oa3a_ d1pvxa_ d1m4wa_ d1f5ja_ d1oa4a_
d1ks5a_ d4ixla1 d1h32a1 d1etea_ d3tgxb_ d1scfa_ d2z3qa1 d3qb1a_
d1hula_ d1m48a_ d4nkqb_ d1eera_ d1v7mv_ d5e4ea_ d2o27a_ d3b5ka_
d2psma1 d4zf7a_ d3uf5a_ d4x51a_ d4ypgc1 d3oq3a_ d1lqsl_ d1n1fa_
d1au1a_ d1wu3i_ d3besl_ d3ux9a_ d1v8fa_ d2ejca_ d3muea1 d1ihoa_
d3q12a1 d4dnxa2 d1m8pa2 d1v47a2 d1jhda2 d1x6va2 d3rbaa_ d2qtra_
d1nupa_ d3l92a_ d1f9aa_ d1qjca_ d2h29a_ d1vlha_ d1od6a_ d1mrza2
d4ruka_ d2el7a_ d1iq0a2 d1irxa2 d1r6ta2 d1ivsa4 d2cyba_ d1rqga2
d1j09a2 d2yxna_ d1f7ua2 d1h3fa1 d1ffya3 d2g36a1 d1nzja_ d1jila_
d1i6la_ d1pfva2 d1h3na3 d3n9ia_ d3sz3a1 d3sw2a_ d1moxc_ d1dx5i3
d1edmb_ d1autl2 d1autl1 d1nqlb_ d1dval1 d1szba2 d1kigl_ d2y5fl_
d1ijqa2 d1gk5a_ d1g1sa2 d1nt0a3 d1xdtr_ d1lmja2 d1lmja1 d1nzia2
d2wphe_ d1klil_ d1pfxl2 d1pfxl1 d1p9ja_ d1g1ta2 d1f7ea_ d4yzub_
d2mgpa1 d1b9wa1 d1b9wa2 d2mgpa2 d1ob1c2 d1n1ia2 d1n1ia1 d2npra2
d2npra1 d1gl4a2 d1f56a_ d1bypa_ d1azca_ d1ws8a_ d3u25a_ d1cuoa_
d1id2a_ d1rkra_ d2iaac_ d4dp1x_ d1nwpa_ d2gima_ d2i7sa_ d1qhqa_
d1qx2a_ d2y5ia_ d1qlsa_ d1psra_ d3n22a_ d2egda_ d4ggfc_ d2wcea_
d2luca_ d4aqia_ d3nsia_ d2lp3a_ d1j55a_ d1a4pa_ d1k8ua_ d1nsha_
d1pvaa_ d3fs7a_ d2pvba_ d1ttxa_ d1rwya_ d2kyfa_ d1rk9a_ d1a75a_
d1tiza1 d1nyaa_ d1g8ia_ d1ggwa_ d1wdcc_ d3fwba_ d1ggza_ d2o5ga_
d1ctaa_ d1m45a_ d3i5gc_ d1sl8a_ d1fpwa_ d2scpa_ d2roaa_ d4aqra_
d3pn7b_ d1br1b_ d2mysb_ d1s6ca_ d1bjfa_ d2obha_ d1jfka_ d1jf0a_
d1qv0a_ d1lkja_ d2mlfc_ d2m97a_ d2d8na_ d4by5a_ d1jbaa_ d4nqga_
d1wdcb_ d2mysc_ d1j7qa_ d1s6ia_ d1uhka2 d1r2ua_ d1dtla_ d3kf9a_
d3pn7c_ d1fi6a_ d1c07a_ d5d67d_ d1tuza_ d1h8ba_ d1eg3a1 d1eg3a2
d2zkmx1 d1snla_ d1gjya_ d1y1xa_ d1hqva_ d1df0a1 d1qxpa2 d4usla_
d1k94a_ d1wkya2 d1uuqa_ d3wh9a_ d4m1ra_ d1xyza_ d4xuya_ d2je8a5
d4bf7a1 d3cuia_ d1ur4a_ d1qw9a2 d1odza_ d2vzsa5 d1bqca_ d2x2ya1
d1hjqa_ d1qnra_ d4y7ea1 d1vjza_ d4k68a_ d2v3ga_ d4hpga_ d1nofa2
d1nq6a_ d1h4pa_ d1aq0a_ d3ziza_ d4bs0a_ d1ecea_ d1h1na_ d1ta3b_
d1w91a2 d1uhva2 d1us3a2 d1egza_ d7a3ha_ d1tvna1 d3nyda1 d1ghsa_
d2cyga1 d4xv0a_ d2c8na2 d1w32a_ d3emca_ d1v6ya1 d1foba_ d2uwfa1
d2fgla_ d3niya1 d2c0ha1 d1rh9a1 d1yq2a5 d1szna2 d1fa2a_ d1pama4
d5do8a1 d1ea9c3 d1hx0a2 d2fhfa5 d1qhoa4 d1avaa2 d1m53a2 d4m8ua1
d2gjpa2 d1lwha2 d4howa1 d1ktba2 d1j0ha3 d3vm5a1 d2x1ia_ d1wzaa2
d1uasa2 d2diea1 d1mxga2 d1ht6a2 d1eswa_ d1iv8a2 d5cpsa_ d1ud2a2

| | | | | | | | |
|---|---|---|---|---|---|---|---|
| d1zjaa1 | d3gbea1 | d1kwga2 | d1wdpa_ | d1gcya2 | d1g5aa2 | d1hvxa2 | d3dhpa2 |
| d2bhua3 | d4do4a1 | d3qgva1 | d1e43a2 | d1g94a2 | d2d3na2 | d1gjwa2 | d1gvia3 |
| d1x1na1 | d1r7aa2 | d1tz7a1 | d4toqa_ | d1wb0a1 | d1w9pa1 | d1eoka_ | d1itxa1 |
| d1kfwa1 | d1goia2 | d1edqa2 | d2gsja_ | d1hjxa1 | d1jnda1 | d1vf8a1 | d3wdpp1 |
| d3wq5a_ | d1gnxa_ | d4gxpa_ | d4hz8a_ | d1e4mm_ | d3aiua_ | d1wcga1 | d1qvba_ |
| d3ahxa_ | d1v02a_ | d2dgaa_ | d1pbga_ | d1vffa1 | d1qoxa_ | d4ha4a_ | d1g4ma1 |
| d1id1a_ | d2hmta_ | d1lssa_ | d1iuka_ | d2csua1 | d3q9ub_ | d2yv1a1 | d3q9na_ |
| d2d59a_ | d1oi7a1 | d2yv2a1 | d1y81a1 | d3nvva1 | d1krha3 | d2h88b1 | d1n62a2 |
| d1t3qa2 | d1kf6b2 | d1zoyb1 | d1jq4a_ | d1dgja2 | d1rm6c2 | d1h6ta1 | d1h6ua1 |
| d9ldta1 | d2ewda3 | d1up7a1 | d2dfda1 | d1s6ya1 | d1vjta1 | d1hyea1 | d1t2da1 |
| d1ojsa3 | d1b8pa1 | d1ldna1 | d1hyha1 | d1civa1 | d2a92a1 | d1ez4a1 | d1i0za1 |
| d1y6ja1 | d1gv0a1 | d1ur5a1 | d1llda1 | d1obba1 | d1npya1 | d3t4ea2 | d1a4ia1 |
| d2hk9a2 | d2egga2 | d1b26a1 | d1bvua1 | d1gq2a1 | d1c1da1 | d1leha1 | d1gtma1 |
| d1euza1 | d1nvta1 | d3dona2 | d1edza1 | d1gpja2 | d1vi2a1 | d1p77a1 | d1nyta1 |
| d1b0aa1 | d1vl6a1 | d1v9la1 | d1etob_ | d1umqa_ | d1g2ha_ | d1kola2 | d1f8fa2 |
| d1qora2 | d1jqba2 | d1vj0a2 | d1cdoa2 | d1gu7a2 | d1d1ta2 | d1xa0a2 | d2dm6a2 |
| d1jvba2 | d1rjwa2 | d1llua2 | d1tt7a2 | d1piwa2 | d1o89a2 | d1iz0a2 | d1e3ja2 |
| d1yb5a2 | d1uufa2 | d1vj1a2 | d1e3ia2 | d3gpdg1 | d1vc2a1 | d2nvwa1 | d1u8fo1 |
| d1ydwa1 | d1hdgo1 | d2g17a1 | d1cf2o1 | d2czca2 | d1r0ka2 | d1obfo1 | d2cvoa1 |
| d1ebfa1 | d1h6da1 | d1i32a1 | d1zh8a1 | d2q49a1 | d1qkia1 | d1j5pa4 | d3pzra1 |
| d1xeaa1 | d1vkoa1 | d1vm6a3 | d3qvsa1 | d2pkqo1 | d1b7go1 | d1gr0a1 | d1p1ja1 |
| d1lc0a1 | d3pwka1 | d2hjsa1 | d1zyao1 | d1vkna1 | d1tlta1 | d1nvmb1 | d3sjua_ |
| d3ojfa1 | d1xu9a1 | d2jjya1 | d1n7ha_ | d1y1pa1 | d1h5qa_ | d1jtva_ | d4fs3a_ |
| d2bkaa1 | d1gy8a_ | d2c5aa1 | d1ulsa_ | d1ooea_ | d2b69a1 | d1y5ma1 | d1spxa_ |
| d1ae1a_ | d1bxka_ | d1ydea1 | d1gega_ | d1hdoa_ | d1o5ia_ | d1e6ua_ | d1snya1 |
| d2rhca_ | d3zv4a_ | d5ceja_ | d2d1ya1 | d3w8ea_ | d2fmua1 | d1x1ta_ | d1rpna_ |
| d3ay7a1 | d1k2wa_ | d1eq2a_ | d2hrba1 | d3ruaa_ | d2ew8a1 | d4hwka_ | d1xgka_ |
| d2bd0a1 | d1yo6a1 | d2a35a1 | d2ntva_ | d1vl8a_ | d1hdca_ | d4bkua_ | d2ag5a1 |
| d2q45a_ | d1nffa_ | d4iy1a_ | d1zbqa1 | d1kewa_ | d1ek6a_ | d1ja9a_ | d2q46a2 |
| d1iy8a_ | d1cyda_ | d2ptga_ | d1hxha_ | d1uzma1 | d3lyla_ | d4m89a_ | d3wyea_ |
| d2bl1a1 | d1w6ua_ | d1sb8a_ | d2q2va_ | d4nr0a_ | d1e6wa_ | d2gasa_ | d3oiga1 |
| d2bgka1 | d1mxha_ | d1vl0a1 | d1rkxa_ | d1r6da_ | d1wmaa1 | d1e7wa_ | d2c07a1 |
| d1xkqa_ | d3tzha_ | d1g0oa_ | d3lz6a_ | d1qyca_ | d1edoa_ | d1gz6a_ | d1oc2a_ |
| d1t2aa_ | d1zema1 | d2xoxa_ | d1z45a2 | d1geea_ | d1p33a_ | d3lt0a_ | d4e6pa_ |
| d2wyua_ | d1db3a_ | d4q9na_ | d1xhla_ | d4o1ma_ | d1fmca_ | d2uvda_ | d2gdza1 |
| d1uaya_ | d2z1ma_ | d2o23a2 | d2fr1a2 | d2fr1a1 | d1qsga_ | d1qyda_ | d4zjua_ |
| d2a4ka1 | d1yb1a_ | d1wvga1 | d1yxma1 | d3f4ba_ | d1mg5a_ | d1xg5a1 | d1r55a1 |
| d3gboa_ | d1atla_ | d1kufa_ | d1buda_ | d4q1la_ | d1quaa_ | d1yp1a_ | d1wnia_ |
| d1ex0a4 | d1g0da4 | d2f4ma1 | d1x3za_ | d1mdwa_ | d1qxpa4 | d1ziva1 | d1zcma1 |
| d1t3ca_ | d1epwa3 | d2a97a_ | d3fiea_ | d3ioqa_ | d1fh0a_ | d1o0ea_ | d1khqa_ |
| d1m6da_ | d2cb5a_ | g8pch.1 | d1s4va_ | d2pnsa_ | d1vsna1 | d1mira_ | d1iwda_ |
| d2oula_ | d7pcka_ | d3bwka_ | d3bcna_ | d2p86a_ | d1pvja_ | d1pcia_ | d1gece_ |
| d1deua_ | d4bs6a_ | d1xkga1 | d1cqda_ | d1jqpa2 | d2c0ya_ | d2bdza_ | d3p5ua_ |
| d2o36a_ | d1ex4a2 | d1c6va_ | d2jk6a2 | d2jk6a1 | d1feca1 | d1feca2 | d1lpfa1 |

d1lpfa2 d1xhca1 d1xhca2 d1dxla2 d1dxla1 d1gera2 d1m6ia2 d1onfa1
d1onfa2 d3lada2 d3lada1 d1w4xa1 d1w4xa2 d1gesa2 d1mo9a1 d1xdia1
d1fl2a2 d1fl2a1 d1fcda1 d1fcda2 d1h6va2 d1h6va1 d1mo9a2 d1gv4a2
d1gv4a1 d2gv8a2 d2gv8a1 d1aoga2 d1hyua1 d1ebda1 d1ebda2 d3dk9a1
d1q1ra2 d1q1ra1 d1gesa1 d1zbfa1 d2z1ha_ d2etja1 d3qioa1 d1jl1a_
d2e4la_ d1jl2a_ d2lsna1 d1io2a_ d1uaxa_ d2yv0x_ d1mu2a1 d1ekea_
d1i39a_ d1pn0a1 d1reoa1 d1ju2a1 d2f5va1 d3c96a1 d2gmha1 d1pj5a2
d1gpea1 d1o5wa1 d1n4wa1 d1k0ia1 d2dw4a2 d2ivda1 d2voua1 d1kdga1
d1s5ja1 d2hbja2 d2igia_ d1wlja_ d1qhta1 d4dlea1 d1tgoa1 d2f96a1
d3tr8a1 d3mxma_ d1q8ia1 d1y97a1 d1w0ha_ d1kfsa1 d2py5a1 d1noya_
d1d5aa1 d1yt3a3 d2guia_ d3mwpa1 d4kb1a_ d2y8ga_ d1xkea1 d1k5db_
d2y8fa_ d2d5ra1 d1b9ra_ d1frra_ d3wcqa_ d1e9ma_ d1l5pa_ d1wria_
d1i7ha_ d3av8a_ d1czpa_ d1rfka_ d3p1ma1 d3b2ga_ d2bt6a2 d1wgqa1
d1v89a1 d2coda1 d1zc3b_ d1wg7a1 d1u5ea1 d1v61a1 d2coca1 d2ys1a1
d1v5pa1 d1ntya2 d1wjma1 d1wi1a1 d1u29a_ d1xx0a1 d1unqa_ d2zkmx3
d1fhoa_ d1v88a1 d2adza1 d1x1fa1 d1p6sa_ d2coaa1 d1btka_ d1v5ma1
d1faoa_ d2x18a_ d1dyna_ d1x1ga1 d4kaxb_ d2j59m1 d1ki1b2 d1xcga2
d1txda2 d1u5da1 d1dbha2 d1w1ha_ d1upqa_ d1v5ua1 d1foea2 d2fjla1
d1eaza_ d2dfka2 d2cofa1 d1kz7a2 d1r7ha_ d1faaa_ d1wika1 d1xfla_
d1ttza1 d2f51a1 d1ti3a_ d1nhoa_ d1sena_ d2i4aa_ d1ep7a_ d1zmaa1
d1fo5a_ d3lefa1 d3diea1 d2yana1 d1z6na1 d1oaza_ d3zywa1 d4aj8a_
d1nm3a1 d4fiwa_ d1wmja_ d4hs1a1 d1iloa_ d1xwaa1 d1wjka1 d2vm1a_
d1fb6a_ d1gh2a_ d1h75a_ d1nw2a_ d1pd212 d1nhya2 d2a2ra2 d1k0dd2
d1f2ea2 d1gsua2 d1f3ba2 d2fhea2 d2pvqa1 d1tw9a2 d1oyja2 d1e6ba2
d1k0ma2 d3ik7a1 d4is0a1 d1r5aa2 d1jlva2 d1m0ua2 d3gtub2 d1z9ha2
d1okta2 d1axda2 d2gsra2 d1tu7a2 d1k3ya2 d1zgma1 d1b48a2 d1gnwa2
d1gwca2 d2gsta2 d1glqa2 d1oe8a2 d1pn9a2 d1v2aa2 d1fw1a2 d1duga2
d1ig8a1 d1czan2 d1czan3 d1czan1 d1czan4 d1bg3a4 d1bg3a3 d1bg3a2
d1bg3a1 d1qqya_ d1gd6a_ d1f6ra_ d1ivma_ d2gv0a_ d1iiza_ d2z2fa_
d1j8xa_ d1ghla_ d1b9oa_ d174la_ d169la_ d2o4wa_ d1p37a_ d1k28a3
d1xjta1 d1jtma_ d2f2qa_ d4e97a1 d1iyka2 d1iyka1 d1rxta1 d1iica2
d2pjsa1 d2i7ra1 d1ecsa_ d1xqaa1 d5cj3a1 d1npba_ d1jifa_ d2p7oa_
d1r9ca_ d1nkia_ d1mpya2 d1kw3b2 d1kw3b1 d4ghga1 d1tfza1 d3isqa1
d3isqa2 d1sqia1 d1sqia2 d1lkda2 d1lkda1 d1mpya1 d1cjxa2 d1sp9b2
d1t47a1 d1t47a2 d1f1ua2 d1sp8a1 d1sp8a2 d1f1ua1 d1f74a_ d3l21a_
d2e1da_ d1to3a1 d1w3ia_ d1sfla_ d1n7ka_ d1vcva1 d4imea_ d1wa3a_
d2ocza1 d1f2ja_ d3m16a_ d2yxga_ d1ub3a_ d3a5fa_ d1fbaa_ d1l6wa_
d1xfba1 d1wx0a1 d1vpxa1 d1ojxa_ d1mxsa_ d2a4aa1 d1xkya1 d3cq0a1
d3s1xa_ d1fdja_ d3cpra1 d4d2ja_ d1s5ja2 d1qhta2 d4dlea2 d1tgoa2
d3py8a2 d1q8ia2 d1kfsa2 d2py5a2 d1d5aa2 d1qsta_ d2beia1 d4m3sa_
d1u6ma1 d1ygha_ d1yk3a1 d3dr6a_ d1xmta_ d1z4ea1 d2ge3a1 d1bo4a_
d2fiaa1 d2jdca1 d1y9wa1 d3to7a_ d1wwza1 d2fl4a1 d2ozua1 d2b5ga_
d2cy2a1 d2p0wa_ d1cm0a_ d4pswa_ d1yrea1 d1yr0a1 d1m4ia_ d2gana1
d1yvka1 d2g3aa1 d1y7ra1 d2euia1 d1q2ya_ d2fsra1 d1xeba_ d2ae6a1

d1cjwa_ d1qsma_ d2aj6a1 d1y9ka1 d1i12a_ d1vkca_ d2atra1 d1n71a_
d2d4pa1 d2fiwa1 d2fe7a1 d1tiqa1 d2ou2a_ d1yx0a1 d2fcka1 d1p0ha_
d2j8ma_ d2pq8a_ d1z4ra1 d1s3za1 d1ufha_ d2hqya1 d2hqya2 d1lrza2
d1ne9a1 d1lrza3 d1f37b_ d1xvqa1 d1pn0a2 d3hy2a_ d2cvba1 d1o8xa_
d1lu4a_ d1we0a1 d2bmxa1 d2a4va1 d1psqa_ d1xiya1 d1q98a_ d1n8ja_
d1zofa1 d2ggta_ d3tjja_ d4je1a1 d1o73a_ d3s9fa_ d2zcta_ d1qmva_
d1jfua_ d2b1ka_ d5dvba1 d2b5xa1 d2v2ga_ d1st9a_ d3tb2a_ d4llra_
d4fh8a1 d1e2ya_ d1knga_ d2h30a_ d1i5ga_ d2z9sa_ d2f8aa1 d1tp9a1
d2b7ka_ d1nm3a2 d3hvva_ d2wfca1 d4kcea_ d1xvwa1 d4gqca1 d3qpma_
d4k1fa_ d1zzoa1 d1gp1a_ d4od7a1 d1z6ma1 d4mcua1 d4dvca1 d1r4wa_
d3l9sa_ d1un2a_ d3rppa_ d1yloa2 d1y7ea2 d1y0ya3 d1lfwa1 d1vixa1
d1fnoa4 d1vhoa2 d1ysja1 d1vhea2 d1z2la1 d2fvga2 d1vgya1 d1r3na1
d2grea2 d1xmba1 g1q7l.1 d1rtqa_ d1ofda3 d1ea0a3 d1ecfa2 d1q15a2
d1ct9a2 d1ao0a2 d1te5a_ d1bnla_ d1dy2a_ d1cr6a2 d1j1ia_ d2rhwa_
d3groa_ d1thta_ d1ei9a_ d1ra6a_ d1muka_ d3gsza_ d1khva_ d1te9a_
d2xwha_ d3uqsa_ d3hkwa_ d3n6ma_ d2cjqa_ d2xyma_ d1teba_ d1te8a_
d1u09a1 d4wfxa_ d3tyqa_ d2pgga1 d3zxha_ d1cgla_ d1rm8a_ d1eaka2
d2jsda_ d1cxva_ d1hv5a_ d1i76a_ d3p0ya1 d1igra1 d1igra2 d1n8yc2
d1n8yc1 d1m6ba2 d1m6ba1 d1dzka_ d4tlja_ d2a2ua_ d4wfva_ d3t1da_
d4iaxa_ d3zq3a_ d2nnda_ d1i4ua_ d1kxoa_ d1bbpa_ d1kt7a_ d1exsa_
d3s26a1 d1qfta_ d4gh7a_ d1z24a1 d1qabe_ d1xkia_ d1ew3a_ d3bx7a_
d4iawa1 d1n0sa_ d1gkab_ d3kzaa_ d2k23a_ d2l9ca1 d2joza1 d1gm6a_
d3kffa_ d3saoa1 d1e5pa_ d1yupa1 d1iiua_ d1h6ua2 d2tbvc_ d1c8nc_
d1x36a_ d1smvc_ d1ng0c_ d4sbvc_ d1opoa_ d1f2nc_ d1cixa_ d1y29a_
d1v7fa_ d2jtba_ d1d1ha_ d1g9pa_ d1lupa_ d1qk7a_ d1mb6a_ d2a2va_
d1i25a_ d1hvwa_ d2mpqa_ d1ie6a_ d1kqha_ d1la4a_ d2mxoa1 d1dl0a_
d1koza_ d1qk6a_ d1emxa_ d1lqra_ d1c6wa_ d1rmka_ d1ixta_ d1eyoa_
d1cnna_ d1fu3a_ d1g1pa_ d1fyga_ d1rypd_ d1irua_ d1rypc_ d3unfk_
d1rypa_ d1j2pa_ d1irui_ d1j2qh_ d3unfh_ d1m4ya_ d1rypb_ d1irul_
d1irug_ d1iru2_ d1q5rh_ d1rypg_ d1g3ka_ d1iruf_ d1q5qa_ d1e94a_
d1irub_ d1ryph_ d3ty6a_ d1iruj_ d1iruk_ d1rype_ d1irud_ d1irue_
d3mi01_ d1rypf_ d1iruh_ d2z3ba_ d1iruc_ d3mka2_ d1iru1_ d1ny721
d1ny722 d1a6ca3 d1a6ca2 d1pgl22 d1pgl21 d1pgl11 d1ny711 d1mqta_
d1d4m3_ d1fmd3_ d2mev1_ g1aym.1 d1ev13_ d4q4w1_ d1r1a3_ d2x5ia_
d1rhi1_ g1r1a.1 g1d4m.1 g1rhi.1 d1b35b_ d1oopa_ d1tme1_ d4iv1a_
g1pvc.1 g1fpn.1 d1fpn1_ d4gb31_ d1d4m1_ d1fmd1_ d1ev11_ d2wzr3_
g1cov.1 d1qqp3_ g1fmd.1 d2x5ic_ d1rhi3_ g1oop.1 d1hxs1_ g1hxs.1
d1fpn3_ d1bev1_ g2mev.1 d1eah3_ d1aym1_ d1pvc3_ d1qqp1_ d1zba3_
d1h8ta_ d3vbhc_ g1b35.1 g1bev.1 d1hxs3_ g1ev1.1 d1bev3_ g1tmf.1
d1tmf1_ d1b35a_ d2mev3_ g1eah.1 g1h8t.1 d1eah1_ d4q4w3_ d1r1a1_
d1aym3_ d1zba1_ d1h8tc_ d1oopc_ d1tme3_ g1qqp.1 d4iv1c_ g1tme.1
d3ubub_ d1egia_ d1v7pa_ d1tdqb_ d3ubua_ d1c3ab_ d1h8ua_ d3bx4b_
d1t8ca1 d1sb2a1 d1qdda_ d2ziba1 d3cada_ d1byfa_ d1j34b_ d1jwia_
d1jzna_ d1c3aa_ d1oz7b_ d1wmza_ d3bdwb_ d1hq8a_ d1fifa1 d1dv8a_

| | | | | | | | |
|---|---|---|---|---|---|---|---|
| d3hupa_ | d1wk1a1 | d1umra_ | d2afpa_ | d3bx4a_ | d1gz2a_ | d1sl6a_ | d1v7pb_ |
| d3g8ka_ | d1ixxa_ | d1j34a_ | d1jwib_ | d1g1sa1 | d1y17a1 | d1ja3a_ | d1fvub_ |
| d3bdwa_ | d1sb2b1 | d1fvua_ | d1umrc_ | d1oz7a_ | d4m17a_ | d3cfwa1 | d1ypqa1 |
| d1g1ta1 | d3fd4a_ | d3l9jc_ | d4dn8a1 | d1v4lb_ | d1rdl1_ | d2msba_ | d4w9ta_ |
| d1jvna1 | d4x2ra1 | d1ka9f_ | d4axka1 | d1thfd_ | d1h5ya_ | d4u28a_ | d2y88a_ |
| d4wd0a_ | d1vzwa1 | d3ia2a_ | d1a88a_ | d4dgqa1 | d1zoia_ | d2flia1 | d1rpxa_ |
| d1tqja_ | d1tqxa_ | d1ux8a_ | d1dlya_ | d4i0va_ | d2bkma_ | d2bmma_ | d2r80b_ |
| d1cqxa1 | d1cg5a_ | d1gcvb_ | d3boma_ | d2h8fb_ | d2raoa_ | d3bj1a_ | d4hrrb_ |
| d2r50a_ | d3d1kb_ | d2aa1b1 | d3zhwa_ | d3hf4b_ | d3gdja_ | d1jebb_ | d3bj1b_ |
| d1x9fb_ | d1wmub_ | d1gcva_ | d3bcqb_ | d3d4xb_ | d1gvha1 | d2dc3a_ | d3hf4a_ |
| d4yu3a_ | d3k8ba_ | d1qpwb_ | d2r80a_ | d3bcqa_ | d1v4wa_ | d3wtgb_ | d1jl7a_ |
| d4esaa_ | d1a9we_ | d3d1ab_ | d1fsla_ | d4h2la_ | d1v4wb_ | d1mwca_ | d1vhba_ |
| d1jeba_ | d1spgb_ | d1h97a_ | d1q1fa_ | d1tu9a1 | d3d1aa_ | d1hdsb_ | d3hyub_ |
| d2zfbb_ | d3fs4a_ | d1oj6a_ | d1shrb_ | d4h2lb_ | d4yu3b_ | d1spga_ | d3hrwa_ |
| d3qqra_ | d3at5a_ | d4wchd_ | d3hyua_ | d1cg5b_ | d1uc3a_ | d1hbra_ | d2raob_ |
| d1outb_ | d4esab_ | d3gdjb_ | d3qqqa_ | d1x9fc_ | d3vrfa_ | d1itha_ | d3mkba_ |
| d3d4xa_ | d3mjpa_ | d1jl6a_ | d3fh9a_ | d1outa_ | d2dc3b_ | d1it2a_ | d3eoka_ |
| d3g46a_ | d1qpwa_ | d3vrfb_ | d1x9fd_ | d1hdsa_ | d2zfba_ | d3mkbb_ | d1or4a_ |
| d3wtga_ | d1a4fa_ | d3fh9b_ | d3bomb_ | d3cy5a_ | d3at5b_ | d1x9fa_ | d2oifa_ |
| d4lmxb_ | d1cpcb_ | d3v57a_ | d1kn1a_ | d1cpca_ | d4f0ta_ | d2vjha_ | d4po5b_ |
| d1eyxa_ | d4f0tb_ | d1b33b_ | d1b8db_ | d4h0mb_ | d1b33a_ | d2vjhb_ | d4lm6b_ |
| d2vmlb_ | d1allb_ | d2vjta_ | d3dbja_ | d2vmla_ | d2vjtb_ | d4f0ub_ | d1f99b_ |
| d4rmpa1 | d1liaa_ | d1eyxb_ | d2uulb_ | d4lmsb_ | d4f0ua_ | d2bv8a_ | d1f99a_ |
| d2bv8b_ | d2c7lb_ | d3dbjb_ | d4rmpb1 | d1b8da_ | d4h0ma_ | d3v57b_ | d1kn1b_ |
| d1liab_ | d2uula_ | d2j96a_ | d1pjqa1 | d1f00i3 | g1gxs.1 | g1wht.1 | d1azwa_ |
| d1mtza_ | d2xqfa_ | d1ea5a_ | d1f6wa_ | d1dx4a_ | d1qe3a_ | d1k4ya_ | d4qwwa_ |
| d2ha2a_ | d3c8da2 | d2pbla1 | d1jkma_ | d1jjia_ | d1jjfa_ | d4iuaa3 | d4bvva_ |
| d5hpga_ | d3k65a_ | d1ki0a3 | d1ki0a2 | d1ki0a1 | d1a0ha1 | d1pmla_ | d3sp8a3 |
| d2feba1 | d2m5ta_ | d1cqqa_ | d1wqsa_ | d5bpea_ | d2ipha_ | d2bhga1 | d3tnta_ |
| d3sjia_ | d3d23a1 | d2fyqa_ | d3tloa_ | d3zvea1 | d2hrva_ | d3q3ya1 | d4asha1 |
| d1e88a1 | d1l6ja5 | d1l6ja4 | d1eaka4 | d1eaka3 | d1l6ja3 | d1h8pa2 | d1h8pa1 |
| d3rp2a_ | g1ucy.1 | d1fuja_ | d2zchp_ | d3w94a_ | d1si5h_ | d1lo6a_ | g2pka.1 |
| d1cgha_ | d1kigh_ | d1a5ia_ | d3s9ba_ | d1hj8a_ | d1ao5a_ | d2anya_ | d1q3xa1 |
| d1gvza_ | d1azza_ | d4gsoa_ | d2f91a1 | d1iaua_ | d1gdna_ | d1eufa_ | g1fiw.1 |
| d1eq9a_ | d4nfea_ | d2rdla_ | d1spja_ | d1elva1 | d1a7sa_ | d1ekbb_ | d1yc0a_ |
| d2aiqa_ | g1fiz.1 | d1fi8a_ | d4k60a_ | d4dgja_ | d1mzaa_ | d1op0a_ | d3tjva_ |
| d1pytd_ | d1sgfg_ | g1gg6.1 | d2eeka_ | g1rtf.1 | d1a0ja_ | d3g01a_ | d1pytc_ |
| d4e7na_ | d1ddja_ | d1z8ga1 | d2hlca_ | d1npma_ | d1brup_ | g1jou.1 | d1sgfa_ |
| d1autc_ | g2hnt.1 | g1kdq.1 | d1orfa_ | d3s69a_ | d4crga_ | d1fxya_ | d1bqya_ |
| d2ftba_ | d1pmpa_ | d2rcqa1 | d1fdqa_ | d4lkpa_ | d1sa8a_ | d1vyfa_ | d1lpja_ |
| d3vg7a1 | d4azqa1 | d1o8va_ | d5bvqa_ | d1p6pa_ | d2qo4a_ | d1opba_ | d1yiva1 |
| d1o1va_ | d4bvma1 | d4tkba_ | d4i3ba_ | d1bwya_ | d1ftpa_ | d4gkca_ | d1cbia_ |
| d3p6da1 | d1kqwa_ | d2a0aa1 | d1m4la_ | d2bk7a_ | d3fjua_ | d1z5ra1 | d1kwma1 |
| d1uwya2 | d1jqga1 | d2c1ca1 | d1dtda_ | d1h8la2 | d3lgia_ | d3i77a_ | d2z9ia2 |

| | | | | | | | |
|---|---|---|---|---|---|---|---|
| d4nsva_ | d1lcya2 | d4jcna_ | d1os8a_ | d1hpga_ | d1p3ca_ | d1l1ja_ | d1qtfa_ |
| d1e69a_ | d1mv5a_ | d3g60a4 | g1ii8.1 | d1ewqa2 | d1ji0a_ | d1qhla_ | d1v43a3 |
| d2wmfa1 | d1vpla_ | d4a82a2 | g1f2t.1 | d2d62a1 | d3tuic1 | d2bbsa1 | d3g60a3 |
| d2ixea_ | d1g6ha_ | d1sgwa1 | d2qi9c_ | d3rlfa1 | d3b60a1 | d3d31a2 | d2pzea1 |
| g1xew.1 | d1jj7a_ | d1oxxk2 | d2onka1 | d1w1wa_ | d1iwea_ | d1qf5a_ | d1vmaa2 |
| d1nija1 | d1np6a_ | d2p67a1 | d3ihla_ | d1okkd2 | d1ls1a2 | d1s1ma2 | d1iona_ |
| d2v40a_ | d3hida_ | d2vo1a1 | d2g0ta1 | d1vcoa2 | d1j8mf2 | d1g3qa_ | d1xjca_ |
| d1p9ba_ | d1qzxa3 | d1ihua2 | d1ihua1 | d2qm8a_ | d1dj2a_ | d4ioja_ | d1cp2a_ |
| d1yrba2 | d1hyqa_ | d3gqba2 | d1skye3 | d1xp8a1 | d1tf7a2 | d1tf7a1 | d1ye8a1 |
| d1skyb3 | d2qe7d2 | d4blta_ | d4huta_ | d3ldaa2 | d4hyya_ | d2i3ba1 | d1cr1a_ |
| d3j9tb2 | d3ntua2 | d1vdza1 | d2z43a_ | d1e9ra_ | d1nlfa_ | d3ziaa2 | d1n0wa_ |
| d1g6oa_ | d3ziad2 | d3j9ta2 | d1pzna2 | d3gqbb2 | d2i49a_ | d1nnfa_ | d1xvxa_ |
| d1ursa_ | d1h3da1 | d1iuda_ | d4m1va1 | d1twya_ | d3e13x_ | d2nxoa1 | d4pfua_ |
| d3ttma_ | d1elja_ | d2qmwa1 | d1us5a_ | d1y3na1 | d1uqwa_ | d4f19a1 | d2esna2 |
| d3thia_ | d3fasa1 | d2uyeb_ | d2xwva1 | d1r9la_ | d2fyia1 | d1pb7a_ | d2ozza1 |
| d1sw5a_ | d1nh8a1 | d1vr5a1 | d2g29a_ | d1xs5a_ | d3lswa_ | d2z23a_ | d3w9va_ |
| d3tcha_ | d1q35a_ | d1z7me1 | d3q27a_ | d1p99a_ | d1ve4a1 | d2i6ea1 | d4mcoa1 |
| d4n8ya_ | d1eu8a_ | d1zbma1 | d1hsla_ | d1ixca2 | d1j1na_ | d1xvya_ | d1y9ua_ |
| d2z22a1 | d1xoca1 | d4oera_ | d1wdna_ | d5f1qa1 | d1xt8a1 | d1i6aa_ | d4gwoa_ |
| d4igta1 | d1pc3a_ | d4mmpa1 | d1o63a_ | d2czla1 | d2onsa2 | d4mija1 | d4ok5a1 |
| d2bmfa1 | d2bmfa2 | d1yksa1 | d1yksa2 | d2qeqa1 | d2jlqa1 | d1a1va2 | d1eh3a_ |
| d3skpa_ | d1dtza1 | d1dtza2 | d1ieja_ | d1jnfa2 | d1ce2a1 | d1jnfa1 | d1b1xa2 |
| d1b1xa1 | d1jw1a1 | d1jw1a2 | d1ryoa_ | d1h76a1 | d1h76a2 | d1etha2 | d1w52x1 |
| d2ppla1 | d1hpla2 | d1lpbb2 | d2oxea1 | d1bu8a2 | d1rp1a2 | d1c4oa1 | d2p6ra3 |
| d2p6ra4 | d1oywa2 | d1oywa3 | d1wrba1 | d1w36b2 | d1w36b1 | d1c4oa2 | d3bora_ |
| d1t6na_ | d2eyqa5 | d2eyqa3 | d2eyqa2 | d2zu6a2 | d1veca_ | d1uaaa1 | d1w36d1 |
| d2g9na1 | d1z3ix2 | d2rb4a_ | d1hv8a1 | d1hv8a2 | d1t5ia_ | d1w36c2 | d2j0sa2 |
| d2j0sa1 | d1fuka_ | d3hjha1 | d1z5za1 | d1qdea_ | d1z63a1 | d1s2ma2 | d1s2ma1 |
| d1wp9a1 | d1wp9a2 | d1gm5a3 | d3dina4 | d3dina3 | d1q0ua_ | d1w36c1 | d1z3ix1 |
| d1nkta3 | d1nkta4 | d1uaaa2 | d1w36d2 | g1qhh.1 | d2fwra2 | d2fwra1 | d3q3ma_ |
| d1za0a1 | d2inca_ | d1jk0a_ | d2incb_ | d1mhyb_ | d3hf1b_ | d1mtyb_ | d1r2fa_ |
| d2uw1a_ | d1mhyd_ | d3n37a_ | d1afra_ | d1oquc_ | d1syya_ | d1mtyd_ | d2o1za_ |
| d1uzra_ | d1smsa_ | d2fkza_ | d1zuja1 | d1s3qa1 | d2fjca1 | d1z6om1 | d1veia_ |
| d4dyya_ | d3gvya_ | d2pyba_ | d4cy9a1 | d3bvfa1 | d1r03a_ | d2cf7a_ | d1nf4a_ |
| d1zs3a1 | d4iwka_ | d3ka8a_ | d3np0x_ | d1n1qa_ | d1lb3a_ | d4e6ka_ | d1z6oa1 |
| d2fzfa1 | d4dyua_ | d3ge4a_ | d1jgca_ | d4am4a_ | d2z6ma_ | d1ji5a_ | d1vlga_ |
| d1lkoa1 | d1krqa_ | d3qz3a_ | d1o9ra_ | d2ffxj_ | d1euma_ | d1yv1a1 | d2v2pa_ |
| d1j30a_ | d4di0a_ | d1dpsa_ | d1oewa_ | d2qzxa_ | d2asia_ | d1psoe_ | d1miqa_ |
| d1smra_ | d2h6ta_ | d1pfza_ | d1izda_ | d3hd8a_ | d1j71a_ | d1dpja_ | d4aa9a_ |
| d1t6ex_ | g1b5f.1 | g1lya.1 | g1htr.1 | d1qdma2 | d1bxoa_ | d1wkra_ | d1tzsa_ |
| d1ibqa_ | d2qzwa_ | d2v76a_ | d1wj1a1 | d1aqca_ | d1qqga2 | d1qqga1 | d1ntva_ |
| d1n3ha_ | d4dbba_ | d3f0wa1 | d1p3ra_ | d1wgua1 | d2cy4a1 | d3hqca_ | d1wvha1 |
| d1xr0b1 | d1bdqa_ | d3ggta_ | d3ixoa_ | d1mrxa_ | d2haha_ | d1tw7a_ | d4njsa_ |
| d1sgua_ | d2p3da_ | d1nsoa_ | d3d3ta_ | d2rkfa_ | d1c6ya_ | d2rspa_ | d3ogpa_ |

| | | | | | | | |
|---|---|---|---|---|---|---|---|
| d3ekwa_ | d2o4na_ | d2j9ja_ | d1baia_ | d3ggua_ | d3lzua_ | d4npua_ | d4j55a_ |
| d2r8na_ | d2fxda_ | d2p3ca_ | d4dqea_ | d3ttpa_ | d3t3ca_ | d2p3aa_ | d2hc0a_ |
| d2fdda_ | d3mwsa_ | d1k8rb_ | d1wgra1 | d1ef5a_ | d1wfya1 | d1e8ya3 | d1wgya1 |
| d1lfda_ | d2cs4a1 | d1wxma1 | d2byea1 | d3kh0a1 | d2byfa1 | d1wxaa1 | d1e7ua3 |
| d1h4ra3 | d4yl8a1 | d2al6a3 | d1wgda1 | d1wgga1 | d4q5eb_ | d1wjna1 | d1se9a_ |
| d3n3kb_ | d1wz0a1 | d1we6a1 | d2m0xa_ | d2gbka_ | d3rt3b1 | d1wh3a1 | d1sifa_ |
| d2ekec2 | d4ii2b1 | d4hk2a1 | d1c3ta_ | d1wgha1 | d1ttna1 | d1j8ca_ | d1wx8a1 |
| d3v6cb1 | d2ma9b_ | d4i6lb1 | d1bt0a_ | d2jvca1 | d1iyfa_ | d2k1fa_ | d1wxva1 |
| d1wf9a1 | d1t0ya_ | d2io0b2 | d1x1ma1 | d2gbma_ | d1zw7a1 | d2m17a_ | d1ud7a_ |
| d1we7a1 | d2lwpa_ | d2gbja_ | d1wjua1 | d1v6ea1 | d1v86a1 | d4eewa_ | d1wiaa1 |
| d4icva_ | d4gu2a_ | d1wx7a1 | d1wy8a1 | d4hjka1 | d2faza1 | d1v5oa1 | d2al3a1 |
| d2cr5a1 | d1wj4a1 | d1s3sg_ | d3ubra_ | d1qo8a1 | d1d4ca1 | d2k3va_ | d1ft5a_ |
| d1qdba_ | d1m1qa_ | d2j7aa_ | d1h21a_ | d1ogyb_ | d4fasa_ | d1jnia_ | d1sp3a_ |
| d1g66a_ | d5ajha1 | d4lu0a_ | d1of8a_ | d3unda1 | d3e9aa1 | d1vr6a2 | d1n8fa_ |
| d2qkfa_ | d1d9ea_ | d3h33a_ | d1rwja_ | d19hca_ | d1os6a_ | d3bxua_ | d3h4na_ |
| d1i77a_ | d3caoa_ | d2ctha_ | d1duwa_ | d1ofwa_ | d2e84a_ | d1hh5a_ | d1gyoa_ |
| d3h34a_ | d2bcgg1 | d2bcgg2 | d1d5ta1 | d1wioa4 | d1ccza2 | d1vcaa1 | d1ic1a1 |
| d2d9qb1 | d2nxyb2 | d1um8a_ | d1ixza_ | d1qvra2 | d1qvra3 | d1qzma_ | d1sxjc2 |
| d1jr3d2 | d2p65a_ | d1l8qa2 | d3hu3a3 | d1sxjd2 | d1njfa_ | d1in4a2 | d1sxja2 |
| d1tuea1 | d2ce7a2 | d1w5sa2 | d1sxjb2 | d1d2na_ | d1ny5a2 | d1ofha_ | d2gnoa2 |
| d2a5yb3 | d1fnna2 | d1u0ja1 | d1lv7a_ | d1iqpa2 | d2fnaa2 | d1ixsb2 | d4gita_ |
| d1sxje2 | d1g41a_ | d1svpa_ | d1mbma_ | d3su6a1 | d1cu1a1 | d4agka_ | d1q31a_ |
| g1n1l.1 | d3e90b_ | d1ep5a_ | d3u1jb_ | d2e3fa_ | d1mm0a_ | d2e3ea_ | d2llda_ |
| d2ny8x_ | d2e3ga_ | d2ny9x_ | d2nz3a_ | d2lj7a_ | d1n4na_ | d2m8bb1 | d2kyqa_ |
| d3psma_ | d1jkza_ | d1jxca_ | d2gl1a_ | d1ugla_ | d4uj0a_ | d2k4ua_ | d1hp2a_ |
| d2lu9a_ | d1baha_ | d2meoa1 | d1wm7a_ | d1hlya_ | d1n8ma_ | d1jlza_ | d1wpda_ |
| d1pvza_ | d1wt7a_ | d1rjia_ | d1wz5a_ | d1m2sa_ | d1pjva_ | d1c49a_ | d1qkya_ |
| d2ktca1 | d1wmta_ | d1du9a_ | d1j5ja_ | d1r1ga_ | d1t0za_ | d2i61a_ | d1t1ta_ |
| d1sn4a_ | d1kv0a_ | d2lkba_ | d1npia_ | d1wwna_ | d1pe4a_ | d2kbka_ | d2li7a_ |
| d1djta_ | d2kbha_ | d1dq7a_ | d1sega_ | d1omya_ | d1zywa1 | d1jzaa_ | d1n9wa1 |
| d1bbua1 | d1e1oa1 | d1c0aa1 | d1l0wa1 | d3a74a1 | d1eova1 | d1iyjb4 | d1iyjb5 |
| d1iyjb3 | d1o7ia_ | d4ipca1 | d1x3ea_ | d4apva_ | d1jb7a1 | d1jb7a2 | d1jb7a3 |
| d3kdfa_ | d1ue1b_ | d1wjja1 | d3eiva_ | d1xjva2 | d1xjva1 | d2ccza2 | d1se8a_ |
| d1l1oc_ | d1jb7b_ | d1qvca_ | d3ulla_ | d1qzga_ | d1miua4 | d1miua5 | d1miua3 |
| d3g48a1 | d1gd7a_ | d1mkha_ | d1jjcb3 | d1pyba_ | d1y14b1 | d2nn6i1 | d1i94l_ |
| d2zjra2 | d1smxa_ | d2qamc2 | d1hh2p1 | d2r7da1 | d1rl2a2 | d1wfqa1 | d3bzka4 |
| d1x6oa2 | d3a0ja_ | d2j01d2 | d2ja9a1 | d4ql5a_ | d1hr0w_ | d1luza_ | d2n3sa_ |
| d2vnud1 | d1h95a_ | d1go3e1 | d2nn6h1 | d1ne3a_ | d2mo1a_ | d1iz6a2 | d1jjga_ |
| d1e3pa2 | d2ix0a3 | d2ix0a2 | d2lxja_ | d1ueba3 | d1ueba2 | d2z0sa1 | d1u0la1 |
| d2ix0a1 | d2eifa2 | d2je6i1 | d3i2za1 | d2c35b1 | d2ahob2 | d1t9ha1 | d3i4oa_ |
| d2qall1 | d1jt8a_ | d2nn6g1 | d1g6pa_ | d3cama_ | d1i94q_ | d1c9oa_ | d1d7qa_ |
| d2ba0a1 | d2vnud2 | d2vnud3 | d1khia2 | d2qalq1 | d1wi5a1 | d1xtda2 | d1ny4a_ |
| d1dgsa2 | d1ckma1 | d1p16a1 | d1x9na2 | d1pfsa_ | d2a1ka_ | d1je5a_ | d1ohea1 |
| d1ohea2 | d2f4da_ | d1rxda_ | d2hxpa_ | d1xria_ | d1fpza_ | d3ezza_ | d4y2ea_ |

d1i9sa_ d3azya_ d3wvja_ d1upsa1 d1ajoa_ d2hyka_ d1ajka_ d2vy0a_
d1o4ya_ d3o5sa_ d3d6ea_ d3atga_ d1dypa_ d1umza_ d3i4ia_ d1c1la_
d4lbqa_ d1a78a_ d1slta_ d4no4a_ d3zsja_ d3duia_ d1g86a_ d1is3a_
d1qmja_ d1hlca_ d1tdha3 d1q3ba3 d1k82a3 g1qmo.1 g1len.1 d3zyra_
d1fnya_ d1n47a_ g2ltn.1 d1avba_ d3usua_ d1dhkb_ d1f9ka_ d1ukga_
d1g8wa_ d1g9fa_ d1wbfa_ d3wcsa_ d1g7ya_ g1loe.1 d1mvqa_ d1hqla_
d1v6ia_ d1dbna_ d1qnwa_ d1fx5a_ d4l8qa_ d3n35a_ d1epwa1 d1eysc_
d2h0ba_ d2erfa_ d1h30a2 d3mw4a1 d1pz7a_ d2r1ba_ d3v64a1 d1d2sa_
d3bopa1 d3mw3a1 d1okqa1 d1okqa2 d1dszb_ d1a6ya_ d3cbba_ d1ynwa1
d1r4ia_ d1hcqa_ d2hana_ d2hanb_ d2enva1 d1kb4b_ d1lo1a_ d1cita_
d1adqa1 d1fnga1 d1uvqb1 d1zs8a1 d2vola1 d2vola2 d1mfbl2 d2jelh2
d1uwel2 d3qq4b1 d1kjvb_ d1k5na1 d1osph2 d4odhl2 d1ow0a1 d1ow0a2
d1bfob2 d3l9ra2 d3jtth1 d2fath2 d4elkb2 d1fn4b2 d2xfxb_ d1pg7x2
d1cqka_ d4k71b2 d1lk2a1 d1eapb2 d4lfhg2 d1i4fa1 d2nw2a2 d1kfah2
d1mhca1 d1h3ua1 d1wejh2 d1h0db2 d1a5fh2 d1kcvh2 d1mjuh2 d1qqda1
d1kcrh2 d1k8ia1 d3huja2 d2nnab1 d1nfdf2 d3mffb2 d1s9va1 d1lk3h2
d1q0xl2 d1fv1b1 d1neza1 d4rfeb2 d1r3ha1 d1a6tb2 d1kjva1 d1pz5b2
d1onqa1 d2bc4a2 d1de4a1 d1k8da1 d1tcra2 d1dn0b2 d1nfde2 d2xwtb2
d1bfoa2 d1mexh2 d1iakb1 d1ypzf2 d4f7ea2 d1q72l2 d2fbjh2 d1fe8h2
d1clzh2 d1fngb1 d1uvqa1 d1vgka1 d1c16a1 d4j12a2 d4x99b2 d1igtb3
d1je6a1 d1q72h2 d1nldh2 d1teth2 d2hmic2 d1i1ca2 d1i1ca1 d1k8ib1
d1q0yh2 d1kfal2 d3frua1 d1pg7w2 d1u58a1 d1igyb4 d1igyb3 d1hyrc1
d1klub1 d1deeb2 d1c12b2 d2bc4b2 d1kcvl2 d3wn5b1 d1lk2b_ d1lk3l2
d1mjul2 d1sm3h2 d1q0xh2 d1zvsa1 d1ydpa1 d1u8ib2 d1ypze2 d1kcuh2
d1op9a_ d1y0la1 d1etzb1 d1egjl1 d2gsib1 d1nj9a1 d4jfhd1 d1aqkl1
d1t2ja_ d1lo4h1 d1zvya1 d3v4vl1 d4enec1 d1kxtb_ d5e8ea1 d2pxya_
d4weud_ d1xiwc_ d2esvd1 d1qnzh_ d1ogae1 d1f4xh1 d4n1hb_ d3ezjb1
d3mlwl1 d1kfah1 d1etza1 d1b4jh1 d3cfic_ d4w68a_ d2arja1 d1ek3a_
d2ok0l1 d1vera_ d1uwgh1 d1blnb1 d1fpth1 d1rzga1 d2giya1 d4m3kb1
d1nc2a1 d2nyyd1 d1ncna_ d1fbih1 d5dxwa_ d4fqqa1 d1dqdh1 d1jfql1
d1kelh1 d1faih1 d1frgl1 d1figl1 d4m6nl1 d1fo0a_ d4hxbl1 d3b5ga_
d3quyd1 d2h9gb1 d1i8kb_ d2dd8h1 d2ck0h1 d1eaja_ d4w6wb1 d2a6ib3
d1t66d1 d2e27h_ d1ob1b1 d1f11b1 d2v7na1 d1ad0a1 d2xt1b1 d1pskh1
d4i13b1 d2op4l1 d1a0ql1 d1uwel1 d1za3a1 d1ncbl1 d4uu9a_ d1bm3h1
d1cloh1 d1nqba2 d1cf8h1 d4y5va_ d1mhph1 d5anma1 d4cdgc1 d4ldeb_
d1y0lb1 d2pcpb1 d1z3gl1 d1dfbl1 d1fh5h1 d3g5xa1 d1tzib1 d4dn3l1
d3zhka1 d2b1hh1 d2uyla1 d1f3dh1 d1nj9b1 d2agjh1 d2h1pl1 d1q72h1
d8fabb1 d1ndmb1 d1rjca1 d4u6hb1 d3upca_ d2fbjl1 d1osph1 d1tvda_
d2cjvh_ d4fhbd1 d1bd2d1 d1rz7h1 d3nn8a_ d1blna1 d1knoa1 d1nfda1
d2bmkb1 d1dqll_ d1igyb1 d1igfl1 d12e8h1 d3utza1 d1nc2b1 d1rihh1
d4mwfb1 d4jvpa1 d1mieh1 d1rhfa1 d1fgnl1 d1sbsh1 d1lp9e1 d1dn0a1
d4lqfl1 d1iaih1 d1kgcd1 d1mh5b1 d1gpol1 d2g60h3 d1f8tl1 d2jixd1
d4grwh_ d1fo0b_ d1bfob1 d1axsa1 d1u9ka_ d1nmbh_ d1h8na1 d1h8na2
d1jguh1 d4eura1 d3sdyl1 d35c8h1 d1w72l1 d1mvub_ d1i9rh1 d1ob1a1

d4wenb_ d1yeeh1 d3ietc1 d1f58h1 d1ad0b1 d1mqkl_ d1nbvh1 d4b50a_
d3r8bb_ d1bafl1 d1mamh1 d4rgne1 d2gsgb_ d35c8l1 d1bafh1 d1ai1h1
d2pcpa1 d2fath1 d2x1pa_ d1orsb1 d1ibgl1 d1shma_ d1a2yb_ d1u0qa_
d2coqa_ d1hilb1 d1qfwi_ d1jv5b_ d1fn4b1 d5e0qa_ d1nsnh1 d1plgl1
d4ocxl1 d1kb5h1 d1rzfh1 d1pg7x1 d2fx7h1 d2oi9c_ d1l7il1 d3eyva1
d1d5il1 d4ffyl_ d1tetl1 d1a6wh_ d1ac6a_ d3dvfa_ d1nfde1 d1mimh1
d1ri8a1 d1jhlh_ d3dura_ d1fskb1 d1f97a1 d3so3b1 d2ak4d1 d1lp9f1
d4z7wf1 d1l7th1 d4y5ya_ d4x7ec1 d1emth1 d1ikfl1 d1nlbh1 d4utal1
d1bjma1 d1eapb1 d1u6ah1 d2dtgd1 d1kj2b_ d1xeda1 d1c1eh1 d1fgvh_
d2bnub1 d2fb4h1 d2dtgb1 d1ktrh_ d1jpth1 d1qkzl1 d4hepg_ d2atpb1
d1flrh1 d2hrpl1 d1mvfa_ d1iaim1 d3k80a1 d2jb5h1 d3zkxc_ d4grwe_
d1it9h1 d1rhhb1 d3w9ec_ d1e4wh1 d1sy6h1 d2j6eh1 d3qxta_ d1jglh1
d1p7ka1 d1tzhb1 d4n8pa1 d1gafl1 d2jelh1 d1wioa2 d4i0cc1 d1cicb1
d1lmka2 d1lmka1 d1iqwh1 d1orqb1 d1ejoh1 d1jnlh1 d4zg1a_ d43c9b_
d1ggbl1 d1wejh1 d3q6fa1 d1f3dj1 d3c6la1 d1bqlh1 d3p9wb_ d1a3rl1
d1h0db1 d2uzil_ d1eo8l1 d1qfwl_ d1n7ml1 d1igja1 d1c5ch1 d2a9mh_
d1rmfh1 d1fj1b1 d4heme_ d1ccza1 d1a5fh1 d1ogad1 d1dlfh_ d1qa9b_
d1vgel1 d3qskb_ d1sjxa_ d4ozfg1 d1sq2n_ d1qd0a_ d1kcvh1 d1c5db1
d3qcul1 d3bn9d1 d1mjuh1 d4y8dc_ d7fabh1 d3dvgb1 d1lo0l1 d1a6tb1
d1gigh1 d1kcrh1 d2hwzl1 d1yjdc1 d3clfl1 d4krna_ d1dl7l_ d1e6ol1
d1mexh1 d3r0ma_ d4kkna_ d1fnsl1 d1oaqh_ d1rzia1 d1ucbh1 d1ncwl1
d4b41a_ d1nfdf1 d2mpah1 d2d7tl1 d4grwf_ d4b5ea_ d1lk3h1 d3u79a_
d4u3xa_ d1ghfl1 d1bvkb_ d1mcww1 d1b88a_ d1jrhh1 d32c2b1 d4wemb_
d1dsfh_ d1op3h1 d1ce1l1 d1iqda1 d1qoka1 d1qoka2 d1qfuh1 d2crya1
d1ay1h1 d1oaql_ d3ab0b_ d1rurl1 d1t4kb1 d1bz7b1 d1n0xh1 d1forl1
d1ucbl1 d4w6yb_ d43c9a_ d2aabl1 d1tzia1 d4n1ca_ d1k6qh1 d3g9ab_
d4tvsa1 d1igmh_ d1pz5b1 d3dx9a1 d3wxwh_ d1nakh1 d1kcul1 d3eaka_
d4qlra1 d1cr9l1 d1d9ka_ d1f4xl1 d1deea1 d2i27n2 d1c12a1 d2orbl1
d1j8hd1 d1mcph1 d1c5da1 d1kenh1 d1bd2e1 d1h5ba_ d4x7fc1 d3upaa_
d1t3fa1 d1indh1 d2dtgc1 d4hc1l1 d1zv5a1 d4wgvb_ d1uwgl1 d4f37k1
d1aqkh1 d1vesa_ d3zkqd_ d1fptl1 d1lo4l1 d1knob1 d3rrqa_ d1ct8b1
d4orzc_ d1mlbb1 d4hgkc1 d1dqdl1 d1bogb1 d4ozhe1 d1dr9a1 d1i7za1
d1dn0b1 d1kell1 d1rzib1 d1ypzf1 d1wtla_ d1hkfa_ d1fail1 d1figh1
d2vyre_ d1sjva_ d1ohqa_ d1bfoa1 d1pkqb1 d1mjul1 d1b4jl1 d1nsnl1
d2arjb1 d1p4bh_ d4aj0a_ d3nn8b_ d2rcja1 d1f2xk_ d4v1dd_ d1adqh1
d2eksa_ d4k3ga_ d1jfqh1 d1uweh1 d1frgh1 d4gftb_ d1uz8b1 d4n1ea_
d1bm3l1 d1clol1 d3mcka1 d2vq1a1 d1ae6h1 d4aq1b1 d1uj3b1 d1nbqa1
d1fh5l1 d2ck0l1 d2ywza_ d1q72l1 d4nbxb_ d1hila1 d4hzlb1 d2j88l1
d1zmya1 d1cd0a_ d1a4ka1 d1pskl1 d1a0qh1 d4krmb_ d1ncbh1 d2fbjh1
d1j8he1 d1i3ua_ d1cf8l1 d1fe8h1 d2p1ya1 d1eeqa_ d2x89a_ d1ol0a_
d2r0lh1 d1ub5a1 d1dfbh1 d3eyvb1 d4jdvb1 d4p2cg_ d2gsia1 d2agjl1
d1u3ha1 d2h1ph1 d1rihl1 d1fskc1 d1boga1 d2a77l1 d1ospl1 d1qfoa_
d3dwta_ d2fx7l1 d1rz7l1 d3stba1 d1pkqa1 d1b6da1 d1dzba2 d1dzba1
d1kj2a_ d4l1ha_ d1rzgb1 d1dqlh_ d12e8l1 d1miel1 d1jmaa_ d1sbsl1

d1fgnh1 d1f58l1 d1mqkh_ d1ndgb1 d3qegl1 d1iail1 d2aw2a1 d1tqbc1
d3fo2a1 d4dkaa_ d1f8th1 d2g60l1 d2ywya_ d1uj3a1 d1yc7a_ d3bd3a_
d4w6xb1 d1nmbl_ d1i8ka_ d1rjlb1 d1ypze1 d1p7kb1 d1kxqe_ d1gpoh1
d2z4qa1 d1w72h1 d1i9rl1 d4qgya1 d4xt1c_ d2qqnh1 d1cica1 d3c08h1
d2cd0a_ d2bdnh1 d1hnga1 d1maml1 d1nldh1 d1cr9h1 d4lcih1 d3bkyh1
d3qg7l1 d1ai1l1 d1kxvc_ d4iosd_ d1l7ih1 d2x1qa_ d1ub6b1 d3k74b_
d1ibgh1 d1teth1 d1igya1 d1hxma1 d2x6ma_ d2e7la_ d5e03a_ d1qfwm_
d2r0kh1 d1igjb1 d1rurh1 d1plgh1 d1mj8h1 d1yuhb1 d3fmgl1 d1rzfl1
d1emtl1 d1ad9b1 d1igtb1 d1nlbl1 d1d5ih1 d3k7ua_ d4ffyh_ d1bwwa_
d3dvga1 d1a6ta1 d1fgvl_ d4qxgl1 d1miml1 d1jhll_ d2hffb1 d2hh0l1
d1qkzh1 d1l7tl1 d1afvh1 d1iaii1 d2cjuh_ d1um5h1 d2ghwb2 d2ghwb1
d1kgce1 d2fgwh1 d1ikfh1 d1dvfd_ d2i24n1 d2xtjb_ d1za3b1 d2yc1a_
d2hh0h1 d2x1oa_ d1bvka_ d1rjla1 d5e7ba1 d1igta1 d1il1b1 d2fb4l1
d1mvua_ d1jptl1 d32c2a1 d1fvca_ d1iqdb1 d2hrph1 d2p4ab_ d4qo1a_
d1p2cb1 d1wt5c_ d1t4ka1 d5c1mb_ d1it9l1 d1bz7a1 d1b2wh1 d1e4wl1
d1jnll1 d1yedb1 d1za6b1 d3u7aa_ d1igfh1 d3gnml1 d1orsa1 d1wejl1
d1a2ya_ d1hxmb1 d1gafh1 d2j4wh1 d1pz5a1 d1j05a_ d1a3rh1 d4fqll1
d1qfwh_ d1eo8h1 d1n7mh1 d4s11a_ d1kegh1 d5da4a_ d1iqwl1 d3whxb1
d1vgeh1 d1deeb1 d1c12b1 d1t2ql1 d1ad9a1 d3gsnb1 d3b2uc1 d1ggbh1
d1t3fb1 d1nakl1 d4ocmc_ d1lila1 d2v7hb3 d2esve1 d1mcoh1 d2qr0b1
d2q3aa_ d1ct8a1 d1kcrl1 d2pxyb_ d2uzih_ d5c3ld_ d3durb_ d1c5cl1
d2ak4e1 d1dl7h_ d1xiwd_ d1mlba1 d1rmfl1 d1i7zb1 d1dlfl_ d2xa3a_
d5bq7b1 d1dqqb1 d3h42l1 d1egjh1 d1kcvl1 d1fe8l1 d2j6el1 d1f3rb2
d1lk3l1 d1sm3h1 d1q0xh1 d1ghfh1 d7fabl1 d1igch1 d1lo0h1 d4idla_
d5c2bl_ d1jrhl1 d1il1a1 d3zhda1 d4kfzc_ d1b0wa_ d1e6oh1 d1mexl1
d4krpb_ d1fnsh1 d1ncwh1 d4hgma_ d4eigb1 d4rzca_ d2mpal1 d4y5xa_
d5en2b1 d3lrga1 d4ej1c1 d1rhha1 d1j05b_ d1forh1 d1q9wb1 d2wzpd_
d2aq2a1 d1h0da1 d3revb1 d2ij0c1 d1tzha1 d4m7jl1 d1k6ql1 d3b9kc1
d1igml_ d3qeua1 d1dsfl_ d1ce1h1 d1kcuh1 d1zvha1 d1zvoa1 d1tqbb1
d1ay1l1 d2qscl1 d2cgrh1 d1nl0h1 d1kenl1 d3eo0a1 d4n9ob1 d1n0xl1
d3k1kc_ d2cqva1 d1fhga_ d2k1ma_ d2dl2a1 d1biha3 d1biha1 d1biha4
d3qp3a1 d1g1ca_ d1gxea_ d1cvsc2 d1cvsc1 d1xmwa2 d1m4ka2 d2otpa2
d1biha2 d1rhfa2 d2fcba2 d2fcba1 d1wwbx_ d1gsma2 d1epfa1 d1epfa2
d2dava1 d1waaa_ d1f97a2 d1x44a1 d1vcaa2 d1fnla2 d1fnla1 d3b5ha2
d3h8na2 d3h8na1 d1jbja1 d1jbja2 d1pd6a_ d1f2qa2 d1f2qa1 d3cx2a_
d1nbqa2 d3b5ha1 d1n26a1 d1xiwb_ d1xaua_ d1fltx_ d2ifga1 d1olla1
d1olla2 d1cs6a4 d1cs6a3 d1cs6a2 d1cs6a1 d2gw5a2 d1sy6a1 d1xiwa_
d1ucta2 d1ucta1 d1ry7b2 d1qz1a3 d1olza1 d2c9aa1 d1wwca_ d2aq2b1
d1ck1a1 d1enfa1 d1i4pa1 d1v1oa1 d1et9a1 d1esfa1 d2g9hd1 d4ohja1
d1klud1 d1bxta1 d1eu3a1 d1ty0a1 d1fnua1 d1c4qa_ d1prtc1 d2wv6d_
d1ltrd_ d4l63b1 d1r4pb_ d1prtd_ d1tl0f_ d2bosa_ d1prtb1 d3chbd_

**Family-7 (a.25.1.1)**

**Positive dataset contains 30 protein sequences.**

d2fkza_ d1zuja1 d1s3qa1 d2fjca1 d1z6om1 d1veia_ d4dyya_ d3gvya_

d2pyba_ d4cy9a1 d3bvfa1 d1r03a_ d2cf7a_ d1zs3a1 d4iwka_ d3ka8a_ d3np0x_ d4e6ka_ d1z6oa1 d2fzfa1 d4dyua_ d3ge4a_ d4am4a_ d2z6ma_ d1vlga_ d3qz3a_ d2ffxj_ d1yv1a1 d2v2pa_ d4di0a_

**Negative dataset contains 4071 protein sequences.**

d1m32a_ d1ibja_ d1c7na_ d4q75a_ d2bkwa1 d1t3ia_ d1n8pa_ d1qz9a_ d1jf9a_ d1eg5a_ d1p3wa_ d3kgwa_ d1cl1a_ d2ctza1 d1vjoa_ d2rfva_ d1gc0a_ d1cs1a_ d1d2fa_ d2ch1a1 d1qgna_ d1pffa_ d1elua_ d1u08a_ d1lc5a_ d1b5pa_ d2ay1a_ d1bw0a_ d3pd6a_ d7aata_ d2csta_ d1j32a_ d1fg7a_ d4wlha_ d1o4sa_ d1v2da_ d1gdea_ d1m6sa_ d1v72a1 d1svva_ d1ajsa_ d1wsta1 d2f8ja2 d1lk9a_ d1vp4a1 d1xi9a1 d2gb3a1 d1xkla_ d2wfla_ d3i2ka1 d1lnsa3 d1mpxa2 d2cfba1 d1dfoa_ d1o69a_ d1bj4a_ d3k28a_ d2bwpa_ d3lg0a_ d2fnua_ d3ou5a_ d1z7da1 d1b9ha_ d1iaya_ d4msoa_ d1bs0a_ d1s0aa_ d1sffa_ d3tqxa_ d1mdoa_ d3qboa_ d3pgya_ d1bjna_ d2dkja_ d1ejia_ d1vefa1 d4wxga_ d3bs8a_ d3gbxa1 d1w23a_ d1ohfc_ d1sefa_ d1sfna_ d1sq4a_ d2tbvc_ d1c8nc_ d1x36a_ d1ng0c_ d4sbvc_ d1opoa_ d1f2nc_ d3lgia_ d3i77a_ d2z9ia2 d4nsva_ d1lcya2 d4jcna_ d1agja_ d1os8a_ d1hpga_ d1p3ca_ d1l1ja_ d1qtfa_ d1ddla_ d1e57b_ d1auyb_ d1qnia1 d1fwxa1 d1js9c_ d1f15b_ d1za7a_ d1laja_ d3ehbb1 d1fftb1 d1gyca3 d1hfua2 d1kbva1 d1kbva2 d1sdda1 d1sdda2 d1gyca1 d1gyca2 d1mzya1 d2bw4a1 d1aoza1 d1aoza2 d1kyaa2 d1kyaa3 d1kyaa1 d1mzya2 d4e9sa1 d1sddb1 d1sddb2 d1hfua1 d1hfua3 d1oe1a2 d1oe1a1 d1v10a2 d1v10a3 d1v10a1 g1f8v.1 d1novc_ d2bbvc_ g1or0.1 g1cp9.1 g1e3a.1 d1a3qa1 d1iknc_ d3brda1 d1oy3c_ d1imhc1 d1gjia1 d1zk9a1 d2cxka1 d2bema_ d1pama1 d1ea9c1 d1qhoa1 d2fhfa2 d2fhfa1 d1k3ia1 d1j0ha1 d1cxla1 d2bhua1 d1edqa1 d1f1sa2 d1ut9a2 d1gvia1 d1txka1 d1adqa1 d1fnga1 d1uvqb1 d1zs8a1 d2vola1 d2vola2 d1mfbl2 d2jelh2 d1uwel2 d3qq4b1 d1kjvb_ d1k5na1 d1osph2 d4odhl2 d1ow0a1 d1ow0a2 d1bfob2 d3l9ra2 d3jtth1 d2fath2 d4elkb2 d1fn4b2 d2xfxb_ d1pg7x2 d1cqka_ d4k71b2 d1lk2a1 d1eapb2 d4lfhg2 d1i4fa1 d2nw2a2 d1kfah2 d1mhca1 d1h3ua1 d1wejh2 d1h0db2 d1a5fh2 d1kcvh2 d1mjuh2 d1qqda1 d1kcrh2 d1k8ia1 d3huja2 d2nnab1 d1nfdf2 d3mffb2 d1s9va1 d1lk3h2 d1q0xl2 d1fv1b1 d1neza1 d4rfeb2 d1r3ha1 d1a6tb2 d1kjva1 d1pz5b2 d1onqa1 d2bc4a2 d1de4a1 d1k8da1 d1tcra2 d1dn0b2 d1nfde2 d2xwtb2 d1bfoa2 d1mexh2 d1iakb1 d1ypzf2 d4f7ea2 d1q72l2 d2fbjh2 d1fe8h2 d1clzh2 d1fngb1 d1uvqa1 d1vgka1 d1c16a1 d4j12a2 d4x99b2 d1igtb3 d1je6a1 d1q72h2 d1nldh2 d1teth2 d2hmic2 d1i1ca2 d1i1ca1 d1k8ib1 d1q0yh2 d1kfal2 d3frua1 d1pg7w2 d1u58a1 d1igyb4 d1igyb3 d1hyrc1 d1klub1 d1deeb2 d1c12b2 d2bc4b2 d2ig2h2 d1kcvl2 d3wn5b1 d1lk2b_ d1lk3l2 d1mjul2 d1sm3h2 d1q0xh2 d1zvsa1 d1ydpa1 d1u8ib2 d1ypze2 d1kcuh2 d2phla2 d2phla1 d1fxza2 d1fxza1 d1lrha_ d1uija1 g1dgw.1 d2v09a_ d1uika2 d1uika1 d1dgwa_ d1ep0a_ d2pa7a1 d1wlta1 d1nxma_ d2c0za1 d1ds6b_ d1fstb_ d1ex0a1 d1g0da1 d1ju8a_ d1tafa_ d1bh9b_ d1tafb_ d1q9ca_ d1h3ob_ d2byka1 d1n1ja_ d4g92b_ d1bh9a_ d1jfia_ d1h3oa_ d2bykb1 d1n1jb_ d1eixa_ d3exra_ d2ffca1 d2fdsa1

d3n3ma1 d2czda_ d1q6oa_ d2yyua_ d1vqta1 d1dbta_ d3ldva1 d1k9ua_
d2opoa_ d1r0ka3 d1j5pa3 d1vkoa2 d1vm6a2 d3qvsa2 d1p1ja2 d3gpdg2
d2q49a2 d1hdgo2 d2g17a2 d2cvoa2 d2czca1 d3pwka2 d1obfo2 d1i32a2
d1zyao2 d2pkqo2 d1b7go2 d2hjsa2 d1cf2o2 d1vkna2 d1nvmb2 d1ux8a_
d1dlya_ d4i0va_ d2bkma_ d1dlwa_ d2bmma_ d2msya_ d2djoa1 d2r5za_
d1au7a1 d1f43a_ d2lmda1 d1akhb_ d1s7ea1 d3nara_ d1zq3p1 d2craa1
d1mija_ d4uuta_ d3a01a_ d1x58a1 d3rkqa1 d3cmya1 d2cqxa1 d2da6a1
d1e3oc1 d1fjla_ d1mh3a1 d1ig7a_ d1x41a1 d1wh7a1 d1gt0c1 d2mw8a_
d1lfup_ d1x2ma1 d1b72a_ d1wi3a1 d1jgga_ d1wjha1 d1x2na1 d1mnmc_
d2cuea1 d3k2aa_ d2hosa_ d2jwta2 d1pufa_ d1octc1 d2hi3a_ d1b8ia_
d4j19a_ d3lnqa_ d1wh5a1 d1b8ib_ d1fexa_ d2cqra1 d1guua_ d1ug2a1
d2cqqa1 d2ajea1 d4a69c_ d2crga1 d2iw5b1 d1xc5a1 d1ofcx1 d2cu7a1
d1wgxa1 d2cjja1 d2ckxa1 d1gvda_ d6paxa1 d6paxa2 d1k78a2 d1hlva1
d1hlva2 d1iufa1 d2iu5a1 d1zk8a1 d2g7sa1 d2fd5a1 d2np5a1 d1z0xa1
d2id6a1 d2d6ya1 d2i10a1 d1vi0a1 d2oi8a1 d3c07a1 d2ns7a1 d2fq4a1
d2yvea1 d1t33a1 d2o7ta1 d1rkta1 d2np3b1 d2g7ga1 d2gena1 d2fbqa1
d2g7la1 d2gfna1 d2g3ba1 d2jj7a1 d2hyja1 d1sgma1 d1ui5a1 d2hkua1
d2id3a1 d4jyka1 d1bl0a2 d1d5ya1 d1d5ya2 d1prtc2 d3lv8a_ d1s3ga1
d1zaka1 d1s4qa_ d3tvta2 d1uf9a_ d1khta_ d1ckea_ d1osna_ d1p5zb_
d4tmka_ d3cm0a_ d1tmka_ d1jaga_ d3h86a_ d1t0hb_ d1nn5a_ d2h92a_
d3kb2a1 d2c95a1 d1yj5a2 d1kgda_ d1ly1a_ d2ak3a1 d1vyva2 d1deka_
d2grja1 d1p3ja1 d1y63a_ d1e4ya1 d1s96a_ d3iija1 d1lvga_ d1nksa_
d1vyua2 d4e22a_ d1qf9a_ d1jjva_ d1q3ta_ d1ki9a_ d1lw7a2 d1teva_
d1kjwa2 d1gsia_ d2iyva2 d1kaga_ d1viaa_ d1g3ma_ d1j99a_ d1t8ta1
d1nsta_ d1fmja_ d1zrha_ d1ls6a_ d2a3ra_ d1aqua_ d2zptx_ d3ckla_
d1texa_ d1q44a_ d2gwha_ d1z0ja1 d3umra2 d3gj0a1 d2gaoa1 d1yzga1
d1x3sa1 d1g7sa4 d1puja_ d1udxa2 d1kk1a3 d2o52a_ d1jwyb_ d1zcaa2
d1zcba2 d2fv8a1 d2cjwa_ d3dz8a1 d1xzpa2 d1vg8a_ d4qxaa_ d2ew1a1
d1x1ra1 d2bcgy_ d3doea1 d3cona_ d3rega_ d3wyga_ d1upta_ d2b6ha1
d1zd9a1 d1lnza2 d1r5ba3 d1n0ua2 d1r2qa_ d2f9la1 d2rhda_ d3q3jb_
d3q85a_ d1efma1 d1r8qa_ d2gf9a1 d2akab1 d1ek0a_ d1z08a1 d2gcoa1
d2dy1a2 d4g01b_ d2cxxa1 d1svia_ d3q72a_ d1ni3a1 d4ac9a4 d2erxa1
d2bmea_ d1wxqa1 d2gf0a1 d4ldja1 d1zunb3 d1d5ca_ d4lv5b_ d1efca3
d1kmqa_ d2g6ba1 d3lrpa_ d1tada2 d2y8ea_ d4rd4a1 d1z06a1 d2heia_
d3a58b_ d1u0la2 d1d2ea3 d1zj6a1 d2ocba_ d3rwoa_ d1mr3f_ d2e9sa1
d4u5xa1 d2iwra_ d2fn4a1 d3bwdd_ d2nzja1 d2atva1 d2ksqa_ d3l43a_
d1mkya2 d1mkya1 d1wmsa_ d1jnya3 d2rmka_ d1f6ba_ d1f60a3 d1gota2
d1jala1 d2fh5b1 d1g16a_ d2wblc_ d1m7ba_ d1wf3a1 d1f5na2 d1t9ha2
d1z6xa1 d1z0aa1 d2fg5a1 d2a5ja1 d2bcjq2 d1s0ua3 d1ky3a_ d4y0va_
d4lhwa_ d2qmea1 d2atxa1 d1egaa1 d1ryha_ d1moza_ d1nrjb_ d2erya1
d3bfka_ d1puia_ d1fzqa_ d3j3az1 d3t5ga_ d1shza2 d3defa_ d2f7sa1
d3raba_ d3tsoa_ d3aq4a_ d2al7a1 d3sfva1 d3bc1a_ d2ncda_ d1sdma_
d3h4sa1 d2heha_ d2grya_ g3kin.1 d1kk8a2 d1br2a2 d1goja_ d1f9va_
d2mysa2 d3t0qa_ d4db1a1 d3gbja_ d1ry6a_ d1v8ka_ d1vava_ d1uaia_

d1k8ka2 d1k8ka1 d3uleb1 d3i33a2 d3i33a1 d1dkgd2 d1dkgd1 d4ci6a2
d1jcfa1 d4a2ba2 d2fxua1 d1yaga2 d1yaga1 d4cbua2 d1bupa2 d3vrda1
d3vrda2 d1fcdc2 d1fcdc1 d1h1oa2 d1m70a2 d1m70a1 d1iqca2 d1iqca1
d1eb7a2 d1nmla2 d1nmla1 d1hroa_ d4gedb_ d3zooa_ d1mz4a_ d1gu2a_
d1f1fa_ d1dvva_ d1c6ra_ d1c75a_ d1e29a_ d1gdva_ d1cnoa_ d1h32b_
d1co6a_ d2bh4x_ d2aiua_ d5cytr_ d1jdla_ d1i8oa_ d2zboa_ d3dmia_
d1ls9a_ d1wvec_ d2yk3a_ d3ph2b_ d1dw0a_ d5aura_ d1ql3a_ d1qn2a_
d1f1ca_ d2nqya_ d1h4ga_ d1hixa_ d1oa2a_ d1olra_ d3b7ma_ d4h7ma1
d3wp3a_ d2vgda_ d1igoa_ d3zsea_ d2nlra_ d3wx5a_ d1oa3a_ d1pvxa_
d1f5ja_ d1oa4a_ d1ks5a_ d4ixla1 d2a6za1 d3a4ua_ d2a6va1 d3sita1
d2ah2a1 d1n1ta1 d1evsa_ d1cnt1_ d1pvhb_ d1z7ca_ d1huwa_ d1bgea_
d1rhga_ d1f6fa_ d1f45b_ d1i1rb_ d1axia_ d1etea_ d3tgxb_ d1scfa_
d2z3qa1 d3qb1a_ d1hula_ d1m48a_ d4nkqb_ d1eera_ d1v7mv_ d5e4ea_
d2o27a_ d3b5ka_ d2psma1 d4zf7a_ d3uf5a_ d1v8fa_ d2ejca_ d3muea1
d1ihoa_ d3q12a1 d3rbaa_ d2qtra_ d1nupa_ d3l92a_ d1f9aa_ d1qjca_
d2h29a_ d1vlha_ d1od6a_ d1lw7a1 d1mrza2 d4ruka_ d3wdpp1 d3wq5a_
d1gnxa_ d4gxpa_ d4hz8a_ d1e4mm_ d3aiua_ d1wcga1 d3ahxa_ d1v02a_
d2dgaa_ d1pbga_ d1vffa1 d1qoxa_ d4ha4a_ d2el7a_ d1iq0a2 d1irxa2
d1r6ta2 d1ivsa4 d2cyba_ d1rqga2 d1j09a2 d2yxna_ d1f7ua2 d1h3fa1
d1ffya3 d2g36a1 d1nzja_ d1jila_ d1n3la_ d1i6la_ d1pfva2 d1h3na3
d3n9ia_ d3sz3a1 d3sw2a_ d1moxc_ d1dx5i3 d1edmb_ d1autl2 d1autl1
d1nqlb_ d1dval1 d1szba2 d1kigl_ d2y5fl_ d1ijqa2 d1gk5a_ d1g1sa2
d1nt0a3 d1xdtr_ d1lmja2 d1lmja1 d1nzia2 d2wphe_ d1klil_ d1pfxl2
d1pfxl1 d1p9ja_ d1g1ta2 d1f7ea_ d4yzub_ d1nuba2 d1m1xb4 d1f56a_
d1bypa_ d1azca_ d1ws8a_ d3u25a_ d1cuoa_ d1id2a_ d1rkra_ d2iaac_
d4dp1x_ d1nwpa_ d2gima_ d2i7sa_ d1qhqa_ d2y5ia_ d1qlsa_ d1psra_
d3n22a_ d2egda_ d4ggfc_ d2wcea_ d2luca_ d4aqia_ d3nsia_ d2lp3a_
d1j55a_ d1a4pa_ d1k8ua_ d1nsha_ d1pvaa_ d3fs7a_ d2pvba_ d1ttxa_
d1rwya_ d2kyfa_ d1rk9a_ d1exra_ d1tiza1 d1nyaa_ d1g8ia_ d1ggwa_
d1wdcc_ d3fwba_ d1ggza_ d2o5ga_ d1ctaa_ d1m45a_ d3i5gc_ d1sl8a_
d1fpwa_ d2scpa_ d2roaa_ d4aqra_ d3pn7b_ d1br1b_ d1s6ca_ d1bjfa_
d2obha_ d1jfka_ d1jf0a_ d1qv0a_ d1lkja_ d2mlfc_ d2m97a_ d2d8na_
d4by5a_ d1jbaa_ d4nqga_ d1wdcb_ d2mysc_ d1j7qa_ d1s6ia_ d1uhka2
d1r2ua_ d1dtla_ d3kf9a_ d3pn7c_ d5d67d_ d1tuza_ d1h8ba_ d1eg3a1
d1eg3a2 d2zkmx1 d1snla_ d1gjya_ d1y1xa_ d1hqva_ d1dvia_ d1df0a1
d1qxpa2 d4usla_ d1k94a_ d3h33a_ d1rwja_ d19hca_ d1os6a_ d3bxua_
d3h4na_ d1i77a_ d3caoa_ d1j0pa_ d2ctha_ d1duwa_ d1ofwa_ d2e84a_
d1hh5a_ d3h34a_ d1wkya2 d1uuqa_ d3wh9a_ d4m1ra_ d1xyza_ d4xuya_
d2je8a5 d4bf7a1 d3cuia_ d1ur4a_ d1qw9a2 d1odza_ d2vzsa5 d1bqca_
d2x2ya1 d1hjqa_ d1qnra_ d4y7ea1 d1vjza_ d4k68a_ d2v3ga_ d4hpga_
d1nofa2 d1nq6a_ d1h4pa_ d1aq0a_ d3ziza_ d4bs0a_ d1ecea_ d1h1na_
d1ta3b_ d1w91a2 d1i1wa_ d1uhva2 d1us3a2 d1egza_ d1tvna1 d3nyda1
d1ghsa_ d2cyga1 d4xv0a_ d2c8na2 d1w32a_ d3emca_ d1v6ya1 d1foba_
d2uwfa1 d2fgla_ d3niya1 d2c0ha1 d1rh9a1 d1yq2a5 d1szna2 d1fa2a_

d1pama4 d5do8a1 d1ea9c3 d1hx0a2 d2fhfa5 d1qhoa4 d1avaa2 d1m53a2
d4m8ua1 d2gjpa2 d1lwha2 d4howa1 d1ktba2 d1j0ha3 d3vm5a1 d2x1ia_
d1wzaa2 d1uasa2 d2diea1 d1mxga2 d1ht6a2 d1eswa_ d1iv8a2 d5cpsa_
d1ud2a2 d1zjaa1 d3gbea1 d1kwga2 d1wdpa_ d1gcya2 d1g5aa2 d1hvxa2
d3dhpa2 d2bhua3 d4do4a1 d3qgva1 d1e43a2 d1g94a2 d2d3na2 d1gjwa2
d1gvia3 d1x1na1 d1r7aa2 d1tz7a1 d4toqa_ d1wb0a1 d1w9pa1 d1eoka_
d1itxa1 d1kfwa1 d1goia2 d1edqa2 d2gsja_ d1hjxa1 d1jnda1 d1vf8a1
d1umqa_ d1g2ha_ d1mixa2 d1e5wa2 d1j19a2 d1isna2 d2al6b2 d3syxa1
d1mkea1 d2p8va_ d1xoda1 d1i7aa_ d1qc6a_ d1egxa_ d1xl4a1 d1n9pa_
d3vsqa_ d2gixa1 d1b9ra_ d1offa_ d1frra_ d3wcqa_ d1e9ma_ d1l5pa_
d1wria_ d1i7ha_ d3av8a_ d1czpa_ d1rfka_ d3p1ma1 d3b2ga_ d2bt6a2
d1wgqa1 d1v89a1 d2coda1 d1zc3b_ d1wg7a1 d1u5ea1 d1v61a1 d2coca1
d2ys1a1 d1v5pa1 d1ntya2 d1wjma1 d1wi1a1 d1u29a_ d1xx0a1 d1unqa_
d2zkmx3 d1fhoa_ d1v88a1 d2adza1 d1x1fa1 d1p6sa_ d2coaa1 d1btka_
d1v5ma1 d1faoa_ d2x18a_ d1dyna_ d1x1ga1 d4kaxb_ d2j59m1 d1ki1b2
d1xcga2 d1txda2 d1u5da1 d1dbha2 d1w1ha_ d1upqa_ d1v5ua1 d1foea2
d2fjla1 d2dfka2 d2cofa1 d1kz7a2 d1h6ua1 d9ldta1 d2ewda3 d1up7a1
d2dfda1 d1s6ya1 d1vjta1 d7mdha1 d1hyea1 d1t2da1 d1ojsa3 d1b8pa1
d1ldna1 d1hyha1 d1civa1 d2a92a1 d1ez4a1 d1i0za1 d1y6ja1 d1gv0a1
d1llda1 d1obba1 d1v8ba1 d1gdha1 d1l7da1 d2dlda1 d1mx3a1 d1ygya1
d1li4a1 d1j4aa1 d2naca1 d1npya1 d3t4ea2 d1a4ia1 d1o0sa1 d2hk9a2
d2egga2 d1b26a1 d1bvua1 d1gq2a1 d1c1da1 d1leha1 d1gtma1 d1euza1
d1nvta1 d3dona2 d1edza1 d1gpja2 d1vi2a1 d1p77a1 d1nyta1 d1b0aa1
d1vl6a1 d1v9la1 d1txga2 d3f47a1 d1mv8a2 d2pv7a2 d1xc2a3 d2ahra2
d4xiya1 d2f1ka2 d1pgja2 d1i36a2 d1np3a2 d1lj8a4 d1teaa3 d1qmga2
d2i76a2 d2g5ca2 d1wdka3 d1dlja2 d3cuma3 d1kola2 d1f8fa2 d1qora2
d1jqba2 d1vj0a2 d1cdoa2 d1gu7a2 d1d1ta2 d1xa0a2 d2dm6a2 d1jvba2
d1rjwa2 d1llua2 d1h2ba2 d1tt7a2 d1piwa2 d1o89a2 d1iz0a2 d1e3ja2
d1yb5a2 d1uufa2 d1vj1a2 d1e3ia2 d3gpdg1 d1vc2a1 d2nvwa1 d1u8fo1
d1ydwa1 d1hdgo1 d2g17a1 d1cf2o1 d2czca2 d1r0ka2 d1obfo1 d2cvoa1
d1ebfa1 d1h6da1 d1i32a1 d1zh8a1 d2q49a1 d1qkia1 d1j5pa4 d3pzra1
d1xeaa1 d1vkoa1 d1vm6a3 d3qvsa1 d2pkqo1 d1b7go1 d1gr0a1 d1p1ja1
d1lc0a1 d1j0xo1 d3pwka1 d2hjsa1 d1zyao1 d1vkna1 d1tlta1 d1nvmb1
d3sjua_ d3ojfa1 d1xu9a1 d2jjya1 d1n7ha_ d1y1pa1 d1h5qa_ d1jtva_
d4fs3a_ d2bkaa1 d1gy8a_ d2c5aa1 d1ulsa_ d1ooea_ d2b69a1 d1y5ma1
d1spxa_ d1ae1a_ d1bxka_ d1ydea1 d1gega_ d1hdoa_ d1o5ia_ d1e6ua_
d1snya1 d2rhca_ d3zv4a_ d5ceja_ d2d1ya1 d3w8ea_ d2fmua1 d1x1ta_
d1rpna_ d3ay7a1 d1k2wa_ d1eq2a_ d2hrba1 d3ruaa_ d2ew8a1 d4hwka_
d1xgka_ d2bd0a1 d1yo6a1 d2a35a1 d2ntva_ d1vl8a_ d1hdca_ d4bkua_
d2ag5a1 d2q45a_ d1nffa_ d4iy1a_ d1zbqa1 d1kewa_ d1ek6a_ d1ja9a_
d2q46a2 d1iy8a_ d1cyda_ d2ptga_ d1hxha_ d1uzma1 d3lyla_ d4m89a_
d3wyea_ d2bl1a1 d1w6ua_ d1sb8a_ d2q2va_ d4nr0a_ d1e6wa_ d2gasa_
d3oiga1 d2bgka1 d1mxha_ d1vl0a1 d1rkxa_ d1r6da_ d1wmaa1 d1e7wa_
d2c07a1 d1xkqa_ d3tzha_ d1g0oa_ d3lz6a_ d1qyca_ d1edoa_ d1gz6a_

d1oc2a_ d1t2aa_ d1zema1 d2xoxa_ d1z45a2 d1geea_ d1p33a_ d3lt0a_
d4e6pa_ d2wyua_ d1db3a_ d4q9na_ d1xhla_ d4o1ma_ d1fmca_ d2uvda_
d2gdza1 d1uaya_ d2z1ma_ d2o23a2 d2fr1a2 d2fr1a1 d1qsga_ d1qyda_
d4zjua_ d2a4ka1 d1yb1a_ d1wvga1 d1yxma1 d3f4ba_ d1mg5a_ d1xg5a1
d1r55a1 d3gboa_ d1atla_ d1kufa_ d1buda_ d4q1la_ d1yp1a_ d1wnia_
d4ekfa_ d2ckga_ d1th0a_ d2bkra_ d1ex0a4 d1g0da4 d2f4ma1 d1x3za_
d3u1ra1 d1g9ka2 d1kapp2 d1t3ca_ d1epwa3 d2a97a_ d3fiea_ g1e1h.1
d3ioqa_ d1fh0a_ d1o0ea_ d1khqa_ d1m6da_ d2cb5a_ d1cs8a_ g8pch.1
d1s4va_ d2pnsa_ d1vsna1 d1mira_ d1iwda_ d2oula_ d7pcka_ d3bwka_
d3bcna_ d2p86a_ d1pvja_ d1pcia_ d1gece_ d1deua_ d4bs6a_ d1xkga1
d1cqda_ d1jqpa2 d2c0ya_ d2bdza_ d3p5ua_ d2o36a_ d1k9xa_ d2jk6a2
d2jk6a1 d1feca1 d1feca2 d1lpfa1 d1lpfa2 d1xhca1 d1xhca2 d1dxla2
d1dxla1 d1gera2 d1m6ia2 d1onfa1 d3lada2 d3lada1 d1w4xa1 d1w4xa2
d1gesa2 d1mo9a1 d1xdia1 d1fl2a2 d1fl2a1 d1fcda1 d1fcda2 d1h6va2
d1h6va1 d1mo9a2 d1gv4a2 d1gv4a1 d2gv8a2 d2gv8a1 d1aoga1 d1aoga2
d1hyua1 d1ebda1 d1ebda2 d3dk9a1 d1q1ra2 d1q1ra1 d1gesa1 d1zbfa1
d2z1ha_ d2etja1 d3qioa1 d1jl1a_ d2e4la_ d1jl2a_ d2lsna1 d1io2a_
d1uaxa_ d2yv0x_ d1mu2a1 d1ekea_ d1i39a_ d1ps9a2 d1lqta1 d1cjca1
d1pn0a1 d1reoa1 d1ju2a1 d2f5va1 d3c96a1 d2gmha1 d1pj5a2 d1gpea1
d1n4wa1 d1k0ia1 d2dw4a2 d2ivda1 d1seza1 d2voua1 d1kdga1 d1s5ja1
d2hbja2 d2igia_ d1wlja_ d1qhta1 d4dlea1 d1tgoa1 d2f96a1 d3tr8a1
d3mxma_ d1q8ia1 d1y97a1 d1w0ha_ d1kfsa1 d2py5a1 d1d5aa1 d1yt3a3
d2guia_ d3mwpa1 d4kb1a_ d1iv0a_ d1nu0a_ d1iuka_ d2csua1 d3q9ub_
d2yv1a1 d3q9na_ d2d59a_ d1oi7a1 d2yv2a1 d1euca1 d1y81a1 d1kv9a1
d3nvva1 d1krha3 d2h88b1 d1t3qa2 d1kf6b2 d1zoyb1 d1jq4a_ d1ffva2
d1dgja2 d1rm6c2 d1r7ha_ d1faaa_ d1wika1 d1xfla_ d1ttza1 d2f51a1
d1ti3a_ d1nhoa_ d1sena_ d2i4aa_ d1ep7a_ d1zmaa1 d1fo5a_ d3lefa1
d3diea1 d2yana1 d1z6na1 d1oaza_ d3zywa1 d4aj8a_ d1nm3a1 d4fiwa_
d1wmja_ d4hs1a1 d1iloa_ d1xwaa1 d1wjka1 d2vm1a_ d1fb6a_ d1gh2a_
d1h75a_ d1nw2a_ d1kx5b_ d3a6na_ d4m6ba2 d2nqbd_ d3wkjd_ d2yfwe_
d4h9na_ d5bnxa_ d1kx5c_ d3r45a_ d1eqzb_ d4jjnb_ d1f66c_ d1eqza_
d4wnnb_ d1kx5a_ d1u35c1 d1id3a_ d2nqbc_ d1kx5d_ d1ku5a_ d1f1ea_
d1b67a_ d2b5ea2 d2b5ea3 d2b5ea1 d2b5ea4 d2kp1a1 d2kp2a1 d1hyua3
d1hyua4 d1j08a2 d1pd212 d1nhya2 d2a2ra2 d1k0dd2 d1f2ea2 d1gsua2
d1f3ba2 d2fhea2 d2pvqa1 d1tw9a2 d1oyja2 d1k0ma2 d3ik7a1 d4is0a1
d1r5aa2 d1jlva2 d1m0ua2 d3gtub2 d1z9ha2 d1okta2 d1axda2 d2gsra2
d1tu7a2 d1k3ya2 d1zgma1 d1b48a2 d1gnwa2 d1gwca2 d2gsta2 d1glqa2
d1oe8a2 d1pn9a2 d1v2aa2 d1fw1a2 d1duga2 d3zxha_ d1cgla_ d1rm8a_
d1eaka2 d2jsda_ d1cxva_ d1hv5a_ d1i76a_ d1a0rp_ d1ig8a1 d1czan2
d1czan1 d1ig8a2 d1czan4 d1bg3a4 d1bg3a3 d1bg3a2 d1bg3a1 d1qqya_
d1gd6a_ d1f6ra_ d1ivma_ d2gv0a_ d1iiza_ d2z2fa_ d1j8xa_ d1ghla_
d1b9oa_ d174la_ d169la_ d2o4wa_ d1p37a_ d1k28a3 d1xjta1 d1jtma_
d2f2qa_ d4e97a1 d2pjsa1 d2i7ra1 d1ecsa_ d1xqaa1 d5cj3a1 d1npba_
d1jifa_ d2p7oa_ d1r9ca_ d1nkia_ d1mpya2 d1kw3b2 d1kw3b1 d4ghga1

d1tfza1 d3isqa1 d3isqa2 d1sqia1 d1sqia2 d1lkda2 d1lkda1 d1mpya1
d1cjxa2 d1cjxa1 d1sp9b2 d1t47a1 d1t47a2 d1f1ua2 d1sp8a1 d1sp8a2
d1f1ua1 d1ro5a1 d4iuaa3 d4bvva_ d5hpga_ d3k65a_ d1ki0a3 d1ki0a2
d1ki0a1 d1a0ha1 d1pmla_ d3sp8a3 d2feba1 d1qsta_ d2beia1 d4m3sa_
d1u6ma1 d1ygha_ d1yk3a1 d3dr6a_ d1xmta_ d1z4ea1 d2ge3a1 d1bo4a_
d2fiaa1 d2jdca1 d1y9wa1 d3to7a_ d1wwza1 d2fl4a1 d2ozua1 d2b5ga_
d2cy2a1 d2p0wa_ d1cm0a_ d4pswa_ d1yrea1 d1yr0a1 d1m4ia_ d2gana1
d1yvka1 d1ghea_ d2g3aa1 d1y7ra1 d2euia1 d1q2ya_ d2fsra1 d1xeba_
d2ae6a1 d1cjwa_ d1qsma_ d2aj6a1 d1y9ka1 d1i12a_ d1vkca_ d2atra1
d1n71a_ d2d4pa1 d2fiwa1 d2fe7a1 d1tiqa1 d2ou2a_ d1yx0a1 d2fcka1
d1p0ha_ d2j8ma_ d2pq8a_ d1z4ra1 d1s3za1 d1ufha_ d1c4qa_ d1prtc1
d2wv6d_ d1prtf_ d1ltrd_ d4l63b1 d1prtd_ d1tl0f_ d2bosa_ d1prtb1
d3chbd_ d1xvqa1 d1pn0a2 d3hy2a_ d2cvba1 d1o8xa_ d1lu4a_ d1we0a1
d2bmxa1 d2a4va1 d1psqa_ d1xiya1 d1q98a_ d1n8ja_ d1zofa1 d2ggta_
d3tjja_ d4je1a1 d3s9fa_ d2zcta_ d1qmva_ d1jfua_ d2b1ka_ d5dvba1
d2b5xa1 d2v2ga_ d1st9a_ d3tb2a_ d4llra_ d4fh8a1 d1e2ya_ d1knga_
d2h30a_ d1i5ga_ d2z9sa_ d2f8aa1 d1tp9a1 d2b7ka_ d1nm3a2 d3hvva_
d2wfca1 d4kcea_ d1xvwa1 d4gqca1 d3qpma_ d4k1fa_ d1zzoa1 d1gp1a_
d1prxa_ d4od7a1 d1z6ma1 d4mcua1 d4dvca1 d1r4wa_ d3l9sa_ d1un2a_
d3rppa_ d2qked_ d4ksoa_ d1t4za1 d1wwja_ d1f74a_ d3l21a_ d2e1da_
d1to3a1 d1w3ia_ d1sfla_ d1n7ka_ d1vcva1 d4imea_ d1wa3a_ d2ocza1
d1f2ja_ d3m16a_ d2yxga_ d1ub3a_ d3a5fa_ d1fbaa_ d1l6wa_ d1xfba1
d1wx0a1 d1vpxa1 d1ojxa_ d1mxsa_ d2a4aa1 d1xkya1 d3cq0a1 d3s1xa_
d1f05a_ d1fdja_ d3cpra1 d4d2ja_ d4aw8a_ d1jgta2 d1ea0a3 d1ecfa2
d1q15a2 d1ct9a2 d1ao0a2 d1te5a_ d1ys1x_ d4hs9a_ d1ispa_ d1ex9a_
d1ku0a_ d3qmma_ d1bbua1 d1e1oa1 d1c0aa1 d1b8aa1 d1l0wa1 d3a74a1
d1eova1 d1lgya_ d4l3wa_ d1llfa_ d1gz7a_ d1ukca_ d1cr6a2 d1svpa_
d1mbma_ d3su6a1 d1cu1a1 d4agka_ d1vcpa_ d1q31a_ g1n1l.1 d3e90b_
d1ep5a_ d3u1jb_ d1xwdc1 d3kj4a_ d1w8aa_ d2ifga3 d1p9ag_ d1xkua_
d3p0ya1 d1igra1 d1igra2 d1n8yc2 d1n8yc1 d1m6ba2 d1m6ba1 d1m4la_
d2bk7a_ d3fjua_ d1z5ra1 d1uwya2 d1jqga1 d2c1ca1 d1dtda_ d1h8la2
d1h6ta2 d1h6ua2 d1lmra_ d1cixa_ d1y29a_ d1v7fa_ d2jtba_ d1d1ha_
d1g9pa_ d1lupa_ d1qk7a_ d1mb6a_ d2a2va_ d1niya_ d1i25a_ d1hvwa_
d2mpqa_ d1ie6a_ d1kqha_ d1la4a_ d2mxoa1 d1koza_ d1qk6a_ d1emxa_
d1lqra_ d1c6wa_ d1rmka_ d1ixta_ d1eyoa_ d1cnna_ d1fu3a_ d1g1pa_
d1f3ka_ d1fyga_ d1rypd_ d1irua_ d1rypc_ d3unfk_ d1rypa_ d1j2pa_
d1irui_ d1j2qh_ d3unfh_ d1m4ya_ d1rypb_ d1irul_ d1irug_ d1iru2_
d1q5rh_ d1rypg_ d1g3ka_ d1iruf_ d1q5qa_ d1e94a_ d1irub_ d1ryph_
d3ty6a_ d1iruj_ d1iruk_ d1rype_ d1irue_ d3mi01_ d1rypf_ d1iruh_
d2z3ba_ d1iruc_ d3mka2_ d1iru1_ d1e88a1 d1l6ja5 d1l6ja4 d1eaka4
d1eaka3 d1h8pa2 d1h8pa1 d1ny721 d1ny722 d1a6ca3 d1a6ca2 d1pgl22
d1pgl21 d1pgl11 d1ny711 d1mqta_ d1d4m3_ d1fmd3_ d2mev1_ g1aym.1
d1ev13_ d4q4w1_ d1r1a3_ d2x5ia_ d1rhi1_ g1r1a.1 g1d4m.1 g1rhi.1
d1b35b_ d1oopa_ d1tme1_ d4iv1a_ g1pvc.1 g1fpn.1 d1fpn1_ d4gb31_

d1d4m1_ d1fmd1_ d1ev11_ d2wzr3_ g1cov.1 d1qqp3_ g1fmd.1 d2x5ic_
d1rhi3_ g1oop.1 d1hxs1_ d1fpn3_ d1bev1_ g2mev.1 d1eah3_ d1aym1_
d1pvc3_ d1qqp1_ d1zba3_ d1h8ta_ d3vbhc_ g1b35.1 g1bev.1 d1hxs3_
g1ev1.1 d1bev3_ g1tmf.1 d1tmf1_ d1b35a_ d2mev3_ g1eah.1 g1h8t.1
d1eah1_ d4q4w3_ d1r1a1_ d1aym3_ d1pvc1_ d1zba1_ d1h8tc_ d1oopc_
d1tme3_ g1qqp.1 d4iv1c_ g1tme.1 d3ubub_ d1egia_ d1qo3c_ d1v7pa_
d1tdqb_ d3ubua_ d1c3ab_ d1h8ua_ d3bx4b_ d1t8ca1 d1sb2a1 d1qdda_
d2ziba1 d3cada_ d1byfa_ d1j34b_ d1jwia_ d1jzna_ d1c3aa_ d1oz7b_
d1wmza_ d3bdwb_ d1hq8a_ d1fifa1 d1dv8a_ d3hupa_ d1wk1a1 d1umra_
d2afpa_ d3bx4a_ d1gz2a_ d1sl6a_ d1v7pb_ d3g8ka_ d1ixxa_ d1j34a_
d1jwib_ d1g1sa1 d1y17a1 d1ja3a_ d1fvub_ d3bdwa_ d1sb2b1 d1fvua_
d1umrc_ d1oz7a_ d4m17a_ d3cfwa1 d1ypqa1 d1g1ta1 d3fd4a_ d3l9jc_
d4dn8a1 d1v4lb_ d1rdl1_ d3ia2a_ d1a88a_ d4dgqa1 d1hkha_ d1zoia_
d4lt9a1 d3tc7a1 d3hoja_ d1i4na_ d3vnda_ d3nl8a1 d1geqa_ d3nava1
d1v5xa_ d4a2ra1 d3tc6a1 d1ujpa_ d4ijba_ d1xcfa_ d1vc4a_ d1rd5a_
d1qopa_ d3b5va_ d2r80b_ d1cqxa1 d1cg5a_ d1gcvb_ d3boma_ d2h8fb_
d2raoa_ d3bj1a_ d4hrrb_ d2r50a_ d3d1kb_ d2aa1b1 d3zhwa_ d3hf4b_
d3gdja_ d1jebb_ d3bj1b_ d1x9fb_ d1wmub_ d1gcva_ d3bcqb_ d3d4xb_
d1gvha1 d2dc3a_ d3hf4a_ d4yu3a_ d3k8ba_ d1qpwb_ d2r80a_ d3bcqa_
d1v4wa_ d3wtgb_ d1jl7a_ d4esaa_ d1a9we_ d3d1ab_ d1fsla_ d4h2la_
d1v4wb_ d1mwca_ d1vhba_ d1jeba_ d1spgb_ d1h97a_ d1q1fa_ d1tu9a1
d3d1aa_ d1hdsb_ d3hyub_ d2zfbb_ d3fs4a_ d1oj6a_ d1shrb_ d4h2lb_
d4yu3b_ d3hrwa_ d3qqra_ d3at5a_ d4wchd_ d3hyua_ d1cg5b_ d1uc3a_
d1hbra_ d2raob_ d1outb_ d4esab_ d3gdjb_ d3qqqa_ d1x9fc_ d3vrfa_
d1itha_ d3mkba_ d3d4xa_ d3mjpa_ d1jl6a_ d3fh9a_ d1outa_ d2dc3b_
d1it2a_ d3eoka_ d3g46a_ d1qpwa_ d3vrfb_ d1ch4a_ d1x9fd_ d1hdsa_
d2zfba_ d3mkbb_ d1or4a_ d3wtga_ d1a4fa_ d3fh9b_ d3bomb_ d3cy5a_
d3at5b_ d1x9fa_ d2oifa_ d4lmxb_ d1cpcb_ d3v57a_ d1kn1a_ d1cpca_
d4f0ta_ d2vjha_ d4po5b_ d1eyxa_ d1alla_ d4f0tb_ d1b33b_ d1b8db_
d4h0mb_ d1b33a_ d2vjhb_ d4lm6b_ d2vmlb_ d1allb_ d2vjta_ d3dbja_
d2vmla_ d2vjtb_ d4f0ub_ d1f99b_ d4rmpa1 d1liaa_ d1eyxb_ d2uulb_
d4lmsb_ d4f0ua_ d2bv8a_ d1f99a_ d2bv8b_ d2c7lb_ d3dbjb_ d4rmpb1
d1b8da_ d4h0ma_ d3v57b_ d1liab_ d2uula_ d2j96a_ g1gxs.1 g1wht.1
d1mtza_ d2xqfa_ d1f6wa_ d1dx4a_ d1qe3a_ d1k4ya_ d4qwwa_ d2ha2a_
d3c8da2 d2pbla1 d1jkma_ d1jjia_ d1lzla_ d1jjfa_ d2qvba_ d2v9za_
d4kafa1 d1s5ja2 d1qhta2 d4dlea2 d1tgoa2 d3py8a2 d1q8ia2 d1kfsa2
d2py5a2 d1d5aa2 d2m5ta_ d1cqqa_ d1wqsa_ d5bpea_ d2ipha_ d1lvoa_
d2bhga1 d3tnta_ d3sjia_ d3d23a1 d2fyqa_ d3tloa_ d3zvea1 d2hrva_
d3q3ya1 d4asha1 d1cg2a1 d1yloa2 d1y7ea2 d1y0ya3 d1lfwa1 d1vixa1
d1fnoa4 d1ysja1 d1vhea2 d1z2la1 d2fvga2 d1vgya1 d1r3na1 d2grea2
d1xmba1 g1q7l.1 d1rtqa_ d3rp2a_ g1ucy.1 d1fuja_ d2zchp_ d3w94a_
d1si5h_ d1lo6a_ g2pka.1 d1cgha_ d1kigh_ d1a5ia_ d3s9ba_ d1hj8a_
d1ao5a_ d2anya_ d1q3xa1 d1gvza_ d1azza_ d4gsoa_ d2f91a1 d1iaua_
d1gdna_ d1eufa_ g1fiw.1 d1eq9a_ d4nfea_ d2rdla_ d1spja_ d1elva1

d1a7sa_ d1ekbb_ d1pfxc_ d1yc0a_ d2aiqa_ g1fiz.1 d1fi8a_ d4k60a_
d4dgja_ d1mzaa_ d1op0a_ d3tjva_ d1pytd_ d1sgfg_ g1gg6.1 d2eeka_
g1rtf.1 d1a0ja_ d3g01a_ d1pytc_ d4e7na_ d1ddja_ d1z8ga1 d2hlca_
d1npma_ d1brup_ g1jou.1 d1sgfa_ d1autc_ g2hnt.1 g1kdq.1 d1orfa_
d3s69a_ d4crga_ d1fxya_ d1bqya_ d2ftba_ d1pmpa_ d2rcqa1 d1fdqa_
d4lkpa_ d1sa8a_ d1vyfa_ d1lpja_ d3vg7a1 d4azqa1 d1o8va_ d5bvqa_
d1p6pa_ d2qo4a_ d1opba_ d1yiva1 d1o1va_ d4bvma1 d4tkba_ d4i3ba_
d1bwya_ d1ftpa_ d1ggla_ d4gkca_ d1cbia_ d3p6da1 d1kqwa_ d2a0aa1
d1jx4a2 d3gv5b1 d1t94a2 d3bq0a2 d4lu0a_ d1of8a_ d3unda1 d3e9aa1
d1vr6a2 d1n8fa_ d2qkfa_ d1d9ea_ d1o60a_ d1e69a_ d1mv5a_ d3g60a4
g1ii8.1 d1ewqa2 d1ji0a_ d1qhla_ d1v43a3 d2wmfa1 d1vpla_ d1g2912
d4a82a2 g1f2t.1 d2d62a1 d3tuic1 d2bbsa1 d3g60a3 d2ixea_ d1g6ha_
d1sgwa1 d2qi9c_ d3rlfa1 d3b60a1 d3d31a2 d2pzea1 g1xew.1 d1jj7a_
d1oxxk2 d2onka1 d1w1wa_ d1iwea_ d1qf5a_ d1vmaa2 d1nija1 d1np6a_
d2p67a1 d3ihla_ d1okkd2 d1ls1a2 d1s1ma2 d1iona_ d2v40a_ d1dj3a_
d3hida_ d2vo1a1 d2g0ta1 d1vcoa2 d1j8mf2 d1g3qa_ d1xjca_ d1p9ba_
d1qzxa3 d1ihua1 d2qm8a_ d1dj2a_ d4ioja_ d1cp2a_ d1yrba2 d1hyqa_
d3gqba2 d1skye3 d1xp8a1 d1tf7a2 d1tf7a1 d1ye8a1 d1skyb3 d2qe7d2
d4blta_ d4huta_ d3ldaa2 d4hyya_ d2i3ba1 d1cr1a_ d3j9tb2 d3ntua2
d1vdza1 d2z43a_ d1e9ra_ d1nlfa_ d3ziaa2 d1n0wa_ d3ziad2 d3j9ta2
d1pzna2 d3gqbb2 d2i49a_ d1nnfa_ d1xvxa_ d1ursa_ d1h3da1 d1iuda_
d4m1va1 d1twya_ d3e13x_ d2nxoa1 d4pfua_ d3ttma_ d1elja_ d2qmwa1
d1us5a_ d1y3na1 d1jeta_ d1uqwa_ d4f19a1 d2esna2 d3thia_ d3fasa1
d2uyeb_ d2xwva1 d1r9la_ d2fyia1 d1pb7a_ d2ozza1 d1sw5a_ d1nh8a1
d1vr5a1 d2g29a_ d1xs5a_ d3lswa_ d2z23a_ d3w9va_ d3tcha_ d1q35a_
d1z7me1 d3q27a_ d1p99a_ d1ve4a1 d2i6ea1 d4mcoa1 d4n8ya_ d1eu8a_
d1zbma1 d1ixca2 d1j1na_ d1xvya_ d1y9ua_ d2z22a1 d1xoca1 d4oera_
d1wdna_ d5f1qa1 d1xt8a1 d1i6aa_ d4gwoa_ d4igta1 d1pc3a_ d4mmpa1
d1o63a_ d2czla1 d2onsa2 d4mija1 d1eh3a_ d3skpa_ d1dtza1 d1dtza2
d1ieja_ d1jnfa2 d1ce2a1 d1jnfa1 d1b1xa1 d1jw1a1 d1jw1a2 d1ryoa_
d1h76a1 d1h76a2 d1c4oa1 d2p6ra3 d2p6ra4 d1oywa2 d1oywa3 d1wrba1
d1w36b2 d1w36b1 d1c4oa2 d3bora_ d1t6na_ d2eyqa5 d2eyqa3 d2eyqa2
d2zu6a2 d1veca_ d1uaaa1 d1w36d1 d2g9na1 d1z3ix2 d2rb4a_ d1hv8a1
d1hv8a2 d1t5ia_ d1w36c2 d2j0sa2 d2j0sa1 d1fuka_ d3hjha1 d1z5za1
d1z63a1 d1s2ma2 d1s2ma1 d1wp9a1 d1wp9a2 d1gm5a3 d1gm5a4 d3dina4
d3dina3 d1q0ua_ d1w36c1 d1z3ix1 d1nkta3 d1nkta4 d1uaaa2 d1w36d2
g1qhh.1 d2fwra2 d2fwra1 d1oewa_ d2qzxa_ d2asia_ d1miqa_ d1smra_
d2h6ta_ d1pfza_ d1izda_ d3hd8a_ d1j71a_ d1dpja_ d4aa9a_ d1t6ex_
g1b5f.1 g1lya.1 g1htr.1 d1qdma2 d1bxoa_ d1wkra_ d1tzsa_ d1ibqa_
d2qzwa_ d2v76a_ d1wj1a1 d1aqca_ d1qqga2 d1qqga1 d1ntva_ d1n3ha_
d4dbba_ d3f0wa1 d1p3ra_ d1wgua1 d2cy4a1 d3hqca_ d1wvha1 d1xr0b1
d1bdqa_ d3ggta_ d3ixoa_ d1mrxa_ d2haha_ d1tw7a_ d4njsa_ d1sgua_
d2p3da_ d1nsoa_ d3d3ta_ d2rkfa_ d1c6ya_ d2rspa_ d3ogpa_ d3ekwa_
d2o4na_ d2j9ja_ d3ggua_ d3lzua_ d4npua_ d4j55a_ d2r8na_ d2fxda_

| | | | | | | | |
|---|---|---|---|---|---|---|---|
| d2p3ca_ | d4dqea_ | d3ttpa_ | d3t3ca_ | d2p3aa_ | d2hc0a_ | d2fdda_ | d3mwsa_ |
| d1sr9a2 | d1rqba2 | d1k8rb_ | d1wgra1 | d1ef5a_ | d1wfya1 | d1e8ya3 | d1wgya1 |
| d1lfda_ | d2cs4a1 | d1wxma1 | d2byea1 | d3kh0a1 | d2byfa1 | d1wxaa1 | d1wgda1 |
| d1wgga1 | d4q5eb_ | d1wjna1 | d1se9a_ | d3n3kb_ | d1wz0a1 | d1we6a1 | d2m0xa_ |
| d2gbka_ | d3rt3b1 | d1wh3a1 | d1sifa_ | d2ekec2 | d4ii2b1 | d4hk2a1 | d1c3ta_ |
| d1m94a_ | d1wgha1 | d1ttna1 | d1j8ca_ | d1wx8a1 | d3v6cb1 | d2ma9b_ | d4i6lb1 |
| d1bt0a_ | d2jvca1 | d1iyfa_ | d2k1fa_ | d1wxva1 | d1wf9a1 | d1t0ya_ | d2io0b2 |
| d1x1ma1 | d2gbma_ | d1zw7a1 | d2m17a_ | d1we7a1 | d2lwpa_ | d2gbja_ | d1wjua1 |
| d1v6ea1 | d1v86a1 | d4eewa_ | d1wiaa1 | d4icva_ | d4gu2a_ | d1wx7a1 | d1wy8a1 |
| d4hjka1 | d2faza1 | d1v5oa1 | d2h1ia1 | d2r8ba1 | d3b5ea1 | d3cn9a_ | d3ubra_ |
| d1qo8a1 | d1d4ca1 | d2k3va_ | d1ft5a_ | d1qdba_ | d1m1qa_ | d2j7aa_ | d1h21a_ |
| d1ogyb_ | d4fasa_ | d1jnia_ | d1oaha_ | d1sp3a_ | d3nqyb_ | d4gera_ | d1bqba_ |
| d1yvca1 | d1yeza1 | d1g66a_ | d1qoza_ | d5ajha1 | d1wioa4 | d1ccza2 | d1vcaa1 |
| d1dr9a2 | d2d9qb1 | d2nxyb2 | d1um8a_ | d1ixza_ | d1qvra2 | d1qvra3 | d1qzma_ |
| d1sxjc2 | d1jr3d2 | d2p65a_ | d1l8qa2 | d3hu3a3 | d1sxjd2 | d1njfa_ | d1in4a2 |
| d1sxja2 | d1tuea1 | d2ce7a2 | d1w5sa2 | d1sxjb2 | d1d2na_ | d1ny5a2 | d1ofha_ |
| d2gnoa2 | d2a5yb3 | d1fnna2 | d1u0ja1 | d1lv7a_ | d1iqpa2 | d2fnaa2 | d1ixsb2 |
| d4gita_ | d1sxje2 | d1g41a_ | d2lj7a_ | d1n4na_ | d2m8bb1 | d2kyqa_ | d3psma_ |
| d1jxca_ | d2gl1a_ | d1ugla_ | d4uj0a_ | d2k4ua_ | d1hp2a_ | d2lu9a_ | d1baha_ |
| d2meoa1 | d1wm7a_ | d1hlya_ | d1n8ma_ | d1jlza_ | d1wpda_ | d1c55a_ | d1pvza_ |
| d1wt7a_ | d1rjia_ | d1wz5a_ | d1m2sa_ | d1pjva_ | d1c49a_ | d1qkya_ | d2ktca1 |
| d1wmta_ | d1du9a_ | d1j5ja_ | d1r1ga_ | d1t0za_ | d2i61a_ | d1t1ta_ | d1sn4a_ |
| d1kv0a_ | d2lkba_ | d1npia_ | d1wwna_ | d1pe4a_ | d2kbka_ | d2li7a_ | d1djta_ |
| d2kbha_ | d1dq7a_ | d1sega_ | d1omya_ | d1zywa1 | d1bnla_ | d1iyjb4 | d1iyjb5 |
| d1iyjb3 | d1o7ia_ | d4ipca1 | d1x3ea_ | d4apva_ | d1jb7a1 | d1jb7a2 | d1jb7a3 |
| d3kdfa_ | d1ue1b_ | d1wjja1 | d3eiva_ | d1xjva2 | d1xjva1 | d2ccza2 | d1se8a_ |
| d1l1oc_ | d1jb7b_ | d1qvca_ | d3ulla_ | d1qzga_ | d1miua4 | d1miua5 | d1miua3 |
| d3g48a1 | d1gd7a_ | d1mkha_ | d1jjcb3 | d1pyba_ | d1y14b1 | d2nn6i1 | d1i94l_ |
| d2zjra2 | d1smxa_ | d2qamc2 | d1hh2p1 | d2r7da1 | d1rl2a2 | d1wfqa1 | d3bzka4 |
| d1x6oa2 | d3a0ja_ | d2j01d2 | d2ja9a1 | d4ql5a_ | d1hr0w_ | d1luza_ | d2n3sa_ |
| d2vnud1 | d1h95a_ | d1go3e1 | d2nn6h1 | d1ne3a_ | d2mo1a_ | d1iz6a2 | d1jjga_ |
| d1e3pa2 | d2ix0a3 | d2ix0a2 | d2lxja_ | d1ueba3 | d1ueba2 | d2z0sa1 | d1u0la1 |
| d2ix0a1 | d2eifa2 | d2je6i1 | d3i2za1 | d2c35b1 | d2ahob2 | d1t9ha1 | d3i4oa_ |
| d2qall1 | d1jt8a_ | d2nn6g1 | d1g6pa_ | d3cama_ | d1i94q_ | d1c9oa_ | d1d7qa_ |
| d2ba0a1 | d2vnud2 | d2vnud3 | d1khia2 | d2qalq1 | d1wi5a1 | d1xtda2 | d1ny4a_ |
| d1dzka_ | d4tlja_ | d2a2ua_ | d4wfva_ | d3t1da_ | d4iaxa_ | d3zq3a_ | d2nnda_ |
| d1i4ua_ | d1kxoa_ | d1bbpa_ | d1kt7a_ | d1exsa_ | d3s26a1 | d1lf7a_ | d1qfta_ |
| d4gh7a_ | d1z24a1 | d1qabe_ | d1xkia_ | d1ew3a_ | d3bx7a_ | d4iawa1 | d1n0sa_ |
| d1gkab_ | d3kzaa_ | d2k23a_ | d2l9ca1 | d2joza1 | d1gm6a_ | d3kffa_ | d3saoa1 |
| d1e5pa_ | d1yupa1 | d1iiua_ | d1f00i3 | d1rpma_ | d1yfoa_ | d1l8ka_ | d2ooqa1 |
| d1g4us2 | d1p15a_ | d1lara2 | d1lara1 | d1wcha_ | d2fh7a2 | d2fh7a1 | d3azya_ |
| d3wvja_ | d1upsa1 | d1ajoa_ | d2hyka_ | d2vy0a_ | d1o4ya_ | d3o5sa_ | d3d6ea_ |
| d3atga_ | d1dypa_ | d1umza_ | d3i4ia_ | d1c1la_ | d4lbqa_ | d1a78a_ | d4no4a_ |
| d3zsja_ | d3duia_ | d1g86a_ | d1is3a_ | d1qmja_ | d1hlca_ | g1qmo.1 | g1len.1 |

| | | | | | | | |
|---|---|---|---|---|---|---|---|
| d3zyra_ | d1fnya_ | d1n47a_ | g2ltn.1 | d1avba_ | d3usua_ | d1dhkb_ | d1f9ka_ |
| d1g8wa_ | d1g9fa_ | d1wbfa_ | d3wcsa_ | d1g7ya_ | g1loe.1 | d1mvqa_ | d1hqla_ |
| d1v6ia_ | d1dbna_ | d1qnwa_ | d1fx5a_ | d4l8qa_ | d1ioaa_ | d3n35a_ | d2h0ba_ |
| d2erfa_ | d1h30a2 | d3mw4a1 | d1pz7a_ | d2r1ba_ | d3v64a1 | d1d2sa_ | d3bopa1 |
| d3mw3a1 | d1okqa1 | d1okqa2 | d1dszb_ | d1a6ya_ | d3cbba_ | d1ynwa1 | d1r4ia_ |
| d1hcqa_ | d2hana_ | d2hanb_ | d2enva1 | d1kb4b_ | d2nllb_ | d1lo1a_ | d1cita_ |
| d2dara2 | d2dara1 | d1wyha2 | d2dloa2 | d1rutx4 | d1rutx3 | d2cuqa2 | d1x4la2 |
| d1x4la1 | d1x3ha2 | d1x3ha1 | d2d8za1 | d1x61a2 | d1x6aa1 | d1x6aa2 | d2cora2 |
| d2cora1 | d1v6ga1 | d1v6ga2 | d2d8za2 | d1x64a1 | d1x64a2 | d1b8ta1 | d2co8a1 |
| d1x63a2 | d2dj7a2 | d2dj7a1 | d2cu8a2 | d2cu8a1 | d1g47a2 | d1g47a1 | d1j2oa1 |
| d1wyha1 | d2dloa1 | d1wiga1 | d1wiga2 | d2cupa2 | d2cupa3 | d1x68a1 | d2d8ya2 |
| d2d8ya1 | d1m3va2 | d1x62a1 | d1x68a2 | d1x61a1 | d1b8ta3 | d1b8ta2 | d2d8xa2 |
| d2d8xa1 | d1x4ka1 | d1x4ka2 | d2cuqa1 | d1m3va1 | d2co8a2 | d1j2oa2 | d1x63a1 |
| d2cura1 | d2cura2 | d1x62a2 | d1op9a_ | d1y0la1 | d1etzb1 | d1egjl1 | d2gsib1 |
| d1nj9a1 | d4jfhd1 | d1aqkl1 | d1t2ja_ | d1lo4h1 | d1zvya1 | d3v4vl1 | d4enec1 |
| d1kxtb_ | d5e8ea1 | d2pxya_ | d4weud_ | d1xiwc_ | d2esvd1 | d1qnzh_ | d1ogae1 |
| d1f4xh1 | d4n1hb_ | d3ezjb1 | d3mlwl1 | d1kfah1 | d1etza1 | d1b4jh1 | d3cfic_ |
| d4w68a_ | d2arja1 | d1ek3a_ | d2ok0l1 | d1vera_ | d1uwgh1 | d1blnb1 | d1fpth1 |
| d1rzga1 | d2giya1 | d4m3kb1 | d1nc2a1 | d2nyyd1 | d1ncna_ | d1fbih1 | d5dxwa_ |
| d4fqqa1 | d1dqdh1 | d1jfql1 | d1kelh1 | d1faih1 | d1frgl1 | d1figl1 | d4m6nl1 |
| d1fo0a_ | d4hxbl1 | d3b5ga_ | d3quyd1 | d2h9gb1 | d1i8kb_ | d2dd8h1 | d2ck0h1 |
| d1eaja_ | d4w6wb1 | d2a6ib3 | d1t66d1 | d2e27h_ | d1ob1b1 | d1f11b1 | d2v7na1 |
| d1ad0a1 | d2xt1b1 | d1pskh1 | d4i13b1 | d2op4l1 | d1a0ql1 | d1uwel1 | d1za3a1 |
| d1ncbl1 | d4uu9a_ | d1bm3h1 | d1cloh1 | d1nqba2 | d1cf8h1 | d4y5va_ | d1mhph1 |
| d5anma1 | d4cdgc1 | d4ldeb_ | d1y0lb1 | d2pcpb1 | d1z3gl1 | d1dfbl1 | d1fh5h1 |
| d3g5xa1 | d1tzib1 | d4dn3l1 | d3zhka1 | d2b1hh1 | d2uyla1 | d1f3dh1 | d1nj9b1 |
| d2agjh1 | d2h1pl1 | d1q72h1 | d8fabb1 | d1ndmb1 | d1rjca1 | d4u6hb1 | d3upca_ |
| d2fbjl1 | d1osph1 | d1tvda_ | d2cjvh_ | d4fhbd1 | d1bd2d1 | d1rz7h1 | d3nn8a_ |
| d1blna1 | d1knoa1 | d1nfda1 | d2bmkb1 | d1dqll_ | d1igyb1 | d1igfl1 | d12e8h1 |
| d3utza1 | d1nc2b1 | d1rihh1 | d4mwfb1 | d4jvpa1 | d1mieh1 | d1rhfa1 | d1fgnl1 |
| d1sbsh1 | d1lp9e1 | d1dn0a1 | d4lqfl1 | d1iaih1 | d1kgcd1 | d1mh5b1 | d1gpol1 |
| d2g60h3 | d1f8tl1 | d2jixd1 | d4grwh_ | d1fo0b_ | d1bfob1 | d1axsa1 | d1u9ka_ |
| d1nmbh_ | d1h8na1 | d1h8na2 | d1jguh1 | d4eura1 | d3sdyl1 | d35c8h1 | d1w72l1 |
| d1mvub_ | d1i9rh1 | d1ob1a1 | d4wenb_ | d1yeeh1 | d3ietc1 | d1f58h1 | d1ad0b1 |
| d1mqkl_ | d1nbvh1 | d4b50a_ | d3r8bb_ | d1bafl1 | d1mamh1 | d4rgne1 | d2gsgb_ |
| d35c8l1 | d1bafh1 | d1ai1h1 | d2pcpa1 | d2fath1 | d2x1pa_ | d1orsb1 | d1ibgl1 |
| d1shma_ | d1a2yb_ | d1u0qa_ | d2coqa_ | d1hilb1 | d1qfwi_ | d1jv5b_ | d1fn4b1 |
| d5e0qa_ | d1nsnh1 | d1plgl1 | d4ocxl1 | d1kb5h1 | d1rzfh1 | d1pg7x1 | d2fx7h1 |
| d2oi9c_ | d1l7il1 | d3eyva1 | d1d5il1 | d4ffyl_ | d1tetl1 | d1a6wh_ | d1ac6a_ |
| d3dvfa_ | d1nfde1 | d1mimh1 | d1ri8a1 | d1jhlh_ | d3dura_ | d1fskb1 | d1f97a1 |
| d3so3b1 | d2ak4d1 | d1lp9f1 | d4z7wf1 | d1l7th1 | d4y5ya_ | d4x7ec1 | d1emth1 |
| d1ikfl1 | d1nlbh1 | d4utal1 | d1bjma1 | d1eapb1 | d1u6ah1 | d2dtgd1 | d1kj2b_ |
| d1xeda1 | d1c1eh1 | d1fgvh_ | d2bnub1 | d2fb4h1 | d2dtgb1 | d1ktrh_ | d1jpth1 |
| d1qkzl1 | d4hepg_ | d2atpb1 | d1flrh1 | d2hrpl1 | d1mvfa_ | d1iaim1 | d3k80a1 |

| | | | | | | | |
|---|---|---|---|---|---|---|---|
| d2jb5h1 | d3zkxc_ | d4grwe_ | d1it9h1 | d1rhhb1 | d3w9ec_ | d1e4wh1 | d1sy6h1 |
| d2j6eh1 | d3qxta_ | d1jglh1 | d1p7ka1 | d1tzhb1 | d4n8pa1 | d1gafl1 | d2jelh1 |
| d1wioa2 | d4i0cc1 | d1cicb1 | d1lmka2 | d1lmka1 | d1iqwh1 | d1orqb1 | d1ejoh1 |
| d1jnlh1 | d4zg1a_ | d43c9b_ | d1ggbl1 | d1wejh1 | d3q6fa1 | d1f3dj1 | d3c6la1 |
| d1bqlh1 | d3p9wb_ | d1a3rl1 | d1h0db1 | d2uzil_ | d1eo8l1 | d1qfwl_ | d1n7ml1 |
| d1igja1 | d1c5ch1 | d2a9mh_ | d1rmfh1 | d1fj1b1 | d4heme_ | d1ccza1 | d1a5fh1 |
| d1ogad1 | d1dlfh_ | d1qa9b_ | d1vgel1 | d3qskb_ | d1sjxa_ | d4ozfg1 | d1sq2n_ |
| d1qd0a_ | d1kcvh1 | d1c5db1 | d3qcul1 | d8faba1 | d3bn9d1 | d1mjuh1 | d4y8dc_ |
| d7fabh1 | d3dvgb1 | d1lo0l1 | d1a6tb1 | d1gigh1 | d1kcrh1 | d2hwzl1 | d1yjdc1 |
| d3clfl1 | d4krna_ | d1dl7l_ | d1e6ol1 | d1mexh1 | d3r0ma_ | d4kkna_ | d1fnsl1 |
| d1oaqh_ | d1rzia1 | d1ucbh1 | d1ncwl1 | d4b41a_ | d1nfdf1 | d2mpah1 | d2d7tl1 |
| d4grwf_ | d4b5ea_ | d1lk3h1 | d3u79a_ | d4u3xa_ | d1ghfl1 | d1bvkb_ | d1mcww1 |
| d1b88a_ | d1jrhh1 | d32c2b1 | d4wemb_ | d1dsfh_ | d1op3h1 | d1ce1l1 | d1iqda1 |
| d1qoka1 | d1qoka2 | d1qfuh1 | d2crya1 | d1ay1h1 | d1oaql_ | d3ab0b_ | d1rurl1 |
| d1t4kb1 | d1bz7b1 | d1n0xh1 | d1forl1 | d1ucbl1 | d4w6yb_ | d43c9a_ | d2aabl1 |
| d1tzia1 | d4n1ca_ | d1k6qh1 | d3g9ab_ | d4tvsa1 | d1igmh_ | d1pz5b1 | d3dx9a1 |
| d3wxwh_ | d1nakh1 | d1kcul1 | d3eaka_ | d4qlra1 | d1cr9l1 | d1d9ka_ | d1f4xl1 |
| d1deea1 | d2i27n2 | d1c12a1 | d2orbl1 | d1j8hd1 | d1mcph1 | d1c5da1 | d1kenh1 |
| d1bd2e1 | d1h5ba_ | d4x7fc1 | d3upaa_ | d1t3fa1 | d1indh1 | d2dtgc1 | d4hc1l1 |
| d1zv5a1 | d4wgvb_ | d1uwgl1 | d4f37k1 | d1aqkh1 | d1vesa_ | d3zkqd_ | d1fptl1 |
| d1lo4l1 | d1knob1 | d3rrqa_ | d1ct8b1 | d4orzc_ | d1mlbb1 | d4hgkc1 | d1dqdl1 |
| d1bogb1 | d4ozhe1 | d1dr9a1 | d1i7za1 | d1dn0b1 | d1kell1 | d1rzib1 | d1ypzf1 |
| d1wtla_ | d1hkfa_ | d1fail1 | d1figh1 | d2vyre_ | d1sjva_ | d1ohqa_ | d1bfoa1 |
| d1pkqb1 | d1mjul1 | d1b4jl1 | d1nsnl1 | d2arjb1 | d1p4bh_ | d4aj0a_ | d3nn8b_ |
| d2rcja1 | d1f2xk_ | d4v1dd_ | d1adqh1 | d2eksa_ | d4k3ga_ | d1jfqh1 | d1uweh1 |
| d1frgh1 | d4gftb_ | d1uz8b1 | d4n1ea_ | d1bm3l1 | d1clol1 | d3mcka1 | d2vq1a1 |
| d1ae6h1 | d4aq1b1 | d1uj3b1 | d1nbqa1 | d1fh5l1 | d2ck0l1 | d2ywza_ | d1q72l1 |
| d4nbxb_ | d1hila1 | d4hzlb1 | d2j88l1 | d1zmya1 | d1cd0a_ | d1a4ka1 | d1pskl1 |
| d1a0qh1 | d4krmb_ | d1ncbh1 | d2fbjh1 | d1j8he1 | d1i3ua_ | d1cf8l1 | d1fe8h1 |
| d2p1ya1 | d1eeqa_ | d2x89a_ | d1ol0a_ | d2r0lh1 | d1ub5a1 | d1dfbh1 | d3eyvb1 |
| d4jdvb1 | d4p2cg_ | d2gsia1 | d2agjl1 | d1u3ha1 | d2h1ph1 | d1rihl1 | d1fskc1 |
| d1boga1 | d2a77l1 | d1ospl1 | d1qfoa_ | d3dwta_ | d2fx7l1 | d1rz7l1 | d3stba1 |
| d1pkqa1 | d1b6da1 | d1dzba2 | d1dzba1 | d1kj2a_ | d4l1ha_ | d1rzgb1 | d1dqlh_ |
| d12e8l1 | d1miel1 | d1jmaa_ | d1sbsl1 | d1fgnh1 | d1f58l1 | d1mqkh_ | d1ndgb1 |
| d3qegl1 | d1iail1 | d2aw2a1 | d1tqbc1 | d3fo2a1 | d4dkaa_ | d1f8th1 | d2g60l1 |
| d2ywya_ | d1uj3a1 | d1yc7a_ | d3bd3a_ | d4w6xb1 | d1nmbl_ | d1i8ka_ | d1rjlb1 |
| d1ypze1 | d1p7kb1 | d1kxqe_ | d1gpoh1 | d2z4qa1 | d1w72h1 | d1i9rl1 | d4qgya1 |
| d4xt1c_ | d2qqnh1 | d1cica1 | d3c08h1 | d2cd0a_ | d2bdnh1 | d1hnga1 | d1maml1 |
| d1nldh1 | d1cr9h1 | d4lcih1 | d3bkyh1 | d3qg7l1 | d1ai1l1 | d1kxvc_ | d4iosd_ |
| d1l7ih1 | d2x1qa_ | d1ub6b1 | d3k74b_ | d1ibgh1 | d1teth1 | d1igya1 | d1hxma1 |
| d2x6ma_ | d2e7la_ | d5e03a_ | d1qfwm_ | d2r0kh1 | d1igjb1 | d1rurh1 | d1plgh1 |
| d1mj8h1 | d1yuhb1 | d3fmgl1 | d1rzfl1 | d1emtl1 | d1ad9b1 | d1igtb1 | d1nlbl1 |
| d1d5ih1 | d3k7ua_ | d4ffyh_ | d3dvga1 | d1a6ta1 | d1fgvl_ | d4qxgl1 | d1miml1 |
| d1jhll_ | d2hffb1 | d2hh0l1 | d1qkzh1 | d1l7tl1 | d1afvh1 | d1iaii1 | d2cjuh_ |

d1um5h1 d2ghwb2 d2ghwb1 d1kgce1 d2fgwh1 d1ikfh1 d1dvfd_ d2i24n1
d2xtjb_ d1za3b1 d2yc1a_ d2hh0h1 d2x1oa_ d1bvka_ d1rjla1 d5e7ba1
d1igta1 d1il1b1 d2fb4l1 d1mvua_ d1jptl1 d32c2a1 d1fvca_ d1iqdb1
d2hrph1 d2p4ab_ d4qo1a_ d1p2cb1 d1wt5c_ d1t4ka1 d5c1mb_ d1it9l1
d1bz7a1 d1b2wh1 d1e4wl1 d1jnll1 d1yedb1 d1za6b1 d3u7aa_ d1igfh1
d3gnml1 d1orsa1 d1wejl1 d1a2ya_ d1hxmb1 d1gafh1 d2j4wh1 d1pz5a1
d1j05a_ d1a3rh1 d4fqll1 d1qfwh_ d1eo8h1 d1n7mh1 d4s11a_ d1kegh1
d5da4a_ d1iqwl1 d3whxb1 d1vgeh1 d1deeb1 d1c12b1 d1t2ql1 d1ad9a1
d3gsnb1 d3b2uc1 d1ggbh1 d1t3fb1 d1nakl1 d4ocmc_ d1lila1 d2v7hb3
d2esve1 d1mcoh1 d2qr0b1 d2q3aa_ d1ct8a1 d1kcrl1 d2pxyb_ d2uzih_
d5c3ld_ d3durb_ d1c5cl1 d2ak4e1 d1dl7h_ d1xiwd_ d1mlba1 d1rmfl1
d1i7zb1 d1dlfl_ d2xa3a_ d5bq7b1 d1dqqb1 d3h42l1 d1egjh1 d1kcvl1
d1fe8l1 d2j6el1 d1f3rb2 d1lk3l1 d1sm3h1 d1q0xh1 d1ghfh1 d7fabl1
d1igch1 d1lo0h1 d4idla_ d5c2bl_ d1jrhl1 d1il1a1 d3zhda1 d4kfzc_
d1b0wa_ d1e6oh1 d1mexl1 d4krpb_ d1fnsh1 d1ncwh1 d4hgma_ d4eigb1
d4rzca_ d2mpal1 d4y5xa_ d5en2b1 d3lrga1 d4ej1c1 d1rhha1 d1j05b_
d1forh1 d1q9wb1 d2wzpd_ d2aq2a1 d1h0da1 d3revb1 d2ij0c1 d1tzha1
d4m7jl1 d1k6ql1 d3b9kc1 d1igml_ d3qeua1 d1dsfl_ d1ce1h1 d1kcuh1
d1zvha1 d1zvoa1 d1tqbb1 d1ay1l1 d2qscl1 d2cgrh1 d1nl0h1 d1kenl1
d3eo0a1 d4n9ob1 d1n0xl1 d3k1kc_ d2aq2b1 d1ck1a1 d1i4pa1 d1v1oa1
d1et9a1 d1esfa1 d2g9hd1 d4ohja1 d1klud1 d1bxta1 d1eu3a1 d1ty0a1
d1fnua1 d2cqva1 d1fhga_ d2k1ma_ d2dl2a1 d1biha3 d1biha1 d1biha4
d3qp3a1 d1g1ca_ d1gxea_ d1cvsc2 d1cvsc1 d1xmwa2 d1m4ka2 d2otpa2
d1biha2 d1rhfa2 d2fcba2 d2fcba1 d1wwbx_ d1gsma2 d1epfa1 d1epfa2
d2dava1 d1waaa_ d1f97a2 d1x44a1 d1vcaa2 d1fnla2 d1fnla1 d3b5ha2
d3h8na2 d3h8na1 d1jbja2 d1pd6a_ d1f2qa2 d1f2qa1 d3cx2a_ d1nbqa2
d3b5ha1 d1n26a1 d1xiwb_ d1xaua_ d1fltx_ d2ifga1 d1olla1 d1olla2
d1cs6a4 d1cs6a3 d1cs6a2 d1cs6a1 d2gw5a2 d1sy6a1 d1xiwa_ d1ucta2
d1ucta1 d1ry7b2 d1gl4b_ d1qz1a3 d1olza1 d2c9aa1 d1wwca_

**Family-8 (a.25.1.2)**

**Positive dataset contains 11 protein sequences.**

d3q3ma_ d1za0a1 d2inca_ d2incb_ d3hf1b_ d2uw1a_ d3n37a_ d1syya_
d2o1za_ d1uzra_ d1smsa_

**Negative dataset contains 4011 protein sequences.**

d1u08a_ d1lc5a_ d1b5pa_ d2ay1a_ d1bw0a_ d3pd6a_ d7aata_ d2csta_
d1j32a_ d1fg7a_ d4wlha_ d1o4sa_ d1v2da_ d1gdea_ d1v72a1 d1svva_
d1ajsa_ d1wsta1 d2f8ja2 d1lk9a_ d1vp4a1 d1xi9a1 d2gb3a1 d1yaaa_
d1xkla_ d2wfla_ d3i2ka1 d1lnsa3 d1mpxa2 d2cfba1 d1o69a_ d1bj4a_
d3k28a_ d2bwpa_ d3lg0a_ d2fnua_ d3ou5a_ d1z7da1 d1b9ha_ d1iaya_
d4msoa_ d1bs0a_ d1s0aa_ d1sffa_ d3tqxa_ d1mdoa_ d3qboa_ d3pgya_
d1bjna_ d2dkja_ d1ejia_ d1vefa1 d4wxga_ d3bs8a_ d3gbxa1 d1w23a_
d2b5aa1 d2ofya1 d4mcxa_ d2xi8a_ d2icta_ d2bnma1 d2ppxa1 d1y7ya1
d1wioa4 d1ccza2 d1vcaa1 d1dr9a2 d1ic1a1 d2d9qb1 d2nxyb2 d1f56a_
d1bypa_ d1azca_ d1ws8a_ d3u25a_ d1cuoa_ d1id2a_ d2iaac_ d4dp1x_

d1nwpa_ d2gima_ d2i7sa_ d1qhqa_ d1gyca3 d1hfua2 d1kbva1 d1kbva2
d1sdda1 d1sdda2 d1gyca2 d1mzya1 d2bw4a1 d1aoza1 d1aoza3 d1aoza2
d1kyaa2 d1kyaa3 d1kyaa1 d1mzya2 d4e9sa1 d1sddb1 d1sddb2 d1hfua1
d1hfua3 d1oe1a2 d1oe1a1 d1v10a2 d1v10a3 d1v10a1 d2dara2 d2dara1
d1wyha2 d2dloa2 d1rutx4 d1rutx3 d2cuqa2 d1x4la2 d1x4la1 d1x3ha2
d1x3ha1 d2d8za1 d1x61a2 d1x6aa1 d1x6aa2 d2cora2 d2cora1 d1v6ga1
d1v6ga2 d2d8za2 d1x64a1 d1x64a2 d1b8ta1 d2co8a1 d1x63a2 d2dj7a2
d2dj7a1 d2cu8a2 d2cu8a1 d1g47a2 d1g47a1 d1j2oa1 d1wyha1 d2dloa1
d1wiga1 d1wiga2 d2cupa2 d2cupa3 d1x68a1 d2d8ya2 d2d8ya1 d1m3va2
d1x62a1 d1x68a2 d1x61a1 d1b8ta3 d1b8ta2 d2d8xa2 d2d8xa1 d1x4ka1
d1x4ka2 d2cuqa1 d1m3va1 d2co8a2 d1x63a1 d2cura1 d2cura2 d1x62a2
d2w0pa_ d2diaa1 d2e9ia2 d2e9ja1 d2dj4a1 d2eeba1 d2diba1 d2dmba1
d2dmca1 d2d7oa1 d2ee6a1 d2d7pa1 d2eeaa1 d2di8a1 d1wlha1 d2d7na1
d2nqca_ d2d7ma1 d2dica1 d3cnka_ d2k3ta1 d2eeca1 d1qfha1 d2bp3a1
d1v05a_ d2ee9a1 d2eeda1 d2di9a1 d2y5ia_ d1qlsa_ d1psra_ d3n22a_
d2egda_ d4ggfc_ d2wcea_ d2luca_ d4aqia_ d3nsia_ d2lp3a_ d1j55a_
d1a4pa_ d1k8ua_ d1nsha_ d2bema_ d1pama1 d1ea9c1 d1qhoa1 d2fhfa2
d2fhfa1 d1k3ia1 d1j0ha1 d1cxla1 d2bhua1 d1edqa1 d1f1sa2 d1ut9a2
d1gvia1 d1txka1 d4alac_ d1pjwa_ d1rera1 d1s6na_ d3uzqb_ d2phla2
d2phla1 d1fxza2 d1fxza1 d1lrha_ d1uija1 g1dgw.1 d2v09a_ d1uika2
d1uika1 d1dgwa_ d1ep0a_ d2pa7a1 d1wlta1 d1nxma_ d2c0za1 d1ds6b_
d1fstb_ d1ju8a_ d1v70a_ d2f4pa1 d1yhfa1 d1tafa_ d1bh9b_ d1jfib_
d1q9ca_ d1h3ob_ d2byka1 d1n1ja_ d4g92b_ d1bh9a_ d1jfia_ d1h3oa_
d2bykb1 d1n1jb_ d1h32a2 d2bjca1 d2hsga1 d1qpza1 d1efaa1 d1ydwa2
d2nvwa2 d1h6da2 d1zh8a2 d1xeaa2 d1tlta2 d1ebfa2 d2ovga_ d1sq8a_
d1rzsa_ d3kz3a_ d3orca_ d3gpdg2 d2q49a2 d1hdgo2 d2g17a2 d2cvoa2
d2czca1 d3pwka2 d1obfo2 d1i32a2 d1zyao2 d2pkqo2 d1b7go2 d2hjsa2
d1cf2o2 d1vkna2 d1nvmb2 d2m9wa1 d3dfxa_ d2msya_ d2djoa1 d2r5za_
d1au7a1 d1f43a_ d2lmda1 d1akhb_ d1s7ea1 d3nara_ d1zq3p1 d2craa1
d1mija_ d4uuta_ d3a01a_ d1x58a1 d3rkqa1 d3cmya1 d2cqxa1 d2da6a1
d1e3oc1 d1fjla_ d1mh3a1 d1ig7a_ d1x41a1 d1wh7a1 d1gt0c1 d2mw8a_
d1lfup_ d1x2ma1 d1b72a_ d1wi3a1 d1jgga_ d1wjha1 d1x2na1 d1mnmc_
d2cuea1 d3k2aa_ d2hosa_ d2jwta2 d1pufa_ d1octc1 d2hi3a_ d1b8ia_
d4j19a_ d3lnqa_ d1wh5a1 d1b8ib_ d1fexa_ d2cqra1 d1ug2a1 d2cqqa1
d2ajea1 d4a69c_ d1h8ac1 d2crga1 d2iw5b1 d1xc5a1 d1ofcx1 d2cu7a1
d1wgxa1 d2cjja1 d2ckxa1 d1gvda_ d6paxa1 d6paxa2 d1k78a2 d1xg1a1
d1hlva1 d1iufa2 d1iufa1 d1bl0a1 d1bl0a2 d1d5ya1 d3lv8a_ d1s3ga1
d1zaka1 d1s4qa_ d3tvta2 d1uf9a_ d1khta_ d1ckea_ d1osna_ d1p5zb_
d4tmka_ d3cm0a_ d1jaga_ d3h86a_ d1t0hb_ d1nn5a_ d2h92a_ d3kb2a1
d2c95a1 d1yj5a2 d1kgda_ d1ly1a_ d2ak3a1 d1vyva2 d1deka_ d2grja1
d1p3ja1 d1y63a_ d1e4ya1 d1s96a_ d3iija1 d1lvga_ d1nksa_ d1vyua2
d4e22a_ d1qf9a_ d1jjva_ d1q3ta_ d1ki9a_ d1lw7a2 d1teva_ d1kjwa2
d1gsia_ d1g3ma_ d1j99a_ d1t8ta1 d1nsta_ d1fmja_ d1zrha_ d1ls6a_
d2a3ra_ d1aqua_ d2zptx_ d3ckla_ d1texa_ d1q44a_ d2gwha_ d1z0ja1

d3umra2 d3gj0a1 d2gaoa1 d1yzga1 d1x3sa1 d1g7sa4 d1puja_ d1udxa2
d1kk1a3 d2o52a_ d1jwyb_ d1zcaa2 d1zcba2 d2fv8a1 d2cjwa_ d3dz8a1
d1xzpa2 d1vg8a_ d4qxaa_ d2ew1a1 d1x1ra1 d2bcgy_ d3doea1 d3cona_
d3rega_ d3wyga_ d1upta_ d2b6ha1 d1zd9a1 d1r5ba3 d1n0ua2 d1r2qa_
d2f9la1 d2rhda_ d3q3jb_ d3q85a_ d1efma1 d1r8qa_ d2gf9a1 d2akab1
d1ek0a_ d1z08a1 d2gcoa1 d2dy1a2 d4g01b_ d2cxxa1 d1svia_ d3q72a_
d1ni3a1 d4ac9a4 d2erxa1 d2bmea_ d1wxqa1 d2gf0a1 d4ldja1 d1zunb3
d1d5ca_ d4lv5b_ d1efca3 d1kmqa_ d2g6ba1 d3lrpa_ d1tada2 d2y8ea_
d4rd4a1 d1z06a1 d2heia_ d3a58b_ d1u0la2 d1d2ea3 d1zj6a1 d2ocba_
d3rwoa_ d1mr3f_ d2e9sa1 d4u5xa1 d2iwra_ d2fn4a1 d3bwdd_ d2nzja1
d2atva1 d2ksqa_ d3l43a_ d1mkya2 d1mkya1 d1wmsa_ d1jnya3 d2rmka_
d1f6ba_ d1f60a3 d1gota2 d1jala1 d2fh5b1 d1g16a_ d2wblc_ d1m7ba_
d1wf3a1 d1f5na2 d1t9ha2 d1z6xa1 d1z0aa1 d2fg5a1 d2a5ja1 d2bcjq2
d1s0ua3 d1ky3a_ d4y0va_ d4lhwa_ d2qmea1 d2atxa1 d1egaa1 d1ryha_
d1moza_ d1nrjb_ d2erya1 d3bfka_ d1puia_ d1fzqa_ d3j3az1 d3t5ga_
d1shza2 d3defa_ d2f7sa1 d3raba_ d3tsoa_ d3aq4a_ d2al7a1 d3sfva1
d3bc1a_ d2ncda_ d1sdma_ d3h4sa1 d2heha_ d2grya_ g3kin.1 d1kk8a2
d1br2a2 d1goja_ d1f9va_ d2mysa2 d1lkxa_ d3t0qa_ d4db1a1 d3gbja_
d1ry6a_ d1v8ka_ d1vava_ d1uaia_ d1y4wa1 d3vrda1 d3vrda2 d1fcdc2
d1h1oa2 d1h1oa1 d1m70a2 d1m70a1 d1iqca2 d1iqca1 d1eb7a2 d1nmla2
d1nmla1 d3h3no1 d3h3no2 d3ezwa2 d1zc6a1 d1zbsa1 d1zbsa2 d1zc6a2
d2ch5a2 d2ch5a1 d1zxoa2 d1zxoa1 d2d0oa3 d2d0oa2 d1nbwa2 d1hroa_
d4gedb_ d3zooa_ d1mz4a_ d1gu2a_ d1f1fa_ d1dvva_ d1c6ra_ d1c75a_
d1e29a_ d1gdva_ d1cnoa_ d1h32b_ d1co6a_ d2bh4x_ d2aiua_ d5cytr_
d1jdla_ d1i8oa_ d2zboa_ d3dmia_ d1ls9a_ d1wvec_ d2yk3a_ d3ph2b_
d1dw0a_ d5aura_ d1qn2a_ d1f1ca_ d4zzva_ d2xspa_ d1eg1a_ d4v20a_
d1ojja_ d4ipma1 d1gpia_ d4csia_ d3pfza_ d3ovwa_ d2yoka_ d2nqya_
d1h4ga_ d1hixa_ d1oa2a_ d1olra_ d3b7ma_ d4h7ma1 d3wp3a_ d2vgda_
d1igoa_ d3zsea_ d2nlra_ d3wx5a_ d1oa3a_ d1pvxa_ d1m4wa_ d1f5ja_
d1oa4a_ d1ks5a_ d4ixla1 d1evsa_ d1cnt1_ d1pvhb_ d1z7ca_ d1huwa_
d1rhga_ d1f6fa_ d1f45b_ d1i1rb_ d1axia_ d1etea_ d3tgxb_ d1scfa_
d2z3qa1 d3qb1a_ d1hula_ d1m48a_ d4nkqb_ d1eera_ d1v7mv_ d5e4ea_
d2o27a_ d3b5ka_ d2psma1 d4zf7a_ d3uf5a_ d4x51a_ d1d9ca_ d4ypgc1
d3oq3a_ d1lqsl_ d1n1fa_ d1au1a_ d1wu3i_ d3besl_ d3ux9a_ d2b7la_
d3rbaa_ d2qtra_ d1nupa_ d3l92a_ d1f9aa_ d1qjca_ d2h29a_ d1vlha_
d1od6a_ d1lw7a1 d4ruka_ d2el7a_ d1iq0a2 d1irxa2 d1r6ta2 d1ivsa4
d2cyba_ d1rqga2 d1j09a2 d2yxna_ d1f7ua2 d1h3fa1 d1ffya3 d2g36a1
d1nzja_ d1jila_ d1n3la_ d1i6la_ d1pfva2 d1h3na3 d3n9ia_ d3sz3a1
d3sw2a_ d1moxc_ d1dx5i3 d1edmb_ d1autl2 d1autl1 d1nqlb_ d1dval1
d1szba2 d1kigl_ d2y5fl_ d1ijqa2 d1gk5a_ d1g1sa2 d1nt0a3 d1xdtr_
d1lmja2 d1lmja1 d1nzia2 d2wphe_ d1klil_ d1pfxl2 d1pfxl1 d1p9ja_
d1g1ta2 d1f7ea_ d4yzub_ d2mgpa1 d1b9wa1 d1b9wa2 d2mgpa2 d1ob1c1
d1ob1c2 d1n1ia2 d1n1ia1 d2npra2 d2npra1 d1s5ja2 d1qhta2 d4dlea2
d1tgoa2 d3py8a2 d1q8ia2 d2py5a2 d1d5aa2 d1pvaa_ d3fs7a_ d2pvba_

d1ttxa_ d1rwya_ d2kyfa_ d1rk9a_ d1a75a_ d1exra_ d1tiza1 d1nyaa_
d1g8ia_ d1ggwa_ d1wdcc_ d3fwba_ d1ggza_ d2o5ga_ d1ctaa_ d1m45a_
d3i5gc_ d1sl8a_ d1fpwa_ d2scpa_ d2roaa_ d4aqra_ d3pn7b_ d1br1b_
d1s6ca_ d1bjfa_ d2obha_ d1jfka_ d1jf0a_ d1qv0a_ d1lkja_ d2mlfc_
d2m97a_ d2d8na_ d4by5a_ d1jbaa_ d4nqga_ d1wdcb_ d2mysc_ d1j7qa_
d1s6ia_ d1uhka2 d1r2ua_ d1dtla_ d3kf9a_ d3pn7c_ d1qjta_ d1fi6a_
d1gjya_ d1y1xa_ d1hqva_ d1dvia_ d1qxpa2 d4usla_ d1k94a_ d3h33a_
d1rwja_ d19hca_ d1os6a_ d3bxua_ d3h4na_ d1i77a_ d3caoa_ d1j0pa_
d2ctha_ d1duwa_ d1ofwa_ d2e84a_ d1hh5a_ d1gyoa_ d3h34a_ d1wkya2
d1uuqa_ d3wh9a_ d4m1ra_ d1xyza_ d4xuya_ d2je8a5 d4bf7a1 d3cuia_
d1ur4a_ d1qw9a2 d1odza_ d2vzsa5 d1bqca_ d2x2ya1 d1hjqa_ d1qnra_
d4y7ea1 d1vjza_ d4k68a_ d2v3ga_ d4hpga_ d1nofa2 d1nq6a_ d1h4pa_
d1aq0a_ d3ziza_ d4bs0a_ d1ecea_ d1h1na_ d1ta3b_ d1w91a2 d1i1wa_
d1uhva2 d1us3a2 d1egza_ d7a3ha_ d1tvna1 d3nyda1 d1ghsa_ d2cyga1
d4xv0a_ d2c8na2 d1w32a_ d3emca_ d1v6ya1 d1foba_ d2uwfa1 d2fgla_
d3niya1 d2c0ha1 d1rh9a1 d1yq2a5 d1szna2 d1fa2a_ d1pama4 d5do8a1
d1ea9c3 d2fhfa5 d1qhoa4 d1avaa2 d1m53a2 d4m8ua1 d2gjpa2 d1lwha2
d4howa1 d1ktba2 d1j0ha3 d3vm5a1 d2x1ia_ d1wzaa2 d1uasa2 d2diea1
d1mxga2 d1ht6a2 d1eswa_ d1iv8a2 d5cpsa_ d1ud2a2 d1zjaa1 d3gbea1
d1kwga2 d1wdpa_ d1gcya2 d1g5aa2 d1hvxa2 d3dhpa2 d2bhua3 d4do4a1
d3qgva1 d1e43a2 d1g94a2 d2d3na2 d1gjwa2 d1gvia3 d1x1na1 d1r7aa2
d1tz7a1 d4toqa_ d1wb0a1 d1w9pa1 d1eoka_ d1itxa1 d1kfwa1 d1goia2
d1edqa2 d2gsja_ d1hjxa1 d1jnda1 d1vf8a1 d3wdpp1 d3wq5a_ d1gnxa_
d4gxpa_ d4hz8a_ d1e4mm_ d3aiua_ d1wcga1 d1qvba_ d3ahxa_ d1v02a_
d2dgaa_ d1pbga_ d1vffa1 d4ha4a_ d1mixa2 d1e5wa2 d1j19a2 d1isna2
d2al6b2 d3syxa1 d1mkea1 d2p8va_ d1xoda1 d1i7aa_ d1evha_ d1qc6a_
d1xl4a1 d3vsqa_ d2gixa1 d1p7ba1 d2v76a_ d1wj1a1 d1aqca_ d1qqga2
d1ntva_ d1n3ha_ d4dbba_ d3f0wa1 d1p3ra_ d1wgua1 d2cy4a1 d3hqca_
d1wvha1 d1xr0b1 d1wgqa1 d1v89a1 d2coda1 d1zc3b_ d1wg7a1 d1u5ea1
d1v61a1 d2coca1 d2ys1a1 d1v5pa1 d1ntya2 d1wjma1 d1wi1a1 d1u29a_
d1xx0a1 d1unqa_ d2zkmx3 d1fhoa_ d1v88a1 d2adza1 d1x1fa1 d1p6sa_
d2coaa1 d1btka_ d1v5ma1 d1faoa_ d2x18a_ d1dyna_ d1x1ga1 d4kaxb_
d2j59m1 d1xcga2 d1txda2 d1u5da1 d1dbha2 d1w1ha_ d1upqa_ d1v5ua1
d1foea2 d2fjla1 d1eaza_ d2dfka2 d2cofa1 d1kz7a2 d9ldta1 d2ewda3
d1up7a1 d2dfda1 d1s6ya1 d1vjta1 d7mdha1 d1hyea1 d1t2da1 d1ojsa3
d1b8pa1 d1ldna1 d1hyha1 d1civa1 d2a92a1 d1ez4a1 d1i0za1 d1y6ja1
d1gv0a1 d1ur5a1 d1llda1 d1obba1 d1v8ba1 d1gdha1 d1l7da1 d2dlda1
d1mx3a1 d1ygya1 d1qp8a1 d1li4a1 d1j4aa1 d2naca1 d1npya1 d3t4ea2
d1a4ia1 d1o0sa1 d2hk9a2 d2egga2 d1b26a1 d1bvua1 d1gq2a1 d1c1da1
d1leha1 d1gtma1 d1euza1 d1nvta1 d3dona2 d1edza1 d1gpja2 d1p77a1
d1nyta1 d1b0aa1 d1vl6a1 d1v9la1 d1txga2 d3f47a1 d1mv8a2 d2pv7a2
d1xc2a3 d2ahra2 d4xiya1 d2f1ka2 d1pgja2 d1i36a2 d1np3a2 d1lj8a4
d1teaa3 d1qmga2 d2i76a2 d2g5ca2 d1wdka3 d1dlja2 d3cuma3 d1kola2
d1f8fa2 d1qora2 d1jqba2 d1vj0a2 d1cdoa2 d1gu7a2 d1d1ta2 d1xa0a2

d2dm6a2 d1jvba2 d1rjwa2 d1llua2 d1h2ba2 d1tt7a2 d1piwa2 d1o89a2
d1iz0a2 d1e3ja2 d1yb5a2 d1uufa2 d1vj1a2 d1e3ia2 d3gpdg1 d1vc2a1
d2nvwa1 d1u8fo1 d1ydwa1 d1hdgo1 d2g17a1 d1cf2o1 d2czca2 d1r0ka2
d1obfo1 d2cvoa1 d1ebfa1 d1h6da1 d1i32a1 d1zh8a1 d2q49a1 d1qkia1
d1j5pa4 d3pzra1 d1xeaa1 d1vkoa1 d1vm6a3 d3qvsa1 d2pkqo1 d1b7go1
d1gr0a1 d1p1ja1 d1lc0a1 d1j0xo1 d3pwka1 d2hjsa1 d1zyao1 d1vkna1
d1tlta1 d1nvmb1 d3sjua_ d3ojfa1 d1xu9a1 d2jjya1 d1n7ha_ d1y1pa1
d1h5qa_ d1jtva_ d4fs3a_ d2bkaa1 d1gy8a_ d2c5aa1 d1ulsa_ d1ooea_
d2b69a1 d1y5ma1 d1spxa_ d1ae1a_ d1bxka_ d1ydea1 d1gega_ d1hdoa_
d1o5ia_ d1e6ua_ d1snya1 d2rhca_ d3zv4a_ d5ceja_ d2d1ya1 d1fjha_
d3w8ea_ d2fmua1 d1x1ta_ d1rpna_ d3ay7a1 d1k2wa_ d1eq2a_ d2hrba1
d3ruaa_ d2ew8a1 d4hwka_ d1xgka_ d2bd0a1 d1yo6a1 d2a35a1 d2ntva_
d1vl8a_ d1hdca_ d4bkua_ d2ag5a1 d2q45a_ d1nffa_ d4iy1a_ d1zbqa1
d1kewa_ d1ek6a_ d1ja9a_ d2q46a2 d1iy8a_ d1cyda_ d2ptga_ d1hxha_
d1uzma1 d3lyla_ d4m89a_ d3wyea_ d2blla1 d1w6ua_ d1sb8a_ d2q2va_
d4nr0a_ d1e6wa_ d2gasa_ d3oiga1 d2bgka1 d1mxha_ d1vl0a1 d1rkxa_
d1r6da_ d1wmaa1 d1e7wa_ d2c07a1 d1xkqa_ d3tzha_ d1g0oa_ d3lz6a_
d1qyca_ d1edoa_ d1gz6a_ d1oc2a_ d1t2aa_ d1zema1 d2xoxa_ d1z45a2
d1geea_ d1p33a_ d3lt0a_ d4e6pa_ d2wyua_ d1db3a_ d4q9na_ d1xhla_
d4o1ma_ d1fmca_ d2uvda_ d2gdza1 d1uaya_ d2z1ma_ d2o23a2 d2fr1a2
d2fr1a1 d1qsga_ d1qyda_ d4zjua_ d2a4ka1 d1yb1a_ d1wvga1 d1yxma1
d3f4ba_ d1mg5a_ d1xg5a1 d1r55a1 d3gboa_ d1atla_ d1kufa_ d1buda_
d4q1la_ d1quaa_ d1yp1a_ d1wnia_ d3nqyb_ d4gera_ d1bqba_ d1xd3a_
d3ifwa_ d1mdwa_ d1ziva1 d1zcma1 d1t3ca_ d1epwa3 d2a97a_ d3fiea_
g1e1h.1 d3ioqa_ d1fh0a_ d1o0ea_ d1khqa_ d1m6da_ d2cb5a_ d1cs8a_
g8pch.1 d1s4va_ d2pnsa_ d1vsna1 d1mira_ d1iwda_ d2oula_ d7pcka_
d3bwka_ d3bcna_ d2p86a_ d1pvja_ d1pcia_ d1gece_ d1deua_ d4bs6a_
d1xkga1 d1cqda_ d1jqpa2 d2c0ya_ d2bdza_ d3p5ua_ d1zoya1 d1qo8a2
d1d4ca2 d1chua2 d1kf6a2 d2jk6a2 d2jk6a1 d1feca1 d1feca2 d1lpfa1
d1lpfa2 d1xhca1 d1xhca2 d1dxla2 d1dxla1 d1gera2 d1m6ia2 d1onfa1
d1onfa2 d3lada2 d3lada1 d1w4xa1 d1w4xa2 d1gesa2 d1mo9a1 d1xdia1
d1fl2a2 d1fl2a1 d1fcda1 d1fcda2 d1h6va2 d1h6va1 d1mo9a2 d1gv4a2
d1gv4a1 d2gv8a2 d2gv8a1 d1aoga1 d1aoga2 d1hyua1 d1ebda1 d1ebda2
d3dk9a1 d1q1ra2 d1q1ra1 d1gesa1 d1g4ma1 d1g4ma2 d1ps9a2 d1o94a2
d1cjca1 d1pn0a1 d1reoa1 d1ju2a1 d2f5va1 d3c96a1 d2gmha1 d1pj5a2
d1gpea1 d1o5wa1 d1n4wa1 d1k0ia1 d2dw4a2 d2ivda1 d2voua1 d1kdga1
d1s5ja1 d2hbja2 d2igia_ d1wlja_ d1qhta1 d4dlea1 d1tgoa1 d2f96a1
d3tr8a1 d3mxma_ d1q8ia1 d1y97a1 d1w0ha_ d2py5a1 d1noya_ d1d5aa1
d1yt3a3 d2guia_ d3mwpa1 d4kb1a_ d1js9c_ d1f15b_ d1za7a_ d1laja_
d1iuka_ d2csua1 d3q9ub_ d2yv1a1 d3q9na_ d2d59a_ d2yv2a1 d1euca1
d1y81a1 d1k8ka2 d1k8ka1 d3uleb1 d3i33a2 d3i33a1 d1bupa1 d1dkgd2
d1dkgd1 d4ci6a2 d1jcfa1 d4a2ba2 d2fxua1 d1yaga2 d1yaga1 d4cbua2
d1bupa2 d1r7ha_ d1faaa_ d1wika1 d1xfla_ d1ttza1 d2f51a1 d1ti3a_
d1nhoa_ d1sena_ d2i4aa_ d1ep7a_ d1zmaa1 d1fo5a_ d3lefa1 d3diea1

d2yana1 d1z6na1 d1oaza_ d3zywa1 d4aj8a_ d1nm3a1 d4fiwa_ d1wmja_
d4hs1a1 d1iloa_ d1xwaa1 d1wjka1 d2vm1a_ d1fb6a_ d1gh2a_ d1h75a_
d1nw2a_ d1kx5b_ d3a6na_ d4m6ba2 d2nqbd_ d3wkjd_ d2yfwe_ d4h9na_
d5bnxa_ d1kx5c_ d3r45a_ d1eqzb_ d4jjnb_ d1f66c_ d1eqza_ d4wnnb_
d1kx5a_ d1u35c1 d1id3a_ d2nqbc_ d1ku5a_ d1f1ea_ d1b67a_ d2b5ea2
d2b5ea3 d2b5ea1 d2b5ea4 d2kp1a1 d2kp2a1 d1hyua3 d1hyua4 d1j08a2
d1pd212 d1nhya2 d2a2ra2 d1k0dd2 d1f2ea2 d1gsua2 d1f3ba2 d2fhea2
d2pvqa1 d1tw9a2 d1oyja2 d1e6ba2 d1k0ma2 d3ik7a1 d4is0a1 d1r5aa2
d1jlva2 d1m0ua2 d3gtub2 d1z9ha2 d1okta2 d1axda2 d2gsra2 d1tu7a2
d1k3ya2 d1zgma1 d1gnwa2 d1gwca2 d2gsta2 d1glqa2 d1oe8a2 d1pn9a2
d1v2aa2 d1fw1a2 d1duga2 d3h1jd1 d3wyha_ d4g9sa1 d3gxra_ d1qqya_
d1gd6a_ d1f6ra_ d1ivma_ d2gv0a_ d1iiza_ d2z2fa_ d1j8xa_ d1ghla_
d1b9oa_ d174la_ d169la_ d2o4wa_ d1p37a_ d1k28a3 d1xjta1 d1jtma_
d2f2qa_ d4e97a1 d2pjsa1 d1klla_ d2i7ra1 d1ecsa_ d1xqaa1 d5cj3a1
d1npba_ d1jifa_ d2p7oa_ d1nkia_ d1mpya2 d1kw3b2 d1kw3b1 d4ghga1
d1tfza1 d3isqa1 d3isqa2 d1sqia1 d1sqia2 d1lkda2 d1lkda1 d1mpya1
d1cjxa2 d1cjxa1 d1sp9b2 d1t47a1 d1t47a2 d1sp8a1 d1sp8a2 d1f1ua1
d1pv8a_ d1w5qa_ d2c1ha_ d1ohla_ d1l6sa_ d1qsta_ d2beia1 d4m3sa_
d1u6ma1 d1ygha_ d1yk3a1 d3dr6a_ d1xmta_ d1z4ea1 d2ge3a1 d1bo4a_
d2fiaa1 d2jdca1 d1y9wa1 d3to7a_ d1wwza1 d2fl4a1 d2ozua1 d2b5ga_
d2cy2a1 d2p0wa_ d1cm0a_ d4pswa_ d1yrea1 d1yr0a1 d1m4ia_ d2gana1
d1yvka1 d1ghea_ d2g3aa1 d1y7ra1 d2euia1 d1q2ya_ d2fsra1 d1xeba_
d2ae6a1 d1qsma_ d2aj6a1 d1y9ka1 d1i12a_ d1vkca_ d2atra1 d1n71a_
d2d4pa1 d2fiwa1 d2fe7a1 d1tiqa1 d2ou2a_ d1yx0a1 d2fcka1 d1p0ha_
d2j8ma_ d2pq8a_ d1z4ra1 d1s3za1 d1ufha_ d1f37b_ d1xvqa1 d1pn0a2
d3hy2a_ d2cvba1 d1o8xa_ d1lu4a_ d1we0a1 d2bmxa1 d2a4va1 d1psqa_
d1xiya1 d1q98a_ d1n8ja_ d1zofa1 d2ggta_ d3tjja_ d4je1a1 d1o73a_
d3s9fa_ d2zcta_ d1qmva_ d2b1ka_ d5dvba1 d2b5xa1 d2v2ga_ d1st9a_
d3tb2a_ d4llra_ d4fh8a1 d1e2ya_ d1knga_ d2h30a_ d1i5ga_ d2z9sa_
d2f8aa1 d1tp9a1 d2b7ka_ d1nm3a2 d3hvva_ d2wfca1 d4kcea_ d1xvwa1
d4gqca1 d3qpma_ d4k1fa_ d1zzoa1 d1gp1a_ d1prxa_ d2m5ta_ d1cqqa_
d1wqsa_ d5bpea_ d2ipha_ d1lvoa_ d2bhga1 d3tnta_ d3sjia_ d3d23a1
d2fyqa_ d3tloa_ d3zvea1 d2hrva_ d3q3ya1 d4asha1 d1jgta2 d1ofda3
d1ea0a3 d1ecfa2 d1q15a2 d1ct9a2 d1te5a_ d1ra6a_ d1muka_ d3gsza_
d1khva_ d1te9a_ d2xwha_ d3uqsa_ d3hkwa_ d3n6ma_ d2cjqa_ d2xyma_
d1teba_ d1te8a_ d1s48a_ d1u09a1 d4wfxa_ d3tyqa_ d2pgga1 d1lgya_
d4l3wa_ d1llfa_ d1gz7a_ d1ukca_ d1c4xa_ d2rhwa_ d3groa_ d1pjaa_
d1ei9a_ d2ftba_ d1pmpa_ d2rcqa1 d1fdqa_ d4lkpa_ d1sa8a_ d1vyfa_
d1lpja_ d3vg7a1 d4azqa1 d1o8va_ d5bvqa_ d1p6pa_ d2qo4a_ d1opba_
d1yiva1 d1o1va_ d4bvma1 d4tkba_ d4i3ba_ d1bwya_ d1ftpa_ d1ggla_
d4gkca_ d1cbia_ d3p6da1 d1kqwa_ d2a0aa1 d3zxha_ d1cgla_ d1rm8a_
d1eaka2 d1hy7a_ d2jsda_ d1hv5a_ d1i76a_ d1g12a_ d3p0ya1 d1igra1
d1igra2 d1n8yc2 d1m6ba2 d1m6ba1 d1m4la_ d2bk7a_ d3fjua_ d1z5ra1
d1kwma1 d1uwya2 d1jqga1 d2c1ca1 d1dtda_ d1h8la2 d2tbvc_ d1c8nc_

| | | | | | | | |
|---|---|---|---|---|---|---|---|
| d1x36a_ | d1smvc_ | d1ng0c_ | d4sbvc_ | d1opoa_ | d1f2nc_ | d1cixa_ | d1y29a_ |
| d1v7fa_ | d2jtba_ | d1d1ha_ | d1g9pa_ | d1lupa_ | d1qk7a_ | d1mb6a_ | d2a2va_ |
| d1niya_ | d1i25a_ | d1hvwa_ | d2mpqa_ | d1ie6a_ | d1kqha_ | d1la4a_ | d2mxoa1 |
| d1dl0a_ | d1koza_ | d1qk6a_ | d1emxa_ | d1lqra_ | d1c6wa_ | d1rmka_ | d1ixta_ |
| d1eyoa_ | d1cnna_ | d1fu3a_ | d1g1pa_ | d1f3ka_ | d1rypd_ | d1irua_ | d1rypc_ |
| d3unfk_ | d1rypa_ | d1j2pa_ | d1irui_ | d1j2qh_ | d3unfh_ | d1m4ya_ | d1rypb_ |
| d1irul_ | d1irug_ | d1iru2_ | d1q5rh_ | d1rypg_ | d1g3ka_ | d1q5qa_ | d1e94a_ |
| d1irub_ | d1ryph_ | d3ty6a_ | d1iruj_ | d1iruk_ | d1rype_ | d1irud_ | d1irue_ |
| d3mi01_ | d1rypf_ | d1iruh_ | d2z3ba_ | d1iruc_ | d3mka2_ | d1iru1_ | d1mqta_ |
| d1d4m3_ | d1fmd3_ | d2mev1_ | g1aym.1 | d1ev13_ | d4q4w1_ | d1r1a3_ | d2x5ia_ |
| g1r1a.1 | g1d4m.1 | g1rhi.1 | d1b35b_ | d1oopa_ | d1tme1_ | d4iv1a_ | g1pvc.1 |
| g1fpn.1 | d1fpn1_ | d4gb31_ | d1d4m1_ | d1fmd1_ | d1ev11_ | d2wzr3_ | g1cov.1 |
| d1qqp3_ | g1fmd.1 | d2x5ic_ | d1rhi3_ | g1oop.1 | d1hxs1_ | g1hxs.1 | d1fpn3_ |
| d1bev1_ | g2mev.1 | d1eah3_ | d1aym1_ | d1pvc3_ | d1qqp1_ | d1zba3_ | d1h8ta_ |
| d3vbhc_ | g1b35.1 | g1bev.1 | d1hxs3_ | g1ev1.1 | d1bev3_ | g1tmf.1 | d1tmf1_ |
| d1b35a_ | d2mev3_ | g1eah.1 | g1h8t.1 | d1eah1_ | d4q4w3_ | d1r1a1_ | d1aym3_ |
| d1pvc1_ | d1zba1_ | d1h8tc_ | d1oopc_ | d1tme3_ | g1qqp.1 | d4iv1c_ | g1tme.1 |
| d3ubub_ | d1egia_ | d1v7pa_ | d1tdqb_ | d3ubua_ | d1c3ab_ | d1h8ua_ | d3bx4b_ |
| d1t8ca1 | d1sb2a1 | d1qdda_ | d2ziba1 | d3cada_ | d1byfa_ | d1j34b_ | d1jwia_ |
| d1jzna_ | d1c3aa_ | d1oz7b_ | d1wmza_ | d3bdwb_ | d1hq8a_ | d1fifa1 | d1dv8a_ |
| d3hupa_ | d1wk1a1 | d1umra_ | d2afpa_ | d3bx4a_ | d1gz2a_ | d1sl6a_ | d1v7pb_ |
| d3g8ka_ | d1ixxa_ | d1j34a_ | d1jwib_ | d1g1sa1 | d1y17a1 | d1ja3a_ | d1fvub_ |
| d3bdwa_ | d1sb2b1 | d1fvua_ | d1umrc_ | d1oz7a_ | d4m17a_ | d3cfwa1 | d1ypqa1 |
| d1g1ta1 | d3fd4a_ | d3l9jc_ | d4dn8a1 | d1v4lb_ | d1rdl1_ | d2msba_ | d3exra_ |
| d2ffca1 | d2fdsa1 | d3n3ma1 | d2czda_ | d1q6oa_ | d2yyua_ | d1vqta1 | d1dbta_ |
| d3ldva1 | d2flia1 | d1rpxa_ | d1tqja_ | d1tqxa_ | d4lt9a1 | d3tc7a1 | d3hoja_ |
| d1i4na_ | d3vnda_ | d3nl8a1 | d1geqa_ | d3nava1 | d1v5xa_ | d4a2ra1 | d3tc6a1 |
| d1ujpa_ | d4ijba_ | d1xcfa_ | d1vc4a_ | d1rd5a_ | d1qopa_ | d3b5va_ | d2r80b_ |
| d1cqxa1 | d1cg5a_ | d1gcvb_ | d3boma_ | d2h8fb_ | d2raoa_ | d3bj1a_ | d4hrrb_ |
| d2r50a_ | d3d1kb_ | d2aa1b1 | d3zhwa_ | d3hf4b_ | d3gdja_ | d1jebb_ | d3bj1b_ |
| d1x9fb_ | d1wmub_ | d1gcva_ | d3bcqb_ | d3d4xb_ | d1gvha1 | d2dc3a_ | d3hf4a_ |
| d4yu3a_ | d3k8ba_ | d1qpwb_ | d2r80a_ | d3bcqa_ | d1v4wa_ | d3wtgb_ | d1jl7a_ |
| d4esaa_ | d3d1ab_ | d1fsla_ | d4h2la_ | d1v4wb_ | d1mwca_ | d1vhba_ | d1jeba_ |
| d1spgb_ | d1h97a_ | d1q1fa_ | d1tu9a1 | d3d1aa_ | d1hdsb_ | d3hyub_ | d2zfbb_ |
| d3fs4a_ | d1oj6a_ | d1shrb_ | d4h2lb_ | d4yu3b_ | d1spga_ | d3hrwa_ | d3qqra_ |
| d3at5a_ | d4wchd_ | d3hyua_ | d1cg5b_ | d1uc3a_ | d1hbra_ | d2raob_ | d1outb_ |
| d4esab_ | d3gdjb_ | d3qqqa_ | d1x9fc_ | d3vrfa_ | d1itha_ | d3mkba_ | d3d4xa_ |
| d3mjpa_ | d1jl6a_ | d3fh9a_ | d1outa_ | d2dc3b_ | d1it2a_ | d3eoka_ | d3g46a_ |
| d1qpwa_ | d3vrfb_ | d1ch4a_ | d1x9fd_ | d1hdsa_ | d2zfba_ | d3mkbb_ | d1or4a_ |
| d3wtga_ | d1a4fa_ | d3fh9b_ | d3bomb_ | d3cy5a_ | d3at5b_ | d1x9fa_ | d2oifa_ |
| d4lmxb_ | d1cpcb_ | d3v57a_ | d1kn1a_ | d1cpca_ | d4f0ta_ | d2vjha_ | d4po5b_ |
| d1eyxa_ | d1alla_ | d4f0tb_ | d1b33b_ | d1b8db_ | d4h0mb_ | d1b33a_ | d2vjhb_ |
| d4lm6b_ | d2vmlb_ | d1allb_ | d2vjta_ | d3dbja_ | d2vmla_ | d2vjtb_ | d4f0ub_ |
| d1f99b_ | d4rmpa1 | d1liaa_ | d1eyxb_ | d2uulb_ | d4lmsb_ | d4f0ua_ | d2bv8a_ |

| | | | | | | | |
|---|---|---|---|---|---|---|---|
| d1f99a_ | d2bv8b_ | d2c7lb_ | d3dbjb_ | d4rmpb1 | d1b8da_ | d4h0ma_ | d3v57b_ |
| d1kn1b_ | d1liab_ | d2uula_ | d2j96a_ | g1gxs.1 | d1hlga_ | d2xqfa_ | d1ea5a_ |
| d1f6wa_ | d1dx4a_ | d1k4ya_ | d4qwwa_ | d2ha2a_ | d1r88a_ | d1sfra_ | d4qeka_ |
| d3c8da2 | d2pbla1 | d1jkma_ | d1lzla_ | d1jjfa_ | d1gl9b1 | d1gkub2 | d1jmxa2 |
| d1pbya1 | d1pbya2 | d1b9ra_ | d1offa_ | d1frra_ | d3wcqa_ | d1e9ma_ | d1wria_ |
| d1i7ha_ | d3av8a_ | d1czpa_ | d1rfka_ | d3p1ma1 | d3b2ga_ | d2bt6a2 | d1xm5a_ |
| d1xaxa_ | d1tvia_ | d4iuaa3 | d4bvva_ | d5hpga_ | d3k65a_ | d1ki0a3 | d1ki0a2 |
| d1ki0a1 | d1a0ha1 | d1pmla_ | d3sp8a3 | d2feba1 | d1gvfa_ | d3qm3a1 | d2fjka_ |
| d1f74a_ | d3l21a_ | d2e1da_ | d1to3a1 | d1w3ia_ | d1sfla_ | d1n7ka_ | d1vcva1 |
| d4imea_ | d1wa3a_ | d2ocza1 | d1f2ja_ | d3m16a_ | d2yxga_ | d1ub3a_ | d3a5fa_ |
| d1fbaa_ | d1l6wa_ | d1xfba1 | d1wx0a1 | d1vpxa1 | d1ojxa_ | d1mxsa_ | d2a4aa1 |
| d1xkya1 | d3cq0a1 | d3s1xa_ | d1f05a_ | d1fdja_ | d3cpra1 | d4d2ja_ | d1e88a1 |
| d1l6ja5 | d1l6ja4 | d1eaka4 | d1eaka3 | d1l6ja3 | d1h8pa2 | d1h8pa1 | d3rp2a_ |
| g1ucy.1 | d1fuja_ | d2zchp_ | d3w94a_ | d1si5h_ | d1lo6a_ | g2pka.1 | d1cgha_ |
| d1kigh_ | d1a5ia_ | d3s9ba_ | d1hj8a_ | d1ao5a_ | d2anya_ | d1q3xa1 | d1gvza_ |
| d1azza_ | d4gsoa_ | d2f91a1 | d1iaua_ | d1gdna_ | d1eufa_ | g1fiw.1 | d1eq9a_ |
| d4nfea_ | d2rdla_ | d1spja_ | d1elva1 | d1a7sa_ | d1ekbb_ | d1pfxc_ | d1yc0a_ |
| d2aiqa_ | g1fiz.1 | d1fi8a_ | d4k60a_ | d4dgja_ | d1mzaa_ | d1op0a_ | d3tjva_ |
| d1pytd_ | d1sgfg_ | g1gg6.1 | d2eeka_ | g1rtf.1 | d1a0ja_ | d3g01a_ | d1pytc_ |
| d4e7na_ | d1ddja_ | d1z8ga1 | d2hlca_ | d1npma_ | d1brup_ | g1jou.1 | d1sgfa_ |
| d1autc_ | g2hnt.1 | g1kdq.1 | d3s69a_ | d4crga_ | d1fxya_ | d1bqya_ | d1svpa_ |
| d3su6a1 | d1cu1a1 | d4agka_ | d1vcpa_ | d1q31a_ | g1n1l.1 | d3e90b_ | d1ep5a_ |
| d3u1jb_ | d1dzka_ | d4tlja_ | d2a2ua_ | d4wfva_ | d3t1da_ | d4iaxa_ | d3zq3a_ |
| d2nnda_ | d1i4ua_ | d1kxoa_ | d1bbpa_ | d1kt7a_ | d1exsa_ | d3s26a1 | d1lf7a_ |
| d1qfta_ | d4gh7a_ | d1z24a1 | d1qabe_ | d1xkia_ | d1ew3a_ | d3bx7a_ | d4iawa1 |
| d1n0sa_ | d1gkab_ | d3kzaa_ | d2k23a_ | d2l9ca1 | d2joza1 | d1gm6a_ | d3kffa_ |
| d3saoa1 | d1e5pa_ | d1yupa1 | d1iiua_ | d3lgia_ | d3i77a_ | d2z9ia2 | d4nsva_ |
| d1lcya2 | d4jcna_ | d1agja_ | d1os8a_ | d1hpga_ | d1p3ca_ | d1l1ja_ | d1qtfa_ |
| d1e69a_ | d1mv5a_ | d3g60a4 | g1ii8.1 | d1ewqa2 | d1ji0a_ | d1qhla_ | d1v43a3 |
| d2wmfa1 | d1vpla_ | d1g2912 | d4a82a2 | g1f2t.1 | d2d62a1 | d3tuic1 | d2bbsa1 |
| d3g60a3 | d2ixea_ | d1g6ha_ | d1sgwa1 | d2qi9c_ | d3rlfa1 | d3b60a1 | d3d31a2 |
| d2pzea1 | g1xew.1 | d1jj7a_ | d1oxxk2 | d2onka1 | d1w1wa_ | d1qf5a_ | d1vmaa2 |
| d1nija1 | d1np6a_ | d2p67a1 | d3ihla_ | d1okkd2 | d1ls1a2 | d1s1ma2 | d1iona_ |
| d2v40a_ | d1dj3a_ | d3hida_ | d2vo1a1 | d2g0ta1 | d1vcoa2 | d1j8mf2 | d1g3qa_ |
| d1xjca_ | d1p9ba_ | d1qzxa3 | d1ihua2 | d1ihua1 | d2qm8a_ | d1dj2a_ | d4ioja_ |
| d1cp2a_ | d1yrba2 | d1hyqa_ | d3gqba2 | d1skye3 | d1xp8a1 | d1tf7a2 | d1tf7a1 |
| d1ye8a1 | d1skyb3 | d2qe7d2 | d4blta_ | d4huta_ | d3ldaa2 | d4hyya_ | d2i3ba1 |
| d1cr1a_ | d3j9tb2 | d3ntua2 | d1vdza1 | d2z43a_ | d1e9ra_ | d1nlfa_ | d3ziaa2 |
| d1n0wa_ | d1g6oa_ | d3ziad2 | d3j9ta2 | d1pzna2 | d3gqbb2 | d2i49a_ | d1nnfa_ |
| d1xvxa_ | d1ursa_ | d1h3da1 | d1iuda_ | d4m1va1 | d1twya_ | d3e13x_ | d2nxoa1 |
| d4pfua_ | d3ttma_ | d1elja_ | d2qmwa1 | d1us5a_ | d1y3na1 | d1jeta_ | d1uqwa_ |
| d4f19a1 | d2esna2 | d3thia_ | d3fasa1 | d2uyeb_ | d2xwva1 | d1r9la_ | d2fyia1 |
| d1pb7a_ | d2ozza1 | d1sw5a_ | d1nh8a1 | d1vr5a1 | d2g29a_ | d1xs5a_ | d3lswa_ |
| d2z23a_ | d3w9va_ | d3tcha_ | d1q35a_ | d1z7me1 | d3q27a_ | d1p99a_ | d1ve4a1 |

d2i6ea1 d4mcoa1 d4n8ya_ d1eu8a_ d1zbma1 d1hsla_ d1ixca2 d1j1na_
d1xvya_ d1y9ua_ d2z22a1 d1xoca1 d4oera_ d1wdna_ d5f1qa1 d1xt8a1
d1i6aa_ d4gwoa_ d4igta1 d1pc3a_ d4mmpa1 d1o63a_ d2czla1 d2onsa2
d4mija1 d4ok5a1 d2bmfa1 d2bmfa2 d1yksa1 d1yksa2 d1jr6a_ d2qeqa1
d2jlqa1 d1eh3a_ d3skpa_ d1dtza2 d1ieja_ d1jnfa2 d1ce2a1 d1jnfa1
d1b1xa2 d1b1xa1 d1jw1a1 d1jw1a2 d1ryoa_ d1h76a1 d1h76a2 d1c4oa1
d2p6ra3 d2p6ra4 d1oywa2 d1oywa3 d1wrba1 d1w36b2 d1w36b1 d1c4oa2
d3bora_ d1t6na_ d2eyqa5 d2eyqa3 d2eyqa2 d2zu6a2 d1veca_ d1uaaa1
d1w36d1 d2g9na1 d1z3ix2 d2rb4a_ d1hv8a1 d1hv8a2 d1t5ia_ d1w36c2
d2j0sa2 d2j0sa1 d1fuka_ d3hjha1 d1z5za1 d1qdea_ d1z63a1 d1s2ma2
d1s2ma1 d1wp9a1 d1wp9a2 d1gm5a3 d1gm5a4 d3dina4 d3dina3 d1q0ua_
d1w36c1 d1z3ix1 d1nkta3 d1nkta4 d1uaaa2 d1w36d2 g1qhh.1 d2fwra2
d2fwra1 d1oewa_ d2qzxa_ d2asia_ d1psoe_ d1miqa_ d1smra_ d2h6ta_
d1pfza_ d1izda_ d3hd8a_ d1j71a_ d1dpja_ d4aa9a_ d1t6ex_ g1b5f.1
g1lya.1 g1htr.1 d1qdma2 d1bxoa_ d1wkra_ d1tzsa_ d2qzwa_ d1bdqa_
d3ggta_ d3ixoa_ d1mrxa_ d2haha_ d1tw7a_ d4njsa_ d1sgua_ d2p3da_
d1nsoa_ d3d3ta_ d2rkfa_ d1c6ya_ d2rspa_ d3ogpa_ d3ekwa_ d2o4na_
d2j9ja_ d1baia_ d3ggua_ d3lzua_ d4npua_ d4j55a_ d2r8na_ d2fxda_
d2p3ca_ d4dqea_ d3ttpa_ d3t3ca_ d2p3aa_ d2hc0a_ d2fdda_ d3mwsa_
d1k8rb_ d1wgra1 d1ef5a_ d1wfya1 d1e8ya3 d1wgya1 d1lfda_ d2cs4a1
d1wxma1 d2byea1 d3kh0a1 d2byfa1 d1wxaa1 d1e7ua3 d4yl8a1 d2al6a3
d1wgda1 d1wgga1 d4q5eb_ d1wjna1 d1se9a_ d3n3kb_ d1wz0a1 d1we6a1
d2m0xa_ d2gbka_ d3rt3b1 d1wh3a1 d1sifa_ d2ekec2 d4ii2b1 d4hk2a1
d1c3ta_ d1m94a_ d1wgha1 d1ttna1 d1j8ca_ d1wx8a1 d3v6cb1 d2ma9b_
d4i6lb1 d1bt0a_ d2jvca1 d1iyfa_ d2k1fa_ d1wxva1 d1wf9a1 d1t0ya_
d2io0b2 d1x1ma1 d2gbma_ d1zw7a1 d2m17a_ d1we7a1 d2lwpa_ d2gbja_
d1wjua1 d1v6ea1 d1v86a1 d4eewa_ d1wiaa1 d4icva_ d4gu2a_ d1wx7a1
d1wy8a1 d4hjka1 d2faza1 d1v5oa1 d2al3a1 d2cr5a1 d1wj4a1 d1s3sg_
d3u1ra1 d1g9ka2 d1kapp2 d1g66a_ d5ajha1 d4lu0a_ d1of8a_ d3unda1
d3e9aa1 d1vr6a2 d1n8fa_ d2qkfa_ d1d9ea_ d1o60a_ g1y43.1 d1um8a_
d1ixza_ d1qvra2 d1qvra3 d1qzma_ d1sxjc2 d1jr3d2 d2p65a_ d1l8qa2
d1g8pa_ d3hu3a3 d1sxjd2 d1njfa_ d1in4a2 d1sxja2 d1tuea1 d2ce7a2
d1w5sa2 d1sxjb2 d1d2na_ d1ny5a2 d1ofha_ d2gnoa2 d2a5yb3 d1fnna2
d1u0ja1 d1lv7a_ d1iqpa2 d2fnaa2 d1ixsb2 d4gita_ d1sxje2 d1g41a_
d2e3fa_ d1mm0a_ d2e3ea_ d2llda_ d2ny8x_ d2e3ga_ d2ny9x_ d2nz3a_
d2lj7a_ d1n4na_ d2m8bb1 d2kyqa_ d3psma_ d1jkza_ d1jxca_ d2gl1a_
d1ugla_ d4uj0a_ d2k4ua_ d1hp2a_ d2lu9a_ d1baha_ d2meoa1 d1wm7a_
d1n8ma_ d1jlza_ d1wpda_ d1c55a_ d1pvza_ d1wt7a_ d1rjia_ d1wz5a_
d1m2sa_ d1pjva_ d1c49a_ d1qkya_ d2ktca1 d1wmta_ d1du9a_ d1j5ja_
d1r1ga_ d1t0za_ d2i61a_ d1t1ta_ d1sn4a_ d1kv0a_ d2lkba_ d1npia_
d1wwna_ d1pe4a_ d2kbka_ d2li7a_ d1djta_ d2kbha_ d1dq7a_ d1sega_
d1omya_ d1zywa1 d1jzaa_ d1ixra2 d1iyjb4 d1iyjb5 d1iyjb3 d1o7ia_
d4ipca1 d1x3ea_ d4apva_ d1jb7a1 d1jb7a2 d1jb7a3 d3kdfa_ d1ue1b_
d1wjja1 d3eiva_ d1xjva2 d1xjva1 d2ccza2 d1se8a_ d1l1oc_ d1jb7b_

| | | | | | | | |
|---|---|---|---|---|---|---|---|
| d1qvca_ | d3ulla_ | d1qzga_ | d1miua4 | d1miua5 | d1miua3 | d1y14b1 | d2nn6i1 |
| d1i94l_ | d2zjra2 | d1smxa_ | d2qamc2 | d1hh2p1 | d2r7da1 | d1rl2a2 | d1wfqa1 |
| d3bzka4 | d1x6oa2 | d3a0ja_ | d2j01d2 | d2ja9a1 | d4ql5a_ | d1hr0w_ | d1luza_ |
| d2n3sa_ | d2vnud1 | d1h95a_ | d1go3e1 | d2nn6h1 | d2mo1a_ | d1iz6a2 | d1jjga_ |
| d1e3pa2 | d2ix0a3 | d2ix0a2 | d2lxja_ | d1ueba3 | d1ueba2 | d2z0sa1 | d1u0la1 |
| d2ix0a1 | d2eifa2 | d2je6i1 | d3i2za1 | d2c35b1 | d2ahob2 | d1t9ha1 | d3i4oa_ |
| d2qall1 | d1jt8a_ | d2nn6g1 | d1g6pa_ | d3cama_ | d1i94q_ | d1c9oa_ | d1d7qa_ |
| d2ba0a1 | d2vnud2 | d2vnud3 | d1khia2 | d2qalq1 | d1wi5a1 | d1xtda2 | d1ny4a_ |
| d1pfsa_ | d2a1ka_ | d1rpma_ | d2shpa1 | d1yfoa_ | d1l8ka_ | d2ooqa1 | d1g4us2 |
| d1p15a_ | d1lara2 | d1lara1 | d1wcha_ | d2fh7a2 | d2fh7a1 | d1ohea1 | d1ohea2 |
| d2f4da_ | d1rxda_ | d2hxpa_ | d1xria_ | d1d5ra2 | d1fpza_ | d3ezza_ | d4y2ea_ |
| d1c1la_ | d4lbqa_ | d1a78a_ | d1slta_ | d4no4a_ | d3zsja_ | d3duia_ | d1is3a_ |
| d1qmja_ | d1hlca_ | d1tdha3 | d1q3ba3 | d1ee8a3 | d1k82a3 | g1qmo.1 | g1len.1 |
| d3zyra_ | d1fnya_ | d1n47a_ | g2ltn.1 | d1avba_ | d3usua_ | d1dhkb_ | d1f9ka_ |
| d1ukga_ | d1g8wa_ | d1g9fa_ | d1wbfa_ | d3wcsa_ | d1g7ya_ | g1loe.1 | d1mvqa_ |
| d1hqla_ | d1v6ia_ | d1qnwa_ | d1fx5a_ | d4l8qa_ | d1ioaa_ | d3n35a_ | d3ubra_ |
| d1qo8a1 | d1d4ca1 | d2k3va_ | d1ft5a_ | d1qdba_ | d2j7aa_ | d1h21a_ | d1ogyb_ |
| d4fasa_ | d1jnia_ | d1oaha_ | d1sp3a_ | d2h0ba_ | d2erfa_ | d1h30a2 | d3mw4a1 |
| d1pz7a_ | d2r1ba_ | d3v64a1 | d1d2sa_ | d3bopa1 | d3mw3a1 | d1okqa1 | d1okqa2 |
| d1dszb_ | d1a6ya_ | d3cbba_ | d1ynwa1 | d1r4ia_ | d1hcqa_ | d2hana_ | d2hanb_ |
| d2enva1 | d1kb4b_ | d2nllb_ | d1lo1a_ | d1adqa1 | d1fnga1 | d1uvqb1 | d1zs8a1 |
| d2vola1 | d2vola2 | d1mfbl2 | d2jelh2 | d1uwel2 | d3qq4b1 | d1kjvb_ | d1k5na1 |
| d1osph2 | d4odhl2 | d1ow0a1 | d1ow0a2 | d1bfob2 | d3l9ra2 | d3jtth1 | d2fath2 |
| d4elkb2 | d1fn4b2 | d2xfxb_ | d1pg7x2 | d1cqka_ | d4k71b2 | d1lk2a1 | d1eapb2 |
| d4lfhg2 | d1i4fa1 | d2nw2a2 | d1kfah2 | d1mhca1 | d1h3ua1 | d1wejh2 | d1h0db2 |
| d1a5fh2 | d1kcvh2 | d1mjuh2 | d1qqda1 | d1kcrh2 | d1k8ia1 | d3huja2 | d2nnab1 |
| d1nfdf2 | d3mffb2 | d1s9va1 | d1lk3h2 | d1q0xl2 | d1fv1b1 | d1neza1 | d4rfeb2 |
| d1r3ha1 | d1a6tb2 | d1kjva1 | d1pz5b2 | d1onqa1 | d2bc4a2 | d1de4a1 | d1k8da1 |
| d1tcra2 | d1dn0b2 | d1nfde2 | d2xwtb2 | d1bfoa2 | d1mexh2 | d1iakb1 | d1ypzf2 |
| d4f7ea2 | d1q72l2 | d2fbjh2 | d1fe8h2 | d1clzh2 | d1fngb1 | d1uvqa1 | d1vgka1 |
| d1c16a1 | d4j12a2 | d4x99b2 | d1igtb3 | d1je6a1 | d1q72h2 | d1teth2 | d2hmic2 |
| d1i1ca2 | d1i1ca1 | d1k8ib1 | d1q0yh2 | d1kfal2 | d3frua1 | d1pg7w2 | d1u58a1 |
| d1igyb4 | d1igyb3 | d1hyrc1 | d1klub1 | d1deeb2 | d1c12b2 | d2bc4b2 | d2ig2h2 |
| d1kcvl2 | d3wn5b1 | d1lk2b_ | d1lk3l2 | d1mjul2 | d1sm3h2 | d1q0xh2 | d1zvsa1 |
| d1ydpa1 | d1u8ib2 | d1ypze2 | d1kcuh2 | d1op9a_ | d1y0la1 | d1etzb1 | d1egjl1 |
| d2gsib1 | d1nj9a1 | d4jfhd1 | d1aqkl1 | d1t2ja_ | d1lo4h1 | d1zvya1 | d3v4vl1 |
| d4enec1 | d1kxtb_ | d5e8ea1 | d2pxya_ | d4weud_ | d1xiwc_ | d2esvd1 | d1qnzh_ |
| d1ogae1 | d1f4xh1 | d4n1hb_ | d3ezjb1 | d3mlwl1 | d1kfah1 | d1etza1 | d1b4jh1 |
| d3cfic_ | d4w68a_ | d2arja1 | d1ek3a_ | d2ok0l1 | d1vera_ | d1uwgh1 | d1blnb1 |
| d1fpth1 | d1rzga1 | d2giya1 | d4m3kb1 | d1nc2a1 | d2nyyd1 | d1ncna_ | d1fbih1 |
| d5dxwa_ | d4fqqa1 | d1dqdh1 | d1jfql1 | d1kelh1 | d1faih1 | d1frgl1 | d1figl1 |
| d4m6nl1 | d1fo0a_ | d4hxbl1 | d3b5ga_ | d3quyd1 | d2h9gb1 | d1i8kb_ | d2dd8h1 |
| d2ck0h1 | d1eaja_ | d4w6wb1 | d2a6ib3 | d1t66d1 | d2e27h_ | d1ob1b1 | d1f11b1 |
| d2v7na1 | d1ad0a1 | d2xt1b1 | d1pskh1 | d4i13b1 | d2op4l1 | d1a0ql1 | d1uwel1 |

d1za3a1 d1ncbl1 d4uu9a_ d1bm3h1 d1cloh1 d1nqba2 d1cf8h1 d4y5va_
d1mhph1 d5anma1 d4cdgc1 d4ldeb_ d1y0lb1 d2pcpb1 d1z3gl1 d1dfbl1
d1fh5h1 d3g5xa1 d1tzib1 d4dn3l1 d3zhka1 d2b1hh1 d2uyla1 d1f3dh1
d1nj9b1 d2agjh1 d2h1pl1 d1q72h1 d8fabb1 d1ndmb1 d1rjca1 d4u6hb1
d3upca_ d2fbjl1 d1osph1 d1tvda_ d2cjvh_ d4fhbd1 d1bd2d1 d1rz7h1
d3nn8a_ d1blna1 d1knoa1 d1nfda1 d2bmkb1 d1dqll_ d1igyb1 d1igfl1
d12e8h1 d3utza1 d1nc2b1 d1rihh1 d4mwfb1 d4jvpa1 d1mieh1 d1rhfa1
d1fgnl1 d1sbsh1 d1lp9e1 d1dn0a1 d4lqfl1 d1iaih1 d1kgcd1 d1mh5b1
d1gpol1 d2g60h3 d1f8tl1 d2jixd1 d4grwh_ d1fo0b_ d1bfob1 d1axsa1
d1u9ka_ d1nmbh_ d1h8na1 d1h8na2 d1jguh1 d4eura1 d3sdyl1 d35c8h1
d1w72l1 d1mvub_ d1i9rh1 d1ob1a1 d4wenb_ d1yeeh1 d3ietc1 d1f58h1
d1ad0b1 d1mqkl_ d1nbvh1 d4b50a_ d3r8bb_ d1bafl1 d1mamh1 d4rgne1
d2gsgb_ d35c8l1 d1bafh1 d1ai1h1 d2pcpa1 d2fath1 d2x1pa_ d1orsb1
d1ibgl1 d1shma_ d1a2yb_ d1u0qa_ d2coqa_ d1hilb1 d1qfwi_ d1jv5b_
d1fn4b1 d5e0qa_ d1nsnh1 d1plgl1 d4ocxl1 d1kb5h1 d1rzfh1 d1pg7x1
d2fx7h1 d2oi9c_ d1l7il1 d3eyva1 d1d5il1 d4ffyl_ d1tetl1 d1a6wh_
d1ac6a_ d3dvfa_ d1nfde1 d1mimh1 d1ri8a1 d1jhlh_ d3dura_ d1fskb1
d1f97a1 d3so3b1 d2ak4d1 d1lp9f1 d4z7wf1 d1l7th1 d4y5ya_ d4x7ec1
d1emth1 d1ikfl1 d1nlbh1 d4utal1 d1bjma1 d1eapb1 d1u6ah1 d2dtgd1
d1kj2b_ d1xeda1 d1c1eh1 d1fgvh_ d2bnub1 d2fb4h1 d2dtgb1 d1ktrh_
d1jpth1 d1qkzl1 d4hepg_ d2atpb1 d1flrh1 d2hrpl1 d1mvfa_ d1iaim1
d3k80a1 d2jb5h1 d3zkxc_ d4grwe_ d1it9h1 d1rhhb1 d3w9ec_ d1e4wh1
d1sy6h1 d2j6eh1 d3qxta_ d1jglh1 d1p7ka1 d1tzhb1 d4n8pa1 d1gafl1
d2jelh1 d1wioa2 d4i0cc1 d1cicb1 d1lmka2 d1lmka1 d1iqwh1 d1orqb1
d1ejoh1 d1jnlh1 d4zg1a_ d43c9b_ d1ggbl1 d1wejh1 d3q6fa1 d1f3dj1
d3c6la1 d1bqlh1 d3p9wb_ d1a3rl1 d1h0db1 d2uzil_ d1eo8l1 d1qfwl_
d1n7ml1 d1igja1 d1c5ch1 d2a9mh_ d1rmfh1 d1fj1b1 d4heme_ d1ccza1
d1a5fh1 d1ogad1 d1dlfh_ d1qa9b_ d1vgel1 d3qskb_ d1sjxa_ d4ozfg1
d1sq2n_ d1qd0a_ d1kcvh1 d1c5db1 d3qcul1 d8faba1 d3bn9d1 d1mjuh1
d4y8dc_ d7fabh1 d3dvgb1 d1lo0l1 d1a6tb1 d1gigh1 d1kcrh1 d2hwzl1
d1yjdc1 d3clfl1 d4krna_ d1dl7l_ d1e6ol1 d1mexh1 d3r0ma_ d4kkna_
d1fnsl1 d1oaqh_ d1rzia1 d1ucbh1 d1ncwl1 d4b41a_ d1nfdf1 d2mpah1
d2d7tl1 d4grwf_ d4b5ea_ d1lk3h1 d3u79a_ d4u3xa_ d1ghfl1 d1bvkb_
d1mcww1 d1b88a_ d1jrhh1 d32c2b1 d4wemb_ d1dsfh_ d1op3h1 d1ce1l1
d1iqda1 d1qoka1 d1qoka2 d1qfuh1 d2crya1 d1ay1h1 d1oaql_ d3ab0b_
d1rurl1 d1t4kb1 d1bz7b1 d1n0xh1 d1forl1 d1ucbl1 d4w6yb_ d43c9a_
d2aabl1 d1tzia1 d4n1ca_ d1k6qh1 d3g9ab_ d4tvsa1 d1igmh_ d1pz5b1
d3dx9a1 d3wxwh_ d1nakh1 d1kcul1 d3eaka_ d4qlra1 d1cr9l1 d1d9ka_
d1f4xl1 d1deea1 d2i27n2 d1c12a1 d2orbl1 d1j8hd1 d1mcph1 d1c5da1
d1kenh1 d1bd2e1 d1h5ba_ d4x7fc1 d3upaa_ d1t3fa1 d1indh1 d2dtgc1
d4hc1l1 d1zv5a1 d4wgvb_ d1uwgl1 d4f37k1 d1aqkh1 d1vesa_ d3zkqd_
d1fptl1 d1lo4l1 d1knob1 d3rrqa_ d1ct8b1 d4orzc_ d1mlbb1 d4hgkc1
d1dqdl1 d1bogb1 d4ozhe1 d1dr9a1 d1i7za1 d1dn0b1 d1kell1 d1rzib1
d1ypzf1 d1wtla_ d1hkfa_ d1fail1 d1figh1 d2vyre_ d1sjva_ d1ohqa_

| | | | | | | | |
|---|---|---|---|---|---|---|---|
| d1bfoa1 | d1pkqb1 | d1mjul1 | d1b4jl1 | d1nsnl1 | d2arjb1 | d1p4bh_ | d4aj0a_ |
| d3nn8b_ | d2rcja1 | d1f2xk_ | d4v1dd_ | d1adqh1 | d2eksa_ | d4k3ga_ | d1jfqh1 |
| d1uweh1 | d1frgh1 | d4gftb_ | d1uz8b1 | d4n1ea_ | d1bm3l1 | d1clol1 | d3mcka1 |
| d2vq1a1 | d1ae6h1 | d4aq1b1 | d1uj3b1 | d1nbqa1 | d1fh5l1 | d2ck0l1 | d2ywza_ |
| d1q72l1 | d4nbxb_ | d1hila1 | d4hzlb1 | d2j88l1 | d1zmya1 | d1cd0a_ | d1a4ka1 |
| d1pskl1 | d1a0qh1 | d4krmb_ | d1ncbh1 | d2fbjh1 | d1j8he1 | d1i3ua_ | d1cf8l1 |
| d1fe8h1 | d2p1ya1 | d1eeqa_ | d2x89a_ | d1ol0a_ | d2r0lh1 | d1ub5a1 | d1dfbh1 |
| d3eyvb1 | d4jdvb1 | d4p2cg_ | d2gsia1 | d2agjl1 | d1u3ha1 | d2h1ph1 | d1rihl1 |
| d1fskc1 | d1boga1 | d2a77l1 | d1ospl1 | d1qfoa_ | d3dwta_ | d2fx7l1 | d1rz7l1 |
| d3stba1 | d1pkqa1 | d1b6da1 | d1dzba2 | d1dzba1 | d1kj2a_ | d4l1ha_ | d1rzgb1 |
| d1dqlh_ | d12e8l1 | d1miel1 | d1jmaa_ | d1sbsl1 | d1fgnh1 | d1f58l1 | d1mqkh_ |
| d1ndgb1 | d3qegl1 | d1iail1 | d2aw2a1 | d1tqbc1 | d3fo2a1 | d4dkaa_ | d1f8th1 |
| d2g60l1 | d2ywya_ | d1uj3a1 | d1yc7a_ | d3bd3a_ | d4w6xb1 | d1nmbl_ | d1i8ka_ |
| d1rjlb1 | d1ypze1 | d1p7kb1 | d1kxqe_ | d1gpoh1 | d2z4qa1 | d1w72h1 | d1i9rl1 |
| d4qgya1 | d4xt1c_ | d2qqnh1 | d1cica1 | d3c08h1 | d2cd0a_ | d2bdnh1 | d1hnga1 |
| d1maml1 | d1nldh1 | d1cr9h1 | d4lcih1 | d3bkyh1 | d3qg7l1 | d1ai1l1 | d1kxvc_ |
| d4iosd_ | d1l7ih1 | d2x1qa_ | d1ub6b1 | d3k74b_ | d1ibgh1 | d1teth1 | d1igya1 |
| d1hxma1 | d2x6ma_ | d2e7la_ | d5e03a_ | d1qfwm_ | d2r0kh1 | d1igjb1 | d1rurh1 |
| d1plgh1 | d1mj8h1 | d1yuhb1 | d3fmgl1 | d1rzfl1 | d1emtl1 | d1ad9b1 | d1igtb1 |
| d1nlbl1 | d1d5ih1 | d3k7ua_ | d4ffyh_ | d1bwwa_ | d3dvga1 | d1a6ta1 | d1fgvl_ |
| d4qxgl1 | d1miml1 | d1jhll_ | d2hffb1 | d2hh0l1 | d1qkzh1 | d1l7tl1 | d1afvh1 |
| d1iaii1 | d2cjuh_ | d1um5h1 | d2ghwb2 | d2ghwb1 | d1kgce1 | d2fgwh1 | d1ikfh1 |
| d1dvfd_ | d2i24n1 | d2xtjb_ | d1za3b1 | d2yc1a_ | d2hh0h1 | d2x1oa_ | d1bvka_ |
| d1rjla1 | d5e7ba1 | d1igta1 | d1il1b1 | d2fb4l1 | d1mvua_ | d1jptl1 | d32c2a1 |
| d1fvca_ | d1iqdb1 | d2hrph1 | d2p4ab_ | d4qo1a_ | d1p2cb1 | d1wt5c_ | d1t4ka1 |
| d5c1mb_ | d1it9l1 | d1bz7a1 | d1b2wh1 | d1e4wl1 | d1jnll1 | d1yedb1 | d1za6b1 |
| d3u7aa_ | d1igfh1 | d3gnml1 | d1orsa1 | d1wejl1 | d1a2ya_ | d1hxmb1 | d1gafh1 |
| d2j4wh1 | d1pz5a1 | d1j05a_ | d1a3rh1 | d4fqll1 | d1qfwh_ | d1eo8h1 | d1n7mh1 |
| d4s11a_ | d1kegh1 | d5da4a_ | d1iqwl1 | d3whxb1 | d1vgeh1 | d1deeb1 | d1c12b1 |
| d1t2ql1 | d1ad9a1 | d3gsnb1 | d3b2uc1 | d1ggbh1 | d1t3fb1 | d1nakl1 | d4ocmc_ |
| d1lila1 | d2v7hb3 | d2esve1 | d1mcoh1 | d2qr0b1 | d2q3aa_ | d1ct8a1 | d1kcrl1 |
| d2pxyb_ | d2uzih_ | d5c3ld_ | d3durb_ | d1c5cl1 | d2ak4e1 | d1dl7h_ | d1xiwd_ |
| d1mlba1 | d1rmfl1 | d1i7zb1 | d1dlfl_ | d2xa3a_ | d5bq7b1 | d1dqqb1 | d3h42l1 |
| d1egjh1 | d1kcvl1 | d1fe8l1 | d2j6el1 | d1f3rb2 | d1lk3l1 | d1sm3h1 | d1q0xh1 |
| d1ghfh1 | d7fabl1 | d1igch1 | d1lo0h1 | d4idla_ | d5c2bl_ | d1jrhl1 | d3zhda1 |
| d4kfzc_ | d1b0wa_ | d1e6oh1 | d1mexl1 | d4krpb_ | d1fnsh1 | d1ncwh1 | d4hgma_ |
| d4eigb1 | d4rzca_ | d2mpal1 | d4y5xa_ | d5en2b1 | d3lrga1 | d4ej1c1 | d1rhha1 |
| d1j05b_ | d1forh1 | d1q9wb1 | d2wzpd_ | d2aq2a1 | d1h0da1 | d3revb1 | d2ij0c1 |
| d1tzha1 | d4m7jl1 | d1k6ql1 | d3b9kc1 | d1igml_ | d3qeua1 | d1dsfl_ | d1ce1h1 |
| d1kcuh1 | d1zvha1 | d1zvoa1 | d1tqbb1 | d1ay1l1 | d2qscl1 | d2cgrh1 | d1nl0h1 |
| d1kenl1 | d3eo0a1 | d4n9ob1 | d1n0xl1 | d3k1kc_ | d1c4qa_ | d1prtc1 | d2wv6d_ |
| d1prtf_ | d1ltrd_ | d4l63b1 | d1r4pb_ | d1tl0f_ | d2bosa_ | d1prtb1 | d3chbd_ |
| d2aq2b1 | d1enfa1 | d1i4pa1 | d1v1oa1 | d1et9a1 | d1esfa1 | d2g9hd1 | d4ohja1 |
| d1klud1 | d1bxta1 | d1eu3a1 | d1ty0a1 | d1fnua1 | d2cqva1 | d1fhga_ | d2k1ma_ |

d1biha3 d1biha1 d1biha4 d3qp3a1 d1g1ca_ d1gxea_ d1cvsc2 d1cvsc1
d1xmwa2 d1m4ka2 d2otpa2 d1biha2 d1rhfa2 d2fcba2 d2fcba1 d1wwbx_
d1gsma2 d1epfa1 d1epfa2 d2dava1 d1waaa_ d1f97a2 d1x44a1 d1vcaa2
d1fnla2 d1fnla1 d3b5ha2 d3h8na2 d3h8na1 d1jbja1 d1jbja2 d1pd6a_
d1f2qa2 d1f2qa1 d3cx2a_ d1nbqa2 d3b5ha1 d1n26a1 d1xiwb_ d1xaua_
d1fltx_ d2ifga1 d1olla1 d1olla2 d1cs6a4 d1cs6a3 d1cs6a2 d1cs6a1
d2gw5a2 d1sy6a1 d1xiwa_ d1ucta2 d1ucta1 d1ry7b2 d1gl4b_ d1qz1a3
d1olza1 d2c9aa1 d1wwca_

**Family-9 (a.26.1.1)**

**Positive dataset contains 2 protein sequences.**

d1z7ca_ d1huwa_

**Negative dataset contains 4058 protein sequences.**

d1m32a_ d1ibja_ d1c7na_ d1iuga_ d4q75a_ d2bkwa1 d1t3ia_ d1n8pa_
d1qz9a_ d1jf9a_ d1eg5a_ d1p3wa_ d3kgwa_ d1cl1a_ d2ctza1 d1vjoa_
d2rfva_ d1gc0a_ d1cs1a_ d1d2fa_ d2ch1a1 d1qgna_ d1pffa_ d1elua_
d1u08a_ d1lc5a_ d1b5pa_ d2ay1a_ d1bw0a_ d3pd6a_ d7aata_ d2csta_
d1fg7a_ d4wlha_ d1o4sa_ d1v2da_ d1gdea_ d1m6sa_ d1v72a1 d1svva_
d1ajsa_ d1wsta1 d2f8ja2 d1lk9a_ d1vp4a1 d1xi9a1 d2gb3a1 d1yaaa_
d1xkla_ d2wfla_ d1xkta1 d2hfka_ d1jmkc_ d5d3ka_ d1i26a_ d1ddla_
d1auyb_ d1qnia1 d1fwxa1 d1f56a_ d1bypa_ d1azca_ d1ws8a_ d3u25a_
d1cuoa_ d1id2a_ d1rkra_ d2iaac_ d4dp1x_ d1nwpa_ d2gima_ d2i7sa_
d1qhqa_ d3ehbb1 d1fftb1 d1gyca3 d1hfua2 d1kbva1 d1kbva2 d1sdda1
d1sdda2 d1gyca1 d1gyca2 d1mzya1 d2bw4a1 d1aoza1 d1aoza3 d1aoza2
d1kyaa2 d1kyaa3 d1kyaa1 d1mzya2 d4e9sa1 d1sddb1 d1sddb2 d1hfua1
d1hfua3 d1oe1a2 d1oe1a1 d1v10a2 d1v10a3 d1v10a1 g1f8v.1 d1novc_
d1h4ra3 d4yl8a1 d2al6a3 d2cfba1 d1dfoa_ d1o69a_ d1bj4a_ d3k28a_
d2bwpa_ d3lg0a_ d2fnua_ d3ou5a_ d1z7da1 d1b9ha_ d1iaya_ d4msoa_
d1bs0a_ d1s0aa_ d1sffa_ d3tqxa_ d1mdoa_ d3qboa_ d3pgya_ d1bjna_
d2dkja_ d1ejia_ d1vefa1 d4wxga_ d3bs8a_ d3gbxa1 d1w23a_ d1a3qa1
d1iknc_ d3brda1 d1p7hl1 d1oy3c_ d1imhc1 d1zk9a1 d2cxka1 d2bema_
d1pama1 d1ea9c1 d1qhoa1 d2fhfa2 d2fhfa1 d1k3ia1 d1j0ha1 d1cxla1
d2bhua1 d1edqa1 d1f1sa2 d1ut9a2 d1gvia1 d1txka1 d4alac_ d1pjwa_
d1rera1 d1s6na_ d3uzqb_ d2phla2 d2phla1 d1fxza2 d1fxza1 d1uija1
g1dgw.1 d2v09a_ d1uika2 d1uika1 d1dgwa_ d1v70a_ d2f4pa1 d1yhfa1
d2b5ea2 d2b5ea3 d2b5ea1 d2b5ea4 d2kp1a1 d2kp2a1 d1hyua3 d1hyua4
d1j08a1 d1j08a2 d1cg2a1 d1yloa2 d1y7ea2 d1y0ya3 d1lfwa1 d1vixa1
d1fnoa4 d1vhoa2 d1ysja1 d1vhea2 d1z2la1 d2fvga2 d1vgya1 d1r3na1
d2grea2 d1xmba1 d1rtqa_ d2bjca1 d2hsga1 d1qpza1 d1efaa1 d1mqta_
d1d4m3_ d1fmd3_ d2mev1_ g1aym.1 d1ev13_ d4q4w1_ d1r1a3_ d2x5ia_
d1rhi1_ g1r1a.1 g1d4m.1 g1rhi.1 d1b35b_ d1oopa_ d1tme1_ d4iv1a_
g1pvc.1 g1fpn.1 d1fpn1_ d4gb31_ d1d4m1_ d1fmd1_ d2wzr3_ g1cov.1
d1qqp3_ g1fmd.1 d2x5ic_ d1rhi3_ g1oop.1 d1hxs1_ g1hxs.1 d1fpn3_
d1bev1_ g2mev.1 d1eah3_ d1aym1_ d1pvc3_ d1qqp1_ d1zba3_ d1h8ta_
d3vbhc_ g1b35.1 g1bev.1 d1hxs3_ g1ev1.1 d1bev3_ g1tmf.1 d1tmf1_

| | | | | | | | |
|---|---|---|---|---|---|---|---|
| d1b35a_ | d2mev3_ | g1eah.1 | g1h8t.1 | d1eah1_ | d4q4w3_ | d1r1a1_ | d1aym3_ |
| d1pvc1_ | d1zba1_ | d1h8tc_ | d1oopc_ | d1tme3_ | g1qqp.1 | d4iv1c_ | g1tme.1 |
| d1r0ka3 | d1j5pa3 | d1vkoa2 | d1vm6a2 | d3qvsa2 | d1p1ja2 | d2ovga_ | d1sq8a_ |
| d1rzsa_ | d3kz3a_ | d3orca_ | d1ux8a_ | d1dlya_ | d4i0va_ | d2bkma_ | d1dlwa_ |
| d2bmma_ | d2msya_ | d2djoa1 | d2r5za_ | d1au7a1 | d1f43a_ | d2lmda1 | d1akhb_ |
| d1s7ea1 | d3nara_ | d1zq3p1 | d2craa1 | d1mija_ | d4uuta_ | d3a01a_ | d1x58a1 |
| d3rkqa1 | d3cmya1 | d2cqxa1 | d2da6a1 | d1e3oc1 | d1fjla_ | d1mh3a1 | d1ig7a_ |
| d1x41a1 | d1wh7a1 | d1gt0c1 | d2mw8a_ | d1lfup_ | d1x2ma1 | d1b72a_ | d1wi3a1 |
| d1jgga_ | d1wjha1 | d1x2na1 | d1mnmc_ | d2cuea1 | d3k2aa_ | d2hosa_ | d2jwta2 |
| d1pufa_ | d1octc1 | d2hi3a_ | d1b8ia_ | d4j19a_ | d3lnqa_ | d1wh5a1 | d1b8ib_ |
| d1hcra_ | d1gdta1 | d1u78a2 | d6paxa1 | d6paxa2 | d1k78a1 | d1k78a2 | d1xg1a1 |
| d1itya_ | d1hlva1 | d1iufa2 | d1iufa1 | d2dara2 | d2dara1 | d1wyha2 | d2dloa2 |
| d1rutx4 | d1rutx3 | d2cuqa2 | d1x4la2 | d1x4la1 | d1x3ha2 | d1x3ha1 | d2d8za1 |
| d1x61a2 | d1x6aa1 | d1x6aa2 | d2cora2 | d2cora1 | d1v6ga1 | d1v6ga2 | d2d8za2 |
| d1x64a1 | d1x64a2 | d1b8ta1 | d2co8a1 | d1x63a2 | d2dj7a2 | d2dj7a1 | d2cu8a2 |
| d2cu8a1 | d1g47a2 | d1j2oa1 | d1wyha1 | d2dloa1 | d1wiga1 | d1wiga2 | d2cupa2 |
| d2cupa3 | d1x68a1 | d2d8ya2 | d2d8ya1 | d1m3va2 | d1x62a1 | d1x68a2 | d1x61a1 |
| d1b8ta3 | d1b8ta2 | d2d8xa2 | d2d8xa1 | d1x4ka1 | d1x4ka2 | d2cuqa1 | d1m3va1 |
| d2co8a2 | d1j2oa2 | d1x63a1 | d2cura1 | d2cura2 | d1x62a2 | d2iu5a1 | d1zk8a1 |
| d2g7sa1 | d2fd5a1 | d2np5a1 | d1z0xa1 | d2id6a1 | d2d6ya1 | d2i10a1 | d1vi0a1 |
| d2oi8a1 | d3c07a1 | d2ns7a1 | d2fq4a1 | d2yvea1 | d1t33a1 | d2o7ta1 | d1rkta1 |
| d2np3b1 | d2g7ga1 | d2gena1 | d2fbqa1 | d2g7la1 | d2gfna1 | d2g3ba1 | d2jj7a1 |
| d2hyja1 | d1sgma1 | d1ui5a1 | d2hkua1 | d2id3a1 | d4jyka1 | d3lv8a_ | d1s3ga1 |
| d1zaka1 | d1s4qa_ | d3tvta2 | d1uf9a_ | d1khta_ | d1ckea_ | d1osna_ | d1p5zb_ |
| d4tmka_ | d3cm0a_ | d1tmka_ | d1jaga_ | d3h86a_ | d1t0hb_ | d1nn5a_ | d2h92a_ |
| d3kb2a1 | d2c95a1 | d1yj5a2 | d1kgda_ | d1ly1a_ | d2ak3a1 | d1vyva2 | d1deka_ |
| d2grja1 | d1p3ja1 | d1y63a_ | d1e4ya1 | d1s96a_ | d3iija1 | d1lvga_ | d1nksa_ |
| d1vyua2 | d4e22a_ | d1qf9a_ | d1jjva_ | d1q3ta_ | d1ki9a_ | d1lw7a2 | d1teva_ |
| d1kjwa2 | d2iyva2 | d1kaga_ | d1viaa_ | d1e6ca_ | d1x6va3 | d1g3ma_ | d1j99a_ |
| d1t8ta1 | d1nsta_ | d1fmja_ | d1zrha_ | d1ls6a_ | d2a3ra_ | d1aqua_ | d2zptx_ |
| d3ckla_ | d1texa_ | d1q44a_ | d2gwha_ | d1z0ja1 | d3umra2 | d3gj0a1 | d2gaoa1 |
| d1yzga1 | d1x3sa1 | d1g7sa4 | d1puja_ | d1udxa2 | d1kk1a3 | d2o52a_ | d1jwyb_ |
| d1zcaa2 | d1zcba2 | d2fv8a1 | d2cjwa_ | d3dz8a1 | d1xzpa2 | d1vg8a_ | d4qxaa_ |
| d2ew1a1 | d1x1ra1 | d2bcgy_ | d3doea1 | d3cona_ | d3rega_ | d3wyga_ | d1upta_ |
| d2b6ha1 | d1zd9a1 | d1lnza2 | d1r5ba3 | d1n0ua2 | d1r2qa_ | d2f9la1 | d2rhda_ |
| d3q3jb_ | d3q85a_ | d1efma1 | d1r8qa_ | d2gf9a1 | d2akab1 | d1ek0a_ | d1z08a1 |
| d2gcoa1 | d2dy1a2 | d4g01b_ | d2cxxa1 | d1svia_ | d3q72a_ | d1ni3a1 | d4ac9a4 |
| d2erxa1 | d2bmea_ | d1wxqa1 | d2gf0a1 | d4ldja1 | d1zunb3 | d1d5ca_ | d4lv5b_ |
| d1efca3 | d1kmqa_ | d2g6ba1 | d3lrpa_ | d1tada2 | d2y8ea_ | d4rd4a1 | d1z06a1 |
| d2heia_ | d3a58b_ | d1u0la2 | d1d2ea3 | d1zj6a1 | d2ocba_ | d3rwoa_ | d1mr3f_ |
| d2e9sa1 | d4u5xa1 | d2iwra_ | d2fn4a1 | d3bwdd_ | d2nzja1 | d2atva1 | d2ksqa_ |
| d3l43a_ | d1mkya2 | d1mkya1 | d1wmsa_ | d1jnya3 | d2rmka_ | d1f6ba_ | d1f60a3 |
| d1gota2 | d1jala1 | d2fh5b1 | d1g16a_ | d2wblc_ | d1m7ba_ | d1wf3a1 | d1f5na2 |
| d1t9ha2 | d1z6xa1 | d1z0aa1 | d2fg5a1 | d2a5ja1 | d2bcjq2 | d1ky3a_ | d4y0va_ |

d4lhwa_ d2qmea1 d2atxa1 d1egaa1 d1ryha_ d1moza_ d1nrjb_ d2erya1
d3bfka_ d1puia_ d1fzqa_ d3j3az1 d3t5ga_ d1shza2 d3defa_ d2f7sa1
d3raba_ d3tsoa_ d3aq4a_ d2al7a1 d3sfva1 d3bc1a_ d2ncda_ d1sdma_
d3h4sa1 d2heha_ d2grya_ g3kin.1 d1br2a2 d1goja_ d1f9va_ d2mysa2
d1lkxa_ d3t0qa_ d4db1a1 d3gbja_ d1ry6a_ d1v8ka_ d1k8ka2 d1k8ka1
d3uleb1 d3i33a2 d3i33a1 d1bupa1 d1dkgd2 d1dkgd1 d4ci6a2 d1jcfa1
d4a2ba2 d2fxua1 d1yaga2 d1yaga1 d4cbua2 d1bupa2 d1g99a1 d2e1za2
d2e1za1 d1saza1 d1saza2 d1ig8a1 d1czan2 d1czan3 d1czan1 d1ig8a2
d1czan4 d1bg3a3 d1bg3a2 d1bg3a1 d1nira1 d1qksa1 d3h1jd1 d2d0oa3
d2d0oa2 d1nbwa2 d1hroa_ d4gedb_ d3zooa_ d1mz4a_ d1gu2a_ d1f1fa_
d1dvva_ d1c6ra_ d1c75a_ d1e29a_ d1gdva_ d1cnoa_ d1h32b_ d1co6a_
d2bh4x_ d2aiua_ d5cytr_ d1jdla_ d1i8oa_ d2zboa_ d3dmia_ d1ls9a_
d1wvec_ d2yk3a_ d3ph2b_ d1dw0a_ d5aura_ d1ql3a_ d1qn2a_ d1f1ca_
d4zzva_ d2xspa_ d4v20a_ d1ojja_ d4ipma1 d1gpia_ d4csia_ d3pfza_
d3ovwa_ d2yoka_ d2nqya_ d1h4ga_ d1hixa_ d1oa2a_ d1olra_ d3b7ma_
d4h7ma1 d3wp3a_ d2vgda_ d1igoa_ d3zsea_ d2nlra_ d3wx5a_ d1oa3a_
d1pvxa_ d1m4wa_ d1f5ja_ d1oa4a_ d4ixla1 d2ah2a1 d1n1ta1 d1g66a_
d5ajha1 d2b7la_ d3rbaa_ d2qtra_ d1nupa_ d3l92a_ d1f9aa_ d1qjca_
d2h29a_ d1vlha_ d1od6a_ d1lw7a1 d1mrza2 d4ruka_ d2el7a_ d1iq0a2
d1irxa2 d1r6ta2 d1ivsa4 d2cyba_ d1rqga2 d1j09a2 d2yxna_ d1f7ua2
d1h3fa1 d1ffya3 d2g36a1 d1nzja_ d1jila_ d1i6la_ d1pfva2 d1h3na3
d3n9ia_ d3sz3a1 d3sw2a_ d1moxc_ d1dx5i3 d1edmb_ d1autl2 d1autl1
d1nqlb_ d1dval1 d1szba2 d1kigl_ d2y5fl_ d1ijqa2 d1gk5a_ d1g1sa2
d1nt0a3 d1xdtr_ d1lmja2 d1lmja1 d1nzia2 d2wphe_ d1klil_ d1pfxl2
d1pfxl1 d1p9ja_ d1g1ta2 d1f7ea_ d4yzub_ d2mgpa1 d1b9wa2 d2mgpa2
d1ob1c1 d1ob1c2 d1n1ia2 d1n1ia1 d2npra2 d2npra1 d1qx2a_ d2y5ia_
d1qlsa_ d1psra_ d3n22a_ d2egda_ d4ggfc_ d2wcea_ d2luca_ d4aqia_
d3nsia_ d2lp3a_ d1j55a_ d1a4pa_ d1k8ua_ d1nsha_ d1pvaa_ d3fs7a_
d2pvba_ d1ttxa_ d1rwya_ d2kyfa_ d1rk9a_ d1a75a_ d1exra_ d1tiza1
d1nyaa_ d1g8ia_ d1ggwa_ d1wdcc_ d3fwba_ d1ggza_ d2o5ga_ d1ctaa_
d1m45a_ d3i5gc_ d1sl8a_ d1fpwa_ d2scpa_ d2roaa_ d4aqra_ d3pn7b_
d1br1b_ d2mysb_ d1s6ca_ d1bjfa_ d2obha_ d1jfka_ d1jf0a_ d1qv0a_
d1lkja_ d2mlfc_ d2m97a_ d2d8na_ d4by5a_ d1jbaa_ d4nqga_ d1wdcb_
d2mysc_ d1j7qa_ d1s6ia_ d1uhka2 d1r2ua_ d1dtla_ d3kf9a_ d3pn7c_
d5d67d_ d1tuza_ d1h8ba_ d1eg3a2 d2zkmx1 d1snla_ d1gjya_ d1y1xa_
d1hqva_ d1dvia_ d1df0a1 d1qxpa2 d4usla_ d1k94a_ d1wkya2 d1uuqa_
d3wh9a_ d4m1ra_ d1xyza_ d4xuya_ d2je8a5 d4bf7a1 d3cuia_ d1ur4a_
d1qw9a2 d1odza_ d2vzsa5 d1bqca_ d2x2ya1 d1hjqa_ d1qnra_ d4y7ea1
d1vjza_ d4k68a_ d2v3ga_ d4hpga_ d1nofa2 d1nq6a_ d1h4pa_ d1aq0a_
d3ziza_ d4bs0a_ d1ecea_ d1h1na_ d1ta3b_ d1w91a2 d1i1wa_ d1uhva2
d1us3a2 d1egza_ d7a3ha_ d1tvna1 d3nyda1 d1ghsa_ d2cyga1 d4xv0a_
d2c8na2 d1w32a_ d3emca_ d1v6ya1 d1foba_ d2uwfa1 d2fgla_ d3niya1
d2c0ha1 d1rh9a1 d1yq2a5 d1szna2 d1fa2a_ d1pama4 d5do8a1 d1ea9c3
d1hx0a2 d2fhfa5 d1qhoa4 d1avaa2 d1m53a2 d4m8ua1 d2gjpa2 d1lwha2

d4howa1 d1ktba2 d1j0ha3 d3vm5a1 d2x1ia_ d1wzaa2 d1uasa2 d2diea1
d1mxga2 d1ht6a2 d1eswa_ d1iv8a2 d5cpsa_ d1ud2a2 d1zjaa1 d3gbea1
d1kwga2 d1wdpa_ d1gcya2 d1g5aa2 d1hvxa2 d3dhpa2 d2bhua3 d4do4a1
d3qgva1 d1e43a2 d1g94a2 d2d3na2 d1gjwa2 d1gvia3 d1x1na1 d1r7aa2
d1tz7a1 d4c7ga2 d2gjxa1 d1nowa1 d1yhta1 d4toqa_ d1wb0a1 d1w9pa1
d1eoka_ d1itxa1 d1kfwa1 d1goia2 d1edqa2 d2gsja_ d1hjxa1 d1jnda1
d1vf8a1 d3wdpp1 d3wq5a_ d1gnxa_ d4gxpa_ d4hz8a_ d1e4mm_ d3aiua_
d1wcga1 d1qvba_ d3ahxa_ d1v02a_ d2dgaa_ d1pbga_ d1vffa1 d1qoxa_
d4ha4a_ d1mixa2 d1e5wa2 d1j19a2 d2al6b2 d3syxa1 d2p8va_ d1xoda1
d1i7aa_ d1evha_ d1qc6a_ d1egxa_ d2y8ga_ d1xkea1 d1k5db_ d2y8fa_
d1offa_ d1frra_ d3wcqa_ d1e9ma_ d1l5pa_ d1wria_ d1i7ha_ d3av8a_
d1czpa_ d1rfka_ d3p1ma1 d3b2ga_ d2bt6a2 d1wgqa1 d1v89a1 d2coda1
d1zc3b_ d1wg7a1 d1u5ea1 d1v61a1 d2coca1 d2ys1a1 d1v5pa1 d1ntya2
d1wjma1 d1wi1a1 d1u29a_ d1xx0a1 d1unqa_ d2zkmx3 d1fhoa_ d1v88a1
d2adza1 d1x1fa1 d1p6sa_ d2coaa1 d1btka_ d1v5ma1 d1faoa_ d2x18a_
d1dyna_ d1x1ga1 d4kaxb_ d2j59m1 d1ki1b2 d1xcga2 d1txda2 d1u5da1
d1w1ha_ d1upqa_ d1v5ua1 d1foea2 d2fjla1 d1eaza_ d2dfka2 d2cofa1
d1kz7a2 d1h6ua1 d2ewda3 d1up7a1 d2dfda1 d1s6ya1 d1vjta1 d7mdha1
d1hyea1 d1t2da1 d1ojsa3 d1b8pa1 d1ldna1 d1hyha1 d1civa1 d2a92a1
d1ez4a1 d1i0za1 d1y6ja1 d1gv0a1 d1ur5a1 d1llda1 d1obba1 d1v8ba1
d1gdha1 d2dlda1 d1mx3a1 d1ygya1 d1qp8a1 d1li4a1 d1j4aa1 d2naca1
d1npya1 d3t4ea2 d1a4ia1 d1o0sa1 d2hk9a2 d2egga2 d1b26a1 d1bvua1
d1gq2a1 d1c1da1 d1leha1 d1gtma1 d1euza1 d3dona2 d1edza1 d1gpja2
d1vi2a1 d1p77a1 d1nyta1 d1b0aa1 d1vl6a1 d1v9la1 d1etob_ d1umqa_
d1g2ha_ d1kola2 d1f8fa2 d1qora2 d1jqba2 d1cdoa2 d1gu7a2 d1d1ta2
d1xa0a2 d2dm6a2 d1jvba2 d1rjwa2 d1llua2 d1h2ba2 d1tt7a2 d1piwa2
d1o89a2 d1iz0a2 d1e3ja2 d1yb5a2 d1uufa2 d1vj1a2 d1e3ia2 d3gpdg1
d1vc2a1 d2nvwa1 d1u8fo1 d1ydwa1 d1hdgo1 d2g17a1 d1cf2o1 d2czca2
d1r0ka2 d1obfo1 d2cvoa1 d1ebfa1 d1h6da1 d1i32a1 d1zh8a1 d2q49a1
d1qkia1 d3pzra1 d1xeaa1 d1vkoa1 d1vm6a3 d3qvsa1 d2pkqo1 d1b7go1
d1gr0a1 d1p1ja1 d1lc0a1 d1j0xo1 d3pwka1 d2hjsa1 d1zyao1 d1vkna1
d1tlta1 d1nvmb1 d3sjua_ d3ojfa1 d1xu9a1 d2jjya1 d1n7ha_ d1y1pa1
d1h5qa_ d1jtva_ d4fs3a_ d2bkaa1 d1gy8a_ d2c5aa1 d1ulsa_ d1ooea_
d2b69a1 d1y5ma1 d1spxa_ d1ae1a_ d1bxka_ d1ydea1 d1gega_ d1hdoa_
d1o5ia_ d1e6ua_ d1snya1 d2rhca_ d3zv4a_ d5ceja_ d2d1ya1 d1fjha_
d3w8ea_ d2fmua1 d1x1ta_ d1rpna_ d3ay7a1 d1k2wa_ d1eq2a_ d2hrba1
d3ruaa_ d2ew8a1 d4hwka_ d1xgka_ d2bd0a1 d1yo6a1 d2a35a1 d2ntva_
d1vl8a_ d1hdca_ d4bkua_ d2ag5a1 d2q45a_ d1nffa_ d4iy1a_ d1zbqa1
d1kewa_ d1ek6a_ d1ja9a_ d2q46a2 d1iy8a_ d1cyda_ d2ptga_ d1hxha_
d1uzma1 d3lyla_ d4m89a_ d3wyea_ d2blla1 d1w6ua_ d1sb8a_ d2q2va_
d4nr0a_ d1e6wa_ d2gasa_ d3oiga1 d2bgka1 d1mxha_ d1vl0a1 d1rkxa_
d1r6da_ d1wmaa1 d1e7wa_ d2c07a1 d1xkqa_ d3tzha_ d1g0oa_ d3lz6a_
d1qyca_ d1edoa_ d1oc2a_ d1t2aa_ d1zema1 d2xoxa_ d1z45a2 d1geea_
d1p33a_ d3lt0a_ d4e6pa_ d2wyua_ d1db3a_ d4q9na_ d1xhla_ d4o1ma_

d1fmca_ d2uvda_ d2gdza1 d1uaya_ d2z1ma_ d2o23a2 d2fr1a2 d2fr1a1
d1qsga_ d1qyda_ d4zjua_ d2a4ka1 d1yb1a_ d1wvga1 d1yxma1 d3f4ba_
d1mg5a_ d1xg5a1 d1r55a1 d3gboa_ d1atla_ d1kufa_ d1buda_ d4q1la_
d1yp1a_ d1wnia_ d2gfoa1 d1vjva_ d2ayna1 d3nhea_ d3nqyb_ d4gera_
d1bqba_ d1ex0a4 d1g0da4 d2f4ma1 d1x3za_ d3ioqa_ d1fh0a_ d1o0ea_
d1m6da_ d2cb5a_ d1cs8a_ g8pch.1 d1s4va_ d2pnsa_ d1vsna1 d1mira_
d1iwda_ d2oula_ d7pcka_ d3bwka_ d3bcna_ d2p86a_ d1pvja_ d1pcia_
d1gece_ d1deua_ d4bs6a_ d1xkga1 d1cqda_ d1jqpa2 d2c0ya_ d2bdza_
d3p5ua_ d2o36a_ d1k9xa_ d1zoya1 d1qo8a2 d1d4ca2 d1jnra2 d1kf6a2
d2jk6a2 d2jk6a1 d1feca1 d1feca2 d1lpfa1 d1lpfa2 d1xhca1 d1xhca2
d1dxla2 d1dxla1 d1gera2 d1m6ia2 d1onfa1 d1onfa2 d3lada2 d3lada1
d1w4xa1 d1w4xa2 d1gesa2 d1mo9a1 d1xdia1 d1fl2a2 d1fl2a1 d1fcda1
d1fcda2 d1h6va2 d1h6va1 d1mo9a2 d1gv4a2 d1gv4a1 d2gv8a2 d2gv8a1
d1aoga1 d1aoga2 d1hyua1 d1ebda1 d1ebda2 d3dk9a1 d1q1ra2 d1q1ra1
d1gesa1 d1zbfa1 d2z1ha_ d2etja1 d3qioa1 d1jl1a_ d2e4la_ d1jl2a_
d2lsna1 d1uaxa_ d2yv0x_ d1mu2a1 d1ekea_ d1i39a_ d1pn0a1 d1reoa1
d1ju2a1 d2f5va1 d3c96a1 d2gmha1 d1pj5a2 d1gpea1 d1o5wa1 d1n4wa1
d1k0ia1 d2dw4a2 d2ivda1 d2voua1 d1kdga1 d1s5ja1 d2hbja2 d2igia_
d1wlja_ d1qhta1 d4dlea1 d1tgoa1 d2f96a1 d3tr8a1 d3mxma_ d1q8ia1
d1y97a1 d1w0ha_ d1kfsa1 d2py5a1 d1d5aa1 d1yt3a3 d2guia_ d3mwpa1
d4kb1a_ d1id1a_ d2hmta_ d1lssa_ d1iv0a_ d1nu0a_ d2v76a_ d1wj1a1
d1qqga2 d1qqga1 d1ntva_ d1n3ha_ d4dbba_ d3f0wa1 d1p3ra_ d1wgua1
d2cy4a1 d3hqca_ d1wvha1 d1xr0b1 d2av4a1 d3nvva1 d2h88b1 d1n62a2
d1t3qa2 d1kf6b2 d1zoyb1 d1jq4a_ d1ffva2 d1dgja2 d1rm6c2 d1r7ha_
d1faaa_ d1wika1 d1xfla_ d1ttza1 d2f51a1 d1ti3a_ d1nhoa_ d1sena_
d2i4aa_ d1ep7a_ d1zmaa1 d1fo5a_ d3lefa1 d3diea1 d2yana1 d1z6na1
d1oaza_ d3zywa1 d4aj8a_ d1nm3a1 d4fiwa_ d1wmja_ d4hs1a1 d1iloa_
d1xwaa1 d1wjka1 d2vm1a_ d1fb6a_ d1gh2a_ d1h75a_ d1nw2a_ d1kx5b_
d3a6na_ d4m6ba2 d2nqbd_ d3wkjd_ d2yfwe_ d4h9na_ d5bnxa_ d1kx5c_
d3r45a_ d1eqzb_ d4jjnb_ d1f66c_ d1eqza_ d4wnnb_ d1kx5a_ d1u35c1
d1id3a_ d2nqbc_ d1kx5d_ d1tafa_ d1bh9b_ d1tafb_ d1jfib_ d1q9ca_
d1h3ob_ d2byka1 d1n1ja_ d4g92b_ d1bh9a_ d1h3oa_ d2bykb1 d1n1jb_
d1pd212 d1nhya2 d2a2ra2 d1k0dd2 d1f2ea2 d1gsua2 d1f3ba2 d2fhea2
d2pvqa1 d1tw9a2 d1oyja2 d1e6ba2 d1k0ma2 d3ik7a1 d4is0a1 d1r5aa2
d1jlva2 d1m0ua2 d3gtub2 d1z9ha2 d1okta2 d1axda2 d2gsra2 d1tu7a2
d1k3ya2 d1zgma1 d1b48a2 d1gnwa2 d1gwca2 d2gsta2 d1glqa2 d1oe8a2
d1pn9a2 d1v2aa2 d1fw1a2 d1duga2 d1iqca2 d1iqca1 d1eb7a2 d1eb7a1
d1nmla2 d4ilya_ d4olta_ d1qgia_ d1txga2 d3f47a1 d1mv8a2 d2pv7a2
d1xc2a3 d2ahra2 d4xiya1 d2f1ka2 d1pgja2 d1i36a2 d1np3a2 d1lj8a4
d1teaa3 d1qmga2 d2i76a2 d2g5ca2 d1wdka3 d1dlja2 d3cuma3 d1gd6a_
d1f6ra_ d1ivma_ d2gv0a_ d1iiza_ d2z2fa_ d1j8xa_ d1ghla_ d1b9oa_
d3w3ea_ d4tx7a_ d3cqla_ d4msta_ d3iwra_ d2z37a_ d4dyga_ d2za0a_
d2c21a1 d1f9za_ d1mpya2 d1kw3b2 d4ghga1 d1tfza1 d3isqa1 d3isqa2
d1sqia1 d1sqia2 d1lkda2 d1lkda1 d1mpya1 d1cjxa2 d1cjxa1 d1sp9b2

d1t47a1 d1t47a2 d1f1ua2 d1sp8a1 d1sp8a2 d1f1ua1 d1ro5a1 d1s5ja2
d1qhta2 d4dlea2 d1tgoa2 d3py8a2 d1q8ia2 d2py5a2 d1d5aa2 d1qsta_
d2beia1 d4m3sa_ d1u6ma1 d1ygha_ d1yk3a1 d3dr6a_ d1xmta_ d1z4ea1
d2ge3a1 d1bo4a_ d2fiaa1 d2jdca1 d1y9wa1 d3to7a_ d1wwza1 d2fl4a1
d2ozua1 d2b5ga_ d2cy2a1 d2p0wa_ d1cm0a_ d4pswa_ d1yrea1 d1yr0a1
d1m4ia_ d2gana1 d1yvka1 d1ghea_ d2g3aa1 d1y7ra1 d2euia1 d1q2ya_
d2fsra1 d1xeba_ d2ae6a1 d1cjwa_ d1qsma_ d2aj6a1 d1y9ka1 d1i12a_
d1vkca_ d2atra1 d1n71a_ d2d4pa1 d2fiwa1 d2fe7a1 d1tiqa1 d2ou2a_
d1yx0a1 d2fcka1 d1p0ha_ d2j8ma_ d2pq8a_ d1z4ra1 d1s3za1 d1ufha_
d1dosa_ d3qm3a1 d2fjka_ d1xvqa1 d1pn0a2 d3hy2a_ d2cvba1 d1o8xa_
d1lu4a_ d1we0a1 d2bmxa1 d2a4va1 d1psqa_ d1xiya1 d1q98a_ d1n8ja_
d1zofa1 d2ggta_ d3tjja_ d4je1a1 d1o73a_ d3s9fa_ d2zcta_ d1qmva_
d1jfua_ d2b1ka_ d5dvba1 d2b5xa1 d2v2ga_ d1st9a_ d3tb2a_ d4llra_
d4fh8a1 d1e2ya_ d1knga_ d2h30a_ d1i5ga_ d2z9sa_ d2f8aa1 d1tp9a1
d2b7ka_ d1nm3a2 d3hvva_ d2wfca1 d4kcea_ d1xvwa1 d4gqca1 d3qpma_
d4k1fa_ d1zzoa1 d1gp1a_ d1prxa_ d2qked_ d4ksoa_ d1t4za1 d1wwja_
d1e88a1 d1l6ja5 d1l6ja4 d1eaka3 d1l6ja3 d1h8pa2 d1h8pa1 d1etha2
d1w52x1 d2ppla1 d1hpla2 d1lpbb2 d2oxea1 d1bu8a2 d1rp1a2 d1ys1x_
d4hs9a_ d1ex9a_ d1ku0a_ d3qmma_ d1bnla_ d1dy2a_ d1ra6a_ d1muka_
d3gsza_ d1khva_ d1te9a_ d2xwha_ d3uqsa_ d3hkwa_ d3n6ma_ d2cjqa_
d2xyma_ d1teba_ d1te8a_ d1s48a_ d1u09a1 d4wfxa_ d3tyqa_ d2pgga1
d4qmea2 d2xdta2 d1z5ha2 d4wfia_ d4cg1a1 d3visa_ d1cr6a2 d1c4xa_
d1j1ia_ d2rhwa_ d3groa_ d1pjaa_ d1ei9a_ d1svpa_ d1mbma_ d3su6a1
d1cu1a1 d4agka_ d1vcpa_ d1q31a_ g1n1l.1 d3e90b_ d3u1jb_ d3zxha_
d1cgla_ d1rm8a_ d1eaka2 d1hy7a_ d2jsda_ d1cxva_ d1hv5a_ d1i76a_
d1eb6a_ d3p0ya1 d1igra1 d1igra2 d1n8yc2 d1n8yc1 d1m6ba2 d1m6ba1
d4dv8a2 d1j7na1 d1m4la_ d2bk7a_ d3fjua_ d1z5ra1 d1kwma1 d1uwya2
d1jqga1 d2c1ca1 d1dtda_ d1h8la2 d1ohfc_ d2tbvc_ d1c8nc_ d1x36a_
d1smvc_ d1ng0c_ d4sbvc_ d1f2nc_ d1cixa_ d1y29a_ d1v7fa_ d2jtba_
d1d1ha_ d1g9pa_ d1lupa_ d1qk7a_ d1mb6a_ d2a2va_ d1niya_ d1i25a_
d2mpqa_ d1ie6a_ d1kqha_ d1la4a_ d2mxoa1 d1dl0a_ d1koza_ d1qk6a_
d1emxa_ d1lqra_ d1c6wa_ d1rmka_ d1ixta_ d1eyoa_ d1cnna_ d1g1pa_
d1f3ka_ d1fyga_ d1rypd_ d1irua_ d1rypc_ d3unfk_ d1rypa_ d1j2pa_
d1irui_ d1j2qh_ d3unfh_ d1m4ya_ d1rypb_ d1irul_ d1irug_ d1iru2_
d1q5rh_ d1rypg_ d1g3ka_ d1iruf_ d1q5qa_ d1e94a_ d1irub_ d1ryph_
d3ty6a_ d1iruj_ d1iruk_ d1rype_ d1irud_ d1irue_ d3mi01_ d1rypf_
d1iruh_ d2z3ba_ d3mka2_ d1iru1_ d1ny721 d1ny722 d1a6ca3 d1a6ca2
d1a6ca1 d1pgl22 d1pgl21 d1pgl11 d1ofda3 d1ea0a3 d1ecfa2 d1q15a2
d1ct9a2 d1ao0a2 d1te5a_ d1y14b1 d2nn6i1 d1i94l_ d2zjra2 d1smxa_
d2qamc2 d1hh2p1 d2r7da1 d1rl2a2 d1wfqa1 d3bzka4 d1x6oa2 d3a0ja_
d2j01d2 d2ja9a1 d4ql5a_ d1hr0w_ d1luza_ d2n3sa_ d2vnud1 d1h95a_
d1go3e1 d2nn6h1 d1ne3a_ d2mo1a_ d1iz6a2 d1jjga_ d1e3pa2 d2ix0a3
d2ix0a2 d2lxja_ d1ueba3 d1ueba2 d2z0sa1 d1u0la1 d2ix0a1 d2eifa2
d2je6i1 d3i2za1 d2c35b1 d2ahob2 d1t9ha1 d3i4oa_ d2qall1 d1jt8a_

d2nn6g1 d1g6pa_ d3cama_ d1i94q_ d1c9oa_ d1d7qa_ d2ba0a1 d2vnud2
d2vnud3 d2qalq1 d1wi5a1 d1xtda2 d1ny4a_ d4w9ta_ d1jvna1 d4x2ra1
d1qo2a_ d4axka1 d1thfd_ d1h5ya_ d4u28a_ d2y88a_ d4wd0a_ d1vzwa1
d3exra_ d2ffca1 d2fdsa1 d3n3ma1 d2czda_ d1q6oa_ d2yyua_ d1vqta1
d1dbta_ d3ldva1 d4lt9a1 d3tc7a1 d3hoja_ d1i4na_ d3vnda_ d3nl8a1
d1geqa_ d3nava1 d4a2ra1 d3tc6a1 d1ujpa_ d4ijba_ d1xcfa_ d1vc4a_
d1rd5a_ d1qopa_ d3b5va_ d2r80b_ d1cqxa1 d1cg5a_ d1gcvb_ d3boma_
d2h8fb_ d2raoa_ d3bj1a_ d4hrrb_ d2r50a_ d3d1kb_ d2aa1b1 d3zhwa_
d3hf4b_ d3gdja_ d1jebb_ d3bj1b_ d1x9fb_ d1wmub_ d1gcva_ d3bcqb_
d3d4xb_ d1gvha1 d2dc3a_ d3hf4a_ d4yu3a_ d3k8ba_ d1qpwb_ d2r80a_
d3bcqa_ d1v4wa_ d3wtgb_ d1jl7a_ d4esaa_ d1a9we_ d3d1ab_ d1fsla_
d4h2la_ d1v4wb_ d1mwca_ d1vhba_ d1jeba_ d1spgb_ d1h97a_ d1q1fa_
d1tu9a1 d3d1aa_ d1hdsb_ d3hyub_ d2zfbb_ d3fs4a_ d1oj6a_ d1shrb_
d4h2lb_ d4yu3b_ d1spga_ d3hrwa_ d3qqra_ d3at5a_ d4wchd_ d3hyua_
d1cg5b_ d1uc3a_ d1hbra_ d2raob_ d1outb_ d4esab_ d3gdjb_ d3qqqa_
d1x9fc_ d3vrfa_ d1itha_ d3mkba_ d3d4xa_ d3mjpa_ d1jl6a_ d3fh9a_
d1outa_ d2dc3b_ d1it2a_ d3eoka_ d3g46a_ d1qpwa_ d3vrfb_ d1ch4a_
d1x9fd_ d1hdsa_ d2zfba_ d3mkbb_ d1or4a_ d3wtga_ d1a4fa_ d3fh9b_
d3bomb_ d3cy5a_ d3at5b_ d1x9fa_ d2oifa_ d4lmxb_ d1cpcb_ d3v57a_
d1kn1a_ d1cpca_ d4f0ta_ d2vjha_ d4po5b_ d1eyxa_ d1alla_ d4f0tb_
d1b33b_ d1b8db_ d4h0mb_ d1b33a_ d2vjhb_ d4lm6b_ d2vmlb_ d1allb_
d2vjta_ d3dbja_ d2vmla_ d2vjtb_ d4f0ub_ d1f99b_ d4rmpa1 d1liaa_
d1eyxb_ d2uulb_ d4lmsb_ d4f0ua_ d2bv8a_ d1f99a_ d2bv8b_ d2c7lb_
d3dbjb_ d4rmpb1 d1b8da_ d4h0ma_ d3v57b_ d1liab_ d2uula_ d2j96a_
d1azwa_ d1k8qa_ d2xqfa_ d1ea5a_ d1f6wa_ d1dx4a_ d1qe3a_ d1k4ya_
d4qwwa_ d2ha2a_ d2h0ba_ d2erfa_ d1h30a1 d3mw4a1 d1pz7a_ d2r1ba_
d3v64a1 d1d2sa_ d3bopa1 d3mw3a1 d1okqa1 d1okqa2 d4iuaa3 d4bvva_
d5hpga_ d3k65a_ d1ki0a3 d1ki0a2 d1ki0a1 d1a0ha1 d1pmla_ d3sp8a3
d2feba1 d1xova2 d1f74a_ d3l21a_ d2e1da_ d1to3a1 d1w3ia_ d1sfla_
d1n7ka_ d1vcva1 d4imea_ d1wa3a_ d2ocza1 d1f2ja_ d3m16a_ d2yxga_
d3a5fa_ d1fbaa_ d1l6wa_ d1xfba1 d1wx0a1 d1vpxa1 d1ojxa_ d1mxsa_
d2a4aa1 d1xkya1 d3cq0a1 d3s1xa_ d1f05a_ d1fdja_ d3cpra1 d4d2ja_
d4mh8a_ d1mu2a2 d4i7ga1 d3rp2a_ g1ucy.1 d1fuja_ d2zchp_ d3w94a_
d1si5h_ d1lo6a_ g2pka.1 d1cgha_ d1kigh_ d1a5ia_ d3s9ba_ d1hj8a_
d1ao5a_ d2anya_ d1q3xa1 d1gvza_ d1azza_ d4gsoa_ d2f91a1 d1iaua_
d1gdna_ d1eufa_ g1fiw.1 d1eq9a_ d4nfea_ d2rdla_ d1spja_ d1elva1
d1a7sa_ d1ekbb_ d1pfxc_ d1yc0a_ d2aiqa_ g1fiz.1 d1fi8a_ d4k60a_
d4dgja_ d1mzaa_ d1op0a_ d3tjva_ d1pytd_ d1sgfg_ g1gg6.1 d2eeka_
g1rtf.1 d1a0ja_ d3g01a_ d1pytc_ d4e7na_ d1ddja_ d1z8ga1 d2hlca_
d1npma_ d1brup_ g1jou.1 d1sgfa_ d1autc_ g2hnt.1 g1kdq.1 d1orfa_
d3s69a_ d4crga_ d1fxya_ d1bqya_ d2ftba_ d1pmpa_ d2rcqa1 d4lkpa_
d1sa8a_ d1vyfa_ d1lpja_ d3vg7a1 d4azqa1 d1o8va_ d5bvqa_ d1p6pa_
d2qo4a_ d1opba_ d1yiva1 d1o1va_ d4bvma1 d4tkba_ d4i3ba_ d1bwya_
d1ftpa_ d1ggla_ d4gkca_ d1cbia_ d3p6da1 d1kqwa_ d2a0aa1 d1dzka_

| | | | | | | | |
|---|---|---|---|---|---|---|---|
| d4tlja_ | d2a2ua_ | d4wfva_ | d3t1da_ | d4iaxa_ | d3zq3a_ | d2nnda_ | d1i4ua_ |
| d1bbpa_ | d1kt7a_ | d1exsa_ | d3s26a1 | d1lf7a_ | d1qfta_ | d4gh7a_ | d1z24a1 |
| d1qabe_ | d1xkia_ | d1ew3a_ | d3bx7a_ | d4iawa1 | d1n0sa_ | d1gkab_ | d3kzaa_ |
| d2k23a_ | d2l9ca1 | d2joza1 | d1gm6a_ | d3kffa_ | d3saoa1 | d1e5pa_ | d1yupa1 |
| d1iiua_ | d3lgia_ | d3i77a_ | d2z9ia2 | d4nsva_ | d1lcya2 | d4jcna_ | d1agja_ |
| d1os8a_ | d1hpga_ | d1l1ja_ | d1qtfa_ | d1e69a_ | d1mv5a_ | d3g60a4 | g1ii8.1 |
| d1ewqa2 | d1ji0a_ | d1v43a3 | d2wmfa1 | d1vpla_ | d1g2912 | d4a82a2 | g1f2t.1 |
| d2d62a1 | d3tuic1 | d2bbsa1 | d3g60a3 | d2ixea_ | d1g6ha_ | d1sgwa1 | d2qi9c_ |
| d3rlfa1 | d3b60a1 | d3d31a2 | d2pzea1 | g1xew.1 | d1jj7a_ | d1oxxk2 | d2onka1 |
| d1w1wa_ | d1iwea_ | d1qf5a_ | d1vmaa2 | d1nija1 | d1np6a_ | d2p67a1 | d3ihla_ |
| d1okkd2 | d1ls1a2 | d1s1ma2 | d1iona_ | d2v40a_ | d1dj3a_ | d3hida_ | d2vo1a1 |
| d2g0ta1 | d1vcoa2 | d1j8mf2 | d1g3qa_ | d1xjca_ | d1p9ba_ | d1qzxa3 | d1ihua2 |
| d1ihua1 | d2qm8a_ | d1dj2a_ | d4ioja_ | d1cp2a_ | d1yrba2 | d1hyqa_ | d3gqba2 |
| d1skye3 | d1xp8a1 | d1tf7a2 | d1tf7a1 | d1ye8a1 | d1skyb3 | d2qe7d2 | d4blta_ |
| d4huta_ | d3ldaa2 | d4hyya_ | d2i3ba1 | d1cr1a_ | d3j9tb2 | d3ntua2 | d1vdza1 |
| d2z43a_ | d1e9ra_ | d1nlfa_ | d3ziaa2 | d1n0wa_ | d3ziad2 | d3j9ta2 | d1pzna2 |
| d3gqbb2 | d2i49a_ | d1nnfa_ | d1xvxa_ | d1ursa_ | d1iuda_ | d4m1va1 | d1twya_ |
| d3e13x_ | d2nxoa1 | d4pfua_ | d3ttma_ | d1elja_ | d2qmwa1 | d1us5a_ | d1y3na1 |
| d1jeta_ | d1uqwa_ | d4f19a1 | d2esna2 | d3thia_ | d3fasa1 | d2uyeb_ | d2xwva1 |
| d1r9la_ | d2fyia1 | d1pb7a_ | d2ozza1 | d1sw5a_ | d1nh8a1 | d1vr5a1 | d2g29a_ |
| d1xs5a_ | d3lswa_ | d2z23a_ | d3w9va_ | d3tcha_ | d1q35a_ | d1z7me1 | d3q27a_ |
| d1p99a_ | d1ve4a1 | d2i6ea1 | d4mcoa1 | d4n8ya_ | d1eu8a_ | d1zbma1 | d1hsla_ |
| d1ixca2 | d1j1na_ | d1xvya_ | d1y9ua_ | d2z22a1 | d1xoca1 | d4oera_ | d1wdna_ |
| d5f1qa1 | d1xt8a1 | d1i6aa_ | d4gwoa_ | d4igta1 | d1pc3a_ | d4mmpa1 | d1o63a_ |
| d2czla1 | d2onsa2 | d4mija1 | d1eh3a_ | d3skpa_ | d1dtza1 | d1dtza2 | d1ieja_ |
| d1jnfa2 | d1ce2a1 | d1jnfa1 | d1b1xa2 | d1b1xa1 | d1jw1a1 | d1jw1a2 | d1ryoa_ |
| d1h76a1 | d1h76a2 | d1c4oa1 | d2p6ra3 | d2p6ra4 | d1oywa2 | d1oywa3 | d1wrba1 |
| d1w36b2 | d1w36b1 | d1c4oa2 | d3bora_ | d1t6na_ | d2eyqa5 | d2eyqa3 | d2eyqa2 |
| d2zu6a2 | d1veca_ | d1uaaa1 | d1w36d1 | d2g9na1 | d1z3ix2 | d2rb4a_ | d1hv8a1 |
| d1hv8a2 | d1t5ia_ | d1w36c2 | d2j0sa2 | d2j0sa1 | d1fuka_ | d3hjha1 | d1z5za1 |
| d1z63a1 | d1s2ma2 | d1s2ma1 | d1wp9a1 | d1wp9a2 | d1gm5a3 | d1gm5a4 | d3dina4 |
| d3dina3 | d1q0ua_ | d1w36c1 | d1z3ix1 | d1nkta3 | d1nkta4 | d1uaaa2 | d1w36d2 |
| g1qhh.1 | d2fwra2 | d2fwra1 | d3q3ma_ | d1za0a1 | d1mxra_ | d2inca_ | d1jk0a_ |
| d2incb_ | d1mhyb_ | d3hf1b_ | d1mtyb_ | d1r2fa_ | d2uw1a_ | d3n37a_ | d1afra_ |
| d1oquc_ | d1syya_ | d1mtyd_ | d2o1za_ | d1uzra_ | d1smsa_ | d2fkza_ | d1zuja1 |
| d1s3qa1 | d2fjca1 | d1z6om1 | d1veia_ | d4dyya_ | d1jiga_ | d3gvya_ | d2pyba_ |
| d4cy9a1 | d3bvfa1 | d1r03a_ | d2cf7a_ | d1nf4a_ | d1zs3a1 | d4iwka_ | d3ka8a_ |
| d3np0x_ | d1lb3a_ | d4e6ka_ | d1z6oa1 | d2fzfa1 | d4dyua_ | d3ge4a_ | d1jgca_ |
| d4am4a_ | d2z6ma_ | d1ji5a_ | d1vlga_ | d1lkoa1 | d1krqa_ | d3qz3a_ | d1o9ra_ |
| d2ffxj_ | d1euma_ | d1yv1a1 | d2v2pa_ | d1j30a_ | d4di0a_ | d1dpsa_ | d2cbia2 |
| d1gqia1 | d2choa2 | d1oewa_ | d2qzxa_ | d2asia_ | d1psoe_ | d1miqa_ | d1smra_ |
| d2h6ta_ | d1pfza_ | d1izda_ | d3hd8a_ | d1j71a_ | d1dpja_ | d4aa9a_ | d1t6ex_ |
| g1b5f.1 | g1lya.1 | g1htr.1 | d1qdma2 | d1bxoa_ | d1wkra_ | d1tzsa_ | d1ibqa_ |
| d2qzwa_ | d1ex4a2 | d1bdqa_ | d3ggta_ | d3ixoa_ | d1mrxa_ | d2haha_ | d1tw7a_ |

d4njsa_ d1sgua_ d2p3da_ d1nsoa_ d3d3ta_ d2rkfa_ d1c6ya_ d2rspa_
d3ogpa_ d3ekwa_ d2o4na_ d2j9ja_ d1baia_ d3ggua_ d3lzua_ d4npua_
d4j55a_ d2r8na_ d2fxda_ d2p3ca_ d4dqea_ d3ttpa_ d3t3ca_ d2p3aa_
d2hc0a_ d2fdda_ d3mwsa_ d1k8rb_ d1wgra1 d1ef5a_ d1wfya1 d1e8ya3
d1wgya1 d1lfda_ d2cs4a1 d1wxma1 d2byea1 d3kh0a1 d2byfa1 d1wxaa1
d1e7ua3 d1wgda1 d1wgga1 d4q5eb_ d1wjna1 d1se9a_ d3n3kb_ d1wz0a1
d1we6a1 d2m0xa_ d2gbka_ d3rt3b1 d1wh3a1 d1sifa_ d2ekec2 d4ii2b1
d4hk2a1 d1c3ta_ d1m94a_ d1wgha1 d1ttna1 d1j8ca_ d1wx8a1 d3v6cb1
d2ma9b_ d4i6lb1 d1bt0a_ d2jvca1 d1iyfa_ d2k1fa_ d1wxva1 d1wf9a1
d1t0ya_ d2io0b2 d1x1ma1 d2gbma_ d1zw7a1 d2m17a_ d1we7a1 d2lwpa_
d2gbja_ d1wjua1 d1v6ea1 d1v86a1 d4eewa_ d1wiaa1 d4icva_ d4gu2a_
d1wx7a1 d1wy8a1 d4hjka1 d2faza1 d1v5oa1 d1lgya_ d4l3wa_ d1llfa_
d1gz7a_ d1ukca_ d2bcgg1 d2bcgg2 d1dszb_ d1a6ya_ d3cbba_ d1ynwa1
d1r4ia_ d1hcqa_ d2hana_ d2hanb_ d2enva1 d1kb4b_ d2nllb_ d1lo1a_
d1cita_ d1um8a_ d1qvra2 d1qvra3 d1qzma_ d1sxjc2 d1jr3d2 d2p65a_
d1l8qa2 d1g8pa_ d3hu3a3 d1sxjd2 d1njfa_ d1in4a2 d1sxja2 d1tuea1
d2ce7a2 d1w5sa2 d1sxjb2 d1d2na_ d1ny5a2 d1ofha_ d2gnoa2 d2a5yb3
d1fnna2 d1u0ja1 d1lv7a_ d1iqpa2 d2fnaa2 d1ixsb2 d4gita_ d1sxje2
d1g41a_ d2e3fa_ d2e3ea_ d2llda_ d2ny8x_ d2e3ga_ d2ny9x_ d2nz3a_
d2lj7a_ d2m8bb1 d2kyqa_ d3psma_ d1jkza_ d1jxca_ d2gl1a_ d1ugla_
d4uj0a_ d2k4ua_ d1hp2a_ d2lu9a_ d1baha_ d2meoa1 d1wm7a_ d1hlya_
d1n8ma_ d1jlza_ d1wpda_ d1c55a_ d1pvza_ d1wt7a_ d1rjia_ d1wz5a_
d1m2sa_ d1pjva_ d1c49a_ d1qkya_ d2ktca1 d1wmta_ d1du9a_ d1j5ja_
d1r1ga_ d1e3oc2 d1ic8a2 d1t0za_ d2i61a_ d1t1ta_ d1sn4a_ d1kv0a_
d2lkba_ d1npia_ d1wwna_ d1pe4a_ d2kbka_ d2li7a_ d2kbha_ d1dq7a_
d1sega_ d1omya_ d1zywa1 d1jzaa_ d2jcqa_ d1n9wa1 d1bbua1 d1c0aa1
d1b8aa1 d1l0wa1 d3a74a1 d1eova1 d1iyjb4 d1iyjb5 d1iyjb3 d1o7ia_
d4ipca1 d1x3ea_ d4apva_ d1jb7a1 d1jb7a2 d1jb7a3 d3kdfa_ d1ue1b_
d1wjja1 d3eiva_ d1xjva2 d1xjva1 d2ccza2 d1se8a_ d1l1oc_ d1jb7b_
d1qvca_ d3ulla_ d1qzga_ d1miua4 d1miua5 d1miua3 d3ubub_ d1egia_
d1qo3c_ d1v7pa_ d1tdqb_ d3ubua_ d1c3ab_ d1h8ua_ d3bx4b_ d1t8ca1
d1sb2a1 d1qdda_ d2ziba1 d3cada_ d1byfa_ d1j34b_ d1jwia_ d1jzna_
d1c3aa_ d1wmza_ d3bdwb_ d1hq8a_ d1fifa1 d1dv8a_ d3hupa_ d1wk1a1
d1umra_ d2afpa_ d3bx4a_ d1gz2a_ d1sl6a_ d1v7pb_ d3g8ka_ d1ixxa_
d1j34a_ d1jwib_ d1g1sa1 d1y17a1 d1ja3a_ d1fvub_ d3bdwa_ d1sb2b1
d1fvua_ d1umrc_ d1oz7a_ d4m17a_ d3cfwa1 d1ypqa1 d1g1ta1 d3fd4a_
d3l9jc_ d4dn8a1 d1v4lb_ d1rdl1_ d2msba_ d1dgsa2 d1fvia1 d1p16a1
d1x9na2 d1rpma_ d2shpa1 d1yfoa_ d1l8ka_ d2ooqa1 d1g4us2 d1p15a_
d1lara2 d1lara1 d1wcha_ d2fh7a2 d2fh7a1 d1ohea1 d1ohea2 d2f4da_
d1rxda_ d2hxpa_ d1xria_ d1d5ra2 d1fpza_ d3ezza_ d4y2ea_ d1i9sa_
d3azya_ d3wvja_ d1upsa1 d1ajoa_ d2hyka_ d1ajka_ d2vy0a_ d1o4ya_
d3o5sa_ d3d6ea_ d3atga_ d1dypa_ d1umza_ d3i4ia_ d1tdha3 d1q3ba3
d1ee8a3 d1k82a3 g1qmo.1 g1len.1 d3zyra_ d1fnya_ g2ltn.1 d1avba_
d3usua_ d1dhkb_ d1f9ka_ d1ukga_ d1g8wa_ d1g9fa_ d1wbfa_ d3wcsa_

d1g7ya_ g1loe.1 d1mvqa_ d1hqla_ d1v6ia_ d1dbna_ d1qnwa_ d1fx5a_
d4l8qa_ d1ioaa_ d3n35a_ d3h33a_ d1rwja_ d19hca_ d1os6a_ d3bxua_
d3h4na_ d1i77a_ d3caoa_ d1j0pa_ d2ctha_ d1duwa_ d1ofwa_ d2e84a_
d1hh5a_ d3h34a_ d1wioa4 d1ccza2 d1dr9a2 d1ic1a1 d2d9qb1 d2nxyb2
d1adqa1 d1fnga1 d1uvqb1 d1zs8a1 d2vola1 d2vola2 d1mfbl2 d2jelh2
d1uwel2 d3qq4b1 d1kjvb_ d1k5na1 d1osph2 d4odhl2 d1ow0a1 d1ow0a2
d1bfob2 d3l9ra2 d3jtth1 d2fath2 d4elkb2 d1fn4b2 d2xfxb_ d1pg7x2
d1cqka_ d4k71b2 d1lk2a1 d1eapb2 d4lfhg2 d1i4fa1 d2nw2a2 d1kfah2
d1mhca1 d1h3ua1 d1wejh2 d1h0db2 d1a5fh2 d1kcvh2 d1mjuh2 d1qqda1
d1kcrh2 d1k8ia1 d3huja2 d2nnab1 d1nfdf2 d3mffb2 d1s9va1 d1lk3h2
d1q0xl2 d1fv1b1 d1neza1 d4rfeb2 d1r3ha1 d1a6tb2 d1kjva1 d1pz5b2
d1onqa1 d2bc4a2 d1de4a1 d1k8da1 d1tcra2 d1dn0b2 d1nfde2 d2xwtb2
d1bfoa2 d1mexh2 d1iakb1 d1ypzf2 d4f7ea2 d1q72l2 d2fbjh2 d1fe8h2
d1clzh2 d1fngb1 d1uvqa1 d1vgka1 d1c16a1 d4j12a2 d4x99b2 d1igtb3
d1je6a1 d1q72h2 d1nldh2 d1teth2 d2hmic2 d1i1ca2 d1i1ca1 d1k8ib1
d1q0yh2 d1kfal2 d3frua1 d1pg7w2 d1u58a1 d1igyb4 d1igyb3 d1hyrc1
d1klub1 d1deeb2 d1c12b2 d2bc4b2 d2ig2h2 d1kcvl2 d3wn5b1 d1lk2b_
d1lk3l2 d1mjul2 d1sm3h2 d1q0xh2 d1zvsa1 d1ydpa1 d1u8ib2 d1ypze2
d1kcuh2 d1op9a_ d1y0la1 d1etzb1 d1egjl1 d2gsib1 d1nj9a1 d4jfhd1
d1aqkl1 d1t2ja_ d1lo4h1 d1zvya1 d3v4vl1 d4enec1 d1kxtb_ d5e8ea1
d2pxya_ d4weud_ d1xiwc_ d2esvd1 d1qnzh_ d1ogae1 d1f4xh1 d4n1hb_
d3ezjb1 d3mlwl1 d1kfah1 d1etza1 d1b4jh1 d3cfic_ d4w68a_ d2arja1
d1ek3a_ d2ok0l1 d1vera_ d1uwgh1 d1blnb1 d1fpth1 d1rzga1 d2giya1
d4m3kb1 d1nc2a1 d2nyyd1 d1ncna_ d1fbih1 d5dxwa_ d4fqqa1 d1dqdh1
d1jfql1 d1kelh1 d1faih1 d1frgl1 d1figl1 d4m6nl1 d1fo0a_ d4hxbl1
d3b5ga_ d3quyd1 d2h9gb1 d1i8kb_ d2dd8h1 d2ck0h1 d1eaja_ d4w6wb1
d2a6ib3 d1t66d1 d2e27h_ d1ob1b1 d1f11b1 d2v7na1 d1ad0a1 d2xt1b1
d1pskh1 d4i13b1 d2op4l1 d1a0ql1 d1uwel1 d1za3a1 d1ncbl1 d4uu9a_
d1bm3h1 d1cloh1 d1nqba2 d1cf8h1 d4y5va_ d1mhph1 d5anma1 d4cdgc1
d4ldeb_ d1y0lb1 d2pcpb1 d1z3gl1 d1dfbl1 d1fh5h1 d3g5xa1 d1tzib1
d4dn3l1 d3zhka1 d2b1hh1 d2uyla1 d1f3dh1 d1nj9b1 d2agjh1 d2h1pl1
d1q72h1 d8fabb1 d1ndmb1 d1rjca1 d4u6hb1 d3upca_ d2fbjl1 d1osph1
d1tvda_ d2cjvh_ d4fhbd1 d1bd2d1 d1rz7h1 d3nn8a_ d1blna1 d1knoa1
d1nfda1 d2bmkb1 d1dqll_ d1igyb1 d1igfl1 d12e8h1 d3utza1 d1nc2b1
d1rihh1 d4mwfb1 d4jvpa1 d1mieh1 d1rhfa1 d1fgnl1 d1sbsh1 d1lp9e1
d1dn0a1 d4lqfl1 d1iaih1 d1kgcd1 d1mh5b1 d1gpol1 d2g60h3 d1f8tl1
d2jixd1 d4grwh_ d1fo0b_ d1bfob1 d1axsa1 d1u9ka_ d1nmbh_ d1h8na1
d1h8na2 d1jguh1 d4eura1 d3sdyl1 d35c8h1 d1w72l1 d1mvub_ d1i9rh1
d1ob1a1 d4wenb_ d1yeeh1 d3ietc1 d1f58h1 d1ad0b1 d1mqkl_ d1nbvh1
d4b50a_ d3r8bb_ d1bafl1 d1mamh1 d4rgne1 d2gsgb_ d35c8l1 d1bafh1
d1ai1h1 d2pcpa1 d2fath1 d2x1pa_ d1orsb1 d1ibgl1 d1shma_ d1a2yb_
d1u0qa_ d2coqa_ d1hilb1 d1qfwi_ d1jv5b_ d1fn4b1 d5e0qa_ d1nsnh1
d1plgl1 d4ocxl1 d1kb5h1 d1rzfh1 d1pg7x1 d2fx7h1 d2oi9c_ d1l7il1
d3eyva1 d1d5il1 d4ffyl_ d1tetl1 d1a6wh_ d1ac6a_ d3dvfa_ d1nfde1

d1mimh1 d1ri8a1 d1jhlh_ d3dura_ d1fskb1 d1f97a1 d3so3b1 d2ak4d1
d1lp9f1 d4z7wf1 d1l7th1 d4y5ya_ d4x7ec1 d1emth1 d1ikfl1 d1nlbh1
d4utal1 d1bjma1 d1eapb1 d1u6ah1 d2dtgd1 d1kj2b_ d1xeda1 d1c1eh1
d1fgvh_ d2bnub1 d2fb4h1 d2dtgb1 d1ktrh_ d1jpth1 d1qkzl1 d4hepg_
d2atpb1 d1flrh1 d2hrpl1 d1mvfa_ d1iaim1 d3k80a1 d2jb5h1 d3zkxc_
d4grwe_ d1it9h1 d1rhhb1 d3w9ec_ d1e4wh1 d1sy6h1 d2j6eh1 d3qxta_
d1jglh1 d1p7ka1 d1tzhb1 d4n8pa1 d1gafl1 d2jelh1 d1wioa2 d4i0cc1
d1cicb1 d1lmka2 d1lmka1 d1iqwh1 d1orqb1 d1ejoh1 d1jnlh1 d4zg1a_
d43c9b_ d1ggbl1 d1wejh1 d3q6fa1 d1f3dj1 d3c6la1 d1bqlh1 d3p9wb_
d1a3rl1 d1h0db1 d2uzil_ d1eo8l1 d1qfwl_ d1n7ml1 d1igja1 d1c5ch1
d2a9mh_ d1rmfh1 d1fj1b1 d4heme_ d1ccza1 d1a5fh1 d1ogad1 d1dlfh_
d1qa9b_ d1vgel1 d3qskb_ d1sjxa_ d4ozfg1 d1sq2n_ d1qd0a_ d1kcvh1
d1c5db1 d3qcul1 d8faba1 d3bn9d1 d1mjuh1 d4y8dc_ d7fabh1 d3dvgb1
d1lo0l1 d1a6tb1 d1gigh1 d1kcrh1 d2hwzl1 d1yjdc1 d3clfl1 d4krna_
d1dl7l_ d1e6ol1 d1mexh1 d3r0ma_ d4kkna_ d1fnsl1 d1oaqh_ d1rzia1
d1ucbh1 d1ncwl1 d4b41a_ d1nfdf1 d2mpah1 d2d7tl1 d4grwf_ d4b5ea_
d1lk3h1 d3u79a_ d4u3xa_ d1ghfl1 d1bvkb_ d1mcww1 d1b88a_ d1jrhh1
d32c2b1 d4wemb_ d1dsfh_ d1op3h1 d1cell1 d1iqda1 d1qoka1 d1qoka2
d1qfuh1 d2crya1 d1ay1h1 d1oaql_ d3ab0b_ d1rurl1 d1t4kb1 d1bz7b1
d1n0xh1 d1forl1 d1ucbl1 d4w6yb_ d43c9a_ d2aabl1 d1tzia1 d4n1ca_
d1k6qh1 d3g9ab_ d4tvsa1 d1igmh_ d1pz5b1 d3dx9a1 d3wxwh_ d1nakh1
d1kcul1 d3eaka_ d4qlra1 d1cr9l1 d1d9ka_ d1f4xl1 d1deea1 d2i27n2
d1c12a1 d2orbl1 d1j8hd1 d1mcph1 d1c5da1 d1kenh1 d1bd2e1 d1h5ba_
d4x7fc1 d3upaa_ d1t3fa1 d1indh1 d2dtgc1 d4hc1l1 d1zv5a1 d4wgvb_
d1uwgl1 d4f37k1 d1aqkh1 d1vesa_ d3zkqd_ d1fptl1 d1lo4l1 d1knob1
d3rrqa_ d1ct8b1 d4orzc_ d1mlbb1 d4hgkc1 d1dqdl1 d1bogb1 d4ozhe1
d1dr9a1 d1i7za1 d1dn0b1 d1kell1 d1rzib1 d1ypzf1 d1wtla_ d1hkfa_
d1fail1 d1figh1 d2vyre_ d1sjva_ d1ohqa_ d1bfoa1 d1pkqb1 d1mjul1
d1b4jl1 d1nsnl1 d2arjb1 d1p4bh_ d4aj0a_ d3nn8b_ d2rcja1 d1f2xk_
d4v1dd_ d1adqh1 d2eksa_ d4k3ga_ d1jfqh1 d1uweh1 d1frgh1 d4gftb_
d1uz8b1 d4n1ea_ d1bm3l1 d1clol1 d3mcka1 d2vq1a1 d1ae6h1 d4aq1b1
d1uj3b1 d1nbqa1 d1fh5l1 d2ck0l1 d2ywza_ d1q72l1 d4nbxb_ d1hila1
d4hzlb1 d2j88l1 d1zmya1 d1cd0a_ d1a4ka1 d1pskl1 d1a0qh1 d4krmb_
d1ncbh1 d2fbjh1 d1j8he1 d1i3ua_ d1cf8l1 d1fe8h1 d2p1ya1 d2x89a_
d1ol0a_ d2r0lh1 d1ub5a1 d1dfbh1 d3eyvb1 d4jdvb1 d4p2cg_ d2gsia1
d2agjl1 d1u3ha1 d2h1ph1 d1rihl1 d1fskc1 d1boga1 d2a77l1 d1ospl1
d1qfoa_ d3dwta_ d2fx7l1 d1rz7l1 d3stba1 d1pkqa1 d1b6da1 d1dzba2
d1dzba1 d1kj2a_ d4l1ha_ d1rzgb1 d1dqlh_ d12e8l1 d1miel1 d1jmaa_
d1sbsl1 d1fgnh1 d1f58l1 d1mqkh_ d1ndgb1 d3qegl1 d1iail1 d2aw2a1
d1tqbc1 d3fo2a1 d4dkaa_ d1f8th1 d2g60l1 d2ywya_ d1uj3a1 d1yc7a_
d3bd3a_ d4w6xb1 d1nmbl_ d1i8ka_ d1rjlb1 d1ypze1 d1p7kb1 d1kxqe_
d1gpoh1 d2z4qa1 d1w72h1 d1i9rl1 d4qgya1 d4xt1c_ d2qqnh1 d1cica1
d3c08h1 d2cd0a_ d2bdnh1 d1hnga1 d1maml1 d1nldh1 d1cr9h1 d4lcih1
d3bkyh1 d3qg7l1 d1ai1l1 d1kxvc_ d4iosd_ d1l7ih1 d2x1qa_ d1ub6b1

d3k74b_ d1ibgh1 d1teth1 d1igya1 d1hxma1 d2x6ma_ d2e7la_ d5e03a_ d1qfwm_ d2r0kh1 d1igjb1 d1rurh1 d1plgh1 d1mj8h1 d1yuhb1 d3fmgl1 d1rzfl1 d1emtl1 d1ad9b1 d1igtb1 d1nlbl1 d1d5ih1 d3k7ua_ d4ffyh_ d1bwwa_ d3dvga1 d1a6ta1 d1fgvl_ d4qxgl1 d1miml1 d1jhll_ d2hffb1 d2hh0l1 d1qkzh1 d1l7tl1 d1afvh1 d1iaii1 d2cjuh_ d1um5h1 d2ghwb2 d2ghwb1 d1kgce1 d2fgwh1 d1ikfh1 d1dvfd_ d2i24n1 d2xtjb_ d1za3b1 d2yc1a_ d2hh0h1 d2x1oa_ d1bvka_ d1rjla1 d5e7ba1 d1igta1 d1il1b1 d2fb4l1 d1mvua_ d1jptl1 d32c2a1 d1fvca_ d1iqdb1 d2hrph1 d2p4ab_ d4qo1a_ d1p2cb1 d1wt5c_ d1t4ka1 d5c1mb_ d1it9l1 d1bz7a1 d1b2wh1 d1e4wl1 d1jnll1 d1yedb1 d1za6b1 d3u7aa_ d1igfh1 d3gnml1 d1orsa1 d1wejl1 d1a2ya_ d1hxmb1 d1gafh1 d2j4wh1 d1pz5a1 d1j05a_ d1a3rh1 d4fqll1 d1qfwh_ d1eo8h1 d1n7mh1 d4s11a_ d1kegh1 d5da4a_ d1iqwl1 d3whxb1 d1vgeh1 d1deeb1 d1c12b1 d1t2ql1 d1ad9a1 d3gsnb1 d3b2uc1 d1ggbh1 d1t3fb1 d1nakl1 d4ocmc_ d1lila1 d2v7hb3 d2esve1 d1mcoh1 d2qr0b1 d2q3aa_ d1ct8a1 d1kcrl1 d2pxyb_ d2uzih_ d5c3ld_ d3durb_ d1c5cl1 d2ak4e1 d1dl7h_ d1xiwd_ d1mlba1 d1rmfl1 d1i7zb1 d1dlfl_ d2xa3a_ d5bq7b1 d1dqqb1 d3h42l1 d1egjh1 d1kcvl1 d1fe8l1 d2j6el1 d1f3rb2 d1lk3l1 d1sm3h1 d1q0xh1 d1ghfh1 d7fabl1 d1igch1 d1lo0h1 d4idla_ d5c2bl_ d1jrhl1 d1il1a1 d3zhda1 d4kfzc_ d1b0wa_ d1e6oh1 d1mexl1 d4krpb_ d1fnsh1 d1ncwh1 d4hgma_ d4eigb1 d4rzca_ d2mpal1 d4y5xa_ d5en2b1 d3lrga1 d4ej1c1 d1rhha1 d1j05b_ d1forh1 d1q9wb1 d2wzpd_ d2aq2a1 d1h0da1 d3revb1 d2ij0c1 d1tzha1 d4m7jl1 d1k6ql1 d3b9kc1 d1igml_ d3qeua1 d1dsfl_ d1ce1h1 d1kcuh1 d1zvha1 d1zvoa1 d1tqbb1 d1ay1l1 d2qscl1 d2cgrh1 d1nl0h1 d1kenl1 d3eo0a1 d4n9ob1 d1n0xl1 d3k1kc_ d2cqva1 d1fhga_ d2k1ma_ d2dl2a1 d1biha3 d1biha1 d1biha4 d3qp3a1 d1g1ca_ d1gxea_ d1cvsc2 d1cvsc1 d1xmwa2 d1m4ka2 d2otpa2 d1biha2 d1rhfa2 d2fcba2 d2fcba1 d1wwbx_ d1gsma2 d1epfa1 d1epfa2 d2dava1 d1waaa_ d1f97a2 d1x44a1 d1vcaa2 d1fnla2 d1fnla1 d3b5ha2 d3h8na2 d3h8na1 d1jbja1 d1jbja2 d1pd6a_ d1f2qa2 d1f2qa1 d3cx2a_ d1nbqa2 d3b5ha1 d1n26a1 d1xiwb_ d1xaua_ d1fltx_ d2ifga1 d1olla1 d1olla2 d1cs6a4 d1cs6a3 d1cs6a2 d1cs6a1 d2gw5a2 d1sy6a1 d1xiwa_ d1ucta2 d1ucta1 d1ry7b2 d1gl4b_ d1qz1a3 d1olza1 d2c9aa1 d1wwca_ d2aq2b1 d1ck1a1 d1enfa1 d1i4pa1 d1v1oa1 d1et9a1 d1esfa1 d2g9hd1 d4ohja1 d1klud1 d1bxta1 d1eu3a1 d1ty0a1 d1fnua1 d1c4qa_ d1prtc1 d2wv6d_ d1prtf_ d1ltrd_ d4l63b1 d1prtd_ d1tl0f_ d2bosa_ d1prtb1 d3chbd_

**Family-10 (a.26.1.2)**

**Positive dataset contains 10 protein sequences.**

d3tgxb_ d2z3qa1 d3qb1a_ d4nkqb_ d5e4ea_ d2o27a_ d3b5ka_ d2psma1 d4zf7a_ d3uf5a_

**Negative dataset contains 4076 protein sequences.**

d1m32a_ d1ibja_ d1c7na_ d1iuga_ d4q75a_ d2bkwa1 d1t3ia_ d1n8pa_ d1qz9a_ d1jf9a_ d1eg5a_ d1p3wa_ d3kgwa_ d1cl1a_ d2ctza1 d1vjoa_ d2rfva_ d1gc0a_ d1cs1a_ d1d2fa_ d2ch1a1 d1qgna_ d1pffa_ d1elua_

d1ax4a_ d2v1pa1 d2ez1a_ d1u08a_ d1lc5a_ d1b5pa_ d2ay1a_ d3pd6a_
d7aata_ d2csta_ d1j32a_ d1fg7a_ d4wlha_ d1o4sa_ d1v2da_ d1gdea_
d1m6sa_ d1v72a1 d1svva_ d1ajsa_ d1wsta1 d2f8ja2 d1lk9a_ d1vp4a1
d1xi9a1 d2gb3a1 d1yaaa_ d1xkla_ d2wfla_ d3i2ka1 d1lnsa3 d1mpxa2
d1xkta1 d2hfka_ d1jmkc_ d5d3ka_ d2cfba1 d1dfoa_ d1o69a_ d1bj4a_
d3k28a_ d2bwpa_ d3lg0a_ d2fnua_ d3ou5a_ d1z7da1 d1b9ha_ d1iaya_
d4msoa_ d1bs0a_ d1s0aa_ d1sffa_ d3tqxa_ d1mdoa_ d3qboa_ d3pgya_
d1bjna_ d2dkja_ d1ejia_ d1vefa1 d4wxga_ d3bs8a_ d3gbxa1 d1w23a_
d4nmwa_ d1r0ka3 d1vkoa2 d1vm6a2 d3qvsa2 d1gr0a2 d1p1ja2 d3lgia_
d3i77a_ d2z9ia2 d4nsva_ d1lcya2 d4jcna_ d1agja_ d1os8a_ d1hpga_
d1p3ca_ d1l1ja_ d1ddla_ d1e57b_ d1auyb_ d1qnia1 d1fwxa1 d1f56a_
d1bypa_ d1azca_ d1ws8a_ d3u25a_ d1cuoa_ d1id2a_ d1rkra_ d2iaac_
d4dp1x_ d1nwpa_ d2gima_ d2i7sa_ d1qhqa_ d1gyca3 d1hfua2 d1kbva1
d1kbva2 d1sdda1 d1sdda2 d1gyca1 d1gyca2 d1mzya1 d2bw4a1 d1aoza1
d1aoza3 d1aoza2 d1kyaa2 d1kyaa3 d1kyaa1 d1mzya2 d4e9sa1 d1sddb1
d1sddb2 d1hfua1 d1hfua3 d1oe1a2 d1oe1a1 d1v10a2 d1v10a3 d1v10a1
d2dara2 d2dara1 d1wyha2 d2dloa2 d1rutx4 d1rutx3 d2cuqa2 d1x4la2
d1x4la1 d1x3ha2 d1x3ha1 d2d8za1 d1x61a2 d1x6aa1 d1x6aa2 d2cora2
d2cora1 d1v6ga1 d1v6ga2 d2d8za2 d1x64a1 d1x64a2 d1b8ta1 d2co8a1
d1x63a2 d2dj7a2 d2dj7a1 d2cu8a2 d2cu8a1 d1g47a2 d1g47a1 d1j2oa1
d1wyha1 d2dloa1 d1wiga1 d1wiga2 d2cupa2 d2cupa3 d1x68a1 d2d8ya2
d2d8ya1 d1m3va2 d1x62a1 d1x68a2 d1x61a1 d1b8ta3 d1b8ta2 d2d8xa2
d2d8xa1 d1x4ka1 d1x4ka2 d2cuqa1 d1m3va1 d2co8a2 d1j2oa2 d1x63a1
d2cura1 d2cura2 d1x62a2 d1ny721 d1ny722 d1a6ca3 d1a6ca2 d1a6ca1
d1pgl22 d1pgl21 d1pgl11 d1ny711 d3sxwa1 d4luka_ d4qgna_ d1vr3a1
d2bema_ d1pama1 d1ea9c1 d1qhoa1 d2fhfa2 d2fhfa1 d1k3ia1 d1j0ha1
d1cxla1 d2bhua1 d1edqa1 d1f1sa2 d1ut9a2 d1gvia1 d1txka1 d2phla2
d1fxza2 d1fxza1 d1lrha_ d1uija1 g1dgw.1 d2v09a_ d1uika2 d1uika1
d1dgwa_ d2pa7a1 d1wlta1 d1nxma_ d2c0za1 d1ktja_ d1ds6b_ d1fstb_
d1g0da1 d3us3a3 d3us3a1 d1yloa2 d1y7ea2 d1y0ya3 d1lfwa1 d1vixa1
d1fnoa4 d1vhoa2 d1ysja1 d1vhea2 d1z2la1 d2fvga2 d1vgya1 d1r3na1
d2grea2 d1xmba1 g1q7l.1 d1rtqa_ d1h4ba_ d2opoa_ d1au7a2 d1ic8a2
d3gpdg2 d2q49a2 d1hdgo2 d2g17a2 d2cvoa2 d2czca1 d3pwka2 d1obfo2
d1i32a2 d1zyao2 d2pkqo2 d1b7go2 d2hjsa2 d1cf2o2 d1vkna2 d1nvmb2
d1ux8a_ d1dlya_ d4i0va_ d2bkma_ d1dlwa_ d2bmma_ d2msya_ d2djoa1
d2r5za_ d1au7a1 d1f43a_ d2lmda1 d1akhb_ d1s7ea1 d3nara_ d1zq3p1
d2craa1 d1mija_ d4uuta_ d3a01a_ d1x58a1 d3rkqa1 d3cmya1 d2cqxa1
d2da6a1 d1e3oc1 d1fjla_ d1mh3a1 d1ig7a_ d1x41a1 d1wh7a1 d1gt0c1
d2mw8a_ d1lfup_ d1x2ma1 d1b72a_ d1wi3a1 d1wjha1 d1x2na1 d1mnmc_
d2cuea1 d3k2aa_ d2hosa_ d2jwta2 d1pufa_ d1octc1 d2hi3a_ d1b8ia_
d4j19a_ d3lnqa_ d1wh5a1 d1b8ib_ d1hlva1 d1iufa2 d1iufa1 d2iu5a1
d1zk8a1 d2g7sa1 d2fd5a1 d2np5a1 d1z0xa1 d2id6a1 d2d6ya1 d2i10a1
d1vi0a1 d2oi8a1 d3c07a1 d2ns7a1 d2fq4a1 d2yvea1 d1t33a1 d2o7ta1
d2np3b1 d2g7ga1 d2gena1 d2fbqa1 d2g7la1 d2gfna1 d2g3ba1 d2jj7a1

d2hyja1 d1sgma1 d1ui5a1 d2hkua1 d2id3a1 d4jyka1 d1bl0a1 d1bl0a2
d1d5ya1 d3ubub_ d1egia_ d1qo3c_ d1v7pa_ d1tdqb_ d3ubua_ d1c3ab_
d1h8ua_ d3bx4b_ d1t8ca1 d1sb2a1 d1qdda_ d2ziba1 d3cada_ d1byfa_
d1j34b_ d1jwia_ d1jzna_ d1c3aa_ d1oz7b_ d1wmza_ d3bdwb_ d1hq8a_
d1fifa1 d1dv8a_ d3hupa_ d1wk1a1 d1umra_ d2afpa_ d3bx4a_ d1gz2a_
d1sl6a_ d1v7pb_ d3g8ka_ d1ixxa_ d1j34a_ d1jwib_ d1g1sa1 d1y17a1
d1ja3a_ d1fvub_ d3bdwa_ d1sb2b1 d1fvua_ d1umrc_ d1oz7a_ d4m17a_
d3cfwa1 d1ypqa1 d1g1ta1 d3fd4a_ d3l9jc_ d4dn8a1 d1v4lb_ d1rdl1_
d2msba_ d3lv8a_ d1s3ga1 d1zaka1 d1s4qa_ d3tvta2 d1uf9a_ d1khta_
d1ckea_ d1osna_ d1p5zb_ d4tmka_ d3cm0a_ d1tmka_ d1jaga_ d3h86a_
d1t0hb_ d1nn5a_ d2h92a_ d3kb2a1 d2c95a1 d1yj5a2 d1kgda_ d1ly1a_
d2ak3a1 d1vyva2 d1deka_ d2grja1 d1p3ja1 d1y63a_ d1e4ya1 d1s96a_
d3iija1 d1lvga_ d1nksa_ d1vyua2 d4e22a_ d1qf9a_ d1jjva_ d1q3ta_
d1ki9a_ d1lw7a2 d1teva_ d1kjwa2 d1gsia_ d1g3ma_ d1j99a_ d1t8ta1
d1nsta_ d1zrha_ d1ls6a_ d2a3ra_ d1aqua_ d2zptx_ d3ckla_ d1texa_
d1q44a_ d2gwha_ d1z0ja1 d3umra2 d3gj0a1 d2gaoa1 d1yzga1 d1x3sa1
d1g7sa4 d1puja_ d1udxa2 d1kk1a3 d2o52a_ d1jwyb_ d1zcaa2 d1zcba2
d2fv8a1 d2cjwa_ d3dz8a1 d1xzpa2 d1vg8a_ d4qxaa_ d2ew1a1 d1x1ra1
d2bcgy_ d3doea1 d3cona_ d3rega_ d3wyga_ d1upta_ d2b6ha1 d1zd9a1
d1lnza2 d1r5ba3 d1n0ua2 d1r2qa_ d2f9la1 d2rhda_ d3q3jb_ d3q85a_
d1efma1 d1r8qa_ d2gf9a1 d2akab1 d1ek0a_ d1z08a1 d2gcoa1 d2dy1a2
d4g01b_ d2cxxa1 d1svia_ d3q72a_ d1ni3a1 d4ac9a4 d2erxa1 d2bmea_
d1wxqa1 d2gf0a1 d4ldja1 d1zunb3 d1d5ca_ d4lv5b_ d1efca3 d1kmqa_
d2g6ba1 d3lrpa_ d1tada2 d2y8ea_ d4rd4a1 d1z06a1 d2heia_ d3a58b_
d1u0la2 d1d2ea3 d1zj6a1 d2ocba_ d3rwoa_ d1mr3f_ d2e9sa1 d4u5xa1
d2iwra_ d2fn4a1 d3bwdd_ d2nzja1 d2atva1 d2ksqa_ d3l43a_ d1mkya2
d1mkya1 d1wmsa_ d1jnya3 d2rmka_ d1f6ba_ d1f60a3 d1gota2 d1jala1
d2fh5b1 d1g16a_ d2wblc_ d1m7ba_ d1wf3a1 d1f5na2 d1t9ha2 d1z6xa1
d1z0aa1 d2fg5a1 d2a5ja1 d2bcjq2 d1s0ua3 d1ky3a_ d4y0va_ d4lhwa_
d2qmea1 d2atxa1 d1egaa1 d1ryha_ d1moza_ d1nrjb_ d2erya1 d3bfka_
d1puia_ d1fzqa_ d3j3az1 d3t5ga_ d1shza2 d3defa_ d2f7sa1 d3raba_
d3tsoa_ d3aq4a_ d2al7a1 d3sfva1 d3bc1a_ d2ncda_ d1sdma_ d3h4sa1
d2heha_ d2grya_ g3kin.1 d1kk8a2 d1br2a2 d1goja_ d1f9va_ d2mysa2
d1lkxa_ d3t0qa_ d4db1a1 d3gbja_ d1ry6a_ d1v8ka_ d1vava_ d1uaia_
d1k8ka2 d1k8ka1 d3uleb1 d3i33a2 d3i33a1 d1dkgd2 d1dkgd1 d4ci6a2
d1jcfa1 d4a2ba2 d2fxua1 d1yaga2 d1yaga1 d4cbua2 d1bupa2 d1g99a2
d2e1za2 d2e1za1 d1saza1 d1saza2 d1ig8a1 d1czan2 d1czan1 d1ig8a2
d1czan4 d1bg3a4 d1bg3a3 d1bg3a2 d1bg3a1 d1nira1 d1qksa1 d1hroa_
d4gedb_ d3zooa_ d1mz4a_ d1gu2a_ d1f1fa_ d1dvva_ d1c6ra_ d1c75a_
d1e29a_ d1gdva_ d1cnoa_ d1h32b_ d1co6a_ d2bh4x_ d2aiua_ d5cytr_
d1jdla_ d1i8oa_ d2zboa_ d3dmia_ d1ls9a_ d1wvec_ d2yk3a_ d3ph2b_
d1dw0a_ d5aura_ d1ql3a_ d1qn2a_ d1f1ca_ d2nqya_ d1h4ga_ d1hixa_
d1oa2a_ d1olra_ d3b7ma_ d4h7ma1 d3wp3a_ d2vgda_ d1igoa_ d3zsea_
d2nlra_ d3wx5a_ d1oa3a_ d1pvxa_ d1f5ja_ d1oa4a_ d1ks5a_ d4ixla1

d1h32a2 d1v8fa_ d2ejca_ d3muea1 d3q12a1 d3rbaa_ d2qtra_ d1nupa_
d3l92a_ d1f9aa_ d1qjca_ d2h29a_ d1vlha_ d1od6a_ d1lw7a1 d1mrza2
d4ruka_ d2el7a_ d1iq0a2 d1irxa2 d1r6ta2 d1ivsa4 d2cyba_ d1rqga2
d1j09a2 d2yxna_ d1f7ua2 d1h3fa1 d1ffya3 d2g36a1 d1nzja_ d1jila_
d1n3la_ d1pfva2 d1h3na3 d3n9ia_ d3sz3a1 d3sw2a_ d1moxc_ d1dx5i3
d1edmb_ d1autl2 d1autl1 d1dval1 d1szba2 d1kigl_ d2y5fl_ d1ijqa2
d1gk5a_ d1g1sa2 d1nt0a3 d1xdtr_ d1lmja2 d1lmja1 d1nzia2 d2wphe_
d1klil_ d1pfxl2 d1pfxl1 d1p9ja_ d1g1ta2 d1f7ea_ d4yzub_ d2mgpa1
d1b9wa1 d1b9wa2 d2mgpa2 d1ob1c1 d1ob1c2 d1n1ia2 d2npra2 d2npra1
d1m1xb4 d2y5ia_ d1qlsa_ d1psra_ d3n22a_ d2egda_ d4ggfc_ d2wcea_
d2luca_ d4aqia_ d3nsia_ d2lp3a_ d1j55a_ d1a4pa_ d1k8ua_ d1nsha_
d1pvaa_ d3fs7a_ d2pvba_ d1ttxa_ d1rwya_ d2kyfa_ d1rk9a_ d1a75a_
d1exra_ d1tiza1 d1nyaa_ d1ggwa_ d1wdcc_ d3fwba_ d1ggza_ d2o5ga_
d1ctaa_ d1m45a_ d3i5gc_ d1sl8a_ d1fpwa_ d2scpa_ d2roaa_ d4aqra_
d3pn7b_ d1br1b_ d2mysb_ d1s6ca_ d1bjfa_ d2obha_ d1jfka_ d1jf0a_
d1qv0a_ d1lkja_ d2mlfc_ d2m97a_ d2d8na_ d4by5a_ d1jbaa_ d4nqga_
d1wdcb_ d2mysc_ d1j7qa_ d1s6ia_ d1uhka2 d1r2ua_ d1dtla_ d3kf9a_
d3pn7c_ d1qjta_ d1fi6a_ d1c07a_ d5d67d_ d1tuza_ d1h8ba_ d1eg3a1
d2zkmx1 d1snla_ d1gjya_ d1y1xa_ d1hqva_ d1dvia_ d1df0a1 d1qxpa2
d4usla_ d1wkya2 d1uuqa_ d3wh9a_ d4m1ra_ d1xyza_ d4xuya_ d2je8a5
d4bf7a1 d3cuia_ d1ur4a_ d1qw9a2 d1odza_ d2vzsa5 d1bqca_ d2x2ya1
d1hjqa_ d1qnra_ d4y7ea1 d4k68a_ d2v3ga_ d4hpga_ d1nofa2 d1nq6a_
d1h4pa_ d1aq0a_ d3ziza_ d4bs0a_ d1ecea_ d1h1na_ d1ta3b_ d1w91a2
d1i1wa_ d1uhva2 d1us3a2 d1egza_ d7a3ha_ d1tvna1 d3nyda1 d1ghsa_
d2cyga1 d4xv0a_ d2c8na2 d1w32a_ d3emca_ d1v6ya1 d1foba_ d2uwfa1
d2fgla_ d3niya1 d2c0ha1 d1rh9a1 d1yq2a5 d1szna2 d1fa2a_ d1pama4
d5do8a1 d1ea9c3 d1hx0a2 d2fhfa5 d1qhoa4 d1avaa2 d1m53a2 d4m8ua1
d2gjpa2 d1lwha2 d4howa1 d1ktba2 d1j0ha3 d3vm5a1 d2x1ia_ d1wzaa2
d1uasa2 d2diea1 d1mxga2 d1ht6a2 d1eswa_ d1iv8a2 d5cpsa_ d1ud2a2
d1zjaa1 d3gbea1 d1kwga2 d1wdpa_ d1gcya2 d1g5aa2 d1hvxa2 d3dhpa2
d2bhua3 d4do4a1 d3qgva1 d1e43a2 d1g94a2 d2d3na2 d1gjwa2 d1gvia3
d1x1na1 d1r7aa2 d1tz7a1 d4toqa_ d1wb0a1 d1w9pa1 d1eoka_ d1itxa1
d1kfwa1 d1goia2 d1edqa2 d2gsja_ d1hjxa1 d1jnda1 d1vf8a1 d3wdpp1
d3wq5a_ d1gnxa_ d4gxpa_ d4hz8a_ d1e4mm_ d3aiua_ d1wcga1 d1qvba_
d3ahxa_ d1v02a_ d2dgaa_ d1pbga_ d1vffa1 d1qoxa_ d4ha4a_ d1mixa2
d1j19a2 d1isna2 d2al6b2 d3syxa1 d1mkea1 d2p8va_ d1xoda1 d1i7aa_
d1evha_ d1egxa_ d1b9ra_ d1offa_ d1frra_ d3wcqa_ d1e9ma_ d1l5pa_
d1wria_ d1i7ha_ d3av8a_ d1rfka_ d3p1ma1 d3b2ga_ d2bt6a2 d1wgqa1
d1v89a1 d2coda1 d1zc3b_ d1wg7a1 d1u5ea1 d1v61a1 d2coca1 d2ys1a1
d1v5pa1 d1ntya2 d1wjma1 d1wi1a1 d1u29a_ d1xx0a1 d1unqa_ d2zkmx3
d1fhoa_ d1v88a1 d2adza1 d1x1fa1 d1p6sa_ d2coaa1 d1btka_ d1v5ma1
d1faoa_ d2x18a_ d1dyna_ d1x1ga1 d4kaxb_ d2j59m1 d1xcga2 d1txda2
d1u5da1 d1dbha2 d1w1ha_ d1upqa_ d1v5ua1 d1foea2 d2fjla1 d1eaza_
d2dfka2 d2cofa1 d1kz7a2 d1h6ta1 d1h6ua1 d9ldta1 d2ewda3 d1up7a1

d2dfda1 d1s6ya1 d1vjta1 d7mdha1 d1hyea1 d1t2da1 d1ojsa3 d1b8pa1
d1ldna1 d1hyha1 d1civa1 d2a92a1 d1ez4a1 d1i0za1 d1y6ja1 d1gv0a1
d1ur5a1 d1llda1 d1obba1 d1v8ba1 d1gdha1 d2dlda1 d1mx3a1 d1ygya1
d1qp8a1 d1li4a1 d1j4aa1 d2naca1 d1npya1 d3t4ea2 d1a4ia1 d1o0sa1
d2hk9a2 d2egga2 d1b26a1 d1bvua1 d1gq2a1 d1c1da1 d1leha1 d1gtma1
d1euza1 d1nvta1 d3dona2 d1edza1 d1gpja2 d1p77a1 d1nyta1 d1b0aa1
d1vl6a1 d1v9la1 d1txga2 d3f47a1 d2pv7a2 d1xc2a3 d2ahra2 d4xiya1
d2f1ka2 d1pgja2 d1i36a2 d1np3a2 d1lj8a4 d1teaa3 d1qmga2 d2i76a2
d2g5ca2 d1wdka3 d1dlja2 d3cuma3 d1kola2 d1f8fa2 d1qora2 d1jqba2
d1vj0a2 d1cdoa2 d1gu7a2 d1d1ta2 d1xa0a2 d2dm6a2 d1jvba2 d1rjwa2
d1llua2 d1h2ba2 d1tt7a2 d1piwa2 d1o89a2 d1iz0a2 d1e3ja2 d1yb5a2
d1uufa2 d1vj1a2 d1e3ia2 d3gpdg1 d2nvwa1 d1u8fo1 d1ydwa1 d1hdgo1
d2g17a1 d1cf2o1 d2czca2 d1r0ka2 d1obfo1 d2cvoa1 d1ebfa1 d1h6da1
d1i32a1 d1zh8a1 d2q49a1 d1qkia1 d1j5pa4 d3pzra1 d1xeaa1 d1vkoa1
d1vm6a3 d3qvsa1 d2pkqo1 d1b7go1 d1gr0a1 d1p1ja1 d1lc0a1 d1j0xo1
d3pwka1 d2hjsa1 d1zyao1 d1vkna1 d1tlta1 d1nvmb1 d3sjua_ d3ojfa1
d1xu9a1 d2jjya1 d1n7ha_ d1y1pa1 d1h5qa_ d1jtva_ d4fs3a_ d2bkaa1
d1gy8a_ d2c5aa1 d1ulsa_ d1ooea_ d2b69a1 d1y5ma1 d1spxa_ d1ae1a_
d1bxka_ d1ydea1 d1gega_ d1hdoa_ d1o5ia_ d1e6ua_ d1snya1 d2rhca_
d3zv4a_ d5ceja_ d2d1ya1 d1fjha_ d3w8ea_ d2fmua1 d1x1ta_ d1rpna_
d3ay7a1 d1k2wa_ d1eq2a_ d2hrba1 d3ruaa_ d2ew8a1 d4hwka_ d1xgka_
d2bd0a1 d1yo6a1 d2a35a1 d2ntva_ d1vl8a_ d1hdca_ d4bkua_ d2ag5a1
d2q45a_ d1nffa_ d4iy1a_ d1zbqa1 d1kewa_ d1ek6a_ d1ja9a_ d2q46a2
d1iy8a_ d1cyda_ d2ptga_ d1hxha_ d1uzma1 d3lyla_ d4m89a_ d3wyea_
d2blla1 d1w6ua_ d1sb8a_ d2q2va_ d4nr0a_ d1e6wa_ d2gasa_ d3oiga1
d2bgka1 d1mxha_ d1vl0a1 d1rkxa_ d1r6da_ d1wmaa1 d1e7wa_ d2c07a1
d1xkqa_ d3tzha_ d1g0oa_ d3lz6a_ d1qyca_ d1edoa_ d1gz6a_ d1oc2a_
d1t2aa_ d1zema1 d2xoxa_ d1z45a2 d1geea_ d1p33a_ d3lt0a_ d4e6pa_
d2wyua_ d1db3a_ d4q9na_ d1xhla_ d4o1ma_ d1fmca_ d2uvda_ d2gdza1
d1uaya_ d2z1ma_ d2o23a2 d2fr1a2 d2fr1a1 d1qsga_ d1qyda_ d4zjua_
d2a4ka1 d1yb1a_ d1wvga1 d1yxma1 d3f4ba_ d1mg5a_ d1xg5a1 d1r55a1
d3gboa_ d1atla_ d1kufa_ d1buda_ d4q1la_ d1yp1a_ d1wnia_ d1xd3a_
d3ifwa_ d3u1ra1 d1g9ka2 d1kapp2 d2w0pa_ d2diaa1 d2e9ia2 d2e9ja1
d2dj4a1 d2eeba1 d2diba1 d2dmba1 d2dmca1 d2d7oa1 d2ee6a1 d2d7pa1
d2eeaa1 d2di8a1 d1wlha1 d2d7na1 d2nqca_ d2d7ma1 d2dica1 d3cnka_
d2k3ta1 d2eeca1 d1qfha2 d2bp3a1 d1v05a_ d2ee9a1 d2eeda1 d2di9a1
d3ioqa_ d1fh0a_ d1o0ea_ d1khqa_ d1m6da_ d2cb5a_ g8pch.1 d1s4va_
d2pnsa_ d1vsna1 d1mira_ d1iwda_ d2oula_ d7pcka_ d3bwka_ d3bcna_
d2p86a_ d1pvja_ d1pcia_ d1gece_ d1deua_ d4bs6a_ d1xkga1 d1cqda_
d1jqpa2 d2c0ya_ d2bdza_ d3p5ua_ d2o36a_ d1k9xa_ d1zoya1 d1qo8a2
d1d4ca2 d1chua2 d1jnra2 d2jk6a2 d2jk6a1 d1feca1 d1feca2 d1lpfa1
d1lpfa2 d1xhca1 d1xhca2 d1dxla2 d1dxla1 d1gera2 d1m6ia2 d1onfa1
d1onfa2 d3lada2 d3lada1 d1w4xa1 d1w4xa2 d1gesa2 d1mo9a1 d1xdia1
d1fl2a2 d1fl2a1 d1fcda1 d1fcda2 d1h6va2 d1h6va1 d1mo9a2 d1gv4a2

d1gv4a1 d2gv8a2 d2gv8a1 d1aoga1 d1aoga2 d1hyua1 d1ebda1 d1ebda2
d3dk9a1 d1q1ra2 d1q1ra1 d1gesa1 d1w5qa_ d2c1ha_ d1ohla_ d1l6sa_
d1zbfa1 d2z1ha_ d2etja1 d3qioa1 d1jl1a_ d2e4la_ d1jl2a_ d2lsna1
d1io2a_ d1uaxa_ d2yv0x_ d1mu2a1 d1ekea_ d1i39a_ d1ps9a2 d1o94a2
d1lqta1 d1pn0a1 d1reoa1 d1ju2a1 d2f5va1 d3c96a1 d2gmha1 d1pj5a2
d1gpea1 d1o5wa1 d1n4wa1 d1k0ia1 d2dw4a2 d2ivda1 d2voua1 d1kdga1
d1s5ja1 d2hbja2 d2igia_ d1wlja_ d1qhta1 d4dlea1 d2f96a1 d3tr8a1
d3mxma_ d1q8ia1 d1y97a1 d1w0ha_ d1kfsa1 d2py5a1 d1noya_ d1d5aa1
d1yt3a3 d2guia_ d3mwpa1 d4kb1a_ d2v76a_ d1wj1a1 d1aqca_ d1qqga2
d1qqga1 d1ntva_ d1n3ha_ d4dbba_ d3f0wa1 d1p3ra_ d1wgua1 d2cy4a1
d3hqca_ d1wvha1 d1xr0b1 d1v58a1 d1t3ba1 d4ilfa2 d4npba2 d1tq5a1
d1jmxa2 d1jmxa1 d1pbya1 d1r7ha_ d1faaa_ d1wika1 d1xfla_ d1ttza1
d2f51a1 d1ti3a_ d1nhoa_ d1sena_ d2i4aa_ d1ep7a_ d1zmaa1 d1fo5a_
d3lefa1 d3diea1 d2yana1 d1z6na1 d1oaza_ d3zywa1 d4aj8a_ d4fiwa_
d1wmja_ d4hs1a1 d1iloa_ d1xwaa1 d1wjka1 d2vm1a_ d1fb6a_ d1gh2a_
d1h75a_ d1nw2a_ d1kx5b_ d3a6na_ d4m6ba2 d2nqbd_ d3wkjd_ d2yfwe_
d4h9na_ d5bnxa_ d1kx5c_ d3r45a_ d1eqzb_ d4jjnb_ d1f66c_ d1eqza_
d4wnnb_ d1kx5a_ d1u35c1 d1id3a_ d2nqbc_ d1kx5d_ d1ku5a_ d1f1ea_
d2b5ea2 d2b5ea3 d2b5ea1 d2b5ea4 d2kp1a1 d2kp2a1 d1hyua3 d1hyua4
d1j08a1 d1j08a2 d1pd212 d1nhya2 d2a2ra2 d1k0dd2 d1f2ea2 d1gsua2
d1f3ba2 d2fhea2 d2pvqa1 d1tw9a2 d1oyja2 d1e6ba2 d1k0ma2 d3ik7a1
d4is0a1 d1r5aa2 d1jlva2 d1m0ua2 d3gtub2 d1z9ha2 d1okta2 d1axda2
d2gsra2 d1tu7a2 d1k3ya2 d1zgma1 d1b48a2 d1gnwa2 d1gwca2 d2gsta2
d1glqa2 d1oe8a2 d1pn9a2 d1v2aa2 d1fw1a2 d1duga2 d1qqya_ d1gd6a_
d1ivma_ d2gv0a_ d1iiza_ d2z2fa_ d1j8xa_ d1ghla_ d1b9oa_ d174la_
d169la_ d2o4wa_ d1p37a_ d1k28a3 d1xjta1 d1jtma_ d2f2qa_ d4e97a1
d3w3ea_ d4tx7a_ d3cqla_ d4msta_ d3iwra_ d1dxja_ d2z37a_ d4dyga_
d1mpya2 d1kw3b2 d1kw3b1 d4ghga1 d1tfza1 d3isqa1 d3isqa2 d1sqia1
d1sqia2 d1lkda2 d1lkda1 d1mpya1 d1cjxa2 d1cjxa1 d1sp9b2 d1t47a1
d1t47a2 d1f1ua2 d1sp8a1 d1sp8a2 d1f1ua1 d1s5ja2 d1qhta2 d4dlea2
d1tgoa2 d3py8a2 d1q8ia2 d1kfsa2 d2py5a2 d1qsta_ d2beia1 d4m3sa_
d1u6ma1 d1ygha_ d1yk3a1 d3dr6a_ d1xmta_ d1z4ea1 d2ge3a1 d1bo4a_
d2fiaa1 d2jdca1 d1y9wa1 d3to7a_ d1wwza1 d2fl4a1 d2ozua1 d2b5ga_
d2cy2a1 d2p0wa_ d1cm0a_ d4pswa_ d1yrea1 d1yr0a1 d1m4ia_ d2gana1
d1yvka1 d1ghea_ d2g3aa1 d1y7ra1 d2euia1 d1q2ya_ d2fsra1 d1xeba_
d2ae6a1 d1cjwa_ d1qsma_ d2aj6a1 d1y9ka1 d1i12a_ d1vkca_ d2atra1
d1n71a_ d2d4pa1 d2fiwa1 d2fe7a1 d1tiqa1 d2ou2a_ d1yx0a1 d2fcka1
d2j8ma_ d2pq8a_ d1z4ra1 d1s3za1 d1ufha_ d1c4qa_ d1prtc1 d2wv6d_
d1prtf_ d1ltrd_ d4l63b1 d1r4pb_ d1prtd_ d1tl0f_ d2bosa_ d1prtb1
d3chbd_ d1xvqa1 d1pn0a2 d3hy2a_ d2cvba1 d1o8xa_ d1lu4a_ d1we0a1
d2bmxa1 d2a4va1 d1psqa_ d1xiya1 d1q98a_ d1n8ja_ d1zofa1 d2ggta_
d3tjja_ d4je1a1 d1o73a_ d3s9fa_ d2zcta_ d1qmva_ d1jfua_ d2b1ka_
d5dvba1 d2b5xa1 d2v2ga_ d1st9a_ d3tb2a_ d4llra_ d4fh8a1 d1e2ya_
d1knga_ d2h30a_ d1i5ga_ d2z9sa_ d2f8aa1 d1tp9a1 d2b7ka_ d1nm3a2

| | | | | | | | |
|---|---|---|---|---|---|---|---|
| d3hvva_ | d2wfca1 | d4kcea_ | d1xvwa1 | d4gqca1 | d3qpma_ | d4k1fa_ | d1zzoa1 |
| d1gp1a_ | d2m5ta_ | d1cqqa_ | d1wqsa_ | d5bpea_ | d2ipha_ | d1lvoa_ | d2bhga1 |
| d3tnta_ | d3sjia_ | d3d23a1 | d2fyqa_ | d3tloa_ | d3zvea1 | d2hrva_ | d3q3ya1 |
| d4asha1 | d4aw8a_ | d1jgta2 | d1ofda3 | d1ea0a3 | d1ecfa2 | d1q15a2 | d1ct9a2 |
| d1te5a_ | d1ys1x_ | d4hs9a_ | d1ispa_ | d1ex9a_ | d3qmma_ | d1ra6a_ | d1muka_ |
| d3gsza_ | d1khva_ | d1te9a_ | d2xwha_ | d3uqsa_ | d3hkwa_ | d3n6ma_ | d2cjqa_ |
| d2xyma_ | d1teba_ | d1te8a_ | d1s48a_ | d1u09a1 | d4wfxa_ | d3tyqa_ | d2pgga1 |
| d3rp2a_ | g1ucy.1 | d1fuja_ | d2zchp_ | d3w94a_ | d1si5h_ | d1lo6a_ | g2pka.1 |
| d1cgha_ | d1kigh_ | d1a5ia_ | d3s9ba_ | d1hj8a_ | d1ao5a_ | d2anya_ | d1q3xa1 |
| d1gvza_ | d1azza_ | d4gsoa_ | d2f91a1 | d1iaua_ | d1gdna_ | d1eufa_ | g1fiw.1 |
| d1eq9a_ | d4nfea_ | d2rdla_ | d1spja_ | d1elva1 | d1a7sa_ | d1ekbb_ | d1pfxc_ |
| d1yc0a_ | d2aiqa_ | g1fiz.1 | d1fi8a_ | d4k60a_ | d4dgja_ | d1mzaa_ | d1op0a_ |
| d3tjva_ | d1pytd_ | d1sgfg_ | g1gg6.1 | d2eeka_ | g1rtf.1 | d1a0ja_ | d3g01a_ |
| d1pytc_ | d4e7na_ | d1ddja_ | d1z8ga1 | d2hlca_ | d1npma_ | d1brup_ | g1jou.1 |
| d1sgfa_ | d1autc_ | g2hnt.1 | g1kdq.1 | d1orfa_ | d3s69a_ | d4crga_ | d1fxya_ |
| d1bqya_ | d1lgya_ | d4l3wa_ | d1gz7a_ | d1ukca_ | d3ia2a_ | d1a88a_ | d4dgqa1 |
| d1hkha_ | d1zoia_ | d3zxha_ | d1cgla_ | d1rm8a_ | d1eaka2 | d1hy7a_ | d2jsda_ |
| d1cxva_ | d1hv5a_ | d1i76a_ | d3p0ya1 | d1igra1 | d1igra2 | d1n8yc2 | d1n8yc1 |
| d1m6ba2 | d1m6ba1 | d1m4la_ | d2bk7a_ | d3fjua_ | d1z5ra1 | d1kwma1 | d1uwya2 |
| d1jqga1 | d2c1ca1 | d1dtda_ | d1h8la2 | d2tbvc_ | d1c8nc_ | d1x36a_ | d1smvc_ |
| d4sbvc_ | d1opoa_ | d1f2nc_ | d1cixa_ | d1y29a_ | d1v7fa_ | d2jtba_ | d1d1ha_ |
| d1g9pa_ | d1lupa_ | d1qk7a_ | d1mb6a_ | d2a2va_ | d1niya_ | d1i25a_ | d1hvwa_ |
| d2mpqa_ | d1kqha_ | d1la4a_ | d2mxoa1 | d1dl0a_ | d1koza_ | d1qk6a_ | d1emxa_ |
| d1lqra_ | d1c6wa_ | d1rmka_ | d1ixta_ | d1eyoa_ | d1cnna_ | d1fu3a_ | d1g1pa_ |
| d1f3ka_ | d1fyga_ | d1rypd_ | d1irua_ | d1rypc_ | d3unfk_ | d1rypa_ | d1j2pa_ |
| d1irui_ | d1j2qh_ | d3unfh_ | d1m4ya_ | d1rypb_ | d1irul_ | d1irug_ | d1iru2_ |
| d1q5rh_ | d1rypg_ | d1g3ka_ | d1iruf_ | d1q5qa_ | d1e94a_ | d1irub_ | d1ryph_ |
| d3ty6a_ | d1iruj_ | d1iruk_ | d1rype_ | d1irud_ | d1irue_ | d3mi01_ | d1rypf_ |
| d2z3ba_ | d1iruc_ | d3mka2_ | d1iru1_ | g1gk9.1 | g1or0.1 | g1e3a.1 | d1mqta_ |
| d1d4m3_ | d1fmd3_ | d2mev1_ | g1aym.1 | d1ev13_ | d4q4w1_ | d1r1a3_ | d2x5ia_ |
| d1rhi1_ | g1r1a.1 | g1d4m.1 | g1rhi.1 | d1b35b_ | d1oopa_ | d1tme1_ | d4iv1a_ |
| g1pvc.1 | g1fpn.1 | d1fpn1_ | d4gb31_ | d1d4m1_ | d1fmd1_ | d1ev11_ | d2wzr3_ |
| g1cov.1 | d1qqp3_ | g1fmd.1 | d2x5ic_ | d1rhi3_ | g1oop.1 | d1hxs1_ | g1hxs.1 |
| d1fpn3_ | d1bev1_ | g2mev.1 | d1eah3_ | d1aym1_ | d1pvc3_ | d1qqp1_ | d1zba3_ |
| d1h8ta_ | d3vbhc_ | g1b35.1 | g1bev.1 | d1hxs3_ | g1ev1.1 | d1bev3_ | g1tmf.1 |
| d1tmf1_ | d2mev3_ | g1eah.1 | g1h8t.1 | d1eah1_ | d4q4w3_ | d1r1a1_ | d1aym3_ |
| d1pvc1_ | d1zba1_ | d1h8tc_ | d1oopc_ | d1tme3_ | g1qqp.1 | d4iv1c_ | g1tme.1 |
| d1p8ba_ | d4w9ta_ | d4x2ra1 | d1ka9f_ | d1qo2a_ | d4axka1 | d1thfd_ | d1h5ya_ |
| d4u28a_ | d2y88a_ | d4wd0a_ | d1vzwa1 | d1eixa_ | d3exra_ | d2ffca1 | d2fdsa1 |
| d3n3ma1 | d2czda_ | d1q6oa_ | d2yyua_ | d1vqta1 | d1dbta_ | d3ldva1 | d4lt9a1 |
| d3tc7a1 | d3hoja_ | d1i4na_ | d3vnda_ | d3nl8a1 | d1geqa_ | d3nava1 | d1v5xa_ |
| d4a2ra1 | d3tc6a1 | d4ijba_ | d1xcfa_ | d1vc4a_ | d1rd5a_ | d1qopa_ | d3b5va_ |
| d2r80b_ | d1cqxa1 | d1cg5a_ | d1gcvb_ | d3boma_ | d2h8fb_ | d2raoa_ | d3bj1a_ |
| d4hrrb_ | d2r50a_ | d3d1kb_ | d2aa1b1 | d3zhwa_ | d3hf4b_ | d3gdja_ | d1jebb_ |

| | | | | | | | |
|---|---|---|---|---|---|---|---|
| d3bj1b_ | d1x9fb_ | d1wmub_ | d1gcva_ | d3bcqb_ | d3d4xb_ | d1gvha1 | d2dc3a_ |
| d3hf4a_ | d4yu3a_ | d3k8ba_ | d1qpwb_ | d2r80a_ | d3bcqa_ | d1v4wa_ | d3wtgb_ |
| d1jl7a_ | d4esaa_ | d1a9we_ | d3d1ab_ | d1fsla_ | d4h2la_ | d1v4wb_ | d1mwca_ |
| d1vhba_ | d1jeba_ | d1spgb_ | d1h97a_ | d1q1fa_ | d1tu9a1 | d3d1aa_ | d1hdsb_ |
| d3hyub_ | d2zfbb_ | d3fs4a_ | d1oj6a_ | d1shrb_ | d4h2lb_ | d4yu3b_ | d1spga_ |
| d3hrwa_ | d3qqra_ | d3at5a_ | d4wchd_ | d3hyua_ | d1cg5b_ | d1uc3a_ | d1hbra_ |
| d2raob_ | d1outb_ | d4esab_ | d3gdjb_ | d3qqqa_ | d1x9fc_ | d3vrfa_ | d1itha_ |
| d3mkba_ | d3d4xa_ | d3mjpa_ | d1jl6a_ | d3fh9a_ | d1outa_ | d2dc3b_ | d1it2a_ |
| d3eoka_ | d3g46a_ | d1qpwa_ | d3vrfb_ | d1ch4a_ | d1x9fd_ | d1hdsa_ | d2zfba_ |
| d3mkbb_ | d1or4a_ | d3wtga_ | d1a4fa_ | d3fh9b_ | d3bomb_ | d3cy5a_ | d3at5b_ |
| d1x9fa_ | d2oifa_ | d4lmxb_ | d1cpcb_ | d3v57a_ | d1kn1a_ | d1cpca_ | d4f0ta_ |
| d2vjha_ | d4po5b_ | d1eyxa_ | d1alla_ | d4f0tb_ | d1b33b_ | d1b8db_ | d4h0mb_ |
| d1b33a_ | d2vjhb_ | d4lm6b_ | d2vmlb_ | d1allb_ | d2vjta_ | d3dbja_ | d2vmla_ |
| d2vjtb_ | d4f0ub_ | d1f99b_ | d4rmpa1 | d1liaa_ | d2uulb_ | d4lmsb_ | d4f0ua_ |
| d2bv8a_ | d1f99a_ | d2bv8b_ | d2c7lb_ | d3dbjb_ | d4rmpb1 | d1b8da_ | d4h0ma_ |
| d3v57b_ | d1kn1b_ | d1liab_ | d2uula_ | d2j96a_ | g1gxs.1 | g1wht.1 | d3c8da2 |
| d2pbla1 | d1jkma_ | d1jjia_ | d1jjfa_ | d1gl9b1 | d1gkub2 | d2qvba_ | d2v9za_ |
| d4kafa1 | d1xm5a_ | d1xaxa_ | d1tvia_ | d4iuaa3 | d4bvva_ | d5hpga_ | d3k65a_ |
| d1ki0a3 | d1ki0a2 | d1ki0a1 | d1a0ha1 | d1pmla_ | d3sp8a3 | d2feba1 | d1mdwa_ |
| d1qxpa4 | d1ziva1 | d1zcma1 | d1f74a_ | d3l21a_ | d2e1da_ | d1to3a1 | d1w3ia_ |
| d1sfla_ | d1n7ka_ | d1vcva1 | d4imea_ | d1wa3a_ | d2ocza1 | d1f2ja_ | d3m16a_ |
| d2yxga_ | d1ub3a_ | d3a5fa_ | d1l6wa_ | d1xfba1 | d1wx0a1 | d1vpxa1 | d1ojxa_ |
| d1mxsa_ | d2a4aa1 | d1xkya1 | d3cq0a1 | d3s1xa_ | d1f05a_ | d1fdja_ | d3cpra1 |
| d4d2ja_ | d1e88a1 | d1l6ja5 | d1l6ja4 | d1eaka4 | d1eaka3 | d1h8pa2 | d1h8pa1 |
| d1mbma_ | d3su6a1 | d1cu1a1 | d4agka_ | d1vcpa_ | d1q31a_ | g1n1l.1 | d3e90b_ |
| d1ep5a_ | d3u1jb_ | d1dzka_ | d4tlja_ | d2a2ua_ | d4wfva_ | d3t1da_ | d4iaxa_ |
| d3zq3a_ | d2nnda_ | d1i4ua_ | d1kxoa_ | d1bbpa_ | d1kt7a_ | d1exsa_ | d3s26a1 |
| d1lf7a_ | d1qfta_ | d4gh7a_ | d1z24a1 | d1qabe_ | d1xkia_ | d3bx7a_ | d4iawa1 |
| d1n0sa_ | d1gkab_ | d3kzaa_ | d2k23a_ | d2l9ca1 | d2joza1 | d1gm6a_ | d3kffa_ |
| d3saoa1 | d1e5pa_ | d1yupa1 | d1iiua_ | d4lu0a_ | d1of8a_ | d3unda1 | d3e9aa1 |
| d1vr6a2 | d1n8fa_ | d2qkfa_ | d1o60a_ | d1e69a_ | d1mv5a_ | d3g60a4 | g1ii8.1 |
| d1ewqa2 | d1ji0a_ | d1qhla_ | d1v43a3 | d2wmfa1 | d1vpla_ | d4a82a2 | g1f2t.1 |
| d2d62a1 | d3tuic1 | d2bbsa1 | d3g60a3 | d2ixea_ | d1g6ha_ | d1sgwa1 | d2qi9c_ |
| d3rlfa1 | d3b60a1 | d3d31a2 | d2pzea1 | g1xew.1 | d1jj7a_ | d1oxxk2 | d2onka1 |
| d1w1wa_ | d1iwea_ | d1qf5a_ | d1vmaa2 | d1nija1 | d1np6a_ | d2p67a1 | d3ihla_ |
| d1okkd2 | d1ls1a2 | d1s1ma2 | d1iona_ | d2v40a_ | d1dj3a_ | d3hida_ | d2vo1a1 |
| d2g0ta1 | d1vcoa2 | d1j8mf2 | d1g3qa_ | d1xjca_ | d1p9ba_ | d1ihua2 | d1ihua1 |
| d2qm8a_ | d1dj2a_ | d4ioja_ | d1cp2a_ | d1yrba2 | d1hyqa_ | d3gqba2 | d1xp8a1 |
| d1tf7a2 | d1tf7a1 | d1ye8a1 | d1skyb3 | d2qe7d2 | d4blta_ | d4huta_ | d3ldaa2 |
| d4hyya_ | d2i3ba1 | d1cr1a_ | d3j9tb2 | d3ntua2 | d1vdza1 | d2z43a_ | d1e9ra_ |
| d1nlfa_ | d3ziaa2 | d1n0wa_ | d1g6oa_ | d3ziad2 | d3j9ta2 | d1pzna2 | d3gqbb2 |
| d2i49a_ | d1nnfa_ | d1xvxa_ | d1ursa_ | d1h3da1 | d1iuda_ | d4m1va1 | d1twya_ |
| d3e13x_ | d2nxoa1 | d4pfua_ | d3ttma_ | d1elja_ | d2qmwa1 | d1us5a_ | d1y3na1 |
| d1jeta_ | d1uqwa_ | d4f19a1 | d2esna2 | d3thia_ | d3fasa1 | d2uyeb_ | d2xwva1 |

d1r9la_ d2fyia1 d1pb7a_ d2ozza1 d1sw5a_ d1nh8a1 d1vr5a1 d2g29a_
d1xs5a_ d3lswa_ d2z23a_ d3w9va_ d3tcha_ d1q35a_ d1z7me1 d3q27a_
d1p99a_ d1ve4a1 d2i6ea1 d4mcoa1 d4n8ya_ d1zbma1 d1hsla_ d1ixca2
d1j1na_ d1xvya_ d1y9ua_ d2z22a1 d1xoca1 d4oera_ d1wdna_ d5f1qa1
d1xt8a1 d1i6aa_ d4gwoa_ d4igta1 d1pc3a_ d4mmpa1 d1o63a_ d2czla1
d2onsa2 d4mija1 d4ok5a1 d2bmfa1 d2bmfa2 d1yksa1 d1yksa2 d1jr6a_
d2qeqa1 d2jlqa1 d1eh3a_ d3skpa_ d1dtza1 d1dtza2 d1ieja_ d1jnfa2
d1jnfa1 d1b1xa2 d1b1xa1 d1jw1a1 d1jw1a2 d1ryoa_ d1h76a1 d1h76a2
d1c4oa1 d2p6ra3 d2p6ra4 d1oywa2 d1oywa3 d1wrba1 d1w36b2 d1w36b1
d1c4oa2 d3bora_ d1t6na_ d2eyqa5 d2eyqa3 d2eyqa2 d2zu6a2 d1veca_
d1uaaa1 d1w36d1 d2g9na1 d1z3ix2 d2rb4a_ d1hv8a1 d1hv8a2 d1t5ia_
d1w36c2 d2j0sa2 d2j0sa1 d1fuka_ d3hjha1 d1z5za1 d1qdea_ d1z63a1
d1s2ma2 d1s2ma1 d1wp9a1 d1wp9a2 d1gm5a3 d1gm5a4 d3dina4 d3dina3
d1q0ua_ d1w36c1 d1z3ix1 d1nkta4 d1uaaa2 d1w36d2 g1qhh.1 d2fwra2
d2fwra1 d2v8ta_ d3q3ma_ d1za0a1 d2inca_ d1jk0a_ d2incb_ d1mhyb_
d3hf1b_ d1mtyb_ d1r2fa_ d2uw1a_ d1mhyd_ d3n37a_ d1afra_ d1oquc_
d1syya_ d1mtyd_ d2o1za_ d1uzra_ d1smsa_ d2fkza_ d1zuja1 d1s3qa1
d2fjca1 d1z6om1 d1veia_ d4dyya_ d1jiga_ d3gvya_ d2pyba_ d4cy9a1
d3bvfa1 d1r03a_ d2cf7a_ d1nf4a_ d1zs3a1 d4iwka_ d3ka8a_ d3np0x_
d1n1qa_ d1lb3a_ d4e6ka_ d1z6oa1 d2fzfa1 d4dyua_ d3ge4a_ d1jgca_
d4am4a_ d2z6ma_ d1ji5a_ d1vlga_ d1lkoa1 d3qz3a_ d1o9ra_ d2ffxj_
d1euma_ d1yv1a1 d2v2pa_ d1j30a_ d4di0a_ d1dpsa_ d1oewa_ d2qzxa_
d2asia_ d1psoe_ d1miqa_ d1smra_ d2h6ta_ d1pfza_ d1izda_ d3hd8a_
d1j71a_ d1dpja_ d4aa9a_ d1t6ex_ g1b5f.1 g1lya.1 g1htr.1 d1qdma2
d1bxoa_ d1wkra_ d1tzsa_ d1ibqa_ d2qzwa_ d1ex4a2 d1c6va_ d1bdqa_
d3ggta_ d3ixoa_ d1mrxa_ d2haha_ d1tw7a_ d4njsa_ d1sgua_ d2p3da_
d1nsoa_ d3d3ta_ d2rkfa_ d1c6ya_ d2rspa_ d3ogpa_ d3ekwa_ d2o4na_
d2j9ja_ d1baia_ d3ggua_ d3lzua_ d4npua_ d4j55a_ d2r8na_ d2fxda_
d2p3ca_ d4dqea_ d3ttpa_ d3t3ca_ d2p3aa_ d2hc0a_ d2fdda_ d3mwsa_
d1k8rb_ d1wgra1 d1ef5a_ d1wfya1 d1e8ya3 d1wgya1 d2cs4a1 d1wxma1
d2byea1 d3kh0a1 d2byfa1 d1wxaa1 d1e7ua3 d1h4ra3 d4yl8a1 d2al6a3
g1qmo.1 g1len.1 d3zyra_ d1fnya_ d1n47a_ g2ltn.1 d1avba_ d3usua_
d1dhkb_ d1f9ka_ d1ukga_ d1g8wa_ d1g9fa_ d3wcsa_ d1g7ya_ g1loe.1
d1mvqa_ d1hqla_ d1v6ia_ d1dbna_ d1qnwa_ d1fx5a_ d4l8qa_ d1ioaa_
d3n35a_ d1wgda1 d1wgga1 d4q5eb_ d1wjna1 d1se9a_ d3n3kb_ d1wz0a1
d1we6a1 d2m0xa_ d2gbka_ d3rt3b1 d1wh3a1 d1sifa_ d2ekec2 d4ii2b1
d4hk2a1 d1c3ta_ d1m94a_ d1wgha1 d1ttna1 d1j8ca_ d1wx8a1 d3v6cb1
d2ma9b_ d4i6lb1 d1bt0a_ d2jvca1 d2k1fa_ d1wxva1 d1wf9a1 d1t0ya_
d2io0b2 d1x1ma1 d2gbma_ d1zw7a1 d2m17a_ d1ud7a_ d1we7a1 d2lwpa_
d2gbja_ d1wjua1 d1v6ea1 d1v86a1 d4eewa_ d1wiaa1 d4icva_ d4gu2a_
d1wx7a1 d1wy8a1 d4hjka1 d2faza1 d1v5oa1 d3ruib_ d1eo6a_ d3waob_
d2r2qa1 d3m95a_ d3wana_ d1jx4a2 d3gv5b1 d1t94a2 d3bq0a2 d1yvca1
d1yeza1 d2h0ba_ d2erfa_ d1h30a2 d3mw4a1 d1pz7a_ d2r1ba_ d3v64a1
d1d2sa_ d3bopa1 d3mw3a1 d1okqa1 d1okqa2 d2ftba_ d1pmpa_ d2rcqa1

d1fdqa_ d4lkpa_ d1sa8a_ d1vyfa_ d1lpja_ d3vg7a1 d4azqa1 d5bvqa_
d1p6pa_ d2qo4a_ d1opba_ d1yiva1 d1o1va_ d4bvma1 d4tkba_ d4i3ba_
d1bwya_ d1ftpa_ d1ggla_ d4gkca_ d1cbia_ d3p6da1 d1kqwa_ d2a0aa1
d2bcgg1 d2bcgg2 d1d5ta1 d1um8a_ d1ixza_ d1qvra2 d1qvra3 d1qzma_
d1sxjc2 d1jr3d2 d2p65a_ d1l8qa2 d1g8pa_ d3hu3a3 d1sxjd2 d1njfa_
d1in4a2 d1sxja2 d1tuea1 d2ce7a2 d1w5sa2 d1sxjb2 d1d2na_ d1ny5a2
d1ofha_ d2gnoa2 d2a5yb3 d1fnna2 d1u0ja1 d1lv7a_ d1iqpa2 d2fnaa2
d1ixsb2 d4gita_ d1sxje2 d1g41a_ d2e3fa_ d1mm0a_ d2e3ea_ d2llda_
d2ny8x_ d2e3ga_ d2ny9x_ d2nz3a_ d2lj7a_ d1n4na_ d2m8bb1 d2kyqa_
d3psma_ d1jkza_ d1jxca_ d2gl1a_ d1ugla_ d4uj0a_ d2k4ua_ d1hp2a_
d2lu9a_ d1baha_ d2meoa1 d1wm7a_ d1hlya_ d1n8ma_ d1jlza_ d1wpda_
d1c55a_ d1pvza_ d1wt7a_ d1wz5a_ d1m2sa_ d1pjva_ d1c49a_ d1qkya_
d2ktca1 d1wmta_ d1du9a_ d1j5ja_ d1r1ga_ d1t0za_ d2i61a_ d1t1ta_
d1sn4a_ d1kv0a_ d2lkba_ d1npia_ d1wwna_ d1pe4a_ d2kbka_ d2li7a_
d1djta_ d2kbha_ d1dq7a_ d1sega_ d1omya_ d1zywa1 d1n9wa1 d1bbua1
d1e1oa1 d1c0aa1 d1l0wa1 d3a74a1 d1eova1 d1iyjb4 d1iyjb5 d1iyjb3
d1o7ia_ d4ipca1 d1x3ea_ d4apva_ d1jb7a1 d1jb7a2 d1jb7a3 d3kdfa_
d1ue1b_ d1wjja1 d3eiva_ d1xjva2 d1xjva1 d2ccza2 d1se8a_ d1l1oc_
d1jb7b_ d1qvca_ d3ulla_ d1qzga_ d1miua4 d1miua5 d1miua3 d3g48a1
d1gd7a_ d1mkha_ d1jjcb3 d1pyba_ d1y14b1 d2nn6i1 d1i94l_ d2zjra2
d1smxa_ d2qamc2 d1hh2p1 d2r7da1 d1rl2a2 d1wfqa1 d3bzka4 d1x6oa2
d3a0ja_ d2j01d2 d2ja9a1 d4ql5a_ d1hr0w_ d1luza_ d2n3sa_ d2vnud1
d1h95a_ d1go3e1 d2nn6h1 d1ne3a_ d2mo1a_ d1iz6a2 d1jjga_ d1e3pa2
d2ix0a3 d2ix0a2 d2lxja_ d1ueba3 d1ueba2 d2z0sa1 d1u0la1 d2ix0a1
d2eifa2 d2je6i1 d3i2za1 d2c35b1 d2ahob2 d1t9ha1 d3i4oa_ d2qall1
d1jt8a_ d2nn6g1 d1g6pa_ d3cama_ d1i94q_ d1c9oa_ d1d7qa_ d2ba0a1
d2vnud2 d2vnud3 d1khia2 d2qalq1 d1wi5a1 d1xtda2 d1ny4a_ d1dgsa2
d1fvia1 d1ckma1 d1p16a1 d1x9na2 d1f00i3 d1rpma_ d2shpa1 d1yfoa_
d1l8ka_ d2ooqa1 d1g4us2 d1p15a_ d1lara2 d1wcha_ d2fh7a2 d2fh7a1
d1ohea1 d1ohea2 d2f4da_ d1rxda_ d2hxpa_ d1xria_ d1d5ra2 d1fpza_
d3ezza_ d4y2ea_ d3azya_ d3wvja_ d1upsa1 d1ajoa_ d2hyka_ d1ajka_
d2vy0a_ d1o4ya_ d3o5sa_ d3d6ea_ d3atga_ d1dypa_ d1umza_ d3i4ia_
d1c1la_ d4lbqa_ d1a78a_ d1slta_ d4no4a_ d3zsja_ d3duia_ d1is3a_
d1qmja_ d1hlca_ d1tdha3 d1q3ba3 d1ee8a3 d1k82a3 d3h33a_ d1rwja_
d19hca_ d1os6a_ d3bxua_ d3h4na_ d1i77a_ d3caoa_ d1j0pa_ d2ctha_
d1duwa_ d1ofwa_ d2e84a_ d1hh5a_ d3h34a_ d1wioa4 d1vcaa1 d1dr9a2
d1ic1a1 d2d9qb1 d2nxyb2 d1adqa1 d1fnga1 d1uvqb1 d1zs8a1 d2vola1
d2vola2 d1mfbl2 d2jelh2 d1uwel2 d3qq4b1 d1kjvb_ d1k5na1 d1osph2
d4odhl2 d1ow0a1 d1ow0a2 d1bfob2 d3l9ra2 d3jtth1 d2fath2 d4elkb2
d1fn4b2 d2xfxb_ d1pg7x2 d1cqka_ d4k71b2 d1lk2a1 d1eapb2 d4lfhg2
d1i4fa1 d2nw2a2 d1kfah2 d1mhca1 d1h3ua1 d1wejh2 d1h0db2 d1a5fh2
d1kcvh2 d1mjuh2 d1qqda1 d1kcrh2 d1k8ia1 d3huja2 d2nnab1 d1nfdf2
d3mffb2 d1s9va1 d1lk3h2 d1q0xl2 d1fv1b1 d1neza1 d4rfeb2 d1r3ha1
d1a6tb2 d1kjva1 d1pz5b2 d1onqa1 d2bc4a2 d1de4a1 d1k8da1 d1tcra2

d1dn0b2 d1nfde2 d2xwtb2 d1bfoa2 d1mexh2 d1iakb1 d1ypzf2 d4f7ea2
d1q72l2 d2fbjh2 d1fe8h2 d1clzh2 d1fngb1 d1uvqa1 d1vgka1 d1c16a1
d4j12a2 d4x99b2 d1igtb3 d1je6a1 d1q72h2 d1nldh2 d1teth2 d2hmic2
d1i1ca2 d1i1ca1 d1k8ib1 d1q0yh2 d1kfal2 d3frua1 d1pg7w2 d1u58a1
d1igyb4 d1igyb3 d1hyrc1 d1klub1 d1deeb2 d1c12b2 d2bc4b2 d2ig2h2
d1kcvl2 d3wn5b1 d1lk2b_ d1lk3l2 d1mjul2 d1sm3h2 d1q0xh2 d1zvsa1
d1ydpa1 d1u8ib2 d1ypze2 d1kcuh2 d1op9a_ d1y0la1 d1etzb1 d1egjl1
d2gsib1 d1nj9a1 d4jfhd1 d1aqkl1 d1t2ja_ d1lo4h1 d1zvya1 d3v4vl1
d4enec1 d1kxtb_ d5e8ea1 d2pxya_ d4weud_ d1xiwc_ d2esvd1 d1qnzh_
d1ogae1 d1f4xh1 d4n1hb_ d3ezjb1 d3mlwl1 d1kfah1 d1etza1 d1b4jh1
d3cfic_ d4w68a_ d2arja1 d1ek3a_ d2ok0l1 d1vera_ d1uwgh1 d1blnb1
d1fpth1 d1rzga1 d2giya1 d4m3kb1 d1nc2a1 d2nyyd1 d1ncna_ d1fbih1
d5dxwa_ d4fqqa1 d1dqdh1 d1jfql1 d1kelh1 d1faih1 d1frgl1 d1figl1
d4m6nl1 d1fo0a_ d4hxbl1 d3b5ga_ d3quyd1 d2h9gb1 d1i8kb_ d2dd8h1
d2ck0h1 d1eaja_ d4w6wb1 d2a6ib3 d1t66d1 d2e27h_ d1ob1b1 d1f11b1
d2v7na1 d1ad0a1 d2xt1b1 d1pskh1 d4i13b1 d2op4l1 d1a0ql1 d1uwel1
d1za3a1 d1ncbl1 d4uu9a_ d1bm3h1 d1cloh1 d1nqba2 d1cf8h1 d4y5va_
d1mhph1 d5anma1 d4cdgc1 d4ldeb_ d1y0lb1 d2pcpb1 d1z3gl1 d1dfbl1
d1fh5h1 d3g5xa1 d1tzib1 d4dn3l1 d3zhka1 d2b1hh1 d2uyla1 d1f3dh1
d1nj9b1 d2agjh1 d2h1pl1 d1q72h1 d8fabb1 d1ndmb1 d1rjca1 d4u6hb1
d3upca_ d2fbjl1 d1osph1 d1tvda_ d2cjvh_ d4fhbd1 d1bd2d1 d1rz7h1
d3nn8a_ d1blna1 d1knoa1 d1nfda1 d2bmkb1 d1dqll_ d1igyb1 d1igfl1
d12e8h1 d3utza1 d1nc2b1 d1rihh1 d4mwfb1 d4jvpa1 d1mieh1 d1rhfa1
d1fgnl1 d1sbsh1 d1lp9e1 d1dn0a1 d4lqfl1 d1iaih1 d1kgcd1 d1mh5b1
d1gpol1 d2g60h3 d1f8tl1 d2jixd1 d4grwh_ d1fo0b_ d1bfob1 d1axsa1
d1u9ka_ d1nmbh_ d1h8na1 d1h8na2 d1jguh1 d4eura1 d3sdyl1 d35c8h1
d1w72l1 d1mvub_ d1i9rh1 d1ob1a1 d4wenb_ d1yeeh1 d3ietc1 d1f58h1
d1ad0b1 d1mqkl_ d1nbvh1 d4b50a_ d3r8bb_ d1bafl1 d1mamh1 d4rgne1
d2gsgb_ d35c8l1 d1bafh1 d1ai1h1 d2pcpa1 d2fath1 d2x1pa_ d1orsb1
d1ibgl1 d1shma_ d1a2yb_ d1u0qa_ d2coqa_ d1hilb1 d1qfwi_ d1jv5b_
d1fn4b1 d5e0qa_ d1nsnh1 d1plgl1 d4ocxl1 d1kb5h1 d1rzfh1 d1pg7x1
d2fx7h1 d2oi9c_ d1l7il1 d3eyva1 d1d5il1 d4ffyl_ d1tetl1 d1a6wh_
d1ac6a_ d3dvfa_ d1nfde1 d1mimh1 d1ri8a1 d1jhlh_ d3dura_ d1fskb1
d1f97a1 d3so3b1 d2ak4d1 d1lp9f1 d4z7wf1 d1l7th1 d4y5ya_ d4x7ec1
d1emth1 d1ikfl1 d1nlbh1 d4utal1 d1bjma1 d1eapb1 d1u6ah1 d2dtgd1
d1kj2b_ d1xeda1 d1c1eh1 d1fgvh_ d2bnub1 d2fb4h1 d2dtgb1 d1ktrh_
d1jpth1 d1qkzl1 d4hepg_ d2atpb1 d1flrh1 d2hrpl1 d1mvfa_ d1iaim1
d3k80a1 d2jb5h1 d3zkxc_ d4grwe_ d1it9h1 d1rhhb1 d3w9ec_ d1e4wh1
d1sy6h1 d2j6eh1 d3qxta_ d1jglh1 d1p7ka1 d1tzhb1 d4n8pa1 d1gafl1
d2jelh1 d1wioa2 d4i0cc1 d1cicb1 d1lmka2 d1lmka1 d1iqwh1 d1orqb1
d1ejoh1 d1jnlh1 d4zg1a_ d43c9b_ d1ggbl1 d1wejh1 d3q6fa1 d1f3dj1
d3c6la1 d1bqlh1 d3p9wb_ d1a3rl1 d1h0db1 d2uzil_ d1eo8l1 d1qfwl_
d1n7ml1 d1igja1 d1c5ch1 d2a9mh_ d1rmfh1 d1fj1b1 d4heme_ d1ccza1
d1a5fh1 d1ogad1 d1dlfh_ d1qa9b_ d1vgel1 d3qskb_ d1sjxa_ d4ozfg1

d1sq2n_ d1qd0a_ d1kcvh1 d1c5db1 d3qcul1 d8faba1 d3bn9d1 d1mjuh1
d4y8dc_ d7fabh1 d3dvgb1 d1lo0l1 d1a6tb1 d1gigh1 d1kcrh1 d2hwzl1
d1yjdc1 d3clfl1 d4krna_ d1dl7l_ d1e6ol1 d1mexh1 d3r0ma_ d4kkna_
d1fnsl1 d1oaqh_ d1rzia1 d1ucbh1 d1ncwl1 d4b41a_ d1nfdf1 d2mpah1
d2d7tl1 d4grwf_ d4b5ea_ d1lk3h1 d3u79a_ d4u3xa_ d1ghfl1 d1bvkb_
d1mcww1 d1b88a_ d1jrhh1 d32c2b1 d4wemb_ d1dsfh_ d1op3h1 d1ce1l1
d1iqda1 d1qoka1 d1qoka2 d1qfuh1 d2crya1 d1ay1h1 d1oaql_ d3ab0b_
d1rurl1 d1t4kb1 d1bz7b1 d1n0xh1 d1forl1 d1ucbl1 d4w6yb_ d43c9a_
d2aabl1 d1tzia1 d4n1ca_ d1k6qh1 d3g9ab_ d4tvsa1 d1igmh_ d1pz5b1
d3dx9a1 d3wxwh_ d1nakh1 d1kcul1 d3eaka_ d4qlra1 d1cr9l1 d1d9ka_
d1f4xl1 d1deea1 d2i27n2 d1c12a1 d2orbl1 d1j8hd1 d1mcph1 d1c5da1
d1kenh1 d1bd2e1 d1h5ba_ d4x7fc1 d3upaa_ d1t3fa1 d1indh1 d2dtgc1
d4hc1l1 d1zv5a1 d4wgvb_ d1uwgl1 d4f37k1 d1aqkh1 d1vesa_ d3zkqd_
d1fptl1 d1lo4l1 d1knob1 d3rrqa_ d4orzc_ d1mlbb1 d4hgkc1 d1dqdl1
d1bogb1 d4ozhe1 d1dr9a1 d1i7za1 d1dn0b1 d1kell1 d1rzib1 d1ypzf1
d1wtla_ d1hkfa_ d1fail1 d1figh1 d2vyre_ d1sjva_ d1ohqa_ d1bfoa1
d1pkqb1 d1mjul1 d1b4jl1 d1nsnl1 d2arjb1 d1p4bh_ d4aj0a_ d3nn8b_
d2rcja1 d1f2xk_ d4v1dd_ d1adqh1 d2eksa_ d4k3ga_ d1jfqh1 d1uweh1
d1frgh1 d4gftb_ d1uz8b1 d4n1ea_ d1bm3l1 d1clol1 d3mcka1 d2vq1a1
d1ae6h1 d4aq1b1 d1uj3b1 d1nbqa1 d1fh5l1 d2ck0l1 d2ywza_ d1q72l1
d4nbxb_ d1hila1 d4hzlb1 d2j88l1 d1zmya1 d1cd0a_ d1a4ka1 d1pskl1
d1a0qh1 d4krmb_ d1ncbh1 d2fbjh1 d1j8he1 d1i3ua_ d1cf8l1 d1fe8h1
d2p1ya1 d1eeqa_ d2x89a_ d1ol0a_ d2r0lh1 d1ub5a1 d1dfbh1 d3eyvb1
d4jdvb1 d4p2cg_ d2gsia1 d2agjl1 d1u3ha1 d2h1ph1 d1rihl1 d1fskc1
d1boga1 d2a77l1 d1ospl1 d1qfoa_ d3dwta_ d2fx7l1 d1rz7l1 d3stba1
d1pkqa1 d1b6da1 d1dzba2 d1dzba1 d1kj2a_ d4l1ha_ d1rzgb1 d1dqlh_
d12e8l1 d1miel1 d1jmaa_ d1sbsl1 d1fgnh1 d1f58l1 d1mqkh_ d1ndgb1
d3qegl1 d1iail1 d2aw2a1 d1tqbc1 d3fo2a1 d4dkaa_ d1f8th1 d2g60l1
d2ywya_ d1uj3a1 d1yc7a_ d3bd3a_ d4w6xb1 d1nmbl_ d1i8ka_ d1rjlb1
d1ypze1 d1p7kb1 d1kxqe_ d1gpoh1 d2z4qa1 d1w72h1 d1i9rl1 d4qgya1
d4xt1c_ d2qqnh1 d1cica1 d3c08h1 d2cd0a_ d2bdnh1 d1hnga1 d1maml1
d1nldh1 d1cr9h1 d4lcih1 d3bkyh1 d3qg7l1 d1ai1l1 d1kxvc_ d4iosd_
d1l7ih1 d2x1qa_ d1ub6b1 d3k74b_ d1ibgh1 d1teth1 d1igya1 d1hxma1
d2x6ma_ d2e7la_ d5e03a_ d1qfwm_ d2r0kh1 d1igjb1 d1rurh1 d1plgh1
d1mj8h1 d1yuhb1 d3fmgl1 d1rzfl1 d1emtl1 d1ad9b1 d1igtb1 d1nlbl1
d1d5ih1 d3k7ua_ d4ffyh_ d1bwwa_ d3dvga1 d1a6ta1 d1fgvl_ d4qxgl1
d1miml1 d1jhll_ d2hffb1 d2hh0l1 d1qkzh1 d1l7tl1 d1afvh1 d1iaii1
d2cjuh_ d1um5h1 d2ghwb2 d2ghwb1 d1kgce1 d2fgwh1 d1ikfh1 d1dvfd_
d2i24n1 d2xtjb_ d1za3b1 d2yc1a_ d2hh0h1 d2x1oa_ d1bvka_ d1rjla1
d5e7ba1 d1igta1 d1il1b1 d2fb4l1 d1mvua_ d1jptl1 d32c2a1 d1fvca_
d1iqdb1 d2hrph1 d2p4ab_ d4qo1a_ d1p2cb1 d1wt5c_ d1t4ka1 d5c1mb_
d1it9l1 d1bz7a1 d1b2wh1 d1e4wl1 d1jnll1 d1yedb1 d1za6b1 d3u7aa_
d1igfh1 d3gnml1 d1orsa1 d1wejl1 d1a2ya_ d1hxmb1 d1gafh1 d2j4wh1
d1pz5a1 d1j05a_ d1a3rh1 d4fqll1 d1qfwh_ d1eo8h1 d1n7mh1 d4s11a_

d1kegh1 d5da4a_ d1iqwl1 d3whxb1 d1vgeh1 d1deeb1 d1c12b1 d1t2ql1 d1ad9a1 d3gsnb1 d3b2uc1 d1ggbh1 d1t3fb1 d1nakl1 d4ocmc_ d1lila1 d2v7hb3 d2esve1 d1mcoh1 d2qr0b1 d2q3aa_ d1ct8a1 d1kcrl1 d2pxyb_ d2uzih_ d5c3ld_ d3durb_ d1c5cl1 d2ak4e1 d1dl7h_ d1xiwd_ d1mlba1 d1rmfl1 d1i7zb1 d1dlfl_ d2xa3a_ d5bq7b1 d1dqqb1 d3h42l1 d1egjh1 d1kcvl1 d1fe8l1 d2j6el1 d1f3rb2 d1lk3l1 d1sm3h1 d1q0xh1 d1ghfh1 d7fabl1 d1igch1 d1lo0h1 d4idla_ d5c2bl_ d1jrhl1 d1il1a1 d3zhda1 d4kfzc_ d1b0wa_ d1e6oh1 d1mexl1 d4krpb_ d1fnsh1 d1ncwh1 d4hgma_ d4eigb1 d4rzca_ d2mpal1 d4y5xa_ d5en2b1 d3lrga1 d4ej1c1 d1rhha1 d1j05b_ d1forh1 d1q9wb1 d2wzpd_ d2aq2a1 d1h0da1 d3revb1 d2ij0c1 d1tzha1 d4m7jl1 d1k6ql1 d3b9kc1 d1igml_ d3qeua1 d1dsfl_ d1ce1h1 d1kcuh1 d1zvha1 d1zvoa1 d1tqbb1 d1ay1l1 d2qscl1 d2cgrh1 d1nl0h1 d1kenl1 d3eo0a1 d4n9ob1 d1n0xl1 d3k1kc_ d2m9wa1 d3dfxa_ d2aq2b1 d1ck1a1 d1enfa1 d1i4pa1 d1v1oa1 d1et9a1 d1esfa1 d2g9hd1 d4ohja1 d1klud1 d1bxta1 d1ty0a1 d1fnua1 d2qaln1 d2cqva1 d1fhga_ d2k1ma_ d2dl2a1 d1biha3 d1biha1 d1biha4 d3qp3a1 d1g1ca_ d1gxea_ d1cvsc2 d1cvsc1 d1xmwa2 d1m4ka2 d2otpa2 d1biha2 d1rhfa2 d2fcba2 d2fcba1 d1wwbx_ d1gsma2 d1epfa1 d1epfa2 d2dava1 d1waaa_ d1f97a2 d1x44a1 d1vcaa2 d1fnla2 d1fnla1 d3b5ha2 d3h8na2 d3h8na1 d1jbja1 d1jbja2 d1pd6a_ d1f2qa2 d1f2qa1 d3cx2a_ d1nbqa2 d3b5ha1 d1n26a1 d1xiwb_ d1xaua_ d1fltx_ d2ifga1 d1olla1 d1olla2 d1cs6a4 d1cs6a3 d1cs6a2 d1cs6a1 d2gw5a2 d1sy6a1 d1xiwa_ d1ucta2 d1ucta1 d1ry7b2 d1gl4b_ d1qz1a3 d1olza1 d2c9aa1 d1wwca_

**Family-11 (a.26.1.3)**

**Positive dataset contains 6 protein sequences.**

d4x51a_ d4ypgc1 d3oq3a_ d1wu3i_ d3besl_ d3ux9a_

**Negative dataset contains 3997 protein sequences.**

d1m32a_ d1ibja_ d1c7na_ d1iuga_ d4q75a_ d2bkwa1 d1t3ia_ d1n8pa_ d1qz9a_ d1jf9a_ d1eg5a_ d1p3wa_ d3kgwa_ d1cl1a_ d2ctza1 d1vjoa_ d2rfva_ d1gc0a_ d1cs1a_ d1d2fa_ d2ch1a1 d1qgna_ d1pffa_ d1ax4a_ d1c7ga_ d2v1pa1 d2ez1a_ d1u08a_ d1lc5a_ d1b5pa_ d2ay1a_ d1bw0a_ d3pd6a_ d7aata_ d2csta_ d1j32a_ d1fg7a_ d4wlha_ d1o4sa_ d1v2da_ d1gdea_ d1m6sa_ d1v72a1 d1svva_ d1ajsa_ d1wsta1 d2f8ja2 d1lk9a_ d1vp4a1 d1xi9a1 d2gb3a1 d1yaaa_ d2bnma2 d3i2ka1 d1lnsa3 d2cfba1 d1o69a_ d1bj4a_ d3k28a_ d2bwpa_ d3lg0a_ d2fnua_ d3ou5a_ d1z7da1 d1b9ha_ d1iaya_ d4msoa_ d1bs0a_ d1s0aa_ d1sffa_ d3tqxa_ d1mdoa_ d3qboa_ d3pgya_ d1bjna_ d2dkja_ d1ejia_ d1vefa1 d4wxga_ d3bs8a_ d3gbxa1 d1w23a_ d1r0ka3 d1j5pa3 d1vkoa2 d1vm6a2 d3qvsa2 d1gr0a2 d1p1ja2 d1f56a_ d1bypa_ d1azca_ d1ws8a_ d3u25a_ d1cuoa_ d1id2a_ d1rkra_ d2iaac_ d4dp1x_ d1nwpa_ d2gima_ d2i7sa_ d1qhqa_ d3ehbb1 d1fftb1 d1gyca3 d1hfua2 d1kbva2 d1sdda1 d1sdda2 d1gyca1 d1gyca2 d1mzya1 d2bw4a1 d1aoza1 d1aoza3 d1aoza2 d1kyaa2 d1kyaa3 d1kyaa1 d1mzya2 d4e9sa1 d1sddb1 d1sddb2 d1hfua1 d1hfua3 d1oe1a2 d1oe1a1 d1v10a2 d1v10a3 d1v10a1 d1rypd_ d1irua_ d1rypc_ d3unfk_ d1rypa_

| | | | | | | | |
|---|---|---|---|---|---|---|---|
| d1j2pa_ | d1irui_ | d1j2qh_ | d3unfh_ | d1m4ya_ | d1rypb_ | d1irul_ | d1irug_ |
| d1iru2_ | d1q5rh_ | d1rypg_ | d1g3ka_ | d1iruf_ | d1q5qa_ | d1e94a_ | d1irub_ |
| d1ryph_ | d3ty6a_ | d1iruj_ | d1iruk_ | d1rype_ | d1irud_ | d1irue_ | d3mi01_ |
| d1rypf_ | d2z3ba_ | d1iruc_ | d3mka2_ | d1iru1_ | d1a3qa1 | d1iknc_ | d3brda1 |
| d1p7hl1 | d1oy3c_ | d1imhc1 | d1zk9a1 | d2cxka1 | d2bema_ | d1ea9c1 | d1qhoa1 |
| d2fhfa2 | d2fhfa1 | d1k3ia1 | d1j0ha1 | d1cxla1 | d2bhua1 | d1edqa1 | d1f1sa2 |
| d1ut9a2 | d1gvia1 | d1txka1 | d4alac_ | d1pjwa_ | d1s6na_ | d3uzqb_ | d1jgta2 |
| d1ofda3 | d1ea0a3 | d1ecfa2 | d1q15a2 | d1ao0a2 | d1te5a_ | d1ep0a_ | d2pa7a1 |
| d1wlta1 | d2c0za1 | d1nepa_ | d1ktja_ | d1ds6b_ | d1fstb_ | d1ex0a1 | d1g0da1 |
| d4w9ta_ | d4x2ra1 | d1ka9f_ | d1qo2a_ | d4axka1 | d1thfd_ | d1h5ya_ | d4u28a_ |
| d2y88a_ | d4wd0a_ | d1vzwa1 | d2bjca1 | d2hsga1 | d1qpza1 | d1efaa1 | d1au7a2 |
| d1e3oc2 | d2ovga_ | d1sq8a_ | d1rzsa_ | d3kz3a_ | d3orca_ | d3gpdg2 | d2q49a2 |
| d1hdgo2 | d2g17a2 | d2cvoa2 | d2czca1 | d3pwka2 | d1obfo2 | d1i32a2 | d1zyao2 |
| d2pkqo2 | d1b7go2 | d2hjsa2 | d1cf2o2 | d1vkna2 | d1nvmb2 | d2msya_ | d2djoa1 |
| d2r5za_ | d1au7a1 | d1f43a_ | d2lmda1 | d1akhb_ | d1s7ea1 | d3nara_ | d1zq3p1 |
| d2craa1 | d1mija_ | d4uuta_ | d3a01a_ | d1x58a1 | d3rkqa1 | d3cmya1 | d2cqxa1 |
| d2da6a1 | d1e3oc1 | d1fjla_ | d1mh3a1 | d1ig7a_ | d1x41a1 | d1wh7a1 | d1gt0c1 |
| d2mw8a_ | d1x2ma1 | d1b72a_ | d1wi3a1 | d1jgga_ | d1wjha1 | d1x2na1 | d1mnmc_ |
| d2cuea1 | d3k2aa_ | d2hosa_ | d2jwta2 | d1pufa_ | d1octc1 | d2hi3a_ | d1b8ia_ |
| d4j19a_ | d3lnqa_ | d1wh5a1 | d1b8ib_ | d1fexa_ | d2cqra1 | d1guua_ | d1ug2a1 |
| d2cqqa1 | d2ajea1 | d4a69c_ | d1h8ac1 | d2crga1 | d2iw5b1 | d1xc5a1 | d1ofcx1 |
| d2cu7a1 | d1wgxa1 | d2cjja1 | d2ckxa1 | d1gvda_ | d1hcra_ | d1tc3c_ | d1u78a2 |
| d6paxa1 | d6paxa2 | d1k78a1 | d1k78a2 | d1hlva1 | d1hlva2 | d1iufa2 | d1iufa1 |
| d2dara2 | d2dara1 | d1wyha2 | d2dloa2 | d1rutx4 | d1rutx3 | d2cuqa2 | d1x4la2 |
| d1x4la1 | d1x3ha2 | d1x3ha1 | d2d8za1 | d1x61a2 | d1x6aa1 | d1x6aa2 | d2cora2 |
| d2cora1 | d1v6ga1 | d1v6ga2 | d2d8za2 | d1x64a1 | d1x64a2 | d1b8ta1 | d2co8a1 |
| d1x63a2 | d2dj7a2 | d2dj7a1 | d2cu8a2 | d2cu8a1 | d1g47a2 | d1g47a1 | d1j2oa1 |
| d1wyha1 | d2dloa1 | d1wiga1 | d1wiga2 | d2cupa2 | d2cupa3 | d1x68a1 | d2d8ya2 |
| d2d8ya1 | d1m3va2 | d1x62a1 | d1x68a2 | d1x61a1 | d1b8ta3 | d1b8ta2 | d2d8xa2 |
| d2d8xa1 | d1x4ka1 | d1x4ka2 | d2cuqa1 | d1m3va1 | d2co8a2 | d1x63a1 | d2cura1 |
| d2cura2 | d1x62a2 | d2iu5a1 | d1zk8a1 | d2g7sa1 | d2fd5a1 | d2np5a1 | d1z0xa1 |
| d2id6a1 | d2d6ya1 | d2i10a1 | d2oi8a1 | d3c07a1 | d2ns7a1 | d2fq4a1 | d2yvea1 |
| d1t33a1 | d2o7ta1 | d1rkta1 | d2np3b1 | d2g7ga1 | d2gena1 | d2fbqa1 | d2g7la1 |
| d2gfna1 | d2g3ba1 | d2jj7a1 | d2hyja1 | d1sgma1 | d1ui5a1 | d2hkua1 | d2id3a1 |
| d4jyka1 | d3lv8a_ | d1s3ga1 | d1zaka1 | d1s4qa_ | d3tvta2 | d1uf9a_ | d1khta_ |
| d1ckea_ | d1osna_ | d1p5zb_ | d4tmka_ | d3cm0a_ | d1tmka_ | d1jaga_ | d3h86a_ |
| d1t0hb_ | d1nn5a_ | d2h92a_ | d3kb2a1 | d2c95a1 | d1yj5a2 | d1kgda_ | d1ly1a_ |
| d2ak3a1 | d1vyva2 | d1deka_ | d2grja1 | d1p3ja1 | d1y63a_ | d1e4ya1 | d1s96a_ |
| d3iija1 | d1lvga_ | d1nksa_ | d1vyua2 | d4e22a_ | d1qf9a_ | d1jjva_ | d1q3ta_ |
| d1ki9a_ | d1lw7a2 | d1teva_ | d1kjwa2 | d1gsia_ | d2iyva2 | d1kaga_ | d1viaa_ |
| d1e6ca_ | d1g3ma_ | d1j99a_ | d1t8ta1 | d1nsta_ | d1fmja_ | d1zrha_ | d1ls6a_ |
| d2a3ra_ | d1aqua_ | d2zptx_ | d3ckla_ | d1texa_ | d1q44a_ | d2gwha_ | d1z0ja1 |
| d3umra2 | d3gj0a1 | d2gaoa1 | d1yzga1 | d1x3sa1 | d1g7sa4 | d1puja_ | d1udxa2 |
| d1kk1a3 | d2o52a_ | d1jwyb_ | d1zcaa2 | d1zcba2 | d2fv8a1 | d2cjwa_ | d3dz8a1 |

d1xzpa2 d1vg8a_ d4qxaa_ d2ew1a1 d1x1ra1 d2bcgy_ d3doea1 d3cona_
d3rega_ d3wyga_ d1upta_ d2b6ha1 d1zd9a1 d1lnza2 d1r5ba3 d1n0ua2
d1r2qa_ d2f9la1 d2rhda_ d3q3jb_ d3q85a_ d1efma1 d1r8qa_ d2gf9a1
d2akab1 d1ek0a_ d1z08a1 d2gcoa1 d2dy1a2 d4g01b_ d2cxxa1 d1svia_
d3q72a_ d1ni3a1 d4ac9a4 d2erxa1 d2bmea_ d1wxqa1 d2gf0a1 d4ldja1
d1zunb3 d1d5ca_ d4lv5b_ d1efca3 d1kmqa_ d2g6ba1 d3lrpa_ d1tada2
d2y8ea_ d4rd4a1 d1z06a1 d2heia_ d3a58b_ d1u0la2 d1d2ea3 d1zj6a1
d2ocba_ d3rwoa_ d1mr3f_ d2e9sa1 d4u5xa1 d2iwra_ d2fn4a1 d3bwdd_
d2nzja1 d2atva1 d2ksqa_ d3l43a_ d1mkya2 d1mkya1 d1wmsa_ d1jnya3
d2rmka_ d1f6ba_ d1f60a3 d1gota2 d1jala1 d2fh5b1 d1g16a_ d2wblc_
d1m7ba_ d1wf3a1 d1f5na2 d1t9ha2 d1z6xa1 d1z0aa1 d2fg5a1 d2a5ja1
d2bcjq2 d1s0ua3 d1ky3a_ d4y0va_ d4lhwa_ d2qmea1 d2atxa1 d1egaa1
d1ryha_ d1moza_ d1nrjb_ d2erya1 d3bfka_ d1puia_ d1fzqa_ d3j3az1
d3t5ga_ d1shza2 d3defa_ d2f7sa1 d3raba_ d3tsoa_ d3aq4a_ d2al7a1
d3sfva1 d3bc1a_ d2ncda_ d1sdma_ d3h4sa1 d2heha_ d2grya_ g3kin.1
d1kk8a2 d1br2a2 d1goja_ d1f9va_ d2mysa2 d1lkxa_ d3t0qa_ d4db1a1
d3gbja_ d1ry6a_ d1k8ka2 d1k8ka1 d3uleb1 d3i33a2 d3i33a1 d1bupa1
d1dkgd2 d1dkgd1 d4ci6a2 d1jcfa1 d4a2ba2 d2fxua1 d1yaga2 d1yaga1
d4cbua2 d1bupa2 d3vrda1 d3vrda2 d1fcdc2 d1fcdc1 d1h1oa2 d1m70a2
d1m70a1 d1iqca2 d1iqca1 d1eb7a1 d1nmla2 d1nmla1 d1nira1 d1qksa1
d1hroa_ d4gedb_ d3zooa_ d1mz4a_ d1gu2a_ d1f1fa_ d1dvva_ d1c6ra_
d1c75a_ d1e29a_ d1gdva_ d1cnoa_ d1h32b_ d1co6a_ d2bh4x_ d2aiua_
d5cytr_ d1jdla_ d1i8oa_ d2zboa_ d3dmia_ d1ls9a_ d1wvec_ d2yk3a_
d3ph2b_ d1dw0a_ d5aura_ d1ql3a_ d1qn2a_ d1f1ca_ d4zzva_ d2xspa_
d1eg1a_ d4v20a_ d1ojja_ d4ipma1 d4csia_ d3pfza_ d3ovwa_ d2yoka_
d2nqya_ d1h4ga_ d1hixa_ d1oa2a_ d1olra_ d3b7ma_ d4h7ma1 d3wp3a_
d2vgda_ d1igoa_ d3zsea_ d2nlra_ d3wx5a_ d1oa3a_ d1pvxa_ d1m4wa_
d1f5ja_ d1oa4a_ d4ixla1 d4dnxa2 d1m8pa2 d1g8fa2 d1jhda2 d1x6va2
d2el7a_ d1iq0a2 d1irxa2 d1r6ta2 d1ivsa4 d2cyba_ d1rqga2 d1j09a2
d2yxna_ d1f7ua2 d1h3fa1 d1ffya3 d2g36a1 d1nzja_ d1jila_ d1n3la_
d1i6la_ d1pfva2 d1h3na3 d3n9ia_ d3sz3a1 d1cwva5 d3sw2a_ d1moxc_
d1dx5i3 d1edmb_ d1autl2 d1autl1 d1nqlb_ d1dval1 d1szba2 d1kigl_
d2y5fl_ d1ijqa2 d1gk5a_ d1g1sa2 d1nt0a3 d1xdtr_ d1lmja2 d1lmja1
d1nzia2 d2wphe_ d1klil_ d1pfxl2 d1pfxl1 d1p9ja_ d1g1ta2 d1f7ea_
d4yzub_ d1lr7a1 d2y5ia_ d1qlsa_ d1psra_ d3n22a_ d2egda_ d4ggfc_
d2wcea_ d2luca_ d4aqia_ d3nsia_ d2lp3a_ d1j55a_ d1a4pa_ d1k8ua_
d1nsha_ d1exra_ d1tiza1 d1nyaa_ d1g8ia_ d1ggwa_ d1wdcc_ d3fwba_
d1ggza_ d2o5ga_ d1ctaa_ d1m45a_ d3i5gc_ d1sl8a_ d1fpwa_ d2scpa_
d2roaa_ d4aqra_ d3pn7b_ d1br1b_ d2mysb_ d1s6ca_ d1bjfa_ d2obha_
d1jfka_ d1jf0a_ d1qv0a_ d1lkja_ d2mlfc_ d2m97a_ d2d8na_ d4by5a_
d1jbaa_ d4nqga_ d1wdcb_ d2mysc_ d1j7qa_ d1s6ia_ d1uhka2 d1r2ua_
d1dtla_ d3kf9a_ d3pn7c_ d1qjta_ d1fi6a_ d1c07a_ d5d67d_ d1tuza_
d1h8ba_ d1eg3a1 d1eg3a2 d2zkmx1 d1snla_ d1gjya_ d1y1xa_ d1hqva_
d1dvia_ d1df0a1 d1qxpa2 d4usla_ d1k94a_ d1wkya2 d1uuqa_ d3wh9a_

d4m1ra_ d1xyza_ d4xuya_ d2je8a5 d4bf7a1 d3cuia_ d1ur4a_ d1qw9a2
d1odza_ d2vzsa5 d1bqca_ d2x2ya1 d1hjqa_ d1qnra_ d4y7ea1 d1vjza_
d4k68a_ d2v3ga_ d4hpga_ d1nofa2 d1nq6a_ d1h4pa_ d1aq0a_ d3ziza_
d4bs0a_ d1ecea_ d1h1na_ d1ta3b_ d1w91a2 d1i1wa_ d1uhva2 d1us3a2
d1egza_ d7a3ha_ d1tvna1 d3nyda1 d1ghsa_ d2cyga1 d4xv0a_ d2c8na2
d1w32a_ d3emca_ d1v6ya1 d1foba_ d2uwfa1 d2fgla_ d3niya1 d2c0ha1
d1rh9a1 d1yq2a5 d1szna2 d1fa2a_ d1pama4 d5do8a1 d1ea9c3 d1hx0a2
d2fhfa5 d1qhoa4 d1avaa2 d1m53a2 d4m8ua1 d2gjpa2 d1lwha2 d4howa1
d1ktba2 d1j0ha3 d3vm5a1 d2x1ia_ d1wzaa2 d1uasa2 d2diea1 d1mxga2
d1ht6a2 d1eswa_ d1iv8a2 d5cpsa_ d1ud2a2 d1zjaa1 d3gbea1 d1kwga2
d1wdpa_ d1gcya2 d1g5aa2 d1hvxa2 d3dhpa2 d2bhua3 d4do4a1 d3qgva1
d1e43a2 d1g94a2 d2d3na2 d1gjwa2 d1gvia3 d1x1na1 d1r7aa2 d1tz7a1
d2x42a1 d1tr9a1 d3abza1 d4c7ga2 d2gjxa1 d1jaka1 d1yhta1 d4toqa_
d1wb0a1 d1w9pa1 d1eoka_ d1itxa1 d1kfwa1 d1goia2 d1edqa2 d2gsja_
d1hjxa1 d1jnda1 d1vf8a1 d1umqa_ d1g2ha_ d1e5wa2 d1j19a2 d1isna2
d2al6b2 d1g4ma1 d1g4ma2 d1id1a_ d2hmta_ d2v76a_ d1wj1a1 d1aqca_
d1qqga2 d1qqga1 d1ntva_ d1n3ha_ d4dbba_ d3f0wa1 d1p3ra_ d1wgua1
d2cy4a1 d3hqca_ d1wvha1 d1xr0b1 d1wgqa1 d1v89a1 d2coda1 d1zc3b_
d1wg7a1 d1u5ea1 d1v61a1 d2coca1 d2ys1a1 d1v5pa1 d1ntya2 d1wjma1
d1wi1a1 d1u29a_ d1xx0a1 d1unqa_ d2zkmx3 d1fhoa_ d1v88a1 d2adza1
d1x1fa1 d1p6sa_ d2coaa1 d1btka_ d1v5ma1 d1faoa_ d2x18a_ d1x1ga1
d4kaxb_ d2j59m1 d1ki1b2 d1xcga2 d1txda2 d1u5da1 d1dbha2 d1w1ha_
d1upqa_ d1v5ua1 d1foea2 d2fjla1 d1eaza_ d2dfka2 d2cofa1 d1kz7a2
d9ldta1 d2ewda3 d1up7a1 d2dfda1 d1s6ya1 d1vjta1 d7mdha1 d1hyea1
d1t2da1 d1ojsa3 d1ldna1 d1hyha1 d1civa1 d2a92a1 d1ez4a1 d1i0za1
d1y6ja1 d1gv0a1 d1ur5a1 d1llda1 d1obba1 d1v8ba1 d1gdha1 d1l7da1
d2dlda1 d1mx3a1 d1ygya1 d1qp8a1 d1j4aa1 d2naca1 d1npya1 d3t4ea2
d1a4ia1 d1o0sa1 d2hk9a2 d2egga2 d1b26a1 d1bvua1 d1gq2a1 d1c1da1
d1leha1 d1gtma1 d1euza1 d1nvta1 d3dona2 d1edza1 d1gpja2 d1vi2a1
d1p77a1 d1nyta1 d1b0aa1 d1vl6a1 d1v9la1 d1txga2 d3f47a1 d1mv8a2
d2pv7a2 d1xc2a3 d2ahra2 d4xiya1 d2f1ka2 d1pgja2 d1i36a2 d1np3a2
d1lj8a4 d1teaa3 d1qmga2 d2i76a2 d2g5ca2 d1wdka3 d1dlja2 d3cuma3
d1kola2 d1f8fa2 d1qora2 d1jqba2 d1vj0a2 d1cdoa2 d1gu7a2 d1d1ta2
d1xa0a2 d2dm6a2 d1jvba2 d1rjwa2 d1llua2 d1h2ba2 d1tt7a2 d1piwa2
d1o89a2 d1iz0a2 d1e3ja2 d1yb5a2 d1uufa2 d1vj1a2 d3gpdg1 d1vc2a1
d2nvwa1 d1u8fo1 d1ydwa1 d1hdgo1 d2g17a1 d1cf2o1 d2czca2 d1r0ka2
d1obfo1 d2cvoa1 d1ebfa1 d1h6da1 d1i32a1 d1zh8a1 d2q49a1 d1qkia1
d1j5pa4 d3pzra1 d1xeaa1 d1vkoa1 d1vm6a3 d3qvsa1 d2pkqo1 d1b7go1
d1gr0a1 d1p1ja1 d1lc0a1 d1j0xo1 d3pwka1 d2hjsa1 d1zyao1 d1vkna1
d1tlta1 d1nvmb1 d3sjua_ d3ojfa1 d1xu9a1 d2jjya1 d1n7ha_ d1y1pa1
d1h5qa_ d1jtva_ d4fs3a_ d2bkaa1 d1gy8a_ d2c5aa1 d1ulsa_ d1ooea_
d2b69a1 d1y5ma1 d1spxa_ d1ae1a_ d1bxka_ d1ydea1 d1gega_ d1hdoa_
d1o5ia_ d1e6ua_ d1snya1 d2rhca_ d3zv4a_ d5ceja_ d2d1ya1 d1fjha_
d3w8ea_ d2fmua1 d1x1ta_ d1rpna_ d3ay7a1 d1k2wa_ d1eq2a_ d2hrba1

| | | | | | | | |
|---|---|---|---|---|---|---|---|
| d3ruaa_ | d2ew8a1 | d4hwka_ | d1xgka_ | d2bd0a1 | d1yo6a1 | d2a35a1 | d2ntva_ |
| d1vl8a_ | d1hdca_ | d4bkua_ | d2ag5a1 | d2q45a_ | d1nffa_ | d4iy1a_ | d1zbqa1 |
| d1kewa_ | d1ek6a_ | d1ja9a_ | d2q46a2 | d1iy8a_ | d1cyda_ | d2ptga_ | d1hxha_ |
| d1uzma1 | d3lyla_ | d4m89a_ | d3wyea_ | d2blla1 | d1w6ua_ | d1sb8a_ | d2q2va_ |
| d4nr0a_ | d1e6wa_ | d2gasa_ | d3oiga1 | d2bgka1 | d1mxha_ | d1vl0a1 | d1rkxa_ |
| d1r6da_ | d1wmaa1 | d1e7wa_ | d2c07a1 | d1xkqa_ | d3tzha_ | d1g0oa_ | d3lz6a_ |
| d1qyca_ | d1edoa_ | d1gz6a_ | d1oc2a_ | d1t2aa_ | d1zema1 | d2xoxa_ | d1z45a2 |
| d1geea_ | d1p33a_ | d3lt0a_ | d4e6pa_ | d2wyua_ | d1db3a_ | d4q9na_ | d1xhla_ |
| d4o1ma_ | d1fmca_ | d2uvda_ | d2gdza1 | d1uaya_ | d2z1ma_ | d2o23a2 | d2fr1a2 |
| d2fr1a1 | d1qsga_ | d1qyda_ | d4zjua_ | d2a4ka1 | d1yb1a_ | d1wvga1 | d1yxma1 |
| d3f4ba_ | d1mg5a_ | d1xg5a1 | d2gfoa1 | d1vjva_ | d2ayna1 | d3nhea_ | d4ekfa_ |
| d2ckga_ | d1th0a_ | d2bkra_ | d1cmxa_ | d1xd3a_ | d3ifwa_ | d1e2ta_ | d4c5pa_ |
| d2bsza1 | d4bgfa_ | d1w4ta1 | d1mdwa_ | d1qxpa4 | d1ziva1 | d1zcma1 | d3ioqa_ |
| d1fh0a_ | d1o0ea_ | d1khqa_ | d1m6da_ | d2cb5a_ | d1cs8a_ | g8pch.1 | d1s4va_ |
| d2pnsa_ | d1vsna1 | d1mira_ | d1iwda_ | d2oula_ | d7pcka_ | d3bwka_ | d3bcna_ |
| d2p86a_ | d1pvja_ | d1pcia_ | d1gece_ | d1deua_ | d4bs6a_ | d1xkga1 | d1cqda_ |
| d1jqpa2 | d2c0ya_ | d2bdza_ | d3p5ua_ | d1zoya1 | d1qo8a2 | d1d4ca2 | d1chua2 |
| d1jnra2 | d1kf6a2 | d2jk6a2 | d2jk6a1 | d1feca1 | d1feca2 | d1lpfa1 | d1lpfa2 |
| d1xhca1 | d1xhca2 | d1dxla2 | d1dxla1 | d1gera2 | d1m6ia2 | d1onfa1 | d1onfa2 |
| d3lada2 | d3lada1 | d1w4xa1 | d1w4xa2 | d1gesa2 | d1mo9a1 | d1xdia1 | d1fl2a2 |
| d1fl2a1 | d1fcda1 | d1fcda2 | d1h6va2 | d1h6va1 | d1mo9a2 | d1gv4a2 | d1gv4a1 |
| d2gv8a2 | d2gv8a1 | d1aoga1 | d1aoga2 | d1hyua1 | d1ebda1 | d1ebda2 | d3dk9a1 |
| d1q1ra2 | d1q1ra1 | d1gesa1 | d1w5qa_ | d2c1ha_ | d1ohla_ | d1l6sa_ | d1zbfa1 |
| d2z1ha_ | d2etja1 | d3qioa1 | d1jl1a_ | d2e4la_ | d1jl2a_ | d2lsna1 | d1io2a_ |
| d1uaxa_ | d2yv0x_ | d1mu2a1 | d1ekea_ | d1i39a_ | d1pn0a1 | d1reoa1 | d1ju2a1 |
| d2f5va1 | d3c96a1 | d2gmha1 | d1pj5a2 | d1gpea1 | d1o5wa1 | d1n4wa1 | d1k0ia1 |
| d2dw4a2 | d2ivda1 | d1seza1 | d2voua1 | d1kdga1 | d1s5ja1 | d2hbja2 | d2igia_ |
| d1wlja_ | d1qhta1 | d4dlea1 | d1tgoa1 | d2f96a1 | d3tr8a1 | d3mxma_ | d1q8ia1 |
| d1y97a1 | d1w0ha_ | d2py5a1 | d1noya_ | d1d5aa1 | d1yt3a3 | d2guia_ | d3mwpa1 |
| d4kb1a_ | d1offa_ | d1frra_ | d3wcqa_ | d1e9ma_ | d1l5pa_ | d1wria_ | d1i7ha_ |
| d3av8a_ | d1czpa_ | d1rfka_ | d3p1ma1 | d3b2ga_ | d2bt6a2 | d2av4a1 | d3nvva1 |
| d1krha3 | d2h88b1 | d1n62a2 | d1t3qa2 | d1kf6b2 | d1zoyb1 | d1ffva2 | d1dgja2 |
| d1rm6c2 | d1r7ha_ | d1faaa_ | d1wika1 | d1xfla_ | d1ttza1 | d2f51a1 | d1ti3a_ |
| d1nhoa_ | d1sena_ | d2i4aa_ | d1ep7a_ | d1zmaa1 | d1fo5a_ | d3lefa1 | d3diea1 |
| d2yana1 | d1z6na1 | d1oaza_ | d3zywa1 | d4aj8a_ | d1nm3a1 | d4fiwa_ | d1wmja_ |
| d4hs1a1 | d1iloa_ | d1xwaa1 | d1wjka1 | d2vm1a_ | d1fb6a_ | d1gh2a_ | d1h75a_ |
| d1nw2a_ | d1kx5b_ | d3a6na_ | d4m6ba2 | d2nqbd_ | d3wkjd_ | d2yfwe_ | d4h9na_ |
| d5bnxa_ | d1kx5c_ | d3r45a_ | d1eqzb_ | d4jjnb_ | d1f66c_ | d1eqza_ | d4wnnb_ |
| d1kx5a_ | d1u35c1 | d1id3a_ | d2nqbc_ | d1kx5d_ | d1ku5a_ | d1f1ea_ | d1b67a_ |
| d1tafa_ | d1bh9b_ | d1jfib_ | d1q9ca_ | d1h3ob_ | d2byka1 | d1n1ja_ | d4g92b_ |
| d1bh9a_ | d1jfia_ | d1h3oa_ | d2bykb1 | d1n1jb_ | d1pd212 | d1nhya2 | d2a2ra2 |
| d1k0dd2 | d1f2ea2 | d1gsua2 | d1f3ba2 | d2fhea2 | d2pvqa1 | d1tw9a2 | d1oyja2 |
| d1e6ba2 | d3ik7a1 | d4is0a1 | d1r5aa2 | d1jlva2 | d1m0ua2 | d3gtub2 | d1z9ha2 |
| d1okta2 | d1axda2 | d2gsra2 | d1tu7a2 | d1k3ya2 | d1zgma1 | d1b48a2 | d1gnwa2 |

| | | | | | | | |
|---|---|---|---|---|---|---|---|
| d1gwca2 | d2gsta2 | d1glqa2 | d1oe8a2 | d1pn9a2 | d1v2aa2 | d1fw1a2 | d1duga2 |
| d1g7ea_ | d1ig8a1 | d1czan2 | d1czan3 | d1czan1 | d1ig8a2 | d1czan4 | d1bg3a4 |
| d1bg3a2 | d1bg3a1 | g1f8v.1 | d1novc_ | d2bbvc_ | d4ilya_ | d1chka_ | d4olta_ |
| d1qqya_ | d1f6ra_ | d1ivma_ | d2gv0a_ | d1iiza_ | d2z2fa_ | d1j8xa_ | d1ghla_ |
| d1b9oa_ | d174la_ | d169la_ | d2o4wa_ | d1p37a_ | d1k28a3 | d1xjta1 | d1jtma_ |
| d2f2qa_ | d4e97a1 | d3w3ea_ | d4tx7a_ | d3cqla_ | d4msta_ | d3iwra_ | d2z37a_ |
| d4dyga_ | d1mpya2 | d1kw3b2 | d1kw3b1 | d4ghga1 | d1tfza1 | d3isqa1 | d3isqa2 |
| d1sqia1 | d1sqia2 | d1lkda2 | d1lkda1 | d1mpya1 | d1cjxa2 | d1cjxa1 | d1sp9b2 |
| d1t47a1 | d1t47a2 | d1sp8a1 | d1sp8a2 | d1f1ua1 | d1f74a_ | d3l21a_ | d2e1da_ |
| d1to3a1 | d1w3ia_ | d1sfla_ | d1vcva1 | d4imea_ | d1wa3a_ | d2ocza1 | d1f2ja_ |
| d3m16a_ | d2yxga_ | d1ub3a_ | d3a5fa_ | d1fbaa_ | d1l6wa_ | d1xfba1 | d1wx0a1 |
| d1vpxa1 | d1ojxa_ | d1mxsa_ | d2a4aa1 | d1xkya1 | d3cq0a1 | d3s1xa_ | d1f05a_ |
| d1fdja_ | d3cpra1 | d4d2ja_ | d1s5ja2 | d1qhta2 | d4dlea2 | d1tgoa2 | d3py8a2 |
| d1q8ia2 | d1kfsa2 | d2py5a2 | d1d5aa2 | d1qsta_ | d2beia1 | d4m3sa_ | d1u6ma1 |
| d1ygha_ | d1yk3a1 | d3dr6a_ | d1xmta_ | d1z4ea1 | d2ge3a1 | d1bo4a_ | d2fiaa1 |
| d2jdca1 | d1y9wa1 | d3to7a_ | d1wwza1 | d2fl4a1 | d2ozua1 | d2b5ga_ | d2cy2a1 |
| d2p0wa_ | d1cm0a_ | d4pswa_ | d1yrea1 | d1yr0a1 | d1m4ia_ | d2gana1 | d1yvka1 |
| d1ghea_ | d2g3aa1 | d1y7ra1 | d2euia1 | d1q2ya_ | d2fsra1 | d1xeba_ | d2ae6a1 |
| d1cjwa_ | d1qsma_ | d2aj6a1 | d1y9ka1 | d1i12a_ | d1vkca_ | d2atra1 | d1n71a_ |
| d2d4pa1 | d2fiwa1 | d2fe7a1 | d1tiqa1 | d2ou2a_ | d1yx0a1 | d2fcka1 | d1p0ha_ |
| d2j8ma_ | d2pq8a_ | d1z4ra1 | d1s3za1 | d1ufha_ | d1gvfa_ | d3qm3a1 | d2fjka_ |
| d1f37b_ | d1xvqa1 | d1pn0a2 | d3hy2a_ | d2cvba1 | d1lu4a_ | d1we0a1 | d2bmxa1 |
| d2a4va1 | d1psqa_ | d1xiya1 | d1q98a_ | d1n8ja_ | d1zofa1 | d2ggta_ | d3tjja_ |
| d4je1a1 | d1o73a_ | d3s9fa_ | d2zcta_ | d1qmva_ | d1jfua_ | d2b1ka_ | d5dvba1 |
| d2b5xa1 | d2v2ga_ | d1st9a_ | d3tb2a_ | d4llra_ | d4fh8a1 | d1e2ya_ | d1knga_ |
| d2h30a_ | d1i5ga_ | d2z9sa_ | d2f8aa1 | d1tp9a1 | d2b7ka_ | d1nm3a2 | d3hvva_ |
| d2wfca1 | d4kcea_ | d1xvwa1 | d4gqca1 | d3qpma_ | d4k1fa_ | d1zzoa1 | d1gp1a_ |
| d1prxa_ | d4od7a1 | d1z6ma1 | d4mcua1 | d4dvca1 | d3l9sa_ | d1un2a_ | d3rppa_ |
| d1zsqa1 | d1rmka_ | d1ixta_ | d1eyoa_ | d1fu3a_ | d1g1pa_ | d1f3ka_ | d1fyga_ |
| d2phla2 | d2phla1 | d1fxza2 | d1fxza1 | d1lrha_ | d1uija1 | g1dgw.1 | d2v09a_ |
| d1uika2 | d1uika1 | d1dgwa_ | d1l6ja5 | d1l6ja4 | d1eaka4 | d1eaka3 | d1l6ja3 |
| d1h8pa2 | d1h8pa1 | d1w52x1 | d2ppla1 | d1hpla2 | d1lpbb2 | d2oxea1 | d1bu8a2 |
| d1rp1a2 | d1ra6a_ | d3gsza_ | d1khva_ | d1te9a_ | d2xwha_ | d3uqsa_ | d3hkwa_ |
| d3n6ma_ | d2cjqa_ | d2xyma_ | d1teba_ | d1te8a_ | d1s48a_ | d1u09a1 | d4wfxa_ |
| d3tyqa_ | d2pgga1 | d2h1ia1 | d2r8ba1 | d3b5ea1 | d3cn9a_ | d1auoa_ | d2hqya1 |
| d2hqya2 | d1lrza2 | d1ne9a1 | d1ne9a2 | d1svpa_ | d3su6a1 | d1cu1a1 | d4agka_ |
| d1vcpa_ | d1q31a_ | g1n1l.1 | d3e90b_ | d1ep5a_ | d3u1jb_ | d3zxha_ | d1cgla_ |
| d1rm8a_ | d1eaka2 | d1hy7a_ | d2jsda_ | d1cxva_ | d1hv5a_ | d1eb6a_ | d4dv8a2 |
| d1dzka_ | d4tlja_ | d2a2ua_ | d4wfva_ | d3t1da_ | d4iaxa_ | d3zq3a_ | d2nnda_ |
| d1i4ua_ | d1kxoa_ | d1bbpa_ | d1kt7a_ | d1exsa_ | d3s26a1 | d1lf7a_ | d1qfta_ |
| d4gh7a_ | d1z24a1 | d1qabe_ | d1xkia_ | d3bx7a_ | d4iawa1 | d1n0sa_ | d1gkab_ |
| d3kzaa_ | d2k23a_ | d2l9ca1 | d2joza1 | d1gm6a_ | d3kffa_ | d3saoa1 | d1e5pa_ |
| d1yupa1 | d1iiua_ | d2tbvc_ | d1c8nc_ | d1x36a_ | d1smvc_ | d1ng0c_ | d4sbvc_ |
| d1opoa_ | d1f2nc_ | d1cixa_ | d1y29a_ | d1v7fa_ | d2jtba_ | d1d1ha_ | d1g9pa_ |

| | | | | | | | |
|---|---|---|---|---|---|---|---|
| d1lupa_ | d1qk7a_ | d1mb6a_ | d2a2va_ | d1niya_ | d1i25a_ | d1hvwa_ | d2mpqa_ |
| d1ie6a_ | d1kqha_ | d1la4a_ | d2mxoa1 | d1koza_ | d1qk6a_ | d1emxa_ | d1lqra_ |
| d1c6wa_ | d1js9c_ | d1za7a_ | d1laja_ | d3lgia_ | d3i77a_ | d2z9ia2 | d4nsva_ |
| d1lcya2 | d4jcna_ | d1agja_ | d1os8a_ | d1hpga_ | d1p3ca_ | d1l1ja_ | d1qtfa_ |
| d1ny721 | d1ny722 | d1a6ca3 | d1a6ca2 | d1pgl22 | d1pgl21 | d1pgl11 | d1ny711 |
| d1mqta_ | d1d4m3_ | d1fmd3_ | d2mev1_ | g1aym.1 | d1ev13_ | d4q4w1_ | d1r1a3_ |
| d2x5ia_ | d1rhi1_ | g1r1a.1 | g1d4m.1 | g1rhi.1 | d1b35b_ | d1oopa_ | d1tme1_ |
| d4iv1a_ | g1pvc.1 | g1fpn.1 | d1fpn1_ | d4gb31_ | d1d4m1_ | d1fmd1_ | d1ev11_ |
| d2wzr3_ | g1cov.1 | d1qqp3_ | g1fmd.1 | d2x5ic_ | d1rhi3_ | g1oop.1 | d1hxs1_ |
| g1hxs.1 | d1fpn3_ | d1bev1_ | g2mev.1 | d1eah3_ | d1aym1_ | d1pvc3_ | d1zba3_ |
| d1h8ta_ | d3vbhc_ | g1b35.1 | g1bev.1 | d1hxs3_ | g1ev1.1 | d1bev3_ | g1tmf.1 |
| d1tmf1_ | d1b35a_ | d2mev3_ | g1eah.1 | g1h8t.1 | d1eah1_ | d4q4w3_ | d1r1a1_ |
| d1aym3_ | d1pvc1_ | d1zba1_ | d1h8tc_ | d1oopc_ | d1tme3_ | g1qqp.1 | d4iv1c_ |
| g1tme.1 | d1y14b1 | d2nn6i1 | d1i94l_ | d2zjra2 | d1smxa_ | d2qamc2 | d1hh2p1 |
| d2r7da1 | d1rl2a2 | d1wfqa1 | d3bzka4 | d1x6oa2 | d3a0ja_ | d2j01d2 | d2ja9a1 |
| d4ql5a_ | d1hr0w_ | d1luza_ | d2n3sa_ | d2vnud1 | d1h95a_ | d1go3e1 | d2nn6h1 |
| d2mo1a_ | d1iz6a2 | d1jjga_ | d1e3pa2 | d2ix0a3 | d2ix0a2 | d2lxja_ | d1ueba3 |
| d1ueba2 | d2z0sa1 | d1u0la1 | d2ix0a1 | d2eifa2 | d2je6i1 | d3i2za1 | d2c35b1 |
| d2ahob2 | d1t9ha1 | d3i4oa_ | d2qall1 | d1jt8a_ | d2nn6g1 | d1g6pa_ | d3cama_ |
| d1i94q_ | d1c9oa_ | d1d7qa_ | d2ba0a1 | d2vnud2 | d2vnud3 | d1khia2 | d2qalq1 |
| d1wi5a1 | d1xtda2 | d1ny4a_ | d1eixa_ | d3exra_ | d2ffca1 | d2fdsa1 | d3n3ma1 |
| d2czda_ | d1q6oa_ | d2yyua_ | d1vqta1 | d1dbta_ | d3ldva1 | d3syxa1 | d1mkea1 |
| d2p8va_ | d1xoda1 | d1i7aa_ | d1evha_ | d1egxa_ | d1ux8a_ | d1dlya_ | d4i0va_ |
| d2bkma_ | d1dlwa_ | d2bmma_ | d2r80b_ | d1cqxa1 | d1cg5a_ | d1gcvb_ | d3boma_ |
| d2h8fb_ | d2raoa_ | d3bj1a_ | d4hrrb_ | d2r50a_ | d3d1kb_ | d2aa1b1 | d3zhwa_ |
| d3hf4b_ | d3gdja_ | d1jebb_ | d3bj1b_ | d1x9fb_ | d1wmub_ | d1gcva_ | d3bcqb_ |
| d3d4xb_ | d1gvha1 | d2dc3a_ | d3hf4a_ | d4yu3a_ | d3k8ba_ | d1qpwb_ | d2r80a_ |
| d3bcqa_ | d1v4wa_ | d3wtgb_ | d1jl7a_ | d4esaa_ | d1a9we_ | d3d1ab_ | d1fsla_ |
| d4h2la_ | d1v4wb_ | d1mwca_ | d1vhba_ | d1jeba_ | d1spgb_ | d1h97a_ | d1q1fa_ |
| d1tu9a1 | d3d1aa_ | d1hdsb_ | d3hyub_ | d2zfbb_ | d3fs4a_ | d1oj6a_ | d1shrb_ |
| d4h2lb_ | d4yu3b_ | d1spga_ | d3hrwa_ | d3qqra_ | d3at5a_ | d4wchd_ | d3hyua_ |
| d1cg5b_ | d1uc3a_ | d1hbra_ | d2raob_ | d1outb_ | d4esab_ | d3gdjb_ | d3qqqa_ |
| d1x9fc_ | d3vrfa_ | d1itha_ | d3mkba_ | d3d4xa_ | d3mjpa_ | d1jl6a_ | d3fh9a_ |
| d1outa_ | d2dc3b_ | d1it2a_ | d3eoka_ | d3g46a_ | d1qpwa_ | d3vrfb_ | d1ch4a_ |
| d1x9fd_ | d1hdsa_ | d2zfba_ | d3mkbb_ | d1or4a_ | d3wtga_ | d1a4fa_ | d3fh9b_ |
| d3bomb_ | d3cy5a_ | d3at5b_ | d1x9fa_ | d2oifa_ | d4lmxb_ | d1cpcb_ | d3v57a_ |
| d1kn1a_ | d1cpca_ | d4f0ta_ | d2vjha_ | d4po5b_ | d1eyxa_ | d1alla_ | d4f0tb_ |
| d1b33b_ | d4h0mb_ | d1b33a_ | d2vjhb_ | d4lm6b_ | d2vmlb_ | d1allb_ | d2vjta_ |
| d3dbja_ | d2vmla_ | d2vjtb_ | d4f0ub_ | d1f99b_ | d4rmpa1 | d1liaa_ | d1eyxb_ |
| d2uulb_ | d4lmsb_ | d4f0ua_ | d2bv8a_ | d1f99a_ | d2bv8b_ | d2c7lb_ | d3dbjb_ |
| d4rmpb1 | d1b8da_ | d4h0ma_ | d3v57b_ | d1kn1b_ | d1liab_ | d2uula_ | d2j96a_ |
| d2qked_ | d4ksoa_ | d1t4za1 | d1wwja_ | d3ia2a_ | d1a88a_ | d4dgqa1 | d1zoia_ |
| g1gxs.1 | g1wht.1 | d2xqfa_ | d1ea5a_ | d1f6wa_ | d1qe3a_ | d1k4ya_ | d4qwwa_ |
| d2ha2a_ | d1r88a_ | d1sfra_ | d4qeka_ | d1gl9b1 | d1gkub2 | d1jmxa4 | d1pbya3 |

d1pbya4 d4iuaa3 d4bvva_ d5hpga_ d3k65a_ d1ki0a3 d1ki0a2 d1ki0a1
d1a0ha1 d1pmla_ d3sp8a3 d2feba1 d1xova2 d2m5ta_ d1cqqa_ d1wqsa_
d5bpea_ d2ipha_ d1lvoa_ d2bhga1 d3tnta_ d3sjia_ d3d23a1 d2fyqa_
d3tloa_ d3zvea1 d2hrva_ d3q3ya1 d4asha1 d1g8fa3 d3rp2a_ g1ucy.1
d1fuja_ d2zchp_ d3w94a_ d1si5h_ d1lo6a_ g2pka.1 d1cgha_ d1kigh_
d1a5ia_ d3s9ba_ d1hj8a_ d1ao5a_ d2anya_ d1q3xa1 d1gvza_ d1azza_
d4gsoa_ d2f91a1 d1iaua_ d1gdna_ d1eufa_ g1fiw.1 d1eq9a_ d4nfea_
d2rdla_ d1spja_ d1elva1 d1a7sa_ d1ekbb_ d1pfxc_ d1yc0a_ d2aiqa_
g1fiz.1 d1fi8a_ d4k60a_ d4dgja_ d1mzaa_ d1op0a_ d3tjva_ d1pytd_
d1sgfg_ g1gg6.1 d2eeka_ g1rtf.1 d1a0ja_ d3g01a_ d1pytc_ d4e7na_
d1ddja_ d1z8ga1 d2hlca_ d1npma_ d1brup_ g1jou.1 d1sgfa_ d1autc_
g2hnt.1 g1kdq.1 d1orfa_ d3s69a_ d4crga_ d1fxya_ d1bqya_ d2ftba_
d1pmpa_ d2rcqa1 d1fdqa_ d4lkpa_ d1sa8a_ d1vyfa_ d1lpja_ d3vg7a1
d4azqa1 d1o8va_ d5bvqa_ d1p6pa_ d2qo4a_ d1opba_ d1yiva1 d1o1va_
d4bvma1 d4tkba_ d4i3ba_ d1bwya_ d1ggla_ d4gkca_ d1cbia_ d3p6da1
d1kqwa_ d2a0aa1 d2bk7a_ d3fjua_ d1z5ra1 d1kwma1 d1uwya2 d1jqga1
d2c1ca1 d1dtda_ d1h8la2 d1e69a_ d1mv5a_ d3g60a4 g1ii8.1 d1ewqa2
d1ji0a_ d1qhla_ d1v43a3 d2wmfa1 d1vpla_ d1g2912 d4a82a2 g1f2t.1
d2d62a1 d3tuic1 d2bbsa1 d3g60a3 d2ixea_ d1g6ha_ d1sgwa1 d2qi9c_
d3rlfa1 d3b60a1 d3d31a2 d2pzea1 g1xew.1 d1jj7a_ d1oxxk2 d2onka1
d1w1wa_ d1iwea_ d1qf5a_ d1vmaa2 d1nija1 d1np6a_ d2p67a1 d3ihla_
d1okkd2 d1ls1a2 d1s1ma2 d1iona_ d2v40a_ d3hida_ d2vo1a1 d2g0ta1
d1vcoa2 d1j8mf2 d1g3qa_ d1xjca_ d1p9ba_ d1qzxa3 d1ihua2 d1ihua1
d2qm8a_ d1dj2a_ d4ioja_ d1cp2a_ d1yrba2 d1hyqa_ d3gqba2 d1skye3
d1xp8a1 d1tf7a2 d1tf7a1 d1ye8a1 d1skyb3 d2qe7d2 d4blta_ d4huta_
d3ldaa2 d4hyya_ d2i3ba1 d1cr1a_ d3j9tb2 d3ntua2 d1vdza1 d2z43a_
d1e9ra_ d1nlfa_ d3ziaa2 d1n0wa_ d1g6oa_ d3ziad2 d3j9ta2 d1pzna2
d3gqbb2 d2i49a_ d1nnfa_ d1xvxa_ d1ursa_ d1h3da1 d1iuda_ d4m1va1
d1twya_ d3e13x_ d2nxoa1 d4pfua_ d3ttma_ d1elja_ d2qmwa1 d1us5a_
d1y3na1 d1jeta_ d1uqwa_ d4f19a1 d2esna2 d3thia_ d3fasa1 d2uyeb_
d2xwva1 d1r9la_ d2fyia1 d1pb7a_ d2ozza1 d1sw5a_ d1nh8a1 d1vr5a1
d2g29a_ d1xs5a_ d3lswa_ d2z23a_ d3w9va_ d3tcha_ d1q35a_ d1z7me1
d3q27a_ d1p99a_ d1ve4a1 d2i6ea1 d4mcoa1 d4n8ya_ d1eu8a_ d1zbma1
d1hsla_ d1ixca2 d1j1na_ d1xvya_ d1y9ua_ d2z22a1 d1xoca1 d4oera_
d1wdna_ d5f1qa1 d1xt8a1 d1i6aa_ d4gwoa_ d4igta1 d1pc3a_ d4mmpa1
d1o63a_ d2czla1 d2onsa2 d4mija1 d1eh3a_ d3skpa_ d1dtza1 d1dtza2
d1ieja_ d1jnfa2 d1ce2a1 d1jnfa1 d1b1xa2 d1b1xa1 d1jw1a1 d1jw1a2
d1ryoa_ d1h76a1 d1h76a2 d1c4oa1 d2p6ra3 d2p6ra4 d1oywa2 d1oywa3
d1wrba1 d1w36b2 d1w36b1 d1c4oa2 d3bora_ d1t6na_ d2eyqa5 d2eyqa3
d2eyqa2 d2zu6a2 d1veca_ d1uaaa1 d1w36d1 d2g9na1 d1z3ix2 d2rb4a_
d1hv8a1 d1hv8a2 d1t5ia_ d1w36c2 d2j0sa2 d2j0sa1 d1fuka_ d3hjha1
d1z5za1 d1qdea_ d1z63a1 d1s2ma2 d1s2ma1 d1wp9a1 d1wp9a2 d1gm5a3
d1gm5a4 d3dina4 d3dina3 d1q0ua_ d1w36c1 d1z3ix1 d1nkta3 d1nkta4
d1uaaa2 d1w36d2 g1qhh.1 d2fwra2 d2fwra1 d3q3ma_ d1za0a1 d1mxra_

d2inca_ d1jk0a_ d2incb_ d1mhyb_ d3hf1b_ d1mtyb_ d1r2fa_ d2uw1a_
d1mhyd_ d3n37a_ d1oquc_ d1syya_ d1mtyd_ d2o1za_ d1uzra_ d1smsa_
d2fkza_ d1zuja1 d1s3qa1 d2fjca1 d1z6om1 d1veia_ d4dyya_ d1jiga_
d3gvya_ d2pyba_ d4cy9a1 d3bvfa1 d1r03a_ d2cf7a_ d1nf4a_ d1zs3a1
d4iwka_ d3ka8a_ d3np0x_ d1n1qa_ d1lb3a_ d4e6ka_ d1z6oa1 d2fzfa1
d4dyua_ d3ge4a_ d1jgca_ d4am4a_ d2z6ma_ d1ji5a_ d1vlga_ d1lkoa1
d3qz3a_ d1o9ra_ d2ffxj_ d1euma_ d1yv1a1 d2v2pa_ d1j30a_ d4di0a_
d1dpsa_ d1oewa_ d2qzxa_ d2asia_ d1psoe_ d1miqa_ d1smra_ d2h6ta_
d1pfza_ d1izda_ d3hd8a_ d1j71a_ d1dpja_ d4aa9a_ d1t6ex_ g1b5f.1
g1lya.1 g1htr.1 d1bxoa_ d1wkra_ d1tzsa_ d1ibqa_ d2qzwa_ d1ex4a2
d1c6va_ d1h4ra3 d4yl8a1 d2al6a3 d1wgda1 d1wgga1 d4q5eb_ d1wjna1
d1se9a_ d3n3kb_ d1wz0a1 d1we6a1 d2m0xa_ d2gbka_ d3rt3b1 d1wh3a1
d1sifa_ d2ekec2 d4ii2b1 d4hk2a1 d1c3ta_ d1m94a_ d1wgha1 d1ttna1
d1j8ca_ d1wx8a1 d3v6cb1 d2ma9b_ d4i6lb1 d1bt0a_ d2jvca1 d1iyfa_
d2k1fa_ d1wxva1 d1wf9a1 d1t0ya_ d2io0b2 d1x1ma1 d2gbma_ d1zw7a1
d2m17a_ d1ud7a_ d1we7a1 d2lwpa_ d2gbja_ d1wjua1 d1v6ea1 d1v86a1
d4eewa_ d1wiaa1 d4icva_ d4gu2a_ d1wx7a1 d1wy8a1 d4hjka1 d2faza1
d1v5oa1 d3ubra_ d1d4ca1 d2k3va_ d1ft5a_ d1qdba_ d1m1qa_ d2j7aa_
d1h21a_ d1ogyb_ d4fasa_ d1jnia_ d1oaha_ d1sp3a_ d3h33a_ d1rwja_
d19hca_ d1os6a_ d3bxua_ d3h4na_ d1i77a_ d3caoa_ d1j0pa_ d2ctha_
d1duwa_ d1ofwa_ d2e84a_ d1gyoa_ d3h34a_ d1iyka2 d1rxta1 d1iica2
d1iica1 d1wioa4 d1ccza2 d1vcaa1 d1dr9a2 d1ic1a1 d2d9qb1 d2nxyb2
d1um8a_ d1qvra2 d1qvra3 d1qzma_ d1sxjc2 d1jr3d2 d2p65a_ d1l8qa2
d1g8pa_ d3hu3a3 d1sxjd2 d1njfa_ d1in4a2 d1sxja2 d1tuea1 d2ce7a2
d1w5sa2 d1sxjb2 d1d2na_ d1ny5a2 d1ofha_ d2gnoa2 d2a5yb3 d1fnna2
d1u0ja1 d1lv7a_ d1iqpa2 d2fnaa2 d1ixsb2 d4gita_ d1sxje2 d1g41a_
d2e3fa_ d1mm0a_ d2e3ea_ d2llda_ d2ny8x_ d2e3ga_ d2ny9x_ d2nz3a_
d2lj7a_ d1n4na_ d2m8bb1 d2kyqa_ d3psma_ d1jkza_ d1jxca_ d2gl1a_
d1ugla_ d4uj0a_ d2k4ua_ d1hp2a_ d2lu9a_ d1baha_ d2meoa1 d1wm7a_
d1hlya_ d1n8ma_ d1jlza_ d1wpda_ d1pvza_ d1wt7a_ d1rjia_ d1wz5a_
d1m2sa_ d1pjva_ d1c49a_ d1qkya_ d2ktca1 d1wmta_ d1du9a_ d1j5ja_
d1r1ga_ d1t0za_ d2i61a_ d1t1ta_ d1sn4a_ d1kv0a_ d2lkba_ d1npia_
d1wwna_ d1pe4a_ d2kbka_ d2li7a_ d1djta_ d2kbha_ d1sega_ d1omya_
d1zywa1 d1jzaa_ d1iyjb4 d1iyjb5 d1iyjb3 d1o7ia_ d4ipca1 d1x3ea_
d4apva_ d1jb7a1 d1jb7a2 d1jb7a3 d3kdfa_ d1ue1b_ d1wjja1 d3eiva_
d1xjva2 d1xjva1 d2ccza2 d1se8a_ d1l1oc_ d1jb7b_ d1qvca_ d3ulla_
d1qzga_ d1miua4 d1miua5 d1miua3 d3g48a1 d1gd7a_ d1mkha_ d1jjcb3
d1pyba_ d3ubub_ d1egia_ d1qo3c_ d1v7pa_ d1tdqb_ d3ubua_ d1c3ab_
d1h8ua_ d3bx4b_ d1t8ca1 d1sb2a1 d1qdda_ d2ziba1 d3cada_ d1byfa_
d1j34b_ d1jwia_ d1jzna_ d1c3aa_ d1oz7b_ d1wmza_ d3bdwb_ d1hq8a_
d1fifa1 d1dv8a_ d3hupa_ d1wk1a1 d1umra_ d2afpa_ d3bx4a_ d1gz2a_
d1sl6a_ d1v7pb_ d3g8ka_ d1ixxa_ d1j34a_ d1jwib_ d1g1sa1 d1y17a1
d1ja3a_ d1fvub_ d3bdwa_ d1sb2b1 d1fvua_ d1oz7a_ d4m17a_ d3cfwa1
d1ypqa1 d1g1ta1 d3fd4a_ d3l9jc_ d4dn8a1 d1v4lb_ d1rdl1_ d2msba_

d1xwdc1 d3kj4a_ d1ozna_ d1w8aa_ d2ifga3 d1xkua_ d1rpma_ d2shpa1
d1yfoa_ d1l8ka_ d2ooqa1 d1g4us2 d1lara2 d1lara1 d1wcha_ d2fh7a2
d2fh7a1 d1ohea1 d1ohea2 d2f4da_ d1rxda_ d2hxpa_ d1xria_ d1d5ra2
d1fpza_ d3ezza_ d4y2ea_ d1i9sa_ d1c1la_ d4lbqa_ d1a78a_ d1slta_
d4no4a_ d3zsja_ d3duia_ d1g86a_ d1qmja_ d1hlca_ d1tdha3 d1q3ba3
d1ee8a3 g1qmo.1 g1len.1 d3zyra_ d1fnya_ d1n47a_ g2ltn.1 d1avba_
d3usua_ d1dhkb_ d1f9ka_ d1ukga_ d1g8wa_ d1g9fa_ d1wbfa_ d3wcsa_
d1g7ya_ g1loe.1 d1mvqa_ d1hqla_ d1v6ia_ d1dbna_ d1qnwa_ d1fx5a_
d4l8qa_ d1ioaa_ d3n35a_ d1eysc_ d2h0ba_ d2erfa_ d1h30a2 d1h30a1
d3mw4a1 d1pz7a_ d2r1ba_ d3v64a1 d1d2sa_ d3bopa1 d3mw3a1 d1okqa1
d1okqa2 d1dszb_ d1a6ya_ d3cbba_ d1ynwa1 d1r4ia_ d1hcqa_ d2hana_
d2hanb_ d2enva1 d1kb4b_ d2nllb_ d1lo1a_ d1adqa1 d1fnga1 d1uvqb1
d1zs8a1 d2vola1 d2vola2 d1mfbl2 d2jelh2 d1uwel2 d3qq4b1 d1kjvb_
d1k5na1 d1osph2 d4odhl2 d1ow0a1 d1ow0a2 d1bfob2 d3l9ra2 d3jtth1
d2fath2 d4elkb2 d1fn4b2 d2xfxb_ d1pg7x2 d1cqka_ d4k71b2 d1lk2a1
d1eapb2 d4lfhg2 d1i4fa1 d2nw2a2 d1kfah2 d1mhca1 d1h3ua1 d1wejh2
d1h0db2 d1a5fh2 d1kcvh2 d1mjuh2 d1qqda1 d1kcrh2 d1k8ia1 d3huja2
d2nnab1 d1nfdf2 d3mffb2 d1s9va1 d1lk3h2 d1q0xl2 d1fv1b1 d1neza1
d4rfeb2 d1r3ha1 d1a6tb2 d1kjva1 d1pz5b2 d1onqa1 d2bc4a2 d1de4a1
d1k8da1 d1tcra2 d1dn0b2 d1nfde2 d2xwtb2 d1bfoa2 d1mexh2 d1iakb1
d1ypzf2 d4f7ea2 d1q72l2 d2fbjh2 d1fe8h2 d1clzh2 d1fngb1 d1uvqa1
d1vgka1 d1c16a1 d4j12a2 d4x99b2 d1igtb3 d1je6a1 d1q72h2 d1nldh2
d1teth2 d2hmic2 d1i1ca2 d1i1ca1 d1k8ib1 d1q0yh2 d1kfal2 d3frua1
d1pg7w2 d1u58a1 d1igyb4 d1igyb3 d1hyrc1 d1klub1 d1deeb2 d1c12b2
d2bc4b2 d2ig2h2 d1kcvl2 d3wn5b1 d1lk2b_ d1lk3l2 d1mjul2 d1sm3h2
d1q0xh2 d1zvsa1 d1ydpa1 d1u8ib2 d1ypze2 d1kcuh2 d1op9a_ d1y0la1
d1etzb1 d1egjl1 d2gsib1 d1nj9a1 d4jfhd1 d1aqkl1 d1t2ja_ d1lo4h1
d1zvya1 d3v4vl1 d4enec1 d1kxtb_ d5e8ea1 d2pxya_ d4weud_ d1xiwc_
d2esvd1 d1qnzh_ d1ogae1 d1f4xh1 d4n1hb_ d3ezjb1 d3mlwl1 d1kfah1
d1etza1 d1b4jh1 d3cfic_ d4w68a_ d2arja1 d1ek3a_ d2ok0l1 d1vera_
d1uwgh1 d1blnb1 d1fpth1 d1rzga1 d2giya1 d4m3kb1 d1nc2a1 d2nyyd1
d1ncna_ d1fbih1 d5dxwa_ d4fqqa1 d1dqdh1 d1jfql1 d1kelh1 d1faih1
d1frgl1 d1figl1 d4m6nl1 d1fo0a_ d4hxbl1 d3b5ga_ d3quyd1 d2h9gb1
d1i8kb_ d2dd8h1 d2ck0h1 d1eaja_ d4w6wb1 d2a6ib3 d1t66d1 d2e27h_
d1ob1b1 d1f11b1 d2v7na1 d2xt1b1 d1pskh1 d4i13b1 d2op4l1 d1a0ql1
d1uwel1 d1za3a1 d1ncbl1 d4uu9a_ d1bm3h1 d1cloh1 d1nqba2 d1cf8h1
d4y5va_ d1mhph1 d5anma1 d4cdgc1 d4ldeb_ d1y0lb1 d2pcpb1 d1z3gl1
d1dfbl1 d1fh5h1 d3g5xa1 d1tzib1 d4dn3l1 d3zhka1 d2b1hh1 d2uyla1
d1f3dh1 d1nj9b1 d2agjh1 d2h1pl1 d1q72h1 d8fabb1 d1ndmb1 d1rjca1
d4u6hb1 d3upca_ d2fbjl1 d1osph1 d1tvda_ d2cjvh_ d4fhbd1 d1bd2d1
d1rz7h1 d3nn8a_ d1blna1 d1knoa1 d1nfda1 d2bmkb1 d1dqll_ d1igyb1
d1igfl1 d12e8h1 d3utza1 d1nc2b1 d1rihh1 d4mwfb1 d4jvpa1 d1mieh1
d1rhfa1 d1fgnl1 d1sbsh1 d1lp9e1 d1dn0a1 d4lqfl1 d1iaih1 d1kgcd1
d1mh5b1 d1gpol1 d2g60h3 d1f8tl1 d2jixd1 d4grwh_ d1fo0b_ d1bfob1

d1axsa1 d1u9ka_ d1nmbh_ d1h8na1 d1h8na2 d1jguh1 d4eura1 d3sdyl1
d35c8h1 d1w72l1 d1mvub_ d1i9rh1 d1ob1a1 d4wenb_ d1yeeh1 d3ietc1
d1f58h1 d1ad0b1 d1mqkl_ d1nbvh1 d4b50a_ d3r8bb_ d1bafl1 d1mamh1
d4rgne1 d2gsgb_ d35c8l1 d1bafh1 d1ai1h1 d2pcpa1 d2fath1 d2x1pa_
d1orsb1 d1ibgl1 d1shma_ d1a2yb_ d1u0qa_ d2coqa_ d1hilb1 d1qfwi_
d1jv5b_ d1fn4b1 d5e0qa_ d1nsnh1 d1plgl1 d4ocxl1 d1kb5h1 d1rzfh1
d1pg7x1 d2fx7h1 d2oi9c_ d1l7il1 d3eyva1 d1d5il1 d4ffyl_ d1tetl1
d1a6wh_ d1ac6a_ d3dvfa_ d1nfde1 d1mimh1 d1ri8a1 d1jhlh_ d3dura_
d1fskb1 d1f97a1 d3so3b1 d2ak4d1 d1lp9f1 d4z7wf1 d1l7th1 d4y5ya_
d4x7ec1 d1emth1 d1ikfl1 d1nlbh1 d4utal1 d1bjma1 d1eapb1 d1u6ah1
d2dtgd1 d1kj2b_ d1xeda1 d1c1eh1 d1fgvh_ d2bnub1 d2fb4h1 d2dtgb1
d1ktrh_ d1jpth1 d1qkzl1 d4hepg_ d2atpb1 d1flrh1 d2hrpl1 d1mvfa_
d1iaim1 d3k80a1 d2jb5h1 d3zkxc_ d4grwe_ d1it9h1 d1rhhb1 d3w9ec_
d1e4wh1 d1sy6h1 d2j6eh1 d3qxta_ d1jglh1 d1p7ka1 d1tzhb1 d4n8pa1
d1gafl1 d2jelh1 d1wioa2 d4i0cc1 d1cicb1 d1lmka2 d1lmka1 d1iqwh1
d1orqb1 d1ejoh1 d1jnlh1 d4zg1a_ d43c9b_ d1ggbl1 d1wejh1 d3q6fa1
d1f3dj1 d3c6la1 d1bqlh1 d3p9wb_ d1a3rl1 d1h0db1 d2uzil_ d1eo8l1
d1qfwl_ d1n7ml1 d1igja1 d1c5ch1 d2a9mh_ d1rmfh1 d1fj1b1 d4heme_
d1ccza1 d1a5fh1 d1ogad1 d1dlfh_ d1qa9b_ d1vgel1 d3qskb_ d1sjxa_
d4ozfg1 d1sq2n_ d1qd0a_ d1kcvh1 d1c5db1 d3qcul1 d8faba1 d3bn9d1
d1mjuh1 d4y8dc_ d7fabh1 d3dvgb1 d1lo0l1 d1a6tb1 d1gigh1 d1kcrh1
d2hwzl1 d1yjdc1 d3clfl1 d4krna_ d1dl7l_ d1e6ol1 d1mexh1 d3r0ma_
d4kkna_ d1fnsl1 d1oaqh_ d1rzia1 d1ucbh1 d1ncwl1 d4b41a_ d1nfdf1
d2mpah1 d2d7tl1 d4grwf_ d4b5ea_ d1lk3h1 d3u79a_ d4u3xa_ d1ghfl1
d1bvkb_ d1mcww1 d1b88a_ d1jrhh1 d32c2b1 d4wemb_ d1dsfh_ d1op3h1
d1ce1l1 d1iqda1 d1qoka1 d1qoka2 d1qfuh1 d2crya1 d1ay1h1 d1oaql_
d3ab0b_ d1rurl1 d1t4kb1 d1bz7b1 d1n0xh1 d1forl1 d1ucbl1 d4w6yb_
d43c9a_ d2aabl1 d1tzia1 d4n1ca_ d1k6qh1 d3g9ab_ d4tvsa1 d1igmh_
d1pz5b1 d3dx9a1 d3wxwh_ d1nakh1 d1kcul1 d3eaka_ d4qlra1 d1cr9l1
d1d9ka_ d1f4xl1 d1deea1 d2i27n2 d1c12a1 d2orbl1 d1j8hd1 d1mcph1
d1c5da1 d1kenh1 d1bd2e1 d1h5ba_ d4x7fc1 d3upaa_ d1t3fa1 d1indh1
d2dtgc1 d4hc1l1 d1zv5a1 d4wgvb_ d1uwgl1 d4f37k1 d1aqkh1 d1vesa_
d3zkqd_ d1fptl1 d1lo4l1 d1knob1 d3rrqa_ d1ct8b1 d4orzc_ d1mlbb1
d4hgkc1 d1dqdl1 d1bogb1 d4ozhe1 d1dr9a1 d1i7za1 d1dn0b1 d1kell1
d1rzib1 d1ypzf1 d1wtla_ d1hkfa_ d1fail1 d1figh1 d2vyre_ d1sjva_
d1ohqa_ d1bfoa1 d1pkqb1 d1mjul1 d1b4jl1 d1nsnl1 d2arjb1 d1p4bh_
d4aj0a_ d3nn8b_ d2rcja1 d1f2xk_ d4v1dd_ d1adqh1 d2eksa_ d4k3ga_
d1jfqh1 d1uweh1 d1frgh1 d4gftb_ d1uz8b1 d4n1ea_ d1bm3l1 d1clol1
d3mcka1 d2vq1a1 d1ae6h1 d4aq1b1 d1uj3b1 d1nbqa1 d1fh5l1 d2ck0l1
d2ywza_ d1q72l1 d4nbxb_ d1hila1 d4hzlb1 d2j88l1 d1zmya1 d1cd0a_
d1a4ka1 d1pskl1 d1a0qh1 d4krmb_ d1ncbh1 d2fbjh1 d1j8he1 d1i3ua_
d1cf8l1 d1fe8h1 d2p1ya1 d1eeqa_ d2x89a_ d1ol0a_ d2r0lh1 d1ub5a1
d1dfbh1 d3eyvb1 d4jdvb1 d4p2cg_ d2gsia1 d2agjl1 d1u3ha1 d2h1ph1
d1rihl1 d1fskc1 d1boga1 d2a77l1 d1ospl1 d1qfoa_ d3dwta_ d2fx7l1

d1rz7l1 d3stba1 d1pkqa1 d1b6da1 d1dzba2 d1dzba1 d1kj2a_ d4l1ha_
d1rzgb1 d1dqlh_ d12e8l1 d1miel1 d1jmaa_ d1sbsl1 d1fgnh1 d1f58l1
d1mqkh_ d1ndgb1 d3qegl1 d1iail1 d2aw2a1 d1tqbc1 d3fo2a1 d4dkaa_
d1f8th1 d2g60l1 d2ywya_ d1uj3a1 d1yc7a_ d3bd3a_ d4w6xb1 d1nmbl_
d1i8ka_ d1rjlb1 d1ypze1 d1p7kb1 d1kxqe_ d1gpoh1 d2z4qa1 d1w72h1
d1i9rl1 d4qgya1 d4xt1c_ d2qqnh1 d1cica1 d3c08h1 d2cd0a_ d2bdnh1
d1hnga1 d1maml1 d1nldh1 d1cr9h1 d4lcih1 d3bkyh1 d3qg7l1 d1ai1l1
d1kxvc_ d4iosd_ d1l7ih1 d2x1qa_ d1ub6b1 d3k74b_ d1ibgh1 d1teth1
d1igya1 d1hxma1 d2x6ma_ d2e7la_ d5e03a_ d1qfwm_ d2r0kh1 d1igjb1
d1rurh1 d1plgh1 d1mj8h1 d1yuhb1 d3fmgl1 d1rzfl1 d1emtl1 d1ad9b1
d1igtb1 d1nlbl1 d1d5ih1 d3k7ua_ d4ffyh_ d1bwwa_ d3dvga1 d1a6ta1
d1fgvl_ d4qxgl1 d1miml1 d1jhll_ d2hffb1 d2hh0l1 d1qkzh1 d1l7tl1
d1afvh1 d1iaii1 d2cjuh_ d1um5h1 d2ghwb2 d2ghwb1 d1kgce1 d2fgwh1
d1ikfh1 d1dvfd_ d2i24n1 d2xtjb_ d1za3b1 d2yc1a_ d2hh0h1 d2x1oa_
d1bvka_ d1rjla1 d5e7ba1 d1igta1 d1il1b1 d2fb4l1 d1mvua_ d1jptl1
d32c2a1 d1fvca_ d1iqdb1 d2hrph1 d2p4ab_ d4qo1a_ d1p2cb1 d1wt5c_
d1t4ka1 d5c1mb_ d1it9l1 d1bz7a1 d1b2wh1 d1e4wl1 d1jnll1 d1yedb1
d1za6b1 d3u7aa_ d1igfh1 d3gnml1 d1orsa1 d1wejl1 d1a2ya_ d1hxmb1
d1gafh1 d2j4wh1 d1pz5a1 d1j05a_ d1a3rh1 d4fqll1 d1qfwh_ d1eo8h1
d1n7mh1 d4s11a_ d1kegh1 d5da4a_ d1iqwl1 d3whxb1 d1vgeh1 d1deeb1
d1c12b1 d1t2ql1 d1ad9a1 d3gsnb1 d3b2uc1 d1ggbh1 d1t3fb1 d1nakl1
d4ocmc_ d1lila1 d2v7hb3 d2esve1 d1mcoh1 d2qr0b1 d2q3aa_ d1ct8a1
d1kcrl1 d2pxyb_ d2uzih_ d5c3ld_ d3durb_ d1c5cl1 d2ak4e1 d1dl7h_
d1xiwd_ d1mlba1 d1rmfl1 d1i7zb1 d1dlfl_ d2xa3a_ d5bq7b1 d1dqqb1
d3h42l1 d1egjh1 d1kcvl1 d1fe8l1 d2j6el1 d1f3rb2 d1lk3l1 d1sm3h1
d1q0xh1 d1ghfh1 d7fabl1 d1igch1 d1lo0h1 d4idla_ d5c2bl_ d1jrhl1
d1il1a1 d3zhda1 d4kfzc_ d1b0wa_ d1e6oh1 d1mexl1 d4krpb_ d1fnsh1
d1ncwh1 d4hgma_ d4eigb1 d4rzca_ d2mpal1 d4y5xa_ d5en2b1 d3lrga1
d4ej1c1 d1rhha1 d1j05b_ d1forh1 d1q9wb1 d2wzpd_ d2aq2a1 d1h0da1
d3revb1 d2ij0c1 d1tzha1 d4m7jl1 d1k6ql1 d3b9kc1 d1iglm_ d3qeua1
d1dsfl_ d1ce1h1 d1kcuh1 d1zvha1 d1zvoa1 d1tqbb1 d1ay1l1 d2qscl1
d2cgrh1 d1nl0h1 d1kenl1 d3eo0a1 d4n9ob1 d1n0xl1 d3k1kc_ d1c4qa_
d1prtc1 d2wv6d_ d1ltrd_ d4l63b1 d1r4pb_ d1prtd_ d1tl0f_ d2bosa_
d1prtb1 d3chbd_ d2aq2b1 d1ck1a1 d1enfa1 d1i4pa1 d1v1oa1 d1et9a1
d1esfa1 d2g9hd1 d4ohja1 d1klud1 d1bxta1 d1ty0a1 d1fnua1 d2cqva1
d1fhga_ d2k1ma_ d1biha3 d1biha1 d1biha4 d3qp3a1 d1g1ca_ d1gxea_
d1cvsc2 d1cvsc1 d1xmwa2 d1m4ka2 d2otpa2 d1biha2 d1rhfa2 d2fcba2
d2fcba1 d1wwbx_ d1gsma2 d1epfa1 d1epfa2 d2dava1 d1waaa_ d1f97a2
d1x44a1 d1vcaa2 d1fnla2 d1fnla1 d3b5ha2 d3h8na2 d3h8na1 d1jbja1
d1jbja2 d1pd6a_ d1f2qa2 d1f2qa1 d3cx2a_ d1nbqa2 d3b5ha1 d1n26a1
d1xiwb_ d1xaua_ d1fltx_ d2ifga1 d1olla1 d1olla2 d1cs6a4 d1cs6a3
d1cs6a2 d1cs6a1 d2gw5a2 d1sy6a1 d1xiwa_ d1ucta2 d1ucta1 d1ry7b2
d1gl4b_ d1qz1a3 d1olza1 d2c9aa1 d1wwca_

**Family-12 (a.3.1.1)**

**Positive dataset contains 10 protein sequences.**

d4gedb_ d3zooa_ d2bh4x_ d2aiua_ d2zboa_ d3dmia_ d1wvec_ d2yk3a_ d3ph2b_ d5aura_

**Negative dataset contains 4078 protein sequences.**

d1m32a_ d1ibja_ d1iuga_ d4q75a_ d2bkwa1 d1t3ia_ d1n8pa_ d1qz9a_ d1jf9a_ d1eg5a_ d1p3wa_ d3kgwa_ d1cl1a_ d2ctza1 d1vjoa_ d2rfva_ d1gc0a_ d1cs1a_ d1d2fa_ d2ch1a1 d1qgna_ d1pffa_ d1elua_ d1orva2 d1xfda2 d1u08a_ d1lc5a_ d1b5pa_ d2ay1a_ d1bw0a_ d3pd6a_ d7aata_ d2csta_ d1fg7a_ d4wlha_ d1o4sa_ d1v2da_ d1gdea_ d1m6sa_ d1v72a1 d1svva_ d1ajsa_ d1wsta1 d2f8ja2 d1lk9a_ d1vp4a1 d1xi9a1 d2gb3a1 d1yaaa_ d1xkla_ d2wfla_ d1xkta1 d2hfka_ d1jmkc_ d5d3ka_ d2cfba1 d1dfoa_ d1o69a_ d1bj4a_ d3k28a_ d2bwpa_ d3lg0a_ d2fnua_ d3ou5a_ d1z7da1 d1b9ha_ d1iaya_ d4msoa_ d1bs0a_ d1s0aa_ d1sffa_ d3tqxa_ d1mdoa_ d3qboa_ d3pgya_ d1bjna_ d2dkja_ d1ejia_ d1vefa1 d4wxga_ d3bs8a_ d3gbxa1 d1w23a_ d1sefa_ d1sfna_ d1rc6a_ d1r0ka3 d1j5pa3 d1vkoa2 d1vm6a2 d3qvsa2 d1gr0a2 d1p1ja2 d1ddla_ d1e57b_ d1auyb_ d1f56a_ d1bypa_ d1azca_ d1ws8a_ d3u25a_ d1cuoa_ d1id2a_ d1rkra_ d2iaac_ d4dp1x_ d1nwpa_ d2gima_ d2i7sa_ d1qhqa_ d3ehbb1 d1fftb1 d1gyca3 d1hfua2 d1kbva1 d1kbva2 d1sdda1 d1sdda2 d1gyca1 d1gyca2 d1mzya1 d2bw4a1 d1aoza1 d1aoza3 d1aoza2 d1kyaa2 d1kyaa3 d1kyaa1 d1mzya2 d4e9sa1 d1sddb1 d1sddb2 d1hfua1 d1hfua3 d1oe1a2 d1oe1a1 d1v10a2 d1v10a3 d1v10a1 g1f8v.1 d1novc_ d2bbvc_ g1or0.1 g1cp9.1 g1e3a.1 d1iknc_ d3brda1 d1p7hl1 d1oy3c_ d1imhc1 d1gjia1 d1zk9a1 d2cxka1 d2bema_ d1pama1 d1ea9c1 d1qhoa1 d2fhfa2 d2fhfa1 d1k3ia1 d1j0ha1 d1cxla1 d2bhua1 d1edqa1 d1f1sa2 d1ut9a2 d1gvia1 d1txka1 d4alac_ d1pjwa_ d1s6na_ d3uzqb_ d2phla1 d1fxza2 d1fxza1 d1lrha_ d1uija1 g1dgw.1 d2v09a_ d1uika2 d1uika1 d1dgwa_ d1ktja_ d1ds6b_ d1fstb_ d1ex0a1 d1g0da1 d1p8ba_ d1ku5a_ d1b67a_ d1tafa_ d1bh9b_ d1tafb_ d1jfib_ d1q9ca_ d1h3ob_ d2byka1 d1n1ja_ d4g92b_ d1bh9a_ d1jfia_ d1h3oa_ d2bykb1 d1ydwa2 d2nvwa2 d1h6da2 d1zh8a2 d1qkia2 d1xeaa2 d1tlta2 d1e3oc2 d1ic8a2 d2ovga_ d1sq8a_ d1rzsa_ d3kz3a_ d3orca_ d3gpdg2 d2q49a2 d1hdgo2 d2g17a2 d2cvoa2 d2czca1 d3pwka2 d1obfo2 d1i32a2 d1zyao2 d2pkqo2 d1b7go2 d2hjsa2 d1cf2o2 d1vkna2 d1nvmb2 d2msya_ d2djoa1 d2r5za_ d1au7a1 d1f43a_ d2lmda1 d1akhb_ d1s7ea1 d3nara_ d1zq3p1 d2craa1 d1mija_ d4uuta_ d3a01a_ d1x58a1 d3rkqa1 d3cmya1 d2cqxa1 d2da6a1 d1e3oc1 d1fjla_ d1mh3a1 d1ig7a_ d1x41a1 d1wh7a1 d1gt0c1 d2mw8a_ d1lfup_ d1x2ma1 d1b72a_ d1wi3a1 d1wjha1 d1x2na1 d1mnmc_ d2cuea1 d3k2aa_ d2hosa_ d2jwta2 d1pufa_ d1octc1 d2hi3a_ d1b8ia_ d4j19a_ d3lnqa_ d1wh5a1 d1b8ib_ d1fexa_ d2cqra1 d1guua_ d1ug2a1 d2cqqa1 d2ajea1 d4a69c_ d1h8ac1 d2crga1 d2iw5b1 d1xc5a1 d1ofcx1 d2cu7a1 d1wgxa1 d2cjja1 d2ckxa1 d1gvda_ d1hcra_ d1tc3c_ d1gdta1 d1u78a2 d1hlva2 d1iufa2 d1iufa1 d2iu5a1 d1zk8a1 d2g7sa1 d2fd5a1 d2np5a1 d1z0xa1 d2id6a1 d2d6ya1 d2i10a1 d1vi0a1 d2oi8a1 d3c07a1 d2ns7a1 d2fq4a1 d2yvea1 d1t33a1 d2o7ta1

d1rkta1 d2np3b1 d2g7ga1 d2gena1 d2fbqa1 d2g7la1 d2gfna1 d2g3ba1
d2jj7a1 d2hyja1 d1sgma1 d1ui5a1 d2hkua1 d2id3a1 d4jyka1 d1bl0a1
d1bl0a2 d1d5ya2 d3lv8a_ d1s3ga1 d1zaka1 d1s4qa_ d3tvta2 d1uf9a_
d1khta_ d1ckea_ d1osna_ d1p5zb_ d4tmka_ d3cm0a_ d1tmka_ d1jaga_
d3h86a_ d1t0hb_ d1nn5a_ d2h92a_ d3kb2a1 d2c95a1 d1yj5a2 d1kgda_
d1ly1a_ d1vyva2 d1deka_ d2grja1 d1p3ja1 d1y63a_ d1e4ya1 d1s96a_
d3iija1 d1lvga_ d1nksa_ d1vyua2 d4e22a_ d1qf9a_ d1jjva_ d1q3ta_
d1ki9a_ d1lw7a2 d1teva_ d1kjwa2 d1gsia_ d1m7ga_ d1x6va3 d1g3ma_
d1j99a_ d1t8ta1 d1nsta_ d1fmja_ d1zrha_ d1ls6a_ d2a3ra_ d1aqua_
d2zptx_ d3ckla_ d1texa_ d1q44a_ d2gwha_ d1z0ja1 d3umra2 d3gj0a1
d2gaoa1 d1yzga1 d1x3sa1 d1g7sa4 d1puja_ d1udxa2 d1kk1a3 d2o52a_
d1jwyb_ d1zcaa2 d1zcba2 d2fv8a1 d2cjwa_ d3dz8a1 d1xzpa2 d1vg8a_
d4qxaa_ d2ew1a1 d1x1ra1 d2bcgy_ d3doea1 d3cona_ d3rega_ d3wyga_
d1upta_ d2b6ha1 d1zd9a1 d1lnza2 d1r5ba3 d1n0ua2 d1r2qa_ d2f9la1
d2rhda_ d3q3jb_ d3q85a_ d1efma1 d1r8qa_ d2gf9a1 d2akab1 d1ek0a_
d1z08a1 d2gcoa1 d2dy1a2 d4g01b_ d2cxxa1 d1svia_ d3q72a_ d1ni3a1
d4ac9a4 d2erxa1 d2bmea_ d1wxqa1 d2gf0a1 d4ldja1 d1zunb3 d1d5ca_
d4lv5b_ d1efca3 d1kmqa_ d2g6ba1 d3lrpa_ d1tada2 d2y8ea_ d4rd4a1
d1z06a1 d2heia_ d3a58b_ d1u0la2 d1d2ea3 d1zj6a1 d2ocba_ d3rwoa_
d1mr3f_ d2e9sa1 d4u5xa1 d2iwra_ d2fn4a1 d3bwdd_ d2nzja1 d2atva1
d2ksqa_ d3l43a_ d1mkya2 d1mkya1 d1wmsa_ d1jnya3 d2rmka_ d1f6ba_
d1f60a3 d1gota2 d1jala1 d2fh5b1 d1g16a_ d2wblc_ d1m7ba_ d1wf3a1
d1f5na2 d1t9ha2 d1z6xa1 d1z0aa1 d2fg5a1 d2a5ja1 d2bcjq2 d1s0ua3
d1ky3a_ d4y0va_ d4lhwa_ d2qmea1 d2atxa1 d1egaa1 d1ryha_ d1moza_
d1nrjb_ d2erya1 d3bfka_ d1puia_ d3j3az1 d3t5ga_ d1shza2 d3defa_
d2f7sa1 d3raba_ d3tsoa_ d3aq4a_ d2al7a1 d3sfva1 d3bc1a_ d2ncda_
d1sdma_ d3h4sa1 d2heha_ d2grya_ g3kin.1 d1kk8a2 d1br2a2 d1goja_
d1f9va_ d2mysa2 d1lkxa_ d3t0qa_ d4db1a1 d3gbja_ d1ry6a_ d1v8ka_
d1k8ka2 d3uleb1 d3i33a2 d3i33a1 d1bupa1 d1dkgd2 d1dkgd1 d4ci6a2
d1jcfa1 d4a2ba2 d2fxua1 d1yaga2 d1yaga1 d4cbua2 d1bupa2 d1ig8a1
d1czan2 d1czan3 d1czan1 d1ig8a2 d1czan4 d1bg3a4 d1bg3a3 d1bg3a2
d2d0oa3 d2d0oa2 d1nbwa3 d4zzva_ d2xspa_ d4v20a_ d1ojja_ d4ipma1
d1gpia_ d4csia_ d3pfza_ d3ovwa_ d2yoka_ d2nqya_ d1h4ga_ d1hixa_
d1oa2a_ d1olra_ d3b7ma_ d4h7ma1 d3wp3a_ d2vgda_ d1igoa_ d3zsea_
d2nlra_ d3wx5a_ d1oa3a_ d1pvxa_ d1m4wa_ d1f5ja_ d1oa4a_ d1ks5a_
d4ixla1 d2ah2a1 d1evsa_ d1cnt1_ d1pvhb_ d1z7ca_ d1huwa_ d1bgea_
d1rhga_ d1f6fa_ d1f45b_ d1axia_ d1etea_ d3tgxb_ d1scfa_ d2z3qa1
d3qb1a_ d1hula_ d1m48a_ d4nkqb_ d1eera_ d1v7mv_ d5e4ea_ d2o27a_
d3b5ka_ d2psma1 d4zf7a_ d3uf5a_ d4x51a_ d1d9ca_ d4ypgc1 d3oq3a_
d1lqsl_ d1n1fa_ d1au1a_ d1wu3i_ d3besl_ d3ux9a_ d4dnxa2 d1m8pa2
d1v47a2 d1g8fa2 d1x6va2 d2el7a_ d1iq0a2 d1irxa2 d1r6ta2 d1ivsa4
d2cyba_ d1rqga2 d1j09a2 d2yxna_ d1f7ua2 d1h3fa1 d2g36a1 d1nzja_
d1jila_ d1n3la_ d1i6la_ d1pfva2 d1h3na3 d3n9ia_ d3sz3a1 d3sw2a_
d1moxc_ d1dx5i3 d1edmb_ d1autl2 d1autl1 d1nqlb_ d1dval1 d1szba2

| | | | | | | | |
|---|---|---|---|---|---|---|---|
| d1kigl_ | d2y5fl_ | d1ijqa2 | d1gk5a_ | d1g1sa2 | d1nt0a3 | d1lmja2 | d1lmja1 |
| d1nzia2 | d2wphe_ | d1klil_ | d1pfxl2 | d1pfxl1 | d1p9ja_ | d1g1ta2 | d1f7ea_ |
| d4yzub_ | d1gl4a2 | d1qx2a_ | d2y5ia_ | d1psra_ | d3n22a_ | d2egda_ | d4ggfc_ |
| d2wcea_ | d2luca_ | d4aqia_ | d3nsia_ | d2lp3a_ | d1j55a_ | d1a4pa_ | d1k8ua_ |
| d1nsha_ | d1exra_ | d1tiza1 | d1nyaa_ | d1g8ia_ | d1ggwa_ | d1wdcc_ | d3fwba_ |
| d1ggza_ | d2o5ga_ | d1ctaa_ | d1m45a_ | d3i5gc_ | d1sl8a_ | d1fpwa_ | d2scpa_ |
| d2roaa_ | d4aqra_ | d3pn7b_ | d1br1b_ | d2mysb_ | d1s6ca_ | d1bjfa_ | d2obha_ |
| d1jfka_ | d1jf0a_ | d1qv0a_ | d1lkja_ | d2mlfc_ | d2m97a_ | d2d8na_ | d4by5a_ |
| d1jbaa_ | d4nqga_ | d1wdcb_ | d2mysc_ | d1j7qa_ | d1s6ia_ | d1uhka2 | d1r2ua_ |
| d1dtla_ | d3kf9a_ | d3pn7c_ | d1qjta_ | d1fi6a_ | d5d67d_ | d1tuza_ | d1h8ba_ |
| d1eg3a2 | d2zkmx1 | d1snla_ | d1y1xa_ | d1hqva_ | d1dvia_ | d1df0a1 | d1qxpa2 |
| d4usla_ | d1k94a_ | d1wkya2 | d1uuqa_ | d3wh9a_ | d4m1ra_ | d1xyza_ | d4xuya_ |
| d2je8a5 | d4bf7a1 | d3cuia_ | d1ur4a_ | d1odza_ | d2vzsa5 | d1bqca_ | d2x2ya1 |
| d1hjqa_ | d1qnra_ | d4y7ea1 | d1vjza_ | d4k68a_ | d2v3ga_ | d4hpga_ | d1nofa2 |
| d1nq6a_ | d1h4pa_ | d1aq0a_ | d3ziza_ | d4bs0a_ | d1ecea_ | d1h1na_ | d1ta3b_ |
| d1w91a2 | d1i1wa_ | d1uhva2 | d1us3a2 | d1egza_ | d7a3ha_ | d1tvna1 | d3nyda1 |
| d1ghsa_ | d2cyga1 | d4xv0a_ | d2c8na2 | d1w32a_ | d3emca_ | d1v6ya1 | d1foba_ |
| d2uwfa1 | d2fgla_ | d3niya1 | d2c0ha1 | d1rh9a1 | d1yq2a5 | d1szna2 | d1fa2a_ |
| d1pama4 | d5do8a1 | d1ea9c3 | d1hx0a2 | d2fhfa5 | d1qhoa4 | d1avaa2 | d1m53a2 |
| d4m8ua1 | d2gjpa2 | d1lwha2 | d4howa1 | d1ktba2 | d1j0ha3 | d3vm5a1 | d2x1ia_ |
| d1wzaa2 | d1uasa2 | d2diea1 | d1mxga2 | d1ht6a2 | d1eswa_ | d1iv8a2 | d5cpsa_ |
| d1ud2a2 | d1zjaa1 | d3gbea1 | d1kwga2 | d1wdpa_ | d1gcya2 | d1g5aa2 | d1hvxa2 |
| d3dhpa2 | d2bhua3 | d4do4a1 | d3qgva1 | d1e43a2 | d1g94a2 | d2d3na2 | d1gjwa2 |
| d1gvia3 | d1x1na1 | d1r7aa2 | d1tz7a1 | d4toqa_ | d1wb0a1 | d1w9pa1 | d1eoka_ |
| d1itxa1 | d1kfwa1 | d1goia2 | d1edqa2 | d2gsja_ | d1hjxa1 | d1jnda1 | d1vf8a1 |
| d3wdpp1 | d3wq5a_ | d1gnxa_ | d4gxpa_ | d4hz8a_ | d1e4mm_ | d3aiua_ | d1wcga1 |
| d1qvba_ | d3ahxa_ | d1v02a_ | d2dgaa_ | d1pbga_ | d1vffa1 | d1qoxa_ | d4ha4a_ |
| d2w0pa_ | d2diaa1 | d2e9ia2 | d2e9ja1 | d2dj4a1 | d2eeba1 | d2diba1 | d2dmba1 |
| d2dmca1 | d2d7oa1 | d2ee6a1 | d2d7pa1 | d2eeaa1 | d2di8a1 | d1wlha1 | d2d7na1 |
| d2nqca_ | d2d7ma1 | d2dica1 | d3cnka_ | d2k3ta1 | d2eeca1 | d1qfha2 | d2bp3a1 |
| d1v05a_ | d2ee9a1 | d2eeda1 | d2di9a1 | d3syxa1 | d1mkea1 | d2p8va_ | d1xoda1 |
| d1i7aa_ | d1evha_ | d1qc6a_ | d1egxa_ | d1id1a_ | d2hmta_ | d2v76a_ | d1wj1a1 |
| d1aqca_ | d1qqga1 | d1ntva_ | d1n3ha_ | d4dbba_ | d3f0wa1 | d1p3ra_ | d1wgua1 |
| d2cy4a1 | d3hqca_ | d1wvha1 | d1xr0b1 | d1wgqa1 | d1v89a1 | d2coda1 | d1zc3b_ |
| d1wg7a1 | d1u5ea1 | d1v61a1 | d2coca1 | d2ys1a1 | d1v5pa1 | d1ntya2 | d1wjma1 |
| d1wi1a1 | d1u29a_ | d1xx0a1 | d1unqa_ | d2zkmx3 | d1fhoa_ | d1v88a1 | d2adza1 |
| d1x1fa1 | d1p6sa_ | d2coaa1 | d1btka_ | d1v5ma1 | d1faoa_ | d2x18a_ | d1dyna_ |
| d1x1ga1 | d4kaxb_ | d2j59m1 | d1ki1b2 | d1xcga2 | d1txda2 | d1u5da1 | d1dbha2 |
| d1w1ha_ | d1upqa_ | d1v5ua1 | d2fjla1 | d1eaza_ | d2dfka2 | d2cofa1 | d1kz7a2 |
| d1h6ta1 | d1h6ua1 | d9ldta1 | d2ewda3 | d1up7a1 | d2dfda1 | d1s6ya1 | d1vjta1 |
| d7mdha1 | d1hyea1 | d1t2da1 | d1ojsa3 | d1b8pa1 | d1ldna1 | d1hyha1 | d1civa1 |
| d2a92a1 | d1ez4a1 | d1i0za1 | d1y6ja1 | d1gv0a1 | d1ur5a1 | d1llda1 | d1obba1 |
| d1v8ba1 | d1gdha1 | d1l7da1 | d2dlda1 | d1mx3a1 | d1ygya1 | d1qp8a1 | d1li4a1 |
| d1j4aa1 | d2naca1 | d1etob_ | d1umqa_ | d1g2ha_ | d1kola2 | d1f8fa2 | d1qora2 |

d1jqba2 d1vj0a2 d1cdoa2 d1gu7a2 d1d1ta2 d1xa0a2 d2dm6a2 d1jvba2
d1rjwa2 d1llua2 d1h2ba2 d1tt7a2 d1piwa2 d1o89a2 d1iz0a2 d1e3ja2
d1yb5a2 d1uufa2 d1vj1a2 d1e3ia2 d3gpdg1 d1vc2a1 d2nvwa1 d1u8fo1
d1ydwa1 d1hdgo1 d2g17a1 d1cf2o1 d2czca2 d1r0ka2 d1obfo1 d2cvoa1
d1ebfa1 d1h6da1 d1i32a1 d1zh8a1 d2q49a1 d1qkia1 d3pzra1 d1xeaa1
d1vkoa1 d1vm6a3 d3qvsa1 d2pkqo1 d1b7go1 d1gr0a1 d1p1ja1 d1lc0a1
d1j0xo1 d3pwka1 d2hjsa1 d1zyao1 d1vkna1 d1tlta1 d1nvmb1 d3sjua_
d3ojfa1 d1xu9a1 d2jjya1 d1n7ha_ d1y1pa1 d1h5qa_ d1jtva_ d4fs3a_
d2bkaa1 d1gy8a_ d2c5aa1 d1ulsa_ d1ooea_ d2b69a1 d1y5ma1 d1ae1a_
d1bxka_ d1ydea1 d1gega_ d1hdoa_ d1o5ia_ d1e6ua_ d1snya1 d2rhca_
d3zv4a_ d5ceja_ d2d1ya1 d1fjha_ d3w8ea_ d2fmua1 d1x1ta_ d1rpna_
d3ay7a1 d1k2wa_ d1eq2a_ d2hrba1 d3ruaa_ d2ew8a1 d4hwka_ d1xgka_
d2bd0a1 d1yo6a1 d2a35a1 d2ntva_ d1vl8a_ d1hdca_ d4bkua_ d2ag5a1
d2q45a_ d1nffa_ d4iy1a_ d1zbqa1 d1kewa_ d1ek6a_ d1ja9a_ d2q46a2
d1iy8a_ d1cyda_ d2ptga_ d1hxha_ d1uzma1 d3lyla_ d4m89a_ d3wyea_
d2blla1 d1w6ua_ d1sb8a_ d2q2va_ d4nr0a_ d1e6wa_ d2gasa_ d3oiga1
d2bgka1 d1mxha_ d1vl0a1 d1rkxa_ d1r6da_ d1wmaa1 d1e7wa_ d2c07a1
d1xkqa_ d3tzha_ d1g0oa_ d3lz6a_ d1qyca_ d1edoa_ d1gz6a_ d1oc2a_
d1t2aa_ d1zema1 d2xoxa_ d1z45a2 d1geea_ d1p33a_ d3lt0a_ d4e6pa_
d2wyua_ d1db3a_ d4q9na_ d1xhla_ d4o1ma_ d1fmca_ d2uvda_ d2gdza1
d1uaya_ d2z1ma_ d2o23a2 d2fr1a2 d2fr1a1 d1qsga_ d1qyda_ d4zjua_
d2a4ka1 d1yb1a_ d1wvga1 d1yxma1 d3f4ba_ d1mg5a_ d1xg5a1 d1r55a1
d3gboa_ d1atla_ d1kufa_ d1buda_ d4q1la_ d1yp1a_ d1wnia_ d4ekfa_
d2ckga_ d1th0a_ d2bkra_ d3u1ra1 d1g9ka2 d1kapp2 d3ioqa_ d1fh0a_
d1o0ea_ d1khqa_ d1m6da_ d2cb5a_ g8pch.1 d1s4va_ d2pnsa_ d1vsna1
d1mira_ d1iwda_ d2oula_ d7pcka_ d3bwka_ d3bcna_ d2p86a_ d1pvja_
d1pcia_ d1gece_ d1deua_ d4bs6a_ d1xkga1 d1cqda_ d1jqpa2 d2c0ya_
d2bdza_ d3p5ua_ d2o36a_ d1k9xa_ d1zoya1 d1qo8a2 d1d4ca2 d1jnra2
d1kf6a2 d2jk6a2 d2jk6a1 d1feca1 d1feca2 d1lpfa1 d1lpfa2 d1xhca1
d1xhca2 d1dxla2 d1dxla1 d1gera2 d1m6ia2 d1onfa1 d1onfa2 d3lada2
d3lada1 d1w4xa1 d1w4xa2 d1gesa2 d1mo9a1 d1xdia1 d1fl2a2 d1fl2a1
d1fcda1 d1fcda2 d1h6va2 d1h6va1 d1mo9a2 d1gv4a2 d1gv4a1 d2gv8a2
d2gv8a1 d1aoga1 d1aoga2 d1hyua1 d1ebda1 d1ebda2 d3dk9a1 d1q1ra2
d1q1ra1 d1gesa1 d1zbfa1 d2z1ha_ d2etja1 d3qioa1 d1jl1a_ d2e4la_
d1jl2a_ d2lsna1 d1io2a_ d1uaxa_ d2yv0x_ d1mu2a1 d1ekea_ d1pn0a1
d1reoa1 d1ju2a1 d2f5va1 d3c96a1 d2gmha1 d1gpea1 d1o5wa1 d1n4wa1
d1k0ia1 d2dw4a2 d2ivda1 d1seza1 d2voua1 d1kdga1 d1s5ja1 d2hbja2
d2igia_ d1wlja_ d1qhta1 d4dlea1 d1tgoa1 d2f96a1 d3tr8a1 d3mxma_
d1q8ia1 d1y97a1 d1w0ha_ d1kfsa1 d2py5a1 d1noya_ d1d5aa1 d1yt3a3
d2guia_ d3mwpa1 d4kb1a_ d1iv0a_ d1nu0a_ d4qmea2 d2xdta2 d1z5ha2
d3nvva1 d1krha3 d2h88b1 d1n62a2 d1t3qa2 d1zoyb1 d1jq4a_ d1ffva2
d1dgja2 d1rm6c2 d1r7ha_ d1faaa_ d1wika1 d1xfla_ d1ttza1 d2f51a1
d1ti3a_ d1nhoa_ d1sena_ d2i4aa_ d1zmaa1 d1fo5a_ d3lefa1 d3diea1
d2yana1 d1z6na1 d1oaza_ d3zywa1 d4aj8a_ d1nm3a1 d4fiwa_ d1wmja_

d4hs1a1 d1iloa_ d1xwaa1 d1wjka1 d2vm1a_ d1fb6a_ d1gh2a_ d1h75a_
d1nw2a_ d1kx5b_ d3a6na_ d4m6ba2 d2nqbd_ d3wkjd_ d2yfwe_ d4h9na_
d5bnxa_ d1kx5c_ d3r45a_ d1eqzb_ d4jjnb_ d1f66c_ d1eqza_ d4wnnb_
d1kx5a_ d1u35c1 d1id3a_ d2nqbc_ d1kx5d_ d3us3a3 d3us3a1 d1um8a_
d1qvra2 d1qvra3 d1qzma_ d1sxjc2 d1jr3d2 d2p65a_ d1l8qa2 d1g8pa_
d3hu3a3 d1sxjd2 d1njfa_ d1in4a2 d1sxja2 d1tuea1 d2ce7a2 d1w5sa2
d1sxjb2 d1d2na_ d1ny5a2 d1ofha_ d2gnoa2 d2a5yb3 d1fnna2 d1u0ja1
d1lv7a_ d1iqpa2 d2fnaa2 d1ixsb2 d4gita_ d1sxje2 d1g41a_ d1pd212
d1nhya2 d2a2ra2 d1k0dd2 d1f2ea2 d1f3ba2 d2fhea2 d2pvqa1 d1tw9a2
d1oyja2 d1e6ba2 d1k0ma2 d3ik7a1 d4is0a1 d1r5aa2 d1jlva2 d1m0ua2
d3gtub2 d1z9ha2 d1okta2 d1axda2 d2gsra2 d1tu7a2 d1k3ya2 d1zgma1
d1b48a2 d1gnwa2 d1gwca2 d2gsta2 d1glqa2 d1oe8a2 d1pn9a2 d1v2aa2
d1fw1a2 d1duga2 d2trcp_ d3t4ea2 d1a4ia1 d1o0sa1 d2hk9a2 d2egga2
d1b26a1 d1bvua1 d1gq2a1 d1c1da1 d1leha1 d1gtma1 d1euza1 d1nvta1
d3dona2 d1edza1 d1gpja2 d1vi2a1 d1p77a1 d1nyta1 d1b0aa1 d1vl6a1
d1v9la1 d4ilya_ d4olta_ d1qgia_ d1txga2 d3f47a1 d1mv8a2 d2pv7a2
d1xc2a3 d2ahra2 d4xiya1 d2f1ka2 d1pgja2 d1i36a2 d1np3a2 d1lj8a4
d1teaa3 d1qmga2 d2i76a2 d2g5ca2 d1wdka3 d1dlja2 d3cuma3 d1qqya_
d1f6ra_ d1ivma_ d2gv0a_ d1iiza_ d2z2fa_ d1j8xa_ d1ghla_ d1b9oa_
d174la_ d169la_ d2o4wa_ d1p37a_ d1k28a3 d1xjta1 d1jtma_ d2f2qa_
d4e97a1 d2za0a_ d2c21a1 d2pjsa1 d1klla_ d2i7ra1 d1ecsa_ d1xqaa1
d5cj3a1 d1npba_ d1jifa_ d2p7oa_ d1r9ca_ d1mpya2 d1kw3b2 d1kw3b1
d4ghga1 d1tfza1 d3isqa1 d3isqa2 d1sqia1 d1sqia2 d1lkda2 d1lkda1
d1mpya1 d1cjxa1 d1sp9b2 d1t47a1 d1t47a2 d1f1ua2 d1sp8a1 d1sp8a2
d1f1ua1 d1ro5a1 d1s5ja2 d1qhta2 d4dlea2 d1tgoa2 d3py8a2 d1q8ia2
d2py5a2 d1d5aa2 d1qsta_ d2beia1 d4m3sa_ d1u6ma1 d1ygha_ d1yk3a1
d3dr6a_ d1xmta_ d1z4ea1 d2ge3a1 d1bo4a_ d2fiaa1 d2jdca1 d1y9wa1
d3to7a_ d1wwza1 d2fl4a1 d2ozua1 d2b5ga_ d2cy2a1 d2p0wa_ d1cm0a_
d4pswa_ d1yrea1 d1yr0a1 d1m4ia_ d2gana1 d1yvka1 d1ghea_ d2g3aa1
d1y7ra1 d2euia1 d1q2ya_ d2fsra1 d1xeba_ d2ae6a1 d1cjwa_ d1qsma_
d2aj6a1 d1y9ka1 d1i12a_ d1vkca_ d2atra1 d1n71a_ d2d4pa1 d2fiwa1
d2fe7a1 d1tiqa1 d2ou2a_ d1yx0a1 d2fcka1 d1p0ha_ d2j8ma_ d2pq8a_
d1z4ra1 d1s3za1 d1ufha_ d2hqya1 d2hqya2 d1lrza2 d1ne9a1 d1ne9a2
d1f37b_ d1xvqa1 d1pn0a2 d3hy2a_ d2cvba1 d1o8xa_ d1lu4a_ d1we0a1
d2bmxa1 d2a4va1 d1psqa_ d1xiya1 d1q98a_ d1n8ja_ d1zofa1 d2ggta_
d3tjja_ d4je1a1 d1o73a_ d3s9fa_ d2zcta_ d1qmva_ d1jfua_ d2b1ka_
d5dvba1 d2b5xa1 d2v2ga_ d1st9a_ d3tb2a_ d4llra_ d4fh8a1 d1e2ya_
d1knga_ d2h30a_ d1i5ga_ d2z9sa_ d2f8aa1 d1tp9a1 d2b7ka_ d1nm3a2
d3hvva_ d2wfca1 d4kcea_ d1xvwa1 d4gqca1 d3qpma_ d4k1fa_ d1zzoa1
d1gp1a_ d1prxa_ d1rmka_ d1ixta_ d1eyoa_ d1cnna_ d1fu3a_ d1g1pa_
d1f3ka_ d1fyga_ d4mh8a_ d1mu2a2 d4i7ga1 d1ofda3 d1ea0a3 d1ecfa2
d1q15a2 d1ct9a2 d1ao0a2 d1te5a_ d1ys1x_ d4hs9a_ d1ispa_ d1ex9a_
d3qmma_ d1ra6a_ d3gsza_ d1khva_ d1te9a_ d2xwha_ d3uqsa_ d3hkwa_
d3n6ma_ d2cjqa_ d2xyma_ d1teba_ d1te8a_ d1s48a_ d1u09a1 d4wfxa_

| | | | | | | | |
|---|---|---|---|---|---|---|---|
| d3tyqa_ | d2pgga1 | d1lgya_ | d4l3wa_ | d1llfa_ | d1ukca_ | d1cr6a2 | d3groa_ |
| d1thta_ | d1ei9a_ | d2ftba_ | d1pmpa_ | d2rcqa1 | d1fdqa_ | d4lkpa_ | d1sa8a_ |
| d1vyfa_ | d1lpja_ | d3vg7a1 | d4azqa1 | d1o8va_ | d5bvqa_ | d1p6pa_ | d2qo4a_ |
| d1opba_ | d1yiva1 | d1o1va_ | d4bvma1 | d4tkba_ | d4i3ba_ | d1bwya_ | d1ftpa_ |
| d1ggla_ | d4gkca_ | d1cbia_ | d3p6da1 | d1kqwa_ | d2a0aa1 | d2ddfa_ | d3zxha_ |
| d1cgla_ | d1rm8a_ | d1eaka2 | d1hy7a_ | d2jsda_ | d1cxva_ | d1hv5a_ | d1i76a_ |
| d3p0ya1 | d1igra1 | d1igra2 | d1n8yc2 | d1n8yc1 | d1m6ba2 | d1m6ba1 | d1dzka_ |
| d4tlja_ | d2a2ua_ | d4wfva_ | d3t1da_ | d4iaxa_ | d3zq3a_ | d2nnda_ | d1i4ua_ |
| d1kxoa_ | d1bbpa_ | d1kt7a_ | d1exsa_ | d3s26a1 | d1lf7a_ | d1qfta_ | d4gh7a_ |
| d1z24a1 | d1qabe_ | d1xkia_ | d1ew3a_ | d3bx7a_ | d4iawa1 | d1n0sa_ | d1gkab_ |
| d3kzaa_ | d2k23a_ | d2l9ca1 | d2joza1 | d1gm6a_ | d3kffa_ | d3saoa1 | d1e5pa_ |
| d1yupa1 | d1iiua_ | d1h6ua2 | d2tbvc_ | d1c8nc_ | d1x36a_ | d1smvc_ | d1ng0c_ |
| d4sbvc_ | d1opoa_ | d1cixa_ | d1y29a_ | d1v7fa_ | d2jtba_ | d1d1ha_ | d1g9pa_ |
| d1lupa_ | d1qk7a_ | d1mb6a_ | d2a2va_ | d1niya_ | d1i25a_ | d1hvwa_ | d2mpqa_ |
| d1ie6a_ | d1kqha_ | d1la4a_ | d2mxoa1 | d1dl0a_ | d1koza_ | d1qk6a_ | d1emxa_ |
| d1lqra_ | d1c6wa_ | d1js9c_ | d1f15b_ | d1za7a_ | d1laja_ | d1rypd_ | d1irua_ |
| d1rypc_ | d3unfk_ | d1rypa_ | d1j2pa_ | d1irui_ | d1j2qh_ | d3unfh_ | d1m4ya_ |
| d1rypb_ | d1irul_ | d1irug_ | d1iru2_ | d1q5rh_ | d1rypg_ | d1g3ka_ | d1q5qa_ |
| d1e94a_ | d1irub_ | d1ryph_ | d3ty6a_ | d1iruj_ | d1iruk_ | d1rype_ | d1irud_ |
| d1irue_ | d3mi01_ | d1rypf_ | d1iruh_ | d2z3ba_ | d1iruc_ | d3mka2_ | d1iru1_ |
| d1ny721 | d1a6ca3 | d1a6ca2 | d1a6ca1 | d1pgl22 | d1pgl21 | d1pgl11 | d1ny711 |
| d1mqta_ | d1d4m3_ | d1fmd3_ | d2mev1_ | g1aym.1 | d1ev13_ | d4q4w1_ | d1r1a3_ |
| d2x5ia_ | d1rhi1_ | g1r1a.1 | g1d4m.1 | g1rhi.1 | d1b35b_ | d1oopa_ | d1tme1_ |
| d4iv1a_ | g1pvc.1 | g1fpn.1 | d1fpn1_ | d4gb31_ | d1d4m1_ | d1fmd1_ | d1ev11_ |
| d2wzr3_ | g1cov.1 | d1qqp3_ | g1fmd.1 | d2x5ic_ | d1rhi3_ | g1oop.1 | d1hxs1_ |
| g1hxs.1 | d1fpn3_ | d1bev1_ | g2mev.1 | d1eah3_ | d1aym1_ | d1pvc3_ | d1zba3_ |
| d1h8ta_ | d3vbhc_ | g1b35.1 | g1bev.1 | d1hxs3_ | g1ev1.1 | d1bev3_ | g1tmf.1 |
| d1tmf1_ | d1b35a_ | d2mev3_ | g1eah.1 | g1h8t.1 | d1eah1_ | d4q4w3_ | d1r1a1_ |
| d1aym3_ | d1pvc1_ | d1zba1_ | d1h8tc_ | d1oopc_ | d1tme3_ | g1qqp.1 | d4iv1c_ |
| g1tme.1 | d3ubub_ | d1egia_ | d1qo3c_ | d1v7pa_ | d1tdqb_ | d3ubua_ | d1c3ab_ |
| d1h8ua_ | d3bx4b_ | d1t8ca1 | d1sb2a1 | d1qdda_ | d2ziba1 | d3cada_ | d1byfa_ |
| d1j34b_ | d1jwia_ | d1jzna_ | d1c3aa_ | d1wmza_ | d3bdwb_ | d1hq8a_ | d1fifa1 |
| d1dv8a_ | d3hupa_ | d1wk1a1 | d1umra_ | d2afpa_ | d3bx4a_ | d1gz2a_ | d1sl6a_ |
| d1v7pb_ | d3g8ka_ | d1ixxa_ | d1j34a_ | d1jwib_ | d1g1sa1 | d1y17a1 | d1ja3a_ |
| d1fvub_ | d3bdwa_ | d1sb2b1 | d1fvua_ | d1umrc_ | d1oz7a_ | d4m17a_ | d3cfwa1 |
| d1ypqa1 | d1g1ta1 | d3fd4a_ | d3l9jc_ | d4dn8a1 | d1v4lb_ | d1rdl1_ | d2msba_ |
| d4w9ta_ | d4x2ra1 | d1ka9f_ | d1qo2a_ | d4axka1 | d1thfd_ | d1h5ya_ | d4u28a_ |
| d2y88a_ | d4wd0a_ | d1vzwa1 | d1eixa_ | d3exra_ | d2ffca1 | d2fdsa1 | d3n3ma1 |
| d2czda_ | d1q6oa_ | d2yyua_ | d1vqta1 | d1dbta_ | d3ldva1 | d2flia1 | d1h1ya_ |
| d1tqja_ | d1tqxa_ | d4lt9a1 | d3tc7a1 | d3hoja_ | d1i4na_ | d3vnda_ | d3nl8a1 |
| d1geqa_ | d3nava1 | d1v5xa_ | d4a2ra1 | d3tc6a1 | d1ujpa_ | d4ijba_ | d1xcfa_ |
| d1vc4a_ | d1rd5a_ | d1qopa_ | d3b5va_ | d2r80b_ | d1cqxa1 | d1cg5a_ | d1gcvb_ |
| d3boma_ | d2h8fb_ | d2raoa_ | d3bj1a_ | d4hrrb_ | d2r50a_ | d3d1kb_ | d2aa1b1 |
| d3zhwa_ | d3hf4b_ | d3gdja_ | d1jebb_ | d3bj1b_ | d1x9fb_ | d1wmub_ | d1gcva_ |

| | | | | | | | |
|---|---|---|---|---|---|---|---|
| d3bcqb_ | d3d4xb_ | d1gvha1 | d2dc3a_ | d3hf4a_ | d4yu3a_ | d3k8ba_ | d1qpwb_ |
| d2r80a_ | d3bcqa_ | d1v4wa_ | d3wtgb_ | d1jl7a_ | d4esaa_ | d1a9we_ | d3d1ab_ |
| d1fsla_ | d4h2la_ | d1v4wb_ | d1mwca_ | d1vhba_ | d1jeba_ | d1spgb_ | d1h97a_ |
| d1q1fa_ | d1tu9a1 | d3d1aa_ | d1hdsb_ | d3hyub_ | d2zfbb_ | d3fs4a_ | d1oj6a_ |
| d1shrb_ | d4h2lb_ | d4yu3b_ | d1spga_ | d3hrwa_ | d3qqra_ | d3at5a_ | d4wchd_ |
| d3hyua_ | d1cg5b_ | d1uc3a_ | d1hbra_ | d2raob_ | d1outb_ | d4esab_ | d3gdjb_ |
| d3qqqa_ | d1x9fc_ | d3vrfa_ | d1itha_ | d3mkba_ | d3d4xa_ | d3mjpa_ | d1jl6a_ |
| d3fh9a_ | d1outa_ | d2dc3b_ | d1it2a_ | d3eoka_ | d3g46a_ | d1qpwa_ | d3vrfb_ |
| d1ch4a_ | d1x9fd_ | d1hdsa_ | d2zfba_ | d3mkbb_ | d1or4a_ | d3wtga_ | d1a4fa_ |
| d3fh9b_ | d3bomb_ | d3cy5a_ | d3at5b_ | d1x9fa_ | d2oifa_ | d4lmxb_ | d1cpcb_ |
| d3v57a_ | d1kn1a_ | d1cpca_ | d4f0ta_ | d2vjha_ | d4po5b_ | d1eyxa_ | d1alla_ |
| d4f0tb_ | d1b33b_ | d1b8db_ | d4h0mb_ | d1b33a_ | d2vjhb_ | d4lm6b_ | d2vmlb_ |
| d1allb_ | d2vjta_ | d3dbja_ | d2vmla_ | d2vjtb_ | d4f0ub_ | d1f99b_ | d4rmpa1 |
| d1liaa_ | d1eyxb_ | d2uulb_ | d4lmsb_ | d4f0ua_ | d2bv8a_ | d1f99a_ | d2bv8b_ |
| d2c7lb_ | d3dbjb_ | d4rmpb1 | d1b8da_ | d4h0ma_ | d3v57b_ | d1liab_ | d2uula_ |
| d2j96a_ | d1kyqa1 | g1gxs.1 | g1wht.1 | d1hlga_ | d2xqfa_ | d1ea5a_ | d1f6wa_ |
| d1dx4a_ | d1qe3a_ | d4qwwa_ | d2ha2a_ | d1r88a_ | d1sfra_ | d4qeka_ | d3c8da2 |
| d2pbla1 | d1jkma_ | d1lzla_ | d1jjfa_ | d1jmxa4 | d1jmxa3 | d1pbya4 | d1b9ra_ |
| d1offa_ | d1frra_ | d3wcqa_ | d1e9ma_ | d1l5pa_ | d1wria_ | d1i7ha_ | d3av8a_ |
| d1czpa_ | d1rfka_ | d3p1ma1 | d3b2ga_ | d2bt6a2 | d4iuaa3 | d4bvva_ | d5hpga_ |
| d3k65a_ | d1ki0a3 | d1ki0a1 | d1a0ha1 | d1pmla_ | d3sp8a3 | d2feba1 | d1mdwa_ |
| d1qxpa4 | d1ziva1 | d1zcma1 | d1f74a_ | d3l21a_ | d2e1da_ | d1to3a1 | d1w3ia_ |
| d1sfla_ | d1vcva1 | d4imea_ | d1wa3a_ | d2ocza1 | d1f2ja_ | d3m16a_ | d2yxga_ |
| d1ub3a_ | d3a5fa_ | d1fbaa_ | d1l6wa_ | d1xfba1 | d1wx0a1 | d1vpxa1 | d1ojxa_ |
| d1mxsa_ | d2a4aa1 | d1xkya1 | d3cq0a1 | d3s1xa_ | d1f05a_ | d1fdja_ | d3cpra1 |
| d4d2ja_ | d1yloa2 | d1y7ea2 | d1y0ya3 | d1lfwa1 | d1vixa1 | d1fnoa4 | d1vhoa2 |
| d1ysja1 | d1vhea2 | d1z2la1 | d2fvga2 | d1vgya1 | d1r3na1 | d2grea2 | d1xmba1 |
| g1q7l.1 | d1rtqa_ | d3rp2a_ | g1ucy.1 | d1fuja_ | d2zchp_ | d3w94a_ | d1si5h_ |
| d1lo6a_ | g2pka.1 | d1cgha_ | d1kigh_ | d1a5ia_ | d3s9ba_ | d1hj8a_ | d1ao5a_ |
| d2anya_ | d1q3xa1 | d1gvza_ | d1azza_ | d4gsoa_ | d2f91a1 | d1iaua_ | d1gdna_ |
| d1eufa_ | g1fiw.1 | d1eq9a_ | d4nfea_ | d2rdla_ | d1spja_ | d1elva1 | d1a7sa_ |
| d1ekbb_ | d1pfxc_ | d1yc0a_ | d2aiqa_ | g1fiz.1 | d1fi8a_ | d4k60a_ | d4dgja_ |
| d1mzaa_ | d1op0a_ | d3tjva_ | d1pytd_ | d1sgfg_ | g1gg6.1 | d2eeka_ | g1rtf.1 |
| d1a0ja_ | d3g01a_ | d1pytc_ | d4e7na_ | d1ddja_ | d1z8ga1 | d2hlca_ | d1npma_ |
| d1brup_ | g1jou.1 | d1sgfa_ | d1autc_ | g2hnt.1 | g1kdq.1 | d3s69a_ | d4crga_ |
| d1fxya_ | d1bqya_ | d1svpa_ | d1mbma_ | d3su6a1 | d1cu1a1 | d4agka_ | d1vcpa_ |
| d1q31a_ | g1n1l.1 | d3e90b_ | d1ep5a_ | d3u1jb_ | d1m4la_ | d2bk7a_ | d3fjua_ |
| d1z5ra1 | d1kwma1 | d1uwya2 | d1jqga1 | d2c1ca1 | d1dtda_ | d1h8la2 | d3lgia_ |
| d3i77a_ | d2z9ia2 | d4nsva_ | d1lcya2 | d4jcna_ | d1agja_ | d1os8a_ | d1hpga_ |
| d1p3ca_ | d1qtfa_ | d1e69a_ | d1mv5a_ | d3g60a4 | g1ii8.1 | d1ewqa2 | d1ji0a_ |
| d1qhla_ | d1v43a3 | d2wmfa1 | d1vpla_ | d1g2912 | d4a82a2 | g1f2t.1 | d2d62a1 |
| d3tuic1 | d2bbsa1 | d3g60a3 | d2ixea_ | d1g6ha_ | d1sgwa1 | d2qi9c_ | d3rlfa1 |
| d3b60a1 | d3d31a2 | d2pzea1 | g1xew.1 | d1jj7a_ | d2onka1 | d1w1wa_ | d1iwea_ |
| d1qf5a_ | d1vmaa2 | d1nija1 | d2p67a1 | d3ihla_ | d1okkd2 | d1ls1a2 | d1s1ma2 |

d1iona_ d2v40a_ d1dj3a_ d3hida_ d2vo1a1 d2g0ta1 d1vcoa2 d1j8mf2
d1g3qa_ d1xjca_ d1p9ba_ d1qzxa3 d1ihua2 d1ihua1 d2qm8a_ d1dj2a_
d4ioja_ d1cp2a_ d1yrba2 d1hyqa_ d3gqba2 d1skye3 d1xp8a1 d1tf7a2
d1tf7a1 d1ye8a1 d1skyb3 d2qe7d2 d4blta_ d4huta_ d3ldaa2 d4hyya_
d2i3ba1 d1cr1a_ d3j9tb2 d3ntua2 d1vdza1 d2z43a_ d1e9ra_ d1nlfa_
d3ziaa2 d1n0wa_ d3ziad2 d3j9ta2 d1pzna2 d3gqbb2 d2i49a_ d1nnfa_
d1xvxa_ d1ursa_ d1h3da1 d1iuda_ d4m1va1 d1twya_ d3e13x_ d2nxoa1
d4pfua_ d3ttma_ d1elja_ d2qmwa1 d1us5a_ d1y3na1 d1jeta_ d1uqwa_
d4f19a1 d2esna2 d3thia_ d3fasa1 d2uyeb_ d2xwva1 d1r9la_ d2fyia1
d1pb7a_ d2ozza1 d1sw5a_ d1nh8a1 d1vr5a1 d2g29a_ d1xs5a_ d3lswa_
d2z23a_ d3w9va_ d3tcha_ d1q35a_ d1z7me1 d3q27a_ d1p99a_ d1ve4a1
d2i6ea1 d4mcoa1 d4n8ya_ d1eu8a_ d1zbma1 d1hsla_ d1ixca2 d1j1na_
d1xvya_ d1y9ua_ d2z22a1 d1xoca1 d4oera_ d1wdna_ d5f1qa1 d1xt8a1
d1i6aa_ d4gwoa_ d4igta1 d1pc3a_ d4mmpa1 d1o63a_ d2czla1 d2onsa2
d4mija1 d1eh3a_ d3skpa_ d1dtza1 d1dtza2 d1ieja_ d1jnfa2 d1ce2a1
d1jnfa1 d1b1xa2 d1b1xa1 d1jw1a1 d1jw1a2 d1ryoa_ d1h76a1 d1h76a2
d1c4oa1 d2p6ra3 d2p6ra4 d1oywa3 d1wrba1 d1w36b2 d1w36b1 d1c4oa2
d3bora_ d1t6na_ d2eyqa5 d2eyqa3 d2eyqa2 d2zu6a2 d1veca_ d1uaaa1
d1w36d1 d2g9na1 d1z3ix2 d2rb4a_ d1hv8a1 d1hv8a2 d1t5ia_ d1w36c2
d2j0sa2 d2j0sa1 d1fuka_ d3hjha1 d1z5za1 d1qdea_ d1z63a1 d1s2ma2
d1s2ma1 d1wp9a1 d1wp9a2 d1gm5a3 d1gm5a4 d3dina4 d3dina3 d1q0ua_
d1w36c1 d1z3ix1 d1nkta3 d1nkta4 d1uaaa2 d1w36d2 g1qhh.1 d2fwra2
d2fwra1 d3q3ma_ d1za0a1 d1mxra_ d2inca_ d1jk0a_ d2incb_ d1mhyb_
d3hf1b_ d1mtyb_ d2uw1a_ d1mhyd_ d3n37a_ d1afra_ d1oquc_ d1syya_
d1mtyd_ d2o1za_ d1uzra_ d1smsa_ d2fkza_ d1zuja1 d1s3qa1 d2fjca1
d1z6om1 d1veia_ d4dyya_ d3gvya_ d2pyba_ d4cy9a1 d3bvfa1 d1r03a_
d2cf7a_ d1nf4a_ d1zs3a1 d4iwka_ d3ka8a_ d3np0x_ d1n1qa_ d1lb3a_
d4e6ka_ d1z6oa1 d2fzfa1 d4dyua_ d3ge4a_ d1jgca_ d4am4a_ d2z6ma_
d1ji5a_ d1vlga_ d1lkoa1 d1krqa_ d3qz3a_ d1o9ra_ d2ffxj_ d1euma_
d1yv1a1 d2v2pa_ d1j30a_ d4di0a_ d1dpsa_ d1oewa_ d2qzxa_ d2asia_
d1psoe_ d1miqa_ d1smra_ d2h6ta_ d1pfza_ d1izda_ d3hd8a_ d1j71a_
d1dpja_ d4aa9a_ d1t6ex_ g1b5f.1 g1lya.1 g1htr.1 d1qdma2 d1bxoa_
d1wkra_ d1tzsa_ d1ibqa_ d2qzwa_ d1ex4a2 d1bdqa_ d3ggta_ d3ixoa_
d1mrxa_ d2haha_ d1tw7a_ d4njsa_ d1sgua_ d2p3da_ d1nsoa_ d3d3ta_
d2rkfa_ d1c6ya_ d3ogpa_ d3ekwa_ d2o4na_ d2j9ja_ d1baia_ d3ggua_
d3lzua_ d4npua_ d4j55a_ d2r8na_ d2fxda_ d2p3ca_ d4dqea_ d3ttpa_
d3t3ca_ d2p3aa_ d2hc0a_ d2fdda_ d3mwsa_ d1k8rb_ d1wgra1 d1ef5a_
d1wfya1 d1e8ya3 d1wgya1 d1lfda_ d2cs4a1 d1wxma1 d2byea1 d3kh0a1
d2byfa1 d1wxaa1 d1e7ua3 d1h4ra3 d4yl8a1 d2al6a3 d1wgda1 d1wgga1
d4q5eb_ d1wjna1 d1se9a_ d3n3kb_ d1wz0a1 d1we6a1 d2m0xa_ d2gbka_
d3rt3b1 d1wh3a1 d1sifa_ d2ekec2 d4ii2b1 d4hk2a1 d1c3ta_ d1m94a_
d1wgha1 d1ttna1 d1j8ca_ d1wx8a1 d3v6cb1 d2ma9b_ d4i6lb1 d1bt0a_
d2jvca1 d1iyfa_ d2k1fa_ d1wxva1 d1wf9a1 d1t0ya_ d2io0b2 d1x1ma1
d2gbma_ d1zw7a1 d2m17a_ d1ud7a_ d1we7a1 d2lwpa_ d2gbja_ d1wjua1

d1v6ea1 d1v86a1 d4eewa_ d1wiaa1 d4icva_ d4gu2a_ d1wx7a1 d1wy8a1
d4hjka1 d2faza1 d1v5oa1 d3ruib_ d3waob_ d2r2qa1 d3m95a_ d3wana_
d1epwa1 d4lu0a_ d1of8a_ d3unda1 d3e9aa1 d1vr6a2 d1n8fa_ d2qkfa_
d1d9ea_ d1wioa4 d1ccza2 d1vcaa1 d1dr9a2 d1ic1a1 d2d9qb1 d2nxyb2
d1adqa1 d1fnga1 d1uvqb1 d1zs8a1 d2vola1 d2vola2 d1mfbl2 d2jelh2
d1uwel2 d3qq4b1 d1kjvb_ d1k5na1 d1osph2 d4odhl2 d1ow0a1 d1ow0a2
d1bfob2 d3l9ra2 d3jtth1 d2fath2 d4elkb2 d1fn4b2 d2xfxb_ d1pg7x2
d1cqka_ d4k71b2 d1lk2a1 d1eapb2 d4lfhg2 d1i4fa1 d2nw2a2 d1kfah2
d1mhca1 d1h3ua1 d1wejh2 d1h0db2 d1a5fh2 d1kcvh2 d1mjuh2 d1qqda1
d1kcrh2 d1k8ia1 d3huja2 d2nnab1 d1nfdf2 d3mffb2 d1s9va1 d1lk3h2
d1q0xl2 d1fv1b1 d1neza1 d4rfeb2 d1r3ha1 d1a6tb2 d1kjva1 d1pz5b2
d1onqa1 d2bc4a2 d1de4a1 d1k8da1 d1tcra2 d1dn0b2 d1nfde2 d2xwtb2
d1bfoa2 d1mexh2 d1iakb1 d1ypzf2 d4f7ea2 d1q72l2 d2fbjh2 d1fe8h2
d1clzh2 d1fngb1 d1uvqa1 d1vgka1 d1c16a1 d4j12a2 d4x99b2 d1igtb3
d1je6a1 d1q72h2 d1nldh2 d1teth2 d2hmic2 d1i1ca2 d1i1ca1 d1k8ib1
d1q0yh2 d1kfal2 d3frua1 d1pg7w2 d1u58a1 d1igyb4 d1igyb3 d1hyrc1
d1klub1 d1deeb2 d1c12b2 d2bc4b2 d2ig2h2 d1kcvl2 d3wn5b1 d1lk2b_
d1lk3l2 d1mjul2 d1sm3h2 d1q0xh2 d1zvsa1 d1ydpa1 d1u8ib2 d1ypze2
d1kcuh2 d2lj7a_ d1n4na_ d2m8bb1 d2kyqa_ d3psma_ d1jxca_ d2gl1a_
d1ugla_ d4uj0a_ d2k4ua_ d1hp2a_ d2lu9a_ d1baha_ d2meoa1 d1wm7a_
d1hlya_ d1n8ma_ d1jlza_ d1wpda_ d1c55a_ d1pvza_ d1wt7a_ d1rjia_
d1wz5a_ d1m2sa_ d1pjva_ d1c49a_ d1qkya_ d2ktca1 d1wmta_ d1du9a_
d1j5ja_ d1r1ga_ d1t0za_ d2i61a_ d1t1ta_ d1sn4a_ d1kv0a_ d2lkba_
d1npia_ d1wwna_ d1pe4a_ d2kbka_ d2li7a_ d1djta_ d2kbha_ d1dq7a_
d1sega_ d1omya_ d1zywa1 d1jzaa_ d1n9wa1 d1bbua1 d1e1oa1 d1c0aa1
d1b8aa1 d1l0wa1 d3a74a1 d1eova1 d1iyjb4 d1iyjb5 d1iyjb3 d1o7ia_
d4ipca1 d1x3ea_ d4apva_ d1jb7a1 d1jb7a2 d1jb7a3 d3kdfa_ d1ue1b_
d1wjja1 d3eiva_ d1xjva2 d1xjva1 d2ccza2 d1se8a_ d1l1oc_ d1jb7b_
d1qvca_ d3ulla_ d1qzga_ d1miua4 d1miua5 d1miua3 d3g48a1 d1gd7a_
d1mkha_ d1jjcb3 d1y14b1 d2nn6i1 d1i94l_ d2zjra2 d1smxa_ d2qamc2
d1hh2p1 d2r7da1 d1rl2a2 d1wfqa1 d3bzka4 d1x6oa2 d3a0ja_ d2j01d2
d2ja9a1 d4ql5a_ d1hr0w_ d1luza_ d2n3sa_ d2vnud1 d1h95a_ d1go3e1
d2nn6h1 d1ne3a_ d2mo1a_ d1iz6a2 d1jjga_ d1e3pa2 d2ix0a3 d2ix0a2
d2lxja_ d1ueba3 d1ueba2 d2z0sa1 d1u0la1 d2ix0a1 d2eifa2 d2je6i1
d3i2za1 d2c35b1 d2ahob2 d1t9ha1 d3i4oa_ d2qall1 d1jt8a_ d2nn6g1
d1g6pa_ d3cama_ d1i94q_ d1c9oa_ d1d7qa_ d2ba0a1 d2vnud2 d2vnud3
d1khia2 d2qalq1 d1wi5a1 d1xtda2 d2shpa1 d1yfoa_ d1l8ka_ d2ooqa1
d1g4us2 d1p15a_ d1lara2 d1lara1 d1wcha_ d2fh7a2 d2fh7a1 d1ohea1
d1ohea2 d2f4da_ d1rxda_ d2hxpa_ d1xria_ d1d5ra2 d1fpza_ d3ezza_
d4y2ea_ d1i9sa_ d3azya_ d3wvja_ d1upsa1 d1ajoa_ d2hyka_ d2vy0a_
d1o4ya_ d3o5sa_ d3d6ea_ d3atga_ d1dypa_ d1umza_ d3i4ia_ d1c1la_
d4lbqa_ d1a78a_ d1slta_ d4no4a_ d3zsja_ d3duia_ d1g86a_ d1is3a_
d1qmja_ d1hlca_ d1tdha3 d1q3ba3 d1k82a3 g1qmo.1 g1len.1 d3zyra_
d1fnya_ d1n47a_ g2ltn.1 d1avba_ d3usua_ d1dhkb_ d1f9ka_ d1ukga_

d1g8wa_ d1g9fa_ d3wcsa_ d1g7ya_ g1loe.1 d1mvqa_ d1hqla_ d1v6ia_
d1dbna_ d1qnwa_ d1fx5a_ d4l8qa_ d1ioaa_ d3n35a_ d3ubra_ d1qo8a1
d1d4ca1 d2k3va_ d1ft5a_ d1qdba_ d1m1qa_ d2j7aa_ d1h21a_ d1ogyb_
d4fasa_ d1jnia_ d1sp3a_ d3h33a_ d1rwja_ d19hca_ d1os6a_ d3bxua_
d3h4na_ d1i77a_ d3caoa_ d1j0pa_ d2ctha_ d1duwa_ d1ofwa_ d2e84a_
d1hh5a_ d3h34a_ d1dszb_ d1a6ya_ d3cbba_ d1ynwa1 d1r4ia_ d1hcqa_
d2hana_ d2hanb_ d2enva1 d1kb4b_ d2nllb_ d1lo1a_ d1cita_ d2dara2
d2dara1 d1wyha2 d2dloa2 d1rutx4 d1rutx3 d2cuqa2 d1x4la2 d1x4la1
d1x3ha2 d1x3ha1 d2d8za1 d1x61a2 d1x6aa1 d1x6aa2 d2cora2 d2cora1
d1v6ga1 d1v6ga2 d2d8za2 d1x64a1 d1x64a2 d1b8ta1 d2co8a1 d1x63a2
d2dj7a2 d2dj7a1 d2cu8a2 d2cu8a1 d1g47a2 d1g47a1 d1j2oa1 d1wyha1
d2dloa1 d1wiga1 d1wiga2 d2cupa2 d2cupa3 d1x68a1 d2d8ya2 d2d8ya1
d1m3va2 d1x62a1 d1x68a2 d1x61a1 d1b8ta3 d1b8ta2 d2d8xa2 d2d8xa1
d1x4ka1 d1x4ka2 d2cuqa1 d1m3va1 d2co8a2 d1j2oa2 d1x63a1 d2cura1
d2cura2 d1x62a2 d1op9a_ d1y0la1 d1etzb1 d1egjl1 d2gsib1 d1nj9a1
d4jfhd1 d1aqkl1 d1t2ja_ d1lo4h1 d1zvya1 d3v4vl1 d4enec1 d1kxtb_
d5e8ea1 d2pxya_ d4weud_ d1xiwc_ d2esvd1 d1qnzh_ d1ogae1 d1f4xh1
d4n1hb_ d3ezjb1 d3mlwl1 d1kfah1 d1etza1 d1b4jh1 d3cfic_ d4w68a_
d2arja1 d1ek3a_ d2ok0l1 d1vera_ d1uwgh1 d1blnb1 d1fpth1 d1rzga1
d2giya1 d4m3kb1 d1nc2a1 d2nyyd1 d1ncna_ d1fbih1 d5dxwa_ d4fqqa1
d1dqdh1 d1jfql1 d1kelh1 d1faih1 d1frgl1 d1figl1 d4m6nl1 d1fo0a_
d4hxbl1 d3b5ga_ d3quyd1 d2h9gb1 d1i8kb_ d2dd8h1 d2ck0h1 d1eaja_
d4w6wb1 d2a6ib3 d1t66d1 d2e27h_ d1ob1b1 d1f11b1 d2v7na1 d1ad0a1
d2xt1b1 d1pskh1 d4i13b1 d2op4l1 d1a0ql1 d1uwel1 d1za3a1 d1ncbl1
d4uu9a_ d1bm3h1 d1cloh1 d1nqba2 d1cf8h1 d4y5va_ d1mhph1 d5anma1
d4cdgc1 d4ldeb_ d1y0lb1 d2pcpb1 d1z3gl1 d1dfbl1 d1fh5h1 d3g5xa1
d1tzib1 d4dn3l1 d3zhka1 d2b1hh1 d2uyla1 d1f3dh1 d1nj9b1 d2agjh1
d2h1pl1 d1q72h1 d8fabb1 d1ndmb1 d1rjca1 d4u6hb1 d3upca_ d2fbjl1
d1osph1 d1tvda_ d2cjvh_ d4fhbd1 d1bd2d1 d1rz7h1 d3nn8a_ d1blna1
d1knoa1 d1nfda1 d2bmkb1 d1dqll_ d1igyb1 d1igfl1 d12e8h1 d3utza1
d1nc2b1 d1rihh1 d4mwfb1 d4jvpa1 d1mieh1 d1rhfa1 d1fgnl1 d1sbsh1
d1lp9e1 d1dn0a1 d4lqfl1 d1iaih1 d1kgcd1 d1mh5b1 d1gpol1 d2g60h3
d1f8tl1 d2jixd1 d4grwh_ d1fo0b_ d1bfob1 d1axsa1 d1u9ka_ d1nmbh_
d1h8na1 d1h8na2 d1jguh1 d4eura1 d3sdyl1 d35c8h1 d1w72l1 d1mvub_
d1i9rh1 d1ob1a1 d4wenb_ d1yeeh1 d3ietc1 d1f58h1 d1ad0b1 d1mqkl_
d1nbvh1 d4b50a_ d3r8bb_ d1bafl1 d1mamh1 d4rgne1 d2gsgb_ d35c8l1
d1bafh1 d1ai1h1 d2pcpa1 d2fath1 d2x1pa_ d1orsb1 d1ibgl1 d1shma_
d1a2yb_ d1u0qa_ d2coqa_ d1hilb1 d1qfwi_ d1jv5b_ d1fn4b1 d5e0qa_
d1nsnh1 d1plgl1 d4ocxl1 d1kb5h1 d1rzfh1 d1pg7x1 d2fx7h1 d2oi9c_
d1l7il1 d3eyva1 d1d5il1 d4ffyl_ d1tetl1 d1a6wh_ d1ac6a_ d3dvfa_
d1nfde1 d1mimh1 d1ri8a1 d1jhlh_ d3dura_ d1fskb1 d1f97a1 d3so3b1
d2ak4d1 d1lp9f1 d4z7wf1 d1l7th1 d4y5ya_ d4x7ec1 d1emth1 d1ikfl1
d1nlbh1 d4utal1 d1bjma1 d1eapb1 d1u6ah1 d2dtgd1 d1kj2b_ d1xeda1
d1c1eh1 d1fgvh_ d2bnub1 d2fb4h1 d2dtgb1 d1ktrh_ d1jpth1 d1qkzl1

d4hepg_ d2atpb1 d1flrh1 d2hrpl1 d1mvfa_ d1iaim1 d3k80a1 d2jb5h1
d3zkxc_ d4grwe_ d1it9h1 d1rhhb1 d3w9ec_ d1e4wh1 d1sy6h1 d2j6eh1
d3qxta_ d1jglh1 d1p7ka1 d1tzhb1 d4n8pa1 d1gafl1 d2jelh1 d1wioa2
d4i0cc1 d1cicb1 d1lmka2 d1lmka1 d1iqwh1 d1orqb1 d1ejoh1 d1jnlh1
d4zg1a_ d43c9b_ d1ggbl1 d1wejh1 d3q6fa1 d1f3dj1 d3c6la1 d1bqlh1
d3p9wb_ d1a3rl1 d1h0db1 d2uzil_ d1eo8l1 d1qfwl_ d1n7ml1 d1igja1
d1c5ch1 d2a9mh_ d1rmfh1 d1fj1b1 d4heme_ d1ccza1 d1a5fh1 d1ogad1
d1dlfh_ d1qa9b_ d1vgel1 d3qskb_ d1sjxa_ d4ozfg1 d1sq2n_ d1qd0a_
d1kcvh1 d1c5db1 d3qcul1 d3bn9d1 d1mjuh1 d4y8dc_ d7fabh1 d3dvgb1
d1lo0l1 d1a6tb1 d1gigh1 d1kcrh1 d2hwzl1 d1yjdc1 d3clfl1 d4krna_
d1dl7l_ d1e6ol1 d1mexh1 d3r0ma_ d4kkna_ d1fnsl1 d1oaqh_ d1rzia1
d1ucbh1 d1ncwl1 d4b41a_ d1nfdf1 d2mpah1 d2d7tl1 d4grwf_ d4b5ea_
d1lk3h1 d3u79a_ d4u3xa_ d1ghfl1 d1bvkb_ d1mcww1 d1b88a_ d1jrhh1
d32c2b1 d4wemb_ d1dsfh_ d1op3h1 d1cel1l d1iqda1 d1qoka1 d1qoka2
d1qfuh1 d2crya1 d1ay1h1 d1oaql_ d3ab0b_ d1rurl1 d1t4kb1 d1bz7b1
d1n0xh1 d1forl1 d1ucbl1 d4w6yb_ d43c9a_ d2aabl1 d1tzia1 d4n1ca_
d1k6qh1 d3g9ab_ d4tvsa1 d1igmh_ d1pz5b1 d3dx9a1 d3wxwh_ d1nakh1
d1kcul1 d3eaka_ d4qlra1 d1cr9l1 d1d9ka_ d1f4xl1 d1deea1 d2i27n2
d1c12a1 d2orbl1 d1j8hd1 d1mcph1 d1c5da1 d1kenh1 d1bd2e1 d1h5ba_
d4x7fc1 d3upaa_ d1t3fa1 d1indh1 d2dtgc1 d4hc1l1 d1zv5a1 d4wgvb_
d1uwgl1 d4f37k1 d1aqkh1 d1vesa_ d3zkqd_ d1fptl1 d1lo4l1 d1knob1
d3rrqa_ d1ct8b1 d4orzc_ d1mlbb1 d4hgkc1 d1dqdl1 d1bogb1 d4ozhe1
d1dr9a1 d1i7za1 d1dn0b1 d1kell1 d1rzib1 d1ypzf1 d1wtla_ d1hkfa_
d1fail1 d1figh1 d2vyre_ d1sjva_ d1ohqa_ d1bfoa1 d1pkqb1 d1mjul1
d1b4jl1 d1nsnl1 d2arjb1 d1p4bh_ d4aj0a_ d3nn8b_ d2rcja1 d1f2xk_
d4v1dd_ d1adqh1 d2eksa_ d4k3ga_ d1jfqh1 d1uweh1 d1frgh1 d4gftb_
d1uz8b1 d4n1ea_ d1bm3l1 d1clol1 d3mcka1 d2vq1a1 d1ae6h1 d4aq1b1
d1uj3b1 d1nbqa1 d1fh5l1 d2ck0l1 d2ywza_ d1q72l1 d4nbxb_ d1hila1
d4hzlb1 d2j88l1 d1zmya1 d1cd0a_ d1a4ka1 d1pskl1 d1a0qh1 d4krmb_
d1ncbh1 d2fbjh1 d1j8he1 d1i3ua_ d1cf8l1 d1fe8h1 d2p1ya1 d1eeqa_
d2x89a_ d1ol0a_ d2r0lh1 d1ub5a1 d1dfbh1 d3eyvb1 d4jdvb1 d4p2cg_
d2gsia1 d2agjl1 d1u3ha1 d2h1ph1 d1rihl1 d1fskc1 d1boga1 d2a77l1
d1ospl1 d1qfoa_ d3dwta_ d2fx7l1 d1rz7l1 d3stba1 d1pkqa1 d1b6da1
d1dzba2 d1dzba1 d1kj2a_ d4l1ha_ d1rzgb1 d1dqlh_ d12e8l1 d1miel1
d1jmaa_ d1sbsl1 d1fgnh1 d1f58l1 d1mqkh_ d1ndgb1 d3qegl1 d1iail1
d2aw2a1 d1tqbc1 d3fo2a1 d4dkaa_ d1f8th1 d2g60l1 d2ywya_ d1uj3a1
d1yc7a_ d3bd3a_ d4w6xb1 d1nmbl_ d1i8ka_ d1rjlb1 d1ypze1 d1p7kb1
d1kxqe_ d1gpoh1 d2z4qa1 d1w72h1 d1i9rl1 d4qgya1 d4xt1c_ d2qqnh1
d1cica1 d3c08h1 d2cd0a_ d2bdnh1 d1hnga1 d1maml1 d1nldh1 d1cr9h1
d4lcih1 d3bkyh1 d3qg7l1 d1ai1l1 d1kxvc_ d4iosd_ d1l7ih1 d2x1qa_
d1ub6b1 d3k74b_ d1ibgh1 d1teth1 d1igya1 d1hxma1 d2x6ma_ d2e7la_
d5e03a_ d1qfwm_ d2r0kh1 d1igjb1 d1rurh1 d1plgh1 d1mj8h1 d1yuhb1
d3fmgl1 d1rzfl1 d1emtl1 d1ad9b1 d1igtb1 d1nlbl1 d1d5ih1 d3k7ua_
d4ffyh_ d1bwwa_ d3dvga1 d1a6ta1 d1fgvl_ d4qxgl1 d1miml1 d1jhll_

d2hffb1 d2hh0l1 d1qkzh1 d1l7tl1 d1afvh1 d1iaii1 d2cjuh_ d1um5h1 d2ghwb2 d2ghwb1 d1kgce1 d2fgwh1 d1ikfh1 d1dvfd_ d2i24n1 d2xtjb_ d1za3b1 d2yc1a_ d2hh0h1 d2x1oa_ d1bvka_ d1rjla1 d5e7ba1 d1igta1 d1il1b1 d2fb4l1 d1mvua_ d1jptl1 d32c2a1 d1fvca_ d1iqdb1 d2hrph1 d2p4ab_ d4qo1a_ d1p2cb1 d1wt5c_ d1t4ka1 d5c1mb_ d1it9l1 d1bz7a1 d1b2wh1 d1e4wl1 d1jnll1 d1yedb1 d1za6b1 d3u7aa_ d1igfh1 d3gnml1 d1orsa1 d1wejl1 d1a2ya_ d1hxmb1 d1gafh1 d2j4wh1 d1pz5a1 d1j05a_ d1a3rh1 d4fqll1 d1qfwh_ d1eo8h1 d1n7mh1 d4s11a_ d1kegh1 d5da4a_ d1iqwl1 d3whxb1 d1vgeh1 d1deeb1 d1c12b1 d1t2ql1 d1ad9a1 d3gsnb1 d3b2uc1 d1ggbh1 d1t3fb1 d1nakl1 d4ocmc_ d1lila1 d2v7hb3 d2esve1 d1mcoh1 d2qr0b1 d2q3aa_ d1ct8a1 d1kcrl1 d2pxyb_ d2uzih_ d5c3ld_ d3durb_ d1c5cl1 d2ak4e1 d1dl7h_ d1xiwd_ d1mlba1 d1rmfl1 d1i7zb1 d1dlfl_ d2xa3a_ d5bq7b1 d1dqqb1 d3h42l1 d1egjh1 d1kcvl1 d1fe8l1 d2j6el1 d1f3rb2 d1lk3l1 d1sm3h1 d1q0xh1 d1ghfh1 d7fabl1 d1igch1 d1lo0h1 d4idla_ d5c2bl_ d1jrhl1 d1il1a1 d3zhda1 d4kfzc_ d1b0wa_ d1e6oh1 d1mexl1 d4krpb_ d1fnsh1 d1ncwh1 d4hgma_ d4eigb1 d4rzca_ d2mpal1 d4y5xa_ d5en2b1 d3lrga1 d4ej1c1 d1rhha1 d1j05b_ d1forh1 d1q9wb1 d2wzpd_ d2aq2a1 d1h0da1 d3revb1 d2ij0c1 d1tzha1 d4m7jl1 d1k6ql1 d3b9kc1 d1igml_ d3qeua1 d1dsfl_ d1ce1h1 d1kcuh1 d1zvha1 d1zvoa1 d1tqbb1 d1ay1l1 d2qscl1 d2cgrh1 d1nl0h1 d1kenl1 d3eo0a1 d4n9ob1 d1n0xl1 d3k1kc_ d2aq2b1 d1ck1a1 d1enfa1 d1i4pa1 d1v1oa1 d1et9a1 d1esfa1 d2g9hd1 d4ohja1 d1klud1 d1bxta1 d1eu3a1 d1ty0a1 d2cqva1 d2k1ma_ d2dl2a1 d1biha3 d1biha1 d1biha4 d3qp3a1 d1g1ca_ d1gxea_ d1cvsc2 d1cvsc1 d1xmwa2 d1m4ka2 d2otpa2 d1biha2 d1rhfa2 d2fcba2 d2fcba1 d1wwbx_ d1gsma2 d1epfa1 d1epfa2 d2dava1 d1waaa_ d1f97a2 d1x44a1 d1vcaa2 d1fnla2 d1fnla1 d3b5ha2 d3h8na2 d3h8na1 d1jbja1 d1jbja2 d1pd6a_ d1f2qa2 d1f2qa1 d3cx2a_ d1nbqa2 d3b5ha1 d1n26a1 d1xiwb_ d1xaua_ d1fltx_ d2ifga1 d1olla1 d1olla2 d1cs6a4 d1cs6a3 d1cs6a2 d1cs6a1 d2gw5a2 d1sy6a1 d1xiwa_ d1ucta2 d1ucta1 d1ry7b2 d1gl4b_ d1qz1a3 d1olza1 d2c9aa1 d1wwca_

**Family-13 (a.39.1.2)**

**Positive dataset contains 9 protein sequences.**

d2y5ia_ d3n22a_ d2egda_ d4ggfc_ d2wcea_ d2luca_ d4aqia_ d3nsia_ d2lp3a_

**Negative dataset contains 3965 protein sequences.**

d1orva2 d1xfda2 d1m32a_ d1ibja_ d1iuga_ d4q75a_ d2bkwa1 d1t3ia_ d1n8pa_ d1qz9a_ d1jf9a_ d1eg5a_ d1p3wa_ d3kgwa_ d1cl1a_ d2ctza1 d1vjoa_ d2rfva_ d1gc0a_ d1cs1a_ d1d2fa_ d2ch1a1 d1qgna_ d1pffa_ d1elua_ d1u08a_ d1lc5a_ d2ay1a_ d1bw0a_ d3pd6a_ d7aata_ d2csta_ d1j32a_ d1fg7a_ d4wlha_ d1o4sa_ d1v2da_ d1gdea_ d1m6sa_ d1v72a1 d1svva_ d1ajsa_ d1wsta1 d2f8ja2 d1lk9a_ d1vp4a1 d1xi9a1 d2gb3a1 d1yaaa_ d3i2ka1 d1mpxa2 d1xkta1 d2hfka_ d1jmkc_ d5d3ka_ d2cfba1 d1dfoa_ d1o69a_ d1bj4a_ d3k28a_ d2bwpa_ d3lg0a_ d2fnua_ d3ou5a_ d1z7da1 d1b9ha_ d1iaya_ d4msoa_ d1bs0a_ d1s0aa_ d1sffa_ d3tqxa_

| | | | | | | | |
|---|---|---|---|---|---|---|---|
| d1mdoa_ | d3qboa_ | d3pgya_ | d2dkja_ | d1ejia_ | d1vefa1 | d4wxga_ | d3bs8a_ |
| d3gbxa1 | d1w23a_ | d1i26a_ | d3lgia_ | d3i77a_ | d2z9ia2 | d4nsva_ | d1lcya2 |
| d4jcna_ | d1os8a_ | d1hpga_ | d1p3ca_ | d1l1ja_ | d1qtfa_ | d1cixa_ | d1y29a_ |
| d1v7fa_ | d2jtba_ | d1d1ha_ | d1g9pa_ | d1lupa_ | d1qk7a_ | d1mb6a_ | d2a2va_ |
| d1niya_ | d1i25a_ | d1hvwa_ | d2mpqa_ | d1ie6a_ | d1kqha_ | d1la4a_ | d2mxoa1 |
| d1dl0a_ | d1koza_ | d1qk6a_ | d1emxa_ | d1c6wa_ | d1f56a_ | d1bypa_ | d1azca_ |
| d1ws8a_ | d3u25a_ | d1cuoa_ | d1id2a_ | d1rkra_ | d2iaac_ | d4dp1x_ | d1nwpa_ |
| d2gima_ | d2i7sa_ | d1qhqa_ | d3ehbb1 | d1fftb1 | d1gyca3 | d1hfua2 | d1kbva1 |
| d1kbva2 | d1sdda1 | d1sdda2 | d1gyca1 | d1gyca2 | d1mzya1 | d2bw4a1 | d1aoza1 |
| d1aoza3 | d1aoza2 | d1kyaa3 | d1kyaa1 | d1mzya2 | d4e9sa1 | d1sddb1 | d1sddb2 |
| d1hfua1 | d1hfua3 | d1oe1a2 | d1oe1a1 | d1v10a2 | d1v10a3 | d1v10a1 | g1f8v.1 |
| d1novc_ | d2bbvc_ | d3sxwa1 | d1j3qa_ | d4luka_ | d1a3qa1 | d1iknc_ | d3brda1 |
| d1p7hl1 | d1imhc1 | d1gjia1 | d1zk9a1 | d2cxka1 | d2bema_ | d1pama1 | d1ea9c1 |
| d1qhoa1 | d2fhfa2 | d2fhfa1 | d1k3ia1 | d1j0ha1 | d1cxla1 | d2bhua1 | d1edqa1 |
| d1f1sa2 | d1ut9a2 | d1gvia1 | d1txka1 | d1adqa1 | d1fnga1 | d1uvqb1 | d1zs8a1 |
| d2vola1 | d2vola2 | d1mfbl2 | d2jelh2 | d1uwel2 | d3qq4b1 | d1kjvb_ | d1k5na1 |
| d1osph2 | d4odhl2 | d1ow0a1 | d1ow0a2 | d1bfob2 | d3l9ra2 | d3jtth1 | d2fath2 |
| d4elkb2 | d1fn4b2 | d2xfxb_ | d1pg7x2 | d1cqka_ | d4k71b2 | d1lk2a1 | d1eapb2 |
| d4lfhg2 | d1i4fa1 | d2nw2a2 | d1kfah2 | d1mhca1 | d1h3ua1 | d1wejh2 | d1h0db2 |
| d1a5fh2 | d1kcvh2 | d1mjuh2 | d1qqda1 | d1kcrh2 | d1k8ia1 | d3huja2 | d2nnab1 |
| d1nfdf2 | d3mffb2 | d1s9va1 | d1lk3h2 | d1q0xl2 | d1fv1b1 | d1neza1 | d4rfeb2 |
| d1r3ha1 | d1a6tb2 | d1kjva1 | d1pz5b2 | d1onqa1 | d2bc4a2 | d1de4a1 | d1k8da1 |
| d1tcra2 | d1dn0b2 | d1nfde2 | d2xwtb2 | d1bfoa2 | d1mexh2 | d1iakb1 | d1ypzf2 |
| d4f7ea2 | d1q72l2 | d2fbjh2 | d1fe8h2 | d1clzh2 | d1fngb1 | d1uvqa1 | d1vgka1 |
| d1c16a1 | d4j12a2 | d4x99b2 | d1igtb3 | d1je6a1 | d1q72h2 | d1nldh2 | d1teth2 |
| d2hmic2 | d1i1ca2 | d1i1ca1 | d1k8ib1 | d1q0yh2 | d1kfal2 | d3frua1 | d1pg7w2 |
| d1u58a1 | d1igyb4 | d1igyb3 | d1hyrc1 | d1klub1 | d1deeb2 | d1c12b2 | d2bc4b2 |
| d2ig2h2 | d1kcvl2 | d3wn5b1 | d1lk2b_ | d1lk3l2 | d1mjul2 | d1sm3h2 | d1q0xh2 |
| d1zvsa1 | d1ydpa1 | d1u8ib2 | d1ypze2 | d1kcuh2 | d4alac_ | d1rera1 | d1s6na_ |
| d3uzqb_ | d1jgta2 | d1ofda3 | d1ea0a3 | d1ecfa2 | d1ct9a2 | d1ao0a2 | d1te5a_ |
| d1ep0a_ | d2pa7a1 | d1wlta1 | d1nxma_ | d2c0za1 | d1ds6b_ | d1fstb_ | d4mh8a_ |
| d1mu2a2 | d4i7ga1 | d1tafa_ | d1bh9b_ | d1jfib_ | d1q9ca_ | d1h3ob_ | d2byka1 |
| d1n1ja_ | d4g92b_ | d1bh9a_ | d1jfia_ | d1h3oa_ | d2bykb1 | d1n1jb_ | d2bjca1 |
| d2hsga1 | d1qpza1 | d1efaa1 | d1r0ka3 | d1j5pa3 | d1vkoa2 | d1vm6a2 | d3qvsa2 |
| d1p1ja2 | d2ovga_ | d1sq8a_ | d1rzsa_ | d3kz3a_ | d3gpdg2 | d2q49a2 | d1hdgo2 |
| d2g17a2 | d2cvoa2 | d2czca1 | d3pwka2 | d1obfo2 | d1i32a2 | d1zyao2 | d2pkqo2 |
| d1b7go2 | d2hjsa2 | d1cf2o2 | d1vkna2 | d1nvmb2 | d2msya_ | d2djoa1 | d2r5za_ |
| d1au7a1 | d1f43a_ | d2lmda1 | d1akhb_ | d1s7ea1 | d3nara_ | d1zq3p1 | d2craa1 |
| d1mija_ | d4uuta_ | d3a01a_ | d1x58a1 | d3rkqa1 | d3cmya1 | d2cqxa1 | d2da6a1 |
| d1e3oc1 | d1fjla_ | d1mh3a1 | d1ig7a_ | d1x41a1 | d1wh7a1 | d1gt0c1 | d2mw8a_ |
| d1lfup_ | d1x2ma1 | d1b72a_ | d1wi3a1 | d1jgga_ | d1wjha1 | d1x2na1 | d1mnmc_ |
| d2cuea1 | d3k2aa_ | d2hosa_ | d2jwta2 | d1pufa_ | d1octc1 | d2hi3a_ | d1b8ia_ |
| d4j19a_ | d3lnqa_ | d1wh5a1 | d1b8ib_ | d1fexa_ | d2cqra1 | d1guua_ | d1ug2a1 |
| d2cqqa1 | d2ajea1 | d4a69c_ | d1h8ac1 | d2crga1 | d2iw5b1 | d1xc5a1 | d2cu7a1 |

d1wgxa1 d2cjja1 d2ckxa1 d1gvda_ d1hcra_ d1gdta1 d1u78a2 d6paxa2
d1k78a1 d1k78a2 d1hlva1 d1iufa2 d1iufa1 d1igna2 d2iu5a1 d1zk8a1
d2g7sa1 d2fd5a1 d2np5a1 d1z0xa1 d2id6a1 d2d6ya1 d2i10a1 d1vi0a1
d2oi8a1 d3c07a1 d2ns7a1 d2fq4a1 d2yvea1 d1t33a1 d2o7ta1 d1rkta1
d2np3b1 d2g7ga1 d2gena1 d2fbqa1 d2g7la1 d2gfna1 d2g3ba1 d2jj7a1
d2hyja1 d1sgma1 d1ui5a1 d2hkua1 d2id3a1 d4jyka1 d1bl0a1 d1d5ya1
d1d5ya2 d3lv8a_ d1s3ga1 d1zaka1 d1s4qa_ d3tvta2 d1uf9a_ d1khta_
d1ckea_ d1osna_ d1p5zb_ d4tmka_ d3cm0a_ d1tmka_ d1jaga_ d3h86a_
d1t0hb_ d1nn5a_ d2h92a_ d3kb2a1 d2c95a1 d1yj5a2 d1kgda_ d1ly1a_
d2ak3a1 d1vyva2 d1deka_ d2grja1 d1p3ja1 d1y63a_ d1e4ya1 d1s96a_
d3iija1 d1lvga_ d1nksa_ d1vyua2 d4e22a_ d1qf9a_ d1jjva_ d1q3ta_
d1ki9a_ d1lw7a2 d1teva_ d1kjwa2 d2iyva2 d1viaa_ d1e6ca_ d1g3ma_
d1t8ta1 d1nsta_ d1fmja_ d1zrha_ d1ls6a_ d2a3ra_ d1aqua_ d2zptx_
d3ckla_ d1texa_ d1q44a_ d2gwha_ d1odfa_ d4gi7a_ d2jeoa1 d1rz3a_
d1z0ja1 d3umra2 d3gj0a1 d2gaoa1 d1yzga1 d1x3sa1 d1g7sa4 d1puja_
d1udxa2 d1kk1a3 d2o52a_ d1jwyb_ d1zcaa2 d1zcba2 d2fv8a1 d2cjwa_
d3dz8a1 d1xzpa2 d1vg8a_ d4qxaa_ d2ew1a1 d1x1ra1 d2bcgy_ d3doea1
d3cona_ d3rega_ d3wyga_ d1upta_ d2b6ha1 d1zd9a1 d1lnza2 d1r5ba3
d1n0ua2 d1r2qa_ d2f9la1 d2rhda_ d3q3jb_ d3q85a_ d1efma1 d1r8qa_
d2gf9a1 d2akab1 d1ek0a_ d1z08a1 d2gcoa1 d2dy1a2 d4g01b_ d2cxxa1
d1svia_ d3q72a_ d1ni3a1 d4ac9a4 d2erxa1 d2bmea_ d1wxqa1 d2gf0a1
d4ldja1 d1zunb3 d1d5ca_ d4lv5b_ d1efca3 d1kmqa_ d2g6ba1 d3lrpa_
d1tada2 d2y8ea_ d4rd4a1 d1z06a1 d2heia_ d3a58b_ d1u0la2 d1d2ea3
d1zj6a1 d2ocba_ d3rwoa_ d1mr3f_ d2e9sa1 d4u5xa1 d2iwra_ d2fn4a1
d3bwdd_ d2nzja1 d2atva1 d2ksqa_ d3l43a_ d1mkya2 d1mkya1 d1wmsa_
d1jnya3 d2rmka_ d1f6ba_ d1f60a3 d1gota2 d1jala1 d2fh5b1 d1g16a_
d2wblc_ d1m7ba_ d1wf3a1 d1f5na2 d1t9ha2 d1z6xa1 d1z0aa1 d2fg5a1
d2a5ja1 d2bcjq2 d1s0ua3 d1ky3a_ d4y0va_ d4lhwa_ d2qmea1 d2atxa1
d1egaa1 d1ryha_ d1moza_ d1nrjb_ d2erya1 d3bfka_ d1puia_ d3j3az1
d3t5ga_ d1shza2 d3defa_ d2f7sa1 d3raba_ d3tsoa_ d3aq4a_ d2al7a1
d3sfva1 d3bc1a_ d2ncda_ d1sdma_ d3h4sa1 d2heha_ d2grya_ g3kin.1
d1kk8a2 d1br2a2 d1f9va_ d2mysa2 d1lkxa_ d3t0qa_ d4db1a1 d3gbja_
d1ry6a_ d1v8ka_ d1k8ka2 d1k8ka1 d3uleb1 d3i33a2 d3i33a1 d1bupa1
d1dkgd2 d1dkgd1 d4ci6a2 d1jcfa1 d4a2ba2 d2fxua1 d1yaga2 d1yaga1
d4cbua2 d1bupa2 d3vrda1 d3vrda2 d1fcdc2 d1fcdc1 d1h1oa2 d1h1oa1
d1m70a1 d1iqca2 d1iqca1 d1eb7a2 d1eb7a1 d1nmla1 d1nira1 d1qksa1
d3h1jd1 d1hroa_ d4gedb_ d3zooa_ d1mz4a_ d1gu2a_ d1f1fa_ d1dvva_
d1c6ra_ d1c75a_ d1e29a_ d1gdva_ d1cnoa_ d1h32b_ d2bh4x_ d2aiua_
d5cytr_ d1jdla_ d1i8oa_ d2zboa_ d3dmia_ d1ls9a_ d1wvec_ d2yk3a_
d3ph2b_ d1dw0a_ d5aura_ d1ql3a_ d1qn2a_ d1f1ca_ d4zzva_ d2xspa_
d1eg1a_ d4v20a_ d4ipma1 d1gpia_ d4csia_ d3pfza_ d3ovwa_ d2yoka_
d2nqya_ d1h4ga_ d1oa2a_ d1olra_ d3b7ma_ d4h7ma1 d3wp3a_ d2vgda_
d1igoa_ d3zsea_ d2nlra_ d3wx5a_ d1oa3a_ d1pvxa_ d1m4wa_ d1f5ja_
d1oa4a_ d1ks5a_ d4ixla1 d3sita1 d1h32a2 d1evsa_ d1cnt1_ d1pvhb_

d1z7ca_ d1huwa_ d1bgea_ d1rhga_ d1f6fa_ d1f45b_ d1i1rb_ d1axia_
d1etea_ d3tgxb_ d2z3qa1 d3qb1a_ d1hula_ d1m48a_ d4nkqb_ d1eera_
d1v7mv_ d5e4ea_ d2o27a_ d3b5ka_ d2psma1 d4zf7a_ d3uf5a_ d4x51a_
d1d9ca_ d4ypgc1 d3oq3a_ d1lqsl_ d1n1fa_ d1au1a_ d1wu3i_ d3besl_
d3ux9a_ d1v8fa_ d2ejca_ d3muea1 d3q12a1 d4dnxa2 d1m8pa2 d1g8fa2
d1jhda2 d1x6va2 d3rbaa_ d2qtra_ d1nupa_ d3l92a_ d1f9aa_ d1qjca_
d2h29a_ d1vlha_ d1od6a_ d1lw7a1 d1mrza2 d4ruka_ d2el7a_ d1iq0a2
d1irxa2 d1r6ta2 d1ivsa4 d2cyba_ d1rqga2 d1j09a2 d2yxna_ d1f7ua2
d1h3fa1 d1ffya3 d2g36a1 d1nzja_ d1jila_ d1n3la_ d1pfva2 d1h3na3
d3n9ia_ d3sz3a1 d1cmxa_ d1xd3a_ d3ifwa_ d3sw2a_ d1moxc_ d1dx5i3
d1edmb_ d1autl2 d1autl1 d1nqlb_ d1dval1 d1szba2 d1kigl_ d2y5fl_
d1ijqa2 d1gk5a_ d1g1sa2 d1nt0a3 d1xdtr_ d1lmja2 d1lmja1 d1nzia2
d2wphe_ d1klil_ d1pfxl2 d1pfxl1 d1p9ja_ d1g1ta2 d1f7ea_ d4yzub_
d1s5ja2 d1qhta2 d4dlea2 d1tgoa2 d3py8a2 d1q8ia2 d2py5a2 d1d5aa2
d1sfsa_ d1jfxa_ d1wkya2 d1uuqa_ d3wh9a_ d4m1ra_ d4xuya_ d2je8a5
d4bf7a1 d3cuia_ d1ur4a_ d1qw9a2 d1odza_ d2vzsa5 d1bqca_ d2x2ya1
d1hjqa_ d1qnra_ d4y7ea1 d1vjza_ d4k68a_ d2v3ga_ d4hpga_ d1nofa2
d1nq6a_ d1h4pa_ d1aq0a_ d3ziza_ d4bs0a_ d1ecea_ d1h1na_ d1ta3b_
d1w91a2 d1i1wa_ d1uhva2 d1us3a2 d1egza_ d7a3ha_ d1tvna1 d3nyda1
d1ghsa_ d2cyga1 d4xv0a_ d2c8na2 d1w32a_ d3emca_ d1v6ya1 d1foba_
d2uwfa1 d2fgla_ d3niya1 d2c0ha1 d1rh9a1 d1yq2a5 d1szna2 d1fa2a_
d1pama4 d5do8a1 d1ea9c3 d1hx0a2 d2fhfa5 d1qhoa4 d1avaa2 d1m53a2
d4m8ua1 d2gjpa2 d1lwha2 d4howa1 d1ktba2 d1j0ha3 d3vm5a1 d2x1ia_
d1wzaa2 d1uasa2 d2diea1 d1mxga2 d1ht6a2 d1eswa_ d1iv8a2 d5cpsa_
d1ud2a2 d1zjaa1 d3gbea1 d1kwga2 d1wdpa_ d1gcya2 d1g5aa2 d1hvxa2
d3dhpa2 d2bhua3 d4do4a1 d3qgva1 d1e43a2 d1g94a2 d2d3na2 d1gjwa2
d1gvia3 d1x1na1 d1r7aa2 d1tz7a1 d2x42a1 d1tr9a1 d3abza1 d4toqa_
d1wb0a1 d1w9pa1 d1eoka_ d1itxa1 d1kfwa1 d1goia2 d1edqa2 d2gsja_
d1hjxa1 d1jnda1 d1vf8a1 d3wdpp1 d3wq5a_ d4gxpa_ d4hz8a_ d1e4mm_
d3aiua_ d1wcga1 d1qvba_ d3ahxa_ d1v02a_ d2dgaa_ d1pbga_ d1vffa1
d1qoxa_ d4ha4a_ d3syxa1 d1mkea1 d2p8va_ d1xoda1 d1i7aa_ d1evha_
d1qc6a_ d1egxa_ d2v76a_ d1wj1a1 d1aqca_ d1qqga2 d1qqga1 d1n3ha_
d4dbba_ d3f0wa1 d1p3ra_ d1wgua1 d2cy4a1 d3hqca_ d1wvha1 d1xr0b1
d3nvva1 d1krha3 d2h88b1 d1t3qa2 d1kf6b2 d1zoyb1 d1jq4a_ d1ffva2
d1dgja2 d1rm6c2 d1h6ta1 d9ldta1 d2ewda3 d1up7a1 d2dfda1 d1s6ya1
d1vjta1 d7mdha1 d1hyea1 d1t2da1 d1ojsa3 d1b8pa1 d1hyha1 d1civa1
d2a92a1 d1ez4a1 d1i0za1 d1y6ja1 d1gv0a1 d1ur5a1 d1llda1 d1obba1
d1v8ba1 d1gdha1 d1l7da1 d2dlda1 d1mx3a1 d1ygya1 d1qp8a1 d1j4aa1
d2naca1 d1npya1 d3t4ea2 d1a4ia1 d1o0sa1 d2hk9a2 d2egga2 d1b26a1
d1bvua1 d1gq2a1 d1c1da1 d1leha1 d1gtma1 d1euza1 d1nvta1 d3dona2
d1edza1 d1gpja2 d1vi2a1 d1p77a1 d1nyta1 d1b0aa1 d1vl6a1 d1v9la1
d1txga2 d3f47a1 d1mv8a2 d2pv7a2 d1xc2a3 d2ahra2 d4xiya1 d2f1ka2
d1pgja2 d1i36a2 d1np3a2 d1lj8a4 d1teaa3 d1qmga2 d2i76a2 d2g5ca2
d1wdka3 d1dlja2 d3cuma3 d1kola2 d1f8fa2 d1qora2 d1jqba2 d1vj0a2

d1cdoa2 d1gu7a2 d1d1ta2 d1xa0a2 d2dm6a2 d1jvba2 d1rjwa2 d1h2ba2
d1tt7a2 d1piwa2 d1o89a2 d1iz0a2 d1e3ja2 d1yb5a2 d1uufa2 d1vj1a2
d1e3ia2 d3gpdg1 d1vc2a1 d2nvwa1 d1u8fo1 d1ydwa1 d1hdgo1 d2g17a1
d1cf2o1 d2czca2 d1r0ka2 d1obfo1 d2cvoa1 d1ebfa1 d1h6da1 d1i32a1
d1zh8a1 d2q49a1 d1qkia1 d1j5pa4 d3pzra1 d1xeaa1 d1vkoa1 d1vm6a3
d3qvsa1 d2pkqo1 d1gr0a1 d1p1ja1 d1lc0a1 d1j0xo1 d3pwka1 d2hjsa1
d1zyao1 d1vkna1 d1tlta1 d1nvmb1 d3sjua_ d3ojfa1 d1xu9a1 d2jjya1
d1n7ha_ d1y1pa1 d1h5qa_ d1jtva_ d4fs3a_ d2bkaa1 d1gy8a_ d2c5aa1
d1ulsa_ d1ooea_ d2b69a1 d1y5ma1 d1spxa_ d1ae1a_ d1bxka_ d1ydea1
d1gega_ d1hdoa_ d1e6ua_ d1snya1 d2rhca_ d3zv4a_ d5ceja_ d2d1ya1
d1fjha_ d3w8ea_ d2fmua1 d1x1ta_ d1rpna_ d3ay7a1 d1k2wa_ d1eq2a_
d2hrba1 d3ruaa_ d2ew8a1 d4hwka_ d1xgka_ d2bd0a1 d1yo6a1 d2a35a1
d2ntva_ d1vl8a_ d1hdca_ d4bkua_ d2ag5a1 d2q45a_ d1nffa_ d4iy1a_
d1zbqa1 d1kewa_ d1ek6a_ d1ja9a_ d2q46a2 d1iy8a_ d1cyda_ d2ptga_
d1hxha_ d1uzma1 d3lyla_ d4m89a_ d3wyea_ d2blla1 d1w6ua_ d1sb8a_
d2q2va_ d4nr0a_ d1e6wa_ d2gasa_ d3oiga1 d2bgka1 d1mxha_ d1vl0a1
d1rkxa_ d1r6da_ d1wmaa1 d1e7wa_ d2c07a1 d1xkqa_ d3tzha_ d1g0oa_
d3lz6a_ d1qyca_ d1edoa_ d1gz6a_ d1oc2a_ d1t2aa_ d1zema1 d2xoxa_
d1z45a2 d1geea_ d1p33a_ d3lt0a_ d4e6pa_ d2wyua_ d1db3a_ d4q9na_
d1xhla_ d4o1ma_ d1fmca_ d2uvda_ d2gdza1 d1uaya_ d2z1ma_ d2o23a2
d2fr1a2 d2fr1a1 d1qsga_ d1qyda_ d4zjua_ d2a4ka1 d1yb1a_ d1wvga1
d1yxma1 d3f4ba_ d1mg5a_ d1xg5a1 d1r55a1 d3gboa_ d1atla_ d1buda_
d4q1la_ d1quaa_ d1yp1a_ d1wnia_ d1e2ta_ d4c5pa_ d2bsza1 d4bgfa_
d1w4ta1 d1g0da4 d2f4ma1 d1x3za_ d1mdwa_ d1qxpa4 d1ziva1 d1zcma1
d3ioqa_ d1fh0a_ d1o0ea_ d1m6da_ d2cb5a_ d1cs8a_ g8pch.1 d1s4va_
d2pnsa_ d1vsna1 d1mira_ d1iwda_ d2oula_ d7pcka_ d3bwka_ d3bcna_
d2p86a_ d1pvja_ d1pcia_ d1gece_ d1deua_ d4bs6a_ d1xkga1 d1cqda_
d1jqpa2 d2c0ya_ d2bdza_ d3p5ua_ d2o36a_ d1k9xa_ d1zoya1 d1d4ca2
d1chua2 d1jnra2 d1kf6a2 d2jk6a2 d2jk6a1 d1feca1 d1feca2 d1lpfa1
d1lpfa2 d1xhca1 d1xhca2 d1dxla2 d1dxla1 d1gera2 d1m6ia2 d1onfa1
d1onfa2 d3lada2 d3lada1 d1w4xa1 d1w4xa2 d1gesa2 d1mo9a1 d1xdia1
d1fl2a2 d1fl2a1 d1fcda1 d1fcda2 d1h6va1 d1mo9a2 d1gv4a2 d1gv4a1
d2gv8a2 d2gv8a1 d1aoga1 d1aoga2 d1hyua1 d1ebda1 d1ebda2 d3dk9a1
d1q1ra2 d1q1ra1 d1gesa1 d1zbfa1 d2z1ha_ d2etja1 d3qioa1 d1jl1a_
d2e4la_ d1jl2a_ d2lsna1 d1io2a_ d1uaxa_ d2yv0x_ d1mu2a1 d1ekea_
d1i39a_ d1pn0a1 d1reoa1 d1ju2a1 d2f5va1 d3c96a1 d2gmha1 d1pj5a2
d1gpea1 d1o5wa1 d1n4wa1 d1k0ia1 d2dw4a2 d2ivda1 d1seza1 d2voua1
d1kdga1 d1s5ja1 d2hbja2 d2igia_ d1wlja_ d1qhta1 d4dlea1 d1tgoa1
d2f96a1 d3tr8a1 d3mxma_ d1q8ia1 d1y97a1 d1w0ha_ d1kfsa1 d2py5a1
d1noya_ d1d5aa1 d1yt3a3 d2guia_ d3mwpa1 d4kb1a_ d1iv0a_ d1nu0a_
d2d5ra1 d1js9c_ d1f15b_ d1za7a_ d1laja_ d3ljqa_ g1k2x.1 d1b9ra_
d1offa_ d1frra_ d3wcqa_ d1e9ma_ d1wria_ d1i7ha_ d3av8a_ d1czpa_
d1rfka_ d3p1ma1 d3b2ga_ d2bt6a2 d1jmxa4 d1jmxa3 d1pbya3 d1r7ha_
d1faaa_ d1wika1 d1xfla_ d1ttza1 d2f51a1 d1ti3a_ d1nhoa_ d1sena_

d2i4aa_ d1ep7a_ d1zmaa1 d1fo5a_ d3lefa1 d3diea1 d2yana1 d1z6na1
d1oaza_ d3zywa1 d4aj8a_ d1nm3a1 d4fiwa_ d1wmja_ d4hs1a1 d1iloa_
d1xwaa1 d1wjka1 d2vm1a_ d1fb6a_ d1gh2a_ d1h75a_ d1nw2a_ d1kx5b_
d3a6na_ d4m6ba2 d2nqbd_ d3wkjd_ d2yfwe_ d4h9na_ d5bnxa_ d1kx5c_
d3r45a_ d1eqzb_ d4jjnb_ d1f66c_ d1eqza_ d4wnnb_ d1kx5a_ d1u35c1
d1id3a_ d2nqbc_ d1kx5d_ d2b5ea2 d2b5ea3 d2b5ea1 d2b5ea4 d2kp1a1
d2kp2a1 d1hyua3 d1hyua4 d1j08a1 d1j08a2 d1pd212 d1nhya2 d2a2ra2
d1k0dd2 d1f2ea2 d1gsua2 d1f3ba2 d2fhea2 d2pvqa1 d1tw9a2 d1oyja2
d1e6ba2 d1k0ma2 d3ik7a1 d4is0a1 d1jlva2 d1m0ua2 d3gtub2 d1z9ha2
d1okta2 d1axda2 d2gsra2 d1tu7a2 d1k3ya2 d1zgma1 d1b48a2 d1gnwa2
d1gwca2 d2gsta2 d1glqa2 d1oe8a2 d1pn9a2 d1v2aa2 d1fw1a2 d1duga2
d1ig8a1 d1czan2 d1czan3 d1czan1 d1ig8a2 d1czan4 d1bg3a4 d1bg3a3
d1bg3a2 d1qusa_ d1sefa_ d1sfna_ d1rc6a_ d1qqya_ d1gd6a_ d1f6ra_
d1ivma_ d2gv0a_ d1iiza_ d2z2fa_ d1j8xa_ d1ghla_ d1b9oa_ d174la_
d169la_ d2o4wa_ d1p37a_ d1k28a3 d1xjta1 d1jtma_ d2f2qa_ d4e97a1
d1g4ma1 d1g4ma2 d2pjsa1 d2i7ra1 d1ecsa_ d1xqaa1 d5cj3a1 d1npba_
d1jifa_ d2p7oa_ d1r9ca_ d1nkia_ d1mpya2 d1kw3b2 d1kw3b1 d4ghga1
d1tfza1 d3isqa1 d3isqa2 d1sqia1 d1sqia2 d1lkda2 d1lkda1 d1mpya1
d1cjxa2 d1cjxa1 d1sp9b2 d1t47a1 d1t47a2 d1f1ua2 d1sp8a1 d1sp8a2
d1pv8a_ d1w5qa_ d2c1ha_ d1ohla_ d1l6sa_ d1qsta_ d2beia1 d4m3sa_
d1u6ma1 d1ygha_ d1yk3a1 d3dr6a_ d1xmta_ d1z4ea1 d2ge3a1 d1bo4a_
d2fiaa1 d2jdca1 d1y9wa1 d3to7a_ d1wwza1 d2fl4a1 d2ozua1 d2b5ga_
d2cy2a1 d2p0wa_ d1cm0a_ d4pswa_ d1yrea1 d1yr0a1 d1m4ia_ d2gana1
d1yvka1 d1ghea_ d2g3aa1 d1y7ra1 d2euia1 d1q2ya_ d2fsra1 d1xeba_
d2ae6a1 d1qsma_ d2aj6a1 d1y9ka1 d1i12a_ d1vkca_ d2atra1 d1n71a_
d2d4pa1 d2fiwa1 d2fe7a1 d1tiqa1 d2ou2a_ d1yx0a1 d2fcka1 d1p0ha_
d2j8ma_ d2pq8a_ d1z4ra1 d1s3za1 d1ufha_ d1c4qa_ d1prtc1 d2wv6d_
d1prtf_ d1ltrd_ d4l63b1 d1r4pb_ d1prtd_ d1tl0f_ d2bosa_ d1prtb1
d3chbd_ d1xvqa1 d1pn0a2 d3hy2a_ d2cvba1 d1o8xa_ d1lu4a_ d1we0a1
d2bmxa1 d2a4va1 d1psqa_ d1xiya1 d1q98a_ d1n8ja_ d1zofa1 d2ggta_
d3tjja_ d4je1a1 d1o73a_ d3s9fa_ d2zcta_ d1qmva_ d2b1ka_ d5dvba1
d2b5xa1 d2v2ga_ d1st9a_ d3tb2a_ d4llra_ d4fh8a1 d1e2ya_ d1knga_
d2h30a_ d1i5ga_ d2z9sa_ d2f8aa1 d1tp9a1 d2b7ka_ d1nm3a2 d3hvva_
d2wfca1 d4kcea_ d1xvwa1 d4gqca1 d3qpma_ d4k1fa_ d1zzoa1 d1gp1a_
d1prxa_ d4od7a1 d1z6ma1 d4mcua1 d4dvca1 d3l9sa_ d1un2a_ d3rppa_
d1zsqa1 d1f74a_ d3l21a_ d2e1da_ d1to3a1 d1w3ia_ d1sfla_ d1n7ka_
d1vcva1 d4imea_ d1wa3a_ d2ocza1 d1f2ja_ d3m16a_ d2yxga_ d3a5fa_
d1fbaa_ d1l6wa_ d1xfba1 d1wx0a1 d1vpxa1 d1ojxa_ d1mxsa_ d2a4aa1
d1xkya1 d3cq0a1 d3s1xa_ d1f05a_ d1fdja_ d3cpra1 d4d2ja_ d2phla2
d2phla1 d1fxza2 d1fxza1 d1lrha_ d1uija1 g1dgw.1 d2v09a_ d1uika2
d1uika1 d1dgwa_ d1e88a1 d1l6ja5 d1l6ja4 d1eaka4 d1eaka3 d1l6ja3
d1h8pa2 d1h8pa1 d1etha2 d1w52x1 d2ppla1 d1hpla2 d1lpbb2 d2oxea1
d1bu8a2 d1rp1a2 d1ys1x_ d4hs9a_ d1ex9a_ d1ku0a_ d3qmma_ d2h1ia1
d2r8ba1 d3b5ea1 d3cn9a_ d1lgya_ d4l3wa_ d1gz7a_ d1ukca_ d2hqya1

d2hqya2 d1lrza2 d1lrza3 d1ne9a2 d1cr6a2 d1ixra2 d3groa_ d1pjaa_
d1thta_ d1ra6a_ d1muka_ d3gsza_ d1khva_ d1te9a_ d2xwha_ d3uqsa_
d3hkwa_ d3n6ma_ d2cjqa_ d2xyma_ d1teba_ d1te8a_ d1s48a_ d1u09a1
d4wfxa_ d3tyqa_ d2pgga1 d3zxha_ d1rm8a_ d1eaka2 d1hy7a_ d2jsda_
d1cxva_ d1hv5a_ d1i76a_ d3p0ya1 d1igra1 d1igra2 d1n8yc1 d1m6ba2
d1m6ba1 d1dzka_ d4tlja_ d2a2ua_ d4wfva_ d3t1da_ d4iaxa_ d3zq3a_
d2nnda_ d1i4ua_ d1kxoa_ d1bbpa_ d1kt7a_ d1exsa_ d3s26a1 d1qfta_
d4gh7a_ d1z24a1 d1qabe_ d1xkia_ d1ew3a_ d3bx7a_ d4iawa1 d1n0sa_
d1gkab_ d3kzaa_ d2k23a_ d2l9ca1 d2joza1 d1gm6a_ d3kffa_ d3saoa1
d1e5pa_ d1yupa1 d1iiua_ d2tbvc_ d1c8nc_ d1x36a_ d1smvc_ d1ng0c_
d4sbvc_ d1f2nc_ d1ddla_ d1e57b_ d1rmka_ d1ixta_ d1eyoa_ d1cnna_
d1fu3a_ d1g1pa_ d1f3ka_ d1fyga_ d1rypd_ d1irua_ d1rypc_ d3unfk_
d1rypa_ d1j2pa_ d1irui_ d1j2qh_ d3unfh_ d1m4ya_ d1rypb_ d1irul_
d1irug_ d1iru2_ d1q5rh_ d1rypg_ d1g3ka_ d1iruf_ d1q5qa_ d1e94a_
d1irub_ d1ryph_ d3ty6a_ d1iruj_ d1iruk_ d1rype_ d1irud_ d1irue_
d3mi01_ d1rypf_ d1iruh_ d2z3ba_ d3mka2_ d1iru1_ d1mqta_ d1d4m3_
d1fmd3_ d2mev1_ g1aym.1 d1ev13_ d4q4w1_ d1r1a3_ d2x5ia_ d1rhi1_
g1r1a.1 g1d4m.1 g1rhi.1 d1b35b_ d1oopa_ d1tme1_ d4iv1a_ g1pvc.1
g1fpn.1 d1fpn1_ d4gb31_ d1d4m1_ d1fmd1_ d1ev11_ d2wzr3_ g1cov.1
d1qqp3_ g1fmd.1 d2x5ic_ d1rhi3_ g1oop.1 d1hxs1_ g1hxs.1 d1fpn3_
d1bev1_ g2mev.1 d1eah3_ d1aym1_ d1qqp1_ d1zba3_ d1h8ta_ d3vbhc_
g1b35.1 g1bev.1 d1hxs3_ g1ev1.1 d1bev3_ g1tmf.1 d1tmf1_ d1b35a_
d2mev3_ g1eah.1 g1h8t.1 d1eah1_ d4q4w3_ d1r1a1_ d1aym3_ d1pvc1_
d1zba1_ d1h8tc_ d1oopc_ d1tme3_ g1qqp.1 d4iv1c_ g1tme.1 d3ubub_
d1egia_ d1v7pa_ d1tdqb_ d3ubua_ d1c3ab_ d1h8ua_ d3bx4b_ d1t8ca1
d1sb2a1 d1qdda_ d2ziba1 d3cada_ d1byfa_ d1j34b_ d1jwia_ d1jzna_
d1c3aa_ d1oz7b_ d1wmza_ d3bdwb_ d1hq8a_ d1fifa1 d1dv8a_ d3hupa_
d1wk1a1 d1umra_ d2afpa_ d3bx4a_ d1gz2a_ d1sl6a_ d1v7pb_ d3g8ka_
d1ixxa_ d1j34a_ d1jwib_ d1g1sa1 d1y17a1 d1ja3a_ d1fvub_ d3bdwa_
d1sb2b1 d1fvua_ d1umrc_ d1oz7a_ d4m17a_ d3cfwa1 d1ypqa1 d1g1ta1
d3fd4a_ d3l9jc_ d4dn8a1 d1v4lb_ d1rdl1_ d2msba_ d1eixa_ d3exra_
d2ffca1 d2fdsa1 d3n3ma1 d2czda_ d1q6oa_ d2yyua_ d1vqta1 d1dbta_
d3ldva1 d2flia1 d1h1ya_ d1tqja_ d1tqxa_ d4lt9a1 d3tc7a1 d3hoja_
d1i4na_ d3vnda_ d3nl8a1 d1geqa_ d3nava1 d4a2ra1 d3tc6a1 d1ujpa_
d4ijba_ d1xcfa_ d1vc4a_ d1rd5a_ d1qopa_ d3b5va_ d2r80b_ d1cqxa1
d1cg5a_ d1gcvb_ d3boma_ d2h8fb_ d2raoa_ d3bj1a_ d4hrrb_ d2r50a_
d3d1kb_ d2aa1b1 d3zhwa_ d3hf4b_ d3gdja_ d1jebb_ d3bj1b_ d1x9fb_
d1wmub_ d1gcva_ d3bcqb_ d3d4xb_ d1gvha1 d2dc3a_ d3hf4a_ d4yu3a_
d3k8ba_ d1qpwb_ d2r80a_ d3bcqa_ d1v4wa_ d3wtgb_ d1jl7a_ d4esaa_
d1a9we_ d3d1ab_ d1fsla_ d4h2la_ d1v4wb_ d1mwca_ d1vhba_ d1jeba_
d1spgb_ d1h97a_ d1q1fa_ d1tu9a1 d3d1aa_ d1hdsb_ d3hyub_ d2zfbb_
d3fs4a_ d1oj6a_ d1shrb_ d4h2lb_ d4yu3b_ d1spga_ d3hrwa_ d3qqra_
d3at5a_ d4wchd_ d3hyua_ d1cg5b_ d1uc3a_ d1hbra_ d2raob_ d1outb_
d4esab_ d3gdjb_ d3qqqa_ d1x9fc_ d3vrfa_ d1itha_ d3mkba_ d3d4xa_

| | | | | | | | |
|---|---|---|---|---|---|---|---|
| d3mjpa_ | d1jl6a_ | d3fh9a_ | d1outa_ | d2dc3b_ | d1it2a_ | d3eoka_ | d3g46a_ |
| d1qpwa_ | d3vrfb_ | d1ch4a_ | d1x9fd_ | d1hdsa_ | d2zfba_ | d3mkbb_ | d1or4a_ |
| d3wtga_ | d1a4fa_ | d3fh9b_ | d3bomb_ | d3cy5a_ | d3at5b_ | d1x9fa_ | d2oifa_ |
| d4lmxb_ | d1cpcb_ | d3v57a_ | d1kn1a_ | d1cpca_ | d4f0ta_ | d2vjha_ | d4po5b_ |
| d1alla_ | d4f0tb_ | d1b33b_ | d1b8db_ | d4h0mb_ | d1b33a_ | d2vjhb_ | d4lm6b_ |
| d2vmlb_ | d1allb_ | d2vjta_ | d3dbja_ | d2vmla_ | d2vjtb_ | d4f0ub_ | d1f99b_ |
| d4rmpa1 | d1liaa_ | d1eyxb_ | d2uulb_ | d4lmsb_ | d4f0ua_ | d2bv8a_ | d1f99a_ |
| d2bv8b_ | d2c7lb_ | d3dbjb_ | d4rmpb1 | d1b8da_ | d4h0ma_ | d3v57b_ | d1kn1b_ |
| d1liab_ | d2uula_ | d2j96a_ | d1kyqa1 | d3ia2a_ | d1a88a_ | d4dgqa1 | d1zoia_ |
| g1gxs.1 | d1azwa_ | d1mtza_ | d2xqfa_ | d1ea5a_ | d1f6wa_ | d1qe3a_ | d1k4ya_ |
| d4qwwa_ | d2ha2a_ | d3c8da2 | d2pbla1 | d1jkma_ | d1jjia_ | d1lzla_ | d2i49a_ |
| d1nnfa_ | d1xvxa_ | d1ursa_ | d1h3da1 | d1iuda_ | d4m1va1 | d1twya_ | d3e13x_ |
| d2nxoa1 | d4pfua_ | d3ttma_ | d1elja_ | d2qmwa1 | d1us5a_ | d1y3na1 | d1jeta_ |
| d1uqwa_ | d4f19a1 | d2esna2 | d3thia_ | d3fasa1 | d2uyeb_ | d2xwva1 | d1r9la_ |
| d2fyia1 | d1pb7a_ | d2ozza1 | d1sw5a_ | d1nh8a1 | d1vr5a1 | d2g29a_ | d1xs5a_ |
| d3lswa_ | d2z23a_ | d3w9va_ | d3tcha_ | d1q35a_ | d1z7me1 | d3q27a_ | d1p99a_ |
| d1ve4a1 | d2i6ea1 | d4mcoa1 | d4n8ya_ | d1eu8a_ | d1zbma1 | d1hsla_ | d1ixca2 |
| d1j1na_ | d1xvya_ | d1y9ua_ | d2z22a1 | d1xoca1 | d4oera_ | d1wdna_ | d5f1qa1 |
| d1xt8a1 | d1i6aa_ | d4gwoa_ | d4igta1 | d1pc3a_ | d4mmpa1 | d1o63a_ | d2czla1 |
| d2onsa2 | d4mija1 | d2qvba_ | d2v9za_ | d4kafa1 | d4iuaa3 | d4bvva_ | d5hpga_ |
| d3k65a_ | d1ki0a3 | d1ki0a2 | d1ki0a1 | d1a0ha1 | d3sp8a3 | d2feba1 | d3u1ra1 |
| d1g9ka2 | d1kapp2 | d2m5ta_ | d1cqqa_ | d1wqsa_ | d5bpea_ | d2ipha_ | d1lvoa_ |
| d2bhga1 | d3tnta_ | d3sjia_ | d3d23a1 | d2fyqa_ | d3tloa_ | d3zvea1 | d2hrva_ |
| d3q3ya1 | d4asha1 | d1cg2a1 | d1yloa2 | d1y7ea2 | d1y0ya3 | d1lfwa1 | d1vixa1 |
| d1fnoa4 | d1vhoa2 | d1ysja1 | d1vhea2 | d1z2la1 | d2fvga2 | d1vgya1 | d1r3na1 |
| d2grea2 | d1xmba1 | g1q7l.1 | d1rtqa_ | d3rp2a_ | g1ucy.1 | d1fuja_ | d2zchp_ |
| d3w94a_ | d1si5h_ | d1lo6a_ | g2pka.1 | d1cgha_ | d1kigh_ | d1a5ia_ | d3s9ba_ |
| d1hj8a_ | d1ao5a_ | d2anya_ | d1q3xa1 | d1gvza_ | d1azza_ | d4gsoa_ | d2f91a1 |
| d1iaua_ | d1gdna_ | d1eufa_ | g1fiw.1 | d1eq9a_ | d4nfea_ | d2rdla_ | d1spja_ |
| d1elva1 | d1a7sa_ | d1ekbb_ | d1pfxc_ | d1yc0a_ | d2aiqa_ | g1fiz.1 | d1fi8a_ |
| d4k60a_ | d4dgja_ | d1mzaa_ | d1op0a_ | d3tjva_ | d1pytd_ | d1sgfg_ | g1gg6.1 |
| d2eeka_ | g1rtf.1 | d1a0ja_ | d3g01a_ | d1pytc_ | d4e7na_ | d1ddja_ | d1z8ga1 |
| d2hlca_ | d1npma_ | g1jou.1 | d1sgfa_ | d1autc_ | g2hnt.1 | g1kdq.1 | d1orfa_ |
| d3s69a_ | d4crga_ | d1fxya_ | d1bqya_ | d2ftba_ | d1pmpa_ | d2rcqa1 | d1fdqa_ |
| d4lkpa_ | d1sa8a_ | d1vyfa_ | d1lpja_ | d3vg7a1 | d4azqa1 | d1o8va_ | d5bvqa_ |
| d1p6pa_ | d2qo4a_ | d1opba_ | d1yiva1 | d4bvma1 | d4tkba_ | d4i3ba_ | d1bwya_ |
| d1ftpa_ | d1ggla_ | d4gkca_ | d1cbia_ | d3p6da1 | d1kqwa_ | d2a0aa1 | d1m4la_ |
| d2bk7a_ | d3fjua_ | d1z5ra1 | d1kwma1 | d1uwya2 | d1jqga1 | d2c1ca1 | d1dtda_ |
| d2h0ba_ | d2erfa_ | d1h30a2 | d1h30a1 | d3mw4a1 | d1pz7a_ | d2r1ba_ | d3v64a1 |
| d1d2sa_ | d3bopa1 | d3mw3a1 | d1okqa1 | d1okqa2 | d1e69a_ | d1mv5a_ | d3g60a4 |
| g1ii8.1 | d1ewqa2 | d1ji0a_ | d1qhla_ | d1v43a3 | d2wmfa1 | d1vpla_ | d1g2912 |
| d4a82a2 | g1f2t.1 | d2d62a1 | d3tuic1 | d2bbsa1 | d3g60a3 | d2ixea_ | d1g6ha_ |
| d1sgwa1 | d2qi9c_ | d3rlfa1 | d3b60a1 | d3d31a2 | d2pzea1 | g1xew.1 | d1jj7a_ |
| d1oxxk2 | d2onka1 | d1w1wa_ | d3gqba2 | d1skye3 | d1xp8a1 | d1tf7a2 | d1tf7a1 |

d1ye8a1 d1skyb3 d2qe7d2 d4blta_ d4huta_ d3ldaa2 d4hyya_ d2i3ba1
d1cr1a_ d3j9tb2 d3ntua2 d1vdza1 d2z43a_ d1e9ra_ d1nlfa_ d3ziaa2
d1n0wa_ d1g6oa_ d3ziad2 d3j9ta2 d1pzna2 d3gqbb2 d1gl9b1 d1gkub2
d1eh3a_ d3skpa_ d1dtza1 d1dtza2 d1ieja_ d1jnfa2 d1ce2a1 d1jnfa1
d1b1xa1 d1jw1a1 d1jw1a2 d1ryoa_ d1h76a1 d1h76a2 d1c4oa1 d2p6ra3
d2p6ra4 d1oywa2 d1oywa3 d1wrba1 d1w36b2 d1w36b1 d1c4oa2 d3bora_
d1t6na_ d2eyqa5 d2eyqa3 d2eyqa2 d2zu6a2 d1veca_ d1uaaa1 d1w36d1
d2g9na1 d1z3ix2 d2rb4a_ d1hv8a1 d1hv8a2 d1t5ia_ d1w36c2 d2j0sa2
d2j0sa1 d1fuka_ d3hjha1 d1z5za1 d1qdea_ d1z63a1 d1s2ma2 d1s2ma1
d1wp9a1 d1wp9a2 d1gm5a3 d1gm5a4 d3dina4 d3dina3 d1q0ua_ d1w36c1
d1z3ix1 d1nkta3 d1nkta4 d1uaaa2 d1w36d2 g1qhh.1 d2fwra2 d2fwra1
d3q3ma_ d1za0a1 d1mxra_ d2inca_ d1jk0a_ d2incb_ d1mhyb_ d3hf1b_
d1mtyb_ d1r2fa_ d2uw1a_ d1mhyd_ d3n37a_ d1afra_ d1syya_ d1mtyd_
d2o1za_ d1uzra_ d1smsa_ d2fkza_ d1zuja1 d1s3qa1 d2fjca1 d1z6om1
d1veia_ d4dyya_ d1jiga_ d3gvya_ d2pyba_ d4cy9a1 d3bvfa1 d1r03a_
d2cf7a_ d1nf4a_ d1zs3a1 d4iwka_ d3ka8a_ d3np0x_ d1n1qa_ d1lb3a_
d4e6ka_ d1z6oa1 d2fzfa1 d4dyua_ d3ge4a_ d1jgca_ d4am4a_ d2z6ma_
d1ji5a_ d1vlga_ d1lkoa1 d3qz3a_ d1o9ra_ d2ffxj_ d1euma_ d1yv1a1
d2v2pa_ d1j30a_ d4di0a_ d1dpsa_ d1oewa_ d2qzxa_ d2asia_ d1psoe_
d1smra_ d2h6ta_ d1pfza_ d1izda_ d3hd8a_ d1j71a_ d1dpja_ d4aa9a_
d1t6ex_ g1b5f.1 g1lya.1 g1htr.1 d1qdma2 d1bxoa_ d1wkra_ d1tzsa_
d1ibqa_ d2qzwa_ d1ex4a2 d1c6va_ d1k8rb_ d1wgra1 d1ef5a_ d1wfya1
d1e8ya3 d1wgya1 d2cs4a1 d1wxma1 d2byea1 d3kh0a1 d2byfa1 d1wxaa1
d1e7ua3 g1qmo.1 g1len.1 d3zyra_ d1fnya_ d1n47a_ g2ltn.1 d1avba_
d3usua_ d1dhkb_ d1f9ka_ d1ukga_ d1g8wa_ d1g9fa_ d1wbfa_ d3wcsa_
d1g7ya_ g1loe.1 d1mvqa_ d1hqla_ d1v6ia_ d1dbna_ d1qnwa_ d1fx5a_
d4l8qa_ d1ioaa_ d3n35a_ d1wgda1 d1wgga1 d4q5eb_ d1wjna1 d1se9a_
d3n3kb_ d1wz0a1 d1we6a1 d2m0xa_ d2gbka_ d3rt3b1 d1wh3a1 d1sifa_
d2ekec2 d4ii2b1 d4hk2a1 d1c3ta_ d1m94a_ d1wgha1 d1ttna1 d1j8ca_
d1wx8a1 d3v6cb1 d2ma9b_ d4i6lb1 d1bt0a_ d2jvca1 d2k1fa_ d1wxva1
d1wf9a1 d1t0ya_ d2io0b2 d1x1ma1 d2gbma_ d1zw7a1 d2m17a_ d1ud7a_
d1we7a1 d2lwpa_ d2gbja_ d1wjua1 d1v6ea1 d1v86a1 d4eewa_ d1wiaa1
d4icva_ d4gu2a_ d1wx7a1 d1wy8a1 d4hjka1 d2faza1 d1v5oa1 d2al3a1
d2cr5a1 d1wj4a1 d3ubra_ d1qo8a1 d1d4ca1 d2k3va_ d1ft5a_ d1qdba_
d1m1qa_ d2j7aa_ d1h21a_ d1ogyb_ d4fasa_ d1jnia_ d1oaha_ d1sp3a_
d1wioa4 d1ccza2 d1vcaa1 d1dr9a2 d2d9qb1 d2nxyb2 d1um8a_ d1ixza_
d1qvra2 d1qvra3 d1qzma_ d1sxjc2 d1jr3d2 d2p65a_ d1l8qa2 d1g8pa_
d3hu3a3 d1sxjd2 d1njfa_ d1in4a2 d1sxja2 d1tuea1 d2ce7a2 d1w5sa2
d1sxjb2 d1d2na_ d1ny5a2 d1ofha_ d2gnoa2 d2a5yb3 d1fnna2 d1u0ja1
d1lv7a_ d2fnaa2 d1ixsb2 d4gita_ d1sxje2 d1g41a_ d1svpa_ d1mbma_
d3su6a1 d1cu1a1 d4agka_ d1vcpa_ d1q31a_ g1n1l.1 d3e90b_ d1ep5a_
d3u1jb_ d2lj7a_ d2m8bb1 d2kyqa_ d3psma_ d1jkza_ d1jxca_ d2gl1a_
d1ugla_ d4uj0a_ d2k4ua_ d1hp2a_ d2lu9a_ d1baha_ d2meoa1 d1wm7a_
d1hlya_ d1n8ma_ d1jlza_ d1wpda_ d1c55a_ d1pvza_ d1wt7a_ d1rjia_

d1wz5a_ d1m2sa_ d1pjva_ d1c49a_ d1qkya_ d2ktca1 d1wmta_ d1du9a_
d1j5ja_ d1r1ga_ d1t0za_ d2i61a_ d1t1ta_ d1sn4a_ d1kv0a_ d2lkba_
d1npia_ d1wwna_ d1pe4a_ d2kbka_ d2li7a_ d1djta_ d2kbha_ d1dq7a_
d1sega_ d1omya_ d1zywa1 d1jzaa_ d1n9wa1 d1bbua1 d1e1oa1 d1b8aa1
d1l0wa1 d3a74a1 d1eova1 d1t61c1 d1iyjb4 d1iyjb5 d1iyjb3 d1o7ia_
d4ipca1 d1x3ea_ d4apva_ d1jb7a1 d1jb7a2 d1jb7a3 d3kdfa_ d1ue1b_
d1wjja1 d3eiva_ d1xjva2 d1xjva1 d2ccza2 d1se8a_ d1l1oc_ d1jb7b_
d1qvca_ d3ulla_ d1qzga_ d1miua4 d1miua5 d1miua3 d3g48a1 d1gd7a_
d1mkha_ d1jjcb3 d1pyba_ d1y14b1 d2nn6i1 d1i94l_ d2zjra2 d1smxa_
d2qamc2 d1hh2p1 d2r7da1 d1rl2a2 d1wfqa1 d3bzka4 d1x6oa2 d3a0ja_
d2j01d2 d2ja9a1 d4ql5a_ d1hr0w_ d1luza_ d2n3sa_ d2vnud1 d1h95a_
d2nn6h1 d1ne3a_ d2mo1a_ d1iz6a2 d1jjga_ d1e3pa2 d2ix0a3 d2ix0a2
d2lxja_ d1ueba3 d1ueba2 d2z0sa1 d1u0la1 d2ix0a1 d2eifa2 d2je6i1
d3i2za1 d2c35b1 d2ahob2 d1t9ha1 d3i4oa_ d2qall1 d1jt8a_ d2nn6g1
d1g6pa_ d3cama_ d1i94q_ d1c9oa_ d1d7qa_ d2ba0a1 d2vnud2 d2vnud3
d1khia2 d2qalq1 d1wi5a1 d1xtda2 d1ny4a_ d1rpma_ d2shpa1 d1yfoa_
d1l8ka_ d2ooqa1 d1g4us2 d1lara2 d1lara1 d1wcha_ d2fh7a2 d2fh7a1
d1ohea1 d1ohea2 d2f4da_ d1rxda_ d2hxpa_ d1xria_ d1d5ra2 d3ezza_
d4y2ea_ d1i9sa_ d3azya_ d3wvja_ d1upsa1 d1ajoa_ d2hyka_ d1ajka_
d2vy0a_ d1o4ya_ d3o5sa_ d3d6ea_ d3atga_ d1dypa_ d1umza_ d3i4ia_
d1c1la_ d4lbqa_ d1slta_ d4no4a_ d3zsja_ d3duia_ d1g86a_ d1is3a_
d1qmja_ d1hlca_ d1tdha3 d1q3ba3 d1k82a3 d4qmea2 d2xdta2 d1z5ha2
d1epwa1 d3h33a_ d1rwja_ d19hca_ d1os6a_ d3bxua_ d3h4na_ d1i77a_
d3caoa_ d1j0pa_ d2ctha_ d1duwa_ d1ofwa_ d2e84a_ d1hh5a_ d3h34a_
d1dszb_ d1a6ya_ d3cbba_ d1ynwa1 d1r4ia_ d1hcqa_ d2hana_ d2hanb_
d2enva1 d1kb4b_ d2nllb_ d1lo1a_ d2dara2 d2dara1 d1wyha2 d2dloa2
d1rutx4 d1rutx3 d2cuqa2 d1x4la2 d1x4la1 d1x3ha2 d1x3ha1 d2d8za1
d1x61a2 d1x6aa1 d1x6aa2 d2cora2 d2cora1 d1v6ga1 d1v6ga2 d2d8za2
d1x64a1 d1x64a2 d1b8ta1 d2co8a1 d1x63a2 d2dj7a2 d2dj7a1 d2cu8a2
d2cu8a1 d1g47a2 d1g47a1 d1j2oa1 d1wyha1 d2dloa1 d1wiga1 d1wiga2
d2cupa2 d2cupa3 d1x68a1 d2d8ya2 d2d8ya1 d1m3va2 d1x62a1 d1x68a2
d1x61a1 d1b8ta3 d2d8xa2 d2d8xa1 d1x4ka1 d1x4ka2 d2cuqa1 d1m3va1
d2co8a2 d1j2oa2 d1x63a1 d2cura1 d2cura2 d1x62a2 d1op9a_ d1y0la1
d1etzb1 d1egjl1 d2gsib1 d1nj9a1 d4jfhd1 d1aqkl1 d1t2ja_ d1lo4h1
d1zvya1 d3v4vl1 d4enec1 d1kxtb_ d5e8ea1 d2pxya_ d4weud_ d1xiwc_
d2esvd1 d1qnzh_ d1ogae1 d1f4xh1 d4n1hb_ d3ezjb1 d3mlwl1 d1kfah1
d1etza1 d1b4jh1 d3cfic_ d4w68a_ d2arja1 d1ek3a_ d2ok0l1 d1vera_
d1uwgh1 d1blnb1 d1fpth1 d1rzga1 d2giya1 d4m3kb1 d1nc2a1 d2nyyd1
d1ncna_ d1fbih1 d5dxwa_ d4fqqa1 d1dqdh1 d1jfql1 d1kelh1 d1faih1
d1frgl1 d1figl1 d4m6nl1 d1fo0a_ d4hxbl1 d3b5ga_ d3quyd1 d2h9gb1
d1i8kb_ d2dd8h1 d2ck0h1 d1eaja_ d4w6wb1 d2a6ib3 d1t66d1 d2e27h_
d1ob1b1 d1f11b1 d2v7na1 d1ad0a1 d2xt1b1 d1pskh1 d4i13b1 d2op4l1
d1a0ql1 d1uwel1 d1za3a1 d1ncbl1 d4uu9a_ d1bm3h1 d1cloh1 d1nqba2
d1cf8h1 d4y5va_ d1mhph1 d5anma1 d4cdgc1 d4ldeb_ d1y0lb1 d2pcpb1

d1z3gl1 d1dfbl1 d1fh5h1 d3g5xa1 d1tzib1 d4dn3l1 d3zhka1 d2b1hh1
d2uyla1 d1f3dh1 d1nj9b1 d2agjh1 d2h1pl1 d1q72h1 d8fabb1 d1ndmb1
d1rjca1 d4u6hb1 d3upca_ d2fbjl1 d1osph1 d1tvda_ d2cjvh_ d4fhbd1
d1bd2d1 d1rz7h1 d3nn8a_ d1knoa1 d1nfda1 d2bmkb1 d1dqll_ d1igyb1
d1igfl1 d12e8h1 d3utza1 d1nc2b1 d1rihh1 d4mwfb1 d4jvpa1 d1mieh1
d1rhfa1 d1fgnl1 d1sbsh1 d1lp9e1 d1dn0a1 d4lqfl1 d1iaih1 d1kgcd1
d1mh5b1 d1gpol1 d2g60h3 d1f8tl1 d2jixd1 d4grwh_ d1fo0b_ d1bfob1
d1axsa1 d1u9ka_ d1nmbh_ d1h8na1 d1h8na2 d1jguh1 d4eura1 d3sdyl1
d35c8h1 d1w72l1 d1mvub_ d1i9rh1 d1ob1a1 d4wenb_ d1yeeh1 d3ietc1
d1f58h1 d1ad0b1 d1mqkl_ d1nbvh1 d4b50a_ d3r8bb_ d1bafl1 d1mamh1
d4rgne1 d2gsgb_ d35c8l1 d1bafh1 d1ai1h1 d2pcpa1 d2fath1 d2x1pa_
d1orsb1 d1ibgl1 d1shma_ d1a2yb_ d1u0qa_ d2coqa_ d1hilb1 d1qfwi_
d1jv5b_ d1fn4b1 d5e0qa_ d1nsnh1 d1plgl1 d4ocxl1 d1kb5h1 d1rzfh1
d1pg7x1 d2fx7h1 d2oi9c_ d1l7il1 d3eyva1 d1d5il1 d4ffyl_ d1tetl1
d1a6wh_ d1ac6a_ d3dvfa_ d1nfde1 d1mimh1 d1ri8a1 d1jhlh_ d3dura_
d1fskb1 d1f97a1 d3so3b1 d2ak4d1 d1lp9f1 d4z7wf1 d1l7th1 d4y5ya_
d4x7ec1 d1emth1 d1ikfl1 d1nlbh1 d4utal1 d1bjma1 d1eapb1 d1u6ah1
d2dtgd1 d1kj2b_ d1xeda1 d1c1eh1 d1fgvh_ d2bnub1 d2fb4h1 d2dtgb1
d1ktrh_ d1jpth1 d1qkzl1 d4hepg_ d2atpb1 d1flrh1 d2hrpl1 d1mvfa_
d1iaim1 d3k80a1 d2jb5h1 d3zkxc_ d4grwe_ d1it9h1 d1rhhb1 d3w9ec_
d1e4wh1 d1sy6h1 d2j6eh1 d3qxta_ d1jglh1 d1p7ka1 d1tzhb1 d4n8pa1
d1gafl1 d2jelh1 d1wioa2 d4i0cc1 d1cicb1 d1lmka2 d1lmka1 d1iqwh1
d1orqb1 d1ejoh1 d1jnlh1 d4zg1a_ d43c9b_ d1ggbl1 d1wejh1 d3q6fa1
d1f3dj1 d3c6la1 d1bqlh1 d3p9wb_ d1a3rl1 d1h0db1 d2uzil_ d1eo8l1
d1qfwl_ d1n7ml1 d1igja1 d1c5ch1 d2a9mh_ d1rmfh1 d1fj1b1 d4heme_
d1ccza1 d1a5fh1 d1ogad1 d1dlfh_ d1qa9b_ d1vgel1 d3qskb_ d1sjxa_
d4ozfg1 d1sq2n_ d1qd0a_ d1kcvh1 d1c5db1 d3qcul1 d8faba1 d3bn9d1
d1mjuh1 d4y8dc_ d7fabh1 d3dvgb1 d1lo0l1 d1a6tb1 d1gigh1 d1kcrh1
d2hwzl1 d1yjdc1 d3clfl1 d4krna_ d1dl7l_ d1e6ol1 d1mexh1 d3r0ma_
d4kkna_ d1fnsl1 d1oaqh_ d1rzia1 d1ucbh1 d1ncwl1 d4b41a_ d1nfdf1
d2mpah1 d2d7tl1 d4grwf_ d4b5ea_ d1lk3h1 d3u79a_ d4u3xa_ d1ghfl1
d1bvkb_ d1mcww1 d1b88a_ d1jrhh1 d32c2b1 d4wemb_ d1dsfh_ d1op3h1
d1ce1l1 d1iqda1 d1qoka1 d1qoka2 d1qfuh1 d2crya1 d1ay1h1 d1oaql_
d3ab0b_ d1rurl1 d1t4kb1 d1bz7b1 d1n0xh1 d1forl1 d1ucbl1 d4w6yb_
d43c9a_ d2aabl1 d1tzia1 d4n1ca_ d1k6qh1 d3g9ab_ d4tvsa1 d1igmh_
d1pz5b1 d3dx9a1 d3wxwh_ d1nakh1 d1kcul1 d3eaka_ d4qlra1 d1cr9l1
d1d9ka_ d1f4xl1 d1deea1 d2i27n2 d1c12a1 d2orbl1 d1j8hd1 d1mcph1
d1c5da1 d1kenh1 d1bd2e1 d1h5ba_ d4x7fc1 d3upaa_ d1t3fa1 d1indh1
d2dtgc1 d4hc1l1 d1zv5a1 d4wgvb_ d1uwgl1 d4f37k1 d1aqkh1 d1vesa_
d3zkqd_ d1fptl1 d1lo4l1 d1knob1 d3rrqa_ d1ct8b1 d4orzc_ d1mlbb1
d4hgkc1 d1dqdl1 d1bogb1 d4ozhe1 d1dr9a1 d1i7za1 d1dn0b1 d1kell1
d1rzib1 d1ypzf1 d1wtla_ d1hkfa_ d1fail1 d1figh1 d2vyre_ d1sjva_
d1ohqa_ d1bfoa1 d1pkqb1 d1mjul1 d1b4jl1 d1nsnl1 d2arjb1 d1p4bh_
d4aj0a_ d3nn8b_ d2rcja1 d1f2xk_ d4v1dd_ d1adqh1 d2eksa_ d4k3ga_

d1jfqh1 d1uweh1 d1frgh1 d4gftb_ d1uz8b1 d4n1ea_ d1bm3l1 d1clol1
d3mcka1 d2vq1a1 d1ae6h1 d4aq1b1 d1uj3b1 d1nbqa1 d1fh5l1 d2ck0l1
d2ywza_ d1q72l1 d4nbxb_ d1hila1 d4hzlb1 d2j88l1 d1zmya1 d1cd0a_
d1a4ka1 d1pskl1 d1a0qh1 d4krmb_ d1ncbh1 d2fbjh1 d1j8he1 d1i3ua_
d1cf8l1 d1fe8h1 d2p1ya1 d1eeqa_ d2x89a_ d1ol0a_ d2r0lh1 d1ub5a1
d1dfbh1 d3eyvb1 d4jdvb1 d4p2cg_ d2gsia1 d2agjl1 d1u3ha1 d2h1ph1
d1rihl1 d1fskc1 d1boga1 d2a77l1 d1ospl1 d1qfoa_ d3dwta_ d2fx7l1
d1rz7l1 d3stba1 d1pkqa1 d1b6da1 d1dzba2 d1dzba1 d1kj2a_ d4l1ha_
d1rzgb1 d1dqlh_ d12e8l1 d1miel1 d1jmaa_ d1sbsl1 d1fgnh1 d1f58l1
d1mqkh_ d1ndgb1 d3qegl1 d1iail1 d2aw2a1 d1tqbc1 d3fo2a1 d4dkaa_
d1f8th1 d2g60l1 d2ywya_ d1uj3a1 d1yc7a_ d3bd3a_ d4w6xb1 d1nmbl_
d1i8ka_ d1rjlb1 d1ypze1 d1p7kb1 d1kxqe_ d1gpoh1 d2z4qa1 d1w72h1
d1i9rl1 d4qgya1 d4xt1c_ d2qqnh1 d1cica1 d3c08h1 d2cd0a_ d2bdnh1
d1hnga1 d1maml1 d1nldh1 d1cr9h1 d4lcih1 d3bkyh1 d3qg7l1 d1ail1l
d1kxvc_ d4iosd_ d1l7ih1 d2x1qa_ d1ub6b1 d3k74b_ d1ibgh1 d1teth1
d1igya1 d1hxma1 d2x6ma_ d2e7la_ d5e03a_ d1qfwm_ d2r0kh1 d1igjb1
d1rurh1 d1plgh1 d1mj8h1 d1yuhb1 d3fmgl1 d1rzfl1 d1emtl1 d1ad9b1
d1igtb1 d1nlbl1 d1d5ih1 d3k7ua_ d4ffyh_ d1bwwa_ d3dvga1 d1a6ta1
d1fgvl_ d4qxgl1 d1miml1 d1jhll_ d2hffb1 d2hh0l1 d1qkzh1 d1l7tl1
d1afvh1 d1iaii1 d2cjuh_ d1um5h1 d2ghwb2 d2ghwb1 d1kgce1 d2fgwh1
d1ikfh1 d1dvfd_ d2i24n1 d2xtjb_ d1za3b1 d2yc1a_ d2hh0h1 d2x1oa_
d1bvka_ d1rjla1 d5e7ba1 d1igta1 d1il1b1 d2fb4l1 d1mvua_ d1jptl1
d32c2a1 d1fvca_ d1iqdb1 d2hrph1 d2p4ab_ d4qo1a_ d1p2cb1 d1wt5c_
d1t4ka1 d5c1mb_ d1it9l1 d1bz7a1 d1b2wh1 d1e4wl1 d1jnll1 d1yedb1
d1za6b1 d3u7aa_ d1igfh1 d3gnml1 d1orsa1 d1wejl1 d1a2ya_ d1hxmb1
d1gafh1 d2j4wh1 d1pz5a1 d1j05a_ d1a3rh1 d4fqll1 d1qfwh_ d1eo8h1
d1n7mh1 d4s11a_ d1kegh1 d5da4a_ d1iqwl1 d3whxb1 d1vgeh1 d1deeb1
d1c12b1 d1t2ql1 d1ad9a1 d3gsnb1 d3b2uc1 d1ggbh1 d1t3fb1 d1nakl1
d4ocmc_ d1lila1 d2v7hb3 d2esve1 d1mcoh1 d2qr0b1 d2q3aa_ d1ct8a1
d1kcrl1 d2pxyb_ d2uzih_ d5c3ld_ d3durb_ d1c5cl1 d2ak4e1 d1dl7h_
d1xiwd_ d1mlba1 d1rmfl1 d1i7zb1 d1dlfl_ d2xa3a_ d5bq7b1 d1dqqb1
d3h42l1 d1egjh1 d1kcvl1 d1fe8l1 d2j6el1 d1f3rb2 d1lk3l1 d1sm3h1
d1q0xh1 d1ghfh1 d7fabl1 d1igch1 d1lo0h1 d4idla_ d5c2bl_ d1jrhl1
d1il1a1 d3zhda1 d4kfzc_ d1b0wa_ d1e6oh1 d1mexl1 d4krpb_ d1fnsh1
d1ncwh1 d4hgma_ d4eigb1 d4rzca_ d2mpal1 d4y5xa_ d5en2b1 d3lrga1
d4ej1c1 d1rhha1 d1j05b_ d1forh1 d1q9wb1 d2wzpd_ d2aq2a1 d1h0da1
d3revb1 d2ij0c1 d1tzha1 d4m7jl1 d1k6ql1 d3b9kc1 d1igml_ d3qeua1
d1dsfl_ d1ce1h1 d1kcuh1 d1zvha1 d1zvoa1 d1tqbb1 d1ay1l1 d2qscl1
d2cgrh1 d1nl0h1 d1kenl1 d3eo0a1 d4n9ob1 d1n0xl1 d3k1kc_ d2cqva1
d1fhga_ d2k1ma_ d1biha3 d1biha1 d1biha4 d3qp3a1 d1g1ca_ d1gxea_
d1cvsc2 d1cvsc1 d1xmwa2 d1m4ka2 d2otpa2 d1biha2 d1rhfa2 d2fcba2
d2fcba1 d1wwbx_ d1gsma2 d1epfa1 d1epfa2 d2dava1 d1waaa_ d1f97a2
d1x44a1 d1vcaa2 d1fnla2 d1fnla1 d3b5ha2 d3h8na2 d3h8na1 d1jbja1
d1jbja2 d1pd6a_ d1f2qa2 d1f2qa1 d3cx2a_ d1nbqa2 d3b5ha1 d1n26a1

d1xiwb_ d1xaua_ d1fltx_ d2ifga1 d1olla1 d1olla2 d1cs6a4 d1cs6a3 d1cs6a2 d1cs6a1 d2gw5a2 d1sy6a1 d1xiwa_ d1ucta2 d1ucta1 d1ry7b2 d1gl4b_ d1qz1a3 d1olza1 d2c9aa1 d1wwca_

**Family-14 (a.39.1.5)**

**Positive dataset contains 19 protein sequences.**

d1tiza1 d3fwba_ d2o5ga_ d3i5gc_ d1sl8a_ d2roaa_ d4aqra_ d3pn7b_ d2obha_ d2mlfc_ d2m97a_ d2d8na_ d4by5a_ d4nqga_ d1s6ia_ d1uhka2 d1r2ua_ d3kf9a_ d3pn7c_

**Negative dataset contains 4019 protein sequences.**

d1m32a_ d1ibja_ d1c7na_ d1iuga_ d4q75a_ d2bkwa1 d1t3ia_ d1n8pa_ d1qz9a_ d1jf9a_ d1eg5a_ d3kgwa_ d1cl1a_ d2ctza1 d1vjoa_ d2rfva_ d1gc0a_ d1cs1a_ d1d2fa_ d2ch1a1 d1qgna_ d1pffa_ d1elua_ d1ax4a_ d2v1pa1 d2ez1a_ d1u08a_ d1lc5a_ d1b5pa_ d2ay1a_ d1bw0a_ d3pd6a_ d7aata_ d2csta_ d1j32a_ d1fg7a_ d4wlha_ d1o4sa_ d1v2da_ d1gdea_ d1m6sa_ d1v72a1 d1svva_ d1ajsa_ d1wsta1 d2f8ja2 d1vp4a1 d1xi9a1 d2gb3a1 d1yaaa_ d3i2ka1 d1lnsa3 d2cfba1 d1o69a_ d1bj4a_ d3k28a_ d2bwpa_ d3lg0a_ d2fnua_ d3ou5a_ d1z7da1 d1b9ha_ d1iaya_ d4msoa_ d1bs0a_ d1s0aa_ d1sffa_ d3tqxa_ d1mdoa_ d3qboa_ d3pgya_ d1bjna_ d2dkja_ d1ejia_ d1vefa1 d4wxga_ d3bs8a_ d3gbxa1 d1w23a_ d2b5aa1 d2ofya1 d4mcxa_ d2xi8a_ d2icta_ d2bnma1 d2ppxa1 d1y7ya1 d1wioa4 d1ccza2 d1vcaa1 d1dr9a2 d1ic1a1 d2d9qb1 d2nxyb2 d1ddla_ d1e57b_ d1auyb_ d1f56a_ d1bypa_ d1azca_ d1ws8a_ d3u25a_ d1cuoa_ d1id2a_ d1rkra_ d2iaac_ d4dp1x_ d1nwpa_ d2gima_ d2i7sa_ d1qhqa_ d3ehbb1 d1fftb1 d1gyca3 d1hfua2 d1kbva1 d1kbva2 d1sdda1 d1sdda2 d1gyca1 d1gyca2 d1mzya1 d2bw4a1 d1aoza1 d1aoza3 d1aoza2 d1kyaa2 d1kyaa3 d1kyaa1 d1mzya2 d4e9sa1 d1sddb1 d1sddb2 d1hfua1 d1hfua3 d1oe1a2 d1oe1a1 d1v10a2 d1v10a3 d1v10a1 d1rypd_ d1irua_ d1rypc_ d3unfk_ d1rypa_ d1j2pa_ d1irui_ d1j2qh_ d3unfh_ d1m4ya_ d1rypb_ d1irug_ d1iru2_ d1q5rh_ d1rypg_ d1g3ka_ d1iruf_ d1q5qa_ d1e94a_ d1irub_ d1ryph_ d3ty6a_ d1iruj_ d1iruk_ d1rype_ d1irud_ d1irue_ d3mi01_ d1rypf_ d1iruh_ d2z3ba_ d1iruc_ d3mka2_ d1iru1_ d1a3qa1 d1iknc_ d3brda1 d1p7hl1 d1oy3c_ d1imhc1 d1gjia1 d1zk9a1 d2cxka1 d2bema_ d1pama1 d1ea9c1 d1qhoa1 d2fhfa2 d2fhfa1 d1k3ia1 d1j0ha1 d1cxla1 d2bhua1 d1edqa1 d1f1sa2 d1ut9a2 d1gvia1 d1txka1 d2phla2 d2phla1 d1fxza2 d1lrha_ d1uija1 g1dgw.1 d2v09a_ d1uika2 d1uika1 d1dgwa_ d1ep0a_ d2pa7a1 d1wlta1 d2c0za1 d1ds6b_ d1fstb_ d1ex0a1 d1eixa_ d3exra_ d2ffca1 d2fdsa1 d3n3ma1 d2czda_ d1q6oa_ d2yyua_ d1vqta1 d1dbta_ d3ldva1 d1au7a2 d1ic8a2 d2ovga_ d1sq8a_ d1rzsa_ d3kz3a_ d3orca_ d2q49a2 d1hdgo2 d2g17a2 d2cvoa2 d2czca1 d3pwka2 d1obfo2 d1i32a2 d1zyao2 d2pkqo2 d1b7go2 d2hjsa2 d1cf2o2 d1vkna2 d1nvmb2 d2msya_ d2djoa1 d2r5za_ d1au7a1 d1f43a_ d2lmda1 d1akhb_ d1s7ea1 d3nara_ d1zq3p1 d2craa1 d1mija_ d4uuta_ d3a01a_ d1x58a1 d3rkqa1 d3cmya1 d2cqxa1 d2da6a1 d1e3oc1 d1fjla_ d1mh3a1 d1ig7a_ d1x41a1 d1wh7a1 d1gt0c1 d2mw8a_ d1lfup_ d1x2ma1 d1b72a_ d1wi3a1 d1jgga_

d1wjha1 d1x2na1 d1mnmc_ d2cuea1 d3k2aa_ d2hosa_ d2jwta2 d1pufa_
d1octc1 d2hi3a_ d1b8ia_ d4j19a_ d3lnqa_ d1wh5a1 d1b8ib_ d1fexa_
d2cqra1 d1guua_ d1ug2a1 d2cqqa1 d2ajea1 d4a69c_ d2crga1 d2iw5b1
d1xc5a1 d1ofcx1 d2cu7a1 d1wgxa1 d2cjja1 d2ckxa1 d1gvda_ d1hcra_
d1tc3c_ d1gdta1 d1u78a2 d1xg1a1 d1itya_ d1hlva2 d1iufa2 d1iufa1
d2dara2 d2dara1 d1wyha2 d2dloa2 d1rutx4 d1rutx3 d2cuqa2 d1x4la2
d1x4la1 d1x3ha2 d1x3ha1 d2d8za1 d1x61a2 d1x6aa1 d1x6aa2 d2cora2
d2cora1 d1v6ga1 d1v6ga2 d2d8za2 d1x64a1 d1x64a2 d1b8ta1 d2co8a1
d1x63a2 d2dj7a2 d2dj7a1 d2cu8a2 d2cu8a1 d1g47a2 d1g47a1 d1j2oa1
d1wyha1 d2dloa1 d1wiga1 d1wiga2 d2cupa2 d2cupa3 d1x68a1 d2d8ya2
d2d8ya1 d1m3va2 d1x62a1 d1x68a2 d1x61a1 d1b8ta3 d1b8ta2 d2d8xa2
d2d8xa1 d1x4ka1 d1x4ka2 d2cuqa1 d2co8a2 d1j2oa2 d1x63a1 d2cura1
d2cura2 d1x62a2 d3ubub_ d1egia_ d1qo3c_ d1v7pa_ d1tdqb_ d3ubua_
d1c3ab_ d1h8ua_ d3bx4b_ d1t8ca1 d1sb2a1 d1qdda_ d2ziba1 d3cada_
d1byfa_ d1j34b_ d1jwia_ d1jzna_ d1c3aa_ d1oz7b_ d1wmza_ d3bdwb_
d1hq8a_ d1fifa1 d1dv8a_ d3hupa_ d1wk1a1 d1umra_ d2afpa_ d3bx4a_
d1gz2a_ d1sl6a_ d1v7pb_ d3g8ka_ d1ixxa_ d1j34a_ d1jwib_ d1g1sa1
d1y17a1 d1ja3a_ d1fvub_ d3bdwa_ d1sb2b1 d1fvua_ d1umrc_ d1oz7a_
d4m17a_ d3cfwa1 d1ypqa1 d1g1ta1 d3fd4a_ d3l9jc_ d4dn8a1 d1v4lb_
d1rdl1_ d2msba_ d3lv8a_ d1s3ga1 d1zaka1 d1s4qa_ d3tvta2 d1uf9a_
d1khta_ d1ckea_ d1osna_ d1p5zb_ d4tmka_ d3cm0a_ d1tmka_ d1jaga_
d3h86a_ d1t0hb_ d1nn5a_ d2h92a_ d3kb2a1 d2c95a1 d1yj5a2 d1kgda_
d1ly1a_ d2ak3a1 d1vyva2 d1deka_ d2grja1 d1p3ja1 d1y63a_ d1e4ya1
d1s96a_ d3iija1 d1lvga_ d1nksa_ d1vyua2 d4e22a_ d1qf9a_ d1jjva_
d1q3ta_ d1ki9a_ d1lw7a2 d1teva_ d1kjwa2 d1gsia_ d2iyva2 d1viaa_
d1e6ca_ d1odfa_ d1uj2a_ d4gi7a_ d2jeoa1 d1z0ja1 d3umra2 d3gj0a1
d2gaoa1 d1yzga1 d1x3sa1 d1g7sa4 d1puja_ d1udxa2 d1kk1a3 d2o52a_
d1jwyb_ d1zcaa2 d1zcba2 d2fv8a1 d2cjwa_ d3dz8a1 d1xzpa2 d1vg8a_
d4qxaa_ d2ew1a1 d1x1ra1 d2bcgy_ d3doea1 d3cona_ d3rega_ d3wyga_
d1upta_ d2b6ha1 d1zd9a1 d1lnza2 d1r5ba3 d1n0ua2 d1r2qa_ d2f9la1
d2rhda_ d3q3jb_ d3q85a_ d1efma1 d1r8qa_ d2gf9a1 d2akab1 d1ek0a_
d1z08a1 d2gcoa1 d2dy1a2 d4g01b_ d2cxxa1 d1svia_ d3q72a_ d1ni3a1
d4ac9a4 d2erxa1 d2bmea_ d1wxqa1 d2gf0a1 d4ldja1 d1zunb3 d1d5ca_
d4lv5b_ d1efca3 d1kmqa_ d2g6ba1 d3lrpa_ d1tada2 d2y8ea_ d4rd4a1
d1z06a1 d2heia_ d3a58b_ d1u0la2 d1d2ea3 d1zj6a1 d2ocba_ d3rwoa_
d1mr3f_ d2e9sa1 d4u5xa1 d2iwra_ d2fn4a1 d3bwdd_ d2nzja1 d2atva1
d2ksqa_ d3l43a_ d1mkya2 d1mkya1 d1wmsa_ d1jnya3 d2rmka_ d1f6ba_
d1f60a3 d1gota2 d1jala1 d2fh5b1 d1g16a_ d2wblc_ d1m7ba_ d1wf3a1
d1f5na2 d1t9ha2 d1z6xa1 d1z0aa1 d2fg5a1 d2a5ja1 d2bcjq2 d1s0ua3
d1ky3a_ d4y0va_ d4lhwa_ d2qmea1 d2atxa1 d1egaa1 d1ryha_ d1moza_
d1nrjb_ d2erya1 d3bfka_ d1puia_ d3j3az1 d3t5ga_ d1shza2 d3defa_
d2f7sa1 d3raba_ d3tsoa_ d3aq4a_ d2al7a1 d3sfva1 d3bc1a_ d2ncda_
d1sdma_ d3h4sa1 d2heha_ d2grya_ g3kin.1 d1kk8a2 d1br2a2 d1goja_
d1f9va_ d2mysa2 d1lkxa_ d3t0qa_ d4db1a1 d3gbja_ d1ry6a_ d1v8ka_

d1vava_ d1uaia_ d1k8ka2 d1k8ka1 d3uleb1 d3i33a2 d3i33a1 d1bupa1
d1dkgd2 d1dkgd1 d4ci6a2 d1jcfa1 d4a2ba2 d2fxua1 d1yaga2 d1yaga1
d4cbua2 d1bupa2 d3vrda1 d3vrda2 d1fcdc2 d1fcdc1 d1h1oa1 d1m70a2
d1m70a1 d1ig8a1 d1czan2 d1czan3 d1czan1 d1ig8a2 d1czan4 d1bg3a4
d1bg3a3 d1bg3a2 d1bg3a1 d1nira1 d1qksa1 d1hroa_ d4gedb_ d3zooa_
d1mz4a_ d1gu2a_ d1f1fa_ d1dvva_ d1c6ra_ d1c75a_ d1e29a_ d1gdva_
d1cnoa_ d1h32b_ d1co6a_ d2bh4x_ d2aiua_ d5cytr_ d1jdla_ d1i8oa_
d2zboa_ d3dmia_ d1ls9a_ d1wvec_ d2yk3a_ d3ph2b_ d1dw0a_ d5aura_
d1ql3a_ d1qn2a_ d1f1ca_ d4zzva_ d2xspa_ d4v20a_ d1ojja_ d4ipma1
d1gpia_ d4csia_ d3pfza_ d3ovwa_ d2yoka_ d2nqya_ d1h4ga_ d1hixa_
d1oa2a_ d1olra_ d3b7ma_ d4h7ma1 d3wp3a_ d2vgda_ d1igoa_ d3zsea_
d2nlra_ d3wx5a_ d1oa3a_ d1pvxa_ d1f5ja_ d1oa4a_ d1ks5a_ d4ixla1
d1h32a2 d1cnt1_ d1pvhb_ d1z7ca_ d1huwa_ d1bgea_ d1rhga_ d1f6fa_
d1f45b_ d1i1rb_ d1axia_ d1etea_ d3tgxb_ d1scfa_ d2z3qa1 d3qb1a_
d1hula_ d4nkqb_ d1eera_ d1v7mv_ d5e4ea_ d2o27a_ d3b5ka_ d2psma1
d4zf7a_ d3uf5a_ d4x51a_ d1d9ca_ d4ypgc1 d3oq3a_ d1lqsl_ d1n1fa_
d1au1a_ d1wu3i_ d3besl_ d3ux9a_ d1v8fa_ d2ejca_ d3muea1 d1ihoa_
d3q12a1 d3rbaa_ d2qtra_ d1nupa_ d3l92a_ d1f9aa_ d1qjca_ d2h29a_
d1vlha_ d1od6a_ d1lw7a1 d1mrza2 d4ruka_ d2el7a_ d1iq0a2 d1irxa2
d1r6ta2 d1ivsa4 d2cyba_ d1rqga2 d1j09a2 d2yxna_ d1f7ua2 d1h3fa1
d2g36a1 d1nzja_ d1jila_ d1n3la_ d1i6la_ d1pfva2 d1h3na3 d3n9ia_
d3sz3a1 d3sw2a_ d1moxc_ d1dx5i3 d1edmb_ d1autl2 d1autl1 d1dval1
d1szba2 d1kigl_ d2y5fl_ d1ijqa2 d1gk5a_ d1g1sa2 d1nt0a3 d1xdtr_
d1lmja2 d1lmja1 d1nzia2 d2wphe_ d1klil_ d1pfxl2 d1pfxl1 d1p9ja_
d1g1ta2 d1f7ea_ d4yzub_ d2mgpa1 d1b9wa1 d2mgpa2 d1ob1c1 d1ob1c2
d1n1ia2 d1n1ia1 d2npra2 d2npra1 d1gl4a2 d1m1xb5 d1s5ja2 d1qhta2
d4dlea2 d1tgoa2 d3py8a2 d1q8ia2 d1kfsa2 d2py5a2 d1d5aa2 d1sfsa_
d1wkya2 d1uuqa_ d3wh9a_ d4m1ra_ d1xyza_ d4xuya_ d2je8a5 d4bf7a1
d3cuia_ d1ur4a_ d1qw9a2 d1odza_ d2vzsa5 d1bqca_ d2x2ya1 d1hjqa_
d1qnra_ d4y7ea1 d1vjza_ d4k68a_ d2v3ga_ d4hpga_ d1nofa2 d1nq6a_
d1h4pa_ d1aq0a_ d3ziza_ d4bs0a_ d1ecea_ d1h1na_ d1ta3b_ d1w91a2
d1i1wa_ d1uhva2 d1us3a2 d1egza_ d7a3ha_ d1tvna1 d3nyda1 d1ghsa_
d2cyga1 d4xv0a_ d2c8na2 d1w32a_ d3emca_ d1v6ya1 d1foba_ d2uwfa1
d2fgla_ d3niya1 d2c0ha1 d1rh9a1 d1yq2a5 d1szna2 d1fa2a_ d1pama4
d5do8a1 d1ea9c3 d1hx0a2 d2fhfa5 d1qhoa4 d1avaa2 d1m53a2 d4m8ua1
d2gjpa2 d1lwha2 d4howa1 d1ktba2 d1j0ha3 d3vm5a1 d2x1ia_ d1wzaa2
d1uasa2 d2diea1 d1mxga2 d1ht6a2 d1eswa_ d1iv8a2 d5cpsa_ d1ud2a2
d1zjaa1 d3gbea1 d1kwga2 d1wdpa_ d1gcya2 d1g5aa2 d1hvxa2 d3dhpa2
d2bhua3 d4do4a1 d3qgva1 d1e43a2 d1g94a2 d2d3na2 d1gjwa2 d1gvia3
d1x1na1 d1r7aa2 d1tz7a1 d4c7ga2 d2gjxa1 d1nowa1 d1yhta1 d4toqa_
d1wb0a1 d1w9pa1 d1eoka_ d1itxa1 d1kfwa1 d1goia2 d1edqa2 d2gsja_
d1hjxa1 d1jnda1 d1vf8a1 d3wdpp1 d3wq5a_ d4gxpa_ d4hz8a_ d1e4mm_
d3aiua_ d1wcga1 d1qvba_ d3ahxa_ d1v02a_ d2dgaa_ d1pbga_ d1vffa1
d1qoxa_ d4ha4a_ d1mixa2 d1j19a2 d1isna2 d2al6b2 d1g4ma1 d1g4ma2

| | | | | | | | |
|---|---|---|---|---|---|---|---|
| d2y8ga_ | d1xkea1 | d1k5db_ | d2y8fa_ | d2v76a_ | d1wj1a1 | d1aqca_ | d1qqga2 |
| d1qqga1 | d1n3ha_ | d4dbba_ | d3f0wa1 | d1p3ra_ | d1wgua1 | d2cy4a1 | d3hqca_ |
| d1wvha1 | d1xr0b1 | d1wgqa1 | d1v89a1 | d2coda1 | d1zc3b_ | d1wg7a1 | d1u5ea1 |
| d1v61a1 | d2coca1 | d2ys1a1 | d1v5pa1 | d1ntya2 | d1wjma1 | d1wi1a1 | d1u29a_ |
| d1xx0a1 | d1unqa_ | d2zkmx3 | d1fhoa_ | d1v88a1 | d2adza1 | d1x1fa1 | d1p6sa_ |
| d2coaa1 | d1btka_ | d1v5ma1 | d1faoa_ | d2x18a_ | d1dyna_ | d1x1ga1 | d4kaxb_ |
| d2j59m1 | d1ki1b2 | d1xcga2 | d1txda2 | d1u5da1 | d1dbha2 | d1w1ha_ | d1upqa_ |
| d1v5ua1 | d1foea2 | d2fjla1 | d1eaza_ | d2dfka2 | d2cofa1 | d1kz7a2 | d1h6ta1 |
| d9ldta1 | d2ewda3 | d1up7a1 | d2dfda1 | d1s6ya1 | d1vjta1 | d7mdha1 | d1hyea1 |
| d1t2da1 | d1ojsa3 | d1b8pa1 | d1ldna1 | d1hyha1 | d1civa1 | d2a92a1 | d1ez4a1 |
| d1i0za1 | d1y6ja1 | d1gv0a1 | d1ur5a1 | d1llda1 | d1obba1 | d1v8ba1 | d1gdha1 |
| d2dlda1 | d1mx3a1 | d1ygya1 | d1qp8a1 | d1li4a1 | d1j4aa1 | d2naca1 | d1npya1 |
| d3t4ea2 | d1a4ia1 | d1o0sa1 | d2hk9a2 | d2egga2 | d1b26a1 | d1bvua1 | d1gq2a1 |
| d1c1da1 | d1leha1 | d1gtma1 | d1euza1 | d1nvta1 | d3dona2 | d1edza1 | d1gpja2 |
| d1vi2a1 | d1p77a1 | d1nyta1 | d1vl6a1 | d1v9la1 | d1txga2 | d3f47a1 | d1mv8a2 |
| d2pv7a2 | d1xc2a3 | d2ahra2 | d4xiya1 | d2f1ka2 | d1pgja2 | d1i36a2 | d1np3a2 |
| d1lj8a4 | d1teaa3 | d2i76a2 | d2g5ca2 | d1wdka3 | d1dlja2 | d3cuma3 | d1kola2 |
| d1f8fa2 | d1qora2 | d1jqba2 | d1vj0a2 | d1cdoa2 | d1gu7a2 | d1d1ta2 | d1xa0a2 |
| d2dm6a2 | d1jvba2 | d1rjwa2 | d1llua2 | d1h2ba2 | d1tt7a2 | d1piwa2 | d1o89a2 |
| d1iz0a2 | d1e3ja2 | d1yb5a2 | d1uufa2 | d1vj1a2 | d1e3ia2 | d3gpdg1 | d1vc2a1 |
| d2nvwa1 | d1u8fo1 | d1ydwa1 | d1hdgo1 | d2g17a1 | d1cf2o1 | d2czca2 | d1r0ka2 |
| d1obfo1 | d2cvoa1 | d1ebfa1 | d1h6da1 | d1i32a1 | d1zh8a1 | d2q49a1 | d1qkia1 |
| d1j5pa4 | d3pzra1 | d1xeaa1 | d1vkoa1 | d1vm6a3 | d3qvsa1 | d2pkqo1 | d1b7go1 |
| d1gr0a1 | d1p1ja1 | d1lc0a1 | d1j0xo1 | d3pwka1 | d2hjsa1 | d1zyao1 | d1vkna1 |
| d1tlta1 | d1nvmb1 | d3sjua_ | d3ojfa1 | d1xu9a1 | d2jjya1 | d1n7ha_ | d1y1pa1 |
| d1h5qa_ | d1jtva_ | d4fs3a_ | d2bkaa1 | d1gy8a_ | d2c5aa1 | d1ulsa_ | d1ooea_ |
| d2b69a1 | d1y5ma1 | d1spxa_ | d1ae1a_ | d1bxka_ | d1ydea1 | d1gega_ | d1hdoa_ |
| d1o5ia_ | d1e6ua_ | d1snya1 | d2rhca_ | d3zv4a_ | d5ceja_ | d2d1ya1 | d1fjha_ |
| d3w8ea_ | d2fmua1 | d1x1ta_ | d1rpna_ | d3ay7a1 | d1k2wa_ | d1eq2a_ | d2hrba1 |
| d3ruaa_ | d2ew8a1 | d4hwka_ | d1xgka_ | d2bd0a1 | d1yo6a1 | d2a35a1 | d2ntva_ |
| d1vl8a_ | d1hdca_ | d4bkua_ | d2ag5a1 | d2q45a_ | d1nffa_ | d4iy1a_ | d1zbqa1 |
| d1kewa_ | d1ek6a_ | d1ja9a_ | d2q46a2 | d1iy8a_ | d1cyda_ | d2ptga_ | d1hxha_ |
| d1uzma1 | d3lyla_ | d4m89a_ | d3wyea_ | d2blla1 | d1w6ua_ | d1sb8a_ | d2q2va_ |
| d4nr0a_ | d1e6wa_ | d2gasa_ | d3oiga1 | d2bgka1 | d1mxha_ | d1vl0a1 | d1rkxa_ |
| d1r6da_ | d1wmaa1 | d1e7wa_ | d2c07a1 | d1xkqa_ | d3tzha_ | d1g0oa_ | d3lz6a_ |
| d1qyca_ | d1edoa_ | d1oc2a_ | d1t2aa_ | d1zema1 | d2xoxa_ | d1z45a2 | d1geea_ |
| d1p33a_ | d3lt0a_ | d4e6pa_ | d2wyua_ | d1db3a_ | d4q9na_ | d1xhla_ | d4o1ma_ |
| d1fmca_ | d2uvda_ | d2gdza1 | d1uaya_ | d2z1ma_ | d2o23a2 | d2fr1a2 | d2fr1a1 |
| d1qsga_ | d1qyda_ | d4zjua_ | d2a4ka1 | d1yb1a_ | d1wvga1 | d1yxma1 | d3f4ba_ |
| d1mg5a_ | d1xg5a1 | d1r55a1 | d3gboa_ | d1atla_ | d1kufa_ | d4q1la_ | d1quaa_ |
| d1yp1a_ | d1wnia_ | d1ex0a4 | d1g0da4 | d2f4ma1 | d1x3za_ | d1mdwa_ | d1ziva1 |
| d1zcma1 | d1t3ca_ | d2a97a_ | d3fiea_ | g1e1h.1 | d3ioqa_ | d1fh0a_ | d1o0ea_ |
| d1khqa_ | d1m6da_ | d2cb5a_ | d1cs8a_ | g8pch.1 | d1s4va_ | d2pnsa_ | d1vsna1 |
| d1mira_ | d1iwda_ | d2oula_ | d7pcka_ | d3bwka_ | d3bcna_ | d2p86a_ | d1pvja_ |

d1pcia_ d1gece_ d1deua_ d4bs6a_ d1xkga1 d1cqda_ d1jqpa2 d2c0ya_
d2bdza_ d3p5ua_ d2o36a_ d1zoya1 d1qo8a2 d1d4ca2 d1chua2 d1jnra2
d1kf6a2 d2jk6a2 d2jk6a1 d1feca1 d1feca2 d1lpfa1 d1lpfa2 d1xhca1
d1xhca2 d1dxla2 d1dxla1 d1gera2 d1m6ia2 d1onfa1 d1onfa2 d3lada2
d3lada1 d1w4xa1 d1w4xa2 d1gesa2 d1mo9a1 d1xdia1 d1fl2a2 d1fl2a1
d1fcda1 d1fcda2 d1h6va2 d1h6va1 d1mo9a2 d1gv4a2 d1gv4a1 d2gv8a2
d2gv8a1 d1aoga1 d1aoga2 d1hyua1 d1ebda1 d1ebda2 d3dk9a1 d1q1ra2
d1q1ra1 d1gesa1 d3syxa1 d1mkea1 d2p8va_ d1xoda1 d1i7aa_ d1evha_
d1qc6a_ d1ps9a2 d1o94a2 d1lqta1 d1pn0a1 d1reoa1 d1ju2a1 d2f5va1
d3c96a1 d2gmha1 d1pj5a2 d1gpea1 d1o5wa1 d1n4wa1 d1k0ia1 d2dw4a2
d2ivda1 d1seza1 d2voua1 d1kdga1 d1s5ja1 d2hbja2 d2igia_ d1wlja_
d1qhta1 d4dlea1 d1tgoa1 d2f96a1 d3tr8a1 d3mxma_ d1q8ia1 d1y97a1
d1w0ha_ d1kfsa1 d2py5a1 d1noya_ d1d5aa1 d1yt3a3 d2guia_ d3mwpa1
d4kb1a_ d2hmta_ d1lssa_ d1js9c_ d1f15b_ d1za7a_ d1iuka_ d2csua1
d3q9ub_ d2yv1a1 d3q9na_ d2d59a_ d1oi7a1 d2yv2a1 d1euca1 d1y81a1
d3nvva1 d1krha3 d2h88b1 d1n62a2 d1t3qa2 d1kf6b2 d1zoyb1 d1jq4a_
d1ffva2 d1dgja2 d1rm6c2 d1jmxa2 d1jmxa1 d1pbya2 d1r7ha_ d1faaa_
d1wika1 d1xfla_ d1ttza1 d2f51a1 d1ti3a_ d1nhoa_ d1sena_ d2i4aa_
d1ep7a_ d1zmaa1 d1fo5a_ d3lefa1 d3diea1 d2yana1 d1z6na1 d1oaza_
d3zywa1 d4aj8a_ d1nm3a1 d4fiwa_ d1wmja_ d4hs1a1 d1iloa_ d1xwaa1
d1wjka1 d2vm1a_ d1fb6a_ d1gh2a_ d1h75a_ d1nw2a_ d1kx5b_ d3a6na_
d4m6ba2 d2nqbd_ d3wkjd_ d2yfwe_ d4h9na_ d5bnxa_ d1kx5c_ d3r45a_
d1eqzb_ d4jjnb_ d1f66c_ d1eqza_ d4wnnb_ d1kx5a_ d1u35c1 d1id3a_
d2nqbc_ d1tafa_ d1bh9b_ d1tafb_ d1jfib_ d1q9ca_ d2byka1 d1n1ja_
d4g92b_ d1bh9a_ d1jfia_ d1h3oa_ d2bykb1 d1n1jb_ d1pd212 d1nhya2
d2a2ra2 d1k0dd2 d1f2ea2 d1gsua2 d2fhea2 d2pvqa1 d1tw9a2 d1oyja2
d1e6ba2 d1k0ma2 d3ik7a1 d4is0a1 d1r5aa2 d1jlva2 d1m0ua2 d3gtub2
d1z9ha2 d1okta2 d1axda2 d2gsra2 d1tu7a2 d1k3ya2 d1zgma1 d1b48a2
d1gnwa2 d1gwca2 d2gsta2 d1glqa2 d1oe8a2 d1pn9a2 d1v2aa2 d1fw1a2
d1duga2 d1a0rp_ d1iqca2 d1iqca1 d1eb7a1 d1nmla2 d1nmla1 d4ilya_
d4olta_ d1qgia_ d1qqya_ d1gd6a_ d1f6ra_ d1ivma_ d2gv0a_ d1iiza_
d2z2fa_ d1j8xa_ d1ghla_ d1b9oa_ d174la_ d169la_ d2o4wa_ d1p37a_
d1k28a3 d1xjta1 d1jtma_ d2f2qa_ d4e97a1 d1mpya2 d1kw3b2 d1kw3b1
d4ghga1 d1tfza1 d3isqa1 d3isqa2 d1sqia1 d1sqia2 d1lkda2 d1lkda1
d1mpya1 d1cjxa2 d1sp9b2 d1t47a1 d1t47a2 d1f1ua2 d1sp8a1 d1sp8a2
d1f1ua1 d1etob_ d1umqa_ d4iuaa3 d4bvva_ d5hpga_ d3k65a_ d1ki0a3
d1ki0a2 d1ki0a1 d1a0ha1 d1pmla_ d3sp8a3 d2feba1 d1qsta_ d2beia1
d4m3sa_ d1u6ma1 d1ygha_ d1yk3a1 d3dr6a_ d1xmta_ d1z4ea1 d2ge3a1
d1bo4a_ d2fiaa1 d2jdca1 d1y9wa1 d3to7a_ d1wwza1 d2fl4a1 d2ozua1
d2b5ga_ d2cy2a1 d2p0wa_ d1cm0a_ d4pswa_ d1yrea1 d1yr0a1 d1m4ia_
d2gana1 d1yvka1 d1ghea_ d2g3aa1 d1y7ra1 d2euia1 d1q2ya_ d2fsra1
d1xeba_ d2ae6a1 d1cjwa_ d1qsma_ d2aj6a1 d1y9ka1 d1i12a_ d1vkca_
d2atra1 d1n71a_ d2d4pa1 d2fiwa1 d2fe7a1 d1tiqa1 d2ou2a_ d1yx0a1
d2fcka1 d1p0ha_ d2j8ma_ d2pq8a_ d1z4ra1 d1s3za1 d1ufha_ d1xvqa1

d1pn0a2 d3hy2a_ d2cvba1 d1o8xa_ d1lu4a_ d1we0a1 d2bmxa1 d2a4va1
d1psqa_ d1xiya1 d1q98a_ d1n8ja_ d1zofa1 d2ggta_ d3tjja_ d4je1a1
d1o73a_ d3s9fa_ d2zcta_ d1qmva_ d1jfua_ d2b1ka_ d5dvba1 d2b5xa1
d2v2ga_ d1st9a_ d3tb2a_ d4llra_ d4fh8a1 d1knga_ d2h30a_ d1i5ga_
d2z9sa_ d2f8aa1 d1tp9a1 d2b7ka_ d1nm3a2 d3hvva_ d2wfca1 d4kcea_
d1xvwa1 d4gqca1 d3qpma_ d4k1fa_ d1zzoa1 d1gp1a_ d1prxa_ d4od7a1
d1z6ma1 d4mcua1 d4dvca1 d1r4wa_ d3l9sa_ d1un2a_ d3rppa_ d1rw1a_
d1z3ea1 d3l78a_ d1f74a_ d3l21a_ d2e1da_ d1to3a1 d1w3ia_ d1sfla_
d1n7ka_ d1vcva1 d4imea_ d1wa3a_ d2ocza1 d1f2ja_ d3m16a_ d2yxga_
d1ub3a_ d3a5fa_ d1fbaa_ d1l6wa_ d1xfba1 d1wx0a1 d1vpxa1 d1ojxa_
d2a4aa1 d1xkya1 d3cq0a1 d3s1xa_ d1f05a_ d1fdja_ d3cpra1 d4d2ja_
d1jgta2 d1ofda3 d1ea0a3 d1ecfa2 d1q15a2 d1ao0a2 d1te5a_ d1ra6a_
d1muka_ d3gsza_ d1khva_ d1te9a_ d2xwha_ d3uqsa_ d3hkwa_ d3n6ma_
d2cjqa_ d2xyma_ d1teba_ d1te8a_ d1u09a1 d4wfxa_ d3tyqa_ d2pgga1
d1lgya_ d4l3wa_ d1llfa_ d1gz7a_ d1ukca_ d1svpa_ d3su6a1 d1cu1a1
d4agka_ d1vcpa_ d1q31a_ g1n1l.1 d3e90b_ d1ep5a_ d3u1jb_ d3zxha_
d1cgla_ d1rm8a_ d1eaka2 d1hy7a_ d2jsda_ d1cxva_ d1hv5a_ d1i76a_
d3p0ya1 d1igra1 d1igra2 d1n8yc2 d1n8yc1 d1m6ba2 d1m6ba1 d1m4la_
d2bk7a_ d3fjua_ d1z5ra1 d1kwma1 d1uwya2 d2c1ca1 d1dtda_ d1h8la2
d2tbvc_ d1c8nc_ d1x36a_ d1smvc_ d1ng0c_ d4sbvc_ d1opoa_ d1f2nc_
d1cixa_ d1y29a_ d1v7fa_ d2jtba_ d1d1ha_ d1g9pa_ d1lupa_ d1qk7a_
d1mb6a_ d2a2va_ d1niya_ d1i25a_ d1hvwa_ d2mpqa_ d1ie6a_ d1kqha_
d1la4a_ d2mxoa1 d1koza_ d1qk6a_ d1emxa_ d1lqra_ d1c6wa_ d1rmka_
d1ixta_ d1eyoa_ d1cnna_ d1fu3a_ d1g1pa_ d1fyga_ d3lgia_ d3i77a_
d2z9ia2 d4nsva_ d1lcya2 d4jcna_ d1agja_ d1os8a_ d1hpga_ d1p3ca_
d1l1ja_ d1qtfa_ d1ny721 d1ny722 d1a6ca3 d1a6ca2 d1a6ca1 d1pgl22
d1pgl21 d1pgl11 d1ny711 d1mqta_ d1d4m3_ d1fmd3_ d2mev1_ g1aym.1
d1ev13_ d4q4w1_ d1r1a3_ d2x5ia_ d1rhi1_ g1r1a.1 g1d4m.1 g1rhi.1
d1b35b_ d1oopa_ d1tme1_ d4iv1a_ g1pvc.1 g1fpn.1 d1fpn1_ d4gb31_
d1d4m1_ d1fmd1_ d1ev11_ d2wzr3_ g1cov.1 d1qqp3_ g1fmd.1 d2x5ic_
d1rhi3_ g1oop.1 d1hxs1_ d1fpn3_ d1bev1_ g2mev.1 d1eah3_ d1aym1_
d1pvc3_ d1qqp1_ d1zba3_ d1h8ta_ d3vbhc_ g1b35.1 g1bev.1 d1hxs3_
g1ev1.1 d1bev3_ g1tmf.1 d1tmf1_ d1b35a_ d2mev3_ g1eah.1 g1h8t.1
d1eah1_ d4q4w3_ d1r1a1_ d1aym3_ d1pvc1_ d1zba1_ d1h8tc_ d1oopc_
d1tme3_ g1qqp.1 d4iv1c_ g1tme.1 d1p8ba_ d4w9ta_ d1jvna1 d4x2ra1
d1ka9f_ d4axka1 d1thfd_ d1h5ya_ d4u28a_ d2y88a_ d4wd0a_ d1vzwa1
d3ia2a_ d1a88a_ d4dgqa1 d1hkha_ d1zoia_ d4lt9a1 d3tc7a1 d3hoja_
d1i4na_ d3vnda_ d3nl8a1 d3nava1 d1v5xa_ d4a2ra1 d3tc6a1 d1ujpa_
d4ijba_ d1xcfa_ d1vc4a_ d1rd5a_ d1qopa_ d3b5va_ d2r80b_ d1cqxa1
d1gcvb_ d3boma_ d2h8fb_ d2raoa_ d3bj1a_ d4hrrb_ d2r50a_ d3d1kb_
d2aa1b1 d3zhwa_ d3hf4b_ d3gdja_ d1jebb_ d3bj1b_ d1x9fb_ d1wmub_
d1gcva_ d3bcqb_ d3d4xb_ d1gvha1 d2dc3a_ d3hf4a_ d4yu3a_ d3k8ba_
d1qpwb_ d2r80a_ d3bcqa_ d1v4wa_ d3wtgb_ d1jl7a_ d4esaa_ d1a9we_
d3d1ab_ d1fsla_ d4h2la_ d1v4wb_ d1mwca_ d1vhba_ d1jeba_ d1spgb_

d1h97a_ d1q1fa_ d1tu9a1 d3d1aa_ d1hdsb_ d3hyub_ d2zfbb_ d3fs4a_
d1oj6a_ d1shrb_ d4h2lb_ d4yu3b_ d1spga_ d3hrwa_ d3qqra_ d3at5a_
d4wchd_ d3hyua_ d1cg5b_ d1uc3a_ d1hbra_ d2raob_ d1outb_ d4esab_
d3gdjb_ d3qqqa_ d1x9fc_ d3vrfa_ d1itha_ d3mkba_ d3d4xa_ d3mjpa_
d1jl6a_ d3fh9a_ d1outa_ d2dc3b_ d1it2a_ d3eoka_ d3g46a_ d1qpwa_
d3vrfb_ d1ch4a_ d1x9fd_ d1hdsa_ d2zfba_ d3mkbb_ d1or4a_ d3wtga_
d1a4fa_ d3fh9b_ d3bomb_ d3cy5a_ d3at5b_ d1x9fa_ d2oifa_ d4lmxb_
d1cpcb_ d3v57a_ d1kn1a_ d1cpca_ d4f0ta_ d2vjha_ d4po5b_ d1eyxa_
d1alla_ d4f0tb_ d1b33b_ d4h0mb_ d1b33a_ d2vjhb_ d4lm6b_ d2vmlb_
d1allb_ d2vjta_ d3dbja_ d2vmla_ d2vjtb_ d4f0ub_ d1f99b_ d4rmpa1
d1liaa_ d1eyxb_ d2uulb_ d4lmsb_ d4f0ua_ d2bv8a_ d1f99a_ d2bv8b_
d2c7lb_ d3dbjb_ d4rmpb1 d1b8da_ d4h0ma_ d3v57b_ d1kn1b_ d1liab_
d2uula_ d2j96a_ g1gxs.1 g1wht.1 d2xqfa_ d1ea5a_ d1f6wa_ d1dx4a_
d1qe3a_ d1k4ya_ d4qwwa_ d2ha2a_ d2qvba_ d2v9za_ d4kafa1 d1b9ra_
d1offa_ d1frra_ d3wcqa_ d1e9ma_ d1l5pa_ d1wria_ d1i7ha_ d3av8a_
d1rfka_ d3p1ma1 d3b2ga_ d2bt6a2 d1w5qa_ d2c1ha_ d1ohla_ d1l6sa_
d2m5ta_ d1cqqa_ d1wqsa_ d5bpea_ d2ipha_ d1lvoa_ d2bhga1 d3tnta_
d3sjia_ d3d23a1 d2fyqa_ d3tloa_ d3zvea1 d2hrva_ d3q3ya1 d4asha1
d1e88a1 d1l6ja4 d1eaka4 d1eaka3 d1l6ja3 d1h8pa2 d1h8pa1 d3rp2a_
g1ucy.1 d1fuja_ d2zchp_ d3w94a_ d1si5h_ d1lo6a_ g2pka.1 d1cgha_
d1kigh_ d1a5ia_ d3s9ba_ d1hj8a_ d1ao5a_ d2anya_ d1q3xa1 d1gvza_
d1azza_ d4gsoa_ d2f91a1 d1iaua_ d1gdna_ d1eufa_ g1fiw.1 d1eq9a_
d4nfea_ d2rdla_ d1spja_ d1elva1 d1a7sa_ d1ekbb_ d1pfxc_ d1yc0a_
d2aiqa_ g1fiz.1 d1fi8a_ d4k60a_ d4dgja_ d1mzaa_ d1op0a_ d3tjva_
d1pytd_ d1sgfg_ g1gg6.1 d2eeka_ d1a0ja_ d3g01a_ d1pytc_ d4e7na_
d1ddja_ d1z8ga1 d2hlca_ d1npma_ d1brup_ g1jou.1 d1sgfa_ d1autc_
g2hnt.1 g1kdq.1 d1orfa_ d3s69a_ d4crga_ d1fxya_ d1bqya_ d2ftba_
d1pmpa_ d2rcqa1 d1fdqa_ d4lkpa_ d1sa8a_ d1vyfa_ d1lpja_ d3vg7a1
d4azqa1 d1o8va_ d5bvqa_ d1p6pa_ d2qo4a_ d1opba_ d1yiva1 d1o1va_
d4bvma1 d4tkba_ d4i3ba_ d1bwya_ d1ftpa_ d1ggla_ d4gkca_ d1cbia_
d3p6da1 d1kqwa_ d2a0aa1 d1dzka_ d4tlja_ d2a2ua_ d4wfva_ d3t1da_
d4iaxa_ d3zq3a_ d2nnda_ d1i4ua_ d1kxoa_ d1bbpa_ d1kt7a_ d1exsa_
d3s26a1 d1lf7a_ d1qfta_ d4gh7a_ d1z24a1 d1qabe_ d1xkia_ d1ew3a_
d3bx7a_ d4iawa1 d1n0sa_ d1gkab_ d3kzaa_ d2k23a_ d2l9ca1 d2joza1
d1gm6a_ d3kffa_ d3saoa1 d1e5pa_ d1yupa1 d1iiua_ d1e69a_ d1mv5a_
d3g60a4 d1ewqa2 d1ji0a_ d1qhla_ d1v43a3 d2wmfa1 d1vpla_ d1g2912
d4a82a2 g1f2t.1 d2d62a1 d3tuic1 d2bbsa1 d3g60a3 d2ixea_ d1g6ha_
d1sgwa1 d2qi9c_ d3rlfa1 d3b60a1 d3d31a2 d2pzea1 g1xew.1 d1jj7a_
d1oxxk2 d2onka1 d1w1wa_ d1iwea_ d1qf5a_ d1vmaa2 d1nija1 d2p67a1
d3ihla_ d1okkd2 d1ls1a2 d1s1ma2 d1iona_ d2v40a_ d1dj3a_ d3hida_
d2vo1a1 d2g0ta1 d1vcoa2 d1j8mf2 d1g3qa_ d1xjca_ d1p9ba_ d1qzxa3
d1ihua2 d1ihua1 d2qm8a_ d1dj2a_ d4ioja_ d1cp2a_ d1yrba2 d1hyqa_
d3gqba2 d1skye3 d1xp8a1 d1tf7a2 d1tf7a1 d1ye8a1 d1skyb3 d2qe7d2
d4blta_ d4huta_ d3ldaa2 d4hyya_ d2i3ba1 d1cr1a_ d3j9tb2 d3ntua2

d1vdza1 d2z43a_ d1e9ra_ d1nlfa_ d3ziaa2 d1n0wa_ d1g6oa_ d3ziad2
d3j9ta2 d1pzna2 d3gqbb2 d2i49a_ d1nnfa_ d1xvxa_ d1ursa_ d1h3da1
d1iuda_ d4m1va1 d1twya_ d3e13x_ d2nxoa1 d4pfua_ d3ttma_ d1elja_
d2qmwa1 d1us5a_ d1y3na1 d1jeta_ d1uqwa_ d4f19a1 d2esna2 d3thia_
d3fasa1 d2uyeb_ d2xwva1 d1r9la_ d2fyia1 d1pb7a_ d2ozza1 d1sw5a_
d1nh8a1 d1vr5a1 d2g29a_ d1xs5a_ d3lswa_ d2z23a_ d3w9va_ d3tcha_
d1q35a_ d1z7me1 d3q27a_ d1p99a_ d1ve4a1 d2i6ea1 d4mcoa1 d4n8ya_
d1eu8a_ d1zbma1 d1hsla_ d1ixca2 d1j1na_ d1xvya_ d1y9ua_ d2z22a1
d1xoca1 d4oera_ d1wdna_ d5f1qa1 d1xt8a1 d1i6aa_ d4gwoa_ d4igta1
d1pc3a_ d4mmpa1 d1o63a_ d2czla1 d2onsa2 d4mija1 d4ok5a1 d2bmfa1
d2bmfa2 d1yksa1 d1yksa2 d2qeqa1 d2jlqa1 d1a1va2 d1eh3a_ d3skpa_
d1dtza1 d1dtza2 d1ieja_ d1jnfa2 d1ce2a1 d1jnfa1 d1b1xa2 d1b1xa1
d1jw1a1 d1jw1a2 d1ryoa_ d1h76a1 d1h76a2 d1c4oa1 d2p6ra3 d2p6ra4
d1oywa2 d1oywa3 d1wrba1 d1w36b2 d1w36b1 d1c4oa2 d3bora_ d1t6na_
d2eyqa5 d2eyqa3 d2eyqa2 d2zu6a2 d1veca_ d1uaaa1 d1w36d1 d2g9na1
d1z3ix2 d2rb4a_ d1hv8a1 d1hv8a2 d1t5ia_ d1w36c2 d2j0sa2 d2j0sa1
d1fuka_ d3hjha1 d1z5za1 d1qdea_ d1z63a1 d1s2ma2 d1s2ma1 d1wp9a1
d1wp9a2 d1gm5a3 d1gm5a4 d3dina4 d3dina3 d1q0ua_ d1w36c1 d1z3ix1
d1nkta3 d1uaaa2 d1w36d2 g1qhh.1 d2fwra2 d2fwra1 d2v8ta_ d3q3ma_
d1za0a1 d1mxra_ d2inca_ d1jk0a_ d2incb_ d1mhyb_ d3hf1b_ d1mtyb_
d1r2fa_ d2uw1a_ d1mhyd_ d3n37a_ d1afra_ d1oquc_ d1syya_ d1mtyd_
d2o1za_ d1uzra_ d1smsa_ d2fkza_ d1zuja1 d1s3qa1 d2fjca1 d1z6om1
d1veia_ d4dyya_ d1jiga_ d3gvya_ d2pyba_ d4cy9a1 d3bvfa1 d1r03a_
d2cf7a_ d1nf4a_ d1zs3a1 d4iwka_ d3ka8a_ d3np0x_ d1n1qa_ d1lb3a_
d4e6ka_ d1z6oa1 d2fzfa1 d4dyua_ d3ge4a_ d1jgca_ d4am4a_ d2z6ma_
d1ji5a_ d1vlga_ d1lkoa1 d1krqa_ d3qz3a_ d1o9ra_ d2ffxj_ d1euma_
d1yv1a1 d2v2pa_ d1j30a_ d4di0a_ d1oewa_ d2qzxa_ d2asia_ d1psoe_
d1miqa_ d2h6ta_ d1pfza_ d1izda_ d3hd8a_ d1j71a_ d1dpja_ d4aa9a_
d1t6ex_ g1b5f.1 g1lya.1 g1htr.1 d1qdma2 d1bxoa_ d1wkra_ d1tzsa_
d1ibqa_ d2qzwa_ d1ex4a2 d1bdqa_ d3ggta_ d3ixoa_ d1mrxa_ d2haha_
d1tw7a_ d4njsa_ d1sgua_ d2p3da_ d1nsoa_ d3d3ta_ d2rkfa_ d1c6ya_
d2rspa_ d3ogpa_ d3ekwa_ d2o4na_ d2j9ja_ d1baia_ d3ggua_ d3lzua_
d4npua_ d4j55a_ d2r8na_ d2fxda_ d2p3ca_ d4dqea_ d3ttpa_ d3t3ca_
d2p3aa_ d2hc0a_ d2fdda_ d3mwsa_ d1js3a_ d3rcha_ d3k40a_ d1k8rb_
d1wgra1 d1ef5a_ d1wfya1 d1e8ya3 d1wgya1 d1lfda_ d2cs4a1 d1wxma1
d2byea1 d3kh0a1 d2byfa1 d1wxaa1 d1e7ua3 d1wgda1 d1wgga1 d4q5eb_
d1wjna1 d1se9a_ d3n3kb_ d1wz0a1 d1we6a1 d2m0xa_ d2gbka_ d3rt3b1
d1wh3a1 d1sifa_ d2ekec2 d4ii2b1 d4hk2a1 d1c3ta_ d1m94a_ d1wgha1
d1ttna1 d1j8ca_ d1wx8a1 d3v6cb1 d2ma9b_ d4i6lb1 d1bt0a_ d2jvca1
d1iyfa_ d2k1fa_ d1wxva1 d1wf9a1 d1t0ya_ d2io0b2 d1x1ma1 d2gbma_
d1zw7a1 d2m17a_ d1we7a1 d2lwpa_ d2gbja_ d1wjua1 d1v6ea1 d1v86a1
d4eewa_ d1wiaa1 d4icva_ d4gu2a_ d1wx7a1 d1wy8a1 d4hjka1 d2faza1
d1v5oa1 d3ruib_ d3waob_ d2r2qa1 d3m95a_ d3wana_ d2al3a1 d2cr5a1
d1wj4a1 d3ubra_ d1qo8a1 d1d4ca1 d2k3va_ d1ft5a_ d1qdba_ d1m1qa_

d2j7aa_ d1h21a_ d1ogyb_ d4fasa_ d1jnia_ d1sp3a_ d4lu0a_ d1of8a_
d3unda1 d3e9aa1 d1vr6a2 d1n8fa_ d2qkfa_ d1o60a_ d3h33a_ d1rwja_
d19hca_ d1os6a_ d3bxua_ d3h4na_ d1i77a_ d3caoa_ d1j0pa_ d2ctha_
d1duwa_ d1ofwa_ d2e84a_ d1hh5a_ d3h34a_ g1y43.1 d1um8a_ d1ixza_
d1qvra2 d1qvra3 d1sxjc2 d1jr3d2 d2p65a_ d1l8qa2 d1g8pa_ d3hu3a3
d1sxjd2 d1njfa_ d1in4a2 d1sxja2 d1tuea1 d2ce7a2 d1w5sa2 d1sxjb2
d1d2na_ d1ny5a2 d1ofha_ d2gnoa2 d2a5yb3 d1fnna2 d1u0ja1 d1lv7a_
d1iqpa2 d2fnaa2 d1ixsb2 d4gita_ d1sxje2 d1g41a_ d2e3fa_ d2e3ea_
d2llda_ d2ny8x_ d2e3ga_ d2ny9x_ d2nz3a_ d2lj7a_ d2m8bb1 d2kyqa_
d3psma_ d1jkza_ d1jxca_ d2gl1a_ d1ugla_ d4uj0a_ d2k4ua_ d1hp2a_
d2lu9a_ d1baha_ d2meoa1 d1wm7a_ d1hlya_ d1n8ma_ d1jlza_ d1wpda_
d1c55a_ d1pvza_ d1wt7a_ d1wz5a_ d1m2sa_ d1pjva_ d1c49a_ d1qkya_
d2ktca1 d1wmta_ d1du9a_ d1j5ja_ d1r1ga_ d1t0za_ d2i61a_ d1t1ta_
d1sn4a_ d1kv0a_ d2lkba_ d1npia_ d1wwna_ d1pe4a_ d2kbka_ d2li7a_
d1djta_ d2kbha_ d1dq7a_ d1sega_ d1omya_ d1zywa1 d1jzaa_ d1bbua1
d1e1oa1 d1c0aa1 d1b8aa1 d1l0wa1 d3a74a1 d1eova1 d1t61c1 d1iyjb4
d1iyjb5 d1iyjb3 d1o7ia_ d4ipca1 d1x3ea_ d4apva_ d1jb7a1 d1jb7a2
d1jb7a3 d3kdfa_ d1ue1b_ d1wjja1 d3eiva_ d1xjva2 d1xjva1 d2ccza2
d1se8a_ d1l1oc_ d1jb7b_ d1qvca_ d1qzga_ d1miua4 d1miua5 d1miua3
d3g48a1 d1gd7a_ d1jjcb3 d1pyba_ d1y14b1 d2nn6i1 d1i94l_ d2zjra2
d1smxa_ d2qamc2 d1hh2p1 d2r7da1 d1rl2a2 d1wfqa1 d3bzka4 d1x6oa2
d3a0ja_ d2j01d2 d2ja9a1 d4ql5a_ d1hr0w_ d1luza_ d2n3sa_ d2vnud1
d1h95a_ d1go3e1 d2nn6h1 d1ne3a_ d2mo1a_ d1iz6a2 d1jjga_ d1e3pa2
d2ix0a3 d2ix0a2 d2lxja_ d1ueba3 d1ueba2 d2z0sa1 d1u0la1 d2ix0a1
d2eifa2 d2je6i1 d3i2za1 d2c35b1 d2ahob2 d1t9ha1 d3i4oa_ d2qall1
d1jt8a_ d2nn6g1 d3cama_ d1i94q_ d1c9oa_ d1d7qa_ d2ba0a1 d2vnud2
d2vnud3 d1khia2 d2qalq1 d1wi5a1 d1xtda2 d1ny4a_ d1dgsa2 d1fvia1
d1ckma1 d1p16a1 d1x9na2 d1rpma_ d2shpa1 d1yfoa_ d1l8ka_ d2ooqa1
d1g4us2 d1p15a_ d1lara2 d1lara1 d1wcha_ d2fh7a2 d2fh7a1 d1ohea1
d1ohea2 d2f4da_ d1rxda_ d2hxpa_ d1xria_ d1d5ra2 d1fpza_ d3ezza_
d4y2ea_ d1i9sa_ d3azya_ d3wvja_ d1upsa1 d1ajoa_ d2hyka_ d1ajka_
d2vy0a_ d1o4ya_ d3o5sa_ d3d6ea_ d3atga_ d1dypa_ d3i4ia_ d1c1la_
d4lbqa_ d1a78a_ d1slta_ d4no4a_ d3zsja_ d3duia_ d1g86a_ d1qmja_
d1hlca_ d1tdha3 d1q3ba3 d1ee8a3 d1k82a3 g1qmo.1 g1len.1 d3zyra_
d1fnya_ d1n47a_ g2ltn.1 d1avba_ d3usua_ d1dhkb_ d1f9ka_ d1ukga_
d1g8wa_ d1g9fa_ d1wbfa_ d3wcsa_ d1g7ya_ g1loe.1 d1mvqa_ d1hqla_
d1v6ia_ d1dbna_ d1qnwa_ d1fx5a_ d4l8qa_ d1ioaa_ d3n35a_ d1epwa1
d1eysc_ d2h0ba_ d2erfa_ d1h30a2 d3mw4a1 d1pz7a_ d2r1ba_ d3v64a1
d1d2sa_ d3bopa1 d3mw3a1 d1okqa1 d1okqa2 d1dszb_ d1a6ya_ d3cbba_
d1ynwa1 d1r4ia_ d1hcqa_ d2hana_ d2hanb_ d2enva1 d1kb4b_ d2nllb_
d1lo1a_ d1cita_ d1adqa1 d1fnga1 d1uvqb1 d1zs8a1 d2vola1 d2vola2
d1mfbl2 d2jelh2 d1uwel2 d3qq4b1 d1kjvb_ d1k5na1 d1osph2 d4odhl2
d1ow0a1 d1ow0a2 d1bfob2 d3l9ra2 d3jtth1 d2fath2 d4elkb2 d1fn4b2
d2xfxb_ d1pg7x2 d1cqka_ d4k71b2 d1lk2a1 d1eapb2 d4lfhg2 d1i4fa1

d2nw2a2 d1kfah2 d1mhca1 d1h3ua1 d1wejh2 d1h0db2 d1a5fh2 d1kcvh2
d1mjuh2 d1qqda1 d1kcrh2 d1k8ia1 d3huja2 d2nnab1 d1nfdf2 d3mffb2
d1s9va1 d1lk3h2 d1q0xl2 d1fv1b1 d1neza1 d4rfeb2 d1r3ha1 d1a6tb2
d1kjva1 d1pz5b2 d1onqa1 d2bc4a2 d1de4a1 d1k8da1 d1tcra2 d1dn0b2
d1nfde2 d2xwtb2 d1bfoa2 d1mexh2 d1iakb1 d1ypzf2 d4f7ea2 d1q72l2
d2fbjh2 d1fe8h2 d1clzh2 d1fngb1 d1uvqa1 d1vgka1 d1c16a1 d4j12a2
d4x99b2 d1igtb3 d1je6a1 d1q72h2 d1nldh2 d1teth2 d2hmic2 d1i1ca2
d1i1ca1 d1k8ib1 d1q0yh2 d1kfal2 d3frua1 d1pg7w2 d1u58a1 d1igyb4
d1igyb3 d1hyrc1 d1klub1 d1deeb2 d1c12b2 d2bc4b2 d2ig2h2 d1kcvl2
d3wn5b1 d1lk2b_ d1lk3l2 d1mjul2 d1sm3h2 d1q0xh2 d1zvsa1 d1ydpa1
d1u8ib2 d1ypze2 d1kcuh2 d1op9a_ d1y0la1 d1etzb1 d1egjl1 d2gsib1
d1nj9a1 d4jfhd1 d1aqkl1 d1t2ja_ d1lo4h1 d1zvya1 d3v4vl1 d4enec1
d1kxtb_ d5e8ea1 d2pxya_ d4weud_ d1xiwc_ d2esvd1 d1qnzh_ d1ogae1
d1f4xh1 d4n1hb_ d3ezjb1 d3mlwl1 d1kfah1 d1etza1 d1b4jh1 d3cfic_
d4w68a_ d2arja1 d1ek3a_ d2ok0l1 d1vera_ d1uwgh1 d1blnb1 d1fpth1
d1rzga1 d2giya1 d4m3kb1 d1nc2a1 d2nyyd1 d1ncna_ d1fbih1 d5dxwa_
d4fqqa1 d1dqdh1 d1jfql1 d1kelh1 d1faih1 d1frgl1 d1figl1 d4m6nl1
d1fo0a_ d4hxbl1 d3b5ga_ d3quyd1 d2h9gb1 d1i8kb_ d2dd8h1 d2ck0h1
d1eaja_ d4w6wb1 d2a6ib3 d1t66d1 d2e27h_ d1ob1b1 d1f11b1 d2v7na1
d1ad0a1 d2xt1b1 d1pskh1 d4i13b1 d2op4l1 d1a0ql1 d1uwel1 d1za3a1
d1ncbl1 d4uu9a_ d1bm3h1 d1cloh1 d1nqba2 d1cf8h1 d4y5va_ d1mhph1
d5anma1 d4cdgc1 d4ldeb_ d1y0lb1 d2pcpb1 d1z3gl1 d1dfbl1 d1fh5h1
d3g5xa1 d1tzib1 d4dn3l1 d3zhka1 d2b1hh1 d2uyla1 d1f3dh1 d1nj9b1
d2agjh1 d2h1pl1 d1q72h1 d8fabb1 d1ndmb1 d1rjca1 d4u6hb1 d3upca_
d2fbjl1 d1osph1 d1tvda_ d2cjvh_ d4fhbd1 d1bd2d1 d1rz7h1 d3nn8a_
d1blna1 d1knoa1 d1nfda1 d2bmkb1 d1dqll_ d1igyb1 d1igfl1 d12e8h1
d3utza1 d1nc2b1 d1rihh1 d4mwfb1 d4jvpa1 d1mieh1 d1rhfa1 d1fgnl1
d1sbsh1 d1lp9e1 d1dn0a1 d4lqfl1 d1iaih1 d1kgcd1 d1mh5b1 d1gpol1
d2g60h3 d1f8tl1 d2jixd1 d4grwh_ d1fo0b_ d1bfob1 d1axsa1 d1u9ka_
d1nmbh_ d1h8na1 d1h8na2 d1jguh1 d4eura1 d3sdyl1 d35c8h1 d1w72l1
d1mvub_ d1i9rh1 d1ob1a1 d4wenb_ d1yeeh1 d3ietc1 d1f58h1 d1ad0b1
d1mqkl_ d1nbvh1 d4b50a_ d3r8bb_ d1bafl1 d1mamh1 d4rgne1 d2gsgb_
d35c8l1 d1bafh1 d1ai1h1 d2pcpa1 d2fath1 d2x1pa_ d1orsb1 d1ibgl1
d1shma_ d1a2yb_ d1u0qa_ d2coqa_ d1hilb1 d1qfwi_ d1jv5b_ d1fn4b1
d5e0qa_ d1nsnh1 d1plgl1 d4ocxl1 d1kb5h1 d1rzfh1 d1pg7x1 d2fx7h1
d2oi9c_ d1l7il1 d3eyva1 d1d5il1 d4ffyl_ d1tetl1 d1a6wh_ d1ac6a_
d3dvfa_ d1nfde1 d1mimh1 d1ri8a1 d1jhlh_ d3dura_ d1fskb1 d1f97a1
d3so3b1 d2ak4d1 d1lp9f1 d4z7wf1 d1l7th1 d4y5ya_ d4x7ec1 d1emth1
d1ikfl1 d1nlbh1 d4utal1 d1bjma1 d1eapb1 d1u6ah1 d2dtgd1 d1kj2b_
d1xeda1 d1c1eh1 d1fgvh_ d2bnub1 d2fb4h1 d2dtgb1 d1ktrh_ d1jpth1
d1qkzl1 d4hepg_ d2atpb1 d1flrh1 d2hrpl1 d1mvfa_ d1iaim1 d3k80a1
d2jb5h1 d3zkxc_ d4grwe_ d1it9h1 d1rhhb1 d3w9ec_ d1e4wh1 d1sy6h1
d2j6eh1 d3qxta_ d1jglh1 d1p7ka1 d1tzhb1 d4n8pa1 d1gafl1 d2jelh1
d1wioa2 d4i0cc1 d1cicb1 d1lmka2 d1lmka1 d1iqwh1 d1orqb1 d1ejoh1

d1jnlh1 d4zg1a_ d43c9b_ d1ggbl1 d1wejh1 d3q6fa1 d1f3dj1 d3c6la1
d1bqlh1 d3p9wb_ d1a3rl1 d1h0db1 d2uzil_ d1eo8l1 d1qfwl_ d1n7ml1
d1igja1 d1c5ch1 d2a9mh_ d1rmfh1 d1fj1b1 d4heme_ d1ccza1 d1a5fh1
d1ogad1 d1dlfh_ d1qa9b_ d1vgel1 d3qskb_ d1sjxa_ d4ozfg1 d1sq2n_
d1qd0a_ d1kcvh1 d1c5db1 d3qcul1 d3bn9d1 d1mjuh1 d4y8dc_ d7fabh1
d3dvgb1 d1lo0l1 d1a6tb1 d1gigh1 d1kcrh1 d2hwzl1 d1yjdc1 d3clfl1
d4krna_ d1dl7l_ d1e6ol1 d1mexh1 d3r0ma_ d4kkna_ d1fnsl1 d1oaqh_
d1rzia1 d1ucbh1 d1ncwl1 d4b41a_ d1nfdf1 d2mpah1 d2d7tl1 d4grwf_
d4b5ea_ d1lk3h1 d3u79a_ d4u3xa_ d1ghfl1 d1bvkb_ d1mcww1 d1b88a_
d1jrhh1 d32c2b1 d4wemb_ d1dsfh_ d1op3h1 d1ce1l1 d1iqda1 d1qoka1
d1qoka2 d1qfuh1 d2crya1 d1ay1h1 d1oaql_ d3ab0b_ d1rurl1 d1t4kb1
d1bz7b1 d1n0xh1 d1forl1 d1ucbl1 d4w6yb_ d43c9a_ d2aabl1 d1tzia1
d4n1ca_ d1k6qh1 d3g9ab_ d4tvsa1 d1igmh_ d1pz5b1 d3dx9a1 d3wxwh_
d1nakh1 d1kcul1 d3eaka_ d4qlra1 d1cr9l1 d1d9ka_ d1f4xl1 d1deea1
d2i27n2 d1c12a1 d2orbl1 d1j8hd1 d1mcph1 d1c5da1 d1kenh1 d1bd2e1
d1h5ba_ d4x7fc1 d3upaa_ d1t3fa1 d1indh1 d2dtgc1 d4hc1l1 d1zv5a1
d4wgvb_ d1uwgl1 d4f37k1 d1aqkh1 d1vesa_ d3zkqd_ d1fptl1 d1lo4l1
d1knob1 d3rrqa_ d1ct8b1 d4orzc_ d1mlbb1 d4hgkc1 d1dqdl1 d1bogb1
d4ozhe1 d1dr9a1 d1i7za1 d1dn0b1 d1kell1 d1rzib1 d1ypzf1 d1wtla_
d1hkfa_ d1fail1 d1figh1 d2vyre_ d1sjva_ d1ohqa_ d1bfoa1 d1pkqb1
d1mjul1 d1b4jl1 d1nsnl1 d2arjb1 d1p4bh_ d4aj0a_ d3nn8b_ d2rcja1
d1f2xk_ d4v1dd_ d1adqh1 d2eksa_ d4k3ga_ d1jfqh1 d1uweh1 d1frgh1
d4gftb_ d1uz8b1 d4n1ea_ d1bm3l1 d1clol1 d3mcka1 d2vq1a1 d1ae6h1
d4aq1b1 d1uj3b1 d1nbqa1 d1fh5l1 d2ck0l1 d2ywza_ d1q72l1 d4nbxb_
d1hila1 d4hzlb1 d2j88l1 d1zmya1 d1cd0a_ d1a4ka1 d1pskl1 d1a0qh1
d4krmb_ d1ncbh1 d2fbjh1 d1j8he1 d1i3ua_ d1cf8l1 d1fe8h1 d2p1ya1
d1eeqa_ d2x89a_ d1ol0a_ d2r0lh1 d1ub5a1 d1dfbh1 d3eyvb1 d4jdvb1
d4p2cg_ d2gsia1 d2agjl1 d1u3ha1 d2h1ph1 d1rihl1 d1fskc1 d1boga1
d2a77l1 d1ospl1 d1qfoa_ d3dwta_ d2fx7l1 d1rz7l1 d3stba1 d1pkqa1
d1b6da1 d1dzba2 d1dzba1 d1kj2a_ d4l1ha_ d1rzgb1 d1dqlh_ d12e8l1
d1miel1 d1jmaa_ d1sbsl1 d1fgnh1 d1f58l1 d1mqkh_ d1ndgb1 d3qegl1
d1iail1 d2aw2a1 d1tqbc1 d3fo2a1 d4dkaa_ d1f8th1 d2g60l1 d2ywya_
d1uj3a1 d1yc7a_ d3bd3a_ d4w6xb1 d1nmbl_ d1i8ka_ d1rjlb1 d1ypze1
d1p7kb1 d1kxqe_ d1gpoh1 d2z4qa1 d1w72h1 d1i9rl1 d4qgya1 d4xt1c_
d2qqnh1 d1cica1 d3c08h1 d2cd0a_ d2bdnh1 d1hnga1 d1maml1 d1nldh1
d1cr9h1 d4lcih1 d3bkyh1 d3qg7l1 d1ai1l1 d1kxvc_ d4iosd_ d1l7ih1
d2x1qa_ d1ub6b1 d3k74b_ d1ibgh1 d1teth1 d1igya1 d1hxma1 d2x6ma_
d2e7la_ d5e03a_ d1qfwm_ d2r0kh1 d1igjb1 d1rurh1 d1plgh1 d1mj8h1
d1yuhb1 d3fmgl1 d1rzfl1 d1emtl1 d1ad9b1 d1igtb1 d1nlbl1 d1d5ih1
d3k7ua_ d4ffyh_ d1bwwa_ d3dvga1 d1a6ta1 d1fgvl_ d4qxgl1 d1miml1
d1jhll_ d2hffb1 d2hh0l1 d1qkzh1 d1l7tl1 d1afvh1 d1iaii1 d2cjuh_
d1um5h1 d2ghwb2 d2ghwb1 d1kgce1 d2fgwh1 d1ikfh1 d1dvfd_ d2i24n1
d2xtjb_ d1za3b1 d2yc1a_ d2hh0h1 d2x1oa_ d1bvka_ d1rjla1 d5e7ba1
d1igta1 d1il1b1 d2fb4l1 d1mvua_ d1jptl1 d32c2a1 d1fvca_ d1iqdb1

d2hrph1 d2p4ab_ d4qo1a_ d1p2cb1 d1wt5c_ d1t4ka1 d5c1mb_ d1it9l1
d1bz7a1 d1b2wh1 d1e4wl1 d1jnll1 d1yedb1 d1za6b1 d3u7aa_ d1igfh1
d3gnml1 d1orsa1 d1wejl1 d1a2ya_ d1hxmb1 d1gafh1 d2j4wh1 d1pz5a1
d1j05a_ d1a3rh1 d4fqll1 d1qfwh_ d1eo8h1 d1n7mh1 d4s11a_ d1kegh1
d5da4a_ d1iqwl1 d3whxb1 d1vgeh1 d1deeb1 d1c12b1 d1t2ql1 d1ad9a1
d3gsnb1 d3b2uc1 d1ggbh1 d1t3fb1 d1nakl1 d4ocmc_ d1lila1 d2v7hb3
d2esve1 d1mcoh1 d2qr0b1 d2q3aa_ d1ct8a1 d1kcrl1 d2pxyb_ d2uzih_
d5c3ld_ d3durb_ d1c5cl1 d2ak4e1 d1dl7h_ d1xiwd_ d1mlba1 d1rmfl1
d1i7zb1 d1dlfl_ d2xa3a_ d5bq7b1 d1dqqb1 d3h42l1 d1egjh1 d1kcvl1
d1fe8l1 d2j6el1 d1f3rb2 d1lk3l1 d1sm3h1 d1q0xh1 d1ghfh1 d7fabl1
d1igch1 d1lo0h1 d4idla_ d5c2bl_ d1jrhl1 d1il1a1 d3zhda1 d4kfzc_
d1b0wa_ d1e6oh1 d1mexl1 d4krpb_ d1fnsh1 d1ncwh1 d4hgma_ d4eigb1
d4rzca_ d2mpal1 d4y5xa_ d5en2b1 d3lrga1 d4ej1c1 d1rhha1 d1j05b_
d1forh1 d1q9wb1 d2wzpd_ d2aq2a1 d1h0da1 d3revb1 d2ij0c1 d1tzha1
d4m7jl1 d1k6ql1 d3b9kc1 d1igml_ d3qeua1 d1dsfl_ d1ce1h1 d1kcuh1
d1zvha1 d1zvoa1 d1tqbb1 d1ayll1 d2qscl1 d2cgrh1 d1nl0h1 d1kenl1
d3eo0a1 d4n9ob1 d1n0xl1 d3k1kc_ d1c4qa_ d1prtc1 d2wv6d_ d1prtf_
d4l63b1 d1r4pb_ d1prtd_ d1tl0f_ d2bosa_ d1prtb1 d3chbd_ d2aq2b1
d1ck1a1 d1enfa1 d1i4pa1 d1v1oa1 d1et9a1 d1esfa1 d2g9hd1 d4ohja1
d1klud1 d1bxta1 d1eu3a1 d1ty0a1 d1fnua1 d2cqva1 d1fhga_ d2k1ma_
d2dl2a1 d1biha3 d1biha1 d1biha4 d3qp3a1 d1g1ca_ d1gxea_ d1cvsc2
d1cvsc1 d1xmwa2 d1m4ka2 d2otpa2 d1biha2 d1rhfa2 d2fcba2 d2fcba1
d1wwbx_ d1gsma2 d1epfa1 d1epfa2 d2dava1 d1waaa_ d1f97a2 d1x44a1
d1vcaa2 d1fnla2 d1fnla1 d3b5ha2 d3h8na2 d3h8na1 d1jbja1 d1jbja2
d1pd6a_ d1f2qa2 d1f2qa1 d3cx2a_ d1nbqa2 d3b5ha1 d1n26a1 d1xiwb_
d1xaua_ d1fltx_ d2ifga1 d1olla1 d1cs6a4 d1cs6a3 d1cs6a2 d1cs6a1
d2gw5a2 d1sy6a1 d1xiwa_ d1ucta2 d1ucta1 d1ry7b2 d1gl4b_ d1qz1a3
d1olza1 d2c9aa1 d1wwca_

**Family-15 (a.39.1.8)**

**Positive dataset contains 2 protein sequences.**

d1y1xa_ d4usla_

**Negative dataset contains 4047 protein sequences.**

d1xfda2 d1m32a_ d1ibja_ d1c7na_ d1iuga_ d4q75a_ d2bkwa1 d1t3ia_
d1n8pa_ d1qz9a_ d1jf9a_ d1eg5a_ d1p3wa_ d3kgwa_ d2ctza1 d1vjoa_
d2rfva_ d1gc0a_ d1cs1a_ d1d2fa_ d2ch1a1 d1qgna_ d1pffa_ d1elua_
d1u08a_ d1lc5a_ d2ay1a_ d1bw0a_ d3pd6a_ d7aata_ d2csta_ d1j32a_
d1fg7a_ d4wlha_ d1o4sa_ d1v2da_ d1gdea_ d1m6sa_ d1v72a1 d1svva_
d1ajsa_ d1wsta1 d2f8ja2 d1lk9a_ d1vp4a1 d1xi9a1 d2gb3a1 d1yaaa_
d2bnma2 d1xkta1 d2hfka_ d1jmkc_ d5d3ka_ d1ax4a_ d2v1pa1 d2ez1a_
d1i26a_ d1ddla_ d1e57b_ d1f56a_ d1bypa_ d1azca_ d1ws8a_ d3u25a_
d1cuoa_ d1id2a_ d1rkra_ d2iaac_ d4dp1x_ d1nwpa_ d2gima_ d2i7sa_
d1qhqa_ d1gyca3 d1hfua2 d1kbva1 d1kbva2 d1sdda1 d1sdda2 d1gyca1
d1gyca2 d1mzya1 d2bw4a1 d1aoza1 d1aoza3 d1kyaa2 d1kyaa3 d1kyaa1
d1mzya2 d4e9sa1 d1sddb1 d1sddb2 d1hfua1 d1hfua3 d1oe1a2 d1oe1a1

d1v10a2 d1v10a3 d1v10a1 g1f8v.1 d1novc_ d2bbvc_ d2cfba1 d1dfoa_
d1o69a_ d1bj4a_ d3k28a_ d2bwpa_ d3lg0a_ d2fnua_ d3ou5a_ d1z7da1
d1b9ha_ d1iaya_ d4msoa_ d1bs0a_ d1s0aa_ d1sffa_ d3tqxa_ d1mdoa_
d3qboa_ d3pgya_ d1bjna_ d2dkja_ d1ejia_ d1vefa1 d4wxga_ d3bs8a_
d3gbxa1 d1w23a_ d1a3qa1 d1iknc_ d3brda1 d1p7hl1 d1oy3c_ d1imhc1
d1gjia1 d1zk9a1 d2cxka1 d2bema_ d1pama1 d1ea9c1 d1qhoa1 d2fhfa2
d2fhfa1 d1k3ia1 d1j0ha1 d1cxla1 d2bhua1 d1f1sa2 d1ut9a2 d1gvia1
d1txka1 d4alac_ d1pjwa_ d1s6na_ d3uzqb_ d2phla2 d2phla1 d1fxza2
d1fxza1 d1uija1 g1dgw.1 d2v09a_ d1uika2 d1uika1 d1dgwa_ d1ep0a_
d2pa7a1 d1wlta1 d1nxma_ d2c0za1 d1ds6b_ d1fstb_ d1ex0a1 d1g0da1
d4mh8a_ d1mu2a2 d4i7ga1 d1tafa_ d1bh9b_ d1tafb_ d1jfib_ d1h3ob_
d2byka1 d1n1ja_ d4g92b_ d1bh9a_ d1jfia_ d1h3oa_ d2bykb1 d1n1jb_
d2bjca1 d2hsga1 d1qpza1 d1efaa1 d1r0ka3 d1vkoa2 d1vm6a2 d3qvsa2
d1gr0a2 d1p1ja2 d2ovga_ d1sq8a_ d1rzsa_ d3kz3a_ d3orca_ d3gpdg2
d2q49a2 d1hdgo2 d2g17a2 d2cvoa2 d2czca1 d3pwka2 d1obfo2 d1zyao2
d2pkqo2 d1b7go2 d2hjsa2 d1cf2o2 d1vkna2 d1nvmb2 d1ux8a_ d4i0va_
d2bkma_ d1dlwa_ d2bmma_ d2msya_ d2djoa1 d2r5za_ d1au7a1 d1f43a_
d2lmda1 d1akhb_ d1s7ea1 d3nara_ d1zq3p1 d2craa1 d1mija_ d4uuta_
d3a01a_ d1x58a1 d3rkqa1 d3cmya1 d2cqxa1 d2da6a1 d1e3oc1 d1fjla_
d1mh3a1 d1ig7a_ d1x41a1 d1wh7a1 d1gt0c1 d2mw8a_ d1lfup_ d1x2ma1
d1b72a_ d1wi3a1 d1jgga_ d1wjha1 d1x2na1 d1mnmc_ d2cuea1 d3k2aa_
d2hosa_ d2jwta2 d1pufa_ d1octc1 d2hi3a_ d1b8ia_ d4j19a_ d3lnqa_
d1wh5a1 d1b8ib_ d1fexa_ d2cqra1 d1guua_ d1ug2a1 d2cqqa1 d2ajea1
d4a69c_ d1h8ac1 d2crga1 d2iw5b1 d1xc5a1 d1ofcx1 d2cu7a1 d1wgxa1
d2cjja1 d2ckxa1 d1gvda_ d6paxa2 d1k78a1 d1k78a2 d1hlva2 d1iufa2
d1iufa1 d2iu5a1 d1zk8a1 d2g7sa1 d2fd5a1 d2np5a1 d1z0xa1 d2id6a1
d2d6ya1 d2i10a1 d1vi0a1 d2oi8a1 d3c07a1 d2ns7a1 d2fq4a1 d2yvea1
d1t33a1 d2o7ta1 d1rkta1 d2np3b1 d2g7ga1 d2gena1 d2fbqa1 d2g7la1
d2gfna1 d2g3ba1 d2jj7a1 d2hyja1 d1sgma1 d1ui5a1 d2hkua1 d2id3a1
d4jyka1 d3lv8a_ d1s3ga1 d1zaka1 d1s4qa_ d3tvta2 d1uf9a_ d1khta_
d1ckea_ d1osna_ d1p5zb_ d4tmka_ d3cm0a_ d1jaga_ d3h86a_ d1t0hb_
d1nn5a_ d2h92a_ d3kb2a1 d2c95a1 d1yj5a2 d1kgda_ d1ly1a_ d2ak3a1
d1vyva2 d1deka_ d2grja1 d1p3ja1 d1y63a_ d1e4ya1 d1s96a_ d3iija1
d1lvga_ d1nksa_ d1vyua2 d4e22a_ d1qf9a_ d1jjva_ d1q3ta_ d1ki9a_
d1lw7a2 d1teva_ d1kjwa2 d1gsia_ d1g3ma_ d1j99a_ d1t8ta1 d1nsta_
d1fmja_ d1zrha_ d1ls6a_ d2a3ra_ d1aqua_ d2zptx_ d3ckla_ d1texa_
d1q44a_ d2gwha_ d1z0ja1 d3umra2 d3gj0a1 d2gaoa1 d1yzga1 d1x3sa1
d1g7sa4 d1puja_ d1udxa2 d1kk1a3 d2o52a_ d1jwyb_ d1zcaa2 d1zcba2
d2fv8a1 d2cjwa_ d3dz8a1 d1xzpa2 d1vg8a_ d4qxaa_ d2ew1a1 d1x1ra1
d2bcgy_ d3doea1 d3cona_ d3rega_ d3wyga_ d1upta_ d2b6ha1 d1zd9a1
d1lnza2 d1r5ba3 d1n0ua2 d1r2qa_ d2f9la1 d2rhda_ d3q3jb_ d3q85a_
d1efma1 d1r8qa_ d2gf9a1 d2akab1 d1ek0a_ d1z08a1 d2gcoa1 d2dy1a2
d4g01b_ d2cxxa1 d1svia_ d3q72a_ d1ni3a1 d4ac9a4 d2erxa1 d2bmea_
d1wxqa1 d2gf0a1 d4ldja1 d1zunb3 d1d5ca_ d4lv5b_ d1efca3 d1kmqa_

| | | | | | | | |
|---|---|---|---|---|---|---|---|
| d2g6ba1 | d3lrpa_ | d1tada2 | d2y8ea_ | d4rd4a1 | d1z06a1 | d2heia_ | d3a58b_ |
| d1u0la2 | d1d2ea3 | d1zj6a1 | d2ocba_ | d3rwoa_ | d1mr3f_ | d2e9sa1 | d4u5xa1 |
| d2iwra_ | d2fn4a1 | d3bwdd_ | d2nzja1 | d2atva1 | d2ksqa_ | d3l43a_ | d1mkya2 |
| d1mkya1 | d1wmsa_ | d1jnya3 | d2rmka_ | d1f6ba_ | d1f60a3 | d1gota2 | d1jala1 |
| d2fh5b1 | d1g16a_ | d2wblc_ | d1m7ba_ | d1wf3a1 | d1f5na2 | d1t9ha2 | d1z6xa1 |
| d1z0aa1 | d2fg5a1 | d2a5ja1 | d2bcjq2 | d1ky3a_ | d4y0va_ | d4lhwa_ | d2qmea1 |
| d2atxa1 | d1egaa1 | d1ryha_ | d1moza_ | d1nrjb_ | d2erya1 | d3bfka_ | d1puia_ |
| d1fzqa_ | d3j3az1 | d3t5ga_ | d1shza2 | d3defa_ | d2f7sa1 | d3raba_ | d3tsoa_ |
| d3aq4a_ | d2al7a1 | d3sfva1 | d3bc1a_ | d2ncda_ | d1sdma_ | d3h4sa1 | d2heha_ |
| d2grya_ | g3kin.1 | d1kk8a2 | d1br2a2 | d1goja_ | d1f9va_ | d2mysa2 | d3t0qa_ |
| d4db1a1 | d3gbja_ | d1ry6a_ | d1v8ka_ | d1k8ka2 | d1k8ka1 | d3uleb1 | d3i33a2 |
| d3i33a1 | d1bupa1 | d1dkgd2 | d1dkgd1 | d4ci6a2 | d1jcfa1 | d4a2ba2 | d2fxua1 |
| d1yaga2 | d1yaga1 | d4cbua2 | d1bupa2 | d1g99a2 | d2e1za2 | d2e1za1 | d1saza1 |
| d1saza2 | d1iqca1 | d1eb7a2 | d1eb7a1 | d1nmla2 | d1nmla1 | d3h1jd1 | d1hroa_ |
| d4gedb_ | d3zooa_ | d1mz4a_ | d1gu2a_ | d1f1fa_ | d1dvva_ | d1c6ra_ | d1c75a_ |
| d1e29a_ | d1gdva_ | d1cnoa_ | d1h32b_ | d1co6a_ | d2bh4x_ | d2aiua_ | d5cytr_ |
| d1jdla_ | d1i8oa_ | d2zboa_ | d3dmia_ | d1ls9a_ | d1wvec_ | d2yk3a_ | d3ph2b_ |
| d1dw0a_ | d5aura_ | d1ql3a_ | d1qn2a_ | d1f1ca_ | d4zzva_ | d2xspa_ | d1eg1a_ |
| d4v20a_ | d1ojja_ | d4ipma1 | d1gpia_ | d4csia_ | d3pfza_ | d2yoka_ | d2nqya_ |
| d1h4ga_ | d1hixa_ | d1oa2a_ | d1olra_ | d3b7ma_ | d4h7ma1 | d3wp3a_ | d2vgda_ |
| d1igoa_ | d3zsea_ | d2nlra_ | d3wx5a_ | d1oa3a_ | d1pvxa_ | d1m4wa_ | d1f5ja_ |
| d1oa4a_ | d1ks5a_ | d4ixla1 | d3sita1 | d1h32a2 | d1evsa_ | d1cnt1_ | d1pvhb_ |
| d1z7ca_ | d1huwa_ | d1bgea_ | d1rhga_ | d1f6fa_ | d1f45b_ | d1i1rb_ | d1axia_ |
| d1etea_ | d3tgxb_ | d1scfa_ | d2z3qa1 | d3qb1a_ | d1hula_ | d1m48a_ | d4nkqb_ |
| d1eera_ | d1v7mv_ | d5e4ea_ | d2o27a_ | d3b5ka_ | d2psma1 | d4zf7a_ | d3uf5a_ |
| d4x51a_ | d1d9ca_ | d4ypgc1 | d3oq3a_ | d1lqsl_ | d1n1fa_ | d1au1a_ | d1wu3i_ |
| d3besl_ | d3ux9a_ | d2b5ea2 | d2b5ea3 | d2b5ea1 | d2b5ea4 | d2kp1a1 | d2kp2a1 |
| d1hyua3 | d1hyua4 | d1j08a1 | d1j08a2 | d3rbaa_ | d2qtra_ | d1nupa_ | d3l92a_ |
| d1f9aa_ | d2h29a_ | d1vlha_ | d1od6a_ | d1lw7a1 | d1mrza2 | d4ruka_ | d2el7a_ |
| d1iq0a2 | d1irxa2 | d1r6ta2 | d1ivsa4 | d2cyba_ | d1rqga2 | d1j09a2 | d2yxna_ |
| d1f7ua2 | d1h3fa1 | d1ffya3 | d2g36a1 | d1nzja_ | d1jila_ | d1n3la_ | d1i6la_ |
| d1pfva2 | d1h3na3 | d3n9ia_ | d3sz3a1 | d1m4la_ | d2bk7a_ | d3fjua_ | d1z5ra1 |
| d1kwma1 | d1uwya2 | d2c1ca1 | d1dtda_ | d1h8la2 | d3sw2a_ | d1moxc_ | d1dx5i3 |
| d1edmb_ | d1autl2 | d1autl1 | d1nqlb_ | d1dval1 | d1szba2 | d1kigl_ | d2y5fl_ |
| d1gk5a_ | d1g1sa2 | d1nt0a3 | d1xdtr_ | d1lmja2 | d1lmja1 | d1nzia2 | d2wphe_ |
| d1klil_ | d1pfxl2 | d1pfxl1 | d1p9ja_ | d1g1ta2 | d1f7ea_ | d4yzub_ | d2mgpa1 |
| d1b9wa1 | d1b9wa2 | d2mgpa2 | d1ob1c1 | d1ob1c2 | d1n1ia2 | d1n1ia1 | d2npra2 |
| d2npra1 | d1m1xb4 | d1m1xb5 | d1s5ja2 | d1qhta2 | d4dlea2 | d1tgoa2 | d3py8a2 |
| d1q8ia2 | d2py5a2 | d1d5aa2 | d1sfsa_ | d1jfxa_ | d1wkya2 | d1uuqa_ | d3wh9a_ |
| d4m1ra_ | d1xyza_ | d4xuya_ | d2je8a5 | d4bf7a1 | d3cuia_ | d1ur4a_ | d1qw9a2 |
| d1odza_ | d2vzsa5 | d1bqca_ | d2x2ya1 | d1hjqa_ | d1qnra_ | d4y7ea1 | d1vjza_ |
| d4k68a_ | d2v3ga_ | d4hpga_ | d1nofa2 | d1nq6a_ | d1h4pa_ | d1aq0a_ | d3ziza_ |
| d4bs0a_ | d1ecea_ | d1h1na_ | d1ta3b_ | d1w91a2 | d1i1wa_ | d1uhva2 | d1egza_ |
| d7a3ha_ | d1tvna1 | d3nyda1 | d1ghsa_ | d2cyga1 | d4xv0a_ | d2c8na2 | d1w32a_ |

d3emca_ d1v6ya1 d1foba_ d2uwfa1 d2fgla_ d3niya1 d2c0ha1 d1rh9a1
d1yq2a5 d1szna2 d1fa2a_ d1pama4 d5do8a1 d1ea9c3 d2fhfa5 d1qhoa4
d1avaa2 d1m53a2 d4m8ua1 d2gjpa2 d1lwha2 d4howa1 d1ktba2 d1j0ha3
d3vm5a1 d2x1ia_ d1wzaa2 d1uasa2 d2diea1 d1mxga2 d1ht6a2 d1eswa_
d1iv8a2 d5cpsa_ d1ud2a2 d1zjaa1 d3gbea1 d1kwga2 d1wdpa_ d1gcya2
d1g5aa2 d1hvxa2 d3dhpa2 d2bhua3 d4do4a1 d3qgva1 d1e43a2 d1g94a2
d2d3na2 d1gjwa2 d1gvia3 d1x1na1 d1r7aa2 d1tz7a1 d4c7ga2 d2gjxa1
d1nowa1 d1yhta1 d4toqa_ d1wb0a1 d1w9pa1 d1eoka_ d1itxa1 d1kfwa1
d1goia2 d1edqa2 d2gsja_ d1hjxa1 d1vf8a1 d3wdpp1 d3wq5a_ d4gxpa_
d4hz8a_ d1e4mm_ d3aiua_ d1wcga1 d1qvba_ d3ahxa_ d1v02a_ d2dgaa_
d1pbga_ d1vffa1 d1qoxa_ d4ha4a_ d2w0pa_ d2diaa1 d2e9ia2 d2e9ja1
d2dj4a1 d2eeba1 d2diba1 d2dmba1 d2dmca1 d2d7oa1 d2ee6a1 d2d7pa1
d2eeaa1 d2di8a1 d1wlha1 d2d7na1 d2nqca_ d2d7ma1 d2dica1 d3cnka_
d2k3ta1 d2eeca1 d1qfha1 d1qfha2 d2bp3a1 d1v05a_ d2ee9a1 d2eeda1
d2di9a1 d1xl4a1 d3vsqa_ d2gixa1 d1p7ba1 d1b9ra_ d1offa_ d1frra_
d3wcqa_ d1e9ma_ d1l5pa_ d1wria_ d3av8a_ d1czpa_ d1rfka_ d3p1ma1
d3b2ga_ d2bt6a2 d1wgqa1 d1v89a1 d2coda1 d1zc3b_ d1wg7a1 d1u5ea1
d1v61a1 d2coca1 d2ys1a1 d1v5pa1 d1ntya2 d1wjma1 d1wi1a1 d1u29a_
d1xx0a1 d1unqa_ d2zkmx3 d1fhoa_ d1v88a1 d2adza1 d1x1fa1 d1p6sa_
d2coaa1 d1btka_ d1v5ma1 d2x18a_ d1dyna_ d1x1ga1 d4kaxb_ d2j59m1
d1ki1b2 d1xcga2 d1txda2 d1u5da1 d1dbha2 d1w1ha_ d1upqa_ d1v5ua1
d1foea2 d2fjla1 d1eaza_ d2dfka2 d2cofa1 d1kz7a2 d9ldta1 d2ewda3
d1up7a1 d2dfda1 d1s6ya1 d1vjta1 d7mdha1 d1hyea1 d1t2da1 d1ojsa3
d1b8pa1 d1ldna1 d1hyha1 d1civa1 d2a92a1 d1ez4a1 d1i0za1 d1y6ja1
d1gv0a1 d1ur5a1 d1llda1 d1obba1 d1v8ba1 d1gdha1 d1l7da1 d2dlda1
d1mx3a1 d1ygya1 d1qp8a1 d1li4a1 d2naca1 d1npya1 d3t4ea2 d1a4ia1
d1o0sa1 d2hk9a2 d2egga2 d1b26a1 d1bvua1 d1gq2a1 d1c1da1 d1leha1
d1gtma1 d1euza1 d1nvta1 d3dona2 d1edza1 d1gpja2 d1p77a1 d1nyta1
d1b0aa1 d1vl6a1 d1v9la1 d1txga2 d3f47a1 d1mv8a2 d2pv7a2 d1xc2a3
d2ahra2 d4xiya1 d2f1ka2 d1pgja2 d1i36a2 d1np3a2 d1lj8a4 d1teaa3
d1qmga2 d2i76a2 d2g5ca2 d1wdka3 d1dlja2 d3cuma3 d1kola2 d1f8fa2
d1qora2 d1jqba2 d1vj0a2 d1cdoa2 d1gu7a2 d1d1ta2 d1xa0a2 d2dm6a2
d1jvba2 d1rjwa2 d1llua2 d1h2ba2 d1tt7a2 d1piwa2 d1o89a2 d1iz0a2
d1e3ja2 d1yb5a2 d1uufa2 d1vj1a2 d1e3ia2 d3gpdg1 d1vc2a1 d2nvwa1
d1u8fo1 d1ydwa1 d1hdgo1 d2g17a1 d1cf2o1 d2czca2 d1r0ka2 d1obfo1
d2cvoa1 d1ebfa1 d1h6da1 d1i32a1 d1zh8a1 d2q49a1 d1qkia1 d1j5pa4
d3pzra1 d1xeaa1 d1vkoa1 d1vm6a3 d3qvsa1 d2pkqo1 d1b7go1 d1gr0a1
d1p1ja1 d1lc0a1 d1j0xo1 d3pwka1 d2hjsa1 d1zyao1 d1vkna1 d1tlta1
d1nvmb1 d3sjua_ d3ojfa1 d1xu9a1 d2jjya1 d1n7ha_ d1y1pa1 d1h5qa_
d1jtva_ d4fs3a_ d2bkaa1 d1gy8a_ d2c5aa1 d1ulsa_ d1ooea_ d2b69a1
d1y5ma1 d1spxa_ d1ae1a_ d1bxka_ d1ydea1 d1gega_ d1hdoa_ d1o5ia_
d1e6ua_ d1snya1 d2rhca_ d3zv4a_ d5ceja_ d2d1ya1 d1fjha_ d3w8ea_
d2fmua1 d1x1ta_ d1rpna_ d3ay7a1 d1k2wa_ d1eq2a_ d2hrba1 d3ruaa_
d2ew8a1 d4hwka_ d1xgka_ d2bd0a1 d1yo6a1 d2a35a1 d2ntva_ d1vl8a_

d1hdca_ d4bkua_ d2ag5a1 d2q45a_ d1nffa_ d4iy1a_ d1zbqa1 d1kewa_
d1ek6a_ d1ja9a_ d2q46a2 d1iy8a_ d1cyda_ d2ptga_ d1hxha_ d1uzma1
d3lyla_ d4m89a_ d3wyea_ d2blla1 d1w6ua_ d1sb8a_ d2q2va_ d4nr0a_
d1e6wa_ d2gasa_ d3oiga1 d2bgka1 d1mxha_ d1vl0a1 d1rkxa_ d1r6da_
d1wmaa1 d1e7wa_ d2c07a1 d1xkqa_ d3tzha_ d1g0oa_ d3lz6a_ d1qyca_
d1edoa_ d1gz6a_ d1oc2a_ d1t2aa_ d1zema1 d2xoxa_ d1z45a2 d1geea_
d1p33a_ d3lt0a_ d4e6pa_ d2wyua_ d1db3a_ d4q9na_ d1xhla_ d4o1ma_
d1fmca_ d2uvda_ d2gdza1 d1uaya_ d2z1ma_ d2o23a2 d2fr1a2 d2fr1a1
d1qsga_ d1qyda_ d4zjua_ d2a4ka1 d1yb1a_ d1wvga1 d1yxma1 d3f4ba_
d1mg5a_ d1xg5a1 d1r55a1 d3gboa_ d1atla_ d1buda_ d4q1la_ d1quaa_
d1yp1a_ d1wnia_ g1y43.1 d4ekfa_ d2ckga_ d1th0a_ d2bkra_ d1xd3a_
d3ifwa_ d1e2ta_ d4c5pa_ d2bsza1 d4bgfa_ d1w4ta1 d3u1ra1 d1g9ka2
d1kapp2 d1k7ia2 d1t3ca_ d1epwa3 d2a97a_ d3fiea_ g1e1h.1 d3ioqa_
d1fh0a_ d1o0ea_ d1khqa_ d1m6da_ d2cb5a_ d1cs8a_ g8pch.1 d1s4va_
d2pnsa_ d1vsna1 d1mira_ d1iwda_ d2oula_ d7pcka_ d3bwka_ d3bcna_
d2p86a_ d1pvja_ d1pcia_ d1gece_ d1deua_ d4bs6a_ d1xkga1 d1cqda_
d1jqpa2 d2c0ya_ d2bdza_ d3p5ua_ d1zoya1 d1qo8a2 d1d4ca2 d1chua2
d1jnra2 d1kf6a2 d2jk6a2 d2jk6a1 d1feca1 d1feca2 d1lpfa1 d1lpfa2
d1xhca1 d1xhca2 d1dxla2 d1dxla1 d1gera2 d1m6ia2 d1onfa1 d1onfa2
d3lada2 d3lada1 d1w4xa1 d1w4xa2 d1gesa2 d1mo9a1 d1xdia1 d1fl2a2
d1fl2a1 d1fcda1 d1fcda2 d1h6va2 d1h6va1 d1mo9a2 d1gv4a2 d1gv4a1
d2gv8a2 d2gv8a1 d1aoga1 d1aoga2 d1hyua1 d1ebda1 d1ebda2 d3dk9a1
d1q1ra2 d1q1ra1 d1gesa1 d1zbfa1 d2z1ha_ d2etja1 d3qioa1 d1jl1a_
d2e4la_ d1jl2a_ d2lsna1 d1io2a_ d1uaxa_ d2yv0x_ d1mu2a1 d1ekea_
d1i39a_ d1o94a2 d1lqta1 d1cjca1 d1pn0a1 d1ju2a1 d2f5va1 d3c96a1
d2gmha1 d1pj5a2 d1gpea1 d1o5wa1 d1n4wa1 d1k0ia1 d2dw4a2 d2ivda1
d1seza1 d2voua1 d1kdga1 d1js9c_ d1f15b_ d1za7a_ d2v76a_ d1wj1a1
d1aqca_ d1qqga2 d1qqga1 d1ntva_ d1n3ha_ d4dbba_ d3f0wa1 d1wgua1
d2cy4a1 d3hqca_ d1wvha1 d1xr0b1 d3nvva1 d1krha3 d2h88b1 d1n62a2
d1t3qa2 d1kf6b2 d1zoyb1 d1jq4a_ d1ffva2 d1dgja2 d1rm6c2 d1r7ha_
d1faaa_ d1wika1 d1xfla_ d1ttza1 d2f51a1 d1ti3a_ d1nhoa_ d1sena_
d2i4aa_ d1ep7a_ d1zmaa1 d1fo5a_ d3lefa1 d3diea1 d2yana1 d1z6na1
d1oaza_ d3zywa1 d4aj8a_ d1nm3a1 d4fiwa_ d1wmja_ d4hs1a1 d1iloa_
d1xwaa1 d1wjka1 d2vm1a_ d1fb6a_ d1gh2a_ d1h75a_ d1nw2a_ d1kx5b_
d3a6na_ d4m6ba2 d2nqbd_ d3wkjd_ d2yfwe_ d4h9na_ d5bnxa_ d1kx5c_
d3r45a_ d1eqzb_ d4jjnb_ d1f66c_ d1eqza_ d4wnnb_ d1kx5a_ d1u35c1
d1id3a_ d2nqbc_ d1kx5d_ d1ku5a_ d1b67a_ d1um8a_ d1ixza_ d1qvra2
d1qvra3 d1qzma_ d1sxjc2 d1jr3d2 d2p65a_ d1l8qa2 d1g8pa_ d3hu3a3
d1sxjd2 d1njfa_ d1in4a2 d1sxja2 d1tuea1 d2ce7a2 d1w5sa2 d1sxjb2
d1d2na_ d1ny5a2 d1ofha_ d2gnoa2 d2a5yb3 d1fnna2 d1u0ja1 d1lv7a_
d1iqpa2 d2fnaa2 d1ixsb2 d4gita_ d1sxje2 d1g41a_ d1pd212 d1nhya2
d2a2ra2 d1k0dd2 d1f2ea2 d1gsua2 d1f3ba2 d2fhea2 d2pvqa1 d1tw9a2
d1oyja2 d1e6ba2 d1k0ma2 d3ik7a1 d4is0a1 d1r5aa2 d1jlva2 d1m0ua2
d3gtub2 d1z9ha2 d1okta2 d1axda2 d2gsra2 d1tu7a2 d1k3ya2 d1zgma1

d1b48a2 d1gnwa2 d1gwca2 d2gsta2 d1glqa2 d1oe8a2 d1pn9a2 d1v2aa2
d1fw1a2 d1duga2 d3vrda1 d3vrda2 d1fcdc2 d1fcdc1 d1h1oa2 d1h1oa1
d1m70a2 d1a0rp_ d1czan2 d1czan3 d1czan1 d1ig8a2 d1czan4 d1bg3a4
d1bg3a3 d1bg3a2 d1bg3a1 d1qusa_ d1qqya_ d1gd6a_ d1ivma_ d2gv0a_
d1iiza_ d2z2fa_ d1j8xa_ d1ghla_ d1b9oa_ d1iyka1 d1rxta1 d1iica2
d1iica1 d2za0a_ d2c21a1 d1f9za_ d2pjsa1 d1klla_ d2i7ra1 d1ecsa_
d1xqaa1 d5cj3a1 d1npba_ d1jifa_ d2p7oa_ d1r9ca_ d1mpya2 d1kw3b2
d1kw3b1 d4ghga1 d1tfza1 d3isqa1 d3isqa2 d1sqia1 d1sqia2 d1lkda2
d1lkda1 d1mpya1 d1cjxa2 d1sp9b2 d1t47a1 d1t47a2 d1f1ua2 d1sp8a1
d1sp8a2 d1f1ua1 d4iuaa3 d4bvva_ d5hpga_ d3k65a_ d1ki0a3 d1ki0a2
d1ki0a1 d1a0ha1 d1pmla_ d3sp8a3 d2feba1 d1qsta_ d2beia1 d4m3sa_
d1u6ma1 d1ygha_ d1yk3a1 d3dr6a_ d1xmta_ d1z4ea1 d2ge3a1 d1bo4a_
d2fiaa1 d2jdca1 d1y9wa1 d3to7a_ d1wwza1 d2fl4a1 d2ozua1 d2b5ga_
d2cy2a1 d2p0wa_ d1cm0a_ d4pswa_ d1yrea1 d1yr0a1 d1m4ia_ d2gana1
d1yvka1 d1ghea_ d2g3aa1 d1y7ra1 d2euia1 d1q2ya_ d2fsra1 d1xeba_
d2ae6a1 d1cjwa_ d1qsma_ d2aj6a1 d1y9ka1 d1i12a_ d1vkca_ d2atra1
d1n71a_ d2d4pa1 d2fiwa1 d2fe7a1 d1tiqa1 d2ou2a_ d1yx0a1 d2fcka1
d1p0ha_ d2j8ma_ d2pq8a_ d1z4ra1 d1s3za1 d1ufha_ d2hqya1 d2hqya2
d1ne9a1 d1lrza3 d1ne9a2 d1f37b_ d1xvqa1 d1pn0a2 d3hy2a_ d2cvba1
d1o8xa_ d1lu4a_ d1we0a1 d2bmxa1 d2a4va1 d1psqa_ d1xiya1 d1q98a_
d1n8ja_ d1zofa1 d2ggta_ d3tjja_ d4je1a1 d3s9fa_ d2zcta_ d1qmva_
d1jfua_ d2b1ka_ d5dvba1 d2b5xa1 d2v2ga_ d1st9a_ d3tb2a_ d4llra_
d4fh8a1 d1e2ya_ d1knga_ d2h30a_ d1i5ga_ d2z9sa_ d2f8aa1 d1tp9a1
d2b7ka_ d1nm3a2 d3hvva_ d2wfca1 d4kcea_ d1xvwa1 d4gqca1 d3qpma_
d4k1fa_ d1zzoa1 d1gp1a_ d1prxa_ d4od7a1 d1z6ma1 d4mcua1 d4dvca1
d1r4wa_ d3l9sa_ d3rppa_ d3kasa1 d3bi1a3 d1e88a1 d1l6ja4 d1eaka4
d1eaka3 d1l6ja3 d1h8pa2 d1h8pa1 d1etha2 d1w52x1 d2ppla1 d1hpla2
d1lpbb2 d2oxea1 d1rp1a2 d1ys1x_ d4hs9a_ d1ispa_ d1ex9a_ d3qmma_
d1mixa2 d1e5wa2 d1isna2 d2al6b2 d1lgya_ d4l3wa_ d1llfa_ d1gz7a_
d1ukca_ d1j1ia_ d2rhwa_ d3ia2a_ d1a88a_ d4dgqa1 d1hkha_ d1zoia_
d3zxha_ d1cgla_ d1rm8a_ d1eaka2 d2jsda_ d1cxva_ d1hv5a_ d1i76a_
d3p0ya1 d1igra1 d1igra2 d1n8yc2 d1n8yc1 d1m6ba2 d1m6ba1 d1dzka_
d4tlja_ d2a2ua_ d4wfva_ d3t1da_ d4iaxa_ d3zq3a_ d2nnda_ d1i4ua_
d1kxoa_ d1bbpa_ d1kt7a_ d1exsa_ d3s26a1 d1lf7a_ d1qfta_ d4gh7a_
d1z24a1 d1qabe_ d1xkia_ d3bx7a_ d4iawa1 d1n0sa_ d1gkab_ d3kzaa_
d2k23a_ d2l9ca1 d2joza1 d1gm6a_ d3kffa_ d3saoa1 d1e5pa_ d1yupa1
d1iiua_ d1jgta2 d1ofda3 d1ea0a3 d1ecfa2 d1q15a2 d1ct9a2 d1te5a_
d1c8nc_ d1x36a_ d1smvc_ d1ng0c_ d4sbvc_ d1opoa_ d1f2nc_ d1cixa_
d1y29a_ d1v7fa_ d2jtba_ d1d1ha_ d1g9pa_ d1lupa_ d1qk7a_ d1mb6a_
d2a2va_ d1niya_ d1i25a_ d1hvwa_ d2mpqa_ d1ie6a_ d1kqha_ d1la4a_
d2mxoa1 d1koza_ d1qk6a_ d1emxa_ d1lqra_ d1c6wa_ d1rmka_ d1ixta_
d1eyoa_ d1cnna_ d1fu3a_ d1f3ka_ d1fyga_ d1rypd_ d1irua_ d1rypc_
d3unfk_ d1rypa_ d1j2pa_ d1irui_ d1j2qh_ d3unfh_ d1m4ya_ d1rypb_
d1irug_ d1iru2_ d1q5rh_ d1rypg_ d1g3ka_ d1iruf_ d1q5qa_ d1e94a_

| | | | | | | | |
|---|---|---|---|---|---|---|---|
| d1irub_ | d1ryph_ | d3ty6a_ | d1iruj_ | d1iruk_ | d1rype_ | d1irud_ | d1irue_ |
| d3mi01_ | d1rypf_ | d1iruh_ | d2z3ba_ | d1iruc_ | d3mka2_ | d1iru1_ | d1ny721 |
| d1ny722 | d1a6ca3 | d1a6ca2 | d1a6ca1 | d1pgl22 | d1pgl11 | d1ny711 | d1mqta_ |
| d1d4m3_ | d1fmd3_ | d2mev1_ | g1aym.1 | d1ev13_ | d4q4w1_ | d1r1a3_ | d2x5ia_ |
| d1rhi1_ | g1r1a.1 | g1d4m.1 | g1rhi.1 | d1b35b_ | d1oopa_ | d1tme1_ | d4iv1a_ |
| g1pvc.1 | g1fpn.1 | d1fpn1_ | d4gb31_ | d1d4m1_ | d1fmd1_ | d1ev11_ | d2wzr3_ |
| g1cov.1 | d1qqp3_ | g1fmd.1 | d2x5ic_ | d1rhi3_ | g1oop.1 | d1hxs1_ | d1fpn3_ |
| d1bev1_ | g2mev.1 | d1eah3_ | d1aym1_ | d1pvc3_ | d1qqp1_ | d1zba3_ | d1h8ta_ |
| d3vbhc_ | g1b35.1 | g1bev.1 | d1hxs3_ | g1ev1.1 | d1bev3_ | g1tmf.1 | d1tmf1_ |
| d1b35a_ | d2mev3_ | g1eah.1 | g1h8t.1 | d1eah1_ | d4q4w3_ | d1r1a1_ | d1aym3_ |
| d1pvc1_ | d1zba1_ | d1h8tc_ | d1oopc_ | d1tme3_ | g1qqp.1 | d4iv1c_ | g1tme.1 |
| d3ubub_ | d1egia_ | d1qo3c_ | d1v7pa_ | d1tdqb_ | d3ubua_ | d1c3ab_ | d1h8ua_ |
| d3bx4b_ | d1t8ca1 | d1sb2a1 | d1qdda_ | d2ziba1 | d3cada_ | d1j34b_ | d1jwia_ |
| d1jzna_ | d1c3aa_ | d1oz7b_ | d1wmza_ | d3bdwb_ | d1hq8a_ | d1fifa1 | d1dv8a_ |
| d3hupa_ | d1wk1a1 | d1umra_ | d2afpa_ | d3bx4a_ | d1gz2a_ | d1sl6a_ | d1v7pb_ |
| d3g8ka_ | d1ixxa_ | d1j34a_ | d1jwib_ | d1g1sa1 | d1y17a1 | d1ja3a_ | d1fvub_ |
| d3bdwa_ | d1sb2b1 | d1fvua_ | d1umrc_ | d1oz7a_ | d4m17a_ | d3cfwa1 | d1ypqa1 |
| d1g1ta1 | d3fd4a_ | d3l9jc_ | d4dn8a1 | d1v4lb_ | d1rdl1_ | d2msba_ | d4w9ta_ |
| d1jvna1 | d4x2ra1 | d1ka9f_ | d1qo2a_ | d4axka1 | d1h5ya_ | d4u28a_ | d2y88a_ |
| d4wd0a_ | d1vzwa1 | d2flia1 | d1h1ya_ | d1tqja_ | d1tqxa_ | d4lt9a1 | d3tc7a1 |
| d3hoja_ | d1i4na_ | d3vnda_ | d3nl8a1 | d1geqa_ | d3nava1 | d1v5xa_ | d4a2ra1 |
| d3tc6a1 | d1ujpa_ | d4ijba_ | d1xcfa_ | d1vc4a_ | d1rd5a_ | d1qopa_ | d3b5va_ |
| d2r80b_ | d1cqxa1 | d1cg5a_ | d1gcvb_ | d3boma_ | d2h8fb_ | d2raoa_ | d3bj1a_ |
| d4hrrb_ | d2r50a_ | d3d1kb_ | d2aa1b1 | d3zhwa_ | d3hf4b_ | d3gdja_ | d1jebb_ |
| d3bj1b_ | d1x9fb_ | d1wmub_ | d1gcva_ | d3bcqb_ | d3d4xb_ | d1gvha1 | d2dc3a_ |
| d3hf4a_ | d4yu3a_ | d3k8ba_ | d1qpwb_ | d2r80a_ | d3bcqa_ | d1v4wa_ | d3wtgb_ |
| d1jl7a_ | d4esaa_ | d1a9we_ | d3d1ab_ | d1fsla_ | d4h2la_ | d1v4wb_ | d1mwca_ |
| d1vhba_ | d1jeba_ | d1spgb_ | d1h97a_ | d1q1fa_ | d1tu9a1 | d3d1aa_ | d1hdsb_ |
| d3hyub_ | d2zfbb_ | d3fs4a_ | d1oj6a_ | d1shrb_ | d4h2lb_ | d4yu3b_ | d1spga_ |
| d3hrwa_ | d3qqra_ | d3at5a_ | d4wchd_ | d3hyua_ | d1cg5b_ | d1uc3a_ | d1hbra_ |
| d2raob_ | d1outb_ | d4esab_ | d3gdjb_ | d3qqqa_ | d1x9fc_ | d3vrfa_ | d1itha_ |
| d3mkba_ | d3d4xa_ | d3mjpa_ | d1jl6a_ | d3fh9a_ | d1outa_ | d2dc3b_ | d1it2a_ |
| d3eoka_ | d3g46a_ | d1qpwa_ | d3vrfb_ | d1ch4a_ | d1x9fd_ | d1hdsa_ | d2zfba_ |
| d3mkbb_ | d1or4a_ | d3wtga_ | d1a4fa_ | d3fh9b_ | d3bomb_ | d3cy5a_ | d3at5b_ |
| d1x9fa_ | d2oifa_ | d4lmxb_ | d1cpcb_ | d3v57a_ | d1kn1a_ | d1cpca_ | d4f0ta_ |
| d2vjha_ | d4po5b_ | d1eyxa_ | d1alla_ | d4f0tb_ | d1b8db_ | d4h0mb_ | d1b33a_ |
| d2vjhb_ | d4lm6b_ | d2vmlb_ | d1allb_ | d2vjta_ | d3dbja_ | d2vmla_ | d2vjtb_ |
| d4f0ub_ | d1f99b_ | d4rmpa1 | d1liaa_ | d1eyxb_ | d2uulb_ | d4lmsb_ | d4f0ua_ |
| d2bv8a_ | d1f99a_ | d2bv8b_ | d2c7lb_ | d3dbjb_ | d4rmpb1 | d1b8da_ | d4h0ma_ |
| d3v57b_ | d1kn1b_ | d1liab_ | d2uula_ | d2j96a_ | d1ra6a_ | d1muka_ | d3gsza_ |
| d1khva_ | d1te9a_ | d2xwha_ | d3uqsa_ | d3hkwa_ | d3n6ma_ | d2cjqa_ | d2xyma_ |
| d1teba_ | d1te8a_ | d1u09a1 | d4wfxa_ | d3tyqa_ | d2pgga1 | d2xqfa_ | d1ea5a_ |
| d1f6wa_ | d1dx4a_ | d1qe3a_ | d4qwwa_ | d2ha2a_ | d3c8da2 | d2pbla1 | d1jkma_ |
| d1jjia_ | d1jjfa_ | d2h0ba_ | d2erfa_ | d1h30a2 | d3mw4a1 | d1pz7a_ | d2r1ba_ |

d3v64a1 d1d2sa_ d3bopa1 d3mw3a1 d1okqa1 d1okqa2 d1jmxa4 d1pbya3
d1pbya4 d1ex0a4 d1g0da4 d2f4ma1 d1x3za_ d1pv8a_ d1w5qa_ d2c1ha_
d1ohla_ d1l6sa_ d1f74a_ d3l21a_ d2e1da_ d1to3a1 d1w3ia_ d1sfla_
d1n7ka_ d1vcva1 d4imea_ d1wa3a_ d2ocza1 d1f2ja_ d3m16a_ d2yxga_
d1ub3a_ d3a5fa_ d1fbaa_ d1l6wa_ d1xfba1 d1wx0a1 d1vpxa1 d1ojxa_
d1mxsa_ d2a4aa1 d1xkya1 d3cq0a1 d3s1xa_ d1f05a_ d1fdja_ d3cpra1
d4d2ja_ d1cg2a1 d1yloa2 d1y7ea2 d1y0ya3 d1lfwa1 d1vixa1 d1fnoa4
d1vhoa2 d1ysja1 d1vhea2 d1z2la1 d2fvga2 d1vgya1 d1r3na1 d2grea2
d1xmba1 d1rtqa_ d3rp2a_ g1ucy.1 d1fuja_ d2zchp_ d3w94a_ d1si5h_
d1lo6a_ g2pka.1 d1cgha_ d1kigh_ d1a5ia_ d3s9ba_ d1hj8a_ d1ao5a_
d2anya_ d1q3xa1 d1gvza_ d4gsoa_ d2f91a1 d1iaua_ d1gdna_ d1eufa_
g1fiw.1 d1eq9a_ d4nfea_ d2rdla_ d1spja_ d1elva1 d1a7sa_ d1ekbb_
d1pfxc_ d1yc0a_ d2aiqa_ g1fiz.1 d1fi8a_ d4k60a_ d4dgja_ d1mzaa_
d1op0a_ d3tjva_ d1pytd_ d1sgfg_ g1gg6.1 d2eeka_ g1rtf.1 d1a0ja_
d3g01a_ d1pytc_ d4e7na_ d1ddja_ d1z8ga1 d2hlca_ d1npma_ d1brup_
g1jou.1 d1sgfa_ d1autc_ g2hnt.1 g1kdq.1 d1orfa_ d3s69a_ d4crga_
d1fxya_ d1bqya_ d1svpa_ d1mbma_ d3su6a1 d1cu1a1 d4agka_ d1vcpa_
d1q31a_ g1n1l.1 d3e90b_ d1ep5a_ d3u1jb_ d3gv5b1 d1t94a2 d3bq0a2
d3lgia_ d3i77a_ d2z9ia2 d4nsva_ d1lcya2 d4jcna_ d1agja_ d1os8a_
d1hpga_ d1l1ja_ d1qtfa_ d1e69a_ d1mv5a_ d3g60a4 g1ii8.1 d1ewqa2
d1qhla_ d1v43a3 d2wmfa1 d1vpla_ d1g2912 d4a82a2 g1f2t.1 d2d62a1
d3tuic1 d2bbsa1 d3g60a3 d2ixea_ d1g6ha_ d1sgwa1 d2qi9c_ d3rlfa1
d3b60a1 d3d31a2 d2pzea1 g1xew.1 d1jj7a_ d1oxxk2 d2onka1 d1w1wa_
d1iwea_ d1qf5a_ d1vmaa2 d1nija1 d1np6a_ d2p67a1 d3ihla_ d1okkd2
d1ls1a2 d1s1ma2 d1iona_ d2v40a_ d3hida_ d2vo1a1 d2g0ta1 d1vcoa2
d1j8mf2 d1g3qa_ d1xjca_ d1p9ba_ d1qzxa3 d1ihua2 d1ihua1 d2qm8a_
d1dj2a_ d4ioja_ d1cp2a_ d1yrba2 d1hyqa_ d3gqba2 d1skye3 d1xp8a1
d1tf7a2 d1tf7a1 d1ye8a1 d1skyb3 d2qe7d2 d4blta_ d4huta_ d3ldaa2
d4hyya_ d2i3ba1 d1cr1a_ d3j9tb2 d3ntua2 d1vdza1 d2z43a_ d1e9ra_
d1nlfa_ d3ziaa2 d1n0wa_ d1g6oa_ d3ziad2 d3j9ta2 d1pzna2 d3gqbb2
d2i49a_ d1nnfa_ d1xvxa_ d1ursa_ d1h3da1 d1iuda_ d4m1va1 d1twya_
d3e13x_ d2nxoa1 d4pfua_ d3ttma_ d1elja_ d2qmwa1 d1us5a_ d1y3na1
d1jeta_ d1uqwa_ d4f19a1 d2esna2 d3thia_ d3fasa1 d2uyeb_ d2xwva1
d1r9la_ d2fyia1 d1pb7a_ d2ozza1 d1sw5a_ d1nh8a1 d1vr5a1 d2g29a_
d1xs5a_ d3lswa_ d2z23a_ d3w9va_ d3tcha_ d1q35a_ d1z7me1 d3q27a_
d1p99a_ d1ve4a1 d2i6ea1 d4mcoa1 d4n8ya_ d1eu8a_ d1zbma1 d1hsla_
d1ixca2 d1j1na_ d1xvya_ d1y9ua_ d2z22a1 d1xoca1 d4oera_ d1wdna_
d5f1qa1 d1xt8a1 d1i6aa_ d4gwoa_ d4igta1 d1pc3a_ d4mmpa1 d1o63a_
d2czla1 d2onsa2 d4mija1 d4ok5a1 d2bmfa1 d2bmfa2 d1yksa1 d1yksa2
d1jr6a_ d2qeqa1 d2jlqa1 d1a1va2 d1eh3a_ d3skpa_ d1dtza1 d1dtza2
d1ieja_ d1jnfa2 d1ce2a1 d1jnfa1 d1b1xa2 d1b1xa1 d1jw1a1 d1jw1a2
d1ryoa_ d1h76a1 d1h76a2 d1c4oa1 d2p6ra3 d2p6ra4 d1oywa2 d1oywa3
d1wrba1 d1w36b2 d1w36b1 d1c4oa2 d3bora_ d1t6na_ d2eyqa5 d2eyqa3
d2eyqa2 d2zu6a2 d1veca_ d1uaaa1 d1w36d1 d2g9na1 d1z3ix2 d2rb4a_

d1hv8a1 d1hv8a2 d1t5ia_ d1w36c2 d2j0sa2 d2j0sa1 d1fuka_ d3hjha1
d1z5za1 d1qdea_ d1z63a1 d1s2ma2 d1s2ma1 d1wp9a1 d1wp9a2 d1gm5a3
d1gm5a4 d3dina4 d3dina3 d1q0ua_ d1w36c1 d1z3ix1 d1nkta4 d1uaaa2
d1w36d2 g1qhh.1 d2fwra2 d2fwra1 d3q3ma_ d1za0a1 d1mxra_ d2inca_
d1jk0a_ d2incb_ d1mhyb_ d3hf1b_ d1r2fa_ d2uw1a_ d1mhyd_ d3n37a_
d1afra_ d1oquc_ d1syya_ d1mtyd_ d2o1za_ d1uzra_ d1smsa_ d2fkza_
d1zuja1 d1s3qa1 d2fjca1 d1z6om1 d1veia_ d4dyya_ d1jiga_ d3gvya_
d2pyba_ d4cy9a1 d3bvfa1 d1r03a_ d2cf7a_ d1nf4a_ d1zs3a1 d4iwka_
d3ka8a_ d3np0x_ d1n1qa_ d1lb3a_ d4e6ka_ d1z6oa1 d2fzfa1 d4dyua_
d3ge4a_ d1jgca_ d4am4a_ d2z6ma_ d1ji5a_ d1vlga_ d1lkoa1 d1krqa_
d3qz3a_ d1o9ra_ d2ffxj_ d1yv1a1 d2v2pa_ d1j30a_ d4di0a_ d1dpsa_
d1ex4a2 d1c6va_ d1bdqa_ d3ggta_ d3ixoa_ d1mrxa_ d2haha_ d1tw7a_
d4njsa_ d1sgua_ d2p3da_ d1nsoa_ d3d3ta_ d2rkfa_ d1c6ya_ d2rspa_
d3ogpa_ d3ekwa_ d2o4na_ d2j9ja_ d1baia_ d3ggua_ d3lzua_ d4npua_
d4j55a_ d2r8na_ d2fxda_ d2p3ca_ d4dqea_ d3ttpa_ d3t3ca_ d2p3aa_
d2hc0a_ d2fdda_ d3mwsa_ d1sr9a2 d1rqba2 d1h4ra3 d4yl8a1 d2al6a3
d1wgda1 d1wgga1 d4q5eb_ d1wjna1 d1se9a_ d3n3kb_ d1wz0a1 d1we6a1
d2m0xa_ d2gbka_ d3rt3b1 d1wh3a1 d1sifa_ d2ekec2 d4ii2b1 d4hk2a1
d1c3ta_ d1m94a_ d1wgha1 d1ttna1 d1j8ca_ d1wx8a1 d3v6cb1 d2ma9b_
d4i6lb1 d1bt0a_ d2jvca1 d1iyfa_ d2k1fa_ d1wxva1 d1wf9a1 d1t0ya_
d2io0b2 d1x1ma1 d2gbma_ d1zw7a1 d2m17a_ d1we7a1 d2lwpa_ d2gbja_
d1wjua1 d1v6ea1 d1v86a1 d4eewa_ d1wiaa1 d4icva_ d4gu2a_ d1wx7a1
d1wy8a1 d4hjka1 d2faza1 d1v5oa1 d4lu0a_ d1of8a_ d3unda1 d3e9aa1
d1vr6a2 d2qkfa_ d1d9ea_ d1o60a_ d2ftba_ d1pmpa_ d2rcqa1 d1fdqa_
d4lkpa_ d1sa8a_ d1vyfa_ d1lpja_ d3vg7a1 d4azqa1 d1o8va_ d5bvqa_
d1p6pa_ d2qo4a_ d1opba_ d1yiva1 d1o1va_ d4bvma1 d4tkba_ d4i3ba_
d1bwya_ d1ggla_ d4gkca_ d1cbia_ d3p6da1 d1kqwa_ d2a0aa1 d1wioa4
d1ccza2 d1vcaa1 d1dr9a2 d1ic1a1 d2d9qb1 d2nxyb2 d1adqa1 d1fnga1
d1uvqb1 d1zs8a1 d2vola1 d2vola2 d1mfbl2 d2jelh2 d1uwel2 d3qq4b1
d1kjvb_ d1k5na1 d1osph2 d4odhl2 d1ow0a1 d1ow0a2 d1bfob2 d3l9ra2
d3jtth1 d2fath2 d4elkb2 d1fn4b2 d2xfxb_ d1pg7x2 d1cqka_ d4k71b2
d1lk2a1 d1eapb2 d4lfhg2 d1i4fa1 d2nw2a2 d1kfah2 d1mhca1 d1h3ua1
d1wejh2 d1h0db2 d1a5fh2 d1kcvh2 d1mjuh2 d1qqda1 d1kcrh2 d1k8ia1
d3huja2 d2nnab1 d1nfdf2 d3mffb2 d1s9va1 d1lk3h2 d1q0xl2 d1fv1b1
d1neza1 d4rfeb2 d1r3ha1 d1a6tb2 d1kjva1 d1pz5b2 d1onqa1 d2bc4a2
d1de4a1 d1k8da1 d1tcra2 d1dn0b2 d1nfde2 d2xwtb2 d1bfoa2 d1mexh2
d1iakb1 d1ypzf2 d4f7ea2 d1q72l2 d2fbjh2 d1fe8h2 d1clzh2 d1fngb1
d1uvqa1 d1vgka1 d1c16a1 d4j12a2 d4x99b2 d1igtb3 d1je6a1 d1q72h2
d1nldh2 d1teth2 d2hmic2 d1i1ca2 d1i1ca1 d1k8ib1 d1q0yh2 d1kfal2
d3frua1 d1pg7w2 d1u58a1 d1igyb4 d1igyb3 d1hyrc1 d1klub1 d1deeb2
d1c12b2 d2bc4b2 d2ig2h2 d1kcvl2 d3wn5b1 d1lk2b_ d1lk3l2 d1mjul2
d1sm3h2 d1q0xh2 d1zvsa1 d1ydpa1 d1u8ib2 d1ypze2 d1kcuh2 d2lj7a_
d1n4na_ d2m8bb1 d2kyqa_ d3psma_ d1jkza_ d1jxca_ d2gl1a_ d1ugla_
d4uj0a_ d2k4ua_ d1hp2a_ d2lu9a_ d1baha_ d2meoa1 d1wm7a_ d1hlya_

d1n8ma_ d1jlza_ d1wpda_ d1c55a_ d1pvza_ d1wt7a_ d1wz5a_ d1m2sa_
d1pjva_ d1c49a_ d1qkya_ d2ktca1 d1wmta_ d1du9a_ d1j5ja_ d1r1ga_
d1t0za_ d2i61a_ d1t1ta_ d1sn4a_ d1kv0a_ d2lkba_ d1npia_ d1wwna_
d1pe4a_ d2kbka_ d2li7a_ d1djta_ d2kbha_ d1dq7a_ d1sega_ d1omya_
d1zywa1 d1jzaa_ d2jcqa_ d1n9wa1 d1bbua1 d1e1oa1 d1b8aa1 d1l0wa1
d3a74a1 d1eova1 d1t61c1 d1iyjb4 d1iyjb5 d1iyjb3 d1o7ia_ d4ipca1
d1x3ea_ d4apva_ d1jb7a1 d1jb7a2 d1jb7a3 d3kdfa_ d1ue1b_ d1wjja1
d3eiva_ d1xjva2 d1xjva1 d2ccza2 d1se8a_ d1l1oc_ d1jb7b_ d1qvca_
d3ulla_ d1qzga_ d1miua4 d1miua5 d1miua3 d1y14b1 d2nn6i1 d1i94l_
d2zjra2 d1smxa_ d2qamc2 d1hh2p1 d2r7da1 d1rl2a2 d1wfqa1 d3bzka4
d1x6oa2 d3a0ja_ d2j01d2 d2ja9a1 d4ql5a_ d1hr0w_ d1luza_ d2n3sa_
d2vnud1 d1h95a_ d1go3e1 d2nn6h1 d2mo1a_ d1iz6a2 d1jjga_ d1e3pa2
d2ix0a3 d2ix0a2 d2lxja_ d1ueba3 d1ueba2 d2z0sa1 d1u0la1 d2ix0a1
d2eifa2 d2je6i1 d3i2za1 d2c35b1 d2ahob2 d1t9ha1 d3i4oa_ d2qall1
d1jt8a_ d2nn6g1 d1g6pa_ d3cama_ d1i94q_ d1c9oa_ d1d7qa_ d2ba0a1
d2vnud2 d2vnud3 d1khia2 d2qalq1 d1wi5a1 d1xtda2 d1ny4a_ d1dgsa2
d1fvia1 d1ckma1 d1p16a1 d1x9na2 d2shpa1 d1yfoa_ d1l8ka_ d2ooqa1
d1g4us2 d1p15a_ d1lara2 d1lara1 d1wcha_ d2fh7a2 d2fh7a1 d1ohea1
d1ohea2 d2f4da_ d1rxda_ d2hxpa_ d1xria_ d1d5ra2 d1fpza_ d3ezza_
d4y2ea_ d1i9sa_ d1eb6a_ d3azya_ d3wvja_ d1upsa1 d1ajoa_ d2hyka_
d1ajka_ d2vy0a_ d1o4ya_ d3o5sa_ d3d6ea_ d3atga_ d1dypa_ d3i4ia_
g1qmo.1 g1len.1 d3zyra_ d1fnya_ d1n47a_ g2ltn.1 d1avba_ d3usua_
d1dhkb_ d1f9ka_ d1ukga_ d1g8wa_ d1g9fa_ d1wbfa_ d3wcsa_ d1g7ya_
g1loe.1 d1mvqa_ d1hqla_ d1v6ia_ d1dbna_ d1qnwa_ d1fx5a_ d4l8qa_
d1ioaa_ d3n35a_ d3ubra_ d1qo8a1 d1d4ca1 d2k3va_ d1ft5a_ d1qdba_
d1m1qa_ d2j7aa_ d1h21a_ d1ogyb_ d4fasa_ d1jnia_ d1oaha_ d1sp3a_
d3h33a_ d1rwja_ d19hca_ d1os6a_ d3bxua_ d3h4na_ d1i77a_ d3caoa_
d1j0pa_ d2ctha_ d1duwa_ d1ofwa_ d2e84a_ d1hh5a_ d3h34a_ d1dszb_
d1a6ya_ d3cbba_ d1ynwa1 d1r4ia_ d1hcqa_ d2hana_ d2hanb_ d2enva1
d1kb4b_ d2nllb_ d1lo1a_ d1cita_ d2dara2 d2dara1 d1wyha2 d2dloa2
d1rutx4 d1rutx3 d2cuqa2 d1x4la2 d1x4la1 d1x3ha2 d1x3ha1 d2d8za1
d1x61a2 d1x6aa1 d1x6aa2 d2cora2 d2cora1 d1v6ga1 d1v6ga2 d2d8za2
d1x64a1 d1x64a2 d1b8ta1 d2co8a1 d1x63a2 d2dj7a2 d2dj7a1 d2cu8a2
d2cu8a1 d1g47a2 d1g47a1 d1j2oa1 d1wyha1 d2dloa1 d1wiga1 d1wiga2
d2cupa2 d2cupa3 d1x68a1 d2d8ya2 d2d8ya1 d1m3va2 d1x62a1 d1x68a2
d1x61a1 d1b8ta3 d1b8ta2 d2d8xa2 d2d8xa1 d1x4ka1 d1x4ka2 d2cuqa1
d1m3va1 d2co8a2 d1j2oa2 d1x63a1 d2cura1 d2cura2 d1x62a2 d1op9a_
d1y0la1 d1etzb1 d1egjl1 d2gsib1 d1nj9a1 d4jfhd1 d1aqkl1 d1t2ja_
d1lo4h1 d1zvya1 d3v4vl1 d4enec1 d1kxtb_ d5e8ea1 d2pxya_ d4weud_
d1xiwc_ d2esvd1 d1qnzh_ d1ogae1 d1f4xh1 d4n1hb_ d3ezjb1 d3mlwl1
d1kfah1 d1etza1 d1b4jh1 d3cfic_ d4w68a_ d2arja1 d1ek3a_ d2ok0l1
d1vera_ d1uwgh1 d1blnb1 d1fpth1 d1rzga1 d2giya1 d4m3kb1 d1nc2a1
d2nyyd1 d1ncna_ d1fbih1 d5dxwa_ d4fqqa1 d1dqdh1 d1jfql1 d1kelh1
d1faih1 d1frgl1 d1figl1 d4m6nl1 d1fo0a_ d4hxbl1 d3b5ga_ d3quyd1

d2h9gb1 d1i8kb_ d2dd8h1 d2ck0h1 d1eaja_ d4w6wb1 d2a6ib3 d1t66d1
d2e27h_ d1ob1b1 d1f11b1 d2v7na1 d1ad0a1 d2xt1b1 d1pskh1 d4i13b1
d2op4l1 d1a0ql1 d1uwel1 d1za3a1 d1ncbl1 d4uu9a_ d1bm3h1 d1cloh1
d1nqba2 d1cf8h1 d4y5va_ d1mhph1 d5anma1 d4cdgc1 d4ldeb_ d1y0lb1
d2pcpb1 d1z3gl1 d1dfbl1 d1fh5h1 d3g5xa1 d1tzib1 d4dn3l1 d3zhka1
d2b1hh1 d2uyla1 d1f3dh1 d1nj9b1 d2agjh1 d2h1pl1 d1q72h1 d8fabb1
d1ndmb1 d1rjca1 d4u6hb1 d3upca_ d2fbjl1 d1osph1 d1tvda_ d2cjvh_
d4fhbd1 d1bd2d1 d1rz7h1 d3nn8a_ d1knoa1 d1nfda1 d2bmkb1 d1dqll_
d1igyb1 d1igfl1 d12e8h1 d3utza1 d1nc2b1 d1rihh1 d4mwfb1 d4jvpa1
d1mieh1 d1rhfa1 d1fgnl1 d1sbsh1 d1lp9e1 d1dn0a1 d4lqfl1 d1iaih1
d1kgcd1 d1mh5b1 d1gpol1 d2g60h3 d1f8tl1 d2jixd1 d4grwh_ d1fo0b_
d1bfob1 d1axsa1 d1u9ka_ d1nmbh_ d1h8na1 d1h8na2 d1jguh1 d4eura1
d3sdyl1 d35c8h1 d1w72l1 d1mvub_ d1i9rh1 d1ob1a1 d4wenb_ d1yeeh1
d3ietc1 d1f58h1 d1ad0b1 d1mqkl_ d1nbvh1 d4b50a_ d3r8bb_ d1bafl1
d1mamh1 d4rgne1 d2gsgb_ d35c8l1 d1bafh1 d1ai1h1 d2pcpa1 d2fath1
d2x1pa_ d1orsb1 d1ibgl1 d1shma_ d1a2yb_ d1u0qa_ d2coqa_ d1hilb1
d1qfwi_ d1jv5b_ d1fn4b1 d5e0qa_ d1nsnh1 d1plgl1 d4ocxl1 d1kb5h1
d1rzfh1 d1pg7x1 d2fx7h1 d2oi9c_ d1l7il1 d3eyva1 d1d5il1 d4ffyl_
d1tetl1 d1a6wh_ d1ac6a_ d3dvfa_ d1nfde1 d1mimh1 d1ri8a1 d1jhlh_
d3dura_ d1fskb1 d1f97a1 d3so3b1 d2ak4d1 d1lp9f1 d4z7wf1 d1l7th1
d4y5ya_ d4x7ec1 d1emth1 d1ikfl1 d1nlbh1 d4utal1 d1bjma1 d1eapb1
d1u6ah1 d2dtgd1 d1kj2b_ d1xeda1 d1c1eh1 d1fgvh_ d2bnub1 d2fb4h1
d2dtgb1 d1ktrh_ d1jpth1 d1qkzl1 d4hepg_ d2atpb1 d1flrh1 d2hrpl1
d1mvfa_ d1iaim1 d3k80a1 d2jb5h1 d3zkxc_ d4grwe_ d1it9h1 d1rhhb1
d3w9ec_ d1e4wh1 d1sy6h1 d2j6eh1 d3qxta_ d1jglh1 d1p7ka1 d1tzhb1
d4n8pa1 d1gafl1 d2jelh1 d1wioa2 d4i0cc1 d1cicb1 d1lmka2 d1lmka1
d1iqwh1 d1orqb1 d1ejoh1 d1jnlh1 d4zg1a_ d43c9b_ d1ggbl1 d1wejh1
d3q6fa1 d1f3dj1 d3c6la1 d1bqlh1 d3p9wb_ d1a3rl1 d1h0db1 d2uzil_
d1eo8l1 d1qfwl_ d1n7ml1 d1igja1 d1c5ch1 d2a9mh_ d1rmfh1 d1fj1b1
d4heme_ d1ccza1 d1a5fh1 d1ogad1 d1dlfh_ d1qa9b_ d1vgel1 d3qskb_
d1sjxa_ d4ozfg1 d1sq2n_ d1qd0a_ d1kcvh1 d1c5db1 d3qcul1 d8faba1
d3bn9d1 d1mjuh1 d4y8dc_ d7fabh1 d3dvgb1 d1lo0l1 d1a6tb1 d1gigh1
d1kcrh1 d2hwzl1 d1yjdc1 d3clfl1 d4krna_ d1dl7l_ d1e6ol1 d1mexh1
d3r0ma_ d4kkna_ d1fnsl1 d1oaqh_ d1rzia1 d1ucbh1 d1ncwl1 d4b41a_
d1nfdf1 d2mpah1 d2d7tl1 d4grwf_ d4b5ea_ d1lk3h1 d3u79a_ d4u3xa_
d1ghfl1 d1bvkb_ d1mcww1 d1b88a_ d1jrhh1 d32c2b1 d4wemb_ d1dsfh_
d1op3h1 d1ce1l1 d1iqda1 d1qoka1 d1qoka2 d1qfuh1 d2crya1 d1ay1h1
d1oaql_ d3ab0b_ d1rurl1 d1t4kb1 d1bz7b1 d1n0xh1 d1forl1 d1ucbl1
d4w6yb_ d43c9a_ d2aabl1 d1tzia1 d4n1ca_ d1k6qh1 d3g9ab_ d4tvsa1
d1igmh_ d1pz5b1 d3dx9a1 d3wxwh_ d1nakh1 d1kcul1 d3eaka_ d4qlra1
d1cr9l1 d1d9ka_ d1f4xl1 d1deea1 d2i27n2 d1c12a1 d2orbl1 d1j8hd1
d1mcph1 d1c5da1 d1kenh1 d1bd2e1 d1h5ba_ d4x7fc1 d3upaa_ d1t3fa1
d1indh1 d2dtgc1 d4hc1l1 d1zv5a1 d4wgvb_ d1uwgl1 d4f37k1 d1aqkh1
d1vesa_ d3zkqd_ d1fptl1 d1lo4l1 d1knob1 d3rrqa_ d1ct8b1 d4orzc_

d1mlbb1 d4hgkc1 d1dqdl1 d1bogb1 d4ozhe1 d1dr9a1 d1i7za1 d1dn0b1
d1kell1 d1rzib1 d1ypzf1 d1wtla_ d1hkfa_ d1fail1 d1figh1 d2vyre_
d1sjva_ d1ohqa_ d1bfoa1 d1pkqb1 d1mjul1 d1b4jl1 d1nsnl1 d2arjb1
d1p4bh_ d4aj0a_ d3nn8b_ d2rcja1 d1f2xk_ d4v1dd_ d1adqh1 d2eksa_
d4k3ga_ d1jfqh1 d1uweh1 d1frgh1 d4gftb_ d1uz8b1 d4n1ea_ d1bm3l1
d1clol1 d3mcka1 d2vq1a1 d1ae6h1 d4aq1b1 d1uj3b1 d1nbqa1 d1fh5l1
d2ck0l1 d2ywza_ d1q72l1 d4nbxb_ d1hila1 d4hzlb1 d2j88l1 d1zmya1
d1cd0a_ d1a4ka1 d1pskl1 d1a0qh1 d4krmb_ d1ncbh1 d2fbjh1 d1j8he1
d1i3ua_ d1cf8l1 d1fe8h1 d2p1ya1 d1eeqa_ d2x89a_ d1ol0a_ d2r0lh1
d1ub5a1 d1dfbh1 d3eyvb1 d4jdvb1 d4p2cg_ d2gsia1 d2agjl1 d1u3ha1
d2h1ph1 d1rihl1 d1fskc1 d1boga1 d2a77l1 d1ospl1 d1qfoa_ d3dwta_
d2fx7l1 d1rz7l1 d3stba1 d1pkqa1 d1b6da1 d1dzba2 d1dzba1 d1kj2a_
d4l1ha_ d1rzgb1 d1dqlh_ d12e8l1 d1miel1 d1jmaa_ d1sbsl1 d1fgnh1
d1f58l1 d1mqkh_ d1ndgb1 d3qegl1 d1iail1 d2aw2a1 d1tqbc1 d3fo2a1
d4dkaa_ d1f8th1 d2g60l1 d2ywya_ d1uj3a1 d1yc7a_ d3bd3a_ d4w6xb1
d1nmbl_ d1i8ka_ d1rjlb1 d1ypze1 d1p7kb1 d1kxqe_ d1gpoh1 d2z4qa1
d1w72h1 d1i9rl1 d4qgya1 d4xt1c_ d2qqnh1 d1cica1 d3c08h1 d2cd0a_
d2bdnh1 d1hnga1 d1maml1 d1nldh1 d1cr9h1 d4lcih1 d3bkyh1 d3qg7l1
d1ai1l1 d1kxvc_ d4iosd_ d1l7ih1 d2x1qa_ d1ub6b1 d3k74b_ d1ibgh1
d1teth1 d1igya1 d1hxma1 d2x6ma_ d2e7la_ d5e03a_ d1qfwm_ d2r0kh1
d1igjb1 d1rurh1 d1plgh1 d1mj8h1 d1yuhb1 d3fmgl1 d1rzfl1 d1emtl1
d1ad9b1 d1igtb1 d1nlbl1 d1d5ih1 d3k7ua_ d4ffyh_ d1bwwa_ d3dvga1
d1a6ta1 d1fgvl_ d4qxgl1 d1miml1 d1jhll_ d2hffb1 d2hh0l1 d1qkzh1
d1l7tl1 d1afvh1 d1iaii1 d2cjuh_ d1um5h1 d2ghwb2 d2ghwb1 d1kgce1
d2fgwh1 d1ikfh1 d1dvfd_ d2i24n1 d2xtjb_ d1za3b1 d2yc1a_ d2hh0h1
d2x1oa_ d1bvka_ d1rjla1 d5e7ba1 d1igta1 d1il1b1 d2fb4l1 d1mvua_
d1jptl1 d32c2a1 d1fvca_ d1iqdb1 d2hrph1 d2p4ab_ d4qo1a_ d1p2cb1
d1wt5c_ d1t4ka1 d5c1mb_ d1it9l1 d1bz7a1 d1b2wh1 d1e4wl1 d1jnll1
d1yedb1 d1za6b1 d3u7aa_ d1igfh1 d3gnml1 d1orsa1 d1wejl1 d1a2ya_
d1hxmb1 d1gafh1 d2j4wh1 d1pz5a1 d1j05a_ d1a3rh1 d4fqll1 d1qfwh_
d1eo8h1 d1n7mh1 d4s11a_ d1kegh1 d5da4a_ d1iqwl1 d3whxb1 d1vgeh1
d1deeb1 d1c12b1 d1t2ql1 d1ad9a1 d3gsnb1 d3b2uc1 d1ggbh1 d1t3fb1
d1nakl1 d4ocmc_ d1lila1 d2v7hb3 d2esve1 d1mcoh1 d2qr0b1 d2q3aa_
d1ct8a1 d1kcrl1 d2pxyb_ d2uzih_ d5c3ld_ d3durb_ d1c5cl1 d2ak4e1
d1dl7h_ d1xiwd_ d1mlba1 d1rmfl1 d1i7zb1 d1dlfl_ d2xa3a_ d5bq7b1
d1dqqb1 d3h42l1 d1egjh1 d1kcvl1 d1fe8l1 d2j6el1 d1f3rb2 d1lk3l1
d1sm3h1 d1q0xh1 d1ghfh1 d7fabl1 d1igch1 d1lo0h1 d4idla_ d5c2bl_
d1jrhl1 d1il1a1 d3zhda1 d4kfzc_ d1b0wa_ d1e6oh1 d1mexl1 d4krpb_
d1fnsh1 d1ncwh1 d4hgma_ d4eigb1 d4rzca_ d2mpal1 d4y5xa_ d5en2b1
d3lrga1 d4ej1c1 d1rhha1 d1j05b_ d1forh1 d1q9wb1 d2wzpd_ d2aq2a1
d1h0da1 d3revb1 d2ij0c1 d1tzha1 d4m7jl1 d1k6ql1 d3b9kc1 d1igml_
d3qeua1 d1dsfl_ d1ce1h1 d1kcuh1 d1zvha1 d1zvoa1 d1tqbb1 d1ay1l1
d2qscl1 d2cgrh1 d1nl0h1 d1kenl1 d3eo0a1 d4n9ob1 d1n0xl1 d3k1kc_
d1prtc1 d2wv6d_ d1prtf_ d1ltrd_ d4l63b1 d1r4pb_ d1prtd_ d1tl0f_

d2bosa_ d1prtb1 d3chbd_ d2aq2b1 d1ck1a1 d1enfa1 d1i4pa1 d1v1oa1
d1et9a1 d1esfa1 d2g9hd1 d4ohja1 d1klud1 d1eu3a1 d1ty0a1 d1fnua1
d2cqva1 d1fhga_ d2k1ma_ d2dl2a1 d1biha3 d1biha1 d1biha4 d3qp3a1
d1g1ca_ d1gxea_ d1cvsc2 d1cvsc1 d1xmwa2 d1m4ka2 d2otpa2 d1biha2
d1rhfa2 d2fcba2 d2fcba1 d1wwbx_ d1gsma2 d1epfa1 d1epfa2 d2dava1
d1waaa_ d1f97a2 d1x44a1 d1vcaa2 d1fnla2 d1fnla1 d3b5ha2 d3h8na2
d3h8na1 d1jbja1 d1jbja2 d1pd6a_ d1f2qa2 d1f2qa1 d3cx2a_ d1nbqa2
d3b5ha1 d1n26a1 d1xiwb_ d1xaua_ d1fltx_ d2ifga1 d1olla1 d1olla2
d1cs6a4 d1cs6a3 d1cs6a2 d1cs6a1 d2gw5a2 d1sy6a1 d1xiwa_ d1ucta2
d1ucta1 d1ry7b2 d1gl4b_ d1qz1a3 d1olza1 d2c9aa1 d1wwca_

**Family-16 (a.4.1.1)**

**Positive dataset contains 30 protein sequences.**

d2msya_ d2djoa1 d2r5za_ d2lmda1 d1s7ea1 d3nara_ d1zq3p1 d2craa1
d4uuta_ d3a01a_ d1x58a1 d3rkqa1 d3cmya1 d2cqxa1 d2da6a1 d1x41a1
d1wh7a1 d2mw8a_ d1x2ma1 d1wi3a1 d1wjha1 d1x2na1 d2cuea1 d3k2aa_
d2hosa_ d2jwta2 d2hi3a_ d4j19a_ d3lnqa_ d1wh5a1

**Negative dataset contains 3931 protein sequences.**

d1m32a_ d1ibja_ d1c7na_ d1iuga_ d4q75a_ d2bkwa1 d1t3ia_ d1n8pa_
d1qz9a_ d1jf9a_ d1eg5a_ d1p3wa_ d3kgwa_ d1cl1a_ d2ctza1 d2rfva_
d1gc0a_ d1cs1a_ d1d2fa_ d2ch1a1 d1qgna_ d1pffa_ d1elua_ d1orva2
d1xfda2 d1u08a_ d1lc5a_ d1b5pa_ d1bw0a_ d3pd6a_ d7aata_ d2csta_
d1j32a_ d1fg7a_ d4wlha_ d1o4sa_ d1v2da_ d1gdea_ d1m6sa_ d1v72a1
d1svva_ d1ajsa_ d1wsta1 d2f8ja2 d1lk9a_ d1vp4a1 d1xi9a1 d2gb3a1
d1yaaa_ d2bnma2 d3rcha_ d3k40a_ d1pmma_ d2cfba1 d1o69a_ d1bj4a_
d3k28a_ d2bwpa_ d3lg0a_ d2fnua_ d3ou5a_ d1z7da1 d1b9ha_ d1iaya_
d4msoa_ d1bs0a_ d1s0aa_ d1sffa_ d3tqxa_ d1mdoa_ d3qboa_ d3pgya_
d1bjna_ d2dkja_ d1ejia_ d1vefa1 d4wxga_ d3bs8a_ d3gbxa1 d1w23a_
d1sefa_ d1sfna_ d1sq4a_ d1ddla_ d1e57b_ d1f56a_ d1bypa_ d1azca_
d1ws8a_ d3u25a_ d1cuoa_ d1id2a_ d1rkra_ d2iaac_ d4dp1x_ d1nwpa_
d2gima_ d2i7sa_ d1qhqa_ d3ehbb1 d1fftb1 d1gyca3 d1hfua2 d1kbva1
d1kbva2 d1sdda1 d1sdda2 d1gyca1 d1gyca2 d1mzya1 d2bw4a1 d1aoza1
d1aoza3 d1aoza2 d1kyaa2 d1kyaa3 d1mzya2 d4e9sa1 d1sddb1 d1sddb2
d1hfua1 d1hfua3 d1oe1a2 d1oe1a1 d1v10a2 d1v10a3 d1v10a1 d1a3qa1
d1iknc_ d3brda1 d1p7hl1 d1imhc1 d1gjia1 d1zk9a1 d2cxka1 d2bema_
d1pama1 d1ea9c1 d1qhoa1 d2fhfa2 d2fhfa1 d1k3ia1 d1j0ha1 d1cxla1
d2bhua1 d1edqa1 d1f1sa2 d1ut9a2 d1gvia1 d1txka1 d4alac_ d1pjwa_
d1rera1 d1s6na_ d3uzqb_ d2phla2 d2phla1 d1fxza2 d1fxza1 d1lrha_
d1uija1 g1dgw.1 d2v09a_ d1uika2 d1uika1 d1dgwa_ d1ep0a_ d2pa7a1
d1wlta1 d1nxma_ d2c0za1 d1ds6b_ d1fstb_ d1ex0a1 d1g0da1 d1tafa_
d1bh9b_ d1tafb_ d1q9ca_ d1h3ob_ d2byka1 d1n1ja_ d4g92b_ d1bh9a_
d1jfia_ d1h3oa_ d2bykb1 d1n1jb_ d3gpdg2 d2q49a2 d1hdgo2 d2g17a2
d2cvoa2 d2czca1 d3pwka2 d1obfo2 d1i32a2 d1zyao2 d2pkqo2 d1b7go2
d2hjsa2 d1cf2o2 d1vkna2 d1nvmb2 d1ux8a_ d1dlya_ d4i0va_ d2bkma_
d1dlwa_ d2bmma_ d3lv8a_ d1s3ga1 d1zaka1 d1s4qa_ d3tvta2 d1uf9a_

d1khta_ d1ckea_ d1osna_ d1p5zb_ d4tmka_ d3cm0a_ d1tmka_ d1jaga_
d3h86a_ d1t0hb_ d1nn5a_ d2h92a_ d3kb2a1 d2c95a1 d1yj5a2 d1kgda_
d1ly1a_ d2ak3a1 d1vyva2 d1deka_ d2grja1 d1p3ja1 d1y63a_ d1e4ya1
d1s96a_ d3iija1 d1lvga_ d1nksa_ d1vyua2 d4e22a_ d1qf9a_ d1jjva_
d1q3ta_ d1ki9a_ d1lw7a2 d1teva_ d1kjwa2 d1gsia_ d2iyva2 d1viaa_
d1e6ca_ d1x6va3 d1odfa_ d4gi7a_ d2jeoa1 d1rz3a_ d1z0ja1 d3umra2
d3gj0a1 d2gaoa1 d1yzga1 d1x3sa1 d1g7sa4 d1puja_ d1udxa2 d1kk1a3
d2o52a_ d1jwyb_ d1zcaa2 d1zcba2 d2fv8a1 d2cjwa_ d3dz8a1 d1xzpa2
d1vg8a_ d4qxaa_ d2ew1a1 d1x1ra1 d2bcgy_ d3doea1 d3cona_ d3rega_
d3wyga_ d1upta_ d2b6ha1 d1zd9a1 d1lnza2 d1r5ba3 d1n0ua2 d1r2qa_
d2f9la1 d2rhda_ d3q3jb_ d3q85a_ d1efma1 d1r8qa_ d2gf9a1 d2akab1
d1ek0a_ d1z08a1 d2gcoa1 d2dy1a2 d4g01b_ d2cxxa1 d1svia_ d3q72a_
d1ni3a1 d4ac9a4 d2erxa1 d2bmea_ d1wxqa1 d2gf0a1 d4ldja1 d1zunb3
d1d5ca_ d4lv5b_ d1efca3 d1kmqa_ d2g6ba1 d3lrpa_ d1tada2 d2y8ea_
d4rd4a1 d1z06a1 d2heia_ d3a58b_ d1u0la2 d1d2ea3 d1zj6a1 d2ocba_
d3rwoa_ d1mr3f_ d2e9sa1 d4u5xa1 d2iwra_ d2fn4a1 d3bwdd_ d2nzja1
d2atva1 d2ksqa_ d3l43a_ d1mkya2 d1mkya1 d1wmsa_ d1jnya3 d2rmka_
d1f6ba_ d1f60a3 d1gota2 d1jala1 d2fh5b1 d1g16a_ d2wblc_ d1m7ba_
d1wf3a1 d1f5na2 d1t9ha2 d1z6xa1 d1z0aa1 d2fg5a1 d2a5ja1 d2bcjq2
d1s0ua3 d1ky3a_ d4y0va_ d4lhwa_ d2qmea1 d2atxa1 d1egaa1 d1ryha_
d1moza_ d1nrjb_ d2erya1 d3bfka_ d1puia_ d3j3az1 d3t5ga_ d1shza2
d3defa_ d2f7sa1 d3raba_ d3tsoa_ d3aq4a_ d2al7a1 d3sfva1 d3bc1a_
d2ncda_ d1sdma_ d3h4sa1 d2heha_ d2grya_ g3kin.1 d1kk8a2 d1br2a2
d1f9va_ d2mysa2 d1lkxa_ d3t0qa_ d4db1a1 d3gbja_ d1ry6a_ d1v8ka_
d1y4wa1 d3vrda1 d3vrda2 d1fcdc1 d1h1oa2 d1h1oa1 d1m70a2 d1m70a1
d1ig8a1 d1czan2 d1czan3 d1czan1 d1ig8a2 d1czan4 d1bg3a4 d1bg3a3
d1bg3a1 d1nira1 d1qksa1 d1zc6a1 d1zbsa1 d1zbsa2 d1zc6a2 d2ch5a2
d2ch5a1 d1zxoa2 d1zxoa1 d1hroa_ d4gedb_ d3zooa_ d1mz4a_ d1gu2a_
d1f1fa_ d1dvva_ d1c6ra_ d1c75a_ d1e29a_ d1gdva_ d1cnoa_ d1h32b_
d1co6a_ d2bh4x_ d2aiua_ d5cytr_ d1jdla_ d1i8oa_ d2zboa_ d3dmia_
d1ls9a_ d1wvec_ d2yk3a_ d3ph2b_ d1dw0a_ d5aura_ d1ql3a_ d1qn2a_
d1f1ca_ d4zzva_ d2xspa_ d4v20a_ d1ojja_ d4ipma1 d1gpia_ d4csia_
d3pfza_ d3ovwa_ d2yoka_ d2nqya_ d1h4ga_ d1hixa_ d1oa2a_ d1olra_
d3b7ma_ d4h7ma1 d3wp3a_ d2vgda_ d1igoa_ d3zsea_ d2nlra_ d3wx5a_
d1oa3a_ d1pvxa_ d1m4wa_ d1f5ja_ d1oa4a_ d4ixla1 d2a6za1 d3a4ua_
d2a6va1 d1evsa_ d1pvhb_ d1z7ca_ d1huwa_ d1bgea_ d1rhga_ d1f6fa_
d1f45b_ d1i1rb_ d1axia_ d1etea_ d3tgxb_ d1scfa_ d2z3qa1 d3qb1a_
d1hula_ d1m48a_ d4nkqb_ d1eera_ d1v7mv_ d5e4ea_ d2o27a_ d3b5ka_
d2psma1 d4zf7a_ d3uf5a_ d4x51a_ d1d9ca_ d4ypgc1 d3oq3a_ d1lqsl_
d1n1fa_ d1au1a_ d1wu3i_ d3besl_ d3ux9a_ d1v8fa_ d2ejca_ d3muea1
d1ihoa_ d3q12a1 d3rbaa_ d2qtra_ d1nupa_ d3l92a_ d1f9aa_ d1qjca_
d2h29a_ d1vlha_ d1lw7a1 d1mrza2 d4ruka_ d2el7a_ d1iq0a2 d1irxa2
d1ivsa4 d2cyba_ d1rqga2 d1j09a2 d2yxna_ d1f7ua2 d1h3fa1 d1ffya3
d2g36a1 d1nzja_ d1jila_ d1n3la_ d1i6la_ d1pfva2 d1h3na3 d3n9ia_

d3sz3a1 d3sw2a_ d1dx5i3 d1edmb_ d1autl2 d1autl1 d1nqlb_ d1dval1
d1szba2 d1kigl_ d2y5fl_ d1ijqa2 d1gk5a_ d1g1sa2 d1nt0a3 d1xdtr_
d1lmja2 d1lmja1 d1nzia2 d2wphe_ d1klil_ d1pfxl2 d1pfxl1 d1p9ja_
d1g1ta2 d1f7ea_ d4yzub_ d2mgpa1 d1b9wa1 d1b9wa2 d2mgpa2 d1ob1c1
d1ob1c2 d1n1ia2 d1n1ia1 d2npra2 d2npra1 d1m1xb4 d1qx2a_ d1ig5a_
d2y5ia_ d1qlsa_ d1psra_ d3n22a_ d2egda_ d4ggfc_ d2wcea_ d2luca_
d4aqia_ d3nsia_ d2lp3a_ d1j55a_ d1a4pa_ d1nsha_ d1pvaa_ d3fs7a_
d1ttxa_ d1rwya_ d2kyfa_ d1rk9a_ d1a75a_ d1exra_ d1tiza1 d1nyaa_
d1g8ia_ d1ggwa_ d1wdcc_ d3fwba_ d1ggza_ d2o5ga_ d1ctaa_ d1m45a_
d3i5gc_ d1sl8a_ d1fpwa_ d2scpa_ d2roaa_ d4aqra_ d3pn7b_ d1br1b_
d2mysb_ d1s6ca_ d1bjfa_ d2obha_ d1jfka_ d1jf0a_ d1qv0a_ d1lkja_
d2mlfc_ d2m97a_ d2d8na_ d4by5a_ d1jbaa_ d4nqga_ d1wdcb_ d2mysc_
d1j7qa_ d1s6ia_ d1uhka2 d1r2ua_ d1dtla_ d3kf9a_ d3pn7c_ d1qjta_
d1fi6a_ d1c07a_ d5d67d_ d1tuza_ d1eg3a1 d1eg3a2 d2zkmx1 d1snla_
d1gjya_ d1y1xa_ d1hqva_ d1dvia_ d1df0a1 d1qxpa2 d4usla_ d1k94a_
d1sfsa_ d1jfxa_ d1wkya2 d1uuqa_ d3wh9a_ d4m1ra_ d1xyza_ d4xuya_
d2je8a5 d4bf7a1 d3cuia_ d1ur4a_ d1qw9a2 d1odza_ d2vzsa5 d1bqca_
d2x2ya1 d1hjqa_ d1qnra_ d4y7ea1 d1vjza_ d4k68a_ d2v3ga_ d4hpga_
d1nofa2 d1nq6a_ d1h4pa_ d1aq0a_ d3ziza_ d4bs0a_ d1ecea_ d1h1na_
d1ta3b_ d1w91a2 d1uhva2 d1us3a2 d1egza_ d7a3ha_ d1tvna1 d3nyda1
d1ghsa_ d2cyga1 d4xv0a_ d2c8na2 d1w32a_ d3emca_ d1v6ya1 d1foba_
d2uwfa1 d2fgla_ d3niya1 d2c0ha1 d1rh9a1 d1yq2a5 d1szna2 d1fa2a_
d1pama4 d5do8a1 d1ea9c3 d1hx0a2 d2fhfa5 d1qhoa4 d1avaa2 d1m53a2
d4m8ua1 d2gjpa2 d1lwha2 d4howa1 d1ktba2 d1j0ha3 d3vm5a1 d2x1ia_
d1wzaa2 d1uasa2 d2diea1 d1mxga2 d1ht6a2 d1eswa_ d1iv8a2 d5cpsa_
d1ud2a2 d1zjaa1 d3gbea1 d1kwga2 d1wdpa_ d1gcya2 d1g5aa2 d1hvxa2
d3dhpa2 d2bhua3 d4do4a1 d3qgva1 d1e43a2 d1g94a2 d2d3na2 d1gjwa2
d1gvia3 d1x1na1 d1r7aa2 d1tz7a1 d2x42a1 d1tr9a1 d3abza1 d4toqa_
d1wb0a1 d1w9pa1 d1itxa1 d1kfwa1 d1goia2 d1edqa2 d2gsja_ d1hjxa1
d1jnda1 d1vf8a1 d3wdpp1 d3wq5a_ d1gnxa_ d4gxpa_ d4hz8a_ d1e4mm_
d3aiua_ d1wcga1 d1qvba_ d3ahxa_ d1v02a_ d2dgaa_ d1pbga_ d1vffa1
d1qoxa_ d4ha4a_ d1mixa2 d1e5wa2 d1j19a2 d2al6b2 d3syxa1 d1mkea1
d2p8va_ d1xoda1 d1i7aa_ d1evha_ d1qc6a_ d1b9ra_ d1offa_ d1frra_
d3wcqa_ d1e9ma_ d1wria_ d1i7ha_ d3av8a_ d1czpa_ d1rfka_ d3p1ma1
d3b2ga_ d2bt6a2 d1wgqa1 d1v89a1 d2coda1 d1zc3b_ d1wg7a1 d1u5ea1
d1v61a1 d2coca1 d2ys1a1 d1v5pa1 d1ntya2 d1wjma1 d1wi1a1 d1u29a_
d1xx0a1 d1unqa_ d2zkmx3 d1fhoa_ d1v88a1 d2adza1 d1x1fa1 d1p6sa_
d2coaa1 d1btka_ d1v5ma1 d2x18a_ d1dyna_ d1x1ga1 d4kaxb_ d2j59m1
d1ki1b2 d1xcga2 d1txda2 d1u5da1 d1dbha2 d1w1ha_ d1upqa_ d1v5ua1
d1foea2 d2fjla1 d1eaza_ d2dfka2 d2cofa1 d1kz7a2 d1d4ua2 d9ldta1
d2ewda3 d1up7a1 d2dfda1 d1s6ya1 d1vjta1 d7mdha1 d1hyea1 d1t2da1
d1ojsa3 d1b8pa1 d1ldna1 d1hyha1 d1civa1 d2a92a1 d1ez4a1 d1i0za1
d1y6ja1 d1gv0a1 d1ur5a1 d1llda1 d1obba1 d1v8ba1 d1gdha1 d2dlda1
d1mx3a1 d1ygya1 d1qp8a1 d1li4a1 d1j4aa1 d2naca1 d1npya1 d3t4ea2

d1a4ia1 d1o0sa1 d2hk9a2 d2egga2 d1b26a1 d1bvua1 d1gq2a1 d1c1da1
d1leha1 d1gtma1 d1euza1 d1nvta1 d3dona2 d1edza1 d1gpja2 d1vi2a1
d1p77a1 d1b0aa1 d1vl6a1 d1v9la1 d1txga2 d3f47a1 d1mv8a2 d2pv7a2
d1xc2a3 d2ahra2 d4xiya1 d2f1ka2 d1pgja2 d1i36a2 d1np3a2 d1lj8a4
d1teaa3 d1qmga2 d2i76a2 d2g5ca2 d1wdka3 d1dlja2 d3cuma3 d1kola2
d1f8fa2 d1qora2 d1jqba2 d1vj0a2 d1cdoa2 d1gu7a2 d1d1ta2 d1xa0a2
d2dm6a2 d1jvba2 d1rjwa2 d1llua2 d1h2ba2 d1tt7a2 d1piwa2 d1o89a2
d1iz0a2 d1e3ja2 d1yb5a2 d1uufa2 d1vj1a2 d1e3ia2 d3gpdg1 d1vc2a1
d2nvwa1 d1u8fo1 d1ydwa1 d1hdgo1 d2g17a1 d1cf2o1 d2czca2 d1r0ka2
d1obfo1 d2cvoa1 d1ebfa1 d1h6da1 d1i32a1 d1zh8a1 d2q49a1 d1qkia1
d1j5pa4 d3pzra1 d1xeaa1 d1vkoa1 d1vm6a3 d3qvsa1 d2pkqo1 d1b7go1
d1gr0a1 d1p1ja1 d1lc0a1 d1j0xo1 d3pwka1 d2hjsa1 d1zyao1 d1vkna1
d1tlta1 d1nvmb1 d3sjua_ d3ojfa1 d1xu9a1 d2jjya1 d1n7ha_ d1y1pa1
d1h5qa_ d1jtva_ d4fs3a_ d2bkaa1 d1gy8a_ d2c5aa1 d1ulsa_ d1ooea_
d2b69a1 d1y5ma1 d1spxa_ d1ae1a_ d1bxka_ d1ydea1 d1gega_ d1hdoa_
d1o5ia_ d1e6ua_ d1snya1 d2rhca_ d3zv4a_ d5ceja_ d2d1ya1 d1fjha_
d3w8ea_ d2fmua1 d1x1ta_ d1rpna_ d3ay7a1 d1k2wa_ d1eq2a_ d2hrba1
d3ruaa_ d2ew8a1 d4hwka_ d1xgka_ d2bd0a1 d1yo6a1 d2a35a1 d2ntva_
d1vl8a_ d1hdca_ d4bkua_ d2ag5a1 d2q45a_ d1nffa_ d4iy1a_ d1zbqa1
d1kewa_ d1ek6a_ d1ja9a_ d2q46a2 d1iy8a_ d1cyda_ d2ptga_ d1hxha_
d1uzma1 d3lyla_ d4m89a_ d3wyea_ d2blla1 d1w6ua_ d1sb8a_ d2q2va_
d4nr0a_ d1e6wa_ d2gasa_ d3oiga1 d2bgka1 d1mxha_ d1vl0a1 d1rkxa_
d1r6da_ d1wmaa1 d1e7wa_ d2c07a1 d1xkqa_ d3tzha_ d1g0oa_ d3lz6a_
d1qyca_ d1edoa_ d1gz6a_ d1oc2a_ d1t2aa_ d1zema1 d2xoxa_ d1z45a2
d1geea_ d1p33a_ d3lt0a_ d4e6pa_ d2wyua_ d1db3a_ d4q9na_ d1xhla_
d4o1ma_ d1fmca_ d2uvda_ d2gdza1 d1uaya_ d2z1ma_ d2o23a2 d2fr1a2
d2fr1a1 d1qsga_ d4zjua_ d2a4ka1 d1yb1a_ d1wvga1 d1yxma1 d3f4ba_
d1mg5a_ d1xg5a1 d4ekfa_ d2ckga_ d1th0a_ d2bkra_ d4c5pa_ d2bsza1
d4bgfa_ d1w4ta1 d1qxpa4 d1ziva1 d1zcma1 d1t3ca_ d1epwa3 d2a97a_
d3fiea_ d3ioqa_ d1fh0a_ d1o0ea_ d1khqa_ d1m6da_ d2cb5a_ g8pch.1
d1s4va_ d2pnsa_ d1vsna1 d1mira_ d1iwda_ d2oula_ d7pcka_ d3bwka_
d3bcna_ d2p86a_ d1pvja_ d1pcia_ d1gece_ d1deua_ d4bs6a_ d1xkga1
d1cqda_ d1jqpa2 d2c0ya_ d2bdza_ d3p5ua_ d2o36a_ d1k9xa_ d1ex4a2
d1c6va_ d2jk6a2 d2jk6a1 d1feca1 d1feca2 d1lpfa1 d1lpfa2 d1xhca1
d1xhca2 d1dxla2 d1dxla1 d1gera2 d1m6ia2 d1onfa1 d1onfa2 d3lada2
d3lada1 d1w4xa1 d1w4xa2 d1gesa2 d1mo9a1 d1xdia1 d1fl2a2 d1fl2a1
d1fcda1 d1fcda2 d1h6va1 d1mo9a2 d1gv4a2 d1gv4a1 d2gv8a2 d2gv8a1
d1aoga1 d1aoga2 d1hyua1 d1ebda1 d1ebda2 d3dk9a1 d1q1ra2 d1q1ra1
d1gesa1 d1zbfa1 d2z1ha_ d2etja1 d3qioa1 d1jl1a_ d2e4la_ d1jl2a_
d2lsna1 d1io2a_ d1uaxa_ d2yv0x_ d1mu2a1 d1ekea_ d1i39a_ d1pn0a1
d1reoa1 d1ju2a1 d2f5va1 d3c96a1 d2gmha1 d1gpea1 d1o5wa1 d1n4wa1
d1k0ia1 d2dw4a2 d2ivda1 d1seza1 d2voua1 d1kdga1 d1s5ja1 d2hbja2
d2igia_ d1wlja_ d1qhta1 d4dlea1 d1tgoa1 d2f96a1 d3tr8a1 d3mxma_
d1q8ia1 d1y97a1 d1w0ha_ d1kfsa1 d2py5a1 d1noya_ d1d5aa1 d1yt3a3

| | | | | | | | |
|---|---|---|---|---|---|---|---|
| d2guia_ | d3mwpa1 | d4kb1a_ | d1iv0a_ | d3ljqa_ | g1k2x.1 | d1ix2a_ | d3nvva1 |
| d1krha3 | d2h88b1 | d1t3qa2 | d1kf6b2 | d1zoyb1 | d1jq4a_ | d1ffva2 | d1dgja2 |
| d1rm6c2 | d1k8ka2 | d1k8ka1 | d3uleb1 | d3i33a2 | d3i33a1 | d1bupa1 | d1dkgd2 |
| d1dkgd1 | d4ci6a2 | d1jcfa1 | d4a2ba2 | d2fxua1 | d1yaga2 | d1yaga1 | d4cbua2 |
| d1bupa2 | d1r7ha_ | d1faaa_ | d1wika1 | d1xfla_ | d1ttza1 | d2f51a1 | d1ti3a_ |
| d1nhoa_ | d1sena_ | d2i4aa_ | d1ep7a_ | d1zmaa1 | d1fo5a_ | d3lefa1 | d3diea1 |
| d2yana1 | d1z6na1 | d1oaza_ | d3zywa1 | d4aj8a_ | d1nm3a1 | d4fiwa_ | d1wmja_ |
| d4hs1a1 | d1iloa_ | d1xwaa1 | d1wjka1 | d2vm1a_ | d1fb6a_ | d1gh2a_ | d1h75a_ |
| d1nw2a_ | d1kx5b_ | d3a6na_ | d4m6ba2 | d2nqbd_ | d3wkjd_ | d2yfwe_ | d4h9na_ |
| d5bnxa_ | d1kx5c_ | d3r45a_ | d1eqzb_ | d4jjnb_ | d1f66c_ | d1eqza_ | d4wnnb_ |
| d1kx5a_ | d1u35c1 | d1id3a_ | d2nqbc_ | d1kx5d_ | d3us3a3 | d3us3a1 | d2b5ea2 |
| d2b5ea3 | d2b5ea1 | d2b5ea4 | d2kp1a1 | d2kp2a1 | d1hyua3 | d1hyua4 | d1j08a1 |
| d1j08a2 | d1pd212 | d1nhya2 | d2a2ra2 | d1k0dd2 | d1f2ea2 | d1gsua2 | d1f3ba2 |
| d2fhea2 | d2pvqa1 | d1tw9a2 | d1oyja2 | d1e6ba2 | d3ik7a1 | d4is0a1 | d1r5aa2 |
| d1jlva2 | d1m0ua2 | d3gtub2 | d1z9ha2 | d1okta2 | d1axda2 | d2gsra2 | d1tu7a2 |
| d1k3ya2 | d1zgma1 | d1b48a2 | d1gnwa2 | d1gwca2 | d2gsta2 | d1glqa2 | d1oe8a2 |
| d1pn9a2 | d1v2aa2 | d1fw1a2 | d1duga2 | d1g7ea_ | d1qqya_ | d1gd6a_ | d1f6ra_ |
| d1ivma_ | d2gv0a_ | d1iiza_ | d2z2fa_ | d1j8xa_ | d1ghla_ | d1b9oa_ | d174la_ |
| d169la_ | d2o4wa_ | d1p37a_ | d1k28a3 | d1xjta1 | d1jtma_ | d2f2qa_ | d4e97a1 |
| d1g4ma1 | d1g4ma2 | d2za0a_ | d2c21a1 | d2pjsa1 | d2i7ra1 | d1ecsa_ | d1xqaa1 |
| d5cj3a1 | d1npba_ | d1jifa_ | d2p7oa_ | d1r9ca_ | d1nkia_ | d2h0va_ | d1iyka2 |
| d1rxta1 | d1iica2 | d1iica1 | d1qsta_ | d2beia1 | d4m3sa_ | d1u6ma1 | d1ygha_ |
| d1yk3a1 | d3dr6a_ | d1xmta_ | d1z4ea1 | d2ge3a1 | d1bo4a_ | d2fiaa1 | d2jdca1 |
| d1y9wa1 | d3to7a_ | d1wwza1 | d2fl4a1 | d2ozua1 | d2b5ga_ | d2cy2a1 | d2p0wa_ |
| d1cm0a_ | d4pswa_ | d1yrea1 | d1yr0a1 | d1m4ia_ | d2gana1 | d1yvka1 | d1ghea_ |
| d2g3aa1 | d1y7ra1 | d2euia1 | d1q2ya_ | d2fsra1 | d1xeba_ | d2ae6a1 | d1cjwa_ |
| d1qsma_ | d2aj6a1 | d1y9ka1 | d1i12a_ | d1vkca_ | d2atra1 | d1n71a_ | d2d4pa1 |
| d2fiwa1 | d2fe7a1 | d1tiqa1 | d2ou2a_ | d1yx0a1 | d2fcka1 | d1p0ha_ | d2j8ma_ |
| d2pq8a_ | d1z4ra1 | d1s3za1 | d1ufha_ | d1c4qa_ | d1prtc1 | d2wv6d_ | d1prtf_ |
| d1ltrd_ | d4l63b1 | d1r4pb_ | d1prtd_ | d1tl0f_ | d2bosa_ | d1prtb1 | d3chbd_ |
| d1xvqa1 | d1pn0a2 | d3hy2a_ | d2cvba1 | d1o8xa_ | d1lu4a_ | d1we0a1 | d2bmxa1 |
| d2a4va1 | d1psqa_ | d1xiya1 | d1q98a_ | d1n8ja_ | d1zofa1 | d2ggta_ | d3tjja_ |
| d4je1a1 | d1o73a_ | d3s9fa_ | d2zcta_ | d1qmva_ | d1jfua_ | d2b1ka_ | d5dvba1 |
| d2b5xa1 | d2v2ga_ | d1st9a_ | d3tb2a_ | d4llra_ | d4fh8a1 | d1knga_ | d2h30a_ |
| d1i5ga_ | d2z9sa_ | d2f8aa1 | d1tp9a1 | d2b7ka_ | d1nm3a2 | d3hvva_ | d2wfca1 |
| d4kcea_ | d1xvwa1 | d4gqca1 | d3qpma_ | d4k1fa_ | d1zzoa1 | d1gp1a_ | d1prxa_ |
| d1zsqa1 | d2qked_ | d4ksoa_ | d1t4za1 | d1wwja_ | d3kasa1 | d3bi1a3 | d1e88a1 |
| d1l6ja5 | d1l6ja4 | d1eaka4 | d1eaka3 | d1l6ja3 | d1h8pa2 | d1h8pa1 | d1etha2 |
| d1w52x1 | d2ppla1 | d1hpla2 | d1lpbb2 | d2oxea1 | d1bu8a2 | d1rp1a2 | d1lgya_ |
| d4l3wa_ | d1gz7a_ | d1ukca_ | d1cr6a2 | d1bvsa3 | d3groa_ | d1pjaa_ | d1ei9a_ |
| d1svpa_ | d1mbma_ | d3su6a1 | d1cu1a1 | d4agka_ | d1q31a_ | g1n1l.1 | d3e90b_ |
| d1ep5a_ | d3u1jb_ | d3zxha_ | d1cgla_ | d1rm8a_ | d1eaka2 | d1hy7a_ | d2jsda_ |
| d1cxva_ | d1hv5a_ | d1i76a_ | d3p0ya1 | d1igra1 | d1igra2 | d1n8yc2 | d1n8yc1 |
| d1m6ba2 | d1m6ba1 | d4dv8a2 | d2tbvc_ | d1c8nc_ | d1x36a_ | d1smvc_ | d1ng0c_ |

d4sbvc_ d1opoa_ d1f2nc_ d1cixa_ d1y29a_ d1v7fa_ d2jtba_ d1d1ha_
d1g9pa_ d1lupa_ d1qk7a_ d1mb6a_ d2a2va_ d1niya_ d1i25a_ d1hvwa_
d2mpqa_ d1ie6a_ d1kqha_ d1la4a_ d2mxoa1 d1dl0a_ d1koza_ d1qk6a_
d1emxa_ d1lqra_ d1c6wa_ d1rmka_ d1ixta_ d1eyoa_ d1cnna_ d1g1pa_
d1f3ka_ d1fyga_ d1rypd_ d1irua_ d1rypc_ d3unfk_ d1rypa_ d1j2pa_
d1irui_ d1j2qh_ d3unfh_ d1m4ya_ d1rypb_ d1irul_ d1irug_ d1iru2_
d1q5rh_ d1rypg_ d1g3ka_ d1q5qa_ d1e94a_ d1irub_ d1ryph_ d3ty6a_
d1iruj_ d1iruk_ d1rype_ d1irud_ d1irue_ d3mi01_ d1rypf_ d1iruh_
d2z3ba_ d1iruc_ d3mka2_ d1iru1_ d1ny721 d1ny722 d1a6ca2 d1a6ca1
d1pgl22 d1pgl21 d1pgl11 d1ny711 d1mqta_ d1d4m3_ d1fmd3_ d2mev1_
g1aym.1 d1ev13_ d4q4w1_ d1r1a3_ d2x5ia_ d1rhi1_ g1r1a.1 g1d4m.1
g1rhi.1 d1b35b_ d1oopa_ d1tme1_ d4iv1a_ g1pvc.1 g1fpn.1 d1fpn1_
d4gb31_ d1d4m1_ d1fmd1_ d2wzr3_ g1cov.1 d1qqp3_ g1fmd.1 d2x5ic_
d1rhi3_ g1oop.1 d1hxs1_ g1hxs.1 d1fpn3_ d1bev1_ g2mev.1 d1eah3_
d1aym1_ d1pvc3_ d1qqp1_ d1zba3_ d1h8ta_ d3vbhc_ g1b35.1 g1bev.1
d1hxs3_ g1ev1.1 d1bev3_ g1tmf.1 d1tmf1_ d1b35a_ d2mev3_ g1eah.1
g1h8t.1 d1eah1_ d4q4w3_ d1r1a1_ d1aym3_ d1pvc1_ d1zba1_ d1h8tc_
d1oopc_ d1tme3_ g1qqp.1 d4iv1c_ g1tme.1 d3ubub_ d1egia_ d1qo3c_
d1v7pa_ d1tdqb_ d3ubua_ d1c3ab_ d1h8ua_ d3bx4b_ d1t8ca1 d1sb2a1
d1qdda_ d2ziba1 d3cada_ d1byfa_ d1j34b_ d1jwia_ d1jzna_ d1c3aa_
d1oz7b_ d1wmza_ d3bdwb_ d1hq8a_ d1fifa1 d1dv8a_ d3hupa_ d1wk1a1
d2afpa_ d3bx4a_ d1gz2a_ d1sl6a_ d1v7pb_ d3g8ka_ d1ixxa_ d1j34a_
d1jwib_ d1g1sa1 d1y17a1 d1ja3a_ d1fvub_ d3bdwa_ d1sb2b1 d1fvua_
d1umrc_ d1oz7a_ d4m17a_ d3cfwa1 d1ypqa1 d1g1ta1 d3fd4a_ d3l9jc_
d4dn8a1 d1v4lb_ d1rdl1_ d2msba_ d4w9ta_ d1jvna1 d4x2ra1 d1ka9f_
d1qo2a_ d4axka1 d1thfd_ d4u28a_ d2y88a_ d4wd0a_ d1vzwa1 d3ia2a_
d1a88a_ d4dgqa1 d1zoia_ d2flia1 d1rpxa_ d1tqja_ d1tqxa_ d4lt9a1
d3tc7a1 d3hoja_ d1i4na_ d3vnda_ d3nl8a1 d1geqa_ d3nava1 d4a2ra1
d3tc6a1 d1ujpa_ d4ijba_ d1xcfa_ d1vc4a_ d1rd5a_ d1qopa_ d3b5va_
d2r80b_ d1cqxa1 d1cg5a_ d1gcvb_ d3boma_ d2h8fb_ d2raoa_ d3bj1a_
d4hrrb_ d2r50a_ d3d1kb_ d2aa1b1 d3zhwa_ d3hf4b_ d3gdja_ d1jebb_
d3bj1b_ d1x9fb_ d1wmub_ d1gcva_ d3bcqb_ d3d4xb_ d1gvha1 d2dc3a_
d3hf4a_ d4yu3a_ d3k8ba_ d1qpwb_ d2r80a_ d3bcqa_ d1v4wa_ d3wtgb_
d1jl7a_ d4esaa_ d1a9we_ d3d1ab_ d1fsla_ d4h2la_ d1v4wb_ d1mwca_
d1vhba_ d1jeba_ d1spgb_ d1h97a_ d1q1fa_ d1tu9a1 d3d1aa_ d1hdsb_
d3hyub_ d2zfbb_ d3fs4a_ d1oj6a_ d1shrb_ d4h2lb_ d4yu3b_ d1spga_
d3hrwa_ d3qqra_ d3at5a_ d4wchd_ d3hyua_ d1cg5b_ d1uc3a_ d1hbra_
d2raob_ d1outb_ d4esab_ d3gdjb_ d3qqqa_ d1x9fc_ d3vrfa_ d1itha_
d3mkba_ d3d4xa_ d3mjpa_ d1jl6a_ d3fh9a_ d1outa_ d2dc3b_ d1it2a_
d3eoka_ d3g46a_ d1qpwa_ d3vrfb_ d1x9fd_ d1hdsa_ d2zfba_ d3mkbb_
d1or4a_ d3wtga_ d1a4fa_ d3fh9b_ d3bomb_ d3cy5a_ d3at5b_ d1x9fa_
d2oifa_ d4lmxb_ d1cpcb_ d3v57a_ d1kn1a_ d1cpca_ d4f0ta_ d2vjha_
d4po5b_ d1eyxa_ d1alla_ d4f0tb_ d1b33b_ d1b8db_ d4h0mb_ d2vjhb_
d4lm6b_ d2vmlb_ d1allb_ d2vjta_ d3dbja_ d2vmla_ d2vjtb_ d4f0ub_

| | | | | | | | |
|---|---|---|---|---|---|---|---|
| d1f99b_ | d4rmpa1 | d1liaa_ | d1eyxb_ | d2uulb_ | d4lmsb_ | d4f0ua_ | d2bv8a_ |
| d1f99a_ | d2bv8b_ | d2c7lb_ | d3dbjb_ | d4rmpb1 | d1b8da_ | d4h0ma_ | d3v57b_ |
| d1kn1b_ | d1liab_ | d2uula_ | d2j96a_ | d1iwea_ | d1qf5a_ | d1vmaa2 | d1nija1 |
| d1np6a_ | d2p67a1 | d3ihla_ | d1okkd2 | d1ls1a2 | d1s1ma2 | d1iona_ | d2v40a_ |
| d1dj3a_ | d3hida_ | d2vo1a1 | d2g0ta1 | d1vcoa2 | d1j8mf2 | d1g3qa_ | d1xjca_ |
| d1p9ba_ | d1qzxa3 | d1ihua2 | d1ihua1 | d2qm8a_ | d1dj2a_ | d4ioja_ | d1cp2a_ |
| d1yrba2 | d1azwa_ | d1mtza_ | d1hlga_ | d2xqfa_ | d1ea5a_ | d1f6wa_ | d1dx4a_ |
| d1qe3a_ | d1k4ya_ | d4qwwa_ | d2ha2a_ | d3c8da2 | d2pbla1 | d1jkma_ | d1lzla_ |
| d1jjfa_ | d2h0ba_ | d2erfa_ | d1h30a2 | d3mw4a1 | d1pz7a_ | d2r1ba_ | d3v64a1 |
| d1d2sa_ | d3bopa1 | d3mw3a1 | d1okqa1 | d1okqa2 | d2m5ta_ | d1cqqa_ | d1wqsa_ |
| d5bpea_ | d2ipha_ | d1lvoa_ | d2bhga1 | d3tnta_ | d3sjia_ | d3d23a1 | d2fyqa_ |
| d3tloa_ | d3zvea1 | d3q3ya1 | d4asha1 | d4iuaa3 | d4bvva_ | d5hpga_ | d3k65a_ |
| d1ki0a3 | d1ki0a2 | d1ki0a1 | d1a0ha1 | d1pmla_ | d3sp8a3 | d2feba1 | d1xova2 |
| d1f74a_ | d3l21a_ | d2e1da_ | d1to3a1 | d1w3ia_ | d1sfla_ | d1vcva1 | d4imea_ |
| d1wa3a_ | d2ocza1 | d1f2ja_ | d3m16a_ | d2yxga_ | d1ub3a_ | d3a5fa_ | d1fbaa_ |
| d1l6wa_ | d1xfba1 | d1wx0a1 | d1vpxa1 | d1ojxa_ | d1mxsa_ | d2a4aa1 | d1xkya1 |
| d3cq0a1 | d3s1xa_ | d1f05a_ | d1fdja_ | d3cpra1 | d4d2ja_ | d1cg2a1 | d1yloa2 |
| d1y7ea2 | d1y0ya3 | d1lfwa1 | d1vixa1 | d1fnoa4 | d1vhoa2 | d1ysja1 | d1vhea2 |
| d1z2la1 | d2fvga2 | d1vgya1 | d1r3na1 | d2grea2 | d1xmba1 | g1q7l.1 | d1rtqa_ |
| d3rp2a_ | g1ucy.1 | d1fuja_ | d2zchp_ | d3w94a_ | d1si5h_ | d1lo6a_ | g2pka.1 |
| d1cgha_ | d1kigh_ | d1a5ia_ | d3s9ba_ | d1hj8a_ | d1ao5a_ | d2anya_ | d1q3xa1 |
| d1gvza_ | d4gsoa_ | d2f91a1 | d1iaua_ | d1gdna_ | d1eufa_ | g1fiw.1 | d1eq9a_ |
| d4nfea_ | d2rdla_ | d1spja_ | d1elva1 | d1a7sa_ | d1ekbb_ | d1pfxc_ | d1yc0a_ |
| d2aiqa_ | g1fiz.1 | d1fi8a_ | d4k60a_ | d4dgja_ | d1mzaa_ | d1op0a_ | d3tjva_ |
| d1pytd_ | d1sgfg_ | g1gg6.1 | d2eeka_ | g1rtf.1 | d1a0ja_ | d3g01a_ | d1pytc_ |
| d4e7na_ | d1ddja_ | d1z8ga1 | d2hlca_ | d1npma_ | d1brup_ | g1jou.1 | d1sgfa_ |
| d1autc_ | g2hnt.1 | g1kdq.1 | d1orfa_ | d3s69a_ | d4crga_ | d1fxya_ | d1bqya_ |
| d2ftba_ | d1pmpa_ | d2rcqa1 | d1fdqa_ | d4lkpa_ | d1sa8a_ | d1vyfa_ | d1lpja_ |
| d3vg7a1 | d4azqa1 | d1o8va_ | d5bvqa_ | d1p6pa_ | d2qo4a_ | d1opba_ | d1yiva1 |
| d1o1va_ | d4bvma1 | d4tkba_ | d4i3ba_ | d1bwya_ | d1ftpa_ | d1ggla_ | d4gkca_ |
| d1cbia_ | d3p6da1 | d1kqwa_ | d2a0aa1 | d1dzka_ | d4tlja_ | d2a2ua_ | d4wfva_ |
| d3t1da_ | d4iaxa_ | d3zq3a_ | d2nnda_ | d1i4ua_ | d1kxoa_ | d1bbpa_ | d1kt7a_ |
| d1exsa_ | d3s26a1 | d1lf7a_ | d1qfta_ | d4gh7a_ | d1z24a1 | d1qabe_ | d1xkia_ |
| d1ew3a_ | d3bx7a_ | d4iawa1 | d1n0sa_ | d3kzaa_ | d2k23a_ | d2l9ca1 | d2joza1 |
| d1gm6a_ | d3kffa_ | d3saoa1 | d1e5pa_ | d1yupa1 | d1iiua_ | d3lgia_ | d3i77a_ |
| d2z9ia2 | d4nsva_ | d1lcya2 | d4jcna_ | d1os8a_ | d1hpga_ | d1p3ca_ | d1l1ja_ |
| d1qtfa_ | d1e69a_ | d1mv5a_ | d3g60a4 | g1ii8.1 | d1ewqa2 | d1ji0a_ | d1qhla_ |
| d1v43a3 | d2wmfa1 | d1vpla_ | d1g2912 | d4a82a2 | g1f2t.1 | d2d62a1 | d3tuic1 |
| d2bbsa1 | d3g60a3 | d2ixea_ | d1g6ha_ | d1sgwa1 | d2qi9c_ | d3rlfa1 | d3b60a1 |
| d3d31a2 | d2pzea1 | g1xew.1 | d1jj7a_ | d1oxxk2 | d2onka1 | d1w1wa_ | d1rw1a_ |
| d1z3ea1 | d3l78a_ | d3gqba2 | d1skye3 | d1xp8a1 | d1tf7a2 | d1tf7a1 | d1ye8a1 |
| d1skyb3 | d2qe7d2 | d4blta_ | d4huta_ | d3ldaa2 | d4hyya_ | d2i3ba1 | d1cr1a_ |
| d3j9tb2 | d3ntua2 | d1vdza1 | d2z43a_ | d1e9ra_ | d1nlfa_ | d3ziaa2 | d1n0wa_ |
| d1g6oa_ | d3ziad2 | d3j9ta2 | d1pzna2 | d3gqbb2 | d2i49a_ | d1nnfa_ | d1xvxa_ |

d1h3da1 d1iuda_ d4m1va1 d1twya_ d3e13x_ d2nxoa1 d4pfua_ d3ttma_
d1elja_ d2qmwa1 d1us5a_ d1y3na1 d1jeta_ d1uqwa_ d4f19a1 d2esna2
d3thia_ d3fasa1 d2uyeb_ d2xwva1 d1r9la_ d2fyia1 d1pb7a_ d2ozza1
d1sw5a_ d1nh8a1 d1vr5a1 d2g29a_ d1xs5a_ d3lswa_ d2z23a_ d3w9va_
d3tcha_ d1q35a_ d1z7me1 d3q27a_ d1p99a_ d1ve4a1 d2i6ea1 d4mcoa1
d4n8ya_ d1eu8a_ d1zbma1 d1hsla_ d1ixca2 d1j1na_ d1xvya_ d1y9ua_
d2z22a1 d1xoca1 d4oera_ d1wdna_ d5f1qa1 d1xt8a1 d1i6aa_ d4gwoa_
d4igta1 d1pc3a_ d4mmpa1 d1o63a_ d2czla1 d2onsa2 d4mija1 d1eh3a_
d3skpa_ d1dtza1 d1dtza2 d1ieja_ d1jnfa2 d1ce2a1 d1jnfa1 d1b1xa2
d1b1xa1 d1jw1a1 d1jw1a2 d1ryoa_ d1h76a1 d1h76a2 d1c4oa1 d2p6ra3
d2p6ra4 d1oywa2 d1oywa3 d1wrba1 d1w36b2 d1w36b1 d1c4oa2 d3bora_
d1t6na_ d2eyqa5 d2eyqa3 d2eyqa2 d2zu6a2 d1veca_ d1uaaa1 d1w36d1
d2g9na1 d1z3ix2 d2rb4a_ d1hv8a1 d1hv8a2 d1t5ia_ d1w36c2 d2j0sa2
d2j0sa1 d1fuka_ d3hjha1 d1z5za1 d1qdea_ d1z63a1 d1s2ma2 d1s2ma1
d1wp9a1 d1wp9a2 d1gm5a3 d1gm5a4 d3dina4 d3dina3 d1q0ua_ d1w36c1
d1z3ix1 d1nkta3 d1uaaa2 d1w36d2 g1qhh.1 d2fwra2 d2fwra1 d3q3ma_
d1za0a1 d1mxra_ d2inca_ d1jk0a_ d2incb_ d1mhyb_ d3hf1b_ d1mtyb_
d1r2fa_ d2uw1a_ d1mhyd_ d3n37a_ d1afra_ d1oquc_ d1syya_ d1mtyd_
d2o1za_ d1uzra_ d1smsa_ d2fkza_ d1zuja1 d1s3qa1 d2fjca1 d1z6om1
d1veia_ d4dyya_ d1jiga_ d3gvya_ d2pyba_ d4cy9a1 d3bvfa1 d1r03a_
d2cf7a_ d1nf4a_ d1zs3a1 d4iwka_ d3ka8a_ d3np0x_ d1n1qa_ d1lb3a_
d4e6ka_ d1z6oa1 d2fzfa1 d4dyua_ d3ge4a_ d1jgca_ d4am4a_ d2z6ma_
d1ji5a_ d1vlga_ d1lkoa1 d1krqa_ d3qz3a_ d1o9ra_ d2ffxj_ d1euma_
d1yv1a1 d2v2pa_ d1j30a_ d4di0a_ d1dpsa_ d1zx5a1 d1oewa_ d2qzxa_
d2asia_ d1psoe_ d1miqa_ d1smra_ d2h6ta_ d1pfza_ d1izda_ d3hd8a_
d1j71a_ d1dpja_ d4aa9a_ d1t6ex_ g1b5f.1 g1lya.1 g1htr.1 d1qdma2
d1bxoa_ d1wkra_ d1tzsa_ d1ibqa_ d2qzwa_ d1bdqa_ d3ggta_ d3ixoa_
d1mrxa_ d2haha_ d1tw7a_ d4njsa_ d1sgua_ d2p3da_ d1nsoa_ d3d3ta_
d2rkfa_ d1c6ya_ d2rspa_ d3ogpa_ d3ekwa_ d2o4na_ d2j9ja_ d1baia_
d3ggua_ d3lzua_ d4npua_ d4j55a_ d2r8na_ d2fxda_ d2p3ca_ d4dqea_
d3ttpa_ d3t3ca_ d2p3aa_ d2hc0a_ d2fdda_ d3mwsa_ d1um8a_ d1ixza_
d1qvra2 d1qvra3 d1sxjc2 d1jr3d2 d2p65a_ d1l8qa2 d1g8pa_ d3hu3a3
d1sxjd2 d1njfa_ d1in4a2 d1sxja2 d1tuea1 d2ce7a2 d1w5sa2 d1sxjb2
d1d2na_ d1ny5a2 d1ofha_ d2gnoa2 d2a5yb3 d1fnna2 d1u0ja1 d1lv7a_
d1iqpa2 d2fnaa2 d1ixsb2 d4gita_ d1sxje2 d1g41a_ d1h4ra3 d4yl8a1
d2al6a3 g1qmo.1 g1len.1 d3zyra_ d1fnya_ d1n47a_ g2ltn.1 d1avba_
d3usua_ d1dhkb_ d1f9ka_ d1ukga_ d1g8wa_ d1g9fa_ d1wbfa_ d3wcsa_
d1g7ya_ g1loe.1 d1mvqa_ d1hqla_ d1v6ia_ d1dbna_ d1qnwa_ d1fx5a_
d4l8qa_ d3n35a_ d1wgda1 d1wgga1 d4q5eb_ d1wjna1 d1se9a_ d3n3kb_
d1wz0a1 d1we6a1 d2m0xa_ d2gbka_ d3rt3b1 d1wh3a1 d1sifa_ d2ekec2
d4ii2b1 d4hk2a1 d1c3ta_ d1m94a_ d1wgha1 d1ttna1 d1j8ca_ d1wx8a1
d3v6cb1 d2ma9b_ d4i6lb1 d1bt0a_ d2jvca1 d1iyfa_ d2k1fa_ d1wxva1
d1wf9a1 d1t0ya_ d2io0b2 d1x1ma1 d2gbma_ d1zw7a1 d2m17a_ d1ud7a_
d1we7a1 d2lwpa_ d2gbja_ d1wjua1 d1v6ea1 d1v86a1 d4eewa_ d1wiaa1

| | | | | | | | |
|---|---|---|---|---|---|---|---|
| d4icva_ | d4gu2a_ | d1wx7a1 | d1wy8a1 | d4hjka1 | d2faza1 | d1v5oa1 | d3ruib_ |
| d1eo6a_ | d3waob_ | d2r2qa1 | d3m95a_ | d3wana_ | d2al3a1 | d2cr5a1 | d1wj4a1 |
| d3nqyb_ | d4gera_ | d1bqba_ | d1dszb_ | d1a6ya_ | d3cbba_ | d1ynwa1 | d1r4ia_ |
| d1hcqa_ | d2hana_ | d2hanb_ | d2enva1 | d1kb4b_ | d2nllb_ | d1lo1a_ | d1cita_ |
| d1adqa1 | d1fnga1 | d1uvqb1 | d1zs8a1 | d2vola1 | d2vola2 | d1mfbl2 | d2jelh2 |
| d1uwel2 | d3qq4b1 | d1kjvb_ | d1k5na1 | d1osph2 | d4odhl2 | d1ow0a1 | d1ow0a2 |
| d1bfob2 | d3l9ra2 | d3jtth1 | d2fath2 | d4elkb2 | d1fn4b2 | d2xfxb_ | d1pg7x2 |
| d1cqka_ | d4k71b2 | d1lk2a1 | d1eapb2 | d4lfhg2 | d1i4fa1 | d2nw2a2 | d1kfah2 |
| d1mhca1 | d1h3ua1 | d1wejh2 | d1h0db2 | d1a5fh2 | d1kcvh2 | d1mjuh2 | d1qqda1 |
| d1kcrh2 | d1k8ia1 | d3huja2 | d2nnab1 | d1nfdf2 | d3mffb2 | d1s9va1 | d1lk3h2 |
| d1q0xl2 | d1fv1b1 | d1neza1 | d4rfeb2 | d1r3ha1 | d1a6tb2 | d1kjva1 | d1pz5b2 |
| d1onqa1 | d2bc4a2 | d1de4a1 | d1k8da1 | d1tcra2 | d1dn0b2 | d1nfde2 | d2xwtb2 |
| d1bfoa2 | d1mexh2 | d1iakb1 | d1ypzf2 | d4f7ea2 | d1q72l2 | d2fbjh2 | d1fe8h2 |
| d1clzh2 | d1fngb1 | d1uvqa1 | d1vgka1 | d1c16a1 | d4j12a2 | d4x99b2 | d1igtb3 |
| d1je6a1 | d1q72h2 | d1nldh2 | d1teth2 | d2hmic2 | d1i1ca2 | d1i1ca1 | d1k8ib1 |
| d1q0yh2 | d1kfal2 | d3frua1 | d1pg7w2 | d1u58a1 | d1igyb4 | d1igyb3 | d1hyrc1 |
| d1klub1 | d1deeb2 | d1c12b2 | d2bc4b2 | d2ig2h2 | d1kcvl2 | d3wn5b1 | d1lk2b_ |
| d1lk3l2 | d1mjul2 | d1sm3h2 | d1q0xh2 | d1zvsa1 | d1ydpa1 | d1u8ib2 | d1ypze2 |
| d1kcuh2 | d2lj7a_ | d1n4na_ | d2m8bb1 | d2kyqa_ | d3psma_ | d1jkza_ | d1jxca_ |
| d2gl1a_ | d4uj0a_ | d2k4ua_ | d1hp2a_ | d2lu9a_ | d1baha_ | d2meoa1 | d1wm7a_ |
| d1hlya_ | d1n8ma_ | d1jlza_ | d1wpda_ | d1c55a_ | d1pvza_ | d1wt7a_ | d1rjia_ |
| d1wz5a_ | d1m2sa_ | d1pjva_ | d1c49a_ | d1qkya_ | d2ktca1 | d1wmta_ | d1du9a_ |
| d1j5ja_ | d1r1ga_ | d1t0za_ | d2i61a_ | d1t1ta_ | d1kv0a_ | d2lkba_ | d1npia_ |
| d1wwna_ | d1pe4a_ | d2kbka_ | d2li7a_ | d1djta_ | d2kbha_ | d1dq7a_ | d1sega_ |
| d1omya_ | d1zywa1 | d1jzaa_ | d1t61c1 | d1iyjb4 | d1iyjb5 | d1iyjb3 | d1o7ia_ |
| d4ipca1 | d1x3ea_ | d4apva_ | d1jb7a1 | d1jb7a2 | d1jb7a3 | d3kdfa_ | d1ue1b_ |
| d1wjja1 | d3eiva_ | d1xjva2 | d1xjva1 | d2ccza2 | d1se8a_ | d1l1oc_ | d1jb7b_ |
| d1qvca_ | d3ulla_ | d1qzga_ | d1miua4 | d1miua5 | d1miua3 | d1y14b1 | d2nn6i1 |
| d1i94l_ | d2zjra2 | d1smxa_ | d2qamc2 | d1hh2p1 | d2r7da1 | d1rl2a2 | d1wfqa1 |
| d3bzka4 | d1x6oa2 | d3a0ja_ | d2j01d2 | d2ja9a1 | d4ql5a_ | d1hr0w_ | d1luza_ |
| d2n3sa_ | d2vnud1 | d1h95a_ | d1go3e1 | d2nn6h1 | d2mo1a_ | d1iz6a2 | d1jjga_ |
| d1e3pa2 | d2ix0a3 | d2ix0a2 | d2lxja_ | d1ueba3 | d1ueba2 | d2z0sa1 | d1u0la1 |
| d2ix0a1 | d2eifa2 | d2je6i1 | d3i2za1 | d2c35b1 | d2ahob2 | d1t9ha1 | d3i4oa_ |
| d2qall1 | d1jt8a_ | d2nn6g1 | d1g6pa_ | d3cama_ | d1i94q_ | d1c9oa_ | d1d7qa_ |
| d2ba0a1 | d2vnud2 | d2vnud3 | d1khia2 | d2qalq1 | d1wi5a1 | d1xtda2 | d1ny4a_ |
| d1pfsa_ | d2a1ka_ | d1rpma_ | d2shpa1 | d1yfoa_ | d1l8ka_ | d2ooqa1 | d1g4us2 |
| d1p15a_ | d1lara2 | d1lara1 | d1wcha_ | d2fh7a2 | d2fh7a1 | d1ohea2 | d2f4da_ |
| d1rxda_ | d2hxpa_ | d1xria_ | d1d5ra2 | d1fpza_ | d3ezza_ | d4y2ea_ | d1i9sa_ |
| d3azya_ | d3wvja_ | d1upsa1 | d1ajoa_ | d2hyka_ | d1ajka_ | d2vy0a_ | d1o4ya_ |
| d3o5sa_ | d3d6ea_ | d3atga_ | d1dypa_ | d1umza_ | d3i4ia_ | d1tdha3 | d1q3ba3 |
| d1ee8a3 | d1k82a3 | d4qmea2 | d2xdta2 | d1z5ha2 | d3ubra_ | d1qo8a1 | d1d4ca1 |
| d2k3va_ | d1ft5a_ | d1qdba_ | d1m1qa_ | d2j7aa_ | d1h21a_ | d1ogyb_ | d4fasa_ |
| d1jnia_ | d1oaha_ | d1sp3a_ | d3h33a_ | d1rwja_ | d19hca_ | d1os6a_ | d3bxua_ |
| d3h4na_ | d1i77a_ | d3caoa_ | d1j0pa_ | d2ctha_ | d1duwa_ | d1ofwa_ | d2e84a_ |

d1hh5a_ d1gyoa_ d3h34a_ d1wioa4 d1ccza2 d1vcaa1 d1dr9a2 d2d9qb1
d2nxyb2 d2dara2 d2dara1 d1wyha2 d2dloa2 d1rutx4 d1rutx3 d2cuqa2
d1x4la2 d1x4la1 d1x3ha2 d1x3ha1 d2d8za1 d1x61a2 d1x6aa1 d1x6aa2
d2cora2 d2cora1 d1v6ga1 d1v6ga2 d2d8za2 d1x64a1 d1x64a2 d1b8ta1
d2co8a1 d1x63a2 d2dj7a2 d2dj7a1 d2cu8a2 d2cu8a1 d1g47a2 d1g47a1
d1j2oa1 d1wyha1 d2dloa1 d1wiga1 d1wiga2 d2cupa2 d2cupa3 d1x68a1
d2d8ya2 d2d8ya1 d1m3va2 d1x62a1 d1x68a2 d1x61a1 d1b8ta3 d1b8ta2
d2d8xa2 d2d8xa1 d1x4ka1 d1x4ka2 d2cuqa1 d1m3va1 d2co8a2 d1x63a1
d2cura1 d2cura2 d1x62a2 d1op9a_ d1y0la1 d1etzb1 d1egjl1 d2gsib1
d1nj9a1 d4jfhd1 d1aqkl1 d1t2ja_ d1lo4h1 d1zvya1 d3v4vl1 d4enec1
d1kxtb_ d5e8ea1 d2pxya_ d4weud_ d1xiwc_ d2esvd1 d1qnzh_ d1ogae1
d1f4xh1 d4n1hb_ d3ezjb1 d3mlwl1 d1kfah1 d1etza1 d1b4jh1 d3cfic_
d4w68a_ d2arja1 d1ek3a_ d2ok0l1 d1vera_ d1uwgh1 d1blnb1 d1fpth1
d1rzga1 d2giya1 d4m3kb1 d1nc2a1 d2nyyd1 d1ncna_ d1fbih1 d5dxwa_
d4fqqa1 d1dqdh1 d1jfql1 d1kelh1 d1faih1 d1frgl1 d1figl1 d4m6nl1
d1fo0a_ d4hxbl1 d3b5ga_ d3quyd1 d2h9gb1 d1i8kb_ d2dd8h1 d2ck0h1
d1eaja_ d4w6wb1 d2a6ib3 d1t66d1 d2e27h_ d1ob1b1 d1f11b1 d2v7na1
d1ad0a1 d2xt1b1 d1pskh1 d4i13b1 d2op4l1 d1a0ql1 d1uwel1 d1za3a1
d1ncbl1 d4uu9a_ d1bm3h1 d1cloh1 d1nqba2 d1cf8h1 d4y5va_ d1mhph1
d5anma1 d4cdgc1 d4ldeb_ d1y0lb1 d2pcpb1 d1z3gl1 d1dfbl1 d1fh5h1
d3g5xa1 d1tzib1 d4dn3l1 d3zhka1 d2b1hh1 d2uyla1 d1f3dh1 d1nj9b1
d2agjh1 d2h1pl1 d1q72h1 d8fabb1 d1ndmb1 d1rjca1 d4u6hb1 d3upca_
d2fbjl1 d1osph1 d1tvda_ d2cjvh_ d4fhbd1 d1bd2d1 d1rz7h1 d3nn8a_
d1blna1 d1knoa1 d1nfda1 d2bmkb1 d1dqll_ d1igyb1 d1igfl1 d12e8h1
d3utza1 d1nc2b1 d1rihh1 d4mwfb1 d4jvpa1 d1mieh1 d1rhfa1 d1fgnl1
d1sbsh1 d1lp9e1 d1dn0a1 d4lqfl1 d1iaih1 d1kgcd1 d1mh5b1 d1gpol1
d2g60h3 d1f8tl1 d2jixd1 d4grwh_ d1fo0b_ d1bfob1 d1axsa1 d1u9ka_
d1nmbh_ d1h8na1 d1h8na2 d1jguh1 d4eura1 d3sdyl1 d35c8h1 d1w72l1
d1mvub_ d1i9rh1 d1ob1a1 d4wenb_ d1yeeh1 d3ietc1 d1f58h1 d1ad0b1
d1mqkl_ d1nbvh1 d4b50a_ d3r8bb_ d1bafl1 d1mamh1 d4rgne1 d2gsgb_
d35c8l1 d1bafh1 d1ai1h1 d2pcpa1 d2fath1 d2x1pa_ d1orsb1 d1ibgl1
d1shma_ d1a2yb_ d1u0qa_ d2coqa_ d1hilb1 d1qfwi_ d1jv5b_ d1fn4b1
d5e0qa_ d1nsnh1 d1plgl1 d4ocxl1 d1kb5h1 d1rzfh1 d1pg7x1 d2fx7h1
d2oi9c_ d1l7il1 d3eyva1 d1d5il1 d4ffyl_ d1tetl1 d1a6wh_ d1ac6a_
d3dvfa_ d1nfde1 d1mimh1 d1ri8a1 d1jhlh_ d3dura_ d1fskb1 d1f97a1
d3so3b1 d2ak4d1 d1lp9f1 d4z7wf1 d1l7th1 d4y5ya_ d4x7ec1 d1emth1
d1ikfl1 d1nlbh1 d4utal1 d1bjma1 d1eapb1 d1u6ah1 d2dtgd1 d1kj2b_
d1xeda1 d1c1eh1 d1fgvh_ d2bnub1 d2fb4h1 d2dtgb1 d1ktrh_ d1jpth1
d1qkzl1 d4hepg_ d2atpb1 d1flrh1 d2hrpl1 d1mvfa_ d1iaim1 d3k80a1
d2jb5h1 d3zkxc_ d4grwe_ d1it9h1 d1rhhb1 d3w9ec_ d1e4wh1 d1sy6h1
d2j6eh1 d3qxta_ d1jglh1 d1p7ka1 d1tzhb1 d4n8pa1 d1gafl1 d2jelh1
d1wioa2 d4i0cc1 d1cicb1 d1lmka2 d1lmka1 d1iqwh1 d1orqb1 d1ejoh1
d1jnlh1 d4zg1a_ d43c9b_ d1ggbl1 d1wejh1 d3q6fa1 d1f3dj1 d3c6la1
d1bqlh1 d3p9wb_ d1a3rl1 d1h0db1 d2uzil_ d1eo8l1 d1qfwl_ d1n7ml1

d1igja1 d1c5ch1 d2a9mh_ d1rmfh1 d1fj1b1 d4heme_ d1ccza1 d1a5fh1
d1ogad1 d1dlfh_ d1qa9b_ d1vgel1 d3qskb_ d1sjxa_ d4ozfg1 d1sq2n_
d1qd0a_ d1kcvh1 d1c5db1 d3qcul1 d8faba1 d3bn9d1 d1mjuh1 d4y8dc_
d7fabh1 d3dvgb1 d1lo0l1 d1a6tb1 d1gigh1 d1kcrh1 d2hwzl1 d1yjdc1
d3clfl1 d4krna_ d1dl7l_ d1e6ol1 d1mexh1 d3r0ma_ d4kkna_ d1fnsl1
d1oaqh_ d1rzia1 d1ucbh1 d1ncwl1 d4b41a_ d1nfdf1 d2mpah1 d2d7tl1
d4grwf_ d4b5ea_ d1lk3h1 d3u79a_ d4u3xa_ d1ghfl1 d1bvkb_ d1mcww1
d1b88a_ d1jrhh1 d32c2b1 d4wemb_ d1dsfh_ d1op3h1 d1ce1l1 d1iqda1
d1qoka1 d1qoka2 d1qfuh1 d2crya1 d1ay1h1 d1oaql_ d3ab0b_ d1rurl1
d1t4kb1 d1bz7b1 d1n0xh1 d1forl1 d1ucbl1 d4w6yb_ d43c9a_ d2aabl1
d1tzia1 d4n1ca_ d1k6qh1 d3g9ab_ d4tvsa1 d1igmh_ d1pz5b1 d3dx9a1
d3wxwh_ d1nakh1 d1kcul1 d3eaka_ d4qlra1 d1cr9l1 d1d9ka_ d1f4xl1
d1deea1 d2i27n2 d1c12a1 d2orbl1 d1j8hd1 d1mcph1 d1c5da1 d1kenh1
d1bd2e1 d1h5ba_ d4x7fc1 d3upaa_ d1t3fa1 d1indh1 d2dtgc1 d4hc1l1
d1zv5a1 d4wgvb_ d1uwgl1 d4f37k1 d1aqkh1 d1vesa_ d3zkqd_ d1fptl1
d1lo4l1 d1knob1 d3rrqa_ d1ct8b1 d4orzc_ d1mlbb1 d4hgkc1 d1dqdl1
d1bogb1 d4ozhe1 d1dr9a1 d1i7za1 d1dn0b1 d1kell1 d1rzib1 d1ypzf1
d1wtla_ d1hkfa_ d1fail1 d1figh1 d2vyre_ d1sjva_ d1ohqa_ d1bfoa1
d1pkqb1 d1mjul1 d1b4jl1 d1nsnl1 d2arjb1 d1p4bh_ d4aj0a_ d3nn8b_
d2rcja1 d1f2xk_ d4v1dd_ d1adqh1 d2eksa_ d4k3ga_ d1jfqh1 d1uweh1
d1frgh1 d4gftb_ d1uz8b1 d4n1ea_ d1bm3l1 d1clol1 d3mcka1 d2vq1a1
d1ae6h1 d4aq1b1 d1uj3b1 d1nbqa1 d1fh5l1 d2ck0l1 d2ywza_ d1q72l1
d4nbxb_ d1hila1 d4hzlb1 d2j88l1 d1zmya1 d1cd0a_ d1a4ka1 d1pskl1
d1a0qh1 d4krmb_ d1ncbh1 d2fbjh1 d1j8he1 d1i3ua_ d1cf8l1 d1fe8h1
d2p1ya1 d1eeqa_ d2x89a_ d1ol0a_ d2r0lh1 d1ub5a1 d1dfbh1 d3eyvb1
d4jdvb1 d4p2cg_ d2gsia1 d2agjl1 d1u3ha1 d2h1ph1 d1rihl1 d1fskc1
d1boga1 d2a77l1 d1ospl1 d1qfoa_ d3dwta_ d2fx7l1 d1rz7l1 d3stba1
d1pkqa1 d1b6da1 d1dzba2 d1dzba1 d1kj2a_ d4l1ha_ d1rzgb1 d1dqlh_
d12e8l1 d1miel1 d1jmaa_ d1sbsl1 d1fgnh1 d1f58l1 d1mqkh_ d1ndgb1
d3qegl1 d1iail1 d2aw2a1 d1tqbc1 d3fo2a1 d4dkaa_ d1f8th1 d2g60l1
d2ywya_ d1uj3a1 d1yc7a_ d3bd3a_ d4w6xb1 d1nmbl_ d1i8ka_ d1rjlb1
d1ypze1 d1p7kb1 d1kxqe_ d1gpoh1 d2z4qa1 d1w72h1 d1i9rl1 d4qgya1
d4xt1c_ d2qqnh1 d1cica1 d3c08h1 d2cd0a_ d2bdnh1 d1hnga1 d1maml1
d1nldh1 d1cr9h1 d4lcih1 d3bkyh1 d3qg7l1 d1ai1l1 d1kxvc_ d4iosd_
d1l7ih1 d2x1qa_ d1ub6b1 d3k74b_ d1ibgh1 d1teth1 d1igya1 d1hxma1
d2x6ma_ d2e7la_ d5e03a_ d1qfwm_ d2r0kh1 d1igjb1 d1rurh1 d1plgh1
d1mj8h1 d1yuhb1 d3fmgl1 d1rzfl1 d1emtl1 d1ad9b1 d1igtb1 d1nlbl1
d1d5ih1 d3k7ua_ d4ffyh_ d1bwwa_ d3dvga1 d1a6ta1 d1fgvl_ d4qxgl1
d1miml1 d1jhll_ d2hffb1 d2hh0l1 d1qkzh1 d1l7tl1 d1afvh1 d1iaii1
d2cjuh_ d1um5h1 d2ghwb2 d2ghwb1 d1kgce1 d2fgwh1 d1ikfh1 d1dvfd_
d2i24n1 d2xtjb_ d1za3b1 d2yc1a_ d2hh0h1 d2x1oa_ d1bvka_ d1rjla1
d5e7ba1 d1igta1 d1il1b1 d2fb4l1 d1mvua_ d1jptl1 d32c2a1 d1fvca_
d1iqdb1 d2hrph1 d2p4ab_ d4qo1a_ d1p2cb1 d1wt5c_ d1t4ka1 d5c1mb_
d1it9l1 d1bz7a1 d1b2wh1 d1e4wl1 d1jnll1 d1yedb1 d1za6b1 d3u7aa_

d1igfh1 d3gnml1 d1orsa1 d1wejl1 d1a2ya_ d1hxmb1 d1gafh1 d2j4wh1 d1pz5a1 d1j05a_ d1a3rh1 d4fqll1 d1qfwh_ d1eo8h1 d1n7mh1 d4s11a_ d1kegh1 d5da4a_ d1iqwl1 d3whxb1 d1vgeh1 d1deeb1 d1c12b1 d1t2ql1 d1ad9a1 d3gsnb1 d3b2uc1 d1ggbh1 d1t3fb1 d1nakl1 d4ocmc_ d1lila1 d2v7hb3 d2esve1 d1mcoh1 d2qr0b1 d2q3aa_ d1ct8a1 d1kcrl1 d2pxyb_ d2uzih_ d5c3ld_ d3durb_ d2ak4e1 d1dl7h_ d1xiwd_ d1mlba1 d1rmfl1 d1i7zb1 d1dlfl_ d2xa3a_ d5bq7b1 d1dqqb1 d3h42l1 d1egjh1 d1kcvl1 d1fe8l1 d2j6el1 d1f3rb2 d1lk3l1 d1sm3h1 d1q0xh1 d1ghfh1 d7fabl1 d1igch1 d1lo0h1 d4idla_ d5c2bl_ d1jrhl1 d1il1a1 d3zhda1 d4kfzc_ d1b0wa_ d1e6oh1 d1mexl1 d4krpb_ d1fnsh1 d1ncwh1 d4hgma_ d4eigb1 d4rzca_ d2mpal1 d4y5xa_ d5en2b1 d3lrga1 d4ej1c1 d1rhha1 d1j05b_ d1forh1 d1q9wb1 d2wzpd_ d2aq2a1 d1h0da1 d3revb1 d2ij0c1 d1tzha1 d4m7jl1 d1k6ql1 d3b9kc1 d1igml_ d3qeua1 d1dsfl_ d1ce1h1 d1kcuh1 d1zvha1 d1zvoa1 d1tqbb1 d1ay1l1 d2qscl1 d2cgrh1 d1nl0h1 d1kenl1 d3eo0a1 d4n9ob1 d1n0xl1 d3k1kc_ d2m9wa1 d3dfxa_ d2aq2b1 d1ck1a1 d1enfa1 d1i4pa1 d1v1oa1 d1et9a1 d1esfa1 d2g9hd1 d4ohja1 d1klud1 d1bxta1 d1eu3a1 d1ty0a1 d1fnua1 d2cqva1 d1fhga_ d2k1ma_ d2dl2a1 d1biha3 d1biha1 d1biha4 d3qp3a1 d1g1ca_ d1gxea_ d1cvsc2 d1cvsc1 d1xmwa2 d1m4ka2 d2otpa2 d1biha2 d1rhfa2 d2fcba2 d2fcba1 d1wwbx_ d1gsma2 d1epfa1 d1epfa2 d2dava1 d1waaa_ d1f97a2 d1x44a1 d1vcaa2 d1fnla2 d1fnla1 d3b5ha2 d3h8na2 d3h8na1 d1jbja1 d1jbja2 d1pd6a_ d1f2qa2 d1f2qa1 d3cx2a_ d1nbqa2 d3b5ha1 d1n26a1 d1xiwb_ d1xaua_ d1fltx_ d2ifga1 d1olla1 d1olla2 d1cs6a4 d1cs6a3 d1cs6a2 d1cs6a1 d2gw5a2 d1sy6a1 d1xiwa_ d1ucta2 d1ucta1 d1ry7b2 d1gl4b_ d1qz1a3 d1olza1 d2c9aa1 d1wwca_

**Family-17 (b.1.1.1)**

**Positive dataset contains 319 protein sequences.**

d1y0la1 d2gsib1 d4jfhd1 d1t2ja_ d1zvya1 d3v4vl1 d4enec1 d5e8ea1 d2pxya_ d4weud_ d1xiwc_ d2esvd1 d4n1hb_ d3ezjb1 d3mlwl1 d3cfic_ d4w68a_ d2arja1 d2ok0l1 d1vera_ d2giya1 d4m3kb1 d2nyyd1 d5dxwa_ d4fqqa1 d4m6nl1 d4hxbl1 d3b5ga_ d3quyd1 d2h9gb1 d2dd8h1 d4w6wb1 d2a6ib3 d1t66d1 d2e27h_ d2v7na1 d2xt1b1 d4i13b1 d2op4l1 d1za3a1 d4uu9a_ d4y5va_ d5anma1 d4cdgc1 d4ldeb_ d1y0lb1 d1z3gl1 d3g5xa1 d1tzib1 d4dn3l1 d3zhka1 d2b1hh1 d2uyla1 d2agjh1 d1rjca1 d4u6hb1 d3upca_ d2cjvh_ d4fhbd1 d3nn8a_ d2bmkb1 d3utza1 d4mwfb1 d4jvpa1 d4lqfl1 d2g60h3 d2jixd1 d4grwh_ d1u9ka_ d1nmbh_ d4eura1 d3sdyl1 d1w72l1 d4wenb_ d3ietc1 d4b50a_ d3r8bb_ d4rgne1 d2gsgb_ d2fath1 d2x1pa_ d1shma_ d1u0qa_ d2coqa_ d5e0qa_ d4ocxl1 d2fx7h1 d2oi9c_ d3eyva1 d4ffyl_ d3dvfa_ d1ri8a1 d3dura_ d3so3b1 d2ak4d1 d4z7wf1 d4y5ya_ d4x7ec1 d4utal1 d1u6ah1 d2dtgd1 d1xeda1 d2bnub1 d2dtgb1 d4hepg_ d2atpb1 d3k80a1 d2jb5h1 d3zkxc_ d4grwe_ d3w9ec_ d1sy6h1 d2j6eh1 d3qxta_ d1tzhb1 d4n8pa1 d4i0cc1 d4zg1a_ d3q6fa1 d3c6la1 d3p9wb_ d2uzil_ d2a9mh_ d4heme_ d3qskb_ d1sjxa_ d4ozfg1 d1sq2n_ d3qcul1 d3bn9d1 d4y8dc_ d3dvgb1 d2hwzl1 d1yjdc1 d3clfl1 d4krna_

d3r0ma_ d4kkna_ d4b41a_ d2d7tl1 d4grwf_ d4b5ea_ d3u79a_ d4u3xa_
d4wemb_ d2crya1 d3ab0b_ d1t4kb1 d4w6yb_ d2aabl1 d1tzia1 d4n1ca_
d3g9ab_ d4tvsa1 d3dx9a1 d3wxwh_ d3eaka_ d4qlra1 d2i27n2 d2orbl1
d4x7fc1 d3upaa_ d1t3fa1 d2dtgc1 d4hc1l1 d1zv5a1 d4wgvb_ d4f37k1
d1vesa_ d3zkqd_ d3rrqa_ d4orzc_ d4hgkc1 d4ozhe1 d1ypzf1 d2vyre_
d1sjva_ d2arjb1 d4aj0a_ d3nn8b_ d2rcja1 d4v1dd_ d2eksa_ d4k3ga_
d4gftb_ d1uz8b1 d4n1ea_ d3mcka1 d2vq1a1 d4aq1b1 d1uj3b1 d2ywza_
d4nbxb_ d4hzlb1 d2j88l1 d1zmya1 d4krmb_ d2p1ya1 d2x89a_ d2r0lh1
d3eyvb1 d4jdvb1 d4p2cg_ d2gsia1 d2agjl1 d1u3ha1 d2a77l1 d3dwta_
d2fx7l1 d3stba1 d4l1ha_ d3qegl1 d2aw2a1 d1tqbc1 d3fo2a1 d4dkaa_
d2g60l1 d2ywya_ d1uj3a1 d1yc7a_ d3bd3a_ d4w6xb1 d1rjlb1 d1ypze1
d2z4qa1 d1w72h1 d4qgya1 d4xt1c_ d2qqnh1 d3c08h1 d2bdnh1 d4lcih1
d3bkyh1 d3qg7l1 d4iosd_ d2x1qa_ d3k74b_ d2x6ma_ d2e7la_ d5e03a_
d2r0kh1 d3fmgl1 d3k7ua_ d4ffyh_ d3dvga1 d4qxgl1 d2hffb1 d2hh0l1
d2cjuh_ d1um5h1 d2ghwb2 d2ghwb1 d2i24n1 d2xtjb_ d1za3b1 d2yc1a_
d2hh0h1 d2x1oa_ d1rjla1 d5e7ba1 d2p4ab_ d4qo1a_ d1wt5c_ d1t4ka1
d5c1mb_ d1za6b1 d3u7aa_ d3gnml1 d2j4wh1 d4fqll1 d4s11a_ d5da4a_
d3whxb1 d1t2ql1 d3gsnb1 d3b2uc1 d1t3fb1 d4ocmc_ d2v7hb3 d2esve1
d2qr0b1 d2q3aa_ d2pxyb_ d2uzih_ d5c3ld_ d3durb_ d2ak4e1 d1xiwd_
d2xa3a_ d5bq7b1 d3h42l1 d2j6el1 d4idla_ d5c2bl_ d3zhda1 d4kfzc_
d4krpb_ d4hgma_ d4eigb1 d4rzca_ d4y5xa_ d5en2b1 d3lrga1 d4ej1c1
d2wzpd_ d2aq2a1 d3revb1 d2ij0c1 d1tzha1 d4m7jl1 d3b9kc1 d3qeua1
d1zvha1 d1zvoa1 d1tqbb1 d2qscl1 d3eo0a1 d4n9ob1 d3k1kc_

**Negative dataset contains 3167 protein sequences.**

d1xfda2 d1m32a_ d1ibja_ d1c7na_ d1iuga_ d4q75a_ d2bkwa1 d1t3ia_
d1n8pa_ d1qz9a_ d1jf9a_ d1eg5a_ d1p3wa_ d3kgwa_ d1cl1a_ d2ctza1
d2rfva_ d1gc0a_ d1cs1a_ d1d2fa_ d2ch1a1 d1qgna_ d1pffa_ d1elua_
d1u08a_ d1lc5a_ d1b5pa_ d2ay1a_ d1bw0a_ d3pd6a_ d7aata_ d2csta_
d1fg7a_ d4wlha_ d1o4sa_ d1v2da_ d1gdea_ d1m6sa_ d1v72a1 d1svva_
d1ajsa_ d1wsta1 d2f8ja2 d1lk9a_ d1vp4a1 d1xi9a1 d2gb3a1 d1yaaa_
d1xkla_ d2wfla_ d1tq5a1 d2b5aa1 d2ofya1 d4mcxa_ d2xi8a_ d2icta_
d2bnma1 d2ppxa1 d1y7ya1 d1sefa_ d1sfna_ d1rc6a_ d3lgia_ d3i77a_
d2z9ia2 d4nsva_ d1lcya2 d4jcna_ d1agja_ d1hpga_ d1p3ca_ d1l1ja_
d1qtfa_ d1ddla_ d1e57b_ d1dzka_ d4tlja_ d2a2ua_ d4wfva_ d3t1da_
d4iaxa_ d3zq3a_ d2nnda_ d1i4ua_ d1kxoa_ d1bbpa_ d1kt7a_ d1exsa_
d3s26a1 d1lf7a_ d1qfta_ d4gh7a_ d1z24a1 d1qabe_ d1xkia_ d1ew3a_
d3bx7a_ d4iawa1 d1n0sa_ d1gkab_ d3kzaa_ d2k23a_ d2l9ca1 d2joza1
d1gm6a_ d3kffa_ d3saoa1 d1e5pa_ d1yupa1 d1iiua_ d2wo3b_ d1shxa_
d1f56a_ d1bypa_ d1azca_ d1ws8a_ d3u25a_ d1cuoa_ d1id2a_ d1rkra_
d2iaac_ d4dp1x_ d1nwpa_ d2gima_ d2i7sa_ d1qhqa_ d3ehbb1 d1fftb1
d1gyca3 d1hfua2 d1kbva1 d1kbva2 d1sdda1 d1sdda2 d1gyca1 d1gyca2
d1mzya1 d2bw4a1 d1aoza1 d1aoza3 d1aoza2 d1kyaa2 d1kyaa3 d1kyaa1
d1mzya2 d4e9sa1 d1sddb1 d1sddb2 d1hfua1 d1hfua3 d1oe1a2 d1oe1a1
d1v10a2 d1v10a3 d1v10a1 g1f8v.1 d2bbvc_ d2cfba1 d1dfoa_ d1o69a_

| | | | | | | | |
|---|---|---|---|---|---|---|---|
| d1bj4a_ | d3k28a_ | d2bwpa_ | d3lg0a_ | d2fnua_ | d3ou5a_ | d1z7da1 | d1b9ha_ |
| d1iaya_ | d4msoa_ | d1bs0a_ | d1s0aa_ | d1sffa_ | d3tqxa_ | d3qboa_ | d3pgya_ |
| d1bjna_ | d2dkja_ | d1ejia_ | d1vefa1 | d4wxga_ | d3bs8a_ | d3gbxa1 | d1w23a_ |
| d1a3qa1 | d1iknc_ | d3brda1 | d1p7hl1 | d1oy3c_ | d1imhc1 | d1zk9a1 | d2cxka1 |
| d2bema_ | d1pama1 | d1ea9c1 | d1qhoa1 | d2fhfa2 | d2fhfa1 | d1k3ia1 | d1j0ha1 |
| d1cxla1 | d2bhua1 | d1edqa1 | d1f1sa2 | d1ut9a2 | d1gvia1 | d1txka1 | d1zx5a1 |
| d1qwra_ | d2phla1 | d1fxza2 | d1fxza1 | d1lrha_ | d1uija1 | g1dgw.1 | d2v09a_ |
| d1uika2 | d1uika1 | d1dgwa_ | d1ep0a_ | d2pa7a1 | d1wlta1 | d1nxma_ | d2c0za1 |
| d1ds6b_ | d1fstb_ | d4alac_ | d1pjwa_ | d1s6na_ | d3uzqb_ | d1cg2a1 | d1yloa2 |
| d1y7ea2 | d1y0ya3 | d1lfwa1 | d1vixa1 | d1fnoa4 | d1vhoa2 | d1ysja1 | d1vhea2 |
| d1z2la1 | d2fvga2 | d1vgya1 | d1r3na1 | d2grea2 | d1xmba1 | d1rtqa_ | d1ydwa2 |
| d2nvwa2 | d1zh8a2 | d1qkia2 | d1xeaa2 | d1tlta2 | d1g7ea_ | d2ovga_ | d1sq8a_ |
| d1rzsa_ | d3kz3a_ | d3orca_ | d3gpdg2 | d2q49a2 | d1hdgo2 | d2g17a2 | d2cvoa2 |
| d2czca1 | d3pwka2 | d1obfo2 | d1i32a2 | d1zyao2 | d2pkqo2 | d1b7go2 | d2hjsa2 |
| d1cf2o2 | d1vkna2 | d1nvmb2 | d2msya_ | d2djoa1 | d2r5za_ | d1au7a1 | d1f43a_ |
| d2lmda1 | d1akhb_ | d1s7ea1 | d3nara_ | d1zq3p1 | d2craa1 | d1mija_ | d4uuta_ |
| d3a01a_ | d1x58a1 | d3rkqa1 | d3cmya1 | d2cqxa1 | d2da6a1 | d1e3oc1 | d1fjla_ |
| d1mh3a1 | d1ig7a_ | d1x41a1 | d1wh7a1 | d1gt0c1 | d2mw8a_ | d1lfup_ | d1x2ma1 |
| d1b72a_ | d1wi3a1 | d1jgga_ | d1wjha1 | d1x2na1 | d1mnmc_ | d2cuea1 | d3k2aa_ |
| d2hosa_ | d2jwta2 | d1pufa_ | d1octc1 | d2hi3a_ | d1b8ia_ | d4j19a_ | d3lnqa_ |
| d1wh5a1 | d1b8ib_ | d1fexa_ | d2cqra1 | d1ug2a1 | d2cqqa1 | d2ajea1 | d4a69c_ |
| d1h8ac1 | d2crga1 | d2iw5b1 | d1xc5a1 | d1ofcx1 | d2cu7a1 | d1wgxa1 | d2cjja1 |
| d2ckxa1 | d1gvda_ | d1xg1a1 | d1itya_ | d1hlva1 | d1iufa2 | d1iufa1 | d2iu5a1 |
| d1zk8a1 | d2g7sa1 | d2fd5a1 | d2np5a1 | d1z0xa1 | d2id6a1 | d2d6ya1 | d2i10a1 |
| d1vi0a1 | d2oi8a1 | d3c07a1 | d2ns7a1 | d2fq4a1 | d2yvea1 | d1t33a1 | d2o7ta1 |
| d1rkta1 | d2np3b1 | d2g7ga1 | d2gena1 | d2fbqa1 | d2g7la1 | d2gfna1 | d2g3ba1 |
| d2jj7a1 | d2hyja1 | d1sgma1 | d1ui5a1 | d2hkua1 | d2id3a1 | d4jyka1 | d3lv8a_ |
| d1s3ga1 | d1zaka1 | d1s4qa_ | d3tvta2 | d1uf9a_ | d1khta_ | d1ckea_ | d1osna_ |
| d1p5zb_ | d4tmka_ | d3cm0a_ | d1tmka_ | d1jaga_ | d3h86a_ | d1t0hb_ | d1nn5a_ |
| d2h92a_ | d3kb2a1 | d2c95a1 | d1yj5a2 | d1kgda_ | d1ly1a_ | d2ak3a1 | d1vyva2 |
| d1deka_ | d2grja1 | d1p3ja1 | d1y63a_ | d1e4ya1 | d1s96a_ | d3iija1 | d1lvga_ |
| d1nksa_ | d1vyua2 | d4e22a_ | d1qf9a_ | d1jjva_ | d1q3ta_ | d1ki9a_ | d1lw7a2 |
| d1teva_ | d1kjwa2 | d1gsia_ | d1g3ma_ | d1j99a_ | d1t8ta1 | d1nsta_ | d1fmja_ |
| d1zrha_ | d1ls6a_ | d2a3ra_ | d1aqua_ | d2zptx_ | d3ckla_ | d1texa_ | d1q44a_ |
| d2gwha_ | d1z0ja1 | d3umra2 | d3gj0a1 | d2gaoa1 | d1yzga1 | d1x3sa1 | d1g7sa4 |
| d1puja_ | d1udxa2 | d1kk1a3 | d2o52a_ | d1jwyb_ | d1zcaa2 | d1zcba2 | d2fv8a1 |
| d2cjwa_ | d3dz8a1 | d1xzpa2 | d1vg8a_ | d4qxaa_ | d2ew1a1 | d1x1ra1 | d2bcgy_ |
| d3doea1 | d3cona_ | d3rega_ | d3wyga_ | d1upta_ | d2b6ha1 | d1zd9a1 | d1r5ba3 |
| d1n0ua2 | d1r2qa_ | d2f9la1 | d2rhda_ | d3q3jb_ | d3q85a_ | d1efma1 | d1r8qa_ |
| d2gf9a1 | d2akab1 | d1ek0a_ | d1z08a1 | d2gcoa1 | d2dy1a2 | d4g01b_ | d2cxxa1 |
| d1svia_ | d3q72a_ | d1ni3a1 | d4ac9a4 | d2erxa1 | d2bmea_ | d1wxqa1 | d2gf0a1 |
| d4ldja1 | d1zunb3 | d1d5ca_ | d4lv5b_ | d1efca3 | d1kmqa_ | d2g6ba1 | d3lrpa_ |
| d1tada2 | d2y8ea_ | d4rd4a1 | d1z06a1 | d2heia_ | d3a58b_ | d1u0la2 | d1d2ea3 |
| d1zj6a1 | d2ocba_ | d3rwoa_ | d1mr3f_ | d2e9sa1 | d4u5xa1 | d2iwra_ | d2fn4a1 |

d3bwdd_ d2nzja1 d2atva1 d2ksqa_ d3l43a_ d1mkya2 d1mkya1 d1wmsa_
d1jnya3 d2rmka_ d1f6ba_ d1f60a3 d1gota2 d1jala1 d2fh5b1 d1g16a_
d2wblc_ d1m7ba_ d1wf3a1 d1f5na2 d1t9ha2 d1z6xa1 d1z0aa1 d2fg5a1
d2a5ja1 d2bcjq2 d1s0ua3 d1ky3a_ d4y0va_ d4lhwa_ d2qmea1 d2atxa1
d1egaa1 d1ryha_ d1moza_ d1nrjb_ d2erya1 d3bfka_ d1puia_ d1fzqa_
d3j3az1 d3t5ga_ d1shza2 d3defa_ d2f7sa1 d3raba_ d3tsoa_ d3aq4a_
d2al7a1 d3sfva1 d3bc1a_ d2ncda_ d1sdma_ d3h4sa1 d2heha_ d2grya_
g3kin.1 d1br2a2 d1goja_ d1f9va_ d2mysa2 d1lkxa_ d3t0qa_ d4db1a1
d3gbja_ d1ry6a_ d1v8ka_ d1k8ka2 d1k8ka1 d3uleb1 d3i33a2 d3i33a1
d1bupa1 d1dkgd2 d1dkgd1 d4ci6a2 d1jcfa1 d4a2ba2 d2fxua1 d1yaga2
d1yaga1 d4cbua2 d1bupa2 d3vrda1 d3vrda2 d1fcdc2 d1fcdc1 d1h1oa2
d1h1oa1 d1m70a2 d1iqca2 d1iqca1 d1eb7a1 d1nmla2 d1nmla1 d1nira1
d1qksa1 d3h1jd1 d2d0oa3 d2d0oa2 d1nbwa3 d1hroa_ d4gedb_ d3zooa_
d1mz4a_ d1gu2a_ d1f1fa_ d1dvva_ d1c6ra_ d1c75a_ d1e29a_ d1gdva_
d1cnoa_ d1h32b_ d1co6a_ d2bh4x_ d2aiua_ d5cytr_ d1jdla_ d1i8oa_
d2zboa_ d3dmia_ d1ls9a_ d1wvec_ d2yk3a_ d3ph2b_ d1dw0a_ d5aura_
d1ql3a_ d1qn2a_ d1f1ca_ d4zzva_ d2xspa_ d1eg1a_ d4v20a_ d1ojja_
d4ipma1 d1gpia_ d4csia_ d3pfza_ d3ovwa_ d2yoka_ d1prtc2 d1evsa_
d1cnt1_ d1pvhb_ d1z7ca_ d1huwa_ d1bgea_ d1rhga_ d1f6fa_ d1i1rb_
d1axia_ d1etea_ d3tgxb_ d1scfa_ d2z3qa1 d3qb1a_ d1hula_ d1m48a_
d4nkqb_ d1eera_ d1v7mv_ d5e4ea_ d2o27a_ d3b5ka_ d2psma1 d4zf7a_
d3uf5a_ d4x51a_ d1d9ca_ d4ypgc1 d3oq3a_ d1lqsl_ d1n1fa_ d1au1a_
d1wu3i_ d3besl_ d3ux9a_ d2ejca_ d3muea1 d1ihoa_ d3q12a1 d4dnxa2
d1m8pa2 d1g8fa2 d1jhda2 d1x6va2 d2el7a_ d1iq0a2 d1irxa2 d1r6ta2
d1ivsa4 d2cyba_ d1rqga2 d1j09a2 d2yxna_ d1f7ua2 d1h3fa1 d1ffya3
d2g36a1 d1nzja_ d1jila_ d1n3la_ d1pfva2 d1h3na3 d3n9ia_ d3sz3a1
d3sw2a_ d1moxc_ d1dx5i3 d1edmb_ d1autl2 d1autl1 d1nqlb_ d1dval1
d1szba2 d1kigl_ d2y5fl_ d1ijqa2 d1gk5a_ d1g1sa2 d1nt0a3 d1xdtr_
d1lmja2 d1lmja1 d1nzia2 d2wphe_ d1klil_ d1pfxl2 d1pfxl1 d1p9ja_
d1g1ta2 d1f7ea_ d4yzub_ d1m1xb5 d2y5ia_ d1qlsa_ d1psra_ d3n22a_
d2egda_ d4ggfc_ d2wcea_ d2luca_ d4aqia_ d3nsia_ d2lp3a_ d1j55a_
d1a4pa_ d1k8ua_ d1nsha_ d1pvaa_ d3fs7a_ d2pvba_ d1ttxa_ d1rwya_
d2kyfa_ d1rk9a_ d1exra_ d1tiza1 d1g8ia_ d1ggwa_ d1wdcc_ d3fwba_
d1ggza_ d2o5ga_ d1ctaa_ d1m45a_ d3i5gc_ d1sl8a_ d1fpwa_ d2scpa_
d2roaa_ d4aqra_ d3pn7b_ d1br1b_ d2mysb_ d1s6ca_ d1bjfa_ d2obha_
d1jfka_ d1jf0a_ d1qv0a_ d1lkja_ d2mlfc_ d2m97a_ d2d8na_ d4by5a_
d1jbaa_ d4nqga_ d1wdcb_ d2mysc_ d1j7qa_ d1s6ia_ d1uhka2 d1r2ua_
d1dtla_ d3kf9a_ d3pn7c_ d5d67d_ d1tuza_ d1h8ba_ d1eg3a1 d1eg3a2
d2zkmx1 d1snla_ d1gjya_ d1y1xa_ d1hqva_ d1dvia_ d1df0a1 d1qxpa2
d4usla_ d1k94a_ d1wkya2 d1uuqa_ d3wh9a_ d4m1ra_ d1xyza_ d4xuya_
d2je8a5 d4bf7a1 d3cuia_ d1ur4a_ d1qw9a2 d1odza_ d2vzsa5 d1bqca_
d2x2ya1 d1hjqa_ d1qnra_ d4y7ea1 d4k68a_ d2v3ga_ d4hpga_ d1nofa2
d1nq6a_ d1h4pa_ d1aq0a_ d3ziza_ d4bs0a_ d1ecea_ d1h1na_ d1ta3b_
d1w91a2 d1i1wa_ d1uhva2 d1us3a2 d1egza_ d7a3ha_ d1tvna1 d3nyda1

d1ghsa_ d2cyga1 d4xv0a_ d2c8na2 d1w32a_ d3emca_ d1v6ya1 d1foba_
d2uwfa1 d2fgla_ d3niya1 d2c0ha1 d1rh9a1 d1yq2a5 d1szna2 d1fa2a_
d1pama4 d5do8a1 d1ea9c3 d1hx0a2 d2fhfa5 d1qhoa4 d1avaa2 d1m53a2
d4m8ua1 d2gjpa2 d1lwha2 d4howa1 d1ktba2 d1j0ha3 d3vm5a1 d2x1ia_
d1wzaa2 d1uasa2 d2diea1 d1mxga2 d1ht6a2 d1eswa_ d1iv8a2 d5cpsa_
d1ud2a2 d1zjaa1 d3gbea1 d1kwga2 d1wdpa_ d1gcya2 d1g5aa2 d1hvxa2
d3dhpa2 d2bhua3 d4do4a1 d3qgva1 d1e43a2 d1g94a2 d2d3na2 d1gjwa2
d1gvia3 d1x1na1 d1r7aa2 d1tz7a1 d4c7ga2 d2gjxa1 d1nowa1 d1yhta1
d4toqa_ d1wb0a1 d1w9pa1 d1eoka_ d1itxa1 d1kfwa1 d1goia2 d1edqa2
d2gsja_ d1hjxa1 d1jnda1 d1vf8a1 d1etob_ d1umqa_ d1e5wa2 d1j19a2
d1isna2 d2al6b2 d3syxa1 d1mkea1 d2p8va_ d1xoda1 d1i7aa_ d1evha_
d1qc6a_ d1egxa_ d2y8ga_ d1xkea1 d1k5db_ d2y8fa_ d1iuka_ d2csua1
d3q9ub_ d2yv1a1 d3q9na_ d2d59a_ d1oi7a1 d2yv2a1 d1euca1 d1y81a1
d1wgqa1 d1v89a1 d2coda1 d1zc3b_ d1wg7a1 d1u5ea1 d1v61a1 d2coca1
d2ys1a1 d1v5pa1 d1ntya2 d1wjma1 d1wi1a1 d1u29a_ d1xx0a1 d1unqa_
d2zkmx3 d1fhoa_ d1v88a1 d2adza1 d1x1fa1 d1p6sa_ d2coaa1 d1btka_
d1v5ma1 d1faoa_ d2x18a_ d1dyna_ d1x1ga1 d4kaxb_ d2j59m1 d1xcga2
d1txda2 d1u5da1 d1dbha2 d1w1ha_ d1upqa_ d1v5ua1 d1foea2 d2fjla1
d1eaza_ d2dfka2 d2cofa1 d1kz7a2 d9ldta1 d2ewda3 d1up7a1 d2dfda1
d1s6ya1 d1vjta1 d7mdha1 d1hyea1 d1t2da1 d1ojsa3 d1b8pa1 d1ldna1
d1hyha1 d1civa1 d2a92a1 d1ez4a1 d1i0za1 d1y6ja1 d1gv0a1 d1ur5a1
d1llda1 d1obba1 d1v8ba1 d1gdha1 d1l7da1 d2dlda1 d1mx3a1 d1ygya1
d1li4a1 d1j4aa1 d2naca1 d1npya1 d3t4ea2 d1a4ia1 d1o0sa1 d2hk9a2
d2egga2 d1b26a1 d1bvua1 d1gq2a1 d1c1da1 d1leha1 d1gtma1 d1euza1
d1nvta1 d3dona2 d1edza1 d1vi2a1 d1p77a1 d1nyta1 d1b0aa1 d1vl6a1
d1v9la1 d3wdpp1 d3wq5a_ d1gnxa_ d4gxpa_ d4hz8a_ d1e4mm_ d3aiua_
d1wcga1 d1qvba_ d3ahxa_ d1v02a_ d2dgaa_ d1pbga_ d1vffa1 d1qoxa_
d4ha4a_ d1kola2 d1f8fa2 d1qora2 d1jqba2 d1vj0a2 d1cdoa2 d1gu7a2
d1d1ta2 d1xa0a2 d2dm6a2 d1jvba2 d1rjwa2 d1llua2 d1h2ba2 d1tt7a2
d1piwa2 d1o89a2 d1iz0a2 d1e3ja2 d1yb5a2 d1uufa2 d1e3ia2 d3gpdg1
d1vc2a1 d2nvwa1 d1u8fo1 d1ydwa1 d1hdgo1 d2g17a1 d1cf2o1 d2czca2
d1r0ka2 d1obfo1 d2cvoa1 d1ebfa1 d1h6da1 d1i32a1 d1zh8a1 d2q49a1
d1qkia1 d3pzra1 d1xeaa1 d1vkoa1 d1vm6a3 d3qvsa1 d2pkqo1 d1b7go1
d1gr0a1 d1p1ja1 d1lc0a1 d1j0xo1 d3pwka1 d2hjsa1 d1zyao1 d1vkna1
d1tlta1 d1nvmb1 d3sjua_ d3ojfa1 d1xu9a1 d2jjya1 d1n7ha_ d1y1pa1
d1h5qa_ d1jtva_ d4fs3a_ d2bkaa1 d1gy8a_ d2c5aa1 d1ulsa_ d1ooea_
d2b69a1 d1y5ma1 d1spxa_ d1ae1a_ d1bxka_ d1ydea1 d1gega_ d1hdoa_
d1o5ia_ d1e6ua_ d1snya1 d2rhca_ d3zv4a_ d5ceja_ d2d1ya1 d1fjha_
d3w8ea_ d2fmua1 d1x1ta_ d1rpna_ d3ay7a1 d1k2wa_ d1eq2a_ d2hrba1
d3ruaa_ d2ew8a1 d4hwka_ d1xgka_ d2bd0a1 d1yo6a1 d2a35a1 d2ntva_
d1vl8a_ d1hdca_ d4bkua_ d2ag5a1 d2q45a_ d1nffa_ d4iy1a_ d1zbqa1
d1kewa_ d1ek6a_ d1ja9a_ d2q46a2 d1iy8a_ d1cyda_ d2ptga_ d1hxha_
d1uzma1 d3lyla_ d4m89a_ d3wyea_ d2blla1 d1w6ua_ d1sb8a_ d2q2va_
d4nr0a_ d1e6wa_ d2gasa_ d3oiga1 d2bgka1 d1mxha_ d1vl0a1 d1rkxa_

| | | | | | | | |
|---|---|---|---|---|---|---|---|
| d1r6da_ | d1wmaa1 | d1e7wa_ | d2c07a1 | d1xkqa_ | d3tzha_ | d1g0oa_ | d3lz6a_ |
| d1qyca_ | d1edoa_ | d1gz6a_ | d1oc2a_ | d1t2aa_ | d1zema1 | d2xoxa_ | d1z45a2 |
| d1geea_ | d1p33a_ | d3lt0a_ | d4e6pa_ | d2wyua_ | d1db3a_ | d4q9na_ | d1xhla_ |
| d4o1ma_ | d1fmca_ | d2uvda_ | d2gdza1 | d1uaya_ | d2z1ma_ | d2o23a2 | d2fr1a2 |
| d2fr1a1 | d1qsga_ | d1qyda_ | d4zjua_ | d2a4ka1 | d1yb1a_ | d1wvga1 | d1yxma1 |
| d3f4ba_ | d1mg5a_ | d1xg5a1 | d2gfoa1 | d1vjva_ | d2ayna1 | d3nhea_ | d1cmxa_ |
| d1xd3a_ | d3ifwa_ | d1mdwa_ | d1ziva1 | d1zcma1 | d1t3ca_ | d1epwa3 | d2a97a_ |
| d3fiea_ | g1e1h.1 | d3ioqa_ | d1fh0a_ | d1o0ea_ | d1khqa_ | d1m6da_ | d2cb5a_ |
| d1cs8a_ | g8pch.1 | d1s4va_ | d2pnsa_ | d1vsna1 | d1mira_ | d1iwda_ | d2oula_ |
| d7pcka_ | d3bwka_ | d3bcna_ | d2p86a_ | d1pvja_ | d1pcia_ | d1gece_ | d1deua_ |
| d4bs6a_ | d1xkga1 | d1cqda_ | d1jqpa2 | d2c0ya_ | d2bdza_ | d3p5ua_ | d1ex4a2 |
| d1c6va_ | d2jk6a2 | d2jk6a1 | d1feca1 | d1feca2 | d1lpfa1 | d1lpfa2 | d1xhca1 |
| d1xhca2 | d1dxla2 | d1dxla1 | d1gera2 | d1m6ia2 | d1onfa2 | d3lada2 | d3lada1 |
| d1w4xa1 | d1w4xa2 | d1gesa2 | d1mo9a1 | d1xdia1 | d1fl2a2 | d1fl2a1 | d1fcda1 |
| d1fcda2 | d1h6va2 | d1h6va1 | d1mo9a2 | d1gv4a2 | d1gv4a1 | d2gv8a2 | d2gv8a1 |
| d1aoga1 | d1aoga2 | d1hyua1 | d1ebda1 | d1ebda2 | d3dk9a1 | d1q1ra2 | d1q1ra1 |
| d1gesa1 | d1g4ma1 | d1g4ma2 | d1ps9a2 | d1o94a2 | d1lqta1 | d1pn0a1 | d1reoa1 |
| d1ju2a1 | d2f5va1 | d3c96a1 | d2gmha1 | d1pj5a2 | d1gpea1 | d1n4wa1 | d1k0ia1 |
| d2dw4a2 | d2ivda1 | d1seza1 | d2voua1 | d1kdga1 | d1s5ja1 | d2hbja2 | d2igia_ |
| d1wlja_ | d1qhta1 | d4dlea1 | d1tgoa1 | d2f96a1 | d3tr8a1 | d3mxma_ | d1q8ia1 |
| d1y97a1 | d1w0ha_ | d1kfsa1 | d2py5a1 | d1noya_ | d1d5aa1 | d1yt3a3 | d2guia_ |
| d3mwpa1 | d4kb1a_ | d1xl4a1 | d3vsqa_ | d2gixa1 | d1p7ba1 | d1b9ra_ | d1offa_ |
| d1frra_ | d3wcqa_ | d1e9ma_ | d1l5pa_ | d1wria_ | d1i7ha_ | d3av8a_ | d1czpa_ |
| d1rfka_ | d3p1ma1 | d3b2ga_ | d2bt6a2 | d2av4a1 | d3nvva1 | d1krha3 | d2h88b1 |
| d1n62a2 | d1t3qa2 | d1kf6b2 | d1zoyb1 | d1jq4a_ | d1dgja2 | d1rm6c2 | d1jmxa2 |
| d1jmxa1 | d1pbya2 | d1r7ha_ | d1faaa_ | d1wika1 | d1xfla_ | d1ttza1 | d2f51a1 |
| d1ti3a_ | d1nhoa_ | d1sena_ | d2i4aa_ | d1ep7a_ | d1zmaa1 | d1fo5a_ | d3lefa1 |
| d3diea1 | d2yana1 | d1z6na1 | d1oaza_ | d3zywa1 | d4aj8a_ | d1nm3a1 | d4fiwa_ |
| d1wmja_ | d4hs1a1 | d1iloa_ | d1xwaa1 | d1wjka1 | d2vm1a_ | d1fb6a_ | d1gh2a_ |
| d1h75a_ | d1nw2a_ | d1kx5b_ | d3a6na_ | d4m6ba2 | d2nqbd_ | d3wkjd_ | d2yfwe_ |
| d4h9na_ | d5bnxa_ | d1kx5c_ | d3r45a_ | d1eqzb_ | d4jjnb_ | d1f66c_ | d1eqza_ |
| d4wnnb_ | d1kx5a_ | d1u35c1 | d1id3a_ | d2nqbc_ | d1kx5d_ | d1ku5a_ | d1f1ea_ |
| d2b5ea2 | d2b5ea3 | d2b5ea1 | d2b5ea4 | d2kp1a1 | d2kp2a1 | d1hyua3 | d1hyua4 |
| d1j08a1 | d1pd212 | d1nhya2 | d2a2ra2 | d1k0dd2 | d1f2ea2 | d1gsua2 | d1f3ba2 |
| d2fhea2 | d2pvqa1 | d1tw9a2 | d1oyja2 | d1e6ba2 | d3ik7a1 | d4is0a1 | d1r5aa2 |
| d1jlva2 | d1m0ua2 | d3gtub2 | d1z9ha2 | d1okta2 | d1axda2 | d2gsra2 | d1tu7a2 |
| d1k3ya2 | d1zgma1 | d1b48a2 | d1gnwa2 | d1gwca2 | d2gsta2 | d1glqa2 | d1oe8a2 |
| d1pn9a2 | d1v2aa2 | d1fw1a2 | d1duga2 | d3zxha_ | d1cgla_ | d1rm8a_ | d1eaka2 |
| d1hy7a_ | d2jsda_ | d1cxva_ | d1hv5a_ | d2lj7a_ | d2m8bb1 | d2kyqa_ | d3psma_ |
| d1jkza_ | d1jxca_ | d2gl1a_ | d1ugla_ | d4uj0a_ | d174la_ | d169la_ | d2o4wa_ |
| d1p37a_ | d1k28a3 | d1xjta1 | d1jtma_ | d2f2qa_ | d4e97a1 | d2za0a_ | d2c21a1 |
| d1f9za_ | d2pjsa1 | d1klla_ | d2i7ra1 | d1ecsa_ | d1xqaa1 | d5cj3a1 | d1npba_ |
| d1jifa_ | d2p7oa_ | d1r9ca_ | d1nkia_ | d1mpya2 | d1kw3b2 | d1kw3b1 | d4ghga1 |
| d1tfza1 | d3isqa1 | d3isqa2 | d1sqia1 | d1sqia2 | d1lkda2 | d1lkda1 | d1cjxa2 |

d1cjxa1 d1sp9b2 d1t47a1 d1t47a2 d1f1ua2 d1sp8a1 d1sp8a2 d1f1ua1
d1s5ja2 d1qhta2 d4dlea2 d1tgoa2 d3py8a2 d1q8ia2 d1kfsa2 d2py5a2
d1d5aa2 d1qsta_ d2beia1 d4m3sa_ d1u6ma1 d1ygha_ d1yk3a1 d3dr6a_
d1xmta_ d1z4ea1 d2ge3a1 d1bo4a_ d2fiaa1 d2jdca1 d1y9wa1 d3to7a_
d1wwza1 d2fl4a1 d2ozua1 d2b5ga_ d2cy2a1 d2p0wa_ d1cm0a_ d4pswa_
d1yrea1 d1yr0a1 d1m4ia_ d2gana1 d1yvka1 d1ghea_ d2g3aa1 d1y7ra1
d2euia1 d1q2ya_ d2fsra1 d1xeba_ d2ae6a1 d1cjwa_ d1qsma_ d2aj6a1
d1y9ka1 d1i12a_ d1vkca_ d2atra1 d1n71a_ d2d4pa1 d2fiwa1 d2fe7a1
d1tiqa1 d2ou2a_ d1yx0a1 d2fcka1 d1p0ha_ d2j8ma_ d2pq8a_ d1z4ra1
d1s3za1 d1ufha_ d2nqya_ d1h4ga_ d1hixa_ d1oa2a_ d1olra_ d3b7ma_
d4h7ma1 d3wp3a_ d2vgda_ d1igoa_ d3zsea_ d2nlra_ d3wx5a_ d1oa3a_
d1pvxa_ d1m4wa_ d1f5ja_ d1oa4a_ d1ks5a_ d4ixla1 d1xvqa1 d1pn0a2
d3hy2a_ d2cvba1 d1o8xa_ d1lu4a_ d1we0a1 d2bmxa1 d2a4va1 d1psqa_
d1xiya1 d1q98a_ d1n8ja_ d1zofa1 d2ggta_ d3tjja_ d4je1a1 d1o73a_
d3s9fa_ d2zcta_ d1qmva_ d1jfua_ d2b1ka_ d5dvba1 d2b5xa1 d2v2ga_
d1st9a_ d3tb2a_ d4llra_ d4fh8a1 d1e2ya_ d1knga_ d2h30a_ d1i5ga_
d2z9sa_ d2f8aa1 d1tp9a1 d2b7ka_ d1nm3a2 d3hvva_ d2wfca1 d4kcea_
d1xvwa1 d4gqca1 d3qpma_ d4k1fa_ d1zzoa1 d1gp1a_ d1prxa_ d4od7a1
d1z6ma1 d4mcua1 d4dvca1 d1r4wa_ d3l9sa_ d1un2a_ d3rppa_ d1rmka_
d1ixta_ d1eyoa_ d1cnna_ d1g1pa_ d1f3ka_ d1fyga_ d2m5ta_ d1cqqa_
d1wqsa_ d5bpea_ d2ipha_ d1lvoa_ d2bhga1 d3tnta_ d3sjia_ d3d23a1
d2fyqa_ d3tloa_ d3zvea1 d2hrva_ d3q3ya1 d4asha1 d1e88a1 d1l6ja5
d1l6ja4 d1eaka4 d1eaka3 d1l6ja3 d1h8pa2 d1etha2 d1w52x1 d2ppla1
d1hpla2 d1lpbb2 d2oxea1 d1bu8a2 d1rp1a2 d1ys1x_ d4hs9a_ d1ex9a_
d1ku0a_ d3qmma_ d1dy2a_ d3groa_ d1pjaa_ d1thta_ d1ra6a_ d1muka_
d3gsza_ d1khva_ d1te9a_ d2xwha_ d3uqsa_ d3hkwa_ d3n6ma_ d2cjqa_
d2xyma_ d1teba_ d1te8a_ d1s48a_ d1u09a1 d4wfxa_ d3tyqa_ d2pgga1
d1xwdc1 d3kj4a_ d1w8aa_ d2ifga3 d1p9ag_ d1xkua_ d4dv8a2 d2bk7a_
d3fjua_ d1z5ra1 d1kwma1 d1uwya2 d1jqga1 d2c1ca1 d1dtda_ d1h8la2
d1ohfc_ d2tbvc_ d1c8nc_ d1x36a_ d1smvc_ d1ng0c_ d4sbvc_ d1opoa_
d1f2nc_ d1cixa_ d1y29a_ d1v7fa_ d2jtba_ d1d1ha_ d1g9pa_ d1lupa_
d1qk7a_ d1mb6a_ d2a2va_ d1niya_ d1i25a_ d1hvwa_ d2mpqa_ d1ie6a_
d1la4a_ d2mxoa1 d1dl0a_ d1koza_ d1qk6a_ d1emxa_ d1lqra_ d1c6wa_
d1js9c_ d1za7a_ d1laja_ d1rypd_ d1irua_ d1rypc_ d3unfk_ d1rypa_
d1j2pa_ d1irui_ d1j2qh_ d3unfh_ d1m4ya_ d1rypb_ d1irul_ d1irug_
d1iru2_ d1q5rh_ d1rypg_ d1g3ka_ d1iruf_ d1q5qa_ d1e94a_ d1irub_
d1ryph_ d3ty6a_ d1iruj_ d1iruk_ d1rype_ d1irud_ d1irue_ d3mi01_
d1rypf_ d1iruh_ d2z3ba_ d1iruc_ d3mka2_ d1iru1_ d1ny721 d1ny722
d1a6ca3 d1a6ca2 d1a6ca1 d1pgl22 d1pgl11 d1ny711 d1mqta_ d1d4m3_
d1fmd3_ d2mev1_ g1aym.1 d1ev13_ d4q4w1_ d1r1a3_ d2x5ia_ d1rhi1_
g1r1a.1 g1d4m.1 g1rhi.1 d1b35b_ d1oopa_ d1tme1_ d4iv1a_ g1pvc.1
g1fpn.1 d1fpn1_ d4gb31_ d1d4m1_ d1fmd1_ d1ev11_ d2wzr3_ g1cov.1
d1qqp3_ g1fmd.1 d2x5ic_ d1rhi3_ g1oop.1 d1hxs1_ d1fpn3_ d1bev1_
g2mev.1 d1eah3_ d1aym1_ d1pvc3_ d1qqp1_ d1zba3_ d1h8ta_ d3vbhc_

g1b35.1 g1bev.1 d1hxs3_ g1ev1.1 d1bev3_ g1tmf.1 d1tmf1_ d1b35a_
d2mev3_ g1eah.1 g1h8t.1 d1eah1_ d4q4w3_ d1r1a1_ d1aym3_ d1pvc1_
d1zba1_ d1h8tc_ d1oopc_ d1tme3_ g1qqp.1 d4iv1c_ g1tme.1 d1y14b1
d2nn6i1 d1i94l_ d2zjra2 d1smxa_ d2qamc2 d1hh2p1 d2r7da1 d1rl2a2
d1wfqa1 d3bzka4 d1x6oa2 d3a0ja_ d2j01d2 d2ja9a1 d4ql5a_ d1hr0w_
d1luza_ d2n3sa_ d2vnud1 d1h95a_ d1go3e1 d2nn6h1 d1ne3a_ d2mo1a_
d1iz6a2 d1jjga_ d1e3pa2 d2ix0a3 d2ix0a2 d2lxja_ d1ueba3 d1ueba2
d2z0sa1 d1u0la1 d2ix0a1 d2eifa2 d2je6i1 d3i2za1 d2c35b1 d2ahob2
d1t9ha1 d3i4oa_ d2qall1 d1jt8a_ d2nn6g1 d1g6pa_ d3cama_ d1i94q_
d1c9oa_ d1d7qa_ d2ba0a1 d2vnud2 d2vnud3 d1khia2 d2qalq1 d1wi5a1
d1xtda2 d1ny4a_ d4w9ta_ d1jvna1 d4x2ra1 d1ka9f_ d1qo2a_ d4axka1
d1thfd_ d4u28a_ d2y88a_ d4wd0a_ d1vzwa1 d1eixa_ d3exra_ d2ffca1
d2fdsa1 d3n3ma1 d2czda_ d2yyua_ d1vqta1 d1dbta_ d3ldva1 d4lt9a1
d3tc7a1 d3hoja_ d1i4na_ d3vnda_ d3nl8a1 d1geqa_ d3nava1 d1v5xa_
d4a2ra1 d3tc6a1 d1ujpa_ d4ijba_ d1xcfa_ d1vc4a_ d1rd5a_ d1qopa_
d3b5va_ d2r80b_ d1cqxa1 d1cg5a_ d1gcvb_ d3boma_ d2h8fb_ d2raoa_
d3bj1a_ d4hrrb_ d2r50a_ d3d1kb_ d2aa1b1 d3zhwa_ d3hf4b_ d3gdja_
d1jebb_ d3bj1b_ d1x9fb_ d1wmub_ d1gcva_ d3bcqb_ d3d4xb_ d1gvha1
d2dc3a_ d3hf4a_ d4yu3a_ d3k8ba_ d1qpwb_ d2r80a_ d3bcqa_ d1v4wa_
d3wtgb_ d1jl7a_ d4esaa_ d1a9we_ d3d1ab_ d1fsla_ d4h2la_ d1v4wb_
d1mwca_ d1vhba_ d1jeba_ d1spgb_ d1h97a_ d1q1fa_ d1tu9a1 d3d1aa_
d1hdsb_ d3hyub_ d2zfbb_ d3fs4a_ d1oj6a_ d1shrb_ d4h2lb_ d4yu3b_
d1spga_ d3hrwa_ d3qqra_ d3at5a_ d4wchd_ d3hyua_ d1cg5b_ d1uc3a_
d1hbra_ d2raob_ d1outb_ d4esab_ d3gdjb_ d3qqqa_ d1x9fc_ d3vrfa_
d1itha_ d3mkba_ d3d4xa_ d3mjpa_ d1jl6a_ d3fh9a_ d1outa_ d2dc3b_
d1it2a_ d3eoka_ d3g46a_ d1qpwa_ d3vrfb_ d1ch4a_ d1x9fd_ d1hdsa_
d2zfba_ d3mkbb_ d1or4a_ d3wtga_ d1a4fa_ d3fh9b_ d3bomb_ d3cy5a_
d3at5b_ d1x9fa_ d2oifa_ d4lmxb_ d1cpcb_ d3v57a_ d1kn1a_ d1cpca_
d4f0ta_ d2vjha_ d4po5b_ d1eyxa_ d1alla_ d4f0tb_ d1b33b_ d1b8db_
d4h0mb_ d1b33a_ d2vjhb_ d4lm6b_ d2vmlb_ d1allb_ d2vjta_ d3dbja_
d2vmla_ d2vjtb_ d4f0ub_ d1f99b_ d4rmpa1 d1liaa_ d1eyxb_ d2uulb_
d4lmsb_ d4f0ua_ d2bv8a_ d1f99a_ d2bv8b_ d2c7lb_ d3dbjb_ d4rmpb1
d1b8da_ d4h0ma_ d3v57b_ d1kn1b_ d1liab_ d2uula_ d2j96a_ d1f00i3
d1azwa_ d1mtza_ d1k8qa_ d2xqfa_ d1ea5a_ d1f6wa_ d1dx4a_ d1qe3a_
d1k4ya_ d4qwwa_ d2ha2a_ d1sfra_ d1f0na_ d4qeka_ d3c8da2 d2pbla1
d1jjia_ d1lzla_ d1jjfa_ d1gl9b1 d1gkub1 d1jmxa4 d1jmxa3 d1pbya4
d1pv8a_ d1w5qa_ d2c1ha_ d1l6sa_ d1dosa_ d1gvfa_ d3qm3a1 d2fjka_
d1f74a_ d3l21a_ d2e1da_ d1to3a1 d1w3ia_ d1sfla_ d1n7ka_ d1vcva1
d4imea_ d1wa3a_ d2ocza1 d1f2ja_ d3m16a_ d2yxga_ d1ub3a_ d3a5fa_
d1fbaa_ d1l6wa_ d1xfba1 d1wx0a1 d1vpxa1 d1ojxa_ d1mxsa_ d2a4aa1
d1xkya1 d3cq0a1 d3s1xa_ d1f05a_ d1fdja_ d3cpra1 d4d2ja_ d4mh8a_
d1mu2a2 d4i7ga1 d3rp2a_ g1ucy.1 d1fuja_ d2zchp_ d3w94a_ d1si5h_
d1lo6a_ g2pka.1 d1cgha_ d1kigh_ d1a5ia_ d3s9ba_ d1hj8a_ d1ao5a_
d2anya_ d1q3xa1 d1gvza_ d1azza_ d4gsoa_ d2f91a1 d1iaua_ d1gdna_

d1eufa_ g1fiw.1 d1eq9a_ d4nfea_ d2rdla_ d1spja_ d1elva1 d1a7sa_
d1ekbb_ d1pfxc_ d1yc0a_ d2aiqa_ g1fiz.1 d4k60a_ d4dgja_ d1mzaa_
d1op0a_ d3tjva_ d1pytd_ d1sgfg_ g1gg6.1 d2eeka_ g1rtf.1 d1a0ja_
d3g01a_ d1pytc_ d4e7na_ d1ddja_ d1z8ga1 d2hlca_ d1npma_ d1brup_
g1jou.1 d1sgfa_ d1autc_ g2hnt.1 g1kdq.1 d1orfa_ d3s69a_ d4crga_
d1fxya_ d1bqya_ d1svpa_ d1mbma_ d3su6a1 d1cu1a1 d4agka_ d1vcpa_
d1q31a_ g1n1l.1 d3e90b_ d1ep5a_ d3u1jb_ d1jx4a2 d3gv5b1 d1t94a2
d3bq0a2 d4lu0a_ d3unda1 d3e9aa1 d1vr6a2 d1n8fa_ d2qkfa_ d1d9ea_
d1o60a_ d1e69a_ d1mv5a_ d3g60a4 g1ii8.1 d1ewqa2 d1ji0a_ d1qhla_
d1v43a3 d2wmfa1 d1vpla_ d4a82a2 g1f2t.1 d2d62a1 d3tuic1 d2bbsa1
d3g60a3 d2ixea_ d1g6ha_ d1sgwa1 d2qi9c_ d3rlfa1 d3b60a1 d3d31a2
d2pzea1 g1xew.1 d1jj7a_ d1oxxk2 d2onka1 d1w1wa_ d1iwea_ d1qf5a_
d1vmaa2 d1nija1 d1np6a_ d2p67a1 d3ihla_ d1okkd2 d1ls1a2 d1s1ma2
d1iona_ d2v40a_ d1dj3a_ d3hida_ d2vo1a1 d2g0ta1 d1vcoa2 d1j8mf2
d1g3qa_ d1xjca_ d1p9ba_ d1qzxa3 d1ihua2 d1ihua1 d2qm8a_ d1dj2a_
d4ioja_ d1cp2a_ d1yrba2 d1hyqa_ d3gqba2 d1skye3 d1xp8a1 d1tf7a2
d1tf7a1 d1ye8a1 d1skyb3 d2qe7d2 d4blta_ d4huta_ d3ldaa2 d4hyya_
d2i3ba1 d1cr1a_ d3j9tb2 d3ntua2 d1vdza1 d2z43a_ d1e9ra_ d1nlfa_
d3ziaa2 d1n0wa_ d1g6oa_ d3ziad2 d3j9ta2 d1pzna2 d3gqbb2 d2i49a_
d1nnfa_ d1xvxa_ d1ursa_ d1h3da1 d1iuda_ d4m1va1 d1twya_ d3e13x_
d2nxoa1 d4pfua_ d3ttma_ d1elja_ d2qmwa1 d1us5a_ d1y3na1 d1jeta_
d1uqwa_ d4f19a1 d2esna2 d3thia_ d3fasa1 d2uyeb_ d2xwva1 d1r9la_
d2fyia1 d1pb7a_ d2ozza1 d1sw5a_ d1nh8a1 d1vr5a1 d2g29a_ d1xs5a_
d3lswa_ d2z23a_ d3w9va_ d3tcha_ d1q35a_ d1z7me1 d3q27a_ d1p99a_
d1ve4a1 d2i6ea1 d4mcoa1 d4n8ya_ d1eu8a_ d1zbma1 d1hsla_ d1ixca2
d1j1na_ d1xvya_ d1y9ua_ d2z22a1 d1xoca1 d4oera_ d1wdna_ d5f1qa1
d1xt8a1 d1i6aa_ d4gwoa_ d4igta1 d1pc3a_ d4mmpa1 d1o63a_ d2czla1
d2onsa2 d4mija1 d4ok5a1 d2bmfa1 d2bmfa2 d1yksa1 d1yksa2 d1jr6a_
d2qeqa1 d2jlqa1 d1a1va2 d1eh3a_ d3skpa_ d1dtza1 d1dtza2 d1ieja_
d1jnfa2 d1ce2a1 d1jnfa1 d1b1xa2 d1b1xa1 d1jw1a1 d1jw1a2 d1ryoa_
d1h76a1 d1h76a2 d1c4oa1 d2p6ra3 d2p6ra4 d1oywa2 d1oywa3 d1wrba1
d1w36b2 d1w36b1 d1c4oa2 d3bora_ d1t6na_ d2eyqa5 d2eyqa3 d2eyqa2
d2zu6a2 d1veca_ d1uaaa1 d1w36d1 d2g9na1 d1z3ix2 d2rb4a_ d1hv8a1
d1hv8a2 d1t5ia_ d1w36c2 d2j0sa2 d2j0sa1 d1fuka_ d3hjha1 d1z5za1
d1qdea_ d1z63a1 d1s2ma2 d1s2ma1 d1wp9a1 d1wp9a2 d1gm5a3 d1gm5a4
d3dina4 d3dina3 d1q0ua_ d1w36c1 d1z3ix1 d1nkta3 d1nkta4 d1uaaa2
d1w36d2 g1qhh.1 d2fwra2 d2fwra1 d3q3ma_ d1za0a1 d2inca_ d1jk0a_
d2incb_ d1mhyb_ d3hf1b_ d1mtyb_ d1r2fa_ d2uw1a_ d1mhyd_ d3n37a_
d1afra_ d1oquc_ d1syya_ d1mtyd_ d2o1za_ d1uzra_ d1smsa_ d2fkza_
d1zuja1 d1s3qa1 d2fjca1 d1z6om1 d1veia_ d4dyya_ d1jiga_ d3gvya_
d2pyba_ d4cy9a1 d3bvfa1 d1r03a_ d2cf7a_ d1nf4a_ d1zs3a1 d4iwka_
d3ka8a_ d3np0x_ d1n1qa_ d1lb3a_ d4e6ka_ d1z6oa1 d2fzfa1 d4dyua_
d3ge4a_ d1jgca_ d4am4a_ d2z6ma_ d1ji5a_ d1vlga_ d1lkoa1 d1krqa_
d3qz3a_ d1o9ra_ d2ffxj_ d1euma_ d1yv1a1 d2v2pa_ d1j30a_ d4di0a_

d1dpsa_ d1oewa_ d2qzxa_ d2asia_ d1psoe_ d1miqa_ d1smra_ d2h6ta_
d1pfza_ d1izda_ d3hd8a_ d1j71a_ d1dpja_ d4aa9a_ d1t6ex_ g1b5f.1
g1lya.1 g1htr.1 d1qdma2 d1bxoa_ d1wkra_ d1tzsa_ d2qzwa_ d2v76a_
d1wj1a1 d1aqca_ d1qqga1 d1ntva_ d1n3ha_ d4dbba_ d3f0wa1 d1p3ra_
d1wgua1 d2cy4a1 d3hqca_ d1wvha1 d1xr0b1 d1bdqa_ d3ggta_ d3ixoa_
d1mrxa_ d2haha_ d1tw7a_ d4njsa_ d1sgua_ d2p3da_ d1nsoa_ d3d3ta_
d2rkfa_ d1c6ya_ d2rspa_ d3ogpa_ d3ekwa_ d2o4na_ d2j9ja_ d1baia_
d3ggua_ d3lzua_ d4npua_ d4j55a_ d2r8na_ d2fxda_ d2p3ca_ d4dqea_
d3ttpa_ d3t3ca_ d2p3aa_ d2hc0a_ d2fdda_ d3mwsa_ d1wgda1 d1wgga1
d4q5eb_ d1wjna1 d1se9a_ d3n3kb_ d1wz0a1 d1we6a1 d2m0xa_ d2gbka_
d3rt3b1 d1wh3a1 d1sifa_ d2ekec2 d4ii2b1 d4hk2a1 d1m94a_ d1wgha1
d1ttna1 d1j8ca_ d1wx8a1 d3v6cb1 d2ma9b_ d4i6lb1 d1bt0a_ d2jvca1
d1iyfa_ d2k1fa_ d1wxva1 d1wf9a1 d1t0ya_ d2io0b2 d1x1ma1 d2gbma_
d1zw7a1 d2m17a_ d1ud7a_ d1we7a1 d2lwpa_ d2gbja_ d1wjua1 d1v6ea1
d1v86a1 d4eewa_ d1wiaa1 d4icva_ d4gu2a_ d1wx7a1 d1wy8a1 d4hjka1
d2faza1 d1v5oa1 d2al3a1 d2cr5a1 d1wj4a1 d1s3sg_ d3ubra_ d1qo8a1
d1d4ca1 d2k3va_ d1ft5a_ d1qdba_ d1m1qa_ d2j7aa_ d1h21a_ d1ogyb_
d4fasa_ d1jnia_ d1oaha_ d1sp3a_ d1yvca1 d1yeza1 d1g66a_ d5ajha1
d2ftba_ d1pmpa_ d2rcqa1 d1fdqa_ d4lkpa_ d1sa8a_ d1vyfa_ d1lpja_
d3vg7a1 d4azqa1 d1o8va_ d5bvqa_ d1p6pa_ d2qo4a_ d1opba_ d1yiva1
d1o1va_ d4bvma1 d4tkba_ d4i3ba_ d1bwya_ d1ggla_ d4gkca_ d1cbia_
d3p6da1 d1kqwa_ d2a0aa1 g1y43.1 d1um8a_ d1ixza_ d1qvra2 d1qvra3
d1qzma_ d1sxjc2 d1jr3d2 d2p65a_ d1l8qa2 d1g8pa_ d3hu3a3 d1sxjd2
d1njfa_ d1in4a2 d1sxja2 d1tuea1 d2ce7a2 d1w5sa2 d1sxjb2 d1d2na_
d1ny5a2 d2gnoa2 d2a5yb3 d1fnna2 d1u0ja1 d1lv7a_ d1iqpa2 d2fnaa2
d1ixsb2 d4gita_ d1sxje2 d1g41a_ d2e3fa_ d1mm0a_ d2e3ea_ d2llda_
d2ny8x_ d2e3ga_ d2ny9x_ d2nz3a_ d3ia2a_ d4dgqa1 d1hkha_ d1zoia_
d2k4ua_ d1hp2a_ d2lu9a_ d1baha_ d2meoa1 d1wm7a_ d1hlya_ d1n8ma_
d1jlza_ d1wpda_ d1c55a_ d1pvza_ d1wt7a_ d1rjia_ d1wz5a_ d1m2sa_
d1pjva_ d1c49a_ d1qkya_ d2ktca1 d1wmta_ d1du9a_ d1j5ja_ d1r1ga_
d1t0za_ d2i61a_ d1t1ta_ d1sn4a_ d1kv0a_ d2lkba_ d1npia_ d1wwna_
d1pe4a_ d2kbka_ d2li7a_ d1djta_ d2kbha_ d1dq7a_ d1sega_ d1omya_
d1zywa1 d1jzaa_ d2jcqa_ d1n9wa1 d1bbua1 d1e1oa1 d1c0aa1 d1l0wa1
d3a74a1 d1eova1 d1t61c1 d1iyjb4 d1iyjb5 d1iyjb3 d1o7ia_ d4ipca1
d1x3ea_ d4apva_ d1jb7a1 d1jb7a2 d1jb7a3 d3kdfa_ d1ue1b_ d1wjja1
d3eiva_ d1xjva2 d1xjva1 d2ccza2 d1se8a_ d1l1oc_ d1jb7b_ d1qvca_
d3ulla_ d1qzga_ d1miua4 d1miua5 d1miua3 d3g48a1 d1gd7a_ d1mkha_
d1jjcb3 d1pyba_ d3ubub_ d1egia_ d1qo3c_ d1v7pa_ d1tdqb_ d3ubua_
d1c3ab_ d1h8ua_ d3bx4b_ d1t8ca1 d1sb2a1 d1qdda_ d2ziba1 d3cada_
d1byfa_ d1j34b_ d1jwia_ d1jzna_ d1c3aa_ d1oz7b_ d1wmza_ d3bdwb_
d1hq8a_ d1fifa1 d1dv8a_ d3hupa_ d1wk1a1 d1umra_ d2afpa_ d3bx4a_
d1gz2a_ d1sl6a_ d1v7pb_ d3g8ka_ d1ixxa_ d1j34a_ d1jwib_ d1g1sa1
d1y17a1 d1ja3a_ d1fvub_ d3bdwa_ d1sb2b1 d1fvua_ d1oz7a_ d4m17a_
d3cfwa1 d1ypqa1 d1g1ta1 d3fd4a_ d3l9jc_ d4dn8a1 d1v4lb_ d1rdl1_

d2msba_ d1dgsa2 d1fvia1 d1ckma1 d1x9na2 d2a1ka_ d1je5a_ d1rpma_ d2shpa1 d1yfoa_ d1l8ka_ d2ooqa1 d1g4us2 d1lara2 d1lara1 d1wcha_ d2fh7a2 d2fh7a1 d1ohea2 d2f4da_ d1rxda_ d2hxpa_ d1xria_ d1d5ra2 d1fpza_ d3ezza_ d4y2ea_ d1i9sa_ d3azya_ d3wvja_ d1upsa1 d1ajoa_ d2hyka_ d1ajka_ d2vy0a_ d1o4ya_ d3o5sa_ d3d6ea_ d3atga_ d1dypa_ d1umza_ d3i4ia_ d1c1la_ d4lbqa_ d1slta_ d4no4a_ d3zsja_ d3duia_ d1g86a_ d1is3a_ d1qmja_ d1hlca_ d1tdha3 d1q3ba3 d1k82a3 g1qmo.1 g1len.1 d3zyra_ d1fnya_ d1n47a_ g2ltn.1 d1avba_ d3usua_ d1dhkb_ d1f9ka_ d1ukga_ d1g8wa_ d1g9fa_ d3wcsa_ d1g7ya_ g1loe.1 d1mvqa_ d1hqla_ d1v6ia_ d1dbna_ d1qnwa_ d1fx5a_ d4l8qa_ d1ioaa_ d3n35a_ d1epwa1 d3h33a_ d1rwja_ d19hca_ d1os6a_ d3bxua_ d3h4na_ d1i77a_ d3caoa_ d1j0pa_ d2ctha_ d1ofwa_ d2e84a_ d1hh5a_ d1gyoa_ d3h34a_ d1dszb_ d1a6ya_ d3cbba_ d1ynwa1 d1r4ia_ d1hcqa_ d2hana_ d2hanb_ d2enva1 d1kb4b_ d2nllb_ d1lo1a_ d1cita_ d2dara2 d2dara1 d1wyha2 d2dloa2 d1rutx4 d1rutx3 d2cuqa2 d1x4la2 d1x4la1 d1x3ha2 d1x3ha1 d2d8za1 d1x61a2 d1x6aa1 d1x6aa2 d2cora2 d2cora1 d1v6ga1 d1v6ga2 d2d8za2 d1x64a1 d1x64a2 d1b8ta1 d2co8a1 d1x63a2 d2dj7a2 d2dj7a1 d2cu8a2 d2cu8a1 d1g47a2 d1g47a1 d1wyha1 d2dloa1 d1wiga1 d1wiga2 d2cupa2 d2cupa3 d1x68a1 d2d8ya2 d2d8ya1 d1m3va2 d1x62a1 d1x68a2 d1x61a1 d1b8ta3 d1b8ta2 d2d8xa2 d2d8xa1 d1x4ka1 d1x4ka2 d2cuqa1 d1m3va1 d2co8a2 d1j2oa2 d1x63a1 d2cura1 d2cura2 d1x62a2 d2aq2b1 d1ck1a1 d1enfa1 d1i4pa1 d1v1oa1 d1et9a1 d1esfa1 d2g9hd1 d4ohja1 d1klud1 d1eu3a1 d1ty0a1 d1fnua1 d1c4qa_ d2wv6d_ d1prtf_ d1ltrd_ d4l63b1 d1r4pb_ d1prtd_ d1tl0f_ d2bosa_ d1prtb1 d3chbd_

**Family-18 (b.1.1.2)**

**Positive dataset contains 31 protein sequences.**

d1zs8a1 d2vola1 d2vola2 d3qq4b1 d4odhl2 d3l9ra2 d3jtth1 d2fath2 d4elkb2 d2xfxb_ d4k71b2 d4lfhg2 d2nw2a2 d3huja2 d2nnab1 d3mffb2 d4rfeb2 d2bc4a2 d2xwtb2 d1ypzf2 d4f7ea2 d1vgka1 d4j12a2 d4x99b2 d1u58a1 d2bc4b2 d3wn5b1 d1zvsa1 d1ydpa1 d1u8ib2 d1ypze2

**Negative dataset contains 3194 protein sequences.**

d1m32a_ d1ibja_ d1c7na_ d1iuga_ d4q75a_ d2bkwa1 d1t3ia_ d1n8pa_ d1qz9a_ d1jf9a_ d1p3wa_ d3kgwa_ d1cl1a_ d2ctza1 d1vjoa_ d2rfva_ d1gc0a_ d1cs1a_ d1d2fa_ d2ch1a1 d1qgna_ d1pffa_ d1elua_ d1c7ga_ d2v1pa1 d2ez1a_ d1u08a_ d1lc5a_ d1b5pa_ d1bw0a_ d3pd6a_ d7aata_ d2csta_ d1j32a_ d1fg7a_ d4wlha_ d1o4sa_ d1v2da_ d1gdea_ d1m6sa_ d1v72a1 d1svva_ d1ajsa_ d1wsta1 d2f8ja2 d1lk9a_ d1vp4a1 d1xi9a1 d2gb3a1 d1yaaa_ d3i2ka1 d1lnsa3 d1mpxa2 d1xkta1 d2hfka_ d5d3ka_ d2cfba1 d1dfoa_ d1o69a_ d1bj4a_ d3k28a_ d2bwpa_ d3lg0a_ d2fnua_ d3ou5a_ d1z7da1 d1b9ha_ d1iaya_ d4msoa_ d1bs0a_ d1s0aa_ d1sffa_ d3tqxa_ d1mdoa_ d3qboa_ d3pgya_ d1bjna_ d2dkja_ d1ejia_ d1vefa1 d4wxga_ d3bs8a_ d3gbxa1 d1w23a_ d1e57b_ d1auyb_ d1qnia1 d1fwxa1 d1f56a_ d1bypa_ d1azca_ d1ws8a_ d3u25a_ d1cuoa_ d1id2a_ d1rkra_ d2iaac_ d4dp1x_ d1nwpa_ d2gima_ d2i7sa_ d1qhqa_ d3ehbb1 d1fftb1

d1gyca3 d1hfua2 d1kbva1 d1kbva2 d1sdda1 d1sdda2 d1gyca1 d1gyca2
d1mzya1 d2bw4a1 d1aoza1 d1aoza3 d1aoza2 d1kyaa2 d1kyaa3 d1kyaa1
d1mzya2 d4e9sa1 d1sddb1 d1sddb2 d1hfua1 d1hfua3 d1oe1a2 d1oe1a1
d1v10a2 d1v10a3 d1v10a1 d2bema_ d1pama1 d1ea9c1 d1qhoa1 d2fhfa2
d2fhfa1 d1k3ia1 d1j0ha1 d1cxla1 d2bhua1 d1edqa1 d1f1sa2 d1ut9a2
d1gvia1 d1txka1 d4alac_ d1rera1 d1s6na_ d3uzqb_ d2phla2 d2phla1
d1fxza2 d1lrha_ d1uija1 g1dgw.1 d2v09a_ d1uika2 d1uika1 d1dgwa_
d1ep0a_ d2pa7a1 d1wlta1 d1nxma_ d2c0za1 d1nepa_ d1ktja_ d1ds6b_
d1fstb_ d1p8ba_ d1tafa_ d1bh9b_ d1tafb_ d1jfib_ d1q9ca_ d1h3ob_
d2byka1 d1n1ja_ d4g92b_ d1jfia_ d1h3oa_ d2bykb1 d1n1jb_ d1eixa_
d3exra_ d2ffca1 d2fdsa1 d3n3ma1 d2czda_ d1q6oa_ d2yyua_ d1vqta1
d1dbta_ d3ldva1 d2bjca1 d2hsga1 d1qpza1 d1efaa1 d1r0ka3 d1j5pa3
d1vkoa2 d1vm6a2 d3qvsa2 d1gr0a2 d1p1ja2 d2ovga_ d1sq8a_ d1rzsa_
d3kz3a_ d3orca_ d3gpdg2 d2q49a2 d1hdgo2 d2g17a2 d2cvoa2 d2czca1
d3pwka2 d1obfo2 d1i32a2 d1zyao2 d2pkqo2 d1b7go2 d2hjsa2 d1cf2o2
d1vkna2 d1nvmb2 d2m9wa1 d3dfxa_ d2msya_ d2djoa1 d2r5za_ d1au7a1
d1f43a_ d2lmda1 d1akhb_ d1s7ea1 d3nara_ d1zq3p1 d2craa1 d1mija_
d4uuta_ d3a01a_ d1x58a1 d3rkqa1 d3cmya1 d2cqxa1 d2da6a1 d1e3oc1
d1fjla_ d1mh3a1 d1ig7a_ d1x41a1 d1wh7a1 d1gt0c1 d2mw8a_ d1lfup_
d1x2ma1 d1b72a_ d1wi3a1 d1jgga_ d1wjha1 d1x2na1 d1mnmc_ d2cuea1
d3k2aa_ d2hosa_ d2jwta2 d1pufa_ d1octc1 d2hi3a_ d1b8ia_ d4j19a_
d3lnqa_ d1wh5a1 d1b8ib_ d2cqra1 d1guua_ d1ug2a1 d2cqqa1 d2ajea1
d4a69c_ d1h8ac1 d2crga1 d2iw5b1 d1xc5a1 d1ofcx1 d2cu7a1 d1wgxa1
d2cjja1 d2ckxa1 d1gvda_ d6paxa1 d1k78a1 d1k78a2 d1xg1a1 d1hlva1
d1iufa2 d1iufa1 d2iu5a1 d1zk8a1 d2g7sa1 d2fd5a1 d2np5a1 d1z0xa1
d2id6a1 d2d6ya1 d2i10a1 d1vi0a1 d2oi8a1 d3c07a1 d2ns7a1 d2fq4a1
d2yvea1 d1t33a1 d2o7ta1 d2np3b1 d2g7ga1 d2gena1 d2fbqa1 d2g7la1
d2gfna1 d2g3ba1 d2jj7a1 d2hyja1 d1sgma1 d1ui5a1 d2hkua1 d2id3a1
d4jyka1 d3lv8a_ d1s3ga1 d1zaka1 d1s4qa_ d3tvta2 d1uf9a_ d1khta_
d1ckea_ d1osna_ d1p5zb_ d4tmka_ d3cm0a_ d1jaga_ d3h86a_ d1t0hb_
d1nn5a_ d2h92a_ d3kb2a1 d2c95a1 d1yj5a2 d1kgda_ d1ly1a_ d2ak3a1
d1vyva2 d1deka_ d2grja1 d1p3ja1 d1y63a_ d1e4ya1 d1s96a_ d3iija1
d1lvga_ d1nksa_ d1vyua2 d4e22a_ d1qf9a_ d1jjva_ d1q3ta_ d1ki9a_
d1lw7a2 d1teva_ d1kjwa2 d1gsia_ d2iyva2 d1kaga_ d1viaa_ d1e6ca_
d1x6va3 d1g3ma_ d1j99a_ d1t8ta1 d1nsta_ d1zrha_ d1ls6a_ d2a3ra_
d1aqua_ d2zptx_ d3ckla_ d1texa_ d1q44a_ d2gwha_ d1odfa_ d1uj2a_
d4gi7a_ d2jeoa1 d1z0ja1 d3umra2 d3gj0a1 d2gaoa1 d1yzga1 d1x3sa1
d1g7sa4 d1puja_ d1udxa2 d1kk1a3 d2o52a_ d1jwyb_ d1zcaa2 d1zcba2
d2fv8a1 d2cjwa_ d3dz8a1 d1xzpa2 d1vg8a_ d4qxaa_ d2ew1a1 d1x1ra1
d2bcgy_ d3doea1 d3cona_ d3rega_ d3wyga_ d1upta_ d2b6ha1 d1zd9a1
d1lnza2 d1r5ba3 d1n0ua2 d1r2qa_ d2f9la1 d2rhda_ d3q3jb_ d3q85a_
d1efma1 d1r8qa_ d2gf9a1 d2akab1 d1ek0a_ d1z08a1 d2gcoa1 d2dy1a2
d4g01b_ d2cxxa1 d1svia_ d3q72a_ d1ni3a1 d4ac9a4 d2erxa1 d2bmea_
d1wxqa1 d2gf0a1 d4ldja1 d1zunb3 d1d5ca_ d4lv5b_ d1efca3 d1kmqa_

d2g6ba1 d3lrpa_ d1tada2 d2y8ea_ d4rd4a1 d1z06a1 d2heia_ d3a58b_
d1u0la2 d1d2ea3 d1zj6a1 d2ocba_ d3rwoa_ d1mr3f_ d2e9sa1 d4u5xa1
d2iwra_ d2fn4a1 d3bwdd_ d2nzja1 d2atva1 d2ksqa_ d3l43a_ d1mkya2
d1mkya1 d1wmsa_ d1jnya3 d2rmka_ d1f60a3 d1gota2 d1jala1 d2fh5b1
d1g16a_ d2wblc_ d1m7ba_ d1wf3a1 d1f5na2 d1t9ha2 d1z6xa1 d1z0aa1
d2fg5a1 d2a5ja1 d2bcjq2 d1s0ua3 d1ky3a_ d4y0va_ d4lhwa_ d2qmea1
d2atxa1 d1egaa1 d1ryha_ d1moza_ d1nrjb_ d2erya1 d3bfka_ d1puia_
d1fzqa_ d3j3az1 d3t5ga_ d1shza2 d3defa_ d2f7sa1 d3raba_ d3tsoa_
d3aq4a_ d2al7a1 d3sfva1 d3bc1a_ d2ncda_ d1sdma_ d3h4sa1 d2heha_
d2grya_ g3kin.1 d1kk8a2 d1br2a2 d1goja_ d1f9va_ d2mysa2 d1lkxa_
d3t0qa_ d4db1a1 d3gbja_ d1ry6a_ d1v8ka_ d1vava_ d1uaia_ d1k8ka2
d1k8ka1 d3uleb1 d3i33a2 d3i33a1 d1bupa1 d1dkgd2 d1dkgd1 d4ci6a2
d1jcfa1 d4a2ba2 d2fxua1 d1yaga2 d1yaga1 d4cbua2 d1g99a1 d2e1za2
d2e1za1 d1saza1 d1saza2 d1ig8a1 d1czan2 d1czan3 d1czan1 d1czan4
d1bg3a4 d1bg3a3 d1bg3a2 d1bg3a1 d1nira1 d1qksa1 d1hroa_ d4gedb_
d3zooa_ d1mz4a_ d1gu2a_ d1f1fa_ d1dvva_ d1c6ra_ d1c75a_ d1e29a_
d1gdva_ d1cnoa_ d1h32b_ d1co6a_ d2bh4x_ d2aiua_ d5cytr_ d1jdla_
d1i8oa_ d2zboa_ d3dmia_ d1ls9a_ d1wvec_ d2yk3a_ d3ph2b_ d1dw0a_
d5aura_ d1ql3a_ d1qn2a_ d1f1ca_ d4zzva_ d2xspa_ d1eg1a_ d4v20a_
d1ojja_ d4ipma1 d4csia_ d3pfza_ d3ovwa_ d2yoka_ d2nqya_ d1h4ga_
d1hixa_ d1oa2a_ d1olra_ d3b7ma_ d4h7ma1 d3wp3a_ d2vgda_ d3zsea_
d2nlra_ d3wx5a_ d1oa3a_ d1pvxa_ d1m4wa_ d1f5ja_ d1oa4a_ d1ks5a_
d4ixla1 d1evsa_ d1cnt1_ d1pvhb_ d1z7ca_ d1huwa_ d1bgea_ d1rhga_
d1f45b_ d1i1rb_ d1axia_ d1etea_ d3tgxb_ d1scfa_ d2z3qa1 d3qb1a_
d1hula_ d1m48a_ d4nkqb_ d1eera_ d1v7mv_ d5e4ea_ d2o27a_ d3b5ka_
d2psma1 d4zf7a_ d3uf5a_ d4x51a_ d1d9ca_ d4ypgc1 d3oq3a_ d1lqsl_
d1n1fa_ d1au1a_ d1wu3i_ d3besl_ d3ux9a_ d2ejca_ d3muea1 d1ihoa_
d3q12a1 d3rbaa_ d2qtra_ d1nupa_ d3l92a_ d1f9aa_ d1qjca_ d2h29a_
d1vlha_ d1od6a_ d1lw7a1 d4ruka_ d2el7a_ d1iq0a2 d1irxa2 d1r6ta2
d1ivsa4 d2cyba_ d1rqga2 d1j09a2 d2yxna_ d1h3fa1 d1ffya3 d2g36a1
d1nzja_ d1jila_ d1n3la_ d1i6la_ d1pfva2 d1h3na3 d3n9ia_ d3sz3a1
d3sw2a_ d1moxc_ d1dx5i3 d1edmb_ d1autl2 d1autl1 d1nqlb_ d1dval1
d1szba2 d1kigl_ d2y5fl_ d1ijqa2 d1gk5a_ d1g1sa2 d1nt0a3 d1xdtr_
d1lmja2 d1lmja1 d1nzia2 d2wphe_ d1pfxl2 d1pfxl1 d1p9ja_ d1g1ta2
d1f7ea_ d4yzub_ d1qx2a_ d2y5ia_ d1qlsa_ d1psra_ d3n22a_ d2egda_
d4ggfc_ d2wcea_ d2luca_ d4aqia_ d3nsia_ d2lp3a_ d1j55a_ d1a4pa_
d1k8ua_ d1pvaa_ d3fs7a_ d1ttxa_ d1rwya_ d2kyfa_ d1rk9a_ d1a75a_
d1exra_ d1tiza1 d1nyaa_ d1g8ia_ d1ggwa_ d1wdcc_ d3fwba_ d1ggza_
d2o5ga_ d1ctaa_ d1m45a_ d3i5gc_ d1sl8a_ d1fpwa_ d2scpa_ d2roaa_
d4aqra_ d3pn7b_ d1br1b_ d2mysb_ d1s6ca_ d1bjfa_ d2obha_ d1jfka_
d1jf0a_ d1qv0a_ d1lkja_ d2mlfc_ d2m97a_ d2d8na_ d4by5a_ d1jbaa_
d4nqga_ d1wdcb_ d2mysc_ d1j7qa_ d1s6ia_ d1uhka2 d1r2ua_ d1dtla_
d3kf9a_ d3pn7c_ d1qjta_ d1fi6a_ d5d67d_ d1tuza_ d1h8ba_ d1eg3a2
d2zkmx1 d1snla_ d1gjya_ d1y1xa_ d1dvia_ d1df0a1 d1qxpa2 d4usla_

d1k94a_ d1sfsa_ d1jfxa_ d1wkya2 d1uuqa_ d3wh9a_ d4m1ra_ d1xyza_
d4xuya_ d2je8a5 d4bf7a1 d3cuia_ d1ur4a_ d1odza_ d2vzsa5 d1bqca_
d2x2ya1 d1hjqa_ d1qnra_ d4y7ea1 d1vjza_ d4k68a_ d2v3ga_ d4hpga_
d1nofa2 d1nq6a_ d1h4pa_ d1aq0a_ d3ziza_ d4bs0a_ d1ecea_ d1h1na_
d1ta3b_ d1w91a2 d1i1wa_ d1uhva2 d1us3a2 d1egza_ d7a3ha_ d1tvna1
d3nyda1 d1ghsa_ d2cyga1 d4xv0a_ d2c8na2 d1w32a_ d3emca_ d1v6ya1
d1foba_ d2uwfa1 d2fgla_ d3niya1 d2c0ha1 d1rh9a1 d1yq2a5 d1szna2
d1fa2a_ d1pama4 d5do8a1 d1ea9c3 d1hx0a2 d2fhfa5 d1qhoa4 d1avaa2
d1m53a2 d4m8ua1 d2gjpa2 d1lwha2 d4howa1 d1ktba2 d1j0ha3 d3vm5a1
d2x1ia_ d1wzaa2 d1uasa2 d2diea1 d1mxga2 d1ht6a2 d1eswa_ d1iv8a2
d5cpsa_ d1ud2a2 d1zjaa1 d3gbea1 d1kwga2 d1wdpa_ d1gcya2 d1g5aa2
d1hvxa2 d3dhpa2 d2bhua3 d4do4a1 d3qgva1 d1e43a2 d1g94a2 d2d3na2
d1gjwa2 d1gvia3 d1x1na1 d1r7aa2 d1tz7a1 d4c7ga2 d2gjxa1 d1nowa1
d1jaka1 d1yhta1 d4toqa_ d1wb0a1 d1w9pa1 d1eoka_ d1itxa1 d1kfwa1
d1goia2 d1edqa2 d2gsja_ d1hjxa1 d1jnda1 d1vf8a1 d3wdpp1 d3wq5a_
d1gnxa_ d4gxpa_ d4hz8a_ d1e4mm_ d3aiua_ d1wcga1 d1qvba_ d3ahxa_
d1v02a_ d2dgaa_ d1pbga_ d1vffa1 d1qoxa_ d4ha4a_ d1t77a2 d1mixa2
d1e5wa2 d1j19a2 d1isna2 d2al6b2 d3syxa1 d1mkea1 d2p8va_ d1xoda1
d1i7aa_ d1evha_ d1qc6a_ d1egxa_ d1iuka_ d2csua1 d3q9ub_ d2yv1a1
d3q9na_ d2d59a_ d2yv2a1 d1euca1 d1y81a1 d1wgqa1 d1v89a1 d2coda1
d1zc3b_ d1wg7a1 d1u5ea1 d1v61a1 d2coca1 d2ys1a1 d1v5pa1 d1ntya2
d1wjma1 d1wi1a1 d1u29a_ d1xx0a1 d1unqa_ d2zkmx3 d1fhoa_ d1v88a1
d2adza1 d1x1fa1 d1p6sa_ d2coaa1 d1btka_ d1v5ma1 d1faoa_ d2x18a_
d1dyna_ d1x1ga1 d4kaxb_ d2j59m1 d1ki1b2 d1xcga2 d1txda2 d1u5da1
d1dbha2 d1w1ha_ d1upqa_ d1v5ua1 d1foea2 d2fjla1 d1eaza_ d2dfka2
d2cofa1 d1kz7a2 d9ldta1 d2ewda3 d1up7a1 d2dfda1 d1s6ya1 d1vjta1
d7mdha1 d1hyea1 d1t2da1 d1ojsa3 d1b8pa1 d1ldna1 d1hyha1 d1civa1
d2a92a1 d1ez4a1 d1i0za1 d1y6ja1 d1gv0a1 d1ur5a1 d1llda1 d1v8ba1
d1gdha1 d1l7da1 d1mx3a1 d1ygya1 d1qp8a1 d1li4a1 d1j4aa1 d2naca1
d1npya1 d3t4ea2 d1a4ia1 d1o0sa1 d2hk9a2 d2egga2 d1b26a1 d1bvua1
d1gq2a1 d1c1da1 d1leha1 d1gtma1 d1euza1 d1nvta1 d3dona2 d1edza1
d1gpja2 d1p77a1 d1nyta1 d1b0aa1 d1vl6a1 d1v9la1 d1txga2 d3f47a1
d2pv7a2 d1xc2a3 d2ahra2 d4xiya1 d2f1ka2 d1pgja2 d1i36a2 d1np3a2
d1lj8a4 d1teaa3 d1qmga2 d2i76a2 d2g5ca2 d1wdka3 d1dlja2 d3cuma3
d1kola2 d1f8fa2 d1qora2 d1jqba2 d1vj0a2 d1cdoa2 d1gu7a2 d1d1ta2
d1xa0a2 d2dm6a2 d1jvba2 d1rjwa2 d1llua2 d1h2ba2 d1tt7a2 d1piwa2
d1o89a2 d1iz0a2 d1e3ja2 d1yb5a2 d1uufa2 d1vj1a2 d1e3ia2 d3gpdg1
d1vc2a1 d2nvwa1 d1u8fo1 d1ydwa1 d1hdgo1 d2g17a1 d1cf2o1 d2czca2
d1r0ka2 d1obfo1 d2cvoa1 d1ebfa1 d1h6da1 d1i32a1 d1zh8a1 d2q49a1
d1qkia1 d1j5pa4 d3pzra1 d1xeaa1 d1vkoa1 d1vm6a3 d3qvsa1 d2pkqo1
d1b7go1 d1gr0a1 d1p1ja1 d1lc0a1 d1j0xo1 d3pwka1 d2hjsa1 d1zyao1
d1vkna1 d1tlta1 d1nvmb1 d3sjua_ d3ojfa1 d1xu9a1 d2jjya1 d1n7ha_
d1y1pa1 d1h5qa_ d1jtva_ d4fs3a_ d2bkaa1 d1gy8a_ d2c5aa1 d1ulsa_
d1ooea_ d2b69a1 d1y5ma1 d1spxa_ d1ae1a_ d1bxka_ d1ydea1 d1gega_

d1hdoa_ d1o5ia_ d1e6ua_ d1snya1 d2rhca_ d3zv4a_ d5ceja_ d2d1ya1
d1fjha_ d3w8ea_ d2fmua1 d1x1ta_ d1rpna_ d3ay7a1 d1k2wa_ d1eq2a_
d2hrba1 d3ruaa_ d2ew8a1 d4hwka_ d1xgka_ d2bd0a1 d1yo6a1 d2a35a1
d2ntva_ d1vl8a_ d1hdca_ d4bkua_ d2ag5a1 d2q45a_ d1nffa_ d4iy1a_
d1zbqa1 d1kewa_ d1ek6a_ d1ja9a_ d2q46a2 d1iy8a_ d1cyda_ d2ptga_
d1hxha_ d1uzma1 d3lyla_ d4m89a_ d3wyea_ d2blla1 d1w6ua_ d1sb8a_
d2q2va_ d4nr0a_ d1e6wa_ d2gasa_ d3oiga1 d2bgka1 d1mxha_ d1vl0a1
d1rkxa_ d1r6da_ d1wmaa1 d1e7wa_ d2c07a1 d1xkqa_ d3tzha_ d1g0oa_
d3lz6a_ d1qyca_ d1edoa_ d1gz6a_ d1oc2a_ d1t2aa_ d1zema1 d2xoxa_
d1z45a2 d1geea_ d1p33a_ d3lt0a_ d4e6pa_ d2wyua_ d1db3a_ d4q9na_
d1xhla_ d4o1ma_ d1fmca_ d2uvda_ d2gdza1 d1uaya_ d2z1ma_ d2o23a2
d2fr1a2 d2fr1a1 d1qsga_ d1qyda_ d4zjua_ d2a4ka1 d1yb1a_ d1wvga1
d1yxma1 d3f4ba_ d1mg5a_ d1xg5a1 d1r55a1 d3gboa_ d1atla_ d1kufa_
d1buda_ d4q1la_ d1quaa_ d1yp1a_ d1wnia_ d1cmxa_ d1xd3a_ d3ifwa_
d1e2ta_ d4c5pa_ d2bsza1 d4bgfa_ d1w4ta1 d1g0da4 d2f4ma1 d1x3za_
d3ioqa_ d1fh0a_ d1o0ea_ d1khqa_ d1m6da_ d2cb5a_ g8pch.1 d1s4va_
d2pnsa_ d1vsna1 d1mira_ d1iwda_ d2oula_ d7pcka_ d3bwka_ d3bcna_
d2p86a_ d1pvja_ d1pcia_ d1gece_ d1deua_ d4bs6a_ d1xkga1 d1cqda_
d1jqpa2 d2c0ya_ d2bdza_ d3p5ua_ d1zoya1 d1qo8a2 d1d4ca2 d1jnra2
d1kf6a2 d2jk6a2 d2jk6a1 d1feca1 d1feca2 d1lpfa1 d1lpfa2 d1xhca1
d1xhca2 d1dxla2 d1dxla1 d1gera2 d1m6ia2 d1onfa1 d1onfa2 d3lada2
d3lada1 d1w4xa1 d1w4xa2 d1gesa2 d1mo9a1 d1xdia1 d1fl2a2 d1fl2a1
d1fcda1 d1fcda2 d1h6va2 d1h6va1 d1mo9a2 d1gv4a2 d1gv4a1 d2gv8a2
d2gv8a1 d1aoga1 d1aoga2 d1hyua1 d1ebda1 d1ebda2 d3dk9a1 d1q1ra2
d1q1ra1 d1gesa1 d1zbfa1 d2z1ha_ d2etja1 d3qioa1 d1jl1a_ d2e4la_
d1jl2a_ d2lsna1 d1io2a_ d1uaxa_ d2yv0x_ d1mu2a1 d1ekea_ d1i39a_
d1pn0a1 d1reoa1 d1ju2a1 d2f5va1 d3c96a1 d2gmha1 d1pj5a2 d1gpea1
d1o5wa1 d1n4wa1 d2dw4a2 d2ivda1 d1seza1 d2voua1 d1kdga1 d1s5ja1
d2hbja2 d2igia_ d1wlja_ d1qhta1 d4dlea1 d1tgoa1 d2f96a1 d3tr8a1
d3mxma_ d1q8ia1 d1y97a1 d1w0ha_ d1kfsa1 d2py5a1 d1noya_ d1d5aa1
d1yt3a3 d2guia_ d3mwpa1 d4kb1a_ d1iv0a_ d2d5ra1 d1b9ra_ d1offa_
d1frra_ d3wcqa_ d1e9ma_ d1l5pa_ d1wria_ d1i7ha_ d3av8a_ d1czpa_
d1rfka_ d3p1ma1 d3b2ga_ d2bt6a2 d3nvva1 d1krha3 d2h88b1 d1n62a2
d1t3qa2 d1kf6b2 d1zoyb1 d1jq4a_ d1ffva2 d1dgja2 d1rm6c2 d1jmxa2
d1jmxa1 d1pbya1 d1r7ha_ d1faaa_ d1wika1 d1xfla_ d1ttza1 d2f51a1
d1ti3a_ d1nhoa_ d1sena_ d2i4aa_ d1ep7a_ d1zmaa1 d1fo5a_ d3lefa1
d3diea1 d2yana1 d1z6na1 d1oaza_ d3zywa1 d4aj8a_ d1nm3a1 d4fiwa_
d1wmja_ d4hs1a1 d1iloa_ d1xwaa1 d1wjka1 d2vm1a_ d1fb6a_ d1gh2a_
d1h75a_ d1nw2a_ d1kx5b_ d3a6na_ d4m6ba2 d2nqbd_ d3wkjd_ d2yfwe_
d4h9na_ d5bnxa_ d1kx5c_ d3r45a_ d1eqzb_ d4jjnb_ d1f66c_ d1eqza_
d4wnnb_ d1kx5a_ d1u35c1 d1id3a_ d2nqbc_ d1kx5d_ d1f1ea_ d1b67a_
d2b5ea2 d2b5ea3 d2b5ea1 d2b5ea4 d2kp1a1 d2kp2a1 d1hyua3 d1hyua4
d1j08a1 d1pd212 d1nhya2 d2a2ra2 d1k0dd2 d1f2ea2 d1gsua2 d1f3ba2
d2fhea2 d2pvqa1 d1tw9a2 d1oyja2 d1e6ba2 d1k0ma2 d3ik7a1 d4is0a1

d1r5aa2 d1jlva2 d1m0ua2 d3gtub2 d1z9ha2 d1okta2 d1axda2 d2gsra2
d1tu7a2 d1k3ya2 d1zgma1 d1b48a2 d1gnwa2 d1gwca2 d2gsta2 d1glqa2
d1oe8a2 d1pn9a2 d1v2aa2 d1fw1a2 d1duga2 d3vrda1 d3vrda2 d1fcdc1
d1h1oa2 d1h1oa1 d1m70a2 d1m70a1 d1a0rp_ d1iqca2 d1iqca1 d1eb7a2
d1eb7a1 d1nmla2 d4ilya_ d4olta_ d1qgia_ d1qqya_ d1gd6a_ d1ivma_
d2gv0a_ d1iiza_ d2z2fa_ d1j8xa_ d1ghla_ d1b9oa_ d174la_ d169la_
d2o4wa_ d1p37a_ d1k28a3 d1xjta1 d1jtma_ d2f2qa_ d4e97a1 d3w3ea_
d4tx7a_ d3cqla_ d4msta_ d3iwra_ d2z37a_ d4dyga_ d2k4ua_ d1hp2a_
d2lu9a_ d1baha_ d2meoa1 d1wm7a_ d1hlya_ d1n8ma_ d1wpda_ d1c55a_
d1pvza_ d1wt7a_ d1rjia_ d1wz5a_ d1m2sa_ d1pjva_ d1c49a_ d1qkya_
d2ktca1 d1wmta_ d1du9a_ d1j5ja_ d1r1ga_ d2pjsa1 d1klla_ d2i7ra1
d1ecsa_ d1xqaa1 d5cj3a1 d1npba_ d1jifa_ d2p7oa_ d1nkia_ d1ro5a1
d1s5ja2 d1qhta2 d4dlea2 d1tgoa2 d3py8a2 d1q8ia2 d1kfsa2 d2py5a2
d1d5aa2 d1qsta_ d2beia1 d4m3sa_ d1u6ma1 d1ygha_ d1yk3a1 d3dr6a_
d1xmta_ d1z4ea1 d2ge3a1 d1bo4a_ d2fiaa1 d2jdca1 d1y9wa1 d3to7a_
d1wwza1 d2fl4a1 d2ozua1 d2b5ga_ d2cy2a1 d2p0wa_ d1cm0a_ d4pswa_
d1yrea1 d1yr0a1 d1m4ia_ d2gana1 d1yvka1 d1ghea_ d2g3aa1 d1y7ra1
d2euia1 d1q2ya_ d2fsra1 d1xeba_ d2ae6a1 d1cjwa_ d1qsma_ d2aj6a1
d1y9ka1 d1i12a_ d1vkca_ d2atra1 d1n71a_ d2d4pa1 d2fiwa1 d2fe7a1
d1tiqa1 d2ou2a_ d1yx0a1 d2fcka1 d1p0ha_ d2j8ma_ d2pq8a_ d1z4ra1
d1s3za1 d1ufha_ d2hqya1 d2hqya2 d1lrza2 d1ne9a1 d1lrza3 d1xvqa1
d1pn0a2 d3hy2a_ d2cvba1 d1o8xa_ d1lu4a_ d1we0a1 d2bmxa1 d2a4va1
d1psqa_ d1xiya1 d1q98a_ d1n8ja_ d1zofa1 d2ggta_ d3tjja_ d4je1a1
d1o73a_ d3s9fa_ d2zcta_ d1qmva_ d1jfua_ d2b1ka_ d5dvba1 d2b5xa1
d2v2ga_ d3tb2a_ d4llra_ d4fh8a1 d1e2ya_ d1knga_ d2h30a_ d1i5ga_
d2z9sa_ d2f8aa1 d1tp9a1 d2b7ka_ d1nm3a2 d3hvva_ d2wfca1 d4kcea_
d1xvwa1 d4gqca1 d3qpma_ d4k1fa_ d1zzoa1 d1gp1a_ d1prxa_ d4od7a1
d1z6ma1 d4mcua1 d4dvca1 d3l9sa_ d1un2a_ d3rppa_ d1zsqa1 d2m5ta_
d1cqqa_ d1wqsa_ d5bpea_ d2ipha_ d1lvoa_ d2bhga1 d3tnta_ d3sjia_
d3d23a1 d2fyqa_ d3tloa_ d3zvea1 d3q3ya1 d4asha1 d1cg2a1 d1yloa2
d1y7ea2 d1y0ya3 d1lfwa1 d1vixa1 d1fnoa4 d1vhoa2 d1ysja1 d1vhea2
d1z2la1 d2fvga2 d1vgya1 d1r3na1 d2grea2 d1xmba1 d1rtqa_ d1w52x1
d2ppla1 d1hpla2 d1lpbb2 d2oxea1 d1bu8a2 d1rp1a2 d1ys1x_ d4hs9a_
d1ispa_ d1ex9a_ d3qmma_ d1lgya_ d4l3wa_ d1llfa_ d1gz7a_ d1ukca_
d4wfia_ d4cg1a1 d3visa_ d1t61c1 d2ftba_ d1pmpa_ d2rcqa1 d1fdqa_
d4lkpa_ d1sa8a_ d1vyfa_ d1lpja_ d3vg7a1 d4azqa1 d1o8va_ d5bvqa_
d1p6pa_ d2qo4a_ d1opba_ d1yiva1 d1o1va_ d4bvma1 d4tkba_ d4i3ba_
d1bwya_ d1ftpa_ d1ggla_ d4gkca_ d3p6da1 d1kqwa_ d2a0aa1 d3zxha_
d1cgla_ d1rm8a_ d1eaka2 d1hy7a_ d2jsda_ d1cxva_ d1hv5a_ d1i76a_
d1eb6a_ d3p0ya1 d1igra1 d1igra2 d1n8yc1 d1m6ba2 d1m6ba1 d1m4la_
d2bk7a_ d3fjua_ d1z5ra1 d1kwma1 d1uwya2 d2c1ca1 d1dtda_ d1h8la2
d1ohfc_ d1cixa_ d1y29a_ d1v7fa_ d2jtba_ d1d1ha_ d1g9pa_ d1lupa_
d1qk7a_ d1mb6a_ d2a2va_ d1niya_ d1i25a_ d1hvwa_ d2mpqa_ d1ie6a_
d1kqha_ d1la4a_ d2mxoa1 d1koza_ d1qk6a_ d1emxa_ d1lqra_ d1c6wa_

d1rmka_ d1ixta_ d1eyoa_ d1cnna_ d1fu3a_ d1g1pa_ d1f3ka_ d1fyga_
d1rypd_ d1irua_ d1rypc_ d3unfk_ d1rypa_ d1j2pa_ d1irui_ d1j2qh_
d3unfh_ d1m4ya_ d1rypb_ d1irul_ d1irug_ d1iru2_ d1q5rh_ d1rypg_
d1g3ka_ d1iruf_ d1q5qa_ d1e94a_ d1irub_ d1ryph_ d3ty6a_ d1iruj_
d1iruk_ d1rype_ d1irud_ d1irue_ d3mi01_ d1rypf_ d2z3ba_ d1iruc_
d3mka2_ d1iru1_ d1e88a1 d1l6ja5 d1l6ja4 d1eaka4 d1eaka3 d1l6ja3
d1h8pa2 d1mqta_ d1d4m3_ d1fmd3_ d2mev1_ g1aym.1 d1ev13_ d4q4w1_
d1r1a3_ d2x5ia_ g1r1a.1 g1d4m.1 g1rhi.1 d1b35b_ d1oopa_ d1tme1_
d4iv1a_ g1pvc.1 g1fpn.1 d1fpn1_ d4gb31_ d1d4m1_ d1fmd1_ d1ev11_
d2wzr3_ g1cov.1 d1qqp3_ g1fmd.1 d2x5ic_ d1rhi3_ g1oop.1 d1hxs1_
g1hxs.1 d1fpn3_ d1bev1_ g2mev.1 d1eah3_ d1aym1_ d1pvc3_ d1qqp1_
d1zba3_ d1h8ta_ d3vbhc_ g1b35.1 g1bev.1 d1hxs3_ g1ev1.1 d1bev3_
g1tmf.1 d1tmf1_ d1b35a_ d2mev3_ g1eah.1 g1h8t.1 d1eah1_ d4q4w3_
d1r1a1_ d1aym3_ d1pvc1_ d1zba1_ d1h8tc_ d1oopc_ d1tme3_ g1qqp.1
d4iv1c_ g1tme.1 d3ubub_ d1egia_ d1qo3c_ d1v7pa_ d1tdqb_ d3ubua_
d1c3ab_ d1h8ua_ d3bx4b_ d1t8ca1 d1sb2a1 d1qdda_ d2ziba1 d3cada_
d1byfa_ d1j34b_ d1jwia_ d1jzna_ d1c3aa_ d1oz7b_ d1wmza_ d3bdwb_
d1hq8a_ d1dv8a_ d3hupa_ d1wk1a1 d1umra_ d2afpa_ d3bx4a_ d1gz2a_
d1sl6a_ d1v7pb_ d3g8ka_ d1ixxa_ d1j34a_ d1jwib_ d1g1sa1 d1y17a1
d1ja3a_ d1fvub_ d3bdwa_ d1sb2b1 d1fvua_ d1umrc_ d1oz7a_ d4m17a_
d3cfwa1 d1ypqa1 d1g1ta1 d3fd4a_ d3l9jc_ d4dn8a1 d1v4lb_ d1rdl1_
d2msba_ d4w9ta_ d1jvna1 d4x2ra1 d1ka9f_ d1qo2a_ d4axka1 d1thfd_
d4u28a_ d2y88a_ d4wd0a_ d1vzwa1 d1ix2a_ d4lt9a1 d3tc7a1 d3hoja_
d1i4na_ d3vnda_ d3nl8a1 d1geqa_ d3nava1 d1v5xa_ d4a2ra1 d3tc6a1
d1ujpa_ d4ijba_ d1xcfa_ d1vc4a_ d1rd5a_ d3b5va_ d2r80b_ d1cqxa1
d1cg5a_ d1gcvb_ d3boma_ d2h8fb_ d2raoa_ d3bj1a_ d4hrrb_ d2r50a_
d3d1kb_ d2aa1b1 d3zhwa_ d3hf4b_ d3gdja_ d1jebb_ d3bj1b_ d1x9fb_
d1wmub_ d1gcva_ d3bcqb_ d3d4xb_ d1gvha1 d2dc3a_ d3hf4a_ d4yu3a_
d3k8ba_ d1qpwb_ d2r80a_ d3bcqa_ d1v4wa_ d3wtgb_ d1jl7a_ d4esaa_
d1a9we_ d3d1ab_ d1fsla_ d4h2la_ d1v4wb_ d1mwca_ d1vhba_ d1jeba_
d1spgb_ d1h97a_ d1q1fa_ d1tu9a1 d3d1aa_ d1hdsb_ d3hyub_ d2zfbb_
d3fs4a_ d1oj6a_ d1shrb_ d4h2lb_ d4yu3b_ d1spga_ d3hrwa_ d3qqra_
d3at5a_ d4wchd_ d3hyua_ d1cg5b_ d1uc3a_ d1hbra_ d2raob_ d1outb_
d4esab_ d3gdjb_ d3qqqa_ d1x9fc_ d3vrfa_ d1itha_ d3mkba_ d3d4xa_
d3mjpa_ d1jl6a_ d3fh9a_ d1outa_ d2dc3b_ d1it2a_ d3eoka_ d3g46a_
d1qpwa_ d3vrfb_ d1ch4a_ d1x9fd_ d1hdsa_ d2zfba_ d3mkbb_ d1or4a_
d3wtga_ d1a4fa_ d3fh9b_ d3bomb_ d3cy5a_ d3at5b_ d1x9fa_ d2oifa_
d4lmxb_ d1cpcb_ d3v57a_ d1kn1a_ d1cpca_ d4f0ta_ d2vjha_ d4po5b_
d1eyxa_ d1alla_ d4f0tb_ d1b33b_ d1b8db_ d4h0mb_ d1b33a_ d2vjhb_
d4lm6b_ d2vmlb_ d1allb_ d2vjta_ d3dbja_ d2vmla_ d2vjtb_ d4f0ub_
d1f99b_ d4rmpa1 d1liaa_ d1eyxb_ d2uulb_ d4lmsb_ d4f0ua_ d2bv8a_
d1f99a_ d2bv8b_ d2c7lb_ d3dbjb_ d4rmpb1 d1b8da_ d4h0ma_ d3v57b_
d1kn1b_ d1liab_ d2uula_ d2j96a_ d3ia2a_ d1a88a_ d4dgqa1 d1hkha_
d1zoia_ d2xqfa_ d1ea5a_ d1f6wa_ d1dx4a_ d1qe3a_ d1k4ya_ d4qwwa_

d2ha2a_ d1sfra_ d1f0na_ d4qeka_ d3c8da2 d2pbla1 d1jkma_ d1lzla_
d1jjfa_ d2qvba_ d2v9za_ d4kafa1 d4iuaa3 d4bvva_ d5hpga_ d3k65a_
d1ki0a3 d1ki0a2 d1ki0a1 d1a0ha1 d1pmla_ d3sp8a3 d2feba1 d1f74a_
d3l21a_ d2e1da_ d1to3a1 d1w3ia_ d1sfla_ d1n7ka_ d1vcva1 d4imea_
d1wa3a_ d2ocza1 d1f2ja_ d3m16a_ d2yxga_ d1ub3a_ d3a5fa_ d1fbaa_
d1l6wa_ d1xfba1 d1wx0a1 d1vpxa1 d1ojxa_ d1mxsa_ d2a4aa1 d1xkya1
d3cq0a1 d3s1xa_ d1f05a_ d1fdja_ d3cpra1 d4d2ja_ d4mh8a_ d4i7ga1
d3rp2a_ g1ucy.1 d1fuja_ d2zchp_ d3w94a_ d1si5h_ d1lo6a_ g2pka.1
d1cgha_ d1kigh_ d1a5ia_ d3s9ba_ d1hj8a_ d1ao5a_ d2anya_ d1q3xa1
d1gvza_ d1azza_ d4gsoa_ d2f91a1 d1iaua_ d1eufa_ g1fiw.1 d1eq9a_
d4nfea_ d2rdla_ d1spja_ d1elva1 d1a7sa_ d1ekbb_ d1pfxc_ d1yc0a_
d2aiqa_ g1fiz.1 d1fi8a_ d4k60a_ d4dgja_ d1mzaa_ d1op0a_ d3tjva_
d1pytd_ d1sgfg_ g1gg6.1 d2eeka_ g1rtf.1 d1a0ja_ d3g01a_ d1pytc_
d4e7na_ d1ddja_ d1z8ga1 d2hlca_ d1npma_ d1brup_ g1jou.1 d1sgfa_
d1autc_ g2hnt.1 g1kdq.1 d1orfa_ d3s69a_ d4crga_ d1fxya_ d1bqya_
d1svpa_ d1mbma_ d3su6a1 d1cu1a1 d4agka_ d1vcpa_ d1q31a_ g1n1l.1
d3e90b_ d1ep5a_ d3u1jb_ d1jx4a2 d3gv5b1 d1t94a2 d3bq0a2 d3lgia_
d3i77a_ d2z9ia2 d4nsva_ d1lcya2 d4jcna_ d1agja_ d1os8a_ d1hpga_
d1p3ca_ d1l1ja_ d1qtfa_ d1e69a_ d1mv5a_ d3g60a4 g1ii8.1 d1ewqa2
d1ji0a_ d1v43a3 d2wmfa1 d1vpla_ d1g2912 d4a82a2 g1f2t.1 d2d62a1
d3tuic1 d2bbsa1 d3g60a3 d2ixea_ d1g6ha_ d1sgwa1 d2qi9c_ d3rlfa1
d3b60a1 d3d31a2 d2pzea1 g1xew.1 d1jj7a_ d1oxxk2 d2onka1 d1w1wa_
d1iwea_ d1qf5a_ d1vmaa2 d1nija1 d1np6a_ d2p67a1 d3ihla_ d1ls1a2
d1s1ma2 d1iona_ d2v40a_ d1dj3a_ d3hida_ d2vo1a1 d2g0ta1 d1vcoa2
d1j8mf2 d1g3qa_ d1xjca_ d1p9ba_ d1qzxa3 d1ihua2 d1ihua1 d2qm8a_
d1dj2a_ d4ioja_ d1cp2a_ d1yrba2 d1hyqa_ d3gqba2 d1xp8a1 d1tf7a2
d1tf7a1 d1ye8a1 d1skyb3 d2qe7d2 d4blta_ d4huta_ d3ldaa2 d4hyya_
d2i3ba1 d1cr1a_ d3j9tb2 d3ntua2 d1vdza1 d2z43a_ d1e9ra_ d1nlfa_
d3ziaa2 d1n0wa_ d1g6oa_ d3ziad2 d3j9ta2 d1pzna2 d3gqbb2 d2i49a_
d1nnfa_ d1xvxa_ d1ursa_ d1h3da1 d1iuda_ d4m1va1 d1twya_ d3e13x_
d2nxoa1 d4pfua_ d3ttma_ d1elja_ d2qmwa1 d1us5a_ d1y3na1 d1jeta_
d1uqwa_ d4f19a1 d2esna2 d3thia_ d3fasa1 d2uyeb_ d2xwva1 d1r9la_
d2fyia1 d1pb7a_ d2ozza1 d1sw5a_ d1nh8a1 d1vr5a1 d2g29a_ d1xs5a_
d3lswa_ d2z23a_ d3w9va_ d3tcha_ d1q35a_ d1z7me1 d3q27a_ d1p99a_
d1ve4a1 d2i6ea1 d4mcoa1 d4n8ya_ d1eu8a_ d1zbma1 d1hsla_ d1ixca2
d1j1na_ d1xvya_ d1y9ua_ d2z22a1 d1xoca1 d4oera_ d1wdna_ d5f1qa1
d1xt8a1 d1i6aa_ d4gwoa_ d4igta1 d1pc3a_ d4mmpa1 d1o63a_ d2czla1
d2onsa2 d4mija1 d4ok5a1 d2bmfa1 d2bmfa2 d1yksa1 d1yksa2 d1jr6a_
d2qeqa1 d2jlqa1 d1a1va2 d1eh3a_ d3skpa_ d1dtza1 d1ieja_ d1jnfa2
d1ce2a1 d1jnfa1 d1b1xa2 d1b1xa1 d1jw1a1 d1jw1a2 d1ryoa_ d1h76a1
d1h76a2 d1c4oa1 d2p6ra3 d2p6ra4 d1oywa2 d1oywa3 d1wrba1 d1w36b2
d1w36b1 d1c4oa2 d3bora_ d1t6na_ d2eyqa5 d2eyqa3 d2eyqa2 d2zu6a2
d1veca_ d1uaaa1 d1w36d1 d2g9na1 d1z3ix2 d2rb4a_ d1hv8a1 d1hv8a2
d1t5ia_ d1w36c2 d2j0sa2 d2j0sa1 d1fuka_ d3hjha1 d1z5za1 d1z63a1

d1s2ma2 d1s2ma1 d1wp9a1 d1wp9a2 d1gm5a3 d1gm5a4 d3dina4 d3dina3
d1q0ua_ d1w36c1 d1z3ix1 d1nkta3 d1nkta4 d1uaaa2 d1w36d2 g1qhh.1
d2fwra2 d2fwra1 d3q3ma_ d1za0a1 d1mxra_ d2inca_ d1jk0a_ d2incb_
d1mhyb_ d3hf1b_ d1mtyb_ d1r2fa_ d2uw1a_ d1mhyd_ d3n37a_ d1oquc_
d1syya_ d1mtyd_ d2o1za_ d1uzra_ d1smsa_ d2fkza_ d1zuja1 d1s3qa1
d2fjca1 d1z6om1 d1veia_ d4dyya_ d1jiga_ d3gvya_ d2pyba_ d4cy9a1
d3bvfa1 d1r03a_ d2cf7a_ d1nf4a_ d1zs3a1 d4iwka_ d3ka8a_ d3np0x_
d1n1qa_ d1lb3a_ d4e6ka_ d1z6oa1 d2fzfa1 d4dyua_ d3ge4a_ d1jgca_
d4am4a_ d2z6ma_ d1ji5a_ d1vlga_ d1lkoa1 d1krqa_ d3qz3a_ d1o9ra_
d2ffxj_ d1yv1a1 d2v2pa_ d1j30a_ d4di0a_ d1dpsa_ d1oewa_ d2qzxa_
d2asia_ d1psoe_ d1smra_ d2h6ta_ d1pfza_ d1izda_ d3hd8a_ d1j71a_
d1dpja_ d4aa9a_ d1t6ex_ g1b5f.1 g1lya.1 g1htr.1 d1qdma2 d1bxoa_
d1wkra_ d1tzsa_ d1ibqa_ d2qzwa_ d1ex4a2 d1c6va_ d2v76a_ d1wj1a1
d1aqca_ d1qqga2 d1qqga1 d1ntva_ d1n3ha_ d4dbba_ d3f0wa1 d1p3ra_
d1wgua1 d2cy4a1 d3hqca_ d1wvha1 d1xr0b1 d1bdqa_ d3ggta_ d3ixoa_
d1mrxa_ d2haha_ d1tw7a_ d4njsa_ d1sgua_ d2p3da_ d1nsoa_ d3d3ta_
d2rkfa_ d1c6ya_ d2rspa_ d3ogpa_ d3ekwa_ d2o4na_ d2j9ja_ d1baia_
d3ggua_ d3lzua_ d4npua_ d4j55a_ d2r8na_ d2fxda_ d2p3ca_ d4dqea_
d3ttpa_ d3t3ca_ d2p3aa_ d2hc0a_ d2fdda_ d3mwsa_ d1wgda1 d1wgga1
d4q5eb_ d1wjna1 d1se9a_ d3n3kb_ d1wz0a1 d1we6a1 d2m0xa_ d2gbka_
d3rt3b1 d1wh3a1 d1sifa_ d2ekec2 d4ii2b1 d4hk2a1 d1c3ta_ d1m94a_
d1wgha1 d1ttna1 d1wx8a1 d3v6cb1 d2ma9b_ d4i6lb1 d1bt0a_ d2jvca1
d1iyfa_ d2k1fa_ d1wxva1 d1wf9a1 d1t0ya_ d2io0b2 d1x1ma1 d2gbma_
d1zw7a1 d2m17a_ d1ud7a_ d1we7a1 d2lwpa_ d2gbja_ d1wjua1 d1v6ea1
d1v86a1 d4eewa_ d1wiaa1 d4icva_ d4gu2a_ d1wx7a1 d1wy8a1 d4hjka1
d2faza1 d1v5oa1 d2al3a1 d2cr5a1 d1wj4a1 d1s3sg_ d3h33a_ d1rwja_
d19hca_ d1os6a_ d3bxua_ d3h4na_ d1i77a_ d3caoa_ d2ctha_ d1duwa_
d1ofwa_ d2e84a_ d1hh5a_ d1gyoa_ d3h34a_ d2bcgg1 d2bcgg2 d1d5ta1
d1iyka2 d1iyka1 d1rxta1 d1iica1 d1um8a_ d1ixza_ d1qvra2 d1qvra3
d1qzma_ d1sxjc2 d1jr3d2 d2p65a_ d1l8qa2 d1g8pa_ d3hu3a3 d1sxjd2
d1njfa_ d1in4a2 d1sxja2 d1tuea1 d2ce7a2 d1w5sa2 d1sxjb2 d1d2na_
d1ny5a2 d2gnoa2 d2a5yb3 d1fnna2 d1u0ja1 d1lv7a_ d1iqpa2 d2fnaa2
d1ixsb2 d4gita_ d1sxje2 d1g41a_ d2e3fa_ d1mm0a_ d2e3ea_ d2llda_
d2ny8x_ d2e3ga_ d2ny9x_ d2nz3a_ d2lj7a_ d1n4na_ d2m8bb1 d2kyqa_
d3psma_ d1jkza_ d1jxca_ d2gl1a_ d1ugla_ d4uj0a_ d1js9c_ d1f15b_
d1za7a_ d1laja_ d1t0za_ d2i61a_ d1t1ta_ d1sn4a_ d1kv0a_ d2lkba_
d1npia_ d1wwna_ d1pe4a_ d2kbka_ d2li7a_ d1djta_ d2kbha_ d1dq7a_
d1sega_ d1omya_ d1zywa1 d1jzaa_ d1n9wa1 d1bbua1 d1e1oa1 d1b8aa1
d1l0wa1 d3a74a1 d1eova1 d1bvsa3 d1iyjb4 d1iyjb5 d1iyjb3 d1o7ia_
d4ipca1 d1x3ea_ d4apva_ d1jb7a1 d1jb7a2 d1jb7a3 d3kdfa_ d1ue1b_
d1wjja1 d3eiva_ d1xjva2 d1xjva1 d2ccza2 d1se8a_ d1l1oc_ d1jb7b_
d3ulla_ d1qzga_ d1miua4 d1miua5 d1miua3 d1y14b1 d2nn6i1 d1i94l_
d2zjra2 d1smxa_ d2qamc2 d1hh2p1 d2r7da1 d1rl2a2 d1wfqa1 d3bzka4
d1x6oa2 d3a0ja_ d2j01d2 d2ja9a1 d4ql5a_ d1hr0w_ d1luza_ d2n3sa_

d2vnud1 d1h95a_ d1go3e1 d2nn6h1 d2mo1a_ d1iz6a2 d1jjga_ d1e3pa2
d2ix0a3 d2ix0a2 d2lxja_ d1ueba3 d1ueba2 d2z0sa1 d1u0la1 d2ix0a1
d2eifa2 d2je6i1 d3i2za1 d2c35b1 d2ahob2 d1t9ha1 d3i4oa_ d2qall1
d1jt8a_ d2nn6g1 d1g6pa_ d3cama_ d1i94q_ d1c9oa_ d1d7qa_ d2ba0a1
d2vnud2 d2vnud3 d1khia2 d2qalq1 d1wi5a1 d1xtda2 d1ny4a_ d1dzka_
d4tlja_ d2a2ua_ d4wfva_ d3t1da_ d4iaxa_ d3zq3a_ d2nnda_ d1i4ua_
d1kxoa_ d1bbpa_ d1kt7a_ d1exsa_ d3s26a1 d1lf7a_ d1qfta_ d4gh7a_
d1z24a1 d1qabe_ d1xkia_ d3bx7a_ d4iawa1 d1n0sa_ d1gkab_ d3kzaa_
d2k23a_ d2l9ca1 d2joza1 d1gm6a_ d3kffa_ d3saoa1 d1e5pa_ d1yupa1
d1iiua_ d1xwdc1 d3kj4a_ d1ozna_ d1w8aa_ d2ifga3 d1xkua_ d1rpma_
d2shpa1 d1yfoa_ d1l8ka_ d2ooqa1 d1p15a_ d1lara2 d1lara1 d1wcha_
d2fh7a2 d2fh7a1 d1ohea1 d1ohea2 d2f4da_ d1rxda_ d2hxpa_ d1xria_
d1d5ra2 d3ezza_ d4y2ea_ d1i9sa_ d3azya_ d3wvja_ d1upsa1 d1ajoa_
d2hyka_ d1ajka_ d2vy0a_ d1o4ya_ d3o5sa_ d3d6ea_ d3atga_ d1dypa_
d1umza_ d3i4ia_ d4lbqa_ d1a78a_ d1slta_ d4no4a_ d3zsja_ d3duia_
d1g86a_ d1is3a_ d1qmja_ d1hlca_ d1tdha3 d1q3ba3 d1k82a3 g1qmo.1
g1len.1 d3zyra_ d1fnya_ d1n47a_ g2ltn.1 d1avba_ d3usua_ d1dhkb_
d1f9ka_ d1ukga_ d1g8wa_ d1g9fa_ d1wbfa_ d3wcsa_ d1g7ya_ d1mvqa_
d1hqla_ d1v6ia_ d1dbna_ d1qnwa_ d1fx5a_ d4l8qa_ d1ioaa_ d3n35a_
d3ubra_ d1qo8a1 d1d4ca1 d2k3va_ d1ft5a_ d1qdba_ d1m1qa_ d2j7aa_
d1h21a_ d1ogyb_ d4fasa_ d1oaha_ d1sp3a_ d1eysc_ d2h0ba_ d2erfa_
d1h30a2 d3mw4a1 d1pz7a_ d2r1ba_ d3v64a1 d1d2sa_ d3bopa1 d3mw3a1
d1okqa1 d1okqa2 d1dszb_ d1a6ya_ d3cbba_ d1ynwa1 d1r4ia_ d1hcqa_
d2hana_ d2hanb_ d2enva1 d1kb4b_ d2nllb_ d1lo1a_ d1cita_ d2dara2
d2dara1 d1wyha2 d2dloa2 d1rutx4 d1rutx3 d2cuqa2 d1x4la2 d1x4la1
d1x3ha2 d1x3ha1 d2d8za1 d1x61a2 d1x6aa1 d1x6aa2 d2cora2 d2cora1
d1v6ga1 d1v6ga2 d2d8za2 d1x64a1 d1x64a2 d1b8ta1 d2co8a1 d1x63a2
d2dj7a2 d2dj7a1 d2cu8a2 d2cu8a1 d1g47a2 d1g47a1 d1j2oa1 d1wyha1
d2dloa1 d1wiga1 d1wiga2 d2cupa2 d2cupa3 d1x68a1 d2d8ya2 d2d8ya1
d1m3va2 d1x62a1 d1x68a2 d1x61a1 d1b8ta3 d1b8ta2 d2d8xa2 d2d8xa1
d1x4ka1 d1x4ka2 d2cuqa1 d1m3va1 d2co8a2 d1j2oa2 d1x63a1 d2cura1
d2cura2 d1x62a2 d2aq2b1 d1enfa1 d1i4pa1 d1v1oa1 d1et9a1 d1esfa1
d2g9hd1 d4ohja1 d1klud1 d1bxta1 d1eu3a1 d1ty0a1 d1fnua1 d1c4qa_
d2wv6d_ d1prtf_ d1ltrd_ d4l63b1 d1r4pb_ d1prtd_ d1tl0f_ d2bosa_
d1prtb1 d3chbd_

**Family-19 (b.1.1.3)**

**Positive dataset contains 2 protein sequences.**

d2d9qb1 d2nxyb2

**Negative dataset contains 3175 protein sequences.**

d1m32a_ d1ibja_ d1c7na_ d1iuga_ d4q75a_ d2bkwa1 d1t3ia_ d1n8pa_
d1qz9a_ d1jf9a_ d1eg5a_ d1p3wa_ d3kgwa_ d1cl1a_ d2ctza1 d1vjoa_
d2rfva_ d1gc0a_ d1cs1a_ d1d2fa_ d2ch1a1 d1qgna_ d1pffa_ d1tq5a1
d1u08a_ d1b5pa_ d2ay1a_ d1bw0a_ d3pd6a_ d7aata_ d2csta_ d1j32a_
d1fg7a_ d4wlha_ d1o4sa_ d1v2da_ d1gdea_ d1m6sa_ d1v72a1 d1svva_

| | | | | | | | |
|---|---|---|---|---|---|---|---|
| d1ajsa_ | d1wsta1 | d2f8ja2 | d1lk9a_ | d1vp4a1 | d1xi9a1 | d2gb3a1 | d1yaaa_ |
| d2bnma2 | d3i2ka1 | d1lnsa3 | d1mpxa2 | d2cfba1 | d1dfoa_ | d1o69a_ | d1bj4a_ |
| d3k28a_ | d2bwpa_ | d3lg0a_ | d2fnua_ | d3ou5a_ | d1z7da1 | d1b9ha_ | d1iaya_ |
| d4msoa_ | d1bs0a_ | d1s0aa_ | d1sffa_ | d3tqxa_ | d1mdoa_ | d3qboa_ | d3pgya_ |
| d1bjna_ | d2dkja_ | d1ejia_ | d1vefa1 | d4wxga_ | d3bs8a_ | d3gbxa1 | d1w23a_ |
| d1fwxa1 | d1ibya_ | d1f56a_ | d1bypa_ | d1azca_ | d1ws8a_ | d3u25a_ | d1cuoa_ |
| d1id2a_ | d1rkra_ | d2iaac_ | d4dp1x_ | d1nwpa_ | d2gima_ | d2i7sa_ | d1qhqa_ |
| d3ehbb1 | d1fftb1 | d1gyca3 | d1hfua2 | d1kbva1 | d1kbva2 | d1sdda1 | d1sdda2 |
| d1gyca1 | d1gyca2 | d1mzya1 | d2bw4a1 | d1aoza1 | d1aoza3 | d1aoza2 | d1kyaa2 |
| d1kyaa1 | d1mzya2 | d4e9sa1 | d1sddb1 | d1sddb2 | d1hfua1 | d1hfua3 | d1oe1a2 |
| d1oe1a1 | d1v10a2 | d1v10a3 | d1v10a1 | g1f8v.1 | d2bbvc_ | d3sxwa1 | d4luka_ |
| d1a3qa1 | d1iknc_ | d3brda1 | d1oy3c_ | d1imhc1 | d1gjia1 | d1zk9a1 | d2cxka1 |
| d2h0va_ | d1ofda3 | d1ea0a3 | d1ecfa2 | d1q15a2 | d1ct9a2 | d1ao0a2 | d1te5a_ |
| d1ep0a_ | d2pa7a1 | d1wlta1 | d1nxma_ | d2c0za1 | d1nepa_ | d1ktja_ | d1ds6b_ |
| d1fstb_ | d1ex0a1 | d2b5ea2 | d2b5ea3 | d2b5ea1 | d2b5ea4 | d2kp1a1 | d2kp2a1 |
| d1hyua3 | d1hyua4 | d1j08a2 | d3nvva1 | d1krha3 | d2h88b1 | d1t3qa2 | d1kf6b2 |
| d1zoyb1 | d1jq4a_ | d1ffva2 | d1dgja2 | d1rm6c2 | d1ydwa2 | d2nvwa2 | d1zh8a2 |
| d1qkia2 | d1xeaa2 | d1tlta2 | d1au7a2 | d1ic8a2 | d2ovga_ | d1sq8a_ | d1rzsa_ |
| d3kz3a_ | d3orca_ | d3gpdg2 | d2q49a2 | d1hdgo2 | d2g17a2 | d2cvoa2 | d2czca1 |
| d3pwka2 | d1obfo2 | d1zyao2 | d2pkqo2 | d1b7go2 | d2hjsa2 | d1cf2o2 | d1vkna2 |
| d1nvmb2 | d1a0rp_ | d2msya_ | d2djoa1 | d2r5za_ | d1au7a1 | d2lmda1 | d1akhb_ |
| d1s7ea1 | d3nara_ | d1zq3p1 | d2craa1 | d1mija_ | d4uuta_ | d3a01a_ | d1x58a1 |
| d3rkqa1 | d3cmya1 | d2cqxa1 | d2da6a1 | d1e3oc1 | d1fjla_ | d1mh3a1 | d1ig7a_ |
| d1x41a1 | d1wh7a1 | d1gt0c1 | d2mw8a_ | d1lfup_ | d1x2ma1 | d1b72a_ | d1wi3a1 |
| d1jgga_ | d1wjha1 | d1x2na1 | d1mnmc_ | d2cuea1 | d3k2aa_ | d2hosa_ | d2jwta2 |
| d1pufa_ | d1octc1 | d2hi3a_ | d1b8ia_ | d4j19a_ | d3lnqa_ | d1wh5a1 | d1b8ib_ |
| d1fexa_ | d2cqra1 | d1guua_ | d1ug2a1 | d2cqqa1 | d2ajea1 | d4a69c_ | d1h8ac1 |
| d2crga1 | d2iw5b1 | d1xc5a1 | d1ofcx1 | d2cu7a1 | d1wgxa1 | d2cjja1 | d2ckxa1 |
| d1gvda_ | d1hcra_ | d1tc3c_ | d1gdta1 | d1u78a2 | d6paxa1 | d6paxa2 | d1k78a1 |
| d1k78a2 | d1hlva1 | d1hlva2 | d1iufa1 | d3lv8a_ | d1s3ga1 | d1zaka1 | d1s4qa_ |
| d3tvta2 | d1uf9a_ | d1khta_ | d1ckea_ | d1osna_ | d1p5zb_ | d3cm0a_ | d1tmka_ |
| d1jaga_ | d3h86a_ | d1t0hb_ | d1nn5a_ | d2h92a_ | d3kb2a1 | d2c95a1 | d1yj5a2 |
| d1kgda_ | d1ly1a_ | d2ak3a1 | d1vyva2 | d1deka_ | d2grja1 | d1p3ja1 | d1y63a_ |
| d1e4ya1 | d1s96a_ | d3iija1 | d1lvga_ | d1nksa_ | d1vyua2 | d4e22a_ | d1qf9a_ |
| d1jjva_ | d1q3ta_ | d1ki9a_ | d1lw7a2 | d1teva_ | d1kjwa2 | d1gsia_ | d2iyva2 |
| d1kaga_ | d1e6ca_ | d1z0ja1 | d3umra2 | d3gj0a1 | d2gaoa1 | d1yzga1 | d1x3sa1 |
| d1g7sa4 | d1puja_ | d1udxa2 | d1kk1a3 | d2o52a_ | d1jwyb_ | d1zcaa2 | d1zcba2 |
| d2fv8a1 | d2cjwa_ | d3dz8a1 | d1xzpa2 | d1vg8a_ | d4qxaa_ | d2ew1a1 | d1x1ra1 |
| d2bcgy_ | d3doea1 | d3cona_ | d3rega_ | d3wyga_ | d1upta_ | d2b6ha1 | d1zd9a1 |
| d1lnza2 | d1r5ba3 | d1r2qa_ | d2f9la1 | d2rhda_ | d3q3jb_ | d3q85a_ | d1efma1 |
| d1r8qa_ | d2gf9a1 | d2akab1 | d1ek0a_ | d1z08a1 | d2gcoa1 | d2dy1a2 | d4g01b_ |
| d2cxxa1 | d1svia_ | d3q72a_ | d1ni3a1 | d4ac9a4 | d2erxa1 | d2bmea_ | d1wxqa1 |
| d2gf0a1 | d4ldja1 | d1zunb3 | d1d5ca_ | d4lv5b_ | d1efca3 | d1kmqa_ | d2g6ba1 |
| d3lrpa_ | d1tada2 | d2y8ea_ | d4rd4a1 | d1z06a1 | d2heia_ | d3a58b_ | d1u0la2 |

d1d2ea3 d1zj6a1 d2ocba_ d3rwoa_ d1mr3f_ d2e9sa1 d4u5xa1 d2iwra_
d2fn4a1 d3bwdd_ d2nzja1 d2atva1 d2ksqa_ d3l43a_ d1mkya2 d1mkya1
d1wmsa_ d1jnya3 d2rmka_ d1f6ba_ d1f60a3 d1gota2 d1jala1 d2fh5b1
d1g16a_ d2wblc_ d1m7ba_ d1wf3a1 d1f5na2 d1t9ha2 d1z6xa1 d1z0aa1
d2fg5a1 d2a5ja1 d2bcjq2 d1s0ua3 d1ky3a_ d4y0va_ d4lhwa_ d2qmea1
d2atxa1 d1egaa1 d1ryha_ d1moza_ d1nrjb_ d2erya1 d3bfka_ d1puia_
d1fzqa_ d3j3az1 d3t5ga_ d1shza2 d3defa_ d2f7sa1 d3raba_ d3tsoa_
d3aq4a_ d2al7a1 d3sfva1 d3bc1a_ d2ncda_ d1sdma_ d3h4sa1 d2heha_
d2grya_ g3kin.1 d1kk8a2 d1br2a2 d1goja_ d1f9va_ d2mysa2 d1lkxa_
d3t0qa_ d4db1a1 d3gbja_ d1ry6a_ d1v8ka_ d1vava_ d1uaia_ d1y4wa1
d1ig8a1 d1czan2 d1czan3 d1czan1 d1ig8a2 d1czan4 d1bg3a4 d1bg3a3
d1bg3a2 d3h1jd1 d2d0oa3 d2d0oa2 d1nbwa2 d1hroa_ d4gedb_ d3zooa_
d1mz4a_ d1gu2a_ d1f1fa_ d1dvva_ d1c6ra_ d1c75a_ d1e29a_ d1gdva_
d1cnoa_ d1h32b_ d1co6a_ d2bh4x_ d2aiua_ d5cytr_ d1jdla_ d1i8oa_
d2zboa_ d3dmia_ d1ls9a_ d1wvec_ d2yk3a_ d3ph2b_ d1dw0a_ d5aura_
d1ql3a_ d1qn2a_ d1f1ca_ d4zzva_ d2xspa_ d1eg1a_ d4v20a_ d1ojja_
d4ipma1 d4csia_ d3pfza_ d3ovwa_ d2yoka_ d2nqya_ d1hixa_ d1oa2a_
d1olra_ d3b7ma_ d4h7ma1 d3wp3a_ d2vgda_ d1igoa_ d3zsea_ d2nlra_
d3wx5a_ d1oa3a_ d1pvxa_ d1m4wa_ d1f5ja_ d1oa4a_ d1ks5a_ d4ixla1
d1h32a1 d1evsa_ d1cnt1_ d1pvhb_ d1z7ca_ d1huwa_ d1bgea_ d1rhga_
d1f6fa_ d1f45b_ d1i1rb_ d1axia_ d1etea_ d3tgxb_ d1scfa_ d2z3qa1
d3qb1a_ d1hula_ d1m48a_ d4nkqb_ d1eera_ d1v7mv_ d5e4ea_ d2o27a_
d3b5ka_ d2psma1 d4zf7a_ d3uf5a_ d4x51a_ d1d9ca_ d4ypgc1 d3oq3a_
d1lqsl_ d1n1fa_ d1wu3i_ d3besl_ d3ux9a_ d1v8fa_ d2ejca_ d3muea1
d1ihoa_ d3q12a1 d3wdpp1 d3wq5a_ d1gnxa_ d4gxpa_ d4hz8a_ d1e4mm_
d3aiua_ d1wcga1 d1qvba_ d3ahxa_ d1v02a_ d2dgaa_ d1pbga_ d1vffa1
d1qoxa_ d4ha4a_ d2el7a_ d1iq0a2 d1irxa2 d1r6ta2 d1ivsa4 d2cyba_
d1rqga2 d1j09a2 d2yxna_ d1f7ua2 d1h3fa1 d1ffya3 d2g36a1 d1nzja_
d1jila_ d1n3la_ d1pfva2 d1h3na3 d3n9ia_ d3sz3a1 d1cmxa_ d1xd3a_
d3ifwa_ d3sw2a_ d1moxc_ d1dx5i3 d1edmb_ d1autl2 d1autl1 d1nqlb_
d1dval1 d1szba2 d1kigl_ d2y5fl_ d1ijqa2 d1gk5a_ d1g1sa2 d1nt0a3
d1xdtr_ d1lmja2 d1lmja1 d1nzia2 d2wphe_ d1klil_ d1pfxl2 d1pfxl1
d1p9ja_ d1g1ta2 d1f7ea_ d4yzub_ d2mgpa1 d1b9wa1 d1b9wa2 d2mgpa2
d1ob1c2 d1n1ia2 d1n1ia1 d2npra2 d2npra1 d2y5ia_ d1qlsa_ d1psra_
d3n22a_ d2egda_ d4ggfc_ d2wcea_ d2luca_ d4aqia_ d3nsia_ d2lp3a_
d1j55a_ d1a4pa_ d1k8ua_ d1pvaa_ d3fs7a_ d2pvba_ d1ttxa_ d1rwya_
d2kyfa_ d1rk9a_ d1a75a_ d1exra_ d1tiza1 d1nyaa_ d1g8ia_ d1ggwa_
d1wdcc_ d3fwba_ d1ggza_ d2o5ga_ d1ctaa_ d1m45a_ d3i5gc_ d1sl8a_
d1fpwa_ d2scpa_ d2roaa_ d4aqra_ d3pn7b_ d1br1b_ d2mysb_ d1s6ca_
d1bjfa_ d2obha_ d1jfka_ d1jf0a_ d1qv0a_ d1lkja_ d2mlfc_ d2m97a_
d2d8na_ d4by5a_ d1jbaa_ d4nqga_ d1wdcb_ d2mysc_ d1j7qa_ d1s6ia_
d1uhka2 d1r2ua_ d1dtla_ d3kf9a_ d3pn7c_ d5d67d_ d1tuza_ d1h8ba_
d1eg3a1 d2zkmx1 d1snla_ d1gjya_ d1y1xa_ d1hqva_ d1dvia_ d1df0a1
d1qxpa2 d4usla_ d1k94a_ d1wkya2 d1uuqa_ d3wh9a_ d4m1ra_ d1xyza_

d4xuya_ d2je8a5 d4bf7a1 d3cuia_ d1ur4a_ d1qw9a2 d1odza_ d2vzsa5
d1bqca_ d2x2ya1 d1hjqa_ d1qnra_ d4y7ea1 d1vjza_ d4k68a_ d2v3ga_
d4hpga_ d1nofa2 d1nq6a_ d1h4pa_ d1aq0a_ d3ziza_ d4bs0a_ d1ecea_
d1h1na_ d1ta3b_ d1w91a2 d1i1wa_ d1uhva2 d1us3a2 d1egza_ d7a3ha_
d1tvna1 d3nyda1 d1ghsa_ d2cyga1 d4xv0a_ d2c8na2 d1w32a_ d3emca_
d1v6ya1 d1foba_ d2uwfa1 d2fgla_ d3niya1 d2c0ha1 d1rh9a1 d1yq2a5
d1szna2 d1fa2a_ d1pama4 d5do8a1 d1ea9c3 d2fhfa5 d1qhoa4 d1avaa2
d1m53a2 d4m8ua1 d2gjpa2 d1lwha2 d4howa1 d1ktba2 d1j0ha3 d3vm5a1
d2x1ia_ d1wzaa2 d1uasa2 d2diea1 d1mxga2 d1ht6a2 d1eswa_ d1iv8a2
d5cpsa_ d1ud2a2 d1zjaa1 d3gbea1 d1kwga2 d1wdpa_ d1gcya2 d1g5aa2
d1hvxa2 d3dhpa2 d2bhua3 d4do4a1 d3qgva1 d1e43a2 d1g94a2 d2d3na2
d1gjwa2 d1gvia3 d1x1na1 d1r7aa2 d1tz7a1 g1apy.1 d3ljqa_ g1k2x.1
d4toqa_ d1wb0a1 d1w9pa1 d1eoka_ d1itxa1 d1kfwa1 d1goia2 d1edqa2
d2gsja_ d1hjxa1 d1jnda1 d1vf8a1 d1umqa_ d1g2ha_ d1t77a2 d1mixa2
d1e5wa2 d1j19a2 d1isna2 d2al6b2 d1g4ma1 d2hmta_ d1lssa_ d1b9ra_
d1offa_ d1frra_ d3wcqa_ d1e9ma_ d1l5pa_ d1wria_ d1i7ha_ d3av8a_
d1czpa_ d1rfka_ d3p1ma1 d3b2ga_ d2bt6a2 d1wgqa1 d1v89a1 d2coda1
d1zc3b_ d1wg7a1 d1u5ea1 d1v61a1 d2coca1 d2ys1a1 d1v5pa1 d1ntya2
d1wjma1 d1wi1a1 d1u29a_ d1xx0a1 d1unqa_ d2zkmx3 d1fhoa_ d1v88a1
d2adza1 d1x1fa1 d1p6sa_ d2coaa1 d1btka_ d1v5ma1 d1faoa_ d2x18a_
d1dyna_ d1x1ga1 d4kaxb_ d2j59m1 d1xcga2 d1txda2 d1u5da1 d1dbha2
d1w1ha_ d1upqa_ d1v5ua1 d1foea2 d2fjla1 d1eaza_ d2dfka2 d2cofa1
d1kz7a2 d1h6ua1 d9ldta1 d2ewda3 d1up7a1 d2dfda1 d1s6ya1 d1vjta1
d7mdha1 d1hyea1 d1t2da1 d1ojsa3 d1b8pa1 d1ldna1 d1hyha1 d1civa1
d2a92a1 d1ez4a1 d1i0za1 d1y6ja1 d1gv0a1 d1ur5a1 d1llda1 d1v8ba1
d1gdha1 d1l7da1 d2dlda1 d1mx3a1 d1ygya1 d1qp8a1 d1j4aa1 d2naca1
d1npya1 d3t4ea2 d1a4ia1 d1o0sa1 d2hk9a2 d2egga2 d1b26a1 d1bvua1
d1gq2a1 d1c1da1 d1leha1 d1gtma1 d1euza1 d1nvta1 d3dona2 d1gpja2
d1vi2a1 d1p77a1 d1nyta1 d1b0aa1 d1vl6a1 d1v9la1 d1txga2 d3f47a1
d1mv8a2 d2pv7a2 d1xc2a3 d2ahra2 d4xiya1 d2f1ka2 d1pgja2 d1i36a2
d1np3a2 d1lj8a4 d1teaa3 d1qmga2 d2i76a2 d2g5ca2 d1wdka3 d1dlja2
d3cuma3 d1kola2 d1f8fa2 d1qora2 d1jqba2 d1vj0a2 d1cdoa2 d1gu7a2
d1d1ta2 d1xa0a2 d2dm6a2 d1jvba2 d1llua2 d1h2ba2 d1tt7a2 d1piwa2
d1o89a2 d1iz0a2 d1e3ja2 d1yb5a2 d1uufa2 d1vj1a2 d1e3ia2 d3gpdg1
d1vc2a1 d2nvwa1 d1u8fo1 d1ydwa1 d1hdgo1 d2g17a1 d1cf2o1 d2czca2
d1r0ka2 d1obfo1 d2cvoa1 d1ebfa1 d1h6da1 d1i32a1 d1zh8a1 d2q49a1
d1qkia1 d1j5pa4 d3pzra1 d1xeaa1 d1vkoa1 d1vm6a3 d3qvsa1 d2pkqo1
d1b7go1 d1gr0a1 d1p1ja1 d1lc0a1 d1j0xo1 d3pwka1 d2hjsa1 d1zyao1
d1vkna1 d1tlta1 d1nvmb1 d3sjua_ d3ojfa1 d1xu9a1 d2jjya1 d1n7ha_
d1y1pa1 d1h5qa_ d1jtva_ d4fs3a_ d2bkaa1 d1gy8a_ d2c5aa1 d1ulsa_
d1ooea_ d2b69a1 d1y5ma1 d1spxa_ d1ae1a_ d1bxka_ d1ydea1 d1gega_
d1hdoa_ d1o5ia_ d1e6ua_ d1snya1 d2rhca_ d3zv4a_ d5ceja_ d2d1ya1
d1fjha_ d3w8ea_ d2fmua1 d1x1ta_ d1rpna_ d3ay7a1 d1k2wa_ d1eq2a_
d2hrba1 d3ruaa_ d2ew8a1 d4hwka_ d1xgka_ d2bd0a1 d1yo6a1 d2a35a1

d2ntva_ d1vl8a_ d1hdca_ d4bkua_ d2ag5a1 d2q45a_ d1nffa_ d4iy1a_
d1zbqa1 d1kewa_ d1ek6a_ d1ja9a_ d2q46a2 d1iy8a_ d1cyda_ d2ptga_
d1hxha_ d1uzma1 d3lyla_ d4m89a_ d3wyea_ d2blla1 d1w6ua_ d1sb8a_
d2q2va_ d4nr0a_ d1e6wa_ d2gasa_ d3oiga1 d2bgka1 d1mxha_ d1vl0a1
d1rkxa_ d1r6da_ d1wmaa1 d1e7wa_ d2c07a1 d1xkqa_ d3tzha_ d1g0oa_
d3lz6a_ d1qyca_ d1edoa_ d1gz6a_ d1oc2a_ d1t2aa_ d1zema1 d2xoxa_
d1z45a2 d1geea_ d3lt0a_ d4e6pa_ d2wyua_ d1db3a_ d4q9na_ d1xhla_
d4o1ma_ d1fmca_ d2uvda_ d2gdza1 d1uaya_ d2z1ma_ d2o23a2 d2fr1a2
d2fr1a1 d1qsga_ d1qyda_ d4zjua_ d2a4ka1 d1yb1a_ d1wvga1 d1yxma1
d3f4ba_ d1mg5a_ d1xg5a1 d1r55a1 d3gboa_ d1kufa_ d1buda_ d4q1la_
d1quaa_ d1yp1a_ d1wnia_ d2gfoa1 d1vjva_ d2ayna1 d3nhea_ d3nqyb_
d4gera_ d1bqba_ d1e2ta_ d4c5pa_ d2bsza1 d4bgfa_ d1w4ta1 d1ex0a4
d1g0da4 d2f4ma1 d1x3za_ d3u1ra1 d1g9ka2 d1k7ia2 d1t3ca_ d1epwa3
d2a97a_ d3fiea_ g1e1h.1 d3ioqa_ d1fh0a_ d1o0ea_ d1khqa_ d1m6da_
d2cb5a_ d1cs8a_ g8pch.1 d1s4va_ d2pnsa_ d1vsna1 d1mira_ d1iwda_
d2oula_ d7pcka_ d3bwka_ d3bcna_ d2p86a_ d1pvja_ d1pcia_ d1gece_
d1deua_ d4bs6a_ d1xkga1 d1cqda_ d1jqpa2 d2c0ya_ d2bdza_ d3p5ua_
d2o36a_ d1ex4a2 d1c6va_ d2jk6a2 d2jk6a1 d1feca1 d1feca2 d1lpfa1
d1lpfa2 d1xhca1 d1xhca2 d1dxla2 d1dxla1 d1gera2 d1m6ia2 d1onfa1
d1onfa2 d3lada2 d3lada1 d1w4xa1 d1w4xa2 d1gesa2 d1mo9a1 d1xdia1
d1fl2a2 d1fl2a1 d1fcda1 d1fcda2 d1h6va2 d1h6va1 d1mo9a2 d1gv4a2
d1gv4a1 d2gv8a2 d2gv8a1 d1aoga1 d1aoga2 d1hyua1 d1ebda1 d1ebda2
d3dk9a1 d1q1ra2 d1q1ra1 d1gesa1 d1w5qa_ d2c1ha_ d1ohla_ d1l6sa_
d1zbfa1 d2z1ha_ d2etja1 d3qioa1 d1jl1a_ d2e4la_ d1jl2a_ d2lsna1
d1io2a_ d1uaxa_ d2yv0x_ d1mu2a1 d1ekea_ d1i39a_ d1ps9a2 d1o94a2
d1cjca1 d1pn0a1 d1reoa1 d1ju2a1 d2f5va1 d3c96a1 d2gmha1 d1pj5a2
d1gpea1 d1o5wa1 d1n4wa1 d1k0ia1 d2dw4a2 d2ivda1 d2voua1 d1kdga1
d1s5ja1 d2hbja2 d2igia_ d1wlja_ d1qhta1 d4dlea1 d1tgoa1 d2f96a1
d3tr8a1 d3mxma_ d1q8ia1 d1y97a1 d1w0ha_ d1kfsa1 d2py5a1 d1noya_
d1yt3a3 d2guia_ d3mwpa1 d4kb1a_ d1xl4a1 d3vsqa_ d2gixa1 d1p7ba1
d2v76a_ d1wj1a1 d1aqca_ d1qqga2 d1qqga1 d1ntva_ d1n3ha_ d4dbba_
d3f0wa1 d1p3ra_ d1wgua1 d2cy4a1 d3hqca_ d1wvha1 d1xr0b1 d1v58a1
d1t3ba1 d4ilfa2 d4npba2 d1jmxa4 d1jmxa3 d1pbya4 d1k8ka2 d1k8ka1
d3uleb1 d3i33a2 d3i33a1 d1bupa1 d1dkgd2 d1dkgd1 d4ci6a2 d1jcfa1
d4a2ba2 d2fxua1 d1yaga2 d1yaga1 d4cbua2 d1r7ha_ d1faaa_ d1wika1
d1xfla_ d1ttza1 d2f51a1 d1ti3a_ d1nhoa_ d1sena_ d2i4aa_ d1ep7a_
d1zmaa1 d1fo5a_ d3lefa1 d3diea1 d2yana1 d1z6na1 d1oaza_ d3zywa1
d4aj8a_ d1nm3a1 d4fiwa_ d1wmja_ d4hs1a1 d1iloa_ d1xwaa1 d1wjka1
d2vm1a_ d1fb6a_ d1gh2a_ d1h75a_ d1nw2a_ d1kx5b_ d3a6na_ d4m6ba2
d2nqbd_ d3wkjd_ d2yfwe_ d4h9na_ d5bnxa_ d1kx5c_ d3r45a_ d1eqzb_
d4jjnb_ d1f66c_ d1eqza_ d4wnnb_ d1kx5a_ d1u35c1 d1id3a_ d2nqbc_
d1kx5d_ d1ku5a_ d1f1ea_ d1b67a_ d1tafa_ d1bh9b_ d1tafb_ d1jfib_
d1h3ob_ d2byka1 d1n1ja_ d4g92b_ d1bh9a_ d1jfia_ d1h3oa_ d2bykb1
d1n1jb_ d1pd212 d1nhya2 d2a2ra2 d1k0dd2 d1f2ea2 d1gsua2 d1f3ba2

d2fhea2 d2pvqa1 d1tw9a2 d1oyja2 d1e6ba2 d1k0ma2 d3ik7a1 d4is0a1
d1r5aa2 d1jlva2 d1m0ua2 d3gtub2 d1z9ha2 d1okta2 d1axda2 d2gsra2
d1tu7a2 d1k3ya2 d1zgma1 d1gnwa2 d1gwca2 d2gsta2 d1glqa2 d1oe8a2
d1pn9a2 d1v2aa2 d1fw1a2 d1duga2 d1y14b1 d2nn6i1 d1i94l_ d2zjra2
d1smxa_ d2qamc2 d1hh2p1 d2r7da1 d1rl2a2 d1wfqa1 d3bzka4 d1x6oa2
d3a0ja_ d2j01d2 d2ja9a1 d4ql5a_ d1hr0w_ d1luza_ d2n3sa_ d2vnud1
d1h95a_ d1go3e1 d2nn6h1 d1ne3a_ d2mo1a_ d1iz6a2 d1jjga_ d1e3pa2
d2ix0a3 d2ix0a2 d2lxja_ d1ueba3 d1ueba2 d2z0sa1 d1u0la1 d2ix0a1
d2eifa2 d2je6i1 d3i2za1 d2c35b1 d2ahob2 d1t9ha1 d3i4oa_ d2qall1
d1jt8a_ d2nn6g1 d1g6pa_ d3cama_ d1i94q_ d1c9oa_ d1d7qa_ d2ba0a1
d2vnud2 d2vnud3 d1khia2 d2qalq1 d1wi5a1 d1xtda2 d1ny4a_ d1qsaa2
d4ilya_ d1chka_ d4olta_ d1qqya_ d1gd6a_ d1f6ra_ d2gv0a_ d1iiza_
d2z2fa_ d1j8xa_ d1ghla_ d1b9oa_ d174la_ d169la_ d2o4wa_ d1p37a_
d1k28a3 d1xjta1 d1jtma_ d2f2qa_ d4e97a1 d2pjsa1 d1klla_ d2i7ra1
d1ecsa_ d1xqaa1 d5cj3a1 d1npba_ d2p7oa_ d1r9ca_ d1nkia_ d1mpya2
d1kw3b2 d1kw3b1 d4ghga1 d1tfza1 d3isqa1 d3isqa2 d1sqia1 d1sqia2
d1lkda2 d1lkda1 d1mpya1 d1cjxa2 d1cjxa1 d1sp9b2 d1t47a1 d1t47a2
d1f1ua2 d1sp8a1 d1sp8a2 d1f1ua1 d2bema_ d1pama1 d1ea9c1 d1qhoa1
d2fhfa2 d2fhfa1 d1k3ia1 d1j0ha1 d1cxla1 d2bhua1 d1edqa1 d1f1sa2
d1ut9a2 d1gvia1 d1txka1 d1s5ja2 d1qhta2 d4dlea2 d1tgoa2 d3py8a2
d1q8ia2 d1kfsa2 d2py5a2 d1d5aa2 d1qsta_ d2beia1 d4m3sa_ d1u6ma1
d1ygha_ d1yk3a1 d3dr6a_ d1xmta_ d1z4ea1 d2ge3a1 d1bo4a_ d2fiaa1
d2jdca1 d1y9wa1 d3to7a_ d1wwza1 d2fl4a1 d2ozua1 d2b5ga_ d2cy2a1
d2p0wa_ d1cm0a_ d4pswa_ d1yrea1 d1yr0a1 d1m4ia_ d2gana1 d1yvka1
d1ghea_ d2g3aa1 d1y7ra1 d2euia1 d1q2ya_ d2fsra1 d1xeba_ d2ae6a1
d1cjwa_ d1qsma_ d2aj6a1 d1y9ka1 d1i12a_ d1vkca_ d2atra1 d1n71a_
d2d4pa1 d2fiwa1 d2fe7a1 d1tiqa1 d2ou2a_ d1yx0a1 d2fcka1 d1p0ha_
d2j8ma_ d2pq8a_ d1z4ra1 d1s3za1 d1ufha_ d2hqya1 d2hqya2 d1lrza2
d1ne9a1 d1lrza3 d1xvqa1 d1pn0a2 d3hy2a_ d2cvba1 d1o8xa_ d1lu4a_
d1we0a1 d2bmxa1 d2a4va1 d1psqa_ d1xiya1 d1q98a_ d1n8ja_ d1zofa1
d2ggta_ d3tjja_ d4je1a1 d1o73a_ d3s9fa_ d2zcta_ d1qmva_ d1jfua_
d2b1ka_ d5dvba1 d2b5xa1 d2v2ga_ d1st9a_ d3tb2a_ d4llra_ d4fh8a1
d1e2ya_ d1knga_ d2h30a_ d1i5ga_ d2z9sa_ d2f8aa1 d1tp9a1 d2b7ka_
d1nm3a2 d3hvva_ d2wfca1 d4kcea_ d1xvwa1 d4gqca1 d3qpma_ d4k1fa_
d1zzoa1 d1gp1a_ d1prxa_ d4od7a1 d1z6ma1 d4mcua1 d4dvca1 d1r4wa_
d3l9sa_ d3rppa_ d2qked_ d4ksoa_ d1t4za1 d1wwja_ d2m5ta_ d1cqqa_
d1wqsa_ d5bpea_ d2ipha_ d1lvoa_ d2bhga1 d3tnta_ d3sjia_ d3d23a1
d2fyqa_ d3tloa_ d3zvea1 d3q3ya1 d4asha1 d2phla2 d2phla1 d1fxza2
d1fxza1 d1uija1 g1dgw.1 d2v09a_ d1uika2 d1uika1 d1dgwa_ d1e88a1
d1l6ja5 d1l6ja4 d1eaka4 d1eaka3 d1l6ja3 d1h8pa2 d1ra6a_ d1muka_
d3gsza_ d1khva_ d1te9a_ d2xwha_ d3uqsa_ d3hkwa_ d3n6ma_ d2cjqa_
d2xyma_ d1teba_ d1te8a_ d1s48a_ d1u09a1 d4wfxa_ d3tyqa_ d2pgga1
d2h1ia1 d2r8ba1 d3b5ea1 d3cn9a_ d1lgya_ d4l3wa_ d1llfa_ d1ukca_
d1cr6a2 d1t61c1 d2ftba_ d1pmpa_ d2rcqa1 d1fdqa_ d4lkpa_ d1sa8a_

d1vyfa_ d1lpja_ d3vg7a1 d4azqa1 d1o8va_ d5bvqa_ d1p6pa_ d2qo4a_
d1opba_ d1yiva1 d1o1va_ d4bvma1 d4tkba_ d4i3ba_ d1bwya_ d1ftpa_
d1ggla_ d4gkca_ d1cbia_ d3p6da1 d1kqwa_ d2a0aa1 d3zxha_ d1cgla_
d1rm8a_ d1eaka2 d1hy7a_ d2jsda_ d1cxva_ d1hv5a_ d1i76a_ d3p0ya1
d1igra1 d1igra2 d1n8yc2 d1n8yc1 d1m6ba2 d1m6ba1 d4dv8a2 d2bk7a_
d3fjua_ d1z5ra1 d1kwma1 d1uwya2 d1jqga1 d2c1ca1 d1dtda_ d1h8la2
d2tbvc_ d1c8nc_ d1x36a_ d1smvc_ d1ng0c_ d4sbvc_ d1f2nc_ d1cixa_
d1y29a_ d1v7fa_ d2jtba_ d1d1ha_ d1g9pa_ d1lupa_ d1qk7a_ d1mb6a_
d2a2va_ d1niya_ d1hvwa_ d2mpqa_ d1ie6a_ d1kqha_ d1la4a_ d2mxoa1
d1dl0a_ d1koza_ d1qk6a_ d1emxa_ d1lqra_ d1c6wa_ d1rmka_ d1ixta_
d1eyoa_ d1fu3a_ d1g1pa_ d1f3ka_ d1fyga_ d1rypd_ d1rypc_ d3unfk_
d1rypa_ d1j2pa_ d1irui_ d1j2qh_ d3unfh_ d1m4ya_ d1rypb_ d1irul_
d1irug_ d1iru2_ d1q5rh_ d1rypg_ d1g3ka_ d1iruf_ d1q5qa_ d1e94a_
d1irub_ d1ryph_ d3ty6a_ d1iruj_ d1iruk_ d1rype_ d1irud_ d1irue_
d3mi01_ d1rypf_ d1iruh_ d2z3ba_ d1iruc_ d3mka2_ d1iru1_ g1gk9.1
g1cp9.1 g1e3a.1 d1mqta_ d1d4m3_ d1fmd3_ d2mev1_ g1aym.1 d1ev13_
d4q4w1_ d1r1a3_ d2x5ia_ d1rhi1_ g1r1a.1 g1d4m.1 g1rhi.1 d1b35b_
d1oopa_ d1tme1_ d4iv1a_ g1pvc.1 g1fpn.1 d1fpn1_ d4gb31_ d1d4m1_
d1fmd1_ d1ev11_ d2wzr3_ g1cov.1 d1qqp3_ g1fmd.1 d2x5ic_ d1rhi3_
g1oop.1 d1hxs1_ d1fpn3_ d1bev1_ g2mev.1 d1eah3_ d1aym1_ d1pvc3_
d1qqp1_ d1zba3_ d1h8ta_ d3vbhc_ g1b35.1 g1bev.1 d1hxs3_ g1ev1.1
d1bev3_ g1tmf.1 d1tmf1_ d1b35a_ d2mev3_ g1eah.1 g1h8t.1 d1eah1_
d4q4w3_ d1r1a1_ d1aym3_ d1pvc1_ d1zba1_ d1h8tc_ d1oopc_ d1tme3_
g1qqp.1 d4iv1c_ g1tme.1 d1p8ba_ d4w9ta_ d1jvna1 d4x2ra1 d1qo2a_
d4axka1 d1thfd_ d1h5ya_ d4u28a_ d2y88a_ d4wd0a_ d1vzwa1 d3exra_
d2ffca1 d2fdsa1 d3n3ma1 d2czda_ d1q6oa_ d2yyua_ d1vqta1 d1dbta_
d3ldva1 d4lt9a1 d3tc7a1 d3hoja_ d1i4na_ d3vnda_ d3nl8a1 d1geqa_
d3nava1 d1v5xa_ d4a2ra1 d3tc6a1 d1ujpa_ d4ijba_ d1xcfa_ d1vc4a_
d1rd5a_ d1qopa_ d3b5va_ d2r80b_ d1cqxa1 d1cg5a_ d1gcvb_ d3boma_
d2h8fb_ d2raoa_ d3bj1a_ d4hrrb_ d2r50a_ d3d1kb_ d2aa1b1 d3zhwa_
d3hf4b_ d3gdja_ d1jebb_ d3bj1b_ d1x9fb_ d1wmub_ d1gcva_ d3bcqb_
d3d4xb_ d1gvha1 d2dc3a_ d3hf4a_ d4yu3a_ d3k8ba_ d1qpwb_ d2r80a_
d3bcqa_ d1v4wa_ d3wtgb_ d1jl7a_ d4esaa_ d3d1ab_ d1fsla_ d4h2la_
d1v4wb_ d1mwca_ d1vhba_ d1jeba_ d1spgb_ d1h97a_ d1q1fa_ d1tu9a1
d3d1aa_ d1hdsb_ d3hyub_ d2zfbb_ d3fs4a_ d1oj6a_ d1shrb_ d4h2lb_
d4yu3b_ d1spga_ d3hrwa_ d3qqra_ d3at5a_ d4wchd_ d3hyua_ d1cg5b_
d1uc3a_ d1hbra_ d2raob_ d1outb_ d4esab_ d3gdjb_ d3qqqa_ d1x9fc_
d3vrfa_ d1itha_ d3mkba_ d3d4xa_ d3mjpa_ d1jl6a_ d3fh9a_ d1outa_
d2dc3b_ d1it2a_ d3eoka_ d3g46a_ d1qpwa_ d3vrfb_ d1ch4a_ d1x9fd_
d1hdsa_ d2zfba_ d3mkbb_ d1or4a_ d3wtga_ d1a4fa_ d3fh9b_ d3bomb_
d3cy5a_ d3at5b_ d1x9fa_ d2oifa_ d4lmxb_ d1cpcb_ d3v57a_ d1kn1a_
d1cpca_ d4f0ta_ d2vjha_ d4po5b_ d1eyxa_ d1alla_ d4f0tb_ d1b8db_
d4h0mb_ d1b33a_ d2vjhb_ d4lm6b_ d2vmlb_ d1allb_ d2vjta_ d3dbja_
d2vmla_ d2vjtb_ d4f0ub_ d1f99b_ d4rmpa1 d1liaa_ d1eyxb_ d2uulb_

d4lmsb_ d4f0ua_ d2bv8a_ d1f99a_ d2bv8b_ d2c7lb_ d3dbjb_ d4rmpb1
d1b8da_ d4h0ma_ d3v57b_ d1kn1b_ d1liab_ d2uula_ d2j96a_ g1gxs.1
g1wht.1 d1azwa_ d1mtza_ d2xqfa_ d1ea5a_ d1f6wa_ d1dx4a_ d1qe3a_
d1k4ya_ d4qwwa_ d2ha2a_ d1r88a_ d1sfra_ d1f0na_ d4qeka_ d3c8da2
d2pbla1 d1jkma_ d1jjia_ d1lzla_ d1jjfa_ d2i49a_ d1nnfa_ d1xvxa_
d1ursa_ d1h3da1 d1iuda_ d4m1va1 d1twya_ d3e13x_ d2nxoa1 d4pfua_
d3ttma_ d1elja_ d2qmwa1 d1us5a_ d1y3na1 d1jeta_ d1uqwa_ d4f19a1
d2esna2 d3thia_ d3fasa1 d2uyeb_ d2xwva1 d1r9la_ d2fyia1 d1pb7a_
d2ozza1 d1sw5a_ d1nh8a1 d1vr5a1 d2g29a_ d1xs5a_ d3lswa_ d2z23a_
d3w9va_ d3tcha_ d1q35a_ d1z7me1 d3q27a_ d1p99a_ d1ve4a1 d2i6ea1
d4mcoa1 d4n8ya_ d1eu8a_ d1zbma1 d1hsla_ d1ixca2 d1j1na_ d1xvya_
d1y9ua_ d2z22a1 d1xoca1 d4oera_ d1wdna_ d5f1qa1 d1xt8a1 d1i6aa_
d4gwoa_ d4igta1 d1pc3a_ d4mmpa1 d1o63a_ d2czla1 d2onsa2 d4mija1
d2qvba_ d2v9za_ d4kafa1 d4iuaa3 d4bvva_ d5hpga_ d3k65a_ d1ki0a3
d1ki0a2 d1ki0a1 d1a0ha1 d1pmla_ d3sp8a3 d2feba1 d1mdwa_ d1qxpa4
d1ziva1 d1zcma1 d1f74a_ d3l21a_ d2e1da_ d1to3a1 d1w3ia_ d1sfla_
d1n7ka_ d1vcva1 d4imea_ d1wa3a_ d2ocza1 d1f2ja_ d3m16a_ d2yxga_
d1ub3a_ d3a5fa_ d1fbaa_ d1l6wa_ d1xfba1 d1wx0a1 d1vpxa1 d1ojxa_
d1mxsa_ d2a4aa1 d1xkya1 d3cq0a1 d3s1xa_ d1f05a_ d1fdja_ d3cpra1
d4d2ja_ d1yloa2 d1y7ea2 d1y0ya3 d1lfwa1 d1vixa1 d1fnoa4 d1vhoa2
d1ysja1 d1vhea2 d1z2la1 d2fvga2 d1vgya1 d1r3na1 d2grea2 d1xmba1
g1q7l.1 d1rtqa_ d3rp2a_ g1ucy.1 d1fuja_ d2zchp_ d3w94a_ d1si5h_
d1lo6a_ g2pka.1 d1cgha_ d1kigh_ d1a5ia_ d3s9ba_ d1hj8a_ d1ao5a_
d2anya_ d1q3xa1 d1gvza_ d1azza_ d4gsoa_ d2f91a1 d1iaua_ d1gdna_
d1eufa_ g1fiw.1 d1eq9a_ d4nfea_ d2rdla_ d1spja_ d1elva1 d1a7sa_
d1ekbb_ d1pfxc_ d1yc0a_ d2aiqa_ g1fiz.1 d1fi8a_ d4k60a_ d4dgja_
d1mzaa_ d1op0a_ d3tjva_ d1pytd_ d1sgfg_ g1gg6.1 d2eeka_ g1rtf.1
d1a0ja_ d3g01a_ d1pytc_ d4e7na_ d1ddja_ d1z8ga1 d2hlca_ d1npma_
d1brup_ g1jou.1 d1sgfa_ d1autc_ g2hnt.1 g1kdq.1 d1orfa_ d3s69a_
d4crga_ d1fxya_ d1bqya_ d1svpa_ d3su6a1 d1cu1a1 d4agka_ d1vcpa_
d1q31a_ g1n1l.1 d3e90b_ d1ep5a_ d3u1jb_ d1dzka_ d4tlja_ d2a2ua_
d4wfva_ d3t1da_ d4iaxa_ d3zq3a_ d2nnda_ d1i4ua_ d1kxoa_ d1bbpa_
d1kt7a_ d1exsa_ d3s26a1 d1lf7a_ d1qfta_ d4gh7a_ d1z24a1 d1qabe_
d1xkia_ d1ew3a_ d3bx7a_ d4iawa1 d1n0sa_ d1gkab_ d3kzaa_ d2k23a_
d2l9ca1 d2joza1 d1gm6a_ d3kffa_ d3saoa1 d1e5pa_ d1yupa1 d1iiua_
d3lgia_ d3i77a_ d2z9ia2 d4nsva_ d1lcya2 d4jcna_ d1agja_ d1os8a_
d1hpga_ d1p3ca_ d1l1ja_ d1qtfa_ d1e69a_ d1mv5a_ d3g60a4 g1ii8.1
d1ewqa2 d1ji0a_ d1v43a3 d2wmfa1 d1vpla_ d1g2912 d4a82a2 g1f2t.1
d2d62a1 d3tuic1 d2bbsa1 d3g60a3 d2ixea_ d1g6ha_ d1sgwa1 d2qi9c_
d3rlfa1 d3b60a1 d3d31a2 d2pzea1 g1xew.1 d1jj7a_ d1oxxk2 d2onka1
d1w1wa_ d1iwea_ d1qf5a_ d1vmaa2 d1nija1 d1np6a_ d2p67a1 d3ihla_
d1ls1a2 d1s1ma2 d1iona_ d2v40a_ d1dj3a_ d3hida_ d2vo1a1 d2g0ta1
d1vcoa2 d1j8mf2 d1g3qa_ d1xjca_ d1p9ba_ d1qzxa3 d1ihua2 d1ihua1
d2qm8a_ d1dj2a_ d4ioja_ d1cp2a_ d1yrba2 d1hyqa_ d3gqba2 d1skye3

d1xp8a1 d1tf7a2 d1tf7a1 d1ye8a1 d1skyb3 d2qe7d2 d4blta_ d4huta_
d3ldaa2 d4hyya_ d2i3ba1 d1cr1a_ d3j9tb2 d3ntua2 d1vdza1 d2z43a_
d1e9ra_ d1nlfa_ d3ziaa2 d1n0wa_ d1g6oa_ d3ziad2 d3j9ta2 d3gqbb2
d1gkub1 d1gkub2 d4ok5a1 d2bmfa1 d2bmfa2 d1yksa1 d1yksa2 d1jr6a_
d2qeqa1 d2jlqa1 d1a1va2 d1eh3a_ d3skpa_ d1dtza2 d1ieja_ d1jnfa2
d1ce2a1 d1jnfa1 d1b1xa2 d1b1xa1 d1jw1a1 d1jw1a2 d1ryoa_ d1h76a1
d1h76a2 d1c4oa1 d2p6ra3 d2p6ra4 d1oywa2 d1oywa3 d1wrba1 d1w36b2
d1w36b1 d1c4oa2 d3bora_ d1t6na_ d2eyqa5 d2eyqa3 d2eyqa2 d2zu6a2
d1veca_ d1uaaa1 d1w36d1 d2g9na1 d1z3ix2 d2rb4a_ d1hv8a1 d1hv8a2
d1t5ia_ d1w36c2 d2j0sa2 d2j0sa1 d1fuka_ d3hjha1 d1z5za1 d1qdea_
d1z63a1 d1s2ma2 d1s2ma1 d1wp9a1 d1wp9a2 d1gm5a3 d1gm5a4 d3dina4
d3dina3 d1q0ua_ d1w36c1 d1z3ix1 d1nkta3 d1uaaa2 d1w36d2 g1qhh.1
d2fwra2 d2fwra1 d3q3ma_ d1za0a1 d1mxra_ d2inca_ d1jk0a_ d2incb_
d1mhyb_ d3hf1b_ d1mtyb_ d1r2fa_ d2uw1a_ d1mhyd_ d3n37a_ d1afra_
d1oquc_ d1syya_ d1mtyd_ d2o1za_ d1uzra_ d1smsa_ d2fkza_ d1zuja1
d1s3qa1 d2fjca1 d1z6om1 d1veia_ d4dyya_ d1jiga_ d3gvya_ d2pyba_
d4cy9a1 d3bvfa1 d1r03a_ d2cf7a_ d1nf4a_ d1zs3a1 d4iwka_ d3ka8a_
d3np0x_ d1n1qa_ d1lb3a_ d4e6ka_ d1z6oa1 d2fzfa1 d4dyua_ d3ge4a_
d1jgca_ d4am4a_ d2z6ma_ d1ji5a_ d1vlga_ d1lkoa1 d1krqa_ d3qz3a_
d1o9ra_ d2ffxj_ d1euma_ d1yv1a1 d2v2pa_ d1j30a_ d4di0a_ d1dpsa_
d1oewa_ d2qzxa_ d2asia_ d1psoe_ d1miqa_ d1smra_ d2h6ta_ d1pfza_
d1izda_ d3hd8a_ d1j71a_ d1dpja_ d4aa9a_ d1t6ex_ g1b5f.1 g1lya.1
g1htr.1 d1qdma2 d1bxoa_ d1wkra_ d1tzsa_ d1ibqa_ d2qzwa_ d1bdqa_
d3ggta_ d3ixoa_ d1mrxa_ d2haha_ d1tw7a_ d4njsa_ d1sgua_ d2p3da_
d1nsoa_ d3d3ta_ d2rkfa_ d1c6ya_ d2rspa_ d3ogpa_ d3ekwa_ d2o4na_
d2j9ja_ d1baia_ d3ggua_ d3lzua_ d4npua_ d4j55a_ d2r8na_ d2fxda_
d2p3ca_ d4dqea_ d3ttpa_ d3t3ca_ d2p3aa_ d2hc0a_ d2fdda_ d3mwsa_
d1k8rb_ d1wgra1 d1ef5a_ d1wfya1 d1e8ya3 d1wgya1 d1lfda_ d2cs4a1
d1wxma1 d2byea1 d3kh0a1 d2byfa1 d1wxaa1 d1wgda1 d1wgga1 d4q5eb_
d1wjna1 d1se9a_ d3n3kb_ d1wz0a1 d1we6a1 d2m0xa_ d2gbka_ d3rt3b1
d1wh3a1 d1sifa_ d2ekec2 d4ii2b1 d4hk2a1 d1c3ta_ d1m94a_ d1wgha1
d1ttna1 d1wx8a1 d3v6cb1 d2ma9b_ d4i6lb1 d1bt0a_ d2jvca1 d1iyfa_
d2k1fa_ d1wxva1 d1wf9a1 d1t0ya_ d2io0b2 d1x1ma1 d2gbma_ d1zw7a1
d2m17a_ d1ud7a_ d1we7a1 d2lwpa_ d2gbja_ d1wjua1 d1v6ea1 d1v86a1
d4eewa_ d1wiaa1 d4icva_ d4gu2a_ d1wx7a1 d1wy8a1 d4hjka1 d2faza1
d1v5oa1 d3ruib_ d3waob_ d2r2qa1 d3m95a_ d3wana_ d2al3a1 d2cr5a1
d1wj4a1 d1s3sg_ d1g66a_ d1qoza_ d5ajha1 d4lu0a_ d1of8a_ d3unda1
d3e9aa1 d1vr6a2 d1n8fa_ d2qkfa_ d1d9ea_ d2bcgg1 d2bcgg2 d1d5ta1
d1um8a_ d1ixza_ d1qvra2 d1qvra3 d1sxjc2 d1jr3d2 d2p65a_ d1l8qa2
d1g8pa_ d3hu3a3 d1sxjd2 d1njfa_ d1in4a2 d1sxja2 d1tuea1 d2ce7a2
d1w5sa2 d1sxjb2 d1d2na_ d1ny5a2 d1ofha_ d2gnoa2 d2a5yb3 d1fnna2
d1u0ja1 d1lv7a_ d1iqpa2 d2fnaa2 d1ixsb2 d4gita_ d1sxje2 d1g41a_
d2e3fa_ d2e3ea_ d2llda_ d2ny8x_ d2e3ga_ d2ny9x_ d2nz3a_ d2lj7a_
d2m8bb1 d2kyqa_ d3psma_ d1jkza_ d1jxca_ d2gl1a_ d1ugla_ d4uj0a_

d2k4ua_ d1hp2a_ d2lu9a_ d1baha_ d2meoa1 d1wm7a_ d1hlya_ d1n8ma_
d1jlza_ d1wpda_ d1c55a_ d1pvza_ d1wt7a_ d1rjia_ d1wz5a_ d1m2sa_
d1pjva_ d1c49a_ d1qkya_ d2ktca1 d1wmta_ d1j5ja_ d1r1ga_ d1t0za_
d2i61a_ d1t1ta_ d1sn4a_ d1kv0a_ d2lkba_ d1npia_ d1wwna_ d1pe4a_
d2kbka_ d2li7a_ d1djta_ d2kbha_ d1dq7a_ d1sega_ d1omya_ d1zywa1
d1n9wa1 d1e1oa1 d1c0aa1 d1b8aa1 d1l0wa1 d3a74a1 d1eova1 d1bvsa3
d1ixra2 d1iyjb4 d1iyjb5 d1iyjb3 d1o7ia_ d4ipca1 d1x3ea_ d4apva_
d1jb7a1 d1jb7a2 d1jb7a3 d3kdfa_ d1ue1b_ d1wjja1 d3eiva_ d1xjva2
d1xjva1 d2ccza2 d1se8a_ d1l1oc_ d1jb7b_ d1qvca_ d3ulla_ d1qzga_
d1miua4 d1miua5 d1miua3 d3g48a1 d1gd7a_ d1jjcb3 d1pyba_ d3ubub_
d1egia_ d1qo3c_ d1v7pa_ d1tdqb_ d3ubua_ d1c3ab_ d1h8ua_ d3bx4b_
d1t8ca1 d1sb2a1 d1qdda_ d2ziba1 d3cada_ d1byfa_ d1j34b_ d1jwia_
d1jzna_ d1c3aa_ d1oz7b_ d1wmza_ d3bdwb_ d1hq8a_ d1fifa1 d1dv8a_
d3hupa_ d1wk1a1 d1umra_ d2afpa_ d3bx4a_ d1gz2a_ d1sl6a_ d1v7pb_
d3g8ka_ d1ixxa_ d1j34a_ d1jwib_ d1g1sa1 d1y17a1 d1ja3a_ d1fvub_
d3bdwa_ d1sb2b1 d1fvua_ d1umrc_ d1oz7a_ d4m17a_ d3cfwa1 d1ypqa1
d1g1ta1 d3fd4a_ d3l9jc_ d4dn8a1 d1v4lb_ d1rdl1_ d2msba_ d1dgsa2
d1fvia1 d1p16a1 d1x9na2 d1pfsa_ d2a1ka_ d1je5a_ d1rpma_ d1yfoa_
d1l8ka_ d2ooqa1 d1g4us2 d1p15a_ d1lara2 d1lara1 d1wcha_ d2fh7a2
d2fh7a1 d1ohea1 d2f4da_ d1rxda_ d2hxpa_ d1xria_ d1d5ra2 d1fpza_
d3ezza_ d4y2ea_ d1i9sa_ d3azya_ d3wvja_ d1upsa1 d1ajoa_ d2hyka_
d1ajka_ d2vy0a_ d3o5sa_ d3d6ea_ d3atga_ d1dypa_ d1umza_ d3i4ia_
d1c1la_ d4lbqa_ d1a78a_ d1slta_ d4no4a_ d3zsja_ d3duia_ d1g86a_
d1is3a_ d1qmja_ d1tdha3 d1q3ba3 d1ee8a3 g1qmo.1 g1len.1 d3zyra_
d1fnya_ d1n47a_ g2ltn.1 d1avba_ d3usua_ d1dhkb_ d1f9ka_ d1ukga_
d1g8wa_ d1g9fa_ d1wbfa_ d3wcsa_ d1g7ya_ g1loe.1 d1mvqa_ d1hqla_
d1v6ia_ d1dbna_ d1fx5a_ d4l8qa_ d1ioaa_ d3n35a_ d3h33a_ d1rwja_
d19hca_ d1os6a_ d3bxua_ d3h4na_ d1i77a_ d3caoa_ d2ctha_ d1duwa_
d1ofwa_ d2e84a_ d1hh5a_ d1gyoa_ d3h34a_ d1dszb_ d1a6ya_ d3cbba_
d1ynwa1 d1r4ia_ d1hcqa_ d2hana_ d2hanb_ d2enva1 d1kb4b_ d2nllb_
d1lo1a_ d1cita_ d2dara2 d2dara1 d1wyha2 d2dloa2 d1rutx4 d1rutx3
d2cuqa2 d1x4la2 d1x4la1 d1x3ha2 d1x3ha1 d2d8za1 d1x61a2 d1x6aa1
d1x6aa2 d2cora2 d2cora1 d1v6ga1 d1v6ga2 d2d8za2 d1x64a1 d1x64a2
d1b8ta1 d2co8a1 d1x63a2 d2dj7a2 d2dj7a1 d2cu8a2 d2cu8a1 d1g47a2
d1g47a1 d1j2oa1 d1wyha1 d2dloa1 d1wiga1 d1wiga2 d2cupa2 d2cupa3
d1x68a1 d2d8ya2 d2d8ya1 d1m3va2 d1x62a1 d1x68a2 d1x61a1 d1b8ta3
d2d8xa2 d2d8xa1 d1x4ka1 d1x4ka2 d2cuqa1 d1m3va1 d2co8a2 d1j2oa2
d1x63a1 d2cura1 d2cura2 d1x62a2 d2aq2b1 d1ck1a1 d1enfa1 d1i4pa1
d1v1oa1 d1et9a1 d1esfa1 d2g9hd1 d4ohja1 d1klud1 d1eu3a1 d1ty0a1
d1fnua1 d1r0ka3 d1vkoa2 d1vm6a2 d3qvsa2 d1gr0a2 d1p1ja2

**Family-20 (b.1.1.4)**

**Positive dataset contains 21 protein sequences.**

d2cqva1 d2k1ma_ d3qp3a1 d1xmwa2 d2otpa2 d2dava1 d1waaa_ d1x44a1
d3b5ha2 d3h8na2 d3h8na1 d1pd6a_ d3cx2a_ d3b5ha1 d1xiwb_ d1xaua_

d2ifga1 d2gw5a2 d1sy6a1 d1xiwa_ d2c9aa1

**Negative dataset contains 3151 protein sequences.**

d1m32a_ d1ibja_ d1c7na_ d1iuga_ d4q75a_ d2bkwa1 d1t3ia_ d1n8pa_
d1qz9a_ d1jf9a_ d1eg5a_ d1p3wa_ d3kgwa_ d1cl1a_ d2ctza1 d1vjoa_
d2rfva_ d1gc0a_ d1cs1a_ d1d2fa_ d2ch1a1 d1qgna_ d1pffa_ d1elua_
d1ax4a_ d1c7ga_ d2v1pa1 d2ez1a_ d1u08a_ d1lc5a_ d1b5pa_ d2ay1a_
d1bw0a_ d3pd6a_ d7aata_ d2csta_ d1j32a_ d1fg7a_ d4wlha_ d1v2da_
d1gdea_ d1m6sa_ d1v72a1 d1svva_ d1ajsa_ d1wsta1 d2f8ja2 d1lk9a_
d1vp4a1 d1xi9a1 d2gb3a1 d1yaaa_ d1xkta1 d2hfka_ d1jmkc_ d5d3ka_
d2cfba1 d1dfoa_ d1o69a_ d1bj4a_ d3k28a_ d2bwpa_ d3lg0a_ d2fnua_
d3ou5a_ d1z7da1 d1b9ha_ d1iaya_ d4msoa_ d1bs0a_ d1s0aa_ d1sffa_
d3tqxa_ d3qboa_ d3pgya_ d1bjna_ d2dkja_ d1ejia_ d1vefa1 d4wxga_
d3bs8a_ d3gbxa1 d1w23a_ d3lgia_ d3i77a_ d2z9ia2 d4nsva_ d1lcya2
d4jcna_ d1agja_ d1os8a_ d1hpga_ d1p3ca_ d1l1ja_ d1f56a_ d1bypa_
d1azca_ d1ws8a_ d3u25a_ d1cuoa_ d1id2a_ d1rkra_ d2iaac_ d4dp1x_
d1nwpa_ d2gima_ d2i7sa_ d1qhqa_ d1gyca3 d1hfua2 d1kbva1 d1kbva2
d1sdda1 d1sdda2 d1gyca1 d1gyca2 d1mzya1 d2bw4a1 d1aoza1 d1aoza3
d1aoza2 d1kyaa2 d1kyaa3 d1kyaa1 d1mzya2 d4e9sa1 d1sddb1 d1sddb2
d1hfua1 d1hfua3 d1oe1a2 d1oe1a1 d1v10a2 d1v10a3 d1v10a1 g1f8v.1
d1novc_ d2bema_ d1pama1 d1ea9c1 d1qhoa1 d2fhfa2 d2fhfa1 d1k3ia1
d1cxla1 d2bhua1 d1edqa1 d1f1sa2 d1ut9a2 d1gvia1 d1txka1 d4alac_
d1rera1 d1s6na_ d3uzqb_ d2phla2 d2phla1 d1fxza2 d1fxza1 d1lrha_
d1uija1 g1dgw.1 d2v09a_ d1uika2 d1uika1 d1dgwa_ d1ds6b_ d1fstb_
d1ex0a1 d1g0da1 d1tafa_ d1bh9b_ d1tafb_ d1jfib_ d1h3ob_ d2byka1
d1n1ja_ d4g92b_ d1bh9a_ d1jfia_ d1h3oa_ d2bykb1 d1n1jb_ d1ydwa2
d2nvwa2 d1zh8a2 d1qkia2 d1xeaa2 d1tlta2 d1e3oc2 d1ic8a2 d2ovga_
d1sq8a_ d1rzsa_ d3kz3a_ d3orca_ d3gpdg2 d2q49a2 d1hdgo2 d2g17a2
d2cvoa2 d2czca1 d3pwka2 d1obfo2 d1i32a2 d1zyao2 d2pkqo2 d1b7go2
d2hjsa2 d1cf2o2 d1vkna2 d1nvmb2 d1ux8a_ d1dlya_ d4i0va_ d2bkma_
d1dlwa_ d2bmma_ d2msya_ d2djoa1 d2r5za_ d1au7a1 d1f43a_ d2lmda1
d1akhb_ d1s7ea1 d3nara_ d1zq3p1 d2craa1 d1mija_ d4uuta_ d3a01a_
d1x58a1 d3rkqa1 d3cmya1 d2cqxa1 d2da6a1 d1e3oc1 d1fjla_ d1mh3a1
d1ig7a_ d1x41a1 d1wh7a1 d1gt0c1 d2mw8a_ d1lfup_ d1x2ma1 d1b72a_
d1wi3a1 d1wjha1 d1x2na1 d1mnmc_ d2cuea1 d3k2aa_ d2hosa_ d2jwta2
d1pufa_ d1octc1 d2hi3a_ d1b8ia_ d4j19a_ d3lnqa_ d1wh5a1 d1b8ib_
d1fexa_ d2cqra1 d1guua_ d1ug2a1 d2cqqa1 d2ajea1 d4a69c_ d1h8ac1
d2crga1 d2iw5b1 d1xc5a1 d1ofcx1 d2cu7a1 d1wgxa1 d2cjja1 d2ckxa1
d1hcra_ d1tc3c_ d1gdta1 d1u78a2 d1xg1a1 d1itya_ d2iu5a1 d1zk8a1
d2g7sa1 d2fd5a1 d2np5a1 d1z0xa1 d2id6a1 d2d6ya1 d2i10a1 d1vi0a1
d2oi8a1 d3c07a1 d2ns7a1 d2fq4a1 d2yvea1 d1t33a1 d2o7ta1 d1rkta1
d2np3b1 d2g7ga1 d2gena1 d2fbqa1 d2g7la1 d2gfna1 d2g3ba1 d2jj7a1
d2hyja1 d1sgma1 d1ui5a1 d2hkua1 d2id3a1 d4jyka1 d1bl0a1 d1bl0a2
d1d5ya1 d3ubub_ d1egia_ d1qo3c_ d1v7pa_ d1tdqb_ d3ubua_ d1c3ab_
d1h8ua_ d3bx4b_ d1t8ca1 d1sb2a1 d1qdda_ d2ziba1 d3cada_ d1byfa_

d1j34b_ d1jwia_ d1jzna_ d1c3aa_ d1wmza_ d3bdwb_ d1hq8a_ d1fifa1
d1dv8a_ d3hupa_ d1wk1a1 d1umra_ d2afpa_ d3bx4a_ d1gz2a_ d1sl6a_
d1v7pb_ d3g8ka_ d1ixxa_ d1j34a_ d1jwib_ d1g1sa1 d1y17a1 d1ja3a_
d1fvub_ d3bdwa_ d1sb2b1 d1fvua_ d1umrc_ d1oz7a_ d4m17a_ d3cfwa1
d1ypqa1 d1g1ta1 d3fd4a_ d3l9jc_ d4dn8a1 d1v4lb_ d1rdl1_ d2msba_
d3lv8a_ d1s3ga1 d1zaka1 d1s4qa_ d3tvta2 d1uf9a_ d1khta_ d1ckea_
d1osna_ d1p5zb_ d4tmka_ d3cm0a_ d1tmka_ d1jaga_ d3h86a_ d1t0hb_
d1nn5a_ d2h92a_ d3kb2a1 d2c95a1 d1yj5a2 d1kgda_ d1ly1a_ d1vyva2
d1deka_ d2grja1 d1p3ja1 d1y63a_ d1e4ya1 d1s96a_ d3iija1 d1lvga_
d1nksa_ d1vyua2 d4e22a_ d1qf9a_ d1jjva_ d1q3ta_ d1ki9a_ d1lw7a2
d1teva_ d1kjwa2 d1gsia_ d2iyva2 d1kaga_ d1e6ca_ d1g3ma_ d1j99a_
d1t8ta1 d1nsta_ d1fmja_ d1zrha_ d1ls6a_ d2a3ra_ d1aqua_ d2zptx_
d3ckla_ d1texa_ d1q44a_ d2gwha_ d1uj2a_ d4gi7a_ d2jeoa1 d1rz3a_
d1z0ja1 d3umra2 d3gj0a1 d2gaoa1 d1yzga1 d1x3sa1 d1g7sa4 d1puja_
d1udxa2 d1kk1a3 d2o52a_ d1jwyb_ d1zcaa2 d1zcba2 d2fv8a1 d2cjwa_
d3dz8a1 d1xzpa2 d1vg8a_ d4qxaa_ d2ew1a1 d1x1ra1 d2bcgy_ d3doea1
d3cona_ d3rega_ d3wyga_ d1upta_ d2b6ha1 d1zd9a1 d1lnza2 d1r5ba3
d1n0ua2 d1r2qa_ d2f9la1 d2rhda_ d3q3jb_ d3q85a_ d1efma1 d1r8qa_
d2gf9a1 d2akab1 d1ek0a_ d1z08a1 d2gcoa1 d2dy1a2 d4g01b_ d2cxxa1
d1svia_ d3q72a_ d1ni3a1 d4ac9a4 d2erxa1 d2bmea_ d1wxqa1 d2gf0a1
d4ldja1 d1zunb3 d1d5ca_ d4lv5b_ d1efca3 d1kmqa_ d2g6ba1 d3lrpa_
d1tada2 d2y8ea_ d4rd4a1 d1z06a1 d2heia_ d3a58b_ d1u0la2 d1d2ea3
d1zj6a1 d2ocba_ d3rwoa_ d1mr3f_ d2e9sa1 d4u5xa1 d2iwra_ d2fn4a1
d3bwdd_ d2nzja1 d2atva1 d2ksqa_ d3l43a_ d1mkya2 d1mkya1 d1wmsa_
d1jnya3 d2rmka_ d1f6ba_ d1f60a3 d1gota2 d1jala1 d2fh5b1 d1g16a_
d2wblc_ d1m7ba_ d1wf3a1 d1f5na2 d1t9ha2 d1z6xa1 d1z0aa1 d2fg5a1
d2a5ja1 d2bcjq2 d1s0ua3 d1ky3a_ d4y0va_ d4lhwa_ d2qmea1 d2atxa1
d1egaa1 d1ryha_ d1moza_ d1nrjb_ d2erya1 d3bfka_ d1puia_ d3j3az1
d3t5ga_ d1shza2 d3defa_ d2f7sa1 d3raba_ d3tsoa_ d3aq4a_ d2al7a1
d3sfva1 d3bc1a_ d2ncda_ d1sdma_ d3h4sa1 d2heha_ d2grya_ g3kin.1
d1br2a2 d1goja_ d1f9va_ d2mysa2 d1lkxa_ d3t0qa_ d4db1a1 d3gbja_
d1ry6a_ d1v8ka_ d1k8ka2 d1k8ka1 d3uleb1 d3i33a2 d3i33a1 d1bupa1
d1dkgd2 d1dkgd1 d4ci6a2 d1jcfa1 d4a2ba2 d2fxua1 d1yaga2 d1yaga1
d4cbua2 d1bupa2 d1g99a1 d2e1za2 d2e1za1 d1saza1 d1saza2 d1iqca2
d1iqca1 d1eb7a1 d1nmla2 d1nmla1 d3h3no1 d3h3no2 d3ezwa2 d3h1jd1
d1hroa_ d4gedb_ d3zooa_ d1mz4a_ d1gu2a_ d1f1fa_ d1dvva_ d1c6ra_
d1c75a_ d1e29a_ d1gdva_ d1cnoa_ d1h32b_ d1co6a_ d2bh4x_ d2aiua_
d5cytr_ d1jdla_ d1i8oa_ d2zboa_ d3dmia_ d1ls9a_ d1wvec_ d2yk3a_
d3ph2b_ d1dw0a_ d5aura_ d1ql3a_ d1qn2a_ d1f1ca_ d4zzva_ d2xspa_
d4v20a_ d1ojja_ d4ipma1 d1gpia_ d4csia_ d3pfza_ d3ovwa_ d2yoka_
d2nqya_ d1h4ga_ d1oa2a_ d1olra_ d3b7ma_ d4h7ma1 d3wp3a_ d2vgda_
d1igoa_ d3zsea_ d2nlra_ d3wx5a_ d1oa3a_ d1pvxa_ d1m4wa_ d1f5ja_
d1oa4a_ d1ks5a_ d4ixla1 d1evsa_ d1cnt1_ d1pvhb_ d1z7ca_ d1huwa_
d1bgea_ d1rhga_ d1f6fa_ d1f45b_ d1i1rb_ d1etea_ d3tgxb_ d1scfa_

d2z3qa1 d3qb1a_ d1hula_ d1m48a_ d4nkqb_ d1v7mv_ d5e4ea_ d2o27a_
d3b5ka_ d2psma1 d4zf7a_ d3uf5a_ d4x51a_ d1d9ca_ d4ypgc1 d3oq3a_
d1lqsl_ d1n1fa_ d1au1a_ d1wu3i_ d3besl_ d3ux9a_ d1v8fa_ d2ejca_
d3muea1 d3q12a1 d3rbaa_ d2qtra_ d1nupa_ d3l92a_ d1f9aa_ d1qjca_
d2h29a_ d1vlha_ d1od6a_ d1lw7a1 d1mrza2 d4ruka_ d2el7a_ d1iq0a2
d1r6ta2 d1ivsa4 d2cyba_ d1rqga2 d1j09a2 d2yxna_ d1f7ua2 d1h3fa1
d1ffya3 d2g36a1 d1nzja_ d1jila_ d1n3la_ d1i6la_ d1pfva2 d1h3na3
d3n9ia_ d3sz3a1 d3sw2a_ d1moxc_ d1dx5i3 d1edmb_ d1autl2 d1autl1
d1nqlb_ d1dval1 d1szba2 d1kigl_ d2y5fl_ d1ijqa2 d1gk5a_ d1g1sa2
d1nt0a3 d1xdtr_ d1lmja2 d1lmja1 d1nzia2 d2wphe_ d1klil_ d1pfxl2
d1pfxl1 d1p9ja_ d1g1ta2 d1f7ea_ d4yzub_ d1gl4a2 d1qx2a_ d2y5ia_
d1qlsa_ d1psra_ d3n22a_ d2egda_ d4ggfc_ d2wcea_ d2luca_ d4aqia_
d3nsia_ d2lp3a_ d1j55a_ d1a4pa_ d1k8ua_ d1nsha_ d1pvaa_ d3fs7a_
d2pvba_ d1ttxa_ d2kyfa_ d1rk9a_ d1a75a_ d1exra_ d1tiza1 d1nyaa_
d1g8ia_ d1ggwa_ d1wdcc_ d3fwba_ d1ggza_ d2o5ga_ d1ctaa_ d1m45a_
d3i5gc_ d1sl8a_ d1fpwa_ d2scpa_ d2roaa_ d4aqra_ d3pn7b_ d1br1b_
d1s6ca_ d1bjfa_ d2obha_ d1jfka_ d1jf0a_ d1qv0a_ d1lkja_ d2mlfc_
d2m97a_ d2d8na_ d4by5a_ d1jbaa_ d4nqga_ d1wdcb_ d2mysc_ d1j7qa_
d1s6ia_ d1uhka2 d1r2ua_ d1dtla_ d3kf9a_ d3pn7c_ d5d67d_ d1tuza_
d1h8ba_ d1eg3a1 d1eg3a2 d2zkmx1 d1snla_ d1gjya_ d1y1xa_ d1hqva_
d1dvia_ d1df0a1 d1qxpa2 d4usla_ d1k94a_ d1wkya2 d1uuqa_ d3wh9a_
d4m1ra_ d1xyza_ d4xuya_ d2je8a5 d4bf7a1 d3cuia_ d1ur4a_ d1qw9a2
d1odza_ d2vzsa5 d1bqca_ d2x2ya1 d1qnra_ d4y7ea1 d1vjza_ d4k68a_
d2v3ga_ d4hpga_ d1nofa2 d1nq6a_ d1h4pa_ d1aq0a_ d3ziza_ d4bs0a_
d1ecea_ d1h1na_ d1ta3b_ d1w91a2 d1i1wa_ d1uhva2 d1us3a2 d1egza_
d7a3ha_ d1tvna1 d3nyda1 d1ghsa_ d2cyga1 d4xv0a_ d2c8na2 d1w32a_
d3emca_ d1v6ya1 d1foba_ d2uwfa1 d2fgla_ d3niya1 d2c0ha1 d1rh9a1
d1yq2a5 d1szna2 d1fa2a_ d1pama4 d5do8a1 d1ea9c3 d1hx0a2 d2fhfa5
d1qhoa4 d1avaa2 d1m53a2 d4m8ua1 d2gjpa2 d1lwha2 d4howa1 d1ktba2
d1j0ha3 d3vm5a1 d2x1ia_ d1wzaa2 d1uasa2 d2diea1 d1mxga2 d1ht6a2
d1eswa_ d1iv8a2 d5cpsa_ d1ud2a2 d1zjaa1 d3gbea1 d1kwga2 d1wdpa_
d1gcya2 d1g5aa2 d1hvxa2 d3dhpa2 d2bhua3 d4do4a1 d3qgva1 d1e43a2
d1g94a2 d2d3na2 d1gjwa2 d1gvia3 d1x1na1 d1r7aa2 d1tz7a1 g1apy.1
d3ljqa_ d4toqa_ d1wb0a1 d1w9pa1 d1eoka_ d1itxa1 d1kfwa1 d1goia2
d1edqa2 d2gsja_ d1hjxa1 d1jnda1 d1vf8a1 d3wdpp1 d3wq5a_ d4gxpa_
d4hz8a_ d1e4mm_ d3aiua_ d1wcga1 d1qvba_ d3ahxa_ d1v02a_ d2dgaa_
d1pbga_ d1vffa1 d1qoxa_ d4ha4a_ d2w0pa_ d2diaa1 d2e9ia2 d2e9ja1
d2dj4a1 d2eeba1 d2diba1 d2dmba1 d2dmca1 d2d7oa1 d2ee6a1 d2d7pa1
d2eeaa1 d2di8a1 d1wlha1 d2d7na1 d2nqca_ d2d7ma1 d2dica1 d3cnka_
d2k3ta1 d2eeca1 d1qfha1 d2bp3a1 d1v05a_ d2ee9a1 d2eeda1 d2di9a1
d3syxa1 d1mkea1 d2p8va_ d1xoda1 d1i7aa_ d1evha_ d1qc6a_ d1egxa_
d1b9ra_ d1offa_ d1frra_ d3wcqa_ d1l5pa_ d1wria_ d1i7ha_ d3av8a_
d1czpa_ d1rfka_ d3p1ma1 d3b2ga_ d2bt6a2 d1wgqa1 d1v89a1 d2coda1
d1zc3b_ d1wg7a1 d1u5ea1 d1v61a1 d2coca1 d2ys1a1 d1v5pa1 d1ntya2

d1wjma1 d1wi1a1 d1u29a_ d1xx0a1 d1unqa_ d2zkmx3 d1fhoa_ d1v88a1
d2adza1 d1x1fa1 d1p6sa_ d2coaa1 d1btka_ d1v5ma1 d1faoa_ d2x18a_
d1dyna_ d1x1ga1 d4kaxb_ d2j59m1 d1ki1b2 d1xcga2 d1txda2 d1u5da1
d1dbha2 d1w1ha_ d1upqa_ d1v5ua1 d1foea2 d2fjla1 d1eaza_ d2dfka2
d2cofa1 d1kz7a2 d1h6ta1 d9ldta1 d2ewda3 d1up7a1 d2dfda1 d1s6ya1
d1vjta1 d1hyea1 d1t2da1 d1ojsa3 d1b8pa1 d1ldna1 d1hyha1 d1civa1
d2a92a1 d1ez4a1 d1i0za1 d1y6ja1 d1gv0a1 d1ur5a1 d1llda1 d1obba1
d1v8ba1 d1gdha1 d1l7da1 d2dlda1 d1mx3a1 d1ygya1 d1qp8a1 d1li4a1
d2naca1 d1npya1 d3t4ea2 d1a4ia1 d1o0sa1 d2hk9a2 d2egga2 d1b26a1
d1bvua1 d1gq2a1 d1c1da1 d1gtma1 d1euza1 d1nvta1 d3dona2 d1edza1
d1gpja2 d1vi2a1 d1p77a1 d1nyta1 d1b0aa1 d1vl6a1 d1v9la1 d1txga2
d3f47a1 d1mv8a2 d2pv7a2 d1xc2a3 d2ahra2 d4xiya1 d2f1ka2 d1pgja2
d1i36a2 d1np3a2 d1lj8a4 d1teaa3 d1qmga2 d2i76a2 d2g5ca2 d1wdka3
d1dlja2 d3cuma3 d1kola2 d1f8fa2 d1qora2 d1vj0a2 d1cdoa2 d1gu7a2
d1d1ta2 d1xa0a2 d2dm6a2 d1jvba2 d1rjwa2 d1llua2 d1h2ba2 d1tt7a2
d1piwa2 d1o89a2 d1iz0a2 d1e3ja2 d1yb5a2 d1uufa2 d1vj1a2 d1e3ia2
d3gpdg1 d1vc2a1 d2nvwa1 d1u8fo1 d1ydwa1 d1hdgo1 d2g17a1 d1cf2o1
d2czca2 d1r0ka2 d1obfo1 d2cvoa1 d1ebfa1 d1h6da1 d1i32a1 d1zh8a1
d2q49a1 d1qkia1 d1j5pa4 d3pzra1 d1xeaa1 d1vkoa1 d1vm6a3 d3qvsa1
d2pkqo1 d1b7go1 d1gr0a1 d1p1ja1 d1lc0a1 d1j0xo1 d3pwka1 d2hjsa1
d1zyao1 d1vkna1 d1tlta1 d1nvmb1 d3sjua_ d3ojfa1 d1xu9a1 d2jjya1
d1n7ha_ d1y1pa1 d1h5qa_ d1jtva_ d4fs3a_ d2bkaa1 d1gy8a_ d2c5aa1
d1ulsa_ d1ooea_ d2b69a1 d1y5ma1 d1spxa_ d1ae1a_ d1bxka_ d1ydea1
d1gega_ d1hdoa_ d1o5ia_ d1e6ua_ d1snya1 d2rhca_ d3zv4a_ d5ceja_
d2d1ya1 d1fjha_ d3w8ea_ d2fmua1 d1x1ta_ d1rpna_ d3ay7a1 d1k2wa_
d1eq2a_ d2hrba1 d3ruaa_ d2ew8a1 d4hwka_ d1xgka_ d2bd0a1 d1yo6a1
d2a35a1 d2ntva_ d1vl8a_ d1hdca_ d4bkua_ d2ag5a1 d2q45a_ d1nffa_
d4iy1a_ d1zbqa1 d1kewa_ d1ek6a_ d1ja9a_ d2q46a2 d1iy8a_ d1cyda_
d2ptga_ d1hxha_ d1uzma1 d3lyla_ d4m89a_ d3wyea_ d2blla1 d1w6ua_
d1sb8a_ d2q2va_ d4nr0a_ d1e6wa_ d2gasa_ d3oiga1 d2bgka1 d1mxha_
d1vl0a1 d1rkxa_ d1r6da_ d1wmaa1 d1e7wa_ d2c07a1 d1xkqa_ d3tzha_
d1g0oa_ d3lz6a_ d1qyca_ d1edoa_ d1gz6a_ d1oc2a_ d1t2aa_ d1zema1
d2xoxa_ d1z45a2 d1geea_ d1p33a_ d3lt0a_ d4e6pa_ d2wyua_ d1db3a_
d4q9na_ d1xhla_ d4o1ma_ d1fmca_ d2uvda_ d2gdza1 d1uaya_ d2z1ma_
d2o23a2 d2fr1a2 d2fr1a1 d1qsga_ d1qyda_ d4zjua_ d2a4ka1 d1yb1a_
d1wvga1 d1yxma1 d3f4ba_ d1mg5a_ d1xg5a1 d2gfoa1 d1vjva_ d2ayna1
d3nhea_ d3nqyb_ d4gera_ d1bqba_ d1mdwa_ d1qxpa4 d1ziva1 d1zcma1
d3ioqa_ d1fh0a_ d1o0ea_ d1khqa_ d1m6da_ d2cb5a_ g8pch.1 d1s4va_
d2pnsa_ d1vsna1 d1mira_ d1iwda_ d2oula_ d7pcka_ d3bwka_ d3bcna_
d2p86a_ d1pvja_ d1pcia_ d1gece_ d1deua_ d4bs6a_ d1xkga1 d1cqda_
d1jqpa2 d2c0ya_ d2bdza_ d3p5ua_ d1zoya1 d1qo8a2 d1d4ca2 d1jnra2
d1kf6a2 d2jk6a2 d2jk6a1 d1feca1 d1feca2 d1lpfa1 d1lpfa2 d1xhca1
d1xhca2 d1dxla2 d1dxla1 d1gera2 d1m6ia2 d1onfa1 d1onfa2 d3lada2
d3lada1 d1w4xa1 d1w4xa2 d1gesa2 d1mo9a1 d1xdia1 d1fl2a2 d1fl2a1

d1fcda1 d1fcda2 d1h6va2 d1h6va1 d1mo9a2 d1gv4a2 d1gv4a1 d2gv8a2
d2gv8a1 d1aoga1 d1aoga2 d1hyua1 d1ebda1 d1ebda2 d3dk9a1 d1q1ra2
d1q1ra1 d1gesa1 d1zbfa1 d2z1ha_ d2etja1 d3qioa1 d1jl1a_ d2e4la_
d1jl2a_ d2lsna1 d1io2a_ d1uaxa_ d2yv0x_ d1mu2a1 d1ekea_ d1i39a_
d1pn0a1 d1reoa1 d1ju2a1 d2f5va1 d3c96a1 d2gmha1 d1gpea1 d1o5wa1
d1n4wa1 d1k0ia1 d2dw4a2 d2ivda1 d1seza1 d2voua1 d1kdga1 d2bcgg1
d2bcgg2 d1d5ta1 d1iv0a_ d2v76a_ d1wj1a1 d1aqca_ d1qqga2 d1qqga1
d1ntva_ d1n3ha_ d4dbba_ d3f0wa1 d1p3ra_ d1wgua1 d2cy4a1 d3hqca_
d1wvha1 d1xr0b1 d3nvva1 d1krha3 d2h88b1 d1t3qa2 d1kf6b2 d1zoyb1
d1jq4a_ d1ffva2 d1dgja2 d1rm6c2 d1jmxa1 d1pbya1 d1pbya2 d1r7ha_
d1faaa_ d1wika1 d1xfla_ d1ttza1 d2f51a1 d1ti3a_ d1nhoa_ d1sena_
d2i4aa_ d1ep7a_ d1zmaa1 d1fo5a_ d3lefa1 d3diea1 d2yana1 d1z6na1
d1oaza_ d3zywa1 d4aj8a_ d1nm3a1 d4fiwa_ d1wmja_ d4hs1a1 d1iloa_
d1xwaa1 d1wjka1 d2vm1a_ d1fb6a_ d1gh2a_ d1h75a_ d1nw2a_ d1kx5b_
d3a6na_ d4m6ba2 d2nqbd_ d3wkjd_ d2yfwe_ d4h9na_ d5bnxa_ d1kx5c_
d3r45a_ d1eqzb_ d4jjnb_ d1f66c_ d1eqza_ d4wnnb_ d1kx5a_ d1u35c1
d1id3a_ d2nqbc_ d1kx5d_ d1ku5a_ d1f1ea_ d2b5ea2 d2b5ea3 d2b5ea1
d2b5ea4 d2kp1a1 d2kp2a1 d1hyua3 d1hyua4 d1j08a1 d1pd212 d1nhya2
d2a2ra2 d1k0dd2 d1f2ea2 d1gsua2 d1f3ba2 d2fhea2 d2pvqa1 d1tw9a2
d1oyja2 d1e6ba2 d1k0ma2 d3ik7a1 d4is0a1 d1r5aa2 d1jlva2 d1m0ua2
d3gtub2 d1z9ha2 d1okta2 d1axda2 d2gsra2 d1tu7a2 d1k3ya2 d1zgma1
d1b48a2 d1gnwa2 d1gwca2 d2gsta2 d1glqa2 d1oe8a2 d1pn9a2 d1v2aa2
d1fw1a2 d1duga2 d3vrda1 d3vrda2 d1fcdc2 d1h1oa2 d1h1oa1 d1m70a2
d1m70a1 d1g7ea_ d1ig8a1 d1czan2 d1czan3 d1czan1 d1ig8a2 d1czan4
d1bg3a4 d1bg3a3 d1bg3a1 d1qsaa2 d4ilya_ d1chka_ d4olta_ d3wyha_
d4g9sa1 d3gxra_ d1qqya_ d1gd6a_ d1ivma_ d2gv0a_ d1iiza_ d2z2fa_
d1j8xa_ d1ghla_ d1b9oa_ d3w3ea_ d4tx7a_ d3cqla_ d4msta_ d3iwra_
d2z37a_ d4dyga_ d1mpya2 d1kw3b2 d1kw3b1 d4ghga1 d1tfza1 d3isqa1
d3isqa2 d1sqia1 d1sqia2 d1lkda2 d1lkda1 d1mpya1 d1cjxa2 d1sp9b2
d1t47a1 d1t47a2 d1f1ua2 d1sp8a1 d1sp8a2 d1f1ua1 d1umqa_ d1g2ha_
d1ro5a1 d1s5ja2 d1qhta2 d4dlea2 d1tgoa2 d3py8a2 d1q8ia2 d1kfsa2
d2py5a2 d1d5aa2 d1qsta_ d2beia1 d4m3sa_ d1u6ma1 d1ygha_ d1yk3a1
d3dr6a_ d1xmta_ d1z4ea1 d2ge3a1 d1bo4a_ d2fiaa1 d2jdca1 d1y9wa1
d3to7a_ d1wwza1 d2fl4a1 d2ozua1 d2b5ga_ d2cy2a1 d2p0wa_ d1cm0a_
d4pswa_ d1yrea1 d1yr0a1 d1m4ia_ d2gana1 d1yvka1 d1ghea_ d2g3aa1
d1y7ra1 d2euia1 d1q2ya_ d2fsra1 d1xeba_ d2ae6a1 d1cjwa_ d1qsma_
d2aj6a1 d1y9ka1 d1i12a_ d1vkca_ d2atra1 d1n71a_ d2d4pa1 d2fiwa1
d2fe7a1 d1tiqa1 d2ou2a_ d1yx0a1 d2fcka1 d1p0ha_ d2j8ma_ d2pq8a_
d1z4ra1 d1s3za1 d1ufha_ d1c4qa_ d1prtc1 d2wv6d_ d1prtf_ d1ltrd_
d4l63b1 d1r4pb_ d1prtd_ d1tl0f_ d2bosa_ d1prtb1 d1xvqa1 d1pn0a2
d3hy2a_ d2cvba1 d1o8xa_ d1lu4a_ d1we0a1 d2bmxa1 d2a4va1 d1psqa_
d1xiya1 d1q98a_ d1n8ja_ d1zofa1 d2ggta_ d3tjja_ d4je1a1 d1o73a_
d3s9fa_ d2zcta_ d1qmva_ d1jfua_ d2b1ka_ d5dvba1 d2b5xa1 d2v2ga_
d1st9a_ d3tb2a_ d4llra_ d4fh8a1 d1e2ya_ d1knga_ d2h30a_ d1i5ga_

d2z9sa_ d2f8aa1 d1tp9a1 d2b7ka_ d1nm3a2 d3hvva_ d2wfca1 d4kcea_
d1xvwa1 d4gqca1 d3qpma_ d4k1fa_ d1zzoa1 d1gp1a_ d1prxa_ d1rmka_
d1ixta_ d1eyoa_ d1cnna_ d1fu3a_ d1f3ka_ d1fyga_ d1cg2a1 d1yloa2
d1y7ea2 d1y0ya3 d1lfwa1 d1vixa1 d1fnoa4 d1vhoa2 d1ysja1 d1z2la1
d2fvga2 d1vgya1 d1r3na1 d2grea2 d1xmba1 g1q7l.1 d1rtqa_ d1ys1x_
d4hs9a_ d1ispa_ d1ku0a_ d3qmma_ d1ra6a_ d1muka_ d3gsza_ d1khva_
d1te9a_ d2xwha_ d3uqsa_ d3hkwa_ d3n6ma_ d2cjqa_ d2xyma_ d1teba_
d1te8a_ d1s48a_ d1u09a1 d4wfxa_ d3tyqa_ d2pgga1 d3rp2a_ g1ucy.1
d1fuja_ d2zchp_ d3w94a_ d1si5h_ d1lo6a_ g2pka.1 d1cgha_ d1kigh_
d1a5ia_ d3s9ba_ d1hj8a_ d1ao5a_ d2anya_ d1q3xa1 d1gvza_ d1azza_
d4gsoa_ d2f91a1 d1iaua_ d1gdna_ d1eufa_ g1fiw.1 d1eq9a_ d4nfea_
d2rdla_ d1spja_ d1elva1 d1a7sa_ d1ekbb_ d1pfxc_ d1yc0a_ d2aiqa_
g1fiz.1 d1fi8a_ d4k60a_ d4dgja_ d1mzaa_ d1op0a_ d3tjva_ d1pytd_
d1sgfg_ g1gg6.1 d2eeka_ g1rtf.1 d1a0ja_ d3g01a_ d1pytc_ d4e7na_
d1ddja_ d1z8ga1 d2hlca_ d1npma_ g1jou.1 d1sgfa_ d1autc_ g2hnt.1
g1kdq.1 d1orfa_ d3s69a_ d4crga_ d1fxya_ d1bqya_ d1lgya_ d4l3wa_
d1llfa_ d1gz7a_ d1ukca_ d2hqya1 d2hqya2 d1ne9a1 d1lrza3 d1ne9a2
d3groa_ d1pjaa_ d1thta_ d1svpa_ d1mbma_ d3su6a1 d1cu1a1 d4agka_
d1vcpa_ d1q31a_ g1n1l.1 d3e90b_ d1ep5a_ d3u1jb_ d3zxha_ d1cgla_
d1rm8a_ d1eaka2 d1hy7a_ d2jsda_ d1cxva_ d1hv5a_ d1i76a_ d3p0ya1
d1igra1 d1igra2 d1n8yc2 d1n8yc1 d1m6ba2 d1m6ba1 d4dv8a2 d1m4la_
d2bk7a_ d3fjua_ d1z5ra1 d1uwya2 d1jqga1 d2c1ca1 d1dtda_ d1h8la2
d2tbvc_ d1c8nc_ d1x36a_ d1smvc_ d1ng0c_ d4sbvc_ d1f2nc_ d1cixa_
d1y29a_ d1v7fa_ d2jtba_ d1d1ha_ d1g9pa_ d1qk7a_ d1mb6a_ d2a2va_
d1niya_ d1i25a_ d1hvwa_ d2mpqa_ d1ie6a_ d1kqha_ d1la4a_ d2mxoa1
d1dl0a_ d1koza_ d1qk6a_ d1emxa_ d1lqra_ d1c6wa_ d1js9c_ d1f15b_
d1za7a_ d1laja_ d1rypd_ d1irua_ d1rypc_ d3unfk_ d1rypa_ d1j2pa_
d1irui_ d1j2qh_ d3unfh_ d1m4ya_ d1rypb_ d1irul_ d1iru2_ d1q5rh_
d1rypg_ d1g3ka_ d1iruf_ d1q5qa_ d1e94a_ d1irub_ d1ryph_ d3ty6a_
d1iruj_ d1iruk_ d1rype_ d1irud_ d1irue_ d3mi01_ d1rypf_ d1iruh_
d2z3ba_ d1iruc_ d3mka2_ d1iru1_ d1ny721 d1ny722 d1a6ca3 d1a6ca2
d1a6ca1 d1pgl22 d1pgl21 d1pgl11 d1mqta_ d1d4m3_ d1fmd3_ d2mev1_
g1aym.1 d1ev13_ d4q4w1_ d1r1a3_ d2x5ia_ d1rhi1_ g1r1a.1 g1d4m.1
g1rhi.1 d1b35b_ d1oopa_ d1tme1_ d4iv1a_ g1pvc.1 g1fpn.1 d1fpn1_
d4gb31_ d1d4m1_ d1fmd1_ d1ev11_ d2wzr3_ g1cov.1 d1qqp3_ g1fmd.1
d2x5ic_ d1rhi3_ g1oop.1 d1hxs1_ g1hxs.1 d1fpn3_ d1bev1_ g2mev.1
d1eah3_ d1aym1_ d1pvc3_ d1qqp1_ d1zba3_ d1h8ta_ d3vbhc_ g1b35.1
g1bev.1 d1hxs3_ g1ev1.1 d1bev3_ g1tmf.1 d1tmf1_ d1b35a_ d2mev3_
g1eah.1 g1h8t.1 d1eah1_ d4q4w3_ d1r1a1_ d1aym3_ d1pvc1_ d1zba1_
d1h8tc_ d1oopc_ d1tme3_ g1qqp.1 d4iv1c_ d1ju8a_ d3ia2a_ d1a88a_
d4dgqa1 d1hkha_ d1zoia_ d4lt9a1 d3tc7a1 d3hoja_ d1i4na_ d3vnda_
d3nl8a1 d1geqa_ d3nava1 d4a2ra1 d3tc6a1 d1ujpa_ d4ijba_ d1xcfa_
d1vc4a_ d1rd5a_ d1qopa_ d3b5va_ d2r80b_ d1cqxa1 d1cg5a_ d1gcvb_
d3boma_ d2h8fb_ d2raoa_ d3bj1a_ d4hrrb_ d2r50a_ d3d1kb_ d2aa1b1

d3zhwa_ d3hf4b_ d3gdja_ d1jebb_ d3bj1b_ d1x9fb_ d1wmub_ d1gcva_
d3bcqb_ d3d4xb_ d1gvha1 d2dc3a_ d3hf4a_ d4yu3a_ d3k8ba_ d1qpwb_
d2r80a_ d3bcqa_ d1v4wa_ d3wtgb_ d1jl7a_ d4esaa_ d1a9we_ d3d1ab_
d1fsla_ d4h2la_ d1v4wb_ d1mwca_ d1vhba_ d1jeba_ d1spgb_ d1h97a_
d1q1fa_ d1tu9a1 d3d1aa_ d1hdsb_ d3hyub_ d2zfbb_ d3fs4a_ d1oj6a_
d1shrb_ d4h2lb_ d4yu3b_ d1spga_ d3hrwa_ d3qqra_ d3at5a_ d4wchd_
d3hyua_ d1cg5b_ d1uc3a_ d1hbra_ d2raob_ d1outb_ d4esab_ d3gdjb_
d3qqqa_ d1x9fc_ d3vrfa_ d1itha_ d3mkba_ d3d4xa_ d3mjpa_ d1jl6a_
d3fh9a_ d1outa_ d2dc3b_ d1it2a_ d3eoka_ d3g46a_ d1qpwa_ d3vrfb_
d1ch4a_ d1x9fd_ d1hdsa_ d2zfba_ d3mkbb_ d1or4a_ d3wtga_ d1a4fa_
d3fh9b_ d3bomb_ d3cy5a_ d3at5b_ d1x9fa_ d2oifa_ d4lmxb_ d1cpcb_
d3v57a_ d1kn1a_ d1cpca_ d4f0ta_ d2vjha_ d4po5b_ d1eyxa_ d1alla_
d4f0tb_ d1b33b_ d1b8db_ d4h0mb_ d1b33a_ d2vjhb_ d4lm6b_ d2vmlb_
d2vjta_ d3dbja_ d2vmla_ d2vjtb_ d4f0ub_ d1f99b_ d4rmpa1 d1liaa_
d1eyxb_ d2uulb_ d4lmsb_ d4f0ua_ d2bv8a_ d1f99a_ d2bv8b_ d2c7lb_
d3dbjb_ d4rmpb1 d1b8da_ d4h0ma_ d3v57b_ d1kn1b_ d1liab_ d2uula_
d2j96a_ g1gxs.1 g1wht.1 d1mtza_ d2xqfa_ d1ea5a_ d1f6wa_ d1dx4a_
d1qe3a_ d1k4ya_ d4qwwa_ d2ha2a_ d3c8da2 d2pbla1 d1jkma_ d1jjia_
d1jjfa_ d2qvba_ d2v9za_ d4kafa1 d4iuaa3 d4bvva_ d5hpga_ d3k65a_
d1ki0a3 d1ki0a2 d1ki0a1 d1a0ha1 d1pmla_ d3sp8a3 d2feba1 d1dosa_
d1gvfa_ d3qm3a1 d2fjka_ d1f74a_ d3l21a_ d2e1da_ d1to3a1 d1w3ia_
d1sfla_ d1n7ka_ d1vcva1 d4imea_ d1wa3a_ d2ocza1 d1f2ja_ d3m16a_
d2yxga_ d1ub3a_ d3a5fa_ d1fbaa_ d1l6wa_ d1xfba1 d1wx0a1 d1vpxa1
d1ojxa_ d1mxsa_ d2a4aa1 d1xkya1 d3cq0a1 d3s1xa_ d1f05a_ d1fdja_
d3cpra1 d4d2ja_ d4mh8a_ d1mu2a2 d4i7ga1 d2ftba_ d1pmpa_ d2rcqa1
d1fdqa_ d4lkpa_ d1sa8a_ d1vyfa_ d1lpja_ d3vg7a1 d4azqa1 d1o8va_
d5bvqa_ d1p6pa_ d2qo4a_ d1opba_ d1yiva1 d1o1va_ d4bvma1 d4tkba_
d4i3ba_ d1bwya_ d1ftpa_ d1ggla_ d4gkca_ d1cbia_ d3p6da1 d1kqwa_
d2a0aa1 d1dzka_ d4tlja_ d2a2ua_ d4wfva_ d3t1da_ d4iaxa_ d3zq3a_
d2nnda_ d1i4ua_ d1kxoa_ d1bbpa_ d1kt7a_ d1exsa_ d3s26a1 d1lf7a_
d1qfta_ d4gh7a_ d1z24a1 d1qabe_ d1xkia_ d3bx7a_ d4iawa1 d1n0sa_
d1gkab_ d3kzaa_ d2k23a_ d2l9ca1 d2joza1 d1gm6a_ d3kffa_ d3saoa1
d1e5pa_ d1yupa1 d1iiua_ d4lu0a_ d1of8a_ d3unda1 d3e9aa1 d1vr6a2
d1n8fa_ d2qkfa_ d1d9ea_ d1o60a_ d1e69a_ d1mv5a_ d3g60a4 g1ii8.1
d1ewqa2 d1ji0a_ d1v43a3 d2wmfa1 d1vpla_ d1g2912 d4a82a2 g1f2t.1
d2d62a1 d3tuic1 d2bbsa1 d3g60a3 d2ixea_ d1g6ha_ d1sgwa1 d2qi9c_
d3rlfa1 d3b60a1 d3d31a2 d2pzea1 g1xew.1 d1jj7a_ d1oxxk2 d2onka1
d1w1wa_ d3gqba2 d1skye3 d1xp8a1 d1tf7a2 d1tf7a1 d1ye8a1 d1skyb3
d2qe7d2 d4blta_ d4huta_ d3ldaa2 d4hyya_ d2i3ba1 d1cr1a_ d3j9tb2
d3ntua2 d1vdza1 d2z43a_ d1e9ra_ d1nlfa_ d3ziaa2 d1n0wa_ d3ziad2
d3j9ta2 d1pzna2 d3gqbb2 d2i49a_ d1nnfa_ d1xvxa_ d1ursa_ d1h3da1
d1iuda_ d4m1va1 d1twya_ d3e13x_ d2nxoa1 d4pfua_ d3ttma_ d1elja_
d2qmwa1 d1us5a_ d1y3na1 d1jeta_ d1uqwa_ d4f19a1 d2esna2 d3thia_
d3fasa1 d2uyeb_ d2xwva1 d1r9la_ d2fyia1 d1pb7a_ d2ozza1 d1sw5a_

d1nh8a1 d1vr5a1 d2g29a_ d1xs5a_ d3lswa_ d2z23a_ d3w9va_ d3tcha_
d1q35a_ d1z7me1 d3q27a_ d1p99a_ d1ve4a1 d2i6ea1 d4mcoa1 d4n8ya_
d1eu8a_ d1zbma1 d1ixca2 d1j1na_ d1xvya_ d1y9ua_ d2z22a1 d1xoca1
d4oera_ d1wdna_ d5f1qa1 d1xt8a1 d1i6aa_ d4gwoa_ d4igta1 d1pc3a_
d4mmpa1 d1o63a_ d2czla1 d2onsa2 d4mija1 d4ok5a1 d2bmfa1 d2bmfa2
d1yksa1 d1yksa2 d2qeqa1 d2jlqa1 d1a1va2 d1eh3a_ d3skpa_ d1dtza1
d1dtza2 d1ieja_ d1jnfa2 d1jnfa1 d1b1xa2 d1b1xa1 d1jw1a1 d1jw1a2
d1ryoa_ d1h76a1 d1h76a2 d1c4oa1 d2p6ra3 d2p6ra4 d1oywa2 d1oywa3
d1wrba1 d1w36b2 d1w36b1 d1c4oa2 d3bora_ d1t6na_ d2eyqa5 d2eyqa3
d2eyqa2 d2zu6a2 d1veca_ d1uaaa1 d1w36d1 d2g9na1 d1z3ix2 d2rb4a_
d1hv8a1 d1hv8a2 d1t5ia_ d1w36c2 d2j0sa2 d2j0sa1 d1fuka_ d3hjha1
d1z5za1 d1qdea_ d1z63a1 d1s2ma2 d1s2ma1 d1wp9a1 d1wp9a2 d1gm5a3
d1gm5a4 d3dina4 d3dina3 d1q0ua_ d1w36c1 d1z3ix1 d1nkta3 d1nkta4
d1uaaa2 d1w36d2 g1qhh.1 d2fwra2 d2fwra1 d3q3ma_ d1za0a1 d1mxra_
d2inca_ d1jk0a_ d2incb_ d1mhyb_ d3hf1b_ d1r2fa_ d2uw1a_ d1mhyd_
d3n37a_ d1afra_ d1oquc_ d1syya_ d1mtyd_ d2o1za_ d1uzra_ d1smsa_
d2fkza_ d1zuja1 d1s3qa1 d2fjca1 d1z6om1 d1veia_ d4dyya_ d1jiga_
d3gvya_ d2pyba_ d4cy9a1 d3bvfa1 d1r03a_ d2cf7a_ d1nf4a_ d1zs3a1
d4iwka_ d3ka8a_ d3np0x_ d1n1qa_ d1lb3a_ d4e6ka_ d1z6oa1 d2fzfa1
d4dyua_ d3ge4a_ d1jgca_ d4am4a_ d2z6ma_ d1ji5a_ d1vlga_ d1lkoa1
d1krqa_ d3qz3a_ d1o9ra_ d2ffxj_ d1euma_ d1yv1a1 d2v2pa_ d1j30a_
d4di0a_ d1oewa_ d2qzxa_ d2asia_ d1psoe_ d1miqa_ d2h6ta_ d1pfza_
d1izda_ d3hd8a_ d1j71a_ d1dpja_ d4aa9a_ d1t6ex_ g1b5f.1 g1lya.1
g1htr.1 d1qdma2 d1bxoa_ d1wkra_ d1tzsa_ d1ibqa_ d2qzwa_ d1bdqa_
d3ggta_ d3ixoa_ d1mrxa_ d2haha_ d1tw7a_ d4njsa_ d1sgua_ d2p3da_
d1nsoa_ d3d3ta_ d2rkfa_ d1c6ya_ d2rspa_ d3ogpa_ d3ekwa_ d2o4na_
d2j9ja_ d1baia_ d3ggua_ d3lzua_ d4npua_ d4j55a_ d2r8na_ d2fxda_
d2p3ca_ d4dqea_ d3ttpa_ d3t3ca_ d2p3aa_ d2hc0a_ d2fdda_ d3mwsa_
d1k8rb_ d1wgra1 d1ef5a_ d1wfya1 d1wgya1 d1lfda_ d2cs4a1 d1wxma1
d2byea1 d3kh0a1 d2byfa1 d1wxaa1 d1e7ua3 d1wgda1 d1wgga1 d4q5eb_
d1wjna1 d1se9a_ d3n3kb_ d1wz0a1 d1we6a1 d2m0xa_ d2gbka_ d3rt3b1
d1wh3a1 d1sifa_ d2ekec2 d4ii2b1 d4hk2a1 d1c3ta_ d1m94a_ d1wgha1
d1ttna1 d1j8ca_ d1wx8a1 d3v6cb1 d2ma9b_ d4i6lb1 d1bt0a_ d2jvca1
d1iyfa_ d2k1fa_ d1wxva1 d1wf9a1 d1t0ya_ d2io0b2 d1x1ma1 d2gbma_
d1zw7a1 d2m17a_ d1ud7a_ d1we7a1 d2lwpa_ d2gbja_ d1wjua1 d1v6ea1
d1v86a1 d4eewa_ d1wiaa1 d4icva_ d4gu2a_ d1wx7a1 d1wy8a1 d4hjka1
d2faza1 d1v5oa1 d3ruib_ d1eo6a_ d3waob_ d2r2qa1 d3m95a_ d3wana_
d2al3a1 d2cr5a1 d1wj4a1 d1s3sg_ d1qoza_ d5ajha1 d2h0ba_ d2erfa_
d1h30a2 d1h30a1 d3mw4a1 d1pz7a_ d2r1ba_ d3v64a1 d1d2sa_ d3bopa1
d3mw3a1 d1okqa1 d1s5ja1 d2hbja2 d2igia_ d1wlja_ d1qhta1 d4dlea1
d1tgoa1 d2f96a1 d3tr8a1 d3mxma_ d1q8ia1 d1y97a1 d1w0ha_ d1kfsa1
d2py5a1 d1d5aa1 d1yt3a3 d2guia_ d3mwpa1 d4kb1a_ d1e88a1 d1l6ja5
d1l6ja4 d1eaka4 d1eaka3 d1l6ja3 d1h8pa2 d1um8a_ d1ixza_ d1qvra2
d1qvra3 d1qzma_ d1sxjc2 d1jr3d2 d2p65a_ d1l8qa2 d1g8pa_ d3hu3a3

d1sxjd2 d1njfa_ d1in4a2 d1sxja2 d1tuea1 d2ce7a2 d1w5sa2 d1sxjb2
d1d2na_ d1ny5a2 d1ofha_ d2gnoa2 d2a5yb3 d1fnna2 d1u0ja1 d1iqpa2
d2fnaa2 d1ixsb2 d4gita_ d1sxje2 d1g41a_ d2e3fa_ d1mm0a_ d2e3ea_
d2llda_ d2ny8x_ d2e3ga_ d2ny9x_ d2nz3a_ d2lj7a_ d1n4na_ d2m8bb1
d2kyqa_ d3psma_ d1jkza_ d1jxca_ d2gl1a_ d1ugla_ d4uj0a_ d2k4ua_
d1hp2a_ d2lu9a_ d1baha_ d2meoa1 d1wm7a_ d1hlya_ d1n8ma_ d1jlza_
d1wpda_ d1pvza_ d1wt7a_ d1rjia_ d1wz5a_ d1m2sa_ d1pjva_ d1c49a_
d1qkya_ d2ktca1 d1wmta_ d1du9a_ d1j5ja_ d1r1ga_ d1t0za_ d2i61a_
d1t1ta_ d1sn4a_ d1kv0a_ d2lkba_ d1npia_ d1wwna_ d1pe4a_ d2kbka_
d2li7a_ d1djta_ d2kbha_ d1dq7a_ d1sega_ d1omya_ d1zywa1 d1jzaa_
d1t61c1 d1iyjb4 d1iyjb5 d1iyjb3 d1o7ia_ d4ipca1 d1x3ea_ d4apva_
d1jb7a1 d1jb7a2 d1jb7a3 d3kdfa_ d1ue1b_ d1wjja1 d3eiva_ d1xjva2
d1xjva1 d2ccza2 d1se8a_ d1l1oc_ d1jb7b_ d1qvca_ d3ulla_ d1qzga_
d1miua4 d1miua5 d1miua3 d1y14b1 d2nn6i1 d1i94l_ d2zjra2 d1smxa_
d2qamc2 d1hh2p1 d2r7da1 d1rl2a2 d1wfqa1 d3bzka4 d1x6oa2 d3a0ja_
d2j01d2 d2ja9a1 d4ql5a_ d1hr0w_ d1luza_ d2n3sa_ d2vnud1 d1h95a_
d1go3e1 d2nn6h1 d1ne3a_ d2mo1a_ d1iz6a2 d1jjga_ d1e3pa2 d2ix0a3
d2ix0a2 d2lxja_ d1ueba3 d1ueba2 d2z0sa1 d1u0la1 d2ix0a1 d2eifa2
d2je6i1 d3i2za1 d2c35b1 d2ahob2 d1t9ha1 d3i4oa_ d2qall1 d1jt8a_
d2nn6g1 d1g6pa_ d3cama_ d1i94q_ d1c9oa_ d1d7qa_ d2ba0a1 d2vnud2
d2vnud3 d1khia2 d2qalq1 d1wi5a1 d1xtda2 d1ny4a_ d1fvia1 d1ckma1
d1p16a1 d1x9na2 d1cwva5 d1ohea1 d1ohea2 d2f4da_ d1rxda_ d2hxpa_
d1xria_ d1d5ra2 d1fpza_ d3ezza_ d4y2ea_ d1i9sa_ d3azya_ d3wvja_
d1upsa1 d1ajoa_ d2hyka_ d1ajka_ d2vy0a_ d3o5sa_ d3d6ea_ d3atga_
d1dypa_ d1umza_ d3i4ia_ d1c1la_ d4lbqa_ d1a78a_ d1slta_ d4no4a_
d3zsja_ d3duia_ d1g86a_ d1qmja_ d1hlca_ d1tdha3 d1q3ba3 d1ee8a3
d1k82a3 g1qmo.1 g1len.1 d3zyra_ d1fnya_ d1n47a_ g2ltn.1 d1avba_
d3usua_ d1dhkb_ d1f9ka_ d1ukga_ d1g8wa_ d1g9fa_ d3wcsa_ d1g7ya_
g1loe.1 d1mvqa_ d1hqla_ d1v6ia_ d1dbna_ d1qnwa_ d1fx5a_ d4l8qa_
d1ioaa_ d3n35a_ d3ubra_ d1qo8a1 d1d4ca1 d2k3va_ d1ft5a_ d1qdba_
d1m1qa_ d2j7aa_ d1h21a_ d1ogyb_ d4fasa_ d1jnia_ d1oaha_ d1sp3a_
d3h33a_ d1rwja_ d19hca_ d1os6a_ d3bxua_ d3h4na_ d1i77a_ d3caoa_
d1j0pa_ d2ctha_ d1duwa_ d1ofwa_ d2e84a_ d1hh5a_ d1gyoa_ d3h34a_
d1dszb_ d1a6ya_ d3cbba_ d1ynwa1 d1r4ia_ d1hcqa_ d2hana_ d2hanb_
d2enva1 d1kb4b_ d2nllb_ d1lo1a_ d1cita_ d2dara2 d2dara1 d1wyha2
d2dloa2 d1rutx4 d1rutx3 d2cuqa2 d1x4la2 d1x4la1 d1x3ha2 d1x3ha1
d2d8za1 d1x61a2 d1x6aa1 d1x6aa2 d2cora2 d2cora1 d1v6ga1 d1v6ga2
d2d8za2 d1x64a1 d1x64a2 d1b8ta1 d2co8a1 d1x63a2 d2dj7a2 d2dj7a1
d2cu8a2 d2cu8a1 d1g47a2 d1g47a1 d1j2oa1 d1wyha1 d2dloa1 d1wiga1
d1wiga2 d2cupa2 d2cupa3 d1x68a1 d2d8ya2 d2d8ya1 d1m3va2 d1x62a1
d1x68a2 d1x61a1 d1b8ta3 d1b8ta2 d2d8xa2 d2d8xa1 d1x4ka1 d1x4ka2
d2cuqa1 d1m3va1 d2co8a2 d1j2oa2 d1x63a1 d2cura1 d2cura2 d1x62a2
d2aq2b1 d1enfa1 d1i4pa1 d1v1oa1 d1et9a1 d1esfa1 d2g9hd1 d4ohja1
d1klud1 d1bxta1 d1eu3a1 d1ty0a1 d1fnua1 d2qaln1 d1d4ua2

**Family-21 (b.6.1.1)**

**Positive dataset contains 6 protein sequences.**

d1ws8a_ d3u25a_ d2iaac_ d4dp1x_ d2gima_ d2i7sa_

**Negative dataset contains 3996 protein sequences.**

d1m32a_ d1ibja_ d1c7na_ d1iuga_ d4q75a_ d2bkwa1 d1t3ia_ d1qz9a_
d1jf9a_ d1eg5a_ d1p3wa_ d3kgwa_ d1cl1a_ d2ctza1 d1vjoa_ d2rfva_
d1gc0a_ d1cs1a_ d1d2fa_ d2ch1a1 d1qgna_ d1pffa_ d1elua_ d1u08a_
d1lc5a_ d1b5pa_ d2ay1a_ d1bw0a_ d3pd6a_ d7aata_ d2csta_ d1j32a_
d4wlha_ d1o4sa_ d1v2da_ d1gdea_ d1m6sa_ d1v72a1 d1svva_ d1ajsa_
d1wsta1 d2f8ja2 d1lk9a_ d1vp4a1 d1xi9a1 d2gb3a1 d1yaaa_ d1xkla_
d2wfla_ d2cfba1 d1dfoa_ d1o69a_ d1bj4a_ d3k28a_ d2bwpa_ d3lg0a_
d2fnua_ d3ou5a_ d1z7da1 d1b9ha_ d1iaya_ d4msoa_ d1bs0a_ d1s0aa_
d1sffa_ d3tqxa_ d1mdoa_ d3qboa_ d3pgya_ d1bjna_ d2dkja_ d1ejia_
d1vefa1 d4wxga_ d3bs8a_ d3gbxa1 d1w23a_ d4nmwa_ d1cixa_ d1y29a_
d1v7fa_ d2jtba_ d1g9pa_ d1lupa_ d1qk7a_ d1mb6a_ d2a2va_ d1niya_
d1i25a_ d1hvwa_ d2mpqa_ d1ie6a_ d1kqha_ d1la4a_ d2mxoa1 d1dl0a_
d1koza_ d1qk6a_ d1emxa_ d1lqra_ d1c6wa_ g1f8v.1 d1novc_ d2bbvc_
d1ny721 d1ny722 d1a6ca3 d1a6ca2 d1a6ca1 d1pgl22 d1pgl21 d1pgl11
d1ny711 d3sxwa1 d1j3qa_ d4luka_ d1a3qa1 d1iknc_ d3brda1 d1p7hl1
d1oy3c_ d1imhc1 d1gjia1 d1zk9a1 d2cxka1 d2bema_ d1pama1 d1ea9c1
d1qhoa1 d2fhfa2 d2fhfa1 d1k3ia1 d1j0ha1 d1cxla1 d2bhua1 d1edqa1
d1f1sa2 d1ut9a2 d1gvia1 d1txka1 d2phla1 d1fxza2 d1fxza1 d1lrha_
d1uija1 g1dgw.1 d2v09a_ d1uika2 d1uika1 d1dgwa_ d1ep0a_ d2pa7a1
d1wlta1 d1nxma_ d2c0za1 d1ds6b_ d1fstb_ d1g0da1 d4w9ta_ d1jvna1
d4x2ra1 d1ka9f_ d4axka1 d1thfd_ d1h5ya_ d4u28a_ d2y88a_ d4wd0a_
d1vzwa1 d2bjca1 d2hsga1 d1qpza1 d1k9ua_ d2opoa_ d1ydwa2 d2nvwa2
d1h6da2 d1zh8a2 d1xeaa2 d1tlta2 d1r0ka3 d1j5pa3 d1vkoa2 d1vm6a2
d3qvsa2 d1gr0a2 d1p1ja2 d3gpdg2 d2q49a2 d1hdgo2 d2g17a2 d2cvoa2
d2czca1 d3pwka2 d1obfo2 d1i32a2 d1zyao2 d2pkqo2 d1b7go2 d2hjsa2
d1cf2o2 d1vkna2 d1nvmb2 d1ux8a_ d1dlya_ d4i0va_ d2bkma_ d1dlwa_
d2bmma_ d2msya_ d2djoa1 d2r5za_ d1au7a1 d1f43a_ d2lmda1 d1akhb_
d1s7ea1 d3nara_ d1zq3p1 d2craa1 d1mija_ d4uuta_ d3a01a_ d1x58a1
d3rkqa1 d3cmya1 d2cqxa1 d2da6a1 d1e3oc1 d1fjla_ d1mh3a1 d1ig7a_
d1x41a1 d1wh7a1 d1gt0c1 d2mw8a_ d1lfup_ d1x2ma1 d1b72a_ d1wi3a1
d1wjha1 d1x2na1 d1mnmc_ d2cuea1 d3k2aa_ d2hosa_ d2jwta2 d1pufa_
d1octc1 d2hi3a_ d1b8ia_ d4j19a_ d3lnqa_ d1wh5a1 d1b8ib_ d1fexa_
d2cqra1 d1guua_ d1ug2a1 d2cqqa1 d2ajea1 d4a69c_ d1h8ac1 d2crga1
d2iw5b1 d1xc5a1 d1ofcx1 d2cu7a1 d1wgxa1 d2cjja1 d2ckxa1 d1hcra_
d1tc3c_ d1u78a2 d1xg1a1 d3lv8a_ d1s3ga1 d1zaka1 d1s4qa_ d3tvta2
d1uf9a_ d1khta_ d1ckea_ d1osna_ d1p5zb_ d4tmka_ d3cm0a_ d1tmka_
d1jaga_ d3h86a_ d1t0hb_ d1nn5a_ d2h92a_ d3kb2a1 d2c95a1 d1yj5a2
d1kgda_ d1ly1a_ d2ak3a1 d1vyva2 d1deka_ d2grja1 d1p3ja1 d1y63a_
d1e4ya1 d1s96a_ d3iija1 d1lvga_ d1nksa_ d1vyua2 d4e22a_ d1qf9a_
d1jjva_ d1q3ta_ d1ki9a_ d1lw7a2 d1teva_ d1kjwa2 d1z0ja1 d3umra2

| | | | | | | | |
|---|---|---|---|---|---|---|---|
| d3gj0a1 | d2gaoa1 | d1yzga1 | d1x3sa1 | d1g7sa4 | d1puja_ | d1udxa2 | d1kk1a3 |
| d2o52a_ | d1jwyb_ | d1zcaa2 | d1zcba2 | d2fv8a1 | d2cjwa_ | d3dz8a1 | d1xzpa2 |
| d1vg8a_ | d4qxaa_ | d2ew1a1 | d1x1ra1 | d2bcgy_ | d3doea1 | d3cona_ | d3rega_ |
| d3wyga_ | d1upta_ | d2b6ha1 | d1zd9a1 | d1lnza2 | d1r5ba3 | d1n0ua2 | d1r2qa_ |
| d2f9la1 | d2rhda_ | d3q3jb_ | d3q85a_ | d1efma1 | d1r8qa_ | d2gf9a1 | d2akab1 |
| d1ek0a_ | d1z08a1 | d2gcoa1 | d2dy1a2 | d4g01b_ | d2cxxa1 | d1svia_ | d3q72a_ |
| d1ni3a1 | d4ac9a4 | d2erxa1 | d2bmea_ | d1wxqa1 | d2gf0a1 | d4ldja1 | d1zunb3 |
| d1d5ca_ | d4lv5b_ | d1efca3 | d1kmqa_ | d2g6ba1 | d3lrpa_ | d1tada2 | d2y8ea_ |
| d4rd4a1 | d1z06a1 | d2heia_ | d3a58b_ | d1u0la2 | d1d2ea3 | d1zj6a1 | d2ocba_ |
| d3rwoa_ | d1mr3f_ | d2e9sa1 | d4u5xa1 | d2iwra_ | d2fn4a1 | d3bwdd_ | d2nzja1 |
| d2atva1 | d2ksqa_ | d3l43a_ | d1mkya2 | d1mkya1 | d1wmsa_ | d1jnya3 | d2rmka_ |
| d1f6ba_ | d1f60a3 | d1gota2 | d1jala1 | d2fh5b1 | d1g16a_ | d2wblc_ | d1m7ba_ |
| d1wf3a1 | d1f5na2 | d1t9ha2 | d1z6xa1 | d1z0aa1 | d2fg5a1 | d2a5ja1 | d2bcjq2 |
| d1s0ua3 | d1ky3a_ | d4y0va_ | d4lhwa_ | d2qmea1 | d2atxa1 | d1egaa1 | d1ryha_ |
| d1moza_ | d1nrjb_ | d2erya1 | d3bfka_ | d1puia_ | d3j3az1 | d3t5ga_ | d1shza2 |
| d3defa_ | d2f7sa1 | d3raba_ | d3tsoa_ | d3aq4a_ | d2al7a1 | d3sfva1 | d3bc1a_ |
| d2ncda_ | d1sdma_ | d3h4sa1 | d2heha_ | d2grya_ | g3kin.1 | d1kk8a2 | d1br2a2 |
| d1goja_ | d1f9va_ | d2mysa2 | d1lkxa_ | d3t0qa_ | d4db1a1 | d3gbja_ | d1v8ka_ |
| d1y4wa1 | d3vrda1 | d3vrda2 | d1fcdc1 | d1h1oa2 | d1h1oa1 | d1m70a2 | d1m70a1 |
| d1ig8a1 | d1czan2 | d1czan1 | d1ig8a2 | d1czan4 | d1bg3a4 | d1bg3a3 | d1bg3a2 |
| d1bg3a1 | d3h3no1 | d3h3no2 | d3ezwa2 | d1hroa_ | d4gedb_ | d3zooa_ | d1mz4a_ |
| d1gu2a_ | d1f1fa_ | d1dvva_ | d1c6ra_ | d1c75a_ | d1e29a_ | d1gdva_ | d1cnoa_ |
| d1co6a_ | d2bh4x_ | d2aiua_ | d5cytr_ | d1jdla_ | d1i8oa_ | d2zboa_ | d3dmia_ |
| d1ls9a_ | d1wvec_ | d2yk3a_ | d3ph2b_ | d1dw0a_ | d5aura_ | d1ql3a_ | d1qn2a_ |
| d1f1ca_ | d4zzva_ | d2xspa_ | d1eg1a_ | d4v20a_ | d1ojja_ | d4ipma1 | d1gpia_ |
| d4csia_ | d3pfza_ | d3ovwa_ | d2yoka_ | d2nqya_ | d1h4ga_ | d1hixa_ | d1oa2a_ |
| d1olra_ | d3b7ma_ | d4h7ma1 | d3wp3a_ | d2vgda_ | d1igoa_ | d3zsea_ | d2nlra_ |
| d3wx5a_ | d1oa3a_ | d1pvxa_ | d1m4wa_ | d1f5ja_ | d1oa4a_ | d4ixla1 | d2ah2a1 |
| d1evsa_ | d1cnt1_ | d1pvhb_ | d1z7ca_ | d1huwa_ | d1bgea_ | d1rhga_ | d1f6fa_ |
| d1f45b_ | d1i1rb_ | d1axia_ | d1etea_ | d3tgxb_ | d2z3qa1 | d3qb1a_ | d1hula_ |
| d1m48a_ | d4nkqb_ | d1eera_ | d1v7mv_ | d5e4ea_ | d2o27a_ | d3b5ka_ | d2psma1 |
| d4zf7a_ | d3uf5a_ | d4x51a_ | d1d9ca_ | d4ypgc1 | d3oq3a_ | d1lqsl_ | d1n1fa_ |
| d1au1a_ | d1wu3i_ | d3besl_ | d3ux9a_ | d1v8fa_ | d2ejca_ | d3muea1 | d3q12a1 |
| d3rbaa_ | d2qtra_ | d1nupa_ | d3l92a_ | d1f9aa_ | d1qjca_ | d2h29a_ | d1vlha_ |
| d1od6a_ | d1lw7a1 | d4ruka_ | d3wdpp1 | d3wq5a_ | d1gnxa_ | d4gxpa_ | d4hz8a_ |
| d1e4mm_ | d3aiua_ | d1wcga1 | d1qvba_ | d3ahxa_ | d1v02a_ | d2dgaa_ | d1pbga_ |
| d1vffa1 | d1qoxa_ | d4ha4a_ | d2el7a_ | d1iq0a2 | d1irxa2 | d1r6ta2 | d1ivsa4 |
| d2cyba_ | d1rqga2 | d1j09a2 | d2yxna_ | d1f7ua2 | d1h3fa1 | d1ffya3 | d2g36a1 |
| d1nzja_ | d1jila_ | d1n3la_ | d1i6la_ | d1pfva2 | d1h3na3 | d3n9ia_ | d3sz3a1 |
| d3sw2a_ | d1moxc_ | d1dx5i3 | d1edmb_ | d1autl2 | d1autl1 | d1dval1 | d1szba2 |
| d1kigl_ | d2y5fl_ | d1ijqa2 | d1gk5a_ | d1g1sa2 | d1nt0a3 | d1xdtr_ | d1lmja2 |
| d1lmja1 | d1nzia2 | d2wphe_ | d1klil_ | d1pfxl2 | d1pfxl1 | d1p9ja_ | d1g1ta2 |
| d1f7ea_ | d4yzub_ | d2mgpa1 | d1b9wa1 | d1b9wa2 | d2mgpa2 | d1ob1c2 | d1n1ia2 |
| d1n1ia1 | d2npra2 | d2npra1 | d1m1xb4 | d2y5ia_ | d1qlsa_ | d3n22a_ | d2egda_ |

d4ggfc_ d2wcea_ d2luca_ d4aqia_ d3nsia_ d2lp3a_ d1j55a_ d1a4pa_
d1k8ua_ d1nsha_ d1exra_ d1tiza1 d1nyaa_ d1g8ia_ d1ggwa_ d1wdcc_
d3fwba_ d1ggza_ d2o5ga_ d1ctaa_ d1m45a_ d3i5gc_ d1sl8a_ d1fpwa_
d2scpa_ d2roaa_ d4aqra_ d3pn7b_ d1br1b_ d2mysb_ d1s6ca_ d1bjfa_
d2obha_ d1jfka_ d1jf0a_ d1qv0a_ d1lkja_ d2mlfc_ d2m97a_ d2d8na_
d4by5a_ d1jbaa_ d4nqga_ d1wdcb_ d2mysc_ d1j7qa_ d1s6ia_ d1uhka2
d1r2ua_ d1dtla_ d3kf9a_ d3pn7c_ d1qjta_ d1c07a_ d1gjya_ d1y1xa_
d1hqva_ d1dvia_ d1df0a1 d1qxpa2 d4usla_ d1k94a_ d1sfsa_ d1jfxa_
d1wkya2 d1uuqa_ d3wh9a_ d4m1ra_ d1xyza_ d4xuya_ d2je8a5 d4bf7a1
d3cuia_ d1ur4a_ d1qw9a2 d1odza_ d2vzsa5 d1bqca_ d2x2ya1 d1hjqa_
d1qnra_ d4y7ea1 d1vjza_ d4k68a_ d2v3ga_ d4hpga_ d1nofa2 d1nq6a_
d1h4pa_ d1aq0a_ d3ziza_ d4bs0a_ d1ecea_ d1h1na_ d1ta3b_ d1w91a2
d1i1wa_ d1uhva2 d1us3a2 d1egza_ d7a3ha_ d1tvna1 d3nyda1 d1ghsa_
d2cyga1 d4xv0a_ d2c8na2 d1w32a_ d3emca_ d1v6ya1 d1foba_ d2uwfa1
d2fgla_ d3niya1 d2c0ha1 d1rh9a1 d1yq2a5 d1szna2 d1fa2a_ d1pama4
d5do8a1 d1ea9c3 d1hx0a2 d2fhfa5 d1qhoa4 d1avaa2 d1m53a2 d4m8ua1
d2gjpa2 d1lwha2 d4howa1 d1ktba2 d1j0ha3 d3vm5a1 d2x1ia_ d1wzaa2
d1uasa2 d2diea1 d1mxga2 d1ht6a2 d1eswa_ d1iv8a2 d5cpsa_ d1ud2a2
d1zjaa1 d3gbea1 d1kwga2 d1wdpa_ d1gcya2 d1g5aa2 d1hvxa2 d3dhpa2
d2bhua3 d4do4a1 d3qgva1 d1e43a2 d1g94a2 d2d3na2 d1gjwa2 d1gvia3
d1x1na1 d1r7aa2 d1tz7a1 d3c8da2 d2pbla1 d1jkma_ d1jjia_ d1jjfa_
d4c7ga2 d2gjxa1 d1jaka1 d1yhta1 d4toqa_ d1wb0a1 d1w9pa1 d1eoka_
d1itxa1 d1kfwa1 d1goia2 d1edqa2 d2gsja_ d1hjxa1 d1jnda1 d1vf8a1
d1etob_ d1g2ha_ d1h6ua1 d1g4ma1 d1g4ma2 d1xl4a1 d1n9pa_ d3vsqa_
d2gixa1 d1b9ra_ d1offa_ d3wcqa_ d1e9ma_ d1l5pa_ d1wria_ d1i7ha_
d3av8a_ d1czpa_ d1rfka_ d3p1ma1 d3b2ga_ d2bt6a2 d1wgqa1 d1v89a1
d2coda1 d1zc3b_ d1wg7a1 d1u5ea1 d1v61a1 d2coca1 d2ys1a1 d1v5pa1
d1ntya2 d1wjma1 d1wi1a1 d1u29a_ d1xx0a1 d1unqa_ d2zkmx3 d1fhoa_
d1v88a1 d2adza1 d1x1fa1 d1p6sa_ d2coaa1 d1btka_ d1v5ma1 d1faoa_
d2x18a_ d1dyna_ d1x1ga1 d4kaxb_ d2j59m1 d1xcga2 d1txda2 d1u5da1
d1dbha2 d1w1ha_ d1upqa_ d1v5ua1 d1foea2 d2fjla1 d1eaza_ d2dfka2
d2cofa1 d1kz7a2 d1d4ua2 d9ldta1 d2ewda3 d1up7a1 d2dfda1 d1s6ya1
d1vjta1 d7mdha1 d1hyea1 d1t2da1 d1ojsa3 d1b8pa1 d1ldna1 d1hyha1
d1civa1 d2a92a1 d1ez4a1 d1i0za1 d1y6ja1 d1gv0a1 d1llda1 d1obba1
d1v8ba1 d1gdha1 d1l7da1 d2dlda1 d1mx3a1 d1ygya1 d1qp8a1 d1j4aa1
d2naca1 d1npya1 d3t4ea2 d1a4ia1 d1o0sa1 d2hk9a2 d2egga2 d1b26a1
d1bvua1 d1gq2a1 d1c1da1 d1leha1 d1gtma1 d1euza1 d1nvta1 d3dona2
d1edza1 d1vi2a1 d1p77a1 d1nyta1 d1b0aa1 d1vl6a1 d1v9la1 d1txga2
d3f47a1 d1mv8a2 d2pv7a2 d1xc2a3 d2ahra2 d4xiya1 d2f1ka2 d1pgja2
d1i36a2 d1np3a2 d1lj8a4 d1teaa3 d1qmga2 d2i76a2 d2g5ca2 d1wdka3
d1dlja2 d3cuma3 d1kola2 d1f8fa2 d1qora2 d1jqba2 d1vj0a2 d1cdoa2
d1gu7a2 d1d1ta2 d1xa0a2 d2dm6a2 d1jvba2 d1rjwa2 d1llua2 d1h2ba2
d1tt7a2 d1piwa2 d1o89a2 d1iz0a2 d1e3ja2 d1yb5a2 d1uufa2 d1vj1a2
d3gpdg1 d1vc2a1 d2nvwa1 d1u8fo1 d1ydwa1 d1hdgo1 d2g17a1 d1cf2o1

d2czca2 d1r0ka2 d1obfo1 d2cvoa1 d1ebfa1 d1h6da1 d1i32a1 d1zh8a1
d2q49a1 d1qkia1 d1j5pa4 d3pzra1 d1xeaa1 d1vkoa1 d1vm6a3 d3qvsa1
d2pkqo1 d1b7go1 d1gr0a1 d1p1ja1 d1lc0a1 d1j0xo1 d3pwka1 d2hjsa1
d1zyao1 d1vkna1 d1tlta1 d1nvmb1 d3sjua_ d3ojfa1 d1xu9a1 d2jjya1
d1n7ha_ d1y1pa1 d1h5qa_ d1jtva_ d4fs3a_ d2bkaa1 d1gy8a_ d2c5aa1
d1ulsa_ d1ooea_ d2b69a1 d1y5ma1 d1spxa_ d1ae1a_ d1bxka_ d1ydea1
d1gega_ d1hdoa_ d1o5ia_ d1e6ua_ d1snya1 d2rhca_ d3zv4a_ d5ceja_
d2d1ya1 d1fjha_ d3w8ea_ d2fmua1 d1x1ta_ d1rpna_ d3ay7a1 d1k2wa_
d1eq2a_ d2hrba1 d3ruaa_ d2ew8a1 d4hwka_ d1xgka_ d2bd0a1 d1yo6a1
d2a35a1 d2ntva_ d1vl8a_ d1hdca_ d4bkua_ d2ag5a1 d2q45a_ d1nffa_
d4iy1a_ d1zbqa1 d1kewa_ d1ek6a_ d1ja9a_ d2q46a2 d1iy8a_ d1cyda_
d2ptga_ d1hxha_ d1uzma1 d3lyla_ d4m89a_ d3wyea_ d2blla1 d1w6ua_
d1sb8a_ d2q2va_ d4nr0a_ d1e6wa_ d2gasa_ d3oiga1 d2bgka1 d1mxha_
d1vl0a1 d1rkxa_ d1r6da_ d1wmaa1 d1e7wa_ d2c07a1 d1xkqa_ d3tzha_
d1g0oa_ d3lz6a_ d1qyca_ d1edoa_ d1gz6a_ d1oc2a_ d1t2aa_ d1zema1
d2xoxa_ d1z45a2 d1p33a_ d3lt0a_ d4e6pa_ d2wyua_ d1db3a_ d4q9na_
d1xhla_ d4o1ma_ d1fmca_ d2uvda_ d2gdza1 d1uaya_ d2z1ma_ d2o23a2
d2fr1a2 d2fr1a1 d1qsga_ d1qyda_ d4zjua_ d2a4ka1 d1yb1a_ d1wvga1
d1yxma1 d3f4ba_ d1mg5a_ d1xg5a1 d1r55a1 d3gboa_ d1atla_ d1kufa_
d1buda_ d4q1la_ d1quaa_ d1yp1a_ d1wnia_ d3nqyb_ d4gera_ d1bqba_
d1ex0a4 d1g0da4 d2f4ma1 d1x3za_ d1qxpa4 d1ziva1 d1zcma1 d1t3ca_
d1epwa3 d2a97a_ d3fiea_ g1e1h.1 d3ioqa_ d1fh0a_ d1o0ea_ d1khqa_
d1m6da_ d2cb5a_ d1cs8a_ g8pch.1 d1s4va_ d2pnsa_ d1vsna1 d1mira_
d1iwda_ d2oula_ d7pcka_ d3bwka_ d3bcna_ d2p86a_ d1pvja_ d1pcia_
d1gece_ d1deua_ d4bs6a_ d1xkga1 d1cqda_ d1jqpa2 d2c0ya_ d2bdza_
d3p5ua_ d1zoya1 d1qo8a2 d1d4ca2 d1jnra2 d1kf6a2 d2jk6a2 d2jk6a1
d1feca1 d1feca2 d1lpfa1 d1lpfa2 d1xhca1 d1xhca2 d1dxla2 d1dxla1
d1gera2 d1m6ia2 d1onfa1 d1onfa2 d3lada2 d3lada1 d1w4xa1 d1w4xa2
d1gesa2 d1mo9a1 d1xdia1 d1fl2a2 d1fl2a1 d1fcda1 d1fcda2 d1h6va2
d1h6va1 d1mo9a2 d1gv4a2 d1gv4a1 d2gv8a2 d2gv8a1 d1aoga1 d1aoga2
d1hyua1 d1ebda1 d1ebda2 d3dk9a1 d1q1ra2 d1q1ra1 d1pv8a_ d1w5qa_
d2c1ha_ d1ohla_ d1zbfa1 d2z1ha_ d2etja1 d3qioa1 d1jl1a_ d2e4la_
d1jl2a_ d2lsna1 d1io2a_ d1uaxa_ d2yv0x_ d1mu2a1 d1ekea_ d1i39a_
d1pn0a1 d1reoa1 d1ju2a1 d2f5va1 d3c96a1 d2gmha1 d1pj5a2 d1gpea1
d1o5wa1 d1n4wa1 d2dw4a2 d2ivda1 d1seza1 d2voua1 d1kdga1 d1s5ja1
d2hbja2 d2igia_ d1wlja_ d1qhta1 d4dlea1 d1tgoa1 d2f96a1 d3tr8a1
d3mxma_ d1q8ia1 d1y97a1 d1w0ha_ d1kfsa1 d2py5a1 d1noya_ d1d5aa1
d1yt3a3 d2guia_ d3mwpa1 d4kb1a_ d2v76a_ d1wj1a1 d1aqca_ d1qqga2
d1qqga1 d1ntva_ d1n3ha_ d4dbba_ d3f0wa1 d1p3ra_ d1wgua1 d2cy4a1
d3hqca_ d1wvha1 d1xr0b1 d3nvva1 d1krha3 d2h88b1 d1t3qa2 d1kf6b2
d1zoyb1 d1jq4a_ d1ffva2 d1dgja2 d1rm6c2 d1k8ka2 d1k8ka1 d3uleb1
d3i33a2 d3i33a1 d1bupa1 d1dkgd2 d1dkgd1 d4ci6a2 d1jcfa1 d4a2ba2
d2fxua1 d1yaga2 d1yaga1 d4cbua2 d1bupa2 d1r7ha_ d1faaa_ d1wika1
d1xfla_ d1ttza1 d2f51a1 d1ti3a_ d1nhoa_ d1sena_ d2i4aa_ d1ep7a_

d1zmaa1 d1fo5a_ d3lefa1 d3diea1 d2yana1 d1z6na1 d1oaza_ d3zywa1
d4aj8a_ d1nm3a1 d4fiwa_ d1wmja_ d4hs1a1 d1iloa_ d1xwaa1 d1wjka1
d2vm1a_ d1fb6a_ d1gh2a_ d1h75a_ d1nw2a_ d1kx5b_ d3a6na_ d4m6ba2
d2nqbd_ d3wkjd_ d2yfwe_ d4h9na_ d5bnxa_ d1kx5c_ d3r45a_ d1eqzb_
d4jjnb_ d1f66c_ d1eqza_ d4wnnb_ d1kx5a_ d1u35c1 d1id3a_ d2nqbc_
d1kx5d_ d1um8a_ d1ixza_ d1qvra2 d1qvra3 d1qzma_ d1sxjc2 d1jr3d2
d2p65a_ d1l8qa2 d3hu3a3 d1sxjd2 d1njfa_ d1in4a2 d1sxja2 d1tuea1
d2ce7a2 d1w5sa2 d1sxjb2 d1d2na_ d1ny5a2 d1ofha_ d2gnoa2 d2a5yb3
d1fnna2 d1u0ja1 d1lv7a_ d1iqpa2 d2fnaa2 d1ixsb2 d4gita_ d1sxje2
d1g41a_ d1pd212 d1nhya2 d2a2ra2 d1k0dd2 d1f2ea2 d1gsua2 d1f3ba2
d2fhea2 d2pvqa1 d1tw9a2 d1oyja2 d1e6ba2 d1k0ma2 d3ik7a1 d4is0a1
d1r5aa2 d1jlva2 d1m0ua2 d3gtub2 d1z9ha2 d1okta2 d1axda2 d2gsra2
d1tu7a2 d1k3ya2 d1zgma1 d1b48a2 d1gnwa2 d1gwca2 d2gsta2 d1glqa2
d1oe8a2 d1pn9a2 d1v2aa2 d1fw1a2 d1duga2 d4ilya_ d4olta_ d1qgia_
d1sefa_ d1sfna_ d1sq4a_ d1qqya_ d1gd6a_ d1f6ra_ d1ivma_ d2gv0a_
d1iiza_ d2z2fa_ d1j8xa_ d1ghla_ d1b9oa_ d174la_ d169la_ d2o4wa_
d1p37a_ d1k28a3 d1xjta1 d1jtma_ d2f2qa_ d4e97a1 d3w3ea_ d4tx7a_
d3cqla_ d4msta_ d3iwra_ d2z37a_ d4dyga_ d2pjsa1 d1klla_ d2i7ra1
d1ecsa_ d1xqaa1 d5cj3a1 d1jifa_ d2p7oa_ d1r9ca_ d1nkia_ d1mpya2
d1kw3b2 d1kw3b1 d4ghga1 d1tfza1 d3isqa1 d3isqa2 d1sqia1 d1sqia2
d1lkda2 d1lkda1 d1mpya1 d1cjxa2 d1sp9b2 d1t47a1 d1t47a2 d1f1ua2
d1sp8a1 d1sp8a2 d1f1ua1 d4iuaa3 d4bvva_ d5hpga_ d3k65a_ d1ki0a3
d1ki0a2 d1ki0a1 d1a0ha1 d1pmla_ d3sp8a3 d2feba1 d1qsta_ d2beia1
d4m3sa_ d1u6ma1 d1ygha_ d1yk3a1 d3dr6a_ d1xmta_ d1z4ea1 d2ge3a1
d1bo4a_ d2fiaa1 d2jdca1 d1y9wa1 d3to7a_ d1wwza1 d2fl4a1 d2ozua1
d2b5ga_ d2cy2a1 d2p0wa_ d1cm0a_ d4pswa_ d1yrea1 d1yr0a1 d1m4ia_
d2gana1 d1yvka1 d1ghea_ d2g3aa1 d1y7ra1 d2euia1 d1q2ya_ d2fsra1
d1xeba_ d2ae6a1 d1cjwa_ d1qsma_ d2aj6a1 d1y9ka1 d1i12a_ d1vkca_
d2atra1 d1n71a_ d2d4pa1 d2fiwa1 d2fe7a1 d1tiqa1 d2ou2a_ d1yx0a1
d2fcka1 d1p0ha_ d2j8ma_ d2pq8a_ d1z4ra1 d1s3za1 d1ufha_ d1xvqa1
d1pn0a2 d3hy2a_ d2cvba1 d1o8xa_ d1lu4a_ d1we0a1 d2bmxa1 d2a4va1
d1psqa_ d1xiya1 d1q98a_ d1n8ja_ d1zofa1 d2ggta_ d3tjja_ d4je1a1
d3s9fa_ d2zcta_ d1qmva_ d1jfua_ d2b1ka_ d5dvba1 d2b5xa1 d2v2ga_
d1st9a_ d3tb2a_ d4llra_ d4fh8a1 d1e2ya_ d1knga_ d2h30a_ d1i5ga_
d2z9sa_ d2f8aa1 d1tp9a1 d2b7ka_ d1nm3a2 d3hvva_ d2wfca1 d4kcea_
d1xvwa1 d4gqca1 d3qpma_ d4k1fa_ d1zzoa1 d1gp1a_ d1prxa_ d4od7a1
d1z6ma1 d4mcua1 d4dvca1 d1r4wa_ d3l9sa_ d1un2a_ d3rppa_ d3l21a_
d2e1da_ d1to3a1 d1w3ia_ d1sfla_ d1n7ka_ d1vcva1 d4imea_ d1wa3a_
d2ocza1 d1f2ja_ d3m16a_ d2yxga_ d1ub3a_ d3a5fa_ d1fbaa_ d1l6wa_
d1xfba1 d1wx0a1 d1vpxa1 d1ojxa_ d1mxsa_ d2a4aa1 d1xkya1 d3cq0a1
d3s1xa_ d1f05a_ d1fdja_ d3cpra1 d4d2ja_ d4mh8a_ d4i7ga1 d1etha2
d1w52x1 d2ppla1 d1hpla2 d1lpbb2 d2oxea1 d1bu8a2 d1rp1a2 d1ys1x_
d4hs9a_ d1ispa_ d1ku0a_ d3qmma_ d1bnla_ d1dy2a_ d1ra6a_ d1muka_
d3gsza_ d1khva_ d1te9a_ d2xwha_ d3uqsa_ d3hkwa_ d3n6ma_ d2cjqa_

d2xyma_ d1teba_ d1te8a_ d1s48a_ d1u09a1 d4wfxa_ d3tyqa_ d2pgga1
d1lgya_ d4l3wa_ d1llfa_ d1gz7a_ d1ukca_ d1svpa_ d1mbma_ d3su6a1
d1cu1a1 d4agka_ d1vcpa_ d1q31a_ g1n1l.1 d3e90b_ d1ep5a_ d3u1jb_
d3zxha_ d1cgla_ d1rm8a_ d1eaka2 d1hy7a_ d2jsda_ d1cxva_ d1hv5a_
d3p0ya1 d1igra1 d1igra2 d1n8yc2 d1m6ba2 d1m6ba1 d1m4la_ d2bk7a_
d3fjua_ d1z5ra1 d1uwya2 d1jqga1 d2c1ca1 d1dtda_ d1h8la2 d1ddla_
d1e57b_ d1auyb_ d1js9c_ d1f15b_ d1za7a_ d1laja_ d1rypd_ d1irua_
d1rypc_ d3unfk_ d1rypa_ d1j2pa_ d1irui_ d1j2qh_ d3unfh_ d1m4ya_
d1rypb_ d1irul_ d1irug_ d1iru2_ d1q5rh_ d1rypg_ d1iruf_ d1q5qa_
d1e94a_ d1irub_ d1ryph_ d3ty6a_ d1iruj_ d1iruk_ d1rype_ d1irud_
d1irue_ d3mi01_ d1rypf_ d1iruh_ d2z3ba_ d1iruc_ d3mka2_ d1iru1_
d1e88a1 d1l6ja5 d1l6ja4 d1eaka4 d1eaka3 d1h8pa2 d1h8pa1 g1gk9.1
g1or0.1 g1e3a.1 d1mqta_ d1d4m3_ d1fmd3_ d2mev1_ g1aym.1 d1ev13_
d4q4w1_ d1r1a3_ d2x5ia_ d1rhi1_ g1r1a.1 g1d4m.1 g1rhi.1 d1b35b_
d1oopa_ d1tme1_ d4iv1a_ g1pvc.1 g1fpn.1 d1fpn1_ d4gb31_ d1d4m1_
d1fmd1_ d1ev11_ d2wzr3_ g1cov.1 d1qqp3_ g1fmd.1 d2x5ic_ d1rhi3_
g1oop.1 d1hxs1_ g1hxs.1 d1fpn3_ d1bev1_ g2mev.1 d1eah3_ d1aym1_
d1pvc3_ d1qqp1_ d1zba3_ d1h8ta_ d3vbhc_ g1b35.1 g1bev.1 d1hxs3_
g1ev1.1 d1bev3_ g1tmf.1 d1tmf1_ d1b35a_ d2mev3_ g1eah.1 g1h8t.1
d1eah1_ d4q4w3_ d1r1a1_ d1aym3_ d1pvc1_ d1zba1_ d1h8tc_ d1oopc_
d1tme3_ g1qqp.1 d4iv1c_ g1tme.1 d3ubub_ d1egia_ d1qo3c_ d1v7pa_
d1tdqb_ d3ubua_ d1c3ab_ d1h8ua_ d3bx4b_ d1t8ca1 d1sb2a1 d1qdda_
d2ziba1 d3cada_ d1byfa_ d1j34b_ d1jwia_ d1jzna_ d1c3aa_ d1oz7b_
d1wmza_ d3bdwb_ d1hq8a_ d1fifa1 d1dv8a_ d3hupa_ d1wk1a1 d1umra_
d2afpa_ d3bx4a_ d1gz2a_ d1sl6a_ d1v7pb_ d3g8ka_ d1ixxa_ d1j34a_
d1jwib_ d1g1sa1 d1y17a1 d1ja3a_ d1fvub_ d3bdwa_ d1sb2b1 d1fvua_
d1umrc_ d1oz7a_ d4m17a_ d3cfwa1 d1ypqa1 d1g1ta1 d3fd4a_ d3l9jc_
d4dn8a1 d1v4lb_ d1rdl1_ d2msba_ d4lt9a1 d3tc7a1 d3hoja_ d1i4na_
d3vnda_ d3nl8a1 d1geqa_ d3nava1 d1v5xa_ d4a2ra1 d3tc6a1 d1ujpa_
d4ijba_ d1xcfa_ d1vc4a_ d1rd5a_ d1qopa_ d3b5va_ d2r80b_ d1cqxa1
d1cg5a_ d1gcvb_ d3boma_ d2h8fb_ d2raoa_ d3bj1a_ d4hrrb_ d2r50a_
d3d1kb_ d2aa1b1 d3zhwa_ d3hf4b_ d3gdja_ d1jebb_ d3bj1b_ d1x9fb_
d1wmub_ d1gcva_ d3bcqb_ d3d4xb_ d2dc3a_ d3hf4a_ d4yu3a_ d3k8ba_
d1qpwb_ d2r80a_ d3bcqa_ d1v4wa_ d3wtgb_ d1jl7a_ d4esaa_ d1a9we_
d3d1ab_ d1fsla_ d4h2la_ d1v4wb_ d1mwca_ d1vhba_ d1jeba_ d1spgb_
d1h97a_ d1q1fa_ d1tu9a1 d3d1aa_ d1hdsb_ d3hyub_ d2zfbb_ d3fs4a_
d1oj6a_ d1shrb_ d4h2lb_ d4yu3b_ d1spga_ d3hrwa_ d3qqra_ d3at5a_
d4wchd_ d3hyua_ d1cg5b_ d1uc3a_ d1hbra_ d2raob_ d1outb_ d4esab_
d3gdjb_ d3qqqa_ d1x9fc_ d3vrfa_ d1itha_ d3mkba_ d3d4xa_ d3mjpa_
d1jl6a_ d3fh9a_ d1outa_ d2dc3b_ d1it2a_ d3eoka_ d3g46a_ d1qpwa_
d3vrfb_ d1ch4a_ d1x9fd_ d1hdsa_ d2zfba_ d3mkbb_ d1or4a_ d3wtga_
d1a4fa_ d3fh9b_ d3bomb_ d3cy5a_ d3at5b_ d1x9fa_ d2oifa_ d4lmxb_
d1cpcb_ d3v57a_ d1kn1a_ d1cpca_ d4f0ta_ d2vjha_ d4po5b_ d1eyxa_
d1alla_ d4f0tb_ d1b33b_ d4h0mb_ d1b33a_ d2vjhb_ d4lm6b_ d2vmlb_

d1allb_ d2vjta_ d3dbja_ d2vmla_ d2vjtb_ d4f0ub_ d1f99b_ d4rmpa1
d1liaa_ d1eyxb_ d2uulb_ d4lmsb_ d4f0ua_ d2bv8a_ d1f99a_ d2bv8b_
d2c7lb_ d3dbjb_ d4rmpb1 d1b8da_ d4h0ma_ d3v57b_ d1kn1b_ d1liab_
d2uula_ d2j96a_ d1kyqa1 d1azwa_ d1mtza_ d1k8qa_ d2xqfa_ d1ea5a_
d1f6wa_ d1dx4a_ d1qe3a_ d1k4ya_ d4qwwa_ d2ha2a_ d2shpa1 d1yfoa_
d1l8ka_ d2ooqa1 d1g4us2 d1p15a_ d1lara2 d1lara1 d1wcha_ d2fh7a2
d2fh7a1 d1gl9b1 d1gkub1 d1jmxa1 d1pbya1 d1pbya2 d1jmxa4 d1jmxa3
d1pbya4 d1s5ja2 d1qhta2 d4dlea2 d1tgoa2 d3py8a2 d1q8ia2 d1kfsa2
d2py5a2 d1d5aa2 d1dosa_ d3qm3a1 d2fjka_ d2m5ta_ d1cqqa_ d1wqsa_
d5bpea_ d2ipha_ d1lvoa_ d2bhga1 d3tnta_ d3sjia_ d3d23a1 d2fyqa_
d3tloa_ d3zvea1 d2hrva_ d3q3ya1 d4asha1 d1cg2a1 d1yloa2 d1y7ea2
d1y0ya3 d1lfwa1 d1vixa1 d1fnoa4 d1vhoa2 d1ysja1 d1vhea2 d1z2la1
d2fvga2 d1vgya1 d2grea2 d1xmba1 g1q7l.1 d1rtqa_ d3rp2a_ g1ucy.1
d1fuja_ d2zchp_ d3w94a_ d1si5h_ d1lo6a_ g2pka.1 d1cgha_ d1kigh_
d1a5ia_ d3s9ba_ d1hj8a_ d1ao5a_ d2anya_ d1q3xa1 d1gvza_ d1azza_
d4gsoa_ d2f91a1 d1iaua_ d1gdna_ d1eufa_ g1fiw.1 d1eq9a_ d4nfea_
d2rdla_ d1spja_ d1elva1 d1a7sa_ d1ekbb_ d1pfxc_ d1yc0a_ d2aiqa_
g1fiz.1 d1fi8a_ d4k60a_ d4dgja_ d1mzaa_ d1op0a_ d3tjva_ d1pytd_
d1sgfg_ g1gg6.1 d2eeka_ g1rtf.1 d1a0ja_ d3g01a_ d1pytc_ d4e7na_
d1ddja_ d1z8ga1 d2hlca_ d1npma_ d1brup_ g1jou.1 d1sgfa_ d1autc_
g2hnt.1 g1kdq.1 d1orfa_ d3s69a_ d4crga_ d1fxya_ d1bqya_ d2ftba_
d1pmpa_ d2rcqa1 d1fdqa_ d4lkpa_ d1sa8a_ d1vyfa_ d1lpja_ d3vg7a1
d4azqa1 d1o8va_ d5bvqa_ d1p6pa_ d2qo4a_ d1opba_ d1yiva1 d1o1va_
d4bvma1 d4tkba_ d4i3ba_ d1ftpa_ d1ggla_ d4gkca_ d1cbia_ d3p6da1
d1kqwa_ d2a0aa1 d1dzka_ d4tlja_ d2a2ua_ d4wfva_ d3t1da_ d4iaxa_
d3zq3a_ d2nnda_ d1i4ua_ d1kxoa_ d1bbpa_ d1kt7a_ d1exsa_ d3s26a1
d1lf7a_ d4gh7a_ d1z24a1 d1qabe_ d1xkia_ d1ew3a_ d3bx7a_ d4iawa1
d1n0sa_ d1gkab_ d3kzaa_ d2k23a_ d2l9ca1 d2joza1 d1gm6a_ d3kffa_
d3saoa1 d1e5pa_ d1yupa1 d1iiua_ d4lu0a_ d1of8a_ d3unda1 d3e9aa1
d1vr6a2 d1n8fa_ d2qkfa_ d1d9ea_ d1o60a_ d1e69a_ d1mv5a_ d3g60a4
g1ii8.1 d1ewqa2 d1ji0a_ d1qhla_ d1v43a3 d2wmfa1 d1vpla_ d1g2912
d4a82a2 g1f2t.1 d2d62a1 d3tuic1 d2bbsa1 d3g60a3 d2ixea_ d1g6ha_
d1sgwa1 d2qi9c_ d3rlfa1 d3b60a1 d3d31a2 d2pzea1 g1xew.1 d1jj7a_
d1oxxk2 d2onka1 d1w1wa_ d1iwea_ d1qf5a_ d1vmaa2 d1nija1 d1np6a_
d2p67a1 d3ihla_ d1okkd2 d1ls1a2 d1s1ma2 d1iona_ d2v40a_ d3hida_
d2vo1a1 d2g0ta1 d1vcoa2 d1j8mf2 d1g3qa_ d1xjca_ d1p9ba_ d1qzxa3
d1ihua2 d1ihua1 d2qm8a_ d1dj2a_ d4ioja_ d1cp2a_ d1yrba2 d1hyqa_
d3gqba2 d1skye3 d1xp8a1 d1tf7a2 d1tf7a1 d1ye8a1 d1skyb3 d2qe7d2
d4blta_ d4huta_ d3ldaa2 d4hyya_ d2i3ba1 d1cr1a_ d3j9tb2 d3ntua2
d1vdza1 d2z43a_ d1e9ra_ d1nlfa_ d3ziaa2 d1n0wa_ d1g6oa_ d3ziad2
d3j9ta2 d3gqbb2 d2i49a_ d1nnfa_ d1xvxa_ d1ursa_ d1h3da1 d1iuda_
d4m1va1 d1twya_ d3e13x_ d2nxoa1 d4pfua_ d3ttma_ d1elja_ d2qmwa1
d1us5a_ d1y3na1 d1jeta_ d1uqwa_ d4f19a1 d2esna2 d3thia_ d3fasa1
d2uyeb_ d2xwva1 d1r9la_ d2fyia1 d1pb7a_ d2ozza1 d1sw5a_ d1nh8a1

d1vr5a1 d2g29a_ d1xs5a_ d3lswa_ d2z23a_ d3w9va_ d3tcha_ d1q35a_
d1z7me1 d3q27a_ d1p99a_ d1ve4a1 d2i6ea1 d4mcoa1 d4n8ya_ d1eu8a_
d1zbma1 d1ixca2 d1j1na_ d1xvya_ d1y9ua_ d2z22a1 d1xoca1 d4oera_
d1wdna_ d5f1qa1 d1xt8a1 d1i6aa_ d4gwoa_ d4igta1 d1pc3a_ d4mmpa1
d1o63a_ d2czla1 d2onsa2 d4mija1 d1eh3a_ d3skpa_ d1dtza1 d1dtza2
d1ieja_ d1jnfa2 d1ce2a1 d1jnfa1 d1b1xa2 d1b1xa1 d1jw1a1 d1jw1a2
d1ryoa_ d1h76a1 d1h76a2 d1c4oa1 d2p6ra3 d2p6ra4 d1oywa2 d1oywa3
d1wrba1 d1w36b2 d1w36b1 d1c4oa2 d3bora_ d1t6na_ d2eyqa5 d2eyqa3
d2eyqa2 d2zu6a2 d1veca_ d1uaaa1 d1w36d1 d2g9na1 d1z3ix2 d2rb4a_
d1hv8a1 d1hv8a2 d1t5ia_ d1w36c2 d2j0sa2 d2j0sa1 d3hjha1 d1z5za1
d1qdea_ d1z63a1 d1s2ma2 d1s2ma1 d1wp9a1 d1wp9a2 d1gm5a3 d1gm5a4
d3dina4 d3dina3 d1q0ua_ d1w36c1 d1z3ix1 d1nkta3 d1nkta4 d1uaaa2
d1w36d2 g1qhh.1 d2fwra2 d2fwra1 d2v8ta_ d3q3ma_ d1za0a1 d2inca_
d1jk0a_ d2incb_ d1mhyb_ d3hf1b_ d1mtyb_ d1r2fa_ d2uw1a_ d1mhyd_
d3n37a_ d1afra_ d1oquc_ d1syya_ d1mtyd_ d2o1za_ d1uzra_ d1smsa_
d2fkza_ d1zuja1 d1s3qa1 d2fjca1 d1z6om1 d1veia_ d4dyya_ d1jiga_
d3gvya_ d2pyba_ d4cy9a1 d3bvfa1 d1r03a_ d2cf7a_ d1nf4a_ d1zs3a1
d4iwka_ d3ka8a_ d3np0x_ d1n1qa_ d1lb3a_ d4e6ka_ d1z6oa1 d2fzfa1
d4dyua_ d3ge4a_ d1jgca_ d4am4a_ d2z6ma_ d1ji5a_ d1vlga_ d1lkoa1
d3qz3a_ d1o9ra_ d2ffxj_ d1euma_ d1yv1a1 d2v2pa_ d1j30a_ d4di0a_
d1dpsa_ d2qzxa_ d2asia_ d1psoe_ d1miqa_ d1smra_ d2h6ta_ d1pfza_
d1izda_ d3hd8a_ d1j71a_ d1dpja_ d4aa9a_ d1t6ex_ g1b5f.1 g1lya.1
g1htr.1 d1qdma2 d1bxoa_ d1wkra_ d1tzsa_ d1ibqa_ d2qzwa_ d1ex4a2
d1c6va_ d1bdqa_ d3ggta_ d3ixoa_ d1mrxa_ d2haha_ d1tw7a_ d4njsa_
d1sgua_ d2p3da_ d1nsoa_ d3d3ta_ d2rkfa_ d1c6ya_ d3ogpa_ d3ekwa_
d2o4na_ d2j9ja_ d1baia_ d3ggua_ d3lzua_ d4npua_ d4j55a_ d2r8na_
d2fxda_ d2p3ca_ d4dqea_ d3ttpa_ d3t3ca_ d2p3aa_ d2hc0a_ d2fdda_
d3mwsa_ d1h4ra3 d4yl8a1 d2al6a3 d1wgda1 d1wgga1 d4q5eb_ d1wjna1
d1se9a_ d3n3kb_ d1wz0a1 d1we6a1 d2m0xa_ d2gbka_ d3rt3b1 d1wh3a1
d1sifa_ d2ekec2 d4ii2b1 d4hk2a1 d1c3ta_ d1m94a_ d1wgha1 d1ttna1
d1j8ca_ d1wx8a1 d3v6cb1 d2ma9b_ d4i6lb1 d1bt0a_ d2jvca1 d1iyfa_
d2k1fa_ d1wxva1 d1wf9a1 d1t0ya_ d2io0b2 d1x1ma1 d2gbma_ d1zw7a1
d2m17a_ d1ud7a_ d1we7a1 d2lwpa_ d2gbja_ d1wjua1 d1v6ea1 d1v86a1
d4eewa_ d1wiaa1 d4icva_ d4gu2a_ d1wx7a1 d1wy8a1 d4hjka1 d2faza1
d1v5oa1 d2al3a1 d2cr5a1 d1wj4a1 d1s3sg_ d1jx4a2 d3gv5b1 d1t94a2
d3bq0a2 d1qoza_ d5ajha1 d3h33a_ d1rwja_ d19hca_ d1os6a_ d3bxua_
d3h4na_ d1i77a_ d3caoa_ d1j0pa_ d2ctha_ d1duwa_ d1ofwa_ d2e84a_
d1hh5a_ d1gyoa_ d3h34a_ d1iyka2 d1iyka1 d1rxta1 d1iica1 d1adqa1
d1fnga1 d1uvqb1 d1zs8a1 d2vola1 d2vola2 d1mfbl2 d2jelh2 d1uwel2
d3qq4b1 d1kjvb_ d1k5na1 d1osph2 d4odhl2 d1ow0a1 d1ow0a2 d1bfob2
d3l9ra2 d3jtth1 d2fath2 d4elkb2 d1fn4b2 d2xfxb_ d1pg7x2 d4k71b2
d1lk2a1 d1eapb2 d4lfhg2 d1i4fa1 d2nw2a2 d1kfah2 d1mhca1 d1h3ua1
d1wejh2 d1h0db2 d1a5fh2 d1kcvh2 d1mjuh2 d1qqda1 d1kcrh2 d1k8ia1
d3huja2 d2nnab1 d1nfdf2 d3mffb2 d1s9va1 d1lk3h2 d1q0xl2 d1fv1b1

d1neza1 d4rfeb2 d1r3ha1 d1a6tb2 d1kjva1 d1pz5b2 d1onqa1 d2bc4a2
d1de4a1 d1k8da1 d1tcra2 d1dn0b2 d1nfde2 d2xwtb2 d1bfoa2 d1mexh2
d1iakb1 d1ypzf2 d4f7ea2 d1q72l2 d2fbjh2 d1fe8h2 d1clzh2 d1fngb1
d1uvqa1 d1vgka1 d1c16a1 d4j12a2 d4x99b2 d1igtb3 d1je6a1 d1q72h2
d1nldh2 d1teth2 d2hmic2 d1i1ca2 d1i1ca1 d1k8ib1 d1q0yh2 d1kfal2
d3frua1 d1pg7w2 d1u58a1 d1igyb4 d1igyb3 d1hyrc1 d1klub1 d1deeb2
d1c12b2 d2bc4b2 d2ig2h2 d1kcvl2 d3wn5b1 d1lk2b_ d1lk3l2 d1mjul2
d1sm3h2 d1q0xh2 d1zvsa1 d1ydpa1 d1u8ib2 d1ypze2 d1kcuh2 d2e3fa_
d1mm0a_ d2e3ea_ d2llda_ d2ny8x_ d2e3ga_ d2ny9x_ d2nz3a_ d2lj7a_
d1n4na_ d2m8bb1 d2kyqa_ d3psma_ d1jxca_ d2gl1a_ d1ugla_ d4uj0a_
d2k4ua_ d1hp2a_ d2lu9a_ d1baha_ d2meoa1 d1wm7a_ d1hlya_ d1n8ma_
d1jlza_ d1wpda_ d1c55a_ d1pvza_ d1wt7a_ d1rjia_ d1wz5a_ d1m2sa_
d1pjva_ d1c49a_ d1qkya_ d2ktca1 d1wmta_ d1du9a_ d1j5ja_ d1r1ga_
d1xwdc1 d3kj4a_ d1ozna_ d1w8aa_ d2ifga3 d1xkua_ d1t0za_ d2i61a_
d1t1ta_ d1sn4a_ d1kv0a_ d2lkba_ d1npia_ d1wwna_ d1pe4a_ d2kbka_
d2li7a_ d1djta_ d2kbha_ d1dq7a_ d1sega_ d1omya_ d1zywa1 d1jzaa_
d1n9wa1 d1bbua1 d1c0aa1 d1b8aa1 d1l0wa1 d3a74a1 d1eova1 d1bvsa3
d1iyjb4 d1iyjb5 d1iyjb3 d1o7ia_ d4ipca1 d1x3ea_ d4apva_ d1jb7a1
d1jb7a2 d1jb7a3 d3kdfa_ d1ue1b_ d1wjja1 d3eiva_ d1xjva2 d1xjva1
d2ccza2 d1se8a_ d1l1oc_ d1jb7b_ d1qvca_ d3ulla_ d1qzga_ d1miua4
d1miua5 d1miua3 d1y14b1 d2nn6i1 d1i94l_ d2zjra2 d1smxa_ d2qamc2
d1hh2p1 d2r7da1 d1rl2a2 d1wfqa1 d3bzka4 d1x6oa2 d3a0ja_ d2j01d2
d2ja9a1 d4ql5a_ d1hr0w_ d1luza_ d2n3sa_ d2vnud1 d1h95a_ d1go3e1
d2nn6h1 d1ne3a_ d2mo1a_ d1iz6a2 d1jjga_ d1e3pa2 d2ix0a3 d2ix0a2
d2lxja_ d1ueba3 d1ueba2 d2z0sa1 d1u0la1 d2ix0a1 d2eifa2 d2je6i1
d3i2za1 d2c35b1 d2ahob2 d1t9ha1 d3i4oa_ d2qall1 d1jt8a_ d2nn6g1
d1g6pa_ d3cama_ d1i94q_ d1c9oa_ d1d7qa_ d2ba0a1 d2vnud2 d2vnud3
d1khia2 d2qalq1 d1wi5a1 d1xtda2 d1ny4a_ d1dgsa2 d1fvia1 d1ckma1
d1x9na2 d1pfsa_ d2a1ka_ d1je5a_ d3azya_ d3wvja_ d1upsa1 d1ajoa_
d2hyka_ d1ajka_ d2vy0a_ d1o4ya_ d3o5sa_ d3d6ea_ d3atga_ d1umza_
d3i4ia_ d1c1la_ d4lbqa_ d1a78a_ d1slta_ d4no4a_ d3zsja_ d3duia_
d1g86a_ d1is3a_ d1hlca_ d1tdha3 d1q3ba3 d1ee8a3 d1k82a3 g1qmo.1
g1len.1 d3zyra_ d1fnya_ d1n47a_ g2ltn.1 d1avba_ d3usua_ d1dhkb_
d1f9ka_ d1ukga_ d1g8wa_ d1g9fa_ d1wbfa_ d3wcsa_ d1g7ya_ g1loe.1
d1mvqa_ d1hqla_ d1v6ia_ d1dbna_ d1qnwa_ d1fx5a_ d4l8qa_ d1ioaa_
d3n35a_ d3ubra_ d1qo8a1 d1d4ca1 d2k3va_ d1ft5a_ d1qdba_ d1m1qa_
d2j7aa_ d1h21a_ d1ogyb_ d4fasa_ d1jnia_ d1sp3a_ d2h0ba_ d2erfa_
d1h30a2 d1h30a1 d3mw4a1 d1pz7a_ d2r1ba_ d3v64a1 d3bopa1 d3mw3a1
d1okqa1 d1okqa2 d1wioa4 d1ccza2 d1vcaa1 d1dr9a2 d2d9qb1 d2nxyb2
d2dara2 d2dara1 d1wyha2 d2dloa2 d1rutx4 d1rutx3 d2cuqa2 d1x4la2
d1x4la1 d1x3ha2 d1x3ha1 d2d8za1 d1x61a2 d1x6aa1 d1x6aa2 d2cora2
d2cora1 d1v6ga1 d1v6ga2 d2d8za2 d1x64a1 d1x64a2 d1b8ta1 d2co8a1
d1x63a2 d2dj7a2 d2dj7a1 d2cu8a2 d2cu8a1 d1g47a2 d1g47a1 d1j2oa1
d1wyha1 d2dloa1 d1wiga1 d1wiga2 d2cupa2 d2cupa3 d1x68a1 d2d8ya2

d2d8ya1 d1m3va2 d1x62a1 d1x68a2 d1x61a1 d1b8ta3 d1b8ta2 d2d8xa2
d2d8xa1 d1x4ka1 d1x4ka2 d2cuqa1 d1m3va1 d2co8a2 d1j2oa2 d1x63a1
d2cura1 d2cura2 d1x62a2 d1op9a_ d1y0la1 d1etzb1 d1egjl1 d2gsib1
d1nj9a1 d4jfhd1 d1aqkl1 d1t2ja_ d1lo4h1 d1zvya1 d3v4vl1 d4enec1
d1kxtb_ d5e8ea1 d2pxya_ d4weud_ d1xiwc_ d2esvd1 d1qnzh_ d1ogae1
d1f4xh1 d4n1hb_ d3ezjb1 d3mlwl1 d1kfah1 d1etza1 d1b4jh1 d3cfic_
d4w68a_ d2arja1 d1ek3a_ d2ok0l1 d1vera_ d1uwgh1 d1blnb1 d1fpth1
d1rzga1 d2giya1 d4m3kb1 d1nc2a1 d2nyyd1 d1ncna_ d1fbih1 d5dxwa_
d4fqqa1 d1dqdh1 d1jfql1 d1kelh1 d1faih1 d1frgl1 d1figl1 d4m6nl1
d1fo0a_ d4hxbl1 d3b5ga_ d3quyd1 d2h9gb1 d1i8kb_ d2dd8h1 d2ck0h1
d1eaja_ d4w6wb1 d2a6ib3 d1t66d1 d2e27h_ d1ob1b1 d1f11b1 d2v7na1
d1ad0a1 d2xt1b1 d1pskh1 d4i13b1 d2op4l1 d1a0ql1 d1uwel1 d1za3a1
d1ncbl1 d4uu9a_ d1bm3h1 d1cloh1 d1nqba2 d1cf8h1 d4y5va_ d1mhph1
d5anma1 d4cdgc1 d4ldeb_ d1y0lb1 d2pcpb1 d1z3gl1 d1dfbl1 d1fh5h1
d3g5xa1 d1tzib1 d4dn3l1 d3zhka1 d2b1hh1 d2uyla1 d1f3dh1 d1nj9b1
d2agjh1 d2h1pl1 d1q72h1 d8fabb1 d1ndmb1 d1rjca1 d4u6hb1 d3upca_
d2fbjl1 d1osph1 d1tvda_ d2cjvh_ d4fhbd1 d1bd2d1 d1rz7h1 d3nn8a_
d1blna1 d1knoa1 d1nfda1 d2bmkb1 d1dqll_ d1igyb1 d1igfl1 d12e8h1
d3utza1 d1nc2b1 d1rihh1 d4mwfb1 d4jvpa1 d1mieh1 d1rhfa1 d1fgnl1
d1sbsh1 d1lp9e1 d1dn0a1 d4lqfl1 d1iaih1 d1kgcd1 d1mh5b1 d1gpol1
d2g60h3 d1f8tl1 d2jixd1 d4grwh_ d1fo0b_ d1bfob1 d1axsa1 d1u9ka_
d1nmbh_ d1h8na1 d1h8na2 d1jguh1 d4eura1 d3sdyl1 d35c8h1 d1w72l1
d1mvub_ d1i9rh1 d1ob1a1 d4wenb_ d1yeeh1 d3ietc1 d1f58h1 d1ad0b1
d1mqkl_ d1nbvh1 d4b50a_ d3r8bb_ d1bafl1 d1mamh1 d4rgne1 d2gsgb_
d35c8l1 d1bafh1 d1ai1h1 d2pcpa1 d2fath1 d2x1pa_ d1orsb1 d1ibgl1
d1shma_ d1a2yb_ d1u0qa_ d2coqa_ d1hilb1 d1qfwi_ d1jv5b_ d1fn4b1
d5e0qa_ d1nsnh1 d1plgl1 d4ocxl1 d1kb5h1 d1rzfh1 d1pg7x1 d2fx7h1
d2oi9c_ d1l7il1 d3eyva1 d1d5il1 d4ffyl_ d1tetl1 d1a6wh_ d1ac6a_
d3dvfa_ d1nfde1 d1mimh1 d1ri8a1 d1jhlh_ d3dura_ d1fskb1 d1f97a1
d3so3b1 d2ak4d1 d1lp9f1 d4z7wf1 d1l7th1 d4y5ya_ d4x7ec1 d1emth1
d1ikfl1 d1nlbh1 d4utal1 d1bjma1 d1eapb1 d1u6ah1 d2dtgd1 d1kj2b_
d1xeda1 d1c1eh1 d1fgvh_ d2bnub1 d2fb4h1 d2dtgb1 d1ktrh_ d1jpth1
d1qkzl1 d4hepg_ d2atpb1 d1flrh1 d2hrpl1 d1mvfa_ d1iaim1 d3k80a1
d2jb5h1 d3zkxc_ d4grwe_ d1it9h1 d1rhhb1 d3w9ec_ d1e4wh1 d1sy6h1
d2j6eh1 d3qxta_ d1jglh1 d1p7ka1 d1tzhb1 d4n8pa1 d1gafl1 d2jelh1
d1wioa2 d4i0cc1 d1cicb1 d1lmka2 d1lmka1 d1iqwh1 d1orqb1 d1ejoh1
d1jnlh1 d4zg1a_ d43c9b_ d1ggbl1 d1wejh1 d3q6fa1 d1f3dj1 d3c6la1
d1bqlh1 d3p9wb_ d1a3rl1 d1h0db1 d2uzil_ d1eo8l1 d1qfwl_ d1n7ml1
d1igja1 d1c5ch1 d2a9mh_ d1rmfh1 d1fj1b1 d4heme_ d1ccza1 d1a5fh1
d1ogad1 d1dlfh_ d1qa9b_ d1vgel1 d3qskb_ d1sjxa_ d4ozfg1 d1sq2n_
d1qd0a_ d1kcvh1 d1c5db1 d3qcul1 d8faba1 d3bn9d1 d1mjuh1 d4y8dc_
d7fabh1 d3dvgb1 d1lo0l1 d1a6tb1 d1gigh1 d1kcrh1 d2hwzl1 d1yjdc1
d3clfl1 d4krna_ d1dl7l_ d1e6ol1 d1mexh1 d3r0ma_ d4kkna_ d1fnsl1
d1oaqh_ d1rzia1 d1ucbh1 d1ncwl1 d4b41a_ d1nfdf1 d2mpah1 d2d7tl1

d4grwf_ d4b5ea_ d1lk3h1 d3u79a_ d4u3xa_ d1ghfl1 d1bvkb_ d1mcww1
d1b88a_ d1jrhh1 d32c2b1 d4wemb_ d1dsfh_ d1op3h1 d1cell1 d1iqda1
d1qoka1 d1qoka2 d1qfuh1 d2crya1 d1ay1h1 d1oaql_ d3ab0b_ d1rurl1
d1t4kb1 d1bz7b1 d1n0xh1 d1forl1 d1ucbl1 d4w6yb_ d43c9a_ d2aabl1
d1tzia1 d4n1ca_ d1k6qh1 d3g9ab_ d4tvsa1 d1igmh_ d1pz5b1 d3dx9a1
d3wxwh_ d1nakh1 d1kcul1 d3eaka_ d4qlra1 d1cr9l1 d1d9ka_ d1f4xl1
d1deea1 d2i27n2 d1c12a1 d2orbl1 d1j8hd1 d1mcph1 d1c5da1 d1kenh1
d1bd2e1 d1h5ba_ d4x7fc1 d3upaa_ d1t3fa1 d1indh1 d2dtgc1 d4hc1l1
d1zv5a1 d4wgvb_ d1uwgl1 d4f37k1 d1aqkh1 d1vesa_ d3zkqd_ d1fptl1
d1lo4l1 d1knob1 d3rrqa_ d1ct8b1 d4orzc_ d1mlbb1 d4hgkc1 d1dqdl1
d1bogb1 d4ozhe1 d1dr9a1 d1i7za1 d1dn0b1 d1kell1 d1rzib1 d1ypzf1
d1wtla_ d1hkfa_ d1fail1 d1figh1 d2vyre_ d1sjva_ d1ohqa_ d1bfoa1
d1pkqb1 d1mjul1 d1b4jl1 d1nsnl1 d2arjb1 d1p4bh_ d4aj0a_ d3nn8b_
d2rcja1 d1f2xk_ d4v1dd_ d1adqh1 d2eksa_ d4k3ga_ d1jfqh1 d1uweh1
d1frgh1 d4gftb_ d1uz8b1 d4n1ea_ d1bm3l1 d1clol1 d3mcka1 d2vq1a1
d1ae6h1 d4aq1b1 d1uj3b1 d1nbqa1 d1fh5l1 d2ck0l1 d2ywza_ d1q72l1
d4nbxb_ d1hila1 d4hzlb1 d2j88l1 d1zmya1 d1cd0a_ d1a4ka1 d1pskl1
d1a0qh1 d4krmb_ d1ncbh1 d2fbjh1 d1j8he1 d1i3ua_ d1cf8l1 d1fe8h1
d2p1ya1 d1eeqa_ d2x89a_ d1ol0a_ d2r0lh1 d1ub5a1 d1dfbh1 d3eyvb1
d4jdvb1 d4p2cg_ d2gsia1 d2agjl1 d1u3ha1 d2h1ph1 d1rihl1 d1fskc1
d1boga1 d2a77l1 d1ospl1 d1qfoa_ d3dwta_ d2fx7l1 d1rz7l1 d3stba1
d1pkqa1 d1b6da1 d1dzba2 d1dzba1 d1kj2a_ d4l1ha_ d1rzgb1 d1dqlh_
d12e8l1 d1miel1 d1jmaa_ d1sbsl1 d1fgnh1 d1f58l1 d1mqkh_ d1ndgb1
d3qegl1 d1iail1 d2aw2a1 d1tqbc1 d3fo2a1 d4dkaa_ d1f8th1 d2g60l1
d2ywya_ d1uj3a1 d1yc7a_ d3bd3a_ d4w6xb1 d1nmbl_ d1i8ka_ d1rjlb1
d1ypze1 d1p7kb1 d1kxqe_ d1gpoh1 d2z4qa1 d1w72h1 d1i9rl1 d4qgya1
d4xt1c_ d2qqnh1 d1cica1 d3c08h1 d2cd0a_ d2bdnh1 d1hnga1 d1maml1
d1nldh1 d1cr9h1 d4lcih1 d3bkyh1 d3qg7l1 d1ai1l1 d1kxvc_ d4iosd_
d1l7ih1 d2x1qa_ d1ub6b1 d3k74b_ d1ibgh1 d1teth1 d1igya1 d1hxma1
d2x6ma_ d2e7la_ d5e03a_ d1qfwm_ d2r0kh1 d1igjb1 d1rurh1 d1plgh1
d1mj8h1 d1yuhb1 d3fmgl1 d1rzfl1 d1emtl1 d1ad9b1 d1igtb1 d1nlbl1
d1d5ih1 d3k7ua_ d4ffyh_ d1bwwa_ d3dvga1 d1a6ta1 d1fgvl_ d4qxgl1
d1miml1 d1jhll_ d2hffb1 d2hh0l1 d1qkzh1 d1l7tl1 d1afvh1 d1iaii1
d2cjuh_ d1um5h1 d2ghwb2 d2ghwb1 d1kgce1 d2fgwh1 d1ikfh1 d1dvfd_
d2i24n1 d2xtjb_ d1za3b1 d2yc1a_ d2hh0h1 d2x1oa_ d1bvka_ d1rjla1
d5e7ba1 d1igta1 d1il1b1 d2fb4l1 d1mvua_ d1jptl1 d32c2a1 d1fvca_
d1iqdb1 d2hrph1 d2p4ab_ d4qo1a_ d1p2cb1 d1wt5c_ d1t4ka1 d5c1mb_
d1it9l1 d1bz7a1 d1b2wh1 d1e4wl1 d1jnll1 d1yedb1 d1za6b1 d3u7aa_
d1igfh1 d3gnml1 d1orsa1 d1wejl1 d1a2ya_ d1hxmb1 d1gafh1 d2j4wh1
d1pz5a1 d1j05a_ d1a3rh1 d4fqll1 d1qfwh_ d1eo8h1 d1n7mh1 d4s11a_
d1kegh1 d5da4a_ d1iqwl1 d3whxb1 d1vgeh1 d1deeb1 d1c12b1 d1t2ql1
d1ad9a1 d3gsnb1 d3b2uc1 d1ggbh1 d1t3fb1 d1nakl1 d4ocmc_ d1lila1
d2v7hb3 d2esve1 d1mcoh1 d2qr0b1 d2q3aa_ d1ct8a1 d1kcrl1 d2pxyb_
d2uzih_ d5c3ld_ d3durb_ d1c5cl1 d2ak4e1 d1dl7h_ d1xiwd_ d1mlba1

d1rmfl1 d1i7zb1 d1dlfl_ d2xa3a_ d5bq7b1 d1dqqb1 d3h42l1 d1egjh1
d1kcvl1 d1fe8l1 d2j6el1 d1f3rb2 d1lk3l1 d1sm3h1 d1q0xh1 d1ghfh1
d7fabl1 d1igch1 d1lo0h1 d4idla_ d5c2bl_ d1jrhl1 d1il1a1 d3zhda1
d4kfzc_ d1b0wa_ d1e6oh1 d1mexl1 d4krpb_ d1fnsh1 d1ncwh1 d4hgma_
d4eigb1 d4rzca_ d2mpal1 d4y5xa_ d5en2b1 d3lrga1 d4ej1c1 d1rhha1
d1j05b_ d1forh1 d1q9wb1 d2wzpd_ d2aq2a1 d1h0da1 d3revb1 d2ij0c1
d1tzha1 d4m7jl1 d1k6ql1 d3b9kc1 d1igml_ d3qeua1 d1dsfl_ d1ce1h1
d1kcuh1 d1zvha1 d1zvoa1 d1tqbb1 d1ay1l1 d2qscl1 d2cgrh1 d1nl0h1
d1kenl1 d3eo0a1 d4n9ob1 d1n0xl1 d3k1kc_ d1c4qa_ d2wv6d_ d1prtf_
d1ltrd_ d4l63b1 d1r4pb_ d1prtd_ d1tl0f_ d2bosa_ d1prtb1 d3chbd_
d2aq2b1 d1ck1a1 d1enfa1 d1i4pa1 d1v1oa1 d1et9a1 d1esfa1 d2g9hd1
d4ohja1 d1klud1 d1bxta1 d1eu3a1 d1ty0a1 d1fnua1 d2cqva1 d1fhga_
d2k1ma_ d2dl2a1 d1biha3 d1biha1 d1biha4 d3qp3a1 d1g1ca_ d1cvsc2
d1cvsc1 d1xmwa2 d1m4ka2 d2otpa2 d1biha2 d1rhfa2 d2fcba2 d2fcba1
d1wwbx_ d1gsma2 d1epfa1 d1epfa2 d2dava1 d1waaa_ d1f97a2 d1x44a1
d1vcaa2 d1fnla2 d1fnla1 d3b5ha2 d3h8na2 d3h8na1 d1jbja1 d1jbja2
d1pd6a_ d1f2qa2 d1f2qa1 d3cx2a_ d1nbqa2 d3b5ha1 d1n26a1 d1xiwb_
d1xaua_ d1fltx_ d2ifga1 d1olla1 d1olla2 d1cs6a4 d1cs6a3 d1cs6a2
d1cs6a1 d2gw5a2 d1sy6a1 d1xiwa_ d1ucta2 d1ucta1 d1ry7b2 d1gl4b_
d1qz1a3 d1olza1 d2c9aa1 d1wwca_

**Family-22 (b.1.18.2)**

**Positive dataset contains 6 protein sequences.**

d2bema_ d2fhfa2 d2fhfa1 d1cxla1 d2bhua1 d1txka1

**Negative dataset contains 4067 protein sequences.**

d1m32a_ d1ibja_ d1iuga_ d4q75a_ d2bkwa1 d1t3ia_ d1n8pa_ d1qz9a_
d1jf9a_ d1eg5a_ d1p3wa_ d3kgwa_ d1cl1a_ d2ctza1 d1vjoa_ d2rfva_
d1gc0a_ d1cs1a_ d1d2fa_ d2ch1a1 d1qgna_ d1pffa_ d1elua_ d2xlba_
d3m83a1 d4nmwa_ d3i2ka1 d1lnsa3 d1mpxa2 d2cfba1 d1dfoa_ d1o69a_
d1bj4a_ d3k28a_ d2bwpa_ d3lg0a_ d2fnua_ d3ou5a_ d1z7da1 d1b9ha_
d1iaya_ d4msoa_ d1bs0a_ d1s0aa_ d1sffa_ d3tqxa_ d1mdoa_ d3qboa_
d3pgya_ d1bjna_ d2dkja_ d1ejia_ d1vefa1 d4wxga_ d3bs8a_ d3gbxa1
d1w23a_ d1u08a_ d1lc5a_ d1b5pa_ d2ay1a_ d1bw0a_ d3pd6a_ d7aata_
d2csta_ d1j32a_ d4wlha_ d1o4sa_ d1v2da_ d1gdea_ d1m6sa_ d1v72a1
d1svva_ d1ajsa_ d1wsta1 d2f8ja2 d1lk9a_ d1vp4a1 d1xi9a1 d2gb3a1
d1yaaa_ d1r0ka3 d1j5pa3 d1vkoa2 d1vm6a2 d3qvsa2 d1gr0a2 d1p1ja2
d1e57b_ d1auyb_ d1qnia1 d1ibya_ d1f56a_ d1bypa_ d1azca_ d1ws8a_
d3u25a_ d1cuoa_ d1id2a_ d1rkra_ d2iaac_ d4dp1x_ d1nwpa_ d2gima_
d2i7sa_ d1qhqa_ d3ehbb1 d1gyca3 d1hfua2 d1kbva1 d1kbva2 d1sdda1
d1sdda2 d1gyca1 d1gyca2 d1mzya1 d2bw4a1 d1aoza1 d1aoza3 d1aoza2
d1kyaa2 d1kyaa3 d1kyaa1 d1mzya2 d4e9sa1 d1sddb1 d1sddb2 d1hfua1
d1hfua3 d1oe1a2 d1oe1a1 d1v10a2 d1v10a3 d1v10a1 d1rypd_ d1irua_
d1rypc_ d3unfk_ d1rypa_ d1j2pa_ d1irui_ d1j2qh_ d3unfh_ d1m4ya_
d1rypb_ d1irul_ d1irug_ d1iru2_ d1q5rh_ d1rypg_ d1g3ka_ d1iruf_
d1q5qa_ d1e94a_ d1irub_ d1ryph_ d3ty6a_ d1iruj_ d1iruk_ d1rype_

d1irud_ d1irue_ d3mi01_ d1rypf_ d1iruh_ d2z3ba_ d1iruc_ d3mka2_
d1iru1_ d4qgna_ d1vr3a1 d1adqa1 d1fnga1 d1uvqb1 d1zs8a1 d2vola1
d2vola2 d1mfbl2 d2jelh2 d1uwel2 d3qq4b1 d1kjvb_ d1k5na1 d1osph2
d4odhl2 d1ow0a1 d1ow0a2 d1bfob2 d3l9ra2 d3jtth1 d2fath2 d4elkb2
d1fn4b2 d2xfxb_ d1pg7x2 d1cqka_ d4k71b2 d1lk2a1 d1eapb2 d4lfhg2
d1i4fa1 d2nw2a2 d1kfah2 d1mhca1 d1h3ua1 d1wejh2 d1h0db2 d1a5fh2
d1kcvh2 d1mjuh2 d1qqda1 d1kcrh2 d1k8ia1 d3huja2 d2nnab1 d1nfdf2
d3mffb2 d1s9va1 d1lk3h2 d1q0xl2 d1fv1b1 d1neza1 d4rfeb2 d1r3ha1
d1a6tb2 d1kjva1 d1pz5b2 d1onqa1 d2bc4a2 d1de4a1 d1k8da1 d1tcra2
d1dn0b2 d1nfde2 d2xwtb2 d1bfoa2 d1mexh2 d1iakb1 d1ypzf2 d4f7ea2
d1q72l2 d2fbjh2 d1fe8h2 d1clzh2 d1fngb1 d1uvqa1 d1vgka1 d1c16a1
d4j12a2 d4x99b2 d1igtb3 d1je6a1 d1q72h2 d1nldh2 d1teth2 d2hmic2
d1i1ca2 d1i1ca1 d1k8ib1 d1q0yh2 d1kfal2 d3frua1 d1pg7w2 d1u58a1
d1igyb4 d1igyb3 d1hyrc1 d1klub1 d1deeb2 d1c12b2 d2bc4b2 d2ig2h2
d1kcvl2 d3wn5b1 d1lk2b_ d1lk3l2 d1mjul2 d1sm3h2 d1q0xh2 d1zvsa1
d1ydpa1 d1u8ib2 d1ypze2 d1kcuh2 d1zx5a1 d2phla2 d2phla1 d1fxza2
d1fxza1 d1lrha_ d1uija1 g1dgw.1 d2v09a_ d1uika2 d1uika1 d1dgwa_
d1ep0a_ d2pa7a1 d1wlta1 d1nxma_ d2c0za1 d2b5ea2 d2b5ea3 d2b5ea1
d2b5ea4 d2kp1a1 d2kp2a1 d1hyua3 d1hyua4 d1j08a2 d1mqta_ d1d4m3_
d1fmd3_ d2mev1_ g1aym.1 d1ev13_ d4q4w1_ d1r1a3_ d2x5ia_ d1rhi1_
g1r1a.1 g1d4m.1 d1b35b_ d1oopa_ d1tme1_ d4iv1a_ g1pvc.1 g1fpn.1
d1fpn1_ d4gb31_ d1d4m1_ d1fmd1_ d1ev11_ d2wzr3_ g1cov.1 d1qqp3_
g1fmd.1 d2x5ic_ d1rhi3_ g1oop.1 d1hxs1_ g1hxs.1 d1fpn3_ d1bev1_
g2mev.1 d1eah3_ d1aym1_ d1pvc3_ d1qqp1_ d1zba3_ d1h8ta_ d3vbhc_
g1b35.1 g1bev.1 d1hxs3_ g1ev1.1 d1bev3_ g1tmf.1 d1tmf1_ d1b35a_
d2mev3_ g1eah.1 g1h8t.1 d1eah1_ d4q4w3_ d1r1a1_ d1aym3_ d1pvc1_
d1zba1_ d1h8tc_ d1oopc_ d1tme3_ g1qqp.1 d4iv1c_ g1tme.1 d1au7a2
d1e3oc2 d2ovga_ d1sq8a_ d1rzsa_ d3kz3a_ d3orca_ d3gpdg2 d2q49a2
d1hdgo2 d2g17a2 d2cvoa2 d2czca1 d3pwka2 d1obfo2 d1i32a2 d1zyao2
d2pkqo2 d1b7go2 d2hjsa2 d1cf2o2 d1vkna2 d1nvmb2 d2m9wa1 d3dfxa_
d2msya_ d2djoa1 d2r5za_ d1f43a_ d2lmda1 d1akhb_ d1s7ea1 d3nara_
d1zq3p1 d2craa1 d1mija_ d4uuta_ d3a01a_ d1x58a1 d3rkqa1 d3cmya1
d2cqxa1 d2da6a1 d1e3oc1 d1fjla_ d1mh3a1 d1ig7a_ d1x41a1 d1wh7a1
d1gt0c1 d2mw8a_ d1lfup_ d1x2ma1 d1b72a_ d1wi3a1 d1jgga_ d1wjha1
d1x2na1 d1mnmc_ d2cuea1 d3k2aa_ d2hosa_ d2jwta2 d1pufa_ d1octc1
d2hi3a_ d1b8ia_ d4j19a_ d3lnqa_ d1wh5a1 d1b8ib_ d1hcra_ d1tc3c_
d1gdta1 d1u78a2 d1xg1a1 d1itya_ d1hlva2 d1iufa2 d1iufa1 d2iu5a1
d1zk8a1 d2g7sa1 d2fd5a1 d2np5a1 d1z0xa1 d2id6a1 d2d6ya1 d2i10a1
d1vi0a1 d2oi8a1 d3c07a1 d2ns7a1 d2fq4a1 d2yvea1 d1t33a1 d2o7ta1
d1rkta1 d2np3b1 d2g7ga1 d2gena1 d2fbqa1 d2g7la1 d2gfna1 d2g3ba1
d2jj7a1 d2hyja1 d1sgma1 d1ui5a1 d2hkua1 d2id3a1 d4jyka1 d3lv8a_
d1s3ga1 d1zaka1 d1s4qa_ d3tvta2 d1uf9a_ d1khta_ d1ckea_ d1osna_
d1p5zb_ d4tmka_ d3cm0a_ d1jaga_ d3h86a_ d1t0hb_ d1nn5a_ d2h92a_
d3kb2a1 d2c95a1 d1yj5a2 d1kgda_ d1ly1a_ d2ak3a1 d1vyva2 d1deka_

d2grja1 d1p3ja1 d1y63a_ d1e4ya1 d1s96a_ d3iija1 d1lvga_ d1nksa_
d1vyua2 d4e22a_ d1qf9a_ d1jjva_ d1q3ta_ d1ki9a_ d1lw7a2 d1teva_
d1kjwa2 d1gsia_ d2iyva2 d1kaga_ d1viaa_ d1e6ca_ d1x6va3 d1g3ma_
d1j99a_ d1t8ta1 d1nsta_ d1zrha_ d1ls6a_ d2a3ra_ d1aqua_ d2zptx_
d3ckla_ d1texa_ d1q44a_ d2gwha_ d1odfa_ d1uj2a_ d4gi7a_ d2jeoa1
d1rz3a_ d1z0ja1 d3umra2 d3gj0a1 d2gaoa1 d1yzga1 d1x3sa1 d1g7sa4
d1puja_ d1udxa2 d1kk1a3 d2o52a_ d1jwyb_ d1zcaa2 d1zcba2 d2fv8a1
d2cjwa_ d3dz8a1 d1xzpa2 d1vg8a_ d4qxaa_ d2ew1a1 d1x1ra1 d2bcgy_
d3doea1 d3cona_ d3rega_ d3wyga_ d1upta_ d2b6ha1 d1zd9a1 d1lnza2
d1r5ba3 d1n0ua2 d1r2qa_ d2f9la1 d2rhda_ d3q3jb_ d3q85a_ d1efma1
d1r8qa_ d2gf9a1 d2akab1 d1ek0a_ d1z08a1 d2gcoa1 d2dy1a2 d4g01b_
d2cxxa1 d1svia_ d3q72a_ d1ni3a1 d4ac9a4 d2erxa1 d2bmea_ d1wxqa1
d2gf0a1 d4ldja1 d1zunb3 d1d5ca_ d4lv5b_ d1efca3 d1kmqa_ d2g6ba1
d3lrpa_ d1tada2 d2y8ea_ d4rd4a1 d1z06a1 d2heia_ d3a58b_ d1u0la2
d1d2ea3 d1zj6a1 d2ocba_ d3rwoa_ d1mr3f_ d2e9sa1 d4u5xa1 d2iwra_
d2fn4a1 d3bwdd_ d2nzja1 d2atva1 d2ksqa_ d3l43a_ d1mkya2 d1mkya1
d1wmsa_ d1jnya3 d2rmka_ d1f6ba_ d1f60a3 d1gota2 d1jala1 d2fh5b1
d1g16a_ d2wblc_ d1m7ba_ d1wf3a1 d1t9ha2 d1z6xa1 d1z0aa1 d2fg5a1
d2a5ja1 d2bcjq2 d1s0ua3 d1ky3a_ d4y0va_ d4lhwa_ d2qmea1 d2atxa1
d1egaa1 d1ryha_ d1moza_ d1nrjb_ d2erya1 d3bfka_ d1puia_ d1fzqa_
d3j3az1 d3t5ga_ d1shza2 d3defa_ d2f7sa1 d3raba_ d3tsoa_ d3aq4a_
d2al7a1 d3sfva1 d3bc1a_ d2ncda_ d1sdma_ d3h4sa1 d2heha_ d2grya_
g3kin.1 d1br2a2 d1goja_ d1f9va_ d2mysa2 d1lkxa_ d3t0qa_ d4db1a1
d3gbja_ d1ry6a_ d1v8ka_ d1kv9a1 d1k8ka2 d1k8ka1 d3uleb1 d3i33a2
d3i33a1 d1bupa1 d1dkgd2 d4ci6a2 d1jcfa1 d4a2ba2 d2fxua1 d1yaga2
d1yaga1 d4cbua2 d1bupa2 d3vrda1 d3vrda2 d1fcdc2 d1h1oa2 d1h1oa1
d1m70a2 d1m70a1 d1ig8a1 d1czan2 d1czan3 d1czan1 d1ig8a2 d1czan4
d1bg3a4 d1bg3a2 d1bg3a1 d1nira1 d1qksa1 d2d0oa3 d2d0oa2 d1nbwa2
d1hroa_ d4gedb_ d3zooa_ d1mz4a_ d1gu2a_ d1f1fa_ d1dvva_ d1c6ra_
d1c75a_ d1e29a_ d1gdva_ d1cnoa_ d1h32b_ d1co6a_ d2bh4x_ d2aiua_
d5cytr_ d1jdla_ d1i8oa_ d2zboa_ d3dmia_ d1ls9a_ d1wvec_ d2yk3a_
d3ph2b_ d1dw0a_ d5aura_ d1ql3a_ d1qn2a_ d1f1ca_ d4zzva_ d2xspa_
d1eg1a_ d4v20a_ d1ojja_ d4ipma1 d1gpia_ d4csia_ d3pfza_ d3ovwa_
d2yoka_ d2nqya_ d1h4ga_ d1oa2a_ d1olra_ d3b7ma_ d4h7ma1 d3wp3a_
d2vgda_ d1igoa_ d3zsea_ d2nlra_ d3wx5a_ d1oa3a_ d1pvxa_ d1m4wa_
d1f5ja_ d1oa4a_ d1ks5a_ d4ixla1 d3sita1 d1g66a_ d5ajha1 d1evsa_
d1cnt1_ d1pvhb_ d1z7ca_ d1huwa_ d1bgea_ d1rhga_ d1f6fa_ d1f45b_
d1axia_ d1etea_ d3tgxb_ d1scfa_ d2z3qa1 d3qb1a_ d1hula_ d1m48a_
d4nkqb_ d1eera_ d1v7mv_ d5e4ea_ d2o27a_ d3b5ka_ d2psma1 d4zf7a_
d3uf5a_ d4x51a_ d1d9ca_ d4ypgc1 d3oq3a_ d1lqsl_ d1n1fa_ d1au1a_
d1wu3i_ d3besl_ d3ux9a_ d3rbaa_ d2qtra_ d1nupa_ d3l92a_ d1f9aa_
d1qjca_ d2h29a_ d1vlha_ d1od6a_ d1lw7a1 d1mrza2 d4ruka_ d2el7a_
d1iq0a2 d1r6ta2 d1ivsa4 d2cyba_ d1rqga2 d1j09a2 d2yxna_ d1f7ua2
d1h3fa1 d1ffya3 d2g36a1 d1nzja_ d1jila_ d1n3la_ d1i6la_ d1pfva2

d1h3na3 d3n9ia_ d3sz3a1 d3sw2a_ d1moxc_ d1dx5i3 d1edmb_ d1autl2
d1autl1 d1nqlb_ d1dval1 d1szba2 d1kigl_ d2y5fl_ d1ijqa2 d1gk5a_
d1g1sa2 d1nt0a3 d1xdtr_ d1lmja2 d1lmja1 d1nzia2 d2wphe_ d1klil_
d1pfxl2 d1pfxl1 d1p9ja_ d1g1ta2 d1f7ea_ d4yzub_ d1lr7a1 d2mgpa1
d1b9wa1 d1b9wa2 d2mgpa2 d1ob1c2 d1n1ia2 d1n1ia1 d2npra2 d2npra1
d2h1ia1 d2r8ba1 d3b5ea1 d3cn9a_ d1auoa_ d2y5ia_ d1psra_ d3n22a_
d2egda_ d4ggfc_ d2wcea_ d2luca_ d4aqia_ d3nsia_ d2lp3a_ d1j55a_
d1a4pa_ d1k8ua_ d1nsha_ d3fs7a_ d2pvba_ d1ttxa_ d1rwya_ d2kyfa_
d1rk9a_ d1a75a_ d1exra_ d1tiza1 d1nyaa_ d1g8ia_ d1ggwa_ d1wdcc_
d3fwba_ d1ggza_ d2o5ga_ d1ctaa_ d1m45a_ d3i5gc_ d1sl8a_ d1fpwa_
d2scpa_ d2roaa_ d4aqra_ d3pn7b_ d1br1b_ d2mysb_ d1s6ca_ d1bjfa_
d2obha_ d1jfka_ d1qv0a_ d1lkja_ d2mlfc_ d2m97a_ d2d8na_ d4by5a_
d1jbaa_ d4nqga_ d1wdcb_ d2mysc_ d1j7qa_ d1s6ia_ d1uhka2 d1r2ua_
d1dtla_ d3kf9a_ d3pn7c_ d1fi6a_ d1c07a_ d1gjya_ d1y1xa_ d1hqva_
d1dvia_ d1df0a1 d4usla_ d1k94a_ d1jfxa_ d1wkya2 d1uuqa_ d3wh9a_
d4m1ra_ d1xyza_ d4xuya_ d2je8a5 d4bf7a1 d3cuia_ d1ur4a_ d1qw9a2
d1odza_ d2vzsa5 d1bqca_ d2x2ya1 d1hjqa_ d1qnra_ d4y7ea1 d1vjza_
d4k68a_ d2v3ga_ d4hpga_ d1nofa2 d1nq6a_ d1h4pa_ d1aq0a_ d3ziza_
d4bs0a_ d1ecea_ d1h1na_ d1ta3b_ d1w91a2 d1i1wa_ d1uhva2 d1us3a2
d1egza_ d7a3ha_ d1tvna1 d3nyda1 d1ghsa_ d2cyga1 d4xv0a_ d2c8na2
d1w32a_ d3emca_ d1v6ya1 d1foba_ d2uwfa1 d2fgla_ d3niya1 d2c0ha1
d1rh9a1 d1yq2a5 d1szna2 d1fa2a_ d1pama4 d5do8a1 d1ea9c3 d1hx0a2
d2fhfa5 d1qhoa4 d1avaa2 d1m53a2 d4m8ua1 d2gjpa2 d1lwha2 d4howa1
d1ktba2 d1j0ha3 d3vm5a1 d2x1ia_ d1wzaa2 d1uasa2 d2diea1 d1mxga2
d1ht6a2 d1eswa_ d1iv8a2 d5cpsa_ d1ud2a2 d1zjaa1 d3gbea1 d1kwga2
d1wdpa_ d1gcya2 d1g5aa2 d1hvxa2 d3dhpa2 d2bhua3 d4do4a1 d3qgva1
d1e43a2 d1g94a2 d2d3na2 d1gjwa2 d1gvia3 d1x1na1 d1r7aa2 d1tz7a1
d4c7ga2 d2gjxa1 d1nowa1 d1yhta1 d4toqa_ d1wb0a1 d1w9pa1 d1eoka_
d1itxa1 d1kfwa1 d1goia2 d1edqa2 d2gsja_ d1hjxa1 d1vf8a1 d3wdpp1
d3wq5a_ d1gnxa_ d4gxpa_ d4hz8a_ d1e4mm_ d3aiua_ d1wcga1 d1qvba_
d3ahxa_ d1v02a_ d2dgaa_ d1pbga_ d1vffa1 d1qoxa_ d4ha4a_ d1mixa2
d1e5wa2 d1j19a2 d1isna2 d2al6b2 d1offa_ d1frra_ d3wcqa_ d1e9ma_
d1l5pa_ d1wria_ d1i7ha_ d3av8a_ d1czpa_ d1rfka_ d3p1ma1 d3b2ga_
d2bt6a2 d1wgqa1 d1v89a1 d2coda1 d1zc3b_ d1wg7a1 d1u5ea1 d1v61a1
d2coca1 d2ys1a1 d1v5pa1 d1ntya2 d1wjma1 d1wi1a1 d1u29a_ d1xx0a1
d1unqa_ d2zkmx3 d1fhoa_ d1v88a1 d2adza1 d1x1fa1 d1p6sa_ d2coaa1
d1btka_ d1v5ma1 d1faoa_ d2x18a_ d1dyna_ d1x1ga1 d4kaxb_ d2j59m1
d1ki1b2 d1xcga2 d1txda2 d1u5da1 d1dbha2 d1w1ha_ d1upqa_ d1v5ua1
d1foea2 d2fjla1 d1eaza_ d2dfka2 d2cofa1 d1kz7a2 d9ldta1 d2ewda3
d1up7a1 d2dfda1 d1s6ya1 d1vjta1 d7mdha1 d1hyea1 d1t2da1 d1ojsa3
d1b8pa1 d1ldna1 d1hyha1 d1civa1 d2a92a1 d1ez4a1 d1i0za1 d1y6ja1
d1gv0a1 d1ur5a1 d1llda1 d1obba1 d1v8ba1 d1gdha1 d1l7da1 d2dlda1
d1mx3a1 d1ygya1 d1qp8a1 d1li4a1 d1j4aa1 d2naca1 d1npya1 d3t4ea2
d1a4ia1 d1o0sa1 d2hk9a2 d2egga2 d1b26a1 d1bvua1 d1gq2a1 d1c1da1

d1leha1 d1gtma1 d1nvta1 d3dona2 d1edza1 d1gpja2 d1vi2a1 d1p77a1
d1nyta1 d1b0aa1 d1vl6a1 d1v9la1 d1txga2 d3f47a1 d1mv8a2 d2pv7a2
d1xc2a3 d2ahra2 d4xiya1 d2f1ka2 d1pgja2 d1i36a2 d1np3a2 d1lj8a4
d1teaa3 d1qmga2 d2i76a2 d2g5ca2 d1wdka3 d1dlja2 d3cuma3 d1kola2
d1f8fa2 d1qora2 d1jqba2 d1vj0a2 d1cdoa2 d1gu7a2 d1d1ta2 d1xa0a2
d2dm6a2 d1jvba2 d1rjwa2 d1llua2 d1h2ba2 d1tt7a2 d1piwa2 d1o89a2
d1iz0a2 d1e3ja2 d1yb5a2 d1uufa2 d1e3ia2 d3gpdg1 d1vc2a1 d2nvwa1
d1u8fo1 d1ydwa1 d1hdgo1 d2g17a1 d1cf2o1 d2czca2 d1r0ka2 d1obfo1
d2cvoa1 d1ebfa1 d1h6da1 d1zh8a1 d2q49a1 d1qkia1 d1j5pa4 d3pzra1
d1xeaa1 d1vkoa1 d1vm6a3 d3qvsa1 d2pkqo1 d1b7go1 d1gr0a1 d1p1ja1
d1lc0a1 d1j0xo1 d3pwka1 d2hjsa1 d1zyao1 d1vkna1 d1tlta1 d1nvmb1
d3sjua_ d3ojfa1 d1xu9a1 d2jjya1 d1n7ha_ d1y1pa1 d1h5qa_ d1jtva_
d4fs3a_ d2bkaa1 d1gy8a_ d2c5aa1 d1ulsa_ d1ooea_ d2b69a1 d1y5ma1
d1spxa_ d1ae1a_ d1bxka_ d1ydea1 d1gega_ d1hdoa_ d1o5ia_ d1e6ua_
d1snya1 d2rhca_ d3zv4a_ d5ceja_ d2d1ya1 d1fjha_ d3w8ea_ d2fmua1
d1x1ta_ d1rpna_ d3ay7a1 d1k2wa_ d1eq2a_ d2hrba1 d3ruaa_ d2ew8a1
d4hwka_ d1xgka_ d2bd0a1 d1yo6a1 d2a35a1 d2ntva_ d1vl8a_ d1hdca_
d4bkua_ d2ag5a1 d2q45a_ d1nffa_ d4iy1a_ d1zbqa1 d1kewa_ d1ek6a_
d1ja9a_ d2q46a2 d1iy8a_ d1cyda_ d2ptga_ d1hxha_ d1uzma1 d3lyla_
d4m89a_ d3wyea_ d2blla1 d1w6ua_ d1sb8a_ d2q2va_ d4nr0a_ d1e6wa_
d2gasa_ d3oiga1 d2bgka1 d1mxha_ d1vl0a1 d1rkxa_ d1r6da_ d1wmaa1
d1e7wa_ d2c07a1 d1xkqa_ d3tzha_ d1g0oa_ d3lz6a_ d1qyca_ d1edoa_
d1gz6a_ d1oc2a_ d1t2aa_ d1zema1 d2xoxa_ d1z45a2 d1geea_ d1p33a_
d3lt0a_ d4e6pa_ d2wyua_ d1db3a_ d4q9na_ d1xhla_ d4o1ma_ d1fmca_
d2uvda_ d2gdza1 d2z1ma_ d2o23a2 d2fr1a2 d2fr1a1 d1qsga_ d1qyda_
d4zjua_ d2a4ka1 d1yb1a_ d1wvga1 d1yxma1 d3f4ba_ d1mg5a_ d1xg5a1
d1r55a1 d3gboa_ d1atla_ d1kufa_ d4q1la_ d1quaa_ d1yp1a_ d1wnia_
d3nqyb_ d4gera_ d4c5pa_ d2bsza1 d4bgfa_ d1w4ta1 d3ioqa_ d1fh0a_
d1o0ea_ d1khqa_ d1m6da_ d2cb5a_ d1cs8a_ g8pch.1 d1s4va_ d2pnsa_
d1vsna1 d1mira_ d1iwda_ d2oula_ d7pcka_ d3bwka_ d3bcna_ d2p86a_
d1pvja_ d1pcia_ d1gece_ d1deua_ d4bs6a_ d1xkga1 d1cqda_ d1jqpa2
d2c0ya_ d2bdza_ d3p5ua_ d2o36a_ d1k9xa_ d1zoya1 d1qo8a2 d1d4ca2
d1chua2 d1jnra2 d2jk6a2 d2jk6a1 d1feca1 d1feca2 d1lpfa1 d1lpfa2
d1xhca1 d1xhca2 d1dxla2 d1dxla1 d1gera2 d1m6ia2 d1onfa1 d1onfa2
d3lada2 d3lada1 d1w4xa1 d1w4xa2 d1gesa2 d1mo9a1 d1xdia1 d1fl2a2
d1fl2a1 d1fcda1 d1fcda2 d1h6va2 d1h6va1 d1mo9a2 d1gv4a2 d1gv4a1
d2gv8a2 d2gv8a1 d1aoga2 d1hyua1 d1ebda1 d1ebda2 d3dk9a1 d1q1ra2
d1q1ra1 d1gesa1 d1zbfa1 d2z1ha_ d2etja1 d3qioa1 d1jl1a_ d2e4la_
d2lsna1 d1io2a_ d1uaxa_ d2yv0x_ d1mu2a1 d1ekea_ d1i39a_ d1ps9a2
d1o94a2 d1cjca1 d1pn0a1 d1reoa1 d1ju2a1 d2f5va1 d3c96a1 d2gmha1
d1pj5a2 d1gpea1 d1o5wa1 d1n4wa1 d2dw4a2 d2ivda1 d1seza1 d2voua1
d1kdga1 d3ljqa_ g1k2x.1 d2v76a_ d1wj1a1 d1qqga2 d1qqga1 d1ntva_
d1n3ha_ d4dbba_ d3f0wa1 d1p3ra_ d1wgua1 d2cy4a1 d3hqca_ d1wvha1
d1xr0b1 d1v58a1 d1t3ba1 d4ilfa2 d4npba2 d2av4a1 d3nvva1 d1krha3

d2h88b1 d1n62a2 d1t3qa2 d1kf6b2 d1zoyb1 d1ffva2 d1dgja2 d1rm6c2
d1faaa_ d1wika1 d1xfla_ d1ttza1 d2f51a1 d1ti3a_ d1nhoa_ d1sena_
d2i4aa_ d1ep7a_ d1zmaa1 d1fo5a_ d3lefa1 d3diea1 d2yana1 d1z6na1
d1oaza_ d3zywa1 d4aj8a_ d1nm3a1 d4fiwa_ d1wmja_ d4hs1a1 d1iloa_
d1xwaa1 d1wjka1 d2vm1a_ d1fb6a_ d1gh2a_ d1h75a_ d1nw2a_ d1kx5b_
d3a6na_ d4m6ba2 d2nqbd_ d3wkjd_ d2yfwe_ d4h9na_ d5bnxa_ d1kx5c_
d3r45a_ d1eqzb_ d4jjnb_ d1f66c_ d1eqza_ d4wnnb_ d1kx5a_ d1u35c1
d1id3a_ d2nqbc_ d1kx5d_ d1tafa_ d1bh9b_ d1tafb_ d1q9ca_ d1h3ob_
d2byka1 d1n1ja_ d4g92b_ d1bh9a_ d1jfia_ d1h3oa_ d2bykb1 d1n1jb_
d1pd212 d1nhya2 d2a2ra2 d1k0dd2 d1f2ea2 d1gsua2 d1f3ba2 d2fhea2
d2pvqa1 d1tw9a2 d1oyja2 d1e6ba2 d1k0ma2 d3ik7a1 d4is0a1 d1r5aa2
d1jlva2 d1m0ua2 d3gtub2 d1z9ha2 d1okta2 d1axda2 d2gsra2 d1tu7a2
d1k3ya2 d1zgma1 d1b48a2 d1gnwa2 d1gwca2 d2gsta2 d1glqa2 d1oe8a2
d1pn9a2 d1v2aa2 d1fw1a2 d1iqca2 d1iqca1 d1eb7a1 d1nmla2 d1nmla1
d1qusa_ d1qqya_ d1f6ra_ d1ivma_ d2gv0a_ d1iiza_ d2z2fa_ d1j8xa_
d1ghla_ d1b9oa_ d174la_ d169la_ d2o4wa_ d1p37a_ d1k28a3 d1xjta1
d1jtma_ d2f2qa_ d4e97a1 d3w3ea_ d4tx7a_ d3cqla_ d4msta_ d3iwra_
d1dxja_ d2z37a_ d4dyga_ d2pjsa1 d1klla_ d2i7ra1 d1ecsa_ d1xqaa1
d5cj3a1 d1jifa_ d2p7oa_ d1r9ca_ d1nkia_ d1mpya2 d1kw3b2 d1kw3b1
d4ghga1 d1tfza1 d3isqa1 d3isqa2 d1sqia1 d1sqia2 d1lkda2 d1lkda1
d1mpya1 d1cjxa2 d1sp9b2 d1t47a1 d1t47a2 d1f1ua2 d1sp8a1 d1sp8a2
d1f1ua1 d1s5ja2 d1qhta2 d4dlea2 d1tgoa2 d3py8a2 d1q8ia2 d1kfsa2
d2py5a2 d1d5aa2 d1qsta_ d2beia1 d4m3sa_ d1u6ma1 d1ygha_ d1yk3a1
d3dr6a_ d1xmta_ d1z4ea1 d2ge3a1 d1bo4a_ d2fiaa1 d2jdca1 d1y9wa1
d3to7a_ d1wwza1 d2fl4a1 d2ozua1 d2b5ga_ d2cy2a1 d2p0wa_ d1cm0a_
d4pswa_ d1yrea1 d1yr0a1 d1m4ia_ d2gana1 d1yvka1 d1ghea_ d2g3aa1
d1y7ra1 d2euia1 d1q2ya_ d2fsra1 d1xeba_ d2ae6a1 d1cjwa_ d1qsma_
d2aj6a1 d1y9ka1 d1i12a_ d1vkca_ d2atra1 d1n71a_ d2d4pa1 d2fiwa1
d2fe7a1 d1tiqa1 d2ou2a_ d1yx0a1 d2fcka1 d1p0ha_ d2j8ma_ d2pq8a_
d1z4ra1 d1s3za1 d1c4qa_ d1prtc1 d2wv6d_ d1prtf_ d4l63b1 d1r4pb_
d1prtd_ d1tl0f_ d2bosa_ d1prtb1 d3chbd_ d1xvqa1 d1pn0a2 d3hy2a_
d2cvba1 d1o8xa_ d1lu4a_ d1we0a1 d2bmxa1 d2a4va1 d1psqa_ d1xiya1
d1q98a_ d1n8ja_ d1zofa1 d2ggta_ d3tjja_ d4je1a1 d1o73a_ d3s9fa_
d2zcta_ d1qmva_ d1jfua_ d2b1ka_ d5dvba1 d2b5xa1 d2v2ga_ d3tb2a_
d4llra_ d4fh8a1 d1e2ya_ d1knga_ d2h30a_ d1i5ga_ d2z9sa_ d2f8aa1
d1tp9a1 d2b7ka_ d1nm3a2 d3hvva_ d2wfca1 d4kcea_ d1xvwa1 d4gqca1
d3qpma_ d4k1fa_ d1zzoa1 d1gp1a_ d1prxa_ d2qked_ d4ksoa_ d1t4za1
d1wwja_ d4mh8a_ d4i7ga1 d1w52x1 d2ppla1 d1hpla2 d1lpbb2 d2oxea1
d1bu8a2 d1rp1a2 d1ys1x_ d4hs9a_ d1ispa_ d1ex9a_ d1ku0a_ d3qmma_
d3rp2a_ g1ucy.1 d1fuja_ d2zchp_ d3w94a_ d1si5h_ d1lo6a_ g2pka.1
d1cgha_ d1kigh_ d1a5ia_ d3s9ba_ d1hj8a_ d1ao5a_ d2anya_ d1q3xa1
d1gvza_ d1azza_ d4gsoa_ d2f91a1 d1iaua_ d1gdna_ d1eufa_ g1fiw.1
d1eq9a_ d4nfea_ d2rdla_ d1spja_ d1elva1 d1a7sa_ d1ekbb_ d1pfxc_
d1yc0a_ d2aiqa_ g1fiz.1 d1fi8a_ d4k60a_ d4dgja_ d1mzaa_ d1op0a_

| | | | | | | | |
|---|---|---|---|---|---|---|---|
| d3tjva_ | d1pytd_ | d1sgfg_ | g1gg6.1 | d2eeka_ | g1rtf.1 | d1a0ja_ | d3g01a_ |
| d1pytc_ | d4e7na_ | d1ddja_ | d1z8ga1 | d2hlca_ | d1npma_ | d1brup_ | g1jou.1 |
| d1sgfa_ | d1autc_ | g2hnt.1 | g1kdq.1 | d1orfa_ | d3s69a_ | d4crga_ | d1fxya_ |
| d1bqya_ | d1lgya_ | d4l3wa_ | d1llfa_ | d1gz7a_ | d1ukca_ | d3groa_ | d1thta_ |
| d1ei9a_ | d1ra6a_ | d1muka_ | d3gsza_ | d1khva_ | d1te9a_ | d2xwha_ | d3uqsa_ |
| d3hkwa_ | d3n6ma_ | d2cjqa_ | d2xyma_ | d1teba_ | d1te8a_ | d1s48a_ | d1u09a1 |
| d4wfxa_ | d3tyqa_ | d2pgga1 | d3zxha_ | d1cgla_ | d1rm8a_ | d1eaka2 | d1hy7a_ |
| d2jsda_ | d1hv5a_ | d1i76a_ | d1dzka_ | d4tlja_ | d2a2ua_ | d4wfva_ | d3t1da_ |
| d4iaxa_ | d3zq3a_ | d2nnda_ | d1i4ua_ | d1kxoa_ | d1bbpa_ | d1kt7a_ | d1exsa_ |
| d3s26a1 | d1lf7a_ | d1qfta_ | d4gh7a_ | d1z24a1 | d1qabe_ | d1xkia_ | d1ew3a_ |
| d3bx7a_ | d4iawa1 | d1n0sa_ | d1gkab_ | d3kzaa_ | d2k23a_ | d2l9ca1 | d2joza1 |
| d1gm6a_ | d3kffa_ | d3saoa1 | d1e5pa_ | d1yupa1 | d1iiua_ | d2tbvc_ | d1c8nc_ |
| d1x36a_ | d1smvc_ | d1ng0c_ | d4sbvc_ | d1f2nc_ | d1cixa_ | d1y29a_ | d1v7fa_ |
| d2jtba_ | d1d1ha_ | d1g9pa_ | d1lupa_ | d1qk7a_ | d1mb6a_ | d2a2va_ | d1niya_ |
| d1i25a_ | d1hvwa_ | d2mpqa_ | d1ie6a_ | d1kqha_ | d1la4a_ | d2mxoa1 | d1dl0a_ |
| d1koza_ | d1qk6a_ | d1emxa_ | d1lqra_ | d1c6wa_ | d1rmka_ | d1ixta_ | d1eyoa_ |
| d1cnna_ | d1fu3a_ | d1g1pa_ | d1f3ka_ | d1fyga_ | d4lu0a_ | d1of8a_ | d3unda1 |
| d3e9aa1 | d1vr6a2 | d1n8fa_ | d2qkfa_ | d1d9ea_ | d1o60a_ | d1e88a1 | d1l6ja5 |
| d1l6ja4 | d1eaka4 | d1eaka3 | d1l6ja3 | d1h8pa1 | d1ny721 | d1ny722 | d1a6ca2 |
| d1a6ca1 | d1pgl22 | d1pgl21 | d1pgl11 | d1ny711 | d1jgta2 | d1ofda3 | d1ea0a3 |
| d1ecfa2 | d1q15a2 | d1ct9a2 | d1te5a_ | d1y14b1 | d2nn6i1 | d1i94l_ | d2zjra2 |
| d1smxa_ | d2qamc2 | d1hh2p1 | d2r7da1 | d1rl2a2 | d1wfqa1 | d3bzka4 | d1x6oa2 |
| d3a0ja_ | d2j01d2 | d2ja9a1 | d4ql5a_ | d1hr0w_ | d1luza_ | d2n3sa_ | d2vnud1 |
| d1h95a_ | d1go3e1 | d2nn6h1 | d1ne3a_ | d2mo1a_ | d1iz6a2 | d1jjga_ | d1e3pa2 |
| d2ix0a3 | d2ix0a2 | d2lxja_ | d1ueba3 | d1ueba2 | d2z0sa1 | d1u0la1 | d2ix0a1 |
| d2eifa2 | d2je6i1 | d3i2za1 | d2c35b1 | d2ahob2 | d1t9ha1 | d3i4oa_ | d2qall1 |
| d1jt8a_ | d2nn6g1 | d1g6pa_ | d3cama_ | d1i94q_ | d1d7qa_ | d2ba0a1 | d2vnud2 |
| d2vnud3 | d1khia2 | d2qalq1 | d1wi5a1 | d1xtda2 | d1ny4a_ | d4w9ta_ | d1jvna1 |
| d4x2ra1 | d1ka9f_ | d1qo2a_ | d4axka1 | d1h5ya_ | d4u28a_ | d2y88a_ | d4wd0a_ |
| d1vzwa1 | d1eixa_ | d3exra_ | d2ffca1 | d2fdsa1 | d3n3ma1 | d2czda_ | d1q6oa_ |
| d2yyua_ | d1vqta1 | d1dbta_ | d3ldva1 | d4lt9a1 | d3tc7a1 | d3hoja_ | d1i4na_ |
| d3vnda_ | d3nl8a1 | d1geqa_ | d3nava1 | d4a2ra1 | d3tc6a1 | d1ujpa_ | d4ijba_ |
| d1xcfa_ | d1vc4a_ | d1rd5a_ | d1qopa_ | d3b5va_ | d2r80b_ | d1cqxa1 | d1cg5a_ |
| d1gcvb_ | d3boma_ | d2h8fb_ | d2raoa_ | d3bj1a_ | d4hrrb_ | d2r50a_ | d3d1kb_ |
| d2aa1b1 | d3zhwa_ | d3hf4b_ | d3gdja_ | d1jebb_ | d3bj1b_ | d1x9fb_ | d1wmub_ |
| d1gcva_ | d3bcqb_ | d3d4xb_ | d2dc3a_ | d3hf4a_ | d4yu3a_ | d3k8ba_ | d1qpwb_ |
| d2r80a_ | d3bcqa_ | d1v4wa_ | d3wtgb_ | d1jl7a_ | d4esaa_ | d1a9we_ | d3d1ab_ |
| d1fsla_ | d4h2la_ | d1v4wb_ | d1mwca_ | d1vhba_ | d1jeba_ | d1spgb_ | d1h97a_ |
| d1q1fa_ | d1tu9a1 | d3d1aa_ | d1hdsb_ | d3hyub_ | d2zfbb_ | d3fs4a_ | d1oj6a_ |
| d1shrb_ | d4h2lb_ | d4yu3b_ | d1spga_ | d3hrwa_ | d3qqra_ | d3at5a_ | d4wchd_ |
| d3hyua_ | d1cg5b_ | d1uc3a_ | d1hbra_ | d2raob_ | d1outb_ | d4esab_ | d3gdjb_ |
| d3qqqa_ | d1x9fc_ | d3vrfa_ | d1itha_ | d3mkba_ | d3d4xa_ | d3mjpa_ | d1jl6a_ |
| d3fh9a_ | d1outa_ | d2dc3b_ | d1it2a_ | d3eoka_ | d3g46a_ | d1qpwa_ | d3vrfb_ |
| d1ch4a_ | d1x9fd_ | d1hdsa_ | d2zfba_ | d3mkbb_ | d1or4a_ | d3wtga_ | d1a4fa_ |

d3fh9b_ d3bomb_ d3cy5a_ d3at5b_ d1x9fa_ d2oifa_ d4lmxb_ d1cpcb_
d3v57a_ d1kn1a_ d1cpca_ d4f0ta_ d2vjha_ d4po5b_ d1eyxa_ d1alla_
d4f0tb_ d1b33b_ d1b8db_ d4h0mb_ d1b33a_ d2vjhb_ d4lm6b_ d2vmlb_
d1allb_ d2vjta_ d3dbja_ d2vmla_ d2vjtb_ d4f0ub_ d1f99b_ d4rmpa1
d1liaa_ d1eyxb_ d2uulb_ d4lmsb_ d4f0ua_ d2bv8a_ d1f99a_ d2bv8b_
d2c7lb_ d3dbjb_ d4rmpb1 d1b8da_ d4h0ma_ d3v57b_ d1kn1b_ d1liab_
d2uula_ d2j96a_ d1azwa_ d1mtza_ d2xqfa_ d1ea5a_ d1f6wa_ d1dx4a_
d1qe3a_ d1k4ya_ d4qwwa_ d2ha2a_ d1r88a_ d1sfra_ d1f0na_ d4qeka_
d2qvba_ d2v9za_ d4kafa1 d4iuaa3 d4bvva_ d5hpga_ d3k65a_ d1ki0a3
d1ki0a2 d1ki0a1 d1a0ha1 d1pmla_ d3sp8a3 d2feba1 d1xova2 d1f74a_
d3l21a_ d2e1da_ d1to3a1 d1w3ia_ d1sfla_ d1n7ka_ d1vcva1 d4imea_
d1wa3a_ d2ocza1 d1f2ja_ d3m16a_ d2yxga_ d1ub3a_ d3a5fa_ d1fbaa_
d1l6wa_ d1xfba1 d1wx0a1 d1vpxa1 d1ojxa_ d1mxsa_ d2a4aa1 d1xkya1
d3cq0a1 d3s1xa_ d1f05a_ d1fdja_ d3cpra1 d4d2ja_ d1cg2a1 d1yloa2
d1y7ea2 d1y0ya3 d1lfwa1 d1vixa1 d1fnoa4 d1vhoa2 d1ysja1 d1vhea2
d1z2la1 d2fvga2 d1r3na1 d2grea2 d1xmba1 g1q7l.1 d1rtqa_ d2ftba_
d1pmpa_ d2rcqa1 d1fdqa_ d4lkpa_ d1sa8a_ d1vyfa_ d1lpja_ d3vg7a1
d4azqa1 d1o8va_ d5bvqa_ d1p6pa_ d2qo4a_ d1opba_ d1yiva1 d1o1va_
d4bvma1 d4tkba_ d4i3ba_ d1bwya_ d1ftpa_ d1ggla_ d4gkca_ d1cbia_
d3p6da1 d1kqwa_ d2a0aa1 d1m4la_ d2bk7a_ d3fjua_ d1z5ra1 d1kwma1
d1uwya2 d2c1ca1 d1dtda_ d1h8la2 d3lgia_ d3i77a_ d2z9ia2 d4nsva_
d1lcya2 d4jcna_ d1agja_ d1os8a_ d1hpga_ d1l1ja_ d1qtfa_ d1e69a_
d1mv5a_ d3g60a4 g1ii8.1 d1ewqa2 d1ji0a_ d1v43a3 d2wmfa1 d1vpla_
d1g2912 d4a82a2 g1f2t.1 d2d62a1 d3tuic1 d2bbsa1 d3g60a3 d2ixea_
d1g6ha_ d1sgwa1 d2qi9c_ d3rlfa1 d3b60a1 d3d31a2 d2pzea1 g1xew.1
d1jj7a_ d1oxxk2 d2onka1 d1w1wa_ d1iwea_ d1qf5a_ d1vmaa2 d1nija1
d1np6a_ d2p67a1 d3ihla_ d1okkd2 d1ls1a2 d1s1ma2 d1iona_ d2v40a_
d1dj3a_ d3hida_ d2vo1a1 d2g0ta1 d1vcoa2 d1j8mf2 d1g3qa_ d1xjca_
d1p9ba_ d1qzxa3 d1ihua2 d1ihua1 d2qm8a_ d1dj2a_ d4ioja_ d1cp2a_
d1yrba2 d3gqba2 d1skye3 d1xp8a1 d1tf7a2 d1tf7a1 d1ye8a1 d1skyb3
d2qe7d2 d4blta_ d4huta_ d3ldaa2 d4hyya_ d2i3ba1 d1cr1a_ d3j9tb2
d3ntua2 d1vdza1 d2z43a_ d1e9ra_ d1nlfa_ d3ziaa2 d1n0wa_ d1g6oa_
d3ziad2 d3j9ta2 d1pzna2 d3gqbb2 d2i49a_ d1nnfa_ d1xvxa_ d1ursa_
d1h3da1 d1iuda_ d4m1va1 d1twya_ d3e13x_ d2nxoa1 d4pfua_ d3ttma_
d1elja_ d2qmwa1 d1us5a_ d1y3na1 d1jeta_ d1uqwa_ d4f19a1 d2esna2
d3thia_ d3fasa1 d2uyeb_ d2xwva1 d1r9la_ d2fyia1 d2ozza1 d1sw5a_
d1nh8a1 d1vr5a1 d2g29a_ d1xs5a_ d3lswa_ d2z23a_ d3w9va_ d3tcha_
d1q35a_ d1z7me1 d3q27a_ d1p99a_ d1ve4a1 d2i6ea1 d4mcoa1 d4n8ya_
d1eu8a_ d1zbma1 d1hsla_ d1ixca2 d1j1na_ d1xvya_ d1y9ua_ d2z22a1
d1xoca1 d4oera_ d1wdna_ d5f1qa1 d1xt8a1 d1i6aa_ d4gwoa_ d4igta1
d1pc3a_ d4mmpa1 d1o63a_ d2czla1 d2onsa2 d4mija1 d4ok5a1 d2bmfa1
d2bmfa2 d1yksa1 d1yksa2 d1jr6a_ d2qeqa1 d2jlqa1 d1a1va2 d1eh3a_
d3skpa_ d1dtza2 d1ieja_ d1jnfa2 d1ce2a1 d1jnfa1 d1b1xa2 d1b1xa1
d1jw1a1 d1jw1a2 d1ryoa_ d1h76a1 d1h76a2 d1c4oa1 d2p6ra3 d2p6ra4

d1oywa2 d1oywa3 d1wrba1 d1w36b2 d1w36b1 d1c4oa2 d3bora_ d1t6na_
d2eyqa5 d2eyqa3 d2eyqa2 d2zu6a2 d1veca_ d1w36d1 d2g9na1 d1z3ix2
d2rb4a_ d1hv8a1 d1hv8a2 d1t5ia_ d1w36c2 d2j0sa2 d2j0sa1 d1fuka_
d3hjha1 d1z5za1 d1qdea_ d1z63a1 d1s2ma2 d1s2ma1 d1wp9a1 d1wp9a2
d1gm5a3 d1gm5a4 d3dina4 d3dina3 d1q0ua_ d1w36c1 d1z3ix1 d1nkta3
d1nkta4 d1uaaa2 d1w36d2 g1qhh.1 d2fwra2 d2fwra1 d3q3ma_ d1za0a1
d1mxra_ d2inca_ d1jk0a_ d2incb_ d1mhyb_ d3hf1b_ d1mtyb_ d2uw1a_
d1mhyd_ d3n37a_ d1afra_ d1oquc_ d1syya_ d1mtyd_ d2o1za_ d1uzra_
d1smsa_ d2fkza_ d1zuja1 d1s3qa1 d2fjca1 d1z6om1 d1veia_ d4dyya_
d1jiga_ d3gvya_ d2pyba_ d4cy9a1 d3bvfa1 d1r03a_ d2cf7a_ d1nf4a_
d1zs3a1 d4iwka_ d3ka8a_ d3np0x_ d1n1qa_ d1lb3a_ d4e6ka_ d1z6oa1
d2fzfa1 d4dyua_ d3ge4a_ d1jgca_ d4am4a_ d2z6ma_ d1ji5a_ d1vlga_
d1lkoa1 d1krqa_ d3qz3a_ d1o9ra_ d2ffxj_ d1yv1a1 d2v2pa_ d1j30a_
d4di0a_ d1dpsa_ d1oewa_ d2qzxa_ d2asia_ d1psoe_ d1miqa_ d1smra_
d2h6ta_ d1pfza_ d1izda_ d3hd8a_ d1j71a_ d1dpja_ d4aa9a_ d1t6ex_
g1b5f.1 g1lya.1 d1qdma2 d1bxoa_ d1wkra_ d1tzsa_ d1ibqa_ d2qzwa_
d1bdqa_ d3ggta_ d3ixoa_ d1mrxa_ d2haha_ d1tw7a_ d4njsa_ d1sgua_
d2p3da_ d1nsoa_ d3d3ta_ d2rkfa_ d1c6ya_ d2rspa_ d3ogpa_ d3ekwa_
d2o4na_ d2j9ja_ d1baia_ d3ggua_ d3lzua_ d4npua_ d4j55a_ d2r8na_
d2fxda_ d2p3ca_ d4dqea_ d3ttpa_ d3t3ca_ d2p3aa_ d2hc0a_ d2fdda_
d3mwsa_ d3rcha_ d3k40a_ d1pmma_ d4yl8a1 d2al6a3 d1wgda1 d1wgga1
d4q5eb_ d1wjna1 d1se9a_ d3n3kb_ d1wz0a1 d1we6a1 d2m0xa_ d2gbka_
d3rt3b1 d1wh3a1 d1sifa_ d2ekec2 d4ii2b1 d4hk2a1 d1c3ta_ d1m94a_
d1wgha1 d1ttna1 d1j8ca_ d1wx8a1 d3v6cb1 d2ma9b_ d4i6lb1 d1bt0a_
d2jvca1 d1iyfa_ d2k1fa_ d1wxva1 d1wf9a1 d2io0b2 d1x1ma1 d2gbma_
d1zw7a1 d2m17a_ d1ud7a_ d1we7a1 d2lwpa_ d2gbja_ d1wjua1 d1v6ea1
d1v86a1 d4eewa_ d1wiaa1 d4icva_ d4gu2a_ d1wx7a1 d1wy8a1 d4hjka1
d2faza1 d1v5oa1 d3ruib_ d1eo6a_ d3waob_ d2r2qa1 d3m95a_ d3wana_
d2al3a1 d2cr5a1 d1wj4a1 d1s3sg_ d3ubra_ d1qo8a1 d1d4ca1 d2k3va_
d1ft5a_ d1qdba_ d1m1qa_ d2j7aa_ d1h21a_ d1ogyb_ d4fasa_ d1jnia_
d1oaha_ d1sp3a_ d3h33a_ d1rwja_ d19hca_ d1os6a_ d3bxua_ d3h4na_
d1i77a_ d3caoa_ d1j0pa_ d2ctha_ d1duwa_ d1ofwa_ d2e84a_ d1hh5a_
d1gyoa_ d3h34a_ d1iyka2 d1iyka1 d1rxta1 d1iica2 d1dszb_ d1a6ya_
d3cbba_ d1ynwa1 d1r4ia_ d1hcqa_ d2hana_ d2hanb_ d2enva1 d1kb4b_
d2nllb_ d1lo1a_ d1um8a_ d1ixza_ d1qvra2 d1qvra3 d1qzma_ d1sxjc2
d1jr3d2 d2p65a_ d1l8qa2 d1g8pa_ d3hu3a3 d1sxjd2 d1in4a2 d1sxja2
d1tuea1 d2ce7a2 d1w5sa2 d1sxjb2 d1d2na_ d1ny5a2 d1ofha_ d2gnoa2
d2a5yb3 d1fnna2 d1u0ja1 d1lv7a_ d1iqpa2 d2fnaa2 d1ixsb2 d4gita_
d1sxje2 d1g41a_ d1svpa_ d1mbma_ d3su6a1 d1cu1a1 d4agka_ d1vcpa_
d1q31a_ g1n1l.1 d3e90b_ d3u1jb_ d2lj7a_ d1n4na_ d2m8bb1 d2kyqa_
d3psma_ d1jkza_ d1jxca_ d2gl1a_ d1ugla_ d4uj0a_ d2k4ua_ d1hp2a_
d2lu9a_ d1baha_ d2meoa1 d1wm7a_ d1hlya_ d1n8ma_ d1jlza_ d1wpda_
d1c55a_ d1pvza_ d1wt7a_ d1rjia_ d1wz5a_ d1m2sa_ d1pjva_ d1c49a_
d1qkya_ d2ktca1 d1wmta_ d1du9a_ d1j5ja_ d1r1ga_ d1t0za_ d2i61a_

d1t1ta_ d1sn4a_ d1kv0a_ d2lkba_ d1npia_ d1wwna_ d1pe4a_ d2kbka_
d2li7a_ d1djta_ d2kbha_ d1dq7a_ d1sega_ d1omya_ d1zywa1 d1jzaa_
d2jcqa_ d1n9wa1 d1bbua1 d1e1oa1 d1b8aa1 d1l0wa1 d3a74a1 d1eova1
d1t61c1 d1iyjb4 d1iyjb5 d1iyjb3 d1o7ia_ d4ipca1 d1x3ea_ d4apva_
d1jb7a1 d1jb7a2 d1jb7a3 d3kdfa_ d1ue1b_ d1wjja1 d3eiva_ d1xjva2
d1xjva1 d2ccza2 d1se8a_ d1l1oc_ d1jb7b_ d1qvca_ d1qzga_ d1miua4
d1miua5 d1miua3 d3g48a1 d1gd7a_ d1mkha_ d1jjcb3 d3ubub_ d1egia_
d1qo3c_ d1v7pa_ d1tdqb_ d3ubua_ d1c3ab_ d1h8ua_ d3bx4b_ d1t8ca1
d1sb2a1 d1qdda_ d2ziba1 d3cada_ d1byfa_ d1j34b_ d1jwia_ d1jzna_
d1c3aa_ d1oz7b_ d1wmza_ d3bdwb_ d1hq8a_ d1fifa1 d1dv8a_ d3hupa_
d1wk1a1 d1umra_ d2afpa_ d3bx4a_ d1gz2a_ d1sl6a_ d1v7pb_ d3g8ka_
d1ixxa_ d1j34a_ d1jwib_ d1g1sa1 d1y17a1 d1ja3a_ d1fvub_ d3bdwa_
d1sb2b1 d1fvua_ d1umrc_ d1oz7a_ d4m17a_ d3cfwa1 d1ypqa1 d1g1ta1
d3fd4a_ d3l9jc_ d4dn8a1 d1v4lb_ d1rdl1_ d2msba_ d1prtb2 d1rpma_
d2shpa1 d1yfoa_ d1l8ka_ d2ooqa1 d1g4us2 d1p15a_ d1lara2 d1lara1
d1wcha_ d2fh7a2 d2fh7a1 d1ohea2 d2f4da_ d1rxda_ d2hxpa_ d1xria_
d1d5ra2 d1fpza_ d3ezza_ d4y2ea_ d1i9sa_ d3azya_ d3wvja_ d1upsa1
d1ajoa_ d2hyka_ d1ajka_ d2vy0a_ d1o4ya_ d3o5sa_ d3d6ea_ d3atga_
d1dypa_ d1umza_ d3i4ia_ d1c1la_ d4lbqa_ d1a78a_ d1slta_ d4no4a_
d3zsja_ d3duia_ d1g86a_ d1is3a_ d1qmja_ d1hlca_ g1qmo.1 g1len.1
d3zyra_ d1fnya_ d1n47a_ g2ltn.1 d1avba_ d3usua_ d1dhkb_ d1f9ka_
d1ukga_ d1g8wa_ d1g9fa_ d1wbfa_ d3wcsa_ d1g7ya_ g1loe.1 d1mvqa_
d1hqla_ d1v6ia_ d1dbna_ d1qnwa_ d1fx5a_ d4l8qa_ d1ioaa_ d3n35a_
d1epwa1 d2h0ba_ d2erfa_ d1h30a2 d3mw4a1 d1pz7a_ d2r1ba_ d3v64a1
d1d2sa_ d3bopa1 d3mw3a1 d1okqa1 d1okqa2 d1wioa4 d1ccza2 d1vcaa1
d1dr9a2 d2d9qb1 d2nxyb2 d2dara2 d2dara1 d1wyha2 d2dloa2 d1rutx4
d1rutx3 d2cuqa2 d1x4la2 d1x4la1 d1x3ha2 d1x3ha1 d2d8za1 d1x61a2
d1x6aa1 d1x6aa2 d2cora2 d2cora1 d1v6ga1 d1v6ga2 d2d8za2 d1x64a1
d1x64a2 d1b8ta1 d2co8a1 d1x63a2 d2dj7a2 d2dj7a1 d2cu8a2 d2cu8a1
d1g47a2 d1g47a1 d1j2oa1 d1wyha1 d2dloa1 d1wiga1 d1wiga2 d2cupa2
d2cupa3 d1x68a1 d2d8ya2 d2d8ya1 d1m3va2 d1x62a1 d1x68a2 d1x61a1
d1b8ta3 d1b8ta2 d2d8xa2 d2d8xa1 d1x4ka1 d1x4ka2 d2cuqa1 d1m3va1
d2co8a2 d1j2oa2 d1x63a1 d2cura1 d2cura2 d1x62a2 d1op9a_ d1y0la1
d1etzb1 d1egjl1 d2gsib1 d1nj9a1 d4jfhd1 d1aqkl1 d1t2ja_ d1lo4h1
d1zvya1 d3v4vl1 d4enec1 d1kxtb_ d5e8ea1 d2pxya_ d4weud_ d1xiwc_
d2esvd1 d1qnzh_ d1ogae1 d1f4xh1 d4n1hb_ d3ezjb1 d3mlwl1 d1kfah1
d1etza1 d1b4jh1 d3cfic_ d4w68a_ d2arja1 d1ek3a_ d2ok0l1 d1vera_
d1uwgh1 d1blnb1 d1fpth1 d1rzga1 d2giya1 d4m3kb1 d1nc2a1 d2nyyd1
d1ncna_ d1fbih1 d5dxwa_ d4fqqa1 d1dqdh1 d1jfql1 d1kelh1 d1faih1
d1frgl1 d1figl1 d4m6nl1 d1fo0a_ d4hxbl1 d3b5ga_ d3quyd1 d2h9gb1
d1i8kb_ d2dd8h1 d2ck0h1 d1eaja_ d4w6wb1 d2a6ib3 d1t66d1 d2e27h_
d1ob1b1 d1f11b1 d2v7na1 d1ad0a1 d2xt1b1 d1pskh1 d4i13b1 d2op4l1
d1a0ql1 d1uwel1 d1za3a1 d1ncbl1 d4uu9a_ d1bm3h1 d1cloh1 d1nqba2
d1cf8h1 d4y5va_ d1mhph1 d5anma1 d4cdgc1 d4ldeb_ d1y0lb1 d2pcpb1

d1z3gl1 d1dfbl1 d1fh5h1 d3g5xa1 d1tzib1 d4dn3l1 d3zhka1 d2b1hh1
d2uyla1 d1f3dh1 d1nj9b1 d2agjh1 d2h1pl1 d1q72h1 d8fabb1 d1ndmb1
d1rjca1 d4u6hb1 d3upca_ d2fbjl1 d1osph1 d1tvda_ d2cjvh_ d4fhbd1
d1bd2d1 d1rz7h1 d3nn8a_ d1blna1 d1knoa1 d1nfda1 d2bmkb1 d1dqll_
d1igyb1 d1igfl1 d12e8h1 d3utza1 d1nc2b1 d1rihh1 d4mwfb1 d4jvpa1
d1mieh1 d1rhfa1 d1fgnl1 d1sbsh1 d1lp9e1 d1dn0a1 d4lqfl1 d1iaih1
d1kgcd1 d1mh5b1 d1gpol1 d2g60h3 d1f8tl1 d2jixd1 d4grwh_ d1fo0b_
d1bfob1 d1axsa1 d1u9ka_ d1nmbh_ d1h8na1 d1h8na2 d1jguh1 d4eura1
d3sdyl1 d35c8h1 d1w72l1 d1mvub_ d1i9rh1 d1ob1a1 d4wenb_ d1yeeh1
d3ietc1 d1f58h1 d1ad0b1 d1mqkl_ d1nbvh1 d4b50a_ d3r8bb_ d1bafl1
d1mamh1 d4rgne1 d2gsgb_ d35c8l1 d1bafh1 d1ai1h1 d2pcpa1 d2fath1
d2x1pa_ d1orsb1 d1ibgl1 d1shma_ d1a2yb_ d1u0qa_ d2coqa_ d1hilb1
d1qfwi_ d1jv5b_ d1fn4b1 d5e0qa_ d1nsnh1 d1plgl1 d4ocxl1 d1kb5h1
d1rzfh1 d1pg7x1 d2fx7h1 d2oi9c_ d1l7il1 d3eyva1 d1d5il1 d4ffyl_
d1tetl1 d1a6wh_ d1ac6a_ d3dvfa_ d1nfde1 d1mimh1 d1ri8a1 d1jhlh_
d3dura_ d1fskb1 d1f97a1 d3so3b1 d2ak4d1 d1lp9f1 d4z7wf1 d1l7th1
d4y5ya_ d4x7ec1 d1emth1 d1ikfl1 d1nlbh1 d4utal1 d1bjma1 d1eapb1
d1u6ah1 d2dtgd1 d1kj2b_ d1xeda1 d1c1eh1 d1fgvh_ d2bnub1 d2fb4h1
d2dtgb1 d1ktrh_ d1jpth1 d1qkzl1 d4hepg_ d2atpb1 d1flrh1 d2hrpl1
d1mvfa_ d1iaim1 d3k80a1 d2jb5h1 d3zkxc_ d4grwe_ d1it9h1 d1rhhb1
d3w9ec_ d1e4wh1 d1sy6h1 d2j6eh1 d3qxta_ d1jglh1 d1p7ka1 d1tzhb1
d4n8pa1 d1gafl1 d2jelh1 d1wioa2 d4i0cc1 d1cicb1 d1lmka2 d1lmka1
d1iqwh1 d1orqb1 d1ejoh1 d1jnlh1 d4zg1a_ d43c9b_ d1ggbl1 d1wejh1
d3q6fa1 d1f3dj1 d3c6la1 d1bqlh1 d3p9wb_ d1a3rl1 d1h0db1 d2uzil_
d1eo8l1 d1qfwl_ d1n7ml1 d1igja1 d1c5ch1 d2a9mh_ d1rmfh1 d1fj1b1
d4heme_ d1ccza1 d1a5fh1 d1ogad1 d1dlfh_ d1qa9b_ d1vgel1 d3qskb_
d1sjxa_ d4ozfg1 d1sq2n_ d1qd0a_ d1kcvh1 d1c5db1 d3qcul1 d3bn9d1
d1mjuh1 d4y8dc_ d7fabh1 d3dvgb1 d1lo0l1 d1a6tb1 d1gigh1 d1kcrh1
d2hwzl1 d1yjdc1 d3clfl1 d4krna_ d1dl7l_ d1e6ol1 d1mexh1 d3r0ma_
d4kkna_ d1fnsl1 d1oaqh_ d1rzia1 d1ucbh1 d1ncwl1 d4b41a_ d1nfdf1
d2mpah1 d2d7tl1 d4grwf_ d4b5ea_ d1lk3h1 d3u79a_ d4u3xa_ d1ghfl1
d1bvkb_ d1mcww1 d1b88a_ d1jrhh1 d32c2b1 d4wemb_ d1dsfh_ d1op3h1
d1cell1 d1iqda1 d1qoka1 d1qoka2 d1qfuh1 d2crya1 d1ay1h1 d1oaql_
d3ab0b_ d1rurl1 d1t4kb1 d1bz7b1 d1n0xh1 d1forl1 d1ucbl1 d4w6yb_
d43c9a_ d2aabl1 d1tzia1 d4n1ca_ d1k6qh1 d3g9ab_ d4tvsa1 d1igmh_
d1pz5b1 d3dx9a1 d3wxwh_ d1nakh1 d1kcul1 d3eaka_ d4qlra1 d1cr9l1
d1d9ka_ d1f4xl1 d1deea1 d2i27n2 d1c12a1 d2orbl1 d1j8hd1 d1mcph1
d1c5da1 d1kenh1 d1bd2e1 d1h5ba_ d4x7fc1 d3upaa_ d1t3fa1 d1indh1
d2dtgc1 d4hc1l1 d1zv5a1 d4wgvb_ d1uwgl1 d4f37k1 d1aqkh1 d1vesa_
d3zkqd_ d1fptl1 d1lo4l1 d1knob1 d3rrqa_ d1ct8b1 d4orzc_ d1mlbb1
d4hgkc1 d1dqdl1 d1bogb1 d4ozhe1 d1dr9a1 d1i7za1 d1dn0b1 d1kell1
d1rzib1 d1ypzf1 d1wtla_ d1hkfa_ d1fail1 d1figh1 d2vyre_ d1sjva_
d1ohqa_ d1bfoa1 d1pkqb1 d1mjul1 d1b4jl1 d1nsnl1 d2arjb1 d1p4bh_
d4aj0a_ d3nn8b_ d2rcja1 d1f2xk_ d4v1dd_ d1adqh1 d2eksa_ d4k3ga_

d1jfqh1 d1uweh1 d1frgh1 d4gftb_ d1uz8b1 d4n1ea_ d1bm3l1 d1clol1
d3mcka1 d2vq1a1 d1ae6h1 d4aq1b1 d1uj3b1 d1nbqa1 d1fh5l1 d2ck0l1
d2ywza_ d1q72l1 d4nbxb_ d1hila1 d4hzlb1 d2j88l1 d1zmya1 d1cd0a_
d1a4ka1 d1pskl1 d1a0qh1 d4krmb_ d1ncbh1 d2fbjh1 d1j8he1 d1i3ua_
d1cf8l1 d1fe8h1 d2p1ya1 d1eeqa_ d2x89a_ d1ol0a_ d2r0lh1 d1ub5a1
d1dfbh1 d3eyvb1 d4jdvb1 d4p2cg_ d2gsia1 d2agjl1 d1u3ha1 d2h1ph1
d1rihl1 d1fskc1 d1boga1 d2a77l1 d1ospl1 d1qfoa_ d3dwta_ d2fx7l1
d1rz7l1 d3stba1 d1pkqa1 d1b6da1 d1dzba2 d1dzba1 d1kj2a_ d4l1ha_
d1rzgb1 d1dqlh_ d12e8l1 d1miel1 d1jmaa_ d1sbsl1 d1fgnh1 d1f58l1
d1mqkh_ d1ndgb1 d3qegl1 d1iail1 d2aw2a1 d1tqbc1 d3fo2a1 d4dkaa_
d1f8th1 d2g60l1 d2ywya_ d1uj3a1 d1yc7a_ d3bd3a_ d4w6xb1 d1nmbl_
d1i8ka_ d1rjlb1 d1ypze1 d1p7kb1 d1kxqe_ d1gpoh1 d2z4qa1 d1w72h1
d1i9rl1 d4qgya1 d4xt1c_ d2qqnh1 d1cica1 d3c08h1 d2cd0a_ d2bdnh1
d1hnga1 d1maml1 d1nldh1 d1cr9h1 d4lcih1 d3bkyh1 d3qg7l1 d1ai1l1
d1kxvc_ d4iosd_ d1l7ih1 d2x1qa_ d1ub6b1 d3k74b_ d1ibgh1 d1teth1
d1igya1 d1hxma1 d2x6ma_ d2e7la_ d5e03a_ d1qfwm_ d2r0kh1 d1igjb1
d1rurh1 d1plgh1 d1mj8h1 d1yuhb1 d3fmgl1 d1rzfl1 d1emtl1 d1ad9b1
d1igtb1 d1nlbl1 d1d5ih1 d3k7ua_ d4ffyh_ d1bwwa_ d3dvga1 d1a6ta1
d1fgvl_ d4qxgl1 d1miml1 d1jhll_ d2hffb1 d2hh0l1 d1qkzh1 d1l7tl1
d1afvh1 d1iaii1 d2cjuh_ d1um5h1 d2ghwb2 d2ghwb1 d1kgce1 d2fgwh1
d1ikfh1 d1dvfd_ d2i24n1 d2xtjb_ d1za3b1 d2yc1a_ d2hh0h1 d2x1oa_
d1bvka_ d1rjla1 d5e7ba1 d1igta1 d1il1b1 d2fb4l1 d1mvua_ d1jptl1
d32c2a1 d1fvca_ d1iqdb1 d2hrph1 d2p4ab_ d4qo1a_ d1p2cb1 d1wt5c_
d1t4ka1 d5c1mb_ d1it9l1 d1bz7a1 d1b2wh1 d1e4wl1 d1jnll1 d1yedb1
d1za6b1 d3u7aa_ d1igfh1 d3gnml1 d1orsa1 d1wejl1 d1a2ya_ d1hxmb1
d1gafh1 d2j4wh1 d1pz5a1 d1j05a_ d1a3rh1 d4fqll1 d1qfwh_ d1eo8h1
d1n7mh1 d4s11a_ d1kegh1 d5da4a_ d1iqwl1 d3whxb1 d1vgeh1 d1deeb1
d1c12b1 d1t2ql1 d1ad9a1 d3gsnb1 d3b2uc1 d1ggbh1 d1t3fb1 d1nakl1
d4ocmc_ d1lila1 d2v7hb3 d2esve1 d1mcoh1 d2qr0b1 d2q3aa_ d1ct8a1
d1kcrl1 d2pxyb_ d2uzih_ d5c3ld_ d3durb_ d1c5cl1 d2ak4e1 d1dl7h_
d1xiwd_ d1mlba1 d1rmfl1 d1i7zb1 d1dlfl_ d2xa3a_ d5bq7b1 d1dqqb1
d3h42l1 d1egjh1 d1kcvl1 d1fe8l1 d2j6el1 d1f3rb2 d1lk3l1 d1sm3h1
d1q0xh1 d1ghfh1 d7fabl1 d1igch1 d1lo0h1 d4idla_ d5c2bl_ d1jrhl1
d1il1a1 d3zhda1 d4kfzc_ d1b0wa_ d1e6oh1 d1mexl1 d4krpb_ d1fnsh1
d1ncwh1 d4hgma_ d4eigb1 d4rzca_ d2mpal1 d4y5xa_ d5en2b1 d3lrga1
d4ej1c1 d1rhha1 d1j05b_ d1forh1 d1q9wb1 d2wzpd_ d2aq2a1 d1h0da1
d3revb1 d2ij0c1 d1tzha1 d4m7jl1 d1k6ql1 d3b9kc1 d1igml_ d3qeua1
d1dsfl_ d1ce1h1 d1kcuh1 d1zvha1 d1zvoa1 d1tqbb1 d1ay1l1 d2qscl1
d2cgrh1 d1nl0h1 d1kenl1 d3eo0a1 d4n9ob1 d1n0xl1 d3k1kc_ d2aq2b1
d1ck1a1 d1enfa1 d1i4pa1 d1v1oa1 d1et9a1 d1esfa1 d2g9hd1 d4ohja1
d1klud1 d1bxta1 d1eu3a1 d1ty0a1 d2cqva1 d1fhga_ d2k1ma_ d2dl2a1
d1biha3 d1biha1 d1biha4 d3qp3a1 d1g1ca_ d1gxea_ d1cvsc2 d1cvsc1
d1xmwa2 d1m4ka2 d2otpa2 d1biha2 d1rhfa2 d2fcba2 d2fcba1 d1wwbx_
d1gsma2 d1epfa1 d1epfa2 d2dava1 d1waaa_ d1f97a2 d1x44a1 d1vcaa2

d1fnla2 d1fnla1 d3b5ha2 d3h8na2 d3h8na1 d1jbja1 d1jbja2 d1pd6a_
d1f2qa2 d1f2qa1 d3cx2a_ d1nbqa2 d3b5ha1 d1n26a1 d1xiwb_ d1xaua_
d1fltx_ d2ifga1 d1olla1 d1olla2 d1cs6a4 d1cs6a3 d1cs6a2 d1cs6a1
d2gw5a2 d1sy6a1 d1xiwa_ d1ucta2 d1ucta1 d1ry7b2 d1gl4b_ d1qz1a3
d1olza1 d2c9aa1 d1wwca_

**Family-23 (b.121.4.1)**

**Positive dataset contains 11 protein sequences.**

d4q4w1_ d2x5ia_ d4iv1a_ d4gb31_ d2wzr3_ d2x5ic_ d1zba3_ d3vbhc_
d4q4w3_ d1zba1_ d4iv1c_

**Negative dataset contains 3919 protein sequences.**

d1m32a_ d1ibja_ d1c7na_ d1iuga_ d4q75a_ d2bkwa1 d1t3ia_ d1n8pa_
d1qz9a_ d1jf9a_ d1eg5a_ d1p3wa_ d3kgwa_ d1cl1a_ d2ctza1 d1vjoa_
d2rfva_ d1gc0a_ d1cs1a_ d2ch1a1 d1qgna_ d1pffa_ d1elua_ d1xfda2
d1u08a_ d1lc5a_ d1b5pa_ d2ay1a_ d1bw0a_ d3pd6a_ d7aata_ d2csta_
d1fg7a_ d4wlha_ d1o4sa_ d1v2da_ d1gdea_ d1m6sa_ d1v72a1 d1svva_
d1ajsa_ d1wsta1 d2f8ja2 d1lk9a_ d1vp4a1 d1xi9a1 d2gb3a1 d1yaaa_
d1xkta1 d2hfka_ d1jmkc_ d5d3ka_ d2cfba1 d1dfoa_ d1o69a_ d1bj4a_
d3k28a_ d2bwpa_ d3lg0a_ d2fnua_ d3ou5a_ d1z7da1 d1b9ha_ d1iaya_
d4msoa_ d1bs0a_ d1s0aa_ d1sffa_ d3tqxa_ d1mdoa_ d3qboa_ d3pgya_
d1bjna_ d2dkja_ d1ejia_ d1vefa1 d4wxga_ d3bs8a_ d3gbxa1 d1w23a_
d1sefa_ d1sfna_ d1rc6a_ d3lgia_ d3i77a_ d2z9ia2 d4nsva_ d1lcya2
d4jcna_ d1agja_ d1os8a_ d1hpga_ d1p3ca_ d1l1ja_ d1dzka_ d4tlja_
d2a2ua_ d4wfva_ d3t1da_ d4iaxa_ d3zq3a_ d2nnda_ d1i4ua_ d1kxoa_
d1bbpa_ d1kt7a_ d1exsa_ d3s26a1 d1lf7a_ d1qfta_ d4gh7a_ d1z24a1
d1qabe_ d1xkia_ d1ew3a_ d3bx7a_ d4iawa1 d1n0sa_ d1gkab_ d3kzaa_
d2k23a_ d2l9ca1 d2joza1 d1gm6a_ d3kffa_ d3saoa1 d1e5pa_ d1yupa1
d1iiua_ d2wo3b_ d1shxa_ d3p0ya1 d1igra1 d1igra2 d1n8yc1 d1m6ba2
d1m6ba1 d1f56a_ d1bypa_ d1azca_ d1ws8a_ d3u25a_ d1cuoa_ d1id2a_
d1rkra_ d2iaac_ d4dp1x_ d1nwpa_ d2gima_ d2i7sa_ d1qhqa_ d1gyca3
d1hfua2 d1kbva1 d1kbva2 d1sdda1 d1sdda2 d1gyca1 d1gyca2 d1mzya1
d2bw4a1 d1aoza1 d1aoza3 d1aoza2 d1kyaa2 d1kyaa3 d1kyaa1 d1mzya2
d4e9sa1 d1sddb1 d1sddb2 d1hfua1 d1hfua3 d1oe1a2 d1v10a2 d1v10a3
d1v10a1 d2dara2 d2dara1 d1wyha2 d2dloa2 d1rutx4 d1rutx3 d2cuqa2
d1x4la2 d1x4la1 d1x3ha2 d1x3ha1 d2d8za1 d1x61a2 d1x6aa1 d1x6aa2
d2cora2 d2cora1 d1v6ga1 d1v6ga2 d2d8za2 d1x64a1 d1x64a2 d1b8ta1
d2co8a1 d1x63a2 d2dj7a2 d2dj7a1 d2cu8a2 d2cu8a1 d1g47a2 d1g47a1
d1j2oa1 d1wyha1 d2dloa1 d1wiga1 d1wiga2 d2cupa2 d2cupa3 d1x68a1
d2d8ya2 d2d8ya1 d1m3va2 d1x62a1 d1x68a2 d1x61a1 d1b8ta3 d1b8ta2
d2d8xa2 d2d8xa1 d1x4ka1 d1x4ka2 d2cuqa1 d1m3va1 d2co8a2 d1x63a1
d2cura1 d2cura2 d1x62a2 d2bema_ d1pama1 d1ea9c1 d1qhoa1 d2fhfa2
d2fhfa1 d1k3ia1 d1j0ha1 d1cxla1 d2bhua1 d1edqa1 d1f1sa2 d1ut9a2
d1gvia1 d1txka1 d1zx5a1 d1qwra_ d2phla2 d2phla1 d1fxza2 d1fxza1
d1lrha_ d1uija1 g1dgw.1 d2v09a_ d1uika2 d1uika1 d1nepa_ d1ktja_
d1ds6b_ d1fstb_ d1ex0a1 d1v70a_ d2f4pa1 d1yhfa1 d1eixa_ d3exra_

d2ffca1 d2fdsa1 d3n3ma1 d2czda_ d2yyua_ d1vqta1 d1dbta_ d3ldva1
d2bjca1 d2hsga1 d1qpza1 d1ydwa2 d2nvwa2 d1h6da2 d1zh8a2 d1xeaa2
d1tlta2 d1r0ka3 d1j5pa3 d1vkoa2 d1vm6a2 d3qvsa2 d1p1ja2 d2ovga_
d1sq8a_ d1rzsa_ d3kz3a_ d3orca_ d3gpdg2 d2q49a2 d1hdgo2 d2g17a2
d2cvoa2 d2czca1 d3pwka2 d1obfo2 d1i32a2 d1zyao2 d2pkqo2 d2hjsa2
d1cf2o2 d1vkna2 d1nvmb2 d2msya_ d2djoa1 d2r5za_ d1au7a1 d1f43a_
d2lmda1 d1akhb_ d1s7ea1 d3nara_ d1zq3p1 d2craa1 d1mija_ d4uuta_
d3a01a_ d1x58a1 d3rkqa1 d3cmya1 d2cqxa1 d2da6a1 d1e3oc1 d1fjla_
d1mh3a1 d1ig7a_ d1x41a1 d1wh7a1 d1gt0c1 d2mw8a_ d1lfup_ d1x2ma1
d1b72a_ d1wi3a1 d1jgga_ d1wjha1 d1x2na1 d1mnmc_ d2cuea1 d3k2aa_
d2hosa_ d2jwta2 d1pufa_ d1octc1 d2hi3a_ d1b8ia_ d4j19a_ d3lnqa_
d1wh5a1 d1b8ib_ d1fexa_ d2cqra1 d1guua_ d1ug2a1 d2cqqa1 d2ajea1
d4a69c_ d1h8ac1 d2crga1 d2iw5b1 d1xc5a1 d1ofcx1 d2cu7a1 d1wgxa1
d2cjja1 d2ckxa1 d1gvda_ d1hcra_ d1tc3c_ d1u78a2 d2iu5a1 d1zk8a1
d2g7sa1 d2fd5a1 d2np5a1 d1z0xa1 d2id6a1 d2d6ya1 d2i10a1 d2oi8a1
d3c07a1 d2ns7a1 d2fq4a1 d2yvea1 d1t33a1 d2o7ta1 d1rkta1 d2np3b1
d2g7ga1 d2gena1 d2fbqa1 d2g7la1 d2gfna1 d2g3ba1 d2jj7a1 d2hyja1
d1sgma1 d1ui5a1 d2hkua1 d2id3a1 d4jyka1 d3lv8a_ d1s3ga1 d1zaka1
d1s4qa_ d3tvta2 d1uf9a_ d1khta_ d1ckea_ d1osna_ d1p5zb_ d4tmka_
d3cm0a_ d1tmka_ d1jaga_ d3h86a_ d1t0hb_ d1nn5a_ d2h92a_ d3kb2a1
d2c95a1 d1yj5a2 d1kgda_ d1ly1a_ d2ak3a1 d1vyva2 d1deka_ d2grja1
d1p3ja1 d1y63a_ d1e4ya1 d1s96a_ d3iija1 d1lvga_ d1nksa_ d1vyua2
d4e22a_ d1qf9a_ d1jjva_ d1q3ta_ d1ki9a_ d1lw7a2 d1teva_ d1kjwa2
d1gsia_ d1m7ga_ d1x6va3 d1z0ja1 d3umra2 d3gj0a1 d2gaoa1 d1yzga1
d1x3sa1 d1g7sa4 d1puja_ d1udxa2 d1kk1a3 d2o52a_ d1jwyb_ d1zcaa2
d1zcba2 d2fv8a1 d2cjwa_ d3dz8a1 d1xzpa2 d1vg8a_ d4qxaa_ d2ew1a1
d1x1ra1 d2bcgy_ d3doea1 d3cona_ d3rega_ d3wyga_ d1upta_ d2b6ha1
d1zd9a1 d1lnza2 d1r5ba3 d1n0ua2 d2f9la1 d2rhda_ d3q3jb_ d3q85a_
d1efma1 d1r8qa_ d2gf9a1 d2akab1 d1ek0a_ d1z08a1 d2gcoa1 d2dy1a2
d4g01b_ d2cxxa1 d1svia_ d3q72a_ d1ni3a1 d4ac9a4 d2erxa1 d2bmea_
d1wxqa1 d2gf0a1 d4ldja1 d1zunb3 d1d5ca_ d4lv5b_ d1efca3 d1kmqa_
d2g6ba1 d3lrpa_ d1tada2 d2y8ea_ d4rd4a1 d1z06a1 d2heia_ d3a58b_
d1u0la2 d1d2ea3 d1zj6a1 d2ocba_ d3rwoa_ d1mr3f_ d2e9sa1 d4u5xa1
d2iwra_ d2fn4a1 d3bwdd_ d2nzja1 d2atva1 d2ksqa_ d3l43a_ d1mkya2
d1mkya1 d1wmsa_ d1jnya3 d2rmka_ d1f6ba_ d1f60a3 d1gota2 d1jala1
d2fh5b1 d1g16a_ d2wblc_ d1m7ba_ d1wf3a1 d1f5na2 d1t9ha2 d1z6xa1
d1z0aa1 d2fg5a1 d2a5ja1 d2bcjq2 d1s0ua3 d1ky3a_ d4y0va_ d4lhwa_
d2qmea1 d2atxa1 d1egaa1 d1ryha_ d1moza_ d1nrjb_ d2erya1 d3bfka_
d1puia_ d1fzqa_ d3j3az1 d3t5ga_ d1shza2 d3defa_ d2f7sa1 d3raba_
d3tsoa_ d3aq4a_ d2al7a1 d3sfva1 d3bc1a_ d1sdma_ d3h4sa1 d2heha_
d2grya_ g3kin.1 d1kk8a2 d1br2a2 d1goja_ d1f9va_ d2mysa2 d1lkxa_
d3t0qa_ d4db1a1 d3gbja_ d1ry6a_ d1v8ka_ d1k8ka2 d1k8ka1 d3uleb1
d3i33a2 d3i33a1 d1bupa1 d1dkgd2 d1dkgd1 d4ci6a2 d1jcfa1 d4a2ba2
d2fxua1 d1yaga2 d1yaga1 d4cbua2 d1bupa2 d1g99a1 d2e1za2 d2e1za1

d1saza1 d1saza2 d1iqca2 d1eb7a2 d1eb7a1 d1nmla2 d1nmla1 d1nira1
d1qksa1 d2d0oa3 d2d0oa2 d1nbwa2 d1hroa_ d4gedb_ d3zooa_ d1mz4a_
d1gu2a_ d1f1fa_ d1dvva_ d1c6ra_ d1c75a_ d1e29a_ d1gdva_ d1cnoa_
d1h32b_ d1co6a_ d2bh4x_ d2aiua_ d5cytr_ d1jdla_ d1i8oa_ d2zboa_
d3dmia_ d1ls9a_ d1wvec_ d2yk3a_ d3ph2b_ d1dw0a_ d5aura_ d1ql3a_
d1qn2a_ d1f1ca_ d4zzva_ d2xspa_ d1eg1a_ d4v20a_ d1ojja_ d4ipma1
d1gpia_ d4csia_ d3pfza_ d3ovwa_ d2yoka_ d2nqya_ d1h4ga_ d1hixa_
d1oa2a_ d1olra_ d3b7ma_ d4h7ma1 d3wp3a_ d2vgda_ d1igoa_ d3zsea_
d2nlra_ d3wx5a_ d1oa3a_ d1pvxa_ d1m4wa_ d1f5ja_ d1oa4a_ d1ks5a_
d4ixla1 d2ah2a1 d1h32a1 d1fwxa1 d1ibya_ d1evsa_ d1pvhb_ d1z7ca_
d1huwa_ d1bgea_ d1rhga_ d1f6fa_ d1f45b_ d1i1rb_ d1axia_ d1etea_
d3tgxb_ d1scfa_ d2z3qa1 d3qb1a_ d1hula_ d1m48a_ d4nkqb_ d1eera_
d1v7mv_ d5e4ea_ d2o27a_ d3b5ka_ d2psma1 d4zf7a_ d3uf5a_ d4x51a_
d1d9ca_ d4ypgc1 d3oq3a_ d1lqsl_ d1n1fa_ d1au1a_ d1wu3i_ d3besl_
d3ux9a_ d1v8fa_ d2ejca_ d3muea1 d3q12a1 d4dnxa2 d1m8pa2 d1g8fa2
d1jhda2 d1x6va2 d3rbaa_ d2qtra_ d1nupa_ d3l92a_ d1f9aa_ d1qjca_
d2h29a_ d1vlha_ d1od6a_ d1lw7a1 d4ruka_ d2el7a_ d1iq0a2 d1irxa2
d1r6ta2 d1ivsa4 d2cyba_ d1rqga2 d1j09a2 d2yxna_ d1f7ua2 d1h3fa1
d1ffya3 d2g36a1 d1nzja_ d1jila_ d1n3la_ d1i6la_ d1pfva2 d1h3na3
d3n9ia_ d3sz3a1 d3sw2a_ d1moxc_ d1edmb_ d1autl2 d1autl1 d1nqlb_
d1dval1 d1szba2 d1kigl_ d2y5fl_ d1ijqa2 d1gk5a_ d1g1sa2 d1nt0a3
d1xdtr_ d1lmja2 d1lmja1 d1nzia2 d2wphe_ d1klil_ d1pfxl2 d1pfxl1
d1p9ja_ d1g1ta2 d1f7ea_ d4yzub_ d1m1xb4 d2y5ia_ d1qlsa_ d1psra_
d3n22a_ d2egda_ d4ggfc_ d2wcea_ d2luca_ d4aqia_ d3nsia_ d2lp3a_
d1j55a_ d1a4pa_ d1nsha_ d1exra_ d1tiza1 d1nyaa_ d1g8ia_ d1ggwa_
d1wdcc_ d3fwba_ d1ggza_ d2o5ga_ d1ctaa_ d1m45a_ d3i5gc_ d1sl8a_
d1fpwa_ d2scpa_ d2roaa_ d4aqra_ d3pn7b_ d1br1b_ d2mysb_ d1s6ca_
d1bjfa_ d2obha_ d1jfka_ d1jf0a_ d1qv0a_ d1lkja_ d2mlfc_ d2m97a_
d2d8na_ d4by5a_ d1jbaa_ d4nqga_ d1wdcb_ d2mysc_ d1j7qa_ d1s6ia_
d1uhka2 d1r2ua_ d1dtla_ d3kf9a_ d3pn7c_ d1qjta_ d1fi6a_ d1c07a_
d5d67d_ d1tuza_ d1eg3a1 d1eg3a2 d2zkmx1 d1snla_ d1sfsa_ d1wkya2
d1uuqa_ d3wh9a_ d4m1ra_ d4xuya_ d2je8a5 d4bf7a1 d3cuia_ d1ur4a_
d1qw9a2 d1odza_ d2vzsa5 d1bqca_ d2x2ya1 d1hjqa_ d1qnra_ d4y7ea1
d1vjza_ d4k68a_ d2v3ga_ d4hpga_ d1nofa2 d1nq6a_ d1h4pa_ d1aq0a_
d3ziza_ d4bs0a_ d1ecea_ d1h1na_ d1ta3b_ d1w91a2 d1i1wa_ d1uhva2
d1us3a2 d1egza_ d7a3ha_ d1tvna1 d3nyda1 d1ghsa_ d2cyga1 d4xv0a_
d2c8na2 d1w32a_ d3emca_ d1v6ya1 d1foba_ d2uwfa1 d2fgla_ d3niya1
d2c0ha1 d1rh9a1 d1yq2a5 d1szna2 d1fa2a_ d1pama4 d5do8a1 d1ea9c3
d1hx0a2 d2fhfa5 d1qhoa4 d1avaa2 d1m53a2 d4m8ua1 d2gjpa2 d1lwha2
d4howa1 d1ktba2 d1j0ha3 d3vm5a1 d2x1ia_ d1wzaa2 d1uasa2 d2diea1
d1mxga2 d1ht6a2 d1eswa_ d1iv8a2 d5cpsa_ d1ud2a2 d1zjaa1 d3gbea1
d1kwga2 d1wdpa_ d1gcya2 d1g5aa2 d1hvxa2 d3dhpa2 d2bhua3 d4do4a1
d3qgva1 d1e43a2 d1g94a2 d2d3na2 d1gjwa2 d1gvia3 d1x1na1 d1r7aa2
d1tz7a1 d2x42a1 d1tr9a1 d3abza1 d4toqa_ d1wb0a1 d1w9pa1 d1eoka_

d1itxa1 d1kfwa1 d1goia2 d1edqa2 d2gsja_ d1hjxa1 d1jnda1 d1vf8a1
d3wdpp1 d3wq5a_ d1gnxa_ d4gxpa_ d4hz8a_ d3aiua_ d1wcga1 d1qvba_
d3ahxa_ d1v02a_ d2dgaa_ d1pbga_ d1vffa1 d1qoxa_ d4ha4a_ d1mixa2
d1e5wa2 d1isna2 d2al6b2 d1g4ma2 d1id1a_ d2hmta_ d1lssa_ d2v76a_
d1wj1a1 d1aqca_ d1qqga2 d1qqga1 d1n3ha_ d4dbba_ d3f0wa1 d1p3ra_
d1wgua1 d2cy4a1 d3hqca_ d1wvha1 d1xr0b1 d1wgqa1 d1v89a1 d2coda1
d1zc3b_ d1wg7a1 d1u5ea1 d1v61a1 d2coca1 d2ys1a1 d1v5pa1 d1ntya2
d1wjma1 d1wi1a1 d1u29a_ d1xx0a1 d1unqa_ d2zkmx3 d1fhoa_ d1v88a1
d2adza1 d1x1fa1 d1p6sa_ d2coaa1 d1btka_ d1v5ma1 d1faoa_ d2x18a_
d1dyna_ d1x1ga1 d4kaxb_ d2j59m1 d1ki1b2 d1xcga2 d1txda2 d1u5da1
d1dbha2 d1w1ha_ d1upqa_ d1v5ua1 d1foea2 d2fjla1 d1eaza_ d2dfka2
d2cofa1 d1kz7a2 d1g8fa3 d9ldta1 d2ewda3 d1up7a1 d2dfda1 d1s6ya1
d1vjta1 d7mdha1 d1hyea1 d1t2da1 d1ojsa3 d1b8pa1 d1ldna1 d1hyha1
d1civa1 d2a92a1 d1ez4a1 d1i0za1 d1y6ja1 d1gv0a1 d1ur5a1 d1llda1
d1obba1 d1v8ba1 d1gdha1 d1l7da1 d2dlda1 d1mx3a1 d1ygya1 d1qp8a1
d1li4a1 d1j4aa1 d2naca1 d1npya1 d3t4ea2 d1a4ia1 d1o0sa1 d2hk9a2
d2egga2 d1b26a1 d1bvua1 d1gq2a1 d1c1da1 d1leha1 d1gtma1 d1nvta1
d3dona2 d1edza1 d1gpja2 d1vi2a1 d1p77a1 d1nyta1 d1b0aa1 d1vl6a1
d1v9la1 d1kola2 d1f8fa2 d1qora2 d1vj0a2 d1cdoa2 d1gu7a2 d1d1ta2
d1xa0a2 d2dm6a2 d1jvba2 d1rjwa2 d1llua2 d1h2ba2 d1tt7a2 d1piwa2
d1o89a2 d1iz0a2 d1e3ja2 d1yb5a2 d1uufa2 d1vj1a2 d1e3ia2 d3gpdg1
d1vc2a1 d2nvwa1 d1u8fo1 d1ydwa1 d1hdgo1 d2g17a1 d1cf2o1 d2czca2
d1r0ka2 d1obfo1 d2cvoa1 d1ebfa1 d1h6da1 d1i32a1 d1zh8a1 d2q49a1
d1qkia1 d1j5pa4 d3pzra1 d1xeaa1 d1vkoa1 d1vm6a3 d3qvsa1 d2pkqo1
d1b7go1 d1gr0a1 d1p1ja1 d1lc0a1 d1j0xo1 d3pwka1 d2hjsa1 d1zyao1
d1vkna1 d1tlta1 d1nvmb1 d3sjua_ d3ojfa1 d1xu9a1 d2jjya1 d1n7ha_
d1y1pa1 d1h5qa_ d1jtva_ d4fs3a_ d2bkaa1 d1gy8a_ d2c5aa1 d1ulsa_
d1ooea_ d2b69a1 d1y5ma1 d1spxa_ d1ae1a_ d1bxka_ d1ydea1 d1gega_
d1hdoa_ d1o5ia_ d1e6ua_ d1snya1 d2rhca_ d3zv4a_ d5ceja_ d2d1ya1
d3w8ea_ d2fmua1 d1x1ta_ d1rpna_ d3ay7a1 d1k2wa_ d1eq2a_ d2hrba1
d3ruaa_ d2ew8a1 d4hwka_ d1xgka_ d2bd0a1 d1yo6a1 d2a35a1 d2ntva_
d1vl8a_ d1hdca_ d4bkua_ d2ag5a1 d2q45a_ d1nffa_ d4iy1a_ d1zbqa1
d1kewa_ d1ek6a_ d1ja9a_ d2q46a2 d1iy8a_ d1cyda_ d2ptga_ d1hxha_
d1uzma1 d3lyla_ d4m89a_ d3wyea_ d2blla1 d1w6ua_ d1sb8a_ d2q2va_
d4nr0a_ d1e6wa_ d2gasa_ d3oiga1 d2bgka1 d1mxha_ d1vl0a1 d1rkxa_
d1r6da_ d1wmaa1 d1e7wa_ d2c07a1 d1xkqa_ d3tzha_ d1g0oa_ d3lz6a_
d1qyca_ d1edoa_ d1gz6a_ d1oc2a_ d1t2aa_ d1zema1 d2xoxa_ d1z45a2
d1geea_ d1p33a_ d3lt0a_ d4e6pa_ d2wyua_ d1db3a_ d4q9na_ d1xhla_
d4o1ma_ d1fmca_ d2uvda_ d2gdza1 d1uaya_ d2z1ma_ d2o23a2 d2fr1a2
d2fr1a1 d1qsga_ d1qyda_ d4zjua_ d2a4ka1 d1yb1a_ d1wvga1 d1yxma1
d3f4ba_ d1mg5a_ d1xg5a1 d3nqyb_ d4gera_ d1bqba_ d4c5pa_ d2bsza1
d4bgfa_ d1w4ta1 d1mdwa_ d1qxpa4 d1ziva1 d1zcma1 d3ioqa_ d1fh0a_
d1o0ea_ d1m6da_ d2cb5a_ d1cs8a_ g8pch.1 d1s4va_ d2pnsa_ d1vsna1
d1mira_ d1iwda_ d2oula_ d7pcka_ d3bwka_ d3bcna_ d2p86a_ d1pvja_

| | | | | | | | |
|---|---|---|---|---|---|---|---|
| d1pcia_ | d1gece_ | d1deua_ | d4bs6a_ | d1xkga1 | d1cqda_ | d1jqpa2 | d2c0ya_ |
| d2bdza_ | d3p5ua_ | d2o36a_ | d2jk6a2 | d2jk6a1 | d1feca1 | d1feca2 | d1lpfa1 |
| d1lpfa2 | d1xhca1 | d1xhca2 | d1dxla2 | d1dxla1 | d1m6ia2 | d1onfa1 | d1onfa2 |
| d3lada2 | d3lada1 | d1w4xa1 | d1w4xa2 | d1gesa2 | d1mo9a1 | d1xdia1 | d1fl2a2 |
| d1fl2a1 | d1fcda1 | d1fcda2 | d1h6va2 | d1h6va1 | d1mo9a2 | d1gv4a2 | d1gv4a1 |
| d2gv8a2 | d2gv8a1 | d1aoga1 | d1aoga2 | d1hyua1 | d1ebda1 | d1ebda2 | d3dk9a1 |
| d1q1ra2 | d1q1ra1 | d1gesa1 | d1pn0a1 | d1reoa1 | d1ju2a1 | d2f5va1 | d3c96a1 |
| d2gmha1 | d1gpea1 | d1o5wa1 | d1n4wa1 | d1k0ia1 | d2dw4a2 | d2ivda1 | d1seza1 |
| d2voua1 | d1kdga1 | d2bcgg1 | d2bcgg2 | d1d5ta1 | d2y8ga_ | d1xkea1 | d1k5db_ |
| d2y8fa_ | d1b9ra_ | d1offa_ | d1frra_ | d3wcqa_ | d1e9ma_ | d1l5pa_ | d1wria_ |
| d1i7ha_ | d3av8a_ | d1czpa_ | d1rfka_ | d3p1ma1 | d3b2ga_ | d2bt6a2 | d1jmxa4 |
| d1pbya3 | d1pbya4 | d1jmxa2 | d1jmxa1 | d1pbya1 | d1r7ha_ | d1faaa_ | d1wika1 |
| d1xfla_ | d1ttza1 | d2f51a1 | d1ti3a_ | d1nhoa_ | d1sena_ | d2i4aa_ | d1ep7a_ |
| d1zmaa1 | d1fo5a_ | d3lefa1 | d3diea1 | d2yana1 | d1z6na1 | d1oaza_ | d3zywa1 |
| d4aj8a_ | d1nm3a1 | d4fiwa_ | d1wmja_ | d4hs1a1 | d1iloa_ | d1xwaa1 | d1wjka1 |
| d2vm1a_ | d1fb6a_ | d1gh2a_ | d1h75a_ | d1nw2a_ | d1kx5b_ | d3a6na_ | d4m6ba2 |
| d2nqbd_ | d3wkjd_ | d2yfwe_ | d4h9na_ | d5bnxa_ | d1kx5c_ | d3r45a_ | d1eqzb_ |
| d4jjnb_ | d1f66c_ | d1eqza_ | d4wnnb_ | d1kx5a_ | d1u35c1 | d1id3a_ | d2nqbc_ |
| d1kx5d_ | d3us3a3 | d3us3a1 | d1tafa_ | d1bh9b_ | d1tafb_ | d1jfib_ | d1q9ca_ |
| d1h3ob_ | d2byka1 | d1n1ja_ | d4g92b_ | d1bh9a_ | d1jfia_ | d1h3oa_ | d2bykb1 |
| d1pd212 | d1nhya2 | d2a2ra2 | d1k0dd2 | d1f2ea2 | d1gsua2 | d1f3ba2 | d2fhea2 |
| d2pvqa1 | d1tw9a2 | d1oyja2 | d1e6ba2 | d3ik7a1 | d4is0a1 | d1r5aa2 | d1jlva2 |
| d1m0ua2 | d3gtub2 | d1z9ha2 | d1okta2 | d1axda2 | d2gsra2 | d1tu7a2 | d1k3ya2 |
| d1zgma1 | d1b48a2 | d1gnwa2 | d1gwca2 | d2gsta2 | d1glqa2 | d1oe8a2 | d1pn9a2 |
| d1v2aa2 | d1fw1a2 | d1duga2 | d3vrda1 | d3vrda2 | d1fcdc2 | d1fcdc1 | d1h1oa2 |
| d1h1oa1 | d1m70a2 | d1ovna2 | d1a0rp_ | d1ig8a1 | d1czan2 | d1czan3 | d1czan1 |
| d1ig8a2 | d1czan4 | d1bg3a4 | d1bg3a3 | d1bg3a1 | d1qqya_ | d1gd6a_ | d1ivma_ |
| d2gv0a_ | d1iiza_ | d2z2fa_ | d1j8xa_ | d1ghla_ | d1b9oa_ | d174la_ | d169la_ |
| d2o4wa_ | d1p37a_ | d1k28a3 | d1xjta1 | d1jtma_ | d2f2qa_ | d4e97a1 | d2za0a_ |
| d2c21a1 | d1f9za_ | d1s5ja2 | d1qhta2 | d4dlea2 | d1tgoa2 | d3py8a2 | d1q8ia2 |
| d2py5a2 | d1d5aa2 | d1qsta_ | d2beia1 | d4m3sa_ | d1u6ma1 | d1ygha_ | d1yk3a1 |
| d3dr6a_ | d1xmta_ | d1z4ea1 | d2ge3a1 | d1bo4a_ | d2fiaa1 | d2jdca1 | d1y9wa1 |
| d3to7a_ | d1wwza1 | d2fl4a1 | d2ozua1 | d2b5ga_ | d2cy2a1 | d2p0wa_ | d1cm0a_ |
| d4pswa_ | d1yrea1 | d1yr0a1 | d1m4ia_ | d2gana1 | d1yvka1 | d2g3aa1 | d1y7ra1 |
| d2euia1 | d1q2ya_ | d2fsra1 | d1xeba_ | d2ae6a1 | d1cjwa_ | d1qsma_ | d2aj6a1 |
| d1y9ka1 | d1i12a_ | d1vkca_ | d2atra1 | d1n71a_ | d2d4pa1 | d2fiwa1 | d2fe7a1 |
| d1tiqa1 | d2ou2a_ | d1yx0a1 | d2fcka1 | d1p0ha_ | d2j8ma_ | d2pq8a_ | d1z4ra1 |
| d1s3za1 | d1ufha_ | d1ax4a_ | d1c7ga_ | d2v1pa1 | d2ez1a_ | d1xvqa1 | d1pn0a2 |
| d3hy2a_ | d2cvba1 | d1o8xa_ | d1lu4a_ | d1we0a1 | d2bmxa1 | d2a4va1 | d1psqa_ |
| d1xiya1 | d1q98a_ | d1n8ja_ | d1zofa1 | d2ggta_ | d3tjja_ | d4je1a1 | d3s9fa_ |
| d2zcta_ | d1qmva_ | d1jfua_ | d2b1ka_ | d5dvba1 | d2b5xa1 | d2v2ga_ | d1st9a_ |
| d3tb2a_ | d4llra_ | d4fh8a1 | d1e2ya_ | d1knga_ | d2h30a_ | d1i5ga_ | d2z9sa_ |
| d2f8aa1 | d1tp9a1 | d2b7ka_ | d1nm3a2 | d3hvva_ | d2wfca1 | d4kcea_ | d1xvwa1 |
| d4gqca1 | d3qpma_ | d4k1fa_ | d1zzoa1 | d1gp1a_ | d1prxa_ | d1rmka_ | d1ixta_ |

d1eyoa_ d1cnna_ d1fu3a_ d1g1pa_ d1f3ka_ d3kasa1 d3bi1a3 d1e88a1
d1l6ja4 d1eaka4 d1eaka3 d1l6ja3 d1h8pa2 d1h8pa1 d1etha2 d1w52x1
d2ppla1 d1hpla2 d2oxea1 d1bu8a2 d1rp1a2 d1ra6a_ d1muka_ d3gsza_
d1khva_ d1te9a_ d2xwha_ d3uqsa_ d3hkwa_ d3n6ma_ d2cjqa_ d2xyma_
d1teba_ d1te8a_ d1u09a1 d4wfxa_ d3tyqa_ d2pgga1 d1c4xa_ d1j1ia_
d2rhwa_ d3groa_ d1pjaa_ d1thta_ d1svpa_ d1mbma_ d3su6a1 d1cu1a1
d4agka_ d1vcpa_ d1q31a_ g1n1l.1 d3e90b_ d3u1jb_ d3zxha_ d1cgla_
d1rm8a_ d1eaka2 d1hy7a_ d2jsda_ d1cxva_ d1hv5a_ d1i76a_ d4qmea2
d2xdta2 d1z5ha2 d4dv8a2 d1j7na1 d1jx4a2 d3gv5b1 d1t94a2 d3bq0a2
d1cixa_ d1y29a_ d1v7fa_ d2jtba_ d1d1ha_ d1g9pa_ d1lupa_ d1qk7a_
d1mb6a_ d2a2va_ d1niya_ d1i25a_ d1hvwa_ d2mpqa_ d1ie6a_ d1kqha_
d1la4a_ d2mxoa1 d1dl0a_ d1koza_ d1qk6a_ d1emxa_ d1lqra_ d1c6wa_
g1apy.1 d3ljqa_ g1k2x.1 d1rypd_ d1irua_ d1rypc_ d3unfk_ d1rypa_
d1j2pa_ d1irui_ d1j2qh_ d3unfh_ d1m4ya_ d1rypb_ d1irul_ d1irug_
d1iru2_ d1q5rh_ d1rypg_ d1g3ka_ d1iruf_ d1q5qa_ d1e94a_ d1irub_
d1ryph_ d3ty6a_ d1iruj_ d1iruk_ d1rype_ d1irud_ d1irue_ d3mi01_
d1rypf_ d1iruh_ d2z3ba_ d1iruc_ d3mka2_ d1iru1_ d1jgta2 d1ofda3
d1ea0a3 d1ecfa2 d1q15a2 d1ct9a2 d1te5a_ d1y14b1 d2nn6i1 d1i94l_
d2zjra2 d1smxa_ d2qamc2 d1hh2p1 d2r7da1 d1rl2a2 d1wfqa1 d3bzka4
d1x6oa2 d3a0ja_ d2j01d2 d2ja9a1 d4ql5a_ d1hr0w_ d1luza_ d2n3sa_
d2vnud1 d1h95a_ d1go3e1 d2nn6h1 d1ne3a_ d2mo1a_ d1iz6a2 d1jjga_
d1e3pa2 d2ix0a3 d2ix0a2 d2lxja_ d1ueba3 d1ueba2 d2z0sa1 d1u0la1
d2ix0a1 d2eifa2 d2je6i1 d3i2za1 d2c35b1 d2ahob2 d1t9ha1 d3i4oa_
d2qall1 d1jt8a_ d2nn6g1 d1g6pa_ d3cama_ d1i94q_ d1d7qa_ d2ba0a1
d2vnud2 d2vnud3 d1khia2 d2qalq1 d1wi5a1 d1xtda2 d1ny4a_ d3ia2a_
d1a88a_ d4dgqa1 d1zoia_ d2flia1 d1h1ya_ d1tqja_ d1tqxa_ d4lt9a1
d3tc7a1 d3hoja_ d3vnda_ d3nl8a1 d1geqa_ d3nava1 d1v5xa_ d4a2ra1
d3tc6a1 d1ujpa_ d4ijba_ d1xcfa_ d1vc4a_ d1rd5a_ d1qopa_ d3b5va_
d2r80b_ d1cqxa1 d1cg5a_ d1gcvb_ d3boma_ d2h8fb_ d2raoa_ d3bj1a_
d4hrrb_ d2r50a_ d3d1kb_ d2aa1b1 d3zhwa_ d3hf4b_ d3gdja_ d1jebb_
d3bj1b_ d1x9fb_ d1wmub_ d1gcva_ d3bcqb_ d3d4xb_ d2dc3a_ d3hf4a_
d4yu3a_ d3k8ba_ d1qpwb_ d2r80a_ d3bcqa_ d1v4wa_ d3wtgb_ d1jl7a_
d4esaa_ d1a9we_ d3d1ab_ d1fsla_ d4h2la_ d1v4wb_ d1mwca_ d1vhba_
d1jeba_ d1spgb_ d1h97a_ d1q1fa_ d1tu9a1 d3d1aa_ d1hdsb_ d3hyub_
d2zfbb_ d3fs4a_ d1oj6a_ d1shrb_ d4h2lb_ d4yu3b_ d1spga_ d3hrwa_
d3qqra_ d3at5a_ d4wchd_ d3hyua_ d1cg5b_ d1uc3a_ d1hbra_ d2raob_
d1outb_ d4esab_ d3gdjb_ d3qqqa_ d1x9fc_ d3vrfa_ d1itha_ d3mkba_
d3d4xa_ d3mjpa_ d1jl6a_ d3fh9a_ d1outa_ d2dc3b_ d1it2a_ d3eoka_
d3g46a_ d1qpwa_ d3vrfb_ d1ch4a_ d1x9fd_ d1hdsa_ d2zfba_ d3mkbb_
d1or4a_ d3wtga_ d1a4fa_ d3fh9b_ d3bomb_ d3cy5a_ d3at5b_ d1x9fa_
d2oifa_ d4lmxb_ d1cpcb_ d3v57a_ d1kn1a_ d1cpca_ d4f0ta_ d2vjha_
d4po5b_ d1eyxa_ d1alla_ d4f0tb_ d1b33b_ d1b8db_ d4h0mb_ d1b33a_
d2vjhb_ d4lm6b_ d2vmlb_ d1allb_ d2vjta_ d3dbja_ d2vmla_ d2vjtb_
d4f0ub_ d1f99b_ d4rmpa1 d1liaa_ d1eyxb_ d2uulb_ d4lmsb_ d4f0ua_

d2bv8a_ d1f99a_ d2bv8b_ d2c7lb_ d3dbjb_ d4rmpb1 d1b8da_ d4h0ma_
d3v57b_ d1kn1b_ d1liab_ d2uula_ d2j96a_ d1azwa_ d1hlga_ d1r88a_
d1sfra_ d4qeka_ d3c8da2 d2pbla1 d1jkma_ d1jjia_ d1lzla_ d1jjfa_
d4iuaa3 d4bvva_ d5hpga_ d3k65a_ d1ki0a3 d1ki0a2 d1ki0a1 d1a0ha1
d1pmla_ d3sp8a3 d2feba1 d1f74a_ d3l21a_ d2e1da_ d1to3a1 d1w3ia_
d1sfla_ d1n7ka_ d1vcva1 d4imea_ d1wa3a_ d2ocza1 d1f2ja_ d3m16a_
d2yxga_ d3a5fa_ d1fbaa_ d1l6wa_ d1xfba1 d1wx0a1 d1vpxa1 d1ojxa_
d1mxsa_ d2a4aa1 d1xkya1 d3cq0a1 d3s1xa_ d1f05a_ d1fdja_ d3cpra1
d4d2ja_ d1cg2a1 d1yloa2 d1y7ea2 d1y0ya3 d1lfwa1 d1vixa1 d1fnoa4
d1vhoa2 d1ysja1 d1vhea2 d1z2la1 d2fvga2 d1vgya1 d1r3na1 d2grea2
d1xmba1 g1q7l.1 d3rp2a_ g1ucy.1 d1fuja_ d2zchp_ d3w94a_ d1si5h_
d1lo6a_ g2pka.1 d1cgha_ d1kigh_ d1a5ia_ d3s9ba_ d1hj8a_ d1ao5a_
d2anya_ d1q3xa1 d1gvza_ d1azza_ d4gsoa_ d2f91a1 d1iaua_ d1gdna_
d1eufa_ g1fiw.1 d1eq9a_ d4nfea_ d2rdla_ d1spja_ d1elva1 d1a7sa_
d1ekbb_ d1pfxc_ d1yc0a_ d2aiqa_ g1fiz.1 d1fi8a_ d4k60a_ d4dgja_
d1mzaa_ d1op0a_ d3tjva_ d1pytd_ d1sgfg_ g1gg6.1 d2eeka_ g1rtf.1
d1a0ja_ d3g01a_ d1pytc_ d4e7na_ d1ddja_ d1z8ga1 d2hlca_ d1npma_
d1brup_ g1jou.1 d1sgfa_ d1autc_ g2hnt.1 g1kdq.1 d1orfa_ d3s69a_
d4crga_ d1fxya_ d1bqya_ d2ftba_ d1pmpa_ d2rcqa1 d1fdqa_ d4lkpa_
d1sa8a_ d1vyfa_ d1lpja_ d3vg7a1 d4azqa1 d1o8va_ d5bvqa_ d1p6pa_
d2qo4a_ d1opba_ d1yiva1 d1o1va_ d4bvma1 d4tkba_ d4i3ba_ d1bwya_
d1ftpa_ d1ggla_ d4gkca_ d1cbia_ d3p6da1 d1kqwa_ d2a0aa1 d2bk7a_
d3fjua_ d1z5ra1 d1kwma1 d1uwya2 d1jqga1 d2c1ca1 d1dtda_ d1h8la2
d4lu0a_ d3unda1 d3e9aa1 d1vr6a2 d1n8fa_ d2qkfa_ d1d9ea_ d1o60a_
d1e69a_ d1mv5a_ d3g60a4 g1ii8.1 d1ewqa2 d1ji0a_ d1qhla_ d1v43a3
d2wmfa1 d1vpla_ d1g2912 d4a82a2 g1f2t.1 d2d62a1 d3tuic1 d2bbsa1
d3g60a3 d2ixea_ d1g6ha_ d1sgwa1 d2qi9c_ d3rlfa1 d3b60a1 d3d31a2
d2pzea1 g1xew.1 d1jj7a_ d1oxxk2 d2onka1 d1w1wa_ d1iwea_ d1qf5a_
d1vmaa2 d1nija1 d1np6a_ d2p67a1 d3ihla_ d1okkd2 d1ls1a2 d1s1ma2
d1iona_ d2v40a_ d1dj3a_ d3hida_ d2vo1a1 d2g0ta1 d1vcoa2 d1j8mf2
d1g3qa_ d1xjca_ d1p9ba_ d1qzxa3 d1ihua2 d2qm8a_ d1dj2a_ d4ioja_
d1cp2a_ d1yrba2 d1hyqa_ d3gqba2 d1skye3 d1xp8a1 d1tf7a2 d1tf7a1
d1ye8a1 d1skyb3 d2qe7d2 d4blta_ d4huta_ d3ldaa2 d4hyya_ d2i3ba1
d1cr1a_ d3j9tb2 d3ntua2 d1vdza1 d2z43a_ d1e9ra_ d1nlfa_ d3ziaa2
d1n0wa_ d1g6oa_ d3ziad2 d3j9ta2 d1pzna2 d3gqbb2 d2i49a_ d1nnfa_
d1xvxa_ d1ursa_ d1h3da1 d1iuda_ d4m1va1 d1twya_ d3e13x_ d2nxoa1
d4pfua_ d3ttma_ d1elja_ d2qmwa1 d1us5a_ d1y3na1 d1jeta_ d1uqwa_
d4f19a1 d2esna2 d3thia_ d3fasa1 d2uyeb_ d2xwva1 d1r9la_ d2fyia1
d1pb7a_ d2ozza1 d1sw5a_ d1nh8a1 d1vr5a1 d2g29a_ d1xs5a_ d3lswa_
d2z23a_ d3w9va_ d3tcha_ d1q35a_ d1z7me1 d3q27a_ d1p99a_ d1ve4a1
d2i6ea1 d4mcoa1 d4n8ya_ d1eu8a_ d1zbma1 d1hsla_ d1ixca2 d1j1na_
d1xvya_ d1y9ua_ d2z22a1 d1xoca1 d4oera_ d1wdna_ d5f1qa1 d1xt8a1
d1i6aa_ d4gwoa_ d4igta1 d1pc3a_ d4mmpa1 d2czla1 d2onsa2 d4mija1
d4ok5a1 d2bmfa1 d2bmfa2 d1yksa1 d1yksa2 d1jr6a_ d2qeqa1 d2jlqa1

d1a1va2 d1eh3a_ d3skpa_ d1dtza1 d1dtza2 d1ieja_ d1jnfa2 d1ce2a1
d1jnfa1 d1b1xa2 d1b1xa1 d1jw1a1 d1ryoa_ d1h76a1 d1h76a2 d1c4oa1
d2p6ra3 d2p6ra4 d1oywa2 d1oywa3 d1wrba1 d1w36b2 d1w36b1 d1c4oa2
d3bora_ d1t6na_ d2eyqa5 d2eyqa3 d2eyqa2 d2zu6a2 d1veca_ d1uaaa1
d1w36d1 d2g9na1 d1z3ix2 d2rb4a_ d1hv8a1 d1hv8a2 d1t5ia_ d1w36c2
d2j0sa2 d2j0sa1 d1fuka_ d3hjha1 d1z5za1 d1qdea_ d1z63a1 d1s2ma2
d1s2ma1 d1wp9a1 d1wp9a2 d1gm5a3 d1gm5a4 d3dina4 d3dina3 d1q0ua_
d1w36c1 d1z3ix1 d1nkta3 d1uaaa2 d1w36d2 g1qhh.1 d2fwra2 d2fwra1
d3q3ma_ d1za0a1 d1mxra_ d2inca_ d1jk0a_ d2incb_ d1mhyb_ d3hf1b_
d1mtyb_ d1r2fa_ d2uw1a_ d1mhyd_ d3n37a_ d1afra_ d1oquc_ d1syya_
d1mtyd_ d2o1za_ d1uzra_ d1smsa_ d2fkza_ d1zuja1 d1s3qa1 d2fjca1
d1z6om1 d1veia_ d4dyya_ d1jiga_ d3gvya_ d2pyba_ d4cy9a1 d3bvfa1
d1r03a_ d2cf7a_ d1nf4a_ d1zs3a1 d4iwka_ d3ka8a_ d3np0x_ d1n1qa_
d1lb3a_ d4e6ka_ d1z6oa1 d2fzfa1 d4dyua_ d3ge4a_ d1jgca_ d4am4a_
d2z6ma_ d1ji5a_ d1vlga_ d1krqa_ d3qz3a_ d1o9ra_ d2ffxj_ d1euma_
d1yv1a1 d2v2pa_ d1j30a_ d4di0a_ d1dpsa_ d1oewa_ d2qzxa_ d2asia_
d1psoe_ d1miqa_ d1smra_ d2h6ta_ d1pfza_ d1izda_ d3hd8a_ d1j71a_
d1dpja_ d4aa9a_ d1t6ex_ g1b5f.1 g1lya.1 g1htr.1 d1qdma2 d1bxoa_
d1wkra_ d1tzsa_ d1ibqa_ d2qzwa_ d1bdqa_ d3ggta_ d3ixoa_ d1mrxa_
d2haha_ d1tw7a_ d4njsa_ d1sgua_ d2p3da_ d1nsoa_ d3d3ta_ d2rkfa_
d1c6ya_ d2rspa_ d3ogpa_ d3ekwa_ d2o4na_ d2j9ja_ d1baia_ d3ggua_
d3lzua_ d4npua_ d4j55a_ d2r8na_ d2fxda_ d2p3ca_ d4dqea_ d3ttpa_
d3t3ca_ d2p3aa_ d2hc0a_ d2fdda_ d3mwsa_ d1k8rb_ d1wgra1 d1ef5a_
d1wfya1 d1e8ya3 d1wgya1 d2cs4a1 d1wxma1 d2byea1 d3kh0a1 d2byfa1
d1wxaa1 d1e7ua3 d4yl8a1 d2al6a3 d1l7ya_ d1wgda1 d1wgga1 d4q5eb_
d1wjna1 d1se9a_ d3n3kb_ d1wz0a1 d1we6a1 d2m0xa_ d2gbka_ d3rt3b1
d1wh3a1 d1sifa_ d2ekec2 d4ii2b1 d4hk2a1 d1c3ta_ d1m94a_ d1wgha1
d1ttna1 d1wx8a1 d3v6cb1 d2ma9b_ d4i6lb1 d1bt0a_ d2jvca1 d1iyfa_
d2k1fa_ d1wxva1 d1wf9a1 d1t0ya_ d2io0b2 d1x1ma1 d2gbma_ d1zw7a1
d2m17a_ d1ud7a_ d1we7a1 d2lwpa_ d2gbja_ d1wjua1 d1v6ea1 d1v86a1
d4eewa_ d1wiaa1 d4icva_ d4gu2a_ d1wx7a1 d1wy8a1 d4hjka1 d2faza1
d1v5oa1 d2al3a1 d2cr5a1 d1wj4a1 d1s3sg_ d1g66a_ d1qoza_ d5ajha1
d3h33a_ d1rwja_ d19hca_ d1os6a_ d3bxua_ d3h4na_ d1i77a_ d3caoa_
d1j0pa_ d2ctha_ d1ofwa_ d2e84a_ d1hh5a_ d1gyoa_ d3h34a_ d1s5ja1
d2hbja2 d2igia_ d1wlja_ d1qhta1 d4dlea1 d1tgoa1 d2f96a1 d3tr8a1
d3mxma_ d1q8ia1 d1y97a1 d1w0ha_ d1kfsa1 d2py5a1 d1noya_ d1d5aa1
d1yt3a3 d2guia_ d3mwpa1 d4kb1a_ d1wioa4 d1ccza2 d1dr9a2 d1ic1a1
d2d9qb1 d2nxyb2 d1um8a_ d1ixza_ d1qvra2 d1qvra3 d1qzma_ d1sxjc2
d1jr3d2 d2p65a_ d1l8qa2 d3hu3a3 d1sxjd2 d1njfa_ d1in4a2 d1sxja2
d1tuea1 d2ce7a2 d1w5sa2 d1sxjb2 d1d2na_ d1ny5a2 d1ofha_ d2gnoa2
d2a5yb3 d1fnna2 d1u0ja1 d1lv7a_ d1iqpa2 d2fnaa2 d1ixsb2 d4gita_
d1sxje2 d1g41a_ d2lj7a_ d1n4na_ d2m8bb1 d2kyqa_ d3psma_ d1jxca_
d2gl1a_ d1ugla_ d4uj0a_ d2k4ua_ d1hp2a_ d2lu9a_ d1baha_ d2meoa1
d1wm7a_ d1hlya_ d1n8ma_ d1jlza_ d1wpda_ d1c55a_ d1pvza_ d1wt7a_

d1rjia_ d1wz5a_ d1m2sa_ d1pjva_ d1c49a_ d1qkya_ d2ktca1 d1wmta_
d1du9a_ d1j5ja_ d1r1ga_ d1t0za_ d2i61a_ d1t1ta_ d1sn4a_ d1kv0a_
d2lkba_ d1npia_ d1wwna_ d1pe4a_ d2kbka_ d2li7a_ d1djta_ d2kbha_
d1dq7a_ d1sega_ d1omya_ d1zywa1 d1jzaa_ d1bnla_ d1t61c1 d1iyjb4
d1iyjb5 d1iyjb3 d1o7ia_ d4ipca1 d1x3ea_ d4apva_ d1jb7a1 d1jb7a2
d1jb7a3 d3kdfa_ d1ue1b_ d1wjja1 d3eiva_ d1xjva2 d1xjva1 d2ccza2
d1se8a_ d1l1oc_ d1jb7b_ d1qvca_ d3ulla_ d1qzga_ d1miua4 d1miua5
d1miua3 d3g48a1 d1gd7a_ d1mkha_ d1jjcb3 d1pyba_ d3ubub_ d1egia_
d1qo3c_ d1v7pa_ d1tdqb_ d3ubua_ d1c3ab_ d1h8ua_ d3bx4b_ d1t8ca1
d1sb2a1 d1qdda_ d2ziba1 d3cada_ d1byfa_ d1j34b_ d1jwia_ d1jzna_
d1c3aa_ d1oz7b_ d1wmza_ d3bdwb_ d1hq8a_ d1fifa1 d1dv8a_ d3hupa_
d1wk1a1 d1umra_ d2afpa_ d3bx4a_ d1gz2a_ d1sl6a_ d1v7pb_ d3g8ka_
d1ixxa_ d1j34a_ d1jwib_ d1g1sa1 d1y17a1 d1ja3a_ d1fvub_ d3bdwa_
d1sb2b1 d1fvua_ d1umrc_ d1oz7a_ d4m17a_ d3cfwa1 d1ypqa1 d1g1ta1
d3fd4a_ d3l9jc_ d4dn8a1 d1v4lb_ d1rdl1_ d2msba_ d1dgsa2 d1ckma1
d1p16a1 d1x9na2 d1pfsa_ d2a1ka_ d1rpma_ d2shpa1 d1yfoa_ d1l8ka_
d2ooqa1 d1g4us2 d1p15a_ d1lara2 d1lara1 d1wcha_ d2fh7a2 d2fh7a1
d1ohea1 d1ohea2 d2f4da_ d1rxda_ d2hxpa_ d1xria_ d1fpza_ d3ezza_
d4y2ea_ d1i9sa_ d4alac_ d1pjwa_ d1s6na_ d3uzqb_ d3azya_ d3wvja_
d1upsa1 d1ajoa_ d2hyka_ d1ajka_ d2vy0a_ d1o4ya_ d3o5sa_ d3d6ea_
d3atga_ d1dypa_ d1umza_ d3i4ia_ d1c1la_ d4lbqa_ d1a78a_ d1slta_
d4no4a_ d3zsja_ d3duia_ d1is3a_ d1qmja_ d1hlca_ d1tdha3 d1q3ba3
d1ee8a3 d1k82a3 g1qmo.1 g1len.1 d3zyra_ d1fnya_ d1n47a_ g2ltn.1
d1avba_ d3usua_ d1dhkb_ d1f9ka_ d1ukga_ d1g8wa_ d1g9fa_ d1wbfa_
d3wcsa_ d1g7ya_ g1loe.1 d1mvqa_ d1hqla_ d1v6ia_ d1dbna_ d1qnwa_
d1fx5a_ d4l8qa_ d1ioaa_ d3n35a_ d3ubra_ d1qo8a1 d1d4ca1 d2k3va_
d1ft5a_ d1qdba_ d1m1qa_ d2j7aa_ d1h21a_ d1ogyb_ d4fasa_ d1jnia_
d1oaha_ d1sp3a_ d2h0ba_ d2erfa_ d1h30a2 d1h30a1 d3mw4a1 d1pz7a_
d2r1ba_ d3v64a1 d1d2sa_ d3bopa1 d3mw3a1 d1okqa1 d1okqa2 d1dszb_
d1a6ya_ d3cbba_ d1ynwa1 d1r4ia_ d1hcqa_ d2hana_ d2hanb_ d2enva1
d1kb4b_ d2nllb_ d1lo1a_ d1cita_ d1adqa1 d1fnga1 d1uvqb1 d1zs8a1
d2vola1 d2vola2 d1mfbl2 d2jelh2 d1uwel2 d3qq4b1 d1kjvb_ d1k5na1
d1osph2 d4odhl2 d1ow0a1 d1ow0a2 d1bfob2 d3l9ra2 d3jtth1 d2fath2
d4elkb2 d1fn4b2 d2xfxb_ d1pg7x2 d1cqka_ d4k71b2 d1lk2a1 d1eapb2
d4lfhg2 d1i4fa1 d2nw2a2 d1kfah2 d1mhca1 d1h3ua1 d1wejh2 d1h0db2
d1a5fh2 d1kcvh2 d1mjuh2 d1qqda1 d1kcrh2 d1k8ia1 d3huja2 d2nnab1
d1nfdf2 d3mffb2 d1s9va1 d1lk3h2 d1q0xl2 d1fv1b1 d1neza1 d4rfeb2
d1r3ha1 d1a6tb2 d1kjva1 d1pz5b2 d1onqa1 d2bc4a2 d1de4a1 d1k8da1
d1tcra2 d1dn0b2 d1nfde2 d2xwtb2 d1bfoa2 d1mexh2 d1iakb1 d1ypzf2
d4f7ea2 d1q72l2 d2fbjh2 d1fe8h2 d1clzh2 d1fngb1 d1uvqa1 d1vgka1
d1c16a1 d4j12a2 d4x99b2 d1igtb3 d1je6a1 d1q72h2 d1nldh2 d1teth2
d2hmic2 d1i1ca2 d1i1ca1 d1k8ib1 d1q0yh2 d1kfal2 d3frua1 d1pg7w2
d1u58a1 d1igyb4 d1igyb3 d1hyrc1 d1klub1 d1deeb2 d1c12b2 d2bc4b2
d2ig2h2 d1kcvl2 d3wn5b1 d1lk2b_ d1lk3l2 d1mjul2 d1sm3h2 d1q0xh2

d1zvsa1 d1ydpa1 d1u8ib2 d1ypze2 d1op9a_ d1y0la1 d1etzb1 d1egjl1
d2gsib1 d1nj9a1 d4jfhd1 d1aqkl1 d1t2ja_ d1lo4h1 d1zvya1 d3v4vl1
d4enec1 d1kxtb_ d5e8ea1 d2pxya_ d4weud_ d1xiwc_ d2esvd1 d1qnzh_
d1ogae1 d1f4xh1 d4n1hb_ d3ezjb1 d3mlwl1 d1kfah1 d1etza1 d1b4jh1
d3cfic_ d4w68a_ d2arja1 d1ek3a_ d2ok0l1 d1vera_ d1uwgh1 d1blnb1
d1fpth1 d1rzga1 d2giya1 d4m3kb1 d1nc2a1 d2nyyd1 d1ncna_ d1fbih1
d5dxwa_ d4fqqa1 d1dqdh1 d1jfql1 d1kelh1 d1faih1 d1frgl1 d1figl1
d4m6nl1 d1fo0a_ d4hxbl1 d3b5ga_ d3quyd1 d2h9gb1 d1i8kb_ d2dd8h1
d2ck0h1 d1eaja_ d4w6wb1 d2a6ib3 d1t66d1 d2e27h_ d1ob1b1 d1f11b1
d2v7na1 d1ad0a1 d2xt1b1 d1pskh1 d4i13b1 d2op4l1 d1a0ql1 d1uwel1
d1za3a1 d1ncbl1 d4uu9a_ d1bm3h1 d1cloh1 d1nqba2 d1cf8h1 d4y5va_
d1mhph1 d5anma1 d4cdgc1 d4ldeb_ d1y0lb1 d2pcpb1 d1z3gl1 d1dfbl1
d1fh5h1 d3g5xa1 d1tzib1 d4dn3l1 d3zhka1 d2b1hh1 d2uyla1 d1f3dh1
d1nj9b1 d2agjh1 d2h1pl1 d1q72h1 d8fabb1 d1ndmb1 d1rjca1 d4u6hb1
d3upca_ d2fbjl1 d1osph1 d1tvda_ d2cjvh_ d4fhbd1 d1bd2d1 d1rz7h1
d3nn8a_ d1knoa1 d1nfda1 d2bmkb1 d1dqll_ d1igyb1 d1igfl1 d12e8h1
d3utza1 d1nc2b1 d1rihh1 d4mwfb1 d4jvpa1 d1mieh1 d1rhfa1 d1fgnl1
d1sbsh1 d1lp9e1 d1dn0a1 d4lqfl1 d1iaih1 d1kgcd1 d1mh5b1 d1gpol1
d2g60h3 d1f8tl1 d2jixd1 d4grwh_ d1fo0b_ d1bfob1 d1axsa1 d1u9ka_
d1nmbh_ d1h8na1 d1h8na2 d1jguh1 d4eura1 d3sdyl1 d35c8h1 d1w72l1
d1mvub_ d1i9rh1 d1ob1a1 d4wenb_ d1yeeh1 d3ietc1 d1f58h1 d1ad0b1
d1mqkl_ d1nbvh1 d4b50a_ d3r8bb_ d1bafl1 d1mamh1 d4rgne1 d2gsgb_
d35c8l1 d1bafh1 d1ai1h1 d2pcpa1 d2fath1 d2x1pa_ d1orsb1 d1ibgl1
d1shma_ d1a2yb_ d1u0qa_ d2coqa_ d1hilb1 d1qfwi_ d1jv5b_ d1fn4b1
d5e0qa_ d1nsnh1 d1plgl1 d4ocxl1 d1kb5h1 d1rzfh1 d1pg7x1 d2fx7h1
d2oi9c_ d1l7il1 d3eyva1 d1d5il1 d4ffyl_ d1tetl1 d1a6wh_ d1ac6a_
d3dvfa_ d1nfde1 d1mimh1 d1ri8a1 d1jhlh_ d3dura_ d1fskb1 d1f97a1
d3so3b1 d2ak4d1 d1lp9f1 d4z7wf1 d1l7th1 d4y5ya_ d4x7ec1 d1emth1
d1ikfl1 d1nlbh1 d4utal1 d1bjma1 d1eapb1 d1u6ah1 d2dtgd1 d1kj2b_
d1xeda1 d1c1eh1 d1fgvh_ d2bnub1 d2fb4h1 d2dtgb1 d1ktrh_ d1jpth1
d1qkzl1 d4hepg_ d2atpb1 d1flrh1 d2hrpl1 d1mvfa_ d1iaim1 d3k80a1
d2jb5h1 d3zkxc_ d4grwe_ d1it9h1 d1rhhb1 d3w9ec_ d1e4wh1 d1sy6h1
d2j6eh1 d3qxta_ d1jglh1 d1p7ka1 d1tzhb1 d4n8pa1 d1gafl1 d2jelh1
d1wioa2 d4i0cc1 d1cicb1 d1lmka2 d1lmka1 d1iqwh1 d1orqb1 d1ejoh1
d1jnlh1 d4zg1a_ d43c9b_ d1ggbl1 d1wejh1 d3q6fa1 d1f3dj1 d3c6la1
d1bqlh1 d3p9wb_ d1a3rl1 d1h0db1 d2uzil_ d1eo8l1 d1qfwl_ d1n7ml1
d1igja1 d1c5ch1 d2a9mh_ d1rmfh1 d1fj1b1 d4heme_ d1ccza1 d1a5fh1
d1ogad1 d1dlfh_ d1qa9b_ d1vgel1 d3qskb_ d1sjxa_ d4ozfg1 d1sq2n_
d1qd0a_ d1kcvh1 d1c5db1 d3qcul1 d8faba1 d3bn9d1 d1mjuh1 d4y8dc_
d7fabh1 d3dvgb1 d1lo0l1 d1a6tb1 d1gigh1 d1kcrh1 d2hwzl1 d1yjdc1
d3clfl1 d4krna_ d1dl7l_ d1e6ol1 d1mexh1 d3r0ma_ d4kkna_ d1fnsl1
d1oaqh_ d1rzia1 d1ucbh1 d1ncwl1 d4b41a_ d1nfdf1 d2mpah1 d2d7tl1
d4grwf_ d4b5ea_ d1lk3h1 d3u79a_ d4u3xa_ d1ghfl1 d1bvkb_ d1mcww1
d1b88a_ d1jrhh1 d32c2b1 d4wemb_ d1dsfh_ d1op3h1 d1ce1l1 d1iqda1

d1qoka1 d1qoka2 d1qfuh1 d2crya1 d1ay1h1 d1oaql_ d3ab0b_ d1rurl1
d1t4kb1 d1bz7b1 d1n0xh1 d1forl1 d1ucbl1 d4w6yb_ d43c9a_ d2aabl1
d1tzia1 d4n1ca_ d1k6qh1 d3g9ab_ d4tvsa1 d1igmh_ d1pz5b1 d3dx9a1
d3wxwh_ d1nakh1 d1kcul1 d3eaka_ d4qlra1 d1cr9l1 d1d9ka_ d1f4xl1
d1deea1 d2i27n2 d1c12a1 d2orbl1 d1j8hd1 d1mcph1 d1c5da1 d1kenh1
d1bd2e1 d1h5ba_ d4x7fc1 d3upaa_ d1t3fa1 d1indh1 d2dtgc1 d4hc1l1
d1zv5a1 d4wgvb_ d1uwgl1 d4f37k1 d1aqkh1 d1vesa_ d3zkqd_ d1fptl1
d1lo4l1 d1knob1 d3rrqa_ d1ct8b1 d4orzc_ d1mlbb1 d4hgkc1 d1dqdl1
d1bogb1 d4ozhe1 d1dr9a1 d1i7za1 d1dn0b1 d1kell1 d1rzib1 d1ypzf1
d1wtla_ d1hkfa_ d1fail1 d1figh1 d2vyre_ d1sjva_ d1ohqa_ d1bfoa1
d1pkqb1 d1mjul1 d1b4jl1 d1nsnl1 d2arjb1 d1p4bh_ d4aj0a_ d3nn8b_
d2rcja1 d1f2xk_ d4v1dd_ d1adqh1 d2eksa_ d4k3ga_ d1jfqh1 d1uweh1
d1frgh1 d4gftb_ d1uz8b1 d4n1ea_ d1bm3l1 d1clol1 d3mcka1 d2vq1a1
d1ae6h1 d4aq1b1 d1uj3b1 d1nbqa1 d1fh5l1 d2ck0l1 d2ywza_ d1q72l1
d4nbxb_ d1hila1 d4hzlb1 d2j88l1 d1zmya1 d1cd0a_ d1a4ka1 d1pskl1
d1a0qh1 d4krmb_ d1ncbh1 d2fbjh1 d1j8he1 d1i3ua_ d1cf8l1 d1fe8h1
d2p1ya1 d1eeqa_ d2x89a_ d1ol0a_ d2r0lh1 d1ub5a1 d1dfbh1 d3eyvb1
d4jdvb1 d4p2cg_ d2gsia1 d2agjl1 d1u3ha1 d2h1ph1 d1rihl1 d1fskc1
d1boga1 d2a77l1 d1ospl1 d1qfoa_ d3dwta_ d2fx7l1 d1rz7l1 d3stba1
d1pkqa1 d1b6da1 d1dzba2 d1dzba1 d1kj2a_ d4l1ha_ d1rzgb1 d1dqlh_
d12e8l1 d1miel1 d1jmaa_ d1sbsl1 d1fgnh1 d1f58l1 d1mqkh_ d1ndgb1
d3qegl1 d1iail1 d2aw2a1 d1tqbc1 d3fo2a1 d4dkaa_ d1f8th1 d2g60l1
d2ywya_ d1uj3a1 d1yc7a_ d3bd3a_ d4w6xb1 d1nmbl_ d1i8ka_ d1rjlb1
d1ypze1 d1p7kb1 d1kxqe_ d1gpoh1 d2z4qa1 d1w72h1 d1i9rl1 d4qgya1
d4xt1c_ d2qqnh1 d1cica1 d3c08h1 d2cd0a_ d2bdnh1 d1hnga1 d1maml1
d1nldh1 d1cr9h1 d4lcih1 d3bkyh1 d3qg7l1 d1ai1l1 d1kxvc_ d4iosd_
d1l7ih1 d2x1qa_ d1ub6b1 d3k74b_ d1ibgh1 d1teth1 d1igya1 d1hxma1
d2x6ma_ d2e7la_ d5e03a_ d1qfwm_ d2r0kh1 d1igjb1 d1rurh1 d1plgh1
d1mj8h1 d1yuhb1 d3fmgl1 d1rzfl1 d1emtl1 d1ad9b1 d1igtb1 d1nlbl1
d1d5ih1 d3k7ua_ d4ffyh_ d1bwwa_ d3dvga1 d1a6ta1 d1fgvl_ d4qxgl1
d1miml1 d1jhll_ d2hffb1 d2hh0l1 d1qkzh1 d1l7tl1 d1afvh1 d1iaii1
d2cjuh_ d1um5h1 d2ghwb2 d2ghwb1 d1kgce1 d2fgwh1 d1ikfh1 d1dvfd_
d2i24n1 d2xtjb_ d1za3b1 d2yc1a_ d2hh0h1 d2x1oa_ d1bvka_ d1rjla1
d5e7ba1 d1igta1 d1il1b1 d2fb4l1 d1mvua_ d1jptl1 d32c2a1 d1fvca_
d1iqdb1 d2hrph1 d2p4ab_ d4qo1a_ d1p2cb1 d1wt5c_ d1t4ka1 d5c1mb_
d1it9l1 d1bz7a1 d1b2wh1 d1e4wl1 d1jnll1 d1yedb1 d1za6b1 d3u7aa_
d1igfh1 d3gnml1 d1orsa1 d1wejl1 d1a2ya_ d1hxmb1 d1gafh1 d2j4wh1
d1pz5a1 d1j05a_ d1a3rh1 d4fqll1 d1qfwh_ d1eo8h1 d1n7mh1 d4s11a_
d1kegh1 d5da4a_ d1iqwl1 d3whxb1 d1vgeh1 d1deeb1 d1c12b1 d1t2ql1
d1ad9a1 d3gsnb1 d3b2uc1 d1ggbh1 d1t3fb1 d1nakl1 d4ocmc_ d1lila1
d2v7hb3 d2esve1 d1mcoh1 d2qr0b1 d2q3aa_ d1ct8a1 d1kcrl1 d2pxyb_
d2uzih_ d5c3ld_ d3durb_ d1c5cl1 d2ak4e1 d1dl7h_ d1xiwd_ d1mlba1
d1rmfl1 d1i7zb1 d1dlfl_ d2xa3a_ d5bq7b1 d1dqqb1 d3h42l1 d1egjh1
d1kcvl1 d1fe8l1 d2j6el1 d1f3rb2 d1lk3l1 d1sm3h1 d1q0xh1 d1ghfh1

d7fabl1 d1igch1 d1lo0h1 d4idla_ d5c2bl_ d1jrhl1 d1il1a1 d3zhda1
d4kfzc_ d1b0wa_ d1e6oh1 d1mexl1 d4krpb_ d1fnsh1 d1ncwh1 d4hgma_
d4eigb1 d4rzca_ d2mpal1 d4y5xa_ d5en2b1 d3lrga1 d4ej1c1 d1rhha1
d1j05b_ d1forh1 d1q9wb1 d2wzpd_ d2aq2a1 d1h0da1 d3revb1 d2ij0c1
d1tzha1 d4m7jl1 d1k6ql1 d3b9kc1 d1igml_ d3qeua1 d1dsfl_ d1ce1h1
d1kcuh1 d1zvha1 d1zvoa1 d1tqbb1 d1ay1l1 d2qscl1 d2cgrh1 d1nl0h1
d1kenl1 d3eo0a1 d4n9ob1 d1n0xl1 d3k1kc_ d1c4qa_ d2wv6d_ d1prtf_
d1ltrd_ d4l63b1 d1r4pb_ d1prtd_ d1tl0f_ d2bosa_ d1prtb1 d3chbd_
d2cqva1 d1fhga_ d2k1ma_ d2dl2a1 d1biha3 d1biha1 d1biha4 d3qp3a1
d1g1ca_ d1gxea_ d1cvsc2 d1cvsc1 d1xmwa2 d1m4ka2 d2otpa2 d1biha2
d1rhfa2 d2fcba2 d2fcba1 d1wwbx_ d1gsma2 d1epfa1 d1epfa2 d2dava1
d1waaa_ d1f97a2 d1x44a1 d1vcaa2 d1fnla2 d1fnla1 d3b5ha2 d3h8na2
d3h8na1 d1jbja1 d1jbja2 d1pd6a_ d1f2qa2 d1f2qa1 d3cx2a_ d1nbqa2
d3b5ha1 d1n26a1 d1xiwb_ d1xaua_ d1fltx_ d2ifga1 d1olla1 d1olla2
d1cs6a4 d1cs6a3 d1cs6a2 d1cs6a1 d2gw5a2 d1sy6a1 d1xiwa_ d1ucta2
d1ucta1 d1ry7b2 d1gl4b_ d1qz1a3 d1olza1 d2c9aa1 d1wwca_

**Family-24 (b.121.4.7)**

**Positive dataset contains 1 protein sequences.**

d1x36a_

**Negative dataset contains 4032 protein sequences.**

d1m32a_ d1ibja_ d1iuga_ d4q75a_ d2bkwa1 d1t3ia_ d1n8pa_ d1qz9a_
d1jf9a_ d1eg5a_ d1p3wa_ d3kgwa_ d1cl1a_ d2ctza1 d1vjoa_ d2rfva_
d1gc0a_ d1cs1a_ d1d2fa_ d2ch1a1 d1qgna_ d1pffa_ d1elua_ d1u08a_
d1lc5a_ d1b5pa_ d2ay1a_ d1bw0a_ d3pd6a_ d7aata_ d2csta_ d1j32a_
d1fg7a_ d4wlha_ d1o4sa_ d1v2da_ d1gdea_ d1m6sa_ d1v72a1 d1svva_
d1ajsa_ d1wsta1 d2f8ja2 d1lk9a_ d1vp4a1 d1xi9a1 d2gb3a1 d3rcha_
d3k40a_ d1pmma_ d1xkta1 d2hfka_ d5d3ka_ d2cfba1 d1o69a_ d1bj4a_
d3k28a_ d2bwpa_ d3lg0a_ d2fnua_ d3ou5a_ d1z7da1 d1b9ha_ d1iaya_
d4msoa_ d1bs0a_ d1s0aa_ d1sffa_ d3tqxa_ d1mdoa_ d3qboa_ d3pgya_
d1bjna_ d2dkja_ d1ejia_ d1vefa1 d4wxga_ d3bs8a_ d3gbxa1 d1w23a_
d1sefa_ d1sfna_ d1rc6a_ d3lgia_ d3i77a_ d2z9ia2 d4nsva_ d1lcya2
d4jcna_ d1os8a_ d1hpga_ d1p3ca_ d1l1ja_ d1qtfa_ d1f56a_ d1bypa_
d1azca_ d1ws8a_ d3u25a_ d1cuoa_ d1id2a_ d1rkra_ d2iaac_ d4dp1x_
d1nwpa_ d2gima_ d2i7sa_ d1qhqa_ d3ehbb1 d1gyca3 d1hfua2 d1kbva1
d1kbva2 d1sdda1 d1sdda2 d1gyca1 d1gyca2 d1mzya1 d2bw4a1 d1aoza1
d1aoza3 d1aoza2 d1kyaa3 d1kyaa1 d1mzya2 d4e9sa1 d1sddb1 d1sddb2
d1hfua1 d1hfua3 d1oe1a2 d1oe1a1 d1v10a2 d1v10a3 d1v10a1 d2bema_
d1pama1 d1ea9c1 d1qhoa1 d2fhfa2 d2fhfa1 d1k3ia1 d1j0ha1 d1cxla1
d2bhua1 d1edqa1 d1f1sa2 d1ut9a2 d1gvia1 d1txka1 d1adqa1 d1fnga1
d1uvqb1 d1zs8a1 d2vola1 d2vola2 d1mfbl2 d2jelh2 d1uwel2 d3qq4b1
d1kjvb_ d1k5na1 d1osph2 d4odhl2 d1ow0a1 d1ow0a2 d1bfob2 d3l9ra2
d3jtth1 d2fath2 d4elkb2 d1fn4b2 d2xfxb_ d1pg7x2 d1cqka_ d4k71b2
d1lk2a1 d1eapb2 d4lfhg2 d1i4fa1 d2nw2a2 d1kfah2 d1mhca1 d1h3ua1
d1wejh2 d1h0db2 d1a5fh2 d1kcvh2 d1mjuh2 d1qqda1 d1kcrh2 d1k8ia1

d3huja2 d2nnab1 d1nfdf2 d3mffb2 d1s9va1 d1lk3h2 d1q0xl2 d1fv1b1
d1neza1 d4rfeb2 d1r3ha1 d1a6tb2 d1kjva1 d1pz5b2 d1onqa1 d2bc4a2
d1de4a1 d1k8da1 d1tcra2 d1dn0b2 d1nfde2 d2xwtb2 d1bfoa2 d1mexh2
d1iakb1 d1ypzf2 d4f7ea2 d1q72l2 d2fbjh2 d1fe8h2 d1clzh2 d1fngb1
d1uvqa1 d1vgka1 d1c16a1 d4j12a2 d4x99b2 d1igtb3 d1je6a1 d1q72h2
d1nldh2 d1teth2 d2hmic2 d1i1ca2 d1i1ca1 d1k8ib1 d1q0yh2 d1kfal2
d3frua1 d1pg7w2 d1u58a1 d1igyb4 d1igyb3 d1hyrc1 d1klub1 d1deeb2
d1c12b2 d2bc4b2 d2ig2h2 d1kcvl2 d3wn5b1 d1lk2b_ d1lk3l2 d1mjul2
d1sm3h2 d1q0xh2 d1zvsa1 d1ydpa1 d1u8ib2 d1ypze2 d1kcuh2 d2phla2
d2phla1 d1fxza2 d1fxza1 d1uija1 g1dgw.1 d2v09a_ d1uika2 d1uika1
d1dgwa_ d1ep0a_ d2pa7a1 d1wlta1 d1nxma_ d2c0za1 d1ds6b_ d1fstb_
d1tafa_ d1bh9b_ d1tafb_ d1jfib_ d1q9ca_ d1h3ob_ d2byka1 d1n1ja_
d4g92b_ d1jfia_ d1h3oa_ d2bykb1 d1n1jb_ d1h32a1 d2bjca1 d2hsga1
d1qpza1 d1efaa1 d1h4ba_ d2opoa_ d1r0ka3 d1vkoa2 d1vm6a2 d3qvsa2
d1gr0a2 d1p1ja2 d2ovga_ d1sq8a_ d1rzsa_ d3kz3a_ d3orca_ d3gpdg2
d2q49a2 d1hdgo2 d2g17a2 d2cvoa2 d2czca1 d3pwka2 d1obfo2 d1zyao2
d2pkqo2 d1b7go2 d2hjsa2 d1cf2o2 d1vkna2 d1nvmb2 d1ux8a_ d1dlya_
d4i0va_ d2bkma_ d1dlwa_ d2bmma_ d2msya_ d2djoa1 d2r5za_ d1au7a1
d1f43a_ d2lmda1 d1akhb_ d1s7ea1 d3nara_ d1zq3p1 d2craa1 d1mija_
d4uuta_ d3a01a_ d1x58a1 d3rkqa1 d3cmya1 d2cqxa1 d2da6a1 d1e3oc1
d1fjla_ d1mh3a1 d1x41a1 d1wh7a1 d1gt0c1 d2mw8a_ d1lfup_ d1x2ma1
d1b72a_ d1wi3a1 d1jgga_ d1wjha1 d1x2na1 d1mnmc_ d2cuea1 d3k2aa_
d2hosa_ d2jwta2 d1pufa_ d1octc1 d2hi3a_ d1b8ia_ d4j19a_ d3lnqa_
d1wh5a1 d1b8ib_ d1hcra_ d1tc3c_ d1gdta1 d1u78a2 d6paxa1 d1k78a1
d1k78a2 d1bl0a1 d1bl0a2 d1d5ya1 d1prtc2 d1prtb2 d3lv8a_ d1s3ga1
d1s4qa_ d3tvta2 d1uf9a_ d1khta_ d1ckea_ d1osna_ d1p5zb_ d4tmka_
d3cm0a_ d1tmka_ d1jaga_ d3h86a_ d1t0hb_ d1nn5a_ d2h92a_ d3kb2a1
d2c95a1 d1yj5a2 d1kgda_ d1ly1a_ d2ak3a1 d1vyva2 d1deka_ d2grja1
d1p3ja1 d1y63a_ d1e4ya1 d1s96a_ d3iija1 d1lvga_ d1nksa_ d1vyua2
d4e22a_ d1qf9a_ d1jjva_ d1q3ta_ d1ki9a_ d1lw7a2 d1teva_ d1kjwa2
d1gsia_ d1g3ma_ d1t8ta1 d1nsta_ d1fmja_ d1zrha_ d1ls6a_ d2a3ra_
d1aqua_ d2zptx_ d3ckla_ d1texa_ d1q44a_ d2gwha_ d1z0ja1 d3umra2
d3gj0a1 d2gaoa1 d1yzga1 d1x3sa1 d1g7sa4 d1puja_ d1udxa2 d1kk1a3
d2o52a_ d1jwyb_ d1zcaa2 d1zcba2 d2fv8a1 d2cjwa_ d3dz8a1 d1xzpa2
d1vg8a_ d4qxaa_ d2ew1a1 d1x1ra1 d2bcgy_ d3doea1 d3cona_ d3rega_
d3wyga_ d1upta_ d2b6ha1 d1zd9a1 d1lnza2 d1r5ba3 d1n0ua2 d1r2qa_
d2f9la1 d2rhda_ d3q3jb_ d3q85a_ d1efma1 d1r8qa_ d2gf9a1 d2akab1
d1ek0a_ d1z08a1 d2gcoa1 d2dy1a2 d4g01b_ d2cxxa1 d1svia_ d3q72a_
d1ni3a1 d4ac9a4 d2erxa1 d2bmea_ d1wxqa1 d2gf0a1 d4ldja1 d1zunb3
d1d5ca_ d4lv5b_ d1efca3 d1kmqa_ d2g6ba1 d3lrpa_ d1tada2 d2y8ea_
d4rd4a1 d1z06a1 d2heia_ d3a58b_ d1u0la2 d1d2ea3 d1zj6a1 d2ocba_
d3rwoa_ d1mr3f_ d2e9sa1 d4u5xa1 d2iwra_ d2fn4a1 d3bwdd_ d2nzja1
d2atva1 d2ksqa_ d3l43a_ d1mkya2 d1mkya1 d1wmsa_ d1jnya3 d2rmka_
d1f6ba_ d1f60a3 d1gota2 d1jala1 d2fh5b1 d1g16a_ d2wblc_ d1m7ba_

d1wf3a1 d1f5na2 d1t9ha2 d1z6xa1 d1z0aa1 d2fg5a1 d2a5ja1 d2bcjq2
d1s0ua3 d1ky3a_ d4y0va_ d4lhwa_ d2qmea1 d2atxa1 d1egaa1 d1ryha_
d1moza_ d1nrjb_ d2erya1 d3bfka_ d1puia_ d1fzqa_ d3j3az1 d3t5ga_
d1shza2 d3defa_ d2f7sa1 d3raba_ d3tsoa_ d3aq4a_ d2al7a1 d3sfva1
d3bc1a_ d2ncda_ d1sdma_ d3h4sa1 d2heha_ d2grya_ g3kin.1 d1kk8a2
d1br2a2 d1f9va_ d2mysa2 d1lkxa_ d3t0qa_ d4db1a1 d3gbja_ d1ry6a_
d1v8ka_ d1k8ka2 d1k8ka1 d3uleb1 d3i33a2 d3i33a1 d1dkgd2 d1dkgd1
d4ci6a2 d1jcfa1 d4a2ba2 d2fxua1 d1yaga2 d1yaga1 d4cbua2 d1bupa2
d1g99a2 d2e1za2 d2e1za1 d1saza1 d1saza2 d1iqca2 d1iqca1 d1eb7a2
d1eb7a1 d1nmla1 d1nira1 d1qksa1 d2d0oa3 d2d0oa2 d1nbwa3 d1hroa_
d4gedb_ d3zooa_ d1mz4a_ d1gu2a_ d1f1fa_ d1dvva_ d1c6ra_ d1c75a_
d1e29a_ d1gdva_ d1cnoa_ d1h32b_ d1co6a_ d2bh4x_ d2aiua_ d5cytr_
d1jdla_ d1i8oa_ d2zboa_ d3dmia_ d1ls9a_ d1wvec_ d2yk3a_ d3ph2b_
d1dw0a_ d5aura_ d1ql3a_ d1qn2a_ d1f1ca_ d4zzva_ d2xspa_ d4v20a_
d1ojja_ d4ipma1 d1gpia_ d4csia_ d3pfza_ d3ovwa_ d2yoka_ d2nqya_
d1h4ga_ d1hixa_ d1oa2a_ d1olra_ d3b7ma_ d4h7ma1 d3wp3a_ d2vgda_
d1igoa_ d3zsea_ d2nlra_ d3wx5a_ d1oa3a_ d1pvxa_ d1m4wa_ d1f5ja_
d1oa4a_ d1ks5a_ d4ixla1 d1evsa_ d1cnt1_ d1pvhb_ d1z7ca_ d1huwa_
d1bgea_ d1rhga_ d1f6fa_ d1f45b_ d1i1rb_ d4x51a_ d1d9ca_ d4ypgc1
d3oq3a_ d1lqsl_ d1n1fa_ d1au1a_ d1wu3i_ d3besl_ d3ux9a_ d1v8fa_
d2ejca_ d3muea1 d3q12a1 d4dnxa2 d1v47a2 d1g8fa2 d1jhda2 d1x6va2
d3rbaa_ d2qtra_ d1nupa_ d3l92a_ d1qjca_ d2h29a_ d1vlha_ d1od6a_
d1lw7a1 d1mrza2 d4ruka_ d3sw2a_ d1moxc_ d1dx5i3 d1edmb_ d1autl2
d1autl1 d1nqlb_ d1dval1 d1szba2 d1kigl_ d2y5fl_ d1ijqa2 d1gk5a_
d1g1sa2 d1nt0a3 d1xdtr_ d1lmja2 d1lmja1 d1nzia2 d2wphe_ d1klil_
d1pfxl2 d1pfxl1 d1p9ja_ d1g1ta2 d1f7ea_ d4yzub_ d2mgpa1 d1b9wa1
d2mgpa2 d1ob1c1 d1ob1c2 d1n1ia2 d1n1ia1 d2npra2 d2npra1 d1qx2a_
d1ig5a_ d2y5ia_ d1qlsa_ d1psra_ d3n22a_ d2egda_ d4ggfc_ d2wcea_
d2luca_ d4aqia_ d3nsia_ d2lp3a_ d1j55a_ d1a4pa_ d1nsha_ d1pvaa_
d3fs7a_ d2pvba_ d1ttxa_ d1rwya_ d2kyfa_ d1rk9a_ d1a75a_ d1exra_
d1tiza1 d1nyaa_ d1g8ia_ d1ggwa_ d1wdcc_ d3fwba_ d1ggza_ d2o5ga_
d1ctaa_ d1m45a_ d3i5gc_ d1sl8a_ d1fpwa_ d2scpa_ d2roaa_ d4aqra_
d3pn7b_ d1br1b_ d2mysb_ d1s6ca_ d1bjfa_ d2obha_ d1jfka_ d1jf0a_
d1qv0a_ d1lkja_ d2mlfc_ d2m97a_ d2d8na_ d4by5a_ d1jbaa_ d4nqga_
d1wdcb_ d2mysc_ d1j7qa_ d1s6ia_ d1uhka2 d1r2ua_ d1dtla_ d3kf9a_
d3pn7c_ d1qjta_ d1fi6a_ d1c07a_ d5d67d_ d1tuza_ d1h8ba_ d1eg3a1
d1eg3a2 d2zkmx1 d1snla_ d1gjya_ d1y1xa_ d1hqva_ d1dvia_ d1df0a1
d1qxpa2 d4usla_ d1k94a_ d1wkya2 d1uuqa_ d3wh9a_ d4m1ra_ d1xyza_
d4xuya_ d2je8a5 d4bf7a1 d3cuia_ d1ur4a_ d1qw9a2 d1odza_ d2vzsa5
d1bqca_ d2x2ya1 d1hjqa_ d1qnra_ d4y7ea1 d1vjza_ d4k68a_ d2v3ga_
d4hpga_ d1nofa2 d1nq6a_ d1h4pa_ d1aq0a_ d3ziza_ d4bs0a_ d1ecea_
d1h1na_ d1ta3b_ d1w91a2 d1i1wa_ d1uhva2 d1us3a2 d1egza_ d7a3ha_
d1tvna1 d3nyda1 d1ghsa_ d2cyga1 d4xv0a_ d2c8na2 d1w32a_ d3emca_
d1v6ya1 d1foba_ d2uwfa1 d2fgla_ d3niya1 d2c0ha1 d1rh9a1 d1yq2a5

d1szna2 d1fa2a_ d1pama4 d5do8a1 d1ea9c3 d1hx0a2 d2fhfa5 d1qhoa4
d1avaa2 d1m53a2 d4m8ua1 d2gjpa2 d1lwha2 d4howa1 d1ktba2 d1j0ha3
d3vm5a1 d2x1ia_ d1wzaa2 d1uasa2 d2diea1 d1mxga2 d1ht6a2 d1eswa_
d1iv8a2 d5cpsa_ d1ud2a2 d1zjaa1 d3gbea1 d1kwga2 d1wdpa_ d1gcya2
d1g5aa2 d1hvxa2 d3dhpa2 d2bhua3 d4do4a1 d3qgva1 d1e43a2 d1g94a2
d2d3na2 d1gjwa2 d1gvia3 d1x1na1 d1r7aa2 d1tz7a1 d4toqa_ d1wb0a1
d1w9pa1 d1eoka_ d1itxa1 d1kfwa1 d1goia2 d2gsja_ d1hjxa1 d1jnda1
d1vf8a1 d3wdpp1 d3wq5a_ d4gxpa_ d4hz8a_ d1e4mm_ d3aiua_ d1wcga1
d1qvba_ d3ahxa_ d1v02a_ d2dgaa_ d1pbga_ d1vffa1 d1qoxa_ d4ha4a_
d2w0pa_ d2diaa1 d2e9ia2 d2e9ja1 d2dj4a1 d2eeba1 d2diba1 d2dmba1
d2dmca1 d2d7oa1 d2ee6a1 d2d7pa1 d2eeaa1 d2di8a1 d1wlha1 d2d7na1
d2nqca_ d2d7ma1 d2dica1 d3cnka_ d2k3ta1 d2eeca1 d1qfha2 d2bp3a1
d1v05a_ d2ee9a1 d2eeda1 d2di9a1 d1g4ma1 d1g4ma2 d1id1a_ d2hmta_
d1offa_ d1frra_ d3wcqa_ d1e9ma_ d1l5pa_ d1wria_ d1i7ha_ d3av8a_
d1czpa_ d1rfka_ d3p1ma1 d3b2ga_ d2bt6a2 d1wgqa1 d1v89a1 d2coda1
d1zc3b_ d1wg7a1 d1u5ea1 d1v61a1 d2coca1 d2ys1a1 d1v5pa1 d1ntya2
d1wjma1 d1wi1a1 d1u29a_ d1xx0a1 d1unqa_ d2zkmx3 d1fhoa_ d1v88a1
d2adza1 d1x1fa1 d1p6sa_ d2coaa1 d1btka_ d1v5ma1 d1faoa_ d2x18a_
d1dyna_ d1x1ga1 d4kaxb_ d2j59m1 d1ki1b2 d1xcga2 d1txda2 d1u5da1
d1dbha2 d1w1ha_ d1upqa_ d1v5ua1 d1foea2 d2fjla1 d1eaza_ d2dfka2
d2cofa1 d1kz7a2 d1h6ua1 d9ldta1 d2ewda3 d1up7a1 d2dfda1 d1s6ya1
d1vjta1 d7mdha1 d1hyea1 d1t2da1 d1ojsa3 d1b8pa1 d1ldna1 d1hyha1
d1civa1 d2a92a1 d1ez4a1 d1i0za1 d1y6ja1 d1gv0a1 d1ur5a1 d1llda1
d1obba1 d1v8ba1 d1gdha1 d1l7da1 d2dlda1 d1mx3a1 d1ygya1 d1qp8a1
d1li4a1 d1j4aa1 d2naca1 d1npya1 d3t4ea2 d1a4ia1 d1o0sa1 d2hk9a2
d2egga2 d1b26a1 d1bvua1 d1gq2a1 d1c1da1 d1leha1 d1gtma1 d1euza1
d1nvta1 d3dona2 d1edza1 d1gpja2 d1vi2a1 d1p77a1 d1nyta1 d1b0aa1
d1vl6a1 d1v9la1 d1txga2 d3f47a1 d1mv8a2 d2pv7a2 d1xc2a3 d2ahra2
d4xiya1 d2f1ka2 d1pgja2 d1i36a2 d1np3a2 d1lj8a4 d1teaa3 d1qmga2
d2i76a2 d2g5ca2 d1wdka3 d1dlja2 d3cuma3 d1kola2 d1f8fa2 d1qora2
d1jqba2 d1vj0a2 d1cdoa2 d1gu7a2 d1d1ta2 d1xa0a2 d2dm6a2 d1jvba2
d1rjwa2 d1llua2 d1h2ba2 d1tt7a2 d1piwa2 d1o89a2 d1iz0a2 d1e3ja2
d1yb5a2 d1uufa2 d1e3ia2 d3gpdg1 d1vc2a1 d2nvwa1 d1u8fo1 d1ydwa1
d1hdgo1 d2g17a1 d1cf2o1 d2czca2 d1r0ka2 d1obfo1 d2cvoa1 d1ebfa1
d1h6da1 d1i32a1 d1zh8a1 d2q49a1 d1qkia1 d1j5pa4 d3pzra1 d1xeaa1
d1vkoa1 d1vm6a3 d3qvsa1 d2pkqo1 d1b7go1 d1gr0a1 d1p1ja1 d1lc0a1
d1j0xo1 d3pwka1 d2hjsa1 d1zyao1 d1vkna1 d1tlta1 d1nvmb1 d3sjua_
d3ojfa1 d1xu9a1 d2jjya1 d1n7ha_ d1y1pa1 d1jtva_ d4fs3a_ d2bkaa1
d1gy8a_ d2c5aa1 d1ulsa_ d1ooea_ d2b69a1 d1y5ma1 d1spxa_ d1ae1a_
d1bxka_ d1ydea1 d1gega_ d1hdoa_ d1o5ia_ d1e6ua_ d1snya1 d2rhca_
d3zv4a_ d5ceja_ d2d1ya1 d1fjha_ d3w8ea_ d2fmua1 d1x1ta_ d1rpna_
d3ay7a1 d1k2wa_ d1eq2a_ d2hrba1 d3ruaa_ d2ew8a1 d4hwka_ d1xgka_
d2bd0a1 d1yo6a1 d2a35a1 d2ntva_ d1vl8a_ d1hdca_ d4bkua_ d2ag5a1
d2q45a_ d1nffa_ d4iy1a_ d1zbqa1 d1kewa_ d1ek6a_ d1ja9a_ d2q46a2

| | | | | | | | |
|---|---|---|---|---|---|---|---|
| d1iy8a_ | d1cyda_ | d2ptga_ | d1hxha_ | d1uzma1 | d3lyla_ | d4m89a_ | d3wyea_ |
| d2blla1 | d1w6ua_ | d1sb8a_ | d2q2va_ | d4nr0a_ | d1e6wa_ | d2gasa_ | d3oiga1 |
| d2bgka1 | d1mxha_ | d1vl0a1 | d1rkxa_ | d1r6da_ | d1wmaa1 | d1e7wa_ | d2c07a1 |
| d1xkqa_ | d3tzha_ | d1g0oa_ | d3lz6a_ | d1qyca_ | d1edoa_ | d1gz6a_ | d1oc2a_ |
| d1t2aa_ | d1zema1 | d2xoxa_ | d1z45a2 | d1geea_ | d1p33a_ | d3lt0a_ | d4e6pa_ |
| d2wyua_ | d1db3a_ | d4q9na_ | d1xhla_ | d4o1ma_ | d1fmca_ | d2uvda_ | d2gdza1 |
| d1uaya_ | d2z1ma_ | d2o23a2 | d2fr1a2 | d2fr1a1 | d1qsga_ | d1qyda_ | d4zjua_ |
| d2a4ka1 | d1yb1a_ | d1wvga1 | d1yxma1 | d3f4ba_ | d1mg5a_ | d1xg5a1 | d1r55a1 |
| d3gboa_ | d1atla_ | d1kufa_ | d1buda_ | d4q1la_ | d1quaa_ | d1yp1a_ | d1wnia_ |
| d4ekfa_ | d2ckga_ | d1th0a_ | d2bkra_ | d1e2ta_ | d4c5pa_ | d2bsza1 | d4bgfa_ |
| d1w4ta1 | d1ex0a4 | d1g0da4 | d2f4ma1 | d1x3za_ | d1mdwa_ | d1ziva1 | d1zcma1 |
| d1t3ca_ | d2a97a_ | d3fiea_ | g1e1h.1 | d3ioqa_ | d1fh0a_ | d1o0ea_ | d1khqa_ |
| d1m6da_ | d2cb5a_ | d1cs8a_ | g8pch.1 | d1s4va_ | d2pnsa_ | d1vsna1 | d1mira_ |
| d1iwda_ | d2oula_ | d7pcka_ | d3bwka_ | d3bcna_ | d2p86a_ | d1pvja_ | d1pcia_ |
| d1gece_ | d1deua_ | d4bs6a_ | d1xkga1 | d1cqda_ | d1jqpa2 | d2c0ya_ | d2bdza_ |
| d3p5ua_ | d2o36a_ | d1k9xa_ | d1zoya1 | d1qo8a2 | d1d4ca2 | d1chua2 | d1jnra2 |
| d2jk6a2 | d2jk6a1 | d1feca1 | d1feca2 | d1lpfa1 | d1lpfa2 | d1xhca1 | d1xhca2 |
| d1dxla2 | d1dxla1 | d1m6ia2 | d1onfa1 | d1onfa2 | d3lada2 | d3lada1 | d1w4xa1 |
| d1w4xa2 | d1gesa2 | d1mo9a1 | d1xdia1 | d1fl2a2 | d1fl2a1 | d1fcda1 | d1fcda2 |
| d1h6va2 | d1h6va1 | d1mo9a2 | d1gv4a2 | d1gv4a1 | d2gv8a2 | d2gv8a1 | d1aoga1 |
| d1aoga2 | d1hyua1 | d1ebda1 | d1ebda2 | d3dk9a1 | d1q1ra2 | d1q1ra1 | d1gesa1 |
| d1zbfa1 | d2z1ha_ | d2etja1 | d3qioa1 | d1jl1a_ | d2e4la_ | d1jl2a_ | d2lsna1 |
| d1io2a_ | d1uaxa_ | d2yv0x_ | d1mu2a1 | d1i39a_ | d1ps9a2 | d1o94a2 | d1cjca1 |
| d1pn0a1 | d1reoa1 | d1ju2a1 | d2f5va1 | d3c96a1 | d2gmha1 | d1pj5a2 | d1gpea1 |
| d1o5wa1 | d1n4wa1 | d1k0ia1 | d2dw4a2 | d2ivda1 | d1seza1 | d2voua1 | d1kdga1 |
| d1s5ja1 | d2hbja2 | d2igia_ | d1wlja_ | d1qhta1 | d4dlea1 | d1tgoa1 | d2f96a1 |
| d3tr8a1 | d3mxma_ | d1q8ia1 | d1y97a1 | d1w0ha_ | d1kfsa1 | d2py5a1 | d1noya_ |
| d1d5aa1 | d1yt3a3 | d2guia_ | d3mwpa1 | d4kb1a_ | d2v76a_ | d1wj1a1 | d1aqca_ |
| d1qqga2 | d1qqga1 | d1ntva_ | d1n3ha_ | d4dbba_ | d3f0wa1 | d1p3ra_ | d1wgua1 |
| d2cy4a1 | d3hqca_ | d1wvha1 | d1xr0b1 | d3nvva1 | d1krha3 | d2h88b1 | d1n62a2 |
| d1t3qa2 | d1zoyb1 | d1jq4a_ | d1ffva2 | d1dgja2 | d1rm6c2 | d1r7ha_ | d1faaa_ |
| d1wika1 | d1xfla_ | d1ttza1 | d2f51a1 | d1ti3a_ | d1nhoa_ | d1sena_ | d2i4aa_ |
| d1ep7a_ | d1zmaa1 | d1fo5a_ | d3lefa1 | d3diea1 | d2yana1 | d1z6na1 | d1oaza_ |
| d3zywa1 | d4aj8a_ | d1nm3a1 | d4fiwa_ | d1wmja_ | d4hs1a1 | d1iloa_ | d1xwaa1 |
| d1wjka1 | d2vm1a_ | d1fb6a_ | d1gh2a_ | d1h75a_ | d1nw2a_ | d1kx5b_ | d3a6na_ |
| d4m6ba2 | d2nqbd_ | d3wkjd_ | d2yfwe_ | d4h9na_ | d5bnxa_ | d1kx5c_ | d3r45a_ |
| d1eqzb_ | d4jjnb_ | d1f66c_ | d1eqza_ | d4wnnb_ | d1kx5a_ | d1u35c1 | d1id3a_ |
| d2nqbc_ | d1kx5d_ | d2b5ea2 | d2b5ea3 | d2b5ea1 | d2b5ea4 | d2kp1a1 | d2kp2a1 |
| d1hyua4 | d1j08a1 | d1j08a2 | d1pd212 | d1nhya2 | d2a2ra2 | d1k0dd2 | d1f2ea2 |
| d1gsua2 | d1f3ba2 | d2fhea2 | d2pvqa1 | d1tw9a2 | d1oyja2 | d1k0ma2 | d3ik7a1 |
| d4is0a1 | d1r5aa2 | d1jlva2 | d1m0ua2 | d3gtub2 | d1z9ha2 | d1okta2 | d1axda2 |
| d2gsra2 | d1tu7a2 | d1k3ya2 | d1zgma1 | d1b48a2 | d1gnwa2 | d1gwca2 | d2gsta2 |
| d1glqa2 | d1oe8a2 | d1pn9a2 | d1v2aa2 | d1fw1a2 | d1duga2 | d3vrda1 | d3vrda2 |
| d1fcdc1 | d1h1oa2 | d1h1oa1 | d1m70a2 | d1m70a1 | d3zxha_ | d1cgla_ | d1rm8a_ |

d1eaka2 d1hy7a_ d2jsda_ d1cxva_ d1hv5a_ d1i76a_ d1ig8a1 d1czan2
d1czan3 d1czan1 d1ig8a2 d1czan4 d1bg3a4 d1bg3a3 d1bg3a2 d1bg3a1
d1qusa_ d1qqya_ d1gd6a_ d1f6ra_ d1ivma_ d2gv0a_ d1iiza_ d2z2fa_
d1j8xa_ d1ghla_ d1b9oa_ d174la_ d169la_ d2o4wa_ d1p37a_ d1k28a3
d1xjta1 d1jtma_ d2f2qa_ d4e97a1 d1iyka2 d1iyka1 d1rxta1 d1iica1
d2za0a_ d2c21a1 d2pjsa1 d1klla_ d2i7ra1 d1ecsa_ d1xqaa1 d5cj3a1
d1npba_ d1jifa_ d2p7oa_ d1r9ca_ d1mpya2 d1kw3b2 d1kw3b1 d4ghga1
d1tfza1 d3isqa1 d3isqa2 d1sqia1 d1sqia2 d1lkda2 d1lkda1 d1mpya1
d1cjxa1 d1sp9b2 d1t47a1 d1t47a2 d1f1ua2 d1sp8a1 d1sp8a2 d1f1ua1
d2h0va_ d4iuaa3 d4bvva_ d5hpga_ d3k65a_ d1ki0a3 d1ki0a2 d1ki0a1
d1a0ha1 d1pmla_ d3sp8a3 d2feba1 d1qsta_ d2beia1 d4m3sa_ d1u6ma1
d1ygha_ d1yk3a1 d3dr6a_ d1xmta_ d1z4ea1 d2ge3a1 d2fiaa1 d2jdca1
d1y9wa1 d3to7a_ d1wwza1 d2fl4a1 d2ozua1 d2b5ga_ d2cy2a1 d2p0wa_
d1cm0a_ d4pswa_ d1yrea1 d1yr0a1 d1m4ia_ d2gana1 d1yvka1 d1ghea_
d2g3aa1 d1y7ra1 d2euia1 d1q2ya_ d2fsra1 d1xeba_ d2ae6a1 d1cjwa_
d1qsma_ d2aj6a1 d1y9ka1 d1i12a_ d1vkca_ d2atra1 d1n71a_ d2d4pa1
d2fiwa1 d2fe7a1 d1tiqa1 d2ou2a_ d1yx0a1 d2fcka1 d1p0ha_ d2j8ma_
d2pq8a_ d1z4ra1 d1s3za1 d1ufha_ d2hqya1 d2hqya2 d1ne9a1 d1lrza3
d1ne9a2 d1xvqa1 d1pn0a2 d3hy2a_ d2cvba1 d1o8xa_ d1lu4a_ d1we0a1
d2bmxa1 d2a4va1 d1psqa_ d1xiya1 d1q98a_ d1n8ja_ d1zofa1 d2ggta_
d3tjja_ d4je1a1 d1o73a_ d3s9fa_ d2zcta_ d1qmva_ d1jfua_ d2b1ka_
d5dvba1 d2b5xa1 d2v2ga_ d1st9a_ d3tb2a_ d4llra_ d4fh8a1 d1e2ya_
d1knga_ d2h30a_ d1i5ga_ d2z9sa_ d2f8aa1 d1tp9a1 d2b7ka_ d1nm3a2
d3hvva_ d2wfca1 d4kcea_ d1xvwa1 d4gqca1 d3qpma_ d4k1fa_ d1zzoa1
d1gp1a_ d1prxa_ d1rw1a_ d1z3ea1 d3l78a_ d1cg2a1 d1yloa2 d1y7ea2
d1y0ya3 d1lfwa1 d1vixa1 d1fnoa4 d1vhoa2 d1ysja1 d1vhea2 d1z2la1
d2fvga2 d1vgya1 d1r3na1 d2grea2 d1xmba1 g1q7l.1 d1rtqa_ d1etha2
d1w52x1 d2ppla1 d1hpla2 d1lpbb2 d2oxea1 d1bu8a2 d1rp1a2 d1ys1x_
d4hs9a_ d1ex9a_ d1ku0a_ d3qmma_ d1lgya_ d4l3wa_ d1llfa_ d1gz7a_
d1ukca_ d1cr6a2 d1bvsa3 d1ixra2 d3groa_ d1pjaa_ d1thta_ d2ftba_
d1pmpa_ d2rcqa1 d1fdqa_ d4lkpa_ d1sa8a_ d1vyfa_ d1lpja_ d3vg7a1
d4azqa1 d1o8va_ d5bvqa_ d1p6pa_ d2qo4a_ d1opba_ d1yiva1 d1o1va_
d4bvma1 d4tkba_ d4i3ba_ d1bwya_ d1ftpa_ d1ggla_ d4gkca_ d1cbia_
d3p6da1 d1kqwa_ d2a0aa1 d1xwdc1 d3kj4a_ d1w8aa_ d2ifga3 d1p9ag_
d1xkua_ d3p0ya1 d1igra1 d1igra2 d1n8yc2 d1n8yc1 d1m6ba2 d1m6ba1
d4dv8a2 d2bk7a_ d3fjua_ d1z5ra1 d1kwma1 d1uwya2 d1jqga1 d2c1ca1
d1dtda_ d1h8la2 d1h6ta2 d1h6ua2 d1i26a_ d1cixa_ d1y29a_ d1v7fa_
d2jtba_ d1d1ha_ d1g9pa_ d1lupa_ d1qk7a_ d1mb6a_ d2a2va_ d1niya_
d1i25a_ d2mpqa_ d1ie6a_ d1kqha_ d1la4a_ d2mxoa1 d1dl0a_ d1koza_
d1qk6a_ d1emxa_ d1lqra_ d1c6wa_ d1rmka_ d1ixta_ d1eyoa_ d1fu3a_
d1g1pa_ d1f3ka_ d1fyga_ d1rypd_ d1irua_ d1rypc_ d3unfk_ d1rypa_
d1j2pa_ d1irui_ d1j2qh_ d3unfh_ d1m4ya_ d1rypb_ d1irul_ d1irug_
d1iru2_ d1q5rh_ d1rypg_ d1iruf_ d1q5qa_ d1e94a_ d1irub_ d1ryph_
d3ty6a_ d1iruj_ d1iruk_ d1rype_ d1irud_ d1irue_ d3mi01_ d1rypf_

d1iruh_ d2z3ba_ d1iruc_ d3mka2_ d1iru1_ g1gk9.1 g1or0.1 g1e3a.1
d1jgta2 d1ea0a3 d1ecfa2 d1q15a2 d1ct9a2 d1ao0a2 d1te5a_ d1y14b1
d2nn6i1 d1i94l_ d2zjra2 d1smxa_ d2qamc2 d1hh2p1 d2r7da1 d1rl2a2
d1wfqa1 d3bzka4 d1x6oa2 d3a0ja_ d2j01d2 d2ja9a1 d4ql5a_ d1hr0w_
d1luza_ d2n3sa_ d2vnud1 d1h95a_ d1go3e1 d2nn6h1 d1ne3a_ d2mo1a_
d1iz6a2 d1jjga_ d1e3pa2 d2ix0a3 d2ix0a2 d2lxja_ d1ueba3 d1ueba2
d2z0sa1 d1u0la1 d2ix0a1 d2eifa2 d2je6i1 d3i2za1 d2c35b1 d2ahob2
d1t9ha1 d3i4oa_ d2qall1 d1jt8a_ d2nn6g1 d1g6pa_ d3cama_ d1i94q_
d1c9oa_ d1d7qa_ d2ba0a1 d2vnud2 d2vnud3 d1khia2 d2qalq1 d1wi5a1
d1xtda2 d1ny4a_ d4w9ta_ d1jvna1 d4x2ra1 d1ka9f_ d1qo2a_ d4axka1
d1h5ya_ d4u28a_ d2y88a_ d4wd0a_ d1vzwa1 d1eixa_ d3exra_ d2ffca1
d2fdsa1 d3n3ma1 d2czda_ d1q6oa_ d2yyua_ d1vqta1 d1dbta_ d3ldva1
d3syxa1 d1mkea1 d2p8va_ d1xoda1 d1i7aa_ d1qc6a_ d1egxa_ d4lt9a1
d3tc7a1 d3hoja_ d1i4na_ d3vnda_ d3nl8a1 d1geqa_ d3nava1 d1v5xa_
d4a2ra1 d3tc6a1 d1ujpa_ d4ijba_ d1xcfa_ d1vc4a_ d1rd5a_ d3b5va_
d2r80b_ d1cqxa1 d1cg5a_ d1gcvb_ d3boma_ d2h8fb_ d2raoa_ d3bj1a_
d4hrrb_ d2r50a_ d3d1kb_ d2aa1b1 d3zhwa_ d3hf4b_ d3gdja_ d1jebb_
d3bj1b_ d1x9fb_ d1wmub_ d1gcva_ d3bcqb_ d3d4xb_ d2dc3a_ d3hf4a_
d4yu3a_ d3k8ba_ d1qpwb_ d2r80a_ d3bcqa_ d1v4wa_ d3wtgb_ d1jl7a_
d4esaa_ d1a9we_ d3d1ab_ d1fsla_ d4h2la_ d1v4wb_ d1mwca_ d1vhba_
d1jeba_ d1spgb_ d1h97a_ d1q1fa_ d1tu9a1 d3d1aa_ d1hdsb_ d3hyub_
d2zfbb_ d3fs4a_ d1oj6a_ d1shrb_ d4h2lb_ d4yu3b_ d1spga_ d3hrwa_
d3qqra_ d3at5a_ d4wchd_ d3hyua_ d1cg5b_ d1uc3a_ d1hbra_ d2raob_
d1outb_ d4esab_ d3gdjb_ d3qqqa_ d1x9fc_ d3vrfa_ d1itha_ d3mkba_
d3d4xa_ d3mjpa_ d1jl6a_ d3fh9a_ d1outa_ d2dc3b_ d1it2a_ d3eoka_
d3g46a_ d1qpwa_ d3vrfb_ d1ch4a_ d1x9fd_ d1hdsa_ d2zfba_ d3mkbb_
d1or4a_ d3wtga_ d1a4fa_ d3fh9b_ d3bomb_ d3cy5a_ d3at5b_ d1x9fa_
d2oifa_ d4lmxb_ d3v57a_ d1kn1a_ d1cpca_ d4f0ta_ d2vjha_ d4po5b_
d1eyxa_ d1alla_ d4f0tb_ d1b33b_ d1b8db_ d4h0mb_ d1b33a_ d2vjhb_
d4lm6b_ d2vmlb_ d1allb_ d2vjta_ d3dbja_ d2vmla_ d2vjtb_ d4f0ub_
d1f99b_ d4rmpa1 d1liaa_ d1eyxb_ d2uulb_ d4lmsb_ d4f0ua_ d2bv8a_
d1f99a_ d2bv8b_ d2c7lb_ d3dbjb_ d4rmpb1 d1b8da_ d4h0ma_ d3v57b_
d1kn1b_ d1liab_ d2uula_ d2j96a_ d3nqyb_ d4gera_ d1bqba_ d1k8qa_
d2xqfa_ d1ea5a_ d1f6wa_ d1dx4a_ d1qe3a_ d1k4ya_ d4qwwa_ d2ha2a_
d3c8da2 d2pbla1 d1jkma_ d1jjia_ d1jjfa_ d2m5ta_ d1wqsa_ d5bpea_
d2ipha_ d1lvoa_ d2bhga1 d3tnta_ d3sjia_ d3d23a1 d2fyqa_ d3tloa_
d3zvea1 d2hrva_ d3q3ya1 d4asha1 d1s5ja2 d1qhta2 d4dlea2 d1tgoa2
d3py8a2 d1q8ia2 d2py5a2 d1d5aa2 d3u1ra1 d1g9ka2 d1kapp2 d1k7ia2
d1f74a_ d3l21a_ d2e1da_ d1to3a1 d1w3ia_ d1sfla_ d1n7ka_ d1vcva1
d4imea_ d1wa3a_ d2ocza1 d1f2ja_ d3m16a_ d2yxga_ d1ub3a_ d3a5fa_
d1fbaa_ d1l6wa_ d1xfba1 d1wx0a1 d1vpxa1 d1ojxa_ d2a4aa1 d1xkya1
d3cq0a1 d3s1xa_ d1f05a_ d1fdja_ d3cpra1 d4d2ja_ d4mh8a_ d1mu2a2
d4i7ga1 d3rp2a_ g1ucy.1 d1fuja_ d2zchp_ d3w94a_ d1si5h_ d1lo6a_
g2pka.1 d1cgha_ d1kigh_ d1a5ia_ d3s9ba_ d1hj8a_ d1ao5a_ d2anya_

d1q3xa1 d1gvza_ d1azza_ d4gsoa_ d2f91a1 d1iaua_ d1gdna_ d1eufa_
g1fiw.1 d1eq9a_ d4nfea_ d2rdla_ d1spja_ d1elva1 d1a7sa_ d1ekbb_
d1pfxc_ d1yc0a_ d2aiqa_ g1fiz.1 d1fi8a_ d4k60a_ d4dgja_ d1mzaa_
d1op0a_ d3tjva_ d1pytd_ d1sgfg_ g1gg6.1 d2eeka_ g1rtf.1 d1a0ja_
d3g01a_ d1pytc_ d4e7na_ d1ddja_ d1z8ga1 d2hlca_ d1npma_ d1brup_
g1jou.1 d1sgfa_ d1autc_ g2hnt.1 g1kdq.1 d3s69a_ d4crga_ d1fxya_
d1bqya_ d1ra6a_ d1muka_ d3gsza_ d1khva_ d1te9a_ d2xwha_ d3uqsa_
d3hkwa_ d3n6ma_ d2cjqa_ d2xyma_ d1teba_ d1te8a_ d1s48a_ d1u09a1
d4wfxa_ d3tyqa_ d2pgga1 d1dzka_ d4tlja_ d2a2ua_ d4wfva_ d3t1da_
d4iaxa_ d3zq3a_ d2nnda_ d1i4ua_ d1kxoa_ d1bbpa_ d1kt7a_ d1exsa_
d3s26a1 d1lf7a_ d1qfta_ d4gh7a_ d1z24a1 d1qabe_ d1xkia_ d1ew3a_
d3bx7a_ d4iawa1 d1n0sa_ d3kzaa_ d2k23a_ d2l9ca1 d2joza1 d1gm6a_
d3kffa_ d3saoa1 d1e5pa_ d1yupa1 d1iiua_ d4lu0a_ d3unda1 d3e9aa1
d1vr6a2 d1n8fa_ d2qkfa_ d1d9ea_ d1o60a_ d1e69a_ d1mv5a_ d3g60a4
g1ii8.1 d1ewqa2 d1ji0a_ d1qhla_ d1v43a3 d2wmfa1 d1vpla_ d1g2912
d4a82a2 g1f2t.1 d2d62a1 d3tuic1 d2bbsa1 d3g60a3 d2ixea_ d1g6ha_
d1sgwa1 d2qi9c_ d3rlfa1 d3b60a1 d3d31a2 d2pzea1 g1xew.1 d1jj7a_
d1oxxk2 d2onka1 d1w1wa_ d1iwea_ d1qf5a_ d1vmaa2 d1nija1 d1np6a_
d2p67a1 d3ihla_ d1okkd2 d1ls1a2 d1s1ma2 d1iona_ d2v40a_ d3hida_
d2vo1a1 d2g0ta1 d1vcoa2 d1j8mf2 d1g3qa_ d1xjca_ d1p9ba_ d1qzxa3
d1ihua2 d1ihua1 d2qm8a_ d1dj2a_ d4ioja_ d1cp2a_ d1yrba2 d1hyqa_
d3gqba2 d1skye3 d1xp8a1 d1tf7a2 d1tf7a1 d1ye8a1 d1skyb3 d2qe7d2
d4blta_ d4huta_ d3ldaa2 d4hyya_ d2i3ba1 d1cr1a_ d3j9tb2 d3ntua2
d1vdza1 d2z43a_ d1e9ra_ d1nlfa_ d3ziaa2 d1n0wa_ d1g6oa_ d3ziad2
d3j9ta2 d3gqbb2 d2i49a_ d1nnfa_ d1xvxa_ d1ursa_ d1h3da1 d1iuda_
d4m1va1 d1twya_ d3e13x_ d2nxoa1 d4pfua_ d3ttma_ d1elja_ d2qmwa1
d1us5a_ d1y3na1 d1jeta_ d1uqwa_ d4f19a1 d2esna2 d3thia_ d3fasa1
d2uyeb_ d2xwva1 d1r9la_ d2fyia1 d1pb7a_ d2ozza1 d1sw5a_ d1nh8a1
d1vr5a1 d2g29a_ d1xs5a_ d3lswa_ d2z23a_ d3w9va_ d3tcha_ d1z7me1
d3q27a_ d1p99a_ d1ve4a1 d2i6ea1 d4mcoa1 d4n8ya_ d1eu8a_ d1zbma1
d1hsla_ d1ixca2 d1j1na_ d1xvya_ d1y9ua_ d2z22a1 d1xoca1 d4oera_
d1wdna_ d5f1qa1 d1xt8a1 d1i6aa_ d4gwoa_ d4igta1 d1pc3a_ d4mmpa1
d1o63a_ d2czla1 d2onsa2 d4mija1 d4ok5a1 d2bmfa1 d2bmfa2 d1yksa1
d1yksa2 d1jr6a_ d2qeqa1 d2jlqa1 d1eh3a_ d3skpa_ d1dtza1 d1ieja_
d1jnfa2 d1ce2a1 d1jnfa1 d1b1xa2 d1b1xa1 d1jw1a1 d1jw1a2 d1ryoa_
d1h76a1 d1h76a2 d1c4oa1 d2p6ra3 d2p6ra4 d1oywa2 d1oywa3 d1wrba1
d1w36b2 d1w36b1 d1c4oa2 d3bora_ d1t6na_ d2eyqa5 d2eyqa3 d2eyqa2
d2zu6a2 d1veca_ d1uaaa1 d1w36d1 d2g9na1 d1z3ix2 d2rb4a_ d1hv8a1
d1hv8a2 d1t5ia_ d1w36c2 d2j0sa2 d2j0sa1 d3hjha1 d1z5za1 d1qdea_
d1z63a1 d1s2ma2 d1s2ma1 d1wp9a1 d1wp9a2 d1gm5a3 d1gm5a4 d3dina4
d3dina3 d1q0ua_ d1w36c1 d1z3ix1 d1nkta3 d1nkta4 d1uaaa2 d1w36d2
g1qhh.1 d2fwra2 d2fwra1 d3q3ma_ d1za0a1 d1mxra_ d2inca_ d1jk0a_
d2incb_ d1mhyb_ d3hf1b_ d1mtyb_ d1r2fa_ d2uw1a_ d1mhyd_ d3n37a_
d1afra_ d1syya_ d1mtyd_ d2o1za_ d1uzra_ d1smsa_ d2fkza_ d1zuja1

d1s3qa1 d2fjca1 d1z6om1 d1veia_ d4dyya_ d1jiga_ d3gvya_ d2pyba_
d4cy9a1 d3bvfa1 d1r03a_ d2cf7a_ d1nf4a_ d1zs3a1 d4iwka_ d3ka8a_
d3np0x_ d1n1qa_ d1lb3a_ d4e6ka_ d1z6oa1 d2fzfa1 d4dyua_ d3ge4a_
d1jgca_ d4am4a_ d2z6ma_ d1ji5a_ d1vlga_ d1lkoa1 d3qz3a_ d1o9ra_
d2ffxj_ d1euma_ d1yv1a1 d2v2pa_ d1j30a_ d4di0a_ d1dpsa_ d1oewa_
d2qzxa_ d2asia_ d1psoe_ d1smra_ d2h6ta_ d1pfza_ d1izda_ d3hd8a_
d1j71a_ d1dpja_ d4aa9a_ d1t6ex_ g1b5f.1 g1lya.1 g1htr.1 d1qdma2
d1bxoa_ d1wkra_ d1tzsa_ d1ibqa_ d2qzwa_ d1ex4a2 d1c6va_ d1bdqa_
d3ggta_ d3ixoa_ d1mrxa_ d2haha_ d1tw7a_ d4njsa_ d1sgua_ d2p3da_
d1nsoa_ d3d3ta_ d2rkfa_ d1c6ya_ d2rspa_ d3ogpa_ d3ekwa_ d2o4na_
d2j9ja_ d1baia_ d3ggua_ d3lzua_ d4npua_ d4j55a_ d2r8na_ d2fxda_
d2p3ca_ d4dqea_ d3ttpa_ d3t3ca_ d2p3aa_ d2hc0a_ d2fdda_ d3mwsa_
d1sr9a2 d1rqba2 d1k8rb_ d1wgra1 d1ef5a_ d1wfya1 d1e8ya3 d1wgya1
d2cs4a1 d1wxma1 d2byea1 d3kh0a1 d2byfa1 d1wxaa1 d1e7ua3 d1l7ya_
d1wgda1 d1wgga1 d4q5eb_ d1wjna1 d1se9a_ d3n3kb_ d1wz0a1 d1we6a1
d2m0xa_ d2gbka_ d3rt3b1 d1wh3a1 d1sifa_ d2ekec2 d4ii2b1 d4hk2a1
d1c3ta_ d1wgha1 d1ttna1 d1j8ca_ d1wx8a1 d3v6cb1 d2ma9b_ d4i6lb1
d1bt0a_ d2jvca1 d1iyfa_ d2k1fa_ d1wxva1 d1wf9a1 d1t0ya_ d2io0b2
d1x1ma1 d2gbma_ d1zw7a1 d2m17a_ d1ud7a_ d1we7a1 d2lwpa_ d2gbja_
d1wjua1 d1v6ea1 d1v86a1 d4eewa_ d1wiaa1 d4icva_ d4gu2a_ d1wx7a1
d1wy8a1 d4hjka1 d2faza1 d1v5oa1 d3ruib_ d1eo6a_ d3waob_ d2r2qa1
d3m95a_ d3wana_ d2al3a1 d2cr5a1 d1wj4a1 d1s3sg_ d3ubra_ d1d4ca1
d2k3va_ d1ft5a_ d1qdba_ d1m1qa_ d2j7aa_ d1h21a_ d1ogyb_ d4fasa_
d1jnia_ d1oaha_ d1sp3a_ d2h0ba_ d2erfa_ d1h30a2 d3mw4a1 d1pz7a_
d2r1ba_ d3v64a1 d1d2sa_ d3bopa1 d3mw3a1 d1okqa1 d1okqa2 d1ccza2
d1vcaa1 d1dr9a2 d1ic1a1 d2d9qb1 d2nxyb2 d1um8a_ d1ixza_ d1qvra2
d1qvra3 d1qzma_ d1sxjc2 d1jr3d2 d2p65a_ d1l8qa2 d1g8pa_ d3hu3a3
d1sxjd2 d1njfa_ d1in4a2 d1sxja2 d1tuea1 d2ce7a2 d1w5sa2 d1sxjb2
d1d2na_ d1ny5a2 d1ofha_ d2gnoa2 d2a5yb3 d1fnna2 d1u0ja1 d1lv7a_
d1iqpa2 d2fnaa2 d1ixsb2 d4gita_ d1sxje2 d2e3fa_ d1mm0a_ d2e3ea_
d2llda_ d2ny8x_ d2e3ga_ d2ny9x_ d2nz3a_ d2k4ua_ d1hp2a_ d2lu9a_
d1baha_ d2meoa1 d1wm7a_ d1hlya_ d1n8ma_ d1jlza_ d1wpda_ d1c55a_
d1pvza_ d1wt7a_ d1rjia_ d1wz5a_ d1m2sa_ d1pjva_ d1c49a_ d1qkya_
d2ktca1 d1wmta_ d1j5ja_ d1r1ga_ d1t0za_ d2i61a_ d1t1ta_ d1sn4a_
d1kv0a_ d2lkba_ d1npia_ d1wwna_ d1pe4a_ d2kbka_ d2li7a_ d1djta_
d2kbha_ d1dq7a_ d1sega_ d1omya_ d1zywa1 d1jzaa_ d1n9wa1 d1e1oa1
d1c0aa1 d1b8aa1 d1l0wa1 d3a74a1 d1eova1 d1t61c1 d1iyjb4 d1iyjb5
d1iyjb3 d1o7ia_ d4ipca1 d1x3ea_ d4apva_ d1jb7a1 d1jb7a2 d1jb7a3
d3kdfa_ d1ue1b_ d1wjja1 d3eiva_ d1xjva2 d1xjva1 d2ccza2 d1se8a_
d1l1oc_ d1jb7b_ d1qvca_ d3ulla_ d1qzga_ d1miua4 d1miua5 d1miua3
d3g48a1 d1gd7a_ d1mkha_ d1jjcb3 d1pyba_ d3ubub_ d1egia_ d1qo3c_
d1v7pa_ d1tdqb_ d3ubua_ d1c3ab_ d1h8ua_ d3bx4b_ d1t8ca1 d1sb2a1
d1qdda_ d2ziba1 d3cada_ d1byfa_ d1j34b_ d1jwia_ d1jzna_ d1c3aa_
d1oz7b_ d1wmza_ d3bdwb_ d1hq8a_ d1fifa1 d1dv8a_ d3hupa_ d1wk1a1

d1umra_ d2afpa_ d3bx4a_ d1gz2a_ d1sl6a_ d1v7pb_ d3g8ka_ d1ixxa_
d1j34a_ d1jwib_ d1g1sa1 d1y17a1 d1ja3a_ d1fvub_ d3bdwa_ d1sb2b1
d1fvua_ d1umrc_ d1oz7a_ d4m17a_ d3cfwa1 d1ypqa1 d1g1ta1 d3fd4a_
d3l9jc_ d4dn8a1 d1v4lb_ d2msba_ d1dgsa2 d1fvia1 d1p16a1 d1x9na2
d1pfsa_ d2a1ka_ d1rpma_ d2shpa1 d1yfoa_ d1l8ka_ d2ooqa1 d1g4us2
d1p15a_ d1lara2 d1lara1 d1wcha_ d2fh7a2 d2fh7a1 d1ohea2 d2f4da_
d1rxda_ d2hxpa_ d1xria_ d1d5ra2 d1fpza_ d3ezza_ d4y2ea_ d1i9sa_
d3azya_ d3wvja_ d1upsa1 d1ajoa_ d2hyka_ d2vy0a_ d1o4ya_ d3o5sa_
d3d6ea_ d3atga_ d1dypa_ d1umza_ d3i4ia_ d1c1la_ d4lbqa_ d1a78a_
d1slta_ d4no4a_ d3zsja_ d3duia_ d1g86a_ d1is3a_ d1qmja_ d1hlca_
d1tdha3 d1q3ba3 d1ee8a3 d1k82a3 g1qmo.1 g1len.1 d3zyra_ d1fnya_
d1n47a_ g2ltn.1 d1avba_ d3usua_ d1dhkb_ d1f9ka_ d1ukga_ d1g8wa_
d1g9fa_ d1wbfa_ d3wcsa_ g1loe.1 d1mvqa_ d1hqla_ d1v6ia_ d1dbna_
d1qnwa_ d1fx5a_ d4l8qa_ d1ioaa_ d3n35a_ d3h33a_ d1rwja_ d19hca_
d1os6a_ d3bxua_ d3h4na_ d1i77a_ d3caoa_ d1j0pa_ d2ctha_ d1duwa_
d1ofwa_ d2e84a_ d1hh5a_ d3h34a_ d1dszb_ d1a6ya_ d3cbba_ d1ynwa1
d1r4ia_ d1hcqa_ d2hana_ d2hanb_ d2enva1 d1kb4b_ d2nllb_ d1lo1a_
d2dara2 d2dara1 d1wyha2 d2dloa2 d1rutx4 d1rutx3 d2cuqa2 d1x4la2
d1x4la1 d1x3ha2 d1x3ha1 d2d8za1 d1x61a2 d1x6aa1 d1x6aa2 d2cora2
d2cora1 d1v6ga1 d1v6ga2 d2d8za2 d1x64a1 d1x64a2 d1b8ta1 d2co8a1
d1x63a2 d2dj7a2 d2dj7a1 d2cu8a2 d2cu8a1 d1g47a2 d1g47a1 d1j2oa1
d1wyha1 d2dloa1 d1wiga1 d1wiga2 d2cupa2 d2cupa3 d1x68a1 d2d8ya2
d2d8ya1 d1m3va2 d1x62a1 d1x68a2 d1x61a1 d1b8ta3 d1b8ta2 d2d8xa2
d2d8xa1 d1x4ka1 d1x4ka2 d2cuqa1 d1m3va1 d2co8a2 d1j2oa2 d1x63a1
d2cura1 d2cura2 d1x62a2 d1op9a_ d1y0la1 d1etzb1 d1egjl1 d2gsib1
d1nj9a1 d4jfhd1 d1aqkl1 d1t2ja_ d1lo4h1 d1zvya1 d3v4vl1 d4enec1
d1kxtb_ d5e8ea1 d2pxya_ d4weud_ d1xiwc_ d2esvd1 d1qnzh_ d1ogae1
d1f4xh1 d4n1hb_ d3ezjb1 d3mlwl1 d1kfah1 d1etza1 d1b4jh1 d3cfic_
d4w68a_ d2arja1 d1ek3a_ d2ok0l1 d1vera_ d1uwgh1 d1blnb1 d1fpth1
d1rzga1 d2giya1 d4m3kb1 d1nc2a1 d2nyyd1 d1ncna_ d1fbih1 d5dxwa_
d4fqqa1 d1dqdh1 d1jfql1 d1kelh1 d1faih1 d1frgl1 d1figl1 d4m6nl1
d1fo0a_ d4hxbl1 d3b5ga_ d3quyd1 d2h9gb1 d1i8kb_ d2dd8h1 d2ck0h1
d1eaja_ d4w6wb1 d2a6ib3 d1t66d1 d2e27h_ d1ob1b1 d1f11b1 d2v7na1
d1ad0a1 d2xt1b1 d1pskh1 d4i13b1 d2op4l1 d1a0ql1 d1uwel1 d1za3a1
d1ncbl1 d4uu9a_ d1bm3h1 d1cloh1 d1nqba2 d1cf8h1 d4y5va_ d1mhph1
d5anma1 d4cdgc1 d4ldeb_ d1y0lb1 d2pcpb1 d1z3gl1 d1dfbl1 d1fh5h1
d3g5xa1 d1tzib1 d4dn3l1 d3zhka1 d2b1hh1 d2uyla1 d1f3dh1 d1nj9b1
d2agjh1 d2h1pl1 d1q72h1 d8fabb1 d1ndmb1 d1rjca1 d4u6hb1 d3upca_
d2fbjl1 d1osph1 d1tvda_ d2cjvh_ d4fhbd1 d1bd2d1 d1rz7h1 d3nn8a_
d1blna1 d1knoa1 d1nfda1 d2bmkb1 d1dqll_ d1igyb1 d1igfl1 d12e8h1
d3utza1 d1nc2b1 d1rihh1 d4mwfb1 d4jvpa1 d1mieh1 d1rhfa1 d1fgnl1
d1sbsh1 d1lp9e1 d1dn0a1 d4lqfl1 d1iaih1 d1kgcd1 d1mh5b1 d1gpol1
d2g60h3 d1f8tl1 d2jixd1 d4grwh_ d1fo0b_ d1bfob1 d1axsa1 d1u9ka_
d1nmbh_ d1h8na1 d1h8na2 d1jguh1 d4eura1 d3sdyl1 d35c8h1 d1w72l1

d1mvub_ d1i9rh1 d1ob1a1 d4wenb_ d1yeeh1 d3ietc1 d1f58h1 d1ad0b1
d1mqkl_ d1nbvh1 d4b50a_ d3r8bb_ d1bafl1 d1mamh1 d4rgne1 d2gsgb_
d35c8l1 d1bafh1 d1ai1h1 d2pcpa1 d2fath1 d2x1pa_ d1orsb1 d1ibgl1
d1shma_ d1a2yb_ d1u0qa_ d2coqa_ d1hilb1 d1qfwi_ d1jv5b_ d1fn4b1
d5e0qa_ d1nsnh1 d1plgl1 d4ocxl1 d1kb5h1 d1rzfh1 d1pg7x1 d2fx7h1
d2oi9c_ d1l7il1 d3eyva1 d1d5il1 d4ffyl_ d1tetl1 d1a6wh_ d1ac6a_
d3dvfa_ d1nfde1 d1mimh1 d1ri8a1 d1jhlh_ d3dura_ d1fskb1 d1f97a1
d3so3b1 d2ak4d1 d1lp9f1 d4z7wf1 d1l7th1 d4y5ya_ d4x7ec1 d1emth1
d1ikfl1 d1nlbh1 d4utal1 d1bjma1 d1eapb1 d1u6ah1 d2dtgd1 d1kj2b_
d1xeda1 d1c1eh1 d1fgvh_ d2bnub1 d2fb4h1 d2dtgb1 d1ktrh_ d1jpth1
d1qkzl1 d4hepg_ d2atpb1 d1flrh1 d2hrpl1 d1mvfa_ d1iaim1 d3k80a1
d2jb5h1 d3zkxc_ d4grwe_ d1it9h1 d1rhhb1 d3w9ec_ d1e4wh1 d1sy6h1
d2j6eh1 d3qxta_ d1jglh1 d1p7ka1 d1tzhb1 d4n8pa1 d1gafl1 d2jelh1
d1wioa2 d4i0cc1 d1cicb1 d1lmka2 d1lmka1 d1iqwh1 d1orqb1 d1ejoh1
d1jnlh1 d4zg1a_ d43c9b_ d1ggbl1 d1wejh1 d3q6fa1 d1f3dj1 d3c6la1
d1bqlh1 d3p9wb_ d1a3rl1 d1h0db1 d2uzil_ d1eo8l1 d1qfwl_ d1n7ml1
d1igja1 d1c5ch1 d2a9mh_ d1rmfh1 d1fj1b1 d4heme_ d1ccza1 d1a5fh1
d1ogad1 d1dlfh_ d1qa9b_ d1vgel1 d3qskb_ d1sjxa_ d4ozfg1 d1sq2n_
d1qd0a_ d1kcvh1 d1c5db1 d3qcul1 d8faba1 d3bn9d1 d1mjuh1 d4y8dc_
d7fabh1 d3dvgb1 d1lo0l1 d1a6tb1 d1gigh1 d1kcrh1 d2hwzl1 d1yjdc1
d3clfl1 d4krna_ d1dl7l_ d1e6ol1 d1mexh1 d3r0ma_ d4kkna_ d1fnsl1
d1oaqh_ d1rzia1 d1ucbh1 d1ncwl1 d4b41a_ d1nfdf1 d2mpah1 d2d7tl1
d4grwf_ d4b5ea_ d1lk3h1 d3u79a_ d4u3xa_ d1ghfl1 d1bvkb_ d1mcww1
d1b88a_ d1jrhh1 d32c2b1 d4wemb_ d1dsfh_ d1op3h1 d1ce1l1 d1iqda1
d1qoka1 d1qoka2 d1qfuh1 d2crya1 d1ay1h1 d1oaql_ d3ab0b_ d1rurl1
d1t4kb1 d1bz7b1 d1n0xh1 d1forl1 d1ucbl1 d4w6yb_ d43c9a_ d2aabl1
d1tzia1 d4n1ca_ d1k6qh1 d3g9ab_ d4tvsa1 d1igmh_ d1pz5b1 d3dx9a1
d3wxwh_ d1nakh1 d1kcul1 d3eaka_ d4qlra1 d1cr9l1 d1d9ka_ d1f4xl1
d1deea1 d2i27n2 d1c12a1 d2orbl1 d1j8hd1 d1mcph1 d1c5da1 d1kenh1
d1bd2e1 d1h5ba_ d4x7fc1 d3upaa_ d1t3fa1 d1indh1 d2dtgc1 d4hc1l1
d1zv5a1 d4wgvb_ d1uwgl1 d4f37k1 d1aqkh1 d1vesa_ d3zkqd_ d1fptl1
d1lo4l1 d1knob1 d3rrqa_ d1ct8b1 d4orzc_ d1mlbb1 d4hgkc1 d1dqdl1
d1bogb1 d4ozhe1 d1dr9a1 d1i7za1 d1dn0b1 d1kell1 d1rzib1 d1ypzf1
d1wtla_ d1hkfa_ d1fail1 d1figh1 d2vyre_ d1sjva_ d1ohqa_ d1bfoa1
d1pkqb1 d1mjul1 d1b4jl1 d1nsnl1 d2arjb1 d1p4bh_ d4aj0a_ d3nn8b_
d2rcja1 d1f2xk_ d4v1dd_ d1adqh1 d2eksa_ d4k3ga_ d1jfqh1 d1uweh1
d1frgh1 d4gftb_ d1uz8b1 d4n1ea_ d1bm3l1 d1clol1 d3mcka1 d2vq1a1
d1ae6h1 d4aq1b1 d1uj3b1 d1nbqa1 d1fh5l1 d2ck0l1 d2ywza_ d1q72l1
d4nbxb_ d1hila1 d4hzlb1 d2j88l1 d1zmya1 d1cd0a_ d1a4ka1 d1pskl1
d1a0qh1 d4krmb_ d1ncbh1 d2fbjh1 d1j8he1 d1i3ua_ d1cf8l1 d1fe8h1
d2p1ya1 d1eeqa_ d2x89a_ d1ol0a_ d2r0lh1 d1ub5a1 d1dfbh1 d3eyvb1
d4jdvb1 d4p2cg_ d2gsia1 d2agjl1 d1u3ha1 d2h1ph1 d1rihl1 d1fskc1
d1boga1 d2a77l1 d1ospl1 d1qfoa_ d3dwta_ d2fx7l1 d1rz7l1 d3stba1
d1pkqa1 d1b6da1 d1dzba2 d1dzba1 d1kj2a_ d4l1ha_ d1rzgb1 d1dqlh_

d12e8l1 d1miel1 d1jmaa_ d1sbsl1 d1fgnh1 d1f58l1 d1mqkh_ d1ndgb1
d3qegl1 d1iail1 d2aw2a1 d1tqbc1 d3fo2a1 d4dkaa_ d1f8th1 d2g60l1
d2ywya_ d1uj3a1 d1yc7a_ d3bd3a_ d4w6xb1 d1nmbl_ d1i8ka_ d1rjlb1
d1ypze1 d1p7kb1 d1kxqe_ d1gpoh1 d2z4qa1 d1w72h1 d1i9rl1 d4qgya1
d4xt1c_ d2qqnh1 d1cica1 d3c08h1 d2cd0a_ d2bdnh1 d1hnga1 d1maml1
d1nldh1 d1cr9h1 d4lcih1 d3bkyh1 d3qg7l1 d1ai1l1 d1kxvc_ d4iosd_
d1l7ih1 d2x1qa_ d1ub6b1 d3k74b_ d1ibgh1 d1teth1 d1igya1 d1hxma1
d2x6ma_ d2e7la_ d5e03a_ d1qfwm_ d2r0kh1 d1igjb1 d1rurh1 d1plgh1
d1mj8h1 d1yuhb1 d3fmgl1 d1rzfl1 d1emtl1 d1ad9b1 d1igtb1 d1nlbl1
d1d5ih1 d3k7ua_ d4ffyh_ d1bwwa_ d3dvga1 d1a6ta1 d1fgvl_ d4qxgl1
d1miml1 d1jhll_ d2hffb1 d2hh0l1 d1qkzh1 d1l7tl1 d1afvh1 d1iaii1
d2cjuh_ d1um5h1 d2ghwb2 d2ghwb1 d1kgce1 d2fgwh1 d1ikfh1 d1dvfd_
d2i24n1 d2xtjb_ d1za3b1 d2yc1a_ d2hh0h1 d2x1oa_ d1bvka_ d1rjla1
d5e7ba1 d1igta1 d1il1b1 d2fb4l1 d1mvua_ d1jptl1 d32c2a1 d1fvca_
d1iqdb1 d2hrph1 d2p4ab_ d4qo1a_ d1p2cb1 d1wt5c_ d1t4ka1 d5c1mb_
d1it9l1 d1bz7a1 d1b2wh1 d1e4wl1 d1jnll1 d1yedb1 d1za6b1 d3u7aa_
d1igfh1 d3gnml1 d1orsa1 d1wejl1 d1a2ya_ d1hxmb1 d1gafh1 d2j4wh1
d1pz5a1 d1j05a_ d1a3rh1 d4fqll1 d1qfwh_ d1eo8h1 d1n7mh1 d4s11a_
d1kegh1 d5da4a_ d1iqwl1 d3whxb1 d1vgeh1 d1deeb1 d1c12b1 d1t2ql1
d1ad9a1 d3gsnb1 d3b2uc1 d1ggbh1 d1t3fb1 d1nakl1 d4ocmc_ d1lila1
d2v7hb3 d2esve1 d1mcoh1 d2qr0b1 d2q3aa_ d1ct8a1 d1kcrl1 d2pxyb_
d2uzih_ d5c3ld_ d3durb_ d1c5cl1 d2ak4e1 d1dl7h_ d1xiwd_ d1mlba1
d1rmfl1 d1i7zb1 d1dlfl_ d2xa3a_ d5bq7b1 d1dqqb1 d3h42l1 d1egjh1
d1kcvl1 d1fe8l1 d2j6el1 d1f3rb2 d1lk3l1 d1sm3h1 d1q0xh1 d1ghfh1
d7fabl1 d1igch1 d1lo0h1 d4idla_ d5c2bl_ d1jrhl1 d3zhda1 d4kfzc_
d1b0wa_ d1e6oh1 d1mexl1 d4krpb_ d1fnsh1 d1ncwh1 d4hgma_ d4eigb1
d4rzca_ d2mpal1 d4y5xa_ d5en2b1 d3lrga1 d4ej1c1 d1rhha1 d1j05b_
d1forh1 d1q9wb1 d2wzpd_ d2aq2a1 d1h0da1 d3revb1 d2ij0c1 d1tzha1
d4m7jl1 d1k6ql1 d3b9kc1 d1igml_ d3qeua1 d1dsfl_ d1ce1h1 d1kcuh1
d1zvha1 d1zvoa1 d1tqbb1 d1ay1l1 d2qscl1 d2cgrh1 d1nl0h1 d1kenl1
d3eo0a1 d4n9ob1 d1n0xl1 d3k1kc_ d2aq2b1 d1ck1a1 d1enfa1 d1i4pa1
d1v1oa1 d1et9a1 d1esfa1 d2g9hd1 d4ohja1 d1klud1 d1eu3a1 d1ty0a1
d1fnua1 d2cqva1 d1fhga_ d2k1ma_ d2dl2a1 d1biha3 d1biha1 d1biha4
d3qp3a1 d1g1ca_ d1gxea_ d1cvsc2 d1cvsc1 d1xmwa2 d1m4ka2 d2otpa2
d1biha2 d1rhfa2 d2fcba2 d2fcba1 d1wwbx_ d1gsma2 d1epfa1 d1epfa2
d2dava1 d1waaa_ d1f97a2 d1x44a1 d1vcaa2 d1fnla2 d1fnla1 d3b5ha2
d3h8na2 d3h8na1 d1jbja1 d1jbja2 d1pd6a_ d1f2qa2 d1f2qa1 d3cx2a_
d1nbqa2 d3b5ha1 d1n26a1 d1xiwb_ d1xaua_ d1fltx_ d2ifga1 d1olla1
d1olla2 d1cs6a4 d1cs6a3 d1cs6a2 d1cs6a1 d2gw5a2 d1sy6a1 d1xiwa_
d1ucta2 d1ucta1 d1ry7b2 d1gl4b_ d1qz1a3 d1olza1 d2c9aa1 d1wwca_

**Family-25 (b.29.1.1)**

**Positive dataset contains 5 protein sequences.**

d3zyra_ d3usua_ d3wcsa_ d4l8qa_ d3n35a_

**Negative dataset contains 3967 protein sequences.**

d1orva2 d1xfda2 d1m32a_ d1ibja_ d1c7na_ d1iuga_ d4q75a_ d2bkwa1
d1t3ia_ d1n8pa_ d1qz9a_ d1jf9a_ d1eg5a_ d1p3wa_ d3kgwa_ d1cl1a_
d2ctza1 d1vjoa_ d2rfva_ d1gc0a_ d1cs1a_ d1d2fa_ d2ch1a1 d1qgna_
d1pffa_ d1elua_ d1sefa_ d1sfna_ d1sq4a_ d3gv5b1 d1t94a2 d3bq0a2
d3rcha_ d3k40a_ d1pmma_ d2cfba1 d1dfoa_ d1o69a_ d1bj4a_ d3k28a_
d2bwpa_ d3lg0a_ d2fnua_ d3ou5a_ d1z7da1 d1b9ha_ d1iaya_ d4msoa_
d1bs0a_ d1s0aa_ d1sffa_ d3tqxa_ d1mdoa_ d3qboa_ d3pgya_ d1bjna_
d2dkja_ d1ejia_ d1vefa1 d4wxga_ d3bs8a_ d3gbxa1 d1w23a_ d1u08a_
d1lc5a_ d2ay1a_ d1bw0a_ d3pd6a_ d7aata_ d2csta_ d1j32a_ d1fg7a_
d4wlha_ d1o4sa_ d1v2da_ d1gdea_ d1m6sa_ d1v72a1 d1svva_ d1ajsa_
d1wsta1 d2f8ja2 d1lk9a_ d1vp4a1 d1xi9a1 d2gb3a1 d1yaaa_ d1m8pa3
d1ddla_ d1e57b_ d1auyb_ d1fwxa1 d1ibya_ d2wo3b_ d1shxa_ d1f56a_
d1bypa_ d1azca_ d1ws8a_ d3u25a_ d1cuoa_ d1id2a_ d1rkra_ d2iaac_
d4dp1x_ d1nwpa_ d2gima_ d2i7sa_ d1qhqa_ d1gyca3 d1hfua2 d1kbva1
d1kbva2 d1sdda1 d1sdda2 d1gyca2 d1mzya1 d2bw4a1 d1aoza1 d1aoza3
d1aoza2 d1kyaa2 d1kyaa3 d1kyaa1 d1mzya2 d4e9sa1 d1sddb1 d1sddb2
d1hfua1 d1hfua3 d1oe1a2 d1oe1a1 d1v10a2 d1v10a3 d1v10a1 d2dara2
d2dara1 d1wyha2 d2dloa2 d1rutx4 d1rutx3 d2cuqa2 d1x4la2 d1x4la1
d1x3ha2 d1x3ha1 d2d8za1 d1x61a2 d1x6aa1 d1x6aa2 d2cora2 d2cora1
d1v6ga1 d1v6ga2 d2d8za2 d1x64a1 d1x64a2 d1b8ta1 d2co8a1 d1x63a2
d2dj7a2 d2dj7a1 d2cu8a2 d2cu8a1 d1g47a2 d1g47a1 d1j2oa1 d1wyha1
d2dloa1 d1wiga1 d1wiga2 d2cupa2 d2cupa3 d1x68a1 d2d8ya2 d2d8ya1
d1m3va2 d1x62a1 d1x68a2 d1x61a1 d1b8ta3 d1b8ta2 d2d8xa2 d2d8xa1
d1x4ka1 d1x4ka2 d2cuqa1 d1m3va1 d2co8a2 d1j2oa2 d1x63a1 d2cura1
d2cura2 d1x62a2 d2y5ia_ d1qlsa_ d1psra_ d3n22a_ d2egda_ d4ggfc_
d2wcea_ d2luca_ d4aqia_ d3nsia_ d2lp3a_ d1j55a_ d1a4pa_ d1nsha_
d1a3qa1 d1iknc_ d3brda1 d1oy3c_ d1imhc1 d1gjia1 d1zk9a1 d2cxka1
d2bema_ d1pama1 d1ea9c1 d1qhoa1 d2fhfa2 d2fhfa1 d1k3ia1 d1j0ha1
d1cxla1 d2bhua1 d1edqa1 d1f1sa2 d1ut9a2 d1gvia1 d1txka1 d4alac_
d1pjwa_ d1rera1 d1s6na_ d3uzqb_ d2phla2 d2phla1 d1fxza2 d1fxza1
d1lrha_ d1uija1 g1dgw.1 d2v09a_ d1uika2 d1uika1 d1dgwa_ d1ep0a_
d2pa7a1 d1wlta1 d1nxma_ d2c0za1 d1ktja_ d1fstb_ d1ex0a1 d1g0da1
d1ju8a_ d4w9ta_ d1jvna1 d4x2ra1 d1ka9f_ d1qo2a_ d4axka1 d1thfd_
d4u28a_ d2y88a_ d4wd0a_ d1vzwa1 d4mh8a_ d1mu2a2 d4i7ga1 d2bjca1
d2hsga1 d1qpza1 d1efaa1 d1r0ka3 d1j5pa3 d1vkoa2 d1vm6a2 d3qvsa2
d1gr0a2 d2ovga_ d1sq8a_ d1rzsa_ d3kz3a_ d3orca_ d3gpdg2 d2q49a2
d1hdgo2 d2g17a2 d2cvoa2 d2czca1 d3pwka2 d1obfo2 d1i32a2 d1zyao2
d2pkqo2 d1b7go2 d2hjsa2 d1cf2o2 d1vkna2 d1nvmb2 d2msya_ d2djoa1
d2r5za_ d1au7a1 d1f43a_ d2lmda1 d1akhb_ d1s7ea1 d3nara_ d1zq3p1
d2craa1 d1mija_ d4uuta_ d3a01a_ d1x58a1 d3rkqa1 d3cmya1 d2cqxa1
d2da6a1 d1e3oc1 d1fjla_ d1mh3a1 d1ig7a_ d1x41a1 d1wh7a1 d1gt0c1
d2mw8a_ d1lfup_ d1x2ma1 d1b72a_ d1wi3a1 d1jgga_ d1wjha1 d1x2na1
d1mnmc_ d2cuea1 d3k2aa_ d2hosa_ d2jwta2 d1pufa_ d1octc1 d2hi3a_
d1b8ia_ d4j19a_ d3lnqa_ d1wh5a1 d1b8ib_ d1fexa_ d2cqra1 d1guua_

d1ug2a1 d2cqqa1 d2ajea1 d4a69c_ d1h8ac1 d2crga1 d2iw5b1 d1xc5a1
d1ofcx1 d2cu7a1 d1wgxa1 d2cjja1 d2ckxa1 d1gvda_ d1hcra_ d1gdta1
d1u78a2 d1xg1a1 d1itya_ d1hlva1 d1hlva2 d1iufa1 d1igna2 d1bl0a1
d1d5ya1 d1d5ya2 d3lv8a_ d1s3ga1 d1zaka1 d1s4qa_ d3tvta2 d1uf9a_
d1khta_ d1ckea_ d1osna_ d1p5zb_ d4tmka_ d3cm0a_ d1tmka_ d1jaga_
d3h86a_ d1t0hb_ d1nn5a_ d2h92a_ d3kb2a1 d2c95a1 d1yj5a2 d1kgda_
d1ly1a_ d2ak3a1 d1vyva2 d1deka_ d2grja1 d1p3ja1 d1y63a_ d1e4ya1
d1s96a_ d3iija1 d1lvga_ d1nksa_ d1vyua2 d4e22a_ d1qf9a_ d1jjva_
d1q3ta_ d1ki9a_ d1lw7a2 d1teva_ d1kjwa2 d2iyva2 d1viaa_ d1e6ca_
d1g3ma_ d1j99a_ d1t8ta1 d1nsta_ d1fmja_ d1zrha_ d1ls6a_ d2a3ra_
d1aqua_ d2zptx_ d3ckla_ d1texa_ d1q44a_ d2gwha_ d1k6ma1 d1z0ja1
d3umra2 d3gj0a1 d2gaoa1 d1yzga1 d1x3sa1 d1g7sa4 d1puja_ d1udxa2
d1kk1a3 d2o52a_ d1jwyb_ d1zcaa2 d1zcba2 d2fv8a1 d2cjwa_ d3dz8a1
d1xzpa2 d1vg8a_ d4qxaa_ d2ew1a1 d1x1ra1 d2bcgy_ d3doea1 d3cona_
d3rega_ d3wyga_ d1upta_ d2b6ha1 d1zd9a1 d1lnza2 d1r5ba3 d1n0ua2
d1r2qa_ d2f9la1 d2rhda_ d3q3jb_ d3q85a_ d1efma1 d1r8qa_ d2gf9a1
d2akab1 d1ek0a_ d1z08a1 d2gcoa1 d2dy1a2 d4g01b_ d2cxxa1 d1svia_
d3q72a_ d1ni3a1 d4ac9a4 d2erxa1 d2bmea_ d1wxqa1 d2gf0a1 d4ldja1
d1zunb3 d1d5ca_ d4lv5b_ d1efca3 d1kmqa_ d2g6ba1 d3lrpa_ d1tada2
d2y8ea_ d4rd4a1 d1z06a1 d2heia_ d3a58b_ d1u0la2 d1d2ea3 d1zj6a1
d2ocba_ d3rwoa_ d1mr3f_ d2e9sa1 d4u5xa1 d2iwra_ d2fn4a1 d3bwdd_
d2nzja1 d2atva1 d2ksqa_ d3l43a_ d1mkya2 d1mkya1 d1wmsa_ d1jnya3
d2rmka_ d1f6ba_ d1f60a3 d1gota2 d1jala1 d2fh5b1 d1g16a_ d2wblc_
d1m7ba_ d1wf3a1 d1f5na2 d1t9ha2 d1z6xa1 d1z0aa1 d2fg5a1 d2a5ja1
d2bcjq2 d1s0ua3 d1ky3a_ d4y0va_ d4lhwa_ d2qmea1 d2atxa1 d1egaa1
d1ryha_ d1moza_ d1nrjb_ d2erya1 d3bfka_ d1puia_ d1fzqa_ d3j3az1
d3t5ga_ d1shza2 d3defa_ d2f7sa1 d3raba_ d3tsoa_ d3aq4a_ d2al7a1
d3sfva1 d3bc1a_ d2ncda_ d1sdma_ d3h4sa1 d2heha_ d2grya_ g3kin.1
d1kk8a2 d1goja_ d1f9va_ d2mysa2 d1lkxa_ d3t0qa_ d4db1a1 d3gbja_
d1ry6a_ d1v8ka_ d1k8ka2 d1k8ka1 d3uleb1 d3i33a2 d3i33a1 d1dkgd2
d1dkgd1 d4ci6a2 d1jcfa1 d4a2ba2 d2fxua1 d1yaga2 d1yaga1 d4cbua2
d1bupa2 d3vrda1 d3vrda2 d1fcdc2 d1fcdc1 d1h1oa2 d1m70a2 d1m70a1
d1ig8a1 d1czan2 d1czan3 d1czan1 d1ig8a2 d1czan4 d1bg3a4 d1bg3a3
d1bg3a2 d3h3no1 d3h3no2 d3ezwa2 d1zc6a1 d1zbsa1 d1zbsa2 d1zc6a2
d2ch5a2 d2ch5a1 d1zxoa2 d1zxoa1 d2d0oa3 d2d0oa2 d1nbwa3 d1hroa_
d4gedb_ d3zooa_ d1mz4a_ d1gu2a_ d1f1fa_ d1dvva_ d1c6ra_ d1c75a_
d1e29a_ d1gdva_ d1cnoa_ d1h32b_ d1co6a_ d2bh4x_ d2aiua_ d5cytr_
d1jdla_ d1i8oa_ d2zboa_ d3dmia_ d1ls9a_ d1wvec_ d2yk3a_ d3ph2b_
d1dw0a_ d5aura_ d1ql3a_ d1qn2a_ d1f1ca_ d1evsa_ d1cnt1_ d1pvhb_
d1z7ca_ d1huwa_ d1bgea_ d1rhga_ d1f6fa_ d1f45b_ d1i1rb_ d1axia_
d1etea_ d3tgxb_ d1scfa_ d2z3qa1 d3qb1a_ d1hula_ d1m48a_ d4nkqb_
d1eera_ d1v7mv_ d5e4ea_ d2o27a_ d3b5ka_ d2psma1 d4zf7a_ d3uf5a_
d4x51a_ d1d9ca_ d4ypgc1 d3oq3a_ d1lqsl_ d1n1fa_ d1au1a_ d1wu3i_
d3besl_ d3ux9a_ d1v8fa_ d2ejca_ d3muea1 d1ihoa_ d3q12a1 d4dnxa2

d1m8pa2 d1g8fa2 d1jhda2 d1x6va2 d2b7la_ d3rbaa_ d2qtra_ d1nupa_
d3l92a_ d1f9aa_ d1qjca_ d2h29a_ d1vlha_ d1od6a_ d1lw7a1 d4ruka_
d2el7a_ d1iq0a2 d1irxa2 d1r6ta2 d1ivsa4 d2cyba_ d1rqga2 d1j09a2
d2yxna_ d1h3fa1 d1ffya3 d2g36a1 d1nzja_ d1jila_ d1n3la_ d1i6la_
d1pfva2 d1h3na3 d3n9ia_ d3sz3a1 d3sw2a_ d1moxc_ d1dx5i3 d1edmb_
d1autl2 d1autl1 d1nqlb_ d1dval1 d1szba2 d1kigl_ d2y5fl_ d1ijqa2
d1gk5a_ d1g1sa2 d1nt0a3 d1xdtr_ d1lmja2 d1lmja1 d1nzia2 d2wphe_
d1klil_ d1pfxl2 d1pfxl1 d1p9ja_ d1g1ta2 d1f7ea_ d4yzub_ d1qx2a_
d1ig5a_ d1s5ja2 d1qhta2 d4dlea2 d1tgoa2 d3py8a2 d1q8ia2 d1kfsa2
d2py5a2 d1exra_ d1tiza1 d1nyaa_ d1g8ia_ d1ggwa_ d1wdcc_ d3fwba_
d1ggza_ d2o5ga_ d1ctaa_ d1m45a_ d3i5gc_ d1sl8a_ d1fpwa_ d2scpa_
d2roaa_ d4aqra_ d3pn7b_ d1br1b_ d2mysb_ d1s6ca_ d1bjfa_ d2obha_
d1jfka_ d1jf0a_ d1qv0a_ d1lkja_ d2mlfc_ d2m97a_ d2d8na_ d4by5a_
d1jbaa_ d4nqga_ d1wdcb_ d2mysc_ d1j7qa_ d1s6ia_ d1uhka2 d1r2ua_
d1dtla_ d3kf9a_ d3pn7c_ d1qjta_ d1c07a_ d1gjya_ d1y1xa_ d1hqva_
d1dvia_ d1qxpa2 d4usla_ d1k94a_ d1wkya2 d1uuqa_ d3wh9a_ d4m1ra_
d1xyza_ d4xuya_ d2je8a5 d4bf7a1 d3cuia_ d1ur4a_ d1qw9a2 d1odza_
d2vzsa5 d1bqca_ d2x2ya1 d1hjqa_ d1qnra_ d4y7ea1 d1vjza_ d4k68a_
d2v3ga_ d4hpga_ d1nofa2 d1nq6a_ d1h4pa_ d1aq0a_ d3ziza_ d4bs0a_
d1ecea_ d1h1na_ d1ta3b_ d1w91a2 d1i1wa_ d1uhva2 d1us3a2 d1egza_
d7a3ha_ d1tvna1 d3nyda1 d1ghsa_ d2cyga1 d4xv0a_ d2c8na2 d1w32a_
d3emca_ d1v6ya1 d1foba_ d2uwfa1 d2fgla_ d3niya1 d2c0ha1 d1rh9a1
d1yq2a5 d1szna2 d1fa2a_ d1pama4 d5do8a1 d1ea9c3 d2fhfa5 d1qhoa4
d1avaa2 d1m53a2 d4m8ua1 d2gjpa2 d1lwha2 d4howa1 d1ktba2 d1j0ha3
d3vm5a1 d2x1ia_ d1wzaa2 d1uasa2 d2diea1 d1mxga2 d1ht6a2 d1eswa_
d1iv8a2 d5cpsa_ d1ud2a2 d1zjaa1 d3gbea1 d1kwga2 d1wdpa_ d1gcya2
d1g5aa2 d1hvxa2 d3dhpa2 d2bhua3 d4do4a1 d3qgva1 d1e43a2 d1g94a2
d2d3na2 d1gjwa2 d1gvia3 d1x1na1 d1r7aa2 d1tz7a1 d4toqa_ d1wb0a1
d1w9pa1 d1itxa1 d1kfwa1 d1goia2 d1edqa2 d2gsja_ d1hjxa1 d1jnda1
d1vf8a1 d3wdpp1 d3wq5a_ d1gnxa_ d4gxpa_ d4hz8a_ d1e4mm_ d3aiua_
d1wcga1 d3ahxa_ d1v02a_ d2dgaa_ d1pbga_ d1vffa1 d1qoxa_ d4ha4a_
d1mixa2 d1e5wa2 d1isna2 d2al6b2 d1g4ma1 d2hmta_ d1lssa_ d1iuka_
d2csua1 d3q9ub_ d2yv1a1 d3q9na_ d2d59a_ d1oi7a1 d2yv2a1 d1y81a1
d1wgqa1 d1v89a1 d2coda1 d1zc3b_ d1wg7a1 d1u5ea1 d1v61a1 d2coca1
d2ys1a1 d1v5pa1 d1ntya2 d1wjma1 d1wi1a1 d1u29a_ d1xx0a1 d1unqa_
d2zkmx3 d1fhoa_ d1v88a1 d2adza1 d1x1fa1 d1p6sa_ d2coaa1 d1v5ma1
d1faoa_ d2x18a_ d1dyna_ d1x1ga1 d4kaxb_ d2j59m1 d1ki1b2 d1xcga2
d1txda2 d1u5da1 d1dbha2 d1w1ha_ d1upqa_ d1v5ua1 d1foea2 d2fjla1
d1eaza_ d2dfka2 d2cofa1 d1kz7a2 d9ldta1 d2ewda3 d1up7a1 d2dfda1
d1s6ya1 d1vjta1 d7mdha1 d1hyea1 d1t2da1 d1ojsa3 d1b8pa1 d1ldna1
d1hyha1 d1civa1 d2a92a1 d1ez4a1 d1i0za1 d1y6ja1 d1gv0a1 d1ur5a1
d1llda1 d1obba1 d1v8ba1 d1gdha1 d1l7da1 d2dlda1 d1mx3a1 d1ygya1
d1qp8a1 d1j4aa1 d2naca1 d1npya1 d3t4ea2 d1a4ia1 d1o0sa1 d2hk9a2
d2egga2 d1b26a1 d1bvua1 d1gq2a1 d1c1da1 d1leha1 d1gtma1 d1euza1

d1nvta1 d3dona2 d1edza1 d1vi2a1 d1p77a1 d1nyta1 d1b0aa1 d1vl6a1
d1v9la1 d1txga2 d3f47a1 d1mv8a2 d2pv7a2 d1xc2a3 d2ahra2 d4xiya1
d2f1ka2 d1pgja2 d1i36a2 d1np3a2 d1lj8a4 d1teaa3 d1qmga2 d2i76a2
d2g5ca2 d1wdka3 d1dlja2 d3cuma3 d1kola2 d1f8fa2 d1qora2 d1jqba2
d1vj0a2 d1cdoa2 d1gu7a2 d1d1ta2 d1xa0a2 d2dm6a2 d1jvba2 d1rjwa2
d1h2ba2 d1tt7a2 d1piwa2 d1o89a2 d1iz0a2 d1e3ja2 d1yb5a2 d1uufa2
d1vj1a2 d1e3ia2 d3gpdg1 d1vc2a1 d2nvwa1 d1u8fo1 d1ydwa1 d1hdgo1
d2g17a1 d1cf2o1 d2czca2 d1r0ka2 d1obfo1 d2cvoa1 d1ebfa1 d1h6da1
d1i32a1 d1zh8a1 d2q49a1 d1qkia1 d1j5pa4 d3pzra1 d1xeaa1 d1vkoa1
d1vm6a3 d3qvsa1 d2pkqo1 d1b7go1 d1gr0a1 d1p1ja1 d1lc0a1 d1j0xo1
d3pwka1 d2hjsa1 d1zyao1 d1vkna1 d1tlta1 d1nvmb1 d3sjua_ d3ojfa1
d1xu9a1 d2jjya1 d1n7ha_ d1y1pa1 d1h5qa_ d1jtva_ d4fs3a_ d2bkaa1
d1gy8a_ d2c5aa1 d1ulsa_ d1ooea_ d2b69a1 d1y5ma1 d1spxa_ d1ae1a_
d1bxka_ d1ydea1 d1gega_ d1hdoa_ d1o5ia_ d1e6ua_ d1snya1 d2rhca_
d3zv4a_ d5ceja_ d2d1ya1 d1fjha_ d3w8ea_ d2fmua1 d1x1ta_ d1rpna_
d3ay7a1 d1k2wa_ d1eq2a_ d2hrba1 d3ruaa_ d2ew8a1 d4hwka_ d1xgka_
d2bd0a1 d1yo6a1 d2a35a1 d2ntva_ d1vl8a_ d1hdca_ d4bkua_ d2ag5a1
d2q45a_ d1nffa_ d4iy1a_ d1zbqa1 d1kewa_ d1ek6a_ d1ja9a_ d2q46a2
d1iy8a_ d1cyda_ d2ptga_ d1hxha_ d1uzma1 d3lyla_ d4m89a_ d3wyea_
d2blla1 d1w6ua_ d1sb8a_ d2q2va_ d4nr0a_ d1e6wa_ d2gasa_ d3oiga1
d2bgka1 d1mxha_ d1vl0a1 d1rkxa_ d1r6da_ d1wmaa1 d1e7wa_ d2c07a1
d1xkqa_ d3tzha_ d1g0oa_ d3lz6a_ d1qyca_ d1edoa_ d1gz6a_ d1oc2a_
d1t2aa_ d1zema1 d2xoxa_ d1z45a2 d1geea_ d1p33a_ d3lt0a_ d4e6pa_
d2wyua_ d1db3a_ d4q9na_ d1xhla_ d4o1ma_ d1fmca_ d2uvda_ d2gdza1
d1uaya_ d2z1ma_ d2o23a2 d2fr1a2 d2fr1a1 d1qsga_ d1qyda_ d4zjua_
d2a4ka1 d1yb1a_ d1wvga1 d1yxma1 d3f4ba_ d1mg5a_ d1xg5a1 d1xd3a_
d3ifwa_ d1ex0a4 d1g0da4 d2f4ma1 d1x3za_ d1mdwa_ d1qxpa4 d1ziva1
d1zcma1 d1t3ca_ d1epwa3 d2a97a_ d3fiea_ g1e1h.1 d3ioqa_ d1fh0a_
d1o0ea_ d1khqa_ d1m6da_ d2cb5a_ d1cs8a_ g8pch.1 d1s4va_ d2pnsa_
d1vsna1 d1mira_ d1iwda_ d2oula_ d7pcka_ d3bwka_ d3bcna_ d2p86a_
d1pvja_ d1pcia_ d1gece_ d1deua_ d4bs6a_ d1xkga1 d1cqda_ d1jqpa2
d2c0ya_ d2bdza_ d3p5ua_ d2o36a_ d1k9xa_ d1ex4a2 d1c6va_ d2jk6a2
d2jk6a1 d1feca1 d1feca2 d1lpfa1 d1lpfa2 d1xhca1 d1xhca2 d1dxla2
d1dxla1 d1gera2 d1m6ia2 d1onfa1 d1onfa2 d3lada2 d3lada1 d1w4xa1
d1w4xa2 d1gesa2 d1mo9a1 d1xdia1 d1fl2a2 d1fl2a1 d1fcda1 d1fcda2
d1h6va2 d1h6va1 d1mo9a2 d1gv4a2 d1gv4a1 d2gv8a2 d2gv8a1 d1aoga1
d1aoga2 d1hyua1 d1ebda1 d1ebda2 d3dk9a1 d1q1ra2 d1q1ra1 d1gesa1
d1zbfa1 d2z1ha_ d2etja1 d3qioa1 d1jl1a_ d2e4la_ d1jl2a_ d2lsna1
d1io2a_ d1uaxa_ d2yv0x_ d1mu2a1 d1ekea_ d1i39a_ d1pn0a1 d1reoa1
d1ju2a1 d2f5va1 d3c96a1 d2gmha1 d1pj5a2 d1gpea1 d1n4wa1 d1k0ia1
d2dw4a2 d2ivda1 d1seza1 d2voua1 d1kdga1 d1s5ja1 d2hbja2 d2igia_
d1wlja_ d1qhta1 d4dlea1 d1tgoa1 d2f96a1 d3tr8a1 d3mxma_ d1q8ia1
d1y97a1 d1w0ha_ d1kfsa1 d2py5a1 d1noya_ d1d5aa1 d1yt3a3 d2guia_
d3mwpa1 d4kb1a_ d1b9ra_ d1offa_ d1frra_ d3wcqa_ d1e9ma_ d1l5pa_

d1wria_ d1i7ha_ d3av8a_ d1czpa_ d1rfka_ d3p1ma1 d3b2ga_ d2bt6a2
d1v58a1 d1t3ba1 d4ilfa2 d4npba2 d1jmxa4 d1jmxa3 d1pbya3 d1jmxa1
d1pbya1 d1pbya2 d1r7ha_ d1faaa_ d1wika1 d1xfla_ d1ttza1 d2f51a1
d1ti3a_ d1nhoa_ d1sena_ d2i4aa_ d1zmaa1 d1fo5a_ d3lefa1 d3diea1
d2yana1 d1z6na1 d1oaza_ d3zywa1 d4aj8a_ d1nm3a1 d4fiwa_ d1wmja_
d4hs1a1 d1iloa_ d1xwaa1 d1wjka1 d2vm1a_ d1fb6a_ d1gh2a_ d1h75a_
d1nw2a_ d1kx5b_ d3a6na_ d4m6ba2 d2nqbd_ d3wkjd_ d2yfwe_ d4h9na_
d5bnxa_ d1kx5c_ d3r45a_ d1eqzb_ d4jjnb_ d1f66c_ d1eqza_ d4wnnb_
d1kx5a_ d1u35c1 d1id3a_ d2nqbc_ d1kx5d_ d2b5ea2 d2b5ea3 d2b5ea1
d2b5ea4 d2kp1a1 d2kp2a1 d1hyua3 d1hyua4 d1j08a1 d1j08a2 d1pd212
d1nhya2 d2a2ra2 d1k0dd2 d1f2ea2 d1gsua2 d1f3ba2 d2fhea2 d2pvqa1
d1tw9a2 d1oyja2 d1e6ba2 d3ik7a1 d4is0a1 d1r5aa2 d1jlva2 d1m0ua2
d3gtub2 d1z9ha2 d1okta2 d1axda2 d2gsra2 d1tu7a2 d1k3ya2 d1zgma1
d1b48a2 d1gnwa2 d1gwca2 d2gsta2 d1glqa2 d1oe8a2 d1pn9a2 d1v2aa2
d1fw1a2 d1duga2 d1nira1 g1f8v.1 d1novc_ d2bbvc_ d4ilya_ d4olta_
d1qgia_ d1qqya_ d1gd6a_ d1f6ra_ d1ivma_ d2gv0a_ d1iiza_ d2z2fa_
d1j8xa_ d1ghla_ d1b9oa_ d3w3ea_ d4tx7a_ d3cqla_ d4msta_ d3iwra_
d2z37a_ d4dyga_ d2za0a_ d2c21a1 d2pjsa1 d1klla_ d2i7ra1 d1ecsa_
d1xqaa1 d5cj3a1 d1jifa_ d2p7oa_ d1r9ca_ d1nkia_ d4iuaa3 d4bvva_
d5hpga_ d3k65a_ d1ki0a3 d1ki0a2 d1ki0a1 d1a0ha1 d3sp8a3 d2feba1
d1qsta_ d2beia1 d4m3sa_ d1u6ma1 d1ygha_ d1yk3a1 d3dr6a_ d1xmta_
d1z4ea1 d2ge3a1 d1bo4a_ d2fiaa1 d2jdca1 d1y9wa1 d3to7a_ d1wwza1
d2fl4a1 d2ozua1 d2b5ga_ d2cy2a1 d2p0wa_ d1cm0a_ d4pswa_ d1yrea1
d1yr0a1 d1m4ia_ d2gana1 d1yvka1 d1ghea_ d2g3aa1 d1y7ra1 d2euia1
d1q2ya_ d2fsra1 d1xeba_ d2ae6a1 d1cjwa_ d1qsma_ d2aj6a1 d1y9ka1
d1i12a_ d1vkca_ d2atra1 d1n71a_ d2d4pa1 d2fiwa1 d2fe7a1 d1tiqa1
d2ou2a_ d1yx0a1 d2fcka1 d1p0ha_ d2j8ma_ d2pq8a_ d1z4ra1 d1s3za1
d1ufha_ d1dosa_ d1gvfa_ d3qm3a1 d2fjka_ d1xvqa1 d1pn0a2 d3hy2a_
d2cvba1 d1o8xa_ d1lu4a_ d1we0a1 d2bmxa1 d2a4va1 d1psqa_ d1xiya1
d1q98a_ d1n8ja_ d1zofa1 d2ggta_ d3tjja_ d4je1a1 d1o73a_ d3s9fa_
d2zcta_ d1qmva_ d1jfua_ d2b1ka_ d5dvba1 d2b5xa1 d2v2ga_ d1st9a_
d3tb2a_ d4llra_ d4fh8a1 d1e2ya_ d1knga_ d2h30a_ d1i5ga_ d2z9sa_
d2f8aa1 d1tp9a1 d2b7ka_ d1nm3a2 d3hvva_ d2wfca1 d4kcea_ d1xvwa1
d4gqca1 d3qpma_ d4k1fa_ d1zzoa1 d1gp1a_ d4od7a1 d1z6ma1 d4mcua1
d4dvca1 d3l9sa_ d1un2a_ d3rppa_ d1cg2a1 d1yloa2 d1y7ea2 d1y0ya3
d1lfwa1 d1vixa1 d1fnoa4 d1vhoa2 d1ysja1 d1vhea2 d1z2la1 d2fvga2
d1vgya1 d1r3na1 d2grea2 d1xmba1 d1rtqa_ d1w52x1 d2ppla1 d1hpla2
d1lpbb2 d2oxea1 d1bu8a2 d1rp1a2 d1ys1x_ d4hs9a_ d1ex9a_ d1ku0a_
d3qmma_ d1ra6a_ d1muka_ d3gsza_ d1khva_ d1te9a_ d2xwha_ d3uqsa_
d3hkwa_ d3n6ma_ d2cjqa_ d2xyma_ d1teba_ d1te8a_ d1u09a1 d4wfxa_
d3tyqa_ d2pgga1 d1gyta2 d1lgya_ d4l3wa_ d1llfa_ d1gz7a_ d1ukca_
d2hqya1 d2hqya2 d1lrza2 d1ne9a1 d1lrza3 d3groa_ d1pjaa_ d1ei9a_
d1svpa_ d3su6a1 d1cu1a1 d4agka_ d1vcpa_ d1q31a_ g1n1l.1 d3e90b_
d1ep5a_ d3u1jb_ d3zxha_ d1cgla_ d1rm8a_ d1eaka2 d1hy7a_ d2jsda_

d1cxva_ d1hv5a_ d1i76a_ d3p0ya1 d1igra1 d1igra2 d1n8yc2 d1n8yc1
d1m6ba2 d1m6ba1 d1dzka_ d4tlja_ d2a2ua_ d4wfva_ d3t1da_ d4iaxa_
d3zq3a_ d2nnda_ d1i4ua_ d1kxoa_ d1bbpa_ d1kt7a_ d1exsa_ d3s26a1
d1lf7a_ d1qfta_ d4gh7a_ d1z24a1 d1qabe_ d1xkia_ d1ew3a_ d3bx7a_
d4iawa1 d1n0sa_ d1gkab_ d3kzaa_ d2k23a_ d2l9ca1 d2joza1 d1gm6a_
d3kffa_ d3saoa1 d1e5pa_ d1yupa1 d1iiua_ d1c8nc_ d1x36a_ d1smvc_
d1ng0c_ d4sbvc_ d1opoa_ d1f2nc_ d1cixa_ d1y29a_ d1v7fa_ d2jtba_
d1d1ha_ d1g9pa_ d1qk7a_ d1mb6a_ d2a2va_ d1niya_ d1i25a_ d1hvwa_
d2mpqa_ d1ie6a_ d1kqha_ d1la4a_ d2mxoa1 d1dl0a_ d1koza_ d1qk6a_
d1emxa_ d1lqra_ d1c6wa_ d1js9c_ d1f15b_ d1za7a_ d1laja_ d1rypd_
d1irua_ d1rypc_ d3unfk_ d1rypa_ d1j2pa_ d1irui_ d1j2qh_ d3unfh_
d1m4ya_ d1rypb_ d1irul_ d1irug_ d1iru2_ d1q5rh_ d1rypg_ d1g3ka_
d1iruf_ d1q5qa_ d1e94a_ d1irub_ d1ryph_ d3ty6a_ d1iruj_ d1iruk_
d1rype_ d1irud_ d1irue_ d3mi01_ d1rypf_ d1iruh_ d2z3ba_ d1iruc_
d3mka2_ d1iru1_ d1ny721 d1a6ca3 d1a6ca2 d1a6ca1 d1pgl22 d1pgl21
d1pgl11 d1ny711 d1mqta_ d1d4m3_ d1fmd3_ d2mev1_ g1aym.1 d1ev13_
d4q4w1_ d1r1a3_ d2x5ia_ d1rhi1_ g1r1a.1 g1d4m.1 g1rhi.1 d1b35b_
d1oopa_ d1tme1_ d4iv1a_ g1pvc.1 g1fpn.1 d1fpn1_ d4gb31_ d1d4m1_
d1fmd1_ d1ev11_ d2wzr3_ d1qqp3_ g1fmd.1 d2x5ic_ d1rhi3_ g1oop.1
d1hxs1_ g1hxs.1 d1fpn3_ d1bev1_ g2mev.1 d1eah3_ d1aym1_ d1pvc3_
d1qqp1_ d1zba3_ d1h8ta_ d3vbhc_ g1b35.1 g1bev.1 d1hxs3_ g1ev1.1
d1bev3_ g1tmf.1 d1tmf1_ d1b35a_ d2mev3_ g1eah.1 g1h8t.1 d1eah1_
d4q4w3_ d1r1a1_ d1aym3_ d1pvc1_ d1zba1_ d1h8tc_ d1oopc_ d1tme3_
g1qqp.1 d4iv1c_ g1tme.1 d1y14b1 d2nn6i1 d1i94l_ d2zjra2 d1smxa_
d2qamc2 d1hh2p1 d2r7da1 d1rl2a2 d1wfqa1 d3bzka4 d1x6oa2 d3a0ja_
d2j01d2 d2ja9a1 d4ql5a_ d1hr0w_ d1luza_ d2n3sa_ d2vnud1 d1h95a_
d1go3e1 d2nn6h1 d1ne3a_ d2mo1a_ d1iz6a2 d1jjga_ d1e3pa2 d2ix0a3
d2ix0a2 d2lxja_ d1ueba3 d1ueba2 d2z0sa1 d1u0la1 d2ix0a1 d2eifa2
d2je6i1 d3i2za1 d2c35b1 d2ahob2 d1t9ha1 d3i4oa_ d2qall1 d1jt8a_
d2nn6g1 d1g6pa_ d3cama_ d1i94q_ d1c9oa_ d1d7qa_ d2ba0a1 d2vnud2
d2vnud3 d1khia2 d2qalq1 d1wi5a1 d1xtda2 d1ny4a_ d1eixa_ d3exra_
d2ffca1 d2fdsa1 d3n3ma1 d2czda_ d2yyua_ d1vqta1 d1dbta_ d3ldva1
d1ux8a_ d4i0va_ d2bkma_ d1dlwa_ d2bmma_ d2r80b_ d1cqxa1 d1cg5a_
d1gcvb_ d3boma_ d2h8fb_ d2raoa_ d3bj1a_ d4hrrb_ d2r50a_ d3d1kb_
d2aa1b1 d3zhwa_ d3hf4b_ d3gdja_ d1jebb_ d3bj1b_ d1x9fb_ d1wmub_
d1gcva_ d3bcqb_ d3d4xb_ d1gvha1 d2dc3a_ d3hf4a_ d4yu3a_ d3k8ba_
d1qpwb_ d2r80a_ d3bcqa_ d1v4wa_ d3wtgb_ d1jl7a_ d4esaa_ d1a9we_
d3d1ab_ d1fsla_ d4h2la_ d1v4wb_ d1mwca_ d1vhba_ d1jeba_ d1spgb_
d1h97a_ d1q1fa_ d1tu9a1 d3d1aa_ d1hdsb_ d3hyub_ d2zfbb_ d3fs4a_
d1oj6a_ d1shrb_ d4h2lb_ d4yu3b_ d1spga_ d3hrwa_ d3qqra_ d3at5a_
d4wchd_ d3hyua_ d1cg5b_ d1uc3a_ d1hbra_ d2raob_ d1outb_ d4esab_
d3gdjb_ d3qqqa_ d1x9fc_ d3vrfa_ d1itha_ d3mkba_ d3d4xa_ d3mjpa_
d1jl6a_ d3fh9a_ d1outa_ d2dc3b_ d1it2a_ d3eoka_ d3g46a_ d1qpwa_
d3vrfb_ d1ch4a_ d1x9fd_ d1hdsa_ d2zfba_ d3mkbb_ d1or4a_ d3wtga_

d1a4fa_ d3fh9b_ d3bomb_ d3cy5a_ d3at5b_ d1x9fa_ d2oifa_ d4lmxb_
d1cpcb_ d3v57a_ d1kn1a_ d1cpca_ d4f0ta_ d2vjha_ d4po5b_ d1eyxa_
d1alla_ d4f0tb_ d1b33b_ d1b8db_ d4h0mb_ d1b33a_ d2vjhb_ d4lm6b_
d2vmlb_ d1allb_ d2vjta_ d3dbja_ d2vmla_ d2vjtb_ d4f0ub_ d1f99b_
d4rmpa1 d1liaa_ d1eyxb_ d2uulb_ d4lmsb_ d4f0ua_ d2bv8a_ d1f99a_
d2bv8b_ d2c7lb_ d3dbjb_ d4rmpb1 d1b8da_ d4h0ma_ d3v57b_ d1kn1b_
d1liab_ d2uula_ d2j96a_ d3ia2a_ d1a88a_ d4dgqa1 d1hkha_ d1zoia_
d1mtza_ d1hlga_ d2xqfa_ d1ea5a_ d1f6wa_ d1dx4a_ d1qe3a_ d1k4ya_
d4qwwa_ d2ha2a_ d2qvba_ d2v9za_ d4kafa1 d1pv8a_ d1w5qa_ d2c1ha_
d1ohla_ d1l6sa_ d1xova2 d1f74a_ d3l21a_ d2e1da_ d1to3a1 d1w3ia_
d1sfla_ d1n7ka_ d1vcva1 d4imea_ d1wa3a_ d2ocza1 d1f2ja_ d3m16a_
d2yxga_ d3a5fa_ d1fbaa_ d1l6wa_ d1xfba1 d1wx0a1 d1vpxa1 d1ojxa_
d1mxsa_ d2a4aa1 d1xkya1 d3cq0a1 d3s1xa_ d1f05a_ d1fdja_ d3cpra1
d4d2ja_ d1e88a1 d1l6ja5 d1eaka4 d1eaka3 d1l6ja3 d1h8pa2 d1h8pa1
d3rp2a_ g1ucy.1 d1fuja_ d2zchp_ d3w94a_ d1si5h_ d1lo6a_ g2pka.1
d1cgha_ d1kigh_ d1a5ia_ d3s9ba_ d1hj8a_ d1ao5a_ d2anya_ d1q3xa1
d1gvza_ d1azza_ d4gsoa_ d2f91a1 d1iaua_ d1gdna_ d1eufa_ g1fiw.1
d1eq9a_ d4nfea_ d2rdla_ d1spja_ d1elva1 d1a7sa_ d1ekbb_ d1pfxc_
d1yc0a_ d2aiqa_ g1fiz.1 d1fi8a_ d4k60a_ d4dgja_ d1mzaa_ d1op0a_
d3tjva_ d1pytd_ d1sgfg_ g1gg6.1 d2eeka_ g1rtf.1 d1a0ja_ d3g01a_
d1pytc_ d4e7na_ d1ddja_ d1z8ga1 d2hlca_ d1npma_ d1brup_ g1jou.1
d1sgfa_ d1autc_ g2hnt.1 g1kdq.1 d3s69a_ d4crga_ d1fxya_ d1bqya_
d2ftba_ d1pmpa_ d2rcqa1 d1fdqa_ d4lkpa_ d1sa8a_ d1vyfa_ d1lpja_
d3vg7a1 d4azqa1 d5bvqa_ d1p6pa_ d2qo4a_ d1opba_ d1yiva1 d1o1va_
d4bvma1 d4tkba_ d4i3ba_ d1bwya_ d1ftpa_ d1ggla_ d4gkca_ d1cbia_
d3p6da1 d1kqwa_ d2a0aa1 d1m4la_ d2bk7a_ d3fjua_ d1z5ra1 d1kwma1
d1uwya2 d1jqga1 d2c1ca1 d1h8la2 d1e69a_ d1mv5a_ d3g60a4 g1ii8.1
d1ewqa2 d1ji0a_ d1qhla_ d1v43a3 d2wmfa1 d1vpla_ d1g2912 d4a82a2
g1f2t.1 d2d62a1 d3tuic1 d2bbsa1 d3g60a3 d2ixea_ d1g6ha_ d1sgwa1
d2qi9c_ d3rlfa1 d3b60a1 d3d31a2 d2pzea1 g1xew.1 d1jj7a_ d1oxxk2
d2onka1 d1w1wa_ d1iwea_ d1qf5a_ d1vmaa2 d1nija1 d1np6a_ d2p67a1
d3ihla_ d1okkd2 d1ls1a2 d1s1ma2 d1iona_ d2v40a_ d1dj3a_ d3hida_
d2vo1a1 d2g0ta1 d1vcoa2 d1j8mf2 d1g3qa_ d1xjca_ d1p9ba_ d1qzxa3
d1ihua2 d1ihua1 d2qm8a_ d1dj2a_ d4ioja_ d1cp2a_ d1yrba2 d3gqba2
d1skye3 d1xp8a1 d1tf7a2 d1tf7a1 d1ye8a1 d1skyb3 d2qe7d2 d4blta_
d4huta_ d3ldaa2 d4hyya_ d2i3ba1 d1cr1a_ d3j9tb2 d3ntua2 d1vdza1
d2z43a_ d1e9ra_ d1nlfa_ d3ziaa2 d1n0wa_ d1g6oa_ d3ziad2 d3j9ta2
d1pzna2 d3gqbb2 d2i49a_ d1nnfa_ d1xvxa_ d1ursa_ d1h3da1 d1iuda_
d4m1va1 d1twya_ d3e13x_ d2nxoa1 d4pfua_ d3ttma_ d1elja_ d2qmwa1
d1us5a_ d1y3na1 d1jeta_ d1uqwa_ d4f19a1 d2esna2 d3thia_ d3fasa1
d2uyeb_ d2xwva1 d1r9la_ d2fyia1 d1pb7a_ d2ozza1 d1sw5a_ d1nh8a1
d1vr5a1 d2g29a_ d1xs5a_ d3lswa_ d2z23a_ d3w9va_ d3tcha_ d1q35a_
d1z7me1 d3q27a_ d1p99a_ d1ve4a1 d2i6ea1 d4mcoa1 d4n8ya_ d1eu8a_
d1zbma1 d1hsla_ d1ixca2 d1j1na_ d1xvya_ d1y9ua_ d2z22a1 d1xoca1

d4oera_ d1wdna_ d5f1qa1 d1xt8a1 d4gwoa_ d4igta1 d1pc3a_ d4mmpa1
d1o63a_ d2czla1 d2onsa2 d4mija1 d4ok5a1 d2bmfa1 d2bmfa2 d1yksa1
d1yksa2 d2qeqa1 d2jlqa1 d1a1va2 d1eh3a_ d3skpa_ d1dtza1 d1dtza2
d1ieja_ d1jnfa2 d1ce2a1 d1jnfa1 d1b1xa2 d1b1xa1 d1jw1a1 d1jw1a2
d1ryoa_ d1h76a1 d1h76a2 d1c4oa1 d2p6ra3 d2p6ra4 d1oywa2 d1oywa3
d1wrba1 d1w36b2 d1w36b1 d1c4oa2 d3bora_ d1t6na_ d2eyqa5 d2eyqa3
d2eyqa2 d2zu6a2 d1veca_ d1uaaa1 d1w36d1 d2g9na1 d1z3ix2 d2rb4a_
d1hv8a1 d1hv8a2 d1t5ia_ d1w36c2 d2j0sa2 d2j0sa1 d1fuka_ d3hjha1
d1z5za1 d1qdea_ d1z63a1 d1s2ma2 d1s2ma1 d1wp9a1 d1wp9a2 d1gm5a3
d1gm5a4 d3dina4 d3dina3 d1q0ua_ d1w36c1 d1z3ix1 d1nkta3 d1nkta4
d1uaaa2 d1w36d2 g1qhh.1 d2fwra2 d2fwra1 d3q3ma_ d1za0a1 d1mxra_
d2inca_ d1jk0a_ d2incb_ d1mhyb_ d3hf1b_ d1mtyb_ d1r2fa_ d2uw1a_
d1mhyd_ d3n37a_ d1afra_ d1oquc_ d1syya_ d1mtyd_ d2o1za_ d1uzra_
d1smsa_ d2fkza_ d1zuja1 d1s3qa1 d2fjca1 d1z6om1 d1veia_ d4dyya_
d1jiga_ d3gvya_ d2pyba_ d4cy9a1 d3bvfa1 d1r03a_ d2cf7a_ d1nf4a_
d1zs3a1 d4iwka_ d3ka8a_ d3np0x_ d1n1qa_ d1lb3a_ d4e6ka_ d1z6oa1
d2fzfa1 d4dyua_ d3ge4a_ d1jgca_ d4am4a_ d2z6ma_ d1ji5a_ d1vlga_
d1lkoa1 d1krqa_ d3qz3a_ d1o9ra_ d2ffxj_ d1euma_ d1yv1a1 d2v2pa_
d1j30a_ d4di0a_ d1dpsa_ d1oewa_ d2qzxa_ d2asia_ d1psoe_ d1miqa_
d1smra_ d2h6ta_ d1pfza_ d1izda_ d3hd8a_ d1dpja_ d4aa9a_ d1t6ex_
g1b5f.1 g1lya.1 g1htr.1 d1qdma2 d1bxoa_ d1wkra_ d1tzsa_ d1ibqa_
d2qzwa_ d2v76a_ d1wj1a1 d1aqca_ d1qqga2 d1ntva_ d1n3ha_ d4dbba_
d3f0wa1 d1p3ra_ d1wgua1 d2cy4a1 d3hqca_ d1wvha1 d1xr0b1 d1bdqa_
d3ggta_ d3ixoa_ d1mrxa_ d2haha_ d1tw7a_ d4njsa_ d1sgua_ d2p3da_
d1nsoa_ d3d3ta_ d2rkfa_ d1c6ya_ d2rspa_ d3ogpa_ d3ekwa_ d2o4na_
d2j9ja_ d1baia_ d3ggua_ d3lzua_ d4npua_ d4j55a_ d2r8na_ d2fxda_
d2p3ca_ d4dqea_ d3ttpa_ d3t3ca_ d2p3aa_ d2hc0a_ d2fdda_ d3mwsa_
d1wgda1 d1wgga1 d4q5eb_ d1wjna1 d1se9a_ d3n3kb_ d1wz0a1 d1we6a1
d2m0xa_ d2gbka_ d3rt3b1 d1wh3a1 d1sifa_ d2ekec2 d4ii2b1 d4hk2a1
d1c3ta_ d1m94a_ d1wgha1 d1ttna1 d1j8ca_ d1wx8a1 d3v6cb1 d2ma9b_
d4i6lb1 d2jvca1 d1iyfa_ d2k1fa_ d1wxva1 d1wf9a1 d1t0ya_ d2io0b2
d1x1ma1 d2gbma_ d1zw7a1 d2m17a_ d1ud7a_ d1we7a1 d2lwpa_ d2gbja_
d1wjua1 d1v6ea1 d1v86a1 d4eewa_ d1wiaa1 d4icva_ d4gu2a_ d1wx7a1
d1wy8a1 d4hjka1 d2faza1 d1v5oa1 d3ruib_ d3waob_ d2r2qa1 d3m95a_
d3wana_ d2al3a1 d2cr5a1 d1wj4a1 d1yvca1 d1yeza1 d4wfia_ d4cg1a1
d3visa_ d1iyka2 d1iyka1 d1rxta1 d1iica1 d1dszb_ d1a6ya_ d3cbba_
d1ynwa1 d1r4ia_ d1hcqa_ d2hana_ d2hanb_ d2enva1 d1kb4b_ d2nllb_
d1lo1a_ d1cita_ d1um8a_ d1ixza_ d1qvra2 d1qvra3 d1qzma_ d1sxjc2
d1jr3d2 d2p65a_ d1l8qa2 d3hu3a3 d1sxjd2 d1njfa_ d1in4a2 d1sxja2
d1tuea1 d2ce7a2 d1w5sa2 d1sxjb2 d1d2na_ d1ny5a2 d1ofha_ d2gnoa2
d2a5yb3 d1fnna2 d1u0ja1 d1lv7a_ d1iqpa2 d2fnaa2 d1ixsb2 d4gita_
d1sxje2 d1g41a_ d2e3fa_ d1mm0a_ d2e3ea_ d2llda_ d2ny8x_ d2e3ga_
d2ny9x_ d2nz3a_ d2lj7a_ d1n4na_ d2m8bb1 d2kyqa_ d3psma_ d1jkza_
d1jxca_ d2gl1a_ d1ugla_ d4uj0a_ d2k4ua_ d1hp2a_ d2lu9a_ d1baha_

| | | | | | | | |
|---|---|---|---|---|---|---|---|
| d2meoa1 | d1wm7a_ | d1hlya_ | d1n8ma_ | d1jlza_ | d1wpda_ | d1c55a_ | d1pvza_ |
| d1wt7a_ | d1rjia_ | d1wz5a_ | d1m2sa_ | d1pjva_ | d1c49a_ | d1qkya_ | d2ktca1 |
| d1wmta_ | d1du9a_ | d1j5ja_ | d1r1ga_ | d1e3oc2 | d1ic8a2 | d1t0za_ | d2i61a_ |
| d1t1ta_ | d1sn4a_ | d1kv0a_ | d2lkba_ | d1npia_ | d1wwna_ | d1pe4a_ | d2kbka_ |
| d2li7a_ | d1djta_ | d2kbha_ | d1dq7a_ | d1sega_ | d1omya_ | d1zywa1 | d1jzaa_ |
| d1n9wa1 | d1bbua1 | d1e1oa1 | d1b8aa1 | d1l0wa1 | d3a74a1 | d1eova1 | d1t61c1 |
| d1iyjb4 | d1iyjb5 | d1iyjb3 | d1o7ia_ | d4ipca1 | d1x3ea_ | d4apva_ | d1jb7a1 |
| d1jb7a2 | d1jb7a3 | d3kdfa_ | d1ue1b_ | d1wjja1 | d3eiva_ | d1xjva2 | d1xjva1 |
| d2ccza2 | d1se8a_ | d1l1oc_ | d1jb7b_ | d1qvca_ | d3ulla_ | d1qzga_ | d1miua4 |
| d1miua5 | d1miua3 | d3g48a1 | d1gd7a_ | d1mkha_ | d1jjcb3 | d1pyba_ | d3ubub_ |
| d1egia_ | d1v7pa_ | d1tdqb_ | d3ubua_ | d1c3ab_ | d1h8ua_ | d3bx4b_ | d1t8ca1 |
| d1sb2a1 | d1qdda_ | d2ziba1 | d3cada_ | d1byfa_ | d1j34b_ | d1jwia_ | d1jzna_ |
| d1c3aa_ | d1oz7b_ | d1wmza_ | d3bdwb_ | d1hq8a_ | d1fifa1 | d1dv8a_ | d3hupa_ |
| d1wk1a1 | d1umra_ | d2afpa_ | d3bx4a_ | d1gz2a_ | d1sl6a_ | d1v7pb_ | d3g8ka_ |
| d1ixxa_ | d1j34a_ | d1jwib_ | d1g1sa1 | d1y17a1 | d1ja3a_ | d1fvub_ | d3bdwa_ |
| d1sb2b1 | d1fvua_ | d1umrc_ | d1oz7a_ | d4m17a_ | d3cfwa1 | d1ypqa1 | d1g1ta1 |
| d3fd4a_ | d3l9jc_ | d4dn8a1 | d1v4lb_ | d1rdl1_ | d2msba_ | d1dgsa2 | d1fvia1 |
| d1p16a1 | d1x9na2 | d2shpa1 | d1yfoa_ | d1l8ka_ | d2ooqa1 | d1g4us2 | d1p15a_ |
| d1lara2 | d1lara1 | d1wcha_ | d2fh7a2 | d2fh7a1 | d1ohea2 | d2f4da_ | d1rxda_ |
| d2hxpa_ | d1xria_ | d1d5ra2 | d1fpza_ | d3ezza_ | d4y2ea_ | d1i9sa_ | d1tdha3 |
| d1q3ba3 | d1ee8a3 | d1k82a3 | d3ubra_ | d1qo8a1 | d1d4ca1 | d2k3va_ | d1ft5a_ |
| d1qdba_ | d1m1qa_ | d2j7aa_ | d1h21a_ | d1ogyb_ | d4fasa_ | d1jnia_ | d1sp3a_ |
| d3h33a_ | d1rwja_ | d19hca_ | d1os6a_ | d3bxua_ | d3h4na_ | d1i77a_ | d3caoa_ |
| d1j0pa_ | d2ctha_ | d1duwa_ | d1ofwa_ | d2e84a_ | d1hh5a_ | d3h34a_ | d1wioa4 |
| d1ccza2 | d1vcaa1 | d1dr9a2 | d1ic1a1 | d2d9qb1 | d2nxyb2 | d1adqa1 | d1fnga1 |
| d1uvqb1 | d1zs8a1 | d2vola1 | d2vola2 | d1mfbl2 | d2jelh2 | d1uwel2 | d3qq4b1 |
| d1kjvb_ | d1k5na1 | d1osph2 | d4odhl2 | d1ow0a1 | d1ow0a2 | d1bfob2 | d3l9ra2 |
| d3jtth1 | d2fath2 | d4elkb2 | d1fn4b2 | d2xfxb_ | d1pg7x2 | d1cqka_ | d4k71b2 |
| d1lk2a1 | d1eapb2 | d4lfhg2 | d1i4fa1 | d2nw2a2 | d1kfah2 | d1mhca1 | d1h3ua1 |
| d1wejh2 | d1h0db2 | d1a5fh2 | d1kcvh2 | d1mjuh2 | d1qqda1 | d1kcrh2 | d1k8ia1 |
| d3huja2 | d2nnab1 | d1nfdf2 | d3mffb2 | d1s9va1 | d1lk3h2 | d1q0xl2 | d1fv1b1 |
| d1neza1 | d4rfeb2 | d1r3ha1 | d1a6tb2 | d1kjva1 | d1pz5b2 | d1onqa1 | d2bc4a2 |
| d1de4a1 | d1k8da1 | d1tcra2 | d1dn0b2 | d1nfde2 | d2xwtb2 | d1bfoa2 | d1mexh2 |
| d1iakb1 | d1ypzf2 | d4f7ea2 | d1q72l2 | d2fbjh2 | d1fe8h2 | d1clzh2 | d1fngb1 |
| d1uvqa1 | d1vgka1 | d1c16a1 | d4j12a2 | d4x99b2 | d1igtb3 | d1je6a1 | d1q72h2 |
| d1nldh2 | d1teth2 | d2hmic2 | d1i1ca2 | d1i1ca1 | d1k8ib1 | d1q0yh2 | d1kfal2 |
| d3frua1 | d1pg7w2 | d1u58a1 | d1igyb4 | d1igyb3 | d1hyrc1 | d1klub1 | d1deeb2 |
| d1c12b2 | d2bc4b2 | d2ig2h2 | d1kcvl2 | d3wn5b1 | d1lk2b_ | d1lk3l2 | d1mjul2 |
| d1sm3h2 | d1q0xh2 | d1zvsa1 | d1ydpa1 | d1u8ib2 | d1ypze2 | d1kcuh2 | d1op9a_ |
| d1y0la1 | d1etzb1 | d1egjl1 | d2gsib1 | d1nj9a1 | d4jfhd1 | d1aqkl1 | d1t2ja_ |
| d1lo4h1 | d1zvya1 | d3v4vl1 | d4enec1 | d1kxtb_ | d5e8ea1 | d2pxya_ | d4weud_ |
| d1xiwc_ | d2esvd1 | d1qnzh_ | d1ogae1 | d1f4xh1 | d4n1hb_ | d3ezjb1 | d3mlwl1 |
| d1kfah1 | d1etza1 | d1b4jh1 | d3cfic_ | d4w68a_ | d2arja1 | d1ek3a_ | d2ok0l1 |
| d1vera_ | d1uwgh1 | d1blnb1 | d1fpth1 | d1rzga1 | d2giya1 | d4m3kb1 | d1nc2a1 |

d2nyyd1 d1ncna_ d1fbih1 d5dxwa_ d4fqqa1 d1dqdh1 d1jfql1 d1kelh1
d1faih1 d1frgl1 d1figl1 d4m6nl1 d1fo0a_ d4hxbl1 d3b5ga_ d3quyd1
d2h9gb1 d1i8kb_ d2dd8h1 d2ck0h1 d1eaja_ d4w6wb1 d2a6ib3 d1t66d1
d2e27h_ d1ob1b1 d1f11b1 d2v7na1 d1ad0a1 d2xt1b1 d1pskh1 d4i13b1
d2op4l1 d1a0ql1 d1uwel1 d1za3a1 d1ncbl1 d4uu9a_ d1bm3h1 d1cloh1
d1nqba2 d1cf8h1 d4y5va_ d1mhph1 d5anma1 d4cdgc1 d4ldeb_ d1y0lb1
d2pcpb1 d1z3gl1 d1dfbl1 d1fh5h1 d3g5xa1 d1tzib1 d4dn3l1 d3zhka1
d2b1hh1 d2uyla1 d1f3dh1 d1nj9b1 d2agjh1 d2h1pl1 d1q72h1 d8fabb1
d1ndmb1 d1rjca1 d4u6hb1 d3upca_ d2fbjl1 d1osph1 d1tvda_ d2cjvh_
d4fhbd1 d1bd2d1 d1rz7h1 d3nn8a_ d1blna1 d1knoa1 d1nfda1 d2bmkb1
d1dqll_ d1igyb1 d1igfl1 d12e8h1 d3utza1 d1nc2b1 d1rihh1 d4mwfb1
d4jvpa1 d1mieh1 d1rhfa1 d1fgnl1 d1sbsh1 d1lp9e1 d1dn0a1 d4lqfl1
d1iaih1 d1kgcd1 d1mh5b1 d1gpol1 d2g60h3 d1f8tl1 d2jixd1 d4grwh_
d1fo0b_ d1bfob1 d1axsa1 d1u9ka_ d1nmbh_ d1h8na1 d1h8na2 d1jguh1
d4eura1 d3sdyl1 d35c8h1 d1w72l1 d1mvub_ d1i9rh1 d1ob1a1 d4wenb_
d1yeeh1 d3ietc1 d1f58h1 d1ad0b1 d1mqkl_ d1nbvh1 d4b50a_ d3r8bb_
d1bafl1 d1mamh1 d4rgne1 d2gsgb_ d35c8l1 d1bafh1 d1ai1h1 d2pcpa1
d2fath1 d2x1pa_ d1orsb1 d1ibgl1 d1shma_ d1a2yb_ d1u0qa_ d2coqa_
d1hilb1 d1qfwi_ d1jv5b_ d1fn4b1 d5e0qa_ d1nsnh1 d1plgl1 d4ocxl1
d1kb5h1 d1rzfh1 d1pg7x1 d2fx7h1 d2oi9c_ d1l7il1 d3eyva1 d1d5il1
d4ffyl_ d1tetl1 d1a6wh_ d1ac6a_ d3dvfa_ d1nfde1 d1mimh1 d1ri8a1
d1jhlh_ d3dura_ d1fskb1 d1f97a1 d3so3b1 d2ak4d1 d1lp9f1 d4z7wf1
d1l7th1 d4y5ya_ d4x7ec1 d1emth1 d1ikfl1 d1nlbh1 d4utal1 d1bjma1
d1eapb1 d1u6ah1 d2dtgd1 d1kj2b_ d1xeda1 d1c1eh1 d1fgvh_ d2bnub1
d2fb4h1 d2dtgb1 d1ktrh_ d1jpth1 d1qkzl1 d4hepg_ d2atpb1 d1flrh1
d2hrpl1 d1mvfa_ d1iaim1 d3k80a1 d2jb5h1 d3zkxc_ d4grwe_ d1it9h1
d1rhhb1 d3w9ec_ d1e4wh1 d1sy6h1 d2j6eh1 d3qxta_ d1jglh1 d1p7ka1
d1tzhb1 d4n8pa1 d1gafl1 d2jelh1 d1wioa2 d4i0cc1 d1cicb1 d1lmka2
d1lmka1 d1iqwh1 d1orqb1 d1ejoh1 d1jnlh1 d4zg1a_ d43c9b_ d1wejh1
d3q6fa1 d1f3dj1 d3c6la1 d1bqlh1 d3p9wb_ d1a3rl1 d1h0db1 d2uzil_
d1eo8l1 d1qfwl_ d1n7ml1 d1igja1 d1c5ch1 d2a9mh_ d1rmfh1 d1fj1b1
d4heme_ d1ccza1 d1a5fh1 d1ogad1 d1dlfh_ d1qa9b_ d1vgel1 d3qskb_
d1sjxa_ d4ozfg1 d1sq2n_ d1qd0a_ d1kcvh1 d1c5db1 d3qcul1 d8faba1
d3bn9d1 d1mjuh1 d4y8dc_ d7fabh1 d3dvgb1 d1lo0l1 d1a6tb1 d1gigh1
d1kcrh1 d2hwzl1 d1yjdc1 d3clfl1 d4krna_ d1dl7l_ d1e6ol1 d1mexh1
d3r0ma_ d4kkna_ d1fnsl1 d1oaqh_ d1rzia1 d1ucbh1 d1ncwl1 d4b41a_
d1nfdf1 d2mpah1 d2d7tl1 d4grwf_ d4b5ea_ d1lk3h1 d3u79a_ d4u3xa_
d1ghfl1 d1bvkb_ d1mcww1 d1b88a_ d1jrhh1 d32c2b1 d4wemb_ d1dsfh_
d1op3h1 d1cel1l d1iqda1 d1qoka1 d1qoka2 d1qfuh1 d2crya1 d1ay1h1
d1oaql_ d3ab0b_ d1rurl1 d1t4kb1 d1bz7b1 d1n0xh1 d1forl1 d1ucbl1
d4w6yb_ d43c9a_ d2aabl1 d1tzia1 d4n1ca_ d1k6qh1 d3g9ab_ d4tvsa1
d1igmh_ d1pz5b1 d3dx9a1 d3wxwh_ d1nakh1 d1kcul1 d3eaka_ d4qlra1
d1cr9l1 d1d9ka_ d1f4xl1 d1deea1 d2i27n2 d1c12a1 d2orbl1 d1j8hd1
d1mcph1 d1c5da1 d1kenh1 d1bd2e1 d1h5ba_ d4x7fc1 d3upaa_ d1t3fa1

d1indh1 d2dtgc1 d4hc1l1 d1zv5a1 d4wgvb_ d1uwgl1 d4f37k1 d1aqkh1
d1vesa_ d3zkqd_ d1fptl1 d1lo4l1 d1knob1 d3rrqa_ d1ct8b1 d4orzc_
d1mlbb1 d4hgkc1 d1dqdl1 d1bogb1 d4ozhe1 d1dr9a1 d1i7za1 d1dn0b1
d1kell1 d1rzib1 d1ypzf1 d1wtla_ d1hkfa_ d1fail1 d1figh1 d2vyre_
d1sjva_ d1ohqa_ d1bfoa1 d1pkqb1 d1mjul1 d1b4jl1 d1nsnl1 d2arjb1
d1p4bh_ d4aj0a_ d3nn8b_ d2rcja1 d1f2xk_ d4v1dd_ d1adqh1 d2eksa_
d4k3ga_ d1jfqh1 d1uweh1 d1frgh1 d4gftb_ d1uz8b1 d4n1ea_ d1bm3l1
d1clol1 d3mcka1 d2vq1a1 d1ae6h1 d4aq1b1 d1uj3b1 d1nbqa1 d1fh5l1
d2ck0l1 d2ywza_ d1q72l1 d4nbxb_ d1hila1 d4hzlb1 d2j88l1 d1zmya1
d1cd0a_ d1a4ka1 d1pskl1 d1a0qh1 d4krmb_ d1ncbh1 d2fbjh1 d1j8he1
d1i3ua_ d1cf8l1 d1fe8h1 d2p1ya1 d1eeqa_ d2x89a_ d1ol0a_ d2r0lh1
d1ub5a1 d1dfbh1 d3eyvb1 d4jdvb1 d4p2cg_ d2gsia1 d2agjl1 d1u3ha1
d2h1ph1 d1rihl1 d1fskc1 d1boga1 d2a77l1 d1ospl1 d1qfoa_ d3dwta_
d2fx7l1 d1rz7l1 d3stba1 d1pkqa1 d1b6da1 d1dzba2 d1dzba1 d1kj2a_
d4l1ha_ d1rzgb1 d1dqlh_ d12e8l1 d1miel1 d1jmaa_ d1sbsl1 d1fgnh1
d1f58l1 d1mqkh_ d1ndgb1 d3qegl1 d1iail1 d2aw2a1 d1tqbc1 d3fo2a1
d4dkaa_ d1f8th1 d2g60l1 d2ywya_ d1uj3a1 d1yc7a_ d3bd3a_ d4w6xb1
d1nmbl_ d1i8ka_ d1rjlb1 d1ypze1 d1p7kb1 d1kxqe_ d1gpoh1 d2z4qa1
d1w72h1 d1i9rl1 d4qgya1 d4xt1c_ d2qqnh1 d1cica1 d3c08h1 d2cd0a_
d2bdnh1 d1hnga1 d1maml1 d1nldh1 d1cr9h1 d4lcih1 d3bkyh1 d3qg7l1
d1ai1l1 d1kxvc_ d4iosd_ d1l7ih1 d2x1qa_ d1ub6b1 d3k74b_ d1ibgh1
d1teth1 d1igya1 d1hxma1 d2x6ma_ d2e7la_ d5e03a_ d1qfwm_ d2r0kh1
d1igjb1 d1rurh1 d1plgh1 d1mj8h1 d1yuhb1 d3fmgl1 d1rzfl1 d1emtl1
d1ad9b1 d1igtb1 d1nlbl1 d1d5ih1 d3k7ua_ d4ffyh_ d1bwwa_ d3dvga1
d1a6ta1 d1fgvl_ d4qxgl1 d1miml1 d1jhll_ d2hffb1 d2hh0l1 d1qkzh1
d1l7tl1 d1afvh1 d1iaii1 d2cjuh_ d1um5h1 d2ghwb2 d2ghwb1 d1kgce1
d2fgwh1 d1ikfh1 d1dvfd_ d2i24n1 d2xtjb_ d1za3b1 d2yc1a_ d2hh0h1
d2x1oa_ d1bvka_ d1rjla1 d5e7ba1 d1igta1 d1il1b1 d2fb4l1 d1mvua_
d1jptl1 d32c2a1 d1fvca_ d1iqdb1 d2hrph1 d2p4ab_ d4qo1a_ d1p2cb1
d1wt5c_ d1t4ka1 d5c1mb_ d1it9l1 d1bz7a1 d1b2wh1 d1e4wl1 d1jnll1
d1yedb1 d1za6b1 d3u7aa_ d1igfh1 d3gnml1 d1orsa1 d1wejl1 d1a2ya_
d1hxmb1 d1gafh1 d2j4wh1 d1pz5a1 d1j05a_ d1a3rh1 d4fqll1 d1qfwh_
d1eo8h1 d1n7mh1 d4s11a_ d1kegh1 d5da4a_ d1iqwl1 d3whxb1 d1vgeh1
d1deeb1 d1c12b1 d1t2ql1 d1ad9a1 d3gsnb1 d3b2uc1 d1ggbh1 d1t3fb1
d1nakl1 d4ocmc_ d1lila1 d2v7hb3 d2esve1 d1mcoh1 d2qr0b1 d2q3aa_
d1ct8a1 d1kcrl1 d2pxyb_ d2uzih_ d5c3ld_ d3durb_ d1c5cl1 d2ak4e1
d1dl7h_ d1xiwd_ d1mlba1 d1rmfl1 d1i7zb1 d1dlfl_ d2xa3a_ d5bq7b1
d1dqqb1 d3h42l1 d1egjh1 d1kcvl1 d1fe8l1 d2j6el1 d1f3rb2 d1lk3l1
d1sm3h1 d1q0xh1 d1ghfh1 d7fabl1 d1igch1 d1lo0h1 d4idla_ d5c2bl_
d1jrhl1 d1il1a1 d3zhda1 d4kfzc_ d1b0wa_ d1e6oh1 d1mexl1 d4krpb_
d1fnsh1 d1ncwh1 d4hgma_ d4eigb1 d4rzca_ d2mpal1 d4y5xa_ d5en2b1
d3lrga1 d4ej1c1 d1rhha1 d1j05b_ d1forh1 d1q9wb1 d2wzpd_ d2aq2a1
d1h0da1 d3revb1 d2ij0c1 d1tzha1 d4m7jl1 d1k6ql1 d3b9kc1 d1igml_
d3qeua1 d1dsfl_ d1ce1h1 d1kcuh1 d1zvha1 d1zvoa1 d1tqbb1 d1ay1l1

d2qscl1 d2cgrh1 d1nl0h1 d1kenl1 d3eo0a1 d4n9ob1 d1n0xl1 d3k1kc_
d2cqva1 d1fhga_ d2k1ma_ d2dl2a1 d1biha3 d1biha1 d1biha4 d3qp3a1
d1g1ca_ d1gxea_ d1cvsc2 d1cvsc1 d1xmwa2 d1m4ka2 d2otpa2 d1biha2
d1rhfa2 d2fcba2 d2fcba1 d1wwbx_ d1gsma2 d1epfa1 d1epfa2 d2dava1
d1waaa_ d1f97a2 d1x44a1 d1vcaa2 d1fnla2 d1fnla1 d3b5ha2 d3h8na2
d3h8na1 d1jbja1 d1jbja2 d1pd6a_ d1f2qa2 d1f2qa1 d3cx2a_ d1nbqa2
d3b5ha1 d1n26a1 d1xiwb_ d1xaua_ d1fltx_ d2ifga1 d1olla1 d1olla2
d1cs6a4 d1cs6a3 d1cs6a2 d1cs6a1 d2gw5a2 d1sy6a1 d1xiwa_ d1ucta2
d1ucta1 d1ry7b2 d1gl4b_ d1qz1a3 d1olza1 d2c9aa1 d1wwca_ d2aq2b1
d1ck1a1 d1enfa1 d1i4pa1 d1v1oa1 d1et9a1 d1esfa1 d2g9hd1 d4ohja1
d1klud1 d1bxta1 d1ty0a1 d1fnua1 d1c4qa_ d2wv6d_ d1prtf_ d1ltrd_
d4l63b1 d1r4pb_ d1prtd_ d1tl0f_ d2bosa_ d1prtb1 d3chbd_

**Family-26 (b.29.1.11)**

**Positive dataset contains 8 protein sequences.**

d2nqya_ d3b7ma_ d4h7ma1 d3wp3a_ d2vgda_ d3zsea_ d3wx5a_ d4ixla1

**Negative dataset contains 3978 protein sequences.**

d1xfda2 d1m32a_ d1ibja_ d1c7na_ d1iuga_ d4q75a_ d2bkwa1 d1t3ia_
d1n8pa_ d1qz9a_ d1jf9a_ d1eg5a_ d1p3wa_ d3kgwa_ d2ctza1 d1vjoa_
d2rfva_ d1gc0a_ d1cs1a_ d1d2fa_ d2ch1a1 d1qgna_ d1pffa_ d1elua_
d1u08a_ d1lc5a_ d1b5pa_ d2ay1a_ d1bw0a_ d3pd6a_ d7aata_ d2csta_
d1j32a_ d1fg7a_ d4wlha_ d1o4sa_ d1v2da_ d1gdea_ d1m6sa_ d1v72a1
d1svva_ d1ajsa_ d1wsta1 d2f8ja2 d1lk9a_ d1vp4a1 d1xi9a1 d2gb3a1
d1yaaa_ d3i2ka1 d1lnsa3 d1mpxa2 d2cfba1 d1dfoa_ d1o69a_ d1bj4a_
d3k28a_ d2bwpa_ d3lg0a_ d2fnua_ d3ou5a_ d1z7da1 d1b9ha_ d1iaya_
d4msoa_ d1bs0a_ d1s0aa_ d1sffa_ d3tqxa_ d1mdoa_ d3qboa_ d3pgya_
d1bjna_ d2dkja_ d1ejia_ d1vefa1 d4wxga_ d3bs8a_ d3gbxa1 d1w23a_
d2b5aa1 d2ofya1 d4mcxa_ d2xi8a_ d2icta_ d2bnma1 d2ppxa1 d1y7ya1
d1ddla_ d1e57b_ d1f56a_ d1bypa_ d1azca_ d1ws8a_ d3u25a_ d1cuoa_
d1id2a_ d1rkra_ d2iaac_ d4dp1x_ d1nwpa_ d2gima_ d2i7sa_ d1qhqa_
d1gyca3 d1hfua2 d1kbva1 d1kbva2 d1sdda1 d1sdda2 d1gyca1 d1gyca2
d1mzya1 d2bw4a1 d1aoza1 d1aoza3 d1aoza2 d1kyaa2 d1kyaa3 d1kyaa1
d1mzya2 d4e9sa1 d1sddb1 d1sddb2 d1hfua1 d1hfua3 d1oe1a2 d1oe1a1
d1v10a2 d1v10a3 d1v10a1 g1gk9.1 g1or0.1 g1e3a.1 d1iknc_ d3brda1
d1p7hl1 d1oy3c_ d1imhc1 d1gjia1 d1zk9a1 d2cxka1 d2h0va_ d1adqa1
d1fnga1 d1uvqb1 d1zs8a1 d2vola1 d2vola2 d1mfbl2 d2jelh2 d1uwel2
d3qq4b1 d1kjvb_ d1k5na1 d1osph2 d4odhl2 d1ow0a1 d1ow0a2 d1bfob2
d3l9ra2 d3jtth1 d2fath2 d4elkb2 d1fn4b2 d2xfxb_ d1pg7x2 d1cqka_
d4k71b2 d1lk2a1 d1eapb2 d4lfhg2 d1i4fa1 d2nw2a2 d1kfah2 d1mhca1
d1h3ua1 d1wejh2 d1h0db2 d1a5fh2 d1kcvh2 d1mjuh2 d1qqda1 d1kcrh2
d1k8ia1 d3huja2 d2nnab1 d1nfdf2 d3mffb2 d1s9va1 d1lk3h2 d1q0xl2
d1fv1b1 d1neza1 d4rfeb2 d1r3ha1 d1a6tb2 d1kjva1 d1pz5b2 d1onqa1
d2bc4a2 d1de4a1 d1k8da1 d1tcra2 d1dn0b2 d1nfde2 d2xwtb2 d1bfoa2
d1mexh2 d1iakb1 d1ypzf2 d4f7ea2 d1q72l2 d2fbjh2 d1fe8h2 d1clzh2
d1fngb1 d1uvqa1 d1vgka1 d1c16a1 d4j12a2 d4x99b2 d1igtb3 d1je6a1

d1q72h2 d1nldh2 d1teth2 d2hmic2 d1i1ca2 d1i1ca1 d1k8ib1 d1q0yh2
d1kfal2 d3frua1 d1pg7w2 d1u58a1 d1igyb4 d1igyb3 d1hyrc1 d1klub1
d1deeb2 d1c12b2 d2bc4b2 d2ig2h2 d1kcvl2 d3wn5b1 d1lk2b_ d1lk3l2
d1mjul2 d1sm3h2 d1q0xh2 d1zvsa1 d1ydpa1 d1u8ib2 d1ypze2 d1kcuh2
d4alac_ d1rera1 d1s6na_ d3uzqb_ d2phla2 d2phla1 d1fxza2 d1fxza1
d1uija1 g1dgw.1 d2v09a_ d1uika2 d1uika1 d1dgwa_ d1ep0a_ d2pa7a1
d1wlta1 d1nxma_ d2c0za1 d1ktja_ d1ex0a1 d3us3a3 d3us3a1 d4mh8a_
d1mu2a2 d4i7ga1 d2bjca1 d2hsga1 d1qpza1 d1efaa1 d1ydwa2 d2nvwa2
d1zh8a2 d1qkia2 d1xeaa2 d1tlta2 d1r0ka3 d1j5pa3 d1vkoa2 d1vm6a2
d3qvsa2 d1gr0a2 d2ovga_ d1sq8a_ d1rzsa_ d3kz3a_ d3orca_ d3gpdg2
d2q49a2 d1hdgo2 d2g17a2 d2cvoa2 d2czca1 d3pwka2 d1obfo2 d1i32a2
d1zyao2 d2pkqo2 d1b7go2 d2hjsa2 d1cf2o2 d1vkna2 d1nvmb2 d4lt9a1
d3tc7a1 d3hoja_ d1i4na_ d3vnda_ d3nl8a1 d3nava1 d1v5xa_ d4a2ra1
d3tc6a1 d1ujpa_ d4ijba_ d1xcfa_ d1vc4a_ d1rd5a_ d1qopa_ d3b5va_
d2msya_ d2djoa1 d2r5za_ d1au7a1 d1f43a_ d2lmda1 d1akhb_ d1s7ea1
d3nara_ d1zq3p1 d2craa1 d1mija_ d4uuta_ d3a01a_ d1x58a1 d3rkqa1
d3cmya1 d2cqxa1 d2da6a1 d1e3oc1 d1fjla_ d1mh3a1 d1ig7a_ d1x41a1
d1wh7a1 d1gt0c1 d2mw8a_ d1lfup_ d1x2ma1 d1b72a_ d1wi3a1 d1wjha1
d1x2na1 d1mnmc_ d2cuea1 d3k2aa_ d2hosa_ d2jwta2 d1pufa_ d1octc1
d2hi3a_ d1b8ia_ d4j19a_ d3lnqa_ d1wh5a1 d1b8ib_ d1fexa_ d2cqra1
d1guua_ d1ug2a1 d2cqqa1 d2ajea1 d4a69c_ d1h8ac1 d2crga1 d2iw5b1
d1xc5a1 d2cu7a1 d1wgxa1 d2cjja1 d2ckxa1 d1gvda_ d1hcra_ d1tc3c_
d1gdta1 d1u78a2 d6paxa1 d6paxa2 d1k78a2 d2iu5a1 d1zk8a1 d2g7sa1
d2fd5a1 d2np5a1 d1z0xa1 d2id6a1 d2d6ya1 d2i10a1 d1vi0a1 d2oi8a1
d3c07a1 d2ns7a1 d2fq4a1 d2yvea1 d1t33a1 d2o7ta1 d1rkta1 d2np3b1
d2g7ga1 d2gena1 d2fbqa1 d2g7la1 d2gfna1 d2g3ba1 d2jj7a1 d2hyja1
d1sgma1 d1ui5a1 d2hkua1 d2id3a1 d4jyka1 d1bl0a2 d1d5ya1 d1d5ya2
d3lv8a_ d1s3ga1 d1zaka1 d1s4qa_ d3tvta2 d1uf9a_ d1khta_ d1ckea_
d1osna_ d1p5zb_ d4tmka_ d3cm0a_ d1jaga_ d3h86a_ d1t0hb_ d1nn5a_
d2h92a_ d3kb2a1 d2c95a1 d1yj5a2 d1kgda_ d1ly1a_ d2ak3a1 d1vyva2
d1deka_ d2grja1 d1p3ja1 d1y63a_ d1e4ya1 d1s96a_ d3iija1 d1lvga_
d1nksa_ d1vyua2 d4e22a_ d1qf9a_ d1jjva_ d1q3ta_ d1ki9a_ d1lw7a2
d1teva_ d1kjwa2 d1gsia_ d2iyva2 d1viaa_ d1e6ca_ d1z0ja1 d3umra2
d3gj0a1 d2gaoa1 d1yzga1 d1x3sa1 d1g7sa4 d1puja_ d1udxa2 d1kk1a3
d2o52a_ d1jwyb_ d1zcaa2 d1zcba2 d2fv8a1 d2cjwa_ d3dz8a1 d1xzpa2
d1vg8a_ d4qxaa_ d2ew1a1 d1x1ra1 d2bcgy_ d3doea1 d3cona_ d3rega_
d3wyga_ d1upta_ d2b6ha1 d1zd9a1 d1r5ba3 d1n0ua2 d1r2qa_ d2f9la1
d2rhda_ d3q3jb_ d3q85a_ d1efma1 d1r8qa_ d2gf9a1 d2akab1 d1ek0a_
d1z08a1 d2gcoa1 d2dy1a2 d4g01b_ d2cxxa1 d1svia_ d3q72a_ d1ni3a1
d4ac9a4 d2erxa1 d2bmea_ d1wxqa1 d2gf0a1 d4ldja1 d1zunb3 d1d5ca_
d4lv5b_ d1efca3 d1kmqa_ d2g6ba1 d3lrpa_ d1tada2 d2y8ea_ d4rd4a1
d1z06a1 d2heia_ d3a58b_ d1u0la2 d1d2ea3 d1zj6a1 d2ocba_ d3rwoa_
d1mr3f_ d2e9sa1 d4u5xa1 d2iwra_ d2fn4a1 d3bwdd_ d2nzja1 d2atva1
d2ksqa_ d3l43a_ d1mkya2 d1mkya1 d1wmsa_ d1jnya3 d2rmka_ d1f6ba_

d1f60a3 d1gota2 d1jala1 d2fh5b1 d1g16a_ d2wblc_ d1m7ba_ d1wf3a1
d1f5na2 d1t9ha2 d1z6xa1 d1z0aa1 d2fg5a1 d2a5ja1 d2bcjq2 d1s0ua3
d1ky3a_ d4y0va_ d4lhwa_ d2qmea1 d2atxa1 d1egaa1 d1ryha_ d1moza_
d1nrjb_ d2erya1 d3bfka_ d1puia_ d1fzqa_ d3j3az1 d3t5ga_ d1shza2
d3defa_ d2f7sa1 d3raba_ d3tsoa_ d3aq4a_ d2al7a1 d3sfva1 d3bc1a_
d2ncda_ d1sdma_ d3h4sa1 d2heha_ d2grya_ g3kin.1 d1kk8a2 d1br2a2
d1goja_ d1f9va_ d2mysa2 d1lkxa_ d3t0qa_ d4db1a1 d3gbja_ d1ry6a_
d1v8ka_ d1kb0a1 d3vrda1 d3vrda2 d1fcdc2 d1h1oa2 d1h1oa1 d1m70a2
d1m70a1 d1iqca2 d1iqca1 d1eb7a1 d1nmla2 d1nmla1 d1nira1 d1qksa1
d1hroa_ d4gedb_ d3zooa_ d1mz4a_ d1gu2a_ d1f1fa_ d1dvva_ d1c6ra_
d1c75a_ d1e29a_ d1gdva_ d1cnoa_ d1h32b_ d1co6a_ d2bh4x_ d2aiua_
d5cytr_ d1jdla_ d1i8oa_ d2zboa_ d3dmia_ d1ls9a_ d1wvec_ d2yk3a_
d3ph2b_ d1dw0a_ d5aura_ d1ql3a_ d1qn2a_ d1f1ca_ d1prtb2 d1evsa_
d1cnt1_ d1pvhb_ d1z7ca_ d1huwa_ d1bgea_ d1rhga_ d1f6fa_ d1f45b_
d1i1rb_ d1axia_ d1etea_ d3tgxb_ d1scfa_ d2z3qa1 d3qb1a_ d1hula_
d1m48a_ d4nkqb_ d1eera_ d1v7mv_ d5e4ea_ d2o27a_ d3b5ka_ d2psma1
d4zf7a_ d3uf5a_ d4x51a_ d1d9ca_ d4ypgc1 d3oq3a_ d1n1fa_ d1au1a_
d1wu3i_ d3besl_ d3ux9a_ d3rbaa_ d2qtra_ d1nupa_ d3l92a_ d1f9aa_
d1qjca_ d2h29a_ d1vlha_ d1od6a_ d1lw7a1 d4ruka_ d2el7a_ d1iq0a2
d1irxa2 d1r6ta2 d1ivsa4 d2cyba_ d1rqga2 d1j09a2 d2yxna_ d1f7ua2
d1h3fa1 d1ffya3 d2g36a1 d1nzja_ d1jila_ d1n3la_ d1pfva2 d1h3na3
d3n9ia_ d3sz3a1 d3sw2a_ d1moxc_ d1dx5i3 d1edmb_ d1autl2 d1autl1
d1nqlb_ d1dval1 d1szba2 d1kigl_ d2y5fl_ d1ijqa2 d1gk5a_ d1g1sa2
d1nt0a3 d1xdtr_ d1lmja2 d1lmja1 d1nzia2 d2wphe_ d1klil_ d1pfxl2
d1pfxl1 d1p9ja_ d1g1ta2 d1f7ea_ d4yzub_ d1nuba2 d2mgpa1 d1b9wa1
d1b9wa2 d2mgpa2 d1ob1c1 d1ob1c2 d1n1ia2 d1n1ia1 d2npra2 d2npra1
d1m1xb4 d2y5ia_ d1qlsa_ d1psra_ d3n22a_ d2egda_ d4ggfc_ d2wcea_
d2luca_ d4aqia_ d3nsia_ d2lp3a_ d1j55a_ d1a4pa_ d1k8ua_ d1nsha_
d1pvaa_ d3fs7a_ d2pvba_ d1ttxa_ d1rwya_ d2kyfa_ d1rk9a_ d1a75a_
d1exra_ d1tiza1 d1nyaa_ d1g8ia_ d1ggwa_ d1wdcc_ d3fwba_ d1ggza_
d2o5ga_ d1ctaa_ d1m45a_ d3i5gc_ d1sl8a_ d1fpwa_ d2scpa_ d2roaa_
d4aqra_ d3pn7b_ d1br1b_ d2mysb_ d1s6ca_ d1bjfa_ d2obha_ d1jfka_
d1jf0a_ d1qv0a_ d1lkja_ d2mlfc_ d2m97a_ d2d8na_ d4by5a_ d1jbaa_
d4nqga_ d1wdcb_ d2mysc_ d1j7qa_ d1s6ia_ d1uhka2 d1r2ua_ d1dtla_
d3kf9a_ d3pn7c_ d5d67d_ d1tuza_ d1h8ba_ d1eg3a1 d1eg3a2 d2zkmx1
d1snla_ d1gjya_ d1y1xa_ d1hqva_ d1dvia_ d1qxpa2 d4usla_ d1k94a_
d1wkya2 d1uuqa_ d3wh9a_ d4m1ra_ d1xyza_ d4xuya_ d2je8a5 d4bf7a1
d3cuia_ d1ur4a_ d1qw9a2 d1odza_ d2vzsa5 d1bqca_ d2x2ya1 d1hjqa_
d1qnra_ d4y7ea1 d1vjza_ d4k68a_ d2v3ga_ d4hpga_ d1nofa2 d1nq6a_
d1h4pa_ d1aq0a_ d3ziza_ d4bs0a_ d1ecea_ d1h1na_ d1ta3b_ d1w91a2
d1i1wa_ d1uhva2 d1us3a2 d1egza_ d7a3ha_ d1tvna1 d3nyda1 d1ghsa_
d2cyga1 d4xv0a_ d2c8na2 d1w32a_ d3emca_ d1v6ya1 d1foba_ d2uwfa1
d2fgla_ d3niya1 d2c0ha1 d1rh9a1 d1yq2a5 d1szna2 d1fa2a_ d1pama4
d5do8a1 d1hx0a2 d2fhfa5 d1qhoa4 d1avaa2 d1m53a2 d4m8ua1 d2gjpa2

d1lwha2 d4howa1 d1ktba2 d1j0ha3 d3vm5a1 d2x1ia_ d1wzaa2 d1uasa2
d2diea1 d1mxga2 d1ht6a2 d1eswa_ d1iv8a2 d5cpsa_ d1ud2a2 d1zjaa1
d3gbea1 d1kwga2 d1wdpa_ d1gcya2 d1g5aa2 d1hvxa2 d3dhpa2 d2bhua3
d4do4a1 d3qgva1 d1e43a2 d1g94a2 d2d3na2 d1gjwa2 d1gvia3 d1x1na1
d1r7aa2 d1tz7a1 g1apy.1 d3ljqa_ d4toqa_ d1wb0a1 d1w9pa1 d1eoka_
d1itxa1 d1kfwa1 d1goia2 d1edqa2 d2gsja_ d1hjxa1 d1jnda1 d1vf8a1
d3wdpp1 d3wq5a_ d1gnxa_ d4gxpa_ d4hz8a_ d1e4mm_ d3aiua_ d1wcga1
d1qvba_ d3ahxa_ d1v02a_ d2dgaa_ d1pbga_ d1vffa1 d1qoxa_ d4ha4a_
d1mixa2 d1e5wa2 d1j19a2 d1isna2 d2al6b2 d3syxa1 d1mkea1 d2p8va_
d1xoda1 d1i7aa_ d1qc6a_ d1egxa_ d1id1a_ d2hmta_ d2v76a_ d1wj1a1
d1aqca_ d1qqga2 d1qqga1 d1ntva_ d1n3ha_ d4dbba_ d3f0wa1 d1p3ra_
d1wgua1 d2cy4a1 d3hqca_ d1wvha1 d1xr0b1 d3nvva1 d1krha3 d2h88b1
d1n62a2 d1t3qa2 d1kf6b2 d1zoyb1 d1jq4a_ d1ffva2 d1dgja2 d1rm6c2
d1h6ta1 d9ldta1 d2ewda3 d1up7a1 d2dfda1 d1s6ya1 d1vjta1 d7mdha1
d1hyea1 d1t2da1 d1ojsa3 d1ldna1 d1hyha1 d1civa1 d2a92a1 d1ez4a1
d1i0za1 d1y6ja1 d1gv0a1 d1ur5a1 d1llda1 d1obba1 d1v8ba1 d1gdha1
d1l7da1 d2dlda1 d1mx3a1 d1ygya1 d1qp8a1 d1li4a1 d1j4aa1 d2naca1
d1npya1 d3t4ea2 d1a4ia1 d1o0sa1 d2hk9a2 d2egga2 d1b26a1 d1bvua1
d1gq2a1 d1c1da1 d1leha1 d1gtma1 d1euza1 d1nvta1 d3dona2 d1edza1
d1gpja2 d1p77a1 d1nyta1 d1b0aa1 d1vl6a1 d1v9la1 d1txga2 d3f47a1
d1mv8a2 d2pv7a2 d1xc2a3 d2ahra2 d4xiya1 d2f1ka2 d1pgja2 d1i36a2
d1np3a2 d1lj8a4 d1teaa3 d1qmga2 d2i76a2 d2g5ca2 d1wdka3 d1dlja2
d3cuma3 d1kola2 d1f8fa2 d1qora2 d1jqba2 d1vj0a2 d1cdoa2 d1gu7a2
d1d1ta2 d1xa0a2 d2dm6a2 d1jvba2 d1rjwa2 d1llua2 d1h2ba2 d1tt7a2
d1piwa2 d1o89a2 d1iz0a2 d1e3ja2 d1yb5a2 d1uufa2 d1vj1a2 d1e3ia2
d1vc2a1 d2nvwa1 d1u8fo1 d1ydwa1 d1hdgo1 d2g17a1 d1cf2o1 d2czca2
d1r0ka2 d1obfo1 d2cvoa1 d1ebfa1 d1h6da1 d1i32a1 d1zh8a1 d2q49a1
d1qkia1 d1j5pa4 d3pzra1 d1xeaa1 d1vkoa1 d1vm6a3 d3qvsa1 d2pkqo1
d1b7go1 d1gr0a1 d1p1ja1 d1lc0a1 d1j0xo1 d3pwka1 d2hjsa1 d1zyao1
d1vkna1 d1tlta1 d1nvmb1 d3sjua_ d3ojfa1 d1xu9a1 d2jjya1 d1n7ha_
d1y1pa1 d1h5qa_ d1jtva_ d4fs3a_ d2bkaa1 d1gy8a_ d2c5aa1 d1ulsa_
d1ooea_ d2b69a1 d1y5ma1 d1spxa_ d1ae1a_ d1bxka_ d1ydea1 d1gega_
d1hdoa_ d1o5ia_ d1e6ua_ d1snya1 d2rhca_ d3zv4a_ d5ceja_ d2d1ya1
d1fjha_ d3w8ea_ d2fmua1 d1x1ta_ d1rpna_ d3ay7a1 d1k2wa_ d1eq2a_
d2hrba1 d3ruaa_ d2ew8a1 d4hwka_ d1xgka_ d2bd0a1 d1yo6a1 d2a35a1
d2ntva_ d1vl8a_ d1hdca_ d4bkua_ d2ag5a1 d2q45a_ d1nffa_ d4iy1a_
d1zbqa1 d1kewa_ d1ek6a_ d1ja9a_ d2q46a2 d1iy8a_ d1cyda_ d2ptga_
d1hxha_ d1uzma1 d3lyla_ d4m89a_ d3wyea_ d2blla1 d1w6ua_ d1sb8a_
d2q2va_ d4nr0a_ d1e6wa_ d2gasa_ d3oiga1 d2bgka1 d1mxha_ d1vl0a1
d1rkxa_ d1r6da_ d1wmaa1 d1e7wa_ d2c07a1 d1xkqa_ d3tzha_ d1g0oa_
d3lz6a_ d1qyca_ d1edoa_ d1gz6a_ d1oc2a_ d1t2aa_ d1zema1 d2xoxa_
d1z45a2 d1geea_ d1p33a_ d3lt0a_ d4e6pa_ d2wyua_ d1db3a_ d4q9na_
d1xhla_ d4o1ma_ d1fmca_ d2uvda_ d2gdza1 d1uaya_ d2z1ma_ d2o23a2
d2fr1a2 d2fr1a1 d1qsga_ d1qyda_ d4zjua_ d2a4ka1 d1yb1a_ d1wvga1

d1yxma1 d3f4ba_ d1mg5a_ d1xg5a1 d3nqyb_ d4gera_ d1ex0a4 d2f4ma1
d1x3za_ d3u1ra1 d1g9ka2 d1k7ia2 d3ioqa_ d1fh0a_ d1khqa_ d1m6da_
d2cb5a_ d1cs8a_ g8pch.1 d1s4va_ d2pnsa_ d1vsna1 d1mira_ d1iwda_
d2oula_ d7pcka_ d3bwka_ d3bcna_ d2p86a_ d1pvja_ d1pcia_ d1gece_
d1deua_ d4bs6a_ d1xkga1 d1cqda_ d1jqpa2 d2c0ya_ d2bdza_ d3p5ua_
d2o36a_ d1k9xa_ d1zoya1 d1qo8a2 d1d4ca2 d1chua2 d1jnra2 d1kf6a2
d2jk6a2 d2jk6a1 d1feca1 d1feca2 d1lpfa1 d1lpfa2 d1xhca1 d1xhca2
d1dxla2 d1dxla1 d1gera2 d1m6ia2 d1onfa1 d3lada2 d3lada1 d1w4xa1
d1w4xa2 d1gesa2 d1mo9a1 d1xdia1 d1fl2a2 d1fl2a1 d1fcda1 d1fcda2
d1h6va2 d1h6va1 d1mo9a2 d1gv4a2 d1gv4a1 d2gv8a2 d2gv8a1 d1aoga1
d1aoga2 d1hyua1 d1ebda1 d1ebda2 d3dk9a1 d1q1ra2 d1q1ra1 d1gesa1
d1zbfa1 d2z1ha_ d2etja1 d3qioa1 d1jl1a_ d2e4la_ d1jl2a_ d2lsna1
d1io2a_ d1uaxa_ d2yv0x_ d1mu2a1 d1ekea_ d1o94a2 d1lqta1 d1cjca1
d1s5ja1 d2hbja2 d2igia_ d1wlja_ d1qhta1 d4dlea1 d1tgoa1 d2f96a1
d3tr8a1 d3mxma_ d1q8ia1 d1y97a1 d1w0ha_ d1kfsa1 d2py5a1 d1d5aa1
d1yt3a3 d2guia_ d3mwpa1 d4kb1a_ d1b9ra_ d1offa_ d1frra_ d3wcqa_
d1l5pa_ d1wria_ d1i7ha_ d3av8a_ d1czpa_ d1rfka_ d3p1ma1 d3b2ga_
d2bt6a2 d1v58a1 d1t3ba1 d4ilfa2 d4npba2 d1wgqa1 d1v89a1 d2coda1
d1zc3b_ d1wg7a1 d1u5ea1 d1v61a1 d2coca1 d2ys1a1 d1v5pa1 d1ntya2
d1wjma1 d1wi1a1 d1u29a_ d1xx0a1 d1unqa_ d2zkmx3 d1fhoa_ d1v88a1
d2adza1 d1x1fa1 d1p6sa_ d2coaa1 d1btka_ d1v5ma1 d1faoa_ d2x18a_
d1dyna_ d1x1ga1 d4kaxb_ d2j59m1 d1xcga2 d1txda2 d1u5da1 d1dbha2
d1w1ha_ d1upqa_ d1v5ua1 d1foea2 d2fjla1 d1eaza_ d2dfka2 d2cofa1
d1kz7a2 d1r7ha_ d1faaa_ d1wika1 d1xfla_ d1ttza1 d2f51a1 d1ti3a_
d1nhoa_ d1sena_ d2i4aa_ d1ep7a_ d1zmaa1 d1fo5a_ d3lefa1 d3diea1
d2yana1 d1z6na1 d1oaza_ d3zywa1 d4aj8a_ d1nm3a1 d4fiwa_ d1wmja_
d4hs1a1 d1iloa_ d1xwaa1 d1wjka1 d2vm1a_ d1fb6a_ d1gh2a_ d1h75a_
d1nw2a_ d1kx5b_ d3a6na_ d4m6ba2 d2nqbd_ d3wkjd_ d2yfwe_ d4h9na_
d5bnxa_ d1kx5c_ d3r45a_ d1eqzb_ d4jjnb_ d1f66c_ d1eqza_ d4wnnb_
d1kx5a_ d1u35c1 d1id3a_ d2nqbc_ d1ku5a_ d1f1ea_ d1b67a_ d1tafa_
d1bh9b_ d1tafb_ d1jfib_ d1q9ca_ d1h3ob_ d2byka1 d1n1ja_ d4g92b_
d1bh9a_ d1jfia_ d1h3oa_ d2bykb1 d1pd212 d1nhya2 d2a2ra2 d1k0dd2
d1f2ea2 d1gsua2 d1f3ba2 d2fhea2 d2pvqa1 d1tw9a2 d1oyja2 d1e6ba2
d3ik7a1 d4is0a1 d1r5aa2 d1jlva2 d1m0ua2 d3gtub2 d1z9ha2 d1okta2
d1axda2 d2gsra2 d1tu7a2 d1k3ya2 d1zgma1 d1b48a2 d1gnwa2 d1gwca2
d2gsta2 d1glqa2 d1oe8a2 d1pn9a2 d1v2aa2 d1fw1a2 d1duga2 d1g99a1
d2e1za2 d2e1za1 d1saza1 d1saza2 d2trcp_ d1ig8a1 d1czan2 d1czan3
d1czan1 d1ig8a2 d1czan4 d1bg3a4 d1bg3a3 d1bg3a2 d1bg3a1 d1qqya_
d1gd6a_ d1f6ra_ d1ivma_ d2gv0a_ d1iiza_ d2z2fa_ d1j8xa_ d1ghla_
d1b9oa_ d174la_ d169la_ d2o4wa_ d1p37a_ d1k28a3 d1xjta1 d1jtma_
d2f2qa_ d4e97a1 d1g4ma1 d1g4ma2 d4qgna_ d1vr3a1 d2pjsa1 d2i7ra1
d1ecsa_ d1xqaa1 d5cj3a1 d1npba_ d1jifa_ d2p7oa_ d1r9ca_ d1nkia_
d1mpya2 d1kw3b2 d1kw3b1 d4ghga1 d1tfza1 d3isqa1 d3isqa2 d1sqia1
d1sqia2 d1lkda2 d1lkda1 d1mpya1 d1cjxa2 d1cjxa1 d1sp9b2 d1t47a1

d1t47a2 d1f1ua2 d1sp8a1 d1sp8a2 d1f1ua1 d2bema_ d1pama1 d1ea9c1
d1qhoa1 d2fhfa2 d2fhfa1 d1k3ia1 d1j0ha1 d1cxla1 d2bhua1 d1edqa1
d1f1sa2 d1ut9a2 d1gvia1 d1txka1 d1ro5a1 d4iuaa3 d4bvva_ d5hpga_
d3k65a_ d1ki0a3 d1ki0a2 d1ki0a1 d1a0ha1 d1pmla_ d3sp8a3 d2feba1
d1qsta_ d2beia1 d4m3sa_ d1u6ma1 d1yk3a1 d3dr6a_ d1xmta_ d1z4ea1
d2ge3a1 d1bo4a_ d2fiaa1 d2jdca1 d1y9wa1 d3to7a_ d1wwza1 d2fl4a1
d2ozua1 d2b5ga_ d2cy2a1 d2p0wa_ d1cm0a_ d4pswa_ d1yrea1 d1yr0a1
d1m4ia_ d2gana1 d1yvka1 d1ghea_ d2g3aa1 d1y7ra1 d2euia1 d1q2ya_
d2fsra1 d1xeba_ d2ae6a1 d1cjwa_ d1qsma_ d2aj6a1 d1y9ka1 d1i12a_
d1vkca_ d2atra1 d1n71a_ d2d4pa1 d2fiwa1 d2fe7a1 d1tiqa1 d2ou2a_
d1yx0a1 d2fcka1 d1p0ha_ d2j8ma_ d2pq8a_ d1z4ra1 d1s3za1 d1ufha_
d2hqya1 d2hqya2 d1lrza2 d1ne9a1 d1lrza3 d1xvqa1 d1pn0a2 d3hy2a_
d2cvba1 d1o8xa_ d1lu4a_ d1we0a1 d2bmxa1 d2a4va1 d1psqa_ d1xiya1
d1q98a_ d1n8ja_ d1zofa1 d2ggta_ d3tjja_ d4je1a1 d1o73a_ d3s9fa_
d2zcta_ d1qmva_ d1jfua_ d2b1ka_ d5dvba1 d2b5xa1 d2v2ga_ d3tb2a_
d4llra_ d4fh8a1 d1e2ya_ d1knga_ d2h30a_ d1i5ga_ d2z9sa_ d2f8aa1
d1tp9a1 d2b7ka_ d1nm3a2 d3hvva_ d2wfca1 d4kcea_ d1xvwa1 d4gqca1
d3qpma_ d4k1fa_ d1zzoa1 d1gp1a_ d1prxa_ d1rmka_ d1ixta_ d1eyoa_
d1cnna_ d1fu3a_ d1g1pa_ d1f3ka_ d1fyga_ d2m5ta_ d1cqqa_ d1wqsa_
d5bpea_ d2ipha_ d1lvoa_ d2bhga1 d3tnta_ d3sjia_ d3d23a1 d2fyqa_
d3tloa_ d3zvea1 d2hrva_ d3q3ya1 d4asha1 d4aw8a_ d1etha2 d1w52x1
d2ppla1 d1hpla2 d1lpbb2 d2oxea1 d1rp1a2 d1ys1x_ d4hs9a_ d1ispa_
d1ku0a_ d3qmma_ d1ra6a_ d1muka_ d3gsza_ d1khva_ d1te9a_ d2xwha_
d3uqsa_ d3hkwa_ d3n6ma_ d2cjqa_ d2xyma_ d1teba_ d1te8a_ d1u09a1
d4wfxa_ d3tyqa_ d2pgga1 d2h1ia1 d2r8ba1 d3b5ea1 d3cn9a_ d1lgya_
d4l3wa_ d1llfa_ d1gz7a_ d1ukca_ d1cr6a2 d1ixra2 d1mbma_ d3su6a1
d1cu1a1 d4agka_ d1vcpa_ d1q31a_ g1n1l.1 d3e90b_ d1ep5a_ d3u1jb_
d3zxha_ d1cgla_ d1rm8a_ d1eaka2 d1hy7a_ d2jsda_ d1cxva_ d1hv5a_
d1i76a_ d3p0ya1 d1igra2 d1n8yc2 d1n8yc1 d1m6ba2 d1m6ba1 d1m4la_
d2bk7a_ d3fjua_ d1z5ra1 d1kwma1 d1uwya2 d1jqga1 d2c1ca1 d1dtda_
d1jgta2 d1ea0a3 d1ecfa2 d1q15a2 d1ct9a2 d1ao0a2 d1te5a_ d2tbvc_
d1c8nc_ d1x36a_ d1smvc_ d1ng0c_ d4sbvc_ d1opoa_ d1f2nc_ d1cixa_
d1y29a_ d1v7fa_ d2jtba_ d1d1ha_ d1g9pa_ d1lupa_ d1qk7a_ d1mb6a_
d2a2va_ d1niya_ d1i25a_ d1hvwa_ d2mpqa_ d1ie6a_ d1la4a_ d2mxoa1
d1dl0a_ d1koza_ d1qk6a_ d1emxa_ d1lqra_ d1c6wa_ d1js9c_ d1f15b_
d1za7a_ d1laja_ d1rypd_ d1irua_ d1rypc_ d3unfk_ d1rypa_ d1j2pa_
d1irui_ d1j2qh_ d3unfh_ d1m4ya_ d1rypb_ d1irul_ d1irug_ d1iru2_
d1q5rh_ d1rypg_ d1g3ka_ d1iruf_ d1q5qa_ d1e94a_ d1irub_ d1ryph_
d3ty6a_ d1iruj_ d1iruk_ d1rype_ d1irud_ d1irue_ d3mi01_ d1rypf_
d1iruh_ d2z3ba_ d3mka2_ d1iru1_ d1ny721 d1a6ca3 d1a6ca2 d1a6ca1
d1pgl22 d1pgl21 d1pgl11 d1ny711 d1mqta_ d1d4m3_ d1fmd3_ d2mev1_
g1aym.1 d1ev13_ d4q4w1_ d1r1a3_ d2x5ia_ g1r1a.1 g1d4m.1 g1rhi.1
d1b35b_ d1oopa_ d1tme1_ d4iv1a_ g1pvc.1 g1fpn.1 d1fpn1_ d4gb31_
d1d4m1_ d1fmd1_ d1ev11_ d2wzr3_ g1cov.1 d1qqp3_ g1fmd.1 d2x5ic_

| | | | | | | | |
|---|---|---|---|---|---|---|---|
| d1rhi3_ | g1oop.1 | d1hxs1_ | g1hxs.1 | d1fpn3_ | d1bev1_ | g2mev.1 | d1eah3_ |
| d1aym1_ | d1pvc3_ | d1qqp1_ | d1zba3_ | d1h8ta_ | d3vbhc_ | g1b35.1 | g1bev.1 |
| d1hxs3_ | g1ev1.1 | d1bev3_ | g1tmf.1 | d1tmf1_ | d1b35a_ | d2mev3_ | g1eah.1 |
| g1h8t.1 | d1eah1_ | d4q4w3_ | d1r1a1_ | d1aym3_ | d1pvc1_ | d1zba1_ | d1h8tc_ |
| d1oopc_ | d1tme3_ | g1qqp.1 | d4iv1c_ | g1tme.1 | d1y14b1 | d2nn6i1 | d1i94l_ |
| d2zjra2 | d1smxa_ | d2qamc2 | d1hh2p1 | d2r7da1 | d1rl2a2 | d1wfqa1 | d3bzka4 |
| d1x6oa2 | d3a0ja_ | d2j01d2 | d2ja9a1 | d4ql5a_ | d1hr0w_ | d1luza_ | d2n3sa_ |
| d2vnud1 | d1h95a_ | d1go3e1 | d2nn6h1 | d1ne3a_ | d2mo1a_ | d1iz6a2 | d1jjga_ |
| d1e3pa2 | d2ix0a3 | d2ix0a2 | d2lxja_ | d1ueba3 | d1ueba2 | d2z0sa1 | d1u0la1 |
| d2ix0a1 | d2eifa2 | d2je6i1 | d3i2za1 | d2c35b1 | d2ahob2 | d1t9ha1 | d3i4oa_ |
| d2qall1 | d1jt8a_ | d2nn6g1 | d1g6pa_ | d3cama_ | d1i94q_ | d1c9oa_ | d1d7qa_ |
| d2ba0a1 | d2vnud2 | d2vnud3 | d2qalq1 | d1wi5a1 | d1xtda2 | d1ny4a_ | d4w9ta_ |
| d1jvna1 | d4x2ra1 | d1ka9f_ | d1qo2a_ | d4axka1 | d1thfd_ | d4u28a_ | d2y88a_ |
| d4wd0a_ | d1vzwa1 | d1ix2a_ | d1ux8a_ | d1dlya_ | d4i0va_ | d2bkma_ | d1dlwa_ |
| d2bmma_ | d2r80b_ | d1cqxa1 | d1cg5a_ | d1gcvb_ | d3boma_ | d2h8fb_ | d2raoa_ |
| d3bj1a_ | d4hrrb_ | d2r50a_ | d3d1kb_ | d2aa1b1 | d3zhwa_ | d3hf4b_ | d3gdja_ |
| d1jebb_ | d3bj1b_ | d1x9fb_ | d1wmub_ | d1gcva_ | d3bcqb_ | d3d4xb_ | d1gvha1 |
| d2dc3a_ | d3hf4a_ | d4yu3a_ | d3k8ba_ | d1qpwb_ | d2r80a_ | d3bcqa_ | d1v4wa_ |
| d3wtgb_ | d1jl7a_ | d4esaa_ | d1a9we_ | d3d1ab_ | d1fsla_ | d4h2la_ | d1v4wb_ |
| d1mwca_ | d1vhba_ | d1jeba_ | d1spgb_ | d1h97a_ | d1q1fa_ | d1tu9a1 | d3d1aa_ |
| d1hdsb_ | d3hyub_ | d2zfbb_ | d3fs4a_ | d1oj6a_ | d1shrb_ | d4h2lb_ | d4yu3b_ |
| d1spga_ | d3hrwa_ | d3qqra_ | d3at5a_ | d4wchd_ | d3hyua_ | d1cg5b_ | d1uc3a_ |
| d1hbra_ | d2raob_ | d1outb_ | d4esab_ | d3gdjb_ | d3qqqa_ | d1x9fc_ | d3vrfa_ |
| d1itha_ | d3mkba_ | d3d4xa_ | d3mjpa_ | d1jl6a_ | d3fh9a_ | d1outa_ | d2dc3b_ |
| d1it2a_ | d3eoka_ | d3g46a_ | d1qpwa_ | d3vrfb_ | d1ch4a_ | d1x9fd_ | d1hdsa_ |
| d2zfba_ | d3mkbb_ | d1or4a_ | d3wtga_ | d1a4fa_ | d3fh9b_ | d3bomb_ | d3cy5a_ |
| d3at5b_ | d1x9fa_ | d2oifa_ | d4lmxb_ | d1cpcb_ | d3v57a_ | d1kn1a_ | d1cpca_ |
| d4f0ta_ | d2vjha_ | d4po5b_ | d1eyxa_ | d1alla_ | d4f0tb_ | d1b33b_ | d1b8db_ |
| d4h0mb_ | d1b33a_ | d2vjhb_ | d4lm6b_ | d2vmlb_ | d1allb_ | d2vjta_ | d3dbja_ |
| d2vmla_ | d2vjtb_ | d4f0ub_ | d1f99b_ | d4rmpa1 | d1liaa_ | d1eyxb_ | d2uulb_ |
| d4lmsb_ | d4f0ua_ | d2bv8a_ | d1f99a_ | d2bv8b_ | d2c7lb_ | d3dbjb_ | d4rmpb1 |
| d1b8da_ | d4h0ma_ | d3v57b_ | d1kn1b_ | d2uula_ | d2j96a_ | d3w3ea_ | d4tx7a_ |
| d3cqla_ | d4msta_ | d3iwra_ | d1dxja_ | d2z37a_ | d4dyga_ | d1k8qa_ | d2xqfa_ |
| d1ea5a_ | d1f6wa_ | d1dx4a_ | d1qe3a_ | d4qwwa_ | d2ha2a_ | d1sfra_ | d1f0na_ |
| d4qeka_ | d1xm5a_ | d1xaxa_ | d1tvia_ | d1pv8a_ | d1w5qa_ | d2c1ha_ | d1ohla_ |
| d1dosa_ | d3qm3a1 | d2fjka_ | d1f74a_ | d3l21a_ | d2e1da_ | d1to3a1 | d1w3ia_ |
| d1sfla_ | d1n7ka_ | d1vcva1 | d4imea_ | d1wa3a_ | d2ocza1 | d1f2ja_ | d3m16a_ |
| d2yxga_ | d1ub3a_ | d3a5fa_ | d1fbaa_ | d1l6wa_ | d1xfba1 | d1wx0a1 | d1vpxa1 |
| d1ojxa_ | d1mxsa_ | d2a4aa1 | d1xkya1 | d3cq0a1 | d3s1xa_ | d1f05a_ | d1fdja_ |
| d3cpra1 | d4d2ja_ | d1yloa2 | d1y7ea2 | d1y0ya3 | d1lfwa1 | d1vixa1 | d1fnoa4 |
| d1vhoa2 | d1ysja1 | d1vhea2 | d1z2la1 | d2fvga2 | d1vgya1 | d1r3na1 | d2grea2 |
| d1xmba1 | g1q7l.1 | d1rtqa_ | d3rp2a_ | g1ucy.1 | d1fuja_ | d2zchp_ | d3w94a_ |
| d1si5h_ | d1lo6a_ | g2pka.1 | d1cgha_ | d1kigh_ | d1a5ia_ | d3s9ba_ | d1hj8a_ |
| d1ao5a_ | d2anya_ | d1q3xa1 | d1gvza_ | d1azza_ | d4gsoa_ | d2f91a1 | d1iaua_ |

d1gdna_ d1eufa_ g1fiw.1 d1eq9a_ d4nfea_ d2rdla_ d1spja_ d1elva1
d1a7sa_ d1ekbb_ d1pfxc_ d1yc0a_ d2aiqa_ g1fiz.1 d1fi8a_ d4k60a_
d4dgja_ d1mzaa_ d1op0a_ d3tjva_ d1pytd_ d1sgfg_ g1gg6.1 d2eeka_
g1rtf.1 d1a0ja_ d3g01a_ d1pytc_ d4e7na_ d1ddja_ d1z8ga1 d2hlca_
d1npma_ d1brup_ g1jou.1 d1sgfa_ d1autc_ g2hnt.1 g1kdq.1 d1orfa_
d3s69a_ d4crga_ d1fxya_ d1bqya_ d2ftba_ d1pmpa_ d2rcqa1 d1fdqa_
d4lkpa_ d1sa8a_ d1vyfa_ d1lpja_ d3vg7a1 d4azqa1 d1o8va_ d5bvqa_
d1p6pa_ d2qo4a_ d1opba_ d1yiva1 d1o1va_ d4bvma1 d4tkba_ d4i3ba_
d1bwya_ d1ftpa_ d4gkca_ d1cbia_ d3p6da1 d1kqwa_ d2a0aa1 d1jx4a2
d3gv5b1 d1t94a2 d3bq0a2 d3lgia_ d3i77a_ d2z9ia2 d4nsva_ d1lcya2
d4jcna_ d1agja_ d1os8a_ d1hpga_ d1p3ca_ d1qtfa_ d1e69a_ d1mv5a_
d3g60a4 g1ii8.1 d1ewqa2 d1qhla_ d1v43a3 d2wmfa1 d1vpla_ d1g2912
d4a82a2 g1f2t.1 d2d62a1 d3tuic1 d2bbsa1 d3g60a3 d2ixea_ d1g6ha_
d1sgwa1 d2qi9c_ d3rlfa1 d3b60a1 d3d31a2 d2pzea1 g1xew.1 d1jj7a_
d1oxxk2 d2onka1 d1w1wa_ d1iwea_ d1qf5a_ d1vmaa2 d1nija1 d1np6a_
d2p67a1 d3ihla_ d1okkd2 d1ls1a2 d1s1ma2 d1iona_ d2v40a_ d1dj3a_
d3hida_ d2vo1a1 d2g0ta1 d1vcoa2 d1j8mf2 d1xjca_ d1p9ba_ d1qzxa3
d1ihua2 d1ihua1 d2qm8a_ d1dj2a_ d4ioja_ d1cp2a_ d1yrba2 d1hyqa_
d3gqba2 d1skye3 d1xp8a1 d1tf7a2 d1tf7a1 d1ye8a1 d1skyb3 d2qe7d2
d4blta_ d4huta_ d3ldaa2 d4hyya_ d2i3ba1 d1cr1a_ d3j9tb2 d3ntua2
d1vdza1 d2z43a_ d1e9ra_ d1nlfa_ d3ziaa2 d1n0wa_ d3ziad2 d3j9ta2
d1pzna2 d3gqbb2 d2i49a_ d1nnfa_ d1xvxa_ d1ursa_ d1h3da1 d1iuda_
d4m1va1 d1twya_ d3e13x_ d2nxoa1 d4pfua_ d3ttma_ d1elja_ d2qmwa1
d1us5a_ d1y3na1 d1jeta_ d1uqwa_ d4f19a1 d2esna2 d3thia_ d3fasa1
d2uyeb_ d2xwva1 d1r9la_ d2fyia1 d1pb7a_ d2ozza1 d1sw5a_ d1nh8a1
d1vr5a1 d2g29a_ d1xs5a_ d3lswa_ d2z23a_ d3w9va_ d3tcha_ d1q35a_
d1z7me1 d3q27a_ d1p99a_ d1ve4a1 d2i6ea1 d4mcoa1 d4n8ya_ d1zbma1
d1hsla_ d1ixca2 d1j1na_ d1xvya_ d1y9ua_ d2z22a1 d1xoca1 d4oera_
d1wdna_ d5f1qa1 d1xt8a1 d1i6aa_ d4gwoa_ d4igta1 d1pc3a_ d4mmpa1
d1o63a_ d2czla1 d2onsa2 d4mija1 d1eh3a_ d3skpa_ d1dtza1 d1dtza2
d1ieja_ d1jnfa2 d1ce2a1 d1jnfa1 d1b1xa2 d1b1xa1 d1jw1a1 d1jw1a2
d1ryoa_ d1h76a1 d1h76a2 d1c4oa1 d2p6ra3 d2p6ra4 d1oywa2 d1oywa3
d1wrba1 d1w36b2 d1w36b1 d1c4oa2 d3bora_ d1t6na_ d2eyqa5 d2eyqa3
d2eyqa2 d2zu6a2 d1veca_ d1uaaa1 d1w36d1 d2g9na1 d1z3ix2 d2rb4a_
d1hv8a1 d1hv8a2 d1t5ia_ d1w36c2 d2j0sa2 d2j0sa1 d3hjha1 d1z5za1
d1qdea_ d1z63a1 d1s2ma2 d1s2ma1 d1wp9a1 d1wp9a2 d1gm5a3 d1gm5a4
d3dina4 d3dina3 d1q0ua_ d1w36c1 d1z3ix1 d1nkta3 d1nkta4 d1uaaa2
d1w36d2 g1qhh.1 d2fwra2 d2fwra1 d2fkza_ d1zuja1 d1s3qa1 d2fjca1
d1z6om1 d1veia_ d4dyya_ d1jiga_ d3gvya_ d2pyba_ d4cy9a1 d3bvfa1
d1r03a_ d2cf7a_ d1nf4a_ d1zs3a1 d4iwka_ d3ka8a_ d3np0x_ d1n1qa_
d1lb3a_ d4e6ka_ d1z6oa1 d2fzfa1 d4dyua_ d3ge4a_ d1jgca_ d4am4a_
d2z6ma_ d1ji5a_ d1vlga_ d1lkoa1 d3qz3a_ d1o9ra_ d2ffxj_ d1euma_
d1yv1a1 d2v2pa_ d1j30a_ d4di0a_ d1dpsa_ d2cbia2 d1gqia1 d2choa2
d1oewa_ d2qzxa_ d2asia_ d1psoe_ d1miqa_ d1smra_ d2h6ta_ d1pfza_

d1izda_ d3hd8a_ d1j71a_ d1dpja_ d4aa9a_ d1t6ex_ g1b5f.1 g1lya.1
g1htr.1 d1qdma2 d1bxoa_ d1wkra_ d1tzsa_ d2qzwa_ d1bdqa_ d3ggta_
d3ixoa_ d1mrxa_ d2haha_ d1tw7a_ d4njsa_ d1sgua_ d2p3da_ d1nsoa_
d3d3ta_ d2rkfa_ d1c6ya_ d3ogpa_ d3ekwa_ d2o4na_ d2j9ja_ d1baia_
d3ggua_ d3lzua_ d4npua_ d4j55a_ d2r8na_ d2fxda_ d2p3ca_ d4dqea_
d3ttpa_ d3t3ca_ d2p3aa_ d2hc0a_ d2fdda_ d3mwsa_ d1k8rb_ d1wgra1
d1ef5a_ d1wfya1 d1wgya1 d1lfda_ d2cs4a1 d1wxma1 d2byea1 d3kh0a1
d2byfa1 d1wxaa1 d1e7ua3 d1l7ya_ d1wgda1 d1wgga1 d4q5eb_ d1wjna1
d1se9a_ d3n3kb_ d1wz0a1 d1we6a1 d2m0xa_ d2gbka_ d3rt3b1 d1wh3a1
d1sifa_ d2ekec2 d4ii2b1 d4hk2a1 d1c3ta_ d1m94a_ d1wgha1 d1ttna1
d1j8ca_ d1wx8a1 d3v6cb1 d2ma9b_ d4i6lb1 d1bt0a_ d2jvca1 d1iyfa_
d2k1fa_ d1wxva1 d1wf9a1 d1t0ya_ d2io0b2 d1x1ma1 d2gbma_ d1zw7a1
d2m17a_ d1ud7a_ d1we7a1 d2lwpa_ d2gbja_ d1wjua1 d1v6ea1 d1v86a1
d4eewa_ d1wiaa1 d4icva_ d4gu2a_ d1wx7a1 d1wy8a1 d4hjka1 d2faza1
d1v5oa1 d3ruib_ d1eo6a_ d3waob_ d2r2qa1 d3m95a_ d3wana_ d1yvca1
d1yeza1 d1g66a_ d5ajha1 d4lu0a_ d1of8a_ d3unda1 d3e9aa1 d1vr6a2
d2qkfa_ d1d9ea_ d1o60a_ d1um8a_ d1ixza_ d1qvra2 d1qvra3 d1qzma_
d1sxjc2 d1jr3d2 d2p65a_ d1l8qa2 d1g8pa_ d3hu3a3 d1sxjd2 d1njfa_
d1in4a2 d1sxja2 d1tuea1 d2ce7a2 d1w5sa2 d1sxjb2 d1d2na_ d1ny5a2
d1ofha_ d2gnoa2 d2a5yb3 d1fnna2 d1u0ja1 d1lv7a_ d1iqpa2 d2fnaa2
d1ixsb2 d4gita_ d1sxje2 d1g41a_ d2lj7a_ d1n4na_ d2m8bb1 d2kyqa_
d3psma_ d1jkza_ d1jxca_ d2gl1a_ d1ugla_ d4uj0a_ d2k4ua_ d1hp2a_
d2lu9a_ d1baha_ d2meoa1 d1wm7a_ d1hlya_ d1n8ma_ d1jlza_ d1wpda_
d1c55a_ d1pvza_ d1wt7a_ d1rjia_ d1wz5a_ d1m2sa_ d1pjva_ d1c49a_
d1qkya_ d2ktca1 d1wmta_ d1du9a_ d1j5ja_ d1r1ga_ d1t0za_ d2i61a_
d1t1ta_ d1sn4a_ d1kv0a_ d2lkba_ d1npia_ d1wwna_ d1pe4a_ d2kbka_
d2li7a_ d1djta_ d2kbha_ d1dq7a_ d1sega_ d1omya_ d1zywa1 d1jzaa_
d2jcqa_ d1dy2a_ d1t61c1 d1iyjb4 d1iyjb5 d1iyjb3 d1o7ia_ d4ipca1
d1x3ea_ d4apva_ d1jb7a1 d1jb7a2 d1jb7a3 d3kdfa_ d1ue1b_ d1wjja1
d3eiva_ d1xjva2 d1xjva1 d2ccza2 d1se8a_ d1l1oc_ d1jb7b_ d1qvca_
d3ulla_ d1qzga_ d1miua4 d1miua5 d1miua3 d3g48a1 d1gd7a_ d1jjcb3
d1pyba_ d3ubub_ d1egia_ d1qo3c_ d1v7pa_ d1tdqb_ d3ubua_ d1c3ab_
d1h8ua_ d3bx4b_ d1t8ca1 d1sb2a1 d1qdda_ d2ziba1 d3cada_ d1byfa_
d1j34b_ d1jwia_ d1jzna_ d1c3aa_ d1oz7b_ d1wmza_ d3bdwb_ d1hq8a_
d1fifa1 d1dv8a_ d3hupa_ d1wk1a1 d1umra_ d2afpa_ d3bx4a_ d1gz2a_
d1sl6a_ d1v7pb_ d3g8ka_ d1ixxa_ d1j34a_ d1jwib_ d1g1sa1 d1y17a1
d1ja3a_ d1fvub_ d3bdwa_ d1sb2b1 d1fvua_ d1umrc_ d1oz7a_ d4m17a_
d3cfwa1 d1ypqa1 d1g1ta1 d3fd4a_ d3l9jc_ d4dn8a1 d1v4lb_ d1rdl1_
d2msba_ d1dzka_ d4tlja_ d2a2ua_ d4wfva_ d3t1da_ d4iaxa_ d3zq3a_
d2nnda_ d1i4ua_ d1kxoa_ d1bbpa_ d1kt7a_ d1exsa_ d3s26a1 d1lf7a_
d4gh7a_ d1z24a1 d1qabe_ d1xkia_ d1ew3a_ d3bx7a_ d4iawa1 d1n0sa_
d1gkab_ d3kzaa_ d2k23a_ d2l9ca1 d2joza1 d1gm6a_ d3kffa_ d3saoa1
d1e5pa_ d1yupa1 d1iiua_ d1pfsa_ d2a1ka_ d1je5a_ d2shpa1 d1yfoa_
d1l8ka_ d2ooqa1 d1g4us2 d1p15a_ d1lara2 d1lara1 d1wcha_ d2fh7a2

d2fh7a1 d1ohea1 d1ohea2 d2f4da_ d1rxda_ d2hxpa_ d1xria_ d1d5ra2
d1fpza_ d3ezza_ d4y2ea_ d1i9sa_ d1tdha3 d1q3ba3 d1k82a3 d4qmea2
d2xdta2 d1z5ha2 d3ubra_ d1qo8a1 d1d4ca1 d2k3va_ d1ft5a_ d1qdba_
d1m1qa_ d2j7aa_ d1h21a_ d1ogyb_ d4fasa_ d1jnia_ d1oaha_ d1sp3a_
d1eysc_ d3h33a_ d1rwja_ d19hca_ d1os6a_ d3bxua_ d3h4na_ d1i77a_
d3caoa_ d1j0pa_ d2ctha_ d1duwa_ d1ofwa_ d2e84a_ d1hh5a_ d3h34a_
d2dara2 d2dara1 d1wyha2 d2dloa2 d1rutx4 d1rutx3 d2cuqa2 d1x4la2
d1x4la1 d1x3ha2 d1x3ha1 d2d8za1 d1x61a2 d1x6aa1 d1x6aa2 d2cora2
d2cora1 d1v6ga1 d1v6ga2 d2d8za2 d1x64a1 d1x64a2 d1b8ta1 d2co8a1
d1x63a2 d2dj7a2 d2dj7a1 d2cu8a2 d2cu8a1 d1g47a2 d1g47a1 d1j2oa1
d1wyha1 d2dloa1 d1wiga1 d1wiga2 d2cupa2 d2cupa3 d1x68a1 d2d8ya2
d2d8ya1 d1m3va2 d1x62a1 d1x68a2 d1x61a1 d1b8ta3 d1b8ta2 d2d8xa2
d2d8xa1 d1x4ka1 d1x4ka2 d2cuqa1 d1m3va1 d2co8a2 d1j2oa2 d1x63a1
d2cura1 d2cura2 d1x62a2 d1op9a_ d1y0la1 d1etzb1 d1egjl1 d2gsib1
d1nj9a1 d4jfhd1 d1aqkl1 d1t2ja_ d1lo4h1 d1zvya1 d3v4vl1 d4enec1
d1kxtb_ d5e8ea1 d2pxya_ d4weud_ d1xiwc_ d2esvd1 d1qnzh_ d1ogae1
d1f4xh1 d4n1hb_ d3ezjb1 d3mlwl1 d1kfah1 d1etza1 d1b4jh1 d3cfic_
d4w68a_ d2arja1 d1ek3a_ d2ok0l1 d1vera_ d1uwgh1 d1blnb1 d1fpth1
d1rzga1 d2giya1 d4m3kb1 d1nc2a1 d2nyyd1 d1ncna_ d1fbih1 d5dxwa_
d4fqqa1 d1dqdh1 d1jfql1 d1kelh1 d1faih1 d1frgl1 d1figl1 d4m6nl1
d1fo0a_ d4hxbl1 d3b5ga_ d3quyd1 d2h9gb1 d1i8kb_ d2dd8h1 d2ck0h1
d1eaja_ d4w6wb1 d2a6ib3 d1t66d1 d2e27h_ d1ob1b1 d1f11b1 d2v7na1
d2xt1b1 d1pskh1 d4i13b1 d2op4l1 d1a0ql1 d1uwel1 d1za3a1 d1ncbl1
d4uu9a_ d1bm3h1 d1cloh1 d1nqba2 d1cf8h1 d4y5va_ d1mhph1 d5anma1
d4cdgc1 d4ldeb_ d1y0lb1 d2pcpb1 d1z3gl1 d1dfbl1 d1fh5h1 d3g5xa1
d1tzib1 d4dn3l1 d3zhka1 d2b1hh1 d2uyla1 d1f3dh1 d1nj9b1 d2agjh1
d2h1pl1 d1q72h1 d8fabb1 d1ndmb1 d1rjca1 d4u6hb1 d3upca_ d2fbjl1
d1osph1 d1tvda_ d2cjvh_ d4fhbd1 d1bd2d1 d1rz7h1 d3nn8a_ d1blna1
d1knoa1 d1nfda1 d2bmkb1 d1dqll_ d1igyb1 d1igfl1 d12e8h1 d3utza1
d1nc2b1 d1rihh1 d4mwfb1 d4jvpa1 d1mieh1 d1rhfa1 d1fgnl1 d1sbsh1
d1lp9e1 d1dn0a1 d4lqfl1 d1iaih1 d1kgcd1 d1mh5b1 d1gpol1 d2g60h3
d1f8tl1 d2jixd1 d4grwh_ d1fo0b_ d1bfob1 d1axsa1 d1u9ka_ d1nmbh_
d1h8na1 d1h8na2 d1jguh1 d4eura1 d3sdyl1 d35c8h1 d1w72l1 d1mvub_
d1i9rh1 d1ob1a1 d4wenb_ d1yeeh1 d3ietc1 d1f58h1 d1ad0b1 d1mqkl_
d1nbvh1 d4b50a_ d3r8bb_ d1bafl1 d1mamh1 d4rgne1 d2gsgb_ d35c8l1
d1bafh1 d1ai1h1 d2pcpa1 d2fath1 d2x1pa_ d1orsb1 d1ibgl1 d1shma_
d1a2yb_ d1u0qa_ d2coqa_ d1hilb1 d1qfwi_ d1jv5b_ d1fn4b1 d5e0qa_
d1nsnh1 d1plgl1 d4ocxl1 d1kb5h1 d1rzfh1 d1pg7x1 d2fx7h1 d2oi9c_
d1l7il1 d3eyva1 d1d5il1 d4ffyl_ d1tetl1 d1a6wh_ d1ac6a_ d3dvfa_
d1nfde1 d1mimh1 d1ri8a1 d1jhlh_ d3dura_ d1fskb1 d1f97a1 d3so3b1
d2ak4d1 d1lp9f1 d4z7wf1 d1l7th1 d4y5ya_ d4x7ec1 d1emth1 d1ikfl1
d1nlbh1 d4utal1 d1bjma1 d1eapb1 d1u6ah1 d2dtgd1 d1kj2b_ d1xeda1
d1c1eh1 d1fgvh_ d2bnub1 d2fb4h1 d2dtgb1 d1ktrh_ d1jpth1 d1qkzl1
d4hepg_ d2atpb1 d1flrh1 d2hrpl1 d1mvfa_ d1iaim1 d3k80a1 d2jb5h1

| | | | | | | | |
|---|---|---|---|---|---|---|---|
| d3zkxc_ | d4grwe_ | d1it9h1 | d1rhhb1 | d3w9ec_ | d1e4wh1 | d1sy6h1 | d2j6eh1 |
| d3qxta_ | d1jglh1 | d1p7ka1 | d1tzhb1 | d4n8pa1 | d1gafl1 | d2jelh1 | d1wioa2 |
| d4i0cc1 | d1cicb1 | d1lmka2 | d1lmka1 | d1iqwh1 | d1orqb1 | d1ejoh1 | d1jnlh1 |
| d4zg1a_ | d43c9b_ | d1ggbl1 | d1wejh1 | d3q6fa1 | d1f3dj1 | d3c6la1 | d1bqlh1 |
| d3p9wb_ | d1a3rl1 | d1h0db1 | d2uzil_ | d1eo8l1 | d1qfwl_ | d1n7ml1 | d1igja1 |
| d1c5ch1 | d2a9mh_ | d1rmfh1 | d1fj1b1 | d4heme_ | d1ccza1 | d1a5fh1 | d1ogad1 |
| d1dlfh_ | d1qa9b_ | d1vgel1 | d3qskb_ | d1sjxa_ | d4ozfg1 | d1sq2n_ | d1qd0a_ |
| d1kcvh1 | d1c5db1 | d3qcul1 | d8faba1 | d3bn9d1 | d1mjuh1 | d4y8dc_ | d7fabh1 |
| d3dvgb1 | d1lo0l1 | d1a6tb1 | d1gigh1 | d1kcrh1 | d2hwzl1 | d1yjdc1 | d3clfl1 |
| d4krna_ | d1dl7l_ | d1e6ol1 | d1mexh1 | d3r0ma_ | d4kkna_ | d1fnsl1 | d1oaqh_ |
| d1rzia1 | d1ucbh1 | d1ncwl1 | d4b41a_ | d1nfdf1 | d2mpah1 | d2d7tl1 | d4grwf_ |
| d4b5ea_ | d1lk3h1 | d3u79a_ | d4u3xa_ | d1ghfl1 | d1bvkb_ | d1mcww1 | d1b88a_ |
| d1jrhh1 | d32c2b1 | d4wemb_ | d1dsfh_ | d1op3h1 | d1cell1 | d1iqda1 | d1qoka1 |
| d1qoka2 | d1qfuh1 | d2crya1 | d1ay1h1 | d1oaql_ | d3ab0b_ | d1rurl1 | d1t4kb1 |
| d1bz7b1 | d1n0xh1 | d1forl1 | d1ucbl1 | d4w6yb_ | d43c9a_ | d2aabl1 | d1tzia1 |
| d4n1ca_ | d1k6qh1 | d3g9ab_ | d4tvsa1 | d1igmh_ | d1pz5b1 | d3dx9a1 | d3wxwh_ |
| d1nakh1 | d1kcul1 | d3eaka_ | d4qlra1 | d1cr9l1 | d1d9ka_ | d1f4xl1 | d1deea1 |
| d2i27n2 | d1c12a1 | d2orbl1 | d1j8hd1 | d1mcph1 | d1c5da1 | d1kenh1 | d1bd2e1 |
| d1h5ba_ | d4x7fc1 | d3upaa_ | d1t3fa1 | d1indh1 | d2dtgc1 | d4hc1l1 | d1zv5a1 |
| d4wgvb_ | d1uwgl1 | d4f37k1 | d1aqkh1 | d1vesa_ | d3zkqd_ | d1fptl1 | d1lo4l1 |
| d1knob1 | d3rrqa_ | d1ct8b1 | d4orzc_ | d1mlbb1 | d4hgkc1 | d1dqdl1 | d1bogb1 |
| d4ozhe1 | d1dr9a1 | d1i7za1 | d1dn0b1 | d1kell1 | d1rzib1 | d1ypzf1 | d1wtla_ |
| d1hkfa_ | d1fail1 | d1figh1 | d2vyre_ | d1sjva_ | d1ohqa_ | d1bfoa1 | d1pkqb1 |
| d1mjul1 | d1b4jl1 | d1nsnl1 | d2arjb1 | d1p4bh_ | d4aj0a_ | d3nn8b_ | d2rcja1 |
| d1f2xk_ | d4v1dd_ | d1adqh1 | d2eksa_ | d4k3ga_ | d1jfqh1 | d1uweh1 | d1frgh1 |
| d4gftb_ | d1uz8b1 | d4n1ea_ | d1bm3l1 | d1clol1 | d3mcka1 | d2vq1a1 | d1ae6h1 |
| d4aq1b1 | d1uj3b1 | d1nbqa1 | d1fh5l1 | d2ck0l1 | d2ywza_ | d1q72l1 | d4nbxb_ |
| d1hila1 | d4hzlb1 | d2j88l1 | d1zmya1 | d1cd0a_ | d1a4ka1 | d1pskl1 | d1a0qh1 |
| d4krmb_ | d1ncbh1 | d2fbjh1 | d1j8he1 | d1i3ua_ | d1cf8l1 | d1fe8h1 | d2p1ya1 |
| d1eeqa_ | d2x89a_ | d1ol0a_ | d2r0lh1 | d1ub5a1 | d1dfbh1 | d3eyvb1 | d4jdvb1 |
| d4p2cg_ | d2gsia1 | d2agjl1 | d1u3ha1 | d2h1ph1 | d1rihl1 | d1fskc1 | d1boga1 |
| d2a77l1 | d1ospl1 | d1qfoa_ | d3dwta_ | d2fx7l1 | d1rz7l1 | d3stba1 | d1pkqa1 |
| d1b6da1 | d1dzba2 | d1dzba1 | d1kj2a_ | d4l1ha_ | d1rzgb1 | d1dqlh_ | d12e8l1 |
| d1miel1 | d1jmaa_ | d1sbsl1 | d1fgnh1 | d1f58l1 | d1mqkh_ | d1ndgb1 | d3qegl1 |
| d1iail1 | d2aw2a1 | d1tqbc1 | d3fo2a1 | d4dkaa_ | d1f8th1 | d2g60l1 | d2ywya_ |
| d1uj3a1 | d1yc7a_ | d3bd3a_ | d4w6xb1 | d1nmbl_ | d1i8ka_ | d1rjlb1 | d1ypze1 |
| d1p7kb1 | d1kxqe_ | d1gpoh1 | d2z4qa1 | d1w72h1 | d1i9rl1 | d4qgya1 | d4xt1c_ |
| d2qqnh1 | d1cica1 | d3c08h1 | d2cd0a_ | d2bdnh1 | d1hnga1 | d1maml1 | d1nldh1 |
| d1cr9h1 | d4lcih1 | d3bkyh1 | d3qg7l1 | d1ai1l1 | d1kxvc_ | d4iosd_ | d1l7ih1 |
| d2x1qa_ | d1ub6b1 | d3k74b_ | d1ibgh1 | d1teth1 | d1igya1 | d1hxma1 | d2x6ma_ |
| d2e7la_ | d5e03a_ | d1qfwm_ | d2r0kh1 | d1igjb1 | d1rurh1 | d1plgh1 | d1mj8h1 |
| d1yuhb1 | d3fmgl1 | d1rzfl1 | d1emtl1 | d1ad9b1 | d1igtb1 | d1nlbl1 | d1d5ih1 |
| d3k7ua_ | d4ffyh_ | d1bwwa_ | d3dvga1 | d1a6ta1 | d1fgvl_ | d4qxgl1 | d1miml1 |
| d1jhll_ | d2hffb1 | d2hh0l1 | d1qkzh1 | d1l7tl1 | d1afvh1 | d1iaii1 | d2cjuh_ |

d1um5h1 d2ghwb2 d2ghwb1 d1kgce1 d2fgwh1 d1ikfh1 d1dvfd_ d2i24n1
d2xtjb_ d1za3b1 d2yc1a_ d2hh0h1 d2x1oa_ d1bvka_ d1rjla1 d5e7ba1
d1igta1 d1il1b1 d2fb4l1 d1mvua_ d1jptl1 d32c2a1 d1fvca_ d1iqdb1
d2hrph1 d2p4ab_ d4qo1a_ d1p2cb1 d1wt5c_ d1t4ka1 d5c1mb_ d1it9l1
d1bz7a1 d1b2wh1 d1e4wl1 d1jnll1 d1yedb1 d1za6b1 d3u7aa_ d1igfh1
d3gnml1 d1orsa1 d1wejl1 d1a2ya_ d1hxmb1 d1gafh1 d2j4wh1 d1pz5a1
d1j05a_ d1a3rh1 d4fqll1 d1qfwh_ d1eo8h1 d1n7mh1 d4s11a_ d1kegh1
d5da4a_ d1iqwl1 d3whxb1 d1vgeh1 d1deeb1 d1c12b1 d1t2ql1 d1ad9a1
d3gsnb1 d3b2uc1 d1ggbh1 d1t3fb1 d1nakl1 d4ocmc_ d1lila1 d2v7hb3
d2esve1 d1mcoh1 d2qr0b1 d2q3aa_ d1ct8a1 d1kcrl1 d2pxyb_ d2uzih_
d5c3ld_ d3durb_ d1c5cl1 d2ak4e1 d1dl7h_ d1xiwd_ d1mlba1 d1rmfl1
d1i7zb1 d1dlfl_ d2xa3a_ d5bq7b1 d1dqqb1 d3h42l1 d1egjh1 d1kcvl1
d1fe8l1 d2j6el1 d1f3rb2 d1lk3l1 d1sm3h1 d1q0xh1 d1ghfh1 d7fabl1
d1igch1 d1lo0h1 d4idla_ d5c2bl_ d1jrhl1 d1il1a1 d3zhda1 d4kfzc_
d1b0wa_ d1e6oh1 d1mexl1 d4krpb_ d1fnsh1 d1ncwh1 d4hgma_ d4eigb1
d4rzca_ d2mpal1 d4y5xa_ d5en2b1 d3lrga1 d4ej1c1 d1rhha1 d1j05b_
d1forh1 d1q9wb1 d2wzpd_ d2aq2a1 d1h0da1 d3revb1 d2ij0c1 d1tzha1
d4m7jl1 d1k6ql1 d3b9kc1 d1igml_ d3qeua1 d1dsfl_ d1ce1h1 d1kcuh1
d1zvha1 d1zvoa1 d1tqbb1 d1ay1l1 d2qscl1 d2cgrh1 d1nl0h1 d1kenl1
d3eo0a1 d4n9ob1 d1n0xl1 d3k1kc_ d2m9wa1 d3dfxa_ d2aq2b1 d1ck1a1
d1enfa1 d1i4pa1 d1v1oa1 d1et9a1 d1esfa1 d2g9hd1 d4ohja1 d1klud1
d1bxta1 d1eu3a1 d1ty0a1 d1fnua1 d2cqva1 d1fhga_ d2k1ma_ d2dl2a1
d1biha3 d1biha1 d1biha4 d3qp3a1 d1g1ca_ d1gxea_ d1cvsc2 d1cvsc1
d1xmwa2 d1m4ka2 d2otpa2 d1biha2 d1rhfa2 d2fcba2 d2fcba1 d1gsma2
d1epfa1 d1epfa2 d2dava1 d1waaa_ d1f97a2 d1x44a1 d1vcaa2 d1fnla2
d1fnla1 d3b5ha2 d3h8na2 d3h8na1 d1jbja1 d1jbja2 d1pd6a_ d1f2qa2
d1f2qa1 d3cx2a_ d1nbqa2 d3b5ha1 d1n26a1 d1xiwb_ d1xaua_ d1fltx_
d2ifga1 d1olla1 d1olla2 d1cs6a4 d1cs6a3 d1cs6a2 d1cs6a1 d2gw5a2
d1sy6a1 d1xiwa_ d1ucta2 d1ucta1 d1ry7b2 d1gl4b_ d1qz1a3 d1olza1
d2c9aa1 d1wwca_

**Family-27 (b.29.1.2)**

**Positive dataset contains 9 protein sequences.**

d3azya_ d3wvja_ d1upsa1 d2hyka_ d2vy0a_ d3o5sa_ d3d6ea_ d3atga_
d3i4ia_

**Negative dataset contains 3988 protein sequences.**

d1m32a_ d1ibja_ d1c7na_ d1iuga_ d4q75a_ d2bkwa1 d1t3ia_ d1n8pa_
d1qz9a_ d1jf9a_ d1p3wa_ d3kgwa_ d1cl1a_ d2ctza1 d1vjoa_ d2rfva_
d1gc0a_ d1cs1a_ d1d2fa_ d2ch1a1 d1qgna_ d1pffa_ d1elua_ d1u08a_
d1lc5a_ d2ay1a_ d1bw0a_ d3pd6a_ d7aata_ d2csta_ d1j32a_ d1fg7a_
d4wlha_ d1o4sa_ d1v2da_ d1gdea_ d1m6sa_ d1v72a1 d1svva_ d1ajsa_
d1wsta1 d2f8ja2 d1lk9a_ d1vp4a1 d1xi9a1 d2gb3a1 d1yaaa_ d2bnma2
d1jx4a2 d3gv5b1 d1t94a2 d3bq0a2 d3i2ka1 d1lnsa3 d1mpxa2 d1xkta1
d2hfka_ d1jmkc_ d5d3ka_ d4nmwa_ d1ddla_ d1e57b_ d1auyb_ d1f56a_
d1bypa_ d1azca_ d1ws8a_ d3u25a_ d1cuoa_ d1id2a_ d1rkra_ d2iaac_

| | | | | | | | |
|---|---|---|---|---|---|---|---|
| d4dp1x_ | d1nwpa_ | d2gima_ | d2i7sa_ | d1qhqa_ | d3ehbb1 | d1fftb1 | d1gyca3 |
| d1hfua2 | d1kbva1 | d1kbva2 | d1sdda1 | d1sdda2 | d1gyca2 | d1mzya1 | d2bw4a1 |
| d1aoza1 | d1aoza3 | d1aoza2 | d1kyaa2 | d1kyaa3 | d1kyaa1 | d1mzya2 | d4e9sa1 |
| d1sddb1 | d1sddb2 | d1hfua1 | d1hfua3 | d1oe1a2 | d1oe1a1 | d1v10a2 | d1v10a3 |
| d1v10a1 | d2b5ea2 | d2b5ea3 | d2b5ea1 | d2b5ea4 | d2kp1a1 | d2kp2a1 | d1hyua3 |
| d1hyua4 | d1j08a1 | d1j08a2 | d2cfba1 | d1dfoa_ | d1o69a_ | d1bj4a_ | d3k28a_ |
| d2bwpa_ | d3lg0a_ | d2fnua_ | d3ou5a_ | d1z7da1 | d1b9ha_ | d4msoa_ | d1bs0a_ |
| d1s0aa_ | d1sffa_ | d3tqxa_ | d1mdoa_ | d3qboa_ | d3pgya_ | d1bjna_ | d2dkja_ |
| d1ejia_ | d1vefa1 | d4wxga_ | d3bs8a_ | d3gbxa1 | d1w23a_ | d1a3qa1 | d1iknc_ |
| d3brda1 | d1p7hl1 | d1oy3c_ | d1imhc1 | d1zk9a1 | d2cxka1 | d2h0va_ | d1zx5a1 |
| d1mqta_ | d1d4m3_ | d1fmd3_ | d2mev1_ | g1aym.1 | d1ev13_ | d4q4w1_ | d1r1a3_ |
| d2x5ia_ | d1rhi1_ | g1r1a.1 | g1d4m.1 | g1rhi.1 | d1b35b_ | d1oopa_ | d1tme1_ |
| d4iv1a_ | g1pvc.1 | g1fpn.1 | d1fpn1_ | d4gb31_ | d1d4m1_ | d1fmd1_ | d1ev11_ |
| d2wzr3_ | g1cov.1 | d1qqp3_ | g1fmd.1 | d2x5ic_ | d1rhi3_ | g1oop.1 | d1hxs1_ |
| g1hxs.1 | d1fpn3_ | d1bev1_ | g2mev.1 | d1eah3_ | d1aym1_ | d1pvc3_ | d1qqp1_ |
| d1zba3_ | d1h8ta_ | d3vbhc_ | g1b35.1 | g1bev.1 | d1hxs3_ | g1ev1.1 | d1bev3_ |
| g1tmf.1 | d1tmf1_ | d1b35a_ | d2mev3_ | g1eah.1 | g1h8t.1 | d1eah1_ | d4q4w3_ |
| d1r1a1_ | d1aym3_ | d1zba1_ | d1h8tc_ | d1oopc_ | d1tme3_ | g1qqp.1 | d4iv1c_ |
| g1tme.1 | d1ep0a_ | d2pa7a1 | d1wlta1 | d1nxma_ | d2c0za1 | d1ds6b_ | d1fstb_ |
| d1ex0a1 | d1g0da1 | d1ju8a_ | d1v70a_ | d2f4pa1 | d1yhfa1 | d1tafa_ | d1bh9b_ |
| d1tafb_ | d1jfib_ | d1q9ca_ | d1h3ob_ | d2byka1 | d1n1ja_ | d4g92b_ | d1bh9a_ |
| d1jfia_ | d1h3oa_ | d2bykb1 | d2bjca1 | d2hsga1 | d1qpza1 | d1r0ka3 | d1j5pa3 |
| d1vkoa2 | d1vm6a2 | d3qvsa2 | d1gr0a2 | d3gpdg2 | d2q49a2 | d1hdgo2 | d2g17a2 |
| d2cvoa2 | d2czca1 | d3pwka2 | d1obfo2 | d1i32a2 | d1zyao2 | d2pkqo2 | d1b7go2 |
| d2hjsa2 | d1cf2o2 | d1vkna2 | d1ux8a_ | d1dlya_ | d4i0va_ | d2bkma_ | d1dlwa_ |
| d2bmma_ | d2msya_ | d2djoa1 | d2r5za_ | d1au7a1 | d1f43a_ | d2lmda1 | d1akhb_ |
| d1s7ea1 | d3nara_ | d1zq3p1 | d2craa1 | d1mija_ | d4uuta_ | d3a01a_ | d1x58a1 |
| d3rkqa1 | d3cmya1 | d2cqxa1 | d2da6a1 | d1e3oc1 | d1fjla_ | d1mh3a1 | d1ig7a_ |
| d1x41a1 | d1wh7a1 | d1gt0c1 | d2mw8a_ | d1lfup_ | d1x2ma1 | d1b72a_ | d1wi3a1 |
| d1jgga_ | d1wjha1 | d1x2na1 | d1mnmc_ | d2cuea1 | d3k2aa_ | d2hosa_ | d2jwta2 |
| d1pufa_ | d1octc1 | d2hi3a_ | d1b8ia_ | d4j19a_ | d3lnqa_ | d1wh5a1 | d1b8ib_ |
| d1fexa_ | d2cqra1 | d1guua_ | d1ug2a1 | d2cqqa1 | d2ajea1 | d4a69c_ | d1h8ac1 |
| d2crga1 | d2iw5b1 | d1xc5a1 | d1ofcx1 | d2cu7a1 | d1wgxa1 | d2cjja1 | d2ckxa1 |
| d1igna2 | d2iu5a1 | d1zk8a1 | d2g7sa1 | d2fd5a1 | d2np5a1 | d1z0xa1 | d2id6a1 |
| d2d6ya1 | d2i10a1 | d2oi8a1 | d3c07a1 | d2ns7a1 | d2fq4a1 | d2yvea1 | d1t33a1 |
| d2o7ta1 | d1rkta1 | d2np3b1 | d2g7ga1 | d2gena1 | d2fbqa1 | d2g7la1 | d2gfna1 |
| d2g3ba1 | d2jj7a1 | d2hyja1 | d1sgma1 | d1ui5a1 | d2hkua1 | d2id3a1 | d4jyka1 |
| d3lv8a_ | d1s3ga1 | d1zaka1 | d1s4qa_ | d3tvta2 | d1uf9a_ | d1khta_ | d1ckea_ |
| d1osna_ | d1p5zb_ | d3cm0a_ | d1tmka_ | d1jaga_ | d3h86a_ | d1t0hb_ | d1nn5a_ |
| d2h92a_ | d3kb2a1 | d2c95a1 | d1yj5a2 | d1kgda_ | d1ly1a_ | d2ak3a1 | d1vyva2 |
| d1deka_ | d2grja1 | d1p3ja1 | d1y63a_ | d1e4ya1 | d1s96a_ | d3iija1 | d1lvga_ |
| d1nksa_ | d1vyua2 | d4e22a_ | d1qf9a_ | d1jjva_ | d1q3ta_ | d1ki9a_ | d1lw7a2 |
| d1teva_ | d1kjwa2 | d1gsia_ | d2iyva2 | d1kaga_ | d1viaa_ | d1e6ca_ | d1g3ma_ |
| d1j99a_ | d1t8ta1 | d1nsta_ | d1zrha_ | d1ls6a_ | d2a3ra_ | d1aqua_ | d2zptx_ |

| | | | | | | | |
|---|---|---|---|---|---|---|---|
| d3ckla_ | d1texa_ | d1q44a_ | d2gwha_ | d1z0ja1 | d3umra2 | d3gj0a1 | d2gaoa1 |
| d1yzga1 | d1x3sa1 | d1g7sa4 | d1puja_ | d1udxa2 | d1kk1a3 | d2o52a_ | d1jwyb_ |
| d1zcaa2 | d1zcba2 | d2fv8a1 | d2cjwa_ | d3dz8a1 | d1xzpa2 | d1vg8a_ | d4qxaa_ |
| d2ew1a1 | d1x1ra1 | d2bcgy_ | d3doea1 | d3cona_ | d3rega_ | d3wyga_ | d1upta_ |
| d2b6ha1 | d1zd9a1 | d1lnza2 | d1r5ba3 | d1n0ua2 | d1r2qa_ | d2f9la1 | d2rhda_ |
| d3q3jb_ | d3q85a_ | d1efma1 | d1r8qa_ | d2gf9a1 | d2akab1 | d1ek0a_ | d1z08a1 |
| d2gcoa1 | d2dy1a2 | d4g01b_ | d2cxxa1 | d1svia_ | d3q72a_ | d1ni3a1 | d4ac9a4 |
| d2erxa1 | d2bmea_ | d1wxqa1 | d2gf0a1 | d4ldja1 | d1zunb3 | d4lv5b_ | d1efca3 |
| d1kmqa_ | d2g6ba1 | d3lrpa_ | d1tada2 | d2y8ea_ | d4rd4a1 | d1z06a1 | d2heia_ |
| d3a58b_ | d1u0la2 | d1d2ea3 | d1zj6a1 | d2ocba_ | d3rwoa_ | d1mr3f_ | d2e9sa1 |
| d4u5xa1 | d2iwra_ | d2fn4a1 | d3bwdd_ | d2nzja1 | d2atva1 | d2ksqa_ | d3l43a_ |
| d1mkya2 | d1mkya1 | d1wmsa_ | d1jnya3 | d2rmka_ | d1f6ba_ | d1f60a3 | d1gota2 |
| d1jala1 | d2fh5b1 | d1g16a_ | d2wblc_ | d1m7ba_ | d1wf3a1 | d1f5na2 | d1t9ha2 |
| d1z6xa1 | d1z0aa1 | d2fg5a1 | d2a5ja1 | d2bcjq2 | d1s0ua3 | d1ky3a_ | d4y0va_ |
| d4lhwa_ | d2qmea1 | d2atxa1 | d1egaa1 | d1ryha_ | d1moza_ | d1nrjb_ | d2erya1 |
| d3bfka_ | d1puia_ | d1fzqa_ | d3j3az1 | d3t5ga_ | d1shza2 | d3defa_ | d2f7sa1 |
| d3raba_ | d3tsoa_ | d3aq4a_ | d2al7a1 | d3sfva1 | d3bc1a_ | d2ncda_ | d1sdma_ |
| d3h4sa1 | d2heha_ | d2grya_ | g3kin.1 | d1kk8a2 | d1br2a2 | d1goja_ | d1f9va_ |
| d2mysa2 | d1lkxa_ | d3t0qa_ | d4db1a1 | d3gbja_ | d1ry6a_ | d1v8ka_ | d1k8ka2 |
| d1k8ka1 | d3uleb1 | d3i33a2 | d3i33a1 | d1bupa1 | d1dkgd2 | d1dkgd1 | d4ci6a2 |
| d1jcfa1 | d4a2ba2 | d2fxua1 | d1yaga2 | d1yaga1 | d4cbua2 | d1bupa2 | d1ig8a1 |
| d1czan2 | d1czan3 | d1czan1 | d1ig8a2 | d1czan4 | d1bg3a4 | d1bg3a2 | d1bg3a1 |
| d3h1jd1 | d2d0oa3 | d2d0oa2 | d1nbwa3 | d1hroa_ | d4gedb_ | d3zooa_ | d1mz4a_ |
| d1gu2a_ | d1f1fa_ | d1dvva_ | d1c6ra_ | d1c75a_ | d1e29a_ | d1gdva_ | d1cnoa_ |
| d1h32b_ | d1co6a_ | d2bh4x_ | d2aiua_ | d5cytr_ | d1jdla_ | d1i8oa_ | d2zboa_ |
| d3dmia_ | d1ls9a_ | d1wvec_ | d2yk3a_ | d3ph2b_ | d1dw0a_ | d5aura_ | d1ql3a_ |
| d1qn2a_ | d1f1ca_ | d1prtc2 | d1cnt1_ | d1pvhb_ | d1z7ca_ | d1huwa_ | d1bgea_ |
| d1rhga_ | d1f6fa_ | d1f45b_ | d1i1rb_ | d1axia_ | d1etea_ | d3tgxb_ | d1scfa_ |
| d2z3qa1 | d3qb1a_ | d1hula_ | d1m48a_ | d4nkqb_ | d1v7mv_ | d5e4ea_ | d2o27a_ |
| d3b5ka_ | d2psma1 | d4zf7a_ | d3uf5a_ | d1v8fa_ | d2ejca_ | d3muea1 | d1ihoa_ |
| d3q12a1 | d4dnxa2 | d1v47a2 | d1g8fa2 | d1jhda2 | d1x6va2 | d3rbaa_ | d2qtra_ |
| d1nupa_ | d3l92a_ | d1f9aa_ | d1qjca_ | d2h29a_ | d1vlha_ | d1od6a_ | d1lw7a1 |
| d1mrza2 | d4ruka_ | d2el7a_ | d1iq0a2 | d1irxa2 | d1r6ta2 | d2cyba_ | d1rqga2 |
| d1j09a2 | d2yxna_ | d1f7ua2 | d1h3fa1 | d1ffya3 | d2g36a1 | d1nzja_ | d1jila_ |
| d1n3la_ | d1i6la_ | d1pfva2 | d1h3na3 | d3n9ia_ | d3sz3a1 | d3sw2a_ | d1moxc_ |
| d1dx5i3 | d1edmb_ | d1autl2 | d1autl1 | d1nqlb_ | d1dval1 | d1szba2 | d1kigl_ |
| d2y5fl_ | d1ijqa2 | d1gk5a_ | d1g1sa2 | d1xdtr_ | d1lmja2 | d1lmja1 | d1nzia2 |
| d2wphe_ | d1klil_ | d1pfxl2 | d1pfxl1 | d1p9ja_ | d1g1ta2 | d1f7ea_ | d4yzub_ |
| d2mgpa1 | d1b9wa2 | d2mgpa2 | d1ob1c1 | d1ob1c2 | d1n1ia2 | d1n1ia1 | d2npra2 |
| d2npra1 | d1gl4a2 | d1qx2a_ | d1ig5a_ | d2y5ia_ | d1qlsa_ | d1psra_ | d3n22a_ |
| d2egda_ | d4ggfc_ | d2wcea_ | d2luca_ | d4aqia_ | d3nsia_ | d2lp3a_ | d1j55a_ |
| d1a4pa_ | d1k8ua_ | d1nsha_ | d1pvaa_ | d3fs7a_ | d2pvba_ | d1ttxa_ | d1rwya_ |
| d2kyfa_ | d1rk9a_ | d1a75a_ | d1exra_ | d1tiza1 | d1nyaa_ | d1g8ia_ | d1ggwa_ |
| d1wdcc_ | d3fwba_ | d1ggza_ | d2o5ga_ | d1ctaa_ | d1m45a_ | d3i5gc_ | d1sl8a_ |

d1fpwa_ d2scpa_ d2roaa_ d4aqra_ d3pn7b_ d1br1b_ d2mysb_ d1s6ca_
d1bjfa_ d2obha_ d1jfka_ d1jf0a_ d1qv0a_ d1lkja_ d2mlfc_ d2m97a_
d2d8na_ d4by5a_ d1jbaa_ d4nqga_ d2mysc_ d1j7qa_ d1s6ia_ d1uhka2
d1r2ua_ d1dtla_ d3kf9a_ d3pn7c_ d1qjta_ d1fi6a_ d1c07a_ d5d67d_
d1tuza_ d1h8ba_ d1eg3a1 d1eg3a2 d2zkmx1 d1snla_ d1gjya_ d1y1xa_
d1dvia_ d1df0a1 d1qxpa2 d4usla_ d1k94a_ d1sfsa_ d1wkya2 d1uuqa_
d3wh9a_ d4m1ra_ d1xyza_ d4xuya_ d2je8a5 d4bf7a1 d3cuia_ d1ur4a_
d1odza_ d2vzsa5 d1bqca_ d2x2ya1 d1hjqa_ d1qnra_ d4y7ea1 d1vjza_
d4k68a_ d2v3ga_ d4hpga_ d1nofa2 d1nq6a_ d1h4pa_ d1aq0a_ d3ziza_
d4bs0a_ d1ecea_ d1h1na_ d1ta3b_ d1w91a2 d1i1wa_ d1uhva2 d1us3a2
d1egza_ d7a3ha_ d1tvna1 d3nyda1 d1ghsa_ d2cyga1 d4xv0a_ d2c8na2
d1w32a_ d3emca_ d1v6ya1 d1foba_ d2uwfa1 d2fgla_ d3niya1 d2c0ha1
d1rh9a1 d1yq2a5 d1szna2 d1fa2a_ d1pama4 d5do8a1 d1ea9c3 d1hx0a2
d2fhfa5 d1qhoa4 d1avaa2 d1m53a2 d4m8ua1 d2gjpa2 d1lwha2 d4howa1
d1ktba2 d1j0ha3 d3vm5a1 d2x1ia_ d1wzaa2 d1uasa2 d2diea1 d1mxga2
d1ht6a2 d1eswa_ d1iv8a2 d5cpsa_ d1ud2a2 d1zjaa1 d3gbea1 d1kwga2
d1wdpa_ d1gcya2 d1g5aa2 d1hvxa2 d3dhpa2 d2bhua3 d4do4a1 d3qgva1
d1e43a2 d1g94a2 d2d3na2 d1gjwa2 d1gvia3 d1x1na1 d1r7aa2 d1tz7a1
d4toqa_ d1wb0a1 d1w9pa1 d1eoka_ d1itxa1 d1kfwa1 d1goia2 d2gsja_
d1hjxa1 d1jnda1 d1vf8a1 d1etob_ d1umqa_ d1g2ha_ d1mixa2 d1e5wa2
d1j19a2 d1isna2 d2al6b2 d1g4ma1 d1g4ma2 d1xl4a1 d3vsqa_ d2gixa1
d1p7ba1 d1iuka_ d2csua1 d3q9ub_ d2yv1a1 d3q9na_ d2d59a_ d1oi7a1
d2yv2a1 d1euca1 d1y81a1 d1wgqa1 d1v89a1 d2coda1 d1zc3b_ d1wg7a1
d1u5ea1 d1v61a1 d2coca1 d2ys1a1 d1v5pa1 d1ntya2 d1wjma1 d1wi1a1
d1u29a_ d1xx0a1 d1unqa_ d2zkmx3 d1fhoa_ d1v88a1 d2adza1 d1x1fa1
d1p6sa_ d2coaa1 d1btka_ d1v5ma1 d1faoa_ d2x18a_ d1dyna_ d1x1ga1
d4kaxb_ d2j59m1 d1xcga2 d1txda2 d1u5da1 d1dbha2 d1w1ha_ d1upqa_
d1v5ua1 d1foea2 d2fjla1 d1eaza_ d2dfka2 d2cofa1 d1kz7a2 d9ldta1
d2ewda3 d1up7a1 d2dfda1 d1s6ya1 d1vjta1 d7mdha1 d1hyea1 d1t2da1
d1ojsa3 d1b8pa1 d1ldna1 d1hyha1 d1civa1 d2a92a1 d1ez4a1 d1i0za1
d1y6ja1 d1gv0a1 d1ur5a1 d1llda1 d1obba1 d1v8ba1 d1gdha1 d1l7da1
d2dlda1 d1mx3a1 d1ygya1 d1qp8a1 d1li4a1 d2naca1 d1npya1 d3t4ea2
d1o0sa1 d2hk9a2 d2egga2 d1b26a1 d1bvua1 d1gq2a1 d1c1da1 d1leha1
d1gtma1 d1euza1 d1nvta1 d3dona2 d1edza1 d1gpja2 d1vi2a1 d1p77a1
d1nyta1 d1b0aa1 d1vl6a1 d1v9la1 d1txga2 d3f47a1 d1mv8a2 d2pv7a2
d1xc2a3 d2ahra2 d4xiya1 d2f1ka2 d1pgja2 d1i36a2 d1lj8a4 d1teaa3
d1qmga2 d2i76a2 d2g5ca2 d1wdka3 d1dlja2 d3cuma3 d1kola2 d1f8fa2
d1qora2 d1jqba2 d1vj0a2 d1cdoa2 d1gu7a2 d1d1ta2 d1xa0a2 d2dm6a2
d1jvba2 d1rjwa2 d1llua2 d1h2ba2 d1tt7a2 d1piwa2 d1o89a2 d1iz0a2
d1e3ja2 d1yb5a2 d1uufa2 d1vj1a2 d1e3ia2 d2aq2b1 d1ck1a1 d1enfa1
d1i4pa1 d1v1oa1 d1et9a1 d1esfa1 d2g9hd1 d4ohja1 d1klud1 d1bxta1
d1eu3a1 d1ty0a1 d1fnua1 d3gpdg1 d1vc2a1 d2nvwa1 d1u8fo1 d1ydwa1
d1hdgo1 d2g17a1 d1cf2o1 d2czca2 d1r0ka2 d1obfo1 d2cvoa1 d1ebfa1
d1h6da1 d1i32a1 d1zh8a1 d2q49a1 d1qkia1 d1j5pa4 d3pzra1 d1xeaa1

d1vkoa1 d1vm6a3 d3qvsa1 d2pkqo1 d1b7go1 d1gr0a1 d1p1ja1 d1lc0a1
d1j0xo1 d3pwka1 d2hjsa1 d1zyao1 d1vkna1 d1tlta1 d1nvmb1 d3sjua_
d3ojfa1 d1xu9a1 d2jjya1 d1n7ha_ d1y1pa1 d1h5qa_ d1jtva_ d4fs3a_
d2bkaa1 d1gy8a_ d2c5aa1 d1ulsa_ d1ooea_ d2b69a1 d1y5ma1 d1spxa_
d1ae1a_ d1bxka_ d1ydea1 d1gega_ d1hdoa_ d1o5ia_ d1e6ua_ d1snya1
d2rhca_ d3zv4a_ d5ceja_ d2d1ya1 d1fjha_ d3w8ea_ d2fmua1 d1x1ta_
d1rpna_ d3ay7a1 d1k2wa_ d1eq2a_ d2hrba1 d3ruaa_ d2ew8a1 d4hwka_
d1xgka_ d2bd0a1 d1yo6a1 d2a35a1 d2ntva_ d1vl8a_ d1hdca_ d4bkua_
d2ag5a1 d2q45a_ d1nffa_ d4iy1a_ d1zbqa1 d1kewa_ d1ek6a_ d1ja9a_
d2q46a2 d1iy8a_ d1cyda_ d2ptga_ d1hxha_ d1uzma1 d3lyla_ d4m89a_
d3wyea_ d2blla1 d1w6ua_ d1sb8a_ d2q2va_ d4nr0a_ d1e6wa_ d2gasa_
d3oiga1 d2bgka1 d1mxha_ d1vl0a1 d1r6da_ d1wmaa1 d1e7wa_ d2c07a1
d1xkqa_ d3tzha_ d1g0oa_ d3lz6a_ d1qyca_ d1edoa_ d1gz6a_ d1oc2a_
d1t2aa_ d1zema1 d2xoxa_ d1z45a2 d1geea_ d1p33a_ d3lt0a_ d4e6pa_
d2wyua_ d1db3a_ d4q9na_ d1xhla_ d4o1ma_ d1fmca_ d2uvda_ d2gdza1
d1uaya_ d2z1ma_ d2o23a2 d2fr1a2 d2fr1a1 d1qsga_ d1qyda_ d4zjua_
d2a4ka1 d1yb1a_ d1wvga1 d1yxma1 d3f4ba_ d1mg5a_ d1xg5a1 d1r55a1
d3gboa_ d1atla_ d1kufa_ d1buda_ d4q1la_ d1quaa_ d1yp1a_ d1wnia_
d3nqyb_ d4gera_ d1bqba_ d4c5pa_ d2bsza1 d4bgfa_ d1w4ta1 d1ex0a4
d1g0da4 d2f4ma1 d1x3za_ d3u1ra1 d1g9ka2 d1k7ia2 d2w0pa_ d2diaa1
d2e9ia2 d2e9ja1 d2dj4a1 d2eeba1 d2diba1 d2dmba1 d2dmca1 d2d7oa1
d2ee6a1 d2d7pa1 d2eeaa1 d2di8a1 d1wlha1 d2d7na1 d2nqca_ d2d7ma1
d2dica1 d3cnka_ d2k3ta1 d2eeca1 d1qfha1 d2bp3a1 d1v05a_ d2ee9a1
d2eeda1 d2di9a1 d3ioqa_ d1fh0a_ d1o0ea_ d1khqa_ d1m6da_ d2cb5a_
d1cs8a_ g8pch.1 d1s4va_ d2pnsa_ d1vsna1 d1mira_ d1iwda_ d2oula_
d7pcka_ d3bwka_ d3bcna_ d2p86a_ d1pvja_ d1pcia_ d1gece_ d1deua_
d4bs6a_ d1xkga1 d1cqda_ d1jqpa2 d2c0ya_ d2bdza_ d3p5ua_ d2o36a_
d1k9xa_ d1ex4a2 d1c6va_ d2jk6a2 d2jk6a1 d1feca1 d1feca2 d1lpfa1
d1lpfa2 d1xhca1 d1xhca2 d1dxla2 d1dxla1 d1gera2 d1m6ia2 d1onfa2
d3lada2 d3lada1 d1w4xa1 d1w4xa2 d1gesa2 d1mo9a1 d1xdia1 d1fl2a2
d1fl2a1 d1fcda1 d1fcda2 d1h6va2 d1h6va1 d1mo9a2 d1gv4a2 d1gv4a1
d2gv8a2 d2gv8a1 d1aoga1 d1aoga2 d1hyua1 d1ebda1 d1ebda2 d3dk9a1
d1q1ra2 d1q1ra1 d1gesa1 d1pv8a_ d1w5qa_ d2c1ha_ d1ohla_ d1zbfa1
d2z1ha_ d2etja1 d3qioa1 d1jl1a_ d2e4la_ d1jl2a_ d2lsna1 d1uaxa_
d2yv0x_ d1mu2a1 d1ekea_ d1i39a_ d1pn0a1 d1reoa1 d1ju2a1 d2f5va1
d3c96a1 d2gmha1 d1pj5a2 d1gpea1 d1o5wa1 d1n4wa1 d1k0ia1 d2dw4a2
d2ivda1 d1seza1 d2voua1 d1kdga1 d1s5ja1 d2hbja2 d2igia_ d1wlja_
d1qhta1 d4dlea1 d1tgoa1 d2f96a1 d3tr8a1 d3mxma_ d1q8ia1 d1y97a1
d1w0ha_ d1kfsa1 d2py5a1 d1d5aa1 d1yt3a3 d2guia_ d3mwpa1 d4kb1a_
d2v76a_ d1wj1a1 d1aqca_ d1qqga2 d1qqga1 d1ntva_ d1n3ha_ d4dbba_
d3f0wa1 d1p3ra_ d1wgua1 d2cy4a1 d3hqca_ d1wvha1 d1xr0b1 d1jmxa2
d1jmxa1 d1pbya1 d1r7ha_ d1faaa_ d1wika1 d1xfla_ d1ttza1 d2f51a1
d1ti3a_ d1nhoa_ d1sena_ d2i4aa_ d1ep7a_ d1zmaa1 d1fo5a_ d3lefa1
d3diea1 d2yana1 d1z6na1 d1oaza_ d3zywa1 d4aj8a_ d1nm3a1 d4fiwa_

d1wmja_ d4hs1a1 d1iloa_ d1xwaa1 d1wjka1 d2vm1a_ d1gh2a_ d1h75a_
d1nw2a_ d1kx5b_ d3a6na_ d4m6ba2 d2nqbd_ d3wkjd_ d2yfwe_ d4h9na_
d5bnxa_ d1kx5c_ d3r45a_ d1eqzb_ d4jjnb_ d1f66c_ d1eqza_ d4wnnb_
d1kx5a_ d1u35c1 d1id3a_ d2nqbc_ d1kx5d_ d3us3a3 d3us3a1 d1um8a_
d1ixza_ d1qvra2 d1qvra3 d1sxjc2 d1jr3d2 d2p65a_ d1l8qa2 d1g8pa_
d3hu3a3 d1sxjd2 d1njfa_ d1in4a2 d1sxja2 d1tuea1 d2ce7a2 d1w5sa2
d1sxjb2 d1d2na_ d1ny5a2 d1ofha_ d2gnoa2 d2a5yb3 d1fnna2 d1u0ja1
d1lv7a_ d1iqpa2 d2fnaa2 d1ixsb2 d4gita_ d1sxje2 d1g41a_ d1pd212
d1nhya2 d2a2ra2 d1k0dd2 d1f2ea2 d1gsua2 d1f3ba2 d2fhea2 d2pvqa1
d1tw9a2 d1oyja2 d1e6ba2 d1k0ma2 d3ik7a1 d4is0a1 d1r5aa2 d1jlva2
d1m0ua2 d3gtub2 d1z9ha2 d1okta2 d1axda2 d2gsra2 d1tu7a2 d1k3ya2
d1zgma1 d1b48a2 d1gnwa2 d1gwca2 d2gsta2 d1glqa2 d1oe8a2 d1pn9a2
d1v2aa2 d1fw1a2 d1duga2 d2m9wa1 d3dfxa_ d4ilya_ d1chka_ d4olta_
d1qqya_ d1gd6a_ d1ivma_ d2gv0a_ d1iiza_ d2z2fa_ d1j8xa_ d1ghla_
d1b9oa_ d174la_ d169la_ d2o4wa_ d1p37a_ d1k28a3 d1xjta1 d1jtma_
d2f2qa_ d4e97a1 d3w3ea_ d4tx7a_ d3cqla_ d4msta_ d3iwra_ d2z37a_
d4dyga_ d1mpya2 d1kw3b2 d1kw3b1 d4ghga1 d1tfza1 d3isqa1 d3isqa2
d1sqia1 d1sqia2 d1lkda2 d1lkda1 d1mpya1 d1cjxa2 d1cjxa1 d1sp9b2
d1t47a1 d1t47a2 d1f1ua2 d1sp8a1 d1sp8a2 d2bema_ d1pama1 d1ea9c1
d1qhoa1 d2fhfa2 d2fhfa1 d1k3ia1 d1j0ha1 d1cxla1 d2bhua1 d1edqa1
d1f1sa2 d1ut9a2 d1gvia1 d1txka1 d1s5ja2 d1qhta2 d4dlea2 d1tgoa2
d3py8a2 d1q8ia2 d1kfsa2 d2py5a2 d1d5aa2 d1qsta_ d2beia1 d4m3sa_
d1u6ma1 d1ygha_ d1yk3a1 d3dr6a_ d1xmta_ d1z4ea1 d2ge3a1 d1bo4a_
d2fiaa1 d2jdca1 d1y9wa1 d3to7a_ d1wwza1 d2fl4a1 d2ozua1 d2b5ga_
d2cy2a1 d2p0wa_ d1cm0a_ d4pswa_ d1yrea1 d1yr0a1 d1m4ia_ d2gana1
d1yvka1 d1ghea_ d2g3aa1 d1y7ra1 d2euia1 d1q2ya_ d2fsra1 d1xeba_
d2ae6a1 d1cjwa_ d1qsma_ d2aj6a1 d1y9ka1 d1i12a_ d1vkca_ d2atra1
d1n71a_ d2d4pa1 d2fiwa1 d2fe7a1 d1tiqa1 d2ou2a_ d1yx0a1 d2fcka1
d1p0ha_ d2j8ma_ d2pq8a_ d1z4ra1 d1s3za1 d1ufha_ d2hqya1 d2hqya2
d1lrza2 d1ne9a1 d1lrza3 d4alac_ d1pjwa_ d1rera1 d1s6na_ d3uzqb_
d4od7a1 d1z6ma1 d4mcua1 d4dvca1 d1r4wa_ d3l9sa_ d1un2a_ d3rppa_
d3kasa1 d3bi1a3 d1etha2 d1w52x1 d2ppla1 d1lpbb2 d2oxea1 d1bu8a2
d1rp1a2 d1ys1x_ d4hs9a_ d1ispa_ d1ex9a_ d1ku0a_ d3qmma_ d1bnla_
d1dy2a_ d2h1ia1 d2r8ba1 d3b5ea1 d3cn9a_ d1auoa_ d1lgya_ d4l3wa_
d1llfa_ d1ukca_ d1c4xa_ d1j1ia_ d2rhwa_ d3groa_ d1pjaa_ d1thta_
d2ftba_ d1pmpa_ d2rcqa1 d1fdqa_ d4lkpa_ d1sa8a_ d1vyfa_ d1lpja_
d3vg7a1 d4azqa1 d1o8va_ d5bvqa_ d1p6pa_ d2qo4a_ d1opba_ d1yiva1
d1o1va_ d4bvma1 d4tkba_ d4i3ba_ d1bwya_ d1ftpa_ d4gkca_ d1cbia_
d3p6da1 d1kqwa_ d2a0aa1 d3zxha_ d1cgla_ d1rm8a_ d1eaka2 d1hy7a_
d2jsda_ d1cxva_ d1hv5a_ d1i76a_ d3p0ya1 d1igra1 d1igra2 d1n8yc2
d1n8yc1 d1m6ba2 d1m6ba1 d1dzka_ d4tlja_ d2a2ua_ d4wfva_ d3t1da_
d4iaxa_ d3zq3a_ d2nnda_ d1i4ua_ d1kxoa_ d1bbpa_ d1kt7a_ d1exsa_
d3s26a1 d1lf7a_ d1qfta_ d4gh7a_ d1z24a1 d1qabe_ d1xkia_ d1ew3a_
d3bx7a_ d4iawa1 d1n0sa_ d1gkab_ d3kzaa_ d2k23a_ d2l9ca1 d2joza1

d1gm6a_ d3kffa_ d3saoa1 d1e5pa_ d1yupa1 d1iiua_ d2tbvc_ d1c8nc_
d1x36a_ d1smvc_ d1ng0c_ d4sbvc_ d1opoa_ d1cixa_ d1y29a_ d1v7fa_
d2jtba_ d1d1ha_ d1g9pa_ d1lupa_ d1qk7a_ d1mb6a_ d2a2va_ d1niya_
d1i25a_ d1hvwa_ d2mpqa_ d1ie6a_ d1kqha_ d1la4a_ d2mxoa1 d1koza_
d1qk6a_ d1emxa_ d1lqra_ d1c6wa_ d1rmka_ d1ixta_ d1eyoa_ d1cnna_
d1fu3a_ d1g1pa_ d1f3ka_ d1fyga_ d1rypd_ d1irua_ d1rypc_ d3unfk_
d1rypa_ d1j2pa_ d1irui_ d1j2qh_ d3unfh_ d1m4ya_ d1rypb_ d1irul_
d1irug_ d1iru2_ d1q5rh_ d1rypg_ d1g3ka_ d1iruf_ d1q5qa_ d1e94a_
d1irub_ d1ryph_ d3ty6a_ d1iruj_ d1iruk_ d1rype_ d1irud_ d1irue_
d3mi01_ d1rypf_ d1iruh_ d2z3ba_ d1iruc_ d3mka2_ d1iru1_ g1gk9.1
g1cp9.1 g1e3a.1 d1jgta2 d1ofda3 d1ea0a3 d1ecfa2 d1q15a2 d1ct9a2
d1ao0a2 d1te5a_ d3ubub_ d1egia_ d1qo3c_ d1v7pa_ d1tdqb_ d3ubua_
d1c3ab_ d1h8ua_ d3bx4b_ d1t8ca1 d1sb2a1 d1qdda_ d2ziba1 d3cada_
d1j34b_ d1jwia_ d1jzna_ d1c3aa_ d1oz7b_ d1wmza_ d3bdwb_ d1hq8a_
d1fifa1 d1dv8a_ d3hupa_ d1wk1a1 d1umra_ d2afpa_ d3bx4a_ d1gz2a_
d1sl6a_ d1v7pb_ d3g8ka_ d1ixxa_ d1j34a_ d1jwib_ d1g1sa1 d1y17a1
d1ja3a_ d1fvub_ d3bdwa_ d1sb2b1 d1fvua_ d1umrc_ d1oz7a_ d4m17a_
d3cfwa1 d1ypqa1 d1g1ta1 d3fd4a_ d3l9jc_ d4dn8a1 d1v4lb_ d1rdl1_
d2msba_ d4w9ta_ d1jvna1 d4x2ra1 d1ka9f_ d1qo2a_ d4axka1 d1h5ya_
d4u28a_ d2y88a_ d4wd0a_ d1vzwa1 d3ia2a_ d1a88a_ d4dgqa1 d1hkha_
d1zoia_ d2flia1 d1rpxa_ d1tqja_ d1tqxa_ d3syxa1 d1mkea1 d2p8va_
d1xoda1 d1i7aa_ d1qc6a_ d1egxa_ d4lt9a1 d3tc7a1 d3hoja_ d3vnda_
d3nl8a1 d1geqa_ d3nava1 d1v5xa_ d4a2ra1 d3tc6a1 d1ujpa_ d4ijba_
d1xcfa_ d1vc4a_ d1rd5a_ d1qopa_ d3b5va_ d2r80b_ d1cqxa1 d1cg5a_
d1gcvb_ d3boma_ d2h8fb_ d2raoa_ d3bj1a_ d4hrrb_ d2r50a_ d3d1kb_
d2aa1b1 d3zhwa_ d3hf4b_ d3gdja_ d1jebb_ d3bj1b_ d1x9fb_ d1wmub_
d1gcva_ d3bcqb_ d3d4xb_ d1gvha1 d2dc3a_ d3hf4a_ d4yu3a_ d3k8ba_
d1qpwb_ d2r80a_ d3bcqa_ d1v4wa_ d3wtgb_ d1jl7a_ d4esaa_ d3d1ab_
d1fsla_ d4h2la_ d1v4wb_ d1mwca_ d1vhba_ d1jeba_ d1spgb_ d1h97a_
d1q1fa_ d1tu9a1 d3d1aa_ d1hdsb_ d3hyub_ d2zfbb_ d3fs4a_ d1oj6a_
d1shrb_ d4h2lb_ d4yu3b_ d1spga_ d3hrwa_ d3qqra_ d3at5a_ d4wchd_
d3hyua_ d1cg5b_ d1uc3a_ d1hbra_ d2raob_ d1outb_ d4esab_ d3gdjb_
d3qqqa_ d1x9fc_ d3vrfa_ d1itha_ d3mkba_ d3d4xa_ d3mjpa_ d1jl6a_
d3fh9a_ d1outa_ d2dc3b_ d1it2a_ d3eoka_ d3g46a_ d1qpwa_ d3vrfb_
d1ch4a_ d1x9fd_ d1hdsa_ d2zfba_ d3mkbb_ d1or4a_ d3wtga_ d1a4fa_
d3fh9b_ d3bomb_ d3cy5a_ d3at5b_ d1x9fa_ d2oifa_ d4lmxb_ d1cpcb_
d3v57a_ d1kn1a_ d1cpca_ d4f0ta_ d2vjha_ d4po5b_ d1eyxa_ d1alla_
d4f0tb_ d1b33b_ d1b8db_ d4h0mb_ d1b33a_ d2vjhb_ d4lm6b_ d2vmlb_
d1allb_ d2vjta_ d3dbja_ d2vmla_ d2vjtb_ d4f0ub_ d1f99b_ d4rmpa1
d1liaa_ d1eyxb_ d2uulb_ d4lmsb_ d4f0ua_ d2bv8a_ d1f99a_ d2bv8b_
d2c7lb_ d3dbjb_ d4rmpb1 d1b8da_ d4h0ma_ d3v57b_ d1liab_ d2uula_
d2j96a_ d1pjqa1 d1cwva5 d4ekfa_ d2ckga_ d1th0a_ d2bkra_ d1mtza_
d1hlga_ d2xqfa_ d1ea5a_ d1f6wa_ d1dx4a_ d1qe3a_ d1k4ya_ d4qwwa_
d2ha2a_ d3c8da2 d2pbla1 d1jkma_ d1jjia_ d1jjfa_ d1gkub1 d1gkub2

d2qvba_ d2v9za_ d4kafa1 d2m5ta_ d1cqqa_ d1wqsa_ d5bpea_ d2ipha_
d1lvoa_ d2bhga1 d3tnta_ d3sjia_ d3d23a1 d2fyqa_ d3tloa_ d3zvea1
d2hrva_ d3q3ya1 d4asha1 d1b9ra_ d1offa_ d1frra_ d3wcqa_ d1e9ma_
d1l5pa_ d1wria_ d1i7ha_ d3av8a_ d1czpa_ d1rfka_ d3p1ma1 d3b2ga_
d2bt6a2 d4iuaa3 d4bvva_ d5hpga_ d3k65a_ d1ki0a3 d1ki0a2 d1ki0a1
d1a0ha1 d1pmla_ d3sp8a3 d2feba1 d1mdwa_ d1qxpa4 d1ziva1 d1zcma1
d1f74a_ d3l21a_ d2e1da_ d1to3a1 d1w3ia_ d1sfla_ d1n7ka_ d1vcva1
d4imea_ d1wa3a_ d2ocza1 d1f2ja_ d3m16a_ d2yxga_ d1ub3a_ d3a5fa_
d1fbaa_ d1l6wa_ d1xfba1 d1wx0a1 d1vpxa1 d1mxsa_ d2a4aa1 d1xkya1
d3cq0a1 d3s1xa_ d1f05a_ d1fdja_ d3cpra1 d4d2ja_ d1e88a1 d1l6ja5
d1l6ja4 d1eaka4 d1eaka3 d1l6ja3 d1h8pa2 d3rp2a_ g1ucy.1 d1fuja_
d2zchp_ d3w94a_ d1si5h_ d1lo6a_ g2pka.1 d1cgha_ d1kigh_ d1a5ia_
d3s9ba_ d1hj8a_ d1ao5a_ d2anya_ d1q3xa1 d1gvza_ d1azza_ d4gsoa_
d2f91a1 d1iaua_ d1gdna_ d1eufa_ g1fiw.1 d1eq9a_ d4nfea_ d2rdla_
d1spja_ d1elva1 d1a7sa_ d1ekbb_ d1pfxc_ d1yc0a_ d2aiqa_ g1fiz.1
d1fi8a_ d4k60a_ d4dgja_ d1mzaa_ d1op0a_ d3tjva_ d1pytd_ d1sgfg_
g1gg6.1 d2eeka_ g1rtf.1 d1a0ja_ d3g01a_ d1pytc_ d4e7na_ d1ddja_
d1z8ga1 d2hlca_ d1npma_ d1brup_ g1jou.1 d1sgfa_ d1autc_ g2hnt.1
g1kdq.1 d1orfa_ d3s69a_ d4crga_ d1fxya_ d1bqya_ d1svpa_ d1mbma_
d3su6a1 d1cu1a1 d4agka_ d1vcpa_ d1q31a_ g1n1l.1 d3e90b_ d1ep5a_
d3u1jb_ d1m4la_ d2bk7a_ d3fjua_ d1z5ra1 d1kwma1 d1jqga1 d2c1ca1
d1dtda_ d1h8la2 d4lu0a_ d1of8a_ d3unda1 d3e9aa1 d1vr6a2 d2qkfa_
d1d9ea_ d1o60a_ d1e69a_ d1mv5a_ d3g60a4 g1ii8.1 d1ewqa2 d1ji0a_
d1qhla_ d1v43a3 d2wmfa1 d1vpla_ d4a82a2 g1f2t.1 d2d62a1 d3tuic1
d2bbsa1 d3g60a3 d2ixea_ d1g6ha_ d1sgwa1 d2qi9c_ d3rlfa1 d3b60a1
d3d31a2 d2pzea1 g1xew.1 d1jj7a_ d1oxxk2 d2onka1 d1w1wa_ d1iwea_
d1vmaa2 d1nija1 d1np6a_ d2p67a1 d3ihla_ d1okkd2 d1ls1a2 d1s1ma2
d1iona_ d2v40a_ d1dj3a_ d3hida_ d2vo1a1 d2g0ta1 d1vcoa2 d1j8mf2
d1g3qa_ d1xjca_ d1p9ba_ d1qzxa3 d1ihua2 d1ihua1 d2qm8a_ d1dj2a_
d4ioja_ d1cp2a_ d1yrba2 d1hyqa_ d3gqba2 d1skye3 d1xp8a1 d1tf7a2
d1tf7a1 d1ye8a1 d1skyb3 d2qe7d2 d4blta_ d4huta_ d3ldaa2 d4hyya_
d2i3ba1 d1cr1a_ d3j9tb2 d3ntua2 d1vdza1 d2z43a_ d1e9ra_ d3ziaa2
d1n0wa_ d1g6oa_ d3ziad2 d3j9ta2 d1pzna2 d3gqbb2 d2i49a_ d1nnfa_
d1xvxa_ d1ursa_ d1h3da1 d1iuda_ d4m1va1 d1twya_ d3e13x_ d2nxoa1
d4pfua_ d3ttma_ d1elja_ d2qmwa1 d1us5a_ d1y3na1 d1jeta_ d1uqwa_
d4f19a1 d2esna2 d3thia_ d3fasa1 d2uyeb_ d2xwva1 d1r9la_ d2fyia1
d1pb7a_ d2ozza1 d1sw5a_ d1nh8a1 d1vr5a1 d2g29a_ d1xs5a_ d3lswa_
d2z23a_ d3w9va_ d3tcha_ d1q35a_ d1z7me1 d3q27a_ d1p99a_ d1ve4a1
d2i6ea1 d4mcoa1 d4n8ya_ d1eu8a_ d1zbma1 d1hsla_ d1ixca2 d1j1na_
d1xvya_ d1y9ua_ d2z22a1 d1xoca1 d4oera_ d1wdna_ d5f1qa1 d1xt8a1
d4gwoa_ d4igta1 d1pc3a_ d4mmpa1 d1o63a_ d2czla1 d2onsa2 d4mija1
d4ok5a1 d2bmfa1 d2bmfa2 d1yksa1 d1yksa2 d2qeqa1 d2jlqa1 d1a1va2
d1eh3a_ d3skpa_ d1dtza1 d1dtza2 d1ieja_ d1jnfa2 d1ce2a1 d1jnfa1
d1b1xa2 d1b1xa1 d1jw1a1 d1jw1a2 d1ryoa_ d1h76a1 d1h76a2 d1c4oa1

| | | | | | | | |
|---|---|---|---|---|---|---|---|
| d2p6ra3 | d2p6ra4 | d1oywa2 | d1oywa3 | d1wrba1 | d1w36b2 | d1w36b1 | d1c4oa2 |
| d3bora_ | d1t6na_ | d2eyqa5 | d2eyqa3 | d2eyqa2 | d2zu6a2 | d1veca_ | d1uaaa1 |
| d1w36d1 | d2g9na1 | d1z3ix2 | d2rb4a_ | d1hv8a1 | d1hv8a2 | d1t5ia_ | d1w36c2 |
| d2j0sa2 | d2j0sa1 | d1fuka_ | d3hjha1 | d1z5za1 | d1qdea_ | d1z63a1 | d1s2ma2 |
| d1s2ma1 | d1wp9a1 | d1wp9a2 | d1gm5a3 | d1gm5a4 | d3dina4 | d3dina3 | d1q0ua_ |
| d1w36c1 | d1z3ix1 | d1nkta3 | d1nkta4 | d1uaaa2 | d1w36d2 | g1qhh.1 | d2fwra2 |
| d2fwra1 | d3q3ma_ | d1za0a1 | d1mxra_ | d2inca_ | d2incb_ | d1mhyb_ | d3hf1b_ |
| d1mtyb_ | d1r2fa_ | d2uw1a_ | d1mhyd_ | d3n37a_ | d1afra_ | d1oquc_ | d1syya_ |
| d1mtyd_ | d2o1za_ | d1uzra_ | d1smsa_ | d2fkza_ | d1zuja1 | d1s3qa1 | d2fjca1 |
| d1z6om1 | d1veia_ | d4dyya_ | d1jiga_ | d3gvya_ | d2pyba_ | d4cy9a1 | d3bvfa1 |
| d1r03a_ | d2cf7a_ | d1nf4a_ | d1zs3a1 | d4iwka_ | d3ka8a_ | d3np0x_ | d1n1qa_ |
| d1lb3a_ | d4e6ka_ | d1z6oa1 | d2fzfa1 | d4dyua_ | d3ge4a_ | d1jgca_ | d4am4a_ |
| d2z6ma_ | d1ji5a_ | d1vlga_ | d1lkoa1 | d1krqa_ | d3qz3a_ | d1o9ra_ | d2ffxj_ |
| d1yv1a1 | d2v2pa_ | d1j30a_ | d4di0a_ | d1dpsa_ | d1oewa_ | d2qzxa_ | d2asia_ |
| d1psoe_ | d1miqa_ | d1smra_ | d2h6ta_ | d1pfza_ | d1izda_ | d3hd8a_ | d1j71a_ |
| d1dpja_ | d4aa9a_ | d1t6ex_ | g1b5f.1 | g1lya.1 | g1htr.1 | d1qdma2 | d1bxoa_ |
| d1wkra_ | d1tzsa_ | d1ibqa_ | d2qzwa_ | d1bdqa_ | d3ggta_ | d3ixoa_ | d1mrxa_ |
| d2haha_ | d1tw7a_ | d4njsa_ | d1sgua_ | d2p3da_ | d1nsoa_ | d3d3ta_ | d2rkfa_ |
| d1c6ya_ | d2rspa_ | d3ogpa_ | d3ekwa_ | d2o4na_ | d2j9ja_ | d1baia_ | d3ggua_ |
| d3lzua_ | d4npua_ | d4j55a_ | d2r8na_ | d2fxda_ | d2p3ca_ | d4dqea_ | d3ttpa_ |
| d3t3ca_ | d2p3aa_ | d2hc0a_ | d2fdda_ | d3mwsa_ | d1sr9a2 | d1rqba2 | d1k8rb_ |
| d1wgra1 | d1ef5a_ | d1wfya1 | d1e8ya3 | d1wgya1 | d1lfda_ | d2cs4a1 | d1wxma1 |
| d2byea1 | d3kh0a1 | d2byfa1 | d1wxaa1 | d1h4ra3 | d4yl8a1 | d2al6a3 | d1wgda1 |
| d1wgga1 | d4q5eb_ | d1wjna1 | d1se9a_ | d3n3kb_ | d1wz0a1 | d1we6a1 | d2m0xa_ |
| d2gbka_ | d3rt3b1 | d1wh3a1 | d1sifa_ | d2ekec2 | d4ii2b1 | d4hk2a1 | d1c3ta_ |
| d1m94a_ | d1wgha1 | d1ttna1 | d1j8ca_ | d1wx8a1 | d3v6cb1 | d2ma9b_ | d4i6lb1 |
| d1bt0a_ | d2jvca1 | d1iyfa_ | d2k1fa_ | d1wxva1 | d1wf9a1 | d1t0ya_ | d2io0b2 |
| d1x1ma1 | d2gbma_ | d1zw7a1 | d2m17a_ | d1ud7a_ | d1we7a1 | d2lwpa_ | d2gbja_ |
| d1wjua1 | d1v6ea1 | d1v86a1 | d4eewa_ | d1wiaa1 | d4icva_ | d4gu2a_ | d1wx7a1 |
| d1wy8a1 | d4hjka1 | d2faza1 | d1v5oa1 | d1yvca1 | d1yeza1 | d2bcgg1 | d2bcgg2 |
| d1d5ta1 | d1dszb_ | d1a6ya_ | d3cbba_ | d1ynwa1 | d1r4ia_ | d1hcqa_ | d2hana_ |
| d2hanb_ | d2enva1 | d1kb4b_ | d2nllb_ | d1lo1a_ | d1cita_ | d1adqa1 | d1fnga1 |
| d1uvqb1 | d1zs8a1 | d2vola1 | d2vola2 | d1mfbl2 | d2jelh2 | d1uwel2 | d3qq4b1 |
| d1kjvb_ | d1k5na1 | d1osph2 | d4odhl2 | d1ow0a1 | d1ow0a2 | d1bfob2 | d3l9ra2 |
| d3jtth1 | d2fath2 | d4elkb2 | d1fn4b2 | d2xfxb_ | d1pg7x2 | d1cqka_ | d4k71b2 |
| d1lk2a1 | d1eapb2 | d4lfhg2 | d1i4fa1 | d2nw2a2 | d1kfah2 | d1mhca1 | d1h3ua1 |
| d1wejh2 | d1h0db2 | d1a5fh2 | d1kcvh2 | d1mjuh2 | d1qqda1 | d1kcrh2 | d1k8ia1 |
| d3huja2 | d2nnab1 | d1nfdf2 | d3mffb2 | d1s9va1 | d1lk3h2 | d1q0xl2 | d1fv1b1 |
| d1neza1 | d4rfeb2 | d1r3ha1 | d1a6tb2 | d1kjva1 | d1pz5b2 | d1onqa1 | d2bc4a2 |
| d1de4a1 | d1k8da1 | d1tcra2 | d1dn0b2 | d1nfde2 | d2xwtb2 | d1bfoa2 | d1mexh2 |
| d1iakb1 | d1ypzf2 | d4f7ea2 | d1q72l2 | d2fbjh2 | d1fe8h2 | d1clzh2 | d1fngb1 |
| d1uvqa1 | d1vgka1 | d1c16a1 | d4j12a2 | d4x99b2 | d1igtb3 | d1je6a1 | d1q72h2 |
| d1nldh2 | d1teth2 | d2hmic2 | d1i1ca2 | d1i1ca1 | d1k8ib1 | d1q0yh2 | d1kfal2 |
| d3frua1 | d1pg7w2 | d1u58a1 | d1igyb4 | d1igyb3 | d1hyrc1 | d1klub1 | d1deeb2 |

d1c12b2 d2bc4b2 d2ig2h2 d1kcvl2 d3wn5b1 d1lk2b_ d1lk3l2 d1mjul2
d1sm3h2 d1q0xh2 d1zvsa1 d1ydpa1 d1u8ib2 d1ypze2 d1kcuh2 d2lj7a_
d1n4na_ d2m8bb1 d2kyqa_ d3psma_ d1jkza_ d1jxca_ d2gl1a_ d1ugla_
d4uj0a_ d2k4ua_ d1hp2a_ d2lu9a_ d1baha_ d2meoa1 d1wm7a_ d1hlya_
d1n8ma_ d1jlza_ d1wpda_ d1c55a_ d1pvza_ d1wt7a_ d1rjia_ d1wz5a_
d1m2sa_ d1pjva_ d1c49a_ d2ktca1 d1wmta_ d1du9a_ d1j5ja_ d1r1ga_
d1t0za_ d2i61a_ d1t1ta_ d1sn4a_ d1kv0a_ d2lkba_ d1npia_ d1wwna_
d1pe4a_ d2kbka_ d2li7a_ d1djta_ d2kbha_ d1dq7a_ d1sega_ d1omya_
d1zywa1 d1jzaa_ d1n9wa1 d1bbua1 d1e1oa1 d1c0aa1 d1l0wa1 d3a74a1
d1eova1 d1t61c1 d1iyjb4 d1iyjb5 d1iyjb3 d1o7ia_ d4ipca1 d1x3ea_
d4apva_ d1jb7a1 d1jb7a2 d1jb7a3 d3kdfa_ d1ue1b_ d1wjja1 d3eiva_
d1xjva2 d1xjva1 d2ccza2 d1se8a_ d1l1oc_ d1qvca_ d3ulla_ d1qzga_
d1miua4 d1miua5 d1miua3 d1y14b1 d2nn6i1 d1i94l_ d2zjra2 d1smxa_
d2qamc2 d1hh2p1 d2r7da1 d1rl2a2 d1wfqa1 d3bzka4 d1x6oa2 d3a0ja_
d2j01d2 d2ja9a1 d4ql5a_ d1hr0w_ d1luza_ d2n3sa_ d2vnud1 d1h95a_
d1go3e1 d2nn6h1 d1ne3a_ d2mo1a_ d1iz6a2 d1jjga_ d1e3pa2 d2ix0a3
d2ix0a2 d2lxja_ d1ueba3 d1ueba2 d2z0sa1 d1u0la1 d2ix0a1 d2eifa2
d2je6i1 d3i2za1 d2c35b1 d2ahob2 d1t9ha1 d3i4oa_ d2qall1 d1jt8a_
d2nn6g1 d1g6pa_ d3cama_ d1i94q_ d1c9oa_ d1d7qa_ d2ba0a1 d2vnud2
d2vnud3 d1khia2 d2qalq1 d1wi5a1 d1xtda2 d1ny4a_ d1dgsa2 d1fvia1
d1p16a1 d1x9na2 d2a1ka_ d1je5a_ d1rpma_ d2shpa1 d1yfoa_ d1l8ka_
d2ooqa1 d1g4us2 d1p15a_ d1lara2 d1lara1 d1wcha_ d2fh7a2 d2fh7a1
d1tdha3 d1q3ba3 d1ee8a3 d1k82a3 d3ubra_ d1qo8a1 d1d4ca1 d2k3va_
d1ft5a_ d1qdba_ d1m1qa_ d2j7aa_ d1h21a_ d1ogyb_ d4fasa_ d1jnia_
d1oaha_ d1sp3a_ d3h33a_ d1rwja_ d19hca_ d3bxua_ d3h4na_ d1i77a_
d3caoa_ d1j0pa_ d2ctha_ d1duwa_ d1ofwa_ d2e84a_ d1hh5a_ d1gyoa_
d3h34a_ d1wioa4 d1ccza2 d1vcaa1 d1dr9a2 d1ic1a1 d2d9qb1 d2nxyb2
d2dara2 d2dara1 d1wyha2 d2dloa2 d1rutx4 d1rutx3 d2cuqa2 d1x4la2
d1x4la1 d1x3ha2 d1x3ha1 d2d8za1 d1x61a2 d1x6aa1 d1x6aa2 d2cora2
d2cora1 d1v6ga1 d1v6ga2 d2d8za2 d1x64a1 d1x64a2 d1b8ta1 d2co8a1
d1x63a2 d2dj7a2 d2dj7a1 d2cu8a2 d2cu8a1 d1g47a1 d1j2oa1 d1wyha1
d2dloa1 d1wiga1 d1wiga2 d2cupa2 d2cupa3 d1x68a1 d2d8ya2 d2d8ya1
d1m3va2 d1x62a1 d1x68a2 d1x61a1 d1b8ta3 d1b8ta2 d2d8xa2 d2d8xa1
d1x4ka1 d1x4ka2 d2cuqa1 d1m3va1 d2co8a2 d1j2oa2 d1x63a1 d2cura1
d2cura2 d1x62a2 d1op9a_ d1y0la1 d1etzb1 d1egjl1 d2gsib1 d1nj9a1
d4jfhd1 d1aqkl1 d1t2ja_ d1lo4h1 d1zvya1 d3v4vl1 d4enec1 d1kxtb_
d5e8ea1 d2pxya_ d4weud_ d1xiwc_ d2esvd1 d1qnzh_ d1ogae1 d1f4xh1
d4n1hb_ d3ezjb1 d3mlwl1 d1kfah1 d1etza1 d1b4jh1 d3cfic_ d4w68a_
d2arja1 d1ek3a_ d2ok0l1 d1vera_ d1uwgh1 d1blnb1 d1fpth1 d1rzga1
d2giya1 d4m3kb1 d1nc2a1 d2nyyd1 d1ncna_ d1fbih1 d5dxwa_ d4fqqa1
d1dqdh1 d1jfql1 d1kelh1 d1faih1 d1frgl1 d1figl1 d4m6nl1 d1fo0a_
d4hxbl1 d3b5ga_ d3quyd1 d2h9gb1 d1i8kb_ d2dd8h1 d2ck0h1 d1eaja_
d4w6wb1 d2a6ib3 d1t66d1 d2e27h_ d1ob1b1 d1f11b1 d2v7na1 d1ad0a1
d2xt1b1 d1pskh1 d4i13b1 d2op4l1 d1a0ql1 d1uwel1 d1za3a1 d1ncbl1

| | | | | | | | |
|---|---|---|---|---|---|---|---|
| d4uu9a_ | d1bm3h1 | d1cloh1 | d1nqba2 | d1cf8h1 | d4y5va_ | d1mhph1 | d5anma1 |
| d4cdgc1 | d4ldeb_ | d1y0lb1 | d2pcpb1 | d1z3gl1 | d1dfbl1 | d1fh5h1 | d3g5xa1 |
| d1tzib1 | d4dn3l1 | d3zhka1 | d2b1hh1 | d2uyla1 | d1f3dh1 | d1nj9b1 | d2agjh1 |
| d2h1pl1 | d1q72h1 | d8fabb1 | d1ndmb1 | d1rjca1 | d4u6hb1 | d3upca_ | d2fbjl1 |
| d1osph1 | d1tvda_ | d2cjvh_ | d4fhbd1 | d1bd2d1 | d1rz7h1 | d3nn8a_ | d1blna1 |
| d1knoa1 | d1nfda1 | d2bmkb1 | d1dqll_ | d1igyb1 | d1igfl1 | d12e8h1 | d3utza1 |
| d1nc2b1 | d1rihh1 | d4mwfb1 | d4jvpa1 | d1mieh1 | d1rhfa1 | d1fgnl1 | d1sbsh1 |
| d1lp9e1 | d1dn0a1 | d4lqfl1 | d1iaih1 | d1kgcd1 | d1mh5b1 | d1gpol1 | d2g60h3 |
| d1f8tl1 | d2jixd1 | d4grwh_ | d1fo0b_ | d1bfob1 | d1axsa1 | d1u9ka_ | d1nmbh_ |
| d1h8na1 | d1h8na2 | d1jguh1 | d4eura1 | d3sdyl1 | d35c8h1 | d1w72l1 | d1mvub_ |
| d1i9rh1 | d1ob1a1 | d4wenb_ | d1yeeh1 | d3ietc1 | d1f58h1 | d1ad0b1 | d1mqkl_ |
| d1nbvh1 | d4b50a_ | d3r8bb_ | d1bafl1 | d1mamh1 | d4rgne1 | d2gsgb_ | d35c8l1 |
| d1bafh1 | d1ai1h1 | d2pcpa1 | d2fath1 | d2x1pa_ | d1orsb1 | d1ibgl1 | d1shma_ |
| d1a2yb_ | d1u0qa_ | d2coqa_ | d1hilb1 | d1qfwi_ | d1jv5b_ | d1fn4b1 | d5e0qa_ |
| d1nsnh1 | d1plgl1 | d4ocxl1 | d1kb5h1 | d1rzfh1 | d1pg7x1 | d2fx7h1 | d2oi9c_ |
| d1l7il1 | d3eyva1 | d1d5il1 | d4ffyl_ | d1tetl1 | d1a6wh_ | d1ac6a_ | d3dvfa_ |
| d1nfde1 | d1mimh1 | d1ri8a1 | d1jhlh_ | d3dura_ | d1fskb1 | d1f97a1 | d3so3b1 |
| d2ak4d1 | d1lp9f1 | d4z7wf1 | d1l7th1 | d4y5ya_ | d4x7ec1 | d1emth1 | d1ikfl1 |
| d1nlbh1 | d4utal1 | d1bjma1 | d1eapb1 | d1u6ah1 | d2dtgd1 | d1kj2b_ | d1xeda1 |
| d1c1eh1 | d1fgvh_ | d2bnub1 | d2fb4h1 | d2dtgb1 | d1ktrh_ | d1jpth1 | d1qkzl1 |
| d4hepg_ | d2atpb1 | d1flrh1 | d2hrpl1 | d1mvfa_ | d1iaim1 | d3k80a1 | d2jb5h1 |
| d3zkxc_ | d4grwe_ | d1it9h1 | d1rhhb1 | d3w9ec_ | d1e4wh1 | d1sy6h1 | d2j6eh1 |
| d3qxta_ | d1jglh1 | d1p7ka1 | d1tzhb1 | d4n8pa1 | d1gafl1 | d2jelh1 | d1wioa2 |
| d4i0cc1 | d1cicb1 | d1lmka2 | d1lmka1 | d1iqwh1 | d1orqb1 | d1ejoh1 | d1jnlh1 |
| d4zg1a_ | d43c9b_ | d1ggbl1 | d1wejh1 | d3q6fa1 | d1f3dj1 | d3c6la1 | d1bqlh1 |
| d3p9wb_ | d1a3rl1 | d1h0db1 | d2uzil_ | d1eo8l1 | d1qfwl_ | d1n7ml1 | d1igja1 |
| d1c5ch1 | d2a9mh_ | d1rmfh1 | d1fj1b1 | d4heme_ | d1ccza1 | d1a5fh1 | d1ogad1 |
| d1dlfh_ | d1qa9b_ | d1vgel1 | d3qskb_ | d1sjxa_ | d4ozfg1 | d1sq2n_ | d1qd0a_ |
| d1kcvh1 | d1c5db1 | d3qcul1 | d3bn9d1 | d1mjuh1 | d4y8dc_ | d7fabh1 | d3dvgb1 |
| d1lo0l1 | d1a6tb1 | d1gigh1 | d1kcrh1 | d2hwzl1 | d1yjdc1 | d3clfl1 | d4krna_ |
| d1dl7l_ | d1e6ol1 | d1mexh1 | d3r0ma_ | d4kkna_ | d1fnsl1 | d1oaqh_ | d1rzia1 |
| d1ucbh1 | d1ncwl1 | d4b41a_ | d1nfdf1 | d2mpah1 | d2d7tl1 | d4grwf_ | d4b5ea_ |
| d1lk3h1 | d3u79a_ | d4u3xa_ | d1ghfl1 | d1bvkb_ | d1mcww1 | d1b88a_ | d1jrhh1 |
| d32c2b1 | d4wemb_ | d1dsfh_ | d1op3h1 | d1cel1l | d1iqda1 | d1qoka1 | d1qoka2 |
| d1qfuh1 | d2crya1 | d1ay1h1 | d1oaql_ | d3ab0b_ | d1rurl1 | d1t4kb1 | d1bz7b1 |
| d1n0xh1 | d1forl1 | d1ucbl1 | d4w6yb_ | d43c9a_ | d2aabl1 | d1tzia1 | d4n1ca_ |
| d1k6qh1 | d3g9ab_ | d4tvsa1 | d1igmh_ | d1pz5b1 | d3dx9a1 | d3wxwh_ | d1nakh1 |
| d1kcul1 | d3eaka_ | d4qlra1 | d1cr9l1 | d1d9ka_ | d1f4xl1 | d1deea1 | d2i27n2 |
| d1c12a1 | d2orbl1 | d1j8hd1 | d1mcph1 | d1c5da1 | d1kenh1 | d1bd2e1 | d1h5ba_ |
| d4x7fc1 | d3upaa_ | d1t3fa1 | d1indh1 | d2dtgc1 | d4hc1l1 | d1zv5a1 | d4wgvb_ |
| d1uwgl1 | d4f37k1 | d1aqkh1 | d1vesa_ | d3zkqd_ | d1fptl1 | d1lo4l1 | d1knob1 |
| d3rrqa_ | d1ct8b1 | d4orzc_ | d1mlbb1 | d4hgkc1 | d1dqdl1 | d1bogb1 | d4ozhe1 |
| d1dr9a1 | d1i7za1 | d1dn0b1 | d1kell1 | d1rzib1 | d1ypzf1 | d1wtla_ | d1hkfa_ |
| d1fail1 | d1figh1 | d2vyre_ | d1sjva_ | d1ohqa_ | d1bfoa1 | d1pkqb1 | d1mjul1 |

d1b4jl1 d1nsnl1 d2arjb1 d1p4bh_ d4aj0a_ d3nn8b_ d2rcja1 d1f2xk_
d4v1dd_ d1adqh1 d2eksa_ d4k3ga_ d1jfqh1 d1uweh1 d1frgh1 d4gftb_
d1uz8b1 d4n1ea_ d1bm3l1 d1clol1 d3mcka1 d2vq1a1 d1ae6h1 d4aq1b1
d1uj3b1 d1nbqa1 d1fh5l1 d2ck0l1 d2ywza_ d1q72l1 d4nbxb_ d1hila1
d4hzlb1 d2j88l1 d1zmya1 d1cd0a_ d1a4ka1 d1pskl1 d1a0qh1 d4krmb_
d1ncbh1 d2fbjh1 d1j8he1 d1i3ua_ d1cf8l1 d1fe8h1 d2p1ya1 d1eeqa_
d2x89a_ d1ol0a_ d2r0lh1 d1ub5a1 d1dfbh1 d3eyvb1 d4jdvb1 d4p2cg_
d2gsia1 d2agjl1 d1u3ha1 d2h1ph1 d1rihl1 d1fskc1 d1boga1 d2a77l1
d1ospl1 d1qfoa_ d3dwta_ d2fx7l1 d1rz7l1 d3stba1 d1pkqa1 d1b6da1
d1dzba2 d1dzba1 d1kj2a_ d4l1ha_ d1rzgb1 d1dqlh_ d12e8l1 d1miel1
d1jmaa_ d1sbsl1 d1fgnh1 d1f58l1 d1mqkh_ d1ndgb1 d3qegl1 d1iail1
d2aw2a1 d1tqbc1 d3fo2a1 d4dkaa_ d1f8th1 d2g60l1 d2ywya_ d1uj3a1
d1yc7a_ d3bd3a_ d4w6xb1 d1nmbl_ d1i8ka_ d1rjlb1 d1ypze1 d1p7kb1
d1kxqe_ d1gpoh1 d2z4qa1 d1w72h1 d1i9rl1 d4qgya1 d4xt1c_ d2qqnh1
d1cica1 d3c08h1 d2cd0a_ d2bdnh1 d1hnga1 d1maml1 d1nldh1 d1cr9h1
d4lcih1 d3bkyh1 d3qg7l1 d1ai1l1 d1kxvc_ d4iosd_ d1l7ih1 d2x1qa_
d1ub6b1 d3k74b_ d1ibgh1 d1teth1 d1igya1 d1hxma1 d2x6ma_ d2e7la_
d5e03a_ d1qfwm_ d2r0kh1 d1igjb1 d1rurh1 d1plgh1 d1mj8h1 d1yuhb1
d3fmgl1 d1rzfl1 d1emtl1 d1ad9b1 d1igtb1 d1nlbl1 d1d5ih1 d3k7ua_
d4ffyh_ d1bwwa_ d3dvga1 d1a6ta1 d1fgvl_ d4qxgl1 d1miml1 d1jhll_
d2hffb1 d2hh0l1 d1qkzh1 d1l7tl1 d1afvh1 d1iaii1 d2cjuh_ d1um5h1
d2ghwb2 d2ghwb1 d1kgce1 d2fgwh1 d1ikfh1 d1dvfd_ d2i24n1 d2xtjb_
d1za3b1 d2yc1a_ d2hh0h1 d2x1oa_ d1bvka_ d1rjla1 d5e7ba1 d1igta1
d1il1b1 d2fb4l1 d1mvua_ d1jptl1 d32c2a1 d1fvca_ d1iqdb1 d2hrph1
d2p4ab_ d4qo1a_ d1p2cb1 d1wt5c_ d1t4ka1 d5c1mb_ d1it9l1 d1bz7a1
d1b2wh1 d1e4wl1 d1jnll1 d1yedb1 d1za6b1 d3u7aa_ d1igfh1 d3gnml1
d1orsa1 d1wejl1 d1a2ya_ d1hxmb1 d1gafh1 d2j4wh1 d1pz5a1 d1j05a_
d1a3rh1 d4fqll1 d1qfwh_ d1eo8h1 d1n7mh1 d4s11a_ d1kegh1 d5da4a_
d1iqwl1 d3whxb1 d1vgeh1 d1deeb1 d1c12b1 d1t2ql1 d1ad9a1 d3gsnb1
d3b2uc1 d1ggbh1 d1t3fb1 d1nakl1 d4ocmc_ d1lila1 d2v7hb3 d2esve1
d1mcoh1 d2qr0b1 d2q3aa_ d1ct8a1 d1kcrl1 d2pxyb_ d2uzih_ d5c3ld_
d3durb_ d1c5cl1 d2ak4e1 d1dl7h_ d1xiwd_ d1mlba1 d1rmfl1 d1i7zb1
d1dlfl_ d2xa3a_ d5bq7b1 d1dqqb1 d3h42l1 d1egjh1 d1kcvl1 d1fe8l1
d2j6el1 d1f3rb2 d1lk3l1 d1sm3h1 d1q0xh1 d1ghfh1 d7fabl1 d1igch1
d1lo0h1 d4idla_ d5c2bl_ d1jrhl1 d1il1a1 d3zhda1 d4kfzc_ d1b0wa_
d1e6oh1 d1mexl1 d4krpb_ d1fnsh1 d1ncwh1 d4hgma_ d4eigb1 d4rzca_
d2mpal1 d4y5xa_ d5en2b1 d3lrga1 d4ej1c1 d1rhha1 d1j05b_ d1forh1
d1q9wb1 d2wzpd_ d2aq2a1 d1h0da1 d3revb1 d2ij0c1 d1tzha1 d4m7jl1
d1k6ql1 d3b9kc1 d1igml_ d3qeua1 d1dsfl_ d1ce1h1 d1kcuh1 d1zvha1
d1zvoa1 d1tqbb1 d1ay1l1 d2qscl1 d2cgrh1 d1nl0h1 d1kenl1 d3eo0a1
d4n9ob1 d1n0xl1 d3k1kc_ d2cqva1 d1fhga_ d2k1ma_ d2dl2a1 d1biha3
d1biha1 d1biha4 d3qp3a1 d1g1ca_ d1gxea_ d1cvsc2 d1cvsc1 d1xmwa2
d1m4ka2 d2otpa2 d1biha2 d1rhfa2 d2fcba2 d2fcba1 d1wwbx_ d1epfa1
d1epfa2 d2dava1 d1waaa_ d1f97a2 d1x44a1 d1vcaa2 d1fnla2 d1fnla1

d3b5ha2 d3h8na2 d3h8na1 d1jbja1 d1jbja2 d1pd6a_ d1f2qa2 d1f2qa1 d3cx2a_ d1nbqa2 d3b5ha1 d1n26a1 d1xiwb_ d1xaua_ d1fltx_ d2ifga1 d1olla1 d1olla2 d1cs6a4 d1cs6a3 d1cs6a2 d1cs6a1 d2gw5a2 d1sy6a1 d1xiwa_ d1ucta2 d1ucta1 d1ry7b2 d1gl4b_ d1qz1a3 d1olza1 d2c9aa1 d1wwca_ d1c4qa_ d1prtc1 d2wv6d_ d1prtf_ d1ltrd_ d4l63b1 d1r4pb_ d1prtd_ d1tl0f_ d2bosa_ d3chbd_

**Family-28 (b.29.1.3)**

**Positive dataset contains 4 protein sequences.**

d4lbqa_ d4no4a_ d3zsja_ d3duia_

**Negative dataset contains 3931 protein sequences.**

d1orva2 d1xfda2 d2xlba_ d3m83a1 d1u08a_ d1lc5a_ d1b5pa_ d2ay1a_ d1bw0a_ d3pd6a_ d7aata_ d2csta_ d1j32a_ d4wlha_ d1o4sa_ d1v2da_ d1gdea_ d1m6sa_ d1v72a1 d1svva_ d1ajsa_ d1wsta1 d2f8ja2 d1lk9a_ d1vp4a1 d1xi9a1 d2gb3a1 d1yaaa_ d2cfba1 d1dfoa_ d1o69a_ d1bj4a_ d3k28a_ d2bwpa_ d3lg0a_ d2fnua_ d3ou5a_ d1z7da1 d1b9ha_ d1iaya_ d4msoa_ d1bs0a_ d1s0aa_ d1sffa_ d3tqxa_ d1mdoa_ d3qboa_ d3pgya_ d2dkja_ d1ejia_ d1vefa1 d4wxga_ d3bs8a_ d3gbxa1 d1w23a_ d2b5aa1 d2ofya1 d4mcxa_ d2xi8a_ d2icta_ d2bnma1 d2ppxa1 d1y7ya1 d1sefa_ d1sfna_ d1sq4a_ d1c8nc_ d1x36a_ d1smvc_ d1ng0c_ d4sbvc_ d1opoa_ d1f2nc_ d1ddla_ d1e57b_ d1auyb_ d1f56a_ d1bypa_ d1azca_ d1ws8a_ d3u25a_ d1cuoa_ d1id2a_ d1rkra_ d2iaac_ d4dp1x_ d1nwpa_ d2gima_ d2i7sa_ d1qhqa_ d1gyca3 d1hfua2 d1kbva1 d1kbva2 d1sdda1 d1sdda2 d1gyca1 d1gyca2 d1mzya1 d2bw4a1 d1aoza1 d1aoza3 d1aoza2 d1kyaa2 d1kyaa3 d1kyaa1 d1mzya2 d4e9sa1 d1sddb1 d1sddb2 d1hfua1 d1hfua3 d1oe1a2 d1oe1a1 d1v10a2 d1v10a3 d1v10a1 d1rypd_ d1irua_ d1rypc_ d3unfk_ d1rypa_ d1j2pa_ d1irui_ d1j2qh_ d3unfh_ d1m4ya_ d1rypb_ d1irul_ d1irug_ d1iru2_ d1q5rh_ d1rypg_ d1g3ka_ d1iruf_ d1q5qa_ d1e94a_ d1irub_ d1ryph_ d3ty6a_ d1iruj_ d1iruk_ d1rype_ d1irud_ d1irue_ d3mi01_ d1rypf_ d1iruh_ d2z3ba_ d3mka2_ d1iru1_ d2bema_ d1pama1 d1ea9c1 d1qhoa1 d2fhfa2 d2fhfa1 d1k3ia1 d1j0ha1 d1cxla1 d2bhua1 d1edqa1 d1f1sa2 d1ut9a2 d1gvia1 d1txka1 d2phla2 d2phla1 d1fxza2 d1fxza1 d1lrha_ d1uija1 g1dgw.1 d2v09a_ d1uika2 d1uika1 d1dgwa_ d2pa7a1 d1wlta1 d1nxma_ d2c0za1 d1ds6b_ d1fstb_ d1ex0a1 d1g0da1 d4mh8a_ d1mu2a2 d4i7ga1 d1tafa_ d1tafb_ d1jfib_ d1q9ca_ d1h3ob_ d2byka1 d1n1ja_ d4g92b_ d1bh9a_ d1jfia_ d1h3oa_ d2bykb1 d1n1jb_ d1ydwa2 d2nvwa2 d1h6da2 d1zh8a2 d1xeaa2 d1tlta2 d1r0ka3 d1j5pa3 d1vkoa2 d1vm6a2 d3qvsa2 d1gr0a2 d1p1ja2 d2ovga_ d1sq8a_ d1rzsa_ d3kz3a_ d3orca_ d3gpdg2 d2q49a2 d1hdgo2 d2g17a2 d2cvoa2 d2czca1 d3pwka2 d1obfo2 d1i32a2 d1zyao2 d2pkqo2 d1b7go2 d2hjsa2 d1cf2o2 d1vkna2 d1nvmb2 d4lt9a1 d3tc7a1 d3hoja_ d1i4na_ d3vnda_ d3nl8a1 d1geqa_ d3nava1 d1v5xa_ d4a2ra1 d3tc6a1 d1ujpa_ d4ijba_ d1xcfa_ d1vc4a_ d1rd5a_ d1qopa_ d3b5va_ d2msya_ d2djoa1 d2r5za_ d1au7a1 d1f43a_ d2lmda1 d1akhb_ d1s7ea1 d3nara_ d1zq3p1 d2craa1 d1mija_ d4uuta_ d3a01a_ d1x58a1 d3rkqa1 d3cmya1 d2cqxa1 d2da6a1

d1e3oc1 d1fjla_ d1mh3a1 d1x41a1 d1wh7a1 d1gt0c1 d2mw8a_ d1lfup_
d1x2ma1 d1b72a_ d1wi3a1 d1jgga_ d1wjha1 d1x2na1 d1mnmc_ d2cuea1
d3k2aa_ d2hosa_ d2jwta2 d1pufa_ d1octc1 d2hi3a_ d1b8ia_ d4j19a_
d3lnqa_ d1wh5a1 d1b8ib_ d1fexa_ d2cqra1 d1guua_ d1ug2a1 d2cqqa1
d2ajea1 d4a69c_ d1h8ac1 d2crga1 d2iw5b1 d1xc5a1 d1ofcx1 d2cu7a1
d1wgxa1 d2cjja1 d2ckxa1 d1gvda_ d1hcra_ d1tc3c_ d1gdta1 d1u78a2
d6paxa1 d6paxa2 d1k78a1 d1xg1a1 d1hlva1 d1hlva2 d1iufa1 d1igna2
d1bl0a1 d1bl0a2 d1d5ya1 d3lv8a_ d1s3ga1 d1zaka1 d1s4qa_ d3tvta2
d1uf9a_ d1khta_ d1ckea_ d1osna_ d1p5zb_ d4tmka_ d3cm0a_ d1tmka_
d1jaga_ d3h86a_ d1t0hb_ d1nn5a_ d2h92a_ d3kb2a1 d2c95a1 d1yj5a2
d1kgda_ d1ly1a_ d2ak3a1 d1vyva2 d1deka_ d2grja1 d1p3ja1 d1y63a_
d1e4ya1 d1s96a_ d3iija1 d1lvga_ d1nksa_ d1vyua2 d4e22a_ d1qf9a_
d1q3ta_ d1ki9a_ d1lw7a2 d1teva_ d1kjwa2 d1gsia_ d2iyva2 d1viaa_
d1e6ca_ d1odfa_ d1uj2a_ d4gi7a_ d2jeoa1 d1z0ja1 d3umra2 d3gj0a1
d2gaoa1 d1yzga1 d1x3sa1 d1g7sa4 d1puja_ d1udxa2 d1kk1a3 d2o52a_
d1jwyb_ d1zcaa2 d1zcba2 d2fv8a1 d2cjwa_ d3dz8a1 d1xzpa2 d1vg8a_
d4qxaa_ d2ew1a1 d1x1ra1 d2bcgy_ d3doea1 d3cona_ d3rega_ d3wyga_
d1upta_ d2b6ha1 d1zd9a1 d1lnza2 d1r5ba3 d1n0ua2 d1r2qa_ d2f9la1
d2rhda_ d3q3jb_ d3q85a_ d1efma1 d1r8qa_ d2gf9a1 d2akab1 d1ek0a_
d1z08a1 d2gcoa1 d2dy1a2 d4g01b_ d2cxxa1 d1svia_ d3q72a_ d1ni3a1
d4ac9a4 d2erxa1 d2bmea_ d1wxqa1 d2gf0a1 d4ldja1 d1zunb3 d1d5ca_
d4lv5b_ d1efca3 d1kmqa_ d2g6ba1 d3lrpa_ d1tada2 d2y8ea_ d4rd4a1
d1z06a1 d2heia_ d3a58b_ d1u0la2 d1d2ea3 d1zj6a1 d2ocba_ d3rwoa_
d1mr3f_ d2e9sa1 d4u5xa1 d2iwra_ d2fn4a1 d3bwdd_ d2nzja1 d2atva1
d2ksqa_ d3l43a_ d1mkya2 d1mkya1 d1wmsa_ d1jnya3 d2rmka_ d1f6ba_
d1f60a3 d1gota2 d1jala1 d2fh5b1 d1g16a_ d2wblc_ d1m7ba_ d1wf3a1
d1f5na2 d1t9ha2 d1z6xa1 d1z0aa1 d2fg5a1 d2a5ja1 d2bcjq2 d1s0ua3
d1ky3a_ d4y0va_ d4lhwa_ d2qmea1 d2atxa1 d1egaa1 d1ryha_ d1moza_
d1nrjb_ d2erya1 d3bfka_ d1puia_ d1fzqa_ d3j3az1 d3t5ga_ d1shza2
d3defa_ d2f7sa1 d3raba_ d3tsoa_ d3aq4a_ d2al7a1 d3sfva1 d3bc1a_
d2ncda_ d1sdma_ d3h4sa1 d2heha_ d2grya_ g3kin.1 d1kk8a2 d1br2a2
d1goja_ d1f9va_ d2mysa2 d1lkxa_ d3t0qa_ d4db1a1 d3gbja_ d1ry6a_
d1v8ka_ d1k8ka2 d1k8ka1 d3uleb1 d3i33a2 d3i33a1 d1bupa1 d1dkgd2
d1dkgd1 d4ci6a2 d1jcfa1 d4a2ba2 d2fxua1 d1yaga2 d1yaga1 d4cbua2
d1g99a1 d2e1za2 d2e1za1 d1saza1 d1saza2 d1ig8a1 d1czan2 d1czan3
d1czan1 d1ig8a2 d1czan4 d1bg3a4 d1bg3a2 d1bg3a1 d1nira1 d1qksa1
d1zc6a1 d1zbsa1 d1zbsa2 d1zc6a2 d2ch5a2 d2ch5a1 d1zxoa2 d1zxoa1
d1hroa_ d4gedb_ d3zooa_ d1mz4a_ d1gu2a_ d1f1fa_ d1dvva_ d1c6ra_
d1c75a_ d1e29a_ d1gdva_ d1cnoa_ d1h32b_ d2bh4x_ d2aiua_ d5cytr_
d1jdla_ d1i8oa_ d2zboa_ d3dmia_ d1ls9a_ d1wvec_ d2yk3a_ d3ph2b_
d1dw0a_ d5aura_ d1ql3a_ d1qn2a_ d1f1ca_ d1evsa_ d1cnt1_ d1pvhb_
d1z7ca_ d1huwa_ d1bgea_ d1rhga_ d1f6fa_ d1f45b_ d1i1rb_ d1axia_
d4x51a_ d1d9ca_ d4ypgc1 d3oq3a_ d1lqsl_ d1n1fa_ d1au1a_ d1wu3i_
d3besl_ d3ux9a_ d1v8fa_ d2ejca_ d3muea1 d3q12a1 d2b7la_ d3rbaa_

| | | | | | | | |
|---|---|---|---|---|---|---|---|
| d2qtra_ | d1nupa_ | d3l92a_ | d1f9aa_ | d1qjca_ | d2h29a_ | d1vlha_ | d1od6a_ |
| d1mrza2 | d4ruka_ | d2el7a_ | d1iq0a2 | d1irxa2 | d1r6ta2 | d1ivsa4 | d2cyba_ |
| d1rqga2 | d1j09a2 | d2yxna_ | d1f7ua2 | d1h3fa1 | d1ffya3 | d2g36a1 | d1jila_ |
| d1n3la_ | d1i6la_ | d1pfva2 | d1h3na3 | d3n9ia_ | d3sz3a1 | d3sw2a_ | d1moxc_ |
| d1dx5i3 | d1edmb_ | d1autl2 | d1autl1 | d1nqlb_ | d1dval1 | d1szba2 | d1kigl_ |
| d2y5fl_ | d1ijqa2 | d1gk5a_ | d1g1sa2 | d1nt0a3 | d1xdtr_ | d1lmja2 | d1lmja1 |
| d1nzia2 | d2wphe_ | d1klil_ | d1pfxl2 | d1pfxl1 | d1p9ja_ | d1g1ta2 | d1f7ea_ |
| d4yzub_ | d2mgpa1 | d1b9wa1 | d1b9wa2 | d2mgpa2 | d1ob1c1 | d1ob1c2 | d1n1ia1 |
| d2npra2 | d2npra1 | d1qx2a_ | d1ig5a_ | d2y5ia_ | d1psra_ | d3n22a_ | d2egda_ |
| d4ggfc_ | d2wcea_ | d2luca_ | d4aqia_ | d3nsia_ | d2lp3a_ | d1j55a_ | d1a4pa_ |
| d1k8ua_ | d1nsha_ | d1pvaa_ | d3fs7a_ | d2pvba_ | d1ttxa_ | d1rwya_ | d2kyfa_ |
| d1rk9a_ | d1exra_ | d1tiza1 | d1nyaa_ | d1g8ia_ | d1wdcc_ | d3fwba_ | d1ggza_ |
| d2o5ga_ | d1ctaa_ | d1m45a_ | d3i5gc_ | d1sl8a_ | d1fpwa_ | d2scpa_ | d2roaa_ |
| d4aqra_ | d3pn7b_ | d1br1b_ | d2mysb_ | d1s6ca_ | d1bjfa_ | d2obha_ | d1jfka_ |
| d1jf0a_ | d1qv0a_ | d1lkja_ | d2mlfc_ | d2m97a_ | d2d8na_ | d4by5a_ | d1jbaa_ |
| d4nqga_ | d1wdcb_ | d2mysc_ | d1j7qa_ | d1s6ia_ | d1uhka2 | d1r2ua_ | d1dtla_ |
| d3kf9a_ | d3pn7c_ | d5d67d_ | d1tuza_ | d1h8ba_ | d1eg3a2 | d2zkmx1 | d1snla_ |
| d1gjya_ | d1y1xa_ | d1hqva_ | d1dvia_ | d1df0a1 | d4usla_ | d1k94a_ | d1sfsa_ |
| d1jfxa_ | d1wkya2 | d1uuqa_ | d3wh9a_ | d4m1ra_ | d1xyza_ | d4xuya_ | d2je8a5 |
| d4bf7a1 | d3cuia_ | d1ur4a_ | d1qw9a2 | d1odza_ | d2vzsa5 | d1bqca_ | d2x2ya1 |
| d1hjqa_ | d1qnra_ | d4y7ea1 | d1vjza_ | d4k68a_ | d2v3ga_ | d4hpga_ | d1nofa2 |
| d1nq6a_ | d1h4pa_ | d1aq0a_ | d3ziza_ | d4bs0a_ | d1ecea_ | d1h1na_ | d1ta3b_ |
| d1w91a2 | d1i1wa_ | d1uhva2 | d1us3a2 | d1egza_ | d7a3ha_ | d1tvna1 | d3nyda1 |
| d1ghsa_ | d2cyga1 | d4xv0a_ | d2c8na2 | d1w32a_ | d3emca_ | d1v6ya1 | d1foba_ |
| d2uwfa1 | d2fgla_ | d3niya1 | d2c0ha1 | d1rh9a1 | d1yq2a5 | d1szna2 | d1fa2a_ |
| d1pama4 | d5do8a1 | d1ea9c3 | d2fhfa5 | d1qhoa4 | d1avaa2 | d1m53a2 | d4m8ua1 |
| d2gjpa2 | d1lwha2 | d4howa1 | d1ktba2 | d1j0ha3 | d3vm5a1 | d2x1ia_ | d1wzaa2 |
| d1uasa2 | d2diea1 | d1mxga2 | d1ht6a2 | d1eswa_ | d1iv8a2 | d5cpsa_ | d1ud2a2 |
| d1zjaa1 | d3gbea1 | d1kwga2 | d1wdpa_ | d1gcya2 | d1g5aa2 | d1hvxa2 | d3dhpa2 |
| d2bhua3 | d4do4a1 | d3qgva1 | d1e43a2 | d1g94a2 | d2d3na2 | d1gjwa2 | d1gvia3 |
| d1x1na1 | d1r7aa2 | d1tz7a1 | d4toqa_ | d1wb0a1 | d1w9pa1 | d1eoka_ | d1itxa1 |
| d1kfwa1 | d1goia2 | d1edqa2 | d2gsja_ | d1hjxa1 | d1jnda1 | d1vf8a1 | d1etob_ |
| d1umqa_ | d1mixa2 | d1e5wa2 | d1j19a2 | d1isna2 | d2al6b2 | d1g4ma1 | d1g4ma2 |
| d2hmta_ | d1lssa_ | d1b9ra_ | d1offa_ | d1frra_ | d3wcqa_ | d1e9ma_ | d1l5pa_ |
| d1wria_ | d1i7ha_ | d3av8a_ | d1czpa_ | d1rfka_ | d3p1ma1 | d3b2ga_ | d2bt6a2 |
| d3nvva1 | d1krha3 | d2h88b1 | d1n62a2 | d1t3qa2 | d1kf6b2 | d1zoyb1 | d1jq4a_ |
| d1ffva2 | d1dgja2 | d1rm6c2 | d1h6ta1 | d1h6ua1 | d9ldta1 | d2ewda3 | d1up7a1 |
| d2dfda1 | d1s6ya1 | d1vjta1 | d7mdha1 | d1hyea1 | d1t2da1 | d1ojsa3 | d1ldna1 |
| d1hyha1 | d1civa1 | d2a92a1 | d1ez4a1 | d1i0za1 | d1y6ja1 | d1gv0a1 | d1ur5a1 |
| d1llda1 | d1obba1 | d1v8ba1 | d1gdha1 | d1l7da1 | d2dlda1 | d1mx3a1 | d1ygya1 |
| d1qp8a1 | d1li4a1 | d1j4aa1 | d2naca1 | d1txga2 | d3f47a1 | d2pv7a2 | d1xc2a3 |
| d2ahra2 | d4xiya1 | d2f1ka2 | d1pgja2 | d1i36a2 | d1np3a2 | d1lj8a4 | d1teaa3 |
| d1qmga2 | d2i76a2 | d2g5ca2 | d1wdka3 | d1dlja2 | d3cuma3 | d1kola2 | d1f8fa2 |
| d1qora2 | d1jqba2 | d1vj0a2 | d1cdoa2 | d1gu7a2 | d1d1ta2 | d1xa0a2 | d2dm6a2 |

d1jvba2 d1rjwa2 d1llua2 d1tt7a2 d1piwa2 d1o89a2 d1iz0a2 d1e3ja2
d1yb5a2 d1uufa2 d1vj1a2 d1e3ia2 d3gpdg1 d1vc2a1 d2nvwa1 d1u8fo1
d1ydwa1 d1hdgo1 d2g17a1 d1cf2o1 d2czca2 d1r0ka2 d1obfo1 d2cvoa1
d1ebfa1 d1h6da1 d1i32a1 d1zh8a1 d2q49a1 d1qkia1 d1j5pa4 d3pzra1
d1xeaa1 d1vkoa1 d1vm6a3 d3qvsa1 d2pkqo1 d1b7go1 d1gr0a1 d1p1ja1
d1lc0a1 d1j0xo1 d3pwka1 d2hjsa1 d1zyao1 d1vkna1 d1tlta1 d1nvmb1
d3sjua_ d3ojfa1 d1xu9a1 d2jjya1 d1n7ha_ d1y1pa1 d1h5qa_ d1jtva_
d4fs3a_ d2bkaa1 d1gy8a_ d2c5aa1 d1ulsa_ d1ooea_ d2b69a1 d1y5ma1
d1spxa_ d1ae1a_ d1bxka_ d1ydea1 d1gega_ d1hdoa_ d1o5ia_ d1e6ua_
d1snya1 d2rhca_ d3zv4a_ d5ceja_ d2d1ya1 d1fjha_ d3w8ea_ d2fmua1
d1x1ta_ d1rpna_ d3ay7a1 d1k2wa_ d1eq2a_ d2hrba1 d3ruaa_ d2ew8a1
d4hwka_ d1xgka_ d2bd0a1 d1yo6a1 d2a35a1 d2ntva_ d1vl8a_ d1hdca_
d4bkua_ d2ag5a1 d2q45a_ d1nffa_ d4iy1a_ d1zbqa1 d1kewa_ d1ek6a_
d1ja9a_ d2q46a2 d1iy8a_ d1cyda_ d2ptga_ d1hxha_ d1uzma1 d3lyla_
d4m89a_ d3wyea_ d2blla1 d1w6ua_ d1sb8a_ d2q2va_ d4nr0a_ d1e6wa_
d2gasa_ d3oiga1 d2bgka1 d1mxha_ d1vl0a1 d1rkxa_ d1r6da_ d1wmaa1
d1e7wa_ d2c07a1 d1xkqa_ d3tzha_ d1g0oa_ d3lz6a_ d1qyca_ d1edoa_
d1gz6a_ d1oc2a_ d1t2aa_ d1zema1 d2xoxa_ d1z45a2 d1geea_ d1p33a_
d3lt0a_ d4e6pa_ d2wyua_ d1db3a_ d4q9na_ d1xhla_ d4o1ma_ d1fmca_
d2uvda_ d2gdza1 d1uaya_ d2z1ma_ d2o23a2 d2fr1a2 d2fr1a1 d1qsga_
d1qyda_ d4zjua_ d2a4ka1 d1yb1a_ d1wvga1 d1yxma1 d3f4ba_ d1mg5a_
d1xg5a1 d1r55a1 d3gboa_ d1atla_ d1kufa_ d4q1la_ d1quaa_ d1yp1a_
d1wnia_ d3nqyb_ d4gera_ d1bqba_ d1ex0a4 d1g0da4 d2f4ma1 d1x3za_
d1mdwa_ d1qxpa4 d1ziva1 d1zcma1 d3ioqa_ d1fh0a_ d1o0ea_ d1khqa_
d1m6da_ d2cb5a_ d1cs8a_ g8pch.1 d1s4va_ d2pnsa_ d1vsna1 d1mira_
d1iwda_ d2oula_ d7pcka_ d3bwka_ d3bcna_ d2p86a_ d1pvja_ d1pcia_
d1deua_ d4bs6a_ d1xkga1 d1cqda_ d1jqpa2 d2c0ya_ d2bdza_ d3p5ua_
d1zoya1 d1qo8a2 d1d4ca2 d1chua2 d1jnra2 d1kf6a2 d2jk6a2 d2jk6a1
d1feca1 d1feca2 d1lpfa1 d1lpfa2 d1xhca1 d1xhca2 d1dxla2 d1dxla1
d1gera2 d1m6ia2 d1onfa1 d1onfa2 d3lada2 d3lada1 d1w4xa1 d1w4xa2
d1gesa2 d1mo9a1 d1xdia1 d1fl2a2 d1fl2a1 d1fcda1 d1fcda2 d1h6va2
d1h6va1 d1mo9a2 d1gv4a2 d1gv4a1 d2gv8a2 d2gv8a1 d1aoga1 d1aoga2
d1hyua1 d1ebda1 d1ebda2 d3dk9a1 d1q1ra2 d1q1ra1 d1gesa1 d1zbfa1
d2z1ha_ d2etja1 d3qioa1 d1jl1a_ d2e4la_ d1jl2a_ d2lsna1 d1io2a_
d1uaxa_ d2yv0x_ d1ekea_ d1i39a_ d1pn0a1 d1reoa1 d1ju2a1 d2f5va1
d3c96a1 d2gmha1 d1pj5a2 d1gpea1 d1n4wa1 d1k0ia1 d2dw4a2 d2ivda1
d1seza1 d2voua1 d1kdga1 d1s5ja1 d2hbja2 d2igia_ d1wlja_ d1qhta1
d4dlea1 d1tgoa1 d2f96a1 d3tr8a1 d3mxma_ d1q8ia1 d1y97a1 d1w0ha_
d1kfsa1 d2py5a1 d1noya_ d1d5aa1 d1yt3a3 d2guia_ d3mwpa1 d4kb1a_
d2v76a_ d1wj1a1 d1aqca_ d1qqga1 d1ntva_ d1n3ha_ d4dbba_ d3f0wa1
d1p3ra_ d1wgua1 d2cy4a1 d3hqca_ d1wvha1 d1xr0b1 d1tq5a1 d1jmxa4
d1pbya3 d1pbya4 d1r7ha_ d1faaa_ d1wika1 d1xfla_ d1ttza1 d2f51a1
d1ti3a_ d1nhoa_ d1sena_ d2i4aa_ d1ep7a_ d1zmaa1 d3lefa1 d3diea1
d2yana1 d1z6na1 d1oaza_ d3zywa1 d4aj8a_ d1nm3a1 d4fiwa_ d1wmja_

d4hs1a1 d1iloa_ d1xwaa1 d1wjka1 d2vm1a_ d1fb6a_ d1gh2a_ d1h75a_
d1nw2a_ d1kx5b_ d3a6na_ d4m6ba2 d2nqbd_ d3wkjd_ d2yfwe_ d4h9na_
d5bnxa_ d1kx5c_ d3r45a_ d1eqzb_ d4jjnb_ d1f66c_ d1eqza_ d4wnnb_
d1kx5a_ d1u35c1 d1id3a_ d2nqbc_ d1kx5d_ d3us3a3 d3us3a1 d2b5ea2
d2b5ea3 d2b5ea1 d2b5ea4 d2kp1a1 d2kp2a1 d1hyua3 d1hyua4 d1j08a1
d1j08a2 d1pd212 d1nhya2 d2a2ra2 d1k0dd2 d1f2ea2 d1gsua2 d1f3ba2
d2fhea2 d2pvqa1 d1tw9a2 d1oyja2 d1e6ba2 d1k0ma2 d3ik7a1 d4is0a1
d1r5aa2 d1jlva2 d1m0ua2 d3gtub2 d1z9ha2 d1okta2 d1axda2 d2gsra2
d1tu7a2 d1k3ya2 d1zgma1 d1b48a2 d1gnwa2 d1gwca2 d2gsta2 d1glqa2
d1oe8a2 d1pn9a2 d1v2aa2 d1fw1a2 d1duga2 d1g7ea_ d1dosa_ d3qm3a1
d2fjka_ d1m32a_ d1ibja_ d1c7na_ d1iuga_ d4q75a_ d2bkwa1 d1t3ia_
d1n8pa_ d1qz9a_ d1jf9a_ d1p3wa_ d3kgwa_ d1cl1a_ d2ctza1 d1vjoa_
d2rfva_ d1gc0a_ d1cs1a_ d1d2fa_ d2ch1a1 d1qgna_ d1pffa_ d1elua_
d1npya1 d3t4ea2 d1a4ia1 d1o0sa1 d2hk9a2 d2egga2 d1b26a1 d1bvua1
d1gq2a1 d1c1da1 d1leha1 d1gtma1 d1euza1 d1nvta1 d3dona2 d1edza1
d1gpja2 d1vi2a1 d1p77a1 d1nyta1 d1b0aa1 d1vl6a1 d1v9la1 d3h1jd1
d1qsaa2 d4ilya_ d4olta_ d1qgia_ d1qqya_ d1gd6a_ d1f6ra_ d1ivma_
d2gv0a_ d1iiza_ d2z2fa_ d1j8xa_ d1ghla_ d1b9oa_ d174la_ d169la_
d2o4wa_ d1p37a_ d1k28a3 d1xjta1 d1jtma_ d2f2qa_ d4e97a1 d1s5ja2
d1qhta2 d4dlea2 d1tgoa2 d3py8a2 d1q8ia2 d1kfsa2 d2py5a2 d1d5aa2
d1qsta_ d2beia1 d4m3sa_ d1u6ma1 d1yk3a1 d3dr6a_ d1xmta_ d1z4ea1
d2ge3a1 d1bo4a_ d2fiaa1 d2jdca1 d1y9wa1 d3to7a_ d1wwza1 d2fl4a1
d2ozua1 d2b5ga_ d2cy2a1 d2p0wa_ d1cm0a_ d4pswa_ d1yrea1 d1yr0a1
d1m4ia_ d2gana1 d1yvka1 d1ghea_ d2g3aa1 d1y7ra1 d2euia1 d1q2ya_
d2fsra1 d1xeba_ d2ae6a1 d1cjwa_ d1qsma_ d2aj6a1 d1y9ka1 d1i12a_
d1vkca_ d2atra1 d1n71a_ d2d4pa1 d2fiwa1 d2fe7a1 d1tiqa1 d2ou2a_
d1yx0a1 d2fcka1 d1p0ha_ d2j8ma_ d2pq8a_ d1z4ra1 d1s3za1 d1ufha_
d2hqya1 d2hqya2 d1ne9a1 d1lrza3 d1ne9a2 d1xvqa1 d1pn0a2 d3hy2a_
d2cvba1 d1o8xa_ d1lu4a_ d1we0a1 d2bmxa1 d2a4va1 d1psqa_ d1xiya1
d1q98a_ d1n8ja_ d1zofa1 d2ggta_ d3tjja_ d4je1a1 d1o73a_ d3s9fa_
d2zcta_ d1qmva_ d1jfua_ d2b1ka_ d5dvba1 d2b5xa1 d2v2ga_ d3tb2a_
d4llra_ d4fh8a1 d1e2ya_ d1knga_ d2h30a_ d1i5ga_ d2z9sa_ d2f8aa1
d1tp9a1 d2b7ka_ d1nm3a2 d3hvva_ d2wfca1 d4kcea_ d1xvwa1 d4gqca1
d3qpma_ d4k1fa_ d1zzoa1 d1gp1a_ d1prxa_ d1f74a_ d3l21a_ d2e1da_
d1to3a1 d1w3ia_ d1sfla_ d1n7ka_ d1vcva1 d4imea_ d1wa3a_ d2ocza1
d1f2ja_ d3m16a_ d2yxga_ d3a5fa_ d1fbaa_ d1l6wa_ d1xfba1 d1wx0a1
d1vpxa1 d1ojxa_ d1mxsa_ d2a4aa1 d1xkya1 d3cq0a1 d3s1xa_ d1f05a_
d1fdja_ d3cpra1 d4d2ja_ d1cg2a1 d1yloa2 d1y7ea2 d1y0ya3 d1lfwa1
d1vixa1 d1fnoa4 d1vhoa2 d1ysja1 d1vhea2 d1z2la1 d2fvga2 d1vgya1
d1r3na1 d2grea2 d1xmba1 d1rtqa_ d1ys1x_ d4hs9a_ d1ispa_ d1ku0a_
d3qmma_ d1ra6a_ d1muka_ d3gsza_ d1khva_ d1te9a_ d2xwha_ d3uqsa_
d3hkwa_ d3n6ma_ d2cjqa_ d2xyma_ d1teba_ d1te8a_ d1u09a1 d4wfxa_
d3tyqa_ d2pgga1 d2h1ia1 d2r8ba1 d3b5ea1 d3cn9a_ d3ia2a_ d1a88a_
d4dgqa1 d1hkha_ d1zoia_ d3zxha_ d1cgla_ d1rm8a_ d1eaka2 d1hy7a_

d2jsda_ d1cxva_ d1hv5a_ d1i76a_ d1eb6a_ d3p0ya1 d1igra1 d1igra2
d1n8yc2 d1n8yc1 d1m6ba2 d1m6ba1 d1dzka_ d4tlja_ d2a2ua_ d4wfva_
d3t1da_ d4iaxa_ d3zq3a_ d2nnda_ d1i4ua_ d1kxoa_ d1bbpa_ d1kt7a_
d1exsa_ d3s26a1 d1lf7a_ d1qfta_ d4gh7a_ d1z24a1 d1qabe_ d1xkia_
d1ew3a_ d3bx7a_ d4iawa1 d1gkab_ d3kzaa_ d2k23a_ d2l9ca1 d2joza1
d1gm6a_ d3kffa_ d3saoa1 d1e5pa_ d1yupa1 d1iiua_ d1i26a_ d1cixa_
d1y29a_ d1v7fa_ d2jtba_ d1d1ha_ d1g9pa_ d1lupa_ d1qk7a_ d1mb6a_
d2a2va_ d1niya_ d1i25a_ d1hvwa_ d2mpqa_ d1kqha_ d1la4a_ d2mxoa1
d1dl0a_ d1koza_ d1qk6a_ d1emxa_ d1lqra_ d1c6wa_ d1rmka_ d1ixta_
d1eyoa_ d1cnna_ d1g1pa_ d1f3ka_ d1fyga_ g1f8v.1 d1novc_ g1gk9.1
g1cp9.1 g1e3a.1 d1mqta_ d1d4m3_ d1fmd3_ d2mev1_ g1aym.1 d1ev13_
d4q4w1_ d1r1a3_ d2x5ia_ d1rhi1_ g1r1a.1 g1d4m.1 g1rhi.1 d1b35b_
d1oopa_ d1tme1_ d4iv1a_ g1pvc.1 g1fpn.1 d1fpn1_ d4gb31_ d1d4m1_
d1fmd1_ d1ev11_ d2wzr3_ g1cov.1 d1qqp3_ g1fmd.1 d2x5ic_ d1rhi3_
g1oop.1 d1hxs1_ g1hxs.1 d1fpn3_ d1bev1_ g2mev.1 d1eah3_ d1aym1_
d1pvc3_ d1qqp1_ d1zba3_ d1h8ta_ d3vbhc_ g1b35.1 g1bev.1 d1hxs3_
g1ev1.1 d1bev3_ g1tmf.1 d1tmf1_ d1b35a_ d2mev3_ g1eah.1 g1h8t.1
d1eah1_ d4q4w3_ d1r1a1_ d1aym3_ d1pvc1_ d1zba1_ d1h8tc_ d1oopc_
d1tme3_ g1qqp.1 d4iv1c_ g1tme.1 d1y14b1 d2nn6i1 d1i94l_ d2zjra2
d1smxa_ d2qamc2 d1hh2p1 d2r7da1 d1rl2a2 d1wfqa1 d3bzka4 d1x6oa2
d3a0ja_ d2j01d2 d2ja9a1 d4ql5a_ d1hr0w_ d1luza_ d2n3sa_ d2vnud1
d1h95a_ d1go3e1 d2nn6h1 d1ne3a_ d2mo1a_ d1iz6a2 d1jjga_ d1e3pa2
d2ix0a3 d2ix0a2 d2lxja_ d1ueba3 d1ueba2 d2z0sa1 d1u0la1 d2ix0a1
d2eifa2 d2je6i1 d3i2za1 d2c35b1 d2ahob2 d1t9ha1 d3i4oa_ d2qall1
d1jt8a_ d2nn6g1 d1g6pa_ d3cama_ d1i94q_ d1c9oa_ d1d7qa_ d2ba0a1
d2vnud2 d2vnud3 d1khia2 d2qalq1 d1wi5a1 d1xtda2 d1ny4a_ d4w9ta_
d1jvna1 d4x2ra1 d1qo2a_ d4axka1 d1thfd_ d1h5ya_ d4u28a_ d2y88a_
d4wd0a_ d1vzwa1 d1eixa_ d3exra_ d2ffca1 d2fdsa1 d3n3ma1 d2czda_
d1q6oa_ d2yyua_ d1vqta1 d1dbta_ d3ldva1 d1ux8a_ d1dlya_ d4i0va_
d2bkma_ d1dlwa_ d2bmma_ d2r80b_ d1cqxa1 d1cg5a_ d1gcvb_ d3boma_
d2h8fb_ d2raoa_ d3bj1a_ d4hrrb_ d2r50a_ d3d1kb_ d2aa1b1 d3zhwa_
d3hf4b_ d3gdja_ d1jebb_ d3bj1b_ d1x9fb_ d1wmub_ d1gcva_ d3bcqb_
d3d4xb_ d1gvha1 d2dc3a_ d3hf4a_ d4yu3a_ d3k8ba_ d1qpwb_ d2r80a_
d3bcqa_ d1v4wa_ d3wtgb_ d1jl7a_ d4esaa_ d1a9we_ d3d1ab_ d1fsla_
d4h2la_ d1v4wb_ d1mwca_ d1vhba_ d1jeba_ d1spgb_ d1h97a_ d1q1fa_
d1tu9a1 d3d1aa_ d1hdsb_ d3hyub_ d2zfbb_ d3fs4a_ d1oj6a_ d1shrb_
d4h2lb_ d4yu3b_ d1spga_ d3hrwa_ d3qqra_ d3at5a_ d4wchd_ d3hyua_
d1cg5b_ d1uc3a_ d1hbra_ d2raob_ d1outb_ d4esab_ d3gdjb_ d3qqqa_
d1x9fc_ d3vrfa_ d1itha_ d3mkba_ d3d4xa_ d3mjpa_ d1jl6a_ d3fh9a_
d1outa_ d2dc3b_ d1it2a_ d3eoka_ d3g46a_ d1qpwa_ d3vrfb_ d1ch4a_
d1x9fd_ d1hdsa_ d2zfba_ d3mkbb_ d3wtga_ d1a4fa_ d3fh9b_ d3bomb_
d3cy5a_ d3at5b_ d1x9fa_ d2oifa_ d4lmxb_ d1cpcb_ d3v57a_ d1kn1a_
d1cpca_ d4f0ta_ d2vjha_ d4po5b_ d1eyxa_ d1alla_ d4f0tb_ d1b8db_
d4h0mb_ d1b33a_ d2vjhb_ d4lm6b_ d2vmlb_ d1allb_ d2vjta_ d3dbja_

d2vmla_ d2vjtb_ d4f0ub_ d1f99b_ d4rmpa1 d1liaa_ d1eyxb_ d2uulb_
d4lmsb_ d4f0ua_ d2bv8a_ d1f99a_ d2bv8b_ d2c7lb_ d3dbjb_ d4rmpb1
d1b8da_ d4h0ma_ d3v57b_ d1kn1b_ d1liab_ d2uula_ d2j96a_ d2xqfa_
d1ea5a_ d1dx4a_ d1qe3a_ d1k4ya_ d4qwwa_ d2ha2a_ d3c8da2 d2pbla1
d1jkma_ d1jjia_ d1lzla_ d1jjfa_ d1xm5a_ d1xaxa_ d1tvia_ d4iuaa3
d4bvva_ d5hpga_ d3k65a_ d1ki0a3 d1ki0a2 d1ki0a1 d1a0ha1 d1pmla_
d3sp8a3 d2feba1 d3u1ra1 d1kapp2 d1k7ia2 d2m5ta_ d1cqqa_ d1wqsa_
d5bpea_ d2ipha_ d2bhga1 d3tnta_ d3sjia_ d3d23a1 d2fyqa_ d3tloa_
d3zvea1 d2hrva_ d3q3ya1 d4asha1 d1e88a1 d1l6ja4 d1eaka4 d1eaka3
d1l6ja3 d1h8pa2 d1h8pa1 d3rp2a_ g1ucy.1 d1fuja_ d2zchp_ d3w94a_
d1si5h_ d1lo6a_ g2pka.1 d1cgha_ d1kigh_ d1a5ia_ d3s9ba_ d1hj8a_
d1ao5a_ d2anya_ d1q3xa1 d1gvza_ d1azza_ d4gsoa_ d2f91a1 d1iaua_
d1gdna_ d1eufa_ g1fiw.1 d1eq9a_ d4nfea_ d2rdla_ d1spja_ d1elva1
d1a7sa_ d1ekbb_ d1pfxc_ d1yc0a_ d2aiqa_ g1fiz.1 d1fi8a_ d4k60a_
d4dgja_ d1mzaa_ d1op0a_ d3tjva_ d1pytd_ d1sgfg_ g1gg6.1 d2eeka_
g1rtf.1 d1a0ja_ d3g01a_ d1pytc_ d4e7na_ d1ddja_ d1z8ga1 d2hlca_
d1npma_ d1brup_ g1jou.1 d1sgfa_ d1autc_ g2hnt.1 g1kdq.1 d3s69a_
d4crga_ d1fxya_ d1bqya_ d2ftba_ d1pmpa_ d2rcqa1 d1fdqa_ d4lkpa_
d1sa8a_ d1vyfa_ d1lpja_ d3vg7a1 d4azqa1 d1o8va_ d5bvqa_ d1p6pa_
d2qo4a_ d1opba_ d1yiva1 d1o1va_ d4bvma1 d4tkba_ d4i3ba_ d1bwya_
d1ftpa_ d4gkca_ d1cbia_ d3p6da1 d1kqwa_ d2a0aa1 d1m4la_ d2bk7a_
d3fjua_ d1z5ra1 d1kwma1 d1uwya2 d1jqga1 d2c1ca1 d1dtda_ d1h8la2
d3lgia_ d3i77a_ d2z9ia2 d4nsva_ d1lcya2 d4jcna_ d1os8a_ d1hpga_
d1p3ca_ d1l1ja_ d1qtfa_ d1e69a_ d1mv5a_ d3g60a4 g1ii8.1 d1ewqa2
d1ji0a_ d1qhla_ d1v43a3 d2wmfa1 d1vpla_ d1g2912 d4a82a2 g1f2t.1
d2d62a1 d3tuic1 d2bbsa1 d3g60a3 d2ixea_ d1g6ha_ d1sgwa1 d2qi9c_
d3rlfa1 d3b60a1 d3d31a2 d2pzea1 g1xew.1 d1jj7a_ d1oxxk2 d2onka1
d1w1wa_ d1iwea_ d1qf5a_ d1vmaa2 d1nija1 d1np6a_ d2p67a1 d3ihla_
d1okkd2 d1ls1a2 d1s1ma2 d1iona_ d2v40a_ d3hida_ d2vo1a1 d2g0ta1
d1vcoa2 d1j8mf2 d1g3qa_ d1xjca_ d1p9ba_ d1qzxa3 d1ihua2 d1ihua1
d2qm8a_ d1dj2a_ d4ioja_ d1cp2a_ d1yrba2 d1hyqa_ d3gqba2 d1skye3
d1xp8a1 d1tf7a2 d1tf7a1 d1ye8a1 d1skyb3 d2qe7d2 d4blta_ d4huta_
d3ldaa2 d4hyya_ d2i3ba1 d1cr1a_ d3j9tb2 d3ntua2 d1vdza1 d2z43a_
d1e9ra_ d3ziaa2 d1n0wa_ d1g6oa_ d3ziad2 d3j9ta2 d1pzna2 d3gqbb2
d2i49a_ d1nnfa_ d1xvxa_ d1ursa_ d1h3da1 d1iuda_ d4m1va1 d1twya_
d3e13x_ d2nxoa1 d4pfua_ d3ttma_ d1elja_ d2qmwa1 d1us5a_ d1y3na1
d1jeta_ d1uqwa_ d4f19a1 d2esna2 d3thia_ d3fasa1 d2uyeb_ d2xwva1
d1r9la_ d2fyia1 d1pb7a_ d2ozza1 d1sw5a_ d1nh8a1 d1vr5a1 d2g29a_
d1xs5a_ d3lswa_ d2z23a_ d3w9va_ d3tcha_ d1q35a_ d1z7me1 d3q27a_
d1p99a_ d1ve4a1 d2i6ea1 d4mcoa1 d4n8ya_ d1eu8a_ d1zbma1 d1hsla_
d1ixca2 d1j1na_ d1xvya_ d1y9ua_ d2z22a1 d1xoca1 d4oera_ d1wdna_
d5f1qa1 d1xt8a1 d1i6aa_ d4gwoa_ d4igta1 d1pc3a_ d4mmpa1 d1o63a_
d2czla1 d2onsa2 d4mija1 d4ok5a1 d2bmfa1 d2bmfa2 d1yksa1 d1yksa2
d1jr6a_ d2qeqa1 d2jlqa1 d1a1va2 d1eh3a_ d3skpa_ d1dtza2 d1ieja_

d1jnfa2 d1ce2a1 d1jnfa1 d1b1xa2 d1b1xa1 d1jw1a1 d1jw1a2 d1ryoa_
d1h76a1 d1h76a2 d1c4oa1 d2p6ra3 d2p6ra4 d1oywa3 d1wrba1 d1w36b2
d1w36b1 d1c4oa2 d3bora_ d1t6na_ d2eyqa5 d2eyqa3 d2eyqa2 d2zu6a2
d1veca_ d1uaaa1 d1w36d1 d2g9na1 d1z3ix2 d2rb4a_ d1hv8a1 d1hv8a2
d1t5ia_ d1w36c2 d2j0sa2 d2j0sa1 d1fuka_ d3hjha1 d1z5za1 d1qdea_
d1z63a1 d1s2ma2 d1s2ma1 d1wp9a1 d1wp9a2 d1gm5a3 d1gm5a4 d3dina4
d3dina3 d1q0ua_ d1w36c1 d1z3ix1 d1nkta3 d1nkta4 d1uaaa2 d1w36d2
g1qhh.1 d2fwra2 d2fwra1 d3q3ma_ d1za0a1 d1mxra_ d2inca_ d1jk0a_
d2incb_ d1mhyb_ d3hf1b_ d1mtyb_ d1r2fa_ d2uw1a_ d1mhyd_ d3n37a_
d1afra_ d1oquc_ d1syya_ d1mtyd_ d2o1za_ d1uzra_ d1smsa_ d2fkza_
d1zuja1 d1s3qa1 d2fjca1 d1z6om1 d1veia_ d4dyya_ d1jiga_ d3gvya_
d2pyba_ d4cy9a1 d3bvfa1 d1r03a_ d2cf7a_ d1nf4a_ d1zs3a1 d4iwka_
d3ka8a_ d3np0x_ d1n1qa_ d1lb3a_ d4e6ka_ d1z6oa1 d2fzfa1 d4dyua_
d3ge4a_ d1jgca_ d4am4a_ d2z6ma_ d1ji5a_ d1vlga_ d1lkoa1 d1krqa_
d3qz3a_ d1o9ra_ d2ffxj_ d1euma_ d1yv1a1 d2v2pa_ d1j30a_ d4di0a_
d1dpsa_ d1oewa_ d2qzxa_ d2asia_ d1psoe_ d1smra_ d2h6ta_ d1pfza_
d1izda_ d3hd8a_ d1j71a_ d1dpja_ d4aa9a_ d1t6ex_ g1b5f.1 g1lya.1
g1htr.1 d1qdma2 d1bxoa_ d1wkra_ d1tzsa_ d1ibqa_ d2qzwa_ d1bdqa_
d3ggta_ d3ixoa_ d1mrxa_ d2haha_ d1tw7a_ d4njsa_ d1sgua_ d2p3da_
d1nsoa_ d3d3ta_ d2rkfa_ d1c6ya_ d2rspa_ d3ogpa_ d3ekwa_ d2o4na_
d2j9ja_ d1baia_ d3ggua_ d3lzua_ d4npua_ d4j55a_ d2r8na_ d2fxda_
d2p3ca_ d4dqea_ d3ttpa_ d3t3ca_ d2p3aa_ d2hc0a_ d2fdda_ d3mwsa_
d1k8rb_ d1wgra1 d1ef5a_ d1wfya1 d1e8ya3 d1wgya1 d1lfda_ d2cs4a1
d1wxma1 d2byea1 d3kh0a1 d2byfa1 d1wxaa1 d1e7ua3 d1wgda1 d1wgga1
d4q5eb_ d1wjna1 d1se9a_ d3n3kb_ d1wz0a1 d1we6a1 d2m0xa_ d2gbka_
d3rt3b1 d1wh3a1 d1sifa_ d2ekec2 d4ii2b1 d4hk2a1 d1c3ta_ d1m94a_
d1wgha1 d1ttna1 d1j8ca_ d1wx8a1 d3v6cb1 d2ma9b_ d4i6lb1 d2jvca1
d1iyfa_ d2k1fa_ d1wxva1 d1wf9a1 d1t0ya_ d2io0b2 d1x1ma1 d2gbma_
d1zw7a1 d2m17a_ d1ud7a_ d1we7a1 d2lwpa_ d2gbja_ d1wjua1 d1v6ea1
d1v86a1 d4eewa_ d1wiaa1 d4icva_ d4gu2a_ d1wx7a1 d1wy8a1 d4hjka1
d2faza1 d1v5oa1 d2al3a1 d2cr5a1 d1wj4a1 d1wgqa1 d1v89a1 d2coda1
d1zc3b_ d1wg7a1 d1u5ea1 d1v61a1 d2coca1 d2ys1a1 d1v5pa1 d1ntya2
d1wjma1 d1wi1a1 d1u29a_ d1xx0a1 d1unqa_ d2zkmx3 d1fhoa_ d1v88a1
d2adza1 d1x1fa1 d1p6sa_ d2coaa1 d1btka_ d1v5ma1 d1faoa_ d2x18a_
d1dyna_ d1x1ga1 d4kaxb_ d2j59m1 d1xcga2 d1txda2 d1u5da1 d1dbha2
d1w1ha_ d1upqa_ d1v5ua1 d1foea2 d2fjla1 d1eaza_ d2dfka2 d2cofa1
d1kz7a2 d2dara2 d2dara1 d1wyha2 d2dloa2 d1rutx4 d1rutx3 d2cuqa2
d1x4la2 d1x4la1 d1x3ha2 d1x3ha1 d2d8za1 d1x61a2 d1x6aa1 d1x6aa2
d2cora2 d2cora1 d1v6ga1 d1v6ga2 d2d8za2 d1x64a1 d1x64a2 d1b8ta1
d2co8a1 d1x63a2 d2dj7a2 d2dj7a1 d2cu8a2 d2cu8a1 d1g47a2 d1g47a1
d1j2oa1 d1wyha1 d2dloa1 d1wiga1 d1wiga2 d2cupa2 d2cupa3 d1x68a1
d2d8ya2 d2d8ya1 d1m3va2 d1x62a1 d1x68a2 d1x61a1 d1b8ta3 d1b8ta2
d2d8xa2 d2d8xa1 d1x4ka1 d1x4ka2 d2cuqa1 d2co8a2 d1j2oa2 d1x63a1
d2cura1 d2cura2 d1x62a2 d2bcgg1 d2bcgg2 d1d5ta1 d1dszb_ d1a6ya_

d3cbba_ d1ynwa1 d1r4ia_ d1hcqa_ d2hana_ d2hanb_ d2enva1 d1kb4b_
d2nllb_ d1lo1a_ d1cita_ d1um8a_ d1ixza_ d1qvra2 d1qvra3 d1qzma_
d1sxjc2 d1jr3d2 d2p65a_ d1g8pa_ d3hu3a3 d1sxjd2 d1njfa_ d1in4a2
d1sxja2 d1tuea1 d2ce7a2 d1w5sa2 d1sxjb2 d1d2na_ d1ny5a2 d1ofha_
d2gnoa2 d2a5yb3 d1fnna2 d1u0ja1 d1lv7a_ d1iqpa2 d2fnaa2 d1ixsb2
d4gita_ d1sxje2 d1g41a_ d1svpa_ d1mbma_ d3su6a1 d1cu1a1 d4agka_
d1vcpa_ d1q31a_ g1n1l.1 d3e90b_ d1ep5a_ d3u1jb_ d2k4ua_ d1hp2a_
d2lu9a_ d1baha_ d2meoa1 d1wm7a_ d1hlya_ d1n8ma_ d1jlza_ d1wpda_
d1c55a_ d1pvza_ d1wt7a_ d1wz5a_ d1m2sa_ d1pjva_ d1c49a_ d1qkya_
d2ktca1 d1wmta_ d1du9a_ d1j5ja_ d1r1ga_ d1c4qa_ d2wv6d_ d1prtf_
d1ltrd_ d4l63b1 d1r4pb_ d1prtd_ d1tl0f_ d2bosa_ d1prtb1 d3chbd_
d1t0za_ d2i61a_ d1t1ta_ d1sn4a_ d1kv0a_ d2lkba_ d1npia_ d1wwna_
d1pe4a_ d2kbka_ d2li7a_ d1djta_ d2kbha_ d1dq7a_ d1sega_ d1omya_
d1zywa1 d1jzaa_ d2jcqa_ d1bnla_ d1bvsa3 d1ixra2 d1iyjb4 d1iyjb5
d1iyjb3 d1o7ia_ d4ipca1 d1x3ea_ d4apva_ d1jb7a2 d1jb7a3 d3kdfa_
d1ue1b_ d1wjja1 d3eiva_ d1xjva2 d1xjva1 d2ccza2 d1se8a_ d1l1oc_
d1jb7b_ d1qvca_ d3ulla_ d1qzga_ d1miua4 d1miua5 d1miua3 d3g48a1
d1gd7a_ d1jjcb3 d1pyba_ d3ubub_ d1egia_ d1v7pa_ d1tdqb_ d3ubua_
d1c3ab_ d1h8ua_ d3bx4b_ d1t8ca1 d1sb2a1 d1qdda_ d2ziba1 d3cada_
d1byfa_ d1j34b_ d1jwia_ d1jzna_ d1c3aa_ d1oz7b_ d1wmza_ d3bdwb_
d1hq8a_ d1fifa1 d1dv8a_ d3hupa_ d1wk1a1 d1umra_ d2afpa_ d3bx4a_
d1gz2a_ d1sl6a_ d1v7pb_ d3g8ka_ d1ixxa_ d1j34a_ d1jwib_ d1g1sa1
d1y17a1 d1ja3a_ d1fvub_ d3bdwa_ d1sb2b1 d1fvua_ d1umrc_ d1oz7a_
d4m17a_ d3cfwa1 d1ypqa1 d1g1ta1 d3fd4a_ d3l9jc_ d4dn8a1 d1v4lb_
d1rdl1_ d2msba_ d1prtb2 d1pfsa_ d2a1ka_ d1rpma_ d2shpa1 d1yfoa_
d1l8ka_ d2ooqa1 d1g4us2 d1p15a_ d1lara1 d1wcha_ d2fh7a2 d2fh7a1
d1ohea1 d1ohea2 d2f4da_ d1rxda_ d2hxpa_ d1xria_ d1d5ra2 d1fpza_
d3ezza_ d4y2ea_ d3ubra_ d1qo8a1 d1d4ca1 d2k3va_ d1ft5a_ d1qdba_
d1m1qa_ d2j7aa_ d1h21a_ d1ogyb_ d4fasa_ d1oaha_ d1sp3a_ d3h33a_
d1rwja_ d19hca_ d1os6a_ d3bxua_ d3h4na_ d1i77a_ d3caoa_ d1j0pa_
d2ctha_ d1duwa_ d1ofwa_ d2e84a_ d1hh5a_ d3h34a_ d1wioa4 d1ccza2
d1vcaa1 d1dr9a2 d2d9qb1 d2nxyb2 d1adqa1 d1fnga1 d1uvqb1 d1zs8a1
d2vola1 d2vola2 d1mfbl2 d2jelh2 d1uwel2 d3qq4b1 d1kjvb_ d1k5na1
d1osph2 d4odhl2 d1ow0a1 d1ow0a2 d1bfob2 d3l9ra2 d3jtth1 d2fath2
d4elkb2 d1fn4b2 d2xfxb_ d1pg7x2 d1cqka_ d4k71b2 d1lk2a1 d1eapb2
d4lfhg2 d1i4fa1 d2nw2a2 d1kfah2 d1mhca1 d1h3ua1 d1wejh2 d1h0db2
d1a5fh2 d1kcvh2 d1mjuh2 d1qqda1 d1kcrh2 d1k8ia1 d3huja2 d2nnab1
d1nfdf2 d3mffb2 d1s9va1 d1lk3h2 d1q0xl2 d1fv1b1 d1neza1 d4rfeb2
d1r3ha1 d1a6tb2 d1kjva1 d1pz5b2 d1onqa1 d2bc4a2 d1de4a1 d1k8da1
d1tcra2 d1dn0b2 d1nfde2 d2xwtb2 d1bfoa2 d1mexh2 d1iakb1 d1ypzf2
d4f7ea2 d1q72l2 d2fbjh2 d1fe8h2 d1clzh2 d1fngb1 d1uvqa1 d1vgka1
d1c16a1 d4j12a2 d4x99b2 d1igtb3 d1je6a1 d1q72h2 d1nldh2 d1teth2
d2hmic2 d1i1ca2 d1i1ca1 d1k8ib1 d1q0yh2 d1kfal2 d3frua1 d1pg7w2
d1u58a1 d1igyb4 d1igyb3 d1hyrc1 d1klub1 d1deeb2 d1c12b2 d2bc4b2

d2ig2h2 d1kcvl2 d3wn5b1 d1lk2b_ d1lk3l2 d1mjul2 d1sm3h2 d1q0xh2
d1zvsa1 d1ydpa1 d1u8ib2 d1ypze2 d1kcuh2 d1op9a_ d1y0la1 d1etzb1
d1egjl1 d2gsib1 d1nj9a1 d4jfhd1 d1aqkl1 d1t2ja_ d1lo4h1 d1zvya1
d3v4vl1 d4enec1 d1kxtb_ d5e8ea1 d2pxya_ d4weud_ d1xiwc_ d2esvd1
d1qnzh_ d1ogae1 d1f4xh1 d4n1hb_ d3ezjb1 d3mlwl1 d1kfah1 d1etza1
d1b4jh1 d3cfic_ d4w68a_ d2arja1 d1ek3a_ d2ok0l1 d1vera_ d1uwgh1
d1blnb1 d1fpth1 d1rzga1 d2giya1 d4m3kb1 d1nc2a1 d2nyyd1 d1ncna_
d1fbih1 d5dxwa_ d4fqqa1 d1dqdh1 d1jfql1 d1kelh1 d1faih1 d1frgl1
d1figl1 d4m6nl1 d1fo0a_ d4hxbl1 d3b5ga_ d3quyd1 d2h9gb1 d1i8kb_
d2dd8h1 d2ck0h1 d1eaja_ d4w6wb1 d2a6ib3 d1t66d1 d2e27h_ d1ob1b1
d1f11b1 d2v7na1 d1ad0a1 d2xt1b1 d1pskh1 d4i13b1 d2op4l1 d1a0ql1
d1uwel1 d1za3a1 d1ncbl1 d4uu9a_ d1bm3h1 d1cloh1 d1nqba2 d1cf8h1
d4y5va_ d1mhph1 d5anma1 d4cdgc1 d4ldeb_ d1y0lb1 d2pcpb1 d1z3gl1
d1dfbl1 d1fh5h1 d3g5xa1 d1tzib1 d4dn3l1 d3zhka1 d2b1hh1 d2uyla1
d1f3dh1 d1nj9b1 d2agjh1 d2h1pl1 d1q72h1 d8fabb1 d1ndmb1 d1rjca1
d4u6hb1 d3upca_ d2fbjl1 d1osph1 d1tvda_ d2cjvh_ d4fhbd1 d1bd2d1
d1rz7h1 d3nn8a_ d1knoa1 d1nfda1 d2bmkb1 d1dqll_ d1igyb1 d1igfl1
d12e8h1 d3utza1 d1nc2b1 d1rihh1 d4mwfb1 d4jvpa1 d1mieh1 d1rhfa1
d1fgnl1 d1sbsh1 d1lp9e1 d1dn0a1 d4lqfl1 d1iaih1 d1kgcd1 d1mh5b1
d1gpol1 d2g60h3 d1f8tl1 d2jixd1 d4grwh_ d1fo0b_ d1bfob1 d1axsa1
d1u9ka_ d1nmbh_ d1h8na1 d1h8na2 d1jguh1 d4eura1 d3sdyl1 d35c8h1
d1w72l1 d1mvub_ d1i9rh1 d1ob1a1 d4wenb_ d1yeeh1 d3ietc1 d1f58h1
d1ad0b1 d1mqkl_ d1nbvh1 d4b50a_ d3r8bb_ d1bafl1 d1mamh1 d4rgne1
d2gsgb_ d35c8l1 d1bafh1 d1ai1h1 d2pcpa1 d2fath1 d2x1pa_ d1orsb1
d1ibgl1 d1shma_ d1a2yb_ d1u0qa_ d2coqa_ d1hilb1 d1qfwi_ d1jv5b_
d1fn4b1 d5e0qa_ d1nsnh1 d1plgl1 d4ocxl1 d1kb5h1 d1rzfh1 d1pg7x1
d2fx7h1 d2oi9c_ d1l7il1 d3eyva1 d1d5il1 d4ffyl_ d1tetl1 d1a6wh_
d1ac6a_ d3dvfa_ d1nfde1 d1mimh1 d1ri8a1 d1jhlh_ d3dura_ d1fskb1
d1f97a1 d3so3b1 d2ak4d1 d1lp9f1 d4z7wf1 d1l7th1 d4y5ya_ d4x7ec1
d1emth1 d1ikfl1 d1nlbh1 d4utal1 d1bjma1 d1eapb1 d1u6ah1 d2dtgd1
d1kj2b_ d1xeda1 d1c1eh1 d1fgvh_ d2bnub1 d2fb4h1 d2dtgb1 d1ktrh_
d1jpth1 d1qkzl1 d4hepg_ d2atpb1 d1flrh1 d2hrpl1 d1mvfa_ d1iaim1
d3k80a1 d2jb5h1 d3zkxc_ d4grwe_ d1it9h1 d1rhhb1 d3w9ec_ d1e4wh1
d1sy6h1 d2j6eh1 d3qxta_ d1jglh1 d1p7ka1 d1tzhb1 d4n8pa1 d1gafl1
d2jelh1 d1wioa2 d4i0cc1 d1cicb1 d1lmka2 d1lmka1 d1iqwh1 d1orqb1
d1ejoh1 d1jnlh1 d4zg1a_ d43c9b_ d1ggbl1 d1wejh1 d3q6fa1 d1f3dj1
d3c6la1 d1bqlh1 d3p9wb_ d1a3rl1 d1h0db1 d2uzil_ d1eo8l1 d1qfwl_
d1n7ml1 d1igja1 d1c5ch1 d2a9mh_ d1rmfh1 d1fj1b1 d4heme_ d1ccza1
d1a5fh1 d1ogad1 d1dlfh_ d1qa9b_ d1vgel1 d3qskb_ d1sjxa_ d4ozfg1
d1sq2n_ d1qd0a_ d1kcvh1 d1c5db1 d3qcul1 d8faba1 d3bn9d1 d1mjuh1
d4y8dc_ d7fabh1 d3dvgb1 d1lo0l1 d1a6tb1 d1gigh1 d1kcrh1 d2hwzl1
d1yjdc1 d3clfl1 d4krna_ d1dl7l_ d1e6ol1 d1mexh1 d3r0ma_ d4kkna_
d1fnsl1 d1oaqh_ d1rzia1 d1ucbh1 d1ncwl1 d4b41a_ d1nfdf1 d2mpah1
d2d7tl1 d4grwf_ d4b5ea_ d1lk3h1 d3u79a_ d4u3xa_ d1ghfl1 d1bvkb_

d1mcww1 d1b88a_ d1jrhh1 d32c2b1 d4wemb_ d1dsfh_ d1op3h1 d1ce1l1
d1iqda1 d1qoka1 d1qoka2 d1qfuh1 d2crya1 d1ay1h1 d1oaql_ d3ab0b_
d1rurl1 d1t4kb1 d1bz7b1 d1n0xh1 d1forl1 d1ucbl1 d4w6yb_ d43c9a_
d2aabl1 d1tzia1 d4n1ca_ d1k6qh1 d3g9ab_ d4tvsa1 d1igmh_ d1pz5b1
d3dx9a1 d3wxwh_ d1nakh1 d1kcul1 d3eaka_ d4qlra1 d1cr9l1 d1d9ka_
d1f4xl1 d1deea1 d2i27n2 d1c12a1 d2orbl1 d1j8hd1 d1mcph1 d1c5da1
d1kenh1 d1bd2e1 d1h5ba_ d4x7fc1 d3upaa_ d1t3fa1 d1indh1 d2dtgc1
d4hc1l1 d1zv5a1 d4wgvb_ d1uwgl1 d4f37k1 d1aqkh1 d1vesa_ d3zkqd_
d1fptl1 d1lo4l1 d1knob1 d3rrqa_ d1ct8b1 d4orzc_ d1mlbb1 d4hgkc1
d1dqdl1 d1bogb1 d4ozhe1 d1dr9a1 d1i7za1 d1dn0b1 d1kell1 d1rzib1
d1ypzf1 d1wtla_ d1hkfa_ d1fail1 d1figh1 d2vyre_ d1sjva_ d1ohqa_
d1bfoa1 d1pkqb1 d1mjul1 d1b4jl1 d1nsnl1 d2arjb1 d1p4bh_ d4aj0a_
d3nn8b_ d2rcja1 d1f2xk_ d4v1dd_ d1adqh1 d2eksa_ d4k3ga_ d1jfqh1
d1uweh1 d1frgh1 d4gftb_ d1uz8b1 d4n1ea_ d1bm3l1 d1clol1 d3mcka1
d2vq1a1 d1ae6h1 d4aq1b1 d1uj3b1 d1nbqa1 d1fh5l1 d2ck0l1 d2ywza_
d1q72l1 d4nbxb_ d1hila1 d4hzlb1 d2j88l1 d1zmya1 d1cd0a_ d1a4ka1
d1pskl1 d1a0qh1 d4krmb_ d1ncbh1 d2fbjh1 d1j8he1 d1i3ua_ d1cf8l1
d1fe8h1 d2p1ya1 d1eeqa_ d2x89a_ d1ol0a_ d2r0lh1 d1ub5a1 d1dfbh1
d3eyvb1 d4jdvb1 d4p2cg_ d2gsia1 d2agjl1 d1u3ha1 d2h1ph1 d1rihl1
d1fskc1 d1boga1 d2a77l1 d1ospl1 d1qfoa_ d3dwta_ d2fx7l1 d1rz7l1
d3stba1 d1pkqa1 d1b6da1 d1dzba2 d1dzba1 d1kj2a_ d4l1ha_ d1rzgb1
d1dqlh_ d12e8l1 d1miel1 d1jmaa_ d1sbsl1 d1fgnh1 d1f58l1 d1mqkh_
d1ndgb1 d3qegl1 d1iail1 d2aw2a1 d1tqbc1 d3fo2a1 d4dkaa_ d1f8th1
d2g60l1 d2ywya_ d1uj3a1 d1yc7a_ d3bd3a_ d4w6xb1 d1nmbl_ d1i8ka_
d1rjlb1 d1ypze1 d1p7kb1 d1kxqe_ d1gpoh1 d2z4qa1 d1w72h1 d1i9rl1
d4qgya1 d4xt1c_ d2qqnh1 d1cica1 d3c08h1 d2cd0a_ d2bdnh1 d1hnga1
d1maml1 d1nldh1 d1cr9h1 d4lcih1 d3bkyh1 d3qg7l1 d1ai1l1 d1kxvc_
d4iosd_ d1l7ih1 d2x1qa_ d1ub6b1 d3k74b_ d1ibgh1 d1teth1 d1igya1
d1hxma1 d2x6ma_ d2e7la_ d5e03a_ d1qfwm_ d2r0kh1 d1igjb1 d1rurh1
d1plgh1 d1mj8h1 d1yuhb1 d3fmgl1 d1rzfl1 d1emtl1 d1ad9b1 d1igtb1
d1nlbl1 d1d5ih1 d3k7ua_ d4ffyh_ d1bwwa_ d3dvga1 d1a6ta1 d1fgvl_
d4qxgl1 d1miml1 d1jhll_ d2hffb1 d2hh0l1 d1qkzh1 d1l7tl1 d1afvh1
d1iaii1 d2cjuh_ d1um5h1 d2ghwb2 d2ghwb1 d1kgce1 d2fgwh1 d1ikfh1
d1dvfd_ d2i24n1 d2xtjb_ d1za3b1 d2yc1a_ d2hh0h1 d2x1oa_ d1bvka_
d1rjla1 d5e7ba1 d1igta1 d1il1b1 d2fb4l1 d1mvua_ d1jptl1 d32c2a1
d1fvca_ d1iqdb1 d2hrph1 d2p4ab_ d4qo1a_ d1p2cb1 d1wt5c_ d1t4ka1
d5c1mb_ d1it9l1 d1bz7a1 d1b2wh1 d1e4wl1 d1jnll1 d1yedb1 d1za6b1
d3u7aa_ d1igfh1 d3gnml1 d1orsa1 d1wejl1 d1a2ya_ d1hxmb1 d1gafh1
d2j4wh1 d1pz5a1 d1j05a_ d1a3rh1 d4fqll1 d1qfwh_ d1eo8h1 d1n7mh1
d4s11a_ d1kegh1 d5da4a_ d1iqwl1 d3whxb1 d1vgeh1 d1deeb1 d1c12b1
d1t2ql1 d1ad9a1 d3gsnb1 d3b2uc1 d1ggbh1 d1t3fb1 d1nakl1 d4ocmc_
d1lila1 d2v7hb3 d2esve1 d1mcoh1 d2qr0b1 d2q3aa_ d1ct8a1 d1kcrl1
d2pxyb_ d2uzih_ d5c3ld_ d3durb_ d1c5cl1 d2ak4e1 d1dl7h_ d1xiwd_
d1mlba1 d1rmfl1 d1i7zb1 d1dlfl_ d2xa3a_ d5bq7b1 d1dqqb1 d3h42l1

d1egjh1 d1kcvl1 d1fe8l1 d2j6el1 d1f3rb2 d1lk3l1 d1sm3h1 d1q0xh1
d1ghfh1 d7fabl1 d1igch1 d1lo0h1 d4idla_ d5c2bl_ d1jrhl1 d1il1a1
d3zhda1 d4kfzc_ d1b0wa_ d1e6oh1 d1mexl1 d4krpb_ d1fnsh1 d1ncwh1
d4hgma_ d4eigb1 d4rzca_ d2mpal1 d4y5xa_ d5en2b1 d3lrga1 d4ej1c1
d1rhha1 d1j05b_ d1forh1 d1q9wb1 d2wzpd_ d2aq2a1 d1h0da1 d3revb1
d2ij0c1 d1tzha1 d4m7jl1 d1k6ql1 d3b9kc1 d1igml_ d3qeua1 d1dsfl_
d1ce1h1 d1kcuh1 d1zvha1 d1zvoa1 d1tqbb1 d1ay1l1 d2qscl1 d2cgrh1
d1nl0h1 d1kenl1 d3eo0a1 d4n9ob1 d1n0xl1 d3k1kc_ d1d4ua2 d2aq2b1
d1enfa1 d1i4pa1 d1v1oa1 d1et9a1 d1esfa1 d2g9hd1 d4ohja1 d1klud1
d1bxta1 d1eu3a1 d1ty0a1 d1fnua1 d2cqva1 d1fhga_ d2k1ma_ d2dl2a1
d1biha3 d1biha1 d1biha4 d3qp3a1 d1g1ca_ d1gxea_ d1cvsc2 d1cvsc1
d1xmwa2 d1m4ka2 d2otpa2 d1biha2 d1rhfa2 d2fcba2 d2fcba1 d1wwbx_
d1gsma2 d1epfa1 d1epfa2 d2dava1 d1waaa_ d1f97a2 d1x44a1 d1vcaa2
d1fnla2 d1fnla1 d3b5ha2 d3h8na2 d3h8na1 d1jbja1 d1jbja2 d1pd6a_
d1f2qa2 d1f2qa1 d3cx2a_ d1nbqa2 d3b5ha1 d1n26a1 d1xiwb_ d1xaua_
d1fltx_ d2ifga1 d1olla1 d1olla2 d1cs6a4 d1cs6a3 d1cs6a2 d1cs6a1
d2gw5a2 d1sy6a1 d1xiwa_ d1ucta2 d1ucta1 d1ry7b2 d1gl4b_ d1qz1a3
d1olza1 d2c9aa1 d1wwca_

**Family-29 (b.40.2.1)**

**Positive dataset contains 3 protein sequences.**

d2wv6d_ d4l63b1 d1tl0f_

**Negative dataset contains 4102 protein sequences.**

d1ibja_ d1c7na_ d1iuga_ d4q75a_ d2bkwa1 d1t3ia_ d1n8pa_ d1qz9a_
d1jf9a_ d1eg5a_ d1p3wa_ d3kgwa_ d1cl1a_ d2ctza1 d1vjoa_ d2rfva_
d1gc0a_ d1cs1a_ d1d2fa_ d2ch1a1 d1qgna_ d1pffa_ d1elua_ d1u08a_
d1lc5a_ d1b5pa_ d2ay1a_ d1bw0a_ d3pd6a_ d7aata_ d2csta_ d1j32a_
d1fg7a_ d4wlha_ d1o4sa_ d1v2da_ d1gdea_ d1m6sa_ d1v72a1 d1svva_
d1wsta1 d2f8ja2 d1lk9a_ d1vp4a1 d1xi9a1 d2gb3a1 d1yaaa_ d1sefa_
d1sfna_ d1rc6a_ d1qnia1 d1fwxa1 d1f56a_ d1bypa_ d1azca_ d1ws8a_
d3u25a_ d1cuoa_ d1id2a_ d1rkra_ d2iaac_ d4dp1x_ d1nwpa_ d2gima_
d2i7sa_ d1qhqa_ d3ehbb1 d1fftb1 d1gyca3 d1hfua2 d1kbva1 d1kbva2
d1sdda1 d1sdda2 d1gyca1 d1mzya1 d2bw4a1 d1aoza1 d1aoza3 d1aoza2
d1kyaa2 d1kyaa3 d1kyaa1 d1mzya2 d4e9sa1 d1sddb1 d1sddb2 d1hfua1
d1hfua3 d1oe1a2 d1oe1a1 d1v10a2 d1v10a3 d1v10a1 d1s5ja2 d1qhta2
d4dlea2 d1tgoa2 d3py8a2 d1q8ia2 d1kfsa2 d2py5a2 d1d5aa2 d2cfba1
d1dfoa_ d1o69a_ d1bj4a_ d3k28a_ d2bwpa_ d3lg0a_ d2fnua_ d3ou5a_
d1z7da1 d1b9ha_ d1iaya_ d4msoa_ d1bs0a_ d1s0aa_ d1sffa_ d3tqxa_
d1mdoa_ d3qboa_ d3pgya_ d1bjna_ d2dkja_ d1ejia_ d1vefa1 d4wxga_
d3bs8a_ d3gbxa1 d1w23a_ d1a3qa1 d1iknc_ d3brda1 d1oy3c_ d1imhc1
d1gjia1 d1zk9a1 d2cxka1 d2bema_ d1pama1 d1ea9c1 d1qhoa1 d2fhfa2
d2fhfa1 d1k3ia1 d1j0ha1 d1cxla1 d2bhua1 d1edqa1 d1f1sa2 d1ut9a2
d1gvia1 d1txka1 d1zx5a1 d2phla2 d2phla1 d1fxza2 d1fxza1 d1lrha_
d1uija1 g1dgw.1 d2v09a_ d1uika2 d1uika1 d1dgwa_ d1ep0a_ d2pa7a1
d1wlta1 d2c0za1 d1zbfa1 d2z1ha_ d2etja1 d3qioa1 d1jl1a_ d2e4la_

d1jl2a_ d2lsna1 d1io2a_ d1uaxa_ d2yv0x_ d1mu2a1 d1ekea_ d1ds6b_
d1fstb_ d1g0da1 d2b5ea2 d2b5ea3 d2b5ea1 d2b5ea4 d2kp1a1 d2kp2a1
d1hyua3 d1hyua4 d1j08a1 d1j08a2 d2bjca1 d2hsga1 d1qpza1 d1efaa1
d1k9ua_ d2opoa_ d1e3oc2 d1ic8a2 d2ovga_ d1sq8a_ d1rzsa_ d3kz3a_
d3orca_ d3gpdg2 d2q49a2 d1hdgo2 d2g17a2 d2cvoa2 d2czca1 d3pwka2
d1obfo2 d1i32a2 d1zyao2 d2pkqo2 d1b7go2 d2hjsa2 d1cf2o2 d1vkna2
d4lt9a1 d3tc7a1 d3hoja_ d1i4na_ d3vnda_ d3nl8a1 d1geqa_ d3nava1
d1v5xa_ d4a2ra1 d3tc6a1 d1ujpa_ d4ijba_ d1xcfa_ d1vc4a_ d1rd5a_
d1qopa_ d3b5va_ d2msya_ d2djoa1 d2r5za_ d1au7a1 d1f43a_ d2lmda1
d1akhb_ d1s7ea1 d3nara_ d1zq3p1 d2craa1 d1mija_ d4uuta_ d3a01a_
d1x58a1 d3rkqa1 d3cmya1 d2cqxa1 d2da6a1 d1e3oc1 d1fjla_ d1mh3a1
d1ig7a_ d1x41a1 d1wh7a1 d1gt0c1 d2mw8a_ d1lfup_ d1x2ma1 d1b72a_
d1wi3a1 d1jgga_ d1wjha1 d1x2na1 d1mnmc_ d2cuea1 d3k2aa_ d2hosa_
d2jwta2 d1pufa_ d1octc1 d2hi3a_ d1b8ia_ d4j19a_ d3lnqa_ d1wh5a1
d1b8ib_ d1fexa_ d2cqra1 d1guua_ d1ug2a1 d2cqqa1 d2ajea1 d4a69c_
d1h8ac1 d2crga1 d2iw5b1 d1xc5a1 d2cu7a1 d1wgxa1 d2cjja1 d2ckxa1
d1gvda_ d1tc3c_ d1gdta1 d1u78a2 d1igna2 d2iu5a1 d1zk8a1 d2g7sa1
d2fd5a1 d2np5a1 d1z0xa1 d2id6a1 d2d6ya1 d2i10a1 d2oi8a1 d3c07a1
d2ns7a1 d2fq4a1 d2yvea1 d1t33a1 d2o7ta1 d1rkta1 d2np3b1 d2g7ga1
d2gena1 d2fbqa1 d2g7la1 d2gfna1 d2g3ba1 d2jj7a1 d2hyja1 d1sgma1
d1ui5a1 d2hkua1 d2id3a1 d4jyka1 d1bl0a1 d1d5ya1 d1d5ya2 d3ubub_
d1egia_ d1qo3c_ d1v7pa_ d1tdqb_ d3ubua_ d1c3ab_ d1h8ua_ d3bx4b_
d1t8ca1 d1sb2a1 d1qdda_ d2ziba1 d3cada_ d1byfa_ d1j34b_ d1jwia_
d1jzna_ d1c3aa_ d1oz7b_ d1wmza_ d3bdwb_ d1hq8a_ d1dv8a_ d3hupa_
d1wk1a1 d1umra_ d2afpa_ d3bx4a_ d1gz2a_ d1sl6a_ d1v7pb_ d3g8ka_
d1ixxa_ d1j34a_ d1jwib_ d1g1sa1 d1y17a1 d1ja3a_ d1fvub_ d3bdwa_
d1sb2b1 d1fvua_ d1umrc_ d1oz7a_ d4m17a_ d3cfwa1 d1ypqa1 d1g1ta1
d3fd4a_ d3l9jc_ d4dn8a1 d1v4lb_ d1rdl1_ d2msba_ d3lv8a_ d1s3ga1
d1zaka1 d1s4qa_ d3tvta2 d1uf9a_ d1khta_ d1ckea_ d1osna_ d1p5zb_
d4tmka_ d3cm0a_ d1tmka_ d1jaga_ d3h86a_ d1t0hb_ d1nn5a_ d2h92a_
d3kb2a1 d2c95a1 d1yj5a2 d1kgda_ d1ly1a_ d2ak3a1 d1vyva2 d1deka_
d2grja1 d1p3ja1 d1y63a_ d1e4ya1 d1s96a_ d3iija1 d1lvga_ d1nksa_
d1vyua2 d4e22a_ d1qf9a_ d1jjva_ d1q3ta_ d1ki9a_ d1lw7a2 d1teva_
d1kjwa2 d1gsia_ d1j99a_ d1t8ta1 d1nsta_ d1fmja_ d1zrha_ d1ls6a_
d2a3ra_ d1aqua_ d2zptx_ d3ckla_ d1texa_ d1q44a_ d2gwha_ d1odfa_
d1uj2a_ d4gi7a_ d2jeoa1 d1rz3a_ d1z0ja1 d3umra2 d3gj0a1 d2gaoa1
d1yzga1 d1x3sa1 d1g7sa4 d1puja_ d1udxa2 d1kk1a3 d2o52a_ d1jwyb_
d1zcaa2 d1zcba2 d2fv8a1 d2cjwa_ d3dz8a1 d1xzpa2 d1vg8a_ d4qxaa_
d2ew1a1 d1x1ra1 d2bcgy_ d3doea1 d3cona_ d3rega_ d3wyga_ d1upta_
d2b6ha1 d1zd9a1 d1r5ba3 d1n0ua2 d1r2qa_ d2f9la1 d2rhda_ d3q3jb_
d3q85a_ d1efma1 d1r8qa_ d2gf9a1 d2akab1 d1ek0a_ d1z08a1 d2gcoa1
d2dy1a2 d4g01b_ d2cxxa1 d1svia_ d3q72a_ d1ni3a1 d4ac9a4 d2erxa1
d2bmea_ d1wxqa1 d2gf0a1 d4ldja1 d1zunb3 d1d5ca_ d4lv5b_ d1efca3
d1kmqa_ d2g6ba1 d3lrpa_ d1tada2 d2y8ea_ d4rd4a1 d1z06a1 d2heia_

d3a58b_ d1u0la2 d1d2ea3 d1zj6a1 d2ocba_ d3rwoa_ d1mr3f_ d2e9sa1
d4u5xa1 d2iwra_ d2fn4a1 d3bwdd_ d2nzja1 d2atva1 d2ksqa_ d3l43a_
d1mkya2 d1mkya1 d1wmsa_ d1jnya3 d2rmka_ d1f6ba_ d1f60a3 d1gota2
d1jala1 d2fh5b1 d1g16a_ d2wblc_ d1m7ba_ d1wf3a1 d1f5na2 d1t9ha2
d1z6xa1 d1z0aa1 d2fg5a1 d2a5ja1 d2bcjq2 d1s0ua3 d1ky3a_ d4y0va_
d4lhwa_ d2qmea1 d2atxa1 d1egaa1 d1ryha_ d1moza_ d1nrjb_ d2erya1
d3bfka_ d1puia_ d1fzqa_ d3j3az1 d3t5ga_ d1shza2 d3defa_ d2f7sa1
d3raba_ d3tsoa_ d3aq4a_ d2al7a1 d3sfva1 d3bc1a_ d2ncda_ d1sdma_
d3h4sa1 d2heha_ d2grya_ g3kin.1 d1kk8a2 d1br2a2 d1goja_ d1f9va_
d2mysa2 d1lkxa_ d3t0qa_ d4db1a1 d3gbja_ d1ry6a_ d1v8ka_ d1kb0a1
d1jmxa2 d1jmxa1 d1pbya1 d3vrda1 d3vrda2 d1fcdc2 d1fcdc1 d1h1oa2
d1m70a2 d1m70a1 d1iqca2 d1iqca1 d1eb7a2 d1nmla2 d1nmla1 d1qksa1
d1zc6a1 d1zbsa1 d1zbsa2 d1zc6a2 d2ch5a2 d2ch5a1 d1zxoa2 d1zxoa1
d1hroa_ d4gedb_ d3zooa_ d1mz4a_ d1gu2a_ d1f1fa_ d1dvva_ d1c6ra_
d1c75a_ d1e29a_ d1gdva_ d1cnoa_ d1h32b_ d1co6a_ d2bh4x_ d2aiua_
d5cytr_ d1jdla_ d1i8oa_ d2zboa_ d3dmia_ d1ls9a_ d1wvec_ d2yk3a_
d3ph2b_ d1dw0a_ d5aura_ d1ql3a_ d1qn2a_ d1f1ca_ d4zzva_ d2xspa_
d1eg1a_ d4v20a_ d4ipma1 d1gpia_ d4csia_ d3pfza_ d3ovwa_ d2yoka_
d2a6za1 d3a4ua_ d2a6va1 d1evsa_ d1cnt1_ d1pvhb_ d1z7ca_ d1huwa_
d1bgea_ d1rhga_ d1f6fa_ d1f45b_ d1i1rb_ d1axia_ d1etea_ d3tgxb_
d1scfa_ d2z3qa1 d3qb1a_ d1m48a_ d4nkqb_ d1eera_ d1v7mv_ d5e4ea_
d2o27a_ d3b5ka_ d2psma1 d4zf7a_ d3uf5a_ d4x51a_ d1d9ca_ d4ypgc1
d3oq3a_ d1lqsl_ d1n1fa_ d1au1a_ d1wu3i_ d3besl_ d3ux9a_ d2b7la_
d3rbaa_ d2qtra_ d1nupa_ d3l92a_ d1f9aa_ d1qjca_ d2h29a_ d1vlha_
d1od6a_ d1lw7a1 d4ruka_ d2el7a_ d1iq0a2 d1irxa2 d1r6ta2 d1ivsa4
d2cyba_ d1rqga2 d1j09a2 d2yxna_ d1f7ua2 d1h3fa1 d1ffya3 d2g36a1
d1nzja_ d1n3la_ d1i6la_ d1pfva2 d1h3na3 d3n9ia_ d3sz3a1 d3sw2a_
d1moxc_ d1edmb_ d1autl2 d1autl1 d1nqlb_ d1dval1 d1szba2 d1kigl_
d2y5fl_ d1ijqa2 d1gk5a_ d1g1sa2 d1nt0a3 d1xdtr_ d1lmja2 d1lmja1
d1nzia2 d2wphe_ d1klil_ d1pfxl2 d1pfxl1 d1p9ja_ d1g1ta2 d1f7ea_
d4yzub_ d1gl4a2 d2y5ia_ d1qlsa_ d1psra_ d3n22a_ d2egda_ d4ggfc_
d2wcea_ d2luca_ d4aqia_ d3nsia_ d2lp3a_ d1a4pa_ d1k8ua_ d1nsha_
d1pvaa_ d3fs7a_ d2pvba_ d1ttxa_ d1rwya_ d2kyfa_ d1rk9a_ d1exra_
d1tiza1 d1nyaa_ d1g8ia_ d1ggwa_ d1wdcc_ d3fwba_ d1ggza_ d2o5ga_
d1ctaa_ d1m45a_ d3i5gc_ d1sl8a_ d1fpwa_ d2scpa_ d2roaa_ d4aqra_
d3pn7b_ d1br1b_ d2mysb_ d1s6ca_ d1bjfa_ d2obha_ d1jfka_ d1jf0a_
d1qv0a_ d1lkja_ d2mlfc_ d2m97a_ d2d8na_ d4by5a_ d1jbaa_ d4nqga_
d1wdcb_ d2mysc_ d1j7qa_ d1s6ia_ d1uhka2 d1r2ua_ d1dtla_ d3kf9a_
d3pn7c_ d1qjta_ d1c07a_ d1wkya2 d1uuqa_ d3wh9a_ d4m1ra_ d1xyza_
d4xuya_ d2je8a5 d4bf7a1 d3cuia_ d1ur4a_ d1qw9a2 d1odza_ d2vzsa5
d1bqca_ d2x2ya1 d1hjqa_ d1qnra_ d4y7ea1 d1vjza_ d4k68a_ d2v3ga_
d4hpga_ d1nofa2 d1nq6a_ d1h4pa_ d1aq0a_ d3ziza_ d4bs0a_ d1ecea_
d1h1na_ d1ta3b_ d1w91a2 d1i1wa_ d1uhva2 d1egza_ d7a3ha_ d1tvna1
d3nyda1 d1ghsa_ d2cyga1 d4xv0a_ d2c8na2 d1w32a_ d3emca_ d1v6ya1

d1foba_ d2uwfa1 d2fgla_ d3niya1 d2c0ha1 d1rh9a1 d1yq2a5 d1szna2
d1fa2a_ d1pama4 d5do8a1 d1ea9c3 d2fhfa5 d1qhoa4 d1avaa2 d1m53a2
d4m8ua1 d2gjpa2 d1lwha2 d4howa1 d1ktba2 d1j0ha3 d3vm5a1 d2x1ia_
d1wzaa2 d1uasa2 d2diea1 d1mxga2 d1ht6a2 d1eswa_ d1iv8a2 d5cpsa_
d1ud2a2 d1zjaa1 d3gbea1 d1kwga2 d1wdpa_ d1gcya2 d1g5aa2 d1hvxa2
d3dhpa2 d2bhua3 d4do4a1 d3qgva1 d1e43a2 d1g94a2 d2d3na2 d1gjwa2
d1gvia3 d1x1na1 d1r7aa2 d1tz7a1 d4toqa_ d1wb0a1 d1w9pa1 d1eoka_
d1itxa1 d1kfwa1 d1goia2 d1edqa2 d2gsja_ d1hjxa1 d1jnda1 d1vf8a1
d1umqa_ d1g2ha_ d1t77a2 d1e5wa2 d1j19a2 d1isna2 d2al6b2 d1g4ma1
d1xl4a1 d3vsqa_ d2gixa1 d1p7ba1 d1b9ra_ d1offa_ d1frra_ d3wcqa_
d1e9ma_ d1l5pa_ d1wria_ d1i7ha_ d3av8a_ d1czpa_ d1rfka_ d3p1ma1
d3b2ga_ d2bt6a2 d1wgqa1 d1v89a1 d2coda1 d1zc3b_ d1wg7a1 d1u5ea1
d1v61a1 d2coca1 d2ys1a1 d1v5pa1 d1ntya2 d1wjma1 d1wi1a1 d1u29a_
d1xx0a1 d1unqa_ d2zkmx3 d1fhoa_ d1v88a1 d2adza1 d1x1fa1 d1p6sa_
d2coaa1 d1btka_ d1v5ma1 d1faoa_ d2x18a_ d1dyna_ d1x1ga1 d4kaxb_
d2j59m1 d1ki1b2 d1xcga2 d1txda2 d1u5da1 d1dbha2 d1w1ha_ d1upqa_
d1v5ua1 d1foea2 d2fjla1 d1eaza_ d2dfka2 d2cofa1 d1kz7a2 d9ldta1
d2ewda3 d1up7a1 d2dfda1 d1s6ya1 d1vjta1 d7mdha1 d1hyea1 d1t2da1
d1ojsa3 d1b8pa1 d1ldna1 d1hyha1 d1civa1 d2a92a1 d1ez4a1 d1i0za1
d1y6ja1 d1gv0a1 d1ur5a1 d1llda1 d1obba1 d1v8ba1 d1gdha1 d1l7da1
d2dlda1 d1ygya1 d1qp8a1 d1li4a1 d1j4aa1 d2naca1 d1npya1 d3t4ea2
d1a4ia1 d1o0sa1 d2hk9a2 d2egga2 d1b26a1 d1bvua1 d1gq2a1 d1c1da1
d1leha1 d1gtma1 d1euza1 d3dona2 d1edza1 d1gpja2 d1vi2a1 d1p77a1
d1nyta1 d1b0aa1 d1vl6a1 d1v9la1 d3wdpp1 d3wq5a_ d1gnxa_ d4gxpa_
d4hz8a_ d1e4mm_ d3aiua_ d1wcga1 d1qvba_ d3ahxa_ d1v02a_ d2dgaa_
d1pbga_ d1vffa1 d1qoxa_ d4ha4a_ d1kola2 d1f8fa2 d1qora2 d1jqba2
d1vj0a2 d1cdoa2 d1gu7a2 d1d1ta2 d1xa0a2 d2dm6a2 d1jvba2 d1rjwa2
d1h2ba2 d1tt7a2 d1piwa2 d1o89a2 d1iz0a2 d1e3ja2 d1yb5a2 d1uufa2
d1vj1a2 d1e3ia2 d3gpdg1 d1vc2a1 d2nvwa1 d1u8fo1 d1ydwa1 d1hdgo1
d2g17a1 d2czca2 d1r0ka2 d1obfo1 d2cvoa1 d1ebfa1 d1h6da1 d1i32a1
d1zh8a1 d2q49a1 d1qkia1 d1j5pa4 d3pzra1 d1xeaa1 d1vkoa1 d1vm6a3
d3qvsa1 d2pkqo1 d1b7go1 d1gr0a1 d1p1ja1 d1lc0a1 d1j0xo1 d3pwka1
d2hjsa1 d1zyao1 d1vkna1 d1tlta1 d1nvmb1 d3sjua_ d3ojfa1 d1xu9a1
d2jjya1 d1n7ha_ d1y1pa1 d1h5qa_ d1jtva_ d4fs3a_ d2bkaa1 d1gy8a_
d2c5aa1 d1ulsa_ d1ooea_ d2b69a1 d1y5ma1 d1spxa_ d1ae1a_ d1bxka_
d1ydea1 d1gega_ d1hdoa_ d1o5ia_ d1e6ua_ d1snya1 d2rhca_ d3zv4a_
d5ceja_ d2d1ya1 d3w8ea_ d2fmua1 d1x1ta_ d1rpna_ d3ay7a1 d1k2wa_
d1eq2a_ d2hrba1 d3ruaa_ d2ew8a1 d4hwka_ d1xgka_ d2bd0a1 d1yo6a1
d2a35a1 d2ntva_ d1vl8a_ d1hdca_ d4bkua_ d2ag5a1 d2q45a_ d1nffa_
d4iy1a_ d1zbqa1 d1kewa_ d1ek6a_ d1ja9a_ d2q46a2 d1iy8a_ d1cyda_
d2ptga_ d1hxha_ d1uzma1 d3lyla_ d4m89a_ d3wyea_ d2blla1 d1w6ua_
d1sb8a_ d2q2va_ d4nr0a_ d1e6wa_ d2gasa_ d3oiga1 d2bgka1 d1mxha_
d1vl0a1 d1rkxa_ d1r6da_ d1wmaa1 d1e7wa_ d2c07a1 d1xkqa_ d3tzha_
d1g0oa_ d3lz6a_ d1qyca_ d1edoa_ d1gz6a_ d1oc2a_ d1t2aa_ d1zema1

d2xoxa_ d1z45a2 d1geea_ d1p33a_ d3lt0a_ d4e6pa_ d2wyua_ d1db3a_
d4q9na_ d1xhla_ d4o1ma_ d1fmca_ d2uvda_ d2gdza1 d1uaya_ d2z1ma_
d2o23a2 d2fr1a2 d2fr1a1 d1qsga_ d1qyda_ d4zjua_ d2a4ka1 d1yb1a_
d1wvga1 d1yxma1 d3f4ba_ d1mg5a_ d1xg5a1 d1r55a1 d3gboa_ d1kufa_
d1buda_ d4q1la_ d1quaa_ d1yp1a_ d1wnia_ d3nqyb_ d4gera_ d1bqba_
d1ex0a4 d1g0da4 d2f4ma1 d1x3za_ d1mdwa_ d1ziva1 d1zcma1 d2w0pa_
d2diaa1 d2e9ia2 d2e9ja1 d2dj4a1 d2eeba1 d2diba1 d2dmba1 d2dmca1
d2d7oa1 d2ee6a1 d2d7pa1 d2eeaa1 d2di8a1 d1wlha1 d2d7na1 d2nqca_
d2d7ma1 d2dica1 d3cnka_ d2k3ta1 d2eeca1 d1qfha2 d2bp3a1 d1v05a_
d2ee9a1 d2eeda1 d2di9a1 d3ioqa_ d1fh0a_ d1o0ea_ d1khqa_ d1m6da_
d2cb5a_ d1cs8a_ g8pch.1 d1s4va_ d2pnsa_ d1vsna1 d1mira_ d1iwda_
d2oula_ d7pcka_ d3bwka_ d3bcna_ d2p86a_ d1pvja_ d1pcia_ d1gece_
d1deua_ d4bs6a_ d1xkga1 d1cqda_ d1jqpa2 d2c0ya_ d2bdza_ d3p5ua_
d2o36a_ d1k9xa_ d1zoya1 d1qo8a2 d1d4ca2 d1chua2 d1kf6a2 d2jk6a2
d2jk6a1 d1feca1 d1feca2 d1lpfa1 d1lpfa2 d1xhca1 d1xhca2 d1dxla2
d1dxla1 d1gera2 d1m6ia2 d1onfa1 d1onfa2 d3lada2 d3lada1 d1w4xa1
d1w4xa2 d1gesa2 d1mo9a1 d1xdia1 d1fl2a2 d1fl2a1 d1fcda1 d1fcda2
d1h6va2 d1h6va1 d1mo9a2 d1gv4a2 d1gv4a1 d2gv8a2 d2gv8a1 d1aoga1
d1aoga2 d1hyua1 d1ebda1 d1ebda2 d3dk9a1 d1q1ra2 d1q1ra1 d1gesa1
d3syxa1 d2p8va_ d1xoda1 d1i7aa_ d1evha_ d1qc6a_ d1egxa_ d1ps9a2
d1o94a2 d1lqta1 d1cjca1 d1reoa1 d1ju2a1 d2f5va1 d3c96a1 d2gmha1
d1pj5a2 d1gpea1 d1o5wa1 d1n4wa1 d1k0ia1 d2dw4a2 d2ivda1 d1seza1
d2voua1 d1kdga1 d1s5ja1 d2hbja2 d2igia_ d1wlja_ d1qhta1 d4dlea1
d1tgoa1 d2f96a1 d3tr8a1 d3mxma_ d1q8ia1 d1y97a1 d1w0ha_ d1kfsa1
d2py5a1 d1noya_ d1d5aa1 d1yt3a3 d2guia_ d3mwpa1 d4kb1a_ d1iuka_
d2csua1 d3q9ub_ d2yv1a1 d3q9na_ d2d59a_ d1oi7a1 d2yv2a1 d1y81a1
d2av4a1 d3nvva1 d1krha3 d2h88b1 d1n62a2 d1t3qa2 d1kf6b2 d1zoyb1
d1jq4a_ d1ffva2 d1dgja2 d1rm6c2 d1k8ka2 d1k8ka1 d3uleb1 d3i33a2
d3i33a1 d1bupa1 d1dkgd1 d4ci6a2 d1jcfa1 d4a2ba2 d2fxua1 d1yaga2
d1yaga1 d4cbua2 d1bupa2 d1r7ha_ d1faaa_ d1wika1 d1xfla_ d1ttza1
d2f51a1 d1ti3a_ d1nhoa_ d1sena_ d2i4aa_ d1ep7a_ d1zmaa1 d1fo5a_
d3lefa1 d3diea1 d2yana1 d1z6na1 d1oaza_ d3zywa1 d4aj8a_ d1nm3a1
d4fiwa_ d1wmja_ d4hs1a1 d1iloa_ d1xwaa1 d1wjka1 d2vm1a_ d1fb6a_
d1gh2a_ d1h75a_ d1nw2a_ d1kx5b_ d3a6na_ d4m6ba2 d2nqbd_ d3wkjd_
d2yfwe_ d4h9na_ d5bnxa_ d1kx5c_ d3r45a_ d1eqzb_ d4jjnb_ d1f66c_
d4wnnb_ d1kx5a_ d1u35c1 d1id3a_ d2nqbc_ d1kx5d_ d3us3a3 d3us3a1
d1um8a_ d1qvra2 d1qvra3 d1qzma_ d1sxjc2 d1jr3d2 d2p65a_ d1l8qa2
d1g8pa_ d3hu3a3 d1sxjd2 d1njfa_ d1in4a2 d1sxja2 d1tuea1 d2ce7a2
d1w5sa2 d1sxjb2 d1d2na_ d1ny5a2 d1ofha_ d2gnoa2 d2a5yb3 d1fnna2
d1u0ja1 d1lv7a_ d1iqpa2 d2fnaa2 d1ixsb2 d4gita_ d1sxje2 d1g41a_
d1pd212 d1nhya2 d2a2ra2 d1k0dd2 d1f2ea2 d1gsua2 d1f3ba2 d2fhea2
d2pvqa1 d1tw9a2 d1oyja2 d1e6ba2 d3ik7a1 d4is0a1 d1r5aa2 d1jlva2
d1m0ua2 d3gtub2 d1z9ha2 d1okta2 d1axda2 d2gsra2 d1tu7a2 d1k3ya2
d1zgma1 d1b48a2 d1gnwa2 d1gwca2 d2gsta2 d1glqa2 d1oe8a2 d1pn9a2

d1v2aa2 d1fw1a2 d1duga2 d1g7ea_ d1ig8a1 d1czan2 d1czan3 d1czan1
d1ig8a2 d1czan4 d1bg3a4 d1bg3a3 d1bg3a2 d1bg3a1 d3wyha_ d4g9sa1
d3gxra_ d1qqya_ d1gd6a_ d1f6ra_ d1ivma_ d2gv0a_ d1iiza_ d2z2fa_
d1j8xa_ d1ghla_ d1b9oa_ d174la_ d169la_ d2o4wa_ d1p37a_ d1k28a3
d1xjta1 d1jtma_ d2f2qa_ d4e97a1 d1iyka2 d1rxta1 d1iica2 d1iica1
d2pjsa1 d1klla_ d2i7ra1 d1ecsa_ d1xqaa1 d5cj3a1 d1jifa_ d2p7oa_
d1r9ca_ d1nkia_ d1mpya2 d1kw3b2 d1kw3b1 d4ghga1 d1tfza1 d3isqa1
d3isqa2 d1sqia1 d1sqia2 d1lkda2 d1lkda1 d1mpya1 d1cjxa1 d1sp9b2
d1t47a1 d1t47a2 d1f1ua2 d1sp8a1 d1sp8a2 d1f1ua1 d4iuaa3 d4bvva_
d5hpga_ d3k65a_ d1ki0a3 d1ki0a2 d1ki0a1 d1a0ha1 d1pmla_ d3sp8a3
d2feba1 d1qsta_ d2beia1 d4m3sa_ d1u6ma1 d1ygha_ d1yk3a1 d3dr6a_
d1xmta_ d1z4ea1 d2ge3a1 d1bo4a_ d2fiaa1 d2jdca1 d1y9wa1 d3to7a_
d1wwza1 d2fl4a1 d2ozua1 d2b5ga_ d2cy2a1 d2p0wa_ d1cm0a_ d4pswa_
d1yrea1 d1yr0a1 d1m4ia_ d2gana1 d1yvka1 d2g3aa1 d1y7ra1 d2euia1
d1q2ya_ d2fsra1 d1xeba_ d2ae6a1 d1cjwa_ d1qsma_ d2aj6a1 d1y9ka1
d1i12a_ d1vkca_ d2atra1 d1n71a_ d2d4pa1 d2fiwa1 d2fe7a1 d1tiqa1
d2ou2a_ d1yx0a1 d2fcka1 d1p0ha_ d2j8ma_ d2pq8a_ d1z4ra1 d1s3za1
d1ufha_ d2hqya1 d2hqya2 d1lrza2 d1ne9a1 d1lrza3 d1xvqa1 d1pn0a2
d3hy2a_ d2cvba1 d1o8xa_ d1lu4a_ d1we0a1 d2bmxa1 d2a4va1 d1psqa_
d1xiya1 d1q98a_ d1n8ja_ d1zofa1 d2ggta_ d3tjja_ d4je1a1 d1o73a_
d3s9fa_ d2zcta_ d1qmva_ d1jfua_ d2b1ka_ d5dvba1 d2b5xa1 d2v2ga_
d1st9a_ d3tb2a_ d4llra_ d4fh8a1 d1e2ya_ d2h30a_ d1i5ga_ d2z9sa_
d2f8aa1 d1tp9a1 d2b7ka_ d1nm3a2 d3hvva_ d2wfca1 d4kcea_ d1xvwa1
d4gqca1 d3qpma_ d4k1fa_ d1zzoa1 d1gp1a_ d1prxa_ d4od7a1 d1z6ma1
d4mcua1 d4dvca1 d1r4wa_ d3l9sa_ d1un2a_ d3rppa_ d1zsqa1 d2m5ta_
d1cqqa_ d1wqsa_ d5bpea_ d2ipha_ d1lvoa_ d2bhga1 d3tnta_ d3sjia_
d3d23a1 d2fyqa_ d3tloa_ d3zvea1 d2hrva_ d3q3ya1 d4asha1 d1e88a1
d1l6ja5 d1eaka4 d1eaka3 d1l6ja3 d1h8pa2 d1h8pa1 d1jgta2 d1ea0a3
d1ecfa2 d1q15a2 d1ct9a2 d1ao0a2 d1te5a_ d1ys1x_ d4hs9a_ d1ispa_
d1ex9a_ d1ku0a_ d3qmma_ d1lgya_ d4l3wa_ d1llfa_ d1gz7a_ d1ukca_
d1ktja_ d3groa_ d1pjaa_ d1thta_ d2ftba_ d1pmpa_ d2rcqa1 d4lkpa_
d1sa8a_ d1vyfa_ d1lpja_ d3vg7a1 d4azqa1 d1o8va_ d5bvqa_ d1p6pa_
d2qo4a_ d1opba_ d1yiva1 d1o1va_ d4bvma1 d4tkba_ d4i3ba_ d1bwya_
d1ftpa_ d1ggla_ d4gkca_ d1cbia_ d3p6da1 d1kqwa_ d2a0aa1 d3zxha_
d1rm8a_ d1eaka2 d1hy7a_ d2jsda_ d1cxva_ d1hv5a_ d1i76a_ d3p0ya1
d1igra1 d1igra2 d1n8yc2 d1n8yc1 d1m6ba2 d1m6ba1 d4dv8a2 d1dzka_
d4tlja_ d2a2ua_ d4wfva_ d3t1da_ d4iaxa_ d3zq3a_ d2nnda_ d1i4ua_
d1kxoa_ d1bbpa_ d1kt7a_ d1exsa_ d3s26a1 d1lf7a_ d1qfta_ d4gh7a_
d1z24a1 d1qabe_ d1xkia_ d1ew3a_ d3bx7a_ d4iawa1 d1n0sa_ d3kzaa_
d2k23a_ d2l9ca1 d2joza1 d1gm6a_ d3kffa_ d3saoa1 d1e5pa_ d1yupa1
d1iiua_ d2tbvc_ d1x36a_ d1smvc_ d1ng0c_ d4sbvc_ d1opoa_ d1f2nc_
d1cixa_ d1y29a_ d1v7fa_ d2jtba_ d1d1ha_ d1g9pa_ d1lupa_ d1qk7a_
d1mb6a_ d2a2va_ d1niya_ d1i25a_ d1hvwa_ d2mpqa_ d1ie6a_ d1la4a_
d2mxoa1 d1dl0a_ d1koza_ d1qk6a_ d1emxa_ d1lqra_ d1c6wa_ d1rypd_

d1irua_ d1rypc_ d3unfk_ d1rypa_ d1j2pa_ d1irui_ d1j2qh_ d3unfh_
d1m4ya_ d1rypb_ d1irul_ d1irug_ d1iru2_ d1q5rh_ d1rypg_ d1g3ka_
d1iruf_ d1q5qa_ d1e94a_ d1irub_ d1ryph_ d3ty6a_ d1iruj_ d1iruk_
d1rype_ d1irud_ d1irue_ d3mi01_ d1rypf_ d1iruh_ d2z3ba_ d1iruc_
d3mka2_ d1iru1_ g1gk9.1 g1cp9.1 g1e3a.1 d1mqta_ d1d4m3_ d1fmd3_
d2mev1_ g1aym.1 d1ev13_ d4q4w1_ d1r1a3_ d2x5ia_ d1rhi1_ g1r1a.1
g1d4m.1 g1rhi.1 d1b35b_ d1oopa_ d1tme1_ d4iv1a_ g1pvc.1 g1fpn.1
d1fpn1_ d4gb31_ d1d4m1_ d1fmd1_ d1ev11_ d2wzr3_ g1cov.1 d1qqp3_
g1fmd.1 d2x5ic_ d1rhi3_ g1oop.1 d1hxs1_ g1hxs.1 d1fpn3_ d1bev1_
d1eah3_ d1aym1_ d1pvc3_ d1qqp1_ d1zba3_ d1h8ta_ d3vbhc_ g1b35.1
g1bev.1 d1hxs3_ g1ev1.1 d1bev3_ g1tmf.1 d1tmf1_ d1b35a_ d2mev3_
g1eah.1 g1h8t.1 d1eah1_ d4q4w3_ d1r1a1_ d1aym3_ d1pvc1_ d1zba1_
d1h8tc_ d1oopc_ d1tme3_ g1qqp.1 d4iv1c_ g1tme.1 d1p8ba_ d1eixa_
d3exra_ d2ffca1 d2fdsa1 d3n3ma1 d2czda_ d1q6oa_ d2yyua_ d1vqta1
d1dbta_ d3ldva1 d1fvia1 d1ckma1 d1p16a1 d1x9na2 d2r80b_ d1cqxa1
d1cg5a_ d1gcvb_ d3boma_ d2h8fb_ d2raoa_ d3bj1a_ d4hrrb_ d2r50a_
d3d1kb_ d2aa1b1 d3zhwa_ d3hf4b_ d3gdja_ d1jebb_ d3bj1b_ d1x9fb_
d1wmub_ d1gcva_ d3bcqb_ d3d4xb_ d2dc3a_ d3hf4a_ d4yu3a_ d3k8ba_
d1qpwb_ d2r80a_ d3bcqa_ d1v4wa_ d3wtgb_ d1jl7a_ d4esaa_ d1a9we_
d3d1ab_ d1fsla_ d4h2la_ d1v4wb_ d1mwca_ d1vhba_ d1jeba_ d1spgb_
d1h97a_ d1q1fa_ d1tu9a1 d3d1aa_ d1hdsb_ d3hyub_ d2zfbb_ d3fs4a_
d1oj6a_ d1shrb_ d4h2lb_ d4yu3b_ d1spga_ d3hrwa_ d3qqra_ d3at5a_
d4wchd_ d3hyua_ d1cg5b_ d1uc3a_ d1hbra_ d2raob_ d1outb_ d4esab_
d3gdjb_ d3qqqa_ d1x9fc_ d3vrfa_ d1itha_ d3mkba_ d3d4xa_ d3mjpa_
d1jl6a_ d3fh9a_ d1outa_ d2dc3b_ d1it2a_ d3eoka_ d3g46a_ d1qpwa_
d3vrfb_ d1ch4a_ d1x9fd_ d1hdsa_ d2zfba_ d3mkbb_ d1or4a_ d3wtga_
d1a4fa_ d3fh9b_ d3bomb_ d3cy5a_ d3at5b_ d1x9fa_ d2oifa_ d4lmxb_
d1cpcb_ d3v57a_ d1kn1a_ d1cpca_ d4f0ta_ d2vjha_ d4po5b_ d1eyxa_
d1alla_ d4f0tb_ d1b33b_ d1b8db_ d4h0mb_ d2vjhb_ d4lm6b_ d2vmlb_
d1allb_ d2vjta_ d3dbja_ d2vmla_ d2vjtb_ d4f0ub_ d1f99b_ d4rmpa1
d1liaa_ d1eyxb_ d2uulb_ d4lmsb_ d4f0ua_ d2bv8a_ d1f99a_ d2bv8b_
d2c7lb_ d3dbjb_ d4rmpb1 d1b8da_ d4h0ma_ d3v57b_ d1kn1b_ d1liab_
d2uula_ d2j96a_ d3ia2a_ d1a88a_ d4dgqa1 d1hkha_ d1zoia_ g1gxs.1
d2xqfa_ d1ea5a_ d1f6wa_ d1dx4a_ d1qe3a_ d1k4ya_ d4qwwa_ d2ha2a_
d1r88a_ d1sfra_ d4qeka_ d3c8da2 d2pbla1 d1jkma_ d1jjia_ d1lzla_
d1jjfa_ d1jmxa4 d1jmxa3 d1pbya4 d1pv8a_ d1w5qa_ d2c1ha_ d1ohla_
d1l6sa_ d1f74a_ d3l21a_ d2e1da_ d1to3a1 d1w3ia_ d1sfla_ d1n7ka_
d1vcva1 d4imea_ d1wa3a_ d2ocza1 d1f2ja_ d3m16a_ d2yxga_ d1ub3a_
d3a5fa_ d1fbaa_ d1l6wa_ d1xfba1 d1wx0a1 d1vpxa1 d1ojxa_ d1mxsa_
d2a4aa1 d1xkya1 d3cq0a1 d3s1xa_ d1f05a_ d1fdja_ d3cpra1 d4d2ja_
d1cg2a1 d1yloa2 d1y7ea2 d1y0ya3 d1lfwa1 d1vixa1 d1fnoa4 d1vhoa2
d1ysja1 d1vhea2 d1z2la1 d2fvga2 d1r3na1 d2grea2 d1xmba1 g1q7l.1
d1rtqa_ d3rp2a_ g1ucy.1 d1fuja_ d2zchp_ d3w94a_ d1si5h_ d1lo6a_
g2pka.1 d1cgha_ d1kigh_ d1a5ia_ d3s9ba_ d1hj8a_ d1ao5a_ d2anya_

d1q3xa1 d1gvza_ d1azza_ d4gsoa_ d2f91a1 d1iaua_ d1gdna_ d1eufa_
g1fiw.1 d1eq9a_ d4nfea_ d2rdla_ d1spja_ d1elva1 d1a7sa_ d1ekbb_
d1pfxc_ d1yc0a_ d2aiqa_ g1fiz.1 d1fi8a_ d4k60a_ d4dgja_ d1mzaa_
d1op0a_ d3tjva_ d1pytd_ d1sgfg_ g1gg6.1 d2eeka_ g1rtf.1 d1a0ja_
d3g01a_ d1pytc_ d4e7na_ d1ddja_ d1z8ga1 d2hlca_ d1npma_ d1brup_
g1jou.1 d1sgfa_ d1autc_ g2hnt.1 g1kdq.1 d1orfa_ d3s69a_ d4crga_
d1fxya_ d1bqya_ d1ra6a_ d3gsza_ d1khva_ d1te9a_ d2xwha_ d3uqsa_
d3hkwa_ d3n6ma_ d2cjqa_ d2xyma_ d1teba_ d1te8a_ d1s48a_ d1u09a1
d4wfxa_ d3tyqa_ d2pgga1 d1m4la_ d2bk7a_ d3fjua_ d1z5ra1 d1kwma1
d1uwya2 d2c1ca1 d1dtda_ d1h8la2 d3lgia_ d3i77a_ d2z9ia2 d4nsva_
d1lcya2 d4jcna_ d1agja_ d1os8a_ d1hpga_ d1p3ca_ d1l1ja_ d1e69a_
d1mv5a_ d3g60a4 g1ii8.1 d1ewqa2 d1ji0a_ d1qhla_ d1v43a3 d2wmfa1
d1vpla_ d1g2912 d4a82a2 g1f2t.1 d2d62a1 d3tuic1 d2bbsa1 d3g60a3
d2ixea_ d1g6ha_ d1sgwa1 d2qi9c_ d3rlfa1 d3b60a1 d3d31a2 d2pzea1
g1xew.1 d1jj7a_ d1oxxk2 d2onka1 d1w1wa_ d1iwea_ d1qf5a_ d1vmaa2
d1nija1 d1np6a_ d2p67a1 d3ihla_ d1okkd2 d1ls1a2 d1s1ma2 d1iona_
d2v40a_ d3hida_ d2vo1a1 d2g0ta1 d1vcoa2 d1j8mf2 d1g3qa_ d1xjca_
d1p9ba_ d1qzxa3 d1ihua2 d1ihua1 d2qm8a_ d1dj2a_ d4ioja_ d1cp2a_
d1yrba2 d1hyqa_ d3gqba2 d1skye3 d1xp8a1 d1tf7a2 d1tf7a1 d1ye8a1
d1skyb3 d2qe7d2 d4blta_ d4huta_ d3ldaa2 d4hyya_ d2i3ba1 d1cr1a_
d3j9tb2 d3ntua2 d1vdza1 d2z43a_ d1e9ra_ d1nlfa_ d3ziaa2 d1n0wa_
d1g6oa_ d3ziad2 d3j9ta2 d1pzna2 d3gqbb2 d2i49a_ d1nnfa_ d1xvxa_
d1ursa_ d1h3da1 d1iuda_ d4m1va1 d1twya_ d3e13x_ d2nxoa1 d4pfua_
d3ttma_ d1elja_ d2qmwa1 d1us5a_ d1y3na1 d1jeta_ d1uqwa_ d4f19a1
d2esna2 d3thia_ d3fasa1 d2uyeb_ d2xwva1 d1r9la_ d2fyia1 d2ozza1
d1sw5a_ d1nh8a1 d1vr5a1 d2g29a_ d1xs5a_ d3lswa_ d2z23a_ d3w9va_
d3tcha_ d1q35a_ d1z7me1 d3q27a_ d1p99a_ d1ve4a1 d2i6ea1 d4mcoa1
d4n8ya_ d1eu8a_ d1zbma1 d1hsla_ d1ixca2 d1j1na_ d1xvya_ d1y9ua_
d2z22a1 d1xoca1 d4oera_ d1wdna_ d5f1qa1 d1xt8a1 d1i6aa_ d4gwoa_
d4igta1 d1pc3a_ d4mmpa1 d1o63a_ d2czla1 d2onsa2 d4mija1 d1eh3a_
d3skpa_ d1dtza1 d1dtza2 d1ieja_ d1jnfa2 d1ce2a1 d1jnfa1 d1b1xa2
d1b1xa1 d1jw1a1 d1jw1a2 d1ryoa_ d1h76a1 d1h76a2 d1etha2 d1w52x1
d2ppla1 d1lpbb2 d2oxea1 d1bu8a2 d1rp1a2 d1c4oa1 d2p6ra3 d2p6ra4
d1oywa2 d1oywa3 d1wrba1 d1w36b2 d1w36b1 d1c4oa2 d3bora_ d1t6na_
d2eyqa5 d2eyqa3 d2eyqa2 d2zu6a2 d1veca_ d1uaaa1 d1w36d1 d2g9na1
d1z3ix2 d2rb4a_ d1hv8a1 d1hv8a2 d1t5ia_ d1w36c2 d2j0sa2 d2j0sa1
d3hjha1 d1z5za1 d1qdea_ d1z63a1 d1s2ma2 d1s2ma1 d1wp9a1 d1wp9a2
d1gm5a3 d1gm5a4 d3dina4 d3dina3 d1q0ua_ d1w36c1 d1z3ix1 d1nkta3
d1nkta4 d1uaaa2 d1w36d2 g1qhh.1 d2fwra2 d2fwra1 d3q3ma_ d1za0a1
d1mxra_ d2inca_ d1jk0a_ d2incb_ d1mhyb_ d3hf1b_ d1mtyb_ d1r2fa_
d2uw1a_ d1mhyd_ d3n37a_ d1afra_ d1syya_ d1mtyd_ d2o1za_ d1uzra_
d1smsa_ d2cbia2 d1gqia1 d2choa2 d1oewa_ d2qzxa_ d2asia_ d1psoe_
d1miqa_ d1smra_ d2h6ta_ d1pfza_ d1izda_ d3hd8a_ d1j71a_ d1dpja_
d4aa9a_ d1t6ex_ g1b5f.1 g1lya.1 g1htr.1 d1qdma2 d1bxoa_ d1wkra_

d1tzsa_ d1ibqa_ d2qzwa_ d1ex4a2 d1c6va_ d2v76a_ d1wj1a1 d1aqca_
d1qqga1 d1ntva_ d1n3ha_ d4dbba_ d3f0wa1 d1p3ra_ d1wgua1 d2cy4a1
d3hqca_ d1wvha1 d1xr0b1 d1bdqa_ d3ggta_ d3ixoa_ d1mrxa_ d2haha_
d1tw7a_ d4njsa_ d1sgua_ d2p3da_ d1nsoa_ d3d3ta_ d2rkfa_ d1c6ya_
d2rspa_ d3ogpa_ d3ekwa_ d2o4na_ d2j9ja_ d1baia_ d3ggua_ d3lzua_
d4npua_ d4j55a_ d2r8na_ d2fxda_ d2p3ca_ d4dqea_ d3ttpa_ d3t3ca_
d2p3aa_ d2hc0a_ d2fdda_ d3mwsa_ d1wgra1 d1ef5a_ d1wfya1 d1e8ya3
d1wgya1 d1lfda_ d2cs4a1 d1wxma1 d2byea1 d3kh0a1 d2byfa1 d1wxaa1
d1e7ua3 d1h4ra3 d4yl8a1 d2al6a3 d1wgda1 d1wgga1 d4q5eb_ d1wjna1
d1se9a_ d3n3kb_ d1wz0a1 d1we6a1 d2m0xa_ d2gbka_ d3rt3b1 d1wh3a1
d1sifa_ d2ekec2 d4ii2b1 d4hk2a1 d1c3ta_ d1m94a_ d1wgha1 d1ttna1
d1j8ca_ d1wx8a1 d3v6cb1 d2ma9b_ d4i6lb1 d1bt0a_ d2jvca1 d1iyfa_
d2k1fa_ d1wxva1 d1wf9a1 d1t0ya_ d2io0b2 d1x1ma1 d2gbma_ d1zw7a1
d2m17a_ d1ud7a_ d1we7a1 d2lwpa_ d2gbja_ d1wjua1 d1v6ea1 d1v86a1
d4eewa_ d1wiaa1 d4icva_ d4gu2a_ d1wx7a1 d1wy8a1 d4hjka1 d2faza1
d1v5oa1 d2fkza_ d1zuja1 d1s3qa1 d2fjca1 d1z6om1 d1veia_ d4dyya_
d1jiga_ d3gvya_ d2pyba_ d4cy9a1 d3bvfa1 d1r03a_ d2cf7a_ d1nf4a_
d1zs3a1 d4iwka_ d3ka8a_ d3np0x_ d1n1qa_ d1lb3a_ d4e6ka_ d1z6oa1
d2fzfa1 d4dyua_ d3ge4a_ d1jgca_ d4am4a_ d2z6ma_ d1ji5a_ d1vlga_
d1lkoa1 d1krqa_ d3qz3a_ d1o9ra_ d2ffxj_ d1yv1a1 d2v2pa_ d1j30a_
d4di0a_ d1dpsa_ d2al3a1 d2cr5a1 d1wj4a1 d1s3sg_ d1tafa_ d1bh9b_
d1tafb_ d1jfib_ d1h3ob_ d2byka1 d1n1ja_ d4g92b_ d1bh9a_ d1jfia_
d1h3oa_ d2bykb1 d1n1jb_ d4lu0a_ d3unda1 d3e9aa1 d1vr6a2 d1n8fa_
d2qkfa_ d1d9ea_ d1o60a_ d3h33a_ d1rwja_ d19hca_ d1os6a_ d3bxua_
d3h4na_ d1i77a_ d3caoa_ d1j0pa_ d2ctha_ d1duwa_ d1ofwa_ d2e84a_
d1hh5a_ d3h34a_ d2bcgg1 d2bcgg2 d1wioa4 d1ccza2 d1vcaa1 d1dr9a2
d1ic1a1 d2d9qb1 d2nxyb2 d2dara2 d2dara1 d1wyha2 d2dloa2 d1rutx4
d1rutx3 d2cuqa2 d1x4la2 d1x4la1 d1x3ha2 d1x3ha1 d2d8za1 d1x61a2
d1x6aa1 d1x6aa2 d2cora2 d2cora1 d1v6ga1 d1v6ga2 d2d8za2 d1x64a1
d1x64a2 d1b8ta1 d2co8a1 d1x63a2 d2dj7a2 d2dj7a1 d2cu8a2 d2cu8a1
d1g47a2 d1g47a1 d1j2oa1 d1wyha1 d2dloa1 d1wiga1 d1wiga2 d2cupa2
d2cupa3 d1x68a1 d2d8ya2 d2d8ya1 d1m3va2 d1x62a1 d1x68a2 d1x61a1
d1b8ta3 d1b8ta2 d2d8xa2 d2d8xa1 d1x4ka1 d1x4ka2 d2cuqa1 d1m3va1
d2co8a2 d1j2oa2 d1x63a1 d2cura1 d2cura2 d1x62a2 d1svpa_ d1mbma_
d3su6a1 d4agka_ d1vcpa_ d1q31a_ g1n1l.1 d3e90b_ d1ep5a_ d3u1jb_
d2e3fa_ d1mm0a_ d2e3ea_ d2llda_ d2ny8x_ d2e3ga_ d2ny9x_ d2nz3a_
d2lj7a_ d1n4na_ d2m8bb1 d2kyqa_ d3psma_ d1jxca_ d2gl1a_ d1ugla_
d4uj0a_ d2k4ua_ d1hp2a_ d2lu9a_ d1baha_ d2meoa1 d1wm7a_ d1hlya_
d1n8ma_ d1jlza_ d1wpda_ d1c55a_ d1pvza_ d1wt7a_ d1wz5a_ d1m2sa_
d1pjva_ d1c49a_ d1qkya_ d2ktca1 d1wmta_ d1du9a_ d1j5ja_ d1r1ga_
d1t0za_ d2i61a_ d1t1ta_ d1sn4a_ d1kv0a_ d2lkba_ d1npia_ d1wwna_
d1pe4a_ d2kbka_ d2li7a_ d1djta_ d2kbha_ d1dq7a_ d1sega_ d1omya_
d1zywa1 d1jzaa_ d1n9wa1 d1bbua1 d1e1oa1 d1c0aa1 d1b8aa1 d1l0wa1
d3a74a1 d1eova1 d1iyjb4 d1iyjb5 d1iyjb3 d1o7ia_ d4ipca1 d1x3ea_

d4apva_ d1jb7a1 d1jb7a2 d1jb7a3 d3kdfa_ d1ue1b_ d1wjja1 d3eiva_
d1xjva2 d1xjva1 d2ccza2 d1se8a_ d1l1oc_ d1jb7b_ d1qvca_ d3ulla_
d1qzga_ d1miua4 d1miua5 d1miua3 d1y14b1 d2nn6i1 d1i94l_ d2zjra2
d1smxa_ d2qamc2 d1hh2p1 d2r7da1 d1rl2a2 d1wfqa1 d3bzka4 d1x6oa2
d3a0ja_ d2j01d2 d2ja9a1 d4ql5a_ d1hr0w_ d1luza_ d2n3sa_ d2vnud1
d1h95a_ d1go3e1 d2nn6h1 d1ne3a_ d2mo1a_ d1iz6a2 d1jjga_ d1e3pa2
d2ix0a3 d2ix0a2 d2lxja_ d1ueba3 d1ueba2 d2z0sa1 d1u0la1 d2ix0a1
d2eifa2 d2je6i1 d3i2za1 d2c35b1 d2ahob2 d1t9ha1 d3i4oa_ d2qall1
d1jt8a_ d2nn6g1 d1g6pa_ d3cama_ d1i94q_ d1c9oa_ d1d7qa_ d2ba0a1
d2vnud2 d2vnud3 d1khia2 d2qalq1 d1wi5a1 d1xtda2 d1ny4a_ d1prtc2
d1rpma_ d2shpa1 d1l8ka_ d2ooqa1 d1g4us2 d1p15a_ d1lara2 d1lara1
d1wcha_ d2fh7a2 d2fh7a1 d3azya_ d3wvja_ d1upsa1 d1ajoa_ d2hyka_
d1ajka_ d2vy0a_ d1o4ya_ d3o5sa_ d3d6ea_ d3atga_ d1dypa_ d1umza_
d3i4ia_ d1c1la_ d4lbqa_ d1a78a_ d1slta_ d4no4a_ d3zsja_ d3duia_
d1is3a_ d1qmja_ d1hlca_ d1tdha3 d1q3ba3 d1ee8a3 d1k82a3 g1qmo.1
g1len.1 d3zyra_ d1fnya_ d1n47a_ g2ltn.1 d1avba_ d3usua_ d1dhkb_
d1f9ka_ d1ukga_ d1g8wa_ d1g9fa_ d1wbfa_ d3wcsa_ d1g7ya_ g1loe.1
d1mvqa_ d1hqla_ d1v6ia_ d1dbna_ d1qnwa_ d1fx5a_ d4l8qa_ d1ioaa_
d3n35a_ d3ubra_ d1qo8a1 d1d4ca1 d2k3va_ d1ft5a_ d1qdba_ d1m1qa_
d2j7aa_ d1ogyb_ d4fasa_ d1jnia_ d1oaha_ d1sp3a_ d2h0ba_ d2erfa_
d1h30a2 d3mw4a1 d1pz7a_ d2r1ba_ d3v64a1 d1d2sa_ d3bopa1 d3mw3a1
d1okqa1 d1okqa2 d1dszb_ d3cbba_ d1ynwa1 d1r4ia_ d1hcqa_ d2hana_
d2hanb_ d2enva1 d1kb4b_ d2nllb_ d1lo1a_ d1cita_ d1adqa1 d1fnga1
d1uvqb1 d1zs8a1 d2vola1 d2vola2 d1mfbl2 d2jelh2 d1uwel2 d3qq4b1
d1kjvb_ d1k5na1 d1osph2 d4odhl2 d1ow0a1 d1ow0a2 d1bfob2 d3l9ra2
d3jtth1 d2fath2 d4elkb2 d1fn4b2 d2xfxb_ d1pg7x2 d1cqka_ d4k71b2
d1lk2a1 d1eapb2 d4lfhg2 d1i4fa1 d2nw2a2 d1kfah2 d1mhca1 d1h3ua1
d1wejh2 d1h0db2 d1a5fh2 d1kcvh2 d1mjuh2 d1qqda1 d1kcrh2 d1k8ia1
d3huja2 d2nnab1 d1nfdf2 d3mffb2 d1s9va1 d1lk3h2 d1q0xl2 d1fv1b1
d1neza1 d4rfeb2 d1r3ha1 d1a6tb2 d1kjva1 d1pz5b2 d1onqa1 d2bc4a2
d1de4a1 d1k8da1 d1tcra2 d1dn0b2 d1nfde2 d2xwtb2 d1bfoa2 d1mexh2
d1iakb1 d1ypzf2 d4f7ea2 d1q72l2 d2fbjh2 d1fe8h2 d1clzh2 d1fngb1
d1uvqa1 d1vgka1 d1c16a1 d4j12a2 d4x99b2 d1igtb3 d1je6a1 d1q72h2
d1nldh2 d1teth2 d2hmic2 d1i1ca2 d1i1ca1 d1k8ib1 d1q0yh2 d1kfal2
d3frua1 d1pg7w2 d1u58a1 d1igyb4 d1igyb3 d1hyrc1 d1klub1 d1deeb2
d1c12b2 d2bc4b2 d1kcvl2 d3wn5b1 d1lk2b_ d1lk3l2 d1mjul2 d1sm3h2
d1q0xh2 d1zvsa1 d1ydpa1 d1u8ib2 d1ypze2 d1kcuh2 d1op9a_ d1y0la1
d1etzb1 d1egjl1 d2gsib1 d1nj9a1 d4jfhd1 d1aqkl1 d1t2ja_ d1lo4h1
d1zvya1 d3v4vl1 d4enec1 d1kxtb_ d5e8ea1 d2pxya_ d4weud_ d1xiwc_
d2esvd1 d1qnzh_ d1ogae1 d1f4xh1 d4n1hb_ d3ezjb1 d3mlwl1 d1kfah1
d1etza1 d1b4jh1 d3cfic_ d4w68a_ d2arja1 d1ek3a_ d2ok0l1 d1vera_
d1uwgh1 d1blnb1 d1fpth1 d1rzga1 d2giya1 d4m3kb1 d1nc2a1 d2nyyd1
d1ncna_ d1fbih1 d5dxwa_ d4fqqa1 d1dqdh1 d1jfql1 d1kelh1 d1faih1
d1frgl1 d1figl1 d4m6nl1 d1fo0a_ d4hxbl1 d3b5ga_ d3quyd1 d2h9gb1

d1i8kb_ d2dd8h1 d2ck0h1 d1eaja_ d4w6wb1 d2a6ib3 d1t66d1 d2e27h_
d1ob1b1 d1f11b1 d2v7na1 d1ad0a1 d2xt1b1 d1pskh1 d4i13b1 d2op4l1
d1a0ql1 d1uwel1 d1za3a1 d1ncbl1 d4uu9a_ d1bm3h1 d1cloh1 d1nqba2
d1cf8h1 d4y5va_ d1mhph1 d5anma1 d4cdgc1 d4ldeb_ d1y0lb1 d2pcpb1
d1z3gl1 d1dfbl1 d1fh5h1 d3g5xa1 d1tzib1 d4dn3l1 d3zhka1 d2b1hh1
d2uyla1 d1f3dh1 d1nj9b1 d2agjh1 d2h1pl1 d1q72h1 d8fabb1 d1ndmb1
d1rjca1 d4u6hb1 d3upca_ d2fbjl1 d1osph1 d1tvda_ d2cjvh_ d4fhbd1
d1bd2d1 d1rz7h1 d3nn8a_ d1knoa1 d1nfda1 d2bmkb1 d1dqll_ d1igyb1
d1igfl1 d12e8h1 d3utza1 d1nc2b1 d1rihh1 d4mwfb1 d4jvpa1 d1mieh1
d1rhfa1 d1fgnl1 d1sbsh1 d1lp9e1 d1dn0a1 d4lqfl1 d1iaih1 d1kgcd1
d1mh5b1 d1gpol1 d2g60h3 d1f8tl1 d2jixd1 d4grwh_ d1fo0b_ d1bfob1
d1axsa1 d1u9ka_ d1nmbh_ d1h8na1 d1h8na2 d1jguh1 d4eura1 d3sdyl1
d35c8h1 d1w72l1 d1mvub_ d1i9rh1 d1ob1a1 d4wenb_ d1yeeh1 d3ietc1
d1f58h1 d1ad0b1 d1mqkl_ d1nbvh1 d4b50a_ d3r8bb_ d1bafl1 d1mamh1
d4rgne1 d2gsgb_ d35c8l1 d1bafh1 d1ai1h1 d2pcpa1 d2fath1 d2x1pa_
d1orsb1 d1ibgl1 d1shma_ d1a2yb_ d1u0qa_ d2coqa_ d1hilb1 d1qfwi_
d1jv5b_ d1fn4b1 d5e0qa_ d1nsnh1 d1plgl1 d4ocxl1 d1kb5h1 d1rzfh1
d1pg7x1 d2fx7h1 d2oi9c_ d1l7il1 d3eyva1 d1d5il1 d4ffyl_ d1tetl1
d1a6wh_ d1ac6a_ d3dvfa_ d1nfde1 d1mimh1 d1ri8a1 d1jhlh_ d3dura_
d1fskb1 d1f97a1 d3so3b1 d2ak4d1 d1lp9f1 d4z7wf1 d1l7th1 d4y5ya_
d4x7ec1 d1emth1 d1ikfl1 d1nlbh1 d4utal1 d1bjma1 d1eapb1 d1u6ah1
d2dtgd1 d1kj2b_ d1xeda1 d1c1eh1 d1fgvh_ d2bnub1 d2fb4h1 d2dtgb1
d1ktrh_ d1jpth1 d1qkzl1 d4hepg_ d2atpb1 d1flrh1 d2hrpl1 d1mvfa_
d1iaim1 d3k80a1 d2jb5h1 d3zkxc_ d4grwe_ d1it9h1 d1rhhb1 d3w9ec_
d1e4wh1 d1sy6h1 d2j6eh1 d3qxta_ d1jglh1 d1p7ka1 d1tzhb1 d4n8pa1
d1gafl1 d2jelh1 d1wioa2 d4i0cc1 d1cicb1 d1lmka2 d1lmka1 d1iqwh1
d1orqb1 d1ejoh1 d1jnlh1 d4zg1a_ d43c9b_ d1ggbl1 d1wejh1 d3q6fa1
d1f3dj1 d3c6la1 d1bqlh1 d3p9wb_ d1a3rl1 d1h0db1 d2uzil_ d1eo8l1
d1qfwl_ d1n7ml1 d1igja1 d1c5ch1 d2a9mh_ d1rmfh1 d1fj1b1 d4heme_
d1ccza1 d1a5fh1 d1ogad1 d1dlfh_ d1qa9b_ d1vgel1 d3qskb_ d1sjxa_
d4ozfg1 d1sq2n_ d1qd0a_ d1kcvh1 d1c5db1 d3qcul1 d8faba1 d3bn9d1
d1mjuh1 d4y8dc_ d7fabh1 d3dvgb1 d1lo0l1 d1a6tb1 d1gigh1 d1kcrh1
d2hwzl1 d1yjdc1 d3clfl1 d4krna_ d1dl7l_ d1e6ol1 d1mexh1 d3r0ma_
d4kkna_ d1fnsl1 d1oaqh_ d1rzia1 d1ucbh1 d1ncwl1 d4b41a_ d1nfdf1
d2mpah1 d2d7tl1 d4grwf_ d4b5ea_ d1lk3h1 d3u79a_ d4u3xa_ d1ghfl1
d1bvkb_ d1mcww1 d1b88a_ d1jrhh1 d32c2b1 d4wemb_ d1dsfh_ d1op3h1
d1ce1l1 d1iqda1 d1qoka1 d1qoka2 d1qfuh1 d2crya1 d1ay1h1 d1oaql_
d3ab0b_ d1rurl1 d1t4kb1 d1bz7b1 d1n0xh1 d1forl1 d1ucbl1 d4w6yb_
d43c9a_ d2aabl1 d1tzia1 d4n1ca_ d1k6qh1 d3g9ab_ d4tvsa1 d1igmh_
d1pz5b1 d3dx9a1 d3wxwh_ d1nakh1 d1kcul1 d3eaka_ d4qlra1 d1cr9l1
d1d9ka_ d1f4xl1 d1deea1 d2i27n2 d1c12a1 d2orbl1 d1j8hd1 d1mcph1
d1c5da1 d1kenh1 d1bd2e1 d1h5ba_ d4x7fc1 d3upaa_ d1t3fa1 d1indh1
d2dtgc1 d4hc1l1 d1zv5a1 d4wgvb_ d1uwgl1 d4f37k1 d1aqkh1 d1vesa_
d3zkqd_ d1fptl1 d1lo4l1 d1knob1 d3rrqa_ d1ct8b1 d4orzc_ d1mlbb1

d4hgkc1 d1dqdl1 d1bogb1 d4ozhe1 d1dr9a1 d1i7za1 d1dn0b1 d1kell1
d1rzib1 d1ypzf1 d1wtla_ d1hkfa_ d1fail1 d1figh1 d2vyre_ d1sjva_
d1ohqa_ d1bfoa1 d1pkqb1 d1mjul1 d1b4jl1 d1nsnl1 d2arjb1 d1p4bh_
d4aj0a_ d3nn8b_ d2rcja1 d1f2xk_ d4v1dd_ d1adqh1 d2eksa_ d4k3ga_
d1jfqh1 d1uweh1 d1frgh1 d4gftb_ d1uz8b1 d4n1ea_ d1bm3l1 d1clol1
d3mcka1 d2vq1a1 d1ae6h1 d4aq1b1 d1uj3b1 d1nbqa1 d1fh5l1 d2ck0l1
d2ywza_ d1q72l1 d4nbxb_ d1hila1 d4hzlb1 d2j88l1 d1zmya1 d1cd0a_
d1a4ka1 d1pskl1 d1a0qh1 d4krmb_ d1ncbh1 d2fbjh1 d1j8he1 d1i3ua_
d1cf8l1 d1fe8h1 d2p1ya1 d1eeqa_ d2x89a_ d1ol0a_ d2r0lh1 d1ub5a1
d1dfbh1 d3eyvb1 d4jdvb1 d4p2cg_ d2gsia1 d2agjl1 d1u3ha1 d2h1ph1
d1rihl1 d1fskc1 d1boga1 d2a77l1 d1ospl1 d1qfoa_ d3dwta_ d2fx7l1
d1rz7l1 d3stba1 d1pkqa1 d1b6da1 d1dzba2 d1dzba1 d1kj2a_ d4l1ha_
d1rzgb1 d1dqlh_ d12e8l1 d1miel1 d1jmaa_ d1sbsl1 d1fgnh1 d1f58l1
d1mqkh_ d1ndgb1 d3qegl1 d1iail1 d2aw2a1 d1tqbc1 d3fo2a1 d4dkaa_
d1f8th1 d2g60l1 d2ywya_ d1uj3a1 d1yc7a_ d3bd3a_ d4w6xb1 d1nmbl_
d1i8ka_ d1rjlb1 d1ypze1 d1p7kb1 d1kxqe_ d1gpoh1 d2z4qa1 d1w72h1
d1i9rl1 d4qgya1 d4xt1c_ d2qqnh1 d1cica1 d3c08h1 d2cd0a_ d2bdnh1
d1hnga1 d1maml1 d1nldh1 d1cr9h1 d4lcih1 d3bkyh1 d3qg7l1 d1ai1l1
d1kxvc_ d4iosd_ d1l7ih1 d2x1qa_ d1ub6b1 d3k74b_ d1ibgh1 d1teth1
d1igya1 d1hxma1 d2x6ma_ d2e7la_ d5e03a_ d1qfwm_ d2r0kh1 d1igjb1
d1rurh1 d1plgh1 d1mj8h1 d1yuhb1 d3fmgl1 d1rzfl1 d1emtl1 d1ad9b1
d1igtb1 d1nlbl1 d1d5ih1 d3k7ua_ d4ffyh_ d1bwwa_ d3dvga1 d1a6ta1
d1fgvl_ d4qxgl1 d1miml1 d1jhll_ d2hffb1 d2hh0l1 d1qkzh1 d1l7tl1
d1afvh1 d1iaii1 d2cjuh_ d1um5h1 d2ghwb2 d2ghwb1 d1kgce1 d2fgwh1
d1ikfh1 d1dvfd_ d2i24n1 d2xtjb_ d1za3b1 d2yc1a_ d2hh0h1 d2x1oa_
d1bvka_ d1rjla1 d5e7ba1 d1igta1 d1il1b1 d2fb4l1 d1mvua_ d1jptl1
d32c2a1 d1fvca_ d1iqdb1 d2hrph1 d2p4ab_ d4qo1a_ d1p2cb1 d1wt5c_
d1t4ka1 d5c1mb_ d1it9l1 d1bz7a1 d1b2wh1 d1e4wl1 d1jnll1 d1yedb1
d1za6b1 d3u7aa_ d1igfh1 d3gnml1 d1orsa1 d1wejl1 d1a2ya_ d1hxmb1
d1gafh1 d2j4wh1 d1pz5a1 d1j05a_ d1a3rh1 d4fqll1 d1qfwh_ d1eo8h1
d1n7mh1 d4s11a_ d1kegh1 d5da4a_ d1iqwl1 d3whxb1 d1vgeh1 d1deeb1
d1c12b1 d1t2ql1 d1ad9a1 d3gsnb1 d3b2uc1 d1ggbh1 d1t3fb1 d1nakl1
d4ocmc_ d1lila1 d2v7hb3 d2esve1 d1mcoh1 d2qr0b1 d2q3aa_ d1ct8a1
d1kcrl1 d2pxyb_ d2uzih_ d5c3ld_ d3durb_ d1c5cl1 d2ak4e1 d1dl7h_
d1xiwd_ d1mlba1 d1rmfl1 d1i7zb1 d1dlfl_ d2xa3a_ d5bq7b1 d1dqqb1
d3h42l1 d1egjh1 d1kcvl1 d1fe8l1 d2j6el1 d1f3rb2 d1lk3l1 d1sm3h1
d1q0xh1 d1ghfh1 d7fabl1 d1igch1 d1lo0h1 d4idla_ d5c2bl_ d1jrhl1
d1il1a1 d3zhda1 d4kfzc_ d1b0wa_ d1e6oh1 d1mexl1 d4krpb_ d1fnsh1
d1ncwh1 d4hgma_ d4eigb1 d4rzca_ d2mpal1 d4y5xa_ d5en2b1 d3lrga1
d4ej1c1 d1rhha1 d1j05b_ d1forh1 d1q9wb1 d2wzpd_ d2aq2a1 d1h0da1
d3revb1 d2ij0c1 d1tzha1 d4m7jl1 d1k6ql1 d3b9kc1 d1igml_ d3qeua1
d1dsfl_ d1ce1h1 d1kcuh1 d1zvha1 d1zvoa1 d1tqbb1 d1ay1l1 d2qscl1
d2cgrh1 d1nl0h1 d1kenl1 d3eo0a1 d4n9ob1 d1n0xl1 d3k1kc_ d2cqva1
d1fhga_ d2k1ma_ d2dl2a1 d1biha3 d1biha1 d1biha4 d3qp3a1 d1g1ca_

d1gxea_ d1cvsc2 d1cvsc1 d1xmwa2 d1m4ka2 d2otpa2 d1biha2 d1rhfa2 d2fcba2 d2fcba1 d1wwbx_ d1gsma2 d1epfa1 d1epfa2 d2dava1 d1waaa_ d1f97a2 d1x44a1 d1vcaa2 d1fnla2 d1fnla1 d3b5ha2 d3h8na2 d3h8na1 d1jbja1 d1jbja2 d1pd6a_ d1f2qa2 d1f2qa1 d3cx2a_ d1nbqa2 d3b5ha1 d1n26a1 d1xiwb_ d1xaua_ d1fltx_ d2ifga1 d1olla1 d1olla2 d1cs6a4 d1cs6a3 d1cs6a2 d1cs6a1 d2gw5a2 d1sy6a1 d1xiwa_ d1ucta2 d1ucta1 d1ry7b2 d1gl4b_ d1qz1a3 d1olza1 d2c9aa1 d1wwca_

**Family-30 (b.40.2.2)**

**Positive dataset contains 6 protein sequences.**

d2aq2b1 d1v1oa1 d1esfa1 d2g9hd1 d4ohja1 d1ty0a1

**Negative dataset contains 4066 protein sequences.**

d1xfda2 d1m32a_ d1ibja_ d1c7na_ d1iuga_ d4q75a_ d2bkwa1 d1t3ia_ d1qz9a_ d1jf9a_ d1eg5a_ d1p3wa_ d3kgwa_ d1cl1a_ d2ctza1 d1vjoa_ d2rfva_ d1gc0a_ d1cs1a_ d1d2fa_ d2ch1a1 d1qgna_ d1pffa_ d1elua_ d1u08a_ d1lc5a_ d1b5pa_ d2ay1a_ d1bw0a_ d3pd6a_ d7aata_ d2csta_ d1fg7a_ d4wlha_ d1o4sa_ d1v2da_ d1gdea_ d1m6sa_ d1v72a1 d1svva_ d1ajsa_ d1wsta1 d2f8ja2 d1lk9a_ d1vp4a1 d1xi9a1 d2gb3a1 d1yaaa_ d1xkla_ d2wfla_ d3i2ka1 d1lnsa3 d1mpxa2 d1r0ka3 d1j5pa3 d1vkoa2 d1vm6a2 d3qvsa2 d1p1ja2 d3lgia_ d3i77a_ d2z9ia2 d4nsva_ d1lcya2 d4jcna_ d1agja_ d1os8a_ d1hpga_ d1l1ja_ d1qtfa_ d1f56a_ d1bypa_ d1azca_ d1ws8a_ d3u25a_ d1cuoa_ d1id2a_ d1rkra_ d2iaac_ d4dp1x_ d1nwpa_ d2gima_ d2i7sa_ d1qhqa_ d3ehbb1 d1fftb1 d1gyca3 d1hfua2 d1kbva1 d1kbva2 d1sdda1 d1sdda2 d1gyca1 d1gyca2 d1mzya1 d2bw4a1 d1aoza1 d1aoza3 d1aoza2 d1kyaa2 d1kyaa3 d1kyaa1 d1mzya2 d4e9sa1 d1sddb1 d1sddb2 d1hfua1 d1hfua3 d1oe1a2 d1oe1a1 d1v10a2 d1v10a3 d1v10a1 d2dara2 d2dara1 d1wyha2 d2dloa2 d1rutx4 d1rutx3 d2cuqa2 d1x4la2 d1x4la1 d1x3ha2 d1x3ha1 d2d8za1 d1x61a2 d1x6aa1 d1x6aa2 d2cora2 d2cora1 d1v6ga1 d1v6ga2 d2d8za2 d1x64a1 d1x64a2 d1b8ta1 d2co8a1 d1x63a2 d2dj7a2 d2dj7a1 d2cu8a2 d2cu8a1 d1g47a2 d1g47a1 d1j2oa1 d1wyha1 d2dloa1 d1wiga1 d1wiga2 d2cupa2 d2cupa3 d1x68a1 d2d8ya2 d2d8ya1 d1m3va2 d1x62a1 d1x68a2 d1x61a1 d1b8ta3 d1b8ta2 d2d8xa2 d2d8xa1 d1x4ka1 d1x4ka2 d2cuqa1 d1m3va1 d2co8a2 d1j2oa2 d1x63a1 d2cura1 d2cura2 d1x62a2 d1xkta1 d2hfka_ d1jmkc_ d5d3ka_ d2cfba1 d1dfoa_ d1o69a_ d1bj4a_ d3k28a_ d2bwpa_ d3lg0a_ d2fnua_ d3ou5a_ d1z7da1 d1b9ha_ d1iaya_ d4msoa_ d1bs0a_ d1s0aa_ d1sffa_ d3tqxa_ d1mdoa_ d3qboa_ d3pgya_ d2dkja_ d1ejia_ d1vefa1 d4wxga_ d3bs8a_ d3gbxa1 d1w23a_ g1gk9.1 g1or0.1 g1e3a.1 d1iknc_ d3brda1 d1p7hl1 d1oy3c_ d1imhc1 d1gjia1 d1zk9a1 d2cxka1 d2bema_ d1pama1 d1ea9c1 d1qhoa1 d2fhfa2 d2fhfa1 d1k3ia1 d1j0ha1 d1cxla1 d2bhua1 d1edqa1 d1f1sa2 d1ut9a2 d1gvia1 d1txka1 d1zx5a1 d2phla2 d2phla1 d1fxza2 d1fxza1 d1lrha_ d1uija1 g1dgw.1 d2v09a_ d1uika2 d1uika1 d1dgwa_ d1ogpa1 d1ktja_ d1ds6b_ d1fstb_ d1ex0a1 d4alac_ d1pjwa_ d1s6na_ d3uzqb_ d1tafa_ d1tafb_ d1jfib_ d1q9ca_ d1h3ob_ d2byka1 d1n1ja_ d4g92b_ d1bh9a_ d1jfia_ d1h3oa_ d2bykb1 d1n1jb_ d2bjca1

d2hsga1 d1qpza1 d1efaa1 d1ydwa2 d2nvwa2 d1h6da2 d1zh8a2 d1xeaa2
d1tlta2 d1au7a2 d1ic8a2 d3gpdg2 d2q49a2 d1hdgo2 d2g17a2 d2cvoa2
d2czca1 d3pwka2 d1obfo2 d1i32a2 d1zyao2 d2pkqo2 d1b7go2 d2hjsa2
d1cf2o2 d1vkna2 d1nvmb2 d2msya_ d2djoa1 d2r5za_ d1f43a_ d2lmda1
d1akhb_ d1s7ea1 d3nara_ d1zq3p1 d2craa1 d1mija_ d4uuta_ d3a01a_
d1x58a1 d3rkqa1 d3cmya1 d2cqxa1 d2da6a1 d1e3oc1 d1fjla_ d1mh3a1
d1ig7a_ d1x41a1 d1wh7a1 d1gt0c1 d2mw8a_ d1lfup_ d1x2ma1 d1b72a_
d1wi3a1 d1jgga_ d1wjha1 d1x2na1 d1mnmc_ d2cuea1 d3k2aa_ d2hosa_
d2jwta2 d1pufa_ d1octc1 d2hi3a_ d1b8ia_ d4j19a_ d3lnqa_ d1wh5a1
d1b8ib_ d1fexa_ d2cqra1 d1guua_ d1ug2a1 d2cqqa1 d2ajea1 d4a69c_
d2crga1 d2iw5b1 d1xc5a1 d1ofcx1 d2cu7a1 d1wgxa1 d2cjja1 d2ckxa1
d1gvda_ d1xg1a1 d1itya_ d1hlva2 d1iufa2 d1iufa1 d2iu5a1 d1zk8a1
d2g7sa1 d2fd5a1 d2np5a1 d1z0xa1 d2id6a1 d2d6ya1 d2i10a1 d2oi8a1
d3c07a1 d2ns7a1 d2fq4a1 d2yvea1 d1t33a1 d2o7ta1 d1rkta1 d2np3b1
d2g7ga1 d2gena1 d2fbqa1 d2g7la1 d2gfna1 d2g3ba1 d2jj7a1 d2hyja1
d1sgma1 d1ui5a1 d2hkua1 d2id3a1 d4jyka1 d1bl0a1 d1d5ya1 d1d5ya2
d3lv8a_ d1s3ga1 d1zaka1 d1s4qa_ d3tvta2 d1uf9a_ d1khta_ d1ckea_
d1osna_ d1p5zb_ d4tmka_ d3cm0a_ d1tmka_ d1jaga_ d3h86a_ d1t0hb_
d1nn5a_ d2h92a_ d3kb2a1 d2c95a1 d1yj5a2 d1kgda_ d1ly1a_ d2ak3a1
d1vyva2 d1deka_ d2grja1 d1p3ja1 d1y63a_ d1e4ya1 d1s96a_ d3iija1
d1lvga_ d1nksa_ d1vyua2 d4e22a_ d1qf9a_ d1jjva_ d1q3ta_ d1ki9a_
d1lw7a2 d1teva_ d1gsia_ d2iyva2 d1kaga_ d1viaa_ d1e6ca_ d1g3ma_
d1j99a_ d1t8ta1 d1nsta_ d1fmja_ d1zrha_ d1ls6a_ d2a3ra_ d1aqua_
d2zptx_ d3ckla_ d1texa_ d1q44a_ d2gwha_ d1odfa_ d1uj2a_ d4gi7a_
d2jeoa1 d1k6ma1 d1z0ja1 d3umra2 d3gj0a1 d2gaoa1 d1yzga1 d1x3sa1
d1g7sa4 d1puja_ d1udxa2 d1kk1a3 d2o52a_ d1jwyb_ d1zcaa2 d1zcba2
d2fv8a1 d2cjwa_ d3dz8a1 d1xzpa2 d1vg8a_ d4qxaa_ d2ew1a1 d1x1ra1
d2bcgy_ d3doea1 d3cona_ d3rega_ d3wyga_ d1upta_ d2b6ha1 d1zd9a1
d1lnza2 d1r5ba3 d1n0ua2 d1r2qa_ d2f9la1 d2rhda_ d3q3jb_ d3q85a_
d1efma1 d1r8qa_ d2gf9a1 d2akab1 d1ek0a_ d1z08a1 d2gcoa1 d2dy1a2
d4g01b_ d2cxxa1 d1svia_ d3q72a_ d1ni3a1 d4ac9a4 d2erxa1 d2bmea_
d1wxqa1 d2gf0a1 d4ldja1 d1zunb3 d1d5ca_ d4lv5b_ d1efca3 d1kmqa_
d2g6ba1 d3lrpa_ d1tada2 d2y8ea_ d4rd4a1 d1z06a1 d2heia_ d3a58b_
d1u0la2 d1d2ea3 d1zj6a1 d2ocba_ d3rwoa_ d1mr3f_ d2e9sa1 d4u5xa1
d2iwra_ d2fn4a1 d3bwdd_ d2nzja1 d2atva1 d2ksqa_ d3l43a_ d1mkya2
d1mkya1 d1wmsa_ d1jnya3 d2rmka_ d1f6ba_ d1f60a3 d1gota2 d1jala1
d2fh5b1 d1g16a_ d2wblc_ d1m7ba_ d1wf3a1 d1f5na2 d1t9ha2 d1z6xa1
d1z0aa1 d2fg5a1 d2a5ja1 d2bcjq2 d1ky3a_ d4y0va_ d4lhwa_ d2qmea1
d2atxa1 d1egaa1 d1ryha_ d1moza_ d1nrjb_ d2erya1 d3bfka_ d1puia_
d1fzqa_ d3j3az1 d3t5ga_ d1shza2 d3defa_ d2f7sa1 d3raba_ d3tsoa_
d3aq4a_ d2al7a1 d3sfva1 d3bc1a_ d1kb0a1 d1k8ka2 d1k8ka1 d3uleb1
d3i33a2 d3i33a1 d1bupa1 d1dkgd1 d4ci6a2 d1jcfa1 d4a2ba2 d2fxua1
d1yaga2 d1yaga1 d4cbua2 d1bupa2 d3vrda1 d3vrda2 d1fcdc2 d1h1oa2
d1h1oa1 d1m70a2 d1m70a1 d1czan2 d1czan3 d1czan1 d1ig8a2 d1czan4

d1bg3a4 d1bg3a3 d1bg3a2 d1bg3a1 d1hroa_ d4gedb_ d3zooa_ d1mz4a_
d1gu2a_ d1f1fa_ d1dvva_ d1c6ra_ d1c75a_ d1e29a_ d1gdva_ d1cnoa_
d1h32b_ d1co6a_ d2bh4x_ d2aiua_ d5cytr_ d1jdla_ d1i8oa_ d2zboa_
d3dmia_ d1ls9a_ d1wvec_ d2yk3a_ d3ph2b_ d1dw0a_ d5aura_ d1ql3a_
d1qn2a_ d1f1ca_ d4zzva_ d2xspa_ d1eg1a_ d4v20a_ d4ipma1 d1gpia_
d4csia_ d3pfza_ d3ovwa_ d2yoka_ d2nqya_ d1h4ga_ d1hixa_ d1oa2a_
d1olra_ d3b7ma_ d4h7ma1 d3wp3a_ d2vgda_ d1igoa_ d3zsea_ d2nlra_
d3wx5a_ d1oa3a_ d1pvxa_ d1m4wa_ d1f5ja_ d1oa4a_ d1ks5a_ d4ixla1
d3sita1 d1evsa_ d1cnt1_ d1pvhb_ d1z7ca_ d1huwa_ d1bgea_ d1rhga_
d1f6fa_ d1f45b_ d1i1rb_ d1axia_ d1etea_ d3tgxb_ d1scfa_ d2z3qa1
d3qb1a_ d1hula_ d4nkqb_ d1eera_ d1v7mv_ d5e4ea_ d2o27a_ d3b5ka_
d2psma1 d4zf7a_ d3uf5a_ d4x51a_ d1d9ca_ d4ypgc1 d3oq3a_ d1lqsl_
d1n1fa_ d1au1a_ d1wu3i_ d3besl_ d3ux9a_ d2ejca_ d3muea1 d1ihoa_
d3q12a1 d4dnxa2 d1v47a2 d1g8fa2 d1jhda2 d1x6va2 d3rbaa_ d2qtra_
d1nupa_ d3l92a_ d1qjca_ d2h29a_ d1vlha_ d1od6a_ d1lw7a1 d1mrza2
d4ruka_ d2el7a_ d1iq0a2 d1irxa2 d1r6ta2 d1ivsa4 d2cyba_ d1rqga2
d2yxna_ d1f7ua2 d1h3fa1 d1ffya3 d2g36a1 d1nzja_ d1jila_ d1n3la_
d1i6la_ d1pfva2 d1h3na3 d3n9ia_ d3sz3a1 d3sw2a_ d1moxc_ d1dx5i3
d1edmb_ d1autl2 d1autl1 d1nqlb_ d1dval1 d1szba2 d1kigl_ d2y5fl_
d1ijqa2 d1gk5a_ d1g1sa2 d1nt0a3 d1xdtr_ d1lmja2 d1lmja1 d1nzia2
d2wphe_ d1klil_ d1pfxl2 d1pfxl1 d1p9ja_ d1g1ta2 d1f7ea_ d4yzub_
d1lr7a1 d1gl4a2 d1m1xb4 d1qx2a_ d2y5ia_ d1qlsa_ d1psra_ d3n22a_
d2egda_ d4ggfc_ d2wcea_ d2luca_ d4aqia_ d3nsia_ d2lp3a_ d1j55a_
d1a4pa_ d1k8ua_ d1nsha_ d3fs7a_ d2pvba_ d1ttxa_ d1rwya_ d2kyfa_
d1rk9a_ d1a75a_ d1exra_ d1tiza1 d1nyaa_ d1g8ia_ d1ggwa_ d1wdcc_
d3fwba_ d1ggza_ d2o5ga_ d1ctaa_ d1m45a_ d3i5gc_ d1sl8a_ d1fpwa_
d2scpa_ d2roaa_ d4aqra_ d3pn7b_ d1br1b_ d2mysb_ d1s6ca_ d1bjfa_
d2obha_ d1jfka_ d1jf0a_ d1qv0a_ d1lkja_ d2mlfc_ d2m97a_ d2d8na_
d4by5a_ d1jbaa_ d4nqga_ d1wdcb_ d2mysc_ d1j7qa_ d1s6ia_ d1uhka2
d1r2ua_ d1dtla_ d3kf9a_ d3pn7c_ d1gjya_ d1y1xa_ d1hqva_ d1dvia_
d1df0a1 d4usla_ d1k94a_ d1wkya2 d1uuqa_ d3wh9a_ d4m1ra_ d1xyza_
d4xuya_ d2je8a5 d4bf7a1 d3cuia_ d1ur4a_ d1qw9a2 d1odza_ d2vzsa5
d1bqca_ d2x2ya1 d1hjqa_ d1qnra_ d4y7ea1 d1vjza_ d4k68a_ d2v3ga_
d4hpga_ d1nofa2 d1nq6a_ d1h4pa_ d1aq0a_ d3ziza_ d4bs0a_ d1ecea_
d1h1na_ d1ta3b_ d1w91a2 d1i1wa_ d1uhva2 d1us3a2 d1egza_ d7a3ha_
d1tvna1 d3nyda1 d1ghsa_ d2cyga1 d4xv0a_ d2c8na2 d1w32a_ d3emca_
d1v6ya1 d1foba_ d2uwfa1 d2fgla_ d3niya1 d2c0ha1 d1rh9a1 d1yq2a5
d1szna2 d1fa2a_ d1pama4 d5do8a1 d1ea9c3 d1hx0a2 d2fhfa5 d1qhoa4
d1avaa2 d1m53a2 d4m8ua1 d2gjpa2 d1lwha2 d4howa1 d1ktba2 d1j0ha3
d3vm5a1 d2x1ia_ d1wzaa2 d1uasa2 d2diea1 d1mxga2 d1ht6a2 d1eswa_
d1iv8a2 d5cpsa_ d1ud2a2 d1zjaa1 d3gbea1 d1kwga2 d1wdpa_ d1gcya2
d1g5aa2 d1hvxa2 d3dhpa2 d2bhua3 d4do4a1 d3qgva1 d1e43a2 d1g94a2
d2d3na2 d1gjwa2 d1gvia3 d1x1na1 d1r7aa2 d1tz7a1 g1apy.1 d3ljqa_
d4toqa_ d1wb0a1 d1w9pa1 d1eoka_ d1itxa1 d1kfwa1 d1goia2 d2gsja_

d1hjxa1 d1jnda1 d1vf8a1 d3wdpp1 d3wq5a_ d1gnxa_ d4gxpa_ d4hz8a_
d1e4mm_ d3aiua_ d1wcga1 d1qvba_ d3ahxa_ d1v02a_ d2dgaa_ d1pbga_
d1vffa1 d1qoxa_ d4ha4a_ d1e5wa2 d1j19a2 d1isna2 d2al6b2 d1g4ma2
d1id1a_ d2hmta_ d1lssa_ d1ix2a_ d3nvva1 d1krha3 d2h88b1 d1t3qa2
d1kf6b2 d1zoyb1 d1jq4a_ d1ffva2 d1dgja2 d1rm6c2 d9ldta1 d2ewda3
d1up7a1 d2dfda1 d1s6ya1 d1vjta1 d7mdha1 d1hyea1 d1t2da1 d1ojsa3
d1b8pa1 d1ldna1 d1hyha1 d1civa1 d2a92a1 d1ez4a1 d1i0za1 d1y6ja1
d1gv0a1 d1ur5a1 d1llda1 d1obba1 d1v8ba1 d1gdha1 d1l7da1 d2dlda1
d1mx3a1 d1ygya1 d1qp8a1 d1li4a1 d1j4aa1 d1npya1 d3t4ea2 d1a4ia1
d1o0sa1 d2hk9a2 d2egga2 d1b26a1 d1bvua1 d1gq2a1 d1c1da1 d1leha1
d1gtma1 d1euza1 d1nvta1 d3dona2 d1edza1 d1vi2a1 d1p77a1 d1nyta1
d1b0aa1 d1vl6a1 d1v9la1 d1txga2 d3f47a1 d1mv8a2 d2pv7a2 d1xc2a3
d2ahra2 d4xiya1 d2f1ka2 d1pgja2 d1i36a2 d1np3a2 d1lj8a4 d1teaa3
d1qmga2 d2i76a2 d2g5ca2 d1wdka3 d1dlja2 d3cuma3 d1kola2 d1f8fa2
d1qora2 d1jqba2 d1vj0a2 d1cdoa2 d1gu7a2 d1d1ta2 d1xa0a2 d2dm6a2
d1jvba2 d1rjwa2 d1llua2 d1h2ba2 d1tt7a2 d1piwa2 d1o89a2 d1iz0a2
d1e3ja2 d1yb5a2 d1uufa2 d1vj1a2 d3gpdg1 d1vc2a1 d2nvwa1 d1u8fo1
d1ydwa1 d1hdgo1 d2g17a1 d1cf2o1 d2czca2 d1r0ka2 d1obfo1 d2cvoa1
d1ebfa1 d1h6da1 d1i32a1 d1zh8a1 d2q49a1 d1qkia1 d3pzra1 d1xeaa1
d1vkoa1 d1vm6a3 d3qvsa1 d2pkqo1 d1b7go1 d1gr0a1 d1p1ja1 d1lc0a1
d1j0xo1 d3pwka1 d2hjsa1 d1zyao1 d1vkna1 d1tlta1 d1nvmb1 d3sjua_
d3ojfa1 d1xu9a1 d2jjya1 d1n7ha_ d1y1pa1 d1h5qa_ d1jtva_ d4fs3a_
d2bkaa1 d1gy8a_ d2c5aa1 d1ulsa_ d1ooea_ d2b69a1 d1y5ma1 d1spxa_
d1ae1a_ d1bxka_ d1ydea1 d1gega_ d1hdoa_ d1o5ia_ d1e6ua_ d1snya1
d2rhca_ d3zv4a_ d5ceja_ d2d1ya1 d1fjha_ d3w8ea_ d2fmua1 d1x1ta_
d1rpna_ d3ay7a1 d1k2wa_ d1eq2a_ d2hrba1 d3ruaa_ d2ew8a1 d4hwka_
d1xgka_ d2bd0a1 d1yo6a1 d2a35a1 d2ntva_ d1vl8a_ d1hdca_ d4bkua_
d2ag5a1 d2q45a_ d1nffa_ d4iy1a_ d1zbqa1 d1kewa_ d1ek6a_ d1ja9a_
d2q46a2 d1iy8a_ d1cyda_ d2ptga_ d1hxha_ d1uzma1 d3lyla_ d4m89a_
d3wyea_ d2blla1 d1w6ua_ d1sb8a_ d2q2va_ d4nr0a_ d1e6wa_ d2gasa_
d3oiga1 d2bgka1 d1vl0a1 d1rkxa_ d1r6da_ d1wmaa1 d1e7wa_ d2c07a1
d1xkqa_ d3tzha_ d1g0oa_ d3lz6a_ d1qyca_ d1edoa_ d1gz6a_ d1oc2a_
d1t2aa_ d1zema1 d2xoxa_ d1z45a2 d1geea_ d1p33a_ d3lt0a_ d4e6pa_
d2wyua_ d1db3a_ d4q9na_ d1xhla_ d4o1ma_ d1fmca_ d2uvda_ d2gdza1
d1uaya_ d2z1ma_ d2o23a2 d2fr1a2 d2fr1a1 d1qsga_ d1qyda_ d4zjua_
d2a4ka1 d1yb1a_ d1wvga1 d1yxma1 d3f4ba_ d1mg5a_ d1xg5a1 d1r55a1
d3gboa_ d1atla_ d1kufa_ d4q1la_ d1quaa_ d1yp1a_ d1wnia_ d3nqyb_
d4gera_ d1bqba_ d1cmxa_ d1xd3a_ d3ifwa_ d1ex0a4 d1g0da4 d2f4ma1
d1x3za_ d3u1ra1 d1kapp2 d1k7ia2 d1t3ca_ d1epwa3 d2a97a_ d3fiea_
d3ioqa_ d1fh0a_ d1o0ea_ d1khqa_ d1m6da_ d2cb5a_ g8pch.1 d1s4va_
d2pnsa_ d1vsna1 d1mira_ d1iwda_ d2oula_ d7pcka_ d3bwka_ d3bcna_
d2p86a_ d1pvja_ d1pcia_ d1gece_ d1deua_ d4bs6a_ d1xkga1 d1cqda_
d1jqpa2 d2c0ya_ d2bdza_ d3p5ua_ d2o36a_ d1ex4a2 d2jk6a2 d2jk6a1
d1feca1 d1feca2 d1lpfa1 d1lpfa2 d1xhca1 d1xhca2 d1dxla2 d1dxla1

d1gera2 d1m6ia2 d1onfa1 d1onfa2 d3lada2 d3lada1 d1w4xa1 d1w4xa2
d1gesa2 d1mo9a1 d1xdia1 d1fl2a2 d1fl2a1 d1fcda1 d1fcda2 d1h6va2
d1h6va1 d1mo9a2 d1gv4a2 d1gv4a1 d2gv8a2 d2gv8a1 d1aoga1 d1aoga2
d1hyua1 d1ebda1 d1ebda2 d3dk9a1 d1q1ra2 d1q1ra1 d1gesa1 d1zbfa1
d2z1ha_ d2etja1 d3qioa1 d2e4la_ d1jl2a_ d2lsna1 d1io2a_ d1uaxa_
d2yv0x_ d1mu2a1 d1ekea_ d1i39a_ d1pn0a1 d1reoa1 d1ju2a1 d2f5va1
d3c96a1 d2gmha1 d1pj5a2 d1gpea1 d1n4wa1 d1k0ia1 d2dw4a2 d2ivda1
d1seza1 d2voua1 d1kdga1 d1s5ja1 d2hbja2 d2igia_ d1wlja_ d1qhta1
d4dlea1 d1tgoa1 d2f96a1 d3tr8a1 d3mxma_ d1q8ia1 d1y97a1 d1w0ha_
d1kfsa1 d2py5a1 d1noya_ d1d5aa1 d1yt3a3 d2guia_ d3mwpa1 d4kb1a_
d1js9c_ d1f15b_ d1za7a_ d1laja_ d2v76a_ d1wj1a1 d1aqca_ d1qqga2
d1ntva_ d1n3ha_ d4dbba_ d3f0wa1 d1p3ra_ d1wgua1 d2cy4a1 d3hqca_
d1wvha1 d1xr0b1 d1tq5a1 d1wgqa1 d1v89a1 d2coda1 d1zc3b_ d1wg7a1
d1u5ea1 d1v61a1 d2coca1 d2ys1a1 d1v5pa1 d1ntya2 d1wjma1 d1wi1a1
d1u29a_ d1xx0a1 d1unqa_ d2zkmx3 d1fhoa_ d1v88a1 d2adza1 d1x1fa1
d1p6sa_ d2coaa1 d1btka_ d1v5ma1 d1faoa_ d2x18a_ d1dyna_ d1x1ga1
d4kaxb_ d2j59m1 d1ki1b2 d1xcga2 d1txda2 d1u5da1 d1dbha2 d1w1ha_
d1upqa_ d1v5ua1 d1foea2 d2fjla1 d2dfka2 d2cofa1 d1kz7a2 d1r7ha_
d1faaa_ d1wika1 d1xfla_ d1ttza1 d2f51a1 d1ti3a_ d1nhoa_ d1sena_
d2i4aa_ d1ep7a_ d1zmaa1 d1fo5a_ d3lefa1 d3diea1 d2yana1 d1z6na1
d1oaza_ d3zywa1 d4aj8a_ d1nm3a1 d4fiwa_ d1wmja_ d4hs1a1 d1iloa_
d1xwaa1 d1wjka1 d2vm1a_ d1fb6a_ d1gh2a_ d1h75a_ d1nw2a_ d1kx5b_
d3a6na_ d4m6ba2 d2nqbd_ d3wkjd_ d2yfwe_ d4h9na_ d5bnxa_ d1kx5c_
d3r45a_ d1eqzb_ d4jjnb_ d1f66c_ d1eqza_ d4wnnb_ d1kx5a_ d1u35c1
d1id3a_ d2nqbc_ d1kx5d_ d2b5ea2 d2b5ea3 d2b5ea1 d2b5ea4 d2kp1a1
d2kp2a1 d1hyua3 d1hyua4 d1j08a1 d1pd212 d1nhya2 d2a2ra2 d1k0dd2
d1f2ea2 d1gsua2 d1f3ba2 d2fhea2 d2pvqa1 d1tw9a2 d1oyja2 d1e6ba2
d1k0ma2 d3ik7a1 d4is0a1 d1r5aa2 d1jlva2 d1m0ua2 d3gtub2 d1z9ha2
d1okta2 d1axda2 d2gsra2 d1tu7a2 d1k3ya2 d1zgma1 d1b48a2 d1gnwa2
d1gwca2 d2gsta2 d1glqa2 d1oe8a2 d1pn9a2 d1v2aa2 d1fw1a2 d1duga2
d1a0rp_ d1dszb_ d1a6ya_ d3cbba_ d1ynwa1 d1r4ia_ d1hcqa_ d2hana_
d2hanb_ d2enva1 d1kb4b_ d2nllb_ d1lo1a_ d1qqya_ d1gd6a_ d1f6ra_
d1ivma_ d2gv0a_ d1iiza_ d2z2fa_ d1j8xa_ d1ghla_ d1b9oa_ d174la_
d169la_ d2o4wa_ d1p37a_ d1k28a3 d1xjta1 d1jtma_ d2f2qa_ d4e97a1
d1iyka2 d1iyka1 d1rxta1 d1iica2 d2za0a_ d2c21a1 d1f9za_ d2pjsa1
d1klla_ d2i7ra1 d1ecsa_ d1xqaa1 d5cj3a1 d1jifa_ d2p7oa_ d1r9ca_
d1nkia_ d1kw3b2 d1kw3b1 d4ghga1 d1tfza1 d3isqa1 d3isqa2 d1sqia1
d1sqia2 d1lkda2 d1lkda1 d1mpya1 d1cjxa2 d1cjxa1 d1sp9b2 d1t47a1
d1t47a2 d1f1ua2 d1sp8a1 d1sp8a2 d1f1ua1 d3ia2a_ d1a88a_ d4dgqa1
d1hkha_ d1zoia_ d1ro5a1 d1pv8a_ d1w5qa_ d2c1ha_ d1ohla_ d1l6sa_
d1qsta_ d2beia1 d4m3sa_ d1u6ma1 d1yk3a1 d3dr6a_ d1xmta_ d1z4ea1
d2ge3a1 d1bo4a_ d2fiaa1 d2jdca1 d1y9wa1 d3to7a_ d1wwza1 d2fl4a1
d2ozua1 d2b5ga_ d2cy2a1 d2p0wa_ d1cm0a_ d4pswa_ d1yrea1 d1yr0a1
d1m4ia_ d2gana1 d1yvka1 d1ghea_ d2g3aa1 d1y7ra1 d2euia1 d1q2ya_

| | | | | | | | |
|---|---|---|---|---|---|---|---|
| d2fsra1 | d1xeba_ | d2ae6a1 | d1cjwa_ | d1qsma_ | d2aj6a1 | d1y9ka1 | d1i12a_ |
| d1vkca_ | d2atra1 | d1n71a_ | d2d4pa1 | d2fiwa1 | d2fe7a1 | d1tiqa1 | d2ou2a_ |
| d1yx0a1 | d2fcka1 | d1p0ha_ | d2j8ma_ | d2pq8a_ | d1z4ra1 | d1s3za1 | d1ufha_ |
| d1ax4a_ | d1c7ga_ | d2v1pa1 | d2ez1a_ | d1xvqa1 | d3hy2a_ | d2cvba1 | d1o8xa_ |
| d1lu4a_ | d1we0a1 | d2bmxa1 | d2a4va1 | d1psqa_ | d1xiya1 | d1q98a_ | d1n8ja_ |
| d1zofa1 | d2ggta_ | d3tjja_ | d4je1a1 | d1o73a_ | d3s9fa_ | d2zcta_ | d1qmva_ |
| d1jfua_ | d2b1ka_ | d5dvba1 | d2b5xa1 | d2v2ga_ | d1st9a_ | d3tb2a_ | d4llra_ |
| d4fh8a1 | d1e2ya_ | d1knga_ | d2h30a_ | d1i5ga_ | d2z9sa_ | d2f8aa1 | d1tp9a1 |
| d2b7ka_ | d1nm3a2 | d3hvva_ | d2wfca1 | d4kcea_ | d1xvwa1 | d4gqca1 | d3qpma_ |
| d4k1fa_ | d1zzoa1 | d1gp1a_ | d1prxa_ | d4od7a1 | d1z6ma1 | d4mcua1 | d4dvca1 |
| d1r4wa_ | d3l9sa_ | d1un2a_ | d3rppa_ | d1e88a1 | d1l6ja5 | d1l6ja4 | d1eaka4 |
| d1eaka3 | d1l6ja3 | d1h8pa2 | d1h8pa1 | d1ys1x_ | d4hs9a_ | d1ispa_ | d1ex9a_ |
| d3qmma_ | d1bnla_ | d1c4xa_ | d2rhwa_ | d2ftba_ | d1pmpa_ | d2rcqa1 | d1fdqa_ |
| d4lkpa_ | d1sa8a_ | d1vyfa_ | d1lpja_ | d3vg7a1 | d4azqa1 | d5bvqa_ | d1p6pa_ |
| d2qo4a_ | d1opba_ | d1yiva1 | d1o1va_ | d4bvma1 | d4tkba_ | d4i3ba_ | d1bwya_ |
| d1ftpa_ | d1ggla_ | d4gkca_ | d1cbia_ | d3p6da1 | d1kqwa_ | d2a0aa1 | d3zxha_ |
| d1cgla_ | d1rm8a_ | d1eaka2 | d1hy7a_ | d2jsda_ | d1cxva_ | d1hv5a_ | d1i76a_ |
| d1g12a_ | d3p0ya1 | d1igra1 | d1igra2 | d1n8yc2 | d1n8yc1 | d1m6ba2 | d1m6ba1 |
| d2bk7a_ | d3fjua_ | d1z5ra1 | d1kwma1 | d1uwya2 | d1jqga1 | d2c1ca1 | d1dtda_ |
| d1h8la2 | d1h6ta2 | d1h6ua2 | d2tbvc_ | d1c8nc_ | d1x36a_ | d1smvc_ | d1ng0c_ |
| d4sbvc_ | d1opoa_ | d1cixa_ | d1y29a_ | d1v7fa_ | d2jtba_ | d1d1ha_ | d1g9pa_ |
| d1lupa_ | d1qk7a_ | d1mb6a_ | d2a2va_ | d1niya_ | d1i25a_ | d1hvwa_ | d2mpqa_ |
| d1ie6a_ | d1kqha_ | d1la4a_ | d2mxoa1 | d1dl0a_ | d1koza_ | d1qk6a_ | d1emxa_ |
| d1lqra_ | d1c6wa_ | d1rmka_ | d1ixta_ | d1eyoa_ | d1cnna_ | d1fu3a_ | d1g1pa_ |
| d1f3ka_ | d1rypd_ | d1irua_ | d1rypc_ | d3unfk_ | d1rypa_ | d1j2pa_ | d1irui_ |
| d1j2qh_ | d3unfh_ | d1m4ya_ | d1rypb_ | d1irul_ | d1irug_ | d1iru2_ | d1q5rh_ |
| d1rypg_ | d1g3ka_ | d1iruf_ | d1q5qa_ | d1e94a_ | d1irub_ | d1ryph_ | d3ty6a_ |
| d1iruj_ | d1iruk_ | d1rype_ | d1irud_ | d1irue_ | d3mi01_ | d1rypf_ | d2z3ba_ |
| d1iruc_ | d3mka2_ | d1iru1_ | d1ny722 | d1a6ca3 | d1a6ca2 | d1a6ca1 | d1pgl22 |
| d1pgl21 | d1pgl11 | d1ny711 | d1mqta_ | d1d4m3_ | d1fmd3_ | d2mev1_ | g1aym.1 |
| d1ev13_ | d4q4w1_ | d1r1a3_ | d2x5ia_ | d1rhi1_ | g1r1a.1 | g1d4m.1 | g1rhi.1 |
| d1b35b_ | d1oopa_ | d1tme1_ | d4iv1a_ | g1pvc.1 | g1fpn.1 | d1fpn1_ | d4gb31_ |
| d1d4m1_ | d1fmd1_ | d2wzr3_ | g1cov.1 | d1qqp3_ | g1fmd.1 | d2x5ic_ | d1rhi3_ |
| g1oop.1 | d1hxs1_ | g1hxs.1 | d1fpn3_ | d1bev1_ | g2mev.1 | d1eah3_ | d1aym1_ |
| d1pvc3_ | d1qqp1_ | d1zba3_ | d1h8ta_ | d3vbhc_ | g1b35.1 | g1bev.1 | d1hxs3_ |
| g1ev1.1 | d1bev3_ | g1tmf.1 | d1tmf1_ | d1b35a_ | d2mev3_ | g1eah.1 | g1h8t.1 |
| d1eah1_ | d4q4w3_ | d1r1a1_ | d1aym3_ | d1pvc1_ | d1zba1_ | d1h8tc_ | d1oopc_ |
| d1tme3_ | g1qqp.1 | d4iv1c_ | g1tme.1 | d1y14b1 | d2nn6i1 | d1i94l_ | d2zjra2 |
| d1smxa_ | d2qamc2 | d1hh2p1 | d2r7da1 | d1rl2a2 | d1wfqa1 | d3bzka4 | d1x6oa2 |
| d3a0ja_ | d2j01d2 | d2ja9a1 | d4ql5a_ | d1hr0w_ | d1luza_ | d2n3sa_ | d2vnud1 |
| d1h95a_ | d1go3e1 | d2nn6h1 | d1ne3a_ | d2mo1a_ | d1iz6a2 | d1jjga_ | d1e3pa2 |
| d2ix0a3 | d2ix0a2 | d2lxja_ | d1ueba3 | d1ueba2 | d2z0sa1 | d1u0la1 | d2ix0a1 |
| d2eifa2 | d2je6i1 | d3i2za1 | d2c35b1 | d2ahob2 | d1t9ha1 | d3i4oa_ | d2qall1 |
| d1jt8a_ | d2nn6g1 | d1g6pa_ | d3cama_ | d1i94q_ | d1d7qa_ | d2ba0a1 | d2vnud2 |

d2vnud3 d1khia2 d2qalq1 d1wi5a1 d1xtda2 d1ny4a_ d4w9ta_ d4x2ra1
d1ka9f_ d1qo2a_ d4axka1 d1thfd_ d1h5ya_ d4u28a_ d2y88a_ d4wd0a_
d1vzwa1 d1eixa_ d3exra_ d2ffca1 d2fdsa1 d3n3ma1 d2czda_ d1q6oa_
d2yyua_ d1vqta1 d1dbta_ d3ldva1 d3syxa1 d1mkea1 d2p8va_ d1xoda1
d1i7aa_ d1evha_ d1egxa_ d1prtb2 d2r80b_ d1cqxa1 d1cg5a_ d1gcvb_
d3boma_ d2h8fb_ d2raoa_ d3bj1a_ d4hrrb_ d2r50a_ d3d1kb_ d2aa1b1
d3zhwa_ d3hf4b_ d3gdja_ d1jebb_ d3bj1b_ d1x9fb_ d1wmub_ d1gcva_
d3bcqb_ d3d4xb_ d1gvha1 d2dc3a_ d3hf4a_ d4yu3a_ d3k8ba_ d1qpwb_
d2r80a_ d3bcqa_ d1v4wa_ d3wtgb_ d1jl7a_ d4esaa_ d1a9we_ d3d1ab_
d4h2la_ d1v4wb_ d1mwca_ d1vhba_ d1jeba_ d1spgb_ d1h97a_ d1q1fa_
d1tu9a1 d3d1aa_ d1hdsb_ d3hyub_ d2zfbb_ d3fs4a_ d1oj6a_ d1shrb_
d4h2lb_ d4yu3b_ d1spga_ d3hrwa_ d3qqra_ d3at5a_ d4wchd_ d3hyua_
d1cg5b_ d1uc3a_ d1hbra_ d2raob_ d1outb_ d4esab_ d3gdjb_ d3qqqa_
d1x9fc_ d3vrfa_ d1itha_ d3mkba_ d3d4xa_ d3mjpa_ d1jl6a_ d3fh9a_
d1outa_ d2dc3b_ d1it2a_ d3eoka_ d3g46a_ d1qpwa_ d3vrfb_ d1ch4a_
d1x9fd_ d1hdsa_ d2zfba_ d3mkbb_ d1or4a_ d3wtga_ d1a4fa_ d3fh9b_
d3bomb_ d3cy5a_ d3at5b_ d1x9fa_ d2oifa_ d4lmxb_ d1cpcb_ d3v57a_
d1kn1a_ d1cpca_ d4f0ta_ d2vjha_ d4po5b_ d1eyxa_ d1alla_ d4f0tb_
d1b33b_ d1b8db_ d4h0mb_ d2vjhb_ d4lm6b_ d2vmlb_ d1allb_ d2vjta_
d3dbja_ d2vmla_ d2vjtb_ d4f0ub_ d1f99b_ d4rmpa1 d1liaa_ d1eyxb_
d2uulb_ d4lmsb_ d4f0ua_ d2bv8a_ d1f99a_ d2bv8b_ d2c7lb_ d3dbjb_
d4rmpb1 d1b8da_ d4h0ma_ d3v57b_ d1kn1b_ d1liab_ d2uula_ d2j96a_
d1f00i3 d1azwa_ d2xqfa_ d1ea5a_ d1f6wa_ d1dx4a_ d1qe3a_ d1k4ya_
d4qwwa_ d2ha2a_ d1r88a_ d1sfra_ d4qeka_ d3c8da2 d2pbla1 d1jkma_
d1jjia_ d1lzla_ d1jjfa_ d1gl9b1 d1gkub2 d2qvba_ d2v9za_ d4kafa1
d2m5ta_ d1wqsa_ d5bpea_ d2ipha_ d1lvoa_ d2bhga1 d3tnta_ d3sjia_
d3d23a1 d2fyqa_ d3tloa_ d3zvea1 d2hrva_ d3q3ya1 d4asha1 d1jmxa4
d1jmxa3 d1pbya4 d4iuaa3 d4bvva_ d5hpga_ d3k65a_ d1ki0a3 d1ki0a1
d1a0ha1 d1pmla_ d3sp8a3 d2feba1 d1f74a_ d3l21a_ d2e1da_ d1to3a1
d1w3ia_ d1sfla_ d1n7ka_ d1vcva1 d4imea_ d1wa3a_ d2ocza1 d1f2ja_
d3m16a_ d2yxga_ d1ub3a_ d3a5fa_ d1fbaa_ d1l6wa_ d1xfba1 d1wx0a1
d1vpxa1 d1ojxa_ d1mxsa_ d2a4aa1 d1xkya1 d3cq0a1 d3s1xa_ d1f05a_
d1fdja_ d3cpra1 d4d2ja_ d1cg2a1 d1yloa2 d1y7ea2 d1y0ya3 d1lfwa1
d1vixa1 d1fnoa4 d1vhoa2 d1ysja1 d1vhea2 d1z2la1 d2fvga2 d1r3na1
d2grea2 d1xmba1 g1q7l.1 d1rtqa_ d3rp2a_ g1ucy.1 d1fuja_ d2zchp_
d3w94a_ d1si5h_ d1lo6a_ g2pka.1 d1cgha_ d1kigh_ d1a5ia_ d3s9ba_
d1hj8a_ d1ao5a_ d2anya_ d1q3xa1 d1gvza_ d1azza_ d4gsoa_ d2f91a1
d1iaua_ d1gdna_ d1eufa_ g1fiw.1 d1eq9a_ d4nfea_ d2rdla_ d1spja_
d1elva1 d1a7sa_ d1ekbb_ d1yc0a_ d2aiqa_ g1fiz.1 d1fi8a_ d4k60a_
d4dgja_ d1mzaa_ d1op0a_ d3tjva_ d1pytd_ d1sgfg_ g1gg6.1 d2eeka_
g1rtf.1 d1a0ja_ d3g01a_ d1pytc_ d4e7na_ d1ddja_ d1z8ga1 d2hlca_
d1npma_ d1brup_ g1jou.1 d1sgfa_ d1autc_ g2hnt.1 g1kdq.1 d1orfa_
d3s69a_ d4crga_ d1fxya_ d1bqya_ d1svpa_ d1mbma_ d3su6a1 d1cu1a1
d4agka_ d1vcpa_ d1q31a_ g1n1l.1 d3e90b_ d3u1jb_ d1dzka_ d4tlja_

d2a2ua_ d4wfva_ d3t1da_ d4iaxa_ d3zq3a_ d2nnda_ d1i4ua_ d1kxoa_
d1bbpa_ d1kt7a_ d1exsa_ d3s26a1 d1lf7a_ d1qfta_ d4gh7a_ d1z24a1
d1qabe_ d1xkia_ d1ew3a_ d3bx7a_ d4iawa1 d1n0sa_ d1gkab_ d3kzaa_
d2k23a_ d2l9ca1 d2joza1 d1gm6a_ d3kffa_ d3saoa1 d1e5pa_ d1yupa1
d1iiua_ d4lu0a_ d1of8a_ d3unda1 d3e9aa1 d1vr6a2 d1n8fa_ d2qkfa_
d1d9ea_ d1e69a_ d1mv5a_ d3g60a4 g1ii8.1 d1ewqa2 d1ji0a_ d1qhla_
d1v43a3 d2wmfa1 d1vpla_ d1g2912 d4a82a2 g1f2t.1 d2d62a1 d3tuic1
d2bbsa1 d3g60a3 d2ixea_ d1g6ha_ d1sgwa1 d2qi9c_ d3rlfa1 d3b60a1
d3d31a2 d2pzea1 g1xew.1 d1jj7a_ d1oxxk2 d2onka1 d1w1wa_ d1iwea_
d1qf5a_ d1vmaa2 d1nija1 d1np6a_ d2p67a1 d3ihla_ d1okkd2 d1ls1a2
d1s1ma2 d1iona_ d2v40a_ d3hida_ d2vo1a1 d2g0ta1 d1vcoa2 d1j8mf2
d1g3qa_ d1xjca_ d1p9ba_ d1qzxa3 d1ihua2 d1ihua1 d2qm8a_ d1dj2a_
d4ioja_ d1cp2a_ d1yrba2 d1hyqa_ d3gqba2 d1skye3 d1xp8a1 d1tf7a2
d1tf7a1 d1ye8a1 d1skyb3 d2qe7d2 d4blta_ d4huta_ d3ldaa2 d4hyya_
d2i3ba1 d1cr1a_ d3j9tb2 d3ntua2 d1vdza1 d2z43a_ d1e9ra_ d1nlfa_
d3ziaa2 d1n0wa_ d1g6oa_ d3ziad2 d3j9ta2 d1pzna2 d3gqbb2 d2i49a_
d1nnfa_ d1xvxa_ d1ursa_ d1h3da1 d1iuda_ d4m1va1 d1twya_ d3e13x_
d2nxoa1 d4pfua_ d3ttma_ d1elja_ d2qmwa1 d1us5a_ d1y3na1 d1jeta_
d1uqwa_ d4f19a1 d2esna2 d3thia_ d3fasa1 d2uyeb_ d2xwva1 d1r9la_
d2fyia1 d1pb7a_ d2ozza1 d1sw5a_ d1nh8a1 d1vr5a1 d2g29a_ d1xs5a_
d3lswa_ d2z23a_ d3w9va_ d3tcha_ d1q35a_ d1z7me1 d3q27a_ d1p99a_
d1ve4a1 d2i6ea1 d4mcoa1 d4n8ya_ d1eu8a_ d1zbma1 d1hsla_ d1ixca2
d1j1na_ d1xvya_ d1y9ua_ d2z22a1 d1xoca1 d4oera_ d1wdna_ d5f1qa1
d1xt8a1 d1i6aa_ d4gwoa_ d4igta1 d1pc3a_ d4mmpa1 d1o63a_ d2czla1
d2onsa2 d4mija1 d4ok5a1 d2bmfa1 d2bmfa2 d1yksa1 d1yksa2 d1jr6a_
d2qeqa1 d2jlqa1 d1a1va2 d1eh3a_ d3skpa_ d1dtza2 d1ieja_ d1jnfa2
d1ce2a1 d1jnfa1 d1b1xa2 d1b1xa1 d1jw1a1 d1jw1a2 d1ryoa_ d1h76a1
d1h76a2 d1c4oa1 d2p6ra3 d2p6ra4 d1oywa2 d1oywa3 d1wrba1 d1w36b2
d1w36b1 d1c4oa2 d3bora_ d1t6na_ d2eyqa5 d2eyqa3 d2eyqa2 d2zu6a2
d1veca_ d1uaaa1 d1w36d1 d2g9na1 d1z3ix2 d2rb4a_ d1hv8a1 d1hv8a2
d1t5ia_ d1w36c2 d2j0sa2 d2j0sa1 d1fuka_ d3hjha1 d1z5za1 d1qdea_
d1z63a1 d1s2ma2 d1s2ma1 d1wp9a1 d1wp9a2 d1gm5a3 d1gm5a4 d3dina4
d3dina3 d1q0ua_ d1w36c1 d1z3ix1 d1nkta3 d1nkta4 d1w36d2 g1qhh.1
d2fwra2 d2fwra1 d2v8ta_ d3q3ma_ d1za0a1 d1mxra_ d2inca_ d1jk0a_
d2incb_ d1mhyb_ d3hf1b_ d1mtyb_ d1r2fa_ d2uw1a_ d1mhyd_ d3n37a_
d1afra_ d1oquc_ d1syya_ d1mtyd_ d2o1za_ d1uzra_ d1smsa_ d2fkza_
d1zuja1 d1s3qa1 d2fjca1 d1z6om1 d1veia_ d4dyya_ d1jiga_ d3gvya_
d2pyba_ d4cy9a1 d3bvfa1 d1r03a_ d2cf7a_ d1nf4a_ d1zs3a1 d4iwka_
d3ka8a_ d3np0x_ d1n1qa_ d1lb3a_ d4e6ka_ d1z6oa1 d2fzfa1 d4dyua_
d3ge4a_ d1jgca_ d4am4a_ d2z6ma_ d1ji5a_ d1vlga_ d1lkoa1 d1krqa_
d3qz3a_ d1o9ra_ d2ffxj_ d1euma_ d1yv1a1 d2v2pa_ d1j30a_ d4di0a_
d1dpsa_ d2cbia2 d1gqia1 d2choa2 d1oewa_ d2qzxa_ d2asia_ d1psoe_
d1miqa_ d1smra_ d2h6ta_ d1pfza_ d1izda_ d3hd8a_ d1dpja_ d4aa9a_
d1t6ex_ g1b5f.1 g1lya.1 g1htr.1 d1qdma2 d1bxoa_ d1wkra_ d1tzsa_

d1ibqa_ d2qzwa_ d1bdqa_ d3ggta_ d3ixoa_ d1mrxa_ d2haha_ d1tw7a_
d4njsa_ d1sgua_ d2p3da_ d1nsoa_ d3d3ta_ d2rkfa_ d1c6ya_ d2rspa_
d3ogpa_ d3ekwa_ d2o4na_ d2j9ja_ d1baia_ d3ggua_ d3lzua_ d4npua_
d4j55a_ d2r8na_ d2fxda_ d2p3ca_ d4dqea_ d3ttpa_ d3t3ca_ d2p3aa_
d2hc0a_ d2fdda_ d3mwsa_ d1wgra1 d1ef5a_ d1wfya1 d1e8ya3 d1wgya1
d1lfda_ d2cs4a1 d1wxma1 d2byea1 d3kh0a1 d2byfa1 d1wxaa1 d1e7ua3
d1h4ra3 d4yl8a1 d2al6a3 d1l7ya_ d1wgda1 d1wgga1 d4q5eb_ d1wjna1
d1se9a_ d3n3kb_ d1wz0a1 d1we6a1 d2m0xa_ d2gbka_ d3rt3b1 d1wh3a1
d1sifa_ d2ekec2 d4ii2b1 d4hk2a1 d1c3ta_ d1m94a_ d1wgha1 d1ttna1
d1j8ca_ d1wx8a1 d3v6cb1 d2ma9b_ d4i6lb1 d1bt0a_ d2jvca1 d1iyfa_
d2k1fa_ d1wxva1 d1wf9a1 d1t0ya_ d2io0b2 d1x1ma1 d2gbma_ d1zw7a1
d2m17a_ d1ud7a_ d1we7a1 d2lwpa_ d2gbja_ d1wjua1 d1v6ea1 d1v86a1
d4eewa_ d1wiaa1 d4icva_ d4gu2a_ d1wx7a1 d1wy8a1 d4hjka1 d2faza1
d1v5oa1 d1g66a_ d1qoza_ d5ajha1 d2h0ba_ d2erfa_ d1h30a2 d3mw4a1
d1pz7a_ d2r1ba_ d3v64a1 d1d2sa_ d3bopa1 d3mw3a1 d1okqa1 d1okqa2
g1y43.1 d1um8a_ d1ixza_ d1qvra2 d1qvra3 d1sxjc2 d1jr3d2 d2p65a_
d1l8qa2 d1g8pa_ d3hu3a3 d1sxjd2 d1njfa_ d1in4a2 d1sxja2 d1tuea1
d2ce7a2 d1w5sa2 d1sxjb2 d1d2na_ d1ny5a2 d1ofha_ d2gnoa2 d2a5yb3
d1fnna2 d1u0ja1 d1lv7a_ d1iqpa2 d2fnaa2 d1ixsb2 d4gita_ d1sxje2
d1g41a_ d2e3fa_ d1mm0a_ d2e3ea_ d2llda_ d2ny8x_ d2e3ga_ d2ny9x_
d2nz3a_ d2lj7a_ d1n4na_ d2m8bb1 d2kyqa_ d3psma_ d1jkza_ d1jxca_
d2gl1a_ d4uj0a_ d2k4ua_ d2lu9a_ d1baha_ d2meoa1 d1wm7a_ d1hlya_
d1n8ma_ d1jlza_ d1wpda_ d1c55a_ d1pvza_ d1wt7a_ d1rjia_ d1wz5a_
d1m2sa_ d1pjva_ d1c49a_ d1qkya_ d2ktca1 d1wmta_ d1du9a_ d1j5ja_
d1r1ga_ d1t0za_ d2i61a_ d1t1ta_ d1sn4a_ d1kv0a_ d2lkba_ d1npia_
d1wwna_ d1pe4a_ d2kbka_ d2li7a_ d1djta_ d2kbha_ d1dq7a_ d1sega_
d1omya_ d1zywa1 d1jzaa_ d2jcqa_ d1n9wa1 d1bbua1 d1e1oa1 d1c0aa1
d1l0wa1 d3a74a1 d1eova1 d1ixra2 d1iyjb4 d1iyjb5 d1iyjb3 d1o7ia_
d4ipca1 d1x3ea_ d4apva_ d1jb7a1 d1jb7a2 d1jb7a3 d3kdfa_ d1ue1b_
d1wjja1 d3eiva_ d1xjva2 d1xjva1 d2ccza2 d1se8a_ d1l1oc_ d1jb7b_
d1qvca_ d3ulla_ d1qzga_ d1miua4 d1miua5 d1miua3 d3g48a1 d1gd7a_
d1mkha_ d1pyba_ d3ubub_ d1egia_ d1qo3c_ d1v7pa_ d1tdqb_ d3ubua_
d1c3ab_ d1h8ua_ d3bx4b_ d1t8ca1 d1sb2a1 d1qdda_ d2ziba1 d3cada_
d1byfa_ d1j34b_ d1jwia_ d1jzna_ d1c3aa_ d1oz7b_ d1wmza_ d3bdwb_
d1hq8a_ d1fifa1 d3hupa_ d1wk1a1 d1umra_ d2afpa_ d3bx4a_ d1gz2a_
d1sl6a_ d1v7pb_ d3g8ka_ d1ixxa_ d1j34a_ d1jwib_ d1g1sa1 d1y17a1
d1ja3a_ d1fvub_ d3bdwa_ d1sb2b1 d1fvua_ d1umrc_ d1oz7a_ d4m17a_
d3cfwa1 d1ypqa1 d1g1ta1 d3fd4a_ d3l9jc_ d4dn8a1 d1v4lb_ d1rdl1_
d2msba_ d1dgsa2 d1fvia1 d1p16a1 d1x9na2 d1pfsa_ d2a1ka_ d1je5a_
d1rpma_ d2shpa1 d1yfoa_ d1l8ka_ d2ooqa1 d1g4us2 d1p15a_ d1lara2
d1lara1 d1wcha_ d2fh7a2 d2fh7a1 d1ohea1 d1ohea2 d2f4da_ d1rxda_
d2hxpa_ d1xria_ d1d5ra2 d1fpza_ d3ezza_ d4y2ea_ d3azya_ d3wvja_
d1upsa1 d1ajoa_ d2hyka_ d2vy0a_ d1o4ya_ d3o5sa_ d3d6ea_ d3atga_
d1dypa_ d1umza_ d3i4ia_ d1c1la_ d4lbqa_ d1a78a_ d1slta_ d4no4a_

d3zsja_ d3duia_ d1g86a_ d1is3a_ d1qmja_ d1hlca_ d1tdha3 d1q3ba3
d1ee8a3 d1k82a3 g1qmo.1 g1len.1 d3zyra_ d1fnya_ d1n47a_ g2ltn.1
d1avba_ d3usua_ d1dhkb_ d1f9ka_ d1ukga_ d1g8wa_ d1g9fa_ d1wbfa_
d3wcsa_ d1g7ya_ g1loe.1 d1mvqa_ d1hqla_ d1v6ia_ d1dbna_ d1qnwa_
d1fx5a_ d4l8qa_ d1ioaa_ d3n35a_ d3h33a_ d1rwja_ d19hca_ d1os6a_
d3bxua_ d3h4na_ d1i77a_ d3caoa_ d1j0pa_ d2ctha_ d1duwa_ d2e84a_
d1hh5a_ d1gyoa_ d3h34a_ d1wioa4 d1ccza2 d1vcaa1 d1dr9a2 d2d9qb1
d2nxyb2 d1adqa1 d1fnga1 d1uvqb1 d1zs8a1 d2vola1 d2vola2 d1mfbl2
d2jelh2 d1uwel2 d3qq4b1 d1kjvb_ d1k5na1 d1osph2 d4odhl2 d1ow0a1
d1ow0a2 d1bfob2 d3l9ra2 d3jtth1 d2fath2 d4elkb2 d1fn4b2 d2xfxb_
d1pg7x2 d1cqka_ d4k71b2 d1lk2a1 d1eapb2 d4lfhg2 d1i4fa1 d2nw2a2
d1kfah2 d1mhca1 d1h3ua1 d1wejh2 d1h0db2 d1a5fh2 d1kcvh2 d1mjuh2
d1qqda1 d1kcrh2 d1k8ia1 d3huja2 d2nnab1 d1nfdf2 d3mffb2 d1s9va1
d1lk3h2 d1q0xl2 d1fv1b1 d1neza1 d4rfeb2 d1r3ha1 d1a6tb2 d1kjva1
d1pz5b2 d1onqa1 d2bc4a2 d1de4a1 d1k8da1 d1tcra2 d1dn0b2 d1nfde2
d2xwtb2 d1bfoa2 d1mexh2 d1iakb1 d1ypzf2 d4f7ea2 d1q72l2 d2fbjh2
d1fe8h2 d1clzh2 d1fngb1 d1uvqa1 d1vgka1 d1c16a1 d4j12a2 d4x99b2
d1igtb3 d1je6a1 d1q72h2 d1nldh2 d1teth2 d2hmic2 d1i1ca2 d1i1ca1
d1k8ib1 d1q0yh2 d1kfal2 d3frua1 d1pg7w2 d1u58a1 d1igyb4 d1igyb3
d1hyrc1 d1klub1 d1deeb2 d1c12b2 d2bc4b2 d2ig2h2 d1kcvl2 d3wn5b1
d1lk2b_ d1lk3l2 d1mjul2 d1sm3h2 d1q0xh2 d1zvsa1 d1ydpa1 d1u8ib2
d1ypze2 d1kcuh2 d1op9a_ d1y0la1 d1etzb1 d1egjl1 d2gsib1 d1nj9a1
d4jfhd1 d1aqkl1 d1t2ja_ d1lo4h1 d1zvya1 d3v4vl1 d4enec1 d1kxtb_
d5e8ea1 d2pxya_ d4weud_ d1xiwc_ d2esvd1 d1qnzh_ d1ogae1 d1f4xh1
d4n1hb_ d3ezjb1 d3mlwl1 d1kfah1 d1etza1 d1b4jh1 d3cfic_ d4w68a_
d2arja1 d1ek3a_ d2ok0l1 d1vera_ d1uwgh1 d1blnb1 d1fpth1 d1rzga1
d2giya1 d4m3kb1 d1nc2a1 d2nyyd1 d1ncna_ d1fbih1 d5dxwa_ d4fqqa1
d1dqdh1 d1jfql1 d1kelh1 d1faih1 d1frgl1 d1figl1 d4m6nl1 d1fo0a_
d4hxbl1 d3b5ga_ d3quyd1 d2h9gb1 d1i8kb_ d2dd8h1 d2ck0h1 d1eaja_
d4w6wb1 d2a6ib3 d1t66d1 d2e27h_ d1ob1b1 d1f11b1 d2v7na1 d1ad0a1
d2xt1b1 d1pskh1 d4i13b1 d2op4l1 d1a0ql1 d1uwel1 d1za3a1 d1ncbl1
d4uu9a_ d1bm3h1 d1cloh1 d1nqba2 d1cf8h1 d4y5va_ d1mhph1 d5anma1
d4cdgc1 d4ldeb_ d1y0lb1 d2pcpb1 d1z3gl1 d1dfbl1 d1fh5h1 d3g5xa1
d1tzib1 d4dn3l1 d3zhka1 d2b1hh1 d2uyla1 d1f3dh1 d1nj9b1 d2agjh1
d2h1pl1 d1q72h1 d8fabb1 d1ndmb1 d1rjca1 d4u6hb1 d3upca_ d2fbjl1
d1osph1 d1tvda_ d2cjvh_ d4fhbd1 d1bd2d1 d1rz7h1 d3nn8a_ d1blna1
d1knoa1 d1nfda1 d2bmkb1 d1dqll_ d1igyb1 d1igfl1 d12e8h1 d3utza1
d1nc2b1 d1rihh1 d4mwfb1 d4jvpa1 d1mieh1 d1rhfa1 d1fgnl1 d1sbsh1
d1lp9e1 d1dn0a1 d4lqfl1 d1iaih1 d1kgcd1 d1mh5b1 d1gpol1 d2g60h3
d1f8tl1 d2jixd1 d4grwh_ d1fo0b_ d1bfob1 d1axsa1 d1u9ka_ d1nmbh_
d1h8na1 d1h8na2 d1jguh1 d4eura1 d3sdyl1 d35c8h1 d1w72l1 d1mvub_
d1i9rh1 d1ob1a1 d4wenb_ d1yeeh1 d3ietc1 d1f58h1 d1ad0b1 d1mqkl_
d1nbvh1 d4b50a_ d3r8bb_ d1bafl1 d1mamh1 d4rgne1 d2gsgb_ d35c8l1
d1bafh1 d1ai1h1 d2pcpa1 d2fath1 d2x1pa_ d1orsb1 d1ibgl1 d1shma_

d1a2yb_ d1u0qa_ d2coqa_ d1hilb1 d1qfwi_ d1jv5b_ d1fn4b1 d5e0qa_
d1nsnh1 d1plgl1 d4ocxl1 d1kb5h1 d1rzfh1 d1pg7x1 d2fx7h1 d2oi9c_
d1l7il1 d3eyva1 d1d5il1 d4ffyl_ d1tetl1 d1a6wh_ d1ac6a_ d3dvfa_
d1nfde1 d1mimh1 d1ri8a1 d1jhlh_ d3dura_ d1fskb1 d1f97a1 d3so3b1
d2ak4d1 d1lp9f1 d4z7wf1 d1l7th1 d4y5ya_ d4x7ec1 d1emth1 d1ikfl1
d1nlbh1 d4utal1 d1bjma1 d1eapb1 d1u6ah1 d2dtgd1 d1kj2b_ d1xeda1
d1c1eh1 d1fgvh_ d2bnub1 d2fb4h1 d2dtgb1 d1ktrh_ d1jpth1 d1qkzl1
d4hepg_ d2atpb1 d1flrh1 d2hrpl1 d1mvfa_ d1iaim1 d3k80a1 d2jb5h1
d3zkxc_ d4grwe_ d1it9h1 d1rhhb1 d3w9ec_ d1e4wh1 d1sy6h1 d2j6eh1
d3qxta_ d1jglh1 d1p7ka1 d1tzhb1 d4n8pa1 d1gafl1 d2jelh1 d1wioa2
d4i0cc1 d1cicb1 d1lmka2 d1lmka1 d1iqwh1 d1orqb1 d1ejoh1 d1jnlh1
d4zg1a_ d43c9b_ d1ggbl1 d1wejh1 d3q6fa1 d1f3dj1 d3c6la1 d1bqlh1
d3p9wb_ d1a3rl1 d1h0db1 d2uzil_ d1eo8l1 d1qfwl_ d1n7ml1 d1igja1
d1c5ch1 d2a9mh_ d1rmfh1 d1fj1b1 d4heme_ d1ccza1 d1a5fh1 d1ogad1
d1dlfh_ d1qa9b_ d1vgel1 d3qskb_ d1sjxa_ d4ozfg1 d1sq2n_ d1qd0a_
d1kcvh1 d1c5db1 d3qcul1 d8faba1 d3bn9d1 d1mjuh1 d4y8dc_ d7fabh1
d3dvgb1 d1lo0l1 d1a6tb1 d1gigh1 d1kcrh1 d2hwzl1 d1yjdc1 d3clfl1
d4krna_ d1dl7l_ d1e6ol1 d1mexh1 d3r0ma_ d4kkna_ d1fnsl1 d1oaqh_
d1rzia1 d1ucbh1 d1ncwl1 d4b41a_ d1nfdf1 d2mpah1 d2d7tl1 d4grwf_
d4b5ea_ d1lk3h1 d3u79a_ d4u3xa_ d1ghfl1 d1bvkb_ d1mcww1 d1b88a_
d1jrhh1 d32c2b1 d4wemb_ d1dsfh_ d1op3h1 d1ce1l1 d1iqda1 d1qoka1
d1qoka2 d1qfuh1 d2crya1 d1ay1h1 d1oaql_ d3ab0b_ d1rurl1 d1t4kb1
d1bz7b1 d1n0xh1 d1forl1 d1ucbl1 d4w6yb_ d43c9a_ d2aabl1 d1tzia1
d4n1ca_ d1k6qh1 d3g9ab_ d4tvsa1 d1igmh_ d1pz5b1 d3dx9a1 d3wxwh_
d1nakh1 d1kcul1 d3eaka_ d4qlra1 d1cr9l1 d1d9ka_ d1f4xl1 d1deea1
d2i27n2 d1c12a1 d2orbl1 d1j8hd1 d1mcph1 d1c5da1 d1kenh1 d1bd2e1
d1h5ba_ d4x7fc1 d3upaa_ d1t3fa1 d1indh1 d2dtgc1 d4hc1l1 d1zv5a1
d4wgvb_ d1uwgl1 d4f37k1 d1aqkh1 d1vesa_ d3zkqd_ d1fptl1 d1lo4l1
d1knob1 d3rrqa_ d1ct8b1 d4orzc_ d1mlbb1 d4hgkc1 d1dqdl1 d1bogb1
d4ozhe1 d1dr9a1 d1i7za1 d1dn0b1 d1kell1 d1rzib1 d1ypzf1 d1wtla_
d1hkfa_ d1fail1 d1figh1 d2vyre_ d1sjva_ d1ohqa_ d1bfoa1 d1pkqb1
d1mjul1 d1b4jl1 d1nsnl1 d2arjb1 d1p4bh_ d4aj0a_ d3nn8b_ d2rcja1
d1f2xk_ d4v1dd_ d1adqh1 d2eksa_ d4k3ga_ d1jfqh1 d1uweh1 d1frgh1
d4gftb_ d1uz8b1 d4n1ea_ d1bm3l1 d1clol1 d3mcka1 d2vq1a1 d1ae6h1
d4aq1b1 d1uj3b1 d1nbqa1 d1fh5l1 d2ck0l1 d2ywza_ d1q72l1 d4nbxb_
d1hila1 d4hzlb1 d2j88l1 d1zmya1 d1cd0a_ d1a4ka1 d1pskl1 d1a0qh1
d4krmb_ d1ncbh1 d2fbjh1 d1j8he1 d1i3ua_ d1cf8l1 d1fe8h1 d2p1ya1
d1eeqa_ d2x89a_ d1ol0a_ d2r0lh1 d1ub5a1 d1dfbh1 d3eyvb1 d4jdvb1
d4p2cg_ d2gsia1 d2agjl1 d1u3ha1 d2h1ph1 d1rihl1 d1fskc1 d1boga1
d2a77l1 d1ospl1 d1qfoa_ d3dwta_ d2fx7l1 d1rz7l1 d3stba1 d1pkqa1
d1b6da1 d1dzba2 d1dzba1 d1kj2a_ d4l1ha_ d1rzgb1 d1dqlh_ d12e8l1
d1miel1 d1jmaa_ d1sbsl1 d1fgnh1 d1f58l1 d1mqkh_ d1ndgb1 d3qegl1
d1iail1 d2aw2a1 d1tqbc1 d3fo2a1 d4dkaa_ d1f8th1 d2g60l1 d2ywya_
d1uj3a1 d1yc7a_ d3bd3a_ d4w6xb1 d1nmbl_ d1i8ka_ d1rjlb1 d1ypze1

d1p7kb1 d1kxqe_ d1gpoh1 d2z4qa1 d1w72h1 d1i9rl1 d4qgya1 d4xt1c_
d2qqnh1 d1cica1 d3c08h1 d2cd0a_ d2bdnh1 d1hnga1 d1maml1 d1nldh1
d1cr9h1 d4lcih1 d3bkyh1 d3qg7l1 d1ai1l1 d1kxvc_ d4iosd_ d1l7ih1
d2x1qa_ d1ub6b1 d3k74b_ d1ibgh1 d1teth1 d1igya1 d1hxma1 d2x6ma_
d2e7la_ d5e03a_ d1qfwm_ d2r0kh1 d1igjb1 d1rurh1 d1plgh1 d1mj8h1
d1yuhb1 d3fmgl1 d1rzfl1 d1emtl1 d1ad9b1 d1igtb1 d1nlbl1 d1d5ih1
d3k7ua_ d4ffyh_ d1bwwa_ d3dvga1 d1a6ta1 d1fgvl_ d4qxgl1 d1miml1
d1jhll_ d2hffb1 d2hh0l1 d1qkzh1 d1l7tl1 d1afvh1 d1iaii1 d2cjuh_
d1um5h1 d2ghwb2 d2ghwb1 d1kgce1 d2fgwh1 d1ikfh1 d1dvfd_ d2i24n1
d2xtjb_ d1za3b1 d2yc1a_ d2hh0h1 d2x1oa_ d1bvka_ d1rjla1 d5e7ba1
d1igta1 d1il1b1 d2fb4l1 d1mvua_ d1jptl1 d32c2a1 d1fvca_ d1iqdb1
d2hrph1 d2p4ab_ d4qo1a_ d1p2cb1 d1wt5c_ d1t4ka1 d5c1mb_ d1it9l1
d1bz7a1 d1b2wh1 d1e4wl1 d1jnll1 d1yedb1 d1za6b1 d3u7aa_ d1igfh1
d3gnml1 d1orsa1 d1wejl1 d1a2ya_ d1hxmb1 d1gafh1 d2j4wh1 d1pz5a1
d1j05a_ d1a3rh1 d4fqll1 d1qfwh_ d1eo8h1 d1n7mh1 d4s11a_ d1kegh1
d5da4a_ d1iqwl1 d3whxb1 d1vgeh1 d1deeb1 d1c12b1 d1t2ql1 d1ad9a1
d3gsnb1 d3b2uc1 d1ggbh1 d1t3fb1 d1nakl1 d4ocmc_ d1lila1 d2v7hb3
d2esve1 d1mcoh1 d2qr0b1 d2q3aa_ d1ct8a1 d1kcrl1 d2pxyb_ d2uzih_
d5c3ld_ d3durb_ d2ak4e1 d1dl7h_ d1xiwd_ d1mlba1 d1rmfl1 d1i7zb1
d1dlfl_ d2xa3a_ d5bq7b1 d1dqqb1 d3h42l1 d1egjh1 d1kcvl1 d1fe8l1
d2j6el1 d1f3rb2 d1lk3l1 d1sm3h1 d1q0xh1 d1ghfh1 d7fabl1 d1igch1
d1lo0h1 d4idla_ d5c2bl_ d1jrhl1 d1il1a1 d3zhda1 d4kfzc_ d1b0wa_
d1e6oh1 d1mexl1 d4krpb_ d1fnsh1 d1ncwh1 d4hgma_ d4eigb1 d4rzca_
d2mpal1 d4y5xa_ d5en2b1 d3lrga1 d4ej1c1 d1rhha1 d1j05b_ d1forh1
d1q9wb1 d2wzpd_ d2aq2a1 d1h0da1 d3revb1 d2ij0c1 d1tzha1 d4m7jl1
d1k6ql1 d3b9kc1 d1igml_ d3qeua1 d1dsfl_ d1ce1h1 d1kcuh1 d1zvha1
d1zvoa1 d1tqbb1 d1ay1l1 d2qscl1 d2cgrh1 d1nl0h1 d1kenl1 d3eo0a1
d4n9ob1 d1n0xl1 d3k1kc_ d1d4ua2 d2cqva1 d1fhga_ d2k1ma_ d2dl2a1
d1biha3 d1biha1 d1biha4 d3qp3a1 d1g1ca_ d1gxea_ d1cvsc2 d1cvsc1
d1xmwa2 d2otpa2 d1biha2 d1rhfa2 d2fcba2 d2fcba1 d1wwbx_ d1gsma2
d1epfa1 d1epfa2 d2dava1 d1waaa_ d1f97a2 d1x44a1 d1vcaa2 d1fnla2
d1fnla1 d3b5ha2 d3h8na2 d3h8na1 d1jbja1 d1jbja2 d1pd6a_ d1f2qa2
d1f2qa1 d3cx2a_ d1nbqa2 d3b5ha1 d1n26a1 d1xiwb_ d1xaua_ d1fltx_
d2ifga1 d1olla1 d1olla2 d1cs6a4 d1cs6a3 d1cs6a2 d1cs6a1 d2gw5a2
d1sy6a1 d1xiwa_ d1ucta2 d1ucta1 d1ry7b2 d1gl4b_ d1qz1a3 d1olza1
d2c9aa1 d1wwca_

**Family-31 (b.40.4.3)**

**Positive dataset contains 11 protein sequences.**

d4ipca1 d1x3ea_ d4apva_ d3kdfa_ d1ue1b_ d1wjja1 d3eiva_ d1xjva2
d1xjva1 d2ccza2 d1se8a_

**Negative dataset contains 3850 protein sequences.**

d1xfda2 d1m32a_ d1ibja_ d1c7na_ d1iuga_ d4q75a_ d2bkwa1 d1t3ia_
d1n8pa_ d1qz9a_ d1jf9a_ d1eg5a_ d1p3wa_ d3kgwa_ d1cl1a_ d2ctza1
d1vjoa_ d2rfva_ d1gc0a_ d1cs1a_ d1d2fa_ d2ch1a1 d1pffa_ d1elua_

d1u08a_ d1lc5a_ d1b5pa_ d1bw0a_ d3pd6a_ d7aata_ d2csta_ d1j32a_
d1fg7a_ d4wlha_ d1o4sa_ d1v2da_ d1gdea_ d1m6sa_ d1v72a1 d1svva_
d1ajsa_ d1wsta1 d2f8ja2 d1lk9a_ d1vp4a1 d1xi9a1 d2gb3a1 d1yaaa_
d1xkla_ d2wfla_ d3i2ka1 d1lnsa3 d1xkta1 d2hfka_ d1jmkc_ d5d3ka_
d2cfba1 d1dfoa_ d1o69a_ d1bj4a_ d3k28a_ d2bwpa_ d3lg0a_ d2fnua_
d3ou5a_ d1z7da1 d1b9ha_ d1iaya_ d4msoa_ d1bs0a_ d1s0aa_ d1sffa_
d3tqxa_ d1mdoa_ d3qboa_ d3pgya_ d1bjna_ d2dkja_ d1ejia_ d1vefa1
d4wxga_ d3bs8a_ d3gbxa1 d1w23a_ d4nmwa_ d2tbvc_ d1c8nc_ d1x36a_
d1smvc_ d1ng0c_ d4sbvc_ d1opoa_ d1f2nc_ d1qnia1 d1fwxa1 d1f56a_
d1bypa_ d1azca_ d1ws8a_ d3u25a_ d1cuoa_ d1id2a_ d1rkra_ d2iaac_
d4dp1x_ d2gima_ d2i7sa_ d1qhqa_ d3ehbb1 d1fftb1 d1gyca3 d1hfua2
d1kbva1 d1kbva2 d1sdda1 d1sdda2 d1gyca1 d1gyca2 d1mzya1 d2bw4a1
d1aoza1 d1aoza3 d1aoza2 d1kyaa2 d1kyaa3 d1kyaa1 d1mzya2 d4e9sa1
d1sddb1 d1sddb2 d1hfua1 d1hfua3 d1oe1a2 d1v10a2 d1v10a3 d1v10a1
d1rypd_ d1irua_ d1rypc_ d3unfk_ d1rypa_ d1j2pa_ d1irui_ d1j2qh_
d3unfh_ d1m4ya_ d1rypb_ d1irul_ d1irug_ d1iru2_ d1q5rh_ d1rypg_
d1g3ka_ d1iruf_ d1q5qa_ d1e94a_ d1irub_ d1ryph_ d3ty6a_ d1iruj_
d1iruk_ d1rype_ d1irud_ d1irue_ d3mi01_ d1rypf_ d1iruh_ d2z3ba_
d1iruc_ d3mka2_ d1iru1_ d3sxwa1 d1j3qa_ d4luka_ d2bema_ d1pama1
d1ea9c1 d1qhoa1 d2fhfa2 d2fhfa1 d1k3ia1 d1j0ha1 d1cxla1 d2bhua1
d1edqa1 d1f1sa2 d1ut9a2 d1gvia1 d1txka1 d2phla2 d2phla1 d1fxza2
d1fxza1 d1lrha_ d1uija1 g1dgw.1 d2v09a_ d1uika2 d1uika1 d1dgwa_
d1ktja_ d1ex0a1 d1g0da1 d1cg2a1 d1yloa2 d1y7ea2 d1y0ya3 d1lfwa1
d1vixa1 d1fnoa4 d1vhoa2 d1ysja1 d1vhea2 d1z2la1 d2fvga2 d1vgya1
d1r3na1 d2grea2 d1xmba1 d1rtqa_ d2bjca1 d2hsga1 d1qpza1 d1k9ua_
d2opoa_ d1r0ka3 d1vkoa2 d1vm6a2 d3qvsa2 d1gr0a2 d1p1ja2 d2ovga_
d1sq8a_ d1rzsa_ d3kz3a_ d3orca_ d3gpdg2 d2q49a2 d1hdgo2 d2g17a2
d2cvoa2 d2czca1 d3pwka2 d1obfo2 d1i32a2 d1zyao2 d2pkqo2 d1b7go2
d2hjsa2 d1cf2o2 d1vkna2 d1nvmb2 d1ux8a_ d1dlya_ d4i0va_ d2bkma_
d2bmma_ d2msya_ d2djoa1 d2r5za_ d1au7a1 d1f43a_ d2lmda1 d1akhb_
d1s7ea1 d3nara_ d1zq3p1 d2craa1 d1mija_ d4uuta_ d3a01a_ d1x58a1
d3rkqa1 d3cmya1 d2cqxa1 d2da6a1 d1e3oc1 d1fjla_ d1mh3a1 d1ig7a_
d1x41a1 d1wh7a1 d1gt0c1 d2mw8a_ d1lfup_ d1x2ma1 d1b72a_ d1wi3a1
d1jgga_ d1wjha1 d1x2na1 d1mnmc_ d2cuea1 d3k2aa_ d2hosa_ d2jwta2
d1pufa_ d1octc1 d2hi3a_ d1b8ia_ d4j19a_ d3lnqa_ d1wh5a1 d1b8ib_
d1fexa_ d2cqra1 d1guua_ d1ug2a1 d2cqqa1 d2ajea1 d4a69c_ d1h8ac1
d2crga1 d2iw5b1 d1xc5a1 d2cu7a1 d1wgxa1 d2cjja1 d2ckxa1 d1gvda_
d1tc3c_ d1gdta1 d1u78a2 d6paxa2 d1k78a1 d1k78a2 d1hlva1 d1hlva2
d1iufa2 d2dara2 d2dara1 d1wyha2 d2dloa2 d1rutx4 d1rutx3 d2cuqa2
d1x4la2 d1x4la1 d1x3ha2 d1x3ha1 d2d8za1 d1x61a2 d1x6aa1 d1x6aa2
d2cora2 d2cora1 d1v6ga1 d1v6ga2 d2d8za2 d1x64a1 d1x64a2 d1b8ta1
d2co8a1 d1x63a2 d2dj7a2 d2dj7a1 d2cu8a2 d2cu8a1 d1g47a2 d1g47a1
d1j2oa1 d1wyha1 d2dloa1 d1wiga1 d1wiga2 d2cupa2 d2cupa3 d1x68a1
d2d8ya2 d2d8ya1 d1m3va2 d1x62a1 d1x68a2 d1x61a1 d1b8ta3 d1b8ta2

d2d8xa2 d2d8xa1 d1x4ka1 d1x4ka2 d2cuqa1 d1m3va1 d2co8a2 d1j2oa2
d1x63a1 d2cura1 d2cura2 d1x62a2 d3lv8a_ d1s3ga1 d1zaka1 d1s4qa_
d3tvta2 d1uf9a_ d1khta_ d1ckea_ d1osna_ d1p5zb_ d4tmka_ d3cm0a_
d1tmka_ d1jaga_ d3h86a_ d1t0hb_ d1nn5a_ d2h92a_ d3kb2a1 d2c95a1
d1yj5a2 d1kgda_ d1ly1a_ d2ak3a1 d1vyva2 d1deka_ d2grja1 d1p3ja1
d1y63a_ d1e4ya1 d1s96a_ d3iija1 d1lvga_ d1nksa_ d1vyua2 d4e22a_
d1qf9a_ d1jjva_ d1q3ta_ d1ki9a_ d1lw7a2 d1teva_ d1kjwa2 d1gsia_
d1g3ma_ d1j99a_ d1t8ta1 d1nsta_ d1zrha_ d1ls6a_ d2a3ra_ d1aqua_
d2zptx_ d3ckla_ d1texa_ d1q44a_ d2gwha_ d1z0ja1 d3umra2 d3gj0a1
d2gaoa1 d1yzga1 d1x3sa1 d1g7sa4 d1puja_ d1udxa2 d1kk1a3 d2o52a_
d1jwyb_ d1zcaa2 d1zcba2 d2fv8a1 d2cjwa_ d3dz8a1 d1xzpa2 d1vg8a_
d4qxaa_ d2ew1a1 d1x1ra1 d2bcgy_ d3doea1 d3cona_ d3rega_ d3wyga_
d1upta_ d2b6ha1 d1zd9a1 d1r5ba3 d1n0ua2 d1r2qa_ d2f9la1 d2rhda_
d3q3jb_ d3q85a_ d1efma1 d1r8qa_ d2gf9a1 d2akab1 d1ek0a_ d1z08a1
d2gcoa1 d2dy1a2 d4g01b_ d2cxxa1 d1svia_ d3q72a_ d1ni3a1 d4ac9a4
d2erxa1 d2bmea_ d1wxqa1 d2gf0a1 d4ldja1 d1zunb3 d1d5ca_ d4lv5b_
d1efca3 d1kmqa_ d2g6ba1 d3lrpa_ d1tada2 d2y8ea_ d4rd4a1 d1z06a1
d2heia_ d3a58b_ d1u0la2 d1d2ea3 d1zj6a1 d2ocba_ d3rwoa_ d1mr3f_
d2e9sa1 d4u5xa1 d2iwra_ d2fn4a1 d3bwdd_ d2nzja1 d2atva1 d2ksqa_
d3l43a_ d1mkya2 d1mkya1 d1wmsa_ d1jnya3 d2rmka_ d1f6ba_ d1f60a3
d1gota2 d1jala1 d2fh5b1 d1g16a_ d2wblc_ d1m7ba_ d1wf3a1 d1f5na2
d1t9ha2 d1z6xa1 d1z0aa1 d2fg5a1 d2a5ja1 d2bcjq2 d1s0ua3 d1ky3a_
d4y0va_ d4lhwa_ d2qmea1 d2atxa1 d1egaa1 d1ryha_ d1moza_ d1nrjb_
d2erya1 d3bfka_ d1puia_ d1fzqa_ d3j3az1 d3t5ga_ d1shza2 d3defa_
d2f7sa1 d3raba_ d3tsoa_ d3aq4a_ d2al7a1 d3sfva1 d3bc1a_ d2ncda_
d1sdma_ d3h4sa1 d2heha_ d2grya_ g3kin.1 d1kk8a2 d1br2a2 d1goja_
d1f9va_ d2mysa2 d1lkxa_ d3t0qa_ d4db1a1 d3gbja_ d1v8ka_ d1k8ka2
d1k8ka1 d3uleb1 d3i33a2 d3i33a1 d1bupa1 d1dkgd2 d1dkgd1 d4ci6a2
d1jcfa1 d4a2ba2 d2fxua1 d1yaga2 d1yaga1 d4cbua2 d1g99a2 d2e1za2
d2e1za1 d1saza1 d1saza2 d1ig8a1 d1czan2 d1czan3 d1ig8a2 d1czan4
d1bg3a4 d1bg3a3 d1bg3a2 d1bg3a1 d3h1jd1 d2d0oa3 d2d0oa2 d1nbwa3
d1hroa_ d4gedb_ d3zooa_ d1mz4a_ d1gu2a_ d1f1fa_ d1dvva_ d1c6ra_
d1c75a_ d1e29a_ d1gdva_ d1cnoa_ d1h32b_ d1co6a_ d2bh4x_ d2aiua_
d5cytr_ d1jdla_ d1i8oa_ d2zboa_ d3dmia_ d1ls9a_ d1wvec_ d2yk3a_
d3ph2b_ d1dw0a_ d5aura_ d1ql3a_ d1qn2a_ d1f1ca_ d4zzva_ d2xspa_
d1eg1a_ d4v20a_ d4ipma1 d1gpia_ d4csia_ d3pfza_ d3ovwa_ d2yoka_
d2nqya_ d1h4ga_ d1hixa_ d1oa2a_ d1olra_ d3b7ma_ d4h7ma1 d3wp3a_
d2vgda_ d1igoa_ d3zsea_ d2nlra_ d3wx5a_ d1oa3a_ d1pvxa_ d1m4wa_
d1f5ja_ d1oa4a_ d1ks5a_ d4ixla1 d1h32a1 d1evsa_ d1cnt1_ d1pvhb_
d1z7ca_ d1huwa_ d1bgea_ d1rhga_ d1f6fa_ d1i1rb_ d1axia_ d1etea_
d3tgxb_ d1scfa_ d2z3qa1 d3qb1a_ d1hula_ d1m48a_ d4nkqb_ d1eera_
d1v7mv_ d5e4ea_ d2o27a_ d3b5ka_ d2psma1 d4zf7a_ d3uf5a_ d4x51a_
d4ypgc1 d3oq3a_ d1lqsl_ d1n1fa_ d1au1a_ d1wu3i_ d3besl_ d3ux9a_
d4dnxa2 d1v47a2 d1g8fa2 d1jhda2 d1x6va2 d3rbaa_ d2qtra_ d1nupa_

d3l92a_ d1f9aa_ d1qjca_ d2h29a_ d1vlha_ d1lw7a1 d1mrza2 d4ruka_
d2el7a_ d1iq0a2 d1irxa2 d1r6ta2 d2cyba_ d1rqga2 d1j09a2 d2yxna_
d1f7ua2 d1h3fa1 d1ffya3 d2g36a1 d1nzja_ d1jila_ d1n3la_ d1i6la_
d1pfva2 d1h3na3 d3n9ia_ d3sz3a1 d3sw2a_ d1moxc_ d1dx5i3 d1edmb_
d1autl2 d1autl1 d1nqlb_ d1dval1 d1szba2 d1kigl_ d2y5fl_ d1ijqa2
d1gk5a_ d1g1sa2 d1nt0a3 d1lmja2 d1lmja1 d1nzia2 d2wphe_ d1klil_
d1pfxl2 d1pfxl1 d1p9ja_ d1g1ta2 d1f7ea_ d4yzub_ d1nuba2 d2mgpa1
d1b9wa1 d2mgpa2 d1ob1c1 d1ob1c2 d1n1ia2 d1n1ia1 d2npra2 d2npra1
d1m1xb5 d1qx2a_ d2y5ia_ d1qlsa_ d1psra_ d3n22a_ d2egda_ d4ggfc_
d2wcea_ d2luca_ d4aqia_ d3nsia_ d2lp3a_ d1j55a_ d1a4pa_ d1nsha_
d1pvaa_ d3fs7a_ d2pvba_ d1ttxa_ d2kyfa_ d1rk9a_ d1a75a_ d1exra_
d1tiza1 d1nyaa_ d1g8ia_ d1ggwa_ d1wdcc_ d3fwba_ d1ggza_ d2o5ga_
d1ctaa_ d3i5gc_ d1sl8a_ d1fpwa_ d2scpa_ d2roaa_ d4aqra_ d3pn7b_
d1br1b_ d2mysb_ d1s6ca_ d1bjfa_ d2obha_ d1jfka_ d1jf0a_ d1qv0a_
d1lkja_ d2mlfc_ d2m97a_ d2d8na_ d4by5a_ d1jbaa_ d4nqga_ d1wdcb_
d2mysc_ d1j7qa_ d1s6ia_ d1uhka2 d1r2ua_ d1dtla_ d3kf9a_ d3pn7c_
d1qjta_ d1fi6a_ d5d67d_ d1tuza_ d1h8ba_ d1eg3a2 d2zkmx1 d1snla_
d1gjya_ d1y1xa_ d1hqva_ d1dvia_ d1df0a1 d1qxpa2 d4usla_ d1jfxa_
d1wkya2 d1uuqa_ d3wh9a_ d4m1ra_ d1xyza_ d4xuya_ d2je8a5 d4bf7a1
d3cuia_ d1ur4a_ d1qw9a2 d1odza_ d2vzsa5 d1bqca_ d2x2ya1 d1hjqa_
d1qnra_ d4y7ea1 d1vjza_ d4k68a_ d2v3ga_ d4hpga_ d1nofa2 d1nq6a_
d1h4pa_ d1aq0a_ d3ziza_ d4bs0a_ d1ecea_ d1h1na_ d1ta3b_ d1w91a2
d1i1wa_ d1uhva2 d1egza_ d7a3ha_ d1tvna1 d3nyda1 d1ghsa_ d2cyga1
d4xv0a_ d2c8na2 d1w32a_ d3emca_ d1v6ya1 d1foba_ d2uwfa1 d2fgla_
d3niya1 d2c0ha1 d1rh9a1 d1yq2a5 d1szna2 d1fa2a_ d1pama4 d5do8a1
d1ea9c3 d1hx0a2 d2fhfa5 d1qhoa4 d1avaa2 d1m53a2 d4m8ua1 d2gjpa2
d1lwha2 d4howa1 d1ktba2 d1j0ha3 d3vm5a1 d2x1ia_ d1wzaa2 d2diea1
d1mxga2 d1ht6a2 d1eswa_ d1iv8a2 d5cpsa_ d1ud2a2 d1zjaa1 d3gbea1
d1kwga2 d1wdpa_ d1gcya2 d1g5aa2 d1hvxa2 d3dhpa2 d2bhua3 d4do4a1
d3qgva1 d1e43a2 d1g94a2 d2d3na2 d1gjwa2 d1gvia3 d1x1na1 d1r7aa2
d1tz7a1 d4toqa_ d1wb0a1 d1w9pa1 d1eoka_ d1itxa1 d1kfwa1 d1goia2
d1edqa2 d2gsja_ d1hjxa1 d1jnda1 d1vf8a1 d1etob_ d1umqa_ d1g2ha_
d1g4ma1 d1g4ma2 d2y8ga_ d1xkea1 d2y8fa_ d2v76a_ d1wj1a1 d1aqca_
d1qqga2 d1ntva_ d1n3ha_ d4dbba_ d3f0wa1 d1p3ra_ d1wgua1 d2cy4a1
d3hqca_ d1wvha1 d1xr0b1 d3nvva1 d1krha3 d2h88b1 d1n62a2 d1t3qa2
d1kf6b2 d1zoyb1 d1jq4a_ d1ffva2 d1dgja2 d1rm6c2 d1h6ua1 d9ldta1
d2ewda3 d1up7a1 d2dfda1 d1s6ya1 d1vjta1 d7mdha1 d1hyea1 d1t2da1
d1ojsa3 d1b8pa1 d1ldna1 d1hyha1 d1civa1 d2a92a1 d1ez4a1 d1i0za1
d1y6ja1 d1gv0a1 d1ur5a1 d1llda1 d1obba1 d1v8ba1 d1gdha1 d1l7da1
d2dlda1 d1mx3a1 d1ygya1 d1li4a1 d1j4aa1 d2naca1 d1npya1 d3t4ea2
d1a4ia1 d1o0sa1 d2hk9a2 d2egga2 d1b26a1 d1bvua1 d1gq2a1 d1c1da1
d1leha1 d1gtma1 d1euza1 d1nvta1 d3dona2 d1edza1 d1gpja2 d1vi2a1
d1p77a1 d1nyta1 d1vl6a1 d1v9la1 d1txga2 d3f47a1 d1mv8a2 d2pv7a2
d1xc2a3 d2ahra2 d4xiya1 d2f1ka2 d1pgja2 d1i36a2 d1np3a2 d1lj8a4

d1teaa3 d1qmga2 d2i76a2 d2g5ca2 d1wdka3 d1dlja2 d3cuma3 d1kola2
d1f8fa2 d1qora2 d1jqba2 d1vj0a2 d1cdoa2 d1gu7a2 d1d1ta2 d1xa0a2
d2dm6a2 d1jvba2 d1rjwa2 d1llua2 d1tt7a2 d1piwa2 d1o89a2 d1iz0a2
d1e3ja2 d1yb5a2 d1uufa2 d1vj1a2 d1e3ia2 d3gpdg1 d1vc2a1 d2nvwa1
d1u8fo1 d1ydwa1 d1hdgo1 d2g17a1 d1cf2o1 d2czca2 d1r0ka2 d1obfo1
d2cvoa1 d1ebfa1 d1h6da1 d1i32a1 d1zh8a1 d2q49a1 d1qkia1 d1j5pa4
d3pzra1 d1xeaa1 d1vkoa1 d1vm6a3 d3qvsa1 d2pkqo1 d1b7go1 d1gr0a1
d1p1ja1 d1lc0a1 d1j0xo1 d3pwka1 d2hjsa1 d1zyao1 d1vkna1 d1tlta1
d1nvmb1 d3sjua_ d3ojfa1 d1xu9a1 d2jjya1 d1n7ha_ d1y1pa1 d1h5qa_
d1jtva_ d4fs3a_ d2bkaa1 d1gy8a_ d2c5aa1 d1ulsa_ d1ooea_ d2b69a1
d1y5ma1 d1spxa_ d1ae1a_ d1bxka_ d1ydea1 d1gega_ d1hdoa_ d1o5ia_
d1e6ua_ d1snya1 d2rhca_ d3zv4a_ d5ceja_ d2d1ya1 d1fjha_ d3w8ea_
d2fmua1 d1x1ta_ d1rpna_ d3ay7a1 d1k2wa_ d1eq2a_ d2hrba1 d3ruaa_
d2ew8a1 d4hwka_ d1xgka_ d2bd0a1 d1yo6a1 d2a35a1 d2ntva_ d1vl8a_
d1hdca_ d4bkua_ d2ag5a1 d2q45a_ d1nffa_ d4iy1a_ d1zbqa1 d1kewa_
d1ek6a_ d1ja9a_ d2q46a2 d1iy8a_ d1cyda_ d2ptga_ d1hxha_ d1uzma1
d3lyla_ d4m89a_ d3wyea_ d2blla1 d1w6ua_ d1sb8a_ d2q2va_ d4nr0a_
d1e6wa_ d2gasa_ d3oiga1 d2bgka1 d1mxha_ d1vl0a1 d1rkxa_ d1r6da_
d1wmaa1 d1e7wa_ d2c07a1 d1xkqa_ d3tzha_ d1g0oa_ d3lz6a_ d1qyca_
d1edoa_ d1oc2a_ d1t2aa_ d1zema1 d2xoxa_ d1z45a2 d1geea_ d1p33a_
d3lt0a_ d4e6pa_ d2wyua_ d1db3a_ d4q9na_ d1xhla_ d4o1ma_ d1fmca_
d2uvda_ d2gdza1 d1uaya_ d2z1ma_ d2o23a2 d2fr1a2 d2fr1a1 d1qsga_
d1qyda_ d4zjua_ d2a4ka1 d1yb1a_ d1wvga1 d1yxma1 d3f4ba_ d1mg5a_
d1xg5a1 d1e2ta_ d4c5pa_ d2bsza1 d4bgfa_ d1w4ta1 d3u1ra1 d1g9ka2
d1kapp2 d1k7ia2 d1t3ca_ d1epwa3 d2a97a_ d3fiea_ d3ioqa_ d1fh0a_
d1o0ea_ d1khqa_ d1m6da_ d2cb5a_ d1cs8a_ g8pch.1 d1s4va_ d2pnsa_
d1vsna1 d1mira_ d1iwda_ d2oula_ d7pcka_ d3bwka_ d3bcna_ d2p86a_
d1pvja_ d1pcia_ d1gece_ d1deua_ d4bs6a_ d1xkga1 d1cqda_ d1jqpa2
d2c0ya_ d2bdza_ d3p5ua_ d2jk6a2 d2jk6a1 d1feca1 d1feca2 d1lpfa1
d1lpfa2 d1xhca1 d1xhca2 d1dxla2 d1dxla1 d1gera2 d1m6ia2 d1onfa1
d1onfa2 d3lada2 d3lada1 d1w4xa1 d1w4xa2 d1gesa2 d1mo9a1 d1xdia1
d1fl2a2 d1fl2a1 d1fcda1 d1fcda2 d1h6va2 d1h6va1 d1mo9a2 d1gv4a2
d1gv4a1 d2gv8a2 d2gv8a1 d1aoga1 d1aoga2 d1hyua1 d1ebda1 d1ebda2
d3dk9a1 d1q1ra2 d1gesa1 d1pv8a_ d1w5qa_ d2c1ha_ d1l6sa_ d1zbfa1
d2z1ha_ d2etja1 d3qioa1 d1jl1a_ d2e4la_ d1jl2a_ d2lsna1 d1uaxa_
d2yv0x_ d1mu2a1 d1ekea_ d1i39a_ d1pn0a1 d1reoa1 d1ju2a1 d2f5va1
d3c96a1 d2gmha1 d1pj5a2 d1gpea1 d1n4wa1 d1k0ia1 d2dw4a2 d2ivda1
d1seza1 d2voua1 d1kdga1 d1s5ja1 d2hbja2 d2igia_ d1wlja_ d1qhta1
d4dlea1 d1tgoa1 d2f96a1 d3tr8a1 d3mxma_ d1q8ia1 d1y97a1 d1w0ha_
d1kfsa1 d2py5a1 d1noya_ d1d5aa1 d1yt3a3 d2guia_ d3mwpa1 d4kb1a_
d1iv0a_ d1nu0a_ d1js9c_ d1f15b_ d1za7a_ d1laja_ d1r7ha_ d1faaa_
d1wika1 d1xfla_ d1ttza1 d2f51a1 d1ti3a_ d1nhoa_ d1sena_ d2i4aa_
d1ep7a_ d1zmaa1 d1fo5a_ d3lefa1 d3diea1 d2yana1 d1z6na1 d1oaza_
d3zywa1 d4aj8a_ d1nm3a1 d4fiwa_ d1wmja_ d4hs1a1 d1iloa_ d1xwaa1

| | | | | | | | |
|---|---|---|---|---|---|---|---|
| d1wjka1 | d2vm1a_ | d1fb6a_ | d1gh2a_ | d1h75a_ | d1nw2a_ | d1kx5b_ | d3a6na_ |
| d4m6ba2 | d2nqbd_ | d3wkjd_ | d2yfwe_ | d4h9na_ | d5bnxa_ | d1kx5c_ | d3r45a_ |
| d1eqzb_ | d4jjnb_ | d1f66c_ | d1eqza_ | d4wnnb_ | d1kx5a_ | d1u35c1 | d1id3a_ |
| d2nqbc_ | d1kx5d_ | d1tafa_ | d1bh9b_ | d1tafb_ | d1jfib_ | d1q9ca_ | d1h3ob_ |
| d2byka1 | d1n1ja_ | d4g92b_ | d1bh9a_ | d1jfia_ | d1h3oa_ | d2bykb1 | d1pd212 |
| d1nhya2 | d2a2ra2 | d1k0dd2 | d1f2ea2 | d1gsua2 | d1f3ba2 | d2fhea2 | d2pvqa1 |
| d1tw9a2 | d1oyja2 | d1e6ba2 | d1k0ma2 | d3ik7a1 | d4is0a1 | d1r5aa2 | d1jlva2 |
| d1m0ua2 | d3gtub2 | d1z9ha2 | d1okta2 | d1axda2 | d2gsra2 | d1tu7a2 | d1k3ya2 |
| d1zgma1 | d1b48a2 | d1gnwa2 | d1gwca2 | d2gsta2 | d1glqa2 | d1oe8a2 | d1v2aa2 |
| d1fw1a2 | d1duga2 | d3vrda1 | d3vrda2 | d1fcdc2 | d1fcdc1 | d1h1oa2 | d1h1oa1 |
| d1m70a1 | d1iqca2 | d1iqca1 | d1eb7a2 | d1eb7a1 | d1nmla1 | g1f8v.1 | d1novc_ |
| d2bbvc_ | d1qusa_ | d1sefa_ | d1sfna_ | d1sq4a_ | d3wyha_ | d4g9sa1 | d3gxra_ |
| d1gd6a_ | d1f6ra_ | d1ivma_ | d2gv0a_ | d1iiza_ | d2z2fa_ | d1j8xa_ | d1ghla_ |
| d1b9oa_ | d174la_ | d169la_ | d2o4wa_ | d1p37a_ | d1k28a3 | d1xjta1 | d1jtma_ |
| d2f2qa_ | d4e97a1 | d2pjsa1 | d1klla_ | d2i7ra1 | d1ecsa_ | d1xqaa1 | d5cj3a1 |
| d1npba_ | d1jifa_ | d2p7oa_ | d1nkia_ | d1mpya2 | d1kw3b2 | d4ghga1 | d1tfza1 |
| d3isqa1 | d3isqa2 | d1sqia1 | d1sqia2 | d1lkda2 | d1lkda1 | d1mpya1 | d1cjxa2 |
| d1cjxa1 | d1sp9b2 | d1t47a1 | d1t47a2 | d1f1ua2 | d1sp8a1 | d1sp8a2 | d1f1ua1 |
| d1s5ja2 | d1qhta2 | d4dlea2 | d1tgoa2 | d3py8a2 | d1q8ia2 | d1kfsa2 | d2py5a2 |
| d1d5aa2 | d1qsta_ | d2beia1 | d4m3sa_ | d1u6ma1 | d1ygha_ | d1yk3a1 | d3dr6a_ |
| d1xmta_ | d1z4ea1 | d2ge3a1 | d1bo4a_ | d2fiaa1 | d2jdca1 | d1y9wa1 | d3to7a_ |
| d1wwza1 | d2fl4a1 | d2ozua1 | d2b5ga_ | d2cy2a1 | d2p0wa_ | d1cm0a_ | d4pswa_ |
| d1yrea1 | d1yr0a1 | d1m4ia_ | d2gana1 | d1yvka1 | d1ghea_ | d2g3aa1 | d1y7ra1 |
| d2euia1 | d1q2ya_ | d2fsra1 | d1xeba_ | d2ae6a1 | d1cjwa_ | d1qsma_ | d2aj6a1 |
| d1y9ka1 | d1i12a_ | d1vkca_ | d2atra1 | d1n71a_ | d2d4pa1 | d2fiwa1 | d2fe7a1 |
| d1tiqa1 | d2ou2a_ | d1yx0a1 | d2fcka1 | d1p0ha_ | d2j8ma_ | d2pq8a_ | d1z4ra1 |
| d1s3za1 | d1ufha_ | d1xvqa1 | d1pn0a2 | d3hy2a_ | d2cvba1 | d1o8xa_ | d1we0a1 |
| d2bmxa1 | d2a4va1 | d1psqa_ | d1xiya1 | d1q98a_ | d1n8ja_ | d1zofa1 | d2ggta_ |
| d3tjja_ | d4je1a1 | d1o73a_ | d3s9fa_ | d2zcta_ | d1qmva_ | d1jfua_ | d2b1ka_ |
| d5dvba1 | d2b5xa1 | d2v2ga_ | d1st9a_ | d3tb2a_ | d4llra_ | d4fh8a1 | d1e2ya_ |
| d1knga_ | d2h30a_ | d1i5ga_ | d2z9sa_ | d2f8aa1 | d1tp9a1 | d2b7ka_ | d1nm3a2 |
| d3hvva_ | d2wfca1 | d4kcea_ | d1xvwa1 | d4gqca1 | d3qpma_ | d4k1fa_ | d1zzoa1 |
| d1gp1a_ | d1prxa_ | d4od7a1 | d1z6ma1 | d4mcua1 | d4dvca1 | d1r4wa_ | d3l9sa_ |
| d1un2a_ | d3rppa_ | d1e88a1 | d1l6ja4 | d1eaka4 | d1eaka3 | d1l6ja3 | d1h8pa2 |
| d1h8pa1 | d1jgta2 | d1ea0a3 | d1ecfa2 | d1q15a2 | d1ct9a2 | d1ao0a2 | d1te5a_ |
| d1ra6a_ | d1muka_ | d3gsza_ | d1khva_ | d1te9a_ | d2xwha_ | d3uqsa_ | d3hkwa_ |
| d3n6ma_ | d2cjqa_ | d2xyma_ | d1teba_ | d1te8a_ | d1s48a_ | d1u09a1 | d4wfxa_ |
| d3tyqa_ | d2pgga1 | d1lgya_ | d4l3wa_ | d1llfa_ | d1gz7a_ | d1ukca_ | d4wfia_ |
| d4cg1a1 | d3visa_ | d1cr6a2 | d3groa_ | d1pjaa_ | d1ei9a_ | d1svpa_ | d1mbma_ |
| d3su6a1 | d1cu1a1 | d4agka_ | d1vcpa_ | d1q31a_ | g1n1l.1 | d3e90b_ | d1ep5a_ |
| d3u1jb_ | d3zxha_ | d1cgla_ | d1rm8a_ | d1eaka2 | d1hy7a_ | d2jsda_ | d1cxva_ |
| d1hv5a_ | d1i76a_ | d3p0ya1 | d1igra1 | d1n8yc2 | d1n8yc1 | d1m6ba2 | d1m6ba1 |
| d1dzka_ | d4tlja_ | d2a2ua_ | d4wfva_ | d3t1da_ | d4iaxa_ | d3zq3a_ | d2nnda_ |
| d1i4ua_ | d1kxoa_ | d1bbpa_ | d1kt7a_ | d1exsa_ | d3s26a1 | d1lf7a_ | d1qfta_ |

| | | | | | | | |
|---|---|---|---|---|---|---|---|
| d4gh7a_ | d1z24a1 | d1qabe_ | d1xkia_ | d3bx7a_ | d4iawa1 | d1n0sa_ | d1gkab_ |
| d3kzaa_ | d2k23a_ | d2l9ca1 | d2joza1 | d1gm6a_ | d3kffa_ | d3saoa1 | d1e5pa_ |
| d1yupa1 | d1iiua_ | d1lmra_ | g1apy.1 | d3ljqa_ | d4lu0a_ | d1of8a_ | d3unda1 |
| d3e9aa1 | d1vr6a2 | d1n8fa_ | d2qkfa_ | d1d9ea_ | d1mqta_ | d1d4m3_ | d1fmd3_ |
| d2mev1_ | g1aym.1 | d1ev13_ | d4q4w1_ | d1r1a3_ | d2x5ia_ | d1rhi1_ | g1r1a.1 |
| g1d4m.1 | g1rhi.1 | d1b35b_ | d1oopa_ | d1tme1_ | d4iv1a_ | g1pvc.1 | g1fpn.1 |
| d1fpn1_ | d4gb31_ | d1d4m1_ | d1fmd1_ | d1ev11_ | d2wzr3_ | g1cov.1 | d1qqp3_ |
| g1fmd.1 | d2x5ic_ | d1rhi3_ | g1oop.1 | d1hxs1_ | g1hxs.1 | d1fpn3_ | d1bev1_ |
| g2mev.1 | d1eah3_ | d1aym1_ | d1pvc3_ | d1qqp1_ | d1zba3_ | d1h8ta_ | d3vbhc_ |
| g1b35.1 | g1bev.1 | d1hxs3_ | g1ev1.1 | d1bev3_ | g1tmf.1 | d1tmf1_ | d1b35a_ |
| d2mev3_ | g1eah.1 | g1h8t.1 | d1eah1_ | d4q4w3_ | d1r1a1_ | d1aym3_ | d1pvc1_ |
| d1zba1_ | d1h8tc_ | d1oopc_ | d1tme3_ | g1qqp.1 | d4iv1c_ | d4w9ta_ | d1jvna1 |
| d4x2ra1 | d1ka9f_ | d4axka1 | d1thfd_ | d1h5ya_ | d4u28a_ | d2y88a_ | d4wd0a_ |
| d1vzwa1 | d3ia2a_ | d4dgqa1 | d1hkha_ | d1zoia_ | d4lt9a1 | d3tc7a1 | d3hoja_ |
| d3vnda_ | d3nl8a1 | d1geqa_ | d3nava1 | d1v5xa_ | d4a2ra1 | d3tc6a1 | d1ujpa_ |
| d4ijba_ | d1xcfa_ | d1vc4a_ | d1rd5a_ | d1qopa_ | d3b5va_ | d2r80b_ | d1cqxa1 |
| d1cg5a_ | d1gcvb_ | d3boma_ | d2h8fb_ | d2raoa_ | d3bj1a_ | d4hrrb_ | d2r50a_ |
| d3d1kb_ | d2aa1b1 | d3zhwa_ | d3hf4b_ | d3gdja_ | d1jebb_ | d3bj1b_ | d1x9fb_ |
| d1wmub_ | d1gcva_ | d3bcqb_ | d3d4xb_ | d1gvha1 | d2dc3a_ | d3hf4a_ | d4yu3a_ |
| d3k8ba_ | d1qpwb_ | d2r80a_ | d3bcqa_ | d1v4wa_ | d3wtgb_ | d1jl7a_ | d4esaa_ |
| d1a9we_ | d3d1ab_ | d1fsla_ | d4h2la_ | d1v4wb_ | d1mwca_ | d1vhba_ | d1jeba_ |
| d1spgb_ | d1h97a_ | d1q1fa_ | d1tu9a1 | d3d1aa_ | d1hdsb_ | d3hyub_ | d2zfbb_ |
| d3fs4a_ | d1oj6a_ | d1shrb_ | d4h2lb_ | d4yu3b_ | d1spga_ | d3hrwa_ | d3qqra_ |
| d3at5a_ | d4wchd_ | d3hyua_ | d1cg5b_ | d1uc3a_ | d1hbra_ | d2raob_ | d1outb_ |
| d4esab_ | d3gdjb_ | d3qqqa_ | d1x9fc_ | d3vrfa_ | d1itha_ | d3mkba_ | d3d4xa_ |
| d3mjpa_ | d1jl6a_ | d3fh9a_ | d1outa_ | d2dc3b_ | d1it2a_ | d3eoka_ | d3g46a_ |
| d1qpwa_ | d3vrfb_ | d1ch4a_ | d1x9fd_ | d1hdsa_ | d2zfba_ | d3mkbb_ | d3wtga_ |
| d1a4fa_ | d3fh9b_ | d3bomb_ | d3cy5a_ | d3at5b_ | d1x9fa_ | d2oifa_ | d4lmxb_ |
| d1cpcb_ | d3v57a_ | d1kn1a_ | d1cpca_ | d4f0ta_ | d2vjha_ | d4po5b_ | d1eyxa_ |
| d1alla_ | d4f0tb_ | d1b33b_ | d1b8db_ | d4h0mb_ | d1b33a_ | d2vjhb_ | d4lm6b_ |
| d2vmlb_ | d1allb_ | d2vjta_ | d3dbja_ | d2vmla_ | d2vjtb_ | d4f0ub_ | d1f99b_ |
| d4rmpa1 | d1liaa_ | d1eyxb_ | d2uulb_ | d4lmsb_ | d4f0ua_ | d2bv8a_ | d1f99a_ |
| d2bv8b_ | d2c7lb_ | d3dbjb_ | d4rmpb1 | d1b8da_ | d4h0ma_ | d3v57b_ | d1kn1b_ |
| d1liab_ | d2uula_ | d2j96a_ | g1gxs.1 | d2xqfa_ | d1ea5a_ | d1f6wa_ | d1dx4a_ |
| d1qe3a_ | d1k4ya_ | d4qwwa_ | d2ha2a_ | d1b9ra_ | d1offa_ | d3wcqa_ | d1e9ma_ |
| d1l5pa_ | d1wria_ | d1i7ha_ | d3av8a_ | d1czpa_ | d1rfka_ | d3p1ma1 | d3b2ga_ |
| d2bt6a2 | d4iuaa3 | d4bvva_ | d5hpga_ | d3k65a_ | d1ki0a3 | d1ki0a2 | d1ki0a1 |
| d1a0ha1 | d1pmla_ | d3sp8a3 | d2feba1 | d1f74a_ | d3l21a_ | d2e1da_ | d1to3a1 |
| d1w3ia_ | d1sfla_ | d1n7ka_ | d1vcva1 | d4imea_ | d1wa3a_ | d2ocza1 | d1f2ja_ |
| d3m16a_ | d2yxga_ | d1ub3a_ | d3a5fa_ | d1fbaa_ | d1l6wa_ | d1xfba1 | d1wx0a1 |
| d1vpxa1 | d1ojxa_ | d1mxsa_ | d2a4aa1 | d1xkya1 | d3cq0a1 | d3s1xa_ | d1f05a_ |
| d1fdja_ | d3cpra1 | d4d2ja_ | d4mh8a_ | d4i7ga1 | d3rp2a_ | g1ucy.1 | d1fuja_ |
| d2zchp_ | d3w94a_ | d1si5h_ | d1lo6a_ | g2pka.1 | d1cgha_ | d1kigh_ | d1a5ia_ |
| d3s9ba_ | d1hj8a_ | d1ao5a_ | d2anya_ | d1q3xa1 | d1gvza_ | d1azza_ | d4gsoa_ |

d2f91a1 d1iaua_ d1gdna_ d1eufa_ g1fiw.1 d1eq9a_ d4nfea_ d2rdla_
d1spja_ d1elva1 d1a7sa_ d1ekbb_ d1pfxc_ d1yc0a_ d2aiqa_ g1fiz.1
d1fi8a_ d4k60a_ d4dgja_ d1mzaa_ d1op0a_ d3tjva_ d1pytd_ d1sgfg_
g1gg6.1 d2eeka_ g1rtf.1 d1a0ja_ d3g01a_ d1pytc_ d4e7na_ d1ddja_
d1z8ga1 d2hlca_ d1npma_ d1brup_ g1jou.1 d1sgfa_ d1autc_ g2hnt.1
g1kdq.1 d1orfa_ d3s69a_ d4crga_ d1fxya_ d1bqya_ d2ftba_ d1pmpa_
d2rcqa1 d1fdqa_ d4lkpa_ d1sa8a_ d1vyfa_ d1lpja_ d3vg7a1 d4azqa1
d1o8va_ d5bvqa_ d1p6pa_ d2qo4a_ d1opba_ d1yiva1 d1o1va_ d4bvma1
d4tkba_ d4i3ba_ d1bwya_ d1ftpa_ d4gkca_ d1cbia_ d3p6da1 d1kqwa_
d2a0aa1 d1m4la_ d2bk7a_ d3fjua_ d1z5ra1 d1kwma1 d1uwya2 d1jqga1
d2c1ca1 d1dtda_ d1h8la2 d3lgia_ d3i77a_ d2z9ia2 d4nsva_ d1lcya2
d4jcna_ d1agja_ d1os8a_ d1hpga_ d1p3ca_ d1l1ja_ d1qtfa_ d1e69a_
d1mv5a_ d3g60a4 g1ii8.1 d1ewqa2 d1ji0a_ d1qhla_ d1v43a3 d2wmfa1
d1vpla_ d1g2912 d4a82a2 g1f2t.1 d2d62a1 d3tuic1 d2bbsa1 d3g60a3
d2ixea_ d1g6ha_ d1sgwa1 d2qi9c_ d3rlfa1 d3b60a1 d3d31a2 d2pzea1
g1xew.1 d1jj7a_ d1oxxk2 d2onka1 d1w1wa_ d1iwea_ d1qf5a_ d1vmaa2
d1nija1 d1np6a_ d2p67a1 d3ihla_ d1okkd2 d1ls1a2 d1s1ma2 d1iona_
d2v40a_ d1dj3a_ d3hida_ d2vo1a1 d2g0ta1 d1vcoa2 d1j8mf2 d1g3qa_
d1xjca_ d1qzxa3 d1ihua2 d1ihua1 d2qm8a_ d1dj2a_ d4ioja_ d1cp2a_
d1yrba2 d1hyqa_ d3gqba2 d1skye3 d1xp8a1 d1tf7a2 d1tf7a1 d1ye8a1
d1skyb3 d2qe7d2 d4blta_ d4huta_ d3ldaa2 d4hyya_ d2i3ba1 d1cr1a_
d3j9tb2 d3ntua2 d1vdza1 d2z43a_ d1e9ra_ d1nlfa_ d3ziaa2 d1n0wa_
d1g6oa_ d3ziad2 d3j9ta2 d1pzna2 d3gqbb2 d2i49a_ d1nnfa_ d1xvxa_
d1ursa_ d1h3da1 d1iuda_ d4m1va1 d1twya_ d3e13x_ d2nxoa1 d4pfua_
d3ttma_ d1elja_ d2qmwa1 d1us5a_ d1y3na1 d1jeta_ d1uqwa_ d4f19a1
d2esna2 d3thia_ d3fasa1 d2uyeb_ d2xwva1 d1r9la_ d2fyia1 d1pb7a_
d2ozza1 d1sw5a_ d1nh8a1 d1vr5a1 d2g29a_ d1xs5a_ d3lswa_ d2z23a_
d3w9va_ d3tcha_ d1q35a_ d1z7me1 d3q27a_ d1p99a_ d1ve4a1 d2i6ea1
d4mcoa1 d4n8ya_ d1eu8a_ d1zbma1 d1hsla_ d1ixca2 d1j1na_ d1xvya_
d1y9ua_ d2z22a1 d1xoca1 d4oera_ d1wdna_ d5f1qa1 d1xt8a1 d4gwoa_
d4igta1 d1pc3a_ d4mmpa1 d1o63a_ d2czla1 d2onsa2 d4mija1 d1eh3a_
d3skpa_ d1dtza1 d1dtza2 d1ieja_ d1jnfa2 d1ce2a1 d1jnfa1 d1b1xa2
d1jw1a1 d1jw1a2 d1ryoa_ d1h76a1 d1h76a2 d1c4oa1 d2p6ra3 d2p6ra4
d1oywa2 d1oywa3 d1wrba1 d1w36b2 d1w36b1 d1c4oa2 d3bora_ d1t6na_
d2eyqa5 d2eyqa3 d2eyqa2 d2zu6a2 d1veca_ d1uaaa1 d1w36d1 d2g9na1
d1z3ix2 d2rb4a_ d1hv8a1 d1hv8a2 d1t5ia_ d1w36c2 d2j0sa2 d2j0sa1
d1fuka_ d3hjha1 d1z5za1 d1qdea_ d1z63a1 d1s2ma2 d1s2ma1 d1wp9a1
d1wp9a2 d1gm5a3 d1gm5a4 d3dina4 d3dina3 d1q0ua_ d1w36c1 d1z3ix1
d1nkta3 d1nkta4 d1uaaa2 d1w36d2 g1qhh.1 d2fwra2 d2fwra1 d2v8ta_
d3q3ma_ d1za0a1 d1mxra_ d2inca_ d1jk0a_ d2incb_ d1mhyb_ d3hf1b_
d1mtyb_ d1r2fa_ d2uw1a_ d1mhyd_ d3n37a_ d1oquc_ d1syya_ d1mtyd_
d2o1za_ d1uzra_ d1smsa_ d2fkza_ d1zuja1 d1s3qa1 d2fjca1 d1z6om1
d1veia_ d4dyya_ d1jiga_ d3gvya_ d2pyba_ d4cy9a1 d3bvfa1 d1r03a_
d2cf7a_ d1nf4a_ d1zs3a1 d4iwka_ d3ka8a_ d3np0x_ d1n1qa_ d1lb3a_

d4e6ka_ d1z6oa1 d2fzfa1 d4dyua_ d3ge4a_ d4am4a_ d2z6ma_ d1ji5a_
d1vlga_ d1lkoa1 d1krqa_ d3qz3a_ d1o9ra_ d2ffxj_ d1euma_ d1yv1a1
d2v2pa_ d1j30a_ d4di0a_ d1dpsa_ d1oewa_ d2qzxa_ d2asia_ d1psoe_
d1smra_ d2h6ta_ d1pfza_ d1izda_ d3hd8a_ d1j71a_ d1dpja_ d4aa9a_
d1t6ex_ g1b5f.1 g1lya.1 g1htr.1 d1qdma2 d1bxoa_ d1wkra_ d1tzsa_
d1ibqa_ d2qzwa_ d1bdqa_ d3ggta_ d3ixoa_ d1mrxa_ d2haha_ d1tw7a_
d4njsa_ d1sgua_ d2p3da_ d1nsoa_ d3d3ta_ d2rkfa_ d1c6ya_ d2rspa_
d3ogpa_ d3ekwa_ d2o4na_ d2j9ja_ d1baia_ d3ggua_ d3lzua_ d4npua_
d4j55a_ d2r8na_ d2fxda_ d2p3ca_ d4dqea_ d3ttpa_ d3t3ca_ d2p3aa_
d2hc0a_ d2fdda_ d3mwsa_ d1k8rb_ d1wgra1 d1ef5a_ d1wfya1 d1e8ya3
d1wgya1 d1lfda_ d2cs4a1 d1wxma1 d2byea1 d3kh0a1 d2byfa1 d1wxaa1
d1e7ua3 d1h4ra3 d4yl8a1 d2al6a3 d1wgda1 d1wgga1 d4q5eb_ d1wjna1
d1se9a_ d3n3kb_ d1wz0a1 d1we6a1 d2m0xa_ d2gbka_ d3rt3b1 d1wh3a1
d1sifa_ d2ekec2 d4ii2b1 d4hk2a1 d1c3ta_ d1m94a_ d1wgha1 d1ttna1
d1j8ca_ d1wx8a1 d3v6cb1 d2ma9b_ d4i6lb1 d1bt0a_ d2jvca1 d1iyfa_
d2k1fa_ d1wxva1 d1wf9a1 d1t0ya_ d2io0b2 d1x1ma1 d2gbma_ d1zw7a1
d2m17a_ d1ud7a_ d1we7a1 d2lwpa_ d2gbja_ d1wjua1 d1v6ea1 d1v86a1
d4eewa_ d1wiaa1 d4icva_ d4gu2a_ d1wx7a1 d1wy8a1 d4hjka1 d2faza1
d1v5oa1 d1dszb_ d3cbba_ d1ynwa1 d1r4ia_ d1hcqa_ d2hana_ d2hanb_
d2enva1 d1kb4b_ d2nllb_ d1lo1a_ d1cita_ d1ixza_ d1qvra2 d1qvra3
d1qzma_ d1sxjc2 d1jr3d2 d2p65a_ d1l8qa2 d1g8pa_ d3hu3a3 d1sxjd2
d1njfa_ d1in4a2 d1sxja2 d1tuea1 d2ce7a2 d1w5sa2 d1sxjb2 d1d2na_
d1ny5a2 d1ofha_ d2gnoa2 d2a5yb3 d1fnna2 d1u0ja1 d1lv7a_ d1iqpa2
d2fnaa2 d1ixsb2 d4gita_ d1sxje2 d1g41a_ d2lj7a_ d1n4na_ d2m8bb1
d2kyqa_ d3psma_ d1jkza_ d1jxca_ d2gl1a_ d1ugla_ d4uj0a_ d2k4ua_
d1hp2a_ d2lu9a_ d1baha_ d2meoa1 d1wm7a_ d1hlya_ d1n8ma_ d1jlza_
d1wpda_ d1c55a_ d1pvza_ d1wt7a_ d1rjia_ d1wz5a_ d1m2sa_ d1pjva_
d1c49a_ d1qkya_ d2ktca1 d1wmta_ d1j5ja_ d1r1ga_ d1t0za_ d2i61a_
d1t1ta_ d1sn4a_ d1kv0a_ d2lkba_ d1npia_ d1wwna_ d1pe4a_ d2kbka_
d2li7a_ d1djta_ d2kbha_ d1dq7a_ d1sega_ d1omya_ d1zywa1 d1jzaa_
d2jcqa_ d1bnla_ d1dy2a_ d1t61c1 d3ubub_ d1qo3c_ d1v7pa_ d1tdqb_
d3ubua_ d1c3ab_ d1h8ua_ d3bx4b_ d1t8ca1 d1sb2a1 d1qdda_ d2ziba1
d3cada_ d1byfa_ d1j34b_ d1jwia_ d1jzna_ d1c3aa_ d1oz7b_ d1wmza_
d3bdwb_ d1hq8a_ d1fifa1 d1dv8a_ d3hupa_ d1wk1a1 d1umra_ d2afpa_
d3bx4a_ d1gz2a_ d1sl6a_ d1v7pb_ d3g8ka_ d1ixxa_ d1j34a_ d1jwib_
d1g1sa1 d1y17a1 d1ja3a_ d1fvub_ d3bdwa_ d1sb2b1 d1fvua_ d1umrc_
d1oz7a_ d4m17a_ d3cfwa1 d1ypqa1 d1g1ta1 d3fd4a_ d3l9jc_ d4dn8a1
d1v4lb_ d1rdl1_ d2msba_ d1rpma_ d2shpa1 d1yfoa_ d1l8ka_ d2ooqa1
d1g4us2 d1p15a_ d1lara2 d1lara1 d1wcha_ d2fh7a2 d2fh7a1 d1ohea1
d1ohea2 d2f4da_ d1rxda_ d2hxpa_ d1xria_ d1fpza_ d3ezza_ d4y2ea_
d1i9sa_ d3azya_ d3wvja_ d1upsa1 d1ajoa_ d2hyka_ d1ajka_ d2vy0a_
d1o4ya_ d3o5sa_ d3d6ea_ d3atga_ d1dypa_ d1umza_ d3i4ia_ d1c1la_
d4lbqa_ d1a78a_ d1slta_ d4no4a_ d3zsja_ d3duia_ d1g86a_ d1is3a_
d1qmja_ d1hlca_ d1tdha3 d1q3ba3 d1ee8a3 d1k82a3 g1qmo.1 g1len.1

d3zyra_ d1n47a_ g2ltn.1 d1avba_ d3usua_ d1dhkb_ d1f9ka_ d1ukga_
d1g8wa_ d1g9fa_ d1wbfa_ d3wcsa_ d1g7ya_ g1loe.1 d1mvqa_ d1hqla_
d1v6ia_ d1dbna_ d1qnwa_ d1fx5a_ d4l8qa_ d1ioaa_ d3n35a_ d3ubra_
d1qo8a1 d1d4ca1 d2k3va_ d1ft5a_ d1qdba_ d1m1qa_ d2j7aa_ d1h21a_
d1ogyb_ d4fasa_ d1jnia_ d1oaha_ d1sp3a_ d1eysc_ d2h0ba_ d2erfa_
d1h30a1 d3mw4a1 d1pz7a_ d2r1ba_ d3v64a1 d1d2sa_ d3bopa1 d3mw3a1
d1okqa1 d1okqa2 d1wioa4 d1ccza2 d1vcaa1 d1dr9a2 d1ic1a1 d2d9qb1
d2nxyb2 d1adqa1 d1fnga1 d1uvqb1 d1zs8a1 d2vola1 d2vola2 d1mfbl2
d2jelh2 d1uwel2 d3qq4b1 d1kjvb_ d1k5na1 d1osph2 d4odhl2 d1ow0a1
d1ow0a2 d1bfob2 d3l9ra2 d3jtth1 d2fath2 d4elkb2 d1fn4b2 d2xfxb_
d1pg7x2 d1cqka_ d4k71b2 d1lk2a1 d1eapb2 d4lfhg2 d1i4fa1 d2nw2a2
d1kfah2 d1mhca1 d1h3ua1 d1wejh2 d1h0db2 d1a5fh2 d1kcvh2 d1mjuh2
d1qqda1 d1kcrh2 d1k8ia1 d3huja2 d2nnab1 d1nfdf2 d3mffb2 d1s9va1
d1lk3h2 d1q0xl2 d1fv1b1 d1neza1 d4rfeb2 d1r3ha1 d1a6tb2 d1kjva1
d1pz5b2 d1onqa1 d2bc4a2 d1de4a1 d1k8da1 d1tcra2 d1dn0b2 d1nfde2
d2xwtb2 d1bfoa2 d1mexh2 d1iakb1 d1ypzf2 d4f7ea2 d1q72l2 d2fbjh2
d1fe8h2 d1clzh2 d1fngb1 d1uvqa1 d1vgka1 d1c16a1 d4j12a2 d4x99b2
d1igtb3 d1je6a1 d1q72h2 d1nldh2 d1teth2 d2hmic2 d1i1ca2 d1i1ca1
d1k8ib1 d1q0yh2 d1kfal2 d3frua1 d1pg7w2 d1u58a1 d1igyb4 d1igyb3
d1hyrc1 d1klub1 d1deeb2 d1c12b2 d2bc4b2 d2ig2h2 d1kcvl2 d3wn5b1
d1lk2b_ d1lk3l2 d1mjul2 d1sm3h2 d1q0xh2 d1zvsa1 d1ydpa1 d1u8ib2
d1ypze2 d1kcuh2 d1op9a_ d1y0la1 d1etzb1 d1egjl1 d2gsib1 d1nj9a1
d4jfhd1 d1aqkl1 d1t2ja_ d1lo4h1 d1zvya1 d3v4vl1 d4enec1 d1kxtb_
d5e8ea1 d2pxya_ d4weud_ d1xiwc_ d2esvd1 d1qnzh_ d1ogae1 d1f4xh1
d4n1hb_ d3ezjb1 d3mlwl1 d1kfah1 d1etza1 d1b4jh1 d3cfic_ d4w68a_
d2arja1 d1ek3a_ d2ok0l1 d1vera_ d1uwgh1 d1blnb1 d1fpth1 d1rzga1
d2giya1 d4m3kb1 d1nc2a1 d2nyyd1 d1ncna_ d1fbih1 d5dxwa_ d4fqqa1
d1dqdh1 d1jfql1 d1kelh1 d1faih1 d1frgl1 d1figl1 d4m6nl1 d1fo0a_
d4hxbl1 d3b5ga_ d3quyd1 d2h9gb1 d1i8kb_ d2dd8h1 d2ck0h1 d1eaja_
d4w6wb1 d2a6ib3 d1t66d1 d2e27h_ d1ob1b1 d1f11b1 d2v7na1 d1ad0a1
d2xt1b1 d1pskh1 d4i13b1 d2op4l1 d1a0ql1 d1uwel1 d1za3a1 d1ncbl1
d4uu9a_ d1bm3h1 d1cloh1 d1nqba2 d1cf8h1 d4y5va_ d1mhph1 d5anma1
d4cdgc1 d4ldeb_ d1y0lb1 d2pcpb1 d1z3gl1 d1dfbl1 d1fh5h1 d3g5xa1
d1tzib1 d4dn3l1 d3zhka1 d2b1hh1 d2uyla1 d1f3dh1 d1nj9b1 d2agjh1
d2h1pl1 d1q72h1 d8fabb1 d1ndmb1 d1rjca1 d4u6hb1 d3upca_ d2fbjl1
d1osph1 d1tvda_ d2cjvh_ d4fhbd1 d1bd2d1 d1rz7h1 d3nn8a_ d1blna1
d1knoa1 d1nfda1 d2bmkb1 d1dqll_ d1igyb1 d1igfl1 d12e8h1 d3utza1
d1nc2b1 d1rihh1 d4mwfb1 d4jvpa1 d1mieh1 d1rhfa1 d1fgnl1 d1sbsh1
d1lp9e1 d1dn0a1 d4lqfl1 d1iaih1 d1kgcd1 d1mh5b1 d1gpol1 d2g60h3
d1f8tl1 d2jixd1 d4grwh_ d1fo0b_ d1bfob1 d1axsa1 d1u9ka_ d1nmbh_
d1h8na1 d1h8na2 d1jguh1 d4eura1 d3sdyl1 d35c8h1 d1w72l1 d1mvub_
d1i9rh1 d1ob1a1 d4wenb_ d1yeeh1 d3ietc1 d1f58h1 d1ad0b1 d1mqkl_
d1nbvh1 d4b50a_ d3r8bb_ d1bafl1 d1mamh1 d4rgne1 d2gsgb_ d35c8l1
d1bafh1 d1ai1h1 d2pcpa1 d2fath1 d2x1pa_ d1orsb1 d1ibgl1 d1shma_

d1a2yb_ d1u0qa_ d2coqa_ d1hilb1 d1qfwi_ d1jv5b_ d1fn4b1 d5e0qa_
d1nsnh1 d1plgl1 d4ocxl1 d1kb5h1 d1rzfh1 d1pg7x1 d2fx7h1 d2oi9c_
d1l7il1 d3eyva1 d1d5il1 d4ffyl_ d1tetl1 d1a6wh_ d1ac6a_ d3dvfa_
d1nfde1 d1mimh1 d1ri8a1 d1jhlh_ d3dura_ d1fskb1 d1f97a1 d3so3b1
d2ak4d1 d1lp9f1 d4z7wf1 d1l7th1 d4y5ya_ d4x7ec1 d1emth1 d1ikfl1
d1nlbh1 d4utal1 d1bjma1 d1eapb1 d1u6ah1 d2dtgd1 d1kj2b_ d1xeda1
d1c1eh1 d1fgvh_ d2bnub1 d2fb4h1 d2dtgb1 d1ktrh_ d1jpth1 d1qkzl1
d4hepg_ d2atpb1 d1flrh1 d2hrpl1 d1mvfa_ d1iaim1 d3k80a1 d2jb5h1
d3zkxc_ d4grwe_ d1it9h1 d1rhhb1 d3w9ec_ d1e4wh1 d1sy6h1 d2j6eh1
d3qxta_ d1jglh1 d1p7ka1 d1tzhb1 d4n8pa1 d1gafl1 d2jelh1 d1wioa2
d4i0cc1 d1cicb1 d1lmka2 d1lmka1 d1iqwh1 d1orqb1 d1ejoh1 d1jnlh1
d4zg1a_ d43c9b_ d1ggbl1 d1wejh1 d3q6fa1 d1f3dj1 d3c6la1 d1bqlh1
d3p9wb_ d1a3rl1 d1h0db1 d2uzil_ d1eo8l1 d1qfwl_ d1n7ml1 d1igja1
d1c5ch1 d2a9mh_ d1fj1b1 d4heme_ d1ccza1 d1a5fh1 d1ogad1 d1dlfh_
d1qa9b_ d1vgel1 d3qskb_ d1sjxa_ d4ozfg1 d1sq2n_ d1qd0a_ d1kcvh1
d1c5db1 d3qcul1 d8faba1 d3bn9d1 d1mjuh1 d4y8dc_ d7fabh1 d3dvgb1
d1lo0l1 d1a6tb1 d1gigh1 d1kcrh1 d2hwzl1 d1yjdc1 d3clfl1 d4krna_
d1dl7l_ d1e6ol1 d1mexh1 d3r0ma_ d4kkna_ d1fnsl1 d1oaqh_ d1rzia1
d1ucbh1 d1ncwl1 d4b41a_ d1nfdf1 d2mpah1 d2d7tl1 d4grwf_ d4b5ea_
d1lk3h1 d3u79a_ d4u3xa_ d1ghfl1 d1bvkb_ d1mcww1 d1b88a_ d1jrhh1
d32c2b1 d4wemb_ d1dsfh_ d1op3h1 d1cell1 d1iqda1 d1qoka1 d1qoka2
d1qfuh1 d2crya1 d1ay1h1 d1oaql_ d3ab0b_ d1rurl1 d1t4kb1 d1bz7b1
d1n0xh1 d1forl1 d1ucbl1 d4w6yb_ d43c9a_ d2aabl1 d1tzia1 d4n1ca_
d1k6qh1 d3g9ab_ d4tvsa1 d1igmh_ d1pz5b1 d3dx9a1 d3wxwh_ d1nakh1
d1kcul1 d3eaka_ d4qlra1 d1cr9l1 d1d9ka_ d1f4xl1 d1deea1 d2i27n2
d1c12a1 d2orbl1 d1j8hd1 d1mcph1 d1c5da1 d1kenh1 d1bd2e1 d1h5ba_
d4x7fc1 d3upaa_ d1t3fa1 d1indh1 d2dtgc1 d4hc1l1 d1zv5a1 d4wgvb_
d1uwgl1 d4f37k1 d1aqkh1 d1vesa_ d3zkqd_ d1fptl1 d1lo4l1 d1knob1
d3rrqa_ d1ct8b1 d4orzc_ d1mlbb1 d4hgkc1 d1dqdl1 d1bogb1 d4ozhe1
d1dr9a1 d1i7za1 d1dn0b1 d1kell1 d1rzib1 d1ypzf1 d1wtla_ d1hkfa_
d1fail1 d1figh1 d2vyre_ d1sjva_ d1ohqa_ d1bfoa1 d1pkqb1 d1mjul1
d1b4jl1 d1nsnl1 d2arjb1 d1p4bh_ d4aj0a_ d3nn8b_ d2rcja1 d1f2xk_
d4v1dd_ d1adqh1 d2eksa_ d4k3ga_ d1jfqh1 d1uweh1 d1frgh1 d4gftb_
d1uz8b1 d4n1ea_ d1bm3l1 d1clol1 d3mcka1 d2vq1a1 d1ae6h1 d4aq1b1
d1uj3b1 d1nbqa1 d1fh5l1 d2ck0l1 d2ywza_ d1q72l1 d4nbxb_ d1hila1
d4hzlb1 d2j88l1 d1zmya1 d1cd0a_ d1a4ka1 d1pskl1 d1a0qh1 d4krmb_
d1ncbh1 d2fbjh1 d1j8he1 d1i3ua_ d1cf8l1 d1fe8h1 d2p1ya1 d1eeqa_
d2x89a_ d1ol0a_ d2r0lh1 d1ub5a1 d1dfbh1 d3eyvb1 d4jdvb1 d4p2cg_
d2gsia1 d2agjl1 d1u3ha1 d2h1ph1 d1rihl1 d1fskc1 d1boga1 d2a77l1
d1ospl1 d1qfoa_ d3dwta_ d2fx7l1 d1rz7l1 d3stba1 d1pkqa1 d1b6da1
d1dzba2 d1dzba1 d1kj2a_ d4l1ha_ d1rzgb1 d1dqlh_ d12e8l1 d1miel1
d1jmaa_ d1sbsl1 d1fgnh1 d1f58l1 d1mqkh_ d1ndgb1 d3qegl1 d1iail1
d2aw2a1 d1tqbc1 d3fo2a1 d4dkaa_ d1f8th1 d2g60l1 d2ywya_ d1uj3a1
d1yc7a_ d3bd3a_ d4w6xb1 d1nmbl_ d1i8ka_ d1rjlb1 d1ypze1 d1p7kb1

d1kxqe_ d1gpoh1 d2z4qa1 d1w72h1 d1i9rl1 d4qgya1 d4xt1c_ d2qqnh1
d1cica1 d3c08h1 d2cd0a_ d2bdnh1 d1hnga1 d1maml1 d1nldh1 d1cr9h1
d4lcih1 d3bkyh1 d3qg7l1 d1ai1l1 d1kxvc_ d4iosd_ d1l7ih1 d2x1qa_
d1ub6b1 d3k74b_ d1ibgh1 d1teth1 d1igya1 d1hxma1 d2x6ma_ d2e7la_
d5e03a_ d1qfwm_ d2r0kh1 d1igjb1 d1rurh1 d1plgh1 d1mj8h1 d1yuhb1
d3fmgl1 d1rzfl1 d1emtl1 d1ad9b1 d1igtb1 d1nlbl1 d1d5ih1 d3k7ua_
d4ffyh_ d1bwwa_ d3dvga1 d1a6ta1 d1fgvl_ d4qxgl1 d1miml1 d1jhll_
d2hffb1 d2hh0l1 d1qkzh1 d1l7tl1 d1afvh1 d1iaii1 d2cjuh_ d1um5h1
d2ghwb2 d2ghwb1 d1kgce1 d2fgwh1 d1ikfh1 d1dvfd_ d2i24n1 d2xtjb_
d1za3b1 d2yc1a_ d2hh0h1 d2x1oa_ d1bvka_ d1rjla1 d5e7ba1 d1igta1
d1il1b1 d2fb4l1 d1mvua_ d1jptl1 d32c2a1 d1fvca_ d1iqdb1 d2hrph1
d2p4ab_ d4qo1a_ d1p2cb1 d1wt5c_ d1t4ka1 d5c1mb_ d1it9l1 d1bz7a1
d1b2wh1 d1e4wl1 d1jnll1 d1yedb1 d1za6b1 d3u7aa_ d1igfh1 d3gnml1
d1orsa1 d1wejl1 d1a2ya_ d1hxmb1 d1gafh1 d2j4wh1 d1pz5a1 d1j05a_
d1a3rh1 d4fqll1 d1qfwh_ d1eo8h1 d1n7mh1 d4s11a_ d1kegh1 d5da4a_
d1iqwl1 d3whxb1 d1vgeh1 d1deeb1 d1c12b1 d1t2ql1 d1ad9a1 d3gsnb1
d3b2uc1 d1ggbh1 d1t3fb1 d1nakl1 d4ocmc_ d1lila1 d2v7hb3 d2esve1
d1mcoh1 d2qr0b1 d2q3aa_ d1ct8a1 d1kcrl1 d2pxyb_ d2uzih_ d5c3ld_
d3durb_ d1c5cl1 d2ak4e1 d1dl7h_ d1xiwd_ d1mlba1 d1rmfl1 d1i7zb1
d1dlfl_ d2xa3a_ d5bq7b1 d1dqqb1 d3h42l1 d1egjh1 d1kcvl1 d1fe8l1
d2j6el1 d1f3rb2 d1lk3l1 d1sm3h1 d1q0xh1 d1ghfh1 d7fabl1 d1igch1
d1lo0h1 d4idla_ d5c2bl_ d1jrhl1 d1il1a1 d3zhda1 d4kfzc_ d1b0wa_
d1e6oh1 d1mexl1 d4krpb_ d1fnsh1 d1ncwh1 d4hgma_ d4eigb1 d4rzca_
d2mpal1 d4y5xa_ d5en2b1 d3lrga1 d4ej1c1 d1rhha1 d1j05b_ d1forh1
d1q9wb1 d2wzpd_ d2aq2a1 d1h0da1 d3revb1 d2ij0c1 d1tzha1 d4m7jl1
d1k6ql1 d3b9kc1 d1igml_ d3qeua1 d1dsfl_ d1ce1h1 d1kcuh1 d1zvha1
d1zvoa1 d1tqbb1 d1ay1l1 d2qscl1 d2cgrh1 d1nl0h1 d1kenl1 d3eo0a1
d4n9ob1 d1n0xl1 d3k1kc_ d1c4qa_ d1prtc1 d2wv6d_ d1prtf_ d1ltrd_
d4l63b1 d1r4pb_ d1tl0f_ d2bosa_ d1prtb1 d3chbd_ d2aq2b1 d1ck1a1
d1enfa1 d1i4pa1 d1v1oa1 d1et9a1 d1esfa1 d2g9hd1 d4ohja1 d1klud1
d1bxta1 d1eu3a1 d1ty0a1 d1fnua1 d2cqva1 d1fhga_ d2k1ma_ d2dl2a1
d1biha3 d1biha1 d1biha4 d3qp3a1 d1g1ca_ d1cvsc2 d1cvsc1 d1xmwa2
d1m4ka2 d2otpa2 d1biha2 d1rhfa2 d2fcba2 d2fcba1 d1wwbx_ d1gsma2
d1epfa1 d1epfa2 d2dava1 d1waaa_ d1f97a2 d1x44a1 d1vcaa2 d1fnla2
d1fnla1 d3b5ha2 d3h8na2 d3h8na1 d1jbja1 d1jbja2 d1pd6a_ d1f2qa2
d1f2qa1 d3cx2a_ d1nbqa2 d3b5ha1 d1n26a1 d1xiwb_ d1xaua_ d1fltx_
d2ifga1 d1olla1 d1olla2 d1cs6a4 d1cs6a3 d1cs6a2 d1cs6a1 d2gw5a2
d1sy6a1 d1xiwa_ d1ucta2 d1ucta1 d1ry7b2 d1gl4b_ d1qz1a3 d1olza1
d2c9aa1 d1wwca_

**Family-32 (b.40.4.5)**

**Positive dataset contains 41 protein sequences.**

d1y14b1 d2nn6i1 d1i94l_ d2zjra2 d1smxa_ d2qamc2 d2r7da1 d1wfqa1
d3bzka4 d1x6oa2 d3a0ja_ d2j01d2 d2ja9a1 d4ql5a_ d2n3sa_ d2vnud1
d2nn6h1 d2mo1a_ d2ix0a3 d2ix0a2 d2lxja_ d1ueba3 d1ueba2 d2z0sa1

d1u0la1 d2ix0a1 d2je6i1 d3i2za1 d2c35b1 d2ahob2 d1t9ha1 d3i4oa_
d2qall1 d2nn6g1 d3cama_ d2ba0a1 d2vnud2 d2vnud3 d2qalq1 d1wi5a1
d1xtda2

**Negative dataset contains 3939 protein sequences.**

d1c7ga_ d2v1pa1 d2ez1a_ d4nmwa_ d2bnma2 d1xkla_ d2wfla_ d1js3a_
d3rcha_ d3k40a_ d2cfba1 d1o69a_ d1bj4a_ d3k28a_ d2bwpa_ d3lg0a_
d2fnua_ d3ou5a_ d1z7da1 d1b9ha_ d1iaya_ d4msoa_ d1bs0a_ d1s0aa_
d1sffa_ d3tqxa_ d1mdoa_ d3qboa_ d3pgya_ d1bjna_ d2dkja_ d1ejia_
d1vefa1 d4wxga_ d3bs8a_ d3gbxa1 d1w23a_ d1qnia1 d1fwxa1 d2wo3b_
d1shxa_ d1f56a_ d1bypa_ d1azca_ d1ws8a_ d3u25a_ d1cuoa_ d1id2a_
d1rkra_ d2iaac_ d4dp1x_ d1nwpa_ d2gima_ d2i7sa_ d1qhqa_ d3ehbb1
d1fftb1 d1gyca3 d1hfua2 d1kbva1 d1kbva2 d1sdda1 d1sdda2 d1gyca1
d1gyca2 d1mzya1 d2bw4a1 d1aoza1 d1aoza3 d1aoza2 d1kyaa2 d1kyaa1
d1mzya2 d4e9sa1 d1sddb1 d1sddb2 d1hfua1 d1hfua3 d1oe1a2 d1oe1a1
d1v10a2 d1v10a3 d1v10a1 d2dara2 d2dara1 d1wyha2 d2dloa2 d1rutx4
d1rutx3 d2cuqa2 d1x4la2 d1x4la1 d1x3ha2 d1x3ha1 d2d8za1 d1x61a2
d1x6aa1 d1x6aa2 d2cora2 d2cora1 d1v6ga1 d1v6ga2 d2d8za2 d1x64a1
d1x64a2 d1b8ta1 d2co8a1 d1x63a2 d2dj7a2 d2dj7a1 d2cu8a2 d2cu8a1
d1g47a2 d1g47a1 d1j2oa1 d1wyha1 d2dloa1 d1wiga1 d1wiga2 d2cupa2
d2cupa3 d1x68a1 d2d8ya2 d2d8ya1 d1m3va2 d1x62a1 d1x68a2 d1x61a1
d1b8ta3 d1b8ta2 d2d8xa2 d2d8xa1 d1x4ka1 d1x4ka2 d2cuqa1 d1m3va1
d2co8a2 d1j2oa2 d1x63a1 d2cura1 d2cura2 d1x62a2 d1iknc_ d3brda1
d1p7hl1 d1oy3c_ d1imhc1 d1gjia1 d1zk9a1 d2cxka1 d2bema_ d1pama1
d1ea9c1 d1qhoa1 d2fhfa2 d2fhfa1 d1k3ia1 d1j0ha1 d1cxla1 d2bhua1
d1edqa1 d1f1sa2 d1ut9a2 d1gvia1 d1txka1 d4alac_ d1pjwa_ d1rera1
d1s6na_ d3uzqb_ d2phla2 d2phla1 d1fxza2 d1fxza1 d1lrha_ d1uija1
g1dgw.1 d2v09a_ d1uika2 d1uika1 d1dgwa_ d2pa7a1 d1wlta1 d1nxma_
d2c0za1 d1nepa_ d1ds6b_ d1fstb_ d1ex0a1 d1g0da1 d3us3a3 d3us3a1
d1eixa_ d3exra_ d2ffca1 d2fdsa1 d3n3ma1 d2czda_ d2yyua_ d1vqta1
d1dbta_ d3ldva1 d2bjca1 d2hsga1 d1qpza1 d1k9ua_ d2opoa_ d1r0ka3
d1j5pa3 d1vkoa2 d1vm6a2 d3qvsa2 d1gr0a2 d1p1ja2 d2ovga_ d1sq8a_
d1rzsa_ d3kz3a_ d3orca_ d3gpdg2 d2q49a2 d1hdgo2 d2g17a2 d2cvoa2
d2czca1 d3pwka2 d1obfo2 d1i32a2 d1zyao2 d2pkqo2 d1b7go2 d2hjsa2
d1cf2o2 d1vkna2 d1nvmb2 d1ux8a_ d1dlya_ d4i0va_ d2bkma_ d1dlwa_
d2bmma_ d2msya_ d2djoa1 d2r5za_ d1au7a1 d1f43a_ d2lmda1 d1akhb_
d1s7ea1 d3nara_ d1zq3p1 d2craa1 d1mija_ d4uuta_ d3a01a_ d1x58a1
d3rkqa1 d3cmya1 d2cqxa1 d2da6a1 d1e3oc1 d1mh3a1 d1ig7a_ d1x41a1
d1wh7a1 d1gt0c1 d2mw8a_ d1lfup_ d1x2ma1 d1b72a_ d1wi3a1 d1jgga_
d1wjha1 d1x2na1 d1mnmc_ d2cuea1 d3k2aa_ d2hosa_ d2jwta2 d1pufa_
d1octc1 d2hi3a_ d1b8ia_ d4j19a_ d3lnqa_ d1wh5a1 d1b8ib_ d1fexa_
d2cqra1 d1guua_ d1ug2a1 d2cqqa1 d2ajea1 d4a69c_ d2crga1 d2iw5b1
d1xc5a1 d1ofcx1 d2cu7a1 d1wgxa1 d2cjja1 d2ckxa1 d1gvda_ d6paxa1
d6paxa2 d1k78a2 d1igna1 d1prtc2 d3lv8a_ d1s3ga1 d1zaka1 d1s4qa_
d3tvta2 d1uf9a_ d1khta_ d1ckea_ d1osna_ d1p5zb_ d4tmka_ d3cm0a_

d1jaga_ d3h86a_ d1t0hb_ d1nn5a_ d2h92a_ d3kb2a1 d2c95a1 d1yj5a2
d1kgda_ d1ly1a_ d2ak3a1 d1vyva2 d1deka_ d2grja1 d1p3ja1 d1y63a_
d1e4ya1 d1s96a_ d3iija1 d1lvga_ d1nksa_ d1vyua2 d4e22a_ d1qf9a_
d1jjva_ d1q3ta_ d1ki9a_ d1lw7a2 d1teva_ d1kjwa2 d1gsia_ d2iyva2
d1kaga_ d1viaa_ d1e6ca_ d1x6va3 d1g3ma_ d1t8ta1 d1nsta_ d1fmja_
d1zrha_ d1ls6a_ d2a3ra_ d1aqua_ d2zptx_ d3ckla_ d1texa_ d1q44a_
d2gwha_ d1odfa_ d1uj2a_ d4gi7a_ d2jeoa1 d1rz3a_ d1mdwa_ d1qxpa4
d1ziva1 d1zcma1 d1z0ja1 d3umra2 d3gj0a1 d2gaoa1 d1yzga1 d1x3sa1
d1g7sa4 d1puja_ d1udxa2 d1kk1a3 d2o52a_ d1jwyb_ d1zcaa2 d1zcba2
d2fv8a1 d2cjwa_ d3dz8a1 d1xzpa2 d1vg8a_ d4qxaa_ d2ew1a1 d1x1ra1
d2bcgy_ d3doea1 d3cona_ d3rega_ d3wyga_ d1upta_ d2b6ha1 d1zd9a1
d1lnza2 d1r5ba3 d1n0ua2 d1r2qa_ d2f9la1 d2rhda_ d3q3jb_ d3q85a_
d1efma1 d1r8qa_ d2gf9a1 d2akab1 d1ek0a_ d1z08a1 d2gcoa1 d2dy1a2
d4g01b_ d2cxxa1 d1svia_ d3q72a_ d1ni3a1 d4ac9a4 d2erxa1 d2bmea_
d1wxqa1 d2gf0a1 d4ldja1 d1zunb3 d1d5ca_ d4lv5b_ d1efca3 d1kmqa_
d2g6ba1 d3lrpa_ d1tada2 d2y8ea_ d4rd4a1 d1z06a1 d2heia_ d3a58b_
d1u0la2 d1d2ea3 d1zj6a1 d2ocba_ d3rwoa_ d1mr3f_ d2e9sa1 d4u5xa1
d2iwra_ d2fn4a1 d3bwdd_ d2nzja1 d2atva1 d2ksqa_ d3l43a_ d1mkya2
d1mkya1 d1wmsa_ d1jnya3 d2rmka_ d1f6ba_ d1f60a3 d1gota2 d1jala1
d2fh5b1 d1g16a_ d2wblc_ d1m7ba_ d1wf3a1 d1f5na2 d1t9ha2 d1z6xa1
d1z0aa1 d2fg5a1 d2a5ja1 d2bcjq2 d1s0ua3 d1ky3a_ d4y0va_ d4lhwa_
d2qmea1 d2atxa1 d1egaa1 d1ryha_ d1moza_ d1nrjb_ d2erya1 d3bfka_
d1puia_ d1fzqa_ d3j3az1 d3t5ga_ d1shza2 d3defa_ d2f7sa1 d3raba_
d3tsoa_ d3aq4a_ d2al7a1 d3sfva1 d3bc1a_ d2ncda_ d1sdma_ d3h4sa1
d2heha_ d2grya_ g3kin.1 d1kk8a2 d1br2a2 d1goja_ d1f9va_ d2mysa2
d1lkxa_ d3t0qa_ d4db1a1 d3gbja_ d1ry6a_ d1v8ka_ d1y4wa1 d3vrda1
d3vrda2 d1fcdc1 d1h1oa2 d1h1oa1 d1m70a2 d1m70a1 d1ig8a1 d1czan2
d1czan3 d1czan1 d1ig8a2 d1czan4 d1bg3a4 d1bg3a3 d1bg3a1 d1nira1
d1qksa1 d1hroa_ d4gedb_ d3zooa_ d1mz4a_ d1gu2a_ d1f1fa_ d1dvva_
d1c6ra_ d1c75a_ d1e29a_ d1gdva_ d1cnoa_ d1h32b_ d1co6a_ d2bh4x_
d2aiua_ d5cytr_ d1jdla_ d1i8oa_ d2zboa_ d3dmia_ d1ls9a_ d1wvec_
d2yk3a_ d3ph2b_ d1dw0a_ d5aura_ d1ql3a_ d1qn2a_ d1f1ca_ d2nqya_
d1h4ga_ d1hixa_ d1oa2a_ d3b7ma_ d4h7ma1 d3wp3a_ d2vgda_ d1igoa_
d3zsea_ d2nlra_ d3wx5a_ d1oa3a_ d1pvxa_ d1m4wa_ d1f5ja_ d1oa4a_
d1ks5a_ d4ixla1 d2ah2a1 d1n1ta1 d1evsa_ d1cnt1_ d1pvhb_ d1z7ca_
d1huwa_ d1bgea_ d1rhga_ d1f6fa_ d1f45b_ d1i1rb_ d1axia_ d1etea_
d3tgxb_ d1scfa_ d2z3qa1 d3qb1a_ d1hula_ d1m48a_ d4nkqb_ d1eera_
d5e4ea_ d2o27a_ d3b5ka_ d2psma1 d4zf7a_ d3uf5a_ d4x51a_ d1d9ca_
d4ypgc1 d3oq3a_ d1n1fa_ d1au1a_ d1wu3i_ d3besl_ d3ux9a_ d1mixa2
d1e5wa2 d1j19a2 d1isna2 d2al6b2 d4dnxa2 d1v47a2 d1g8fa2 d1jhda2
d1x6va2 d2el7a_ d1irxa2 d1r6ta2 d1ivsa4 d2cyba_ d1rqga2 d1j09a2
d2yxna_ d1f7ua2 d1h3fa1 d1ffya3 d2g36a1 d1nzja_ d1jila_ d1n3la_
d1i6la_ d1pfva2 d1h3na3 d3n9ia_ d3sz3a1 d3sw2a_ d1moxc_ d1dx5i3
d1edmb_ d1autl2 d1autl1 d1nqlb_ d1dval1 d1szba2 d1kigl_ d2y5fl_

d1ijqa2 d1gk5a_ d1g1sa2 d1nt0a3 d1xdtr_ d1lmja2 d1lmja1 d1nzia2
d2wphe_ d1klil_ d1pfxl2 d1pfxl1 d1p9ja_ d1g1ta2 d1f7ea_ d4yzub_
d2mgpa1 d1b9wa1 d1b9wa2 d2mgpa2 d1ob1c1 d1ob1c2 d1n1ia2 d1n1ia1
d2npra2 d2npra1 d2y5ia_ d1qlsa_ d1psra_ d3n22a_ d2egda_ d4ggfc_
d2wcea_ d2luca_ d4aqia_ d3nsia_ d2lp3a_ d1j55a_ d1a4pa_ d1k8ua_
d1nsha_ d1exra_ d1tiza1 d1nyaa_ d1g8ia_ d1ggwa_ d1wdcc_ d3fwba_
d1ggza_ d2o5ga_ d1ctaa_ d1m45a_ d3i5gc_ d1sl8a_ d1fpwa_ d2scpa_
d2roaa_ d4aqra_ d3pn7b_ d1br1b_ d2mysb_ d1s6ca_ d1bjfa_ d2obha_
d1jfka_ d1jf0a_ d1qv0a_ d1lkja_ d2mlfc_ d2m97a_ d2d8na_ d4by5a_
d1jbaa_ d4nqga_ d1wdcb_ d2mysc_ d1j7qa_ d1s6ia_ d1uhka2 d1r2ua_
d1dtla_ d3kf9a_ d3pn7c_ d1qjta_ d1fi6a_ d1c07a_ d5d67d_ d1tuza_
d1eg3a1 d1eg3a2 d2zkmx1 d1snla_ d1y1xa_ d1hqva_ d1dvia_ d1df0a1
d1qxpa2 d4usla_ d1k94a_ d1sfsa_ d1wkya2 d1uuqa_ d3wh9a_ d4m1ra_
d1xyza_ d4xuya_ d2je8a5 d4bf7a1 d3cuia_ d1ur4a_ d1qw9a2 d1odza_
d2vzsa5 d1bqca_ d2x2ya1 d1hjqa_ d1qnra_ d4y7ea1 d1vjza_ d4k68a_
d2v3ga_ d4hpga_ d1nofa2 d1nq6a_ d1h4pa_ d1aq0a_ d3ziza_ d4bs0a_
d1ecea_ d1h1na_ d1ta3b_ d1w91a2 d1i1wa_ d1uhva2 d1us3a2 d1egza_
d7a3ha_ d1tvna1 d3nyda1 d1ghsa_ d2cyga1 d4xv0a_ d2c8na2 d1w32a_
d3emca_ d1v6ya1 d1foba_ d2uwfa1 d2fgla_ d3niya1 d2c0ha1 d1rh9a1
d1yq2a5 d1szna2 d1fa2a_ d1pama4 d5do8a1 d1ea9c3 d1hx0a2 d2fhfa5
d1qhoa4 d1avaa2 d1m53a2 d4m8ua1 d2gjpa2 d1lwha2 d4howa1 d1ktba2
d1j0ha3 d3vm5a1 d2x1ia_ d1wzaa2 d1uasa2 d2diea1 d1mxga2 d1ht6a2
d1eswa_ d1iv8a2 d5cpsa_ d1ud2a2 d1zjaa1 d3gbea1 d1kwga2 d1wdpa_
d1gcya2 d1g5aa2 d1hvxa2 d3dhpa2 d2bhua3 d4do4a1 d3qgva1 d1e43a2
d1g94a2 d2d3na2 d1gjwa2 d1gvia3 d1x1na1 d1r7aa2 d1tz7a1 d4c7ga2
d2gjxa1 d1nowa1 d1yhta1 d4toqa_ d1wb0a1 d1w9pa1 d1eoka_ d1itxa1
d1kfwa1 d1goia2 d1edqa2 d2gsja_ d1hjxa1 d1vf8a1 d3wdpp1 d3wq5a_
d1gnxa_ d4gxpa_ d4hz8a_ d1e4mm_ d3aiua_ d1wcga1 d1qvba_ d3ahxa_
d1v02a_ d2dgaa_ d1pbga_ d1vffa1 d1qoxa_ d4ha4a_ d2w0pa_ d2diaa1
d2e9ia2 d2e9ja1 d2dj4a1 d2eeba1 d2diba1 d2dmba1 d2dmca1 d2d7oa1
d2ee6a1 d2d7pa1 d2eeaa1 d2di8a1 d1wlha1 d2d7na1 d2nqca_ d2d7ma1
d2dica1 d3cnka_ d2k3ta1 d2eeca1 d1qfha1 d1qfha2 d2bp3a1 d1v05a_
d2ee9a1 d2eeda1 d2di9a1 d3syxa1 d2p8va_ d1xoda1 d1i7aa_ d1evha_
d1qc6a_ d1egxa_ d1eh3a_ d3skpa_ d1dtza1 d1dtza2 d1jnfa2 d1ce2a1
d1jnfa1 d1b1xa2 d1b1xa1 d1jw1a1 d1jw1a2 d1ryoa_ d1h76a1 d1h76a2
d1iuka_ d2csua1 d3q9ub_ d2yv1a1 d3q9na_ d2d59a_ d1oi7a1 d2yv2a1
d1y81a1 d1wgqa1 d1v89a1 d2coda1 d1zc3b_ d1wg7a1 d1u5ea1 d1v61a1
d2coca1 d2ys1a1 d1v5pa1 d1ntya2 d1wjma1 d1wi1a1 d1u29a_ d1xx0a1
d1unqa_ d2zkmx3 d1fhoa_ d1v88a1 d2adza1 d1x1fa1 d1p6sa_ d2coaa1
d1btka_ d1v5ma1 d1faoa_ d2x18a_ d1dyna_ d1x1ga1 d4kaxb_ d2j59m1
d1xcga2 d1txda2 d1u5da1 d1dbha2 d1w1ha_ d1upqa_ d1v5ua1 d1foea2
d2fjla1 d1eaza_ d2dfka2 d2cofa1 d1kz7a2 d1h6ta1 d9ldta1 d2ewda3
d1up7a1 d2dfda1 d1s6ya1 d1vjta1 d7mdha1 d1hyea1 d1t2da1 d1ojsa3
d1b8pa1 d1ldna1 d1hyha1 d1civa1 d2a92a1 d1ez4a1 d1i0za1 d1y6ja1

d1gv0a1 d1ur5a1 d1llda1 d1obba1 d1npya1 d3t4ea2 d1o0sa1 d2hk9a2
d2egga2 d1b26a1 d1bvua1 d1gq2a1 d1c1da1 d1leha1 d1gtma1 d1euza1
d1nvta1 d3dona2 d1edza1 d1gpja2 d1vi2a1 d1p77a1 d1nyta1 d1b0aa1
d1vl6a1 d1v9la1 d1txga2 d3f47a1 d1mv8a2 d2pv7a2 d1xc2a3 d2ahra2
d4xiya1 d2f1ka2 d1pgja2 d1i36a2 d1np3a2 d1lj8a4 d1teaa3 d1qmga2
d2i76a2 d2g5ca2 d1wdka3 d1dlja2 d3cuma3 d1kola2 d1f8fa2 d1qora2
d1jqba2 d1vj0a2 d1cdoa2 d1gu7a2 d1d1ta2 d1xa0a2 d2dm6a2 d1jvba2
d1rjwa2 d1llua2 d1h2ba2 d1tt7a2 d1piwa2 d1o89a2 d1iz0a2 d1e3ja2
d1yb5a2 d1uufa2 d1e3ia2 d1vc2a1 d2nvwa1 d1u8fo1 d1ydwa1 d1hdgo1
d2g17a1 d1cf2o1 d2czca2 d1r0ka2 d1obfo1 d2cvoa1 d1ebfa1 d1h6da1
d1i32a1 d1zh8a1 d2q49a1 d1qkia1 d1j5pa4 d3pzra1 d1xeaa1 d1vkoa1
d1vm6a3 d3qvsa1 d2pkqo1 d1b7go1 d1gr0a1 d1p1ja1 d1lc0a1 d1j0xo1
d3pwka1 d2hjsa1 d1zyao1 d1vkna1 d1tlta1 d1nvmb1 d3sjua_ d3ojfa1
d1xu9a1 d2jjya1 d1n7ha_ d1y1pa1 d1h5qa_ d1jtva_ d4fs3a_ d2bkaa1
d1gy8a_ d2c5aa1 d1ulsa_ d1ooea_ d2b69a1 d1y5ma1 d1spxa_ d1ae1a_
d1bxka_ d1ydea1 d1gega_ d1hdoa_ d1o5ia_ d1e6ua_ d1snya1 d2rhca_
d3zv4a_ d5ceja_ d2d1ya1 d1fjha_ d3w8ea_ d2fmua1 d1x1ta_ d1rpna_
d3ay7a1 d1k2wa_ d1eq2a_ d2hrba1 d3ruaa_ d2ew8a1 d4hwka_ d1xgka_
d2bd0a1 d1yo6a1 d2a35a1 d2ntva_ d1vl8a_ d1hdca_ d4bkua_ d2ag5a1
d2q45a_ d1nffa_ d4iy1a_ d1zbqa1 d1kewa_ d1ek6a_ d1ja9a_ d2q46a2
d1iy8a_ d1cyda_ d2ptga_ d1hxha_ d1uzma1 d3lyla_ d4m89a_ d3wyea_
d2blla1 d1w6ua_ d1sb8a_ d2q2va_ d4nr0a_ d1e6wa_ d2gasa_ d3oiga1
d2bgka1 d1mxha_ d1vl0a1 d1rkxa_ d1r6da_ d1wmaa1 d1e7wa_ d2c07a1
d1xkqa_ d3tzha_ d1g0oa_ d3lz6a_ d1qyca_ d1edoa_ d1gz6a_ d1oc2a_
d1t2aa_ d1zema1 d2xoxa_ d1z45a2 d1geea_ d1p33a_ d3lt0a_ d4e6pa_
d2wyua_ d1db3a_ d4q9na_ d1xhla_ d4o1ma_ d1fmca_ d2uvda_ d2gdza1
d1uaya_ d2z1ma_ d2o23a2 d2fr1a2 d2fr1a1 d1qsga_ d1qyda_ d4zjua_
d2a4ka1 d1yb1a_ d1wvga1 d1yxma1 d3f4ba_ d1mg5a_ d1xg5a1 d1r55a1
d3gboa_ d1atla_ d1kufa_ d1buda_ d4q1la_ d1yp1a_ d1wnia_ d3u1ra1
d1kapp2 d1k7ia2 d1t3ca_ d1epwa3 d2a97a_ d3fiea_ d3ioqa_ d1fh0a_
d1o0ea_ d1khqa_ d1m6da_ d2cb5a_ g8pch.1 d1s4va_ d2pnsa_ d1vsna1
d1mira_ d1iwda_ d2oula_ d7pcka_ d3bwka_ d3bcna_ d2p86a_ d1pvja_
d1pcia_ d1gece_ d1deua_ d4bs6a_ d1xkga1 d1cqda_ d1jqpa2 d2c0ya_
d2bdza_ d3p5ua_ d2o36a_ d1k9xa_ d1zoya1 d1qo8a2 d1d4ca2 d1chua2
d1jnra2 d1kf6a2 d2jk6a2 d2jk6a1 d1feca1 d1feca2 d1lpfa1 d1lpfa2
d1xhca1 d1xhca2 d1dxla2 d1dxla1 d1gera2 d1m6ia2 d1onfa1 d1onfa2
d3lada2 d3lada1 d1w4xa1 d1w4xa2 d1mo9a1 d1xdia1 d1fl2a2 d1fl2a1
d1fcda1 d1fcda2 d1h6va2 d1h6va1 d1mo9a2 d1gv4a2 d1gv4a1 d2gv8a2
d2gv8a1 d1aoga1 d1aoga2 d1hyua1 d1ebda1 d1ebda2 d3dk9a1 d1q1ra2
d1q1ra1 d1gesa1 d1pv8a_ d1w5qa_ d2c1ha_ d1ohla_ d1zbfa1 d2z1ha_
d2etja1 d3qioa1 d1jl1a_ d2e4la_ d1jl2a_ d2lsna1 d1io2a_ d1uaxa_
d2yv0x_ d1mu2a1 d1i39a_ d1pn0a1 d1reoa1 d1ju2a1 d2f5va1 d3c96a1
d2gmha1 d1pj5a2 d1gpea1 d1n4wa1 d1k0ia1 d2dw4a2 d2ivda1 d1seza1
d2voua1 d1kdga1 d1s5ja1 d2hbja2 d2igia_ d1wlja_ d1qhta1 d4dlea1

d2f96a1 d3tr8a1 d3mxma_ d1q8ia1 d1y97a1 d1w0ha_ d1kfsa1 d2py5a1
d1noya_ d1d5aa1 d1yt3a3 d2guia_ d3mwpa1 d4kb1a_ d2y8ga_ d1xkea1
d2y8fa_ d1iv0a_ d1nu0a_ d1b9ra_ d1offa_ d1frra_ d3wcqa_ d1e9ma_
d1l5pa_ d1wria_ d1i7ha_ d3av8a_ d1czpa_ d1rfka_ d3p1ma1 d3b2ga_
d2bt6a2 d1v58a1 d1t3ba1 d4ilfa2 d4npba2 d1tq5a1 d3nvva1 d1krha3
d2h88b1 d1n62a2 d1t3qa2 d1kf6b2 d1zoyb1 d1jq4a_ d1ffva2 d1dgja2
d1rm6c2 d1jmxa1 d1pbya1 d1pbya2 d1r7ha_ d1faaa_ d1wika1 d1xfla_
d1ttza1 d2f51a1 d1ti3a_ d1nhoa_ d1sena_ d2i4aa_ d1ep7a_ d1zmaa1
d1fo5a_ d3lefa1 d3diea1 d2yana1 d1z6na1 d1oaza_ d3zywa1 d4aj8a_
d1nm3a1 d4fiwa_ d1wmja_ d4hs1a1 d1iloa_ d1xwaa1 d1wjka1 d2vm1a_
d1fb6a_ d1gh2a_ d1h75a_ d1nw2a_ d1kx5b_ d3a6na_ d4m6ba2 d2nqbd_
d3wkjd_ d2yfwe_ d4h9na_ d5bnxa_ d1kx5c_ d3r45a_ d1eqzb_ d4jjnb_
d1f66c_ d1eqza_ d4wnnb_ d1u35c1 d1id3a_ d2nqbc_ d1kx5d_ d1ku5a_
d1f1ea_ d1b67a_ d1tafa_ d1bh9b_ d1tafb_ d1jfib_ d1q9ca_ d1h3ob_
d2byka1 d1n1ja_ d4g92b_ d1jfia_ d1h3oa_ d2bykb1 d1n1jb_ d1pd212
d1nhya2 d2a2ra2 d1k0dd2 d1f2ea2 d1gsua2 d1f3ba2 d2fhea2 d2pvqa1
d1tw9a2 d1oyja2 d1e6ba2 d1k0ma2 d3ik7a1 d4is0a1 d1r5aa2 d1jlva2
d1m0ua2 d3gtub2 d1z9ha2 d1okta2 d1axda2 d2gsra2 d1tu7a2 d1k3ya2
d1zgma1 d1gnwa2 d1gwca2 d2gsta2 d1glqa2 d1oe8a2 d1pn9a2 d1v2aa2
d1fw1a2 d1duga2 d1g7ea_ d1a0rp_ d1iqca2 d1iqca1 d1eb7a2 d1eb7a1
d1nmla1 d4ilya_ d4olta_ d1qgia_ d3wyha_ d4g9sa1 d3gxra_ d1qqya_
d1f6ra_ d1ivma_ d2gv0a_ d1iiza_ d2z2fa_ d1j8xa_ d1ghla_ d1b9oa_
d174la_ d169la_ d2o4wa_ d1p37a_ d1k28a3 d1xjta1 d1jtma_ d2f2qa_
d4e97a1 d1iyka2 d1rxta1 d1iica2 d1iica1 d2za0a_ d2c21a1 d1f9za_
d2pjsa1 d1klla_ d2i7ra1 d1ecsa_ d1xqaa1 d5cj3a1 d1jifa_ d2p7oa_
d1r9ca_ d1nkia_ d1mpya2 d1kw3b2 d4ghga1 d1tfza1 d3isqa1 d3isqa2
d1sqia1 d1sqia2 d1lkda2 d1lkda1 d1mpya1 d1cjxa2 d1cjxa1 d1sp9b2
d1t47a1 d1t47a2 d1f1ua2 d1sp8a1 d1sp8a2 d1f1ua1 d1s5ja2 d1qhta2
d4dlea2 d1tgoa2 d3py8a2 d1q8ia2 d1kfsa2 d2py5a2 d1d5aa2 d1qsta_
d2beia1 d4m3sa_ d1u6ma1 d1ygha_ d1yk3a1 d3dr6a_ d1xmta_ d1z4ea1
d2ge3a1 d1bo4a_ d2fiaa1 d2jdca1 d1y9wa1 d3to7a_ d1wwza1 d2fl4a1
d2ozua1 d2b5ga_ d2cy2a1 d2p0wa_ d1cm0a_ d4pswa_ d1yrea1 d1yr0a1
d1m4ia_ d2gana1 d1yvka1 d1ghea_ d2g3aa1 d1y7ra1 d2euia1 d2fsra1
d1xeba_ d2ae6a1 d1cjwa_ d1qsma_ d2aj6a1 d1y9ka1 d1i12a_ d1vkca_
d2atra1 d1n71a_ d2d4pa1 d2fiwa1 d2fe7a1 d1tiqa1 d2ou2a_ d1yx0a1
d2fcka1 d1p0ha_ d2j8ma_ d2pq8a_ d1z4ra1 d1s3za1 d1ufha_ d1xvqa1
d1pn0a2 d3hy2a_ d2cvba1 d1o8xa_ d1lu4a_ d1we0a1 d2bmxa1 d2a4va1
d1psqa_ d1xiya1 d1q98a_ d1n8ja_ d1zofa1 d2ggta_ d3tjja_ d4je1a1
d3s9fa_ d2zcta_ d1qmva_ d1jfua_ d2b1ka_ d5dvba1 d2b5xa1 d2v2ga_
d1st9a_ d3tb2a_ d4llra_ d4fh8a1 d1e2ya_ d1knga_ d2h30a_ d1i5ga_
d2z9sa_ d2f8aa1 d1tp9a1 d2b7ka_ d1nm3a2 d3hvva_ d2wfca1 d4kcea_
d1xvwa1 d4gqca1 d3qpma_ d4k1fa_ d1zzoa1 d1gp1a_ d1prxa_ d1f74a_
d3l21a_ d2e1da_ d1to3a1 d1w3ia_ d1sfla_ d1n7ka_ d1vcva1 d4imea_
d1wa3a_ d2ocza1 d1f2ja_ d3m16a_ d2yxga_ d1ub3a_ d3a5fa_ d1fbaa_

d1l6wa_ d1xfba1 d1wx0a1 d1vpxa1 d1ojxa_ d2a4aa1 d1xkya1 d3cq0a1
d3s1xa_ d1f05a_ d1fdja_ d3cpra1 d4d2ja_ d1cg2a1 d1yloa2 d1y7ea2
d1y0ya3 d1lfwa1 d1vixa1 d1fnoa4 d1vhoa2 d1ysja1 d1vhea2 d1z2la1
d2fvga2 d1vgya1 d2grea2 d1xmba1 g1q7l.1 d1rtqa_ d1jgta2 d1ofda3
d1ecfa2 d1q15a2 d1ct9a2 d1ao0a2 d1te5a_ d1ra6a_ d1muka_ d3gsza_
d1khva_ d1te9a_ d2xwha_ d3uqsa_ d3hkwa_ d3n6ma_ d2cjqa_ d2xyma_
d1teba_ d1te8a_ d1u09a1 d4wfxa_ d3tyqa_ d2pgga1 d1gyta2 d1cr6a2
d3groa_ d1thta_ d1ei9a_ d2ftba_ d1pmpa_ d2rcqa1 d1fdqa_ d4lkpa_
d1sa8a_ d1vyfa_ d1lpja_ d3vg7a1 d4azqa1 d1o8va_ d5bvqa_ d1p6pa_
d2qo4a_ d1opba_ d1yiva1 d1o1va_ d4bvma1 d4tkba_ d4i3ba_ d1bwya_
d1ftpa_ d1ggla_ d4gkca_ d1cbia_ d3p6da1 d1kqwa_ d2a0aa1 d3zxha_
d1cgla_ d1rm8a_ d1eaka2 d1hy7a_ d2jsda_ d1cxva_ d1hv5a_ d1i76a_
d1u08a_ d1lc5a_ d1b5pa_ d2ay1a_ d3pd6a_ d7aata_ d2csta_ d1j32a_
d1fg7a_ d4wlha_ d1o4sa_ d1v2da_ d1gdea_ d1m6sa_ d1v72a1 d1svva_
d1ajsa_ d1wsta1 d2f8ja2 d1lk9a_ d1vp4a1 d1xi9a1 d2gb3a1 d1yaaa_
d3p0ya1 d1igra1 d1igra2 d1n8yc2 d1n8yc1 d1m6ba2 d4dv8a2 d2bk7a_
d3fjua_ d1z5ra1 d1kwma1 d1uwya2 d1jqga1 d2c1ca1 d1dtda_ d1h8la2
d1h6ta2 d1h6ua2 d2tbvc_ d1c8nc_ d1x36a_ d1smvc_ d1ng0c_ d4sbvc_
d1opoa_ d1f2nc_ d1cixa_ d1y29a_ d1v7fa_ d2jtba_ d1g9pa_ d1lupa_
d1qk7a_ d1mb6a_ d2a2va_ d1niya_ d1i25a_ d1hvwa_ d2mpqa_ d1ie6a_
d1kqha_ d1la4a_ d2mxoa1 d1dl0a_ d1koza_ d1qk6a_ d1emxa_ d1lqra_
d1c6wa_ d1rmka_ d1ixta_ d1eyoa_ d1cnna_ d1fu3a_ d1g1pa_ d1fyga_
d1rypd_ d1irua_ d1rypc_ d3unfk_ d1rypa_ d1j2pa_ d1irui_ d1j2qh_
d3unfh_ d1m4ya_ d1rypb_ d1irul_ d1irug_ d1iru2_ d1q5rh_ d1rypg_
d1g3ka_ d1iruf_ d1q5qa_ d1e94a_ d1irub_ d1ryph_ d3ty6a_ d1iruj_
d1iruk_ d1rype_ d1irud_ d1irue_ d3mi01_ d1rypf_ d1iruh_ d2z3ba_
d1iruc_ d3mka2_ g1gk9.1 g1or0.1 g1e3a.1 d1mqta_ d1d4m3_ d1fmd3_
d2mev1_ g1aym.1 d1ev13_ d4q4w1_ d1r1a3_ d2x5ia_ d1rhi1_ g1r1a.1
g1d4m.1 g1rhi.1 d1b35b_ d1oopa_ d4iv1a_ g1pvc.1 g1fpn.1 d1fpn1_
d4gb31_ d1d4m1_ d1fmd1_ d1ev11_ d2wzr3_ g1cov.1 d1qqp3_ g1fmd.1
d2x5ic_ d1rhi3_ g1oop.1 d1hxs1_ g1hxs.1 d1fpn3_ d1bev1_ g2mev.1
d1eah3_ d1aym1_ d1pvc3_ d1qqp1_ d1zba3_ d1h8ta_ d3vbhc_ g1b35.1
g1bev.1 d1hxs3_ g1ev1.1 d1bev3_ g1tmf.1 d1tmf1_ d1b35a_ d2mev3_
g1eah.1 g1h8t.1 d1eah1_ d4q4w3_ d1r1a1_ d1aym3_ d1pvc1_ d1zba1_
d1h8tc_ d1oopc_ d1tme3_ g1qqp.1 d4iv1c_ g1tme.1 d1p8ba_ d3ia2a_
d1a88a_ d4dgqa1 d1hkha_ d1zoia_ d4lt9a1 d3tc7a1 d3hoja_ d1i4na_
d3vnda_ d3nl8a1 d1geqa_ d3nava1 d1v5xa_ d4a2ra1 d3tc6a1 d1ujpa_
d4ijba_ d1xcfa_ d1vc4a_ d1rd5a_ d1qopa_ d3b5va_ d2r80b_ d1cqxa1
d1cg5a_ d1gcvb_ d3boma_ d2h8fb_ d2raoa_ d3bj1a_ d4hrrb_ d2r50a_
d3d1kb_ d2aa1b1 d3zhwa_ d3hf4b_ d3gdja_ d1jebb_ d3bj1b_ d1x9fb_
d1wmub_ d1gcva_ d3bcqb_ d3d4xb_ d1gvha1 d2dc3a_ d3hf4a_ d4yu3a_
d3k8ba_ d1qpwb_ d2r80a_ d3bcqa_ d1v4wa_ d3wtgb_ d1jl7a_ d4esaa_
d1a9we_ d3d1ab_ d1fsla_ d4h2la_ d1v4wb_ d1mwca_ d1vhba_ d1jeba_
d1spgb_ d1h97a_ d1q1fa_ d1tu9a1 d3d1aa_ d1hdsb_ d3hyub_ d2zfbb_

d3fs4a_ d1oj6a_ d1shrb_ d4h2lb_ d4yu3b_ d1spga_ d3hrwa_ d3qqra_
d3at5a_ d4wchd_ d3hyua_ d1cg5b_ d1uc3a_ d1hbra_ d2raob_ d1outb_
d4esab_ d3gdjb_ d3qqqa_ d1x9fc_ d3vrfa_ d1itha_ d3mkba_ d3d4xa_
d3mjpa_ d1jl6a_ d3fh9a_ d1outa_ d2dc3b_ d1it2a_ d3eoka_ d3g46a_
d1qpwa_ d3vrfb_ d1ch4a_ d1x9fd_ d1hdsa_ d2zfba_ d3mkbb_ d1or4a_
d3wtga_ d1a4fa_ d3fh9b_ d3bomb_ d3cy5a_ d3at5b_ d1x9fa_ d2oifa_
d4lmxb_ d1cpcb_ d3v57a_ d1kn1a_ d1cpca_ d4f0ta_ d2vjha_ d4po5b_
d1eyxa_ d1alla_ d4f0tb_ d1b33b_ d1b8db_ d4h0mb_ d2vjhb_ d4lm6b_
d2vmlb_ d1allb_ d2vjta_ d3dbja_ d2vmla_ d2vjtb_ d4f0ub_ d1f99b_
d4rmpa1 d1liaa_ d1eyxb_ d2uulb_ d4lmsb_ d4f0ua_ d2bv8a_ d1f99a_
d2bv8b_ d2c7lb_ d3dbjb_ d4rmpb1 d1b8da_ d4h0ma_ d3v57b_ d1kn1b_
d1liab_ d2uula_ d2j96a_ g1wht.1 d2xqfa_ d1ea5a_ d1f6wa_ d1dx4a_
d1qe3a_ d1k4ya_ d4qwwa_ d2ha2a_ d2ejca_ d3muea1 d1ihoa_ d3q12a1
d1xm5a_ d1xaxa_ d1tvia_ d4iuaa3 d4bvva_ d5hpga_ d3k65a_ d1ki0a3
d1ki0a2 d1ki0a1 d1pmla_ d3sp8a3 d2feba1 d1dosa_ d3qm3a1 d2fjka_
d1e88a1 d1l6ja5 d1l6ja4 d1eaka4 d1eaka3 d1l6ja3 d1h8pa1 d3rp2a_
g1ucy.1 d1fuja_ d2zchp_ d3w94a_ d1si5h_ d1lo6a_ g2pka.1 d1cgha_
d1kigh_ d1a5ia_ d3s9ba_ d1hj8a_ d1ao5a_ d2anya_ d1q3xa1 d1gvza_
d1azza_ d4gsoa_ d2f91a1 d1iaua_ d1gdna_ d1eufa_ g1fiw.1 d1eq9a_
d4nfea_ d2rdla_ d1spja_ d1elva1 d1a7sa_ d1ekbb_ d1pfxc_ d1yc0a_
d2aiqa_ g1fiz.1 d1fi8a_ d4k60a_ d4dgja_ d1mzaa_ d1op0a_ d3tjva_
d1pytd_ d1sgfg_ g1gg6.1 d2eeka_ g1rtf.1 d1a0ja_ d3g01a_ d1pytc_
d4e7na_ d1ddja_ d1z8ga1 d2hlca_ d1npma_ d1brup_ g1jou.1 d1sgfa_
d1autc_ g2hnt.1 g1kdq.1 d1orfa_ d3s69a_ d4crga_ d1fxya_ d1bqya_
d1svpa_ d1mbma_ d3su6a1 d1cu1a1 d4agka_ d1vcpa_ d1q31a_ g1n1l.1
d3e90b_ d1ep5a_ d3u1jb_ d1jx4a2 d3gv5b1 d1t94a2 d3bq0a2 d3lgia_
d3i77a_ d2z9ia2 d4nsva_ d1lcya2 d4jcna_ d1os8a_ d1hpga_ d1p3ca_
d1l1ja_ d1qtfa_ d1e69a_ d1mv5a_ d3g60a4 g1ii8.1 d1ewqa2 d1ji0a_
d1qhla_ d1v43a3 d2wmfa1 d1vpla_ d1g2912 d4a82a2 g1f2t.1 d2d62a1
d3tuic1 d2bbsa1 d3g60a3 d2ixea_ d1g6ha_ d1sgwa1 d2qi9c_ d3rlfa1
d3b60a1 d3d31a2 d2pzea1 g1xew.1 d1jj7a_ d1oxxk2 d2onka1 d1w1wa_
d1iwea_ d1qf5a_ d1vmaa2 d1nija1 d2p67a1 d3ihla_ d1okkd2 d1ls1a2
d1s1ma2 d1iona_ d2v40a_ d1dj3a_ d3hida_ d2vo1a1 d2g0ta1 d1vcoa2
d1j8mf2 d1g3qa_ d1xjca_ d1p9ba_ d1qzxa3 d1ihua2 d1ihua1 d2qm8a_
d1dj2a_ d4ioja_ d1cp2a_ d1yrba2 d1hyqa_ d3gqba2 d1skye3 d1xp8a1
d1tf7a2 d1tf7a1 d1ye8a1 d1skyb3 d2qe7d2 d4blta_ d4huta_ d3ldaa2
d4hyya_ d2i3ba1 d1cr1a_ d3j9tb2 d3ntua2 d1vdza1 d2z43a_ d1e9ra_
d1nlfa_ d3ziaa2 d1g6oa_ d3ziad2 d3j9ta2 d1pzna2 d3gqbb2 d2i49a_
d1nnfa_ d1xvxa_ d1ursa_ d1h3da1 d1iuda_ d4m1va1 d1twya_ d3e13x_
d2nxoa1 d4pfua_ d3ttma_ d1elja_ d2qmwa1 d1us5a_ d1y3na1 d1jeta_
d1uqwa_ d4f19a1 d2esna2 d3thia_ d3fasa1 d2uyeb_ d2xwva1 d1r9la_
d2fyia1 d1pb7a_ d2ozza1 d1sw5a_ d1nh8a1 d1vr5a1 d2g29a_ d1xs5a_
d3lswa_ d2z23a_ d3w9va_ d3tcha_ d1z7me1 d3q27a_ d1p99a_ d1ve4a1
d2i6ea1 d4mcoa1 d4n8ya_ d1eu8a_ d1zbma1 d1hsla_ d1ixca2 d1j1na_

d1xvya_ d1y9ua_ d2z22a1 d1xoca1 d4oera_ d1wdna_ d5f1qa1 d1xt8a1
d1i6aa_ d4gwoa_ d4igta1 d1pc3a_ d4mmpa1 d1o63a_ d2czla1 d2onsa2
d4mija1 d1m8pa3 d1etha2 d1w52x1 d2ppla1 d1hpla2 d1lpbb2 d2oxea1
d1bu8a2 d1rp1a2 d1c4oa1 d2p6ra3 d2p6ra4 d1oywa2 d1oywa3 d1wrba1
d1w36b2 d1w36b1 d1c4oa2 d3bora_ d1t6na_ d2eyqa5 d2eyqa3 d2eyqa2
d2zu6a2 d1veca_ d1uaaa1 d1w36d1 d2g9na1 d1z3ix2 d2rb4a_ d1hv8a1
d1hv8a2 d1t5ia_ d1w36c2 d2j0sa2 d2j0sa1 d3hjha1 d1z5za1 d1qdea_
d1z63a1 d1s2ma2 d1s2ma1 d1wp9a1 d1wp9a2 d1gm5a3 d1gm5a4 d3dina4
d3dina3 d1q0ua_ d1w36c1 d1z3ix1 d1nkta3 d1nkta4 d1uaaa2 d1w36d2
g1qhh.1 d2fwra2 d2fwra1 d3q3ma_ d1za0a1 d1mxra_ d2inca_ d2incb_
d1mhyb_ d3hf1b_ d1mtyb_ d1r2fa_ d2uw1a_ d1mhyd_ d3n37a_ d1afra_
d1oquc_ d1syya_ d1mtyd_ d2o1za_ d1uzra_ d1smsa_ d2fkza_ d1zuja1
d1s3qa1 d2fjca1 d1z6om1 d1veia_ d4dyya_ d1jiga_ d3gvya_ d2pyba_
d4cy9a1 d3bvfa1 d1r03a_ d2cf7a_ d1nf4a_ d1zs3a1 d4iwka_ d3ka8a_
d3np0x_ d1n1qa_ d1lb3a_ d4e6ka_ d1z6oa1 d2fzfa1 d4dyua_ d3ge4a_
d1jgca_ d4am4a_ d2z6ma_ d1ji5a_ d1vlga_ d1lkoa1 d1krqa_ d3qz3a_
d1o9ra_ d2ffxj_ d1euma_ d1yv1a1 d2v2pa_ d1j30a_ d4di0a_ d1dpsa_
d1oewa_ d2qzxa_ d2asia_ d1psoe_ d1miqa_ d1smra_ d2h6ta_ d1pfza_
d1izda_ d3hd8a_ d1j71a_ d1dpja_ d4aa9a_ d1t6ex_ g1b5f.1 g1lya.1
g1htr.1 d1qdma2 d1bxoa_ d1wkra_ d1tzsa_ d1ibqa_ d2qzwa_ d2v76a_
d1wj1a1 d1aqca_ d1qqga2 d1ntva_ d1n3ha_ d4dbba_ d3f0wa1 d1p3ra_
d1wgua1 d2cy4a1 d3hqca_ d1wvha1 d1xr0b1 d1bdqa_ d3ggta_ d3ixoa_
d1mrxa_ d2haha_ d1tw7a_ d4njsa_ d1sgua_ d2p3da_ d1nsoa_ d3d3ta_
d2rkfa_ d1c6ya_ d2rspa_ d3ogpa_ d3ekwa_ d2o4na_ d2j9ja_ d3ggua_
d3lzua_ d4npua_ d4j55a_ d2r8na_ d2fxda_ d2p3ca_ d4dqea_ d3ttpa_
d3t3ca_ d2p3aa_ d2hc0a_ d2fdda_ d3mwsa_ d1k8rb_ d1wgra1 d1ef5a_
d1wfya1 d1e8ya3 d1wgya1 d1lfda_ d2cs4a1 d1wxma1 d2byea1 d3kh0a1
d2byfa1 d1wxaa1 d1e7ua3 d3ruib_ d1eo6a_ d3waob_ d2r2qa1 d3m95a_
d3wana_ d2al3a1 d2cr5a1 d1wj4a1 d1s3sg_ d3ubra_ d1qo8a1 d1d4ca1
d2k3va_ d1ft5a_ d1qdba_ d1m1qa_ d2j7aa_ d1h21a_ d1ogyb_ d4fasa_
d1jnia_ d1oaha_ d1sp3a_ d1g66a_ d5ajha1 d4lu0a_ d1of8a_ d3unda1
d3e9aa1 d1vr6a2 d2qkfa_ d1d9ea_ d1o60a_ d3h33a_ d1rwja_ d19hca_
d1os6a_ d3bxua_ d3h4na_ d1i77a_ d1j0pa_ d2ctha_ d1duwa_ d1ofwa_
d2e84a_ d1hh5a_ d1gyoa_ d3h34a_ d1dszb_ d1a6ya_ d3cbba_ d1ynwa1
d1r4ia_ d1hcqa_ d2hana_ d2hanb_ d2enva1 d1kb4b_ d2nllb_ d1lo1a_
d1cita_ d1um8a_ d1ixza_ d1qvra2 d1qvra3 d1qzma_ d1sxjc2 d1jr3d2
d2p65a_ d1g8pa_ d3hu3a3 d1sxjd2 d1njfa_ d1in4a2 d1sxja2 d1tuea1
d2ce7a2 d1w5sa2 d1sxjb2 d1d2na_ d1ny5a2 d1ofha_ d2gnoa2 d2a5yb3
d1fnna2 d1u0ja1 d1lv7a_ d1iqpa2 d2fnaa2 d1ixsb2 d4gita_ d1sxje2
d1g41a_ d1k8ka2 d1k8ka1 d3uleb1 d3i33a2 d3i33a1 d1bupa1 d1dkgd2
d1dkgd1 d4ci6a2 d1jcfa1 d4a2ba2 d2fxua1 d1yaga2 d4cbua2 d1bupa2
d2lj7a_ d1n4na_ d2m8bb1 d2kyqa_ d3psma_ d1jkza_ d1jxca_ d2gl1a_
d1ugla_ d4uj0a_ d2k4ua_ d1hp2a_ d2lu9a_ d1baha_ d2meoa1 d1wm7a_
d1hlya_ d1n8ma_ d1jlza_ d1wpda_ d1c55a_ d1pvza_ d1wt7a_ d1rjia_

d1wz5a_ d1m2sa_ d1pjva_ d1c49a_ d1qkya_ d2ktca1 d1wmta_ d1du9a_
d1j5ja_ d1r1ga_ d1t0za_ d2i61a_ d1t1ta_ d1kv0a_ d2lkba_ d1npia_
d1wwna_ d1pe4a_ d2kbka_ d2li7a_ d1djta_ d2kbha_ d1dq7a_ d1sega_
d1omya_ d1zywa1 d1jzaa_ d1bnla_ d1dy2a_ d3ubub_ d1egia_ d1qo3c_
d1v7pa_ d1tdqb_ d3ubua_ d1c3ab_ d1h8ua_ d3bx4b_ d1t8ca1 d1sb2a1
d1qdda_ d2ziba1 d3cada_ d1byfa_ d1j34b_ d1jwia_ d1jzna_ d1c3aa_
d1wmza_ d3bdwb_ d1hq8a_ d1fifa1 d1dv8a_ d3hupa_ d1wk1a1 d1umra_
d2afpa_ d3bx4a_ d1gz2a_ d1sl6a_ d1v7pb_ d3g8ka_ d1ixxa_ d1j34a_
d1jwib_ d1g1sa1 d1y17a1 d1ja3a_ d1fvub_ d3bdwa_ d1sb2b1 d1fvua_
d1umrc_ d1oz7a_ d4m17a_ d3cfwa1 d1ypqa1 d1g1ta1 d3fd4a_ d3l9jc_
d4dn8a1 d1v4lb_ d1rdl1_ d2msba_ d1dzka_ d4tlja_ d2a2ua_ d4wfva_
d3t1da_ d4iaxa_ d3zq3a_ d2nnda_ d1i4ua_ d1bbpa_ d1kt7a_ d1exsa_
d3s26a1 d1lf7a_ d1qfta_ d4gh7a_ d1z24a1 d1qabe_ d1xkia_ d1ew3a_
d3bx7a_ d4iawa1 d1n0sa_ d1gkab_ d3kzaa_ d2k23a_ d2l9ca1 d2joza1
d1gm6a_ d3kffa_ d3saoa1 d1e5pa_ d1yupa1 d1iiua_ d1cwva5 d1rpma_
d2shpa1 d1l8ka_ d2ooqa1 d1g4us2 d1p15a_ d1lara2 d1lara1 d1wcha_
d2fh7a2 d2fh7a1 d1ohea1 d1ohea2 d2f4da_ d1rxda_ d2hxpa_ d1xria_
d1d5ra2 d1fpza_ d3ezza_ d4y2ea_ d3azya_ d3wvja_ d1upsa1 d2hyka_
d1ajka_ d2vy0a_ d1o4ya_ d3o5sa_ d3d6ea_ d3atga_ d1dypa_ d1umza_
d3i4ia_ d1tdha3 d1q3ba3 d1ee8a3 d1k82a3 g1qmo.1 g1len.1 d3zyra_
d1fnya_ d1n47a_ g2ltn.1 d1avba_ d3usua_ d1dhkb_ d1f9ka_ d1ukga_
d1g8wa_ d1g9fa_ d1wbfa_ d3wcsa_ d1g7ya_ d1mvqa_ d1hqla_ d1v6ia_
d1dbna_ d1qnwa_ d1fx5a_ d4l8qa_ d1ioaa_ d3n35a_ d2h0ba_ d2erfa_
d1h30a2 d1h30a1 d3mw4a1 d1pz7a_ d2r1ba_ d3v64a1 d1d2sa_ d3bopa1
d3mw3a1 d1okqa1 d1okqa2 d1wioa4 d1ccza2 d1vcaa1 d1dr9a2 d1ic1a1
d2d9qb1 d2nxyb2 d1adqa1 d1fnga1 d1uvqb1 d1zs8a1 d2vola1 d2vola2
d1mfbl2 d2jelh2 d1uwel2 d3qq4b1 d1kjvb_ d1k5na1 d1osph2 d4odhl2
d1ow0a1 d1ow0a2 d1bfob2 d3l9ra2 d3jtth1 d2fath2 d4elkb2 d1fn4b2
d2xfxb_ d1pg7x2 d1cqka_ d4k71b2 d1lk2a1 d1eapb2 d4lfhg2 d1i4fa1
d2nw2a2 d1kfah2 d1mhca1 d1h3ua1 d1wejh2 d1h0db2 d1a5fh2 d1kcvh2
d1mjuh2 d1qqda1 d1kcrh2 d1k8ia1 d3huja2 d2nnab1 d1nfdf2 d3mffb2
d1s9va1 d1lk3h2 d1q0xl2 d1fv1b1 d1neza1 d4rfeb2 d1r3ha1 d1a6tb2
d1kjva1 d1pz5b2 d1onqa1 d2bc4a2 d1de4a1 d1k8da1 d1tcra2 d1dn0b2
d1nfde2 d2xwtb2 d1bfoa2 d1mexh2 d1iakb1 d1ypzf2 d4f7ea2 d1q72l2
d2fbjh2 d1fe8h2 d1clzh2 d1fngb1 d1uvqa1 d1vgka1 d1c16a1 d4j12a2
d4x99b2 d1igtb3 d1je6a1 d1q72h2 d1nldh2 d1teth2 d2hmic2 d1i1ca2
d1i1ca1 d1q0yh2 d1kfal2 d3frua1 d1pg7w2 d1u58a1 d1igyb4 d1igyb3
d1hyrc1 d1klub1 d1deeb2 d1c12b2 d2bc4b2 d2ig2h2 d1kcvl2 d3wn5b1
d1lk2b_ d1lk3l2 d1mjul2 d1sm3h2 d1q0xh2 d1zvsa1 d1ydpa1 d1u8ib2
d1ypze2 d1kcuh2 d1op9a_ d1y0la1 d1etzb1 d1egjl1 d2gsib1 d1nj9a1
d4jfhd1 d1aqkl1 d1t2ja_ d1lo4h1 d1zvya1 d3v4vl1 d4enec1 d1kxtb_
d5e8ea1 d2pxya_ d4weud_ d1xiwc_ d2esvd1 d1qnzh_ d1ogae1 d1f4xh1
d4n1hb_ d3ezjb1 d3mlwl1 d1kfah1 d1etza1 d1b4jh1 d3cfic_ d4w68a_
d2arja1 d1ek3a_ d2ok0l1 d1vera_ d1uwgh1 d1blnb1 d1fpth1 d1rzga1

d2giya1 d4m3kb1 d1nc2a1 d2nyyd1 d1ncna_ d1fbih1 d5dxwa_ d4fqqa1
d1dqdh1 d1jfql1 d1kelh1 d1faih1 d1frgl1 d1figl1 d4m6nl1 d1fo0a_
d4hxbl1 d3b5ga_ d3quyd1 d2h9gb1 d1i8kb_ d2dd8h1 d2ck0h1 d1eaja_
d4w6wb1 d2a6ib3 d1t66d1 d2e27h_ d1ob1b1 d1f11b1 d2v7na1 d1ad0a1
d2xt1b1 d1pskh1 d4i13b1 d2op4l1 d1a0ql1 d1uwel1 d1za3a1 d1ncbl1
d4uu9a_ d1bm3h1 d1cloh1 d1nqba2 d1cf8h1 d4y5va_ d1mhph1 d5anma1
d4cdgc1 d4ldeb_ d1y0lb1 d2pcpb1 d1z3gl1 d1dfbl1 d1fh5h1 d3g5xa1
d1tzib1 d4dn3l1 d3zhka1 d2b1hh1 d2uyla1 d1f3dh1 d1nj9b1 d2agjh1
d2h1pl1 d1q72h1 d8fabb1 d1ndmb1 d1rjca1 d4u6hb1 d3upca_ d2fbjl1
d1osph1 d1tvda_ d2cjvh_ d4fhbd1 d1bd2d1 d1rz7h1 d3nn8a_ d1knoa1
d1nfda1 d2bmkb1 d1dqll_ d1igyb1 d1igfl1 d12e8h1 d3utza1 d1nc2b1
d1rihh1 d4mwfb1 d4jvpa1 d1mieh1 d1rhfa1 d1fgnl1 d1sbsh1 d1lp9e1
d1dn0a1 d4lqfl1 d1iaih1 d1kgcd1 d1mh5b1 d1gpol1 d2g60h3 d1f8tl1
d2jixd1 d4grwh_ d1fo0b_ d1bfob1 d1axsa1 d1u9ka_ d1nmbh_ d1h8na1
d1h8na2 d1jguh1 d4eura1 d3sdyl1 d35c8h1 d1w72l1 d1mvub_ d1i9rh1
d1ob1a1 d4wenb_ d1yeeh1 d3ietc1 d1f58h1 d1ad0b1 d1mqkl_ d1nbvh1
d4b50a_ d3r8bb_ d1bafl1 d1mamh1 d4rgne1 d2gsgb_ d35c8l1 d1bafh1
d1ai1h1 d2pcpa1 d2fath1 d2x1pa_ d1orsb1 d1ibgl1 d1shma_ d1a2yb_
d1u0qa_ d2coqa_ d1hilb1 d1qfwi_ d1jv5b_ d1fn4b1 d5e0qa_ d1nsnh1
d1plgl1 d4ocxl1 d1kb5h1 d1rzfh1 d1pg7x1 d2fx7h1 d2oi9c_ d1l7il1
d3eyva1 d1d5il1 d4ffyl_ d1tetl1 d1a6wh_ d1ac6a_ d3dvfa_ d1nfde1
d1mimh1 d1ri8a1 d1jhlh_ d3dura_ d1fskb1 d1f97a1 d3so3b1 d2ak4d1
d1lp9f1 d4z7wf1 d1l7th1 d4y5ya_ d4x7ec1 d1emth1 d1ikfl1 d1nlbh1
d4utal1 d1bjma1 d1eapb1 d1u6ah1 d2dtgd1 d1kj2b_ d1xeda1 d1c1eh1
d1fgvh_ d2bnub1 d2fb4h1 d2dtgb1 d1ktrh_ d1jpth1 d1qkzl1 d4hepg_
d2atpb1 d1flrh1 d2hrpl1 d1mvfa_ d1iaim1 d3k80a1 d2jb5h1 d3zkxc_
d4grwe_ d1it9h1 d1rhhb1 d3w9ec_ d1e4wh1 d1sy6h1 d2j6eh1 d3qxta_
d1jglh1 d1p7ka1 d1tzhb1 d4n8pa1 d1gafl1 d2jelh1 d1wioa2 d4i0cc1
d1cicb1 d1lmka2 d1lmka1 d1iqwh1 d1orqb1 d1ejoh1 d1jnlh1 d4zg1a_
d43c9b_ d1ggbl1 d1wejh1 d3q6fa1 d1f3dj1 d3c6la1 d1bqlh1 d3p9wb_
d1a3rl1 d1h0db1 d2uzil_ d1eo8l1 d1qfwl_ d1n7ml1 d1igja1 d1c5ch1
d2a9mh_ d1rmfh1 d1fj1b1 d4heme_ d1ccza1 d1a5fh1 d1ogad1 d1dlfh_
d1qa9b_ d1vgel1 d3qskb_ d1sjxa_ d4ozfg1 d1sq2n_ d1qd0a_ d1kcvh1
d1c5db1 d3qcul1 d8faba1 d3bn9d1 d1mjuh1 d4y8dc_ d7fabh1 d3dvgb1
d1lo0l1 d1a6tb1 d1gigh1 d1kcrh1 d2hwzl1 d1yjdc1 d3clfl1 d4krna_
d1dl7l_ d1e6ol1 d1mexh1 d3r0ma_ d4kkna_ d1fnsl1 d1oaqh_ d1rzia1
d1ucbh1 d1ncwl1 d4b41a_ d1nfdf1 d2mpah1 d2d7tl1 d4grwf_ d4b5ea_
d1lk3h1 d3u79a_ d4u3xa_ d1ghfl1 d1bvkb_ d1mcww1 d1b88a_ d1jrhh1
d32c2b1 d4wemb_ d1dsfh_ d1op3h1 d1ce1l1 d1iqda1 d1qoka1 d1qoka2
d1qfuh1 d2crya1 d1ay1h1 d1oaql_ d3ab0b_ d1rurl1 d1t4kb1 d1bz7b1
d1n0xh1 d1forl1 d1ucbl1 d4w6yb_ d43c9a_ d2aabl1 d1tzia1 d4n1ca_
d1k6qh1 d3g9ab_ d4tvsa1 d1igmh_ d1pz5b1 d3dx9a1 d3wxwh_ d1nakh1
d1kcul1 d3eaka_ d4qlra1 d1cr9l1 d1d9ka_ d1f4xl1 d1deea1 d2i27n2
d1c12a1 d2orbl1 d1j8hd1 d1mcph1 d1c5da1 d1kenh1 d1bd2e1 d1h5ba_

d4x7fc1 d3upaa_ d1t3fa1 d1indh1 d2dtgc1 d4hc1l1 d1zv5a1 d4wgvb_

d1uwgl1 d4f37k1 d1aqkh1 d1vesa_ d3zkqd_ d1fptl1 d1lo4l1 d1knob1

d3rrqa_ d1ct8b1 d4orzc_ d1mlbb1 d4hgkc1 d1dqdl1 d1bogb1 d4ozhe1

d1dr9a1 d1i7za1 d1dn0b1 d1kell1 d1rzib1 d1ypzf1 d1wtla_ d1hkfa_

d1fail1 d1figh1 d2vyre_ d1sjva_ d1ohqa_ d1bfoa1 d1pkqb1 d1mjul1

d1b4jl1 d1nsnl1 d2arjb1 d1p4bh_ d4aj0a_ d3nn8b_ d2rcja1 d1f2xk_

d4v1dd_ d1adqh1 d2eksa_ d4k3ga_ d1jfqh1 d1uweh1 d1frgh1 d4gftb_

d1uz8b1 d4n1ea_ d1bm3l1 d1clol1 d3mcka1 d2vq1a1 d1ae6h1 d4aq1b1

d1uj3b1 d1nbqa1 d1fh5l1 d2ck0l1 d2ywza_ d1q72l1 d4nbxb_ d1hila1

d4hzlb1 d2j88l1 d1zmya1 d1cd0a_ d1a4ka1 d1pskl1 d1a0qh1 d4krmb_

d1ncbh1 d2fbjh1 d1j8he1 d1i3ua_ d1cf8l1 d1fe8h1 d2p1ya1 d1eeqa_

d2x89a_ d1ol0a_ d2r0lh1 d1ub5a1 d1dfbh1 d3eyvb1 d4jdvb1 d4p2cg_

d2gsia1 d2agjl1 d1u3ha1 d2h1ph1 d1rihl1 d1fskc1 d1boga1 d2a77l1

d1ospl1 d1qfoa_ d3dwta_ d2fx7l1 d1rz7l1 d3stba1 d1pkqa1 d1b6da1

d1dzba2 d1dzba1 d1kj2a_ d4l1ha_ d1rzgb1 d1dqlh_ d12e8l1 d1miel1

d1jmaa_ d1sbsl1 d1fgnh1 d1f58l1 d1mqkh_ d1ndgb1 d3qegl1 d1iail1

d2aw2a1 d1tqbc1 d3fo2a1 d4dkaa_ d1f8th1 d2g60l1 d2ywya_ d1uj3a1

d1yc7a_ d3bd3a_ d4w6xb1 d1nmbl_ d1i8ka_ d1rjlb1 d1ypze1 d1p7kb1

d1kxqe_ d1gpoh1 d2z4qa1 d1w72h1 d1i9rl1 d4qgya1 d4xt1c_ d2qqnh1

d1cica1 d3c08h1 d2cd0a_ d2bdnh1 d1hnga1 d1maml1 d1nldh1 d1cr9h1

d4lcih1 d3bkyh1 d3qg7l1 d1ai1l1 d1kxvc_ d4iosd_ d1l7ih1 d2x1qa_

d1ub6b1 d3k74b_ d1ibgh1 d1teth1 d1igya1 d1hxma1 d2x6ma_ d2e7la_

d5e03a_ d1qfwm_ d2r0kh1 d1igjb1 d1rurh1 d1plgh1 d1mj8h1 d1yuhb1

d3fmgl1 d1rzfl1 d1emtl1 d1ad9b1 d1igtb1 d1nlbl1 d1d5ih1 d3k7ua_

d4ffyh_ d1bwwa_ d3dvga1 d1a6ta1 d1fgvl_ d4qxgl1 d1miml1 d1jhll_

d2hffb1 d2hh0l1 d1qkzh1 d1l7tl1 d1afvh1 d1iaii1 d2cjuh_ d1um5h1

d2ghwb2 d2ghwb1 d1kgce1 d2fgwh1 d1ikfh1 d1dvfd_ d2i24n1 d2xtjb_

d1za3b1 d2yc1a_ d2hh0h1 d2x1oa_ d1bvka_ d1rjla1 d5e7ba1 d1igta1

d1il1b1 d2fb4l1 d1mvua_ d1jptl1 d32c2a1 d1fvca_ d1iqdb1 d2hrph1

d2p4ab_ d4qo1a_ d1p2cb1 d1wt5c_ d1t4ka1 d5c1mb_ d1it9l1 d1bz7a1

d1b2wh1 d1e4wl1 d1jnll1 d1yedb1 d1za6b1 d3u7aa_ d1igfh1 d3gnml1

d1orsa1 d1wejl1 d1a2ya_ d1hxmb1 d1gafh1 d2j4wh1 d1pz5a1 d1j05a_

d1a3rh1 d4fqll1 d1qfwh_ d1eo8h1 d1n7mh1 d4s11a_ d1kegh1 d5da4a_

d1iqwl1 d3whxb1 d1vgeh1 d1deeb1 d1c12b1 d1t2ql1 d1ad9a1 d3gsnb1

d3b2uc1 d1ggbh1 d1t3fb1 d1nakl1 d4ocmc_ d1lila1 d2v7hb3 d2esve1

d1mcoh1 d2qr0b1 d2q3aa_ d1ct8a1 d1kcrl1 d2pxyb_ d2uzih_ d5c3ld_

d3durb_ d1c5cl1 d2ak4e1 d1dl7h_ d1xiwd_ d1mlba1 d1rmfl1 d1i7zb1

d1dlfl_ d2xa3a_ d5bq7b1 d1dqqb1 d3h42l1 d1egjh1 d1kcvl1 d1fe8l1

d2j6el1 d1f3rb2 d1lk3l1 d1sm3h1 d1q0xh1 d1ghfh1 d7fabl1 d1igch1

d1lo0h1 d4idla_ d5c2bl_ d1jrhl1 d1il1a1 d3zhda1 d4kfzc_ d1b0wa_

d1e6oh1 d1mexl1 d4krpb_ d1fnsh1 d1ncwh1 d4hgma_ d4eigb1 d4rzca_

d2mpal1 d4y5xa_ d5en2b1 d3lrga1 d4ej1c1 d1rhha1 d1j05b_ d1forh1

d1q9wb1 d2wzpd_ d2aq2a1 d1h0da1 d3revb1 d2ij0c1 d1tzha1 d4m7jl1

d1k6ql1 d3b9kc1 d1igml_ d3qeua1 d1dsfl_ d1ce1h1 d1kcuh1 d1zvha1

d1zvoa1 d1tqbb1 d1ay1l1 d2qscl1 d2cgrh1 d1nl0h1 d1kenl1 d3eo0a1 d4n9ob1 d1n0xl1 d3k1kc_ d1c4qa_ d1prtc1 d2wv6d_ d1prtf_ d1ltrd_ d4l63b1 d1prtd_ d1tl0f_ d2bosa_ d1prtb1 d3chbd_ d2aq2b1 d1ck1a1 d1enfa1 d1i4pa1 d1v1oa1 d1et9a1 d1esfa1 d2g9hd1 d4ohja1 d1klud1 d1bxta1 d1eu3a1 d1ty0a1 d1fnua1 d2cqva1 d1fhga_ d2k1ma_ d2dl2a1 d1biha3 d1biha1 d1biha4 d3qp3a1 d1g1ca_ d1gxea_ d1cvsc2 d1cvsc1 d1xmwa2 d1m4ka2 d2otpa2 d1biha2 d1rhfa2 d2fcba2 d2fcba1 d1wwbx_ d1gsma2 d1epfa1 d1epfa2 d2dava1 d1waaa_ d1f97a2 d1x44a1 d1vcaa2 d1fnla2 d1fnla1 d3b5ha2 d3h8na2 d3h8na1 d1jbja1 d1jbja2 d1pd6a_ d1f2qa2 d1f2qa1 d3cx2a_ d1nbqa2 d3b5ha1 d1n26a1 d1xiwb_ d1xaua_ d1fltx_ d2ifga1 d1olla1 d1olla2 d1cs6a4 d1cs6a3 d1cs6a2 d1cs6a1 d2gw5a2 d1sy6a1 d1xiwa_ d1ucta2 d1ucta1 d1ry7b2 d1gl4b_ d1qz1a3 d1olza1 d2c9aa1 d1wwca_

**Family-33 (b.47.1.1)**

**Positive dataset contains 5 protein sequences.**

d3lgia_ d3i77a_ d2z9ia2 d4nsva_ d4jcna_

**Negative dataset contains 3928 protein sequences.**

d1m32a_ d1ibja_ d1c7na_ d4q75a_ d2bkwa1 d1t3ia_ d1n8pa_ d1qz9a_ d1jf9a_ d1eg5a_ d1p3wa_ d3kgwa_ d1cl1a_ d2ctza1 d1vjoa_ d2rfva_ d1gc0a_ d1cs1a_ d1d2fa_ d2ch1a1 d1qgna_ d1pffa_ d1elua_ d1ax4a_ d1c7ga_ d2v1pa1 d2ez1a_ d1u08a_ d1lc5a_ d1b5pa_ d2ay1a_ d1bw0a_ d3pd6a_ d7aata_ d2csta_ d1j32a_ d1fg7a_ d4wlha_ d1o4sa_ d1v2da_ d1gdea_ d1m6sa_ d1v72a1 d1svva_ d1ajsa_ d1wsta1 d2f8ja2 d1lk9a_ d1vp4a1 d1xi9a1 d2gb3a1 d1yaaa_ d1xkla_ d2wfla_ d3i2ka1 d1lnsa3 d1xkta1 d2hfka_ d5d3ka_ d2cfba1 d1dfoa_ d1o69a_ d1bj4a_ d3k28a_ d2bwpa_ d3lg0a_ d2fnua_ d3ou5a_ d1z7da1 d1b9ha_ d1iaya_ d4msoa_ d1bs0a_ d1s0aa_ d1sffa_ d3tqxa_ d1mdoa_ d3qboa_ d3pgya_ d1bjna_ d2dkja_ d1ejia_ d1vefa1 d4wxga_ d3bs8a_ d3gbxa1 d1w23a_ d1r0ka3 d1vkoa2 d1vm6a2 d3qvsa2 d1gr0a2 d1p1ja2 d1ddla_ d1auyb_ d1f56a_ d1bypa_ d1azca_ d1ws8a_ d3u25a_ d1cuoa_ d1id2a_ d1rkra_ d2iaac_ d4dp1x_ d1nwpa_ d2gima_ d2i7sa_ d1qhqa_ d1gyca3 d1hfua2 d1kbva1 d1kbva2 d1sdda1 d1sdda2 d1gyca2 d1mzya1 d2bw4a1 d1aoza1 d1aoza3 d1aoza2 d1kyaa2 d1kyaa3 d1kyaa1 d1mzya2 d4e9sa1 d1sddb1 d1sddb2 d1hfua1 d1hfua3 d1oe1a2 d1oe1a1 d1v10a2 d1v10a3 d1v10a1 g1f8v.1 d1novc_ d2bbvc_ g1or0.1 g1cp9.1 g1e3a.1 d1a3qa1 d1iknc_ d3brda1 d1p7hl1 d1oy3c_ d1imhc1 d1gjia1 d1zk9a1 d2cxka1 d2bema_ d1pama1 d1ea9c1 d1qhoa1 d2fhfa2 d2fhfa1 d1k3ia1 d1j0ha1 d1cxla1 d2bhua1 d1edqa1 d1f1sa2 d1ut9a2 d1gvia1 d1txka1 d2phla2 d2phla1 d1fxza2 d1fxza1 d1lrha_ d1uija1 g1dgw.1 d2v09a_ d1uika2 d1uika1 d1dgwa_ d1ep0a_ d2pa7a1 d1wlta1 d1nxma_ d2c0za1 d1ds6b_ d1fstb_ d2b5ea2 d2b5ea3 d2b5ea1 d2b5ea4 d2kp1a1 d2kp2a1 d1hyua3 d1hyua4 d1j08a1 d1j08a2 d1ydwa2 d2nvwa2 d1zh8a2 d1qkia2 d1xeaa2 d1tlta2 d1au7a2 d1e3oc2 d2ovga_ d1sq8a_ d1rzsa_ d3kz3a_ d3orca_ d3gpdg2 d2q49a2 d1hdgo2 d2g17a2 d2cvoa2 d2czca1 d3pwka2 d1obfo2 d1i32a2 d1zyao2

d2pkqo2 d1b7go2 d2hjsa2 d1cf2o2 d1vkna2 d1nvmb2 d1ux8a_ d4i0va_
d2bkma_ d1dlwa_ d2bmma_ d2msya_ d2djoa1 d2r5za_ d1au7a1 d1f43a_
d2lmda1 d1akhb_ d1s7ea1 d3nara_ d1zq3p1 d2craa1 d1mija_ d4uuta_
d3a01a_ d1x58a1 d3rkqa1 d3cmya1 d2cqxa1 d2da6a1 d1e3oc1 d1fjla_
d1mh3a1 d1ig7a_ d1x41a1 d1wh7a1 d1gt0c1 d2mw8a_ d1x2ma1 d1b72a_
d1wi3a1 d1jgga_ d1wjha1 d1x2na1 d1mnmc_ d2cuea1 d3k2aa_ d2hosa_
d2jwta2 d1pufa_ d1octc1 d2hi3a_ d1b8ia_ d4j19a_ d3lnqa_ d1wh5a1
d1b8ib_ d1fexa_ d2cqra1 d1guua_ d1ug2a1 d2cqqa1 d2ajea1 d4a69c_
d1h8ac1 d2crga1 d2iw5b1 d1xc5a1 d1ofcx1 d2cu7a1 d1wgxa1 d2cjja1
d2ckxa1 d1gvda_ d1hcra_ d1gdta1 d1u78a2 d2iu5a1 d1zk8a1 d2g7sa1
d2fd5a1 d2np5a1 d1z0xa1 d2id6a1 d2d6ya1 d2i10a1 d1vi0a1 d2oi8a1
d3c07a1 d2ns7a1 d2fq4a1 d2yvea1 d1t33a1 d2o7ta1 d1rkta1 d2np3b1
d2g7ga1 d2gena1 d2fbqa1 d2g7la1 d2gfna1 d2g3ba1 d2jj7a1 d2hyja1
d1sgma1 d1ui5a1 d2hkua1 d2id3a1 d4jyka1 d1v8fa_ d2ejca_ d3muea1
d1ihoa_ d3q12a1 d3lv8a_ d1zaka1 d1s4qa_ d3tvta2 d1uf9a_ d1khta_
d1ckea_ d1osna_ d1p5zb_ d4tmka_ d3cm0a_ d1tmka_ d1jaga_ d3h86a_
d1t0hb_ d1nn5a_ d2h92a_ d3kb2a1 d2c95a1 d1yj5a2 d1kgda_ d1ly1a_
d2ak3a1 d1vyva2 d1deka_ d2grja1 d1p3ja1 d1y63a_ d1e4ya1 d1s96a_
d3iija1 d1lvga_ d1nksa_ d1vyua2 d4e22a_ d1qf9a_ d1jjva_ d1q3ta_
d1ki9a_ d1lw7a2 d1teva_ d1kjwa2 d1gsia_ d2iyva2 d1kaga_ d1e6ca_
d1m7ga_ d1x6va3 d1z0ja1 d3umra2 d3gj0a1 d2gaoa1 d1yzga1 d1x3sa1
d1g7sa4 d1puja_ d1udxa2 d1kk1a3 d2o52a_ d1jwyb_ d1zcaa2 d1zcba2
d2fv8a1 d2cjwa_ d3dz8a1 d1xzpa2 d1vg8a_ d4qxaa_ d2ew1a1 d1x1ra1
d2bcgy_ d3doea1 d3cona_ d3rega_ d3wyga_ d1upta_ d2b6ha1 d1zd9a1
d1lnza2 d1r5ba3 d1n0ua2 d1r2qa_ d2f9la1 d2rhda_ d3q3jb_ d3q85a_
d1efma1 d1r8qa_ d2gf9a1 d2akab1 d1ek0a_ d1z08a1 d2gcoa1 d2dy1a2
d4g01b_ d2cxxa1 d1svia_ d3q72a_ d1ni3a1 d4ac9a4 d2erxa1 d2bmea_
d1wxqa1 d2gf0a1 d4ldja1 d1zunb3 d1d5ca_ d4lv5b_ d1efca3 d1kmqa_
d2g6ba1 d3lrpa_ d1tada2 d2y8ea_ d4rd4a1 d1z06a1 d2heia_ d3a58b_
d1u0la2 d1d2ea3 d1zj6a1 d2ocba_ d3rwoa_ d1mr3f_ d2e9sa1 d4u5xa1
d2iwra_ d2fn4a1 d3bwdd_ d2nzja1 d2atva1 d2ksqa_ d3l43a_ d1mkya2
d1mkya1 d1wmsa_ d1jnya3 d2rmka_ d1f6ba_ d1f60a3 d1gota2 d1jala1
d2fh5b1 d1g16a_ d2wblc_ d1m7ba_ d1wf3a1 d1f5na2 d1t9ha2 d1z6xa1
d1z0aa1 d2fg5a1 d2a5ja1 d2bcjq2 d1s0ua3 d1ky3a_ d4y0va_ d4lhwa_
d2qmea1 d2atxa1 d1egaa1 d1ryha_ d1moza_ d1nrjb_ d2erya1 d3bfka_
d1puia_ d1fzqa_ d3j3az1 d3t5ga_ d1shza2 d3defa_ d2f7sa1 d3raba_
d3tsoa_ d3aq4a_ d2al7a1 d3sfva1 d3bc1a_ d2ncda_ d1sdma_ d3h4sa1
d2heha_ d2grya_ g3kin.1 d1br2a2 d1goja_ d1f9va_ d2mysa2 d1lkxa_
d3t0qa_ d4db1a1 d3gbja_ d1ry6a_ d1v8ka_ d1s5ja2 d1qhta2 d4dlea2
d1tgoa2 d3py8a2 d1q8ia2 d1kfsa2 d2py5a2 d1d5aa2 d1k8ka2 d1k8ka1
d3uleb1 d3i33a2 d3i33a1 d1bupa1 d1dkgd2 d1dkgd1 d4ci6a2 d1jcfa1
d4a2ba2 d2fxua1 d1yaga2 d1yaga1 d4cbua2 d1czan2 d1czan3 d1czan1
d1ig8a2 d1czan4 d1bg3a4 d1bg3a3 d1bg3a2 d1bg3a1 d3h3no1 d3h3no2
d3ezwa2 d1hroa_ d4gedb_ d3zooa_ d1mz4a_ d1gu2a_ d1f1fa_ d1dvva_

d1c6ra_ d1c75a_ d1e29a_ d1gdva_ d1cnoa_ d1h32b_ d1co6a_ d2bh4x_
d2aiua_ d5cytr_ d1jdla_ d1i8oa_ d2zboa_ d3dmia_ d1ls9a_ d1wvec_
d2yk3a_ d3ph2b_ d1dw0a_ d5aura_ d1ql3a_ d1qn2a_ d1f1ca_ d4zzva_
d2xspa_ d4v20a_ d1ojja_ d4ipma1 d1gpia_ d4csia_ d3pfza_ d3ovwa_
d2yoka_ d2nqya_ d1h4ga_ d1hixa_ d1oa2a_ d1olra_ d3b7ma_ d4h7ma1
d3wp3a_ d2vgda_ d1igoa_ d3zsea_ d2nlra_ d3wx5a_ d1oa3a_ d1pvxa_
d1m4wa_ d1f5ja_ d1oa4a_ d1ks5a_ d4ixla1 d2a6za1 d3a4ua_ d2a6va1
d1h32a2 d1evsa_ d1cnt1_ d1pvhb_ d1z7ca_ d1huwa_ d1bgea_ d1rhga_
d1f6fa_ d1f45b_ d1i1rb_ d1axia_ d1etea_ d3tgxb_ d1scfa_ d2z3qa1
d3qb1a_ d1hula_ d1m48a_ d4nkqb_ d1eera_ d5e4ea_ d2o27a_ d3b5ka_
d2psma1 d4zf7a_ d3uf5a_ d4x51a_ d1d9ca_ d4ypgc1 d3oq3a_ d1lqsl_
d1n1fa_ d1au1a_ d1wu3i_ d3besl_ d3ux9a_ d1mixa2 d1e5wa2 d1isna2
d2al6b2 d4dnxa2 d1v47a2 d1g8fa2 d1jhda2 d1x6va2 d2el7a_ d1iq0a2
d1irxa2 d1r6ta2 d1ivsa4 d2cyba_ d1rqga2 d1j09a2 d2yxna_ d1f7ua2
d1ffya3 d2g36a1 d1nzja_ d1jila_ d1n3la_ d1i6la_ d1pfva2 d1h3na3
d3n9ia_ d3sz3a1 d3sw2a_ d1moxc_ d1dx5i3 d1edmb_ d1autl2 d1autl1
d1nqlb_ d1dval1 d1szba2 d1kigl_ d2y5fl_ d1ijqa2 d1gk5a_ d1g1sa2
d1nt0a3 d1xdtr_ d1lmja2 d1lmja1 d1nzia2 d2wphe_ d1klil_ d1pfxl2
d1pfxl1 d1p9ja_ d1g1ta2 d1f7ea_ d4yzub_ d2mgpa1 d1b9wa1 d2mgpa2
d1ob1c1 d1ob1c2 d1n1ia2 d1n1ia1 d2npra2 d2npra1 d2y5ia_ d1qlsa_
d1psra_ d3n22a_ d2egda_ d4ggfc_ d2wcea_ d2luca_ d4aqia_ d3nsia_
d2lp3a_ d1j55a_ d1a4pa_ d1k8ua_ d1nsha_ d1pvaa_ d3fs7a_ d2pvba_
d1ttxa_ d1rwya_ d2kyfa_ d1rk9a_ d1a75a_ d1exra_ d1tiza1 d1nyaa_
d1g8ia_ d1ggwa_ d1wdcc_ d3fwba_ d1ggza_ d2o5ga_ d1ctaa_ d1m45a_
d3i5gc_ d1sl8a_ d1fpwa_ d2scpa_ d2roaa_ d4aqra_ d3pn7b_ d1br1b_
d2mysb_ d1s6ca_ d1bjfa_ d2obha_ d1jfka_ d1jf0a_ d1qv0a_ d1lkja_
d2mlfc_ d2m97a_ d2d8na_ d4by5a_ d1jbaa_ d4nqga_ d1wdcb_ d2mysc_
d1j7qa_ d1s6ia_ d1uhka2 d1r2ua_ d1dtla_ d3kf9a_ d3pn7c_ d5d67d_
d1tuza_ d1h8ba_ d1eg3a1 d2zkmx1 d1snla_ d1gjya_ d1y1xa_ d1hqva_
d1df0a1 d1qxpa2 d4usla_ d1k94a_ d1wkya2 d1uuqa_ d3wh9a_ d4m1ra_
d1xyza_ d4xuya_ d2je8a5 d4bf7a1 d3cuia_ d1ur4a_ d1qw9a2 d1odza_
d2vzsa5 d1bqca_ d2x2ya1 d1hjqa_ d1qnra_ d4y7ea1 d1vjza_ d4k68a_
d2v3ga_ d4hpga_ d1nofa2 d1nq6a_ d1h4pa_ d1aq0a_ d3ziza_ d4bs0a_
d1ecea_ d1h1na_ d1ta3b_ d1w91a2 d1i1wa_ d1uhva2 d1us3a2 d1egza_
d7a3ha_ d1tvna1 d3nyda1 d1ghsa_ d2cyga1 d4xv0a_ d2c8na2 d1w32a_
d3emca_ d1v6ya1 d1foba_ d2uwfa1 d2fgla_ d3niya1 d2c0ha1 d1rh9a1
d1yq2a5 d1szna2 d1fa2a_ d1pama4 d5do8a1 d1ea9c3 d1hx0a2 d2fhfa5
d1qhoa4 d1avaa2 d1m53a2 d4m8ua1 d2gjpa2 d1lwha2 d4howa1 d1ktba2
d1j0ha3 d3vm5a1 d2x1ia_ d1wzaa2 d1uasa2 d2diea1 d1mxga2 d1ht6a2
d1eswa_ d1iv8a2 d5cpsa_ d1ud2a2 d1zjaa1 d3gbea1 d1kwga2 d1wdpa_
d1gcya2 d1g5aa2 d1hvxa2 d3dhpa2 d2bhua3 d4do4a1 d3qgva1 d1e43a2
d1g94a2 d2d3na2 d1gjwa2 d1gvia3 d1x1na1 d1r7aa2 d1tz7a1 d4toqa_
d1wb0a1 d1w9pa1 d1eoka_ d1itxa1 d1kfwa1 d1goia2 d1edqa2 d2gsja_
d1hjxa1 d1jnda1 d1vf8a1 d3wdpp1 d3wq5a_ d1gnxa_ d4gxpa_ d4hz8a_

| | | | | | | | |
|---|---|---|---|---|---|---|---|
| d1e4mm_ | d3aiua_ | d1wcga1 | d3ahxa_ | d1v02a_ | d2dgaa_ | d1pbga_ | d1vffa1 |
| d1qoxa_ | d4ha4a_ | d2w0pa_ | d2diaa1 | d2e9ia2 | d2e9ja1 | d2dj4a1 | d2eeba1 |
| d2diba1 | d2dmba1 | d2dmca1 | d2d7oa1 | d2ee6a1 | d2d7pa1 | d2eeaa1 | d2di8a1 |
| d1wlha1 | d2d7na1 | d2nqca_ | d2d7ma1 | d2dica1 | d3cnka_ | d2k3ta1 | d2eeca1 |
| d1qfha1 | d2bp3a1 | d1v05a_ | d2ee9a1 | d2eeda1 | d2di9a1 | d3syxa1 | d1mkea1 |
| d2p8va_ | d1xoda1 | d1i7aa_ | d1qc6a_ | d1egxa_ | d2hmta_ | d1lssa_ | d1b9ra_ |
| d1offa_ | d1frra_ | d3wcqa_ | d1e9ma_ | d1l5pa_ | d1wria_ | d1i7ha_ | d3av8a_ |
| d1czpa_ | d1rfka_ | d3p1ma1 | d3b2ga_ | d2bt6a2 | d1wgqa1 | d1v89a1 | d2coda1 |
| d1zc3b_ | d1wg7a1 | d1u5ea1 | d1v61a1 | d2coca1 | d2ys1a1 | d1v5pa1 | d1ntya2 |
| d1wjma1 | d1wi1a1 | d1u29a_ | d1xx0a1 | d1unqa_ | d2zkmx3 | d1fhoa_ | d1v88a1 |
| d2adza1 | d1x1fa1 | d1p6sa_ | d2coaa1 | d1btka_ | d1v5ma1 | d1faoa_ | d2x18a_ |
| d1dyna_ | d1x1ga1 | d4kaxb_ | d2j59m1 | d1ki1b2 | d1xcga2 | d1txda2 | d1u5da1 |
| d1dbha2 | d1w1ha_ | d1upqa_ | d1v5ua1 | d1foea2 | d2fjla1 | d1eaza_ | d2dfka2 |
| d2cofa1 | d1kz7a2 | d9ldta1 | d2ewda3 | d1up7a1 | d2dfda1 | d1s6ya1 | d1vjta1 |
| d7mdha1 | d1hyea1 | d1t2da1 | d1ojsa3 | d1b8pa1 | d1ldna1 | d1hyha1 | d1civa1 |
| d2a92a1 | d1ez4a1 | d1i0za1 | d1y6ja1 | d1gv0a1 | d1ur5a1 | d1llda1 | d1obba1 |
| d1v8ba1 | d1gdha1 | d1l7da1 | d2dlda1 | d1mx3a1 | d1ygya1 | d1qp8a1 | d1li4a1 |
| d2naca1 | d1npya1 | d3t4ea2 | d1a4ia1 | d1o0sa1 | d2hk9a2 | d2egga2 | d1b26a1 |
| d1bvua1 | d1gq2a1 | d1c1da1 | d1leha1 | d1gtma1 | d1euza1 | d1nvta1 | d3dona2 |
| d1edza1 | d1gpja2 | d1vi2a1 | d1p77a1 | d1nyta1 | d1vl6a1 | d1v9la1 | d1txga2 |
| d3f47a1 | d2pv7a2 | d1xc2a3 | d2ahra2 | d4xiya1 | d2f1ka2 | d1pgja2 | d1i36a2 |
| d1np3a2 | d1lj8a4 | d1teaa3 | d1qmga2 | d2i76a2 | d2g5ca2 | d1wdka3 | d1dlja2 |
| d3cuma3 | d1kola2 | d1f8fa2 | d1qora2 | d1jqba2 | d1vj0a2 | d1cdoa2 | d1gu7a2 |
| d1d1ta2 | d1xa0a2 | d2dm6a2 | d1jvba2 | d1rjwa2 | d1llua2 | d1tt7a2 | d1piwa2 |
| d1o89a2 | d1iz0a2 | d1e3ja2 | d1yb5a2 | d1uufa2 | d1vj1a2 | d1e3ia2 | d3gpdg1 |
| d1vc2a1 | d2nvwa1 | d1u8fo1 | d1ydwa1 | d1hdgo1 | d2g17a1 | d1cf2o1 | d2czca2 |
| d1r0ka2 | d1obfo1 | d2cvoa1 | d1ebfa1 | d1h6da1 | d1i32a1 | d1zh8a1 | d2q49a1 |
| d1qkia1 | d3pzra1 | d1xeaa1 | d1vkoa1 | d1vm6a3 | d3qvsa1 | d2pkqo1 | d1b7go1 |
| d1gr0a1 | d1p1ja1 | d1lc0a1 | d1j0xo1 | d3pwka1 | d2hjsa1 | d1zyao1 | d1vkna1 |
| d1tlta1 | d1nvmb1 | d3sjua_ | d3ojfa1 | d1xu9a1 | d2jjya1 | d1n7ha_ | d1y1pa1 |
| d1h5qa_ | d1jtva_ | d4fs3a_ | d2bkaa1 | d1gy8a_ | d2c5aa1 | d1ulsa_ | d1ooea_ |
| d2b69a1 | d1y5ma1 | d1spxa_ | d1ae1a_ | d1bxka_ | d1ydea1 | d1gega_ | d1hdoa_ |
| d1o5ia_ | d1e6ua_ | d1snya1 | d2rhca_ | d3zv4a_ | d5ceja_ | d2d1ya1 | d1fjha_ |
| d3w8ea_ | d2fmua1 | d1x1ta_ | d1rpna_ | d3ay7a1 | d1k2wa_ | d1eq2a_ | d2hrba1 |
| d3ruaa_ | d2ew8a1 | d4hwka_ | d1xgka_ | d2bd0a1 | d1yo6a1 | d2a35a1 | d2ntva_ |
| d1vl8a_ | d1hdca_ | d4bkua_ | d2ag5a1 | d2q45a_ | d1nffa_ | d4iy1a_ | d1zbqa1 |
| d1kewa_ | d1ek6a_ | d1ja9a_ | d2q46a2 | d1iy8a_ | d1cyda_ | d2ptga_ | d1hxha_ |
| d1uzma1 | d3lyla_ | d4m89a_ | d3wyea_ | d2blla1 | d1w6ua_ | d1sb8a_ | d2q2va_ |
| d4nr0a_ | d1e6wa_ | d2gasa_ | d3oiga1 | d2bgka1 | d1mxha_ | d1vl0a1 | d1rkxa_ |
| d1r6da_ | d1wmaa1 | d1e7wa_ | d2c07a1 | d1xkqa_ | d3tzha_ | d1g0oa_ | d3lz6a_ |
| d1qyca_ | d1edoa_ | d1gz6a_ | d1oc2a_ | d1t2aa_ | d1zema1 | d2xoxa_ | d1z45a2 |
| d1geea_ | d1p33a_ | d3lt0a_ | d4e6pa_ | d2wyua_ | d1db3a_ | d4q9na_ | d1xhla_ |
| d4o1ma_ | d1fmca_ | d2uvda_ | d2gdza1 | d1uaya_ | d2z1ma_ | d2o23a2 | d2fr1a2 |
| d2fr1a1 | d1qsga_ | d1qyda_ | d4zjua_ | d2a4ka1 | d1yb1a_ | d1wvga1 | d1yxma1 |

d3f4ba_ d1mg5a_ d1xg5a1 d3nqyb_ d4gera_ d1bqba_ d1t3ca_ d2a97a_
d3fiea_ g1e1h.1 d3ioqa_ d1fh0a_ d1o0ea_ d1khqa_ d1m6da_ d2cb5a_
d1cs8a_ g8pch.1 d1s4va_ d2pnsa_ d1vsna1 d1mira_ d1iwda_ d2oula_
d7pcka_ d3bwka_ d3bcna_ d2p86a_ d1pvja_ d1pcia_ d1gece_ d1deua_
d4bs6a_ d1xkga1 d1cqda_ d1jqpa2 d2c0ya_ d2bdza_ d3p5ua_ d2o36a_
d1k9xa_ d1ex4a2 d1c6va_ d2jk6a2 d2jk6a1 d1feca1 d1feca2 d1lpfa1
d1lpfa2 d1xhca1 d1xhca2 d1dxla2 d1dxla1 d1gera2 d1m6ia2 d1onfa1
d1onfa2 d3lada2 d3lada1 d1w4xa1 d1w4xa2 d1gesa2 d1mo9a1 d1xdia1
d1fl2a2 d1fl2a1 d1fcda1 d1fcda2 d1h6va2 d1h6va1 d1mo9a2 d1gv4a2
d1gv4a1 d2gv8a2 d2gv8a1 d1aoga2 d1hyua1 d1ebda1 d1ebda2 d3dk9a1
d1q1ra2 d1q1ra1 d1gesa1 d1zbfa1 d2z1ha_ d2etja1 d3qioa1 d1jl1a_
d2e4la_ d1jl2a_ d2lsna1 d1io2a_ d1uaxa_ d2yv0x_ d1mu2a1 d1ekea_
d1i39a_ d1s5ja1 d2hbja2 d2igia_ d1wlja_ d1qhta1 d4dlea1 d1tgoa1
d2f96a1 d3tr8a1 d3mxma_ d1q8ia1 d1y97a1 d1w0ha_ d1kfsa1 d2py5a1
d1noya_ d1d5aa1 d1yt3a3 d2guia_ d3mwpa1 d4kb1a_ d4qmea2 d2xdta2
d1z5ha2 d3nvva1 d1krha3 d2h88b1 d1n62a2 d1t3qa2 d1kf6b2 d1zoyb1
d1jq4a_ d1ffva2 d1rm6c2 d1jmxa2 d1pbya1 d1pbya2 d1r7ha_ d1faaa_
d1wika1 d1xfla_ d1ttza1 d2f51a1 d1ti3a_ d1nhoa_ d1sena_ d2i4aa_
d1ep7a_ d1zmaa1 d1fo5a_ d3lefa1 d3diea1 d2yana1 d1z6na1 d1oaza_
d3zywa1 d4aj8a_ d1nm3a1 d4fiwa_ d1wmja_ d4hs1a1 d1iloa_ d1xwaa1
d1wjka1 d2vm1a_ d1fb6a_ d1gh2a_ d1h75a_ d1nw2a_ d1kx5b_ d3a6na_
d4m6ba2 d2nqbd_ d3wkjd_ d2yfwe_ d4h9na_ d5bnxa_ d1kx5c_ d3r45a_
d1eqzb_ d4jjnb_ d1f66c_ d1eqza_ d4wnnb_ d1kx5a_ d1u35c1 d1id3a_
d2nqbc_ d1kx5d_ d1ku5a_ d1b67a_ d1tafa_ d1bh9b_ d1tafb_ d1q9ca_
d1h3ob_ d2byka1 d1n1ja_ d4g92b_ d1bh9a_ d1jfia_ d1h3oa_ d2bykb1
d1n1jb_ d1pd212 d1nhya2 d2a2ra2 d1k0dd2 d1f2ea2 d1gsua2 d1f3ba2
d2fhea2 d2pvqa1 d1tw9a2 d1oyja2 d1e6ba2 d1k0ma2 d3ik7a1 d4is0a1
d1r5aa2 d1jlva2 d1m0ua2 d3gtub2 d1z9ha2 d1okta2 d1axda2 d2gsra2
d1tu7a2 d1k3ya2 d1zgma1 d1b48a2 d1gnwa2 d1gwca2 d2gsta2 d1glqa2
d1oe8a2 d1pn9a2 d1v2aa2 d1fw1a2 d1duga2 d1nira1 d4ilya_ d1chka_
d4olta_ d1qqya_ d1gd6a_ d1f6ra_ d1ivma_ d2gv0a_ d1iiza_ d2z2fa_
d1j8xa_ d1ghla_ d174la_ d169la_ d2o4wa_ d1p37a_ d1k28a3 d1xjta1
d2f2qa_ d4e97a1 d1g4ma1 d1g4ma2 d1iyka2 d1iyka1 d1rxta1 d1iica2
d2za0a_ d2c21a1 d1mpya2 d1kw3b2 d1kw3b1 d4ghga1 d1tfza1 d3isqa1
d3isqa2 d1sqia1 d1sqia2 d1lkda2 d1lkda1 d1mpya1 d1cjxa2 d1cjxa1
d1sp9b2 d1t47a1 d1t47a2 d1f1ua2 d1sp8a1 d1sp8a2 d1etob_ d1umqa_
d1w5qa_ d2c1ha_ d1ohla_ d1l6sa_ d1qsta_ d2beia1 d4m3sa_ d1u6ma1
d1ygha_ d1yk3a1 d3dr6a_ d1xmta_ d1z4ea1 d2ge3a1 d1bo4a_ d2fiaa1
d2jdca1 d1y9wa1 d3to7a_ d1wwza1 d2fl4a1 d2ozua1 d2b5ga_ d2cy2a1
d2p0wa_ d1cm0a_ d4pswa_ d1yrea1 d1yr0a1 d1m4ia_ d2gana1 d1yvka1
d1ghea_ d2g3aa1 d1y7ra1 d2euia1 d1q2ya_ d2fsra1 d1xeba_ d2ae6a1
d1cjwa_ d1qsma_ d2aj6a1 d1y9ka1 d1i12a_ d1vkca_ d2atra1 d1n71a_
d2d4pa1 d2fiwa1 d2fe7a1 d1tiqa1 d2ou2a_ d1yx0a1 d2fcka1 d1p0ha_
d2j8ma_ d2pq8a_ d1z4ra1 d1s3za1 d1ufha_ d1xvqa1 d1pn0a2 d3hy2a_

d2cvba1 d1o8xa_ d1we0a1 d2bmxa1 d2a4va1 d1psqa_ d1xiya1 d1q98a_
d1n8ja_ d1zofa1 d2ggta_ d3tjja_ d4je1a1 d1o73a_ d3s9fa_ d2zcta_
d1qmva_ d1jfua_ d2b1ka_ d5dvba1 d2b5xa1 d2v2ga_ d1st9a_ d3tb2a_
d4llra_ d4fh8a1 d1e2ya_ d1knga_ d2h30a_ d1i5ga_ d2z9sa_ d2f8aa1
d1tp9a1 d2b7ka_ d1nm3a2 d3hvva_ d2wfca1 d4kcea_ d1xvwa1 d4gqca1
d3qpma_ d4k1fa_ d1zzoa1 d1gp1a_ d1prxa_ d1yloa2 d1y7ea2 d1y0ya3
d1lfwa1 d1vixa1 d1fnoa4 d1vhoa2 d1ysja1 d1vhea2 d1z2la1 d2fvga2
d1vgya1 d1r3na1 d2grea2 d1xmba1 g1q7l.1 d1rtqa_ d1etha2 d1w52x1
d2ppla1 d1hpla2 d1lpbb2 d2oxea1 d1rp1a2 d1ra6a_ d1muka_ d3gsza_
d1khva_ d1te9a_ d2xwha_ d3uqsa_ d3hkwa_ d3n6ma_ d2cjqa_ d2xyma_
d1teba_ d1te8a_ d1s48a_ d1u09a1 d4wfxa_ d3tyqa_ d2pgga1 d1lgya_
d4l3wa_ d1llfa_ d1gz7a_ d1ukca_ d1cr6a2 d1c4xa_ d1j1ia_ d2rhwa_
d3ia2a_ d1a88a_ d4dgqa1 d1zoia_ d3zxha_ d1cgla_ d1rm8a_ d1eaka2
d1hy7a_ d2jsda_ d1cxva_ d1hv5a_ d1eb6a_ d3p0ya1 d1igra1 d1igra2
d1n8yc2 d1n8yc1 d1m6ba2 d1m6ba1 d1xm5a_ d1xaxa_ d1tvia_ d2tbvc_
d1c8nc_ d1x36a_ d1smvc_ d1ng0c_ d4sbvc_ d1opoa_ d1f2nc_ d1cixa_
d1y29a_ d1v7fa_ d2jtba_ d1d1ha_ d1g9pa_ d1lupa_ d1qk7a_ d1mb6a_
d2a2va_ d1niya_ d1i25a_ d1hvwa_ d2mpqa_ d1ie6a_ d1kqha_ d1la4a_
d2mxoa1 d1koza_ d1qk6a_ d1emxa_ d1lqra_ d1c6wa_ d1rypd_ d1irua_
d1rypc_ d3unfk_ d1rypa_ d1j2pa_ d1irui_ d1j2qh_ d3unfh_ d1m4ya_
d1rypb_ d1irul_ d1irug_ d1iru2_ d1q5rh_ d1rypg_ d1g3ka_ d1iruf_
d1q5qa_ d1e94a_ d1irub_ d1ryph_ d3ty6a_ d1iruj_ d1iruk_ d1rype_
d1irud_ d1irue_ d3mi01_ d1rypf_ d1iruh_ d2z3ba_ d1iruc_ d3mka2_
d1iru1_ d1ny721 d1ny722 d1a6ca3 d1a6ca2 d1a6ca1 d1pgl22 d1pgl21
d1pgl11 d1mqta_ d1d4m3_ d1fmd3_ d2mev1_ g1aym.1 d1ev13_ d4q4w1_
d1r1a3_ d2x5ia_ d1rhi1_ g1r1a.1 g1d4m.1 g1rhi.1 d1b35b_ d1oopa_
d4iv1a_ g1pvc.1 g1fpn.1 d1fpn1_ d4gb31_ d1d4m1_ d1fmd1_ d1ev11_
d2wzr3_ g1cov.1 d1qqp3_ g1fmd.1 d2x5ic_ d1rhi3_ g1oop.1 d1hxs1_
g1hxs.1 d1fpn3_ d1bev1_ g2mev.1 d1eah3_ d1aym1_ d1pvc3_ d1qqp1_
d1zba3_ d1h8ta_ d3vbhc_ g1b35.1 g1bev.1 d1hxs3_ g1ev1.1 d1bev3_
g1tmf.1 d1tmf1_ d1b35a_ d2mev3_ g1eah.1 g1h8t.1 d1eah1_ d4q4w3_
d1r1a1_ d1aym3_ d1pvc1_ d1zba1_ d1h8tc_ d1oopc_ d1tme3_ g1qqp.1
d4iv1c_ g1tme.1 d1y14b1 d2nn6i1 d1i94l_ d2zjra2 d1smxa_ d2qamc2
d1hh2p1 d2r7da1 d1rl2a2 d1wfqa1 d3bzka4 d1x6oa2 d3a0ja_ d2j01d2
d2ja9a1 d4ql5a_ d1hr0w_ d1luza_ d2n3sa_ d2vnud1 d1h95a_ d1go3e1
d2nn6h1 d1ne3a_ d2mo1a_ d1iz6a2 d1jjga_ d1e3pa2 d2ix0a3 d2ix0a2
d2lxja_ d1ueba3 d1ueba2 d2z0sa1 d1u0la1 d2ix0a1 d2eifa2 d2je6i1
d3i2za1 d2c35b1 d2ahob2 d1t9ha1 d3i4oa_ d2qall1 d1jt8a_ d2nn6g1
d1g6pa_ d3cama_ d1i94q_ d1c9oa_ d1d7qa_ d2ba0a1 d2vnud2 d2vnud3
d1khia2 d2qalq1 d1wi5a1 d1xtda2 d1ny4a_ d4w9ta_ d1jvna1 d4x2ra1
d1ka9f_ d1qo2a_ d4axka1 d1thfd_ d4u28a_ d2y88a_ d4wd0a_ d1vzwa1
d4lt9a1 d3tc7a1 d3hoja_ d1i4na_ d3vnda_ d3nl8a1 d3nava1 d1v5xa_
d4a2ra1 d3tc6a1 d1ujpa_ d4ijba_ d1xcfa_ d1vc4a_ d1rd5a_ d1qopa_
d3b5va_ d2r80b_ d1cqxa1 d1cg5a_ d1gcvb_ d3boma_ d2h8fb_ d2raoa_

| | | | | | | | |
|---|---|---|---|---|---|---|---|
| d3bj1a_ | d4hrrb_ | d2r50a_ | d3d1kb_ | d2aa1b1 | d3zhwa_ | d3hf4b_ | d3gdja_ |
| d1jebb_ | d3bj1b_ | d1x9fb_ | d1wmub_ | d1gcva_ | d3bcqb_ | d3d4xb_ | d1gvha1 |
| d2dc3a_ | d3hf4a_ | d4yu3a_ | d3k8ba_ | d1qpwb_ | d2r80a_ | d3bcqa_ | d1v4wa_ |
| d3wtgb_ | d1jl7a_ | d4esaa_ | d1a9we_ | d3d1ab_ | d1fsla_ | d4h2la_ | d1v4wb_ |
| d1mwca_ | d1vhba_ | d1jeba_ | d1spgb_ | d1h97a_ | d1q1fa_ | d1tu9a1 | d3d1aa_ |
| d1hdsb_ | d3hyub_ | d2zfbb_ | d3fs4a_ | d1oj6a_ | d1shrb_ | d4h2lb_ | d4yu3b_ |
| d1spga_ | d3hrwa_ | d3qqra_ | d3at5a_ | d4wchd_ | d3hyua_ | d1cg5b_ | d1uc3a_ |
| d1hbra_ | d2raob_ | d4esab_ | d3gdjb_ | d3qqqa_ | d1x9fc_ | d3vrfa_ | d1itha_ |
| d3mkba_ | d3d4xa_ | d3mjpa_ | d1jl6a_ | d3fh9a_ | d1outa_ | d2dc3b_ | d1it2a_ |
| d3eoka_ | d3g46a_ | d1qpwa_ | d3vrfb_ | d1ch4a_ | d1x9fd_ | d1hdsa_ | d2zfba_ |
| d3mkbb_ | d1or4a_ | d3wtga_ | d1a4fa_ | d3fh9b_ | d3bomb_ | d3cy5a_ | d3at5b_ |
| d1x9fa_ | d2oifa_ | d4lmxb_ | d1cpcb_ | d3v57a_ | d1kn1a_ | d1cpca_ | d4f0ta_ |
| d2vjha_ | d4po5b_ | d1eyxa_ | d1alla_ | d4f0tb_ | d1b33b_ | d1b8db_ | d4h0mb_ |
| d1b33a_ | d2vjhb_ | d4lm6b_ | d2vmlb_ | d1allb_ | d2vjta_ | d3dbja_ | d2vmla_ |
| d2vjtb_ | d4f0ub_ | d1f99b_ | d4rmpa1 | d1liaa_ | d1eyxb_ | d2uulb_ | d4lmsb_ |
| d4f0ua_ | d2bv8a_ | d1f99a_ | d2bv8b_ | d2c7lb_ | d3dbjb_ | d4rmpb1 | d1b8da_ |
| d4h0ma_ | d3v57b_ | d1kn1b_ | d2uula_ | d2j96a_ | g1gxs.1 | g1wht.1 | d1azwa_ |
| d1hlga_ | d2xqfa_ | d1ea5a_ | d1f6wa_ | d1dx4a_ | d1qe3a_ | d1k4ya_ | d4qwwa_ |
| d2ha2a_ | d1sfra_ | d1f0na_ | d4qeka_ | d3c8da2 | d2pbla1 | d1jkma_ | d1jjia_ |
| d1lzla_ | d1jjfa_ | d2qvba_ | d2v9za_ | d4kafa1 | d4iuaa3 | d4bvva_ | d5hpga_ |
| d3k65a_ | d1ki0a3 | d1ki0a2 | d1ki0a1 | d1a0ha1 | d1pmla_ | d3sp8a3 | d2feba1 |
| d1dosa_ | d1gvfa_ | d3qm3a1 | d2fjka_ | d1f74a_ | d3l21a_ | d2e1da_ | d1to3a1 |
| d1w3ia_ | d1sfla_ | d1n7ka_ | d1vcva1 | d4imea_ | d1wa3a_ | d2ocza1 | d1f2ja_ |
| d3m16a_ | d2yxga_ | d1ub3a_ | d3a5fa_ | d1fbaa_ | d1l6wa_ | d1xfba1 | d1wx0a1 |
| d1vpxa1 | d1ojxa_ | d1mxsa_ | d2a4aa1 | d1xkya1 | d3cq0a1 | d3s1xa_ | d1f05a_ |
| d1fdja_ | d3cpra1 | d4d2ja_ | d1e88a1 | d1l6ja5 | d1l6ja4 | d1eaka3 | d1l6ja3 |
| d1h8pa2 | d1h8pa1 | d2ftba_ | d1pmpa_ | d2rcqa1 | d1fdqa_ | d4lkpa_ | d1sa8a_ |
| d1vyfa_ | d1lpja_ | d3vg7a1 | d4azqa1 | d5bvqa_ | d1p6pa_ | d2qo4a_ | d1opba_ |
| d1yiva1 | d1o1va_ | d4bvma1 | d4tkba_ | d4i3ba_ | d1bwya_ | d1ftpa_ | d1ggla_ |
| d4gkca_ | d1cbia_ | d3p6da1 | d1kqwa_ | d2a0aa1 | d1dzka_ | d4tlja_ | d2a2ua_ |
| d4wfva_ | d3t1da_ | d4iaxa_ | d3zq3a_ | d2nnda_ | d1i4ua_ | d1kxoa_ | d1bbpa_ |
| d1kt7a_ | d1exsa_ | d3s26a1 | d1lf7a_ | d1qfta_ | d4gh7a_ | d1z24a1 | d1qabe_ |
| d1xkia_ | d3bx7a_ | d4iawa1 | d1n0sa_ | d1gkab_ | d3kzaa_ | d2k23a_ | d2l9ca1 |
| d2joza1 | d1gm6a_ | d3kffa_ | d3saoa1 | d1e5pa_ | d1yupa1 | d1iiua_ | d4lu0a_ |
| d1of8a_ | d3unda1 | d3e9aa1 | d1vr6a2 | d1n8fa_ | d2qkfa_ | d1o60a_ | d1e69a_ |
| d1mv5a_ | d3g60a4 | g1ii8.1 | d1ewqa2 | d1ji0a_ | d1qhla_ | d1v43a3 | d2wmfa1 |
| d1vpla_ | d1g2912 | d4a82a2 | g1f2t.1 | d2d62a1 | d3tuic1 | d2bbsa1 | d3g60a3 |
| d2ixea_ | d1g6ha_ | d1sgwa1 | d2qi9c_ | d3rlfa1 | d3b60a1 | d3d31a2 | d2pzea1 |
| g1xew.1 | d1jj7a_ | d1oxxk2 | d2onka1 | d1w1wa_ | d3gqba2 | d1xp8a1 | d1tf7a2 |
| d1tf7a1 | d1ye8a1 | d1skyb3 | d2qe7d2 | d4blta_ | d4huta_ | d3ldaa2 | d4hyya_ |
| d2i3ba1 | d1cr1a_ | d3j9tb2 | d3ntua2 | d1vdza1 | d2z43a_ | d1e9ra_ | d1nlfa_ |
| d3ziaa2 | d1n0wa_ | d1g6oa_ | d3ziad2 | d3j9ta2 | d1pzna2 | d3gqbb2 | d2i49a_ |
| d1nnfa_ | d1xvxa_ | d1ursa_ | d1h3da1 | d1iuda_ | d4m1va1 | d1twya_ | d3e13x_ |
| d2nxoa1 | d4pfua_ | d3ttma_ | d1elja_ | d2qmwa1 | d1us5a_ | d1y3na1 | d1jeta_ |

d1uqwa_ d4f19a1 d2esna2 d3thia_ d3fasa1 d2uyeb_ d2xwva1 d1r9la_
d2fyia1 d1pb7a_ d2ozza1 d1sw5a_ d1nh8a1 d1vr5a1 d2g29a_ d1xs5a_
d3lswa_ d2z23a_ d3w9va_ d3tcha_ d1q35a_ d1z7me1 d3q27a_ d1p99a_
d1ve4a1 d2i6ea1 d4mcoa1 d4n8ya_ d1eu8a_ d1zbma1 d1hsla_ d1ixca2
d1j1na_ d1xvya_ d1y9ua_ d2z22a1 d1xoca1 d4oera_ d1wdna_ d5f1qa1
d1xt8a1 d1i6aa_ d4gwoa_ d4igta1 d1pc3a_ d4mmpa1 d1o63a_ d2czla1
d2onsa2 d4mija1 d1eh3a_ d3skpa_ d1dtza1 d1dtza2 d1ieja_ d1jnfa2
d1ce2a1 d1jnfa1 d1b1xa2 d1b1xa1 d1jw1a1 d1jw1a2 d1ryoa_ d1h76a1
d1h76a2 d1c4oa1 d2p6ra3 d2p6ra4 d1oywa2 d1oywa3 d1wrba1 d1w36b2
d1w36b1 d1c4oa2 d3bora_ d1t6na_ d2eyqa5 d2eyqa3 d2eyqa2 d2zu6a2
d1veca_ d1uaaa1 d1w36d1 d2g9na1 d1z3ix2 d2rb4a_ d1hv8a1 d1hv8a2
d1t5ia_ d1w36c2 d2j0sa2 d2j0sa1 d1fuka_ d3hjha1 d1z5za1 d1z63a1
d1s2ma2 d1s2ma1 d1wp9a1 d1wp9a2 d1gm5a3 d1gm5a4 d3dina4 d3dina3
d1q0ua_ d1w36c1 d1z3ix1 d1nkta3 d1nkta4 d1uaaa2 d1w36d2 g1qhh.1
d2fwra2 d2fwra1 d3q3ma_ d1za0a1 d1mxra_ d2inca_ d1jk0a_ d2incb_
d1mhyb_ d3hf1b_ d1mtyb_ d1r2fa_ d2uw1a_ d1mhyd_ d3n37a_ d1afra_
d1oquc_ d1syya_ d1mtyd_ d2o1za_ d1uzra_ d1smsa_ d2cbia2 d1gqia1
d2choa2 d1oewa_ d2qzxa_ d2asia_ d1psoe_ d1miqa_ d1smra_ d2h6ta_
d1pfza_ d1izda_ d3hd8a_ d1j71a_ d1dpja_ d4aa9a_ d1t6ex_ g1b5f.1
g1lya.1 g1htr.1 d1qdma2 d1bxoa_ d1wkra_ d1tzsa_ d1ibqa_ d2qzwa_
d1bdqa_ d3ggta_ d3ixoa_ d1mrxa_ d2haha_ d1tw7a_ d4njsa_ d1sgua_
d2p3da_ d1nsoa_ d3d3ta_ d2rkfa_ d1c6ya_ d3ogpa_ d3ekwa_ d2o4na_
d2j9ja_ d1baia_ d3ggua_ d3lzua_ d4npua_ d4j55a_ d2r8na_ d2fxda_
d2p3ca_ d4dqea_ d3ttpa_ d3t3ca_ d2p3aa_ d2hc0a_ d2fdda_ d3mwsa_
d1k8rb_ d1wgra1 d1ef5a_ d1wfya1 d1e8ya3 d1wgya1 d1lfda_ d2cs4a1
d1wxma1 d2byea1 d3kh0a1 d2byfa1 d1wxaa1 d1e7ua3 d4yl8a1 d2al6a3
d1l7ya_ d1wgda1 d1wgga1 d4q5eb_ d1wjna1 d1se9a_ d3n3kb_ d1wz0a1
d1we6a1 d2m0xa_ d2gbka_ d3rt3b1 d1wh3a1 d1sifa_ d2ekec2 d4ii2b1
d4hk2a1 d1c3ta_ d1m94a_ d1wgha1 d1ttna1 d1j8ca_ d1wx8a1 d3v6cb1
d2ma9b_ d4i6lb1 d1bt0a_ d2jvca1 d1iyfa_ d2k1fa_ d1wxva1 d1wf9a1
d1t0ya_ d2io0b2 d1x1ma1 d2gbma_ d1zw7a1 d2m17a_ d1we7a1 d2lwpa_
d2gbja_ d1wjua1 d1v6ea1 d1v86a1 d4eewa_ d1wiaa1 d4icva_ d4gu2a_
d1wx7a1 d1wy8a1 d4hjka1 d2faza1 d1v5oa1 d2fkza_ d1zuja1 d1s3qa1
d2fjca1 d1z6om1 d1veia_ d4dyya_ d1jiga_ d3gvya_ d2pyba_ d4cy9a1
d3bvfa1 d1r03a_ d2cf7a_ d1nf4a_ d1zs3a1 d4iwka_ d3ka8a_ d3np0x_
d1n1qa_ d1lb3a_ d4e6ka_ d1z6oa1 d2fzfa1 d4dyua_ d3ge4a_ d1jgca_
d4am4a_ d2z6ma_ d1ji5a_ d1vlga_ d1lkoa1 d1krqa_ d3qz3a_ d1o9ra_
d2ffxj_ d1euma_ d1yv1a1 d2v2pa_ d1j30a_ d4di0a_ d1dpsa_ d1jx4a2
d3gv5b1 d1t94a2 d3bq0a2 d1g66a_ d5ajha1 d2h0ba_ d2erfa_ d1h30a1
d3mw4a1 d1pz7a_ d2r1ba_ d3v64a1 d1d2sa_ d3bopa1 d3mw3a1 d1okqa1
d1okqa2 d1wioa4 d1ccza2 d1vcaa1 d1dr9a2 d1ic1a1 d2d9qb1 d2nxyb2
d2dara2 d2dara1 d1wyha2 d2dloa2 d1rutx4 d1rutx3 d2cuqa2 d1x4la2
d1x4la1 d1x3ha2 d1x3ha1 d2d8za1 d1x61a2 d1x6aa1 d1x6aa2 d2cora2
d2cora1 d1v6ga1 d1v6ga2 d2d8za2 d1x64a1 d1x64a2 d1b8ta1 d2co8a1

d1x63a2 d2dj7a2 d2dj7a1 d2cu8a2 d2cu8a1 d1g47a2 d1g47a1 d1j2oa1
d1wyha1 d2dloa1 d1wiga1 d1wiga2 d2cupa2 d2cupa3 d1x68a1 d2d8ya2
d2d8ya1 d1m3va2 d1x62a1 d1x68a2 d1x61a1 d1b8ta3 d1b8ta2 d2d8xa2
d2d8xa1 d1x4ka1 d1x4ka2 d2cuqa1 d1m3va1 d2co8a2 d1j2oa2 d1x63a1
d2cura1 d2cura2 d1x62a2 d2e3fa_ d1mm0a_ d2e3ea_ d2llda_ d2ny8x_
d2e3ga_ d2ny9x_ d2nz3a_ d2lj7a_ d1n4na_ d2m8bb1 d2kyqa_ d3psma_
d1jkza_ d1jxca_ d2gl1a_ d1ugla_ d4uj0a_ d2k4ua_ d1hp2a_ d2lu9a_
d1baha_ d2meoa1 d1wm7a_ d1hlya_ d1n8ma_ d1jlza_ d1wpda_ d1c55a_
d1pvza_ d1wt7a_ d1rjia_ d1wz5a_ d1m2sa_ d1pjva_ d1c49a_ d1qkya_
d2ktca1 d1wmta_ d1du9a_ d1j5ja_ d1r1ga_ d1t0za_ d2i61a_ d1t1ta_
d1sn4a_ d1kv0a_ d2lkba_ d1npia_ d1wwna_ d1pe4a_ d2kbka_ d2li7a_
d1djta_ d2kbha_ d1dq7a_ d1sega_ d1omya_ d1zywa1 d1jzaa_ d1n9wa1
d1bbua1 d1e1oa1 d1c0aa1 d1l0wa1 d3a74a1 d1eova1 d1iyjb4 d1iyjb5
d1iyjb3 d1o7ia_ d4ipca1 d1x3ea_ d4apva_ d1jb7a1 d1jb7a2 d1jb7a3
d3kdfa_ d1ue1b_ d1wjja1 d3eiva_ d1xjva2 d1xjva1 d2ccza2 d1se8a_
d1l1oc_ d1jb7b_ d1qvca_ d1qzga_ d1miua4 d1miua5 d1miua3 d3g48a1
d1gd7a_ d1jjcb3 d1pyba_ d3ubub_ d1egia_ d1qo3c_ d1v7pa_ d1tdqb_
d3ubua_ d1c3ab_ d1h8ua_ d3bx4b_ d1t8ca1 d1sb2a1 d1qdda_ d2ziba1
d3cada_ d1byfa_ d1j34b_ d1jwia_ d1jzna_ d1c3aa_ d1oz7b_ d1wmza_
d3bdwb_ d1hq8a_ d1fifa1 d1dv8a_ d3hupa_ d1wk1a1 d1umra_ d2afpa_
d3bx4a_ d1sl6a_ d1v7pb_ d3g8ka_ d1ixxa_ d1j34a_ d1jwib_ d1g1sa1
d1y17a1 d1ja3a_ d1fvub_ d3bdwa_ d1sb2b1 d1fvua_ d1umrc_ d1oz7a_
d4m17a_ d3cfwa1 d1ypqa1 d1g1ta1 d3fd4a_ d3l9jc_ d4dn8a1 d1v4lb_
d1rdl1_ d2msba_ d1prtc2 d1pfsa_ d2a1ka_ d1je5a_ d1rpma_ d2shpa1
d1yfoa_ d1l8ka_ d2ooqa1 d1g4us2 d1p15a_ d1lara2 d1lara1 d1wcha_
d2fh7a2 d2fh7a1 d1ohea1 d1ohea2 d2f4da_ d1rxda_ d2hxpa_ d1xria_
d1d5ra2 d1fpza_ d3ezza_ d4y2ea_ d3azya_ d3wvja_ d1upsa1 d1ajoa_
d2hyka_ d1ajka_ d2vy0a_ d3o5sa_ d3d6ea_ d3atga_ d1dypa_ d1umza_
d3i4ia_ d1c1la_ d4lbqa_ d1a78a_ d4no4a_ d3zsja_ d3duia_ d1g86a_
d1is3a_ d1qmja_ d1hlca_ d1tdha3 d1q3ba3 d1ee8a3 d1k82a3 g1qmo.1
g1len.1 d3zyra_ d1fnya_ d1n47a_ g2ltn.1 d1avba_ d3usua_ d1dhkb_
d1f9ka_ d1ukga_ d1g8wa_ d1g9fa_ d1wbfa_ d3wcsa_ d1g7ya_ g1loe.1
d1mvqa_ d1hqla_ d1v6ia_ d1dbna_ d1fx5a_ d4l8qa_ d1ioaa_ d3n35a_
d1epwa1 d3h33a_ d1rwja_ d19hca_ d1os6a_ d3bxua_ d3h4na_ d1i77a_
d3caoa_ d1j0pa_ d2ctha_ d1duwa_ d1ofwa_ d2e84a_ d1hh5a_ d1gyoa_
d3h34a_ d1dszb_ d1a6ya_ d3cbba_ d1ynwa1 d1r4ia_ d1hcqa_ d2hana_
d2hanb_ d2enva1 d1kb4b_ d2nllb_ d1lo1a_ d1cita_ d1adqa1 d1fnga1
d1uvqb1 d1zs8a1 d2vola1 d2vola2 d1mfbl2 d2jelh2 d1uwel2 d3qq4b1
d1kjvb_ d1k5na1 d1osph2 d4odhl2 d1ow0a1 d1ow0a2 d1bfob2 d3l9ra2
d3jtth1 d2fath2 d4elkb2 d1fn4b2 d2xfxb_ d1pg7x2 d1cqka_ d4k71b2
d1lk2a1 d1eapb2 d4lfhg2 d1i4fa1 d2nw2a2 d1kfah2 d1mhca1 d1h3ua1
d1wejh2 d1h0db2 d1a5fh2 d1kcvh2 d1mjuh2 d1qqda1 d1kcrh2 d1k8ia1
d3huja2 d2nnab1 d1nfdf2 d3mffb2 d1s9va1 d1lk3h2 d1q0xl2 d1fv1b1
d1neza1 d4rfeb2 d1r3ha1 d1a6tb2 d1kjva1 d1pz5b2 d1onqa1 d2bc4a2

d1de4a1 d1k8da1 d1tcra2 d1dn0b2 d1nfde2 d2xwtb2 d1bfoa2 d1mexh2
d1iakb1 d1ypzf2 d4f7ea2 d1q72l2 d2fbjh2 d1fe8h2 d1clzh2 d1fngb1
d1uvqa1 d1vgka1 d1c16a1 d4j12a2 d4x99b2 d1igtb3 d1je6a1 d1q72h2
d1nldh2 d1teth2 d2hmic2 d1i1ca2 d1i1ca1 d1k8ib1 d1q0yh2 d1kfal2
d3frua1 d1pg7w2 d1u58a1 d1igyb4 d1igyb3 d1hyrc1 d1klub1 d1deeb2
d1c12b2 d2bc4b2 d2ig2h2 d1kcvl2 d3wn5b1 d1lk2b_ d1lk3l2 d1mjul2
d1sm3h2 d1q0xh2 d1zvsa1 d1ydpa1 d1u8ib2 d1ypze2 d1kcuh2 d1op9a_
d1y0la1 d1etzb1 d1egjl1 d2gsib1 d1nj9a1 d4jfhd1 d1aqkl1 d1t2ja_
d1lo4h1 d1zvya1 d3v4vl1 d4enec1 d1kxtb_ d5e8ea1 d2pxya_ d4weud_
d1xiwc_ d2esvd1 d1qnzh_ d1ogae1 d1f4xh1 d4n1hb_ d3ezjb1 d3mlwl1
d1kfah1 d1etza1 d1b4jh1 d3cfic_ d4w68a_ d2arja1 d1ek3a_ d2ok0l1
d1vera_ d1uwgh1 d1blnb1 d1fpth1 d1rzga1 d2giya1 d4m3kb1 d1nc2a1
d2nyyd1 d1ncna_ d1fbih1 d5dxwa_ d4fqqa1 d1dqdh1 d1jfql1 d1kelh1
d1faih1 d1frgl1 d1figl1 d4m6nl1 d1fo0a_ d4hxbl1 d3b5ga_ d3quyd1
d2h9gb1 d1i8kb_ d2dd8h1 d2ck0h1 d1eaja_ d4w6wb1 d2a6ib3 d1t66d1
d2e27h_ d1ob1b1 d1f11b1 d2v7na1 d1ad0a1 d2xt1b1 d1pskh1 d4i13b1
d2op4l1 d1a0ql1 d1uwel1 d1za3a1 d1ncbl1 d4uu9a_ d1bm3h1 d1cloh1
d1nqba2 d1cf8h1 d4y5va_ d1mhph1 d5anma1 d4cdgc1 d4ldeb_ d1y0lb1
d2pcpb1 d1z3gl1 d1dfbl1 d1fh5h1 d3g5xa1 d1tzib1 d4dn3l1 d3zhka1
d2b1hh1 d2uyla1 d1f3dh1 d1nj9b1 d2agjh1 d2h1pl1 d1q72h1 d8fabb1
d1ndmb1 d1rjca1 d4u6hb1 d3upca_ d2fbjl1 d1osph1 d1tvda_ d2cjvh_
d4fhbd1 d1bd2d1 d1rz7h1 d3nn8a_ d1blna1 d1knoa1 d1nfda1 d2bmkb1
d1dqll_ d1igyb1 d1igfl1 d12e8h1 d3utza1 d1nc2b1 d1rihh1 d4mwfb1
d4jvpa1 d1mieh1 d1rhfa1 d1fgnl1 d1sbsh1 d1lp9e1 d1dn0a1 d4lqfl1
d1iaih1 d1kgcd1 d1mh5b1 d1gpol1 d2g60h3 d1f8tl1 d2jixd1 d4grwh_
d1fo0b_ d1bfob1 d1axsa1 d1u9ka_ d1nmbh_ d1h8na1 d1h8na2 d1jguh1
d4eura1 d3sdyl1 d35c8h1 d1w72l1 d1mvub_ d1i9rh1 d1ob1a1 d4wenb_
d1yeeh1 d3ietc1 d1f58h1 d1ad0b1 d1mqkl_ d1nbvh1 d4b50a_ d3r8bb_
d1bafl1 d1mamh1 d4rgne1 d2gsgb_ d35c8l1 d1bafh1 d1ai1h1 d2pcpa1
d2fath1 d2x1pa_ d1orsb1 d1ibgl1 d1shma_ d1a2yb_ d1u0qa_ d2coqa_
d1hilb1 d1qfwi_ d1jv5b_ d1fn4b1 d5e0qa_ d1nsnh1 d1plgl1 d4ocxl1
d1kb5h1 d1rzfh1 d1pg7x1 d2fx7h1 d2oi9c_ d1l7il1 d3eyva1 d1d5il1
d4ffyl_ d1tetl1 d1a6wh_ d1ac6a_ d3dvfa_ d1nfde1 d1mimh1 d1ri8a1
d1jhlh_ d3dura_ d1fskb1 d1f97a1 d3so3b1 d2ak4d1 d1lp9f1 d4z7wf1
d1l7th1 d4y5ya_ d4x7ec1 d1emth1 d1ikfl1 d1nlbh1 d4utal1 d1bjma1
d1eapb1 d1u6ah1 d2dtgd1 d1kj2b_ d1xeda1 d1c1eh1 d1fgvh_ d2bnub1
d2fb4h1 d2dtgb1 d1ktrh_ d1jpth1 d1qkzl1 d4hepg_ d2atpb1 d1flrh1
d2hrpl1 d1mvfa_ d1iaim1 d3k80a1 d2jb5h1 d3zkxc_ d4grwe_ d1it9h1
d1rhhb1 d3w9ec_ d1e4wh1 d1sy6h1 d2j6eh1 d3qxta_ d1jglh1 d1p7ka1
d1tzhb1 d4n8pa1 d1gafl1 d2jelh1 d1wioa2 d4i0cc1 d1cicb1 d1lmka2
d1lmka1 d1iqwh1 d1orqb1 d1ejoh1 d1jnlh1 d4zg1a_ d43c9b_ d1ggbl1
d1wejh1 d3q6fa1 d1f3dj1 d3c6la1 d1bqlh1 d3p9wb_ d1a3rl1 d1h0db1
d2uzil_ d1eo8l1 d1qfwl_ d1n7ml1 d1igja1 d1c5ch1 d2a9mh_ d1rmfh1
d1fj1b1 d4heme_ d1ccza1 d1a5fh1 d1ogad1 d1dlfh_ d1qa9b_ d1vgel1

d3qskb_ d1sjxa_ d4ozfg1 d1sq2n_ d1qd0a_ d1kcvh1 d1c5db1 d3qcul1
d8faba1 d3bn9d1 d1mjuh1 d4y8dc_ d7fabh1 d3dvgb1 d1lo0l1 d1a6tb1
d1gigh1 d1kcrh1 d2hwzl1 d1yjdc1 d3clfl1 d4krna_ d1dl7l_ d1e6ol1
d1mexh1 d3r0ma_ d4kkna_ d1fnsl1 d1oaqh_ d1rzia1 d1ucbh1 d1ncwl1
d4b41a_ d1nfdf1 d2mpah1 d2d7tl1 d4grwf_ d4b5ea_ d1lk3h1 d3u79a_
d4u3xa_ d1ghfl1 d1bvkb_ d1mcww1 d1b88a_ d1jrhh1 d32c2b1 d4wemb_
d1dsfh_ d1op3h1 d1ce1l1 d1iqda1 d1qoka1 d1qoka2 d1qfuh1 d2crya1
d1ay1h1 d1oaql_ d3ab0b_ d1rurl1 d1t4kb1 d1bz7b1 d1n0xh1 d1forl1
d1ucbl1 d4w6yb_ d43c9a_ d2aabl1 d1tzia1 d4n1ca_ d1k6qh1 d3g9ab_
d4tvsa1 d1igmh_ d1pz5b1 d3dx9a1 d3wxwh_ d1nakh1 d1kcul1 d3eaka_
d4qlra1 d1cr9l1 d1d9ka_ d1f4xl1 d1deea1 d2i27n2 d1c12a1 d2orbl1
d1j8hd1 d1mcph1 d1c5da1 d1kenh1 d1bd2e1 d1h5ba_ d4x7fc1 d3upaa_
d1t3fa1 d1indh1 d2dtgc1 d4hc1l1 d1zv5a1 d4wgvb_ d1uwgl1 d4f37k1
d1aqkh1 d1vesa_ d3zkqd_ d1fptl1 d1lo4l1 d1knob1 d3rrqa_ d1ct8b1
d4orzc_ d1mlbb1 d4hgkc1 d1dqdl1 d1bogb1 d4ozhe1 d1dr9a1 d1i7za1
d1dn0b1 d1kell1 d1rzib1 d1ypzf1 d1wtla_ d1hkfa_ d1fail1 d1figh1
d2vyre_ d1sjva_ d1ohqa_ d1bfoa1 d1pkqb1 d1mjul1 d1b4jl1 d1nsnl1
d2arjb1 d1p4bh_ d4aj0a_ d3nn8b_ d2rcja1 d1f2xk_ d4v1dd_ d1adqh1
d2eksa_ d4k3ga_ d1jfqh1 d1uweh1 d1frgh1 d4gftb_ d1uz8b1 d4n1ea_
d1bm3l1 d1clol1 d3mcka1 d2vq1a1 d1ae6h1 d4aq1b1 d1uj3b1 d1nbqa1
d1fh5l1 d2ck0l1 d2ywza_ d1q72l1 d4nbxb_ d1hila1 d4hzlb1 d2j88l1
d1zmya1 d1cd0a_ d1a4ka1 d1pskl1 d1a0qh1 d4krmb_ d1ncbh1 d2fbjh1
d1j8he1 d1i3ua_ d1cf8l1 d1fe8h1 d2p1ya1 d1eeqa_ d2x89a_ d1ol0a_
d2r0lh1 d1ub5a1 d1dfbh1 d3eyvb1 d4jdvb1 d4p2cg_ d2gsia1 d2agjl1
d1u3ha1 d2h1ph1 d1rihl1 d1fskc1 d1boga1 d2a77l1 d1ospl1 d1qfoa_
d3dwta_ d2fx7l1 d1rz7l1 d3stba1 d1pkqa1 d1b6da1 d1dzba2 d1dzba1
d1kj2a_ d4l1ha_ d1rzgb1 d1dqlh_ d12e8l1 d1miel1 d1jmaa_ d1sbsl1
d1fgnh1 d1f58l1 d1mqkh_ d1ndgb1 d3qegl1 d1iail1 d2aw2a1 d1tqbc1
d3fo2a1 d4dkaa_ d1f8th1 d2g60l1 d2ywya_ d1uj3a1 d1yc7a_ d3bd3a_
d4w6xb1 d1nmbl_ d1i8ka_ d1rjlb1 d1ypze1 d1p7kb1 d1kxqe_ d1gpoh1
d2z4qa1 d1w72h1 d1i9rl1 d4qgya1 d4xt1c_ d2qqnh1 d1cica1 d3c08h1
d2cd0a_ d2bdnh1 d1hnga1 d1maml1 d1nldh1 d1cr9h1 d4lcih1 d3bkyh1
d3qg7l1 d1ai1l1 d1kxvc_ d4iosd_ d1l7ih1 d2x1qa_ d1ub6b1 d3k74b_
d1ibgh1 d1teth1 d1igya1 d1hxma1 d2x6ma_ d2e7la_ d5e03a_ d1qfwm_
d2r0kh1 d1igjb1 d1rurh1 d1plgh1 d1mj8h1 d1yuhb1 d3fmgl1 d1rzfl1
d1emtl1 d1ad9b1 d1igtb1 d1nlbl1 d1d5ih1 d3k7ua_ d4ffyh_ d1bwwa_
d3dvga1 d1a6ta1 d1fgvl_ d4qxgl1 d1miml1 d1jhll_ d2hffb1 d2hh0l1
d1qkzh1 d1l7tl1 d1afvh1 d1iaii1 d2cjuh_ d1um5h1 d2ghwb2 d2ghwb1
d1kgce1 d2fgwh1 d1ikfh1 d1dvfd_ d2i24n1 d2xtjb_ d1za3b1 d2yc1a_
d2hh0h1 d2x1oa_ d1bvka_ d1rjla1 d5e7ba1 d1igta1 d1il1b1 d2fb4l1
d1mvua_ d1jptl1 d32c2a1 d1fvca_ d1iqdb1 d2hrph1 d2p4ab_ d4qo1a_
d1p2cb1 d1wt5c_ d1t4ka1 d5c1mb_ d1it9l1 d1bz7a1 d1b2wh1 d1e4wl1
d1jnll1 d1yedb1 d1za6b1 d3u7aa_ d1igfh1 d3gnml1 d1orsa1 d1wejl1
d1a2ya_ d1hxmb1 d1gafh1 d2j4wh1 d1pz5a1 d1j05a_ d1a3rh1 d4fqll1

d1qfwh_ d1eo8h1 d1n7mh1 d4s11a_ d1kegh1 d5da4a_ d1iqwl1 d3whxb1
d1vgeh1 d1deeb1 d1c12b1 d1t2ql1 d1ad9a1 d3gsnb1 d3b2uc1 d1ggbh1
d1t3fb1 d1nakl1 d4ocmc_ d1lila1 d2v7hb3 d2esve1 d1mcoh1 d2qr0b1
d2q3aa_ d1ct8a1 d1kcrl1 d2pxyb_ d2uzih_ d5c3ld_ d3durb_ d1c5cl1
d2ak4e1 d1dl7h_ d1xiwd_ d1mlba1 d1rmfl1 d1i7zb1 d1dlfl_ d2xa3a_
d5bq7b1 d1dqqb1 d3h42l1 d1egjh1 d1kcvl1 d1fe8l1 d2j6el1 d1f3rb2
d1lk3l1 d1sm3h1 d1q0xh1 d1ghfh1 d7fabl1 d1igch1 d1lo0h1 d4idla_
d5c2bl_ d1jrhl1 d1il1a1 d3zhda1 d4kfzc_ d1b0wa_ d1e6oh1 d1mexl1
d4krpb_ d1fnsh1 d1ncwh1 d4hgma_ d4eigb1 d4rzca_ d2mpal1 d4y5xa_
d5en2b1 d3lrga1 d4ej1c1 d1rhha1 d1j05b_ d1forh1 d1q9wb1 d2wzpd_
d2aq2a1 d1h0da1 d3revb1 d2ij0c1 d1tzha1 d4m7jl1 d1k6ql1 d3b9kc1
d1igml_ d3qeua1 d1dsfl_ d1ce1h1 d1kcuh1 d1zvha1 d1zvoa1 d1tqbb1
d1ay1l1 d2qscl1 d2cgrh1 d1nl0h1 d1kenl1 d3eo0a1 d4n9ob1 d1n0xl1
d3k1kc_ d2cqva1 d1fhga_ d2k1ma_ d2dl2a1 d1biha3 d1biha1 d1biha4
d3qp3a1 d1g1ca_ d1gxea_ d1cvsc2 d1cvsc1 d1xmwa2 d1m4ka2 d2otpa2
d1biha2 d1rhfa2 d2fcba2 d2fcba1 d1wwbx_ d1gsma2 d1epfa1 d1epfa2
d2dava1 d1waaa_ d1f97a2 d1x44a1 d1vcaa2 d1fnla2 d1fnla1 d3b5ha2
d3h8na2 d3h8na1 d1jbja1 d1jbja2 d1pd6a_ d1f2qa2 d1f2qa1 d3cx2a_
d1nbqa2 d3b5ha1 d1n26a1 d1xiwb_ d1xaua_ d1fltx_ d2ifga1 d1olla1
d1olla2 d1cs6a4 d1cs6a3 d1cs6a2 d1cs6a1 d2gw5a2 d1sy6a1 d1xiwa_
d1ucta2 d1ucta1 d1ry7b2 d1qz1a3 d1olza1 d2c9aa1 d1wwca_ d2aq2b1
d1ck1a1 d1enfa1 d1i4pa1 d1v1oa1 d1et9a1 d1esfa1 d2g9hd1 d4ohja1
d1klud1 d1bxta1 d1ty0a1 d1fnua1 d1c4qa_ d1prtc1 d2wv6d_ d1prtf_
d1ltrd_ d4l63b1 d1r4pb_ d1prtd_ d1tl0f_ d2bosa_ d1prtb1 d3chbd_

**Family-34 (b.47.1.2)**

**Positive dataset contains 24 protein sequences.**

d2zchp_ d3w94a_ d1si5h_ d3s9ba_ d2anya_ d1q3xa1 d4gsoa_ d2f91a1
d4nfea_ d2rdla_ d1spja_ d1a7sa_ d1yc0a_ d2aiqa_ d4k60a_ d4dgja_
d1op0a_ d3tjva_ d2eeka_ d3g01a_ d4e7na_ d1z8ga1 d3s69a_ d4crga_

**Negative dataset contains 4012 protein sequences.**

d1ibja_ d1c7na_ d1iuga_ d4q75a_ d2bkwa1 d1t3ia_ d1n8pa_ d1qz9a_
d1jf9a_ d1eg5a_ d1p3wa_ d3kgwa_ d1cl1a_ d2ctza1 d1vjoa_ d2rfva_
d1gc0a_ d1cs1a_ d1d2fa_ d2ch1a1 d1qgna_ d1pffa_ d1elua_ d1u08a_
d1lc5a_ d1b5pa_ d2ay1a_ d1bw0a_ d3pd6a_ d7aata_ d2csta_ d1j32a_
d1fg7a_ d4wlha_ d1o4sa_ d1v2da_ d1gdea_ d1m6sa_ d1v72a1 d1svva_
d1ajsa_ d1wsta1 d2f8ja2 d1lk9a_ d1vp4a1 d1xi9a1 d2gb3a1 d1xkla_
d2wfla_ d3i2ka1 d1lnsa3 d1mpxa2 d1xkta1 d2hfka_ d5d3ka_ d2cfba1
d1dfoa_ d1o69a_ d1bj4a_ d3k28a_ d2bwpa_ d3lg0a_ d2fnua_ d3ou5a_
d1z7da1 d1iaya_ d4msoa_ d1bs0a_ d1s0aa_ d1sffa_ d3tqxa_ d1mdoa_
d3qboa_ d3pgya_ d1bjna_ d2dkja_ d1ejia_ d1vefa1 d4wxga_ d3bs8a_
d3gbxa1 d1w23a_ d2b5aa1 d2ofya1 d4mcxa_ d2xi8a_ d2icta_ d2bnma1
d2ppxa1 d1y7ya1 d1r0ka3 d1j5pa3 d1vkoa2 d1vm6a2 d3qvsa2 d1gr0a2
d1p1ja2 d1i26a_ d1fwxa1 d1ibya_ d1f56a_ d1bypa_ d1azca_ d1ws8a_
d3u25a_ d1cuoa_ d1id2a_ d1rkra_ d2iaac_ d4dp1x_ d1nwpa_ d2gima_

d2i7sa_ d1qhqa_ d3ehbb1 d1fftb1 d1gyca3 d1hfua2 d1kbva1 d1kbva2
d1sdda1 d1sdda2 d1gyca1 d1gyca2 d1mzya1 d2bw4a1 d1aoza1 d1aoza3
d1aoza2 d1kyaa2 d1kyaa1 d1mzya2 d4e9sa1 d1sddb1 d1sddb2 d1hfua1
d1hfua3 d1oe1a2 d1oe1a1 d1v10a2 d1v10a3 d1v10a1 d1rypd_ d1irua_
d1rypc_ d3unfk_ d1rypa_ d1j2pa_ d1irui_ d1j2qh_ d3unfh_ d1m4ya_
d1rypb_ d1irul_ d1irug_ d1iru2_ d1q5rh_ d1rypg_ d1g3ka_ d1iruf_
d1q5qa_ d1e94a_ d1irub_ d1ryph_ d3ty6a_ d1iruj_ d1iruk_ d1rype_
d1irud_ d1irue_ d3mi01_ d1rypf_ d1iruh_ d2z3ba_ d1iruc_ d3mka2_
d1iru1_ d1a3qa1 d1iknc_ d3brda1 d1p7hl1 d1oy3c_ d1gjia1 d1zk9a1
d2cxka1 d2bema_ d1pama1 d1ea9c1 d1qhoa1 d2fhfa2 d2fhfa1 d1k3ia1
d1j0ha1 d1cxla1 d2bhua1 d1edqa1 d1f1sa2 d1ut9a2 d1gvia1 d1txka1
d1zx5a1 d2phla2 d2phla1 d1fxza2 d1fxza1 d1lrha_ d1uija1 g1dgw.1
d2v09a_ d1uika2 d1uika1 d1dgwa_ d1ep0a_ d2pa7a1 d1wlta1 d1nxma_
d2c0za1 d1f1ea_ d1b67a_ d2bjca1 d2hsga1 d1qpza1 d1efaa1 d1mqta_
d1d4m3_ d1fmd3_ d2mev1_ g1aym.1 d1ev13_ d4q4w1_ d1r1a3_ d2x5ia_
d1rhi1_ g1r1a.1 g1d4m.1 g1rhi.1 d1b35b_ d1oopa_ d1tme1_ d4iv1a_
g1pvc.1 g1fpn.1 d1fpn1_ d4gb31_ d1d4m1_ d1fmd1_ d1ev11_ d2wzr3_
d1qqp3_ g1fmd.1 d2x5ic_ d1rhi3_ g1oop.1 d1hxs1_ g1hxs.1 d1fpn3_
d1bev1_ g2mev.1 d1eah3_ d1aym1_ d1pvc3_ d1qqp1_ d1zba3_ d1h8ta_
d3vbhc_ g1b35.1 g1bev.1 d1hxs3_ g1ev1.1 d1bev3_ g1tmf.1 d1tmf1_
d1b35a_ d2mev3_ g1eah.1 g1h8t.1 d1eah1_ d4q4w3_ d1r1a1_ d1aym3_
d1pvc1_ d1zba1_ d1h8tc_ d1oopc_ d1tme3_ g1qqp.1 d4iv1c_ g1tme.1
d1au7a2 d1ic8a2 d3gpdg2 d2q49a2 d1hdgo2 d2g17a2 d2cvoa2 d2czca1
d3pwka2 d1obfo2 d1i32a2 d1zyao2 d2pkqo2 d1b7go2 d2hjsa2 d1cf2o2
d1vkna2 d1nvmb2 d2msya_ d2djoa1 d2r5za_ d1au7a1 d1f43a_ d2lmda1
d1akhb_ d1s7ea1 d3nara_ d1zq3p1 d2craa1 d1mija_ d4uuta_ d3a01a_
d1x58a1 d3rkqa1 d3cmya1 d2cqxa1 d2da6a1 d1e3oc1 d1fjla_ d1mh3a1
d1x41a1 d1wh7a1 d1gt0c1 d2mw8a_ d1lfup_ d1x2ma1 d1b72a_ d1wi3a1
d1jgga_ d1wjha1 d1x2na1 d1mnmc_ d2cuea1 d3k2aa_ d2hosa_ d2jwta2
d1pufa_ d1octc1 d2hi3a_ d1b8ia_ d4j19a_ d3lnqa_ d1wh5a1 d1b8ib_
d1fexa_ d2cqra1 d1guua_ d1ug2a1 d2cqqa1 d2ajea1 d4a69c_ d1h8ac1
d2crga1 d2iw5b1 d1xc5a1 d2cu7a1 d1wgxa1 d2cjja1 d2ckxa1 d1gvda_
d1hcra_ d1tc3c_ d1gdta1 d1u78a2 d6paxa1 d6paxa2 d1k78a2 d1xg1a1
d1itya_ d1hlva1 d1hlva2 d1iufa1 d1igna2 d1bl0a1 d1bl0a2 d1d5ya2
d3lv8a_ d1s3ga1 d1zaka1 d1s4qa_ d3tvta2 d1uf9a_ d1khta_ d1ckea_
d1osna_ d1p5zb_ d4tmka_ d3cm0a_ d1jaga_ d3h86a_ d1t0hb_ d1nn5a_
d2h92a_ d3kb2a1 d2c95a1 d1yj5a2 d1kgda_ d1ly1a_ d2ak3a1 d1vyva2
d1deka_ d2grja1 d1p3ja1 d1y63a_ d1e4ya1 d1s96a_ d3iija1 d1lvga_
d1nksa_ d1vyua2 d4e22a_ d1qf9a_ d1jjva_ d1q3ta_ d1ki9a_ d1lw7a2
d1teva_ d1kjwa2 d1gsia_ d2iyva2 d1viaa_ d1e6ca_ d1m7ga_ d1x6va3
d1g3ma_ d1j99a_ d1t8ta1 d1nsta_ d1fmja_ d1zrha_ d1ls6a_ d2a3ra_
d1aqua_ d2zptx_ d3ckla_ d1texa_ d1q44a_ d2gwha_ d1odfa_ d1uj2a_
d4gi7a_ d2jeoa1 d1z0ja1 d3umra2 d3gj0a1 d2gaoa1 d1yzga1 d1x3sa1
d1g7sa4 d1puja_ d1udxa2 d1kk1a3 d2o52a_ d1jwyb_ d1zcaa2 d1zcba2

d2fv8a1 d2cjwa_ d3dz8a1 d1xzpa2 d1vg8a_ d4qxaa_ d2ew1a1 d1x1ra1
d2bcgy_ d3doea1 d3cona_ d3rega_ d3wyga_ d1upta_ d2b6ha1 d1zd9a1
d1lnza2 d1r5ba3 d1n0ua2 d1r2qa_ d2f9la1 d2rhda_ d3q3jb_ d3q85a_
d1efma1 d1r8qa_ d2gf9a1 d2akab1 d1ek0a_ d1z08a1 d2gcoa1 d2dy1a2
d4g01b_ d2cxxa1 d1svia_ d3q72a_ d1ni3a1 d4ac9a4 d2erxa1 d2bmea_
d1wxqa1 d2gf0a1 d4ldja1 d1zunb3 d1d5ca_ d4lv5b_ d1efca3 d1kmqa_
d2g6ba1 d3lrpa_ d1tada2 d2y8ea_ d4rd4a1 d1z06a1 d2heia_ d3a58b_
d1u0la2 d1d2ea3 d1zj6a1 d2ocba_ d3rwoa_ d1mr3f_ d2e9sa1 d4u5xa1
d2iwra_ d2fn4a1 d3bwdd_ d2nzja1 d2atva1 d2ksqa_ d3l43a_ d1mkya2
d1mkya1 d1wmsa_ d1jnya3 d2rmka_ d1f60a3 d1gota2 d1jala1 d2fh5b1
d1g16a_ d2wblc_ d1m7ba_ d1wf3a1 d1f5na2 d1t9ha2 d1z6xa1 d1z0aa1
d2fg5a1 d2a5ja1 d2bcjq2 d1s0ua3 d1ky3a_ d4y0va_ d4lhwa_ d2qmea1
d2atxa1 d1egaa1 d1ryha_ d1moza_ d1nrjb_ d2erya1 d3bfka_ d1puia_
d1fzqa_ d3j3az1 d3t5ga_ d1shza2 d3defa_ d2f7sa1 d3raba_ d3tsoa_
d3aq4a_ d2al7a1 d3sfva1 d3bc1a_ d2ncda_ d1sdma_ d3h4sa1 d2heha_
d2grya_ g3kin.1 d1kk8a2 d1br2a2 d1goja_ d1f9va_ d2mysa2 d1lkxa_
d3t0qa_ d4db1a1 d3gbja_ d1ry6a_ d1v8ka_ d1vava_ d1uaia_ d1k8ka2
d1k8ka1 d3uleb1 d3i33a2 d3i33a1 d1bupa1 d1dkgd2 d1dkgd1 d4ci6a2
d1jcfa1 d4a2ba2 d2fxua1 d1yaga1 d4cbua2 d1bupa2 d1g99a2 d2e1za2
d2e1za1 d1saza1 d1saza2 d1iqca2 d1iqca1 d1eb7a2 d1eb7a1 d1nmla2
d3h1jd1 d1hroa_ d4gedb_ d3zooa_ d1mz4a_ d1gu2a_ d1f1fa_ d1dvva_
d1c6ra_ d1c75a_ d1e29a_ d1gdva_ d1cnoa_ d1h32b_ d1co6a_ d2bh4x_
d2aiua_ d5cytr_ d1jdla_ d1i8oa_ d2zboa_ d3dmia_ d1ls9a_ d1wvec_
d2yk3a_ d3ph2b_ d1dw0a_ d5aura_ d1ql3a_ d1qn2a_ d1f1ca_ d4zzva_
d2xspa_ d1eg1a_ d4v20a_ d1ojja_ d4ipma1 d1gpia_ d4csia_ d3pfza_
d3ovwa_ d2yoka_ d2nqya_ d1h4ga_ d1oa2a_ d1olra_ d3b7ma_ d4h7ma1
d3wp3a_ d2vgda_ d1igoa_ d3zsea_ d2nlra_ d3wx5a_ d1oa3a_ d1pvxa_
d1m4wa_ d1f5ja_ d1oa4a_ d1ks5a_ d4ixla1 d3sita1 d1evsa_ d1cnt1_
d1pvhb_ d1z7ca_ d1huwa_ d1bgea_ d1rhga_ d1f6fa_ d1f45b_ d1i1rb_
d1etea_ d3tgxb_ d1scfa_ d2z3qa1 d3qb1a_ d1hula_ d1m48a_ d4nkqb_
d1eera_ d1v7mv_ d5e4ea_ d2o27a_ d3b5ka_ d2psma1 d4zf7a_ d3uf5a_
d4x51a_ d1d9ca_ d4ypgc1 d3oq3a_ d1lqsl_ d1n1fa_ d1au1a_ d1wu3i_
d3besl_ d3ux9a_ d2b7la_ d3rbaa_ d2qtra_ d1nupa_ d3l92a_ d1f9aa_
d1qjca_ d2h29a_ d1vlha_ d1lw7a1 d1mrza2 d4ruka_ d2el7a_ d1iq0a2
d1irxa2 d1r6ta2 d1ivsa4 d2cyba_ d1rqga2 d1j09a2 d2yxna_ d1f7ua2
d1h3fa1 d1ffya3 d2g36a1 d1jila_ d1n3la_ d1i6la_ d1pfva2 d1h3na3
d3n9ia_ d3sz3a1 d1m4la_ d2bk7a_ d3fjua_ d1z5ra1 d1kwma1 d1jqga1
d2c1ca1 d1dtda_ d1h8la2 d3sw2a_ d1moxc_ d1dx5i3 d1autl2 d1autl1
d1nqlb_ d1dval1 d1szba2 d1kigl_ d2y5fl_ d1ijqa2 d1gk5a_ d1g1sa2
d1nt0a3 d1xdtr_ d1lmja2 d1lmja1 d1nzia2 d2wphe_ d1klil_ d1pfxl2
d1pfxl1 d1p9ja_ d1g1ta2 d1f7ea_ d4yzub_ d2mgpa1 d1b9wa2 d2mgpa2
d1ob1c1 d1ob1c2 d1n1ia2 d1n1ia1 d2npra2 d2npra1 d1qx2a_ d2y5ia_
d1qlsa_ d1psra_ d3n22a_ d2egda_ d4ggfc_ d2wcea_ d2luca_ d4aqia_
d3nsia_ d2lp3a_ d1j55a_ d1a4pa_ d1k8ua_ d1pvaa_ d3fs7a_ d2pvba_

d1ttxa_ d1rwya_ d2kyfa_ d1rk9a_ d1a75a_ d1exra_ d1tiza1 d1nyaa_
d1g8ia_ d1ggwa_ d1wdcc_ d3fwba_ d1ggza_ d2o5ga_ d1ctaa_ d1m45a_
d3i5gc_ d1sl8a_ d1fpwa_ d2scpa_ d2roaa_ d4aqra_ d3pn7b_ d1br1b_
d2mysb_ d1s6ca_ d1bjfa_ d2obha_ d1jfka_ d1jf0a_ d1qv0a_ d1lkja_
d2mlfc_ d2m97a_ d2d8na_ d4by5a_ d1jbaa_ d4nqga_ d1wdcb_ d2mysc_
d1j7qa_ d1s6ia_ d1uhka2 d1r2ua_ d1dtla_ d3kf9a_ d3pn7c_ d1gjya_
d1y1xa_ d1hqva_ d1dvia_ d1df0a1 d1qxpa2 d4usla_ d1k94a_ d1wkya2
d1uuqa_ d3wh9a_ d4m1ra_ d1xyza_ d4xuya_ d2je8a5 d4bf7a1 d3cuia_
d1ur4a_ d1qw9a2 d1odza_ d2vzsa5 d1bqca_ d2x2ya1 d1qnra_ d4y7ea1
d1vjza_ d4k68a_ d2v3ga_ d4hpga_ d1nofa2 d1nq6a_ d1h4pa_ d1aq0a_
d3ziza_ d4bs0a_ d1ecea_ d1h1na_ d1ta3b_ d1w91a2 d1i1wa_ d1uhva2
d1us3a2 d1egza_ d7a3ha_ d1tvna1 d3nyda1 d1ghsa_ d2cyga1 d4xv0a_
d2c8na2 d1w32a_ d3emca_ d1v6ya1 d1foba_ d2uwfa1 d2fgla_ d3niya1
d2c0ha1 d1rh9a1 d1yq2a5 d1szna2 d1fa2a_ d1pama4 d5do8a1 d1ea9c3
d1hx0a2 d2fhfa5 d1qhoa4 d1avaa2 d1m53a2 d4m8ua1 d2gjpa2 d1lwha2
d4howa1 d1ktba2 d1j0ha3 d3vm5a1 d2x1ia_ d1wzaa2 d1uasa2 d2diea1
d1mxga2 d1ht6a2 d1eswa_ d1iv8a2 d5cpsa_ d1ud2a2 d1zjaa1 d3gbea1
d1kwga2 d1wdpa_ d1gcya2 d1g5aa2 d1hvxa2 d3dhpa2 d2bhua3 d4do4a1
d3qgva1 d1e43a2 d1g94a2 d2d3na2 d1gjwa2 d1gvia3 d1x1na1 d1r7aa2
d1tz7a1 d4c7ga2 d2gjxa1 d1nowa1 d1jaka1 d1yhta1 d4toqa_ d1wb0a1
d1w9pa1 d1eoka_ d1itxa1 d1kfwa1 d1goia2 d1edqa2 d2gsja_ d1hjxa1
d1vf8a1 d1etob_ d1umqa_ d1g2ha_ d2w0pa_ d2diaa1 d2e9ia2 d2e9ja1
d2dj4a1 d2eeba1 d2diba1 d2dmba1 d2dmca1 d2d7oa1 d2ee6a1 d2d7pa1
d2eeaa1 d2di8a1 d1wlha1 d2d7na1 d2nqca_ d2d7ma1 d2dica1 d3cnka_
d2k3ta1 d2eeca1 d1qfha1 d2bp3a1 d1v05a_ d2ee9a1 d2eeda1 d2di9a1
d3syxa1 d1mkea1 d2p8va_ d1xoda1 d1i7aa_ d1evha_ d1qc6a_ d2hmta_
d1lssa_ d1b9ra_ d1offa_ d1frra_ d3wcqa_ d1e9ma_ d1l5pa_ d1wria_
d1i7ha_ d3av8a_ d1czpa_ d1rfka_ d3p1ma1 d3b2ga_ d2bt6a2 d3nvva1
d1krha3 d2h88b1 d1n62a2 d1t3qa2 d1kf6b2 d1zoyb1 d1jq4a_ d1dgja2
d1rm6c2 d9ldta1 d2ewda3 d1up7a1 d2dfda1 d1s6ya1 d1vjta1 d7mdha1
d1hyea1 d1t2da1 d1ojsa3 d1b8pa1 d1ldna1 d1hyha1 d1civa1 d2a92a1
d1ez4a1 d1i0za1 d1y6ja1 d1gv0a1 d1ur5a1 d1llda1 d1obba1 d1v8ba1
d1gdha1 d1l7da1 d2dlda1 d1mx3a1 d1ygya1 d1qp8a1 d1li4a1 d2naca1
d1npya1 d3t4ea2 d1a4ia1 d1o0sa1 d2hk9a2 d2egga2 d1b26a1 d1bvua1
d1gq2a1 d1c1da1 d1leha1 d1gtma1 d1euza1 d3dona2 d1edza1 d1gpja2
d1vi2a1 d1p77a1 d1nyta1 d1b0aa1 d1vl6a1 d1v9la1 d3wdpp1 d3wq5a_
d4gxpa_ d4hz8a_ d1e4mm_ d3aiua_ d1wcga1 d1qvba_ d3ahxa_ d1v02a_
d2dgaa_ d1pbga_ d1vffa1 d1qoxa_ d4ha4a_ d1kola2 d1f8fa2 d1qora2
d1jqba2 d1vj0a2 d1cdoa2 d1gu7a2 d1d1ta2 d1xa0a2 d2dm6a2 d1jvba2
d1rjwa2 d1llua2 d1h2ba2 d1tt7a2 d1piwa2 d1o89a2 d1iz0a2 d1e3ja2
d1yb5a2 d1uufa2 d1vj1a2 d1e3ia2 d3gpdg1 d1vc2a1 d2nvwa1 d1u8fo1
d1ydwa1 d1hdgo1 d2g17a1 d1cf2o1 d2czca2 d1r0ka2 d1obfo1 d2cvoa1
d1ebfa1 d1h6da1 d1i32a1 d1zh8a1 d2q49a1 d1qkia1 d1j5pa4 d3pzra1
d1xeaa1 d1vkoa1 d1vm6a3 d3qvsa1 d2pkqo1 d1b7go1 d1gr0a1 d1p1ja1

d1lc0a1 d1j0xo1 d3pwka1 d2hjsa1 d1zyao1 d1vkna1 d1tlta1 d1nvmb1
d3sjua_ d3ojfa1 d1xu9a1 d2jjya1 d1n7ha_ d1y1pa1 d1h5qa_ d1jtva_
d4fs3a_ d2bkaa1 d1gy8a_ d2c5aa1 d1ulsa_ d1ooea_ d2b69a1 d1y5ma1
d1spxa_ d1ae1a_ d1bxka_ d1ydea1 d1gega_ d1hdoa_ d1o5ia_ d1e6ua_
d1snya1 d2rhca_ d3zv4a_ d5ceja_ d2d1ya1 d1fjha_ d3w8ea_ d2fmua1
d1x1ta_ d1rpna_ d3ay7a1 d1k2wa_ d1eq2a_ d2hrba1 d3ruaa_ d2ew8a1
d4hwka_ d1xgka_ d2bd0a1 d1yo6a1 d2a35a1 d2ntva_ d1vl8a_ d1hdca_
d4bkua_ d2ag5a1 d2q45a_ d1nffa_ d4iy1a_ d1zbqa1 d1kewa_ d1ek6a_
d1ja9a_ d2q46a2 d1iy8a_ d1cyda_ d2ptga_ d1hxha_ d1uzma1 d3lyla_
d4m89a_ d3wyea_ d2blla1 d1w6ua_ d1sb8a_ d2q2va_ d4nr0a_ d1e6wa_
d2gasa_ d3oiga1 d2bgka1 d1mxha_ d1vl0a1 d1rkxa_ d1r6da_ d1wmaa1
d1e7wa_ d2c07a1 d1xkqa_ d3tzha_ d1g0oa_ d3lz6a_ d1qyca_ d1edoa_
d1gz6a_ d1oc2a_ d1t2aa_ d1zema1 d2xoxa_ d1z45a2 d1geea_ d1p33a_
d3lt0a_ d4e6pa_ d2wyua_ d1db3a_ d4q9na_ d1xhla_ d4o1ma_ d1fmca_
d2uvda_ d2gdza1 d1uaya_ d2z1ma_ d2o23a2 d2fr1a2 d2fr1a1 d1qsga_
d1qyda_ d4zjua_ d2a4ka1 d1yb1a_ d1wvga1 d1yxma1 d3f4ba_ d1mg5a_
d1xg5a1 d1r55a1 d3gboa_ d1atla_ d1kufa_ d1buda_ d4q1la_ d1yp1a_
d1wnia_ d2gfoa1 d1vjva_ d2ayna1 d3nhea_ d3nqyb_ d4gera_ d1g0da4
d2f4ma1 d1x3za_ d1t3ca_ d1epwa3 d2a97a_ d3fiea_ g1e1h.1 d3ioqa_
d1fh0a_ d1o0ea_ d1khqa_ d1m6da_ d2cb5a_ d1cs8a_ g8pch.1 d1s4va_
d2pnsa_ d1vsna1 d1mira_ d1iwda_ d2oula_ d7pcka_ d3bwka_ d3bcna_
d2p86a_ d1pvja_ d1pcia_ d1gece_ d1deua_ d4bs6a_ d1xkga1 d1cqda_
d1jqpa2 d2c0ya_ d2bdza_ d3p5ua_ d2o36a_ d1k9xa_ d1ex4a2 d1c6va_
d2jk6a2 d2jk6a1 d1feca1 d1feca2 d1lpfa1 d1lpfa2 d1xhca1 d1xhca2
d1dxla2 d1dxla1 d1gera2 d1m6ia2 d1onfa1 d3lada2 d3lada1 d1w4xa1
d1w4xa2 d1gesa2 d1mo9a1 d1xdia1 d1fl2a2 d1fl2a1 d1fcda1 d1fcda2
d1h6va2 d1h6va1 d1mo9a2 d1gv4a2 d1gv4a1 d2gv8a2 d2gv8a1 d1aoga1
d1aoga2 d1hyua1 d1ebda1 d1ebda2 d3dk9a1 d1q1ra2 d1q1ra1 d1gesa1
d1g4ma1 d1ps9a2 d1lqta1 d1cjca1 d1pn0a1 d1reoa1 d1ju2a1 d2f5va1
d3c96a1 d2gmha1 d1gpea1 d1o5wa1 d1n4wa1 d1k0ia1 d2dw4a2 d2ivda1
d1seza1 d2voua1 d1kdga1 d2bcgg1 d2bcgg2 d1wgqa1 d1v89a1 d2coda1
d1zc3b_ d1wg7a1 d1u5ea1 d1v61a1 d2coca1 d2ys1a1 d1v5pa1 d1ntya2
d1wjma1 d1wi1a1 d1u29a_ d1xx0a1 d1unqa_ d2zkmx3 d1fhoa_ d1v88a1
d2adza1 d1x1fa1 d1p6sa_ d2coaa1 d1btka_ d1v5ma1 d1faoa_ d2x18a_
d1dyna_ d1x1ga1 d4kaxb_ d2j59m1 d1ki1b2 d1xcga2 d1txda2 d1u5da1
d1dbha2 d1w1ha_ d1upqa_ d1v5ua1 d1foea2 d2fjla1 d1eaza_ d2dfka2
d2cofa1 d1kz7a2 d1jmxa1 d1pbya1 d1pbya2 d1r7ha_ d1faaa_ d1wika1
d1xfla_ d1ttza1 d2f51a1 d1ti3a_ d1nhoa_ d1sena_ d2i4aa_ d1ep7a_
d1zmaa1 d1fo5a_ d3lefa1 d3diea1 d2yana1 d1z6na1 d1oaza_ d3zywa1
d4aj8a_ d1nm3a1 d4fiwa_ d1wmja_ d4hs1a1 d1iloa_ d1xwaa1 d1wjka1
d2vm1a_ d1fb6a_ d1gh2a_ d1h75a_ d1nw2a_ d1kx5b_ d3a6na_ d4m6ba2
d2nqbd_ d3wkjd_ d2yfwe_ d4h9na_ d5bnxa_ d1kx5c_ d3r45a_ d1eqzb_
d4jjnb_ d1f66c_ d1eqza_ d4wnnb_ d1kx5a_ d1u35c1 d1id3a_ d2nqbc_
d1kx5d_ d3us3a3 d3us3a1 d1tafa_ d1bh9b_ d1tafb_ d1jfib_ d1q9ca_

| | | | | | | | |
|---|---|---|---|---|---|---|---|
| d1h3ob_ | d2byka1 | d1n1ja_ | d4g92b_ | d1bh9a_ | d1jfia_ | d2bykb1 | d1n1jb_ |
| d1pd212 | d1nhya2 | d2a2ra2 | d1k0dd2 | d1f2ea2 | d1gsua2 | d1f3ba2 | d2fhea2 |
| d2pvqa1 | d1tw9a2 | d1oyja2 | d1e6ba2 | d1k0ma2 | d3ik7a1 | d4is0a1 | d1r5aa2 |
| d1jlva2 | d1m0ua2 | d3gtub2 | d1z9ha2 | d1okta2 | d1axda2 | d2gsra2 | d1tu7a2 |
| d1k3ya2 | d1zgma1 | d1b48a2 | d1gnwa2 | d1gwca2 | d2gsta2 | d1glqa2 | d1oe8a2 |
| d1pn9a2 | d1fw1a2 | d1duga2 | d3vrda1 | d3vrda2 | d1fcdc2 | d1fcdc1 | d1h1oa2 |
| d1h1oa1 | d1m70a2 | d2trcp_ | d1ig8a1 | d1czan2 | d1czan1 | d1ig8a2 | d1czan4 |
| d1bg3a4 | d1bg3a3 | d1bg3a2 | d1bg3a1 | d1qsaa2 | d1txga2 | d3f47a1 | d1mv8a2 |
| d2pv7a2 | d1xc2a3 | d2ahra2 | d4xiya1 | d2f1ka2 | d1pgja2 | d1i36a2 | d1np3a2 |
| d1lj8a4 | d1teaa3 | d1qmga2 | d2i76a2 | d2g5ca2 | d1wdka3 | d1dlja2 | d3cuma3 |
| d1qqya_ | d1gd6a_ | d1f6ra_ | d1ivma_ | d2gv0a_ | d1iiza_ | d2z2fa_ | d1j8xa_ |
| d1b9oa_ | d174la_ | d169la_ | d2o4wa_ | d1p37a_ | d1k28a3 | d1xjta1 | d1jtma_ |
| d2f2qa_ | d4e97a1 | d2pjsa1 | d1klla_ | d2i7ra1 | d1ecsa_ | d1xqaa1 | d5cj3a1 |
| d1npba_ | d2p7oa_ | d1r9ca_ | d1nkia_ | d1mpya2 | d1kw3b2 | d1kw3b1 | d4ghga1 |
| d1tfza1 | d3isqa1 | d3isqa2 | d1sqia1 | d1sqia2 | d1lkda2 | d1lkda1 | d1cjxa2 |
| d1cjxa1 | d1sp9b2 | d1t47a1 | d1t47a2 | d1f1ua2 | d1sp8a1 | d1sp8a2 | d1f1ua1 |
| d1s5ja2 | d1qhta2 | d4dlea2 | d1tgoa2 | d3py8a2 | d1q8ia2 | d1kfsa2 | d2py5a2 |
| d1d5aa2 | d1qsta_ | d2beia1 | d4m3sa_ | d1u6ma1 | d1ygha_ | d1yk3a1 | d3dr6a_ |
| d1xmta_ | d1z4ea1 | d2ge3a1 | d2fiaa1 | d2jdca1 | d1y9wa1 | d3to7a_ | d1wwza1 |
| d2fl4a1 | d2ozua1 | d2b5ga_ | d2cy2a1 | d2p0wa_ | d1cm0a_ | d4pswa_ | d1yrea1 |
| d1yr0a1 | d1m4ia_ | d2gana1 | d1yvka1 | d1ghea_ | d2g3aa1 | d1y7ra1 | d2euia1 |
| d1q2ya_ | d2fsra1 | d1xeba_ | d2ae6a1 | d1cjwa_ | d1qsma_ | d2aj6a1 | d1y9ka1 |
| d1i12a_ | d1vkca_ | d2atra1 | d1n71a_ | d2d4pa1 | d2fiwa1 | d2fe7a1 | d1tiqa1 |
| d2ou2a_ | d1yx0a1 | d2fcka1 | d1p0ha_ | d2j8ma_ | d2pq8a_ | d1z4ra1 | d1s3za1 |
| d1ufha_ | d1c4qa_ | d1prtc1 | d2wv6d_ | d1prtf_ | d1ltrd_ | d4l63b1 | d1r4pb_ |
| d1prtd_ | d1tl0f_ | d2bosa_ | d1prtb1 | d3chbd_ | d1xvqa1 | d1pn0a2 | d3hy2a_ |
| d2cvba1 | d1o8xa_ | d1lu4a_ | d1we0a1 | d2bmxa1 | d2a4va1 | d1psqa_ | d1xiya1 |
| d1q98a_ | d1n8ja_ | d1zofa1 | d2ggta_ | d3tjja_ | d4je1a1 | d1o73a_ | d3s9fa_ |
| d2zcta_ | d1qmva_ | d1jfua_ | d2b1ka_ | d5dvba1 | d2b5xa1 | d2v2ga_ | d1st9a_ |
| d3tb2a_ | d4llra_ | d4fh8a1 | d1e2ya_ | d1knga_ | d2h30a_ | d1i5ga_ | d2z9sa_ |
| d2f8aa1 | d1tp9a1 | d2b7ka_ | d1nm3a2 | d3hvva_ | d2wfca1 | d4kcea_ | d1xvwa1 |
| d4gqca1 | d3qpma_ | d4k1fa_ | d1zzoa1 | d1gp1a_ | d4od7a1 | d1z6ma1 | d4mcua1 |
| d4dvca1 | d1r4wa_ | d3l9sa_ | d1un2a_ | d3rppa_ | d3kasa1 | d3bi1a3 | d1e88a1 |
| d1l6ja5 | d1l6ja4 | d1eaka4 | d1eaka3 | d1l6ja3 | d1h8pa2 | d1h8pa1 | d1ogpa1 |
| d1dy2a_ | d1gyta2 | d4l3wa_ | d1llfa_ | d1gz7a_ | d1ukca_ | d1cr6a2 | d1c4xa_ |
| d2rhwa_ | d2ftba_ | d1pmpa_ | d2rcqa1 | d1fdqa_ | d4lkpa_ | d1sa8a_ | d1vyfa_ |
| d1lpja_ | d3vg7a1 | d4azqa1 | d1o8va_ | d5bvqa_ | d1p6pa_ | d2qo4a_ | d1opba_ |
| d1yiva1 | d1o1va_ | d4bvma1 | d4tkba_ | d4i3ba_ | d1bwya_ | d1ftpa_ | d4gkca_ |
| d1cbia_ | d3p6da1 | d1kqwa_ | d2a0aa1 | d3zxha_ | d1cgla_ | d1rm8a_ | d1hy7a_ |
| d2jsda_ | d1cxva_ | d1hv5a_ | d1i76a_ | d1g12a_ | d3p0ya1 | d1igra1 | d1igra2 |
| d1n8yc2 | d1n8yc1 | d1m6ba2 | d1m6ba1 | d1sr9a2 | d1rqba2 | d2tbvc_ | d1c8nc_ |
| d1x36a_ | d1smvc_ | d1ng0c_ | d4sbvc_ | d1opoa_ | d1f2nc_ | d1cixa_ | d1y29a_ |
| d1v7fa_ | d2jtba_ | d1d1ha_ | d1g9pa_ | d1lupa_ | d1qk7a_ | d1mb6a_ | d2a2va_ |
| d1niya_ | d1i25a_ | d1hvwa_ | d2mpqa_ | d1ie6a_ | d1kqha_ | d1la4a_ | d2mxoa1 |

d1dl0a_ d1koza_ d1qk6a_ d1emxa_ d1lqra_ d1c6wa_ d1rmka_ d1eyoa_
d1cnna_ d1fu3a_ d1g1pa_ d1f3ka_ d1fyga_ d4lu0a_ d1of8a_ d3unda1
d3e9aa1 d1vr6a2 d2qkfa_ d1d9ea_ d1o60a_ d1jgta2 d1ofda3 d1ea0a3
d1ecfa2 d1q15a2 d1ct9a2 d1te5a_ d1y14b1 d2nn6i1 d1i94l_ d2zjra2
d1smxa_ d2qamc2 d1hh2p1 d2r7da1 d1rl2a2 d1wfqa1 d3bzka4 d1x6oa2
d3a0ja_ d2j01d2 d2ja9a1 d4ql5a_ d1luza_ d2n3sa_ d2vnud1 d1h95a_
d1go3e1 d2nn6h1 d1ne3a_ d2mo1a_ d1iz6a2 d1jjga_ d1e3pa2 d2ix0a3
d2ix0a2 d2lxja_ d1ueba3 d1ueba2 d2z0sa1 d1u0la1 d2ix0a1 d2eifa2
d2je6i1 d3i2za1 d2c35b1 d2ahob2 d1t9ha1 d3i4oa_ d2qall1 d1jt8a_
d2nn6g1 d1g6pa_ d3cama_ d1i94q_ d1c9oa_ d1d7qa_ d2ba0a1 d2vnud2
d2vnud3 d1khia2 d2qalq1 d1wi5a1 d1xtda2 d1ny4a_ d4w9ta_ d4x2ra1
d1ka9f_ d1qo2a_ d4axka1 d1thfd_ d1h5ya_ d4u28a_ d2y88a_ d4wd0a_
d1vzwa1 d1eixa_ d3exra_ d2ffca1 d2fdsa1 d3n3ma1 d2czda_ d1q6oa_
d2yyua_ d1vqta1 d1dbta_ d3ldva1 d1prtc2 d2r80b_ d1cqxa1 d1cg5a_
d3boma_ d2h8fb_ d2raoa_ d3bj1a_ d4hrrb_ d2r50a_ d3d1kb_ d2aa1b1
d3zhwa_ d3hf4b_ d3gdja_ d1jebb_ d3bj1b_ d1x9fb_ d1wmub_ d1gcva_
d3bcqb_ d3d4xb_ d1gvha1 d2dc3a_ d3hf4a_ d4yu3a_ d3k8ba_ d1qpwb_
d2r80a_ d3bcqa_ d1v4wa_ d3wtgb_ d1jl7a_ d4esaa_ d1a9we_ d3d1ab_
d1fsla_ d4h2la_ d1v4wb_ d1mwca_ d1vhba_ d1jeba_ d1spgb_ d1h97a_
d1q1fa_ d1tu9a1 d3d1aa_ d1hdsb_ d3hyub_ d2zfbb_ d3fs4a_ d1oj6a_
d1shrb_ d4h2lb_ d4yu3b_ d1spga_ d3hrwa_ d3qqra_ d3at5a_ d4wchd_
d3hyua_ d1cg5b_ d1uc3a_ d1hbra_ d2raob_ d1outb_ d4esab_ d3gdjb_
d3qqqa_ d1x9fc_ d3vrfa_ d1itha_ d3mkba_ d3d4xa_ d3mjpa_ d1jl6a_
d3fh9a_ d1outa_ d2dc3b_ d1it2a_ d3eoka_ d3g46a_ d1qpwa_ d3vrfb_
d1ch4a_ d1x9fd_ d1hdsa_ d2zfba_ d3mkbb_ d1or4a_ d3wtga_ d1a4fa_
d3fh9b_ d3bomb_ d3cy5a_ d3at5b_ d1x9fa_ d2oifa_ d4lmxb_ d3v57a_
d1kn1a_ d1cpca_ d4f0ta_ d2vjha_ d4po5b_ d1eyxa_ d1alla_ d4f0tb_
d1b33b_ d1b8db_ d4h0mb_ d1b33a_ d2vjhb_ d4lm6b_ d2vmlb_ d1allb_
d2vjta_ d3dbja_ d2vmla_ d2vjtb_ d4f0ub_ d1f99b_ d4rmpa1 d1liaa_
d1eyxb_ d2uulb_ d4lmsb_ d4f0ua_ d2bv8a_ d1f99a_ d2bv8b_ d2c7lb_
d3dbjb_ d4rmpb1 d1b8da_ d4h0ma_ d3v57b_ d1kn1b_ d1liab_ d2uula_
d2j96a_ g1gxs.1 d4ekfa_ d2ckga_ d1th0a_ d2bkra_ d2xqfa_ d1ea5a_
d1f6wa_ d1dx4a_ d1qe3a_ d1k4ya_ d4qwwa_ d2ha2a_ d1r88a_ d1sfra_
d4qeka_ d3c8da2 d2pbla1 d1jkma_ d1jjia_ d1lzla_ d4iuaa3 d4bvva_
d5hpga_ d3k65a_ d1ki0a3 d1ki0a2 d1ki0a1 d1a0ha1 d1pmla_ d3sp8a3
d2feba1 d1dosa_ d3qm3a1 d2fjka_ d1f74a_ d3l21a_ d2e1da_ d1to3a1
d1w3ia_ d1sfla_ d1vcva1 d4imea_ d1wa3a_ d2ocza1 d1f2ja_ d3m16a_
d2yxga_ d1ub3a_ d3a5fa_ d1fbaa_ d1l6wa_ d1xfba1 d1wx0a1 d1vpxa1
d1ojxa_ d1mxsa_ d2a4aa1 d1xkya1 d3cq0a1 d3s1xa_ d1f05a_ d1fdja_
d3cpra1 d4d2ja_ d1cg2a1 d1yloa2 d1y7ea2 d1y0ya3 d1lfwa1 d1vixa1
d1fnoa4 d1vhoa2 d1ysja1 d1vhea2 d1z2la1 d2fvga2 d1vgya1 d1r3na1
d2grea2 d1xmba1 g1q7l.1 d1rtqa_ d4aw8a_ d1ra6a_ d1muka_ d3gsza_
d1khva_ d1te9a_ d2xwha_ d3uqsa_ d3hkwa_ d3n6ma_ d2cjqa_ d2xyma_
d1teba_ d1te8a_ d1s48a_ d1u09a1 d4wfxa_ d3tyqa_ d2pgga1 d1dzka_

d4tlja_ d2a2ua_ d4wfva_ d3t1da_ d4iaxa_ d3zq3a_ d2nnda_ d1i4ua_
d1kxoa_ d1bbpa_ d1kt7a_ d1exsa_ d3s26a1 d1lf7a_ d1qfta_ d4gh7a_
d1z24a1 d1qabe_ d1xkia_ d1ew3a_ d3bx7a_ d4iawa1 d1n0sa_ d1gkab_
d3kzaa_ d2k23a_ d2l9ca1 d2joza1 d1gm6a_ d3kffa_ d3saoa1 d1e5pa_
d1yupa1 d1iiua_ d1e69a_ d1mv5a_ d3g60a4 g1ii8.1 d1ewqa2 d1ji0a_
d1qhla_ d1v43a3 d2wmfa1 d1vpla_ d1g2912 d4a82a2 g1f2t.1 d2d62a1
d3tuic1 d2bbsa1 d3g60a3 d2ixea_ d1g6ha_ d1sgwa1 d2qi9c_ d3rlfa1
d3b60a1 d3d31a2 d2pzea1 g1xew.1 d1jj7a_ d1oxxk2 d2onka1 d1w1wa_
d1iwea_ d1qf5a_ d1vmaa2 d1nija1 d1np6a_ d2p67a1 d3ihla_ d1okkd2
d1ls1a2 d1s1ma2 d1iona_ d2v40a_ d1dj3a_ d3hida_ d2vo1a1 d2g0ta1
d1vcoa2 d1j8mf2 d1g3qa_ d1xjca_ d1p9ba_ d1qzxa3 d1ihua2 d1ihua1
d2qm8a_ d1dj2a_ d4ioja_ d1cp2a_ d1yrba2 d3gqba2 d1skye3 d1xp8a1
d1tf7a2 d1tf7a1 d1ye8a1 d1skyb3 d2qe7d2 d4blta_ d4huta_ d3ldaa2
d4hyya_ d2i3ba1 d1cr1a_ d3j9tb2 d3ntua2 d1vdza1 d2z43a_ d1e9ra_
d1nlfa_ d3ziaa2 d1n0wa_ d1g6oa_ d3ziad2 d3j9ta2 d1pzna2 d3gqbb2
d2i49a_ d1nnfa_ d1xvxa_ d1ursa_ d1h3da1 d1iuda_ d4m1va1 d1twya_
d3e13x_ d2nxoa1 d4pfua_ d3ttma_ d1elja_ d2qmwa1 d1us5a_ d1y3na1
d1jeta_ d1uqwa_ d4f19a1 d2esna2 d3thia_ d3fasa1 d2uyeb_ d2xwva1
d1r9la_ d2fyia1 d1pb7a_ d2ozza1 d1sw5a_ d1nh8a1 d1vr5a1 d2g29a_
d1xs5a_ d3lswa_ d2z23a_ d3w9va_ d3tcha_ d1q35a_ d1z7me1 d3q27a_
d1p99a_ d1ve4a1 d2i6ea1 d4mcoa1 d4n8ya_ d1eu8a_ d1zbma1 d1hsla_
d1ixca2 d1j1na_ d1xvya_ d1y9ua_ d2z22a1 d1xoca1 d4oera_ d1wdna_
d5f1qa1 d1xt8a1 d1i6aa_ d4gwoa_ d4igta1 d1pc3a_ d4mmpa1 d1o63a_
d2czla1 d2onsa2 d4mija1 d1eh3a_ d3skpa_ d1dtza1 d1dtza2 d1ieja_
d1jnfa2 d1ce2a1 d1jnfa1 d1b1xa2 d1b1xa1 d1jw1a1 d1jw1a2 d1ryoa_
d1h76a1 d1h76a2 d1c4oa1 d2p6ra3 d2p6ra4 d1oywa2 d1oywa3 d1wrba1
d1w36b2 d1w36b1 d1c4oa2 d3bora_ d1t6na_ d2eyqa5 d2eyqa3 d2eyqa2
d2zu6a2 d1veca_ d1uaaa1 d1w36d1 d2g9na1 d1z3ix2 d2rb4a_ d1hv8a1
d1hv8a2 d1t5ia_ d1w36c2 d2j0sa2 d2j0sa1 d1fuka_ d3hjha1 d1z5za1
d1qdea_ d1z63a1 d1s2ma2 d1s2ma1 d1wp9a1 d1wp9a2 d1gm5a3 d1gm5a4
d3dina4 d3dina3 d1q0ua_ d1w36c1 d1z3ix1 d1nkta3 d1nkta4 d1uaaa2
d1w36d2 g1qhh.1 d2fwra2 d2fwra1 d3q3ma_ d1za0a1 d1mxra_ d2inca_
d2incb_ d1mhyb_ d3hf1b_ d1mtyb_ d1r2fa_ d2uw1a_ d1mhyd_ d3n37a_
d1afra_ d1oquc_ d1syya_ d1mtyd_ d2o1za_ d1uzra_ d1smsa_ d2fkza_
d1zuja1 d1s3qa1 d2fjca1 d1z6om1 d1veia_ d4dyya_ d1jiga_ d3gvya_
d2pyba_ d4cy9a1 d3bvfa1 d1r03a_ d2cf7a_ d1nf4a_ d1zs3a1 d4iwka_
d3ka8a_ d3np0x_ d1n1qa_ d1lb3a_ d4e6ka_ d1z6oa1 d2fzfa1 d4dyua_
d3ge4a_ d1jgca_ d4am4a_ d2z6ma_ d1ji5a_ d1vlga_ d1lkoa1 d1krqa_
d3qz3a_ d1o9ra_ d2ffxj_ d1euma_ d1yv1a1 d2v2pa_ d1j30a_ d4di0a_
d1dpsa_ d2cbia2 d2choa2 d1l8na1 d1oewa_ d2qzxa_ d2asia_ d1psoe_
d1miqa_ d2h6ta_ d1pfza_ d1izda_ d3hd8a_ d1j71a_ d1dpja_ d4aa9a_
d1t6ex_ g1b5f.1 g1lya.1 g1htr.1 d1qdma2 d1bxoa_ d1wkra_ d1tzsa_
d1ibqa_ d2qzwa_ d1zoya1 d1d4ca2 d1chua2 d1jnra2 d1kf6a2 d3ggta_
d3ixoa_ d1mrxa_ d2haha_ d1tw7a_ d4njsa_ d1sgua_ d2p3da_ d1nsoa_

d3d3ta_ d2rkfa_ d1c6ya_ d2rspa_ d3ogpa_ d3ekwa_ d2o4na_ d2j9ja_
d1baia_ d3ggua_ d3lzua_ d4npua_ d4j55a_ d2r8na_ d2fxda_ d2p3ca_
d4dqea_ d3ttpa_ d3t3ca_ d2p3aa_ d2hc0a_ d2fdda_ d3mwsa_ d1um8a_
d1ixza_ d1qvra2 d1qvra3 d1qzma_ d1sxjc2 d1jr3d2 d2p65a_ d1l8qa2
d1g8pa_ d3hu3a3 d1sxjd2 d1njfa_ d1in4a2 d1sxja2 d1tuea1 d2ce7a2
d1w5sa2 d1sxjb2 d1d2na_ d1ny5a2 d1ofha_ d2gnoa2 d2a5yb3 d1fnna2
d1u0ja1 d1lv7a_ d1iqpa2 d2fnaa2 d1ixsb2 d4gita_ d1sxje2 d1g41a_
d1js3a_ d3rcha_ d3k40a_ d1k8rb_ d1wgra1 d1ef5a_ d1wfya1 d1e8ya3
d1wgya1 d2cs4a1 d1wxma1 d2byea1 d3kh0a1 d2byfa1 d1wxaa1 d1e7ua3
d1h4ra3 d4yl8a1 d2al6a3 d1wgda1 d1wgga1 d4q5eb_ d1wjna1 d1se9a_
d3n3kb_ d1wz0a1 d1we6a1 d2m0xa_ d2gbka_ d3rt3b1 d1wh3a1 d1sifa_
d2ekec2 d4ii2b1 d4hk2a1 d1c3ta_ d1m94a_ d1wgha1 d1ttna1 d1j8ca_
d1wx8a1 d3v6cb1 d2ma9b_ d4i6lb1 d1bt0a_ d2jvca1 d1iyfa_ d2k1fa_
d1wxva1 d1wf9a1 d1t0ya_ d2io0b2 d1x1ma1 d2gbma_ d1zw7a1 d2m17a_
d1ud7a_ d1we7a1 d2lwpa_ d2gbja_ d1wjua1 d1v6ea1 d1v86a1 d4eewa_
d1wiaa1 d4icva_ d4gu2a_ d1wx7a1 d1wy8a1 d4hjka1 d2faza1 d1v5oa1
d3ruib_ d1eo6a_ d3waob_ d2r2qa1 d3m95a_ d3wana_ d2al3a1 d2cr5a1
d1wj4a1 d3ubra_ d1qo8a1 d1d4ca1 d2k3va_ d1ft5a_ d1qdba_ d1m1qa_
d2j7aa_ d1h21a_ d1ogyb_ d4fasa_ d1oaha_ d1sp3a_ d1s5ja1 d2hbja2
d2igia_ d1wlja_ d1qhta1 d4dlea1 d1tgoa1 d2f96a1 d3tr8a1 d3mxma_
d1q8ia1 d1y97a1 d1w0ha_ d1kfsa1 d2py5a1 d1noya_ d1d5aa1 d1yt3a3
d2guia_ d3mwpa1 d4kb1a_ d1wioa4 d1ccza2 d1vcaa1 d1dr9a2 d2d9qb1
d2nxyb2 d2dara2 d2dara1 d1wyha2 d2dloa2 d1rutx4 d1rutx3 d2cuqa2
d1x4la2 d1x4la1 d1x3ha2 d1x3ha1 d2d8za1 d1x61a2 d1x6aa1 d1x6aa2
d2cora2 d2cora1 d1v6ga1 d1v6ga2 d2d8za2 d1x64a1 d1x64a2 d1b8ta1
d2co8a1 d1x63a2 d2dj7a2 d2dj7a1 d2cu8a2 d2cu8a1 d1g47a2 d1g47a1
d1j2oa1 d1wyha1 d2dloa1 d1wiga1 d1wiga2 d2cupa2 d2cupa3 d1x68a1
d2d8ya2 d2d8ya1 d1m3va2 d1x62a1 d1x68a2 d1x61a1 d1b8ta3 d1b8ta2
d2d8xa2 d2d8xa1 d1x4ka1 d1x4ka2 d2cuqa1 d1m3va1 d2co8a2 d1j2oa2
d1x63a1 d2cura1 d2cura2 d1x62a2 d2e3fa_ d1mm0a_ d2e3ea_ d2llda_
d2ny8x_ d2e3ga_ d2ny9x_ d2nz3a_ d2lj7a_ d1n4na_ d2m8bb1 d2kyqa_
d3psma_ d1jkza_ d1jxca_ d2gl1a_ d1ugla_ d4uj0a_ d2k4ua_ d1hp2a_
d2lu9a_ d1baha_ d2meoa1 d1wm7a_ d1hlya_ d1n8ma_ d1jlza_ d1wpda_
d1c55a_ d1pvza_ d1wt7a_ d1rjia_ d1wz5a_ d1m2sa_ d1pjva_ d1c49a_
d2ktca1 d1wmta_ d1du9a_ d1j5ja_ d1r1ga_ d1t0za_ d2i61a_ d1t1ta_
d1sn4a_ d1kv0a_ d2lkba_ d1npia_ d1wwna_ d1pe4a_ d2kbka_ d2li7a_
d1djta_ d2kbha_ d1dq7a_ d1sega_ d1omya_ d1zywa1 d1jzaa_ d1n9wa1
d1bbua1 d1c0aa1 d1b8aa1 d1l0wa1 d3a74a1 d1eova1 d1iyjb4 d1iyjb5
d1iyjb3 d1o7ia_ d4ipca1 d1x3ea_ d4apva_ d1jb7a1 d1jb7a2 d1jb7a3
d3kdfa_ d1ue1b_ d1wjja1 d3eiva_ d1xjva2 d1xjva1 d2ccza2 d1se8a_
d1l1oc_ d1jb7b_ d1qvca_ d3ulla_ d1qzga_ d1miua4 d1miua5 d1miua3
d3g48a1 d1gd7a_ d1mkha_ d1jjcb3 d1pyba_ d3ubub_ d1egia_ d1qo3c_
d1v7pa_ d1tdqb_ d3ubua_ d1c3ab_ d1h8ua_ d3bx4b_ d1t8ca1 d1sb2a1
d1qdda_ d2ziba1 d3cada_ d1byfa_ d1j34b_ d1jwia_ d1jzna_ d1c3aa_

d1oz7b_ d1wmza_ d3bdwb_ d1hq8a_ d1fifa1 d1dv8a_ d3hupa_ d1wk1a1
d1umra_ d2afpa_ d3bx4a_ d1gz2a_ d1sl6a_ d1v7pb_ d3g8ka_ d1ixxa_
d1j34a_ d1jwib_ d1g1sa1 d1y17a1 d1ja3a_ d1fvub_ d3bdwa_ d1sb2b1
d1fvua_ d1umrc_ d1oz7a_ d4m17a_ d3cfwa1 d1ypqa1 d1g1ta1 d3fd4a_
d3l9jc_ d4dn8a1 d1v4lb_ d1rdl1_ d2msba_ d1dgsa2 d1ckma1 d1p16a1
d1x9na2 d1rpma_ d2shpa1 d1yfoa_ d1l8ka_ d2ooqa1 d1g4us2 d1p15a_
d1lara2 d1lara1 d1wcha_ d2fh7a2 d2fh7a1 d1ohea2 d2f4da_ d1rxda_
d2hxpa_ d1xria_ d1d5ra2 d1fpza_ d3ezza_ d4y2ea_ d1i9sa_ d3azya_
d3wvja_ d1upsa1 d1ajoa_ d2hyka_ d1ajka_ d2vy0a_ d1o4ya_ d3o5sa_
d3d6ea_ d3atga_ d1dypa_ d1umza_ d3i4ia_ d1c1la_ d4lbqa_ d1a78a_
d1slta_ d4no4a_ d3zsja_ d3duia_ d1g86a_ d1is3a_ d1hlca_ d1tdha3
d1q3ba3 d1ee8a3 d1k82a3 g1qmo.1 g1len.1 d3zyra_ d1fnya_ d1n47a_
g2ltn.1 d1avba_ d3usua_ d1dhkb_ d1f9ka_ d1ukga_ d1g8wa_ d1g9fa_
d1wbfa_ d3wcsa_ d1g7ya_ d1mvqa_ d1hqla_ d1v6ia_ d1dbna_ d1qnwa_
d1fx5a_ d4l8qa_ d1ioaa_ d3n35a_ d2h0ba_ d2erfa_ d1h30a1 d3mw4a1
d1pz7a_ d2r1ba_ d3v64a1 d1d2sa_ d3bopa1 d3mw3a1 d1okqa1 d1okqa2
d1dszb_ d1a6ya_ d3cbba_ d1ynwa1 d1r4ia_ d1hcqa_ d2hana_ d2hanb_
d2enva1 d1kb4b_ d2nllb_ d1lo1a_ d1cita_ d1adqa1 d1fnga1 d1uvqb1
d1zs8a1 d2vola1 d2vola2 d1mfbl2 d2jelh2 d1uwel2 d3qq4b1 d1kjvb_
d1k5na1 d1osph2 d4odhl2 d1ow0a1 d1ow0a2 d1bfob2 d3l9ra2 d3jtth1
d2fath2 d4elkb2 d1fn4b2 d2xfxb_ d1pg7x2 d1cqka_ d4k71b2 d1lk2a1
d1eapb2 d4lfhg2 d1i4fa1 d2nw2a2 d1kfah2 d1mhca1 d1h3ua1 d1wejh2
d1h0db2 d1a5fh2 d1kcvh2 d1mjuh2 d1qqda1 d1kcrh2 d1k8ia1 d3huja2
d2nnab1 d1nfdf2 d3mffb2 d1s9va1 d1lk3h2 d1q0xl2 d1fv1b1 d1neza1
d4rfeb2 d1r3ha1 d1a6tb2 d1kjva1 d1pz5b2 d1onqa1 d2bc4a2 d1de4a1
d1k8da1 d1tcra2 d1dn0b2 d1nfde2 d2xwtb2 d1bfoa2 d1mexh2 d1iakb1
d1ypzf2 d4f7ea2 d1q72l2 d2fbjh2 d1fe8h2 d1clzh2 d1fngb1 d1uvqa1
d1vgka1 d1c16a1 d4j12a2 d4x99b2 d1igtb3 d1je6a1 d1q72h2 d1nldh2
d1teth2 d2hmic2 d1i1ca2 d1i1ca1 d1k8ib1 d1q0yh2 d1kfal2 d3frua1
d1pg7w2 d1u58a1 d1igyb4 d1igyb3 d1hyrc1 d1klub1 d1deeb2 d1c12b2
d2bc4b2 d2ig2h2 d1kcvl2 d3wn5b1 d1lk2b_ d1lk3l2 d1mjul2 d1sm3h2
d1q0xh2 d1zvsa1 d1ydpa1 d1u8ib2 d1ypze2 d1kcuh2 d1op9a_ d1y0la1
d1etzb1 d1egjl1 d2gsib1 d1nj9a1 d4jfhd1 d1aqkl1 d1t2ja_ d1lo4h1
d1zvya1 d3v4vl1 d4enec1 d1kxtb_ d5e8ea1 d2pxya_ d4weud_ d1xiwc_
d2esvd1 d1qnzh_ d1ogae1 d1f4xh1 d4n1hb_ d3ezjb1 d3mlwl1 d1kfah1
d1etza1 d1b4jh1 d3cfic_ d4w68a_ d2arja1 d1ek3a_ d2ok0l1 d1vera_
d1uwgh1 d1blnb1 d1fpth1 d1rzga1 d2giya1 d4m3kb1 d1nc2a1 d2nyyd1
d1ncna_ d1fbih1 d5dxwa_ d4fqqa1 d1dqdh1 d1jfql1 d1kelh1 d1faih1
d1frgl1 d1figl1 d4m6nl1 d1fo0a_ d4hxbl1 d3b5ga_ d3quyd1 d2h9gb1
d1i8kb_ d2dd8h1 d2ck0h1 d1eaja_ d4w6wb1 d2a6ib3 d1t66d1 d2e27h_
d1ob1b1 d1f11b1 d2v7na1 d1ad0a1 d2xt1b1 d1pskh1 d4i13b1 d2op4l1
d1a0ql1 d1uwel1 d1za3a1 d1ncbl1 d4uu9a_ d1bm3h1 d1cloh1 d1nqba2
d1cf8h1 d4y5va_ d1mhph1 d5anma1 d4cdgc1 d4ldeb_ d1y0lb1 d2pcpb1
d1z3gl1 d1dfbl1 d1fh5h1 d3g5xa1 d1tzib1 d4dn3l1 d3zhka1 d2b1hh1

d2uyla1 d1f3dh1 d1nj9b1 d2agjh1 d2h1pl1 d1q72h1 d8fabb1 d1ndmb1
d1rjca1 d4u6hb1 d3upca_ d2fbjl1 d1osph1 d1tvda_ d2cjvh_ d4fhbd1
d1bd2d1 d1rz7h1 d3nn8a_ d1blna1 d1knoa1 d1nfda1 d2bmkb1 d1dqll_
d1igyb1 d1igfl1 d12e8h1 d3utza1 d1nc2b1 d1rihh1 d4mwfb1 d4jvpa1
d1mieh1 d1rhfa1 d1fgnl1 d1sbsh1 d1lp9e1 d1dn0a1 d4lqfl1 d1iaih1
d1kgcd1 d1mh5b1 d1gpol1 d2g60h3 d1f8tl1 d2jixd1 d4grwh_ d1fo0b_
d1bfob1 d1axsa1 d1u9ka_ d1nmbh_ d1h8na1 d1h8na2 d1jguh1 d4eura1
d3sdyl1 d35c8h1 d1w72l1 d1mvub_ d1i9rh1 d1ob1a1 d4wenb_ d1yeeh1
d3ietc1 d1f58h1 d1ad0b1 d1mqkl_ d1nbvh1 d4b50a_ d3r8bb_ d1bafl1
d1mamh1 d4rgne1 d2gsgb_ d35c8l1 d1bafh1 d1ai1h1 d2pcpa1 d2fath1
d2x1pa_ d1orsb1 d1ibgl1 d1shma_ d1a2yb_ d1u0qa_ d2coqa_ d1hilb1
d1qfwi_ d1jv5b_ d1fn4b1 d5e0qa_ d1nsnh1 d1plgl1 d4ocxl1 d1kb5h1
d1rzfh1 d1pg7x1 d2fx7h1 d2oi9c_ d1l7il1 d3eyva1 d1d5il1 d4ffyl_
d1tetl1 d1a6wh_ d1ac6a_ d3dvfa_ d1nfde1 d1mimh1 d1ri8a1 d1jhlh_
d3dura_ d1fskb1 d1f97a1 d3so3b1 d2ak4d1 d1lp9f1 d4z7wf1 d1l7th1
d4y5ya_ d4x7ec1 d1emth1 d1ikfl1 d1nlbh1 d4utal1 d1bjma1 d1eapb1
d1u6ah1 d2dtgd1 d1kj2b_ d1xeda1 d1c1eh1 d1fgvh_ d2bnub1 d2fb4h1
d2dtgb1 d1ktrh_ d1jpth1 d1qkzl1 d4hepg_ d2atpb1 d1flrh1 d2hrpl1
d1mvfa_ d1iaim1 d3k80a1 d2jb5h1 d3zkxc_ d4grwe_ d1it9h1 d1rhhb1
d3w9ec_ d1e4wh1 d1sy6h1 d2j6eh1 d3qxta_ d1jglh1 d1p7ka1 d1tzhb1
d4n8pa1 d1gafl1 d2jelh1 d1wioa2 d4i0cc1 d1cicb1 d1lmka2 d1lmka1
d1iqwh1 d1orqb1 d1ejoh1 d1jnlh1 d4zg1a_ d43c9b_ d1ggbl1 d1wejh1
d3q6fa1 d1f3dj1 d3c6la1 d1bqlh1 d3p9wb_ d1a3rl1 d1h0db1 d2uzil_
d1eo8l1 d1qfwl_ d1n7ml1 d1igja1 d1c5ch1 d2a9mh_ d1rmfh1 d1fj1b1
d4heme_ d1ccza1 d1a5fh1 d1ogad1 d1dlfh_ d1qa9b_ d1vgel1 d3qskb_
d1sjxa_ d4ozfg1 d1sq2n_ d1qd0a_ d1kcvh1 d1c5db1 d3qcul1 d8faba1
d3bn9d1 d1mjuh1 d4y8dc_ d7fabh1 d3dvgb1 d1lo0l1 d1a6tb1 d1gigh1
d1kcrh1 d2hwzl1 d1yjdc1 d3clfl1 d4krna_ d1dl7l_ d1e6ol1 d1mexh1
d3r0ma_ d4kkna_ d1fnsl1 d1oaqh_ d1rzia1 d1ucbh1 d1ncwl1 d4b41a_
d1nfdf1 d2mpah1 d2d7tl1 d4grwf_ d4b5ea_ d1lk3h1 d3u79a_ d4u3xa_
d1ghfl1 d1bvkb_ d1mcww1 d1b88a_ d1jrhh1 d32c2b1 d4wemb_ d1dsfh_
d1op3h1 d1ce1l1 d1iqda1 d1qoka1 d1qoka2 d1qfuh1 d2crya1 d1ay1h1
d1oaql_ d3ab0b_ d1rurl1 d1t4kb1 d1bz7b1 d1n0xh1 d1forl1 d1ucbl1
d4w6yb_ d43c9a_ d2aabl1 d1tzia1 d4n1ca_ d1k6qh1 d3g9ab_ d4tvsa1
d1igmh_ d1pz5b1 d3dx9a1 d3wxwh_ d1nakh1 d1kcul1 d3eaka_ d4qlra1
d1cr9l1 d1d9ka_ d1f4xl1 d1deea1 d2i27n2 d1c12a1 d2orbl1 d1j8hd1
d1mcph1 d1c5da1 d1kenh1 d1bd2e1 d1h5ba_ d4x7fc1 d3upaa_ d1t3fa1
d1indh1 d2dtgc1 d4hc1l1 d1zv5a1 d4wgvb_ d1uwgl1 d4f37k1 d1aqkh1
d1vesa_ d3zkqd_ d1fptl1 d1lo4l1 d1knob1 d3rrqa_ d1ct8b1 d4orzc_
d1mlbb1 d4hgkc1 d1dqdl1 d1bogb1 d4ozhe1 d1dr9a1 d1i7za1 d1dn0b1
d1kell1 d1rzib1 d1ypzf1 d1wtla_ d1hkfa_ d1fail1 d1figh1 d2vyre_
d1sjva_ d1ohqa_ d1bfoa1 d1pkqb1 d1mjul1 d1b4jl1 d1nsnl1 d2arjb1
d1p4bh_ d4aj0a_ d3nn8b_ d2rcja1 d1f2xk_ d4v1dd_ d1adqh1 d2eksa_
d4k3ga_ d1jfqh1 d1uweh1 d1frgh1 d4gftb_ d1uz8b1 d4n1ea_ d1bm3l1

| | | | | | | | |
|---|---|---|---|---|---|---|---|
| d1clol1 | d3mcka1 | d2vq1a1 | d1ae6h1 | d4aq1b1 | d1uj3b1 | d1nbqa1 | d1fh5l1 |
| d2ck0l1 | d2ywza_ | d1q72l1 | d4nbxb_ | d1hila1 | d4hzlb1 | d2j88l1 | d1zmya1 |
| d1cd0a_ | d1a4ka1 | d1pskl1 | d1a0qh1 | d4krmb_ | d1ncbh1 | d2fbjh1 | d1j8he1 |
| d1i3ua_ | d1cf8l1 | d1fe8h1 | d2p1ya1 | d1eeqa_ | d2x89a_ | d1ol0a_ | d2r0lh1 |
| d1ub5a1 | d1dfbh1 | d3eyvb1 | d4jdvb1 | d4p2cg_ | d2gsia1 | d2agjl1 | d1u3ha1 |
| d2h1ph1 | d1rihl1 | d1fskc1 | d1boga1 | d2a77l1 | d1ospl1 | d1qfoa_ | d3dwta_ |
| d2fx7l1 | d1rz7l1 | d3stba1 | d1pkqa1 | d1b6da1 | d1dzba2 | d1dzba1 | d1kj2a_ |
| d4l1ha_ | d1rzgb1 | d1dqlh_ | d12e8l1 | d1miel1 | d1jmaa_ | d1sbsl1 | d1fgnh1 |
| d1f58l1 | d1mqkh_ | d1ndgb1 | d3qegl1 | d1iail1 | d2aw2a1 | d1tqbc1 | d3fo2a1 |
| d4dkaa_ | d1f8th1 | d2g60l1 | d2ywya_ | d1uj3a1 | d1yc7a_ | d3bd3a_ | d4w6xb1 |
| d1nmbl_ | d1i8ka_ | d1rjlb1 | d1ypze1 | d1p7kb1 | d1kxqe_ | d1gpoh1 | d2z4qa1 |
| d1w72h1 | d1i9rl1 | d4qgya1 | d4xt1c_ | d2qqnh1 | d1cica1 | d3c08h1 | d2cd0a_ |
| d2bdnh1 | d1hnga1 | d1maml1 | d1nldh1 | d1cr9h1 | d4lcih1 | d3bkyh1 | d3qg7l1 |
| d1ai1l1 | d1kxvc_ | d4iosd_ | d1l7ih1 | d2x1qa_ | d1ub6b1 | d3k74b_ | d1ibgh1 |
| d1teth1 | d1igya1 | d1hxma1 | d2x6ma_ | d2e7la_ | d5e03a_ | d1qfwm_ | d2r0kh1 |
| d1igjb1 | d1rurh1 | d1plgh1 | d1mj8h1 | d1yuhb1 | d3fmgl1 | d1rzfl1 | d1emtl1 |
| d1ad9b1 | d1igtb1 | d1nlbl1 | d1d5ih1 | d3k7ua_ | d4ffyh_ | d1bwwa_ | d3dvga1 |
| d1a6ta1 | d1fgvl_ | d4qxgl1 | d1miml1 | d1jhll_ | d2hffb1 | d2hh0l1 | d1qkzh1 |
| d1l7tl1 | d1afvh1 | d1iaii1 | d2cjuh_ | d1um5h1 | d2ghwb2 | d2ghwb1 | d1kgce1 |
| d2fgwh1 | d1ikfh1 | d1dvfd_ | d2i24n1 | d2xtjb_ | d1za3b1 | d2yc1a_ | d2hh0h1 |
| d2x1oa_ | d1bvka_ | d1rjla1 | d5e7ba1 | d1igta1 | d1il1b1 | d2fb4l1 | d1mvua_ |
| d1jptl1 | d32c2a1 | d1fvca_ | d1iqdb1 | d2hrph1 | d2p4ab_ | d4qo1a_ | d1p2cb1 |
| d1wt5c_ | d1t4ka1 | d5c1mb_ | d1it9l1 | d1bz7a1 | d1b2wh1 | d1e4wl1 | d1jnll1 |
| d1za6b1 | d3u7aa_ | d1igfh1 | d3gnml1 | d1orsa1 | d1wejl1 | d1a2ya_ | d1hxmb1 |
| d1gafh1 | d2j4wh1 | d1pz5a1 | d1j05a_ | d1a3rh1 | d4fqll1 | d1qfwh_ | d1eo8h1 |
| d1n7mh1 | d4s11a_ | d1kegh1 | d5da4a_ | d1iqwl1 | d3whxb1 | d1vgeh1 | d1deeb1 |
| d1c12b1 | d1t2ql1 | d1ad9a1 | d3gsnb1 | d3b2uc1 | d1ggbh1 | d1t3fb1 | d1nakl1 |
| d4ocmc_ | d1lila1 | d2v7hb3 | d2esve1 | d1mcoh1 | d2qr0b1 | d2q3aa_ | d1ct8a1 |
| d1kcrl1 | d2pxyb_ | d2uzih_ | d5c3ld_ | d3durb_ | d1c5cl1 | d2ak4e1 | d1dl7h_ |
| d1xiwd_ | d1mlba1 | d1rmfl1 | d1i7zb1 | d1dlfl_ | d2xa3a_ | d5bq7b1 | d1dqqb1 |
| d3h42l1 | d1egjh1 | d1kcvl1 | d1fe8l1 | d2j6el1 | d1f3rb2 | d1lk3l1 | d1sm3h1 |
| d1q0xh1 | d1ghfh1 | d7fabl1 | d1igch1 | d1lo0h1 | d4idla_ | d5c2bl_ | d1jrhl1 |
| d1il1a1 | d3zhda1 | d4kfzc_ | d1b0wa_ | d1e6oh1 | d1mexl1 | d4krpb_ | d1fnsh1 |
| d1ncwh1 | d4hgma_ | d4eigb1 | d4rzca_ | d2mpal1 | d4y5xa_ | d5en2b1 | d3lrga1 |
| d4ej1c1 | d1rhha1 | d1j05b_ | d1forh1 | d1q9wb1 | d2wzpd_ | d2aq2a1 | d1h0da1 |
| d3revb1 | d2ij0c1 | d1tzha1 | d4m7jl1 | d1k6ql1 | d3b9kc1 | d1igml_ | d3qeua1 |
| d1dsfl_ | d1ce1h1 | d1kcuh1 | d1zvha1 | d1zvoa1 | d1tqbb1 | d1ay1l1 | d2qscl1 |
| d2cgrh1 | d1nl0h1 | d1kenl1 | d3eo0a1 | d4n9ob1 | d1n0xl1 | d3k1kc_ | d1d4ua2 |
| d2aq2b1 | d1ck1a1 | d1enfa1 | d1i4pa1 | d1v1oa1 | d1et9a1 | d1esfa1 | d2g9hd1 |
| d4ohja1 | d1klud1 | d1bxta1 | d1eu3a1 | d1ty0a1 | d1fnua1 | d2cqva1 | d1fhga_ |
| d2k1ma_ | d2dl2a1 | d1biha3 | d1biha1 | d1biha4 | d3qp3a1 | d1g1ca_ | d1gxea_ |
| d1cvsc2 | d1cvsc1 | d1xmwa2 | d1m4ka2 | d2otpa2 | d1biha2 | d1rhfa2 | d2fcba2 |
| d2fcba1 | d1gsma2 | d1epfa1 | d1epfa2 | d2dava1 | d1waaa_ | d1f97a2 | d1x44a1 |
| d1vcaa2 | d1fnla2 | d1fnla1 | d3b5ha2 | d3h8na2 | d3h8na1 | d1jbja1 | d1jbja2 |

d1pd6a_ d1f2qa2 d1f2qa1 d3cx2a_ d1nbqa2 d3b5ha1 d1n26a1 d1xiwb_ d1xaua_ d1fltx_ d2ifga1 d1olla1 d1olla2 d1cs6a4 d1cs6a3 d1cs6a2 d1cs6a1 d2gw5a2 d1sy6a1 d1xiwa_ d1ucta2 d1ucta1 d1ry7b2 d1gl4b_ d1qz1a3 d1olza1 d2c9aa1 d1wwca_

**Family-35 (b.47.1.3)**

**Positive dataset contains 5 protein sequences.**

d3su6a1 d4agka_ d1q31a_ d3e90b_ d3u1jb_

**Negative dataset contains 4000 protein sequences.**

d1m32a_ d1ibja_ d1c7na_ d1iuga_ d4q75a_ d2bkwa1 d1t3ia_ d1n8pa_ d1qz9a_ d1jf9a_ d1p3wa_ d3kgwa_ d1cl1a_ d2ctza1 d1vjoa_ d2rfva_ d1gc0a_ d1cs1a_ d1d2fa_ d2ch1a1 d1qgna_ d1pffa_ d1elua_ d1ax4a_ d1c7ga_ d2v1pa1 d2ez1a_ d1jx4a2 d3gv5b1 d1t94a2 d3bq0a2 d3i2ka1 d1lnsa3 d1mpxa2 d1xkta1 d2hfka_ d5d3ka_ d2cfba1 d1dfoa_ d1o69a_ d1bj4a_ d3k28a_ d2bwpa_ d3lg0a_ d2fnua_ d3ou5a_ d1z7da1 d1b9ha_ d1iaya_ d4msoa_ d1bs0a_ d1s0aa_ d1sffa_ d3tqxa_ d1mdoa_ d3qboa_ d3pgya_ d1bjna_ d2dkja_ d1ejia_ d1vefa1 d4wxga_ d3bs8a_ d3gbxa1 d1w23a_ d1g8fa3 d1ddla_ d1e57b_ d1auyb_ d1qnia1 d1fwxa1 d2wo3b_ d1shxa_ d1f56a_ d1bypa_ d1azca_ d1ws8a_ d3u25a_ d1cuoa_ d1id2a_ d1rkra_ d2iaac_ d4dp1x_ d1nwpa_ d2gima_ d2i7sa_ d1qhqa_ d3ehbb1 d1fftb1 d1gyca3 d1hfua2 d1kbva1 d1kbva2 d1sdda1 d1sdda2 d1gyca1 d1gyca2 d1mzya1 d2bw4a1 d1aoza1 d1aoza3 d1aoza2 d1kyaa2 d1kyaa3 d1kyaa1 d1mzya2 d4e9sa1 d1sddb1 d1sddb2 d1hfua1 d1hfua3 d1oe1a2 d1oe1a1 d1v10a2 d1v10a3 d1v10a1 d1novc_ d2bbvc_ d1a3qa1 d1iknc_ d3brda1 d1oy3c_ d1imhc1 d1gjia1 d1zk9a1 d2cxka1 d2h0va_ d1mqta_ d1d4m3_ d1fmd3_ d2mev1_ g1aym.1 d1ev13_ d4q4w1_ d1r1a3_ d2x5ia_ d1rhi1_ g1r1a.1 g1d4m.1 g1rhi.1 d1b35b_ d1oopa_ d1tme1_ d4iv1a_ g1pvc.1 g1fpn.1 d1fpn1_ d4gb31_ d1d4m1_ d1fmd1_ d2wzr3_ g1cov.1 d1qqp3_ g1fmd.1 d2x5ic_ d1rhi3_ g1oop.1 d1hxs1_ g1hxs.1 d1fpn3_ d1bev1_ g2mev.1 d1eah3_ d1aym1_ d1pvc3_ d1qqp1_ d1zba3_ d1h8ta_ d3vbhc_ g1b35.1 g1bev.1 d1hxs3_ g1ev1.1 d1bev3_ g1tmf.1 d1tmf1_ d1b35a_ d2mev3_ g1eah.1 g1h8t.1 d1eah1_ d4q4w3_ d1r1a1_ d1aym3_ d1pvc1_ d1zba1_ d1h8tc_ d1oopc_ d1tme3_ g1qqp.1 d4iv1c_ g1tme.1 d1nepa_ d1ktja_ d1ds6b_ d1fstb_ d1um8a_ d1ixza_ d1qvra3 d1qzma_ d1sxjc2 d1jr3d2 d2p65a_ d1l8qa2 d1g8pa_ d3hu3a3 d1sxjd2 d1njfa_ d1in4a2 d1sxja2 d1tuea1 d2ce7a2 d1w5sa2 d1sxjb2 d1d2na_ d1ny5a2 d1ofha_ d2gnoa2 d2a5yb3 d1fnna2 d1u0ja1 d1lv7a_ d1iqpa2 d2fnaa2 d1ixsb2 d4gita_ d1sxje2 d1g41a_ d1tafa_ d1bh9b_ d1tafb_ d1jfib_ d1q9ca_ d1h3ob_ d2byka1 d1n1ja_ d4g92b_ d1jfia_ d1h3oa_ d2bykb1 d1n1jb_ d1pd212 d1nhya2 d2a2ra2 d1k0dd2 d1f2ea2 d1gsua2 d1f3ba2 d2fhea2 d2pvqa1 d1tw9a2 d1oyja2 d1e6ba2 d1k0ma2 d3ik7a1 d4is0a1 d1r5aa2 d1jlva2 d1m0ua2 d3gtub2 d1z9ha2 d1okta2 d1axda2 d2gsra2 d1tu7a2 d1k3ya2 d1zgma1 d1b48a2 d1gnwa2 d1gwca2 d2gsta2 d1glqa2 d1oe8a2 d1pn9a2 d1v2aa2 d1fw1a2 d1duga2 d1ydwa2 d2nvwa2 d1h6da2 d1zh8a2 d1qkia2 d1xeaa2 d1tlta2 d1r0ka3 d1vkoa2 d1vm6a2 d3qvsa2

d1gr0a2 d1p1ja2 d4aw8a_ d3gpdg2 d2q49a2 d1hdgo2 d2g17a2 d2cvoa2
d2czca1 d3pwka2 d1obfo2 d1i32a2 d1zyao2 d2pkqo2 d1b7go2 d2hjsa2
d1cf2o2 d1vkna2 d1nvmb2 d1ux8a_ d1dlya_ d4i0va_ d2bkma_ d1dlwa_
d2bmma_ d2msya_ d2djoa1 d2r5za_ d1au7a1 d1f43a_ d2lmda1 d1akhb_
d1s7ea1 d3nara_ d1zq3p1 d2craa1 d1mija_ d4uuta_ d3a01a_ d1x58a1
d3rkqa1 d3cmya1 d2cqxa1 d2da6a1 d1e3oc1 d1fjla_ d1mh3a1 d1ig7a_
d1x41a1 d1wh7a1 d1gt0c1 d2mw8a_ d1x2ma1 d1b72a_ d1wi3a1 d1jgga_
d1wjha1 d1x2na1 d1mnmc_ d2cuea1 d3k2aa_ d2hosa_ d2jwta2 d1pufa_
d1octc1 d2hi3a_ d1b8ia_ d4j19a_ d3lnqa_ d1wh5a1 d1b8ib_ d1tc3c_
d1gdta1 d1u78a2 d1hlva1 d1iufa2 d1iufa1 d1igna1 d2iu5a1 d1zk8a1
d2g7sa1 d2fd5a1 d2np5a1 d1z0xa1 d2id6a1 d2d6ya1 d2i10a1 d1vi0a1
d2oi8a1 d3c07a1 d2ns7a1 d2fq4a1 d2yvea1 d1t33a1 d2o7ta1 d2np3b1
d2g7ga1 d2gena1 d2fbqa1 d2g7la1 d2gfna1 d2g3ba1 d2jj7a1 d2hyja1
d1sgma1 d1ui5a1 d2hkua1 d2id3a1 d4jyka1 d3lv8a_ d1s3ga1 d1zaka1
d1s4qa_ d3tvta2 d1uf9a_ d1khta_ d1ckea_ d1osna_ d1p5zb_ d4tmka_
d3cm0a_ d1tmka_ d1jaga_ d3h86a_ d1t0hb_ d1nn5a_ d2h92a_ d3kb2a1
d2c95a1 d1yj5a2 d1kgda_ d1ly1a_ d1vyva2 d1deka_ d2grja1 d1p3ja1
d1y63a_ d1e4ya1 d1s96a_ d3iija1 d1lvga_ d1nksa_ d1vyua2 d4e22a_
d1qf9a_ d1jjva_ d1q3ta_ d1ki9a_ d1lw7a2 d1teva_ d1kjwa2 d1gsia_
d2iyva2 d1kaga_ d1viaa_ d1g3ma_ d1j99a_ d1t8ta1 d1nsta_ d1fmja_
d1zrha_ d2a3ra_ d1aqua_ d2zptx_ d3ckla_ d1texa_ d1q44a_ d2gwha_
d1z0ja1 d3umra2 d3gj0a1 d2gaoa1 d1yzga1 d1x3sa1 d1g7sa4 d1puja_
d1udxa2 d1kk1a3 d2o52a_ d1jwyb_ d1zcaa2 d1zcba2 d2fv8a1 d2cjwa_
d3dz8a1 d1xzpa2 d1vg8a_ d4qxaa_ d2ew1a1 d1x1ra1 d2bcgy_ d3doea1
d3cona_ d3rega_ d3wyga_ d1upta_ d2b6ha1 d1zd9a1 d1lnza2 d1r5ba3
d1n0ua2 d1r2qa_ d2f9la1 d2rhda_ d3q3jb_ d3q85a_ d1efma1 d1r8qa_
d2gf9a1 d2akab1 d1ek0a_ d1z08a1 d2gcoa1 d2dy1a2 d4g01b_ d2cxxa1
d1svia_ d3q72a_ d1ni3a1 d4ac9a4 d2erxa1 d2bmea_ d1wxqa1 d2gf0a1
d4ldja1 d1zunb3 d1d5ca_ d4lv5b_ d1efca3 d1kmqa_ d2g6ba1 d3lrpa_
d1tada2 d2y8ea_ d4rd4a1 d1z06a1 d2heia_ d3a58b_ d1u0la2 d1d2ea3
d1zj6a1 d2ocba_ d3rwoa_ d1mr3f_ d2e9sa1 d4u5xa1 d2iwra_ d2fn4a1
d3bwdd_ d2nzja1 d2atva1 d2ksqa_ d3l43a_ d1mkya2 d1mkya1 d1wmsa_
d1jnya3 d2rmka_ d1f6ba_ d1f60a3 d1gota2 d1jala1 d2fh5b1 d1g16a_
d2wblc_ d1m7ba_ d1wf3a1 d1f5na2 d1t9ha2 d1z6xa1 d1z0aa1 d2fg5a1
d2a5ja1 d2bcjq2 d1s0ua3 d1ky3a_ d4y0va_ d4lhwa_ d2qmea1 d2atxa1
d1egaa1 d1ryha_ d1moza_ d1nrjb_ d2erya1 d3bfka_ d1puia_ d1fzqa_
d3j3az1 d3t5ga_ d1shza2 d3defa_ d2f7sa1 d3raba_ d3tsoa_ d3aq4a_
d2al7a1 d3sfva1 d3bc1a_ d2ncda_ d1sdma_ d3h4sa1 d2heha_ d2grya_
g3kin.1 d1kk8a2 d1br2a2 d1goja_ d1f9va_ d2mysa2 d1lkxa_ d3t0qa_
d4db1a1 d3gbja_ d1ry6a_ d1v8ka_ d2ovga_ d1sq8a_ d1rzsa_ d3kz3a_
d3orca_ d1y4wa1 d1g99a2 d2e1za2 d2e1za1 d1saza1 d1saza2 d1ig8a1
d1czan2 d1czan1 d1ig8a2 d1czan4 d1bg3a4 d1bg3a3 d1bg3a2 d1bg3a1
d1nira1 d1qksa1 d1hroa_ d4gedb_ d3zooa_ d1mz4a_ d1gu2a_ d1f1fa_
d1dvva_ d1c6ra_ d1c75a_ d1e29a_ d1gdva_ d1cnoa_ d1h32b_ d1co6a_

d2bh4x_ d2aiua_ d5cytr_ d1jdla_ d1i8oa_ d2zboa_ d3dmia_ d1ls9a_
d1wvec_ d2yk3a_ d3ph2b_ d1dw0a_ d5aura_ d1ql3a_ d1qn2a_ d1f1ca_
d2nqya_ d1h4ga_ d1oa2a_ d1olra_ d3b7ma_ d4h7ma1 d3wp3a_ d2vgda_
d1igoa_ d3zsea_ d2nlra_ d3wx5a_ d1oa3a_ d1pvxa_ d1m4wa_ d1f5ja_
d1oa4a_ d1ks5a_ d4ixla1 d1evsa_ d1cnt1_ d1pvhb_ d1z7ca_ d1huwa_
d1bgea_ d1rhga_ d1f6fa_ d1f45b_ d1i1rb_ d1axia_ d1etea_ d3tgxb_
d2z3qa1 d3qb1a_ d1hula_ d1m48a_ d4nkqb_ d1eera_ d1v7mv_ d5e4ea_
d2o27a_ d3b5ka_ d2psma1 d4zf7a_ d3uf5a_ d4x51a_ d1d9ca_ d4ypgc1
d3oq3a_ d1lqsl_ d1n1fa_ d1au1a_ d1wu3i_ d3besl_ d3ux9a_ d4dnxa2
d1m8pa2 d1g8fa2 d1jhda2 d1x6va2 d3rbaa_ d2qtra_ d1nupa_ d3l92a_
d1f9aa_ d1qjca_ d2h29a_ d1vlha_ d1od6a_ d1lw7a1 d1mrza2 d4ruka_
d2el7a_ d1iq0a2 d1irxa2 d1ivsa4 d2cyba_ d1rqga2 d1j09a2 d2yxna_
d1f7ua2 d1h3fa1 d1ffya3 d2g36a1 d1nzja_ d1jila_ d1n3la_ d1i6la_
d1pfva2 d1h3na3 d3n9ia_ d3sz3a1 d3sw2a_ d1moxc_ d1dx5i3 d1edmb_
d1autl2 d1autl1 d1nqlb_ d1dval1 d1szba2 d1kigl_ d2y5fl_ d1ijqa2
d1gk5a_ d1g1sa2 d1nt0a3 d1xdtr_ d1lmja2 d1lmja1 d1nzia2 d2wphe_
d1klil_ d1pfxl2 d1pfxl1 d1p9ja_ d1g1ta2 d1f7ea_ d4yzub_ d2mgpa1
d1b9wa1 d1b9wa2 d2mgpa2 d1ob1c1 d1ob1c2 d1n1ia2 d1n1ia1 d2npra2
d2npra1 d2y5ia_ d1qlsa_ d1psra_ d3n22a_ d2egda_ d4ggfc_ d2wcea_
d2luca_ d4aqia_ d3nsia_ d2lp3a_ d1j55a_ d1a4pa_ d1k8ua_ d1nsha_
d1pvaa_ d3fs7a_ d2pvba_ d1ttxa_ d1rwya_ d2kyfa_ d1rk9a_ d1a75a_
d1exra_ d1tiza1 d1nyaa_ d1g8ia_ d1ggwa_ d1wdcc_ d3fwba_ d1ggza_
d2o5ga_ d1ctaa_ d3i5gc_ d1sl8a_ d1fpwa_ d2scpa_ d2roaa_ d4aqra_
d3pn7b_ d1br1b_ d2mysb_ d1s6ca_ d1bjfa_ d2obha_ d1jfka_ d1jf0a_
d1qv0a_ d1lkja_ d2mlfc_ d2m97a_ d2d8na_ d4by5a_ d1jbaa_ d4nqga_
d1wdcb_ d2mysc_ d1j7qa_ d1s6ia_ d1uhka2 d1r2ua_ d1dtla_ d3kf9a_
d3pn7c_ d5d67d_ d1tuza_ d1h8ba_ d1eg3a1 d2zkmx1 d1snla_ d1jfxa_
d1wkya2 d1uuqa_ d3wh9a_ d4m1ra_ d1xyza_ d4xuya_ d2je8a5 d4bf7a1
d3cuia_ d1ur4a_ d1qw9a2 d1odza_ d2vzsa5 d1bqca_ d2x2ya1 d1hjqa_
d1qnra_ d4y7ea1 d1vjza_ d4k68a_ d2v3ga_ d4hpga_ d1nofa2 d1nq6a_
d1h4pa_ d1aq0a_ d3ziza_ d4bs0a_ d1ecea_ d1h1na_ d1ta3b_ d1w91a2
d1i1wa_ d1uhva2 d1us3a2 d1egza_ d7a3ha_ d1tvna1 d3nyda1 d1ghsa_
d2cyga1 d4xv0a_ d2c8na2 d1w32a_ d3emca_ d1v6ya1 d1foba_ d2uwfa1
d2fgla_ d3niya1 d2c0ha1 d1rh9a1 d1yq2a5 d1szna2 d1fa2a_ d1pama4
d5do8a1 d1ea9c3 d1hx0a2 d2fhfa5 d1qhoa4 d1avaa2 d1m53a2 d4m8ua1
d2gjpa2 d1lwha2 d4howa1 d1ktba2 d1j0ha3 d3vm5a1 d2x1ia_ d1wzaa2
d1uasa2 d2diea1 d1mxga2 d1ht6a2 d1eswa_ d1iv8a2 d5cpsa_ d1ud2a2
d1zjaa1 d3gbea1 d1kwga2 d1wdpa_ d1gcya2 d1g5aa2 d1hvxa2 d3dhpa2
d2bhua3 d4do4a1 d3qgva1 d1e43a2 d1g94a2 d2d3na2 d1gjwa2 d1gvia3
d1x1na1 d1r7aa2 d1tz7a1 g1apy.1 d3ljqa_ d4toqa_ d1wb0a1 d1w9pa1
d1itxa1 d1kfwa1 d1goia2 d1edqa2 d2gsja_ d1hjxa1 d1jnda1 d1vf8a1
d3wdpp1 d3wq5a_ d1gnxa_ d4gxpa_ d4hz8a_ d1e4mm_ d3aiua_ d1wcga1
d3ahxa_ d1v02a_ d2dgaa_ d1pbga_ d1vffa1 d1qoxa_ d4ha4a_ d1mixa2
d1e5wa2 d1j19a2 d1isna2 d2al6b2 d3syxa1 d1mkea1 d2p8va_ d1xoda1

d1i7aa_ d1evha_ d1qc6a_ d1egxa_ d1iuka_ d2csua1 d3q9ub_ d2yv1a1
d3q9na_ d2d59a_ d1oi7a1 d2yv2a1 d1euca1 d1y81a1 d3nvva1 d1krha3
d2h88b1 d1n62a2 d1t3qa2 d1kf6b2 d1zoyb1 d1jq4a_ d1ffva2 d1rm6c2
d9ldta1 d2ewda3 d1up7a1 d2dfda1 d1s6ya1 d1vjta1 d7mdha1 d1hyea1
d1t2da1 d1ojsa3 d1b8pa1 d1ldna1 d1hyha1 d1civa1 d2a92a1 d1ez4a1
d1i0za1 d1y6ja1 d1gv0a1 d1ur5a1 d1llda1 d1obba1 d1v8ba1 d1gdha1
d1l7da1 d2dlda1 d1mx3a1 d1ygya1 d1qp8a1 d1li4a1 d1j4aa1 d2naca1
d1npya1 d3t4ea2 d1a4ia1 d1o0sa1 d2hk9a2 d2egga2 d1b26a1 d1bvua1
d1gq2a1 d1c1da1 d1leha1 d1gtma1 d1euza1 d1nvta1 d3dona2 d1edza1
d1gpja2 d1vi2a1 d1p77a1 d1b0aa1 d1vl6a1 d1v9la1 d1etob_ d1umqa_
d1kola2 d1f8fa2 d1qora2 d1jqba2 d1vj0a2 d1cdoa2 d1gu7a2 d1d1ta2
d1xa0a2 d2dm6a2 d1jvba2 d1rjwa2 d1llua2 d1h2ba2 d1tt7a2 d1piwa2
d1o89a2 d1iz0a2 d1e3ja2 d1yb5a2 d1uufa2 d1e3ia2 d3gpdg1 d1vc2a1
d2nvwa1 d1u8fo1 d1ydwa1 d1hdgo1 d2g17a1 d1cf2o1 d2czca2 d1r0ka2
d1obfo1 d2cvoa1 d1ebfa1 d1h6da1 d1i32a1 d1zh8a1 d2q49a1 d1qkia1
d1j5pa4 d3pzra1 d1xeaa1 d1vkoa1 d1vm6a3 d3qvsa1 d2pkqo1 d1b7go1
d1gr0a1 d1p1ja1 d1lc0a1 d1j0xo1 d3pwka1 d2hjsa1 d1zyao1 d1vkna1
d1tlta1 d1nvmb1 d3sjua_ d3ojfa1 d1xu9a1 d2jjya1 d1n7ha_ d1y1pa1
d1h5qa_ d1jtva_ d4fs3a_ d2bkaa1 d1gy8a_ d2c5aa1 d1ulsa_ d1ooea_
d2b69a1 d1y5ma1 d1spxa_ d1ae1a_ d1bxka_ d1ydea1 d1gega_ d1hdoa_
d1o5ia_ d1e6ua_ d1snya1 d2rhca_ d3zv4a_ d5ceja_ d2d1ya1 d1fjha_
d3w8ea_ d2fmua1 d1x1ta_ d1rpna_ d3ay7a1 d1k2wa_ d1eq2a_ d2hrba1
d3ruaa_ d2ew8a1 d4hwka_ d1xgka_ d2bd0a1 d1yo6a1 d2a35a1 d2ntva_
d1vl8a_ d1hdca_ d4bkua_ d2ag5a1 d2q45a_ d1nffa_ d4iy1a_ d1zbqa1
d1kewa_ d1ek6a_ d1ja9a_ d2q46a2 d1iy8a_ d1cyda_ d2ptga_ d1hxha_
d1uzma1 d3lyla_ d4m89a_ d3wyea_ d2blla1 d1w6ua_ d1sb8a_ d2q2va_
d4nr0a_ d1e6wa_ d2gasa_ d3oiga1 d2bgka1 d1mxha_ d1vl0a1 d1rkxa_
d1r6da_ d1wmaa1 d1e7wa_ d2c07a1 d1xkqa_ d3tzha_ d1g0oa_ d3lz6a_
d1qyca_ d1edoa_ d1gz6a_ d1oc2a_ d1t2aa_ d1zema1 d2xoxa_ d1z45a2
d1geea_ d1p33a_ d3lt0a_ d4e6pa_ d2wyua_ d1db3a_ d4q9na_ d1xhla_
d4o1ma_ d1fmca_ d2uvda_ d2gdza1 d1uaya_ d2z1ma_ d2o23a2 d2fr1a2
d2fr1a1 d1qsga_ d1qyda_ d4zjua_ d2a4ka1 d1yb1a_ d1wvga1 d1yxma1
d3f4ba_ d1mg5a_ d1xg5a1 d1r55a1 d3gboa_ d1atla_ d1buda_ d4q1la_
d1quaa_ d1yp1a_ d1wnia_ g1y43.1 d4ekfa_ d2ckga_ d1th0a_ d2bkra_
d1cmxa_ d1xd3a_ d3ifwa_ d1g0da4 d2f4ma1 d1x3za_ d3u1ra1 d1g9ka2
d1kapp2 d1t3ca_ d1epwa3 d2a97a_ d3fiea_ g1e1h.1 d3ioqa_ d1fh0a_
d1o0ea_ d1khqa_ d1m6da_ d2cb5a_ d1cs8a_ g8pch.1 d1s4va_ d2pnsa_
d1vsna1 d1mira_ d2oula_ d7pcka_ d3bwka_ d3bcna_ d2p86a_ d1pvja_
d1pcia_ d1gece_ d1deua_ d4bs6a_ d1xkga1 d1cqda_ d1jqpa2 d2c0ya_
d2bdza_ d3p5ua_ d2o36a_ d1zoya1 d1qo8a2 d1d4ca2 d1chua2 d1jnra2
d1kf6a2 d2jk6a2 d2jk6a1 d1feca1 d1feca2 d1lpfa1 d1xhca1 d1xhca2
d1dxla2 d1dxla1 d1gera2 d1m6ia2 d1onfa1 d1onfa2 d3lada2 d3lada1
d1w4xa1 d1w4xa2 d1gesa2 d1mo9a1 d1xdia1 d1fl2a2 d1fl2a1 d1fcda1
d1fcda2 d1h6va2 d1h6va1 d1mo9a2 d1gv4a2 d1gv4a1 d2gv8a2 d2gv8a1

d1aoga1 d1aoga2 d1hyua1 d1ebda1 d1ebda2 d3dk9a1 d1q1ra2 d1q1ra1
d1gesa1 d1zbfa1 d2z1ha_ d2etja1 d3qioa1 d1jl1a_ d2e4la_ d1jl2a_
d2lsna1 d1io2a_ d1uaxa_ d2yv0x_ d1mu2a1 d1ekea_ d1i39a_ d1pn0a1
d1reoa1 d1ju2a1 d2f5va1 d3c96a1 d2gmha1 d1pj5a2 d1gpea1 d1o5wa1
d1n4wa1 d1k0ia1 d2dw4a2 d2ivda1 d1seza1 d2voua1 d1s5ja1 d2hbja2
d2igia_ d1wlja_ d1qhta1 d4dlea1 d1tgoa1 d2f96a1 d3tr8a1 d3mxma_
d1q8ia1 d1y97a1 d1w0ha_ d1kfsa1 d2py5a1 d1noya_ d1d5aa1 d1yt3a3
d2guia_ d3mwpa1 d4kb1a_ d2d5ra1 d1offa_ d1frra_ d3wcqa_ d1e9ma_
d1l5pa_ d1wria_ d1i7ha_ d3av8a_ d1czpa_ d1rfka_ d3p1ma1 d3b2ga_
d2bt6a2 d1jmxa4 d1jmxa3 d1pbya3 d1k8ka2 d1k8ka1 d3uleb1 d3i33a2
d3i33a1 d1bupa1 d1dkgd2 d1dkgd1 d4ci6a2 d1jcfa1 d4a2ba2 d2fxua1
d1yaga2 d1yaga1 d4cbua2 d1bupa2 d1r7ha_ d1faaa_ d1wika1 d1xfla_
d1ttza1 d2f51a1 d1ti3a_ d1nhoa_ d1sena_ d2i4aa_ d1ep7a_ d1zmaa1
d1fo5a_ d3lefa1 d3diea1 d2yana1 d1z6na1 d1oaza_ d3zywa1 d4aj8a_
d1nm3a1 d4fiwa_ d1wmja_ d4hs1a1 d1iloa_ d1xwaa1 d1wjka1 d2vm1a_
d1fb6a_ d1gh2a_ d1h75a_ d1nw2a_ d3a6na_ d4m6ba2 d2nqbd_ d3wkjd_
d2yfwe_ d4h9na_ d5bnxa_ d1kx5c_ d3r45a_ d1eqzb_ d4jjnb_ d1f66c_
d1eqza_ d4wnnb_ d1kx5a_ d1u35c1 d1id3a_ d2nqbc_ d1kx5d_ d3us3a3
d3us3a1 d2b5ea2 d2b5ea3 d2b5ea1 d2b5ea4 d2kp1a1 d2kp2a1 d1hyua3
d1hyua4 d1j08a1 d1j08a2 d4mh8a_ d1mu2a2 d4i7ga1 d1ovna2 d1iqca2
d1iqca1 d1eb7a2 d1eb7a1 d1nmla2 d3h3no1 d3h3no2 d3ezwa2 d4ilya_
d1chka_ d4olta_ d1txga2 d3f47a1 d1mv8a2 d2pv7a2 d1xc2a3 d2ahra2
d4xiya1 d2f1ka2 d1pgja2 d1i36a2 d1np3a2 d1lj8a4 d1teaa3 d1qmga2
d2i76a2 d2g5ca2 d1wdka3 d3cuma3 d1qqya_ d1gd6a_ d1f6ra_ d2gv0a_
d1iiza_ d2z2fa_ d1j8xa_ d1ghla_ d1b9oa_ d174la_ d169la_ d2o4wa_
d1p37a_ d1k28a3 d1xjta1 d1jtma_ d2f2qa_ d4e97a1 d1g4ma1 d1g4ma2
d4qgna_ d1vr3a1 d2za0a_ d2c21a1 d1f9za_ d1mpya2 d1kw3b2 d1kw3b1
d4ghga1 d1tfza1 d3isqa1 d3isqa2 d1sqia1 d1sqia2 d1lkda2 d1lkda1
d1mpya1 d1cjxa2 d1cjxa1 d1sp9b2 d1t47a1 d1t47a2 d1f1ua2 d1sp8a1
d1sp8a2 d1f1ua1 d2bema_ d1pama1 d1ea9c1 d1qhoa1 d2fhfa2 d2fhfa1
d1k3ia1 d1j0ha1 d1cxla1 d2bhua1 d1edqa1 d1f1sa2 d1ut9a2 d1gvia1
d1txka1 d1s5ja2 d1qhta2 d4dlea2 d1tgoa2 d3py8a2 d1q8ia2 d1kfsa2
d2py5a2 d1d5aa2 d1qsta_ d2beia1 d4m3sa_ d1u6ma1 d1ygha_ d1yk3a1
d3dr6a_ d1xmta_ d1z4ea1 d2ge3a1 d1bo4a_ d2fiaa1 d2jdca1 d1y9wa1
d3to7a_ d1wwza1 d2fl4a1 d2ozua1 d2b5ga_ d2cy2a1 d2p0wa_ d1cm0a_
d4pswa_ d1yrea1 d1yr0a1 d1m4ia_ d2gana1 d1yvka1 d1ghea_ d2g3aa1
d1y7ra1 d2euia1 d1q2ya_ d2fsra1 d1xeba_ d2ae6a1 d1cjwa_ d1qsma_
d2aj6a1 d1y9ka1 d1i12a_ d1vkca_ d2atra1 d1n71a_ d2d4pa1 d2fiwa1
d2fe7a1 d1tiqa1 d2ou2a_ d1yx0a1 d2fcka1 d1p0ha_ d2j8ma_ d2pq8a_
d1z4ra1 d1s3za1 d1ufha_ d1xvqa1 d1pn0a2 d3hy2a_ d2cvba1 d1o8xa_
d1lu4a_ d1we0a1 d2bmxa1 d2a4va1 d1psqa_ d1xiya1 d1q98a_ d1n8ja_
d1zofa1 d2ggta_ d3tjja_ d4je1a1 d1o73a_ d3s9fa_ d2zcta_ d1jfua_
d2b1ka_ d5dvba1 d2b5xa1 d2v2ga_ d1st9a_ d3tb2a_ d4llra_ d4fh8a1
d1e2ya_ d1knga_ d2h30a_ d1i5ga_ d2z9sa_ d2f8aa1 d1tp9a1 d2b7ka_

| | | | | | | | |
|---|---|---|---|---|---|---|---|
| d1nm3a2 | d3hvva_ | d2wfca1 | d4kcea_ | d1xvwa1 | d4gqca1 | d3qpma_ | d4k1fa_ |
| d1zzoa1 | d1gp1a_ | d1prxa_ | d2qked_ | d4ksoa_ | d1t4za1 | d1wwja_ | d3kasa1 |
| d3bi1a3 | d2phla2 | d2phla1 | d1fxza2 | d1fxza1 | d1lrha_ | d1uija1 | g1dgw.1 |
| d2v09a_ | d1uika2 | d1uika1 | d1dgwa_ | d1e88a1 | d1l6ja5 | d1l6ja4 | d1eaka4 |
| d1eaka3 | d1l6ja3 | d1h8pa1 | d1etha2 | d1w52x1 | d2ppla1 | d1hpla2 | d1lpbb2 |
| d2oxea1 | d1bu8a2 | d1rp1a2 | d1ys1x_ | d4hs9a_ | d1ispa_ | d1ku0a_ | d3qmma_ |
| d1bnla_ | d2h1ia1 | d2r8ba1 | d3b5ea1 | d3cn9a_ | d1lgya_ | d4l3wa_ | d1llfa_ |
| d1gz7a_ | d1ukca_ | d4wfia_ | d4cg1a1 | d3visa_ | d3groa_ | d1thta_ | d1ei9a_ |
| d1ra6a_ | d1muka_ | d3gsza_ | d1khva_ | d1te9a_ | d2xwha_ | d3uqsa_ | d3hkwa_ |
| d3n6ma_ | d2cjqa_ | d2xyma_ | d1teba_ | d1te8a_ | d1u09a1 | d4wfxa_ | d3tyqa_ |
| d2pgga1 | d3zxha_ | d1cgla_ | d1rm8a_ | d1eaka2 | d1hy7a_ | d2jsda_ | d1cxva_ |
| d1hv5a_ | d1g12a_ | d3p0ya1 | d1igra1 | d1igra2 | d1n8yc2 | d1m6ba2 | d1m6ba1 |
| d4dv8a2 | d1m4la_ | d2bk7a_ | d3fjua_ | d1z5ra1 | d1kwma1 | d1jqga1 | d2c1ca1 |
| d1dtda_ | d1h8la2 | d2tbvc_ | d1c8nc_ | d1x36a_ | d1smvc_ | d1ng0c_ | d4sbvc_ |
| d1opoa_ | d1f2nc_ | d1cixa_ | d1y29a_ | d1v7fa_ | d2jtba_ | d1d1ha_ | d1g9pa_ |
| d1lupa_ | d1qk7a_ | d1mb6a_ | d2a2va_ | d1niya_ | d1i25a_ | d1hvwa_ | d2mpqa_ |
| d1ie6a_ | d1kqha_ | d1la4a_ | d2mxoa1 | d1koza_ | d1qk6a_ | d1emxa_ | d1lqra_ |
| d1c6wa_ | d1js9c_ | d1f15b_ | d1za7a_ | d1laja_ | d1rypd_ | d1irua_ | d1rypc_ |
| d3unfk_ | d1rypa_ | d1j2pa_ | d1irui_ | d1j2qh_ | d3unfh_ | d1m4ya_ | d1rypb_ |
| d1irul_ | d1irug_ | d1iru2_ | d1q5rh_ | d1rypg_ | d1g3ka_ | d1iruf_ | d1q5qa_ |
| d1e94a_ | d1irub_ | d1ryph_ | d3ty6a_ | d1iruj_ | d1iruk_ | d1rype_ | d1irud_ |
| d1irue_ | d3mi01_ | d1rypf_ | d1iruh_ | d2z3ba_ | d1iruc_ | d3mka2_ | d1iru1_ |
| d1ny721 | d1ny722 | d1a6ca3 | d1a6ca2 | d1a6ca1 | d1pgl22 | d1pgl11 | d1ny711 |
| d1jgta2 | d1ofda3 | d1ea0a3 | d1ecfa2 | d1q15a2 | d1ct9a2 | d1te5a_ | d1y14b1 |
| d2nn6i1 | d1i94l_ | d2zjra2 | d1smxa_ | d2qamc2 | d1hh2p1 | d2r7da1 | d1rl2a2 |
| d1wfqa1 | d3bzka4 | d1x6oa2 | d3a0ja_ | d2j01d2 | d2ja9a1 | d4ql5a_ | d1hr0w_ |
| d1luza_ | d2n3sa_ | d2vnud1 | d1h95a_ | d1go3e1 | d2nn6h1 | d1ne3a_ | d2mo1a_ |
| d1iz6a2 | d1jjga_ | d1e3pa2 | d2ix0a3 | d2ix0a2 | d2lxja_ | d1ueba3 | d1ueba2 |
| d2z0sa1 | d1u0la1 | d2ix0a1 | d2eifa2 | d2je6i1 | d3i2za1 | d2c35b1 | d2ahob2 |
| d1t9ha1 | d3i4oa_ | d2qall1 | d1jt8a_ | d2nn6g1 | d1g6pa_ | d3cama_ | d1i94q_ |
| d1c9oa_ | d1d7qa_ | d2ba0a1 | d2vnud2 | d2vnud3 | d1khia2 | d2qalq1 | d1wi5a1 |
| d1xtda2 | d4w9ta_ | d4x2ra1 | d1ka9f_ | d1qo2a_ | d4axka1 | d1thfd_ | d1h5ya_ |
| d4u28a_ | d2y88a_ | d4wd0a_ | d1vzwa1 | d4lt9a1 | d3tc7a1 | d3hoja_ | d1i4na_ |
| d3vnda_ | d3nl8a1 | d1geqa_ | d3nava1 | d4a2ra1 | d3tc6a1 | d1ujpa_ | d4ijba_ |
| d1xcfa_ | d1vc4a_ | d1rd5a_ | d1qopa_ | d3b5va_ | d2r80b_ | d1cqxa1 | d1cg5a_ |
| d1gcvb_ | d3boma_ | d2h8fb_ | d2raoa_ | d3bj1a_ | d4hrrb_ | d2r50a_ | d3d1kb_ |
| d2aa1b1 | d3zhwa_ | d3hf4b_ | d3gdja_ | d1jebb_ | d3bj1b_ | d1x9fb_ | d1wmub_ |
| d1gcva_ | d3bcqb_ | d3d4xb_ | d1gvha1 | d2dc3a_ | d3hf4a_ | d4yu3a_ | d3k8ba_ |
| d1qpwb_ | d2r80a_ | d3bcqa_ | d1v4wa_ | d3wtgb_ | d1jl7a_ | d4esaa_ | d1a9we_ |
| d3d1ab_ | d1fsla_ | d4h2la_ | d1v4wb_ | d1mwca_ | d1vhba_ | d1jeba_ | d1spgb_ |
| d1h97a_ | d1q1fa_ | d1tu9a1 | d3d1aa_ | d1hdsb_ | d3hyub_ | d2zfbb_ | d3fs4a_ |
| d1oj6a_ | d1shrb_ | d4h2lb_ | d4yu3b_ | d1spga_ | d3hrwa_ | d3qqra_ | d3at5a_ |
| d4wchd_ | d3hyua_ | d1cg5b_ | d1uc3a_ | d1hbra_ | d2raob_ | d1outb_ | d4esab_ |
| d3gdjb_ | d3qqqa_ | d1x9fc_ | d3vrfa_ | d1itha_ | d3mkba_ | d3d4xa_ | d3mjpa_ |

d1jl6a_ d3fh9a_ d1outa_ d2dc3b_ d1it2a_ d3eoka_ d3g46a_ d1qpwa_
d3vrfb_ d1ch4a_ d1x9fd_ d1hdsa_ d2zfba_ d3mkbb_ d1or4a_ d3wtga_
d1a4fa_ d3fh9b_ d3bomb_ d3cy5a_ d3at5b_ d1x9fa_ d2oifa_ d4lmxb_
d1cpcb_ d3v57a_ d1kn1a_ d1cpca_ d4f0ta_ d2vjha_ d4po5b_ d1eyxa_
d1alla_ d4f0tb_ d1b8db_ d4h0mb_ d1b33a_ d2vjhb_ d4lm6b_ d2vmlb_
d1allb_ d2vjta_ d3dbja_ d2vmla_ d2vjtb_ d4f0ub_ d1f99b_ d4rmpa1
d1liaa_ d1eyxb_ d2uulb_ d4lmsb_ d4f0ua_ d2bv8a_ d1f99a_ d2bv8b_
d2c7lb_ d3dbjb_ d4rmpb1 d1b8da_ d4h0ma_ d3v57b_ d1kn1b_ d1liab_
d2uula_ d2j96a_ d3w3ea_ d4tx7a_ d3cqla_ d4msta_ d3iwra_ d2z37a_
d4dyga_ d2xqfa_ d1ea5a_ d1f6wa_ d1dx4a_ d1qe3a_ d1k4ya_ d4qwwa_
d2ha2a_ d1sfra_ d1f0na_ d4qeka_ d3c8da2 d2pbla1 d1jkma_ d1jjia_
d1lzla_ d1jjfa_ d4iuaa3 d4bvva_ d5hpga_ d3k65a_ d1ki0a3 d1ki0a2
d1ki0a1 d1a0ha1 d1pmla_ d3sp8a3 d2feba1 d1mdwa_ d1ziva1 d1zcma1
d1f74a_ d3l21a_ d2e1da_ d1to3a1 d1w3ia_ d1sfla_ d1n7ka_ d1vcva1
d4imea_ d1wa3a_ d2ocza1 d1f2ja_ d3m16a_ d2yxga_ d1ub3a_ d3a5fa_
d1fbaa_ d1l6wa_ d1xfba1 d1wx0a1 d1vpxa1 d1ojxa_ d1mxsa_ d2a4aa1
d1xkya1 d3cq0a1 d3s1xa_ d1f05a_ d1fdja_ d3cpra1 d4d2ja_ d1cg2a1
d1yloa2 d1y7ea2 d1y0ya3 d1lfwa1 d1vixa1 d1fnoa4 d1vhoa2 d1ysja1
d1vhea2 d1z2la1 d2fvga2 d1vgya1 d1r3na1 d2grea2 d1xmba1 g1q7l.1
d2ftba_ d1pmpa_ d2rcqa1 d1fdqa_ d4lkpa_ d1sa8a_ d1vyfa_ d1lpja_
d3vg7a1 d4azqa1 d1o8va_ d5bvqa_ d1p6pa_ d2qo4a_ d1opba_ d1yiva1
d1o1va_ d4bvma1 d4tkba_ d4i3ba_ d1bwya_ d1ftpa_ d1ggla_ d4gkca_
d3p6da1 d1kqwa_ d2a0aa1 d1dzka_ d4tlja_ d2a2ua_ d4wfva_ d3t1da_
d4iaxa_ d3zq3a_ d2nnda_ d1i4ua_ d1kxoa_ d1bbpa_ d1kt7a_ d1exsa_
d3s26a1 d1lf7a_ d1qfta_ d4gh7a_ d1z24a1 d1qabe_ d1xkia_ d3bx7a_
d4iawa1 d1n0sa_ d1gkab_ d3kzaa_ d2k23a_ d2l9ca1 d2joza1 d1gm6a_
d3kffa_ d3saoa1 d1e5pa_ d1yupa1 d1iiua_ d1e69a_ d1mv5a_ d3g60a4
g1ii8.1 d1ewqa2 d1qhla_ d1v43a3 d2wmfa1 d1vpla_ d1g2912 d4a82a2
g1f2t.1 d2d62a1 d3tuic1 d2bbsa1 d3g60a3 d2ixea_ d1g6ha_ d1sgwa1
d2qi9c_ d3rlfa1 d3b60a1 d3d31a2 d2pzea1 g1xew.1 d1jj7a_ d1oxxk2
d2onka1 d1w1wa_ d1iwea_ d1qf5a_ d1vmaa2 d1nija1 d1np6a_ d2p67a1
d3ihla_ d1okkd2 d1ls1a2 d1s1ma2 d1iona_ d2v40a_ d1dj3a_ d3hida_
d2vo1a1 d2g0ta1 d1vcoa2 d1j8mf2 d1g3qa_ d1xjca_ d1qzxa3 d1ihua2
d1ihua1 d2qm8a_ d1dj2a_ d4ioja_ d1cp2a_ d1yrba2 d1hyqa_ d3gqba2
d1skye3 d1xp8a1 d1tf7a2 d1tf7a1 d1ye8a1 d1skyb3 d2qe7d2 d4blta_
d4huta_ d3ldaa2 d4hyya_ d2i3ba1 d1cr1a_ d3j9tb2 d3ntua2 d1vdza1
d2z43a_ d1e9ra_ d1nlfa_ d3ziaa2 d1n0wa_ d1g6oa_ d3ziad2 d3j9ta2
d1pzna2 d3gqbb2 d2i49a_ d1nnfa_ d1xvxa_ d1ursa_ d1h3da1 d1iuda_
d4m1va1 d1twya_ d3e13x_ d2nxoa1 d4pfua_ d3ttma_ d1elja_ d2qmwa1
d1us5a_ d1y3na1 d1jeta_ d1uqwa_ d4f19a1 d2esna2 d3thia_ d3fasa1
d2uyeb_ d2xwva1 d1r9la_ d2fyia1 d1pb7a_ d2ozza1 d1sw5a_ d1nh8a1
d1vr5a1 d2g29a_ d1xs5a_ d3lswa_ d2z23a_ d3w9va_ d3tcha_ d1q35a_
d1z7me1 d3q27a_ d1p99a_ d1ve4a1 d2i6ea1 d4mcoa1 d4n8ya_ d1eu8a_
d1zbma1 d1hsla_ d1ixca2 d1j1na_ d1xvya_ d1y9ua_ d2z22a1 d1xoca1

d4oera_ d1wdna_ d5f1qa1 d1xt8a1 d1i6aa_ d4gwoa_ d4igta1 d1pc3a_
d4mmpa1 d1o63a_ d2czla1 d2onsa2 d4mija1 d4ok5a1 d2bmfa1 d2bmfa2
d1yksa1 d1yksa2 d1jr6a_ d2qeqa1 d2jlqa1 d1a1va2 d1eh3a_ d3skpa_
d1dtza1 d1dtza2 d1jnfa2 d1ce2a1 d1jnfa1 d1b1xa2 d1b1xa1 d1jw1a1
d1jw1a2 d1ryoa_ d1h76a1 d1h76a2 d1c4oa1 d2p6ra3 d2p6ra4 d1oywa2
d1wrba1 d1w36b2 d1w36b1 d1c4oa2 d3bora_ d1t6na_ d2eyqa5 d2eyqa3
d2eyqa2 d2zu6a2 d1veca_ d1uaaa1 d1w36d1 d2g9na1 d1z3ix2 d2rb4a_
d1hv8a1 d1hv8a2 d1t5ia_ d1w36c2 d2j0sa2 d2j0sa1 d1fuka_ d3hjha1
d1z5za1 d1qdea_ d1z63a1 d1s2ma2 d1s2ma1 d1wp9a1 d1wp9a2 d1gm5a3
d1gm5a4 d3dina4 d3dina3 d1q0ua_ d1w36c1 d1z3ix1 d1nkta3 d1nkta4
d1uaaa2 d1w36d2 g1qhh.1 d2fwra2 d2fwra1 d3q3ma_ d1za0a1 d1mxra_
d2inca_ d2incb_ d1mhyb_ d3hf1b_ d1mtyb_ d1r2fa_ d2uw1a_ d1mhyd_
d3n37a_ d1afra_ d1oquc_ d1syya_ d1mtyd_ d2o1za_ d1uzra_ d1smsa_
d2fkza_ d1zuja1 d1s3qa1 d2fjca1 d1z6om1 d1veia_ d4dyya_ d1jiga_
d3gvya_ d2pyba_ d4cy9a1 d3bvfa1 d1r03a_ d2cf7a_ d1nf4a_ d1zs3a1
d4iwka_ d3ka8a_ d3np0x_ d1n1qa_ d1lb3a_ d4e6ka_ d1z6oa1 d2fzfa1
d4dyua_ d3ge4a_ d1jgca_ d4am4a_ d2z6ma_ d1ji5a_ d1vlga_ d1lkoa1
d1krqa_ d3qz3a_ d1o9ra_ d2ffxj_ d1euma_ d1yv1a1 d2v2pa_ d1j30a_
d4di0a_ d1dpsa_ d1oewa_ d2qzxa_ d2asia_ d1psoe_ d1miqa_ d1smra_
d2h6ta_ d1pfza_ d1izda_ d3hd8a_ d1j71a_ d1dpja_ d4aa9a_ d1t6ex_
g1b5f.1 g1lya.1 g1htr.1 d1qdma2 d1bxoa_ d1wkra_ d1tzsa_ d1ibqa_
d2qzwa_ d1ex4a2 d1c6va_ d2v76a_ d1wj1a1 d1aqca_ d1qqga2 d1qqga1
d1ntva_ d1n3ha_ d4dbba_ d3f0wa1 d1p3ra_ d1wgua1 d2cy4a1 d3hqca_
d1wvha1 d1xr0b1 d1bdqa_ d3ggta_ d3ixoa_ d1mrxa_ d2haha_ d1tw7a_
d4njsa_ d1sgua_ d2p3da_ d1nsoa_ d3d3ta_ d2rkfa_ d1c6ya_ d2rspa_
d3ogpa_ d3ekwa_ d2o4na_ d2j9ja_ d1baia_ d3ggua_ d3lzua_ d4npua_
d4j55a_ d2r8na_ d2fxda_ d2p3ca_ d4dqea_ d3ttpa_ d3t3ca_ d2p3aa_
d2hc0a_ d2fdda_ d3mwsa_ d1sr9a2 d1rqba2 d1wgda1 d1wgga1 d4q5eb_
d1wjna1 d1se9a_ d3n3kb_ d1wz0a1 d1we6a1 d2m0xa_ d2gbka_ d3rt3b1
d1wh3a1 d1sifa_ d2ekec2 d4ii2b1 d4hk2a1 d1m94a_ d1wgha1 d1ttna1
d1j8ca_ d1wx8a1 d3v6cb1 d2ma9b_ d4i6lb1 d1bt0a_ d2jvca1 d1iyfa_
d2k1fa_ d1wxva1 d1wf9a1 d1t0ya_ d2io0b2 d1x1ma1 d2gbma_ d1zw7a1
d2m17a_ d1ud7a_ d1we7a1 d2lwpa_ d2gbja_ d1wjua1 d1v6ea1 d1v86a1
d4eewa_ d1wiaa1 d4icva_ d4gu2a_ d1wx7a1 d1wy8a1 d4hjka1 d2faza1
d1v5oa1 d3ubra_ d1qo8a1 d1d4ca1 d2k3va_ d1ft5a_ d1qdba_ d1m1qa_
d2j7aa_ d1h21a_ d1ogyb_ d4fasa_ d1jnia_ d1oaha_ d1sp3a_ d1wgqa1
d1v89a1 d2coda1 d1zc3b_ d1wg7a1 d1u5ea1 d1v61a1 d2coca1 d2ys1a1
d1v5pa1 d1ntya2 d1wjma1 d1wi1a1 d1u29a_ d1xx0a1 d1unqa_ d2zkmx3
d1fhoa_ d1v88a1 d2adza1 d1x1fa1 d1p6sa_ d2coaa1 d1btka_ d1v5ma1
d1faoa_ d2x18a_ d1dyna_ d1x1ga1 d4kaxb_ d2j59m1 d1ki1b2 d1xcga2
d1txda2 d1u5da1 d1w1ha_ d1upqa_ d1v5ua1 d1foea2 d2fjla1 d1eaza_
d2dfka2 d2cofa1 d1kz7a2 d1g66a_ d5ajha1 d1iyka2 d1rxta1 d1iica2
d1iica1 d1wioa4 d1ccza2 d1vcaa1 d1dr9a2 d2d9qb1 d2nxyb2 d2dara2
d2dara1 d1wyha2 d2dloa2 d1rutx4 d1rutx3 d2cuqa2 d1x4la2 d1x4la1

d1x3ha2 d1x3ha1 d2d8za1 d1x61a2 d1x6aa1 d1x6aa2 d2cora2 d2cora1
d1v6ga1 d1v6ga2 d2d8za2 d1x64a1 d1x64a2 d1b8ta1 d2co8a1 d1x63a2
d2dj7a2 d2dj7a1 d2cu8a2 d2cu8a1 d1g47a2 d1j2oa1 d1wyha1 d2dloa1
d1wiga1 d1wiga2 d2cupa2 d2cupa3 d1x68a1 d2d8ya2 d2d8ya1 d1m3va2
d1x62a1 d1x68a2 d1x61a1 d1b8ta3 d1b8ta2 d2d8xa2 d2d8xa1 d1x4ka1
d1x4ka2 d2cuqa1 d1m3va1 d2co8a2 d1j2oa2 d1x63a1 d2cura1 d2cura2
d1x62a2 d2e3fa_ d2e3ea_ d2llda_ d2ny8x_ d2e3ga_ d2ny9x_ d2nz3a_
d2lj7a_ d1n4na_ d2m8bb1 d2kyqa_ d3psma_ d1jkza_ d1jxca_ d2gl1a_
d1ugla_ d4uj0a_ d2k4ua_ d1hp2a_ d2lu9a_ d1baha_ d2meoa1 d1wm7a_
d1hlya_ d1n8ma_ d1jlza_ d1wpda_ d1c55a_ d1pvza_ d1wt7a_ d1rjia_
d1wz5a_ d1m2sa_ d1pjva_ d1c49a_ d2ktca1 d1wmta_ d1du9a_ d1j5ja_
d1r1ga_ d1t0za_ d2i61a_ d1t1ta_ d1sn4a_ d1kv0a_ d2lkba_ d1npia_
d1wwna_ d1pe4a_ d2kbka_ d2li7a_ d1djta_ d2kbha_ d1dq7a_ d1sega_
d1omya_ d1zywa1 d1jzaa_ d1n9wa1 d1bbua1 d1e1oa1 d1c0aa1 d1l0wa1
d3a74a1 d1eova1 d1t61c1 d1iyjb4 d1iyjb5 d1iyjb3 d1o7ia_ d4ipca1
d1x3ea_ d4apva_ d1jb7a1 d1jb7a2 d1jb7a3 d3kdfa_ d1ue1b_ d1wjja1
d3eiva_ d1xjva2 d1xjva1 d2ccza2 d1se8a_ d1l1oc_ d1jb7b_ d1qvca_
d3ulla_ d1qzga_ d1miua4 d1miua5 d1miua3 d3g48a1 d1gd7a_ d1mkha_
d1jjcb3 d3ubub_ d1egia_ d1qo3c_ d1v7pa_ d1tdqb_ d3ubua_ d1c3ab_
d1h8ua_ d3bx4b_ d1t8ca1 d1sb2a1 d1qdda_ d2ziba1 d3cada_ d1byfa_
d1j34b_ d1jwia_ d1jzna_ d1c3aa_ d1wmza_ d3bdwb_ d1hq8a_ d1fifa1
d1dv8a_ d3hupa_ d1wk1a1 d1umra_ d2afpa_ d3bx4a_ d1gz2a_ d1sl6a_
d1v7pb_ d3g8ka_ d1ixxa_ d1j34a_ d1jwib_ d1g1sa1 d1y17a1 d1ja3a_
d1fvub_ d3bdwa_ d1sb2b1 d1fvua_ d1umrc_ d1oz7a_ d4m17a_ d3cfwa1
d1ypqa1 d1g1ta1 d3fd4a_ d3l9jc_ d4dn8a1 d1v4lb_ d1rdl1_ d2msba_
d2a1ka_ d1je5a_ d1rpma_ d2shpa1 d1yfoa_ d1l8ka_ d2ooqa1 d1g4us2
d1p15a_ d1lara2 d1lara1 d1wcha_ d2fh7a2 d2fh7a1 d1ohea1 d1ohea2
d2f4da_ d1rxda_ d2hxpa_ d1xria_ d1d5ra2 d1fpza_ d3ezza_ d4y2ea_
d1i9sa_ d3azya_ d3wvja_ d1upsa1 d1ajoa_ d2hyka_ d1ajka_ d2vy0a_
d1o4ya_ d3o5sa_ d3d6ea_ d3atga_ d1dypa_ d1umza_ d3i4ia_ d1c1la_
d4lbqa_ d1a78a_ d1slta_ d4no4a_ d3zsja_ d3duia_ d1g86a_ d1qmja_
d1hlca_ d1tdha3 d1q3ba3 d1ee8a3 d1k82a3 g1qmo.1 g1len.1 d3zyra_
d1n47a_ g2ltn.1 d1avba_ d3usua_ d1dhkb_ d1f9ka_ d1ukga_ d1g8wa_
d1g9fa_ d1wbfa_ d3wcsa_ d1g7ya_ g1loe.1 d1mvqa_ d1hqla_ d1v6ia_
d1dbna_ d1qnwa_ d1fx5a_ d4l8qa_ d1ioaa_ d3n35a_ d3h33a_ d1rwja_
d19hca_ d1os6a_ d3bxua_ d3h4na_ d1i77a_ d3caoa_ d1j0pa_ d1duwa_
d1ofwa_ d2e84a_ d1hh5a_ d1gyoa_ d3h34a_ d1a6ya_ d3cbba_ d1ynwa1
d1r4ia_ d1hcqa_ d2hana_ d2hanb_ d2enva1 d1kb4b_ d2nllb_ d1lo1a_
d1cita_ d1adqa1 d1fnga1 d1uvqb1 d1zs8a1 d2vola1 d2vola2 d1mfbl2
d2jelh2 d1uwel2 d3qq4b1 d1kjvb_ d1k5na1 d1osph2 d4odhl2 d1ow0a1
d1ow0a2 d1bfob2 d3l9ra2 d3jtth1 d2fath2 d4elkb2 d1fn4b2 d2xfxb_
d1pg7x2 d1cqka_ d4k71b2 d1lk2a1 d1eapb2 d4lfhg2 d1i4fa1 d2nw2a2
d1kfah2 d1mhca1 d1h3ua1 d1wejh2 d1h0db2 d1a5fh2 d1kcvh2 d1mjuh2
d1qqda1 d1kcrh2 d1k8ia1 d3huja2 d2nnab1 d1nfdf2 d3mffb2 d1s9va1

d1lk3h2 d1q0xl2 d1fv1b1 d1neza1 d4rfeb2 d1r3ha1 d1a6tb2 d1kjva1
d1pz5b2 d1onqa1 d2bc4a2 d1de4a1 d1k8da1 d1tcra2 d1dn0b2 d1nfde2
d2xwtb2 d1bfoa2 d1mexh2 d1iakb1 d1ypzf2 d4f7ea2 d1q72l2 d2fbjh2
d1fe8h2 d1clzh2 d1fngb1 d1uvqa1 d1vgka1 d1c16a1 d4j12a2 d4x99b2
d1igtb3 d1je6a1 d1q72h2 d1nldh2 d1teth2 d2hmic2 d1i1ca2 d1i1ca1
d1k8ib1 d1q0yh2 d1kfal2 d3frua1 d1pg7w2 d1u58a1 d1igyb4 d1igyb3
d1hyrc1 d1klub1 d1deeb2 d1c12b2 d2bc4b2 d2ig2h2 d1kcvl2 d3wn5b1
d1lk2b_ d1lk3l2 d1mjul2 d1sm3h2 d1q0xh2 d1zvsa1 d1ydpa1 d1u8ib2
d1ypze2 d1kcuh2 d1op9a_ d1y0la1 d1etzb1 d1egjl1 d2gsib1 d1nj9a1
d4jfhd1 d1aqkl1 d1t2ja_ d1lo4h1 d1zvya1 d3v4vl1 d4enec1 d1kxtb_
d5e8ea1 d2pxya_ d4weud_ d1xiwc_ d2esvd1 d1qnzh_ d1ogae1 d1f4xh1
d4n1hb_ d3ezjb1 d3mlwl1 d1kfah1 d1etza1 d1b4jh1 d3cfic_ d4w68a_
d2arja1 d1ek3a_ d2ok0l1 d1vera_ d1uwgh1 d1blnb1 d1fpth1 d1rzga1
d2giya1 d4m3kb1 d1nc2a1 d2nyyd1 d1ncna_ d1fbih1 d5dxwa_ d4fqqa1
d1dqdh1 d1jfql1 d1kelh1 d1faih1 d1frgl1 d1figl1 d4m6nl1 d1fo0a_
d4hxbl1 d3b5ga_ d3quyd1 d2h9gb1 d1i8kb_ d2dd8h1 d2ck0h1 d1eaja_
d4w6wb1 d2a6ib3 d1t66d1 d2e27h_ d1ob1b1 d1f11b1 d2v7na1 d1ad0a1
d2xt1b1 d1pskh1 d4i13b1 d2op4l1 d1a0ql1 d1uwel1 d1za3a1 d1ncbl1
d4uu9a_ d1bm3h1 d1cloh1 d1nqba2 d1cf8h1 d4y5va_ d1mhph1 d5anma1
d4cdgc1 d4ldeb_ d1y0lb1 d2pcpb1 d1z3gl1 d1dfbl1 d1fh5h1 d3g5xa1
d1tzib1 d4dn3l1 d3zhka1 d2b1hh1 d2uyla1 d1f3dh1 d1nj9b1 d2agjh1
d2h1pl1 d1q72h1 d8fabb1 d1ndmb1 d1rjca1 d4u6hb1 d3upca_ d2fbjl1
d1osph1 d1tvda_ d2cjvh_ d4fhbd1 d1bd2d1 d1rz7h1 d3nn8a_ d1blna1
d1knoa1 d1nfda1 d2bmkb1 d1dqll_ d1igyb1 d1igfl1 d12e8h1 d3utza1
d1nc2b1 d1rihh1 d4mwfb1 d4jvpa1 d1mieh1 d1rhfa1 d1fgnl1 d1sbsh1
d1lp9e1 d1dn0a1 d4lqfl1 d1iaih1 d1kgcd1 d1mh5b1 d1gpol1 d2g60h3
d1f8tl1 d2jixd1 d4grwh_ d1fo0b_ d1bfob1 d1axsa1 d1u9ka_ d1nmbh_
d1h8na1 d1h8na2 d1jguh1 d4eura1 d3sdyl1 d35c8h1 d1w72l1 d1mvub_
d1i9rh1 d1ob1a1 d4wenb_ d1yeeh1 d3ietc1 d1f58h1 d1ad0b1 d1mqkl_
d1nbvh1 d4b50a_ d3r8bb_ d1bafl1 d1mamh1 d4rgne1 d2gsgb_ d35c8l1
d1bafh1 d1ai1h1 d2pcpa1 d2fath1 d2x1pa_ d1orsb1 d1ibgl1 d1shma_
d1a2yb_ d1u0qa_ d2coqa_ d1hilb1 d1qfwi_ d1jv5b_ d1fn4b1 d5e0qa_
d1nsnh1 d1plgl1 d4ocxl1 d1kb5h1 d1rzfh1 d1pg7x1 d2fx7h1 d2oi9c_
d1l7il1 d3eyva1 d1d5il1 d4ffyl_ d1tetl1 d1a6wh_ d1ac6a_ d3dvfa_
d1nfde1 d1mimh1 d1ri8a1 d1jhlh_ d3dura_ d1fskb1 d1f97a1 d3so3b1
d2ak4d1 d1lp9f1 d4z7wf1 d1l7th1 d4y5ya_ d4x7ec1 d1emth1 d1ikfl1
d1nlbh1 d4utal1 d1bjma1 d1eapb1 d1u6ah1 d2dtgd1 d1kj2b_ d1xeda1
d1c1eh1 d1fgvh_ d2bnub1 d2fb4h1 d2dtgb1 d1ktrh_ d1jpth1 d1qkzl1
d4hepg_ d2atpb1 d1flrh1 d2hrpl1 d1mvfa_ d1iaim1 d3k80a1 d2jb5h1
d3zkxc_ d4grwe_ d1it9h1 d1rhhb1 d3w9ec_ d1e4wh1 d1sy6h1 d2j6eh1
d3qxta_ d1jglh1 d1p7ka1 d1tzhb1 d4n8pa1 d1gafl1 d2jelh1 d1wioa2
d4i0cc1 d1cicb1 d1lmka2 d1lmka1 d1iqwh1 d1orqb1 d1ejoh1 d1jnlh1
d4zg1a_ d43c9b_ d1ggbl1 d1wejh1 d3q6fa1 d1f3dj1 d3c6la1 d1bqlh1
d3p9wb_ d1a3rl1 d1h0db1 d2uzil_ d1eo8l1 d1qfwl_ d1n7ml1 d1igja1

| | | | | | | | |
|---|---|---|---|---|---|---|---|
| d1c5ch1 | d2a9mh_ | d1rmfh1 | d1fj1b1 | d4heme_ | d1ccza1 | d1a5fh1 | d1ogad1 |
| d1dlfh_ | d1qa9b_ | d1vgel1 | d3qskb_ | d1sjxa_ | d4ozfg1 | d1sq2n_ | d1qd0a_ |
| d1kcvh1 | d1c5db1 | d3qcul1 | d3bn9d1 | d1mjuh1 | d4y8dc_ | d7fabh1 | d3dvgb1 |
| d1lo0l1 | d1a6tb1 | d1gigh1 | d1kcrh1 | d2hwzl1 | d1yjdc1 | d3clfl1 | d4krna_ |
| d1dl7l_ | d1e6ol1 | d1mexh1 | d3r0ma_ | d4kkna_ | d1fnsl1 | d1oaqh_ | d1rzia1 |
| d1ucbh1 | d1ncwl1 | d4b41a_ | d1nfdf1 | d2mpah1 | d2d7tl1 | d4grwf_ | d4b5ea_ |
| d1lk3h1 | d3u79a_ | d4u3xa_ | d1ghfl1 | d1bvkb_ | d1mcww1 | d1b88a_ | d1jrhh1 |
| d32c2b1 | d4wemb_ | d1dsfh_ | d1op3h1 | d1ce1l1 | d1iqda1 | d1qoka1 | d1qoka2 |
| d1qfuh1 | d2crya1 | d1ay1h1 | d1oaql_ | d3ab0b_ | d1rurl1 | d1t4kb1 | d1bz7b1 |
| d1n0xh1 | d1forl1 | d1ucbl1 | d4w6yb_ | d43c9a_ | d2aabl1 | d1tzia1 | d4n1ca_ |
| d1k6qh1 | d3g9ab_ | d4tvsa1 | d1igmh_ | d1pz5b1 | d3dx9a1 | d3wxwh_ | d1nakh1 |
| d1kcul1 | d3eaka_ | d4qlra1 | d1cr9l1 | d1d9ka_ | d1f4xl1 | d1deea1 | d2i27n2 |
| d1c12a1 | d2orbl1 | d1j8hd1 | d1mcph1 | d1c5da1 | d1kenh1 | d1bd2e1 | d1h5ba_ |
| d4x7fc1 | d3upaa_ | d1t3fa1 | d1indh1 | d2dtgc1 | d4hc1l1 | d1zv5a1 | d4wgvb_ |
| d1uwgl1 | d4f37k1 | d1aqkh1 | d1vesa_ | d3zkqd_ | d1fptl1 | d1lo4l1 | d1knob1 |
| d3rrqa_ | d1ct8b1 | d4orzc_ | d1mlbb1 | d4hgkc1 | d1dqdl1 | d1bogb1 | d4ozhe1 |
| d1dr9a1 | d1i7za1 | d1dn0b1 | d1kell1 | d1rzib1 | d1ypzf1 | d1wtla_ | d1hkfa_ |
| d1fail1 | d1figh1 | d2vyre_ | d1sjva_ | d1ohqa_ | d1bfoa1 | d1pkqb1 | d1mjul1 |
| d1b4jl1 | d1nsnl1 | d2arjb1 | d1p4bh_ | d4aj0a_ | d3nn8b_ | d2rcja1 | d1f2xk_ |
| d4v1dd_ | d1adqh1 | d2eksa_ | d4k3ga_ | d1jfqh1 | d1uweh1 | d1frgh1 | d4gftb_ |
| d1uz8b1 | d4n1ea_ | d1bm3l1 | d1clol1 | d3mcka1 | d2vq1a1 | d1ae6h1 | d4aq1b1 |
| d1uj3b1 | d1nbqa1 | d1fh5l1 | d2ck0l1 | d2ywza_ | d1q72l1 | d4nbxb_ | d1hila1 |
| d4hzlb1 | d2j88l1 | d1zmya1 | d1cd0a_ | d1a4ka1 | d1pskl1 | d1a0qh1 | d4krmb_ |
| d1ncbh1 | d2fbjh1 | d1j8he1 | d1i3ua_ | d1cf8l1 | d1fe8h1 | d2p1ya1 | d1eeqa_ |
| d2x89a_ | d1ol0a_ | d2r0lh1 | d1ub5a1 | d1dfbh1 | d3eyvb1 | d4jdvb1 | d4p2cg_ |
| d2gsia1 | d2agjl1 | d1u3ha1 | d2h1ph1 | d1rihl1 | d1fskc1 | d1boga1 | d2a77l1 |
| d1ospl1 | d1qfoa_ | d3dwta_ | d2fx7l1 | d1rz7l1 | d3stba1 | d1pkqa1 | d1b6da1 |
| d1dzba2 | d1dzba1 | d1kj2a_ | d4l1ha_ | d1rzgb1 | d1dqlh_ | d12e8l1 | d1miel1 |
| d1jmaa_ | d1sbsl1 | d1fgnh1 | d1f58l1 | d1mqkh_ | d1ndgb1 | d3qegl1 | d1iail1 |
| d2aw2a1 | d1tqbc1 | d3fo2a1 | d4dkaa_ | d1f8th1 | d2g60l1 | d2ywya_ | d1uj3a1 |
| d1yc7a_ | d3bd3a_ | d4w6xb1 | d1nmbl_ | d1i8ka_ | d1rjlb1 | d1ypze1 | d1p7kb1 |
| d1kxqe_ | d1gpoh1 | d2z4qa1 | d1w72h1 | d1i9rl1 | d4qgya1 | d4xt1c_ | d2qqnh1 |
| d1cica1 | d3c08h1 | d2cd0a_ | d2bdnh1 | d1hnga1 | d1maml1 | d1nldh1 | d1cr9h1 |
| d4lcih1 | d3bkyh1 | d3qg7l1 | d1ai1l1 | d1kxvc_ | d4iosd_ | d1l7ih1 | d2x1qa_ |
| d1ub6b1 | d3k74b_ | d1ibgh1 | d1teth1 | d1igya1 | d1hxma1 | d2x6ma_ | d2e7la_ |
| d5e03a_ | d1qfwm_ | d2r0kh1 | d1igjb1 | d1rurh1 | d1plgh1 | d1mj8h1 | d1yuhb1 |
| d3fmgl1 | d1rzfl1 | d1emtl1 | d1ad9b1 | d1igtb1 | d1nlbl1 | d1d5ih1 | d3k7ua_ |
| d4ffyh_ | d1bwwa_ | d3dvga1 | d1a6ta1 | d1fgvl_ | d4qxgl1 | d1miml1 | d1jhll_ |
| d2hffb1 | d2hh0l1 | d1qkzh1 | d1l7tl1 | d1afvh1 | d1iaii1 | d2cjuh_ | d1um5h1 |
| d2ghwb2 | d2ghwb1 | d1kgce1 | d2fgwh1 | d1ikfh1 | d1dvfd_ | d2i24n1 | d2xtjb_ |
| d1za3b1 | d2yc1a_ | d2hh0h1 | d2x1oa_ | d1bvka_ | d1rjla1 | d5e7ba1 | d1igta1 |
| d1il1b1 | d2fb4l1 | d1mvua_ | d1jptl1 | d32c2a1 | d1fvca_ | d1iqdb1 | d2hrph1 |
| d2p4ab_ | d4qo1a_ | d1p2cb1 | d1wt5c_ | d1t4ka1 | d5c1mb_ | d1it9l1 | d1bz7a1 |
| d1b2wh1 | d1e4wl1 | d1jnll1 | d1yedb1 | d1za6b1 | d3u7aa_ | d1igfh1 | d3gnml1 |

d1orsa1 d1wejl1 d1a2ya_ d1hxmb1 d1gafh1 d2j4wh1 d1pz5a1 d1j05a_
d1a3rh1 d4fqll1 d1qfwh_ d1eo8h1 d1n7mh1 d4s11a_ d1kegh1 d5da4a_
d1iqwl1 d3whxb1 d1vgeh1 d1deeb1 d1c12b1 d1t2ql1 d1ad9a1 d3gsnb1
d3b2uc1 d1ggbh1 d1t3fb1 d1nakl1 d4ocmc_ d1lila1 d2v7hb3 d2esve1
d1mcoh1 d2qr0b1 d2q3aa_ d1ct8a1 d1kcrl1 d2pxyb_ d2uzih_ d5c3ld_
d3durb_ d1c5cl1 d2ak4e1 d1dl7h_ d1xiwd_ d1mlba1 d1rmfl1 d1i7zb1
d1dlfl_ d2xa3a_ d5bq7b1 d1dqqb1 d3h42l1 d1egjh1 d1kcvl1 d1fe8l1
d2j6el1 d1f3rb2 d1lk3l1 d1sm3h1 d1q0xh1 d1ghfh1 d7fabl1 d1igch1
d1lo0h1 d4idla_ d5c2bl_ d1jrhl1 d1il1a1 d3zhda1 d4kfzc_ d1b0wa_
d1e6oh1 d1mexl1 d4krpb_ d1fnsh1 d1ncwh1 d4hgma_ d4eigb1 d4rzca_
d2mpal1 d4y5xa_ d5en2b1 d3lrga1 d4ej1c1 d1rhha1 d1j05b_ d1forh1
d1q9wb1 d2wzpd_ d2aq2a1 d1h0da1 d3revb1 d2ij0c1 d1tzha1 d4m7jl1
d1k6ql1 d3b9kc1 d1igml_ d3qeua1 d1dsfl_ d1ce1h1 d1kcuh1 d1zvha1
d1zvoa1 d1tqbb1 d1ay1l1 d2qscl1 d2cgrh1 d1nl0h1 d1kenl1 d3eo0a1
d4n9ob1 d1n0xl1 d3k1kc_ d2m9wa1 d3dfxa_ d2aq2b1 d1ck1a1 d1enfa1
d1i4pa1 d1v1oa1 d1et9a1 d1esfa1 d2g9hd1 d4ohja1 d1klud1 d1bxta1
d1eu3a1 d1ty0a1 d2cqva1 d1fhga_ d2k1ma_ d2dl2a1 d1biha3 d1biha1
d1biha4 d3qp3a1 d1g1ca_ d1gxea_ d1cvsc2 d1cvsc1 d1xmwa2 d1m4ka2
d2otpa2 d1biha2 d1rhfa2 d2fcba2 d2fcba1 d1gsma2 d1epfa1 d1epfa2
d2dava1 d1waaa_ d1f97a2 d1x44a1 d1vcaa2 d1fnla2 d1fnla1 d3b5ha2
d3h8na2 d3h8na1 d1jbja1 d1jbja2 d1pd6a_ d1f2qa2 d1f2qa1 d3cx2a_
d1nbqa2 d3b5ha1 d1n26a1 d1xiwb_ d1xaua_ d1fltx_ d2ifga1 d1olla1
d1olla2 d1cs6a4 d1cs6a3 d1cs6a2 d1cs6a1 d2gw5a2 d1sy6a1 d1xiwa_
d1ucta2 d1ucta1 d1ry7b2 d1gl4b_ d1qz1a3 d1olza1 d2c9aa1 d1wwca_

**Family-36 (b.50.1.1)**

**Positive dataset contains 27 protein sequences.**

d3ggta_ d3ixoa_ d2haha_ d1tw7a_ d4njsa_ d1sgua_ d2p3da_ d3d3ta_
d2rkfa_ d3ogpa_ d3ekwa_ d2o4na_ d2j9ja_ d3ggua_ d3lzua_ d4npua_
d4j55a_ d2r8na_ d2fxda_ d2p3ca_ d4dqea_ d3ttpa_ d3t3ca_ d2p3aa_
d2hc0a_ d2fdda_ d3mwsa_

**Negative dataset contains 4035 protein sequences.**

d1m32a_ d1ibja_ d1c7na_ d1iuga_ d4q75a_ d2bkwa1 d1t3ia_ d1n8pa_
d1qz9a_ d1jf9a_ d1eg5a_ d1p3wa_ d3kgwa_ d1cl1a_ d2ctza1 d1vjoa_
d2rfva_ d1gc0a_ d1cs1a_ d1d2fa_ d2ch1a1 d1qgna_ d1pffa_ d2xlba_
d3m83a1 d1u08a_ d1lc5a_ d1b5pa_ d2ay1a_ d1bw0a_ d3pd6a_ d7aata_
d2csta_ d1j32a_ d1fg7a_ d4wlha_ d1o4sa_ d1v2da_ d1gdea_ d1m6sa_
d1v72a1 d1svva_ d1wsta1 d2f8ja2 d1lk9a_ d1vp4a1 d1xi9a1 d2gb3a1
d1yaaa_ d2bnma2 d3i2ka1 d1lnsa3 d1mpxa2 d1sefa_ d1sfna_ d1rc6a_
d1rmka_ d1ixta_ d1eyoa_ d1cnna_ d1g1pa_ d1f3ka_ d1fyga_ d1cixa_
d1y29a_ d1v7fa_ d2jtba_ d1g9pa_ d1lupa_ d1qk7a_ d1mb6a_ d2a2va_
d1niya_ d1i25a_ d1hvwa_ d2mpqa_ d1ie6a_ d1kqha_ d1la4a_ d2mxoa1
d1dl0a_ d1koza_ d1qk6a_ d1emxa_ d1lqra_ d1c6wa_ d1f56a_ d1bypa_
d1azca_ d1ws8a_ d3u25a_ d1cuoa_ d1id2a_ d1rkra_ d2iaac_ d4dp1x_
d1nwpa_ d2gima_ d2i7sa_ d1qhqa_ d3ehbb1 d1fftb1 d1gyca3 d1hfua2

d1kbva1 d1kbva2 d1sdda1 d1sdda2 d1gyca2 d1mzya1 d2bw4a1 d1aoza1
d1aoza3 d1aoza2 d1kyaa2 d1kyaa3 d1kyaa1 d1mzya2 d4e9sa1 d1sddb1
d1sddb2 d1hfua1 d1hfua3 d1oe1a2 d1oe1a1 d1v10a2 d1v10a3 d1v10a1
d2dara2 d2dara1 d1wyha2 d2dloa2 d1rutx4 d1rutx3 d2cuqa2 d1x4la2
d1x4la1 d1x3ha2 d1x3ha1 d2d8za1 d1x61a2 d1x6aa1 d1x6aa2 d2cora2
d2cora1 d1v6ga1 d1v6ga2 d2d8za2 d1x64a1 d1x64a2 d1b8ta1 d2co8a1
d1x63a2 d2dj7a2 d2dj7a1 d2cu8a2 d2cu8a1 d1g47a1 d1j2oa1 d1wyha1
d2dloa1 d1wiga1 d1wiga2 d2cupa2 d2cupa3 d1x68a1 d2d8ya2 d2d8ya1
d1m3va2 d1x62a1 d1x68a2 d1x61a1 d1b8ta3 d1b8ta2 d2d8xa2 d2d8xa1
d1x4ka1 d1x4ka2 d2cuqa1 d1m3va1 d2co8a2 d1j2oa2 d1x63a1 d2cura1
d2cura2 d1x62a2 d1xkta1 d2hfka_ d5d3ka_ d2cfba1 d1o69a_ d1bj4a_
d3k28a_ d2bwpa_ d3lg0a_ d2fnua_ d3ou5a_ d1z7da1 d1b9ha_ d1iaya_
d4msoa_ d1bs0a_ d1s0aa_ d1sffa_ d3tqxa_ d1mdoa_ d3qboa_ d3pgya_
d1bjna_ d2dkja_ d1ejia_ d1vefa1 d4wxga_ d3bs8a_ d3gbxa1 d1w23a_
d3kasa1 d3bi1a3 d1iknc_ d3brda1 d1p7hl1 d1oy3c_ d1imhc1 d1gjia1
d1zk9a1 d2cxka1 d2bema_ d1ea9c1 d1qhoa1 d2fhfa2 d2fhfa1 d1k3ia1
d1j0ha1 d1cxla1 d2bhua1 d1edqa1 d1f1sa2 d1ut9a2 d1gvia1 d1txka1
d2phla2 d2phla1 d1fxza2 d1lrha_ d1uija1 g1dgw.1 d2v09a_ d1uika2
d1uika1 d1dgwa_ d1ktja_ d1ds6b_ d1fstb_ d1ex0a1 d1ku5a_ d1b67a_
d1eixa_ d3exra_ d2ffca1 d2fdsa1 d3n3ma1 d2czda_ d2yyua_ d1vqta1
d1dbta_ d3ldva1 d1h4ba_ d2opoa_ d1ydwa2 d2nvwa2 d1zh8a2 d1qkia2
d1xeaa2 d1tlta2 d1r0ka3 d1j5pa3 d1vkoa2 d1vm6a2 d3qvsa2 d1p1ja2
d2ovga_ d1sq8a_ d1rzsa_ d3kz3a_ d3orca_ d3gpdg2 d2q49a2 d1hdgo2
d2g17a2 d2cvoa2 d2czca1 d3pwka2 d1obfo2 d1i32a2 d1zyao2 d2pkqo2
d1b7go2 d2hjsa2 d1cf2o2 d1vkna2 d1nvmb2 d4lt9a1 d3tc7a1 d3hoja_
d1i4na_ d3vnda_ d3nl8a1 d1geqa_ d3nava1 d1v5xa_ d4a2ra1 d3tc6a1
d1ujpa_ d4ijba_ d1xcfa_ d1vc4a_ d1rd5a_ d3b5va_ d2msya_ d2djoa1
d2r5za_ d1au7a1 d1f43a_ d2lmda1 d1akhb_ d1s7ea1 d3nara_ d1zq3p1
d2craa1 d1mija_ d4uuta_ d3a01a_ d1x58a1 d3rkqa1 d3cmya1 d2cqxa1
d2da6a1 d1e3oc1 d1fjla_ d1mh3a1 d1ig7a_ d1x41a1 d1wh7a1 d1gt0c1
d2mw8a_ d1lfup_ d1x2ma1 d1b72a_ d1wi3a1 d1jgga_ d1wjha1 d1x2na1
d1mnmc_ d2cuea1 d3k2aa_ d2hosa_ d2jwta2 d1pufa_ d1octc1 d2hi3a_
d1b8ia_ d4j19a_ d3lnqa_ d1wh5a1 d1b8ib_ d1fexa_ d2cqra1 d1guua_
d1ug2a1 d2cqqa1 d2ajea1 d4a69c_ d1h8ac1 d2crga1 d2iw5b1 d1xc5a1
d1ofcx1 d2cu7a1 d1wgxa1 d2cjja1 d2ckxa1 d1gvda_ d1hcra_ d1gdta1
d1u78a2 d2iu5a1 d1zk8a1 d2g7sa1 d2fd5a1 d2np5a1 d1z0xa1 d2id6a1
d2d6ya1 d2i10a1 d1vi0a1 d2oi8a1 d3c07a1 d2ns7a1 d2fq4a1 d2yvea1
d1t33a1 d2o7ta1 d1rkta1 d2np3b1 d2g7ga1 d2gena1 d2fbqa1 d2g7la1
d2gfna1 d2g3ba1 d2jj7a1 d2hyja1 d1sgma1 d1ui5a1 d2hkua1 d2id3a1
d4jyka1 d1prtc2 d1prtb2 d3lv8a_ d1s3ga1 d1zaka1 d1s4qa_ d3tvta2
d1uf9a_ d1khta_ d1ckea_ d1osna_ d1p5zb_ d4tmka_ d3cm0a_ d1tmka_
d1jaga_ d3h86a_ d1t0hb_ d1nn5a_ d2h92a_ d3kb2a1 d2c95a1 d1yj5a2
d1kgda_ d1ly1a_ d2ak3a1 d1vyva2 d1deka_ d2grja1 d1p3ja1 d1y63a_
d1e4ya1 d1s96a_ d3iija1 d1lvga_ d1nksa_ d1vyua2 d4e22a_ d1qf9a_

d1jjva_ d1q3ta_ d1ki9a_ d1lw7a2 d1teva_ d1kjwa2 d1gsia_ d1g3ma_
d1j99a_ d1t8ta1 d1nsta_ d1zrha_ d1ls6a_ d2a3ra_ d1aqua_ d2zptx_
d3ckla_ d1texa_ d1q44a_ d2gwha_ d1uj2a_ d4gi7a_ d2jeoa1 d1rz3a_
d1z0ja1 d3umra2 d3gj0a1 d2gaoa1 d1yzga1 d1x3sa1 d1g7sa4 d1puja_
d1udxa2 d1kk1a3 d2o52a_ d1jwyb_ d1zcaa2 d1zcba2 d2fv8a1 d2cjwa_
d3dz8a1 d1xzpa2 d1vg8a_ d4qxaa_ d2ew1a1 d1x1ra1 d2bcgy_ d3doea1
d3cona_ d3rega_ d3wyga_ d1upta_ d2b6ha1 d1zd9a1 d1lnza2 d1r5ba3
d1n0ua2 d1r2qa_ d2f9la1 d2rhda_ d3q3jb_ d3q85a_ d1efma1 d1r8qa_
d2gf9a1 d2akab1 d1ek0a_ d1z08a1 d2gcoa1 d2dy1a2 d4g01b_ d2cxxa1
d1svia_ d3q72a_ d1ni3a1 d4ac9a4 d2erxa1 d2bmea_ d1wxqa1 d2gf0a1
d4ldja1 d1zunb3 d1d5ca_ d4lv5b_ d1efca3 d1kmqa_ d2g6ba1 d3lrpa_
d1tada2 d2y8ea_ d4rd4a1 d1z06a1 d2heia_ d3a58b_ d1u0la2 d1d2ea3
d1zj6a1 d2ocba_ d3rwoa_ d1mr3f_ d2e9sa1 d4u5xa1 d2iwra_ d2fn4a1
d3bwdd_ d2nzja1 d2atva1 d2ksqa_ d3l43a_ d1mkya2 d1mkya1 d1wmsa_
d1jnya3 d2rmka_ d1f6ba_ d1f60a3 d1gota2 d1jala1 d2fh5b1 d1g16a_
d2wblc_ d1m7ba_ d1wf3a1 d1f5na2 d1t9ha2 d1z6xa1 d1z0aa1 d2fg5a1
d2a5ja1 d2bcjq2 d1s0ua3 d1ky3a_ d4y0va_ d4lhwa_ d2qmea1 d2atxa1
d1egaa1 d1ryha_ d1moza_ d1nrjb_ d2erya1 d3bfka_ d1puia_ d1fzqa_
d3j3az1 d3t5ga_ d1shza2 d3defa_ d2f7sa1 d3raba_ d3tsoa_ d3aq4a_
d2al7a1 d3sfva1 d3bc1a_ d2ncda_ d1sdma_ d3h4sa1 d2heha_ d2grya_
g3kin.1 d1kk8a2 d1br2a2 d1goja_ d1f9va_ d2mysa2 d1lkxa_ d3t0qa_
d4db1a1 d3gbja_ d1ry6a_ d1v8ka_ d1k8ka2 d1k8ka1 d3uleb1 d3i33a2
d3i33a1 d1dkgd2 d1dkgd1 d4ci6a2 d1jcfa1 d4a2ba2 d2fxua1 d1yaga2
d1yaga1 d4cbua2 d1bupa2 d3vrda1 d3vrda2 d1fcdc2 d1h1oa2 d1h1oa1
d1m70a2 d1m70a1 d1ig8a1 d1czan2 d1czan3 d1czan1 d1ig8a2 d1czan4
d1bg3a3 d1bg3a2 d1bg3a1 d3h1jd1 d2d0oa3 d2d0oa2 d1nbwa3 d1hroa_
d4gedb_ d3zooa_ d1mz4a_ d1gu2a_ d1f1fa_ d1dvva_ d1c6ra_ d1c75a_
d1e29a_ d1gdva_ d1cnoa_ d1h32b_ d1co6a_ d2bh4x_ d2aiua_ d5cytr_
d1jdla_ d1i8oa_ d2zboa_ d3dmia_ d1ls9a_ d1wvec_ d2yk3a_ d3ph2b_
d1dw0a_ d5aura_ d1ql3a_ d1qn2a_ d1f1ca_ d2nqya_ d1h4ga_ d1hixa_
d1oa2a_ d1olra_ d3b7ma_ d4h7ma1 d3wp3a_ d2vgda_ d1igoa_ d3zsea_
d2nlra_ d3wx5a_ d1oa3a_ d1pvxa_ d1m4wa_ d1f5ja_ d1ks5a_ d4ixla1
d2ah2a1 d1n1ta1 d1evsa_ d1cnt1_ d1pvhb_ d1z7ca_ d1huwa_ d1bgea_
d1rhga_ d1f6fa_ d1f45b_ d1i1rb_ d1axia_ d1etea_ d3tgxb_ d2z3qa1
d3qb1a_ d1hula_ d1m48a_ d4nkqb_ d1eera_ d1v7mv_ d5e4ea_ d2o27a_
d3b5ka_ d2psma1 d4zf7a_ d3uf5a_ d4x51a_ d4ypgc1 d3oq3a_ d1lqsl_
d1n1fa_ d1au1a_ d1wu3i_ d3besl_ d3ux9a_ d3rbaa_ d2qtra_ d3l92a_
d1f9aa_ d1qjca_ d2h29a_ d1vlha_ d1od6a_ d1lw7a1 d1mrza2 d4ruka_
d2el7a_ d1iq0a2 d1irxa2 d1r6ta2 d1ivsa4 d2cyba_ d1rqga2 d1j09a2
d2yxna_ d1f7ua2 d1h3fa1 d1ffya3 d2g36a1 d1nzja_ d1jila_ d1n3la_
d1pfva2 d1h3na3 d3n9ia_ d3sz3a1 d3sw2a_ d1moxc_ d1dx5i3 d1edmb_
d1autl2 d1autl1 d1nqlb_ d1dval1 d1szba2 d1kigl_ d2y5fl_ d1ijqa2
d1gk5a_ d1g1sa2 d1nt0a3 d1xdtr_ d1lmja2 d1lmja1 d2wphe_ d1klil_
d1pfxl2 d1pfxl1 d1p9ja_ d1g1ta2 d1f7ea_ d4yzub_ d1lr7a1 d2mgpa1

d1b9wa1 d2mgpa2 d1ob1c1 d1ob1c2 d1n1ia2 d1n1ia1 d2npra2 d2npra1
d2y5ia_ d1qlsa_ d1psra_ d3n22a_ d2egda_ d4ggfc_ d2wcea_ d2luca_
d4aqia_ d3nsia_ d2lp3a_ d1j55a_ d1a4pa_ d1k8ua_ d1nsha_ d1pvaa_
d3fs7a_ d2pvba_ d1ttxa_ d2kyfa_ d1rk9a_ d1a75a_ d1exra_ d1tiza1
d1nyaa_ d1g8ia_ d1ggwa_ d1wdcc_ d3fwba_ d1ggza_ d2o5ga_ d1ctaa_
d1m45a_ d3i5gc_ d1sl8a_ d1fpwa_ d2scpa_ d2roaa_ d4aqra_ d3pn7b_
d1br1b_ d2mysb_ d1s6ca_ d1bjfa_ d2obha_ d1jfka_ d1jf0a_ d1qv0a_
d1lkja_ d2mlfc_ d2m97a_ d2d8na_ d4by5a_ d1jbaa_ d4nqga_ d1wdcb_
d2mysc_ d1j7qa_ d1s6ia_ d1uhka2 d1r2ua_ d1dtla_ d3kf9a_ d3pn7c_
d1fi6a_ d1c07a_ d5d67d_ d1tuza_ d1h8ba_ d1eg3a1 d1eg3a2 d2zkmx1
d1snla_ d1sfsa_ d1jfxa_ d1wkya2 d1uuqa_ d3wh9a_ d4m1ra_ d1xyza_
d4xuya_ d2je8a5 d4bf7a1 d3cuia_ d1ur4a_ d1qw9a2 d1odza_ d2vzsa5
d1bqca_ d2x2ya1 d1hjqa_ d1qnra_ d4y7ea1 d1vjza_ d4k68a_ d2v3ga_
d4hpga_ d1nofa2 d1nq6a_ d1h4pa_ d1aq0a_ d3ziza_ d4bs0a_ d1ecea_
d1h1na_ d1ta3b_ d1w91a2 d1i1wa_ d1uhva2 d1egza_ d7a3ha_ d1tvna1
d3nyda1 d1ghsa_ d2cyga1 d4xv0a_ d2c8na2 d1w32a_ d3emca_ d1v6ya1
d1foba_ d2uwfa1 d2fgla_ d3niya1 d2c0ha1 d1rh9a1 d1yq2a5 d1szna2
d1fa2a_ d1pama4 d5do8a1 d1ea9c3 d1hx0a2 d2fhfa5 d1qhoa4 d1avaa2
d1m53a2 d4m8ua1 d2gjpa2 d1lwha2 d4howa1 d1ktba2 d1j0ha3 d3vm5a1
d2x1ia_ d1wzaa2 d1uasa2 d2diea1 d1mxga2 d1ht6a2 d1eswa_ d1iv8a2
d5cpsa_ d1ud2a2 d1zjaa1 d3gbea1 d1kwga2 d1wdpa_ d1gcya2 d1g5aa2
d1hvxa2 d3dhpa2 d2bhua3 d4do4a1 d3qgva1 d1e43a2 d1g94a2 d2d3na2
d1gjwa2 d1gvia3 d1x1na1 d1r7aa2 d1tz7a1 d4c7ga2 d2gjxa1 d1nowa1
d1jaka1 d1yhta1 d4toqa_ d1wb0a1 d1w9pa1 d1eoka_ d1itxa1 d1kfwa1
d1goia2 d1edqa2 d2gsja_ d1hjxa1 d1jnda1 d1vf8a1 d3wdpp1 d3wq5a_
d1gnxa_ d4gxpa_ d4hz8a_ d1e4mm_ d3aiua_ d1wcga1 d1qvba_ d3ahxa_
d1v02a_ d2dgaa_ d1vffa1 d1qoxa_ d4ha4a_ d1mixa2 d1e5wa2 d1j19a2
d1isna2 d2al6b2 d3syxa1 d1mkea1 d2p8va_ d1xoda1 d1i7aa_ d1evha_
d1egxa_ d1xl4a1 d1n9pa_ d3vsqa_ d2gixa1 d2v76a_ d1wj1a1 d1aqca_
d1qqga2 d1qqga1 d1n3ha_ d4dbba_ d3f0wa1 d1p3ra_ d1wgua1 d2cy4a1
d3hqca_ d1wvha1 d1xr0b1 d3nvva1 d1krha3 d2h88b1 d1n62a2 d1t3qa2
d1zoyb1 d1jq4a_ d1ffva2 d1dgja2 d1rm6c2 d1h6ta1 d1h6ua1 d9ldta1
d2ewda3 d1up7a1 d2dfda1 d1s6ya1 d1vjta1 d7mdha1 d1hyea1 d1t2da1
d1ojsa3 d1b8pa1 d1ldna1 d1hyha1 d1civa1 d2a92a1 d1ez4a1 d1i0za1
d1y6ja1 d1gv0a1 d1ur5a1 d1llda1 d1obba1 d1v8ba1 d1gdha1 d2dlda1
d1mx3a1 d1ygya1 d1qp8a1 d1li4a1 d1j4aa1 d2naca1 d1npya1 d3t4ea2
d1a4ia1 d1o0sa1 d2hk9a2 d2egga2 d1b26a1 d1bvua1 d1gq2a1 d1c1da1
d1leha1 d1gtma1 d1nvta1 d3dona2 d1edza1 d1gpja2 d1vi2a1 d1p77a1
d1nyta1 d1b0aa1 d1vl6a1 d1v9la1 d1txga2 d3f47a1 d1mv8a2 d2pv7a2
d1xc2a3 d2ahra2 d4xiya1 d2f1ka2 d1pgja2 d1i36a2 d1np3a2 d1lj8a4
d1teaa3 d1qmga2 d2i76a2 d2g5ca2 d1wdka3 d1dlja2 d3cuma3 d1kola2
d1f8fa2 d1qora2 d1jqba2 d1vj0a2 d1cdoa2 d1gu7a2 d1d1ta2 d1xa0a2
d2dm6a2 d1jvba2 d1rjwa2 d1llua2 d1tt7a2 d1piwa2 d1o89a2 d1iz0a2
d1e3ja2 d1yb5a2 d1uufa2 d1vj1a2 d1e3ia2 d3gpdg1 d1vc2a1 d2nvwa1

d1u8fo1 d1ydwa1 d1hdgo1 d2g17a1 d1cf2o1 d2czca2 d1r0ka2 d1obfo1
d2cvoa1 d1ebfa1 d1h6da1 d1i32a1 d1zh8a1 d2q49a1 d1qkia1 d1j5pa4
d3pzra1 d1xeaa1 d1vkoa1 d1vm6a3 d3qvsa1 d2pkqo1 d1b7go1 d1gr0a1
d1p1ja1 d1lc0a1 d1j0xo1 d3pwka1 d2hjsa1 d1zyao1 d1vkna1 d1tlta1
d1nvmb1 d3sjua_ d3ojfa1 d1xu9a1 d2jjya1 d1n7ha_ d1y1pa1 d1h5qa_
d1jtva_ d4fs3a_ d2bkaa1 d1gy8a_ d2c5aa1 d1ulsa_ d1ooea_ d2b69a1
d1y5ma1 d1spxa_ d1ae1a_ d1bxka_ d1ydea1 d1gega_ d1hdoa_ d1o5ia_
d1e6ua_ d1snya1 d2rhca_ d3zv4a_ d5ceja_ d2d1ya1 d1fjha_ d3w8ea_
d2fmua1 d1x1ta_ d1rpna_ d3ay7a1 d1k2wa_ d1eq2a_ d2hrba1 d3ruaa_
d2ew8a1 d4hwka_ d1xgka_ d2bd0a1 d1yo6a1 d2a35a1 d2ntva_ d1vl8a_
d1hdca_ d4bkua_ d2ag5a1 d2q45a_ d1nffa_ d4iy1a_ d1zbqa1 d1kewa_
d1ek6a_ d1ja9a_ d2q46a2 d1iy8a_ d1cyda_ d2ptga_ d1hxha_ d1uzma1
d3lyla_ d4m89a_ d3wyea_ d2blla1 d1w6ua_ d1sb8a_ d2q2va_ d4nr0a_
d1e6wa_ d2gasa_ d3oiga1 d2bgka1 d1mxha_ d1vl0a1 d1rkxa_ d1r6da_
d1wmaa1 d1e7wa_ d2c07a1 d1xkqa_ d3tzha_ d1g0oa_ d3lz6a_ d1qyca_
d1edoa_ d1gz6a_ d1oc2a_ d1t2aa_ d1zema1 d2xoxa_ d1z45a2 d1p33a_
d3lt0a_ d4e6pa_ d2wyua_ d1db3a_ d4q9na_ d1xhla_ d4o1ma_ d1fmca_
d2uvda_ d2gdza1 d1uaya_ d2z1ma_ d2o23a2 d2fr1a2 d2fr1a1 d1qsga_
d1qyda_ d4zjua_ d2a4ka1 d1yb1a_ d1wvga1 d1yxma1 d3f4ba_ d1mg5a_
d1xg5a1 d2gfoa1 d1vjva_ d2ayna1 d3nhea_ d3u1ra1 d1g9ka2 d1kapp2
d1t3ca_ d1epwa3 d2a97a_ d3fiea_ g1e1h.1 d3ioqa_ d1fh0a_ d1o0ea_
d1khqa_ d1m6da_ d2cb5a_ d1cs8a_ g8pch.1 d1s4va_ d2pnsa_ d1vsna1
d1mira_ d1iwda_ d2oula_ d7pcka_ d3bwka_ d3bcna_ d2p86a_ d1pvja_
d1pcia_ d1gece_ d1deua_ d4bs6a_ d1xkga1 d1cqda_ d1jqpa2 d2c0ya_
d2bdza_ d3p5ua_ d1zoya1 d1qo8a2 d1d4ca2 d1chua2 d1jnra2 d2jk6a2
d2jk6a1 d1feca1 d1feca2 d1lpfa1 d1lpfa2 d1xhca1 d1xhca2 d1dxla2
d1dxla1 d1gera2 d1m6ia2 d1onfa1 d1onfa2 d3lada2 d3lada1 d1w4xa1
d1w4xa2 d1gesa2 d1mo9a1 d1xdia1 d1fl2a1 d1fcda1 d1fcda2 d1h6va2
d1h6va1 d1mo9a2 d1gv4a2 d1gv4a1 d2gv8a2 d2gv8a1 d1aoga1 d1aoga2
d1hyua1 d1ebda1 d1ebda2 d3dk9a1 d1q1ra2 d1q1ra1 d1gesa1 d1pv8a_
d1w5qa_ d2c1ha_ d1ohla_ d1zbfa1 d2z1ha_ d2etja1 d3qioa1 d1jl1a_
d2e4la_ d1jl2a_ d2lsna1 d1io2a_ d1uaxa_ d2yv0x_ d1mu2a1 d1ekea_
d1i39a_ d1pn0a1 d1reoa1 d1ju2a1 d2f5va1 d3c96a1 d2gmha1 d1pj5a2
d1o5wa1 d1n4wa1 d1k0ia1 d2dw4a2 d2ivda1 d1seza1 d2voua1 d1kdga1
d1s5ja1 d2hbja2 d2igia_ d1wlja_ d1qhta1 d4dlea1 d1tgoa1 d2f96a1
d3tr8a1 d3mxma_ d1y97a1 d1w0ha_ d1kfsa1 d2py5a1 d1noya_ d1d5aa1
d1yt3a3 d2guia_ d3mwpa1 d4kb1a_ d2hmta_ d1lssa_ d1iuka_ d2csua1
d3q9ub_ d2yv1a1 d3q9na_ d2d59a_ d1oi7a1 d2yv2a1 d1y81a1 d1wgqa1
d1v89a1 d2coda1 d1zc3b_ d1wg7a1 d1u5ea1 d1v61a1 d2coca1 d2ys1a1
d1v5pa1 d1ntya2 d1wjma1 d1wi1a1 d1u29a_ d1xx0a1 d1unqa_ d2zkmx3
d1fhoa_ d1v88a1 d2adza1 d1x1fa1 d1p6sa_ d2coaa1 d1btka_ d1v5ma1
d1faoa_ d2x18a_ d1dyna_ d1x1ga1 d4kaxb_ d2j59m1 d1ki1b2 d1xcga2
d1txda2 d1u5da1 d1w1ha_ d1upqa_ d1v5ua1 d1foea2 d2fjla1 d1eaza_
d2dfka2 d2cofa1 d1kz7a2 d1jmxa2 d1jmxa1 d1pbya1 d1r7ha_ d1faaa_

d1wika1 d1xfla_ d1ttza1 d2f51a1 d1ti3a_ d1nhoa_ d1sena_ d2i4aa_
d1ep7a_ d1zmaa1 d1fo5a_ d3lefa1 d3diea1 d2yana1 d1z6na1 d1oaza_
d3zywa1 d4aj8a_ d1nm3a1 d4fiwa_ d1wmja_ d4hs1a1 d1iloa_ d1xwaa1
d1wjka1 d2vm1a_ d1fb6a_ d1gh2a_ d1h75a_ d1nw2a_ d1kx5b_ d3a6na_
d4m6ba2 d2nqbd_ d3wkjd_ d2yfwe_ d4h9na_ d5bnxa_ d1kx5c_ d3r45a_
d1eqzb_ d4jjnb_ d1f66c_ d1eqza_ d4wnnb_ d1kx5a_ d1u35c1 d1id3a_
d2nqbc_ d1kx5d_ d3us3a3 d3us3a1 d2b5ea2 d2b5ea3 d2b5ea1 d2b5ea4
d2kp1a1 d2kp2a1 d1hyua3 d1hyua4 d1j08a1 d1j08a2 d1pd212 d1nhya2
d2a2ra2 d1k0dd2 d1f2ea2 d1gsua2 d1f3ba2 d2fhea2 d2pvqa1 d1tw9a2
d1oyja2 d1e6ba2 d1k0ma2 d3ik7a1 d4is0a1 d1r5aa2 d1jlva2 d1m0ua2
d3gtub2 d1z9ha2 d1okta2 d1axda2 d2gsra2 d1tu7a2 d1k3ya2 d1zgma1
d1b48a2 d1gwca2 d2gsta2 d1glqa2 d1oe8a2 d1pn9a2 d1v2aa2 d1fw1a2
d1duga2 g1f8v.1 d1novc_ d2bbvc_ d1qqya_ d1gd6a_ d1ivma_ d2gv0a_
d1iiza_ d2z2fa_ d1j8xa_ d1ghla_ d1b9oa_ d174la_ d169la_ d2o4wa_
d1p37a_ d1k28a3 d1xjta1 d1jtma_ d2f2qa_ d4e97a1 d2za0a_ d2c21a1
d2pjsa1 d1klla_ d2i7ra1 d1xqaa1 d5cj3a1 d1npba_ d1jifa_ d2p7oa_
d1r9ca_ d1nkia_ d1mpya2 d1kw3b2 d1kw3b1 d4ghga1 d1tfza1 d3isqa1
d3isqa2 d1sqia1 d1sqia2 d1lkda2 d1lkda1 d1mpya1 d1cjxa2 d1cjxa1
d1sp9b2 d1t47a1 d1t47a2 d1f1ua2 d1sp8a1 d1sp8a2 d1f1ua1 d1etob_
d1umqa_ d1g2ha_ d1iyka2 d1rxta1 d1iica2 d1iica1 d1qsta_ d2beia1
d4m3sa_ d1u6ma1 d1ygha_ d1yk3a1 d3dr6a_ d1xmta_ d1z4ea1 d2ge3a1
d1bo4a_ d2fiaa1 d2jdca1 d1y9wa1 d3to7a_ d1wwza1 d2fl4a1 d2ozua1
d2b5ga_ d2cy2a1 d2p0wa_ d4pswa_ d1yrea1 d1yr0a1 d1m4ia_ d2gana1
d1yvka1 d1ghea_ d2g3aa1 d1y7ra1 d2euia1 d1q2ya_ d2fsra1 d1xeba_
d2ae6a1 d1cjwa_ d1qsma_ d2aj6a1 d1y9ka1 d1i12a_ d1vkca_ d2atra1
d1n71a_ d2d4pa1 d2fiwa1 d2fe7a1 d1tiqa1 d2ou2a_ d1yx0a1 d2fcka1
d1p0ha_ d2j8ma_ d2pq8a_ d1z4ra1 d1s3za1 d1ufha_ d2hqya1 d2hqya2
d1lrza2 d1lrza3 d1ne9a2 d1f37b_ d1xvqa1 d1pn0a2 d3hy2a_ d2cvba1
d1o8xa_ d1lu4a_ d1we0a1 d2bmxa1 d2a4va1 d1psqa_ d1xiya1 d1q98a_
d1n8ja_ d1zofa1 d2ggta_ d3tjja_ d4je1a1 d1o73a_ d3s9fa_ d2zcta_
d1qmva_ d1jfua_ d2b1ka_ d5dvba1 d2b5xa1 d2v2ga_ d3tb2a_ d4llra_
d4fh8a1 d1e2ya_ d1knga_ d2h30a_ d1i5ga_ d2z9sa_ d2f8aa1 d1tp9a1
d2b7ka_ d1nm3a2 d3hvva_ d2wfca1 d4kcea_ d1xvwa1 d4gqca1 d3qpma_
d4k1fa_ d1zzoa1 d1gp1a_ d1prxa_ d1zsqa1 d2qked_ d4ksoa_ d1t4za1
d1wwja_ d1f74a_ d3l21a_ d2e1da_ d1to3a1 d1w3ia_ d1sfla_ d1n7ka_
d1vcva1 d4imea_ d1wa3a_ d2ocza1 d1f2ja_ d3m16a_ d2yxga_ d1ub3a_
d3a5fa_ d1fbaa_ d1l6wa_ d1xfba1 d1wx0a1 d1vpxa1 d1ojxa_ d1mxsa_
d2a4aa1 d1xkya1 d3cq0a1 d3s1xa_ d1fdja_ d3cpra1 d4d2ja_ d4mh8a_
d1mu2a2 d4i7ga1 d1etha2 d1w52x1 d2ppla1 d1hpla2 d1lpbb2 d2oxea1
d1bu8a2 d1rp1a2 d1ys1x_ d4hs9a_ d1ispa_ d1ex9a_ d1ku0a_ d3qmma_
d1lgya_ d4l3wa_ d1llfa_ d1gz7a_ d1ukca_ d4wfia_ d4cg1a1 d3visa_
d1c4xa_ d1j1ia_ d2rhwa_ d1ra6a_ d1muka_ d3gsza_ d1khva_ d1te9a_
d2xwha_ d3uqsa_ d3hkwa_ d3n6ma_ d2cjqa_ d2xyma_ d1teba_ d1te8a_
d1s48a_ d1u09a1 d4wfxa_ d3tyqa_ d2pgga1 d3zxha_ d1cgla_ d1rm8a_

d1eaka2 d1hy7a_ d2jsda_ d1cxva_ d1hv5a_ d1i76a_ d1m4la_ d2bk7a_
d3fjua_ d1z5ra1 d1kwma1 d1uwya2 d1jqga1 d2c1ca1 d1dtda_ d1h8la2
d2tbvc_ d1c8nc_ d1x36a_ d1smvc_ d1ng0c_ d4sbvc_ d1opoa_ d1f2nc_
d1e57b_ d1auyb_ d1f15b_ d1za7a_ d1laja_ d1rypd_ d1irua_ d1rypc_
d3unfk_ d1rypa_ d1j2pa_ d1irui_ d1j2qh_ d3unfh_ d1m4ya_ d1rypb_
d1irul_ d1irug_ d1iru2_ d1q5rh_ d1rypg_ d1g3ka_ d1q5qa_ d1e94a_
d1irub_ d1ryph_ d3ty6a_ d1iruj_ d1iruk_ d1rype_ d1irud_ d1irue_
d3mi01_ d1rypf_ d1iruh_ d2z3ba_ d1iruc_ d3mka2_ d1iru1_ d1ny721
d1ny722 d1a6ca3 d1a6ca2 d1pgl22 d1pgl21 d1pgl11 d1ny711 d1mqta_
d1d4m3_ d1fmd3_ d2mev1_ g1aym.1 d1ev13_ d4q4w1_ d1r1a3_ d2x5ia_
d1rhi1_ g1r1a.1 g1d4m.1 g1rhi.1 d1b35b_ d1oopa_ d1tme1_ d4iv1a_
g1pvc.1 g1fpn.1 d1fpn1_ d4gb31_ d1d4m1_ d1fmd1_ d2wzr3_ g1cov.1
d1qqp3_ g1fmd.1 d2x5ic_ d1rhi3_ g1oop.1 d1hxs1_ g1hxs.1 d1fpn3_
d1bev1_ g2mev.1 d1eah3_ d1aym1_ d1pvc3_ d1qqp1_ d1zba3_ d1h8ta_
d3vbhc_ g1b35.1 g1bev.1 d1hxs3_ g1ev1.1 d1bev3_ g1tmf.1 d1tmf1_
d1b35a_ d2mev3_ g1eah.1 g1h8t.1 d1eah1_ d4q4w3_ d1r1a1_ d1aym3_
d1pvc1_ d1zba1_ d1h8tc_ d1oopc_ d1tme3_ g1qqp.1 d4iv1c_ g1tme.1
d3ubub_ d1egia_ d1qo3c_ d1v7pa_ d1tdqb_ d3ubua_ d1c3ab_ d1h8ua_
d3bx4b_ d1t8ca1 d1sb2a1 d1qdda_ d2ziba1 d3cada_ d1byfa_ d1j34b_
d1jwia_ d1jzna_ d1c3aa_ d1oz7b_ d1wmza_ d3bdwb_ d1hq8a_ d1fifa1
d1dv8a_ d3hupa_ d1wk1a1 d1umra_ d2afpa_ d3bx4a_ d1gz2a_ d1sl6a_
d1v7pb_ d3g8ka_ d1ixxa_ d1j34a_ d1jwib_ d1g1sa1 d1y17a1 d1ja3a_
d1fvub_ d3bdwa_ d1sb2b1 d1fvua_ d1umrc_ d1oz7a_ d4m17a_ d3cfwa1
d1ypqa1 d1g1ta1 d3fd4a_ d3l9jc_ d4dn8a1 d1v4lb_ d1rdl1_ d2msba_
d3ia2a_ d1a88a_ d4dgqa1 d1hkha_ d1zoia_ d1ux8a_ d1dlya_ d4i0va_
d2bkma_ d1dlwa_ d2bmma_ d2r80b_ d1cqxa1 d1cg5a_ d1gcvb_ d3boma_
d2h8fb_ d2raoa_ d3bj1a_ d4hrrb_ d2r50a_ d3d1kb_ d2aa1b1 d3zhwa_
d3hf4b_ d3gdja_ d1jebb_ d3bj1b_ d1x9fb_ d1wmub_ d1gcva_ d3bcqb_
d3d4xb_ d1gvha1 d2dc3a_ d3hf4a_ d4yu3a_ d3k8ba_ d1qpwb_ d2r80a_
d3bcqa_ d1v4wa_ d3wtgb_ d1jl7a_ d4esaa_ d1a9we_ d3d1ab_ d1fsla_
d4h2la_ d1v4wb_ d1mwca_ d1vhba_ d1jeba_ d1spgb_ d1h97a_ d1q1fa_
d1tu9a1 d3d1aa_ d1hdsb_ d3hyub_ d2zfbb_ d3fs4a_ d1oj6a_ d1shrb_
d4h2lb_ d4yu3b_ d1spga_ d3hrwa_ d3qqra_ d3at5a_ d4wchd_ d3hyua_
d1cg5b_ d1uc3a_ d1hbra_ d2raob_ d1outb_ d4esab_ d3gdjb_ d3qqqa_
d1x9fc_ d3vrfa_ d1itha_ d3mkba_ d3d4xa_ d3mjpa_ d1jl6a_ d3fh9a_
d1outa_ d2dc3b_ d1it2a_ d3eoka_ d3g46a_ d1qpwa_ d3vrfb_ d1x9fd_
d1hdsa_ d2zfba_ d3mkbb_ d1or4a_ d3wtga_ d1a4fa_ d3fh9b_ d3bomb_
d3cy5a_ d3at5b_ d1x9fa_ d2oifa_ d4lmxb_ d1cpcb_ d3v57a_ d1kn1a_
d1cpca_ d4f0ta_ d2vjha_ d4po5b_ d1eyxa_ d1alla_ d4f0tb_ d1b33b_
d1b8db_ d4h0mb_ d1b33a_ d2vjhb_ d4lm6b_ d2vmlb_ d1allb_ d2vjta_
d3dbja_ d2vmla_ d2vjtb_ d4f0ub_ d1f99b_ d4rmpa1 d1liaa_ d1eyxb_
d2uulb_ d4lmsb_ d4f0ua_ d2bv8a_ d1f99a_ d2bv8b_ d2c7lb_ d3dbjb_
d4rmpb1 d1b8da_ d4h0ma_ d3v57b_ d1liab_ d2uula_ d2j96a_ d1azwa_
d1mtza_ d1sfra_ d1f0na_ d4qeka_ d3c8da2 d2pbla1 d1jkma_ d1jjia_

d1lzla_ d1gkub1 d1gkub2 d2qvba_ d2v9za_ d4kafa1 d1b9ra_ d1offa_
d1frra_ d3wcqa_ d1e9ma_ d1l5pa_ d1wria_ d1i7ha_ d3av8a_ d1czpa_
d1rfka_ d3p1ma1 d3b2ga_ d2bt6a2 d4iuaa3 d4bvva_ d5hpga_ d3k65a_
d1ki0a3 d1ki0a2 d1ki0a1 d1a0ha1 d1pmla_ d3sp8a3 d2feba1 d2m5ta_
d1cqqa_ d1wqsa_ d5bpea_ d2ipha_ d1lvoa_ d2bhga1 d3tnta_ d3sjia_
d3d23a1 d2fyqa_ d3tloa_ d3zvea1 d3q3ya1 d4asha1 d1cg2a1 d1yloa2
d1y7ea2 d1y0ya3 d1lfwa1 d1vixa1 d1fnoa4 d1vhoa2 d1ysja1 d1vhea2
d1z2la1 d2fvga2 d1r3na1 d2grea2 d1xmba1 g1q7l.1 d1rtqa_ d3rp2a_
g1ucy.1 d1fuja_ d2zchp_ d3w94a_ d1si5h_ d1lo6a_ g2pka.1 d1cgha_
d1kigh_ d1a5ia_ d3s9ba_ d1hj8a_ d1ao5a_ d2anya_ d1q3xa1 d1gvza_
d1azza_ d4gsoa_ d2f91a1 d1iaua_ d1gdna_ d1eufa_ g1fiw.1 d1eq9a_
d4nfea_ d2rdla_ d1spja_ d1elva1 d1a7sa_ d1ekbb_ d1pfxc_ d1yc0a_
d2aiqa_ g1fiz.1 d1fi8a_ d4k60a_ d4dgja_ d1mzaa_ d1op0a_ d3tjva_
d1pytd_ d1sgfg_ g1gg6.1 d2eeka_ g1rtf.1 d1a0ja_ d3g01a_ d1pytc_
d4e7na_ d1ddja_ d1z8ga1 d2hlca_ d1npma_ g1jou.1 d1sgfa_ d1autc_
g2hnt.1 g1kdq.1 d1orfa_ d3s69a_ d4crga_ d1fxya_ d1bqya_ d2ftba_
d1pmpa_ d2rcqa1 d1fdqa_ d4lkpa_ d1sa8a_ d1vyfa_ d1lpja_ d3vg7a1
d4azqa1 d1o8va_ d5bvqa_ d1p6pa_ d2qo4a_ d1opba_ d1yiva1 d1o1va_
d4bvma1 d4tkba_ d4i3ba_ d1bwya_ d1ggla_ d4gkca_ d1cbia_ d3p6da1
d1kqwa_ d2a0aa1 d1dzka_ d4tlja_ d2a2ua_ d4wfva_ d3t1da_ d4iaxa_
d3zq3a_ d2nnda_ d1i4ua_ d1kxoa_ d1bbpa_ d1kt7a_ d1exsa_ d3s26a1
d1lf7a_ d1qfta_ d4gh7a_ d1z24a1 d1qabe_ d1xkia_ d1ew3a_ d3bx7a_
d4iawa1 d1n0sa_ d1gkab_ d3kzaa_ d2k23a_ d2l9ca1 d2joza1 d1gm6a_
d3kffa_ d3saoa1 d1e5pa_ d1yupa1 d3lgia_ d3i77a_ d2z9ia2 d4nsva_
d1lcya2 d4jcna_ d1agja_ d1os8a_ d1hpga_ d1p3ca_ d1l1ja_ d1qtfa_
d1e69a_ d1mv5a_ d3g60a4 g1ii8.1 d1ewqa2 d1ji0a_ d1qhla_ d1v43a3
d2wmfa1 d1vpla_ d1g2912 d4a82a2 g1f2t.1 d2d62a1 d3tuic1 d2bbsa1
d3g60a3 d2ixea_ d1g6ha_ d1sgwa1 d2qi9c_ d3rlfa1 d3b60a1 d3d31a2
d2pzea1 g1xew.1 d1oxxk2 d2onka1 d1w1wa_ d1iwea_ d1qf5a_ d1vmaa2
d1nija1 d1np6a_ d2p67a1 d3ihla_ d1okkd2 d1ls1a2 d1s1ma2 d1iona_
d2v40a_ d1dj3a_ d3hida_ d2vo1a1 d2g0ta1 d1vcoa2 d1j8mf2 d1g3qa_
d1xjca_ d1p9ba_ d1qzxa3 d1ihua2 d1ihua1 d2qm8a_ d1dj2a_ d4ioja_
d1cp2a_ d1yrba2 d1hyqa_ d3gqba2 d1skye3 d1xp8a1 d1tf7a2 d1tf7a1
d1ye8a1 d1skyb3 d2qe7d2 d4blta_ d4huta_ d3ldaa2 d4hyya_ d2i3ba1
d1cr1a_ d3j9tb2 d3ntua2 d1vdza1 d2z43a_ d1e9ra_ d1nlfa_ d3ziaa2
d1n0wa_ d3ziad2 d3j9ta2 d1pzna2 d3gqbb2 d2i49a_ d1nnfa_ d1xvxa_
d1ursa_ d1h3da1 d1iuda_ d4m1va1 d1twya_ d3e13x_ d2nxoa1 d4pfua_
d3ttma_ d1elja_ d2qmwa1 d1us5a_ d1y3na1 d1jeta_ d1uqwa_ d4f19a1
d2esna2 d3thia_ d3fasa1 d2uyeb_ d2xwva1 d1r9la_ d2fyia1 d1pb7a_
d2ozza1 d1sw5a_ d1nh8a1 d1vr5a1 d2g29a_ d1xs5a_ d3lswa_ d2z23a_
d3w9va_ d3tcha_ d1q35a_ d1z7me1 d3q27a_ d1p99a_ d1ve4a1 d2i6ea1
d4mcoa1 d4n8ya_ d1eu8a_ d1zbma1 d1hsla_ d1ixca2 d1j1na_ d1xvya_
d1y9ua_ d2z22a1 d1xoca1 d4oera_ d1wdna_ d5f1qa1 d1xt8a1 d1i6aa_
d4gwoa_ d4igta1 d1pc3a_ d4mmpa1 d1o63a_ d2czla1 d2onsa2 d4mija1

d4ok5a1 d2bmfa1 d2bmfa2 d1yksa1 d1yksa2 d1jr6a_ d2qeqa1 d2jlqa1
d1eh3a_ d3skpa_ d1dtza1 d1ieja_ d1jnfa2 d1ce2a1 d1jnfa1 d1b1xa2
d1b1xa1 d1jw1a1 d1jw1a2 d1ryoa_ d1h76a1 d1h76a2 d1c4oa1 d2p6ra3
d2p6ra4 d1oywa2 d1oywa3 d1wrba1 d1w36b2 d1w36b1 d1c4oa2 d3bora_
d1t6na_ d2eyqa5 d2eyqa3 d2eyqa2 d2zu6a2 d1veca_ d1uaaa1 d1w36d1
d2g9na1 d1z3ix2 d2rb4a_ d1hv8a1 d1hv8a2 d1t5ia_ d1w36c2 d2j0sa2
d2j0sa1 d1fuka_ d3hjha1 d1z5za1 d1z63a1 d1s2ma2 d1s2ma1 d1wp9a1
d1wp9a2 d1gm5a3 d1gm5a4 d3dina4 d3dina3 d1q0ua_ d1w36c1 d1z3ix1
d1nkta3 d1nkta4 d1uaaa2 d1w36d2 g1qhh.1 d2fwra2 d2fwra1 d2fkza_
d1zuja1 d1s3qa1 d2fjca1 d1z6om1 d1veia_ d4dyya_ d1jiga_ d3gvya_
d2pyba_ d4cy9a1 d3bvfa1 d1r03a_ d2cf7a_ d1nf4a_ d1zs3a1 d4iwka_
d3ka8a_ d3np0x_ d1n1qa_ d1lb3a_ d4e6ka_ d1z6oa1 d2fzfa1 d4dyua_
d3ge4a_ d1jgca_ d4am4a_ d2z6ma_ d1ji5a_ d1vlga_ d1lkoa1 d1krqa_
d3qz3a_ d1o9ra_ d2ffxj_ d1euma_ d1yv1a1 d2v2pa_ d1j30a_ d4di0a_
d1dpsa_ d1k8rb_ d1wgra1 d1ef5a_ d1wfya1 d1wgya1 d1lfda_ d2cs4a1
d1wxma1 d2byea1 d3kh0a1 d2byfa1 d1wxaa1 d1e7ua3 d1wgda1 d1wgga1
d4q5eb_ d1wjna1 d3n3kb_ d1wz0a1 d1we6a1 d2m0xa_ d2gbka_ d3rt3b1
d1wh3a1 d1sifa_ d2ekec2 d4ii2b1 d4hk2a1 d1c3ta_ d1m94a_ d1wgha1
d1ttna1 d1j8ca_ d1wx8a1 d3v6cb1 d2ma9b_ d4i6lb1 d1bt0a_ d2jvca1
d1iyfa_ d2k1fa_ d1wxva1 d1wf9a1 d1t0ya_ d2io0b2 d1x1ma1 d2gbma_
d1zw7a1 d2m17a_ d1ud7a_ d1we7a1 d2lwpa_ d2gbja_ d1wjua1 d1v6ea1
d1v86a1 d4eewa_ d1wiaa1 d4icva_ d4gu2a_ d1wx7a1 d1wy8a1 d4hjka1
d2faza1 d1v5oa1 d3ruib_ d1eo6a_ d3waob_ d2r2qa1 d3m95a_ d3wana_
d1yvca1 d1yeza1 d3h33a_ d1rwja_ d19hca_ d1os6a_ d3bxua_ d3h4na_
d1i77a_ d3caoa_ d1j0pa_ d2ctha_ d1duwa_ d1ofwa_ d2e84a_ d1hh5a_
d1gyoa_ d3h34a_ d1dszb_ d1a6ya_ d3cbba_ d1ynwa1 d1r4ia_ d1hcqa_
d2hana_ d2hanb_ d2enva1 d1kb4b_ d2nllb_ d1lo1a_ d1cita_ d1um8a_
d1ixza_ d1qvra2 d1qvra3 d1qzma_ d1sxjc2 d1jr3d2 d2p65a_ d1l8qa2
d3hu3a3 d1sxjd2 d1njfa_ d1in4a2 d1sxja2 d1tuea1 d2ce7a2 d1w5sa2
d1sxjb2 d1d2na_ d1ny5a2 d1ofha_ d2gnoa2 d2a5yb3 d1fnna2 d1u0ja1
d1lv7a_ d1iqpa2 d2fnaa2 d1ixsb2 d4gita_ d1sxje2 d1g41a_ d1mbma_
d3su6a1 d1cu1a1 d4agka_ d1vcpa_ d1q31a_ g1n1l.1 d3e90b_ d1ep5a_
d3u1jb_ d2e3fa_ d1mm0a_ d2e3ea_ d2llda_ d2ny8x_ d2e3ga_ d2ny9x_
d2nz3a_ d2lj7a_ d2m8bb1 d2kyqa_ d3psma_ d1jkza_ d1jxca_ d2gl1a_
d1ugla_ d4uj0a_ d2k4ua_ d1hp2a_ d2lu9a_ d1baha_ d2meoa1 d1wm7a_
d1hlya_ d1n8ma_ d1jlza_ d1wpda_ d1c55a_ d1pvza_ d1wt7a_ d1rjia_
d1wz5a_ d1m2sa_ d1pjva_ d1qkya_ d2ktca1 d1wmta_ d1du9a_ d1j5ja_
d1r1ga_ d1xwdc1 d3kj4a_ d1ozna_ d1w8aa_ d2ifga3 d1xkua_ d1t0za_
d2i61a_ d1t1ta_ d1sn4a_ d1kv0a_ d2lkba_ d1npia_ d1wwna_ d1pe4a_
d2kbka_ d2li7a_ d2kbha_ d1dq7a_ d1sega_ d1omya_ d1zywa1 d1jzaa_
d1n9wa1 d1bbua1 d1e1oa1 d1c0aa1 d1l0wa1 d3a74a1 d1eova1 d1t61c1
d1iyjb4 d1iyjb5 d1iyjb3 d1o7ia_ d4ipca1 d1x3ea_ d4apva_ d1jb7a1
d1jb7a2 d1jb7a3 d3kdfa_ d1ue1b_ d1wjja1 d3eiva_ d1xjva2 d1xjva1
d2ccza2 d1se8a_ d1l1oc_ d1jb7b_ d1qvca_ d3ulla_ d1qzga_ d1miua5

d1miua3 d1y14b1 d2nn6i1 d1i94l_ d2zjra2 d1smxa_ d2qamc2 d1hh2p1
d2r7da1 d1rl2a2 d1wfqa1 d3bzka4 d1x6oa2 d3a0ja_ d2j01d2 d2ja9a1
d4ql5a_ d1hr0w_ d1luza_ d2n3sa_ d2vnud1 d1h95a_ d1go3e1 d2nn6h1
d1ne3a_ d2mo1a_ d1iz6a2 d1jjga_ d1e3pa2 d2ix0a3 d2ix0a2 d2lxja_
d1ueba3 d1ueba2 d2z0sa1 d1u0la1 d2ix0a1 d2eifa2 d2je6i1 d3i2za1
d2c35b1 d2ahob2 d1t9ha1 d3i4oa_ d2qall1 d1jt8a_ d2nn6g1 d1g6pa_
d3cama_ d1i94q_ d1c9oa_ d1d7qa_ d2ba0a1 d2vnud2 d2vnud3 d1khia2
d2qalq1 d1wi5a1 d1xtda2 d1ny4a_ d1fvia1 d1ckma1 d1p16a1 d1x9na2
d1pfsa_ d2a1ka_ d1ohea1 d1ohea2 d2f4da_ d1rxda_ d2hxpa_ d1xria_
d1d5ra2 d1fpza_ d3ezza_ d4y2ea_ d1i9sa_ d3azya_ d3wvja_ d1upsa1
d1ajoa_ d2hyka_ d2vy0a_ d1o4ya_ d3o5sa_ d3d6ea_ d3atga_ d1dypa_
d1umza_ d3i4ia_ d4lbqa_ d1a78a_ d1slta_ d4no4a_ d3zsja_ d3duia_
d1g86a_ d1is3a_ d1qmja_ d1hlca_ d1tdha3 d1q3ba3 d1ee8a3 d1k82a3
g1qmo.1 g1len.1 d3zyra_ d1fnya_ d1n47a_ g2ltn.1 d1avba_ d3usua_
d1dhkb_ d1f9ka_ d1ukga_ d1g8wa_ d1g9fa_ d1wbfa_ d3wcsa_ d1g7ya_
g1loe.1 d1mvqa_ d1hqla_ d1v6ia_ d1dbna_ d1fx5a_ d4l8qa_ d1ioaa_
d3n35a_ d3ubra_ d1qo8a1 d1d4ca1 d2k3va_ d1ft5a_ d1qdba_ d1m1qa_
d2j7aa_ d1h21a_ d1ogyb_ d4fasa_ d1oaha_ d1sp3a_ d2h0ba_ d2erfa_
d1h30a2 d1h30a1 d3mw4a1 d1pz7a_ d2r1ba_ d3v64a1 d1d2sa_ d3bopa1
d3mw3a1 d1okqa1 d1okqa2 d1wioa4 d1ccza2 d1vcaa1 d1ic1a1 d2d9qb1
d2nxyb2 d1adqa1 d1fnga1 d1uvqb1 d1zs8a1 d2vola1 d2vola2 d1mfbl2
d2jelh2 d1uwel2 d3qq4b1 d1kjvb_ d1k5na1 d1osph2 d4odhl2 d1ow0a1
d1ow0a2 d1bfob2 d3l9ra2 d3jtth1 d2fath2 d4elkb2 d1fn4b2 d2xfxb_
d1pg7x2 d1cqka_ d4k71b2 d1lk2a1 d1eapb2 d4lfhg2 d1i4fa1 d2nw2a2
d1kfah2 d1mhca1 d1h3ua1 d1wejh2 d1h0db2 d1a5fh2 d1kcvh2 d1mjuh2
d1qqda1 d1kcrh2 d1k8ia1 d3huja2 d2nnab1 d1nfdf2 d3mffb2 d1s9va1
d1lk3h2 d1q0xl2 d1fv1b1 d1neza1 d4rfeb2 d1r3ha1 d1a6tb2 d1kjva1
d1pz5b2 d1onqa1 d2bc4a2 d1de4a1 d1k8da1 d1tcra2 d1dn0b2 d1nfde2
d2xwtb2 d1bfoa2 d1mexh2 d1iakb1 d1ypzf2 d4f7ea2 d1q72l2 d2fbjh2
d1fe8h2 d1clzh2 d1fngb1 d1uvqa1 d1vgka1 d1c16a1 d4j12a2 d4x99b2
d1igtb3 d1je6a1 d1q72h2 d1nldh2 d1teth2 d2hmic2 d1i1ca2 d1i1ca1
d1k8ib1 d1q0yh2 d1kfal2 d3frua1 d1pg7w2 d1u58a1 d1igyb4 d1igyb3
d1hyrc1 d1klub1 d1deeb2 d1c12b2 d2bc4b2 d1kcvl2 d3wn5b1 d1lk2b_
d1lk3l2 d1mjul2 d1sm3h2 d1q0xh2 d1zvsa1 d1ydpa1 d1u8ib2 d1ypze2
d1kcuh2 d1op9a_ d1y0la1 d1etzb1 d1egjl1 d2gsib1 d1nj9a1 d4jfhd1
d1aqkl1 d1t2ja_ d1lo4h1 d1zvya1 d3v4vl1 d4enec1 d1kxtb_ d5e8ea1
d2pxya_ d4weud_ d1xiwc_ d2esvd1 d1qnzh_ d1ogae1 d1f4xh1 d4n1hb_
d3ezjb1 d3mlwl1 d1kfah1 d1etza1 d1b4jh1 d3cfic_ d4w68a_ d2arja1
d1ek3a_ d2ok0l1 d1vera_ d1uwgh1 d1blnb1 d1fpth1 d1rzga1 d2giya1
d4m3kb1 d1nc2a1 d2nyyd1 d1ncna_ d1fbih1 d5dxwa_ d4fqqa1 d1dqdh1
d1jfql1 d1kelh1 d1faih1 d1frgl1 d1figl1 d4m6nl1 d1fo0a_ d4hxbl1
d3b5ga_ d3quyd1 d2h9gb1 d1i8kb_ d2dd8h1 d2ck0h1 d1eaja_ d4w6wb1
d2a6ib3 d1t66d1 d2e27h_ d1ob1b1 d1f11b1 d2v7na1 d1ad0a1 d2xt1b1
d1pskh1 d4i13b1 d2op4l1 d1a0ql1 d1uwel1 d1za3a1 d1ncbl1 d4uu9a_

| | | | | | | | |
|---|---|---|---|---|---|---|---|
| d1bm3h1 | d1cloh1 | d1nqba2 | d1cf8h1 | d4y5va_ | d1mhph1 | d5anma1 | d4cdgc1 |
| d4ldeb_ | d1y0lb1 | d2pcpb1 | d1z3gl1 | d1dfbl1 | d1fh5h1 | d3g5xa1 | d1tzib1 |
| d4dn3l1 | d3zhka1 | d2b1hh1 | d2uyla1 | d1f3dh1 | d1nj9b1 | d2agjh1 | d2h1pl1 |
| d1q72h1 | d8fabb1 | d1ndmb1 | d1rjca1 | d4u6hb1 | d3upca_ | d2fbjl1 | d1osph1 |
| d1tvda_ | d2cjvh_ | d4fhbd1 | d1bd2d1 | d1rz7h1 | d3nn8a_ | d1blna1 | d1knoa1 |
| d1nfda1 | d2bmkb1 | d1dqll_ | d1igyb1 | d1igfl1 | d12e8h1 | d3utza1 | d1nc2b1 |
| d1rihh1 | d4mwfb1 | d4jvpa1 | d1mieh1 | d1rhfa1 | d1fgnl1 | d1sbsh1 | d1lp9e1 |
| d1dn0a1 | d4lqfl1 | d1iaih1 | d1kgcd1 | d1mh5b1 | d1gpol1 | d2g60h3 | d1f8tl1 |
| d2jixd1 | d4grwh_ | d1fo0b_ | d1bfob1 | d1axsa1 | d1u9ka_ | d1nmbh_ | d1h8na1 |
| d1h8na2 | d1jguh1 | d4eura1 | d3sdyl1 | d35c8h1 | d1w72l1 | d1mvub_ | d1i9rh1 |
| d1ob1a1 | d4wenb_ | d1yeeh1 | d3ietc1 | d1f58h1 | d1ad0b1 | d1mqkl_ | d1nbvh1 |
| d4b50a_ | d3r8bb_ | d1bafl1 | d1mamh1 | d4rgne1 | d2gsgb_ | d35c8l1 | d1bafh1 |
| d1ai1h1 | d2pcpa1 | d2fath1 | d2x1pa_ | d1orsb1 | d1ibgl1 | d1shma_ | d1a2yb_ |
| d1u0qa_ | d2coqa_ | d1hilb1 | d1qfwi_ | d1jv5b_ | d1fn4b1 | d5e0qa_ | d1nsnh1 |
| d1plgl1 | d4ocxl1 | d1kb5h1 | d1rzfh1 | d1pg7x1 | d2fx7h1 | d2oi9c_ | d1l7il1 |
| d3eyva1 | d1d5il1 | d4ffyl_ | d1tetl1 | d1a6wh_ | d1ac6a_ | d3dvfa_ | d1nfde1 |
| d1mimh1 | d1ri8a1 | d1jhlh_ | d3dura_ | d1fskb1 | d1f97a1 | d3so3b1 | d2ak4d1 |
| d1lp9f1 | d4z7wf1 | d1l7th1 | d4y5ya_ | d4x7ec1 | d1emth1 | d1ikfl1 | d1nlbh1 |
| d4utal1 | d1bjma1 | d1eapb1 | d1u6ah1 | d2dtgd1 | d1kj2b_ | d1xeda1 | d1c1eh1 |
| d1fgvh_ | d2bnub1 | d2fb4h1 | d2dtgb1 | d1ktrh_ | d1jpth1 | d1qkzl1 | d4hepg_ |
| d2atpb1 | d1flrh1 | d2hrpl1 | d1mvfa_ | d1iaim1 | d3k80a1 | d2jb5h1 | d3zkxc_ |
| d4grwe_ | d1it9h1 | d1rhhb1 | d3w9ec_ | d1e4wh1 | d1sy6h1 | d2j6eh1 | d3qxta_ |
| d1jglh1 | d1p7ka1 | d1tzhb1 | d4n8pa1 | d1gafl1 | d2jelh1 | d1wioa2 | d4i0cc1 |
| d1cicb1 | d1lmka2 | d1lmka1 | d1iqwh1 | d1orqb1 | d1ejoh1 | d1jnlh1 | d4zg1a_ |
| d43c9b_ | d1ggbl1 | d1wejh1 | d3q6fa1 | d1f3dj1 | d3c6la1 | d1bqlh1 | d3p9wb_ |
| d1a3rl1 | d1h0db1 | d2uzil_ | d1eo8l1 | d1qfwl_ | d1n7ml1 | d1igja1 | d1c5ch1 |
| d2a9mh_ | d1rmfh1 | d1fj1b1 | d4heme_ | d1ccza1 | d1a5fh1 | d1ogad1 | d1dlfh_ |
| d1qa9b_ | d1vgel1 | d3qskb_ | d1sjxa_ | d4ozfg1 | d1sq2n_ | d1qd0a_ | d1kcvh1 |
| d1c5db1 | d3qcul1 | d8faba1 | d3bn9d1 | d1mjuh1 | d4y8dc_ | d7fabh1 | d3dvgb1 |
| d1lo0l1 | d1a6tb1 | d1gigh1 | d1kcrh1 | d2hwzl1 | d1yjdc1 | d3clfl1 | d4krna_ |
| d1dl7l_ | d1e6ol1 | d1mexh1 | d3r0ma_ | d4kkna_ | d1fnsl1 | d1oaqh_ | d1rzia1 |
| d1ucbh1 | d1ncwl1 | d4b41a_ | d1nfdf1 | d2mpah1 | d2d7tl1 | d4grwf_ | d4b5ea_ |
| d1lk3h1 | d3u79a_ | d4u3xa_ | d1ghfl1 | d1bvkb_ | d1mcww1 | d1b88a_ | d1jrhh1 |
| d32c2b1 | d4wemb_ | d1dsfh_ | d1op3h1 | d1ce1l1 | d1iqda1 | d1qoka1 | d1qoka2 |
| d1qfuh1 | d2crya1 | d1ay1h1 | d1oaql_ | d3ab0b_ | d1rurl1 | d1t4kb1 | d1bz7b1 |
| d1n0xh1 | d1forl1 | d1ucbl1 | d4w6yb_ | d43c9a_ | d2aabl1 | d1tzia1 | d4n1ca_ |
| d1k6qh1 | d3g9ab_ | d4tvsa1 | d1igmh_ | d1pz5b1 | d3dx9a1 | d3wxwh_ | d1nakh1 |
| d1kcul1 | d3eaka_ | d4qlra1 | d1cr9l1 | d1d9ka_ | d1f4xl1 | d1deea1 | d2i27n2 |
| d1c12a1 | d2orbl1 | d1j8hd1 | d1mcph1 | d1c5da1 | d1kenh1 | d1bd2e1 | d1h5ba_ |
| d4x7fc1 | d3upaa_ | d1t3fa1 | d1indh1 | d2dtgc1 | d4hc1l1 | d1zv5a1 | d4wgvb_ |
| d1uwgl1 | d4f37k1 | d1aqkh1 | d1vesa_ | d3zkqd_ | d1fptl1 | d1lo4l1 | d1knob1 |
| d3rrqa_ | d1ct8b1 | d4orzc_ | d1mlbb1 | d4hgkc1 | d1dqdl1 | d1bogb1 | d4ozhe1 |
| d1dr9a1 | d1i7za1 | d1dn0b1 | d1kell1 | d1rzib1 | d1ypzf1 | d1wtla_ | d1hkfa_ |
| d1fail1 | d1figh1 | d2vyre_ | d1sjva_ | d1ohqa_ | d1bfoa1 | d1pkqb1 | d1mjul1 |

| | | | | | | | |
|---|---|---|---|---|---|---|---|
| d1b4jl1 | d1nsnl1 | d2arjb1 | d1p4bh_ | d4aj0a_ | d3nn8b_ | d2rcja1 | d1f2xk_ |
| d4v1dd_ | d1adqh1 | d2eksa_ | d4k3ga_ | d1jfqh1 | d1uweh1 | d1frgh1 | d4gftb_ |
| d1uz8b1 | d4n1ea_ | d1bm3l1 | d1clol1 | d3mcka1 | d2vq1a1 | d1ae6h1 | d4aq1b1 |
| d1uj3b1 | d1nbqa1 | d1fh5l1 | d2ck0l1 | d2ywza_ | d1q72l1 | d4nbxb_ | d1hila1 |
| d4hzlb1 | d2j88l1 | d1zmya1 | d1cd0a_ | d1a4ka1 | d1pskl1 | d1a0qh1 | d4krmb_ |
| d1ncbh1 | d2fbjh1 | d1j8he1 | d1i3ua_ | d1cf8l1 | d1fe8h1 | d2p1ya1 | d1eeqa_ |
| d2x89a_ | d1ol0a_ | d2r0lh1 | d1ub5a1 | d1dfbh1 | d3eyvb1 | d4jdvb1 | d4p2cg_ |
| d2gsia1 | d2agjl1 | d1u3ha1 | d2h1ph1 | d1rihl1 | d1fskc1 | d1boga1 | d2a77l1 |
| d1ospl1 | d1qfoa_ | d3dwta_ | d2fx7l1 | d1rz7l1 | d3stba1 | d1pkqa1 | d1b6da1 |
| d1dzba2 | d1dzba1 | d1kj2a_ | d4l1ha_ | d1rzgb1 | d1dqlh_ | d12e8l1 | d1miel1 |
| d1jmaa_ | d1sbsl1 | d1fgnh1 | d1f58l1 | d1mqkh_ | d1ndgb1 | d3qegl1 | d1iail1 |
| d2aw2a1 | d1tqbc1 | d3fo2a1 | d4dkaa_ | d1f8th1 | d2g60l1 | d2ywya_ | d1uj3a1 |
| d1yc7a_ | d3bd3a_ | d4w6xb1 | d1nmbl_ | d1i8ka_ | d1rjlb1 | d1ypze1 | d1p7kb1 |
| d1kxqe_ | d1gpoh1 | d2z4qa1 | d1w72h1 | d1i9rl1 | d4qgya1 | d4xt1c_ | d2qqnh1 |
| d1cica1 | d3c08h1 | d2cd0a_ | d2bdnh1 | d1hnga1 | d1maml1 | d1nldh1 | d1cr9h1 |
| d4lcih1 | d3bkyh1 | d3qg7l1 | d1ai1l1 | d1kxvc_ | d4iosd_ | d1l7ih1 | d2x1qa_ |
| d1ub6b1 | d3k74b_ | d1ibgh1 | d1teth1 | d1igya1 | d1hxma1 | d2x6ma_ | d2e7la_ |
| d5e03a_ | d1qfwm_ | d2r0kh1 | d1igjb1 | d1rurh1 | d1plgh1 | d1mj8h1 | d1yuhb1 |
| d3fmgl1 | d1rzfl1 | d1emtl1 | d1ad9b1 | d1igtb1 | d1nlbl1 | d1d5ih1 | d3k7ua_ |
| d4ffyh_ | d1bwwa_ | d3dvga1 | d1a6ta1 | d1fgvl_ | d4qxgl1 | d1miml1 | d1jhll_ |
| d2hffb1 | d2hh0l1 | d1qkzh1 | d1l7tl1 | d1afvh1 | d1iaii1 | d2cjuh_ | d1um5h1 |
| d2ghwb2 | d2ghwb1 | d1kgce1 | d2fgwh1 | d1ikfh1 | d1dvfd_ | d2i24n1 | d2xtjb_ |
| d1za3b1 | d2yc1a_ | d2hh0h1 | d2x1oa_ | d1bvka_ | d1rjla1 | d5e7ba1 | d1igta1 |
| d1il1b1 | d2fb4l1 | d1mvua_ | d1jptl1 | d32c2a1 | d1fvca_ | d1iqdb1 | d2hrph1 |
| d2p4ab_ | d4qo1a_ | d1p2cb1 | d1wt5c_ | d1t4ka1 | d5c1mb_ | d1it9l1 | d1bz7a1 |
| d1b2wh1 | d1e4wl1 | d1jnll1 | d1yedb1 | d1za6b1 | d3u7aa_ | d1igfh1 | d3gnml1 |
| d1orsa1 | d1wejl1 | d1a2ya_ | d1hxmb1 | d1gafh1 | d2j4wh1 | d1pz5a1 | d1j05a_ |
| d1a3rh1 | d4fqll1 | d1qfwh_ | d1eo8h1 | d1n7mh1 | d4s11a_ | d1kegh1 | d5da4a_ |
| d1iqwl1 | d3whxb1 | d1vgeh1 | d1deeb1 | d1c12b1 | d1t2ql1 | d1ad9a1 | d3gsnb1 |
| d3b2uc1 | d1ggbh1 | d1t3fb1 | d1nakl1 | d4ocmc_ | d1lila1 | d2v7hb3 | d2esve1 |
| d1mcoh1 | d2qr0b1 | d2q3aa_ | d1ct8a1 | d1kcrl1 | d2pxyb_ | d2uzih_ | d5c3ld_ |
| d3durb_ | d1c5cl1 | d2ak4e1 | d1dl7h_ | d1xiwd_ | d1mlba1 | d1rmfl1 | d1i7zb1 |
| d1dlfl_ | d2xa3a_ | d5bq7b1 | d1dqqb1 | d3h42l1 | d1egjh1 | d1kcvl1 | d1fe8l1 |
| d2j6el1 | d1f3rb2 | d1lk3l1 | d1sm3h1 | d1q0xh1 | d1ghfh1 | d7fabl1 | d1igch1 |
| d1lo0h1 | d4idla_ | d5c2bl_ | d1jrhl1 | d1il1a1 | d3zhda1 | d4kfzc_ | d1b0wa_ |
| d1e6oh1 | d1mexl1 | d4krpb_ | d1fnsh1 | d1ncwh1 | d4hgma_ | d4eigb1 | d4rzca_ |
| d2mpal1 | d4y5xa_ | d5en2b1 | d3lrga1 | d4ej1c1 | d1rhha1 | d1j05b_ | d1forh1 |
| d1q9wb1 | d2wzpd_ | d2aq2a1 | d1h0da1 | d3revb1 | d2ij0c1 | d1tzha1 | d4m7jl1 |
| d1k6ql1 | d3b9kc1 | d1igml_ | d3qeua1 | d1dsfl_ | d1ce1h1 | d1kcuh1 | d1zvha1 |
| d1zvoa1 | d1tqbb1 | d1ay1l1 | d2qscl1 | d2cgrh1 | d1nl0h1 | d1kenl1 | d3eo0a1 |
| d4n9ob1 | d1n0xl1 | d3k1kc_ | d1c4qa_ | d1prtc1 | d2wv6d_ | d1prtf_ | d1ltrd_ |
| d4l63b1 | d1r4pb_ | d1prtd_ | d1tl0f_ | d2bosa_ | d1prtb1 | d2aq2b1 | d1ck1a1 |
| d1enfa1 | d1i4pa1 | d1v1oa1 | d1et9a1 | d1esfa1 | d2g9hd1 | d4ohja1 | d1klud1 |
| d1bxta1 | d1eu3a1 | d1ty0a1 | d1fnua1 | d2cqva1 | d1fhga_ | d2k1ma_ | d2dl2a1 |

d1biha3 d1biha1 d1biha4 d3qp3a1 d1g1ca_ d1gxea_ d1cvsc2 d1cvsc1 d1xmwa2 d1m4ka2 d2otpa2 d1biha2 d1rhfa2 d2fcba2 d2fcba1 d1wwbx_ d1gsma2 d1epfa1 d1epfa2 d2dava1 d1waaa_ d1f97a2 d1x44a1 d1vcaa2 d1fnla2 d1fnla1 d3b5ha2 d3h8na2 d3h8na1 d1jbja1 d1jbja2 d1pd6a_ d1f2qa2 d1f2qa1 d3cx2a_ d1nbqa2 d3b5ha1 d1n26a1 d1xiwb_ d1xaua_ d1fltx_ d2ifga1 d1olla1 d1olla2 d1cs6a4 d1cs6a3 d1cs6a2 d1cs6a1 d2gw5a2 d1sy6a1 d1xiwa_ d1ucta2 d1ucta1 d1ry7b2 d1gl4b_ d1qz1a3 d1olza1 d2c9aa1 d1wwca_

**Family-37 (b.50.1.2)**

**Positive dataset contains 9 protein sequences.**

d2qzxa_ d2asia_ d2h6ta_ d3hd8a_ d4aa9a_ d1t6ex_ d1wkra_ d1tzsa_ d2qzwa_

**Negative dataset contains 4045 protein sequences.**

d1m32a_ d1ibja_ d1c7na_ d1iuga_ d4q75a_ d2bkwa1 d1t3ia_ d1n8pa_ d1qz9a_ d1jf9a_ d1eg5a_ d1p3wa_ d3kgwa_ d1cl1a_ d2ctza1 d2rfva_ d1gc0a_ d1cs1a_ d1d2fa_ d2ch1a1 d1qgna_ d1pffa_ d1elua_ d2xlba_ d3m83a1 d1u08a_ d1lc5a_ d1b5pa_ d2ay1a_ d1bw0a_ d3pd6a_ d7aata_ d2csta_ d1j32a_ d1fg7a_ d4wlha_ d1o4sa_ d1v2da_ d1gdea_ d1m6sa_ d1v72a1 d1svva_ d1ajsa_ d1wsta1 d2f8ja2 d1lk9a_ d1vp4a1 d1xi9a1 d2gb3a1 d1yaaa_ d2cfba1 d1dfoa_ d1o69a_ d1bj4a_ d3k28a_ d2bwpa_ d3lg0a_ d2fnua_ d3ou5a_ d1z7da1 d1iaya_ d4msoa_ d1bs0a_ d1s0aa_ d1sffa_ d3tqxa_ d1mdoa_ d3qboa_ d3pgya_ d1bjna_ d2dkja_ d1ejia_ d1vefa1 d4wxga_ d3bs8a_ d3gbxa1 d1w23a_ d1qnia1 d1ibya_ d1f56a_ d1bypa_ d1azca_ d1ws8a_ d3u25a_ d1cuoa_ d1id2a_ d1rkra_ d2iaac_ d4dp1x_ d1nwpa_ d2gima_ d2i7sa_ d1qhqa_ d3ehbb1 d1fftb1 d1hfua2 d1kbva1 d1kbva2 d1sdda1 d1sdda2 d1gyca1 d1gyca2 d1mzya1 d2bw4a1 d1aoza1 d1aoza3 d1aoza2 d1kyaa2 d1kyaa3 d1kyaa1 d1mzya2 d4e9sa1 d1sddb1 d1sddb2 d1hfua1 d1hfua3 d1oe1a2 d1oe1a1 d1v10a2 d1v10a3 d1v10a1 d2dara2 d2dara1 d1wyha2 d2dloa2 d1rutx4 d1rutx3 d2cuqa2 d1x4la2 d1x4la1 d1x3ha2 d1x3ha1 d2d8za1 d1x61a2 d1x6aa1 d1x6aa2 d2cora2 d2cora1 d1v6ga1 d1v6ga2 d2d8za2 d1x64a1 d1x64a2 d1b8ta1 d2co8a1 d1x63a2 d2dj7a2 d2dj7a1 d2cu8a2 d2cu8a1 d1g47a2 d1g47a1 d1j2oa1 d1wyha1 d2dloa1 d1wiga1 d1wiga2 d2cupa2 d2cupa3 d1x68a1 d2d8ya2 d2d8ya1 d1x62a1 d1x68a2 d1x61a1 d1b8ta3 d1b8ta2 d2d8xa2 d2d8xa1 d1x4ka1 d1x4ka2 d2cuqa1 d1m3va1 d2co8a2 d1j2oa2 d1x63a1 d2cura1 d2cura2 d1x62a2 d3sxwa1 d4luka_ d2bema_ d1pama1 d1ea9c1 d1qhoa1 d2fhfa2 d2fhfa1 d1k3ia1 d1j0ha1 d1cxla1 d2bhua1 d1edqa1 d1f1sa2 d1ut9a2 d1gvia1 d1txka1 d4alac_ d1rera1 d1s6na_ d3uzqb_ d1jgta2 d1ofda3 d1ea0a3 d1ecfa2 d1ct9a2 d1ao0a2 d1te5a_ d1ep0a_ d2pa7a1 d1wlta1 d1nxma_ d2c0za1 d1nepa_ d1ds6b_ d1fstb_ d1ex0a1 d1g0da1 d4w9ta_ d4x2ra1 d1ka9f_ d1qo2a_ d4axka1 d1thfd_ d1h5ya_ d4u28a_ d2y88a_ d4wd0a_ d1vzwa1 d1tafa_ d1bh9b_ d1tafb_ d1jfib_ d1h3ob_ d2byka1 d1n1ja_ d4g92b_ d1bh9a_ d1jfia_ d1h3oa_ d2bykb1 d1n1jb_ d2bjca1 d2hsga1 d1qpza1 d1ydwa2 d2nvwa2 d1h6da2 d1zh8a2

| | | | | | | | |
|---|---|---|---|---|---|---|---|
| d1qkia2 | d1xeaa2 | d1tlta2 | d2ovga_ | d1sq8a_ | d1rzsa_ | d3kz3a_ | d3orca_ |
| d2q49a2 | d1hdgo2 | d2g17a2 | d2cvoa2 | d2czca1 | d3pwka2 | d1obfo2 | d1i32a2 |
| d1zyao2 | d2pkqo2 | d1b7go2 | d2hjsa2 | d1cf2o2 | d1vkna2 | d1nvmb2 | d1ux8a_ |
| d4i0va_ | d2bkma_ | d1dlwa_ | d2bmma_ | d2msya_ | d2djoa1 | d2r5za_ | d1au7a1 |
| d1f43a_ | d2lmda1 | d1akhb_ | d1s7ea1 | d3nara_ | d1zq3p1 | d2craa1 | d1mija_ |
| d4uuta_ | d3a01a_ | d1x58a1 | d3rkqa1 | d3cmya1 | d2cqxa1 | d2da6a1 | d1e3oc1 |
| d1fjla_ | d1mh3a1 | d1ig7a_ | d1x41a1 | d1wh7a1 | d1gt0c1 | d2mw8a_ | d1lfup_ |
| d1x2ma1 | d1b72a_ | d1wi3a1 | d1jgga_ | d1wjha1 | d1x2na1 | d1mnmc_ | d2cuea1 |
| d3k2aa_ | d2hosa_ | d2jwta2 | d1pufa_ | d1octc1 | d2hi3a_ | d1b8ia_ | d4j19a_ |
| d3lnqa_ | d1wh5a1 | d1b8ib_ | d1fexa_ | d2cqra1 | d1ug2a1 | d2cqqa1 | d2ajea1 |
| d4a69c_ | d1h8ac1 | d2crga1 | d2iw5b1 | d1xc5a1 | d1ofcx1 | d2cu7a1 | d1wgxa1 |
| d2cjja1 | d2ckxa1 | d1gvda_ | d1tc3c_ | d1gdta1 | d1u78a2 | d1xg1a1 | d1hlva1 |
| d1hlva2 | d1iufa2 | d1iufa1 | d2iu5a1 | d1zk8a1 | d2g7sa1 | d2fd5a1 | d2np5a1 |
| d1z0xa1 | d2id6a1 | d2d6ya1 | d2i10a1 | d1vi0a1 | d2oi8a1 | d3c07a1 | d2ns7a1 |
| d2fq4a1 | d2yvea1 | d1t33a1 | d2o7ta1 | d1rkta1 | d2np3b1 | d2g7ga1 | d2gena1 |
| d2fbqa1 | d2g7la1 | d2gfna1 | d2g3ba1 | d2jj7a1 | d2hyja1 | d1sgma1 | d1ui5a1 |
| d2hkua1 | d2id3a1 | d4jyka1 | d1bl0a1 | d1bl0a2 | d1d5ya1 | d1prtb2 | d3lv8a_ |
| d1s3ga1 | d1zaka1 | d1s4qa_ | d3tvta2 | d1uf9a_ | d1khta_ | d1ckea_ | d1osna_ |
| d1p5zb_ | d4tmka_ | d3cm0a_ | d1tmka_ | d1jaga_ | d3h86a_ | d1t0hb_ | d1nn5a_ |
| d2h92a_ | d3kb2a1 | d2c95a1 | d1yj5a2 | d1kgda_ | d1ly1a_ | d2ak3a1 | d1vyva2 |
| d1deka_ | d2grja1 | d1p3ja1 | d1y63a_ | d1e4ya1 | d1s96a_ | d3iija1 | d1lvga_ |
| d1nksa_ | d1vyua2 | d4e22a_ | d1qf9a_ | d1jjva_ | d1q3ta_ | d1ki9a_ | d1lw7a2 |
| d1teva_ | d1kjwa2 | d1gsia_ | d1x6va3 | d1g3ma_ | d1j99a_ | d1t8ta1 | d1nsta_ |
| d1fmja_ | d1zrha_ | d1ls6a_ | d2a3ra_ | d2zptx_ | d3ckla_ | d1texa_ | d1q44a_ |
| d2gwha_ | d1odfa_ | d1uj2a_ | d4gi7a_ | d2jeoa1 | d1rz3a_ | d1k6ma1 | d1z0ja1 |
| d3umra2 | d3gj0a1 | d2gaoa1 | d1yzga1 | d1x3sa1 | d1g7sa4 | d1puja_ | d1udxa2 |
| d1kk1a3 | d2o52a_ | d1jwyb_ | d1zcaa2 | d1zcba2 | d2fv8a1 | d2cjwa_ | d3dz8a1 |
| d1xzpa2 | d1vg8a_ | d4qxaa_ | d2ew1a1 | d1x1ra1 | d2bcgy_ | d3doea1 | d3cona_ |
| d3rega_ | d3wyga_ | d1upta_ | d2b6ha1 | d1zd9a1 | d1lnza2 | d1r5ba3 | d1n0ua2 |
| d1r2qa_ | d2f9la1 | d2rhda_ | d3q3jb_ | d3q85a_ | d1efma1 | d1r8qa_ | d2gf9a1 |
| d2akab1 | d1ek0a_ | d1z08a1 | d2gcoa1 | d2dy1a2 | d4g01b_ | d2cxxa1 | d1svia_ |
| d3q72a_ | d1ni3a1 | d4ac9a4 | d2erxa1 | d2bmea_ | d1wxqa1 | d2gf0a1 | d4ldja1 |
| d1zunb3 | d1d5ca_ | d4lv5b_ | d1efca3 | d1kmqa_ | d2g6ba1 | d3lrpa_ | d1tada2 |
| d2y8ea_ | d4rd4a1 | d1z06a1 | d2heia_ | d3a58b_ | d1u0la2 | d1d2ea3 | d1zj6a1 |
| d2ocba_ | d3rwoa_ | d1mr3f_ | d2e9sa1 | d4u5xa1 | d2iwra_ | d2fn4a1 | d3bwdd_ |
| d2nzja1 | d2atva1 | d2ksqa_ | d3l43a_ | d1mkya2 | d1mkya1 | d1wmsa_ | d1jnya3 |
| d2rmka_ | d1f6ba_ | d1f60a3 | d1gota2 | d1jala1 | d2fh5b1 | d1g16a_ | d2wblc_ |
| d1m7ba_ | d1wf3a1 | d1f5na2 | d1t9ha2 | d1z6xa1 | d1z0aa1 | d2fg5a1 | d2a5ja1 |
| d2bcjq2 | d1s0ua3 | d1ky3a_ | d4y0va_ | d4lhwa_ | d2qmea1 | d2atxa1 | d1egaa1 |
| d1ryha_ | d1moza_ | d1nrjb_ | d2erya1 | d3bfka_ | d1puia_ | d3j3az1 | d3t5ga_ |
| d1shza2 | d3defa_ | d2f7sa1 | d3raba_ | d3tsoa_ | d3aq4a_ | d2al7a1 | d3sfva1 |
| d3bc1a_ | d2ncda_ | d1sdma_ | d3h4sa1 | d2heha_ | d2grya_ | g3kin.1 | d1kk8a2 |
| d1br2a2 | d1goja_ | d1f9va_ | d2mysa2 | d1lkxa_ | d3t0qa_ | d4db1a1 | d3gbja_ |
| d1ry6a_ | d1v8ka_ | d1k8ka2 | d1k8ka1 | d3uleb1 | d3i33a2 | d3i33a1 | d1bupa1 |

d1dkgd2 d1dkgd1 d4ci6a2 d1jcfa1 d4a2ba2 d2fxua1 d1yaga2 d1yaga1
d4cbua2 d1bupa2 d3vrda1 d3vrda2 d1fcdc2 d1h1oa2 d1h1oa1 d1m70a2
d1m70a1 d1iqca2 d1iqca1 d1eb7a1 d1nmla2 d1nmla1 d1hroa_ d4gedb_
d3zooa_ d1mz4a_ d1gu2a_ d1f1fa_ d1dvva_ d1c6ra_ d1c75a_ d1e29a_
d1gdva_ d1cnoa_ d1h32b_ d1co6a_ d2bh4x_ d2aiua_ d5cytr_ d1jdla_
d1i8oa_ d2zboa_ d3dmia_ d1ls9a_ d1wvec_ d2yk3a_ d3ph2b_ d1dw0a_
d5aura_ d1ql3a_ d1qn2a_ d1f1ca_ d2nqya_ d1h4ga_ d1hixa_ d1oa2a_
d1olra_ d3b7ma_ d4h7ma1 d3wp3a_ d2vgda_ d1igoa_ d3zsea_ d2nlra_
d3wx5a_ d1oa3a_ d1pvxa_ d1m4wa_ d1f5ja_ d1oa4a_ d1ks5a_ d4ixla1
d2ah2a1 d1evsa_ d1cnt1_ d1pvhb_ d1z7ca_ d1huwa_ d1bgea_ d1rhga_
d1f6fa_ d1f45b_ d1i1rb_ d1axia_ d1etea_ d3tgxb_ d1scfa_ d2z3qa1
d3qb1a_ d1hula_ d1m48a_ d4nkqb_ d1eera_ d1v7mv_ d5e4ea_ d2o27a_
d3b5ka_ d2psma1 d4zf7a_ d3uf5a_ d4x51a_ d1d9ca_ d4ypgc1 d3oq3a_
d1n1fa_ d1au1a_ d1wu3i_ d3besl_ d3ux9a_ d1v8fa_ d2ejca_ d3muea1
d1ihoa_ d3q12a1 d4dnxa2 d1m8pa2 d1v47a2 d1jhda2 d1x6va2 d3rbaa_
d2qtra_ d1nupa_ d3l92a_ d1f9aa_ d1qjca_ d2h29a_ d1vlha_ d1od6a_
d1lw7a1 d1mrza2 d4ruka_ d2el7a_ d1iq0a2 d1irxa2 d1r6ta2 d1ivsa4
d2cyba_ d1rqga2 d1j09a2 d2yxna_ d1f7ua2 d1h3fa1 d1ffya3 d2g36a1
d1nzja_ d1jila_ d1n3la_ d1i6la_ d1pfva2 d1h3na3 d3n9ia_ d3sz3a1
d3sw2a_ d1moxc_ d1dx5i3 d1edmb_ d1autl2 d1autl1 d1nqlb_ d1dval1
d1szba2 d1kigl_ d2y5fl_ d1ijqa2 d1gk5a_ d1g1sa2 d1nt0a3 d1xdtr_
d1lmja2 d1lmja1 d1nzia2 d2wphe_ d1klil_ d1pfxl2 d1pfxl1 d1p9ja_
d1g1ta2 d1f7ea_ d4yzub_ d1m1xb4 d1qx2a_ d1ig5a_ d2y5ia_ d1qlsa_
d1psra_ d3n22a_ d2egda_ d4ggfc_ d2wcea_ d2luca_ d4aqia_ d3nsia_
d2lp3a_ d1j55a_ d1a4pa_ d1k8ua_ d1nsha_ d1pvaa_ d3fs7a_ d1ttxa_
d1rwya_ d2kyfa_ d1rk9a_ d1a75a_ d1exra_ d1tiza1 d1nyaa_ d1g8ia_
d1ggwa_ d1wdcc_ d3fwba_ d1ggza_ d2o5ga_ d1ctaa_ d1m45a_ d3i5gc_
d1sl8a_ d1fpwa_ d2scpa_ d2roaa_ d4aqra_ d3pn7b_ d1br1b_ d2mysb_
d1s6ca_ d1bjfa_ d2obha_ d1jfka_ d1jf0a_ d1qv0a_ d1lkja_ d2mlfc_
d2m97a_ d2d8na_ d4by5a_ d1jbaa_ d4nqga_ d1wdcb_ d2mysc_ d1j7qa_
d1s6ia_ d1uhka2 d1r2ua_ d1dtla_ d3kf9a_ d3pn7c_ d1qjta_ d1fi6a_
d1c07a_ d5d67d_ d1tuza_ d1h8ba_ d1eg3a1 d1eg3a2 d2zkmx1 d1snla_
d1gjya_ d1y1xa_ d1hqva_ d1dvia_ d1df0a1 d1qxpa2 d4usla_ d1k94a_
d1wkya2 d1uuqa_ d3wh9a_ d4m1ra_ d1xyza_ d4xuya_ d2je8a5 d4bf7a1
d3cuia_ d1ur4a_ d1qw9a2 d1odza_ d2vzsa5 d1bqca_ d2x2ya1 d1hjqa_
d1qnra_ d4y7ea1 d4k68a_ d2v3ga_ d4hpga_ d1nofa2 d1nq6a_ d1h4pa_
d1aq0a_ d3ziza_ d4bs0a_ d1ecea_ d1h1na_ d1ta3b_ d1w91a2 d1i1wa_
d1uhva2 d1us3a2 d1egza_ d7a3ha_ d1tvna1 d3nyda1 d1ghsa_ d2cyga1
d4xv0a_ d2c8na2 d1w32a_ d3emca_ d1v6ya1 d1foba_ d2uwfa1 d2fgla_
d3niya1 d2c0ha1 d1rh9a1 d1yq2a5 d1szna2 d1fa2a_ d1pama4 d5do8a1
d1ea9c3 d1hx0a2 d2fhfa5 d1qhoa4 d1avaa2 d1m53a2 d4m8ua1 d2gjpa2
d1lwha2 d4howa1 d1ktba2 d1j0ha3 d3vm5a1 d2x1ia_ d1wzaa2 d1uasa2
d2diea1 d1mxga2 d1ht6a2 d1eswa_ d1iv8a2 d5cpsa_ d1ud2a2 d1zjaa1
d3gbea1 d1kwga2 d1wdpa_ d1gcya2 d1g5aa2 d1hvxa2 d3dhpa2 d2bhua3

d4do4a1 d3qgva1 d1e43a2 d1g94a2 d2d3na2 d1gjwa2 d1gvia3 d1x1na1
d1r7aa2 d1tz7a1 g1apy.1 d3ljqa_ d4c7ga2 d2gjxa1 d1nowa1 d1jaka1
d1yhta1 d4toqa_ d1wb0a1 d1w9pa1 d1eoka_ d1itxa1 d1kfwa1 d1goia2
d1edqa2 d2gsja_ d1hjxa1 d1jnda1 d1vf8a1 d1etob_ d1umqa_ d1mixa2
d1e5wa2 d1j19a2 d1isna2 d2al6b2 d3syxa1 d1mkea1 d2p8va_ d1xoda1
d1i7aa_ d1evha_ d1qc6a_ d1egxa_ d1id1a_ d2hmta_ d1lssa_ d1iuka_
d2csua1 d3q9ub_ d2yv1a1 d3q9na_ d2d59a_ d2yv2a1 d1euca1 d1y81a1
d1wgqa1 d1v89a1 d2coda1 d1zc3b_ d1wg7a1 d1u5ea1 d1v61a1 d2coca1
d2ys1a1 d1v5pa1 d1ntya2 d1wjma1 d1wi1a1 d1u29a_ d1xx0a1 d1unqa_
d2zkmx3 d1fhoa_ d1v88a1 d2adza1 d1x1fa1 d1p6sa_ d2coaa1 d1btka_
d1v5ma1 d1faoa_ d2x18a_ d1dyna_ d1x1ga1 d4kaxb_ d2j59m1 d1ki1b2
d1xcga2 d1txda2 d1u5da1 d1dbha2 d1w1ha_ d1upqa_ d1v5ua1 d1foea2
d2fjla1 d1eaza_ d2dfka2 d2cofa1 d1kz7a2 d1h6ua1 d9ldta1 d2ewda3
d1up7a1 d2dfda1 d1s6ya1 d1vjta1 d7mdha1 d1hyea1 d1t2da1 d1ojsa3
d1b8pa1 d1ldna1 d1hyha1 d1civa1 d2a92a1 d1ez4a1 d1i0za1 d1y6ja1
d1gv0a1 d1ur5a1 d1llda1 d1obba1 d1npya1 d3t4ea2 d1a4ia1 d1o0sa1
d2hk9a2 d2egga2 d1b26a1 d1bvua1 d1gq2a1 d1c1da1 d1leha1 d1gtma1
d1nvta1 d3dona2 d1edza1 d1gpja2 d1vi2a1 d1p77a1 d1nyta1 d1b0aa1
d1vl6a1 d1v9la1 d1txga2 d3f47a1 d1mv8a2 d2pv7a2 d1xc2a3 d2ahra2
d4xiya1 d2f1ka2 d1pgja2 d1i36a2 d1np3a2 d1lj8a4 d1teaa3 d1qmga2
d2i76a2 d2g5ca2 d1wdka3 d3cuma3 d1kola2 d1f8fa2 d1qora2 d1jqba2
d1vj0a2 d1cdoa2 d1gu7a2 d1d1ta2 d1xa0a2 d2dm6a2 d1jvba2 d1rjwa2
d1llua2 d1tt7a2 d1piwa2 d1o89a2 d1iz0a2 d1e3ja2 d1yb5a2 d1uufa2
d1vj1a2 d1e3ia2 d3gpdg1 d1vc2a1 d2nvwa1 d1u8fo1 d1ydwa1 d1hdgo1
d2g17a1 d1cf2o1 d2czca2 d1r0ka2 d1obfo1 d2cvoa1 d1ebfa1 d1h6da1
d1i32a1 d1zh8a1 d2q49a1 d1qkia1 d3pzra1 d1xeaa1 d1vkoa1 d1vm6a3
d3qvsa1 d2pkqo1 d1b7go1 d1gr0a1 d1p1ja1 d1lc0a1 d1j0xo1 d3pwka1
d2hjsa1 d1zyao1 d1vkna1 d1tlta1 d1nvmb1 d3sjua_ d3ojfa1 d1xu9a1
d2jjya1 d1n7ha_ d1y1pa1 d1h5qa_ d1jtva_ d4fs3a_ d2bkaa1 d1gy8a_
d2c5aa1 d1ulsa_ d1ooea_ d2b69a1 d1y5ma1 d1spxa_ d1ae1a_ d1bxka_
d1ydea1 d1gega_ d1hdoa_ d1o5ia_ d1e6ua_ d1snya1 d2rhca_ d3zv4a_
d5ceja_ d2d1ya1 d1fjha_ d3w8ea_ d2fmua1 d1x1ta_ d1rpna_ d3ay7a1
d1k2wa_ d1eq2a_ d2hrba1 d3ruaa_ d2ew8a1 d4hwka_ d1xgka_ d2bd0a1
d1yo6a1 d2a35a1 d2ntva_ d1vl8a_ d1hdca_ d4bkua_ d2ag5a1 d2q45a_
d1nffa_ d4iy1a_ d1zbqa1 d1kewa_ d1ek6a_ d1ja9a_ d2q46a2 d1iy8a_
d1cyda_ d2ptga_ d1hxha_ d1uzma1 d3lyla_ d4m89a_ d3wyea_ d2blla1
d1w6ua_ d1sb8a_ d2q2va_ d4nr0a_ d1e6wa_ d2gasa_ d3oiga1 d2bgka1
d1mxha_ d1vl0a1 d1rkxa_ d1r6da_ d1wmaa1 d1e7wa_ d2c07a1 d1xkqa_
d3tzha_ d1g0oa_ d3lz6a_ d1qyca_ d1edoa_ d1gz6a_ d1oc2a_ d1t2aa_
d1zema1 d2xoxa_ d1z45a2 d1geea_ d1p33a_ d3lt0a_ d4e6pa_ d2wyua_
d1db3a_ d4q9na_ d1xhla_ d4o1ma_ d1fmca_ d2uvda_ d2gdza1 d1uaya_
d2z1ma_ d2o23a2 d2fr1a2 d2fr1a1 d1qsga_ d4zjua_ d2a4ka1 d1yb1a_
d1wvga1 d1yxma1 d3f4ba_ d1mg5a_ d1xg5a1 d4ekfa_ d2ckga_ d1th0a_
d2bkra_ d1cmxa_ d1xd3a_ d3ifwa_ d1ex0a4 d2f4ma1 d1x3za_ d1t3ca_

d1epwa3 d2a97a_ d3fiea_ d3ioqa_ d1fh0a_ d1o0ea_ d1khqa_ d1m6da_
d2cb5a_ g8pch.1 d1s4va_ d2pnsa_ d1vsna1 d1mira_ d1iwda_ d2oula_
d7pcka_ d3bwka_ d3bcna_ d2p86a_ d1pvja_ d1pcia_ d1gece_ d1deua_
d4bs6a_ d1xkga1 d1cqda_ d1jqpa2 d2c0ya_ d2bdza_ d3p5ua_ d2o36a_
d1k9xa_ d1zoya1 d1qo8a2 d1d4ca2 d1chua2 d1jnra2 d1kf6a2 d2jk6a2
d2jk6a1 d1feca1 d1feca2 d1lpfa1 d1lpfa2 d1xhca1 d1xhca2 d1dxla2
d1dxla1 d1gera2 d1m6ia2 d1onfa1 d1onfa2 d3lada2 d3lada1 d1w4xa1
d1w4xa2 d1gesa2 d1mo9a1 d1xdia1 d1fl2a2 d1fl2a1 d1fcda1 d1fcda2
d1h6va2 d1h6va1 d1mo9a2 d1gv4a2 d1gv4a1 d2gv8a2 d2gv8a1 d1aoga1
d1aoga2 d1hyua1 d1ebda1 d1ebda2 d3dk9a1 d1q1ra2 d1q1ra1 d1gesa1
d1pv8a_ d1w5qa_ d2c1ha_ d1ohla_ d1l6sa_ d1zbfa1 d2z1ha_ d2etja1
d3qioa1 d1jl1a_ d2e4la_ d1jl2a_ d2lsna1 d1io2a_ d1uaxa_ d2yv0x_
d1mu2a1 d1ekea_ d1i39a_ d1ps9a2 d1o94a2 d1cjca1 d1pn0a1 d1reoa1
d1ju2a1 d2f5va1 d3c96a1 d2gmha1 d1pj5a2 d1gpea1 d1o5wa1 d1n4wa1
d1k0ia1 d2dw4a2 d2ivda1 d1seza1 d2voua1 d1kdga1 d1s5ja1 d2hbja2
d2igia_ d1wlja_ d1qhta1 d4dlea1 d1tgoa1 d2f96a1 d3tr8a1 d3mxma_
d1q8ia1 d1y97a1 d1w0ha_ d2py5a1 d1noya_ d1d5aa1 d1yt3a3 d2guia_
d3mwpa1 d4kb1a_ d1b9ra_ d1offa_ d1frra_ d3wcqa_ d1e9ma_ d1l5pa_
d1wria_ d1i7ha_ d3av8a_ d1rfka_ d3p1ma1 d3b2ga_ d2bt6a2 d1mpya2
d1kw3b2 d1kw3b1 d4ghga1 d1tfza1 d3isqa1 d3isqa2 d1sqia1 d1sqia2
d1lkda2 d1lkda1 d1mpya1 d1cjxa2 d1cjxa1 d1sp9b2 d1t47a1 d1t47a2
d1f1ua2 d1sp8a1 d1sp8a2 d1jmxa3 d1pbya3 d1pbya4 d1r7ha_ d1faaa_
d1wika1 d1xfla_ d1ttza1 d2f51a1 d1ti3a_ d1nhoa_ d1sena_ d2i4aa_
d1ep7a_ d1zmaa1 d3lefa1 d3diea1 d2yana1 d1z6na1 d1oaza_ d3zywa1
d4aj8a_ d1nm3a1 d4fiwa_ d1wmja_ d4hs1a1 d1iloa_ d1xwaa1 d1wjka1
d2vm1a_ d1fb6a_ d1gh2a_ d1h75a_ d1nw2a_ d1kx5b_ d3a6na_ d4m6ba2
d2nqbd_ d3wkjd_ d2yfwe_ d4h9na_ d5bnxa_ d1kx5c_ d3r45a_ d1eqzb_
d4jjnb_ d1f66c_ d1eqza_ d4wnnb_ d1kx5a_ d1u35c1 d1id3a_ d2nqbc_
d1kx5d_ d2b5ea2 d2b5ea3 d2b5ea1 d2b5ea4 d2kp1a1 d2kp2a1 d1hyua3
d1hyua4 d1j08a2 d1pd212 d1nhya2 d2a2ra2 d1k0dd2 d1f2ea2 d1gsua2
d1f3ba2 d2fhea2 d2pvqa1 d1tw9a2 d1oyja2 d1e6ba2 d3ik7a1 d4is0a1
d1r5aa2 d1jlva2 d1m0ua2 d3gtub2 d1z9ha2 d1okta2 d1axda2 d2gsra2
d1tu7a2 d1k3ya2 d1zgma1 d1b48a2 d1gnwa2 d1gwca2 d2gsta2 d1glqa2
d1oe8a2 d1pn9a2 d1v2aa2 d1fw1a2 d1duga2 d1g99a2 d2e1za2 d2e1za1
d1saza1 d1saza2 d1ovna2 d1ig8a1 d1czan2 d1czan3 d1czan1 d1ig8a2
d1bg3a4 d1bg3a3 d1bg3a2 d1bg3a1 g1f8v.1 d1novc_ d2bbvc_ d1qsaa2
d1qqya_ d1gd6a_ d1ivma_ d2gv0a_ d1iiza_ d2z2fa_ d1j8xa_ d1ghla_
d1b9oa_ d169la_ d2o4wa_ d1p37a_ d1k28a3 d1xjta1 d1jtma_ d2f2qa_
d4e97a1 d1iyka2 d1rxta1 d1iica2 d1iica1 d1y14b1 d2nn6i1 d1i94l_
d2zjra2 d1smxa_ d2qamc2 d1hh2p1 d2r7da1 d1rl2a2 d1wfqa1 d3bzka4
d1x6oa2 d3a0ja_ d2j01d2 d2ja9a1 d4ql5a_ d1hr0w_ d1luza_ d2n3sa_
d2vnud1 d1h95a_ d1go3e1 d2nn6h1 d2mo1a_ d1iz6a2 d1jjga_ d1e3pa2
d2ix0a3 d2ix0a2 d2lxja_ d1ueba3 d1ueba2 d2z0sa1 d1u0la1 d2ix0a1
d2eifa2 d2je6i1 d3i2za1 d2c35b1 d2ahob2 d1t9ha1 d3i4oa_ d2qall1

d1jt8a_ d2nn6g1 d1g6pa_ d3cama_ d1i94q_ d1c9oa_ d1d7qa_ d2ba0a1
d2vnud2 d2vnud3 d1khia2 d2qalq1 d1wi5a1 d1xtda2 d1ny4a_ d4iuaa3
d4bvva_ d5hpga_ d3k65a_ d1ki0a3 d1ki0a2 d1ki0a1 d1a0ha1 d1pmla_
d3sp8a3 d2feba1 d1qsta_ d2beia1 d4m3sa_ d1u6ma1 d1ygha_ d1yk3a1
d3dr6a_ d1xmta_ d1z4ea1 d2ge3a1 d1bo4a_ d2fiaa1 d2jdca1 d1y9wa1
d3to7a_ d1wwza1 d2fl4a1 d2ozua1 d2b5ga_ d2cy2a1 d2p0wa_ d1cm0a_
d4pswa_ d1yrea1 d1yr0a1 d1m4ia_ d2gana1 d1yvka1 d1ghea_ d2g3aa1
d1y7ra1 d2euia1 d1q2ya_ d2fsra1 d1xeba_ d2ae6a1 d1cjwa_ d1qsma_
d2aj6a1 d1y9ka1 d1i12a_ d1vkca_ d2atra1 d1n71a_ d2d4pa1 d2fiwa1
d2fe7a1 d1tiqa1 d2ou2a_ d1yx0a1 d2fcka1 d1p0ha_ d2j8ma_ d2pq8a_
d1z4ra1 d1s3za1 d1ufha_ d2hqya1 d2hqya2 d1lrza2 d1lrza3 d1ne9a2
d1xvqa1 d1pn0a2 d3hy2a_ d2cvba1 d1o8xa_ d1lu4a_ d1we0a1 d2bmxa1
d2a4va1 d1psqa_ d1xiya1 d1q98a_ d1n8ja_ d1zofa1 d2ggta_ d3tjja_
d4je1a1 d3s9fa_ d2zcta_ d1qmva_ d1jfua_ d2b1ka_ d5dvba1 d2b5xa1
d2v2ga_ d1st9a_ d3tb2a_ d4llra_ d4fh8a1 d1e2ya_ d1knga_ d2h30a_
d1i5ga_ d2z9sa_ d2f8aa1 d1tp9a1 d2b7ka_ d1nm3a2 d3hvva_ d2wfca1
d4kcea_ d1xvwa1 d4gqca1 d3qpma_ d4k1fa_ d1zzoa1 d1gp1a_ d1prxa_
d2qked_ d4ksoa_ d1t4za1 d1wwja_ d1e88a1 d1l6ja5 d1l6ja4 d1eaka4
d1eaka3 d1l6ja3 d1h8pa2 d1h8pa1 d1etha2 d1w52x1 d2ppla1 d1lpbb2
d2oxea1 d1bu8a2 d1rp1a2 d1ys1x_ d4hs9a_ d1ispa_ d1ex9a_ d1ku0a_
d3qmma_ d1lgya_ d4l3wa_ d1llfa_ d1gz7a_ d1ukca_ d1cr6a2 d1bvsa3
d3groa_ d1pjaa_ d1ei9a_ d1svpa_ d1mbma_ d3su6a1 d1cu1a1 d4agka_
d1q31a_ g1n1l.1 d3e90b_ d1ep5a_ d3u1jb_ d2ddfa_ d3zxha_ d1cgla_
d1eaka2 d1hy7a_ d2jsda_ d1cxva_ d1hv5a_ d1i76a_ d3p0ya1 d1igra1
d1igra2 d1n8yc2 d1n8yc1 d1m6ba2 d1m6ba1 d1dzka_ d4tlja_ d2a2ua_
d4wfva_ d3t1da_ d4iaxa_ d3zq3a_ d2nnda_ d1i4ua_ d1kxoa_ d1bbpa_
d1kt7a_ d1exsa_ d3s26a1 d1lf7a_ d1qfta_ d4gh7a_ d1z24a1 d1qabe_
d1xkia_ d3bx7a_ d4iawa1 d1n0sa_ d1gkab_ d3kzaa_ d2k23a_ d2l9ca1
d2joza1 d1gm6a_ d3kffa_ d3saoa1 d1e5pa_ d1yupa1 d1iiua_ d1h6ua2
d2tbvc_ d1c8nc_ d1x36a_ d1smvc_ d1ng0c_ d4sbvc_ d1opoa_ d1f2nc_
d1rmka_ d1ixta_ d1eyoa_ d1cnna_ d1fu3a_ d1g1pa_ d1f3ka_ d1fyga_
d1rypd_ d1irua_ d1rypc_ d3unfk_ d1rypa_ d1j2pa_ d1irui_ d1j2qh_
d3unfh_ d1m4ya_ d1rypb_ d1irul_ d1irug_ d1iru2_ d1q5rh_ d1rypg_
d1g3ka_ d1iruf_ d1q5qa_ d1e94a_ d1irub_ d1ryph_ d3ty6a_ d1iruj_
d1iruk_ d1rype_ d1irud_ d1irue_ d3mi01_ d1rypf_ d1iruh_ d2z3ba_
d1iruc_ d3mka2_ d1iru1_ d1ny721 d1a6ca3 d1a6ca2 d1a6ca1 d1pgl22
d1pgl21 d1pgl11 d1ny711 d1mqta_ d1d4m3_ d1fmd3_ d2mev1_ g1aym.1
d1ev13_ d4q4w1_ d1r1a3_ d2x5ia_ d1rhi1_ g1r1a.1 g1d4m.1 g1rhi.1
d1b35b_ d1oopa_ d1tme1_ d4iv1a_ g1pvc.1 g1fpn.1 d1fpn1_ d4gb31_
d1d4m1_ d1fmd1_ d1ev11_ d2wzr3_ g1cov.1 d1qqp3_ g1fmd.1 d2x5ic_
d1rhi3_ g1oop.1 d1hxs1_ g1hxs.1 d1fpn3_ d1bev1_ g2mev.1 d1eah3_
d1aym1_ d1pvc3_ d1qqp1_ d1zba3_ d1h8ta_ d3vbhc_ g1b35.1 g1bev.1
d1hxs3_ g1ev1.1 d1bev3_ g1tmf.1 d1tmf1_ d1b35a_ d2mev3_ g1eah.1
g1h8t.1 d1eah1_ d4q4w3_ d1r1a1_ d1aym3_ d1pvc1_ d1zba1_ d1h8tc_

d1oopc_ d1tme3_ g1qqp.1 d4iv1c_ d1p8ba_ d1eixa_ d3exra_ d2ffca1
d2fdsa1 d3n3ma1 d2czda_ d1q6oa_ d2yyua_ d1vqta1 d1dbta_ d3ldva1
d2flia1 d1h1ya_ d1tqja_ d1tqxa_ d4lt9a1 d3tc7a1 d3hoja_ d3vnda_
d3nl8a1 d1geqa_ d3nava1 d1v5xa_ d4a2ra1 d3tc6a1 d1ujpa_ d4ijba_
d1xcfa_ d1vc4a_ d1rd5a_ d1qopa_ d3b5va_ d2r80b_ d1cqxa1 d1cg5a_
d1gcvb_ d3boma_ d2h8fb_ d2raoa_ d3bj1a_ d4hrrb_ d2r50a_ d3d1kb_
d2aa1b1 d3zhwa_ d3hf4b_ d3gdja_ d1jebb_ d3bj1b_ d1x9fb_ d1wmub_
d1gcva_ d3bcqb_ d3d4xb_ d1gvha1 d2dc3a_ d3hf4a_ d4yu3a_ d3k8ba_
d1qpwb_ d2r80a_ d3bcqa_ d1v4wa_ d3wtgb_ d1jl7a_ d4esaa_ d1a9we_
d3d1ab_ d1fsla_ d4h2la_ d1v4wb_ d1mwca_ d1vhba_ d1jeba_ d1spgb_
d1h97a_ d1q1fa_ d1tu9a1 d3d1aa_ d1hdsb_ d3hyub_ d2zfbb_ d3fs4a_
d1oj6a_ d1shrb_ d4h2lb_ d4yu3b_ d1spga_ d3hrwa_ d3qqra_ d3at5a_
d4wchd_ d3hyua_ d1cg5b_ d1uc3a_ d1hbra_ d2raob_ d1outb_ d4esab_
d3gdjb_ d3qqqa_ d1x9fc_ d3vrfa_ d1itha_ d3mkba_ d3d4xa_ d3mjpa_
d1jl6a_ d3fh9a_ d1outa_ d2dc3b_ d1it2a_ d3eoka_ d3g46a_ d1qpwa_
d3vrfb_ d1ch4a_ d1x9fd_ d1hdsa_ d2zfba_ d3mkbb_ d1or4a_ d3wtga_
d1a4fa_ d3fh9b_ d3bomb_ d3cy5a_ d3at5b_ d1x9fa_ d2oifa_ d4lmxb_
d1cpcb_ d3v57a_ d1kn1a_ d1cpca_ d4f0ta_ d2vjha_ d4po5b_ d1eyxa_
d1alla_ d4f0tb_ d1b33b_ d1b8db_ d4h0mb_ d1b33a_ d2vjhb_ d4lm6b_
d2vmlb_ d1allb_ d2vjta_ d3dbja_ d2vmla_ d2vjtb_ d4f0ub_ d1f99b_
d4rmpa1 d1liaa_ d1eyxb_ d2uulb_ d4lmsb_ d4f0ua_ d2bv8a_ d1f99a_
d2bv8b_ d2c7lb_ d3dbjb_ d4rmpb1 d1b8da_ d4h0ma_ d3v57b_ d1kn1b_
d1liab_ d2uula_ d2j96a_ d1hlga_ d2xqfa_ d1ea5a_ d1f6wa_ d1dx4a_
d1qe3a_ d1k4ya_ d4qwwa_ d2ha2a_ d3c8da2 d2pbla1 d1jkma_ d1jjia_
d1lzla_ d1gl9b1 d1gkub1 d2m5ta_ d1cqqa_ d1wqsa_ d5bpea_ d2ipha_
d1lvoa_ d2bhga1 d3tnta_ d3sjia_ d3d23a1 d2fyqa_ d3tloa_ d3zvea1
d3q3ya1 d4asha1 d1s5ja2 d1qhta2 d4dlea2 d1tgoa2 d3py8a2 d1q8ia2
d2py5a2 d1d5aa2 d1xova2 d1f74a_ d3l21a_ d2e1da_ d1to3a1 d1w3ia_
d1sfla_ d1n7ka_ d1vcva1 d4imea_ d1wa3a_ d2ocza1 d1f2ja_ d3m16a_
d2yxga_ d1ub3a_ d3a5fa_ d1fbaa_ d1l6wa_ d1xfba1 d1wx0a1 d1vpxa1
d1ojxa_ d1mxsa_ d2a4aa1 d1xkya1 d3cq0a1 d3s1xa_ d1f05a_ d1fdja_
d3cpra1 d4d2ja_ d1cg2a1 d1yloa2 d1y7ea2 d1y0ya3 d1lfwa1 d1vixa1
d1fnoa4 d1vhoa2 d1ysja1 d1vhea2 d1z2la1 d2fvga2 d1vgya1 d1r3na1
d2grea2 d1xmba1 d1rtqa_ d3rp2a_ g1ucy.1 d1fuja_ d2zchp_ d3w94a_
d1si5h_ d1lo6a_ g2pka.1 d1cgha_ d1kigh_ d1a5ia_ d3s9ba_ d1hj8a_
d1ao5a_ d2anya_ d1q3xa1 d1gvza_ d1azza_ d4gsoa_ d2f91a1 d1iaua_
d1gdna_ d1eufa_ g1fiw.1 d1eq9a_ d4nfea_ d2rdla_ d1spja_ d1elva1
d1a7sa_ d1ekbb_ d1pfxc_ d1yc0a_ d2aiqa_ g1fiz.1 d1fi8a_ d4k60a_
d4dgja_ d1mzaa_ d1op0a_ d3tjva_ d1pytd_ d1sgfg_ g1gg6.1 d2eeka_
g1rtf.1 d1a0ja_ d3g01a_ d1pytc_ d4e7na_ d1ddja_ d1z8ga1 d2hlca_
d1npma_ d1brup_ g1jou.1 d1sgfa_ d1autc_ g2hnt.1 g1kdq.1 d1orfa_
d3s69a_ d4crga_ d1fxya_ d1bqya_ d2ftba_ d1pmpa_ d2rcqa1 d1fdqa_
d4lkpa_ d1sa8a_ d1vyfa_ d1lpja_ d3vg7a1 d4azqa1 d1o8va_ d5bvqa_
d1p6pa_ d2qo4a_ d1opba_ d1yiva1 d1o1va_ d4bvma1 d4tkba_ d4i3ba_

| | | | | | | | |
|---|---|---|---|---|---|---|---|
| d1bwya_ | d1ftpa_ | d1ggla_ | d4gkca_ | d1cbia_ | d3p6da1 | d1kqwa_ | d2a0aa1 |
| d1m4la_ | d2bk7a_ | d3fjua_ | d1z5ra1 | d1kwma1 | d1uwya2 | d2c1ca1 | d1dtda_ |
| d1h8la2 | d3lgia_ | d3i77a_ | d2z9ia2 | d4nsva_ | d1lcya2 | d4jcna_ | d1agja_ |
| d1os8a_ | d1hpga_ | d1l1ja_ | d1qtfa_ | d1e69a_ | d1mv5a_ | d3g60a4 | g1ii8.1 |
| d1ewqa2 | d1ji0a_ | d1v43a3 | d2wmfa1 | d1vpla_ | d1g2912 | d4a82a2 | g1f2t.1 |
| d2d62a1 | d3tuic1 | d2bbsa1 | d3g60a3 | d2ixea_ | d1g6ha_ | d1sgwa1 | d2qi9c_ |
| d3rlfa1 | d3b60a1 | d3d31a2 | d2pzea1 | g1xew.1 | d1jj7a_ | d1oxxk2 | d2onka1 |
| d1w1wa_ | d1iwea_ | d1qf5a_ | d1vmaa2 | d1nija1 | d1np6a_ | d2p67a1 | d3ihla_ |
| d1okkd2 | d1ls1a2 | d1s1ma2 | d1iona_ | d2v40a_ | d1dj3a_ | d3hida_ | d2vo1a1 |
| d2g0ta1 | d1vcoa2 | d1j8mf2 | d1g3qa_ | d1xjca_ | d1p9ba_ | d1qzxa3 | d1ihua2 |
| d1ihua1 | d2qm8a_ | d1dj2a_ | d4ioja_ | d1yrba2 | d1hyqa_ | d3gqba2 | d1skye3 |
| d1xp8a1 | d1tf7a2 | d1tf7a1 | d1ye8a1 | d1skyb3 | d2qe7d2 | d4blta_ | d4huta_ |
| d3ldaa2 | d4hyya_ | d2i3ba1 | d1cr1a_ | d3j9tb2 | d3ntua2 | d1vdza1 | d2z43a_ |
| d1e9ra_ | d1nlfa_ | d3ziaa2 | d1n0wa_ | d1g6oa_ | d3ziad2 | d3j9ta2 | d1pzna2 |
| d3gqbb2 | d2i49a_ | d1nnfa_ | d1xvxa_ | d1ursa_ | d1h3da1 | d1iuda_ | d4m1va1 |
| d1twya_ | d3e13x_ | d2nxoa1 | d4pfua_ | d3ttma_ | d1elja_ | d2qmwa1 | d1us5a_ |
| d1y3na1 | d1jeta_ | d1uqwa_ | d4f19a1 | d2esna2 | d3thia_ | d3fasa1 | d2uyeb_ |
| d2xwva1 | d1r9la_ | d2fyia1 | d1pb7a_ | d2ozza1 | d1sw5a_ | d1nh8a1 | d1vr5a1 |
| d2g29a_ | d1xs5a_ | d3lswa_ | d2z23a_ | d3w9va_ | d3tcha_ | d1q35a_ | d1z7me1 |
| d3q27a_ | d1p99a_ | d1ve4a1 | d2i6ea1 | d4mcoa1 | d4n8ya_ | d1eu8a_ | d1zbma1 |
| d1hsla_ | d1ixca2 | d1j1na_ | d1xvya_ | d1y9ua_ | d2z22a1 | d1xoca1 | d4oera_ |
| d1wdna_ | d5f1qa1 | d1xt8a1 | d1i6aa_ | d4gwoa_ | d4igta1 | d1pc3a_ | d4mmpa1 |
| d1o63a_ | d2czla1 | d2onsa2 | d4mija1 | d4ok5a1 | d2bmfa1 | d2bmfa2 | d1yksa1 |
| d1yksa2 | d1jr6a_ | d2qeqa1 | d2jlqa1 | d1a1va2 | d1eh3a_ | d3skpa_ | d1dtza1 |
| d1dtza2 | d1ieja_ | d1jnfa2 | d1ce2a1 | d1jnfa1 | d1b1xa2 | d1jw1a1 | d1jw1a2 |
| d1ryoa_ | d1h76a1 | d1h76a2 | d1c4oa1 | d2p6ra3 | d2p6ra4 | d1oywa2 | d1oywa3 |
| d1wrba1 | d1w36b2 | d1w36b1 | d1c4oa2 | d3bora_ | d1t6na_ | d2eyqa5 | d2eyqa3 |
| d2eyqa2 | d2zu6a2 | d1veca_ | d1w36d1 | d2g9na1 | d1z3ix2 | d2rb4a_ | d1hv8a1 |
| d1hv8a2 | d1t5ia_ | d1w36c2 | d2j0sa2 | d2j0sa1 | d1fuka_ | d3hjha1 | d1z5za1 |
| d1qdea_ | d1z63a1 | d1s2ma2 | d1s2ma1 | d1wp9a1 | d1wp9a2 | d1gm5a3 | d1gm5a4 |
| d3dina4 | d3dina3 | d1q0ua_ | d1w36c1 | d1z3ix1 | d1nkta3 | d1nkta4 | d1uaaa2 |
| d1w36d2 | g1qhh.1 | d2fwra2 | d2fwra1 | d3q3ma_ | d1za0a1 | d1mxra_ | d2inca_ |
| d1jk0a_ | d2incb_ | d1mhyb_ | d3hf1b_ | d1mtyb_ | d1r2fa_ | d2uw1a_ | d1mhyd_ |
| d3n37a_ | d1afra_ | d1oquc_ | d1syya_ | d1mtyd_ | d2o1za_ | d1uzra_ | d1smsa_ |
| d2fkza_ | d1zuja1 | d1s3qa1 | d2fjca1 | d1z6om1 | d1veia_ | d4dyya_ | d1jiga_ |
| d3gvya_ | d2pyba_ | d4cy9a1 | d3bvfa1 | d1r03a_ | d2cf7a_ | d1nf4a_ | d1zs3a1 |
| d4iwka_ | d3ka8a_ | d3np0x_ | d1lb3a_ | d4e6ka_ | d1z6oa1 | d2fzfa1 | d4dyua_ |
| d3ge4a_ | d1jgca_ | d4am4a_ | d2z6ma_ | d1ji5a_ | d1vlga_ | d1lkoa1 | d1krqa_ |
| d3qz3a_ | d1o9ra_ | d2ffxj_ | d1euma_ | d1yv1a1 | d2v2pa_ | d1j30a_ | d4di0a_ |
| d1dpsa_ | d2cbia2 | d1gqia1 | d2choa2 | d3nvva1 | d1krha3 | d2h88b1 | d1n62a2 |
| d1t3qa2 | d1zoyb1 | d1jq4a_ | d1ffva2 | d1dgja2 | d1rm6c2 | d1ex4a2 | d1c6va_ |
| d1k8rb_ | d1wgra1 | d1ef5a_ | d1wfya1 | d1e8ya3 | d1wgya1 | d2cs4a1 | d1wxma1 |
| d2byea1 | d3kh0a1 | d2byfa1 | d1wxaa1 | d1e7ua3 | d1h4ra3 | d4yl8a1 | d2al6a3 |
| d1wgda1 | d1wgga1 | d4q5eb_ | d1wjna1 | d1se9a_ | d3n3kb_ | d1wz0a1 | d1we6a1 |

d2m0xa_ d2gbka_ d3rt3b1 d1wh3a1 d1sifa_ d2ekec2 d4ii2b1 d4hk2a1
d1c3ta_ d1m94a_ d1wgha1 d1ttna1 d1j8ca_ d1wx8a1 d3v6cb1 d2ma9b_
d4i6lb1 d1bt0a_ d2jvca1 d1iyfa_ d2k1fa_ d1wxva1 d1wf9a1 d1t0ya_
d2io0b2 d1x1ma1 d2gbma_ d1zw7a1 d2m17a_ d1ud7a_ d1we7a1 d2lwpa_
d2gbja_ d1wjua1 d1v6ea1 d1v86a1 d4eewa_ d1wiaa1 d4icva_ d4gu2a_
d1wx7a1 d1wy8a1 d4hjka1 d2faza1 d1v5oa1 d3ubra_ d1qo8a1 d1d4ca1
d2k3va_ d1ft5a_ d1qdba_ d1m1qa_ d2j7aa_ d1h21a_ d1ogyb_ d4fasa_
d1jnia_ d1oaha_ d1sp3a_ d4lu0a_ d1of8a_ d3unda1 d3e9aa1 d1vr6a2
d1n8fa_ d2qkfa_ d1d9ea_ d3h33a_ d1rwja_ d19hca_ d1os6a_ d3bxua_
d3h4na_ d1i77a_ d3caoa_ d1j0pa_ d2ctha_ d1duwa_ d2e84a_ d1hh5a_
d1gyoa_ d3h34a_ d2bcgg1 d2bcgg2 d1c4xa_ d1j1ia_ d2rhwa_ d1wioa4
d1ccza2 d1vcaa1 d1dr9a2 d2d9qb1 d2nxyb2 d1um8a_ d1ixza_ d1qvra2
d1qvra3 d1qzma_ d1sxjc2 d1jr3d2 d2p65a_ d1l8qa2 d1g8pa_ d3hu3a3
d1sxjd2 d1in4a2 d1sxja2 d1tuea1 d2ce7a2 d1w5sa2 d1sxjb2 d1d2na_
d1ny5a2 d1ofha_ d2gnoa2 d2a5yb3 d1fnna2 d1u0ja1 d1lv7a_ d1iqpa2
d2fnaa2 d1ixsb2 d4gita_ d1sxje2 d1g41a_ d2e3fa_ d1mm0a_ d2e3ea_
d2llda_ d2ny8x_ d2e3ga_ d2ny9x_ d2nz3a_ d2lj7a_ d1n4na_ d2m8bb1
d2kyqa_ d3psma_ d1jkza_ d1jxca_ d2gl1a_ d1ugla_ d4uj0a_ d2k4ua_
d1hp2a_ d2lu9a_ d1baha_ d2meoa1 d1wm7a_ d1hlya_ d1n8ma_ d1jlza_
d1wpda_ d1c55a_ d1pvza_ d1wt7a_ d1rjia_ d1wz5a_ d1m2sa_ d1pjva_
d1c49a_ d2ktca1 d1wmta_ d1du9a_ d1j5ja_ d1r1ga_ d1c4qa_ d1prtc1
d2wv6d_ d1prtf_ d1ltrd_ d4l63b1 d1r4pb_ d1tl0f_ d2bosa_ d1prtb1
d3chbd_ d1t0za_ d2i61a_ d1t1ta_ d1sn4a_ d1kv0a_ d2lkba_ d1npia_
d1wwna_ d1pe4a_ d2kbka_ d2li7a_ d1djta_ d2kbha_ d1dq7a_ d1sega_
d1omya_ d1zywa1 d1jzaa_ d1t61c1 d1iyjb4 d1iyjb5 d1iyjb3 d1o7ia_
d4ipca1 d1x3ea_ d4apva_ d1jb7a1 d1jb7a2 d1jb7a3 d3kdfa_ d1ue1b_
d1wjja1 d3eiva_ d1xjva2 d1xjva1 d2ccza2 d1se8a_ d1l1oc_ d1jb7b_
d1qvca_ d3ulla_ d1qzga_ d1miua4 d1miua5 d1miua3 d3g48a1 d1gd7a_
d1mkha_ d1pyba_ d3ubub_ d1egia_ d1qo3c_ d1v7pa_ d1tdqb_ d3ubua_
d1c3ab_ d1h8ua_ d3bx4b_ d1t8ca1 d1sb2a1 d1qdda_ d2ziba1 d3cada_
d1byfa_ d1j34b_ d1jwia_ d1jzna_ d1c3aa_ d1wmza_ d3bdwb_ d1hq8a_
d1fifa1 d1dv8a_ d3hupa_ d1wk1a1 d1umra_ d2afpa_ d3bx4a_ d1gz2a_
d1sl6a_ d1v7pb_ d3g8ka_ d1ixxa_ d1j34a_ d1jwib_ d1g1sa1 d1y17a1
d1ja3a_ d1fvub_ d3bdwa_ d1sb2b1 d1fvua_ d1umrc_ d1oz7a_ d4m17a_
d3cfwa1 d1ypqa1 d1g1ta1 d3fd4a_ d3l9jc_ d4dn8a1 d1v4lb_ d1rdl1_
d2msba_ d1dgsa2 d1fvia1 d1ckma1 d1x9na2 d1xwdc1 d3kj4a_ d1ozna_
d1w8aa_ d2ifga3 d1xkua_ d2shpa1 d1yfoa_ d1l8ka_ d2ooqa1 d1g4us2
d1p15a_ d1lara2 d1lara1 d1wcha_ d2fh7a2 d2fh7a1 d1ohea1 d1ohea2
d2f4da_ d1rxda_ d2hxpa_ d1xria_ d1d5ra2 d1fpza_ d3ezza_ d4y2ea_
d1eb6a_ d3azya_ d3wvja_ d1upsa1 d1ajoa_ d2hyka_ d1ajka_ d2vy0a_
d1o4ya_ d3o5sa_ d3d6ea_ d3atga_ d1dypa_ d1umza_ d3i4ia_ d1c1la_
d4lbqa_ d1a78a_ d1slta_ d4no4a_ d3zsja_ d3duia_ d1g86a_ d1is3a_
d1qmja_ d1hlca_ g1qmo.1 g1len.1 d3zyra_ d1fnya_ d1n47a_ g2ltn.1
d1avba_ d3usua_ d1dhkb_ d1f9ka_ d1ukga_ d1g8wa_ d1g9fa_ d1wbfa_

d3wcsa_ d1g7ya_ g1loe.1 d1mvqa_ d1hqla_ d1v6ia_ d1dbna_ d1qnwa_
d1fx5a_ d4l8qa_ d1ioaa_ d3n35a_ d2h0ba_ d2erfa_ d1h30a2 d3mw4a1
d1pz7a_ d2r1ba_ d3v64a1 d1d2sa_ d3bopa1 d3mw3a1 d1okqa1 d1okqa2
d1dszb_ d1a6ya_ d3cbba_ d1ynwa1 d1r4ia_ d1hcqa_ d2hana_ d2hanb_
d2enva1 d1kb4b_ d2nllb_ d1lo1a_ d1cita_ d1adqa1 d1fnga1 d1uvqb1
d1zs8a1 d2vola1 d2vola2 d1mfbl2 d2jelh2 d1uwel2 d3qq4b1 d1kjvb_
d1k5na1 d1osph2 d4odhl2 d1ow0a1 d1ow0a2 d1bfob2 d3l9ra2 d3jtth1
d2fath2 d4elkb2 d1fn4b2 d2xfxb_ d1pg7x2 d1cqka_ d4k71b2 d1lk2a1
d1eapb2 d4lfhg2 d1i4fa1 d2nw2a2 d1kfah2 d1mhca1 d1h3ua1 d1wejh2
d1h0db2 d1a5fh2 d1kcvh2 d1mjuh2 d1qqda1 d1kcrh2 d1k8ia1 d3huja2
d2nnab1 d1nfdf2 d3mffb2 d1s9va1 d1lk3h2 d1q0xl2 d1fv1b1 d1neza1
d4rfeb2 d1r3ha1 d1a6tb2 d1kjva1 d1pz5b2 d1onqa1 d2bc4a2 d1de4a1
d1k8da1 d1tcra2 d1dn0b2 d1nfde2 d2xwtb2 d1bfoa2 d1mexh2 d1iakb1
d1ypzf2 d4f7ea2 d1q72l2 d2fbjh2 d1fe8h2 d1clzh2 d1fngb1 d1uvqa1
d1vgka1 d1c16a1 d4j12a2 d4x99b2 d1igtb3 d1je6a1 d1q72h2 d1nldh2
d1teth2 d2hmic2 d1i1ca2 d1i1ca1 d1k8ib1 d1q0yh2 d1kfal2 d3frua1
d1pg7w2 d1u58a1 d1igyb4 d1igyb3 d1hyrc1 d1klub1 d1deeb2 d1c12b2
d2bc4b2 d2ig2h2 d1kcvl2 d3wn5b1 d1lk2b_ d1lk3l2 d1mjul2 d1sm3h2
d1q0xh2 d1zvsa1 d1ydpa1 d1u8ib2 d1ypze2 d1kcuh2 d1op9a_ d1y0la1
d1etzb1 d1egjl1 d2gsib1 d1nj9a1 d4jfhd1 d1aqkl1 d1t2ja_ d1lo4h1
d1zvya1 d3v4vl1 d4enec1 d1kxtb_ d5e8ea1 d2pxya_ d4weud_ d1xiwc_
d2esvd1 d1qnzh_ d1ogae1 d1f4xh1 d4n1hb_ d3ezjb1 d3mlwl1 d1kfah1
d1etza1 d1b4jh1 d3cfic_ d4w68a_ d2arja1 d1ek3a_ d2ok0l1 d1vera_
d1uwgh1 d1blnb1 d1fpth1 d1rzga1 d2giya1 d4m3kb1 d1nc2a1 d2nyyd1
d1ncna_ d1fbih1 d5dxwa_ d4fqqa1 d1dqdh1 d1jfql1 d1kelh1 d1faih1
d1frgl1 d1figl1 d4m6nl1 d1fo0a_ d4hxbl1 d3b5ga_ d3quyd1 d2h9gb1
d1i8kb_ d2dd8h1 d2ck0h1 d1eaja_ d4w6wb1 d2a6ib3 d1t66d1 d2e27h_
d1ob1b1 d1f11b1 d2v7na1 d1ad0a1 d2xt1b1 d1pskh1 d4i13b1 d2op4l1
d1a0ql1 d1uwel1 d1za3a1 d1ncbl1 d4uu9a_ d1bm3h1 d1cloh1 d1nqba2
d1cf8h1 d4y5va_ d1mhph1 d5anma1 d4cdgc1 d4ldeb_ d1y0lb1 d2pcpb1
d1z3gl1 d1dfbl1 d1fh5h1 d3g5xa1 d1tzib1 d4dn3l1 d3zhka1 d2b1hh1
d2uyla1 d1f3dh1 d1nj9b1 d2agjh1 d2h1pl1 d1q72h1 d8fabb1 d1ndmb1
d1rjca1 d4u6hb1 d3upca_ d2fbjl1 d1osph1 d1tvda_ d2cjvh_ d4fhbd1
d1bd2d1 d1rz7h1 d3nn8a_ d1blna1 d1knoa1 d1nfda1 d2bmkb1 d1dqll_
d1igyb1 d1igfl1 d12e8h1 d3utza1 d1nc2b1 d1rihh1 d4mwfb1 d4jvpa1
d1mieh1 d1rhfa1 d1fgnl1 d1sbsh1 d1lp9e1 d1dn0a1 d4lqfl1 d1iaih1
d1kgcd1 d1mh5b1 d1gpol1 d2g60h3 d1f8tl1 d2jixd1 d4grwh_ d1fo0b_
d1bfob1 d1axsa1 d1u9ka_ d1nmbh_ d1h8na1 d1h8na2 d1jguh1 d4eura1
d3sdyl1 d35c8h1 d1w72l1 d1mvub_ d1i9rh1 d1ob1a1 d4wenb_ d1yeeh1
d3ietc1 d1f58h1 d1ad0b1 d1mqkl_ d1nbvh1 d4b50a_ d3r8bb_ d1bafl1
d1mamh1 d4rgne1 d2gsgb_ d35c8l1 d1bafh1 d1ai1h1 d2pcpa1 d2fath1
d2x1pa_ d1orsb1 d1ibgl1 d1shma_ d1a2yb_ d1u0qa_ d2coqa_ d1hilb1
d1qfwi_ d1jv5b_ d1fn4b1 d5e0qa_ d1nsnh1 d1plgl1 d4ocxl1 d1kb5h1
d1rzfh1 d1pg7x1 d2fx7h1 d2oi9c_ d1l7il1 d3eyva1 d1d5il1 d4ffyl_

d1tetl1 d1a6wh_ d1ac6a_ d3dvfa_ d1nfde1 d1mimh1 d1ri8a1 d1jhlh_
d3dura_ d1fskb1 d1f97a1 d3so3b1 d2ak4d1 d1lp9f1 d4z7wf1 d1l7th1
d4y5ya_ d4x7ec1 d1emth1 d1ikfl1 d1nlbh1 d4utal1 d1bjma1 d1eapb1
d1u6ah1 d2dtgd1 d1kj2b_ d1xeda1 d1c1eh1 d1fgvh_ d2bnub1 d2fb4h1
d2dtgb1 d1ktrh_ d1jpth1 d1qkzl1 d4hepg_ d2atpb1 d1flrh1 d2hrpl1
d1mvfa_ d1iaim1 d3k80a1 d2jb5h1 d3zkxc_ d4grwe_ d1it9h1 d1rhhb1
d3w9ec_ d1e4wh1 d1sy6h1 d2j6eh1 d3qxta_ d1jglh1 d1p7ka1 d1tzhb1
d4n8pa1 d1gafl1 d2jelh1 d1wioa2 d4i0cc1 d1cicb1 d1lmka2 d1lmka1
d1iqwh1 d1orqb1 d1ejoh1 d1jnlh1 d4zg1a_ d43c9b_ d1ggbl1 d1wejh1
d3q6fa1 d1f3dj1 d3c6la1 d1bqlh1 d3p9wb_ d1a3rl1 d1h0db1 d2uzil_
d1eo8l1 d1qfwl_ d1n7ml1 d1igja1 d1c5ch1 d2a9mh_ d1fj1b1 d4heme_
d1ccza1 d1a5fh1 d1ogad1 d1dlfh_ d1qa9b_ d1vgel1 d3qskb_ d1sjxa_
d4ozfg1 d1sq2n_ d1qd0a_ d1kcvh1 d1c5db1 d3qcul1 d8faba1 d3bn9d1
d1mjuh1 d4y8dc_ d7fabh1 d3dvgb1 d1lo0l1 d1a6tb1 d1gigh1 d1kcrh1
d2hwzl1 d1yjdc1 d3clfl1 d4krna_ d1dl7l_ d1e6ol1 d1mexh1 d3r0ma_
d4kkna_ d1fnsl1 d1oaqh_ d1rzia1 d1ucbh1 d1ncwl1 d4b41a_ d1nfdf1
d2mpah1 d2d7tl1 d4grwf_ d4b5ea_ d1lk3h1 d3u79a_ d4u3xa_ d1ghfl1
d1bvkb_ d1mcww1 d1b88a_ d1jrhh1 d32c2b1 d4wemb_ d1dsfh_ d1op3h1
d1ce1l1 d1iqda1 d1qoka1 d1qoka2 d1qfuh1 d2crya1 d1ay1h1 d1oaql_
d3ab0b_ d1rurl1 d1t4kb1 d1bz7b1 d1n0xh1 d1forl1 d1ucbl1 d4w6yb_
d43c9a_ d2aabl1 d1tzia1 d4n1ca_ d1k6qh1 d3g9ab_ d4tvsa1 d1igmh_
d1pz5b1 d3dx9a1 d3wxwh_ d1nakh1 d1kcul1 d3eaka_ d4qlra1 d1cr9l1
d1d9ka_ d1f4xl1 d1deea1 d2i27n2 d1c12a1 d2orbl1 d1j8hd1 d1mcph1
d1c5da1 d1kenh1 d1bd2e1 d1h5ba_ d4x7fc1 d3upaa_ d1t3fa1 d1indh1
d2dtgc1 d4hc1l1 d1zv5a1 d4wgvb_ d1uwgl1 d4f37k1 d1aqkh1 d1vesa_
d3zkqd_ d1fptl1 d1lo4l1 d1knob1 d3rrqa_ d1ct8b1 d4orzc_ d1mlbb1
d4hgkc1 d1dqdl1 d1bogb1 d4ozhe1 d1dr9a1 d1i7za1 d1dn0b1 d1kell1
d1rzib1 d1ypzf1 d1wtla_ d1hkfa_ d1fail1 d1figh1 d2vyre_ d1sjva_
d1ohqa_ d1bfoa1 d1pkqb1 d1mjul1 d1b4jl1 d1nsnl1 d2arjb1 d1p4bh_
d4aj0a_ d3nn8b_ d2rcja1 d1f2xk_ d4v1dd_ d1adqh1 d2eksa_ d4k3ga_
d1jfqh1 d1uweh1 d1frgh1 d4gftb_ d1uz8b1 d4n1ea_ d1bm3l1 d1clol1
d3mcka1 d2vq1a1 d1ae6h1 d4aq1b1 d1uj3b1 d1nbqa1 d1fh5l1 d2ck0l1
d2ywza_ d1q72l1 d4nbxb_ d1hila1 d4hzlb1 d2j88l1 d1zmya1 d1cd0a_
d1a4ka1 d1pskl1 d1a0qh1 d4krmb_ d1ncbh1 d2fbjh1 d1j8he1 d1i3ua_
d1cf8l1 d1fe8h1 d2p1ya1 d1eeqa_ d2x89a_ d1ol0a_ d2r0lh1 d1ub5a1
d1dfbh1 d3eyvb1 d4jdvb1 d4p2cg_ d2gsia1 d2agjl1 d1u3ha1 d2h1ph1
d1rihl1 d1fskc1 d1boga1 d2a77l1 d1ospl1 d1qfoa_ d3dwta_ d2fx7l1
d1rz7l1 d3stba1 d1pkqa1 d1b6da1 d1dzba2 d1dzba1 d1kj2a_ d4l1ha_
d1rzgb1 d1dqlh_ d12e8l1 d1miel1 d1jmaa_ d1sbsl1 d1fgnh1 d1f58l1
d1mqkh_ d1ndgb1 d3qegl1 d1iail1 d2aw2a1 d1tqbc1 d3fo2a1 d4dkaa_
d1f8th1 d2g60l1 d2ywya_ d1uj3a1 d1yc7a_ d3bd3a_ d4w6xb1 d1nmbl_
d1i8ka_ d1rjlb1 d1ypze1 d1p7kb1 d1kxqe_ d1gpoh1 d2z4qa1 d1w72h1
d1i9rl1 d4qgya1 d4xt1c_ d2qqnh1 d1cica1 d3c08h1 d2cd0a_ d2bdnh1
d1hnga1 d1maml1 d1nldh1 d1cr9h1 d4lcih1 d3bkyh1 d3qg7l1 d1ai1l1

d1kxvc_ d4iosd_ d1l7ih1 d2x1qa_ d1ub6b1 d3k74b_ d1ibgh1 d1teth1
d1igya1 d1hxma1 d2x6ma_ d2e7la_ d5e03a_ d1qfwm_ d2r0kh1 d1igjb1
d1rurh1 d1plgh1 d1mj8h1 d1yuhb1 d3fmgl1 d1rzfl1 d1emtl1 d1ad9b1
d1igtb1 d1nlbl1 d1d5ih1 d3k7ua_ d4ffyh_ d1bwwa_ d3dvga1 d1a6ta1
d1fgvl_ d4qxgl1 d1miml1 d1jhll_ d2hffb1 d2hh0l1 d1qkzh1 d1l7tl1
d1afvh1 d1iaii1 d2cjuh_ d1um5h1 d2ghwb2 d2ghwb1 d1kgce1 d2fgwh1
d1ikfh1 d1dvfd_ d2i24n1 d2xtjb_ d1za3b1 d2yc1a_ d2hh0h1 d2x1oa_
d1bvka_ d1rjla1 d5e7ba1 d1igta1 d1il1b1 d2fb4l1 d1mvua_ d1jptl1
d32c2a1 d1fvca_ d1iqdb1 d2hrph1 d2p4ab_ d4qo1a_ d1p2cb1 d1wt5c_
d1t4ka1 d5c1mb_ d1it9l1 d1bz7a1 d1b2wh1 d1e4wl1 d1jnll1 d1yedb1
d1za6b1 d3u7aa_ d1igfh1 d3gnml1 d1orsa1 d1wejl1 d1a2ya_ d1hxmb1
d1gafh1 d2j4wh1 d1pz5a1 d1j05a_ d1a3rh1 d4fqll1 d1qfwh_ d1eo8h1
d1n7mh1 d4s11a_ d1kegh1 d5da4a_ d1iqwl1 d3whxb1 d1vgeh1 d1deeb1
d1c12b1 d1t2ql1 d1ad9a1 d3gsnb1 d3b2uc1 d1ggbh1 d1t3fb1 d1nakl1
d4ocmc_ d1lila1 d2v7hb3 d2esve1 d1mcoh1 d2qr0b1 d2q3aa_ d1ct8a1
d1kcrl1 d2pxyb_ d2uzih_ d5c3ld_ d3durb_ d1c5cl1 d2ak4e1 d1dl7h_
d1xiwd_ d1mlba1 d1rmfl1 d1i7zb1 d1dlfl_ d2xa3a_ d5bq7b1 d1dqqb1
d3h42l1 d1egjh1 d1kcvl1 d1fe8l1 d2j6el1 d1f3rb2 d1lk3l1 d1sm3h1
d1q0xh1 d1ghfh1 d7fabl1 d1igch1 d1lo0h1 d4idla_ d5c2bl_ d1jrhl1
d1il1a1 d3zhda1 d4kfzc_ d1b0wa_ d1e6oh1 d1mexl1 d4krpb_ d1fnsh1
d1ncwh1 d4hgma_ d4eigb1 d4rzca_ d2mpal1 d4y5xa_ d5en2b1 d3lrga1
d4ej1c1 d1rhha1 d1j05b_ d1forh1 d1q9wb1 d2wzpd_ d2aq2a1 d1h0da1
d3revb1 d2ij0c1 d1tzha1 d4m7jl1 d1k6ql1 d3b9kc1 d1igml_ d3qeua1
d1dsfl_ d1ce1h1 d1kcuh1 d1zvha1 d1zvoa1 d1tqbb1 d1ay1l1 d2qscl1
d2cgrh1 d1nl0h1 d1kenl1 d3eo0a1 d4n9ob1 d1n0xl1 d3k1kc_ d2m9wa1
d3dfxa_ d2aq2b1 d1ck1a1 d1enfa1 d1i4pa1 d1v1oa1 d1et9a1 d1esfa1
d2g9hd1 d4ohja1 d1klud1 d1bxta1 d1ty0a1 d1fnua1 d2qaln1 d2cqva1
d1fhga_ d2k1ma_ d2dl2a1 d1biha3 d1biha1 d1biha4 d3qp3a1 d1g1ca_
d1gxea_ d1cvsc2 d1cvsc1 d1xmwa2 d2otpa2 d1biha2 d1rhfa2 d2fcba2
d2fcba1 d1wwbx_ d1gsma2 d1epfa1 d1epfa2 d2dava1 d1waaa_ d1f97a2
d1x44a1 d1vcaa2 d1fnla2 d1fnla1 d3b5ha2 d3h8na2 d3h8na1 d1jbja1
d1jbja2 d1pd6a_ d1f2qa2 d1f2qa1 d3cx2a_ d1nbqa2 d3b5ha1 d1n26a1
d1xiwb_ d1xaua_ d1fltx_ d2ifga1 d1olla1 d1olla2 d1cs6a4 d1cs6a3
d1cs6a2 d1cs6a1 d2gw5a2 d1sy6a1 d1xiwa_ d1ucta2 d1ucta1 d1ry7b2
d1gl4b_ d1qz1a3 d1olza1 d2c9aa1 d1wwca_

**Family-38 (b.55.1.1)**

**Positive dataset contains 36 protein sequences.**

d1wgqa1 d1v89a1 d2coda1 d1zc3b_ d1wg7a1 d1u5ea1 d1v61a1 d2coca1
d2ys1a1 d1v5pa1 d1ntya2 d1wjma1 d1wi1a1 d1u29a_ d1xx0a1 d1unqa_
d2zkmx3 d1v88a1 d2adza1 d1x1fa1 d1p6sa_ d2coaa1 d1v5ma1 d2x18a_
d1x1ga1 d4kaxb_ d2j59m1 d1xcga2 d1txda2 d1u5da1 d1w1ha_ d1upqa_
d1v5ua1 d2fjla1 d2dfka2 d2cofa1

**Negative dataset contains 4028 protein sequences.**

d1ax4a_ d2v1pa1 d2ez1a_ d1u08a_ d1lc5a_ d2ay1a_ d1bw0a_ d3pd6a_

d7aata_ d2csta_ d1j32a_ d1fg7a_ d4wlha_ d1o4sa_ d1v2da_ d1gdea_
d1m6sa_ d1v72a1 d1svva_ d1ajsa_ d1wsta1 d2f8ja2 d1lk9a_ d1vp4a1
d1xi9a1 d2gb3a1 d1yaaa_ d2bnma2 d3i2ka1 d1mpxa2 d1js9c_ d1f15b_
d1za7a_ d1laja_ d3ehbb1 d1fftb1 d1gyca3 d1hfua2 d1kbva1 d1kbva2
d1sdda1 d1sdda2 d1gyca1 d1gyca2 d1mzya1 d2bw4a1 d1aoza1 d1aoza3
d1aoza2 d1kyaa2 d1kyaa3 d1kyaa1 d1mzya2 d4e9sa1 d1sddb1 d1sddb2
d1hfua1 d1hfua3 d1oe1a2 d1oe1a1 d1v10a2 d1v10a3 d1v10a1 d2dara2
d2dara1 d1wyha2 d2dloa2 d1rutx4 d1rutx3 d2cuqa2 d1x4la2 d1x4la1
d1x3ha2 d1x3ha1 d2d8za1 d1x61a2 d1x6aa1 d1x6aa2 d2cora2 d2cora1
d1v6ga1 d1v6ga2 d2d8za2 d1x64a1 d1x64a2 d1b8ta1 d2co8a1 d1x63a2
d2dj7a2 d2dj7a1 d2cu8a2 d2cu8a1 d1g47a2 d1g47a1 d1j2oa1 d1wyha1
d2dloa1 d1wiga1 d1wiga2 d2cupa2 d2cupa3 d1x68a1 d2d8ya2 d2d8ya1
d1m3va2 d1x62a1 d1x68a2 d1x61a1 d1b8ta3 d1b8ta2 d2d8xa2 d2d8xa1
d1x4ka1 d1x4ka2 d2cuqa1 d1m3va1 d2co8a2 d1j2oa2 d1x63a1 d2cura1
d2cura2 d1x62a2 d2cfba1 d1dfoa_ d1o69a_ d1bj4a_ d3k28a_ d2bwpa_
d3lg0a_ d2fnua_ d3ou5a_ d1z7da1 d1b9ha_ d1iaya_ d4msoa_ d1bs0a_
d1s0aa_ d1sffa_ d3tqxa_ d1mdoa_ d3qboa_ d3pgya_ d1bjna_ d2dkja_
d1ejia_ d1vefa1 d4wxga_ d3bs8a_ d3gbxa1 d1w23a_ d1a3qa1 d1iknc_
d3brda1 d1p7hl1 d1oy3c_ d1gjia1 d1zk9a1 d2cxka1 d2bema_ d1pama1
d1ea9c1 d1qhoa1 d2fhfa2 d2fhfa1 d1k3ia1 d1j0ha1 d1cxla1 d2bhua1
d1edqa1 d1f1sa2 d1ut9a2 d1gvia1 d1txka1 d4alac_ d1pjwa_ d1rera1
d1s6na_ d3uzqb_ d2phla1 d1fxza2 d1fxza1 d1lrha_ d1uija1 g1dgw.1
d2v09a_ d1uika2 d1uika1 d1dgwa_ d1ep0a_ d2pa7a1 d1wlta1 d1nxma_
d2c0za1 d1ex0a1 d1g0da1 d1ju8a_ d2bjca1 d2hsga1 d1qpza1 d1mqta_
d1d4m3_ d1fmd3_ d2mev1_ g1aym.1 d1ev13_ d4q4w1_ d1r1a3_ d2x5ia_
d1rhi1_ g1r1a.1 g1d4m.1 g1rhi.1 d1b35b_ d1oopa_ d1tme1_ d4iv1a_
g1pvc.1 g1fpn.1 d1fpn1_ d4gb31_ d1d4m1_ d1fmd1_ d1ev11_ d2wzr3_
g1cov.1 d1qqp3_ g1fmd.1 d2x5ic_ d1rhi3_ g1oop.1 d1hxs1_ g1hxs.1
d1fpn3_ d1bev1_ g2mev.1 d1eah3_ d1aym1_ d1pvc3_ d1qqp1_ d1zba3_
d1h8ta_ d3vbhc_ g1b35.1 g1bev.1 g1ev1.1 d1bev3_ g1tmf.1 d1tmf1_
d1b35a_ d2mev3_ g1eah.1 g1h8t.1 d1eah1_ d4q4w3_ d1r1a1_ d1aym3_
d1pvc1_ d1zba1_ d1h8tc_ d1oopc_ d1tme3_ g1qqp.1 d4iv1c_ g1tme.1
d1r0ka3 d1j5pa3 d1vkoa2 d1vm6a2 d3qvsa2 d1gr0a2 d1p1ja2 d2ovga_
d1sq8a_ d1rzsa_ d3kz3a_ d3orca_ d3gpdg2 d2q49a2 d1hdgo2 d2g17a2
d2cvoa2 d2czca1 d3pwka2 d1obfo2 d1i32a2 d1zyao2 d2pkqo2 d1b7go2
d2hjsa2 d1cf2o2 d1vkna2 d1nvmb2 d1ux8a_ d4i0va_ d2bkma_ d1dlwa_
d2bmma_ d2msya_ d2djoa1 d2r5za_ d1au7a1 d1f43a_ d2lmda1 d1akhb_
d1s7ea1 d3nara_ d1zq3p1 d2craa1 d1mija_ d4uuta_ d3a01a_ d1x58a1
d3rkqa1 d3cmya1 d2cqxa1 d2da6a1 d1e3oc1 d1fjla_ d1mh3a1 d1ig7a_
d1x41a1 d1wh7a1 d1gt0c1 d2mw8a_ d1lfup_ d1x2ma1 d1b72a_ d1wi3a1
d1jgga_ d1wjha1 d1x2na1 d1mnmc_ d2cuea1 d3k2aa_ d2hosa_ d2jwta2
d1pufa_ d1octc1 d2hi3a_ d1b8ia_ d4j19a_ d3lnqa_ d1wh5a1 d1b8ib_
d1fexa_ d2cqra1 d1guua_ d1ug2a1 d2cqqa1 d2ajea1 d4a69c_ d1h8ac1
d2crga1 d2iw5b1 d1xc5a1 d1ofcx1 d2cu7a1 d1wgxa1 d2cjja1 d2ckxa1

d1gvda_ d1hcra_ d1tc3c_ d1gdta1 d1u78a2 d6paxa1 d6paxa2 d1k78a1
d2r80b_ d1cqxa1 d1cg5a_ d1gcvb_ d3boma_ d2h8fb_ d2raoa_ d3bj1a_
d4hrrb_ d2r50a_ d3d1kb_ d2aa1b1 d3zhwa_ d3hf4b_ d3gdja_ d1jebb_
d3bj1b_ d1x9fb_ d1wmub_ d1gcva_ d3bcqb_ d3d4xb_ d1gvha1 d2dc3a_
d3hf4a_ d4yu3a_ d3k8ba_ d1qpwb_ d2r80a_ d3bcqa_ d1v4wa_ d3wtgb_
d1jl7a_ d4esaa_ d3d1ab_ d1fsla_ d4h2la_ d1v4wb_ d1mwca_ d1vhba_
d1jeba_ d1spgb_ d1h97a_ d1q1fa_ d1tu9a1 d3d1aa_ d1hdsb_ d3hyub_
d2zfbb_ d3fs4a_ d1oj6a_ d1shrb_ d4h2lb_ d4yu3b_ d1spga_ d3hrwa_
d3qqra_ d3at5a_ d4wchd_ d3hyua_ d1cg5b_ d1uc3a_ d1hbra_ d2raob_
d1outb_ d4esab_ d3gdjb_ d3qqqa_ d1x9fc_ d3vrfa_ d1itha_ d3mkba_
d3d4xa_ d3mjpa_ d1jl6a_ d3fh9a_ d1outa_ d2dc3b_ d1it2a_ d3eoka_
d3g46a_ d1qpwa_ d3vrfb_ d1ch4a_ d1x9fd_ d1hdsa_ d2zfba_ d3mkbb_
d1or4a_ d3wtga_ d1a4fa_ d3fh9b_ d3bomb_ d3cy5a_ d3at5b_ d1x9fa_
d2oifa_ d1hlva1 d1iufa2 d1iufa1 d1bl0a1 d1bl0a2 d1d5ya2 d3lv8a_
d1s3ga1 d1zaka1 d1s4qa_ d3tvta2 d1uf9a_ d1khta_ d1ckea_ d1osna_
d1p5zb_ d4tmka_ d3cm0a_ d1tmka_ d1jaga_ d3h86a_ d1t0hb_ d1nn5a_
d2h92a_ d3kb2a1 d2c95a1 d1yj5a2 d1kgda_ d1ly1a_ d2ak3a1 d1vyva2
d2grja1 d1p3ja1 d1y63a_ d1e4ya1 d1s96a_ d3iija1 d1lvga_ d1nksa_
d1vyua2 d4e22a_ d1qf9a_ d1jjva_ d1q3ta_ d1ki9a_ d1lw7a2 d1teva_
d1kjwa2 d1gsia_ d2iyva2 d1viaa_ d1e6ca_ d1g3ma_ d1j99a_ d1t8ta1
d1nsta_ d1fmja_ d1zrha_ d1ls6a_ d2a3ra_ d1aqua_ d2zptx_ d3ckla_
d1texa_ d1q44a_ d2gwha_ d1odfa_ d1uj2a_ d4gi7a_ d2jeoa1 d1rz3a_
d1z0ja1 d3umra2 d3gj0a1 d2gaoa1 d1yzga1 d1x3sa1 d1g7sa4 d1puja_
d1udxa2 d1kk1a3 d2o52a_ d1jwyb_ d1zcaa2 d1zcba2 d2fv8a1 d2cjwa_
d3dz8a1 d1xzpa2 d1vg8a_ d4qxaa_ d2ew1a1 d1x1ra1 d2bcgy_ d3doea1
d3cona_ d3rega_ d3wyga_ d1upta_ d2b6ha1 d1zd9a1 d1lnza2 d1r5ba3
d1r2qa_ d2f9la1 d2rhda_ d3q3jb_ d3q85a_ d1efma1 d1r8qa_ d2gf9a1
d2akab1 d1ek0a_ d1z08a1 d2gcoa1 d2dy1a2 d4g01b_ d2cxxa1 d1svia_
d3q72a_ d1ni3a1 d4ac9a4 d2erxa1 d2bmea_ d1wxqa1 d2gf0a1 d4ldja1
d1zunb3 d1d5ca_ d4lv5b_ d1efca3 d1kmqa_ d2g6ba1 d3lrpa_ d1tada2
d2y8ea_ d4rd4a1 d1z06a1 d2heia_ d3a58b_ d1u0la2 d1d2ea3 d1zj6a1
d2ocba_ d3rwoa_ d1mr3f_ d2e9sa1 d4u5xa1 d2iwra_ d2fn4a1 d3bwdd_
d2nzja1 d2atva1 d2ksqa_ d3l43a_ d1mkya2 d1mkya1 d1wmsa_ d1jnya3
d2rmka_ d1f6ba_ d1f60a3 d1gota2 d1jala1 d2fh5b1 d1g16a_ d2wblc_
d1m7ba_ d1wf3a1 d1f5na2 d1t9ha2 d1z6xa1 d1z0aa1 d2fg5a1 d2a5ja1
d2bcjq2 d1s0ua3 d1ky3a_ d4y0va_ d4lhwa_ d2qmea1 d2atxa1 d1egaa1
d1ryha_ d1moza_ d1nrjb_ d2erya1 d3bfka_ d1puia_ d1fzqa_ d3j3az1
d3t5ga_ d1shza2 d3defa_ d2f7sa1 d3raba_ d3tsoa_ d3aq4a_ d2al7a1
d3sfva1 d3bc1a_ d2ncda_ d1sdma_ d3h4sa1 d2heha_ d2grya_ g3kin.1
d1kk8a2 d1br2a2 d1goja_ d1f9va_ d2mysa2 d3t0qa_ d4db1a1 d3gbja_
d1ry6a_ d1v8ka_ d1k8ka2 d1k8ka1 d3uleb1 d3i33a2 d3i33a1 d1dkgd2
d1dkgd1 d4ci6a2 d1jcfa1 d4a2ba2 d2fxua1 d1yaga2 d1yaga1 d4cbua2
d1bupa2 d1g99a1 d2e1za2 d2e1za1 d1saza1 d1saza2 d1iqca2 d1eb7a2
d1eb7a1 d1nmla2 d1nmla1 d2d0oa3 d2d0oa2 d1nbwa3 d1hroa_ d4gedb_

d3zooa_ d1mz4a_ d1gu2a_ d1f1fa_ d1dvva_ d1c6ra_ d1c75a_ d1e29a_
d1gdva_ d1cnoa_ d1h32b_ d1co6a_ d2bh4x_ d2aiua_ d5cytr_ d1jdla_
d1i8oa_ d2zboa_ d3dmia_ d1ls9a_ d1wvec_ d2yk3a_ d3ph2b_ d1dw0a_
d5aura_ d1ql3a_ d1qn2a_ d1f1ca_ d4zzva_ d2xspa_ d1eg1a_ d4v20a_
d1ojja_ d4ipma1 d4csia_ d3pfza_ d3ovwa_ d2yoka_ d2nqya_ d1h4ga_
d1hixa_ d1oa2a_ d1olra_ d3b7ma_ d4h7ma1 d3wp3a_ d2vgda_ d1igoa_
d3zsea_ d2nlra_ d3wx5a_ d1oa3a_ d1pvxa_ d1m4wa_ d1f5ja_ d1oa4a_
d1ks5a_ d4ixla1 d2ah2a1 d1n1ta1 d1evsa_ d1cnt1_ d1pvhb_ d1z7ca_
d1huwa_ d1bgea_ d1rhga_ d1f6fa_ d1f45b_ d1i1rb_ d1axia_ d1etea_
d3tgxb_ d1scfa_ d2z3qa1 d3qb1a_ d1hula_ d1m48a_ d4nkqb_ d1eera_
d1v7mv_ d5e4ea_ d2o27a_ d3b5ka_ d2psma1 d4zf7a_ d3uf5a_ d4x51a_
d1d9ca_ d4ypgc1 d3oq3a_ d1lqsl_ d1n1fa_ d1au1a_ d1wu3i_ d3besl_
d3ux9a_ d1v8fa_ d2ejca_ d3muea1 d3q12a1 d2el7a_ d1iq0a2 d1irxa2
d1ivsa4 d2cyba_ d1rqga2 d1j09a2 d2yxna_ d1f7ua2 d1h3fa1 d1ffya3
d2g36a1 d1nzja_ d1jila_ d1n3la_ d1i6la_ d1pfva2 d1h3na3 d3n9ia_
d3sz3a1 d3sw2a_ d1moxc_ d1dx5i3 d1edmb_ d1autl2 d1autl1 d1nqlb_
d1dval1 d1szba2 d1kigl_ d2y5fl_ d1ijqa2 d1gk5a_ d1g1sa2 d1nt0a3
d1xdtr_ d1lmja2 d1lmja1 d1nzia2 d2wphe_ d1klil_ d1pfxl2 d1pfxl1
d1p9ja_ d1g1ta2 d1f7ea_ d4yzub_ d1f56a_ d1bypa_ d1azca_ d1ws8a_
d3u25a_ d1cuoa_ d1id2a_ d1rkra_ d2iaac_ d4dp1x_ d1nwpa_ d2gima_
d2i7sa_ d1qhqa_ d2y5ia_ d1qlsa_ d1psra_ d3n22a_ d2egda_ d4ggfc_
d2wcea_ d2luca_ d4aqia_ d3nsia_ d2lp3a_ d1j55a_ d1k8ua_ d1nsha_
d1pvaa_ d3fs7a_ d1ttxa_ d1rwya_ d2kyfa_ d1rk9a_ d1a75a_ d1exra_
d1tiza1 d1nyaa_ d1g8ia_ d1ggwa_ d1wdcc_ d3fwba_ d1ggza_ d2o5ga_
d1ctaa_ d1m45a_ d3i5gc_ d1sl8a_ d1fpwa_ d2scpa_ d2roaa_ d4aqra_
d3pn7b_ d1br1b_ d2mysb_ d1s6ca_ d1bjfa_ d2obha_ d1jfka_ d1jf0a_
d1qv0a_ d1lkja_ d2mlfc_ d2m97a_ d2d8na_ d4by5a_ d1jbaa_ d4nqga_
d1wdcb_ d2mysc_ d1j7qa_ d1s6ia_ d1uhka2 d1r2ua_ d1dtla_ d3kf9a_
d3pn7c_ d1qjta_ d1fi6a_ d1c07a_ d5d67d_ d1tuza_ d1h8ba_ d1eg3a1
d2zkmx1 d1snla_ d1gjya_ d1y1xa_ d1hqva_ d1dvia_ d1df0a1 d4usla_
d1k94a_ d1wkya2 d1uuqa_ d3wh9a_ d4m1ra_ d1xyza_ d4xuya_ d2je8a5
d4bf7a1 d3cuia_ d1ur4a_ d1qw9a2 d1odza_ d2vzsa5 d1bqca_ d2x2ya1
d1hjqa_ d1qnra_ d4y7ea1 d1vjza_ d4k68a_ d2v3ga_ d4hpga_ d1nofa2
d1nq6a_ d1h4pa_ d1aq0a_ d3ziza_ d4bs0a_ d1ecea_ d1h1na_ d1ta3b_
d1w91a2 d1i1wa_ d1uhva2 d1us3a2 d1egza_ d7a3ha_ d1tvna1 d3nyda1
d1ghsa_ d2cyga1 d4xv0a_ d2c8na2 d1w32a_ d3emca_ d1v6ya1 d1foba_
d2uwfa1 d2fgla_ d3niya1 d2c0ha1 d1rh9a1 d1yq2a5 d1szna2 d1fa2a_
d1pama4 d5do8a1 d1ea9c3 d1hx0a2 d2fhfa5 d1qhoa4 d1avaa2 d1m53a2
d4m8ua1 d2gjpa2 d1lwha2 d4howa1 d1ktba2 d1j0ha3 d3vm5a1 d2x1ia_
d1wzaa2 d1uasa2 d2diea1 d1mxga2 d1ht6a2 d1eswa_ d1iv8a2 d5cpsa_
d1ud2a2 d1zjaa1 d3gbea1 d1kwga2 d1wdpa_ d1gcya2 d1g5aa2 d1hvxa2
d3dhpa2 d2bhua3 d4do4a1 d3qgva1 d1e43a2 d1g94a2 d2d3na2 d1gjwa2
d1gvia3 d1x1na1 d1r7aa2 d1tz7a1 d4toqa_ d1wb0a1 d1w9pa1 d1eoka_
d1itxa1 d1kfwa1 d1goia2 d1edqa2 d2gsja_ d1hjxa1 d1jnda1 d1vf8a1

d3wdpp1 d3wq5a_ d1gnxa_ d4gxpa_ d4hz8a_ d1e4mm_ d3aiua_ d1wcga1
d3ahxa_ d1v02a_ d2dgaa_ d1pbga_ d1vffa1 d1qoxa_ d4ha4a_ d2csua1
d3q9ub_ d2yv1a1 d3q9na_ d2d59a_ d1oi7a1 d2yv2a1 d1euca1 d1y81a1
d3nvva1 d1krha3 d2h88b1 d1n62a2 d1t3qa2 d1kf6b2 d1zoyb1 d1jq4a_
d1ffva2 d1dgja2 d1rm6c2 d9ldta1 d2ewda3 d1up7a1 d2dfda1 d1s6ya1
d1vjta1 d7mdha1 d1hyea1 d1t2da1 d1ojsa3 d1b8pa1 d1ldna1 d1hyha1
d1civa1 d2a92a1 d1ez4a1 d1y6ja1 d1gv0a1 d1ur5a1 d1llda1 d1obba1
d1v8ba1 d1gdha1 d1l7da1 d2dlda1 d1mx3a1 d1ygya1 d1qp8a1 d1li4a1
d1j4aa1 d1npya1 d3t4ea2 d1a4ia1 d1o0sa1 d2hk9a2 d2egga2 d1b26a1
d1bvua1 d1gq2a1 d1c1da1 d1leha1 d1gtma1 d1euza1 d1nvta1 d3dona2
d1edza1 d1vi2a1 d1p77a1 d1nyta1 d1b0aa1 d1vl6a1 d1v9la1 d1txga2
d3f47a1 d2pv7a2 d1xc2a3 d2ahra2 d4xiya1 d2f1ka2 d1pgja2 d1i36a2
d1np3a2 d1lj8a4 d1teaa3 d1qmga2 d2i76a2 d2g5ca2 d1wdka3 d1dlja2
d3cuma3 d1f8fa2 d1qora2 d1jqba2 d1vj0a2 d1cdoa2 d1gu7a2 d1d1ta2
d1xa0a2 d2dm6a2 d1jvba2 d1rjwa2 d1llua2 d1h2ba2 d1tt7a2 d1piwa2
d1o89a2 d1iz0a2 d1e3ja2 d1yb5a2 d1uufa2 d1vj1a2 d1e3ia2 d3gpdg1
d1vc2a1 d2nvwa1 d1u8fo1 d1ydwa1 d1hdgo1 d2g17a1 d1cf2o1 d2czca2
d1r0ka2 d1obfo1 d2cvoa1 d1ebfa1 d1h6da1 d1i32a1 d1zh8a1 d2q49a1
d1qkia1 d1j5pa4 d3pzra1 d1xeaa1 d1vkoa1 d1vm6a3 d3qvsa1 d2pkqo1
d1b7go1 d1gr0a1 d1p1ja1 d1lc0a1 d1j0xo1 d3pwka1 d2hjsa1 d1zyao1
d1vkna1 d1tlta1 d1nvmb1 d3sjua_ d3ojfa1 d1xu9a1 d2jjya1 d1n7ha_
d1y1pa1 d1h5qa_ d1jtva_ d4fs3a_ d2bkaa1 d1gy8a_ d2c5aa1 d1ulsa_
d1ooea_ d2b69a1 d1y5ma1 d1spxa_ d1ae1a_ d1bxka_ d1ydea1 d1gega_
d1hdoa_ d1o5ia_ d1e6ua_ d1snya1 d2rhca_ d3zv4a_ d5ceja_ d2d1ya1
d1fjha_ d3w8ea_ d2fmua1 d1x1ta_ d1rpna_ d3ay7a1 d1k2wa_ d1eq2a_
d2hrba1 d3ruaa_ d2ew8a1 d4hwka_ d1xgka_ d2bd0a1 d1yo6a1 d2a35a1
d2ntva_ d1vl8a_ d1hdca_ d4bkua_ d2ag5a1 d2q45a_ d1nffa_ d4iy1a_
d1zbqa1 d1kewa_ d1ek6a_ d1ja9a_ d2q46a2 d1iy8a_ d1cyda_ d2ptga_
d1hxha_ d1uzma1 d3lyla_ d4m89a_ d3wyea_ d2blla1 d1w6ua_ d1sb8a_
d2q2va_ d4nr0a_ d1e6wa_ d2gasa_ d3oiga1 d2bgka1 d1mxha_ d1vl0a1
d1rkxa_ d1r6da_ d1wmaa1 d1e7wa_ d2c07a1 d1xkqa_ d3tzha_ d1g0oa_
d3lz6a_ d1qyca_ d1edoa_ d1gz6a_ d1oc2a_ d1t2aa_ d1zema1 d2xoxa_
d1z45a2 d1geea_ d1p33a_ d3lt0a_ d4e6pa_ d2wyua_ d1db3a_ d4q9na_
d1xhla_ d4o1ma_ d1fmca_ d2uvda_ d2gdza1 d1uaya_ d2z1ma_ d2o23a2
d2fr1a2 d2fr1a1 d1qsga_ d1qyda_ d4zjua_ d2a4ka1 d1yb1a_ d1wvga1
d1yxma1 d3f4ba_ d1mg5a_ d1xg5a1 d1r55a1 d3gboa_ d1atla_ d1kufa_
d1buda_ d4q1la_ d1quaa_ d1yp1a_ d1wnia_ d4ekfa_ d2ckga_ d1th0a_
d2bkra_ d1qxpa4 d1ziva1 d1zcma1 d3ioqa_ d1fh0a_ d1o0ea_ d1khqa_
d1m6da_ d2cb5a_ d1cs8a_ g8pch.1 d1s4va_ d2pnsa_ d1vsna1 d1mira_
d1iwda_ d2oula_ d7pcka_ d3bwka_ d3bcna_ d2p86a_ d1pvja_ d1pcia_
d1gece_ d1deua_ d4bs6a_ d1xkga1 d1cqda_ d1jqpa2 d2c0ya_ d2bdza_
d3p5ua_ d1zoya1 d1qo8a2 d1d4ca2 d1chua2 d1jnra2 d1kf6a2 d2jk6a2
d2jk6a1 d1feca1 d1feca2 d1lpfa1 d1lpfa2 d1xhca1 d1xhca2 d1dxla2
d1dxla1 d1gera2 d1m6ia2 d1onfa1 d1onfa2 d3lada2 d3lada1 d1w4xa1

d1w4xa2 d1gesa2 d1mo9a1 d1xdia1 d1fl2a2 d1fl2a1 d1fcda1 d1fcda2
d1h6va2 d1h6va1 d1mo9a2 d1gv4a2 d1gv4a1 d2gv8a2 d2gv8a1 d1aoga1
d1aoga2 d1hyua1 d1ebda1 d1ebda2 d3dk9a1 d1q1ra2 d1q1ra1 d1gesa1
d1zbfa1 d2z1ha_ d2etja1 d3qioa1 d1jl1a_ d2e4la_ d1jl2a_ d2lsna1
d1io2a_ d1uaxa_ d2yv0x_ d1mu2a1 d1ekea_ d1i39a_ d1ps9a2 d1o94a2
d1cjca1 d1pn0a1 d1reoa1 d1ju2a1 d2f5va1 d3c96a1 d2gmha1 d1pj5a2
d1o5wa1 d1n4wa1 d1k0ia1 d2dw4a2 d2ivda1 d1seza1 d2voua1 d1kdga1
d1s5ja1 d2hbja2 d2igia_ d1wlja_ d1qhta1 d4dlea1 d1tgoa1 d2f96a1
d3tr8a1 d3mxma_ d1q8ia1 d1y97a1 d1w0ha_ d1kfsa1 d2py5a1 d1noya_
d1d5aa1 d1yt3a3 d2guia_ d3mwpa1 d4kb1a_ g1apy.1 d3ljqa_ g1k2x.1
d1v58a1 d1t3ba1 d4ilfa2 d4npba2 d4iuaa3 d4bvva_ d5hpga_ d3k65a_
d1ki0a3 d1ki0a2 d1ki0a1 d1a0ha1 d1pmla_ d3sp8a3 d2feba1 d1r7ha_
d1faaa_ d1wika1 d1xfla_ d1ttza1 d2f51a1 d1ti3a_ d1nhoa_ d1sena_
d2i4aa_ d1ep7a_ d1zmaa1 d1fo5a_ d3lefa1 d3diea1 d2yana1 d1z6na1
d1oaza_ d3zywa1 d4aj8a_ d1nm3a1 d4fiwa_ d1wmja_ d4hs1a1 d1iloa_
d1xwaa1 d1wjka1 d2vm1a_ d1fb6a_ d1gh2a_ d1h75a_ d1nw2a_ d1kx5b_
d3a6na_ d4m6ba2 d2nqbd_ d3wkjd_ d2yfwe_ d4h9na_ d5bnxa_ d1kx5c_
d3r45a_ d1eqzb_ d4jjnb_ d1f66c_ d1eqza_ d4wnnb_ d1kx5a_ d1u35c1
d1id3a_ d2nqbc_ d1tafa_ d1bh9b_ d1tafb_ d1jfib_ d1h3ob_ d2byka1
d1n1ja_ d4g92b_ d1bh9a_ d1jfia_ d1h3oa_ d2bykb1 d1n1jb_ d1pd212
d1nhya2 d2a2ra2 d1k0dd2 d1f2ea2 d1gsua2 d1f3ba2 d2fhea2 d2pvqa1
d1tw9a2 d1oyja2 d1e6ba2 d1k0ma2 d3ik7a1 d4is0a1 d1r5aa2 d1jlva2
d1m0ua2 d3gtub2 d1z9ha2 d1okta2 d1axda2 d2gsra2 d1tu7a2 d1k3ya2
d1zgma1 d1gnwa2 d1gwca2 d2gsta2 d1glqa2 d1oe8a2 d1pn9a2 d1v2aa2
d1fw1a2 d1duga2 d3vrda1 d3vrda2 d1fcdc2 d1fcdc1 d1h1oa2 d1h1oa1
d1m70a1 d1ig8a1 d1czan2 d1czan3 d1czan1 d1czan4 d1bg3a4 d1bg3a3
d1bg3a2 d1bg3a1 d4ilya_ d1chka_ d4olta_ d1qqya_ d1gd6a_ d1f6ra_
d1ivma_ d2gv0a_ d1iiza_ d2z2fa_ d1j8xa_ d1ghla_ d1b9oa_ d174la_
d169la_ d2o4wa_ d1p37a_ d1k28a3 d1xjta1 d1jtma_ d2f2qa_ d4e97a1
d2pjsa1 d2i7ra1 d1ecsa_ d1xqaa1 d5cj3a1 d1npba_ d1jifa_ d2p7oa_
d1r9ca_ d1nkia_ d1mpya2 d1kw3b2 d1kw3b1 d4ghga1 d1tfza1 d3isqa1
d3isqa2 d1sqia1 d1sqia2 d1lkda2 d1lkda1 d1mpya1 d1cjxa2 d1cjxa1
d1sp9b2 d1t47a1 d1t47a2 d1f1ua2 d1sp8a1 d1sp8a2 d1pv8a_ d1w5qa_
d2c1ha_ d1l6sa_ d1qsta_ d2beia1 d4m3sa_ d1u6ma1 d1ygha_ d1yk3a1
d3dr6a_ d1xmta_ d1z4ea1 d2ge3a1 d1bo4a_ d2fiaa1 d2jdca1 d1y9wa1
d3to7a_ d1wwza1 d2fl4a1 d2ozua1 d2b5ga_ d2cy2a1 d2p0wa_ d1cm0a_
d4pswa_ d1yrea1 d1yr0a1 d1m4ia_ d2gana1 d1yvka1 d1ghea_ d2g3aa1
d1y7ra1 d2euia1 d1q2ya_ d2fsra1 d1xeba_ d2ae6a1 d1cjwa_ d1qsma_
d2aj6a1 d1y9ka1 d1i12a_ d1vkca_ d2atra1 d1n71a_ d2d4pa1 d2fiwa1
d2fe7a1 d1tiqa1 d2ou2a_ d1yx0a1 d2fcka1 d1p0ha_ d2j8ma_ d2pq8a_
d1z4ra1 d1s3za1 d1ufha_ d2hqya1 d2hqya2 d1ne9a1 d1lrza3 d1ne9a2
d1xvqa1 d1pn0a2 d3hy2a_ d2cvba1 d1o8xa_ d1lu4a_ d1we0a1 d2bmxa1
d2a4va1 d1psqa_ d1xiya1 d1q98a_ d1n8ja_ d1zofa1 d2ggta_ d3tjja_
d4je1a1 d1o73a_ d3s9fa_ d2zcta_ d1qmva_ d1jfua_ d2b1ka_ d5dvba1

d2b5xa1 d2v2ga_ d1st9a_ d3tb2a_ d4llra_ d4fh8a1 d1e2ya_ d1knga_
d2h30a_ d1i5ga_ d2z9sa_ d2f8aa1 d1tp9a1 d2b7ka_ d1nm3a2 d3hvva_
d2wfca1 d4kcea_ d1xvwa1 d4gqca1 d3qpma_ d4k1fa_ d1zzoa1 d1gp1a_
d1prxa_ d4od7a1 d1z6ma1 d4mcua1 d4dvca1 d1r4wa_ d3l9sa_ d3rppa_
d2qked_ d4ksoa_ d1t4za1 d1wwja_ d1f74a_ d3l21a_ d2e1da_ d1to3a1
d1w3ia_ d1sfla_ d1n7ka_ d1vcva1 d4imea_ d1wa3a_ d2ocza1 d1f2ja_
d3m16a_ d2yxga_ d1ub3a_ d3a5fa_ d1fbaa_ d1l6wa_ d1xfba1 d1wx0a1
d1vpxa1 d1ojxa_ d2a4aa1 d1xkya1 d3cq0a1 d3s1xa_ d1f05a_ d1fdja_
d3cpra1 d4d2ja_ d1cg2a1 d1yloa2 d1y7ea2 d1y0ya3 d1lfwa1 d1vixa1
d1fnoa4 d1vhoa2 d1ysja1 d1vhea2 d1z2la1 d2fvga2 d1vgya1 d1r3na1
d2grea2 d1xmba1 g1q7l.1 d1rtqa_ d1w52x1 d2ppla1 d1hpla2 d1lpbb2
d2oxea1 d1bu8a2 d1rp1a2 d1ys1x_ d4hs9a_ d1ispa_ d1ex9a_ d1ku0a_
d3qmma_ d1ra6a_ d1muka_ d3gsza_ d1khva_ d1te9a_ d2xwha_ d3uqsa_
d3hkwa_ d3n6ma_ d2cjqa_ d2xyma_ d1teba_ d1te8a_ d1s48a_ d1u09a1
d4wfxa_ d3tyqa_ d2pgga1 d1lgya_ d4l3wa_ d1llfa_ d1gz7a_ d1ukca_
d2ftba_ d1pmpa_ d2rcqa1 d1fdqa_ d4lkpa_ d1sa8a_ d1vyfa_ d1lpja_
d3vg7a1 d4azqa1 d1o8va_ d5bvqa_ d1p6pa_ d2qo4a_ d1opba_ d1yiva1
d1o1va_ d4bvma1 d4tkba_ d4i3ba_ d1bwya_ d1ftpa_ d1ggla_ d4gkca_
d1cbia_ d3p6da1 d1kqwa_ d2a0aa1 d3p0ya1 d1igra1 d1igra2 d1n8yc2
d1n8yc1 d1m6ba2 d1m6ba1 d4dv8a2 d1dzka_ d4tlja_ d2a2ua_ d4wfva_
d3t1da_ d4iaxa_ d3zq3a_ d2nnda_ d1i4ua_ d1kxoa_ d1bbpa_ d1kt7a_
d1exsa_ d3s26a1 d1lf7a_ d1qfta_ d4gh7a_ d1z24a1 d1qabe_ d1xkia_
d1ew3a_ d3bx7a_ d4iawa1 d1n0sa_ d1gkab_ d3kzaa_ d2k23a_ d2l9ca1
d2joza1 d1gm6a_ d3kffa_ d3saoa1 d1e5pa_ d1yupa1 d1iiua_ d2tbvc_
d1x36a_ d1smvc_ d1ng0c_ d4sbvc_ d1opoa_ d1f2nc_ d1cixa_ d1y29a_
d1v7fa_ d2jtba_ d1g9pa_ d1lupa_ d1qk7a_ d1mb6a_ d2a2va_ d1niya_
d1i25a_ d1hvwa_ d2mpqa_ d1ie6a_ d1kqha_ d1la4a_ d2mxoa1 d1dl0a_
d1koza_ d1qk6a_ d1emxa_ d1lqra_ d1c6wa_ d1rmka_ d1ixta_ d1eyoa_
d1cnna_ d1fu3a_ d1g1pa_ d1f3ka_ d1fyga_ d1rypd_ d1irua_ d1rypc_
d3unfk_ d1rypa_ d1j2pa_ d1irui_ d1j2qh_ d3unfh_ d1m4ya_ d1rypb_
d1irul_ d1irug_ d1iru2_ d1q5rh_ d1rypg_ d1g3ka_ d1q5qa_ d1e94a_
d1irub_ d1ryph_ d3ty6a_ d1iruj_ d1iruk_ d1rype_ d1irud_ d1irue_
d3mi01_ d1rypf_ d1iruh_ d2z3ba_ d1iruc_ d3mka2_ d1iru1_ g1or0.1
g1cp9.1 g1e3a.1 d1jgta2 d1ofda3 d1ea0a3 d1q15a2 d1ct9a2 d1ao0a2
d1te5a_ d1y14b1 d2nn6i1 d1i94l_ d2zjra2 d1smxa_ d2qamc2 d1hh2p1
d2r7da1 d1rl2a2 d1wfqa1 d3bzka4 d1x6oa2 d3a0ja_ d2j01d2 d2ja9a1
d4ql5a_ d1hr0w_ d1luza_ d2n3sa_ d2vnud1 d1h95a_ d1go3e1 d2nn6h1
d1ne3a_ d2mo1a_ d1iz6a2 d1jjga_ d1e3pa2 d2ix0a3 d2ix0a2 d2lxja_
d1ueba3 d1ueba2 d2z0sa1 d1u0la1 d2ix0a1 d2eifa2 d2je6i1 d3i2za1
d2c35b1 d2ahob2 d1t9ha1 d3i4oa_ d2qall1 d1jt8a_ d2nn6g1 d1g6pa_
d3cama_ d1i94q_ d1c9oa_ d1d7qa_ d2ba0a1 d2vnud2 d2vnud3 d1khia2
d2qalq1 d1wi5a1 d1xtda2 d1ny4a_ d4w9ta_ d1jvna1 d4x2ra1 d1qo2a_
d4axka1 d1thfd_ d1h5ya_ d4u28a_ d2y88a_ d4wd0a_ d1vzwa1 d3ia2a_
d4dgqa1 d1hkha_ d1zoia_ d2flia1 d1rpxa_ d1tqja_ d1tqxa_ d4lt9a1

| | | | | | | | |
|---|---|---|---|---|---|---|---|
| d3tc7a1 | d3hoja_ | d1i4na_ | d3vnda_ | d3nl8a1 | d3nava1 | d1v5xa_ | d4a2ra1 |
| d3tc6a1 | d1ujpa_ | d4ijba_ | d1xcfa_ | d1vc4a_ | d1rd5a_ | d1qopa_ | d3b5va_ |
| d3gv5b1 | d1t94a2 | d3bq0a2 | d4lmxb_ | d1cpcb_ | d3v57a_ | d1kn1a_ | d1cpca_ |
| d4f0ta_ | d2vjha_ | d4po5b_ | d1eyxa_ | d1alla_ | d4f0tb_ | d1b33b_ | d1b8db_ |
| d4h0mb_ | d1b33a_ | d2vjhb_ | d4lm6b_ | d2vmlb_ | d1allb_ | d2vjta_ | d3dbja_ |
| d2vmla_ | d2vjtb_ | d4f0ub_ | d1f99b_ | d4rmpa1 | d1liaa_ | d1eyxb_ | d2uulb_ |
| d4lmsb_ | d4f0ua_ | d2bv8a_ | d1f99a_ | d2bv8b_ | d2c7lb_ | d3dbjb_ | d4rmpb1 |
| d1b8da_ | d4h0ma_ | d3v57b_ | d1kn1b_ | d2uula_ | d2j96a_ | g1gxs.1 | d2xqfa_ |
| d1ea5a_ | d1f6wa_ | d1dx4a_ | d1k4ya_ | d4qwwa_ | d2ha2a_ | d3c8da2 | d2pbla1 |
| d1jkma_ | d1jjia_ | d1jjfa_ | d1gkub1 | d1gkub2 | d2qvba_ | d2v9za_ | d4kafa1 |
| d1b9ra_ | d1frra_ | d3wcqa_ | d1e9ma_ | d1l5pa_ | d1wria_ | d1i7ha_ | d3av8a_ |
| d1czpa_ | d1rfka_ | d3p1ma1 | d3b2ga_ | d2bt6a2 | d1s5ja2 | d1qhta2 | d4dlea2 |
| d1tgoa2 | d3py8a2 | d1q8ia2 | d1kfsa2 | d2py5a2 | d1d5aa2 | d2m5ta_ | d1cqqa_ |
| d1wqsa_ | d5bpea_ | d2ipha_ | d2bhga1 | d3tnta_ | d3sjia_ | d3d23a1 | d2fyqa_ |
| d3tloa_ | d3zvea1 | d2hrva_ | d3q3ya1 | d4asha1 | d1e88a1 | d1l6ja5 | d1l6ja4 |
| d1eaka4 | d1eaka3 | d1l6ja3 | d1h8pa2 | d3rp2a_ | g1ucy.1 | d1fuja_ | d2zchp_ |
| d3w94a_ | d1si5h_ | d1lo6a_ | g2pka.1 | d1cgha_ | d1kigh_ | d1a5ia_ | d3s9ba_ |
| d1hj8a_ | d1ao5a_ | d2anya_ | d1q3xa1 | d1gvza_ | d1azza_ | d4gsoa_ | d2f91a1 |
| d1iaua_ | d1gdna_ | d1eufa_ | g1fiw.1 | d1eq9a_ | d4nfea_ | d2rdla_ | d1spja_ |
| d1elva1 | d1a7sa_ | d1ekbb_ | d1pfxc_ | d1yc0a_ | d2aiqa_ | g1fiz.1 | d1fi8a_ |
| d4k60a_ | d4dgja_ | d1mzaa_ | d1op0a_ | d3tjva_ | d1pytd_ | d1sgfg_ | g1gg6.1 |
| d2eeka_ | g1rtf.1 | d1a0ja_ | d3g01a_ | d1pytc_ | d4e7na_ | d1ddja_ | d1z8ga1 |
| d1npma_ | d1brup_ | g1jou.1 | d1sgfa_ | d1autc_ | g2hnt.1 | g1kdq.1 | d1orfa_ |
| d3s69a_ | d4crga_ | d1fxya_ | d1bqya_ | d1svpa_ | d1mbma_ | d3su6a1 | d1cu1a1 |
| d4agka_ | d1vcpa_ | d1q31a_ | g1n1l.1 | d3e90b_ | d1ep5a_ | d3u1jb_ | d2bk7a_ |
| d3fjua_ | d1z5ra1 | d1kwma1 | d1uwya2 | d1jqga1 | d2c1ca1 | d1dtda_ | d1h8la2 |
| d3lgia_ | d3i77a_ | d2z9ia2 | d4nsva_ | d1lcya2 | d4jcna_ | d1agja_ | d1os8a_ |
| d1hpga_ | d1p3ca_ | d1l1ja_ | d1qtfa_ | d1e69a_ | d1mv5a_ | d3g60a4 | g1ii8.1 |
| d1ewqa2 | d1ji0a_ | d1qhla_ | d1v43a3 | d2wmfa1 | d1vpla_ | d1g2912 | d4a82a2 |
| g1f2t.1 | d2d62a1 | d3tuic1 | d2bbsa1 | d3g60a3 | d2ixea_ | d1g6ha_ | d1sgwa1 |
| d2qi9c_ | d3rlfa1 | d3b60a1 | d3d31a2 | d2pzea1 | g1xew.1 | d1jj7a_ | d1oxxk2 |
| d2onka1 | d1w1wa_ | d1iwea_ | d1qf5a_ | d1vmaa2 | d1nija1 | d1np6a_ | d2p67a1 |
| d3ihla_ | d1okkd2 | d1ls1a2 | d1s1ma2 | d1iona_ | d2v40a_ | d1dj3a_ | d3hida_ |
| d2vo1a1 | d2g0ta1 | d1vcoa2 | d1j8mf2 | d1g3qa_ | d1xjca_ | d1p9ba_ | d1qzxa3 |
| d1ihua2 | d2qm8a_ | d1dj2a_ | d4ioja_ | d1cp2a_ | d1yrba2 | d1hyqa_ | d3gqba2 |
| d1xp8a1 | d1tf7a2 | d1tf7a1 | d1ye8a1 | d1skyb3 | d2qe7d2 | d4blta_ | d4huta_ |
| d3ldaa2 | d4hyya_ | d2i3ba1 | d1cr1a_ | d3j9tb2 | d3ntua2 | d1vdza1 | d2z43a_ |
| d1e9ra_ | d1nlfa_ | d3ziaa2 | d1n0wa_ | d1g6oa_ | d3ziad2 | d3j9ta2 | d1pzna2 |
| d3gqbb2 | d2i49a_ | d1nnfa_ | d1xvxa_ | d1ursa_ | d1h3da1 | d1iuda_ | d4m1va1 |
| d1twya_ | d3e13x_ | d2nxoa1 | d4pfua_ | d3ttma_ | d1elja_ | d2qmwa1 | d1us5a_ |
| d1y3na1 | d1jeta_ | d1uqwa_ | d4f19a1 | d2esna2 | d3thia_ | d3fasa1 | d2uyeb_ |
| d2xwva1 | d1r9la_ | d2fyia1 | d1pb7a_ | d2ozza1 | d1sw5a_ | d1nh8a1 | d1vr5a1 |
| d2g29a_ | d1xs5a_ | d3lswa_ | d2z23a_ | d3w9va_ | d3tcha_ | d1q35a_ | d1z7me1 |
| d3q27a_ | d1p99a_ | d1ve4a1 | d2i6ea1 | d4mcoa1 | d4n8ya_ | d1eu8a_ | d1zbma1 |

d1hsla_ d1ixca2 d1j1na_ d1xvya_ d1y9ua_ d2z22a1 d1xoca1 d4oera_
d5f1qa1 d1xt8a1 d1i6aa_ d4gwoa_ d4igta1 d1pc3a_ d4mmpa1 d1o63a_
d2czla1 d2onsa2 d4mija1 d1eh3a_ d3skpa_ d1dtza1 d1ieja_ d1jnfa2
d1ce2a1 d1jnfa1 d1b1xa2 d1b1xa1 d1jw1a1 d1jw1a2 d1ryoa_ d1h76a1
d1h76a2 d1c4oa1 d2p6ra3 d2p6ra4 d1oywa2 d1oywa3 d1wrba1 d1w36b2
d1w36b1 d1c4oa2 d3bora_ d1t6na_ d2eyqa5 d2eyqa3 d2eyqa2 d2zu6a2
d1veca_ d1uaaa1 d1w36d1 d2g9na1 d1z3ix2 d2rb4a_ d1hv8a1 d1t5ia_
d1w36c2 d2j0sa2 d2j0sa1 d1fuka_ d3hjha1 d1z5za1 d1qdea_ d1z63a1
d1s2ma2 d1s2ma1 d1wp9a1 d1wp9a2 d1gm5a3 d1gm5a4 d3dina4 d3dina3
d1q0ua_ d1w36c1 d1z3ix1 d1nkta3 d1nkta4 d1uaaa2 d1w36d2 g1qhh.1
d2fwra2 d2fwra1 d3q3ma_ d1za0a1 d1mxra_ d2inca_ d1jk0a_ d2incb_
d1mhyb_ d3hf1b_ d1mtyb_ d1r2fa_ d2uw1a_ d1mhyd_ d3n37a_ d1afra_
d1oquc_ d1syya_ d1mtyd_ d2o1za_ d1uzra_ d1smsa_ d2fkza_ d1zuja1
d1s3qa1 d2fjca1 d1z6om1 d1veia_ d4dyya_ d1jiga_ d3gvya_ d2pyba_
d4cy9a1 d3bvfa1 d1r03a_ d2cf7a_ d1nf4a_ d1zs3a1 d4iwka_ d3ka8a_
d3np0x_ d1n1qa_ d1lb3a_ d4e6ka_ d1z6oa1 d2fzfa1 d4dyua_ d3ge4a_
d1jgca_ d4am4a_ d2z6ma_ d1ji5a_ d1vlga_ d1lkoa1 d3qz3a_ d1o9ra_
d2ffxj_ d1euma_ d1yv1a1 d2v2pa_ d1j30a_ d4di0a_ d1dpsa_ d2cbia2
d2choa2 d1l8na1 d1oewa_ d2qzxa_ d2asia_ d1psoe_ d1smra_ d2h6ta_
d1pfza_ d1izda_ d3hd8a_ d1j71a_ d1dpja_ d4aa9a_ d1t6ex_ g1b5f.1
g1lya.1 g1htr.1 d1qdma2 d1bxoa_ d1wkra_ d1tzsa_ d1ibqa_ d2qzwa_
d3ggta_ d3ixoa_ d1mrxa_ d2haha_ d1tw7a_ d4njsa_ d1sgua_ d2p3da_
d1nsoa_ d3d3ta_ d2rkfa_ d1c6ya_ d2rspa_ d3ogpa_ d3ekwa_ d2o4na_
d2j9ja_ d1baia_ d3ggua_ d3lzua_ d4npua_ d4j55a_ d2r8na_ d2fxda_
d2p3ca_ d4dqea_ d3ttpa_ d3t3ca_ d2p3aa_ d2hc0a_ d2fdda_ d3mwsa_
d1k8rb_ d1wgra1 d1ef5a_ d1wfya1 d1e8ya3 d1wgya1 d2cs4a1 d1wxma1
d2byea1 d3kh0a1 d2byfa1 d1wxaa1 d1e7ua3 d1h4ra3 d4yl8a1 d2al6a3
d1wgda1 d1wgga1 d4q5eb_ d1wjna1 d1se9a_ d3n3kb_ d1wz0a1 d1we6a1
d2m0xa_ d2gbka_ d3rt3b1 d1wh3a1 d1sifa_ d2ekec2 d4ii2b1 d4hk2a1
d1c3ta_ d1m94a_ d1wgha1 d1ttna1 d1wx8a1 d3v6cb1 d2ma9b_ d4i6lb1
d1bt0a_ d2jvca1 d1iyfa_ d2k1fa_ d1wxva1 d1wf9a1 d1t0ya_ d2io0b2
d1x1ma1 d2gbma_ d1zw7a1 d2m17a_ d1ud7a_ d1we7a1 d2lwpa_ d2gbja_
d1wjua1 d1v6ea1 d1v86a1 d4eewa_ d1wiaa1 d4icva_ d4gu2a_ d1wx7a1
d1wy8a1 d4hjka1 d2faza1 d1v5oa1 d3ruib_ d1eo6a_ d3waob_ d2r2qa1
d3m95a_ d3wana_ d3nqyb_ d4gera_ d1yvca1 d1yeza1 d1g66a_ d1qoza_
d5ajha1 d4lu0a_ d1of8a_ d3unda1 d3e9aa1 d1vr6a2 d2qkfa_ d1d9ea_
d1o60a_ d1wioa4 d1ccza2 d1vcaa1 d1dr9a2 d2d9qb1 d2nxyb2 d1um8a_
d1ixza_ d1qvra2 d1qvra3 d1qzma_ d1sxjc2 d1jr3d2 d2p65a_ d1l8qa2
d1g8pa_ d3hu3a3 d1sxjd2 d1njfa_ d1in4a2 d1sxja2 d1tuea1 d2ce7a2
d1w5sa2 d1sxjb2 d1d2na_ d1ny5a2 d2gnoa2 d2a5yb3 d1fnna2 d1u0ja1
d1lv7a_ d1iqpa2 d2fnaa2 d1ixsb2 d4gita_ d1sxje2 d1g41a_ d2lj7a_
d1n4na_ d2m8bb1 d2kyqa_ d3psma_ d1jkza_ d1jxca_ d2gl1a_ d4uj0a_
d2k4ua_ d1hp2a_ d2lu9a_ d1baha_ d2meoa1 d1wm7a_ d1hlya_ d1n8ma_
d1jlza_ d1wpda_ d1c55a_ d1pvza_ d1wt7a_ d1rjia_ d1wz5a_ d1m2sa_

| | | | | | | | |
|---|---|---|---|---|---|---|---|
| d1pjva_ | d1c49a_ | d1qkya_ | d2ktca1 | d1wmta_ | d1du9a_ | d1j5ja_ | d1r1ga_ |
| d1t0za_ | d2i61a_ | d1t1ta_ | d1sn4a_ | d1kv0a_ | d2lkba_ | d1npia_ | d1wwna_ |
| d1pe4a_ | d2kbka_ | d2li7a_ | d1djta_ | d2kbha_ | d1dq7a_ | d1sega_ | d1omya_ |
| d1zywa1 | d1jzaa_ | d1n9wa1 | d1bbua1 | d1e1oa1 | d1b8aa1 | d1l0wa1 | d3a74a1 |
| d1eova1 | d2h0ba_ | d2erfa_ | d1h30a1 | d3mw4a1 | d1pz7a_ | d2r1ba_ | d3v64a1 |
| d1d2sa_ | d3bopa1 | d3mw3a1 | d1okqa1 | d1okqa2 | d1iyjb4 | d1iyjb5 | d1iyjb3 |
| d1o7ia_ | d4ipca1 | d1x3ea_ | d4apva_ | d1jb7a1 | d1jb7a2 | d1jb7a3 | d3kdfa_ |
| d1ue1b_ | d1wjja1 | d3eiva_ | d1xjva2 | d1xjva1 | d2ccza2 | d1se8a_ | d1l1oc_ |
| d1jb7b_ | d1qvca_ | d3ulla_ | d1qzga_ | d1miua4 | d1miua5 | d1miua3 | d3g48a1 |
| d1gd7a_ | d1mkha_ | d1jjcb3 | d3ubub_ | d1egia_ | d1qo3c_ | d1v7pa_ | d1tdqb_ |
| d3ubua_ | d1c3ab_ | d1h8ua_ | d3bx4b_ | d1t8ca1 | d1sb2a1 | d1qdda_ | d2ziba1 |
| d3cada_ | d1byfa_ | d1j34b_ | d1jwia_ | d1jzna_ | d1c3aa_ | d1oz7b_ | d1wmza_ |
| d3bdwb_ | d1hq8a_ | d1fifa1 | d1dv8a_ | d3hupa_ | d1wk1a1 | d1umra_ | d2afpa_ |
| d3bx4a_ | d1gz2a_ | d1sl6a_ | d1v7pb_ | d3g8ka_ | d1ixxa_ | d1j34a_ | d1jwib_ |
| d1g1sa1 | d1y17a1 | d1ja3a_ | d1fvub_ | d3bdwa_ | d1sb2b1 | d1fvua_ | d1umrc_ |
| d1oz7a_ | d4m17a_ | d3cfwa1 | d1ypqa1 | d1g1ta1 | d3fd4a_ | d3l9jc_ | d4dn8a1 |
| d1v4lb_ | d1rdl1_ | d2msba_ | d1dgsa2 | d1fvia1 | d1ckma1 | d1x9na2 | d1pfsa_ |
| d2a1ka_ | d1je5a_ | d1rpma_ | d2shpa1 | d1yfoa_ | d2ooqa1 | d1g4us2 | d1p15a_ |
| d1lara2 | d1lara1 | d1wcha_ | d2fh7a2 | d2fh7a1 | d1ohea1 | d1ohea2 | d2f4da_ |
| d1rxda_ | d2hxpa_ | d1xria_ | d1fpza_ | d3ezza_ | d4y2ea_ | d1i9sa_ | d3azya_ |
| d3wvja_ | d1upsa1 | d1ajoa_ | d2hyka_ | d2vy0a_ | d1o4ya_ | d3o5sa_ | d3d6ea_ |
| d3atga_ | d1dypa_ | d1umza_ | d3i4ia_ | d1c1la_ | d4lbqa_ | d1slta_ | d4no4a_ |
| d3zsja_ | d3duia_ | d1g86a_ | d1is3a_ | d1qmja_ | d1hlca_ | d1tdha3 | d1q3ba3 |
| d1ee8a3 | g1len.1 | d3zyra_ | d1fnya_ | d1n47a_ | g2ltn.1 | d1avba_ | d3usua_ |
| d1dhkb_ | d1f9ka_ | d1ukga_ | d1g8wa_ | d1g9fa_ | d1wbfa_ | d3wcsa_ | d1g7ya_ |
| g1loe.1 | d1mvqa_ | d1hqla_ | d1v6ia_ | d1dbna_ | d1qnwa_ | d1fx5a_ | d4l8qa_ |
| d1ioaa_ | d3n35a_ | d3ubra_ | d1qo8a1 | d1d4ca1 | d2k3va_ | d1ft5a_ | d1qdba_ |
| d1m1qa_ | d2j7aa_ | d1h21a_ | d1ogyb_ | d4fasa_ | d1jnia_ | d1oaha_ | d1sp3a_ |
| d3h33a_ | d1rwja_ | d19hca_ | d1os6a_ | d3bxua_ | d3h4na_ | d1i77a_ | d3caoa_ |
| d2ctha_ | d1duwa_ | d1ofwa_ | d2e84a_ | d1hh5a_ | d1gyoa_ | d3h34a_ | d1dszb_ |
| d1a6ya_ | d3cbba_ | d1ynwa1 | d1r4ia_ | d2hana_ | d2hanb_ | d2enva1 | d1kb4b_ |
| d2nllb_ | d1lo1a_ | d1cita_ | d1adqa1 | d1fnga1 | d1uvqb1 | d1zs8a1 | d2vola1 |
| d2vola2 | d1mfbl2 | d2jelh2 | d1uwel2 | d3qq4b1 | d1kjvb_ | d1k5na1 | d1osph2 |
| d4odhl2 | d1ow0a1 | d1ow0a2 | d1bfob2 | d3l9ra2 | d3jtth1 | d2fath2 | d4elkb2 |
| d1fn4b2 | d2xfxb_ | d1pg7x2 | d1cqka_ | d4k71b2 | d1lk2a1 | d1eapb2 | d4lfhg2 |
| d1i4fa1 | d2nw2a2 | d1kfah2 | d1mhca1 | d1h3ua1 | d1wejh2 | d1h0db2 | d1a5fh2 |
| d1kcvh2 | d1mjuh2 | d1qqda1 | d1kcrh2 | d1k8ia1 | d3huja2 | d2nnab1 | d1nfdf2 |
| d3mffb2 | d1s9va1 | d1lk3h2 | d1q0xl2 | d1fv1b1 | d1neza1 | d4rfeb2 | d1r3ha1 |
| d1a6tb2 | d1kjva1 | d1pz5b2 | d1onqa1 | d2bc4a2 | d1de4a1 | d1k8da1 | d1tcra2 |
| d1dn0b2 | d1nfde2 | d2xwtb2 | d1bfoa2 | d1mexh2 | d1iakb1 | d1ypzf2 | d4f7ea2 |
| d1q72l2 | d2fbjh2 | d1fe8h2 | d1clzh2 | d1fngb1 | d1uvqa1 | d1vgka1 | d1c16a1 |
| d4j12a2 | d4x99b2 | d1igtb3 | d1je6a1 | d1q72h2 | d1nldh2 | d1teth2 | d2hmic2 |
| d1i1ca2 | d1i1ca1 | d1k8ib1 | d1q0yh2 | d1kfal2 | d3frua1 | d1pg7w2 | d1u58a1 |
| d1igyb4 | d1igyb3 | d1hyrc1 | d1klub1 | d1deeb2 | d1c12b2 | d2bc4b2 | d2ig2h2 |

d1kcvl2 d3wn5b1 d1lk2b_ d1lk3l2 d1mjul2 d1sm3h2 d1q0xh2 d1zvsa1

d1ydpa1 d1u8ib2 d1ypze2 d1kcuh2 d1op9a_ d1y0la1 d1etzb1 d1egjl1

d2gsib1 d1nj9a1 d4jfhd1 d1aqkl1 d1t2ja_ d1lo4h1 d1zvya1 d3v4vl1

d4enec1 d1kxtb_ d5e8ea1 d2pxya_ d4weud_ d1xiwc_ d2esvd1 d1qnzh_

d1ogae1 d1f4xh1 d4n1hb_ d3ezjb1 d3mlwl1 d1kfah1 d1etza1 d1b4jh1

d3cfic_ d4w68a_ d2arja1 d1ek3a_ d2ok0l1 d1vera_ d1uwgh1 d1blnb1

d1fpth1 d1rzga1 d2giya1 d4m3kb1 d1nc2a1 d2nyyd1 d1ncna_ d1fbih1

d5dxwa_ d4fqqa1 d1dqdh1 d1jfql1 d1kelh1 d1faih1 d1frgl1 d1figl1

d4m6nl1 d1fo0a_ d4hxbl1 d3b5ga_ d3quyd1 d2h9gb1 d1i8kb_ d2dd8h1

d2ck0h1 d1eaja_ d4w6wb1 d2a6ib3 d1t66d1 d2e27h_ d1ob1b1 d1f11b1

d2v7na1 d1ad0a1 d2xt1b1 d1pskh1 d4i13b1 d2op4l1 d1a0ql1 d1uwel1

d1za3a1 d1ncbl1 d4uu9a_ d1bm3h1 d1cloh1 d1nqba2 d1cf8h1 d4y5va_

d1mhph1 d5anma1 d4cdgc1 d4ldeb_ d1y0lb1 d2pcpb1 d1z3gl1 d1dfbl1

d1fh5h1 d3g5xa1 d1tzib1 d4dn3l1 d3zhka1 d2b1hh1 d2uyla1 d1f3dh1

d1nj9b1 d2agjh1 d2h1pl1 d1q72h1 d8fabb1 d1ndmb1 d1rjca1 d4u6hb1

d3upca_ d2fbjl1 d1osph1 d1tvda_ d2cjvh_ d4fhbd1 d1bd2d1 d1rz7h1

d3nn8a_ d1blna1 d1knoa1 d1nfda1 d2bmkb1 d1dqll_ d1igyb1 d1igfl1

d12e8h1 d3utza1 d1nc2b1 d1rihh1 d4mwfb1 d4jvpa1 d1mieh1 d1rhfa1

d1fgnl1 d1sbsh1 d1lp9e1 d1dn0a1 d4lqfl1 d1iaih1 d1kgcd1 d1mh5b1

d1gpol1 d2g60h3 d1f8tl1 d2jixd1 d4grwh_ d1fo0b_ d1bfob1 d1axsa1

d1u9ka_ d1nmbh_ d1h8na1 d1h8na2 d1jguh1 d4eura1 d3sdyl1 d35c8h1

d1w72l1 d1mvub_ d1i9rh1 d1ob1a1 d4wenb_ d1yeeh1 d3ietc1 d1f58h1

d1ad0b1 d1mqkl_ d1nbvh1 d4b50a_ d3r8bb_ d1bafl1 d1mamh1 d4rgne1

d2gsgb_ d35c8l1 d1bafh1 d1ai1h1 d2pcpa1 d2fath1 d2x1pa_ d1orsb1

d1ibgl1 d1shma_ d1a2yb_ d1u0qa_ d2coqa_ d1hilb1 d1qfwi_ d1jv5b_

d1fn4b1 d5e0qa_ d1nsnh1 d1plgl1 d4ocxl1 d1kb5h1 d1rzfh1 d1pg7x1

d2fx7h1 d2oi9c_ d1l7il1 d3eyva1 d1d5il1 d4ffyl_ d1tetl1 d1a6wh_

d1ac6a_ d3dvfa_ d1nfde1 d1mimh1 d1ri8a1 d1jhlh_ d3dura_ d1fskb1

d1f97a1 d3so3b1 d2ak4d1 d1lp9f1 d4z7wf1 d1l7th1 d4y5ya_ d4x7ec1

d1emth1 d1ikfl1 d1nlbh1 d4utal1 d1bjma1 d1eapb1 d1u6ah1 d2dtgd1

d1kj2b_ d1xeda1 d1c1eh1 d1fgvh_ d2bnub1 d2fb4h1 d2dtgb1 d1ktrh_

d1jpth1 d1qkzl1 d4hepg_ d2atpb1 d1flrh1 d2hrpl1 d1mvfa_ d1iaim1

d3k80a1 d2jb5h1 d3zkxc_ d4grwe_ d1it9h1 d1rhhb1 d3w9ec_ d1e4wh1

d1sy6h1 d2j6eh1 d3qxta_ d1jglh1 d1p7ka1 d1tzhb1 d4n8pa1 d1gafl1

d2jelh1 d1wioa2 d4i0cc1 d1cicb1 d1lmka2 d1lmka1 d1iqwh1 d1orqb1

d1ejoh1 d1jnlh1 d4zg1a_ d43c9b_ d1ggbl1 d1wejh1 d3q6fa1 d1f3dj1

d3c6la1 d1bqlh1 d3p9wb_ d1a3rl1 d1h0db1 d2uzil_ d1eo8l1 d1qfwl_

d1n7ml1 d1igja1 d1c5ch1 d2a9mh_ d1fj1b1 d4heme_ d1ccza1 d1a5fh1

d1ogad1 d1dlfh_ d1qa9b_ d1vgel1 d3qskb_ d1sjxa_ d4ozfg1 d1sq2n_

d1qd0a_ d1kcvh1 d1c5db1 d3qcul1 d8faba1 d3bn9d1 d1mjuh1 d4y8dc_

d7fabh1 d3dvgb1 d1lo0l1 d1a6tb1 d1gigh1 d1kcrh1 d2hwzl1 d1yjdc1

d3clfl1 d4krna_ d1dl7l_ d1e6ol1 d1mexh1 d3r0ma_ d4kkna_ d1fnsl1

d1oaqh_ d1rzia1 d1ucbh1 d1ncwl1 d4b41a_ d1nfdf1 d2mpah1 d2d7tl1

d4grwf_ d4b5ea_ d1lk3h1 d3u79a_ d4u3xa_ d1ghfl1 d1bvkb_ d1mcww1

d1b88a_ d1jrhh1 d32c2b1 d4wemb_ d1dsfh_ d1op3h1 d1ce1l1 d1iqda1
d1qoka1 d1qoka2 d1qfuh1 d2crya1 d1ay1h1 d1oaql_ d3ab0b_ d1rurl1
d1t4kb1 d1bz7b1 d1n0xh1 d1forl1 d1ucbl1 d4w6yb_ d43c9a_ d2aabl1
d1tzia1 d4n1ca_ d1k6qh1 d3g9ab_ d4tvsa1 d1igmh_ d1pz5b1 d3dx9a1
d3wxwh_ d1nakh1 d1kcul1 d3eaka_ d4qlra1 d1cr9l1 d1d9ka_ d1f4xl1
d1deea1 d2i27n2 d1c12a1 d2orbl1 d1j8hd1 d1mcph1 d1c5da1 d1kenh1
d1bd2e1 d1h5ba_ d4x7fc1 d3upaa_ d1t3fa1 d1indh1 d2dtgc1 d4hc1l1
d1zv5a1 d4wgvb_ d1uwgl1 d4f37k1 d1aqkh1 d1vesa_ d3zkqd_ d1fptl1
d1lo4l1 d1knob1 d3rrqa_ d1ct8b1 d4orzc_ d1mlbb1 d4hgkc1 d1dqdl1
d1bogb1 d4ozhe1 d1dr9a1 d1i7za1 d1dn0b1 d1kell1 d1rzib1 d1ypzf1
d1wtla_ d1hkfa_ d1fail1 d1figh1 d2vyre_ d1sjva_ d1ohqa_ d1bfoa1
d1pkqb1 d1mjul1 d1b4jl1 d1nsnl1 d2arjb1 d1p4bh_ d4aj0a_ d3nn8b_
d2rcja1 d1f2xk_ d4v1dd_ d1adqh1 d2eksa_ d4k3ga_ d1jfqh1 d1uweh1
d1frgh1 d4gftb_ d1uz8b1 d4n1ea_ d1bm3l1 d1clol1 d3mcka1 d2vq1a1
d1ae6h1 d4aq1b1 d1uj3b1 d1nbqa1 d1fh5l1 d2ck0l1 d2ywza_ d1q72l1
d4nbxb_ d1hila1 d4hzlb1 d2j88l1 d1zmya1 d1cd0a_ d1a4ka1 d1pskl1
d1a0qh1 d4krmb_ d1ncbh1 d2fbjh1 d1j8he1 d1i3ua_ d1cf8l1 d1fe8h1
d2p1ya1 d1eeqa_ d2x89a_ d1ol0a_ d2r0lh1 d1ub5a1 d1dfbh1 d3eyvb1
d4jdvb1 d4p2cg_ d2gsia1 d2agjl1 d1u3ha1 d2h1ph1 d1rihl1 d1fskc1
d1boga1 d2a77l1 d1ospl1 d1qfoa_ d3dwta_ d2fx7l1 d1rz7l1 d3stba1
d1pkqa1 d1b6da1 d1dzba2 d1dzba1 d1kj2a_ d4l1ha_ d1rzgb1 d1dqlh_
d12e8l1 d1miel1 d1jmaa_ d1sbsl1 d1fgnh1 d1f58l1 d1mqkh_ d1ndgb1
d3qegl1 d1iail1 d2aw2a1 d1tqbc1 d3fo2a1 d4dkaa_ d1f8th1 d2g60l1
d2ywya_ d1uj3a1 d1yc7a_ d3bd3a_ d4w6xb1 d1nmbl_ d1i8ka_ d1rjlb1
d1ypze1 d1p7kb1 d1kxqe_ d1gpoh1 d2z4qa1 d1w72h1 d1i9rl1 d4qgya1
d4xt1c_ d2qqnh1 d1cica1 d3c08h1 d2cd0a_ d2bdnh1 d1hnga1 d1maml1
d1nldh1 d1cr9h1 d4lcih1 d3bkyh1 d3qg7l1 d1ai1l1 d1kxvc_ d4iosd_
d1l7ih1 d2x1qa_ d1ub6b1 d3k74b_ d1ibgh1 d1teth1 d1igya1 d1hxma1
d2x6ma_ d2e7la_ d5e03a_ d1qfwm_ d2r0kh1 d1igjb1 d1rurh1 d1plgh1
d1mj8h1 d1yuhb1 d3fmgl1 d1rzfl1 d1emtl1 d1ad9b1 d1igtb1 d1nlbl1
d1d5ih1 d3k7ua_ d4ffyh_ d1bwwa_ d3dvga1 d1a6ta1 d1fgvl_ d4qxgl1
d1miml1 d1jhll_ d2hffb1 d2hh0l1 d1qkzh1 d1l7tl1 d1afvh1 d1iaii1
d2cjuh_ d1um5h1 d2ghwb2 d2ghwb1 d1kgce1 d2fgwh1 d1ikfh1 d1dvfd_
d2i24n1 d2xtjb_ d1za3b1 d2yc1a_ d2hh0h1 d2x1oa_ d1bvka_ d1rjla1
d5e7ba1 d1igta1 d1il1b1 d2fb4l1 d1mvua_ d1jptl1 d32c2a1 d1fvca_
d1iqdb1 d2hrph1 d2p4ab_ d4qo1a_ d1p2cb1 d1wt5c_ d1t4ka1 d5c1mb_
d1it9l1 d1bz7a1 d1b2wh1 d1e4wl1 d1jnll1 d1yedb1 d1za6b1 d3u7aa_
d1igfh1 d3gnml1 d1orsa1 d1wejl1 d1a2ya_ d1hxmb1 d1gafh1 d2j4wh1
d1pz5a1 d1j05a_ d1a3rh1 d4fqll1 d1qfwh_ d1eo8h1 d1n7mh1 d4s1la_
d1kegh1 d5da4a_ d1iqwl1 d3whxb1 d1vgeh1 d1deeb1 d1c12b1 d1t2ql1
d1ad9a1 d3gsnb1 d3b2uc1 d1ggbh1 d1t3fb1 d1nakl1 d4ocmc_ d1lila1
d2v7hb3 d2esve1 d1mcoh1 d2qr0b1 d2q3aa_ d1ct8a1 d1kcrl1 d2pxyb_
d2uzih_ d5c3ld_ d3durb_ d1c5cl1 d2ak4e1 d1dl7h_ d1xiwd_ d1mlba1
d1rmfl1 d1i7zb1 d1dlfl_ d2xa3a_ d5bq7b1 d1dqqb1 d3h42l1 d1egjh1

d1kcvl1 d1fe8l1 d2j6el1 d1f3rb2 d1lk3l1 d1sm3h1 d1q0xh1 d1ghfh1 d7fabl1 d1igch1 d1lo0h1 d4idla_ d5c2bl_ d1jrhl1 d1il1a1 d3zhda1 d4kfzc_ d1b0wa_ d1e6oh1 d1mexl1 d4krpb_ d1fnsh1 d1ncwh1 d4hgma_ d4eigb1 d4rzca_ d2mpal1 d4y5xa_ d5en2b1 d3lrga1 d4ej1c1 d1rhha1 d1j05b_ d1forh1 d1q9wb1 d2wzpd_ d2aq2a1 d1h0da1 d3revb1 d2ij0c1 d1tzha1 d4m7jl1 d1k6ql1 d3b9kc1 d1igml_ d3qeua1 d1dsfl_ d1ce1h1 d1kcuh1 d1zvha1 d1zvoa1 d1tqbb1 d1ay1l1 d2qscl1 d2cgrh1 d1nl0h1 d1kenl1 d3eo0a1 d4n9ob1 d1n0xl1 d3k1kc_ d2cqva1 d1fhga_ d2k1ma_ d2dl2a1 d1biha3 d1biha1 d1biha4 d3qp3a1 d1g1ca_ d1gxea_ d1cvsc2 d1cvsc1 d1xmwa2 d1m4ka2 d2otpa2 d1biha2 d1rhfa2 d2fcba2 d2fcba1 d1wwbx_ d1gsma2 d1epfa1 d1epfa2 d2dava1 d1waaa_ d1f97a2 d1x44a1 d1vcaa2 d1fnla2 d1fnla1 d3b5ha2 d3h8na2 d3h8na1 d1jbja1 d1jbja2 d1pd6a_ d1f2qa2 d1f2qa1 d3cx2a_ d1nbqa2 d3b5ha1 d1n26a1 d1xiwb_ d1xaua_ d1fltx_ d2ifga1 d1olla1 d1olla2 d1cs6a4 d1cs6a3 d1cs6a2 d1cs6a1 d2gw5a2 d1sy6a1 d1xiwa_ d1ucta2 d1ucta1 d1ry7b2 d1gl4b_ d1qz1a3 d1olza1 d2c9aa1 d1wwca_ d2aq2b1 d1ck1a1 d1enfa1 d1i4pa1 d1v1oa1 d1et9a1 d1esfa1 d2g9hd1 d4ohja1 d1klud1 d1bxta1 d1ty0a1 d1fnua1 d1c4qa_ d1prtc1 d2wv6d_ d1prtf_ d1ltrd_ d4l63b1 d1r4pb_ d1tl0f_ d2bosa_ d1prtb1 d3chbd_

**Family-39 (b.6.1.3)**

**Positive dataset contains 11 protein sequences.**

d1sdda1 d1sdda2 d1mzya1 d2bw4a1 d1mzya2 d4e9sa1 d1sddb1 d1sddb2 d1v10a2 d1v10a3 d1v10a1

**Negative dataset contains 4039 protein sequences.**

d1m32a_ d1ibja_ d1c7na_ d1iuga_ d4q75a_ d2bkwa1 d1t3ia_ d1n8pa_ d1qz9a_ d1jf9a_ d1eg5a_ d1p3wa_ d3kgwa_ d1cl1a_ d2ctza1 d1vjoa_ d2rfva_ d1gc0a_ d1cs1a_ d1d2fa_ d2ch1a1 d1pffa_ d1elua_ d1ax4a_ d2v1pa1 d2ez1a_ d1u08a_ d1b5pa_ d2ay1a_ d1bw0a_ d3pd6a_ d7aata_ d2csta_ d1j32a_ d1fg7a_ d4wlha_ d1o4sa_ d1v2da_ d1gdea_ d1m6sa_ d1v72a1 d1svva_ d1ajsa_ d1wsta1 d2f8ja2 d1lk9a_ d1vp4a1 d1xi9a1 d2gb3a1 d1yaaa_ d3i2ka1 d1mpxa2 d2cfba1 d1dfoa_ d1o69a_ d1bj4a_ d3k28a_ d2bwpa_ d3lg0a_ d2fnua_ d3ou5a_ d1z7da1 d1b9ha_ d1iaya_ d4msoa_ d1s0aa_ d1sffa_ d3tqxa_ d1mdoa_ d3qboa_ d3pgya_ d1bjna_ d2dkja_ d1ejia_ d1vefa1 d4wxga_ d3bs8a_ d3gbxa1 d1w23a_ d1h6ua2 d2tbvc_ d1c8nc_ d1x36a_ d1smvc_ d1ng0c_ d4sbvc_ d1opoa_ d1f2nc_ d1rmka_ d1ixta_ d1eyoa_ d1fu3a_ d1g1pa_ d1f3ka_ d1fyga_ g1f8v.1 d1novc_ d2bbvc_ g1gk9.1 g1cp9.1 g1e3a.1 d1a3qa1 d1iknc_ d3brda1 d1p7hl1 d1oy3c_ d1imhc1 d1gjia1 d1zk9a1 d2cxka1 d2bema_ d1pama1 d1qhoa1 d2fhfa2 d2fhfa1 d1k3ia1 d1j0ha1 d1cxla1 d2bhua1 d1edqa1 d1f1sa2 d1ut9a2 d1gvia1 d1txka1 d1adqa1 d1fnga1 d1uvqb1 d1zs8a1 d2vola1 d2vola2 d1mfbl2 d2jelh2 d1uwel2 d3qq4b1 d1kjvb_ d1k5na1 d1osph2 d4odhl2 d1ow0a1 d1ow0a2 d1bfob2 d3l9ra2 d3jtth1 d2fath2 d4elkb2 d1fn4b2 d2xfxb_ d1pg7x2 d1cqka_ d4k71b2 d1lk2a1 d1eapb2 d4lfhg2 d1i4fa1 d2nw2a2 d1kfah2 d1mhca1 d1h3ua1 d1wejh2 d1h0db2

d1a5fh2 d1kcvh2 d1mjuh2 d1qqda1 d1kcrh2 d1k8ia1 d3huja2 d2nnab1
d1nfdf2 d3mffb2 d1s9va1 d1lk3h2 d1q0xl2 d1fv1b1 d1neza1 d4rfeb2
d1r3ha1 d1a6tb2 d1kjva1 d1pz5b2 d1onqa1 d2bc4a2 d1de4a1 d1k8da1
d1tcra2 d1dn0b2 d2xwtb2 d1bfoa2 d1mexh2 d1iakb1 d1ypzf2 d4f7ea2
d1q72l2 d2fbjh2 d1fe8h2 d1clzh2 d1fngb1 d1uvqa1 d1vgka1 d1c16a1
d4j12a2 d4x99b2 d1igtb3 d1je6a1 d1q72h2 d1nldh2 d1teth2 d2hmic2
d1i1ca2 d1i1ca1 d1k8ib1 d1q0yh2 d1kfal2 d3frua1 d1pg7w2 d1u58a1
d1igyb4 d1igyb3 d1hyrc1 d1klub1 d1deeb2 d1c12b2 d2bc4b2 d2ig2h2
d1kcvl2 d3wn5b1 d1lk2b_ d1lk3l2 d1mjul2 d1sm3h2 d1q0xh2 d1zvsa1
d1ydpa1 d1u8ib2 d1ypze2 d1kcuh2 d1zx5a1 d1qwra_ d2phla2 d2phla1
d1fxza2 d1fxza1 d1lrha_ d1uija1 g1dgw.1 d2v09a_ d1uika2 d1uika1
d1dgwa_ d1ogpa1 d1ktja_ d1ds6b_ d1fstb_ d2b5ea2 d2b5ea3 d2b5ea1
d2b5ea4 d2kp1a1 d2kp2a1 d1hyua3 d1hyua4 d1j08a1 d1j08a2 d1eixa_
d3exra_ d2ffca1 d2fdsa1 d3n3ma1 d2czda_ d1q6oa_ d2yyua_ d1vqta1
d1dbta_ d3ldva1 d1mqta_ d1d4m3_ d1fmd3_ d2mev1_ g1aym.1 d1ev13_
d4q4w1_ d1r1a3_ d2x5ia_ d1rhi1_ g1r1a.1 g1d4m.1 g1rhi.1 d1b35b_
d1oopa_ d1tme1_ d4iv1a_ g1pvc.1 g1fpn.1 d1fpn1_ d4gb31_ d1d4m1_
d1fmd1_ d1ev11_ d2wzr3_ g1cov.1 d1qqp3_ d2x5ic_ d1rhi3_ g1oop.1
d1hxs1_ g1hxs.1 d1fpn3_ d1bev1_ g2mev.1 d1eah3_ d1aym1_ d1pvc3_
d1qqp1_ d1zba3_ d1h8ta_ d3vbhc_ g1b35.1 g1bev.1 d1hxs3_ g1ev1.1
d1bev3_ g1tmf.1 d1tmf1_ d1b35a_ d2mev3_ g1eah.1 g1h8t.1 d1eah1_
d4q4w3_ d1r1a1_ d1aym3_ d1pvc1_ d1zba1_ d1h8tc_ d1oopc_ d1tme3_
g1qqp.1 d4iv1c_ g1tme.1 d2ovga_ d1sq8a_ d1rzsa_ d3kz3a_ d3orca_
d3gpdg2 d2q49a2 d1hdgo2 d2g17a2 d2cvoa2 d2czca1 d3pwka2 d1obfo2
d1i32a2 d1zyao2 d2pkqo2 d1b7go2 d2hjsa2 d1cf2o2 d1vkna2 d1nvmb2
d2m9wa1 d3dfxa_ d2msya_ d2djoa1 d2r5za_ d1au7a1 d1f43a_ d2lmda1
d1akhb_ d1s7ea1 d3nara_ d1zq3p1 d2craa1 d1mija_ d4uuta_ d3a01a_
d1x58a1 d3rkqa1 d3cmya1 d2cqxa1 d2da6a1 d1e3oc1 d1fjla_ d1mh3a1
d1ig7a_ d1x41a1 d1wh7a1 d1gt0c1 d2mw8a_ d1lfup_ d1x2ma1 d1b72a_
d1wi3a1 d1wjha1 d1x2na1 d1mnmc_ d2cuea1 d3k2aa_ d2hosa_ d2jwta2
d1pufa_ d1octc1 d2hi3a_ d1b8ia_ d4j19a_ d3lnqa_ d1wh5a1 d1b8ib_
d1fexa_ d2cqra1 d1guua_ d1ug2a1 d2cqqa1 d2ajea1 d4a69c_ d2crga1
d2iw5b1 d1xc5a1 d1ofcx1 d2cu7a1 d1wgxa1 d2cjja1 d2ckxa1 d1gvda_
d1tc3c_ d1gdta1 d1u78a2 d1hlva2 d1iufa2 d1iufa1 d2iu5a1 d1zk8a1
d2g7sa1 d2fd5a1 d2np5a1 d1z0xa1 d2id6a1 d2d6ya1 d2i10a1 d1vi0a1
d2oi8a1 d3c07a1 d2ns7a1 d2fq4a1 d2yvea1 d1t33a1 d2o7ta1 d1rkta1
d2np3b1 d2g7ga1 d2gena1 d2fbqa1 d2g7la1 d2gfna1 d2g3ba1 d2jj7a1
d2hyja1 d1sgma1 d1ui5a1 d2hkua1 d2id3a1 d4jyka1 d1ex4a2 d1c6va_
d3lv8a_ d1s3ga1 d1zaka1 d1s4qa_ d3tvta2 d1uf9a_ d1khta_ d1ckea_
d1osna_ d1p5zb_ d4tmka_ d3cm0a_ d1tmka_ d1jaga_ d3h86a_ d1t0hb_
d1nn5a_ d2h92a_ d3kb2a1 d2c95a1 d1yj5a2 d1ly1a_ d2ak3a1 d1vyva2
d1deka_ d2grja1 d1p3ja1 d1y63a_ d1e4ya1 d1s96a_ d3iija1 d1lvga_
d1nksa_ d1vyua2 d4e22a_ d1qf9a_ d1jjva_ d1q3ta_ d1ki9a_ d1lw7a2
d1teva_ d1kjwa2 d1gsia_ d1x6va3 d1g3ma_ d1j99a_ d1t8ta1 d1nsta_

d1zrha_ d1ls6a_ d2a3ra_ d1aqua_ d2zptx_ d3ckla_ d1texa_ d1q44a_
d2gwha_ d1odfa_ d1uj2a_ d4gi7a_ d2jeoa1 d1rz3a_ d1z0ja1 d3umra2
d3gj0a1 d2gaoa1 d1yzga1 d1x3sa1 d1g7sa4 d1puja_ d1udxa2 d1kk1a3
d2o52a_ d1jwyb_ d1zcaa2 d1zcba2 d2fv8a1 d2cjwa_ d3dz8a1 d1xzpa2
d1vg8a_ d4qxaa_ d2ew1a1 d1x1ra1 d2bcgy_ d3doea1 d3cona_ d3rega_
d3wyga_ d1upta_ d2b6ha1 d1zd9a1 d1lnza2 d1r5ba3 d1n0ua2 d1r2qa_
d2f9la1 d2rhda_ d3q3jb_ d3q85a_ d1efma1 d1r8qa_ d2gf9a1 d2akab1
d1ek0a_ d1z08a1 d2gcoa1 d2dy1a2 d4g01b_ d2cxxa1 d1svia_ d3q72a_
d1ni3a1 d4ac9a4 d2erxa1 d2bmea_ d1wxqa1 d2gf0a1 d4ldja1 d1zunb3
d1d5ca_ d4lv5b_ d1efca3 d1kmqa_ d2g6ba1 d3lrpa_ d1tada2 d2y8ea_
d4rd4a1 d1z06a1 d2heia_ d3a58b_ d1u0la2 d1d2ea3 d1zj6a1 d2ocba_
d3rwoa_ d1mr3f_ d2e9sa1 d4u5xa1 d2iwra_ d2fn4a1 d3bwdd_ d2nzja1
d2atva1 d2ksqa_ d3l43a_ d1mkya2 d1mkya1 d1wmsa_ d1jnya3 d2rmka_
d1f6ba_ d1f60a3 d1gota2 d1jala1 d2fh5b1 d1g16a_ d2wblc_ d1m7ba_
d1wf3a1 d1f5na2 d1t9ha2 d1z6xa1 d1z0aa1 d2fg5a1 d2a5ja1 d2bcjq2
d1s0ua3 d1ky3a_ d4y0va_ d4lhwa_ d2qmea1 d2atxa1 d1egaa1 d1ryha_
d1moza_ d1nrjb_ d2erya1 d3bfka_ d1puia_ d3j3az1 d3t5ga_ d1shza2
d3defa_ d2f7sa1 d3raba_ d3tsoa_ d3aq4a_ d2al7a1 d3sfva1 d3bc1a_
d2ncda_ d1sdma_ d3h4sa1 d2heha_ d2grya_ g3kin.1 d1kk8a2 d1br2a2
d1goja_ d1f9va_ d2mysa2 d1lkxa_ d3t0qa_ d4db1a1 d3gbja_ d1ry6a_
d1v8ka_ d1kb0a1 d1k8ka2 d1k8ka1 d3uleb1 d3i33a2 d3i33a1 d1bupa1
d1dkgd2 d1dkgd1 d4ci6a2 d1jcfa1 d4a2ba2 d2fxua1 d1yaga2 d1yaga1
d4cbua2 d1bupa2 d3vrda1 d3vrda2 d1fcdc2 d1fcdc1 d1h1oa2 d1h1oa1
d1m70a2 d1iqca2 d1iqca1 d1eb7a2 d1nmla2 d1nmla1 d1nira1 d1qksa1
d3h1jd1 d2d0oa3 d2d0oa2 d1nbwa3 d1hroa_ d4gedb_ d3zooa_ d1mz4a_
d1gu2a_ d1f1fa_ d1dvva_ d1c6ra_ d1c75a_ d1e29a_ d1gdva_ d1cnoa_
d1h32b_ d1co6a_ d2bh4x_ d2aiua_ d5cytr_ d1jdla_ d1i8oa_ d2zboa_
d3dmia_ d1ls9a_ d1wvec_ d2yk3a_ d3ph2b_ d1dw0a_ d5aura_ d1ql3a_
d1qn2a_ d1f1ca_ d4zzva_ d2xspa_ d1eg1a_ d4v20a_ d1ojja_ d4ipma1
d4csia_ d3pfza_ d3ovwa_ d2yoka_ d2nqya_ d1h4ga_ d1hixa_ d1oa2a_
d1olra_ d3b7ma_ d4h7ma1 d3wp3a_ d2vgda_ d1igoa_ d3zsea_ d2nlra_
d3wx5a_ d1oa3a_ d1pvxa_ d1m4wa_ d1f5ja_ d1oa4a_ d1ks5a_ d4ixla1
d1h32a1 d1evsa_ d1pvhb_ d1z7ca_ d1huwa_ d1bgea_ d1rhga_ d1f6fa_
d1f45b_ d1i1rb_ d1axia_ d1etea_ d3tgxb_ d1scfa_ d2z3qa1 d3qb1a_
d1hula_ d1m48a_ d4nkqb_ d1eera_ d5e4ea_ d2o27a_ d3b5ka_ d2psma1
d4zf7a_ d3uf5a_ d4x51a_ d1d9ca_ d4ypgc1 d3oq3a_ d1n1fa_ d1au1a_
d1wu3i_ d3besl_ d3ux9a_ d3rbaa_ d2qtra_ d1nupa_ d3l92a_ d1f9aa_
d1qjca_ d2h29a_ d1vlha_ d1lw7a1 d1mrza2 d4ruka_ d2el7a_ d1iq0a2
d1r6ta2 d1ivsa4 d2cyba_ d1rqga2 d1j09a2 d2yxna_ d1f7ua2 d1h3fa1
d1ffya3 d2g36a1 d1nzja_ d1jila_ d1n3la_ d1i6la_ d1pfva2 d1h3na3
d3n9ia_ d3sz3a1 d2bk7a_ d3fjua_ d1z5ra1 d1kwma1 d1uwya2 d1jqga1
d2c1ca1 d1dtda_ d1h8la2 d3sw2a_ d1moxc_ d1dx5i3 d1edmb_ d1autl2
d1autl1 d1dval1 d1szba2 d1kigl_ d2y5fl_ d1ijqa2 d1gk5a_ d1g1sa2
d1nt0a3 d1xdtr_ d1lmja2 d1lmja1 d1nzia2 d2wphe_ d1klil_ d1pfxl2

d1pfxl1 d1p9ja_ d1g1ta2 d1f7ea_ d4yzub_ d2mgpa1 d1b9wa1 d1b9wa2
d2mgpa2 d1ob1c1 d1n1ia2 d1n1ia1 d2npra2 d2npra1 d1m1xb5 d2y5ia_
d1qlsa_ d1psra_ d3n22a_ d2egda_ d4ggfc_ d2wcea_ d2luca_ d4aqia_
d3nsia_ d2lp3a_ d1j55a_ d1a4pa_ d1k8ua_ d1nsha_ d1pvaa_ d3fs7a_
d2pvba_ d1ttxa_ d1rwya_ d2kyfa_ d1rk9a_ d1a75a_ d1exra_ d1tiza1
d1nyaa_ d1g8ia_ d1ggwa_ d1wdcc_ d3fwba_ d1ggza_ d2o5ga_ d1ctaa_
d1m45a_ d3i5gc_ d1sl8a_ d1fpwa_ d2scpa_ d2roaa_ d4aqra_ d3pn7b_
d1br1b_ d2mysb_ d1s6ca_ d1bjfa_ d2obha_ d1jfka_ d1jf0a_ d1qv0a_
d1lkja_ d2mlfc_ d2m97a_ d2d8na_ d4by5a_ d1jbaa_ d4nqga_ d1wdcb_
d2mysc_ d1j7qa_ d1s6ia_ d1uhka2 d1r2ua_ d1dtla_ d3kf9a_ d3pn7c_
d5d67d_ d1tuza_ d1h8ba_ d1eg3a1 d1eg3a2 d2zkmx1 d1snla_ d1gjya_
d1y1xa_ d1hqva_ d1dvia_ d1df0a1 d1qxpa2 d4usla_ d1k94a_ d1wkya2
d1uuqa_ d3wh9a_ d4m1ra_ d1xyza_ d4xuya_ d2je8a5 d4bf7a1 d3cuia_
d1ur4a_ d1qw9a2 d1odza_ d2vzsa5 d1bqca_ d2x2ya1 d1hjqa_ d1qnra_
d4y7ea1 d1vjza_ d4k68a_ d2v3ga_ d4hpga_ d1nofa2 d1nq6a_ d1h4pa_
d1aq0a_ d3ziza_ d4bs0a_ d1ecea_ d1h1na_ d1ta3b_ d1w91a2 d1i1wa_
d1uhva2 d1us3a2 d1egza_ d7a3ha_ d1tvna1 d3nyda1 d1ghsa_ d2cyga1
d4xv0a_ d2c8na2 d1w32a_ d3emca_ d1v6ya1 d1foba_ d2uwfa1 d2fgla_
d3niya1 d2c0ha1 d1rh9a1 d1yq2a5 d1szna2 d1fa2a_ d1pama4 d5do8a1
d1ea9c3 d1hx0a2 d2fhfa5 d1qhoa4 d1avaa2 d1m53a2 d4m8ua1 d2gjpa2
d1lwha2 d4howa1 d1ktba2 d1j0ha3 d3vm5a1 d2x1ia_ d1wzaa2 d1uasa2
d2diea1 d1mxga2 d1ht6a2 d1eswa_ d1iv8a2 d5cpsa_ d1ud2a2 d1zjaa1
d3gbea1 d1kwga2 d1wdpa_ d1gcya2 d1g5aa2 d1hvxa2 d3dhpa2 d2bhua3
d4do4a1 d3qgva1 d1e43a2 d1g94a2 d2d3na2 d1gjwa2 d1gvia3 d1x1na1
d1r7aa2 d1tz7a1 d4toqa_ d1wb0a1 d1w9pa1 d1eoka_ d1itxa1 d1kfwa1
d1goia2 d1edqa2 d2gsja_ d1hjxa1 d1jnda1 d1vf8a1 d3wdpp1 d3wq5a_
d1gnxa_ d4gxpa_ d4hz8a_ d1e4mm_ d3aiua_ d1wcga1 d3ahxa_ d1v02a_
d2dgaa_ d1pbga_ d1vffa1 d1qoxa_ d4ha4a_ d1e5wa2 d1j19a2 d1isna2
d2al6b2 d3syxa1 d1mkea1 d2p8va_ d1xoda1 d1i7aa_ d1evha_ d1qc6a_
d1eh3a_ d3skpa_ d1dtza1 d1dtza2 d1ieja_ d1jnfa2 d1ce2a1 d1jnfa1
d1b1xa2 d1b1xa1 d1jw1a1 d1jw1a2 d1ryoa_ d1h76a1 d1h76a2 d2v76a_
d1wj1a1 d1aqca_ d1qqga2 d1qqga1 d1ntva_ d1n3ha_ d4dbba_ d3f0wa1
d1p3ra_ d1wgua1 d2cy4a1 d3hqca_ d1wvha1 d1xr0b1 d3nvva1 d1krha3
d2h88b1 d1n62a2 d1t3qa2 d1kf6b2 d1zoyb1 d1jq4a_ d1ffva2 d1dgja2
d1rm6c2 d9ldta1 d2ewda3 d1up7a1 d2dfda1 d1s6ya1 d1vjta1 d7mdha1
d1hyea1 d1t2da1 d1ojsa3 d1b8pa1 d1ldna1 d1hyha1 d1civa1 d2a92a1
d1ez4a1 d1i0za1 d1y6ja1 d1gv0a1 d1ur5a1 d1llda1 d1obba1 d1v8ba1
d1gdha1 d2dlda1 d1mx3a1 d1ygya1 d1qp8a1 d1li4a1 d1j4aa1 d2naca1
d1txga2 d3f47a1 d1mv8a2 d2pv7a2 d1xc2a3 d2ahra2 d4xiya1 d2f1ka2
d1pgja2 d1i36a2 d1np3a2 d1lj8a4 d1teaa3 d1qmga2 d2i76a2 d2g5ca2
d1wdka3 d1dlja2 d3cuma3 d1kola2 d1f8fa2 d1qora2 d1jqba2 d1vj0a2
d1cdoa2 d1gu7a2 d1d1ta2 d1xa0a2 d2dm6a2 d1jvba2 d1rjwa2 d1llua2
d1tt7a2 d1piwa2 d1o89a2 d1iz0a2 d1e3ja2 d1yb5a2 d1uufa2 d1vj1a2
d1e3ia2 d3gpdg1 d1vc2a1 d2nvwa1 d1u8fo1 d1ydwa1 d1hdgo1 d2g17a1

d1cf2o1 d2czca2 d1r0ka2 d1obfo1 d2cvoa1 d1ebfa1 d1h6da1 d1i32a1
d1zh8a1 d2q49a1 d1qkia1 d1j5pa4 d3pzra1 d1xeaa1 d1vkoa1 d1vm6a3
d3qvsa1 d2pkqo1 d1b7go1 d1gr0a1 d1p1ja1 d1lc0a1 d1j0xo1 d3pwka1
d2hjsa1 d1zyao1 d1vkna1 d1tlta1 d1nvmb1 d3sjua_ d3ojfa1 d1xu9a1
d2jjya1 d1n7ha_ d1y1pa1 d1h5qa_ d1jtva_ d4fs3a_ d2bkaa1 d1gy8a_
d2c5aa1 d1ulsa_ d1ooea_ d2b69a1 d1y5ma1 d1spxa_ d1ae1a_ d1bxka_
d1ydea1 d1gega_ d1hdoa_ d1o5ia_ d1e6ua_ d1snya1 d2rhca_ d3zv4a_
d5ceja_ d2d1ya1 d1fjha_ d3w8ea_ d2fmua1 d1x1ta_ d1rpna_ d3ay7a1
d1k2wa_ d1eq2a_ d2hrba1 d3ruaa_ d2ew8a1 d4hwka_ d1xgka_ d2bd0a1
d1yo6a1 d2a35a1 d2ntva_ d1vl8a_ d1hdca_ d4bkua_ d2ag5a1 d2q45a_
d1nffa_ d4iy1a_ d1zbqa1 d1kewa_ d1ek6a_ d1ja9a_ d2q46a2 d1iy8a_
d1cyda_ d2ptga_ d1hxha_ d1uzma1 d3lyla_ d4m89a_ d3wyea_ d2blla1
d1w6ua_ d1sb8a_ d2q2va_ d4nr0a_ d1e6wa_ d2gasa_ d3oiga1 d2bgka1
d1mxha_ d1vl0a1 d1rkxa_ d1r6da_ d1wmaa1 d1e7wa_ d2c07a1 d1xkqa_
d3tzha_ d1g0oa_ d3lz6a_ d1qyca_ d1edoa_ d1gz6a_ d1oc2a_ d1t2aa_
d1zema1 d2xoxa_ d1z45a2 d1geea_ d1p33a_ d3lt0a_ d4e6pa_ d2wyua_
d1db3a_ d4q9na_ d1xhla_ d4o1ma_ d1fmca_ d2uvda_ d2gdza1 d1uaya_
d2z1ma_ d2o23a2 d2fr1a2 d2fr1a1 d1qsga_ d1qyda_ d4zjua_ d2a4ka1
d1yb1a_ d1wvga1 d1yxma1 d3f4ba_ d1mg5a_ d1xg5a1 d1r55a1 d3gboa_
d1atla_ d1kufa_ d4q1la_ d1quaa_ d1yp1a_ d1wnia_ d4ekfa_ d2ckga_
d1th0a_ d2bkra_ d1rypd_ d1irua_ d1rypc_ d3unfk_ d1rypa_ d1j2pa_
d1irui_ d1j2qh_ d3unfh_ d1m4ya_ d1rypb_ d1irug_ d1iru2_ d1q5rh_
d1rypg_ d1g3ka_ d1iruf_ d1q5qa_ d1e94a_ d1irub_ d1ryph_ d3ty6a_
d1iruj_ d1iruk_ d1rype_ d1irud_ d1irue_ d3mi01_ d1rypf_ d1iruh_
d2z3ba_ d1iruc_ d3mka2_ d1iru1_ d1g0da4 d2f4ma1 d1x3za_ d3u1ra1
d1g9ka2 d1k7ia2 d1t3ca_ d1epwa3 d2a97a_ d3fiea_ g1e1h.1 d3ioqa_
d1fh0a_ d1o0ea_ d1m6da_ d2cb5a_ d1cs8a_ g8pch.1 d1s4va_ d2pnsa_
d1vsna1 d1mira_ d1iwda_ d2oula_ d7pcka_ d3bwka_ d3bcna_ d2p86a_
d1pvja_ d1pcia_ d1gece_ d1deua_ d4bs6a_ d1xkga1 d1cqda_ d1jqpa2
d2c0ya_ d2bdza_ d3p5ua_ d1zoya1 d1qo8a2 d1d4ca2 d1jnra2 d1kf6a2
d2jk6a2 d2jk6a1 d1feca1 d1feca2 d1lpfa1 d1lpfa2 d1xhca1 d1xhca2
d1dxla2 d1dxla1 d1gera2 d1m6ia2 d1onfa1 d3lada2 d3lada1 d1w4xa1
d1w4xa2 d1gesa2 d1mo9a1 d1xdia1 d1fl2a2 d1fl2a1 d1fcda1 d1fcda2
d1h6va2 d1h6va1 d1mo9a2 d1gv4a2 d1gv4a1 d2gv8a2 d2gv8a1 d1aoga1
d1aoga2 d1hyua1 d1ebda1 d1ebda2 d3dk9a1 d1q1ra2 d1q1ra1 d1gesa1
d1zbfa1 d2z1ha_ d2etja1 d3qioa1 d1jl1a_ d2e4la_ d1jl2a_ d2lsna1
d1uaxa_ d2yv0x_ d1mu2a1 d1ekea_ d1i39a_ d1pn0a1 d1reoa1 d1ju2a1
d2f5va1 d3c96a1 d2gmha1 d1pj5a2 d1o5wa1 d1n4wa1 d1k0ia1 d2dw4a2
d2ivda1 d1seza1 d2voua1 d1kdga1 d1s5ja1 d2hbja2 d2igia_ d1wlja_
d1qhta1 d4dlea1 d1tgoa1 d2f96a1 d3tr8a1 d3mxma_ d1q8ia1 d1y97a1
d1w0ha_ d1kfsa1 d2py5a1 d1d5aa1 d1yt3a3 d2guia_ d3mwpa1 d4kb1a_
d2y8ga_ d1xkea1 d2y8fa_ d1iv0a_ d1nu0a_ d1iuka_ d2csua1 d3q9ub_
d2yv1a1 d3q9na_ d2d59a_ d1oi7a1 d2yv2a1 d1euca1 d1y81a1 d1wgqa1
d1v89a1 d2coda1 d1zc3b_ d1wg7a1 d1u5ea1 d1v61a1 d2coca1 d2ys1a1

d1v5pa1 d1ntya2 d1wjma1 d1wi1a1 d1u29a_ d1xx0a1 d1unqa_ d2zkmx3
d1fhoa_ d1v88a1 d2adza1 d1x1fa1 d1p6sa_ d2coaa1 d1btka_ d1v5ma1
d1faoa_ d2x18a_ d1dyna_ d1x1ga1 d4kaxb_ d2j59m1 d1ki1b2 d1xcga2
d1txda2 d1u5da1 d1dbha2 d1w1ha_ d1upqa_ d1v5ua1 d2fjla1 d1eaza_
d2dfka2 d2cofa1 d1kz7a2 d1jmxa2 d1jmxa1 d1pbya2 d1r7ha_ d1faaa_
d1wika1 d1xfla_ d1ttza1 d2f51a1 d1ti3a_ d1nhoa_ d1sena_ d2i4aa_
d1ep7a_ d1zmaa1 d3lefa1 d3diea1 d2yana1 d1z6na1 d1oaza_ d3zywa1
d4aj8a_ d1nm3a1 d4fiwa_ d1wmja_ d4hs1a1 d1iloa_ d1xwaa1 d1wjka1
d2vm1a_ d1fb6a_ d1gh2a_ d1h75a_ d1nw2a_ d1kx5b_ d3a6na_ d4m6ba2
d2nqbd_ d3wkjd_ d2yfwe_ d4h9na_ d5bnxa_ d1kx5c_ d3r45a_ d1eqzb_
d4jjnb_ d1f66c_ d1eqza_ d4wnnb_ d1kx5a_ d1u35c1 d1id3a_ d2nqbc_
d1kx5d_ d1ku5a_ d1f1ea_ d1tafa_ d1bh9b_ d1tafb_ d1q9ca_ d1h3ob_
d2byka1 d1n1ja_ d4g92b_ d1bh9a_ d1jfia_ d1h3oa_ d2bykb1 d1n1jb_
d1pd212 d1nhya2 d2a2ra2 d1k0dd2 d1f2ea2 d1gsua2 d1f3ba2 d2fhea2
d2pvqa1 d1tw9a2 d1oyja2 d1e6ba2 d1k0ma2 d3ik7a1 d4is0a1 d1r5aa2
d1jlva2 d1m0ua2 d3gtub2 d1z9ha2 d1okta2 d1axda2 d2gsra2 d1tu7a2
d1k3ya2 d1zgma1 d1gnwa2 d1gwca2 d2gsta2 d1glqa2 d1oe8a2 d1pn9a2
d1v2aa2 d1fw1a2 d1duga2 d1ovna2 d1npya1 d3t4ea2 d1a4ia1 d1o0sa1
d2hk9a2 d2egga2 d1b26a1 d1bvua1 d1gq2a1 d1c1da1 d1leha1 d1gtma1
d1euza1 d1nvta1 d3dona2 d1edza1 d1gpja2 d1p77a1 d1nyta1 d1b0aa1
d1vl6a1 d1v9la1 d1qsaa2 d1qqya_ d1gd6a_ d1ivma_ d2gv0a_ d1iiza_
d2z2fa_ d1j8xa_ d1ghla_ d1b9oa_ d174la_ d169la_ d2o4wa_ d1p37a_
d1k28a3 d1xjta1 d1jtma_ d2f2qa_ d4e97a1 d1mpya2 d1kw3b2 d4ghga1
d1tfza1 d3isqa1 d3isqa2 d1sqia1 d1sqia2 d1lkda2 d1lkda1 d1mpya1
d1cjxa2 d1cjxa1 d1sp9b2 d1t47a1 d1t47a2 d1f1ua2 d1sp8a1 d1sp8a2
d1f1ua1 d1etob_ d1umqa_ d1iyka2 d1rxta1 d1iica2 d1iica1 d1qsta_
d2beia1 d4m3sa_ d1u6ma1 d1ygha_ d1yk3a1 d3dr6a_ d1xmta_ d1z4ea1
d2ge3a1 d1bo4a_ d2fiaa1 d2jdca1 d1y9wa1 d3to7a_ d1wwza1 d2fl4a1
d2ozua1 d2b5ga_ d2cy2a1 d2p0wa_ d1cm0a_ d4pswa_ d1yrea1 d1yr0a1
d1m4ia_ d2gana1 d1yvka1 d1ghea_ d2g3aa1 d1y7ra1 d2euia1 d1q2ya_
d2fsra1 d1xeba_ d2ae6a1 d1cjwa_ d1qsma_ d2aj6a1 d1y9ka1 d1i12a_
d1vkca_ d2atra1 d1n71a_ d2d4pa1 d2fiwa1 d2fe7a1 d1tiqa1 d2ou2a_
d1yx0a1 d2fcka1 d1p0ha_ d2j8ma_ d2pq8a_ d1z4ra1 d1s3za1 d1ufha_
d1dosa_ d3qm3a1 d2fjka_ d1xvqa1 d1pn0a2 d3hy2a_ d2cvba1 d1o8xa_
d1lu4a_ d1we0a1 d2bmxa1 d2a4va1 d1psqa_ d1xiya1 d1q98a_ d1n8ja_
d1zofa1 d2ggta_ d3tjja_ d4je1a1 d1o73a_ d3s9fa_ d2zcta_ d1qmva_
d1jfua_ d2b1ka_ d5dvba1 d2b5xa1 d2v2ga_ d1st9a_ d3tb2a_ d4llra_
d4fh8a1 d1e2ya_ d1knga_ d2h30a_ d1i5ga_ d2z9sa_ d2f8aa1 d1tp9a1
d2b7ka_ d1nm3a2 d3hvva_ d2wfca1 d4kcea_ d1xvwa1 d4gqca1 d3qpma_
d4k1fa_ d1zzoa1 d1gp1a_ d1prxa_ d4od7a1 d1z6ma1 d4mcua1 d4dvca1
d1r4wa_ d3l9sa_ d3rppa_ d2qked_ d4ksoa_ d1t4za1 d1wwja_ d1f74a_
d3l21a_ d2e1da_ d1to3a1 d1w3ia_ d1sfla_ d1n7ka_ d1vcva1 d4imea_
d1wa3a_ d2ocza1 d1f2ja_ d3m16a_ d2yxga_ d3a5fa_ d1fbaa_ d1l6wa_
d1xfba1 d1wx0a1 d1vpxa1 d1ojxa_ d1mxsa_ d2a4aa1 d1xkya1 d3cq0a1

d3s1xa_ d1f05a_ d1fdja_ d3cpra1 d4d2ja_ d1cg2a1 d1yloa2 d1y7ea2
d1y0ya3 d1lfwa1 d1vixa1 d1fnoa4 d1ysja1 d1vhea2 d1z2la1 d2fvga2
d1vgya1 d1r3na1 d2grea2 d1xmba1 g1q7l.1 d1rtqa_ d1ys1x_ d4hs9a_
d1ex9a_ d1ku0a_ d3qmma_ d1ra6a_ d1muka_ d3gsza_ d1khva_ d1te9a_
d2xwha_ d3uqsa_ d3hkwa_ d3n6ma_ d2cjqa_ d2xyma_ d1teba_ d1te8a_
d1s48a_ d1u09a1 d4wfxa_ d3tyqa_ d2pgga1 d1lgya_ d4l3wa_ d1llfa_
d1gz7a_ d1ukca_ d2hqya1 d2hqya2 d1lrza2 d1lrza3 d1ne9a2 d1ixra2
d3ia2a_ d1a88a_ d4dgqa1 d1hkha_ d1zoia_ d3zxha_ d1cgla_ d1rm8a_
d1hy7a_ d2jsda_ d1cxva_ d1hv5a_ d1i76a_ d3p0ya1 d1igra1 d1igra2
d1n8yc2 d1m6ba2 d1m6ba1 d1sr9a2 d1rqba2 d1i26a_ d1cixa_ d1y29a_
d1v7fa_ d2jtba_ d1d1ha_ d1g9pa_ d1qk7a_ d1mb6a_ d2a2va_ d1niya_
d1i25a_ d1hvwa_ d2mpqa_ d1ie6a_ d1kqha_ d1la4a_ d2mxoa1 d1dl0a_
d1koza_ d1qk6a_ d1emxa_ d1lqra_ d1c6wa_ d1js9c_ d1za7a_ d1laja_
d4lu0a_ d3unda1 d3e9aa1 d1vr6a2 d1n8fa_ d2qkfa_ d1d9ea_ d1o60a_
d1e88a1 d1l6ja5 d1l6ja4 d1eaka4 d1eaka3 d1l6ja3 d1h8pa2 d1ny721
d1a6ca3 d1a6ca2 d1a6ca1 d1pgl22 d1pgl21 d1pgl11 d1ny711 d1jgta2
d1ofda3 d1ea0a3 d1ecfa2 d1ct9a2 d1ao0a2 d1te5a_ d3ubub_ d1egia_
d1qo3c_ d1v7pa_ d1tdqb_ d3ubua_ d1c3ab_ d1h8ua_ d3bx4b_ d1t8ca1
d1sb2a1 d1qdda_ d2ziba1 d3cada_ d1byfa_ d1j34b_ d1jwia_ d1jzna_
d1c3aa_ d1oz7b_ d1wmza_ d3bdwb_ d1hq8a_ d1fifa1 d1dv8a_ d3hupa_
d1wk1a1 d1umra_ d2afpa_ d3bx4a_ d1gz2a_ d1sl6a_ d1v7pb_ d3g8ka_
d1ixxa_ d1j34a_ d1jwib_ d1g1sa1 d1y17a1 d1ja3a_ d1fvub_ d3bdwa_
d1sb2b1 d1fvua_ d1umrc_ d1oz7a_ d4m17a_ d3cfwa1 d1ypqa1 d1g1ta1
d3fd4a_ d3l9jc_ d4dn8a1 d1v4lb_ d1rdl1_ d2msba_ d1ix2a_ d4lt9a1
d3tc7a1 d3hoja_ d1i4na_ d3vnda_ d3nl8a1 d1geqa_ d3nava1 d1v5xa_
d4a2ra1 d3tc6a1 d1ujpa_ d4ijba_ d1xcfa_ d1rd5a_ d1qopa_ d3b5va_
d2r80b_ d1cqxa1 d1cg5a_ d1gcvb_ d3boma_ d2h8fb_ d2raoa_ d3bj1a_
d4hrrb_ d2r50a_ d3d1kb_ d2aa1b1 d3zhwa_ d3hf4b_ d3gdja_ d1jebb_
d3bj1b_ d1x9fb_ d1wmub_ d1gcva_ d3bcqb_ d3d4xb_ d1gvha1 d2dc3a_
d3hf4a_ d4yu3a_ d3k8ba_ d1qpwb_ d2r80a_ d3bcqa_ d1v4wa_ d3wtgb_
d1jl7a_ d4esaa_ d1a9we_ d3d1ab_ d1fsla_ d4h2la_ d1v4wb_ d1mwca_
d1vhba_ d1jeba_ d1spgb_ d1h97a_ d1q1fa_ d1tu9a1 d3d1aa_ d1hdsb_
d3hyub_ d2zfbb_ d3fs4a_ d1oj6a_ d1shrb_ d4h2lb_ d4yu3b_ d1spga_
d3hrwa_ d3qqra_ d3at5a_ d4wchd_ d3hyua_ d1cg5b_ d1uc3a_ d1hbra_
d2raob_ d1outb_ d4esab_ d3gdjb_ d3qqqa_ d1x9fc_ d3vrfa_ d1itha_
d3mkba_ d3d4xa_ d3mjpa_ d1jl6a_ d3fh9a_ d1outa_ d2dc3b_ d1it2a_
d3eoka_ d3g46a_ d1qpwa_ d3vrfb_ d1ch4a_ d1x9fd_ d1hdsa_ d2zfba_
d3mkbb_ d1or4a_ d3wtga_ d1a4fa_ d3fh9b_ d3bomb_ d3cy5a_ d3at5b_
d1x9fa_ d2oifa_ d4lmxb_ d1cpcb_ d3v57a_ d1kn1a_ d1cpca_ d4f0ta_
d2vjha_ d4po5b_ d1eyxa_ d1alla_ d4f0tb_ d1b33b_ d1b8db_ d4h0mb_
d2vjhb_ d4lm6b_ d2vmlb_ d1allb_ d2vjta_ d3dbja_ d2vmla_ d2vjtb_
d4f0ub_ d1f99b_ d4rmpa1 d1liaa_ d1eyxb_ d2uulb_ d4lmsb_ d4f0ua_
d2bv8a_ d1f99a_ d2bv8b_ d2c7lb_ d3dbjb_ d4rmpb1 d1b8da_ d4h0ma_
d3v57b_ d1kn1b_ d1liab_ d2uula_ d2j96a_ d1kyqa1 g1gxs.1 g1wht.1

d1azwa_ d1mtza_ d2xqfa_ d1ea5a_ d1f6wa_ d1dx4a_ d1qe3a_ d1k4ya_
d4qwwa_ d2ha2a_ d3c8da2 d2pbla1 d1jkma_ d1jjia_ d1jjfa_ d1jmxa4
d1jmxa3 d1pbya3 d1b9ra_ d1offa_ d1frra_ d3wcqa_ d1e9ma_ d1l5pa_
d1wria_ d1i7ha_ d3av8a_ d1rfka_ d3p1ma1 d3b2ga_ d2bt6a2 d4iuaa3
d4bvva_ d5hpga_ d3k65a_ d1ki0a3 d1ki0a2 d1ki0a1 d1a0ha1 d1pmla_
d3sp8a3 d2feba1 d1mdwa_ d1qxpa4 d1ziva1 d1zcma1 d2m5ta_ d1wqsa_
d5bpea_ d2ipha_ d1lvoa_ d2bhga1 d3tnta_ d3sjia_ d3d23a1 d2fyqa_
d3tloa_ d3zvea1 d2hrva_ d3q3ya1 d4asha1 d4mh8a_ d1mu2a2 d4i7ga1
d3rp2a_ g1ucy.1 d1fuja_ d2zchp_ d3w94a_ d1si5h_ d1lo6a_ g2pka.1
d1cgha_ d1kigh_ d1a5ia_ d3s9ba_ d1hj8a_ d1ao5a_ d2anya_ d1q3xa1
d1gvza_ d1azza_ d4gsoa_ d2f91a1 d1iaua_ d1gdna_ d1eufa_ g1fiw.1
d1eq9a_ d4nfea_ d2rdla_ d1spja_ d1elva1 d1a7sa_ d1ekbb_ d1pfxc_
d1yc0a_ d2aiqa_ g1fiz.1 d4k60a_ d4dgja_ d1mzaa_ d1op0a_ d3tjva_
d1pytd_ d1sgfg_ g1gg6.1 d2eeka_ g1rtf.1 d1a0ja_ d3g01a_ d1pytc_
d4e7na_ d1ddja_ d1z8ga1 d2hlca_ d1npma_ d1brup_ g1jou.1 d1sgfa_
d1autc_ g2hnt.1 g1kdq.1 d1orfa_ d3s69a_ d4crga_ d1fxya_ d1bqya_
d2ftba_ d1pmpa_ d2rcqa1 d1fdqa_ d4lkpa_ d1sa8a_ d1vyfa_ d1lpja_
d3vg7a1 d4azqa1 d1o8va_ d5bvqa_ d1p6pa_ d2qo4a_ d1opba_ d1yiva1
d1o1va_ d4bvma1 d4tkba_ d4i3ba_ d1bwya_ d1ftpa_ d1ggla_ d4gkca_
d1cbia_ d3p6da1 d1kqwa_ d2a0aa1 d1dzka_ d4tlja_ d2a2ua_ d4wfva_
d3t1da_ d4iaxa_ d3zq3a_ d2nnda_ d1i4ua_ d1kxoa_ d1bbpa_ d1kt7a_
d1exsa_ d3s26a1 d1lf7a_ d1qfta_ d4gh7a_ d1z24a1 d1qabe_ d1xkia_
d1ew3a_ d3bx7a_ d4iawa1 d1n0sa_ d1gkab_ d3kzaa_ d2k23a_ d2l9ca1
d2joza1 d1gm6a_ d3kffa_ d3saoa1 d1e5pa_ d1yupa1 d1iiua_ d3lgia_
d3i77a_ d2z9ia2 d4nsva_ d1lcya2 d4jcna_ d1agja_ d1os8a_ d1hpga_
d1p3ca_ d1l1ja_ d1e69a_ d1mv5a_ d3g60a4 g1ii8.1 d1ewqa2 d1ji0a_
d1qhla_ d1v43a3 d2wmfa1 d1vpla_ d1g2912 d4a82a2 g1f2t.1 d2d62a1
d3tuic1 d2bbsa1 d3g60a3 d2ixea_ d1sgwa1 d2qi9c_ d3rlfa1 d3b60a1
d3d31a2 d2pzea1 g1xew.1 d1jj7a_ d1oxxk2 d2onka1 d1w1wa_ d1iwea_
d1qf5a_ d1vmaa2 d1nija1 d1np6a_ d2p67a1 d3ihla_ d1ls1a2 d1s1ma2
d1iona_ d2v40a_ d1dj3a_ d3hida_ d2vo1a1 d2g0ta1 d1vcoa2 d1j8mf2
d1g3qa_ d1xjca_ d1p9ba_ d1qzxa3 d1ihua2 d1ihua1 d2qm8a_ d1dj2a_
d4ioja_ d1cp2a_ d1yrba2 d1hyqa_ d3gqba2 d1skye3 d1xp8a1 d1tf7a2
d1tf7a1 d1ye8a1 d1skyb3 d2qe7d2 d4blta_ d4huta_ d3ldaa2 d4hyya_
d2i3ba1 d1cr1a_ d3j9tb2 d3ntua2 d1vdza1 d2z43a_ d1e9ra_ d1nlfa_
d3ziaa2 d1n0wa_ d3ziad2 d3j9ta2 d1pzna2 d3gqbb2 d2i49a_ d1nnfa_
d1xvxa_ d1ursa_ d1iuda_ d4m1va1 d1twya_ d3e13x_ d2nxoa1 d4pfua_
d3ttma_ d1elja_ d2qmwa1 d1us5a_ d1y3na1 d1jeta_ d1uqwa_ d4f19a1
d2esna2 d3thia_ d3fasa1 d2uyeb_ d2xwva1 d1r9la_ d2fyia1 d1pb7a_
d2ozza1 d1sw5a_ d1nh8a1 d1vr5a1 d2g29a_ d1xs5a_ d3lswa_ d2z23a_
d3w9va_ d3tcha_ d1q35a_ d1z7me1 d3q27a_ d1p99a_ d1ve4a1 d2i6ea1
d4mcoa1 d4n8ya_ d1eu8a_ d1zbma1 d1hsla_ d1ixca2 d1j1na_ d1xvya_
d1y9ua_ d2z22a1 d1xoca1 d4oera_ d1wdna_ d5f1qa1 d1xt8a1 d1i6aa_
d4gwoa_ d4igta1 d1pc3a_ d4mmpa1 d1o63a_ d2czla1 d2onsa2 d4mija1

d1m8pa3 d3q3ma_ d1za0a1 d2inca_ d1jk0a_ d2incb_ d1mhyb_ d3hf1b_
d1mtyb_ d1r2fa_ d2uw1a_ d1mhyd_ d3n37a_ d1afra_ d1oquc_ d1syya_
d1mtyd_ d2o1za_ d1uzra_ d1smsa_ d2fkza_ d1zuja1 d1s3qa1 d2fjca1
d1z6om1 d1veia_ d4dyya_ d1jiga_ d3gvya_ d2pyba_ d4cy9a1 d3bvfa1
d1r03a_ d2cf7a_ d1nf4a_ d1zs3a1 d4iwka_ d3ka8a_ d3np0x_ d1lb3a_
d4e6ka_ d1z6oa1 d2fzfa1 d4dyua_ d3ge4a_ d1jgca_ d4am4a_ d2z6ma_
d1ji5a_ d1vlga_ d1lkoa1 d1krqa_ d3qz3a_ d1o9ra_ d2ffxj_ d1euma_
d1yv1a1 d2v2pa_ d1j30a_ d4di0a_ d1dpsa_ d2qzxa_ d2asia_ d1psoe_
d1miqa_ d1smra_ d2h6ta_ d1pfza_ d1izda_ d3hd8a_ d1j71a_ d1dpja_
d4aa9a_ d1t6ex_ g1b5f.1 g1lya.1 g1htr.1 d1qdma2 d1bxoa_ d1wkra_
d1tzsa_ d1ibqa_ d2qzwa_ d3azya_ d3wvja_ d1upsa1 d1ajoa_ d2hyka_
d1ajka_ d2vy0a_ d1o4ya_ d3o5sa_ d3d6ea_ d3atga_ d1dypa_ d1umza_
d3i4ia_ d1bdqa_ d3ggta_ d3ixoa_ d1mrxa_ d2haha_ d1tw7a_ d4njsa_
d1sgua_ d2p3da_ d1nsoa_ d3d3ta_ d2rkfa_ d1c6ya_ d2rspa_ d3ogpa_
d3ekwa_ d2o4na_ d2j9ja_ d1baia_ d3ggua_ d3lzua_ d4npua_ d4j55a_
d2r8na_ d2fxda_ d2p3ca_ d4dqea_ d3ttpa_ d3t3ca_ d2p3aa_ d2hc0a_
d2fdda_ d3mwsa_ d1k8rb_ d1wgra1 d1ef5a_ d1wfya1 d1e8ya3 d1wgya1
d1lfda_ d2cs4a1 d1wxma1 d2byea1 d3kh0a1 d2byfa1 d1wxaa1 d1e7ua3
d1h4ra3 d4yl8a1 d2al6a3 d1wgda1 d1wgga1 d4q5eb_ d1wjna1 d1se9a_
d3n3kb_ d1wz0a1 d1we6a1 d2m0xa_ d2gbka_ d3rt3b1 d1wh3a1 d1sifa_
d2ekec2 d4ii2b1 d4hk2a1 d1c3ta_ d1m94a_ d1wgha1 d1ttna1 d1j8ca_
d1wx8a1 d3v6cb1 d2ma9b_ d4i6lb1 d1bt0a_ d2jvca1 d1iyfa_ d2k1fa_
d1wxva1 d1wf9a1 d1t0ya_ d2io0b2 d1x1ma1 d2gbma_ d1zw7a1 d2m17a_
d1ud7a_ d1we7a1 d2lwpa_ d2gbja_ d1wjua1 d1v6ea1 d1v86a1 d4eewa_
d1wiaa1 d4icva_ d4gu2a_ d1wx7a1 d1wy8a1 d4hjka1 d2faza1 d1v5oa1
d2al3a1 d2cr5a1 d1wj4a1 d1s3sg_ d3ubra_ d1qo8a1 d1d4ca1 d2k3va_
d1ft5a_ d1qdba_ d1m1qa_ d2j7aa_ d1h21a_ d1ogyb_ d4fasa_ d1jnia_
d1oaha_ d1sp3a_ d1ps9a2 d1lqta1 d1cjca1 d2h0ba_ d2erfa_ d1h30a2
d3mw4a1 d1pz7a_ d2r1ba_ d3v64a1 d1d2sa_ d3bopa1 d3mw3a1 d1okqa1
d1okqa2 d1c4xa_ d1j1ia_ d2rhwa_ d1wioa4 d1ccza2 d1vcaa1 d1dr9a2
d1ic1a1 d2d9qb1 d2nxyb2 d1um8a_ d1ixza_ d1qvra2 d1qvra3 d1qzma_
d1sxjc2 d1jr3d2 d2p65a_ d1l8qa2 d1g8pa_ d3hu3a3 d1sxjd2 d1njfa_
d1in4a2 d1sxja2 d1tuea1 d2ce7a2 d1w5sa2 d1sxjb2 d1d2na_ d1ny5a2
d1ofha_ d2gnoa2 d2a5yb3 d1fnna2 d1u0ja1 d1lv7a_ d1iqpa2 d2fnaa2
d1ixsb2 d4gita_ d1sxje2 d2e3fa_ d1mm0a_ d2e3ea_ d2llda_ d2ny8x_
d2e3ga_ d2ny9x_ d2nz3a_ d2lj7a_ d1n4na_ d2m8bb1 d2kyqa_ d3psma_
d1jkza_ d1jxca_ d2gl1a_ d1ugla_ d4uj0a_ d2k4ua_ d1hp2a_ d2lu9a_
d1baha_ d2meoa1 d1wm7a_ d1hlya_ d1n8ma_ d1jlza_ d1wpda_ d1c55a_
d1pvza_ d1wt7a_ d1rjia_ d1wz5a_ d1m2sa_ d1pjva_ d1c49a_ d1qkya_
d2ktca1 d1wmta_ d1du9a_ d1j5ja_ d1r1ga_ d1t0za_ d2i61a_ d1t1ta_
d1sn4a_ d1kv0a_ d2lkba_ d1npia_ d1wwna_ d1pe4a_ d2kbka_ d2li7a_
d2kbha_ d1dq7a_ d1sega_ d1omya_ d1zywa1 d1jzaa_ d1n9wa1 d1bbua1
d1c0aa1 d1b8aa1 d1l0wa1 d3a74a1 d1eova1 d1t61c1 d1iyjb4 d1iyjb5
d1iyjb3 d1o7ia_ d4ipca1 d1x3ea_ d4apva_ d1jb7a1 d1jb7a2 d1jb7a3

d3kdfa_ d1ue1b_ d1wjja1 d3eiva_ d1xjva2 d1xjva1 d2ccza2 d1se8a_
d1l1oc_ d1jb7b_ d1qvca_ d3ulla_ d1qzga_ d1miua4 d1miua5 d1miua3
d1y14b1 d2nn6i1 d1i94l_ d2zjra2 d1smxa_ d2qamc2 d1hh2p1 d2r7da1
d1rl2a2 d1wfqa1 d3bzka4 d1x6oa2 d3a0ja_ d2j01d2 d2ja9a1 d4ql5a_
d1hr0w_ d1luza_ d2n3sa_ d2vnud1 d1h95a_ d1go3e1 d2nn6h1 d1ne3a_
d2mo1a_ d1iz6a2 d1jjga_ d1e3pa2 d2ix0a3 d2ix0a2 d2lxja_ d1ueba3
d1ueba2 d2z0sa1 d1u0la1 d2ix0a1 d2eifa2 d2je6i1 d3i2za1 d2c35b1
d2ahob2 d1t9ha1 d3i4oa_ d2qall1 d1jt8a_ d2nn6g1 d1g6pa_ d3cama_
d1i94q_ d1d7qa_ d2ba0a1 d2vnud2 d2vnud3 d1khia2 d2qalq1 d1wi5a1
d1xtda2 d1ny4a_ d2a1ka_ d1je5a_ d1rpma_ d2shpa1 d1yfoa_ d1l8ka_
d2ooqa1 d1g4us2 d1p15a_ d1lara2 d1lara1 d1wcha_ d2fh7a2 d2fh7a1
d1ohea1 d1ohea2 d2f4da_ d1rxda_ d2hxpa_ d1xria_ d1fpza_ d3ezza_
d4y2ea_ d1i9sa_ d1c1la_ d4lbqa_ d1a78a_ d4no4a_ d3zsja_ d3duia_
d1g86a_ d1is3a_ d1qmja_ d1hlca_ d1tdha3 d1q3ba3 d1k82a3 g1qmo.1
g1len.1 d3zyra_ d1fnya_ d1n47a_ g2ltn.1 d1avba_ d3usua_ d1dhkb_
d1f9ka_ d1ukga_ d1g8wa_ d1g9fa_ d1wbfa_ d3wcsa_ d1g7ya_ g1loe.1
d1mvqa_ d1hqla_ d1v6ia_ d1dbna_ d1fx5a_ d4l8qa_ d1ioaa_ d3n35a_
d3h33a_ d1rwja_ d19hca_ d1os6a_ d3bxua_ d3h4na_ d1i77a_ d3caoa_
d1j0pa_ d1duwa_ d1ofwa_ d2e84a_ d1hh5a_ d1gyoa_ d3h34a_ d1dszb_
d1a6ya_ d3cbba_ d1ynwa1 d1r4ia_ d1hcqa_ d2hana_ d2hanb_ d2enva1
d1kb4b_ d2nllb_ d1lo1a_ d1cita_ d2dara2 d2dara1 d1wyha2 d2dloa2
d1rutx4 d1rutx3 d2cuqa2 d1x4la2 d1x4la1 d1x3ha2 d1x3ha1 d2d8za1
d1x61a2 d1x6aa1 d1x6aa2 d2cora2 d2cora1 d1v6ga1 d1v6ga2 d2d8za2
d1x64a1 d1x64a2 d1b8ta1 d2co8a1 d1x63a2 d2dj7a2 d2dj7a1 d2cu8a2
d2cu8a1 d1g47a2 d1g47a1 d1j2oa1 d1wyha1 d2dloa1 d1wiga1 d1wiga2
d2cupa2 d2cupa3 d1x68a1 d2d8ya2 d2d8ya1 d1m3va2 d1x62a1 d1x68a2
d1x61a1 d1b8ta3 d1b8ta2 d2d8xa2 d2d8xa1 d1x4ka1 d1x4ka2 d2cuqa1
d1m3va1 d2co8a2 d1x63a1 d2cura1 d2cura2 d1x62a2 d1op9a_ d1y0la1
d1etzb1 d1egjl1 d2gsib1 d1nj9a1 d4jfhd1 d1aqkl1 d1t2ja_ d1lo4h1
d1zvya1 d3v4vl1 d4enec1 d1kxtb_ d5e8ea1 d2pxya_ d4weud_ d1xiwc_
d2esvd1 d1qnzh_ d1ogae1 d1f4xh1 d4n1hb_ d3ezjb1 d3mlwl1 d1kfah1
d1etza1 d1b4jh1 d3cfic_ d4w68a_ d2arja1 d1ek3a_ d2ok0l1 d1vera_
d1uwgh1 d1blnb1 d1fpth1 d1rzga1 d2giya1 d4m3kb1 d1nc2a1 d2nyyd1
d1ncna_ d1fbih1 d5dxwa_ d4fqqa1 d1dqdh1 d1jfql1 d1kelh1 d1faih1
d1frgl1 d1figl1 d4m6nl1 d1fo0a_ d4hxbl1 d3b5ga_ d3quyd1 d2h9gb1
d1i8kb_ d2dd8h1 d2ck0h1 d1eaja_ d4w6wb1 d2a6ib3 d1t66d1 d2e27h_
d1ob1b1 d1f11b1 d2v7na1 d1ad0a1 d2xt1b1 d1pskh1 d4i13b1 d2op4l1
d1a0ql1 d1uwel1 d1za3a1 d1ncbl1 d4uu9a_ d1bm3h1 d1cloh1 d1nqba2
d1cf8h1 d4y5va_ d1mhph1 d5anma1 d4cdgc1 d4ldeb_ d1y0lb1 d2pcpb1
d1z3gl1 d1dfbl1 d1fh5h1 d3g5xa1 d1tzib1 d4dn3l1 d3zhka1 d2b1hh1
d2uyla1 d1f3dh1 d1nj9b1 d2agjh1 d2h1pl1 d1q72h1 d8fabb1 d1ndmb1
d1rjca1 d4u6hb1 d3upca_ d2fbjl1 d1osph1 d1tvda_ d2cjvh_ d4fhbd1
d1bd2d1 d1rz7h1 d3nn8a_ d1blna1 d1knoa1 d1nfda1 d2bmkb1 d1dqll_
d1igyb1 d1igfl1 d12e8h1 d3utza1 d1nc2b1 d1rihh1 d4mwfb1 d4jvpa1

d1mieh1 d1rhfa1 d1fgnl1 d1sbsh1 d1lp9e1 d1dn0a1 d4lqfl1 d1iaih1
d1kgcd1 d1mh5b1 d1gpol1 d2g60h3 d1f8tl1 d2jixd1 d4grwh_ d1fo0b_
d1bfob1 d1axsa1 d1u9ka_ d1nmbh_ d1h8na1 d1h8na2 d1jguh1 d4eura1
d3sdyl1 d35c8h1 d1w72l1 d1mvub_ d1i9rh1 d1ob1a1 d4wenb_ d1yeeh1
d3ietc1 d1f58h1 d1ad0b1 d1mqkl_ d1nbvh1 d4b50a_ d3r8bb_ d1bafl1
d1mamh1 d4rgne1 d2gsgb_ d35c8l1 d1bafh1 d1ai1h1 d2pcpa1 d2fath1
d2x1pa_ d1orsb1 d1ibgl1 d1shma_ d1a2yb_ d1u0qa_ d2coqa_ d1hilb1
d1qfwi_ d1jv5b_ d1fn4b1 d5e0qa_ d1nsnh1 d1plgl1 d4ocxl1 d1kb5h1
d1rzfh1 d1pg7x1 d2fx7h1 d2oi9c_ d1l7il1 d3eyva1 d1d5il1 d4ffyl_
d1tetl1 d1a6wh_ d1ac6a_ d3dvfa_ d1nfde1 d1mimh1 d1ri8a1 d1jhlh_
d3dura_ d1fskb1 d1f97a1 d3so3b1 d2ak4d1 d1lp9f1 d4z7wf1 d1l7th1
d4y5ya_ d4x7ec1 d1emth1 d1ikfl1 d1nlbh1 d4utal1 d1bjma1 d1eapb1
d1u6ah1 d2dtgd1 d1kj2b_ d1xeda1 d1c1eh1 d1fgvh_ d2bnub1 d2fb4h1
d2dtgb1 d1ktrh_ d1jpth1 d1qkzl1 d4hepg_ d2atpb1 d1flrh1 d2hrpl1
d1mvfa_ d1iaim1 d3k80a1 d2jb5h1 d3zkxc_ d4grwe_ d1it9h1 d1rhhb1
d3w9ec_ d1e4wh1 d1sy6h1 d2j6eh1 d3qxta_ d1jglh1 d1p7ka1 d1tzhb1
d4n8pa1 d1gafl1 d2jelh1 d1wioa2 d4i0cc1 d1cicb1 d1lmka2 d1lmka1
d1iqwh1 d1orqb1 d1ejoh1 d1jnlh1 d4zg1a_ d43c9b_ d1ggbl1 d1wejh1
d3q6fa1 d1f3dj1 d3c6la1 d1bqlh1 d3p9wb_ d1a3rl1 d1h0db1 d2uzil_
d1eo8l1 d1qfwl_ d1n7ml1 d1igja1 d1c5ch1 d2a9mh_ d1rmfh1 d1fj1b1
d4heme_ d1ccza1 d1a5fh1 d1ogad1 d1dlfh_ d1qa9b_ d1vgel1 d3qskb_
d1sjxa_ d4ozfg1 d1sq2n_ d1qd0a_ d1kcvh1 d1c5db1 d3qcul1 d8faba1
d3bn9d1 d1mjuh1 d4y8dc_ d7fabh1 d3dvgb1 d1lo0l1 d1a6tb1 d1gigh1
d1kcrh1 d2hwzl1 d1yjdc1 d3clfl1 d4krna_ d1dl7l_ d1e6ol1 d1mexh1
d3r0ma_ d4kkna_ d1fnsl1 d1oaqh_ d1rzia1 d1ucbh1 d1ncwl1 d4b41a_
d1nfdf1 d2mpah1 d2d7tl1 d4grwf_ d4b5ea_ d1lk3h1 d3u79a_ d4u3xa_
d1ghfl1 d1bvkb_ d1mcww1 d1b88a_ d1jrhh1 d32c2b1 d4wemb_ d1dsfh_
d1op3h1 d1ce1l1 d1iqda1 d1qoka1 d1qoka2 d1qfuh1 d2crya1 d1ay1h1
d1oaql_ d3ab0b_ d1rurl1 d1t4kb1 d1bz7b1 d1n0xh1 d1forl1 d1ucbl1
d4w6yb_ d43c9a_ d2aabl1 d1tzia1 d4n1ca_ d1k6qh1 d3g9ab_ d4tvsa1
d1igmh_ d1pz5b1 d3dx9a1 d3wxwh_ d1kcul1 d3eaka_ d4qlra1 d1cr9l1
d1d9ka_ d1f4xl1 d1deea1 d2i27n2 d1c12a1 d2orbl1 d1j8hd1 d1mcph1
d1c5da1 d1kenh1 d1bd2e1 d1h5ba_ d4x7fc1 d3upaa_ d1t3fa1 d1indh1
d2dtgc1 d4hc1l1 d1zv5a1 d4wgvb_ d1uwgl1 d4f37k1 d1aqkh1 d1vesa_
d3zkqd_ d1fptl1 d1lo4l1 d1knob1 d3rrqa_ d1ct8b1 d4orzc_ d1mlbb1
d4hgkc1 d1dqdl1 d1bogb1 d4ozhe1 d1dr9a1 d1i7za1 d1dn0b1 d1kell1
d1rzib1 d1ypzf1 d1wtla_ d1hkfa_ d1fail1 d1figh1 d2vyre_ d1sjva_
d1ohqa_ d1bfoa1 d1pkqb1 d1mjul1 d1b4jl1 d1nsnl1 d2arjb1 d1p4bh_
d4aj0a_ d3nn8b_ d2rcja1 d1f2xk_ d4v1dd_ d1adqh1 d2eksa_ d4k3ga_
d1jfqh1 d1uweh1 d1frgh1 d4gftb_ d1uz8b1 d4n1ea_ d1bm3l1 d1clol1
d3mcka1 d2vq1a1 d1ae6h1 d4aq1b1 d1uj3b1 d1nbqa1 d1fh5l1 d2ck0l1
d2ywza_ d1q72l1 d4nbxb_ d1hila1 d4hzlb1 d2j88l1 d1zmya1 d1cd0a_
d1a4ka1 d1pskl1 d1a0qh1 d4krmb_ d1ncbh1 d2fbjh1 d1j8he1 d1i3ua_
d1cf8l1 d1fe8h1 d2p1ya1 d1eeqa_ d2x89a_ d1ol0a_ d2r0lh1 d1ub5a1

d1dfbh1 d3eyvb1 d4jdvb1 d4p2cg_ d2gsia1 d2agjl1 d1u3ha1 d2h1ph1
d1rihl1 d1fskc1 d1boga1 d2a77l1 d1ospl1 d1qfoa_ d3dwta_ d2fx7l1
d1rz7l1 d3stba1 d1pkqa1 d1b6da1 d1dzba2 d1dzba1 d1kj2a_ d4l1ha_
d1rzgb1 d1dqlh_ d12e8l1 d1miel1 d1jmaa_ d1sbsl1 d1fgnh1 d1f58l1
d1mqkh_ d1ndgb1 d3qegl1 d1iail1 d2aw2a1 d1tqbc1 d3fo2a1 d4dkaa_
d1f8th1 d2g60l1 d2ywya_ d1uj3a1 d1yc7a_ d3bd3a_ d4w6xb1 d1nmbl_
d1i8ka_ d1rjlb1 d1ypze1 d1p7kb1 d1kxqe_ d1gpoh1 d2z4qa1 d1w72h1
d1i9rl1 d4qgya1 d4xt1c_ d2qqnh1 d1cica1 d3c08h1 d2cd0a_ d2bdnh1
d1hnga1 d1maml1 d1nldh1 d1cr9h1 d4lcih1 d3bkyh1 d3qg7l1 d1ai1l1
d1kxvc_ d4iosd_ d1l7ih1 d2x1qa_ d1ub6b1 d3k74b_ d1ibgh1 d1teth1
d1igya1 d1hxma1 d2x6ma_ d2e7la_ d5e03a_ d1qfwm_ d2r0kh1 d1igjb1
d1rurh1 d1plgh1 d1mj8h1 d1yuhb1 d3fmgl1 d1rzfl1 d1emtl1 d1ad9b1
d1igtb1 d1nlbl1 d1d5ih1 d3k7ua_ d4ffyh_ d1bwwa_ d3dvga1 d1a6ta1
d1fgvl_ d4qxgl1 d1miml1 d1jhll_ d2hffb1 d2hh0l1 d1qkzh1 d1l7tl1
d1afvh1 d1iaii1 d2cjuh_ d1um5h1 d2ghwb2 d2ghwb1 d1kgce1 d2fgwh1
d1ikfh1 d1dvfd_ d2i24n1 d2xtjb_ d1za3b1 d2yc1a_ d2hh0h1 d2x1oa_
d1bvka_ d1rjla1 d5e7ba1 d1igta1 d1il1b1 d2fb4l1 d1mvua_ d1jptl1
d32c2a1 d1fvca_ d1iqdb1 d2hrph1 d2p4ab_ d4qo1a_ d1p2cb1 d1wt5c_
d1t4ka1 d5c1mb_ d1it9l1 d1bz7a1 d1b2wh1 d1e4wl1 d1jnll1 d1yedb1
d1za6b1 d3u7aa_ d1igfh1 d3gnml1 d1orsa1 d1wejl1 d1a2ya_ d1hxmb1
d1gafh1 d2j4wh1 d1pz5a1 d1j05a_ d1a3rh1 d4fqll1 d1qfwh_ d1eo8h1
d1n7mh1 d4s11a_ d1kegh1 d5da4a_ d1iqwl1 d3whxb1 d1vgeh1 d1deeb1
d1c12b1 d1t2ql1 d1ad9a1 d3gsnb1 d3b2uc1 d1ggbh1 d1t3fb1 d1nakl1
d4ocmc_ d1lila1 d2v7hb3 d2esve1 d1mcoh1 d2qr0b1 d2q3aa_ d1ct8a1
d1kcrl1 d2pxyb_ d2uzih_ d5c3ld_ d3durb_ d1c5cl1 d2ak4e1 d1dl7h_
d1xiwd_ d1mlba1 d1rmfl1 d1i7zb1 d1dlfl_ d2xa3a_ d5bq7b1 d1dqqb1
d3h42l1 d1egjh1 d1kcvl1 d1fe8l1 d2j6el1 d1f3rb2 d1lk3l1 d1sm3h1
d1q0xh1 d1ghfh1 d7fabl1 d1igch1 d1lo0h1 d4idla_ d5c2bl_ d1jrhl1
d1il1a1 d3zhda1 d4kfzc_ d1b0wa_ d1e6oh1 d1mexl1 d4krpb_ d1fnsh1
d1ncwh1 d4hgma_ d4eigb1 d4rzca_ d2mpal1 d4y5xa_ d5en2b1 d3lrga1
d4ej1c1 d1rhha1 d1j05b_ d1forh1 d1q9wb1 d2wzpd_ d2aq2a1 d1h0da1
d3revb1 d2ij0c1 d1tzha1 d4m7jl1 d1k6ql1 d3b9kc1 d1igml_ d3qeua1
d1dsfl_ d1ce1h1 d1kcuh1 d1zvha1 d1zvoa1 d1tqbb1 d1ay1l1 d2qscl1
d2cgrh1 d1nl0h1 d1kenl1 d3eo0a1 d4n9ob1 d1n0xl1 d3k1kc_ d1c4qa_
d1prtc1 d2wv6d_ d1ltrd_ d4l63b1 d1r4pb_ d1prtd_ d1tl0f_ d2bosa_
d1prtb1 d3chbd_ d2aq2b1 d1ck1a1 d1enfa1 d1i4pa1 d1v1oa1 d1et9a1
d1esfa1 d2g9hd1 d4ohja1 d1klud1 d1bxta1 d1eu3a1 d1ty0a1 d1fnua1
d2qaln1 d2cqva1 d1fhga_ d2k1ma_ d2dl2a1 d1biha3 d1biha1 d1biha4
d3qp3a1 d1g1ca_ d1gxea_ d1cvsc2 d1cvsc1 d1xmwa2 d2otpa2 d1biha2
d1rhfa2 d2fcba2 d2fcba1 d1wwbx_ d1gsma2 d1epfa1 d1epfa2 d2dava1
d1waaa_ d1f97a2 d1x44a1 d1vcaa2 d1fnla2 d1fnla1 d3b5ha2 d3h8na2
d3h8na1 d1jbja1 d1jbja2 d1pd6a_ d1f2qa2 d1f2qa1 d3cx2a_ d1nbqa2
d3b5ha1 d1n26a1 d1xiwb_ d1xaua_ d1fltx_ d2ifga1 d1olla1 d1olla2
d1cs6a4 d1cs6a3 d1cs6a2 d1cs6a1 d2gw5a2 d1sy6a1 d1xiwa_ d1ucta2

d1ucta1 d1ry7b2 d1gl4b_ d1qz1a3 d1olza1 d2c9aa1 d1wwca_

**Family-40 (b.60.1.1)**

**Positive dataset contains 19 protein sequences.**

d4tlja_ d4wfva_ d3t1da_ d4iaxa_ d3zq3a_ d2nnda_ d3s26a1 d4gh7a_ d1z24a1 d1xkia_ d3bx7a_ d4iawa1 d3kzaa_ d2k23a_ d2l9ca1 d2joza1 d3kffa_ d3saoa1 d1yupa1

**Negative dataset contains 3923 protein sequences.**

d1m32a_ d1ibja_ d1c7na_ d1iuga_ d4q75a_ d2bkwa1 d1t3ia_ d1n8pa_ d1qz9a_ d1jf9a_ d1eg5a_ d1p3wa_ d3kgwa_ d1cl1a_ d2ctza1 d1vjoa_ d2rfva_ d1gc0a_ d1cs1a_ d1d2fa_ d2ch1a1 d1qgna_ d1pffa_ d1elua_ d1c7ga_ d2v1pa1 d2ez1a_ d1u08a_ d1lc5a_ d1b5pa_ d2ay1a_ d1bw0a_ d3pd6a_ d7aata_ d2csta_ d1j32a_ d1fg7a_ d4wlha_ d1o4sa_ d1v2da_ d1gdea_ d1m6sa_ d1v72a1 d1svva_ d1ajsa_ d1wsta1 d2f8ja2 d1vp4a1 d1xi9a1 d2gb3a1 d1yaaa_ d2ovga_ d1sq8a_ d1rzsa_ d3kz3a_ d3orca_ d1xkta1 d2hfka_ d1jmkc_ d5d3ka_ d1sefa_ d1sfna_ d1sq4a_ d1r0ka3 d1vkoa2 d1vm6a2 d3qvsa2 d1gr0a2 d1p1ja2 d1g8fa3 d1qnia1 d1ibya_ d1f56a_ d1bypa_ d1azca_ d1ws8a_ d3u25a_ d1cuoa_ d1id2a_ d1rkra_ d2iaac_ d4dp1x_ d1nwpa_ d2gima_ d2i7sa_ d1qhqa_ d3ehbb1 d1fftb1 d1gyca3 d1hfua2 d1kbva1 d1kbva2 d1sdda1 d1sdda2 d1gyca1 d1gyca2 d1mzya1 d2bw4a1 d1aoza1 d1aoza3 d1aoza2 d1kyaa2 d1kyaa3 d1kyaa1 d1mzya2 d4e9sa1 d1sddb1 d1sddb2 d1hfua1 d1hfua3 d1oe1a2 d1oe1a1 d1v10a2 d1v10a3 d1v10a1 g1f8v.1 d2bbvc_ d2cfba1 d1dfoa_ d1o69a_ d1bj4a_ d3k28a_ d2bwpa_ d3lg0a_ d2fnua_ d3ou5a_ d1z7da1 d1b9ha_ d1iaya_ d4msoa_ d1bs0a_ d1s0aa_ d1sffa_ d3tqxa_ d1mdoa_ d3qboa_ d3pgya_ d1bjna_ d2dkja_ d1ejia_ d1vefa1 d4wxga_ d3bs8a_ d3gbxa1 d1w23a_ d1a3qa1 d1iknc_ d3brda1 d1oy3c_ d1imhc1 d1gjia1 d1zk9a1 d2cxka1 d2bema_ d1ea9c1 d1qhoa1 d2fhfa2 d2fhfa1 d1k3ia1 d1j0ha1 d1cxla1 d2bhua1 d1edqa1 d1f1sa2 d1ut9a2 d1gvia1 d1txka1 d4alac_ d1rera1 d1s6na_ d3uzqb_ d2phla1 d1fxza2 d1fxza1 d1lrha_ d1uija1 g1dgw.1 d2v09a_ d1uika2 d1uika1 d1dgwa_ d1ds6b_ d1fstb_ d1ex0a1 d1g0da1 d1ju8a_ d1tafa_ d1bh9b_ d1tafb_ d1q9ca_ d1h3ob_ d2byka1 d1n1ja_ d4g92b_ d1bh9a_ d1jfia_ d1h3oa_ d2bykb1 d1n1jb_ d1cg2a1 d1yloa2 d1y7ea2 d1y0ya3 d1lfwa1 d1vixa1 d1fnoa4 d1vhoa2 d1ysja1 d1vhea2 d1z2la1 d2fvga2 d1vgya1 d1r3na1 d2grea2 d1xmba1 g1q7l.1 d2bjca1 d2hsga1 d1qpza1 d1efaa1 d1ydwa2 d2nvwa2 d1zh8a2 d1qkia2 d1xeaa2 d1tlta2 d2b5ea2 d2b5ea3 d2b5ea1 d2b5ea4 d2kp1a1 d2kp2a1 d1hyua3 d1hyua4 d1j08a1 d1j08a2 d1orva2 d1xfda2 d3gpdg2 d2q49a2 d1hdgo2 d2g17a2 d2cvoa2 d2czca1 d3pwka2 d1obfo2 d1i32a2 d1zyao2 d2pkqo2 d1b7go2 d2hjsa2 d1cf2o2 d1vkna2 d1nvmb2 d2msya_ d2djoa1 d2r5za_ d1au7a1 d1f43a_ d2lmda1 d1akhb_ d1s7ea1 d3nara_ d1zq3p1 d2craa1 d1mija_ d4uuta_ d3a01a_ d1x58a1 d3rkqa1 d3cmya1 d2cqxa1 d2da6a1 d1e3oc1 d1fjla_ d1mh3a1 d1x41a1 d1wh7a1 d1gt0c1 d2mw8a_ d1lfup_ d1x2ma1 d1b72a_ d1wi3a1 d1jgga_ d1wjha1 d1x2na1 d1mnmc_ d2cuea1 d3k2aa_ d2hosa_ d2jwta2 d1pufa_ d1octc1 d2hi3a_ d1b8ia_

d4j19a_ d3lnqa_ d1wh5a1 d1b8ib_ d1hcra_ d1tc3c_ d1gdta1 d1u78a2
d6paxa1 d1k78a1 d1k78a2 d2iu5a1 d1zk8a1 d2g7sa1 d2fd5a1 d2np5a1
d1z0xa1 d2id6a1 d2d6ya1 d2i10a1 d1vi0a1 d2oi8a1 d3c07a1 d2ns7a1
d2fq4a1 d2yvea1 d1t33a1 d2o7ta1 d1rkta1 d2np3b1 d2g7ga1 d2gena1
d2fbqa1 d2g7la1 d2gfna1 d2g3ba1 d2jj7a1 d2hyja1 d1sgma1 d1ui5a1
d2hkua1 d2id3a1 d4jyka1 d1bl0a1 d1d5ya1 d1d5ya2 d3lv8a_ d1s3ga1
d1zaka1 d1s4qa_ d3tvta2 d1uf9a_ d1khta_ d1ckea_ d1osna_ d1p5zb_
d4tmka_ d3cm0a_ d1tmka_ d1jaga_ d3h86a_ d1t0hb_ d1nn5a_ d2h92a_
d3kb2a1 d2c95a1 d1yj5a2 d1kgda_ d1ly1a_ d2ak3a1 d1vyva2 d1deka_
d2grja1 d1p3ja1 d1y63a_ d1e4ya1 d1s96a_ d3iija1 d1lvga_ d1nksa_
d1vyua2 d4e22a_ d1qf9a_ d1q3ta_ d1ki9a_ d1lw7a2 d1teva_ d1kjwa2
d1gsia_ d2iyva2 d1kaga_ d1viaa_ d1e6ca_ d1x6va3 d1odfa_ d1uj2a_
d4gi7a_ d2jeoa1 d1k6ma1 d1z0ja1 d3umra2 d3gj0a1 d2gaoa1 d1yzga1
d1x3sa1 d1g7sa4 d1puja_ d1udxa2 d1kk1a3 d2o52a_ d1jwyb_ d1zcaa2
d1zcba2 d2fv8a1 d2cjwa_ d3dz8a1 d1xzpa2 d1vg8a_ d4qxaa_ d2ew1a1
d1x1ra1 d2bcgy_ d3doea1 d3cona_ d3rega_ d3wyga_ d1upta_ d2b6ha1
d1zd9a1 d1lnza2 d1r5ba3 d1n0ua2 d1r2qa_ d2f9la1 d2rhda_ d3q3jb_
d3q85a_ d1efma1 d1r8qa_ d2gf9a1 d2akab1 d1ek0a_ d1z08a1 d2gcoa1
d2dy1a2 d4g01b_ d2cxxa1 d1svia_ d3q72a_ d1ni3a1 d4ac9a4 d2erxa1
d2bmea_ d1wxqa1 d2gf0a1 d4ldja1 d1zunb3 d1d5ca_ d4lv5b_ d1efca3
d1kmqa_ d2g6ba1 d3lrpa_ d1tada2 d2y8ea_ d4rd4a1 d1z06a1 d2heia_
d3a58b_ d1u0la2 d1d2ea3 d1zj6a1 d2ocba_ d3rwoa_ d1mr3f_ d2e9sa1
d4u5xa1 d2iwra_ d2fn4a1 d3bwdd_ d2nzja1 d2atva1 d2ksqa_ d3l43a_
d1mkya2 d1mkya1 d1wmsa_ d1jnya3 d2rmka_ d1f6ba_ d1f60a3 d1gota2
d1jala1 d2fh5b1 d1g16a_ d2wblc_ d1m7ba_ d1wf3a1 d1f5na2 d1t9ha2
d1z6xa1 d1z0aa1 d2fg5a1 d2a5ja1 d2bcjq2 d1s0ua3 d1ky3a_ d4y0va_
d4lhwa_ d2qmea1 d2atxa1 d1egaa1 d1ryha_ d1moza_ d1nrjb_ d2erya1
d3bfka_ d1puia_ d3j3az1 d3t5ga_ d1shza2 d3defa_ d2f7sa1 d3raba_
d3tsoa_ d3aq4a_ d2al7a1 d3sfva1 d3bc1a_ d2ncda_ d1sdma_ d3h4sa1
d2heha_ d2grya_ g3kin.1 d1kk8a2 d1br2a2 d1goja_ d1f9va_ d2mysa2
d1lkxa_ d3t0qa_ d4db1a1 d3gbja_ d1ry6a_ d1v8ka_ d1k8ka2 d1k8ka1
d3uleb1 d3i33a2 d3i33a1 d1bupa1 d1dkgd2 d1dkgd1 d4ci6a2 d1jcfa1
d4a2ba2 d2fxua1 d1yaga2 d1yaga1 d4cbua2 d1bupa2 d1g99a1 d2e1za2
d2e1za1 d1saza1 d1saza2 d1ig8a1 d1czan2 d1czan3 d1czan1 d1ig8a2
d1czan4 d1bg3a4 d1bg3a3 d1bg3a2 d3h3no1 d3h3no2 d3ezwa2 d1hroa_
d4gedb_ d3zooa_ d1mz4a_ d1gu2a_ d1f1fa_ d1dvva_ d1c6ra_ d1c75a_
d1e29a_ d1gdva_ d1cnoa_ d1h32b_ d1co6a_ d2bh4x_ d2aiua_ d5cytr_
d1jdla_ d1i8oa_ d2zboa_ d3dmia_ d1ls9a_ d1wvec_ d2yk3a_ d3ph2b_
d1dw0a_ d5aura_ d1ql3a_ d1qn2a_ d1f1ca_ d2nqya_ d1h4ga_ d1hixa_
d1oa2a_ d1olra_ d3b7ma_ d4h7ma1 d3wp3a_ d2vgda_ d1igoa_ d3zsea_
d2nlra_ d3wx5a_ d1oa3a_ d1pvxa_ d1m4wa_ d1f5ja_ d1oa4a_ d1ks5a_
d4ixla1 d4nmwa_ d1evsa_ d1cnt1_ d1pvhb_ d1z7ca_ d1huwa_ d1bgea_
d1f6fa_ d1f45b_ d1i1rb_ d1axia_ d1etea_ d3tgxb_ d1scfa_ d2z3qa1
d3qb1a_ d1hula_ d4nkqb_ d1eera_ d1v7mv_ d5e4ea_ d2o27a_ d3b5ka_

d2psma1 d4zf7a_ d3uf5a_ d4x51a_ d1d9ca_ d4ypgc1 d3oq3a_ d1lqsl_
d1n1fa_ d1au1a_ d1wu3i_ d3besl_ d3ux9a_ d1v8fa_ d2ejca_ d3muea1
d3q12a1 d3rbaa_ d2qtra_ d1nupa_ d3l92a_ d1f9aa_ d1qjca_ d2h29a_
d1vlha_ d1od6a_ d1mrza2 d4ruka_ d2el7a_ d1iq0a2 d1irxa2 d1r6ta2
d1ivsa4 d2cyba_ d1rqga2 d1j09a2 d2yxna_ d1f7ua2 d1h3fa1 d1ffya3
d2g36a1 d1jila_ d1n3la_ d1i6la_ d1pfva2 d1h3na3 d3n9ia_ d3sz3a1
d3sw2a_ d1moxc_ d1dx5i3 d1edmb_ d1autl2 d1autl1 d1nqlb_ d1dval1
d1szba2 d1kigl_ d2y5fl_ d1ijqa2 d1gk5a_ d1g1sa2 d1nt0a3 d1xdtr_
d1lmja2 d1lmja1 d1nzia2 d2wphe_ d1klil_ d1pfxl2 d1pfxl1 d1p9ja_
d1g1ta2 d1f7ea_ d4yzub_ d1lr7a1 d2y5ia_ d1qlsa_ d1psra_ d3n22a_
d2egda_ d4ggfc_ d2wcea_ d2luca_ d4aqia_ d3nsia_ d2lp3a_ d1j55a_
d1a4pa_ d1k8ua_ d1nsha_ d1pvaa_ d3fs7a_ d2pvba_ d1ttxa_ d1rwya_
d2kyfa_ d1rk9a_ d1a75a_ d1exra_ d1tiza1 d1nyaa_ d1g8ia_ d1ggwa_
d1wdcc_ d3fwba_ d1ggza_ d2o5ga_ d1ctaa_ d1m45a_ d3i5gc_ d1sl8a_
d1fpwa_ d2scpa_ d2roaa_ d4aqra_ d3pn7b_ d1br1b_ d2mysb_ d1s6ca_
d1bjfa_ d2obha_ d1jfka_ d1jf0a_ d1qv0a_ d1lkja_ d2mlfc_ d2m97a_
d2d8na_ d4by5a_ d1jbaa_ d4nqga_ d1wdcb_ d2mysc_ d1j7qa_ d1s6ia_
d1uhka2 d1r2ua_ d1dtla_ d3kf9a_ d3pn7c_ d5d67d_ d1tuza_ d1h8ba_
d1eg3a1 d2zkmx1 d1snla_ d1gjya_ d1y1xa_ d1hqva_ d1dvia_ d1qxpa2
d4usla_ d1k94a_ d1wkya2 d1uuqa_ d3wh9a_ d4m1ra_ d1xyza_ d4xuya_
d2je8a5 d4bf7a1 d3cuia_ d1ur4a_ d1qw9a2 d1odza_ d2vzsa5 d1bqca_
d2x2ya1 d1hjqa_ d1qnra_ d4y7ea1 d1vjza_ d4k68a_ d2v3ga_ d4hpga_
d1nofa2 d1nq6a_ d1h4pa_ d1aq0a_ d3ziza_ d4bs0a_ d1ecea_ d1h1na_
d1ta3b_ d1w91a2 d1i1wa_ d1uhva2 d1us3a2 d1egza_ d7a3ha_ d1tvna1
d3nyda1 d1ghsa_ d2cyga1 d4xv0a_ d2c8na2 d1w32a_ d3emca_ d1v6ya1
d1foba_ d2uwfa1 d2fgla_ d3niya1 d2c0ha1 d1rh9a1 d1yq2a5 d1szna2
d1fa2a_ d1pama4 d5do8a1 d1ea9c3 d1hx0a2 d2fhfa5 d1qhoa4 d1avaa2
d1m53a2 d4m8ua1 d2gjpa2 d1lwha2 d4howa1 d1ktba2 d1j0ha3 d3vm5a1
d2x1ia_ d1wzaa2 d1uasa2 d2diea1 d1mxga2 d1ht6a2 d1eswa_ d1iv8a2
d5cpsa_ d1ud2a2 d1zjaa1 d3gbea1 d1kwga2 d1wdpa_ d1gcya2 d1g5aa2
d1hvxa2 d3dhpa2 d2bhua3 d4do4a1 d3qgva1 d1e43a2 d1g94a2 d2d3na2
d1gjwa2 d1gvia3 d1x1na1 d1r7aa2 d1tz7a1 d2x42a1 d1tr9a1 d3abza1
d4toqa_ d1wb0a1 d1w9pa1 d1eoka_ d1itxa1 d1kfwa1 d1goia2 d1edqa2
d2gsja_ d1hjxa1 d1jnda1 d1vf8a1 d3wdpp1 d3wq5a_ d4gxpa_ d4hz8a_
d1e4mm_ d3aiua_ d1wcga1 d1qvba_ d3ahxa_ d1v02a_ d2dgaa_ d1pbga_
d1vffa1 d1qoxa_ d4ha4a_ d2y8ga_ d1xkea1 d2y8fa_ d2v76a_ d1wj1a1
d1aqca_ d1qqga2 d1qqga1 d1n3ha_ d4dbba_ d3f0wa1 d1p3ra_ d1wgua1
d2cy4a1 d3hqca_ d1wvha1 d1xr0b1 d1wgqa1 d1v89a1 d2coda1 d1zc3b_
d1wg7a1 d1u5ea1 d1v61a1 d2coca1 d2ys1a1 d1v5pa1 d1ntya2 d1wjma1
d1wi1a1 d1u29a_ d1xx0a1 d1unqa_ d2zkmx3 d1fhoa_ d1v88a1 d2adza1
d1x1fa1 d1p6sa_ d2coaa1 d1btka_ d1v5ma1 d1faoa_ d2x18a_ d1dyna_
d1x1ga1 d4kaxb_ d2j59m1 d1ki1b2 d1xcga2 d1txda2 d1u5da1 d1dbha2
d1w1ha_ d1upqa_ d1v5ua1 d1foea2 d2fjla1 d1eaza_ d2dfka2 d2cofa1
d1kz7a2 d1h6ta1 d9ldta1 d2ewda3 d1up7a1 d2dfda1 d1s6ya1 d1vjta1

d7mdha1 d1hyea1 d1t2da1 d1ojsa3 d1b8pa1 d1ldna1 d1hyha1 d1civa1
d2a92a1 d1ez4a1 d1i0za1 d1y6ja1 d1gv0a1 d1ur5a1 d1llda1 d1obba1
d1npya1 d3t4ea2 d1a4ia1 d1o0sa1 d2hk9a2 d2egga2 d1b26a1 d1bvua1
d1gq2a1 d1c1da1 d1leha1 d1gtma1 d1euza1 d1nvta1 d3dona2 d1edza1
d1gpja2 d1vi2a1 d1p77a1 d1nyta1 d1b0aa1 d1vl6a1 d1v9la1 d1txga2
d3f47a1 d1mv8a2 d2pv7a2 d1xc2a3 d2ahra2 d4xiya1 d2f1ka2 d1pgja2
d1i36a2 d1np3a2 d1lj8a4 d1teaa3 d1qmga2 d2i76a2 d2g5ca2 d1wdka3
d1dlja2 d3cuma3 d1kola2 d1f8fa2 d1qora2 d1jqba2 d1vj0a2 d1cdoa2
d1gu7a2 d1d1ta2 d1xa0a2 d2dm6a2 d1rjwa2 d1llua2 d1h2ba2 d1tt7a2
d1piwa2 d1o89a2 d1iz0a2 d1e3ja2 d1yb5a2 d1uufa2 d1vj1a2 d1e3ia2
d3gpdg1 d1vc2a1 d2nvwa1 d1u8fo1 d1ydwa1 d1hdgo1 d2g17a1 d1cf2o1
d2czca2 d1r0ka2 d1obfo1 d2cvoa1 d1ebfa1 d1h6da1 d1i32a1 d1zh8a1
d2q49a1 d1qkia1 d1j5pa4 d3pzra1 d1xeaa1 d1vkoa1 d1vm6a3 d3qvsa1
d2pkqo1 d1b7go1 d1gr0a1 d1p1ja1 d1lc0a1 d1j0xo1 d3pwka1 d2hjsa1
d1zyao1 d1vkna1 d1tlta1 d1nvmb1 d3sjua_ d3ojfa1 d1xu9a1 d2jjya1
d1n7ha_ d1y1pa1 d1h5qa_ d1jtva_ d4fs3a_ d2bkaa1 d1gy8a_ d2c5aa1
d1ulsa_ d1ooea_ d2b69a1 d1y5ma1 d1spxa_ d1ae1a_ d1bxka_ d1ydea1
d1gega_ d1hdoa_ d1o5ia_ d1e6ua_ d1snya1 d2rhca_ d3zv4a_ d5ceja_
d2d1ya1 d1fjha_ d3w8ea_ d2fmua1 d1x1ta_ d1rpna_ d3ay7a1 d1k2wa_
d1eq2a_ d2hrba1 d3ruaa_ d2ew8a1 d4hwka_ d1xgka_ d2bd0a1 d1yo6a1
d2a35a1 d2ntva_ d1vl8a_ d1hdca_ d4bkua_ d2ag5a1 d2q45a_ d1nffa_
d4iy1a_ d1zbqa1 d1kewa_ d1ek6a_ d1ja9a_ d2q46a2 d1iy8a_ d1cyda_
d2ptga_ d1hxha_ d1uzma1 d3lyla_ d4m89a_ d3wyea_ d2blla1 d1w6ua_
d1sb8a_ d2q2va_ d4nr0a_ d1e6wa_ d2gasa_ d3oiga1 d2bgka1 d1mxha_
d1vl0a1 d1rkxa_ d1r6da_ d1wmaa1 d1e7wa_ d2c07a1 d1xkqa_ d3tzha_
d1g0oa_ d3lz6a_ d1qyca_ d1edoa_ d1gz6a_ d1oc2a_ d1t2aa_ d1zema1
d2xoxa_ d1z45a2 d1geea_ d1p33a_ d3lt0a_ d4e6pa_ d2wyua_ d1db3a_
d4q9na_ d1xhla_ d4o1ma_ d1fmca_ d2uvda_ d2gdza1 d1uaya_ d2z1ma_
d2o23a2 d2fr1a2 d2fr1a1 d1qsga_ d1qyda_ d4zjua_ d2a4ka1 d1yb1a_
d1wvga1 d1yxma1 d3f4ba_ d1mg5a_ d1xg5a1 d1r55a1 d3gboa_ d1atla_
d1kufa_ d4q1la_ d1quaa_ d1yp1a_ d1wnia_ d3nqyb_ d4gera_ d1xd3a_
d3ifwa_ d1ex0a4 d1g0da4 d2f4ma1 d1x3za_ d1mdwa_ d1qxpa4 d1ziva1
d1zcma1 d1t3ca_ d2a97a_ d3fiea_ g1e1h.1 d3ioqa_ d1fh0a_ d1o0ea_
d1khqa_ d1m6da_ d2cb5a_ d1cs8a_ g8pch.1 d1s4va_ d2pnsa_ d1vsna1
d1mira_ d1iwda_ d2oula_ d7pcka_ d3bwka_ d3bcna_ d2p86a_ d1pvja_
d1pcia_ d1gece_ d1deua_ d4bs6a_ d1xkga1 d1cqda_ d1jqpa2 d2c0ya_
d2bdza_ d3p5ua_ d1ex4a2 d1c6va_ d2jk6a2 d2jk6a1 d1feca1 d1feca2
d1lpfa1 d1lpfa2 d1xhca1 d1xhca2 d1dxla2 d1dxla1 d1gera2 d1m6ia2
d1onfa1 d1onfa2 d3lada2 d3lada1 d1w4xa1 d1w4xa2 d1gesa2 d1mo9a1
d1xdia1 d1fl2a2 d1fl2a1 d1fcda1 d1fcda2 d1h6va2 d1h6va1 d1mo9a2
d1gv4a2 d1gv4a1 d2gv8a2 d2gv8a1 d1aoga1 d1aoga2 d1hyua1 d1ebda1
d1ebda2 d3dk9a1 d1q1ra2 d1q1ra1 d1gesa1 d1zbfa1 d2z1ha_ d2etja1
d3qioa1 d1jl1a_ d2e4la_ d1jl2a_ d2lsna1 d1io2a_ d1uaxa_ d2yv0x_
d1mu2a1 d1ekea_ d1i39a_ d1o94a2 d1lqta1 d1cjca1 d1pn0a1 d1reoa1

d1ju2a1 d2f5va1 d3c96a1 d2gmha1 d1pj5a2 d1gpea1 d1o5wa1 d1n4wa1
d1k0ia1 d2dw4a2 d2ivda1 d1seza1 d2voua1 d1kdga1 d1s5ja1 d2hbja2
d2igia_ d1wlja_ d1qhta1 d4dlea1 d1tgoa1 d2f96a1 d3tr8a1 d3mxma_
d1q8ia1 d1y97a1 d1w0ha_ d1kfsa1 d2py5a1 d1noya_ d1d5aa1 d1yt3a3
d2guia_ d3mwpa1 d4kb1a_ d1js9c_ d1f15b_ d1za7a_ d1laja_ g1apy.1
d3ljqa_ d4qmea2 d2xdta2 d1z5ha2 d3nvva1 d1krha3 d2h88b1 d1n62a2
d1t3qa2 d1zoyb1 d1jq4a_ d1ffva2 d1dgja2 d1rm6c2 d1jmxa2 d1jmxa1
d1pbya2 d1r7ha_ d1faaa_ d1wika1 d1xfla_ d1ttza1 d2f51a1 d1ti3a_
d1nhoa_ d1sena_ d2i4aa_ d1ep7a_ d1zmaa1 d1fo5a_ d3lefa1 d3diea1
d2yana1 d1z6na1 d1oaza_ d3zywa1 d4aj8a_ d1nm3a1 d4fiwa_ d1wmja_
d4hs1a1 d1iloa_ d1xwaa1 d1wjka1 d2vm1a_ d1fb6a_ d1gh2a_ d1h75a_
d1nw2a_ d1kx5b_ d3a6na_ d4m6ba2 d2nqbd_ d3wkjd_ d2yfwe_ d4h9na_
d5bnxa_ d1kx5c_ d3r45a_ d1eqzb_ d4jjnb_ d1f66c_ d1eqza_ d4wnnb_
d1kx5a_ d1u35c1 d1id3a_ d2nqbc_ d1kx5d_ d3us3a3 d3us3a1 d1um8a_
d1ixza_ d1qvra2 d1qvra3 d1qzma_ d1sxjc2 d1jr3d2 d2p65a_ d1l8qa2
d1g8pa_ d3hu3a3 d1sxjd2 d1njfa_ d1sxja2 d1tuea1 d2ce7a2 d1w5sa2
d1sxjb2 d1d2na_ d1ny5a2 d1ofha_ d2gnoa2 d2a5yb3 d1fnna2 d1u0ja1
d1lv7a_ d1iqpa2 d2fnaa2 d1ixsb2 d4gita_ d1sxje2 d1g41a_ d1pd212
d1nhya2 d2a2ra2 d1k0dd2 d1f2ea2 d1gsua2 d1f3ba2 d2fhea2 d2pvqa1
d1tw9a2 d1oyja2 d1e6ba2 d1k0ma2 d3ik7a1 d4is0a1 d1r5aa2 d1jlva2
d1m0ua2 d3gtub2 d1z9ha2 d1okta2 d1axda2 d2gsra2 d1tu7a2 d1k3ya2
d1zgma1 d1b48a2 d1gnwa2 d1gwca2 d2gsta2 d1glqa2 d1oe8a2 d1pn9a2
d1v2aa2 d1fw1a2 d1duga2 d3vrda1 d3vrda2 d1fcdc2 d1fcdc1 d1h1oa2
d1h1oa1 d1m70a2 d1qusa_ d1qqya_ d1gd6a_ d1f6ra_ d2gv0a_ d1iiza_
d2z2fa_ d1j8xa_ d1ghla_ d1b9oa_ d1iyka2 d1iyka1 d1rxta1 d1iica2
d2pjsa1 d1klla_ d2i7ra1 d1ecsa_ d1xqaa1 d5cj3a1 d1npba_ d1jifa_
d2p7oa_ d1r9ca_ d1mpya2 d1kw3b2 d1kw3b1 d4ghga1 d1tfza1 d3isqa1
d3isqa2 d1sqia1 d1sqia2 d1lkda2 d1lkda1 d1mpya1 d1cjxa1 d1sp9b2
d1t47a1 d1t47a2 d1f1ua2 d1sp8a1 d1sp8a2 d1f1ua1 d4iuaa3 d4bvva_
d5hpga_ d3k65a_ d1ki0a3 d1ki0a2 d1ki0a1 d1a0ha1 d1pmla_ d3sp8a3
d2feba1 d1qsta_ d2beia1 d4m3sa_ d1u6ma1 d1ygha_ d1yk3a1 d3dr6a_
d1xmta_ d1z4ea1 d2ge3a1 d1bo4a_ d2fiaa1 d2jdca1 d1y9wa1 d3to7a_
d1wwza1 d2fl4a1 d2ozua1 d2b5ga_ d2cy2a1 d2p0wa_ d1cm0a_ d4pswa_
d1yrea1 d1yr0a1 d1m4ia_ d2gana1 d1yvka1 d1ghea_ d2g3aa1 d1y7ra1
d2euia1 d1q2ya_ d2fsra1 d1xeba_ d2ae6a1 d1cjwa_ d1qsma_ d2aj6a1
d1y9ka1 d1i12a_ d1vkca_ d2atra1 d1n71a_ d2d4pa1 d2fiwa1 d2fe7a1
d1tiqa1 d2ou2a_ d1yx0a1 d2fcka1 d1p0ha_ d2j8ma_ d2pq8a_ d1z4ra1
d1s3za1 d1ufha_ d1xvqa1 d1pn0a2 d3hy2a_ d2cvba1 d1o8xa_ d1lu4a_
d1we0a1 d2bmxa1 d2a4va1 d1psqa_ d1xiya1 d1q98a_ d1n8ja_ d1zofa1
d2ggta_ d3tjja_ d4je1a1 d1o73a_ d3s9fa_ d2zcta_ d1qmva_ d1jfua_
d2b1ka_ d5dvba1 d2b5xa1 d2v2ga_ d1st9a_ d3tb2a_ d4llra_ d4fh8a1
d1e2ya_ d1knga_ d2h30a_ d1i5ga_ d2z9sa_ d2f8aa1 d1tp9a1 d2b7ka_
d1nm3a2 d3hvva_ d2wfca1 d4kcea_ d1xvwa1 d4gqca1 d3qpma_ d4k1fa_
d1zzoa1 d1gp1a_ d1prxa_ d2m5ta_ d1cqqa_ d1wqsa_ d5bpea_ d2ipha_

d1lvoa_ d2bhga1 d3tnta_ d3sjia_ d3d23a1 d2fyqa_ d3tloa_ d3zvea1
d2hrva_ d3q3ya1 d4asha1 d1e88a1 d1l6ja5 d1l6ja4 d1eaka4 d1eaka3
d1l6ja3 d1h8pa2 d1h8pa1 d1etha2 d1w52x1 d2ppla1 d1hpla2 d1lpbb2
d2oxea1 d1bu8a2 d1rp1a2 d1lgya_ d4l3wa_ d1llfa_ d1gz7a_ d1ukca_
d3ia2a_ d1a88a_ d4dgqa1 d1hkha_ d1zoia_ d3zxha_ d1cgla_ d1rm8a_
d1eaka2 d1hy7a_ d2jsda_ d1cxva_ d1hv5a_ d1i76a_ d3p0ya1 d1igra2
d1n8yc2 d1n8yc1 d1m6ba2 d1m6ba1 d1sr9a2 d1rqba2 d1cixa_ d1y29a_
d1v7fa_ d2jtba_ d1d1ha_ d1g9pa_ d1lupa_ d1qk7a_ d1mb6a_ d2a2va_
d1niya_ d1i25a_ d1hvwa_ d2mpqa_ d1ie6a_ d1kqha_ d1la4a_ d2mxoa1
d1dl0a_ d1koza_ d1qk6a_ d1emxa_ d1lqra_ d1c6wa_ d1rmka_ d1ixta_
d1eyoa_ d1cnna_ d1fu3a_ d1g1pa_ d1f3ka_ d1fyga_ d1rypd_ d1irua_
d1rypc_ d3unfk_ d1rypa_ d1j2pa_ d1irui_ d1j2qh_ d3unfh_ d1m4ya_
d1rypb_ d1irul_ d1irug_ d1iru2_ d1q5rh_ d1rypg_ d1g3ka_ d1iruf_
d1q5qa_ d1irub_ d1ryph_ d3ty6a_ d1iruj_ d1iruk_ d1rype_ d1irud_
d1irue_ d3mi01_ d1rypf_ d1iruh_ d2z3ba_ d1iruc_ d3mka2_ d1iru1_
d1ny721 d1ny722 d1a6ca3 d1a6ca2 d1a6ca1 d1pgl21 d1pgl11 d1ny711
d1mqta_ d1d4m3_ d1fmd3_ d2mev1_ g1aym.1 d1ev13_ d4q4w1_ d1r1a3_
d2x5ia_ d1rhi1_ g1r1a.1 g1d4m.1 g1rhi.1 d1b35b_ d1oopa_ d1tme1_
d4iv1a_ g1pvc.1 g1fpn.1 d1fpn1_ d4gb31_ d1d4m1_ d1fmd1_ d1ev11_
d2wzr3_ d1qqp3_ g1fmd.1 d2x5ic_ d1rhi3_ g1oop.1 d1hxs1_ g1hxs.1
d1fpn3_ d1bev1_ g2mev.1 d1eah3_ d1aym1_ d1pvc3_ d1qqp1_ d1zba3_
d1h8ta_ d3vbhc_ g1b35.1 g1bev.1 d1hxs3_ g1ev1.1 d1bev3_ g1tmf.1
d1tmf1_ d1b35a_ d2mev3_ g1eah.1 g1h8t.1 d1eah1_ d4q4w3_ d1r1a1_
d1aym3_ d1pvc1_ d1zba1_ d1h8tc_ d1oopc_ d1tme3_ g1qqp.1 d4iv1c_
g1tme.1 d3ubub_ d1egia_ d1qo3c_ d1v7pa_ d1tdqb_ d3ubua_ d1c3ab_
d1h8ua_ d3bx4b_ d1t8ca1 d1sb2a1 d1qdda_ d2ziba1 d3cada_ d1byfa_
d1j34b_ d1jwia_ d1jzna_ d1c3aa_ d1oz7b_ d1wmza_ d3bdwb_ d1hq8a_
d1fifa1 d1dv8a_ d3hupa_ d1wk1a1 d1umra_ d2afpa_ d3bx4a_ d1gz2a_
d1sl6a_ d1v7pb_ d3g8ka_ d1ixxa_ d1j34a_ d1jwib_ d1g1sa1 d1y17a1
d1ja3a_ d1fvub_ d3bdwa_ d1sb2b1 d1fvua_ d1umrc_ d1oz7a_ d4m17a_
d3cfwa1 d1ypqa1 d1g1ta1 d3fd4a_ d3l9jc_ d4dn8a1 d1v4lb_ d1rdl1_
d2msba_ d1eixa_ d3exra_ d2ffca1 d2fdsa1 d3n3ma1 d2czda_ d2yyua_
d1vqta1 d1dbta_ d3ldva1 d4lt9a1 d3tc7a1 d3hoja_ d1i4na_ d3vnda_
d3nl8a1 d1geqa_ d3nava1 d1v5xa_ d4a2ra1 d3tc6a1 d1ujpa_ d4ijba_
d1xcfa_ d1vc4a_ d1rd5a_ d1qopa_ d3b5va_ d2r80b_ d1cqxa1 d1cg5a_
d1gcvb_ d3boma_ d2h8fb_ d2raoa_ d3bj1a_ d4hrrb_ d2r50a_ d3d1kb_
d2aa1b1 d3zhwa_ d3hf4b_ d3gdja_ d1jebb_ d3bj1b_ d1x9fb_ d1wmub_
d1gcva_ d3bcqb_ d3d4xb_ d2dc3a_ d3hf4a_ d4yu3a_ d3k8ba_ d1qpwb_
d2r80a_ d3bcqa_ d1v4wa_ d3wtgb_ d1jl7a_ d4esaa_ d1a9we_ d3d1ab_
d1fsla_ d4h2la_ d1v4wb_ d1mwca_ d1vhba_ d1jeba_ d1spgb_ d1h97a_
d1q1fa_ d1tu9a1 d3d1aa_ d1hdsb_ d3hyub_ d2zfbb_ d3fs4a_ d1oj6a_
d1shrb_ d4h2lb_ d4yu3b_ d1spga_ d3hrwa_ d3qqra_ d3at5a_ d4wchd_
d3hyua_ d1cg5b_ d1uc3a_ d1hbra_ d2raob_ d1outb_ d4esab_ d3gdjb_
d3qqqa_ d1x9fc_ d3vrfa_ d1itha_ d3mkba_ d3d4xa_ d3mjpa_ d1jl6a_

d3fh9a_ d1outa_ d2dc3b_ d1it2a_ d3eoka_ d3g46a_ d1qpwa_ d3vrfb_
d1ch4a_ d1x9fd_ d1hdsa_ d2zfba_ d3mkbb_ d1or4a_ d3wtga_ d1a4fa_
d3fh9b_ d3bomb_ d3cy5a_ d3at5b_ d1x9fa_ d2oifa_ d4lmxb_ d1cpcb_
d3v57a_ d1kn1a_ d1cpca_ d4f0ta_ d2vjha_ d4po5b_ d1eyxa_ d1alla_
d4f0tb_ d1b33b_ d1b8db_ d4h0mb_ d2vjhb_ d4lm6b_ d2vmlb_ d1allb_
d2vjta_ d3dbja_ d2vmla_ d2vjtb_ d4f0ub_ d1f99b_ d4rmpa1 d1liaa_
d1eyxb_ d2uulb_ d4lmsb_ d4f0ua_ d2bv8a_ d1f99a_ d2bv8b_ d2c7lb_
d3dbjb_ d4rmpb1 d1b8da_ d4h0ma_ d3v57b_ d1kn1b_ d1liab_ d2uula_
d2j96a_ d1kyqa1 d3w3ea_ d4tx7a_ d3cqla_ d4msta_ d3iwra_ d2z37a_
d4dyga_ d1hlga_ d2xqfa_ d1ea5a_ d1dx4a_ d1qe3a_ d1k4ya_ d4qwwa_
d2ha2a_ d3c8da2 d2pbla1 d1jkma_ d1jjia_ d1lzla_ d1jjfa_ d1b9ra_
d1offa_ d1frra_ d3wcqa_ d1e9ma_ d1l5pa_ d1wria_ d1i7ha_ d3av8a_
d1czpa_ d1rfka_ d3p1ma1 d3b2ga_ d2bt6a2 d1pv8a_ d1w5qa_ d2c1ha_
d1ohla_ d1l6sa_ d1f74a_ d3l21a_ d2e1da_ d1to3a1 d1w3ia_ d1sfla_
d1n7ka_ d1vcva1 d4imea_ d1wa3a_ d2ocza1 d1f2ja_ d3m16a_ d2yxga_
d1ub3a_ d3a5fa_ d1fbaa_ d1l6wa_ d1xfba1 d1wx0a1 d1vpxa1 d1mxsa_
d2a4aa1 d1xkya1 d3cq0a1 d3s1xa_ d1f05a_ d1fdja_ d3cpra1 d4d2ja_
d4mh8a_ d1mu2a2 d4i7ga1 d3rp2a_ g1ucy.1 d1fuja_ d2zchp_ d3w94a_
d1si5h_ d1lo6a_ g2pka.1 d1cgha_ d1kigh_ d1a5ia_ d3s9ba_ d1hj8a_
d1ao5a_ d2anya_ d1q3xa1 d1gvza_ d1azza_ d4gsoa_ d2f91a1 d1iaua_
d1gdna_ d1eufa_ g1fiw.1 d1eq9a_ d4nfea_ d2rdla_ d1spja_ d1elva1
d1a7sa_ d1ekbb_ d1pfxc_ d1yc0a_ d2aiqa_ d1fi8a_ d4k60a_ d4dgja_
d1mzaa_ d1op0a_ d3tjva_ d1pytd_ d1sgfg_ g1gg6.1 d2eeka_ g1rtf.1
d1a0ja_ d3g01a_ d1pytc_ d4e7na_ d1ddja_ d1z8ga1 d2hlca_ d1npma_
d1brup_ g1jou.1 d1sgfa_ d1autc_ g2hnt.1 g1kdq.1 d1orfa_ d3s69a_
d4crga_ d1fxya_ d1bqya_ d1ra6a_ d1muka_ d3gsza_ d1te9a_ d2xwha_
d3uqsa_ d3hkwa_ d3n6ma_ d2cjqa_ d2xyma_ d1teba_ d1te8a_ d1s48a_
d1u09a1 d4wfxa_ d3tyqa_ d2pgga1 d1m4la_ d2bk7a_ d3fjua_ d1z5ra1
d1kwma1 d1jqga1 d2c1ca1 d1dtda_ d1h8la2 d3lgia_ d3i77a_ d2z9ia2
d4nsva_ d1lcya2 d4jcna_ d1agja_ d1os8a_ d1hpga_ d1p3ca_ d1l1ja_
d1qtfa_ d1e69a_ d1mv5a_ d3g60a4 g1ii8.1 d1ewqa2 d1ji0a_ d1v43a3
d2wmfa1 d1vpla_ d1g2912 d4a82a2 g1f2t.1 d2d62a1 d3tuic1 d2bbsa1
d3g60a3 d2ixea_ d1g6ha_ d1sgwa1 d2qi9c_ d3rlfa1 d3b60a1 d3d31a2
d2pzea1 g1xew.1 d1jj7a_ d1oxxk2 d2onka1 d1w1wa_ d1iwea_ d1qf5a_
d1vmaa2 d1nija1 d1np6a_ d2p67a1 d3ihla_ d1okkd2 d1ls1a2 d1s1ma2
d1iona_ d2v40a_ d1dj3a_ d3hida_ d2vo1a1 d2g0ta1 d1vcoa2 d1j8mf2
d1g3qa_ d1xjca_ d1p9ba_ d1qzxa3 d1ihua2 d1ihua1 d2qm8a_ d1dj2a_
d4ioja_ d1cp2a_ d1yrba2 d3gqba2 d1xp8a1 d1tf7a2 d1tf7a1 d1ye8a1
d1skyb3 d2qe7d2 d4blta_ d4huta_ d3ldaa2 d4hyya_ d2i3ba1 d1cr1a_
d3j9tb2 d3ntua2 d1vdza1 d2z43a_ d1e9ra_ d1nlfa_ d3ziaa2 d1n0wa_
d1g6oa_ d3ziad2 d3j9ta2 d1pzna2 d3gqbb2 d2i49a_ d1nnfa_ d1xvxa_
d1ursa_ d1h3da1 d1iuda_ d4m1va1 d1twya_ d3e13x_ d2nxoa1 d4pfua_
d3ttma_ d1elja_ d2qmwa1 d1us5a_ d1y3na1 d1jeta_ d1uqwa_ d4f19a1
d2esna2 d3thia_ d3fasa1 d2uyeb_ d2xwva1 d1r9la_ d2fyia1 d1pb7a_

d2ozza1 d1sw5a_ d1nh8a1 d1vr5a1 d2g29a_ d1xs5a_ d3lswa_ d2z23a_
d3w9va_ d3tcha_ d1q35a_ d1z7me1 d3q27a_ d1p99a_ d1ve4a1 d2i6ea1
d4mcoa1 d4n8ya_ d1eu8a_ d1zbma1 d1hsla_ d1ixca2 d1j1na_ d1xvya_
d1y9ua_ d2z22a1 d1xoca1 d4oera_ d1wdna_ d5f1qa1 d1xt8a1 d4gwoa_
d4igta1 d1pc3a_ d4mmpa1 d1o63a_ d2czla1 d2onsa2 d4mija1 d1eh3a_
d3skpa_ d1dtza1 d1dtza2 d1ieja_ d1jnfa2 d1ce2a1 d1jnfa1 d1b1xa2
d1b1xa1 d1jw1a1 d1jw1a2 d1ryoa_ d1h76a1 d1h76a2 d3q3ma_ d1za0a1
d1mxra_ d2inca_ d1jk0a_ d2incb_ d1mhyb_ d3hf1b_ d1mtyb_ d1r2fa_
d2uw1a_ d1mhyd_ d3n37a_ d1afra_ d1oquc_ d1syya_ d1mtyd_ d2o1za_
d1uzra_ d1smsa_ d2fkza_ d1zuja1 d1s3qa1 d2fjca1 d1z6om1 d1veia_
d4dyya_ d1jiga_ d3gvya_ d2pyba_ d4cy9a1 d3bvfa1 d1r03a_ d2cf7a_
d1nf4a_ d1zs3a1 d4iwka_ d3ka8a_ d3np0x_ d1n1qa_ d1lb3a_ d4e6ka_
d1z6oa1 d2fzfa1 d4dyua_ d3ge4a_ d1jgca_ d4am4a_ d2z6ma_ d1ji5a_
d1vlga_ d1lkoa1 d1krqa_ d3qz3a_ d1o9ra_ d2ffxj_ d1euma_ d1yv1a1
d2v2pa_ d1j30a_ d4di0a_ d1zx5a1 d1oewa_ d2qzxa_ d2asia_ d1psoe_
d1miqa_ d1smra_ d2h6ta_ d1pfza_ d1izda_ d3hd8a_ d1j71a_ d1dpja_
d4aa9a_ d1t6ex_ g1b5f.1 g1lya.1 g1htr.1 d1qdma2 d1bxoa_ d1wkra_
d1tzsa_ d1ibqa_ d2qzwa_ d1zoya1 d1d4ca2 d1chua2 d1jnra2 d1kf6a2
d1bdqa_ d3ggta_ d3ixoa_ d1mrxa_ d2haha_ d1tw7a_ d4njsa_ d1sgua_
d2p3da_ d1nsoa_ d3d3ta_ d2rkfa_ d1c6ya_ d2rspa_ d3ogpa_ d3ekwa_
d2o4na_ d2j9ja_ d1baia_ d3ggua_ d3lzua_ d4npua_ d4j55a_ d2r8na_
d2fxda_ d2p3ca_ d4dqea_ d3ttpa_ d3t3ca_ d2p3aa_ d2hc0a_ d2fdda_
d3mwsa_ d1k8rb_ d1wgra1 d1ef5a_ d1wfya1 d1e8ya3 d1wgya1 d1lfda_
d2cs4a1 d1wxma1 d2byea1 d3kh0a1 d2byfa1 d1wxaa1 d1e7ua3 d1h4ra3
d4yl8a1 d2al6a3 d1wgda1 d1wgga1 d4q5eb_ d1wjna1 d1se9a_ d3n3kb_
d1wz0a1 d1we6a1 d2m0xa_ d2gbka_ d3rt3b1 d1wh3a1 d1sifa_ d2ekec2
d4ii2b1 d4hk2a1 d1c3ta_ d1m94a_ d1wgha1 d1ttna1 d1j8ca_ d1wx8a1
d3v6cb1 d2ma9b_ d4i6lb1 d1bt0a_ d2jvca1 d1iyfa_ d2k1fa_ d1wxva1
d1wf9a1 d1t0ya_ d2io0b2 d1x1ma1 d2gbma_ d1zw7a1 d2m17a_ d1ud7a_
d1we7a1 d2lwpa_ d2gbja_ d1wjua1 d1v6ea1 d1v86a1 d4eewa_ d1wiaa1
d4icva_ d4gu2a_ d1wx7a1 d1wy8a1 d4hjka1 d2faza1 d1v5oa1 d2h0ba_
d2erfa_ d1h30a1 d3mw4a1 d1pz7a_ d2r1ba_ d3v64a1 d1d2sa_ d3bopa1
d3mw3a1 d1okqa1 d1okqa2 d2bcgg1 d2bcgg2 d1d5ta1 d1dszb_ d1a6ya_
d3cbba_ d1ynwa1 d1r4ia_ d1hcqa_ d2hana_ d2hanb_ d2enva1 d1kb4b_
d2nllb_ d1llo1a_ d1cita_ d1adqa1 d1fnga1 d1uvqb1 d1zs8a1 d2vola1
d2vola2 d1mfbl2 d2jelh2 d1uwel2 d3qq4b1 d1kjvb_ d1k5na1 d1osph2
d4odhl2 d1ow0a1 d1ow0a2 d1bfob2 d3l9ra2 d3jtth1 d2fath2 d4elkb2
d1fn4b2 d2xfxb_ d1pg7x2 d1cqka_ d4k71b2 d1lk2a1 d1eapb2 d4lfhg2
d1i4fa1 d2nw2a2 d1kfah2 d1mhca1 d1h3ua1 d1wejh2 d1h0db2 d1a5fh2
d1kcvh2 d1mjuh2 d1qqda1 d1kcrh2 d1k8ia1 d3huja2 d2nnab1 d1nfdf2
d3mffb2 d1s9va1 d1lk3h2 d1q0xl2 d1fv1b1 d1neza1 d4rfeb2 d1r3ha1
d1a6tb2 d1kjva1 d1pz5b2 d1onqa1 d2bc4a2 d1de4a1 d1k8da1 d1tcra2
d1dn0b2 d1nfde2 d2xwtb2 d1bfoa2 d1mexh2 d1iakb1 d1ypzf2 d4f7ea2
d1q72l2 d2fbjh2 d1clzh2 d1fngb1 d1uvqa1 d1vgka1 d1c16a1 d4j12a2

d4x99b2 d1igtb3 d1je6a1 d1q72h2 d1nldh2 d1teth2 d2hmic2 d1i1ca2
d1i1ca1 d1k8ib1 d1q0yh2 d1kfal2 d3frua1 d1pg7w2 d1u58a1 d1igyb4
d1igyb3 d1hyrc1 d1klub1 d1deeb2 d1c12b2 d2bc4b2 d2ig2h2 d1kcvl2
d3wn5b1 d1lk2b_ d1lk3l2 d1mjul2 d1sm3h2 d1q0xh2 d1zvsa1 d1ydpa1
d1u8ib2 d1ypze2 d1kcuh2 d2lj7a_ d1n4na_ d2m8bb1 d2kyqa_ d3psma_
d1jkza_ d1jxca_ d2gl1a_ d1ugla_ d4uj0a_ d2k4ua_ d1hp2a_ d2lu9a_
d1baha_ d2meoa1 d1wm7a_ d1n8ma_ d1jlza_ d1wpda_ d1c55a_ d1pvza_
d1wt7a_ d1rjia_ d1wz5a_ d1m2sa_ d1pjva_ d1c49a_ d1qkya_ d2ktca1
d1wmta_ d1du9a_ d1j5ja_ d1r1ga_ d1t0za_ d2i61a_ d1t1ta_ d1sn4a_
d1kv0a_ d2lkba_ d1npia_ d1wwna_ d1pe4a_ d2kbka_ d2li7a_ d1djta_
d2kbha_ d1dq7a_ d1sega_ d1omya_ d1zywa1 d1jzaa_ d1dy2a_ d1iyjb4
d1iyjb5 d1iyjb3 d1o7ia_ d4ipca1 d1x3ea_ d4apva_ d1jb7a1 d1jb7a2
d1jb7a3 d3kdfa_ d1ue1b_ d1wjja1 d3eiva_ d1xjva2 d1xjva1 d2ccza2
d1se8a_ d1l1oc_ d1jb7b_ d1qvca_ d3ulla_ d1qzga_ d1miua4 d1miua5
d1miua3 d1y14b1 d2nn6i1 d1i94l_ d2zjra2 d1smxa_ d2qamc2 d1hh2p1
d2r7da1 d1rl2a2 d1wfqa1 d3bzka4 d1x6oa2 d3a0ja_ d2j01d2 d2ja9a1
d4ql5a_ d1hr0w_ d1luza_ d2n3sa_ d2vnud1 d1h95a_ d1go3e1 d2nn6h1
d1ne3a_ d2mo1a_ d1iz6a2 d1jjga_ d1e3pa2 d2ix0a3 d2ix0a2 d2lxja_
d1ueba3 d1ueba2 d2z0sa1 d1u0la1 d2ix0a1 d2eifa2 d2je6i1 d3i2za1
d2c35b1 d2ahob2 d1t9ha1 d3i4oa_ d2qall1 d1jt8a_ d2nn6g1 d1g6pa_
d3cama_ d1i94q_ d1c9oa_ d1d7qa_ d2ba0a1 d2vnud2 d2vnud3 d1khia2
d2qalq1 d1wi5a1 d1xtda2 d1dgsa2 d1fvia1 d1ckma1 d1p16a1 d1x9na2
d1rpma_ d2shpa1 d1yfoa_ d1l8ka_ d2ooqa1 d1g4us2 d1p15a_ d1lara2
d1lara1 d1wcha_ d2fh7a2 d2fh7a1 d3azya_ d3wvja_ d1upsa1 d1ajoa_
d2hyka_ d2vy0a_ d1o4ya_ d3o5sa_ d3d6ea_ d3atga_ d1dypa_ d1umza_
d3i4ia_ d1tdha3 d1q3ba3 d1ee8a3 g1qmo.1 g1len.1 d3zyra_ d1fnya_
d1n47a_ g2ltn.1 d1avba_ d3usua_ d1dhkb_ d1f9ka_ d1ukga_ d1g8wa_
d1g9fa_ d3wcsa_ d1g7ya_ g1loe.1 d1mvqa_ d1hqla_ d1v6ia_ d1dbna_
d1qnwa_ d1fx5a_ d4l8qa_ d1ioaa_ d3n35a_ d3ubra_ d1qo8a1 d1d4ca1
d2k3va_ d1ft5a_ d1qdba_ d1m1qa_ d2j7aa_ d1h21a_ d1ogyb_ d4fasa_
d1oaha_ d1sp3a_ d3h33a_ d1rwja_ d19hca_ d1os6a_ d3bxua_ d3h4na_
d1i77a_ d3caoa_ d1j0pa_ d2ctha_ d1duwa_ d2e84a_ d1hh5a_ d1gyoa_
d3h34a_ d1wioa4 d1ccza2 d1vcaa1 d1dr9a2 d1ic1a1 d2d9qb1 d2nxyb2
d2dara2 d2dara1 d1wyha2 d2dloa2 d1rutx4 d1rutx3 d2cuqa2 d1x4la2
d1x4la1 d1x3ha2 d1x3ha1 d2d8za1 d1x61a2 d1x6aa1 d1x6aa2 d2cora2
d2cora1 d1v6ga1 d1v6ga2 d2d8za2 d1x64a1 d1x64a2 d1b8ta1 d2co8a1
d1x63a2 d2dj7a2 d2dj7a1 d2cu8a2 d2cu8a1 d1g47a2 d1g47a1 d1j2oa1
d1wyha1 d2dloa1 d1wiga1 d1wiga2 d2cupa2 d2cupa3 d1x68a1 d2d8ya2
d2d8ya1 d1m3va2 d1x62a1 d1x68a2 d1x61a1 d1b8ta3 d1b8ta2 d2d8xa2
d2d8xa1 d1x4ka1 d1x4ka2 d2cuqa1 d1m3va1 d2co8a2 d1x63a1 d2cura1
d2cura2 d1x62a2 d1op9a_ d1y0la1 d1etzb1 d1egjl1 d2gsib1 d1nj9a1
d4jfhd1 d1aqkl1 d1t2ja_ d1lo4h1 d1zvya1 d3v4vl1 d4enec1 d1kxtb_
d5e8ea1 d2pxya_ d4weud_ d1xiwc_ d2esvd1 d1qnzh_ d1ogae1 d1f4xh1
d4n1hb_ d3ezjb1 d3mlwl1 d1kfah1 d1etza1 d1b4jh1 d3cfic_ d4w68a_

d2arja1 d1ek3a_ d2ok0l1 d1vera_ d1uwgh1 d1blnb1 d1fpth1 d1rzga1
d2giya1 d4m3kb1 d1nc2a1 d2nyyd1 d1ncna_ d1fbih1 d5dxwa_ d4fqqa1
d1dqdh1 d1jfql1 d1kelh1 d1faih1 d1frgl1 d1figl1 d4m6nl1 d1fo0a_
d4hxbl1 d3b5ga_ d3quyd1 d2h9gb1 d1i8kb_ d2dd8h1 d2ck0h1 d1eaja_
d4w6wb1 d2a6ib3 d1t66d1 d2e27h_ d1ob1b1 d1f11b1 d2v7na1 d1ad0a1
d2xt1b1 d1pskh1 d4i13b1 d2op4l1 d1a0ql1 d1uwel1 d1za3a1 d1ncbl1
d4uu9a_ d1bm3h1 d1cloh1 d1nqba2 d1cf8h1 d4y5va_ d1mhph1 d5anma1
d4cdgc1 d4ldeb_ d1y0lb1 d2pcpb1 d1z3gl1 d1dfbl1 d1fh5h1 d3g5xa1
d1tzib1 d4dn3l1 d3zhka1 d2b1hh1 d2uyla1 d1f3dh1 d1nj9b1 d2agjh1
d2h1pl1 d1q72h1 d8fabb1 d1ndmb1 d1rjca1 d4u6hb1 d3upca_ d2fbjl1
d1osph1 d1tvda_ d2cjvh_ d4fhbd1 d1bd2d1 d1rz7h1 d3nn8a_ d1knoa1
d1nfda1 d2bmkb1 d1dqll_ d1igyb1 d1igfl1 d12e8h1 d3utza1 d1nc2b1
d1rihh1 d4mwfb1 d4jvpa1 d1mieh1 d1rhfa1 d1fgnl1 d1sbsh1 d1lp9e1
d1dn0a1 d4lqfl1 d1iaih1 d1kgcd1 d1mh5b1 d1gpol1 d2g60h3 d1f8tl1
d2jixd1 d4grwh_ d1fo0b_ d1bfob1 d1axsa1 d1u9ka_ d1nmbh_ d1h8na1
d1h8na2 d1jguh1 d4eura1 d3sdyl1 d35c8h1 d1w72l1 d1mvub_ d1i9rh1
d1ob1a1 d4wenb_ d1yeeh1 d3ietc1 d1f58h1 d1ad0b1 d1mqkl_ d1nbvh1
d4b50a_ d3r8bb_ d1bafl1 d1mamh1 d4rgne1 d2gsgb_ d35c8l1 d1bafh1
d1ai1h1 d2pcpa1 d2fath1 d2x1pa_ d1orsb1 d1ibgl1 d1shma_ d1a2yb_
d1u0qa_ d2coqa_ d1hilb1 d1qfwi_ d1jv5b_ d1fn4b1 d5e0qa_ d1nsnh1
d1plgl1 d4ocxl1 d1kb5h1 d1rzfh1 d1pg7x1 d2fx7h1 d2oi9c_ d1l7il1
d3eyva1 d1d5il1 d4ffyl_ d1tetl1 d1a6wh_ d1ac6a_ d3dvfa_ d1nfde1
d1mimh1 d1ri8a1 d1jhlh_ d3dura_ d1fskb1 d1f97a1 d3so3b1 d2ak4d1
d1lp9f1 d4z7wf1 d1l7th1 d4y5ya_ d4x7ec1 d1emth1 d1ikfl1 d1nlbh1
d4utal1 d1bjma1 d1eapb1 d1u6ah1 d2dtgd1 d1kj2b_ d1xeda1 d1c1eh1
d1fgvh_ d2bnub1 d2fb4h1 d2dtgb1 d1ktrh_ d1jpth1 d1qkzl1 d4hepg_
d2atpb1 d1flrh1 d2hrpl1 d1mvfa_ d1iaim1 d3k80a1 d2jb5h1 d3zkxc_
d4grwe_ d1it9h1 d1rhhb1 d3w9ec_ d1e4wh1 d1sy6h1 d2j6eh1 d3qxta_
d1jglh1 d1p7ka1 d1tzhb1 d4n8pa1 d1gafl1 d2jelh1 d1wioa2 d4i0cc1
d1cicb1 d1lmka2 d1lmka1 d1iqwh1 d1orqb1 d1ejoh1 d1jnlh1 d4zg1a_
d43c9b_ d1ggbl1 d1wejh1 d3q6fa1 d1f3dj1 d3c6la1 d1bqlh1 d3p9wb_
d1a3rl1 d1h0db1 d2uzil_ d1eo8l1 d1qfwl_ d1n7ml1 d1igja1 d1c5ch1
d2a9mh_ d1rmfh1 d1fj1b1 d4heme_ d1ccza1 d1a5fh1 d1ogad1 d1dlfh_
d1qa9b_ d1vgel1 d3qskb_ d1sjxa_ d4ozfg1 d1sq2n_ d1qd0a_ d1kcvh1
d1c5db1 d3qcul1 d8faba1 d3bn9d1 d1mjuh1 d4y8dc_ d7fabh1 d3dvgb1
d1lo0l1 d1a6tb1 d1gigh1 d1kcrh1 d2hwzl1 d1yjdc1 d3clfl1 d4krna_
d1dl7l_ d1e6ol1 d1mexh1 d3r0ma_ d4kkna_ d1fnsl1 d1oaqh_ d1rzia1
d1ucbh1 d1ncwl1 d4b41a_ d1nfdf1 d2mpah1 d2d7tl1 d4grwf_ d4b5ea_
d1lk3h1 d3u79a_ d4u3xa_ d1ghfl1 d1bvkb_ d1mcww1 d1b88a_ d1jrhh1
d32c2b1 d4wemb_ d1dsfh_ d1op3h1 d1cell1 d1iqda1 d1qoka1 d1qoka2
d1qfuh1 d2crya1 d1ay1h1 d1oaql_ d3ab0b_ d1rurl1 d1t4kb1 d1bz7b1
d1n0xh1 d1forl1 d1ucbl1 d4w6yb_ d43c9a_ d2aabl1 d1tzia1 d4n1ca_
d1k6qh1 d3g9ab_ d4tvsa1 d1igmh_ d1pz5b1 d3dx9a1 d3wxwh_ d1nakh1
d1kcul1 d3eaka_ d4qlra1 d1cr9l1 d1d9ka_ d1f4xl1 d1deea1 d2i27n2

d1c12a1 d2orbl1 d1j8hd1 d1mcph1 d1c5da1 d1kenh1 d1bd2e1 d1h5ba_
d4x7fc1 d3upaa_ d1t3fa1 d1indh1 d2dtgc1 d4hc1l1 d1zv5a1 d4wgvb_
d1uwgl1 d4f37k1 d1aqkh1 d1vesa_ d3zkqd_ d1fptl1 d1lo4l1 d1knob1
d3rrqa_ d1ct8b1 d4orzc_ d1mlbb1 d4hgkc1 d1dqdl1 d1bogb1 d4ozhe1
d1dr9a1 d1i7za1 d1dn0b1 d1kell1 d1rzib1 d1ypzf1 d1wtla_ d1hkfa_
d1fail1 d1figh1 d2vyre_ d1sjva_ d1ohqa_ d1bfoa1 d1pkqb1 d1mjul1
d1b4jl1 d1nsnl1 d2arjb1 d1p4bh_ d4aj0a_ d3nn8b_ d2rcja1 d1f2xk_
d4v1dd_ d1adqh1 d2eksa_ d4k3ga_ d1jfqh1 d1uweh1 d1frgh1 d4gftb_
d1uz8b1 d4n1ea_ d1bm3l1 d1clol1 d3mcka1 d2vq1a1 d1ae6h1 d4aq1b1
d1uj3b1 d1nbqa1 d1fh5l1 d2ck0l1 d2ywza_ d1q72l1 d4nbxb_ d1hila1
d4hzlb1 d2j88l1 d1zmya1 d1cd0a_ d1a4ka1 d1pskl1 d1a0qh1 d4krmb_
d1ncbh1 d2fbjh1 d1j8he1 d1i3ua_ d1cf8l1 d1fe8h1 d2p1ya1 d1eeqa_
d2x89a_ d1ol0a_ d2r0lh1 d1ub5a1 d1dfbh1 d3eyvb1 d4jdvb1 d4p2cg_
d2gsia1 d2agjl1 d1u3ha1 d2h1ph1 d1rihl1 d1fskc1 d1boga1 d2a77l1
d1ospl1 d1qfoa_ d3dwta_ d2fx7l1 d1rz7l1 d3stba1 d1pkqa1 d1b6da1
d1dzba2 d1dzba1 d1kj2a_ d4l1ha_ d1rzgb1 d1dqlh_ d12e8l1 d1miel1
d1jmaa_ d1sbsl1 d1fgnh1 d1f58l1 d1mqkh_ d1ndgb1 d3qegl1 d1iail1
d2aw2a1 d1tqbc1 d3fo2a1 d4dkaa_ d1f8th1 d2g60l1 d2ywya_ d1uj3a1
d1yc7a_ d3bd3a_ d4w6xb1 d1nmbl_ d1i8ka_ d1rjlb1 d1ypze1 d1p7kb1
d1kxqe_ d1gpoh1 d2z4qa1 d1w72h1 d1i9rl1 d4qgya1 d4xt1c_ d2qqnh1
d1cica1 d3c08h1 d2cd0a_ d2bdnh1 d1hnga1 d1maml1 d1nldh1 d1cr9h1
d4lcih1 d3bkyh1 d3qg7l1 d1ai1l1 d1kxvc_ d4iosd_ d1l7ih1 d2x1qa_
d1ub6b1 d3k74b_ d1ibgh1 d1teth1 d1igya1 d1hxma1 d2x6ma_ d2e7la_
d5e03a_ d1qfwm_ d2r0kh1 d1igjb1 d1rurh1 d1plgh1 d1mj8h1 d1yuhb1
d3fmgl1 d1rzfl1 d1emtl1 d1ad9b1 d1igtb1 d1nlbl1 d1d5ih1 d3k7ua_
d4ffyh_ d1bwwa_ d3dvga1 d1a6ta1 d1fgvl_ d4qxgl1 d1miml1 d1jhll_
d2hffb1 d2hh0l1 d1qkzh1 d1l7tl1 d1afvh1 d1iaii1 d2cjuh_ d1um5h1
d2ghwb2 d2ghwb1 d1kgce1 d2fgwh1 d1ikfh1 d1dvfd_ d2i24n1 d2xtjb_
d1za3b1 d2yc1a_ d2hh0h1 d2x1oa_ d1bvka_ d1rjla1 d5e7ba1 d1igta1
d1il1b1 d2fb4l1 d1mvua_ d1jptl1 d32c2a1 d1fvca_ d1iqdb1 d2hrph1
d2p4ab_ d4qo1a_ d1p2cb1 d1wt5c_ d1t4ka1 d5c1mb_ d1it9l1 d1bz7a1
d1b2wh1 d1e4wl1 d1jnll1 d1yedb1 d1za6b1 d3u7aa_ d1igfh1 d3gnml1
d1orsa1 d1wejl1 d1a2ya_ d1hxmb1 d1gafh1 d2j4wh1 d1pz5a1 d1j05a_
d1a3rh1 d4fqll1 d1qfwh_ d1eo8h1 d1n7mh1 d4s11a_ d1kegh1 d5da4a_
d1iqwl1 d3whxb1 d1vgeh1 d1deeb1 d1c12b1 d1t2ql1 d1ad9a1 d3gsnb1
d3b2uc1 d1ggbh1 d1t3fb1 d1nakl1 d4ocmc_ d1lila1 d2v7hb3 d2esve1
d1mcoh1 d2qr0b1 d2q3aa_ d1ct8a1 d1kcrl1 d2pxyb_ d2uzih_ d5c3ld_
d3durb_ d1c5cl1 d2ak4e1 d1dl7h_ d1xiwd_ d1mlba1 d1rmfl1 d1i7zb1
d1dlfl_ d2xa3a_ d5bq7b1 d1dqqb1 d3h42l1 d1egjh1 d1kcvl1 d1fe8l1
d2j6el1 d1f3rb2 d1lk3l1 d1sm3h1 d1q0xh1 d1ghfh1 d7fabl1 d1igch1
d1lo0h1 d4idla_ d5c2bl_ d1jrhl1 d1il1a1 d3zhda1 d4kfzc_ d1b0wa_
d1e6oh1 d1mexl1 d4krpb_ d1fnsh1 d1ncwh1 d4hgma_ d4eigb1 d4rzca_
d2mpal1 d4y5xa_ d5en2b1 d3lrga1 d4ej1c1 d1rhha1 d1j05b_ d1forh1
d1q9wb1 d2wzpd_ d2aq2a1 d1h0da1 d3revb1 d2ij0c1 d1tzha1 d4m7jl1

d1k6ql1 d3b9kc1 d1igml_ d3qeua1 d1dsfl_ d1ce1h1 d1kcuh1 d1zvha1 d1zvoa1 d1tqbb1 d1ay1l1 d2qscl1 d2cgrh1 d1nl0h1 d1kenl1 d3eo0a1 d4n9ob1 d1n0xl1 d3k1kc_ d1c4qa_ d1prtc1 d2wv6d_ d1prtf_ d1ltrd_ d4l63b1 d1r4pb_ d1prtd_ d1tl0f_ d2bosa_ d1prtb1 d3chbd_ d2aq2b1 d1ck1a1 d1enfa1 d1i4pa1 d1v1oa1 d1et9a1 d1esfa1 d2g9hd1 d4ohja1 d1klud1 d1bxta1 d1eu3a1 d1ty0a1 d1fnua1 d2cqva1 d1fhga_ d2k1ma_ d2dl2a1 d1biha3 d1biha1 d1biha4 d3qp3a1 d1g1ca_ d1gxea_ d1cvsc2 d1cvsc1 d1xmwa2 d2otpa2 d1biha2 d1rhfa2 d2fcba2 d2fcba1 d1wwbx_ d1gsma2 d1epfa1 d1epfa2 d2dava1 d1waaa_ d1f97a2 d1x44a1 d1vcaa2 d1fnla2 d1fnla1 d3b5ha2 d3h8na2 d3h8na1 d1jbja1 d1jbja2 d1pd6a_ d1f2qa2 d1f2qa1 d3cx2a_ d1nbqa2 d3b5ha1 d1n26a1 d1xiwb_ d1xaua_ d1fltx_ d2ifga1 d1olla1 d1olla2 d1cs6a4 d1cs6a3 d1cs6a2 d1cs6a1 d2gw5a2 d1sy6a1 d1xiwa_ d1ucta2 d1ucta1 d1ry7b2 d1gl4b_ d1qz1a3 d1olza1 d2c9aa1 d1wwca_

**Family-41 (b.60.1.2)**

**Positive dataset contains 16 protein sequences.**

d2ftba_ d2rcqa1 d4lkpa_ d1sa8a_ d1vyfa_ d3vg7a1 d4azqa1 d5bvqa_ d2qo4a_ d1yiva1 d4bvma1 d4tkba_ d4i3ba_ d4gkca_ d3p6da1 d2a0aa1

**Negative dataset contains 3965 protein sequences.**

d1m32a_ d1ibja_ d1c7na_ d1iuga_ d4q75a_ d2bkwa1 d1t3ia_ d1qz9a_ d1jf9a_ d1eg5a_ d1p3wa_ d3kgwa_ d1cl1a_ d2ctza1 d1vjoa_ d2rfva_ d1gc0a_ d1cs1a_ d1d2fa_ d2ch1a1 d1qgna_ d1pffa_ d1elua_ d1xfda2 d2bnma2 d3i2ka1 d1lnsa3 d1mpxa2 d1xkta1 d2hfka_ d5d3ka_ d2cfba1 d1dfoa_ d1o69a_ d1bj4a_ d3k28a_ d2bwpa_ d3lg0a_ d2fnua_ d3ou5a_ d1z7da1 d1iaya_ d4msoa_ d1bs0a_ d1s0aa_ d1sffa_ d3tqxa_ d1mdoa_ d3qboa_ d3pgya_ d1bjna_ d2dkja_ d1ejia_ d1vefa1 d4wxga_ d3bs8a_ d3gbxa1 d1w23a_ d1m8pa3 d1ddla_ d1e57b_ d1f56a_ d1bypa_ d1azca_ d1ws8a_ d3u25a_ d1cuoa_ d1rkra_ d2iaac_ d4dp1x_ d1nwpa_ d2gima_ d2i7sa_ d1qhqa_ d1gyca3 d1hfua2 d1kbva1 d1kbva2 d1sdda1 d1sdda2 d1gyca2 d1mzya1 d2bw4a1 d1aoza1 d1aoza3 d1aoza2 d1kyaa2 d1kyaa3 d1kyaa1 d1mzya2 d4e9sa1 d1sddb1 d1sddb2 d1hfua1 d1hfua3 d1oe1a2 d1oe1a1 d1v10a2 d1v10a3 d1v10a1 g1f8v.1 d1novc_ d2bbvc_ g1gk9.1 g1cp9.1 g1e3a.1 d4qgna_ d1vr3a1 d2h0va_ d4alac_ d1rera1 d1s6na_ d3uzqb_ d2phla2 d2phla1 d1fxza2 d1fxza1 d1lrha_ d1uija1 g1dgw.1 d2v09a_ d1uika2 d1uika1 d1ktja_ d3us3a3 d3us3a1 d1v70a_ d2f4pa1 d1yhfa1 d4w9ta_ d4x2ra1 d1ka9f_ d1qo2a_ d4axka1 d1thfd_ d1h5ya_ d4u28a_ d2y88a_ d4wd0a_ d1vzwa1 d1bh9b_ d1tafb_ d1jfib_ d1q9ca_ d1h3ob_ d2byka1 d1n1ja_ d4g92b_ d1bh9a_ d1jfia_ d1h3oa_ d2bykb1 d1n1jb_ d4lt9a1 d3tc7a1 d3hoja_ d1i4na_ d3vnda_ d3nl8a1 d1geqa_ d3nava1 d1v5xa_ d4a2ra1 d3tc6a1 d1ujpa_ d4ijba_ d1xcfa_ d1vc4a_ d1rd5a_ d1qopa_ d3b5va_ d2bjca1 d2hsga1 d1qpza1 d1efaa1 d1ydwa2 d2nvwa2 d1h6da2 d1zh8a2 d1xeaa2 d1tlta2 d1r0ka3 d1j5pa3 d1vkoa2 d1vm6a2 d3qvsa2 d1gr0a2 d1p1ja2 d2ovga_ d1sq8a_ d1rzsa_ d3kz3a_ d3orca_ d3gpdg2 d2q49a2 d1hdgo2 d2g17a2 d2cvoa2 d2czca1 d3pwka2

d1obfo2 d1i32a2 d1zyao2 d2pkqo2 d1b7go2 d2hjsa2 d1cf2o2 d1vkna2
d1nvmb2 d2m9wa1 d3dfxa_ d2msya_ d2djoa1 d2r5za_ d1au7a1 d1f43a_
d2lmda1 d1akhb_ d1s7ea1 d3nara_ d1zq3p1 d2craa1 d1mija_ d4uuta_
d3a01a_ d1x58a1 d3rkqa1 d3cmya1 d2cqxa1 d2da6a1 d1e3oc1 d1fjla_
d1ig7a_ d1x41a1 d1wh7a1 d1gt0c1 d2mw8a_ d1lfup_ d1x2ma1 d1b72a_
d1wi3a1 d1jgga_ d1wjha1 d1x2na1 d1mnmc_ d2cuea1 d3k2aa_ d2hosa_
d2jwta2 d1pufa_ d1octc1 d2hi3a_ d1b8ia_ d4j19a_ d3lnqa_ d1wh5a1
d1b8ib_ d1fexa_ d2cqra1 d1guua_ d1ug2a1 d2cqqa1 d2ajea1 d4a69c_
d1h8ac1 d2crga1 d2iw5b1 d1xc5a1 d1ofcx1 d2cu7a1 d1wgxa1 d2cjja1
d2ckxa1 d1gvda_ d1hcra_ d1tc3c_ d1gdta1 d1u78a2 d6paxa1 d6paxa2
d1k78a2 d1hlva1 d1iufa2 d1iufa1 d2iu5a1 d1zk8a1 d2g7sa1 d2fd5a1
d2np5a1 d1z0xa1 d2id6a1 d2d6ya1 d2i10a1 d1vi0a1 d2oi8a1 d3c07a1
d2ns7a1 d2fq4a1 d2yvea1 d1t33a1 d2o7ta1 d1rkta1 d2np3b1 d2g7ga1
d2gena1 d2fbqa1 d2g7la1 d2gfna1 d2g3ba1 d2jj7a1 d2hyja1 d1sgma1
d1ui5a1 d2hkua1 d2id3a1 d4jyka1 d3lv8a_ d1s3ga1 d1zaka1 d1s4qa_
d3tvta2 d1uf9a_ d1khta_ d1ckea_ d1osna_ d1p5zb_ d4tmka_ d3cm0a_
d1jaga_ d3h86a_ d1t0hb_ d1nn5a_ d2h92a_ d3kb2a1 d2c95a1 d1yj5a2
d1kgda_ d1ly1a_ d2ak3a1 d1vyva2 d1deka_ d2grja1 d1p3ja1 d1y63a_
d1e4ya1 d1s96a_ d3iija1 d1lvga_ d1nksa_ d1vyua2 d4e22a_ d1qf9a_
d1jjva_ d1q3ta_ d1ki9a_ d1lw7a2 d1teva_ d1kjwa2 d1gsia_ d2iyva2
d1viaa_ d1e6ca_ d1g3ma_ d1j99a_ d1t8ta1 d1nsta_ d1fmja_ d1zrha_
d1ls6a_ d2a3ra_ d1aqua_ d2zptx_ d3ckla_ d1texa_ d1q44a_ d2gwha_
d1odfa_ d4gi7a_ d2jeoa1 d1rz3a_ d1z0ja1 d3umra2 d3gj0a1 d2gaoa1
d1yzga1 d1x3sa1 d1g7sa4 d1puja_ d1udxa2 d1kk1a3 d2o52a_ d1jwyb_
d1zcaa2 d1zcba2 d2fv8a1 d2cjwa_ d3dz8a1 d1xzpa2 d1vg8a_ d4qxaa_
d2ew1a1 d1x1ra1 d2bcgy_ d3doea1 d3cona_ d3rega_ d3wyga_ d1upta_
d2b6ha1 d1zd9a1 d1lnza2 d1r5ba3 d1n0ua2 d1r2qa_ d2f9la1 d2rhda_
d3q3jb_ d3q85a_ d1efma1 d1r8qa_ d2gf9a1 d2akab1 d1ek0a_ d1z08a1
d2gcoa1 d2dy1a2 d4g01b_ d2cxxa1 d1svia_ d3q72a_ d1ni3a1 d4ac9a4
d2erxa1 d2bmea_ d1wxqa1 d2gf0a1 d4ldja1 d1zunb3 d1d5ca_ d4lv5b_
d1efca3 d1kmqa_ d2g6ba1 d3lrpa_ d1tada2 d2y8ea_ d4rd4a1 d1z06a1
d2heia_ d3a58b_ d1u0la2 d1d2ea3 d1zj6a1 d2ocba_ d3rwoa_ d1mr3f_
d2e9sa1 d4u5xa1 d2iwra_ d2fn4a1 d3bwdd_ d2nzja1 d2atva1 d2ksqa_
d3l43a_ d1mkya2 d1mkya1 d1wmsa_ d1jnya3 d2rmka_ d1f6ba_ d1f60a3
d1gota2 d1jala1 d2fh5b1 d1g16a_ d2wblc_ d1m7ba_ d1wf3a1 d1f5na2
d1t9ha2 d1z6xa1 d1z0aa1 d2fg5a1 d2a5ja1 d2bcjq2 d1s0ua3 d1ky3a_
d4y0va_ d4lhwa_ d2qmea1 d2atxa1 d1egaa1 d1ryha_ d1moza_ d1nrjb_
d2erya1 d3bfka_ d1puia_ d3j3az1 d3t5ga_ d1shza2 d3defa_ d2f7sa1
d3raba_ d3tsoa_ d3aq4a_ d2al7a1 d3sfva1 d3bc1a_ d1sdma_ d3h4sa1
d2heha_ d2grya_ g3kin.1 d1kk8a2 d1br2a2 d1goja_ d1f9va_ d2mysa2
d1lkxa_ d3t0qa_ d4db1a1 d3gbja_ d1ry6a_ d1v8ka_ d1k8ka2 d1k8ka1
d3uleb1 d3i33a2 d3i33a1 d1bupa1 d1dkgd2 d1dkgd1 d4ci6a2 d1jcfa1
d4a2ba2 d2fxua1 d1yaga2 d1yaga1 d4cbua2 d1bupa2 d1g99a2 d2e1za2
d2e1za1 d1saza1 d1saza2 d1ig8a1 d1czan2 d1czan3 d1czan1 d1ig8a2

d1czan4 d1bg3a4 d1bg3a2 d1bg3a1 d3h3no1 d3h3no2 d3ezwa2 d1js9c_
d1f15b_ d1za7a_ d1laja_ d4zzva_ d2xspa_ d1eg1a_ d4v20a_ d1ojja_
d4ipma1 d1gpia_ d4csia_ d3pfza_ d2yoka_ d2nqya_ d1h4ga_ d1hixa_
d1oa2a_ d1olra_ d3b7ma_ d4h7ma1 d3wp3a_ d2vgda_ d1igoa_ d3zsea_
d2nlra_ d3wx5a_ d1oa3a_ d1pvxa_ d1f5ja_ d1oa4a_ d1ks5a_ d4ixla1
d1evsa_ d1cnt1_ d1pvhb_ d1z7ca_ d1huwa_ d1bgea_ d1rhga_ d1f6fa_
d1f45b_ d1i1rb_ d1etea_ d3tgxb_ d2z3qa1 d3qb1a_ d1hula_ d1m48a_
d4nkqb_ d1eera_ d1v7mv_ d5e4ea_ d2o27a_ d3b5ka_ d2psma1 d4zf7a_
d3uf5a_ d4x51a_ d4ypgc1 d3oq3a_ d1lqsl_ d1n1fa_ d1au1a_ d1wu3i_
d3besl_ d3ux9a_ d1v8fa_ d2ejca_ d3muea1 d1ihoa_ d3q12a1 d4dnxa2
d1m8pa2 d1g8fa2 d1jhda2 d1x6va2 d3rbaa_ d2qtra_ d1nupa_ d3l92a_
d1f9aa_ d1qjca_ d2h29a_ d1vlha_ d1od6a_ d1lw7a1 d1mrza2 d4ruka_
d2el7a_ d1iq0a2 d1irxa2 d1r6ta2 d1ivsa4 d2cyba_ d1rqga2 d1j09a2
d2yxna_ d1f7ua2 d1h3fa1 d1ffya3 d2g36a1 d1nzja_ d1n3la_ d1i6la_
d1pfva2 d1h3na3 d3n9ia_ d3sz3a1 d3sw2a_ d1moxc_ d1dx5i3 d1edmb_
d1autl2 d1autl1 d1nqlb_ d1dval1 d1szba2 d1kigl_ d2y5fl_ d1ijqa2
d1gk5a_ d1g1sa2 d1nt0a3 d1xdtr_ d1lmja2 d1lmja1 d1nzia2 d2wphe_
d1klil_ d1pfxl2 d1pfxl1 d1p9ja_ d1g1ta2 d1f7ea_ d4yzub_ d2y5ia_
d1qlsa_ d1psra_ d3n22a_ d2egda_ d4ggfc_ d2wcea_ d2luca_ d4aqia_
d3nsia_ d2lp3a_ d1j55a_ d1a4pa_ d1k8ua_ d1nsha_ d1pvaa_ d3fs7a_
d2pvba_ d1ttxa_ d2kyfa_ d1rk9a_ d1a75a_ d1exra_ d1tiza1 d1nyaa_
d1g8ia_ d1ggwa_ d1wdcc_ d3fwba_ d1ggza_ d2o5ga_ d1ctaa_ d3i5gc_
d1sl8a_ d1fpwa_ d2scpa_ d2roaa_ d4aqra_ d3pn7b_ d1br1b_ d2mysb_
d1s6ca_ d1bjfa_ d2obha_ d1jfka_ d1jf0a_ d1qv0a_ d1lkja_ d2mlfc_
d2m97a_ d2d8na_ d4by5a_ d1jbaa_ d4nqga_ d1wdcb_ d2mysc_ d1j7qa_
d1s6ia_ d1uhka2 d1r2ua_ d1dtla_ d3kf9a_ d3pn7c_ d1fi6a_ d1c07a_
d5d67d_ d1tuza_ d1h8ba_ d1eg3a2 d2zkmx1 d1snla_ d1gjya_ d1y1xa_
d1hqva_ d1dvia_ d1qxpa2 d4usla_ d1k94a_ d1sfsa_ d1wkya2 d1uuqa_
d3wh9a_ d4m1ra_ d1xyza_ d4xuya_ d2je8a5 d4bf7a1 d3cuia_ d1ur4a_
d1qw9a2 d1odza_ d2vzsa5 d1bqca_ d2x2ya1 d1hjqa_ d1qnra_ d4y7ea1
d1vjza_ d4k68a_ d2v3ga_ d4hpga_ d1nofa2 d1nq6a_ d1h4pa_ d1aq0a_
d3ziza_ d4bs0a_ d1ecea_ d1h1na_ d1ta3b_ d1w91a2 d1i1wa_ d1uhva2
d1us3a2 d1egza_ d7a3ha_ d1tvna1 d3nyda1 d1ghsa_ d2cyga1 d4xv0a_
d2c8na2 d1w32a_ d3emca_ d1v6ya1 d1foba_ d2uwfa1 d2fgla_ d3niya1
d2c0ha1 d1rh9a1 d1yq2a5 d1szna2 d1fa2a_ d1pama4 d5do8a1 d1ea9c3
d1hx0a2 d2fhfa5 d1qhoa4 d1avaa2 d1m53a2 d4m8ua1 d2gjpa2 d1lwha2
d4howa1 d1ktba2 d1j0ha3 d3vm5a1 d2x1ia_ d1wzaa2 d1uasa2 d2diea1
d1mxga2 d1ht6a2 d1eswa_ d1iv8a2 d5cpsa_ d1ud2a2 d1zjaa1 d3gbea1
d1kwga2 d1wdpa_ d1gcya2 d1g5aa2 d1hvxa2 d3dhpa2 d2bhua3 d4do4a1
d3qgva1 d1e43a2 d1g94a2 d2d3na2 d1gjwa2 d1gvia3 d1x1na1 d1r7aa2
d1tz7a1 d4toqa_ d1wb0a1 d1w9pa1 d1eoka_ d1itxa1 d1kfwa1 d1goia2
d1edqa2 d2gsja_ d1hjxa1 d1jnda1 d1vf8a1 d3wdpp1 d3wq5a_ d1gnxa_
d4gxpa_ d4hz8a_ d1e4mm_ d3aiua_ d1wcga1 d1qvba_ d3ahxa_ d1v02a_
d2dgaa_ d1pbga_ d1vffa1 d1qoxa_ d4ha4a_ d1t77a2 d3syxa1 d1mkea1

d2p8va_ d1xoda1 d1i7aa_ d1evha_ d1qc6a_ d1egxa_ d1id1a_ d2hmta_
d1lssa_ d1iuka_ d2csua1 d3q9ub_ d2yv1a1 d3q9na_ d2d59a_ d1oi7a1
d2yv2a1 d1y81a1 d1wgqa1 d1v89a1 d2coda1 d1zc3b_ d1wg7a1 d1u5ea1
d1v61a1 d2coca1 d2ys1a1 d1v5pa1 d1ntya2 d1wjma1 d1wi1a1 d1u29a_
d1xx0a1 d1unqa_ d2zkmx3 d1fhoa_ d1v88a1 d2adza1 d1x1fa1 d1p6sa_
d2coaa1 d1btka_ d1v5ma1 d1faoa_ d2x18a_ d1dyna_ d1x1ga1 d4kaxb_
d2j59m1 d1ki1b2 d1xcga2 d1txda2 d1u5da1 d1dbha2 d1w1ha_ d1upqa_
d1v5ua1 d1foea2 d2fjla1 d1eaza_ d2dfka2 d2cofa1 d1kz7a2 d9ldta1
d2ewda3 d1up7a1 d2dfda1 d1s6ya1 d1vjta1 d7mdha1 d1hyea1 d1t2da1
d1ojsa3 d1b8pa1 d1ldna1 d1hyha1 d1civa1 d2a92a1 d1ez4a1 d1i0za1
d1y6ja1 d1gv0a1 d1ur5a1 d1llda1 d1obba1 d1v8ba1 d1gdha1 d1l7da1
d2dlda1 d1mx3a1 d1ygya1 d1qp8a1 d1li4a1 d2naca1 d3t4ea2 d1a4ia1
d1o0sa1 d2hk9a2 d2egga2 d1b26a1 d1bvua1 d1gq2a1 d1c1da1 d1leha1
d1gtma1 d1euza1 d1nvta1 d3dona2 d1edza1 d1gpja2 d1vi2a1 d1p77a1
d1nyta1 d1b0aa1 d1vl6a1 d1v9la1 d1txga2 d3f47a1 d1mv8a2 d2pv7a2
d1xc2a3 d2ahra2 d4xiya1 d2f1ka2 d1pgja2 d1i36a2 d1np3a2 d1lj8a4
d1teaa3 d1qmga2 d2i76a2 d2g5ca2 d1wdka3 d3cuma3 d1kola2 d1f8fa2
d1qora2 d1jqba2 d1vj0a2 d1cdoa2 d1gu7a2 d1d1ta2 d1xa0a2 d2dm6a2
d1jvba2 d1rjwa2 d1llua2 d1h2ba2 d1tt7a2 d1piwa2 d1o89a2 d1iz0a2
d1e3ja2 d1yb5a2 d1uufa2 d1vj1a2 d1e3ia2 d3gpdg1 d1vc2a1 d2nvwa1
d1u8fo1 d1ydwa1 d1hdgo1 d2g17a1 d1cf2o1 d2czca2 d1r0ka2 d1obfo1
d2cvoa1 d1ebfa1 d1h6da1 d1i32a1 d1zh8a1 d2q49a1 d1qkia1 d1j5pa4
d3pzra1 d1xeaa1 d1vkoa1 d1vm6a3 d3qvsa1 d2pkqo1 d1b7go1 d1gr0a1
d1p1ja1 d1lc0a1 d1j0xo1 d3pwka1 d2hjsa1 d1zyao1 d1vkna1 d1tlta1
d1nvmb1 d3sjua_ d3ojfa1 d1xu9a1 d2jjya1 d1n7ha_ d1y1pa1 d1h5qa_
d1jtva_ d4fs3a_ d2bkaa1 d1gy8a_ d2c5aa1 d1ulsa_ d1ooea_ d2b69a1
d1y5ma1 d1spxa_ d1ae1a_ d1bxka_ d1ydea1 d1gega_ d1hdoa_ d1o5ia_
d1e6ua_ d1snya1 d2rhca_ d3zv4a_ d5ceja_ d2d1ya1 d1fjha_ d3w8ea_
d2fmua1 d1x1ta_ d1rpna_ d3ay7a1 d1k2wa_ d1eq2a_ d2hrba1 d3ruaa_
d2ew8a1 d4hwka_ d1xgka_ d2bd0a1 d1yo6a1 d2a35a1 d2ntva_ d1vl8a_
d1hdca_ d4bkua_ d2ag5a1 d2q45a_ d1nffa_ d4iy1a_ d1zbqa1 d1kewa_
d1ek6a_ d1ja9a_ d2q46a2 d1iy8a_ d1cyda_ d2ptga_ d1hxha_ d1uzma1
d3lyla_ d4m89a_ d3wyea_ d2blla1 d1w6ua_ d1sb8a_ d2q2va_ d4nr0a_
d1e6wa_ d2gasa_ d3oiga1 d2bgka1 d1mxha_ d1vl0a1 d1rkxa_ d1r6da_
d1wmaa1 d1e7wa_ d2c07a1 d1xkqa_ d3tzha_ d1g0oa_ d3lz6a_ d1qyca_
d1edoa_ d1gz6a_ d1oc2a_ d1t2aa_ d1zema1 d2xoxa_ d1z45a2 d1geea_
d1p33a_ d3lt0a_ d4e6pa_ d2wyua_ d1db3a_ d4q9na_ d1xhla_ d4o1ma_
d1fmca_ d2uvda_ d2gdza1 d1uaya_ d2z1ma_ d2o23a2 d2fr1a2 d2fr1a1
d1qsga_ d1qyda_ d4zjua_ d2a4ka1 d1yb1a_ d1wvga1 d1yxma1 d3f4ba_
d1mg5a_ d1xg5a1 d1r55a1 d3gboa_ d1atla_ d1kufa_ d1buda_ d4q1la_
d1yp1a_ d1wnia_ d4ekfa_ d2ckga_ d1th0a_ d2bkra_ d1ex0a4 d1g0da4
d2f4ma1 d1x3za_ d3u1ra1 d1kapp2 d1k7ia2 d3ioqa_ d1fh0a_ d1o0ea_
d1khqa_ d1m6da_ d1cs8a_ g8pch.1 d1s4va_ d2pnsa_ d1vsna1 d1mira_
d1iwda_ d2oula_ d7pcka_ d3bwka_ d3bcna_ d2p86a_ d1pvja_ d1pcia_

d1gece_ d1deua_ d4bs6a_ d1xkga1 d1cqda_ d1jqpa2 d2c0ya_ d2bdza_
d3p5ua_ d2o36a_ d1k9xa_ d1ex4a2 d1c6va_ d2jk6a2 d2jk6a1 d1feca1
d1feca2 d1lpfa1 d1lpfa2 d1xhca1 d1xhca2 d1dxla2 d1dxla1 d1gera2
d1m6ia2 d1onfa1 d1onfa2 d3lada2 d3lada1 d1w4xa1 d1w4xa2 d1gesa2
d1mo9a1 d1xdia1 d1fl2a2 d1fl2a1 d1fcda1 d1fcda2 d1h6va2 d1h6va1
d1mo9a2 d1gv4a2 d1gv4a1 d2gv8a2 d2gv8a1 d1aoga1 d1aoga2 d1hyua1
d1ebda1 d1ebda2 d3dk9a1 d1q1ra2 d1q1ra1 d1gesa1 d1g4ma1 d1pn0a1
d1reoa1 d1ju2a1 d2f5va1 d3c96a1 d2gmha1 d1pj5a2 d1gpea1 d1o5wa1
d1n4wa1 d1k0ia1 d2dw4a2 d2ivda1 d1seza1 d2voua1 d1kdga1 d2bcgg1
d2bcgg2 d2flia1 d1rpxa_ d1tqja_ d1tqxa_ d1b9ra_ d1offa_ d1frra_
d3wcqa_ d1e9ma_ d1l5pa_ d1wria_ d1i7ha_ d3av8a_ d1czpa_ d1rfka_
d3p1ma1 d3b2ga_ d2bt6a2 d1mpya2 d1kw3b2 d1kw3b1 d4ghga1 d1tfza1
d3isqa1 d3isqa2 d1sqia1 d1sqia2 d1lkda2 d1lkda1 d1mpya1 d1cjxa2
d1sp9b2 d1t47a1 d1t47a2 d1f1ua2 d1sp8a1 d1sp8a2 d1f1ua1 d1jmxa4
d1pbya3 d1pbya4 d1jmxa2 d1pbya1 d1pbya2 d1faaa_ d1wika1 d1xfla_
d1ttza1 d2f51a1 d1ti3a_ d1nhoa_ d1sena_ d2i4aa_ d1ep7a_ d1zmaa1
d1fo5a_ d3lefa1 d3diea1 d2yana1 d1z6na1 d1oaza_ d3zywa1 d4aj8a_
d1nm3a1 d4fiwa_ d1wmja_ d4hs1a1 d1iloa_ d1xwaa1 d1wjka1 d2vm1a_
d1fb6a_ d1gh2a_ d1h75a_ d1nw2a_ d1kx5b_ d3a6na_ d4m6ba2 d2nqbd_
d3wkjd_ d2yfwe_ d4h9na_ d5bnxa_ d1kx5c_ d3r45a_ d1eqzb_ d4jjnb_
d1f66c_ d1eqza_ d4wnnb_ d1kx5a_ d1u35c1 d1id3a_ d2nqbc_ d1kx5d_
d1ku5a_ d1f1ea_ d2b5ea2 d2b5ea3 d2b5ea1 d2b5ea4 d2kp1a1 d2kp2a1
d1hyua3 d1hyua4 d1j08a1 d1j08a2 d1pd212 d1nhya2 d2a2ra2 d1k0dd2
d1f2ea2 d1gsua2 d1f3ba2 d2fhea2 d2pvqa1 d1tw9a2 d1oyja2 d1e6ba2
d1k0ma2 d3ik7a1 d4is0a1 d1r5aa2 d1jlva2 d1m0ua2 d3gtub2 d1z9ha2
d1okta2 d1axda2 d1tu7a2 d1k3ya2 d1zgma1 d1b48a2 d1gnwa2 d1gwca2
d2gsta2 d1glqa2 d1oe8a2 d1pn9a2 d1v2aa2 d1fw1a2 d1duga2 d1dosa_
d3qm3a1 d2fjka_ d1qusa_ d1qqya_ d1gd6a_ d1f6ra_ d1ivma_ d2gv0a_
d1iiza_ d2z2fa_ d1j8xa_ d1ghla_ d1b9oa_ d174la_ d169la_ d2o4wa_
d1p37a_ d1k28a3 d1xjta1 d1jtma_ d2f2qa_ d4e97a1 d2pjsa1 d1klla_
d2i7ra1 d1ecsa_ d1xqaa1 d5cj3a1 d1npba_ d2p7oa_ d1r9ca_ d1nkia_
d1hroa_ d4gedb_ d3zooa_ d1mz4a_ d1gu2a_ d1f1fa_ d1dvva_ d1c6ra_
d1c75a_ d1e29a_ d1gdva_ d1cnoa_ d1h32b_ d1co6a_ d2bh4x_ d2aiua_
d5cytr_ d1jdla_ d1i8oa_ d2zboa_ d3dmia_ d1ls9a_ d1wvec_ d2yk3a_
d3ph2b_ d1dw0a_ d5aura_ d1ql3a_ d1qn2a_ d1f1ca_ d2bema_ d1pama1
d1ea9c1 d1qhoa1 d2fhfa2 d2fhfa1 d1k3ia1 d1j0ha1 d1cxla1 d2bhua1
d1edqa1 d1f1sa2 d1ut9a2 d1gvia1 d1txka1 d1etob_ d1umqa_ d1g2ha_
d2hqya1 d2hqya2 d1ne9a1 d1lrza3 d1ne9a2 d1xvqa1 d1pn0a2 d3hy2a_
d2cvba1 d1o8xa_ d1lu4a_ d1we0a1 d2bmxa1 d2a4va1 d1psqa_ d1xiya1
d1q98a_ d1n8ja_ d1zofa1 d2ggta_ d3tjja_ d4je1a1 d3s9fa_ d2zcta_
d1qmva_ d1jfua_ d2b1ka_ d5dvba1 d2b5xa1 d2v2ga_ d1st9a_ d3tb2a_
d4llra_ d4fh8a1 d1e2ya_ d1knga_ d2h30a_ d1i5ga_ d2z9sa_ d2f8aa1
d1tp9a1 d2b7ka_ d1nm3a2 d3hvva_ d2wfca1 d4kcea_ d1xvwa1 d4gqca1
d3qpma_ d4k1fa_ d1zzoa1 d1gp1a_ d1prxa_ d4od7a1 d1z6ma1 d4mcua1

d4dvca1 d1r4wa_ d3l9sa_ d3rppa_ d1zsqa1 d1rmka_ d1ixta_ d1eyoa_
d1cnna_ d1fu3a_ d1g1pa_ d1f3ka_ d1fyga_ d1f74a_ d3l21a_ d2e1da_
d1to3a1 d1w3ia_ d1sfla_ d1n7ka_ d1vcva1 d4imea_ d1wa3a_ d2ocza1
d1f2ja_ d3m16a_ d2yxga_ d1ub3a_ d3a5fa_ d1fbaa_ d1l6wa_ d1xfba1
d1wx0a1 d1vpxa1 d1ojxa_ d1mxsa_ d2a4aa1 d1xkya1 d3cq0a1 d3s1xa_
d1f05a_ d1fdja_ d3cpra1 d4d2ja_ d1e88a1 d1l6ja5 d1l6ja4 d1eaka4
d1eaka3 d1h8pa2 d1h8pa1 d1jgta2 d1ofda3 d1ea0a3 d1ecfa2 d1ct9a2
d1ao0a2 d1te5a_ d3rp2a_ g1ucy.1 d1fuja_ d2zchp_ d3w94a_ d1si5h_
d1lo6a_ g2pka.1 d1cgha_ d1kigh_ d1a5ia_ d3s9ba_ d1hj8a_ d1ao5a_
d2anya_ d1q3xa1 d1gvza_ d4gsoa_ d2f91a1 d1iaua_ d1gdna_ d1eufa_
g1fiw.1 d1eq9a_ d4nfea_ d2rdla_ d1spja_ d1elva1 d1a7sa_ d1ekbb_
d1pfxc_ d1yc0a_ d2aiqa_ g1fiz.1 d1fi8a_ d4k60a_ d4dgja_ d1mzaa_
d1op0a_ d3tjva_ d1pytd_ d1sgfg_ g1gg6.1 d2eeka_ g1rtf.1 d1a0ja_
d3g01a_ d1pytc_ d4e7na_ d1ddja_ d1z8ga1 d2hlca_ d1npma_ d1brup_
g1jou.1 d1sgfa_ d1autc_ g2hnt.1 g1kdq.1 d1orfa_ d3s69a_ d4crga_
d1fxya_ d1bqya_ d1lgya_ d4l3wa_ d1llfa_ d1gz7a_ d1ukca_ d3groa_
d1pjaa_ d1ei9a_ d1svpa_ d1mbma_ d3su6a1 d1cu1a1 d4agka_ d1vcpa_
d1q31a_ g1n1l.1 d3e90b_ d1ep5a_ d3u1jb_ d3zxha_ d1cgla_ d1rm8a_
d1eaka2 d1hy7a_ d2jsda_ d1cxva_ d1i76a_ d1eb6a_ d1jx4a2 d3gv5b1
d1t94a2 d3bq0a2 d2tbvc_ d1c8nc_ d1x36a_ d1ng0c_ d4sbvc_ d1opoa_
d1f2nc_ d1cixa_ d1y29a_ d1v7fa_ d2jtba_ d1d1ha_ d1g9pa_ d1lupa_
d1qk7a_ d1mb6a_ d2a2va_ d1niya_ d1hvwa_ d2mpqa_ d1ie6a_ d1kqha_
d1la4a_ d2mxoa1 d1dl0a_ d1koza_ d1qk6a_ d1emxa_ d1lqra_ d1c6wa_
d3ljqa_ g1k2x.1 d1rypd_ d1irua_ d1rypc_ d3unfk_ d1rypa_ d1j2pa_
d1irui_ d1j2qh_ d3unfh_ d1m4ya_ d1rypb_ d1irul_ d1irug_ d1iru2_
d1q5rh_ d1rypg_ d1g3ka_ d1iruf_ d1q5qa_ d1e94a_ d1irub_ d1ryph_
d3ty6a_ d1iruj_ d1iruk_ d1rype_ d1irud_ d1irue_ d3mi01_ d1rypf_
d1iruh_ d2z3ba_ d1iruc_ d3mka2_ d1iru1_ d1ny721 d1ny722 d1a6ca3
d1a6ca1 d1pgl22 d1pgl21 d1pgl11 d1ny711 d1mqta_ d1d4m3_ d1fmd3_
d2mev1_ g1aym.1 d1ev13_ d4q4w1_ d1r1a3_ d2x5ia_ d1rhi1_ g1r1a.1
g1d4m.1 g1rhi.1 d1b35b_ d1oopa_ d1tme1_ d4iv1a_ g1pvc.1 g1fpn.1
d1fpn1_ d4gb31_ d1d4m1_ d1fmd1_ d1ev11_ d2wzr3_ g1cov.1 d1qqp3_
g1fmd.1 d2x5ic_ d1rhi3_ g1oop.1 d1hxs1_ g1hxs.1 d1fpn3_ d1bev1_
g2mev.1 d1eah3_ d1aym1_ d1pvc3_ d1zba3_ d1h8ta_ d3vbhc_ g1b35.1
g1bev.1 d1hxs3_ g1ev1.1 d1bev3_ g1tmf.1 d1tmf1_ d1b35a_ d2mev3_
g1eah.1 g1h8t.1 d1eah1_ d4q4w3_ d1r1a1_ d1aym3_ d1pvc1_ d1zba1_
d1h8tc_ d1oopc_ d1tme3_ g1qqp.1 d4iv1c_ g1tme.1 d3ubub_ d1egia_
d1qo3c_ d1v7pa_ d1tdqb_ d3ubua_ d1c3ab_ d1h8ua_ d3bx4b_ d1t8ca1
d1sb2a1 d1qdda_ d2ziba1 d3cada_ d1byfa_ d1j34b_ d1jwia_ d1jzna_
d1c3aa_ d1oz7b_ d1wmza_ d3bdwb_ d1hq8a_ d1fifa1 d3hupa_ d1wk1a1
d1umra_ d2afpa_ d3bx4a_ d1gz2a_ d1sl6a_ d1v7pb_ d3g8ka_ d1ixxa_
d1j34a_ d1jwib_ d1g1sa1 d1y17a1 d1ja3a_ d1fvub_ d3bdwa_ d1sb2b1
d1fvua_ d1umrc_ d1oz7a_ d4m17a_ d3cfwa1 d1ypqa1 d1g1ta1 d3fd4a_
d3l9jc_ d4dn8a1 d1v4lb_ d1rdl1_ d2msba_ d1rw1a_ d1z3ea1 d3l78a_

d1prtb2 d2r80b_ d1cqxa1 d1cg5a_ d1gcvb_ d3boma_ d2h8fb_ d2raoa_
d3bj1a_ d4hrrb_ d2r50a_ d3d1kb_ d2aa1b1 d3zhwa_ d3hf4b_ d3gdja_
d1jebb_ d3bj1b_ d1x9fb_ d1wmub_ d1gcva_ d3bcqb_ d3d4xb_ d2dc3a_
d3hf4a_ d4yu3a_ d3k8ba_ d1qpwb_ d2r80a_ d3bcqa_ d1v4wa_ d3wtgb_
d1jl7a_ d4esaa_ d1a9we_ d3d1ab_ d1fsla_ d4h2la_ d1v4wb_ d1mwca_
d1vhba_ d1jeba_ d1spgb_ d1h97a_ d1q1fa_ d1tu9a1 d3d1aa_ d1hdsb_
d3hyub_ d2zfbb_ d3fs4a_ d1oj6a_ d1shrb_ d4h2lb_ d4yu3b_ d1spga_
d3hrwa_ d3qqra_ d3at5a_ d4wchd_ d3hyua_ d1cg5b_ d1uc3a_ d1hbra_
d2raob_ d1outb_ d4esab_ d3gdjb_ d3qqqa_ d1x9fc_ d3vrfa_ d1itha_
d3mkba_ d3d4xa_ d3mjpa_ d1jl6a_ d3fh9a_ d1outa_ d2dc3b_ d1it2a_
d3eoka_ d3g46a_ d1qpwa_ d3vrfb_ d1ch4a_ d1x9fd_ d1hdsa_ d2zfba_
d3mkbb_ d1or4a_ d3wtga_ d1a4fa_ d3fh9b_ d3bomb_ d3cy5a_ d3at5b_
d1x9fa_ d2oifa_ d4lmxb_ d1cpcb_ d3v57a_ d1kn1a_ d1cpca_ d4f0ta_
d2vjha_ d4po5b_ d1eyxa_ d1alla_ d4f0tb_ d1b33b_ d1b8db_ d4h0mb_
d1b33a_ d2vjhb_ d4lm6b_ d2vmlb_ d1allb_ d2vjta_ d3dbja_ d2vmla_
d2vjtb_ d4f0ub_ d1f99b_ d4rmpa1 d1liaa_ d1eyxb_ d2uulb_ d4lmsb_
d4f0ua_ d2bv8a_ d1f99a_ d2bv8b_ d2c7lb_ d3dbjb_ d4rmpb1 d1b8da_
d4h0ma_ d3v57b_ d1kn1b_ d1liab_ d2uula_ d2j96a_ d1kyqa1 d3nqyb_
d4gera_ d1bqba_ d2xqfa_ d1f6wa_ d1dx4a_ d1qe3a_ d1k4ya_ d4qwwa_
d2ha2a_ d3c8da2 d2pbla1 d1jkma_ d1jjia_ d1jjfa_ d4iuaa3 d4bvva_
d5hpga_ d3k65a_ d1ki0a3 d1ki0a2 d1ki0a1 d1a0ha1 d1pmla_ d3sp8a3
d2feba1 d1mdwa_ d1ziva1 d1zcma1 d2m5ta_ d1cqqa_ d1wqsa_ d5bpea_
d2ipha_ d1lvoa_ d2bhga1 d3tnta_ d3sjia_ d3d23a1 d2fyqa_ d3tloa_
d3zvea1 d2hrva_ d3q3ya1 d4asha1 d1cg2a1 d1yloa2 d1y7ea2 d1y0ya3
d1lfwa1 d1vixa1 d1fnoa4 d1vhoa2 d1ysja1 d1vhea2 d1z2la1 d2fvga2
d1r3na1 d2grea2 d1xmba1 g1q7l.1 d1rtqa_ d1ra6a_ d1muka_ d3gsza_
d1te9a_ d2xwha_ d3uqsa_ d3hkwa_ d3n6ma_ d2cjqa_ d2xyma_ d1teba_
d1te8a_ d1s48a_ d1u09a1 d4wfxa_ d3tyqa_ d2pgga1 d1m4la_ d2bk7a_
d3fjua_ d1z5ra1 d1kwma1 d1uwya2 d1jqga1 d2c1ca1 d1dtda_ d1h8la2
d3lgia_ d3i77a_ d2z9ia2 d4nsva_ d1lcya2 d4jcna_ d1agja_ d1os8a_
d1hpga_ d1p3ca_ d1l1ja_ d1qtfa_ d1e69a_ d1mv5a_ d3g60a4 g1ii8.1
d1ewqa2 d1ji0a_ d1qhla_ d1v43a3 d2wmfa1 d1vpla_ d1g2912 d4a82a2
d2d62a1 d3tuic1 d2bbsa1 d3g60a3 d2ixea_ d1g6ha_ d1sgwa1 d2qi9c_
d3rlfa1 d3b60a1 d3d31a2 d2pzea1 g1xew.1 d1jj7a_ d1oxxk2 d2onka1
d1w1wa_ d1iwea_ d1qf5a_ d1vmaa2 d1nija1 d1np6a_ d2p67a1 d3ihla_
d1okkd2 d1ls1a2 d1s1ma2 d1iona_ d2v40a_ d3hida_ d2vo1a1 d2g0ta1
d1vcoa2 d1j8mf2 d1g3qa_ d1xjca_ d1p9ba_ d1qzxa3 d1ihua2 d1ihua1
d2qm8a_ d1dj2a_ d4ioja_ d1cp2a_ d1yrba2 d1hyqa_ d3gqba2 d1skye3
d1xp8a1 d1tf7a2 d1tf7a1 d1ye8a1 d1skyb3 d2qe7d2 d4blta_ d4huta_
d3ldaa2 d4hyya_ d2i3ba1 d1cr1a_ d3j9tb2 d3ntua2 d1vdza1 d2z43a_
d1e9ra_ d1nlfa_ d3ziaa2 d1n0wa_ d1g6oa_ d3ziad2 d3j9ta2 d1pzna2
d3gqbb2 d2i49a_ d1nnfa_ d1xvxa_ d1ursa_ d1h3da1 d1iuda_ d4m1va1
d1twya_ d3e13x_ d2nxoa1 d4pfua_ d3ttma_ d1elja_ d2qmwa1 d1y3na1
d1jeta_ d1uqwa_ d4f19a1 d2esna2 d3thia_ d3fasa1 d2uyeb_ d2xwva1

| | | | | | | | |
|---|---|---|---|---|---|---|---|
| d1r9la_ | d2fyia1 | d1pb7a_ | d2ozza1 | d1sw5a_ | d1nh8a1 | d1vr5a1 | d2g29a_ |
| d1xs5a_ | d3lswa_ | d2z23a_ | d3w9va_ | d3tcha_ | d1q35a_ | d1z7me1 | d3q27a_ |
| d1p99a_ | d1ve4a1 | d2i6ea1 | d4mcoa1 | d4n8ya_ | d1eu8a_ | d1zbma1 | d1hsla_ |
| d1ixca2 | d1j1na_ | d1xvya_ | d1y9ua_ | d2z22a1 | d1xoca1 | d4oera_ | d1wdna_ |
| d5f1qa1 | d1xt8a1 | d1i6aa_ | d4gwoa_ | d4igta1 | d1pc3a_ | d4mmpa1 | d1o63a_ |
| d2czla1 | d2onsa2 | d4mija1 | d1eh3a_ | d3skpa_ | d1dtza1 | d1dtza2 | d1ieja_ |
| d1jnfa2 | d1ce2a1 | d1jnfa1 | d1b1xa2 | d1b1xa1 | d1jw1a1 | d1jw1a2 | d1ryoa_ |
| d1h76a1 | d1h76a2 | d1w52x1 | d2ppla1 | d1hpla2 | d1lpbb2 | d2oxea1 | d1bu8a2 |
| d1rp1a2 | d1c4oa1 | d2p6ra3 | d2p6ra4 | d1oywa2 | d1oywa3 | d1wrba1 | d1w36b2 |
| d1w36b1 | d1c4oa2 | d3bora_ | d1t6na_ | d2eyqa5 | d2eyqa3 | d2eyqa2 | d2zu6a2 |
| d1veca_ | d1uaaa1 | d1w36d1 | d2g9na1 | d1z3ix2 | d2rb4a_ | d1hv8a1 | d1hv8a2 |
| d1t5ia_ | d1w36c2 | d2j0sa2 | d2j0sa1 | d1fuka_ | d3hjha1 | d1z5za1 | d1qdea_ |
| d1z63a1 | d1s2ma2 | d1s2ma1 | d1wp9a1 | d1wp9a2 | d1gm5a3 | d1gm5a4 | d3dina4 |
| d3dina3 | d1q0ua_ | d1w36c1 | d1z3ix1 | d1nkta3 | d1uaaa2 | d1w36d2 | g1qhh.1 |
| d2fwra2 | d2fwra1 | d3q3ma_ | d1za0a1 | d1mxra_ | d2inca_ | d1jk0a_ | d2incb_ |
| d1mhyb_ | d3hf1b_ | d1mtyb_ | d1r2fa_ | d2uw1a_ | d3n37a_ | d1afra_ | d1oquc_ |
| d1syya_ | d1mtyd_ | d2o1za_ | d1uzra_ | d1smsa_ | d2fkza_ | d1zuja1 | d1s3qa1 |
| d2fjca1 | d1z6om1 | d1veia_ | d4dyya_ | d1jiga_ | d3gvya_ | d2pyba_ | d4cy9a1 |
| d3bvfa1 | d1r03a_ | d2cf7a_ | d1nf4a_ | d1zs3a1 | d4iwka_ | d3ka8a_ | d3np0x_ |
| d1n1qa_ | d1lb3a_ | d4e6ka_ | d1z6oa1 | d2fzfa1 | d4dyua_ | d3ge4a_ | d1jgca_ |
| d4am4a_ | d2z6ma_ | d1ji5a_ | d1vlga_ | d1lkoa1 | d1krqa_ | d3qz3a_ | d1o9ra_ |
| d2ffxj_ | d1euma_ | d1yv1a1 | d2v2pa_ | d1j30a_ | d4di0a_ | d1dpsa_ | d1oewa_ |
| d2qzxa_ | d2asia_ | d1psoe_ | d1miqa_ | d1smra_ | d2h6ta_ | d1pfza_ | d1izda_ |
| d3hd8a_ | d1j71a_ | d1dpja_ | d4aa9a_ | d1t6ex_ | g1b5f.1 | g1lya.1 | d1qdma2 |
| d1bxoa_ | d1wkra_ | d1tzsa_ | d1ibqa_ | d2qzwa_ | d2v76a_ | d1wj1a1 | d1aqca_ |
| d1qqga2 | d1qqga1 | d1ntva_ | d1n3ha_ | d4dbba_ | d3f0wa1 | d1p3ra_ | d1wgua1 |
| d2cy4a1 | d3hqca_ | d1wvha1 | d1xr0b1 | d1bdqa_ | d3ggta_ | d3ixoa_ | d1mrxa_ |
| d2haha_ | d1tw7a_ | d4njsa_ | d1sgua_ | d2p3da_ | d1nsoa_ | d3d3ta_ | d2rkfa_ |
| d1c6ya_ | d3ogpa_ | d3ekwa_ | d2o4na_ | d2j9ja_ | d1baia_ | d3ggua_ | d3lzua_ |
| d4npua_ | d4j55a_ | d2r8na_ | d2fxda_ | d2p3ca_ | d4dqea_ | d3ttpa_ | d3t3ca_ |
| d2p3aa_ | d2hc0a_ | d2fdda_ | d3mwsa_ | d1um8a_ | d1ixza_ | d1qvra2 | d1qvra3 |
| d1qzma_ | d1sxjc2 | d1jr3d2 | d2p65a_ | d1l8qa2 | d1g8pa_ | d3hu3a3 | d1sxjd2 |
| d1njfa_ | d1in4a2 | d1sxja2 | d1tuea1 | d2ce7a2 | d1w5sa2 | d1sxjb2 | d1d2na_ |
| d1ny5a2 | d1ofha_ | d2gnoa2 | d2a5yb3 | d1fnna2 | d1u0ja1 | d1lv7a_ | d1iqpa2 |
| d2fnaa2 | d1ixsb2 | d4gita_ | d1sxje2 | d1g41a_ | d1k8rb_ | d1wgra1 | d1ef5a_ |
| d1wfya1 | d1e8ya3 | d1wgya1 | d1lfda_ | d2cs4a1 | d1wxma1 | d2byea1 | d3kh0a1 |
| d2byfa1 | d1wxaa1 | d1e7ua3 | d1h4ra3 | d4yl8a1 | d2al6a3 | d1wgda1 | d1wgga1 |
| d4q5eb_ | d1wjna1 | d1se9a_ | d3n3kb_ | d1wz0a1 | d1we6a1 | d2m0xa_ | d2gbka_ |
| d3rt3b1 | d1wh3a1 | d1sifa_ | d2ekec2 | d4ii2b1 | d4hk2a1 | d1c3ta_ | d1m94a_ |
| d1wgha1 | d1ttna1 | d1j8ca_ | d1wx8a1 | d3v6cb1 | d2ma9b_ | d4i6lb1 | d1bt0a_ |
| d2jvca1 | d1iyfa_ | d2k1fa_ | d1wxva1 | d1wf9a1 | d1t0ya_ | d2io0b2 | d1x1ma1 |
| d2gbma_ | d1zw7a1 | d2m17a_ | d1we7a1 | d2lwpa_ | d2gbja_ | d1wjua1 | d1v6ea1 |
| d1v86a1 | d4eewa_ | d1wiaa1 | d4icva_ | d4gu2a_ | d1wx7a1 | d1wy8a1 | d4hjka1 |
| d2faza1 | d1v5oa1 | d2h1ia1 | d2r8ba1 | d3b5ea1 | d3cn9a_ | d2h0ba_ | d2erfa_ |

| | | | | | | | |
|---|---|---|---|---|---|---|---|
| d1h30a1 | d3mw4a1 | d1pz7a_ | d2r1ba_ | d3v64a1 | d1d2sa_ | d3bopa1 | d3mw3a1 |
| d1okqa1 | d1okqa2 | d1s5ja1 | d2hbja2 | d2igia_ | d1wlja_ | d1qhta1 | d4dlea1 |
| d1tgoa1 | d2f96a1 | d3tr8a1 | d3mxma_ | d1q8ia1 | d1y97a1 | d1w0ha_ | d1kfsa1 |
| d2py5a1 | d1d5aa1 | d1yt3a3 | d2guia_ | d3mwpa1 | d4kb1a_ | d1wioa4 | d1ccza2 |
| d1vcaa1 | d1dr9a2 | d1ic1a1 | d2d9qb1 | d2nxyb2 | d1adqa1 | d1fnga1 | d1uvqb1 |
| d1zs8a1 | d2vola1 | d2vola2 | d1mfbl2 | d2jelh2 | d1uwel2 | d3qq4b1 | d1kjvb_ |
| d1k5na1 | d1osph2 | d4odhl2 | d1ow0a1 | d1ow0a2 | d1bfob2 | d3l9ra2 | d3jtth1 |
| d2fath2 | d4elkb2 | d1fn4b2 | d2xfxb_ | d1pg7x2 | d1cqka_ | d4k71b2 | d1lk2a1 |
| d1eapb2 | d4lfhg2 | d1i4fa1 | d2nw2a2 | d1kfah2 | d1mhca1 | d1h3ua1 | d1wejh2 |
| d1h0db2 | d1a5fh2 | d1kcvh2 | d1mjuh2 | d1qqda1 | d1kcrh2 | d1k8ia1 | d3huja2 |
| d2nnab1 | d1nfdf2 | d3mffb2 | d1s9va1 | d1lk3h2 | d1q0xl2 | d1fv1b1 | d1neza1 |
| d4rfeb2 | d1r3ha1 | d1a6tb2 | d1kjva1 | d1pz5b2 | d1onqa1 | d2bc4a2 | d1de4a1 |
| d1k8da1 | d1tcra2 | d1dn0b2 | d1nfde2 | d2xwtb2 | d1bfoa2 | d1mexh2 | d1iakb1 |
| d1ypzf2 | d4f7ea2 | d1q72l2 | d2fbjh2 | d1fe8h2 | d1clzh2 | d1fngb1 | d1uvqa1 |
| d1vgka1 | d1c16a1 | d4j12a2 | d4x99b2 | d1igtb3 | d1je6a1 | d1q72h2 | d1nldh2 |
| d1teth2 | d2hmic2 | d1i1ca2 | d1i1ca1 | d1k8ib1 | d1q0yh2 | d1kfal2 | d3frua1 |
| d1pg7w2 | d1u58a1 | d1igyb4 | d1igyb3 | d1hyrc1 | d1klub1 | d1deeb2 | d1c12b2 |
| d2bc4b2 | d2ig2h2 | d1kcvl2 | d3wn5b1 | d1lk2b_ | d1lk3l2 | d1mjul2 | d1sm3h2 |
| d1q0xh2 | d1zvsa1 | d1ydpa1 | d1u8ib2 | d1ypze2 | d1kcuh2 | d2e3fa_ | d1mm0a_ |
| d2e3ea_ | d2llda_ | d2ny8x_ | d2e3ga_ | d2ny9x_ | d2nz3a_ | d2lj7a_ | d1n4na_ |
| d2m8bb1 | d2kyqa_ | d3psma_ | d1jxca_ | d2gl1a_ | d1ugla_ | d4uj0a_ | d2k4ua_ |
| d1hp2a_ | d2lu9a_ | d1baha_ | d2meoa1 | d1wm7a_ | d1hlya_ | d1n8ma_ | d1jlza_ |
| d1wpda_ | d1c55a_ | d1pvza_ | d1wt7a_ | d1rjia_ | d1wz5a_ | d1m2sa_ | d1pjva_ |
| d1c49a_ | d1qkya_ | d2ktca1 | d1wmta_ | d1du9a_ | d1j5ja_ | d1r1ga_ | d1e3oc2 |
| d1ic8a2 | d1t0za_ | d2i61a_ | d1t1ta_ | d1sn4a_ | d1kv0a_ | d2lkba_ | d1npia_ |
| d1wwna_ | d1pe4a_ | d2kbka_ | d2li7a_ | d1djta_ | d2kbha_ | d1dq7a_ | d1sega_ |
| d1omya_ | d1zywa1 | d1jzaa_ | d2jcqa_ | d1n9wa1 | d1bbua1 | d1e1oa1 | d1c0aa1 |
| d1l0wa1 | d3a74a1 | d1eova1 | d1iyjb4 | d1iyjb5 | d1iyjb3 | d1o7ia_ | d4ipca1 |
| d1x3ea_ | d4apva_ | d1jb7a1 | d1jb7a2 | d1jb7a3 | d3kdfa_ | d1ue1b_ | d1wjja1 |
| d3eiva_ | d1xjva2 | d1xjva1 | d2ccza2 | d1se8a_ | d1l1oc_ | d1jb7b_ | d1qvca_ |
| d3ulla_ | d1qzga_ | d1miua4 | d1miua5 | d1miua3 | d3g48a1 | d1gd7a_ | d1mkha_ |
| d1jjcb3 | d1pyba_ | d1y14b1 | d2nn6i1 | d1i94l_ | d2zjra2 | d1smxa_ | d2qamc2 |
| d1hh2p1 | d2r7da1 | d1rl2a2 | d1wfqa1 | d3bzka4 | d1x6oa2 | d3a0ja_ | d2j01d2 |
| d2ja9a1 | d4ql5a_ | d1hr0w_ | d1luza_ | d2n3sa_ | d2vnud1 | d1h95a_ | d1go3e1 |
| d2nn6h1 | d2mo1a_ | d1iz6a2 | d1jjga_ | d1e3pa2 | d2ix0a3 | d2ix0a2 | d2lxja_ |
| d1ueba3 | d1ueba2 | d2z0sa1 | d1u0la1 | d2ix0a1 | d2eifa2 | d2je6i1 | d3i2za1 |
| d2c35b1 | d2ahob2 | d1t9ha1 | d3i4oa_ | d2qall1 | d1jt8a_ | d2nn6g1 | d1g6pa_ |
| d3cama_ | d1i94q_ | d1c9oa_ | d1d7qa_ | d2ba0a1 | d2vnud2 | d2vnud3 | d1khia2 |
| d2qalq1 | d1wi5a1 | d1xtda2 | d1ny4a_ | d1dgsa2 | d1fvia1 | d1ckma1 | d1p16a1 |
| d1x9na2 | d1rpma_ | d2shpa1 | d1yfoa_ | d1l8ka_ | d2ooqa1 | d1g4us2 | d1p15a_ |
| d1lara2 | d1lara1 | d1wcha_ | d2fh7a2 | d2fh7a1 | d3azya_ | d3wvja_ | d1upsa1 |
| d1ajoa_ | d2hyka_ | d1ajka_ | d2vy0a_ | d1o4ya_ | d3o5sa_ | d3d6ea_ | d3atga_ |
| d1dypa_ | d1umza_ | d3i4ia_ | d1c1la_ | d4lbqa_ | d1a78a_ | d1slta_ | d4no4a_ |
| d3zsja_ | d3duia_ | d1is3a_ | d1qmja_ | d1hlca_ | g1len.1 | d3zyra_ | d1fnya_ |

d1n47a_ g2ltn.1 d1avba_ d3usua_ d1dhkb_ d1f9ka_ d1ukga_ d1g8wa_

d1g9fa_ d1wbfa_ d3wcsa_ d1g7ya_ g1loe.1 d1mvqa_ d1hqla_ d1v6ia_

d1dbna_ d1qnwa_ d1fx5a_ d4l8qa_ d1ioaa_ d3n35a_ d3h33a_ d1rwja_

d19hca_ d1os6a_ d3bxua_ d3h4na_ d1i77a_ d3caoa_ d1j0pa_ d2ctha_

d1duwa_ d2e84a_ d1hh5a_ d1gyoa_ d3h34a_ d1dszb_ d1a6ya_ d3cbba_

d1ynwa1 d1r4ia_ d2hana_ d2hanb_ d2enva1 d1kb4b_ d2nllb_ d1lo1a_

d1cita_ d2dara2 d2dara1 d1wyha2 d2dloa2 d1rutx4 d1rutx3 d2cuqa2

d1x4la2 d1x4la1 d1x3ha2 d1x3ha1 d2d8za1 d1x61a2 d1x6aa1 d1x6aa2

d2cora2 d2cora1 d1v6ga1 d1v6ga2 d2d8za2 d1x64a1 d1x64a2 d1b8ta1

d2co8a1 d1x63a2 d2dj7a2 d2dj7a1 d2cu8a2 d2cu8a1 d1g47a2 d1g47a1

d1j2oa1 d1wyha1 d2dloa1 d1wiga1 d1wiga2 d2cupa2 d2cupa3 d1x68a1

d2d8ya2 d2d8ya1 d1m3va2 d1x62a1 d1x68a2 d1x61a1 d1b8ta3 d1b8ta2

d2d8xa2 d2d8xa1 d1x4ka1 d1x4ka2 d2cuqa1 d1m3va1 d2co8a2 d1j2oa2

d1x63a1 d2cura1 d2cura2 d1x62a2 d1op9a_ d1y0la1 d1etzb1 d1egjl1

d2gsib1 d1nj9a1 d4jfhd1 d1aqkl1 d1t2ja_ d1lo4h1 d1zvya1 d3v4vl1

d4enec1 d1kxtb_ d5e8ea1 d2pxya_ d4weud_ d1xiwc_ d2esvd1 d1qnzh_

d1ogae1 d1f4xh1 d4n1hb_ d3ezjb1 d3mlwl1 d1kfah1 d1etza1 d1b4jh1

d3cfic_ d4w68a_ d2arja1 d1ek3a_ d2ok0l1 d1vera_ d1uwgh1 d1blnb1

d1fpth1 d1rzga1 d2giya1 d4m3kb1 d1nc2a1 d2nyyd1 d1ncna_ d1fbih1

d5dxwa_ d4fqqa1 d1dqdh1 d1jfql1 d1kelh1 d1faih1 d1frgl1 d1figl1

d4m6nl1 d1fo0a_ d4hxbl1 d3b5ga_ d3quyd1 d2h9gb1 d1i8kb_ d2dd8h1

d2ck0h1 d1eaja_ d4w6wb1 d2a6ib3 d1t66d1 d2e27h_ d1ob1b1 d1f11b1

d2v7na1 d2xt1b1 d1pskh1 d4i13b1 d2op4l1 d1a0ql1 d1uwel1 d1za3a1

d1ncbl1 d4uu9a_ d1bm3h1 d1cloh1 d1nqba2 d1cf8h1 d4y5va_ d1mhph1

d5anma1 d4cdgc1 d4ldeb_ d1y0lb1 d2pcpb1 d1z3gl1 d1dfbl1 d1fh5h1

d3g5xa1 d1tzib1 d4dn3l1 d3zhka1 d2b1hh1 d2uyla1 d1f3dh1 d1nj9b1

d2agjh1 d2h1pl1 d1q72h1 d8fabb1 d1ndmb1 d1rjca1 d4u6hb1 d3upca_

d2fbjl1 d1osph1 d1tvda_ d2cjvh_ d4fhbd1 d1bd2d1 d1rz7h1 d3nn8a_

d1blna1 d1knoa1 d1nfda1 d2bmkb1 d1dqll_ d1igyb1 d1igfl1 d12e8h1

d3utza1 d1nc2b1 d1rihh1 d4mwfb1 d4jvpa1 d1mieh1 d1rhfa1 d1fgnl1

d1sbsh1 d1lp9e1 d1dn0a1 d4lqfl1 d1iaih1 d1kgcd1 d1mh5b1 d1gpol1

d2g60h3 d1f8tl1 d2jixd1 d4grwh_ d1fo0b_ d1bfob1 d1axsa1 d1u9ka_

d1nmbh_ d1h8na1 d1h8na2 d1jguh1 d4eura1 d3sdyl1 d35c8h1 d1w72l1

d1mvub_ d1i9rh1 d1ob1a1 d4wenb_ d1yeeh1 d3ietc1 d1f58h1 d1ad0b1

d1mqkl_ d1nbvh1 d4b50a_ d3r8bb_ d1bafl1 d1mamh1 d4rgne1 d2gsgb_

d35c8l1 d1bafh1 d1ai1h1 d2pcpa1 d2fath1 d2x1pa_ d1orsb1 d1ibgl1

d1shma_ d1a2yb_ d1u0qa_ d2coqa_ d1hilb1 d1qfwi_ d1jv5b_ d1fn4b1

d5e0qa_ d1nsnh1 d1plgl1 d4ocxl1 d1kb5h1 d1rzfh1 d1pg7x1 d2fx7h1

d2oi9c_ d1l7il1 d3eyva1 d1d5il1 d4ffyl_ d1tetl1 d1a6wh_ d1ac6a_

d3dvfa_ d1nfde1 d1mimh1 d1ri8a1 d1jhlh_ d3dura_ d1fskb1 d1f97a1

d3so3b1 d2ak4d1 d1lp9f1 d4z7wf1 d1l7th1 d4y5ya_ d4x7ec1 d1emth1

d1ikfl1 d1nlbh1 d4utal1 d1bjma1 d1eapb1 d1u6ah1 d2dtgd1 d1kj2b_

d1xeda1 d1c1eh1 d1fgvh_ d2bnub1 d2fb4h1 d2dtgb1 d1ktrh_ d1jpth1

d1qkzl1 d4hepg_ d2atpb1 d1flrh1 d2hrpl1 d1mvfa_ d1iaim1 d3k80a1

d2jb5h1 d3zkxc_ d4grwe_ d1it9h1 d1rhhb1 d3w9ec_ d1e4wh1 d1sy6h1
d2j6eh1 d3qxta_ d1jglh1 d1p7ka1 d1tzhb1 d4n8pa1 d1gafl1 d2jelh1
d1wioa2 d4i0cc1 d1cicb1 d1lmka2 d1lmka1 d1iqwh1 d1orqb1 d1ejoh1
d1jnlh1 d4zg1a_ d43c9b_ d1ggbl1 d1wejh1 d3q6fa1 d1f3dj1 d3c6la1
d1bqlh1 d3p9wb_ d1a3rl1 d1h0db1 d2uzil_ d1eo8l1 d1qfwl_ d1n7ml1
d1igja1 d1c5ch1 d2a9mh_ d1rmfh1 d1fj1b1 d4heme_ d1ccza1 d1a5fh1
d1ogad1 d1dlfh_ d1qa9b_ d1vgel1 d3qskb_ d1sjxa_ d4ozfg1 d1sq2n_
d1qd0a_ d1kcvh1 d1c5db1 d3qcul1 d8faba1 d3bn9d1 d1mjuh1 d4y8dc_
d7fabh1 d3dvgb1 d1lo0l1 d1a6tb1 d1gigh1 d1kcrh1 d2hwzl1 d1yjdc1
d3clfl1 d4krna_ d1dl7l_ d1e6ol1 d1mexh1 d3r0ma_ d4kkna_ d1fnsl1
d1oaqh_ d1rzia1 d1ucbh1 d1ncwl1 d4b41a_ d1nfdf1 d2mpah1 d2d7tl1
d4grwf_ d4b5ea_ d1lk3h1 d3u79a_ d4u3xa_ d1ghfl1 d1bvkb_ d1mcww1
d1b88a_ d1jrhh1 d32c2b1 d4wemb_ d1dsfh_ d1op3h1 d1ce1l1 d1iqda1
d1qoka1 d1qoka2 d1qfuh1 d2crya1 d1ay1h1 d1oaql_ d3ab0b_ d1rurl1
d1t4kb1 d1bz7b1 d1n0xh1 d1forl1 d1ucbl1 d4w6yb_ d43c9a_ d2aabl1
d1tzia1 d4n1ca_ d1k6qh1 d3g9ab_ d4tvsa1 d1igmh_ d1pz5b1 d3dx9a1
d3wxwh_ d1nakh1 d1kcul1 d3eaka_ d4qlra1 d1cr9l1 d1d9ka_ d1f4xl1
d1deea1 d2i27n2 d1c12a1 d2orbl1 d1j8hd1 d1mcph1 d1c5da1 d1kenh1
d1bd2e1 d1h5ba_ d4x7fc1 d3upaa_ d1t3fa1 d1indh1 d2dtgc1 d4hc1l1
d1zv5a1 d4wgvb_ d1uwgl1 d4f37k1 d1aqkh1 d1vesa_ d3zkqd_ d1fptl1
d1lo4l1 d1knob1 d3rrqa_ d1ct8b1 d4orzc_ d1mlbb1 d4hgkc1 d1dqdl1
d1bogb1 d4ozhe1 d1dr9a1 d1i7za1 d1dn0b1 d1kell1 d1rzib1 d1ypzf1
d1wtla_ d1hkfa_ d1fail1 d1figh1 d2vyre_ d1sjva_ d1ohqa_ d1bfoa1
d1pkqb1 d1mjul1 d1b4jl1 d1nsnl1 d2arjb1 d1p4bh_ d4aj0a_ d3nn8b_
d2rcja1 d1f2xk_ d4v1dd_ d1adqh1 d2eksa_ d4k3ga_ d1jfqh1 d1uweh1
d1frgh1 d4gftb_ d1uz8b1 d4n1ea_ d1bm3l1 d1clol1 d3mcka1 d2vq1a1
d1ae6h1 d4aq1b1 d1uj3b1 d1nbqa1 d1fh5l1 d2ck0l1 d2ywza_ d1q72l1
d4nbxb_ d1hila1 d4hzlb1 d2j88l1 d1zmya1 d1cd0a_ d1a4ka1 d1pskl1
d1a0qh1 d4krmb_ d1ncbh1 d2fbjh1 d1j8he1 d1i3ua_ d1cf8l1 d1fe8h1
d2p1ya1 d1eeqa_ d2x89a_ d1ol0a_ d2r0lh1 d1ub5a1 d1dfbh1 d3eyvb1
d4jdvb1 d4p2cg_ d2gsia1 d2agjl1 d1u3ha1 d2h1ph1 d1rihl1 d1fskc1
d1boga1 d2a77l1 d1ospl1 d1qfoa_ d3dwta_ d2fx7l1 d1rz7l1 d3stba1
d1pkqa1 d1b6da1 d1dzba2 d1dzba1 d1kj2a_ d4l1ha_ d1rzgb1 d1dqlh_
d12e8l1 d1miel1 d1jmaa_ d1sbsl1 d1fgnh1 d1f58l1 d1mqkh_ d1ndgb1
d3qegl1 d1iail1 d2aw2a1 d1tqbc1 d3fo2a1 d4dkaa_ d1f8th1 d2g60l1
d2ywya_ d1uj3a1 d1yc7a_ d3bd3a_ d4w6xb1 d1nmbl_ d1i8ka_ d1rjlb1
d1ypze1 d1p7kb1 d1kxqe_ d1gpoh1 d2z4qa1 d1w72h1 d1i9rl1 d4qgya1
d4xt1c_ d2qqnh1 d1cica1 d3c08h1 d2cd0a_ d2bdnh1 d1hnga1 d1maml1
d1nldh1 d1cr9h1 d4lcih1 d3bkyh1 d3qg7l1 d1ai1l1 d1kxvc_ d4iosd_
d1l7ih1 d2x1qa_ d1ub6b1 d3k74b_ d1ibgh1 d1teth1 d1igya1 d1hxma1
d2x6ma_ d2e7la_ d5e03a_ d1qfwm_ d2r0kh1 d1igjb1 d1rurh1 d1plgh1
d1mj8h1 d1yuhb1 d3fmgl1 d1rzfl1 d1emtl1 d1ad9b1 d1igtb1 d1nlbl1
d1d5ih1 d3k7ua_ d4ffyh_ d1bwwa_ d3dvga1 d1a6ta1 d1fgvl_ d4qxgl1
d1miml1 d1jhll_ d2hffb1 d2hh0l1 d1qkzh1 d1l7tl1 d1afvh1 d1iaii1

d2cjuh_ d1um5h1 d2ghwb2 d2ghwb1 d1kgce1 d2fgwh1 d1ikfh1 d1dvfd_
d2i24n1 d2xtjb_ d1za3b1 d2yc1a_ d2hh0h1 d2x1oa_ d1bvka_ d1rjla1
d5e7ba1 d1igta1 d1il1b1 d2fb4l1 d1mvua_ d1jptl1 d32c2a1 d1fvca_
d1iqdb1 d2hrph1 d2p4ab_ d4qo1a_ d1p2cb1 d1wt5c_ d1t4ka1 d5c1mb_
d1it9l1 d1bz7a1 d1b2wh1 d1e4wl1 d1jnll1 d1yedb1 d1za6b1 d3u7aa_
d1igfh1 d3gnml1 d1orsa1 d1wejl1 d1a2ya_ d1hxmb1 d1gafh1 d2j4wh1
d1pz5a1 d1j05a_ d1a3rh1 d4fqll1 d1qfwh_ d1eo8h1 d1n7mh1 d4s11a_
d1kegh1 d5da4a_ d1iqwl1 d3whxb1 d1vgeh1 d1deeb1 d1c12b1 d1t2ql1
d1ad9a1 d3gsnb1 d3b2uc1 d1ggbh1 d1t3fb1 d1nakl1 d4ocmc_ d1lila1
d2v7hb3 d2esve1 d1mcoh1 d2qr0b1 d2q3aa_ d1ct8a1 d1kcrl1 d2pxyb_
d2uzih_ d5c3ld_ d3durb_ d1c5cl1 d2ak4e1 d1dl7h_ d1xiwd_ d1mlba1
d1rmfl1 d1i7zb1 d1dlfl_ d2xa3a_ d5bq7b1 d1dqqb1 d3h42l1 d1egjh1
d1kcvl1 d1fe8l1 d2j6el1 d1f3rb2 d1lk3l1 d1sm3h1 d1q0xh1 d1ghfh1
d7fabl1 d1igch1 d1lo0h1 d4idla_ d5c2bl_ d1jrhl1 d1il1a1 d3zhda1
d4kfzc_ d1b0wa_ d1e6oh1 d1mexl1 d4krpb_ d1fnsh1 d1ncwh1 d4hgma_
d4eigb1 d4rzca_ d2mpal1 d4y5xa_ d5en2b1 d3lrga1 d4ej1c1 d1rhha1
d1j05b_ d1forh1 d1q9wb1 d2wzpd_ d2aq2a1 d1h0da1 d3revb1 d2ij0c1
d1tzha1 d4m7jl1 d1k6ql1 d3b9kc1 d1igml_ d3qeua1 d1dsfl_ d1ce1h1
d1kcuh1 d1zvha1 d1zvoa1 d1tqbb1 d1ayl11 d2qscl1 d2cgrh1 d1nl0h1
d1kenl1 d3eo0a1 d4n9ob1 d1n0xl1 d3k1kc_ d2cqva1 d1fhga_ d2k1ma_
d2dl2a1 d1biha3 d1biha1 d1biha4 d3qp3a1 d1g1ca_ d1gxea_ d1cvsc2
d1cvsc1 d1xmwa2 d1m4ka2 d2otpa2 d1biha2 d1rhfa2 d2fcba2 d2fcba1
d1wwbx_ d1epfa1 d1epfa2 d2dava1 d1waaa_ d1f97a2 d1x44a1 d1vcaa2
d1fnla2 d1fnla1 d3b5ha2 d3h8na2 d3h8na1 d1jbja1 d1jbja2 d1pd6a_
d1f2qa2 d1f2qa1 d3cx2a_ d1nbqa2 d3b5ha1 d1n26a1 d1xiwb_ d1xaua_
d1fltx_ d2ifga1 d1olla1 d1olla2 d1cs6a4 d1cs6a3 d1cs6a2 d1cs6a1
d2gw5a2 d1sy6a1 d1xiwa_ d1ucta2 d1ucta1 d1ry7b2 d1gl4b_ d1qz1a3
d1olza1 d2c9aa1 d1wwca_ d2aq2b1 d1ck1a1 d1enfa1 d1i4pa1 d1v1oa1
d1et9a1 d1esfa1 d2g9hd1 d4ohja1 d1klud1 d1bxta1 d1eu3a1 d1ty0a1
d1fnua1 d2qaln1 d1c4qa_ d1prtc1 d2wv6d_ d1prtf_ d1ltrd_ d4l63b1
d1r4pb_ d1prtd_ d1tl0f_ d2bosa_ d1prtb1

**Family-42 (b.82.1.2)**

**Positive dataset contains 4 protein sequences.**

d1uija1 d2v09a_ d1uika2 d1uika1

**Negative dataset contains 4033 protein sequences.**

d1m32a_ d1ibja_ d1iuga_ d4q75a_ d2bkwa1 d1t3ia_ d1n8pa_ d1qz9a_
d1jf9a_ d1eg5a_ d1p3wa_ d3kgwa_ d1cl1a_ d2ctza1 d1vjoa_ d2rfva_
d1gc0a_ d1cs1a_ d1d2fa_ d2ch1a1 d1qgna_ d1pffa_ d1elua_ d2xlba_
d3m83a1 d1u08a_ d1lc5a_ d1b5pa_ d2ay1a_ d3pd6a_ d7aata_ d2csta_
d1j32a_ d1fg7a_ d4wlha_ d1o4sa_ d1v2da_ d1gdea_ d1m6sa_ d1v72a1
d1svva_ d1ajsa_ d1wsta1 d2f8ja2 d1lk9a_ d1vp4a1 d1xi9a1 d2gb3a1
d1yaaa_ d3i2ka1 d1lnsa3 d1mpxa2 d1xkta1 d2hfka_ d1jmkc_ d5d3ka_
d3lgia_ d3i77a_ d2z9ia2 d4nsva_ d1lcya2 d4jcna_ d1agja_ d1os8a_
d1hpga_ d1p3ca_ d1l1ja_ d1qtfa_ d1ddla_ d1e57b_ d1auyb_ d1f56a_

d1bypa_ d1azca_ d1ws8a_ d3u25a_ d1cuoa_ d1id2a_ d1rkra_ d2iaac_
d4dp1x_ d1nwpa_ d2gima_ d2i7sa_ d1qhqa_ d3ehbb1 d1fftb1 d1gyca3
d1hfua2 d1kbva1 d1kbva2 d1sdda1 d1sdda2 d1gyca1 d1gyca2 d1mzya1
d2bw4a1 d1aoza1 d1aoza2 d1kyaa2 d1kyaa3 d1kyaa1 d1mzya2 d4e9sa1
d1sddb1 d1sddb2 d1hfua1 d1hfua3 d1oe1a2 d1oe1a1 d1v10a2 d1v10a3
d1v10a1 d2dara2 d2dara1 d1wyha2 d2dloa2 d1rutx4 d1rutx3 d2cuqa2
d1x4la2 d1x4la1 d1x3ha2 d1x3ha1 d2d8za1 d1x61a2 d1x6aa1 d1x6aa2
d2cora2 d2cora1 d1v6ga1 d1v6ga2 d2d8za2 d1x64a1 d1x64a2 d1b8ta1
d2co8a1 d1x63a2 d2dj7a2 d2dj7a1 d2cu8a2 d2cu8a1 d1g47a2 d1g47a1
d1j2oa1 d1wyha1 d2dloa1 d1wiga1 d1wiga2 d2cupa2 d2cupa3 d1x68a1
d2d8ya2 d2d8ya1 d1m3va2 d1x62a1 d1x68a2 d1x61a1 d1b8ta3 d1b8ta2
d2d8xa2 d2d8xa1 d1x4ka1 d1x4ka2 d2cuqa1 d1m3va1 d2co8a2 d1j2oa2
d1x63a1 d2cura1 d2cura2 d1x62a2 d2cfba1 d1dfoa_ d1o69a_ d1bj4a_
d3k28a_ d2bwpa_ d3lg0a_ d2fnua_ d3ou5a_ d1z7da1 d1b9ha_ d1iaya_
d4msoa_ d1s0aa_ d1sffa_ d3tqxa_ d1mdoa_ d3qboa_ d3pgya_ d1bjna_
d2dkja_ d1ejia_ d1vefa1 d4wxga_ d3bs8a_ d3gbxa1 d1w23a_ g1gk9.1
g1cp9.1 g1e3a.1 d2bema_ d1pama1 d1ea9c1 d1qhoa1 d2fhfa2 d2fhfa1
d1k3ia1 d1j0ha1 d1cxla1 d2bhua1 d1edqa1 d1f1sa2 d1ut9a2 d1gvia1
d1txka1 d4alac_ d1pjwa_ d1s6na_ d3uzqb_ d1jgta2 d1ofda3 d1ea0a3
d1q15a2 d1ct9a2 d1ao0a2 d1te5a_ d1ex0a1 d4w9ta_ d1jvna1 d4x2ra1
d1ka9f_ d4axka1 d1thfd_ d1h5ya_ d4u28a_ d2y88a_ d4wd0a_ d1vzwa1
d4mh8a_ d1mu2a2 d4i7ga1 d1k9ua_ d2opoa_ d1au7a2 d1ic8a2 d2ovga_
d1sq8a_ d1rzsa_ d3kz3a_ d3orca_ d2msya_ d2djoa1 d2r5za_ d1au7a1
d1f43a_ d2lmda1 d1akhb_ d1s7ea1 d3nara_ d1zq3p1 d2craa1 d1mija_
d4uuta_ d3a01a_ d1x58a1 d3rkqa1 d3cmya1 d2cqxa1 d2da6a1 d1e3oc1
d1fjla_ d1mh3a1 d1ig7a_ d1x41a1 d1wh7a1 d1gt0c1 d2mw8a_ d1lfup_
d1x2ma1 d1b72a_ d1wi3a1 d1jgga_ d1wjha1 d1x2na1 d1mnmc_ d2cuea1
d3k2aa_ d2hosa_ d2jwta2 d1pufa_ d1octc1 d2hi3a_ d1b8ia_ d4j19a_
d3lnqa_ d1wh5a1 d1b8ib_ d1fexa_ d2cqra1 d1guua_ d1ug2a1 d2cqqa1
d2ajea1 d4a69c_ d1h8ac1 d2crga1 d2iw5b1 d1xc5a1 d1ofcx1 d2cu7a1
d1wgxa1 d2cjja1 d2ckxa1 d1hcra_ d1gdta1 d1u78a2 d6paxa2 d1k78a1
d1k78a2 d1xg1a1 d1hlva2 d1iufa2 d1iufa1 d2iu5a1 d1zk8a1 d2g7sa1
d2fd5a1 d2np5a1 d1z0xa1 d2id6a1 d2d6ya1 d2i10a1 d1vi0a1 d2oi8a1
d3c07a1 d2ns7a1 d2fq4a1 d2yvea1 d1t33a1 d2o7ta1 d1rkta1 d2np3b1
d2g7ga1 d2gena1 d2fbqa1 d2g7la1 d2gfna1 d2g3ba1 d2jj7a1 d2hyja1
d1sgma1 d1ui5a1 d2hkua1 d2id3a1 d4jyka1 d1bl0a1 d1bl0a2 d1d5ya2
d3ubub_ d1egia_ d1qo3c_ d1v7pa_ d1tdqb_ d3ubua_ d1c3ab_ d1h8ua_
d3bx4b_ d1t8ca1 d1sb2a1 d1qdda_ d2ziba1 d3cada_ d1byfa_ d1j34b_
d1jwia_ d1jzna_ d1c3aa_ d1oz7b_ d1wmza_ d3bdwb_ d1hq8a_ d1fifa1
d1dv8a_ d3hupa_ d1wk1a1 d1umra_ d2afpa_ d3bx4a_ d1gz2a_ d1sl6a_
d1v7pb_ d3g8ka_ d1ixxa_ d1j34a_ d1jwib_ d1g1sa1 d1y17a1 d1ja3a_
d1fvub_ d3bdwa_ d1sb2b1 d1fvua_ d1umrc_ d1oz7a_ d4m17a_ d3cfwa1
d1ypqa1 d1g1ta1 d3fd4a_ d3l9jc_ d4dn8a1 d1v4lb_ d2msba_ d3lv8a_
d1s3ga1 d1zaka1 d1s4qa_ d3tvta2 d1uf9a_ d1khta_ d1ckea_ d1osna_

| | | | | | | | |
|---|---|---|---|---|---|---|---|
| d1p5zb_ | d4tmka_ | d3cm0a_ | d1tmka_ | d1jaga_ | d3h86a_ | d1t0hb_ | d1nn5a_ |
| d2h92a_ | d3kb2a1 | d2c95a1 | d1yj5a2 | d1kgda_ | d1ly1a_ | d2ak3a1 | d1vyva2 |
| d1deka_ | d2grja1 | d1p3ja1 | d1y63a_ | d1e4ya1 | d1s96a_ | d3iija1 | d1lvga_ |
| d1nksa_ | d1vyua2 | d4e22a_ | d1qf9a_ | d1jjva_ | d1q3ta_ | d1ki9a_ | d1lw7a2 |
| d1teva_ | d1kjwa2 | d1g3ma_ | d1j99a_ | d1t8ta1 | d1nsta_ | d1fmja_ | d1zrha_ |
| d1ls6a_ | d2a3ra_ | d1aqua_ | d2zptx_ | d3ckla_ | d1texa_ | d1q44a_ | d2gwha_ |
| d1z0ja1 | d3umra2 | d3gj0a1 | d2gaoa1 | d1yzga1 | d1x3sa1 | d1g7sa4 | d1puja_ |
| d1udxa2 | d1kk1a3 | d2o52a_ | d1jwyb_ | d1zcaa2 | d1zcba2 | d2fv8a1 | d2cjwa_ |
| d3dz8a1 | d1xzpa2 | d1vg8a_ | d4qxaa_ | d2ew1a1 | d1x1ra1 | d2bcgy_ | d3doea1 |
| d3cona_ | d3rega_ | d3wyga_ | d2b6ha1 | d1zd9a1 | d1lnza2 | d1r5ba3 | d1n0ua2 |
| d1r2qa_ | d2f9la1 | d2rhda_ | d3q3jb_ | d3q85a_ | d1efma1 | d1r8qa_ | d2gf9a1 |
| d2akab1 | d1ek0a_ | d1z08a1 | d2gcoa1 | d2dy1a2 | d4g01b_ | d2cxxa1 | d1svia_ |
| d3q72a_ | d1ni3a1 | d4ac9a4 | d2erxa1 | d2bmea_ | d1wxqa1 | d2gf0a1 | d4ldja1 |
| d1zunb3 | d1d5ca_ | d4lv5b_ | d1efca3 | d1kmqa_ | d2g6ba1 | d3lrpa_ | d1tada2 |
| d2y8ea_ | d4rd4a1 | d1z06a1 | d2heia_ | d3a58b_ | d1u0la2 | d1d2ea3 | d1zj6a1 |
| d2ocba_ | d3rwoa_ | d1mr3f_ | d2e9sa1 | d4u5xa1 | d2iwra_ | d2fn4a1 | d3bwdd_ |
| d2nzja1 | d2atva1 | d2ksqa_ | d3l43a_ | d1mkya2 | d1mkya1 | d1wmsa_ | d1jnya3 |
| d2rmka_ | d1f6ba_ | d1f60a3 | d1gota2 | d1jala1 | d2fh5b1 | d1g16a_ | d2wblc_ |
| d1m7ba_ | d1wf3a1 | d1f5na2 | d1t9ha2 | d1z6xa1 | d1z0aa1 | d2fg5a1 | d2a5ja1 |
| d2bcjq2 | d1s0ua3 | d1ky3a_ | d4y0va_ | d4lhwa_ | d2qmea1 | d2atxa1 | d1egaa1 |
| d1ryha_ | d1moza_ | d1nrjb_ | d2erya1 | d3bfka_ | d1puia_ | d1fzqa_ | d3j3az1 |
| d3t5ga_ | d1shza2 | d3defa_ | d2f7sa1 | d3raba_ | d3tsoa_ | d3aq4a_ | d2al7a1 |
| d3sfva1 | d3bc1a_ | d2ncda_ | d1sdma_ | d3h4sa1 | d2heha_ | d2grya_ | g3kin.1 |
| d1kk8a2 | d1br2a2 | d1goja_ | d1f9va_ | d2mysa2 | d1lkxa_ | d3t0qa_ | d4db1a1 |
| d3gbja_ | d1ry6a_ | d1v8ka_ | d1k8ka2 | d1k8ka1 | d3uleb1 | d3i33a2 | d3i33a1 |
| d1bupa1 | d1dkgd1 | d4ci6a2 | d1jcfa1 | d4a2ba2 | d2fxua1 | d1yaga2 | d1yaga1 |
| d4cbua2 | d1bupa2 | d1g99a2 | d2e1za2 | d2e1za1 | d1saza1 | d1saza2 | d1ig8a1 |
| d1czan2 | d1czan3 | d1czan1 | d1ig8a2 | d1czan4 | d1bg3a4 | d1bg3a3 | d1bg3a2 |
| d3h3no1 | d3h3no2 | d3ezwa2 | d1zc6a1 | d1zbsa1 | d1zbsa2 | d1zc6a2 | d2ch5a2 |
| d2ch5a1 | d1zxoa2 | d1zxoa1 | d1hroa_ | d4gedb_ | d3zooa_ | d1mz4a_ | d1gu2a_ |
| d1f1fa_ | d1dvva_ | d1c6ra_ | d1c75a_ | d1e29a_ | d1gdva_ | d1cnoa_ | d1h32b_ |
| d1co6a_ | d2bh4x_ | d2aiua_ | d5cytr_ | d1jdla_ | d1i8oa_ | d2zboa_ | d3dmia_ |
| d1ls9a_ | d1wvec_ | d2yk3a_ | d3ph2b_ | d1dw0a_ | d5aura_ | d1ql3a_ | d1qn2a_ |
| d1f1ca_ | d4zzva_ | d2xspa_ | d1eg1a_ | d4v20a_ | d1ojja_ | d4ipma1 | d4csia_ |
| d3pfza_ | d3ovwa_ | d2yoka_ | d2ah2a1 | d1n1ta1 | d1evsa_ | d1cnt1_ | d1pvhb_ |
| d1z7ca_ | d1huwa_ | d1bgea_ | d1f6fa_ | d1f45b_ | d1i1rb_ | d1axia_ | d1etea_ |
| d3tgxb_ | d1scfa_ | d2z3qa1 | d3qb1a_ | d1hula_ | d1m48a_ | d4nkqb_ | d1eera_ |
| d5e4ea_ | d2o27a_ | d3b5ka_ | d2psma1 | d4zf7a_ | d3uf5a_ | d4x51a_ | d1d9ca_ |
| d4ypgc1 | d3oq3a_ | d1lqsl_ | d1n1fa_ | d1au1a_ | d1wu3i_ | d3besl_ | d3ux9a_ |
| d2ejca_ | d3muea1 | d1ihoa_ | d3q12a1 | d2el7a_ | d1iq0a2 | d1irxa2 | d1r6ta2 |
| d1ivsa4 | d2cyba_ | d1rqga2 | d1j09a2 | d2yxna_ | d1f7ua2 | d1h3fa1 | d1ffya3 |
| d2g36a1 | d1nzja_ | d1jila_ | d1n3la_ | d1i6la_ | d1pfva2 | d1h3na3 | d3n9ia_ |
| d3sz3a1 | d3sw2a_ | d1moxc_ | d1dx5i3 | d1edmb_ | d1autl2 | d1autl1 | d1nqlb_ |
| d1dval1 | d1szba2 | d1kigl_ | d2y5fl_ | d1ijqa2 | d1gk5a_ | d1g1sa2 | d1nt0a3 |

d1xdtr_ d1lmja2 d1lmja1 d1nzia2 d2wphe_ d1klil_ d1pfxl2 d1pfxl1
d1p9ja_ d1g1ta2 d1f7ea_ d4yzub_ d2mgpa1 d1b9wa1 d1b9wa2 d2mgpa2
d1ob1c1 d1ob1c2 d1n1ia2 d2npra2 d2npra1 d2y5ia_ d1qlsa_ d1psra_
d3n22a_ d2egda_ d4ggfc_ d2wcea_ d2luca_ d4aqia_ d3nsia_ d2lp3a_
d1j55a_ d1a4pa_ d1k8ua_ d1nsha_ d1exra_ d1tiza1 d1nyaa_ d1ggwa_
d1wdcc_ d3fwba_ d1ggza_ d2o5ga_ d1ctaa_ d1m45a_ d3i5gc_ d1sl8a_
d1fpwa_ d2scpa_ d2roaa_ d4aqra_ d3pn7b_ d1br1b_ d2mysb_ d1s6ca_
d1bjfa_ d2obha_ d1jfka_ d1jf0a_ d1qv0a_ d1lkja_ d2mlfc_ d2m97a_
d2d8na_ d4by5a_ d1jbaa_ d4nqga_ d1wdcb_ d2mysc_ d1j7qa_ d1s6ia_
d1uhka2 d1r2ua_ d1dtla_ d3kf9a_ d3pn7c_ d5d67d_ d1tuza_ d1h8ba_
d1eg3a2 d2zkmx1 d1snla_ d1gjya_ d1y1xa_ d1hqva_ d1dvia_ d1df0a1
d1qxpa2 d4usla_ d1k94a_ d1sfsa_ d1wkya2 d1uuqa_ d3wh9a_ d4m1ra_
d1xyza_ d4xuya_ d2je8a5 d4bf7a1 d3cuia_ d1ur4a_ d1qw9a2 d1odza_
d2vzsa5 d1bqca_ d2x2ya1 d1hjqa_ d1qnra_ d4y7ea1 d1vjza_ d4k68a_
d2v3ga_ d4hpga_ d1nofa2 d1nq6a_ d1h4pa_ d1aq0a_ d3ziza_ d4bs0a_
d1ecea_ d1h1na_ d1ta3b_ d1w91a2 d1i1wa_ d1uhva2 d1us3a2 d1egza_
d7a3ha_ d1tvna1 d3nyda1 d1ghsa_ d2cyga1 d4xv0a_ d2c8na2 d1w32a_
d3emca_ d1v6ya1 d1foba_ d2uwfa1 d2fgla_ d3niya1 d2c0ha1 d1rh9a1
d1yq2a5 d1szna2 d1fa2a_ d1pama4 d5do8a1 d1ea9c3 d1hx0a2 d2fhfa5
d1qhoa4 d1m53a2 d4m8ua1 d2gjpa2 d1lwha2 d4howa1 d1ktba2 d1j0ha3
d3vm5a1 d2x1ia_ d1wzaa2 d1uasa2 d2diea1 d1mxga2 d1ht6a2 d1eswa_
d1iv8a2 d5cpsa_ d1ud2a2 d1zjaa1 d3gbea1 d1kwga2 d1wdpa_ d1gcya2
d1g5aa2 d1hvxa2 d3dhpa2 d2bhua3 d4do4a1 d3qgva1 d1e43a2 d1g94a2
d2d3na2 d1gjwa2 d1gvia3 d1x1na1 d1r7aa2 d1tz7a1 d4c7ga2 d2gjxa1
d1nowa1 d1jaka1 d1yhta1 d3wdpp1 d3wq5a_ d1gnxa_ d4gxpa_ d4hz8a_
d1e4mm_ d3aiua_ d1wcga1 d1qvba_ d3ahxa_ d1v02a_ d2dgaa_ d1pbga_
d1vffa1 d1qoxa_ d4ha4a_ d1t77a2 d1mixa2 d1e5wa2 d1j19a2 d1isna2
d2al6b2 d3syxa1 d1mkea1 d2p8va_ d1xoda1 d1i7aa_ d1evha_ d1egxa_
d1b9ra_ d1offa_ d1frra_ d3wcqa_ d1e9ma_ d1l5pa_ d1wria_ d1i7ha_
d3av8a_ d1czpa_ d1rfka_ d3p1ma1 d3b2ga_ d2bt6a2 d1wgqa1 d1v89a1
d2coda1 d1zc3b_ d1wg7a1 d1u5ea1 d1v61a1 d2coca1 d2ys1a1 d1v5pa1
d1ntya2 d1wjma1 d1wi1a1 d1u29a_ d1xx0a1 d1unqa_ d2zkmx3 d1fhoa_
d1v88a1 d2adza1 d1x1fa1 d1p6sa_ d2coaa1 d1btka_ d1v5ma1 d1faoa_
d2x18a_ d1dyna_ d1x1ga1 d4kaxb_ d2j59m1 d1ki1b2 d1xcga2 d1txda2
d1u5da1 d1dbha2 d1w1ha_ d1upqa_ d1v5ua1 d2fjla1 d1eaza_ d2dfka2
d2cofa1 d1kz7a2 d9ldta1 d2ewda3 d1up7a1 d2dfda1 d1s6ya1 d1vjta1
d7mdha1 d1hyea1 d1t2da1 d1ojsa3 d1b8pa1 d1ldna1 d1hyha1 d1civa1
d2a92a1 d1ez4a1 d1i0za1 d1y6ja1 d1gv0a1 d1ur5a1 d1llda1 d1obba1
d1v8ba1 d1gdha1 d2dlda1 d1mx3a1 d1ygya1 d1qp8a1 d1li4a1 d1j4aa1
d2naca1 d1npya1 d3t4ea2 d1a4ia1 d1o0sa1 d2hk9a2 d2egga2 d1b26a1
d1bvua1 d1gq2a1 d1c1da1 d1leha1 d1gtma1 d1euza1 d1nvta1 d3dona2
d1edza1 d1vi2a1 d1p77a1 d1nyta1 d1b0aa1 d1vl6a1 d1v9la1 d1txga2
d3f47a1 d1mv8a2 d2pv7a2 d1xc2a3 d2ahra2 d4xiya1 d2f1ka2 d1pgja2
d1i36a2 d1np3a2 d1lj8a4 d1teaa3 d1qmga2 d2i76a2 d2g5ca2 d1wdka3

d1dlja2 d3cuma3 d1kola2 d1f8fa2 d1qora2 d1jqba2 d1vj0a2 d1cdoa2
d1gu7a2 d1d1ta2 d1xa0a2 d2dm6a2 d1jvba2 d1rjwa2 d1llua2 d1tt7a2
d1piwa2 d1o89a2 d1iz0a2 d1e3ja2 d1yb5a2 d1uufa2 d1vj1a2 d1e3ia2
d3gpdg1 d1vc2a1 d2nvwa1 d1u8fo1 d1ydwa1 d1hdgo1 d2g17a1 d1cf2o1
d2czca2 d1r0ka2 d1obfo1 d2cvoa1 d1ebfa1 d1h6da1 d1i32a1 d1zh8a1
d2q49a1 d1qkia1 d1j5pa4 d3pzra1 d1xeaa1 d1vkoa1 d1vm6a3 d3qvsa1
d2pkqo1 d1b7go1 d1gr0a1 d1p1ja1 d1lc0a1 d1j0xo1 d3pwka1 d2hjsa1
d1zyao1 d1vkna1 d1tlta1 d1nvmb1 d3sjua_ d3ojfa1 d1xu9a1 d2jjya1
d1n7ha_ d1y1pa1 d1h5qa_ d1jtva_ d4fs3a_ d2bkaa1 d1gy8a_ d2c5aa1
d1ulsa_ d1ooea_ d2b69a1 d1y5ma1 d1spxa_ d1ae1a_ d1bxka_ d1ydea1
d1gega_ d1hdoa_ d1o5ia_ d1e6ua_ d1snya1 d2rhca_ d3zv4a_ d5ceja_
d2d1ya1 d1fjha_ d3w8ea_ d2fmua1 d1x1ta_ d1rpna_ d3ay7a1 d1k2wa_
d1eq2a_ d2hrba1 d3ruaa_ d2ew8a1 d4hwka_ d1xgka_ d2bd0a1 d1yo6a1
d2a35a1 d2ntva_ d1vl8a_ d1hdca_ d4bkua_ d2ag5a1 d2q45a_ d1nffa_
d4iy1a_ d1zbqa1 d1kewa_ d1ek6a_ d1ja9a_ d2q46a2 d1iy8a_ d1cyda_
d2ptga_ d1hxha_ d1uzma1 d3lyla_ d4m89a_ d3wyea_ d2blla1 d1w6ua_
d1sb8a_ d2q2va_ d4nr0a_ d1e6wa_ d2gasa_ d3oiga1 d2bgka1 d1mxha_
d1vl0a1 d1rkxa_ d1r6da_ d1wmaa1 d1e7wa_ d2c07a1 d1xkqa_ d3tzha_
d1g0oa_ d3lz6a_ d1qyca_ d1edoa_ d1gz6a_ d1oc2a_ d1t2aa_ d1zema1
d2xoxa_ d1z45a2 d1geea_ d1p33a_ d3lt0a_ d4e6pa_ d2wyua_ d1db3a_
d4q9na_ d1xhla_ d4o1ma_ d1fmca_ d2uvda_ d2gdza1 d1uaya_ d2z1ma_
d2o23a2 d2fr1a2 d2fr1a1 d1qsga_ d1qyda_ d4zjua_ d2a4ka1 d1yb1a_
d1wvga1 d1yxma1 d3f4ba_ d1mg5a_ d1xg5a1 d1r55a1 d3gboa_ d1atla_
d1buda_ d4q1la_ d1quaa_ d1yp1a_ d1wnia_ d3nqyb_ d4gera_ d1bqba_
d1ex0a4 d2f4ma1 d1x3za_ d3u1ra1 d1g9ka2 d1kapp2 d1k7ia2 d3ioqa_
d1fh0a_ d1o0ea_ d1khqa_ d1m6da_ d2cb5a_ d1cs8a_ g8pch.1 d2pnsa_
d1vsna1 d1mira_ d1iwda_ d2oula_ d7pcka_ d3bwka_ d3bcna_ d2p86a_
d1pvja_ d1pcia_ d1gece_ d1deua_ d4bs6a_ d1xkga1 d1cqda_ d1jqpa2
d2c0ya_ d2bdza_ d3p5ua_ d2o36a_ d1zoya1 d1qo8a2 d1d4ca2 d1chua2
d1jnra2 d1kf6a2 d2jk6a2 d2jk6a1 d1feca1 d1feca2 d1lpfa1 d1lpfa2
d1xhca1 d1xhca2 d1dxla2 d1dxla1 d1gera2 d1m6ia2 d1onfa1 d1onfa2
d3lada2 d3lada1 d1w4xa1 d1w4xa2 d1gesa2 d1mo9a1 d1xdia1 d1fl2a2
d1fl2a1 d1fcda1 d1fcda2 d1h6va2 d1h6va1 d1mo9a2 d1gv4a2 d1gv4a1
d2gv8a2 d2gv8a1 d1aoga1 d1aoga2 d1hyua1 d1ebda1 d1ebda2 d3dk9a1
d1q1ra2 d1q1ra1 d1gesa1 d1zbfa1 d2z1ha_ d2etja1 d3qioa1 d1jl1a_
d2e4la_ d1jl2a_ d2lsna1 d1io2a_ d1uaxa_ d2yv0x_ d1mu2a1 d1ekea_
d1i39a_ d1ps9a2 d1o94a2 d1cjca1 d1pn0a1 d1reoa1 d1ju2a1 d2f5va1
d3c96a1 d2gmha1 d1pj5a2 d1gpea1 d1o5wa1 d1n4wa1 d1k0ia1 d2dw4a2
d2ivda1 d2voua1 d1kdga1 d1s5ja1 d2hbja2 d2igia_ d1wlja_ d1qhta1
d4dlea1 d1tgoa1 d2f96a1 d3tr8a1 d3mxma_ d1q8ia1 d1y97a1 d1w0ha_
d1kfsa1 d2py5a1 d1noya_ d1d5aa1 d1yt3a3 d2guia_ d3mwpa1 d4kb1a_
d1iv0a_ d1nu0a_ d2d5ra1 d1iuka_ d2csua1 d3q9ub_ d2yv1a1 d3q9na_
d2d59a_ d1oi7a1 d2yv2a1 d1euca1 d1y81a1 d3nvva1 d1krha3 d2h88b1
d1n62a2 d1t3qa2 d1kf6b2 d1zoyb1 d1ffva2 d1dgja2 d1rm6c2 d1jmxa1

d1pbya1 d1pbya2 d1r7ha_ d1faaa_ d1wika1 d1xfla_ d1ttza1 d2f51a1
d1ti3a_ d1nhoa_ d1sena_ d2i4aa_ d1ep7a_ d1zmaa1 d1fo5a_ d3lefa1
d3diea1 d2yana1 d1z6na1 d1oaza_ d3zywa1 d4aj8a_ d1nm3a1 d4fiwa_
d1wmja_ d4hs1a1 d1iloa_ d1xwaa1 d1wjka1 d2vm1a_ d1fb6a_ d1gh2a_
d1h75a_ d1nw2a_ d1kx5b_ d3a6na_ d4m6ba2 d2nqbd_ d3wkjd_ d2yfwe_
d4h9na_ d5bnxa_ d1kx5c_ d3r45a_ d1eqzb_ d4jjnb_ d1f66c_ d1eqza_
d4wnnb_ d1kx5a_ d1u35c1 d1id3a_ d2nqbc_ d1kx5d_ d1ku5a_ d1f1ea_
d1b67a_ d1tafa_ d1tafb_ d1jfib_ d1q9ca_ d1h3ob_ d2byka1 d1n1ja_
d4g92b_ d1bh9a_ d1jfia_ d1h3oa_ d2bykb1 d1n1jb_ d1pd212 d1nhya2
d2a2ra2 d1k0dd2 d1f2ea2 d1gsua2 d1f3ba2 d2fhea2 d2pvqa1 d1tw9a2
d1oyja2 d1e6ba2 d3ik7a1 d4is0a1 d1r5aa2 d1jlva2 d1m0ua2 d3gtub2
d1z9ha2 d1okta2 d1axda2 d2gsra2 d1tu7a2 d1k3ya2 d1zgma1 d1b48a2
d1gnwa2 d1gwca2 d2gsta2 d1glqa2 d1oe8a2 d1pn9a2 d1v2aa2 d1fw1a2
d1duga2 d3vrda1 d3vrda2 d1fcdc2 d1fcdc1 d1h1oa2 d1h1oa1 d1m70a2
d1nira1 d1qksa1 d1qsaa2 d1qqya_ d1gd6a_ d1f6ra_ d1ivma_ d2gv0a_
d1iiza_ d2z2fa_ d1j8xa_ d1ghla_ d1b9oa_ d174la_ d169la_ d2o4wa_
d1p37a_ d1k28a3 d1xjta1 d1jtma_ d2f2qa_ d4e97a1 d3w3ea_ d4tx7a_
d3cqla_ d4msta_ d3iwra_ d1dxja_ d2z37a_ d4dyga_ d2za0a_ d2c21a1
d1f9za_ d2pjsa1 d1klla_ d2i7ra1 d1ecsa_ d1xqaa1 d5cj3a1 d1jifa_
d2p7oa_ d1r9ca_ d1nkia_ d1etob_ d1umqa_ d1g2ha_ d2beia1 d4m3sa_
d1u6ma1 d1ygha_ d1yk3a1 d3dr6a_ d1xmta_ d1z4ea1 d2ge3a1 d1bo4a_
d2fiaa1 d2jdca1 d1y9wa1 d3to7a_ d1wwza1 d2fl4a1 d2ozua1 d2b5ga_
d2cy2a1 d2p0wa_ d1cm0a_ d4pswa_ d1yrea1 d1yr0a1 d1m4ia_ d2gana1
d1yvka1 d1ghea_ d2g3aa1 d1y7ra1 d2euia1 d1q2ya_ d2fsra1 d1xeba_
d2ae6a1 d1cjwa_ d1qsma_ d2aj6a1 d1y9ka1 d1i12a_ d1vkca_ d2atra1
d1n71a_ d2d4pa1 d2fiwa1 d2fe7a1 d1tiqa1 d2ou2a_ d1yx0a1 d2fcka1
d1p0ha_ d2j8ma_ d2pq8a_ d1z4ra1 d1s3za1 d1ufha_ d1xvqa1 d1pn0a2
d3hy2a_ d2cvba1 d1o8xa_ d1lu4a_ d1we0a1 d2bmxa1 d2a4va1 d1psqa_
d1xiya1 d1q98a_ d1n8ja_ d1zofa1 d2ggta_ d3tjja_ d4je1a1 d3s9fa_
d2zcta_ d1qmva_ d1jfua_ d2b1ka_ d5dvba1 d2b5xa1 d2v2ga_ d1st9a_
d3tb2a_ d4llra_ d4fh8a1 d1e2ya_ d1knga_ d2h30a_ d1i5ga_ d2z9sa_
d2f8aa1 d1tp9a1 d2b7ka_ d1nm3a2 d3hvva_ d2wfca1 d4kcea_ d1xvwa1
d4gqca1 d3qpma_ d4k1fa_ d1zzoa1 d1gp1a_ d1prxa_ d1rmka_ d1ixta_
d1eyoa_ d1cnna_ d1fu3a_ d1g1pa_ d1f3ka_ d1fyga_ d2m5ta_ d1cqqa_
d1wqsa_ d5bpea_ d2ipha_ d1lvoa_ d2bhga1 d3tnta_ d3sjia_ d3d23a1
d2fyqa_ d3tloa_ d3zvea1 d2hrva_ d3q3ya1 d4asha1 d1e88a1 d1l6ja5
d1l6ja4 d1eaka4 d1l6ja3 d1h8pa2 d1h8pa1 d1etha2 d1w52x1 d2ppla1
d1hpla2 d1lpbb2 d2oxea1 d1bu8a2 d1rp1a2 d1ys1x_ d4hs9a_ d1ispa_
d1ex9a_ d1ku0a_ d3qmma_ d1lgya_ d4l3wa_ d1llfa_ d1ukca_ d1cr6a2
d1svpa_ d1mbma_ d3su6a1 d1cu1a1 d4agka_ d1vcpa_ d1q31a_ g1n1l.1
d3e90b_ d1ep5a_ d3u1jb_ d4qmea2 d2xdta2 d1z5ha2 d3zxha_ d1cgla_
d1rm8a_ d1eaka2 d2jsda_ d1cxva_ d1hv5a_ d1i76a_ d3p0ya1 d1igra1
d1igra2 d1n8yc2 d1m6ba2 d1m6ba1 d1m4la_ d2bk7a_ d3fjua_ d1z5ra1
d1kwma1 d1uwya2 d1jqga1 d2c1ca1 d1dtda_ d1h8la2 d2tbvc_ d1c8nc_

| | | | | | | | |
|---|---|---|---|---|---|---|---|
| d1x36a_ | d1smvc_ | d1ng0c_ | d4sbvc_ | d1opoa_ | d1f2nc_ | d1cixa_ | d1y29a_ |
| d1v7fa_ | d2jtba_ | d1g9pa_ | d1lupa_ | d1qk7a_ | d1mb6a_ | d2a2va_ | d1niya_ |
| d1i25a_ | d1hvwa_ | d2mpqa_ | d1ie6a_ | d1kqha_ | d1la4a_ | d2mxoa1 | d1dl0a_ |
| d1koza_ | d1qk6a_ | d1emxa_ | d1lqra_ | d1c6wa_ | d3ljqa_ | g1k2x.1 | d1rypd_ |
| d1irua_ | d1rypc_ | d3unfk_ | d1rypa_ | d1j2pa_ | d1irui_ | d1j2qh_ | d3unfh_ |
| d1m4ya_ | d1rypb_ | d1irul_ | d1irug_ | d1iru2_ | d1q5rh_ | d1rypg_ | d1g3ka_ |
| d1iruf_ | d1q5qa_ | d1e94a_ | d1irub_ | d1ryph_ | d3ty6a_ | d1iruj_ | d1iruk_ |
| d1rype_ | d1irud_ | d1irue_ | d3mi01_ | d1rypf_ | d2z3ba_ | d1iruc_ | d3mka2_ |
| d1iru1_ | d1ny721 | d1ny722 | d1a6ca3 | d1a6ca2 | d1a6ca1 | d1pgl22 | d1pgl21 |
| d1pgl11 | d1ny711 | d1mqta_ | d1d4m3_ | d1fmd3_ | d2mev1_ | g1aym.1 | d1ev13_ |
| d4q4w1_ | d1r1a3_ | d2x5ia_ | d1rhi1_ | g1r1a.1 | g1d4m.1 | d1b35b_ | d1oopa_ |
| d1tme1_ | d4iv1a_ | g1pvc.1 | g1fpn.1 | d1fpn1_ | d4gb31_ | d1d4m1_ | d1fmd1_ |
| d1ev11_ | d2wzr3_ | g1cov.1 | d1qqp3_ | g1fmd.1 | d2x5ic_ | d1rhi3_ | g1oop.1 |
| d1hxs1_ | g1hxs.1 | d1fpn3_ | d1bev1_ | g2mev.1 | d1eah3_ | d1aym1_ | d1pvc3_ |
| d1qqp1_ | d1zba3_ | d1h8ta_ | d3vbhc_ | g1b35.1 | g1bev.1 | d1hxs3_ | g1ev1.1 |
| d1bev3_ | g1tmf.1 | d1tmf1_ | d1b35a_ | d2mev3_ | g1eah.1 | g1h8t.1 | d1eah1_ |
| d4q4w3_ | d1r1a1_ | d1aym3_ | d1pvc1_ | d1zba1_ | d1h8tc_ | d1oopc_ | d1tme3_ |
| g1qqp.1 | d4iv1c_ | g1tme.1 | d1p8ba_ | d1eixa_ | d3exra_ | d2ffca1 | d2fdsa1 |
| d3n3ma1 | d2czda_ | d1q6oa_ | d2yyua_ | d1vqta1 | d1dbta_ | d3ldva1 | d2flia1 |
| d1rpxa_ | d1tqja_ | d1tqxa_ | d1ux8a_ | d1dlya_ | d4i0va_ | d2bkma_ | d1dlwa_ |
| d2bmma_ | d2r80b_ | d1cqxa1 | d1cg5a_ | d1gcvb_ | d3boma_ | d2h8fb_ | d2raoa_ |
| d3bj1a_ | d4hrrb_ | d2r50a_ | d3d1kb_ | d2aa1b1 | d3zhwa_ | d3hf4b_ | d3gdja_ |
| d1jebb_ | d3bj1b_ | d1x9fb_ | d1wmub_ | d1gcva_ | d3bcqb_ | d3d4xb_ | d2dc3a_ |
| d3hf4a_ | d4yu3a_ | d3k8ba_ | d1qpwb_ | d2r80a_ | d3bcqa_ | d1v4wa_ | d3wtgb_ |
| d1jl7a_ | d4esaa_ | d1a9we_ | d3d1ab_ | d1fsla_ | d4h2la_ | d1v4wb_ | d1mwca_ |
| d1vhba_ | d1jeba_ | d1spgb_ | d1h97a_ | d1q1fa_ | d1tu9a1 | d3d1aa_ | d1hdsb_ |
| d3hyub_ | d2zfbb_ | d3fs4a_ | d1oj6a_ | d1shrb_ | d4h2lb_ | d4yu3b_ | d1spga_ |
| d3hrwa_ | d3qqra_ | d3at5a_ | d4wchd_ | d3hyua_ | d1cg5b_ | d1uc3a_ | d1hbra_ |
| d2raob_ | d1outb_ | d4esab_ | d3gdjb_ | d3qqqa_ | d1x9fc_ | d3vrfa_ | d1itha_ |
| d3mkba_ | d3d4xa_ | d3mjpa_ | d1jl6a_ | d3fh9a_ | d1outa_ | d2dc3b_ | d1it2a_ |
| d3eoka_ | d3g46a_ | d1qpwa_ | d3vrfb_ | d1ch4a_ | d1x9fd_ | d1hdsa_ | d2zfba_ |
| d3mkbb_ | d1or4a_ | d3wtga_ | d1a4fa_ | d3fh9b_ | d3bomb_ | d3cy5a_ | d3at5b_ |
| d1x9fa_ | d2oifa_ | d4lmxb_ | d1cpcb_ | d3v57a_ | d1kn1a_ | d1cpca_ | d4f0ta_ |
| d2vjha_ | d4po5b_ | d1eyxa_ | d4f0tb_ | d1b33b_ | d1b8db_ | d4h0mb_ | d1b33a_ |
| d2vjhb_ | d4lm6b_ | d2vmlb_ | d1allb_ | d2vjta_ | d3dbja_ | d2vmla_ | d2vjtb_ |
| d4f0ub_ | d1f99b_ | d4rmpa1 | d1liaa_ | d1eyxb_ | d2uulb_ | d4lmsb_ | d4f0ua_ |
| d2bv8a_ | d1f99a_ | d2bv8b_ | d2c7lb_ | d3dbjb_ | d4rmpb1 | d1b8da_ | d4h0ma_ |
| d3v57b_ | d1kn1b_ | d1liab_ | d2uula_ | d2j96a_ | d1pjqa1 | d3ia2a_ | d4dgqa1 |
| d1hkha_ | d1zoia_ | d1mtza_ | d2xqfa_ | d1ea5a_ | d1f6wa_ | d1dx4a_ | d1qe3a_ |
| d1k4ya_ | d4qwwa_ | d2ha2a_ | d2i49a_ | d1nnfa_ | d1xvxa_ | d1ursa_ | d1h3da1 |
| d1iuda_ | d4m1va1 | d1twya_ | d3e13x_ | d2nxoa1 | d4pfua_ | d3ttma_ | d1elja_ |
| d2qmwa1 | d1us5a_ | d1y3na1 | d1jeta_ | d1uqwa_ | d4f19a1 | d2esna2 | d3thia_ |
| d3fasa1 | d2uyeb_ | d2xwva1 | d1r9la_ | d2fyia1 | d1pb7a_ | d2ozza1 | d1sw5a_ |
| d1nh8a1 | d1vr5a1 | d2g29a_ | d1xs5a_ | d3lswa_ | d2z23a_ | d3w9va_ | d3tcha_ |

| | | | | | | | |
|---|---|---|---|---|---|---|---|
| d1q35a_ | d1z7me1 | d3q27a_ | d1p99a_ | d1ve4a1 | d2i6ea1 | d4mcoa1 | d4n8ya_ |
| d1eu8a_ | d1zbma1 | d1hsla_ | d1ixca2 | d1j1na_ | d1xvya_ | d1y9ua_ | d2z22a1 |
| d1xoca1 | d4oera_ | d1wdna_ | d5f1qa1 | d1xt8a1 | d4gwoa_ | d4igta1 | d1pc3a_ |
| d4mmpa1 | d1o63a_ | d2czla1 | d2onsa2 | d4mija1 | d1jmxa4 | d1pbya3 | d1pbya4 |
| d4iuaa3 | d4bvva_ | d5hpga_ | d3k65a_ | d1ki0a3 | d1ki0a1 | d1a0ha1 | d1pmla_ |
| d3sp8a3 | d2feba1 | d1f74a_ | d3l21a_ | d2e1da_ | d1to3a1 | d1w3ia_ | d1sfla_ |
| d1n7ka_ | d1vcva1 | d4imea_ | d1wa3a_ | d2ocza1 | d1f2ja_ | d3m16a_ | d2yxga_ |
| d1ub3a_ | d3a5fa_ | d1fbaa_ | d1l6wa_ | d1xfba1 | d1wx0a1 | d1vpxa1 | d1ojxa_ |
| d1mxsa_ | d2a4aa1 | d1xkya1 | d3cq0a1 | d3s1xa_ | d1f05a_ | d1fdja_ | d3cpra1 |
| d4d2ja_ | d1cg2a1 | d1yloa2 | d1y7ea2 | d1y0ya3 | d1vixa1 | d1fnoa4 | d1vhoa2 |
| d1ysja1 | d1vhea2 | d1z2la1 | d2fvga2 | d1vgya1 | d1r3na1 | d2grea2 | d1xmba1 |
| g1q7l.1 | d1rtqa_ | d3rp2a_ | g1ucy.1 | d1fuja_ | d2zchp_ | d3w94a_ | d1si5h_ |
| d1lo6a_ | g2pka.1 | d1cgha_ | d1kigh_ | d1a5ia_ | d3s9ba_ | d1hj8a_ | d1ao5a_ |
| d2anya_ | d1q3xa1 | d1gvza_ | d1azza_ | d4gsoa_ | d2f91a1 | d1iaua_ | d1gdna_ |
| d1eufa_ | g1fiw.1 | d1eq9a_ | d4nfea_ | d2rdla_ | d1spja_ | d1elva1 | d1a7sa_ |
| d1ekbb_ | d1pfxc_ | d1yc0a_ | d2aiqa_ | g1fiz.1 | d1fi8a_ | d4k60a_ | d4dgja_ |
| d1mzaa_ | d1op0a_ | d3tjva_ | d1pytd_ | d1sgfg_ | g1gg6.1 | d2eeka_ | g1rtf.1 |
| d1a0ja_ | d3g01a_ | d1pytc_ | d4e7na_ | d1ddja_ | d1z8ga1 | d2hlca_ | d1npma_ |
| d1brup_ | g1jou.1 | d1sgfa_ | d1autc_ | g2hnt.1 | g1kdq.1 | d1orfa_ | d3s69a_ |
| d4crga_ | d1fxya_ | d1bqya_ | d2ftba_ | d1pmpa_ | d2rcqa1 | d1fdqa_ | d4lkpa_ |
| d1sa8a_ | d1vyfa_ | d1lpja_ | d3vg7a1 | d4azqa1 | d1o8va_ | d5bvqa_ | d1p6pa_ |
| d2qo4a_ | d1opba_ | d1yiva1 | d1o1va_ | d4bvma1 | d4tkba_ | d4i3ba_ | d1bwya_ |
| d1ftpa_ | d1ggla_ | d4gkca_ | d1cbia_ | d3p6da1 | d1kqwa_ | d2a0aa1 | d1jx4a2 |
| d3gv5b1 | d1t94a2 | d3bq0a2 | d1e69a_ | d1mv5a_ | d3g60a4 | g1ii8.1 | d1ewqa2 |
| d1ji0a_ | d1qhla_ | d1v43a3 | d2wmfa1 | d1vpla_ | d1g2912 | d4a82a2 | g1f2t.1 |
| d2d62a1 | d3tuic1 | d2bbsa1 | d3g60a3 | d2ixea_ | d1g6ha_ | d1sgwa1 | d2qi9c_ |
| d3rlfa1 | d3b60a1 | d3d31a2 | d2pzea1 | g1xew.1 | d1jj7a_ | d1oxxk2 | d2onka1 |
| d1w1wa_ | d1iwea_ | d1qf5a_ | d1vmaa2 | d1nija1 | d1np6a_ | d2p67a1 | d3ihla_ |
| d1okkd2 | d1ls1a2 | d1s1ma2 | d1iona_ | d2v40a_ | d1dj3a_ | d3hida_ | d2vo1a1 |
| d2g0ta1 | d1vcoa2 | d1j8mf2 | d1g3qa_ | d1xjca_ | d1p9ba_ | d1qzxa3 | d1ihua2 |
| d1ihua1 | d2qm8a_ | d1dj2a_ | d4ioja_ | d1cp2a_ | d1yrba2 | d3gqba2 | d1skye3 |
| d1xp8a1 | d1tf7a2 | d1tf7a1 | d1ye8a1 | d1skyb3 | d2qe7d2 | d4blta_ | d4huta_ |
| d3ldaa2 | d4hyya_ | d2i3ba1 | d1cr1a_ | d3j9tb2 | d3ntua2 | d1vdza1 | d2z43a_ |
| d1e9ra_ | d1nlfa_ | d3ziaa2 | d1n0wa_ | d1g6oa_ | d3ziad2 | d3j9ta2 | d1pzna2 |
| d3gqbb2 | d1gkub1 | d1gkub2 | d1eh3a_ | d3skpa_ | d1dtza1 | d1dtza2 | d1ieja_ |
| d1jnfa2 | d1ce2a1 | d1jnfa1 | d1b1xa2 | d1b1xa1 | d1jw1a1 | d1jw1a2 | d1ryoa_ |
| d1h76a1 | d1h76a2 | d1c4oa1 | d2p6ra3 | d2p6ra4 | d1oywa2 | d1oywa3 | d1wrba1 |
| d1w36b2 | d1w36b1 | d1c4oa2 | d3bora_ | d1t6na_ | d2eyqa5 | d2eyqa3 | d2eyqa2 |
| d2zu6a2 | d1veca_ | d1uaaa1 | d1w36d1 | d2g9na1 | d1z3ix2 | d2rb4a_ | d1hv8a1 |
| d1hv8a2 | d1t5ia_ | d1w36c2 | d2j0sa2 | d2j0sa1 | d1fuka_ | d3hjha1 | d1z5za1 |
| d1z63a1 | d1s2ma2 | d1s2ma1 | d1wp9a1 | d1wp9a2 | d1gm5a3 | d1gm5a4 | d3dina4 |
| d3dina3 | d1q0ua_ | d1w36c1 | d1z3ix1 | d1nkta3 | d1nkta4 | d1uaaa2 | d1w36d2 |
| g1qhh.1 | d2fwra2 | d2fwra1 | d3q3ma_ | d1za0a1 | d1mxra_ | d2inca_ | d1jk0a_ |
| d2incb_ | d1mhyb_ | d3hf1b_ | d1mtyb_ | d1r2fa_ | d2uw1a_ | d1mhyd_ | d3n37a_ |

d1afra_ d1oquc_ d1syya_ d1mtyd_ d2o1za_ d1uzra_ d1smsa_ d2fkza_
d1zuja1 d1s3qa1 d2fjca1 d1z6om1 d1veia_ d4dyya_ d3gvya_ d2pyba_
d4cy9a1 d3bvfa1 d1r03a_ d2cf7a_ d1nf4a_ d1zs3a1 d4iwka_ d3ka8a_
d3np0x_ d1n1qa_ d1lb3a_ d4e6ka_ d1z6oa1 d2fzfa1 d4dyua_ d3ge4a_
d1jgca_ d4am4a_ d2z6ma_ d1ji5a_ d1vlga_ d1lkoa1 d1krqa_ d3qz3a_
d1o9ra_ d2ffxj_ d1euma_ d1yv1a1 d2v2pa_ d1j30a_ d4di0a_ d1dpsa_
d2cbia2 d1gqia1 d2choa2 d1oewa_ d2qzxa_ d2asia_ d1psoe_ d1miqa_
d1smra_ d2h6ta_ d1pfza_ d1izda_ d3hd8a_ d1j71a_ d1dpja_ d4aa9a_
d1t6ex_ g1b5f.1 g1lya.1 g1htr.1 d1qdma2 d1bxoa_ d1wkra_ d1tzsa_
d1ibqa_ d2qzwa_ d1ex4a2 d1c6va_ d2v76a_ d1wj1a1 d1aqca_ d1qqga2
d1qqga1 d1ntva_ d1n3ha_ d4dbba_ d3f0wa1 d1p3ra_ d1wgua1 d2cy4a1
d3hqca_ d1wvha1 d1xr0b1 d1bdqa_ d3ggta_ d3ixoa_ d1mrxa_ d2haha_
d1tw7a_ d4njsa_ d1sgua_ d2p3da_ d1nsoa_ d3d3ta_ d2rkfa_ d1c6ya_
d2rspa_ d3ogpa_ d3ekwa_ d2o4na_ d2j9ja_ d1baia_ d3ggua_ d3lzua_
d4npua_ d4j55a_ d2r8na_ d2fxda_ d2p3ca_ d4dqea_ d3ttpa_ d3t3ca_
d2p3aa_ d2hc0a_ d2fdda_ d3mwsa_ d1k8rb_ d1wgra1 d1ef5a_ d1wfya1
d1e8ya3 d1wgya1 d1lfda_ d2cs4a1 d1wxma1 d2byea1 d3kh0a1 d2byfa1
d1wxaa1 d1wgda1 d1wgga1 d4q5eb_ d1wjna1 d1se9a_ d3n3kb_ d1wz0a1
d1we6a1 d2m0xa_ d2gbka_ d3rt3b1 d1wh3a1 d1sifa_ d2ekec2 d4ii2b1
d4hk2a1 d1c3ta_ d1m94a_ d1wgha1 d1ttna1 d1j8ca_ d1wx8a1 d3v6cb1
d2ma9b_ d4i6lb1 d1bt0a_ d2jvca1 d2k1fa_ d1wxva1 d1wf9a1 d1t0ya_
d2io0b2 d1x1ma1 d2gbma_ d1zw7a1 d2m17a_ d1ud7a_ d1we7a1 d2lwpa_
d2gbja_ d1wjua1 d1v6ea1 d1v86a1 d4eewa_ d1wiaa1 d4icva_ d4gu2a_
d1wx7a1 d1wy8a1 d4hjka1 d2faza1 d1v5oa1 d3ruib_ d1eo6a_ d3waob_
d2r2qa1 d3m95a_ d3wana_ d3ubra_ d1qo8a1 d1d4ca1 d2k3va_ d1ft5a_
d1qdba_ d1m1qa_ d2j7aa_ d1ogyb_ d4fasa_ d1jnia_ d1oaha_ d1sp3a_
d1g66a_ d5ajha1 d3h33a_ d1rwja_ d19hca_ d1os6a_ d3bxua_ d3h4na_
d1i77a_ d3caoa_ d2ctha_ d1duwa_ d1ofwa_ d2e84a_ d1hh5a_ d1gyoa_
d3h34a_ d1um8a_ d1ixza_ d1qvra2 d1qvra3 d1qzma_ d1sxjc2 d1jr3d2
d2p65a_ d1l8qa2 d3hu3a3 d1sxjd2 d1njfa_ d1in4a2 d1sxja2 d1tuea1
d2ce7a2 d1w5sa2 d1sxjb2 d1d2na_ d1ny5a2 d1ofha_ d2gnoa2 d2a5yb3
d1fnna2 d1u0ja1 d1lv7a_ d1iqpa2 d2fnaa2 d1ixsb2 d4gita_ d1sxje2
d1g41a_ d2e3fa_ d2e3ea_ d2llda_ d2ny8x_ d2e3ga_ d2ny9x_ d2nz3a_
d2lj7a_ d1n4na_ d2m8bb1 d2kyqa_ d3psma_ d1jkza_ d1jxca_ d2gl1a_
d1ugla_ d4uj0a_ d2k4ua_ d1hp2a_ d2lu9a_ d1baha_ d2meoa1 d1wm7a_
d1hlya_ d1n8ma_ d1jlza_ d1wpda_ d1c55a_ d1pvza_ d1wt7a_ d1rjia_
d1wz5a_ d1m2sa_ d1pjva_ d1c49a_ d1qkya_ d2ktca1 d1wmta_ d1du9a_
d1j5ja_ d1r1ga_ d1t0za_ d2i61a_ d1t1ta_ d1sn4a_ d1kv0a_ d2lkba_
d1npia_ d1wwna_ d1pe4a_ d2kbka_ d2li7a_ d1djta_ d2kbha_ d1dq7a_
d1sega_ d1omya_ d1zywa1 d1jzaa_ d1n9wa1 d1bbua1 d1e1oa1 d1c0aa1
d1b8aa1 d1l0wa1 d3a74a1 d1eova1 d1iyjb4 d1iyjb5 d1iyjb3 d1o7ia_
d4ipca1 d1x3ea_ d4apva_ d1jb7a1 d1jb7a2 d1jb7a3 d3kdfa_ d1ue1b_
d1wjja1 d3eiva_ d1xjva2 d1xjva1 d2ccza2 d1se8a_ d1l1oc_ d1jb7b_
d1qvca_ d1qzga_ d1miua4 d1miua5 d1miua3 d3g48a1 d1gd7a_ d1mkha_

d1jjcb3 d1pyba_ d1y14b1 d2nn6i1 d1i94l_ d2zjra2 d1smxa_ d2qamc2
d1hh2p1 d2r7da1 d1rl2a2 d1wfqa1 d3bzka4 d1x6oa2 d3a0ja_ d2j01d2
d2ja9a1 d4ql5a_ d1hr0w_ d1luza_ d2n3sa_ d2vnud1 d1h95a_ d1go3e1
d2nn6h1 d1ne3a_ d2mo1a_ d1iz6a2 d1jjga_ d1e3pa2 d2ix0a3 d2ix0a2
d2lxja_ d1ueba3 d1ueba2 d2z0sa1 d1u0la1 d2ix0a1 d2eifa2 d2je6i1
d3i2za1 d2c35b1 d2ahob2 d1t9ha1 d3i4oa_ d2qall1 d1jt8a_ d2nn6g1
d1g6pa_ d3cama_ d1i94q_ d1c9oa_ d1d7qa_ d2ba0a1 d2vnud2 d2vnud3
d1khia2 d2qalq1 d1wi5a1 d1xtda2 d1ny4a_ d1dzka_ d4tlja_ d2a2ua_
d4wfva_ d3t1da_ d4iaxa_ d3zq3a_ d2nnda_ d1i4ua_ d1kxoa_ d1bbpa_
d1kt7a_ d1exsa_ d3s26a1 d1lf7a_ d1qfta_ d4gh7a_ d1z24a1 d1qabe_
d1xkia_ d3bx7a_ d4iawa1 d1n0sa_ d1gkab_ d3kzaa_ d2k23a_ d2l9ca1
d2joza1 d1gm6a_ d3kffa_ d3saoa1 d1e5pa_ d1yupa1 d1iiua_ d2shpa1
d1yfoa_ d1l8ka_ d2ooqa1 d1g4us2 d1p15a_ d1lara2 d1lara1 d1wcha_
d2fh7a2 d2fh7a1 d1ohea1 d1ohea2 d2f4da_ d1rxda_ d2hxpa_ d1xria_
d1d5ra2 d1fpza_ d3ezza_ d4y2ea_ d1i9sa_ d3azya_ d3wvja_ d1upsa1
d1ajoa_ d2hyka_ d1ajka_ d2vy0a_ d1o4ya_ d3o5sa_ d3d6ea_ d3atga_
d1dypa_ d1umza_ d3i4ia_ d1c1la_ d4lbqa_ d1a78a_ d1slta_ d4no4a_
d3zsja_ d3duia_ d1g86a_ d1is3a_ d1qmja_ d1hlca_ d1tdha3 d1q3ba3
d1ee8a3 d1k82a3 g1qmo.1 g1len.1 d3zyra_ d1fnya_ d1n47a_ g2ltn.1
d1avba_ d3usua_ d1dhkb_ d1f9ka_ d1ukga_ d1g8wa_ d1g9fa_ d1wbfa_
d3wcsa_ g1loe.1 d1mvqa_ d1hqla_ d1v6ia_ d1dbna_ d1qnwa_ d1fx5a_
d4l8qa_ d1ioaa_ d3n35a_ d1epwa1 d1eysc_ d2h0ba_ d2erfa_ d1h30a1
d3mw4a1 d1pz7a_ d2r1ba_ d3v64a1 d1d2sa_ d3bopa1 d3mw3a1 d1okqa1
d1okqa2 d1wioa4 d1ccza2 d1vcaa1 d1dr9a2 d2d9qb1 d2nxyb2 d1adqa1
d1fnga1 d1uvqb1 d1zs8a1 d2vola1 d2vola2 d1mfbl2 d2jelh2 d1uwel2
d3qq4b1 d1kjvb_ d1k5na1 d1osph2 d4odhl2 d1ow0a1 d1ow0a2 d1bfob2
d3l9ra2 d3jtth1 d2fath2 d4elkb2 d1fn4b2 d2xfxb_ d1pg7x2 d1cqka_
d4k71b2 d1lk2a1 d1eapb2 d4lfhg2 d1i4fa1 d2nw2a2 d1kfah2 d1mhca1
d1h3ua1 d1wejh2 d1h0db2 d1a5fh2 d1kcvh2 d1mjuh2 d1qqda1 d1kcrh2
d1k8ia1 d3huja2 d2nnab1 d1nfdf2 d3mffb2 d1s9va1 d1lk3h2 d1q0xl2
d1fv1b1 d1neza1 d4rfeb2 d1r3ha1 d1a6tb2 d1kjva1 d1pz5b2 d1onqa1
d2bc4a2 d1de4a1 d1k8da1 d1tcra2 d1dn0b2 d1nfde2 d2xwtb2 d1bfoa2
d1mexh2 d1iakb1 d1ypzf2 d4f7ea2 d1q72l2 d2fbjh2 d1fe8h2 d1clzh2
d1fngb1 d1uvqa1 d1vgka1 d1c16a1 d4j12a2 d4x99b2 d1igtb3 d1je6a1
d1q72h2 d1nldh2 d1teth2 d2hmic2 d1i1ca2 d1i1ca1 d1k8ib1 d1q0yh2
d1kfal2 d3frua1 d1pg7w2 d1u58a1 d1igyb4 d1igyb3 d1hyrc1 d1klub1
d1deeb2 d1c12b2 d2bc4b2 d2ig2h2 d1kcvl2 d3wn5b1 d1lk2b_ d1lk3l2
d1mjul2 d1sm3h2 d1q0xh2 d1zvsa1 d1ydpa1 d1u8ib2 d1ypze2 d1kcuh2
d1op9a_ d1y0la1 d1etzb1 d1egjl1 d2gsib1 d1nj9a1 d4jfhd1 d1aqkl1
d1t2ja_ d1lo4h1 d1zvya1 d3v4vl1 d4enec1 d1kxtb_ d5e8ea1 d2pxya_
d4weud_ d1xiwc_ d2esvd1 d1qnzh_ d1ogae1 d1f4xh1 d4n1hb_ d3ezjb1
d3mlwl1 d1kfah1 d1etza1 d1b4jh1 d3cfic_ d4w68a_ d2arja1 d1ek3a_
d2ok0l1 d1vera_ d1uwgh1 d1blnb1 d1fpth1 d1rzga1 d2giya1 d4m3kb1
d1nc2a1 d2nyyd1 d1ncna_ d1fbih1 d5dxwa_ d4fqqa1 d1dqdh1 d1jfql1

d1kelh1 d1faih1 d1frgl1 d1figl1 d4m6nl1 d1fo0a_ d4hxbl1 d3b5ga_
d3quyd1 d2h9gb1 d1i8kb_ d2dd8h1 d2ck0h1 d1eaja_ d4w6wb1 d2a6ib3
d1t66d1 d2e27h_ d1ob1b1 d1f11b1 d2v7na1 d1ad0a1 d2xt1b1 d1pskh1
d4i13b1 d2op4l1 d1a0ql1 d1uwel1 d1za3a1 d1ncbl1 d4uu9a_ d1bm3h1
d1cloh1 d1nqba2 d1cf8h1 d4y5va_ d1mhph1 d5anma1 d4cdgc1 d4ldeb_
d1y0lb1 d2pcpb1 d1z3gl1 d1dfbl1 d1fh5h1 d3g5xa1 d1tzib1 d4dn3l1
d3zhka1 d2b1hh1 d2uyla1 d1f3dh1 d1nj9b1 d2agjh1 d2h1pl1 d1q72h1
d8fabb1 d1ndmb1 d1rjca1 d4u6hb1 d3upca_ d2fbjl1 d1osph1 d1tvda_
d2cjvh_ d4fhbd1 d1bd2d1 d1rz7h1 d3nn8a_ d1blna1 d1knoa1 d1nfda1
d2bmkb1 d1dqll_ d1igyb1 d1igfl1 d12e8h1 d3utza1 d1nc2b1 d1rihh1
d4mwfb1 d4jvpa1 d1mieh1 d1rhfa1 d1fgnl1 d1sbsh1 d1lp9e1 d1dn0a1
d4lqfl1 d1iaih1 d1kgcd1 d1mh5b1 d1gpol1 d2g60h3 d1f8tl1 d2jixd1
d4grwh_ d1fo0b_ d1bfob1 d1axsa1 d1u9ka_ d1nmbh_ d1h8na1 d1h8na2
d1jguh1 d4eura1 d3sdyl1 d35c8h1 d1w72l1 d1mvub_ d1i9rh1 d1ob1a1
d4wenb_ d1yeeh1 d3ietc1 d1f58h1 d1ad0b1 d1mqkl_ d1nbvh1 d4b50a_
d3r8bb_ d1bafl1 d1mamh1 d4rgne1 d2gsgb_ d35c8l1 d1bafh1 d1ai1h1
d2pcpa1 d2fath1 d2x1pa_ d1orsb1 d1ibgl1 d1shma_ d1a2yb_ d1u0qa_
d2coqa_ d1hilb1 d1qfwi_ d1jv5b_ d1fn4b1 d5e0qa_ d1nsnh1 d1plgl1
d4ocxl1 d1kb5h1 d1rzfh1 d1pg7x1 d2fx7h1 d2oi9c_ d1l7il1 d3eyva1
d1d5il1 d4ffyl_ d1tetl1 d1a6wh_ d1ac6a_ d3dvfa_ d1nfde1 d1mimh1
d1ri8a1 d1jhlh_ d3dura_ d1fskb1 d1f97a1 d3so3b1 d2ak4d1 d1lp9f1
d4z7wf1 d1l7th1 d4y5ya_ d4x7ec1 d1emth1 d1ikfl1 d1nlbh1 d4utal1
d1bjma1 d1eapb1 d1u6ah1 d2dtgd1 d1kj2b_ d1xeda1 d1c1eh1 d1fgvh_
d2bnub1 d2fb4h1 d2dtgb1 d1ktrh_ d1jpth1 d1qkzl1 d4hepg_ d2atpb1
d1flrh1 d2hrpl1 d1mvfa_ d1iaim1 d3k80a1 d2jb5h1 d3zkxc_ d4grwe_
d1it9h1 d1rhhb1 d3w9ec_ d1e4wh1 d1sy6h1 d2j6eh1 d3qxta_ d1jglh1
d1p7ka1 d1tzhb1 d4n8pa1 d1gafl1 d2jelh1 d1wioa2 d4i0cc1 d1cicb1
d1lmka2 d1lmka1 d1iqwh1 d1orqb1 d1ejoh1 d1jnlh1 d4zg1a_ d43c9b_
d1ggbl1 d1wejh1 d3q6fa1 d1f3dj1 d3c6la1 d1bqlh1 d3p9wb_ d1a3rl1
d1h0db1 d2uzil_ d1eo8l1 d1qfwl_ d1n7ml1 d1igja1 d1c5ch1 d2a9mh_
d1rmfh1 d1fj1b1 d4heme_ d1ccza1 d1a5fh1 d1ogad1 d1dlfh_ d1qa9b_
d1vgel1 d3qskb_ d1sjxa_ d4ozfg1 d1sq2n_ d1qd0a_ d1kcvh1 d1c5db1
d3qcul1 d8faba1 d3bn9d1 d1mjuh1 d4y8dc_ d7fabh1 d3dvgb1 d1lo0l1
d1a6tb1 d1gigh1 d1kcrh1 d2hwzl1 d1yjdc1 d3clfl1 d4krna_ d1dl7l_
d1e6ol1 d1mexh1 d3r0ma_ d4kkna_ d1fnsl1 d1oaqh_ d1rzia1 d1ucbh1
d1ncwl1 d4b41a_ d1nfdf1 d2mpah1 d2d7tl1 d4grwf_ d4b5ea_ d1lk3h1
d3u79a_ d4u3xa_ d1ghfl1 d1bvkb_ d1mcww1 d1b88a_ d1jrhh1 d32c2b1
d4wemb_ d1dsfh_ d1op3h1 d1cell1 d1iqda1 d1qoka1 d1qoka2 d1qfuh1
d2crya1 d1ay1h1 d1oaql_ d3ab0b_ d1rurl1 d1t4kb1 d1bz7b1 d1n0xh1
d1forl1 d1ucbl1 d4w6yb_ d43c9a_ d2aabl1 d1tzia1 d4n1ca_ d1k6qh1
d3g9ab_ d4tvsa1 d1igmh_ d1pz5b1 d3dx9a1 d3wxwh_ d1nakh1 d1kcul1
d3eaka_ d4qlra1 d1cr9l1 d1d9ka_ d1f4xl1 d1deea1 d2i27n2 d1c12a1
d2orbl1 d1j8hd1 d1mcph1 d1c5da1 d1kenh1 d1bd2e1 d1h5ba_ d4x7fc1
d3upaa_ d1t3fa1 d1indh1 d2dtgc1 d4hc1l1 d1zv5a1 d4wgvb_ d1uwgl1

d4f37k1 d1aqkh1 d1vesa_ d3zkqd_ d1fptl1 d1lo4l1 d1knob1 d3rrqa_
d1ct8b1 d4orzc_ d1mlbb1 d4hgkc1 d1dqdl1 d1bogb1 d4ozhe1 d1dr9a1
d1i7za1 d1dn0b1 d1kell1 d1rzib1 d1ypzf1 d1wtla_ d1hkfa_ d1fail1
d1figh1 d2vyre_ d1sjva_ d1ohqa_ d1bfoa1 d1pkqb1 d1mjul1 d1b4jl1
d1nsnl1 d2arjb1 d1p4bh_ d4aj0a_ d3nn8b_ d2rcja1 d1f2xk_ d4v1dd_
d1adqh1 d2eksa_ d4k3ga_ d1jfqh1 d1uweh1 d1frgh1 d4gftb_ d1uz8b1
d4n1ea_ d1bm3l1 d1clol1 d3mcka1 d2vq1a1 d1ae6h1 d4aq1b1 d1uj3b1
d1nbqa1 d1fh5l1 d2ck0l1 d2ywza_ d1q72l1 d4nbxb_ d1hila1 d4hzlb1
d2j88l1 d1zmya1 d1cd0a_ d1a4ka1 d1pskl1 d1a0qh1 d4krmb_ d1ncbh1
d2fbjh1 d1j8he1 d1i3ua_ d1cf8l1 d1fe8h1 d2p1ya1 d1eeqa_ d2x89a_
d1ol0a_ d2r0lh1 d1ub5a1 d1dfbh1 d3eyvb1 d4jdvb1 d4p2cg_ d2gsia1
d2agjl1 d1u3ha1 d2h1ph1 d1rihl1 d1fskc1 d1boga1 d2a77l1 d1ospl1
d1qfoa_ d3dwta_ d2fx7l1 d1rz7l1 d3stba1 d1pkqa1 d1b6da1 d1dzba2
d1dzba1 d1kj2a_ d4l1ha_ d1rzgb1 d1dqlh_ d12e8l1 d1miel1 d1jmaa_
d1sbsl1 d1fgnh1 d1f58l1 d1mqkh_ d1ndgb1 d3qegl1 d1iail1 d2aw2a1
d1tqbc1 d3fo2a1 d4dkaa_ d1f8th1 d2g60l1 d2ywya_ d1uj3a1 d1yc7a_
d3bd3a_ d4w6xb1 d1nmbl_ d1i8ka_ d1rjlb1 d1ypze1 d1p7kb1 d1kxqe_
d1gpoh1 d2z4qa1 d1w72h1 d1i9rl1 d4qgya1 d4xt1c_ d2qqnh1 d1cica1
d3c08h1 d2cd0a_ d2bdnh1 d1hnga1 d1maml1 d1nldh1 d1cr9h1 d4lcih1
d3bkyh1 d3qg7l1 d1ai1l1 d1kxvc_ d4iosd_ d1l7ih1 d2x1qa_ d1ub6b1
d3k74b_ d1ibgh1 d1teth1 d1igya1 d1hxma1 d2x6ma_ d2e7la_ d5e03a_
d1qfwm_ d2r0kh1 d1igjb1 d1rurh1 d1plgh1 d1mj8h1 d1yuhb1 d3fmgl1
d1rzfl1 d1emtl1 d1ad9b1 d1igtb1 d1nlbl1 d1d5ih1 d3k7ua_ d4ffyh_
d1bwwa_ d3dvga1 d1a6ta1 d1fgvl_ d4qxgl1 d1miml1 d1jhll_ d2hffb1
d2hh0l1 d1qkzh1 d1l7tl1 d1afvh1 d1iaii1 d2cjuh_ d1um5h1 d2ghwb2
d2ghwb1 d1kgce1 d2fgwh1 d1ikfh1 d1dvfd_ d2i24n1 d2xtjb_ d1za3b1
d2yc1a_ d2hh0h1 d2x1oa_ d1bvka_ d1rjla1 d5e7ba1 d1igta1 d1il1b1
d2fb4l1 d1mvua_ d1jptl1 d32c2a1 d1fvca_ d1iqdb1 d2hrph1 d2p4ab_
d4qo1a_ d1p2cb1 d1wt5c_ d1t4ka1 d5c1mb_ d1it9l1 d1bz7a1 d1b2wh1
d1e4wl1 d1jnll1 d1yedb1 d1za6b1 d3u7aa_ d1igfh1 d3gnml1 d1orsa1
d1wejl1 d1a2ya_ d1hxmb1 d1gafh1 d2j4wh1 d1pz5a1 d1j05a_ d1a3rh1
d4fqll1 d1qfwh_ d1eo8h1 d1n7mh1 d4s11a_ d1kegh1 d5da4a_ d1iqwl1
d3whxb1 d1vgeh1 d1deeb1 d1c12b1 d1t2ql1 d1ad9a1 d3gsnb1 d3b2uc1
d1ggbh1 d1t3fb1 d1nakl1 d4ocmc_ d1lila1 d2v7hb3 d2esve1 d1mcoh1
d2qr0b1 d2q3aa_ d1ct8a1 d1kcrl1 d2pxyb_ d2uzih_ d5c3ld_ d3durb_
d1c5cl1 d2ak4e1 d1dl7h_ d1xiwd_ d1mlba1 d1rmfl1 d1i7zb1 d1dlfl_
d2xa3a_ d5bq7b1 d1dqqb1 d3h42l1 d1egjh1 d1kcvl1 d1fe8l1 d2j6el1
d1f3rb2 d1lk3l1 d1sm3h1 d1q0xh1 d1ghfh1 d7fabl1 d1igch1 d1lo0h1
d4idla_ d5c2bl_ d1jrhl1 d3zhda1 d4kfzc_ d1b0wa_ d1e6oh1 d1mexl1
d4krpb_ d1fnsh1 d1ncwh1 d4hgma_ d4eigb1 d4rzca_ d2mpal1 d4y5xa_
d5en2b1 d3lrga1 d4ej1c1 d1rhha1 d1j05b_ d1forh1 d1q9wb1 d2wzpd_
d2aq2a1 d1h0da1 d3revb1 d2ij0c1 d1tzha1 d4m7jl1 d1k6ql1 d3b9kc1
d1igml_ d3qeua1 d1dsfl_ d1ce1h1 d1kcuh1 d1zvha1 d1zvoa1 d1tqbb1
d1ay1l1 d2qscl1 d2cgrh1 d1nl0h1 d1kenl1 d3eo0a1 d4n9ob1 d1n0xl1

d3k1kc_ d1c4qa_ d1prtc1 d2wv6d_ d1prtf_ d1ltrd_ d4l63b1 d1r4pb_
d1prtd_ d1tl0f_ d2bosa_ d1prtb1 d2aq2b1 d1ck1a1 d1enfa1 d1i4pa1
d1v1oa1 d1et9a1 d1esfa1 d2g9hd1 d4ohja1 d1klud1 d1bxta1 d1eu3a1
d1ty0a1 d1fnua1 d2cqva1 d1fhga_ d2k1ma_ d2dl2a1 d1biha3 d1biha1
d1biha4 d3qp3a1 d1g1ca_ d1gxea_ d1cvsc2 d1cvsc1 d1xmwa2 d1m4ka2
d2otpa2 d1biha2 d1rhfa2 d2fcba2 d2fcba1 d1wwbx_ d1gsma2 d1epfa1
d1epfa2 d2dava1 d1waaa_ d1f97a2 d1x44a1 d1vcaa2 d1fnla2 d1fnla1
d3b5ha2 d3h8na2 d3h8na1 d1jbja1 d1jbja2 d1pd6a_ d1f2qa2 d1f2qa1
d3cx2a_ d1nbqa2 d3b5ha1 d1n26a1 d1xiwb_ d1xaua_ d1fltx_ d2ifga1
d1olla1 d1olla2 d1cs6a4 d1cs6a3 d1cs6a2 d1cs6a1 d2gw5a2 d1sy6a1
d1xiwa_ d1ucta2 d1ucta1 d1ry7b2 d1gl4b_ d1qz1a3 d1olza1 d2c9aa1
d1wwca_

**Family-43 (c.1.10.1)**

**Positive dataset contains 21 protein sequences.**

d3l21a_ d2e1da_ d1to3a1 d1w3ia_ d1sfla_ d1vcva1 d4imea_ d1wa3a_
d2ocza1 d3m16a_ d2yxga_ d3a5fa_ d1xfba1 d1wx0a1 d1vpxa1 d2a4aa1
d1xkya1 d3cq0a1 d3s1xa_ d3cpra1 d4d2ja_

**Negative dataset contains 3954 protein sequences.**

d1m32a_ d1ibja_ d1c7na_ d1iuga_ d4q75a_ d2bkwa1 d1t3ia_ d1n8pa_
d1qz9a_ d1jf9a_ d1p3wa_ d3kgwa_ d1cl1a_ d2ctza1 d1vjoa_ d2rfva_
d1gc0a_ d1cs1a_ d1d2fa_ d2ch1a1 d1qgna_ d1pffa_ d1elua_ d1xfda2
d1u08a_ d1lc5a_ d1b5pa_ d1bw0a_ d3pd6a_ d7aata_ d2csta_ d1j32a_
d1fg7a_ d4wlha_ d1o4sa_ d1v2da_ d1gdea_ d1m6sa_ d1v72a1 d1svva_
d1ajsa_ d1wsta1 d2f8ja2 d1lk9a_ d1vp4a1 d1xi9a1 d2gb3a1 d1yaaa_
d3i2ka1 d1lnsa3 d1mpxa2 d1xkta1 d2hfka_ d1jmkc_ d5d3ka_ d1c7ga_
d2v1pa1 d2ez1a_ d2bnma2 d1qnia1 d1ibya_ d2wo3b_ d1shxa_ d1js9c_
d1f15b_ d1za7a_ d1laja_ d1gyca3 d1hfua2 d1kbva1 d1kbva2 d1sdda1
d1sdda2 d1gyca1 d1gyca2 d1mzya1 d2bw4a1 d1aoza1 d1aoza3 d1aoza2
d1kyaa2 d1kyaa3 d1kyaa1 d1mzya2 d4e9sa1 d1sddb1 d1sddb2 d1hfua1
d1hfua3 d1oe1a2 d1oe1a1 d1v10a2 d1v10a3 d1v10a1 g1f8v.1 d1novc_
d2bbvc_ d1a3qa1 d1iknc_ d3brda1 d1p7hl1 d1oy3c_ d1gjia1 d1zk9a1
d2cxka1 d2h0va_ d1adqa1 d1fnga1 d1uvqb1 d1zs8a1 d2vola1 d2vola2
d1mfbl2 d2jelh2 d1uwel2 d3qq4b1 d1kjvb_ d1k5na1 d1osph2 d4odhl2
d1ow0a1 d1ow0a2 d1bfob2 d3l9ra2 d3jtth1 d2fath2 d4elkb2 d1fn4b2
d2xfxb_ d1pg7x2 d1cqka_ d4k71b2 d1lk2a1 d1eapb2 d4lfhg2 d1i4fa1
d2nw2a2 d1kfah2 d1mhca1 d1h3ua1 d1wejh2 d1h0db2 d1a5fh2 d1kcvh2
d1mjuh2 d1qqda1 d1kcrh2 d1k8ia1 d3huja2 d2nnab1 d1nfdf2 d3mffb2
d1s9va1 d1lk3h2 d1q0xl2 d1fv1b1 d1neza1 d4rfeb2 d1r3ha1 d1a6tb2
d1kjva1 d1pz5b2 d1onqa1 d2bc4a2 d1de4a1 d1k8da1 d1tcra2 d1dn0b2
d1nfde2 d2xwtb2 d1bfoa2 d1mexh2 d1iakb1 d1ypzf2 d4f7ea2 d1q72l2
d2fbjh2 d1fe8h2 d1clzh2 d1fngb1 d1uvqa1 d1vgka1 d1c16a1 d4j12a2
d4x99b2 d1igtb3 d1je6a1 d1q72h2 d1nldh2 d1teth2 d2hmic2 d1i1ca2
d1i1ca1 d1k8ib1 d1q0yh2 d1kfal2 d3frua1 d1pg7w2 d1u58a1 d1igyb4
d1igyb3 d1hyrc1 d1klub1 d1deeb2 d1c12b2 d2bc4b2 d2ig2h2 d1kcvl2

| | | | | | | | |
|---|---|---|---|---|---|---|---|
| d3wn5b1 | d1lk2b_ | d1lk3l2 | d1mjul2 | d1sm3h2 | d1q0xh2 | d1zvsa1 | d1ydpa1 |
| d1u8ib2 | d1ypze2 | d1kcuh2 | d1zx5a1 | d1qwra_ | d2phla2 | d2phla1 | d1fxza2 |
| d1fxza1 | d1lrha_ | d1uija1 | g1dgw.1 | d2v09a_ | d1uika2 | d1uika1 | d1dgwa_ |
| d1nepa_ | d1ds6b_ | d1fstb_ | d1ex0a1 | d1g0da1 | d3us3a3 | d3us3a1 | d1v70a_ |
| d2f4pa1 | d1yhfa1 | d4mh8a_ | d4i7ga1 | d1au7a2 | d1ic8a2 | d2ovga_ | d1sq8a_ |
| d1rzsa_ | d3kz3a_ | d3orca_ | d3gpdg2 | d2q49a2 | d1hdgo2 | d2g17a2 | d2cvoa2 |
| d2czca1 | d3pwka2 | d1obfo2 | d1i32a2 | d1zyao2 | d2pkqo2 | d1b7go2 | d2hjsa2 |
| d1cf2o2 | d1vkna2 | d1nvmb2 | d2msya_ | d2djoa1 | d2r5za_ | d1au7a1 | d1f43a_ |
| d2lmda1 | d1akhb_ | d1s7ea1 | d3nara_ | d1zq3p1 | d2craa1 | d1mija_ | d4uuta_ |
| d3a01a_ | d1x58a1 | d3rkqa1 | d3cmya1 | d2cqxa1 | d2da6a1 | d1e3oc1 | d1fjla_ |
| d1mh3a1 | d1ig7a_ | d1x41a1 | d1wh7a1 | d1gt0c1 | d2mw8a_ | d1lfup_ | d1x2ma1 |
| d1b72a_ | d1wi3a1 | d1wjha1 | d1x2na1 | d1mnmc_ | d2cuea1 | d3k2aa_ | d2hosa_ |
| d2jwta2 | d1pufa_ | d1octc1 | d2hi3a_ | d1b8ia_ | d4j19a_ | d3lnqa_ | d1wh5a1 |
| d1b8ib_ | d1fexa_ | d2cqra1 | d1guua_ | d1ug2a1 | d2cqqa1 | d2ajea1 | d4a69c_ |
| d2crga1 | d2iw5b1 | d1xc5a1 | d1ofcx1 | d2cu7a1 | d1wgxa1 | d2cjja1 | d2ckxa1 |
| d1gvda_ | d1igna2 | d2iu5a1 | d1zk8a1 | d2g7sa1 | d2fd5a1 | d2np5a1 | d1z0xa1 |
| d2id6a1 | d2d6ya1 | d2i10a1 | d1vi0a1 | d2oi8a1 | d3c07a1 | d2ns7a1 | d2fq4a1 |
| d2yvea1 | d1t33a1 | d2o7ta1 | d1rkta1 | d2np3b1 | d2g7ga1 | d2gena1 | d2fbqa1 |
| d2g7la1 | d2gfna1 | d2g3ba1 | d2jj7a1 | d2hyja1 | d1sgma1 | d1ui5a1 | d2hkua1 |
| d2id3a1 | d4jyka1 | d3lv8a_ | d1s3ga1 | d1zaka1 | d1s4qa_ | d3tvta2 | d1uf9a_ |
| d1khta_ | d1ckea_ | d1osna_ | d1p5zb_ | d4tmka_ | d3cm0a_ | d1tmka_ | d1jaga_ |
| d3h86a_ | d1t0hb_ | d1nn5a_ | d2h92a_ | d3kb2a1 | d2c95a1 | d1yj5a2 | d1kgda_ |
| d1ly1a_ | d2ak3a1 | d1vyva2 | d1deka_ | d2grja1 | d1p3ja1 | d1y63a_ | d1e4ya1 |
| d1s96a_ | d3iija1 | d1lvga_ | d1nksa_ | d1vyua2 | d4e22a_ | d1qf9a_ | d1jjva_ |
| d1q3ta_ | d1ki9a_ | d1lw7a2 | d1teva_ | d1kjwa2 | d2iyva2 | d1kaga_ | d1e6ca_ |
| d1g3ma_ | d1j99a_ | d1t8ta1 | d1nsta_ | d1fmja_ | d1zrha_ | d1ls6a_ | d2a3ra_ |
| d1aqua_ | d2zptx_ | d3ckla_ | d1texa_ | d1q44a_ | d2gwha_ | d1z0ja1 | d3umra2 |
| d3gj0a1 | d2gaoa1 | d1yzga1 | d1x3sa1 | d1g7sa4 | d1puja_ | d1udxa2 | d1kk1a3 |
| d2o52a_ | d1jwyb_ | d1zcaa2 | d1zcba2 | d2fv8a1 | d2cjwa_ | d3dz8a1 | d1xzpa2 |
| d1vg8a_ | d4qxaa_ | d2ew1a1 | d1x1ra1 | d2bcgy_ | d3doea1 | d3cona_ | d3rega_ |
| d3wyga_ | d1upta_ | d2b6ha1 | d1zd9a1 | d1lnza2 | d1r5ba3 | d1n0ua2 | d1r2qa_ |
| d2f9la1 | d2rhda_ | d3q3jb_ | d3q85a_ | d1efma1 | d1r8qa_ | d2gf9a1 | d2akab1 |
| d1ek0a_ | d1z08a1 | d2gcoa1 | d2dy1a2 | d4g01b_ | d2cxxa1 | d1svia_ | d3q72a_ |
| d1ni3a1 | d4ac9a4 | d2erxa1 | d2bmea_ | d1wxqa1 | d2gf0a1 | d4ldja1 | d1zunb3 |
| d1d5ca_ | d4lv5b_ | d1efca3 | d1kmqa_ | d2g6ba1 | d3lrpa_ | d1tada2 | d2y8ea_ |
| d4rd4a1 | d1z06a1 | d2heia_ | d3a58b_ | d1u0la2 | d1d2ea3 | d1zj6a1 | d2ocba_ |
| d3rwoa_ | d1mr3f_ | d2e9sa1 | d4u5xa1 | d2iwra_ | d2fn4a1 | d3bwdd_ | d2nzja1 |
| d2atva1 | d2ksqa_ | d3l43a_ | d1mkya2 | d1mkya1 | d1wmsa_ | d1jnya3 | d2rmka_ |
| d1f6ba_ | d1f60a3 | d1gota2 | d1jala1 | d2fh5b1 | d1g16a_ | d2wblc_ | d1m7ba_ |
| d1wf3a1 | d1f5na2 | d1t9ha2 | d1z6xa1 | d1z0aa1 | d2fg5a1 | d2a5ja1 | d2bcjq2 |
| d1s0ua3 | d1ky3a_ | d4y0va_ | d4lhwa_ | d2qmea1 | d2atxa1 | d1egaa1 | d1ryha_ |
| d1moza_ | d1nrjb_ | d2erya1 | d3bfka_ | d1puia_ | d3j3az1 | d3t5ga_ | d1shza2 |
| d3defa_ | d2f7sa1 | d3raba_ | d3tsoa_ | d3aq4a_ | d2al7a1 | d3sfva1 | d3bc1a_ |
| d2ncda_ | d1sdma_ | d3h4sa1 | d2heha_ | d2grya_ | g3kin.1 | d1kk8a2 | d1br2a2 |

d1goja_ d1f9va_ d2mysa2 d1lkxa_ d3t0qa_ d4db1a1 d3gbja_ d1ry6a_
d1v8ka_ d1k8ka2 d1k8ka1 d3uleb1 d3i33a2 d3i33a1 d1dkgd2 d1dkgd1
d4ci6a2 d1jcfa1 d4a2ba2 d2fxua1 d1yaga2 d1yaga1 d4cbua2 d1bupa2
d1ig8a1 d1czan2 d1czan3 d1czan1 d1ig8a2 d1czan4 d1bg3a4 d1bg3a3
d1bg3a2 d1nira1 d1qksa1 d2d0oa3 d2d0oa2 d1nbwa3 d1hroa_ d4gedb_
d3zooa_ d1mz4a_ d1gu2a_ d1f1fa_ d1dvva_ d1c6ra_ d1c75a_ d1e29a_
d1gdva_ d1cnoa_ d1h32b_ d1co6a_ d2bh4x_ d2aiua_ d5cytr_ d1jdla_
d1i8oa_ d2zboa_ d3dmia_ d1ls9a_ d1wvec_ d2yk3a_ d3ph2b_ d1dw0a_
d5aura_ d1ql3a_ d1qn2a_ d1f1ca_ d2nqya_ d1h4ga_ d1hixa_ d1oa2a_
d1olra_ d3b7ma_ d4h7ma1 d3wp3a_ d2vgda_ d1igoa_ d3zsea_ d2nlra_
d3wx5a_ d1oa3a_ d1pvxa_ d1m4wa_ d1f5ja_ d1oa4a_ d1ks5a_ d4ixla1
d1evsa_ d1cnt1_ d1pvhb_ d1z7ca_ d1huwa_ d1bgea_ d1rhga_ d1f6fa_
d1f45b_ d1i1rb_ d1axia_ d1etea_ d3tgxb_ d1scfa_ d2z3qa1 d3qb1a_
d1hula_ d1m48a_ d4nkqb_ d1eera_ d1v7mv_ d5e4ea_ d2o27a_ d3b5ka_
d2psma1 d4zf7a_ d3uf5a_ d4x51a_ d1d9ca_ d4ypgc1 d3oq3a_ d1n1fa_
d1au1a_ d1wu3i_ d3besl_ d3ux9a_ d1v8fa_ d2ejca_ d3muea1 d1ihoa_
d3q12a1 d4dnxa2 d1v47a2 d1g8fa2 d1jhda2 d1x6va2 d3rbaa_ d2qtra_
d1nupa_ d3l92a_ d1f9aa_ d1qjca_ d2h29a_ d1vlha_ d1od6a_ d1mrza2
d4ruka_ d2el7a_ d1iq0a2 d1r6ta2 d1ivsa4 d2cyba_ d1rqga2 d1j09a2
d2yxna_ d1f7ua2 d1h3fa1 d1ffya3 d2g36a1 d1nzja_ d1jila_ d1n3la_
d1i6la_ d1pfva2 d1h3na3 d3n9ia_ d3sz3a1 d3sw2a_ d1moxc_ d1dx5i3
d1edmb_ d1autl2 d1autl1 d1nqlb_ d1dval1 d1szba2 d1kigl_ d2y5fl_
d1ijqa2 d1gk5a_ d1g1sa2 d1nt0a3 d1xdtr_ d1lmja2 d1lmja1 d1nzia2
d2wphe_ d1klil_ d1pfxl2 d1pfxl1 d1p9ja_ d1f7ea_ d4yzub_ d2mgpa1
d1b9wa1 d2mgpa2 d1ob1c1 d1ob1c2 d1n1ia2 d1n1ia1 d2npra2 d2npra1
d1f56a_ d1bypa_ d1azca_ d1ws8a_ d3u25a_ d1cuoa_ d1id2a_ d1rkra_
d2iaac_ d4dp1x_ d1nwpa_ d2gima_ d2i7sa_ d1qhqa_ d2y5ia_ d1qlsa_
d1psra_ d3n22a_ d2egda_ d4ggfc_ d2wcea_ d2luca_ d4aqia_ d3nsia_
d2lp3a_ d1j55a_ d1a4pa_ d1k8ua_ d1nsha_ d1pvaa_ d3fs7a_ d2pvba_
d1ttxa_ d2kyfa_ d1rk9a_ d1a75a_ d1exra_ d1tiza1 d1nyaa_ d1g8ia_
d1ggwa_ d1wdcc_ d3fwba_ d1ggza_ d2o5ga_ d1ctaa_ d1m45a_ d3i5gc_
d1sl8a_ d1fpwa_ d2scpa_ d2roaa_ d4aqra_ d3pn7b_ d1br1b_ d2mysb_
d1s6ca_ d1bjfa_ d2obha_ d1jfka_ d1jf0a_ d1qv0a_ d1lkja_ d2mlfc_
d2m97a_ d2d8na_ d4by5a_ d1jbaa_ d4nqga_ d1wdcb_ d2mysc_ d1j7qa_
d1s6ia_ d1uhka2 d1r2ua_ d1dtla_ d3kf9a_ d3pn7c_ d1gjya_ d1y1xa_
d1hqva_ d1dvia_ d1qxpa2 d4usla_ d1k94a_ d1wkya2 d1uuqa_ d3wh9a_
d4m1ra_ d1xyza_ d4xuya_ d2je8a5 d4bf7a1 d3cuia_ d1ur4a_ d1qw9a2
d1odza_ d2vzsa5 d1bqca_ d2x2ya1 d1hjqa_ d1qnra_ d4y7ea1 d1vjza_
d4k68a_ d2v3ga_ d4hpga_ d1nofa2 d1nq6a_ d1h4pa_ d1aq0a_ d3ziza_
d4bs0a_ d1ecea_ d1h1na_ d1ta3b_ d1w91a2 d1i1wa_ d1uhva2 d1us3a2
d1egza_ d7a3ha_ d1tvna1 d3nyda1 d1ghsa_ d2cyga1 d4xv0a_ d2c8na2
d1w32a_ d3emca_ d1v6ya1 d1foba_ d2uwfa1 d2fgla_ d3niya1 d2c0ha1
d1rh9a1 d1yq2a5 d1szna2 d1fa2a_ d1pama4 d5do8a1 d1ea9c3 d1hx0a2
d2fhfa5 d1qhoa4 d1avaa2 d1m53a2 d4m8ua1 d2gjpa2 d1lwha2 d4howa1

d1ktba2 d1j0ha3 d3vm5a1 d2x1ia_ d1wzaa2 d1uasa2 d2diea1 d1mxga2
d1ht6a2 d1eswa_ d1iv8a2 d5cpsa_ d1ud2a2 d1zjaa1 d3gbea1 d1kwga2
d1wdpa_ d1gcya2 d1g5aa2 d1hvxa2 d3dhpa2 d2bhua3 d4do4a1 d3qgva1
d1e43a2 d1g94a2 d2d3na2 d1gjwa2 d1gvia3 d1x1na1 d1r7aa2 d1tz7a1
d2x42a1 d1tr9a1 d3abza1 d4toqa_ d1wb0a1 d1w9pa1 d1eoka_ d1itxa1
d1kfwa1 d1goia2 d1edqa2 d2gsja_ d1hjxa1 d1jnda1 d1vf8a1 d1etob_
d1umqa_ d2w0pa_ d2diaa1 d2e9ia2 d2e9ja1 d2dj4a1 d2eeba1 d2diba1
d2dmba1 d2dmca1 d2d7oa1 d2ee6a1 d2d7pa1 d2eeaa1 d2di8a1 d1wlha1
d2d7na1 d2nqca_ d2d7ma1 d2dica1 d3cnka_ d2k3ta1 d2eeca1 d1qfha1
d2bp3a1 d1v05a_ d2ee9a1 d2eeda1 d2di9a1 d1g4ma1 d1g4ma2 d1id1a_
d2hmta_ d1lssa_ d1offa_ d1frra_ d3wcqa_ d1e9ma_ d1l5pa_ d1wria_
d1i7ha_ d3av8a_ d1czpa_ d1rfka_ d3p1ma1 d3b2ga_ d2bt6a2 d1wgqa1
d1v89a1 d2coda1 d1zc3b_ d1wg7a1 d1u5ea1 d1v61a1 d2coca1 d2ys1a1
d1v5pa1 d1ntya2 d1wjma1 d1wi1a1 d1u29a_ d1xx0a1 d1unqa_ d2zkmx3
d1fhoa_ d1v88a1 d2adza1 d1x1fa1 d1p6sa_ d2coaa1 d1btka_ d1v5ma1
d1faoa_ d2x18a_ d1dyna_ d1x1ga1 d4kaxb_ d2j59m1 d1xcga2 d1txda2
d1u5da1 d1dbha2 d1w1ha_ d1upqa_ d1v5ua1 d1foea2 d2fjla1 d1eaza_
d2dfka2 d2cofa1 d1kz7a2 d9ldta1 d2ewda3 d1up7a1 d2dfda1 d1s6ya1
d1vjta1 d7mdha1 d1hyea1 d1t2da1 d1ojsa3 d1ldna1 d1hyha1 d1civa1
d2a92a1 d1ez4a1 d1i0za1 d1y6ja1 d1gv0a1 d1ur5a1 d1llda1 d1obba1
d1v8ba1 d1gdha1 d2dlda1 d1mx3a1 d1ygya1 d1qp8a1 d1li4a1 d1j4aa1
d2naca1 d1npya1 d3t4ea2 d1a4ia1 d1o0sa1 d2hk9a2 d2egga2 d1b26a1
d1bvua1 d1gq2a1 d1c1da1 d1leha1 d1gtma1 d1euza1 d3dona2 d1edza1
d1gpja2 d1vi2a1 d1p77a1 d1nyta1 d1b0aa1 d1vl6a1 d1v9la1 d1txga2
d3f47a1 d1mv8a2 d2pv7a2 d1xc2a3 d2ahra2 d4xiya1 d2f1ka2 d1pgja2
d1i36a2 d1np3a2 d1lj8a4 d1teaa3 d1qmga2 d2i76a2 d2g5ca2 d1wdka3
d1dlja2 d3cuma3 d1kola2 d1f8fa2 d1qora2 d1jqba2 d1vj0a2 d1cdoa2
d1gu7a2 d1d1ta2 d1xa0a2 d2dm6a2 d1jvba2 d1rjwa2 d1llua2 d1h2ba2
d1tt7a2 d1piwa2 d1o89a2 d1iz0a2 d1e3ja2 d1yb5a2 d1uufa2 d1vj1a2
d3gpdg1 d1vc2a1 d2nvwa1 d1u8fo1 d1ydwa1 d1hdgo1 d2g17a1 d1cf2o1
d2czca2 d1r0ka2 d1obfo1 d2cvoa1 d1ebfa1 d1h6da1 d1i32a1 d1zh8a1
d2q49a1 d1qkia1 d1j5pa4 d3pzra1 d1xeaa1 d1vkoa1 d1vm6a3 d3qvsa1
d2pkqo1 d1b7go1 d1gr0a1 d1p1ja1 d1lc0a1 d1j0xo1 d3pwka1 d2hjsa1
d1zyao1 d1vkna1 d1tlta1 d1nvmb1 d3sjua_ d3ojfa1 d1xu9a1 d2jjya1
d1n7ha_ d1y1pa1 d1h5qa_ d1jtva_ d4fs3a_ d2bkaa1 d1gy8a_ d2c5aa1
d1ulsa_ d1ooea_ d2b69a1 d1y5ma1 d1spxa_ d1ae1a_ d1bxka_ d1ydea1
d1gega_ d1hdoa_ d1o5ia_ d1e6ua_ d1snya1 d2rhca_ d3zv4a_ d5ceja_
d2d1ya1 d1fjha_ d3w8ea_ d2fmua1 d1x1ta_ d1rpna_ d3ay7a1 d1k2wa_
d1eq2a_ d2hrba1 d3ruaa_ d2ew8a1 d4hwka_ d1xgka_ d2bd0a1 d1yo6a1
d2a35a1 d2ntva_ d1vl8a_ d1hdca_ d4bkua_ d2ag5a1 d2q45a_ d1nffa_
d4iy1a_ d1zbqa1 d1kewa_ d1ek6a_ d1ja9a_ d2q46a2 d1iy8a_ d1cyda_
d2ptga_ d1hxha_ d1uzma1 d3lyla_ d4m89a_ d3wyea_ d2blla1 d1w6ua_
d1sb8a_ d2q2va_ d4nr0a_ d1e6wa_ d2gasa_ d3oiga1 d2bgka1 d1mxha_
d1vl0a1 d1rkxa_ d1r6da_ d1wmaa1 d1e7wa_ d2c07a1 d1xkqa_ d3tzha_

d1g0oa_ d3lz6a_ d1qyca_ d1edoa_ d1oc2a_ d1t2aa_ d1zema1 d2xoxa_
d1z45a2 d1geea_ d1p33a_ d3lt0a_ d4e6pa_ d2wyua_ d1db3a_ d4q9na_
d1xhla_ d4o1ma_ d1fmca_ d2uvda_ d2gdza1 d1uaya_ d2z1ma_ d2o23a2
d2fr1a2 d2fr1a1 d1qsga_ d1qyda_ d4zjua_ d2a4ka1 d1yb1a_ d1wvga1
d1yxma1 d3f4ba_ d1mg5a_ d1xg5a1 d1r55a1 d3gboa_ d1atla_ d1buda_
d4q1la_ d1quaa_ d1yp1a_ d1wnia_ d4ekfa_ d2ckga_ d1th0a_ d2bkra_
d3u1ra1 d1g9ka2 d1k7ia2 d1mixa2 d1e5wa2 d1isna2 d2al6b2 d3ioqa_
d1fh0a_ d1o0ea_ d1khqa_ d1m6da_ d2cb5a_ g8pch.1 d1s4va_ d2pnsa_
d1vsna1 d1mira_ d1iwda_ d2oula_ d7pcka_ d3bwka_ d3bcna_ d2p86a_
d1pvja_ d1pcia_ d1gece_ d1deua_ d4bs6a_ d1xkga1 d1cqda_ d1jqpa2
d2c0ya_ d2bdza_ d3p5ua_ d2o36a_ d1k9xa_ d1zoya1 d1qo8a2 d1d4ca2
d1chua2 d1jnra2 d1kf6a2 d2jk6a2 d2jk6a1 d1feca1 d1feca2 d1lpfa1
d1lpfa2 d1xhca1 d1xhca2 d1dxla2 d1dxla1 d1gera2 d1m6ia2 d1onfa1
d1onfa2 d3lada2 d3lada1 d1w4xa1 d1w4xa2 d1gesa2 d1mo9a1 d1xdia1
d1fl2a2 d1fl2a1 d1fcda1 d1fcda2 d1h6va2 d1h6va1 d1mo9a2 d1gv4a2
d1gv4a1 d2gv8a2 d2gv8a1 d1aoga1 d1aoga2 d1hyua1 d1ebda1 d1ebda2
d3dk9a1 d1q1ra1 d1gesa1 d3syxa1 d1mkea1 d2p8va_ d1xoda1 d1i7aa_
d1evha_ d1egxa_ d1pn0a1 d1ju2a1 d2f5va1 d3c96a1 d2gmha1 d1pj5a2
d1gpea1 d1o5wa1 d1n4wa1 d1k0ia1 d2dw4a2 d2ivda1 d1seza1 d2voua1
d1kdga1 d1s5ja1 d2hbja2 d2igia_ d1wlja_ d1qhta1 d4dlea1 d1tgoa1
d2f96a1 d3tr8a1 d3mxma_ d1y97a1 d1w0ha_ d1kfsa1 d2py5a1 d1noya_
d1d5aa1 d1yt3a3 d2guia_ d3mwpa1 d4kb1a_ d2av4a1 d1mpya2 d1kw3b2
d1kw3b1 d4ghga1 d1tfza1 d3isqa1 d3isqa2 d1sqia1 d1sqia2 d1lkda2
d1lkda1 d1cjxa2 d1cjxa1 d1sp9b2 d1t47a1 d1t47a2 d1f1ua2 d1sp8a1
d1sp8a2 d1f1ua1 d3nvva1 d1krha3 d2h88b1 d1n62a2 d1t3qa2 d1zoyb1
d1jq4a_ d1ffva2 d1dgja2 d1rm6c2 d1jmxa1 d1pbya1 d1pbya2 d1r7ha_
d1faaa_ d1wika1 d1xfla_ d1ttza1 d2f51a1 d1ti3a_ d1nhoa_ d1sena_
d2i4aa_ d1ep7a_ d1zmaa1 d1fo5a_ d3lefa1 d3diea1 d2yana1 d1z6na1
d1oaza_ d3zywa1 d4aj8a_ d1nm3a1 d4fiwa_ d1wmja_ d4hs1a1 d1iloa_
d1xwaa1 d1wjka1 d2vm1a_ d1fb6a_ d1gh2a_ d1h75a_ d1nw2a_ d1kx5b_
d3a6na_ d4m6ba2 d2nqbd_ d3wkjd_ d2yfwe_ d4h9na_ d5bnxa_ d1kx5c_
d3r45a_ d1eqzb_ d4jjnb_ d1f66c_ d1eqza_ d4wnnb_ d1kx5a_ d1u35c1
d1id3a_ d2nqbc_ d1kx5d_ d1ku5a_ d1f1ea_ d1b67a_ d1tafa_ d1tafb_
d1jfib_ d1q9ca_ d1h3ob_ d2byka1 d1n1ja_ d4g92b_ d1bh9a_ d1jfia_
d1h3oa_ d2bykb1 d1n1jb_ d1pd212 d1nhya2 d2a2ra2 d1k0dd2 d1f2ea2
d1gsua2 d1f3ba2 d2fhea2 d2pvqa1 d1tw9a2 d1oyja2 d1e6ba2 d3ik7a1
d4is0a1 d1r5aa2 d1jlva2 d1m0ua2 d3gtub2 d1z9ha2 d1okta2 d1axda2
d2gsra2 d1tu7a2 d1k3ya2 d1zgma1 d1b48a2 d1gnwa2 d1gwca2 d2gsta2
d1glqa2 d1oe8a2 d1pn9a2 d1v2aa2 d1fw1a2 d1duga2 d1iqca2 d1iqca1
d1eb7a2 d1eb7a1 d1nmla1 d3wyha_ d4g9sa1 d3gxra_ d1qqya_ d1gd6a_
d1ivma_ d2gv0a_ d1iiza_ d2z2fa_ d1j8xa_ d1ghla_ d1b9oa_ d174la_
d169la_ d2o4wa_ d1p37a_ d1k28a3 d1xjta1 d1jtma_ d2f2qa_ d4e97a1
d2pjsa1 d1klla_ d2i7ra1 d1ecsa_ d1xqaa1 d5cj3a1 d1npba_ d1jifa_
d2p7oa_ d1nkia_ d1y14b1 d2nn6i1 d1i94l_ d2zjra2 d1smxa_ d2qamc2

d1hh2p1 d2r7da1 d1rl2a2 d1wfqa1 d3bzka4 d1x6oa2 d3a0ja_ d2j01d2
d2ja9a1 d4ql5a_ d1hr0w_ d1luza_ d2n3sa_ d2vnud1 d1h95a_ d1go3e1
d2nn6h1 d1ne3a_ d2mo1a_ d1iz6a2 d1jjga_ d1e3pa2 d2ix0a3 d2ix0a2
d2lxja_ d1ueba3 d1ueba2 d2z0sa1 d1u0la1 d2ix0a1 d2eifa2 d2je6i1
d3i2za1 d2c35b1 d2ahob2 d1t9ha1 d3i4oa_ d2qall1 d1jt8a_ d2nn6g1
d1g6pa_ d3cama_ d1i94q_ d1c9oa_ d1d7qa_ d2ba0a1 d2vnud2 d2vnud3
d1khia2 d2qalq1 d1wi5a1 d1xtda2 d1ny4a_ d2bema_ d1pama1 d1ea9c1
d1qhoa1 d2fhfa2 d2fhfa1 d1k3ia1 d1j0ha1 d1cxla1 d2bhua1 d1edqa1
d1f1sa2 d1ut9a2 d1gvia1 d1txka1 d1s5ja2 d1qhta2 d4dlea2 d1tgoa2
d3py8a2 d1q8ia2 d1kfsa2 d2py5a2 d1qsta_ d2beia1 d4m3sa_ d1u6ma1
d1ygha_ d1yk3a1 d3dr6a_ d1xmta_ d1z4ea1 d2ge3a1 d1bo4a_ d2fiaa1
d2jdca1 d1y9wa1 d3to7a_ d1wwza1 d2fl4a1 d2ozua1 d2b5ga_ d2cy2a1
d2p0wa_ d1cm0a_ d4pswa_ d1yrea1 d1yr0a1 d1m4ia_ d2gana1 d1yvka1
d1ghea_ d2g3aa1 d1y7ra1 d2euia1 d1q2ya_ d2fsra1 d1xeba_ d2ae6a1
d1qsma_ d2aj6a1 d1y9ka1 d1i12a_ d1vkca_ d2atra1 d1n71a_ d2d4pa1
d2fiwa1 d2fe7a1 d1tiqa1 d2ou2a_ d1yx0a1 d2fcka1 d1p0ha_ d2j8ma_
d2pq8a_ d1z4ra1 d1s3za1 d1ufha_ d1dgsa2 d1fvia1 d1ckma1 d1x9na2
d2hqya1 d2hqya2 d1lrza2 d1ne9a1 d1ne9a2 d1xvqa1 d1pn0a2 d3hy2a_
d2cvba1 d1o8xa_ d1lu4a_ d1we0a1 d2bmxa1 d2a4va1 d1psqa_ d1xiya1
d1q98a_ d1n8ja_ d1zofa1 d2ggta_ d3tjja_ d4je1a1 d1o73a_ d3s9fa_
d2zcta_ d1qmva_ d1jfua_ d2b1ka_ d5dvba1 d2b5xa1 d2v2ga_ d1st9a_
d3tb2a_ d4llra_ d4fh8a1 d1e2ya_ d1knga_ d2h30a_ d1i5ga_ d2z9sa_
d2f8aa1 d1tp9a1 d2b7ka_ d1nm3a2 d3hvva_ d2wfca1 d4kcea_ d1xvwa1
d4gqca1 d3qpma_ d4k1fa_ d1zzoa1 d1gp1a_ d1prxa_ d4od7a1 d1z6ma1
d4mcua1 d4dvca1 d1r4wa_ d3l9sa_ d1un2a_ d3rppa_ d2qked_ d4ksoa_
d1t4za1 d1wwja_ d1e88a1 d1l6ja5 d1l6ja4 d1eaka4 d1eaka3 d1l6ja3
d1h8pa2 d1w52x1 d2ppla1 d1hpla2 d1lpbb2 d2oxea1 d1bu8a2 d1rp1a2
d1n9wa1 d1bbua1 d1e1oa1 d1c0aa1 d1b8aa1 d3a74a1 d1eova1 d1lgya_
d4l3wa_ d1llfa_ d1gz7a_ d1ukca_ d3ia2a_ d4dgqa1 d1hkha_ d1zoia_
d2ddfa_ d3zxha_ d1cgla_ d1rm8a_ d1eaka2 d1hy7a_ d2jsda_ d1cxva_
d1hv5a_ d1i76a_ d3p0ya1 d1igra1 d1igra2 d1n8yc2 d1n8yc1 d1m6ba2
d1m6ba1 d4dv8a2 d2tbvc_ d1c8nc_ d1x36a_ d1smvc_ d1ng0c_ d4sbvc_
d1f2nc_ d1cixa_ d1y29a_ d1v7fa_ d2jtba_ d1d1ha_ d1g9pa_ d1lupa_
d1qk7a_ d2a2va_ d1niya_ d1i25a_ d1hvwa_ d2mpqa_ d1ie6a_ d1kqha_
d1la4a_ d2mxoa1 d1dl0a_ d1koza_ d1qk6a_ d1emxa_ d1lqra_ d1c6wa_
d1rmka_ d1ixta_ d1eyoa_ d1cnna_ d1fu3a_ d1g1pa_ d1fyga_ d1rypd_
d1irua_ d1rypc_ d3unfk_ d1rypa_ d1j2pa_ d1irui_ d1j2qh_ d3unfh_
d1m4ya_ d1rypb_ d1irul_ d1irug_ d1iru2_ d1q5rh_ d1rypg_ d1g3ka_
d1iruf_ d1q5qa_ d1e94a_ d1irub_ d1ryph_ d3ty6a_ d1iruj_ d1iruk_
d1rype_ d1irud_ d1irue_ d3mi01_ d1rypf_ d1iruh_ d2z3ba_ d1iruc_
d3mka2_ d1iru1_ d1mqta_ d1d4m3_ d1fmd3_ d2mev1_ g1aym.1 d1ev13_
d4q4w1_ d1r1a3_ d2x5ia_ d1rhi1_ g1r1a.1 g1d4m.1 g1rhi.1 d1b35b_
d1oopa_ d1tme1_ d4iv1a_ g1pvc.1 g1fpn.1 d1fpn1_ d4gb31_ d1d4m1_
d1fmd1_ d1ev11_ d2wzr3_ g1cov.1 d1qqp3_ g1fmd.1 d2x5ic_ d1rhi3_

g1oop.1 d1hxs1_ d1fpn3_ d1bev1_ g2mev.1 d1eah3_ d1aym1_ d1pvc3_
d1qqp1_ d1zba3_ d1h8ta_ d3vbhc_ g1b35.1 g1bev.1 d1hxs3_ g1ev1.1
d1bev3_ g1tmf.1 d1tmf1_ d1b35a_ d2mev3_ g1eah.1 g1h8t.1 d1eah1_
d4q4w3_ d1r1a1_ d1aym3_ d1pvc1_ d1zba1_ d1h8tc_ d1oopc_ d1tme3_
g1qqp.1 d4iv1c_ g1tme.1 d1p8ba_ d4w9ta_ d1jvna1 d4x2ra1 d1ka9f_
d1qo2a_ d4axka1 d1thfd_ d4u28a_ d2y88a_ d4wd0a_ d1vzwa1 d4lt9a1
d3tc7a1 d3hoja_ d1i4na_ d3vnda_ d3nl8a1 d1geqa_ d3nava1 d1v5xa_
d4a2ra1 d3tc6a1 d1ujpa_ d4ijba_ d1xcfa_ d1vc4a_ d1rd5a_ d1qopa_
d3b5va_ d2r80b_ d1cqxa1 d1cg5a_ d1gcvb_ d3boma_ d2h8fb_ d2raoa_
d3bj1a_ d4hrrb_ d2r50a_ d3d1kb_ d2aa1b1 d3zhwa_ d3hf4b_ d3gdja_
d1jebb_ d3bj1b_ d1x9fb_ d1wmub_ d1gcva_ d3bcqb_ d3d4xb_ d1gvha1
d2dc3a_ d3hf4a_ d4yu3a_ d3k8ba_ d1qpwb_ d2r80a_ d3bcqa_ d1v4wa_
d3wtgb_ d1jl7a_ d4esaa_ d1a9we_ d3d1ab_ d1fsla_ d4h2la_ d1v4wb_
d1mwca_ d1vhba_ d1jeba_ d1spgb_ d1h97a_ d1q1fa_ d1tu9a1 d3d1aa_
d1hdsb_ d3hyub_ d2zfbb_ d3fs4a_ d1oj6a_ d1shrb_ d4h2lb_ d4yu3b_
d1spga_ d3hrwa_ d3qqra_ d3at5a_ d4wchd_ d3hyua_ d1cg5b_ d1uc3a_
d1hbra_ d2raob_ d1outb_ d4esab_ d3gdjb_ d3qqqa_ d1x9fc_ d3vrfa_
d1itha_ d3mkba_ d3d4xa_ d3mjpa_ d1jl6a_ d3fh9a_ d1outa_ d2dc3b_
d1it2a_ d3eoka_ d3g46a_ d1qpwa_ d3vrfb_ d1ch4a_ d1x9fd_ d1hdsa_
d2zfba_ d3mkbb_ d1or4a_ d3wtga_ d1a4fa_ d3fh9b_ d3bomb_ d3cy5a_
d3at5b_ d1x9fa_ d2oifa_ d4lmxb_ d1cpcb_ d3v57a_ d1kn1a_ d1cpca_
d4f0ta_ d2vjha_ d4po5b_ d1eyxa_ d1alla_ d4f0tb_ d1b33b_ d1b8db_
d4h0mb_ d1b33a_ d2vjhb_ d4lm6b_ d2vmlb_ d1allb_ d2vjta_ d3dbja_
d2vmla_ d2vjtb_ d4f0ub_ d1f99b_ d4rmpa1 d1liaa_ d1eyxb_ d2uulb_
d4lmsb_ d4f0ua_ d2bv8a_ d1f99a_ d2bv8b_ d2c7lb_ d3dbjb_ d4rmpb1
d1b8da_ d4h0ma_ d3v57b_ d1kn1b_ d1liab_ d2uula_ d2j96a_ g1gxs.1
d3nqyb_ d4gera_ d1bqba_ d2xqfa_ d1ea5a_ d1f6wa_ d1dx4a_ d1qe3a_
d4qwwa_ d2ha2a_ d3c8da2 d2pbla1 d1jkma_ d1jjia_ d1jjfa_ d2qvba_
d2v9za_ d4kafa1 d1ex0a4 d2f4ma1 d1x3za_ d4iuaa3 d4bvva_ d5hpga_
d3k65a_ d1ki0a2 d1ki0a1 d1a0ha1 d1pmla_ d3sp8a3 d2feba1 d1mdwa_
d1qxpa4 d1ziva1 d1zcma1 d1cg2a1 d1yloa2 d1y7ea2 d1y0ya3 d1lfwa1
d1vixa1 d1fnoa4 d1vhoa2 d1ysja1 d1vhea2 d1z2la1 d2fvga2 d1vgya1
d1r3na1 d2grea2 d1xmba1 d1rtqa_ d3rp2a_ g1ucy.1 d1fuja_ d2zchp_
d3w94a_ d1si5h_ d1lo6a_ g2pka.1 d1cgha_ d1kigh_ d1a5ia_ d3s9ba_
d1hj8a_ d1ao5a_ d2anya_ d1q3xa1 d1gvza_ d1azza_ d4gsoa_ d2f91a1
d1iaua_ d1gdna_ d1eufa_ g1fiw.1 d1eq9a_ d4nfea_ d2rdla_ d1spja_
d1elva1 d1a7sa_ d1ekbb_ d1pfxc_ d1yc0a_ d2aiqa_ g1fiz.1 d1fi8a_
d4k60a_ d4dgja_ d1mzaa_ d1op0a_ d3tjva_ d1pytd_ d1sgfg_ g1gg6.1
d2eeka_ g1rtf.1 d1a0ja_ d3g01a_ d1pytc_ d4e7na_ d1ddja_ d1z8ga1
d2hlca_ d1npma_ d1brup_ g1jou.1 d1sgfa_ d1autc_ g2hnt.1 g1kdq.1
d1orfa_ d3s69a_ d4crga_ d1fxya_ d1bqya_ d2ftba_ d1pmpa_ d2rcqa1
d1fdqa_ d4lkpa_ d1sa8a_ d1vyfa_ d1lpja_ d3vg7a1 d4azqa1 d1o8va_
d5bvqa_ d1p6pa_ d2qo4a_ d1opba_ d1yiva1 d1o1va_ d4bvma1 d4tkba_
d4i3ba_ d1bwya_ d1ftpa_ d1ggla_ d4gkca_ d3p6da1 d1kqwa_ d2a0aa1

d1dzka_ d4tlja_ d2a2ua_ d4wfva_ d3t1da_ d4iaxa_ d3zq3a_ d2nnda_
d1i4ua_ d1kxoa_ d1bbpa_ d1kt7a_ d1exsa_ d3s26a1 d1lf7a_ d1qfta_
d4gh7a_ d1z24a1 d1qabe_ d1xkia_ d1ew3a_ d3bx7a_ d4iawa1 d1n0sa_
d3kzaa_ d2k23a_ d2l9ca1 d2joza1 d1gm6a_ d3kffa_ d3saoa1 d1e5pa_
d1yupa1 d1iiua_ d3lgia_ d3i77a_ d2z9ia2 d4nsva_ d1lcya2 d4jcna_
d1agja_ d1os8a_ d1p3ca_ d1l1ja_ d1qtfa_ d1e69a_ d1mv5a_ d3g60a4
g1ii8.1 d1ewqa2 d1ji0a_ d1qhla_ d1v43a3 d2wmfa1 d1vpla_ d1g2912
d4a82a2 g1f2t.1 d2d62a1 d3tuic1 d2bbsa1 d3g60a3 d2ixea_ d1g6ha_
d1sgwa1 d2qi9c_ d3rlfa1 d3b60a1 d3d31a2 d2pzea1 g1xew.1 d1jj7a_
d1oxxk2 d2onka1 d1w1wa_ d1iwea_ d1qf5a_ d1vmaa2 d1nija1 d2p67a1
d3ihla_ d1okkd2 d1ls1a2 d1s1ma2 d1iona_ d2v40a_ d1dj3a_ d3hida_
d2vo1a1 d2g0ta1 d1vcoa2 d1j8mf2 d1g3qa_ d1xjca_ d1p9ba_ d1qzxa3
d1ihua2 d1ihua1 d2qm8a_ d1dj2a_ d4ioja_ d1cp2a_ d1yrba2 d1hyqa_
d3gqba2 d1skye3 d1xp8a1 d1tf7a2 d1tf7a1 d1ye8a1 d1skyb3 d2qe7d2
d4blta_ d4huta_ d3ldaa2 d4hyya_ d2i3ba1 d1cr1a_ d3j9tb2 d3ntua2
d1vdza1 d2z43a_ d1e9ra_ d1nlfa_ d3ziaa2 d1n0wa_ d1g6oa_ d3ziad2
d3j9ta2 d1pzna2 d3gqbb2 d2i49a_ d1nnfa_ d1xvxa_ d1ursa_ d1h3da1
d1iuda_ d4m1va1 d1twya_ d3e13x_ d2nxoa1 d4pfua_ d3ttma_ d1elja_
d2qmwa1 d1us5a_ d1y3na1 d1jeta_ d1uqwa_ d4f19a1 d2esna2 d3thia_
d3fasa1 d2uyeb_ d2xwva1 d1r9la_ d2fyia1 d1pb7a_ d2ozza1 d1sw5a_
d1nh8a1 d1vr5a1 d2g29a_ d1xs5a_ d3lswa_ d2z23a_ d3w9va_ d3tcha_
d1q35a_ d1z7me1 d3q27a_ d1p99a_ d1ve4a1 d2i6ea1 d4mcoa1 d4n8ya_
d1eu8a_ d1zbma1 d1hsla_ d1ixca2 d1j1na_ d1xvya_ d1y9ua_ d2z22a1
d1xoca1 d4oera_ d1wdna_ d5f1qa1 d1xt8a1 d1i6aa_ d4gwoa_ d4igta1
d1pc3a_ d4mmpa1 d1o63a_ d2czla1 d2onsa2 d4mija1 d4ok5a1 d2bmfa1
d2bmfa2 d1yksa1 d1yksa2 d1jr6a_ d2qeqa1 d2jlqa1 d1eh3a_ d3skpa_
d1dtza1 d1dtza2 d1ieja_ d1jnfa2 d1ce2a1 d1jnfa1 d1b1xa2 d1jw1a1
d1jw1a2 d1ryoa_ d1h76a1 d1h76a2 d1c4oa1 d2p6ra3 d2p6ra4 d1oywa2
d1oywa3 d1wrba1 d1w36b2 d1w36b1 d1c4oa2 d3bora_ d1t6na_ d2eyqa5
d2eyqa3 d2eyqa2 d2zu6a2 d1veca_ d1uaaa1 d1w36d1 d2g9na1 d1z3ix2
d2rb4a_ d1hv8a1 d1hv8a2 d1t5ia_ d1w36c2 d2j0sa2 d2j0sa1 d1fuka_
d3hjha1 d1z5za1 d1qdea_ d1z63a1 d1s2ma2 d1s2ma1 d1wp9a1 d1wp9a2
d1gm5a3 d1gm5a4 d3dina4 d3dina3 d1q0ua_ d1w36c1 d1z3ix1 d1nkta3
d1uaaa2 d1w36d2 g1qhh.1 d2fwra2 d2fwra1 d2v8ta_ d3q3ma_ d1za0a1
d1mxra_ d2inca_ d1jk0a_ d2incb_ d1mhyb_ d3hf1b_ d1mtyb_ d1r2fa_
d2uw1a_ d1mhyd_ d3n37a_ d1afra_ d1oquc_ d1syya_ d1mtyd_ d2o1za_
d1uzra_ d1smsa_ d2fkza_ d1zuja1 d1s3qa1 d2fjca1 d1z6om1 d1veia_
d4dyya_ d1jiga_ d3gvya_ d2pyba_ d4cy9a1 d3bvfa1 d1r03a_ d2cf7a_
d1nf4a_ d1zs3a1 d4iwka_ d3ka8a_ d3np0x_ d1lb3a_ d4e6ka_ d1z6oa1
d2fzfa1 d4dyua_ d3ge4a_ d1jgca_ d4am4a_ d2z6ma_ d1ji5a_ d1vlga_
d1lkoa1 d1krqa_ d3qz3a_ d1o9ra_ d2ffxj_ d1euma_ d1yv1a1 d2v2pa_
d1j30a_ d4di0a_ d1dpsa_ d2cbia2 d1gqia1 d2choa2 d1oewa_ d2qzxa_
d2asia_ d1miqa_ d1smra_ d2h6ta_ d1pfza_ d1izda_ d3hd8a_ d1j71a_
d1dpja_ d4aa9a_ d1t6ex_ g1b5f.1 g1lya.1 g1htr.1 d1qdma2 d1bxoa_

d1wkra_ d1tzsa_ d1ibqa_ d2qzwa_ d1ex4a2 d1c6va_ d1bdqa_ d3ggta_
d3ixoa_ d1mrxa_ d2haha_ d1tw7a_ d4njsa_ d1sgua_ d2p3da_ d1nsoa_
d3d3ta_ d2rkfa_ d1c6ya_ d2rspa_ d3ogpa_ d3ekwa_ d2o4na_ d2j9ja_
d1baia_ d3ggua_ d3lzua_ d4npua_ d4j55a_ d2r8na_ d2fxda_ d2p3ca_
d4dqea_ d3ttpa_ d3t3ca_ d2p3aa_ d2hc0a_ d2fdda_ d3mwsa_ d1js3a_
d3rcha_ d3k40a_ d1k8rb_ d1wgra1 d1ef5a_ d1wfya1 d1e8ya3 d1wgya1
d1lfda_ d2cs4a1 d1wxma1 d2byea1 d3kh0a1 d2byfa1 d1wxaa1 d1e7ua3
d1h4ra3 d4yl8a1 d2al6a3 d1wgda1 d1wgga1 d4q5eb_ d1wjna1 d1se9a_
d3n3kb_ d1wz0a1 d1we6a1 d2m0xa_ d2gbka_ d3rt3b1 d1wh3a1 d1sifa_
d2ekec2 d4ii2b1 d4hk2a1 d1c3ta_ d1m94a_ d1wgha1 d1ttna1 d1wx8a1
d3v6cb1 d2ma9b_ d4i6lb1 d1bt0a_ d2jvca1 d1iyfa_ d2k1fa_ d1wxva1
d1wf9a1 d1t0ya_ d2io0b2 d1x1ma1 d2gbma_ d1zw7a1 d2m17a_ d1ud7a_
d1we7a1 d2lwpa_ d2gbja_ d1wjua1 d1v6ea1 d1v86a1 d4eewa_ d1wiaa1
d4icva_ d4gu2a_ d1wx7a1 d1wy8a1 d4hjka1 d2faza1 d1v5oa1 d1wioa4
d1ccza2 d1vcaa1 d1dr9a2 d1ic1a1 d2d9qb1 d2nxyb2 d1um8a_ d1ixza_
d1qvra2 d1qvra3 d1qzma_ d1sxjc2 d1jr3d2 d2p65a_ d1l8qa2 d1g8pa_
d3hu3a3 d1sxjd2 d1njfa_ d1in4a2 d1sxja2 d1tuea1 d2ce7a2 d1w5sa2
d1sxjb2 d1d2na_ d1ny5a2 d1ofha_ d2gnoa2 d2a5yb3 d1fnna2 d1u0ja1
d1lv7a_ d2fnaa2 d1ixsb2 d4gita_ d1sxje2 d1g41a_ d2e3fa_ d1mm0a_
d2e3ea_ d2llda_ d2ny8x_ d2e3ga_ d2ny9x_ d2nz3a_ d2lj7a_ d1n4na_
d2m8bb1 d2kyqa_ d3psma_ d1jkza_ d1jxca_ d2gl1a_ d1ugla_ d4uj0a_
d2k4ua_ d1hp2a_ d2lu9a_ d1baha_ d2meoa1 d1wm7a_ d1hlya_ d1n8ma_
d1jlza_ d1wpda_ d1pvza_ d1wt7a_ d1rjia_ d1wz5a_ d1m2sa_ d1pjva_
d1c49a_ d1qkya_ d2ktca1 d1wmta_ d1du9a_ d1j5ja_ d1r1ga_ d1t0za_
d2i61a_ d1t1ta_ d1sn4a_ d1kv0a_ d2lkba_ d1npia_ d1wwna_ d1pe4a_
d2kbka_ d2li7a_ d1djta_ d2kbha_ d1dq7a_ d1sega_ d1omya_ d1zywa1
d1jzaa_ d1bnla_ d1dy2a_ d1ixra2 d1iyjb4 d1iyjb5 d1iyjb3 d1o7ia_
d4ipca1 d1x3ea_ d4apva_ d1jb7a1 d1jb7a2 d1jb7a3 d3kdfa_ d1ue1b_
d1wjja1 d3eiva_ d1xjva2 d1xjva1 d2ccza2 d1se8a_ d1l1oc_ d1jb7b_
d1qvca_ d3ulla_ d1qzga_ d1miua4 d1miua5 d1miua3 d3ubub_ d1egia_
d1qo3c_ d1v7pa_ d1tdqb_ d3ubua_ d1c3ab_ d1h8ua_ d3bx4b_ d1t8ca1
d1sb2a1 d1qdda_ d2ziba1 d3cada_ d1byfa_ d1j34b_ d1jwia_ d1jzna_
d1c3aa_ d1oz7b_ d1wmza_ d3bdwb_ d1hq8a_ d1fifa1 d3hupa_ d1wk1a1
d1umra_ d2afpa_ d3bx4a_ d1gz2a_ d1sl6a_ d1v7pb_ d3g8ka_ d1ixxa_
d1j34a_ d1jwib_ d1g1sa1 d1y17a1 d1ja3a_ d1fvub_ d3bdwa_ d1sb2b1
d1fvua_ d1umrc_ d1oz7a_ d4m17a_ d3cfwa1 d1ypqa1 d1g1ta1 d3fd4a_
d3l9jc_ d4dn8a1 d1v4lb_ d1rdl1_ d2msba_ d1prtc2 d1pfsa_ d2a1ka_
d1rpma_ d2shpa1 d1yfoa_ d1l8ka_ d2ooqa1 d1g4us2 d1p15a_ d1lara2
d1wcha_ d2fh7a2 d2fh7a1 d1ohea1 d1ohea2 d2f4da_ d1rxda_ d2hxpa_
d1xria_ d1d5ra2 d1fpza_ d3ezza_ d4y2ea_ d1c1la_ d4lbqa_ d1a78a_
d1slta_ d4no4a_ d3zsja_ d3duia_ d1is3a_ d1qmja_ d1hlca_ g1qmo.1
g1len.1 d3zyra_ d1fnya_ d1n47a_ g2ltn.1 d1avba_ d3usua_ d1dhkb_
d1f9ka_ d1ukga_ d1g8wa_ d1g9fa_ d1wbfa_ d3wcsa_ d1g7ya_ g1loe.1
d1mvqa_ d1hqla_ d1dbna_ d1qnwa_ d1fx5a_ d4l8qa_ d1ioaa_ d3n35a_

d3ubra_ d1qo8a1 d1d4ca1 d2k3va_ d1ft5a_ d1qdba_ d1m1qa_ d2j7aa_
d1h21a_ d1ogyb_ d4fasa_ d1oaha_ d1sp3a_ d3h33a_ d1rwja_ d19hca_
d1os6a_ d3bxua_ d3h4na_ d1i77a_ d3caoa_ d1j0pa_ d2ctha_ d1duwa_
d1ofwa_ d2e84a_ d1hh5a_ d1gyoa_ d3h34a_ d1dszb_ d1a6ya_ d3cbba_
d1ynwa1 d1r4ia_ d1hcqa_ d2hana_ d2hanb_ d2enva1 d1kb4b_ d2nllb_
d1lo1a_ d1cita_ d2dara2 d2dara1 d1wyha2 d2dloa2 d1rutx4 d1rutx3
d2cuqa2 d1x4la2 d1x4la1 d1x3ha2 d1x3ha1 d2d8za1 d1x61a2 d1x6aa1
d1x6aa2 d2cora2 d2cora1 d1v6ga1 d1v6ga2 d2d8za2 d1x64a1 d1x64a2
d1b8ta1 d2co8a1 d1x63a2 d2dj7a2 d2dj7a1 d2cu8a2 d2cu8a1 d1g47a2
d1g47a1 d1wyha1 d2dloa1 d1wiga1 d1wiga2 d2cupa2 d2cupa3 d1x68a1
d2d8ya2 d2d8ya1 d1m3va2 d1x62a1 d1x68a2 d1x61a1 d1b8ta3 d1b8ta2
d2d8xa2 d2d8xa1 d1x4ka1 d1x4ka2 d2cuqa1 d1m3va1 d2co8a2 d1j2oa2
d1x63a1 d2cura1 d2cura2 d1x62a2 d1op9a_ d1y0la1 d1etzb1 d1egjl1
d2gsib1 d1nj9a1 d4jfhd1 d1aqkl1 d1t2ja_ d1lo4h1 d1zvya1 d3v4vl1
d4enec1 d1kxtb_ d5e8ea1 d2pxya_ d4weud_ d1xiwc_ d2esvd1 d1qnzh_
d1ogae1 d1f4xh1 d4n1hb_ d3ezjb1 d3mlwl1 d1kfah1 d1etza1 d1b4jh1
d3cfic_ d4w68a_ d2arja1 d1ek3a_ d2ok0l1 d1vera_ d1uwgh1 d1blnb1
d1fpth1 d1rzga1 d2giya1 d4m3kb1 d1nc2a1 d2nyyd1 d1ncna_ d1fbih1
d5dxwa_ d4fqqa1 d1dqdh1 d1jfql1 d1kelh1 d1faih1 d1frgl1 d1figl1
d4m6nl1 d1fo0a_ d4hxbl1 d3b5ga_ d3quyd1 d2h9gb1 d1i8kb_ d2dd8h1
d2ck0h1 d1eaja_ d4w6wb1 d2a6ib3 d1t66d1 d2e27h_ d1ob1b1 d1f11b1
d2v7na1 d1ad0a1 d2xt1b1 d1pskh1 d4i13b1 d2op4l1 d1a0ql1 d1uwel1
d1za3a1 d1ncbl1 d4uu9a_ d1bm3h1 d1cloh1 d1nqba2 d1cf8h1 d4y5va_
d1mhph1 d5anma1 d4cdgc1 d4ldeb_ d1y0lb1 d2pcpb1 d1z3gl1 d1dfbl1
d1fh5h1 d3g5xa1 d1tzib1 d4dn3l1 d3zhka1 d2b1hh1 d2uyla1 d1f3dh1
d1nj9b1 d2agjh1 d2h1pl1 d1q72h1 d8fabb1 d1ndmb1 d1rjca1 d4u6hb1
d3upca_ d2fbjl1 d1osph1 d1tvda_ d2cjvh_ d4fhbd1 d1bd2d1 d1rz7h1
d3nn8a_ d1blna1 d1knoa1 d1nfda1 d2bmkb1 d1dqll_ d1igyb1 d1igfl1
d12e8h1 d3utza1 d1nc2b1 d1rihh1 d4mwfb1 d4jvpa1 d1mieh1 d1rhfa1
d1fgnl1 d1sbsh1 d1lp9e1 d1dn0a1 d4lqfl1 d1iaih1 d1kgcd1 d1mh5b1
d1gpol1 d2g60h3 d1f8tl1 d2jixd1 d4grwh_ d1fo0b_ d1bfob1 d1axsa1
d1u9ka_ d1nmbh_ d1h8na1 d1h8na2 d1jguh1 d4eura1 d3sdyl1 d35c8h1
d1w72l1 d1mvub_ d1i9rh1 d1ob1a1 d4wenb_ d1yeeh1 d3ietc1 d1f58h1
d1ad0b1 d1mqkl_ d1nbvh1 d4b50a_ d3r8bb_ d1bafl1 d1mamh1 d4rgne1
d2gsgb_ d35c8l1 d1bafh1 d1ai1h1 d2pcpa1 d2fath1 d2x1pa_ d1orsb1
d1ibgl1 d1shma_ d1a2yb_ d1u0qa_ d2coqa_ d1hilb1 d1qfwi_ d1jv5b_
d1fn4b1 d5e0qa_ d1nsnh1 d1plgl1 d4ocxl1 d1kb5h1 d1rzfh1 d1pg7x1
d2fx7h1 d2oi9c_ d1l7il1 d3eyva1 d1d5il1 d4ffyl_ d1tetl1 d1a6wh_
d1ac6a_ d3dvfa_ d1nfde1 d1mimh1 d1ri8a1 d1jhlh_ d3dura_ d1fskb1
d1f97a1 d3so3b1 d2ak4d1 d1lp9f1 d4z7wf1 d1l7th1 d4y5ya_ d4x7ec1
d1emth1 d1ikfl1 d1nlbh1 d4utal1 d1bjma1 d1eapb1 d1u6ah1 d2dtgd1
d1kj2b_ d1xeda1 d1c1eh1 d1fgvh_ d2bnub1 d2fb4h1 d2dtgb1 d1ktrh_
d1jpth1 d1qkzl1 d4hepg_ d2atpb1 d1flrh1 d2hrpl1 d1mvfa_ d1iaim1
d3k80a1 d2jb5h1 d3zkxc_ d4grwe_ d1it9h1 d1rhhb1 d3w9ec_ d1e4wh1

d1sy6h1 d2j6eh1 d3qxta_ d1jglh1 d1p7ka1 d1tzhb1 d4n8pa1 d1gafl1
d2jelh1 d1wioa2 d4i0cc1 d1cicb1 d1lmka2 d1lmka1 d1iqwh1 d1orqb1
d1ejoh1 d1jnlh1 d4zg1a_ d43c9b_ d1ggbl1 d1wejh1 d3q6fa1 d1f3dj1
d3c6la1 d1bqlh1 d3p9wb_ d1a3rl1 d1h0db1 d2uzil_ d1eo8l1 d1qfwl_
d1n7ml1 d1igja1 d1c5ch1 d2a9mh_ d1rmfh1 d1fj1b1 d4heme_ d1ccza1
d1a5fh1 d1ogad1 d1dlfh_ d1qa9b_ d1vgel1 d3qskb_ d1sjxa_ d4ozfg1
d1sq2n_ d1qd0a_ d1kcvh1 d1c5db1 d3qcul1 d8faba1 d3bn9d1 d1mjuh1
d4y8dc_ d7fabh1 d3dvgb1 d1lo0l1 d1a6tb1 d1gigh1 d1kcrh1 d2hwzl1
d1yjdc1 d3clfl1 d4krna_ d1dl7l_ d1e6ol1 d1mexh1 d3r0ma_ d4kkna_
d1fnsl1 d1oaqh_ d1rzia1 d1ucbh1 d1ncwl1 d4b41a_ d1nfdf1 d2mpah1
d2d7tl1 d4grwf_ d4b5ea_ d1lk3h1 d3u79a_ d4u3xa_ d1ghfl1 d1bvkb_
d1mcww1 d1b88a_ d1jrhh1 d32c2b1 d4wemb_ d1dsfh_ d1op3h1 d1ce1l1
d1iqda1 d1qoka1 d1qoka2 d1qfuh1 d2crya1 d1ay1h1 d1oaql_ d3ab0b_
d1rurl1 d1t4kb1 d1bz7b1 d1n0xh1 d1forl1 d1ucbl1 d4w6yb_ d43c9a_
d2aabl1 d1tzia1 d4n1ca_ d1k6qh1 d3g9ab_ d4tvsa1 d1igmh_ d1pz5b1
d3dx9a1 d3wxwh_ d1nakh1 d1kcul1 d3eaka_ d4qlra1 d1cr9l1 d1d9ka_
d1f4xl1 d1deea1 d2i27n2 d1c12a1 d2orbl1 d1j8hd1 d1mcph1 d1c5da1
d1kenh1 d1bd2e1 d1h5ba_ d4x7fc1 d3upaa_ d1t3fa1 d1indh1 d2dtgc1
d4hc1l1 d1zv5a1 d4wgvb_ d1uwgl1 d4f37k1 d1aqkh1 d1vesa_ d3zkqd_
d1fptl1 d1lo4l1 d1knob1 d3rrqa_ d1ct8b1 d4orzc_ d1mlbb1 d4hgkc1
d1dqdl1 d1bogb1 d4ozhe1 d1dr9a1 d1i7za1 d1dn0b1 d1kell1 d1rzib1
d1ypzf1 d1wtla_ d1hkfa_ d1fail1 d1figh1 d2vyre_ d1sjva_ d1ohqa_
d1bfoa1 d1pkqb1 d1mjul1 d1b4jl1 d1nsnl1 d2arjb1 d1p4bh_ d4aj0a_
d3nn8b_ d2rcja1 d1f2xk_ d4v1dd_ d1adqh1 d2eksa_ d4k3ga_ d1jfqh1
d1uweh1 d1frgh1 d4gftb_ d1uz8b1 d4n1ea_ d1bm3l1 d1clol1 d3mcka1
d2vq1a1 d1ae6h1 d4aq1b1 d1uj3b1 d1nbqa1 d1fh5l1 d2ck0l1 d2ywza_
d1q72l1 d4nbxb_ d1hila1 d4hzlb1 d2j88l1 d1zmya1 d1cd0a_ d1a4ka1
d1pskl1 d1a0qh1 d4krmb_ d1ncbh1 d2fbjh1 d1j8he1 d1i3ua_ d1cf8l1
d1fe8h1 d2p1ya1 d1eeqa_ d2x89a_ d1ol0a_ d2r0lh1 d1ub5a1 d1dfbh1
d3eyvb1 d4jdvb1 d4p2cg_ d2gsia1 d2agjl1 d1u3ha1 d2h1ph1 d1rihl1
d1fskc1 d1boga1 d2a77l1 d1ospl1 d1qfoa_ d3dwta_ d2fx7l1 d1rz7l1
d3stba1 d1pkqa1 d1b6da1 d1dzba2 d1dzba1 d1kj2a_ d4l1ha_ d1rzgb1
d1dqlh_ d12e8l1 d1miel1 d1jmaa_ d1sbsl1 d1fgnh1 d1f58l1 d1mqkh_
d1ndgb1 d3qegl1 d1iail1 d2aw2a1 d1tqbc1 d3fo2a1 d4dkaa_ d1f8th1
d2g60l1 d2ywya_ d1uj3a1 d1yc7a_ d3bd3a_ d4w6xb1 d1nmbl_ d1i8ka_
d1rjlb1 d1ypze1 d1p7kb1 d1kxqe_ d1gpoh1 d2z4qa1 d1w72h1 d1i9rl1
d4qgya1 d4xt1c_ d2qqnh1 d1cica1 d3c08h1 d2cd0a_ d2bdnh1 d1hnga1
d1maml1 d1nldh1 d1cr9h1 d4lcih1 d3bkyh1 d3qg7l1 d1ai1l1 d1kxvc_
d4iosd_ d1l7ih1 d2x1qa_ d1ub6b1 d3k74b_ d1ibgh1 d1teth1 d1igya1
d1hxma1 d2x6ma_ d2e7la_ d5e03a_ d1qfwm_ d2r0kh1 d1igjb1 d1rurh1
d1plgh1 d1mj8h1 d1yuhb1 d3fmgl1 d1rzfl1 d1emtl1 d1ad9b1 d1igtb1
d1nlbl1 d1d5ih1 d3k7ua_ d4ffyh_ d1bwwa_ d3dvga1 d1a6ta1 d1fgvl_
d4qxgl1 d1miml1 d1jhll_ d2hffb1 d2hh0l1 d1qkzh1 d1l7tl1 d1afvh1
d1iaii1 d2cjuh_ d1um5h1 d2ghwb2 d2ghwb1 d1kgce1 d2fgwh1 d1ikfh1

d1dvfd_ d2i24n1 d2xtjb_ d1za3b1 d2yc1a_ d2hh0h1 d2x1oa_ d1bvka_
d1rjla1 d5e7ba1 d1igta1 d1il1b1 d2fb4l1 d1mvua_ d32c2a1 d1fvca_
d1iqdb1 d2hrph1 d2p4ab_ d4qo1a_ d1p2cb1 d1wt5c_ d1t4ka1 d5c1mb_
d1it9l1 d1bz7a1 d1b2wh1 d1e4wl1 d1jnll1 d1yedb1 d1za6b1 d3u7aa_
d1igfh1 d3gnml1 d1orsa1 d1wejl1 d1a2ya_ d1hxmb1 d1gafh1 d2j4wh1
d1pz5a1 d1j05a_ d1a3rh1 d4fqll1 d1qfwh_ d1eo8h1 d1n7mh1 d4s11a_
d1kegh1 d5da4a_ d1iqwl1 d3whxb1 d1vgeh1 d1deeb1 d1c12b1 d1t2ql1
d1ad9a1 d3gsnb1 d3b2uc1 d1ggbh1 d1t3fb1 d1nakl1 d4ocmc_ d1lila1
d2v7hb3 d2esve1 d1mcoh1 d2qr0b1 d2q3aa_ d1ct8a1 d1kcrl1 d2pxyb_
d2uzih_ d5c3ld_ d3durb_ d1c5cl1 d2ak4e1 d1dl7h_ d1xiwd_ d1mlba1
d1rmfl1 d1i7zb1 d1dlfl_ d2xa3a_ d5bq7b1 d1dqqb1 d3h42l1 d1egjh1
d1kcvl1 d1fe8l1 d2j6el1 d1f3rb2 d1lk3l1 d1sm3h1 d1q0xh1 d1ghfh1
d7fabl1 d1igch1 d1lo0h1 d4idla_ d5c2bl_ d1jrhl1 d1il1a1 d3zhda1
d4kfzc_ d1b0wa_ d1e6oh1 d1mexl1 d4krpb_ d1fnsh1 d1ncwh1 d4hgma_
d4eigb1 d4rzca_ d2mpal1 d4y5xa_ d5en2b1 d3lrga1 d4ej1c1 d1rhha1
d1j05b_ d1forh1 d1q9wb1 d2wzpd_ d2aq2a1 d1h0da1 d3revb1 d2ij0c1
d1tzha1 d4m7jl1 d1k6ql1 d3b9kc1 d1igml_ d3qeua1 d1dsfl_ d1ce1h1
d1kcuh1 d1zvha1 d1zvoa1 d1tqbb1 d1ay1l1 d2qscl1 d2cgrh1 d1nl0h1
d1kenl1 d3eo0a1 d4n9ob1 d1n0xl1 d3k1kc_ d2aq2b1 d1ck1a1 d1enfa1
d1i4pa1 d1v1oa1 d1et9a1 d1esfa1 d2g9hd1 d4ohja1 d1klud1 d1bxta1
d1eu3a1 d1ty0a1 d1fnua1 d2cqva1 d1fhga_ d2k1ma_ d2dl2a1 d1biha3
d1biha1 d1biha4 d3qp3a1 d1g1ca_ d1gxea_ d1cvsc2 d1cvsc1 d1xmwa2
d1m4ka2 d2otpa2 d1biha2 d1rhfa2 d2fcba2 d2fcba1 d1wwbx_ d1gsma2
d1epfa1 d1epfa2 d2dava1 d1waaa_ d1f97a2 d1x44a1 d1vcaa2 d1fnla2
d1fnla1 d3b5ha2 d3h8na2 d3h8na1 d1jbja1 d1jbja2 d1pd6a_ d1f2qa2
d1f2qa1 d3cx2a_ d1nbqa2 d3b5ha1 d1n26a1 d1xiwb_ d1xaua_ d1fltx_
d2ifga1 d1olla1 d1olla2 d1cs6a4 d1cs6a3 d1cs6a2 d1cs6a1 d2gw5a2
d1sy6a1 d1xiwa_ d1ucta2 d1ucta1 d1ry7b2 d1gl4b_ d1qz1a3 d1olza1
d2c9aa1 d1wwca_

**Family-44 (c.1.2.4)**

**Positive dataset contains 12 protein sequences.**

d4lt9a1 d3tc7a1 d3hoja_ d3vnda_ d3nl8a1 d3nava1 d4a2ra1 d3tc6a1
d4ijba_ d1xcfa_ d1rd5a_ d3b5va_

**Negative dataset contains 4001 protein sequences.**

d1m32a_ d1ibja_ d1c7na_ d1iuga_ d4q75a_ d2bkwa1 d1t3ia_ d1n8pa_
d1qz9a_ d1jf9a_ d1eg5a_ d1p3wa_ d3kgwa_ d1cl1a_ d2ctza1 d1vjoa_
d2rfva_ d1gc0a_ d1cs1a_ d1d2fa_ d2ch1a1 d1qgna_ d1pffa_ d1elua_
d1u08a_ d1lc5a_ d1b5pa_ d2ay1a_ d3pd6a_ d7aata_ d2csta_ d1j32a_
d1fg7a_ d4wlha_ d1o4sa_ d1v2da_ d1gdea_ d1m6sa_ d1v72a1 d1svva_
d1ajsa_ d1wsta1 d2f8ja2 d1lk9a_ d1vp4a1 d1xi9a1 d2gb3a1 d1yaaa_
d1js3a_ d3rcha_ d3k40a_ d1xkta1 d2hfka_ d1jmkc_ d5d3ka_ d1ddla_
d1auyb_ d1f56a_ d1bypa_ d1azca_ d1ws8a_ d3u25a_ d1cuoa_ d1id2a_
d1rkra_ d2iaac_ d4dp1x_ d1nwpa_ d2gima_ d2i7sa_ d1qhqa_ d1gyca3
d1hfua2 d1kbva1 d1kbva2 d1sdda1 d1sdda2 d1gyca1 d1gyca2 d1mzya1

d2bw4a1 d1aoza1 d1aoza3 d1aoza2 d1kyaa2 d1kyaa3 d1kyaa1 d1mzya2
d4e9sa1 d1sddb1 d1sddb2 d1hfua1 d1hfua3 d1oe1a2 d1oe1a1 d1v10a2
d1v10a3 d1v10a1 g1f8v.1 d1novc_ d2bbvc_ g1or0.1 g1cp9.1 g1e3a.1
d4qgna_ d1vr3a1 d2bema_ d1pama1 d1ea9c1 d1qhoa1 d2fhfa2 d2fhfa1
d1k3ia1 d1j0ha1 d1cxla1 d2bhua1 d1edqa1 d1f1sa2 d1gvia1 d1txka1
d4alac_ d1pjwa_ d1rera1 d1s6na_ d3uzqb_ d2phla2 d2phla1 d1fxza2
d1fxza1 d1lrha_ d1uija1 g1dgw.1 d2v09a_ d1uika2 d1uika1 d1fstb_
d1ex0a1 d1g0da1 d1v70a_ d2f4pa1 d1yhfa1 d1yloa2 d1y7ea2 d1y0ya3
d1lfwa1 d1vixa1 d1fnoa4 d1vhoa2 d1ysja1 d1vhea2 d1z2la1 d2fvga2
d1vgya1 d1r3na1 d2grea2 d1xmba1 g1q7l.1 d1rtqa_ d1k9ua_ d2opoa_
d1r0ka3 d1vkoa2 d1vm6a2 d3qvsa2 d1gr0a2 d1p1ja2 d2ovga_ d1sq8a_
d1rzsa_ d3kz3a_ d3orca_ d3gpdg2 d2q49a2 d1hdgo2 d2g17a2 d2cvoa2
d2czca1 d3pwka2 d1i32a2 d1zyao2 d2pkqo2 d1b7go2 d2hjsa2 d1cf2o2
d1vkna2 d1nvmb2 d2msya_ d2djoa1 d2r5za_ d1au7a1 d1f43a_ d2lmda1
d1akhb_ d1s7ea1 d3nara_ d1zq3p1 d2craa1 d1mija_ d4uuta_ d3a01a_
d1x58a1 d3rkqa1 d3cmya1 d2cqxa1 d2da6a1 d1e3oc1 d1fjla_ d1mh3a1
d1x41a1 d1wh7a1 d1gt0c1 d2mw8a_ d1lfup_ d1x2ma1 d1b72a_ d1wi3a1
d1jgga_ d1wjha1 d1x2na1 d1mnmc_ d2cuea1 d3k2aa_ d2hosa_ d2jwta2
d1pufa_ d1octc1 d2hi3a_ d1b8ia_ d4j19a_ d3lnqa_ d1wh5a1 d1b8ib_
d1fexa_ d2cqra1 d1guua_ d1ug2a1 d2cqqa1 d2ajea1 d4a69c_ d2crga1
d2iw5b1 d1xc5a1 d1ofcx1 d2cu7a1 d1wgxa1 d2cjja1 d2ckxa1 d1gvda_
d6paxa1 d6paxa2 d1k78a2 d1xg1a1 d1hlva1 d1hlva2 d1iufa2 d1iufa1
d1igna1 d2iu5a1 d1zk8a1 d2g7sa1 d2fd5a1 d2np5a1 d1z0xa1 d2id6a1
d2d6ya1 d2i10a1 d1vi0a1 d2oi8a1 d3c07a1 d2ns7a1 d2fq4a1 d2yvea1
d1t33a1 d2o7ta1 d2np3b1 d2g7ga1 d2gena1 d2fbqa1 d2g7la1 d2gfna1
d2g3ba1 d2jj7a1 d2hyja1 d1sgma1 d1ui5a1 d2hkua1 d2id3a1 d4jyka1
d1bl0a2 d1d5ya1 d1d5ya2 d3lv8a_ d1s3ga1 d1zaka1 d1s4qa_ d3tvta2
d1uf9a_ d1khta_ d1ckea_ d1osna_ d1p5zb_ d4tmka_ d3cm0a_ d1tmka_
d1jaga_ d3h86a_ d1t0hb_ d1nn5a_ d2h92a_ d3kb2a1 d2c95a1 d1yj5a2
d1kgda_ d1ly1a_ d2ak3a1 d1vyva2 d1deka_ d2grja1 d1p3ja1 d1y63a_
d1e4ya1 d1s96a_ d3iija1 d1lvga_ d1nksa_ d1vyua2 d4e22a_ d1qf9a_
d1jjva_ d1q3ta_ d1ki9a_ d1lw7a2 d1teva_ d1kjwa2 d1gsia_ d1x6va3
d1g3ma_ d1j99a_ d1t8ta1 d1fmja_ d1zrha_ d1ls6a_ d2a3ra_ d1aqua_
d2zptx_ d3ckla_ d1texa_ d1q44a_ d2gwha_ d1odfa_ d1uj2a_ d4gi7a_
d2jeoa1 d1z0ja1 d3umra2 d3gj0a1 d2gaoa1 d1yzga1 d1x3sa1 d1g7sa4
d1puja_ d1udxa2 d1kk1a3 d2o52a_ d1jwyb_ d1zcaa2 d1zcba2 d2fv8a1
d2cjwa_ d3dz8a1 d1xzpa2 d1vg8a_ d4qxaa_ d2ew1a1 d1x1ra1 d2bcgy_
d3doea1 d3cona_ d3rega_ d3wyga_ d1upta_ d2b6ha1 d1zd9a1 d1lnza2
d1r5ba3 d1n0ua2 d1r2qa_ d2f9la1 d2rhda_ d3q3jb_ d3q85a_ d1efma1
d1r8qa_ d2gf9a1 d2akab1 d1ek0a_ d1z08a1 d2gcoa1 d2dy1a2 d4g01b_
d2cxxa1 d1svia_ d3q72a_ d1ni3a1 d4ac9a4 d2erxa1 d2bmea_ d1wxqa1
d2gf0a1 d4ldja1 d1zunb3 d1d5ca_ d4lv5b_ d1efca3 d1kmqa_ d2g6ba1
d3lrpa_ d1tada2 d2y8ea_ d4rd4a1 d1z06a1 d2heia_ d3a58b_ d1u0la2
d1d2ea3 d1zj6a1 d2ocba_ d3rwoa_ d1mr3f_ d2e9sa1 d4u5xa1 d2iwra_

| | | | | | | | |
|---|---|---|---|---|---|---|---|
| d2fn4a1 | d3bwdd_ | d2nzja1 | d2atva1 | d2ksqa_ | d3l43a_ | d1mkya2 | d1mkya1 |
| d1wmsa_ | d1jnya3 | d2rmka_ | d1f6ba_ | d1f60a3 | d1gota2 | d1jala1 | d2fh5b1 |
| d1g16a_ | d2wblc_ | d1m7ba_ | d1wf3a1 | d1f5na2 | d1t9ha2 | d1z6xa1 | d1z0aa1 |
| d2fg5a1 | d2a5ja1 | d2bcjq2 | d1s0ua3 | d1ky3a_ | d4y0va_ | d4lhwa_ | d2qmea1 |
| d2atxa1 | d1egaa1 | d1ryha_ | d1moza_ | d1nrjb_ | d2erya1 | d3bfka_ | d1puia_ |
| d3j3az1 | d3t5ga_ | d1shza2 | d3defa_ | d2f7sa1 | d3raba_ | d3tsoa_ | d3aq4a_ |
| d2al7a1 | d3sfva1 | d3bc1a_ | d2ncda_ | d1sdma_ | d3h4sa1 | d2heha_ | d2grya_ |
| g3kin.1 | d1kk8a2 | d1br2a2 | d1goja_ | d1f9va_ | d2mysa2 | d1lkxa_ | d3t0qa_ |
| d4db1a1 | d3gbja_ | d1ry6a_ | d1v8ka_ | d1k8ka2 | d1k8ka1 | d3uleb1 | d3i33a2 |
| d3i33a1 | d1bupa1 | d1dkgd2 | d1dkgd1 | d4ci6a2 | d1jcfa1 | d4a2ba2 | d2fxua1 |
| d1yaga1 | d4cbua2 | d1bupa2 | d1g99a1 | d2e1za2 | d2e1za1 | d1saza1 | d1saza2 |
| d1iqca2 | d1iqca1 | d1eb7a2 | d1eb7a1 | d1nmla2 | d3h1jd1 | d2d0oa3 | d2d0oa2 |
| d1nbwa3 | d1hroa_ | d4gedb_ | d3zooa_ | d1mz4a_ | d1gu2a_ | d1f1fa_ | d1dvva_ |
| d1c6ra_ | d1c75a_ | d1e29a_ | d1gdva_ | d1cnoa_ | d1h32b_ | d1co6a_ | d2bh4x_ |
| d2aiua_ | d5cytr_ | d1i8oa_ | d2zboa_ | d3dmia_ | d1ls9a_ | d1wvec_ | d2yk3a_ |
| d3ph2b_ | d1dw0a_ | d5aura_ | d1ql3a_ | d1qn2a_ | d1f1ca_ | d2nqya_ | d1h4ga_ |
| d1hixa_ | d1oa2a_ | d1olra_ | d3b7ma_ | d4h7ma1 | d3wp3a_ | d2vgda_ | d3zsea_ |
| d2nlra_ | d3wx5a_ | d1oa3a_ | d1pvxa_ | d1m4wa_ | d1f5ja_ | d1oa4a_ | d1ks5a_ |
| d4ixla1 | d2a6za1 | d3a4ua_ | d2a6va1 | d1evsa_ | d1cnt1_ | d1pvhb_ | d1z7ca_ |
| d1huwa_ | d1bgea_ | d1f6fa_ | d1f45b_ | d1i1rb_ | d1axia_ | d4x51a_ | d1d9ca_ |
| d4ypgc1 | d3oq3a_ | d1lqsl_ | d1n1fa_ | d1au1a_ | d1wu3i_ | d3besl_ | d3ux9a_ |
| d1v8fa_ | d2ejca_ | d3muea1 | d1ihoa_ | d3q12a1 | d4dnxa2 | d1v47a2 | d1g8fa2 |
| d1jhda2 | d1x6va2 | d3rbaa_ | d2qtra_ | d1nupa_ | d3l92a_ | d1f9aa_ | d1qjca_ |
| d2h29a_ | d1vlha_ | d1od6a_ | d1lw7a1 | d1mrza2 | d4ruka_ | d2el7a_ | d1iq0a2 |
| d1irxa2 | d1r6ta2 | d1ivsa4 | d2cyba_ | d1rqga2 | d1j09a2 | d2yxna_ | d1f7ua2 |
| d1h3fa1 | d1ffya3 | d2g36a1 | d1nzja_ | d1n3la_ | d1i6la_ | d1pfva2 | d1h3na3 |
| d3n9ia_ | d3sz3a1 | d3sw2a_ | d1moxc_ | d1dx5i3 | d1edmb_ | d1autl2 | d1autl1 |
| d1nqlb_ | d1dval1 | d1szba2 | d1kigl_ | d2y5fl_ | d1ijqa2 | d1gk5a_ | d1g1sa2 |
| d1nt0a3 | d1xdtr_ | d1lmja2 | d1lmja1 | d1nzia2 | d2wphe_ | d1klil_ | d1pfxl2 |
| d1pfxl1 | d1p9ja_ | d1g1ta2 | d1f7ea_ | d4yzub_ | d2y5ia_ | d1qlsa_ | d3n22a_ |
| d2egda_ | d4ggfc_ | d2wcea_ | d2luca_ | d4aqia_ | d3nsia_ | d2lp3a_ | d1j55a_ |
| d1a4pa_ | d1k8ua_ | d1nsha_ | d1pvaa_ | d3fs7a_ | d2pvba_ | d1ttxa_ | d1rwya_ |
| d2kyfa_ | d1rk9a_ | d1a75a_ | d1exra_ | d1tiza1 | d1nyaa_ | d1g8ia_ | d1ggwa_ |
| d1wdcc_ | d3fwba_ | d1ggza_ | d2o5ga_ | d1ctaa_ | d1m45a_ | d3i5gc_ | d1sl8a_ |
| d1fpwa_ | d2scpa_ | d2roaa_ | d4aqra_ | d3pn7b_ | d1br1b_ | d2mysb_ | d1s6ca_ |
| d1bjfa_ | d2obha_ | d1jfka_ | d1jf0a_ | d1qv0a_ | d1lkja_ | d2mlfc_ | d2m97a_ |
| d2d8na_ | d4by5a_ | d1jbaa_ | d4nqga_ | d1wdcb_ | d2mysc_ | d1j7qa_ | d1s6ia_ |
| d1uhka2 | d1r2ua_ | d1dtla_ | d3kf9a_ | d3pn7c_ | d1qjta_ | d1fi6a_ | d1c07a_ |
| d1sfsa_ | d1wkya2 | d1uuqa_ | d3wh9a_ | d4m1ra_ | d1xyza_ | d4xuya_ | d2je8a5 |
| d4bf7a1 | d3cuia_ | d1ur4a_ | d1odza_ | d2vzsa5 | d1bqca_ | d2x2ya1 | d1hjqa_ |
| d1qnra_ | d4y7ea1 | d1vjza_ | d4k68a_ | d2v3ga_ | d4hpga_ | d1nofa2 | d1nq6a_ |
| d1h4pa_ | d1aq0a_ | d3ziza_ | d4bs0a_ | d1ecea_ | d1h1na_ | d1ta3b_ | d1w91a2 |
| d1i1wa_ | d1uhva2 | d1us3a2 | d1egza_ | d7a3ha_ | d1tvna1 | d3nyda1 | d1ghsa_ |
| d2cyga1 | d4xv0a_ | d2c8na2 | d1w32a_ | d3emca_ | d1v6ya1 | d1foba_ | d2uwfa1 |

d2fgla_ d3niya1 d2c0ha1 d1rh9a1 d1yq2a5 d1szna2 d1fa2a_ d1pama4
d5do8a1 d1ea9c3 d1hx0a2 d2fhfa5 d1qhoa4 d1avaa2 d1m53a2 d4m8ua1
d2gjpa2 d1lwha2 d4howa1 d1ktba2 d1j0ha3 d3vm5a1 d2x1ia_ d1wzaa2
d1uasa2 d2diea1 d1mxga2 d1ht6a2 d1eswa_ d1iv8a2 d5cpsa_ d1ud2a2
d1zjaa1 d3gbea1 d1kwga2 d1wdpa_ d1gcya2 d1g5aa2 d1hvxa2 d3dhpa2
d2bhua3 d4do4a1 d3qgva1 d1e43a2 d1g94a2 d2d3na2 d1gjwa2 d1gvia3
d1x1na1 d1r7aa2 d1tz7a1 d4toqa_ d1wb0a1 d1w9pa1 d1itxa1 d1kfwa1
d1goia2 d1edqa2 d2gsja_ d1hjxa1 d1jnda1 d1vf8a1 d3wdpp1 d3wq5a_
d1gnxa_ d4gxpa_ d4hz8a_ d1e4mm_ d3aiua_ d1wcga1 d1qvba_ d3ahxa_
d1v02a_ d2dgaa_ d1pbga_ d1vffa1 d1qoxa_ d4ha4a_ d1t77a2 d1mixa2
d1e5wa2 d1isna2 d2al6b2 d3syxa1 d1mkea1 d2p8va_ d1xoda1 d1i7aa_
d1evha_ d1qc6a_ d1egxa_ d1id1a_ d2hmta_ d1ix2a_ d3nvva1 d1krha3
d2h88b1 d1n62a2 d1t3qa2 d1kf6b2 d1zoyb1 d1ffva2 d1dgja2 d1rm6c2
d1h6ua1 d9ldta1 d2ewda3 d1up7a1 d2dfda1 d1s6ya1 d1vjta1 d7mdha1
d1hyea1 d1t2da1 d1ojsa3 d1b8pa1 d1ldna1 d1hyha1 d1civa1 d2a92a1
d1ez4a1 d1i0za1 d1y6ja1 d1gv0a1 d1ur5a1 d1llda1 d1obba1 d1v8ba1
d1gdha1 d2dlda1 d1mx3a1 d1ygya1 d1qp8a1 d1li4a1 d1j4aa1 d2naca1
d1npya1 d3t4ea2 d1a4ia1 d1o0sa1 d2hk9a2 d2egga2 d1b26a1 d1bvua1
d1gq2a1 d1c1da1 d1leha1 d1gtma1 d1euza1 d1nvta1 d3dona2 d1edza1
d1gpja2 d1p77a1 d1nyta1 d1b0aa1 d1vl6a1 d1v9la1 d1etob_ d1umqa_
d1kola2 d1f8fa2 d1qora2 d1jqba2 d1vj0a2 d1cdoa2 d1gu7a2 d1d1ta2
d1xa0a2 d2dm6a2 d1jvba2 d1llua2 d1h2ba2 d1tt7a2 d1piwa2 d1o89a2
d1iz0a2 d1e3ja2 d1yb5a2 d1uufa2 d1vj1a2 d1e3ia2 d3gpdg1 d1vc2a1
d2nvwa1 d1u8fo1 d1ydwa1 d1hdgo1 d2g17a1 d1cf2o1 d2czca2 d1r0ka2
d1obfo1 d2cvoa1 d1ebfa1 d1h6da1 d1i32a1 d1zh8a1 d2q49a1 d1qkia1
d1j5pa4 d3pzra1 d1xeaa1 d1vkoa1 d1vm6a3 d3qvsa1 d2pkqo1 d1b7go1
d1gr0a1 d1p1ja1 d1lc0a1 d1j0xo1 d3pwka1 d2hjsa1 d1zyao1 d1vkna1
d1tlta1 d1nvmb1 d3sjua_ d3ojfa1 d1xu9a1 d2jjya1 d1n7ha_ d1y1pa1
d1h5qa_ d1jtva_ d4fs3a_ d2bkaa1 d1gy8a_ d2c5aa1 d1ulsa_ d1ooea_
d2b69a1 d1y5ma1 d1spxa_ d1ae1a_ d1bxka_ d1ydea1 d1gega_ d1hdoa_
d1o5ia_ d1e6ua_ d1snya1 d2rhca_ d3zv4a_ d5ceja_ d2d1ya1 d1fjha_
d3w8ea_ d2fmua1 d1x1ta_ d1rpna_ d3ay7a1 d1k2wa_ d1eq2a_ d2hrba1
d3ruaa_ d2ew8a1 d4hwka_ d1xgka_ d2bd0a1 d1yo6a1 d2a35a1 d2ntva_
d1vl8a_ d1hdca_ d4bkua_ d2ag5a1 d2q45a_ d1nffa_ d4iy1a_ d1zbqa1
d1kewa_ d1ek6a_ d1ja9a_ d2q46a2 d1iy8a_ d1cyda_ d2ptga_ d1hxha_
d1uzma1 d3lyla_ d4m89a_ d3wyea_ d2blla1 d1w6ua_ d1sb8a_ d2q2va_
d4nr0a_ d1e6wa_ d2gasa_ d3oiga1 d2bgka1 d1mxha_ d1vl0a1 d1rkxa_
d1r6da_ d1wmaa1 d1e7wa_ d2c07a1 d1xkqa_ d3tzha_ d1g0oa_ d3lz6a_
d1qyca_ d1edoa_ d1gz6a_ d1oc2a_ d1t2aa_ d1zema1 d2xoxa_ d1z45a2
d1geea_ d1p33a_ d3lt0a_ d4e6pa_ d2wyua_ d4q9na_ d1xhla_ d4o1ma_
d1fmca_ d2uvda_ d2gdza1 d1uaya_ d2z1ma_ d2o23a2 d2fr1a2 d2fr1a1
d1qsga_ d1qyda_ d4zjua_ d2a4ka1 d1yb1a_ d1wvga1 d1yxma1 d3f4ba_
d1mg5a_ d1xg5a1 d1r55a1 d3gboa_ d1atla_ d1kufa_ d1buda_ d4q1la_
d1yp1a_ d1wnia_ d1ex0a4 d2f4ma1 d1x3za_ d3i2ka1 d1mpxa2 d1t3ca_

d1epwa3 d2a97a_ d3fiea_ g1e1h.1 d3ioqa_ d1fh0a_ d1o0ea_ d1khqa_
d1m6da_ d2cb5a_ d1cs8a_ g8pch.1 d1s4va_ d2pnsa_ d1vsna1 d1iwda_
d2oula_ d7pcka_ d3bwka_ d3bcna_ d2p86a_ d1pvja_ d1pcia_ d1gece_
d1deua_ d4bs6a_ d1xkga1 d1cqda_ d1jqpa2 d2c0ya_ d2bdza_ d3p5ua_
d2o36a_ d1k9xa_ d1zoya1 d1qo8a2 d1d4ca2 d1chua2 d1jnra2 d2jk6a2
d2jk6a1 d1feca1 d1feca2 d1lpfa1 d1lpfa2 d1xhca1 d1xhca2 d1dxla2
d1dxla1 d1gera2 d1m6ia2 d1onfa1 d1onfa2 d3lada2 d3lada1 d1w4xa1
d1w4xa2 d1gesa2 d1mo9a1 d1xdia1 d1fl2a2 d1fl2a1 d1fcda1 d1fcda2
d1h6va2 d1h6va1 d1mo9a2 d1gv4a2 d1gv4a1 d2gv8a2 d2gv8a1 d1aoga1
d1aoga2 d1hyua1 d1ebda1 d1ebda2 d3dk9a1 d1q1ra2 d1q1ra1 d1gesa1
d1zbfa1 d2z1ha_ d2etja1 d3qioa1 d1jl1a_ d2e4la_ d1jl2a_ d2lsna1
d1io2a_ d1uaxa_ d2yv0x_ d1mu2a1 d1ekea_ d1i39a_ d1o94a2 d1lqta1
d1cjca1 d1reoa1 d1ju2a1 d2f5va1 d3c96a1 d2gmha1 d1pj5a2 d1gpea1
d1o5wa1 d1n4wa1 d1k0ia1 d2dw4a2 d2ivda1 d1seza1 d2voua1 d1kdga1
d1s5ja1 d2hbja2 d2igia_ d1wlja_ d1qhta1 d4dlea1 d1tgoa1 d2f96a1
d3tr8a1 d3mxma_ d1q8ia1 d1y97a1 d1w0ha_ d1kfsa1 d2py5a1 d1noya_
d1d5aa1 d1yt3a3 d2guia_ d3mwpa1 d4kb1a_ d1iv0a_ d1b9ra_ d1offa_
d1frra_ d3wcqa_ d1e9ma_ d1l5pa_ d1wria_ d1i7ha_ d3av8a_ d1czpa_
d1rfka_ d3p1ma1 d3b2ga_ d2bt6a2 d1wgqa1 d1v89a1 d2coda1 d1zc3b_
d1wg7a1 d1u5ea1 d1v61a1 d2coca1 d2ys1a1 d1v5pa1 d1ntya2 d1wjma1
d1wi1a1 d1u29a_ d1xx0a1 d1unqa_ d2zkmx3 d1fhoa_ d1v88a1 d2adza1
d1x1fa1 d1p6sa_ d2coaa1 d1btka_ d1v5ma1 d1faoa_ d2x18a_ d1dyna_
d1x1ga1 d4kaxb_ d2j59m1 d1ki1b2 d1xcga2 d1txda2 d1u5da1 d1dbha2
d1w1ha_ d1upqa_ d1v5ua1 d1foea2 d2fjla1 d1eaza_ d2dfka2 d2cofa1
d1kz7a2 d1jmxa2 d1jmxa1 d1pbya1 d1r7ha_ d1faaa_ d1wika1 d1xfla_
d1ttza1 d2f51a1 d1ti3a_ d1nhoa_ d1sena_ d2i4aa_ d1ep7a_ d1zmaa1
d1fo5a_ d3lefa1 d3diea1 d2yana1 d1z6na1 d1oaza_ d3zywa1 d4aj8a_
d1nm3a1 d4fiwa_ d1wmja_ d4hs1a1 d1iloa_ d1xwaa1 d1wjka1 d2vm1a_
d1fb6a_ d1gh2a_ d1h75a_ d1nw2a_ d1kx5b_ d3a6na_ d4m6ba2 d2nqbd_
d3wkjd_ d2yfwe_ d4h9na_ d5bnxa_ d1kx5c_ d3r45a_ d1eqzb_ d4jjnb_
d1f66c_ d1eqza_ d4wnnb_ d1kx5a_ d1u35c1 d1id3a_ d2nqbc_ d1kx5d_
d3us3a3 d3us3a1 d2b5ea2 d2b5ea3 d2b5ea1 d2b5ea4 d2kp1a1 d2kp2a1
d1hyua3 d1j08a1 d1j08a2 d1pd212 d1nhya2 d2a2ra2 d1k0dd2 d1f2ea2
d1gsua2 d1f3ba2 d2fhea2 d2pvqa1 d1tw9a2 d1oyja2 d1e6ba2 d1k0ma2
d3ik7a1 d4is0a1 d1r5aa2 d1jlva2 d1m0ua2 d3gtub2 d1z9ha2 d1okta2
d1axda2 d2gsra2 d1tu7a2 d1k3ya2 d1zgma1 d1gnwa2 d1gwca2 d2gsta2
d1glqa2 d1oe8a2 d1pn9a2 d1v2aa2 d1fw1a2 d1duga2 d3vrda1 d3vrda2
d1fcdc2 d1fcdc1 d1h1oa2 d1m70a2 d1m70a1 d1ig8a1 d1czan2 d1czan3
d1czan1 d1ig8a2 d1czan4 d1bg3a4 d1bg3a2 d1bg3a1 d1sefa_ d1sfna_
d1rc6a_ d1qqya_ d1gd6a_ d1ivma_ d2gv0a_ d1iiza_ d2z2fa_ d1j8xa_
d1ghla_ d1b9oa_ d174la_ d169la_ d2o4wa_ d1p37a_ d1k28a3 d1xjta1
d1jtma_ d2f2qa_ d4e97a1 d1a3qa1 d1iknc_ d3brda1 d1p7hl1 d1oy3c_
d1imhc1 d1gjia1 d1zk9a1 d2cxka1 d2pjsa1 d2i7ra1 d1ecsa_ d1xqaa1
d5cj3a1 d1npba_ d1jifa_ d2p7oa_ d1r9ca_ d1nkia_ d1mpya2 d1kw3b2

d1kw3b1 d4ghga1 d1tfza1 d3isqa1 d3isqa2 d1sqia1 d1sqia2 d1lkda2
d1lkda1 d1mpya1 d1cjxa1 d1sp9b2 d1t47a1 d1t47a2 d1f1ua2 d1sp8a1
d1sp8a2 d1f1ua1 d1ro5a1 d1s5ja2 d4dlea2 d1tgoa2 d3py8a2 d1q8ia2
d1kfsa2 d2py5a2 d1d5aa2 d1qsta_ d2beia1 d4m3sa_ d1u6ma1 d1yk3a1
d3dr6a_ d1xmta_ d1z4ea1 d2ge3a1 d1bo4a_ d2fiaa1 d2jdca1 d1y9wa1
d3to7a_ d1wwza1 d2fl4a1 d2ozua1 d2b5ga_ d2cy2a1 d2p0wa_ d1cm0a_
d4pswa_ d1yrea1 d1yr0a1 d1m4ia_ d2gana1 d1yvka1 d1ghea_ d2g3aa1
d1y7ra1 d2euia1 d1q2ya_ d2fsra1 d1xeba_ d2ae6a1 d1cjwa_ d1qsma_
d2aj6a1 d1y9ka1 d1i12a_ d1vkca_ d2atra1 d1n71a_ d2d4pa1 d2fiwa1
d2fe7a1 d1tiqa1 d2ou2a_ d1yx0a1 d2fcka1 d1p0ha_ d2j8ma_ d2pq8a_
d1z4ra1 d1s3za1 d1ufha_ d1dosa_ d1gvfa_ d3qm3a1 d2fjka_ d1xvqa1
d1pn0a2 d3hy2a_ d2cvba1 d1o8xa_ d1lu4a_ d1we0a1 d2bmxa1 d2a4va1
d1psqa_ d1xiya1 d1q98a_ d1n8ja_ d1zofa1 d2ggta_ d3tjja_ d4je1a1
d1o73a_ d3s9fa_ d2zcta_ d1qmva_ d1jfua_ d2b1ka_ d5dvba1 d2b5xa1
d2v2ga_ d1st9a_ d3tb2a_ d4llra_ d4fh8a1 d1e2ya_ d1knga_ d2h30a_
d1i5ga_ d2z9sa_ d2f8aa1 d1tp9a1 d2b7ka_ d1nm3a2 d3hvva_ d2wfca1
d4kcea_ d1xvwa1 d4gqca1 d3qpma_ d4k1fa_ d1zzoa1 d1gp1a_ d2qked_
d4ksoa_ d1t4za1 d1wwja_ d4mh8a_ d1mu2a2 d4i7ga1 d1etha2 d1w52x1
d2ppla1 d1hpla2 d1lpbb2 d2oxea1 d1bu8a2 d1rp1a2 d1ys1x_ d4hs9a_
d1ispa_ d1ex9a_ d3qmma_ d1ra6a_ d1muka_ d3gsza_ d1khva_ d1te9a_
d2xwha_ d3uqsa_ d3hkwa_ d3n6ma_ d2cjqa_ d2xyma_ d1teba_ d1te8a_
d1u09a1 d4wfxa_ d3tyqa_ d2pgga1 d1svpa_ d1mbma_ d3su6a1 d1cu1a1
d4agka_ d1vcpa_ d1q31a_ g1n1l.1 d3e90b_ d3u1jb_ d1xwdc1 d3kj4a_
d1w8aa_ d2ifga3 d1p9ag_ d1xkua_ d1g12a_ d3p0ya1 d1igra1 d1igra2
d1n8yc2 d1n8yc1 d1m6ba2 d1m6ba1 d1dzka_ d4tlja_ d2a2ua_ d4wfva_
d3t1da_ d4iaxa_ d3zq3a_ d2nnda_ d1i4ua_ d1kxoa_ d1bbpa_ d1kt7a_
d1exsa_ d3s26a1 d1lf7a_ d1qfta_ d4gh7a_ d1z24a1 d1qabe_ d1xkia_
d1ew3a_ d3bx7a_ d4iawa1 d1n0sa_ d1gkab_ d3kzaa_ d2k23a_ d2l9ca1
d2joza1 d1gm6a_ d3kffa_ d3saoa1 d1e5pa_ d1yupa1 d1iiua_ d1jgta2
d1ea0a3 d1ecfa2 d1q15a2 d1ct9a2 d1ao0a2 d1te5a_ d1ohfc_ d2tbvc_
d1c8nc_ d1x36a_ d1smvc_ d1ng0c_ d4sbvc_ d1opoa_ d1f2nc_ d1cixa_
d1y29a_ d1v7fa_ d2jtba_ d1d1ha_ d1g9pa_ d1lupa_ d1qk7a_ d1mb6a_
d2a2va_ d1niya_ d1i25a_ d1hvwa_ d2mpqa_ d1ie6a_ d1la4a_ d2mxoa1
d1dl0a_ d1koza_ d1qk6a_ d1emxa_ d1lqra_ d1c6wa_ d1rmka_ d1ixta_
d1eyoa_ d1cnna_ d1g1pa_ d1f3ka_ d1fyga_ d1rypd_ d1irua_ d1rypc_
d3unfk_ d1rypa_ d1j2pa_ d1irui_ d1j2qh_ d3unfh_ d1m4ya_ d1rypb_
d1irul_ d1irug_ d1iru2_ d1q5rh_ d1rypg_ d1iruf_ d1q5qa_ d1e94a_
d1irub_ d1ryph_ d3ty6a_ d1iruj_ d1iruk_ d1rype_ d1irud_ d1irue_
d3mi01_ d1rypf_ d1iruh_ d2z3ba_ d1iruc_ d3mka2_ d1iru1_ d1e88a1
d1l6ja5 d1l6ja4 d1eaka4 d1eaka3 d1l6ja3 d1h8pa1 d1ny721 d1ny722
d1a6ca3 d1a6ca2 d1a6ca1 d1pgl22 d1pgl21 d1pgl11 d1ny711 d1mqta_
d1d4m3_ d1fmd3_ d2mev1_ g1aym.1 d1ev13_ d4q4w1_ d1r1a3_ d2x5ia_
d1rhi1_ g1r1a.1 g1d4m.1 g1rhi.1 d1b35b_ d1oopa_ d1tme1_ d4iv1a_
g1pvc.1 g1fpn.1 d1fpn1_ d4gb31_ d1d4m1_ d1fmd1_ d1ev11_ d2wzr3_

| | | | | | | | |
|---|---|---|---|---|---|---|---|
| g1cov.1 | d1qqp3_ | g1fmd.1 | d2x5ic_ | d1rhi3_ | g1oop.1 | d1hxs1_ | g1hxs.1 |
| d1fpn3_ | d1bev1_ | g2mev.1 | d1eah3_ | d1aym1_ | d1pvc3_ | d1qqp1_ | d1zba3_ |
| d1h8ta_ | d3vbhc_ | g1b35.1 | g1bev.1 | d1hxs3_ | g1ev1.1 | d1bev3_ | g1tmf.1 |
| d1tmf1_ | d2mev3_ | g1eah.1 | g1h8t.1 | d1eah1_ | d4q4w3_ | d1r1a1_ | d1aym3_ |
| d1pvc1_ | d1zba1_ | d1h8tc_ | d1oopc_ | d1tme3_ | g1qqp.1 | d4iv1c_ | g1tme.1 |
| d1y14b1 | d2nn6i1 | d1i94l_ | d2zjra2 | d1smxa_ | d2qamc2 | d1hh2p1 | d2r7da1 |
| d1rl2a2 | d1wfqa1 | d3bzka4 | d1x6oa2 | d3a0ja_ | d2j01d2 | d2ja9a1 | d4ql5a_ |
| d1hr0w_ | d1luza_ | d2n3sa_ | d2vnud1 | d1h95a_ | d1go3e1 | d2nn6h1 | d1ne3a_ |
| d2mo1a_ | d1iz6a2 | d1jjga_ | d1e3pa2 | d2ix0a3 | d2ix0a2 | d2lxja_ | d1ueba3 |
| d1ueba2 | d2z0sa1 | d1u0la1 | d2ix0a1 | d2eifa2 | d2je6i1 | d3i2za1 | d2c35b1 |
| d2ahob2 | d1t9ha1 | d3i4oa_ | d2qall1 | d1jt8a_ | d2nn6g1 | d1g6pa_ | d3cama_ |
| d1i94q_ | d1c9oa_ | d1d7qa_ | d2ba0a1 | d2vnud2 | d2vnud3 | d1khia2 | d2qalq1 |
| d1wi5a1 | d1xtda2 | d1ny4a_ | d3ia2a_ | d1a88a_ | d4dgqa1 | d1hkha_ | d1zoia_ |
| d2r80b_ | d1cqxa1 | d1cg5a_ | d3boma_ | d2h8fb_ | d2raoa_ | d3bj1a_ | d4hrrb_ |
| d2r50a_ | d3d1kb_ | d2aa1b1 | d3zhwa_ | d3hf4b_ | d3gdja_ | d1jebb_ | d3bj1b_ |
| d1x9fb_ | d1wmub_ | d1gcva_ | d3bcqb_ | d3d4xb_ | d1gvha1 | d2dc3a_ | d3hf4a_ |
| d4yu3a_ | d3k8ba_ | d1qpwb_ | d2r80a_ | d3bcqa_ | d1v4wa_ | d3wtgb_ | d1jl7a_ |
| d4esaa_ | d1a9we_ | d3d1ab_ | d1fsla_ | d4h2la_ | d1v4wb_ | d1mwca_ | d1vhba_ |
| d1jeba_ | d1spgb_ | d1h97a_ | d1q1fa_ | d1tu9a1 | d3d1aa_ | d1hdsb_ | d3hyub_ |
| d2zfbb_ | d3fs4a_ | d1oj6a_ | d1shrb_ | d4h2lb_ | d4yu3b_ | d1spga_ | d3hrwa_ |
| d3qqra_ | d3at5a_ | d4wchd_ | d3hyua_ | d1cg5b_ | d1uc3a_ | d1hbra_ | d2raob_ |
| d1outb_ | d4esab_ | d3gdjb_ | d3qqqa_ | d1x9fc_ | d3vrfa_ | d1itha_ | d3mkba_ |
| d3d4xa_ | d3mjpa_ | d1jl6a_ | d3fh9a_ | d1outa_ | d2dc3b_ | d1it2a_ | d3eoka_ |
| d3g46a_ | d1qpwa_ | d3vrfb_ | d1ch4a_ | d1x9fd_ | d1hdsa_ | d2zfba_ | d3mkbb_ |
| d1or4a_ | d3wtga_ | d1a4fa_ | d3fh9b_ | d3bomb_ | d3cy5a_ | d3at5b_ | d1x9fa_ |
| d2oifa_ | g1gxs.1 | g1wht.1 | d2xqfa_ | d1ea5a_ | d1f6wa_ | d1dx4a_ | d1qe3a_ |
| d1k4ya_ | d4qwwa_ | d2ha2a_ | d1gkub1 | d1gkub2 | d2qvba_ | d2v9za_ | d4kafa1 |
| d2m5ta_ | d1wqsa_ | d5bpea_ | d2ipha_ | d1lvoa_ | d2bhga1 | d3tnta_ | d3sjia_ |
| d3d23a1 | d2fyqa_ | d3tloa_ | d3zvea1 | d2hrva_ | d3q3ya1 | d4asha1 | d1jmxa3 |
| d1pbya3 | d1pbya4 | d4iuaa3 | d4bvva_ | d5hpga_ | d3k65a_ | d1ki0a3 | d1ki0a2 |
| d1ki0a1 | d1a0ha1 | d1pmla_ | d3sp8a3 | d2feba1 | d1mdwa_ | d1ziva1 | d1zcma1 |
| d1f74a_ | d3l21a_ | d2e1da_ | d1to3a1 | d1w3ia_ | d1sfla_ | d1n7ka_ | d1vcva1 |
| d4imea_ | d1wa3a_ | d2ocza1 | d1f2ja_ | d3m16a_ | d2yxga_ | d1ub3a_ | d3a5fa_ |
| d1fbaa_ | d1l6wa_ | d1xfba1 | d1wx0a1 | d1vpxa1 | d1ojxa_ | d1mxsa_ | d2a4aa1 |
| d1xkya1 | d3cq0a1 | d3s1xa_ | d1f05a_ | d1fdja_ | d3cpra1 | d4d2ja_ | d1m8pa3 |
| d3rp2a_ | g1ucy.1 | d1fuja_ | d2zchp_ | d3w94a_ | d1si5h_ | d1lo6a_ | g2pka.1 |
| d1cgha_ | d1kigh_ | d1a5ia_ | d3s9ba_ | d1hj8a_ | d1ao5a_ | d2anya_ | d1q3xa1 |
| d1gvza_ | d1azza_ | d4gsoa_ | d2f91a1 | d1iaua_ | d1gdna_ | d1eufa_ | g1fiw.1 |
| d1eq9a_ | d4nfea_ | d2rdla_ | d1spja_ | d1elva1 | d1a7sa_ | d1ekbb_ | d1pfxc_ |
| d1yc0a_ | d2aiqa_ | g1fiz.1 | d1fi8a_ | d4k60a_ | d4dgja_ | d1mzaa_ | d1op0a_ |
| d3tjva_ | d1pytd_ | d1sgfg_ | g1gg6.1 | d2eeka_ | g1rtf.1 | d1a0ja_ | d3g01a_ |
| d1pytc_ | d4e7na_ | d1ddja_ | d1z8ga1 | d2hlca_ | d1npma_ | d1brup_ | g1jou.1 |
| d1sgfa_ | d1autc_ | g2hnt.1 | g1kdq.1 | d1orfa_ | d3s69a_ | d4crga_ | d1fxya_ |
| d1bqya_ | d2ftba_ | d1pmpa_ | d2rcqa1 | d1fdqa_ | d4lkpa_ | d1sa8a_ | d1vyfa_ |

d1lpja_ d3vg7a1 d4azqa1 d1o8va_ d5bvqa_ d1p6pa_ d2qo4a_ d1opba_
d1yiva1 d1o1va_ d4bvma1 d4tkba_ d4i3ba_ d1bwya_ d1ftpa_ d4gkca_
d1cbia_ d3p6da1 d1kqwa_ d2a0aa1 d1m4la_ d2bk7a_ d3fjua_ d1z5ra1
d1kwma1 d1uwya2 d2c1ca1 d1dtda_ d1h8la2 d3lgia_ d3i77a_ d2z9ia2
d4nsva_ d1lcya2 d4jcna_ d1os8a_ d1hpga_ d1p3ca_ d1l1ja_ d1qtfa_
d1e69a_ d1mv5a_ d3g60a4 g1ii8.1 d1ewqa2 d1ji0a_ d1qhla_ d1v43a3
d2wmfa1 d1vpla_ d1g2912 d4a82a2 g1f2t.1 d2d62a1 d3tuic1 d2bbsa1
d3g60a3 d2ixea_ d1g6ha_ d1sgwa1 d2qi9c_ d3rlfa1 d3b60a1 d3d31a2
d2pzea1 g1xew.1 d1jj7a_ d1oxxk2 d2onka1 d1w1wa_ d1iwea_ d1qf5a_
d1vmaa2 d1nija1 d1np6a_ d2p67a1 d3ihla_ d1okkd2 d1ls1a2 d1s1ma2
d1iona_ d2v40a_ d1dj3a_ d3hida_ d2vo1a1 d2g0ta1 d1vcoa2 d1j8mf2
d1g3qa_ d1xjca_ d1p9ba_ d1qzxa3 d1ihua2 d1ihua1 d2qm8a_ d1dj2a_
d4ioja_ d1cp2a_ d1yrba2 d3gqba2 d1skye3 d1xp8a1 d1tf7a2 d1tf7a1
d1ye8a1 d1skyb3 d2qe7d2 d4blta_ d4huta_ d3ldaa2 d4hyya_ d2i3ba1
d1cr1a_ d3j9tb2 d3ntua2 d1vdza1 d2z43a_ d1e9ra_ d1nlfa_ d3ziaa2
d1n0wa_ d1g6oa_ d3ziad2 d3j9ta2 d1pzna2 d3gqbb2 d2i49a_ d1nnfa_
d1xvxa_ d1ursa_ d1h3da1 d1iuda_ d4m1va1 d1twya_ d3e13x_ d2nxoa1
d4pfua_ d3ttma_ d1elja_ d2qmwa1 d1us5a_ d1y3na1 d1jeta_ d1uqwa_
d4f19a1 d2esna2 d3thia_ d3fasa1 d2uyeb_ d2xwva1 d1r9la_ d2fyia1
d1pb7a_ d2ozza1 d1sw5a_ d1nh8a1 d1vr5a1 d2g29a_ d1xs5a_ d3lswa_
d2z23a_ d3w9va_ d3tcha_ d1q35a_ d1z7me1 d3q27a_ d1p99a_ d1ve4a1
d2i6ea1 d4mcoa1 d4n8ya_ d1eu8a_ d1zbma1 d1hsla_ d1ixca2 d1j1na_
d1xvya_ d1y9ua_ d2z22a1 d1xoca1 d4oera_ d1wdna_ d5f1qa1 d1xt8a1
d4gwoa_ d4igta1 d1pc3a_ d4mmpa1 d1o63a_ d2czla1 d2onsa2 d4mija1
d4ok5a1 d2bmfa1 d2bmfa2 d1yksa1 d1yksa2 d2qeqa1 d2jlqa1 d1a1va2
d1eh3a_ d3skpa_ d1dtza1 d1dtza2 d1ieja_ d1jnfa2 d1ce2a1 d1jnfa1
d1b1xa2 d1b1xa1 d1jw1a1 d1jw1a2 d1ryoa_ d1h76a1 d1h76a2 d1c4oa1
d2p6ra3 d2p6ra4 d1oywa2 d1oywa3 d1wrba1 d1w36b2 d1w36b1 d1c4oa2
d3bora_ d1t6na_ d2eyqa5 d2eyqa3 d2eyqa2 d2zu6a2 d1veca_ d1uaaa1
d1w36d1 d2g9na1 d1z3ix2 d2rb4a_ d1hv8a1 d1hv8a2 d1t5ia_ d1w36c2
d2j0sa2 d2j0sa1 d1fuka_ d3hjha1 d1z5za1 d1qdea_ d1z63a1 d1s2ma2
d1s2ma1 d1wp9a1 d1wp9a2 d1gm5a3 d1gm5a4 d3dina4 d3dina3 d1q0ua_
d1w36c1 d1z3ix1 d1nkta3 d1nkta4 d1uaaa2 d1w36d2 g1qhh.1 d2fwra2
d2fwra1 d3q3ma_ d1za0a1 d1mxra_ d2inca_ d1jk0a_ d2incb_ d1mhyb_
d3hf1b_ d1r2fa_ d2uw1a_ d1mhyd_ d3n37a_ d1afra_ d1oquc_ d1syya_
d1mtyd_ d2o1za_ d1uzra_ d1smsa_ d2fkza_ d1zuja1 d1s3qa1 d2fjca1
d1z6om1 d1veia_ d4dyya_ d1jiga_ d3gvya_ d2pyba_ d4cy9a1 d3bvfa1
d1r03a_ d2cf7a_ d1nf4a_ d1zs3a1 d4iwka_ d3ka8a_ d3np0x_ d1n1qa_
d1lb3a_ d4e6ka_ d1z6oa1 d2fzfa1 d4dyua_ d3ge4a_ d1jgca_ d4am4a_
d2z6ma_ d1ji5a_ d1vlga_ d1lkoa1 d1krqa_ d3qz3a_ d1o9ra_ d2ffxj_
d1yv1a1 d2v2pa_ d1j30a_ d4di0a_ d1dpsa_ d1oewa_ d2qzxa_ d2asia_
d1psoe_ d1miqa_ d1smra_ d2h6ta_ d1pfza_ d1izda_ d3hd8a_ d1j71a_
d1dpja_ d4aa9a_ d1t6ex_ g1b5f.1 g1lya.1 d1qdma2 d1bxoa_ d1wkra_
d1tzsa_ d1ibqa_ d2qzwa_ d2v76a_ d1wj1a1 d1aqca_ d1qqga2 d1qqga1

d1ntva_ d1n3ha_ d4dbba_ d3f0wa1 d1p3ra_ d1wgua1 d2cy4a1 d3hqca_
d1wvha1 d1xr0b1 d1bdqa_ d3ggta_ d3ixoa_ d1mrxa_ d2haha_ d1tw7a_
d4njsa_ d1sgua_ d2p3da_ d1nsoa_ d3d3ta_ d2rkfa_ d1c6ya_ d2rspa_
d3ogpa_ d3ekwa_ d2o4na_ d2j9ja_ d1baia_ d3ggua_ d3lzua_ d4npua_
d4j55a_ d2r8na_ d2fxda_ d2p3ca_ d4dqea_ d3ttpa_ d3t3ca_ d2p3aa_
d2hc0a_ d2fdda_ d3mwsa_ d1um8a_ d1ixza_ d1qvra2 d1qvra3 d1qzma_
d1sxjc2 d1jr3d2 d2p65a_ d1l8qa2 d3hu3a3 d1sxjd2 d1njfa_ d1in4a2
d1sxja2 d1tuea1 d2ce7a2 d1w5sa2 d1sxjb2 d1d2na_ d1ny5a2 d1ofha_
d2gnoa2 d2a5yb3 d1fnna2 d1u0ja1 d1lv7a_ d1iqpa2 d2fnaa2 d1ixsb2
d4gita_ d1sxje2 d1g41a_ d1k8rb_ d1wgra1 d1ef5a_ d1wfya1 d1e8ya3
d1wgya1 d1lfda_ d2cs4a1 d1wxma1 d2byea1 d3kh0a1 d2byfa1 d1wxaa1
d1e7ua3 d4yl8a1 d2al6a3 d1wgda1 d1wgga1 d4q5eb_ d1wjna1 d1se9a_
d3n3kb_ d1wz0a1 d1we6a1 d2m0xa_ d2gbka_ d3rt3b1 d1wh3a1 d1sifa_
d2ekec2 d4ii2b1 d4hk2a1 d1c3ta_ d1m94a_ d1wgha1 d1ttna1 d1j8ca_
d1wx8a1 d3v6cb1 d2ma9b_ d4i6lb1 d1bt0a_ d2jvca1 d1iyfa_ d2k1fa_
d1wxva1 d1wf9a1 d1t0ya_ d2io0b2 d1x1ma1 d2gbma_ d1zw7a1 d2m17a_
d1ud7a_ d1we7a1 d2lwpa_ d2gbja_ d1wjua1 d1v6ea1 d1v86a1 d4eewa_
d1wiaa1 d4icva_ d4gu2a_ d1wx7a1 d1wy8a1 d4hjka1 d2faza1 d1v5oa1
d2al3a1 d2cr5a1 d1wj4a1 d1s3sg_ d3ubra_ d1qo8a1 d1d4ca1 d2k3va_
d1ft5a_ d1qdba_ d1m1qa_ d2j7aa_ d1h21a_ d1ogyb_ d4fasa_ d1jnia_
d1oaha_ d1sp3a_ d1g66a_ d5ajha1 d3h33a_ d1rwja_ d19hca_ d1os6a_
d3bxua_ d3h4na_ d3caoa_ d1j0pa_ d2ctha_ d1duwa_ d1ofwa_ d2e84a_
d1hh5a_ d1gyoa_ d3h34a_ d1wioa4 d1ccza2 d1vcaa1 d1dr9a2 d1ic1a1
d2d9qb1 d2nxyb2 d1adqa1 d1fnga1 d1uvqb1 d1zs8a1 d2vola1 d2vola2
d1mfbl2 d2jelh2 d1uwel2 d3qq4b1 d1kjvb_ d1k5na1 d1osph2 d4odhl2
d1ow0a1 d1ow0a2 d1bfob2 d3l9ra2 d3jtth1 d2fath2 d4elkb2 d1fn4b2
d2xfxb_ d1pg7x2 d1cqka_ d4k71b2 d1lk2a1 d1eapb2 d4lfhg2 d1i4fa1
d2nw2a2 d1kfah2 d1mhca1 d1h3ua1 d1wejh2 d1h0db2 d1a5fh2 d1kcvh2
d1mjuh2 d1qqda1 d1kcrh2 d1k8ia1 d3huja2 d2nnab1 d1nfdf2 d3mffb2
d1s9va1 d1lk3h2 d1q0xl2 d1fv1b1 d1neza1 d4rfeb2 d1r3ha1 d1a6tb2
d1kjva1 d1pz5b2 d1onqa1 d2bc4a2 d1de4a1 d1k8da1 d1tcra2 d1dn0b2
d1nfde2 d2xwtb2 d1bfoa2 d1mexh2 d1iakb1 d1ypzf2 d4f7ea2 d1q72l2
d2fbjh2 d1fe8h2 d1clzh2 d1fngb1 d1uvqa1 d1vgka1 d1c16a1 d4j12a2
d4x99b2 d1igtb3 d1je6a1 d1q72h2 d1nldh2 d1teth2 d2hmic2 d1i1ca2
d1i1ca1 d1k8ib1 d1q0yh2 d1kfal2 d3frua1 d1pg7w2 d1u58a1 d1igyb4
d1igyb3 d1hyrc1 d1klub1 d1deeb2 d1c12b2 d2bc4b2 d2ig2h2 d1kcvl2
d3wn5b1 d1lk2b_ d1lk3l2 d1mjul2 d1sm3h2 d1q0xh2 d1zvsa1 d1ydpa1
d1u8ib2 d1ypze2 d1kcuh2 d2e3fa_ d1mm0a_ d2e3ea_ d2llda_ d2ny8x_
d2e3ga_ d2ny9x_ d2nz3a_ d2lj7a_ d1n4na_ d2m8bb1 d2kyqa_ d3psma_
d1jkza_ d1jxca_ d2gl1a_ d1ugla_ d4uj0a_ d2k4ua_ d1hp2a_ d2lu9a_
d1baha_ d2meoa1 d1wm7a_ d1hlya_ d1n8ma_ d1jlza_ d1wpda_ d1c55a_
d1pvza_ d1wt7a_ d1rjia_ d1wz5a_ d1m2sa_ d1pjva_ d1c49a_ d1qkya_
d2ktca1 d1wmta_ d1j5ja_ d1r1ga_ d1t0za_ d2i61a_ d1t1ta_ d1sn4a_
d1kv0a_ d2lkba_ d1npia_ d1wwna_ d1pe4a_ d2kbka_ d2li7a_ d1djta_

d2kbha_ d1dq7a_ d1sega_ d1omya_ d1zywa1 d1jzaa_ d1t61c1 d1iyjb4
d1iyjb5 d1iyjb3 d1o7ia_ d4ipca1 d1x3ea_ d4apva_ d1jb7a1 d1jb7a2
d1jb7a3 d3kdfa_ d1ue1b_ d1wjja1 d3eiva_ d1xjva2 d1xjva1 d2ccza2
d1se8a_ d1l1oc_ d1jb7b_ d1qvca_ d3ulla_ d1qzga_ d1miua4 d1miua5
d1miua3 d3g48a1 d1gd7a_ d1mkha_ d1jjcb3 d1pyba_ d3ubub_ d1egia_
d1v7pa_ d1tdqb_ d3ubua_ d1c3ab_ d1h8ua_ d3bx4b_ d1t8ca1 d1sb2a1
d1qdda_ d2ziba1 d3cada_ d1byfa_ d1j34b_ d1jwia_ d1jzna_ d1c3aa_
d1oz7b_ d1wmza_ d3bdwb_ d1hq8a_ d1fifa1 d1dv8a_ d3hupa_ d1wk1a1
d1umra_ d2afpa_ d3bx4a_ d1gz2a_ d1sl6a_ d1v7pb_ d3g8ka_ d1ixxa_
d1j34a_ d1jwib_ d1g1sa1 d1y17a1 d1ja3a_ d1fvub_ d3bdwa_ d1sb2b1
d1fvua_ d1umrc_ d1oz7a_ d4m17a_ d3cfwa1 d1ypqa1 d1g1ta1 d3fd4a_
d3l9jc_ d4dn8a1 d1v4lb_ d1rdl1_ d2msba_ d1dgsa2 d1ckma1 d1p16a1
d1x9na2 d3zxha_ d1cgla_ d1rm8a_ d1eaka2 d1hy7a_ d2jsda_ d1cxva_
d1hv5a_ d1i76a_ d1rpma_ d2shpa1 d1yfoa_ d1l8ka_ d2ooqa1 d1g4us2
d1lara2 d1lara1 d1wcha_ d2fh7a2 d2fh7a1 d1ohea1 d1ohea2 d2f4da_
d1rxda_ d2hxpa_ d1xria_ d1d5ra2 d1fpza_ d3ezza_ d4y2ea_ d1i9sa_
d3azya_ d3wvja_ d1upsa1 d1ajoa_ d2hyka_ d1ajka_ d2vy0a_ d1o4ya_
d3o5sa_ d3d6ea_ d3atga_ d1umza_ d3i4ia_ d1c1la_ d4lbqa_ d1a78a_
d1slta_ d4no4a_ d3zsja_ d3duia_ d1g86a_ d1is3a_ d1qmja_ d1hlca_
d1tdha3 d1q3ba3 d1ee8a3 g1qmo.1 g1len.1 d3zyra_ d1fnya_ d1n47a_
g2ltn.1 d1avba_ d3usua_ d1dhkb_ d1f9ka_ d1ukga_ d1g8wa_ d1g9fa_
d1wbfa_ d3wcsa_ d1g7ya_ d1mvqa_ d1hqla_ d1v6ia_ d1dbna_ d1qnwa_
d1fx5a_ d4l8qa_ d1ioaa_ d3n35a_ d1epwa1 d2h0ba_ d2erfa_ d1h30a2
d3mw4a1 d1pz7a_ d2r1ba_ d3v64a1 d1d2sa_ d3bopa1 d3mw3a1 d1okqa1
d1okqa2 d1dszb_ d1a6ya_ d3cbba_ d1ynwa1 d1r4ia_ d2hana_ d2hanb_
d2enva1 d1kb4b_ d2nllb_ d1lo1a_ d1cita_ d2dara2 d2dara1 d1wyha2
d2dloa2 d1rutx4 d1rutx3 d2cuqa2 d1x4la2 d1x4la1 d1x3ha2 d1x3ha1
d2d8za1 d1x61a2 d1x6aa1 d1x6aa2 d2cora2 d2cora1 d1v6ga1 d1v6ga2
d2d8za2 d1x64a1 d1x64a2 d1b8ta1 d2co8a1 d1x63a2 d2dj7a2 d2dj7a1
d2cu8a2 d2cu8a1 d1g47a2 d1g47a1 d1j2oa1 d1wyha1 d2dloa1 d1wiga1
d1wiga2 d2cupa2 d2cupa3 d1x68a1 d2d8ya2 d2d8ya1 d1m3va2 d1x62a1
d1x68a2 d1x61a1 d1b8ta3 d1b8ta2 d2d8xa2 d2d8xa1 d1x4ka1 d1x4ka2
d2cuqa1 d1m3va1 d2co8a2 d1x63a1 d2cura1 d2cura2 d1x62a2 d1op9a_
d1y0la1 d1etzb1 d1egjl1 d2gsib1 d1nj9a1 d4jfhd1 d1aqkl1 d1t2ja_
d1lo4h1 d1zvya1 d3v4vl1 d4enec1 d1kxtb_ d5e8ea1 d2pxya_ d4weud_
d1xiwc_ d2esvd1 d1qnzh_ d1ogae1 d1f4xh1 d4n1hb_ d3ezjb1 d3mlwl1
d1kfah1 d1etza1 d1b4jh1 d3cfic_ d4w68a_ d2arja1 d1ek3a_ d2ok0l1
d1vera_ d1uwgh1 d1blnb1 d1fpth1 d1rzga1 d2giya1 d4m3kb1 d1nc2a1
d2nyyd1 d1ncna_ d1fbih1 d5dxwa_ d4fqqa1 d1dqdh1 d1jfql1 d1kelh1
d1faih1 d1frgl1 d1figl1 d4m6nl1 d1fo0a_ d4hxbl1 d3b5ga_ d3quyd1
d2h9gb1 d1i8kb_ d2dd8h1 d2ck0h1 d1eaja_ d4w6wb1 d2a6ib3 d1t66d1
d2e27h_ d1ob1b1 d1f11b1 d2v7na1 d1ad0a1 d2xt1b1 d1pskh1 d4i13b1
d2op4l1 d1a0ql1 d1uwel1 d1za3a1 d1ncbl1 d4uu9a_ d1bm3h1 d1cloh1
d1nqba2 d1cf8h1 d4y5va_ d1mhph1 d5anma1 d4cdgc1 d4ldeb_ d1y0lb1

| | | | | | | | |
|---|---|---|---|---|---|---|---|
| d2pcpb1 | d1z3gl1 | d1dfbl1 | d1fh5h1 | d3g5xa1 | d1tzib1 | d4dn3l1 | d3zhka1 |
| d2b1hh1 | d2uyla1 | d1f3dh1 | d1nj9b1 | d2agjh1 | d2h1pl1 | d1q72h1 | d8fabb1 |
| d1ndmb1 | d1rjca1 | d4u6hb1 | d3upca_ | d2fbjl1 | d1osph1 | d1tvda_ | d2cjvh_ |
| d4fhbd1 | d1bd2d1 | d1rz7h1 | d3nn8a_ | d1blna1 | d1knoa1 | d1nfda1 | d2bmkb1 |
| d1dqll_ | d1igyb1 | d1igfl1 | d12e8h1 | d3utza1 | d1nc2b1 | d1rihh1 | d4mwfb1 |
| d4jvpa1 | d1mieh1 | d1rhfa1 | d1fgnl1 | d1sbsh1 | d1lp9e1 | d1dn0a1 | d4lqfl1 |
| d1iaih1 | d1kgcd1 | d1mh5b1 | d1gpol1 | d2g60h3 | d1f8tl1 | d2jixd1 | d4grwh_ |
| d1fo0b_ | d1bfob1 | d1axsa1 | d1u9ka_ | d1nmbh_ | d1h8na1 | d1h8na2 | d1jguh1 |
| d4eura1 | d3sdyl1 | d35c8h1 | d1w72l1 | d1mvub_ | d1i9rh1 | d1ob1a1 | d4wenb_ |
| d1yeeh1 | d3ietc1 | d1f58h1 | d1ad0b1 | d1mqkl_ | d1nbvh1 | d4b50a_ | d3r8bb_ |
| d1bafl1 | d1mamh1 | d4rgne1 | d2gsgb_ | d35c8l1 | d1bafh1 | d1ai1h1 | d2pcpa1 |
| d2fath1 | d2x1pa_ | d1orsb1 | d1ibgl1 | d1shma_ | d1a2yb_ | d1u0qa_ | d2coqa_ |
| d1hilb1 | d1qfwi_ | d1jv5b_ | d1fn4b1 | d5e0qa_ | d1nsnh1 | d1plgl1 | d4ocxl1 |
| d1kb5h1 | d1rzfh1 | d1pg7x1 | d2fx7h1 | d2oi9c_ | d1l7il1 | d3eyva1 | d1d5il1 |
| d4ffyl_ | d1tetl1 | d1a6wh_ | d1ac6a_ | d3dvfa_ | d1nfde1 | d1mimh1 | d1ri8a1 |
| d1jhlh_ | d3dura_ | d1fskb1 | d1f97a1 | d3so3b1 | d2ak4d1 | d1lp9f1 | d4z7wf1 |
| d1l7th1 | d4y5ya_ | d4x7ec1 | d1emth1 | d1ikfl1 | d1nlbh1 | d4utal1 | d1bjma1 |
| d1eapb1 | d1u6ah1 | d2dtgd1 | d1kj2b_ | d1xeda1 | d1c1eh1 | d1fgvh_ | d2bnub1 |
| d2fb4h1 | d2dtgb1 | d1ktrh_ | d1jpth1 | d1qkzl1 | d4hepg_ | d2atpb1 | d1flrh1 |
| d2hrpl1 | d1mvfa_ | d1iaim1 | d3k80a1 | d2jb5h1 | d3zkxc_ | d4grwe_ | d1it9h1 |
| d1rhhb1 | d3w9ec_ | d1e4wh1 | d1sy6h1 | d2j6eh1 | d3qxta_ | d1jglh1 | d1p7ka1 |
| d1tzhb1 | d4n8pa1 | d1gafl1 | d2jelh1 | d1wioa2 | d4i0cc1 | d1cicb1 | d1lmka2 |
| d1lmka1 | d1iqwh1 | d1orqb1 | d1ejoh1 | d1jnlh1 | d4zg1a_ | d43c9b_ | d1ggbl1 |
| d1wejh1 | d3q6fa1 | d1f3dj1 | d3c6la1 | d1bqlh1 | d3p9wb_ | d1a3rl1 | d1h0db1 |
| d2uzil_ | d1eo8l1 | d1qfwl_ | d1n7ml1 | d1igja1 | d1c5ch1 | d2a9mh_ | d1rmfh1 |
| d1fj1b1 | d4heme_ | d1ccza1 | d1a5fh1 | d1ogad1 | d1dlfh_ | d1qa9b_ | d1vgel1 |
| d3qskb_ | d1sjxa_ | d4ozfg1 | d1sq2n_ | d1qd0a_ | d1kcvh1 | d1c5db1 | d3qcul1 |
| d8faba1 | d3bn9d1 | d1mjuh1 | d4y8dc_ | d7fabh1 | d3dvgb1 | d1lo0l1 | d1a6tb1 |
| d1gigh1 | d1kcrh1 | d2hwzl1 | d1yjdc1 | d3clfl1 | d4krna_ | d1dl7l_ | d1e6ol1 |
| d1mexh1 | d3r0ma_ | d4kkna_ | d1fnsl1 | d1oaqh_ | d1rzia1 | d1ucbh1 | d1ncwl1 |
| d4b41a_ | d1nfdf1 | d2mpah1 | d2d7tl1 | d4grwf_ | d4b5ea_ | d1lk3h1 | d3u79a_ |
| d4u3xa_ | d1ghfl1 | d1bvkb_ | d1mcww1 | d1b88a_ | d1jrhh1 | d32c2b1 | d4wemb_ |
| d1dsfh_ | d1op3h1 | d1ce1l1 | d1iqda1 | d1qoka1 | d1qoka2 | d1qfuh1 | d2crya1 |
| d1ay1h1 | d1oaql_ | d3ab0b_ | d1rurl1 | d1t4kb1 | d1bz7b1 | d1n0xh1 | d1forl1 |
| d1ucbl1 | d4w6yb_ | d43c9a_ | d2aabl1 | d1tzia1 | d4n1ca_ | d1k6qh1 | d3g9ab_ |
| d4tvsa1 | d1igmh_ | d1pz5b1 | d3dx9a1 | d3wxwh_ | d1nakh1 | d1kcul1 | d3eaka_ |
| d4qlra1 | d1cr9l1 | d1d9ka_ | d1f4xl1 | d1deea1 | d2i27n2 | d1c12a1 | d2orbl1 |
| d1j8hd1 | d1mcph1 | d1c5da1 | d1kenh1 | d1bd2e1 | d1h5ba_ | d4x7fc1 | d3upaa_ |
| d1t3fa1 | d1indh1 | d2dtgc1 | d4hc1l1 | d1zv5a1 | d4wgvb_ | d1uwgl1 | d4f37k1 |
| d1aqkh1 | d1vesa_ | d3zkqd_ | d1fptl1 | d1lo4l1 | d1knob1 | d3rrqa_ | d4orzc_ |
| d1mlbb1 | d4hgkc1 | d1dqdl1 | d1bogb1 | d4ozhe1 | d1dr9a1 | d1i7za1 | d1dn0b1 |
| d1kell1 | d1rzib1 | d1ypzf1 | d1wtla_ | d1hkfa_ | d1fail1 | d1figh1 | d2vyre_ |
| d1sjva_ | d1ohqa_ | d1bfoa1 | d1pkqb1 | d1mjul1 | d1b4jl1 | d1nsnl1 | d2arjb1 |
| d1p4bh_ | d4aj0a_ | d3nn8b_ | d2rcja1 | d1f2xk_ | d4v1dd_ | d1adqh1 | d2eksa_ |

d4k3ga_ d1jfqh1 d1uweh1 d1frgh1 d4gftb_ d1uz8b1 d4n1ea_ d1bm3l1
d1clol1 d3mcka1 d2vq1a1 d1ae6h1 d4aq1b1 d1uj3b1 d1nbqa1 d1fh5l1
d2ck0l1 d2ywza_ d1q72l1 d4nbxb_ d1hila1 d4hzlb1 d2j88l1 d1zmya1
d1cd0a_ d1a4ka1 d1pskl1 d1a0qh1 d4krmb_ d1ncbh1 d2fbjh1 d1j8he1
d1i3ua_ d1cf8l1 d1fe8h1 d2p1ya1 d1eeqa_ d2x89a_ d1ol0a_ d2r0lh1
d1ub5a1 d1dfbh1 d3eyvb1 d4jdvb1 d4p2cg_ d2gsia1 d2agjl1 d1u3ha1
d2h1ph1 d1rihl1 d1fskc1 d1boga1 d2a77l1 d1ospl1 d1qfoa_ d3dwta_
d2fx7l1 d1rz7l1 d3stba1 d1pkqa1 d1b6da1 d1dzba2 d1dzba1 d1kj2a_
d4l1ha_ d1rzgb1 d1dqlh_ d12e8l1 d1miel1 d1jmaa_ d1sbsl1 d1fgnh1
d1f58l1 d1mqkh_ d1ndgb1 d3qegl1 d1iail1 d2aw2a1 d1tqbc1 d3fo2a1
d4dkaa_ d1f8th1 d2g60l1 d2ywya_ d1uj3a1 d1yc7a_ d3bd3a_ d4w6xb1
d1nmbl_ d1i8ka_ d1rjlb1 d1ypze1 d1p7kb1 d1kxqe_ d1gpoh1 d2z4qa1
d1w72h1 d1i9rl1 d4qgya1 d4xt1c_ d2qqnh1 d1cica1 d3c08h1 d2cd0a_
d2bdnh1 d1hnga1 d1maml1 d1nldh1 d1cr9h1 d4lcih1 d3bkyh1 d3qg7l1
d1ai1l1 d1kxvc_ d4iosd_ d1l7ih1 d2x1qa_ d1ub6b1 d3k74b_ d1ibgh1
d1teth1 d1igya1 d1hxma1 d2x6ma_ d2e7la_ d5e03a_ d1qfwm_ d2r0kh1
d1igjb1 d1rurh1 d1plgh1 d1mj8h1 d1yuhb1 d3fmgl1 d1rzfl1 d1emtl1
d1ad9b1 d1igtb1 d1nlbl1 d1d5ih1 d3k7ua_ d4ffyh_ d1bwwa_ d3dvga1
d1a6ta1 d1fgvl_ d4qxgl1 d1miml1 d1jhll_ d2hffb1 d2hh0l1 d1qkzh1
d1l7tl1 d1afvh1 d1iaii1 d2cjuh_ d1um5h1 d2ghwb2 d2ghwb1 d1kgce1
d2fgwh1 d1ikfh1 d1dvfd_ d2i24n1 d2xtjb_ d1za3b1 d2yc1a_ d2hh0h1
d2x1oa_ d1bvka_ d1rjla1 d5e7ba1 d1igta1 d1il1b1 d2fb4l1 d1mvua_
d1jptl1 d32c2a1 d1fvca_ d1iqdb1 d2hrph1 d2p4ab_ d4qo1a_ d1p2cb1
d1wt5c_ d1t4ka1 d5c1mb_ d1it9l1 d1bz7a1 d1b2wh1 d1e4wl1 d1jnll1
d1yedb1 d1za6b1 d3u7aa_ d1igfh1 d3gnml1 d1orsa1 d1wejl1 d1a2ya_
d1hxmb1 d1gafh1 d2j4wh1 d1pz5a1 d1j05a_ d1a3rh1 d4fqll1 d1qfwh_
d1eo8h1 d1n7mh1 d4s11a_ d1kegh1 d5da4a_ d1iqwl1 d3whxb1 d1vgeh1
d1deeb1 d1c12b1 d1t2ql1 d1ad9a1 d3gsnb1 d3b2uc1 d1ggbh1 d1t3fb1
d1nakl1 d4ocmc_ d1lila1 d2v7hb3 d2esve1 d1mcoh1 d2qr0b1 d2q3aa_
d1ct8a1 d1kcrl1 d2pxyb_ d2uzih_ d5c3ld_ d3durb_ d1c5cl1 d2ak4e1
d1dl7h_ d1xiwd_ d1mlba1 d1rmfl1 d1i7zb1 d1dlfl_ d2xa3a_ d5bq7b1
d1dqqb1 d3h42l1 d1egjh1 d1kcvl1 d1fe8l1 d2j6el1 d1f3rb2 d1lk3l1
d1sm3h1 d1q0xh1 d1ghfh1 d7fabl1 d1igch1 d1lo0h1 d4idla_ d5c2bl_
d1jrhl1 d1il1a1 d3zhda1 d4kfzc_ d1b0wa_ d1e6oh1 d1mexl1 d4krpb_
d1fnsh1 d1ncwh1 d4hgma_ d4eigb1 d4rzca_ d2mpal1 d4y5xa_ d5en2b1
d3lrga1 d4ej1c1 d1rhha1 d1j05b_ d1forh1 d1q9wb1 d2wzpd_ d2aq2a1
d1h0da1 d3revb1 d2ij0c1 d1tzha1 d4m7jl1 d1k6ql1 d3b9kc1 d1igml_
d3qeua1 d1dsfl_ d1ce1h1 d1kcuh1 d1zvha1 d1zvoa1 d1tqbb1 d1ay1l1
d2qscl1 d2cgrh1 d1nl0h1 d1kenl1 d3eo0a1 d4n9ob1 d1n0xl1 d3k1kc_
d1c4qa_ d1prtc1 d2wv6d_ d1ltrd_ d4l63b1 d1r4pb_ d1prtd_ d1tl0f_
d2bosa_ d1prtb1 d3chbd_ d2aq2b1 d1ck1a1 d1enfa1 d1i4pa1 d1v1oa1
d1et9a1 d1esfa1 d2g9hd1 d4ohja1 d1klud1 d1bxta1 d1eu3a1 d1ty0a1
d1fnua1 d2qaln1 d2cqva1 d1fhga_ d2k1ma_ d2dl2a1 d1biha3 d1biha1
d1biha4 d3qp3a1 d1g1ca_ d1gxea_ d1cvsc2 d1cvsc1 d1xmwa2 d1m4ka2

d2otpa2 d1biha2 d1rhfa2 d2fcba2 d2fcba1 d1wwbx_ d1gsma2 d1epfa1 d1epfa2 d2dava1 d1waaa_ d1f97a2 d1x44a1 d1vcaa2 d1fnla2 d1fnla1 d3b5ha2 d3h8na2 d3h8na1 d1jbja1 d1jbja2 d1pd6a_ d1f2qa2 d1f2qa1 d3cx2a_ d1nbqa2 d3b5ha1 d1n26a1 d1xiwb_ d1xaua_ d1fltx_ d2ifga1 d1olla1 d1olla2 d1cs6a4 d1cs6a3 d1cs6a2 d1cs6a1 d2gw5a2 d1sy6a1 d1xiwa_ d1ucta2 d1ucta1 d1ry7b2 d1gl4b_ d1qz1a3 d1olza1 d2c9aa1 d1wwca_

**Family-45 (c.1.8.1)**

**Positive dataset contains 21 protein sequences.**

d1szna2 d5do8a1 d2fhfa5 d4m8ua1 d2gjpa2 d4howa1 d3vm5a1 d2x1ia_ d1wzaa2 d2diea1 d5cpsa_ d1zjaa1 d3gbea1 d1wdpa_ d3dhpa2 d2bhua3 d4do4a1 d3qgva1 d2d3na2 d1x1na1 d1tz7a1

**Negative dataset contains 3963 protein sequences.**

d1m32a_ d1ibja_ d1c7na_ d1iuga_ d4q75a_ d2bkwa1 d1t3ia_ d1n8pa_ d1qz9a_ d1jf9a_ d1eg5a_ d3kgwa_ d1cl1a_ d2ctza1 d1vjoa_ d2rfva_ d1gc0a_ d1cs1a_ d1d2fa_ d2ch1a1 d1qgna_ d1pffa_ d1elua_ d1c7ga_ d2v1pa1 d2ez1a_ d1u08a_ d1lc5a_ d2ay1a_ d1bw0a_ d3pd6a_ d7aata_ d2csta_ d1j32a_ d1fg7a_ d4wlha_ d1o4sa_ d1v2da_ d1gdea_ d1m6sa_ d1v72a1 d1svva_ d1ajsa_ d1wsta1 d2f8ja2 d1lk9a_ d1vp4a1 d1xi9a1 d2gb3a1 d1yaaa_ d1xkla_ d2wfla_ d3i2ka1 d1lnsa3 d2cfba1 d1dfoa_ d1bj4a_ d3k28a_ d2bwpa_ d3lg0a_ d2fnua_ d3ou5a_ d1z7da1 d1b9ha_ d1iaya_ d4msoa_ d1bs0a_ d1s0aa_ d1sffa_ d3tqxa_ d1mdoa_ d3qboa_ d3pgya_ d1bjna_ d2dkja_ d1ejia_ d1vefa1 d4wxga_ d3bs8a_ d3gbxa1 d1w23a_ d1h6ua2 d2tbvc_ d1c8nc_ d1x36a_ d1smvc_ d1ng0c_ d4sbvc_ d1opoa_ d3lgia_ d3i77a_ d2z9ia2 d4nsva_ d1lcya2 d4jcna_ d1agja_ d1os8a_ d1hpga_ d1p3ca_ d1l1ja_ d1qtfa_ d1ddla_ d1auyb_ d1qnia1 d1ibya_ d1f56a_ d1bypa_ d1azca_ d1ws8a_ d3u25a_ d1cuoa_ d1id2a_ d1rkra_ d2iaac_ d4dp1x_ d1nwpa_ d2gima_ d2i7sa_ d1qhqa_ d3ehbb1 d1fftb1 d1gyca3 d1hfua2 d1kbva1 d1kbva2 d1sdda1 d1sdda2 d1gyca1 d1gyca2 d1mzya1 d2bw4a1 d1aoza1 d1aoza3 d1aoza2 d1kyaa2 d1kyaa3 d1kyaa1 d1mzya2 d4e9sa1 d1sddb1 d1sddb2 d1hfua1 d1hfua3 d1oe1a2 d1oe1a1 d1v10a2 d1v10a3 d1v10a1 d1novc_ d2bbvc_ d1a3qa1 d1iknc_ d3brda1 d1oy3c_ d1imhc1 d1gjia1 d1zk9a1 d2cxka1 d2bema_ d1pama1 d1ea9c1 d1qhoa1 d2fhfa2 d2fhfa1 d1k3ia1 d1j0ha1 d1cxla1 d2bhua1 d1edqa1 d1f1sa2 d1ut9a2 d1gvia1 d1txka1 d4alac_ d1pjwa_ d1rera1 d1s6na_ d3uzqb_ d2phla2 d2phla1 d1fxza2 d1fxza1 d1lrha_ d1uija1 g1dgw.1 d2v09a_ d1uika2 d1uika1 d1dgwa_ d2pa7a1 d1wlta1 d1nxma_ d2c0za1 d1tafa_ d1bh9b_ d1tafb_ d1q9ca_ d1h3ob_ d2byka1 d1n1ja_ d4g92b_ d1bh9a_ d1jfia_ d1h3oa_ d2bykb1 d1n1jb_ d1au7a2 d1e3oc2 d2ovga_ d1sq8a_ d1rzsa_ d3kz3a_ d3orca_ d3gpdg2 d2q49a2 d1hdgo2 d2g17a2 d2cvoa2 d2czca1 d3pwka2 d1i32a2 d1zyao2 d2pkqo2 d1b7go2 d2hjsa2 d1cf2o2 d1vkna2 d1nvmb2 d1ux8a_ d1dlya_ d4i0va_ d2bkma_ d1dlwa_ d2bmma_ d2msya_ d2djoa1 d2r5za_ d1au7a1 d1f43a_ d2lmda1 d1akhb_ d1s7ea1 d3nara_ d1zq3p1 d2craa1 d1mija_ d4uuta_ d3a01a_

d1x58a1 d3rkqa1 d3cmya1 d2cqxa1 d2da6a1 d1e3oc1 d1fjla_ d1ig7a_
d1x41a1 d1wh7a1 d1gt0c1 d2mw8a_ d1lfup_ d1x2ma1 d1b72a_ d1wi3a1
d1jgga_ d1wjha1 d1x2na1 d1mnmc_ d2cuea1 d3k2aa_ d2hosa_ d2jwta2
d1pufa_ d1octc1 d2hi3a_ d1b8ia_ d4j19a_ d3lnqa_ d1wh5a1 d1b8ib_
d1fexa_ d2cqra1 d1guua_ d1ug2a1 d2cqqa1 d2ajea1 d4a69c_ d1h8ac1
d2crga1 d2iw5b1 d1xc5a1 d1ofcx1 d2cu7a1 d1wgxa1 d2cjja1 d2ckxa1
d1gvda_ d1hcra_ d1tc3c_ d1gdta1 d1u78a2 d6paxa1 d6paxa2 d1k78a1
d2iu5a1 d1zk8a1 d2g7sa1 d2fd5a1 d2np5a1 d1z0xa1 d2id6a1 d2d6ya1
d2i10a1 d2oi8a1 d3c07a1 d2ns7a1 d2fq4a1 d2yvea1 d1t33a1 d2o7ta1
d1rkta1 d2np3b1 d2g7ga1 d2gena1 d2fbqa1 d2g7la1 d2gfna1 d2g3ba1
d2jj7a1 d2hyja1 d1sgma1 d1ui5a1 d2hkua1 d2id3a1 d4jyka1 d3ubub_
d1egia_ d1v7pa_ d1tdqb_ d3ubua_ d1c3ab_ d1h8ua_ d3bx4b_ d1t8ca1
d1sb2a1 d1qdda_ d2ziba1 d3cada_ d1byfa_ d1j34b_ d1jwia_ d1jzna_
d1c3aa_ d1oz7b_ d1wmza_ d3bdwb_ d1hq8a_ d1fifa1 d1dv8a_ d3hupa_
d1wk1a1 d1umra_ d2afpa_ d3bx4a_ d1gz2a_ d1sl6a_ d1v7pb_ d3g8ka_
d1ixxa_ d1j34a_ d1jwib_ d1g1sa1 d1y17a1 d1ja3a_ d1fvub_ d3bdwa_
d1sb2b1 d1fvua_ d1umrc_ d1oz7a_ d4m17a_ d3cfwa1 d1ypqa1 d1g1ta1
d3fd4a_ d3l9jc_ d4dn8a1 d1v4lb_ d1rdl1_ d2msba_ d3lv8a_ d1s3ga1
d1zaka1 d1s4qa_ d3tvta2 d1uf9a_ d1khta_ d1ckea_ d1osna_ d1p5zb_
d4tmka_ d3cm0a_ d1jaga_ d3h86a_ d1t0hb_ d1nn5a_ d2h92a_ d3kb2a1
d2c95a1 d1yj5a2 d1kgda_ d1ly1a_ d2ak3a1 d1vyva2 d1deka_ d2grja1
d1p3ja1 d1y63a_ d1e4ya1 d1s96a_ d3iija1 d1lvga_ d1nksa_ d1vyua2
d4e22a_ d1qf9a_ d1jjva_ d1q3ta_ d1ki9a_ d1lw7a2 d1teva_ d1kjwa2
d1gsia_ d2iyva2 d1kaga_ d1viaa_ d1e6ca_ d1x6va3 d1g3ma_ d1j99a_
d1t8ta1 d1nsta_ d1zrha_ d1ls6a_ d2a3ra_ d1aqua_ d2zptx_ d3ckla_
d1texa_ d1q44a_ d2gwha_ d1odfa_ d1uj2a_ d4gi7a_ d2jeoa1 d1z0ja1
d3umra2 d3gj0a1 d2gaoa1 d1yzga1 d1x3sa1 d1g7sa4 d1puja_ d1udxa2
d1kk1a3 d2o52a_ d1jwyb_ d1zcaa2 d1zcba2 d2fv8a1 d2cjwa_ d3dz8a1
d1xzpa2 d1vg8a_ d4qxaa_ d2ew1a1 d1x1ra1 d2bcgy_ d3doea1 d3cona_
d3rega_ d3wyga_ d1upta_ d2b6ha1 d1zd9a1 d1r5ba3 d1n0ua2 d1r2qa_
d2f9la1 d2rhda_ d3q3jb_ d3q85a_ d1efma1 d1r8qa_ d2gf9a1 d2akab1
d1ek0a_ d1z08a1 d2gcoa1 d2dy1a2 d4g01b_ d2cxxa1 d1svia_ d3q72a_
d1ni3a1 d4ac9a4 d2erxa1 d2bmea_ d1wxqa1 d2gf0a1 d4ldja1 d1zunb3
d1d5ca_ d4lv5b_ d1efca3 d1kmqa_ d2g6ba1 d3lrpa_ d1tada2 d2y8ea_
d4rd4a1 d1z06a1 d2heia_ d3a58b_ d1u0la2 d1d2ea3 d1zj6a1 d2ocba_
d3rwoa_ d1mr3f_ d2e9sa1 d4u5xa1 d2iwra_ d2fn4a1 d3bwdd_ d2nzja1
d2atva1 d2ksqa_ d3l43a_ d1mkya2 d1mkya1 d1wmsa_ d1jnya3 d2rmka_
d1f6ba_ d1f60a3 d1gota2 d1jala1 d2fh5b1 d1g16a_ d2wblc_ d1m7ba_
d1wf3a1 d1f5na2 d1t9ha2 d1z6xa1 d1z0aa1 d2fg5a1 d2a5ja1 d2bcjq2
d1s0ua3 d1ky3a_ d4y0va_ d4lhwa_ d2qmea1 d2atxa1 d1egaa1 d1ryha_
d1moza_ d1nrjb_ d2erya1 d3bfka_ d1puia_ d1fzqa_ d3j3az1 d3t5ga_
d1shza2 d3defa_ d2f7sa1 d3raba_ d3tsoa_ d3aq4a_ d2al7a1 d3sfva1
d3bc1a_ d2ncda_ d1sdma_ d3h4sa1 d2heha_ d2grya_ g3kin.1 d1kk8a2
d1br2a2 d1goja_ d1f9va_ d2mysa2 d3t0qa_ d4db1a1 d3gbja_ d1ry6a_

| | | | | | | | |
|---|---|---|---|---|---|---|---|
| d1v8ka_ | d1k8ka2 | d1k8ka1 | d3uleb1 | d3i33a2 | d3i33a1 | d1bupa1 | d1dkgd2 |
| d1dkgd1 | d4ci6a2 | d1jcfa1 | d4a2ba2 | d2fxua1 | d1yaga2 | d1yaga1 | d4cbua2 |
| d1bupa2 | d1ig8a1 | d1czan2 | d1czan3 | d1czan1 | d1ig8a2 | d1czan4 | d1bg3a4 |
| d1bg3a3 | d1bg3a2 | d3h3no1 | d3h3no2 | d3ezwa2 | d3h1jd1 | d1hroa_ | d4gedb_ |
| d3zooa_ | d1mz4a_ | d1gu2a_ | d1f1fa_ | d1dvva_ | d1c6ra_ | d1c75a_ | d1e29a_ |
| d1gdva_ | d1cnoa_ | d1h32b_ | d1co6a_ | d2bh4x_ | d2aiua_ | d5cytr_ | d1jdla_ |
| d1i8oa_ | d2zboa_ | d3dmia_ | d1ls9a_ | d1wvec_ | d2yk3a_ | d3ph2b_ | d1dw0a_ |
| d5aura_ | d1ql3a_ | d1qn2a_ | d1f1ca_ | d4zzva_ | d2xspa_ | d1eg1a_ | d4v20a_ |
| d1ojja_ | d4ipma1 | d1gpia_ | d4csia_ | d3pfza_ | d3ovwa_ | d2yoka_ | d2nqya_ |
| d1h4ga_ | d1hixa_ | d1oa2a_ | d1olra_ | d3b7ma_ | d4h7ma1 | d3wp3a_ | d2vgda_ |
| d1igoa_ | d3zsea_ | d2nlra_ | d3wx5a_ | d1oa3a_ | d1pvxa_ | d1m4wa_ | d1f5ja_ |
| d1oa4a_ | d1ks5a_ | d4ixla1 | d3sita1 | d1evsa_ | d1cnt1_ | d1pvhb_ | d1z7ca_ |
| d1huwa_ | d1bgea_ | d1rhga_ | d1f6fa_ | d1f45b_ | d1i1rb_ | d1axia_ | d1etea_ |
| d3tgxb_ | d2z3qa1 | d3qb1a_ | d1hula_ | d1m48a_ | d4nkqb_ | d1eera_ | d1v7mv_ |
| d5e4ea_ | d2o27a_ | d3b5ka_ | d2psma1 | d4zf7a_ | d3uf5a_ | d4x51a_ | d1d9ca_ |
| d4ypgc1 | d3oq3a_ | d1lqsl_ | d1n1fa_ | d1au1a_ | d1wu3i_ | d3besl_ | d3ux9a_ |
| d4dnxa2 | d1m8pa2 | d1v47a2 | d1jhda2 | d1x6va2 | d3rbaa_ | d2qtra_ | d1nupa_ |
| d3l92a_ | d1f9aa_ | d1qjca_ | d2h29a_ | d1vlha_ | d1od6a_ | d1lw7a1 | d1mrza2 |
| d4ruka_ | d2el7a_ | d1iq0a2 | d1irxa2 | d1r6ta2 | d1ivsa4 | d2cyba_ | d1rqga2 |
| d1j09a2 | d2yxna_ | d1f7ua2 | d1h3fa1 | d1ffya3 | d2g36a1 | d1jila_ | d1n3la_ |
| d1i6la_ | d1pfva2 | d1h3na3 | d3n9ia_ | d3sz3a1 | d3sw2a_ | d1moxc_ | d1edmb_ |
| d1autl2 | d1autl1 | d1nqlb_ | d1dval1 | d1szba2 | d1kigl_ | d2y5fl_ | d1ijqa2 |
| d1gk5a_ | d1g1sa2 | d1nt0a3 | d1xdtr_ | d1lmja2 | d1lmja1 | d1nzia2 | d2wphe_ |
| d1klil_ | d1pfxl2 | d1pfxl1 | d1p9ja_ | d1g1ta2 | d1f7ea_ | d4yzub_ | d1qx2a_ |
| d1ig5a_ | d2y5ia_ | d1qlsa_ | d1psra_ | d3n22a_ | d2egda_ | d4ggfc_ | d2wcea_ |
| d2luca_ | d4aqia_ | d3nsia_ | d2lp3a_ | d1j55a_ | d1a4pa_ | d1k8ua_ | d1nsha_ |
| d1pvaa_ | d3fs7a_ | d1ttxa_ | d1rwya_ | d2kyfa_ | d1rk9a_ | d1a75a_ | d1exra_ |
| d1tiza1 | d1nyaa_ | d1g8ia_ | d1ggwa_ | d1wdcc_ | d3fwba_ | d1ggza_ | d2o5ga_ |
| d1ctaa_ | d1m45a_ | d3i5gc_ | d1sl8a_ | d1fpwa_ | d2scpa_ | d2roaa_ | d4aqra_ |
| d3pn7b_ | d1br1b_ | d2mysb_ | d1s6ca_ | d1bjfa_ | d2obha_ | d1jfka_ | d1jf0a_ |
| d1qv0a_ | d1lkja_ | d2mlfc_ | d2m97a_ | d2d8na_ | d4by5a_ | d1jbaa_ | d4nqga_ |
| d1wdcb_ | d2mysc_ | d1j7qa_ | d1s6ia_ | d1uhka2 | d1r2ua_ | d1dtla_ | d3kf9a_ |
| d3pn7c_ | d1fi6a_ | d1c07a_ | d5d67d_ | d1tuza_ | d1h8ba_ | d1eg3a2 | d2zkmx1 |
| d1snla_ | d1y1xa_ | d1hqva_ | d1dvia_ | d1df0a1 | d1qxpa2 | d4usla_ | d1k94a_ |
| d1etob_ | d1umqa_ | d1g2ha_ | d1mixa2 | d1e5wa2 | d1j19a2 | d1isna2 | d2al6b2 |
| d3syxa1 | d1mkea1 | d2p8va_ | d1xoda1 | d1i7aa_ | d1evha_ | d1qc6a_ | d1egxa_ |
| d1ix2a_ | d3nvva1 | d1krha3 | d2h88b1 | d1n62a2 | d1t3qa2 | d1kf6b2 | d1zoyb1 |
| d1ffva2 | d1dgja2 | d1rm6c2 | d1h6ua1 | d9ldta1 | d2ewda3 | d1up7a1 | d2dfda1 |
| d1s6ya1 | d1vjta1 | d7mdha1 | d1hyea1 | d1t2da1 | d1ojsa3 | d1b8pa1 | d1ldna1 |
| d1hyha1 | d1civa1 | d2a92a1 | d1ez4a1 | d1i0za1 | d1y6ja1 | d1gv0a1 | d1ur5a1 |
| d1llda1 | d1obba1 | d1v8ba1 | d1gdha1 | d1l7da1 | d2dlda1 | d1mx3a1 | d1ygya1 |
| d1qp8a1 | d1j4aa1 | d2naca1 | d1txga2 | d3f47a1 | d1mv8a2 | d2pv7a2 | d1xc2a3 |
| d2ahra2 | d4xiya1 | d2f1ka2 | d1pgja2 | d1i36a2 | d1np3a2 | d1lj8a4 | d1teaa3 |
| d1qmga2 | d2i76a2 | d2g5ca2 | d1wdka3 | d3cuma3 | d1kola2 | d1f8fa2 | d1qora2 |

d1jqba2 d1vj0a2 d1cdoa2 d1gu7a2 d1d1ta2 d1xa0a2 d2dm6a2 d1jvba2
d1rjwa2 d1llua2 d1h2ba2 d1tt7a2 d1piwa2 d1o89a2 d1iz0a2 d1e3ja2
d1yb5a2 d1uufa2 d1e3ia2 d3gpdg1 d1vc2a1 d2nvwa1 d1u8fo1 d1ydwa1
d1hdgo1 d2g17a1 d1cf2o1 d2czca2 d1r0ka2 d1obfo1 d2cvoa1 d1ebfa1
d1h6da1 d1i32a1 d1zh8a1 d2q49a1 d1qkia1 d1j5pa4 d3pzra1 d1xeaa1
d1vkoa1 d1vm6a3 d3qvsa1 d2pkqo1 d1b7go1 d1gr0a1 d1p1ja1 d1lc0a1
d3pwka1 d2hjsa1 d1zyao1 d1vkna1 d1tlta1 d1nvmb1 d3sjua_ d3ojfa1
d1xu9a1 d2jjya1 d1n7ha_ d1y1pa1 d1h5qa_ d1jtva_ d4fs3a_ d2bkaa1
d1gy8a_ d2c5aa1 d1ulsa_ d1ooea_ d2b69a1 d1y5ma1 d1spxa_ d1ae1a_
d1bxka_ d1ydea1 d1gega_ d1hdoa_ d1o5ia_ d1e6ua_ d1snya1 d2rhca_
d3zv4a_ d5ceja_ d2d1ya1 d1fjha_ d3w8ea_ d2fmua1 d1x1ta_ d1rpna_
d3ay7a1 d1k2wa_ d1eq2a_ d2hrba1 d3ruaa_ d2ew8a1 d4hwka_ d1xgka_
d2bd0a1 d1yo6a1 d2a35a1 d2ntva_ d1vl8a_ d1hdca_ d4bkua_ d2ag5a1
d2q45a_ d1nffa_ d4iy1a_ d1zbqa1 d1kewa_ d1ek6a_ d1ja9a_ d2q46a2
d1iy8a_ d1cyda_ d2ptga_ d1hxha_ d1uzma1 d3lyla_ d4m89a_ d3wyea_
d2blla1 d1w6ua_ d1sb8a_ d2q2va_ d4nr0a_ d1e6wa_ d2gasa_ d3oiga1
d2bgka1 d1mxha_ d1vl0a1 d1rkxa_ d1r6da_ d1wmaa1 d1e7wa_ d2c07a1
d1xkqa_ d3tzha_ d1g0oa_ d3lz6a_ d1qyca_ d1edoa_ d1gz6a_ d1oc2a_
d1t2aa_ d1zema1 d2xoxa_ d1z45a2 d1geea_ d1p33a_ d3lt0a_ d4e6pa_
d2wyua_ d1db3a_ d4q9na_ d1xhla_ d4o1ma_ d1fmca_ d2uvda_ d2gdza1
d1uaya_ d2z1ma_ d2o23a2 d2fr1a2 d2fr1a1 d1qsga_ d1qyda_ d4zjua_
d2a4ka1 d1yb1a_ d1wvga1 d1yxma1 d3f4ba_ d1mg5a_ d1xg5a1 d1r55a1
d3gboa_ d1atla_ d1kufa_ d1buda_ d4q1la_ d1yp1a_ d1wnia_ d2cqva1
d1fhga_ d2k1ma_ d2dl2a1 d1biha3 d1biha1 d1biha4 d3qp3a1 d1g1ca_
d1gxea_ d1cvsc2 d1cvsc1 d1xmwa2 d1m4ka2 d2otpa2 d1biha2 d1rhfa2
d2fcba2 d2fcba1 d1wwbx_ d1gsma2 d1epfa1 d1epfa2 d2dava1 d1waaa_
d1f97a2 d1x44a1 d1vcaa2 d1fnla2 d1fnla1 d3b5ha2 d3h8na2 d3h8na1
d1jbja1 d1jbja2 d1pd6a_ d1f2qa2 d1f2qa1 d3cx2a_ d1nbqa2 d3b5ha1
d1n26a1 d1xiwb_ d1xaua_ d1fltx_ d2ifga1 d1olla1 d1olla2 d1cs6a4
d1cs6a3 d1cs6a2 d1cs6a1 d2gw5a2 d1sy6a1 d1xiwa_ d1ucta2 d1ucta1
d1ry7b2 d1gl4b_ d1qz1a3 d1olza1 d2c9aa1 d1wwca_ d3nqyb_ d4gera_
d1bqba_ d1ex0a4 d2f4ma1 d1x3za_ d1t3ca_ d1epwa3 d2a97a_ d3fiea_
g1e1h.1 d3ioqa_ d1fh0a_ d1o0ea_ d1khqa_ d1m6da_ d2cb5a_ d1cs8a_
g8pch.1 d1s4va_ d2pnsa_ d1vsna1 d1mira_ d1iwda_ d2oula_ d7pcka_
d3bwka_ d3bcna_ d2p86a_ d1pvja_ d1pcia_ d1gece_ d1deua_ d4bs6a_
d1xkga1 d1cqda_ d1jqpa2 d2c0ya_ d2bdza_ d3p5ua_ d2o36a_ d1zoya1
d1qo8a2 d1d4ca2 d1chua2 d1jnra2 d2jk6a2 d2jk6a1 d1feca1 d1feca2
d1lpfa1 d1lpfa2 d1xhca1 d1xhca2 d1dxla2 d1dxla1 d1gera2 d1m6ia2
d1onfa1 d1onfa2 d3lada2 d3lada1 d1w4xa1 d1w4xa2 d1gesa2 d1mo9a1
d1xdia1 d1fl2a2 d1fl2a1 d1fcda1 d1fcda2 d1h6va2 d1h6va1 d1mo9a2
d1gv4a2 d1gv4a1 d2gv8a2 d2gv8a1 d1aoga1 d1aoga2 d1hyua1 d1ebda1
d1ebda2 d3dk9a1 d1q1ra2 d1q1ra1 d1gesa1 d1w5qa_ d2c1ha_ d1ohla_
d1l6sa_ d1zbfa1 d2z1ha_ d2etja1 d3qioa1 d1jl1a_ d2e4la_ d1jl2a_
d2lsna1 d1io2a_ d1uaxa_ d2yv0x_ d1mu2a1 d1ekea_ d1i39a_ d1pn0a1

d1reoa1 d1ju2a1 d2f5va1 d3c96a1 d2gmha1 d1pj5a2 d1gpea1 d1o5wa1
d1n4wa1 d1k0ia1 d2dw4a2 d2ivda1 d1seza1 d2voua1 d1s5ja1 d2hbja2
d2igia_ d1wlja_ d1qhta1 d4dlea1 d1tgoa1 d2f96a1 d3tr8a1 d3mxma_
d1q8ia1 d1y97a1 d1w0ha_ d1kfsa1 d2py5a1 d1noya_ d1d5aa1 d1yt3a3
d2guia_ d3mwpa1 d4kb1a_ d2d5ra1 d1b9ra_ d1offa_ d1frra_ d3wcqa_
d1e9ma_ d1wria_ d1i7ha_ d3av8a_ d1czpa_ d1rfka_ d3p1ma1 d3b2ga_
d2bt6a2 d1v58a1 d1t3ba1 d4ilfa2 d4npba2 d1wgqa1 d1v89a1 d2coda1
d1zc3b_ d1wg7a1 d1u5ea1 d1v61a1 d2coca1 d2ys1a1 d1v5pa1 d1ntya2
d1wjma1 d1wi1a1 d1u29a_ d1xx0a1 d1unqa_ d2zkmx3 d1fhoa_ d1v88a1
d2adza1 d1x1fa1 d1p6sa_ d2coaa1 d1btka_ d1v5ma1 d2x18a_ d1dyna_
d1x1ga1 d4kaxb_ d2j59m1 d1ki1b2 d1xcga2 d1txda2 d1u5da1 d1dbha2
d1w1ha_ d1upqa_ d1v5ua1 d1foea2 d2fjla1 d1eaza_ d2dfka2 d2cofa1
d1kz7a2 d1r7ha_ d1faaa_ d1wika1 d1xfla_ d1ttza1 d2f51a1 d1ti3a_
d1nhoa_ d1sena_ d2i4aa_ d1ep7a_ d1zmaa1 d1fo5a_ d3lefa1 d3diea1
d2yana1 d1z6na1 d1oaza_ d3zywa1 d4aj8a_ d1nm3a1 d4fiwa_ d1wmja_
d4hs1a1 d1iloa_ d1xwaa1 d1wjka1 d2vm1a_ d1fb6a_ d1gh2a_ d1h75a_
d1nw2a_ d1kx5b_ d3a6na_ d4m6ba2 d2nqbd_ d3wkjd_ d2yfwe_ d4h9na_
d5bnxa_ d1kx5c_ d3r45a_ d1eqzb_ d4jjnb_ d1f66c_ d1eqza_ d4wnnb_
d1kx5a_ d1u35c1 d1id3a_ d2nqbc_ d1kx5d_ d2b5ea2 d2b5ea3 d2b5ea1
d2b5ea4 d2kp1a1 d2kp2a1 d1hyua3 d1hyua4 d1j08a2 d1pd212 d1nhya2
d2a2ra2 d1k0dd2 d1f2ea2 d1gsua2 d1f3ba2 d2fhea2 d2pvqa1 d1tw9a2
d1oyja2 d1e6ba2 d1k0ma2 d3ik7a1 d4is0a1 d1r5aa2 d1jlva2 d1m0ua2
d3gtub2 d1z9ha2 d1okta2 d1axda2 d2gsra2 d1tu7a2 d1k3ya2 d1zgma1
d1b48a2 d1gnwa2 d1gwca2 d2gsta2 d1glqa2 d1oe8a2 d1pn9a2 d1v2aa2
d1fw1a2 d1duga2 d1nira1 d1npya1 d3t4ea2 d1a4ia1 d1o0sa1 d2hk9a2
d2egga2 d1b26a1 d1bvua1 d1gq2a1 d1c1da1 d1leha1 d1gtma1 d1euza1
d3dona2 d1edza1 d1gpja2 d1vi2a1 d1p77a1 d1nyta1 d1b0aa1 d1vl6a1
d1v9la1 d1qusa_ d1qqya_ d1gd6a_ d1ivma_ d2gv0a_ d1iiza_ d2z2fa_
d1j8xa_ d1ghla_ d1b9oa_ d1iyka2 d1iyka1 d1rxta1 d1iica1 d2k4ua_
d1hp2a_ d2lu9a_ d1baha_ d2meoa1 d1wm7a_ d1hlya_ d1n8ma_ d1jlza_
d1wpda_ d1c55a_ d1pvza_ d1wt7a_ d1rjia_ d1wz5a_ d1m2sa_ d1pjva_
d1c49a_ d1qkya_ d2ktca1 d1wmta_ d1du9a_ d1j5ja_ d1r1ga_ d1mpya2
d1kw3b2 d1kw3b1 d4ghga1 d1tfza1 d3isqa1 d3isqa2 d1sqia1 d1sqia2
d1lkda2 d1lkda1 d1mpya1 d1cjxa2 d1cjxa1 d1sp9b2 d1t47a1 d1t47a2
d1f1ua2 d1sp8a1 d1sp8a2 d1f1ua1 d3ia2a_ d1a88a_ d4dgqa1 d1hkha_
d1zoia_ d1s5ja2 d1qhta2 d4dlea2 d1tgoa2 d3py8a2 d1q8ia2 d1kfsa2
d2py5a2 d1d5aa2 d1qsta_ d2beia1 d4m3sa_ d1u6ma1 d1ygha_ d1yk3a1
d3dr6a_ d1xmta_ d1z4ea1 d2ge3a1 d1bo4a_ d2fiaa1 d2jdca1 d1y9wa1
d3to7a_ d1wwza1 d2fl4a1 d2ozua1 d2b5ga_ d2cy2a1 d2p0wa_ d1cm0a_
d4pswa_ d1yrea1 d1yr0a1 d1m4ia_ d2gana1 d1yvka1 d1ghea_ d2g3aa1
d1y7ra1 d2euia1 d1q2ya_ d2fsra1 d1xeba_ d2ae6a1 d1cjwa_ d1qsma_
d2aj6a1 d1y9ka1 d1i12a_ d1vkca_ d2atra1 d1n71a_ d2d4pa1 d2fiwa1
d2fe7a1 d1tiqa1 d2ou2a_ d1yx0a1 d2fcka1 d1p0ha_ d2j8ma_ d2pq8a_
d1z4ra1 d1s3za1 d1ufha_ d2hqya1 d2hqya2 d1lrza2 d1lrza3 d1ne9a2

d1xvqa1 d1pn0a2 d3hy2a_ d2cvba1 d1o8xa_ d1lu4a_ d1we0a1 d2bmxa1
d2a4va1 d1psqa_ d1xiya1 d1q98a_ d1n8ja_ d1zofa1 d2ggta_ d3tjja_
d4je1a1 d1o73a_ d3s9fa_ d2zcta_ d1qmva_ d1jfua_ d2b1ka_ d5dvba1
d2b5xa1 d2v2ga_ d1st9a_ d3tb2a_ d4llra_ d4fh8a1 d1e2ya_ d1knga_
d2h30a_ d1i5ga_ d2z9sa_ d2f8aa1 d1tp9a1 d2b7ka_ d3hvva_ d2wfca1
d4kcea_ d1xvwa1 d4gqca1 d3qpma_ d4k1fa_ d1zzoa1 d1gp1a_ d1prxa_
d4od7a1 d1z6ma1 d4mcua1 d4dvca1 d1r4wa_ d3l9sa_ d3rppa_ d2qked_
d4ksoa_ d1t4za1 d1wwja_ d2m5ta_ d1wqsa_ d5bpea_ d2ipha_ d1lvoa_
d2bhga1 d3tnta_ d3sjia_ d3d23a1 d2fyqa_ d3tloa_ d3zvea1 d2hrva_
d3q3ya1 d4asha1 d1cg2a1 d1yloa2 d1y7ea2 d1y0ya3 d1lfwa1 d1vixa1
d1fnoa4 d1vhoa2 d1ysja1 d1vhea2 d1z2la1 d2fvga2 d1vgya1 d1r3na1
d2grea2 d1xmba1 d1rtqa_ d1etha2 d1w52x1 d2ppla1 d1hpla2 d1lpbb2
d2oxea1 d1bu8a2 d1rp1a2 d1ra6a_ d3gsza_ d1khva_ d1te9a_ d2xwha_
d3uqsa_ d3hkwa_ d3n6ma_ d2cjqa_ d2xyma_ d1teba_ d1te8a_ d1s48a_
d1u09a1 d4wfxa_ d3tyqa_ d2pgga1 d1lgya_ d4l3wa_ d1llfa_ d1gz7a_
d1ukca_ d4wfia_ d4cg1a1 d3visa_ d1j1ia_ d2rhwa_ d1svpa_ d1mbma_
d3su6a1 d1cu1a1 d4agka_ d1vcpa_ d1q31a_ g1n1l.1 d3e90b_ d1ep5a_
d3u1jb_ d3zxha_ d1cgla_ d1rm8a_ d1eaka2 d1hy7a_ d2jsda_ d1cxva_
d1hv5a_ d1i76a_ d3p0ya1 d1igra1 d1igra2 d1n8yc2 d1n8yc1 d1m6ba1
d4dv8a2 d1jx4a2 d3gv5b1 d1t94a2 d3bq0a2 d1ohfc_ d1lmra_ d1cixa_
d1y29a_ d1v7fa_ d2jtba_ d1d1ha_ d1g9pa_ d1lupa_ d1qk7a_ d1mb6a_
d2a2va_ d1niya_ d1i25a_ d1hvwa_ d2mpqa_ d1ie6a_ d1kqha_ d1la4a_
d2mxoa1 d1koza_ d1qk6a_ d1emxa_ d1lqra_ d1c6wa_ d1rmka_ d1ixta_
d1eyoa_ d1fu3a_ d1g1pa_ d1f3ka_ d1fyga_ d1rypd_ d1irua_ d1rypc_
d3unfk_ d1rypa_ d1j2pa_ d1irui_ d1j2qh_ d3unfh_ d1m4ya_ d1rypb_
d1irul_ d1irug_ d1iru2_ d1q5rh_ d1rypg_ d1g3ka_ d1iruf_ d1q5qa_
d1e94a_ d1irub_ d1ryph_ d3ty6a_ d1iruj_ d1iruk_ d1rype_ d1irud_
d1irue_ d3mi01_ d1rypf_ d1iruh_ d2z3ba_ d1iruc_ d3mka2_ d1iru1_
d1e88a1 d1l6ja5 d1l6ja4 d1eaka4 d1eaka3 d1l6ja3 d1h8pa2 d1h8pa1
d1ny721 d1ny722 d1a6ca3 d1a6ca2 d1a6ca1 d1pgl22 d1pgl21 d1pgl11
d1ny711 d1mqta_ d1d4m3_ d1fmd3_ d2mev1_ g1aym.1 d1ev13_ d4q4w1_
d1r1a3_ d2x5ia_ d1rhi1_ g1r1a.1 g1d4m.1 g1rhi.1 d1b35b_ d1oopa_
d1tme1_ d4iv1a_ g1pvc.1 g1fpn.1 d1fpn1_ d4gb31_ d1d4m1_ d1fmd1_
d1ev11_ d2wzr3_ d1qqp3_ g1fmd.1 d2x5ic_ d1rhi3_ g1oop.1 d1hxs1_
g1hxs.1 d1fpn3_ d1bev1_ g2mev.1 d1eah3_ d1aym1_ d1pvc3_ d1qqp1_
d1zba3_ d1h8ta_ d3vbhc_ g1b35.1 g1bev.1 d1hxs3_ g1ev1.1 d1bev3_
g1tmf.1 d1tmf1_ d1b35a_ d2mev3_ g1eah.1 g1h8t.1 d1eah1_ d4q4w3_
d1r1a1_ d1aym3_ d1pvc1_ d1zba1_ d1h8tc_ d1oopc_ d1tme3_ g1qqp.1
d4iv1c_ g1tme.1 d1ju8a_ d1eixa_ d3exra_ d2ffca1 d2fdsa1 d3n3ma1
d2czda_ d1q6oa_ d2yyua_ d1vqta1 d3ldva1 d4lt9a1 d3tc7a1 d3hoja_
d1i4na_ d3vnda_ d3nl8a1 d1geqa_ d3nava1 d1v5xa_ d4a2ra1 d3tc6a1
d1ujpa_ d4ijba_ d1xcfa_ d1vc4a_ d1rd5a_ d1qopa_ d3b5va_ d2r80b_
d1cqxa1 d1cg5a_ d1gcvb_ d3boma_ d2h8fb_ d2raoa_ d3bj1a_ d4hrrb_
d2r50a_ d3d1kb_ d2aa1b1 d3zhwa_ d3hf4b_ d3gdja_ d1jebb_ d3bj1b_

d1x9fb_ d1wmub_ d1gcva_ d3bcqb_ d3d4xb_ d2dc3a_ d3hf4a_ d4yu3a_
d3k8ba_ d1qpwb_ d2r80a_ d3bcqa_ d1v4wa_ d3wtgb_ d1jl7a_ d4esaa_
d1a9we_ d3d1ab_ d1fsla_ d4h2la_ d1v4wb_ d1mwca_ d1vhba_ d1jeba_
d1spgb_ d1h97a_ d1q1fa_ d1tu9a1 d3d1aa_ d1hdsb_ d3hyub_ d2zfbb_
d3fs4a_ d1oj6a_ d1shrb_ d4h2lb_ d4yu3b_ d1spga_ d3hrwa_ d3qqra_
d3at5a_ d4wchd_ d3hyua_ d1cg5b_ d1uc3a_ d1hbra_ d2raob_ d1outb_
d4esab_ d3gdjb_ d3qqqa_ d1x9fc_ d3vrfa_ d1itha_ d3mkba_ d3d4xa_
d3mjpa_ d1jl6a_ d3fh9a_ d1outa_ d2dc3b_ d1it2a_ d3eoka_ d3g46a_
d1qpwa_ d3vrfb_ d1ch4a_ d1x9fd_ d1hdsa_ d2zfba_ d3mkbb_ d1or4a_
d3wtga_ d1a4fa_ d3fh9b_ d3bomb_ d3cy5a_ d3at5b_ d1x9fa_ d2oifa_
d4lmxb_ d1cpcb_ d3v57a_ d1kn1a_ d1cpca_ d4f0ta_ d2vjha_ d4po5b_
d1eyxa_ d1alla_ d4f0tb_ d1b33b_ d1b8db_ d4h0mb_ d1b33a_ d2vjhb_
d4lm6b_ d2vmlb_ d1allb_ d2vjta_ d3dbja_ d2vmla_ d2vjtb_ d4f0ub_
d1f99b_ d4rmpa1 d1liaa_ d1eyxb_ d2uulb_ d4lmsb_ d4f0ua_ d2bv8a_
d1f99a_ d2bv8b_ d2c7lb_ d3dbjb_ d4rmpb1 d1b8da_ d4h0ma_ d3v57b_
d1kn1b_ d1liab_ d2uula_ d2j96a_ d1cwva5 d1azwa_ d1mtza_ d1sfra_
d1f0na_ d4qeka_ d1gkub1 d1gkub2 d2qvba_ d2v9za_ d4kafa1 d4iuaa3
d4bvva_ d5hpga_ d3k65a_ d1ki0a3 d1ki0a2 d1ki0a1 d1a0ha1 d1pmla_
d3sp8a3 d2feba1 d1f74a_ d3l21a_ d2e1da_ d1to3a1 d1w3ia_ d1sfla_
d1n7ka_ d1vcva1 d4imea_ d1wa3a_ d2ocza1 d1f2ja_ d3m16a_ d2yxga_
d1ub3a_ d3a5fa_ d1fbaa_ d1l6wa_ d1xfba1 d1wx0a1 d1vpxa1 d1ojxa_
d1mxsa_ d2a4aa1 d1xkya1 d3cq0a1 d3s1xa_ d1f05a_ d1fdja_ d3cpra1
d4d2ja_ d4mh8a_ d1mu2a2 d4i7ga1 d3rp2a_ g1ucy.1 d1fuja_ d2zchp_
d3w94a_ d1si5h_ d1lo6a_ g2pka.1 d1cgha_ d1kigh_ d1a5ia_ d3s9ba_
d1hj8a_ d1ao5a_ d2anya_ d1q3xa1 d1gvza_ d1azza_ d4gsoa_ d2f91a1
d1iaua_ d1gdna_ d1eufa_ g1fiw.1 d1eq9a_ d4nfea_ d2rdla_ d1spja_
d1elva1 d1a7sa_ d1ekbb_ d1pfxc_ d1yc0a_ d2aiqa_ g1fiz.1 d4k60a_
d4dgja_ d1mzaa_ d1op0a_ d3tjva_ d1pytd_ d1sgfg_ g1gg6.1 d2eeka_
g1rtf.1 d1a0ja_ d3g01a_ d1pytc_ d4e7na_ d1ddja_ d1z8ga1 d2hlca_
d1npma_ d1brup_ g1jou.1 d1sgfa_ d1autc_ g2hnt.1 g1kdq.1 d1orfa_
d3s69a_ d4crga_ d1fxya_ d1bqya_ d2ftba_ d1pmpa_ d2rcqa1 d1fdqa_
d4lkpa_ d1sa8a_ d1vyfa_ d1lpja_ d3vg7a1 d4azqa1 d1o8va_ d5bvqa_
d1p6pa_ d2qo4a_ d1opba_ d1yiva1 d1o1va_ d4bvma1 d4tkba_ d4i3ba_
d1bwya_ d1ftpa_ d1ggla_ d4gkca_ d1cbia_ d3p6da1 d1kqwa_ d2a0aa1
d1dzka_ d4tlja_ d2a2ua_ d4wfva_ d3t1da_ d4iaxa_ d3zq3a_ d2nnda_
d1i4ua_ d1kxoa_ d1bbpa_ d1kt7a_ d1exsa_ d3s26a1 d1lf7a_ d1qfta_
d4gh7a_ d1z24a1 d1qabe_ d1xkia_ d1ew3a_ d3bx7a_ d4iawa1 d1n0sa_
d1gkab_ d3kzaa_ d2k23a_ d2l9ca1 d2joza1 d1gm6a_ d3kffa_ d3saoa1
d1e5pa_ d1yupa1 d1iiua_ d4lu0a_ d1of8a_ d3unda1 d3e9aa1 d1vr6a2
d1n8fa_ d2qkfa_ d1d9ea_ d1o60a_ d1e69a_ d1mv5a_ d3g60a4 g1ii8.1
d1ewqa2 d1qhla_ d1v43a3 d2wmfa1 d1vpla_ d1g2912 d4a82a2 g1f2t.1
d2d62a1 d3tuic1 d2bbsa1 d3g60a3 d2ixea_ d1g6ha_ d1sgwa1 d2qi9c_
d3rlfa1 d3b60a1 d3d31a2 d2pzea1 g1xew.1 d1jj7a_ d1oxxk2 d2onka1
d1w1wa_ d1iwea_ d1qf5a_ d1vmaa2 d1nija1 d1np6a_ d2p67a1 d3ihla_

d1okkd2 d1ls1a2 d1s1ma2 d1iona_ d2v40a_ d1dj3a_ d3hida_ d2vo1a1
d2g0ta1 d1vcoa2 d1j8mf2 d1xjca_ d1p9ba_ d1qzxa3 d1ihua2 d1ihua1
d2qm8a_ d1dj2a_ d4ioja_ d1cp2a_ d1yrba2 d1hyqa_ d3gqba2 d1skye3
d1xp8a1 d1tf7a2 d1tf7a1 d1ye8a1 d1skyb3 d2qe7d2 d4blta_ d4huta_
d3ldaa2 d4hyya_ d2i3ba1 d1cr1a_ d3j9tb2 d3ntua2 d1vdza1 d2z43a_
d1e9ra_ d1nlfa_ d3ziaa2 d1n0wa_ d1g6oa_ d3ziad2 d3j9ta2 d3gqbb2
d2i49a_ d1nnfa_ d1xvxa_ d1ursa_ d1h3da1 d1iuda_ d4m1va1 d1twya_
d3e13x_ d2nxoa1 d4pfua_ d3ttma_ d1elja_ d2qmwa1 d1us5a_ d1y3na1
d1jeta_ d1uqwa_ d4f19a1 d2esna2 d3thia_ d3fasa1 d2uyeb_ d2xwva1
d1r9la_ d2fyia1 d1pb7a_ d2ozza1 d1sw5a_ d1vr5a1 d2g29a_ d1xs5a_
d3lswa_ d2z23a_ d3w9va_ d3tcha_ d1q35a_ d1z7me1 d3q27a_ d1p99a_
d1ve4a1 d2i6ea1 d4mcoa1 d4n8ya_ d1eu8a_ d1zbma1 d1hsla_ d1ixca2
d1j1na_ d1xvya_ d1y9ua_ d2z22a1 d1xoca1 d4oera_ d1wdna_ d5f1qa1
d1xt8a1 d1i6aa_ d4gwoa_ d4igta1 d1pc3a_ d4mmpa1 d1o63a_ d2czla1
d2onsa2 d4mija1 d1eh3a_ d3skpa_ d1dtza1 d1dtza2 d1ieja_ d1jnfa2
d1ce2a1 d1jnfa1 d1b1xa2 d1b1xa1 d1jw1a1 d1jw1a2 d1ryoa_ d1h76a1
d1h76a2 d1c4oa1 d2p6ra3 d2p6ra4 d1oywa2 d1oywa3 d1wrba1 d1w36b2
d1w36b1 d1c4oa2 d3bora_ d1t6na_ d2eyqa5 d2eyqa3 d2eyqa2 d2zu6a2
d1veca_ d1uaaa1 d1w36d1 d2g9na1 d1z3ix2 d2rb4a_ d1hv8a1 d1hv8a2
d1t5ia_ d1w36c2 d2j0sa2 d2j0sa1 d1fuka_ d3hjha1 d1z5za1 d1qdea_
d1z63a1 d1s2ma2 d1s2ma1 d1wp9a1 d1wp9a2 d1gm5a3 d1gm5a4 d3dina4
d3dina3 d1q0ua_ d1w36c1 d1z3ix1 d1nkta3 d1nkta4 d1uaaa2 d1w36d2
g1qhh.1 d2fwra2 d2fwra1 d2v8ta_ d2fkza_ d1zuja1 d1s3qa1 d2fjca1
d1z6om1 d1veia_ d4dyya_ d1jiga_ d3gvya_ d2pyba_ d4cy9a1 d3bvfa1
d1r03a_ d2cf7a_ d1nf4a_ d1zs3a1 d4iwka_ d3ka8a_ d3np0x_ d1n1qa_
d1lb3a_ d4e6ka_ d1z6oa1 d2fzfa1 d4dyua_ d3ge4a_ d1jgca_ d4am4a_
d2z6ma_ d1ji5a_ d1vlga_ d1lkoa1 d1krqa_ d3qz3a_ d1o9ra_ d2ffxj_
d1euma_ d1yv1a1 d2v2pa_ d1j30a_ d4di0a_ d1dpsa_ d1oewa_ d2qzxa_
d2asia_ d1psoe_ d1miqa_ d1smra_ d2h6ta_ d1izda_ d3hd8a_ d1j71a_
d1dpja_ d4aa9a_ d1t6ex_ g1b5f.1 g1lya.1 g1htr.1 d1qdma2 d1bxoa_
d1wkra_ d1tzsa_ d1ibqa_ d2qzwa_ d1orva2 d1xfda2 d2v76a_ d1wj1a1
d1aqca_ d1qqga2 d1qqga1 d1n3ha_ d4dbba_ d3f0wa1 d1p3ra_ d1wgua1
d2cy4a1 d3hqca_ d1wvha1 d1xr0b1 d1bdqa_ d3ggta_ d3ixoa_ d1mrxa_
d2haha_ d1tw7a_ d4njsa_ d1sgua_ d2p3da_ d1nsoa_ d3d3ta_ d2rkfa_
d1c6ya_ d2rspa_ d3ogpa_ d3ekwa_ d2o4na_ d2j9ja_ d1baia_ d3ggua_
d3lzua_ d4npua_ d4j55a_ d2r8na_ d2fxda_ d2p3ca_ d4dqea_ d3ttpa_
d3t3ca_ d2p3aa_ d2hc0a_ d2fdda_ d3mwsa_ d1um8a_ d1ixza_ d1qvra2
d1qvra3 d1qzma_ d1sxjc2 d1jr3d2 d2p65a_ d1l8qa2 d1g8pa_ d3hu3a3
d1sxjd2 d1njfa_ d1in4a2 d1sxja2 d1tuea1 d2ce7a2 d1w5sa2 d1sxjb2
d1d2na_ d1ny5a2 d1ofha_ d2gnoa2 d2a5yb3 d1fnna2 d1u0ja1 d1lv7a_
d1iqpa2 d2fnaa2 d1ixsb2 d4gita_ d1sxje2 d1g41a_ d1wgda1 d1wgga1
d4q5eb_ d1wjna1 d1se9a_ d3n3kb_ d1wz0a1 d1we6a1 d2m0xa_ d2gbka_
d3rt3b1 d1wh3a1 d1sifa_ d2ekec2 d4ii2b1 d4hk2a1 d1c3ta_ d1m94a_
d1wgha1 d1ttna1 d1j8ca_ d1wx8a1 d3v6cb1 d2ma9b_ d4i6lb1 d1bt0a_

d2jvca1 d1iyfa_ d2k1fa_ d1wxva1 d1wf9a1 d1t0ya_ d2io0b2 d1x1ma1
d2gbma_ d1zw7a1 d2m17a_ d1ud7a_ d1we7a1 d2lwpa_ d2gbja_ d1wjua1
d1v6ea1 d1v86a1 d4eewa_ d1wiaa1 d4icva_ d4gu2a_ d1wx7a1 d1wy8a1
d4hjka1 d2faza1 d1v5oa1 d2h0ba_ d2erfa_ d1h30a2 d1h30a1 d3mw4a1
d1pz7a_ d2r1ba_ d3v64a1 d1d2sa_ d3bopa1 d3mw3a1 d1okqa2 d2bcgg1
d2bcgg2 d1wioa4 d1ccza2 d1vcaa1 d1dr9a2 d1ic1a1 d2d9qb1 d2nxyb2
d2dara2 d2dara1 d1wyha2 d2dloa2 d1rutx4 d1rutx3 d2cuqa2 d1x4la2
d1x4la1 d1x3ha2 d1x3ha1 d2d8za1 d1x61a2 d1x6aa1 d1x6aa2 d2cora2
d2cora1 d1v6ga1 d1v6ga2 d2d8za2 d1x64a1 d1x64a2 d2co8a1 d1x63a2
d2dj7a2 d2dj7a1 d2cu8a2 d2cu8a1 d1g47a2 d1g47a1 d1j2oa1 d1wyha1
d2dloa1 d1wiga1 d1wiga2 d2cupa2 d2cupa3 d1x68a1 d2d8ya2 d2d8ya1
d1m3va2 d1x62a1 d1x68a2 d1x61a1 d1b8ta3 d1b8ta2 d2d8xa2 d2d8xa1
d1x4ka1 d1x4ka2 d2cuqa1 d1m3va1 d2co8a2 d1j2oa2 d1x63a1 d2cura1
d2cura2 d1x62a2 d2lj7a_ d1n4na_ d2m8bb1 d2kyqa_ d3psma_ d1jkza_
d1jxca_ d2gl1a_ d1ugla_ d4uj0a_ d1js9c_ d1f15b_ d1za7a_ d1laja_
d1ex4a2 d1c6va_ d1t0za_ d2i61a_ d1t1ta_ d1sn4a_ d1kv0a_ d2lkba_
d1npia_ d1wwna_ d1pe4a_ d2kbka_ d2li7a_ d1djta_ d2kbha_ d1dq7a_
d1sega_ d1omya_ d1zywa1 d1jzaa_ d1n9wa1 d1bbua1 d1e1oa1 d1c0aa1
d1b8aa1 d1l0wa1 d3a74a1 d1bvsa3 d1ixra2 d1iyjb4 d1iyjb5 d1iyjb3
d1o7ia_ d4ipca1 d1x3ea_ d4apva_ d1jb7a1 d1jb7a2 d1jb7a3 d3kdfa_
d1ue1b_ d1wjja1 d3eiva_ d1xjva2 d1xjva1 d2ccza2 d1se8a_ d1l1oc_
d1jb7b_ d1qvca_ d3ulla_ d1qzga_ d1miua4 d1miua5 d1miua3 d3g48a1
d1gd7a_ d1mkha_ d1jjcb3 d1pyba_ d1y14b1 d2nn6i1 d1i94l_ d2zjra2
d1smxa_ d2qamc2 d1hh2p1 d2r7da1 d1rl2a2 d1wfqa1 d3bzka4 d1x6oa2
d3a0ja_ d2j01d2 d2ja9a1 d4ql5a_ d1hr0w_ d1luza_ d2n3sa_ d2vnud1
d1h95a_ d1go3e1 d2nn6h1 d2mo1a_ d1iz6a2 d1jjga_ d1e3pa2 d2ix0a3
d2ix0a2 d2lxja_ d1ueba3 d1ueba2 d2z0sa1 d1u0la1 d2ix0a1 d2eifa2
d2je6i1 d3i2za1 d2c35b1 d2ahob2 d1t9ha1 d3i4oa_ d2qall1 d1jt8a_
d2nn6g1 d1g6pa_ d3cama_ d1i94q_ d1c9oa_ d1d7qa_ d2ba0a1 d2vnud2
d2vnud3 d1khia2 d2qalq1 d1wi5a1 d1xtda2 d1ny4a_ d1dgsa2 d1fvia1
d1ckma1 d1x9na2 d2a1ka_ d1je5a_ d1rpma_ d2shpa1 d1yfoa_ d1l8ka_
d2ooqa1 d1g4us2 d1lara2 d1lara1 d1wcha_ d2fh7a2 d2fh7a1 d1ohea1
d1ohea2 d2f4da_ d1rxda_ d2hxpa_ d1xria_ d1fpza_ d3ezza_ d4y2ea_
d1i9sa_ d3azya_ d3wvja_ d1upsa1 d1ajoa_ d2hyka_ d1ajka_ d2vy0a_
d1o4ya_ d3o5sa_ d3d6ea_ d3atga_ d1umza_ d3i4ia_ d1c1la_ d4lbqa_
d1a78a_ d1slta_ d4no4a_ d3zsja_ d3duia_ d1g86a_ d1is3a_ d1qmja_
d1hlca_ d1tdha3 d1q3ba3 d1ee8a3 d1k82a3 g1qmo.1 g1len.1 d3zyra_
d1fnya_ d1n47a_ g2ltn.1 d1avba_ d3usua_ d1dhkb_ d1f9ka_ d1ukga_
d1g8wa_ d1g9fa_ d1wbfa_ d3wcsa_ d1g7ya_ d1mvqa_ d1hqla_ d1v6ia_
d1dbna_ d1qnwa_ d1fx5a_ d4l8qa_ d1ioaa_ d3n35a_ d3ubra_ d1qo8a1
d1d4ca1 d2k3va_ d1ft5a_ d1qdba_ d1m1qa_ d2j7aa_ d1h21a_ d1ogyb_
d4fasa_ d1jnia_ d1oaha_ d1sp3a_ d3h33a_ d1rwja_ d19hca_ d1os6a_
d3bxua_ d3h4na_ d1i77a_ d3caoa_ d1j0pa_ d2ctha_ d1duwa_ d2e84a_
d1hh5a_ d1gyoa_ d3h34a_ d1dszb_ d1a6ya_ d3cbba_ d1ynwa1 d1r4ia_

d1hcqa_ d2hana_ d2hanb_ d2enva1 d1kb4b_ d2nllb_ d1lo1a_ d1cita_
d1adqa1 d1fnga1 d1uvqb1 d1zs8a1 d2vola1 d2vola2 d1mfbl2 d2jelh2
d1uwel2 d3qq4b1 d1kjvb_ d1k5na1 d1osph2 d4odhl2 d1ow0a1 d1ow0a2
d1bfob2 d3l9ra2 d3jtth1 d2fath2 d4elkb2 d1fn4b2 d2xfxb_ d1pg7x2
d1cqka_ d4k71b2 d1lk2a1 d1eapb2 d4lfhg2 d1i4fa1 d2nw2a2 d1kfah2
d1mhca1 d1h3ua1 d1wejh2 d1h0db2 d1a5fh2 d1kcvh2 d1mjuh2 d1qqda1
d1kcrh2 d1k8ia1 d3huja2 d2nnab1 d1nfdf2 d3mffb2 d1s9va1 d1lk3h2
d1q0xl2 d1fv1b1 d1neza1 d4rfeb2 d1r3ha1 d1a6tb2 d1kjva1 d1pz5b2
d1onqa1 d2bc4a2 d1de4a1 d1k8da1 d1tcra2 d1dn0b2 d1nfde2 d2xwtb2
d1bfoa2 d1mexh2 d1iakb1 d1ypzf2 d4f7ea2 d1q72l2 d2fbjh2 d1fe8h2
d1clzh2 d1fngb1 d1uvqa1 d1vgka1 d1c16a1 d4j12a2 d4x99b2 d1igtb3
d1je6a1 d1q72h2 d1nldh2 d1teth2 d2hmic2 d1i1ca2 d1i1ca1 d1k8ib1
d1q0yh2 d1kfal2 d3frua1 d1pg7w2 d1u58a1 d1igyb4 d1igyb3 d1hyrc1
d1klub1 d1deeb2 d1c12b2 d2bc4b2 d2ig2h2 d1kcvl2 d3wn5b1 d1lk2b_
d1lk3l2 d1mjul2 d1sm3h2 d1q0xh2 d1zvsa1 d1ydpa1 d1u8ib2 d1ypze2
d1kcuh2 d1op9a_ d1y0la1 d1etzb1 d1egjl1 d2gsib1 d1nj9a1 d4jfhd1
d1aqkl1 d1t2ja_ d1lo4h1 d1zvya1 d3v4vl1 d4enec1 d1kxtb_ d5e8ea1
d2pxya_ d4weud_ d1xiwc_ d2esvd1 d1qnzh_ d1ogae1 d1f4xh1 d4n1hb_
d3ezjb1 d3mlwl1 d1kfah1 d1etza1 d1b4jh1 d3cfic_ d4w68a_ d2arja1
d1ek3a_ d2ok0l1 d1vera_ d1uwgh1 d1blnb1 d1fpth1 d1rzga1 d2giya1
d4m3kb1 d1nc2a1 d2nyyd1 d1ncna_ d1fbih1 d5dxwa_ d4fqqa1 d1dqdh1
d1jfql1 d1kelh1 d1faih1 d1frgl1 d1figl1 d4m6nl1 d1fo0a_ d4hxbl1
d3b5ga_ d3quyd1 d2h9gb1 d1i8kb_ d2dd8h1 d2ck0h1 d1eaja_ d4w6wb1
d2a6ib3 d1t66d1 d2e27h_ d1ob1b1 d1f11b1 d2v7na1 d1ad0a1 d2xt1b1
d1pskh1 d4i13b1 d2op4l1 d1a0ql1 d1uwel1 d1za3a1 d1ncbl1 d4uu9a_
d1bm3h1 d1cloh1 d1nqba2 d1cf8h1 d4y5va_ d1mhph1 d5anma1 d4cdgc1
d4ldeb_ d1y0lb1 d2pcpb1 d1z3gl1 d1dfbl1 d1fh5h1 d3g5xa1 d1tzib1
d4dn3l1 d3zhka1 d2b1hh1 d2uyla1 d1f3dh1 d1nj9b1 d2agjh1 d2h1pl1
d1q72h1 d8fabb1 d1ndmb1 d1rjca1 d4u6hb1 d3upca_ d2fbjl1 d1osph1
d1tvda_ d2cjvh_ d4fhbd1 d1bd2d1 d1rz7h1 d3nn8a_ d1knoa1 d1nfda1
d2bmkb1 d1dqll_ d1igyb1 d1igfl1 d12e8h1 d3utza1 d1nc2b1 d1rihh1
d4mwfb1 d4jvpa1 d1mieh1 d1rhfa1 d1fgnl1 d1sbsh1 d1lp9e1 d1dn0a1
d4lqfl1 d1iaih1 d1kgcd1 d1mh5b1 d1gpol1 d2g60h3 d1f8tl1 d2jixd1
d4grwh_ d1fo0b_ d1bfob1 d1axsa1 d1u9ka_ d1nmbh_ d1h8na1 d1h8na2
d1jguh1 d4eura1 d3sdyl1 d35c8h1 d1w72l1 d1mvub_ d1i9rh1 d1ob1a1
d4wenb_ d1yeeh1 d3ietc1 d1f58h1 d1ad0b1 d1mqkl_ d1nbvh1 d4b50a_
d3r8bb_ d1bafl1 d1mamh1 d4rgne1 d2gsgb_ d35c8l1 d1bafh1 d1ai1h1
d2pcpa1 d2fath1 d2x1pa_ d1orsb1 d1ibgl1 d1shma_ d1a2yb_ d1u0qa_
d2coqa_ d1hilb1 d1qfwi_ d1jv5b_ d1fn4b1 d5e0qa_ d1nsnh1 d1plgl1
d4ocxl1 d1kb5h1 d1rzfh1 d1pg7x1 d2fx7h1 d2oi9c_ d1l7il1 d3eyva1
d1d5il1 d4ffyl_ d1tetl1 d1a6wh_ d1ac6a_ d3dvfa_ d1nfde1 d1mimh1
d1ri8a1 d1jhlh_ d3dura_ d1fskb1 d1f97a1 d3so3b1 d2ak4d1 d1lp9f1
d4z7wf1 d1l7th1 d4y5ya_ d4x7ec1 d1emth1 d1ikfl1 d1nlbh1 d4utal1
d1bjma1 d1eapb1 d1u6ah1 d2dtgd1 d1kj2b_ d1xeda1 d1c1eh1 d1fgvh_

d2bnub1 d2fb4h1 d2dtgb1 d1ktrh_ d1jpth1 d1qkzl1 d4hepg_ d2atpb1
d1flrh1 d2hrpl1 d1mvfa_ d1iaim1 d3k80a1 d2jb5h1 d3zkxc_ d4grwe_
d1it9h1 d1rhhb1 d3w9ec_ d1e4wh1 d1sy6h1 d2j6eh1 d3qxta_ d1jglh1
d1p7ka1 d1tzhb1 d4n8pa1 d1gafl1 d2jelh1 d1wioa2 d4i0cc1 d1cicb1
d1lmka2 d1lmka1 d1iqwh1 d1orqb1 d1ejoh1 d1jnlh1 d4zg1a_ d43c9b_
d1ggbl1 d1wejh1 d3q6fa1 d1f3dj1 d3c6la1 d1bqlh1 d3p9wb_ d1a3rl1
d1h0db1 d2uzil_ d1eo8l1 d1qfwl_ d1n7ml1 d1igja1 d1c5ch1 d2a9mh_
d1rmfh1 d1fj1b1 d4heme_ d1ccza1 d1a5fh1 d1ogad1 d1dlfh_ d1qa9b_
d1vgel1 d3qskb_ d1sjxa_ d4ozfg1 d1sq2n_ d1qd0a_ d1kcvh1 d1c5db1
d3qcul1 d8faba1 d3bn9d1 d1mjuh1 d4y8dc_ d7fabh1 d3dvgb1 d1lo0l1
d1a6tb1 d1gigh1 d1kcrh1 d2hwzl1 d1yjdc1 d3clfl1 d4krna_ d1dl7l_
d1e6ol1 d1mexh1 d3r0ma_ d4kkna_ d1fnsl1 d1oaqh_ d1rzia1 d1ucbh1
d1ncwl1 d4b41a_ d1nfdf1 d2mpah1 d2d7tl1 d4grwf_ d4b5ea_ d1lk3h1
d3u79a_ d4u3xa_ d1ghfl1 d1bvkb_ d1mcww1 d1b88a_ d1jrhh1 d32c2b1
d4wemb_ d1dsfh_ d1op3h1 d1cell1 d1iqda1 d1qoka1 d1qoka2 d1qfuh1
d2crya1 d1ay1h1 d1oaql_ d3ab0b_ d1rurl1 d1t4kb1 d1bz7b1 d1n0xh1
d1forl1 d1ucbl1 d4w6yb_ d43c9a_ d2aabl1 d1tzia1 d4n1ca_ d1k6qh1
d3g9ab_ d4tvsa1 d1igmh_ d1pz5b1 d3dx9a1 d3wxwh_ d1nakh1 d1kcul1
d3eaka_ d4qlra1 d1cr9l1 d1d9ka_ d1f4xl1 d1deea1 d2i27n2 d1c12a1
d2orbl1 d1j8hd1 d1mcph1 d1c5da1 d1kenh1 d1bd2e1 d1h5ba_ d4x7fc1
d3upaa_ d1t3fa1 d1indh1 d2dtgc1 d4hc1l1 d1zv5a1 d4wgvb_ d1uwgl1
d4f37k1 d1aqkh1 d1vesa_ d3zkqd_ d1fptl1 d1lo4l1 d1knob1 d3rrqa_
d1ct8b1 d4orzc_ d1mlbb1 d4hgkc1 d1dqdl1 d1bogb1 d4ozhe1 d1dr9a1
d1i7za1 d1dn0b1 d1kell1 d1rzib1 d1ypzf1 d1wtla_ d1hkfa_ d1fail1
d1figh1 d2vyre_ d1sjva_ d1ohqa_ d1bfoa1 d1pkqb1 d1mjul1 d1b4jl1
d1nsnl1 d2arjb1 d1p4bh_ d4aj0a_ d3nn8b_ d2rcja1 d1f2xk_ d4v1dd_
d1adqh1 d2eksa_ d4k3ga_ d1jfqh1 d1uweh1 d1frgh1 d4gftb_ d1uz8b1
d4n1ea_ d1bm3l1 d1clol1 d3mcka1 d2vq1a1 d1ae6h1 d4aq1b1 d1uj3b1
d1nbqa1 d1fh5l1 d2ck0l1 d2ywza_ d1q72l1 d4nbxb_ d1hila1 d4hzlb1
d2j88l1 d1zmya1 d1cd0a_ d1a4ka1 d1pskl1 d1a0qh1 d4krmb_ d1ncbh1
d2fbjh1 d1j8he1 d1i3ua_ d1cf8l1 d1fe8h1 d2p1ya1 d1eeqa_ d2x89a_
d1ol0a_ d2r0lh1 d1ub5a1 d1dfbh1 d3eyvb1 d4jdvb1 d4p2cg_ d2gsia1
d2agjl1 d1u3ha1 d2h1ph1 d1rihl1 d1fskc1 d1boga1 d2a77l1 d1ospl1
d1qfoa_ d3dwta_ d2fx7l1 d1rz7l1 d3stba1 d1pkqa1 d1b6da1 d1dzba2
d1dzba1 d1kj2a_ d4l1ha_ d1rzgb1 d1dqlh_ d12e8l1 d1miel1 d1jmaa_
d1sbsl1 d1fgnh1 d1f58l1 d1mqkh_ d1ndgb1 d3qegl1 d1iail1 d2aw2a1
d1tqbc1 d3fo2a1 d4dkaa_ d1f8th1 d2g60l1 d2ywya_ d1uj3a1 d1yc7a_
d3bd3a_ d4w6xb1 d1nmbl_ d1i8ka_ d1rjlb1 d1ypze1 d1p7kb1 d1kxqe_
d1gpoh1 d2z4qa1 d1w72h1 d1i9rl1 d4qgya1 d4xt1c_ d2qqnh1 d1cica1
d3c08h1 d2cd0a_ d2bdnh1 d1hnga1 d1maml1 d1nldh1 d1cr9h1 d4lcih1
d3bkyh1 d3qg7l1 d1ai1l1 d1kxvc_ d4iosd_ d1l7ih1 d2x1qa_ d1ub6b1
d3k74b_ d1ibgh1 d1teth1 d1igya1 d1hxma1 d2x6ma_ d2e7la_ d5e03a_
d1qfwm_ d2r0kh1 d1igjb1 d1rurh1 d1plgh1 d1mj8h1 d1yuhb1 d3fmgl1
d1rzfl1 d1emtl1 d1ad9b1 d1igtb1 d1nlbl1 d1d5ih1 d3k7ua_ d4ffyh_

d1bwwa_ d3dvga1 d1a6ta1 d1fgvl_ d4qxgl1 d1miml1 d1jhll_ d2hffb1
d2hh0l1 d1qkzh1 d1l7tl1 d1afvh1 d1iaii1 d2cjuh_ d1um5h1 d2ghwb2
d2ghwb1 d1kgce1 d2fgwh1 d1ikfh1 d1dvfd_ d2i24n1 d2xtjb_ d1za3b1
d2yc1a_ d2hh0h1 d2x1oa_ d1bvka_ d1rjla1 d5e7ba1 d1igta1 d1il1b1
d2fb4l1 d1mvua_ d1jptl1 d32c2a1 d1fvca_ d1iqdb1 d2hrph1 d2p4ab_
d4qo1a_ d1p2cb1 d1wt5c_ d1t4ka1 d5c1mb_ d1it9l1 d1bz7a1 d1b2wh1
d1e4wl1 d1jnll1 d1yedb1 d1za6b1 d3u7aa_ d1igfh1 d3gnml1 d1orsa1
d1wejl1 d1a2ya_ d1hxmb1 d1gafh1 d2j4wh1 d1pz5a1 d1j05a_ d1a3rh1
d4fqll1 d1qfwh_ d1eo8h1 d1n7mh1 d4s11a_ d1kegh1 d5da4a_ d1iqwl1
d3whxb1 d1vgeh1 d1deeb1 d1c12b1 d1t2ql1 d1ad9a1 d3gsnb1 d3b2uc1
d1ggbh1 d1t3fb1 d1nakl1 d4ocmc_ d1lila1 d2v7hb3 d2esve1 d1mcoh1
d2qr0b1 d2q3aa_ d1ct8a1 d1kcrl1 d2pxyb_ d2uzih_ d5c3ld_ d3durb_
d1c5cl1 d2ak4e1 d1dl7h_ d1xiwd_ d1mlba1 d1rmfl1 d1i7zb1 d1dlfl_
d2xa3a_ d5bq7b1 d1dqqb1 d3h42l1 d1egjh1 d1kcvl1 d1fe8l1 d2j6el1
d1f3rb2 d1lk3l1 d1sm3h1 d1q0xh1 d1ghfh1 d7fabl1 d1igch1 d1lo0h1
d4idla_ d5c2bl_ d1jrhl1 d1il1a1 d3zhda1 d4kfzc_ d1b0wa_ d1e6oh1
d1mexl1 d4krpb_ d1fnsh1 d1ncwh1 d4hgma_ d4eigb1 d4rzca_ d2mpal1
d4y5xa_ d5en2b1 d3lrga1 d4ej1c1 d1rhha1 d1j05b_ d1forh1 d1q9wb1
d2wzpd_ d2aq2a1 d1h0da1 d3revb1 d2ij0c1 d1tzha1 d4m7jl1 d1k6ql1
d3b9kc1 d1igml_ d3qeua1 d1dsfl_ d1ce1h1 d1kcuh1 d1zvha1 d1zvoa1
d1tqbb1 d1ay1l1 d2qscl1 d2cgrh1 d1nl0h1 d1kenl1 d3eo0a1 d4n9ob1
d1n0xl1 d3k1kc_ d2aq2b1 d1ck1a1 d1enfa1 d1i4pa1 d1v1oa1 d1et9a1
d1esfa1 d2g9hd1 d4ohja1 d1klud1 d1bxta1 d1ty0a1 d1fnua1 d1c4qa_
d1prtc1 d2wv6d_ d1prtf_ d1ltrd_ d4l63b1 d1r4pb_ d1prtd_ d1tl0f_
d2bosa_ d1prtb1 d3chbd_

**Family-46 (c.1.8.3)**

**Positive dataset contains 32 protein sequences.**

d1wkya2 d3wh9a_ d4m1ra_ d4xuya_ d2je8a5 d4bf7a1 d3cuia_ d1ur4a_
d2vzsa5 d2x2ya1 d4y7ea1 d4k68a_ d2v3ga_ d4hpga_ d3ziza_ d4bs0a_
d1ta3b_ d1w91a2 d1tvna1 d3nyda1 d2cyga1 d4xv0a_ d2c8na2 d1w32a_
d3emca_ d1v6ya1 d2uwfa1 d2fgla_ d3niya1 d2c0ha1 d1rh9a1 d1yq2a5

**Negative dataset contains 4024 protein sequences.**

d1m32a_ d1ibja_ d1c7na_ d1iuga_ d4q75a_ d2bkwa1 d1t3ia_ d1n8pa_
d1qz9a_ d1jf9a_ d1eg5a_ d3kgwa_ d1cl1a_ d2ctza1 d1vjoa_ d2rfva_
d1gc0a_ d1cs1a_ d1d2fa_ d2ch1a1 d1qgna_ d1pffa_ d1elua_ d1u08a_
d1lc5a_ d1b5pa_ d2ay1a_ d1bw0a_ d3pd6a_ d7aata_ d2csta_ d1j32a_
d1fg7a_ d4wlha_ d1o4sa_ d1v2da_ d1gdea_ d1m6sa_ d1v72a1 d1svva_
d1ajsa_ d1wsta1 d2f8ja2 d1lk9a_ d1vp4a1 d1xi9a1 d2gb3a1 d1js3a_
d3rcha_ d3k40a_ d2cfba1 d1dfoa_ d1o69a_ d1bj4a_ d3k28a_ d2bwpa_
d3lg0a_ d2fnua_ d3ou5a_ d1z7da1 d1b9ha_ d1iaya_ d4msoa_ d1s0aa_
d1sffa_ d3tqxa_ d1mdoa_ d3qboa_ d3pgya_ d1bjna_ d2dkja_ d1ejia_
d1vefa1 d4wxga_ d3bs8a_ d3gbxa1 d1w23a_ d1g8fa3 d1f56a_ d1bypa_
d1azca_ d1ws8a_ d3u25a_ d1cuoa_ d1id2a_ d1rkra_ d2iaac_ d4dp1x_
d1nwpa_ d2gima_ d2i7sa_ d1qhqa_ d3ehbb1 d1fftb1 d1gyca3 d1hfua2

d1kbva1 d1kbva2 d1sdda1 d1sdda2 d1gyca1 d1gyca2 d1mzya1 d2bw4a1
d1aoza1 d1aoza3 d1aoza2 d1kyaa2 d1kyaa3 d1mzya2 d4e9sa1 d1sddb1
d1sddb2 d1hfua1 d1hfua3 d1oe1a2 d1oe1a1 d1v10a2 d1v10a3 d1v10a1
g1f8v.1 d2bbvc_ g1or0.1 g1cp9.1 g1e3a.1 d1a3qa1 d1iknc_ d3brda1
d1p7hl1 d1oy3c_ d1imhc1 d1zk9a1 d2cxka1 d2bema_ d1pama1 d1qhoa1
d2fhfa2 d2fhfa1 d1k3ia1 d1j0ha1 d1cxla1 d2bhua1 d1edqa1 d1f1sa2
d1ut9a2 d1gvia1 d1txka1 d2phla2 d2phla1 d1fxza2 d1fxza1 d1lrha_
d1uija1 g1dgw.1 d2v09a_ d1uika2 d1uika1 d1dgwa_ d1ep0a_ d2pa7a1
d1wlta1 d1nxma_ d2c0za1 d1ds6b_ d1fstb_ d3us3a3 d3us3a1 d3u1ra1
d1g9ka2 d1kapp2 d2bjca1 d2hsga1 d1qpza1 d1efaa1 d1ydwa2 d2nvwa2
d1zh8a2 d1qkia2 d1xeaa2 d1tlta2 d1r0ka3 d1vkoa2 d1vm6a2 d3qvsa2
d1gr0a2 d1p1ja2 d2ovga_ d1sq8a_ d1rzsa_ d3kz3a_ d3orca_ d3gpdg2
d2q49a2 d1hdgo2 d2g17a2 d2cvoa2 d2czca1 d3pwka2 d1obfo2 d1i32a2
d1zyao2 d2pkqo2 d1b7go2 d2hjsa2 d1vkna2 d1nvmb2 d1ux8a_ d1dlya_
d4i0va_ d2bkma_ d1dlwa_ d2bmma_ d2msya_ d2djoa1 d2r5za_ d1au7a1
d1f43a_ d2lmda1 d1akhb_ d1s7ea1 d3nara_ d1zq3p1 d2craa1 d1mija_
d4uuta_ d3a01a_ d1x58a1 d3rkqa1 d3cmya1 d2cqxa1 d2da6a1 d1e3oc1
d1fjla_ d1mh3a1 d1ig7a_ d1x41a1 d1wh7a1 d1gt0c1 d2mw8a_ d1lfup_
d1x2ma1 d1b72a_ d1wi3a1 d1wjha1 d1x2na1 d1mnmc_ d2cuea1 d3k2aa_
d2hosa_ d2jwta2 d1pufa_ d1octc1 d2hi3a_ d1b8ia_ d4j19a_ d3lnqa_
d1wh5a1 d1b8ib_ d1fexa_ d2cqra1 d1guua_ d1ug2a1 d2cqqa1 d2ajea1
d4a69c_ d1h8ac1 d2crga1 d2iw5b1 d1xc5a1 d2cu7a1 d1wgxa1 d2cjja1
d2ckxa1 d1gvda_ d1hcra_ d1tc3c_ d1u78a2 d1igna2 d2iu5a1 d1zk8a1
d2g7sa1 d2fd5a1 d2np5a1 d1z0xa1 d2id6a1 d2d6ya1 d2i10a1 d1vi0a1
d2oi8a1 d3c07a1 d2ns7a1 d2fq4a1 d2yvea1 d1t33a1 d2o7ta1 d1rkta1
d2np3b1 d2g7ga1 d2gena1 d2fbqa1 d2g7la1 d2gfna1 d2g3ba1 d2jj7a1
d2hyja1 d1sgma1 d1ui5a1 d2hkua1 d2id3a1 d4jyka1 d1bl0a1 d1bl0a2
d1d5ya1 d3lv8a_ d1s3ga1 d1zaka1 d1s4qa_ d3tvta2 d1uf9a_ d1khta_
d1ckea_ d1osna_ d1p5zb_ d4tmka_ d3cm0a_ d1tmka_ d1jaga_ d3h86a_
d1t0hb_ d2h92a_ d3kb2a1 d2c95a1 d1yj5a2 d1kgda_ d1ly1a_ d2ak3a1
d1vyva2 d1deka_ d2grja1 d1p3ja1 d1y63a_ d1e4ya1 d1s96a_ d3iija1
d1lvga_ d1nksa_ d1vyua2 d4e22a_ d1qf9a_ d1jjva_ d1q3ta_ d1ki9a_
d1lw7a2 d1teva_ d1kjwa2 d1gsia_ d1g3ma_ d1j99a_ d1t8ta1 d1nsta_
d1fmja_ d1zrha_ d1ls6a_ d2a3ra_ d1aqua_ d2zptx_ d3ckla_ d1texa_
d1q44a_ d2gwha_ d1odfa_ d1uj2a_ d4gi7a_ d2jeoa1 d1rz3a_ d1z0ja1
d3umra2 d3gj0a1 d2gaoa1 d1yzga1 d1x3sa1 d1g7sa4 d1puja_ d1udxa2
d1kk1a3 d2o52a_ d1jwyb_ d1zcaa2 d1zcba2 d2fv8a1 d2cjwa_ d3dz8a1
d1xzpa2 d1vg8a_ d4qxaa_ d2ew1a1 d1x1ra1 d2bcgy_ d3doea1 d3cona_
d3rega_ d3wyga_ d1upta_ d2b6ha1 d1zd9a1 d1lnza2 d1r5ba3 d1n0ua2
d1r2qa_ d2f9la1 d2rhda_ d3q3jb_ d3q85a_ d1efma1 d1r8qa_ d2gf9a1
d2akab1 d1ek0a_ d1z08a1 d2gcoa1 d2dy1a2 d4g01b_ d2cxxa1 d1svia_
d3q72a_ d1ni3a1 d4ac9a4 d2erxa1 d2bmea_ d1wxqa1 d2gf0a1 d4ldja1
d1zunb3 d1d5ca_ d4lv5b_ d1efca3 d1kmqa_ d2g6ba1 d3lrpa_ d1tada2
d2y8ea_ d4rd4a1 d1z06a1 d2heia_ d3a58b_ d1u0la2 d1d2ea3 d1zj6a1

d2ocba_ d3rwoa_ d1mr3f_ d2e9sa1 d4u5xa1 d2iwra_ d2fn4a1 d3bwdd_
d2nzja1 d2atva1 d2ksqa_ d3l43a_ d1mkya2 d1mkya1 d1wmsa_ d1jnya3
d2rmka_ d1f6ba_ d1f60a3 d1gota2 d1jala1 d2fh5b1 d1g16a_ d2wblc_
d1m7ba_ d1wf3a1 d1f5na2 d1t9ha2 d1z6xa1 d1z0aa1 d2fg5a1 d2a5ja1
d2bcjq2 d1s0ua3 d1ky3a_ d4y0va_ d4lhwa_ d2qmea1 d2atxa1 d1egaa1
d1ryha_ d1moza_ d1nrjb_ d2erya1 d3bfka_ d1puia_ d3j3az1 d3t5ga_
d1shza2 d3defa_ d2f7sa1 d3raba_ d3tsoa_ d3aq4a_ d2al7a1 d3sfva1
d3bc1a_ d2ncda_ d1sdma_ d3h4sa1 d2heha_ d2grya_ g3kin.1 d1kk8a2
d1br2a2 d1goja_ d1f9va_ d1lkxa_ d3t0qa_ d4db1a1 d3gbja_ d1ry6a_
d1v8ka_ d1ogpa1 d1kb0a1 d1k8ka2 d1k8ka1 d3uleb1 d3i33a2 d3i33a1
d1bupa1 d1dkgd2 d1dkgd1 d4ci6a2 d1jcfa1 d4a2ba2 d2fxua1 d1yaga2
d1yaga1 d4cbua2 d1iqca2 d1iqca1 d1eb7a2 d1nmla2 d1nmla1 d3h3no1
d3h3no2 d3ezwa2 d1hroa_ d4gedb_ d3zooa_ d1mz4a_ d1gu2a_ d1f1fa_
d1dvva_ d1c6ra_ d1c75a_ d1e29a_ d1gdva_ d1cnoa_ d1h32b_ d1co6a_
d2bh4x_ d2aiua_ d5cytr_ d1jdla_ d1i8oa_ d2zboa_ d3dmia_ d1ls9a_
d1wvec_ d2yk3a_ d3ph2b_ d1dw0a_ d5aura_ d1ql3a_ d1qn2a_ d1f1ca_
d2nqya_ d1h4ga_ d1hixa_ d1oa2a_ d1olra_ d3b7ma_ d4h7ma1 d3wp3a_
d2vgda_ d1igoa_ d3zsea_ d2nlra_ d3wx5a_ d1oa3a_ d1pvxa_ d1m4wa_
d1f5ja_ d1oa4a_ d4ixla1 d2a6za1 d3a4ua_ d2a6va1 d2ah2a1 d1evsa_
d1cnt1_ d1pvhb_ d1z7ca_ d1huwa_ d1bgea_ d1rhga_ d1f45b_ d1i1rb_
d1axia_ d1etea_ d3tgxb_ d1scfa_ d2z3qa1 d3qb1a_ d1hula_ d1m48a_
d4nkqb_ d1eera_ d1v7mv_ d5e4ea_ d2o27a_ d3b5ka_ d2psma1 d4zf7a_
d3uf5a_ d4x51a_ d1d9ca_ d4ypgc1 d3oq3a_ d1n1fa_ d1au1a_ d1wu3i_
d3besl_ d3ux9a_ d3rbaa_ d2qtra_ d1nupa_ d3l92a_ d1f9aa_ d1qjca_
d2h29a_ d1vlha_ d1od6a_ d1lw7a1 d1mrza2 d4ruka_ d2el7a_ d1iq0a2
d1irxa2 d1r6ta2 d1ivsa4 d2cyba_ d1rqga2 d1j09a2 d2yxna_ d1f7ua2
d1h3fa1 d1ffya3 d2g36a1 d1nzja_ d1jila_ d1i6la_ d1pfva2 d1h3na3
d3n9ia_ d3sz3a1 d3sw2a_ d1moxc_ d1dx5i3 d1edmb_ d1autl2 d1autl1
d1nqlb_ d1dval1 d1szba2 d1kigl_ d2y5fl_ d1ijqa2 d1gk5a_ d1g1sa2
d1nt0a3 d1xdtr_ d1lmja2 d1lmja1 d1nzia2 d2wphe_ d1klil_ d1pfxl2
d1pfxl1 d1p9ja_ d1g1ta2 d1f7ea_ d4yzub_ d2mgpa1 d1b9wa2 d2mgpa2
d1ob1c1 d1ob1c2 d1n1ia2 d1n1ia1 d2npra2 d2npra1 d1m1xb4 d1qx2a_
d1ig5a_ d2y5ia_ d1psra_ d3n22a_ d2egda_ d4ggfc_ d2wcea_ d2luca_
d4aqia_ d3nsia_ d2lp3a_ d1j55a_ d1a4pa_ d1k8ua_ d1nsha_ d1pvaa_
d3fs7a_ d1ttxa_ d1rwya_ d2kyfa_ d1rk9a_ d1a75a_ d1exra_ d1tiza1
d1nyaa_ d1g8ia_ d1ggwa_ d1wdcc_ d3fwba_ d1ggza_ d2o5ga_ d1ctaa_
d1m45a_ d3i5gc_ d1sl8a_ d1fpwa_ d2scpa_ d2roaa_ d4aqra_ d3pn7b_
d1br1b_ d2mysb_ d1s6ca_ d1bjfa_ d2obha_ d1jfka_ d1jf0a_ d1qv0a_
d1lkja_ d2mlfc_ d2m97a_ d2d8na_ d4by5a_ d1jbaa_ d4nqga_ d1wdcb_
d2mysc_ d1j7qa_ d1s6ia_ d1uhka2 d1r2ua_ d1dtla_ d3kf9a_ d3pn7c_
d5d67d_ d1tuza_ d1h8ba_ d1eg3a1 d1eg3a2 d2zkmx1 d1snla_ d1gjya_
d1y1xa_ d1hqva_ d1dvia_ d1df0a1 d1qxpa2 d4usla_ d1k94a_ d1etob_
d1umqa_ d1e5wa2 d1j19a2 d1isna2 d2al6b2 d3syxa1 d2p8va_ d1xoda1
d1i7aa_ d1evha_ d1qc6a_ d1egxa_ d1b9ra_ d1offa_ d1frra_ d3wcqa_

d1e9ma_ d1l5pa_ d1wria_ d1i7ha_ d3av8a_ d1czpa_ d1rfka_ d3p1ma1
d3b2ga_ d2bt6a2 d1wgqa1 d1v89a1 d2coda1 d1zc3b_ d1wg7a1 d1u5ea1
d1v61a1 d2coca1 d2ys1a1 d1v5pa1 d1ntya2 d1wjma1 d1wi1a1 d1u29a_
d1xx0a1 d1unqa_ d2zkmx3 d1fhoa_ d1v88a1 d2adza1 d1x1fa1 d1p6sa_
d2coaa1 d1btka_ d1v5ma1 d1faoa_ d2x18a_ d1dyna_ d1x1ga1 d4kaxb_
d2j59m1 d1ki1b2 d1xcga2 d1txda2 d1u5da1 d1w1ha_ d1upqa_ d1v5ua1
d1foea2 d2fjla1 d1eaza_ d2dfka2 d2cofa1 d1kz7a2 d1h6ua1 d9ldta1
d2ewda3 d1up7a1 d2dfda1 d1s6ya1 d1vjta1 d7mdha1 d1hyea1 d1t2da1
d1ojsa3 d1b8pa1 d1ldna1 d1hyha1 d1civa1 d2a92a1 d1ez4a1 d1i0za1
d1y6ja1 d1gv0a1 d1ur5a1 d1llda1 d1obba1 d1v8ba1 d1gdha1 d1l7da1
d2dlda1 d1mx3a1 d1ygya1 d1qp8a1 d1li4a1 d1j4aa1 d2naca1 d1npya1
d3t4ea2 d1a4ia1 d1o0sa1 d2hk9a2 d2egga2 d1b26a1 d1bvua1 d1gq2a1
d1c1da1 d1leha1 d1gtma1 d1nvta1 d3dona2 d1edza1 d1gpja2 d1vi2a1
d1p77a1 d1nyta1 d1b0aa1 d1vl6a1 d1v9la1 d1txga2 d3f47a1 d1mv8a2
d2pv7a2 d1xc2a3 d2ahra2 d4xiya1 d2f1ka2 d1pgja2 d1i36a2 d1np3a2
d1lj8a4 d1teaa3 d1qmga2 d2i76a2 d2g5ca2 d1wdka3 d1dlja2 d3cuma3
d1kola2 d1f8fa2 d1qora2 d1jqba2 d1vj0a2 d1cdoa2 d1gu7a2 d1d1ta2
d1xa0a2 d2dm6a2 d1jvba2 d1rjwa2 d1llua2 d1h2ba2 d1tt7a2 d1piwa2
d1o89a2 d1iz0a2 d1e3ja2 d1yb5a2 d1uufa2 d1vj1a2 d1e3ia2 d3gpdg1
d1vc2a1 d2nvwa1 d1u8fo1 d1ydwa1 d1hdgo1 d2g17a1 d1cf2o1 d2czca2
d1r0ka2 d1obfo1 d2cvoa1 d1ebfa1 d1h6da1 d1i32a1 d1zh8a1 d2q49a1
d1qkia1 d1j5pa4 d3pzra1 d1xeaa1 d1vkoa1 d1vm6a3 d3qvsa1 d2pkqo1
d1b7go1 d1gr0a1 d1p1ja1 d1lc0a1 d1j0xo1 d3pwka1 d2hjsa1 d1zyao1
d1vkna1 d1tlta1 d1nvmb1 d3sjua_ d3ojfa1 d1xu9a1 d2jjya1 d1n7ha_
d1y1pa1 d1h5qa_ d1jtva_ d4fs3a_ d2bkaa1 d1gy8a_ d2c5aa1 d1ulsa_
d1ooea_ d2b69a1 d1y5ma1 d1spxa_ d1ae1a_ d1bxka_ d1ydea1 d1gega_
d1hdoa_ d1o5ia_ d1e6ua_ d1snya1 d2rhca_ d3zv4a_ d5ceja_ d2d1ya1
d3w8ea_ d2fmua1 d1x1ta_ d1rpna_ d3ay7a1 d1k2wa_ d1eq2a_ d2hrba1
d3ruaa_ d2ew8a1 d4hwka_ d1xgka_ d2bd0a1 d1yo6a1 d2a35a1 d2ntva_
d1vl8a_ d1hdca_ d4bkua_ d2ag5a1 d2q45a_ d1nffa_ d4iy1a_ d1zbqa1
d1kewa_ d1ek6a_ d1ja9a_ d2q46a2 d1iy8a_ d1cyda_ d2ptga_ d1hxha_
d1uzma1 d3lyla_ d4m89a_ d3wyea_ d2blla1 d1w6ua_ d1sb8a_ d2q2va_
d4nr0a_ d1e6wa_ d2gasa_ d3oiga1 d2bgka1 d1mxha_ d1vl0a1 d1rkxa_
d1r6da_ d1wmaa1 d1e7wa_ d2c07a1 d1xkqa_ d3tzha_ d1g0oa_ d3lz6a_
d1qyca_ d1edoa_ d1gz6a_ d1oc2a_ d1t2aa_ d1zema1 d2xoxa_ d1z45a2
d1geea_ d1p33a_ d3lt0a_ d4e6pa_ d2wyua_ d1db3a_ d4q9na_ d1xhla_
d4o1ma_ d1fmca_ d2uvda_ d2gdza1 d1uaya_ d2z1ma_ d2o23a2 d2fr1a2
d2fr1a1 d1qsga_ d1qyda_ d4zjua_ d2a4ka1 d1yb1a_ d1wvga1 d1yxma1
d3f4ba_ d1mg5a_ d1xg5a1 d1r55a1 d3gboa_ d1kufa_ d1buda_ d4q1la_
d1quaa_ d1yp1a_ d1wnia_ d2gfoa1 d1vjva_ d2ayna1 d3nhea_ d1qxpa4
d1ziva1 d1zcma1 d3ioqa_ d1fh0a_ d1o0ea_ d1khqa_ d1m6da_ d2cb5a_
d1cs8a_ g8pch.1 d1s4va_ d2pnsa_ d1vsna1 d1mira_ d1iwda_ d2oula_
d7pcka_ d3bwka_ d3bcna_ d2p86a_ d1pvja_ d1pcia_ d1gece_ d1deua_
d4bs6a_ d1xkga1 d1cqda_ d1jqpa2 d2c0ya_ d2bdza_ d3p5ua_ d2o36a_

d1zoya1 d1qo8a2 d1d4ca2 d1chua2 d1jnra2 d2jk6a2 d2jk6a1 d1feca1
d1feca2 d1lpfa1 d1lpfa2 d1xhca1 d1xhca2 d1dxla2 d1dxla1 d1gera2
d1m6ia2 d1onfa1 d1onfa2 d3lada2 d3lada1 d1w4xa1 d1w4xa2 d1gesa2
d1mo9a1 d1xdia1 d1fl2a2 d1fl2a1 d1fcda1 d1fcda2 d1h6va2 d1h6va1
d1mo9a2 d1gv4a2 d1gv4a1 d2gv8a2 d2gv8a1 d1aoga1 d1aoga2 d1hyua1
d1ebda1 d1ebda2 d3dk9a1 d1q1ra2 d1q1ra1 d1gesa1 d1zbfa1 d2z1ha_
d2etja1 d3qioa1 d1jl1a_ d2e4la_ d1jl2a_ d2lsna1 d1io2a_ d1uaxa_
d2yv0x_ d1ekea_ d1i39a_ d1o94a2 d1lqta1 d1cjca1 d1s5ja1 d2hbja2
d2igia_ d1wlja_ d1qhta1 d4dlea1 d1tgoa1 d2f96a1 d3tr8a1 d3mxma_
d1q8ia1 d1y97a1 d1w0ha_ d1kfsa1 d2py5a1 d1d5aa1 d1yt3a3 d2guia_
d3mwpa1 d4kb1a_ d1js9c_ d1f15b_ d1za7a_ d1laja_ d2v76a_ d1wj1a1
d1aqca_ d1qqga2 d1qqga1 d1n3ha_ d4dbba_ d3f0wa1 d1p3ra_ d1wgua1
d2cy4a1 d3hqca_ d1wvha1 d1xr0b1 d1v58a1 d1t3ba1 d4ilfa2 d4npba2
d3nvva1 d1krha3 d2h88b1 d1n62a2 d1t3qa2 d1kf6b2 d1zoyb1 d1jq4a_
d1dgja2 d1rm6c2 d1r7ha_ d1faaa_ d1wika1 d1xfla_ d1ttza1 d2f51a1
d1ti3a_ d1nhoa_ d1sena_ d2i4aa_ d1ep7a_ d1zmaa1 d1fo5a_ d3lefa1
d3diea1 d2yana1 d1z6na1 d1oaza_ d3zywa1 d4aj8a_ d1nm3a1 d4fiwa_
d1wmja_ d4hs1a1 d1iloa_ d1xwaa1 d1wjka1 d2vm1a_ d1fb6a_ d1gh2a_
d1h75a_ d1nw2a_ d1kx5b_ d3a6na_ d4m6ba2 d2nqbd_ d3wkjd_ d2yfwe_
d4h9na_ d5bnxa_ d1kx5c_ d3r45a_ d1eqzb_ d4jjnb_ d1f66c_ d1eqza_
d4wnnb_ d1kx5a_ d1u35c1 d2nqbc_ d1kx5d_ d1ku5a_ d1f1ea_ d1tafa_
d1bh9b_ d1tafb_ d1jfib_ d1q9ca_ d1h3ob_ d2byka1 d4g92b_ d1bh9a_
d1jfia_ d1h3oa_ d2bykb1 d1n1jb_ d1pd212 d1nhya2 d2a2ra2 d1k0dd2
d1f2ea2 d1gsua2 d1f3ba2 d2fhea2 d2pvqa1 d1tw9a2 d1oyja2 d1e6ba2
d1k0ma2 d3ik7a1 d4is0a1 d1r5aa2 d1jlva2 d1m0ua2 d3gtub2 d1z9ha2
d1okta2 d1axda2 d2gsra2 d1tu7a2 d1k3ya2 d1zgma1 d1b48a2 d1gnwa2
d1gwca2 d2gsta2 d1glqa2 d1oe8a2 d1pn9a2 d1v2aa2 d1fw1a2 d1duga2
d1ovna2 d1ig8a1 d1czan2 d1czan1 d1ig8a2 d1czan4 d1bg3a4 d1bg3a3
d1bg3a2 d1bg3a1 d1nira1 d1qqya_ d1gd6a_ d1f6ra_ d1ivma_ d2gv0a_
d1iiza_ d2z2fa_ d1j8xa_ d1ghla_ d1b9oa_ d174la_ d169la_ d2o4wa_
d1p37a_ d1k28a3 d1xjta1 d1jtma_ d2f2qa_ d4e97a1 d1g4ma1 d2pjsa1
d1klla_ d2i7ra1 d1ecsa_ d1xqaa1 d5cj3a1 d1jifa_ d2p7oa_ d1r9ca_
d1nkia_ d1mpya2 d1kw3b2 d4ghga1 d1tfza1 d3isqa1 d3isqa2 d1sqia1
d1sqia2 d1lkda2 d1lkda1 d1mpya1 d1cjxa2 d1cjxa1 d1sp9b2 d1t47a1
d1t47a2 d1f1ua2 d1sp8a1 d1sp8a2 d1f1ua1 d1ro5a1 d1s5ja2 d1qhta2
d4dlea2 d1tgoa2 d3py8a2 d1q8ia2 d2py5a2 d1d5aa2 d1qsta_ d2beia1
d4m3sa_ d1u6ma1 d1ygha_ d1yk3a1 d3dr6a_ d1xmta_ d1z4ea1 d2ge3a1
d1bo4a_ d2fiaa1 d2jdca1 d1y9wa1 d3to7a_ d1wwza1 d2fl4a1 d2ozua1
d2b5ga_ d2cy2a1 d2p0wa_ d1cm0a_ d4pswa_ d1yrea1 d1yr0a1 d1m4ia_
d2gana1 d1yvka1 d1ghea_ d2g3aa1 d1y7ra1 d2euia1 d1q2ya_ d2fsra1
d1xeba_ d2ae6a1 d1cjwa_ d1qsma_ d2aj6a1 d1y9ka1 d1i12a_ d1vkca_
d2atra1 d1n71a_ d2d4pa1 d2fiwa1 d2fe7a1 d1tiqa1 d2ou2a_ d1yx0a1
d2fcka1 d1p0ha_ d2j8ma_ d2pq8a_ d1z4ra1 d1s3za1 d1ufha_ d1xvqa1
d1pn0a2 d3hy2a_ d2cvba1 d1o8xa_ d1lu4a_ d1we0a1 d2bmxa1 d2a4va1

d1psqa_ d1xiya1 d1q98a_ d1n8ja_ d1zofa1 d2ggta_ d3tjja_ d4je1a1
d1o73a_ d3s9fa_ d2zcta_ d1qmva_ d1jfua_ d2b1ka_ d5dvba1 d2b5xa1
d2v2ga_ d1st9a_ d3tb2a_ d4llra_ d4fh8a1 d1e2ya_ d1knga_ d2h30a_
d1i5ga_ d2z9sa_ d2f8aa1 d1tp9a1 d2b7ka_ d1nm3a2 d3hvva_ d2wfca1
d4kcea_ d1xvwa1 d4gqca1 d3qpma_ d4k1fa_ d1zzoa1 d1gp1a_ d1prxa_
d4od7a1 d1z6ma1 d4mcua1 d4dvca1 d1r4wa_ d3l9sa_ d1un2a_ d3rppa_
d2qked_ d4ksoa_ d1t4za1 d1wwja_ d2m5ta_ d1cqqa_ d1wqsa_ d5bpea_
d2ipha_ d1lvoa_ d2bhga1 d3tnta_ d3sjia_ d3d23a1 d2fyqa_ d3tloa_
d3zvea1 d3q3ya1 d4asha1 d1cg2a1 d1yloa2 d1y7ea2 d1y0ya3 d1lfwa1
d1vixa1 d1fnoa4 d1vhoa2 d1ysja1 d1vhea2 d1z2la1 d2fvga2 d1vgya1
d1r3na1 d2grea2 d1xmba1 g1q7l.1 d1ofda3 d1ea0a3 d1ecfa2 d1q15a2
d1ct9a2 d1ao0a2 d1te5a_ d1ys1x_ d4hs9a_ d1ispa_ d1ex9a_ d1ku0a_
d3qmma_ d1lgya_ d4l3wa_ d1gz7a_ d1ukca_ d1cr6a2 d3groa_ d1thta_
d1ei9a_ d1ra6a_ d3gsza_ d1khva_ d1te9a_ d2xwha_ d3uqsa_ d3hkwa_
d3n6ma_ d2cjqa_ d2xyma_ d1teba_ d1te8a_ d1s48a_ d1u09a1 d4wfxa_
d3tyqa_ d2pgga1 d3zxha_ d1cgla_ d1rm8a_ d1eaka2 d1hy7a_ d2jsda_
d1cxva_ d1hv5a_ d1i76a_ d3p0ya1 d1igra1 d1igra2 d1n8yc2 d1n8yc1
d1m6ba2 d1m6ba1 d4dv8a2 d1jx4a2 d3gv5b1 d1t94a2 d3bq0a2 d1ohfc_
d2tbvc_ d1c8nc_ d1x36a_ d1smvc_ d1ng0c_ d4sbvc_ d1f2nc_ d1cixa_
d1y29a_ d1v7fa_ d2jtba_ d1d1ha_ d1g9pa_ d1lupa_ d1qk7a_ d1mb6a_
d2a2va_ d1niya_ d1i25a_ d2mpqa_ d1ie6a_ d1kqha_ d1la4a_ d2mxoa1
d1dl0a_ d1koza_ d1qk6a_ d1emxa_ d1lqra_ d1c6wa_ d1rmka_ d1eyoa_
d1cnna_ d1fu3a_ d1g1pa_ d1f3ka_ d1fyga_ d1rypd_ d1irua_ d1rypc_
d3unfk_ d1rypa_ d1j2pa_ d1irui_ d3unfh_ d1m4ya_ d1rypb_ d1irul_
d1irug_ d1iru2_ d1q5rh_ d1rypg_ d1g3ka_ d1iruf_ d1q5qa_ d1e94a_
d1irub_ d1ryph_ d3ty6a_ d1iruj_ d1iruk_ d1rype_ d1irud_ d1irue_
d3mi01_ d1rypf_ d1iruh_ d2z3ba_ d1iruc_ d3mka2_ d1iru1_ d1ny721
d1ny722 d1a6ca3 d1a6ca2 d1a6ca1 d1pgl22 d1pgl21 d1pgl11 d1ny711
d1mqta_ d1d4m3_ d1fmd3_ d2mev1_ g1aym.1 d1ev13_ d4q4w1_ d1r1a3_
d2x5ia_ d1rhi1_ g1r1a.1 g1d4m.1 g1rhi.1 d1b35b_ d1oopa_ d1tme1_
d4iv1a_ g1pvc.1 g1fpn.1 d1fpn1_ d4gb31_ d1d4m1_ d1fmd1_ d1ev11_
d2wzr3_ d1qqp3_ g1fmd.1 d2x5ic_ d1rhi3_ g1oop.1 d1hxs1_ g1hxs.1
d1fpn3_ d1bev1_ g2mev.1 d1eah3_ d1aym1_ d1pvc3_ d1qqp1_ d1zba3_
d1h8ta_ d3vbhc_ g1b35.1 g1bev.1 d1hxs3_ g1ev1.1 d1bev3_ g1tmf.1
d1tmf1_ d1b35a_ d2mev3_ g1eah.1 g1h8t.1 d1eah1_ d4q4w3_ d1r1a1_
d1aym3_ d1pvc1_ d1zba1_ d1h8tc_ d1oopc_ d1tme3_ g1qqp.1 d4iv1c_
g1tme.1 d3ubub_ d1egia_ d1v7pa_ d1tdqb_ d3ubua_ d1c3ab_ d1h8ua_
d3bx4b_ d1t8ca1 d1sb2a1 d1qdda_ d2ziba1 d3cada_ d1byfa_ d1j34b_
d1jwia_ d1jzna_ d1c3aa_ d1oz7b_ d1wmza_ d3bdwb_ d1hq8a_ d1fifa1
d1dv8a_ d3hupa_ d1wk1a1 d1umra_ d2afpa_ d3bx4a_ d1gz2a_ d1sl6a_
d1v7pb_ d3g8ka_ d1ixxa_ d1j34a_ d1jwib_ d1g1sa1 d1y17a1 d1ja3a_
d1fvub_ d3bdwa_ d1sb2b1 d1fvua_ d1umrc_ d1oz7a_ d4m17a_ d3cfwa1
d1ypqa1 d1g1ta1 d3fd4a_ d3l9jc_ d4dn8a1 d1v4lb_ d1rdl1_ d2msba_
d4w9ta_ d4x2ra1 d1ka9f_ d1qo2a_ d4axka1 d1thfd_ d1h5ya_ d4u28a_

d2y88a_ d4wd0a_ d1vzwa1 d1eixa_ d3exra_ d2ffca1 d2fdsa1 d3n3ma1
d2czda_ d1q6oa_ d2yyua_ d1vqta1 d1dbta_ d3ldva1 d2flia1 d1rpxa_
d1tqja_ d1tqxa_ d4lt9a1 d3tc7a1 d3hoja_ d1i4na_ d3vnda_ d3nl8a1
d1geqa_ d3nava1 d1v5xa_ d4a2ra1 d3tc6a1 d1ujpa_ d4ijba_ d1xcfa_
d1vc4a_ d1rd5a_ d1qopa_ d3b5va_ d2r80b_ d1cqxa1 d1cg5a_ d1gcvb_
d3boma_ d2h8fb_ d2raoa_ d3bj1a_ d4hrrb_ d2r50a_ d3d1kb_ d2aa1b1
d3zhwa_ d3hf4b_ d3gdja_ d1jebb_ d3bj1b_ d1x9fb_ d1wmub_ d1gcva_
d3bcqb_ d3d4xb_ d1gvha1 d2dc3a_ d3hf4a_ d4yu3a_ d3k8ba_ d1qpwb_
d2r80a_ d3bcqa_ d1v4wa_ d3wtgb_ d1jl7a_ d4esaa_ d1a9we_ d3d1ab_
d4h2la_ d1v4wb_ d1mwca_ d1vhba_ d1jeba_ d1spgb_ d1h97a_ d1q1fa_
d1tu9a1 d3d1aa_ d1hdsb_ d3hyub_ d2zfbb_ d3fs4a_ d1oj6a_ d1shrb_
d4h2lb_ d4yu3b_ d1spga_ d3hrwa_ d3qqra_ d3at5a_ d4wchd_ d3hyua_
d1cg5b_ d1uc3a_ d1hbra_ d2raob_ d1outb_ d4esab_ d3gdjb_ d3qqqa_
d1x9fc_ d3vrfa_ d1itha_ d3mkba_ d3d4xa_ d3mjpa_ d1jl6a_ d3fh9a_
d1outa_ d2dc3b_ d1it2a_ d3eoka_ d3g46a_ d1qpwa_ d3vrfb_ d1ch4a_
d1x9fd_ d1hdsa_ d2zfba_ d3mkbb_ d1or4a_ d3wtga_ d1a4fa_ d3fh9b_
d3bomb_ d3cy5a_ d3at5b_ d1x9fa_ d2oifa_ d4lmxb_ d1cpcb_ d3v57a_
d1kn1a_ d1cpca_ d4f0ta_ d2vjha_ d4po5b_ d1eyxa_ d1alla_ d4f0tb_
d1b33b_ d1b8db_ d4h0mb_ d1b33a_ d2vjhb_ d4lm6b_ d2vmlb_ d1allb_
d2vjta_ d3dbja_ d2vmla_ d2vjtb_ d4f0ub_ d1f99b_ d4rmpa1 d1liaa_
d1eyxb_ d2uulb_ d4lmsb_ d4f0ua_ d2bv8a_ d1f99a_ d2bv8b_ d2c7lb_
d3dbjb_ d4rmpb1 d1b8da_ d4h0ma_ d3v57b_ d1liab_ d2uula_ d2j96a_
d1pjqa1 g1wht.1 d2xqfa_ d1ea5a_ d1f6wa_ d1dx4a_ d1qe3a_ d1k4ya_
d4qwwa_ d2ha2a_ d1r88a_ d1sfra_ d4qeka_ d1rpma_ d2shpa1 d1yfoa_
d1l8ka_ d2ooqa1 d1g4us2 d1p15a_ d1lara2 d1lara1 d1wcha_ d2fh7a2
d2fh7a1 d2h0ba_ d2erfa_ d1h30a2 d1h30a1 d3mw4a1 d2r1ba_ d3v64a1
d1d2sa_ d3bopa1 d3mw3a1 d1okqa1 d1okqa2 d1jmxa4 d1pbya3 d1pbya4
d4iuaa3 d4bvva_ d5hpga_ d3k65a_ d1ki0a3 d1ki0a2 d1ki0a1 d1pmla_
d3sp8a3 d2feba1 d1gvfa_ d3qm3a1 d2fjka_ d3l21a_ d2e1da_ d1to3a1
d1w3ia_ d1sfla_ d1n7ka_ d1vcva1 d4imea_ d1wa3a_ d2ocza1 d1f2ja_
d3m16a_ d2yxga_ d1ub3a_ d3a5fa_ d1fbaa_ d1l6wa_ d1xfba1 d1wx0a1
d1vpxa1 d1ojxa_ d1mxsa_ d2a4aa1 d1xkya1 d3cq0a1 d3s1xa_ d1f05a_
d1fdja_ d3cpra1 d4d2ja_ d1l6ja5 d1l6ja4 d1eaka4 d1eaka3 d1l6ja3
d1h8pa2 d1h8pa1 d3rp2a_ g1ucy.1 d1fuja_ d2zchp_ d3w94a_ d1si5h_
d1lo6a_ g2pka.1 d1cgha_ d1kigh_ d1a5ia_ d3s9ba_ d1hj8a_ d1ao5a_
d2anya_ d1q3xa1 d1gvza_ d1azza_ d4gsoa_ d2f91a1 d1iaua_ d1gdna_
d1eufa_ g1fiw.1 d1eq9a_ d4nfea_ d2rdla_ d1spja_ d1elva1 d1a7sa_
d1ekbb_ d1pfxc_ d1yc0a_ d2aiqa_ g1fiz.1 d1fi8a_ d4k60a_ d4dgja_
d1mzaa_ d1op0a_ d3tjva_ d1pytd_ d1sgfg_ g1gg6.1 d2eeka_ g1rtf.1
d1a0ja_ d3g01a_ d1pytc_ d4e7na_ d1ddja_ d1z8ga1 d2hlca_ d1npma_
d1brup_ g1jou.1 d1sgfa_ d1autc_ g2hnt.1 g1kdq.1 d3s69a_ d4crga_
d1fxya_ d1bqya_ d2ftba_ d1pmpa_ d2rcqa1 d1fdqa_ d4lkpa_ d1sa8a_
d1vyfa_ d1lpja_ d3vg7a1 d4azqa1 d1o8va_ d5bvqa_ d1p6pa_ d2qo4a_
d1opba_ d1yiva1 d1o1va_ d4bvma1 d4tkba_ d4i3ba_ d1bwya_ d1ggla_

| | | | | | | | |
|---|---|---|---|---|---|---|---|
| d4gkca_ | d1cbia_ | d3p6da1 | d1kqwa_ | d2a0aa1 | d1m4la_ | d2bk7a_ | d3fjua_ |
| d1z5ra1 | d1kwma1 | d1uwya2 | d1jqga1 | d2c1ca1 | d1h8la2 | d3lgia_ | d3i77a_ |
| d2z9ia2 | d4nsva_ | d1lcya2 | d4jcna_ | d1agja_ | d1os8a_ | d1p3ca_ | d1l1ja_ |
| d1qtfa_ | d1e69a_ | d1mv5a_ | d3g60a4 | g1ii8.1 | d1ewqa2 | d1ji0a_ | d1qhla_ |
| d1v43a3 | d2wmfa1 | d1vpla_ | d1g2912 | d4a82a2 | g1f2t.1 | d2d62a1 | d3tuic1 |
| d2bbsa1 | d3g60a3 | d2ixea_ | d1g6ha_ | d1sgwa1 | d2qi9c_ | d3rlfa1 | d3b60a1 |
| d3d31a2 | d2pzea1 | g1xew.1 | d1jj7a_ | d1oxxk2 | d2onka1 | d1w1wa_ | d1iwea_ |
| d1qf5a_ | d1vmaa2 | d1nija1 | d1np6a_ | d2p67a1 | d3ihla_ | d1okkd2 | d1ls1a2 |
| d1s1ma2 | d1iona_ | d2v40a_ | d1dj3a_ | d3hida_ | d2vo1a1 | d2g0ta1 | d1vcoa2 |
| d1j8mf2 | d1g3qa_ | d1xjca_ | d1p9ba_ | d1qzxa3 | d1ihua2 | d1ihua1 | d2qm8a_ |
| d1dj2a_ | d4ioja_ | d1cp2a_ | d1yrba2 | d3gqba2 | d1xp8a1 | d1tf7a2 | d1tf7a1 |
| d1ye8a1 | d1skyb3 | d2qe7d2 | d4blta_ | d4huta_ | d3ldaa2 | d4hyya_ | d2i3ba1 |
| d1cr1a_ | d3j9tb2 | d3ntua2 | d1vdza1 | d2z43a_ | d1e9ra_ | d1nlfa_ | d3ziaa2 |
| d1n0wa_ | d1g6oa_ | d3ziad2 | d3j9ta2 | d1pzna2 | d3gqbb2 | d2i49a_ | d1nnfa_ |
| d1xvxa_ | d1ursa_ | d1h3da1 | d1iuda_ | d4m1va1 | d1twya_ | d3e13x_ | d2nxoa1 |
| d4pfua_ | d3ttma_ | d1elja_ | d2qmwa1 | d1us5a_ | d1y3na1 | d1jeta_ | d1uqwa_ |
| d4f19a1 | d2esna2 | d3thia_ | d3fasa1 | d2uyeb_ | d2xwva1 | d1r9la_ | d2fyia1 |
| d1pb7a_ | d2ozza1 | d1sw5a_ | d1nh8a1 | d1vr5a1 | d2g29a_ | d1xs5a_ | d3lswa_ |
| d2z23a_ | d3w9va_ | d3tcha_ | d1q35a_ | d1z7me1 | d3q27a_ | d1p99a_ | d1ve4a1 |
| d2i6ea1 | d4mcoa1 | d4n8ya_ | d1eu8a_ | d1zbma1 | d1hsla_ | d1ixca2 | d1j1na_ |
| d1xvya_ | d1y9ua_ | d2z22a1 | d1xoca1 | d4oera_ | d1wdna_ | d5f1qa1 | d1xt8a1 |
| d1i6aa_ | d4gwoa_ | d4igta1 | d1pc3a_ | d4mmpa1 | d1o63a_ | d2czla1 | d2onsa2 |
| d4mija1 | d4ok5a1 | d2bmfa1 | d2bmfa2 | d1yksa1 | d1yksa2 | d1jr6a_ | d2qeqa1 |
| d2jlqa1 | d1a1va2 | d1eh3a_ | d3skpa_ | d1dtza2 | d1ieja_ | d1jnfa2 | d1ce2a1 |
| d1jnfa1 | d1b1xa2 | d1b1xa1 | d1jw1a1 | d1jw1a2 | d1ryoa_ | d1h76a1 | d1h76a2 |
| d1etha2 | d1w52x1 | d2ppla1 | d1hpla2 | d1lpbb2 | d2oxea1 | d1bu8a2 | d1rp1a2 |
| d1c4oa1 | d2p6ra3 | d2p6ra4 | d1oywa2 | d1oywa3 | d1wrba1 | d1w36b2 | d1w36b1 |
| d1c4oa2 | d3bora_ | d1t6na_ | d2eyqa5 | d2eyqa3 | d2eyqa2 | d2zu6a2 | d1veca_ |
| d1uaaa1 | d1w36d1 | d2g9na1 | d1z3ix2 | d2rb4a_ | d1hv8a1 | d1hv8a2 | d1t5ia_ |
| d1w36c2 | d2j0sa2 | d2j0sa1 | d1fuka_ | d3hjha1 | d1z5za1 | d1qdea_ | d1z63a1 |
| d1s2ma2 | d1s2ma1 | d1wp9a1 | d1wp9a2 | d1gm5a3 | d1gm5a4 | d3dina4 | d3dina3 |
| d1q0ua_ | d1w36c1 | d1z3ix1 | d1nkta4 | d1uaaa2 | d1w36d2 | g1qhh.1 | d2fwra2 |
| d2fwra1 | d3q3ma_ | d1za0a1 | d1mxra_ | d2inca_ | d1jk0a_ | d2incb_ | d1mhyb_ |
| d3hf1b_ | d1mtyb_ | d1r2fa_ | d2uw1a_ | d1mhyd_ | d3n37a_ | d1afra_ | d1oquc_ |
| d1syya_ | d2o1za_ | d1uzra_ | d1smsa_ | d2fkza_ | d1zuja1 | d1s3qa1 | d2fjca1 |
| d1z6om1 | d1veia_ | d4dyya_ | d1jiga_ | d3gvya_ | d2pyba_ | d4cy9a1 | d3bvfa1 |
| d1r03a_ | d2cf7a_ | d1nf4a_ | d1zs3a1 | d4iwka_ | d3ka8a_ | d3np0x_ | d1n1qa_ |
| d1lb3a_ | d4e6ka_ | d1z6oa1 | d2fzfa1 | d4dyua_ | d3ge4a_ | d1jgca_ | d4am4a_ |
| d2z6ma_ | d1ji5a_ | d1vlga_ | d1lkoa1 | d1krqa_ | d3qz3a_ | d1o9ra_ | d2ffxj_ |
| d1euma_ | d1yv1a1 | d2v2pa_ | d1j30a_ | d4di0a_ | d1dpsa_ | d1oewa_ | d2qzxa_ |
| d2asia_ | d1psoe_ | d1miqa_ | d1smra_ | d2h6ta_ | d1pfza_ | d1izda_ | d3hd8a_ |
| d1j71a_ | d1dpja_ | d4aa9a_ | d1t6ex_ | g1b5f.1 | g1lya.1 | g1htr.1 | d1qdma2 |
| d1bxoa_ | d1wkra_ | d1tzsa_ | d1ibqa_ | d2qzwa_ | d1ex4a2 | d1c6va_ | d1bdqa_ |
| d3ggta_ | d3ixoa_ | d1mrxa_ | d2haha_ | d1tw7a_ | d4njsa_ | d1sgua_ | d2p3da_ |

d1nsoa_ d3d3ta_ d2rkfa_ d1c6ya_ d2rspa_ d3ogpa_ d3ekwa_ d2o4na_
d2j9ja_ d1baia_ d3ggua_ d3lzua_ d4npua_ d4j55a_ d2r8na_ d2fxda_
d2p3ca_ d4dqea_ d3ttpa_ d3t3ca_ d2p3aa_ d2hc0a_ d2fdda_ d3mwsa_
d1um8a_ d1ixza_ d1qvra2 d1qvra3 d1qzma_ d1sxjc2 d1jr3d2 d2p65a_
d1l8qa2 d1g8pa_ d3hu3a3 d1sxjd2 d1njfa_ d1in4a2 d1sxja2 d1tuea1
d2ce7a2 d1w5sa2 d1sxjb2 d1d2na_ d1ny5a2 d2gnoa2 d2a5yb3 d1fnna2
d1u0ja1 d1lv7a_ d1iqpa2 d2fnaa2 d1ixsb2 d4gita_ d1sxje2 d1g41a_
d1k8rb_ d1wgra1 d1ef5a_ d1wfya1 d1e8ya3 d1wgya1 d1lfda_ d2cs4a1
d1wxma1 d2byea1 d3kh0a1 d2byfa1 d1wxaa1 d1e7ua3 d4yl8a1 d2al6a3
d1wgda1 d1wgga1 d4q5eb_ d1wjna1 d1se9a_ d3n3kb_ d1wz0a1 d1we6a1
d2m0xa_ d2gbka_ d3rt3b1 d1wh3a1 d1sifa_ d2ekec2 d4ii2b1 d4hk2a1
d1c3ta_ d1m94a_ d1wgha1 d1ttna1 d1wx8a1 d3v6cb1 d2ma9b_ d4i6lb1
d1bt0a_ d2jvca1 d1iyfa_ d2k1fa_ d1wxva1 d1wf9a1 d1t0ya_ d2io0b2
d1x1ma1 d2gbma_ d1zw7a1 d2m17a_ d1ud7a_ d1we7a1 d2lwpa_ d2gbja_
d1wjua1 d1v6ea1 d1v86a1 d4eewa_ d1wiaa1 d4icva_ d4gu2a_ d1wx7a1
d1wy8a1 d4hjka1 d2faza1 d1v5oa1 d4lu0a_ d3unda1 d3e9aa1 d1vr6a2
d1n8fa_ d2qkfa_ d1d9ea_ d1o60a_ d1pn0a1 d1ju2a1 d2f5va1 d3c96a1
d2gmha1 d1pj5a2 d1gpea1 d1o5wa1 d1n4wa1 d1k0ia1 d2dw4a2 d2ivda1
d1seza1 d2voua1 d1kdga1 d1wioa4 d1ccza2 d1vcaa1 d1ic1a1 d2d9qb1
d2nxyb2 d1adqa1 d1fnga1 d1uvqb1 d1zs8a1 d2vola1 d2vola2 d1mfbl2
d2jelh2 d1uwel2 d3qq4b1 d1kjvb_ d1k5na1 d1osph2 d4odhl2 d1ow0a1
d1ow0a2 d1bfob2 d3l9ra2 d3jtth1 d2fath2 d4elkb2 d1fn4b2 d2xfxb_
d1pg7x2 d1cqka_ d4k71b2 d1lk2a1 d1eapb2 d4lfhg2 d1i4fa1 d2nw2a2
d1kfah2 d1mhca1 d1h3ua1 d1wejh2 d1h0db2 d1a5fh2 d1kcvh2 d1mjuh2
d1qqda1 d1kcrh2 d1k8ia1 d3huja2 d2nnab1 d1nfdf2 d3mffb2 d1s9va1
d1lk3h2 d1q0xl2 d1fv1b1 d1neza1 d4rfeb2 d1r3ha1 d1a6tb2 d1kjva1
d1pz5b2 d1onqa1 d2bc4a2 d1de4a1 d1k8da1 d1tcra2 d1dn0b2 d1nfde2
d2xwtb2 d1bfoa2 d1mexh2 d1iakb1 d1ypzf2 d4f7ea2 d1q72l2 d2fbjh2
d1fe8h2 d1clzh2 d1fngb1 d1uvqa1 d1vgka1 d1c16a1 d4j12a2 d4x99b2
d1igtb3 d1je6a1 d1q72h2 d1nldh2 d1teth2 d2hmic2 d1i1ca2 d1i1ca1
d1k8ib1 d1q0yh2 d1kfal2 d3frua1 d1pg7w2 d1u58a1 d1igyb4 d1igyb3
d1hyrc1 d1klub1 d1deeb2 d1c12b2 d2bc4b2 d2ig2h2 d1kcvl2 d3wn5b1
d1lk2b_ d1lk3l2 d1mjul2 d1sm3h2 d1q0xh2 d1zvsa1 d1ydpa1 d1u8ib2
d1ypze2 d1kcuh2 d1svpa_ d1mbma_ d3su6a1 d1cu1a1 d4agka_ d1vcpa_
d1q31a_ g1n1l.1 d3e90b_ d1ep5a_ d3u1jb_ d2e3fa_ d1mm0a_ d2e3ea_
d2llda_ d2ny8x_ d2e3ga_ d2ny9x_ d2nz3a_ d2lj7a_ d1n4na_ d2m8bb1
d2kyqa_ d3psma_ d1jkza_ d1jxca_ d2gl1a_ d1ugla_ d4uj0a_ d2k4ua_
d1hp2a_ d2lu9a_ d1baha_ d2meoa1 d1wm7a_ d1hlya_ d1n8ma_ d1wpda_
d1c55a_ d1pvza_ d1wt7a_ d1rjia_ d1wz5a_ d1m2sa_ d1pjva_ d1c49a_
d1qkya_ d2ktca1 d1wmta_ d1du9a_ d1j5ja_ d1r1ga_ d1t0za_ d2i61a_
d1t1ta_ d1sn4a_ d1kv0a_ d2lkba_ d1npia_ d1wwna_ d1pe4a_ d2kbka_
d2li7a_ d1djta_ d2kbha_ d1dq7a_ d1sega_ d1omya_ d1zywa1 d1jzaa_
d1n9wa1 d1bbua1 d1e1oa1 d1c0aa1 d1b8aa1 d3a74a1 d1eova1 d1t61c1
d1iyjb4 d1iyjb5 d1iyjb3 d1o7ia_ d4ipca1 d1x3ea_ d4apva_ d1jb7a1

d1jb7a2 d1jb7a3 d3kdfa_ d1ue1b_ d1wjja1 d3eiva_ d1xjva2 d1xjva1
d2ccza2 d1se8a_ d1l1oc_ d1jb7b_ d1qvca_ d3ulla_ d1qzga_ d1miua4
d1miua5 d1miua3 d3g48a1 d1gd7a_ d1mkha_ d1jjcb3 d1pyba_ d1y14b1
d2nn6i1 d1i94l_ d2zjra2 d1smxa_ d2qamc2 d1hh2p1 d2r7da1 d1rl2a2
d1wfqa1 d3bzka4 d1x6oa2 d3a0ja_ d2j01d2 d2ja9a1 d4ql5a_ d1hr0w_
d1luza_ d2n3sa_ d2vnud1 d1h95a_ d1go3e1 d2nn6h1 d1ne3a_ d2mo1a_
d1iz6a2 d1jjga_ d1e3pa2 d2ix0a3 d2ix0a2 d2lxja_ d1ueba3 d1ueba2
d2z0sa1 d1u0la1 d2ix0a1 d2eifa2 d2je6i1 d3i2za1 d2c35b1 d2ahob2
d1t9ha1 d3i4oa_ d2qall1 d1jt8a_ d2nn6g1 d1g6pa_ d3cama_ d1i94q_
d1c9oa_ d1d7qa_ d2ba0a1 d2vnud2 d2vnud3 d1khia2 d2qalq1 d1wi5a1
d1xtda2 d1ny4a_ d1dzka_ d4tlja_ d2a2ua_ d4wfva_ d3t1da_ d4iaxa_
d3zq3a_ d2nnda_ d1i4ua_ d1kxoa_ d1bbpa_ d1kt7a_ d1exsa_ d3s26a1
d1lf7a_ d1qfta_ d4gh7a_ d1z24a1 d1qabe_ d1xkia_ d3bx7a_ d4iawa1
d1n0sa_ d1gkab_ d3kzaa_ d2k23a_ d2l9ca1 d2joza1 d1gm6a_ d3kffa_
d3saoa1 d1e5pa_ d1yupa1 d1iiua_ d2a1ka_ d1je5a_ d1ohea1 d1ohea2
d2f4da_ d1rxda_ d2hxpa_ d1xria_ d1d5ra2 d1fpza_ d3ezza_ d4y2ea_
d3azya_ d3wvja_ d1upsa1 d1ajoa_ d2hyka_ d1ajka_ d2vy0a_ d1o4ya_
d3o5sa_ d3d6ea_ d3atga_ d1dypa_ d1umza_ d3i4ia_ d1c1la_ d4lbqa_
d1a78a_ d1slta_ d4no4a_ d3zsja_ d3duia_ d1is3a_ d1qmja_ d1hlca_
d1tdha3 d1q3ba3 d1ee8a3 d1k82a3 g1qmo.1 g1len.1 d3zyra_ d1fnya_
d1n47a_ g2ltn.1 d1avba_ d3usua_ d1dhkb_ d1f9ka_ d1ukga_ d1g8wa_
d1g9fa_ d1wbfa_ d3wcsa_ d1g7ya_ g1loe.1 d1mvqa_ d1hqla_ d1v6ia_
d1dbna_ d1qnwa_ d1fx5a_ d4l8qa_ d3n35a_ d3ubra_ d1qo8a1 d1d4ca1
d2k3va_ d1ft5a_ d1qdba_ d1m1qa_ d2j7aa_ d1h21a_ d1ogyb_ d4fasa_
d1jnia_ d1oaha_ d1sp3a_ d1eysc_ d3h33a_ d1rwja_ d19hca_ d1os6a_
d3bxua_ d3h4na_ d1i77a_ d3caoa_ d2ctha_ d1duwa_ d1ofwa_ d2e84a_
d1hh5a_ d1gyoa_ d3h34a_ d1dszb_ d1a6ya_ d3cbba_ d1ynwa1 d1r4ia_
d1hcqa_ d2hana_ d2hanb_ d2enva1 d1kb4b_ d2nllb_ d1lo1a_ d1cita_
d2dara2 d2dara1 d1wyha2 d2dloa2 d1rutx4 d1rutx3 d2cuqa2 d1x4la2
d1x4la1 d1x3ha2 d1x3ha1 d2d8za1 d1x61a2 d1x6aa1 d1x6aa2 d2cora2
d2cora1 d1v6ga1 d1v6ga2 d2d8za2 d1x64a1 d1x64a2 d1b8ta1 d2co8a1
d1x63a2 d2dj7a2 d2dj7a1 d2cu8a2 d2cu8a1 d1g47a2 d1g47a1 d1j2oa1
d1wyha1 d2dloa1 d1wiga1 d1wiga2 d2cupa2 d2cupa3 d1x68a1 d2d8ya2
d2d8ya1 d1m3va2 d1x62a1 d1x68a2 d1x61a1 d1b8ta3 d1b8ta2 d2d8xa2
d2d8xa1 d1x4ka1 d1x4ka2 d2cuqa1 d1m3va1 d2co8a2 d1j2oa2 d1x63a1
d2cura1 d2cura2 d1x62a2 d1op9a_ d1y0la1 d1etzb1 d1egjl1 d2gsib1
d1nj9a1 d4jfhd1 d1aqkl1 d1t2ja_ d1lo4h1 d1zvya1 d3v4vl1 d4enec1
d1kxtb_ d5e8ea1 d2pxya_ d4weud_ d1xiwc_ d2esvd1 d1qnzh_ d1ogae1
d1f4xh1 d4n1hb_ d3ezjb1 d3mlwl1 d1kfah1 d1etza1 d1b4jh1 d3cfic_
d4w68a_ d2arja1 d1ek3a_ d2ok0l1 d1vera_ d1uwgh1 d1blnb1 d1fpth1
d1rzga1 d2giya1 d4m3kb1 d1nc2a1 d2nyyd1 d1ncna_ d1fbih1 d5dxwa_
d4fqqa1 d1dqdh1 d1jfql1 d1kelh1 d1faih1 d1frgl1 d1figl1 d4m6nl1
d1fo0a_ d4hxbl1 d3b5ga_ d3quyd1 d2h9gb1 d1i8kb_ d2dd8h1 d2ck0h1
d1eaja_ d4w6wb1 d2a6ib3 d1t66d1 d2e27h_ d1ob1b1 d1f11b1 d2v7na1

d1ad0a1 d2xt1b1 d1pskh1 d4i13b1 d2op4l1 d1a0ql1 d1uwel1 d1za3a1
d1ncbl1 d4uu9a_ d1bm3h1 d1cloh1 d1nqba2 d1cf8h1 d4y5va_ d1mhph1
d5anma1 d4cdgc1 d4ldeb_ d1y0lb1 d2pcpb1 d1z3gl1 d1dfbl1 d1fh5h1
d3g5xa1 d1tzib1 d4dn3l1 d3zhka1 d2b1hh1 d2uyla1 d1f3dh1 d1nj9b1
d2agjh1 d2h1pl1 d1q72h1 d8fabb1 d1ndmb1 d1rjca1 d4u6hb1 d3upca_
d2fbjl1 d1osph1 d1tvda_ d2cjvh_ d4fhbd1 d1bd2d1 d1rz7h1 d3nn8a_
d1blna1 d1knoa1 d1nfda1 d2bmkb1 d1dqll_ d1igyb1 d1igfl1 d12e8h1
d3utza1 d1nc2b1 d1rihh1 d4mwfb1 d4jvpa1 d1mieh1 d1rhfa1 d1fgnl1
d1sbsh1 d1lp9e1 d1dn0a1 d4lqfl1 d1iaih1 d1kgcd1 d1mh5b1 d1gpol1
d2g60h3 d1f8tl1 d2jixd1 d4grwh_ d1fo0b_ d1bfob1 d1axsa1 d1u9ka_
d1nmbh_ d1h8na1 d1h8na2 d1jguh1 d4eura1 d3sdyl1 d35c8h1 d1w72l1
d1mvub_ d1i9rh1 d1ob1a1 d4wenb_ d1yeeh1 d3ietc1 d1f58h1 d1ad0b1
d1mqkl_ d1nbvh1 d4b50a_ d3r8bb_ d1bafl1 d1mamh1 d4rgne1 d2gsgb_
d35c8l1 d1bafh1 d1ai1h1 d2pcpa1 d2fath1 d2x1pa_ d1orsb1 d1ibgl1
d1shma_ d1a2yb_ d1u0qa_ d2coqa_ d1hilb1 d1qfwi_ d1jv5b_ d1fn4b1
d5e0qa_ d1nsnh1 d1plgl1 d4ocxl1 d1kb5h1 d1rzfh1 d1pg7x1 d2fx7h1
d2oi9c_ d1l7il1 d3eyva1 d1d5il1 d4ffyl_ d1tetl1 d1a6wh_ d1ac6a_
d3dvfa_ d1nfde1 d1mimh1 d1ri8a1 d1jhlh_ d3dura_ d1fskb1 d1f97a1
d3so3b1 d2ak4d1 d1lp9f1 d4z7wf1 d1l7th1 d4y5ya_ d4x7ec1 d1emth1
d1ikfl1 d1nlbh1 d4utal1 d1bjma1 d1eapb1 d1u6ah1 d2dtgd1 d1kj2b_
d1xeda1 d1c1eh1 d1fgvh_ d2bnub1 d2fb4h1 d2dtgb1 d1ktrh_ d1jpth1
d1qkzl1 d4hepg_ d2atpb1 d1flrh1 d2hrpl1 d1mvfa_ d1iaim1 d3k80a1
d2jb5h1 d3zkxc_ d4grwe_ d1it9h1 d1rhhb1 d3w9ec_ d1e4wh1 d1sy6h1
d2j6eh1 d3qxta_ d1jglh1 d1p7ka1 d1tzhb1 d4n8pa1 d1gafl1 d2jelh1
d1wioa2 d4i0cc1 d1cicb1 d1lmka2 d1lmka1 d1iqwh1 d1orqb1 d1ejoh1
d1jnlh1 d4zg1a_ d43c9b_ d1ggbl1 d1wejh1 d3q6fa1 d1f3dj1 d3c6la1
d1bqlh1 d3p9wb_ d1a3rl1 d1h0db1 d2uzil_ d1eo8l1 d1qfwl_ d1n7ml1
d1igja1 d1c5ch1 d2a9mh_ d1fj1b1 d4heme_ d1ccza1 d1a5fh1 d1ogad1
d1dlfh_ d1qa9b_ d1vgel1 d3qskb_ d1sjxa_ d4ozfg1 d1sq2n_ d1qd0a_
d1kcvh1 d1c5db1 d3qcul1 d8faba1 d3bn9d1 d1mjuh1 d4y8dc_ d7fabh1
d3dvgb1 d1lo0l1 d1a6tb1 d1gigh1 d1kcrh1 d2hwzl1 d1yjdc1 d3clfl1
d4krna_ d1dl7l_ d1e6ol1 d1mexh1 d3r0ma_ d4kkna_ d1fnsl1 d1oaqh_
d1rzia1 d1ucbh1 d1ncwl1 d4b41a_ d1nfdf1 d2mpah1 d2d7tl1 d4grwf_
d4b5ea_ d1lk3h1 d3u79a_ d4u3xa_ d1ghfl1 d1bvkb_ d1mcww1 d1b88a_
d1jrhh1 d32c2b1 d4wemb_ d1dsfh_ d1op3h1 d1ce1l1 d1iqda1 d1qoka1
d1qoka2 d1qfuh1 d2crya1 d1ay1h1 d1oaql_ d3ab0b_ d1rurl1 d1t4kb1
d1bz7b1 d1n0xh1 d1forl1 d1ucbl1 d4w6yb_ d43c9a_ d2aabl1 d1tzia1
d4n1ca_ d1k6qh1 d3g9ab_ d4tvsa1 d1igmh_ d1pz5b1 d3dx9a1 d3wxwh_
d1nakh1 d1kcul1 d3eaka_ d4qlra1 d1cr9l1 d1d9ka_ d1f4xl1 d1deea1
d2i27n2 d1c12a1 d2orbl1 d1j8hd1 d1mcph1 d1c5da1 d1kenh1 d1bd2e1
d1h5ba_ d4x7fc1 d3upaa_ d1t3fa1 d1indh1 d2dtgc1 d4hc1l1 d1zv5a1
d4wgvb_ d1uwgl1 d4f37k1 d1aqkh1 d1vesa_ d3zkqd_ d1fptl1 d1lo4l1
d1knob1 d3rrqa_ d1ct8b1 d4orzc_ d1mlbb1 d4hgkc1 d1dqdl1 d1bogb1
d4ozhe1 d1dr9a1 d1i7za1 d1dn0b1 d1kell1 d1rzib1 d1ypzf1 d1wtla_

d1hkfa_ d1fail1 d1figh1 d2vyre_ d1sjva_ d1ohqa_ d1bfoa1 d1pkqb1
d1mjul1 d1b4jl1 d1nsnl1 d2arjb1 d1p4bh_ d4aj0a_ d3nn8b_ d2rcja1
d1f2xk_ d4v1dd_ d1adqh1 d2eksa_ d4k3ga_ d1jfqh1 d1uweh1 d1frgh1
d4gftb_ d1uz8b1 d4n1ea_ d1bm3l1 d1clol1 d3mcka1 d2vq1a1 d1ae6h1
d4aq1b1 d1uj3b1 d1nbqa1 d1fh5l1 d2ck0l1 d2ywza_ d1q72l1 d4nbxb_
d1hila1 d4hzlb1 d2j88l1 d1zmya1 d1cd0a_ d1a4ka1 d1pskl1 d1a0qh1
d4krmb_ d1ncbh1 d2fbjh1 d1j8he1 d1i3ua_ d1cf8l1 d1fe8h1 d2p1ya1
d1eeqa_ d2x89a_ d1ol0a_ d2r0lh1 d1ub5a1 d1dfbh1 d3eyvb1 d4jdvb1
d4p2cg_ d2gsia1 d2agjl1 d1u3ha1 d2h1ph1 d1rihl1 d1fskc1 d1boga1
d2a77l1 d1ospl1 d1qfoa_ d3dwta_ d2fx7l1 d1rz7l1 d3stba1 d1pkqa1
d1b6da1 d1dzba2 d1dzba1 d1kj2a_ d4l1ha_ d1rzgb1 d1dqlh_ d12e8l1
d1miel1 d1jmaa_ d1sbsl1 d1fgnh1 d1f58l1 d1mqkh_ d1ndgb1 d3qegl1
d1iail1 d2aw2a1 d1tqbc1 d3fo2a1 d4dkaa_ d1f8th1 d2g60l1 d2ywya_
d1uj3a1 d1yc7a_ d3bd3a_ d4w6xb1 d1nmbl_ d1i8ka_ d1rjlb1 d1ypze1
d1p7kb1 d1kxqe_ d1gpoh1 d2z4qa1 d1w72h1 d1i9rl1 d4qgya1 d4xt1c_
d2qqnh1 d1cica1 d3c08h1 d2cd0a_ d2bdnh1 d1hnga1 d1maml1 d1nldh1
d1cr9h1 d4lcih1 d3bkyh1 d3qg7l1 d1ail1l d1kxvc_ d4iosd_ d1l7ih1
d2x1qa_ d1ub6b1 d3k74b_ d1ibgh1 d1teth1 d1igya1 d1hxma1 d2x6ma_
d2e7la_ d5e03a_ d1qfwm_ d2r0kh1 d1igjb1 d1rurh1 d1plgh1 d1mj8h1
d1yuhb1 d3fmgl1 d1rzfl1 d1emtl1 d1ad9b1 d1igtb1 d1nlbl1 d1d5ih1
d3k7ua_ d4ffyh_ d1bwwa_ d3dvga1 d1a6ta1 d1fgvl_ d4qxgl1 d1miml1
d1jhll_ d2hffb1 d2hh0l1 d1qkzh1 d1l7tl1 d1afvh1 d1iaii1 d2cjuh_
d1um5h1 d2ghwb2 d2ghwb1 d1kgce1 d2fgwh1 d1ikfh1 d1dvfd_ d2i24n1
d2xtjb_ d1za3b1 d2yc1a_ d2hh0h1 d2x1oa_ d1bvka_ d1rjla1 d5e7ba1
d1igta1 d1il1b1 d2fb4l1 d1mvua_ d1jptl1 d32c2a1 d1fvca_ d1iqdb1
d2hrph1 d2p4ab_ d4qo1a_ d1p2cb1 d1wt5c_ d1t4ka1 d5c1mb_ d1it9l1
d1bz7a1 d1b2wh1 d1e4wl1 d1jnll1 d1yedb1 d1za6b1 d3u7aa_ d1igfh1
d3gnml1 d1orsa1 d1wejl1 d1a2ya_ d1hxmb1 d1gafh1 d2j4wh1 d1pz5a1
d1j05a_ d1a3rh1 d4fqll1 d1qfwh_ d1eo8h1 d1n7mh1 d4s11a_ d1kegh1
d5da4a_ d1iqwl1 d3whxb1 d1vgeh1 d1deeb1 d1c12b1 d1t2ql1 d1ad9a1
d3gsnb1 d3b2uc1 d1ggbh1 d1t3fb1 d1nakl1 d4ocmc_ d1lila1 d2v7hb3
d2esve1 d1mcoh1 d2qr0b1 d2q3aa_ d1ct8a1 d1kcrl1 d2pxyb_ d2uzih_
d5c3ld_ d3durb_ d1c5cl1 d2ak4e1 d1dl7h_ d1xiwd_ d1mlba1 d1rmfl1
d1i7zb1 d1dlfl_ d2xa3a_ d5bq7b1 d1dqqb1 d3h42l1 d1egjh1 d1kcvl1
d1fe8l1 d2j6el1 d1f3rb2 d1lk3l1 d1sm3h1 d1q0xh1 d1ghfh1 d7fabl1
d1igch1 d1lo0h1 d4idla_ d5c2bl_ d1jrhl1 d1il1a1 d3zhda1 d4kfzc_
d1b0wa_ d1e6oh1 d1mexl1 d4krpb_ d1fnsh1 d1ncwh1 d4hgma_ d4eigb1
d4rzca_ d2mpal1 d4y5xa_ d5en2b1 d3lrga1 d4ej1c1 d1rhha1 d1j05b_
d1forh1 d1q9wb1 d2wzpd_ d2aq2a1 d1h0da1 d3revb1 d2ij0c1 d1tzha1
d4m7jl1 d1k6ql1 d3b9kc1 d1igml_ d3qeua1 d1dsfl_ d1ce1h1 d1kcuh1
d1zvha1 d1zvoa1 d1tqbb1 d1ay1l1 d2qscl1 d2cgrh1 d1nl0h1 d1kenl1
d3eo0a1 d4n9ob1 d1n0xl1 d3k1kc_ d2aq2b1 d1ck1a1 d1enfa1 d1i4pa1
d1v1oa1 d1et9a1 d1esfa1 d2g9hd1 d4ohja1 d1klud1 d1bxta1 d1eu3a1
d1ty0a1 d1fnua1 d2cqva1 d1fhga_ d2k1ma_ d1biha3 d1biha1 d1biha4

d3qp3a1 d1g1ca_ d1gxea_ d1cvsc2 d1cvsc1 d1xmwa2 d1m4ka2 d2otpa2 d1biha2 d1rhfa2 d2fcba2 d2fcba1 d1wwbx_ d1gsma2 d1epfa1 d1epfa2 d2dava1 d1waaa_ d1f97a2 d1x44a1 d1vcaa2 d1fnla2 d1fnla1 d3b5ha2 d3h8na2 d3h8na1 d1jbja1 d1jbja2 d1pd6a_ d1f2qa2 d1f2qa1 d3cx2a_ d1nbqa2 d3b5ha1 d1n26a1 d1xiwb_ d1xaua_ d1fltx_ d2ifga1 d1olla1 d1olla2 d1cs6a4 d1cs6a3 d1cs6a2 d1cs6a1 d2gw5a2 d1sy6a1 d1xiwa_ d1ucta2 d1ucta1 d1ry7b2 d1gl4b_ d1qz1a3 d1olza1 d2c9aa1 d1wwca_

**Family-47 (c.1.8.4)**

**Positive dataset contains 11 protein sequences.**

d3wdpp1 d3wq5a_ d4gxpa_ d4hz8a_ d3aiua_ d1wcga1 d3ahxa_ d1v02a_ d2dgaa_ d1vffa1 d4ha4a_

**Negative dataset contains 3897 protein sequences.**

d1m32a_ d1ibja_ d1c7na_ d1iuga_ d4q75a_ d2bkwa1 d1t3ia_ d1n8pa_ d1qz9a_ d1jf9a_ d1p3wa_ d3kgwa_ d1cl1a_ d2ctza1 d1vjoa_ d2rfva_ d1gc0a_ d1cs1a_ d1d2fa_ d2ch1a1 d1qgna_ d1pffa_ d1elua_ d1u08a_ d1lc5a_ d2ay1a_ d1bw0a_ d3pd6a_ d7aata_ d2csta_ d1j32a_ d1fg7a_ d4wlha_ d1o4sa_ d1v2da_ d1gdea_ d1m6sa_ d1v72a1 d1svva_ d1ajsa_ d1wsta1 d2f8ja2 d1lk9a_ d1vp4a1 d1xi9a1 d2gb3a1 d1yaaa_ d2bnma2 d3rcha_ d3k40a_ d1pmma_ d2cfba1 d1dfoa_ d1o69a_ d1bj4a_ d3k28a_ d2bwpa_ d3lg0a_ d2fnua_ d3ou5a_ d1z7da1 d1b9ha_ d1iaya_ d4msoa_ d1bs0a_ d1s0aa_ d1sffa_ d3tqxa_ d1mdoa_ d3qboa_ d3pgya_ d1bjna_ d2dkja_ d1ejia_ d1vefa1 d4wxga_ d3bs8a_ d3gbxa1 d1w23a_ d1e57b_ d1auyb_ d1fwxa1 d1ibya_ d1f56a_ d1bypa_ d1azca_ d1ws8a_ d3u25a_ d1cuoa_ d1id2a_ d1rkra_ d2iaac_ d4dp1x_ d1nwpa_ d2gima_ d2i7sa_ d1qhqa_ d3ehbb1 d1fftb1 d1gyca3 d1hfua2 d1kbva1 d1kbva2 d1sdda1 d1sdda2 d1gyca1 d1mzya1 d2bw4a1 d1aoza1 d1aoza3 d1aoza2 d1kyaa2 d1kyaa3 d1kyaa1 d1mzya2 d4e9sa1 d1sddb1 d1sddb2 d1hfua1 d1hfua3 d1oe1a2 d1oe1a1 d1v10a2 d1v10a3 d1v10a1 d1a3qa1 d1iknc_ d3brda1 d1p7hl1 d1oy3c_ d1imhc1 d1gjia1 d1zk9a1 d2cxka1 d2bema_ d1pama1 d1ea9c1 d1qhoa1 d2fhfa2 d2fhfa1 d1k3ia1 d1j0ha1 d1cxla1 d2bhua1 d1edqa1 d1f1sa2 d1ut9a2 d1gvia1 d1txka1 d4alac_ d1pjwa_ d1rera1 d1s6na_ d3uzqb_ d2phla1 d1fxza2 d1fxza1 d1lrha_ d1uija1 g1dgw.1 d2v09a_ d1uika2 d1uika1 d1dgwa_ d1nepa_ d1ktja_ d1ds6b_ d1fstb_ d1ex0a1 d1g0da1 d1ju8a_ d4mh8a_ d1mu2a2 d4i7ga1 d2b5ea2 d2b5ea3 d2b5ea1 d2b5ea4 d2kp1a1 d2kp2a1 d1hyua3 d1hyua4 d1j08a2 d3nvva1 d1krha3 d2h88b1 d1n62a2 d1t3qa2 d1kf6b2 d1zoyb1 d1jq4a_ d1ffva2 d1dgja2 d1rm6c2 d2bjca1 d2hsga1 d1qpza1 d1efaa1 d1ydwa2 d2nvwa2 d1h6da2 d1zh8a2 d1qkia2 d1xeaa2 d1tlta2 d1r0ka3 d1vkoa2 d1vm6a2 d3qvsa2 d1gr0a2 d1p1ja2 d2ovga_ d1sq8a_ d1rzsa_ d3kz3a_ d3orca_ d3gpdg2 d2q49a2 d1hdgo2 d2g17a2 d2cvoa2 d2czca1 d3pwka2 d1obfo2 d1i32a2 d1zyao2 d2pkqo2 d1b7go2 d2hjsa2 d1cf2o2 d1vkna2 d1nvmb2 d1ux8a_ d1dlya_ d4i0va_ d2bkma_ d1dlwa_ d2bmma_ d2msya_ d2djoa1 d2r5za_ d1au7a1 d1f43a_ d2lmda1 d1akhb_ d1s7ea1 d3nara_ d1zq3p1 d2craa1 d1mija_ d4uuta_ d3a01a_ d1x58a1 d3rkqa1 d3cmya1 d2cqxa1

| | | | | | | | |
|---|---|---|---|---|---|---|---|
| d2da6a1 | d1e3oc1 | d1fjla_ | d1mh3a1 | d1ig7a_ | d1x41a1 | d1wh7a1 | d1gt0c1 |
| d2mw8a_ | d1lfup_ | d1x2ma1 | d1b72a_ | d1wi3a1 | d1wjha1 | d1x2na1 | d1mnmc_ |
| d2cuea1 | d3k2aa_ | d2hosa_ | d2jwta2 | d1pufa_ | d1octc1 | d2hi3a_ | d1b8ia_ |
| d4j19a_ | d3lnqa_ | d1wh5a1 | d1b8ib_ | d1fexa_ | d2cqra1 | d1guua_ | d1ug2a1 |
| d2cqqa1 | d2ajea1 | d4a69c_ | d2crga1 | d2iw5b1 | d1xc5a1 | d1ofcx1 | d2cu7a1 |
| d1wgxa1 | d2cjja1 | d2ckxa1 | d1gvda_ | d1bl0a1 | d1bl0a2 | d1d5ya1 | d3lv8a_ |
| d1s3ga1 | d1zaka1 | d1s4qa_ | d3tvta2 | d1uf9a_ | d1khta_ | d1ckea_ | d1osna_ |
| d1p5zb_ | d4tmka_ | d3cm0a_ | d1jaga_ | d3h86a_ | d1t0hb_ | d1nn5a_ | d2h92a_ |
| d3kb2a1 | d2c95a1 | d1yj5a2 | d1kgda_ | d1ly1a_ | d2ak3a1 | d1vyva2 | d1deka_ |
| d2grja1 | d1p3ja1 | d1y63a_ | d1e4ya1 | d1s96a_ | d3iija1 | d1lvga_ | d1nksa_ |
| d1vyua2 | d4e22a_ | d1qf9a_ | d1jjva_ | d1q3ta_ | d1ki9a_ | d1lw7a2 | d1teva_ |
| d1kjwa2 | d1gsia_ | d1g3ma_ | d1j99a_ | d1t8ta1 | d1nsta_ | d1fmja_ | d1zrha_ |
| d1ls6a_ | d2a3ra_ | d1aqua_ | d2zptx_ | d3ckla_ | d1texa_ | d1q44a_ | d2gwha_ |
| d1odfa_ | d1uj2a_ | d4gi7a_ | d2jeoa1 | d1rz3a_ | d1k6ma1 | d1z0ja1 | d3umra2 |
| d3gj0a1 | d2gaoa1 | d1yzga1 | d1x3sa1 | d1g7sa4 | d1puja_ | d1udxa2 | d1kk1a3 |
| d2o52a_ | d1jwyb_ | d1zcaa2 | d1zcba2 | d2fv8a1 | d2cjwa_ | d3dz8a1 | d1xzpa2 |
| d1vg8a_ | d4qxaa_ | d2ew1a1 | d1x1ra1 | d2bcgy_ | d3doea1 | d3cona_ | d3rega_ |
| d3wyga_ | d1upta_ | d2b6ha1 | d1zd9a1 | d1lnza2 | d1r5ba3 | d1n0ua2 | d1r2qa_ |
| d2f9la1 | d2rhda_ | d3q3jb_ | d3q85a_ | d1efma1 | d1r8qa_ | d2gf9a1 | d2akab1 |
| d1ek0a_ | d1z08a1 | d2gcoa1 | d2dy1a2 | d4g01b_ | d2cxxa1 | d1svia_ | d3q72a_ |
| d1ni3a1 | d4ac9a4 | d2erxa1 | d2bmea_ | d1wxqa1 | d2gf0a1 | d4ldja1 | d1zunb3 |
| d1d5ca_ | d4lv5b_ | d1efca3 | d1kmqa_ | d2g6ba1 | d3lrpa_ | d1tada2 | d2y8ea_ |
| d4rd4a1 | d1z06a1 | d2heia_ | d3a58b_ | d1u0la2 | d1d2ea3 | d1zj6a1 | d2ocba_ |
| d3rwoa_ | d1mr3f_ | d2e9sa1 | d4u5xa1 | d2iwra_ | d2fn4a1 | d3bwdd_ | d2nzja1 |
| d2atva1 | d2ksqa_ | d3l43a_ | d1mkya2 | d1mkya1 | d1wmsa_ | d1jnya3 | d2rmka_ |
| d1f6ba_ | d1f60a3 | d1gota2 | d1jala1 | d2fh5b1 | d1g16a_ | d2wblc_ | d1m7ba_ |
| d1wf3a1 | d1f5na2 | d1t9ha2 | d1z6xa1 | d1z0aa1 | d2fg5a1 | d2a5ja1 | d2bcjq2 |
| d1s0ua3 | d1ky3a_ | d4y0va_ | d4lhwa_ | d2qmea1 | d2atxa1 | d1egaa1 | d1ryha_ |
| d1moza_ | d1nrjb_ | d2erya1 | d3bfka_ | d1puia_ | d1fzqa_ | d3j3az1 | d3t5ga_ |
| d1shza2 | d3defa_ | d2f7sa1 | d3raba_ | d3tsoa_ | d3aq4a_ | d2al7a1 | d3sfva1 |
| d3bc1a_ | d2ncda_ | d1sdma_ | d3h4sa1 | d2heha_ | d2grya_ | g3kin.1 | d1kk8a2 |
| d1br2a2 | d1goja_ | d1f9va_ | d2mysa2 | d1lkxa_ | d3t0qa_ | d4db1a1 | d3gbja_ |
| d1ry6a_ | d1v8ka_ | d1kv9a1 | d1k8ka2 | d1k8ka1 | d3uleb1 | d3i33a2 | d3i33a1 |
| d1bupa1 | d1dkgd2 | d1dkgd1 | d4ci6a2 | d1jcfa1 | d4a2ba2 | d2fxua1 | d1yaga2 |
| d4cbua2 | d1bupa2 | d3vrda1 | d3vrda2 | d1fcdc2 | d1fcdc1 | d1h1oa2 | d1h1oa1 |
| d1m70a1 | d1iqca2 | d1iqca1 | d1eb7a2 | d1eb7a1 | d1nmla2 | d3h1jd1 | d1hroa_ |
| d4gedb_ | d3zooa_ | d1mz4a_ | d1gu2a_ | d1f1fa_ | d1dvva_ | d1c6ra_ | d1c75a_ |
| d1e29a_ | d1gdva_ | d1cnoa_ | d1h32b_ | d1co6a_ | d2bh4x_ | d2aiua_ | d5cytr_ |
| d1jdla_ | d1i8oa_ | d2zboa_ | d3dmia_ | d1ls9a_ | d1wvec_ | d2yk3a_ | d3ph2b_ |
| d1dw0a_ | d5aura_ | d1ql3a_ | d1qn2a_ | d1f1ca_ | d4zzva_ | d2xspa_ | d1eg1a_ |
| d4v20a_ | d1ojja_ | d4ipma1 | d1gpia_ | d4csia_ | d3pfza_ | d2yoka_ | d2nqya_ |
| d1h4ga_ | d1hixa_ | d1oa2a_ | d1olra_ | d3b7ma_ | d4h7ma1 | d3wp3a_ | d2vgda_ |
| d1igoa_ | d3zsea_ | d2nlra_ | d3wx5a_ | d1oa3a_ | d1pvxa_ | d1f5ja_ | d1oa4a_ |
| d1ks5a_ | d4ixla1 | d1h32a1 | d3i2ka1 | d1lnsa3 | d1mpxa2 | d1evsa_ | d1pvhb_ |

| | | | | | | | |
|---|---|---|---|---|---|---|---|
| d1z7ca_ | d1huwa_ | d1bgea_ | d1rhga_ | d1f6fa_ | d1f45b_ | d1i1rb_ | d1axia_ |
| d1etea_ | d3tgxb_ | d1scfa_ | d2z3qa1 | d3qb1a_ | d1hula_ | d1m48a_ | d4nkqb_ |
| d1eera_ | d1v7mv_ | d5e4ea_ | d2o27a_ | d3b5ka_ | d2psma1 | d4zf7a_ | d3uf5a_ |
| d4x51a_ | d1d9ca_ | d4ypgc1 | d3oq3a_ | d1lqsl_ | d1n1fa_ | d1au1a_ | d1wu3i_ |
| d3besl_ | d3ux9a_ | d4dnxa2 | d1m8pa2 | d1v47a2 | d1g8fa2 | d1x6va2 | d3rbaa_ |
| d2qtra_ | d1nupa_ | d3l92a_ | d1f9aa_ | d1qjca_ | d2h29a_ | d1vlha_ | d1lw7a1 |
| d1mrza2 | d4ruka_ | d2el7a_ | d1iq0a2 | d1irxa2 | d1r6ta2 | d1ivsa4 | d2cyba_ |
| d1rqga2 | d2yxna_ | d1f7ua2 | d1h3fa1 | d1ffya3 | d2g36a1 | d1nzja_ | d1jila_ |
| d1n3la_ | d1i6la_ | d1pfva2 | d1h3na3 | d3n9ia_ | d3sz3a1 | d3sw2a_ | d1moxc_ |
| d1dx5i3 | d1edmb_ | d1autl2 | d1autl1 | d1nqlb_ | d1dval1 | d1szba2 | d1kigl_ |
| d2y5fl_ | d1ijqa2 | d1gk5a_ | d1g1sa2 | d1nt0a3 | d1xdtr_ | d1lmja2 | d1lmja1 |
| d1nzia2 | d2wphe_ | d1klil_ | d1pfxl2 | d1pfxl1 | d1p9ja_ | d1g1ta2 | d1f7ea_ |
| d4yzub_ | d1m1xb5 | d2y5ia_ | d1qlsa_ | d1psra_ | d3n22a_ | d2egda_ | d4ggfc_ |
| d2wcea_ | d2luca_ | d4aqia_ | d3nsia_ | d2lp3a_ | d1j55a_ | d1a4pa_ | d1k8ua_ |
| d1nsha_ | d1pvaa_ | d3fs7a_ | d2pvba_ | d1ttxa_ | d1rwya_ | d2kyfa_ | d1rk9a_ |
| d1a75a_ | d1exra_ | d1tiza1 | d1nyaa_ | d1g8ia_ | d1ggwa_ | d1wdcc_ | d3fwba_ |
| d1ggza_ | d2o5ga_ | d1ctaa_ | d1m45a_ | d3i5gc_ | d1sl8a_ | d1fpwa_ | d2scpa_ |
| d2roaa_ | d4aqra_ | d3pn7b_ | d1br1b_ | d2mysb_ | d1s6ca_ | d1bjfa_ | d2obha_ |
| d1jfka_ | d1jf0a_ | d1qv0a_ | d1lkja_ | d2mlfc_ | d2m97a_ | d2d8na_ | d4by5a_ |
| d1jbaa_ | d4nqga_ | d1wdcb_ | d2mysc_ | d1j7qa_ | d1s6ia_ | d1uhka2 | d1r2ua_ |
| d1dtla_ | d3kf9a_ | d3pn7c_ | d1gjya_ | d1y1xa_ | d1hqva_ | d1dvia_ | d1df0a1 |
| d4usla_ | d1k94a_ | d1etob_ | d1umqa_ | d1mixa2 | d1e5wa2 | d1j19a2 | d2al6b2 |
| d1g4ma1 | d1g4ma2 | d1id1a_ | d2hmta_ | d1b9ra_ | d1offa_ | d1frra_ | d3wcqa_ |
| d1e9ma_ | d1l5pa_ | d1wria_ | d1i7ha_ | d3av8a_ | d1czpa_ | d1rfka_ | d3p1ma1 |
| d3b2ga_ | d2bt6a2 | d1wgqa1 | d1v89a1 | d2coda1 | d1zc3b_ | d1wg7a1 | d1u5ea1 |
| d1v61a1 | d2coca1 | d2ys1a1 | d1v5pa1 | d1ntya2 | d1wjma1 | d1wi1a1 | d1u29a_ |
| d1xx0a1 | d1unqa_ | d2zkmx3 | d1fhoa_ | d1v88a1 | d2adza1 | d1x1fa1 | d1p6sa_ |
| d2coaa1 | d1btka_ | d1v5ma1 | d1faoa_ | d2x18a_ | d1dyna_ | d1x1ga1 | d4kaxb_ |
| d2j59m1 | d1ki1b2 | d1xcga2 | d1txda2 | d1u5da1 | d1dbha2 | d1w1ha_ | d1upqa_ |
| d1v5ua1 | d1foea2 | d2fjla1 | d1eaza_ | d2dfka2 | d2cofa1 | d1kz7a2 | d9ldta1 |
| d2ewda3 | d1up7a1 | d2dfda1 | d1s6ya1 | d1vjta1 | d7mdha1 | d1hyea1 | d1t2da1 |
| d1ojsa3 | d1b8pa1 | d1ldna1 | d1hyha1 | d1civa1 | d2a92a1 | d1ez4a1 | d1i0za1 |
| d1y6ja1 | d1gv0a1 | d1ur5a1 | d1llda1 | d1obba1 | d1v8ba1 | d1gdha1 | d2dlda1 |
| d1mx3a1 | d1ygya1 | d1qp8a1 | d1li4a1 | d1j4aa1 | d2naca1 | d1npya1 | d3t4ea2 |
| d1a4ia1 | d1o0sa1 | d2hk9a2 | d2egga2 | d1b26a1 | d1bvua1 | d1gq2a1 | d1c1da1 |
| d1gtma1 | d1euza1 | d1nvta1 | d3dona2 | d1edza1 | d1gpja2 | d1vi2a1 | d1p77a1 |
| d1nyta1 | d1b0aa1 | d1vl6a1 | d1v9la1 | d1txga2 | d3f47a1 | d1mv8a2 | d2pv7a2 |
| d1xc2a3 | d2ahra2 | d4xiya1 | d2f1ka2 | d1pgja2 | d1i36a2 | d1np3a2 | d1lj8a4 |
| d1teaa3 | d1qmga2 | d2i76a2 | d2g5ca2 | d1wdka3 | d1dlja2 | d3cuma3 | d1kola2 |
| d1f8fa2 | d1qora2 | d1jqba2 | d1vj0a2 | d1cdoa2 | d1gu7a2 | d1d1ta2 | d1xa0a2 |
| d2dm6a2 | d1jvba2 | d1llua2 | d1h2ba2 | d1tt7a2 | d1piwa2 | d1o89a2 | d1iz0a2 |
| d1e3ja2 | d1yb5a2 | d1uufa2 | d1vj1a2 | d1e3ia2 | d3gpdg1 | d1vc2a1 | d2nvwa1 |
| d1u8fo1 | d1ydwa1 | d1hdgo1 | d2g17a1 | d1cf2o1 | d2czca2 | d1r0ka2 | d1obfo1 |
| d2cvoa1 | d1ebfa1 | d1h6da1 | d1i32a1 | d1zh8a1 | d2q49a1 | d1qkia1 | d1j5pa4 |

d3pzra1 d1xeaa1 d1vkoa1 d1vm6a3 d3qvsa1 d2pkqo1 d1b7go1 d1gr0a1
d1p1ja1 d1lc0a1 d1j0xo1 d3pwka1 d2hjsa1 d1zyao1 d1vkna1 d1tlta1
d1nvmb1 d3sjua_ d3ojfa1 d1xu9a1 d2jjya1 d1n7ha_ d1y1pa1 d1h5qa_
d1jtva_ d4fs3a_ d2bkaa1 d1gy8a_ d2c5aa1 d1ulsa_ d1ooea_ d2b69a1
d1y5ma1 d1spxa_ d1ae1a_ d1bxka_ d1ydea1 d1gega_ d1hdoa_ d1o5ia_
d1e6ua_ d1snya1 d2rhca_ d3zv4a_ d5ceja_ d2d1ya1 d1fjha_ d3w8ea_
d2fmua1 d1x1ta_ d1rpna_ d3ay7a1 d1k2wa_ d1eq2a_ d2hrba1 d3ruaa_
d2ew8a1 d4hwka_ d1xgka_ d2bd0a1 d1yo6a1 d2a35a1 d2ntva_ d1vl8a_
d1hdca_ d4bkua_ d2ag5a1 d2q45a_ d1nffa_ d4iy1a_ d1zbqa1 d1kewa_
d1ek6a_ d1ja9a_ d2q46a2 d1iy8a_ d1cyda_ d2ptga_ d1hxha_ d1uzma1
d3lyla_ d4m89a_ d3wyea_ d2blla1 d1w6ua_ d1sb8a_ d2q2va_ d4nr0a_
d1e6wa_ d2gasa_ d3oiga1 d2bgka1 d1mxha_ d1vl0a1 d1rkxa_ d1r6da_
d1wmaa1 d1e7wa_ d2c07a1 d1xkqa_ d3tzha_ d1g0oa_ d3lz6a_ d1qyca_
d1edoa_ d1gz6a_ d1oc2a_ d1t2aa_ d1zema1 d2xoxa_ d1z45a2 d1geea_
d1p33a_ d3lt0a_ d4e6pa_ d2wyua_ d1db3a_ d4q9na_ d1xhla_ d4o1ma_
d1fmca_ d2uvda_ d2gdza1 d1uaya_ d2z1ma_ d2o23a2 d2fr1a2 d2fr1a1
d1qsga_ d1qyda_ d4zjua_ d2a4ka1 d1yb1a_ d1wvga1 d1yxma1 d3f4ba_
d1mg5a_ d1xg5a1 d1r55a1 d3gboa_ d1atla_ d1buda_ d4q1la_ d1quaa_
d1yp1a_ d1wnia_ d4ekfa_ d2ckga_ d1th0a_ d2bkra_ d3u1ra1 d1kapp2
d1k7ia2 d3ioqa_ d1fh0a_ d1o0ea_ d1m6da_ d2cb5a_ d1cs8a_ g8pch.1
d1s4va_ d2pnsa_ d1vsna1 d1mira_ d1iwda_ d2oula_ d7pcka_ d3bwka_
d3bcna_ d2p86a_ d1pvja_ d1pcia_ d1gece_ d1deua_ d4bs6a_ d1xkga1
d1cqda_ d1jqpa2 d2c0ya_ d2bdza_ d3p5ua_ d2o36a_ d1zoya1 d1qo8a2
d1d4ca2 d1chua2 d1jnra2 d1kf6a2 d2jk6a2 d2jk6a1 d1feca1 d1feca2
d1lpfa1 d1lpfa2 d1xhca1 d1xhca2 d1dxla2 d1dxla1 d1gera2 d1m6ia2
d1onfa1 d3lada2 d3lada1 d1w4xa1 d1w4xa2 d1gesa2 d1mo9a1 d1xdia1
d1fl2a2 d1fl2a1 d1fcda1 d1fcda2 d1h6va2 d1h6va1 d1mo9a2 d1gv4a2
d1gv4a1 d2gv8a2 d2gv8a1 d1aoga1 d1aoga2 d1hyua1 d1ebda1 d1ebda2
d3dk9a1 d1q1ra2 d1q1ra1 d1gesa1 d1zbfa1 d2z1ha_ d2etja1 d3qioa1
d1jl1a_ d2e4la_ d1jl2a_ d2lsna1 d1io2a_ d1uaxa_ d2yv0x_ d1mu2a1
d1ekea_ d1i39a_ d1ps9a2 d1lqta1 d1cjca1 d1reoa1 d1ju2a1 d2f5va1
d3c96a1 d2gmha1 d1pj5a2 d1gpea1 d1o5wa1 d1n4wa1 d1k0ia1 d2dw4a2
d2ivda1 d1seza1 d2voua1 d1kdga1 d1xl4a1 d3vsqa_ d2gixa1 d1p7ba1
d1js9c_ d1f15b_ d1za7a_ d1laja_ d1ix2a_ d1jmxa4 d1jmxa3 d1pbya3
d1jmxa1 d1pbya1 d1pbya2 d1faaa_ d1wika1 d1xfla_ d1ttza1 d2f51a1
d1ti3a_ d1nhoa_ d1sena_ d2i4aa_ d1ep7a_ d1zmaa1 d1fo5a_ d3lefa1
d3diea1 d2yana1 d1z6na1 d1oaza_ d3zywa1 d4aj8a_ d1nm3a1 d4fiwa_
d1wmja_ d4hs1a1 d1iloa_ d1xwaa1 d1wjka1 d2vm1a_ d1fb6a_ d1gh2a_
d1h75a_ d1nw2a_ d1kx5b_ d3a6na_ d4m6ba2 d2nqbd_ d3wkjd_ d2yfwe_
d4h9na_ d5bnxa_ d1kx5c_ d3r45a_ d1eqzb_ d4jjnb_ d1f66c_ d1eqza_
d4wnnb_ d1kx5a_ d1u35c1 d1id3a_ d2nqbc_ d1kx5d_ d1tafa_ d1bh9b_
d1tafb_ d1jfib_ d1h3ob_ d2byka1 d1n1ja_ d4g92b_ d1bh9a_ d1jfia_
d1h3oa_ d2bykb1 d1n1jb_ d1pd212 d1nhya2 d2a2ra2 d1k0dd2 d1f2ea2
d1gsua2 d1f3ba2 d2fhea2 d2pvqa1 d1tw9a2 d1oyja2 d1e6ba2 d1k0ma2

d3ik7a1 d4is0a1 d1r5aa2 d1jlva2 d1m0ua2 d3gtub2 d1z9ha2 d1okta2
d1axda2 d2gsra2 d1tu7a2 d1k3ya2 d1zgma1 d1b48a2 d1gnwa2 d1gwca2
d2gsta2 d1glqa2 d1oe8a2 d1pn9a2 d1v2aa2 d1fw1a2 d1duga2 d1g7ea_
d2trcp_ d1ig8a1 d1czan2 d1czan3 d1czan1 d1ig8a2 d1czan4 d1bg3a4
d1bg3a3 d1bg3a2 d1qsaa2 d3wyha_ d4g9sa1 d3gxra_ d1qqya_ d1f6ra_
d1ivma_ d2gv0a_ d1iiza_ d2z2fa_ d1j8xa_ d1ghla_ d1b9oa_ d174la_
d169la_ d2o4wa_ d1p37a_ d1k28a3 d1xjta1 d1jtma_ d2f2qa_ d4e97a1
d2pjsa1 d1klla_ d2i7ra1 d1ecsa_ d1xqaa1 d5cj3a1 d1jifa_ d2p7oa_
d1r9ca_ d1nkia_ d1mpya2 d1kw3b2 d1kw3b1 d4ghga1 d1tfza1 d3isqa1
d3isqa2 d1sqia1 d1sqia2 d1lkda2 d1lkda1 d1cjxa2 d1cjxa1 d1sp9b2
d1t47a1 d1t47a2 d1f1ua2 d1sp8a1 d1sp8a2 d1f1ua1 d1f74a_ d3l21a_
d2e1da_ d1to3a1 d1w3ia_ d1sfla_ d1n7ka_ d1vcva1 d4imea_ d1wa3a_
d2ocza1 d1f2ja_ d3m16a_ d2yxga_ d1ub3a_ d3a5fa_ d1fbaa_ d1l6wa_
d1xfba1 d1wx0a1 d1vpxa1 d1mxsa_ d2a4aa1 d1xkya1 d3cq0a1 d3s1xa_
d1f05a_ d1fdja_ d3cpra1 d4d2ja_ d1s5ja2 d1qhta2 d4dlea2 d1tgoa2
d3py8a2 d1q8ia2 d1kfsa2 d2py5a2 d1d5aa2 d1qsta_ d2beia1 d4m3sa_
d1u6ma1 d1ygha_ d1yk3a1 d3dr6a_ d1xmta_ d1z4ea1 d2ge3a1 d2fiaa1
d2jdca1 d1y9wa1 d3to7a_ d1wwza1 d2fl4a1 d2ozua1 d2b5ga_ d2cy2a1
d2p0wa_ d1cm0a_ d4pswa_ d1yrea1 d1yr0a1 d1m4ia_ d2gana1 d1yvka1
d1ghea_ d2g3aa1 d1y7ra1 d2euia1 d1q2ya_ d2fsra1 d1xeba_ d2ae6a1
d1cjwa_ d1qsma_ d2aj6a1 d1y9ka1 d1i12a_ d1vkca_ d2atra1 d1n71a_
d2d4pa1 d2fiwa1 d2fe7a1 d1tiqa1 d2ou2a_ d1yx0a1 d2fcka1 d1p0ha_
d2j8ma_ d2pq8a_ d1z4ra1 d1s3za1 d1ufha_ d1prtc1 d2wv6d_ d1prtf_
d1ltrd_ d4l63b1 d1r4pb_ d1prtd_ d1tl0f_ d2bosa_ d1prtb1 d3chbd_
d1xvqa1 d1pn0a2 d3hy2a_ d2cvba1 d1o8xa_ d1lu4a_ d1we0a1 d2bmxa1
d2a4va1 d1psqa_ d1xiya1 d1q98a_ d1n8ja_ d1zofa1 d2ggta_ d3tjja_
d4je1a1 d1o73a_ d3s9fa_ d2zcta_ d1qmva_ d2b1ka_ d5dvba1 d2b5xa1
d2v2ga_ d1st9a_ d3tb2a_ d4llra_ d4fh8a1 d1e2ya_ d1knga_ d2h30a_
d1i5ga_ d2z9sa_ d2f8aa1 d1tp9a1 d2b7ka_ d1nm3a2 d3hvva_ d2wfca1
d4kcea_ d1xvwa1 d4gqca1 d3qpma_ d4k1fa_ d1zzoa1 d1gp1a_ d1prxa_
d4od7a1 d1z6ma1 d4mcua1 d4dvca1 d1r4wa_ d3l9sa_ d1un2a_ d3rppa_
d2m5ta_ d1cqqa_ d1wqsa_ d5bpea_ d2ipha_ d1lvoa_ d2bhga1 d3tnta_
d3sjia_ d3d23a1 d2fyqa_ d3tloa_ d3zvea1 d2hrva_ d3q3ya1 d4asha1
d1l6ja5 d1l6ja4 d1eaka4 d1eaka3 d1l6ja3 d1h8pa2 d1h8pa1 d1etha2
d1w52x1 d2ppla1 d1lpbb2 d2oxea1 d1bu8a2 d1rp1a2 d4qmea2 d2xdta2
d1z5ha2 d1j1ia_ d2rhwa_ d2ftba_ d1pmpa_ d2rcqa1 d1fdqa_ d4lkpa_
d1sa8a_ d1vyfa_ d1lpja_ d3vg7a1 d4azqa1 d1o8va_ d5bvqa_ d1p6pa_
d2qo4a_ d1opba_ d1yiva1 d1o1va_ d4bvma1 d4tkba_ d4i3ba_ d1bwya_
d1ftpa_ d1ggla_ d4gkca_ d1cbia_ d3p6da1 d1kqwa_ d2a0aa1 d1xwdc1
d3kj4a_ d1ozna_ d1w8aa_ d2ifga3 d1xkua_ d1g12a_ d3p0ya1 d1igra1
d1igra2 d1n8yc2 d1n8yc1 d1m6ba2 d1m6ba1 d1dzka_ d4tlja_ d2a2ua_
d4wfva_ d3t1da_ d4iaxa_ d3zq3a_ d2nnda_ d1i4ua_ d1kxoa_ d1bbpa_
d1kt7a_ d1exsa_ d3s26a1 d1lf7a_ d1qfta_ d4gh7a_ d1z24a1 d1qabe_
d1xkia_ d1ew3a_ d3bx7a_ d4iawa1 d1n0sa_ d1gkab_ d3kzaa_ d2k23a_

d2l9ca1 d2joza1 d1gm6a_ d3kffa_ d3saoa1 d1e5pa_ d1yupa1 d1iiua_
d2tbvc_ d1c8nc_ d1x36a_ d1smvc_ d1ng0c_ d4sbvc_ d1opoa_ d1f2nc_
d1cixa_ d1y29a_ d1v7fa_ d2jtba_ d1d1ha_ d1g9pa_ d1qk7a_ d1mb6a_
d2a2va_ d1niya_ d1i25a_ d1hvwa_ d2mpqa_ d1ie6a_ d1kqha_ d1la4a_
d2mxoa1 d1dl0a_ d1koza_ d1qk6a_ d1emxa_ d1lqra_ d1c6wa_ d1rmka_
d1ixta_ d1eyoa_ d1cnna_ d1fu3a_ d1g1pa_ d1f3ka_ d1fyga_ d1rypd_
d1irua_ d1rypc_ d3unfk_ d1rypa_ d1j2pa_ d1irui_ d1j2qh_ d3unfh_
d1m4ya_ d1rypb_ d1irul_ d1irug_ d1iru2_ d1q5rh_ d1rypg_ d1g3ka_
d1iruf_ d1q5qa_ d1e94a_ d1irub_ d1ryph_ d3ty6a_ d1iruj_ d1iruk_
d1rype_ d1irud_ d1irue_ d3mi01_ d1rypf_ d2z3ba_ d1iruc_ d3mka2_
d1iru1_ d1mqta_ d1d4m3_ d1fmd3_ d2mev1_ g1aym.1 d1ev13_ d4q4w1_
d1r1a3_ d2x5ia_ d1rhi1_ g1r1a.1 g1d4m.1 g1rhi.1 d1b35b_ d1oopa_
d1tme1_ d4iv1a_ g1pvc.1 g1fpn.1 d1fpn1_ d4gb31_ d1d4m1_ d1fmd1_
d1ev11_ d2wzr3_ g1cov.1 d1qqp3_ g1fmd.1 d2x5ic_ d1rhi3_ g1oop.1
d1hxs1_ g1hxs.1 d1fpn3_ d1bev1_ d1eah3_ d1aym1_ d1pvc3_ d1qqp1_
d1zba3_ d1h8ta_ d3vbhc_ g1b35.1 g1bev.1 d1hxs3_ g1ev1.1 d1bev3_
g1tmf.1 d1tmf1_ d1b35a_ d2mev3_ g1eah.1 g1h8t.1 d1eah1_ d4q4w3_
d1r1a1_ d1aym3_ d1pvc1_ d1zba1_ d1h8tc_ d1oopc_ d1tme3_ g1qqp.1
d4iv1c_ g1tme.1 d3ubub_ d1egia_ d1qo3c_ d1v7pa_ d1tdqb_ d3ubua_
d1c3ab_ d1h8ua_ d3bx4b_ d1t8ca1 d1sb2a1 d1qdda_ d2ziba1 d3cada_
d1byfa_ d1j34b_ d1jwia_ d1jzna_ d1c3aa_ d1oz7b_ d1wmza_ d3bdwb_
d1hq8a_ d1fifa1 d1dv8a_ d3hupa_ d1wk1a1 d1umra_ d2afpa_ d3bx4a_
d1gz2a_ d1sl6a_ d1v7pb_ d3g8ka_ d1ixxa_ d1j34a_ d1jwib_ d1g1sa1
d1y17a1 d1ja3a_ d1fvub_ d3bdwa_ d1sb2b1 d1fvua_ d1umrc_ d1oz7a_
d4m17a_ d3cfwa1 d1ypqa1 d1g1ta1 d3fd4a_ d3l9jc_ d4dn8a1 d1v4lb_
d1rdl1_ d2msba_ d1eixa_ d3exra_ d2ffca1 d2fdsa1 d3n3ma1 d2czda_
d2yyua_ d1vqta1 d1dbta_ d3ldva1 d4lt9a1 d3tc7a1 d3hoja_ d3vnda_
d3nl8a1 d1geqa_ d3nava1 d1v5xa_ d4a2ra1 d3tc6a1 d1ujpa_ d4ijba_
d1xcfa_ d1vc4a_ d1rd5a_ d1qopa_ d3b5va_ d2r80b_ d1cqxa1 d1cg5a_
d3boma_ d2h8fb_ d2raoa_ d3bj1a_ d4hrrb_ d2r50a_ d3d1kb_ d2aa1b1
d3zhwa_ d3hf4b_ d3gdja_ d1jebb_ d3bj1b_ d1x9fb_ d1wmub_ d1gcva_
d3bcqb_ d3d4xb_ d1gvha1 d2dc3a_ d3hf4a_ d4yu3a_ d3k8ba_ d1qpwb_
d2r80a_ d3bcqa_ d1v4wa_ d3wtgb_ d1jl7a_ d4esaa_ d1a9we_ d3d1ab_
d1fsla_ d4h2la_ d1v4wb_ d1mwca_ d1vhba_ d1jeba_ d1spgb_ d1h97a_
d1q1fa_ d1tu9a1 d3d1aa_ d1hdsb_ d3hyub_ d2zfbb_ d3fs4a_ d1oj6a_
d1shrb_ d4h2lb_ d4yu3b_ d1spga_ d3hrwa_ d3qqra_ d3at5a_ d4wchd_
d3hyua_ d1cg5b_ d1uc3a_ d1hbra_ d2raob_ d1outb_ d4esab_ d3gdjb_
d3qqqa_ d1x9fc_ d3vrfa_ d1itha_ d3mkba_ d3d4xa_ d3mjpa_ d1jl6a_
d3fh9a_ d1outa_ d2dc3b_ d1it2a_ d3eoka_ d3g46a_ d1qpwa_ d3vrfb_
d1ch4a_ d1x9fd_ d1hdsa_ d2zfba_ d3mkbb_ d1or4a_ d3wtga_ d1a4fa_
d3fh9b_ d3bomb_ d3cy5a_ d3at5b_ d1x9fa_ d2oifa_ d4lmxb_ d1cpcb_
d3v57a_ d1kn1a_ d1cpca_ d4f0ta_ d2vjha_ d4po5b_ d1eyxa_ d1alla_
d4f0tb_ d1b33b_ d1b8db_ d4h0mb_ d1b33a_ d2vjhb_ d4lm6b_ d2vmlb_
d1allb_ d2vjta_ d3dbja_ d2vmla_ d2vjtb_ d4f0ub_ d1f99b_ d4rmpa1

d1liaa_ d1eyxb_ d2uulb_ d4lmsb_ d4f0ua_ d2bv8a_ d1f99a_ d2bv8b_
d2c7lb_ d3dbjb_ d4rmpb1 d1b8da_ d4h0ma_ d3v57b_ d1liab_ d2uula_
d2j96a_ g1wht.1 d3nqyb_ d4gera_ d1bqba_ d2xqfa_ d1ea5a_ d1f6wa_
d1dx4a_ d1qe3a_ d1k4ya_ d4qwwa_ d2ha2a_ d1sfra_ d1f0na_ d4qeka_
d1gl9b1 d1gkub1 d4iuaa3 d4bvva_ d5hpga_ d3k65a_ d1ki0a3 d1ki0a2
d1ki0a1 d1a0ha1 d3sp8a3 d2feba1 d1dosa_ d1gvfa_ d3qm3a1 d2fjka_
d1cg2a1 d1yloa2 d1y7ea2 d1y0ya3 d1lfwa1 d1fnoa4 d1vhoa2 d1ysja1
d1vhea2 d1z2la1 d2fvga2 d1vgya1 d1r3na1 d2grea2 d1xmba1 g1q7l.1
d1rtqa_ d3rp2a_ g1ucy.1 d1fuja_ d2zchp_ d3w94a_ d1si5h_ d1lo6a_
g2pka.1 d1cgha_ d1kigh_ d1a5ia_ d3s9ba_ d1hj8a_ d1ao5a_ d2anya_
d1q3xa1 d1gvza_ d1azza_ d4gsoa_ d2f91a1 d1iaua_ d1gdna_ d1eufa_
g1fiw.1 d1eq9a_ d4nfea_ d2rdla_ d1spja_ d1elva1 d1a7sa_ d1ekbb_
d1pfxc_ d1yc0a_ d2aiqa_ g1fiz.1 d1fi8a_ d4k60a_ d4dgja_ d1mzaa_
d1op0a_ d3tjva_ d1pytd_ d1sgfg_ g1gg6.1 d2eeka_ g1rtf.1 d1a0ja_
d3g01a_ d1pytc_ d4e7na_ d1ddja_ d1z8ga1 d2hlca_ d1npma_ d1brup_
g1jou.1 d1sgfa_ d1autc_ g2hnt.1 g1kdq.1 d1orfa_ d3s69a_ d4crga_
d1fxya_ d1bqya_ d1svpa_ d1mbma_ d3su6a1 d1cu1a1 d4agka_ d1vcpa_
d1q31a_ g1n1l.1 d3e90b_ d1ep5a_ d3u1jb_ d1m4la_ d2bk7a_ d3fjua_
d1z5ra1 d1kwma1 d1uwya2 d2c1ca1 d1dtda_ d1h8la2 d3lgia_ d3i77a_
d2z9ia2 d4nsva_ d1lcya2 d4jcna_ d1agja_ d1os8a_ d1hpga_ d1p3ca_
d1l1ja_ d1e69a_ d1mv5a_ d3g60a4 g1ii8.1 d1ewqa2 d1ji0a_ d1v43a3
d2wmfa1 d1vpla_ d1g2912 d4a82a2 g1f2t.1 d2d62a1 d3tuic1 d2bbsa1
d3g60a3 d2ixea_ d1g6ha_ d1sgwa1 d2qi9c_ d3rlfa1 d3b60a1 d3d31a2
d2pzea1 g1xew.1 d1jj7a_ d1oxxk2 d2onka1 d1w1wa_ d1iwea_ d1qf5a_
d1vmaa2 d1nija1 d1np6a_ d2p67a1 d3ihla_ d1okkd2 d1ls1a2 d1s1ma2
d1iona_ d2v40a_ d1dj3a_ d3hida_ d2vo1a1 d2g0ta1 d1vcoa2 d1j8mf2
d1xjca_ d1p9ba_ d1qzxa3 d1ihua2 d1ihua1 d2qm8a_ d1dj2a_ d4ioja_
d1cp2a_ d1yrba2 d1hyqa_ d3gqba2 d1skye3 d1xp8a1 d1tf7a2 d1tf7a1
d1ye8a1 d1skyb3 d2qe7d2 d4blta_ d4huta_ d3ldaa2 d4hyya_ d2i3ba1
d1cr1a_ d3j9tb2 d3ntua2 d1vdza1 d2z43a_ d1e9ra_ d1nlfa_ d3ziaa2
d1n0wa_ d3ziad2 d3j9ta2 d1pzna2 d3gqbb2 d2i49a_ d1nnfa_ d1xvxa_
d1ursa_ d1h3da1 d1iuda_ d4m1va1 d1twya_ d3e13x_ d2nxoa1 d4pfua_
d3ttma_ d1elja_ d2qmwa1 d1us5a_ d1y3na1 d1jeta_ d1uqwa_ d4f19a1
d2esna2 d3thia_ d3fasa1 d2uyeb_ d2xwva1 d1r9la_ d2fyia1 d2ozza1
d1sw5a_ d1nh8a1 d1vr5a1 d2g29a_ d1xs5a_ d3lswa_ d2z23a_ d3w9va_
d3tcha_ d1q35a_ d1z7me1 d3q27a_ d1p99a_ d1ve4a1 d2i6ea1 d4mcoa1
d4n8ya_ d1eu8a_ d1zbma1 d1hsla_ d1ixca2 d1j1na_ d1xvya_ d1y9ua_
d2z22a1 d1xoca1 d4oera_ d1wdna_ d5f1qa1 d1xt8a1 d1i6aa_ d4gwoa_
d4igta1 d1pc3a_ d4mmpa1 d1o63a_ d2czla1 d2onsa2 d4mija1 d1eh3a_
d3skpa_ d1dtza1 d1ieja_ d1jnfa2 d1ce2a1 d1jnfa1 d1b1xa2 d1b1xa1
d1jw1a1 d1jw1a2 d1ryoa_ d1h76a1 d1h76a2 d1c4oa1 d2p6ra3 d2p6ra4
d1oywa2 d1oywa3 d1wrba1 d1w36b2 d1w36b1 d1c4oa2 d3bora_ d1t6na_
d2eyqa5 d2eyqa3 d2eyqa2 d2zu6a2 d1veca_ d1uaaa1 d1w36d1 d2g9na1
d1z3ix2 d2rb4a_ d1hv8a1 d1hv8a2 d1t5ia_ d1w36c2 d2j0sa2 d2j0sa1

d1fuka_ d3hjha1 d1z5za1 d1qdea_ d1z63a1 d1s2ma2 d1s2ma1 d1wp9a1
d1wp9a2 d1gm5a3 d1gm5a4 d3dina4 d3dina3 d1q0ua_ d1w36c1 d1z3ix1
d1nkta3 d1uaaa2 d1w36d2 g1qhh.1 d2fwra2 d2fwra1 d3q3ma_ d1za0a1
d1mxra_ d2inca_ d1jk0a_ d2incb_ d1mhyb_ d3hf1b_ d1mtyb_ d1r2fa_
d2uw1a_ d1mhyd_ d3n37a_ d1oquc_ d1syya_ d1mtyd_ d2o1za_ d1uzra_
d1smsa_ d2fkza_ d1zuja1 d1s3qa1 d2fjca1 d1z6om1 d1veia_ d4dyya_
d1jiga_ d3gvya_ d2pyba_ d4cy9a1 d3bvfa1 d1r03a_ d2cf7a_ d1nf4a_
d1zs3a1 d4iwka_ d3ka8a_ d3np0x_ d1n1qa_ d1lb3a_ d4e6ka_ d1z6oa1
d2fzfa1 d4dyua_ d3ge4a_ d1jgca_ d4am4a_ d2z6ma_ d1ji5a_ d1vlga_
d1lkoa1 d3qz3a_ d1o9ra_ d2ffxj_ d1euma_ d1yv1a1 d2v2pa_ d1j30a_
d4di0a_ d1dpsa_ d1oewa_ d2qzxa_ d2asia_ d1psoe_ d1miqa_ d1smra_
d2h6ta_ d1pfza_ d1izda_ d3hd8a_ d1j71a_ d1dpja_ d4aa9a_ d1t6ex_
g1b5f.1 g1lya.1 g1htr.1 d1qdma2 d1bxoa_ d1wkra_ d1tzsa_ d1ibqa_
d2qzwa_ d2v76a_ d1wj1a1 d1aqca_ d1qqga1 d1ntva_ d1n3ha_ d4dbba_
d3f0wa1 d1p3ra_ d1wgua1 d2cy4a1 d3hqca_ d1wvha1 d1xr0b1 d1bdqa_
d3ggta_ d3ixoa_ d1mrxa_ d2haha_ d1tw7a_ d4njsa_ d1sgua_ d2p3da_
d1nsoa_ d3d3ta_ d2rkfa_ d1c6ya_ d2rspa_ d3ogpa_ d3ekwa_ d2o4na_
d2j9ja_ d3ggua_ d3lzua_ d4npua_ d4j55a_ d2r8na_ d2fxda_ d2p3ca_
d4dqea_ d3ttpa_ d3t3ca_ d2p3aa_ d2hc0a_ d2fdda_ d3mwsa_ d1um8a_
d1ixza_ d1qvra2 d1qvra3 d1qzma_ d1sxjc2 d1jr3d2 d2p65a_ d1l8qa2
d1g8pa_ d3hu3a3 d1sxjd2 d1in4a2 d1sxja2 d1tuea1 d2ce7a2 d1w5sa2
d1sxjb2 d1d2na_ d1ny5a2 d1ofha_ d2gnoa2 d2a5yb3 d1fnna2 d1u0ja1
d1lv7a_ d1iqpa2 d2fnaa2 d1ixsb2 d4gita_ d1sxje2 d1g41a_ d1wgda1
d1wgga1 d4q5eb_ d1wjna1 d1se9a_ d3n3kb_ d1wz0a1 d1we6a1 d2m0xa_
d2gbka_ d3rt3b1 d1wh3a1 d1sifa_ d2ekec2 d4ii2b1 d4hk2a1 d1c3ta_
d1m94a_ d1wgha1 d1ttna1 d1j8ca_ d1wx8a1 d3v6cb1 d2ma9b_ d4i6lb1
d1bt0a_ d2jvca1 d1iyfa_ d2k1fa_ d1wxva1 d1wf9a1 d1t0ya_ d2io0b2
d1x1ma1 d2gbma_ d1zw7a1 d2m17a_ d1we7a1 d2lwpa_ d2gbja_ d1wjua1
d1v6ea1 d1v86a1 d4eewa_ d1wiaa1 d4icva_ d4gu2a_ d1wx7a1 d1wy8a1
d4hjka1 d2faza1 d1v5oa1 d3h33a_ d1rwja_ d19hca_ d1os6a_ d3bxua_
d3h4na_ d1i77a_ d3caoa_ d1j0pa_ d2ctha_ d1duwa_ d2e84a_ d1hh5a_
d1gyoa_ d3h34a_ d1wioa4 d1ccza2 d1vcaa1 d1dr9a2 d1ic1a1 d2d9qb1
d2nxyb2 d1adqa1 d1fnga1 d1uvqb1 d1zs8a1 d2vola1 d2vola2 d1mfbl2
d2jelh2 d1uwel2 d3qq4b1 d1kjvb_ d1k5na1 d1osph2 d4odhl2 d1ow0a1
d1ow0a2 d1bfob2 d3l9ra2 d3jtth1 d2fath2 d4elkb2 d1fn4b2 d2xfxb_
d1pg7x2 d1cqka_ d4k71b2 d1lk2a1 d1eapb2 d4lfhg2 d1i4fa1 d2nw2a2
d1kfah2 d1mhca1 d1h3ua1 d1wejh2 d1h0db2 d1a5fh2 d1kcvh2 d1mjuh2
d1qqda1 d1kcrh2 d1k8ia1 d3huja2 d2nnab1 d1nfdf2 d3mffb2 d1s9va1
d1lk3h2 d1q0xl2 d1fv1b1 d1neza1 d4rfeb2 d1r3ha1 d1a6tb2 d1kjva1
d1pz5b2 d1onqa1 d2bc4a2 d1de4a1 d1k8da1 d1tcra2 d1dn0b2 d1nfde2
d2xwtb2 d1bfoa2 d1mexh2 d1iakb1 d1ypzf2 d4f7ea2 d1q72l2 d2fbjh2
d1fe8h2 d1clzh2 d1fngb1 d1uvqa1 d1vgka1 d1c16a1 d4j12a2 d4x99b2
d1igtb3 d1je6a1 d1q72h2 d1nldh2 d1teth2 d2hmic2 d1i1ca2 d1i1ca1
d1k8ib1 d1q0yh2 d1kfal2 d3frua1 d1pg7w2 d1u58a1 d1igyb4 d1igyb3

d1hyrc1 d1klub1 d1deeb2 d1c12b2 d2bc4b2 d2ig2h2 d1kcvl2 d3wn5b1
d1lk2b_ d1lk3l2 d1mjul2 d1sm3h2 d1q0xh2 d1zvsa1 d1ydpa1 d1u8ib2
d1ypze2 d1kcuh2 d2e3fa_ d2e3ea_ d2llda_ d2ny8x_ d2e3ga_ d2ny9x_
d2nz3a_ d2lj7a_ d1n4na_ d2m8bb1 d2kyqa_ d3psma_ d1jxca_ d2gl1a_
d1ugla_ d4uj0a_ d2k4ua_ d1hp2a_ d2lu9a_ d1baha_ d2meoa1 d1wm7a_
d1hlya_ d1n8ma_ d1jlza_ d1wpda_ d1c55a_ d1pvza_ d1wt7a_ d1rjia_
d1wz5a_ d1m2sa_ d1pjva_ d1c49a_ d1qkya_ d2ktca1 d1wmta_ d1du9a_
d1j5ja_ d1r1ga_ d1t0za_ d2i61a_ d1t1ta_ d1sn4a_ d1kv0a_ d2lkba_
d1npia_ d1wwna_ d1pe4a_ d2kbka_ d2li7a_ d1djta_ d2kbha_ d1dq7a_
d1sega_ d1omya_ d1zywa1 d1jzaa_ d1n9wa1 d1bbua1 d1e1oa1 d1c0aa1
d1b8aa1 d1l0wa1 d3a74a1 d1iyjb4 d1iyjb5 d1iyjb3 d1o7ia_ d4ipca1
d1x3ea_ d4apva_ d1jb7a1 d1jb7a2 d1jb7a3 d3kdfa_ d1ue1b_ d1wjja1
d3eiva_ d1xjva2 d1xjva1 d2ccza2 d1se8a_ d1l1oc_ d1jb7b_ d1qvca_
d3ulla_ d1qzga_ d1miua4 d1miua5 d1miua3 d3g48a1 d1gd7a_ d1mkha_
d1jjcb3 d1pyba_ d1y14b1 d2nn6i1 d1i94l_ d2zjra2 d1smxa_ d2qamc2
d1hh2p1 d2r7da1 d1rl2a2 d1wfqa1 d3bzka4 d1x6oa2 d3a0ja_ d2j01d2
d2ja9a1 d4ql5a_ d1hr0w_ d1luza_ d2n3sa_ d2vnud1 d1h95a_ d1go3e1
d2nn6h1 d1ne3a_ d2mo1a_ d1jjga_ d1e3pa2 d2ix0a3 d2ix0a2 d2lxja_
d1ueba3 d1ueba2 d2z0sa1 d1u0la1 d2ix0a1 d2eifa2 d2je6i1 d3i2za1
d2c35b1 d2ahob2 d1t9ha1 d3i4oa_ d2qall1 d1jt8a_ d2nn6g1 d1g6pa_
d3cama_ d1i94q_ d1c9oa_ d1d7qa_ d2ba0a1 d2vnud2 d2vnud3 d1khia2
d2qalq1 d1wi5a1 d1xtda2 d1ny4a_ d1fvia1 d1ckma1 d1p16a1 d1x9na2
d3zxha_ d1cgla_ d1rm8a_ d1eaka2 d1hy7a_ d2jsda_ d1cxva_ d1hv5a_
d1i76a_ d1rpma_ d2shpa1 d1yfoa_ d1l8ka_ d2ooqa1 d1g4us2 d1p15a_
d1lara2 d1lara1 d1wcha_ d2fh7a2 d2fh7a1 d1ohea1 d1ohea2 d2f4da_
d1rxda_ d2hxpa_ d1xria_ d1d5ra2 d1fpza_ d3ezza_ d4y2ea_ d1i9sa_
d3azya_ d3wvja_ d1upsa1 d1ajoa_ d2hyka_ d1ajka_ d2vy0a_ d1o4ya_
d3o5sa_ d3d6ea_ d3atga_ d1dypa_ d1umza_ d3i4ia_ d1c1la_ d4lbqa_
d1a78a_ d1slta_ d4no4a_ d3zsja_ d3duia_ d1g86a_ d1qmja_ d1hlca_
d1tdha3 d1q3ba3 d1ee8a3 d1k82a3 g1qmo.1 d3zyra_ d1fnya_ d1n47a_
g2ltn.1 d1avba_ d3usua_ d1dhkb_ d1f9ka_ d1ukga_ d1g8wa_ d1g9fa_
d1wbfa_ d3wcsa_ d1g7ya_ g1loe.1 d1mvqa_ d1hqla_ d1v6ia_ d1dbna_
d1qnwa_ d1fx5a_ d4l8qa_ d1ioaa_ d3n35a_ d1epwa1 d2h0ba_ d2erfa_
d1h30a2 d1h30a1 d3mw4a1 d2r1ba_ d3v64a1 d1d2sa_ d3bopa1 d3mw3a1
d1okqa1 d1okqa2 d1dszb_ d1a6ya_ d3cbba_ d1ynwa1 d1r4ia_ d1hcqa_
d2hana_ d2hanb_ d2enva1 d1kb4b_ d2nllb_ d1lo1a_ d1cita_ d2dara2
d2dara1 d1wyha2 d2dloa2 d1rutx4 d1rutx3 d2cuqa2 d1x4la2 d1x4la1
d1x3ha2 d1x3ha1 d2d8za1 d1x61a2 d1x6aa1 d1x6aa2 d2cora2 d2cora1
d1v6ga1 d1v6ga2 d2d8za2 d1x64a1 d1x64a2 d1b8ta1 d2co8a1 d1x63a2
d2dj7a2 d2dj7a1 d2cu8a2 d2cu8a1 d1g47a2 d1g47a1 d1j2oa1 d1wyha1
d2dloa1 d1wiga1 d1wiga2 d2cupa2 d2cupa3 d1x68a1 d2d8ya2 d2d8ya1
d1m3va2 d1x62a1 d1x68a2 d1x61a1 d1b8ta3 d1b8ta2 d2d8xa2 d2d8xa1
d1x4ka1 d1x4ka2 d2cuqa1 d1m3va1 d2co8a2 d1j2oa2 d1x63a1 d2cura1
d2cura2 d1x62a2 d1op9a_ d1y0la1 d1etzb1 d1egjl1 d2gsib1 d1nj9a1

d4jfhd1 d1aqkl1 d1t2ja_ d1lo4h1 d1zvya1 d3v4vl1 d4enec1 d1kxtb_
d5e8ea1 d2pxya_ d4weud_ d1xiwc_ d2esvd1 d1qnzh_ d1ogae1 d1f4xh1
d4n1hb_ d3ezjb1 d3mlwl1 d1kfah1 d1etza1 d1b4jh1 d3cfic_ d4w68a_
d2arja1 d1ek3a_ d2ok0l1 d1vera_ d1uwgh1 d1blnb1 d1fpth1 d1rzga1
d2giya1 d4m3kb1 d1nc2a1 d2nyyd1 d1ncna_ d1fbih1 d5dxwa_ d4fqqa1
d1dqdh1 d1jfql1 d1kelh1 d1faih1 d1frgl1 d1figl1 d4m6nl1 d1fo0a_
d4hxbl1 d3b5ga_ d3quyd1 d2h9gb1 d1i8kb_ d2dd8h1 d2ck0h1 d1eaja_
d4w6wb1 d2a6ib3 d1t66d1 d2e27h_ d1ob1b1 d1f11b1 d2v7na1 d1ad0a1
d2xt1b1 d1pskh1 d4i13b1 d2op4l1 d1a0ql1 d1uwel1 d1za3a1 d1ncbl1
d4uu9a_ d1bm3h1 d1cloh1 d1nqba2 d1cf8h1 d4y5va_ d1mhph1 d5anma1
d4cdgc1 d4ldeb_ d1y0lb1 d2pcpb1 d1z3gl1 d1dfbl1 d1fh5h1 d3g5xa1
d1tzib1 d4dn3l1 d3zhka1 d2b1hh1 d2uyla1 d1f3dh1 d1nj9b1 d2agjh1
d2h1pl1 d1q72h1 d8fabb1 d1ndmb1 d1rjca1 d4u6hb1 d3upca_ d2fbjl1
d1osph1 d1tvda_ d2cjvh_ d4fhbd1 d1bd2d1 d1rz7h1 d3nn8a_ d1blna1
d1knoa1 d1nfda1 d2bmkb1 d1dqll_ d1igyb1 d1igfl1 d12e8h1 d3utza1
d1nc2b1 d1rihh1 d4mwfb1 d4jvpa1 d1mieh1 d1rhfa1 d1fgnl1 d1sbsh1
d1lp9e1 d1dn0a1 d4lqfl1 d1iaih1 d1kgcd1 d1mh5b1 d1gpol1 d2g60h3
d1f8tl1 d2jixd1 d4grwh_ d1fo0b_ d1bfob1 d1axsa1 d1u9ka_ d1nmbh_
d1h8na1 d1h8na2 d1jguh1 d4eura1 d3sdyl1 d35c8h1 d1w72l1 d1mvub_
d1i9rh1 d1ob1a1 d4wenb_ d1yeeh1 d3ietc1 d1f58h1 d1ad0b1 d1mqkl_
d1nbvh1 d4b50a_ d3r8bb_ d1bafl1 d1mamh1 d4rgne1 d2gsgb_ d35c8l1
d1bafh1 d1ai1h1 d2pcpa1 d2fath1 d2x1pa_ d1orsb1 d1ibgl1 d1shma_
d1a2yb_ d1u0qa_ d2coqa_ d1hilb1 d1qfwi_ d1jv5b_ d1fn4b1 d5e0qa_
d1nsnh1 d1plgl1 d4ocxl1 d1kb5h1 d1rzfh1 d1pg7x1 d2fx7h1 d2oi9c_
d1l7il1 d3eyva1 d1d5il1 d4ffyl_ d1tetl1 d1a6wh_ d1ac6a_ d3dvfa_
d1nfde1 d1mimh1 d1ri8a1 d1jhlh_ d3dura_ d1fskb1 d1f97a1 d3so3b1
d2ak4d1 d1lp9f1 d4z7wf1 d1l7th1 d4y5ya_ d4x7ec1 d1emth1 d1ikfl1
d1nlbh1 d4utal1 d1bjma1 d1eapb1 d1u6ah1 d2dtgd1 d1kj2b_ d1xeda1
d1c1eh1 d1fgvh_ d2bnub1 d2fb4h1 d2dtgb1 d1ktrh_ d1jpth1 d1qkzl1
d4hepg_ d2atpb1 d1flrh1 d2hrpl1 d1mvfa_ d1iaim1 d3k80a1 d2jb5h1
d3zkxc_ d4grwe_ d1it9h1 d1rhhb1 d3w9ec_ d1e4wh1 d1sy6h1 d2j6eh1
d3qxta_ d1jglh1 d1p7ka1 d1tzhb1 d4n8pa1 d1gafl1 d2jelh1 d1wioa2
d4i0cc1 d1cicb1 d1lmka2 d1lmka1 d1iqwh1 d1orqb1 d1ejoh1 d1jnlh1
d4zg1a_ d43c9b_ d1ggbl1 d1wejh1 d3q6fa1 d1f3dj1 d3c6la1 d1bqlh1
d3p9wb_ d1a3rl1 d1h0db1 d2uzil_ d1eo8l1 d1qfwl_ d1n7ml1 d1igja1
d1c5ch1 d2a9mh_ d1rmfh1 d1fj1b1 d4heme_ d1ccza1 d1a5fh1 d1ogad1
d1dlfh_ d1qa9b_ d1vgel1 d3qskb_ d1sjxa_ d4ozfg1 d1sq2n_ d1qd0a_
d1kcvh1 d1c5db1 d3qcul1 d8faba1 d3bn9d1 d1mjuh1 d4y8dc_ d7fabh1
d3dvgb1 d1lo0l1 d1a6tb1 d1gigh1 d1kcrh1 d2hwzl1 d1yjdc1 d3clfl1
d4krna_ d1dl7l_ d1e6ol1 d1mexh1 d3r0ma_ d4kkna_ d1fnsl1 d1oaqh_
d1rzia1 d1ucbh1 d1ncwl1 d4b41a_ d1nfdf1 d2mpah1 d2d7tl1 d4grwf_
d4b5ea_ d1lk3h1 d3u79a_ d4u3xa_ d1ghfl1 d1bvkb_ d1mcww1 d1b88a_
d1jrhh1 d32c2b1 d4wemb_ d1dsfh_ d1op3h1 d1ce1l1 d1iqda1 d1qoka1
d1qoka2 d1qfuh1 d2crya1 d1ay1h1 d1oaql_ d3ab0b_ d1rurl1 d1t4kb1

| | | | | | | | |
|---|---|---|---|---|---|---|---|
| d1bz7b1 | d1n0xh1 | d1forl1 | d1ucbl1 | d4w6yb_ | d43c9a_ | d2aabl1 | d1tzia1 |
| d4n1ca_ | d1k6qh1 | d3g9ab_ | d4tvsa1 | d1igmh_ | d1pz5b1 | d3dx9a1 | d3wxwh_ |
| d1kcul1 | d3eaka_ | d4qlra1 | d1cr9l1 | d1d9ka_ | d1f4xl1 | d1deea1 | d2i27n2 |
| d1c12a1 | d2orbl1 | d1j8hd1 | d1mcph1 | d1c5da1 | d1kenh1 | d1bd2e1 | d1h5ba_ |
| d4x7fc1 | d3upaa_ | d1t3fa1 | d1indh1 | d2dtgc1 | d4hc1l1 | d1zv5a1 | d4wgvb_ |
| d1uwgl1 | d4f37k1 | d1aqkh1 | d1vesa_ | d3zkqd_ | d1fptl1 | d1lo4l1 | d1knob1 |
| d3rrqa_ | d1ct8b1 | d4orzc_ | d1mlbb1 | d4hgkc1 | d1dqdl1 | d1bogb1 | d4ozhe1 |
| d1dr9a1 | d1i7za1 | d1dn0b1 | d1kell1 | d1rzib1 | d1ypzf1 | d1wtla_ | d1hkfa_ |
| d1fail1 | d1figh1 | d2vyre_ | d1sjva_ | d1ohqa_ | d1bfoa1 | d1pkqb1 | d1mjul1 |
| d1b4jl1 | d1nsnl1 | d2arjb1 | d1p4bh_ | d4aj0a_ | d3nn8b_ | d2rcja1 | d1f2xk_ |
| d4v1dd_ | d1adqh1 | d2eksa_ | d4k3ga_ | d1jfqh1 | d1uweh1 | d1frgh1 | d4gftb_ |
| d1uz8b1 | d4n1ea_ | d1bm3l1 | d1clol1 | d3mcka1 | d2vq1a1 | d1ae6h1 | d4aq1b1 |
| d1uj3b1 | d1nbqa1 | d1fh5l1 | d2ck0l1 | d2ywza_ | d1q72l1 | d4nbxb_ | d1hila1 |
| d4hzlb1 | d2j88l1 | d1zmya1 | d1cd0a_ | d1a4ka1 | d1pskl1 | d1a0qh1 | d4krmb_ |
| d1ncbh1 | d2fbjh1 | d1j8he1 | d1i3ua_ | d1cf8l1 | d1fe8h1 | d2p1ya1 | d1eeqa_ |
| d2x89a_ | d1ol0a_ | d2r0lh1 | d1ub5a1 | d1dfbh1 | d3eyvb1 | d4jdvb1 | d4p2cg_ |
| d2gsia1 | d2agjl1 | d1u3ha1 | d2h1ph1 | d1rihl1 | d1fskc1 | d1boga1 | d2a77l1 |
| d1ospl1 | d1qfoa_ | d3dwta_ | d2fx7l1 | d1rz7l1 | d3stba1 | d1pkqa1 | d1b6da1 |
| d1dzba2 | d1dzba1 | d1kj2a_ | d4l1ha_ | d1rzgb1 | d1dqlh_ | d12e8l1 | d1miel1 |
| d1jmaa_ | d1sbsl1 | d1fgnh1 | d1f58l1 | d1mqkh_ | d1ndgb1 | d3qegl1 | d1iail1 |
| d2aw2a1 | d1tqbc1 | d3fo2a1 | d4dkaa_ | d1f8th1 | d2g60l1 | d2ywya_ | d1uj3a1 |
| d1yc7a_ | d3bd3a_ | d4w6xb1 | d1nmbl_ | d1i8ka_ | d1rjlb1 | d1ypze1 | d1p7kb1 |
| d1kxqe_ | d1gpoh1 | d2z4qa1 | d1w72h1 | d1i9rl1 | d4qgya1 | d4xt1c_ | d2qqnh1 |
| d1cica1 | d3c08h1 | d2cd0a_ | d2bdnh1 | d1hnga1 | d1maml1 | d1nldh1 | d1cr9h1 |
| d4lcih1 | d3bkyh1 | d3qg7l1 | d1ai1l1 | d1kxvc_ | d4iosd_ | d1l7ih1 | d2x1qa_ |
| d1ub6b1 | d3k74b_ | d1ibgh1 | d1teth1 | d1igya1 | d1hxma1 | d2x6ma_ | d2e7la_ |
| d5e03a_ | d1qfwm_ | d2r0kh1 | d1igjb1 | d1rurh1 | d1plgh1 | d1mj8h1 | d1yuhb1 |
| d3fmgl1 | d1rzfl1 | d1emtl1 | d1ad9b1 | d1igtb1 | d1nlbl1 | d1d5ih1 | d3k7ua_ |
| d4ffyh_ | d1bwwa_ | d3dvga1 | d1a6ta1 | d1fgvl_ | d4qxgl1 | d1miml1 | d1jhll_ |
| d2hffb1 | d2hh0l1 | d1qkzh1 | d1l7tl1 | d1afvh1 | d1iaii1 | d2cjuh_ | d1um5h1 |
| d2ghwb2 | d2ghwb1 | d1kgce1 | d2fgwh1 | d1ikfh1 | d1dvfd_ | d2i24n1 | d2xtjb_ |
| d1za3b1 | d2yc1a_ | d2hh0h1 | d2x1oa_ | d1bvka_ | d1rjla1 | d5e7ba1 | d1igta1 |
| d1il1b1 | d2fb4l1 | d1mvua_ | d1jptl1 | d32c2a1 | d1fvca_ | d1iqdb1 | d2hrph1 |
| d2p4ab_ | d4qo1a_ | d1p2cb1 | d1wt5c_ | d1t4ka1 | d5c1mb_ | d1it9l1 | d1bz7a1 |
| d1b2wh1 | d1e4wl1 | d1jnll1 | d1yedb1 | d1za6b1 | d3u7aa_ | d1igfh1 | d3gnml1 |
| d1orsa1 | d1wejl1 | d1a2ya_ | d1hxmb1 | d1gafh1 | d2j4wh1 | d1pz5a1 | d1j05a_ |
| d1a3rh1 | d4fqll1 | d1qfwh_ | d1eo8h1 | d1n7mh1 | d4s11a_ | d1kegh1 | d5da4a_ |
| d1iqwl1 | d3whxb1 | d1vgeh1 | d1deeb1 | d1c12b1 | d1t2ql1 | d1ad9a1 | d3gsnb1 |
| d3b2uc1 | d1ggbh1 | d1t3fb1 | d1nakl1 | d4ocmc_ | d1lila1 | d2v7hb3 | d2esve1 |
| d1mcoh1 | d2qr0b1 | d2q3aa_ | d1ct8a1 | d1kcrl1 | d2pxyb_ | d2uzih_ | d5c3ld_ |
| d3durb_ | d1c5cl1 | d2ak4e1 | d1dl7h_ | d1xiwd_ | d1mlba1 | d1rmfl1 | d1i7zb1 |
| d1dlfl_ | d2xa3a_ | d5bq7b1 | d1dqqb1 | d3h42l1 | d1egjh1 | d1kcvl1 | d1fe8l1 |
| d2j6el1 | d1f3rb2 | d1lk3l1 | d1sm3h1 | d1q0xh1 | d1ghfh1 | d7fabl1 | d1igch1 |
| d1lo0h1 | d4idla_ | d5c2bl_ | d1jrhl1 | d1il1a1 | d3zhda1 | d4kfzc_ | d1b0wa_ |

d1e6oh1 d1mexl1 d4krpb_ d1fnsh1 d1ncwh1 d4hgma_ d4eigb1 d4rzca_
d2mpal1 d4y5xa_ d5en2b1 d3lrga1 d4ej1c1 d1rhha1 d1j05b_ d1forh1
d1q9wb1 d2wzpd_ d2aq2a1 d1h0da1 d3revb1 d2ij0c1 d1tzha1 d4m7jl1
d1k6ql1 d3b9kc1 d1igml_ d3qeua1 d1dsfl_ d1ce1h1 d1kcuh1 d1zvha1
d1zvoa1 d1tqbb1 d1ay1l1 d2qscl1 d2cgrh1 d1nl0h1 d1kenl1 d3eo0a1
d4n9ob1 d1n0xl1 d3k1kc_ d2m9wa1 d3dfxa_ d2aq2b1 d1enfa1 d1i4pa1
d1v1oa1 d1et9a1 d1esfa1 d2g9hd1 d4ohja1 d1klud1 d1bxta1 d1eu3a1
d1ty0a1 d1fnua1 d2cqva1 d1fhga_ d2k1ma_ d2dl2a1 d1biha3 d1biha1
d1biha4 d3qp3a1 d1g1ca_ d1gxea_ d1cvsc2 d1cvsc1 d1xmwa2 d1m4ka2
d2otpa2 d1biha2 d1rhfa2 d2fcba2 d2fcba1 d1wwbx_ d1gsma2 d1epfa1
d1epfa2 d2dava1 d1waaa_ d1f97a2 d1x44a1 d1vcaa2 d1fnla2 d1fnla1
d3b5ha2 d3h8na2 d3h8na1 d1jbja1 d1jbja2 d1pd6a_ d1f2qa2 d1f2qa1
d3cx2a_ d1nbqa2 d3b5ha1 d1n26a1 d1xiwb_ d1xaua_ d1fltx_ d2ifga1
d1olla1 d1olla2 d1cs6a4 d1cs6a3 d1cs6a2 d1cs6a1 d2gw5a2 d1sy6a1
d1xiwa_ d1ucta2 d1ucta1 d1ry7b2 d1gl4b_ d1qz1a3 d1olza1 d2c9aa1
d1wwca_

**Family-48 (c.1.8.5)**

**Positive dataset contains 5 protein sequences.**

d4toqa_ d1wb0a1 d1w9pa1 d2gsja_ d1vf8a1

**Negative dataset contains 3976 protein sequences.**

d1m32a_ d1ibja_ d1c7na_ d1iuga_ d4q75a_ d2bkwa1 d1t3ia_ d1n8pa_
d1qz9a_ d1jf9a_ d1eg5a_ d3kgwa_ d1cl1a_ d2ctza1 d1vjoa_ d2rfva_
d1gc0a_ d1cs1a_ d1d2fa_ d2ch1a1 d1qgna_ d1pffa_ d1elua_ d1u08a_
d1lc5a_ d2ay1a_ d1bw0a_ d3pd6a_ d7aata_ d2csta_ d1j32a_ d1fg7a_
d4wlha_ d1o4sa_ d1v2da_ d1gdea_ d1m6sa_ d1v72a1 d1svva_ d1ajsa_
d1wsta1 d2f8ja2 d1lk9a_ d1vp4a1 d1xi9a1 d2gb3a1 d1yaaa_ d3i2ka1
d1lnsa3 d1mpxa2 d1xkta1 d2hfka_ d1jmkc_ d5d3ka_ d2cfba1 d1dfoa_
d1o69a_ d1bj4a_ d3k28a_ d2bwpa_ d3lg0a_ d2fnua_ d3ou5a_ d1z7da1
d1b9ha_ d1iaya_ d4msoa_ d1bs0a_ d1s0aa_ d1sffa_ d3tqxa_ d1mdoa_
d3qboa_ d3pgya_ d1bjna_ d2dkja_ d1ejia_ d1vefa1 d4wxga_ d3bs8a_
d3gbxa1 d1w23a_ d1r0ka3 d1j5pa3 d1vkoa2 d1vm6a2 d3qvsa2 d1gr0a2
d1p1ja2 d1i26a_ d1f56a_ d1bypa_ d1azca_ d1ws8a_ d3u25a_ d1cuoa_
d1id2a_ d1rkra_ d2iaac_ d4dp1x_ d1nwpa_ d2gima_ d2i7sa_ d1qhqa_
d1gyca3 d1hfua2 d1kbva1 d1kbva2 d1sdda1 d1sdda2 d1gyca1 d1gyca2
d1mzya1 d2bw4a1 d1aoza1 d1aoza3 d1aoza2 d1kyaa2 d1kyaa3 d1kyaa1
d1mzya2 d4e9sa1 d1sddb1 d1sddb2 d1hfua1 d1hfua3 d1oe1a2 d1oe1a1
d1v10a2 d1v10a3 d1v10a1 g1f8v.1 d1novc_ d2bbvc_ d3sxwa1 d4luka_
d1a3qa1 d1iknc_ d3brda1 d1p7hl1 d1oy3c_ d1imhc1 d1gjia1 d1zk9a1
d2cxka1 d2bema_ d1pama1 d1ea9c1 d1qhoa1 d2fhfa2 d2fhfa1 d1k3ia1
d1j0ha1 d1cxla1 d2bhua1 d1edqa1 d1f1sa2 d1ut9a2 d1gvia1 d1txka1
d4alac_ d1rera1 d1s6na_ d3uzqb_ d2phla2 d2phla1 d1fxza2 d1fxza1
d1lrha_ d1uija1 g1dgw.1 d2v09a_ d1uika2 d1uika1 d1dgwa_ d1ds6b_
d1fstb_ d1ex0a1 d1g0da1 d4mh8a_ d4i7ga1 d1tafa_ d1tafb_ d1jfib_
d1q9ca_ d1h3ob_ d2byka1 d1n1ja_ d4g92b_ d1bh9a_ d1jfia_ d1h3oa_

d2bykb1 d1n1jb_ d2bjca1 d2hsga1 d1qpza1 d1k9ua_ d2opoa_ d1ydwa2
d2nvwa2 d1zh8a2 d1qkia2 d1xeaa2 d1tlta2 d1ebfa2 d1au7a2 d1e3oc2
d2ovga_ d1sq8a_ d1rzsa_ d3kz3a_ d3orca_ d3gpdg2 d2q49a2 d1hdgo2
d2g17a2 d2cvoa2 d2czca1 d3pwka2 d1i32a2 d1zyao2 d2pkqo2 d1b7go2
d2hjsa2 d1cf2o2 d1vkna2 d1nvmb2 d2msya_ d2djoa1 d2r5za_ d1au7a1
d1f43a_ d2lmda1 d1akhb_ d1s7ea1 d3nara_ d1zq3p1 d2craa1 d1mija_
d4uuta_ d3a01a_ d1x58a1 d3rkqa1 d3cmya1 d2cqxa1 d2da6a1 d1e3oc1
d1fjla_ d1mh3a1 d1ig7a_ d1x41a1 d1wh7a1 d1gt0c1 d2mw8a_ d1lfup_
d1x2ma1 d1b72a_ d1wi3a1 d1jgga_ d1wjha1 d1x2na1 d1mnmc_ d2cuea1
d3k2aa_ d2hosa_ d2jwta2 d1pufa_ d1octc1 d2hi3a_ d1b8ia_ d4j19a_
d3lnqa_ d1wh5a1 d1b8ib_ d2cqra1 d1guua_ d1ug2a1 d2cqqa1 d2ajea1
d4a69c_ d1h8ac1 d2crga1 d2iw5b1 d1xc5a1 d1ofcx1 d2cu7a1 d1wgxa1
d2cjja1 d2ckxa1 d1gvda_ d1hcra_ d1tc3c_ d1gdta1 d1u78a2 d1hlva1
d1iufa2 d1iufa1 d2iu5a1 d1zk8a1 d2g7sa1 d2fd5a1 d2np5a1 d1z0xa1
d2id6a1 d2d6ya1 d2i10a1 d1vi0a1 d2oi8a1 d3c07a1 d2ns7a1 d2fq4a1
d2yvea1 d1t33a1 d2o7ta1 d2np3b1 d2g7ga1 d2gena1 d2fbqa1 d2g7la1
d2gfna1 d2g3ba1 d2jj7a1 d2hyja1 d1sgma1 d1ui5a1 d2hkua1 d2id3a1
d4jyka1 d1bl0a1 d1d5ya1 d1d5ya2 d3lv8a_ d1s3ga1 d1zaka1 d1s4qa_
d3tvta2 d1uf9a_ d1khta_ d1ckea_ d1osna_ d1p5zb_ d4tmka_ d3cm0a_
d1tmka_ d1jaga_ d3h86a_ d1t0hb_ d1nn5a_ d2h92a_ d3kb2a1 d2c95a1
d1yj5a2 d1kgda_ d1ly1a_ d2ak3a1 d1vyva2 d1deka_ d2grja1 d1p3ja1
d1y63a_ d1e4ya1 d1s96a_ d3iija1 d1lvga_ d1nksa_ d1vyua2 d4e22a_
d1qf9a_ d1jjva_ d1q3ta_ d1ki9a_ d1lw7a2 d1teva_ d1kjwa2 d1gsia_
d1g3ma_ d1j99a_ d1t8ta1 d1nsta_ d1fmja_ d1zrha_ d1ls6a_ d2a3ra_
d1aqua_ d2zptx_ d3ckla_ d1texa_ d1q44a_ d2gwha_ d1z0ja1 d3umra2
d3gj0a1 d2gaoa1 d1yzga1 d1x3sa1 d1puja_ d1udxa2 d1kk1a3 d2o52a_
d1jwyb_ d1zcaa2 d1zcba2 d2fv8a1 d2cjwa_ d3dz8a1 d1xzpa2 d1vg8a_
d4qxaa_ d2ew1a1 d1x1ra1 d2bcgy_ d3doea1 d3cona_ d3rega_ d3wyga_
d1upta_ d2b6ha1 d1zd9a1 d1lnza2 d1r5ba3 d1n0ua2 d1r2qa_ d2f9la1
d2rhda_ d3q3jb_ d3q85a_ d1efma1 d1r8qa_ d2gf9a1 d2akab1 d1ek0a_
d1z08a1 d2gcoa1 d2dy1a2 d4g01b_ d2cxxa1 d1svia_ d3q72a_ d1ni3a1
d4ac9a4 d2erxa1 d2bmea_ d1wxqa1 d2gf0a1 d4ldja1 d1zunb3 d1d5ca_
d4lv5b_ d1efca3 d1kmqa_ d2g6ba1 d3lrpa_ d1tada2 d2y8ea_ d4rd4a1
d1z06a1 d2heia_ d3a58b_ d1u0la2 d1d2ea3 d1zj6a1 d2ocba_ d3rwoa_
d1mr3f_ d2e9sa1 d4u5xa1 d2iwra_ d2fn4a1 d3bwdd_ d2nzja1 d2atva1
d2ksqa_ d3l43a_ d1mkya2 d1mkya1 d1wmsa_ d1jnya3 d2rmka_ d1f6ba_
d1f60a3 d1gota2 d1jala1 d2fh5b1 d1g16a_ d2wblc_ d1m7ba_ d1wf3a1
d1f5na2 d1t9ha2 d1z6xa1 d1z0aa1 d2fg5a1 d2a5ja1 d2bcjq2 d1s0ua3
d1ky3a_ d4y0va_ d4lhwa_ d2qmea1 d2atxa1 d1egaa1 d1ryha_ d1moza_
d1nrjb_ d2erya1 d3bfka_ d1puia_ d1fzqa_ d3j3az1 d3t5ga_ d1shza2
d3defa_ d2f7sa1 d3raba_ d3tsoa_ d3aq4a_ d2al7a1 d3sfva1 d3bc1a_
d2ncda_ d1sdma_ d3h4sa1 d2heha_ d2grya_ g3kin.1 d1br2a2 d1goja_
d1f9va_ d2mysa2 d1lkxa_ d3t0qa_ d4db1a1 d3gbja_ d1ry6a_ d1v8ka_
d2h0ba_ d2erfa_ d1h30a1 d3mw4a1 d1pz7a_ d2r1ba_ d3v64a1 d1d2sa_

| | | | | | | | |
|---|---|---|---|---|---|---|---|
| d3bopa1 | d3mw3a1 | d1okqa1 | d1okqa2 | d1s5ja2 | d1qhta2 | d4dlea2 | d1tgoa2 |
| d3py8a2 | d1q8ia2 | d2py5a2 | d1d5aa2 | d1k8ka2 | d1k8ka1 | d3uleb1 | d3i33a2 |
| d3i33a1 | d1bupa1 | d1dkgd2 | d1dkgd1 | d4ci6a2 | d1jcfa1 | d4a2ba2 | d2fxua1 |
| d1yaga1 | d4cbua2 | d1bupa2 | d1g99a1 | d2e1za2 | d2e1za1 | d1saza1 | d1saza2 |
| d1ig8a1 | d1czan2 | d1czan3 | d1czan1 | d1ig8a2 | d1czan4 | d1bg3a4 | d1bg3a3 |
| d1bg3a2 | d1bg3a1 | d3h3no1 | d3h3no2 | d3ezwa2 | d3h1jd1 | d2d0oa3 | d2d0oa2 |
| d1nbwa2 | d1hroa_ | d4gedb_ | d3zooa_ | d1mz4a_ | d1gu2a_ | d1f1fa_ | d1dvva_ |
| d1c6ra_ | d1c75a_ | d1e29a_ | d1gdva_ | d1cnoa_ | d1h32b_ | d1co6a_ | d2bh4x_ |
| d2aiua_ | d5cytr_ | d1jdla_ | d1i8oa_ | d2zboa_ | d3dmia_ | d1ls9a_ | d1wvec_ |
| d2yk3a_ | d3ph2b_ | d1dw0a_ | d5aura_ | d1ql3a_ | d1qn2a_ | d1f1ca_ | d4zzva_ |
| d2xspa_ | d1eg1a_ | d4v20a_ | d1ojja_ | d4ipma1 | d4csia_ | d3pfza_ | d3ovwa_ |
| d2yoka_ | d2nqya_ | d1h4ga_ | d1hixa_ | d1oa2a_ | d1olra_ | d3b7ma_ | d4h7ma1 |
| d3wp3a_ | d2vgda_ | d1igoa_ | d3zsea_ | d2nlra_ | d3wx5a_ | d1oa3a_ | d1pvxa_ |
| d1m4wa_ | d1f5ja_ | d1oa4a_ | d1ks5a_ | d4ixla1 | d2ah2a1 | d1n1ta1 | d1h32a2 |
| d1evsa_ | d1cnt1_ | d1pvhb_ | d1z7ca_ | d1huwa_ | d1bgea_ | d1rhga_ | d1f6fa_ |
| d1f45b_ | d1i1rb_ | d1axia_ | d1etea_ | d3tgxb_ | d1scfa_ | d2z3qa1 | d3qb1a_ |
| d1hula_ | d1m48a_ | d4nkqb_ | d1eera_ | d1v7mv_ | d5e4ea_ | d2o27a_ | d3b5ka_ |
| d2psma1 | d4zf7a_ | d3uf5a_ | d4x51a_ | d1d9ca_ | d4ypgc1 | d3oq3a_ | d1lqsl_ |
| d1n1fa_ | d1au1a_ | d1wu3i_ | d3besl_ | d3ux9a_ | d3rbaa_ | d2qtra_ | d1nupa_ |
| d3l92a_ | d1f9aa_ | d1qjca_ | d2h29a_ | d1vlha_ | d1od6a_ | d1lw7a1 | d1mrza2 |
| d4ruka_ | d2el7a_ | d1iq0a2 | d1r6ta2 | d1ivsa4 | d2cyba_ | d1rqga2 | d1j09a2 |
| d2yxna_ | d1f7ua2 | d1h3fa1 | d1ffya3 | d2g36a1 | d1nzja_ | d1jila_ | d1n3la_ |
| d1i6la_ | d1pfva2 | d1h3na3 | d3n9ia_ | d3sz3a1 | d3sw2a_ | d1moxc_ | d1dx5i3 |
| d1edmb_ | d1autl2 | d1autl1 | d1nqlb_ | d1dval1 | d1szba2 | d1kigl_ | d2y5fl_ |
| d1ijqa2 | d1gk5a_ | d1g1sa2 | d1nt0a3 | d1xdtr_ | d1lmja2 | d1lmja1 | d1nzia2 |
| d2wphe_ | d1klil_ | d1pfxl2 | d1pfxl1 | d1p9ja_ | d1g1ta2 | d1f7ea_ | d4yzub_ |
| d2mgpa1 | d1b9wa1 | d1b9wa2 | d2mgpa2 | d1ob1c2 | d1n1ia2 | d1n1ia1 | d2npra2 |
| d2npra1 | d1m1xb4 | d2y5ia_ | d1qlsa_ | d1psra_ | d3n22a_ | d2egda_ | d4ggfc_ |
| d2wcea_ | d2luca_ | d4aqia_ | d3nsia_ | d2lp3a_ | d1j55a_ | d1a4pa_ | d1k8ua_ |
| d1nsha_ | d1pvaa_ | d3fs7a_ | d2pvba_ | d1ttxa_ | d1rwya_ | d2kyfa_ | d1rk9a_ |
| d1a75a_ | d1exra_ | d1tiza1 | d1nyaa_ | d1g8ia_ | d1ggwa_ | d1wdcc_ | d3fwba_ |
| d1ggza_ | d2o5ga_ | d1ctaa_ | d1m45a_ | d3i5gc_ | d1sl8a_ | d1fpwa_ | d2scpa_ |
| d2roaa_ | d4aqra_ | d3pn7b_ | d1br1b_ | d2mysb_ | d1s6ca_ | d1bjfa_ | d2obha_ |
| d1jfka_ | d1jf0a_ | d1qv0a_ | d1lkja_ | d2mlfc_ | d2m97a_ | d2d8na_ | d4by5a_ |
| d1jbaa_ | d4nqga_ | d1wdcb_ | d2mysc_ | d1j7qa_ | d1s6ia_ | d1uhka2 | d1r2ua_ |
| d1dtla_ | d3kf9a_ | d3pn7c_ | d1fi6a_ | d1c07a_ | d5d67d_ | d1tuza_ | d1h8ba_ |
| d1eg3a1 | d2zkmx1 | d1snla_ | d1gjya_ | d1y1xa_ | d1hqva_ | d1dvia_ | d1df0a1 |
| d1qxpa2 | d4usla_ | d1k94a_ | d2w0pa_ | d2diaa1 | d2e9ia2 | d2e9ja1 | d2dj4a1 |
| d2eeba1 | d2diba1 | d2dmba1 | d2dmca1 | d2d7oa1 | d2ee6a1 | d2d7pa1 | d2eeaa1 |
| d2di8a1 | d1wlha1 | d2d7na1 | d2nqca_ | d2d7ma1 | d2dica1 | d3cnka_ | d2k3ta1 |
| d2eeca1 | d1qfha1 | d2bp3a1 | d1v05a_ | d2ee9a1 | d2eeda1 | d2di9a1 | d3syxa1 |
| d1mkea1 | d2p8va_ | d1xoda1 | d1evha_ | d1qc6a_ | d1egxa_ | d1xl4a1 | d3vsqa_ |
| d2gixa1 | d1p7ba1 | d2v76a_ | d1wj1a1 | d1aqca_ | d1qqga2 | d1qqga1 | d1ntva_ |
| d1n3ha_ | d4dbba_ | d3f0wa1 | d1p3ra_ | d1wgua1 | d2cy4a1 | d3hqca_ | d1wvha1 |

d1xr0b1 d1wgqa1 d1v89a1 d2coda1 d1zc3b_ d1wg7a1 d1u5ea1 d1v61a1
d2coca1 d2ys1a1 d1v5pa1 d1ntya2 d1wjma1 d1wi1a1 d1u29a_ d1xx0a1
d1unqa_ d2zkmx3 d1fhoa_ d1v88a1 d2adza1 d1x1fa1 d1p6sa_ d2coaa1
d1btka_ d1v5ma1 d1faoa_ d2x18a_ d1dyna_ d1x1ga1 d4kaxb_ d2j59m1
d1ki1b2 d1xcga2 d1txda2 d1u5da1 d1dbha2 d1w1ha_ d1upqa_ d1v5ua1
d2fjla1 d1eaza_ d2dfka2 d2cofa1 d1kz7a2 d1d4ua2 d9ldta1 d2ewda3
d1up7a1 d2dfda1 d1s6ya1 d1vjta1 d7mdha1 d1hyea1 d1t2da1 d1ojsa3
d1b8pa1 d1ldna1 d1hyha1 d1civa1 d2a92a1 d1ez4a1 d1i0za1 d1y6ja1
d1ur5a1 d1llda1 d1obba1 d1v8ba1 d1gdha1 d2dlda1 d1mx3a1 d1ygya1
d1qp8a1 d1li4a1 d1j4aa1 d2naca1 d1npya1 d3t4ea2 d1a4ia1 d1o0sa1
d2hk9a2 d2egga2 d1b26a1 d1bvua1 d1gq2a1 d1c1da1 d1leha1 d1gtma1
d1euza1 d1nvta1 d3dona2 d1edza1 d1gpja2 d1p77a1 d1nyta1 d1b0aa1
d1vl6a1 d1v9la1 d1txga2 d3f47a1 d1mv8a2 d2pv7a2 d1xc2a3 d2ahra2
d4xiya1 d2f1ka2 d1pgja2 d1i36a2 d1np3a2 d1lj8a4 d1teaa3 d1qmga2
d2i76a2 d2g5ca2 d1wdka3 d1dlja2 d3cuma3 d1kola2 d1f8fa2 d1qora2
d1jqba2 d1vj0a2 d1cdoa2 d1gu7a2 d1d1ta2 d1xa0a2 d2dm6a2 d1jvba2
d1rjwa2 d1llua2 d1h2ba2 d1tt7a2 d1piwa2 d1o89a2 d1iz0a2 d1yb5a2
d1uufa2 d1vj1a2 d1e3ia2 d3gpdg1 d1vc2a1 d2nvwa1 d1u8fo1 d1ydwa1
d1hdgo1 d2g17a1 d1cf2o1 d2czca2 d1r0ka2 d1obfo1 d2cvoa1 d1ebfa1
d1h6da1 d1i32a1 d1zh8a1 d2q49a1 d1qkia1 d1j5pa4 d3pzra1 d1xeaa1
d1vkoa1 d1vm6a3 d3qvsa1 d2pkqo1 d1b7go1 d1gr0a1 d1p1ja1 d1lc0a1
d1j0xo1 d3pwka1 d2hjsa1 d1zyao1 d1vkna1 d1tlta1 d1nvmb1 d3sjua_
d3ojfa1 d1xu9a1 d2jjya1 d1n7ha_ d1y1pa1 d1h5qa_ d1jtva_ d4fs3a_
d2bkaa1 d1gy8a_ d2c5aa1 d1ulsa_ d1ooea_ d2b69a1 d1y5ma1 d1spxa_
d1ae1a_ d1bxka_ d1ydea1 d1gega_ d1hdoa_ d1o5ia_ d1e6ua_ d1snya1
d2rhca_ d3zv4a_ d5ceja_ d2d1ya1 d3w8ea_ d2fmua1 d1x1ta_ d1rpna_
d3ay7a1 d1k2wa_ d1eq2a_ d2hrba1 d3ruaa_ d2ew8a1 d4hwka_ d1xgka_
d2bd0a1 d1yo6a1 d2a35a1 d2ntva_ d1vl8a_ d1hdca_ d4bkua_ d2ag5a1
d2q45a_ d1nffa_ d4iy1a_ d1zbqa1 d1kewa_ d1ek6a_ d1ja9a_ d2q46a2
d1iy8a_ d1cyda_ d2ptga_ d1hxha_ d1uzma1 d3lyla_ d4m89a_ d3wyea_
d2blla1 d1w6ua_ d1sb8a_ d2q2va_ d4nr0a_ d1e6wa_ d2gasa_ d3oiga1
d2bgka1 d1mxha_ d1vl0a1 d1rkxa_ d1r6da_ d1wmaa1 d1e7wa_ d2c07a1
d1xkqa_ d3tzha_ d1g0oa_ d3lz6a_ d1qyca_ d1edoa_ d1gz6a_ d1oc2a_
d1t2aa_ d1zema1 d2xoxa_ d1z45a2 d1geea_ d1p33a_ d3lt0a_ d4e6pa_
d2wyua_ d1db3a_ d4q9na_ d1xhla_ d4o1ma_ d1fmca_ d2uvda_ d2gdza1
d1uaya_ d2z1ma_ d2o23a2 d2fr1a2 d2fr1a1 d1qsga_ d1qyda_ d4zjua_
d2a4ka1 d1yb1a_ d1wvga1 d1yxma1 d3f4ba_ d1mg5a_ d1xg5a1 d1r55a1
d3gboa_ d1atla_ d1kufa_ d1buda_ d4q1la_ d1yp1a_ d1wnia_ d3nqyb_
d4gera_ d1mdwa_ d1qxpa4 d1ziva1 d1zcma1 d1t3ca_ d1epwa3 d2a97a_
d3fiea_ d3ioqa_ d1fh0a_ d1o0ea_ d1khqa_ d1m6da_ d2cb5a_ d1cs8a_
g8pch.1 d1s4va_ d2pnsa_ d1vsna1 d1mira_ d1iwda_ d2oula_ d7pcka_
d3bwka_ d3bcna_ d2p86a_ d1pvja_ d1pcia_ d1deua_ d4bs6a_ d1xkga1
d1cqda_ d1jqpa2 d2c0ya_ d2bdza_ d3p5ua_ d2o36a_ d1k9xa_ d1zoya1
d1qo8a2 d1d4ca2 d1chua2 d1jnra2 d1kf6a2 d2jk6a2 d2jk6a1 d1feca1

d1feca2 d1lpfa1 d1lpfa2 d1xhca1 d1xhca2 d1dxla2 d1dxla1 d1gera2
d1m6ia2 d1onfa1 d1onfa2 d3lada2 d3lada1 d1w4xa1 d1w4xa2 d1gesa2
d1mo9a1 d1xdia1 d1fl2a2 d1fl2a1 d1fcda1 d1fcda2 d1h6va2 d1h6va1
d1mo9a2 d1gv4a2 d1gv4a1 d2gv8a2 d2gv8a1 d1aoga1 d1aoga2 d1hyua1
d1ebda1 d1ebda2 d3dk9a1 d1q1ra2 d1q1ra1 d1gesa1 d1zbfa1 d2z1ha_
d2etja1 d3qioa1 d1jl1a_ d2e4la_ d1jl2a_ d2lsna1 d1io2a_ d1uaxa_
d2yv0x_ d1mu2a1 d1ekea_ d1i39a_ d1pn0a1 d1reoa1 d1ju2a1 d2f5va1
d3c96a1 d2gmha1 d1pj5a2 d1gpea1 d1n4wa1 d1k0ia1 d2dw4a2 d2ivda1
d1seza1 d2voua1 d1kdga1 d1s5ja1 d2hbja2 d2igia_ d1wlja_ d1qhta1
d4dlea1 d1tgoa1 d2f96a1 d3tr8a1 d3mxma_ d1q8ia1 d1y97a1 d1w0ha_
d2py5a1 d1noya_ d1d5aa1 d1yt3a3 d2guia_ d3mwpa1 d4kb1a_ d1nu0a_
d1js9c_ d1f15b_ d1za7a_ d1laja_ d1b9ra_ d1offa_ d1frra_ d3wcqa_
d1e9ma_ d1l5pa_ d1wria_ d1i7ha_ d3av8a_ d1czpa_ d1rfka_ d3p1ma1
d3b2ga_ d2bt6a2 d3nvva1 d1krha3 d2h88b1 d1n62a2 d1t3qa2 d1kf6b2
d1zoyb1 d1jq4a_ d1ffva2 d1dgja2 d1rm6c2 d1r7ha_ d1faaa_ d1wika1
d1xfla_ d1ttza1 d2f51a1 d1ti3a_ d1nhoa_ d1sena_ d2i4aa_ d1ep7a_
d1zmaa1 d1fo5a_ d3lefa1 d3diea1 d2yana1 d1z6na1 d1oaza_ d3zywa1
d4aj8a_ d1nm3a1 d4fiwa_ d1wmja_ d4hs1a1 d1iloa_ d1xwaa1 d1wjka1
d2vm1a_ d1fb6a_ d1gh2a_ d1h75a_ d1nw2a_ d1kx5b_ d3a6na_ d4m6ba2
d2nqbd_ d3wkjd_ d2yfwe_ d4h9na_ d5bnxa_ d1kx5c_ d3r45a_ d1eqzb_
d4jjnb_ d1f66c_ d1eqza_ d4wnnb_ d1kx5a_ d1u35c1 d1id3a_ d2nqbc_
d1kx5d_ d1um8a_ d1ixza_ d1qvra2 d1qvra3 d1qzma_ d1sxjc2 d1jr3d2
d2p65a_ d1l8qa2 d1g8pa_ d3hu3a3 d1sxjd2 d1njfa_ d1in4a2 d1sxja2
d1tuea1 d2ce7a2 d1w5sa2 d1sxjb2 d1d2na_ d1ny5a2 d1ofha_ d2gnoa2
d2a5yb3 d1fnna2 d1u0ja1 d1lv7a_ d1iqpa2 d2fnaa2 d1ixsb2 d4gita_
d1sxje2 d1g41a_ d1pd212 d1nhya2 d2a2ra2 d1k0dd2 d1f2ea2 d1gsua2
d1f3ba2 d2fhea2 d2pvqa1 d1tw9a2 d1oyja2 d1e6ba2 d3ik7a1 d4is0a1
d1r5aa2 d1jlva2 d1m0ua2 d3gtub2 d1z9ha2 d1okta2 d1axda2 d2gsra2
d1tu7a2 d1k3ya2 d1zgma1 d1b48a2 d1gnwa2 d1gwca2 d2gsta2 d1glqa2
d1oe8a2 d1pn9a2 d1v2aa2 d1fw1a2 d1duga2 d1ovna2 d3wyha_ d4g9sa1
d3gxra_ d1qqya_ d1gd6a_ d1f6ra_ d1ivma_ d2gv0a_ d1iiza_ d2z2fa_
d1j8xa_ d1ghla_ d1b9oa_ d2pjsa1 d1klla_ d2i7ra1 d1ecsa_ d1xqaa1
d5cj3a1 d1jifa_ d2p7oa_ d1r9ca_ d1nkia_ d1mpya2 d1kw3b2 d1kw3b1
d4ghga1 d1tfza1 d3isqa1 d3isqa2 d1sqia1 d1sqia2 d1lkda2 d1mpya1
d1cjxa2 d1cjxa1 d1sp9b2 d1t47a1 d1t47a2 d1f1ua2 d1sp8a1 d1sp8a2
d1f1ua1 d1iyka2 d1rxta1 d1iica2 d1iica1 d1qsta_ d2beia1 d4m3sa_
d1u6ma1 d1ygha_ d1yk3a1 d3dr6a_ d1xmta_ d1z4ea1 d2ge3a1 d1bo4a_
d2fiaa1 d2jdca1 d1y9wa1 d3to7a_ d1wwza1 d2fl4a1 d2ozua1 d2b5ga_
d2cy2a1 d2p0wa_ d1cm0a_ d4pswa_ d1yrea1 d1yr0a1 d1m4ia_ d2gana1
d1yvka1 d1ghea_ d2g3aa1 d1y7ra1 d2euia1 d1q2ya_ d2fsra1 d1xeba_
d2ae6a1 d1cjwa_ d1qsma_ d2aj6a1 d1y9ka1 d1i12a_ d1vkca_ d2atra1
d1n71a_ d2d4pa1 d2fiwa1 d2fe7a1 d1tiqa1 d2ou2a_ d1yx0a1 d2fcka1
d2j8ma_ d2pq8a_ d1z4ra1 d1s3za1 d1ufha_ d2hqya1 d2hqya2 d1lrza2
d1ne9a1 d1ne9a2 d1xvqa1 d1pn0a2 d3hy2a_ d2cvba1 d1o8xa_ d1lu4a_

d1we0a1 d2bmxa1 d2a4va1 d1psqa_ d1xiya1 d1q98a_ d1n8ja_ d1zofa1
d2ggta_ d3tjja_ d4je1a1 d1o73a_ d3s9fa_ d2zcta_ d1qmva_ d1jfua_
d2b1ka_ d5dvba1 d2b5xa1 d2v2ga_ d1st9a_ d3tb2a_ d4llra_ d4fh8a1
d1e2ya_ d1knga_ d2h30a_ d1i5ga_ d2z9sa_ d2f8aa1 d1tp9a1 d2b7ka_
d1nm3a2 d3hvva_ d2wfca1 d4kcea_ d1xvwa1 d4gqca1 d3qpma_ d4k1fa_
d1zzoa1 d1gp1a_ d1prxa_ d2m5ta_ d1cqqa_ d1wqsa_ d5bpea_ d2ipha_
d1lvoa_ d2bhga1 d3tnta_ d3sjia_ d3d23a1 d2fyqa_ d3tloa_ d3zvea1
d2hrva_ d3q3ya1 d4asha1 d1e88a1 d1l6ja5 d1l6ja4 d1eaka3 d1l6ja3
d1h8pa2 d1h8pa1 d1ys1x_ d4hs9a_ d1ex9a_ d1ku0a_ d3qmma_ d1mixa2
d1e5wa2 d1j19a2 d1isna2 d2al6b2 d4l3wa_ d1llfa_ d1gz7a_ d1ukca_
d3zxha_ d1cgla_ d1rm8a_ d1eaka2 d1hy7a_ d2jsda_ d1cxva_ d1hv5a_
d1i76a_ d3p0ya1 d1igra1 d1igra2 d1n8yc2 d1n8yc1 d1m6ba1 d1m4la_
d2bk7a_ d3fjua_ d1z5ra1 d1kwma1 d1uwya2 d1jqga1 d2c1ca1 d1dtda_
d2tbvc_ d1c8nc_ d1x36a_ d1smvc_ d1ng0c_ d4sbvc_ d1opoa_ d1f2nc_
d1cixa_ d1y29a_ d1v7fa_ d2jtba_ d1d1ha_ d1g9pa_ d1lupa_ d1qk7a_
d1mb6a_ d2a2va_ d1niya_ d1i25a_ d1hvwa_ d2mpqa_ d1ie6a_ d1la4a_
d2mxoa1 d1dl0a_ d1koza_ d1qk6a_ d1emxa_ d1lqra_ d1c6wa_ d1rmka_
d1ixta_ d1eyoa_ d1cnna_ d1fu3a_ d1g1pa_ d1fyga_ d1rypd_ d1irua_
d1rypc_ d3unfk_ d1rypa_ d1j2pa_ d1irui_ d1j2qh_ d3unfh_ d1m4ya_
d1rypb_ d1irul_ d1irug_ d1iru2_ d1q5rh_ d1rypg_ d1g3ka_ d1iruf_
d1q5qa_ d1e94a_ d1irub_ d1ryph_ d3ty6a_ d1iruj_ d1iruk_ d1rype_
d1irud_ d1irue_ d3mi01_ d1rypf_ d1iruh_ d2z3ba_ d1iruc_ d3mka2_
d1iru1_ d1ny721 d1ny722 d1a6ca3 d1a6ca2 d1pgl22 d1pgl21 d1pgl11
d1ny711 d1mqta_ d1d4m3_ d1fmd3_ d2mev1_ g1aym.1 d1ev13_ d4q4w1_
d1r1a3_ d2x5ia_ d1rhi1_ g1r1a.1 g1d4m.1 g1rhi.1 d1b35b_ d1oopa_
d1tme1_ d4iv1a_ g1pvc.1 g1fpn.1 d1fpn1_ d4gb31_ d1d4m1_ d1fmd1_
d1ev11_ d2wzr3_ g1cov.1 d1qqp3_ g1fmd.1 d2x5ic_ d1rhi3_ g1oop.1
d1hxs1_ g1hxs.1 d1fpn3_ d1bev1_ g2mev.1 d1aym1_ d1pvc3_ d1qqp1_
d1zba3_ d1h8ta_ d3vbhc_ g1b35.1 g1bev.1 d1hxs3_ g1ev1.1 d1bev3_
g1tmf.1 d1tmf1_ d1b35a_ d2mev3_ g1eah.1 g1h8t.1 d1eah1_ d4q4w3_
d1r1a1_ d1aym3_ d1pvc1_ d1zba1_ d1h8tc_ d1oopc_ d1tme3_ g1qqp.1
d4iv1c_ g1tme.1 d1y14b1 d2nn6i1 d1i94l_ d2zjra2 d1smxa_ d2qamc2
d1hh2p1 d2r7da1 d1rl2a2 d1wfqa1 d3bzka4 d1x6oa2 d3a0ja_ d2j01d2
d2ja9a1 d4ql5a_ d1hr0w_ d1luza_ d2n3sa_ d2vnud1 d1h95a_ d1go3e1
d2nn6h1 d1ne3a_ d2mo1a_ d1iz6a2 d1jjga_ d1e3pa2 d2ix0a3 d2ix0a2
d2lxja_ d1ueba3 d1ueba2 d2z0sa1 d1u0la1 d2ix0a1 d2eifa2 d2je6i1
d3i2za1 d2c35b1 d2ahob2 d1t9ha1 d3i4oa_ d2qall1 d1jt8a_ d2nn6g1
d1g6pa_ d3cama_ d1i94q_ d1c9oa_ d1d7qa_ d2ba0a1 d2vnud2 d2vnud3
d1khia2 d2qalq1 d1wi5a1 d1xtda2 d1ny4a_ d2flia1 d1rpxa_ d1tqja_
d1tqxa_ d4lt9a1 d3tc7a1 d3hoja_ d1i4na_ d3vnda_ d3nl8a1 d1geqa_
d3nava1 d1v5xa_ d4a2ra1 d3tc6a1 d1ujpa_ d4ijba_ d1xcfa_ d1vc4a_
d1rd5a_ d1qopa_ d3b5va_ d2r80b_ d1cqxa1 d1cg5a_ d1gcvb_ d3boma_
d2h8fb_ d2raoa_ d3bj1a_ d4hrrb_ d2r50a_ d3d1kb_ d2aa1b1 d3zhwa_
d3hf4b_ d3gdja_ d1jebb_ d3bj1b_ d1x9fb_ d1wmub_ d1gcva_ d3bcqb_

d3d4xb_ d1gvha1 d2dc3a_ d3hf4a_ d4yu3a_ d3k8ba_ d1qpwb_ d2r80a_
d3bcqa_ d1v4wa_ d3wtgb_ d1jl7a_ d4esaa_ d1a9we_ d3d1ab_ d1fsla_
d4h2la_ d1v4wb_ d1mwca_ d1vhba_ d1jeba_ d1spgb_ d1h97a_ d1q1fa_
d1tu9a1 d3d1aa_ d1hdsb_ d3hyub_ d2zfbb_ d3fs4a_ d1oj6a_ d1shrb_
d4h2lb_ d4yu3b_ d1spga_ d3hrwa_ d3qqra_ d3at5a_ d4wchd_ d3hyua_
d1cg5b_ d1uc3a_ d2raob_ d1outb_ d4esab_ d3gdjb_ d3qqqa_ d1x9fc_
d3vrfa_ d1itha_ d3mkba_ d3d4xa_ d3mjpa_ d1jl6a_ d3fh9a_ d1outa_
d2dc3b_ d1it2a_ d3eoka_ d3g46a_ d1qpwa_ d3vrfb_ d1ch4a_ d1x9fd_
d1hdsa_ d2zfba_ d3mkbb_ d1or4a_ d3wtga_ d1a4fa_ d3fh9b_ d3bomb_
d3cy5a_ d3at5b_ d1x9fa_ d2oifa_ d4lmxb_ d1cpcb_ d3v57a_ d1kn1a_
d1cpca_ d4f0ta_ d2vjha_ d4po5b_ d1eyxa_ d1alla_ d4f0tb_ d1b33b_
d1b8db_ d4h0mb_ d1b33a_ d2vjhb_ d4lm6b_ d2vmlb_ d1allb_ d2vjta_
d3dbja_ d2vmla_ d2vjtb_ d4f0ub_ d1f99b_ d4rmpa1 d1liaa_ d1eyxb_
d2uulb_ d4lmsb_ d4f0ua_ d2bv8a_ d1f99a_ d2bv8b_ d2c7lb_ d3dbjb_
d4rmpb1 d1b8da_ d4h0ma_ d3v57b_ d1kn1b_ d1liab_ d2uula_ d2j96a_
g1gxs.1 d1azwa_ d2xqfa_ d1ea5a_ d1f6wa_ d1dx4a_ d1qe3a_ d1k4ya_
d4qwwa_ d2ha2a_ d1r88a_ d1sfra_ d4qeka_ d3c8da2 d2pbla1 d1jkma_
d1jjia_ d1lzla_ d1jjfa_ d2i49a_ d1nnfa_ d1xvxa_ d1ursa_ d1h3da1
d1iuda_ d4m1va1 d1twya_ d3e13x_ d2nxoa1 d4pfua_ d3ttma_ d1elja_
d2qmwa1 d1y3na1 d1jeta_ d1uqwa_ d4f19a1 d2esna2 d3thia_ d3fasa1
d2uyeb_ d2xwva1 d1r9la_ d2fyia1 d1pb7a_ d2ozza1 d1sw5a_ d1nh8a1
d1vr5a1 d2g29a_ d1xs5a_ d3lswa_ d2z23a_ d3w9va_ d3tcha_ d1q35a_
d1z7me1 d3q27a_ d1p99a_ d1ve4a1 d2i6ea1 d4mcoa1 d4n8ya_ d1eu8a_
d1zbma1 d1hsla_ d1ixca2 d1j1na_ d1xvya_ d1y9ua_ d2z22a1 d1xoca1
d4oera_ d1wdna_ d5f1qa1 d1xt8a1 d1i6aa_ d4gwoa_ d4igta1 d1pc3a_
d4mmpa1 d1o63a_ d2czla1 d2onsa2 d4mija1 d2qvba_ d2v9za_ d4kafa1
d1jmxa3 d1pbya3 d1pbya4 d4iuaa3 d4bvva_ d5hpga_ d3k65a_ d1ki0a3
d1ki0a2 d1ki0a1 d1a0ha1 d1pmla_ d3sp8a3 d2feba1 d1dosa_ d3qm3a1
d2fjka_ d1f74a_ d3l21a_ d2e1da_ d1to3a1 d1w3ia_ d1sfla_ d1n7ka_
d1vcva1 d4imea_ d1wa3a_ d2ocza1 d1f2ja_ d3m16a_ d2yxga_ d1ub3a_
d3a5fa_ d1fbaa_ d1l6wa_ d1xfba1 d1wx0a1 d1vpxa1 d1ojxa_ d1mxsa_
d2a4aa1 d1xkya1 d3cq0a1 d3s1xa_ d1fdja_ d3cpra1 d4d2ja_ d1cg2a1
d1yloa2 d1y7ea2 d1y0ya3 d1lfwa1 d1vixa1 d1fnoa4 d1vhoa2 d1ysja1
d1vhea2 d1z2la1 d2fvga2 d1vgya1 d1r3na1 d2grea2 d1xmba1 g1q7l.1
d1rtqa_ d3rp2a_ g1ucy.1 d1fuja_ d2zchp_ d3w94a_ d1si5h_ d1lo6a_
g2pka.1 d1cgha_ d1kigh_ d1a5ia_ d3s9ba_ d1hj8a_ d1ao5a_ d2anya_
d1q3xa1 d1gvza_ d1azza_ d4gsoa_ d2f91a1 d1iaua_ d1gdna_ d1eufa_
g1fiw.1 d1eq9a_ d4nfea_ d2rdla_ d1spja_ d1elva1 d1a7sa_ d1ekbb_
d1yc0a_ d2aiqa_ g1fiz.1 d1fi8a_ d4k60a_ d4dgja_ d1mzaa_ d1op0a_
d3tjva_ d1pytd_ d1sgfg_ g1gg6.1 d2eeka_ g1rtf.1 d1a0ja_ d3g01a_
d1pytc_ d4e7na_ d1ddja_ d1z8ga1 d2hlca_ d1npma_ d1brup_ g1jou.1
d1sgfa_ d1autc_ g2hnt.1 g1kdq.1 d1orfa_ d3s69a_ d4crga_ d1fxya_
d1bqya_ d2ftba_ d1pmpa_ d2rcqa1 d1fdqa_ d4lkpa_ d1sa8a_ d1vyfa_
d1lpja_ d3vg7a1 d4azqa1 d5bvqa_ d1p6pa_ d2qo4a_ d1opba_ d1yiva1

d1o1va_ d4bvma1 d4tkba_ d4i3ba_ d1bwya_ d1ftpa_ d1ggla_ d4gkca_
d1cbia_ d3p6da1 d1kqwa_ d2a0aa1 d1dzka_ d4tlja_ d2a2ua_ d4wfva_
d3t1da_ d4iaxa_ d3zq3a_ d2nnda_ d1i4ua_ d1kxoa_ d1bbpa_ d1kt7a_
d1exsa_ d3s26a1 d1lf7a_ d1qfta_ d4gh7a_ d1z24a1 d1qabe_ d1xkia_
d1ew3a_ d3bx7a_ d4iawa1 d1n0sa_ d1gkab_ d3kzaa_ d2k23a_ d2l9ca1
d2joza1 d1gm6a_ d3kffa_ d3saoa1 d1e5pa_ d1yupa1 d1iiua_ d3lgia_
d3i77a_ d2z9ia2 d4nsva_ d1lcya2 d4jcna_ d1agja_ d1os8a_ d1hpga_
d1l1ja_ d1qtfa_ d1e69a_ d3g60a4 g1ii8.1 d1ewqa2 d1ji0a_ d1qhla_
d1v43a3 d2wmfa1 d1vpla_ d1g2912 d4a82a2 g1f2t.1 d2d62a1 d3tuic1
d2bbsa1 d3g60a3 d2ixea_ d1g6ha_ d1sgwa1 d2qi9c_ d3rlfa1 d3b60a1
d3d31a2 d2pzea1 g1xew.1 d1jj7a_ d1oxxk2 d2onka1 d1w1wa_ d1iwea_
d1qf5a_ d1vmaa2 d1nija1 d1np6a_ d2p67a1 d3ihla_ d1okkd2 d1ls1a2
d1s1ma2 d1iona_ d2v40a_ d1dj3a_ d3hida_ d2vo1a1 d2g0ta1 d1vcoa2
d1j8mf2 d1g3qa_ d1xjca_ d1qzxa3 d1ihua2 d1ihua1 d2qm8a_ d1dj2a_
d4ioja_ d1cp2a_ d1yrba2 d1hyqa_ d3gqba2 d1skye3 d1xp8a1 d1tf7a2
d1tf7a1 d1ye8a1 d1skyb3 d2qe7d2 d4blta_ d4huta_ d3ldaa2 d4hyya_
d2i3ba1 d1cr1a_ d3j9tb2 d3ntua2 d1vdza1 d2z43a_ d1e9ra_ d1nlfa_
d3ziaa2 d1n0wa_ d1g6oa_ d3ziad2 d3j9ta2 d1pzna2 d3gqbb2 d1gkub1
d1gkub2 d4ok5a1 d2bmfa1 d2bmfa2 d1yksa1 d1yksa2 d1jr6a_ d2qeqa1
d2jlqa1 d1eh3a_ d3skpa_ d1dtza1 d1dtza2 d1ieja_ d1jnfa2 d1ce2a1
d1jnfa1 d1b1xa2 d1b1xa1 d1jw1a1 d1jw1a2 d1ryoa_ d1h76a1 d1h76a2
d1c4oa1 d2p6ra3 d2p6ra4 d1oywa2 d1oywa3 d1wrba1 d1w36b2 d1w36b1
d1c4oa2 d3bora_ d1t6na_ d2eyqa5 d2eyqa3 d2eyqa2 d2zu6a2 d1veca_
d1uaaa1 d1w36d1 d2g9na1 d1z3ix2 d2rb4a_ d1hv8a1 d1hv8a2 d1t5ia_
d1w36c2 d2j0sa2 d2j0sa1 d1fuka_ d3hjha1 d1z5za1 d1qdea_ d1z63a1
d1s2ma2 d1s2ma1 d1wp9a1 d1wp9a2 d1gm5a3 d1gm5a4 d3dina4 d3dina3
d1q0ua_ d1w36c1 d1z3ix1 d1nkta3 d1nkta4 d1uaaa2 d1w36d2 g1qhh.1
d2fwra2 d2fwra1 d2v8ta_ d3q3ma_ d1za0a1 d1mxra_ d2inca_ d1jk0a_
d2incb_ d1mhyb_ d3hf1b_ d1mtyb_ d1r2fa_ d2uw1a_ d3n37a_ d1afra_
d1oquc_ d1syya_ d1mtyd_ d2o1za_ d1uzra_ d1smsa_ d2fkza_ d1zuja1
d1s3qa1 d2fjca1 d1z6om1 d1veia_ d4dyya_ d1jiga_ d3gvya_ d2pyba_
d4cy9a1 d3bvfa1 d1r03a_ d2cf7a_ d1nf4a_ d1zs3a1 d4iwka_ d3ka8a_
d3np0x_ d1n1qa_ d1lb3a_ d4e6ka_ d1z6oa1 d2fzfa1 d4dyua_ d3ge4a_
d1jgca_ d4am4a_ d2z6ma_ d1ji5a_ d1vlga_ d1lkoa1 d1krqa_ d3qz3a_
d1o9ra_ d2ffxj_ d1yv1a1 d2v2pa_ d1j30a_ d4di0a_ d1dpsa_ d1oewa_
d2qzxa_ d2asia_ d1psoe_ d1miqa_ d2h6ta_ d1pfza_ d1izda_ d3hd8a_
d1j71a_ d1dpja_ d4aa9a_ d1t6ex_ g1b5f.1 g1lya.1 g1htr.1 d1qdma2
d1bxoa_ d1wkra_ d1tzsa_ d1ibqa_ d2qzwa_ d1ex4a2 d1c6va_ d1bdqa_
d3ggta_ d3ixoa_ d1mrxa_ d2haha_ d1tw7a_ d4njsa_ d1sgua_ d2p3da_
d1nsoa_ d3d3ta_ d2rkfa_ d1c6ya_ d2rspa_ d3ogpa_ d3ekwa_ d2o4na_
d2j9ja_ d3ggua_ d3lzua_ d4npua_ d4j55a_ d2r8na_ d2fxda_ d2p3ca_
d4dqea_ d3ttpa_ d3t3ca_ d2p3aa_ d2hc0a_ d2fdda_ d3mwsa_ d1k8rb_
d1wgra1 d1ef5a_ d1wfya1 d1wgya1 d1lfda_ d2cs4a1 d1wxma1 d2byea1
d3kh0a1 d2byfa1 d1wxaa1 d1e7ua3 d1h4ra3 d4yl8a1 d2al6a3 d1l7ya_

d1wgda1 d1wgga1 d4q5eb_ d1wjna1 d1se9a_ d3n3kb_ d1wz0a1 d1we6a1
d2m0xa_ d2gbka_ d3rt3b1 d1wh3a1 d1sifa_ d2ekec2 d4ii2b1 d4hk2a1
d1c3ta_ d1m94a_ d1wgha1 d1ttna1 d1wx8a1 d3v6cb1 d2ma9b_ d4i6lb1
d1bt0a_ d2jvca1 d1iyfa_ d2k1fa_ d1wxva1 d1wf9a1 d1t0ya_ d2io0b2
d1x1ma1 d2gbma_ d1zw7a1 d2m17a_ d1ud7a_ d1we7a1 d2lwpa_ d2gbja_
d1wjua1 d1v6ea1 d1v86a1 d4eewa_ d1wiaa1 d4icva_ d4gu2a_ d1wx7a1
d1wy8a1 d4hjka1 d2faza1 d1v5oa1 d3ruib_ d1eo6a_ d3waob_ d2r2qa1
d3m95a_ d3wana_ d2bcgg1 d2bcgg2 d2dara2 d2dara1 d1wyha2 d2dloa2
d1rutx4 d1rutx3 d2cuqa2 d1x4la2 d1x4la1 d1x3ha2 d1x3ha1 d2d8za1
d1x61a2 d1x6aa1 d1x6aa2 d2cora2 d2cora1 d1v6ga1 d1v6ga2 d2d8za2
d1x64a1 d1x64a2 d1b8ta1 d2co8a1 d1x63a2 d2dj7a2 d2dj7a1 d2cu8a2
d2cu8a1 d1g47a2 d1j2oa1 d1wyha1 d2dloa1 d1wiga1 d1wiga2 d2cupa2
d2cupa3 d1x68a1 d2d8ya2 d2d8ya1 d1m3va2 d1x62a1 d1x68a2 d1x61a1
d1b8ta3 d1b8ta2 d2d8xa2 d2d8xa1 d1x4ka1 d1x4ka2 d2cuqa1 d1m3va1
d2co8a2 d1j2oa2 d1x63a1 d2cura1 d2cura2 d1x62a2 d2lj7a_ d1n4na_
d2m8bb1 d2kyqa_ d3psma_ d1jkza_ d1jxca_ d2gl1a_ d1ugla_ d4uj0a_
d2k4ua_ d1hp2a_ d2lu9a_ d1baha_ d2meoa1 d1wm7a_ d1hlya_ d1n8ma_
d1jlza_ d1wpda_ d1c55a_ d1pvza_ d1wt7a_ d1rjia_ d1wz5a_ d1m2sa_
d1pjva_ d1c49a_ d1qkya_ d2ktca1 d1wmta_ d1du9a_ d1j5ja_ d1r1ga_
d1c4qa_ d1prtc1 d2wv6d_ d1prtf_ d1ltrd_ d4l63b1 d1r4pb_ d1tl0f_
d2bosa_ d1prtb1 d3chbd_ d1t0za_ d2i61a_ d1t1ta_ d1sn4a_ d1kv0a_
d2lkba_ d1npia_ d1wwna_ d1pe4a_ d2kbka_ d2li7a_ d1djta_ d2kbha_
d1dq7a_ d1sega_ d1omya_ d1zywa1 d1bbua1 d1e1oa1 d1c0aa1 d1b8aa1
d1l0wa1 d3a74a1 d1eova1 d1t61c1 d1iyjb4 d1iyjb5 d1iyjb3 d1o7ia_
d4ipca1 d1x3ea_ d4apva_ d1jb7a1 d1jb7a2 d1jb7a3 d3kdfa_ d1ue1b_
d1wjja1 d3eiva_ d1xjva2 d1xjva1 d2ccza2 d1se8a_ d1l1oc_ d1jb7b_
d1qvca_ d3ulla_ d1qzga_ d1miua4 d1miua5 d1miua3 d3g48a1 d1gd7a_
d1mkha_ d1jjcb3 d1pyba_ d3ubub_ d1egia_ d1qo3c_ d1v7pa_ d1tdqb_
d3ubua_ d1c3ab_ d1h8ua_ d3bx4b_ d1t8ca1 d1sb2a1 d1qdda_ d2ziba1
d3cada_ d1byfa_ d1j34b_ d1jwia_ d1jzna_ d1c3aa_ d1oz7b_ d1wmza_
d3bdwb_ d1hq8a_ d1fifa1 d3hupa_ d1wk1a1 d1umra_ d2afpa_ d3bx4a_
d1gz2a_ d1sl6a_ d1v7pb_ d3g8ka_ d1ixxa_ d1j34a_ d1jwib_ d1g1sa1
d1y17a1 d1ja3a_ d1fvub_ d3bdwa_ d1sb2b1 d1fvua_ d1umrc_ d1oz7a_
d4m17a_ d3cfwa1 d1ypqa1 d1g1ta1 d3fd4a_ d3l9jc_ d4dn8a1 d1v4lb_
d1rdl1_ d2msba_ d1rpma_ d2shpa1 d1yfoa_ d1l8ka_ d2ooqa1 d1g4us2
d1p15a_ d1lara2 d1lara1 d1wcha_ d2fh7a2 d2fh7a1 d1ohea1 d1ohea2
d2f4da_ d1rxda_ d2hxpa_ d1xria_ d1d5ra2 d1fpza_ d3ezza_ d4y2ea_
d1c1la_ d4lbqa_ d1a78a_ d1slta_ d4no4a_ d3zsja_ d3duia_ d1g86a_
d1is3a_ d1qmja_ d1hlca_ d1tdha3 d1q3ba3 d1k82a3 g1qmo.1 g1len.1
d3zyra_ d1fnya_ d1n47a_ g2ltn.1 d1avba_ d3usua_ d1dhkb_ d1f9ka_
d1ukga_ d1g8wa_ d1g9fa_ d1wbfa_ d3wcsa_ d1g7ya_ g1loe.1 d1mvqa_
d1hqla_ d1v6ia_ d1dbna_ d1qnwa_ d1fx5a_ d4l8qa_ d1ioaa_ d3n35a_
d3ubra_ d1qo8a1 d1d4ca1 d2k3va_ d1ft5a_ d1qdba_ d1m1qa_ d2j7aa_
d1h21a_ d1ogyb_ d4fasa_ d1jnia_ d1sp3a_ d3h33a_ d1rwja_ d19hca_

d3bxua_ d3h4na_ d1i77a_ d3caoa_ d1j0pa_ d2ctha_ d1duwa_ d1ofwa_
d2e84a_ d1hh5a_ d1gyoa_ d3h34a_ d1dszb_ d1a6ya_ d3cbba_ d1ynwa1
d1r4ia_ d1hcqa_ d2hana_ d2hanb_ d2enva1 d1kb4b_ d2nllb_ d1lo1a_
d1adqa1 d1fnga1 d1uvqb1 d1zs8a1 d2vola1 d2vola2 d1mfbl2 d2jelh2
d1uwel2 d3qq4b1 d1kjvb_ d1k5na1 d1osph2 d4odhl2 d1ow0a1 d1ow0a2
d1bfob2 d3l9ra2 d3jtth1 d2fath2 d4elkb2 d1fn4b2 d2xfxb_ d1pg7x2
d1cqka_ d4k71b2 d1lk2a1 d1eapb2 d4lfhg2 d1i4fa1 d2nw2a2 d1kfah2
d1mhca1 d1h3ua1 d1wejh2 d1h0db2 d1a5fh2 d1kcvh2 d1mjuh2 d1qqda1
d1kcrh2 d1k8ia1 d3huja2 d2nnab1 d1nfdf2 d3mffb2 d1s9va1 d1lk3h2
d1q0xl2 d1fv1b1 d1neza1 d4rfeb2 d1r3ha1 d1a6tb2 d1kjva1 d1pz5b2
d1onqa1 d2bc4a2 d1de4a1 d1k8da1 d1tcra2 d1dn0b2 d1nfde2 d2xwtb2
d1bfoa2 d1mexh2 d1iakb1 d1ypzf2 d4f7ea2 d1q72l2 d2fbjh2 d1fe8h2
d1clzh2 d1fngb1 d1uvqa1 d1vgka1 d1c16a1 d4j12a2 d4x99b2 d1igtb3
d1je6a1 d1q72h2 d1nldh2 d1teth2 d2hmic2 d1i1ca2 d1i1ca1 d1k8ib1
d1q0yh2 d1kfal2 d3frua1 d1pg7w2 d1u58a1 d1igyb4 d1igyb3 d1hyrc1
d1klub1 d1deeb2 d1c12b2 d2bc4b2 d1kcvl2 d3wn5b1 d1lk2b_ d1lk3l2
d1mjul2 d1sm3h2 d1q0xh2 d1zvsa1 d1ydpa1 d1u8ib2 d1ypze2 d1kcuh2
d1op9a_ d1y0la1 d1etzb1 d1egjl1 d2gsib1 d1nj9a1 d4jfhd1 d1aqkl1
d1t2ja_ d1lo4h1 d1zvya1 d3v4vl1 d4enec1 d1kxtb_ d5e8ea1 d2pxya_
d4weud_ d1xiwc_ d2esvd1 d1qnzh_ d1ogae1 d1f4xh1 d4n1hb_ d3ezjb1
d3mlwl1 d1kfah1 d1etza1 d1b4jh1 d3cfic_ d4w68a_ d2arja1 d1ek3a_
d2ok0l1 d1vera_ d1uwgh1 d1blnb1 d1fpth1 d1rzga1 d2giya1 d4m3kb1
d1nc2a1 d2nyyd1 d1ncna_ d1fbih1 d5dxwa_ d4fqqa1 d1dqdh1 d1jfql1
d1kelh1 d1faih1 d1frgl1 d1figl1 d4m6nl1 d1fo0a_ d4hxbl1 d3b5ga_
d3quyd1 d2h9gb1 d1i8kb_ d2dd8h1 d2ck0h1 d1eaja_ d4w6wb1 d2a6ib3
d1t66d1 d2e27h_ d1ob1b1 d1f11b1 d2v7na1 d1ad0a1 d2xt1b1 d1pskh1
d4i13b1 d2op4l1 d1a0ql1 d1uwel1 d1za3a1 d1ncbl1 d4uu9a_ d1bm3h1
d1cloh1 d1nqba2 d1cf8h1 d4y5va_ d1mhph1 d5anma1 d4cdgc1 d4ldeb_
d1y0lb1 d2pcpb1 d1z3gl1 d1dfbl1 d1fh5h1 d3g5xa1 d1tzib1 d4dn3l1
d3zhka1 d2b1hh1 d2uyla1 d1f3dh1 d1nj9b1 d2agjh1 d2h1pl1 d1q72h1
d8fabb1 d1ndmb1 d1rjca1 d4u6hb1 d3upca_ d2fbjl1 d1osph1 d1tvda_
d2cjvh_ d4fhbd1 d1bd2d1 d1rz7h1 d3nn8a_ d1blna1 d1knoa1 d1nfda1
d2bmkb1 d1dqll_ d1igyb1 d1igfl1 d12e8h1 d3utza1 d1nc2b1 d1rihh1
d4mwfb1 d4jvpa1 d1mieh1 d1rhfa1 d1fgnl1 d1sbsh1 d1lp9e1 d1dn0a1
d4lqfl1 d1iaih1 d1kgcd1 d1mh5b1 d1gpol1 d2g60h3 d1f8tl1 d2jixd1
d4grwh_ d1fo0b_ d1bfob1 d1axsa1 d1u9ka_ d1nmbh_ d1h8na1 d1h8na2
d1jguh1 d4eura1 d3sdyl1 d35c8h1 d1w72l1 d1mvub_ d1i9rh1 d1ob1a1
d4wenb_ d1yeeh1 d3ietc1 d1f58h1 d1ad0b1 d1mqkl_ d1nbvh1 d4b50a_
d3r8bb_ d1bafl1 d1mamh1 d4rgne1 d2gsgb_ d35c8l1 d1bafh1 d1ai1h1
d2pcpa1 d2fath1 d2x1pa_ d1orsb1 d1ibgl1 d1shma_ d1a2yb_ d1u0qa_
d2coqa_ d1hilb1 d1qfwi_ d1jv5b_ d1fn4b1 d5e0qa_ d1nsnh1 d1plgl1
d4ocxl1 d1kb5h1 d1rzfh1 d1pg7x1 d2fx7h1 d2oi9c_ d1l7il1 d3eyva1
d1d5il1 d4ffyl_ d1tetl1 d1a6wh_ d1ac6a_ d3dvfa_ d1nfde1 d1mimh1
d1ri8a1 d1jhlh_ d3dura_ d1fskb1 d1f97a1 d3so3b1 d2ak4d1 d1lp9f1

d4z7wf1 d1l7th1 d4y5ya_ d4x7ec1 d1emth1 d1ikfl1 d1nlbh1 d4utal1
d1bjma1 d1eapb1 d1u6ah1 d2dtgd1 d1kj2b_ d1xeda1 d1c1eh1 d1fgvh_
d2bnub1 d2fb4h1 d2dtgb1 d1ktrh_ d1jpth1 d1qkzl1 d4hepg_ d2atpb1
d1flrh1 d2hrpl1 d1mvfa_ d1iaim1 d3k80a1 d2jb5h1 d3zkxc_ d4grwe_
d1it9h1 d1rhhb1 d3w9ec_ d1e4wh1 d1sy6h1 d2j6eh1 d3qxta_ d1jglh1
d1p7ka1 d1tzhb1 d4n8pa1 d1gafl1 d2jelh1 d1wioa2 d4i0cc1 d1cicb1
d1lmka2 d1lmka1 d1iqwh1 d1orqb1 d1ejoh1 d1jnlh1 d4zg1a_ d43c9b_
d1ggbl1 d1wejh1 d3q6fa1 d1f3dj1 d3c6la1 d1bqlh1 d3p9wb_ d1a3rl1
d1h0db1 d2uzil_ d1eo8l1 d1qfwl_ d1n7ml1 d1igja1 d1c5ch1 d2a9mh_
d1rmfh1 d1fj1b1 d4heme_ d1ccza1 d1a5fh1 d1ogad1 d1dlfh_ d1qa9b_
d1vgel1 d3qskb_ d1sjxa_ d4ozfg1 d1sq2n_ d1qd0a_ d1kcvh1 d1c5db1
d3qcul1 d8faba1 d3bn9d1 d1mjuh1 d4y8dc_ d7fabh1 d3dvgb1 d1lo0l1
d1a6tb1 d1gigh1 d1kcrh1 d2hwzl1 d1yjdc1 d3clfl1 d4krna_ d1dl7l_
d1e6ol1 d1mexh1 d3r0ma_ d4kkna_ d1fnsl1 d1oaqh_ d1rzia1 d1ucbh1
d1ncwl1 d4b41a_ d1nfdf1 d2mpah1 d2d7tl1 d4grwf_ d4b5ea_ d1lk3h1
d3u79a_ d4u3xa_ d1ghfl1 d1bvkb_ d1mcww1 d1jrhh1 d32c2b1 d4wemb_
d1dsfh_ d1op3h1 d1ce1l1 d1iqda1 d1qoka1 d1qoka2 d1qfuh1 d2crya1
d1ay1h1 d1oaql_ d3ab0b_ d1rurl1 d1t4kb1 d1bz7b1 d1n0xh1 d1forl1
d1ucbl1 d4w6yb_ d43c9a_ d2aabl1 d1tzia1 d4n1ca_ d1k6qh1 d3g9ab_
d4tvsa1 d1igmh_ d1pz5b1 d3dx9a1 d3wxwh_ d1nakh1 d1kcul1 d3eaka_
d4qlra1 d1cr9l1 d1d9ka_ d1f4xl1 d1deea1 d2i27n2 d1c12a1 d2orbl1
d1j8hd1 d1mcph1 d1c5da1 d1kenh1 d1bd2e1 d1h5ba_ d4x7fc1 d3upaa_
d1t3fa1 d1indh1 d2dtgc1 d4hc1l1 d1zv5a1 d4wgvb_ d1uwgl1 d4f37k1
d1aqkh1 d1vesa_ d3zkqd_ d1fptl1 d1lo4l1 d1knob1 d3rrqa_ d1ct8b1
d4orzc_ d1mlbb1 d4hgkc1 d1dqdl1 d1bogb1 d4ozhe1 d1dr9a1 d1i7za1
d1dn0b1 d1kell1 d1rzib1 d1ypzf1 d1wtla_ d1hkfa_ d1fail1 d1figh1
d2vyre_ d1sjva_ d1ohqa_ d1bfoa1 d1pkqb1 d1mjul1 d1b4jl1 d1nsnl1
d2arjb1 d1p4bh_ d4aj0a_ d3nn8b_ d2rcja1 d1f2xk_ d4v1dd_ d1adqh1
d2eksa_ d4k3ga_ d1jfqh1 d1uweh1 d1frgh1 d4gftb_ d1uz8b1 d4n1ea_
d1bm3l1 d1clol1 d3mcka1 d2vq1a1 d1ae6h1 d4aq1b1 d1uj3b1 d1nbqa1
d1fh5l1 d2ck0l1 d2ywza_ d1q72l1 d4nbxb_ d1hila1 d4hzlb1 d2j88l1
d1zmya1 d1cd0a_ d1a4ka1 d1pskl1 d1a0qh1 d4krmb_ d1ncbh1 d2fbjh1
d1j8he1 d1i3ua_ d1cf8l1 d1fe8h1 d2p1ya1 d1eeqa_ d2x89a_ d1ol0a_
d2r0lh1 d1ub5a1 d1dfbh1 d3eyvb1 d4jdvb1 d4p2cg_ d2gsia1 d2agjl1
d1u3ha1 d2h1ph1 d1rihl1 d1fskc1 d1boga1 d2a77l1 d1ospl1 d1qfoa_
d3dwta_ d2fx7l1 d1rz7l1 d3stba1 d1pkqa1 d1b6da1 d1dzba2 d1dzba1
d1kj2a_ d4l1ha_ d1rzgb1 d1dqlh_ d12e8l1 d1miel1 d1jmaa_ d1sbsl1
d1fgnh1 d1f58l1 d1mqkh_ d1ndgb1 d3qegl1 d1iail1 d2aw2a1 d1tqbc1
d3fo2a1 d4dkaa_ d1f8th1 d2g60l1 d2ywya_ d1uj3a1 d1yc7a_ d3bd3a_
d4w6xb1 d1nmbl_ d1i8ka_ d1rjlb1 d1ypze1 d1p7kb1 d1kxqe_ d1gpoh1
d2z4qa1 d1w72h1 d1i9rl1 d4qgya1 d4xt1c_ d2qqnh1 d1cica1 d3c08h1
d2cd0a_ d2bdnh1 d1hnga1 d1maml1 d1nldh1 d1cr9h1 d4lcih1 d3bkyh1
d3qg7l1 d1ai1l1 d1kxvc_ d4iosd_ d1l7ih1 d2x1qa_ d1ub6b1 d3k74b_
d1ibgh1 d1teth1 d1igya1 d1hxma1 d2x6ma_ d2e7la_ d5e03a_ d1qfwm_

d2r0kh1 d1igjb1 d1rurh1 d1plgh1 d1mj8h1 d1yuhb1 d3fmgl1 d1rzfl1
d1emtl1 d1ad9b1 d1igtb1 d1nlbl1 d1d5ih1 d3k7ua_ d4ffyh_ d1bwwa_
d3dvga1 d1a6ta1 d1fgvl_ d4qxgl1 d1miml1 d1jhll_ d2hffb1 d2hh0l1
d1qkzh1 d1l7tl1 d1afvh1 d1iaii1 d2cjuh_ d1um5h1 d2ghwb2 d2ghwb1
d1kgce1 d2fgwh1 d1ikfh1 d1dvfd_ d2i24n1 d2xtjb_ d1za3b1 d2yc1a_
d2hh0h1 d2x1oa_ d1bvka_ d1rjla1 d5e7ba1 d1igta1 d1il1b1 d2fb4l1
d1mvua_ d1jptl1 d32c2a1 d1fvca_ d1iqdb1 d2hrph1 d2p4ab_ d4qo1a_
d1p2cb1 d1wt5c_ d1t4ka1 d5c1mb_ d1it9l1 d1bz7a1 d1b2wh1 d1e4wl1
d1jnll1 d1yedb1 d1za6b1 d3u7aa_ d1igfh1 d3gnml1 d1orsa1 d1wejl1
d1a2ya_ d1hxmb1 d1gafh1 d2j4wh1 d1pz5a1 d1j05a_ d1a3rh1 d4fqll1
d1qfwh_ d1eo8h1 d1n7mh1 d4s11a_ d1kegh1 d5da4a_ d1iqwl1 d3whxb1
d1vgeh1 d1deeb1 d1c12b1 d1t2ql1 d1ad9a1 d3gsnb1 d3b2uc1 d1ggbh1
d1t3fb1 d1nakl1 d4ocmc_ d1lila1 d2v7hb3 d2esve1 d1mcoh1 d2qr0b1
d2q3aa_ d1ct8a1 d1kcrl1 d2pxyb_ d2uzih_ d5c3ld_ d3durb_ d1c5cl1
d2ak4e1 d1dl7h_ d1xiwd_ d1mlba1 d1rmfl1 d1i7zb1 d1dlfl_ d2xa3a_
d5bq7b1 d1dqqb1 d3h42l1 d1egjh1 d1kcvl1 d1fe8l1 d2j6el1 d1f3rb2
d1lk3l1 d1sm3h1 d1q0xh1 d1ghfh1 d7fabl1 d1igch1 d1lo0h1 d4idla_
d5c2bl_ d1jrhl1 d1il1a1 d3zhda1 d4kfzc_ d1b0wa_ d1e6oh1 d1mexl1
d4krpb_ d1fnsh1 d1ncwh1 d4hgma_ d4eigb1 d4rzca_ d2mpal1 d4y5xa_
d5en2b1 d3lrga1 d4ej1c1 d1rhha1 d1j05b_ d1forh1 d1q9wb1 d2wzpd_
d2aq2a1 d1h0da1 d3revb1 d2ij0c1 d1tzha1 d4m7jl1 d1k6ql1 d3b9kc1
d1igml_ d3qeua1 d1dsfl_ d1ce1h1 d1kcuh1 d1zvha1 d1zvoa1 d1tqbb1
d1ay1l1 d2qscl1 d2cgrh1 d1nl0h1 d1kenl1 d3eo0a1 d4n9ob1 d1n0xl1
d3k1kc_ d2m9wa1 d3dfxa_ d2aq2b1 d1enfa1 d1i4pa1 d1v1oa1 d1et9a1
d1esfa1 d2g9hd1 d4ohja1 d1klud1 d1bxta1 d1eu3a1 d1ty0a1 d1fnua1
d2qaln1 d2cqva1 d1fhga_ d2k1ma_ d2dl2a1 d1biha3 d1biha1 d1biha4
d3qp3a1 d1g1ca_ d1gxea_ d1cvsc2 d1cvsc1 d1xmwa2 d1m4ka2 d2otpa2
d1biha2 d1rhfa2 d2fcba2 d2fcba1 d1wwbx_ d1gsma2 d1epfa1 d1epfa2
d2dava1 d1waaa_ d1f97a2 d1x44a1 d1vcaa2 d1fnla2 d1fnla1 d3b5ha2
d3h8na2 d3h8na1 d1jbja1 d1jbja2 d1pd6a_ d1f2qa2 d1f2qa1 d3cx2a_
d1nbqa2 d3b5ha1 d1n26a1 d1xiwb_ d1xaua_ d1fltx_ d2ifga1 d1olla1
d1olla2 d1cs6a4 d1cs6a3 d1cs6a2 d1cs6a1 d2gw5a2 d1sy6a1 d1xiwa_
d1ucta2 d1ucta1 d1ry7b2 d1gl4b_ d1qz1a3 d1olza1 d2c9aa1 d1wwca_

**Family-49 (c.2.1.1)**

**Positive dataset contains 5 protein sequences.**

d1xa0a2 d2dm6a2 d1tt7a2 d1piwa2 d1yb5a2

**Negative dataset contains 3781 protein sequences.**

d1ibja_ d1c7na_ d1iuga_ d4q75a_ d2bkwa1 d1t3ia_ d1n8pa_ d1qz9a_
d1jf9a_ d1eg5a_ d1p3wa_ d3kgwa_ d1cl1a_ d2ctza1 d1vjoa_ d2rfva_
d1gc0a_ d1cs1a_ d1d2fa_ d2ch1a1 d1qgna_ d1pffa_ d1elua_ d1u08a_
d1lc5a_ d1b5pa_ d2ay1a_ d1bw0a_ d3pd6a_ d7aata_ d1j32a_ d1fg7a_
d4wlha_ d1o4sa_ d1v2da_ d1gdea_ d1m6sa_ d1v72a1 d1svva_ d1ajsa_
d1wsta1 d2f8ja2 d1lk9a_ d1vp4a1 d1xi9a1 d2gb3a1 d1yaaa_ d3i2ka1
d1lnsa3 d1mpxa2 d2cfba1 d1dfoa_ d1o69a_ d1bj4a_ d3k28a_ d2bwpa_

| | | | | | | | |
|---|---|---|---|---|---|---|---|
| d3lg0a_ | d2fnua_ | d3ou5a_ | d1z7da1 | d1b9ha_ | d1iaya_ | d4msoa_ | d1bs0a_ |
| d1s0aa_ | d1sffa_ | d3tqxa_ | d1mdoa_ | d3qboa_ | d3pgya_ | d1bjna_ | d2dkja_ |
| d1ejia_ | d1vefa1 | d4wxga_ | d3bs8a_ | d3gbxa1 | d1w23a_ | d2b5aa1 | d2ofya1 |
| d4mcxa_ | d2xi8a_ | d2icta_ | d2bnma1 | d2ppxa1 | d1y7ya1 | d1cixa_ | d1y29a_ |
| d1v7fa_ | d2jtba_ | d1d1ha_ | d1g9pa_ | d1lupa_ | d1qk7a_ | d1mb6a_ | d2a2va_ |
| d1niya_ | d1i25a_ | d1hvwa_ | d2mpqa_ | d1ie6a_ | d1kqha_ | d1la4a_ | d2mxoa1 |
| d1dl0a_ | d1koza_ | d1qk6a_ | d1emxa_ | d1lqra_ | d1c6wa_ | d1fwxa1 | d1ibya_ |
| d2wo3b_ | d1shxa_ | d1f56a_ | d1bypa_ | d1azca_ | d1ws8a_ | d3u25a_ | d1cuoa_ |
| d1id2a_ | d1rkra_ | d2iaac_ | d4dp1x_ | d1nwpa_ | d2gima_ | d2i7sa_ | d1qhqa_ |
| d1gyca3 | d1hfua2 | d1kbva1 | d1kbva2 | d1sdda1 | d1sdda2 | d1gyca1 | d1mzya1 |
| d2bw4a1 | d1aoza1 | d1aoza3 | d1aoza2 | d1kyaa2 | d1kyaa3 | d1kyaa1 | d1mzya2 |
| d4e9sa1 | d1sddb1 | d1sddb2 | d1hfua1 | d1hfua3 | d1oe1a2 | d1oe1a1 | d1v10a2 |
| d1v10a3 | d1v10a1 | d1rypd_ | d1irua_ | d1rypc_ | d3unfk_ | d1rypa_ | d1j2pa_ |
| d1irui_ | d1j2qh_ | d3unfh_ | d1m4ya_ | d1rypb_ | d1irul_ | d1irug_ | d1iru2_ |
| d1q5rh_ | d1rypg_ | d1g3ka_ | d1q5qa_ | d1e94a_ | d1irub_ | d1ryph_ | d3ty6a_ |
| d1iruj_ | d1iruk_ | d1rype_ | d1irud_ | d1irue_ | d3mi01_ | d1rypf_ | d1iruh_ |
| d2z3ba_ | d1iruc_ | d3mka2_ | d1iru1_ | d1a3qa1 | d1iknc_ | d3brda1 | d1oy3c_ |
| d1imhc1 | d1gjia1 | d1zk9a1 | d2cxka1 | d2bema_ | d1pama1 | d1ea9c1 | d1qhoa1 |
| d2fhfa2 | d2fhfa1 | d1k3ia1 | d1j0ha1 | d1cxla1 | d2bhua1 | d1edqa1 | d1f1sa2 |
| d1ut9a2 | d1gvia1 | d1txka1 | d1adqa1 | d1fnga1 | d1uvqb1 | d1zs8a1 | d2vola1 |
| d2vola2 | d1mfbl2 | d2jelh2 | d1uwel2 | d3qq4b1 | d1kjvb_ | d1k5na1 | d1osph2 |
| d4odhl2 | d1ow0a1 | d1ow0a2 | d1bfob2 | d3l9ra2 | d3jtth1 | d2fath2 | d4elkb2 |
| d1fn4b2 | d2xfxb_ | d1pg7x2 | d1cqka_ | d4k71b2 | d1lk2a1 | d1eapb2 | d4lfhg2 |
| d1i4fa1 | d2nw2a2 | d1kfah2 | d1mhca1 | d1h3ua1 | d1wejh2 | d1h0db2 | d1a5fh2 |
| d1kcvh2 | d1mjuh2 | d1qqda1 | d1kcrh2 | d1k8ia1 | d3huja2 | d2nnab1 | d1nfdf2 |
| d3mffb2 | d1s9va1 | d1lk3h2 | d1q0xl2 | d1fv1b1 | d1neza1 | d4rfeb2 | d1r3ha1 |
| d1a6tb2 | d1kjva1 | d1pz5b2 | d1onqa1 | d2bc4a2 | d1de4a1 | d1k8da1 | d1tcra2 |
| d1dn0b2 | d1nfde2 | d2xwtb2 | d1bfoa2 | d1mexh2 | d1iakb1 | d1ypzf2 | d4f7ea2 |
| d1q72l2 | d2fbjh2 | d1fe8h2 | d1clzh2 | d1fngb1 | d1uvqa1 | d1vgka1 | d1c16a1 |
| d4j12a2 | d4x99b2 | d1igtb3 | d1je6a1 | d1q72h2 | d1nldh2 | d1teth2 | d2hmic2 |
| d1i1ca2 | d1i1ca1 | d1k8ib1 | d1q0yh2 | d1kfal2 | d3frua1 | d1pg7w2 | d1u58a1 |
| d1igyb4 | d1igyb3 | d1hyrc1 | d1klub1 | d1deeb2 | d1c12b2 | d2bc4b2 | d2ig2h2 |
| d1kcvl2 | d3wn5b1 | d1lk2b_ | d1lk3l2 | d1mjul2 | d1sm3h2 | d1q0xh2 | d1zvsa1 |
| d1ydpa1 | d1u8ib2 | d1ypze2 | d1kcuh2 | d4alac_ | d1pjwa_ | d1rera1 | d1s6na_ |
| d3uzqb_ | d2phla2 | d2phla1 | d1fxza2 | d1fxza1 | d1lrha_ | d1uija1 | g1dgw.1 |
| d2v09a_ | d1uika2 | d1uika1 | d1ds6b_ | d1fstb_ | d1ex0a1 | d1g0da1 | d1ju8a_ |
| d3us3a3 | d3us3a1 | d4w9ta_ | d1jvna1 | d4x2ra1 | d1qo2a_ | d4axka1 | d1thfd_ |
| d1h5ya_ | d4u28a_ | d2y88a_ | d4wd0a_ | d1vzwa1 | d4mh8a_ | d1mu2a2 | d4i7ga1 |
| d1k9ua_ | d2opoa_ | d1r0ka3 | d1j5pa3 | d1vkoa2 | d1vm6a2 | d3qvsa2 | d1gr0a2 |
| d1p1ja2 | d4aw8a_ | d3gpdg2 | d2q49a2 | d1hdgo2 | d2g17a2 | d2cvoa2 | d2czca1 |
| d3pwka2 | d1obfo2 | d1i32a2 | d1zyao2 | d2pkqo2 | d1b7go2 | d2hjsa2 | d1vkna2 |
| d1nvmb2 | d2msya_ | d2djoa1 | d2r5za_ | d1au7a1 | d1f43a_ | d2lmda1 | d1akhb_ |
| d1s7ea1 | d3nara_ | d1zq3p1 | d2craa1 | d1mija_ | d4uuta_ | d3a01a_ | d1x58a1 |
| d3rkqa1 | d3cmya1 | d2cqxa1 | d2da6a1 | d1e3oc1 | d1fjla_ | d1mh3a1 | d1x41a1 |

d1wh7a1 d1gt0c1 d2mw8a_ d1lfup_ d1x2ma1 d1b72a_ d1wi3a1 d1jgga_
d1wjha1 d1x2na1 d1mnmc_ d2cuea1 d3k2aa_ d2hosa_ d2jwta2 d1pufa_
d1octc1 d2hi3a_ d1b8ia_ d4j19a_ d3lnqa_ d1wh5a1 d1b8ib_ d1hcra_
d1tc3c_ d1gdta1 d1u78a2 d1hlva1 d1hlva2 d1iufa2 d1igna1 d2iu5a1
d1zk8a1 d2g7sa1 d2fd5a1 d2np5a1 d1z0xa1 d2id6a1 d2d6ya1 d2i10a1
d1vi0a1 d2oi8a1 d3c07a1 d2ns7a1 d2fq4a1 d2yvea1 d1t33a1 d2o7ta1
d1rkta1 d2np3b1 d2g7ga1 d2gena1 d2fbqa1 d2g7la1 d2gfna1 d2g3ba1
d2jj7a1 d2hyja1 d1sgma1 d1ui5a1 d2hkua1 d2id3a1 d4jyka1 d3lv8a_
d1s3ga1 d1zaka1 d1s4qa_ d3tvta2 d1uf9a_ d1khta_ d1ckea_ d1osna_
d1p5zb_ d4tmka_ d3cm0a_ d1tmka_ d1jaga_ d3h86a_ d1t0hb_ d1nn5a_
d2h92a_ d3kb2a1 d2c95a1 d1yj5a2 d1kgda_ d1ly1a_ d2ak3a1 d1vyva2
d1deka_ d2grja1 d1p3ja1 d1y63a_ d1e4ya1 d1s96a_ d3iija1 d1lvga_
d1vyua2 d4e22a_ d1qf9a_ d1jjva_ d1q3ta_ d1ki9a_ d1lw7a2 d1teva_
d1kjwa2 d1gsia_ d1g3ma_ d1j99a_ d1t8ta1 d1nsta_ d1fmja_ d1zrha_
d1ls6a_ d2a3ra_ d2zptx_ d3ckla_ d1texa_ d1q44a_ d2gwha_ d1z0ja1
d3umra2 d3gj0a1 d2gaoa1 d1yzga1 d1x3sa1 d1g7sa4 d1puja_ d1udxa2
d1kk1a3 d2o52a_ d1jwyb_ d1zcaa2 d1zcba2 d2fv8a1 d2cjwa_ d3dz8a1
d1xzpa2 d1vg8a_ d4qxaa_ d2ew1a1 d1x1ra1 d2bcgy_ d3doea1 d3cona_
d3rega_ d3wyga_ d1upta_ d2b6ha1 d1zd9a1 d1lnza2 d1r5ba3 d1r2qa_
d2f9la1 d2rhda_ d3q3jb_ d3q85a_ d1efma1 d1r8qa_ d2gf9a1 d2akab1
d1ek0a_ d1z08a1 d2gcoa1 d2dy1a2 d4g01b_ d2cxxa1 d1svia_ d3q72a_
d1ni3a1 d4ac9a4 d2erxa1 d2bmea_ d1wxqa1 d2gf0a1 d4ldja1 d1zunb3
d1d5ca_ d4lv5b_ d1efca3 d1kmqa_ d2g6ba1 d3lrpa_ d1tada2 d2y8ea_
d4rd4a1 d1z06a1 d2heia_ d3a58b_ d1u0la2 d1d2ea3 d1zj6a1 d2ocba_
d3rwoa_ d1mr3f_ d2e9sa1 d4u5xa1 d2iwra_ d2fn4a1 d3bwdd_ d2nzja1
d2atva1 d2ksqa_ d3l43a_ d1mkya2 d1mkya1 d1wmsa_ d1jnya3 d2rmka_
d1f6ba_ d1f60a3 d1gota2 d1jala1 d2fh5b1 d1g16a_ d2wblc_ d1m7ba_
d1wf3a1 d1f5na2 d1t9ha2 d1z6xa1 d1z0aa1 d2fg5a1 d2a5ja1 d2bcjq2
d1s0ua3 d1ky3a_ d4y0va_ d4lhwa_ d2qmea1 d2atxa1 d1egaa1 d1ryha_
d1moza_ d1nrjb_ d2erya1 d3bfka_ d1puia_ d1fzqa_ d3j3az1 d3t5ga_
d1shza2 d3defa_ d2f7sa1 d3raba_ d3tsoa_ d3aq4a_ d2al7a1 d3sfva1
d3bc1a_ d2ncda_ d1sdma_ d3h4sa1 d2heha_ d2grya_ g3kin.1 d1kk8a2
d1br2a2 d1f9va_ d2mysa2 d1lkxa_ d3t0qa_ d4db1a1 d3gbja_ d1ry6a_
d1v8ka_ d2ovga_ d1sq8a_ d1rzsa_ d3kz3a_ d3orca_ d1kb0a1 d1k8ka2
d1k8ka1 d3uleb1 d3i33a2 d3i33a1 d1bupa1 d1dkgd2 d1dkgd1 d4ci6a2
d1jcfa1 d4a2ba2 d2fxua1 d1yaga2 d1yaga1 d4cbua2 d1bupa2 d3vrda1
d3vrda2 d1fcdc2 d1h1oa2 d1h1oa1 d1m70a2 d1m70a1 d1ig8a1 d1czan2
d1czan3 d1czan1 d1ig8a2 d1czan4 d1bg3a4 d1bg3a2 d1bg3a1 d1nira1
d1qksa1 d1hroa_ d4gedb_ d3zooa_ d1mz4a_ d1gu2a_ d1f1fa_ d1dvva_
d1c6ra_ d1c75a_ d1e29a_ d1gdva_ d1cnoa_ d1h32b_ d1co6a_ d2bh4x_
d2aiua_ d5cytr_ d1jdla_ d1i8oa_ d2zboa_ d3dmia_ d1ls9a_ d1wvec_
d2yk3a_ d3ph2b_ d1dw0a_ d5aura_ d1ql3a_ d1qn2a_ d1f1ca_ d4zzva_
d2xspa_ d1eg1a_ d4v20a_ d1ojja_ d4ipma1 d1gpia_ d4csia_ d3pfza_
d3ovwa_ d2yoka_ d2nqya_ d1h4ga_ d1hixa_ d1oa2a_ d1olra_ d3b7ma_

d4h7ma1 d3wp3a_ d2vgda_ d1igoa_ d3zsea_ d2nlra_ d3wx5a_ d1oa3a_
d1pvxa_ d1m4wa_ d1f5ja_ d1oa4a_ d1ks5a_ d4ixla1 d1h32a2 d1evsa_
d1cnt1_ d1pvhb_ d1z7ca_ d1huwa_ d1rhga_ d1f6fa_ d1f45b_ d1i1rb_
d1axia_ d1etea_ d3tgxb_ d1scfa_ d2z3qa1 d3qb1a_ d1hula_ d1m48a_
d4nkqb_ d1eera_ d1v7mv_ d5e4ea_ d2o27a_ d3b5ka_ d2psma1 d4zf7a_
d3uf5a_ d4x51a_ d1d9ca_ d4ypgc1 d3oq3a_ d1lqsl_ d1n1fa_ d1au1a_
d1wu3i_ d3besl_ d3ux9a_ d3rbaa_ d2qtra_ d1nupa_ d3l92a_ d1f9aa_
d1qjca_ d2h29a_ d1vlha_ d1od6a_ d1lw7a1 d1mrza2 d4ruka_ d2el7a_
d1iq0a2 d1irxa2 d1r6ta2 d1ivsa4 d2cyba_ d1rqga2 d1j09a2 d2yxna_
d1f7ua2 d1h3fa1 d1ffya3 d2g36a1 d1nzja_ d1jila_ d1n3la_ d1i6la_
d1pfva2 d1h3na3 d3n9ia_ d3sz3a1 d3sw2a_ d1moxc_ d1dx5i3 d1edmb_
d1autl2 d1autl1 d1dval1 d1szba2 d1kigl_ d2y5fl_ d1ijqa2 d1gk5a_
d1g1sa2 d1nt0a3 d1xdtr_ d1lmja2 d1lmja1 d1nzia2 d2wphe_ d1klil_
d1pfxl2 d1pfxl1 d1p9ja_ d1g1ta2 d1f7ea_ d4yzub_ d1nuba2 d2mgpa1
d1b9wa1 d1b9wa2 d2mgpa2 d1ob1c1 d1ob1c2 d1n1ia2 d1n1ia1 d2npra2
d2npra1 d2y5ia_ d1psra_ d3n22a_ d2egda_ d4ggfc_ d2wcea_ d2luca_
d4aqia_ d3nsia_ d2lp3a_ d1j55a_ d1a4pa_ d1k8ua_ d1nsha_ d1pvaa_
d3fs7a_ d2pvba_ d1ttxa_ d1rwya_ d2kyfa_ d1rk9a_ d1a75a_ d1exra_
d1tiza1 d1nyaa_ d1g8ia_ d1ggwa_ d1wdcc_ d3fwba_ d1ggza_ d2o5ga_
d1ctaa_ d1m45a_ d3i5gc_ d1sl8a_ d1fpwa_ d2scpa_ d2roaa_ d4aqra_
d3pn7b_ d1br1b_ d2mysb_ d1s6ca_ d1bjfa_ d2obha_ d1jfka_ d1jf0a_
d1qv0a_ d1lkja_ d2mlfc_ d2m97a_ d2d8na_ d4by5a_ d1jbaa_ d4nqga_
d1wdcb_ d2mysc_ d1j7qa_ d1s6ia_ d1uhka2 d1r2ua_ d1dtla_ d3kf9a_
d3pn7c_ d1fi6a_ d1c07a_ d5d67d_ d1tuza_ d1h8ba_ d1eg3a2 d2zkmx1
d1snla_ d1y1xa_ d1hqva_ d1dvia_ d1df0a1 d1qxpa2 d4usla_ d1k94a_
d1sfsa_ d1jfxa_ d1wkya2 d1uuqa_ d3wh9a_ d4m1ra_ d1xyza_ d4xuya_
d2je8a5 d4bf7a1 d3cuia_ d1ur4a_ d1qw9a2 d1odza_ d2vzsa5 d1bqca_
d2x2ya1 d1hjqa_ d1qnra_ d4y7ea1 d1vjza_ d4k68a_ d2v3ga_ d4hpga_
d1nofa2 d1nq6a_ d1h4pa_ d1aq0a_ d3ziza_ d4bs0a_ d1ecea_ d1h1na_
d1ta3b_ d1w91a2 d1i1wa_ d1uhva2 d1us3a2 d1egza_ d7a3ha_ d1tvna1
d3nyda1 d1ghsa_ d2cyga1 d4xv0a_ d2c8na2 d1w32a_ d3emca_ d1v6ya1
d1foba_ d2uwfa1 d2fgla_ d3niya1 d2c0ha1 d1rh9a1 d1yq2a5 d1szna2
d1fa2a_ d1pama4 d5do8a1 d1ea9c3 d1hx0a2 d2fhfa5 d1qhoa4 d1avaa2
d1m53a2 d4m8ua1 d2gjpa2 d1lwha2 d4howa1 d1ktba2 d1j0ha3 d3vm5a1
d2x1ia_ d1wzaa2 d1uasa2 d2diea1 d1mxga2 d1ht6a2 d1eswa_ d5cpsa_
d1ud2a2 d1zjaa1 d3gbea1 d1kwga2 d1wdpa_ d1gcya2 d1g5aa2 d1hvxa2
d3dhpa2 d2bhua3 d4do4a1 d3qgva1 d1e43a2 d1g94a2 d2d3na2 d1gjwa2
d1gvia3 d1x1na1 d1r7aa2 d1tz7a1 d2x42a1 d1tr9a1 d3abza1 d4toqa_
d1wb0a1 d1w9pa1 d1eoka_ d1itxa1 d1kfwa1 d1goia2 d1edqa2 d2gsja_
d1hjxa1 d1jnda1 d1vf8a1 d3wdpp1 d3wq5a_ d1gnxa_ d4gxpa_ d4hz8a_
d1e4mm_ d3aiua_ d1wcga1 d1qvba_ d3ahxa_ d1v02a_ d2dgaa_ d1pbga_
d1vffa1 d1qoxa_ d4ha4a_ d1mixa2 d1e5wa2 d1j19a2 d1isna2 d2al6b2
d3syxa1 d1mkea1 d2p8va_ d1xoda1 d1i7aa_ d1evha_ d1qc6a_ d2v76a_
d1wj1a1 d1aqca_ d1qqga2 d1qqga1 d1ntva_ d1n3ha_ d4dbba_ d3f0wa1

d1p3ra_ d1wgua1 d2cy4a1 d3hqca_ d1wvha1 d1xr0b1 d1wgqa1 d1v89a1
d2coda1 d1zc3b_ d1wg7a1 d1u5ea1 d1v61a1 d2coca1 d2ys1a1 d1v5pa1
d1ntya2 d1wjma1 d1wi1a1 d1u29a_ d1xx0a1 d1unqa_ d2zkmx3 d1fhoa_
d1v88a1 d2adza1 d1x1fa1 d1p6sa_ d2coaa1 d1v5ma1 d1faoa_ d2x18a_
d1dyna_ d1x1ga1 d4kaxb_ d2j59m1 d1ki1b2 d1xcga2 d1txda2 d1u5da1
d1dbha2 d1w1ha_ d1upqa_ d1v5ua1 d1foea2 d2fjla1 d1eaza_ d2dfka2
d2cofa1 d1kz7a2 d1zsqa1 d1r55a1 d3gboa_ d1atla_ d1kufa_ d1buda_
d4q1la_ d1quaa_ d1yp1a_ d1wnia_ d3u1ra1 d1g9ka2 d1kapp2 d1k7ia2
d1t3ca_ d1epwa3 d2a97a_ d3fiea_ g1e1h.1 d3ioqa_ d1fh0a_ d1o0ea_
d1khqa_ d1m6da_ d2cb5a_ g8pch.1 d1s4va_ d2pnsa_ d1vsna1 d1mira_
d1iwda_ d2oula_ d7pcka_ d3bwka_ d3bcna_ d2p86a_ d1pvja_ d1pcia_
d1gece_ d1deua_ d4bs6a_ d1xkga1 d1cqda_ d1jqpa2 d2c0ya_ d2bdza_
d3p5ua_ d2o36a_ d2jk6a2 d2jk6a1 d1feca1 d1feca2 d1lpfa1 d1lpfa2
d1xhca1 d1xhca2 d1dxla2 d1dxla1 d1gera2 d1m6ia2 d1onfa1 d1onfa2
d3lada2 d3lada1 d1w4xa1 d1w4xa2 d1gesa2 d1mo9a1 d1xdia1 d1fl2a2
d1fl2a1 d1fcda1 d1fcda2 d1h6va2 d1h6va1 d1mo9a2 d1gv4a2 d1gv4a1
d2gv8a2 d2gv8a1 d1aoga1 d1aoga2 d1hyua1 d1ebda1 d1ebda2 d3dk9a1
d1q1ra2 d1q1ra1 d1gesa1 d1zbfa1 d2z1ha_ d2etja1 d3qioa1 d1jl1a_
d2e4la_ d1jl2a_ d2lsna1 d1io2a_ d1uaxa_ d2yv0x_ d1mu2a1 d1ekea_
d1i39a_ d1ps9a2 d1o94a2 d1lqta1 d1pn0a1 d1reoa1 d1ju2a1 d2f5va1
d3c96a1 d2gmha1 d1pj5a2 d1gpea1 d1o5wa1 d1n4wa1 d1k0ia1 d2dw4a2
d2ivda1 d1seza1 d2voua1 d1kdga1 d1s5ja1 d2hbja2 d2igia_ d1wlja_
d1qhta1 d4dlea1 d1tgoa1 d2f96a1 d3tr8a1 d3mxma_ d1q8ia1 d1y97a1
d1w0ha_ d1kfsa1 d2py5a1 d1noya_ d1yt3a3 d2guia_ d3mwpa1 d4kb1a_
d2d5ra1 d1b9ra_ d1offa_ d1frra_ d3wcqa_ d1e9ma_ d1l5pa_ d1wria_
d1i7ha_ d3av8a_ d1czpa_ d1rfka_ d3p1ma1 d3b2ga_ d2bt6a2 d3nvva1
d1krha3 d2h88b1 d1n62a2 d1t3qa2 d1zoyb1 d1jq4a_ d1ffva2 d1dgja2
d1rm6c2 d1faaa_ d1wika1 d1xfla_ d1ttza1 d2f51a1 d1ti3a_ d1nhoa_
d1sena_ d2i4aa_ d1ep7a_ d1zmaa1 d1fo5a_ d3lefa1 d3diea1 d2yana1
d1z6na1 d1oaza_ d3zywa1 d4aj8a_ d1nm3a1 d4fiwa_ d1wmja_ d4hs1a1
d1iloa_ d1xwaa1 d1wjka1 d2vm1a_ d1fb6a_ d1gh2a_ d1h75a_ d1nw2a_
d1kx5b_ d3a6na_ d4m6ba2 d2nqbd_ d3wkjd_ d2yfwe_ d4h9na_ d5bnxa_
d1kx5c_ d3r45a_ d1eqzb_ d4jjnb_ d1f66c_ d1eqza_ d4wnnb_ d1kx5a_
d1u35c1 d1id3a_ d2nqbc_ d1kx5d_ d1f1ea_ d1b67a_ d1pd212 d1nhya2
d2a2ra2 d1k0dd2 d1f2ea2 d1gsua2 d1f3ba2 d2fhea2 d2pvqa1 d1tw9a2
d1oyja2 d1e6ba2 d1k0ma2 d3ik7a1 d4is0a1 d1r5aa2 d1jlva2 d1m0ua2
d3gtub2 d1z9ha2 d1okta2 d1axda2 d2gsra2 d1tu7a2 d1k3ya2 d1zgma1
d1gnwa2 d1gwca2 d2gsta2 d1glqa2 d1oe8a2 d1pn9a2 d1v2aa2 d1fw1a2
d1duga2 d3h3no1 d3h3no2 d3ezwa2 d1gd6a_ d1f6ra_ d1ivma_ d2gv0a_
d1iiza_ d2z2fa_ d1j8xa_ d1ghla_ d1b9oa_ d174la_ d169la_ d2o4wa_
d1p37a_ d1k28a3 d1xjta1 d1jtma_ d2f2qa_ d4e97a1 d1g4ma1 d1g4ma2
d1mpya2 d1kw3b2 d1kw3b1 d4ghga1 d1tfza1 d3isqa1 d3isqa2 d1sqia1
d1sqia2 d1lkda2 d1lkda1 d1mpya1 d1cjxa2 d1cjxa1 d1sp9b2 d1t47a1
d1t47a2 d1sp8a1 d1sp8a2 d1f1ua1 d1s5ja2 d1qhta2 d4dlea2 d1tgoa2

d3py8a2 d1q8ia2 d1kfsa2 d2py5a2 d1d5aa2 d1qsta_ d2beia1 d4m3sa_
d1u6ma1 d1ygha_ d1yk3a1 d3dr6a_ d1xmta_ d1z4ea1 d2ge3a1 d1bo4a_
d2fiaa1 d2jdca1 d1y9wa1 d3to7a_ d1wwza1 d2fl4a1 d2ozua1 d2b5ga_
d2cy2a1 d2p0wa_ d1cm0a_ d4pswa_ d1yrea1 d1yr0a1 d1m4ia_ d2gana1
d1yvka1 d1ghea_ d2g3aa1 d1y7ra1 d2euia1 d1q2ya_ d2fsra1 d1xeba_
d2ae6a1 d1cjwa_ d1qsma_ d2aj6a1 d1y9ka1 d1i12a_ d1vkca_ d2atra1
d1n71a_ d2d4pa1 d2fiwa1 d2fe7a1 d1tiqa1 d2ou2a_ d1yx0a1 d2fcka1
d2j8ma_ d2pq8a_ d1z4ra1 d1s3za1 d1ufha_ d2hqya1 d2hqya2 d1ne9a1
d1lrza3 d1ne9a2 d1xvqa1 d1pn0a2 d3hy2a_ d2cvba1 d1o8xa_ d1lu4a_
d1we0a1 d2bmxa1 d2a4va1 d1psqa_ d1xiya1 d1q98a_ d1n8ja_ d1zofa1
d2ggta_ d3tjja_ d4je1a1 d1o73a_ d3s9fa_ d2zcta_ d1qmva_ d1jfua_
d2b1ka_ d5dvba1 d2b5xa1 d2v2ga_ d1st9a_ d3tb2a_ d4llra_ d4fh8a1
d1e2ya_ d1knga_ d2h30a_ d1i5ga_ d2z9sa_ d2f8aa1 d1tp9a1 d2b7ka_
d1nm3a2 d3hvva_ d2wfca1 d4kcea_ d1xvwa1 d4gqca1 d3qpma_ d4k1fa_
d1zzoa1 d1gp1a_ d1prxa_ d4od7a1 d1z6ma1 d4mcua1 d4dvca1 d1r4wa_
d3l9sa_ d3rppa_ d2qked_ d4ksoa_ d1t4za1 d1wwja_ d2m5ta_ d1wqsa_
d5bpea_ d2ipha_ d1lvoa_ d2bhga1 d3tnta_ d3sjia_ d3d23a1 d2fyqa_
d3tloa_ d3zvea1 d2hrva_ d3q3ya1 d4asha1 d1e88a1 d1l6ja5 d1l6ja4
d1eaka4 d1eaka3 d1l6ja3 d1h8pa2 d1ys1x_ d4hs9a_ d1ispa_ d1ex9a_
d1ku0a_ d3qmma_ d1n9wa1 d1bbua1 d1e1oa1 d1b8aa1 d1l0wa1 d3a74a1
d1eova1 d1ra6a_ d1muka_ d3gsza_ d1khva_ d1te9a_ d2xwha_ d3uqsa_
d3hkwa_ d3n6ma_ d2cjqa_ d2xyma_ d1teba_ d1te8a_ d1s48a_ d1u09a1
d4wfxa_ d3tyqa_ d2pgga1 d1lgya_ d4l3wa_ d1llfa_ d1gz7a_ d1ukca_
d4wfia_ d4cg1a1 d3visa_ d1ixra2 d1svpa_ d1mbma_ d3su6a1 d1cu1a1
d4agka_ d1vcpa_ d1q31a_ g1n1l.1 d3e90b_ d1ep5a_ d3u1jb_ d2ddfa_
d3zxha_ d1cgla_ d1rm8a_ d1eaka2 d1hy7a_ d2jsda_ d1cxva_ d1hv5a_
d1i76a_ d3p0ya1 d1igra1 d1igra2 d1n8yc2 d1m6ba2 d1m6ba1 d1dzka_
d4tlja_ d2a2ua_ d4wfva_ d3t1da_ d4iaxa_ d3zq3a_ d2nnda_ d1i4ua_
d1kxoa_ d1bbpa_ d1kt7a_ d1exsa_ d3s26a1 d1lf7a_ d4gh7a_ d1z24a1
d1qabe_ d1xkia_ d1ew3a_ d3bx7a_ d4iawa1 d1n0sa_ d1gkab_ d3kzaa_
d2k23a_ d2l9ca1 d2joza1 d1gm6a_ d3kffa_ d3saoa1 d1e5pa_ d1yupa1
d1iiua_ d2tbvc_ d1c8nc_ d1x36a_ d1smvc_ d1ng0c_ d4sbvc_ d1opoa_
d1f2nc_ d1ddla_ d1e57b_ d1auyb_ d1rmka_ d1ixta_ d1eyoa_ d1cnna_
d1fu3a_ d1g1pa_ d1f3ka_ d1fyga_ g1f8v.1 d1novc_ d1ny721 d1ny722
d1a6ca2 d1a6ca1 d1pgl22 d1pgl21 d1pgl11 d1ny711 d1mqta_ d1d4m3_
d1fmd3_ d2mev1_ g1aym.1 d1ev13_ d4q4w1_ d1r1a3_ d2x5ia_ d1rhi1_
g1r1a.1 g1d4m.1 g1rhi.1 d1b35b_ d1oopa_ d1tme1_ d4iv1a_ g1pvc.1
g1fpn.1 d1fpn1_ d4gb31_ d1d4m1_ d1fmd1_ d1ev11_ d2wzr3_ d1qqp3_
g1fmd.1 d2x5ic_ d1rhi3_ g1oop.1 d1hxs1_ g1hxs.1 d1fpn3_ d1bev1_
g2mev.1 d1eah3_ d1aym1_ d1pvc3_ d1qqp1_ d1zba3_ d1h8ta_ d3vbhc_
g1b35.1 g1bev.1 d1hxs3_ g1ev1.1 d1bev3_ g1tmf.1 d1tmf1_ d1b35a_
d2mev3_ g1eah.1 g1h8t.1 d1eah1_ d4q4w3_ d1r1a1_ d1aym3_ d1pvc1_
d1zba1_ d1h8tc_ d1oopc_ d1tme3_ g1qqp.1 d4iv1c_ g1tme.1 d3ubub_
d1egia_ d1qo3c_ d1v7pa_ d1tdqb_ d3ubua_ d1c3ab_ d1h8ua_ d3bx4b_

| | | | | | | | |
|---|---|---|---|---|---|---|---|
| d1t8ca1 | d1sb2a1 | d1qdda_ | d2ziba1 | d3cada_ | d1byfa_ | d1j34b_ | d1jwia_ |
| d1jzna_ | d1c3aa_ | d1oz7b_ | d1wmza_ | d3bdwb_ | d1hq8a_ | d1fifa1 | d1dv8a_ |
| d3hupa_ | d1wk1a1 | d1umra_ | d2afpa_ | d3bx4a_ | d1gz2a_ | d1sl6a_ | d1v7pb_ |
| d3g8ka_ | d1ixxa_ | d1j34a_ | d1jwib_ | d1g1sa1 | d1y17a1 | d1ja3a_ | d1fvub_ |
| d3bdwa_ | d1sb2b1 | d1fvua_ | d1umrc_ | d1oz7a_ | d4m17a_ | d3cfwa1 | d1ypqa1 |
| d1g1ta1 | d3fd4a_ | d3l9jc_ | d4dn8a1 | d1v4lb_ | d1rdl1_ | d2msba_ | d1eixa_ |
| d3exra_ | d2ffca1 | d2fdsa1 | d3n3ma1 | d2czda_ | d1q6oa_ | d2yyua_ | d1vqta1 |
| d3ldva1 | d1ux8a_ | d1dlya_ | d4i0va_ | d2bkma_ | d1dlwa_ | d2bmma_ | d2r80b_ |
| d1cqxa1 | d1cg5a_ | d1gcvb_ | d3boma_ | d2h8fb_ | d2raoa_ | d3bj1a_ | d4hrrb_ |
| d2r50a_ | d3d1kb_ | d2aa1b1 | d3zhwa_ | d3hf4b_ | d3gdja_ | d1jebb_ | d3bj1b_ |
| d1x9fb_ | d1wmub_ | d1gcva_ | d3bcqb_ | d3d4xb_ | d2dc3a_ | d3hf4a_ | d4yu3a_ |
| d3k8ba_ | d1qpwb_ | d2r80a_ | d3bcqa_ | d1v4wa_ | d3wtgb_ | d1jl7a_ | d4esaa_ |
| d1a9we_ | d3d1ab_ | d1fsla_ | d4h2la_ | d1v4wb_ | d1mwca_ | d1vhba_ | d1jeba_ |
| d1spgb_ | d1h97a_ | d1q1fa_ | d1tu9a1 | d3d1aa_ | d1hdsb_ | d3hyub_ | d2zfbb_ |
| d3fs4a_ | d1oj6a_ | d1shrb_ | d4h2lb_ | d4yu3b_ | d1spga_ | d3hrwa_ | d3qqra_ |
| d3at5a_ | d4wchd_ | d3hyua_ | d1cg5b_ | d1uc3a_ | d1hbra_ | d2raob_ | d1outb_ |
| d4esab_ | d3gdjb_ | d3qqqa_ | d1x9fc_ | d3vrfa_ | d1itha_ | d3mkba_ | d3d4xa_ |
| d3mjpa_ | d1jl6a_ | d3fh9a_ | d1outa_ | d2dc3b_ | d1it2a_ | d3eoka_ | d3g46a_ |
| d1qpwa_ | d3vrfb_ | d1ch4a_ | d1x9fd_ | d1hdsa_ | d2zfba_ | d3mkbb_ | d1or4a_ |
| d3wtga_ | d1a4fa_ | d3fh9b_ | d3bomb_ | d3cy5a_ | d3at5b_ | d1x9fa_ | d2oifa_ |
| d4lmxb_ | d1cpcb_ | d3v57a_ | d1kn1a_ | d1cpca_ | d4f0ta_ | d2vjha_ | d4po5b_ |
| d1eyxa_ | d1alla_ | d4f0tb_ | d1b33b_ | d1b8db_ | d4h0mb_ | d1b33a_ | d2vjhb_ |
| d4lm6b_ | d2vmlb_ | d1allb_ | d2vjta_ | d3dbja_ | d2vmla_ | d2vjtb_ | d4f0ub_ |
| d1f99b_ | d4rmpa1 | d1liaa_ | d1eyxb_ | d2uulb_ | d4lmsb_ | d4f0ua_ | d2bv8a_ |
| d1f99a_ | d2bv8b_ | d2c7lb_ | d3dbjb_ | d4rmpb1 | d1b8da_ | d4h0ma_ | d3v57b_ |
| d1kn1b_ | d1liab_ | d2uula_ | d2j96a_ | d4dv8a2 | d1j7na1 | d3ia2a_ | d4dgqa1 |
| d1hkha_ | d1zoia_ | g1gxs.1 | g1wht.1 | d2xqfa_ | d1ea5a_ | d1f6wa_ | d1qe3a_ |
| d1k4ya_ | d4qwwa_ | d2ha2a_ | d1sfra_ | d1f0na_ | d4qeka_ | d1gl9b1 | d1gkub1 |
| d2qvba_ | d2v9za_ | d4kafa1 | d1jmxa4 | d1pbya3 | d1pbya4 | d4iuaa3 | d4bvva_ |
| d5hpga_ | d3k65a_ | d1ki0a3 | d1ki0a2 | d1ki0a1 | d1a0ha1 | d1pmla_ | d3sp8a3 |
| d2feba1 | d1mdwa_ | d1qxpa4 | d1ziva1 | d1zcma1 | d1f74a_ | d3l21a_ | d2e1da_ |
| d1to3a1 | d1w3ia_ | d1sfla_ | d1n7ka_ | d1vcva1 | d4imea_ | d1wa3a_ | d2ocza1 |
| d1f2ja_ | d3m16a_ | d2yxga_ | d1ub3a_ | d3a5fa_ | d1fbaa_ | d1l6wa_ | d1xfba1 |
| d1wx0a1 | d1vpxa1 | d1ojxa_ | d1mxsa_ | d2a4aa1 | d1xkya1 | d3cq0a1 | d3s1xa_ |
| d1f05a_ | d1fdja_ | d3cpra1 | d4d2ja_ | d1cg2a1 | d1yloa2 | d1y7ea2 | d1y0ya3 |
| d1lfwa1 | d1vixa1 | d1vhoa2 | d1ysja1 | d1vhea2 | d1z2la1 | d2fvga2 | d1vgya1 |
| d1r3na1 | d2grea2 | d1xmba1 | g1q7l.1 | d1rtqa_ | d3rp2a_ | g1ucy.1 | d1fuja_ |
| d2zchp_ | d3w94a_ | d1si5h_ | d1lo6a_ | g2pka.1 | d1cgha_ | d1kigh_ | d1a5ia_ |
| d3s9ba_ | d1hj8a_ | d1ao5a_ | d2anya_ | d1q3xa1 | d1gvza_ | d1azza_ | d4gsoa_ |
| d2f91a1 | d1iaua_ | d1gdna_ | d1eufa_ | g1fiw.1 | d1eq9a_ | d4nfea_ | d2rdla_ |
| d1spja_ | d1elva1 | d1a7sa_ | d1ekbb_ | d1pfxc_ | d1yc0a_ | d2aiqa_ | g1fiz.1 |
| d4k60a_ | d4dgja_ | d1mzaa_ | d1op0a_ | d3tjva_ | d1pytd_ | d1sgfg_ | g1gg6.1 |
| d2eeka_ | g1rtf.1 | d1a0ja_ | d3g01a_ | d1pytc_ | d4e7na_ | d1ddja_ | d1z8ga1 |
| d2hlca_ | d1npma_ | d1brup_ | g1jou.1 | d1sgfa_ | d1autc_ | g2hnt.1 | g1kdq.1 |

d1orfa_ d3s69a_ d4crga_ d1fxya_ d1bqya_ d2ftba_ d1pmpa_ d2rcqa1
d1fdqa_ d4lkpa_ d1sa8a_ d1vyfa_ d1lpja_ d3vg7a1 d4azqa1 d5bvqa_
d1p6pa_ d2qo4a_ d1opba_ d1yiva1 d1o1va_ d4bvma1 d4tkba_ d4i3ba_
d1bwya_ d1ftpa_ d1ggla_ d4gkca_ d1cbia_ d3p6da1 d1kqwa_ d2a0aa1
d1m4la_ d2bk7a_ d3fjua_ d1z5ra1 d1kwma1 d1jqga1 d2c1ca1 d1dtda_
d1h8la2 d3lgia_ d3i77a_ d2z9ia2 d4nsva_ d1lcya2 d4jcna_ d1agja_
d1os8a_ d1hpga_ d1p3ca_ d1l1ja_ d1e69a_ d1mv5a_ d3g60a4 g1ii8.1
d1ewqa2 d1ji0a_ d1qhla_ d1v43a3 d2wmfa1 d1vpla_ d1g2912 d4a82a2
g1f2t.1 d2d62a1 d3tuic1 d2bbsa1 d3g60a3 d2ixea_ d1g6ha_ d1sgwa1
d2qi9c_ d3rlfa1 d3b60a1 d3d31a2 d2pzea1 g1xew.1 d1jj7a_ d1oxxk2
d2onka1 d1w1wa_ d1iwea_ d1qf5a_ d1vmaa2 d1nija1 d1np6a_ d2p67a1
d3ihla_ d1ls1a2 d1s1ma2 d1iona_ d2v40a_ d1dj3a_ d3hida_ d2vo1a1
d2g0ta1 d1vcoa2 d1j8mf2 d1g3qa_ d1xjca_ d1p9ba_ d1qzxa3 d1ihua2
d1ihua1 d2qm8a_ d1dj2a_ d4ioja_ d1cp2a_ d1yrba2 d1hyqa_ d3gqba2
d1skye3 d1xp8a1 d1tf7a2 d1tf7a1 d1ye8a1 d1skyb3 d2qe7d2 d4blta_
d4huta_ d3ldaa2 d4hyya_ d2i3ba1 d1cr1a_ d3j9tb2 d3ntua2 d1vdza1
d2z43a_ d1e9ra_ d1nlfa_ d3ziaa2 d1n0wa_ d1g6oa_ d3ziad2 d3j9ta2
d1pzna2 d3gqbb2 d2i49a_ d1nnfa_ d1xvxa_ d1ursa_ d1h3da1 d1iuda_
d4m1va1 d1twya_ d3e13x_ d2nxoa1 d4pfua_ d3ttma_ d1elja_ d2qmwa1
d1us5a_ d1y3na1 d1jeta_ d1uqwa_ d4f19a1 d2esna2 d3thia_ d3fasa1
d2uyeb_ d2xwva1 d1r9la_ d2fyia1 d1pb7a_ d2ozza1 d1sw5a_ d1nh8a1
d1vr5a1 d2g29a_ d1xs5a_ d3lswa_ d2z23a_ d3w9va_ d3tcha_ d1q35a_
d1z7me1 d3q27a_ d1p99a_ d1ve4a1 d2i6ea1 d4mcoa1 d4n8ya_ d1eu8a_
d1zbma1 d1hsla_ d1ixca2 d1j1na_ d1xvya_ d1y9ua_ d2z22a1 d1xoca1
d4oera_ d1wdna_ d5f1qa1 d1xt8a1 d1i6aa_ d4gwoa_ d4igta1 d1pc3a_
d4mmpa1 d1o63a_ d2czla1 d2onsa2 d4mija1 d1eh3a_ d3skpa_ d1dtza1
d1ieja_ d1jnfa2 d1ce2a1 d1jnfa1 d1b1xa2 d1b1xa1 d1jw1a1 d1jw1a2
d1ryoa_ d1h76a1 d1h76a2 d3q3ma_ d1za0a1 d1mxra_ d2inca_ d1jk0a_
d2incb_ d1mhyb_ d3hf1b_ d1mtyb_ d1r2fa_ d2uw1a_ d1mhyd_ d3n37a_
d1afra_ d1syya_ d1mtyd_ d2o1za_ d1uzra_ d1smsa_ d2fkza_ d1zuja1
d1s3qa1 d2fjca1 d1z6om1 d1veia_ d4dyya_ d1jiga_ d3gvya_ d2pyba_
d4cy9a1 d3bvfa1 d1r03a_ d2cf7a_ d1nf4a_ d1zs3a1 d4iwka_ d3ka8a_
d3np0x_ d1n1qa_ d1lb3a_ d4e6ka_ d1z6oa1 d2fzfa1 d4dyua_ d3ge4a_
d1jgca_ d4am4a_ d2z6ma_ d1ji5a_ d1vlga_ d1lkoa1 d1krqa_ d3qz3a_
d1o9ra_ d2ffxj_ d1euma_ d1yv1a1 d2v2pa_ d1j30a_ d4di0a_ d1dpsa_
d2cbia2 d1gqia1 d2choa2 d1oewa_ d2qzxa_ d2asia_ d1psoe_ d1miqa_
d2h6ta_ d1pfza_ d1izda_ d3hd8a_ d1j71a_ d1dpja_ d4aa9a_ d1t6ex_
g1b5f.1 g1lya.1 g1htr.1 d1qdma2 d1bxoa_ d1wkra_ d1tzsa_ d1ibqa_
d2qzwa_ d1bdqa_ d3ggta_ d3ixoa_ d1mrxa_ d2haha_ d1tw7a_ d4njsa_
d1sgua_ d2p3da_ d1nsoa_ d3d3ta_ d2rkfa_ d1c6ya_ d2rspa_ d3ogpa_
d3ekwa_ d2o4na_ d2j9ja_ d3ggua_ d3lzua_ d4npua_ d4j55a_ d2r8na_
d2fxda_ d2p3ca_ d4dqea_ d3ttpa_ d3t3ca_ d2p3aa_ d2hc0a_ d2fdda_
d3mwsa_ d1js3a_ d3rcha_ d3k40a_ d1k8rb_ d1wgra1 d1ef5a_ d1wfya1
d1e8ya3 d1wgya1 d2cs4a1 d1wxma1 d2byea1 d3kh0a1 d2byfa1 d1wxaa1

d1e7ua3 d1h4ra3 d4yl8a1 d2al6a3 d1wgda1 d1wgga1 d4q5eb_ d1wjna1
d1se9a_ d3n3kb_ d1wz0a1 d1we6a1 d2m0xa_ d2gbka_ d3rt3b1 d1wh3a1
d1sifa_ d2ekec2 d4ii2b1 d4hk2a1 d1c3ta_ d1wgha1 d1ttna1 d1j8ca_
d1wx8a1 d3v6cb1 d2ma9b_ d4i6lb1 d1bt0a_ d2jvca1 d1iyfa_ d2k1fa_
d1wxva1 d1wf9a1 d1t0ya_ d2io0b2 d1x1ma1 d2gbma_ d1zw7a1 d2m17a_
d1ud7a_ d1we7a1 d2lwpa_ d2gbja_ d1wjua1 d1v6ea1 d1v86a1 d4eewa_
d1wiaa1 d4icva_ d4gu2a_ d1wx7a1 d1wy8a1 d4hjka1 d2faza1 d1v5oa1
d3ubra_ d1qo8a1 d1d4ca1 d2k3va_ d1ft5a_ d1qdba_ d1m1qa_ d2j7aa_
d1h21a_ d1ogyb_ d4fasa_ d1oaha_ d1sp3a_ d4lu0a_ d1of8a_ d3unda1
d3e9aa1 d1vr6a2 d2qkfa_ d1d9ea_ d1o60a_ d2h0ba_ d2erfa_ d1h30a2
d3mw4a1 d1pz7a_ d2r1ba_ d3v64a1 d1d2sa_ d3bopa1 d3mw3a1 d1okqa1
d1okqa2 d1wioa4 d1ccza2 d1dr9a2 d1ic1a1 d2d9qb1 d2nxyb2 d1um8a_
d1qvra2 d1qvra3 d1qzma_ d1sxjc2 d1jr3d2 d2p65a_ d1l8qa2 d1g8pa_
d3hu3a3 d1sxjd2 d1njfa_ d1in4a2 d1sxja2 d1tuea1 d2ce7a2 d1w5sa2
d1sxjb2 d1d2na_ d1ny5a2 d1ofha_ d2gnoa2 d2a5yb3 d1fnna2 d1u0ja1
d1lv7a_ d1iqpa2 d2fnaa2 d1ixsb2 d4gita_ d1sxje2 d1g41a_ d2e3fa_
d1mm0a_ d2e3ea_ d2llda_ d2ny8x_ d2e3ga_ d2ny9x_ d2nz3a_ d2lj7a_
d1n4na_ d2m8bb1 d2kyqa_ d3psma_ d1jxca_ d2gl1a_ d1ugla_ d4uj0a_
d2k4ua_ d1hp2a_ d2lu9a_ d1baha_ d2meoa1 d1wm7a_ d1hlya_ d1n8ma_
d1jlza_ d1wpda_ d1c55a_ d1pvza_ d1wt7a_ d1rjia_ d1wz5a_ d1m2sa_
d1pjva_ d1c49a_ d1qkya_ d2ktca1 d1wmta_ d1du9a_ d1j5ja_ d1r1ga_
d1t0za_ d2i61a_ d1t1ta_ d1sn4a_ d1kv0a_ d2lkba_ d1wwna_ d1pe4a_
d2kbka_ d2li7a_ d1djta_ d2kbha_ d1dq7a_ d1sega_ d1omya_ d1zywa1
d1jzaa_ d2jcqa_ d1bnla_ d1dy2a_ d1t61c1 d1iyjb4 d1iyjb5 d1iyjb3
d1o7ia_ d4ipca1 d1x3ea_ d4apva_ d1jb7a1 d1jb7a2 d1jb7a3 d3kdfa_
d1ue1b_ d1wjja1 d3eiva_ d1xjva2 d1xjva1 d2ccza2 d1se8a_ d1l1oc_
d1jb7b_ d1qvca_ d1qzga_ d1miua4 d1miua5 d1miua3 d3g48a1 d1gd7a_
d1mkha_ d1pyba_ d1y14b1 d2nn6i1 d1i94l_ d2zjra2 d1smxa_ d2qamc2
d1hh2p1 d2r7da1 d1rl2a2 d1wfqa1 d3bzka4 d1x6oa2 d3a0ja_ d2j01d2
d2ja9a1 d4ql5a_ d1luza_ d2n3sa_ d2vnud1 d1h95a_ d1go3e1 d2nn6h1
d1ne3a_ d2mo1a_ d1iz6a2 d1jjga_ d1e3pa2 d2ix0a3 d2ix0a2 d2lxja_
d1ueba3 d1ueba2 d2z0sa1 d1u0la1 d2ix0a1 d2eifa2 d2je6i1 d3i2za1
d2c35b1 d2ahob2 d1t9ha1 d3i4oa_ d2qall1 d1jt8a_ d2nn6g1 d1g6pa_
d3cama_ d1i94q_ d1c9oa_ d1d7qa_ d2ba0a1 d2vnud2 d2vnud3 d1khia2
d2qalq1 d1wi5a1 d1xtda2 d1ny4a_ d1pfsa_ d2a1ka_ d1rpma_ d2shpa1
d1yfoa_ d1l8ka_ d2ooqa1 d1g4us2 d1p15a_ d1lara2 d1lara1 d1wcha_
d2fh7a2 d2fh7a1 d1ohea1 d2f4da_ d1rxda_ d2hxpa_ d1xria_ d1d5ra2
d1fpza_ d3ezza_ d4y2ea_ d1i9sa_ d1c1la_ d4lbqa_ d1a78a_ d1slta_
d4no4a_ d3zsja_ d3duia_ d1g86a_ d1qmja_ d1hlca_ g1qmo.1 g1len.1
d3zyra_ d1n47a_ g2ltn.1 d1avba_ d3usua_ d1dhkb_ d1f9ka_ d1ukga_
d1g8wa_ d1g9fa_ d1wbfa_ d3wcsa_ d1g7ya_ g1loe.1 d1mvqa_ d1hqla_
d1v6ia_ d1dbna_ d1qnwa_ d1fx5a_ d4l8qa_ d1ioaa_ d3n35a_ d1epwa1
d3h33a_ d1rwja_ d19hca_ d1os6a_ d3bxua_ d3h4na_ d1i77a_ d3caoa_
d1j0pa_ d2ctha_ d1ofwa_ d2e84a_ d1hh5a_ d1gyoa_ d3h34a_ d1dszb_

d1a6ya_ d3cbba_ d1ynwa1 d1r4ia_ d1hcqa_ d2hana_ d2hanb_ d2enva1
d1kb4b_ d2nllb_ d1cita_ d2dara2 d2dara1 d1wyha2 d2dloa2 d1rutx4
d1rutx3 d2cuqa2 d1x4la2 d1x4la1 d1x3ha2 d1x3ha1 d2d8za1 d1x61a2
d1x6aa1 d1x6aa2 d2cora2 d2cora1 d1v6ga1 d1v6ga2 d2d8za2 d1x64a1
d1x64a2 d1b8ta1 d2co8a1 d1x63a2 d2dj7a2 d2dj7a1 d2cu8a2 d2cu8a1
d1g47a1 d1j2oa1 d1wyha1 d2dloa1 d1wiga1 d1wiga2 d2cupa2 d2cupa3
d1x68a1 d2d8ya2 d2d8ya1 d1m3va2 d1x62a1 d1x68a2 d1x61a1 d1b8ta3
d1b8ta2 d2d8xa2 d2d8xa1 d1x4ka1 d1x4ka2 d2cuqa1 d1m3va1 d2co8a2
d1j2oa2 d1x63a1 d2cura1 d2cura2 d1x62a2 d1op9a_ d1y0la1 d1etzb1
d1egjl1 d2gsib1 d1nj9a1 d4jfhd1 d1aqkl1 d1t2ja_ d1lo4h1 d1zvya1
d3v4vl1 d4enec1 d1kxtb_ d5e8ea1 d2pxya_ d4weud_ d1xiwc_ d2esvd1
d1qnzh_ d1ogae1 d1f4xh1 d4n1hb_ d3ezjb1 d3mlwl1 d1kfah1 d1etza1
d1b4jh1 d3cfic_ d4w68a_ d2arja1 d1ek3a_ d2ok0l1 d1vera_ d1uwgh1
d1blnb1 d1fpth1 d1rzga1 d2giya1 d4m3kb1 d1nc2a1 d2nyyd1 d1ncna_
d1fbih1 d5dxwa_ d4fqqa1 d1dqdh1 d1jfql1 d1kelh1 d1faih1 d1frgl1
d1figl1 d4m6nl1 d1fo0a_ d4hxbl1 d3b5ga_ d3quyd1 d2h9gb1 d1i8kb_
d2dd8h1 d2ck0h1 d1eaja_ d4w6wb1 d2a6ib3 d1t66d1 d2e27h_ d1ob1b1
d1f11b1 d2v7na1 d1ad0a1 d2xt1b1 d1pskh1 d4i13b1 d2op4l1 d1a0ql1
d1uwel1 d1za3a1 d1ncbl1 d4uu9a_ d1bm3h1 d1cloh1 d1nqba2 d1cf8h1
d4y5va_ d1mhph1 d5anma1 d4cdgc1 d4ldeb_ d1y0lb1 d2pcpb1 d1z3gl1
d1dfbl1 d1fh5h1 d3g5xa1 d1tzib1 d4dn3l1 d3zhka1 d2b1hh1 d2uyla1
d1f3dh1 d1nj9b1 d2agjh1 d2h1pl1 d1q72h1 d8fabb1 d1ndmb1 d1rjca1
d4u6hb1 d3upca_ d2fbjl1 d1osph1 d1tvda_ d2cjvh_ d4fhbd1 d1bd2d1
d1rz7h1 d3nn8a_ d1blna1 d1knoa1 d1nfda1 d2bmkb1 d1dqll_ d1igyb1
d1igfl1 d12e8h1 d3utza1 d1nc2b1 d1rihh1 d4mwfb1 d4jvpa1 d1mieh1
d1rhfa1 d1fgnl1 d1sbsh1 d1lp9e1 d1dn0a1 d4lqfl1 d1iaih1 d1kgcd1
d1mh5b1 d1gpol1 d2g60h3 d1f8tl1 d2jixd1 d4grwh_ d1fo0b_ d1bfob1
d1axsa1 d1u9ka_ d1nmbh_ d1h8na1 d1h8na2 d1jguh1 d4eura1 d3sdyl1
d35c8h1 d1w72l1 d1mvub_ d1i9rh1 d1ob1a1 d4wenb_ d1yeeh1 d3ietc1
d1f58h1 d1ad0b1 d1mqkl_ d1nbvh1 d4b50a_ d3r8bb_ d1bafl1 d1mamh1
d4rgne1 d2gsgb_ d35c8l1 d1bafh1 d1ai1h1 d2pcpa1 d2fath1 d2x1pa_
d1orsb1 d1ibgl1 d1shma_ d1a2yb_ d1u0qa_ d2coqa_ d1hilb1 d1qfwi_
d1jv5b_ d1fn4b1 d5e0qa_ d1nsnh1 d1plgl1 d4ocxl1 d1kb5h1 d1rzfh1
d1pg7x1 d2fx7h1 d2oi9c_ d1l7il1 d3eyva1 d1d5il1 d4ffyl_ d1tetl1
d1a6wh_ d1ac6a_ d3dvfa_ d1nfde1 d1mimh1 d1ri8a1 d1jhlh_ d3dura_
d1fskb1 d1f97a1 d3so3b1 d2ak4d1 d1lp9f1 d4z7wf1 d1l7th1 d4y5ya_
d4x7ec1 d1emth1 d1ikfl1 d1nlbh1 d4utal1 d1bjma1 d1eapb1 d1u6ah1
d2dtgd1 d1kj2b_ d1xeda1 d1c1eh1 d1fgvh_ d2bnub1 d2fb4h1 d2dtgb1
d1ktrh_ d1jpth1 d1qkzl1 d4hepg_ d2atpb1 d1flrh1 d2hrpl1 d1mvfa_
d1iaim1 d3k80a1 d2jb5h1 d3zkxc_ d4grwe_ d1it9h1 d1rhhb1 d3w9ec_
d1e4wh1 d1sy6h1 d2j6eh1 d3qxta_ d1jglh1 d1p7ka1 d1tzhb1 d4n8pa1
d1gafl1 d2jelh1 d1wioa2 d4i0cc1 d1cicb1 d1lmka2 d1lmka1 d1iqwh1
d1orqb1 d1ejoh1 d1jnlh1 d4zg1a_ d43c9b_ d1ggbl1 d1wejh1 d3q6fa1
d1f3dj1 d3c6la1 d1bqlh1 d3p9wb_ d1a3rl1 d1h0db1 d2uzil_ d1eo8l1

| | | | | | | | |
|---|---|---|---|---|---|---|---|
| d1qfwl_ | d1n7ml1 | d1igja1 | d1c5ch1 | d2a9mh_ | d1rmfh1 | d1fj1b1 | d4heme_ |
| d1ccza1 | d1a5fh1 | d1ogad1 | d1dlfh_ | d1qa9b_ | d1vgel1 | d3qskb_ | d1sjxa_ |
| d4ozfg1 | d1sq2n_ | d1qd0a_ | d1kcvh1 | d1c5db1 | d3qcul1 | d8faba1 | d3bn9d1 |
| d1mjuh1 | d4y8dc_ | d7fabh1 | d3dvgb1 | d1lo0l1 | d1a6tb1 | d1gigh1 | d1kcrh1 |
| d2hwzl1 | d1yjdc1 | d3clfl1 | d4krna_ | d1dl7l_ | d1e6ol1 | d1mexh1 | d3r0ma_ |
| d4kkna_ | d1fnsl1 | d1oaqh_ | d1rzia1 | d1ucbh1 | d1ncwl1 | d4b41a_ | d1nfdf1 |
| d2mpah1 | d2d7tl1 | d4grwf_ | d4b5ea_ | d1lk3h1 | d3u79a_ | d4u3xa_ | d1ghfl1 |
| d1bvkb_ | d1mcww1 | d1b88a_ | d1jrhh1 | d32c2b1 | d4wemb_ | d1dsfh_ | d1op3h1 |
| d1ce1l1 | d1iqda1 | d1qoka1 | d1qoka2 | d1qfuh1 | d2crya1 | d1ay1h1 | d1oaql_ |
| d3ab0b_ | d1rurl1 | d1t4kb1 | d1bz7b1 | d1n0xh1 | d1ucbl1 | d4w6yb_ | d43c9a_ |
| d2aabl1 | d1tzia1 | d4n1ca_ | d1k6qh1 | d3g9ab_ | d4tvsa1 | d1igmh_ | d1pz5b1 |
| d3dx9a1 | d3wxwh_ | d1nakh1 | d1kcul1 | d3eaka_ | d4qlra1 | d1cr9l1 | d1d9ka_ |
| d1f4xl1 | d1deea1 | d2i27n2 | d1c12a1 | d2orbl1 | d1j8hd1 | d1mcph1 | d1c5da1 |
| d1kenh1 | d1bd2e1 | d1h5ba_ | d4x7fc1 | d3upaa_ | d1t3fa1 | d1indh1 | d2dtgc1 |
| d4hc1l1 | d1zv5a1 | d4wgvb_ | d1uwgl1 | d4f37k1 | d1aqkh1 | d1vesa_ | d3zkqd_ |
| d1fptl1 | d1lo4l1 | d1knob1 | d3rrqa_ | d1ct8b1 | d4orzc_ | d1mlbb1 | d4hgkc1 |
| d1dqdl1 | d1bogb1 | d4ozhe1 | d1dr9a1 | d1i7za1 | d1dn0b1 | d1kell1 | d1rzib1 |
| d1ypzf1 | d1wtla_ | d1hkfa_ | d1fail1 | d1figh1 | d2vyre_ | d1sjva_ | d1ohqa_ |
| d1bfoa1 | d1pkqb1 | d1mjul1 | d1b4jl1 | d1nsnl1 | d2arjb1 | d1p4bh_ | d4aj0a_ |
| d3nn8b_ | d2rcja1 | d1f2xk_ | d4v1dd_ | d1adqh1 | d2eksa_ | d4k3ga_ | d1jfqh1 |
| d1uweh1 | d1frgh1 | d4gftb_ | d1uz8b1 | d4n1ea_ | d1bm3l1 | d1clol1 | d3mcka1 |
| d2vq1a1 | d1ae6h1 | d4aq1b1 | d1uj3b1 | d1nbqa1 | d1fh5l1 | d2ck0l1 | d2ywza_ |
| d1q72l1 | d4nbxb_ | d1hila1 | d4hzlb1 | d2j88l1 | d1zmya1 | d1cd0a_ | d1a4ka1 |
| d1pskl1 | d1a0qh1 | d4krmb_ | d1ncbh1 | d2fbjh1 | d1j8he1 | d1i3ua_ | d1cf8l1 |
| d1fe8h1 | d2p1ya1 | d1eeqa_ | d2x89a_ | d1ol0a_ | d2r0lh1 | d1ub5a1 | d1dfbh1 |
| d3eyvb1 | d4jdvb1 | d4p2cg_ | d2gsia1 | d2agjl1 | d1u3ha1 | d2h1ph1 | d1rihl1 |
| d1fskc1 | d1boga1 | d2a77l1 | d1ospl1 | d1qfoa_ | d3dwta_ | d2fx7l1 | d1rz7l1 |
| d3stba1 | d1pkqa1 | d1b6da1 | d1dzba2 | d1dzba1 | d1kj2a_ | d4l1ha_ | d1rzgb1 |
| d1dqlh_ | d12e8l1 | d1miel1 | d1jmaa_ | d1sbsl1 | d1fgnh1 | d1f58l1 | d1mqkh_ |
| d1ndgb1 | d3qegl1 | d1iail1 | d2aw2a1 | d1tqbc1 | d3fo2a1 | d4dkaa_ | d1f8th1 |
| d2g60l1 | d2ywya_ | d1uj3a1 | d1yc7a_ | d3bd3a_ | d4w6xb1 | d1nmbl_ | d1i8ka_ |
| d1rjlb1 | d1ypze1 | d1p7kb1 | d1kxqe_ | d1gpoh1 | d2z4qa1 | d1w72h1 | d1i9rl1 |
| d4qgya1 | d4xt1c_ | d2qqnh1 | d1cica1 | d3c08h1 | d2cd0a_ | d2bdnh1 | d1hnga1 |
| d1maml1 | d1nldh1 | d1cr9h1 | d4lcih1 | d3bkyh1 | d3qg7l1 | d1ai1l1 | d1kxvc_ |
| d4iosd_ | d1l7ih1 | d2x1qa_ | d1ub6b1 | d3k74b_ | d1ibgh1 | d1teth1 | d1igya1 |
| d1hxma1 | d2x6ma_ | d2e7la_ | d5e03a_ | d1qfwm_ | d2r0kh1 | d1igjb1 | d1rurh1 |
| d1plgh1 | d1mj8h1 | d1yuhb1 | d3fmgl1 | d1rzfl1 | d1emtl1 | d1ad9b1 | d1igtb1 |
| d1nlbl1 | d1d5ih1 | d3k7ua_ | d4ffyh_ | d1bwwa_ | d3dvga1 | d1a6ta1 | d1fgvl_ |
| d4qxgl1 | d1miml1 | d1jhll_ | d2hffb1 | d2hh0l1 | d1qkzh1 | d1l7tl1 | d1afvh1 |
| d1iaii1 | d2cjuh_ | d1um5h1 | d2ghwb2 | d2ghwb1 | d1kgce1 | d2fgwh1 | d1ikfh1 |
| d1dvfd_ | d2i24n1 | d2xtjb_ | d1za3b1 | d2yc1a_ | d2hh0h1 | d2x1oa_ | d1bvka_ |
| d1rjla1 | d5e7ba1 | d1igta1 | d1il1b1 | d2fb4l1 | d1mvua_ | d1jptl1 | d32c2a1 |
| d1fvca_ | d1iqdb1 | d2hrph1 | d2p4ab_ | d4qo1a_ | d1p2cb1 | d1wt5c_ | d1t4ka1 |
| d5c1mb_ | d1it9l1 | d1bz7a1 | d1b2wh1 | d1e4wl1 | d1jnll1 | d1yedb1 | d1za6b1 |

d3u7aa_ d1igfh1 d3gnml1 d1orsa1 d1wejl1 d1a2ya_ d1hxmb1 d1gafh1
d2j4wh1 d1pz5a1 d1j05a_ d1a3rh1 d4fqll1 d1qfwh_ d1eo8h1 d1n7mh1
d4s11a_ d1kegh1 d5da4a_ d1iqwl1 d3whxb1 d1vgeh1 d1deeb1 d1c12b1
d1t2ql1 d1ad9a1 d3gsnb1 d3b2uc1 d1ggbh1 d1t3fb1 d1nakl1 d4ocmc_
d1lila1 d2v7hb3 d2esve1 d1mcoh1 d2qr0b1 d2q3aa_ d1ct8a1 d1kcrl1
d2pxyb_ d2uzih_ d5c3ld_ d3durb_ d1c5cl1 d2ak4e1 d1dl7h_ d1xiwd_
d1mlba1 d1rmfl1 d1i7zb1 d1dlfl_ d2xa3a_ d5bq7b1 d1dqqb1 d3h42l1
d1egjh1 d1kcvl1 d1fe8l1 d2j6el1 d1f3rb2 d1lk3l1 d1sm3h1 d1q0xh1
d1ghfh1 d7fabl1 d1igch1 d1lo0h1 d4idla_ d5c2bl_ d1jrhl1 d1il1a1
d3zhda1 d4kfzc_ d1b0wa_ d1e6oh1 d1mexl1 d4krpb_ d1fnsh1 d1ncwh1
d4hgma_ d4eigb1 d4rzca_ d2mpal1 d4y5xa_ d5en2b1 d3lrga1 d4ej1c1
d1rhha1 d1j05b_ d1forh1 d1q9wb1 d2wzpd_ d2aq2a1 d1h0da1 d3revb1
d2ij0c1 d1tzha1 d4m7jl1 d1k6ql1 d3b9kc1 d1igml_ d3qeua1 d1dsfl_
d1ce1h1 d1kcuh1 d1zvha1 d1zvoa1 d1tqbb1 d1ay1l1 d2qscl1 d2cgrh1
d1nl0h1 d1kenl1 d3eo0a1 d4n9ob1 d1n0xl1 d3k1kc_ d1c4qa_ d1prtc1
d2wv6d_ d1prtf_ d1ltrd_ d4l63b1 d1r4pb_ d1tl0f_ d2bosa_ d1prtb1
d3chbd_ d2aq2b1 d1ck1a1 d1enfa1 d1i4pa1 d1v1oa1 d1et9a1 d1esfa1
d2g9hd1 d4ohja1 d1klud1 d1bxta1 d1eu3a1 d1ty0a1 d1fnua1 d2cqva1
d1fhga_ d2k1ma_ d2dl2a1 d1biha3 d1biha1 d1biha4 d3qp3a1 d1g1ca_
d1gxea_ d1cvsc2 d1cvsc1 d1xmwa2 d2otpa2 d1biha2 d1rhfa2 d2fcba2
d2fcba1 d1wwbx_ d1gsma2 d1epfa1 d1epfa2 d2dava1 d1waaa_ d1f97a2
d1x44a1 d1vcaa2 d1fnla2 d1fnla1 d3b5ha2 d3h8na2 d3h8na1 d1jbja1
d1jbja2 d1pd6a_ d1f2qa2 d1f2qa1 d3cx2a_ d1nbqa2 d3b5ha1 d1n26a1
d1xiwb_ d1xaua_ d1fltx_ d2ifga1 d1olla1 d1olla2 d1cs6a4 d1cs6a3
d1cs6a2 d1cs6a1 d2gw5a2 d1sy6a1 d1xiwa_ d1ucta2 d1ucta1 d1ry7b2
d1gl4b_ d1qz1a3 d1olza1 d2c9aa1 d1wwca_

**Family-50 (c.2.1.2)**

**Positive dataset contains 78 protein sequences.**

d3sjua_ d3ojfa1 d1xu9a1 d2jjya1 d1y1pa1 d4fs3a_ d2bkaa1 d2c5aa1
d1ulsa_ d2b69a1 d1y5ma1 d1ydea1 d1snya1 d2rhca_ d3zv4a_ d5ceja_
d2d1ya1 d3w8ea_ d2fmua1 d1x1ta_ d3ay7a1 d2hrba1 d3ruaa_ d2ew8a1
d4hwka_ d1xgka_ d2bd0a1 d1yo6a1 d2a35a1 d2ntva_ d1vl8a_ d4bkua_
d2ag5a1 d2q45a_ d4iy1a_ d1zbqa1 d2q46a2 d2ptga_ d1uzma1 d3lyla_
d4m89a_ d3wyea_ d2blla1 d1w6ua_ d1sb8a_ d2q2va_ d4nr0a_ d2gasa_
d3oiga1 d2bgka1 d1vl0a1 d1wmaa1 d2c07a1 d1xkqa_ d3tzha_ d3lz6a_
d1zema1 d2xoxa_ d1z45a2 d3lt0a_ d4e6pa_ d2wyua_ d4q9na_ d1xhla_
d4o1ma_ d2uvda_ d2gdza1 d2z1ma_ d2o23a2 d2fr1a2 d2fr1a1 d4zjua_
d2a4ka1 d1yb1a_ d1wvga1 d1yxma1 d3f4ba_ d1xg5a1

**Negative dataset contains 3851 protein sequences.**

d1m32a_ d1ibja_ d1c7na_ d1iuga_ d4q75a_ d2bkwa1 d1t3ia_ d1n8pa_
d1qz9a_ d1jf9a_ d1eg5a_ d1p3wa_ d3kgwa_ d1cl1a_ d2ctza1 d2rfva_
d1gc0a_ d1cs1a_ d1d2fa_ d2ch1a1 d1qgna_ d1pffa_ d1elua_ d1c7ga_
d2v1pa1 d2ez1a_ d1u08a_ d1lc5a_ d1b5pa_ d2ay1a_ d1bw0a_ d3pd6a_
d7aata_ d2csta_ d1j32a_ d1fg7a_ d4wlha_ d1o4sa_ d1v2da_ d1gdea_

d1m6sa_ d1v72a1 d1svva_ d1ajsa_ d1wsta1 d2f8ja2 d1lk9a_ d1vp4a1
d1xi9a1 d2gb3a1 d2bnma2 d2ovga_ d1sq8a_ d1rzsa_ d3kz3a_ d3orca_
d1xkta1 d2hfka_ d1jmkc_ d5d3ka_ d2cfba1 d1dfoa_ d1o69a_ d1bj4a_
d3k28a_ d2bwpa_ d3lg0a_ d2fnua_ d3ou5a_ d1z7da1 d1b9ha_ d1iaya_
d4msoa_ d1bs0a_ d1s0aa_ d1sffa_ d3tqxa_ d1mdoa_ d3qboa_ d3pgya_
d1bjna_ d2dkja_ d1ejia_ d1vefa1 d4wxga_ d3bs8a_ d3gbxa1 d1w23a_
d2tbvc_ d1c8nc_ d1x36a_ d1smvc_ d1ng0c_ d1opoa_ d1f2nc_ d1ddla_
d1e57b_ d1fwxa1 d1ibya_ d1f56a_ d1bypa_ d1azca_ d1ws8a_ d3u25a_
d1cuoa_ d1id2a_ d1rkra_ d2iaac_ d4dp1x_ d1nwpa_ d2gima_ d2i7sa_
d1qhqa_ d1gyca3 d1hfua2 d1kbva1 d1kbva2 d1sdda1 d1sdda2 d1gyca1
d1gyca2 d1mzya1 d2bw4a1 d1aoza1 d1aoza3 d1aoza2 d1kyaa2 d1kyaa3
d1kyaa1 d1mzya2 d4e9sa1 d1sddb1 d1sddb2 d1hfua1 d1hfua3 d1oe1a2
d1oe1a1 d1v10a2 d1v10a3 d1v10a1 d2w0pa_ d2diaa1 d2e9ia2 d2e9ja1
d2dj4a1 d2eeba1 d2diba1 d2dmba1 d2dmca1 d2d7oa1 d2ee6a1 d2d7pa1
d2eeaa1 d2di8a1 d1wlha1 d2d7na1 d2nqca_ d2d7ma1 d2dica1 d3cnka_
d2k3ta1 d2eeca1 d1qfha2 d2bp3a1 d1v05a_ d2ee9a1 d2eeda1 d2di9a1
d3kasa1 d3bi1a3 d4qgna_ d1vr3a1 d2bema_ d1pama1 d1ea9c1 d1qhoa1
d2fhfa2 d2fhfa1 d1k3ia1 d1cxla1 d2bhua1 d1edqa1 d1f1sa2 d1ut9a2
d1gvia1 d1txka1 d1adqa1 d1fnga1 d1uvqb1 d1zs8a1 d2vola1 d2vola2
d1mfbl2 d2jelh2 d1uwel2 d3qq4b1 d1kjvb_ d1k5na1 d1osph2 d4odhl2
d1ow0a1 d1ow0a2 d1bfob2 d3l9ra2 d3jtth1 d2fath2 d4elkb2 d1fn4b2
d2xfxb_ d1pg7x2 d1cqka_ d4k71b2 d1lk2a1 d1eapb2 d4lfhg2 d1i4fa1
d2nw2a2 d1kfah2 d1mhca1 d1h3ua1 d1wejh2 d1h0db2 d1a5fh2 d1kcvh2
d1mjuh2 d1qqda1 d1kcrh2 d1k8ia1 d3huja2 d2nnab1 d1nfdf2 d3mffb2
d1s9va1 d1lk3h2 d1q0xl2 d1fv1b1 d1neza1 d4rfeb2 d1r3ha1 d1a6tb2
d1kjva1 d1pz5b2 d1onqa1 d2bc4a2 d1de4a1 d1k8da1 d1tcra2 d1dn0b2
d1nfde2 d2xwtb2 d1bfoa2 d1mexh2 d1iakb1 d1ypzf2 d4f7ea2 d1q72l2
d2fbjh2 d1fe8h2 d1clzh2 d1fngb1 d1uvqa1 d1vgka1 d1c16a1 d4j12a2
d4x99b2 d1igtb3 d1je6a1 d1q72h2 d1nldh2 d1teth2 d2hmic2 d1i1ca2
d1i1ca1 d1k8ib1 d1q0yh2 d1kfal2 d3frua1 d1pg7w2 d1u58a1 d1igyb4
d1igyb3 d1hyrc1 d1klub1 d1deeb2 d1c12b2 d2bc4b2 d2ig2h2 d1kcvl2
d3wn5b1 d1lk2b_ d1lk3l2 d1mjul2 d1sm3h2 d1q0xh2 d1zvsa1 d1ydpa1
d1u8ib2 d1ypze2 d1kcuh2 d4alac_ d1pjwa_ d1rera1 d1s6na_ d3uzqb_
d1ofda3 d1ea0a3 d1ecfa2 d1q15a2 d1ct9a2 d1ao0a2 d1te5a_ d1ep0a_
d2pa7a1 d1wlta1 d1nxma_ d2c0za1 d1nepa_ d1ktja_ d1ds6b_ d1fstb_
d1ex0a1 d3us3a3 d3us3a1 d4mh8a_ d1mu2a2 d4i7ga1 d1tafa_ d1bh9b_
d1jfib_ d1q9ca_ d1h3ob_ d2byka1 d1n1ja_ d4g92b_ d1bh9a_ d1jfia_
d1h3oa_ d2bykb1 d1n1jb_ d3nvva1 d1krha3 d2h88b1 d1n62a2 d1t3qa2
d1zoyb1 d1jq4a_ d1ffva2 d1dgja2 d1rm6c2 d1h4ba_ d2opoa_ d1ydwa2
d2nvwa2 d1h6da2 d1zh8a2 d1qkia2 d1xeaa2 d1tlta2 d1r0ka3 d1j5pa3
d1vkoa2 d1vm6a2 d3qvsa2 d1gr0a2 d1p1ja2 d1orva2 d1xfda2 d3gpdg2
d2q49a2 d1hdgo2 d2g17a2 d2cvoa2 d2czca1 d3pwka2 d1obfo2 d1i32a2
d1zyao2 d2pkqo2 d1b7go2 d2hjsa2 d1cf2o2 d1vkna2 d1nvmb2 d4lt9a1
d3tc7a1 d3hoja_ d1i4na_ d3vnda_ d3nl8a1 d1geqa_ d3nava1 d1v5xa_

d4a2ra1 d3tc6a1 d1ujpa_ d4ijba_ d1xcfa_ d1vc4a_ d1rd5a_ d1qopa_
d3b5va_ d2msya_ d2djoa1 d2r5za_ d1au7a1 d1f43a_ d2lmda1 d1akhb_
d1s7ea1 d3nara_ d1zq3p1 d2craa1 d1mija_ d4uuta_ d3a01a_ d1x58a1
d3rkqa1 d3cmya1 d2cqxa1 d2da6a1 d1e3oc1 d1fjla_ d1mh3a1 d1x41a1
d1wh7a1 d1gt0c1 d2mw8a_ d1lfup_ d1x2ma1 d1b72a_ d1wi3a1 d1jgga_
d1wjha1 d1x2na1 d1mnmc_ d2cuea1 d3k2aa_ d2hosa_ d2jwta2 d1pufa_
d1octc1 d2hi3a_ d1b8ia_ d4j19a_ d3lnqa_ d1wh5a1 d1b8ib_ d1fexa_
d2cqra1 d1guua_ d1ug2a1 d2cqqa1 d2ajea1 d4a69c_ d1h8ac1 d2crga1
d2iw5b1 d1xc5a1 d1ofcx1 d2cu7a1 d1wgxa1 d2cjja1 d2ckxa1 d1gvda_
d1hcra_ d1tc3c_ d1gdta1 d1u78a2 d1hlva1 d1hlva2 d1iufa1 d1bl0a1
d1bl0a2 d1d5ya1 d3lv8a_ d1zaka1 d1s4qa_ d3tvta2 d1uf9a_ d1khta_
d1ckea_ d1osna_ d1p5zb_ d4tmka_ d3cm0a_ d1tmka_ d1jaga_ d3h86a_
d1t0hb_ d1nn5a_ d2h92a_ d3kb2a1 d2c95a1 d1yj5a2 d1kgda_ d1ly1a_
d2ak3a1 d1vyva2 d1deka_ d2grja1 d1p3ja1 d1y63a_ d1e4ya1 d1s96a_
d3iija1 d1lvga_ d1nksa_ d1vyua2 d4e22a_ d1qf9a_ d1jjva_ d1q3ta_
d1ki9a_ d1lw7a2 d1teva_ d1kjwa2 d1gsia_ d1x6va3 d1g3ma_ d1j99a_
d1t8ta1 d1nsta_ d1fmja_ d1zrha_ d1ls6a_ d2a3ra_ d2zptx_ d3ckla_
d1texa_ d1q44a_ d2gwha_ d1odfa_ d1uj2a_ d4gi7a_ d2jeoa1 d1z0ja1
d3umra2 d3gj0a1 d2gaoa1 d1yzga1 d1x3sa1 d1g7sa4 d1puja_ d1udxa2
d1kk1a3 d2o52a_ d1jwyb_ d1zcaa2 d1zcba2 d2fv8a1 d2cjwa_ d3dz8a1
d1xzpa2 d1vg8a_ d4qxaa_ d2ew1a1 d1x1ra1 d2bcgy_ d3doea1 d3cona_
d3rega_ d3wyga_ d1upta_ d2b6ha1 d1zd9a1 d1lnza2 d1r5ba3 d1n0ua2
d1r2qa_ d2f9la1 d2rhda_ d3q3jb_ d3q85a_ d1efma1 d1r8qa_ d2gf9a1
d2akab1 d1ek0a_ d1z08a1 d2gcoa1 d2dy1a2 d4g01b_ d2cxxa1 d1svia_
d3q72a_ d1ni3a1 d4ac9a4 d2erxa1 d2bmea_ d1wxqa1 d2gf0a1 d4ldja1
d1zunb3 d1d5ca_ d4lv5b_ d1efca3 d1kmqa_ d2g6ba1 d3lrpa_ d1tada2
d2y8ea_ d4rd4a1 d1z06a1 d2heia_ d3a58b_ d1u0la2 d1d2ea3 d1zj6a1
d2ocba_ d3rwoa_ d1mr3f_ d2e9sa1 d4u5xa1 d2iwra_ d2fn4a1 d3bwdd_
d2nzja1 d2atva1 d2ksqa_ d3l43a_ d1mkya2 d1mkya1 d1wmsa_ d1jnya3
d2rmka_ d1f6ba_ d1f60a3 d1gota2 d1jala1 d2fh5b1 d1g16a_ d2wblc_
d1m7ba_ d1wf3a1 d1f5na2 d1t9ha2 d1z6xa1 d1z0aa1 d2fg5a1 d2a5ja1
d2bcjq2 d1s0ua3 d1ky3a_ d4y0va_ d4lhwa_ d2qmea1 d2atxa1 d1egaa1
d1ryha_ d1moza_ d1nrjb_ d2erya1 d3bfka_ d1puia_ d1fzqa_ d3j3az1
d3t5ga_ d1shza2 d3defa_ d2f7sa1 d3raba_ d3tsoa_ d3aq4a_ d2al7a1
d3sfva1 d3bc1a_ d2ncda_ d1sdma_ d3h4sa1 d2heha_ d2grya_ g3kin.1
d1kk8a2 d1br2a2 d1goja_ d1f9va_ d2mysa2 d3t0qa_ d4db1a1 d3gbja_
d1ry6a_ d1v8ka_ d1s5ja2 d1qhta2 d4dlea2 d1tgoa2 d3py8a2 d1q8ia2
d1kfsa2 d2py5a2 d1d5aa2 d1k8ka2 d1k8ka1 d3uleb1 d3i33a2 d3i33a1
d1bupa1 d1dkgd2 d1dkgd1 d4ci6a2 d1jcfa1 d4a2ba2 d2fxua1 d1yaga1
d4cbua2 d1bupa2 d3vrda1 d3vrda2 d1fcdc2 d1fcdc1 d1h1oa2 d1h1oa1
d1m70a1 d1ig8a1 d1czan2 d1czan3 d1czan1 d1ig8a2 d1czan4 d1bg3a4
d1bg3a3 d1bg3a2 d1bg3a1 d1nira1 d1qksa1 d3h1jd1 d2d0oa3 d2d0oa2
d1nbwa2 d1hroa_ d4gedb_ d3zooa_ d1mz4a_ d1gu2a_ d1f1fa_ d1dvva_
d1c6ra_ d1c75a_ d1e29a_ d1gdva_ d1cnoa_ d1h32b_ d1co6a_ d2bh4x_

d2aiua_ d5cytr_ d1jdla_ d1i8oa_ d2zboa_ d3dmia_ d1ls9a_ d1wvec_
d2yk3a_ d3ph2b_ d1dw0a_ d5aura_ d1ql3a_ d1qn2a_ d1f1ca_ d4zzva_
d2xspa_ d1eg1a_ d4v20a_ d1ojja_ d4ipma1 d4csia_ d3pfza_ d3ovwa_
d2yoka_ d2nqya_ d1h4ga_ d1hixa_ d1oa2a_ d1olra_ d3b7ma_ d4h7ma1
d3wp3a_ d2vgda_ d1igoa_ d3zsea_ d2nlra_ d3wx5a_ d1oa3a_ d1pvxa_
d1m4wa_ d1f5ja_ d1oa4a_ d4ixla1 d2a6za1 d3a4ua_ d2a6va1 d3sita1
d1evsa_ d1cnt1_ d1pvhb_ d1z7ca_ d1huwa_ d1bgea_ d1rhga_ d1f6fa_
d1f45b_ d1i1rb_ d1axia_ d1etea_ d3tgxb_ d1scfa_ d2z3qa1 d3qb1a_
d1hula_ d1m48a_ d4nkqb_ d1eera_ d1v7mv_ d5e4ea_ d2o27a_ d3b5ka_
d2psma1 d4zf7a_ d3uf5a_ d4x51a_ d1d9ca_ d4ypgc1 d3oq3a_ d1lqsl_
d1au1a_ d1wu3i_ d3besl_ d3ux9a_ d3rbaa_ d2qtra_ d1nupa_ d3l92a_
d1f9aa_ d1qjca_ d2h29a_ d1vlha_ d1od6a_ d1lw7a1 d4ruka_ d2el7a_
d1iq0a2 d1irxa2 d1r6ta2 d1ivsa4 d2cyba_ d1rqga2 d1j09a2 d2yxna_
d1f7ua2 d1h3fa1 d2g36a1 d1nzja_ d1jila_ d1n3la_ d1i6la_ d1pfva2
d1h3na3 d3n9ia_ d3sz3a1 d3sw2a_ d1moxc_ d1dx5i3 d1autl2 d1autl1
d1nqlb_ d1dval1 d1szba2 d1kigl_ d2y5fl_ d1ijqa2 d1gk5a_ d1g1sa2
d1nt0a3 d1xdtr_ d1lmja2 d1lmja1 d1nzia2 d2wphe_ d1klil_ d1pfxl2
d1pfxl1 d1p9ja_ d1g1ta2 d1f7ea_ d4yzub_ d1kb0a1 d2mgpa1 d1b9wa1
d1b9wa2 d2mgpa2 d1ob1c1 d1ob1c2 d1n1ia2 d2npra2 d2npra1 d2y5ia_
d1qlsa_ d1psra_ d3n22a_ d2egda_ d4ggfc_ d2wcea_ d2luca_ d4aqia_
d3nsia_ d2lp3a_ d1j55a_ d1a4pa_ d1nsha_ d3fs7a_ d2pvba_ d1ttxa_
d1rwya_ d2kyfa_ d1rk9a_ d1a75a_ d1exra_ d1tiza1 d1nyaa_ d1g8ia_
d1ggwa_ d1wdcc_ d3fwba_ d1ggza_ d2o5ga_ d1ctaa_ d1m45a_ d3i5gc_
d1sl8a_ d1fpwa_ d2scpa_ d2roaa_ d4aqra_ d3pn7b_ d1br1b_ d2mysb_
d1s6ca_ d1bjfa_ d2obha_ d1jfka_ d1jf0a_ d1qv0a_ d1lkja_ d2mlfc_
d2m97a_ d2d8na_ d4by5a_ d1jbaa_ d4nqga_ d2mysc_ d1j7qa_ d1s6ia_
d1uhka2 d1r2ua_ d1dtla_ d3kf9a_ d3pn7c_ d5d67d_ d1tuza_ d1h8ba_
d1eg3a2 d2zkmx1 d1snla_ d1gjya_ d1y1xa_ d1dvia_ d1df0a1 d1qxpa2
d4usla_ d1k94a_ d1sfsa_ d1jfxa_ d1wkya2 d1uuqa_ d3wh9a_ d4m1ra_
d1xyza_ d4xuya_ d2je8a5 d4bf7a1 d3cuia_ d1ur4a_ d1qw9a2 d1odza_
d2vzsa5 d1bqca_ d2x2ya1 d1hjqa_ d1qnra_ d4y7ea1 d1vjza_ d4k68a_
d2v3ga_ d4hpga_ d1nofa2 d1nq6a_ d1h4pa_ d1aq0a_ d3ziza_ d4bs0a_
d1ecea_ d1h1na_ d1ta3b_ d1w91a2 d1i1wa_ d1us3a2 d1egza_ d7a3ha_
d1tvna1 d3nyda1 d1ghsa_ d2cyga1 d4xv0a_ d2c8na2 d1w32a_ d3emca_
d1v6ya1 d1foba_ d2uwfa1 d2fgla_ d3niya1 d2c0ha1 d1rh9a1 d1yq2a5
d1szna2 d1fa2a_ d1pama4 d5do8a1 d1ea9c3 d1hx0a2 d2fhfa5 d1qhoa4
d1avaa2 d1m53a2 d4m8ua1 d2gjpa2 d1lwha2 d4howa1 d1ktba2 d1j0ha3
d3vm5a1 d2x1ia_ d1wzaa2 d1uasa2 d2diea1 d1mxga2 d1ht6a2 d1eswa_
d1iv8a2 d5cpsa_ d1ud2a2 d1zjaa1 d3gbea1 d1kwga2 d1wdpa_ d1gcya2
d1g5aa2 d1hvxa2 d3dhpa2 d2bhua3 d4do4a1 d3qgva1 d1e43a2 d1g94a2
d2d3na2 d1gjwa2 d1gvia3 d1x1na1 d1r7aa2 d1tz7a1 d4toqa_ d1wb0a1
d1w9pa1 d1eoka_ d1itxa1 d1kfwa1 d1goia2 d1edqa2 d2gsja_ d1hjxa1
d1jnda1 d1vf8a1 d3wdpp1 d3wq5a_ d1gnxa_ d4gxpa_ d4hz8a_ d1e4mm_
d3aiua_ d1wcga1 d1qvba_ d3ahxa_ d1v02a_ d2dgaa_ d1pbga_ d1vffa1

d1qoxa_ d4ha4a_ d1mixa2 d1e5wa2 d1j19a2 d1isna2 d2al6b2 d3syxa1
d1mkea1 d2p8va_ d1xoda1 d1i7aa_ d1evha_ d1egxa_ d2y8ga_ d1xkea1
d1k5db_ d2y8fa_ d1b9ra_ d1offa_ d1frra_ d3wcqa_ d1e9ma_ d1l5pa_
d1wria_ d1i7ha_ d3av8a_ d1rfka_ d3p1ma1 d3b2ga_ d2bt6a2 d1wgqa1
d1v89a1 d2coda1 d1zc3b_ d1wg7a1 d1u5ea1 d1v61a1 d2coca1 d2ys1a1
d1v5pa1 d1ntya2 d1wjma1 d1wi1a1 d1u29a_ d1xx0a1 d1unqa_ d2zkmx3
d1fhoa_ d1v88a1 d2adza1 d1x1fa1 d1p6sa_ d2coaa1 d1btka_ d1v5ma1
d1faoa_ d2x18a_ d1dyna_ d1x1ga1 d4kaxb_ d2j59m1 d1ki1b2 d1xcga2
d1txda2 d1u5da1 d1dbha2 d1w1ha_ d1upqa_ d1v5ua1 d1foea2 d2fjla1
d1eaza_ d2dfka2 d2cofa1 d1kz7a2 d1r55a1 d3gboa_ d1atla_ d1kufa_
d1buda_ d4q1la_ d1quaa_ d1yp1a_ d1wnia_ d1ex0a4 d1g0da4 d2f4ma1
d1x3za_ d3u1ra1 d1g9ka2 d1kapp2 d1t3ca_ d2a97a_ d3fiea_ g1e1h.1
d3ioqa_ d1fh0a_ d1o0ea_ d1khqa_ d1m6da_ d2cb5a_ d1cs8a_ g8pch.1
d1s4va_ d2pnsa_ d1vsna1 d1mira_ d1iwda_ d2oula_ d7pcka_ d3bwka_
d3bcna_ d2p86a_ d1pvja_ d1pcia_ d1gece_ d1deua_ d4bs6a_ d1xkga1
d1cqda_ d1jqpa2 d2c0ya_ d2bdza_ d3p5ua_ d1zoya1 d1qo8a2 d1d4ca2
d1jnra2 d1kf6a2 d2jk6a2 d2jk6a1 d1feca1 d1feca2 d1lpfa1 d1lpfa2
d1xhca1 d1xhca2 d1dxla2 d1dxla1 d1gera2 d1m6ia2 d1onfa1 d1onfa2
d3lada2 d3lada1 d1w4xa1 d1w4xa2 d1mo9a1 d1xdia1 d1fl2a2 d1fl2a1
d1fcda1 d1fcda2 d1h6va2 d1h6va1 d1mo9a2 d1gv4a2 d1gv4a1 d2gv8a2
d2gv8a1 d1aoga1 d1aoga2 d1hyua1 d1ebda1 d1ebda2 d3dk9a1 d1q1ra2
d1q1ra1 d1gesa1 d1zbfa1 d2z1ha_ d2etja1 d3qioa1 d1jl1a_ d2e4la_
d1jl2a_ d2lsna1 d1io2a_ d1uaxa_ d2yv0x_ d1mu2a1 d1ekea_ d1i39a_
d1ps9a2 d1o94a2 d1cjca1 d1pn0a1 d1reoa1 d1ju2a1 d2f5va1 d3c96a1
d2gmha1 d1pj5a2 d1gpea1 d1o5wa1 d1n4wa1 d1k0ia1 d2dw4a2 d2ivda1
d1seza1 d2voua1 d1kdga1 d1s5ja1 d2hbja2 d2igia_ d1wlja_ d1qhta1
d4dlea1 d1tgoa1 d2f96a1 d3tr8a1 d3mxma_ d1q8ia1 d1y97a1 d1w0ha_
d1kfsa1 d2py5a1 d1d5aa1 d1yt3a3 d2guia_ d3mwpa1 d4kb1a_ d2v76a_
d1wj1a1 d1aqca_ d1qqga2 d1qqga1 d1ntva_ d1n3ha_ d4dbba_ d3f0wa1
d1p3ra_ d1wgua1 d2cy4a1 d3hqca_ d1wvha1 d1xr0b1 d2av4a1 d1jmxa4
d1jmxa3 d1pbya3 d1jmxa2 d1pbya1 d1pbya2 d1faaa_ d1wika1 d1xfla_
d1ttza1 d2f51a1 d1ti3a_ d1nhoa_ d1sena_ d2i4aa_ d1ep7a_ d1zmaa1
d1fo5a_ d3lefa1 d3diea1 d2yana1 d1z6na1 d1oaza_ d3zywa1 d4aj8a_
d1nm3a1 d4fiwa_ d1wmja_ d4hs1a1 d1iloa_ d1xwaa1 d1wjka1 d2vm1a_
d1fb6a_ d1gh2a_ d1h75a_ d1nw2a_ d1kx5b_ d3a6na_ d4m6ba2 d2nqbd_
d3wkjd_ d2yfwe_ d4h9na_ d5bnxa_ d1kx5c_ d3r45a_ d1eqzb_ d4jjnb_
d1f66c_ d1eqza_ d4wnnb_ d1kx5a_ d1u35c1 d1id3a_ d2nqbc_ d1kx5d_
d1ku5a_ d1f1ea_ d1b67a_ d2b5ea2 d2b5ea3 d2b5ea1 d2b5ea4 d2kp1a1
d2kp2a1 d1hyua3 d1hyua4 d1j08a1 d1j08a2 d1pd212 d1nhya2 d2a2ra2
d1k0dd2 d1f2ea2 d1gsua2 d1f3ba2 d2fhea2 d2pvqa1 d1tw9a2 d1oyja2
d1e6ba2 d1k0ma2 d3ik7a1 d4is0a1 d1r5aa2 d1jlva2 d1m0ua2 d3gtub2
d1z9ha2 d1okta2 d1axda2 d2gsra2 d1tu7a2 d1k3ya2 d1zgma1 d1gnwa2
d1gwca2 d2gsta2 d1glqa2 d1oe8a2 d1pn9a2 d1v2aa2 d1fw1a2 d1duga2
d4ilya_ d4olta_ d1qgia_ d1gd6a_ d1f6ra_ d1ivma_ d2gv0a_ d1iiza_

d2z2fa_ d1j8xa_ d1ghla_ d1b9oa_ d174la_ d169la_ d2o4wa_ d1p37a_
d1k28a3 d1xjta1 d1jtma_ d2f2qa_ d4e97a1 d1g4ma1 d1a3qa1 d1iknc_
d3brda1 d1p7hl1 d1oy3c_ d1gjia1 d1zk9a1 d2cxka1 d2za0a_ d2c21a1
d2pjsa1 d1klla_ d2i7ra1 d1ecsa_ d1xqaa1 d5cj3a1 d1npba_ d1jifa_
d2p7oa_ d1r9ca_ d1nkia_ d1mpya2 d1kw3b2 d1kw3b1 d4ghga1 d1tfza1
d3isqa1 d3isqa2 d1sqia1 d1sqia2 d1lkda2 d1lkda1 d1mpya1 d1cjxa2
d1sp9b2 d1t47a1 d1t47a2 d1f1ua2 d1sp8a1 d1sp8a2 d1f1ua1 d1ro5a1
d1pv8a_ d1w5qa_ d2c1ha_ d1l6sa_ d1qsta_ d2beia1 d4m3sa_ d1u6ma1
d1ygha_ d1yk3a1 d3dr6a_ d1xmta_ d1z4ea1 d2ge3a1 d1bo4a_ d2fiaa1
d2jdca1 d1y9wa1 d3to7a_ d1wwza1 d2fl4a1 d2ozua1 d2b5ga_ d2cy2a1
d2p0wa_ d1cm0a_ d4pswa_ d1yrea1 d1yr0a1 d1m4ia_ d2gana1 d1yvka1
d1ghea_ d2g3aa1 d1y7ra1 d2euia1 d1q2ya_ d2fsra1 d1xeba_ d2ae6a1
d1cjwa_ d1qsma_ d2aj6a1 d1y9ka1 d1i12a_ d1vkca_ d2atra1 d1n71a_
d2d4pa1 d2fiwa1 d2fe7a1 d1tiqa1 d2ou2a_ d1yx0a1 d2fcka1 d1p0ha_
d2j8ma_ d2pq8a_ d1z4ra1 d1s3za1 d1ufha_ d1xvqa1 d1pn0a2 d3hy2a_
d2cvba1 d1o8xa_ d1lu4a_ d1we0a1 d2bmxa1 d2a4va1 d1xiya1 d1q98a_
d1n8ja_ d1zofa1 d2ggta_ d3tjja_ d4je1a1 d1o73a_ d3s9fa_ d2zcta_
d1qmva_ d1jfua_ d2b1ka_ d5dvba1 d2b5xa1 d2v2ga_ d1st9a_ d3tb2a_
d4llra_ d4fh8a1 d1e2ya_ d1knga_ d2h30a_ d1i5ga_ d2z9sa_ d2f8aa1
d1tp9a1 d2b7ka_ d1nm3a2 d3hvva_ d2wfca1 d4kcea_ d1xvwa1 d4gqca1
d3qpma_ d4k1fa_ d1zzoa1 d1gp1a_ d1prxa_ d4od7a1 d1z6ma1 d4mcua1
d4dvca1 d3l9sa_ d1un2a_ d3rppa_ d1rmka_ d1ixta_ d1eyoa_ d1cnna_
d1g1pa_ d1f3ka_ d1fyga_ d2m5ta_ d1cqqa_ d1wqsa_ d5bpea_ d2ipha_
d2bhga1 d3tnta_ d3sjia_ d3d23a1 d2fyqa_ d3tloa_ d3zvea1 d2hrva_
d3q3ya1 d4asha1 d2phla2 d2phla1 d1fxza2 d1fxza1 d1lrha_ d1uija1
g1dgw.1 d2v09a_ d1uika2 d1uika1 d1dgwa_ d1e88a1 d1l6ja5 d1l6ja4
d1eaka4 d1eaka3 d1l6ja3 d1h8pa1 d1etha2 d1w52x1 d2ppla1 d1hpla2
d1lpbb2 d2oxea1 d1bu8a2 d1rp1a2 d1lgya_ d4l3wa_ d1llfa_ d1gz7a_
d1ukca_ d3groa_ d1pjaa_ d1thta_ d3ia2a_ d1a88a_ d4dgqa1 d1zoia_
d3zxha_ d1cgla_ d1rm8a_ d1eaka2 d1hy7a_ d2jsda_ d1cxva_ d1hv5a_
d4dv8a2 d1j7na1 d1dzka_ d4tlja_ d2a2ua_ d4wfva_ d3t1da_ d4iaxa_
d3zq3a_ d2nnda_ d1i4ua_ d1kxoa_ d1bbpa_ d1kt7a_ d1exsa_ d3s26a1
d1lf7a_ d1qfta_ d4gh7a_ d1z24a1 d1qabe_ d1xkia_ d1ew3a_ d3bx7a_
d4iawa1 d1n0sa_ d1gkab_ d3kzaa_ d2k23a_ d2l9ca1 d2joza1 d1gm6a_
d3kffa_ d3saoa1 d1e5pa_ d1yupa1 d1iiua_ d1lmra_ d1cixa_ d1y29a_
d1v7fa_ d2jtba_ d1d1ha_ d1g9pa_ d1lupa_ d1qk7a_ d1mb6a_ d2a2va_
d1niya_ d1i25a_ d1hvwa_ d2mpqa_ d1ie6a_ d1kqha_ d1la4a_ d2mxoa1
d1koza_ d1qk6a_ d1emxa_ d1lqra_ d1c6wa_ d1js9c_ d1f15b_ d1za7a_
d1laja_ d1rypd_ d1irua_ d1rypc_ d3unfk_ d1rypa_ d1j2pa_ d1irui_
d1j2qh_ d3unfh_ d1m4ya_ d1rypb_ d1irul_ d1irug_ d1iru2_ d1q5rh_
d1rypg_ d1g3ka_ d1iruf_ d1q5qa_ d1e94a_ d1irub_ d1ryph_ d3ty6a_
d1iruj_ d1iruk_ d1rype_ d1irud_ d1irue_ d3mi01_ d1rypf_ d1iruh_
d2z3ba_ d3mka2_ d1iru1_ d1mqta_ d1d4m3_ d1fmd3_ d2mev1_ g1aym.1
d1ev13_ d4q4w1_ d1r1a3_ d2x5ia_ d1rhi1_ g1r1a.1 g1d4m.1 g1rhi.1

d1b35b_ d1oopa_ d1tme1_ d4iv1a_ g1pvc.1 g1fpn.1 d1fpn1_ d4gb31_
d1d4m1_ d1fmd1_ d1ev11_ d2wzr3_ g1cov.1 d1qqp3_ g1fmd.1 d2x5ic_
d1rhi3_ g1oop.1 d1hxs1_ g1hxs.1 d1fpn3_ d1bev1_ g2mev.1 d1eah3_
d1aym1_ d1pvc3_ d1qqp1_ d1zba3_ d1h8ta_ d3vbhc_ g1b35.1 g1bev.1
d1hxs3_ g1ev1.1 d1bev3_ g1tmf.1 d1tmf1_ d1b35a_ d2mev3_ g1eah.1
g1h8t.1 d1eah1_ d4q4w3_ d1r1a1_ d1pvc1_ d1zba1_ d1h8tc_ d1oopc_
d1tme3_ g1qqp.1 d4iv1c_ g1tme.1 d1y14b1 d2nn6i1 d1i94l_ d2zjra2
d1smxa_ d2qamc2 d1hh2p1 d2r7da1 d1rl2a2 d1wfqa1 d3bzka4 d1x6oa2
d3a0ja_ d2j01d2 d2ja9a1 d4ql5a_ d1hr0w_ d1luza_ d2n3sa_ d2vnud1
d1h95a_ d1go3e1 d2nn6h1 d2mo1a_ d1iz6a2 d1jjga_ d1e3pa2 d2ix0a3
d2ix0a2 d2lxja_ d1ueba3 d1ueba2 d2z0sa1 d1u0la1 d2ix0a1 d2eifa2
d2je6i1 d3i2za1 d2c35b1 d2ahob2 d1t9ha1 d3i4oa_ d2qall1 d1jt8a_
d2nn6g1 d1g6pa_ d3cama_ d1i94q_ d1c9oa_ d1d7qa_ d2ba0a1 d2vnud2
d2vnud3 d1khia2 d2qalq1 d1wi5a1 d1xtda2 d1ny4a_ d1eixa_ d3exra_
d2ffca1 d2fdsa1 d3n3ma1 d2czda_ d2yyua_ d1vqta1 d1dbta_ d3ldva1
d1ux8a_ d4i0va_ d2bkma_ d1dlwa_ d2bmma_ d2r80b_ d1cqxa1 d1cg5a_
d1gcvb_ d3boma_ d2h8fb_ d2raoa_ d3bj1a_ d4hrrb_ d2r50a_ d3d1kb_
d2aa1b1 d3zhwa_ d3hf4b_ d3gdja_ d1jebb_ d3bj1b_ d1x9fb_ d1wmub_
d1gcva_ d3bcqb_ d3d4xb_ d1gvha1 d2dc3a_ d3hf4a_ d4yu3a_ d3k8ba_
d1qpwb_ d2r80a_ d3bcqa_ d1v4wa_ d3wtgb_ d1jl7a_ d4esaa_ d1a9we_
d3d1ab_ d1fsla_ d4h2la_ d1v4wb_ d1mwca_ d1vhba_ d1jeba_ d1spgb_
d1h97a_ d1q1fa_ d1tu9a1 d3d1aa_ d1hdsb_ d3hyub_ d2zfbb_ d3fs4a_
d1oj6a_ d1shrb_ d4h2lb_ d4yu3b_ d1spga_ d3hrwa_ d3qqra_ d3at5a_
d4wchd_ d3hyua_ d1cg5b_ d1uc3a_ d1hbra_ d2raob_ d1outb_ d4esab_
d3gdjb_ d3qqqa_ d1x9fc_ d3vrfa_ d1itha_ d3mkba_ d3d4xa_ d3mjpa_
d1jl6a_ d3fh9a_ d1outa_ d2dc3b_ d1it2a_ d3eoka_ d3g46a_ d1qpwa_
d3vrfb_ d1ch4a_ d1x9fd_ d1hdsa_ d2zfba_ d3mkbb_ d1or4a_ d3wtga_
d1a4fa_ d3fh9b_ d3bomb_ d3cy5a_ d3at5b_ d1x9fa_ d2oifa_ d4lmxb_
d1cpcb_ d3v57a_ d1kn1a_ d1cpca_ d4f0ta_ d2vjha_ d4po5b_ d1eyxa_
d1alla_ d4f0tb_ d1b33b_ d1b8db_ d4h0mb_ d1b33a_ d2vjhb_ d4lm6b_
d2vmlb_ d1allb_ d2vjta_ d3dbja_ d2vmla_ d2vjtb_ d4f0ub_ d1f99b_
d4rmpa1 d1liaa_ d1eyxb_ d2uulb_ d4lmsb_ d4f0ua_ d2bv8a_ d1f99a_
d2bv8b_ d2c7lb_ d3dbjb_ d4rmpb1 d1b8da_ d4h0ma_ d3v57b_ d1liab_
d2uula_ d2j96a_ d1mtza_ d2xqfa_ d1ea5a_ d1f6wa_ d1dx4a_ d1qe3a_
d1k4ya_ d4qwwa_ d2ha2a_ d1gl9b1 d1gkub1 d2qvba_ d2v9za_ d4kafa1
d4iuaa3 d4bvva_ d5hpga_ d3k65a_ d1ki0a3 d1ki0a2 d1ki0a1 d1pmla_
d3sp8a3 d2feba1 d1f74a_ d3l21a_ d2e1da_ d1to3a1 d1w3ia_ d1sfla_
d1n7ka_ d1vcva1 d4imea_ d1wa3a_ d2ocza1 d1f2ja_ d3m16a_ d2yxga_
d1ub3a_ d3a5fa_ d1fbaa_ d1l6wa_ d1xfba1 d1wx0a1 d1vpxa1 d1ojxa_
d1mxsa_ d2a4aa1 d1xkya1 d3cq0a1 d3s1xa_ d1f05a_ d1fdja_ d3cpra1
d4d2ja_ d1cg2a1 d1yloa2 d1y7ea2 d1y0ya3 d1lfwa1 d1vixa1 d1fnoa4
d1vhoa2 d1ysja1 d1vhea2 d1z2la1 d2fvga2 d1vgya1 d1r3na1 d2grea2
d1xmba1 d1rtqa_ d3rp2a_ g1ucy.1 d1fuja_ d2zchp_ d3w94a_ d1si5h_
d1lo6a_ g2pka.1 d1cgha_ d1kigh_ d1a5ia_ d3s9ba_ d1hj8a_ d1ao5a_

d2anya_ d1q3xa1 d1gvza_ d1azza_ d4gsoa_ d2f91a1 d1iaua_ d1gdna_
d1eufa_ g1fiw.1 d1eq9a_ d4nfea_ d2rdla_ d1spja_ d1elva1 d1a7sa_
d1ekbb_ d1pfxc_ d1yc0a_ d2aiqa_ g1fiz.1 d1fi8a_ d4k60a_ d4dgja_
d1mzaa_ d1op0a_ d3tjva_ d1pytd_ d1sgfg_ g1gg6.1 d2eeka_ g1rtf.1
d1a0ja_ d3g01a_ d1pytc_ d4e7na_ d1ddja_ d1z8ga1 d2hlca_ d1npma_
d1brup_ g1jou.1 d1sgfa_ d1autc_ g2hnt.1 g1kdq.1 d1orfa_ d3s69a_
d4crga_ d1fxya_ d1ra6a_ d1muka_ d3gsza_ d1te9a_ d2xwha_ d3uqsa_
d3hkwa_ d3n6ma_ d2cjqa_ d2xyma_ d1teba_ d1te8a_ d1s48a_ d1u09a1
d4wfxa_ d3tyqa_ d2pgga1 d1m4la_ d2bk7a_ d3fjua_ d1z5ra1 d1kwma1
d1uwya2 d1jqga1 d2c1ca1 d1dtda_ d3lgia_ d3i77a_ d2z9ia2 d4nsva_
d1lcya2 d4jcna_ d1agja_ d1os8a_ d1hpga_ d1p3ca_ d1l1ja_ d1e69a_
d1mv5a_ d3g60a4 g1ii8.1 d1ewqa2 d1ji0a_ d1qhla_ d1v43a3 d2wmfa1
d1vpla_ d1g2912 d4a82a2 g1f2t.1 d2d62a1 d3tuic1 d2bbsa1 d3g60a3
d2ixea_ d1g6ha_ d1sgwa1 d2qi9c_ d3rlfa1 d3b60a1 d3d31a2 d2pzea1
g1xew.1 d1jj7a_ d1oxxk2 d2onka1 d1w1wa_ d1iwea_ d1qf5a_ d1vmaa2
d1nija1 d1np6a_ d2p67a1 d3ihla_ d1okkd2 d1ls1a2 d1s1ma2 d1iona_
d2v40a_ d3hida_ d2vo1a1 d2g0ta1 d1vcoa2 d1j8mf2 d1g3qa_ d1xjca_
d1p9ba_ d1qzxa3 d1ihua2 d1ihua1 d2qm8a_ d1dj2a_ d4ioja_ d1cp2a_
d1yrba2 d1hyqa_ d3gqba2 d1xp8a1 d1tf7a2 d1tf7a1 d1ye8a1 d1skyb3
d2qe7d2 d4blta_ d4huta_ d3ldaa2 d4hyya_ d2i3ba1 d1cr1a_ d3j9tb2
d3ntua2 d1vdza1 d2z43a_ d1e9ra_ d1nlfa_ d3ziaa2 d1n0wa_ d1g6oa_
d3ziad2 d3j9ta2 d1pzna2 d3gqbb2 d2i49a_ d1nnfa_ d1xvxa_ d1ursa_
d1h3da1 d1iuda_ d4m1va1 d1twya_ d3e13x_ d2nxoa1 d4pfua_ d3ttma_
d1elja_ d2qmwa1 d1us5a_ d1y3na1 d1jeta_ d1uqwa_ d4f19a1 d2esna2
d3thia_ d3fasa1 d2uyeb_ d2xwva1 d1r9la_ d2fyia1 d1pb7a_ d2ozza1
d1sw5a_ d1nh8a1 d1vr5a1 d2g29a_ d1xs5a_ d3lswa_ d2z23a_ d3w9va_
d3tcha_ d1q35a_ d1z7me1 d3q27a_ d1p99a_ d1ve4a1 d2i6ea1 d4mcoa1
d4n8ya_ d1eu8a_ d1zbma1 d1hsla_ d1ixca2 d1j1na_ d1xvya_ d1y9ua_
d2z22a1 d1xoca1 d4oera_ d1wdna_ d5f1qa1 d1xt8a1 d4gwoa_ d4igta1
d1pc3a_ d4mmpa1 d1o63a_ d2czla1 d2onsa2 d4mija1 d4ok5a1 d2bmfa1
d2bmfa2 d1yksa1 d1yksa2 d2qeqa1 d2jlqa1 d1a1va2 d1eh3a_ d3skpa_
d1dtza1 d1ieja_ d1jnfa2 d1ce2a1 d1jnfa1 d1b1xa2 d1b1xa1 d1jw1a1
d1jw1a2 d1ryoa_ d1h76a1 d1h76a2 d1c4oa1 d2p6ra3 d2p6ra4 d1oywa2
d1oywa3 d1wrba1 d1w36b2 d1w36b1 d1c4oa2 d3bora_ d1t6na_ d2eyqa5
d2eyqa3 d2eyqa2 d2zu6a2 d1veca_ d1uaaa1 d1w36d1 d2g9na1 d1z3ix2
d2rb4a_ d1hv8a1 d1hv8a2 d1t5ia_ d1w36c2 d2j0sa2 d2j0sa1 d1fuka_
d3hjha1 d1z5za1 d1qdea_ d1z63a1 d1s2ma2 d1s2ma1 d1wp9a1 d1wp9a2
d1gm5a3 d1gm5a4 d3dina4 d3dina3 d1q0ua_ d1w36c1 d1z3ix1 d1nkta4
d1uaaa2 d1w36d2 g1qhh.1 d2fwra2 d2fwra1 d3q3ma_ d1za0a1 d1mxra_
d2inca_ d1jk0a_ d2incb_ d1mhyb_ d3hf1b_ d1mtyb_ d1r2fa_ d2uw1a_
d1mhyd_ d3n37a_ d1afra_ d1oquc_ d1syya_ d1mtyd_ d2o1za_ d1uzra_
d1smsa_ d2fkza_ d1zuja1 d1s3qa1 d2fjca1 d1z6om1 d1veia_ d4dyya_
d1jiga_ d3gvya_ d2pyba_ d4cy9a1 d3bvfa1 d1r03a_ d2cf7a_ d1nf4a_
d1zs3a1 d4iwka_ d3ka8a_ d3np0x_ d1n1qa_ d1lb3a_ d4e6ka_ d1z6oa1

d2fzfa1 d4dyua_ d3ge4a_ d1jgca_ d4am4a_ d2z6ma_ d1ji5a_ d1vlga_
d1lkoa1 d3qz3a_ d1o9ra_ d2ffxj_ d1euma_ d1yv1a1 d2v2pa_ d1j30a_
d4di0a_ d1dpsa_ d1oewa_ d2qzxa_ d2asia_ d1psoe_ d1smra_ d2h6ta_
d1pfza_ d1izda_ d3hd8a_ d1j71a_ d1dpja_ d4aa9a_ d1t6ex_ g1b5f.1
g1lya.1 g1htr.1 d1qdma2 d1bxoa_ d1wkra_ d1tzsa_ d1ibqa_ d2qzwa_
d1bdqa_ d3ggta_ d3ixoa_ d1mrxa_ d2haha_ d1tw7a_ d4njsa_ d1sgua_
d2p3da_ d1nsoa_ d3d3ta_ d2rkfa_ d2rspa_ d3ogpa_ d3ekwa_ d2o4na_
d2j9ja_ d1baia_ d3ggua_ d3lzua_ d4npua_ d4j55a_ d2r8na_ d2fxda_
d2p3ca_ d4dqea_ d3ttpa_ d3t3ca_ d2p3aa_ d2hc0a_ d2fdda_ d3mwsa_
d1wgda1 d1wgga1 d4q5eb_ d1wjna1 d1se9a_ d3n3kb_ d1wz0a1 d1we6a1
d2m0xa_ d2gbka_ d3rt3b1 d1wh3a1 d1sifa_ d2ekec2 d4ii2b1 d4hk2a1
d1c3ta_ d1m94a_ d1wgha1 d1ttna1 d1j8ca_ d1wx8a1 d3v6cb1 d2ma9b_
d4i6lb1 d1bt0a_ d2jvca1 d1iyfa_ d2k1fa_ d1wxva1 d1wf9a1 d1t0ya_
d2io0b2 d1x1ma1 d2gbma_ d1zw7a1 d2m17a_ d1we7a1 d2lwpa_ d2gbja_
d1wjua1 d1v6ea1 d1v86a1 d4eewa_ d1wiaa1 d4icva_ d4gu2a_ d1wx7a1
d1wy8a1 d4hjka1 d2faza1 d1v5oa1 d2al3a1 d2cr5a1 d1wj4a1 d1s3sg_
d3ubra_ d1qo8a1 d1d4ca1 d2k3va_ d1ft5a_ d1qdba_ d1m1qa_ d2j7aa_
d1h21a_ d1ogyb_ d4fasa_ d1jnia_ d1oaha_ d1sp3a_ d1yvca1 d1yeza1
d1qoza_ d5ajha1 d4lu0a_ d1of8a_ d3unda1 d3e9aa1 d1vr6a2 d2qkfa_
d1d9ea_ d1o60a_ d2h0ba_ d2erfa_ d1h30a2 d1h30a1 d3mw4a1 d2r1ba_
d3v64a1 d1d2sa_ d3bopa1 d3mw3a1 d1okqa1 d1okqa2 d1iyka2 d1rxta1
d1iica2 d1iica1 d1dszb_ d3cbba_ d1ynwa1 d1r4ia_ d1hcqa_ d2hana_
d2hanb_ d2enva1 d1kb4b_ d2nllb_ d1lo1a_ d1cita_ d1um8a_ d1ixza_
d1qvra2 d1qvra3 d1qzma_ d1sxjc2 d1jr3d2 d2p65a_ d1l8qa2 d1g8pa_
d3hu3a3 d1sxjd2 d1njfa_ d1in4a2 d1sxja2 d1tuea1 d2ce7a2 d1w5sa2
d1sxjb2 d1d2na_ d1ny5a2 d1ofha_ d2gnoa2 d2a5yb3 d1fnna2 d1u0ja1
d1lv7a_ d1iqpa2 d2fnaa2 d1ixsb2 d4gita_ d1sxje2 d1g41a_ d1svpa_
d3su6a1 d1cu1a1 d4agka_ d1vcpa_ d1q31a_ g1n1l.1 d3e90b_ d1ep5a_
d3u1jb_ d2e3fa_ d1mm0a_ d2e3ea_ d2llda_ d2ny8x_ d2e3ga_ d2ny9x_
d2nz3a_ d2lj7a_ d1n4na_ d2m8bb1 d2kyqa_ d3psma_ d1jkza_ d1jxca_
d2gl1a_ d1ugla_ d4uj0a_ d2k4ua_ d1hp2a_ d2lu9a_ d1baha_ d2meoa1
d1wm7a_ d1hlya_ d1n8ma_ d1jlza_ d1wpda_ d1c55a_ d1pvza_ d1wt7a_
d1wz5a_ d1m2sa_ d1pjva_ d1c49a_ d1qkya_ d2ktca1 d1wmta_ d1du9a_
d1j5ja_ d1r1ga_ d1t0za_ d2i61a_ d1t1ta_ d1sn4a_ d1kv0a_ d2lkba_
d1npia_ d1wwna_ d1pe4a_ d2kbka_ d2li7a_ d1djta_ d2kbha_ d1dq7a_
d1sega_ d1omya_ d1zywa1 d1jzaa_ d1n9wa1 d1bbua1 d1e1oa1 d1b8aa1
d1l0wa1 d3a74a1 d1eova1 d1iyjb4 d1iyjb5 d1iyjb3 d1o7ia_ d4ipca1
d1x3ea_ d4apva_ d1jb7a1 d1jb7a2 d1jb7a3 d3kdfa_ d1ue1b_ d1wjja1
d3eiva_ d1xjva2 d1xjva1 d2ccza2 d1se8a_ d1l1oc_ d1jb7b_ d1qvca_
d1qzga_ d1miua4 d1miua5 d1miua3 d3ubub_ d1egia_ d1qo3c_ d1v7pa_
d1tdqb_ d3ubua_ d1c3ab_ d1h8ua_ d3bx4b_ d1t8ca1 d1sb2a1 d1qdda_
d2ziba1 d3cada_ d1j34b_ d1jwia_ d1jzna_ d1c3aa_ d1oz7b_ d1wmza_
d3bdwb_ d1hq8a_ d1fifa1 d1dv8a_ d3hupa_ d1wk1a1 d1umra_ d2afpa_
d3bx4a_ d1gz2a_ d1sl6a_ d1v7pb_ d3g8ka_ d1ixxa_ d1j34a_ d1jwib_

d1g1sa1 d1y17a1 d1ja3a_ d1fvub_ d3bdwa_ d1sb2b1 d1fvua_ d1umrc_
d1oz7a_ d4m17a_ d3cfwa1 d1ypqa1 d1g1ta1 d3fd4a_ d3l9jc_ d4dn8a1
d1v4lb_ d1rdl1_ d2msba_ d1prtc2 d1prtb2 d1ohea2 d2f4da_ d1rxda_
d2hxpa_ d1xria_ d1d5ra2 d1fpza_ d3ezza_ d4y2ea_ d1i9sa_ d3azya_
d3wvja_ d1upsa1 d1ajoa_ d2hyka_ d1ajka_ d2vy0a_ d1o4ya_ d3o5sa_
d3d6ea_ d3atga_ d1dypa_ d3i4ia_ d1tdha3 d1q3ba3 d1ee8a3 d1k82a3
g1qmo.1 g1len.1 d3zyra_ d1fnya_ d1n47a_ g2ltn.1 d1avba_ d3usua_
d1dhkb_ d1f9ka_ d1ukga_ d1g8wa_ d1g9fa_ d1wbfa_ d3wcsa_ d1g7ya_
g1loe.1 d1mvqa_ d1hqla_ d1v6ia_ d1dbna_ d1fx5a_ d4l8qa_ d1ioaa_
d3n35a_ d1epwa1 d3h33a_ d1rwja_ d19hca_ d1os6a_ d3bxua_ d3h4na_
d1i77a_ d1j0pa_ d2ctha_ d1duwa_ d1ofwa_ d2e84a_ d1hh5a_ d1gyoa_
d3h34a_ d1wioa4 d1ccza2 d1vcaa1 d1dr9a2 d1ic1a1 d2d9qb1 d2nxyb2
d2dara2 d2dara1 d1wyha2 d2dloa2 d1rutx4 d1rutx3 d2cuqa2 d1x4la2
d1x4la1 d1x3ha2 d1x3ha1 d2d8za1 d1x61a2 d1x6aa1 d1x6aa2 d2cora2
d2cora1 d1v6ga1 d1v6ga2 d2d8za2 d1x64a1 d1x64a2 d1b8ta1 d2co8a1
d1x63a2 d2dj7a2 d2dj7a1 d2cu8a2 d2cu8a1 d1g47a2 d1g47a1 d1j2oa1
d1wyha1 d2dloa1 d1wiga1 d1wiga2 d2cupa2 d2cupa3 d1x68a1 d2d8ya2
d2d8ya1 d1m3va2 d1x62a1 d1x68a2 d1x61a1 d1b8ta3 d1b8ta2 d2d8xa2
d2d8xa1 d1x4ka1 d1x4ka2 d2cuqa1 d1m3va1 d2co8a2 d1j2oa2 d1x63a1
d2cura1 d2cura2 d1x62a2 d1op9a_ d1y0la1 d1etzb1 d1egjl1 d2gsib1
d1nj9a1 d4jfhd1 d1aqkl1 d1t2ja_ d1lo4h1 d1zvya1 d3v4vl1 d4enec1
d1kxtb_ d5e8ea1 d2pxya_ d4weud_ d1xiwc_ d2esvd1 d1qnzh_ d1ogae1
d1f4xh1 d4n1hb_ d3ezjb1 d3mlwl1 d1kfah1 d1etza1 d1b4jh1 d3cfic_
d4w68a_ d2arja1 d1ek3a_ d2ok0l1 d1vera_ d1uwgh1 d1blnb1 d1fpth1
d1rzga1 d2giya1 d4m3kb1 d1nc2a1 d2nyyd1 d1ncna_ d1fbih1 d5dxwa_
d4fqqa1 d1dqdh1 d1jfql1 d1kelh1 d1faih1 d1frgl1 d1figl1 d4m6nl1
d1fo0a_ d4hxbl1 d3b5ga_ d3quyd1 d2h9gb1 d1i8kb_ d2dd8h1 d2ck0h1
d1eaja_ d4w6wb1 d2a6ib3 d1t66d1 d2e27h_ d1ob1b1 d1f11b1 d2v7na1
d1ad0a1 d2xt1b1 d1pskh1 d4i13b1 d2op4l1 d1a0ql1 d1uwel1 d1za3a1
d1ncbl1 d4uu9a_ d1bm3h1 d1cloh1 d1nqba2 d1cf8h1 d4y5va_ d1mhph1
d5anma1 d4cdgc1 d4ldeb_ d1y0lb1 d2pcpb1 d1z3gl1 d1dfbl1 d1fh5h1
d3g5xa1 d1tzib1 d4dn3l1 d3zhka1 d2b1hh1 d2uyla1 d1f3dh1 d1nj9b1
d2agjh1 d2h1pl1 d1q72h1 d8fabb1 d1ndmb1 d1rjca1 d4u6hb1 d3upca_
d2fbjl1 d1osph1 d1tvda_ d2cjvh_ d4fhbd1 d1bd2d1 d1rz7h1 d3nn8a_
d1blna1 d1knoa1 d1nfda1 d2bmkb1 d1dqll_ d1igyb1 d1igfl1 d12e8h1
d3utza1 d1nc2b1 d1rihh1 d4mwfb1 d4jvpa1 d1mieh1 d1rhfa1 d1fgnl1
d1sbsh1 d1lp9e1 d1dn0a1 d4lqfl1 d1iaih1 d1kgcd1 d1mh5b1 d1gpol1
d2g60h3 d1f8tl1 d2jixd1 d4grwh_ d1fo0b_ d1bfob1 d1axsa1 d1u9ka_
d1nmbh_ d1h8na1 d1h8na2 d1jguh1 d4eura1 d3sdyl1 d35c8h1 d1w72l1
d1mvub_ d1i9rh1 d1ob1a1 d4wenb_ d1yeeh1 d3ietc1 d1f58h1 d1ad0b1
d1mqkl_ d1nbvh1 d4b50a_ d3r8bb_ d1bafl1 d1mamh1 d4rgne1 d2gsgb_
d35c8l1 d1bafh1 d1ai1h1 d2pcpa1 d2fath1 d2x1pa_ d1orsb1 d1ibgl1
d1shma_ d1a2yb_ d1u0qa_ d2coqa_ d1hilb1 d1qfwi_ d1jv5b_ d1fn4b1
d5e0qa_ d1nsnh1 d1plgl1 d4ocxl1 d1kb5h1 d1rzfh1 d1pg7x1 d2fx7h1

d2oi9c_ d1l7il1 d3eyva1 d1d5il1 d4ffyl_ d1tetl1 d1a6wh_ d1ac6a_
d3dvfa_ d1nfde1 d1mimh1 d1ri8a1 d1jhlh_ d3dura_ d1fskb1 d1f97a1
d3so3b1 d2ak4d1 d1lp9f1 d4z7wf1 d1l7th1 d4y5ya_ d4x7ec1 d1emth1
d1ikfl1 d1nlbh1 d4utal1 d1bjma1 d1eapb1 d1u6ah1 d2dtgd1 d1kj2b_
d1xeda1 d1c1eh1 d1fgvh_ d2bnub1 d2fb4h1 d2dtgb1 d1ktrh_ d1jpth1
d1qkzl1 d4hepg_ d2atpb1 d1flrh1 d2hrpl1 d1mvfa_ d1iaim1 d3k80a1
d2jb5h1 d3zkxc_ d4grwe_ d1it9h1 d1rhhb1 d3w9ec_ d1e4wh1 d1sy6h1
d2j6eh1 d3qxta_ d1jglh1 d1p7ka1 d1tzhb1 d4n8pa1 d1gafl1 d2jelh1
d1wioa2 d4i0cc1 d1cicb1 d1lmka2 d1lmka1 d1iqwh1 d1orqb1 d1ejoh1
d1jnlh1 d4zg1a_ d43c9b_ d1ggbl1 d1wejh1 d3q6fa1 d1f3dj1 d3c6la1
d1bqlh1 d3p9wb_ d1a3rl1 d1h0db1 d2uzil_ d1eo8l1 d1qfwl_ d1n7ml1
d1igja1 d1c5ch1 d2a9mh_ d1rmfh1 d1fj1b1 d4heme_ d1ccza1 d1a5fh1
d1ogad1 d1dlfh_ d1qa9b_ d1vgel1 d3qskb_ d1sjxa_ d4ozfg1 d1sq2n_
d1qd0a_ d1kcvh1 d1c5db1 d3qcul1 d8faba1 d3bn9d1 d1mjuh1 d4y8dc_
d7fabh1 d3dvgb1 d1lo0l1 d1a6tb1 d1gigh1 d1kcrh1 d2hwzl1 d1yjdc1
d3clfl1 d4krna_ d1dl7l_ d1e6ol1 d1mexh1 d3r0ma_ d4kkna_ d1fnsl1
d1oaqh_ d1rzia1 d1ucbh1 d1ncwl1 d4b41a_ d1nfdf1 d2mpah1 d2d7tl1
d4grwf_ d4b5ea_ d1lk3h1 d3u79a_ d4u3xa_ d1ghfl1 d1bvkb_ d1mcww1
d1b88a_ d1jrhh1 d32c2b1 d4wemb_ d1dsfh_ d1op3h1 d1ce1l1 d1iqda1
d1qoka1 d1qoka2 d1qfuh1 d2crya1 d1ay1h1 d1oaql_ d3ab0b_ d1rurl1
d1t4kb1 d1bz7b1 d1n0xh1 d1forl1 d1ucbl1 d4w6yb_ d43c9a_ d2aabl1
d1tzia1 d4n1ca_ d1k6qh1 d3g9ab_ d4tvsa1 d1igmh_ d1pz5b1 d3dx9a1
d3wxwh_ d1nakh1 d1kcul1 d3eaka_ d4qlra1 d1cr9l1 d1d9ka_ d1f4xl1
d1deea1 d2i27n2 d1c12a1 d2orbl1 d1j8hd1 d1mcph1 d1c5da1 d1kenh1
d1bd2e1 d1h5ba_ d4x7fc1 d3upaa_ d1t3fa1 d1indh1 d2dtgc1 d4hc1l1
d1zv5a1 d4wgvb_ d1uwgl1 d4f37k1 d1aqkh1 d1vesa_ d3zkqd_ d1fptl1
d1lo4l1 d1knob1 d3rrqa_ d1ct8b1 d4orzc_ d1mlbb1 d4hgkc1 d1dqdl1
d1bogb1 d4ozhe1 d1dr9a1 d1i7za1 d1dn0b1 d1kell1 d1rzib1 d1ypzf1
d1wtla_ d1hkfa_ d1fail1 d1figh1 d2vyre_ d1sjva_ d1ohqa_ d1bfoa1
d1pkqb1 d1mjul1 d1b4jl1 d1nsnl1 d2arjb1 d1p4bh_ d4aj0a_ d3nn8b_
d2rcja1 d1f2xk_ d4v1dd_ d1adqh1 d2eksa_ d4k3ga_ d1jfqh1 d1uweh1
d1frgh1 d4gftb_ d1uz8b1 d4n1ea_ d1bm3l1 d1clol1 d3mcka1 d2vq1a1
d1ae6h1 d4aq1b1 d1uj3b1 d1nbqa1 d1fh5l1 d2ck0l1 d2ywza_ d1q72l1
d4nbxb_ d1hila1 d4hzlb1 d2j88l1 d1zmya1 d1cd0a_ d1a4ka1 d1pskl1
d1a0qh1 d4krmb_ d1ncbh1 d2fbjh1 d1j8he1 d1i3ua_ d1cf8l1 d1fe8h1
d2p1ya1 d1eeqa_ d2x89a_ d1ol0a_ d2r0lh1 d1ub5a1 d1dfbh1 d3eyvb1
d4jdvb1 d4p2cg_ d2gsia1 d2agjl1 d1u3ha1 d2h1ph1 d1rihl1 d1fskc1
d1boga1 d2a77l1 d1ospl1 d1qfoa_ d3dwta_ d2fx7l1 d1rz7l1 d3stba1
d1pkqa1 d1b6da1 d1dzba2 d1dzba1 d1kj2a_ d4l1ha_ d1rzgb1 d1dqlh_
d12e8l1 d1miel1 d1jmaa_ d1sbsl1 d1fgnh1 d1f58l1 d1mqkh_ d1ndgb1
d3qegl1 d1iail1 d2aw2a1 d1tqbc1 d3fo2a1 d4dkaa_ d1f8th1 d2g60l1
d2ywya_ d1uj3a1 d1yc7a_ d3bd3a_ d4w6xb1 d1nmbl_ d1i8ka_ d1rjlb1
d1ypze1 d1p7kb1 d1kxqe_ d1gpoh1 d2z4qa1 d1w72h1 d1i9rl1 d4qgya1
d4xt1c_ d2qqnh1 d1cica1 d3c08h1 d2cd0a_ d2bdnh1 d1hnga1 d1maml1

d1nldh1 d1cr9h1 d4lcih1 d3bkyh1 d3qg7l1 d1ai1l1 d1kxvc_ d4iosd_
d1l7ih1 d2x1qa_ d1ub6b1 d3k74b_ d1ibgh1 d1teth1 d1igya1 d1hxma1
d2x6ma_ d2e7la_ d5e03a_ d1qfwm_ d2r0kh1 d1igjb1 d1rurh1 d1plgh1
d1mj8h1 d1yuhb1 d3fmgl1 d1rzfl1 d1emtl1 d1ad9b1 d1igtb1 d1nlbl1
d1d5ih1 d3k7ua_ d4ffyh_ d1bwwa_ d3dvga1 d1a6ta1 d1fgvl_ d4qxgl1
d1miml1 d1jhll_ d2hffb1 d2hh0l1 d1qkzh1 d1l7tl1 d1afvh1 d1iaii1
d2cjuh_ d1um5h1 d2ghwb2 d2ghwb1 d1kgce1 d2fgwh1 d1ikfh1 d1dvfd_
d2i24n1 d2xtjb_ d1za3b1 d2yc1a_ d2hh0h1 d2x1oa_ d1bvka_ d1rjla1
d5e7ba1 d1igta1 d1il1b1 d2fb4l1 d1mvua_ d1jptl1 d32c2a1 d1fvca_
d1iqdb1 d2hrph1 d2p4ab_ d4qo1a_ d1p2cb1 d1wt5c_ d1t4ka1 d5c1mb_
d1it9l1 d1bz7a1 d1b2wh1 d1e4wl1 d1jnll1 d1yedb1 d1za6b1 d3u7aa_
d1igfh1 d3gnml1 d1orsa1 d1wejl1 d1a2ya_ d1hxmb1 d1gafh1 d2j4wh1
d1pz5a1 d1j05a_ d1a3rh1 d4fqll1 d1qfwh_ d1eo8h1 d1n7mh1 d4s1la_
d1kegh1 d5da4a_ d1iqwl1 d3whxb1 d1vgeh1 d1deeb1 d1c12b1 d1t2ql1
d1ad9a1 d3gsnb1 d3b2uc1 d1ggbh1 d1t3fb1 d1nakl1 d4ocmc_ d1lila1
d2v7hb3 d2esve1 d1mcoh1 d2qr0b1 d2q3aa_ d1ct8a1 d1kcrl1 d2pxyb_
d2uzih_ d5c3ld_ d3durb_ d2ak4e1 d1dl7h_ d1xiwd_ d1mlba1 d1rmfl1
d1i7zb1 d1dlfl_ d2xa3a_ d5bq7b1 d1dqqb1 d3h42l1 d1egjh1 d1kcvl1
d1fe8l1 d2j6el1 d1f3rb2 d1lk3l1 d1sm3h1 d1q0xh1 d1ghfh1 d7fabl1
d1igch1 d1lo0h1 d4idla_ d5c2bl_ d1jrhl1 d1il1a1 d3zhda1 d4kfzc_
d1b0wa_ d1e6oh1 d1mexl1 d4krpb_ d1fnsh1 d1ncwh1 d4hgma_ d4eigb1
d4rzca_ d2mpal1 d4y5xa_ d5en2b1 d3lrga1 d4ej1c1 d1rhha1 d1j05b_
d1forh1 d1q9wb1 d2wzpd_ d2aq2a1 d1h0da1 d3revb1 d2ij0c1 d1tzha1
d4m7jl1 d1k6ql1 d3b9kc1 d1igml_ d3qeua1 d1dsfl_ d1ce1h1 d1kcuh1
d1zvha1 d1zvoa1 d1tqbb1 d1ay1l1 d2qscl1 d2cgrh1 d1nl0h1 d1kenl1
d3eo0a1 d4n9ob1 d1n0xl1 d3k1kc_ d2cqva1 d2k1ma_ d2dl2a1 d1biha3
d1biha1 d1biha4 d3qp3a1 d1g1ca_ d1gxea_ d1cvsc2 d1cvsc1 d1xmwa2
d1m4ka2 d2otpa2 d1biha2 d1rhfa2 d2fcba2 d2fcba1 d1wwbx_ d1gsma2
d1epfa1 d1epfa2 d2dava1 d1waaa_ d1f97a2 d1x44a1 d1vcaa2 d1fnla2
d1fnla1 d3b5ha2 d3h8na2 d3h8na1 d1jbja1 d1jbja2 d1pd6a_ d1f2qa2
d1f2qa1 d3cx2a_ d1nbqa2 d3b5ha1 d1n26a1 d1xiwb_ d1xaua_ d1fltx_
d2ifga1 d1olla1 d1olla2 d1cs6a4 d1cs6a3 d1cs6a2 d1cs6a1 d2gw5a2
d1sy6a1 d1xiwa_ d1ucta2 d1ucta1 d1ry7b2 d1gl4b_ d1qz1a3 d1olza1
d2c9aa1 d1wwca_ d2aq2b1 d1ck1a1 d1enfa1 d1i4pa1 d1v1oa1 d1et9a1
d1esfa1 d2g9hd1 d4ohja1 d1bxta1 d1eu3a1 d1ty0a1 d1fnua1 d1c4qa_
d1prtc1 d2wv6d_ d1prtf_ d1ltrd_ d4l63b1 d1r4pb_ d1prtd_ d1tl0f_
d2bosa_ d1prtb1 d3chbd_

**Family-51 (c.2.1.3)**

**Positive dataset contains 20 protein sequences.**

d2nvwa1 d1u8fo1 d1ydwa1 d2g17a1 d2czca2 d1r0ka2 d2cvoa1 d1zh8a1
d2q49a1 d3pzra1 d1xeaa1 d1vkoa1 d1vm6a3 d3qvsa1 d2pkqo1 d3pwka1
d2hjsa1 d1zyao1 d1vkna1 d1tlta1

**Negative dataset contains 3802 protein sequences.**

d1m32a_ d1ibja_ d1c7na_ d1iuga_ d4q75a_ d2bkwa1 d1t3ia_ d1n8pa_

| | | | | | | | |
|---|---|---|---|---|---|---|---|
| d1qz9a_ | d1jf9a_ | d1eg5a_ | d1p3wa_ | d3kgwa_ | d1cl1a_ | d2ctza1 | d1vjoa_ |
| d2rfva_ | d1gc0a_ | d1cs1a_ | d1d2fa_ | d2ch1a1 | d1qgna_ | d1pffa_ | d1elua_ |
| d1ax4a_ | d1c7ga_ | d2v1pa1 | d2ez1a_ | d1u08a_ | d1lc5a_ | d2ay1a_ | d1bw0a_ |
| d3pd6a_ | d7aata_ | d2csta_ | d1j32a_ | d1fg7a_ | d4wlha_ | d1o4sa_ | d1v2da_ |
| d1gdea_ | d1m6sa_ | d1v72a1 | d1svva_ | d1ajsa_ | d1wsta1 | d2f8ja2 | d1lk9a_ |
| d1vp4a1 | d1xi9a1 | d2gb3a1 | d1yaaa_ | d3i2ka1 | d1lnsa3 | d1mpxa2 | d2cfba1 |
| d1o69a_ | d1bj4a_ | d3k28a_ | d2bwpa_ | d3lg0a_ | d2fnua_ | d3ou5a_ | d1z7da1 |
| d1b9ha_ | d1iaya_ | d4msoa_ | d1bs0a_ | d1s0aa_ | d1sffa_ | d3tqxa_ | d1mdoa_ |
| d3qboa_ | d3pgya_ | d1bjna_ | d2dkja_ | d1ejia_ | d1vefa1 | d4wxga_ | d3bs8a_ |
| d3gbxa1 | d1w23a_ | d4nmwa_ | d1ddla_ | d1e57b_ | d1auyb_ | d1fwxa1 | d1ibya_ |
| d1f56a_ | d1bypa_ | d1azca_ | d1ws8a_ | d3u25a_ | d1cuoa_ | d1id2a_ | d1rkra_ |
| d2iaac_ | d4dp1x_ | d1nwpa_ | d2gima_ | d2i7sa_ | d1qhqa_ | d3ehbb1 | d1fftb1 |
| d1gyca3 | d1hfua2 | d1kbva1 | d1kbva2 | d1sdda1 | d1sdda2 | d1gyca1 | d1gyca2 |
| d1mzya1 | d2bw4a1 | d1aoza1 | d1aoza3 | d1aoza2 | d1kyaa2 | d1kyaa3 | d1kyaa1 |
| d1mzya2 | d4e9sa1 | d1sddb1 | d1sddb2 | d1hfua1 | d1hfua3 | d1oe1a2 | d1oe1a1 |
| d1v10a2 | d1v10a3 | d1v10a1 | d2dara2 | d2dara1 | d1wyha2 | d2dloa2 | d1rutx4 |
| d1rutx3 | d2cuqa2 | d1x4la2 | d1x4la1 | d1x3ha2 | d1x3ha1 | d2d8za1 | d1x61a2 |
| d1x6aa1 | d1x6aa2 | d2cora2 | d2cora1 | d1v6ga1 | d1v6ga2 | d2d8za2 | d1x64a1 |
| d1x64a2 | d1b8ta1 | d2co8a1 | d1x63a2 | d2dj7a2 | d2dj7a1 | d2cu8a2 | d2cu8a1 |
| d1g47a2 | d1g47a1 | d1wyha1 | d2dloa1 | d1wiga1 | d1wiga2 | d2cupa2 | d2cupa3 |
| d1x68a1 | d2d8ya2 | d2d8ya1 | d1m3va2 | d1x62a1 | d1x68a2 | d1x61a1 | d1b8ta3 |
| d1b8ta2 | d2d8xa2 | d2d8xa1 | d1x4ka1 | d1x4ka2 | d2cuqa1 | d1m3va1 | d2co8a2 |
| d1j2oa2 | d1x63a1 | d2cura1 | d2cura2 | d1x62a2 | d1xkta1 | d2hfka_ | d1jmkc_ |
| d5d3ka_ | d3sxwa1 | d1j3qa_ | d4luka_ | d1a3qa1 | d1iknc_ | d3brda1 | d1p7hl1 |
| d1oy3c_ | d1imhc1 | d1zk9a1 | d2cxka1 | d2bema_ | d1pama1 | d1ea9c1 | d1qhoa1 |
| d2fhfa2 | d2fhfa1 | d1k3ia1 | d1j0ha1 | d1cxla1 | d2bhua1 | d1edqa1 | d1f1sa2 |
| d1ut9a2 | d1gvia1 | d1txka1 | d4alac_ | d1rera1 | d1s6na_ | d3uzqb_ | d2phla2 |
| d2phla1 | d1fxza2 | d1fxza1 | d1lrha_ | d1uija1 | g1dgw.1 | d2v09a_ | d1uika2 |
| d1uika1 | d1dgwa_ | d1zbfa1 | d2z1ha_ | d2etja1 | d3qioa1 | d1jl1a_ | d2e4la_ |
| d1jl2a_ | d2lsna1 | d1io2a_ | d1uaxa_ | d2yv0x_ | d1mu2a1 | d1ekea_ | d1i39a_ |
| d1ds6b_ | d1fstb_ | d2b5ea2 | d2b5ea3 | d2b5ea1 | d2b5ea4 | d2kp1a1 | d2kp2a1 |
| d1hyua3 | d1hyua4 | d1j08a1 | d2bjca1 | d2hsga1 | d1qpza1 | d1efaa1 | d1ydwa2 |
| d2nvwa2 | d1zh8a2 | d1qkia2 | d1xeaa2 | d1tlta2 | d1r0ka3 | d1j5pa3 | d1vkoa2 |
| d1vm6a2 | d3qvsa2 | d1gr0a2 | d1p1ja2 | d2ovga_ | d1sq8a_ | d1rzsa_ | d3kz3a_ |
| d3orca_ | d3gpdg2 | d2q49a2 | d1hdgo2 | d2g17a2 | d2cvoa2 | d2czca1 | d3pwka2 |
| d1obfo2 | d1i32a2 | d1zyao2 | d2pkqo2 | d1b7go2 | d2hjsa2 | d1cf2o2 | d1vkna2 |
| d1nvmb2 | d4lt9a1 | d3tc7a1 | d3hoja_ | d1i4na_ | d3vnda_ | d3nl8a1 | d1geqa_ |
| d3nava1 | d1v5xa_ | d4a2ra1 | d3tc6a1 | d1ujpa_ | d4ijba_ | d1xcfa_ | d1vc4a_ |
| d1rd5a_ | d1qopa_ | d3b5va_ | d2msya_ | d2djoa1 | d2r5za_ | d1au7a1 | d1f43a_ |
| d2lmda1 | d1akhb_ | d1s7ea1 | d3nara_ | d1zq3p1 | d2craa1 | d1mija_ | d4uuta_ |
| d3a01a_ | d1x58a1 | d3rkqa1 | d3cmya1 | d2cqxa1 | d2da6a1 | d1e3oc1 | d1fjla_ |
| d1mh3a1 | d1ig7a_ | d1x41a1 | d1wh7a1 | d1gt0c1 | d2mw8a_ | d1lfup_ | d1x2ma1 |
| d1b72a_ | d1wi3a1 | d1jgga_ | d1wjha1 | d1x2na1 | d1mnmc_ | d2cuea1 | d3k2aa_ |
| d2hosa_ | d2jwta2 | d1pufa_ | d1octc1 | d2hi3a_ | d1b8ia_ | d4j19a_ | d3lnqa_ |

d1wh5a1 d1b8ib_ d1fexa_ d2cqra1 d1guua_ d1ug2a1 d2cqqa1 d2ajea1
d4a69c_ d1h8ac1 d2crga1 d2iw5b1 d1xc5a1 d1ofcx1 d2cu7a1 d1wgxa1
d2cjja1 d2ckxa1 d1hcra_ d1tc3c_ d1gdta1 d1u78a2 d6paxa1 d6paxa2
d1k78a1 d1hlva1 d1hlva2 d1iufa2 d3lv8a_ d1s3ga1 d1zaka1 d1s4qa_
d3tvta2 d1uf9a_ d1khta_ d1ckea_ d1osna_ d1p5zb_ d4tmka_ d3cm0a_
d1tmka_ d1jaga_ d3h86a_ d1t0hb_ d1nn5a_ d2h92a_ d3kb2a1 d2c95a1
d1yj5a2 d1kgda_ d1ly1a_ d2ak3a1 d1vyva2 d1deka_ d2grja1 d1p3ja1
d1y63a_ d1e4ya1 d1s96a_ d3iija1 d1lvga_ d1nksa_ d1vyua2 d4e22a_
d1qf9a_ d1jjva_ d1q3ta_ d1ki9a_ d1lw7a2 d1teva_ d1kjwa2 d1gsia_
d2iyva2 d1viaa_ d1e6ca_ d1g3ma_ d1j99a_ d1t8ta1 d1nsta_ d1zrha_
d1ls6a_ d2a3ra_ d1aqua_ d2zptx_ d3ckla_ d1texa_ d1q44a_ d2gwha_
d1odfa_ d1uj2a_ d4gi7a_ d2jeoa1 d1rz3a_ d1z0ja1 d3umra2 d3gj0a1
d2gaoa1 d1yzga1 d1x3sa1 d1g7sa4 d1puja_ d1udxa2 d1kk1a3 d2o52a_
d1jwyb_ d1zcaa2 d1zcba2 d2fv8a1 d2cjwa_ d3dz8a1 d1xzpa2 d1vg8a_
d4qxaa_ d2ew1a1 d1x1ra1 d2bcgy_ d3doea1 d3cona_ d3rega_ d3wyga_
d1upta_ d2b6ha1 d1zd9a1 d1lnza2 d1r5ba3 d1n0ua2 d1r2qa_ d2f9la1
d2rhda_ d3q3jb_ d3q85a_ d1efma1 d1r8qa_ d2gf9a1 d2akab1 d1ek0a_
d1z08a1 d2gcoa1 d2dy1a2 d4g01b_ d2cxxa1 d1svia_ d3q72a_ d1ni3a1
d4ac9a4 d2erxa1 d2bmea_ d1wxqa1 d2gf0a1 d4ldja1 d1zunb3 d4lv5b_
d1efca3 d1kmqa_ d2g6ba1 d3lrpa_ d1tada2 d2y8ea_ d4rd4a1 d1z06a1
d2heia_ d3a58b_ d1u0la2 d1d2ea3 d1zj6a1 d2ocba_ d3rwoa_ d1mr3f_
d2e9sa1 d4u5xa1 d2iwra_ d2fn4a1 d3bwdd_ d2nzja1 d2atva1 d2ksqa_
d3l43a_ d1mkya2 d1mkya1 d1wmsa_ d1jnya3 d2rmka_ d1f6ba_ d1f60a3
d1gota2 d1jala1 d2fh5b1 d1g16a_ d2wblc_ d1m7ba_ d1wf3a1 d1f5na2
d1t9ha2 d1z6xa1 d1z0aa1 d2fg5a1 d2a5ja1 d2bcjq2 d1s0ua3 d1ky3a_
d4y0va_ d4lhwa_ d2qmea1 d2atxa1 d1egaa1 d1ryha_ d1moza_ d1nrjb_
d2erya1 d3bfka_ d1puia_ d1fzqa_ d3j3az1 d3t5ga_ d1shza2 d3defa_
d2f7sa1 d3raba_ d3tsoa_ d3aq4a_ d2al7a1 d3sfva1 d3bc1a_ d1y4wa1
d3vrda1 d3vrda2 d1fcdc2 d1fcdc1 d1h1oa2 d1m70a2 d1m70a1 d1iqca2
d1iqca1 d1eb7a1 d1nmla2 d1nmla1 d3h1jd1 d1hroa_ d4gedb_ d3zooa_
d1mz4a_ d1gu2a_ d1f1fa_ d1dvva_ d1c6ra_ d1c75a_ d1e29a_ d1gdva_
d1cnoa_ d1h32b_ d1co6a_ d2bh4x_ d2aiua_ d5cytr_ d1jdla_ d1i8oa_
d2zboa_ d3dmia_ d1ls9a_ d1wvec_ d2yk3a_ d3ph2b_ d1dw0a_ d5aura_
d1ql3a_ d1qn2a_ d1f1ca_ d4zzva_ d2xspa_ d1eg1a_ d4v20a_ d1ojja_
d4ipma1 d4csia_ d3pfza_ d3ovwa_ d2yoka_ d2nqya_ d1h4ga_ d1hixa_
d1oa2a_ d1olra_ d3b7ma_ d4h7ma1 d3wp3a_ d2vgda_ d1igoa_ d3zsea_
d2nlra_ d3wx5a_ d1oa3a_ d1pvxa_ d1f5ja_ d1oa4a_ d1ks5a_ d4ixla1
d2ah2a1 d1n1ta1 d1evsa_ d1cnt1_ d1pvhb_ d1z7ca_ d1huwa_ d1bgea_
d1rhga_ d1f6fa_ d1i1rb_ d1axia_ d1etea_ d3tgxb_ d2z3qa1 d3qb1a_
d1hula_ d1m48a_ d4nkqb_ d1eera_ d1v7mv_ d5e4ea_ d2o27a_ d3b5ka_
d2psma1 d4zf7a_ d3uf5a_ d4x51a_ d4ypgc1 d3oq3a_ d1lqsl_ d1n1fa_
d1au1a_ d1wu3i_ d3besl_ d3ux9a_ d2b7la_ d3rbaa_ d2qtra_ d1nupa_
d3l92a_ d1qjca_ d2h29a_ d1vlha_ d1od6a_ d1lw7a1 d1mrza2 d4ruka_
d2el7a_ d1iq0a2 d1irxa2 d1r6ta2 d1ivsa4 d2cyba_ d1rqga2 d1j09a2

d2yxna_ d1f7ua2 d1h3fa1 d1ffya3 d2g36a1 d1nzja_ d1jila_ d1n3la_
d1i6la_ d1pfva2 d1h3na3 d3n9ia_ d3sz3a1 d3sw2a_ d1moxc_ d1dx5i3
d1edmb_ d1autl2 d1autl1 d1dval1 d1szba2 d1kigl_ d2y5fl_ d1ijqa2
d1gk5a_ d1g1sa2 d1nt0a3 d1xdtr_ d1lmja2 d1lmja1 d1nzia2 d2wphe_
d1klil_ d1pfxl2 d1pfxl1 d1p9ja_ d1g1ta2 d1f7ea_ d4yzub_ d2mgpa1
d1b9wa1 d2mgpa2 d1ob1c1 d1ob1c2 d1n1ia2 d1n1ia1 d2npra2 d2npra1
d1m1xb5 d1qx2a_ d1pvaa_ d3fs7a_ d2pvba_ d1ttxa_ d1rwya_ d2kyfa_
d1rk9a_ d1a75a_ d1exra_ d1tiza1 d1nyaa_ d1g8ia_ d1ggwa_ d1wdcc_
d3fwba_ d1ggza_ d2o5ga_ d1ctaa_ d1m45a_ d3i5gc_ d1sl8a_ d1fpwa_
d2scpa_ d2roaa_ d4aqra_ d3pn7b_ d1br1b_ d2mysb_ d1s6ca_ d1bjfa_
d2obha_ d1jfka_ d1jf0a_ d1qv0a_ d1lkja_ d2mlfc_ d2m97a_ d2d8na_
d4by5a_ d1jbaa_ d4nqga_ d1wdcb_ d2mysc_ d1j7qa_ d1s6ia_ d1uhka2
d1r2ua_ d1dtla_ d3kf9a_ d3pn7c_ d1qjta_ d1fi6a_ d1c07a_ d5d67d_
d1tuza_ d1h8ba_ d1eg3a1 d1eg3a2 d2zkmx1 d1snla_ d1gjya_ d1y1xa_
d1hqva_ d1df0a1 d1qxpa2 d4usla_ d1k94a_ d1wkya2 d1uuqa_ d3wh9a_
d4m1ra_ d1xyza_ d4xuya_ d2je8a5 d4bf7a1 d3cuia_ d1ur4a_ d1qw9a2
d1odza_ d2vzsa5 d1bqca_ d2x2ya1 d1hjqa_ d1qnra_ d4y7ea1 d1vjza_
d4k68a_ d2v3ga_ d4hpga_ d1nofa2 d1nq6a_ d1h4pa_ d1aq0a_ d3ziza_
d4bs0a_ d1ecea_ d1h1na_ d1ta3b_ d1w91a2 d1i1wa_ d1uhva2 d1us3a2
d1egza_ d1tvna1 d3nyda1 d1ghsa_ d2cyga1 d4xv0a_ d2c8na2 d1w32a_
d3emca_ d1v6ya1 d1foba_ d2uwfa1 d2fgla_ d3niya1 d2c0ha1 d1rh9a1
d1yq2a5 d1szna2 d1fa2a_ d1pama4 d5do8a1 d1ea9c3 d1hx0a2 d2fhfa5
d1qhoa4 d1avaa2 d1m53a2 d4m8ua1 d2gjpa2 d1lwha2 d4howa1 d1ktba2
d1j0ha3 d3vm5a1 d2x1ia_ d1wzaa2 d1uasa2 d2diea1 d1mxga2 d1ht6a2
d1eswa_ d1iv8a2 d5cpsa_ d1ud2a2 d1zjaa1 d3gbea1 d1kwga2 d1wdpa_
d1gcya2 d1g5aa2 d1hvxa2 d3dhpa2 d2bhua3 d4do4a1 d3qgva1 d1e43a2
d1g94a2 d2d3na2 d1gjwa2 d1gvia3 d1x1na1 d1r7aa2 d1tz7a1 g1apy.1
d3ljqa_ g1k2x.1 d4c7ga2 d2gjxa1 d1nowa1 d1jaka1 d1yhta1 d4toqa_
d1wb0a1 d1w9pa1 d1itxa1 d1kfwa1 d1goia2 d1edqa2 d2gsja_ d1hjxa1
d1jnda1 d1vf8a1 d1etob_ d1umqa_ d1g2ha_ d1mixa2 d1e5wa2 d1j19a2
d1isna2 d2al6b2 d3syxa1 d1mkea1 d2p8va_ d1xoda1 d1evha_ d1qc6a_
d1egxa_ d2v76a_ d1wj1a1 d1qqga2 d1qqga1 d1ntva_ d1n3ha_ d4dbba_
d3f0wa1 d1p3ra_ d1wgua1 d2cy4a1 d3hqca_ d1wvha1 d1xr0b1 d1wgqa1
d1v89a1 d2coda1 d1zc3b_ d1wg7a1 d1u5ea1 d1v61a1 d2coca1 d2ys1a1
d1v5pa1 d1ntya2 d1wjma1 d1wi1a1 d1u29a_ d1xx0a1 d1unqa_ d2zkmx3
d1fhoa_ d1v88a1 d2adza1 d1x1fa1 d1p6sa_ d2coaa1 d1btka_ d1v5ma1
d1faoa_ d2x18a_ d1dyna_ d1x1ga1 d4kaxb_ d2j59m1 d1ki1b2 d1xcga2
d1txda2 d1u5da1 d1dbha2 d1w1ha_ d1upqa_ d1v5ua1 d1foea2 d2fjla1
d1eaza_ d2dfka2 d2cofa1 d1kz7a2 d1h6ua1 d1r55a1 d3gboa_ d1atla_
d1buda_ d4q1la_ d1quaa_ d1yp1a_ d1wnia_ d1cmxa_ d1xd3a_ d3ifwa_
d1rypd_ d1irua_ d1rypc_ d3unfk_ d1rypa_ d1j2pa_ d1irui_ d1j2qh_
d3unfh_ d1m4ya_ d1rypb_ d1irul_ d1irug_ d1iru2_ d1q5rh_ d1rypg_
d1g3ka_ d1q5qa_ d1e94a_ d1irub_ d1ryph_ d3ty6a_ d1iruj_ d1iruk_
d1rype_ d1irud_ d1irue_ d3mi01_ d1rypf_ d1iruh_ d2z3ba_ d1iruc_

d3mka2_ d1iru1_ d3u1ra1 d1g9ka2 d1kapp2 d1t3ca_ d1epwa3 d2a97a_
d3fiea_ d3ioqa_ d1fh0a_ d1o0ea_ d1khqa_ d1m6da_ d2cb5a_ g8pch.1
d1s4va_ d2pnsa_ d1vsna1 d1mira_ d1iwda_ d2oula_ d7pcka_ d3bwka_
d3bcna_ d2p86a_ d1pvja_ d1pcia_ d1gece_ d1deua_ d4bs6a_ d1xkga1
d1cqda_ d1jqpa2 d2c0ya_ d2bdza_ d3p5ua_ d1zoya1 d1qo8a2 d1d4ca2
d1jnra2 d1kf6a2 d2jk6a2 d2jk6a1 d1feca1 d1feca2 d1lpfa1 d1lpfa2
d1xhca1 d1xhca2 d1dxla2 d1dxla1 d1gera2 d1m6ia2 d1onfa1 d1onfa2
d3lada2 d3lada1 d1w4xa1 d1w4xa2 d1gesa2 d1mo9a1 d1xdia1 d1fl2a2
d1fl2a1 d1fcda1 d1fcda2 d1h6va2 d1h6va1 d1mo9a2 d1gv4a2 d1gv4a1
d2gv8a2 d2gv8a1 d1aoga1 d1aoga2 d1hyua1 d1ebda1 d1ebda2 d3dk9a1
d1q1ra2 d1q1ra1 d1gesa1 d1g4ma2 d1o94a2 d1lqta1 d1cjca1 d1pn0a1
d1reoa1 d1ju2a1 d2f5va1 d3c96a1 d2gmha1 d1pj5a2 d1gpea1 d1o5wa1
d1n4wa1 d1k0ia1 d2dw4a2 d2ivda1 d1seza1 d2voua1 d1kdga1 d1s5ja1
d2hbja2 d2igia_ d1wlja_ d1qhta1 d4dlea1 d1tgoa1 d2f96a1 d3tr8a1
d3mxma_ d1q8ia1 d1y97a1 d1w0ha_ d1kfsa1 d2py5a1 d1noya_ d1d5aa1
d1yt3a3 d2guia_ d3mwpa1 d4kb1a_ d1b9ra_ d1offa_ d1frra_ d3wcqa_
d1e9ma_ d1l5pa_ d1wria_ d1i7ha_ d3av8a_ d1czpa_ d1rfka_ d3p1ma1
d3b2ga_ d2bt6a2 d1tq5a1 d3nvva1 d1krha3 d2h88b1 d1n62a2 d1t3qa2
d1kf6b2 d1zoyb1 d1jq4a_ d1ffva2 d1dgja2 d1rm6c2 d1k8ka2 d1k8ka1
d3uleb1 d3i33a2 d3i33a1 d1bupa1 d1dkgd2 d1dkgd1 d4ci6a2 d1jcfa1
d4a2ba2 d2fxua1 d1yaga2 d1yaga1 d4cbua2 d1bupa2 d1r7ha_ d1faaa_
d1wika1 d1xfla_ d1ttza1 d2f51a1 d1ti3a_ d1nhoa_ d1sena_ d2i4aa_
d1ep7a_ d1zmaa1 d1fo5a_ d3lefa1 d3diea1 d2yana1 d1z6na1 d1oaza_
d3zywa1 d4aj8a_ d1nm3a1 d4fiwa_ d1wmja_ d4hs1a1 d1iloa_ d1xwaa1
d1wjka1 d2vm1a_ d1fb6a_ d1gh2a_ d1h75a_ d1nw2a_ d1kx5b_ d3a6na_
d4m6ba2 d2nqbd_ d3wkjd_ d2yfwe_ d4h9na_ d5bnxa_ d1kx5c_ d3r45a_
d1eqzb_ d4jjnb_ d1f66c_ d1eqza_ d4wnnb_ d1kx5a_ d1u35c1 d1id3a_
d2nqbc_ d1kx5d_ d3us3a3 d3us3a1 d1tafa_ d1bh9b_ d1tafb_ d1jfib_
d1h3ob_ d2byka1 d1n1ja_ d4g92b_ d1bh9a_ d1jfia_ d1h3oa_ d2bykb1
d1n1jb_ d1pd212 d1nhya2 d2a2ra2 d1k0dd2 d1f2ea2 d1gsua2 d1f3ba2
d2fhea2 d2pvqa1 d1tw9a2 d1oyja2 d1e6ba2 d1k0ma2 d3ik7a1 d4is0a1
d1r5aa2 d1jlva2 d1m0ua2 d3gtub2 d1z9ha2 d1okta2 d1axda2 d2gsra2
d1tu7a2 d1k3ya2 d1zgma1 d1b48a2 d1gnwa2 d1gwca2 d2gsta2 d1glqa2
d1oe8a2 d1pn9a2 d1v2aa2 d1fw1a2 d1duga2 d1ig8a1 d1czan2 d1czan3
d1czan1 d1ig8a2 d1czan4 d1bg3a4 d1bg3a3 d1bg3a2 d1novc_ d2bbvc_
d1qusa_ d1sefa_ d1sfna_ d1rc6a_ d1qqya_ d1gd6a_ d1f6ra_ d1ivma_
d2gv0a_ d1iiza_ d2z2fa_ d1j8xa_ d1ghla_ d1b9oa_ d174la_ d169la_
d2o4wa_ d1p37a_ d1k28a3 d1xjta1 d1jtma_ d2f2qa_ d4e97a1 d2k4ua_
d1hp2a_ d2lu9a_ d1baha_ d2meoa1 d1wm7a_ d1hlya_ d1n8ma_ d1jlza_
d1wpda_ d1c55a_ d1pvza_ d1wt7a_ d1rjia_ d1wz5a_ d1m2sa_ d1pjva_
d1c49a_ d1qkya_ d2ktca1 d1wmta_ d1du9a_ d1j5ja_ d1r1ga_ d1mpya2
d1kw3b2 d1kw3b1 d4ghga1 d1tfza1 d3isqa1 d3isqa2 d1sqia1 d1sqia2
d1lkda2 d1lkda1 d1mpya1 d1cjxa2 d1cjxa1 d1sp9b2 d1t47a1 d1t47a2
d1sp8a1 d1sp8a2 d1f1ua1 d2h0va_ d1s5ja2 d1qhta2 d4dlea2 d1tgoa2

d3py8a2 d1q8ia2 d1kfsa2 d2py5a2 d1d5aa2 d1qsta_ d2beia1 d4m3sa_
d1u6ma1 d1ygha_ d1yk3a1 d3dr6a_ d1xmta_ d1z4ea1 d2ge3a1 d2fiaa1
d2jdca1 d1y9wa1 d3to7a_ d1wwza1 d2fl4a1 d2ozua1 d2b5ga_ d2cy2a1
d2p0wa_ d1cm0a_ d4pswa_ d1yrea1 d1yr0a1 d1m4ia_ d2gana1 d1yvka1
d1ghea_ d2g3aa1 d1y7ra1 d2euia1 d1q2ya_ d2fsra1 d1xeba_ d2ae6a1
d1cjwa_ d1qsma_ d2aj6a1 d1y9ka1 d1i12a_ d1vkca_ d2atra1 d1n71a_
d2d4pa1 d2fiwa1 d2fe7a1 d1tiqa1 d2ou2a_ d1yx0a1 d2fcka1 d1p0ha_
d2j8ma_ d2pq8a_ d1z4ra1 d1s3za1 d1ufha_ d1prtc1 d2wv6d_ d1prtf_
d1ltrd_ d4l63b1 d1r4pb_ d1prtd_ d1tl0f_ d2bosa_ d1prtb1 d3chbd_
d1xvqa1 d1pn0a2 d3hy2a_ d2cvba1 d1o8xa_ d1lu4a_ d1we0a1 d2bmxa1
d2a4va1 d1psqa_ d1xiya1 d1q98a_ d1n8ja_ d1zofa1 d2ggta_ d3tjja_
d4je1a1 d1o73a_ d3s9fa_ d2zcta_ d1qmva_ d2b1ka_ d5dvba1 d2b5xa1
d2v2ga_ d1st9a_ d3tb2a_ d4llra_ d4fh8a1 d1e2ya_ d1knga_ d2h30a_
d1i5ga_ d2z9sa_ d2f8aa1 d1tp9a1 d2b7ka_ d1nm3a2 d3hvva_ d2wfca1
d4kcea_ d1xvwa1 d4gqca1 d3qpma_ d4k1fa_ d1zzoa1 d1gp1a_ d1prxa_
d4od7a1 d1z6ma1 d4mcua1 d4dvca1 d1r4wa_ d3l9sa_ d1un2a_ d3rppa_
d2m5ta_ d1cqqa_ d1wqsa_ d5bpea_ d2ipha_ d1lvoa_ d2bhga1 d3tnta_
d3sjia_ d3d23a1 d2fyqa_ d3tloa_ d3zvea1 d2hrva_ d3q3ya1 d4asha1
d1cg2a1 d1yloa2 d1y7ea2 d1y0ya3 d1lfwa1 d1vixa1 d1fnoa4 d1vhoa2
d1ysja1 d1vhea2 d1z2la1 d2fvga2 d1r3na1 d2grea2 d1xmba1 g1q7l.1
d1rtqa_ d1w52x1 d2ppla1 d1hpla2 d1lpbb2 d2oxea1 d1bu8a2 d1rp1a2
d1ys1x_ d4hs9a_ d1ispa_ d1ex9a_ d1ku0a_ d3qmma_ d1n9wa1 d1e1oa1
d1c0aa1 d1b8aa1 d1l0wa1 d3a74a1 d1eova1 d1lgya_ d4l3wa_ d1gz7a_
d1ukca_ d1bvsa3 d2ftba_ d1pmpa_ d2rcqa1 d1fdqa_ d4lkpa_ d1sa8a_
d1vyfa_ d1lpja_ d3vg7a1 d4azqa1 d1o8va_ d5bvqa_ d1p6pa_ d2qo4a_
d1opba_ d1yiva1 d1o1va_ d4bvma1 d4tkba_ d4i3ba_ d1bwya_ d1ftpa_
d4gkca_ d1cbia_ d3p6da1 d1kqwa_ d2a0aa1 d3zxha_ d1cgla_ d1rm8a_
d1eaka2 d1hy7a_ d2jsda_ d1cxva_ d1hv5a_ d1i76a_ d1eb6a_ d1m4la_
d2bk7a_ d3fjua_ d1z5ra1 d1kwma1 d1uwya2 d1jqga1 d2c1ca1 d1dtda_
d1jgta2 d1ofda3 d1ecfa2 d1q15a2 d1ct9a2 d1ao0a2 d1te5a_ d1c8nc_
d1x36a_ d1smvc_ d1ng0c_ d4sbvc_ d1opoa_ d1f2nc_ d1cixa_ d1y29a_
d1v7fa_ d2jtba_ d1d1ha_ d1g9pa_ d1lupa_ d1qk7a_ d1mb6a_ d2a2va_
d1niya_ d1i25a_ d1hvwa_ d2mpqa_ d1ie6a_ d1kqha_ d1la4a_ d2mxoa1
d1dl0a_ d1koza_ d1qk6a_ d1emxa_ d1lqra_ d1c6wa_ d1rmka_ d1ixta_
d1eyoa_ d1cnna_ d1fu3a_ d1f3ka_ d1fyga_ d4lu0a_ d1of8a_ d3unda1
d3e9aa1 d1vr6a2 d1n8fa_ d2qkfa_ d1d9ea_ d1o60a_ d1ny721 d1ny722
d1a6ca2 d1a6ca1 d1pgl22 d1pgl21 d1pgl11 d1ny711 d1mqta_ d1d4m3_
d1fmd3_ d2mev1_ g1aym.1 d1ev13_ d4q4w1_ d1r1a3_ d2x5ia_ g1r1a.1
g1d4m.1 g1rhi.1 d1b35b_ d1oopa_ d1tme1_ d4iv1a_ g1pvc.1 g1fpn.1
d1fpn1_ d4gb31_ d1d4m1_ d1fmd1_ d1ev11_ d2wzr3_ g1cov.1 d1qqp3_
g1fmd.1 d2x5ic_ d1rhi3_ g1oop.1 d1hxs1_ g1hxs.1 d1fpn3_ d1bev1_
g2mev.1 d1eah3_ d1aym1_ d1pvc3_ d1qqp1_ d1zba3_ d1h8ta_ d3vbhc_
g1b35.1 g1bev.1 d1hxs3_ g1ev1.1 d1bev3_ g1tmf.1 d1tmf1_ d1b35a_
d2mev3_ g1eah.1 g1h8t.1 d1eah1_ d4q4w3_ d1r1a1_ d1aym3_ d1pvc1_

d1zba1_ d1h8tc_ d1oopc_ d1tme3_ g1qqp.1 d4iv1c_ g1tme.1 d3ubub_
d1egia_ d1v7pa_ d1tdqb_ d3ubua_ d1c3ab_ d1h8ua_ d3bx4b_ d1t8ca1
d1sb2a1 d1qdda_ d2ziba1 d3cada_ d1byfa_ d1j34b_ d1jwia_ d1jzna_
d1c3aa_ d1oz7b_ d1wmza_ d3bdwb_ d1hq8a_ d1fifa1 d1dv8a_ d3hupa_
d1wk1a1 d1umra_ d2afpa_ d3bx4a_ d1gz2a_ d1sl6a_ d1v7pb_ d3g8ka_
d1ixxa_ d1j34a_ d1jwib_ d1g1sa1 d1y17a1 d1ja3a_ d1fvub_ d3bdwa_
d1sb2b1 d1fvua_ d1umrc_ d1oz7a_ d4m17a_ d3cfwa1 d1ypqa1 d1g1ta1
d3fd4a_ d3l9jc_ d4dn8a1 d1v4lb_ d1rdl1_ d2msba_ d4w9ta_ d1jvna1
d4x2ra1 d1ka9f_ d1qo2a_ d4axka1 d1thfd_ d4u28a_ d2y88a_ d4wd0a_
d1vzwa1 d2flia1 d1h1ya_ d1tqja_ d1tqxa_ d1ux8a_ d1dlya_ d4i0va_
d2bkma_ d1dlwa_ d2bmma_ d2r80b_ d1cqxa1 d1cg5a_ d1gcvb_ d3boma_
d2h8fb_ d2raoa_ d3bj1a_ d4hrrb_ d2r50a_ d3d1kb_ d2aa1b1 d3zhwa_
d3hf4b_ d3gdja_ d1jebb_ d3bj1b_ d1x9fb_ d1wmub_ d1gcva_ d3bcqb_
d3d4xb_ d1gvha1 d2dc3a_ d3hf4a_ d4yu3a_ d3k8ba_ d1qpwb_ d2r80a_
d3bcqa_ d1v4wa_ d3wtgb_ d1jl7a_ d4esaa_ d1a9we_ d3d1ab_ d1fsla_
d4h2la_ d1v4wb_ d1mwca_ d1vhba_ d1jeba_ d1spgb_ d1h97a_ d1q1fa_
d1tu9a1 d3d1aa_ d1hdsb_ d3hyub_ d2zfbb_ d3fs4a_ d1oj6a_ d1shrb_
d4h2lb_ d4yu3b_ d1spga_ d3hrwa_ d3qqra_ d3at5a_ d4wchd_ d3hyua_
d1cg5b_ d1uc3a_ d1hbra_ d2raob_ d1outb_ d4esab_ d3gdjb_ d3qqqa_
d1x9fc_ d3vrfa_ d1itha_ d3mkba_ d3d4xa_ d3mjpa_ d1jl6a_ d3fh9a_
d1outa_ d2dc3b_ d1it2a_ d3eoka_ d3g46a_ d1qpwa_ d3vrfb_ d1ch4a_
d1x9fd_ d1hdsa_ d2zfba_ d3mkbb_ d1or4a_ d3wtga_ d1a4fa_ d3fh9b_
d3bomb_ d3cy5a_ d3at5b_ d1x9fa_ d2oifa_ d4lmxb_ d1cpcb_ d3v57a_
d1kn1a_ d1cpca_ d4f0ta_ d2vjha_ d4po5b_ d1eyxa_ d1alla_ d4f0tb_
d1b33b_ d1b8db_ d4h0mb_ d1b33a_ d2vjhb_ d4lm6b_ d2vmlb_ d1allb_
d2vjta_ d3dbja_ d2vmla_ d2vjtb_ d4f0ub_ d1f99b_ d4rmpa1 d1liaa_
d1eyxb_ d2uulb_ d4lmsb_ d4f0ua_ d2bv8a_ d1f99a_ d2bv8b_ d2c7lb_
d3dbjb_ d4rmpb1 d4h0ma_ d3v57b_ d1kn1b_ d1liab_ d2uula_ d2j96a_
g1gxs.1 d1k8qa_ d2xqfa_ d1ea5a_ d1f6wa_ d1dx4a_ d1qe3a_ d1k4ya_
d4qwwa_ d2ha2a_ d3c8da2 d2pbla1 d1jkma_ d1jjia_ d1jjfa_ d1gl9b1
d1gkub1 d1jmxa1 d1pbya1 d1pbya2 d1jmxa4 d1jmxa3 d1pbya4 d4iuaa3
d4bvva_ d5hpga_ d3k65a_ d1ki0a3 d1ki0a2 d1ki0a1 d1a0ha1 d1pmla_
d3sp8a3 d2feba1 d1dosa_ d3qm3a1 d2fjka_ d1f74a_ d3l21a_ d2e1da_
d1to3a1 d1w3ia_ d1sfla_ d1n7ka_ d1vcva1 d4imea_ d1wa3a_ d2occza1
d1f2ja_ d3m16a_ d2yxga_ d1ub3a_ d3a5fa_ d1fbaa_ d1l6wa_ d1xfba1
d1wx0a1 d1vpxa1 d1ojxa_ d2a4aa1 d1xkya1 d3cq0a1 d3s1xa_ d1f05a_
d1fdja_ d3cpra1 d4d2ja_ d1e88a1 d1l6ja5 d1l6ja4 d1eaka4 d1l6ja3
d1h8pa2 d1h8pa1 d3rp2a_ g1ucy.1 d1fuja_ d2zchp_ d3w94a_ d1si5h_
d1lo6a_ g2pka.1 d1cgha_ d1kigh_ d1a5ia_ d3s9ba_ d1hj8a_ d1ao5a_
d2anya_ d1q3xa1 d1gvza_ d1azza_ d4gsoa_ d2f91a1 d1iaua_ d1gdna_
d1eufa_ g1fiw.1 d1eq9a_ d4nfea_ d2rdla_ d1spja_ d1elva1 d1a7sa_
d1ekbb_ d1pfxc_ d1yc0a_ d2aiqa_ g1fiz.1 d4k60a_ d4dgja_ d1mzaa_
d1op0a_ d3tjva_ d1pytd_ d1sgfg_ g1gg6.1 d2eeka_ g1rtf.1 d1a0ja_
d3g01a_ d1pytc_ d4e7na_ d1ddja_ d1z8ga1 d2hlca_ d1npma_ d1brup_

g1jou.1 d1sgfa_ d1autc_ g2hnt.1 g1kdq.1 d1orfa_ d3s69a_ d4crga_
d1fxya_ d1bqya_ d1ra6a_ d1muka_ d3gsza_ d1khva_ d1te9a_ d2xwha_
d3uqsa_ d3hkwa_ d3n6ma_ d2cjqa_ d2xyma_ d1teba_ d1te8a_ d1s48a_
d1u09a1 d4wfxa_ d3tyqa_ d2pgga1 d1dzka_ d4tlja_ d2a2ua_ d4wfva_
d3t1da_ d4iaxa_ d3zq3a_ d2nnda_ d1i4ua_ d1kxoa_ d1bbpa_ d1kt7a_
d1exsa_ d3s26a1 d1lf7a_ d1qfta_ d4gh7a_ d1z24a1 d1qabe_ d1xkia_
d1ew3a_ d3bx7a_ d4iawa1 d1n0sa_ d1gkab_ d3kzaa_ d2k23a_ d2l9ca1
d2joza1 d1gm6a_ d3kffa_ d3saoa1 d1e5pa_ d1yupa1 d1iiua_ d3lgia_
d3i77a_ d2z9ia2 d4nsva_ d1lcya2 d4jcna_ d1agja_ d1os8a_ d1hpga_
d1p3ca_ d1l1ja_ d1qtfa_ d1iwea_ d1qf5a_ d1vmaa2 d1nija1 d1np6a_
d2p67a1 d3ihla_ d1okkd2 d1ls1a2 d1s1ma2 d1iona_ d2v40a_ d3hida_
d2vo1a1 d2g0ta1 d1vcoa2 d1j8mf2 d1g3qa_ d1xjca_ d1p9ba_ d1qzxa3
d1ihua2 d1ihua1 d2qm8a_ d1dj2a_ d4ioja_ d1cp2a_ d1yrba2 d1hyqa_
d3gqba2 d1skye3 d1xp8a1 d1tf7a2 d1tf7a1 d1ye8a1 d1skyb3 d2qe7d2
d4blta_ d4huta_ d3ldaa2 d4hyya_ d2i3ba1 d1cr1a_ d3j9tb2 d3ntua2
d1vdza1 d2z43a_ d1e9ra_ d3ziaa2 d1n0wa_ d1g6oa_ d3ziad2 d3j9ta2
d1pzna2 d3gqbb2 d2i49a_ d1nnfa_ d1xvxa_ d1ursa_ d1h3da1 d1iuda_
d4m1va1 d1twya_ d3e13x_ d2nxoa1 d4pfua_ d3ttma_ d1elja_ d2qmwa1
d1us5a_ d1y3na1 d1jeta_ d1uqwa_ d4f19a1 d2esna2 d3thia_ d3fasa1
d2uyeb_ d2xwva1 d1r9la_ d2fyia1 d1pb7a_ d2ozza1 d1sw5a_ d1nh8a1
d1vr5a1 d2g29a_ d1xs5a_ d3lswa_ d2z23a_ d3w9va_ d3tcha_ d1q35a_
d1z7me1 d3q27a_ d1p99a_ d1ve4a1 d2i6ea1 d4mcoa1 d4n8ya_ d1eu8a_
d1zbma1 d1hsla_ d1ixca2 d1j1na_ d1xvya_ d1y9ua_ d2z22a1 d1xoca1
d4oera_ d1wdna_ d5f1qa1 d1xt8a1 d1i6aa_ d4gwoa_ d4igta1 d1pc3a_
d4mmpa1 d1o63a_ d2czla1 d2onsa2 d4mija1 d4ok5a1 d2bmfa1 d2bmfa2
d1yksa1 d1yksa2 d1jr6a_ d2qeqa1 d2jlqa1 d1a1va2 d1eh3a_ d3skpa_
d1dtza1 d1dtza2 d1ieja_ d1jnfa2 d1ce2a1 d1jnfa1 d1b1xa1 d1jw1a1
d1jw1a2 d1ryoa_ d1h76a1 d1h76a2 d1c4oa1 d2p6ra3 d2p6ra4 d1oywa2
d1oywa3 d1wrba1 d1w36b2 d1w36b1 d1c4oa2 d3bora_ d1t6na_ d2eyqa5
d2eyqa3 d2eyqa2 d2zu6a2 d1veca_ d1uaaa1 d1w36d1 d2g9na1 d1z3ix2
d2rb4a_ d1hv8a1 d1hv8a2 d1t5ia_ d1w36c2 d2j0sa2 d2j0sa1 d1fuka_
d3hjha1 d1z5za1 d1qdea_ d1z63a1 d1s2ma2 d1s2ma1 d1wp9a1 d1wp9a2
d1gm5a3 d1gm5a4 d3dina4 d3dina3 d1q0ua_ d1w36c1 d1z3ix1 d1nkta3
d1nkta4 d1uaaa2 d1w36d2 g1qhh.1 d2fwra2 d2fwra1 d3q3ma_ d1za0a1
d1mxra_ d2inca_ d1jk0a_ d2incb_ d1mhyb_ d3hf1b_ d1mtyb_ d1r2fa_
d2uw1a_ d1mhyd_ d3n37a_ d1afra_ d1syya_ d1mtyd_ d2o1za_ d1uzra_
d1smsa_ d2fkza_ d1zuja1 d1s3qa1 d2fjca1 d1z6om1 d1veia_ d4dyya_
d1jiga_ d3gvya_ d2pyba_ d4cy9a1 d3bvfa1 d1r03a_ d2cf7a_ d1nf4a_
d1zs3a1 d4iwka_ d3ka8a_ d3np0x_ d1n1qa_ d1lb3a_ d4e6ka_ d1z6oa1
d2fzfa1 d4dyua_ d3ge4a_ d1jgca_ d4am4a_ d2z6ma_ d1ji5a_ d1vlga_
d1krqa_ d3qz3a_ d1o9ra_ d2ffxj_ d1euma_ d1yv1a1 d2v2pa_ d1j30a_
d4di0a_ d1dpsa_ d2cbia2 d2choa2 d1l8na1 d1oewa_ d2qzxa_ d2asia_
d1psoe_ d1miqa_ d1smra_ d2h6ta_ d1pfza_ d1izda_ d3hd8a_ d1j71a_
d1dpja_ d4aa9a_ d1t6ex_ g1b5f.1 g1lya.1 g1htr.1 d1qdma2 d1bxoa_

d1wkra_ d1tzsa_ d1ibqa_ d2qzwa_ d1bdqa_ d3ggta_ d3ixoa_ d1mrxa_
d2haha_ d1tw7a_ d4njsa_ d1sgua_ d2p3da_ d1nsoa_ d3d3ta_ d2rkfa_
d1c6ya_ d2rspa_ d3ogpa_ d3ekwa_ d2o4na_ d2j9ja_ d3ggua_ d3lzua_
d4npua_ d4j55a_ d2r8na_ d2fxda_ d2p3ca_ d4dqea_ d3ttpa_ d3t3ca_
d2p3aa_ d2hc0a_ d2fdda_ d3mwsa_ d1wgra1 d1ef5a_ d1wfya1 d1e8ya3
d1wgya1 d1lfda_ d2cs4a1 d1wxma1 d2byea1 d3kh0a1 d2byfa1 d1wxaa1
d1e7ua3 d1h4ra3 d4yl8a1 d2al6a3 d1wgda1 d1wgga1 d4q5eb_ d1wjna1
d1se9a_ d3n3kb_ d1wz0a1 d1we6a1 d2m0xa_ d2gbka_ d3rt3b1 d1wh3a1
d1sifa_ d2ekec2 d4ii2b1 d4hk2a1 d1c3ta_ d1m94a_ d1wgha1 d1ttna1
d1j8ca_ d1wx8a1 d3v6cb1 d2ma9b_ d4i6lb1 d2jvca1 d1iyfa_ d2k1fa_
d1wxva1 d1wf9a1 d1t0ya_ d2io0b2 d1x1ma1 d2gbma_ d1zw7a1 d2m17a_
d1ud7a_ d1we7a1 d2lwpa_ d2gbja_ d1wjua1 d1v6ea1 d1v86a1 d4eewa_
d1wiaa1 d4icva_ d4gu2a_ d1wx7a1 d1wy8a1 d4hjka1 d2faza1 d1v5oa1
d1g66a_ d5ajha1 d1j1ia_ d2rhwa_ d1dszb_ d1a6ya_ d3cbba_ d1ynwa1
d1r4ia_ d1hcqa_ d2hana_ d2hanb_ d2enva1 d1kb4b_ d2nllb_ d1lo1a_
d1cita_ d1um8a_ d1ixza_ d1qvra2 d1qvra3 d1qzma_ d1sxjc2 d1jr3d2
d2p65a_ d1l8qa2 d3hu3a3 d1sxjd2 d1njfa_ d1in4a2 d1sxja2 d1tuea1
d2ce7a2 d1w5sa2 d1sxjb2 d1d2na_ d1ny5a2 d1ofha_ d2gnoa2 d2a5yb3
d1fnna2 d1u0ja1 d1lv7a_ d1iqpa2 d2fnaa2 d1ixsb2 d4gita_ d1sxje2
d1g41a_ d1svpa_ d1mbma_ d3su6a1 d1cu1a1 d4agka_ d1vcpa_ d1q31a_
g1n1l.1 d3e90b_ d1ep5a_ d3u1jb_ d2e3fa_ d2e3ea_ d2llda_ d2ny8x_
d2e3ga_ d2ny9x_ d2nz3a_ d2lj7a_ d1n4na_ d2m8bb1 d2kyqa_ d3psma_
d1jxca_ d2gl1a_ d1ugla_ d4uj0a_ d1js9c_ d1za7a_ d1laja_ d1t0za_
d2i61a_ d1t1ta_ d1sn4a_ d1kv0a_ d2lkba_ d1npia_ d1wwna_ d1pe4a_
d2kbka_ d2li7a_ d1djta_ d2kbha_ d1dq7a_ d1sega_ d1omya_ d1zywa1
d1jzaa_ d1bnla_ d1t61c1 d1iyjb4 d1iyjb5 d1iyjb3 d1o7ia_ d4ipca1
d1x3ea_ d4apva_ d1jb7a1 d1jb7a2 d1jb7a3 d3kdfa_ d1ue1b_ d1wjja1
d3eiva_ d1xjva2 d1xjva1 d2ccza2 d1se8a_ d1l1oc_ d1jb7b_ d1qvca_
d1qzga_ d1miua4 d1miua5 d1miua3 d3g48a1 d1gd7a_ d1mkha_ d1pyba_
d1y14b1 d2nn6i1 d1i94l_ d2zjra2 d1smxa_ d2qamc2 d1hh2p1 d2r7da1
d1rl2a2 d1wfqa1 d3bzka4 d1x6oa2 d3a0ja_ d2j01d2 d2ja9a1 d4ql5a_
d1hr0w_ d1luza_ d2n3sa_ d2vnud1 d1h95a_ d1go3e1 d2nn6h1 d1ne3a_
d2mo1a_ d1iz6a2 d1jjga_ d1e3pa2 d2ix0a3 d2ix0a2 d2lxja_ d1ueba3
d1ueba2 d2z0sa1 d1u0la1 d2ix0a1 d2eifa2 d2je6i1 d3i2za1 d2c35b1
d2ahob2 d1t9ha1 d3i4oa_ d2qall1 d1jt8a_ d2nn6g1 d1g6pa_ d3cama_
d1i94q_ d1d7qa_ d2ba0a1 d2vnud2 d2vnud3 d1khia2 d2qalq1 d1wi5a1
d1xtda2 d1ny4a_ d1dgsa2 d1fvia1 d1ckma1 d1p16a1 d1x9na2 d1pfsa_
d2a1ka_ d1rpma_ d2shpa1 d1yfoa_ d1l8ka_ d2ooqa1 d1g4us2 d1p15a_
d1lara2 d1lara1 d1wcha_ d2fh7a2 d2fh7a1 d1ohea2 d2f4da_ d1rxda_
d2hxpa_ d1xria_ d1d5ra2 d1fpza_ d3ezza_ d4y2ea_ d1i9sa_ d3azya_
d3wvja_ d1upsa1 d2hyka_ d1ajka_ d2vy0a_ d1o4ya_ d3o5sa_ d3d6ea_
d3atga_ d1dypa_ d1umza_ d3i4ia_ d1c1la_ d4lbqa_ d1a78a_ d1slta_
d4no4a_ d3zsja_ d3duia_ d1g86a_ d1is3a_ d1qmja_ d1tdha3 d1q3ba3
d1ee8a3 d1k82a3 g1qmo.1 g1len.1 d3zyra_ d1n47a_ g2ltn.1 d1avba_

d3usua_ d1dhkb_ d1f9ka_ d1ukga_ d1g8wa_ d1g9fa_ d1wbfa_ d3wcsa_
d1g7ya_ g1loe.1 d1mvqa_ d1hqla_ d1v6ia_ d1dbna_ d1qnwa_ d1fx5a_
d4l8qa_ d1ioaa_ d3n35a_ d2h0ba_ d2erfa_ d1h30a2 d3mw4a1 d1pz7a_
d2r1ba_ d3v64a1 d1d2sa_ d3bopa1 d3mw3a1 d1okqa1 d1okqa2 d1wioa4
d1ccza2 d1dr9a2 d1ic1a1 d2d9qb1 d2nxyb2 d1adqa1 d1fnga1 d1uvqb1
d1zs8a1 d2vola1 d2vola2 d1mfbl2 d2jelh2 d1uwel2 d3qq4b1 d1kjvb_
d1k5na1 d1osph2 d4odhl2 d1ow0a1 d1ow0a2 d1bfob2 d3l9ra2 d3jtth1
d2fath2 d4elkb2 d1fn4b2 d2xfxb_ d1pg7x2 d1cqka_ d4k71b2 d1lk2a1
d1eapb2 d4lfhg2 d1i4fa1 d2nw2a2 d1kfah2 d1mhca1 d1h3ua1 d1wejh2
d1h0db2 d1a5fh2 d1kcvh2 d1mjuh2 d1qqda1 d1kcrh2 d1k8ia1 d3huja2
d2nnab1 d1nfdf2 d3mffb2 d1s9va1 d1lk3h2 d1q0xl2 d1fv1b1 d1neza1
d4rfeb2 d1r3ha1 d1a6tb2 d1kjva1 d1pz5b2 d1onqa1 d2bc4a2 d1de4a1
d1k8da1 d1tcra2 d1dn0b2 d1nfde2 d2xwtb2 d1bfoa2 d1mexh2 d1iakb1
d1ypzf2 d4f7ea2 d1q72l2 d2fbjh2 d1fe8h2 d1clzh2 d1fngb1 d1uvqa1
d1vgka1 d1c16a1 d4j12a2 d4x99b2 d1igtb3 d1je6a1 d1q72h2 d1nldh2
d1teth2 d2hmic2 d1i1ca2 d1i1ca1 d1k8ib1 d1q0yh2 d1kfal2 d3frua1
d1pg7w2 d1u58a1 d1igyb4 d1igyb3 d1hyrc1 d1klub1 d1deeb2 d1c12b2
d2bc4b2 d2ig2h2 d1kcvl2 d3wn5b1 d1lk2b_ d1lk3l2 d1mjul2 d1sm3h2
d1q0xh2 d1zvsa1 d1ydpa1 d1u8ib2 d1ypze2 d1kcuh2 d1op9a_ d1y0la1
d1etzb1 d1egjl1 d2gsib1 d1nj9a1 d4jfhd1 d1aqkl1 d1t2ja_ d1lo4h1
d1zvya1 d3v4vl1 d4enec1 d1kxtb_ d5e8ea1 d2pxya_ d4weud_ d1xiwc_
d2esvd1 d1qnzh_ d1ogae1 d1f4xh1 d4n1hb_ d3ezjb1 d3mlwl1 d1kfah1
d1etza1 d1b4jh1 d3cfic_ d4w68a_ d2arja1 d1ek3a_ d2ok0l1 d1vera_
d1uwgh1 d1blnb1 d1fpth1 d1rzga1 d2giya1 d4m3kb1 d1nc2a1 d2nyyd1
d1ncna_ d1fbih1 d5dxwa_ d4fqqa1 d1dqdh1 d1jfql1 d1kelh1 d1faih1
d1frgl1 d1figl1 d4m6nl1 d1fo0a_ d4hxbl1 d3b5ga_ d3quyd1 d2h9gb1
d1i8kb_ d2dd8h1 d2ck0h1 d1eaja_ d4w6wb1 d2a6ib3 d1t66d1 d2e27h_
d1ob1b1 d1f11b1 d2v7na1 d1ad0a1 d2xt1b1 d1pskh1 d4i13b1 d2op4l1
d1a0ql1 d1uwel1 d1za3a1 d1ncbl1 d4uu9a_ d1bm3h1 d1cloh1 d1nqba2
d1cf8h1 d4y5va_ d1mhph1 d5anma1 d4cdgc1 d4ldeb_ d1y0lb1 d2pcpb1
d1z3gl1 d1dfbl1 d1fh5h1 d3g5xa1 d1tzib1 d4dn3l1 d3zhka1 d2b1hh1
d2uyla1 d1f3dh1 d1nj9b1 d2agjh1 d2h1pl1 d1q72h1 d8fabb1 d1ndmb1
d1rjca1 d4u6hb1 d3upca_ d2fbjl1 d1osph1 d1tvda_ d2cjvh_ d4fhbd1
d1bd2d1 d1rz7h1 d3nn8a_ d1knoa1 d1nfda1 d2bmkb1 d1dqll_ d1igyb1
d1igfl1 d12e8h1 d3utza1 d1nc2b1 d1rihh1 d4mwfb1 d4jvpa1 d1mieh1
d1rhfa1 d1fgnl1 d1sbsh1 d1lp9e1 d1dn0a1 d4lqfl1 d1iaih1 d1kgcd1
d1mh5b1 d1gpol1 d2g60h3 d1f8tl1 d2jixd1 d4grwh_ d1fo0b_ d1bfob1
d1axsa1 d1u9ka_ d1nmbh_ d1h8na1 d1h8na2 d1jguh1 d4eura1 d3sdyl1
d35c8h1 d1w72l1 d1mvub_ d1i9rh1 d1ob1a1 d4wenb_ d1yeeh1 d3ietc1
d1f58h1 d1ad0b1 d1mqkl_ d1nbvh1 d4b50a_ d3r8bb_ d1bafl1 d1mamh1
d4rgne1 d2gsgb_ d35c8l1 d1bafh1 d1ai1h1 d2pcpa1 d2fath1 d2x1pa_
d1orsb1 d1ibgl1 d1shma_ d1a2yb_ d1u0qa_ d2coqa_ d1hilb1 d1qfwi_
d1jv5b_ d1fn4b1 d5e0qa_ d1nsnh1 d1plgl1 d4ocxl1 d1kb5h1 d1rzfh1
d1pg7x1 d2fx7h1 d2oi9c_ d1l7il1 d3eyva1 d1d5il1 d4ffyl_ d1tetl1

d1a6wh_ d1ac6a_ d3dvfa_ d1nfde1 d1mimh1 d1ri8a1 d1jhlh_ d3dura_
d1fskb1 d1f97a1 d3so3b1 d2ak4d1 d1lp9f1 d4z7wf1 d1l7th1 d4y5ya_
d4x7ec1 d1emth1 d1ikfl1 d1nlbh1 d4utal1 d1bjma1 d1eapb1 d1u6ah1
d2dtgd1 d1kj2b_ d1xeda1 d1c1eh1 d1fgvh_ d2bnub1 d2fb4h1 d2dtgb1
d1ktrh_ d1jpth1 d1qkzl1 d4hepg_ d2atpb1 d1flrh1 d2hrpl1 d1mvfa_
d1iaim1 d3k80a1 d2jb5h1 d3zkxc_ d4grwe_ d1it9h1 d1rhhb1 d3w9ec_
d1e4wh1 d1sy6h1 d2j6eh1 d3qxta_ d1jglh1 d1p7ka1 d1tzhb1 d4n8pa1
d1gafl1 d2jelh1 d1wioa2 d4i0cc1 d1cicb1 d1lmka2 d1lmka1 d1iqwh1
d1orqb1 d1ejoh1 d1jnlh1 d4zg1a_ d43c9b_ d1ggbl1 d1wejh1 d3q6fa1
d1f3dj1 d3c6la1 d1bqlh1 d3p9wb_ d1a3rl1 d1h0db1 d2uzil_ d1eo8l1
d1qfwl_ d1n7ml1 d1igja1 d1c5ch1 d2a9mh_ d1rmfh1 d1fj1b1 d4heme_
d1ccza1 d1a5fh1 d1ogad1 d1dlfh_ d1qa9b_ d1vgel1 d3qskb_ d1sjxa_
d4ozfg1 d1sq2n_ d1qd0a_ d1kcvh1 d1c5db1 d3qcul1 d8faba1 d3bn9d1
d1mjuh1 d4y8dc_ d7fabh1 d3dvgb1 d1lo0l1 d1a6tb1 d1gigh1 d1kcrh1
d2hwzl1 d1yjdc1 d3clfl1 d4krna_ d1dl7l_ d1e6ol1 d1mexh1 d3r0ma_
d4kkna_ d1fnsl1 d1oaqh_ d1rzia1 d1ucbh1 d1ncwl1 d4b41a_ d1nfdf1
d2mpah1 d2d7tl1 d4grwf_ d4b5ea_ d1lk3h1 d3u79a_ d4u3xa_ d1ghfl1
d1bvkb_ d1mcww1 d1b88a_ d1jrhh1 d32c2b1 d4wemb_ d1dsfh_ d1op3h1
d1ce1l1 d1iqda1 d1qoka1 d1qoka2 d1qfuh1 d2crya1 d1ay1h1 d1oaql_
d3ab0b_ d1rurl1 d1t4kb1 d1bz7b1 d1n0xh1 d1forl1 d1ucbl1 d4w6yb_
d43c9a_ d2aabl1 d1tzia1 d4n1ca_ d1k6qh1 d3g9ab_ d4tvsa1 d1igmh_
d1pz5b1 d3dx9a1 d3wxwh_ d1nakh1 d1kcul1 d3eaka_ d4qlra1 d1cr9l1
d1d9ka_ d1f4xl1 d1deea1 d2i27n2 d1c12a1 d2orbl1 d1j8hd1 d1mcph1
d1c5da1 d1kenh1 d1bd2e1 d1h5ba_ d4x7fc1 d3upaa_ d1t3fa1 d1indh1
d2dtgc1 d4hc1l1 d1zv5a1 d4wgvb_ d1uwgl1 d4f37k1 d1aqkh1 d1vesa_
d3zkqd_ d1fptl1 d1lo4l1 d1knob1 d3rrqa_ d1ct8b1 d4orzc_ d1mlbb1
d4hgkc1 d1dqdl1 d1bogb1 d4ozhe1 d1dr9a1 d1i7za1 d1dn0b1 d1kell1
d1rzib1 d1ypzf1 d1wtla_ d1hkfa_ d1fail1 d1figh1 d2vyre_ d1sjva_
d1ohqa_ d1bfoa1 d1pkqb1 d1mjul1 d1b4jl1 d1nsnl1 d2arjb1 d1p4bh_
d4aj0a_ d3nn8b_ d2rcja1 d1f2xk_ d4v1dd_ d1adqh1 d2eksa_ d4k3ga_
d1jfqh1 d1uweh1 d1frgh1 d4gftb_ d1uz8b1 d4n1ea_ d1bm3l1 d1clol1
d3mcka1 d2vq1a1 d1ae6h1 d4aq1b1 d1uj3b1 d1nbqa1 d1fh5l1 d2ck0l1
d2ywza_ d1q72l1 d4nbxb_ d1hila1 d4hzlb1 d2j88l1 d1zmya1 d1cd0a_
d1a4ka1 d1pskl1 d1a0qh1 d4krmb_ d1ncbh1 d2fbjh1 d1j8he1 d1i3ua_
d1cf8l1 d1fe8h1 d2p1ya1 d1eeqa_ d2x89a_ d1ol0a_ d2r0lh1 d1ub5a1
d1dfbh1 d3eyvb1 d4jdvb1 d4p2cg_ d2gsia1 d2agjl1 d1u3ha1 d2h1ph1
d1rihl1 d1fskc1 d1boga1 d2a77l1 d1ospl1 d1qfoa_ d3dwta_ d2fx7l1
d1rz7l1 d3stba1 d1pkqa1 d1b6da1 d1dzba2 d1dzba1 d1kj2a_ d4l1ha_
d1rzgb1 d1dqlh_ d12e8l1 d1miel1 d1jmaa_ d1sbsl1 d1fgnh1 d1f58l1
d1mqkh_ d1ndgb1 d3qegl1 d1iail1 d2aw2a1 d1tqbc1 d3fo2a1 d4dkaa_
d1f8th1 d2g60l1 d2ywya_ d1uj3a1 d1yc7a_ d3bd3a_ d4w6xb1 d1nmbl_
d1i8ka_ d1rjlb1 d1ypze1 d1p7kb1 d1kxqe_ d1gpoh1 d2z4qa1 d1w72h1
d1i9rl1 d4qgya1 d4xt1c_ d2qqnh1 d1cica1 d3c08h1 d2cd0a_ d2bdnh1
d1hnga1 d1maml1 d1nldh1 d1cr9h1 d4lcih1 d3bkyh1 d3qg7l1 d1ai1l1

d1kxvc_ d4iosd_ d1l7ih1 d2x1qa_ d1ub6b1 d3k74b_ d1ibgh1 d1teth1
d1igya1 d1hxma1 d2x6ma_ d2e7la_ d5e03a_ d1qfwm_ d2r0kh1 d1igjb1
d1rurh1 d1plgh1 d1mj8h1 d1yuhb1 d3fmgl1 d1rzfl1 d1emtl1 d1ad9b1
d1igtb1 d1nlbl1 d1d5ih1 d3k7ua_ d4ffyh_ d1bwwa_ d3dvga1 d1a6ta1
d1fgvl_ d4qxgl1 d1miml1 d1jhll_ d2hffb1 d2hh0l1 d1qkzh1 d1l7tl1
d1afvh1 d1iaii1 d2cjuh_ d1um5h1 d2ghwb2 d2ghwb1 d1kgce1 d2fgwh1
d1ikfh1 d1dvfd_ d2i24n1 d2xtjb_ d1za3b1 d2yc1a_ d2hh0h1 d2x1oa_
d1bvka_ d1rjla1 d5e7ba1 d1igta1 d1il1b1 d2fb4l1 d1mvua_ d1jptl1
d32c2a1 d1fvca_ d1iqdb1 d2hrph1 d2p4ab_ d4qo1a_ d1p2cb1 d1wt5c_
d1t4ka1 d5c1mb_ d1it9l1 d1bz7a1 d1b2wh1 d1e4wl1 d1jnll1 d1yedb1
d1za6b1 d3u7aa_ d1igfh1 d3gnml1 d1orsa1 d1wejl1 d1a2ya_ d1hxmb1
d1gafh1 d2j4wh1 d1pz5a1 d1j05a_ d1a3rh1 d4fqll1 d1qfwh_ d1eo8h1
d1n7mh1 d4s11a_ d1kegh1 d5da4a_ d1iqwl1 d3whxb1 d1vgeh1 d1deeb1
d1c12b1 d1t2ql1 d1ad9a1 d3gsnb1 d3b2uc1 d1ggbh1 d1t3fb1 d1nakl1
d4ocmc_ d1lila1 d2v7hb3 d2esve1 d1mcoh1 d2qr0b1 d2q3aa_ d1ct8a1
d1kcrl1 d2pxyb_ d2uzih_ d5c3ld_ d3durb_ d1c5cl1 d2ak4e1 d1dl7h_
d1xiwd_ d1mlba1 d1rmfl1 d1i7zb1 d1dlfl_ d2xa3a_ d5bq7b1 d1dqqb1
d3h42l1 d1egjh1 d1kcvl1 d1fe8l1 d2j6el1 d1f3rb2 d1lk3l1 d1sm3h1
d1q0xh1 d1ghfh1 d7fabl1 d1igch1 d1lo0h1 d4idla_ d5c2bl_ d1jrhl1
d1il1a1 d3zhda1 d4kfzc_ d1b0wa_ d1e6oh1 d1mexl1 d4krpb_ d1fnsh1
d1ncwh1 d4hgma_ d4eigb1 d4rzca_ d2mpal1 d4y5xa_ d5en2b1 d3lrga1
d4ej1c1 d1rhha1 d1j05b_ d1forh1 d1q9wb1 d2wzpd_ d2aq2a1 d1h0da1
d3revb1 d2ij0c1 d1tzha1 d4m7jl1 d1k6ql1 d3b9kc1 d1igml_ d3qeua1
d1dsfl_ d1ce1h1 d1kcuh1 d1zvha1 d1zvoa1 d1tqbb1 d1ay1l1 d2qscl1
d2cgrh1 d1nl0h1 d1kenl1 d3eo0a1 d4n9ob1 d1n0xl1 d3k1kc_ d2aq2b1
d1enfa1 d1i4pa1 d1v1oa1 d1et9a1 d1esfa1 d2g9hd1 d4ohja1 d1klud1
d1bxta1 d1eu3a1 d1ty0a1 d1fnua1 d2cqva1 d1fhga_ d2k1ma_ d1biha3
d1biha1 d1biha4 d3qp3a1 d1g1ca_ d1gxea_ d1cvsc2 d1cvsc1 d1xmwa2
d1m4ka2 d2otpa2 d1biha2 d1rhfa2 d2fcba2 d2fcba1 d1wwbx_ d1gsma2
d1epfa1 d1epfa2 d2dava1 d1waaa_ d1f97a2 d1x44a1 d1vcaa2 d1fnla2
d1fnla1 d3b5ha2 d3h8na2 d3h8na1 d1jbja1 d1jbja2 d1pd6a_ d1f2qa2
d1f2qa1 d3cx2a_ d1nbqa2 d3b5ha1 d1n26a1 d1xiwb_ d1xaua_ d1fltx_
d2ifga1 d1olla1 d1olla2 d1cs6a4 d1cs6a3 d1cs6a2 d1cs6a1 d2gw5a2
d1sy6a1 d1xiwa_ d1ucta2 d1ucta1 d1ry7b2 d1gl4b_ d1qz1a3 d1olza1
d2c9aa1 d1wwca_

**Family-52 (c.2.1.4)**

**Positive dataset contains 2 protein sequences.**

d1v8ba1 d1ygya1

**Negative dataset contains 3878 protein sequences.**

d1orva2 d1xfda2 d1m32a_ d1ibja_ d1c7na_ d1iuga_ d4q75a_ d2bkwa1
d1t3ia_ d1n8pa_ d1qz9a_ d1jf9a_ d1eg5a_ d3kgwa_ d1cl1a_ d2ctza1
d1vjoa_ d2rfva_ d1gc0a_ d1cs1a_ d1d2fa_ d2ch1a1 d1qgna_ d1pffa_
d1elua_ d1u08a_ d1lc5a_ d1b5pa_ d2ay1a_ d1bw0a_ d3pd6a_ d7aata_
d2csta_ d1j32a_ d1fg7a_ d4wlha_ d1o4sa_ d1v2da_ d1gdea_ d1v72a1

| | | | | | | | |
|---|---|---|---|---|---|---|---|
| d1svva_ | d1ajsa_ | d1wsta1 | d2f8ja2 | d1lk9a_ | d1vp4a1 | d1xi9a1 | d2gb3a1 |
| d1yaaa_ | d1xkla_ | d2wfla_ | d3i2ka1 | d1lnsa3 | d1mpxa2 | d1xkta1 | d2hfka_ |
| d5d3ka_ | d2cfba1 | d1dfoa_ | d1o69a_ | d1bj4a_ | d3k28a_ | d2bwpa_ | d3lg0a_ |
| d2fnua_ | d3ou5a_ | d1z7da1 | d1iaya_ | d4msoa_ | d1bs0a_ | d1s0aa_ | d1sffa_ |
| d3tqxa_ | d1mdoa_ | d3qboa_ | d3pgya_ | d1bjna_ | d2dkja_ | d1ejia_ | d1vefa1 |
| d4wxga_ | d3bs8a_ | d3gbxa1 | d1w23a_ | d1sefa_ | d1sfna_ | d1sq4a_ | d1r0ka3 |
| d1j5pa3 | d1vkoa2 | d1vm6a2 | d3qvsa2 | d1gr0a2 | d1ddla_ | d1auyb_ | d1f56a_ |
| d1bypa_ | d1azca_ | d1ws8a_ | d3u25a_ | d1cuoa_ | d1id2a_ | d1rkra_ | d2iaac_ |
| d4dp1x_ | d1nwpa_ | d2gima_ | d2i7sa_ | d1qhqa_ | d3ehbb1 | d1fftb1 | d1gyca3 |
| d1hfua2 | d1kbva1 | d1kbva2 | d1sdda1 | d1sdda2 | d1gyca1 | d1gyca2 | d1mzya1 |
| d2bw4a1 | d1aoza3 | d1aoza2 | d1kyaa2 | d1kyaa3 | d1kyaa1 | d1mzya2 | d4e9sa1 |
| d1sddb1 | d1sddb2 | d1hfua1 | d1hfua3 | d1oe1a2 | d1oe1a1 | d1v10a2 | d1v10a3 |
| d1v10a1 | d1novc_ | d2bbvc_ | d1a3qa1 | d1iknc_ | d3brda1 | d1p7hl1 | d1oy3c_ |
| d1gjia1 | d1zk9a1 | d2cxka1 | d2bema_ | d1pama1 | d1qhoa1 | d2fhfa2 | d2fhfa1 |
| d1k3ia1 | d1j0ha1 | d1cxla1 | d2bhua1 | d1edqa1 | d1f1sa2 | d1ut9a2 | d1gvia1 |
| d1txka1 | d1zx5a1 | d2phla2 | d2phla1 | d1fxza2 | d1fxza1 | d1uija1 | g1dgw.1 |
| d2v09a_ | d1uika2 | d1uika1 | d1dgwa_ | d1nepa_ | d1ktja_ | d1ds6b_ | d1fstb_ |
| d3us3a3 | d3us3a1 | d4w9ta_ | d1jvna1 | d4x2ra1 | d1ka9f_ | d4axka1 | d1thfd_ |
| d1h5ya_ | d4u28a_ | d2y88a_ | d4wd0a_ | d1vzwa1 | d1tafa_ | d1tafb_ | d1jfib_ |
| d1q9ca_ | d1h3ob_ | d2byka1 | d1n1ja_ | d4g92b_ | d1bh9a_ | d1jfia_ | d1h3oa_ |
| d2bykb1 | d1n1jb_ | d2bjca1 | d2hsga1 | d1qpza1 | d1efaa1 | d1ydwa2 | d2nvwa2 |
| d1h6da2 | d1zh8a2 | d1qkia2 | d1xeaa2 | d1tlta2 | d1ebfa2 | d1au7a2 | d1ic8a2 |
| d2ovga_ | d1sq8a_ | d1rzsa_ | d3kz3a_ | d3orca_ | d3gpdg2 | d2q49a2 | d1hdgo2 |
| d2g17a2 | d2cvoa2 | d2czca1 | d3pwka2 | d1obfo2 | d1zyao2 | d2pkqo2 | d1b7go2 |
| d2hjsa2 | d1cf2o2 | d1vkna2 | d1nvmb2 | d2msya_ | d2djoa1 | d2r5za_ | d1au7a1 |
| d1f43a_ | d2lmda1 | d1akhb_ | d1s7ea1 | d3nara_ | d1zq3p1 | d2craa1 | d1mija_ |
| d4uuta_ | d3a01a_ | d1x58a1 | d3rkqa1 | d3cmya1 | d2cqxa1 | d2da6a1 | d1e3oc1 |
| d1fjla_ | d1mh3a1 | d1x41a1 | d1wh7a1 | d1gt0c1 | d2mw8a_ | d1lfup_ | d1x2ma1 |
| d1b72a_ | d1wi3a1 | d1jgga_ | d1wjha1 | d1x2na1 | d1mnmc_ | d2cuea1 | d3k2aa_ |
| d2hosa_ | d2jwta2 | d1pufa_ | d1octc1 | d2hi3a_ | d1b8ia_ | d4j19a_ | d3lnqa_ |
| d1wh5a1 | d1b8ib_ | d2cqra1 | d1guua_ | d1ug2a1 | d2cqqa1 | d2ajea1 | d4a69c_ |
| d1h8ac1 | d2crga1 | d2iw5b1 | d1xc5a1 | d1ofcx1 | d2cu7a1 | d1wgxa1 | d2cjja1 |
| d2ckxa1 | d1gvda_ | d1hcra_ | d1tc3c_ | d1gdta1 | d1u78a2 | d1hlva2 | d1iufa2 |
| d1iufa1 | d2iu5a1 | d1zk8a1 | d2g7sa1 | d2fd5a1 | d2np5a1 | d1z0xa1 | d2id6a1 |
| d2d6ya1 | d2i10a1 | d1vi0a1 | d2oi8a1 | d3c07a1 | d2ns7a1 | d2fq4a1 | d2yvea1 |
| d1t33a1 | d2o7ta1 | d1rkta1 | d2np3b1 | d2g7ga1 | d2gena1 | d2fbqa1 | d2g7la1 |
| d2gfna1 | d2g3ba1 | d2jj7a1 | d2hyja1 | d1sgma1 | d1ui5a1 | d2hkua1 | d2id3a1 |
| d4jyka1 | d1bl0a1 | d1bl0a2 | d1d5ya1 | d3lv8a_ | d1s3ga1 | d1zaka1 | d1s4qa_ |
| d3tvta2 | d1uf9a_ | d1khta_ | d1ckea_ | d1osna_ | d1p5zb_ | d4tmka_ | d3cm0a_ |
| d1tmka_ | d1jaga_ | d3h86a_ | d1t0hb_ | d1nn5a_ | d2h92a_ | d3kb2a1 | d2c95a1 |
| d1yj5a2 | d1kgda_ | d1ly1a_ | d2ak3a1 | d1vyva2 | d1deka_ | d2grja1 | d1p3ja1 |
| d1y63a_ | d1e4ya1 | d1s96a_ | d3iija1 | d1lvga_ | d1nksa_ | d1vyua2 | d4e22a_ |
| d1qf9a_ | d1jjva_ | d1q3ta_ | d1ki9a_ | d1lw7a2 | d1teva_ | d1kjwa2 | d1gsia_ |
| d2iyva2 | d1viaa_ | d1e6ca_ | d1g3ma_ | d1j99a_ | d1t8ta1 | d1fmja_ | d1zrha_ |

d1ls6a_ d2a3ra_ d1aqua_ d2zptx_ d3ckla_ d1texa_ d1q44a_ d2gwha_
d1odfa_ d1uj2a_ d4gi7a_ d2jeoa1 d1k6ma1 d1z0ja1 d3umra2 d3gj0a1
d2gaoa1 d1yzga1 d1x3sa1 d1g7sa4 d1puja_ d1udxa2 d1kk1a3 d2o52a_
d1jwyb_ d1zcaa2 d1zcba2 d2fv8a1 d2cjwa_ d3dz8a1 d1xzpa2 d1vg8a_
d4qxaa_ d2ew1a1 d1x1ra1 d2bcgy_ d3doea1 d3cona_ d3rega_ d3wyga_
d1upta_ d2b6ha1 d1zd9a1 d1lnza2 d1r5ba3 d1n0ua2 d1r2qa_ d2f9la1
d2rhda_ d3q3jb_ d3q85a_ d1efma1 d1r8qa_ d2gf9a1 d2akab1 d1ek0a_
d1z08a1 d2gcoa1 d2dy1a2 d4g01b_ d2cxxa1 d1svia_ d3q72a_ d1ni3a1
d4ac9a4 d2erxa1 d2bmea_ d1wxqa1 d2gf0a1 d4ldja1 d1zunb3 d1d5ca_
d4lv5b_ d1efca3 d1kmqa_ d2g6ba1 d3lrpa_ d1tada2 d2y8ea_ d4rd4a1
d1z06a1 d2heia_ d3a58b_ d1u0la2 d1d2ea3 d1zj6a1 d2ocba_ d3rwoa_
d1mr3f_ d2e9sa1 d4u5xa1 d2iwra_ d2fn4a1 d3bwdd_ d2nzja1 d2atva1
d2ksqa_ d3l43a_ d1mkya2 d1mkya1 d1wmsa_ d1jnya3 d2rmka_ d1f6ba_
d1f60a3 d1gota2 d1jala1 d2fh5b1 d1g16a_ d2wblc_ d1m7ba_ d1wf3a1
d1f5na2 d1t9ha2 d1z6xa1 d1z0aa1 d2fg5a1 d2a5ja1 d2bcjq2 d1s0ua3
d1ky3a_ d4y0va_ d4lhwa_ d2qmea1 d2atxa1 d1egaa1 d1ryha_ d1moza_
d1nrjb_ d2erya1 d3bfka_ d1puia_ d3j3az1 d3t5ga_ d1shza2 d3defa_
d2f7sa1 d3raba_ d3tsoa_ d3aq4a_ d2al7a1 d3sfva1 d3bc1a_ d2ncda_
d1sdma_ d3h4sa1 d2heha_ d2grya_ g3kin.1 d1kk8a2 d1br2a2 d1goja_
d1f9va_ d2mysa2 d1lkxa_ d3t0qa_ d4db1a1 d3gbja_ d1ry6a_ d1v8ka_
d1k8ka2 d1k8ka1 d3uleb1 d3i33a2 d3i33a1 d1bupa1 d1dkgd2 d1dkgd1
d4ci6a2 d1jcfa1 d4a2ba2 d2fxua1 d1yaga2 d1yaga1 d4cbua2 d1bupa2
d3vrda1 d3vrda2 d1fcdc2 d1fcdc1 d1h1oa2 d1m70a2 d1m70a1 d1ig8a1
d1czan2 d1czan3 d1czan1 d1ig8a2 d1czan4 d1bg3a4 d1bg3a2 d1bg3a1
d3h3no1 d3h3no2 d3ezwa2 d3h1jd1 d2d0oa3 d2d0oa2 d1nbwa2 d1hroa_
d4gedb_ d3zooa_ d1mz4a_ d1gu2a_ d1f1fa_ d1dvva_ d1c6ra_ d1c75a_
d1e29a_ d1gdva_ d1cnoa_ d1h32b_ d1co6a_ d2bh4x_ d2aiua_ d5cytr_
d1jdla_ d1i8oa_ d2zboa_ d3dmia_ d1ls9a_ d1wvec_ d2yk3a_ d3ph2b_
d1dw0a_ d5aura_ d1ql3a_ d1qn2a_ d1f1ca_ d2nqya_ d1h4ga_ d1hixa_
d1oa2a_ d1olra_ d3b7ma_ d4h7ma1 d3wp3a_ d2vgda_ d1igoa_ d3zsea_
d2nlra_ d3wx5a_ d1oa3a_ d1pvxa_ d1m4wa_ d1f5ja_ d1oa4a_ d1ks5a_
d4ixla1 d2ah2a1 d1evsa_ d1cnt1_ d1pvhb_ d1z7ca_ d1huwa_ d1bgea_
d1f6fa_ d1f45b_ d1i1rb_ d1axia_ d1etea_ d3tgxb_ d2z3qa1 d3qb1a_
d1hula_ d1m48a_ d4nkqb_ d1eera_ d1v7mv_ d5e4ea_ d2o27a_ d3b5ka_
d2psma1 d4zf7a_ d3uf5a_ d4x51a_ d4ypgc1 d3oq3a_ d1lqsl_ d1n1fa_
d1au1a_ d1wu3i_ d3besl_ d3ux9a_ d3rbaa_ d2qtra_ d1nupa_ d3l92a_
d1f9aa_ d1qjca_ d2h29a_ d1vlha_ d1od6a_ d1lw7a1 d1mrza2 d4ruka_
d2el7a_ d1iq0a2 d1irxa2 d1r6ta2 d1ivsa4 d2cyba_ d1rqga2 d1j09a2
d2yxna_ d1f7ua2 d1h3fa1 d2g36a1 d1nzja_ d1jila_ d1n3la_ d1i6la_
d1pfva2 d1h3na3 d3n9ia_ d3sz3a1 d3sw2a_ d1moxc_ d1dx5i3 d1edmb_
d1autl2 d1autl1 d1nqlb_ d1dval1 d1szba2 d1kigl_ d2y5fl_ d1ijqa2
d1gk5a_ d1g1sa2 d1nt0a3 d1xdtr_ d1lmja2 d1lmja1 d1nzia2 d2wphe_
d1klil_ d1pfxl2 d1pfxl1 d1p9ja_ d1g1ta2 d1f7ea_ d4yzub_ d2mgpa1
d1b9wa1 d2mgpa2 d1ob1c1 d1ob1c2 d1n1ia2 d1n1ia1 d2npra2 d2npra1

d1m1xb5 d2y5ia_ d1psra_ d3n22a_ d2egda_ d4ggfc_ d2wcea_ d2luca_
d4aqia_ d3nsia_ d2lp3a_ d1j55a_ d1a4pa_ d1k8ua_ d1nsha_ d1pvaa_
d3fs7a_ d2pvba_ d1ttxa_ d1rwya_ d2kyfa_ d1rk9a_ d1a75a_ d1exra_
d1tiza1 d1nyaa_ d1g8ia_ d1ggwa_ d1wdcc_ d3fwba_ d1ggza_ d2o5ga_
d1ctaa_ d3i5gc_ d1sl8a_ d1fpwa_ d2scpa_ d2roaa_ d4aqra_ d3pn7b_
d1br1b_ d2mysb_ d1s6ca_ d1bjfa_ d2obha_ d1jfka_ d1jf0a_ d1qv0a_
d1lkja_ d2mlfc_ d2m97a_ d2d8na_ d4by5a_ d1jbaa_ d4nqga_ d1wdcb_
d2mysc_ d1j7qa_ d1s6ia_ d1uhka2 d1r2ua_ d1dtla_ d3kf9a_ d3pn7c_
d1qjta_ d1c07a_ d5d67d_ d1tuza_ d1h8ba_ d1eg3a2 d2zkmx1 d1snla_
d1gjya_ d1y1xa_ d1hqva_ d1dvia_ d1df0a1 d1qxpa2 d4usla_ d1k94a_
d1wkya2 d1uuqa_ d3wh9a_ d4m1ra_ d4xuya_ d2je8a5 d4bf7a1 d3cuia_
d1ur4a_ d1qw9a2 d1odza_ d2vzsa5 d1bqca_ d2x2ya1 d1hjqa_ d1qnra_
d4y7ea1 d1vjza_ d4k68a_ d2v3ga_ d4hpga_ d1nofa2 d1nq6a_ d1h4pa_
d1aq0a_ d3ziza_ d4bs0a_ d1ecea_ d1h1na_ d1ta3b_ d1w91a2 d1i1wa_
d1uhva2 d1us3a2 d1egza_ d7a3ha_ d1tvna1 d3nyda1 d1ghsa_ d2cyga1
d4xv0a_ d2c8na2 d1w32a_ d3emca_ d1v6ya1 d1foba_ d2uwfa1 d2fgla_
d3niya1 d2c0ha1 d1rh9a1 d1yq2a5 d1szna2 d1fa2a_ d1pama4 d5do8a1
d1ea9c3 d1hx0a2 d2fhfa5 d1qhoa4 d1avaa2 d1m53a2 d4m8ua1 d2gjpa2
d1lwha2 d4howa1 d1ktba2 d1j0ha3 d3vm5a1 d2x1ia_ d1wzaa2 d1uasa2
d2diea1 d1mxga2 d1ht6a2 d1eswa_ d1iv8a2 d5cpsa_ d1ud2a2 d1zjaa1
d3gbea1 d1kwga2 d1wdpa_ d1gcya2 d1g5aa2 d1hvxa2 d3dhpa2 d2bhua3
d4do4a1 d3qgva1 d1e43a2 d1g94a2 d2d3na2 d1gjwa2 d1gvia3 d1x1na1
d1r7aa2 d1tz7a1 d4c7ga2 d2gjxa1 d1nowa1 d1yhta1 d4toqa_ d1wb0a1
d1w9pa1 d1eoka_ d1itxa1 d1kfwa1 d1goia2 d1edqa2 d2gsja_ d1hjxa1
d1vf8a1 d1etob_ d1umqa_ d1g2ha_ d1mixa2 d1e5wa2 d1isna2 d2al6b2
d1g4ma2 d1eh3a_ d3skpa_ d1dtza1 d1ieja_ d1jnfa2 d1ce2a1 d1jnfa1
d1b1xa2 d1b1xa1 d1jw1a1 d1jw1a2 d1ryoa_ d1h76a1 d1h76a2 d1wgqa1
d1v89a1 d2coda1 d1zc3b_ d1wg7a1 d1u5ea1 d1v61a1 d2coca1 d2ys1a1
d1v5pa1 d1ntya2 d1wjma1 d1wi1a1 d1u29a_ d1xx0a1 d1unqa_ d2zkmx3
d1fhoa_ d1v88a1 d2adza1 d1x1fa1 d1p6sa_ d2coaa1 d1btka_ d1v5ma1
d1faoa_ d2x18a_ d1dyna_ d1x1ga1 d4kaxb_ d2j59m1 d1ki1b2 d1xcga2
d1txda2 d1u5da1 d1dbha2 d1w1ha_ d1upqa_ d1v5ua1 d1foea2 d2fjla1
d1eaza_ d2dfka2 d2cofa1 d1kz7a2 d1h6ta1 d1h6ua1 d3wdpp1 d3wq5a_
d1gnxa_ d4gxpa_ d4hz8a_ d1e4mm_ d3aiua_ d1wcga1 d1qvba_ d3ahxa_
d1v02a_ d2dgaa_ d1pbga_ d1vffa1 d1qoxa_ d4ha4a_ d1r55a1 d3gboa_
d1atla_ d1kufa_ d1buda_ d4q1la_ d1quaa_ d1yp1a_ d1wnia_ d2gfoa1
d1vjva_ d2ayna1 d3nhea_ d3nqyb_ d4gera_ d1e2ta_ d4c5pa_ d2bsza1
d4bgfa_ d1w4ta1 d1ex0a4 d2f4ma1 d1x3za_ d1mdwa_ d1qxpa4 d1ziva1
d1zcma1 d2w0pa_ d2diaa1 d2e9ia2 d2e9ja1 d2dj4a1 d2eeba1 d2diba1
d2dmba1 d2dmca1 d2d7oa1 d2ee6a1 d2d7pa1 d2eeaa1 d2di8a1 d1wlha1
d2d7na1 d2nqca_ d2d7ma1 d2dica1 d3cnka_ d2k3ta1 d2eeca1 d1qfha2
d2bp3a1 d1v05a_ d2ee9a1 d2eeda1 d2di9a1 d3ioqa_ d1fh0a_ d1o0ea_
d1khqa_ d1m6da_ d2cb5a_ d1cs8a_ g8pch.1 d1s4va_ d2pnsa_ d1vsna1
d1mira_ d1iwda_ d2oula_ d7pcka_ d3bwka_ d3bcna_ d2p86a_ d1pvja_

d1pcia_ d1gece_ d1deua_ d4bs6a_ d1xkga1 d1cqda_ d1jqpa2 d2c0ya_
d2bdza_ d3p5ua_ d1zoya1 d1qo8a2 d1d4ca2 d1chua2 d1jnra2 d2jk6a2
d2jk6a1 d1feca1 d1feca2 d1lpfa1 d1lpfa2 d1xhca1 d1xhca2 d1dxla2
d1dxla1 d1gera2 d1m6ia2 d1onfa1 d3lada2 d3lada1 d1w4xa1 d1w4xa2
d1gesa2 d1mo9a1 d1xdia1 d1fl2a2 d1fl2a1 d1fcda1 d1fcda2 d1h6va2
d1h6va1 d1mo9a2 d1gv4a2 d1gv4a1 d2gv8a2 d2gv8a1 d1aoga1 d1aoga2
d1hyua1 d1ebda1 d1ebda2 d3dk9a1 d1q1ra2 d1q1ra1 d1gesa1 d1zbfa1
d2z1ha_ d2etja1 d3qioa1 d1jl1a_ d2e4la_ d1jl2a_ d2lsna1 d1io2a_
d1uaxa_ d2yv0x_ d1mu2a1 d1ekea_ d1i39a_ d1pn0a1 d1ju2a1 d2f5va1
d3c96a1 d2gmha1 d1pj5a2 d1gpea1 d1o5wa1 d1n4wa1 d1k0ia1 d2dw4a2
d2ivda1 d1seza1 d2voua1 d1kdga1 d1s5ja1 d2hbja2 d2igia_ d1wlja_
d1qhta1 d4dlea1 d1tgoa1 d2f96a1 d3tr8a1 d3mxma_ d1q8ia1 d1y97a1
d1w0ha_ d1kfsa1 d2py5a1 d1noya_ d1d5aa1 d1yt3a3 d2guia_ d3mwpa1
d4kb1a_ d2v76a_ d1wj1a1 d1aqca_ d1qqga2 d1qqga1 d1n3ha_ d4dbba_
d3f0wa1 d1p3ra_ d1wgua1 d2cy4a1 d3hqca_ d1wvha1 d1xr0b1 d2av4a1
d3nvva1 d1krha3 d2h88b1 d1t3qa2 d1kf6b2 d1zoyb1 d1jq4a_ d1ffva2
d1dgja2 d1rm6c2 d1r7ha_ d1faaa_ d1wika1 d1xfla_ d1ttza1 d2f51a1
d1ti3a_ d1nhoa_ d1sena_ d2i4aa_ d1ep7a_ d1zmaa1 d1fo5a_ d3lefa1
d3diea1 d2yana1 d1z6na1 d1oaza_ d3zywa1 d4aj8a_ d1nm3a1 d4fiwa_
d1wmja_ d4hs1a1 d1iloa_ d1xwaa1 d1wjka1 d2vm1a_ d1fb6a_ d1gh2a_
d1h75a_ d1nw2a_ d1kx5b_ d3a6na_ d4m6ba2 d2nqbd_ d3wkjd_ d2yfwe_
d4h9na_ d5bnxa_ d1kx5c_ d3r45a_ d1eqzb_ d4jjnb_ d1f66c_ d1eqza_
d4wnnb_ d1kx5a_ d1u35c1 d1id3a_ d2nqbc_ d1kx5d_ d1ku5a_ d1f1ea_
d1um8a_ d1ixza_ d1qvra2 d1qvra3 d1qzma_ d1sxjc2 d1jr3d2 d2p65a_
d1l8qa2 d1g8pa_ d3hu3a3 d1sxjd2 d1njfa_ d1in4a2 d1sxja2 d1tuea1
d2ce7a2 d1w5sa2 d1sxjb2 d1ny5a2 d1ofha_ d2gnoa2 d2a5yb3 d1fnna2
d1u0ja1 d1lv7a_ d1iqpa2 d2fnaa2 d1ixsb2 d4gita_ d1sxje2 d1g41a_
d1pd212 d2a2ra2 d1k0dd2 d1f2ea2 d1gsua2 d1f3ba2 d2fhea2 d2pvqa1
d1tw9a2 d1oyja2 d1e6ba2 d1k0ma2 d3ik7a1 d4is0a1 d1r5aa2 d1jlva2
d1m0ua2 d3gtub2 d1z9ha2 d1okta2 d1axda2 d2gsra2 d1tu7a2 d1k3ya2
d1zgma1 d1b48a2 d1gnwa2 d1gwca2 d2gsta2 d1glqa2 d1oe8a2 d1pn9a2
d1v2aa2 d1fw1a2 d1duga2 d1g7ea_ d1nira1 d1qksa1 d1qqya_ d1gd6a_
d1f6ra_ d2gv0a_ d1iiza_ d2z2fa_ d1j8xa_ d1ghla_ d1b9oa_ d174la_
d169la_ d2o4wa_ d1p37a_ d1k28a3 d1xjta1 d1jtma_ d2f2qa_ d4e97a1
d1mpya2 d1kw3b2 d1kw3b1 d4ghga1 d1tfza1 d3isqa1 d3isqa2 d1sqia1
d1sqia2 d1lkda1 d1mpya1 d1cjxa2 d1cjxa1 d1sp9b2 d1t47a1 d1t47a2
d1f1ua2 d1sp8a1 d1sp8a2 d1f1ua1 d4iuaa3 d4bvva_ d5hpga_ d3k65a_
d1ki0a3 d1ki0a2 d1ki0a1 d1a0ha1 d1pmla_ d3sp8a3 d2feba1 d1qsta_
d2beia1 d4m3sa_ d1u6ma1 d1ygha_ d1yk3a1 d3dr6a_ d1xmta_ d1z4ea1
d2ge3a1 d1bo4a_ d2fiaa1 d2jdca1 d1y9wa1 d3to7a_ d1wwza1 d2fl4a1
d2ozua1 d2b5ga_ d2cy2a1 d2p0wa_ d1cm0a_ d4pswa_ d1yrea1 d1yr0a1
d1m4ia_ d2gana1 d1yvka1 d1ghea_ d2g3aa1 d1y7ra1 d2euia1 d1q2ya_
d2fsra1 d1xeba_ d2ae6a1 d1cjwa_ d1qsma_ d2aj6a1 d1y9ka1 d1i12a_
d1vkca_ d2atra1 d1n71a_ d2d4pa1 d2fiwa1 d2fe7a1 d1tiqa1 d2ou2a_

d1yx0a1 d2fcka1 d1p0ha_ d2j8ma_ d2pq8a_ d1z4ra1 d1s3za1 d1ufha_
d1f37b_ d1xvqa1 d1pn0a2 d3hy2a_ d2cvba1 d1o8xa_ d1lu4a_ d1we0a1
d2bmxa1 d2a4va1 d1psqa_ d1xiya1 d1q98a_ d1n8ja_ d1zofa1 d2ggta_
d3tjja_ d4je1a1 d3s9fa_ d2zcta_ d1qmva_ d1jfua_ d2b1ka_ d5dvba1
d2b5xa1 d2v2ga_ d1st9a_ d3tb2a_ d4llra_ d4fh8a1 d1e2ya_ d1knga_
d2h30a_ d1i5ga_ d2z9sa_ d2f8aa1 d1tp9a1 d2b7ka_ d1nm3a2 d3hvva_
d2wfca1 d4kcea_ d1xvwa1 d4gqca1 d3qpma_ d4k1fa_ d1zzoa1 d1gp1a_
d1prxa_ d4od7a1 d1z6ma1 d4mcua1 d4dvca1 d1r4wa_ d3l9sa_ d1un2a_
d3rppa_ d2qked_ d4ksoa_ d1t4za1 d1wwja_ d4mh8a_ d4i7ga1 d1ys1x_
d4hs9a_ d1ex9a_ d1ku0a_ d3qmma_ d1lgya_ d4l3wa_ d1gz7a_ d1ukca_
d1cr6a2 d3groa_ d1pjaa_ d1ei9a_ d1ra6a_ d3gsza_ d1khva_ d1te9a_
d2xwha_ d3uqsa_ d3hkwa_ d3n6ma_ d2cjqa_ d2xyma_ d1teba_ d1te8a_
d1s48a_ d1u09a1 d4wfxa_ d3tyqa_ d2pgga1 d2ddfa_ d3p0ya1 d1igra1
d1igra2 d1n8yc2 d1n8yc1 d1m6ba2 d1m6ba1 d1jx4a2 d3gv5b1 d1t94a2
d3bq0a2 d2tbvc_ d1c8nc_ d1x36a_ d1smvc_ d1ng0c_ d4sbvc_ d1opoa_
d1f2nc_ d1cixa_ d1y29a_ d1v7fa_ d2jtba_ d1d1ha_ d1g9pa_ d1lupa_
d1qk7a_ d1mb6a_ d2a2va_ d1niya_ d1i25a_ d2mpqa_ d1ie6a_ d1kqha_
d1la4a_ d2mxoa1 d1dl0a_ d1koza_ d1qk6a_ d1emxa_ d1lqra_ d1c6wa_
d1rmka_ d1ixta_ d1eyoa_ d1cnna_ d1fu3a_ d1f3ka_ d1fyga_ d3lgia_
d3i77a_ d2z9ia2 d4nsva_ d1lcya2 d4jcna_ d1agja_ d1os8a_ d1hpga_
d1p3ca_ d1l1ja_ d1qtfa_ d1ny721 d1ny722 d1a6ca3 d1a6ca2 d1a6ca1
d1pgl22 d1pgl11 d1ny711 d1mqta_ d1d4m3_ d1fmd3_ d2mev1_ g1aym.1
d1ev13_ d4q4w1_ d1r1a3_ d2x5ia_ d1rhi1_ g1r1a.1 g1d4m.1 g1rhi.1
d1b35b_ d1oopa_ d1tme1_ d4iv1a_ g1pvc.1 g1fpn.1 d1fpn1_ d4gb31_
d1d4m1_ d1fmd1_ d1ev11_ d2wzr3_ g1cov.1 d1qqp3_ g1fmd.1 d2x5ic_
d1rhi3_ g1oop.1 d1hxs1_ g1hxs.1 d1fpn3_ d1bev1_ g2mev.1 d1eah3_
d1aym1_ d1pvc3_ d1qqp1_ d1zba3_ d1h8ta_ d3vbhc_ g1b35.1 g1bev.1
d1hxs3_ g1ev1.1 d1bev3_ g1tmf.1 d1tmf1_ d1b35a_ d2mev3_ g1eah.1
g1h8t.1 d1eah1_ d4q4w3_ d1r1a1_ d1aym3_ d1pvc1_ d1zba1_ d1h8tc_
d1oopc_ d1tme3_ g1qqp.1 d4iv1c_ d1y14b1 d2nn6i1 d1i94l_ d2zjra2
d1smxa_ d2qamc2 d2r7da1 d1rl2a2 d1wfqa1 d3bzka4 d1x6oa2 d3a0ja_
d2j01d2 d2ja9a1 d4ql5a_ d1hr0w_ d1luza_ d2n3sa_ d2vnud1 d1h95a_
d1go3e1 d2nn6h1 d1ne3a_ d2mo1a_ d1iz6a2 d1jjga_ d1e3pa2 d2ix0a3
d2ix0a2 d2lxja_ d1ueba3 d1ueba2 d2z0sa1 d1u0la1 d2ix0a1 d2eifa2
d2je6i1 d3i2za1 d2c35b1 d2ahob2 d1t9ha1 d3i4oa_ d2qall1 d1jt8a_
d2nn6g1 d1g6pa_ d3cama_ d1i94q_ d1c9oa_ d1d7qa_ d2ba0a1 d2vnud2
d2vnud3 d1khia2 d2qalq1 d1wi5a1 d1xtda2 d1ny4a_ d1eixa_ d3exra_
d2ffca1 d2fdsa1 d3n3ma1 d2czda_ d1q6oa_ d2yyua_ d1vqta1 d1dbta_
d3ldva1 d4lt9a1 d3tc7a1 d3hoja_ d1i4na_ d3vnda_ d3nl8a1 d1geqa_
d3nava1 d1v5xa_ d4a2ra1 d3tc6a1 d1ujpa_ d4ijba_ d1xcfa_ d1vc4a_
d1rd5a_ d3b5va_ d2r80b_ d1cqxa1 d1cg5a_ d1gcvb_ d3boma_ d2h8fb_
d2raoa_ d3bj1a_ d4hrrb_ d2r50a_ d3d1kb_ d2aa1b1 d3zhwa_ d3hf4b_
d3gdja_ d1jebb_ d3bj1b_ d1x9fb_ d1wmub_ d1gcva_ d3bcqb_ d3d4xb_
d1gvha1 d2dc3a_ d3hf4a_ d4yu3a_ d3k8ba_ d1qpwb_ d2r80a_ d3bcqa_

d1v4wa_ d3wtgb_ d1jl7a_ d4esaa_ d1a9we_ d3d1ab_ d1fsla_ d4h2la_
d1v4wb_ d1mwca_ d1vhba_ d1jeba_ d1spgb_ d1h97a_ d1q1fa_ d1tu9a1
d3d1aa_ d1hdsb_ d3hyub_ d2zfbb_ d3fs4a_ d1oj6a_ d1shrb_ d4h2lb_
d4yu3b_ d1spga_ d3hrwa_ d3qqra_ d3at5a_ d4wchd_ d3hyua_ d1cg5b_
d1uc3a_ d1hbra_ d2raob_ d1outb_ d4esab_ d3gdjb_ d3qqqa_ d1x9fc_
d3vrfa_ d1itha_ d3mkba_ d3d4xa_ d3mjpa_ d1jl6a_ d3fh9a_ d1outa_
d2dc3b_ d1it2a_ d3eoka_ d3g46a_ d1qpwa_ d3vrfb_ d1x9fd_ d1hdsa_
d2zfba_ d3mkbb_ d1or4a_ d3wtga_ d1a4fa_ d3fh9b_ d3bomb_ d3cy5a_
d3at5b_ d1x9fa_ d2oifa_ d4lmxb_ d1cpcb_ d3v57a_ d1kn1a_ d1cpca_
d4f0ta_ d2vjha_ d4po5b_ d1eyxa_ d1alla_ d4f0tb_ d1b33b_ d1b8db_
d4h0mb_ d1b33a_ d2vjhb_ d4lm6b_ d2vmlb_ d1allb_ d2vjta_ d3dbja_
d2vmla_ d2vjtb_ d4f0ub_ d1f99b_ d4rmpa1 d1liaa_ d1eyxb_ d2uulb_
d4lmsb_ d4f0ua_ d2bv8a_ d1f99a_ d2bv8b_ d2c7lb_ d3dbjb_ d4rmpb1
d1b8da_ d4h0ma_ d3v57b_ d1liab_ d2uula_ d2j96a_ d1cwva5 g1gxs.1
g1wht.1 d1hlga_ d2xqfa_ d1f6wa_ d1dx4a_ d1qe3a_ d1k4ya_ d4qwwa_
d2ha2a_ d1r88a_ d1sfra_ d4qeka_ d1rypd_ d1irua_ d1rypc_ d3unfk_
d1rypa_ d1j2pa_ d1irui_ d1j2qh_ d3unfh_ d1m4ya_ d1rypb_ d1irul_
d1irug_ d1iru2_ d1q5rh_ d1rypg_ d1g3ka_ d1q5qa_ d1e94a_ d1irub_
d1ryph_ d3ty6a_ d1iruj_ d1iruk_ d1rype_ d1irud_ d1irue_ d3mi01_
d1rypf_ d1iruh_ d2z3ba_ d1iruc_ d3mka2_ d1iru1_ d2qvba_ d2v9za_
d4kafa1 d1b9ra_ d1offa_ d1frra_ d3wcqa_ d1e9ma_ d1l5pa_ d1wria_
d1i7ha_ d3av8a_ d1rfka_ d3p1ma1 d3b2ga_ d2bt6a2 d1s5ja2 d1qhta2
d4dlea2 d1tgoa2 d3py8a2 d1q8ia2 d1kfsa2 d2py5a2 d1d5aa2 d1f74a_
d3l21a_ d2e1da_ d1to3a1 d1w3ia_ d1sfla_ d1n7ka_ d1vcva1 d4imea_
d1wa3a_ d2ocza1 d1f2ja_ d3m16a_ d2yxga_ d1ub3a_ d3a5fa_ d1fbaa_
d1l6wa_ d1xfba1 d1wx0a1 d1vpxa1 d1ojxa_ d1mxsa_ d2a4aa1 d1xkya1
d3cq0a1 d3s1xa_ d1f05a_ d1fdja_ d3cpra1 d4d2ja_ d1cg2a1 d1yloa2
d1y7ea2 d1y0ya3 d1lfwa1 d1vixa1 d1fnoa4 d1ysja1 d1vhea2 d1z2la1
d2fvga2 d1vgya1 d1r3na1 d2grea2 d1xmba1 g1q7l.1 d1rtqa_ d3rp2a_
g1ucy.1 d1fuja_ d2zchp_ d3w94a_ d1si5h_ d1lo6a_ g2pka.1 d1cgha_
d1kigh_ d1a5ia_ d3s9ba_ d1hj8a_ d1ao5a_ d2anya_ d1q3xa1 d1gvza_
d1azza_ d4gsoa_ d2f91a1 d1iaua_ d1gdna_ d1eufa_ g1fiw.1 d1eq9a_
d4nfea_ d2rdla_ d1spja_ d1elva1 d1a7sa_ d1ekbb_ d1pfxc_ d1yc0a_
d2aiqa_ g1fiz.1 d1fi8a_ d4k60a_ d4dgja_ d1mzaa_ d1op0a_ d3tjva_
d1pytd_ d1sgfg_ g1gg6.1 d2eeka_ g1rtf.1 d1a0ja_ d3g01a_ d1pytc_
d4e7na_ d1ddja_ d1z8ga1 d2hlca_ d1npma_ g1jou.1 d1sgfa_ d1autc_
g2hnt.1 g1kdq.1 d1orfa_ d3s69a_ d4crga_ d1fxya_ d1bqya_ d2ftba_
d1pmpa_ d2rcqa1 d1fdqa_ d4lkpa_ d1sa8a_ d1vyfa_ d1lpja_ d3vg7a1
d4azqa1 d1o8va_ d5bvqa_ d1p6pa_ d2qo4a_ d1opba_ d1yiva1 d1o1va_
d4bvma1 d4tkba_ d4i3ba_ d1bwya_ d1ggla_ d4gkca_ d1cbia_ d3p6da1
d1kqwa_ d2a0aa1 d1dzka_ d4tlja_ d2a2ua_ d4wfva_ d3t1da_ d4iaxa_
d3zq3a_ d2nnda_ d1i4ua_ d1kxoa_ d1bbpa_ d1kt7a_ d1exsa_ d3s26a1
d1lf7a_ d1qfta_ d4gh7a_ d1z24a1 d1qabe_ d1xkia_ d1ew3a_ d3bx7a_
d4iawa1 d1n0sa_ d1gkab_ d3kzaa_ d2k23a_ d2l9ca1 d2joza1 d1gm6a_

d3kffa_ d3saoa1 d1e5pa_ d1yupa1 d1iiua_ d1e69a_ d1mv5a_ d3g60a4
g1ii8.1 d1ewqa2 d1ji0a_ d1qhla_ d1v43a3 d2wmfa1 d1vpla_ d1g2912
d4a82a2 g1f2t.1 d2d62a1 d3tuic1 d2bbsa1 d3g60a3 d2ixea_ d1g6ha_
d1sgwa1 d2qi9c_ d3rlfa1 d3b60a1 d3d31a2 d2pzea1 g1xew.1 d1jj7a_
d1oxxk2 d2onka1 d1w1wa_ d1iwea_ d1qf5a_ d1vmaa2 d1nija1 d1np6a_
d2p67a1 d3ihla_ d1okkd2 d1ls1a2 d1s1ma2 d1iona_ d2v40a_ d3hida_
d2vo1a1 d2g0ta1 d1vcoa2 d1j8mf2 d1g3qa_ d1xjca_ d1p9ba_ d1qzxa3
d1ihua2 d1ihua1 d2qm8a_ d1dj2a_ d4ioja_ d1cp2a_ d1yrba2 d1hyqa_
d3gqba2 d1skye3 d1xp8a1 d1tf7a2 d1tf7a1 d1ye8a1 d1skyb3 d2qe7d2
d4blta_ d4huta_ d3ldaa2 d4hyya_ d2i3ba1 d1cr1a_ d3j9tb2 d3ntua2
d1vdza1 d2z43a_ d1e9ra_ d1nlfa_ d3ziaa2 d1n0wa_ d1g6oa_ d3ziad2
d3j9ta2 d1pzna2 d3gqbb2 d2i49a_ d1nnfa_ d1xvxa_ d1ursa_ d1h3da1
d1iuda_ d4m1va1 d1twya_ d3e13x_ d2nxoa1 d4pfua_ d3ttma_ d1elja_
d2qmwa1 d1us5a_ d1y3na1 d1jeta_ d1uqwa_ d4f19a1 d2esna2 d3thia_
d3fasa1 d2uyeb_ d2xwva1 d1r9la_ d2fyia1 d1pb7a_ d2ozza1 d1sw5a_
d1nh8a1 d1vr5a1 d2g29a_ d1xs5a_ d3lswa_ d2z23a_ d3w9va_ d3tcha_
d1q35a_ d1z7me1 d3q27a_ d1p99a_ d1ve4a1 d2i6ea1 d4mcoa1 d4n8ya_
d1zbma1 d1hsla_ d1ixca2 d1j1na_ d1xvya_ d1y9ua_ d2z22a1 d1xoca1
d4oera_ d1wdna_ d5f1qa1 d1xt8a1 d1i6aa_ d4gwoa_ d4igta1 d1pc3a_
d4mmpa1 d1o63a_ d2czla1 d2onsa2 d4mija1 d4ok5a1 d2bmfa1 d2bmfa2
d1yksa1 d1yksa2 d1jr6a_ d2qeqa1 d2jlqa1 d1g8fa3 d1c4oa1 d2p6ra3
d2p6ra4 d1oywa2 d1oywa3 d1wrba1 d1w36b2 d1w36b1 d1c4oa2 d3bora_
d1t6na_ d2eyqa5 d2eyqa3 d2eyqa2 d2zu6a2 d1veca_ d1uaaa1 d1w36d1
d2g9na1 d1z3ix2 d2rb4a_ d1hv8a1 d1hv8a2 d1t5ia_ d1w36c2 d2j0sa2
d2j0sa1 d1fuka_ d3hjha1 d1z5za1 d1qdea_ d1z63a1 d1s2ma2 d1s2ma1
d1wp9a1 d1wp9a2 d1gm5a3 d1gm5a4 d3dina4 d3dina3 d1q0ua_ d1w36c1
d1z3ix1 d1nkta3 d1uaaa2 d1w36d2 g1qhh.1 d2fwra2 d2fwra1 d3q3ma_
d1za0a1 d1mxra_ d2inca_ d1jk0a_ d2incb_ d1mhyb_ d3hf1b_ d1mtyb_
d1r2fa_ d2uw1a_ d3n37a_ d1afra_ d1oquc_ d1syya_ d1mtyd_ d2o1za_
d1uzra_ d1smsa_ d2fkza_ d1zuja1 d1s3qa1 d2fjca1 d1z6om1 d1veia_
d4dyya_ d1jiga_ d3gvya_ d2pyba_ d4cy9a1 d3bvfa1 d1r03a_ d2cf7a_
d1nf4a_ d1zs3a1 d4iwka_ d3ka8a_ d3np0x_ d1n1qa_ d1lb3a_ d4e6ka_
d1z6oa1 d2fzfa1 d4dyua_ d3ge4a_ d1jgca_ d4am4a_ d2z6ma_ d1ji5a_
d1vlga_ d1lkoa1 d1krqa_ d3qz3a_ d1o9ra_ d2ffxj_ d1euma_ d1yv1a1
d2v2pa_ d1j30a_ d4di0a_ d1dpsa_ d1oewa_ d2qzxa_ d2asia_ d1psoe_
d1miqa_ d1smra_ d2h6ta_ d1pfza_ d1izda_ d3hd8a_ d1j71a_ d1dpja_
d4aa9a_ d1t6ex_ g1b5f.1 g1lya.1 g1htr.1 d1qdma2 d1wkra_ d1tzsa_
d1ibqa_ d2qzwa_ d1bdqa_ d3ggta_ d3ixoa_ d1mrxa_ d2haha_ d1tw7a_
d4njsa_ d1sgua_ d2p3da_ d1nsoa_ d3d3ta_ d2rkfa_ d1c6ya_ d3ogpa_
d3ekwa_ d2o4na_ d2j9ja_ d1baia_ d3ggua_ d3lzua_ d4npua_ d4j55a_
d2r8na_ d2fxda_ d2p3ca_ d4dqea_ d3ttpa_ d3t3ca_ d2p3aa_ d2hc0a_
d2fdda_ d3mwsa_ d1k8rb_ d1wgra1 d1ef5a_ d1wfya1 d1e8ya3 d1wgya1
d1lfda_ d2cs4a1 d1wxma1 d2byea1 d3kh0a1 d2byfa1 d1wxaa1 d1h4ra3
d4yl8a1 d2al6a3 d1wgda1 d1wgga1 d4q5eb_ d1wjna1 d1se9a_ d3n3kb_

d1wz0a1 d1we6a1 d2m0xa_ d2gbka_ d3rt3b1 d1wh3a1 d1sifa_ d2ekec2
d4ii2b1 d4hk2a1 d1c3ta_ d1m94a_ d1wgha1 d1ttna1 d1j8ca_ d1wx8a1
d3v6cb1 d2ma9b_ d4i6lb1 d1bt0a_ d2jvca1 d1iyfa_ d2k1fa_ d1wxva1
d1wf9a1 d1t0ya_ d2io0b2 d1x1ma1 d2gbma_ d1zw7a1 d2m17a_ d1ud7a_
d1we7a1 d2lwpa_ d2gbja_ d1wjua1 d1v6ea1 d1v86a1 d4eewa_ d1wiaa1
d4icva_ d4gu2a_ d1wx7a1 d1wy8a1 d4hjka1 d2faza1 d1v5oa1 d3ruib_
d1eo6a_ d3waob_ d2r2qa1 d3m95a_ d3wana_ d1yvca1 d1yeza1 d2h0ba_
d2erfa_ d1h30a2 d3mw4a1 d1pz7a_ d2r1ba_ d3v64a1 d1d2sa_ d3bopa1
d3mw3a1 d1okqa1 d1okqa2 d2bcgg1 d2bcgg2 d1d5ta1 d1dszb_ d1a6ya_
d3cbba_ d1ynwa1 d1r4ia_ d1hcqa_ d2hana_ d2hanb_ d2enva1 d1kb4b_
d2nllb_ d1lo1a_ d1cita_ d1adqa1 d1fnga1 d1uvqb1 d1zs8a1 d2vola1
d2vola2 d1mfbl2 d2jelh2 d1uwel2 d3qq4b1 d1kjvb_ d1k5na1 d1osph2
d4odhl2 d1ow0a1 d1ow0a2 d1bfob2 d3l9ra2 d3jtth1 d2fath2 d4elkb2
d1fn4b2 d2xfxb_ d1pg7x2 d1cqka_ d4k71b2 d1lk2a1 d1eapb2 d4lfhg2
d1i4fa1 d2nw2a2 d1kfah2 d1mhca1 d1h3ua1 d1wejh2 d1h0db2 d1a5fh2
d1kcvh2 d1mjuh2 d1qqda1 d1kcrh2 d1k8ia1 d3huja2 d2nnab1 d1nfdf2
d3mffb2 d1s9va1 d1lk3h2 d1q0xl2 d1fv1b1 d1neza1 d4rfeb2 d1r3ha1
d1a6tb2 d1kjva1 d1pz5b2 d1onqa1 d2bc4a2 d1de4a1 d1k8da1 d1tcra2
d1dn0b2 d1nfde2 d2xwtb2 d1bfoa2 d1mexh2 d1iakb1 d1ypzf2 d4f7ea2
d1q72l2 d2fbjh2 d1fe8h2 d1clzh2 d1fngb1 d1uvqa1 d1vgka1 d1c16a1
d4j12a2 d4x99b2 d1igtb3 d1je6a1 d1q72h2 d1nldh2 d1teth2 d2hmic2
d1i1ca2 d1i1ca1 d1k8ib1 d1q0yh2 d1kfal2 d3frua1 d1pg7w2 d1u58a1
d1igyb4 d1igyb3 d1hyrc1 d1klub1 d1deeb2 d1c12b2 d2bc4b2 d2ig2h2
d1kcvl2 d3wn5b1 d1lk2b_ d1lk3l2 d1mjul2 d1sm3h2 d1q0xh2 d1zvsa1
d1ydpa1 d1u8ib2 d1ypze2 d1kcuh2 d1mbma_ d3su6a1 d1cu1a1 d4agka_
d1vcpa_ d1q31a_ g1n1l.1 d3e90b_ d1ep5a_ d3u1jb_ d2e3fa_ d1mm0a_
d2e3ea_ d2llda_ d2ny8x_ d2e3ga_ d2ny9x_ d2nz3a_ d2lj7a_ d1n4na_
d2m8bb1 d2kyqa_ d3psma_ d1jkza_ d1jxca_ d2gl1a_ d1ugla_ d4uj0a_
d2k4ua_ d1hp2a_ d2lu9a_ d1baha_ d2meoa1 d1wm7a_ d1hlya_ d1n8ma_
d1wpda_ d1c55a_ d1pvza_ d1wt7a_ d1rjia_ d1wz5a_ d1m2sa_ d1pjva_
d1c49a_ d1qkya_ d2ktca1 d1wmta_ d1du9a_ d1j5ja_ d1r1ga_ d1t0za_
d2i61a_ d1t1ta_ d1sn4a_ d1kv0a_ d2lkba_ d1npia_ d1wwna_ d1pe4a_
d2kbka_ d2li7a_ d1djta_ d2kbha_ d1dq7a_ d1sega_ d1omya_ d1zywa1
d1jzaa_ d1n9wa1 d1bbua1 d1e1oa1 d1c0aa1 d1b8aa1 d1l0wa1 d3a74a1
d1ixra2 d1iyjb4 d1iyjb5 d1iyjb3 d1o7ia_ d4ipca1 d1x3ea_ d4apva_
d1jb7a1 d1jb7a2 d1jb7a3 d3kdfa_ d1ue1b_ d1wjja1 d3eiva_ d1xjva2
d1xjva1 d2ccza2 d1se8a_ d1l1oc_ d1jb7b_ d1qvca_ d3ulla_ d1qzga_
d1miua4 d1miua5 d1miua3 d3g48a1 d1gd7a_ d1mkha_ d1jjcb3 d1pyba_
d3ubub_ d1egia_ d1qo3c_ d1v7pa_ d1tdqb_ d3ubua_ d1c3ab_ d1h8ua_
d3bx4b_ d1t8ca1 d1sb2a1 d1qdda_ d2ziba1 d3cada_ d1j34b_ d1jwia_
d1jzna_ d1c3aa_ d1oz7b_ d1wmza_ d3bdwb_ d1hq8a_ d1fifa1 d1dv8a_
d3hupa_ d1wk1a1 d1umra_ d2afpa_ d3bx4a_ d1gz2a_ d1sl6a_ d1v7pb_
d3g8ka_ d1ixxa_ d1j34a_ d1jwib_ d1g1sa1 d1y17a1 d1ja3a_ d1fvub_
d3bdwa_ d1sb2b1 d1fvua_ d1umrc_ d1oz7a_ d4m17a_ d3cfwa1 d1ypqa1

d1g1ta1 d3fd4a_ d3l9jc_ d4dn8a1 d1v4lb_ d1rdl1_ d2msba_ d1dgsa2
d1fvia1 d1p16a1 d1x9na2 d1pfsa_ d2a1ka_ d1je5a_ d1rpma_ d2shpa1
d1yfoa_ d1l8ka_ d2ooqa1 d1g4us2 d1p15a_ d1lara1 d1wcha_ d2fh7a2
d2fh7a1 d1ohea1 d1ohea2 d2f4da_ d1rxda_ d2hxpa_ d1xria_ d1fpza_
d3ezza_ d4y2ea_ d1i9sa_ d1d4ua2 d3azya_ d3wvja_ d1upsa1 d1ajoa_
d2hyka_ d1ajka_ d2vy0a_ d1o4ya_ d3o5sa_ d3d6ea_ d3atga_ d1dypa_
d1umza_ d3i4ia_ d1c1la_ d4lbqa_ d1a78a_ d1slta_ d4no4a_ d3zsja_
d3duia_ d1g86a_ d1is3a_ d1hlca_ d1tdha3 d1q3ba3 d1ee8a3 d1k82a3
g1qmo.1 g1len.1 d3zyra_ d1fnya_ d1n47a_ g2ltn.1 d1avba_ d3usua_
d1dhkb_ d1f9ka_ d1ukga_ d1g8wa_ d1g9fa_ d1wbfa_ d3wcsa_ d1g7ya_
d1mvqa_ d1hqla_ d1v6ia_ d1dbna_ d1qnwa_ d1fx5a_ d4l8qa_ d1ioaa_
d3n35a_ d3ubra_ d1d4ca1 d2k3va_ d1ft5a_ d1qdba_ d1m1qa_ d2j7aa_
d1h21a_ d1ogyb_ d4fasa_ d1jnia_ d1oaha_ d1sp3a_ d3h33a_ d1rwja_
d19hca_ d1os6a_ d3bxua_ d3h4na_ d1i77a_ d3caoa_ d2ctha_ d1duwa_
d1ofwa_ d2e84a_ d1hh5a_ d1gyoa_ d3h34a_ d1wioa4 d1ccza2 d1vcaa1
d1dr9a2 d1ic1a1 d2d9qb1 d2nxyb2 d2dara2 d2dara1 d1wyha2 d2dloa2
d1rutx4 d1rutx3 d2cuqa2 d1x4la2 d1x4la1 d1x3ha2 d1x3ha1 d2d8za1
d1x61a2 d1x6aa1 d1x6aa2 d2cora2 d2cora1 d1v6ga1 d1v6ga2 d2d8za2
d1x64a1 d1x64a2 d1b8ta1 d2co8a1 d1x63a2 d2dj7a2 d2dj7a1 d2cu8a2
d2cu8a1 d1g47a2 d1g47a1 d1j2oa1 d1wyha1 d2dloa1 d1wiga1 d1wiga2
d2cupa2 d2cupa3 d1x68a1 d2d8ya2 d2d8ya1 d1m3va2 d1x62a1 d1x68a2
d1x61a1 d1b8ta3 d1b8ta2 d2d8xa2 d2d8xa1 d1x4ka1 d1x4ka2 d2cuqa1
d2co8a2 d1j2oa2 d1x63a1 d2cura1 d2cura2 d1x62a2 d1op9a_ d1y0la1
d1etzb1 d1egjl1 d2gsib1 d1nj9a1 d4jfhd1 d1aqkl1 d1t2ja_ d1lo4h1
d1zvya1 d3v4vl1 d4enec1 d1kxtb_ d5e8ea1 d2pxya_ d4weud_ d1xiwc_
d2esvd1 d1qnzh_ d1ogae1 d1f4xh1 d4n1hb_ d3ezjb1 d3mlwl1 d1kfah1
d1etza1 d1b4jh1 d3cfic_ d4w68a_ d2arja1 d1ek3a_ d2ok0l1 d1vera_
d1uwgh1 d1blnb1 d1fpth1 d1rzga1 d2giya1 d4m3kb1 d1nc2a1 d2nyyd1
d1ncna_ d1fbih1 d5dxwa_ d4fqqa1 d1dqdh1 d1jfql1 d1kelh1 d1faih1
d1frgl1 d1figl1 d4m6nl1 d1fo0a_ d4hxbl1 d3b5ga_ d3quyd1 d2h9gb1
d1i8kb_ d2dd8h1 d2ck0h1 d1eaja_ d4w6wb1 d2a6ib3 d1t66d1 d2e27h_
d1ob1b1 d1f11b1 d2v7na1 d1ad0a1 d2xt1b1 d1pskh1 d4i13b1 d2op4l1
d1a0ql1 d1uwel1 d1za3a1 d1ncbl1 d4uu9a_ d1bm3h1 d1cloh1 d1nqba2
d1cf8h1 d4y5va_ d1mhph1 d5anma1 d4cdgc1 d4ldeb_ d1y0lb1 d2pcpb1
d1z3gl1 d1dfbl1 d1fh5h1 d3g5xa1 d1tzib1 d4dn3l1 d3zhka1 d2b1hh1
d2uyla1 d1f3dh1 d1nj9b1 d2agjh1 d2h1pl1 d1q72h1 d8fabb1 d1ndmb1
d1rjca1 d4u6hb1 d3upca_ d2fbjl1 d1osph1 d1tvda_ d2cjvh_ d4fhbd1
d1bd2d1 d1rz7h1 d3nn8a_ d1blna1 d1knoa1 d1nfda1 d2bmkb1 d1dqll_
d1igyb1 d1igfl1 d12e8h1 d3utza1 d1nc2b1 d1rihh1 d4mwfb1 d4jvpa1
d1mieh1 d1rhfa1 d1fgnl1 d1sbsh1 d1lp9e1 d1dn0a1 d4lqfl1 d1iaih1
d1kgcd1 d1mh5b1 d1gpol1 d2g60h3 d1f8tl1 d2jixd1 d4grwh_ d1fo0b_
d1bfob1 d1axsa1 d1u9ka_ d1nmbh_ d1h8na1 d1h8na2 d1jguh1 d4eura1
d3sdyl1 d35c8h1 d1w72l1 d1mvub_ d1i9rh1 d1ob1a1 d4wenb_ d1yeeh1
d3ietc1 d1f58h1 d1ad0b1 d1mqkl_ d1nbvh1 d4b50a_ d3r8bb_ d1bafl1

| | | | | | | | |
|---|---|---|---|---|---|---|---|
| d1mamh1 | d4rgne1 | d2gsgb_ | d35c8l1 | d1bafh1 | d1ai1h1 | d2pcpa1 | d2fath1 |
| d2x1pa_ | d1orsb1 | d1ibgl1 | d1shma_ | d1a2yb_ | d1u0qa_ | d2coqa_ | d1hilb1 |
| d1qfwi_ | d1jv5b_ | d1fn4b1 | d5e0qa_ | d1nsnh1 | d1plgl1 | d4ocxl1 | d1kb5h1 |
| d1rzfh1 | d1pg7x1 | d2fx7h1 | d2oi9c_ | d1l7il1 | d3eyva1 | d1d5il1 | d4ffyl_ |
| d1tetl1 | d1a6wh_ | d1ac6a_ | d3dvfa_ | d1nfde1 | d1mimh1 | d1ri8a1 | d1jhlh_ |
| d3dura_ | d1fskb1 | d1f97a1 | d3so3b1 | d2ak4d1 | d1lp9f1 | d4z7wf1 | d1l7th1 |
| d4y5ya_ | d4x7ec1 | d1emth1 | d1ikfl1 | d1nlbh1 | d4utal1 | d1bjma1 | d1eapb1 |
| d1u6ah1 | d2dtgd1 | d1kj2b_ | d1xeda1 | d1c1eh1 | d1fgvh_ | d2bnub1 | d2fb4h1 |
| d2dtgb1 | d1ktrh_ | d1jpth1 | d1qkzl1 | d4hepg_ | d2atpb1 | d1flrh1 | d2hrpl1 |
| d1mvfa_ | d1iaim1 | d3k80a1 | d2jb5h1 | d3zkxc_ | d4grwe_ | d1it9h1 | d1rhhb1 |
| d3w9ec_ | d1e4wh1 | d1sy6h1 | d2j6eh1 | d3qxta_ | d1jglh1 | d1p7ka1 | d1tzhb1 |
| d4n8pa1 | d1gafl1 | d2jelh1 | d1wioa2 | d4i0cc1 | d1cicb1 | d1lmka2 | d1lmka1 |
| d1iqwh1 | d1orqb1 | d1ejoh1 | d1jnlh1 | d4zg1a_ | d43c9b_ | d1ggbl1 | d1wejh1 |
| d3q6fa1 | d1f3dj1 | d3c6la1 | d1bqlh1 | d3p9wb_ | d1a3rl1 | d1h0db1 | d2uzil_ |
| d1eo8l1 | d1qfwl_ | d1n7ml1 | d1igja1 | d1c5ch1 | d2a9mh_ | d1rmfh1 | d1fj1b1 |
| d4heme_ | d1ccza1 | d1a5fh1 | d1ogad1 | d1dlfh_ | d1qa9b_ | d1vgel1 | d3qskb_ |
| d1sjxa_ | d4ozfg1 | d1sq2n_ | d1qd0a_ | d1kcvh1 | d1c5db1 | d3qcul1 | d3bn9d1 |
| d1mjuh1 | d4y8dc_ | d7fabh1 | d3dvgb1 | d1lo0l1 | d1a6tb1 | d1gigh1 | d1kcrh1 |
| d2hwzl1 | d1yjdc1 | d3clfl1 | d4krna_ | d1dl7l_ | d1e6ol1 | d1mexh1 | d3r0ma_ |
| d4kkna_ | d1fnsl1 | d1oaqh_ | d1rzia1 | d1ucbh1 | d1ncwl1 | d4b41a_ | d1nfdf1 |
| d2mpah1 | d2d7tl1 | d4grwf_ | d4b5ea_ | d1lk3h1 | d3u79a_ | d4u3xa_ | d1ghfl1 |
| d1bvkb_ | d1mcww1 | d1b88a_ | d1jrhh1 | d32c2b1 | d4wemb_ | d1dsfh_ | d1op3h1 |
| d1cell1 | d1iqda1 | d1qoka1 | d1qoka2 | d1qfuh1 | d2crya1 | d1ay1h1 | d1oaql_ |
| d3ab0b_ | d1rurl1 | d1t4kb1 | d1bz7b1 | d1n0xh1 | d1forl1 | d1ucbl1 | d4w6yb_ |
| d43c9a_ | d2aabl1 | d1tzia1 | d4n1ca_ | d1k6qh1 | d3g9ab_ | d4tvsa1 | d1igmh_ |
| d1pz5b1 | d3dx9a1 | d3wxwh_ | d1nakh1 | d1kcul1 | d3eaka_ | d4qlra1 | d1cr9l1 |
| d1d9ka_ | d1f4xl1 | d1deea1 | d2i27n2 | d1c12a1 | d2orbl1 | d1j8hd1 | d1mcph1 |
| d1c5da1 | d1kenh1 | d1bd2e1 | d1h5ba_ | d4x7fc1 | d3upaa_ | d1t3fa1 | d1indh1 |
| d2dtgc1 | d4hc1l1 | d1zv5a1 | d4wgvb_ | d1uwgl1 | d4f37k1 | d1aqkh1 | d1vesa_ |
| d3zkqd_ | d1fptl1 | d1lo4l1 | d1knob1 | d3rrqa_ | d1ct8b1 | d4orzc_ | d1mlbb1 |
| d4hgkc1 | d1dqdl1 | d1bogb1 | d4ozhe1 | d1dr9a1 | d1i7za1 | d1dn0b1 | d1kell1 |
| d1rzib1 | d1ypzf1 | d1wtla_ | d1hkfa_ | d1fail1 | d1figh1 | d2vyre_ | d1sjva_ |
| d1ohqa_ | d1bfoa1 | d1pkqb1 | d1mjul1 | d1b4jl1 | d1nsnl1 | d2arjb1 | d1p4bh_ |
| d4aj0a_ | d3nn8b_ | d2rcja1 | d1f2xk_ | d4v1dd_ | d1adqh1 | d2eksa_ | d4k3ga_ |
| d1jfqh1 | d1uweh1 | d1frgh1 | d4gftb_ | d1uz8b1 | d4n1ea_ | d1bm3l1 | d1clol1 |
| d3mcka1 | d2vq1a1 | d1ae6h1 | d4aq1b1 | d1uj3b1 | d1nbqa1 | d1fh5l1 | d2ck0l1 |
| d2ywza_ | d1q72l1 | d4nbxb_ | d1hila1 | d4hzlb1 | d2j88l1 | d1zmya1 | d1cd0a_ |
| d1a4ka1 | d1pskl1 | d1a0qh1 | d4krmb_ | d1ncbh1 | d2fbjh1 | d1j8he1 | d1i3ua_ |
| d1cf8l1 | d1fe8h1 | d2p1ya1 | d1eeqa_ | d2x89a_ | d1ol0a_ | d2r0lh1 | d1ub5a1 |
| d1dfbh1 | d3eyvb1 | d4jdvb1 | d4p2cg_ | d2gsia1 | d2agjl1 | d1u3ha1 | d2h1ph1 |
| d1rihl1 | d1fskc1 | d1boga1 | d2a77l1 | d1ospl1 | d1qfoa_ | d3dwta_ | d2fx7l1 |
| d1rz7l1 | d3stba1 | d1pkqa1 | d1b6da1 | d1dzba2 | d1dzba1 | d1kj2a_ | d4l1ha_ |
| d1rzgb1 | d1dqlh_ | d12e8l1 | d1miel1 | d1jmaa_ | d1sbsl1 | d1fgnh1 | d1f58l1 |
| d1mqkh_ | d1ndgb1 | d3qegl1 | d1iail1 | d2aw2a1 | d1tqbc1 | d3fo2a1 | d4dkaa_ |

d1f8th1 d2g60l1 d2ywya_ d1uj3a1 d1yc7a_ d3bd3a_ d4w6xb1 d1nmbl_
d1i8ka_ d1rjlb1 d1ypze1 d1p7kb1 d1kxqe_ d1gpoh1 d2z4qa1 d1w72h1
d1i9rl1 d4qgya1 d4xt1c_ d2qqnh1 d1cica1 d3c08h1 d2cd0a_ d2bdnh1
d1hnga1 d1maml1 d1nldh1 d1cr9h1 d4lcih1 d3bkyh1 d3qg7l1 d1ai1l1
d1kxvc_ d4iosd_ d1l7ih1 d2x1qa_ d1ub6b1 d3k74b_ d1ibgh1 d1teth1
d1igya1 d1hxma1 d2x6ma_ d2e7la_ d5e03a_ d1qfwm_ d2r0kh1 d1igjb1
d1rurh1 d1plgh1 d1mj8h1 d1yuhb1 d3fmgl1 d1rzfl1 d1emtl1 d1ad9b1
d1igtb1 d1nlbl1 d1d5ih1 d3k7ua_ d4ffyh_ d1bwwa_ d3dvga1 d1a6ta1
d1fgvl_ d4qxgl1 d1miml1 d1jhll_ d2hffb1 d2hh0l1 d1qkzh1 d1l7tl1
d1afvh1 d1iaii1 d2cjuh_ d1um5h1 d2ghwb2 d2ghwb1 d1kgce1 d2fgwh1
d1ikfh1 d1dvfd_ d2i24n1 d2xtjb_ d1za3b1 d2yc1a_ d2hh0h1 d2x1oa_
d1bvka_ d1rjla1 d5e7ba1 d1igta1 d1il1b1 d2fb4l1 d1mvua_ d1jptl1
d32c2a1 d1fvca_ d1iqdb1 d2hrph1 d2p4ab_ d4qo1a_ d1p2cb1 d1wt5c_
d1t4ka1 d5c1mb_ d1it9l1 d1bz7a1 d1b2wh1 d1e4wl1 d1jnll1 d1yedb1
d1za6b1 d3u7aa_ d1igfh1 d3gnml1 d1orsa1 d1wejl1 d1a2ya_ d1hxmb1
d1gafh1 d2j4wh1 d1pz5a1 d1j05a_ d1a3rh1 d4fqll1 d1qfwh_ d1eo8h1
d1n7mh1 d4s11a_ d1kegh1 d5da4a_ d1iqwl1 d3whxb1 d1vgeh1 d1deeb1
d1c12b1 d1t2ql1 d1ad9a1 d3gsnb1 d3b2uc1 d1ggbh1 d1t3fb1 d1nakl1
d4ocmc_ d1lila1 d2v7hb3 d2esve1 d1mcoh1 d2qr0b1 d2q3aa_ d1ct8a1
d1kcrl1 d2pxyb_ d2uzih_ d5c3ld_ d3durb_ d1c5cl1 d2ak4e1 d1dl7h_
d1xiwd_ d1mlba1 d1rmfl1 d1i7zb1 d1dlfl_ d2xa3a_ d5bq7b1 d1dqqb1
d3h42l1 d1egjh1 d1kcvl1 d1fe8l1 d2j6el1 d1f3rb2 d1lk3l1 d1sm3h1
d1q0xh1 d1ghfh1 d7fabl1 d1igch1 d1lo0h1 d4idla_ d5c2bl_ d1jrhl1
d1il1a1 d3zhda1 d4kfzc_ d1b0wa_ d1e6oh1 d1mexl1 d4krpb_ d1fnsh1
d1ncwh1 d4hgma_ d4eigb1 d4rzca_ d2mpal1 d4y5xa_ d5en2b1 d3lrga1
d4ej1c1 d1rhha1 d1j05b_ d1forh1 d1q9wb1 d2wzpd_ d2aq2a1 d1h0da1
d3revb1 d2ij0c1 d1tzha1 d4m7jl1 d1k6ql1 d3b9kc1 d1igml_ d3qeua1
d1dsfl_ d1ce1h1 d1kcuh1 d1zvha1 d1zvoa1 d1tqbb1 d1ay1l1 d2qscl1
d2cgrh1 d1nl0h1 d1kenl1 d3eo0a1 d4n9ob1 d1n0xl1 d3k1kc_ d1c4qa_
d1prtc1 d2wv6d_ d1prtf_ d1ltrd_ d4l63b1 d1r4pb_ d1tl0f_ d2bosa_
d1prtb1 d3chbd_ d2aq2b1 d1ck1a1 d1enfa1 d1v1oa1 d1et9a1 d1esfa1
d2g9hd1 d4ohja1 d1klud1 d1bxta1 d1eu3a1 d1ty0a1 d1fnua1 d2cqva1
d1fhga_ d2k1ma_ d2dl2a1 d1biha3 d1biha1 d1biha4 d3qp3a1 d1g1ca_
d1gxea_ d1cvsc2 d1cvsc1 d1xmwa2 d1m4ka2 d2otpa2 d1biha2 d1rhfa2
d2fcba2 d2fcba1 d1wwbx_ d1gsma2 d1epfa1 d1epfa2 d2dava1 d1waaa_
d1f97a2 d1x44a1 d1vcaa2 d1fnla2 d1fnla1 d3b5ha2 d3h8na2 d3h8na1
d1jbja1 d1jbja2 d1pd6a_ d1f2qa2 d1f2qa1 d3cx2a_ d1nbqa2 d3b5ha1
d1n26a1 d1xiwb_ d1xaua_ d1fltx_ d2ifga1 d1olla1 d1olla2 d1cs6a4
d1cs6a3 d1cs6a2 d1cs6a1 d2gw5a2 d1sy6a1 d1xiwa_ d1ucta2 d1ucta1
d1ry7b2 d1gl4b_ d1qz1a3 d1olza1 d2c9aa1 d1wwca_

**Family-53 (c.2.1.5)**

**Positive dataset contains 7 protein sequences.**

d2ewda3 d1up7a1 d2dfda1 d1s6ya1 d1ojsa3 d2a92a1 d1y6ja1

**Negative dataset contains 3755 protein sequences.**

d1m32a_ d1ibja_ d1iuga_ d4q75a_ d2bkwa1 d1t3ia_ d1n8pa_ d1qz9a_
d1jf9a_ d1eg5a_ d1p3wa_ d3kgwa_ d1cl1a_ d2ctza1 d1vjoa_ d2rfva_
d1gc0a_ d1cs1a_ d1d2fa_ d2ch1a1 d1qgna_ d1pffa_ d1elua_ d1u08a_
d1lc5a_ d1b5pa_ d2ay1a_ d1bw0a_ d3pd6a_ d7aata_ d2csta_ d1j32a_
d4wlha_ d1o4sa_ d1v2da_ d1gdea_ d1m6sa_ d1v72a1 d1svva_ d1ajsa_
d1wsta1 d2f8ja2 d1lk9a_ d1vp4a1 d1xi9a1 d2gb3a1 d1yaaa_ d1xkla_
d2wfla_ d3i2ka1 d1lnsa3 d1xkta1 d2hfka_ d1jmkc_ d5d3ka_ d1ohfc_
d1g8fa3 d3p0ya1 d1igra1 d1igra2 d1n8yc2 d1n8yc1 d1m6ba2 d1m6ba1
d1f56a_ d1bypa_ d1azca_ d1ws8a_ d3u25a_ d1cuoa_ d1id2a_ d1rkra_
d2iaac_ d4dp1x_ d1nwpa_ d2gima_ d2i7sa_ d1qhqa_ d3ehbb1 d1fftb1
d1gyca3 d1hfua2 d1kbva1 d1kbva2 d1sdda1 d1sdda2 d1gyca1 d1gyca2
d1mzya1 d2bw4a1 d1aoza1 d1aoza3 d1aoza2 d1kyaa2 d1kyaa3 d1kyaa1
d1mzya2 d4e9sa1 d1sddb1 d1sddb2 d1hfua1 d1hfua3 d1oe1a2 d1oe1a1
d1v10a2 d1v10a3 d1v10a1 d2dara2 d2dara1 d1wyha2 d2dloa2 d1rutx4
d1rutx3 d2cuqa2 d1x4la2 d1x4la1 d1x3ha2 d1x3ha1 d2d8za1 d1x61a2
d1x6aa1 d1x6aa2 d2cora2 d2cora1 d1v6ga1 d1v6ga2 d2d8za2 d1x64a1
d1x64a2 d1b8ta1 d2co8a1 d1x63a2 d2dj7a2 d2dj7a1 d2cu8a2 d2cu8a1
d1g47a2 d1g47a1 d1j2oa1 d1wyha1 d2dloa1 d1wiga1 d1wiga2 d2cupa2
d2cupa3 d1x68a1 d2d8ya2 d2d8ya1 d1x62a1 d1x68a2 d1x61a1 d1b8ta3
d1b8ta2 d2d8xa2 d2d8xa1 d1x4ka1 d1x4ka2 d2cuqa1 d1m3va1 d2co8a2
d1j2oa2 d1x63a1 d2cura1 d2cura2 d1x62a2 d2cfba1 d1dfoa_ d1o69a_
d1bj4a_ d3k28a_ d2bwpa_ d3lg0a_ d2fnua_ d3ou5a_ d1z7da1 d1b9ha_
d4msoa_ d1bs0a_ d1s0aa_ d1sffa_ d3tqxa_ d1mdoa_ d3qboa_ d3pgya_
d1bjna_ d2dkja_ d1ejia_ d1vefa1 d4wxga_ d3bs8a_ d3gbxa1 d1w23a_
d2y5ia_ d1qlsa_ d1psra_ d3n22a_ d2egda_ d4ggfc_ d2wcea_ d2luca_
d4aqia_ d3nsia_ d2lp3a_ d1j55a_ d1a4pa_ d1k8ua_ d1nsha_ d3lgia_
d3i77a_ d2z9ia2 d4nsva_ d1lcya2 d4jcna_ d1agja_ d1os8a_ d1hpga_
d1p3ca_ d1l1ja_ d1qtfa_ d1a3qa1 d1iknc_ d3brda1 d1p7hl1 d1oy3c_
d1imhc1 d1gjia1 d1zk9a1 d2cxka1 d2bema_ d1pama1 d1ea9c1 d1qhoa1
d2fhfa2 d2fhfa1 d1k3ia1 d1j0ha1 d1cxla1 d2bhua1 d1edqa1 d1f1sa2
d1ut9a2 d1txka1 d1mqta_ d1d4m3_ d1fmd3_ d2mev1_ g1aym.1 d1ev13_
d4q4w1_ d1r1a3_ d2x5ia_ d1rhi1_ g1r1a.1 g1d4m.1 g1rhi.1 d1b35b_
d1oopa_ d1tme1_ d4iv1a_ g1pvc.1 g1fpn.1 d1fpn1_ d4gb31_ d1d4m1_
d1fmd1_ d1ev11_ d2wzr3_ g1cov.1 d1qqp3_ g1fmd.1 d2x5ic_ d1rhi3_
g1oop.1 d1hxs1_ g1hxs.1 d1fpn3_ d1bev1_ g2mev.1 d1eah3_ d1aym1_
d1pvc3_ d1zba3_ d1h8ta_ d3vbhc_ g1b35.1 g1bev.1 d1hxs3_ g1ev1.1
d1bev3_ g1tmf.1 d1tmf1_ d1b35a_ d2mev3_ g1eah.1 g1h8t.1 d1eah1_
d4q4w3_ d1r1a1_ d1aym3_ d1pvc1_ d1zba1_ d1h8tc_ d1oopc_ d1tme3_
g1qqp.1 d4iv1c_ g1tme.1 d1nepa_ d1ds6b_ d1fstb_ d1ex0a1 d1ku5a_
d1b67a_ d2b5ea2 d2b5ea3 d2b5ea1 d2b5ea4 d2kp1a1 d2kp2a1 d1hyua3
d1hyua4 d1j08a1 d1j08a2 d2bjca1 d2hsga1 d1qpza1 d1efaa1 d1e3oc2
d1ic8a2 d2ovga_ d1sq8a_ d1rzsa_ d3kz3a_ d3gpdg2 d2q49a2 d1hdgo2
d2g17a2 d2cvoa2 d2czca1 d3pwka2 d1obfo2 d1i32a2 d1zyao2 d2pkqo2
d1b7go2 d2hjsa2 d1cf2o2 d1vkna2 d1nvmb2 d2m9wa1 d3dfxa_ d2msya_

| | | | | | | | |
|---|---|---|---|---|---|---|---|
| d2djoa1 | d2r5za_ | d1au7a1 | d1f43a_ | d2lmda1 | d1akhb_ | d1s7ea1 | d3nara_ |
| d1zq3p1 | d2craa1 | d1mija_ | d4uuta_ | d3a01a_ | d1x58a1 | d3rkqa1 | d3cmya1 |
| d2cqxa1 | d2da6a1 | d1e3oc1 | d1fjla_ | d1mh3a1 | d1ig7a_ | d1x41a1 | d1wh7a1 |
| d1gt0c1 | d2mw8a_ | d1lfup_ | d1x2ma1 | d1b72a_ | d1wi3a1 | d1jgga_ | d1wjha1 |
| d1x2na1 | d1mnmc_ | d2cuea1 | d3k2aa_ | d2hosa_ | d2jwta2 | d1pufa_ | d1octc1 |
| d2hi3a_ | d1b8ia_ | d4j19a_ | d3lnqa_ | d1wh5a1 | d1b8ib_ | d1fexa_ | d2cqra1 |
| d1guua_ | d1ug2a1 | d2cqqa1 | d2ajea1 | d4a69c_ | d1h8ac1 | d2crga1 | d2iw5b1 |
| d1xc5a1 | d1ofcx1 | d2cu7a1 | d1wgxa1 | d2cjja1 | d2ckxa1 | d1gvda_ | d1hcra_ |
| d1tc3c_ | d1u78a2 | d1xg1a1 | d1itya_ | d1bl0a1 | d1d5ya1 | d1d5ya2 | d1prtb2 |
| d3lv8a_ | d1s3ga1 | d1zaka1 | d1s4qa_ | d3tvta2 | d1uf9a_ | d1khta_ | d1ckea_ |
| d1osna_ | d1p5zb_ | d4tmka_ | d3cm0a_ | d1jaga_ | d3h86a_ | d1t0hb_ | d1nn5a_ |
| d2h92a_ | d3kb2a1 | d2c95a1 | d1yj5a2 | d1kgda_ | d1ly1a_ | d2ak3a1 | d1vyva2 |
| d1deka_ | d2grja1 | d1p3ja1 | d1y63a_ | d1e4ya1 | d1s96a_ | d3iija1 | d1lvga_ |
| d1nksa_ | d1vyua2 | d4e22a_ | d1qf9a_ | d1jjva_ | d1q3ta_ | d1ki9a_ | d1lw7a2 |
| d1teva_ | d1kjwa2 | d1gsia_ | d1m7ga_ | d1x6va3 | d1g3ma_ | d1j99a_ | d1t8ta1 |
| d1nsta_ | d1fmja_ | d1zrha_ | d1ls6a_ | d2a3ra_ | d1aqua_ | d2zptx_ | d3ckla_ |
| d1texa_ | d1q44a_ | d2gwha_ | d1uj2a_ | d4gi7a_ | d2jeoa1 | d1rz3a_ | d1z0ja1 |
| d3umra2 | d3gj0a1 | d2gaoa1 | d1yzga1 | d1x3sa1 | d1g7sa4 | d1puja_ | d1udxa2 |
| d1kk1a3 | d2o52a_ | d1jwyb_ | d1zcaa2 | d1zcba2 | d2fv8a1 | d2cjwa_ | d3dz8a1 |
| d1xzpa2 | d1vg8a_ | d4qxaa_ | d2ew1a1 | d1x1ra1 | d2bcgy_ | d3doea1 | d3cona_ |
| d3rega_ | d3wyga_ | d1upta_ | d2b6ha1 | d1zd9a1 | d1lnza2 | d1r5ba3 | d1n0ua2 |
| d1r2qa_ | d2f9la1 | d2rhda_ | d3q3jb_ | d3q85a_ | d1efma1 | d1r8qa_ | d2gf9a1 |
| d2akab1 | d1ek0a_ | d1z08a1 | d2gcoa1 | d2dy1a2 | d4g01b_ | d2cxxa1 | d1svia_ |
| d3q72a_ | d1ni3a1 | d4ac9a4 | d2erxa1 | d2bmea_ | d1wxqa1 | d2gf0a1 | d4ldja1 |
| d1zunb3 | d1d5ca_ | d4lv5b_ | d1efca3 | d1kmqa_ | d2g6ba1 | d3lrpa_ | d1tada2 |
| d2y8ea_ | d4rd4a1 | d1z06a1 | d2heia_ | d3a58b_ | d1u0la2 | d1d2ea3 | d1zj6a1 |
| d2ocba_ | d3rwoa_ | d1mr3f_ | d2e9sa1 | d4u5xa1 | d2iwra_ | d2fn4a1 | d3bwdd_ |
| d2nzja1 | d2atva1 | d2ksqa_ | d3l43a_ | d1mkya2 | d1mkya1 | d1wmsa_ | d1jnya3 |
| d2rmka_ | d1f6ba_ | d1f60a3 | d1gota2 | d1jala1 | d2fh5b1 | d1g16a_ | d2wblc_ |
| d1m7ba_ | d1wf3a1 | d1f5na2 | d1t9ha2 | d1z6xa1 | d1z0aa1 | d2fg5a1 | d2a5ja1 |
| d2bcjq2 | d1ky3a_ | d4y0va_ | d4lhwa_ | d2qmea1 | d2atxa1 | d1egaa1 | d1ryha_ |
| d1moza_ | d1nrjb_ | d2erya1 | d3bfka_ | d1puia_ | d1fzqa_ | d3j3az1 | d3t5ga_ |
| d1shza2 | d3defa_ | d2f7sa1 | d3raba_ | d3tsoa_ | d3aq4a_ | d2al7a1 | d3sfva1 |
| d3bc1a_ | d1sdma_ | d3h4sa1 | d2heha_ | d2grya_ | g3kin.1 | d1kk8a2 | d1br2a2 |
| d1goja_ | d1f9va_ | d2mysa2 | d1lkxa_ | d3t0qa_ | d4db1a1 | d3gbja_ | d1ry6a_ |
| d1v8ka_ | d1vava_ | d1uaia_ | d1y4wa1 | d3vrda1 | d3vrda2 | d1fcdc2 | d1fcdc1 |
| d1h1oa1 | d1m70a2 | d1m70a1 | d1hroa_ | d4gedb_ | d3zooa_ | d1mz4a_ | d1gu2a_ |
| d1f1fa_ | d1dvva_ | d1c6ra_ | d1c75a_ | d1e29a_ | d1gdva_ | d1cnoa_ | d1h32b_ |
| d1co6a_ | d2bh4x_ | d2aiua_ | d5cytr_ | d1jdla_ | d1i8oa_ | d2zboa_ | d3dmia_ |
| d1ls9a_ | d1wvec_ | d2yk3a_ | d3ph2b_ | d1dw0a_ | d5aura_ | d1ql3a_ | d1qn2a_ |
| d1f1ca_ | d2nqya_ | d1h4ga_ | d1oa2a_ | d1olra_ | d3b7ma_ | d4h7ma1 | d3wp3a_ |
| d2vgda_ | d1igoa_ | d3zsea_ | d2nlra_ | d3wx5a_ | d1oa3a_ | d1pvxa_ | d1m4wa_ |
| d1f5ja_ | d1oa4a_ | d1ks5a_ | d4ixla1 | d3sita1 | d2ah2a1 | d1evsa_ | d1cnt1_ |
| d1pvhb_ | d1z7ca_ | d1huwa_ | d1bgea_ | d1rhga_ | d1f6fa_ | d1f45b_ | d1i1rb_ |

d1axia_ d1etea_ d3tgxb_ d2z3qa1 d3qb1a_ d1hula_ d1m48a_ d4nkqb_
d1eera_ d1v7mv_ d5e4ea_ d2o27a_ d3b5ka_ d2psma1 d4zf7a_ d3uf5a_
d4x51a_ d1d9ca_ d4ypgc1 d3oq3a_ d1lqsl_ d1n1fa_ d1au1a_ d1wu3i_
d3besl_ d3ux9a_ d2b7la_ d3rbaa_ d2qtra_ d3l92a_ d1f9aa_ d1qjca_
d2h29a_ d1vlha_ d1od6a_ d1lw7a1 d1mrza2 d4ruka_ d2el7a_ d1iq0a2
d1irxa2 d1r6ta2 d1ivsa4 d2cyba_ d1rqga2 d1j09a2 d2yxna_ d1f7ua2
d1h3fa1 d1ffya3 d2g36a1 d1nzja_ d1jila_ d1n3la_ d1i6la_ d1pfva2
d1h3na3 d3n9ia_ d3sz3a1 d2bk7a_ d3fjua_ d1z5ra1 d1kwma1 d1uwya2
d1jqga1 d2c1ca1 d1dtda_ d1h8la2 d3sw2a_ d1moxc_ d1dx5i3 d1edmb_
d1autl2 d1autl1 d1nqlb_ d1dval1 d1szba2 d1kigl_ d2y5fl_ d1ijqa2
d1gk5a_ d1g1sa2 d1nt0a3 d1xdtr_ d1lmja2 d1lmja1 d1nzia2 d2wphe_
d1klil_ d1pfxl2 d1pfxl1 d1p9ja_ d1g1ta2 d1f7ea_ d4yzub_ d2mgpa1
d1b9wa2 d2mgpa2 d1ob1c1 d1ob1c2 d1n1ia2 d1n1ia1 d2npra2 d2npra1
d1qx2a_ d1ig5a_ d1s5ja2 d1qhta2 d4dlea2 d1tgoa2 d3py8a2 d1q8ia2
d1kfsa2 d2py5a2 d1d5aa2 d1exra_ d1tiza1 d1nyaa_ d1g8ia_ d1ggwa_
d1wdcc_ d3fwba_ d1ggza_ d2o5ga_ d1ctaa_ d1m45a_ d3i5gc_ d1sl8a_
d1fpwa_ d2scpa_ d2roaa_ d4aqra_ d3pn7b_ d1br1b_ d2mysb_ d1s6ca_
d1bjfa_ d2obha_ d1jfka_ d1jf0a_ d1qv0a_ d1lkja_ d2mlfc_ d2m97a_
d2d8na_ d4by5a_ d1jbaa_ d4nqga_ d1wdcb_ d2mysc_ d1j7qa_ d1s6ia_
d1uhka2 d1r2ua_ d1dtla_ d3kf9a_ d3pn7c_ d1fi6a_ d1c07a_ d5d67d_
d1tuza_ d1h8ba_ d1eg3a1 d2zkmx1 d1snla_ d1gjya_ d1y1xa_ d1hqva_
d1dvia_ d1df0a1 d1qxpa2 d4usla_ d1k94a_ d1jfxa_ d1wkya2 d1uuqa_
d3wh9a_ d4m1ra_ d1xyza_ d4xuya_ d2je8a5 d4bf7a1 d3cuia_ d1ur4a_
d1qw9a2 d1odza_ d2vzsa5 d1bqca_ d2x2ya1 d1hjqa_ d1qnra_ d4y7ea1
d1vjza_ d4k68a_ d2v3ga_ d4hpga_ d1nofa2 d1nq6a_ d1h4pa_ d1aq0a_
d3ziza_ d4bs0a_ d1ecea_ d1h1na_ d1ta3b_ d1w91a2 d1i1wa_ d1uhva2
d1us3a2 d1egza_ d7a3ha_ d1tvna1 d3nyda1 d1ghsa_ d2cyga1 d4xv0a_
d2c8na2 d1w32a_ d3emca_ d1v6ya1 d1foba_ d2uwfa1 d2fgla_ d3niya1
d2c0ha1 d1rh9a1 d1yq2a5 d1szna2 d1fa2a_ d1pama4 d5do8a1 d1ea9c3
d1hx0a2 d2fhfa5 d1qhoa4 d1avaa2 d1m53a2 d4m8ua1 d2gjpa2 d1lwha2
d4howa1 d1ktba2 d1j0ha3 d3vm5a1 d2x1ia_ d1wzaa2 d1uasa2 d2diea1
d1mxga2 d1ht6a2 d1eswa_ d1iv8a2 d5cpsa_ d1ud2a2 d1zjaa1 d3gbea1
d1kwga2 d1wdpa_ d1gcya2 d1g5aa2 d1hvxa2 d3dhpa2 d2bhua3 d4do4a1
d3qgva1 d1e43a2 d1g94a2 d2d3na2 d1gjwa2 d1gvia3 d1x1na1 d1r7aa2
d1tz7a1 d3ljqa_ g1k2x.1 d4toqa_ d1wb0a1 d1w9pa1 d1eoka_ d1itxa1
d1kfwa1 d1goia2 d1edqa2 d2gsja_ d1hjxa1 d1jnda1 d1vf8a1 d3wdpp1
d3wq5a_ d1gnxa_ d4gxpa_ d4hz8a_ d3aiua_ d1wcga1 d1qvba_ d3ahxa_
d1v02a_ d2dgaa_ d1pbga_ d1vffa1 d1qoxa_ d4ha4a_ d1bvsa3 d2w0pa_
d2diaa1 d2e9ia2 d2e9ja1 d2dj4a1 d2eeba1 d2diba1 d2dmba1 d2dmca1
d2d7oa1 d2ee6a1 d2d7pa1 d2eeaa1 d2di8a1 d1wlha1 d2d7na1 d2nqca_
d2d7ma1 d2dica1 d3cnka_ d2k3ta1 d2eeca1 d1qfha1 d1qfha2 d2bp3a1
d1v05a_ d2ee9a1 d2eeda1 d2di9a1 d3syxa1 d1mkea1 d2p8va_ d1xoda1
d1i7aa_ d1evha_ d1qc6a_ d1egxa_ d1wgqa1 d1v89a1 d2coda1 d1zc3b_
d1wg7a1 d1u5ea1 d1v61a1 d2coca1 d2ys1a1 d1v5pa1 d1ntya2 d1wjma1

| | | | | | | | |
|---|---|---|---|---|---|---|---|
| d1wi1a1 | d1u29a_ | d1xx0a1 | d1unqa_ | d2zkmx3 | d1fhoa_ | d1v88a1 | d2adza1 |
| d1x1fa1 | d1p6sa_ | d2coaa1 | d1btka_ | d1v5ma1 | d1faoa_ | d2x18a_ | d1dyna_ |
| d1x1ga1 | d4kaxb_ | d2j59m1 | d1ki1b2 | d1xcga2 | d1txda2 | d1u5da1 | d1dbha2 |
| d1w1ha_ | d1upqa_ | d1v5ua1 | d1foea2 | d2fjla1 | d2dfka2 | d2cofa1 | d1kz7a2 |
| d1h6ua1 | d1etob_ | d1umqa_ | d1r55a1 | d3gboa_ | d1atla_ | d1buda_ | d4q1la_ |
| d1quaa_ | d1yp1a_ | d1wnia_ | d4c5pa_ | d2bsza1 | d4bgfa_ | d1w4ta1 | d1t3ca_ |
| d1epwa3 | d2a97a_ | d3fiea_ | d3ioqa_ | d1fh0a_ | d1o0ea_ | d1khqa_ | d1m6da_ |
| d2cb5a_ | d1cs8a_ | g8pch.1 | d1s4va_ | d2pnsa_ | d1vsna1 | d1iwda_ | d2oula_ |
| d7pcka_ | d3bwka_ | d3bcna_ | d2p86a_ | d1pvja_ | d1pcia_ | d1gece_ | d1deua_ |
| d4bs6a_ | d1xkga1 | d1cqda_ | d1jqpa2 | d2c0ya_ | d2bdza_ | d3p5ua_ | d2o36a_ |
| d1k9xa_ | d1zoya1 | d1qo8a2 | d1d4ca2 | d1chua2 | d1jnra2 | d1kf6a2 | d2jk6a2 |
| d2jk6a1 | d1feca1 | d1feca2 | d1lpfa1 | d1lpfa2 | d1xhca1 | d1xhca2 | d1dxla2 |
| d1dxla1 | d1gera2 | d1m6ia2 | d1onfa1 | d1onfa2 | d3lada2 | d3lada1 | d1w4xa1 |
| d1w4xa2 | d1gesa2 | d1mo9a1 | d1xdia1 | d1fl2a2 | d1fl2a1 | d1fcda1 | d1fcda2 |
| d1h6va2 | d1h6va1 | d1mo9a2 | d1gv4a2 | d1gv4a1 | d2gv8a2 | d2gv8a1 | d1aoga1 |
| d1aoga2 | d1hyua1 | d1ebda1 | d1ebda2 | d3dk9a1 | d1q1ra2 | d1q1ra1 | d1zbfa1 |
| d2z1ha_ | d2etja1 | d3qioa1 | d1jl1a_ | d2e4la_ | d1jl2a_ | d2lsna1 | d1io2a_ |
| d1uaxa_ | d2yv0x_ | d1mu2a1 | d1ekea_ | d1i39a_ | d1pn0a1 | d1reoa1 | d1ju2a1 |
| d2f5va1 | d3c96a1 | d2gmha1 | d1pj5a2 | d1gpea1 | d1o5wa1 | d1n4wa1 | d1k0ia1 |
| d2dw4a2 | d2ivda1 | d1seza1 | d2voua1 | d1kdga1 | d1s5ja1 | d2hbja2 | d2igia_ |
| d1wlja_ | d1qhta1 | d4dlea1 | d1tgoa1 | d2f96a1 | d3tr8a1 | d3mxma_ | d1q8ia1 |
| d1y97a1 | d1w0ha_ | d1kfsa1 | d2py5a1 | d1d5aa1 | d1yt3a3 | d2guia_ | d3mwpa1 |
| d4kb1a_ | d2v76a_ | d1wj1a1 | d1aqca_ | d1qqga2 | d1ntva_ | d1n3ha_ | d4dbba_ |
| d3f0wa1 | d1p3ra_ | d1wgua1 | d2cy4a1 | d3hqca_ | d1wvha1 | d1xr0b1 | d1kb0a1 |
| d3nvva1 | d1krha3 | d2h88b1 | d1n62a2 | d1t3qa2 | d1zoyb1 | d1jq4a_ | d1ffva2 |
| d1dgja2 | d1rm6c2 | d1k8ka1 | d3uleb1 | d3i33a2 | d3i33a1 | d1bupa1 | d1dkgd2 |
| d1dkgd1 | d4ci6a2 | d1jcfa1 | d4a2ba2 | d2fxua1 | d1yaga2 | d1yaga1 | d4cbua2 |
| d1bupa2 | d1r7ha_ | d1faaa_ | d1wika1 | d1xfla_ | d1ttza1 | d2f51a1 | d1ti3a_ |
| d1nhoa_ | d1sena_ | d2i4aa_ | d1ep7a_ | d1zmaa1 | d1fo5a_ | d3lefa1 | d3diea1 |
| d2yana1 | d1z6na1 | d1oaza_ | d3zywa1 | d4aj8a_ | d1nm3a1 | d4fiwa_ | d1wmja_ |
| d4hs1a1 | d1iloa_ | d1xwaa1 | d1wjka1 | d2vm1a_ | d1fb6a_ | d1gh2a_ | d1h75a_ |
| d1nw2a_ | d1kx5b_ | d3a6na_ | d4m6ba2 | d2nqbd_ | d3wkjd_ | d2yfwe_ | d4h9na_ |
| d5bnxa_ | d1kx5c_ | d3r45a_ | d1eqzb_ | d4jjnb_ | d1f66c_ | d1eqza_ | d4wnnb_ |
| d1kx5a_ | d1u35c1 | d1id3a_ | d2nqbc_ | d1kx5d_ | d3us3a3 | d3us3a1 | d1tafa_ |
| d1bh9b_ | d1jfib_ | d1q9ca_ | d1h3ob_ | d2byka1 | d1n1ja_ | d4g92b_ | d1bh9a_ |
| d1jfia_ | d1h3oa_ | d2bykb1 | d1n1jb_ | d1pd212 | d1nhya2 | d2a2ra2 | d1k0dd2 |
| d1f2ea2 | d1gsua2 | d1f3ba2 | d2fhea2 | d2pvqa1 | d1tw9a2 | d1oyja2 | d1e6ba2 |
| d1k0ma2 | d3ik7a1 | d4is0a1 | d1r5aa2 | d1jlva2 | d1m0ua2 | d3gtub2 | d1z9ha2 |
| d1okta2 | d1axda2 | d2gsra2 | d1tu7a2 | d1k3ya2 | d1zgma1 | d1b48a2 | d1gnwa2 |
| d1gwca2 | d2gsta2 | d1glqa2 | d1oe8a2 | d1pn9a2 | d1v2aa2 | d1fw1a2 | d1duga2 |
| d2trcp_ | g1f8v.1 | d1novc_ | d2bbvc_ | d1qusa_ | d1qqya_ | d1gd6a_ | d1f6ra_ |
| d1ivma_ | d2gv0a_ | d1iiza_ | d2z2fa_ | d1j8xa_ | d1ghla_ | d1b9oa_ | d174la_ |
| d169la_ | d2o4wa_ | d1p37a_ | d1k28a3 | d1xjta1 | d1jtma_ | d2f2qa_ | d4e97a1 |
| d1iyka2 | d1iyka1 | d1rxta1 | d1iica1 | d2pjsa1 | d1klla_ | d2i7ra1 | d1ecsa_ |

d1xqaa1 d5cj3a1 d1npba_ d2p7oa_ d1r9ca_ d1nkia_ d4iuaa3 d4bvva_
d5hpga_ d3k65a_ d1ki0a3 d1ki0a2 d1ki0a1 d1a0ha1 d1pmla_ d3sp8a3
d2feba1 d1qsta_ d2beia1 d4m3sa_ d1u6ma1 d1ygha_ d1yk3a1 d3dr6a_
d1xmta_ d1z4ea1 d2ge3a1 d1bo4a_ d2fiaa1 d2jdca1 d1y9wa1 d3to7a_
d1wwza1 d2fl4a1 d2ozua1 d2b5ga_ d2cy2a1 d2p0wa_ d1cm0a_ d4pswa_
d1yrea1 d1yr0a1 d1m4ia_ d2gana1 d1yvka1 d1ghea_ d2g3aa1 d1y7ra1
d2euia1 d1q2ya_ d2fsra1 d1xeba_ d2ae6a1 d1cjwa_ d1qsma_ d2aj6a1
d1y9ka1 d1i12a_ d1vkca_ d2atra1 d2d4pa1 d2fiwa1 d2fe7a1 d1tiqa1
d2ou2a_ d1yx0a1 d2fcka1 d1p0ha_ d2j8ma_ d2pq8a_ d1z4ra1 d1s3za1
d1ufha_ d2hqya1 d2hqya2 d1lrza2 d1ne9a1 d1lrza3 d2qked_ d4ksoa_
d1t4za1 d1wwja_ d2m5ta_ d1cqqa_ d1wqsa_ d5bpea_ d2ipha_ d1lvoa_
d2bhga1 d3tnta_ d3sjia_ d3d23a1 d2fyqa_ d3tloa_ d3zvea1 d2hrva_
d3q3ya1 d4asha1 d1e88a1 d1l6ja5 d1l6ja4 d1eaka4 d1eaka3 d1l6ja3
d1h8pa1 d1ys1x_ d4hs9a_ d1ispa_ d1ku0a_ d3qmma_ d1mixa2 d1e5wa2
d1isna2 d2al6b2 d3rp2a_ g1ucy.1 d1fuja_ d2zchp_ d3w94a_ d1si5h_
d1lo6a_ g2pka.1 d1cgha_ d1kigh_ d1a5ia_ d3s9ba_ d1hj8a_ d1ao5a_
d2anya_ d1q3xa1 d1gvza_ d1azza_ d4gsoa_ d2f91a1 d1iaua_ d1gdna_
d1eufa_ g1fiw.1 d1eq9a_ d4nfea_ d2rdla_ d1spja_ d1elva1 d1a7sa_
d1ekbb_ d1pfxc_ d1yc0a_ d2aiqa_ g1fiz.1 d1fi8a_ d4k60a_ d4dgja_
d1mzaa_ d1op0a_ d3tjva_ d1pytd_ d1sgfg_ g1gg6.1 d2eeka_ g1rtf.1
d1a0ja_ d3g01a_ d1pytc_ d4e7na_ d1ddja_ d1z8ga1 d2hlca_ d1npma_
d1brup_ g1jou.1 d1sgfa_ d1autc_ g2hnt.1 g1kdq.1 d1orfa_ d3s69a_
d4crga_ d1fxya_ d1lgya_ d4l3wa_ d1llfa_ d1gz7a_ d1ukca_ d1j1ia_
d2rhwa_ d1ra6a_ d1muka_ d3gsza_ d1te9a_ d2xwha_ d3uqsa_ d3hkwa_
d3n6ma_ d2cjqa_ d2xyma_ d1teba_ d1te8a_ d1s48a_ d1u09a1 d4wfxa_
d3tyqa_ d2pgga1 d1xwdc1 d3kj4a_ d1w8aa_ d2ifga3 d1p9ag_ d1xkua_
d4qmea2 d2xdta2 d1z5ha2 d1dzka_ d4tlja_ d2a2ua_ d4wfva_ d3t1da_
d4iaxa_ d3zq3a_ d2nnda_ d1i4ua_ d1kxoa_ d1bbpa_ d1kt7a_ d1exsa_
d3s26a1 d1lf7a_ d1qfta_ d4gh7a_ d1z24a1 d1qabe_ d1xkia_ d1ew3a_
d3bx7a_ d4iawa1 d1n0sa_ d1gkab_ d3kzaa_ d2k23a_ d2l9ca1 d2joza1
d1gm6a_ d3kffa_ d3saoa1 d1e5pa_ d1yupa1 d1iiua_ d1h6ua2 d2tbvc_
d1c8nc_ d1x36a_ d1smvc_ d1ng0c_ d4sbvc_ d1opoa_ d1f2nc_ d1cixa_
d1y29a_ d1v7fa_ d2jtba_ d1d1ha_ d1g9pa_ d1lupa_ d1qk7a_ d1mb6a_
d2a2va_ d1niya_ d1i25a_ d1hvwa_ d2mpqa_ d1ie6a_ d1kqha_ d1la4a_
d2mxoa1 d1koza_ d1qk6a_ d1emxa_ d1lqra_ d1c6wa_ d1rmka_ d1ixta_
d1eyoa_ d1cnna_ d1fu3a_ d1g1pa_ d1f3ka_ d1fyga_ d1rypd_ d1irua_
d1rypc_ d3unfk_ d1rypa_ d1j2pa_ d1irui_ d1j2qh_ d3unfh_ d1m4ya_
d1rypb_ d1irul_ d1irug_ d1iru2_ d1q5rh_ d1rypg_ d1g3ka_ d1iruf_
d1q5qa_ d1e94a_ d1irub_ d1ryph_ d3ty6a_ d1iruj_ d1iruk_ d1rype_
d1irud_ d1irue_ d3mi01_ d1rypf_ d1iruh_ d2z3ba_ d1iruc_ d3mka2_
d1iru1_ d1ny721 d1a6ca3 d1a6ca2 d1a6ca1 d1pgl22 d1pgl21 d1pgl11
d1ny711 d1jgta2 d1ofda3 d1ea0a3 d1q15a2 d1ct9a2 d1ao0a2 d1te5a_
d3ubub_ d1egia_ d1v7pa_ d1tdqb_ d3ubua_ d1c3ab_ d1h8ua_ d3bx4b_
d1t8ca1 d1sb2a1 d1qdda_ d2ziba1 d3cada_ d1byfa_ d1j34b_ d1jwia_

| | | | | | | | |
|---|---|---|---|---|---|---|---|
| d1jzna_ | d1c3aa_ | d1oz7b_ | d1wmza_ | d3bdwb_ | d1hq8a_ | d1fifa1 | d1dv8a_ |
| d3hupa_ | d1wk1a1 | d1umra_ | d2afpa_ | d3bx4a_ | d1gz2a_ | d1sl6a_ | d1v7pb_ |
| d3g8ka_ | d1ixxa_ | d1j34a_ | d1jwib_ | d1g1sa1 | d1y17a1 | d1ja3a_ | d1fvub_ |
| d3bdwa_ | d1sb2b1 | d1fvua_ | d1umrc_ | d1oz7a_ | d4m17a_ | d3cfwa1 | d1ypqa1 |
| d1g1ta1 | d3fd4a_ | d3l9jc_ | d4dn8a1 | d1v4lb_ | d1rdl1_ | d2msba_ | d1eixa_ |
| d3exra_ | d2ffca1 | d2fdsa1 | d3n3ma1 | d2czda_ | d1q6oa_ | d2yyua_ | d1vqta1 |
| d1dbta_ | d3ldva1 | d1ux8a_ | d1dlya_ | d4i0va_ | d2bkma_ | d1dlwa_ | d2bmma_ |
| d2r80b_ | d1cqxa1 | d1cg5a_ | d1gcvb_ | d3boma_ | d2h8fb_ | d2raoa_ | d3bj1a_ |
| d4hrrb_ | d2r50a_ | d3d1kb_ | d2aa1b1 | d3zhwa_ | d3hf4b_ | d3gdja_ | d1jebb_ |
| d3bj1b_ | d1x9fb_ | d1wmub_ | d1gcva_ | d3bcqb_ | d3d4xb_ | d1gvha1 | d2dc3a_ |
| d3hf4a_ | d4yu3a_ | d3k8ba_ | d1qpwb_ | d2r80a_ | d3bcqa_ | d1v4wa_ | d3wtgb_ |
| d1jl7a_ | d4esaa_ | d1a9we_ | d3d1ab_ | d1fsla_ | d4h2la_ | d1v4wb_ | d1mwca_ |
| d1vhba_ | d1jeba_ | d1spgb_ | d1h97a_ | d1q1fa_ | d1tu9a1 | d3d1aa_ | d1hdsb_ |
| d3hyub_ | d2zfbb_ | d3fs4a_ | d1shrb_ | d4h2lb_ | d4yu3b_ | d1spga_ | d3hrwa_ |
| d3qqra_ | d3at5a_ | d4wchd_ | d3hyua_ | d1cg5b_ | d1uc3a_ | d1hbra_ | d2raob_ |
| d1outb_ | d4esab_ | d3gdjb_ | d3qqqa_ | d1x9fc_ | d3vrfa_ | d1itha_ | d3mkba_ |
| d3d4xa_ | d3mjpa_ | d1jl6a_ | d3fh9a_ | d1outa_ | d2dc3b_ | d1it2a_ | d3eoka_ |
| d3g46a_ | d1qpwa_ | d3vrfb_ | d1ch4a_ | d1x9fd_ | d1hdsa_ | d2zfba_ | d3mkbb_ |
| d1or4a_ | d3wtga_ | d1a4fa_ | d3fh9b_ | d3bomb_ | d3cy5a_ | d3at5b_ | d1x9fa_ |
| d2oifa_ | d4lmxb_ | d1cpcb_ | d3v57a_ | d1kn1a_ | d1cpca_ | d4f0ta_ | d2vjha_ |
| d4po5b_ | d1alla_ | d4f0tb_ | d1b33b_ | d1b8db_ | d4h0mb_ | d1b33a_ | d2vjhb_ |
| d4lm6b_ | d2vmlb_ | d1allb_ | d2vjta_ | d3dbja_ | d2vmla_ | d2vjtb_ | d4f0ub_ |
| d1f99b_ | d4rmpa1 | d1liaa_ | d1eyxb_ | d2uulb_ | d4lmsb_ | d4f0ua_ | d2bv8a_ |
| d1f99a_ | d2bv8b_ | d2c7lb_ | d3dbjb_ | d4rmpb1 | d1b8da_ | d4h0ma_ | d3v57b_ |
| d1kn1b_ | d1liab_ | d2uula_ | d2j96a_ | d1pfsa_ | d2a1ka_ | d1je5a_ | g1wht.1 |
| d2xqfa_ | d1ea5a_ | d1f6wa_ | d1dx4a_ | d1k4ya_ | d4qwwa_ | d2ha2a_ | d2qvba_ |
| d2v9za_ | d4kafa1 | d1b9ra_ | d1offa_ | d1frra_ | d3wcqa_ | d1l5pa_ | d1wria_ |
| d1i7ha_ | d3av8a_ | d1czpa_ | d1rfka_ | d3p1ma1 | d3b2ga_ | d2bt6a2 | d1pv8a_ |
| d1w5qa_ | d2c1ha_ | d1ohla_ | d1l6sa_ | d1dosa_ | d1gvfa_ | d3qm3a1 | d2fjka_ |
| d1f74a_ | d3l21a_ | d2e1da_ | d1to3a1 | d1w3ia_ | d1sfla_ | d1n7ka_ | d1vcva1 |
| d4imea_ | d1wa3a_ | d2ocza1 | d1f2ja_ | d3m16a_ | d2yxga_ | d1ub3a_ | d3a5fa_ |
| d1fbaa_ | d1l6wa_ | d1xfba1 | d1wx0a1 | d1vpxa1 | d1ojxa_ | d1mxsa_ | d2a4aa1 |
| d1xkya1 | d3cq0a1 | d3s1xa_ | d1f05a_ | d1fdja_ | d3cpra1 | d4d2ja_ | d1cg2a1 |
| d1yloa2 | d1y7ea2 | d1y0ya3 | d1lfwa1 | d1vixa1 | d1fnoa4 | d1vhoa2 | d1ysja1 |
| d1vhea2 | d1z2la1 | d2fvga2 | d1vgya1 | d2grea2 | d1xmba1 | g1q7l.1 | d1rtqa_ |
| d2ftba_ | d1pmpa_ | d2rcqa1 | d1fdqa_ | d4lkpa_ | d1sa8a_ | d1vyfa_ | d1lpja_ |
| d3vg7a1 | d4azqa1 | d1o8va_ | d5bvqa_ | d1p6pa_ | d2qo4a_ | d1opba_ | d1yiva1 |
| d1o1va_ | d4bvma1 | d4tkba_ | d4i3ba_ | d1bwya_ | d1ftpa_ | d4gkca_ | d1cbia_ |
| d3p6da1 | d1kqwa_ | d2a0aa1 | d1jx4a2 | d3gv5b1 | d1t94a2 | d3bq0a2 | d1e69a_ |
| d1mv5a_ | d3g60a4 | g1ii8.1 | d1ewqa2 | d1ji0a_ | d1qhla_ | d1v43a3 | d2wmfa1 |
| d1vpla_ | d1g2912 | d4a82a2 | g1f2t.1 | d2d62a1 | d3tuic1 | d2bbsa1 | d3g60a3 |
| d2ixea_ | d1g6ha_ | d1sgwa1 | d2qi9c_ | d3rlfa1 | d3b60a1 | d3d31a2 | d2pzea1 |
| g1xew.1 | d1jj7a_ | d1oxxk2 | d2onka1 | d1w1wa_ | d1iwea_ | d1qf5a_ | d1vmaa2 |
| d1nija1 | d1np6a_ | d2p67a1 | d3ihla_ | d1okkd2 | d1ls1a2 | d1s1ma2 | d1iona_ |

d2v40a_ d3hida_ d2vo1a1 d2g0ta1 d1vcoa2 d1j8mf2 d1g3qa_ d1xjca_
d1p9ba_ d1qzxa3 d1ihua2 d1ihua1 d2qm8a_ d1dj2a_ d4ioja_ d1cp2a_
d1yrba2 d1hyqa_ d3gqba2 d1skye3 d1xp8a1 d1tf7a2 d1tf7a1 d1ye8a1
d1skyb3 d2qe7d2 d4blta_ d4huta_ d3ldaa2 d4hyya_ d2i3ba1 d1cr1a_
d3j9tb2 d3ntua2 d1vdza1 d2z43a_ d1e9ra_ d1nlfa_ d3ziaa2 d1n0wa_
d1g6oa_ d3ziad2 d3j9ta2 d1pzna2 d3gqbb2 d2i49a_ d1nnfa_ d1xvxa_
d1ursa_ d1h3da1 d1iuda_ d4m1va1 d1twya_ d3e13x_ d2nxoa1 d4pfua_
d3ttma_ d1elja_ d2qmwa1 d1us5a_ d1y3na1 d1jeta_ d1uqwa_ d4f19a1
d2esna2 d3thia_ d3fasa1 d2uyeb_ d2xwva1 d1r9la_ d2fyia1 d1pb7a_
d2ozza1 d1sw5a_ d1nh8a1 d1vr5a1 d2g29a_ d1xs5a_ d3lswa_ d2z23a_
d3w9va_ d3tcha_ d1q35a_ d1z7me1 d3q27a_ d1p99a_ d1ve4a1 d2i6ea1
d4mcoa1 d4n8ya_ d1eu8a_ d1zbma1 d1hsla_ d1ixca2 d1j1na_ d1xvya_
d1y9ua_ d2z22a1 d1xoca1 d4oera_ d1wdna_ d5f1qa1 d1xt8a1 d1i6aa_
d4gwoa_ d4igta1 d1pc3a_ d4mmpa1 d1o63a_ d2czla1 d2onsa2 d4mija1
d4ok5a1 d2bmfa1 d2bmfa2 d1yksa1 d1yksa2 d2qeqa1 d2jlqa1 d1a1va2
d1eh3a_ d3skpa_ d1dtza1 d1dtza2 d1ieja_ d1jnfa2 d1ce2a1 d1jnfa1
d1b1xa2 d1b1xa1 d1jw1a1 d1ryoa_ d1h76a1 d1h76a2 d1c4oa1 d2p6ra3
d2p6ra4 d1oywa2 d1oywa3 d1wrba1 d1w36b2 d1w36b1 d1c4oa2 d3bora_
d1t6na_ d2eyqa5 d2eyqa3 d2eyqa2 d2zu6a2 d1veca_ d1uaaa1 d1w36d1
d2g9na1 d1z3ix2 d2rb4a_ d1hv8a1 d1hv8a2 d1t5ia_ d1w36c2 d2j0sa2
d2j0sa1 d1fuka_ d3hjha1 d1z5za1 d1qdea_ d1z63a1 d1s2ma2 d1s2ma1
d1wp9a1 d1wp9a2 d1gm5a3 d1gm5a4 d3dina4 d3dina3 d1q0ua_ d1w36c1
d1z3ix1 d1nkta3 d1uaaa2 d1w36d2 g1qhh.1 d2fwra2 d2fwra1 d2v8ta_
d3q3ma_ d1za0a1 d1mxra_ d2inca_ d1jk0a_ d2incb_ d3hf1b_ d1mtyb_
d1r2fa_ d2uw1a_ d1mhyd_ d3n37a_ d1afra_ d1oquc_ d1syya_ d1mtyd_
d2o1za_ d1uzra_ d1smsa_ d2fkza_ d1zuja1 d1s3qa1 d2fjca1 d1z6om1
d1veia_ d4dyya_ d1jiga_ d3gvya_ d2pyba_ d4cy9a1 d3bvfa1 d1r03a_
d2cf7a_ d1nf4a_ d1zs3a1 d4iwka_ d3ka8a_ d3np0x_ d1n1qa_ d1lb3a_
d4e6ka_ d1z6oa1 d2fzfa1 d4dyua_ d3ge4a_ d1jgca_ d4am4a_ d2z6ma_
d1ji5a_ d1vlga_ d1lkoa1 d3qz3a_ d1o9ra_ d2ffxj_ d1euma_ d1yv1a1
d2v2pa_ d1j30a_ d4di0a_ d1dpsa_ d1oewa_ d2qzxa_ d2asia_ d1psoe_
d1smra_ d2h6ta_ d1pfza_ d1izda_ d3hd8a_ d1j71a_ d1dpja_ d4aa9a_
d1t6ex_ g1b5f.1 g1lya.1 g1htr.1 d1qdma2 d1bxoa_ d1wkra_ d1tzsa_
d1ibqa_ d2qzwa_ d1bdqa_ d3ggta_ d3ixoa_ d1mrxa_ d2haha_ d1tw7a_
d4njsa_ d1sgua_ d2p3da_ d1nsoa_ d3d3ta_ d2rkfa_ d1c6ya_ d2rspa_
d3ogpa_ d3ekwa_ d2o4na_ d2j9ja_ d1baia_ d3ggua_ d3lzua_ d4npua_
d4j55a_ d2r8na_ d2fxda_ d2p3ca_ d4dqea_ d3ttpa_ d3t3ca_ d2p3aa_
d2hc0a_ d2fdda_ d3mwsa_ d1k8rb_ d1wgra1 d1ef5a_ d1wfya1 d1wgya1
d1lfda_ d2cs4a1 d1wxma1 d2byea1 d3kh0a1 d2byfa1 d1wxaa1 d1e7ua3
d1h4ra3 d4yl8a1 d2al6a3 d1wgda1 d1wgga1 d4q5eb_ d1wjna1 d1se9a_
d3n3kb_ d1wz0a1 d1we6a1 d2m0xa_ d2gbka_ d3rt3b1 d1wh3a1 d1sifa_
d2ekec2 d4ii2b1 d4hk2a1 d1c3ta_ d1wgha1 d1ttna1 d1j8ca_ d1wx8a1
d3v6cb1 d2ma9b_ d4i6lb1 d1bt0a_ d2jvca1 d1iyfa_ d2k1fa_ d1wxva1
d1wf9a1 d1t0ya_ d2io0b2 d1x1ma1 d2gbma_ d1zw7a1 d2m17a_ d1ud7a_

d1we7a1 d2lwpa_ d2gbja_ d1wjua1 d1v6ea1 d1v86a1 d4eewa_ d1wiaa1
d4icva_ d4gu2a_ d1wx7a1 d1wy8a1 d4hjka1 d2faza1 d1v5oa1 d3ruib_
d1eo6a_ d3waob_ d2r2qa1 d3m95a_ d3wana_ d1yvca1 d1yeza1 d4lu0a_
d1of8a_ d3unda1 d3e9aa1 d1vr6a2 d1n8fa_ d2qkfa_ d1d9ea_ d2h0ba_
d2erfa_ d1h30a2 d1h30a1 d3mw4a1 d2r1ba_ d3v64a1 d1d2sa_ d3bopa1
d3mw3a1 d1okqa1 d1okqa2 d2bcgg1 d2bcgg2 d1wioa4 d1ccza2 d1vcaa1
d1ic1a1 d2d9qb1 d2nxyb2 d1um8a_ d1ixza_ d1qvra2 d1qvra3 d1qzma_
d1sxjc2 d1jr3d2 d2p65a_ d1l8qa2 d1g8pa_ d3hu3a3 d1sxjd2 d1njfa_
d1in4a2 d1sxja2 d1tuea1 d2ce7a2 d1w5sa2 d1sxjb2 d1d2na_ d1ny5a2
d1ofha_ d2gnoa2 d2a5yb3 d1fnna2 d1u0ja1 d1lv7a_ d1iqpa2 d2fnaa2
d1ixsb2 d4gita_ d1sxje2 d1g41a_ d1mbma_ d3su6a1 d1cu1a1 d4agka_
d1vcpa_ d1q31a_ g1n1l.1 d3e90b_ d1ep5a_ d3u1jb_ d2e3fa_ d1mm0a_
d2e3ea_ d2llda_ d2ny8x_ d2e3ga_ d2ny9x_ d2nz3a_ d3ia2a_ d1a88a_
d4dgqa1 d1hkha_ d1zoia_ d2k4ua_ d1hp2a_ d2lu9a_ d1baha_ d2meoa1
d1wm7a_ d1hlya_ d1n8ma_ d1jlza_ d1wpda_ d1c55a_ d1pvza_ d1wt7a_
d1rjia_ d1wz5a_ d1m2sa_ d1pjva_ d1c49a_ d1qkya_ d2ktca1 d1wmta_
d1du9a_ d1j5ja_ d1r1ga_ d1t0za_ d2i61a_ d1t1ta_ d1sn4a_ d1kv0a_
d2lkba_ d1npia_ d1wwna_ d1pe4a_ d2kbka_ d2li7a_ d1djta_ d2kbha_
d1dq7a_ d1sega_ d1omya_ d1zywa1 d1jzaa_ d1n9wa1 d1bbua1 d1c0aa1
d1b8aa1 d1l0wa1 d3a74a1 d1eova1 d1t61c1 d1iyjb4 d1iyjb5 d1iyjb3
d1o7ia_ d4ipca1 d1x3ea_ d4apva_ d1jb7a1 d1jb7a2 d1jb7a3 d3kdfa_
d1ue1b_ d1wjja1 d3eiva_ d1xjva2 d1xjva1 d2ccza2 d1se8a_ d1l1oc_
d1jb7b_ d1qvca_ d1qzga_ d1miua4 d1miua5 d1miua3 d3g48a1 d1gd7a_
d1mkha_ d1pyba_ d1y14b1 d2nn6i1 d1i94l_ d2zjra2 d1smxa_ d2qamc2
d1hh2p1 d2r7da1 d1rl2a2 d1wfqa1 d3bzka4 d1x6oa2 d3a0ja_ d2j01d2
d2ja9a1 d4ql5a_ d1hr0w_ d1luza_ d2n3sa_ d2vnud1 d1h95a_ d1go3e1
d2nn6h1 d2mo1a_ d1iz6a2 d1jjga_ d1e3pa2 d2ix0a3 d2ix0a2 d2lxja_
d1ueba3 d1ueba2 d2z0sa1 d1u0la1 d2ix0a1 d2eifa2 d2je6i1 d3i2za1
d2c35b1 d2ahob2 d1t9ha1 d3i4oa_ d2qall1 d1jt8a_ d2nn6g1 d1g6pa_
d3cama_ d1i94q_ d1c9oa_ d1d7qa_ d2ba0a1 d2vnud2 d2vnud3 d1khia2
d2qalq1 d1wi5a1 d1xtda2 d1ny4a_ d1dgsa2 d1fvia1 d1ckma1 d1p16a1
d1x9na2 d3zxha_ d1cgla_ d1rm8a_ d1eaka2 d1hy7a_ d2jsda_ d1cxva_
d1hv5a_ d1i76a_ d3azya_ d3wvja_ d1upsa1 d1ajoa_ d2hyka_ d1ajka_
d2vy0a_ d1o4ya_ d3o5sa_ d3d6ea_ d3atga_ d1dypa_ d1umza_ d3i4ia_
d1c1la_ d4lbqa_ d1slta_ d4no4a_ d3zsja_ d3duia_ d1g86a_ d1is3a_
d1qmja_ d1hlca_ d1tdha3 d1q3ba3 d1k82a3 g1qmo.1 g1len.1 d3zyra_
d1fnya_ d1n47a_ g2ltn.1 d1avba_ d3usua_ d1dhkb_ d1f9ka_ d1ukga_
d1g8wa_ d1g9fa_ d1wbfa_ d3wcsa_ d1g7ya_ g1loe.1 d1mvqa_ d1hqla_
d1v6ia_ d1dbna_ d1qnwa_ d1fx5a_ d4l8qa_ d1ioaa_ d3n35a_ d3ubra_
d1qo8a1 d1d4ca1 d2k3va_ d1ft5a_ d1qdba_ d1m1qa_ d2j7aa_ d1h21a_
d1ogyb_ d4fasa_ d1jnia_ d1sp3a_ d3h33a_ d1rwja_ d19hca_ d1os6a_
d3bxua_ d3h4na_ d1i77a_ d3caoa_ d2ctha_ d1duwa_ d1ofwa_ d2e84a_
d1hh5a_ d1gyoa_ d3h34a_ d1a6ya_ d3cbba_ d1ynwa1 d1r4ia_ d1hcqa_
d2hana_ d2hanb_ d2enva1 d1kb4b_ d2nllb_ d1lo1a_ d1cita_ d1adqa1

d1fnga1 d1uvqb1 d1zs8a1 d2vola1 d2vola2 d1mfbl2 d2jelh2 d1uwel2
d3qq4b1 d1kjvb_ d1k5na1 d1osph2 d4odhl2 d1ow0a1 d1ow0a2 d1bfob2
d3l9ra2 d3jtth1 d2fath2 d4elkb2 d1fn4b2 d2xfxb_ d1pg7x2 d1cqka_
d4k71b2 d1lk2a1 d1eapb2 d4lfhg2 d1i4fa1 d2nw2a2 d1kfah2 d1mhca1
d1h3ua1 d1wejh2 d1h0db2 d1a5fh2 d1kcvh2 d1mjuh2 d1qqda1 d1kcrh2
d1k8ia1 d3huja2 d2nnab1 d1nfdf2 d3mffb2 d1s9va1 d1lk3h2 d1q0xl2
d1fv1b1 d1neza1 d4rfeb2 d1r3ha1 d1a6tb2 d1kjva1 d1pz5b2 d1onqa1
d2bc4a2 d1de4a1 d1k8da1 d1tcra2 d1dn0b2 d1nfde2 d2xwtb2 d1bfoa2
d1mexh2 d1iakb1 d1ypzf2 d4f7ea2 d1q72l2 d2fbjh2 d1fe8h2 d1clzh2
d1fngb1 d1uvqa1 d1vgka1 d1c16a1 d4j12a2 d4x99b2 d1igtb3 d1je6a1
d1q72h2 d1nldh2 d1teth2 d2hmic2 d1i1ca2 d1i1ca1 d1k8ib1 d1q0yh2
d1kfal2 d3frua1 d1pg7w2 d1u58a1 d1igyb4 d1igyb3 d1hyrc1 d1klub1
d1deeb2 d1c12b2 d2bc4b2 d2ig2h2 d1kcvl2 d3wn5b1 d1lk2b_ d1lk3l2
d1mjul2 d1sm3h2 d1q0xh2 d1zvsa1 d1ydpa1 d1u8ib2 d1ypze2 d1kcuh2
d1op9a_ d1y0la1 d1etzb1 d1egjl1 d2gsib1 d1nj9a1 d4jfhd1 d1aqkl1
d1t2ja_ d1lo4h1 d1zvya1 d3v4vl1 d4enec1 d1kxtb_ d5e8ea1 d2pxya_
d4weud_ d1xiwc_ d2esvd1 d1qnzh_ d1ogae1 d1f4xh1 d4n1hb_ d3ezjb1
d3mlwl1 d1kfah1 d1etza1 d1b4jh1 d3cfic_ d4w68a_ d2arja1 d1ek3a_
d2ok0l1 d1vera_ d1uwgh1 d1blnb1 d1fpth1 d1rzga1 d2giya1 d4m3kb1
d1nc2a1 d2nyyd1 d1ncna_ d1fbih1 d5dxwa_ d4fqqa1 d1dqdh1 d1jfql1
d1kelh1 d1faih1 d1frgl1 d1figl1 d4m6nl1 d1fo0a_ d4hxbl1 d3b5ga_
d3quyd1 d2h9gb1 d1i8kb_ d2dd8h1 d2ck0h1 d1eaja_ d4w6wb1 d2a6ib3
d1t66d1 d2e27h_ d1ob1b1 d1f11b1 d2v7na1 d1ad0a1 d2xt1b1 d1pskh1
d4i13b1 d2op4l1 d1a0ql1 d1uwel1 d1za3a1 d1ncbl1 d4uu9a_ d1bm3h1
d1cloh1 d1nqba2 d1cf8h1 d4y5va_ d1mhph1 d5anma1 d4cdgc1 d4ldeb_
d1y0lb1 d2pcpb1 d1z3gl1 d1dfbl1 d1fh5h1 d3g5xa1 d1tzib1 d4dn3l1
d3zhka1 d2b1hh1 d2uyla1 d1f3dh1 d1nj9b1 d2agjh1 d2h1pl1 d1q72h1
d8fabb1 d1ndmb1 d1rjca1 d4u6hb1 d3upca_ d2fbjl1 d1osph1 d1tvda_
d2cjvh_ d4fhbd1 d1bd2d1 d1rz7h1 d3nn8a_ d1blna1 d1knoa1 d1nfda1
d2bmkb1 d1dqll_ d1igyb1 d1igfl1 d12e8h1 d3utza1 d1nc2b1 d1rihh1
d4mwfb1 d4jvpa1 d1mieh1 d1rhfa1 d1fgnl1 d1sbsh1 d1lp9e1 d1dn0a1
d4lqfl1 d1iaih1 d1kgcd1 d1mh5b1 d1gpol1 d2g60h3 d1f8tl1 d2jixd1
d4grwh_ d1fo0b_ d1bfob1 d1axsa1 d1u9ka_ d1nmbh_ d1h8na1 d1h8na2
d1jguh1 d4eura1 d3sdyl1 d35c8h1 d1w72l1 d1mvub_ d1i9rh1 d1ob1a1
d4wenb_ d1yeeh1 d3ietc1 d1f58h1 d1ad0b1 d1mqkl_ d1nbvh1 d4b50a_
d3r8bb_ d1bafl1 d1mamh1 d4rgne1 d2gsgb_ d35c8l1 d1bafh1 d1ai1h1
d2pcpa1 d2fath1 d2x1pa_ d1orsb1 d1ibgl1 d1shma_ d1a2yb_ d1u0qa_
d2coqa_ d1hilb1 d1qfwi_ d1jv5b_ d1fn4b1 d5e0qa_ d1nsnh1 d1plgl1
d4ocxl1 d1kb5h1 d1rzfh1 d1pg7x1 d2fx7h1 d2oi9c_ d1l7il1 d3eyva1
d1d5il1 d4ffyl_ d1a6wh_ d1ac6a_ d3dvfa_ d1nfde1 d1mimh1 d1ri8a1
d1jhlh_ d3dura_ d1fskb1 d1f97a1 d3so3b1 d2ak4d1 d1lp9f1 d4z7wf1
d1l7th1 d4y5ya_ d4x7ec1 d1emth1 d1ikfl1 d1nlbh1 d4utal1 d1bjma1
d1eapb1 d1u6ah1 d2dtgd1 d1kj2b_ d1xeda1 d1c1eh1 d1fgvh_ d2bnub1
d2fb4h1 d2dtgb1 d1ktrh_ d1jpth1 d1qkzl1 d4hepg_ d2atpb1 d1flrh1

d2hrpl1 d1mvfa_ d1iaim1 d3k80a1 d2jb5h1 d3zkxc_ d4grwe_ d1it9h1
d1rhhb1 d3w9ec_ d1e4wh1 d1sy6h1 d2j6eh1 d3qxta_ d1jglh1 d1p7ka1
d1tzhb1 d4n8pa1 d1gafl1 d2jelh1 d1wioa2 d4i0cc1 d1cicb1 d1lmka2
d1lmka1 d1iqwh1 d1orqb1 d1ejoh1 d1jnlh1 d4zg1a_ d43c9b_ d1ggbl1
d1wejh1 d3q6fa1 d1f3dj1 d3c6la1 d1bqlh1 d3p9wb_ d1a3rl1 d1h0db1
d2uzil_ d1eo8l1 d1qfwl_ d1n7ml1 d1igja1 d1c5ch1 d2a9mh_ d1rmfh1
d1fj1b1 d4heme_ d1ccza1 d1a5fh1 d1ogad1 d1dlfh_ d1qa9b_ d1vgel1
d3qskb_ d1sjxa_ d4ozfg1 d1sq2n_ d1qd0a_ d1kcvh1 d1c5db1 d3qcul1
d8faba1 d3bn9d1 d1mjuh1 d4y8dc_ d7fabh1 d3dvgb1 d1lo0l1 d1a6tb1
d1gigh1 d1kcrh1 d2hwzl1 d1yjdc1 d3clfl1 d4krna_ d1dl7l_ d1e6ol1
d1mexh1 d3r0ma_ d4kkna_ d1fnsl1 d1oaqh_ d1rzia1 d1ucbh1 d1ncwl1
d4b41a_ d1nfdf1 d2mpah1 d2d7tl1 d4grwf_ d4b5ea_ d1lk3h1 d3u79a_
d4u3xa_ d1ghfl1 d1bvkb_ d1mcww1 d1b88a_ d1jrhh1 d32c2b1 d4wemb_
d1dsfh_ d1op3h1 d1cell1 d1iqda1 d1qoka1 d1qoka2 d1qfuh1 d2crya1
d1ay1h1 d1oaql_ d3ab0b_ d1rurl1 d1t4kb1 d1bz7b1 d1n0xh1 d1forl1
d1ucbl1 d4w6yb_ d43c9a_ d2aabl1 d1tzia1 d4n1ca_ d1k6qh1 d3g9ab_
d4tvsa1 d1igmh_ d1pz5b1 d3dx9a1 d3wxwh_ d1nakh1 d1kcul1 d3eaka_
d4qlra1 d1cr9l1 d1d9ka_ d1f4xl1 d1deea1 d2i27n2 d1c12a1 d2orbl1
d1j8hd1 d1mcph1 d1c5da1 d1kenh1 d1bd2e1 d1h5ba_ d4x7fc1 d3upaa_
d1t3fa1 d1indh1 d2dtgc1 d4hc1l1 d1zv5a1 d4wgvb_ d1uwgl1 d4f37k1
d1aqkh1 d1vesa_ d3zkqd_ d1fptl1 d1lo4l1 d1knob1 d3rrqa_ d1ct8b1
d4orzc_ d1mlbb1 d4hgkc1 d1dqdl1 d1bogb1 d4ozhe1 d1dr9a1 d1i7za1
d1dn0b1 d1kell1 d1rzib1 d1ypzf1 d1wtla_ d1hkfa_ d1fail1 d1figh1
d2vyre_ d1sjva_ d1ohqa_ d1bfoa1 d1pkqb1 d1mjul1 d1b4jl1 d1nsnl1
d2arjb1 d1p4bh_ d4aj0a_ d3nn8b_ d2rcja1 d1f2xk_ d4v1dd_ d1adqh1
d2eksa_ d4k3ga_ d1jfqh1 d1uweh1 d1frgh1 d4gftb_ d1uz8b1 d4n1ea_
d1bm3l1 d1clol1 d3mcka1 d2vq1a1 d1ae6h1 d4aq1b1 d1uj3b1 d1nbqa1
d1fh5l1 d2ck0l1 d2ywza_ d1q72l1 d4nbxb_ d1hila1 d4hzlb1 d2j88l1
d1zmya1 d1cd0a_ d1a4ka1 d1pskl1 d1a0qh1 d4krmb_ d1ncbh1 d2fbjh1
d1j8he1 d1i3ua_ d1cf8l1 d1fe8h1 d2p1ya1 d1eeqa_ d2x89a_ d1ol0a_
d2r0lh1 d1ub5a1 d1dfbh1 d3eyvb1 d4jdvb1 d4p2cg_ d2gsia1 d2agjl1
d1u3ha1 d2h1ph1 d1rihl1 d1fskc1 d1boga1 d2a77l1 d1ospl1 d1qfoa_
d3dwta_ d2fx7l1 d1rz7l1 d3stba1 d1pkqa1 d1b6da1 d1dzba2 d1dzba1
d1kj2a_ d4l1ha_ d1rzgb1 d1dqlh_ d12e8l1 d1miel1 d1jmaa_ d1sbsl1
d1fgnh1 d1f58l1 d1mqkh_ d1ndgb1 d3qegl1 d1iail1 d2aw2a1 d1tqbc1
d3fo2a1 d4dkaa_ d1f8th1 d2g60l1 d2ywya_ d1uj3a1 d1yc7a_ d3bd3a_
d4w6xb1 d1nmbl_ d1i8ka_ d1rjlb1 d1ypze1 d1p7kb1 d1kxqe_ d1gpoh1
d2z4qa1 d1w72h1 d1i9rl1 d4qgya1 d4xt1c_ d2qqnh1 d1cica1 d3c08h1
d2cd0a_ d2bdnh1 d1hnga1 d1maml1 d1nldh1 d1cr9h1 d4lcih1 d3bkyh1
d3qg7l1 d1ai1l1 d1kxvc_ d4iosd_ d1l7ih1 d2x1qa_ d1ub6b1 d3k74b_
d1ibgh1 d1teth1 d1igya1 d1hxma1 d2x6ma_ d2e7la_ d5e03a_ d1qfwm_
d2r0kh1 d1igjb1 d1rurh1 d1plgh1 d1mj8h1 d1yuhb1 d3fmgl1 d1rzfl1
d1emtl1 d1ad9b1 d1igtb1 d1nlbl1 d1d5ih1 d3k7ua_ d4ffyh_ d1bwwa_
d3dvga1 d1a6ta1 d1fgvl_ d4qxgl1 d1miml1 d1jhll_ d2hffb1 d2hh0l1

d1qkzh1 d1l7tl1 d1afvh1 d1iaii1 d2cjuh_ d1um5h1 d2ghwb2 d2ghwb1 d1kgce1 d2fgwh1 d1ikfh1 d1dvfd_ d2i24n1 d2xtjb_ d1za3b1 d2yc1a_ d2hh0h1 d2x1oa_ d1bvka_ d1rjla1 d5e7ba1 d1igta1 d1il1b1 d2fb4l1 d1mvua_ d1jptl1 d32c2a1 d1fvca_ d1iqdb1 d2hrph1 d2p4ab_ d4qo1a_ d1p2cb1 d1wt5c_ d1t4ka1 d5c1mb_ d1it9l1 d1bz7a1 d1b2wh1 d1e4wl1 d1jnll1 d1yedb1 d1za6b1 d3u7aa_ d1igfh1 d3gnml1 d1orsa1 d1wejl1 d1a2ya_ d1hxmb1 d1gafh1 d2j4wh1 d1pz5a1 d1j05a_ d1a3rh1 d4fqll1 d1qfwh_ d1eo8h1 d1n7mh1 d4s11a_ d1kegh1 d5da4a_ d1iqwl1 d3whxb1 d1vgeh1 d1deeb1 d1c12b1 d1t2ql1 d1ad9a1 d3gsnb1 d3b2uc1 d1ggbh1 d1t3fb1 d1nakl1 d4ocmc_ d1lila1 d2v7hb3 d2esve1 d1mcoh1 d2qr0b1 d2q3aa_ d1ct8a1 d1kcrl1 d2pxyb_ d2uzih_ d5c3ld_ d3durb_ d1c5cl1 d2ak4e1 d1dl7h_ d1xiwd_ d1mlba1 d1rmfl1 d1i7zb1 d1dlfl_ d2xa3a_ d5bq7b1 d1dqqb1 d3h42l1 d1egjh1 d1kcvl1 d1fe8l1 d2j6el1 d1f3rb2 d1lk3l1 d1sm3h1 d1q0xh1 d1ghfh1 d7fabl1 d1igch1 d1lo0h1 d4idla_ d5c2bl_ d1jrhl1 d1il1a1 d3zhda1 d4kfzc_ d1b0wa_ d1e6oh1 d1mexl1 d4krpb_ d1fnsh1 d1ncwh1 d4hgma_ d4eigb1 d4rzca_ d2mpal1 d4y5xa_ d5en2b1 d3lrga1 d4ej1c1 d1rhha1 d1j05b_ d1forh1 d1q9wb1 d2wzpd_ d2aq2a1 d1h0da1 d3revb1 d2ij0c1 d1tzha1 d4m7jl1 d1k6ql1 d3b9kc1 d1igml_ d3qeua1 d1dsfl_ d1ce1h1 d1kcuh1 d1zvha1 d1zvoa1 d1tqbb1 d1ay1l1 d2qscl1 d2cgrh1 d1nl0h1 d1kenl1 d3eo0a1 d4n9ob1 d1n0xl1 d3k1kc_ d1c4qa_ d1prtc1 d2wv6d_ d1prtf_ d1ltrd_ d4l63b1 d1r4pb_ d1prtd_ d1tl0f_ d2bosa_ d3chbd_ d2cqva1 d1fhga_ d2k1ma_ d2dl2a1 d1biha3 d1biha1 d1biha4 d3qp3a1 d1g1ca_ d1gxea_ d1cvsc2 d1cvsc1 d1xmwa2 d1m4ka2 d2otpa2 d1biha2 d1rhfa2 d2fcba2 d2fcba1 d1wwbx_ d1gsma2 d1epfa1 d1epfa2 d2dava1 d1waaa_ d1f97a2 d1x44a1 d1vcaa2 d1fnla2 d1fnla1 d3b5ha2 d3h8na2 d3h8na1 d1jbja1 d1jbja2 d1pd6a_ d1f2qa2 d1f2qa1 d3cx2a_ d1nbqa2 d3b5ha1 d1n26a1 d1xiwb_ d1xaua_ d1fltx_ d2ifga1 d1olla1 d1olla2 d1cs6a4 d1cs6a3 d1cs6a2 d1cs6a1 d2gw5a2 d1sy6a1 d1xiwa_ d1ucta2 d1ucta1 d1ry7b2 d1gl4b_ d1qz1a3 d1olza1 d2c9aa1 d1wwca_

**Family-54 (c.2.1.6)**

**Positive dataset contains 12 protein sequences.**

d1txga2 d3f47a1 d2pv7a2 d1xc2a3 d2ahra2 d4xiya1 d2f1ka2 d1teaa3 d2i76a2 d2g5ca2 d1wdka3 d3cuma3

**Negative dataset contains 3793 protein sequences.**

d1m32a_ d1ibja_ d1c7na_ d1iuga_ d4q75a_ d2bkwa1 d1t3ia_ d1n8pa_ d1qz9a_ d1jf9a_ d1p3wa_ d3kgwa_ d1cl1a_ d2ctza1 d1vjoa_ d2rfva_ d1gc0a_ d1cs1a_ d1d2fa_ d2ch1a1 d1qgna_ d1pffa_ d1elua_ d1xkla_ d2wfla_ d3i2ka1 d1lnsa3 d2cfba1 d1dfoa_ d1o69a_ d1bj4a_ d3k28a_ d2bwpa_ d3lg0a_ d2fnua_ d3ou5a_ d1z7da1 d1iaya_ d4msoa_ d1bs0a_ d1s0aa_ d1sffa_ d3tqxa_ d1mdoa_ d3qboa_ d3pgya_ d1bjna_ d2dkja_ d1ejia_ d1vefa1 d4wxga_ d3bs8a_ d3gbxa1 d1w23a_ d3lgia_ d3i77a_ d2z9ia2 d4nsva_ d1lcya2 d4jcna_ d1agja_ d1os8a_ d1hpga_ d1l1ja_ d1qtfa_ d1cixa_ d1y29a_ d1v7fa_ d2jtba_ d1d1ha_ d1g9pa_ d1lupa_

d1qk7a_ d1mb6a_ d2a2va_ d1niya_ d1i25a_ d2mpqa_ d1ie6a_ d1kqha_
d1la4a_ d2mxoa1 d1dl0a_ d1koza_ d1qk6a_ d1emxa_ d1lqra_ d1c6wa_
d1qnia1 d1fwxa1 d1f56a_ d1bypa_ d1azca_ d1ws8a_ d3u25a_ d1cuoa_
d1id2a_ d1rkra_ d2iaac_ d4dp1x_ d1nwpa_ d2gima_ d2i7sa_ d1qhqa_
d1gyca3 d1hfua2 d1kbva1 d1kbva2 d1sdda1 d1sdda2 d1gyca1 d1gyca2
d1mzya1 d2bw4a1 d1aoza1 d1aoza3 d1aoza2 d1kyaa2 d1kyaa3 d1kyaa1
d1mzya2 d4e9sa1 d1sddb1 d1sddb2 d1hfua1 d1hfua3 d1oe1a2 d1oe1a1
d1v10a2 d1v10a3 d1v10a1 d1rypd_ d1irua_ d1rypc_ d3unfk_ d1rypa_
d1j2pa_ d1irui_ d1j2qh_ d3unfh_ d1m4ya_ d1rypb_ d1irul_ d1irug_
d1iru2_ d1q5rh_ d1rypg_ d1g3ka_ d1iruf_ d1q5qa_ d1e94a_ d1irub_
d1ryph_ d3ty6a_ d1iruj_ d1iruk_ d1rype_ d1irud_ d1irue_ d3mi01_
d1rypf_ d1iruh_ d2z3ba_ d3mka2_ d1iru1_ d3sxwa1 d1j3qa_ d4luka_
d1a3qa1 d1iknc_ d3brda1 d1p7hl1 d1oy3c_ d1imhc1 d1gjia1 d1zk9a1
d2cxka1 d2bema_ d1pama1 d1ea9c1 d1qhoa1 d2fhfa2 d2fhfa1 d1k3ia1
d1j0ha1 d1cxla1 d2bhua1 d1edqa1 d1f1sa2 d1ut9a2 d1gvia1 d1txka1
d1adqa1 d1fnga1 d1uvqb1 d1zs8a1 d2vola1 d2vola2 d1mfbl2 d2jelh2
d1uwel2 d3qq4b1 d1kjvb_ d1k5na1 d1osph2 d4odhl2 d1ow0a1 d1ow0a2
d1bfob2 d3l9ra2 d3jtth1 d2fath2 d4elkb2 d1fn4b2 d2xfxb_ d1pg7x2
d1cqka_ d4k71b2 d1lk2a1 d1eapb2 d4lfhg2 d1i4fa1 d2nw2a2 d1kfah2
d1mhca1 d1h3ua1 d1wejh2 d1h0db2 d1a5fh2 d1kcvh2 d1mjuh2 d1qqda1
d1kcrh2 d1k8ia1 d3huja2 d2nnab1 d1nfdf2 d3mffb2 d1s9va1 d1lk3h2
d1q0xl2 d1fv1b1 d1neza1 d4rfeb2 d1r3ha1 d1a6tb2 d1kjva1 d1pz5b2
d1onqa1 d2bc4a2 d1de4a1 d1k8da1 d1tcra2 d1dn0b2 d1nfde2 d2xwtb2
d1bfoa2 d1mexh2 d1iakb1 d1ypzf2 d4f7ea2 d1q72l2 d2fbjh2 d1fe8h2
d1clzh2 d1fngb1 d1uvqa1 d1vgka1 d1c16a1 d4j12a2 d4x99b2 d1igtb3
d1je6a1 d1q72h2 d1nldh2 d1teth2 d2hmic2 d1i1ca2 d1i1ca1 d1k8ib1
d1q0yh2 d1kfal2 d3frua1 d1pg7w2 d1u58a1 d1igyb4 d1igyb3 d1hyrc1
d1klub1 d1deeb2 d1c12b2 d2bc4b2 d2ig2h2 d1kcvl2 d3wn5b1 d1lk2b_
d1lk3l2 d1mjul2 d1sm3h2 d1q0xh2 d1zvsa1 d1ydpa1 d1u8ib2 d1ypze2
d1kcuh2 d1mqta_ d1d4m3_ d1fmd3_ d2mev1_ g1aym.1 d1ev13_ d4q4w1_
d1r1a3_ d2x5ia_ d1rhi1_ g1r1a.1 g1d4m.1 g1rhi.1 d1b35b_ d1oopa_
d1tme1_ d4iv1a_ g1fpn.1 d1fpn1_ d4gb31_ d1d4m1_ d1fmd1_ d1ev11_
d2wzr3_ g1cov.1 d1qqp3_ g1fmd.1 d2x5ic_ d1rhi3_ g1oop.1 d1hxs1_
g1hxs.1 d1fpn3_ d1bev1_ g2mev.1 d1eah3_ d1aym1_ d1pvc3_ d1qqp1_
d1zba3_ d1h8ta_ d3vbhc_ g1b35.1 g1bev.1 d1hxs3_ g1ev1.1 d1bev3_
g1tmf.1 d1tmf1_ d1b35a_ d2mev3_ g1eah.1 g1h8t.1 d1eah1_ d4q4w3_
d1r1a1_ d1aym3_ d1pvc1_ d1zba1_ d1h8tc_ d1oopc_ d1tme3_ g1qqp.1
d4iv1c_ g1tme.1 d1ep0a_ d2pa7a1 d1wlta1 d1nxma_ d2c0za1 d1nepa_
d1ex0a1 d1g0da1 d2b5ea2 d2b5ea3 d2b5ea1 d2b5ea4 d2kp1a1 d2kp2a1
d1hyua3 d1hyua4 d1j08a1 d1j08a2 d3nvva1 d1krha3 d2h88b1 d1n62a2
d1t3qa2 d1kf6b2 d1zoyb1 d1jq4a_ d1dgja2 d1rm6c2 d2bjca1 d2hsga1
d1qpza1 d1efaa1 d1k9ua_ d2opoa_ d1ydwa2 d2nvwa2 d1zh8a2 d1qkia2
d1xeaa2 d1tlta2 d1r0ka3 d1j5pa3 d1vkoa2 d1vm6a2 d3qvsa2 d1gr0a2
d1p1ja2 d2ovga_ d1sq8a_ d1rzsa_ d3kz3a_ d3orca_ d3gpdg2 d2q49a2

d1hdgo2 d2g17a2 d2cvoa2 d2czca1 d3pwka2 d1obfo2 d1i32a2 d1zyao2
d2pkqo2 d1b7go2 d2hjsa2 d1cf2o2 d1vkna2 d1nvmb2 d2msya_ d2djoa1
d2r5za_ d1au7a1 d1f43a_ d2lmda1 d1akhb_ d1s7ea1 d3nara_ d1zq3p1
d2craa1 d1mija_ d4uuta_ d3a01a_ d1x58a1 d3rkqa1 d3cmya1 d2cqxa1
d2da6a1 d1e3oc1 d1fjla_ d1mh3a1 d1ig7a_ d1x41a1 d1wh7a1 d1gt0c1
d2mw8a_ d1lfup_ d1x2ma1 d1b72a_ d1wi3a1 d1wjha1 d1x2na1 d1mnmc_
d2cuea1 d3k2aa_ d2hosa_ d2jwta2 d1pufa_ d1octc1 d2hi3a_ d1b8ia_
d4j19a_ d3lnqa_ d1wh5a1 d1b8ib_ d1fexa_ d2cqra1 d1guua_ d1ug2a1
d2cqqa1 d2ajea1 d4a69c_ d1h8ac1 d2crga1 d2iw5b1 d1xc5a1 d2cu7a1
d1wgxa1 d2cjja1 d2ckxa1 d1gvda_ d1hcra_ d1tc3c_ d1gdta1 d1u78a2
d1xg1a1 d1hlva1 d1iufa2 d1iufa1 d1igna1 d1bl0a1 d1d5ya1 d1d5ya2
d3lv8a_ d1s3ga1 d1zaka1 d1s4qa_ d3tvta2 d1uf9a_ d1khta_ d1ckea_
d1osna_ d1p5zb_ d4tmka_ d3cm0a_ d1tmka_ d1jaga_ d3h86a_ d1t0hb_
d1nn5a_ d2h92a_ d3kb2a1 d2c95a1 d1yj5a2 d1kgda_ d1ly1a_ d2ak3a1
d1vyva2 d1deka_ d2grja1 d1p3ja1 d1y63a_ d1e4ya1 d1s96a_ d3iija1
d1lvga_ d1nksa_ d1vyua2 d4e22a_ d1qf9a_ d1jjva_ d1q3ta_ d1ki9a_
d1teva_ d1kjwa2 d1gsia_ d1g3ma_ d1j99a_ d1t8ta1 d1nsta_ d1fmja_
d1zrha_ d1ls6a_ d2a3ra_ d2zptx_ d3ckla_ d1texa_ d1q44a_ d2gwha_
d1z0ja1 d3umra2 d3gj0a1 d2gaoa1 d1yzga1 d1x3sa1 d1g7sa4 d1puja_
d1udxa2 d1kk1a3 d2o52a_ d1jwyb_ d1zcaa2 d1zcba2 d2fv8a1 d2cjwa_
d3dz8a1 d1xzpa2 d1vg8a_ d4qxaa_ d2ew1a1 d1x1ra1 d2bcgy_ d3doea1
d3cona_ d3rega_ d3wyga_ d1upta_ d2b6ha1 d1zd9a1 d1lnza2 d1r5ba3
d1n0ua2 d1r2qa_ d2f9la1 d2rhda_ d3q3jb_ d3q85a_ d1efma1 d1r8qa_
d2gf9a1 d2akab1 d1ek0a_ d1z08a1 d2gcoa1 d2dy1a2 d4g01b_ d2cxxa1
d1svia_ d3q72a_ d1ni3a1 d4ac9a4 d2erxa1 d2bmea_ d1wxqa1 d2gf0a1
d4ldja1 d1zunb3 d1d5ca_ d4lv5b_ d1efca3 d1kmqa_ d2g6ba1 d3lrpa_
d1tada2 d2y8ea_ d4rd4a1 d1z06a1 d2heia_ d3a58b_ d1u0la2 d1d2ea3
d1zj6a1 d2ocba_ d3rwoa_ d1mr3f_ d2e9sa1 d4u5xa1 d2iwra_ d2fn4a1
d3bwdd_ d2nzja1 d2atva1 d2ksqa_ d3l43a_ d1mkya2 d1mkya1 d1wmsa_
d1jnya3 d2rmka_ d1f60a3 d1gota2 d1jala1 d2fh5b1 d1g16a_ d2wblc_
d1m7ba_ d1wf3a1 d1f5na2 d1t9ha2 d1z6xa1 d1z0aa1 d2fg5a1 d2a5ja1
d2bcjq2 d1s0ua3 d1ky3a_ d4y0va_ d4lhwa_ d2qmea1 d2atxa1 d1egaa1
d1ryha_ d1moza_ d1nrjb_ d2erya1 d3bfka_ d1puia_ d1fzqa_ d3j3az1
d3t5ga_ d1shza2 d3defa_ d2f7sa1 d3raba_ d3tsoa_ d3aq4a_ d2al7a1
d3sfva1 d3bc1a_ d2ncda_ d1sdma_ d3h4sa1 d2heha_ d2grya_ g3kin.1
d1kk8a2 d1br2a2 d1goja_ d1f9va_ d2mysa2 d1lkxa_ d3t0qa_ d4db1a1
d3gbja_ d1v8ka_ d1vava_ d1uaia_ d1jmxa2 d1jmxa1 d1pbya1 d1g99a2
d2e1za2 d2e1za1 d1saza1 d1saza2 d1ig8a1 d1czan2 d1czan3 d1czan1
d1ig8a2 d1czan4 d1bg3a4 d1bg3a3 d1bg3a2 d3h3no1 d3h3no2 d3ezwa2
d3h1jd1 d2d0oa3 d2d0oa2 d1nbwa2 d1hroa_ d4gedb_ d3zooa_ d1mz4a_
d1gu2a_ d1f1fa_ d1dvva_ d1c6ra_ d1c75a_ d1e29a_ d1gdva_ d1cnoa_
d1h32b_ d2bh4x_ d2aiua_ d5cytr_ d1jdla_ d1i8oa_ d2zboa_ d3dmia_
d1ls9a_ d1wvec_ d2yk3a_ d3ph2b_ d1dw0a_ d5aura_ d1ql3a_ d1qn2a_
d1f1ca_ d2nqya_ d1h4ga_ d1hixa_ d1oa2a_ d1olra_ d3b7ma_ d4h7ma1

| | | | | | | | |
|---|---|---|---|---|---|---|---|
| d3wp3a_ | d2vgda_ | d1igoa_ | d3zsea_ | d2nlra_ | d3wx5a_ | d1oa3a_ | d1pvxa_ |
| d1m4wa_ | d1f5ja_ | d1oa4a_ | d1ks5a_ | d4ixla1 | d3sita1 | d1evsa_ | d1cnt1_ |
| d1pvhb_ | d1z7ca_ | d1huwa_ | d1bgea_ | d1f6fa_ | d1f45b_ | d1i1rb_ | d1axia_ |
| d1etea_ | d3tgxb_ | d1scfa_ | d2z3qa1 | d3qb1a_ | d1hula_ | d1m48a_ | d4nkqb_ |
| d1eera_ | d1v7mv_ | d5e4ea_ | d2o27a_ | d3b5ka_ | d2psma1 | d4zf7a_ | d3uf5a_ |
| d4x51a_ | d1d9ca_ | d4ypgc1 | d3oq3a_ | d1lqsl_ | d1au1a_ | d1wu3i_ | d3besl_ |
| d3ux9a_ | d1v8fa_ | d2ejca_ | d3muea1 | d1ihoa_ | d3q12a1 | d2b7la_ | d2el7a_ |
| d1iq0a2 | d1irxa2 | d1r6ta2 | d1ivsa4 | d2cyba_ | d1rqga2 | d1j09a2 | d2yxna_ |
| d1f7ua2 | d1h3fa1 | d1ffya3 | d2g36a1 | d1nzja_ | d1jila_ | d1n3la_ | d1i6la_ |
| d1pfva2 | d1h3na3 | d3n9ia_ | d3sz3a1 | d3sw2a_ | d1moxc_ | d1dx5i3 | d1edmb_ |
| d1autl2 | d1autl1 | d1nqlb_ | d1dval1 | d1szba2 | d1kigl_ | d2y5fl_ | d1ijqa2 |
| d1gk5a_ | d1g1sa2 | d1nt0a3 | d1xdtr_ | d1lmja2 | d1lmja1 | d1nzia2 | d2wphe_ |
| d1klil_ | d1pfxl2 | d1pfxl1 | d1p9ja_ | d1g1ta2 | d1f7ea_ | d4yzub_ | d1lr7a1 |
| d2y5ia_ | d1psra_ | d3n22a_ | d2egda_ | d4ggfc_ | d2wcea_ | d2luca_ | d4aqia_ |
| d3nsia_ | d2lp3a_ | d1j55a_ | d1a4pa_ | d1k8ua_ | d1nsha_ | d1pvaa_ | d3fs7a_ |
| d1ttxa_ | d1rwya_ | d2kyfa_ | d1rk9a_ | d1a75a_ | d1exra_ | d1tiza1 | d1nyaa_ |
| d1g8ia_ | d1ggwa_ | d1wdcc_ | d3fwba_ | d1ggza_ | d2o5ga_ | d1ctaa_ | d1m45a_ |
| d3i5gc_ | d1sl8a_ | d1fpwa_ | d2scpa_ | d2roaa_ | d4aqra_ | d3pn7b_ | d1br1b_ |
| d2mysb_ | d1s6ca_ | d1bjfa_ | d2obha_ | d1jfka_ | d1jf0a_ | d1qv0a_ | d1lkja_ |
| d2mlfc_ | d2m97a_ | d2d8na_ | d4by5a_ | d1jbaa_ | d4nqga_ | d1wdcb_ | d2mysc_ |
| d1j7qa_ | d1s6ia_ | d1uhka2 | d1r2ua_ | d1dtla_ | d3kf9a_ | d3pn7c_ | d5d67d_ |
| d1tuza_ | d1eg3a1 | d1eg3a2 | d2zkmx1 | d1snla_ | d1wkya2 | d1uuqa_ | d3wh9a_ |
| d4m1ra_ | d1xyza_ | d4xuya_ | d2je8a5 | d4bf7a1 | d3cuia_ | d1ur4a_ | d1qw9a2 |
| d1odza_ | d2vzsa5 | d1bqca_ | d2x2ya1 | d1hjqa_ | d1qnra_ | d4y7ea1 | d1vjza_ |
| d4k68a_ | d2v3ga_ | d4hpga_ | d1nofa2 | d1nq6a_ | d1h4pa_ | d1aq0a_ | d3ziza_ |
| d4bs0a_ | d1ecea_ | d1h1na_ | d1ta3b_ | d1w91a2 | d1i1wa_ | d1uhva2 | d1us3a2 |
| d1egza_ | d7a3ha_ | d1tvna1 | d3nyda1 | d1ghsa_ | d2cyga1 | d4xv0a_ | d2c8na2 |
| d1w32a_ | d3emca_ | d1v6ya1 | d1foba_ | d2uwfa1 | d2fgla_ | d3niya1 | d2c0ha1 |
| d1rh9a1 | d1yq2a5 | d1szna2 | d1fa2a_ | d1pama4 | d5do8a1 | d1ea9c3 | d1hx0a2 |
| d2fhfa5 | d1qhoa4 | d1avaa2 | d1m53a2 | d4m8ua1 | d2gjpa2 | d1lwha2 | d4howa1 |
| d1ktba2 | d1j0ha3 | d3vm5a1 | d2x1ia_ | d1wzaa2 | d1uasa2 | d2diea1 | d1mxga2 |
| d1ht6a2 | d1eswa_ | d1iv8a2 | d5cpsa_ | d1ud2a2 | d1zjaa1 | d3gbea1 | d1kwga2 |
| d1wdpa_ | d1gcya2 | d1g5aa2 | d1hvxa2 | d3dhpa2 | d2bhua3 | d4do4a1 | d3qgva1 |
| d1e43a2 | d1g94a2 | d2d3na2 | d1gjwa2 | d1gvia3 | d1x1na1 | d1r7aa2 | d1tz7a1 |
| d4c7ga2 | d2gjxa1 | d1nowa1 | d1jaka1 | d1yhta1 | d4toqa_ | d1wb0a1 | d1w9pa1 |
| d1eoka_ | d1itxa1 | d1kfwa1 | d1edqa2 | d2gsja_ | d1hjxa1 | d1jnda1 | d1vf8a1 |
| d3wdpp1 | d3wq5a_ | d1gnxa_ | d4gxpa_ | d4hz8a_ | d3aiua_ | d1wcga1 | d1qvba_ |
| d3ahxa_ | d1v02a_ | d2dgaa_ | d1pbga_ | d1vffa1 | d1qoxa_ | d4ha4a_ | d1e5wa2 |
| d1j19a2 | d1isna2 | d2al6b2 | d1b9ra_ | d1offa_ | d1frra_ | d3wcqa_ | d1e9ma_ |
| d1l5pa_ | d1wria_ | d1i7ha_ | d3av8a_ | d1czpa_ | d1rfka_ | d3p1ma1 | d3b2ga_ |
| d2bt6a2 | d1jmxa3 | d1pbya3 | d1pbya4 | d1h6ua1 | d1r55a1 | d3gboa_ | d1atla_ |
| d1kufa_ | d1buda_ | d4q1la_ | d1yp1a_ | d1wnia_ | d1mdwa_ | d1ziva1 | d1zcma1 |
| d1t3ca_ | d1epwa3 | d2a97a_ | d3fiea_ | d3ioqa_ | d1fh0a_ | d1o0ea_ | d1khqa_ |
| d1m6da_ | d2cb5a_ | d1cs8a_ | g8pch.1 | d1s4va_ | d2pnsa_ | d1vsna1 | d1mira_ |

d1iwda_ d2oula_ d7pcka_ d3bwka_ d3bcna_ d2p86a_ d1pvja_ d1pcia_
d1gece_ d1deua_ d4bs6a_ d1xkga1 d1cqda_ d1jqpa2 d2c0ya_ d2bdza_
d3p5ua_ d2o36a_ d1k9xa_ d1zoya1 d1qo8a2 d1d4ca2 d1jnra2 d1kf6a2
d2jk6a2 d2jk6a1 d1feca1 d1feca2 d1lpfa1 d1lpfa2 d1xhca1 d1xhca2
d1dxla2 d1dxla1 d1gera2 d1m6ia2 d1onfa1 d3lada2 d3lada1 d1w4xa1
d1w4xa2 d1gesa2 d1mo9a1 d1xdia1 d1fl2a2 d1fl2a1 d1fcda1 d1fcda2
d1h6va2 d1h6va1 d1mo9a2 d1gv4a2 d1gv4a1 d2gv8a2 d2gv8a1 d1aoga1
d1aoga2 d1hyua1 d1ebda1 d1ebda2 d3dk9a1 d1q1ra2 d1q1ra1 d1gesa1
d1pv8a_ d1w5qa_ d2c1ha_ d1ohla_ d1zbfa1 d2z1ha_ d2etja1 d3qioa1
d1jl1a_ d2e4la_ d1jl2a_ d2lsna1 d1uaxa_ d2yv0x_ d1mu2a1 d1ekea_
d1i39a_ d1ps9a2 d1o94a2 d1cjca1 d1pn0a1 d1reoa1 d1ju2a1 d2f5va1
d3c96a1 d2gmha1 d1pj5a2 d1gpea1 d1o5wa1 d1n4wa1 d2dw4a2 d2ivda1
d1seza1 d2voua1 d1kdga1 d1s5ja1 d2hbja2 d2igia_ d1wlja_ d1qhta1
d4dlea1 d1tgoa1 d2f96a1 d3tr8a1 d3mxma_ d1q8ia1 d1y97a1 d1w0ha_
d1kfsa1 d2py5a1 d1noya_ d1d5aa1 d1yt3a3 d2guia_ d3mwpa1 d4kb1a_
d1iv0a_ d1nu0a_ d2d5ra1 d1ix2a_ d2av4a1 d1wgqa1 d1v89a1 d2coda1
d1zc3b_ d1wg7a1 d1u5ea1 d1v61a1 d2coca1 d2ys1a1 d1v5pa1 d1ntya2
d1wjma1 d1wi1a1 d1u29a_ d1xx0a1 d1unqa_ d2zkmx3 d1fhoa_ d1v88a1
d2adza1 d1x1fa1 d1p6sa_ d2coaa1 d1btka_ d1v5ma1 d1faoa_ d2x18a_
d1dyna_ d1x1ga1 d4kaxb_ d2j59m1 d1ki1b2 d1xcga2 d1txda2 d1u5da1
d1dbha2 d1w1ha_ d1upqa_ d1v5ua1 d1foea2 d2fjla1 d1eaza_ d2dfka2
d2cofa1 d1kz7a2 d1k8ka2 d1k8ka1 d3uleb1 d3i33a2 d3i33a1 d1bupa1
d1dkgd2 d1dkgd1 d4ci6a2 d1jcfa1 d4a2ba2 d2fxua1 d1yaga2 d4cbua2
d1bupa2 d1r7ha_ d1faaa_ d1wika1 d1xfla_ d1ttza1 d2f51a1 d1ti3a_
d1nhoa_ d1sena_ d2i4aa_ d1zmaa1 d1fo5a_ d3lefa1 d3diea1 d2yana1
d1z6na1 d1oaza_ d3zywa1 d4aj8a_ d1nm3a1 d4fiwa_ d1wmja_ d4hs1a1
d1iloa_ d1xwaa1 d1wjka1 d2vm1a_ d1fb6a_ d1gh2a_ d1h75a_ d1nw2a_
d1kx5b_ d3a6na_ d4m6ba2 d2nqbd_ d3wkjd_ d2yfwe_ d4h9na_ d5bnxa_
d1kx5c_ d3r45a_ d1eqzb_ d4jjnb_ d1f66c_ d1eqza_ d4wnnb_ d1kx5a_
d1u35c1 d1id3a_ d2nqbc_ d1kx5d_ d1ku5a_ d1f1ea_ d1b67a_ d1tafa_
d1bh9b_ d1tafb_ d1jfib_ d1q9ca_ d1h3ob_ d2byka1 d1n1ja_ d4g92b_
d1bh9a_ d1jfia_ d1h3oa_ d2bykb1 d1pd212 d1nhya2 d2a2ra2 d1k0dd2
d1f2ea2 d1gsua2 d1f3ba2 d2fhea2 d2pvqa1 d1tw9a2 d1oyja2 d1e6ba2
d1k0ma2 d3ik7a1 d4is0a1 d1r5aa2 d1jlva2 d1m0ua2 d3gtub2 d1z9ha2
d1okta2 d1axda2 d2gsra2 d1tu7a2 d1k3ya2 d1zgma1 d1b48a2 d1gnwa2
d1gwca2 d2gsta2 d1glqa2 d1oe8a2 d1pn9a2 d1v2aa2 d1fw1a2 d1duga2
d3vrda1 d3vrda2 d1fcdc2 d1fcdc1 d1h1oa2 d1h1oa1 d1m70a1 d1nira1
d1qksa1 d4ilya_ d1chka_ d4olta_ d1qqya_ d1gd6a_ d1f6ra_ d1ivma_
d2gv0a_ d1iiza_ d2z2fa_ d1j8xa_ d1ghla_ d1b9oa_ d169la_ d2o4wa_
d1p37a_ d1k28a3 d1xjta1 d1jtma_ d2f2qa_ d4e97a1 d1mpya2 d1kw3b2
d4ghga1 d1tfza1 d3isqa1 d3isqa2 d1sqia1 d1sqia2 d1lkda2 d1lkda1
d1mpya1 d1cjxa2 d1cjxa1 d1sp9b2 d1t47a1 d1t47a2 d1f1ua2 d1sp8a1
d1sp8a2 d1f1ua1 d4iuaa3 d4bvva_ d5hpga_ d3k65a_ d1ki0a3 d1ki0a2
d1ki0a1 d1a0ha1 d1pmla_ d3sp8a3 d2feba1 d1qsta_ d2beia1 d4m3sa_

d1u6ma1 d1ygha_ d1yk3a1 d3dr6a_ d1xmta_ d1z4ea1 d2ge3a1 d1bo4a_
d2fiaa1 d2jdca1 d1y9wa1 d3to7a_ d1wwza1 d2fl4a1 d2ozua1 d2b5ga_
d2cy2a1 d2p0wa_ d1cm0a_ d4pswa_ d1yrea1 d1yr0a1 d1m4ia_ d2gana1
d1yvka1 d1ghea_ d2g3aa1 d1y7ra1 d2euia1 d1q2ya_ d2fsra1 d1xeba_
d2ae6a1 d1cjwa_ d1qsma_ d2aj6a1 d1y9ka1 d1i12a_ d1vkca_ d2atra1
d1n71a_ d2d4pa1 d2fiwa1 d2fe7a1 d1tiqa1 d2ou2a_ d1yx0a1 d2fcka1
d1p0ha_ d2j8ma_ d2pq8a_ d1z4ra1 d1s3za1 d1ufha_ d1dgsa2 d1fvia1
d1ckma1 d1x9na2 d1c4qa_ d1prtc1 d2wv6d_ d1prtf_ d1ltrd_ d4l63b1
d1prtd_ d1tl0f_ d2bosa_ d1prtb1 d3chbd_ d1xvqa1 d1pn0a2 d3hy2a_
d2cvba1 d1o8xa_ d1lu4a_ d1we0a1 d2bmxa1 d2a4va1 d1psqa_ d1xiya1
d1q98a_ d1n8ja_ d1zofa1 d2ggta_ d3tjja_ d4je1a1 d3s9fa_ d2zcta_
d1qmva_ d1jfua_ d2b1ka_ d5dvba1 d2b5xa1 d2v2ga_ d1st9a_ d3tb2a_
d4llra_ d4fh8a1 d1e2ya_ d1knga_ d2h30a_ d1i5ga_ d2z9sa_ d2f8aa1
d1tp9a1 d2b7ka_ d1nm3a2 d3hvva_ d2wfca1 d4kcea_ d1xvwa1 d4gqca1
d3qpma_ d4k1fa_ d1zzoa1 d1gp1a_ d1prxa_ d4od7a1 d1z6ma1 d4mcua1
d4dvca1 d3l9sa_ d1un2a_ d3rppa_ d1f74a_ d3l21a_ d2e1da_ d1to3a1
d1w3ia_ d1sfla_ d1n7ka_ d1vcva1 d4imea_ d1wa3a_ d2ocza1 d1f2ja_
d3m16a_ d2yxga_ d1ub3a_ d3a5fa_ d1fbaa_ d1l6wa_ d1xfba1 d1wx0a1
d1vpxa1 d1ojxa_ d1mxsa_ d2a4aa1 d1xkya1 d3cq0a1 d3s1xa_ d1f05a_
d1fdja_ d3cpra1 d4d2ja_ d2phla2 d2phla1 d1fxza2 d1fxza1 d1lrha_
d1uija1 g1dgw.1 d2v09a_ d1uika2 d1uika1 d1dgwa_ d1e88a1 d1l6ja5
d1l6ja4 d1eaka4 d1eaka3 d1l6ja3 d1h8pa1 d1etha2 d1w52x1 d2ppla1
d1hpla2 d1lpbb2 d2oxea1 d1bu8a2 d1rp1a2 d1ys1x_ d4hs9a_ d1ex9a_
d1ku0a_ d3qmma_ d1n9wa1 d1e1oa1 d1c0aa1 d1b8aa1 d1l0wa1 d3a74a1
d1eova1 d3rp2a_ g1ucy.1 d1fuja_ d2zchp_ d3w94a_ d1si5h_ d1lo6a_
g2pka.1 d1cgha_ d1kigh_ d1a5ia_ d3s9ba_ d1hj8a_ d1ao5a_ d2anya_
d1q3xa1 d1gvza_ d1azza_ d4gsoa_ d2f91a1 d1iaua_ d1gdna_ d1eufa_
g1fiw.1 d1eq9a_ d4nfea_ d2rdla_ d1spja_ d1elva1 d1a7sa_ d1ekbb_
d1pfxc_ d1yc0a_ d2aiqa_ g1fiz.1 d1fi8a_ d4k60a_ d4dgja_ d1mzaa_
d1op0a_ d3tjva_ d1pytd_ d1sgfg_ g1gg6.1 d2eeka_ g1rtf.1 d1a0ja_
d3g01a_ d1pytc_ d4e7na_ d1ddja_ d1z8ga1 d2hlca_ d1npma_ d1brup_
g1jou.1 d1sgfa_ d1autc_ g2hnt.1 g1kdq.1 d1orfa_ d3s69a_ d4crga_
d1fxya_ d1bqya_ d4wfia_ d4cg1a1 d3visa_ d1svpa_ d1mbma_ d3su6a1
d1cu1a1 d4agka_ d1vcpa_ d1q31a_ g1n1l.1 d3e90b_ d1ep5a_ d3u1jb_
d3zxha_ d1cgla_ d1rm8a_ d1eaka2 d1hy7a_ d2jsda_ d1cxva_ d1hv5a_
d1i76a_ d1eb6a_ d4dv8a2 d1j7na1 d1dzka_ d4tlja_ d2a2ua_ d4wfva_
d3t1da_ d4iaxa_ d3zq3a_ d2nnda_ d1i4ua_ d1kxoa_ d1bbpa_ d1kt7a_
d1exsa_ d3s26a1 d1lf7a_ d4gh7a_ d1z24a1 d1qabe_ d1xkia_ d1ew3a_
d3bx7a_ d4iawa1 d1n0sa_ d1gkab_ d3kzaa_ d2k23a_ d2l9ca1 d2joza1
d1gm6a_ d3kffa_ d3saoa1 d1e5pa_ d1yupa1 d1iiua_ d2tbvc_ d1c8nc_
d1x36a_ d1smvc_ d1ng0c_ d4sbvc_ d1f2nc_ d1ddla_ d1e57b_ d1js9c_
d1f15b_ d1za7a_ d1laja_ d1novc_ d2bbvc_ d1ny721 d1ny722 d1a6ca3
d1a6ca2 d1a6ca1 d1pgl22 d1pgl21 d1pgl11 d1ny711 d1jgta2 d1ofda3
d1ea0a3 d1ecfa2 d1q15a2 d1ct9a2 d1te5a_ d1y14b1 d2nn6i1 d1i94l_

d2zjra2 d1smxa_ d2qamc2 d1hh2p1 d2r7da1 d1rl2a2 d1wfqa1 d3bzka4
d1x6oa2 d3a0ja_ d2j01d2 d2ja9a1 d4ql5a_ d1hr0w_ d1luza_ d2n3sa_
d2vnud1 d1h95a_ d1go3e1 d2nn6h1 d1ne3a_ d2mo1a_ d1iz6a2 d1jjga_
d1e3pa2 d2ix0a3 d2ix0a2 d2lxja_ d1ueba3 d1ueba2 d2z0sa1 d1u0la1
d2ix0a1 d2eifa2 d2je6i1 d3i2za1 d2c35b1 d2ahob2 d1t9ha1 d3i4oa_
d2qall1 d1jt8a_ d2nn6g1 d1g6pa_ d3cama_ d1i94q_ d1c9oa_ d1d7qa_
d2ba0a1 d2vnud2 d2vnud3 d2qalq1 d1wi5a1 d1xtda2 d1ny4a_ d4w9ta_
d1jvna1 d4x2ra1 d1qo2a_ d4axka1 d1thfd_ d1h5ya_ d4u28a_ d2y88a_
d4wd0a_ d1vzwa1 d1eixa_ d3exra_ d2ffca1 d2fdsa1 d3n3ma1 d2czda_
d1q6oa_ d2yyua_ d1vqta1 d1dbta_ d3ldva1 d4lt9a1 d3tc7a1 d3hoja_
d1i4na_ d3vnda_ d3nl8a1 d1geqa_ d3nava1 d4a2ra1 d3tc6a1 d1ujpa_
d4ijba_ d1xcfa_ d1vc4a_ d1rd5a_ d1qopa_ d3b5va_ d2r80b_ d1cqxa1
d1cg5a_ d1gcvb_ d3boma_ d2h8fb_ d2raoa_ d3bj1a_ d4hrrb_ d2r50a_
d3d1kb_ d2aa1b1 d3zhwa_ d3hf4b_ d3gdja_ d1jebb_ d3bj1b_ d1x9fb_
d1wmub_ d1gcva_ d3bcqb_ d3d4xb_ d1gvha1 d2dc3a_ d3hf4a_ d4yu3a_
d3k8ba_ d1qpwb_ d2r80a_ d3bcqa_ d1v4wa_ d3wtgb_ d1jl7a_ d4esaa_
d1a9we_ d3d1ab_ d1fsla_ d4h2la_ d1v4wb_ d1mwca_ d1vhba_ d1jeba_
d1spgb_ d1h97a_ d1q1fa_ d1tu9a1 d3d1aa_ d1hdsb_ d3hyub_ d2zfbb_
d3fs4a_ d1oj6a_ d1shrb_ d4h2lb_ d4yu3b_ d1spga_ d3hrwa_ d3qqra_
d3at5a_ d4wchd_ d3hyua_ d1cg5b_ d1uc3a_ d1hbra_ d2raob_ d1outb_
d4esab_ d3gdjb_ d3qqqa_ d1x9fc_ d3vrfa_ d1itha_ d3mkba_ d3d4xa_
d3mjpa_ d1jl6a_ d3fh9a_ d1outa_ d2dc3b_ d1it2a_ d3eoka_ d3g46a_
d1qpwa_ d3vrfb_ d1ch4a_ d1x9fd_ d1hdsa_ d2zfba_ d3mkbb_ d1or4a_
d3wtga_ d1a4fa_ d3fh9b_ d3bomb_ d3cy5a_ d3at5b_ d1x9fa_ d2oifa_
d4lmxb_ d1cpcb_ d3v57a_ d1kn1a_ d1cpca_ d4f0ta_ d2vjha_ d4po5b_
d1eyxa_ d1alla_ d4f0tb_ d1b33b_ d1b8db_ d4h0mb_ d2vjhb_ d4lm6b_
d2vmlb_ d1allb_ d2vjta_ d3dbja_ d2vmla_ d2vjtb_ d4f0ub_ d1f99b_
d4rmpa1 d1liaa_ d1eyxb_ d2uulb_ d4lmsb_ d4f0ua_ d2bv8a_ d1f99a_
d2bv8b_ d2c7lb_ d3dbjb_ d4rmpb1 d1b8da_ d4h0ma_ d3v57b_ d1kn1b_
d1liab_ d2uula_ d2j96a_ g1wht.1 d1azwa_ d2xqfa_ d1f6wa_ d1dx4a_
d1qe3a_ d1k4ya_ d4qwwa_ d2ha2a_ d1xm5a_ d1xaxa_ d1tvia_ d1s5ja2
d1qhta2 d4dlea2 d1tgoa2 d3py8a2 d1q8ia2 d1kfsa2 d2py5a2 d1d5aa2
d2m5ta_ d1cqqa_ d1wqsa_ d5bpea_ d2ipha_ d1lvoa_ d2bhga1 d3tnta_
d3sjia_ d3d23a1 d2fyqa_ d3tloa_ d3zvea1 d2hrva_ d3q3ya1 d4asha1
d1cg2a1 d1yloa2 d1y7ea2 d1y0ya3 d1lfwa1 d1vixa1 d1fnoa4 d1vhoa2
d1ysja1 d1z2la1 d2fvga2 d1vgya1 d1r3na1 d2grea2 d1xmba1 g1q7l.1
d1rtqa_ d1ra6a_ d1muka_ d3gsza_ d1khva_ d1te9a_ d2xwha_ d3uqsa_
d3hkwa_ d3n6ma_ d2cjqa_ d2xyma_ d1teba_ d1te8a_ d1s48a_ d1u09a1
d4wfxa_ d3tyqa_ d2pgga1 d1m4la_ d2bk7a_ d3fjua_ d1z5ra1 d1kwma1
d1uwya2 d1jqga1 d2c1ca1 d1h8la2 d4lu0a_ d1of8a_ d3unda1 d3e9aa1
d1vr6a2 d2qkfa_ d1d9ea_ d1o60a_ d1e69a_ d1mv5a_ d3g60a4 g1ii8.1
d1ewqa2 d1ji0a_ d1qhla_ d1v43a3 d2wmfa1 d1vpla_ d1g2912 d4a82a2
g1f2t.1 d2d62a1 d3tuic1 d2bbsa1 d3g60a3 d2ixea_ d1g6ha_ d1sgwa1
d2qi9c_ d3rlfa1 d3b60a1 d3d31a2 d2pzea1 g1xew.1 d1jj7a_ d1oxxk2

d2onka1 d1w1wa_ d1iwea_ d1qf5a_ d1vmaa2 d1nija1 d2p67a1 d3ihla_
d1okkd2 d1ls1a2 d1s1ma2 d1iona_ d2v40a_ d1dj3a_ d3hida_ d2vo1a1
d2g0ta1 d1vcoa2 d1j8mf2 d1g3qa_ d1xjca_ d1p9ba_ d1qzxa3 d1ihua2
d1ihua1 d2qm8a_ d1dj2a_ d4ioja_ d1cp2a_ d1yrba2 d1hyqa_ d3gqba2
d1skye3 d1xp8a1 d1tf7a2 d1tf7a1 d1ye8a1 d1skyb3 d2qe7d2 d4blta_
d4huta_ d3ldaa2 d4hyya_ d2i3ba1 d1cr1a_ d3j9tb2 d3ntua2 d1vdza1
d2z43a_ d1e9ra_ d1nlfa_ d3ziaa2 d1n0wa_ d1g6oa_ d3ziad2 d3j9ta2
d1pzna2 d3gqbb2 d2i49a_ d1nnfa_ d1xvxa_ d1ursa_ d1h3da1 d1iuda_
d4m1va1 d1twya_ d3e13x_ d2nxoa1 d4pfua_ d3ttma_ d1elja_ d2qmwa1
d1us5a_ d1y3na1 d1jeta_ d1uqwa_ d4f19a1 d2esna2 d3thia_ d3fasa1
d2uyeb_ d2xwva1 d1r9la_ d2fyia1 d1pb7a_ d2ozza1 d1sw5a_ d1nh8a1
d1vr5a1 d2g29a_ d1xs5a_ d3lswa_ d2z23a_ d3w9va_ d3tcha_ d1q35a_
d1z7me1 d3q27a_ d1p99a_ d1ve4a1 d2i6ea1 d4mcoa1 d4n8ya_ d1eu8a_
d1zbma1 d1hsla_ d1ixca2 d1j1na_ d1xvya_ d1y9ua_ d2z22a1 d1xoca1
d4oera_ d1wdna_ d5f1qa1 d1xt8a1 d1i6aa_ d4gwoa_ d4igta1 d1pc3a_
d4mmpa1 d2czla1 d2onsa2 d4mija1 d4ok5a1 d2bmfa1 d2bmfa2 d1yksa1
d1yksa2 d1jr6a_ d2qeqa1 d2jlqa1 d1eh3a_ d3skpa_ d1dtza1 d1dtza2
d1ieja_ d1jnfa2 d1ce2a1 d1jnfa1 d1b1xa2 d1jw1a1 d1jw1a2 d1ryoa_
d1h76a1 d1h76a2 d1c4oa1 d2p6ra3 d2p6ra4 d1oywa2 d1oywa3 d1wrba1
d1w36b2 d1w36b1 d1c4oa2 d3bora_ d1t6na_ d2eyqa5 d2eyqa3 d2eyqa2
d2zu6a2 d1veca_ d1uaaa1 d1w36d1 d2g9na1 d1z3ix2 d2rb4a_ d1hv8a1
d1hv8a2 d1t5ia_ d1w36c2 d2j0sa2 d2j0sa1 d1fuka_ d3hjha1 d1z5za1
d1qdea_ d1z63a1 d1s2ma2 d1s2ma1 d1wp9a1 d1wp9a2 d1gm5a3 d1gm5a4
d3dina4 d3dina3 d1q0ua_ d1w36c1 d1z3ix1 d1nkta3 d1nkta4 d1uaaa2
d1w36d2 g1qhh.1 d2fwra2 d2fwra1 d2v8ta_ d3q3ma_ d1za0a1 d1mxra_
d2inca_ d1jk0a_ d2incb_ d1mhyb_ d3hf1b_ d1mtyb_ d1r2fa_ d2uw1a_
d1mhyd_ d3n37a_ d1oquc_ d1syya_ d1mtyd_ d2o1za_ d1uzra_ d1smsa_
d2fkza_ d1zuja1 d1s3qa1 d2fjca1 d1z6om1 d1veia_ d4dyya_ d1jiga_
d3gvya_ d2pyba_ d4cy9a1 d3bvfa1 d1r03a_ d2cf7a_ d1nf4a_ d1zs3a1
d4iwka_ d3ka8a_ d3np0x_ d1n1qa_ d4e6ka_ d1z6oa1 d2fzfa1 d4dyua_
d3ge4a_ d1jgca_ d4am4a_ d2z6ma_ d1ji5a_ d1vlga_ d1lkoa1 d1krqa_
d3qz3a_ d1o9ra_ d2ffxj_ d1euma_ d1yv1a1 d2v2pa_ d1j30a_ d4di0a_
d1dpsa_ d1oewa_ d2qzxa_ d2asia_ d1psoe_ d1miqa_ d1smra_ d2h6ta_
d1pfza_ d1izda_ d3hd8a_ d1j71a_ d1dpja_ d4aa9a_ d1t6ex_ g1b5f.1
g1lya.1 g1htr.1 d1qdma2 d1bxoa_ d1wkra_ d1tzsa_ d1ibqa_ d2qzwa_
d1ex4a2 d1c6va_ d2v76a_ d1wj1a1 d1aqca_ d1qqga2 d1ntva_ d1n3ha_
d4dbba_ d3f0wa1 d1p3ra_ d1wgua1 d2cy4a1 d3hqca_ d1wvha1 d1xr0b1
d1bdqa_ d3ggta_ d3ixoa_ d1mrxa_ d2haha_ d1tw7a_ d4njsa_ d1sgua_
d2p3da_ d1nsoa_ d3d3ta_ d2rkfa_ d1c6ya_ d2rspa_ d3ogpa_ d3ekwa_
d2o4na_ d2j9ja_ d1baia_ d3ggua_ d3lzua_ d4npua_ d4j55a_ d2r8na_
d2fxda_ d2p3ca_ d4dqea_ d3ttpa_ d3t3ca_ d2p3aa_ d2hc0a_ d2fdda_
d3mwsa_ d1k8rb_ d1wgra1 d1ef5a_ d1wfya1 d1e8ya3 d1wgya1 d1lfda_
d2cs4a1 d1wxma1 d2byea1 d3kh0a1 d2byfa1 d1wxaa1 d1e7ua3 d1h4ra3
d4yl8a1 d2al6a3 d1wgda1 d1wgga1 d4q5eb_ d1wjna1 d1se9a_ d3n3kb_

| | | | | | | | |
|---|---|---|---|---|---|---|---|
| d1wz0a1 | d1we6a1 | d2m0xa_ | d2gbka_ | d3rt3b1 | d1wh3a1 | d1sifa_ | d2ekec2 |
| d4ii2b1 | d4hk2a1 | d1c3ta_ | d1m94a_ | d1wgha1 | d1ttna1 | d1j8ca_ | d1wx8a1 |
| d3v6cb1 | d2ma9b_ | d4i6lb1 | d1bt0a_ | d2jvca1 | d1iyfa_ | d2k1fa_ | d1wxva1 |
| d1wf9a1 | d1t0ya_ | d2io0b2 | d1x1ma1 | d2gbma_ | d1zw7a1 | d2m17a_ | d1ud7a_ |
| d1we7a1 | d2lwpa_ | d2gbja_ | d1wjua1 | d1v6ea1 | d1v86a1 | d4eewa_ | d1wiaa1 |
| d4icva_ | d4gu2a_ | d1wx7a1 | d1wy8a1 | d4hjka1 | d2faza1 | d1v5oa1 | d3ruib_ |
| d3waob_ | d2r2qa1 | d3m95a_ | d3wana_ | d3ubra_ | d1qo8a1 | d1d4ca1 | d2k3va_ |
| d1ft5a_ | d1m1qa_ | d2j7aa_ | d1h21a_ | d1ogyb_ | d4fasa_ | d1jnia_ | d1oaha_ |
| d1sp3a_ | d3h33a_ | d1rwja_ | d19hca_ | d1os6a_ | d3bxua_ | d3h4na_ | d1i77a_ |
| d3caoa_ | d2ctha_ | d1duwa_ | d1ofwa_ | d2e84a_ | d1hh5a_ | d1gyoa_ | d3h34a_ |
| d2ftba_ | d1pmpa_ | d2rcqa1 | d1fdqa_ | d4lkpa_ | d1sa8a_ | d1vyfa_ | d1lpja_ |
| d3vg7a1 | d4azqa1 | d1o8va_ | d5bvqa_ | d1p6pa_ | d2qo4a_ | d1opba_ | d1yiva1 |
| d1o1va_ | d4bvma1 | d4tkba_ | d4i3ba_ | d1bwya_ | d1ftpa_ | d1ggla_ | d4gkca_ |
| d1cbia_ | d3p6da1 | d1kqwa_ | d2a0aa1 | d1um8a_ | d1ixza_ | d1qvra2 | d1qvra3 |
| d1sxjc2 | d1jr3d2 | d2p65a_ | d1l8qa2 | d1g8pa_ | d3hu3a3 | d1sxjd2 | d1njfa_ |
| d1in4a2 | d1sxja2 | d1tuea1 | d2ce7a2 | d1w5sa2 | d1sxjb2 | d1d2na_ | d1ny5a2 |
| d1ofha_ | d2gnoa2 | d2a5yb3 | d1fnna2 | d1u0ja1 | d1lv7a_ | d1iqpa2 | d2fnaa2 |
| d1ixsb2 | d4gita_ | d1sxje2 | d1g41a_ | d2lj7a_ | d1n4na_ | d2m8bb1 | d2kyqa_ |
| d3psma_ | d1jkza_ | d1jxca_ | d2gl1a_ | d1ugla_ | d4uj0a_ | d2k4ua_ | d1hp2a_ |
| d2lu9a_ | d1baha_ | d2meoa1 | d1wm7a_ | d1hlya_ | d1n8ma_ | d1jlza_ | d1wpda_ |
| d1c55a_ | d1pvza_ | d1wt7a_ | d1rjia_ | d1wz5a_ | d1m2sa_ | d1pjva_ | d1c49a_ |
| d1qkya_ | d2ktca1 | d1wmta_ | d1du9a_ | d1j5ja_ | d1r1ga_ | d1t0za_ | d2i61a_ |
| d1t1ta_ | d1sn4a_ | d1kv0a_ | d2lkba_ | d1npia_ | d1wwna_ | d1pe4a_ | d2kbka_ |
| d2li7a_ | d2kbha_ | d1dq7a_ | d1sega_ | d1omya_ | d1zywa1 | d1jzaa_ | d1bnla_ |
| d1dy2a_ | d1iyjb4 | d1iyjb5 | d1iyjb3 | d1o7ia_ | d4ipca1 | d1x3ea_ | d4apva_ |
| d1jb7a1 | d1jb7a2 | d1jb7a3 | d3kdfa_ | d1ue1b_ | d1wjja1 | d3eiva_ | d1xjva2 |
| d1xjva1 | d2ccza2 | d1se8a_ | d1l1oc_ | d1jb7b_ | d1qvca_ | d3ulla_ | d1qzga_ |
| d1miua4 | d1miua5 | d1miua3 | d3g48a1 | d1gd7a_ | d1mkha_ | d1jjcb3 | d3ubub_ |
| d1egia_ | d1qo3c_ | d1v7pa_ | d1tdqb_ | d3ubua_ | d1c3ab_ | d1h8ua_ | d3bx4b_ |
| d1t8ca1 | d1sb2a1 | d1qdda_ | d2ziba1 | d3cada_ | d1byfa_ | d1j34b_ | d1jwia_ |
| d1jzna_ | d1c3aa_ | d1wmza_ | d3bdwb_ | d1hq8a_ | d1fifa1 | d1dv8a_ | d3hupa_ |
| d1wk1a1 | d1umra_ | d2afpa_ | d3bx4a_ | d1gz2a_ | d1sl6a_ | d1v7pb_ | d3g8ka_ |
| d1ixxa_ | d1j34a_ | d1jwib_ | d1g1sa1 | d1y17a1 | d1ja3a_ | d1fvub_ | d3bdwa_ |
| d1sb2b1 | d1fvua_ | d1umrc_ | d1oz7a_ | d4m17a_ | d3cfwa1 | d1ypqa1 | d1g1ta1 |
| d3fd4a_ | d3l9jc_ | d4dn8a1 | d1v4lb_ | d1rdl1_ | d2msba_ | d1prtc2 | d1rpma_ |
| d2shpa1 | d1yfoa_ | d1l8ka_ | d2ooqa1 | d1g4us2 | d1p15a_ | d1lara2 | d1lara1 |
| d1wcha_ | d2fh7a2 | d2fh7a1 | d1ohea1 | d1ohea2 | d2f4da_ | d1rxda_ | d2hxpa_ |
| d1xria_ | d1d5ra2 | d1fpza_ | d3ezza_ | d4y2ea_ | d1i9sa_ | d3azya_ | d3wvja_ |
| d1upsa1 | d1ajoa_ | d2hyka_ | d1ajka_ | d2vy0a_ | d1o4ya_ | d3o5sa_ | d3d6ea_ |
| d3atga_ | d1umza_ | d3i4ia_ | d1c1la_ | d4lbqa_ | d1a78a_ | d1slta_ | d4no4a_ |
| d3zsja_ | d3duia_ | d1g86a_ | d1is3a_ | d1qmja_ | d1tdha3 | d1q3ba3 | d1ee8a3 |
| g1qmo.1 | g1len.1 | d3zyra_ | d1fnya_ | d1n47a_ | g2ltn.1 | d1avba_ | d3usua_ |
| d1dhkb_ | d1f9ka_ | d1ukga_ | d1g8wa_ | d1g9fa_ | d1wbfa_ | d3wcsa_ | d1g7ya_ |
| g1loe.1 | d1mvqa_ | d1hqla_ | d1v6ia_ | d1dbna_ | d1qnwa_ | d1fx5a_ | d4l8qa_ |

d1ioaa_ d3n35a_ d2h0ba_ d2erfa_ d1h30a2 d3mw4a1 d1pz7a_ d2r1ba_
d3v64a1 d1d2sa_ d3bopa1 d3mw3a1 d1okqa1 d1okqa2 d2dara2 d2dara1
d1wyha2 d2dloa2 d1rutx4 d1rutx3 d2cuqa2 d1x4la2 d1x4la1 d1x3ha2
d1x3ha1 d2d8za1 d1x61a2 d1x6aa1 d1x6aa2 d2cora2 d2cora1 d1v6ga1
d1v6ga2 d2d8za2 d1x64a1 d1x64a2 d1b8ta1 d2co8a1 d1x63a2 d2dj7a2
d2dj7a1 d2cu8a2 d2cu8a1 d1g47a2 d1g47a1 d1j2oa1 d1wyha1 d2dloa1
d1wiga1 d1wiga2 d2cupa2 d2cupa3 d1x68a1 d2d8ya2 d2d8ya1 d1m3va2
d1x62a1 d1x68a2 d1x61a1 d1b8ta3 d1b8ta2 d2d8xa2 d2d8xa1 d1x4ka1
d1x4ka2 d2cuqa1 d1m3va1 d2co8a2 d1j2oa2 d1x63a1 d2cura1 d2cura2
d1x62a2 d1op9a_ d1y0la1 d1etzb1 d1egjl1 d2gsib1 d1nj9a1 d4jfhd1
d1aqkl1 d1t2ja_ d1lo4h1 d1zvya1 d3v4vl1 d4enec1 d1kxtb_ d5e8ea1
d2pxya_ d4weud_ d1xiwc_ d2esvd1 d1qnzh_ d1ogae1 d1f4xh1 d4n1hb_
d3ezjb1 d3mlwl1 d1kfah1 d1etza1 d1b4jh1 d3cfic_ d4w68a_ d2arja1
d1ek3a_ d2ok0l1 d1vera_ d1uwgh1 d1blnb1 d1fpth1 d1rzga1 d2giya1
d4m3kb1 d1nc2a1 d2nyyd1 d1ncna_ d1fbih1 d5dxwa_ d4fqqa1 d1dqdh1
d1jfql1 d1kelh1 d1faih1 d1frgl1 d1figl1 d4m6nl1 d1fo0a_ d4hxbl1
d3b5ga_ d3quyd1 d2h9gb1 d1i8kb_ d2dd8h1 d2ck0h1 d1eaja_ d4w6wb1
d2a6ib3 d1t66d1 d2e27h_ d1ob1b1 d1f11b1 d2v7na1 d1ad0a1 d2xt1b1
d1pskh1 d4i13b1 d2op4l1 d1a0ql1 d1uwel1 d1za3a1 d1ncbl1 d4uu9a_
d1bm3h1 d1cloh1 d1nqba2 d1cf8h1 d4y5va_ d1mhph1 d5anma1 d4cdgc1
d4ldeb_ d1y0lb1 d2pcpb1 d1z3gl1 d1dfbl1 d1fh5h1 d3g5xa1 d1tzib1
d4dn3l1 d3zhka1 d2b1hh1 d2uyla1 d1f3dh1 d1nj9b1 d2agjh1 d2h1pl1
d1q72h1 d8fabb1 d1ndmb1 d1rjca1 d4u6hb1 d3upca_ d2fbjl1 d1osph1
d1tvda_ d2cjvh_ d4fhbd1 d1bd2d1 d1rz7h1 d3nn8a_ d1blna1 d1knoa1
d1nfda1 d2bmkb1 d1dqll_ d1igyb1 d1igfl1 d12e8h1 d3utza1 d1nc2b1
d1rihh1 d4mwfb1 d4jvpa1 d1mieh1 d1rhfa1 d1fgnl1 d1sbsh1 d1lp9e1
d1dn0a1 d4lqfl1 d1iaih1 d1kgcd1 d1mh5b1 d1gpol1 d2g60h3 d1f8tl1
d2jixd1 d4grwh_ d1fo0b_ d1bfob1 d1axsa1 d1u9ka_ d1nmbh_ d1h8na1
d1h8na2 d1jguh1 d4eura1 d3sdyl1 d35c8h1 d1w72l1 d1mvub_ d1i9rh1
d1ob1a1 d4wenb_ d1yeeh1 d3ietc1 d1f58h1 d1ad0b1 d1mqkl_ d1nbvh1
d4b50a_ d3r8bb_ d1bafl1 d1mamh1 d4rgne1 d2gsgb_ d35c8l1 d1bafh1
d1ai1h1 d2pcpa1 d2fath1 d2x1pa_ d1orsb1 d1ibgl1 d1shma_ d1a2yb_
d1u0qa_ d2coqa_ d1hilb1 d1qfwi_ d1jv5b_ d1fn4b1 d5e0qa_ d1nsnh1
d1plgl1 d4ocxl1 d1kb5h1 d1rzfh1 d1pg7x1 d2fx7h1 d2oi9c_ d1l7il1
d3eyva1 d1d5il1 d4ffyl_ d1tetl1 d1a6wh_ d1ac6a_ d3dvfa_ d1nfde1
d1mimh1 d1ri8a1 d1jhlh_ d3dura_ d1fskb1 d1f97a1 d3so3b1 d2ak4d1
d1lp9f1 d4z7wf1 d1l7th1 d4y5ya_ d4x7ec1 d1emth1 d1ikfl1 d1nlbh1
d4utal1 d1bjma1 d1eapb1 d1u6ah1 d2dtgd1 d1kj2b_ d1xeda1 d1c1eh1
d1fgvh_ d2bnub1 d2fb4h1 d2dtgb1 d1ktrh_ d1jpth1 d1qkzl1 d4hepg_
d2atpb1 d1flrh1 d2hrpl1 d1mvfa_ d1iaim1 d3k80a1 d2jb5h1 d3zkxc_
d4grwe_ d1it9h1 d1rhhb1 d3w9ec_ d1e4wh1 d1sy6h1 d2j6eh1 d3qxta_
d1jglh1 d1p7ka1 d1tzhb1 d4n8pa1 d1gafl1 d2jelh1 d1wioa2 d4i0cc1
d1cicb1 d1lmka2 d1lmka1 d1iqwh1 d1orqb1 d1ejoh1 d1jnlh1 d4zg1a_
d43c9b_ d1ggbl1 d1wejh1 d3q6fa1 d1f3dj1 d3c6la1 d1bqlh1 d3p9wb_

d1a3rl1 d1h0db1 d2uzil_ d1eo8l1 d1qfwl_ d1n7ml1 d1igja1 d1c5ch1
d2a9mh_ d1rmfh1 d1fj1b1 d4heme_ d1ccza1 d1a5fh1 d1ogad1 d1dlfh_
d1qa9b_ d1vgel1 d3qskb_ d1sjxa_ d4ozfg1 d1sq2n_ d1qd0a_ d1kcvh1
d1c5db1 d3qcul1 d8faba1 d3bn9d1 d1mjuh1 d4y8dc_ d7fabh1 d3dvgb1
d1lo0l1 d1a6tb1 d1gigh1 d1kcrh1 d2hwzl1 d1yjdc1 d3clfl1 d4krna_
d1dl7l_ d1e6ol1 d1mexh1 d3r0ma_ d4kkna_ d1fnsl1 d1oaqh_ d1rzia1
d1ucbh1 d1ncwl1 d4b41a_ d1nfdf1 d2mpah1 d2d7tl1 d4grwf_ d4b5ea_
d1lk3h1 d3u79a_ d4u3xa_ d1ghfl1 d1bvkb_ d1mcww1 d1b88a_ d1jrhh1
d32c2b1 d4wemb_ d1dsfh_ d1op3h1 d1ce1l1 d1iqda1 d1qoka1 d1qoka2
d1qfuh1 d2crya1 d1ay1h1 d1oaql_ d3ab0b_ d1rurl1 d1t4kb1 d1bz7b1
d1n0xh1 d1forl1 d1ucbl1 d4w6yb_ d43c9a_ d2aabl1 d1tzia1 d4n1ca_
d1k6qh1 d3g9ab_ d4tvsa1 d1igmh_ d1pz5b1 d3dx9a1 d3wxwh_ d1nakh1
d1kcul1 d3eaka_ d4qlra1 d1cr9l1 d1d9ka_ d1f4xl1 d1deea1 d2i27n2
d1c12a1 d2orbl1 d1j8hd1 d1mcph1 d1c5da1 d1kenh1 d1bd2e1 d1h5ba_
d4x7fc1 d3upaa_ d1t3fa1 d1indh1 d2dtgc1 d4hc1l1 d1zv5a1 d4wgvb_
d1uwgl1 d4f37k1 d1aqkh1 d1vesa_ d3zkqd_ d1fptl1 d1lo4l1 d1knob1
d3rrqa_ d1ct8b1 d4orzc_ d1mlbb1 d4hgkc1 d1dqdl1 d1bogb1 d4ozhe1
d1dr9a1 d1i7za1 d1dn0b1 d1kell1 d1rzib1 d1ypzf1 d1wtla_ d1hkfa_
d1fail1 d1figh1 d2vyre_ d1sjva_ d1ohqa_ d1bfoa1 d1pkqb1 d1mjul1
d1b4jl1 d1nsnl1 d2arjb1 d1p4bh_ d4aj0a_ d3nn8b_ d2rcja1 d1f2xk_
d4v1dd_ d1adqh1 d2eksa_ d4k3ga_ d1jfqh1 d1uweh1 d1frgh1 d4gftb_
d1uz8b1 d4n1ea_ d1bm3l1 d1clol1 d3mcka1 d2vq1a1 d1ae6h1 d4aq1b1
d1uj3b1 d1nbqa1 d1fh5l1 d2ck0l1 d2ywza_ d1q72l1 d4nbxb_ d1hila1
d4hzlb1 d2j88l1 d1zmya1 d1cd0a_ d1a4ka1 d1pskl1 d1a0qh1 d4krmb_
d1ncbh1 d2fbjh1 d1j8he1 d1i3ua_ d1cf8l1 d1fe8h1 d2p1ya1 d1eeqa_
d2x89a_ d1ol0a_ d2r0lh1 d1ub5a1 d1dfbh1 d3eyvb1 d4jdvb1 d4p2cg_
d2gsia1 d2agjl1 d1u3ha1 d2h1ph1 d1rihl1 d1fskc1 d1boga1 d2a77l1
d1ospl1 d1qfoa_ d3dwta_ d2fx7l1 d1rz7l1 d3stba1 d1pkqa1 d1b6da1
d1dzba2 d1dzba1 d1kj2a_ d4l1ha_ d1rzgb1 d1dqlh_ d12e8l1 d1miel1
d1jmaa_ d1sbsl1 d1fgnh1 d1f58l1 d1mqkh_ d1ndgb1 d3qegl1 d1iail1
d2aw2a1 d1tqbc1 d3fo2a1 d4dkaa_ d1f8th1 d2g60l1 d2ywya_ d1uj3a1
d1yc7a_ d3bd3a_ d4w6xb1 d1nmbl_ d1i8ka_ d1rjlb1 d1ypze1 d1p7kb1
d1kxqe_ d1gpoh1 d2z4qa1 d1w72h1 d1i9rl1 d4qgya1 d4xt1c_ d2qqnh1
d1cica1 d3c08h1 d2cd0a_ d2bdnh1 d1hnga1 d1maml1 d1nldh1 d1cr9h1
d4lcih1 d3bkyh1 d3qg7l1 d1ai1l1 d1kxvc_ d4iosd_ d1l7ih1 d2x1qa_
d1ub6b1 d3k74b_ d1ibgh1 d1teth1 d1igya1 d1hxma1 d2x6ma_ d2e7la_
d5e03a_ d1qfwm_ d2r0kh1 d1igjb1 d1rurh1 d1plgh1 d1mj8h1 d1yuhb1
d3fmgl1 d1rzfl1 d1emtl1 d1ad9b1 d1igtb1 d1nlbl1 d1d5ih1 d3k7ua_
d4ffyh_ d1bwwa_ d3dvga1 d1a6ta1 d1fgvl_ d4qxgl1 d1miml1 d1jhll_
d2hffb1 d2hh0l1 d1qkzh1 d1l7tl1 d1afvh1 d1iaii1 d2cjuh_ d1um5h1
d2ghwb2 d2ghwb1 d1kgce1 d2fgwh1 d1ikfh1 d1dvfd_ d2i24n1 d2xtjb_
d1za3b1 d2yc1a_ d2hh0h1 d2x1oa_ d1bvka_ d1rjla1 d5e7ba1 d1igta1
d1il1b1 d2fb4l1 d1mvua_ d1jptl1 d32c2a1 d1fvca_ d1iqdb1 d2hrph1
d2p4ab_ d4qo1a_ d1p2cb1 d1wt5c_ d1t4ka1 d5c1mb_ d1it9l1 d1bz7a1

d1b2wh1 d1e4wl1 d1jnll1 d1yedb1 d1za6b1 d3u7aa_ d1igfh1 d3gnml1
d1orsa1 d1wejl1 d1a2ya_ d1hxmb1 d1gafh1 d2j4wh1 d1pz5a1 d1j05a_
d1a3rh1 d4fqll1 d1qfwh_ d1eo8h1 d1n7mh1 d4s11a_ d1kegh1 d5da4a_
d1iqwl1 d3whxb1 d1vgeh1 d1deeb1 d1c12b1 d1t2ql1 d1ad9a1 d3gsnb1
d3b2uc1 d1ggbh1 d1t3fb1 d1nakl1 d4ocmc_ d1lila1 d2v7hb3 d2esve1
d1mcoh1 d2qr0b1 d2q3aa_ d1ct8a1 d1kcrl1 d2pxyb_ d2uzih_ d5c3ld_
d3durb_ d2ak4e1 d1dl7h_ d1xiwd_ d1mlba1 d1rmfl1 d1i7zb1 d1dlfl_
d2xa3a_ d5bq7b1 d1dqqb1 d3h42l1 d1egjh1 d1kcvl1 d1fe8l1 d2j6el1
d1f3rb2 d1lk3l1 d1sm3h1 d1q0xh1 d1ghfh1 d7fabl1 d1igch1 d1lo0h1
d4idla_ d5c2bl_ d1jrhl1 d1il1a1 d3zhda1 d4kfzc_ d1b0wa_ d1e6oh1
d1mexl1 d4krpb_ d1fnsh1 d1ncwh1 d4hgma_ d4eigb1 d4rzca_ d2mpal1
d4y5xa_ d5en2b1 d3lrga1 d4ej1c1 d1rhha1 d1j05b_ d1forh1 d1q9wb1
d2wzpd_ d2aq2a1 d1h0da1 d3revb1 d2ij0c1 d1tzha1 d4m7jl1 d1k6ql1
d3b9kc1 d1igml_ d3qeua1 d1dsfl_ d1ce1h1 d1kcuh1 d1zvha1 d1zvoa1
d1tqbb1 d1ay1l1 d2qscl1 d2cgrh1 d1nl0h1 d1kenl1 d3eo0a1 d4n9ob1
d1n0xl1 d3k1kc_ d2m9wa1 d3dfxa_ d2aq2b1 d1ck1a1 d1enfa1 d1i4pa1
d1v1oa1 d1et9a1 d1esfa1 d2g9hd1 d4ohja1 d1klud1 d1bxta1 d1eu3a1
d1ty0a1 d1fnua1 d2cqva1 d1fhga_ d2k1ma_ d2dl2a1 d1biha3 d1biha1
d1biha4 d3qp3a1 d1g1ca_ d1gxea_ d1cvsc2 d1cvsc1 d1xmwa2 d1m4ka2
d2otpa2 d1biha2 d1rhfa2 d2fcba2 d2fcba1 d1wwbx_ d1gsma2 d1epfa1
d1epfa2 d2dava1 d1waaa_ d1f97a2 d1x44a1 d1vcaa2 d1fnla2 d1fnla1
d3b5ha2 d3h8na2 d3h8na1 d1jbja1 d1jbja2 d1pd6a_ d1f2qa2 d1f2qa1
d3cx2a_ d1nbqa2 d3b5ha1 d1n26a1 d1xiwb_ d1xaua_ d1fltx_ d2ifga1
d1olla1 d1olla2 d1cs6a4 d1cs6a3 d1cs6a2 d1cs6a1 d2gw5a2 d1sy6a1
d1xiwa_ d1ucta2 d1ucta1 d1ry7b2 d1gl4b_ d1qz1a3 d1olza1 d2c9aa1
d1wwca_

**Family-55 (c.2.1.7)**

**Positive dataset contains 6 protein sequences.**

d3t4ea2 d2hk9a2 d2egga2 d3dona2 d1vl6a1 d1v9la1

**Negative dataset contains 3783 protein sequences.**

d1m32a_ d1ibja_ d1iuga_ d4q75a_ d2bkwa1 d1t3ia_ d1n8pa_ d1qz9a_
d1jf9a_ d1eg5a_ d1p3wa_ d3kgwa_ d1cl1a_ d2ctza1 d1vjoa_ d2rfva_
d1gc0a_ d1cs1a_ d1d2fa_ d2ch1a1 d1qgna_ d1pffa_ d1elua_ d1c7ga_
d2v1pa1 d2ez1a_ d1u08a_ d1lc5a_ d1b5pa_ d2ay1a_ d1bw0a_ d3pd6a_
d7aata_ d2csta_ d1j32a_ d1fg7a_ d4wlha_ d1v2da_ d1gdea_ d1m6sa_
d1v72a1 d1svva_ d1ajsa_ d1wsta1 d2f8ja2 d1lk9a_ d1vp4a1 d1xi9a1
d2gb3a1 d1yaaa_ d3rcha_ d3k40a_ d1pmma_ d2cfba1 d1dfoa_ d1o69a_
d1bj4a_ d3k28a_ d2bwpa_ d3lg0a_ d2fnua_ d3ou5a_ d1z7da1 d1b9ha_
d4msoa_ d1bs0a_ d1s0aa_ d1sffa_ d3tqxa_ d1mdoa_ d3qboa_ d3pgya_
d1bjna_ d2dkja_ d1ejia_ d1vefa1 d4wxga_ d3bs8a_ d3gbxa1 d1w23a_
d4nmwa_ d1i26a_ d3lgia_ d3i77a_ d2z9ia2 d4nsva_ d1lcya2 d4jcna_
d1agja_ d1os8a_ d1hpga_ d1p3ca_ d1qtfa_ d1ddla_ d1e57b_ d1f56a_
d1bypa_ d1azca_ d1ws8a_ d3u25a_ d1cuoa_ d1id2a_ d1rkra_ d2iaac_
d4dp1x_ d1nwpa_ d2gima_ d2i7sa_ d1qhqa_ d3ehbb1 d1fftb1 d1gyca3

d1hfua2 d1kbva1 d1kbva2 d1sdda1 d1sdda2 d1gyca1 d1gyca2 d1mzya1
d2bw4a1 d1aoza1 d1aoza3 d1aoza2 d1kyaa2 d1kyaa1 d1mzya2 d4e9sa1
d1sddb1 d1sddb2 d1hfua1 d1hfua3 d1oe1a2 d1oe1a1 d1v10a2 d1v10a3
d1v10a1 d2dara2 d2dara1 d1wyha2 d2dloa2 d1rutx4 d1rutx3 d2cuqa2
d1x4la2 d1x4la1 d1x3ha2 d1x3ha1 d2d8za1 d1x61a2 d1x6aa1 d1x6aa2
d2cora2 d2cora1 d1v6ga1 d1v6ga2 d2d8za2 d1x64a1 d1x64a2 d1b8ta1
d2co8a1 d1x63a2 d2dj7a2 d2dj7a1 d2cu8a2 d2cu8a1 d1g47a1 d1j2oa1
d1wyha1 d2dloa1 d1wiga1 d1wiga2 d2cupa2 d2cupa3 d1x68a1 d2d8ya2
d2d8ya1 d1m3va2 d1x62a1 d1x68a2 d1x61a1 d1b8ta3 d1b8ta2 d2d8xa2
d2d8xa1 d1x4ka1 d1x4ka2 d2cuqa1 d1m3va1 d2co8a2 d1j2oa2 d1x63a1
d2cura1 d2cura2 d1x62a2 d1a3qa1 d1iknc_ d3brda1 d1oy3c_ d1imhc1
d1gjia1 d1zk9a1 d2cxka1 d4qgna_ d1vr3a1 d2bema_ d1pama1 d1ea9c1
d2fhfa2 d2fhfa1 d1k3ia1 d1j0ha1 d1cxla1 d2bhua1 d1edqa1 d1f1sa2
d1ut9a2 d1gvia1 d1txka1 d4alac_ d1pjwa_ d1rera1 d1s6na_ d3uzqb_
d2phla2 d1fxza2 d1fxza1 d1lrha_ d1uija1 g1dgw.1 d2v09a_ d1uika2
d1uika1 d1dgwa_ d1ep0a_ d2pa7a1 d1wlta1 d2c0za1 d1fstb_ d1ex0a1
d1tafa_ d1bh9b_ d1tafb_ d1jfib_ d1h3ob_ d2byka1 d1n1ja_ d4g92b_
d1bh9a_ d1jfia_ d1h3oa_ d2bykb1 d1n1jb_ d1r0ka3 d1j5pa3 d1vkoa2
d1vm6a2 d3qvsa2 d1gr0a2 d1p1ja2 d2ovga_ d1sq8a_ d1rzsa_ d3kz3a_
d3orca_ d3gpdg2 d2q49a2 d1hdgo2 d2g17a2 d2cvoa2 d2czca1 d3pwka2
d1obfo2 d1i32a2 d1zyao2 d2pkqo2 d1b7go2 d2hjsa2 d1cf2o2 d1vkna2
d1nvmb2 d4lt9a1 d3tc7a1 d3hoja_ d1i4na_ d3vnda_ d3nl8a1 d1geqa_
d3nava1 d1v5xa_ d4a2ra1 d3tc6a1 d1ujpa_ d4ijba_ d1xcfa_ d1vc4a_
d1rd5a_ d1qopa_ d3b5va_ d2msya_ d2djoa1 d2r5za_ d1au7a1 d1f43a_
d2lmda1 d1akhb_ d1s7ea1 d3nara_ d1zq3p1 d2craa1 d1mija_ d4uuta_
d3a01a_ d1x58a1 d3rkqa1 d3cmya1 d2cqxa1 d2da6a1 d1e3oc1 d1fjla_
d1mh3a1 d1ig7a_ d1x41a1 d1wh7a1 d1gt0c1 d2mw8a_ d1x2ma1 d1b72a_
d1wi3a1 d1jgga_ d1wjha1 d1x2na1 d1mnmc_ d2cuea1 d3k2aa_ d2hosa_
d2jwta2 d1pufa_ d1octc1 d2hi3a_ d1b8ia_ d4j19a_ d3lnqa_ d1wh5a1
d1b8ib_ d1fexa_ d2cqra1 d1guua_ d1ug2a1 d2cqqa1 d2ajea1 d4a69c_
d1h8ac1 d2crga1 d2iw5b1 d1xc5a1 d1ofcx1 d2cu7a1 d1wgxa1 d2cjja1
d2ckxa1 d1gvda_ d1igna1 d2iu5a1 d1zk8a1 d2g7sa1 d2fd5a1 d2np5a1
d1z0xa1 d2id6a1 d2d6ya1 d2i10a1 d1vi0a1 d2oi8a1 d3c07a1 d2ns7a1
d2fq4a1 d2yvea1 d1t33a1 d2o7ta1 d2np3b1 d2g7ga1 d2gena1 d2fbqa1
d2g7la1 d2gfna1 d2g3ba1 d2jj7a1 d2hyja1 d1sgma1 d1ui5a1 d2hkua1
d2id3a1 d4jyka1 d3lv8a_ d1s3ga1 d1zaka1 d1s4qa_ d3tvta2 d1uf9a_
d1khta_ d1ckea_ d1osna_ d1p5zb_ d3cm0a_ d1tmka_ d1jaga_ d3h86a_
d1t0hb_ d1nn5a_ d2h92a_ d3kb2a1 d2c95a1 d1yj5a2 d1kgda_ d1ly1a_
d2ak3a1 d1vyva2 d1deka_ d2grja1 d1p3ja1 d1y63a_ d1e4ya1 d1s96a_
d3iija1 d1lvga_ d1nksa_ d1vyua2 d4e22a_ d1qf9a_ d1jjva_ d1q3ta_
d1ki9a_ d1lw7a2 d1teva_ d1kjwa2 d1gsia_ d1x6va3 d1g3ma_ d1j99a_
d1t8ta1 d1nsta_ d1fmja_ d1zrha_ d1ls6a_ d2a3ra_ d2zptx_ d3ckla_
d1texa_ d1q44a_ d2gwha_ d1uj2a_ d4gi7a_ d2jeoa1 d1rz3a_ d1z0ja1
d3umra2 d3gj0a1 d2gaoa1 d1yzga1 d1x3sa1 d1g7sa4 d1puja_ d1udxa2

d1kk1a3 d2o52a_ d1jwyb_ d1zcaa2 d1zcba2 d2fv8a1 d2cjwa_ d3dz8a1
d1xzpa2 d1vg8a_ d4qxaa_ d2ew1a1 d1x1ra1 d2bcgy_ d3doea1 d3cona_
d3rega_ d3wyga_ d1upta_ d2b6ha1 d1zd9a1 d1r5ba3 d1n0ua2 d1r2qa_
d2f9la1 d2rhda_ d3q3jb_ d3q85a_ d1efma1 d1r8qa_ d2gf9a1 d2akab1
d1ek0a_ d1z08a1 d2gcoa1 d2dy1a2 d4g01b_ d2cxxa1 d1svia_ d3q72a_
d1ni3a1 d4ac9a4 d2erxa1 d2bmea_ d1wxqa1 d2gf0a1 d4ldja1 d1zunb3
d1d5ca_ d4lv5b_ d1efca3 d1kmqa_ d2g6ba1 d3lrpa_ d1tada2 d2y8ea_
d4rd4a1 d1z06a1 d2heia_ d3a58b_ d1u0la2 d1d2ea3 d1zj6a1 d2ocba_
d3rwoa_ d1mr3f_ d2e9sa1 d4u5xa1 d2iwra_ d2fn4a1 d3bwdd_ d2nzja1
d2atva1 d2ksqa_ d3l43a_ d1mkya2 d1mkya1 d1wmsa_ d1jnya3 d2rmka_
d1f6ba_ d1f60a3 d1gota2 d1jala1 d2fh5b1 d1g16a_ d2wblc_ d1m7ba_
d1wf3a1 d1f5na2 d1t9ha2 d1z6xa1 d1z0aa1 d2fg5a1 d2a5ja1 d2bcjq2
d1s0ua3 d1ky3a_ d4y0va_ d4lhwa_ d2qmea1 d2atxa1 d1egaa1 d1ryha_
d1moza_ d1nrjb_ d2erya1 d3bfka_ d1puia_ d1fzqa_ d3j3az1 d3t5ga_
d1shza2 d3defa_ d2f7sa1 d3raba_ d3tsoa_ d3aq4a_ d2al7a1 d3sfva1
d3bc1a_ d1sdma_ d3h4sa1 d2heha_ d2grya_ g3kin.1 d1kk8a2 d1br2a2
d1goja_ d1f9va_ d2mysa2 d1lkxa_ d3t0qa_ d4db1a1 d3gbja_ d1ry6a_
d1v8ka_ d1k8ka2 d1k8ka1 d3uleb1 d3i33a2 d3i33a1 d1bupa1 d1dkgd2
d1dkgd1 d4ci6a2 d1jcfa1 d4a2ba2 d2fxua1 d1yaga1 d4cbua2 d1bupa2
d1iqca2 d1iqca1 d1eb7a2 d1eb7a1 d1nmla1 d3h3no1 d3h3no2 d3ezwa2
d1hroa_ d4gedb_ d3zooa_ d1mz4a_ d1gu2a_ d1f1fa_ d1dvva_ d1c6ra_
d1c75a_ d1e29a_ d1gdva_ d1cnoa_ d1h32b_ d1co6a_ d2bh4x_ d2aiua_
d5cytr_ d1jdla_ d1i8oa_ d2zboa_ d3dmia_ d1ls9a_ d1wvec_ d2yk3a_
d3ph2b_ d1dw0a_ d5aura_ d1ql3a_ d1qn2a_ d1f1ca_ d2nqya_ d1h4ga_
d1oa2a_ d1olra_ d3b7ma_ d4h7ma1 d3wp3a_ d2vgda_ d1igoa_ d3zsea_
d2nlra_ d3wx5a_ d1oa3a_ d1pvxa_ d1m4wa_ d1f5ja_ d1oa4a_ d1ks5a_
d4ixla1 d3sita1 d2ah2a1 d1evsa_ d1cnt1_ d1pvhb_ d1z7ca_ d1huwa_
d1bgea_ d1rhga_ d1f6fa_ d1f45b_ d1i1rb_ d1axia_ d1etea_ d3tgxb_
d1scfa_ d2z3qa1 d3qb1a_ d1hula_ d1m48a_ d4nkqb_ d1eera_ d1v7mv_
d5e4ea_ d2o27a_ d3b5ka_ d2psma1 d4zf7a_ d3uf5a_ d4x51a_ d1d9ca_
d4ypgc1 d3oq3a_ d1lqsl_ d1n1fa_ d1au1a_ d1wu3i_ d3besl_ d3ux9a_
d4dnxa2 d1v47a2 d1g8fa2 d1jhda2 d1x6va2 d2el7a_ d1iq0a2 d1irxa2
d1r6ta2 d1ivsa4 d2cyba_ d1rqga2 d1j09a2 d2yxna_ d1f7ua2 d1h3fa1
d1ffya3 d2g36a1 d1nzja_ d1jila_ d1n3la_ d1i6la_ d1pfva2 d1h3na3
d3n9ia_ d3sz3a1 d3sw2a_ d1moxc_ d1dx5i3 d1edmb_ d1autl2 d1autl1
d1nqlb_ d1dval1 d1szba2 d1kigl_ d2y5fl_ d1ijqa2 d1gk5a_ d1g1sa2
d1nt0a3 d1xdtr_ d1lmja2 d1lmja1 d1nzia2 d2wphe_ d1klil_ d1pfxl2
d1pfxl1 d1p9ja_ d1g1ta2 d1f7ea_ d4yzub_ d1lr7a1 d2mgpa1 d1b9wa1
d2mgpa2 d1ob1c1 d1ob1c2 d1n1ia2 d1n1ia1 d2npra2 d2npra1 d2y5ia_
d1qlsa_ d1psra_ d3n22a_ d2egda_ d4ggfc_ d2wcea_ d2luca_ d4aqia_
d3nsia_ d2lp3a_ d1j55a_ d1a4pa_ d1k8ua_ d1nsha_ d1pvaa_ d3fs7a_
d2pvba_ d1ttxa_ d1rwya_ d2kyfa_ d1rk9a_ d1a75a_ d1exra_ d1tiza1
d1nyaa_ d1g8ia_ d1ggwa_ d1wdcc_ d3fwba_ d1ggza_ d2o5ga_ d1ctaa_
d1m45a_ d3i5gc_ d1sl8a_ d1fpwa_ d2scpa_ d2roaa_ d4aqra_ d3pn7b_

d1br1b_ d2mysb_ d1s6ca_ d1bjfa_ d2obha_ d1jfka_ d1jf0a_ d1qv0a_
d1lkja_ d2mlfc_ d2m97a_ d2d8na_ d4by5a_ d1jbaa_ d4nqga_ d1wdcb_
d2mysc_ d1j7qa_ d1s6ia_ d1uhka2 d1r2ua_ d1dtla_ d3kf9a_ d3pn7c_
d1qjta_ d1fi6a_ d1c07a_ d5d67d_ d1tuza_ d1h8ba_ d1eg3a1 d1eg3a2
d2zkmx1 d1snla_ d1gjya_ d1y1xa_ d1dvia_ d1df0a1 d1qxpa2 d4usla_
d1k94a_ d1wkya2 d1uuqa_ d3wh9a_ d4m1ra_ d1xyza_ d4xuya_ d2je8a5
d4bf7a1 d3cuia_ d1ur4a_ d1qw9a2 d1odza_ d2vzsa5 d1bqca_ d2x2ya1
d1hjqa_ d1qnra_ d4y7ea1 d1vjza_ d4k68a_ d2v3ga_ d4hpga_ d1nofa2
d1nq6a_ d1h4pa_ d1aq0a_ d3ziza_ d4bs0a_ d1h1na_ d1ta3b_ d1w91a2
d1i1wa_ d1uhva2 d1us3a2 d1egza_ d7a3ha_ d1tvna1 d3nyda1 d1ghsa_
d2cyga1 d4xv0a_ d2c8na2 d1w32a_ d3emca_ d1v6ya1 d1foba_ d2uwfa1
d2fgla_ d3niya1 d2c0ha1 d1rh9a1 d1yq2a5 d1szna2 d1fa2a_ d1pama4
d5do8a1 d1ea9c3 d1hx0a2 d2fhfa5 d1qhoa4 d1avaa2 d1m53a2 d4m8ua1
d2gjpa2 d1lwha2 d4howa1 d1ktba2 d1j0ha3 d3vm5a1 d2x1ia_ d1wzaa2
d1uasa2 d2diea1 d1mxga2 d1ht6a2 d1iv8a2 d5cpsa_ d1ud2a2 d1zjaa1
d3gbea1 d1kwga2 d1wdpa_ d1gcya2 d1g5aa2 d1hvxa2 d3dhpa2 d2bhua3
d4do4a1 d3qgva1 d1e43a2 d1g94a2 d2d3na2 d1gjwa2 d1gvia3 d1x1na1
d1r7aa2 d1tz7a1 d4c7ga2 d2gjxa1 d1nowa1 d1yhta1 d4toqa_ d1wb0a1
d1w9pa1 d1eoka_ d1itxa1 d1kfwa1 d1edqa2 d2gsja_ d1hjxa1 d1jnda1
d1vf8a1 d3wdpp1 d3wq5a_ d1gnxa_ d4gxpa_ d4hz8a_ d3aiua_ d1wcga1
d1qvba_ d3ahxa_ d1v02a_ d2dgaa_ d1pbga_ d1vffa1 d1qoxa_ d4ha4a_
d1mixa2 d1e5wa2 d1j19a2 d2al6b2 d3syxa1 d1mkea1 d2p8va_ d1xoda1
d1evha_ d1qc6a_ d1egxa_ d1b9ra_ d1offa_ d1frra_ d3wcqa_ d1e9ma_
d1l5pa_ d1wria_ d1i7ha_ d3av8a_ d1czpa_ d1rfka_ d3p1ma1 d3b2ga_
d2bt6a2 d1wgqa1 d1v89a1 d2coda1 d1zc3b_ d1wg7a1 d1u5ea1 d1v61a1
d2coca1 d2ys1a1 d1v5pa1 d1ntya2 d1wjma1 d1wi1a1 d1u29a_ d1xx0a1
d1unqa_ d2zkmx3 d1fhoa_ d1v88a1 d2adza1 d1x1fa1 d1p6sa_ d2coaa1
d1btka_ d1v5ma1 d1faoa_ d2x18a_ d1dyna_ d1x1ga1 d4kaxb_ d2j59m1
d1xcga2 d1txda2 d1u5da1 d1dbha2 d1w1ha_ d1upqa_ d1v5ua1 d1foea2
d2fjla1 d1eaza_ d2dfka2 d2cofa1 d1kz7a2 d1etob_ d1umqa_ d1g2ha_
d1r55a1 d3gboa_ d1atla_ d1kufa_ d1buda_ d4q1la_ d1quaa_ d1yp1a_
d1wnia_ d2gfoa1 d1vjva_ d2ayna1 d3nhea_ d3u1ra1 d1g9ka2 d1kapp2
d3ioqa_ d1fh0a_ d1o0ea_ d1khqa_ d1m6da_ d2cb5a_ g8pch.1 d1s4va_
d2pnsa_ d1vsna1 d1mira_ d1iwda_ d2oula_ d7pcka_ d3bwka_ d3bcna_
d2p86a_ d1pvja_ d1pcia_ d1gece_ d1deua_ d4bs6a_ d1xkga1 d1cqda_
d1jqpa2 d2c0ya_ d2bdza_ d3p5ua_ d2o36a_ d1k9xa_ d1ex4a2 d1c6va_
d2jk6a2 d2jk6a1 d1feca1 d1feca2 d1lpfa1 d1lpfa2 d1xhca1 d1xhca2
d1dxla2 d1dxla1 d1gera2 d1m6ia2 d1onfa1 d1onfa2 d3lada2 d3lada1
d1w4xa1 d1w4xa2 d1gesa2 d1mo9a1 d1xdia1 d1fl2a2 d1fl2a1 d1fcda1
d1fcda2 d1h6va2 d1h6va1 d1mo9a2 d1gv4a2 d1gv4a1 d2gv8a2 d2gv8a1
d1aoga1 d1aoga2 d1hyua1 d1ebda1 d1ebda2 d3dk9a1 d1q1ra2 d1q1ra1
d1gesa1 d1zbfa1 d2z1ha_ d2etja1 d3qioa1 d1jl1a_ d2e4la_ d1jl2a_
d2lsna1 d1io2a_ d1uaxa_ d2yv0x_ d1mu2a1 d1i39a_ d1pn0a1 d1reoa1
d1ju2a1 d2f5va1 d3c96a1 d2gmha1 d1gpea1 d1o5wa1 d1n4wa1 d1k0ia1

d2dw4a2 d2ivda1 d1seza1 d2voua1 d1kdga1 d1s5ja1 d2hbja2 d2igia_
d1wlja_ d1qhta1 d4dlea1 d1tgoa1 d2f96a1 d3tr8a1 d3mxma_ d1q8ia1
d1y97a1 d1w0ha_ d1kfsa1 d2py5a1 d1noya_ d1d5aa1 d1yt3a3 d2guia_
d3mwpa1 d4kb1a_ d1iv0a_ d1nu0a_ d3nvva1 d1krha3 d2h88b1 d1n62a2
d1t3qa2 d1kf6b2 d1zoyb1 d1ffva2 d1dgja2 d1rm6c2 d1jmxa2 d1jmxa1
d1pbya1 d1r7ha_ d1faaa_ d1wika1 d1xfla_ d1ttza1 d2f51a1 d1ti3a_
d1nhoa_ d1sena_ d2i4aa_ d1ep7a_ d1zmaa1 d1fo5a_ d3lefa1 d3diea1
d2yana1 d1z6na1 d1oaza_ d3zywa1 d4aj8a_ d1nm3a1 d4fiwa_ d1wmja_
d4hs1a1 d1iloa_ d1xwaa1 d1wjka1 d2vm1a_ d1fb6a_ d1gh2a_ d1h75a_
d1nw2a_ d1kx5b_ d3a6na_ d4m6ba2 d2nqbd_ d3wkjd_ d2yfwe_ d4h9na_
d5bnxa_ d1kx5c_ d3r45a_ d1eqzb_ d4jjnb_ d1f66c_ d1eqza_ d4wnnb_
d1kx5a_ d1u35c1 d1id3a_ d2nqbc_ d1kx5d_ d2b5ea2 d2b5ea3 d2b5ea1
d2b5ea4 d2kp1a1 d2kp2a1 d1hyua3 d1hyua4 d1j08a1 d1pd212 d1nhya2
d2a2ra2 d1k0dd2 d1f2ea2 d1gsua2 d1f3ba2 d2fhea2 d2pvqa1 d1tw9a2
d1oyja2 d1e6ba2 d1k0ma2 d3ik7a1 d4is0a1 d1r5aa2 d1jlva2 d1m0ua2
d3gtub2 d1z9ha2 d1okta2 d1axda2 d2gsra2 d1tu7a2 d1k3ya2 d1zgma1
d1b48a2 d1gnwa2 d1gwca2 d2gsta2 d1glqa2 d1oe8a2 d1pn9a2 d1v2aa2
d1fw1a2 d1duga2 d1ovna2 d1ig8a1 d1czan3 d1czan1 d1ig8a2 d1czan4
d1bg3a4 d1bg3a3 d1bg3a2 d1bg3a1 d1nira1 d1qksa1 g1f8v.1 d1novc_
d2bbvc_ d3wyha_ d4g9sa1 d3gxra_ d1qqya_ d1f6ra_ d1ivma_ d2gv0a_
d1iiza_ d2z2fa_ d1j8xa_ d1ghla_ d1b9oa_ d174la_ d169la_ d2o4wa_
d1p37a_ d1k28a3 d1xjta1 d1jtma_ d2f2qa_ d4e97a1 d3w3ea_ d4tx7a_
d3cqla_ d4msta_ d3iwra_ d2z37a_ d4dyga_ d2k4ua_ d1hp2a_ d2lu9a_
d1baha_ d2meoa1 d1wm7a_ d1hlya_ d1n8ma_ d1jlza_ d1wpda_ d1c55a_
d1pvza_ d1wt7a_ d1rjia_ d1wz5a_ d1m2sa_ d1pjva_ d1c49a_ d1qkya_
d2ktca1 d1wmta_ d1du9a_ d1j5ja_ d1r1ga_ d2pjsa1 d1klla_ d2i7ra1
d1ecsa_ d1xqaa1 d5cj3a1 d1npba_ d1jifa_ d2p7oa_ d1nkia_ d1mpya2
d1kw3b2 d1kw3b1 d4ghga1 d1tfza1 d3isqa1 d3isqa2 d1sqia1 d1sqia2
d1lkda2 d1lkda1 d1mpya1 d1cjxa2 d1cjxa1 d1sp9b2 d1t47a1 d1t47a2
d1f1ua2 d1sp8a1 d1sp8a2 d1f1ua1 d1iyka2 d1iyka1 d1rxta1 d1iica2
d1qsta_ d2beia1 d4m3sa_ d1u6ma1 d1ygha_ d1yk3a1 d3dr6a_ d1xmta_
d1z4ea1 d2ge3a1 d1bo4a_ d2fiaa1 d2jdca1 d1y9wa1 d3to7a_ d1wwza1
d2fl4a1 d2ozua1 d2b5ga_ d2cy2a1 d2p0wa_ d1cm0a_ d4pswa_ d1yrea1
d1yr0a1 d1m4ia_ d2gana1 d1yvka1 d1ghea_ d2g3aa1 d1y7ra1 d2euia1
d1q2ya_ d2fsra1 d1xeba_ d2ae6a1 d1cjwa_ d1qsma_ d2aj6a1 d1y9ka1
d1i12a_ d1vkca_ d2atra1 d1n71a_ d2d4pa1 d2fiwa1 d2fe7a1 d1tiqa1
d2ou2a_ d1yx0a1 d2fcka1 d1p0ha_ d2j8ma_ d2pq8a_ d1z4ra1 d1s3za1
d1ufha_ d2hqya1 d2hqya2 d1lrza2 d1lrza3 d1ne9a2 d1xvqa1 d1pn0a2
d3hy2a_ d2cvba1 d1o8xa_ d1lu4a_ d1we0a1 d2bmxa1 d2a4va1 d1psqa_
d1xiya1 d1q98a_ d1n8ja_ d1zofa1 d2ggta_ d3tjja_ d4je1a1 d1o73a_
d3s9fa_ d2zcta_ d1qmva_ d1jfua_ d2b1ka_ d5dvba1 d2b5xa1 d2v2ga_
d1st9a_ d3tb2a_ d4llra_ d4fh8a1 d1knga_ d2h30a_ d1i5ga_ d2z9sa_
d2f8aa1 d1tp9a1 d2b7ka_ d1nm3a2 d3hvva_ d2wfca1 d4kcea_ d1xvwa1
d4gqca1 d3qpma_ d4k1fa_ d1zzoa1 d1gp1a_ d1prxa_ d4od7a1 d1z6ma1

d4mcua1 d4dvca1 d1r4wa_ d3l9sa_ d1un2a_ d3rppa_ d1rw1a_ d1z3ea1
d3l78a_ d2qked_ d4ksoa_ d1t4za1 d1wwja_ d1e88a1 d1l6ja5 d1l6ja4
d1eaka4 d1eaka3 d1l6ja3 d1h8pa2 d1etha2 d1w52x1 d2ppla1 d1hpla2
d2oxea1 d1bu8a2 d1rp1a2 d1ys1x_ d4hs9a_ d1ispa_ d1ku0a_ d3qmma_
d1ra6a_ d1muka_ d3gsza_ d1khva_ d1te9a_ d2xwha_ d3uqsa_ d3hkwa_
d3n6ma_ d2cjqa_ d2xyma_ d1teba_ d1te8a_ d1s48a_ d1u09a1 d4wfxa_
d3tyqa_ d2pgga1 d1lgya_ d4l3wa_ d1llfa_ d1gz7a_ d1ukca_ d1j1ia_
d2rhwa_ d3groa_ d1pjaa_ d1thta_ d1svpa_ d1mbma_ d3su6a1 d1cu1a1
d4agka_ d1vcpa_ d1q31a_ g1n1l.1 d3e90b_ d1ep5a_ d3u1jb_ d1xwdc1
d3kj4a_ d1w8aa_ d2ifga3 d1p9ag_ d1xkua_ d1g12a_ d1dzka_ d4tlja_
d2a2ua_ d4wfva_ d3t1da_ d4iaxa_ d3zq3a_ d2nnda_ d1i4ua_ d1kxoa_
d1bbpa_ d1kt7a_ d1exsa_ d3s26a1 d1lf7a_ d1qfta_ d4gh7a_ d1z24a1
d1qabe_ d1xkia_ d1ew3a_ d3bx7a_ d4iawa1 d1n0sa_ d1gkab_ d3kzaa_
d2k23a_ d2l9ca1 d2joza1 d1gm6a_ d3kffa_ d3saoa1 d1e5pa_ d1yupa1
d1iiua_ d2tbvc_ d1c8nc_ d1x36a_ d1smvc_ d1ng0c_ d4sbvc_ d1opoa_
d1f2nc_ d1cixa_ d1y29a_ d1v7fa_ d2jtba_ d1d1ha_ d1g9pa_ d1lupa_
d1qk7a_ d1mb6a_ d2a2va_ d1niya_ d1i25a_ d2mpqa_ d1ie6a_ d1kqha_
d1la4a_ d2mxoa1 d1dl0a_ d1koza_ d1qk6a_ d1emxa_ d1lqra_ d1c6wa_
d1rmka_ d1ixta_ d1eyoa_ d1cnna_ d1fu3a_ d1g1pa_ d1f3ka_ d1rypd_
d1irua_ d1rypc_ d3unfk_ d1rypa_ d1j2pa_ d1irui_ d1j2qh_ d3unfh_
d1m4ya_ d1rypb_ d1irul_ d1irug_ d1iru2_ d1q5rh_ d1rypg_ d1g3ka_
d1iruf_ d1q5qa_ d1e94a_ d1irub_ d1ryph_ d3ty6a_ d1iruj_ d1iruk_
d1rype_ d1irud_ d1irue_ d3mi01_ d1rypf_ d1iruh_ d2z3ba_ d1iruc_
d3mka2_ d1iru1_ d1ny721 d1ny722 d1a6ca3 d1a6ca2 d1a6ca1 d1pgl22
d1pgl21 d1pgl11 d1mqta_ d1d4m3_ d1fmd3_ d2mev1_ g1aym.1 d1ev13_
d4q4w1_ d1r1a3_ d2x5ia_ d1rhi1_ g1r1a.1 g1d4m.1 g1rhi.1 d1b35b_
d1oopa_ d1tme1_ d4iv1a_ g1pvc.1 g1fpn.1 d1fpn1_ d4gb31_ d1d4m1_
d1fmd1_ d2wzr3_ g1cov.1 d1qqp3_ g1fmd.1 d2x5ic_ d1rhi3_ g1oop.1
d1hxs1_ g1hxs.1 d1fpn3_ d1bev1_ g2mev.1 d1eah3_ d1aym1_ d1pvc3_
d1qqp1_ d1zba3_ d1h8ta_ d3vbhc_ g1b35.1 g1bev.1 d1hxs3_ g1ev1.1
d1bev3_ g1tmf.1 d1tmf1_ d1b35a_ d2mev3_ g1eah.1 g1h8t.1 d1eah1_
d4q4w3_ d1r1a1_ d1aym3_ d1pvc1_ d1zba1_ d1h8tc_ d1oopc_ d1tme3_
g1qqp.1 d4iv1c_ g1tme.1 d3ubub_ d1egia_ d1v7pa_ d1tdqb_ d3ubua_
d1c3ab_ d1h8ua_ d3bx4b_ d1t8ca1 d1sb2a1 d1qdda_ d2ziba1 d3cada_
d1byfa_ d1j34b_ d1jwia_ d1jzna_ d1c3aa_ d1oz7b_ d1wmza_ d3bdwb_
d1hq8a_ d1fifa1 d1dv8a_ d3hupa_ d1wk1a1 d1umra_ d2afpa_ d3bx4a_
d1gz2a_ d1sl6a_ d1v7pb_ d3g8ka_ d1ixxa_ d1j34a_ d1jwib_ d1g1sa1
d1y17a1 d1ja3a_ d1fvub_ d3bdwa_ d1sb2b1 d1fvua_ d1umrc_ d1oz7a_
d4m17a_ d3cfwa1 d1ypqa1 d1g1ta1 d3fd4a_ d3l9jc_ d4dn8a1 d1v4lb_
d1rdl1_ d2msba_ d4w9ta_ d1jvna1 d4x2ra1 d1ka9f_ d1qo2a_ d4axka1
d1h5ya_ d4u28a_ d2y88a_ d4wd0a_ d1vzwa1 d1ux8a_ d1dlya_ d4i0va_
d2bkma_ d2bmma_ d2r80b_ d1cqxa1 d1cg5a_ d1gcvb_ d3boma_ d2h8fb_
d2raoa_ d3bj1a_ d4hrrb_ d2r50a_ d3d1kb_ d2aa1b1 d3zhwa_ d3hf4b_
d3gdja_ d1jebb_ d3bj1b_ d1x9fb_ d1wmub_ d1gcva_ d3bcqb_ d3d4xb_

d1gvha1 d2dc3a_ d3hf4a_ d4yu3a_ d3k8ba_ d1qpwb_ d2r80a_ d3bcqa_
d1v4wa_ d3wtgb_ d1jl7a_ d4esaa_ d1a9we_ d3d1ab_ d1fsla_ d4h2la_
d1v4wb_ d1mwca_ d1vhba_ d1jeba_ d1spgb_ d1h97a_ d1q1fa_ d1tu9a1
d3d1aa_ d1hdsb_ d3hyub_ d2zfbb_ d3fs4a_ d1oj6a_ d1shrb_ d4h2lb_
d4yu3b_ d1spga_ d3hrwa_ d3qqra_ d3at5a_ d4wchd_ d3hyua_ d1cg5b_
d1uc3a_ d1hbra_ d2raob_ d1outb_ d4esab_ d3gdjb_ d3qqqa_ d1x9fc_
d3vrfa_ d1itha_ d3mkba_ d3d4xa_ d3mjpa_ d1jl6a_ d3fh9a_ d1outa_
d2dc3b_ d1it2a_ d3eoka_ d3g46a_ d1qpwa_ d3vrfb_ d1ch4a_ d1x9fd_
d1hdsa_ d2zfba_ d3mkbb_ d1or4a_ d3wtga_ d1a4fa_ d3fh9b_ d3bomb_
d3cy5a_ d3at5b_ d1x9fa_ d2oifa_ d4lmxb_ d1cpcb_ d3v57a_ d1kn1a_
d1cpca_ d4f0ta_ d2vjha_ d4po5b_ d1eyxa_ d1alla_ d4f0tb_ d1b33b_
d1b8db_ d4h0mb_ d1b33a_ d2vjhb_ d4lm6b_ d2vmlb_ d1allb_ d2vjta_
d3dbja_ d2vmla_ d2vjtb_ d4f0ub_ d1f99b_ d4rmpa1 d1liaa_ d1eyxb_
d2uulb_ d4lmsb_ d4f0ua_ d2bv8a_ d1f99a_ d2bv8b_ d2c7lb_ d3dbjb_
d4rmpb1 d1b8da_ d4h0ma_ d3v57b_ d1kn1b_ d1liab_ d2uula_ d2j96a_
g1wht.1 d1mtza_ d2xqfa_ d1ea5a_ d1f6wa_ d1dx4a_ d1qe3a_ d1k4ya_
d4qwwa_ d2ha2a_ d1rpma_ d2shpa1 d1yfoa_ d1l8ka_ d2ooqa1 d1g4us2
d1p15a_ d1lara2 d1lara1 d1wcha_ d2fh7a2 d2fh7a1 d2qvba_ d2v9za_
d4kafa1 d1jmxa4 d1pbya3 d1pbya4 d4iuaa3 d4bvva_ d3k65a_ d1ki0a3
d1ki0a2 d1ki0a1 d1a0ha1 d1pmla_ d3sp8a3 d2feba1 d2m5ta_ d1cqqa_
d1wqsa_ d5bpea_ d2ipha_ d1lvoa_ d2bhga1 d3tnta_ d3sjia_ d3d23a1
d2fyqa_ d3tloa_ d3zvea1 d2hrva_ d3q3ya1 d4asha1 d4mh8a_ d1mu2a2
d4i7ga1 d3rp2a_ g1ucy.1 d1fuja_ d2zchp_ d3w94a_ d1si5h_ d1lo6a_
g2pka.1 d1cgha_ d1kigh_ d1a5ia_ d3s9ba_ d1hj8a_ d1ao5a_ d2anya_
d1q3xa1 d1gvza_ d1azza_ d4gsoa_ d2f91a1 d1iaua_ d1gdna_ d1eufa_
g1fiw.1 d1eq9a_ d4nfea_ d2rdla_ d1spja_ d1elva1 d1a7sa_ d1ekbb_
d1pfxc_ d1yc0a_ d2aiqa_ g1fiz.1 d1fi8a_ d4k60a_ d4dgja_ d1mzaa_
d1op0a_ d3tjva_ d1pytd_ d1sgfg_ g1gg6.1 d2eeka_ g1rtf.1 d1a0ja_
d3g01a_ d1pytc_ d4e7na_ d1ddja_ d1z8ga1 d1npma_ d1brup_ g1jou.1
d1sgfa_ d1autc_ g2hnt.1 g1kdq.1 d1orfa_ d3s69a_ d4crga_ d1fxya_
d1bqya_ d2ftba_ d1pmpa_ d2rcqa1 d1fdqa_ d4lkpa_ d1sa8a_ d1vyfa_
d1lpja_ d3vg7a1 d4azqa1 d1o8va_ d5bvqa_ d1p6pa_ d2qo4a_ d1opba_
d1yiva1 d1o1va_ d4bvma1 d4tkba_ d4i3ba_ d1bwya_ d1ftpa_ d4gkca_
d1cbia_ d3p6da1 d1kqwa_ d2a0aa1 d1m4la_ d2bk7a_ d3fjua_ d1z5ra1
d1uwya2 d1jqga1 d2c1ca1 d1dtda_ d1h8la2 d1e69a_ d1mv5a_ d3g60a4
g1ii8.1 d1ewqa2 d1ji0a_ d1qhla_ d1v43a3 d2wmfa1 d1vpla_ d1g2912
d4a82a2 g1f2t.1 d2d62a1 d3tuic1 d2bbsa1 d3g60a3 d2ixea_ d1g6ha_
d1sgwa1 d2qi9c_ d3rlfa1 d3b60a1 d3d31a2 d2pzea1 g1xew.1 d1jj7a_
d1oxxk2 d2onka1 d1w1wa_ d1iwea_ d1qf5a_ d1vmaa2 d1nija1 d1np6a_
d2p67a1 d3ihla_ d1ls1a2 d1s1ma2 d1iona_ d2v40a_ d1dj3a_ d3hida_
d2vo1a1 d2g0ta1 d1vcoa2 d1j8mf2 d1g3qa_ d1xjca_ d1p9ba_ d1qzxa3
d1ihua2 d1ihua1 d2qm8a_ d1dj2a_ d4ioja_ d1cp2a_ d1yrba2 d1hyqa_
d3gqba2 d1skye3 d1xp8a1 d1tf7a2 d1tf7a1 d1ye8a1 d1skyb3 d2qe7d2
d4blta_ d4huta_ d3ldaa2 d4hyya_ d2i3ba1 d1cr1a_ d3j9tb2 d3ntua2

d1vdza1 d2z43a_ d1e9ra_ d1nlfa_ d3ziaa2 d1n0wa_ d1g6oa_ d3ziad2
d3j9ta2 d1pzna2 d3gqbb2 d2i49a_ d1nnfa_ d1xvxa_ d1ursa_ d1h3da1
d1iuda_ d4m1va1 d1twya_ d3e13x_ d2nxoa1 d4pfua_ d3ttma_ d1elja_
d2qmwa1 d1us5a_ d1y3na1 d1jeta_ d1uqwa_ d4f19a1 d2esna2 d3thia_
d3fasa1 d2uyeb_ d2xwva1 d1r9la_ d2fyia1 d1pb7a_ d2ozza1 d1sw5a_
d1nh8a1 d1vr5a1 d2g29a_ d1xs5a_ d3lswa_ d2z23a_ d3w9va_ d3tcha_
d1q35a_ d1z7me1 d3q27a_ d1p99a_ d1ve4a1 d2i6ea1 d4mcoa1 d4n8ya_
d1eu8a_ d1zbma1 d1hsla_ d1ixca2 d1j1na_ d1xvya_ d1y9ua_ d2z22a1
d1xoca1 d4oera_ d1wdna_ d5f1qa1 d1xt8a1 d4gwoa_ d4igta1 d1pc3a_
d4mmpa1 d1o63a_ d2czla1 d2onsa2 d4mija1 d1eh3a_ d3skpa_ d1dtza1
d1dtza2 d1ieja_ d1jnfa2 d1ce2a1 d1jnfa1 d1b1xa2 d1b1xa1 d1jw1a1
d1jw1a2 d1ryoa_ d1h76a1 d1h76a2 d1c4oa1 d2p6ra3 d2p6ra4 d1oywa2
d1oywa3 d1wrba1 d1w36b2 d1w36b1 d1c4oa2 d3bora_ d1t6na_ d2eyqa5
d2eyqa3 d2eyqa2 d2zu6a2 d1veca_ d1uaaa1 d1w36d1 d2g9na1 d1z3ix2
d2rb4a_ d1hv8a1 d1hv8a2 d1t5ia_ d1w36c2 d2j0sa2 d2j0sa1 d3hjha1
d1z5za1 d1qdea_ d1z63a1 d1s2ma2 d1s2ma1 d1wp9a1 d1wp9a2 d1gm5a3
d1gm5a4 d3dina4 d3dina3 d1q0ua_ d1w36c1 d1z3ix1 d1nkta3 d1nkta4
d1uaaa2 d1w36d2 g1qhh.1 d2fwra2 d2fwra1 d2m9wa1 d3dfxa_ d2fkza_
d1zuja1 d1s3qa1 d2fjca1 d1z6om1 d1veia_ d4dyya_ d1jiga_ d3gvya_
d2pyba_ d4cy9a1 d3bvfa1 d1r03a_ d2cf7a_ d1nf4a_ d1zs3a1 d4iwka_
d3ka8a_ d3np0x_ d1n1qa_ d1lb3a_ d4e6ka_ d1z6oa1 d2fzfa1 d4dyua_
d3ge4a_ d1jgca_ d4am4a_ d2z6ma_ d1ji5a_ d1vlga_ d1lkoa1 d1krqa_
d3qz3a_ d1o9ra_ d2ffxj_ d1euma_ d1yv1a1 d2v2pa_ d1j30a_ d4di0a_
d1dpsa_ d1oewa_ d2qzxa_ d2asia_ d1psoe_ d1miqa_ d1smra_ d2h6ta_
d1pfza_ d1izda_ d3hd8a_ d1j71a_ d1dpja_ d4aa9a_ d1t6ex_ g1b5f.1
g1lya.1 g1htr.1 d1qdma2 d1bxoa_ d1wkra_ d1tzsa_ d2qzwa_ d2v76a_
d1wj1a1 d1aqca_ d1qqga2 d1ntva_ d1n3ha_ d4dbba_ d3f0wa1 d1p3ra_
d1wgua1 d2cy4a1 d3hqca_ d1wvha1 d1xr0b1 d1bdqa_ d3ggta_ d3ixoa_
d1mrxa_ d2haha_ d1tw7a_ d4njsa_ d1sgua_ d2p3da_ d1nsoa_ d3d3ta_
d2rkfa_ d1c6ya_ d2rspa_ d3ogpa_ d3ekwa_ d2o4na_ d2j9ja_ d1baia_
d3ggua_ d3lzua_ d4npua_ d4j55a_ d2r8na_ d2fxda_ d2p3ca_ d4dqea_
d3ttpa_ d3t3ca_ d2p3aa_ d2hc0a_ d2fdda_ d3mwsa_ d2za0a_ d2c21a1
d1k8rb_ d1wgra1 d1ef5a_ d1wfya1 d1e8ya3 d1wgya1 d2cs4a1 d1wxma1
d2byea1 d3kh0a1 d2byfa1 d1wxaa1 d1e7ua3 d1wgda1 d1wgga1 d4q5eb_
d1wjna1 d1se9a_ d3n3kb_ d1wz0a1 d1we6a1 d2m0xa_ d2gbka_ d3rt3b1
d1wh3a1 d1sifa_ d2ekec2 d4ii2b1 d4hk2a1 d1c3ta_ d1m94a_ d1wgha1
d1ttna1 d1j8ca_ d1wx8a1 d3v6cb1 d2ma9b_ d4i6lb1 d1bt0a_ d2jvca1
d1iyfa_ d2k1fa_ d1wxva1 d1wf9a1 d1t0ya_ d2io0b2 d1x1ma1 d2gbma_
d1zw7a1 d2m17a_ d1we7a1 d2lwpa_ d2gbja_ d1wjua1 d1v6ea1 d1v86a1
d4eewa_ d1wiaa1 d4icva_ d4gu2a_ d1wx7a1 d1wy8a1 d4hjka1 d2faza1
d1v5oa1 d3ruib_ d3waob_ d2r2qa1 d3m95a_ d3wana_ d1eysc_ d4lu0a_
d1of8a_ d3unda1 d3e9aa1 d1vr6a2 d1n8fa_ d2qkfa_ d1o60a_ d2h0ba_
d2erfa_ d1h30a2 d1h30a1 d3mw4a1 d1pz7a_ d2r1ba_ d3v64a1 d3bopa1
d3mw3a1 d1okqa1 d1okqa2 d1dszb_ d1a6ya_ d3cbba_ d1ynwa1 d1r4ia_

d1hcqa_ d2hana_ d2hanb_ d2enva1 d1kb4b_ d2nllb_ d1lo1a_ d1cita_
d1um8a_ d1ixza_ d1qvra2 d1qvra3 d1qzma_ d1sxjc2 d1jr3d2 d2p65a_
d1l8qa2 d1g8pa_ d3hu3a3 d1sxjd2 d1njfa_ d1in4a2 d1sxja2 d1tuea1
d2ce7a2 d1w5sa2 d1sxjb2 d1d2na_ d1ny5a2 d1ofha_ d2gnoa2 d2a5yb3
d1fnna2 d1u0ja1 d1lv7a_ d1iqpa2 d2fnaa2 d1ixsb2 d4gita_ d1sxje2
d1g41a_ d2e3fa_ d2e3ea_ d2llda_ d2ny8x_ d2e3ga_ d2ny9x_ d2nz3a_
d2lj7a_ d1n4na_ d2m8bb1 d2kyqa_ d3psma_ d1jkza_ d1jxca_ d2gl1a_
d1ugla_ d4uj0a_ d1js9c_ d1f15b_ d1za7a_ d1laja_ d1t0za_ d2i61a_
d1t1ta_ d1sn4a_ d1kv0a_ d2lkba_ d1npia_ d1wwna_ d1pe4a_ d2kbka_
d2li7a_ d1djta_ d2kbha_ d1dq7a_ d1sega_ d1omya_ d1zywa1 d1jzaa_
d1n9wa1 d1bbua1 d1e1oa1 d1c0aa1 d1b8aa1 d1l0wa1 d3a74a1 d1eova1
d1iyjb4 d1iyjb5 d1iyjb3 d1o7ia_ d4ipca1 d1x3ea_ d4apva_ d1jb7a1
d1jb7a2 d1jb7a3 d3kdfa_ d1ue1b_ d1wjja1 d3eiva_ d1xjva2 d1xjva1
d2ccza2 d1se8a_ d1l1oc_ d1jb7b_ d1qvca_ d3ulla_ d1qzga_ d1miua4
d1miua5 d1miua3 d3g48a1 d1gd7a_ d1jjcb3 d1pyba_ d1y14b1 d2nn6i1
d1i94l_ d2zjra2 d1smxa_ d2qamc2 d1hh2p1 d2r7da1 d1rl2a2 d1wfqa1
d3bzka4 d1x6oa2 d3a0ja_ d2j01d2 d2ja9a1 d4ql5a_ d1hr0w_ d1luza_
d2n3sa_ d2vnud1 d1h95a_ d1go3e1 d2nn6h1 d1ne3a_ d2mo1a_ d1iz6a2
d1jjga_ d1e3pa2 d2ix0a3 d2ix0a2 d2lxja_ d1ueba3 d1ueba2 d2z0sa1
d1u0la1 d2ix0a1 d2eifa2 d2je6i1 d3i2za1 d2c35b1 d2ahob2 d1t9ha1
d3i4oa_ d2qall1 d1jt8a_ d2nn6g1 d1g6pa_ d3cama_ d1i94q_ d1c9oa_
d1d7qa_ d2ba0a1 d2vnud2 d2vnud3 d1khia2 d2qalq1 d1wi5a1 d1xtda2
d1dgsa2 d1ckma1 d1p16a1 d1x9na2 d3zxha_ d1cgla_ d1rm8a_ d1eaka2
d2jsda_ d1cxva_ d1hv5a_ d1i76a_ d1ohea1 d1ohea2 d2f4da_ d1rxda_
d2hxpa_ d1xria_ d1d5ra2 d1fpza_ d3ezza_ d4y2ea_ d3azya_ d3wvja_
d1upsa1 d1ajoa_ d2hyka_ d1ajka_ d2vy0a_ d1o4ya_ d3o5sa_ d3d6ea_
d3atga_ d1dypa_ d1umza_ d3i4ia_ d1tdha3 d1q3ba3 d1ee8a3 d1k82a3
g1qmo.1 g1len.1 d3zyra_ d1fnya_ d1n47a_ g2ltn.1 d1avba_ d3usua_
d1dhkb_ d1f9ka_ d1ukga_ d1g8wa_ d1g9fa_ d1wbfa_ d3wcsa_ d1g7ya_
g1loe.1 d1mvqa_ d1hqla_ d1v6ia_ d1dbna_ d1qnwa_ d4l8qa_ d1ioaa_
d3n35a_ d3h33a_ d1rwja_ d19hca_ d1os6a_ d3bxua_ d3h4na_ d1i77a_
d3caoa_ d1j0pa_ d2ctha_ d1duwa_ d1ofwa_ d2e84a_ d1hh5a_ d1gyoa_
d3h34a_ d1wioa4 d1ccza2 d1vcaa1 d1dr9a2 d1ic1a1 d2d9qb1 d2nxyb2
d1adqa1 d1fnga1 d1uvqb1 d1zs8a1 d2vola1 d2vola2 d1mfbl2 d2jelh2
d1uwel2 d3qq4b1 d1kjvb_ d1k5na1 d1osph2 d4odhl2 d1ow0a1 d1ow0a2
d1bfob2 d3l9ra2 d3jtth1 d2fath2 d4elkb2 d1fn4b2 d2xfxb_ d1pg7x2
d1cqka_ d4k71b2 d1lk2a1 d1eapb2 d4lfhg2 d1i4fa1 d2nw2a2 d1kfah2
d1mhca1 d1h3ua1 d1wejh2 d1h0db2 d1a5fh2 d1kcvh2 d1mjuh2 d1qqda1
d1kcrh2 d1k8ia1 d3huja2 d2nnab1 d1nfdf2 d3mffb2 d1s9va1 d1lk3h2
d1q0xl2 d1fv1b1 d1neza1 d4rfeb2 d1r3ha1 d1a6tb2 d1kjva1 d1pz5b2
d1onqa1 d2bc4a2 d1de4a1 d1k8da1 d1tcra2 d1dn0b2 d1nfde2 d2xwtb2
d1bfoa2 d1mexh2 d1iakb1 d1ypzf2 d4f7ea2 d1q72l2 d2fbjh2 d1fe8h2
d1clzh2 d1fngb1 d1uvqa1 d1vgka1 d1c16a1 d4j12a2 d4x99b2 d1igtb3
d1je6a1 d1q72h2 d1nldh2 d1teth2 d2hmic2 d1i1ca2 d1i1ca1 d1k8ib1

d1q0yh2 d1kfal2 d3frua1 d1pg7w2 d1u58a1 d1igyb4 d1igyb3 d1hyrc1
d1klub1 d1deeb2 d1c12b2 d2bc4b2 d2ig2h2 d1kcvl2 d3wn5b1 d1lk2b_
d1lk3l2 d1mjul2 d1sm3h2 d1q0xh2 d1zvsa1 d1ydpa1 d1u8ib2 d1ypze2
d1kcuh2 d1op9a_ d1y0la1 d1etzb1 d1egjl1 d2gsib1 d1nj9a1 d4jfhd1
d1aqkl1 d1t2ja_ d1lo4h1 d1zvya1 d3v4vl1 d4enec1 d1kxtb_ d5e8ea1
d2pxya_ d4weud_ d1xiwc_ d2esvd1 d1qnzh_ d1ogae1 d1f4xh1 d4n1hb_
d3ezjb1 d3mlwl1 d1kfah1 d1etza1 d1b4jh1 d3cfic_ d4w68a_ d2arja1
d1ek3a_ d2ok0l1 d1vera_ d1uwgh1 d1blnb1 d1fpth1 d1rzga1 d2giya1
d4m3kb1 d1nc2a1 d2nyyd1 d1ncna_ d1fbih1 d5dxwa_ d4fqqa1 d1dqdh1
d1jfql1 d1kelh1 d1faih1 d1frgl1 d1figl1 d4m6nl1 d1fo0a_ d4hxbl1
d3b5ga_ d3quyd1 d2h9gb1 d1i8kb_ d2dd8h1 d2ck0h1 d1eaja_ d4w6wb1
d2a6ib3 d1t66d1 d2e27h_ d1ob1b1 d1f11b1 d2v7na1 d2xt1b1 d1pskh1
d4i13b1 d2op4l1 d1a0ql1 d1uwel1 d1za3a1 d1ncbl1 d4uu9a_ d1bm3h1
d1cloh1 d1nqba2 d1cf8h1 d4y5va_ d1mhph1 d5anma1 d4cdgc1 d4ldeb_
d1y0lb1 d2pcpb1 d1z3gl1 d1dfbl1 d1fh5h1 d3g5xa1 d1tzib1 d4dn3l1
d3zhka1 d2b1hh1 d2uyla1 d1f3dh1 d1nj9b1 d2agjh1 d2h1pl1 d1q72h1
d8fabb1 d1ndmb1 d1rjca1 d4u6hb1 d3upca_ d2fbjl1 d1osph1 d1tvda_
d2cjvh_ d4fhbd1 d1bd2d1 d1rz7h1 d3nn8a_ d1blna1 d1knoa1 d1nfda1
d2bmkb1 d1dqll_ d1igyb1 d1igfl1 d12e8h1 d3utza1 d1nc2b1 d1rihh1
d4mwfb1 d4jvpa1 d1mieh1 d1rhfa1 d1fgnl1 d1sbsh1 d1lp9e1 d1dn0a1
d4lqfl1 d1iaih1 d1kgcd1 d1mh5b1 d1gpol1 d2g60h3 d1f8tl1 d2jixd1
d4grwh_ d1fo0b_ d1bfob1 d1axsa1 d1u9ka_ d1nmbh_ d1h8na1 d1h8na2
d1jguh1 d4eura1 d3sdyl1 d35c8h1 d1w72l1 d1mvub_ d1i9rh1 d1ob1a1
d4wenb_ d1yeeh1 d3ietc1 d1f58h1 d1ad0b1 d1mqkl_ d1nbvh1 d4b50a_
d3r8bb_ d1bafl1 d1mamh1 d4rgne1 d2gsgb_ d35c8l1 d1bafh1 d1ai1h1
d2pcpa1 d2fath1 d2x1pa_ d1orsb1 d1ibgl1 d1shma_ d1a2yb_ d1u0qa_
d2coqa_ d1hilb1 d1qfwi_ d1jv5b_ d1fn4b1 d5e0qa_ d1nsnh1 d1plgl1
d4ocxl1 d1kb5h1 d1rzfh1 d1pg7x1 d2fx7h1 d2oi9c_ d1l7il1 d3eyva1
d1d5il1 d4ffyl_ d1tetl1 d1a6wh_ d1ac6a_ d3dvfa_ d1nfde1 d1mimh1
d1ri8a1 d1jhlh_ d3dura_ d1fskb1 d1f97a1 d3so3b1 d2ak4d1 d1lp9f1
d4z7wf1 d1l7th1 d4y5ya_ d4x7ec1 d1emth1 d1ikfl1 d1nlbh1 d4utal1
d1bjma1 d1eapb1 d1u6ah1 d2dtgd1 d1kj2b_ d1xeda1 d1c1eh1 d1fgvh_
d2bnub1 d2fb4h1 d2dtgb1 d1ktrh_ d1jpth1 d1qkzl1 d4hepg_ d2atpb1
d1flrh1 d2hrpl1 d1mvfa_ d1iaim1 d3k80a1 d2jb5h1 d3zkxc_ d4grwe_
d1it9h1 d1rhhb1 d3w9ec_ d1e4wh1 d1sy6h1 d2j6eh1 d3qxta_ d1jglh1
d1p7ka1 d1tzhb1 d4n8pa1 d1gafl1 d2jelh1 d1wioa2 d4i0cc1 d1cicb1
d1lmka2 d1lmka1 d1iqwh1 d1orqb1 d1ejoh1 d1jnlh1 d4zg1a_ d43c9b_
d1ggbl1 d1wejh1 d3q6fa1 d1f3dj1 d3c6la1 d1bqlh1 d3p9wb_ d1a3rl1
d1h0db1 d2uzil_ d1eo8l1 d1qfwl_ d1n7ml1 d1igja1 d1c5ch1 d2a9mh_
d1rmfh1 d1fj1b1 d4heme_ d1ccza1 d1a5fh1 d1ogad1 d1dlfh_ d1qa9b_
d1vgel1 d3qskb_ d1sjxa_ d4ozfg1 d1sq2n_ d1qd0a_ d1kcvh1 d1c5db1
d3qcul1 d8faba1 d3bn9d1 d1mjuh1 d4y8dc_ d7fabh1 d3dvgb1 d1lo0l1
d1a6tb1 d1gigh1 d1kcrh1 d2hwzl1 d1yjdc1 d3clfl1 d4krna_ d1dl7l_
d1e6ol1 d1mexh1 d3r0ma_ d4kkna_ d1fnsl1 d1oaqh_ d1rzia1 d1ucbh1

d1ncwl1 d4b41a_ d1nfdf1 d2mpah1 d2d7tl1 d4grwf_ d4b5ea_ d1lk3h1
d3u79a_ d4u3xa_ d1ghfl1 d1bvkb_ d1mcww1 d1b88a_ d1jrhh1 d32c2b1
d4wemb_ d1dsfh_ d1op3h1 d1ce1l1 d1iqda1 d1qoka1 d1qoka2 d1qfuh1
d2crya1 d1ay1h1 d1oaql_ d3ab0b_ d1rurl1 d1t4kb1 d1bz7b1 d1n0xh1
d1forl1 d1ucbl1 d4w6yb_ d43c9a_ d2aabl1 d1tzia1 d4n1ca_ d1k6qh1
d3g9ab_ d4tvsa1 d1igmh_ d1pz5b1 d3dx9a1 d3wxwh_ d1nakh1 d1kcul1
d3eaka_ d4qlra1 d1cr9l1 d1d9ka_ d1f4xl1 d1deea1 d2i27n2 d1c12a1
d2orbl1 d1j8hd1 d1mcph1 d1c5da1 d1kenh1 d1bd2e1 d1h5ba_ d4x7fc1
d3upaa_ d1t3fa1 d1indh1 d2dtgc1 d4hc1l1 d1zv5a1 d4wgvb_ d1uwgl1
d4f37k1 d1aqkh1 d1vesa_ d3zkqd_ d1fptl1 d1lo4l1 d1knob1 d3rrqa_
d1ct8b1 d4orzc_ d1mlbb1 d4hgkc1 d1dqdl1 d1bogb1 d4ozhe1 d1dr9a1
d1i7za1 d1dn0b1 d1kell1 d1rzib1 d1ypzf1 d1wtla_ d1hkfa_ d1fail1
d1figh1 d2vyre_ d1sjva_ d1ohqa_ d1bfoa1 d1pkqb1 d1mjul1 d1b4jl1
d1nsnl1 d2arjb1 d1p4bh_ d4aj0a_ d3nn8b_ d2rcja1 d1f2xk_ d4v1dd_
d1adqh1 d2eksa_ d4k3ga_ d1jfqh1 d1uweh1 d1frgh1 d4gftb_ d1uz8b1
d4n1ea_ d1bm3l1 d1clol1 d3mcka1 d2vq1a1 d1ae6h1 d4aq1b1 d1uj3b1
d1nbqa1 d1fh5l1 d2ck0l1 d2ywza_ d1q72l1 d4nbxb_ d1hila1 d4hzlb1
d2j88l1 d1zmya1 d1cd0a_ d1a4ka1 d1pskl1 d1a0qh1 d4krmb_ d1ncbh1
d2fbjh1 d1j8he1 d1i3ua_ d1cf8l1 d1fe8h1 d2p1ya1 d1eeqa_ d2x89a_
d1ol0a_ d2r0lh1 d1ub5a1 d1dfbh1 d3eyvb1 d4jdvb1 d4p2cg_ d2gsia1
d2agjl1 d1u3ha1 d2h1ph1 d1rihl1 d1fskc1 d1boga1 d2a77l1 d1ospl1
d1qfoa_ d3dwta_ d2fx7l1 d1rz7l1 d3stba1 d1pkqa1 d1b6da1 d1dzba2
d1dzba1 d1kj2a_ d4l1ha_ d1rzgb1 d1dqlh_ d12e8l1 d1miel1 d1jmaa_
d1sbsl1 d1fgnh1 d1f58l1 d1mqkh_ d1ndgb1 d3qegl1 d1iail1 d2aw2a1
d1tqbc1 d3fo2a1 d4dkaa_ d1f8th1 d2g60l1 d2ywya_ d1uj3a1 d1yc7a_
d3bd3a_ d4w6xb1 d1nmbl_ d1i8ka_ d1rjlb1 d1ypze1 d1p7kb1 d1kxqe_
d1gpoh1 d2z4qa1 d1w72h1 d1i9rl1 d4qgya1 d4xt1c_ d2qqnh1 d1cica1
d3c08h1 d2cd0a_ d2bdnh1 d1hnga1 d1maml1 d1nldh1 d1cr9h1 d4lcih1
d3bkyh1 d3qg7l1 d1ai1l1 d1kxvc_ d4iosd_ d1l7ih1 d2x1qa_ d1ub6b1
d3k74b_ d1ibgh1 d1teth1 d1igya1 d1hxma1 d2x6ma_ d2e7la_ d5e03a_
d1qfwm_ d2r0kh1 d1igjb1 d1rurh1 d1plgh1 d1mj8h1 d1yuhb1 d3fmgl1
d1rzfl1 d1emtl1 d1ad9b1 d1igtb1 d1nlbl1 d1d5ih1 d3k7ua_ d4ffyh_
d1bwwa_ d3dvga1 d1a6ta1 d1fgvl_ d4qxgl1 d1miml1 d1jhll_ d2hffb1
d2hh0l1 d1qkzh1 d1l7tl1 d1afvh1 d1iaii1 d2cjuh_ d1um5h1 d2ghwb2
d2ghwb1 d1kgce1 d2fgwh1 d1ikfh1 d1dvfd_ d2i24n1 d2xtjb_ d1za3b1
d2yc1a_ d2hh0h1 d2x1oa_ d1bvka_ d1rjla1 d5e7ba1 d1igta1 d1il1b1
d2fb4l1 d1mvua_ d1jptl1 d32c2a1 d1fvca_ d1iqdb1 d2hrph1 d2p4ab_
d4qo1a_ d1p2cb1 d1wt5c_ d1t4ka1 d5c1mb_ d1it9l1 d1bz7a1 d1b2wh1
d1e4wl1 d1jnll1 d1yedb1 d1za6b1 d3u7aa_ d1igfh1 d3gnml1 d1orsa1
d1wejl1 d1a2ya_ d1hxmb1 d1gafh1 d2j4wh1 d1pz5a1 d1j05a_ d1a3rh1
d4fqll1 d1qfwh_ d1eo8h1 d1n7mh1 d4s11a_ d1kegh1 d5da4a_ d1iqwl1
d3whxb1 d1vgeh1 d1deeb1 d1c12b1 d1t2ql1 d1ad9a1 d3gsnb1 d3b2uc1
d1ggbh1 d1t3fb1 d1nakl1 d4ocmc_ d1lila1 d2v7hb3 d2esve1 d1mcoh1
d2qr0b1 d2q3aa_ d1ct8a1 d1kcrl1 d2pxyb_ d2uzih_ d5c3ld_ d3durb_

d1c5cl1 d2ak4e1 d1dl7h_ d1xiwd_ d1mlba1 d1rmfl1 d1i7zb1 d1dlfl_
d2xa3a_ d5bq7b1 d1dqqb1 d3h42l1 d1egjh1 d1kcvl1 d1fe8l1 d2j6el1
d1f3rb2 d1lk3l1 d1sm3h1 d1q0xh1 d1ghfh1 d7fabl1 d1igch1 d1lo0h1
d4idla_ d5c2bl_ d1jrhl1 d1il1a1 d3zhda1 d4kfzc_ d1b0wa_ d1e6oh1
d1mexl1 d4krpb_ d1fnsh1 d1ncwh1 d4hgma_ d4eigb1 d4rzca_ d2mpal1
d4y5xa_ d5en2b1 d3lrga1 d4ej1c1 d1rhha1 d1j05b_ d1forh1 d1q9wb1
d2wzpd_ d2aq2a1 d1h0da1 d3revb1 d2ij0c1 d1tzha1 d4m7jl1 d1k6ql1
d3b9kc1 d1igml_ d3qeua1 d1dsfl_ d1ce1h1 d1kcuh1 d1zvha1 d1zvoa1
d1tqbb1 d1ay1l1 d2qscl1 d2cgrh1 d1nl0h1 d1kenl1 d3eo0a1 d4n9ob1
d1n0xl1 d3k1kc_ d2aq2b1 d1ck1a1 d1enfa1 d1i4pa1 d1v1oa1 d1et9a1
d1esfa1 d2g9hd1 d4ohja1 d1klud1 d1eu3a1 d1ty0a1 d1fnua1 d2qaln1
d2cqva1 d1fhga_ d2k1ma_ d2dl2a1 d1biha3 d1biha1 d1biha4 d3qp3a1
d1g1ca_ d1gxea_ d1cvsc2 d1cvsc1 d1xmwa2 d1m4ka2 d2otpa2 d1biha2
d1rhfa2 d2fcba2 d2fcba1 d1wwbx_ d1gsma2 d1epfa1 d1epfa2 d2dava1
d1waaa_ d1f97a2 d1x44a1 d1vcaa2 d1fnla2 d1fnla1 d3b5ha2 d3h8na2
d3h8na1 d1jbja1 d1jbja2 d1pd6a_ d1f2qa2 d1f2qa1 d3cx2a_ d1nbqa2
d3b5ha1 d1n26a1 d1xiwb_ d1xaua_ d1fltx_ d2ifga1 d1olla1 d1olla2
d1cs6a4 d1cs6a3 d1cs6a2 d1cs6a1 d2gw5a2 d1sy6a1 d1xiwa_ d1ucta2
d1ucta1 d1ry7b2 d1gl4b_ d1qz1a3 d1olza1 d2c9aa1 d1wwca_

**Family-56 (c.26.1.1)**

**Positive dataset contains 7 protein sequences.**

d2el7a_ d2cyba_ d1rqga2 d2yxna_ d2g36a1 d3n9ia_ d3sz3a1

**Negative dataset contains 3976 protein sequences.**

d1m32a_ d1ibja_ d1c7na_ d1iuga_ d4q75a_ d2bkwa1 d1t3ia_ d1qz9a_
d1jf9a_ d1eg5a_ d1p3wa_ d3kgwa_ d1cl1a_ d2ctza1 d1vjoa_ d2rfva_
d1gc0a_ d1cs1a_ d1d2fa_ d2ch1a1 d1qgna_ d1pffa_ d1elua_ d1sefa_
d1sfna_ d1sq4a_ d1xkla_ d2wfla_ d3i2ka1 d1lnsa3 d1mpxa2 d2cfba1
d1dfoa_ d1o69a_ d1bj4a_ d3k28a_ d2bwpa_ d3lg0a_ d2fnua_ d3ou5a_
d1z7da1 d1b9ha_ d1iaya_ d4msoa_ d1bs0a_ d1sffa_ d3tqxa_ d1mdoa_
d3qboa_ d3pgya_ d1bjna_ d2dkja_ d1ejia_ d1vefa1 d4wxga_ d3bs8a_
d3gbxa1 d1w23a_ d1u08a_ d1lc5a_ d1b5pa_ d2ay1a_ d1bw0a_ d3pd6a_
d7aata_ d2csta_ d1j32a_ d1fg7a_ d4wlha_ d1o4sa_ d1v2da_ d1gdea_
d1m6sa_ d1v72a1 d1svva_ d1ajsa_ d1wsta1 d2f8ja2 d1lk9a_ d1vp4a1
d1xi9a1 d2gb3a1 d1yaaa_ d1r0ka3 d1j5pa3 d1vkoa2 d1vm6a2 d3qvsa2
d1gr0a2 d1p1ja2 d1lmra_ d1fwxa1 d1ibya_ d1f56a_ d1bypa_ d1azca_
d1ws8a_ d3u25a_ d1cuoa_ d1id2a_ d1rkra_ d2iaac_ d4dp1x_ d1nwpa_
d2gima_ d2i7sa_ d1qhqa_ d1gyca3 d1hfua2 d1kbva1 d1kbva2 d1sdda1
d1sdda2 d1gyca1 d1gyca2 d1mzya1 d2bw4a1 d1aoza1 d1aoza3 d1aoza2
d1kyaa2 d1kyaa3 d1kyaa1 d1mzya2 d4e9sa1 d1sddb1 d1sddb2 d1hfua1
d1hfua3 d1oe1a2 d1oe1a1 d1v10a2 d1v10a3 d1v10a1 d2dara2 d2dara1
d1wyha2 d2dloa2 d1rutx4 d1rutx3 d2cuqa2 d1x4la2 d1x4la1 d1x3ha2
d1x3ha1 d2d8za1 d1x61a2 d1x6aa1 d1x6aa2 d2cora2 d2cora1 d1v6ga1
d1v6ga2 d2d8za2 d1x64a1 d1x64a2 d1b8ta1 d2co8a1 d1x63a2 d2dj7a2
d2dj7a1 d2cu8a2 d2cu8a1 d1g47a2 d1g47a1 d1j2oa1 d1wyha1 d2dloa1

d1wiga1 d1wiga2 d2cupa2 d2cupa3 d1x68a1 d2d8ya2 d2d8ya1 d1m3va2
d1x62a1 d1x68a2 d1x61a1 d1b8ta3 d1b8ta2 d2d8xa2 d2d8xa1 d1x4ka1
d1x4ka2 d2cuqa1 d1m3va1 d2co8a2 d1j2oa2 d1x63a1 d2cura1 d2cura2
d1x62a2 d3sxwa1 d1j3qa_ d4luka_ d1a3qa1 d1iknc_ d3brda1 d1p7hl1
d1oy3c_ d1imhc1 d1zk9a1 d2cxka1 d2bema_ d1pama1 d1ea9c1 d1qhoa1
d2fhfa2 d2fhfa1 d1k3ia1 d1j0ha1 d1cxla1 d2bhua1 d1edqa1 d1f1sa2
d1ut9a2 d1gvia1 d1txka1 d1mqta_ d1d4m3_ d1fmd3_ d2mev1_ g1aym.1
d1ev13_ d4q4w1_ d1r1a3_ d2x5ia_ d1rhi1_ g1r1a.1 g1d4m.1 g1rhi.1
d1b35b_ d1oopa_ d1tme1_ d4iv1a_ g1pvc.1 g1fpn.1 d1fpn1_ d4gb31_
d1d4m1_ d1fmd1_ d1ev11_ d2wzr3_ g1cov.1 d1qqp3_ g1fmd.1 d2x5ic_
d1rhi3_ g1oop.1 d1hxs1_ g1hxs.1 d1fpn3_ d1bev1_ g2mev.1 d1eah3_
d1aym1_ d1pvc3_ d1zba3_ d1h8ta_ d3vbhc_ g1b35.1 g1bev.1 d1hxs3_
g1ev1.1 d1bev3_ g1tmf.1 d1tmf1_ d1b35a_ d2mev3_ g1eah.1 g1h8t.1
d1eah1_ d4q4w3_ d1r1a1_ d1aym3_ d1pvc1_ d1zba1_ d1h8tc_ d1oopc_
d1tme3_ g1qqp.1 d4iv1c_ g1tme.1 d1ktja_ d1ex0a1 d1g0da1 d1v70a_
d2f4pa1 d1yhfa1 d2bjca1 d2hsga1 d1qpza1 d1efaa1 d1ebfa2 d1au7a2
d1e3oc2 d2ovga_ d1sq8a_ d1rzsa_ d3kz3a_ d3orca_ d3gpdg2 d2q49a2
d1hdgo2 d2g17a2 d2cvoa2 d2czca1 d3pwka2 d1obfo2 d1i32a2 d1zyao2
d2pkqo2 d1b7go2 d2hjsa2 d1cf2o2 d1vkna2 d1nvmb2 d1ux8a_ d1dlya_
d4i0va_ d2bkma_ d1dlwa_ d2bmma_ d2msya_ d2djoa1 d2r5za_ d1au7a1
d1f43a_ d2lmda1 d1akhb_ d1s7ea1 d3nara_ d1zq3p1 d2craa1 d1mija_
d4uuta_ d3a01a_ d1x58a1 d3rkqa1 d3cmya1 d2cqxa1 d2da6a1 d1e3oc1
d1mh3a1 d1ig7a_ d1x41a1 d1wh7a1 d1gt0c1 d2mw8a_ d1lfup_ d1x2ma1
d1b72a_ d1wi3a1 d1jgga_ d1wjha1 d1x2na1 d1mnmc_ d2cuea1 d3k2aa_
d2hosa_ d2jwta2 d1pufa_ d1octc1 d2hi3a_ d1b8ia_ d4j19a_ d3lnqa_
d1wh5a1 d1b8ib_ d1fexa_ d2cqra1 d1guua_ d1ug2a1 d2cqqa1 d2ajea1
d4a69c_ d1h8ac1 d2crga1 d2iw5b1 d1xc5a1 d1ofcx1 d2cu7a1 d1wgxa1
d2cjja1 d2ckxa1 d1gvda_ d1hcra_ d1tc3c_ d1gdta1 d1u78a2 d1hlva2
d1iufa2 d1iufa1 d1bl0a2 d1d5ya1 d1d5ya2 d3lv8a_ d1s3ga1 d1zaka1
d1s4qa_ d3tvta2 d1uf9a_ d1khta_ d1osna_ d1p5zb_ d4tmka_ d3cm0a_
d1tmka_ d1jaga_ d3h86a_ d1t0hb_ d1nn5a_ d2h92a_ d3kb2a1 d2c95a1
d1yj5a2 d1kgda_ d1ly1a_ d2ak3a1 d1vyva2 d1deka_ d2grja1 d1p3ja1
d1y63a_ d1e4ya1 d1s96a_ d3iija1 d1lvga_ d1nksa_ d1vyua2 d4e22a_
d1qf9a_ d1jjva_ d1q3ta_ d1ki9a_ d1lw7a2 d1teva_ d1kjwa2 d1gsia_
d2iyva2 d1viaa_ d1e6ca_ d1g3ma_ d1t8ta1 d1nsta_ d1fmja_ d1zrha_
d1ls6a_ d2a3ra_ d1aqua_ d2zptx_ d3ckla_ d1texa_ d1q44a_ d2gwha_
d1odfa_ d1uj2a_ d4gi7a_ d2jeoa1 d1rz3a_ d1k6ma1 d1z0ja1 d3umra2
d3gj0a1 d2gaoa1 d1yzga1 d1x3sa1 d1g7sa4 d1puja_ d1udxa2 d1kk1a3
d2o52a_ d1jwyb_ d1zcaa2 d1zcba2 d2fv8a1 d2cjwa_ d3dz8a1 d1xzpa2
d1vg8a_ d4qxaa_ d2ew1a1 d1x1ra1 d2bcgy_ d3doea1 d3cona_ d3rega_
d3wyga_ d1upta_ d2b6ha1 d1zd9a1 d1lnza2 d1r5ba3 d1n0ua2 d1r2qa_
d2f9la1 d2rhda_ d3q3jb_ d3q85a_ d1efma1 d1r8qa_ d2gf9a1 d2akab1
d1ek0a_ d1z08a1 d2gcoa1 d2dy1a2 d4g01b_ d2cxxa1 d1svia_ d3q72a_
d1ni3a1 d4ac9a4 d2erxa1 d2bmea_ d1wxqa1 d2gf0a1 d4ldja1 d1zunb3

d1d5ca_ d4lv5b_ d1efca3 d1kmqa_ d2g6ba1 d3lrpa_ d1tada2 d2y8ea_
d4rd4a1 d1z06a1 d2heia_ d3a58b_ d1u0la2 d1d2ea3 d1zj6a1 d2ocba_
d3rwoa_ d1mr3f_ d2e9sa1 d4u5xa1 d2iwra_ d2fn4a1 d3bwdd_ d2nzja1
d2atva1 d2ksqa_ d3l43a_ d1mkya2 d1mkya1 d1wmsa_ d1jnya3 d2rmka_
d1f60a3 d1gota2 d1jala1 d2fh5b1 d1g16a_ d2wblc_ d1m7ba_ d1wf3a1
d1f5na2 d1t9ha2 d1z6xa1 d1z0aa1 d2fg5a1 d2a5ja1 d2bcjq2 d1s0ua3
d1ky3a_ d4y0va_ d4lhwa_ d2qmea1 d2atxa1 d1egaa1 d1ryha_ d1moza_
d1nrjb_ d2erya1 d3bfka_ d1puia_ d1fzqa_ d3j3az1 d3t5ga_ d1shza2
d3defa_ d2f7sa1 d3raba_ d3tsoa_ d3aq4a_ d2al7a1 d3sfva1 d3bc1a_
d2ncda_ d1sdma_ d3h4sa1 d2heha_ d2grya_ g3kin.1 d1kk8a2 d1br2a2
d1goja_ d1f9va_ d2mysa2 d1lkxa_ d3t0qa_ d4db1a1 d3gbja_ d1v8ka_
d1k8ka2 d1k8ka1 d3uleb1 d3i33a2 d3i33a1 d1bupa1 d1dkgd2 d1dkgd1
d4ci6a2 d1jcfa1 d4a2ba2 d2fxua1 d1yaga2 d1yaga1 d4cbua2 d1bupa2
d1iqca2 d1iqca1 d1eb7a2 d1eb7a1 d1nmla1 d3h3no1 d3h3no2 d3ezwa2
d1hroa_ d4gedb_ d3zooa_ d1mz4a_ d1gu2a_ d1f1fa_ d1dvva_ d1c6ra_
d1c75a_ d1e29a_ d1gdva_ d1cnoa_ d1h32b_ d1co6a_ d2bh4x_ d2aiua_
d5cytr_ d1jdla_ d1i8oa_ d2zboa_ d3dmia_ d1ls9a_ d1wvec_ d2yk3a_
d3ph2b_ d1dw0a_ d5aura_ d1ql3a_ d1qn2a_ d1f1ca_ d2nqya_ d1h4ga_
d1hixa_ d1oa2a_ d1olra_ d3b7ma_ d4h7ma1 d3wp3a_ d2vgda_ d1igoa_
d3zsea_ d2nlra_ d3wx5a_ d1oa3a_ d1pvxa_ d1f5ja_ d1oa4a_ d1ks5a_
d4ixla1 d3sita1 d1h32a2 d1evsa_ d1cnt1_ d1pvhb_ d1z7ca_ d1huwa_
d1bgea_ d1rhga_ d1f6fa_ d1f45b_ d1i1rb_ d1axia_ d1etea_ d3tgxb_
d1scfa_ d2z3qa1 d3qb1a_ d1m48a_ d4nkqb_ d1eera_ d1v7mv_ d5e4ea_
d2o27a_ d3b5ka_ d2psma1 d4zf7a_ d3uf5a_ d3sw2a_ d1moxc_ d1dx5i3
d1edmb_ d1autl2 d1autl1 d1dval1 d1szba2 d1kigl_ d2y5fl_ d1ijqa2
d1gk5a_ d1g1sa2 d1nt0a3 d1xdtr_ d1lmja2 d1lmja1 d1nzia2 d2wphe_
d1klil_ d1pfxl2 d1pfxl1 d1p9ja_ d1g1ta2 d1f7ea_ d4yzub_ d1m1xb4
d1m1xb5 d2y5ia_ d1qlsa_ d1psra_ d3n22a_ d2egda_ d4ggfc_ d2wcea_
d2luca_ d4aqia_ d3nsia_ d2lp3a_ d1j55a_ d1a4pa_ d1k8ua_ d1pvaa_
d3fs7a_ d2pvba_ d1ttxa_ d1rwya_ d2kyfa_ d1rk9a_ d1exra_ d1tiza1
d1nyaa_ d1g8ia_ d1ggwa_ d1wdcc_ d3fwba_ d1ggza_ d2o5ga_ d1ctaa_
d1m45a_ d3i5gc_ d1sl8a_ d1fpwa_ d2scpa_ d2roaa_ d4aqra_ d3pn7b_
d1br1b_ d2mysb_ d1s6ca_ d1bjfa_ d2obha_ d1jfka_ d1jf0a_ d1qv0a_
d1lkja_ d2mlfc_ d2m97a_ d2d8na_ d4by5a_ d1jbaa_ d4nqga_ d1wdcb_
d2mysc_ d1j7qa_ d1s6ia_ d1uhka2 d1r2ua_ d1dtla_ d3kf9a_ d3pn7c_
d1fi6a_ d1c07a_ d5d67d_ d1tuza_ d1h8ba_ d1eg3a2 d2zkmx1 d1snla_
d1gjya_ d1y1xa_ d1hqva_ d1dvia_ d1df0a1 d1qxpa2 d4usla_ d1k94a_
d1wkya2 d1uuqa_ d3wh9a_ d4m1ra_ d1xyza_ d4xuya_ d2je8a5 d4bf7a1
d3cuia_ d1ur4a_ d1qw9a2 d1odza_ d2vzsa5 d1bqca_ d2x2ya1 d1hjqa_
d1qnra_ d4y7ea1 d1vjza_ d4k68a_ d2v3ga_ d4hpga_ d1nofa2 d1nq6a_
d1h4pa_ d1aq0a_ d3ziza_ d4bs0a_ d1ecea_ d1h1na_ d1ta3b_ d1w91a2
d1i1wa_ d1uhva2 d1us3a2 d1egza_ d7a3ha_ d1tvna1 d3nyda1 d1ghsa_
d2cyga1 d4xv0a_ d2c8na2 d1w32a_ d3emca_ d1v6ya1 d1foba_ d2uwfa1
d2fgla_ d3niya1 d2c0ha1 d1rh9a1 d1yq2a5 d1szna2 d1fa2a_ d1pama4

d5do8a1 d1ea9c3 d1hx0a2 d2fhfa5 d1qhoa4 d1avaa2 d1m53a2 d4m8ua1
d2gjpa2 d1lwha2 d4howa1 d1ktba2 d1j0ha3 d3vm5a1 d2x1ia_ d1wzaa2
d1uasa2 d2diea1 d1mxga2 d1ht6a2 d1eswa_ d1iv8a2 d5cpsa_ d1ud2a2
d1zjaa1 d3gbea1 d1kwga2 d1wdpa_ d1gcya2 d1g5aa2 d1hvxa2 d3dhpa2
d2bhua3 d4do4a1 d3qgva1 d1e43a2 d1g94a2 d2d3na2 d1gjwa2 d1gvia3
d1x1na1 d1r7aa2 d1tz7a1 d4toqa_ d1wb0a1 d1w9pa1 d1eoka_ d1itxa1
d1kfwa1 d1edqa2 d2gsja_ d1hjxa1 d1jnda1 d1vf8a1 d1etob_ d1umqa_
d1g2ha_ d2w0pa_ d2diaa1 d2e9ia2 d2e9ja1 d2dj4a1 d2eeba1 d2diba1
d2dmba1 d2dmca1 d2d7oa1 d2ee6a1 d2d7pa1 d2eeaa1 d2di8a1 d1wlha1
d2d7na1 d2nqca_ d2d7ma1 d2dica1 d3cnka_ d2k3ta1 d2eeca1 d1qfha1
d2bp3a1 d1v05a_ d2ee9a1 d2eeda1 d2di9a1 d3syxa1 d1mkea1 d2p8va_
d1xoda1 d1i7aa_ d1evha_ d1qc6a_ d1egxa_ d1iuka_ d2csua1 d3q9ub_
d2yv1a1 d3q9na_ d2d59a_ d1oi7a1 d2yv2a1 d1euca1 d1y81a1 d1wgqa1
d1v89a1 d2coda1 d1zc3b_ d1wg7a1 d1u5ea1 d1v61a1 d2coca1 d2ys1a1
d1v5pa1 d1ntya2 d1wjma1 d1wi1a1 d1u29a_ d1xx0a1 d1unqa_ d2zkmx3
d1fhoa_ d1v88a1 d2adza1 d1x1fa1 d1p6sa_ d2coaa1 d1btka_ d1v5ma1
d1faoa_ d2x18a_ d1dyna_ d1x1ga1 d4kaxb_ d2j59m1 d1ki1b2 d1xcga2
d1txda2 d1u5da1 d1dbha2 d1w1ha_ d1upqa_ d1v5ua1 d1foea2 d2fjla1
d1eaza_ d2dfka2 d2cofa1 d1kz7a2 d9ldta1 d2ewda3 d1up7a1 d2dfda1
d1s6ya1 d1vjta1 d7mdha1 d1hyea1 d1t2da1 d1ojsa3 d1b8pa1 d1ldna1
d1hyha1 d1civa1 d2a92a1 d1ez4a1 d1i0za1 d1y6ja1 d1gv0a1 d1ur5a1
d1llda1 d1obba1 d1v8ba1 d1gdha1 d2dlda1 d1mx3a1 d1ygya1 d1qp8a1
d1li4a1 d1j4aa1 d2naca1 d1txga2 d3f47a1 d2pv7a2 d1xc2a3 d2ahra2
d4xiya1 d2f1ka2 d1pgja2 d1i36a2 d1np3a2 d1lj8a4 d1teaa3 d1qmga2
d2i76a2 d2g5ca2 d1wdka3 d1dlja2 d3cuma3 d1kola2 d1f8fa2 d1qora2
d1jqba2 d1vj0a2 d1cdoa2 d1gu7a2 d1d1ta2 d1xa0a2 d2dm6a2 d1jvba2
d1rjwa2 d1llua2 d1tt7a2 d1piwa2 d1o89a2 d1iz0a2 d1e3ja2 d1yb5a2
d1uufa2 d1vj1a2 d1e3ia2 d1vc2a1 d2nvwa1 d1u8fo1 d1ydwa1 d1hdgo1
d2g17a1 d1cf2o1 d2czca2 d1r0ka2 d1obfo1 d2cvoa1 d1ebfa1 d1h6da1
d1i32a1 d1zh8a1 d2q49a1 d1qkia1 d1j5pa4 d3pzra1 d1xeaa1 d1vkoa1
d1vm6a3 d3qvsa1 d2pkqo1 d1b7go1 d1gr0a1 d1p1ja1 d1lc0a1 d1j0xo1
d3pwka1 d2hjsa1 d1zyao1 d1vkna1 d1tlta1 d1nvmb1 d3sjua_ d3ojfa1
d1xu9a1 d2jjya1 d1n7ha_ d1y1pa1 d1h5qa_ d1jtva_ d4fs3a_ d2bkaa1
d1gy8a_ d2c5aa1 d1ulsa_ d1ooea_ d2b69a1 d1y5ma1 d1spxa_ d1ae1a_
d1bxka_ d1ydea1 d1gega_ d1hdoa_ d1o5ia_ d1e6ua_ d1snya1 d2rhca_
d3zv4a_ d5ceja_ d2d1ya1 d3w8ea_ d2fmua1 d1x1ta_ d1rpna_ d3ay7a1
d1k2wa_ d1eq2a_ d2hrba1 d3ruaa_ d2ew8a1 d4hwka_ d1xgka_ d2bd0a1
d1yo6a1 d2a35a1 d2ntva_ d1vl8a_ d1hdca_ d4bkua_ d2ag5a1 d2q45a_
d1nffa_ d4iy1a_ d1zbqa1 d1kewa_ d1ek6a_ d1ja9a_ d2q46a2 d1iy8a_
d1cyda_ d2ptga_ d1hxha_ d1uzma1 d3lyla_ d4m89a_ d3wyea_ d2blla1
d1w6ua_ d1sb8a_ d2q2va_ d4nr0a_ d1e6wa_ d2gasa_ d3oiga1 d2bgka1
d1mxha_ d1vl0a1 d1rkxa_ d1r6da_ d1wmaa1 d1e7wa_ d2c07a1 d1xkqa_
d3tzha_ d1g0oa_ d3lz6a_ d1qyca_ d1edoa_ d1gz6a_ d1oc2a_ d1t2aa_
d1zema1 d2xoxa_ d1z45a2 d1geea_ d1p33a_ d3lt0a_ d4e6pa_ d2wyua_

d1db3a_ d4q9na_ d1xhla_ d4o1ma_ d1fmca_ d2uvda_ d2gdza1 d1uaya_
d2z1ma_ d2o23a2 d2fr1a2 d2fr1a1 d1qsga_ d1qyda_ d4zjua_ d2a4ka1
d1yb1a_ d1wvga1 d1yxma1 d3f4ba_ d1mg5a_ d1xg5a1 d2gfoa1 d1vjva_
d2ayna1 d3nhea_ d4ekfa_ d2ckga_ d1th0a_ d2bkra_ d1ex0a4 d2f4ma1
d1x3za_ d1t3ca_ d1epwa3 d2a97a_ d3fiea_ g1e1h.1 d3ioqa_ d1fh0a_
d1o0ea_ d1khqa_ d1m6da_ d2cb5a_ d1cs8a_ g8pch.1 d1s4va_ d2pnsa_
d1vsna1 d1mira_ d1iwda_ d2oula_ d7pcka_ d3bwka_ d3bcna_ d2p86a_
d1pvja_ d1pcia_ d1gece_ d1deua_ d4bs6a_ d1xkga1 d1cqda_ d1jqpa2
d2c0ya_ d2bdza_ d3p5ua_ d1zoya1 d1qo8a2 d1d4ca2 d1chua2 d1jnra2
d2jk6a2 d2jk6a1 d1feca1 d1feca2 d1lpfa1 d1lpfa2 d1xhca1 d1xhca2
d1dxla2 d1dxla1 d1gera2 d1m6ia2 d1onfa1 d1onfa2 d3lada2 d3lada1
d1w4xa1 d1w4xa2 d1gesa2 d1mo9a1 d1xdia1 d1fl2a2 d1fcda1 d1fcda2
d1h6va2 d1h6va1 d1mo9a2 d1gv4a2 d1gv4a1 d2gv8a2 d2gv8a1 d1aoga1
d1aoga2 d1hyua1 d1ebda1 d1ebda2 d3dk9a1 d1q1ra2 d1q1ra1 d1gesa1
d1w5qa_ d2c1ha_ d1ohla_ d1l6sa_ d1zbfa1 d2z1ha_ d2etja1 d3qioa1
d1jl1a_ d2e4la_ d1jl2a_ d2lsna1 d1uaxa_ d2yv0x_ d1mu2a1 d1ekea_
d1i39a_ d1ps9a2 d1lqta1 d1cjca1 d1pn0a1 d1reoa1 d1ju2a1 d2f5va1
d3c96a1 d2gmha1 d1pj5a2 d1gpea1 d1o5wa1 d1n4wa1 d2dw4a2 d2ivda1
d1seza1 d2voua1 d1kdga1 d1s5ja1 d2hbja2 d2igia_ d1wlja_ d4dlea1
d1tgoa1 d2f96a1 d3tr8a1 d3mxma_ d1q8ia1 d1y97a1 d1w0ha_ d1kfsa1
d2py5a1 d1noya_ d1d5aa1 d1yt3a3 d2guia_ d3mwpa1 d4kb1a_ d1iv0a_
d2v76a_ d1wj1a1 d1aqca_ d1qqga2 d1qqga1 d1ntva_ d1n3ha_ d4dbba_
d3f0wa1 d1p3ra_ d1wgua1 d2cy4a1 d3hqca_ d1wvha1 d1xr0b1 d1v58a1
d1t3ba1 d4ilfa2 d4npba2 d2av4a1 d3nvva1 d1krha3 d2h88b1 d1n62a2
d1t3qa2 d1kf6b2 d1zoyb1 d1ffva2 d1dgja2 d1rm6c2 d1r7ha_ d1faaa_
d1wika1 d1xfla_ d1ttza1 d2f51a1 d1ti3a_ d1nhoa_ d1sena_ d2i4aa_
d1ep7a_ d1zmaa1 d1fo5a_ d3lefa1 d3diea1 d2yana1 d1z6na1 d1oaza_
d3zywa1 d4aj8a_ d1nm3a1 d4fiwa_ d1wmja_ d4hs1a1 d1iloa_ d1xwaa1
d1wjka1 d2vm1a_ d1fb6a_ d1gh2a_ d1h75a_ d1nw2a_ d1kx5b_ d3a6na_
d4m6ba2 d2nqbd_ d3wkjd_ d2yfwe_ d4h9na_ d5bnxa_ d1kx5c_ d3r45a_
d1eqzb_ d4jjnb_ d1f66c_ d1eqza_ d4wnnb_ d1kx5a_ d1u35c1 d1id3a_
d2nqbc_ d1kx5d_ d1ku5a_ d1b67a_ d1tafa_ d1tafb_ d1jfib_ d1q9ca_
d1h3ob_ d2byka1 d1n1ja_ d4g92b_ d1bh9a_ d1jfia_ d1h3oa_ d2bykb1
d1n1jb_ d1pd212 d1nhya2 d2a2ra2 d1k0dd2 d1f2ea2 d1gsua2 d1f3ba2
d2fhea2 d2pvqa1 d1tw9a2 d1oyja2 d1e6ba2 d1k0ma2 d3ik7a1 d4is0a1
d1r5aa2 d1jlva2 d1m0ua2 d3gtub2 d1z9ha2 d1okta2 d1axda2 d2gsra2
d1tu7a2 d1k3ya2 d1zgma1 d1b48a2 d1gnwa2 d1gwca2 d2gsta2 d1glqa2
d1oe8a2 d1pn9a2 d1v2aa2 d1fw1a2 d1g7ea_ d1nira1 d1qksa1 d2lj7a_
d2m8bb1 d2kyqa_ d3psma_ d1jkza_ d1jxca_ d2gl1a_ d1ugla_ d4uj0a_
d1qqya_ d1f6ra_ d1ivma_ d2gv0a_ d1iiza_ d2z2fa_ d1j8xa_ d1ghla_
d1b9oa_ d3w3ea_ d4tx7a_ d3cqla_ d4msta_ d3iwra_ d1dxja_ d2z37a_
d4dyga_ d2pjsa1 d1klla_ d2i7ra1 d1ecsa_ d1xqaa1 d5cj3a1 d1npba_
d1jifa_ d2p7oa_ d1nkia_ d1mpya2 d1kw3b2 d1kw3b1 d4ghga1 d1tfza1
d3isqa1 d3isqa2 d1sqia1 d1sqia2 d1lkda2 d1lkda1 d1mpya1 d1cjxa2

d1sp9b2 d1t47a1 d1t47a2 d1f1ua2 d1sp8a1 d1sp8a2 d1f1ua1 d1s5ja2
d1qhta2 d4dlea2 d1tgoa2 d3py8a2 d1q8ia2 d1kfsa2 d2py5a2 d1d5aa2
d1qsta_ d2beia1 d4m3sa_ d1u6ma1 d1ygha_ d1yk3a1 d3dr6a_ d1xmta_
d1z4ea1 d2ge3a1 d1bo4a_ d2fiaa1 d2jdca1 d1y9wa1 d3to7a_ d1wwza1
d2fl4a1 d2ozua1 d2b5ga_ d2cy2a1 d2p0wa_ d1cm0a_ d4pswa_ d1yrea1
d1yr0a1 d1m4ia_ d2gana1 d1yvka1 d1ghea_ d2g3aa1 d1y7ra1 d2euia1
d1q2ya_ d2fsra1 d1xeba_ d2ae6a1 d1cjwa_ d1qsma_ d2aj6a1 d1y9ka1
d1i12a_ d1vkca_ d2atra1 d1n71a_ d2d4pa1 d2fiwa1 d2fe7a1 d1tiqa1
d2ou2a_ d1yx0a1 d2fcka1 d1p0ha_ d2j8ma_ d2pq8a_ d1z4ra1 d1s3za1
d1ufha_ d2hqya1 d2hqya2 d1lrza2 d1lrza3 d1ne9a2 d1xvqa1 d1pn0a2
d3hy2a_ d2cvba1 d1o8xa_ d1we0a1 d2bmxa1 d2a4va1 d1psqa_ d1xiya1
d1q98a_ d1n8ja_ d1zofa1 d2ggta_ d3tjja_ d4je1a1 d1o73a_ d3s9fa_
d2zcta_ d1qmva_ d1jfua_ d2b1ka_ d5dvba1 d2b5xa1 d2v2ga_ d1st9a_
d3tb2a_ d4llra_ d4fh8a1 d1e2ya_ d1knga_ d2h30a_ d1i5ga_ d2z9sa_
d2f8aa1 d1tp9a1 d2b7ka_ d1nm3a2 d3hvva_ d2wfca1 d4kcea_ d1xvwa1
d4gqca1 d3qpma_ d4k1fa_ d1zzoa1 d1gp1a_ d1prxa_ d2m5ta_ d1cqqa_
d1wqsa_ d5bpea_ d2ipha_ d1lvoa_ d2bhga1 d3tnta_ d3sjia_ d3d23a1
d2fyqa_ d3tloa_ d3zvea1 d2hrva_ d3q3ya1 d4asha1 d2phla1 d1fxza2
d1fxza1 d1lrha_ d1uija1 g1dgw.1 d2v09a_ d1uika2 d1uika1 d1dgwa_
d1cg2a1 d1yloa2 d1y7ea2 d1y0ya3 d1lfwa1 d1vixa1 d1fnoa4 d1vhoa2
d1ysja1 d1vhea2 d1z2la1 d2fvga2 d1vgya1 d1r3na1 d2grea2 d1xmba1
g1q7l.1 d1rtqa_ d1etha2 d1w52x1 d2ppla1 d1hpla2 d1lpbb2 d2oxea1
d1rp1a2 d1ys1x_ d4hs9a_ d1ispa_ d1ku0a_ d3qmma_ d1dy2a_ d4wfia_
d4cg1a1 d3visa_ d3groa_ d1pjaa_ d1thta_ d2ftba_ d1pmpa_ d2rcqa1
d1fdqa_ d4lkpa_ d1sa8a_ d1vyfa_ d1lpja_ d3vg7a1 d4azqa1 d1o8va_
d5bvqa_ d1p6pa_ d2qo4a_ d1opba_ d1yiva1 d1o1va_ d4bvma1 d4tkba_
d4i3ba_ d1bwya_ d1ftpa_ d1ggla_ d4gkca_ d3p6da1 d1kqwa_ d2a0aa1
d3zxha_ d1cgla_ d1rm8a_ d1eaka2 d1hy7a_ d2jsda_ d1hv5a_ d1i76a_
d3p0ya1 d1igra1 d1igra2 d1n8yc1 d1m6ba2 d1m6ba1 d4dv8a2 d1m4la_
d2bk7a_ d3fjua_ d1z5ra1 d1kwma1 d1uwya2 d1jqga1 d2c1ca1 d1h8la2
d2tbvc_ d1c8nc_ d1x36a_ d1smvc_ d1ng0c_ d4sbvc_ d1opoa_ d1f2nc_
d1cixa_ d1y29a_ d1v7fa_ d2jtba_ d1d1ha_ d1g9pa_ d1lupa_ d1qk7a_
d1mb6a_ d2a2va_ d1niya_ d1i25a_ d1hvwa_ d2mpqa_ d1ie6a_ d1kqha_
d1la4a_ d2mxoa1 d1dl0a_ d1koza_ d1qk6a_ d1emxa_ d1lqra_ d1c6wa_
d1rmka_ d1ixta_ d1eyoa_ d1cnna_ d1fu3a_ d1g1pa_ d1f3ka_ d1fyga_
d1rypd_ d1irua_ d1rypc_ d3unfk_ d1rypa_ d1j2pa_ d1irui_ d1j2qh_
d3unfh_ d1m4ya_ d1rypb_ d1irul_ d1irug_ d1iru2_ d1q5rh_ d1rypg_
d1g3ka_ d1iruf_ d1q5qa_ d1e94a_ d1irub_ d1ryph_ d3ty6a_ d1iruj_
d1iruk_ d1rype_ d1irud_ d1irue_ d3mi01_ d1rypf_ d1iruh_ d2z3ba_
d1iruc_ d3mka2_ g1gk9.1 g1cp9.1 g1e3a.1 d1jgta2 d1ofda3 d1ea0a3
d1ecfa2 d1ct9a2 d1ao0a2 d1te5a_ d3ubub_ d1egia_ d1qo3c_ d1v7pa_
d1tdqb_ d3ubua_ d1c3ab_ d1h8ua_ d3bx4b_ d1t8ca1 d1sb2a1 d1qdda_
d2ziba1 d3cada_ d1byfa_ d1j34b_ d1jwia_ d1jzna_ d1c3aa_ d1wmza_
d3bdwb_ d1hq8a_ d1fifa1 d1dv8a_ d3hupa_ d1wk1a1 d1umra_ d2afpa_

d3bx4a_ d1gz2a_ d1sl6a_ d1v7pb_ d3g8ka_ d1ixxa_ d1j34a_ d1jwib_
d1g1sa1 d1y17a1 d1ja3a_ d1fvub_ d3bdwa_ d1sb2b1 d1fvua_ d1umrc_
d1oz7a_ d4m17a_ d3cfwa1 d1ypqa1 d1g1ta1 d3fd4a_ d3l9jc_ d4dn8a1
d1v4lb_ d1rdl1_ d2msba_ d4w9ta_ d1jvna1 d4x2ra1 d1ka9f_ d1qo2a_
d4axka1 d1h5ya_ d4u28a_ d2y88a_ d4wd0a_ d1vzwa1 d1eixa_ d3exra_
d2ffca1 d2fdsa1 d3n3ma1 d2czda_ d1q6oa_ d2yyua_ d1vqta1 d1dbta_
d3ldva1 d4lt9a1 d3tc7a1 d3hoja_ d1i4na_ d3vnda_ d3nl8a1 d1geqa_
d3nava1 d1v5xa_ d4a2ra1 d3tc6a1 d1ujpa_ d4ijba_ d1xcfa_ d1vc4a_
d1rd5a_ d1qopa_ d3b5va_ d2r80b_ d1cqxa1 d1cg5a_ d1gcvb_ d3boma_
d2h8fb_ d2raoa_ d3bj1a_ d4hrrb_ d2r50a_ d3d1kb_ d2aa1b1 d3zhwa_
d3hf4b_ d3gdja_ d1jebb_ d3bj1b_ d1x9fb_ d1wmub_ d1gcva_ d3bcqb_
d3d4xb_ d1gvha1 d2dc3a_ d3hf4a_ d4yu3a_ d3k8ba_ d1qpwb_ d2r80a_
d3bcqa_ d1v4wa_ d3wtgb_ d1jl7a_ d4esaa_ d1a9we_ d3d1ab_ d1fsla_
d4h2la_ d1v4wb_ d1mwca_ d1vhba_ d1jeba_ d1spgb_ d1h97a_ d1q1fa_
d1tu9a1 d3d1aa_ d1hdsb_ d3hyub_ d2zfbb_ d3fs4a_ d1oj6a_ d1shrb_
d4h2lb_ d4yu3b_ d1spga_ d3hrwa_ d3qqra_ d3at5a_ d4wchd_ d3hyua_
d1cg5b_ d1uc3a_ d1hbra_ d2raob_ d1outb_ d4esab_ d3gdjb_ d3qqqa_
d1x9fc_ d3vrfa_ d1itha_ d3mkba_ d3d4xa_ d3mjpa_ d1jl6a_ d3fh9a_
d1outa_ d2dc3b_ d1it2a_ d3eoka_ d3g46a_ d1qpwa_ d3vrfb_ d1ch4a_
d1x9fd_ d1hdsa_ d2zfba_ d3mkbb_ d1or4a_ d3wtga_ d1a4fa_ d3fh9b_
d3bomb_ d3cy5a_ d3at5b_ d1x9fa_ d2oifa_ d4lmxb_ d1cpcb_ d3v57a_
d1kn1a_ d1cpca_ d4f0ta_ d2vjha_ d4po5b_ d1eyxa_ d1alla_ d4f0tb_
d1b33b_ d1b8db_ d4h0mb_ d1b33a_ d2vjhb_ d4lm6b_ d2vmlb_ d1allb_
d2vjta_ d3dbja_ d2vmla_ d2vjtb_ d4f0ub_ d1f99b_ d4rmpa1 d1liaa_
d1eyxb_ d2uulb_ d4lmsb_ d4f0ua_ d2bv8a_ d1f99a_ d2bv8b_ d2c7lb_
d3dbjb_ d4rmpb1 d1b8da_ d4h0ma_ d3v57b_ d1liab_ d2uula_ d2j96a_
g1gxs.1 d3nqyb_ d4gera_ d1bqba_ d1r88a_ d1sfra_ d4qeka_ d1b9ra_
d1offa_ d1frra_ d3wcqa_ d1e9ma_ d1l5pa_ d1wria_ d1i7ha_ d3av8a_
d1czpa_ d1rfka_ d3p1ma1 d3b2ga_ d2bt6a2 d4iuaa3 d4bvva_ d5hpga_
d3k65a_ d1ki0a3 d1ki0a2 d1ki0a1 d1a0ha1 d1pmla_ d3sp8a3 d2feba1
d1xova2 d1f74a_ d3l21a_ d2e1da_ d1to3a1 d1w3ia_ d1sfla_ d1vcva1
d4imea_ d1wa3a_ d2ocza1 d1f2ja_ d3m16a_ d2yxga_ d1ub3a_ d3a5fa_
d1fbaa_ d1l6wa_ d1xfba1 d1wx0a1 d1vpxa1 d1ojxa_ d1mxsa_ d2a4aa1
d1xkya1 d3cq0a1 d3s1xa_ d1f05a_ d1fdja_ d3cpra1 d4d2ja_ d1e88a1
d1l6ja5 d1eaka4 d1eaka3 d1l6ja3 d1h8pa2 d1h8pa1 d3rp2a_ g1ucy.1
d1fuja_ d2zchp_ d3w94a_ d1si5h_ d1lo6a_ g2pka.1 d1cgha_ d1kigh_
d1a5ia_ d3s9ba_ d1hj8a_ d1ao5a_ d2anya_ d1q3xa1 d1gvza_ d1azza_
d4gsoa_ d2f91a1 d1iaua_ d1gdna_ d1eufa_ g1fiw.1 d1eq9a_ d4nfea_
d2rdla_ d1spja_ d1elva1 d1a7sa_ d1ekbb_ d1pfxc_ d1yc0a_ d2aiqa_
g1fiz.1 d1fi8a_ d4k60a_ d4dgja_ d1mzaa_ d1op0a_ d3tjva_ d1pytd_
d1sgfg_ g1gg6.1 d2eeka_ g1rtf.1 d1a0ja_ d3g01a_ d1pytc_ d4e7na_
d1ddja_ d1z8ga1 d2hlca_ d1npma_ d1brup_ g1jou.1 d1sgfa_ d1autc_
g2hnt.1 g1kdq.1 d3s69a_ d4crga_ d1fxya_ d1bqya_ d1svpa_ d3su6a1
d1cu1a1 d4agka_ d1vcpa_ d1q31a_ g1n1l.1 d3e90b_ d1ep5a_ d3u1jb_

d1dzka_ d4tlja_ d2a2ua_ d4wfva_ d3t1da_ d4iaxa_ d3zq3a_ d2nnda_
d1i4ua_ d1kxoa_ d1bbpa_ d1kt7a_ d1exsa_ d3s26a1 d1lf7a_ d1qfta_
d4gh7a_ d1z24a1 d1qabe_ d1xkia_ d1ew3a_ d3bx7a_ d4iawa1 d1n0sa_
d1gkab_ d3kzaa_ d2k23a_ d2l9ca1 d2joza1 d1gm6a_ d3kffa_ d3saoa1
d1e5pa_ d1yupa1 d1iiua_ d3lgia_ d3i77a_ d2z9ia2 d4nsva_ d1lcya2
d4jcna_ d1os8a_ d1hpga_ d1p3ca_ d1l1ja_ d1qtfa_ d1e69a_ d1mv5a_
d3g60a4 g1ii8.1 d1ewqa2 d1ji0a_ d1qhla_ d1v43a3 d2wmfa1 d1vpla_
d1g2912 d4a82a2 g1f2t.1 d2d62a1 d3tuic1 d2bbsa1 d3g60a3 d2ixea_
d1g6ha_ d1sgwa1 d2qi9c_ d3rlfa1 d3b60a1 d3d31a2 d2pzea1 g1xew.1
d1jj7a_ d1oxxk2 d2onka1 d1w1wa_ d1iwea_ d1qf5a_ d1vmaa2 d1nija1
d1np6a_ d2p67a1 d3ihla_ d1okkd2 d1ls1a2 d1s1ma2 d1iona_ d2v40a_
d1dj3a_ d3hida_ d2vo1a1 d2g0ta1 d1vcoa2 d1j8mf2 d1g3qa_ d1xjca_
d1p9ba_ d1qzxa3 d1ihua2 d2qm8a_ d1dj2a_ d4ioja_ d1cp2a_ d1yrba2
d1hyqa_ d3gqba2 d1skye3 d1xp8a1 d1tf7a2 d1tf7a1 d1ye8a1 d1skyb3
d2qe7d2 d4blta_ d4huta_ d3ldaa2 d4hyya_ d2i3ba1 d1cr1a_ d3j9tb2
d3ntua2 d1vdza1 d2z43a_ d1e9ra_ d1nlfa_ d3ziaa2 d1n0wa_ d1g6oa_
d3ziad2 d3j9ta2 d1pzna2 d3gqbb2 d2i49a_ d1nnfa_ d1xvxa_ d1ursa_
d1h3da1 d1iuda_ d4m1va1 d1twya_ d3e13x_ d2nxoa1 d4pfua_ d3ttma_
d1elja_ d2qmwa1 d1us5a_ d1y3na1 d1jeta_ d1uqwa_ d4f19a1 d2esna2
d3thia_ d3fasa1 d2uyeb_ d2xwva1 d1r9la_ d2fyia1 d1pb7a_ d2ozza1
d1sw5a_ d1nh8a1 d1vr5a1 d2g29a_ d1xs5a_ d3lswa_ d2z23a_ d3w9va_
d3tcha_ d1q35a_ d1z7me1 d3q27a_ d1p99a_ d1ve4a1 d2i6ea1 d4mcoa1
d4n8ya_ d1eu8a_ d1zbma1 d1hsla_ d1ixca2 d1j1na_ d1xvya_ d1y9ua_
d2z22a1 d1xoca1 d4oera_ d1wdna_ d5f1qa1 d1xt8a1 d1i6aa_ d4gwoa_
d4igta1 d1pc3a_ d4mmpa1 d1o63a_ d2czla1 d2onsa2 d4mija1 d1eh3a_
d3skpa_ d1dtza1 d1ieja_ d1jnfa2 d1ce2a1 d1jnfa1 d1b1xa2 d1b1xa1
d1jw1a1 d1jw1a2 d1ryoa_ d1h76a1 d1h76a2 d1c4oa1 d2p6ra3 d2p6ra4
d1oywa2 d1oywa3 d1wrba1 d1w36b2 d1w36b1 d1c4oa2 d3bora_ d1t6na_
d2eyqa5 d2eyqa3 d2eyqa2 d2zu6a2 d1veca_ d1uaaa1 d1w36d1 d2g9na1
d1z3ix2 d2rb4a_ d1hv8a1 d1hv8a2 d1t5ia_ d1w36c2 d2j0sa2 d2j0sa1
d1fuka_ d3hjha1 d1z5za1 d1qdea_ d1z63a1 d1s2ma2 d1s2ma1 d1wp9a1
d1wp9a2 d1gm5a3 d1gm5a4 d3dina4 d3dina3 d1q0ua_ d1w36c1 d1z3ix1
d1nkta3 d1nkta4 d1uaaa2 d1w36d2 g1qhh.1 d2fwra2 d2fwra1 d2v8ta_
d3q3ma_ d1za0a1 d1mxra_ d2inca_ d1jk0a_ d2incb_ d1mhyb_ d3hf1b_
d1mtyb_ d1r2fa_ d2uw1a_ d1mhyd_ d3n37a_ d1afra_ d1oquc_ d1syya_
d1mtyd_ d2o1za_ d1uzra_ d1smsa_ d2fkza_ d1zuja1 d1s3qa1 d2fjca1
d1z6om1 d1veia_ d4dyya_ d1jiga_ d3gvya_ d2pyba_ d4cy9a1 d3bvfa1
d1r03a_ d2cf7a_ d1nf4a_ d1zs3a1 d4iwka_ d3ka8a_ d3np0x_ d1lb3a_
d4e6ka_ d1z6oa1 d2fzfa1 d4dyua_ d3ge4a_ d1jgca_ d4am4a_ d2z6ma_
d1ji5a_ d1vlga_ d1lkoa1 d1krqa_ d3qz3a_ d1o9ra_ d2ffxj_ d1euma_
d1yv1a1 d2v2pa_ d1j30a_ d4di0a_ d1dpsa_ d2cbia2 d2choa2 d1l8na1
d1oewa_ d2qzxa_ d2asia_ d1psoe_ d1miqa_ d1smra_ d2h6ta_ d1pfza_
d1izda_ d3hd8a_ d1j71a_ d1dpja_ d4aa9a_ d1t6ex_ g1b5f.1 g1lya.1
g1htr.1 d1qdma2 d1bxoa_ d1wkra_ d1tzsa_ d1ibqa_ d2qzwa_ d1ex4a2

d1c6va_ d1bdqa_ d3ggta_ d3ixoa_ d1mrxa_ d2haha_ d1tw7a_ d4njsa_
d1sgua_ d2p3da_ d1nsoa_ d3d3ta_ d2rkfa_ d1c6ya_ d2rspa_ d3ogpa_
d3ekwa_ d2o4na_ d2j9ja_ d1baia_ d3ggua_ d3lzua_ d4npua_ d4j55a_
d2r8na_ d2fxda_ d2p3ca_ d4dqea_ d3ttpa_ d3t3ca_ d2p3aa_ d2hc0a_
d2fdda_ d3mwsa_ d1h4ra3 d4yl8a1 d2al6a3 d1wgda1 d1wgga1 d4q5eb_
d1wjna1 d1se9a_ d3n3kb_ d1wz0a1 d1we6a1 d2m0xa_ d2gbka_ d3rt3b1
d1wh3a1 d1sifa_ d2ekec2 d4ii2b1 d4hk2a1 d1c3ta_ d1m94a_ d1wgha1
d1ttna1 d1j8ca_ d1wx8a1 d3v6cb1 d2ma9b_ d4i6lb1 d1bt0a_ d2jvca1
d1iyfa_ d2k1fa_ d1wxva1 d1wf9a1 d1t0ya_ d2io0b2 d1x1ma1 d2gbma_
d1zw7a1 d2m17a_ d1ud7a_ d1we7a1 d2lwpa_ d2gbja_ d1wjua1 d1v6ea1
d1v86a1 d4eewa_ d1wiaa1 d4icva_ d4gu2a_ d1wx7a1 d1wy8a1 d4hjka1
d2faza1 d1v5oa1 d3ruib_ d1eo6a_ d3waob_ d2r2qa1 d3m95a_ d3wana_
d2al3a1 d2cr5a1 d1wj4a1 d1s3sg_ d1epwa1 d1jx4a2 d3gv5b1 d1t94a2
d3bq0a2 d3h33a_ d1rwja_ d19hca_ d3bxua_ d3h4na_ d1i77a_ d3caoa_
d1j0pa_ d2ctha_ d1duwa_ d1ofwa_ d2e84a_ d1hh5a_ d1gyoa_ d3h34a_
d1dszb_ d1a6ya_ d3cbba_ d1ynwa1 d1r4ia_ d1hcqa_ d2hana_ d2hanb_
d2enva1 d1kb4b_ d2nllb_ d1lo1a_ d1um8a_ d1ixza_ d1qvra2 d1qvra3
d1qzma_ d1sxjc2 d1jr3d2 d2p65a_ d1g8pa_ d3hu3a3 d1sxjd2 d1njfa_
d1in4a2 d1sxja2 d1tuea1 d2ce7a2 d1w5sa2 d1sxjb2 d1d2na_ d1ny5a2
d1ofha_ d2gnoa2 d2a5yb3 d1fnna2 d1u0ja1 d1lv7a_ d1iqpa2 d2fnaa2
d1ixsb2 d4gita_ d1sxje2 d1g41a_ d2e3fa_ d1mm0a_ d2e3ea_ d2llda_
d2ny8x_ d2e3ga_ d2ny9x_ d2nz3a_ d3ia2a_ d1a88a_ d4dgqa1 d1hkha_
d1zoia_ d2k4ua_ d1hp2a_ d2lu9a_ d1baha_ d2meoa1 d1wm7a_ d1hlya_
d1n8ma_ d1jlza_ d1wpda_ d1c55a_ d1pvza_ d1wt7a_ d1rjia_ d1wz5a_
d1m2sa_ d1pjva_ d1c49a_ d1qkya_ d2ktca1 d1wmta_ d1du9a_ d1j5ja_
d1r1ga_ d2jcqa_ d1bbua1 d1e1oa1 d1c0aa1 d1b8aa1 d1l0wa1 d3a74a1
d1eova1 d1iyjb4 d1iyjb5 d1iyjb3 d1o7ia_ d4ipca1 d1x3ea_ d4apva_
d1jb7a1 d1jb7a2 d1jb7a3 d3kdfa_ d1ue1b_ d1wjja1 d3eiva_ d1xjva2
d1xjva1 d2ccza2 d1se8a_ d1l1oc_ d1jb7b_ d1qvca_ d1qzga_ d1miua4
d1miua5 d1miua3 d3g48a1 d1gd7a_ d1mkha_ d1pyba_ d1y14b1 d2nn6i1
d1i94l_ d2zjra2 d1smxa_ d2qamc2 d1hh2p1 d2r7da1 d1rl2a2 d1wfqa1
d3bzka4 d1x6oa2 d3a0ja_ d2j01d2 d2ja9a1 d4ql5a_ d1hr0w_ d1luza_
d2n3sa_ d2vnud1 d1h95a_ d1go3e1 d2nn6h1 d1ne3a_ d2mo1a_ d1iz6a2
d1jjga_ d1e3pa2 d2ix0a3 d2ix0a2 d2lxja_ d1ueba3 d1ueba2 d2z0sa1
d1u0la1 d2ix0a1 d2eifa2 d2je6i1 d3i2za1 d2c35b1 d2ahob2 d1t9ha1
d3i4oa_ d2qall1 d1jt8a_ d2nn6g1 d1g6pa_ d3cama_ d1i94q_ d1d7qa_
d2ba0a1 d2vnud2 d2vnud3 d1khia2 d2qalq1 d1wi5a1 d1xtda2 d1ny4a_
d1dgsa2 d1ckma1 d1p16a1 d1x9na2 d1xwdc1 d3kj4a_ d1ozna_ d1w8aa_
d2ifga3 d1xkua_ d1ohea1 d1ohea2 d2f4da_ d1rxda_ d2hxpa_ d1xria_
d1fpza_ d3ezza_ d4y2ea_ d1i9sa_ d1c1la_ d4lbqa_ d1a78a_ d1slta_
d4no4a_ d3zsja_ d3duia_ d1g86a_ d1is3a_ d1qmja_ d1hlca_ d1tdha3
d1q3ba3 d1ee8a3 d1k82a3 g1qmo.1 g1len.1 d3zyra_ d1fnya_ d1n47a_
g2ltn.1 d1avba_ d3usua_ d1dhkb_ d1f9ka_ d1ukga_ d1g8wa_ d1g9fa_
d1wbfa_ d3wcsa_ d1g7ya_ g1loe.1 d1mvqa_ d1hqla_ d1v6ia_ d1dbna_

d1qnwa_ d1fx5a_ d4l8qa_ d1ioaa_ d3n35a_ d3ubra_ d1qo8a1 d1d4ca1
d2k3va_ d1qdba_ d1m1qa_ d2j7aa_ d1h21a_ d1ogyb_ d4fasa_ d1jnia_
d1oaha_ d1sp3a_ d1eysc_ d2h0ba_ d2erfa_ d1h30a2 d1h30a1 d3mw4a1
d2r1ba_ d3v64a1 d1d2sa_ d3bopa1 d3mw3a1 d1okqa1 d1okqa2 d1wioa4
d1ccza2 d1vcaa1 d1dr9a2 d1ic1a1 d2d9qb1 d2nxyb2 d1adqa1 d1fnga1
d1uvqb1 d1zs8a1 d2vola1 d2vola2 d1mfbl2 d2jelh2 d1uwel2 d3qq4b1
d1kjvb_ d1k5na1 d1osph2 d4odhl2 d1ow0a1 d1ow0a2 d1bfob2 d3l9ra2
d3jtth1 d2fath2 d4elkb2 d1fn4b2 d2xfxb_ d1pg7x2 d1cqka_ d4k71b2
d1lk2a1 d1eapb2 d4lfhg2 d1i4fa1 d2nw2a2 d1kfah2 d1mhca1 d1h3ua1
d1wejh2 d1h0db2 d1a5fh2 d1kcvh2 d1mjuh2 d1qqda1 d1kcrh2 d1k8ia1
d3huja2 d2nnab1 d1nfdf2 d3mffb2 d1s9va1 d1lk3h2 d1q0xl2 d1fv1b1
d1neza1 d4rfeb2 d1r3ha1 d1a6tb2 d1kjva1 d1pz5b2 d1onqa1 d2bc4a2
d1de4a1 d1k8da1 d1tcra2 d1dn0b2 d1nfde2 d2xwtb2 d1bfoa2 d1mexh2
d1iakb1 d1ypzf2 d4f7ea2 d1q72l2 d2fbjh2 d1fe8h2 d1clzh2 d1fngb1
d1uvqa1 d1vgka1 d1c16a1 d4j12a2 d4x99b2 d1igtb3 d1je6a1 d1q72h2
d1nldh2 d1teth2 d2hmic2 d1i1ca2 d1i1ca1 d1k8ib1 d1q0yh2 d1kfal2
d3frua1 d1pg7w2 d1u58a1 d1igyb4 d1igyb3 d1hyrc1 d1klub1 d1deeb2
d1c12b2 d2bc4b2 d2ig2h2 d1kcvl2 d3wn5b1 d1lk2b_ d1lk3l2 d1mjul2
d1sm3h2 d1q0xh2 d1zvsa1 d1ydpa1 d1u8ib2 d1ypze2 d1kcuh2 d1op9a_
d1y0la1 d1etzb1 d1egjl1 d2gsib1 d1nj9a1 d4jfhd1 d1aqkl1 d1t2ja_
d1lo4h1 d1zvya1 d3v4vl1 d4enec1 d1kxtb_ d5e8ea1 d2pxya_ d4weud_
d1xiwc_ d2esvd1 d1qnzh_ d1ogae1 d1f4xh1 d4n1hb_ d3ezjb1 d3mlwl1
d1kfah1 d1etza1 d1b4jh1 d3cfic_ d4w68a_ d2arja1 d1ek3a_ d2ok0l1
d1vera_ d1uwgh1 d1blnb1 d1fpth1 d1rzga1 d2giya1 d4m3kb1 d1nc2a1
d2nyyd1 d1ncna_ d1fbih1 d5dxwa_ d4fqqa1 d1dqdh1 d1jfql1 d1kelh1
d1faih1 d1frgl1 d1figl1 d4m6nl1 d1fo0a_ d4hxbl1 d3b5ga_ d3quyd1
d2h9gb1 d1i8kb_ d2dd8h1 d2ck0h1 d1eaja_ d4w6wb1 d2a6ib3 d1t66d1
d2e27h_ d1ob1b1 d1f11b1 d2v7na1 d1ad0a1 d2xt1b1 d1pskh1 d4i13b1
d2op4l1 d1a0ql1 d1uwel1 d1za3a1 d1ncbl1 d4uu9a_ d1bm3h1 d1cloh1
d1nqba2 d1cf8h1 d4y5va_ d1mhph1 d5anma1 d4cdgc1 d4ldeb_ d1y0lb1
d2pcpb1 d1z3gl1 d1dfbl1 d1fh5h1 d3g5xa1 d1tzib1 d4dn3l1 d3zhka1
d2b1hh1 d2uyla1 d1f3dh1 d1nj9b1 d2agjh1 d2h1pl1 d1q72h1 d8fabb1
d1ndmb1 d1rjca1 d4u6hb1 d3upca_ d2fbjl1 d1osph1 d1tvda_ d2cjvh_
d4fhbd1 d1bd2d1 d1rz7h1 d3nn8a_ d1knoa1 d1nfda1 d2bmkb1 d1dqll_
d1igyb1 d1igfl1 d12e8h1 d3utza1 d1nc2b1 d1rihh1 d4mwfb1 d4jvpa1
d1mieh1 d1rhfa1 d1fgnl1 d1sbsh1 d1lp9e1 d1dn0a1 d4lqfl1 d1iaih1
d1kgcd1 d1mh5b1 d1gpol1 d2g60h3 d1f8tl1 d2jixd1 d4grwh_ d1fo0b_
d1bfob1 d1axsa1 d1u9ka_ d1nmbh_ d1h8na1 d1h8na2 d1jguh1 d4eura1
d3sdyl1 d35c8h1 d1w72l1 d1mvub_ d1i9rh1 d1ob1a1 d4wenb_ d1yeeh1
d3ietc1 d1f58h1 d1ad0b1 d1mqkl_ d1nbvh1 d4b50a_ d3r8bb_ d1bafl1
d1mamh1 d4rgne1 d2gsgb_ d35c8l1 d1bafh1 d1ai1h1 d2pcpa1 d2fath1
d2x1pa_ d1orsb1 d1ibgl1 d1shma_ d1a2yb_ d1u0qa_ d2coqa_ d1hilb1
d1qfwi_ d1jv5b_ d1fn4b1 d5e0qa_ d1nsnh1 d1plgl1 d4ocxl1 d1kb5h1
d1rzfh1 d1pg7x1 d2fx7h1 d2oi9c_ d1l7il1 d3eyva1 d1d5il1 d4ffyl_

d1tetl1 d1a6wh_ d1ac6a_ d3dvfa_ d1nfde1 d1mimh1 d1ri8a1 d1jhlh_
d3dura_ d1fskb1 d1f97a1 d3so3b1 d2ak4d1 d1lp9f1 d4z7wf1 d1l7th1
d4y5ya_ d4x7ec1 d1emth1 d1ikfl1 d1nlbh1 d4utal1 d1bjma1 d1eapb1
d1u6ah1 d2dtgd1 d1kj2b_ d1xeda1 d1c1eh1 d1fgvh_ d2bnub1 d2fb4h1
d2dtgb1 d1ktrh_ d1jpth1 d1qkzl1 d4hepg_ d2atpb1 d1flrh1 d2hrpl1
d1mvfa_ d1iaim1 d3k80a1 d2jb5h1 d3zkxc_ d4grwe_ d1it9h1 d1rhhb1
d3w9ec_ d1e4wh1 d1sy6h1 d2j6eh1 d3qxta_ d1jglh1 d1p7ka1 d1tzhb1
d4n8pa1 d1gafl1 d2jelh1 d1wioa2 d4i0cc1 d1cicb1 d1lmka2 d1lmka1
d1iqwh1 d1orqb1 d1ejoh1 d1jnlh1 d4zg1a_ d43c9b_ d1ggbl1 d1wejh1
d3q6fa1 d1f3dj1 d3c6la1 d1bqlh1 d3p9wb_ d1a3rl1 d1h0db1 d2uzil_
d1eo8l1 d1qfwl_ d1n7ml1 d1igja1 d1c5ch1 d2a9mh_ d1rmfh1 d1fj1b1
d4heme_ d1ccza1 d1a5fh1 d1ogad1 d1dlfh_ d1qa9b_ d1vgel1 d3qskb_
d1sjxa_ d4ozfg1 d1sq2n_ d1qd0a_ d1kcvh1 d1c5db1 d3qcul1 d8faba1
d3bn9d1 d1mjuh1 d4y8dc_ d7fabh1 d3dvgb1 d1lo0l1 d1a6tb1 d1gigh1
d1kcrh1 d2hwzl1 d1yjdc1 d3clfl1 d4krna_ d1dl7l_ d1e6ol1 d1mexh1
d3r0ma_ d4kkna_ d1fnsl1 d1oaqh_ d1rzia1 d1ucbh1 d1ncwl1 d4b41a_
d1nfdf1 d2mpah1 d2d7tl1 d4grwf_ d4b5ea_ d1lk3h1 d3u79a_ d4u3xa_
d1ghfl1 d1bvkb_ d1mcww1 d1b88a_ d1jrhh1 d32c2b1 d4wemb_ d1dsfh_
d1op3h1 d1cel1l1 d1iqda1 d1qoka1 d1qoka2 d1qfuh1 d2crya1 d1ay1h1
d1oaql_ d3ab0b_ d1rurl1 d1t4kb1 d1bz7b1 d1n0xh1 d1forl1 d1ucbl1
d4w6yb_ d43c9a_ d2aabl1 d1tzia1 d4n1ca_ d1k6qh1 d3g9ab_ d4tvsa1
d1igmh_ d1pz5b1 d3dx9a1 d3wxwh_ d1nakh1 d1kcul1 d3eaka_ d4qlra1
d1cr9l1 d1d9ka_ d1f4xl1 d1deea1 d2i27n2 d1c12a1 d2orbl1 d1j8hd1
d1mcph1 d1c5da1 d1kenh1 d1bd2e1 d1h5ba_ d4x7fc1 d3upaa_ d1t3fa1
d1indh1 d2dtgc1 d4hc1l1 d1zv5a1 d4wgvb_ d1uwgl1 d4f37k1 d1aqkh1
d1vesa_ d3zkqd_ d1fptl1 d1lo4l1 d1knob1 d3rrqa_ d1ct8b1 d4orzc_
d1mlbb1 d4hgkc1 d1dqdl1 d1bogb1 d4ozhe1 d1dr9a1 d1i7za1 d1dn0b1
d1kell1 d1rzib1 d1ypzf1 d1wtla_ d1hkfa_ d1fail1 d1figh1 d2vyre_
d1sjva_ d1ohqa_ d1bfoa1 d1pkqb1 d1mjul1 d1b4jl1 d1nsnl1 d2arjb1
d1p4bh_ d4aj0a_ d3nn8b_ d2rcja1 d1f2xk_ d4v1dd_ d1adqh1 d2eksa_
d4k3ga_ d1jfqh1 d1uweh1 d1frgh1 d4gftb_ d1uz8b1 d4n1ea_ d1bm3l1
d1clol1 d3mcka1 d2vq1a1 d1ae6h1 d4aq1b1 d1uj3b1 d1nbqa1 d1fh5l1
d2ck0l1 d2ywza_ d1q72l1 d4nbxb_ d1hila1 d4hzlb1 d2j88l1 d1zmya1
d1cd0a_ d1a4ka1 d1pskl1 d1a0qh1 d4krmb_ d1ncbh1 d2fbjh1 d1j8he1
d1i3ua_ d1cf8l1 d1fe8h1 d2p1ya1 d1eeqa_ d2x89a_ d1ol0a_ d2r0lh1
d1ub5a1 d1dfbh1 d3eyvb1 d4jdvb1 d4p2cg_ d2gsia1 d2agjl1 d1u3ha1
d2h1ph1 d1rihl1 d1fskc1 d1boga1 d2a77l1 d1ospl1 d1qfoa_ d3dwta_
d2fx7l1 d1rz7l1 d3stba1 d1pkqa1 d1b6da1 d1dzba2 d1dzba1 d1kj2a_
d4l1ha_ d1rzgb1 d1dqlh_ d12e8l1 d1miel1 d1jmaa_ d1sbsl1 d1fgnh1
d1f58l1 d1mqkh_ d1ndgb1 d3qegl1 d1iail1 d2aw2a1 d1tqbc1 d3fo2a1
d4dkaa_ d1f8th1 d2g60l1 d2ywya_ d1uj3a1 d1yc7a_ d3bd3a_ d4w6xb1
d1nmbl_ d1i8ka_ d1rjlb1 d1ypze1 d1p7kb1 d1kxqe_ d1gpoh1 d2z4qa1
d1w72h1 d1i9rl1 d4qgya1 d4xt1c_ d2qqnh1 d1cica1 d3c08h1 d2cd0a_
d2bdnh1 d1hnga1 d1maml1 d1nldh1 d1cr9h1 d4lcih1 d3bkyh1 d3qg7l1

d1ai1l1 d1kxvc_ d4iosd_ d1l7ih1 d2x1qa_ d1ub6b1 d3k74b_ d1ibgh1
d1teth1 d1igya1 d1hxma1 d2x6ma_ d2e7la_ d5e03a_ d1qfwm_ d2r0kh1
d1igjb1 d1rurh1 d1plgh1 d1mj8h1 d1yuhb1 d3fmgl1 d1rzfl1 d1emtl1
d1ad9b1 d1igtb1 d1nlbl1 d1d5ih1 d3k7ua_ d4ffyh_ d1bwwa_ d3dvga1
d1a6ta1 d1fgvl_ d4qxgl1 d1miml1 d1jhll_ d2hffb1 d2hh0l1 d1qkzh1
d1l7tl1 d1afvh1 d1iaii1 d2cjuh_ d1um5h1 d2ghwb2 d2ghwb1 d1kgce1
d2fgwh1 d1ikfh1 d1dvfd_ d2i24n1 d2xtjb_ d1za3b1 d2yc1a_ d2hh0h1
d2x1oa_ d1bvka_ d1rjla1 d5e7ba1 d1igta1 d1il1b1 d2fb4l1 d1mvua_
d1jptl1 d32c2a1 d1fvca_ d1iqdb1 d2hrph1 d2p4ab_ d4qo1a_ d1p2cb1
d1wt5c_ d1t4ka1 d5c1mb_ d1it9l1 d1bz7a1 d1b2wh1 d1e4wl1 d1jnll1
d1yedb1 d1za6b1 d3u7aa_ d1igfh1 d3gnml1 d1orsa1 d1wejl1 d1a2ya_
d1hxmb1 d1gafh1 d2j4wh1 d1pz5a1 d1j05a_ d1a3rh1 d4fqll1 d1qfwh_
d1eo8h1 d1n7mh1 d4s11a_ d1kegh1 d5da4a_ d1iqwl1 d3whxb1 d1vgeh1
d1deeb1 d1c12b1 d1t2ql1 d1ad9a1 d3gsnb1 d3b2uc1 d1ggbh1 d1t3fb1
d1nakl1 d4ocmc_ d1lila1 d2v7hb3 d2esve1 d1mcoh1 d2qr0b1 d2q3aa_
d1ct8a1 d1kcrl1 d2pxyb_ d2uzih_ d5c3ld_ d3durb_ d1c5cl1 d2ak4e1
d1dl7h_ d1xiwd_ d1mlba1 d1rmfl1 d1i7zb1 d1dlfl_ d2xa3a_ d5bq7b1
d1dqqb1 d3h42l1 d1egjh1 d1kcvl1 d1fe8l1 d2j6el1 d1f3rb2 d1lk3l1
d1sm3h1 d1q0xh1 d1ghfh1 d7fabl1 d1igch1 d1lo0h1 d4idla_ d5c2bl_
d1jrhl1 d1il1a1 d3zhda1 d4kfzc_ d1b0wa_ d1e6oh1 d1mexl1 d4krpb_
d1fnsh1 d1ncwh1 d4hgma_ d4eigb1 d4rzca_ d2mpal1 d4y5xa_ d5en2b1
d3lrga1 d4ej1c1 d1rhha1 d1j05b_ d1forh1 d1q9wb1 d2wzpd_ d2aq2a1
d1h0da1 d3revb1 d2ij0c1 d1tzha1 d4m7jl1 d1k6ql1 d3b9kc1 d1igml_
d3qeua1 d1dsfl_ d1ce1h1 d1kcuh1 d1zvha1 d1zvoa1 d1tqbb1 d1ay1l1
d2qscl1 d2cgrh1 d1nl0h1 d1kenl1 d3eo0a1 d4n9ob1 d1n0xl1 d3k1kc_
d1c4qa_ d2wv6d_ d1prtf_ d1ltrd_ d4l63b1 d1r4pb_ d1prtd_ d1tl0f_
d2bosa_ d1prtb1 d3chbd_ d2aq2b1 d1ck1a1 d1enfa1 d1i4pa1 d1v1oa1
d1et9a1 d1esfa1 d2g9hd1 d4ohja1 d1klud1 d1bxta1 d1eu3a1 d1ty0a1
d1fnua1 d2cqva1 d1fhga_ d2k1ma_ d2dl2a1 d1biha3 d1biha1 d1biha4
d3qp3a1 d1g1ca_ d1gxea_ d1cvsc2 d1cvsc1 d1xmwa2 d1m4ka2 d2otpa2
d1biha2 d1rhfa2 d2fcba2 d2fcba1 d1wwbx_ d1gsma2 d1epfa1 d1epfa2
d2dava1 d1waaa_ d1f97a2 d1x44a1 d1vcaa2 d1fnla2 d1fnla1 d3b5ha2
d3h8na2 d3h8na1 d1jbja1 d1jbja2 d1pd6a_ d1f2qa2 d1f2qa1 d3cx2a_
d1nbqa2 d3b5ha1 d1n26a1 d1xiwb_ d1xaua_ d1fltx_ d2ifga1 d1olla1
d1olla2 d1cs6a4 d1cs6a3 d1cs6a2 d1cs6a1 d2gw5a2 d1sy6a1 d1xiwa_
d1ucta2 d1ucta1 d1ry7b2 d1gl4b_ d1qz1a3 d1olza1 d2c9aa1 d1wwca_

**Family-57 (c.26.1.3)**

**Positive dataset contains 6 protein sequences.**

d3rbaa_ d2qtra_ d3l92a_ d2h29a_ d1vlha_ d4ruka_

**Negative dataset contains 3978 protein sequences.**

d1m32a_ d1ibja_ d1c7na_ d1iuga_ d4q75a_ d2bkwa1 d1t3ia_ d1n8pa_
d1qz9a_ d1jf9a_ d1eg5a_ d3kgwa_ d1cl1a_ d2ctza1 d1vjoa_ d2rfva_
d1gc0a_ d1cs1a_ d1d2fa_ d2ch1a1 d1qgna_ d1pffa_ d1elua_ d4nmwa_
d2bnma2 d1xkla_ d2wfla_ d3i2ka1 d1lnsa3 d1mpxa2 d1xkta1 d2hfka_

d5d3ka_ d2cfba1 d1dfoa_ d1o69a_ d1bj4a_ d3k28a_ d2bwpa_ d3lg0a_
d2fnua_ d3ou5a_ d1z7da1 d1b9ha_ d1iaya_ d4msoa_ d1bs0a_ d1s0aa_
d1sffa_ d3tqxa_ d1mdoa_ d3qboa_ d3pgya_ d1bjna_ d2dkja_ d1ejia_
d1vefa1 d4wxga_ d3bs8a_ d3gbxa1 d1w23a_ d1sefa_ d1sfna_ d1sq4a_
d1r0ka3 d1j5pa3 d1vkoa2 d1vm6a2 d3qvsa2 d1gr0a2 d3lgia_ d3i77a_
d2z9ia2 d4nsva_ d1lcya2 d4jcna_ d1agja_ d1os8a_ d1hpga_ d1p3ca_
d1l1ja_ d1qtfa_ d1f56a_ d1bypa_ d1azca_ d1ws8a_ d3u25a_ d1cuoa_
d1id2a_ d1rkra_ d2iaac_ d4dp1x_ d1nwpa_ d2gima_ d2i7sa_ d1qhqa_
d3ehbb1 d1fftb1 d1gyca3 d1hfua2 d1kbva1 d1kbva2 d1sdda1 d1sdda2
d1gyca1 d1gyca2 d1mzya1 d2bw4a1 d1aoza1 d1aoza3 d1aoza2 d1kyaa2
d1kyaa1 d1mzya2 d4e9sa1 d1sddb1 d1sddb2 d1hfua1 d1hfua3 d1oe1a2
d1oe1a1 d1v10a2 d1v10a3 d1v10a1 d2w0pa_ d2diaa1 d2e9ia2 d2e9ja1
d2dj4a1 d2eeba1 d2diba1 d2dmba1 d2dmca1 d2d7oa1 d2ee6a1 d2d7pa1
d2eeaa1 d2di8a1 d1wlha1 d2d7na1 d2nqca_ d2d7ma1 d2dica1 d3cnka_
d2k3ta1 d2eeca1 d1qfha1 d2bp3a1 d1v05a_ d2ee9a1 d2eeda1 d2di9a1
d1a3qa1 d1iknc_ d3brda1 d1oy3c_ d1imhc1 d1gjia1 d1zk9a1 d2cxka1
d2bema_ d1pama1 d1ea9c1 d1qhoa1 d2fhfa2 d2fhfa1 d1k3ia1 d1j0ha1
d1cxla1 d2bhua1 d1edqa1 d1f1sa2 d1ut9a2 d1gvia1 d1txka1 d1zx5a1
d2phla2 d2phla1 d1fxza2 d1fxza1 d1uija1 g1dgw.1 d2v09a_ d1uika2
d1uika1 d1dgwa_ d2pa7a1 d1wlta1 d1nxma_ d2c0za1 d1ds6b_ d1fstb_
d1ex0a1 d1g0da1 d1ju8a_ d1eixa_ d3exra_ d2ffca1 d2fdsa1 d3n3ma1
d2czda_ d1q6oa_ d2yyua_ d1vqta1 d1dbta_ d3ldva1 d2bjca1 d2hsga1
d1qpza1 d1efaa1 d1ydwa2 d2nvwa2 d1h6da2 d1zh8a2 d1qkia2 d1xeaa2
d1tlta2 d1au7a2 d1ic8a2 d3gpdg2 d2q49a2 d1hdgo2 d2g17a2 d2cvoa2
d2czca1 d3pwka2 d1obfo2 d1i32a2 d1zyao2 d2pkqo2 d1b7go2 d2hjsa2
d1cf2o2 d1vkna2 d1nvmb2 d2trcp_ d2m9wa1 d3dfxa_ d2msya_ d2djoa1
d2r5za_ d1au7a1 d1f43a_ d2lmda1 d1akhb_ d1s7ea1 d3nara_ d1zq3p1
d2craa1 d1mija_ d4uuta_ d3a01a_ d1x58a1 d3rkqa1 d3cmya1 d2cqxa1
d2da6a1 d1e3oc1 d1fjla_ d1mh3a1 d1ig7a_ d1x41a1 d1wh7a1 d1gt0c1
d2mw8a_ d1lfup_ d1x2ma1 d1b72a_ d1wi3a1 d1jgga_ d1wjha1 d1x2na1
d1mnmc_ d2cuea1 d3k2aa_ d2hosa_ d2jwta2 d1pufa_ d1octc1 d2hi3a_
d1b8ia_ d4j19a_ d3lnqa_ d1wh5a1 d1b8ib_ d1fexa_ d2cqra1 d1guua_
d1ug2a1 d2cqqa1 d2ajea1 d4a69c_ d1h8ac1 d2crga1 d2iw5b1 d1xc5a1
d2cu7a1 d1wgxa1 d2cjja1 d2ckxa1 d1gvda_ d1hcra_ d1gdta1 d1u78a2
d6paxa1 d6paxa2 d1k78a1 d1k78a2 d1bl0a2 d1d5ya1 d1d5ya2 d1gyta2
d3lv8a_ d1s3ga1 d1zaka1 d1s4qa_ d3tvta2 d1uf9a_ d1khta_ d1ckea_
d1osna_ d1p5zb_ d3cm0a_ d1tmka_ d1jaga_ d3h86a_ d1t0hb_ d1nn5a_
d2h92a_ d3kb2a1 d2c95a1 d1yj5a2 d1kgda_ d1ly1a_ d2ak3a1 d1vyva2
d1deka_ d2grja1 d1p3ja1 d1y63a_ d1e4ya1 d1s96a_ d3iija1 d1lvga_
d1nksa_ d1vyua2 d4e22a_ d1qf9a_ d1jjva_ d1q3ta_ d1ki9a_ d1lw7a2
d1teva_ d1kjwa2 d1gsia_ d1x6va3 d1g3ma_ d1j99a_ d1t8ta1 d1nsta_
d1zrha_ d1ls6a_ d2a3ra_ d1aqua_ d2zptx_ d3ckla_ d1texa_ d1q44a_
d2gwha_ d1z0ja1 d3umra2 d3gj0a1 d2gaoa1 d1yzga1 d1x3sa1 d1g7sa4
d1puja_ d1udxa2 d1kk1a3 d2o52a_ d1jwyb_ d1zcaa2 d1zcba2 d2fv8a1

d2cjwa_ d3dz8a1 d1xzpa2 d1vg8a_ d4qxaa_ d2ew1a1 d1x1ra1 d2bcgy_
d3doea1 d3cona_ d3rega_ d3wyga_ d1upta_ d2b6ha1 d1zd9a1 d1lnza2
d1r5ba3 d1n0ua2 d1r2qa_ d2f9la1 d2rhda_ d3q3jb_ d3q85a_ d1efma1
d1r8qa_ d2gf9a1 d2akab1 d1ek0a_ d1z08a1 d2gcoa1 d2dy1a2 d4g01b_
d2cxxa1 d1svia_ d3q72a_ d1ni3a1 d4ac9a4 d2erxa1 d2bmea_ d1wxqa1
d2gf0a1 d4ldja1 d1zunb3 d1d5ca_ d4lv5b_ d1efca3 d1kmqa_ d2g6ba1
d3lrpa_ d1tada2 d2y8ea_ d4rd4a1 d1z06a1 d2heia_ d3a58b_ d1u0la2
d1d2ea3 d1zj6a1 d2ocba_ d3rwoa_ d1mr3f_ d2e9sa1 d4u5xa1 d2iwra_
d2fn4a1 d3bwdd_ d2nzja1 d2atva1 d2ksqa_ d3l43a_ d1mkya2 d1mkya1
d1wmsa_ d1jnya3 d2rmka_ d1f6ba_ d1f60a3 d1gota2 d1jala1 d2fh5b1
d1g16a_ d2wblc_ d1wf3a1 d1f5na2 d1t9ha2 d1z6xa1 d1z0aa1 d2fg5a1
d2a5ja1 d2bcjq2 d1s0ua3 d1ky3a_ d4y0va_ d4lhwa_ d2qmea1 d2atxa1
d1egaa1 d1ryha_ d1moza_ d1nrjb_ d2erya1 d3bfka_ d1puia_ d1fzqa_
d3j3az1 d3t5ga_ d1shza2 d3defa_ d2f7sa1 d3raba_ d3tsoa_ d3aq4a_
d2al7a1 d3sfva1 d3bc1a_ d2ncda_ d1sdma_ d3h4sa1 d2heha_ d2grya_
g3kin.1 d1kk8a2 d1br2a2 d1goja_ d1f9va_ d2mysa2 d1lkxa_ d3t0qa_
d4db1a1 d3gbja_ d1ry6a_ d2ovga_ d1sq8a_ d1rzsa_ d3kz3a_ d3orca_
d1k8ka2 d1k8ka1 d3uleb1 d3i33a2 d3i33a1 d1bupa1 d1dkgd2 d1dkgd1
d4ci6a2 d1jcfa1 d4a2ba2 d2fxua1 d1yaga2 d1yaga1 d4cbua2 d1bupa2
d3vrda1 d3vrda2 d1fcdc2 d1fcdc1 d1h1oa2 d1m70a2 d1m70a1 d1ig8a1
d1czan2 d1czan3 d1czan1 d1ig8a2 d1czan4 d1bg3a4 d1bg3a3 d1bg3a2
d1bg3a1 d1nira1 d1zc6a1 d1zbsa1 d1zbsa2 d1zc6a2 d2ch5a2 d2ch5a1
d1zxoa2 d1zxoa1 d1hroa_ d4gedb_ d3zooa_ d1mz4a_ d1gu2a_ d1f1fa_
d1dvva_ d1c6ra_ d1c75a_ d1e29a_ d1gdva_ d1cnoa_ d1h32b_ d1co6a_
d2bh4x_ d2aiua_ d5cytr_ d1jdla_ d1i8oa_ d2zboa_ d3dmia_ d1ls9a_
d1wvec_ d2yk3a_ d3ph2b_ d1dw0a_ d5aura_ d1ql3a_ d1qn2a_ d1f1ca_
d2nqya_ d1h4ga_ d1hixa_ d1oa2a_ d1olra_ d3b7ma_ d4h7ma1 d3wp3a_
d2vgda_ d1igoa_ d3zsea_ d2nlra_ d3wx5a_ d1oa3a_ d1pvxa_ d1m4wa_
d1f5ja_ d1oa4a_ d1ks5a_ d4ixla1 d3sita1 d1evsa_ d1cnt1_ d1pvhb_
d1z7ca_ d1huwa_ d1bgea_ d1rhga_ d1f6fa_ d1f45b_ d1i1rb_ d1axia_
d1etea_ d3tgxb_ d1scfa_ d2z3qa1 d3qb1a_ d1hula_ d1m48a_ d4nkqb_
d1eera_ d1v7mv_ d5e4ea_ d2o27a_ d3b5ka_ d2psma1 d4zf7a_ d3uf5a_
d4x51a_ d1d9ca_ d4ypgc1 d3oq3a_ d1lqsl_ d1n1fa_ d1au1a_ d1wu3i_
d3besl_ d3ux9a_ d3sw2a_ d1moxc_ d1dx5i3 d1edmb_ d1autl2 d1autl1
d1dval1 d1szba2 d1kigl_ d2y5fl_ d1ijqa2 d1gk5a_ d1g1sa2 d1nt0a3
d1xdtr_ d1lmja2 d1lmja1 d1nzia2 d2wphe_ d1klil_ d1pfxl2 d1pfxl1
d1p9ja_ d1g1ta2 d1f7ea_ d4yzub_ d2mgpa1 d1b9wa1 d1b9wa2 d2mgpa2
d1ob1c2 d1n1ia2 d1n1ia1 d2npra2 d2npra1 d1m1xb5 d2y5ia_ d1qlsa_
d1psra_ d3n22a_ d2egda_ d4ggfc_ d2wcea_ d2luca_ d4aqia_ d3nsia_
d2lp3a_ d1j55a_ d1a4pa_ d1nsha_ d1exra_ d1tiza1 d1nyaa_ d1g8ia_
d1ggwa_ d1wdcc_ d3fwba_ d1ggza_ d2o5ga_ d1ctaa_ d1m45a_ d3i5gc_
d1sl8a_ d1fpwa_ d2scpa_ d2roaa_ d4aqra_ d3pn7b_ d1br1b_ d2mysb_
d1s6ca_ d1bjfa_ d2obha_ d1jfka_ d1jf0a_ d1qv0a_ d1lkja_ d2mlfc_
d2m97a_ d2d8na_ d4by5a_ d1jbaa_ d4nqga_ d1wdcb_ d2mysc_ d1j7qa_

d1s6ia_ d1uhka2 d1r2ua_ d1dtla_ d3kf9a_ d3pn7c_ d1qjta_ d1c07a_
d5d67d_ d1tuza_ d1h8ba_ d1eg3a1 d1eg3a2 d2zkmx1 d1snla_ d1sfsa_
d1wkya2 d1uuqa_ d3wh9a_ d4m1ra_ d1xyza_ d4xuya_ d2je8a5 d4bf7a1
d3cuia_ d1ur4a_ d1qw9a2 d1odza_ d2vzsa5 d1bqca_ d2x2ya1 d1hjqa_
d1qnra_ d4y7ea1 d1vjza_ d4k68a_ d2v3ga_ d4hpga_ d1nofa2 d1nq6a_
d1h4pa_ d1aq0a_ d3ziza_ d4bs0a_ d1ecea_ d1h1na_ d1ta3b_ d1w91a2
d1i1wa_ d1us3a2 d1egza_ d7a3ha_ d1tvna1 d3nyda1 d1ghsa_ d2cyga1
d4xv0a_ d2c8na2 d1w32a_ d3emca_ d1v6ya1 d1foba_ d2uwfa1 d2fgla_
d3niya1 d2c0ha1 d1rh9a1 d1yq2a5 d1szna2 d1fa2a_ d1pama4 d5do8a1
d1ea9c3 d1hx0a2 d2fhfa5 d1qhoa4 d1avaa2 d1m53a2 d4m8ua1 d2gjpa2
d1lwha2 d4howa1 d1ktba2 d1j0ha3 d3vm5a1 d2x1ia_ d1wzaa2 d1uasa2
d2diea1 d1mxga2 d1ht6a2 d1iv8a2 d5cpsa_ d1ud2a2 d1zjaa1 d3gbea1
d1kwga2 d1wdpa_ d1gcya2 d1g5aa2 d1hvxa2 d3dhpa2 d2bhua3 d4do4a1
d3qgva1 d1e43a2 d1g94a2 d2d3na2 d1gjwa2 d1gvia3 d1x1na1 d1r7aa2
d1tz7a1 d4c7ga2 d2gjxa1 d1jaka1 d1yhta1 d4toqa_ d1wb0a1 d1w9pa1
d1eoka_ d1itxa1 d1kfwa1 d1goia2 d1edqa2 d2gsja_ d1hjxa1 d1jnda1
d1vf8a1 d3wdpp1 d3wq5a_ d1gnxa_ d4gxpa_ d4hz8a_ d1e4mm_ d3aiua_
d1wcga1 d1qvba_ d3ahxa_ d1v02a_ d2dgaa_ d1pbga_ d1vffa1 d1qoxa_
d4ha4a_ d1mixa2 d1e5wa2 d1isna2 d2al6b2 d1g4ma1 d2y8ga_ d1xkea1
d2y8fa_ d2v76a_ d1wj1a1 d1aqca_ d1qqga2 d1qqga1 d1ntva_ d1n3ha_
d4dbba_ d3f0wa1 d1p3ra_ d1wgua1 d2cy4a1 d3hqca_ d1wvha1 d1xr0b1
d1wgqa1 d1v89a1 d2coda1 d1zc3b_ d1wg7a1 d1u5ea1 d1v61a1 d2coca1
d2ys1a1 d1v5pa1 d1ntya2 d1wjma1 d1wi1a1 d1u29a_ d1xx0a1 d1unqa_
d2zkmx3 d1fhoa_ d1v88a1 d2adza1 d1x1fa1 d1p6sa_ d2coaa1 d1btka_
d1v5ma1 d1faoa_ d2x18a_ d1dyna_ d1x1ga1 d4kaxb_ d2j59m1 d1ki1b2
d1xcga2 d1txda2 d1u5da1 d1dbha2 d1w1ha_ d1upqa_ d1v5ua1 d1foea2
d2fjla1 d1eaza_ d2dfka2 d2cofa1 d1kz7a2 d9ldta1 d2ewda3 d1up7a1
d2dfda1 d1s6ya1 d1vjta1 d7mdha1 d1hyea1 d1t2da1 d1ojsa3 d1ldna1
d1hyha1 d1civa1 d2a92a1 d1ez4a1 d1i0za1 d1y6ja1 d1gv0a1 d1ur5a1
d1llda1 d1obba1 d1v8ba1 d1gdha1 d1l7da1 d2dlda1 d1mx3a1 d1ygya1
d1qp8a1 d1li4a1 d1j4aa1 d1npya1 d3t4ea2 d1a4ia1 d1o0sa1 d2hk9a2
d2egga2 d1b26a1 d1bvua1 d1gq2a1 d1c1da1 d1leha1 d1gtma1 d1euza1
d1nvta1 d3dona2 d1gpja2 d1vi2a1 d1p77a1 d1nyta1 d1b0aa1 d1vl6a1
d1v9la1 d1txga2 d3f47a1 d1mv8a2 d2pv7a2 d1xc2a3 d2ahra2 d4xiya1
d2f1ka2 d1pgja2 d1i36a2 d1np3a2 d1lj8a4 d1teaa3 d1qmga2 d2i76a2
d2g5ca2 d1wdka3 d1dlja2 d3cuma3 d1kola2 d1f8fa2 d1qora2 d1jqba2
d1vj0a2 d1cdoa2 d1gu7a2 d1d1ta2 d1xa0a2 d2dm6a2 d1jvba2 d1llua2
d1h2ba2 d1tt7a2 d1piwa2 d1o89a2 d1iz0a2 d1e3ja2 d1yb5a2 d1uufa2
d1vj1a2 d1e3ia2 d3gpdg1 d2nvwa1 d1u8fo1 d1ydwa1 d1hdgo1 d2g17a1
d1cf2o1 d2czca2 d1r0ka2 d1obfo1 d2cvoa1 d1ebfa1 d1h6da1 d1i32a1
d1zh8a1 d2q49a1 d1qkia1 d1j5pa4 d3pzra1 d1xeaa1 d1vkoa1 d1vm6a3
d3qvsa1 d2pkqo1 d1b7go1 d1gr0a1 d1p1ja1 d1lc0a1 d1j0xo1 d3pwka1
d2hjsa1 d1zyao1 d1vkna1 d1tlta1 d1nvmb1 d3sjua_ d3ojfa1 d1xu9a1
d2jjya1 d1n7ha_ d1y1pa1 d1h5qa_ d1jtva_ d4fs3a_ d2bkaa1 d1gy8a_

d2c5aa1 d1ulsa_ d1ooea_ d2b69a1 d1y5ma1 d1spxa_ d1ae1a_ d1bxka_
d1ydea1 d1gega_ d1hdoa_ d1o5ia_ d1e6ua_ d1snya1 d2rhca_ d3zv4a_
d5ceja_ d2d1ya1 d1fjha_ d3w8ea_ d2fmua1 d1x1ta_ d1rpna_ d3ay7a1
d1k2wa_ d1eq2a_ d2hrba1 d3ruaa_ d2ew8a1 d4hwka_ d1xgka_ d2bd0a1
d1yo6a1 d2a35a1 d2ntva_ d1vl8a_ d1hdca_ d4bkua_ d2ag5a1 d2q45a_
d1nffa_ d4iy1a_ d1zbqa1 d1kewa_ d1ek6a_ d1ja9a_ d2q46a2 d1iy8a_
d1cyda_ d2ptga_ d1hxha_ d1uzma1 d3lyla_ d4m89a_ d3wyea_ d2blla1
d1w6ua_ d1sb8a_ d2q2va_ d4nr0a_ d1e6wa_ d2gasa_ d3oiga1 d2bgka1
d1mxha_ d1vl0a1 d1rkxa_ d1r6da_ d1wmaa1 d1e7wa_ d2c07a1 d1xkqa_
d3tzha_ d1g0oa_ d3lz6a_ d1qyca_ d1edoa_ d1gz6a_ d1oc2a_ d1t2aa_
d1zema1 d2xoxa_ d1z45a2 d1geea_ d1p33a_ d3lt0a_ d4e6pa_ d2wyua_
d1db3a_ d4q9na_ d1xhla_ d4o1ma_ d1fmca_ d2uvda_ d2gdza1 d1uaya_
d2z1ma_ d2o23a2 d2fr1a2 d2fr1a1 d1qsga_ d1qyda_ d4zjua_ d2a4ka1
d1yb1a_ d1wvga1 d1yxma1 d3f4ba_ d1mg5a_ d1xg5a1 d1cmxa_ d1xd3a_
d3ifwa_ d3u1ra1 d1g9ka2 d1kapp2 d1k7ia2 d1t3ca_ d2a97a_ d3fiea_
g1e1h.1 d3ioqa_ d1fh0a_ d1o0ea_ d1khqa_ d1m6da_ d2cb5a_ d1cs8a_
g8pch.1 d1s4va_ d2pnsa_ d1vsna1 d1iwda_ d2oula_ d7pcka_ d3bwka_
d3bcna_ d2p86a_ d1pvja_ d1pcia_ d1gece_ d1deua_ d4bs6a_ d1xkga1
d1cqda_ d1jqpa2 d2c0ya_ d2bdza_ d3p5ua_ d1ex4a2 d2jk6a2 d2jk6a1
d1feca1 d1feca2 d1lpfa1 d1lpfa2 d1xhca1 d1xhca2 d1dxla2 d1dxla1
d1gera2 d1m6ia2 d1onfa1 d1onfa2 d3lada2 d3lada1 d1w4xa1 d1w4xa2
d1gesa2 d1mo9a1 d1xdia1 d1fl2a2 d1fl2a1 d1fcda1 d1fcda2 d1h6va2
d1h6va1 d1mo9a2 d1gv4a2 d1gv4a1 d2gv8a2 d2gv8a1 d1aoga1 d1aoga2
d1hyua1 d1ebda1 d1ebda2 d3dk9a1 d1q1ra2 d1q1ra1 d1gesa1 d1pv8a_
d1w5qa_ d2c1ha_ d1l6sa_ d1zbfa1 d2z1ha_ d2etja1 d3qioa1 d1jl1a_
d2e4la_ d1jl2a_ d2lsna1 d1io2a_ d1uaxa_ d2yv0x_ d1mu2a1 d1ekea_
d1i39a_ d1pn0a1 d1reoa1 d1ju2a1 d2f5va1 d3c96a1 d2gmha1 d1pj5a2
d1o5wa1 d1n4wa1 d1k0ia1 d2dw4a2 d2ivda1 d1seza1 d2voua1 d1kdga1
d1s5ja1 d2hbja2 d2igia_ d1wlja_ d1qhta1 d4dlea1 d1tgoa1 d2f96a1
d3tr8a1 d3mxma_ d1y97a1 d1w0ha_ d1kfsa1 d2py5a1 d1noya_ d1d5aa1
d1yt3a3 d2guia_ d3mwpa1 d4kb1a_ d1id1a_ d2hmta_ d1b9ra_ d1offa_
d1frra_ d3wcqa_ d1e9ma_ d1wria_ d1i7ha_ d3av8a_ d1czpa_ d1rfka_
d3p1ma1 d3b2ga_ d2bt6a2 d3nvva1 d1krha3 d2h88b1 d1n62a2 d1t3qa2
d1zoyb1 d1jq4a_ d1ffva2 d1dgja2 d1rm6c2 d1jmxa1 d1pbya1 d1pbya2
d1r7ha_ d1faaa_ d1wika1 d1xfla_ d1ttza1 d2f51a1 d1ti3a_ d1nhoa_
d1sena_ d2i4aa_ d1ep7a_ d1zmaa1 d1fo5a_ d3lefa1 d3diea1 d2yana1
d1z6na1 d1oaza_ d3zywa1 d4aj8a_ d1nm3a1 d4fiwa_ d1wmja_ d4hs1a1
d1iloa_ d1xwaa1 d1wjka1 d2vm1a_ d1fb6a_ d1gh2a_ d1h75a_ d1nw2a_
d1kx5b_ d3a6na_ d4m6ba2 d2nqbd_ d3wkjd_ d2yfwe_ d4h9na_ d5bnxa_
d1kx5c_ d3r45a_ d1eqzb_ d4jjnb_ d1f66c_ d1eqza_ d4wnnb_ d1kx5a_
d1u35c1 d1id3a_ d2nqbc_ d1kx5d_ d1tafa_ d1bh9b_ d1tafb_ d1q9ca_
d1h3ob_ d2byka1 d1n1ja_ d4g92b_ d1bh9a_ d1jfia_ d1h3oa_ d2bykb1
d1n1jb_ d1pd212 d1nhya2 d2a2ra2 d1k0dd2 d1f2ea2 d1gsua2 d1f3ba2
d2fhea2 d2pvqa1 d1tw9a2 d1oyja2 d1e6ba2 d1k0ma2 d3ik7a1 d4is0a1

d1r5aa2 d1jlva2 d1m0ua2 d3gtub2 d1z9ha2 d1okta2 d1axda2 d2gsra2
d1tu7a2 d1k3ya2 d1zgma1 d1gnwa2 d1gwca2 d2gsta2 d1glqa2 d1oe8a2
d1pn9a2 d1v2aa2 d1fw1a2 d1duga2 d1iuka_ d2csua1 d3q9ub_ d2yv1a1
d3q9na_ d2d59a_ d1oi7a1 d2yv2a1 d1y81a1 d1qqya_ d1gd6a_ d1ivma_
d2gv0a_ d1iiza_ d2z2fa_ d1j8xa_ d1ghla_ d1b9oa_ d174la_ d169la_
d2o4wa_ d1p37a_ d1k28a3 d1xjta1 d1jtma_ d2f2qa_ d4e97a1 d1iyka2
d1iyka1 d1rxta1 d1iica1 d2pjsa1 d1klla_ d2i7ra1 d1ecsa_ d1xqaa1
d5cj3a1 d1npba_ d1jifa_ d2p7oa_ d1nkia_ d1mpya2 d1kw3b2 d1kw3b1
d4ghga1 d1tfza1 d3isqa1 d3isqa2 d1sqia1 d1sqia2 d1lkda2 d1lkda1
d1mpya1 d1cjxa1 d1sp9b2 d1t47a1 d1t47a2 d1f1ua2 d1sp8a1 d1sp8a2
d1f1ua1 d2h0va_ d4iuaa3 d4bvva_ d5hpga_ d3k65a_ d1ki0a3 d1ki0a2
d1ki0a1 d1a0ha1 d1pmla_ d3sp8a3 d2feba1 d1qsta_ d2beia1 d4m3sa_
d1u6ma1 d1yk3a1 d3dr6a_ d1xmta_ d1z4ea1 d2ge3a1 d1bo4a_ d2fiaa1
d2jdca1 d1y9wa1 d3to7a_ d1wwza1 d2fl4a1 d2ozua1 d2b5ga_ d2cy2a1
d2p0wa_ d1cm0a_ d4pswa_ d1yrea1 d1yr0a1 d1m4ia_ d2gana1 d1yvka1
d1ghea_ d2g3aa1 d1y7ra1 d2euia1 d1q2ya_ d2fsra1 d1xeba_ d2ae6a1
d1cjwa_ d1qsma_ d2aj6a1 d1y9ka1 d1i12a_ d1vkca_ d2atra1 d1n71a_
d2d4pa1 d2fiwa1 d2fe7a1 d1tiqa1 d2ou2a_ d1yx0a1 d2fcka1 d1p0ha_
d2j8ma_ d2pq8a_ d1z4ra1 d1s3za1 d1ufha_ d2hqya1 d2hqya2 d1lrza2
d1ne9a1 d1lrza3 d1xvqa1 d1pn0a2 d3hy2a_ d2cvba1 d1o8xa_ d1lu4a_
d1we0a1 d2bmxa1 d2a4va1 d1psqa_ d1xiya1 d1q98a_ d1n8ja_ d1zofa1
d2ggta_ d3tjja_ d4je1a1 d1o73a_ d3s9fa_ d2zcta_ d1qmva_ d1jfua_
d2b1ka_ d5dvba1 d2b5xa1 d2v2ga_ d3tb2a_ d4llra_ d4fh8a1 d1e2ya_
d1knga_ d2h30a_ d1i5ga_ d2z9sa_ d2f8aa1 d1tp9a1 d2b7ka_ d1nm3a2
d3hvva_ d2wfca1 d4kcea_ d1xvwa1 d4gqca1 d3qpma_ d4k1fa_ d1zzoa1
d1gp1a_ d1prxa_ d4od7a1 d1z6ma1 d4mcua1 d4dvca1 d1r4wa_ d3l9sa_
d3rppa_ d1rmka_ d1ixta_ d1eyoa_ d1cnna_ d1fu3a_ d1g1pa_ d1f3ka_
d1fyga_ d1e88a1 d1l6ja5 d1l6ja4 d1eaka4 d1eaka3 d1h8pa2 d1h8pa1
d1jgta2 d1ea0a3 d1ecfa2 d1q15a2 d1ct9a2 d1ao0a2 d1te5a_ d1ys1x_
d4hs9a_ d1ex9a_ d1ku0a_ d3qmma_ d1ra6a_ d1muka_ d3gsza_ d1khva_
d1te9a_ d2xwha_ d3uqsa_ d3hkwa_ d3n6ma_ d2cjqa_ d2xyma_ d1teba_
d1te8a_ d1s48a_ d1u09a1 d4wfxa_ d3tyqa_ d2pgga1 d2h1ia1 d2r8ba1
d3b5ea1 d3cn9a_ d1auoa_ d1lgya_ d4l3wa_ d1llfa_ d1gz7a_ d1ukca_
d1cr6a2 d1bvsa3 d1ixra2 d1mbma_ d3su6a1 d1cu1a1 d4agka_ d1vcpa_
d1q31a_ g1n1l.1 d3e90b_ d1ep5a_ d3u1jb_ d3zxha_ d1cgla_ d1rm8a_
d1eaka2 d1hy7a_ d2jsda_ d1cxva_ d1hv5a_ d1i76a_ d3p0ya1 d1igra1
d1igra2 d1n8yc2 d1m6ba2 d1m6ba1 d4dv8a2 d1dzka_ d4tlja_ d2a2ua_
d4wfva_ d3t1da_ d4iaxa_ d3zq3a_ d2nnda_ d1i4ua_ d1kxoa_ d1bbpa_
d1kt7a_ d1exsa_ d3s26a1 d1lf7a_ d1qfta_ d4gh7a_ d1z24a1 d1qabe_
d1xkia_ d1ew3a_ d3bx7a_ d4iawa1 d1n0sa_ d1gkab_ d3kzaa_ d2k23a_
d2l9ca1 d2joza1 d1gm6a_ d3kffa_ d3saoa1 d1e5pa_ d1yupa1 d1iiua_
d2tbvc_ d1c8nc_ d1x36a_ d1smvc_ d1ng0c_ d4sbvc_ d1opoa_ d1f2nc_
d1cixa_ d1y29a_ d1v7fa_ d2jtba_ d1d1ha_ d1g9pa_ d1lupa_ d1qk7a_
d1mb6a_ d2a2va_ d1niya_ d1i25a_ d1hvwa_ d2mpqa_ d1ie6a_ d1kqha_

| | | | | | | | |
|---|---|---|---|---|---|---|---|
| d1la4a_ | d2mxoa1 | d1dl0a_ | d1koza_ | d1qk6a_ | d1emxa_ | d1lqra_ | d1c6wa_ |
| d1js9c_ | d1f15b_ | d1za7a_ | d1laja_ | d1rypd_ | d1irua_ | d1rypc_ | d3unfk_ |
| d1rypa_ | d1j2pa_ | d1irui_ | d1j2qh_ | d3unfh_ | d1m4ya_ | d1rypb_ | d1irul_ |
| d1irug_ | d1iru2_ | d1q5rh_ | d1rypg_ | d1g3ka_ | d1iruf_ | d1q5qa_ | d1e94a_ |
| d1irub_ | d1ryph_ | d3ty6a_ | d1iruj_ | d1iruk_ | d1rype_ | d1irud_ | d1irue_ |
| d3mi01_ | d1rypf_ | d1iruh_ | d2z3ba_ | d1iruc_ | d3mka2_ | d1iru1_ | d1ny721 |
| d1ny722 | d1a6ca3 | d1a6ca2 | d1a6ca1 | d1pgl21 | d1pgl11 | d1ny711 | d1mqta_ |
| d1d4m3_ | d1fmd3_ | d2mev1_ | g1aym.1 | d1ev13_ | d4q4w1_ | d1r1a3_ | d2x5ia_ |
| d1rhi1_ | g1r1a.1 | g1d4m.1 | g1rhi.1 | d1b35b_ | d1oopa_ | d1tme1_ | d4iv1a_ |
| g1pvc.1 | g1fpn.1 | d1fpn1_ | d4gb31_ | d1d4m1_ | d1fmd1_ | d1ev11_ | d2wzr3_ |
| g1cov.1 | d1qqp3_ | g1fmd.1 | d2x5ic_ | d1rhi3_ | g1oop.1 | d1hxs1_ | g1hxs.1 |
| d1fpn3_ | g2mev.1 | d1eah3_ | d1aym1_ | d1pvc3_ | d1qqp1_ | d1zba3_ | d1h8ta_ |
| d3vbhc_ | g1b35.1 | g1bev.1 | d1hxs3_ | g1ev1.1 | d1bev3_ | g1tmf.1 | d1tmf1_ |
| d1b35a_ | d2mev3_ | g1eah.1 | g1h8t.1 | d1eah1_ | d4q4w3_ | d1r1a1_ | d1aym3_ |
| d1pvc1_ | d1zba1_ | d1h8tc_ | d1oopc_ | d1tme3_ | g1qqp.1 | d4iv1c_ | g1tme.1 |
| d3ubub_ | d1egia_ | d1qo3c_ | d1v7pa_ | d1tdqb_ | d3ubua_ | d1c3ab_ | d1h8ua_ |
| d3bx4b_ | d1t8ca1 | d1sb2a1 | d1qdda_ | d2ziba1 | d3cada_ | d1byfa_ | d1j34b_ |
| d1jwia_ | d1jzna_ | d1c3aa_ | d1oz7b_ | d1wmza_ | d3bdwb_ | d1hq8a_ | d1fifa1 |
| d1dv8a_ | d3hupa_ | d1wk1a1 | d1umra_ | d2afpa_ | d3bx4a_ | d1gz2a_ | d1sl6a_ |
| d1v7pb_ | d3g8ka_ | d1ixxa_ | d1j34a_ | d1jwib_ | d1g1sa1 | d1y17a1 | d1ja3a_ |
| d3bdwa_ | d1sb2b1 | d1fvua_ | d1umrc_ | d1oz7a_ | d4m17a_ | d3cfwa1 | d1ypqa1 |
| d1g1ta1 | d3fd4a_ | d3l9jc_ | d4dn8a1 | d1v4lb_ | d1rdl1_ | d2msba_ | d2flia1 |
| d1rpxa_ | d1tqja_ | d1tqxa_ | d4lt9a1 | d3tc7a1 | d3hoja_ | d1i4na_ | d3vnda_ |
| d3nl8a1 | d1geqa_ | d3nava1 | d4a2ra1 | d3tc6a1 | d1ujpa_ | d4ijba_ | d1xcfa_ |
| d1vc4a_ | d1rd5a_ | d1qopa_ | d3b5va_ | d2r80b_ | d1cqxa1 | d1cg5a_ | d1gcvb_ |
| d3boma_ | d2h8fb_ | d2raoa_ | d3bj1a_ | d4hrrb_ | d2r50a_ | d3d1kb_ | d2aa1b1 |
| d3zhwa_ | d3hf4b_ | d3gdja_ | d1jebb_ | d3bj1b_ | d1x9fb_ | d1wmub_ | d1gcva_ |
| d3bcqb_ | d3d4xb_ | d1gvha1 | d2dc3a_ | d3hf4a_ | d4yu3a_ | d3k8ba_ | d1qpwb_ |
| d2r80a_ | d3bcqa_ | d1v4wa_ | d3wtgb_ | d1jl7a_ | d4esaa_ | d1a9we_ | d3d1ab_ |
| d1fsla_ | d4h2la_ | d1v4wb_ | d1mwca_ | d1vhba_ | d1jeba_ | d1spgb_ | d1h97a_ |
| d1q1fa_ | d1tu9a1 | d3d1aa_ | d1hdsb_ | d3hyub_ | d2zfbb_ | d3fs4a_ | d1oj6a_ |
| d1shrb_ | d4h2lb_ | d4yu3b_ | d1spga_ | d3hrwa_ | d3qqra_ | d3at5a_ | d4wchd_ |
| d3hyua_ | d1cg5b_ | d1uc3a_ | d1hbra_ | d2raob_ | d4esab_ | d3gdjb_ | d3qqqa_ |
| d1x9fc_ | d3vrfa_ | d1itha_ | d3mkba_ | d3d4xa_ | d3mjpa_ | d1jl6a_ | d3fh9a_ |
| d1outa_ | d2dc3b_ | d1it2a_ | d3eoka_ | d3g46a_ | d1qpwa_ | d3vrfb_ | d1ch4a_ |
| d1x9fd_ | d1hdsa_ | d2zfba_ | d3mkbb_ | d1or4a_ | d3wtga_ | d1a4fa_ | d3fh9b_ |
| d3bomb_ | d3cy5a_ | d3at5b_ | d1x9fa_ | d2oifa_ | d4lmxb_ | d1cpcb_ | d3v57a_ |
| d1kn1a_ | d1cpca_ | d4f0ta_ | d2vjha_ | d4po5b_ | d1eyxa_ | d1alla_ | d4f0tb_ |
| d1b33b_ | d1b8db_ | d4h0mb_ | d1b33a_ | d2vjhb_ | d4lm6b_ | d2vmlb_ | d1allb_ |
| d2vjta_ | d3dbja_ | d2vmla_ | d2vjtb_ | d4f0ub_ | d1f99b_ | d4rmpa1 | d1liaa_ |
| d1eyxb_ | d2uulb_ | d4lmsb_ | d4f0ua_ | d2bv8a_ | d1f99a_ | d2bv8b_ | d2c7lb_ |
| d3dbjb_ | d4rmpb1 | d1b8da_ | d4h0ma_ | d3v57b_ | d1kn1b_ | d1liab_ | d2uula_ |
| d2j96a_ | d3ia2a_ | d1a88a_ | d4dgqa1 | d1hkha_ | d1zoia_ | g1gxs.1 | g1wht.1 |
| d1azwa_ | d1hlga_ | d2xqfa_ | d1f6wa_ | d1dx4a_ | d1qe3a_ | d1k4ya_ | d4qwwa_ |

d2ha2a_ d1r88a_ d1sfra_ d1f0na_ d4qeka_ d3c8da2 d2pbla1 d1jjia_
d1lzla_ d1jjfa_ d1jmxa4 d1jmxa3 d1pbya4 d1s5ja2 d1qhta2 d4dlea2
d1tgoa2 d3py8a2 d1q8ia2 d1kfsa2 d2py5a2 d1d5aa2 d1f74a_ d3l21a_
d2e1da_ d1to3a1 d1w3ia_ d1sfla_ d1vcva1 d4imea_ d1wa3a_ d2ocza1
d1f2ja_ d3m16a_ d2yxga_ d1ub3a_ d3a5fa_ d1fbaa_ d1l6wa_ d1xfba1
d1wx0a1 d1vpxa1 d1ojxa_ d1mxsa_ d2a4aa1 d1xkya1 d3cq0a1 d3s1xa_
d1f05a_ d1fdja_ d3cpra1 d4d2ja_ d1cg2a1 d1yloa2 d1y7ea2 d1y0ya3
d1lfwa1 d1vixa1 d1fnoa4 d1vhoa2 d1ysja1 d1vhea2 d1z2la1 d2fvga2
d1vgya1 d1r3na1 d2grea2 d1xmba1 g1q7l.1 d3rp2a_ g1ucy.1 d1fuja_
d2zchp_ d3w94a_ d1si5h_ d1lo6a_ g2pka.1 d1cgha_ d1kigh_ d1a5ia_
d3s9ba_ d1hj8a_ d1ao5a_ d2anya_ d1q3xa1 d1gvza_ d1azza_ d4gsoa_
d2f91a1 d1iaua_ d1gdna_ d1eufa_ g1fiw.1 d1eq9a_ d4nfea_ d2rdla_
d1spja_ d1elva1 d1a7sa_ d1ekbb_ d1pfxc_ d1yc0a_ d2aiqa_ g1fiz.1
d1fi8a_ d4k60a_ d4dgja_ d1mzaa_ d1op0a_ d3tjva_ d1pytd_ d1sgfg_
g1gg6.1 d2eeka_ g1rtf.1 d1a0ja_ d3g01a_ d1pytc_ d4e7na_ d1ddja_
d1z8ga1 d2hlca_ d1npma_ d1brup_ g1jou.1 d1sgfa_ d1autc_ g2hnt.1
g1kdq.1 d1orfa_ d3s69a_ d4crga_ d1fxya_ d2ftba_ d1pmpa_ d2rcqa1
d1fdqa_ d4lkpa_ d1sa8a_ d1vyfa_ d1lpja_ d3vg7a1 d4azqa1 d1o8va_
d5bvqa_ d1p6pa_ d2qo4a_ d1opba_ d1yiva1 d1o1va_ d4bvma1 d4tkba_
d4i3ba_ d1bwya_ d1ftpa_ d1ggla_ d4gkca_ d1cbia_ d3p6da1 d1kqwa_
d2a0aa1 d1m4la_ d2bk7a_ d3fjua_ d1z5ra1 d1kwma1 d1uwya2 d1jqga1
d2c1ca1 d1dtda_ d1h8la2 d4lu0a_ d3unda1 d3e9aa1 d1vr6a2 d1n8fa_
d2qkfa_ d1d9ea_ d1o60a_ d1e69a_ d1mv5a_ d3g60a4 g1ii8.1 d1ewqa2
d1ji0a_ d1v43a3 d2wmfa1 d1vpla_ d1g2912 d4a82a2 g1f2t.1 d2d62a1
d3tuic1 d2bbsa1 d3g60a3 d2ixea_ d1g6ha_ d1sgwa1 d2qi9c_ d3rlfa1
d3b60a1 d3d31a2 d2pzea1 g1xew.1 d1jj7a_ d1oxxk2 d2onka1 d1w1wa_
d1iwea_ d1qf5a_ d1vmaa2 d1nija1 d1np6a_ d2p67a1 d3ihla_ d1okkd2
d1ls1a2 d1s1ma2 d1iona_ d2v40a_ d1dj3a_ d3hida_ d2vo1a1 d2g0ta1
d1vcoa2 d1j8mf2 d1g3qa_ d1xjca_ d1qzxa3 d1ihua2 d1ihua1 d2qm8a_
d1dj2a_ d4ioja_ d1cp2a_ d1yrba2 d1hyqa_ d3gqba2 d1skye3 d1xp8a1
d1tf7a2 d1tf7a1 d1ye8a1 d1skyb3 d2qe7d2 d4blta_ d4huta_ d3ldaa2
d4hyya_ d2i3ba1 d1cr1a_ d3j9tb2 d3ntua2 d1vdza1 d2z43a_ d1e9ra_
d1nlfa_ d3ziaa2 d1n0wa_ d1g6oa_ d3ziad2 d3j9ta2 d1pzna2 d3gqbb2
d2i49a_ d1nnfa_ d1xvxa_ d1ursa_ d1h3da1 d1iuda_ d4m1va1 d1twya_
d3e13x_ d2nxoa1 d4pfua_ d3ttma_ d1elja_ d2qmwa1 d1us5a_ d1y3na1
d1jeta_ d1uqwa_ d4f19a1 d2esna2 d3thia_ d3fasa1 d2uyeb_ d2xwva1
d1r9la_ d2fyia1 d1pb7a_ d2ozza1 d1sw5a_ d1nh8a1 d1vr5a1 d2g29a_
d1xs5a_ d3lswa_ d2z23a_ d3w9va_ d3tcha_ d1q35a_ d1z7me1 d3q27a_
d1p99a_ d1ve4a1 d2i6ea1 d4mcoa1 d4n8ya_ d1eu8a_ d1zbma1 d1hsla_
d1ixca2 d1j1na_ d1xvya_ d1y9ua_ d2z22a1 d1xoca1 d4oera_ d1wdna_
d5f1qa1 d1xt8a1 d4gwoa_ d4igta1 d1pc3a_ d4mmpa1 d1o63a_ d2czla1
d2onsa2 d4mija1 d4ok5a1 d2bmfa1 d2bmfa2 d1yksa1 d1yksa2 d2qeqa1
d2jlqa1 d1a1va2 d1eh3a_ d3skpa_ d1dtza1 d1dtza2 d1ieja_ d1jnfa2
d1ce2a1 d1jnfa1 d1b1xa2 d1jw1a1 d1jw1a2 d1ryoa_ d1h76a1 d1h76a2

d1etha2 d1w52x1 d2ppla1 d1hpla2 d2oxea1 d1bu8a2 d1rp1a2 d1c4oa1
d2p6ra3 d2p6ra4 d1oywa2 d1wrba1 d1w36b2 d1w36b1 d1c4oa2 d3bora_
d1t6na_ d2eyqa5 d2eyqa3 d2eyqa2 d2zu6a2 d1veca_ d1uaaa1 d1w36d1
d2g9na1 d1z3ix2 d2rb4a_ d1hv8a1 d1hv8a2 d1t5ia_ d1w36c2 d2j0sa2
d2j0sa1 d1fuka_ d3hjha1 d1z5za1 d1qdea_ d1z63a1 d1s2ma2 d1s2ma1
d1wp9a1 d1wp9a2 d1gm5a3 d1gm5a4 d3dina4 d3dina3 d1q0ua_ d1w36c1
d1z3ix1 d1nkta3 d1nkta4 d1uaaa2 d1w36d2 g1qhh.1 d2fwra2 d2fwra1
d3q3ma_ d1za0a1 d1mxra_ d2inca_ d1jk0a_ d2incb_ d1mhyb_ d3hf1b_
d1mtyb_ d1r2fa_ d2uw1a_ d1mhyd_ d3n37a_ d1afra_ d1oquc_ d1syya_
d1mtyd_ d2o1za_ d1uzra_ d1smsa_ d2fkza_ d1zuja1 d1s3qa1 d2fjca1
d1z6om1 d1veia_ d4dyya_ d1jiga_ d3gvya_ d2pyba_ d4cy9a1 d3bvfa1
d1r03a_ d2cf7a_ d1nf4a_ d1zs3a1 d4iwka_ d3ka8a_ d3np0x_ d1n1qa_
d1lb3a_ d4e6ka_ d1z6oa1 d2fzfa1 d4dyua_ d3ge4a_ d1jgca_ d4am4a_
d2z6ma_ d1ji5a_ d1vlga_ d1lkoa1 d1krqa_ d3qz3a_ d1o9ra_ d2ffxj_
d1euma_ d1yv1a1 d2v2pa_ d1j30a_ d4di0a_ d1dpsa_ d1oewa_ d2qzxa_
d2asia_ d1psoe_ d1miqa_ d1smra_ d2h6ta_ d1pfza_ d1izda_ d3hd8a_
d1j71a_ d1dpja_ d4aa9a_ d1t6ex_ g1b5f.1 g1lya.1 g1htr.1 d1qdma2
d1bxoa_ d1wkra_ d1tzsa_ d1ibqa_ d2qzwa_ d1bdqa_ d3ggta_ d3ixoa_
d1mrxa_ d2haha_ d1tw7a_ d4njsa_ d1sgua_ d2p3da_ d1nsoa_ d3d3ta_
d2rkfa_ d1c6ya_ d2rspa_ d3ogpa_ d3ekwa_ d2o4na_ d2j9ja_ d1baia_
d3ggua_ d3lzua_ d4npua_ d4j55a_ d2r8na_ d2fxda_ d2p3ca_ d4dqea_
d3ttpa_ d3t3ca_ d2p3aa_ d2hc0a_ d2fdda_ d3mwsa_ d1sr9a2 d1rqba2
d3rcha_ d3k40a_ d1pmma_ d1h4ra3 d4yl8a1 d2al6a3 d1l7ya_ d1wgda1
d1wgga1 d4q5eb_ d1wjna1 d1se9a_ d3n3kb_ d1wz0a1 d1we6a1 d2m0xa_
d2gbka_ d3rt3b1 d1wh3a1 d1sifa_ d2ekec2 d4ii2b1 d4hk2a1 d1c3ta_
d1m94a_ d1wgha1 d1ttna1 d1j8ca_ d1wx8a1 d3v6cb1 d2ma9b_ d4i6lb1
d1bt0a_ d2jvca1 d1iyfa_ d2k1fa_ d1wxva1 d1wf9a1 d1t0ya_ d2io0b2
d1x1ma1 d2gbma_ d1zw7a1 d2m17a_ d1we7a1 d2lwpa_ d2gbja_ d1wjua1
d1v6ea1 d1v86a1 d4eewa_ d1wiaa1 d4icva_ d4gu2a_ d1wx7a1 d1wy8a1
d4hjka1 d2faza1 d1v5oa1 d3ruib_ d1eo6a_ d3waob_ d2r2qa1 d3m95a_
d3wana_ d3h33a_ d1rwja_ d19hca_ d1os6a_ d3bxua_ d3h4na_ d1i77a_
d3caoa_ d1j0pa_ d2ctha_ d1duwa_ d2e84a_ d1hh5a_ d1gyoa_ d3h34a_
d1dszb_ d1a6ya_ d3cbba_ d1ynwa1 d1r4ia_ d1hcqa_ d2hana_ d2hanb_
d2enva1 d1kb4b_ d2nllb_ d1lo1a_ d1cita_ d1um8a_ d1ixza_ d1qvra2
d1qvra3 d1qzma_ d1sxjc2 d1jr3d2 d2p65a_ d1l8qa2 d1g8pa_ d3hu3a3
d1sxjd2 d1njfa_ d1in4a2 d1sxja2 d1tuea1 d2ce7a2 d1w5sa2 d1sxjb2
d1d2na_ d1ny5a2 d1ofha_ d2gnoa2 d2a5yb3 d1fnna2 d1u0ja1 d1lv7a_
d1iqpa2 d2fnaa2 d1ixsb2 d4gita_ d1sxje2 d2e3fa_ d1mm0a_ d2e3ea_
d2llda_ d2ny8x_ d2e3ga_ d2ny9x_ d2nz3a_ d2lj7a_ d1n4na_ d2m8bb1
d2kyqa_ d3psma_ d1jxca_ d2gl1a_ d1ugla_ d4uj0a_ d2k4ua_ d1hp2a_
d2lu9a_ d1baha_ d2meoa1 d1wm7a_ d1hlya_ d1n8ma_ d1jlza_ d1wpda_
d1c55a_ d1pvza_ d1wt7a_ d1rjia_ d1wz5a_ d1m2sa_ d1pjva_ d1c49a_
d1qkya_ d2ktca1 d1wmta_ d1j5ja_ d1r1ga_ d1t0za_ d2i61a_ d1t1ta_
d1sn4a_ d1kv0a_ d2lkba_ d1npia_ d1wwna_ d1pe4a_ d2kbka_ d2li7a_

d1djta_ d2kbha_ d1dq7a_ d1sega_ d1omya_ d1zywa1 d1jzaa_ d1t61c1
d1iyjb4 d1iyjb5 d1iyjb3 d1o7ia_ d4ipca1 d1x3ea_ d4apva_ d1jb7a1
d1jb7a2 d1jb7a3 d3kdfa_ d1ue1b_ d1wjja1 d3eiva_ d1xjva2 d1xjva1
d2ccza2 d1se8a_ d1l1oc_ d1jb7b_ d1qvca_ d3ulla_ d1qzga_ d1miua4
d1miua5 d1miua3 d1y14b1 d2nn6i1 d1i94l_ d2zjra2 d1smxa_ d2qamc2
d1hh2p1 d2r7da1 d1rl2a2 d1wfqa1 d3bzka4 d1x6oa2 d3a0ja_ d2j01d2
d2ja9a1 d4ql5a_ d1hr0w_ d1luza_ d2n3sa_ d2vnud1 d1h95a_ d1go3e1
d2nn6h1 d1ne3a_ d2mo1a_ d1iz6a2 d1jjga_ d1e3pa2 d2ix0a3 d2ix0a2
d2lxja_ d1ueba3 d1ueba2 d2z0sa1 d1u0la1 d2ix0a1 d2eifa2 d2je6i1
d3i2za1 d2c35b1 d2ahob2 d1t9ha1 d3i4oa_ d2qall1 d1jt8a_ d2nn6g1
d1g6pa_ d3cama_ d1i94q_ d1c9oa_ d1d7qa_ d2ba0a1 d2vnud2 d2vnud3
d1khia2 d2qalq1 d1wi5a1 d1xtda2 d2a1ka_ d1je5a_ d1rpma_ d2shpa1
d1yfoa_ d1l8ka_ d2ooqa1 d1g4us2 d1p15a_ d1lara2 d1lara1 d1wcha_
d2fh7a2 d2fh7a1 d3azya_ d3wvja_ d1upsa1 d1ajoa_ d2hyka_ d1ajka_
d2vy0a_ d3o5sa_ d3d6ea_ d3atga_ d1dypa_ d1umza_ d3i4ia_ d1c1la_
d4lbqa_ d1a78a_ d1slta_ d4no4a_ d3zsja_ d3duia_ d1g86a_ d1is3a_
d1hlca_ d1tdha3 d1q3ba3 d1ee8a3 d1k82a3 g1qmo.1 g1len.1 d3zyra_
d1fnya_ d1n47a_ g2ltn.1 d1avba_ d3usua_ d1dhkb_ d1f9ka_ d1ukga_
d1g8wa_ d1g9fa_ d1wbfa_ d3wcsa_ d1g7ya_ g1loe.1 d1mvqa_ d1hqla_
d1v6ia_ d1dbna_ d1qnwa_ d1fx5a_ d4l8qa_ d1ioaa_ d3n35a_ d2h0ba_
d2erfa_ d1h30a2 d1h30a1 d3mw4a1 d1pz7a_ d2r1ba_ d3v64a1 d3bopa1
d3mw3a1 d1okqa1 d1okqa2 d1wioa4 d1ccza2 d1vcaa1 d1dr9a2 d2d9qb1
d2nxyb2 d1adqa1 d1fnga1 d1uvqb1 d1zs8a1 d2vola1 d2vola2 d1mfbl2
d2jelh2 d1uwel2 d3qq4b1 d1kjvb_ d1k5na1 d1osph2 d4odhl2 d1ow0a1
d1ow0a2 d1bfob2 d3l9ra2 d3jtth1 d2fath2 d4elkb2 d1fn4b2 d2xfxb_
d1pg7x2 d4k71b2 d1lk2a1 d1eapb2 d4lfhg2 d1i4fa1 d2nw2a2 d1kfah2
d1mhca1 d1h3ua1 d1wejh2 d1h0db2 d1a5fh2 d1kcvh2 d1mjuh2 d1qqda1
d1kcrh2 d1k8ia1 d3huja2 d2nnab1 d1nfdf2 d3mffb2 d1s9va1 d1lk3h2
d1q0xl2 d1fv1b1 d1neza1 d4rfeb2 d1r3ha1 d1a6tb2 d1kjva1 d1pz5b2
d1onqa1 d2bc4a2 d1de4a1 d1k8da1 d1tcra2 d1dn0b2 d1nfde2 d2xwtb2
d1bfoa2 d1mexh2 d1iakb1 d1ypzf2 d4f7ea2 d1q72l2 d2fbjh2 d1fe8h2
d1clzh2 d1fngb1 d1uvqa1 d1vgka1 d1c16a1 d4j12a2 d4x99b2 d1igtb3
d1je6a1 d1q72h2 d1nldh2 d1teth2 d2hmic2 d1i1ca2 d1i1ca1 d1k8ib1
d1q0yh2 d1kfal2 d3frua1 d1pg7w2 d1u58a1 d1igyb4 d1igyb3 d1hyrc1
d1klub1 d1deeb2 d1c12b2 d2bc4b2 d2ig2h2 d1kcvl2 d3wn5b1 d1lk2b_
d1lk3l2 d1mjul2 d1sm3h2 d1q0xh2 d1zvsa1 d1ydpa1 d1u8ib2 d1ypze2
d1kcuh2 d1op9a_ d1y0la1 d1etzb1 d1egjl1 d2gsib1 d1nj9a1 d4jfhd1
d1aqkl1 d1t2ja_ d1lo4h1 d1zvya1 d3v4vl1 d4enec1 d1kxtb_ d5e8ea1
d2pxya_ d4weud_ d1xiwc_ d2esvd1 d1qnzh_ d1ogae1 d1f4xh1 d4n1hb_
d3ezjb1 d3mlwl1 d1kfah1 d1etza1 d1b4jh1 d3cfic_ d4w68a_ d2arja1
d1ek3a_ d2ok0l1 d1vera_ d1uwgh1 d1blnb1 d1fpth1 d1rzga1 d2giya1
d4m3kb1 d1nc2a1 d2nyyd1 d1ncna_ d1fbih1 d5dxwa_ d4fqqa1 d1dqdh1
d1jfql1 d1kelh1 d1faih1 d1frgl1 d1figl1 d4m6nl1 d1fo0a_ d4hxbl1
d3b5ga_ d3quyd1 d2h9gb1 d1i8kb_ d2dd8h1 d2ck0h1 d1eaja_ d4w6wb1

d2a6ib3 d1t66d1 d2e27h_ d1ob1b1 d1f11b1 d2v7na1 d1ad0a1 d2xt1b1
d1pskh1 d4i13b1 d2op4l1 d1a0ql1 d1uwel1 d1za3a1 d1ncbl1 d4uu9a_
d1bm3h1 d1cloh1 d1nqba2 d1cf8h1 d4y5va_ d1mhph1 d5anma1 d4cdgc1
d4ldeb_ d1y0lb1 d2pcpb1 d1z3gl1 d1dfbl1 d1fh5h1 d3g5xa1 d1tzib1
d4dn3l1 d3zhka1 d2b1hh1 d2uyla1 d1f3dh1 d1nj9b1 d2agjh1 d2h1pl1
d1q72h1 d8fabb1 d1ndmb1 d1rjca1 d4u6hb1 d3upca_ d2fbjl1 d1osph1
d1tvda_ d2cjvh_ d4fhbd1 d1bd2d1 d1rz7h1 d3nn8a_ d1blna1 d1knoa1
d1nfda1 d2bmkb1 d1dqll_ d1igyb1 d1igfl1 d12e8h1 d3utza1 d1nc2b1
d1rihh1 d4mwfb1 d4jvpa1 d1mieh1 d1rhfa1 d1fgnl1 d1sbsh1 d1lp9e1
d1dn0a1 d4lqfl1 d1iaih1 d1kgcd1 d1mh5b1 d1gpol1 d2g60h3 d1f8tl1
d2jixd1 d4grwh_ d1fo0b_ d1bfob1 d1axsa1 d1u9ka_ d1nmbh_ d1h8na1
d1h8na2 d1jguh1 d4eura1 d3sdyl1 d35c8h1 d1w72l1 d1mvub_ d1i9rh1
d1ob1a1 d4wenb_ d1yeeh1 d3ietc1 d1f58h1 d1ad0b1 d1mqkl_ d1nbvh1
d4b50a_ d3r8bb_ d1bafl1 d1mamh1 d4rgne1 d2gsgb_ d35c8l1 d1bafh1
d1ai1h1 d2pcpa1 d2fath1 d2x1pa_ d1orsb1 d1ibgl1 d1shma_ d1a2yb_
d1u0qa_ d2coqa_ d1hilb1 d1qfwi_ d1jv5b_ d1fn4b1 d5e0qa_ d1nsnh1
d1plgl1 d4ocxl1 d1kb5h1 d1rzfh1 d1pg7x1 d2fx7h1 d2oi9c_ d1l7il1
d3eyva1 d1d5il1 d4ffyl_ d1tetl1 d1a6wh_ d1ac6a_ d3dvfa_ d1nfde1
d1mimh1 d1ri8a1 d1jhlh_ d3dura_ d1fskb1 d1f97a1 d3so3b1 d2ak4d1
d1lp9f1 d4z7wf1 d1l7th1 d4y5ya_ d4x7ec1 d1emth1 d1ikfl1 d1nlbh1
d4utal1 d1bjma1 d1eapb1 d1u6ah1 d2dtgd1 d1kj2b_ d1xeda1 d1c1eh1
d1fgvh_ d2bnub1 d2fb4h1 d2dtgb1 d1ktrh_ d1jpth1 d1qkzl1 d4hepg_
d2atpb1 d1flrh1 d2hrpl1 d1mvfa_ d1iaim1 d3k80a1 d2jb5h1 d3zkxc_
d4grwe_ d1it9h1 d1rhhb1 d3w9ec_ d1e4wh1 d1sy6h1 d2j6eh1 d3qxta_
d1jglh1 d1p7ka1 d1tzhb1 d4n8pa1 d1gafl1 d2jelh1 d1wioa2 d4i0cc1
d1cicb1 d1lmka2 d1lmka1 d1iqwh1 d1orqb1 d1ejoh1 d1jnlh1 d4zg1a_
d43c9b_ d1ggbl1 d1wejh1 d3q6fa1 d1f3dj1 d3c6la1 d1bqlh1 d3p9wb_
d1a3rl1 d1h0db1 d2uzil_ d1eo8l1 d1qfwl_ d1n7ml1 d1igja1 d1c5ch1
d2a9mh_ d1rmfh1 d1fj1b1 d4heme_ d1ccza1 d1a5fh1 d1ogad1 d1dlfh_
d1qa9b_ d1vgel1 d3qskb_ d1sjxa_ d4ozfg1 d1sq2n_ d1qd0a_ d1kcvh1
d1c5db1 d3qcul1 d8faba1 d3bn9d1 d1mjuh1 d4y8dc_ d7fabh1 d3dvgb1
d1lo0l1 d1a6tb1 d1gigh1 d1kcrh1 d2hwzl1 d1yjdc1 d3clfl1 d4krna_
d1dl7l_ d1e6ol1 d1mexh1 d3r0ma_ d4kkna_ d1fnsl1 d1oaqh_ d1rzia1
d1ucbh1 d1ncwl1 d4b41a_ d1nfdf1 d2mpah1 d2d7tl1 d4grwf_ d4b5ea_
d1lk3h1 d3u79a_ d4u3xa_ d1ghfl1 d1bvkb_ d1mcww1 d1b88a_ d1jrhh1
d32c2b1 d4wemb_ d1dsfh_ d1op3h1 d1ce1l1 d1iqda1 d1qoka1 d1qoka2
d1qfuh1 d2crya1 d1ay1h1 d1oaql_ d3ab0b_ d1rurl1 d1t4kb1 d1bz7b1
d1n0xh1 d1forl1 d1ucbl1 d4w6yb_ d43c9a_ d2aabl1 d1tzia1 d4n1ca_
d1k6qh1 d3g9ab_ d4tvsa1 d1igmh_ d1pz5b1 d3dx9a1 d3wxwh_ d1nakh1
d1kcul1 d3eaka_ d4qlra1 d1cr9l1 d1d9ka_ d1f4xl1 d1deea1 d2i27n2
d1c12a1 d2orbl1 d1j8hd1 d1mcph1 d1c5da1 d1kenh1 d1bd2e1 d1h5ba_
d4x7fc1 d3upaa_ d1t3fa1 d1indh1 d2dtgc1 d4hc1l1 d1zv5a1 d4wgvb_
d1uwgl1 d4f37k1 d1aqkh1 d1vesa_ d3zkqd_ d1fptl1 d1lo4l1 d1knob1
d3rrqa_ d1ct8b1 d4orzc_ d1mlbb1 d4hgkc1 d1dqdl1 d1bogb1 d4ozhe1

d1dr9a1 d1i7za1 d1dn0b1 d1kell1 d1rzib1 d1ypzf1 d1wtla_ d1hkfa_
d1fail1 d1figh1 d2vyre_ d1sjva_ d1ohqa_ d1bfoa1 d1pkqb1 d1mjul1
d1b4jl1 d1nsnl1 d2arjb1 d1p4bh_ d4aj0a_ d3nn8b_ d2rcja1 d1f2xk_
d4v1dd_ d1adqh1 d2eksa_ d4k3ga_ d1jfqh1 d1uweh1 d1frgh1 d4gftb_
d1uz8b1 d4n1ea_ d1bm3l1 d1clol1 d3mcka1 d2vq1a1 d1ae6h1 d4aq1b1
d1uj3b1 d1nbqa1 d1fh5l1 d2ck0l1 d2ywza_ d1q72l1 d4nbxb_ d1hila1
d4hzlb1 d2j88l1 d1zmya1 d1cd0a_ d1a4ka1 d1pskl1 d1a0qh1 d4krmb_
d1ncbh1 d2fbjh1 d1j8he1 d1i3ua_ d1cf8l1 d1fe8h1 d2p1ya1 d2x89a_
d1ol0a_ d2r0lh1 d1ub5a1 d1dfbh1 d3eyvb1 d4jdvb1 d4p2cg_ d2gsia1
d2agjl1 d1u3ha1 d2h1ph1 d1rihl1 d1fskc1 d1boga1 d2a77l1 d1ospl1
d1qfoa_ d3dwta_ d2fx7l1 d1rz7l1 d3stba1 d1pkqa1 d1b6da1 d1dzba2
d1dzba1 d1kj2a_ d4l1ha_ d1rzgb1 d1dqlh_ d12e8l1 d1miel1 d1jmaa_
d1sbsl1 d1fgnh1 d1f58l1 d1mqkh_ d1ndgb1 d3qegl1 d1iail1 d2aw2a1
d1tqbc1 d3fo2a1 d4dkaa_ d1f8th1 d2g60l1 d2ywya_ d1uj3a1 d1yc7a_
d3bd3a_ d4w6xb1 d1nmbl_ d1i8ka_ d1rjlb1 d1ypze1 d1p7kb1 d1kxqe_
d1gpoh1 d2z4qa1 d1w72h1 d1i9rl1 d4qgya1 d4xt1c_ d2qqnh1 d1cica1
d3c08h1 d2cd0a_ d2bdnh1 d1hnga1 d1maml1 d1nldh1 d1cr9h1 d4lcih1
d3bkyh1 d3qg7l1 d1ai1l1 d1kxvc_ d4iosd_ d1l7ih1 d2x1qa_ d1ub6b1
d3k74b_ d1ibgh1 d1teth1 d1igya1 d1hxma1 d2x6ma_ d2e7la_ d5e03a_
d1qfwm_ d2r0kh1 d1igjb1 d1rurh1 d1plgh1 d1mj8h1 d1yuhb1 d3fmgl1
d1rzfl1 d1emtl1 d1ad9b1 d1igtb1 d1nlbl1 d1d5ih1 d3k7ua_ d4ffyh_
d1bwwa_ d3dvga1 d1a6ta1 d1fgvl_ d4qxgl1 d1miml1 d1jhll_ d2hffb1
d2hh0l1 d1qkzh1 d1l7tl1 d1afvh1 d1iaii1 d2cjuh_ d1um5h1 d2ghwb2
d2ghwb1 d1kgce1 d2fgwh1 d1ikfh1 d1dvfd_ d2i24n1 d2xtjb_ d1za3b1
d2yc1a_ d2hh0h1 d2x1oa_ d1bvka_ d1rjla1 d5e7ba1 d1igta1 d1il1b1
d2fb4l1 d1mvua_ d1jptl1 d32c2a1 d1fvca_ d1iqdb1 d2hrph1 d2p4ab_
d4qo1a_ d1p2cb1 d1wt5c_ d1t4ka1 d5c1mb_ d1it9l1 d1bz7a1 d1b2wh1
d1e4wl1 d1jnll1 d1yedb1 d1za6b1 d3u7aa_ d1igfh1 d3gnml1 d1orsa1
d1wejl1 d1a2ya_ d1hxmb1 d1gafh1 d2j4wh1 d1pz5a1 d1j05a_ d1a3rh1
d4fqll1 d1qfwh_ d1eo8h1 d1n7mh1 d4s11a_ d1kegh1 d5da4a_ d1iqwl1
d3whxb1 d1vgeh1 d1deeb1 d1c12b1 d1t2ql1 d1ad9a1 d3gsnb1 d3b2uc1
d1ggbh1 d1t3fb1 d1nakl1 d4ocmc_ d1lila1 d2v7hb3 d2esve1 d1mcoh1
d2qr0b1 d2q3aa_ d1ct8a1 d1kcrl1 d2pxyb_ d2uzih_ d5c3ld_ d3durb_
d1c5cl1 d2ak4e1 d1dl7h_ d1xiwd_ d1mlba1 d1rmfl1 d1i7zb1 d1dlfl_
d2xa3a_ d5bq7b1 d1dqqb1 d3h42l1 d1egjh1 d1kcvl1 d1fe8l1 d2j6el1
d1f3rb2 d1lk3l1 d1sm3h1 d1q0xh1 d1ghfh1 d7fabl1 d1igch1 d1lo0h1
d4idla_ d5c2bl_ d1jrhl1 d1il1a1 d3zhda1 d4kfzc_ d1b0wa_ d1e6oh1
d1mexl1 d4krpb_ d1fnsh1 d1ncwh1 d4hgma_ d4eigb1 d4rzca_ d2mpal1
d4y5xa_ d5en2b1 d3lrga1 d4ej1c1 d1rhha1 d1j05b_ d1forh1 d1q9wb1
d2wzpd_ d2aq2a1 d1h0da1 d3revb1 d2ij0c1 d1tzha1 d4m7jl1 d1k6ql1
d3b9kc1 d1igml_ d3qeua1 d1dsfl_ d1ce1h1 d1kcuh1 d1zvha1 d1zvoa1
d1tqbb1 d1ay1l1 d2qscl1 d2cgrh1 d1nl0h1 d1kenl1 d3eo0a1 d4n9ob1
d1n0xl1 d3k1kc_ d2cqva1 d1fhga_ d2k1ma_ d2dl2a1 d1biha3 d1biha1
d1biha4 d3qp3a1 d1g1ca_ d1gxea_ d1cvsc2 d1cvsc1 d1xmwa2 d1m4ka2

d2otpa2 d1biha2 d1rhfa2 d2fcba2 d2fcba1 d1wwbx_ d1gsma2 d1epfa1
d1epfa2 d2dava1 d1waaa_ d1f97a2 d1x44a1 d1vcaa2 d1fnla2 d1fnla1
d3b5ha2 d3h8na2 d3h8na1 d1jbja1 d1jbja2 d1pd6a_ d1f2qa2 d1f2qa1
d3cx2a_ d1nbqa2 d3b5ha1 d1n26a1 d1xiwb_ d1xaua_ d1fltx_ d2ifga1
d1olla1 d1olla2 d1cs6a4 d1cs6a3 d1cs6a2 d1cs6a1 d2gw5a2 d1sy6a1
d1xiwa_ d1ucta2 d1ucta1 d1ry7b2 d1gl4b_ d1qz1a3 d1olza1 d2c9aa1
d1wwca_ d2aq2b1 d1ck1a1 d1enfa1 d1i4pa1 d1v1oa1 d1et9a1 d1esfa1
d2g9hd1 d4ohja1 d1bxta1 d1eu3a1 d1ty0a1 d1fnua1 d2qaln1 d1c4qa_
d1prtc1 d2wv6d_ d1prtf_ d4l63b1 d1r4pb_ d1prtd_ d1tl0f_ d2bosa_
d1prtb1 d3chbd_

**Family-58 (c.3.1.2)**

**Positive dataset contains 6 protein sequences.**

d2f5va1 d3c96a1 d2gmha1 d2dw4a2 d2ivda1 d2voua1

**Negative dataset contains 3870 protein sequences.**

d1m32a_ d1ibja_ d1c7na_ d1iuga_ d4q75a_ d2bkwa1 d1t3ia_ d1n8pa_
d1qz9a_ d1jf9a_ d1eg5a_ d1p3wa_ d3kgwa_ d1cl1a_ d2ctza1 d1vjoa_
d2rfva_ d1gc0a_ d1cs1a_ d1d2fa_ d2ch1a1 d1qgna_ d1pffa_ d1u08a_
d1lc5a_ d2ay1a_ d1bw0a_ d3pd6a_ d7aata_ d2csta_ d1j32a_ d1fg7a_
d4wlha_ d1o4sa_ d1v2da_ d1gdea_ d1m6sa_ d1v72a1 d1svva_ d1ajsa_
d1wsta1 d2f8ja2 d1lk9a_ d1vp4a1 d1xi9a1 d2gb3a1 d1yaaa_ d1jx4a2
d3gv5b1 d1t94a2 d3bq0a2 d3i2ka1 d1mpxa2 d2cfba1 d1o69a_ d1bj4a_
d3k28a_ d2bwpa_ d3lg0a_ d2fnua_ d3ou5a_ d1z7da1 d1b9ha_ d1iaya_
d4msoa_ d1bs0a_ d1s0aa_ d1sffa_ d3tqxa_ d1mdoa_ d3qboa_ d3pgya_
d1bjna_ d2dkja_ d1ejia_ d1vefa1 d4wxga_ d3bs8a_ d3gbxa1 d1w23a_
d2b5aa1 d2ofya1 d4mcxa_ d2xi8a_ d2icta_ d2bnma1 d2ppxa1 d1y7ya1
d1f56a_ d1bypa_ d1azca_ d1ws8a_ d3u25a_ d1cuoa_ d1id2a_ d1rkra_
d2iaac_ d4dp1x_ d1nwpa_ d2gima_ d2i7sa_ d1qhqa_ d3ehbb1 d1fftb1
d1gyca3 d1hfua2 d1kbva1 d1kbva2 d1sdda1 d1sdda2 d1gyca1 d1gyca2
d1mzya1 d2bw4a1 d1aoza1 d1aoza3 d1aoza2 d1kyaa2 d1kyaa3 d1mzya2
d4e9sa1 d1sddb1 d1sddb2 d1hfua1 d1hfua3 d1oe1a2 d1oe1a1 d1v10a2
d1v10a3 d1v10a1 g1f8v.1 d1novc_ d2bbvc_ g1or0.1 g1cp9.1 g1e3a.1
d3sxwa1 d4luka_ d1a3qa1 d1iknc_ d3brda1 d1p7hl1 d1oy3c_ d1gjia1
d1zk9a1 d2cxka1 d2bema_ d1pama1 d1ea9c1 d1qhoa1 d2fhfa2 d2fhfa1
d1k3ia1 d1j0ha1 d1cxla1 d2bhua1 d1edqa1 d1f1sa2 d1ut9a2 d1gvia1
d1txka1 d1adqa1 d1fnga1 d1uvqb1 d1zs8a1 d2vola1 d2vola2 d1mfbl2
d2jelh2 d1uwel2 d3qq4b1 d1kjvb_ d1k5na1 d1osph2 d4odhl2 d1ow0a1
d1ow0a2 d1bfob2 d3l9ra2 d3jtth1 d2fath2 d4elkb2 d1fn4b2 d2xfxb_
d1pg7x2 d1cqka_ d4k71b2 d1lk2a1 d1eapb2 d4lfhg2 d1i4fa1 d2nw2a2
d1kfah2 d1mhca1 d1h3ua1 d1wejh2 d1h0db2 d1a5fh2 d1kcvh2 d1mjuh2
d1qqda1 d1kcrh2 d1k8ia1 d3huja2 d2nnab1 d1nfdf2 d3mffb2 d1s9va1
d1lk3h2 d1q0xl2 d1fv1b1 d1neza1 d4rfeb2 d1r3ha1 d1a6tb2 d1kjva1
d1pz5b2 d1onqa1 d2bc4a2 d1de4a1 d1k8da1 d1tcra2 d1dn0b2 d1nfde2
d2xwtb2 d1bfoa2 d1mexh2 d1iakb1 d1ypzf2 d4f7ea2 d1q72l2 d2fbjh2
d1fe8h2 d1clzh2 d1fngb1 d1uvqa1 d1vgka1 d1c16a1 d4j12a2 d4x99b2

d1igtb3 d1je6a1 d1q72h2 d1nldh2 d1teth2 d2hmic2 d1i1ca2 d1i1ca1
d1k8ib1 d1q0yh2 d1kfal2 d3frua1 d1pg7w2 d1u58a1 d1igyb4 d1igyb3
d1hyrc1 d1klub1 d1deeb2 d1c12b2 d2bc4b2 d2ig2h2 d1kcvl2 d3wn5b1
d1lk2b_ d1lk3l2 d1mjul2 d1sm3h2 d1q0xh2 d1zvsa1 d1ydpa1 d1u8ib2
d1ypze2 d1kcuh2 d1zx5a1 d1qwra_ d2phla2 d2phla1 d1fxza2 d1fxza1
d1lrha_ d1uija1 g1dgw.1 d2v09a_ d1uika2 d1uika1 d1dgwa_ d1ep0a_
d2pa7a1 d1wlta1 d1nxma_ d2c0za1 d1g0da1 d4mh8a_ d1mu2a2 d4i7ga1
d1tafa_ d1bh9b_ d1tafb_ d1jfib_ d1q9ca_ d1h3ob_ d2byka1 d4g92b_
d1bh9a_ d1jfia_ d1h3oa_ d2bykb1 d1n1jb_ d2bjca1 d2hsga1 d1qpza1
d1efaa1 d1ydwa2 d2nvwa2 d1h6da2 d1zh8a2 d1qkia2 d1xeaa2 d1tlta2
d1au7a2 d1ic8a2 d3gpdg2 d2q49a2 d1hdgo2 d2g17a2 d2cvoa2 d2czca1
d3pwka2 d1obfo2 d1i32a2 d1zyao2 d2pkqo2 d1b7go2 d2hjsa2 d1cf2o2
d1vkna2 d1nvmb2 d2m9wa1 d3dfxa_ d2msya_ d2djoa1 d2r5za_ d1au7a1
d1f43a_ d2lmda1 d1akhb_ d1s7ea1 d3nara_ d1zq3p1 d2craa1 d1mija_
d4uuta_ d3a01a_ d1x58a1 d3rkqa1 d3cmya1 d2cqxa1 d2da6a1 d1e3oc1
d1fjla_ d1mh3a1 d1ig7a_ d1x41a1 d1wh7a1 d1gt0c1 d2mw8a_ d1lfup_
d1x2ma1 d1b72a_ d1wi3a1 d1jgga_ d1wjha1 d1x2na1 d1mnmc_ d2cuea1
d3k2aa_ d2hosa_ d2jwta2 d1pufa_ d1octc1 d2hi3a_ d1b8ia_ d4j19a_
d3lnqa_ d1wh5a1 d1b8ib_ d1fexa_ d2cqra1 d1guua_ d1ug2a1 d2cqqa1
d2ajea1 d4a69c_ d1h8ac1 d2crga1 d2iw5b1 d1xc5a1 d1ofcx1 d2cu7a1
d1wgxa1 d2cjja1 d2ckxa1 d1gvda_ d1hcra_ d1tc3c_ d1gdta1 d1u78a2
d6paxa1 d6paxa2 d1k78a1 d1igna2 d1prtb2 d3lv8a_ d1s3ga1 d1zaka1
d1s4qa_ d3tvta2 d1uf9a_ d1khta_ d1ckea_ d1osna_ d1p5zb_ d4tmka_
d3cm0a_ d1tmka_ d1jaga_ d3h86a_ d1t0hb_ d1nn5a_ d2h92a_ d3kb2a1
d2c95a1 d1yj5a2 d1kgda_ d1ly1a_ d2ak3a1 d1vyva2 d1deka_ d2grja1
d1p3ja1 d1y63a_ d1e4ya1 d1s96a_ d3iija1 d1lvga_ d1nksa_ d1vyua2
d4e22a_ d1qf9a_ d1jjva_ d1q3ta_ d1ki9a_ d1lw7a2 d1teva_ d1kjwa2
d1gsia_ d2iyva2 d1kaga_ d1viaa_ d1e6ca_ d1g3ma_ d1j99a_ d1t8ta1
d1nsta_ d1fmja_ d1zrha_ d1ls6a_ d2a3ra_ d1aqua_ d2zptx_ d3ckla_
d1texa_ d2gwha_ d1odfa_ d1uj2a_ d4gi7a_ d2jeoa1 d1z0ja1 d3umra2
d3gj0a1 d2gaoa1 d1yzga1 d1x3sa1 d1g7sa4 d1puja_ d1udxa2 d1kk1a3
d2o52a_ d1jwyb_ d1zcaa2 d1zcba2 d2fv8a1 d2cjwa_ d3dz8a1 d1xzpa2
d1vg8a_ d4qxaa_ d2ew1a1 d1x1ra1 d2bcgy_ d3doea1 d3cona_ d3rega_
d3wyga_ d1upta_ d2b6ha1 d1zd9a1 d1lnza2 d1r5ba3 d1n0ua2 d1r2qa_
d2f9la1 d2rhda_ d3q3jb_ d3q85a_ d1efma1 d1r8qa_ d2gf9a1 d2akab1
d1ek0a_ d1z08a1 d2gcoa1 d2dy1a2 d4g01b_ d2cxxa1 d1svia_ d3q72a_
d1ni3a1 d4ac9a4 d2erxa1 d2bmea_ d1wxqa1 d2gf0a1 d4ldja1 d1zunb3
d1d5ca_ d4lv5b_ d1efca3 d1kmqa_ d2g6ba1 d3lrpa_ d1tada2 d2y8ea_
d4rd4a1 d1z06a1 d2heia_ d3a58b_ d1u0la2 d1d2ea3 d1zj6a1 d2ocba_
d3rwoa_ d1mr3f_ d2e9sa1 d4u5xa1 d2iwra_ d2fn4a1 d3bwdd_ d2nzja1
d2atva1 d2ksqa_ d3l43a_ d1mkya2 d1mkya1 d1wmsa_ d1jnya3 d2rmka_
d1f6ba_ d1f60a3 d1gota2 d1jala1 d2fh5b1 d1g16a_ d2wblc_ d1m7ba_
d1wf3a1 d1f5na2 d1t9ha2 d1z6xa1 d1z0aa1 d2fg5a1 d2a5ja1 d2bcjq2
d1s0ua3 d1ky3a_ d4y0va_ d4lhwa_ d2qmea1 d2atxa1 d1egaa1 d1ryha_

d1moza_ d1nrjb_ d2erya1 d3bfka_ d1puia_ d1fzqa_ d3j3az1 d3t5ga_
d1shza2 d3defa_ d2f7sa1 d3raba_ d3tsoa_ d3aq4a_ d2al7a1 d3sfva1
d3bc1a_ d2ncda_ d1sdma_ d3h4sa1 d2heha_ d2grya_ g3kin.1 d1kk8a2
d1br2a2 d1goja_ d1f9va_ d2mysa2 d1lkxa_ d3t0qa_ d4db1a1 d3gbja_
d1ry6a_ d1k8ka2 d1k8ka1 d3uleb1 d3i33a2 d3i33a1 d1bupa1 d1dkgd2
d1dkgd1 d4ci6a2 d1jcfa1 d4a2ba2 d2fxua1 d1yaga2 d1yaga1 d4cbua2
d1bupa2 d3vrda1 d3vrda2 d1fcdc2 d1fcdc1 d1h1oa2 d1h1oa1 d1m70a2
d1ig8a1 d1czan2 d1czan3 d1czan1 d1ig8a2 d1czan4 d1bg3a4 d1bg3a3
d1bg3a2 d1nira1 d1qksa1 d3h1jd1 d2d0oa3 d2d0oa2 d1nbwa3 d1hroa_
d4gedb_ d3zooa_ d1mz4a_ d1gu2a_ d1f1fa_ d1dvva_ d1c6ra_ d1c75a_
d1e29a_ d1gdva_ d1cnoa_ d1h32b_ d1co6a_ d2bh4x_ d2aiua_ d5cytr_
d1jdla_ d1i8oa_ d2zboa_ d3dmia_ d1ls9a_ d1wvec_ d2yk3a_ d3ph2b_
d1dw0a_ d5aura_ d1ql3a_ d1qn2a_ d1f1ca_ d2nqya_ d1h4ga_ d1oa2a_
d1olra_ d3b7ma_ d4h7ma1 d3wp3a_ d2vgda_ d1igoa_ d3zsea_ d2nlra_
d3wx5a_ d1oa3a_ d1pvxa_ d1m4wa_ d1f5ja_ d1oa4a_ d1ks5a_ d4ixla1
d2ah2a1 d1n1ta1 d1evsa_ d1cnt1_ d1pvhb_ d1z7ca_ d1huwa_ d1bgea_
d1rhga_ d1f6fa_ d1f45b_ d1i1rb_ d1axia_ d1etea_ d3tgxb_ d1scfa_
d2z3qa1 d3qb1a_ d1hula_ d1m48a_ d4nkqb_ d1eera_ d1v7mv_ d5e4ea_
d2o27a_ d3b5ka_ d2psma1 d4zf7a_ d3uf5a_ d4x51a_ d1d9ca_ d4ypgc1
d3oq3a_ d1lqsl_ d1n1fa_ d1au1a_ d1wu3i_ d3besl_ d3ux9a_ d1v8fa_
d2ejca_ d3muea1 d1ihoa_ d3q12a1 d4dnxa2 d1m8pa2 d1g8fa2 d1jhda2
d1x6va2 d3rbaa_ d2qtra_ d1nupa_ d3l92a_ d1f9aa_ d1qjca_ d2h29a_
d1vlha_ d1od6a_ d1lw7a1 d1mrza2 d4ruka_ d3wdpp1 d3wq5a_ d1gnxa_
d4gxpa_ d4hz8a_ d1e4mm_ d3aiua_ d1wcga1 d1qvba_ d3ahxa_ d1v02a_
d2dgaa_ d1pbga_ d1vffa1 d1qoxa_ d4ha4a_ d2el7a_ d1iq0a2 d1irxa2
d1r6ta2 d1ivsa4 d2cyba_ d1rqga2 d1j09a2 d2yxna_ d1f7ua2 d1h3fa1
d1ffya3 d2g36a1 d1nzja_ d1n3la_ d1i6la_ d1pfva2 d1h3na3 d3n9ia_
d3sz3a1 d3sw2a_ d1moxc_ d1dx5i3 d1edmb_ d1autl2 d1autl1 d1nqlb_
d1dval1 d1szba2 d1kigl_ d2y5fl_ d1ijqa2 d1gk5a_ d1g1sa2 d1nt0a3
d1lmja2 d1lmja1 d1nzia2 d2wphe_ d1klil_ d1pfxl2 d1pfxl1 d1p9ja_
d1g1ta2 d1f7ea_ d4yzub_ d1nuba2 d1m1xb5 d1qx2a_ d2y5ia_ d1qlsa_
d1psra_ d3n22a_ d2egda_ d4ggfc_ d2wcea_ d2luca_ d4aqia_ d3nsia_
d2lp3a_ d1j55a_ d1a4pa_ d1k8ua_ d1nsha_ d1exra_ d1tiza1 d1nyaa_
d1g8ia_ d1ggwa_ d1wdcc_ d3fwba_ d1ggza_ d2o5ga_ d1ctaa_ d1m45a_
d3i5gc_ d1sl8a_ d1fpwa_ d2scpa_ d2roaa_ d4aqra_ d3pn7b_ d1br1b_
d2mysb_ d1s6ca_ d1bjfa_ d2obha_ d1jfka_ d1jf0a_ d1qv0a_ d1lkja_
d2mlfc_ d2m97a_ d2d8na_ d4by5a_ d1jbaa_ d4nqga_ d1wdcb_ d2mysc_
d1j7qa_ d1s6ia_ d1uhka2 d1r2ua_ d1dtla_ d3kf9a_ d3pn7c_ d1gjya_
d1y1xa_ d1hqva_ d1dvia_ d1df0a1 d1qxpa2 d4usla_ d1k94a_ d1wkya2
d1uuqa_ d3wh9a_ d4m1ra_ d4xuya_ d2je8a5 d4bf7a1 d3cuia_ d1ur4a_
d1qw9a2 d1odza_ d2vzsa5 d1bqca_ d2x2ya1 d1hjqa_ d1qnra_ d4y7ea1
d1vjza_ d4k68a_ d2v3ga_ d4hpga_ d1nofa2 d1nq6a_ d1h4pa_ d1aq0a_
d3ziza_ d4bs0a_ d1ecea_ d1h1na_ d1ta3b_ d1w91a2 d1i1wa_ d1uhva2
d1us3a2 d1egza_ d7a3ha_ d1tvna1 d3nyda1 d1ghsa_ d2cyga1 d4xv0a_

d2c8na2 d1w32a_ d3emca_ d1v6ya1 d1foba_ d2uwfa1 d2fgla_ d3niya1
d2c0ha1 d1rh9a1 d1yq2a5 d1szna2 d1fa2a_ d1pama4 d5do8a1 d1ea9c3
d1hx0a2 d2fhfa5 d1qhoa4 d1avaa2 d1m53a2 d4m8ua1 d2gjpa2 d1lwha2
d4howa1 d1ktba2 d1j0ha3 d3vm5a1 d2x1ia_ d1wzaa2 d1uasa2 d2diea1
d1mxga2 d1ht6a2 d1eswa_ d1iv8a2 d5cpsa_ d1ud2a2 d1zjaa1 d3gbea1
d1kwga2 d1wdpa_ d1gcya2 d1g5aa2 d1hvxa2 d3dhpa2 d2bhua3 d4do4a1
d3qgva1 d1e43a2 d1g94a2 d2d3na2 d1gjwa2 d1gvia3 d1x1na1 d1r7aa2
d1tz7a1 d4c7ga2 d2gjxa1 d1jaka1 d1yhta1 d4toqa_ d1wb0a1 d1w9pa1
d1eoka_ d1itxa1 d1kfwa1 d1edqa2 d2gsja_ d1hjxa1 d1jnda1 d1vf8a1
d1umqa_ d1g2ha_ d1mixa2 d1e5wa2 d1j19a2 d1isna2 d2al6b2 d1g4ma1
d1g4ma2 d2y8ga_ d1xkea1 d2y8fa_ d1b9ra_ d1offa_ d1frra_ d3wcqa_
d1e9ma_ d1l5pa_ d1wria_ d1i7ha_ d3av8a_ d1czpa_ d1rfka_ d3p1ma1
d3b2ga_ d2bt6a2 d3nvva1 d1krha3 d2h88b1 d1n62a2 d1t3qa2 d1zoyb1
d1jq4a_ d1ffva2 d1dgja2 d1rm6c2 d9ldta1 d2ewda3 d1up7a1 d2dfda1
d1s6ya1 d1vjta1 d7mdha1 d1hyea1 d1t2da1 d1ojsa3 d1b8pa1 d1ldna1
d1hyha1 d1civa1 d2a92a1 d1ez4a1 d1i0za1 d1y6ja1 d1gv0a1 d1ur5a1
d1llda1 d1obba1 d1v8ba1 d1gdha1 d2dlda1 d1mx3a1 d1ygya1 d1qp8a1
d1li4a1 d1j4aa1 d2naca1 d1npya1 d3t4ea2 d1a4ia1 d1o0sa1 d2hk9a2
d2egga2 d1b26a1 d1bvua1 d1gq2a1 d1c1da1 d1leha1 d1gtma1 d1euza1
d1nvta1 d3dona2 d1edza1 d1gpja2 d1vi2a1 d1p77a1 d1b0aa1 d1vl6a1
d1v9la1 d1txga2 d3f47a1 d1mv8a2 d2pv7a2 d1xc2a3 d2ahra2 d4xiya1
d2f1ka2 d1pgja2 d1i36a2 d1np3a2 d1lj8a4 d1teaa3 d1qmga2 d2i76a2
d2g5ca2 d1wdka3 d1dlja2 d3cuma3 d1kola2 d1f8fa2 d1qora2 d1jqba2
d1vj0a2 d1cdoa2 d1gu7a2 d1d1ta2 d1xa0a2 d2dm6a2 d1jvba2 d1rjwa2
d1llua2 d1h2ba2 d1tt7a2 d1piwa2 d1o89a2 d1iz0a2 d1e3ja2 d1yb5a2
d1uufa2 d1vj1a2 d1e3ia2 d3gpdg1 d2nvwa1 d1u8fo1 d1ydwa1 d1hdgo1
d2g17a1 d1cf2o1 d2czca2 d1r0ka2 d1obfo1 d2cvoa1 d1ebfa1 d1h6da1
d1i32a1 d1zh8a1 d2q49a1 d1qkia1 d1j5pa4 d3pzra1 d1xeaa1 d1vkoa1
d1vm6a3 d3qvsa1 d2pkqo1 d1b7go1 d1gr0a1 d1p1ja1 d1lc0a1 d1j0xo1
d3pwka1 d2hjsa1 d1zyao1 d1vkna1 d1tlta1 d1nvmb1 d3sjua_ d3ojfa1
d1xu9a1 d2jjya1 d1n7ha_ d1y1pa1 d1h5qa_ d1jtva_ d4fs3a_ d2bkaa1
d1gy8a_ d2c5aa1 d1ulsa_ d1ooea_ d2b69a1 d1y5ma1 d1spxa_ d1ae1a_
d1bxka_ d1ydea1 d1gega_ d1hdoa_ d1o5ia_ d1e6ua_ d1snya1 d2rhca_
d3zv4a_ d5ceja_ d2d1ya1 d1fjha_ d3w8ea_ d2fmua1 d1x1ta_ d1rpna_
d3ay7a1 d1k2wa_ d1eq2a_ d2hrba1 d3ruaa_ d2ew8a1 d4hwka_ d1xgka_
d2bd0a1 d1yo6a1 d2a35a1 d2ntva_ d1vl8a_ d1hdca_ d4bkua_ d2ag5a1
d2q45a_ d1nffa_ d4iy1a_ d1zbqa1 d1kewa_ d1ek6a_ d1ja9a_ d2q46a2
d1iy8a_ d1cyda_ d2ptga_ d1hxha_ d1uzma1 d3lyla_ d4m89a_ d3wyea_
d2bl1a1 d1w6ua_ d1sb8a_ d2q2va_ d4nr0a_ d1e6wa_ d2gasa_ d3oiga1
d2bgka1 d1mxha_ d1vl0a1 d1rkxa_ d1r6da_ d1wmaa1 d1e7wa_ d2c07a1
d1xkqa_ d3tzha_ d1g0oa_ d3lz6a_ d1qyca_ d1edoa_ d1gz6a_ d1oc2a_
d1t2aa_ d1zema1 d2xoxa_ d1z45a2 d1geea_ d1p33a_ d3lt0a_ d4e6pa_
d2wyua_ d1db3a_ d4q9na_ d1xhla_ d4o1ma_ d1fmca_ d2uvda_ d2gdza1
d1uaya_ d2z1ma_ d2o23a2 d2fr1a2 d2fr1a1 d1qsga_ d1qyda_ d4zjua_

d2a4ka1 d1yb1a_ d1wvga1 d1yxma1 d3f4ba_ d1mg5a_ d1xg5a1 d1r55a1
d3gboa_ d1atla_ d1kufa_ d1buda_ d4q1la_ d1yp1a_ d1wnia_ d2gfoa1
d1vjva_ d2ayna1 d3nhea_ d4ekfa_ d2ckga_ d1th0a_ d2bkra_ d1xd3a_
d3ifwa_ d1ex0a4 d1g0da4 d2f4ma1 d1x3za_ d1mdwa_ d1qxpa4 d1ziva1
d1zcma1 d3ioqa_ d1fh0a_ d1o0ea_ d1m6da_ d2cb5a_ d1cs8a_ g8pch.1
d1s4va_ d2pnsa_ d1vsna1 d1mira_ d1iwda_ d2oula_ d7pcka_ d3bwka_
d3bcna_ d2p86a_ d1pvja_ d1pcia_ d1gece_ d1deua_ d4bs6a_ d1xkga1
d1cqda_ d1jqpa2 d2c0ya_ d2bdza_ d3p5ua_ d2o36a_ d1k9xa_ d1ex4a2
d1c6va_ d1zbfa1 d2z1ha_ d2etja1 d3qioa1 d1jl1a_ d2e4la_ d2lsna1
d1io2a_ d1uaxa_ d2yv0x_ d1mu2a1 d1ekea_ d1i39a_ d1iv0a_ d1nu0a_
d2d5ra1 d2v76a_ d1wj1a1 d1aqca_ d1qqga2 d1qqga1 d1ntva_ d1n3ha_
d4dbba_ d3f0wa1 d1p3ra_ d1wgua1 d2cy4a1 d3hqca_ d1wvha1 d1xr0b1
d1r7ha_ d1faaa_ d1wika1 d1xfla_ d1ttza1 d2f51a1 d1ti3a_ d1nhoa_
d1sena_ d2i4aa_ d1ep7a_ d1zmaa1 d1fo5a_ d3lefa1 d3diea1 d2yana1
d1z6na1 d1oaza_ d3zywa1 d4aj8a_ d1nm3a1 d4fiwa_ d1wmja_ d4hs1a1
d1iloa_ d1xwaa1 d1wjka1 d2vm1a_ d1fb6a_ d1gh2a_ d1h75a_ d1nw2a_
d1kx5b_ d3a6na_ d4m6ba2 d2nqbd_ d3wkjd_ d2yfwe_ d4h9na_ d5bnxa_
d1kx5c_ d3r45a_ d1eqzb_ d4jjnb_ d1f66c_ d1eqza_ d4wnnb_ d1kx5a_
d1u35c1 d1id3a_ d2nqbc_ d1kx5d_ d1f1ea_ d1b67a_ d2b5ea2 d2b5ea3
d2b5ea1 d2b5ea4 d2kp1a1 d2kp2a1 d1hyua4 d1j08a1 d1j08a2 d1pd212
d1nhya2 d2a2ra2 d1k0dd2 d1f2ea2 d1gsua2 d1f3ba2 d2fhea2 d2pvqa1
d1tw9a2 d1oyja2 d1e6ba2 d1k0ma2 d3ik7a1 d4is0a1 d1r5aa2 d1jlva2
d1m0ua2 d3gtub2 d1z9ha2 d1okta2 d1axda2 d2gsra2 d1tu7a2 d1k3ya2
d1zgma1 d1b48a2 d1gnwa2 d1gwca2 d2gsta2 d1glqa2 d1oe8a2 d1pn9a2
d1v2aa2 d1fw1a2 d1duga2 d2trcp_ d1iqca2 d1iqca1 d1eb7a2 d1eb7a1
d1nmla1 d174la_ d169la_ d2o4wa_ d1p37a_ d1k28a3 d1xjta1 d1jtma_
d2f2qa_ d4e97a1 d2za0a_ d2c21a1 d2pjsa1 d1klla_ d2i7ra1 d1ecsa_
d1xqaa1 d5cj3a1 d1jifa_ d2p7oa_ d1r9ca_ d1nkia_ d1mpya2 d1kw3b2
d1kw3b1 d4ghga1 d1tfza1 d3isqa1 d3isqa2 d1sqia1 d1sqia2 d1lkda2
d1lkda1 d1mpya1 d1cjxa2 d1cjxa1 d1sp9b2 d1t47a1 d1t47a2 d1f1ua2
d1sp8a1 d1sp8a2 d1f1ua1 d1iyka2 d1rxta1 d1iica2 d1iica1 d1qsta_
d2beia1 d4m3sa_ d1u6ma1 d1ygha_ d1yk3a1 d3dr6a_ d1xmta_ d1z4ea1
d2ge3a1 d1bo4a_ d2fiaa1 d2jdca1 d1y9wa1 d3to7a_ d1wwza1 d2fl4a1
d2ozua1 d2b5ga_ d2cy2a1 d2p0wa_ d1cm0a_ d4pswa_ d1yrea1 d1yr0a1
d1m4ia_ d2gana1 d1yvka1 d1ghea_ d2g3aa1 d1y7ra1 d2euia1 d1q2ya_
d2fsra1 d1xeba_ d2ae6a1 d1cjwa_ d1qsma_ d2aj6a1 d1y9ka1 d1i12a_
d1vkca_ d2atra1 d1n71a_ d2d4pa1 d2fiwa1 d2fe7a1 d1tiqa1 d2ou2a_
d1yx0a1 d2fcka1 d1p0ha_ d2j8ma_ d2pq8a_ d1z4ra1 d1s3za1 d1ufha_
d1xvqa1 d1pn0a2 d3hy2a_ d2cvba1 d1o8xa_ d1lu4a_ d1we0a1 d2bmxa1
d2a4va1 d1psqa_ d1xiya1 d1q98a_ d1n8ja_ d1zofa1 d2ggta_ d3tjja_
d4je1a1 d1o73a_ d3s9fa_ d2zcta_ d1qmva_ d1jfua_ d2b1ka_ d5dvba1
d2b5xa1 d2v2ga_ d3tb2a_ d4llra_ d4fh8a1 d1e2ya_ d1knga_ d2h30a_
d1i5ga_ d2z9sa_ d2f8aa1 d1tp9a1 d2b7ka_ d1nm3a2 d3hvva_ d2wfca1
d4kcea_ d1xvwa1 d4gqca1 d3qpma_ d4k1fa_ d1zzoa1 d1gp1a_ d1prxa_

d1e88a1 d1l6ja4 d1eaka4 d1eaka3 d1l6ja3 d1h8pa2 d1h8pa1 d1jgta2
d1ofda3 d1ea0a3 d1ecfa2 d1q15a2 d1ct9a2 d1te5a_ d1ra6a_ d1muka_
d3gsza_ d1khva_ d1te9a_ d2xwha_ d3uqsa_ d3hkwa_ d3n6ma_ d2cjqa_
d2xyma_ d1teba_ d1te8a_ d1u09a1 d4wfxa_ d3tyqa_ d2pgga1 d2h1ia1
d2r8ba1 d3b5ea1 d3cn9a_ d1auoa_ d1lgya_ d4l3wa_ d1gz7a_ d1ukca_
d1j1ia_ d2rhwa_ d3groa_ d1pjaa_ d1thta_ d2ftba_ d1pmpa_ d2rcqa1
d1fdqa_ d4lkpa_ d1sa8a_ d1vyfa_ d1lpja_ d3vg7a1 d4azqa1 d1o8va_
d5bvqa_ d1p6pa_ d2qo4a_ d1opba_ d1yiva1 d1o1va_ d4bvma1 d4tkba_
d4i3ba_ d1bwya_ d1ftpa_ d1ggla_ d4gkca_ d1cbia_ d3p6da1 d1kqwa_
d2a0aa1 d3zxha_ d1cgla_ d1rm8a_ d1eaka2 d1hy7a_ d2jsda_ d1cxva_
d1hv5a_ d1i76a_ d1g12a_ d3p0ya1 d1igra1 d1igra2 d1n8yc2 d1n8yc1
d1m6ba2 d1m6ba1 d2bk7a_ d3fjua_ d1z5ra1 d1kwma1 d1uwya2 d1jqga1
d2c1ca1 d1dtda_ d1h8la2 d1h6ta2 d2tbvc_ d1c8nc_ d1x36a_ d1smvc_
d1ng0c_ d4sbvc_ d1opoa_ d1f2nc_ d1cixa_ d1y29a_ d1v7fa_ d2jtba_
d1d1ha_ d1g9pa_ d1lupa_ d1qk7a_ d1mb6a_ d2a2va_ d1niya_ d1i25a_
d1hvwa_ d2mpqa_ d1ie6a_ d1kqha_ d1la4a_ d2mxoa1 d1dl0a_ d1koza_
d1qk6a_ d1emxa_ d1lqra_ d1c6wa_ d1rmka_ d1ixta_ d1eyoa_ d1fu3a_
d1g1pa_ d1f3ka_ d1fyga_ d1rypd_ d1irua_ d1rypc_ d3unfk_ d1rypa_
d1j2pa_ d1irui_ d1j2qh_ d3unfh_ d1m4ya_ d1rypb_ d1irul_ d1irug_
d1iru2_ d1q5rh_ d1rypg_ d1g3ka_ d1iruf_ d1q5qa_ d1e94a_ d1irub_
d1ryph_ d3ty6a_ d1iruj_ d1iruk_ d1rype_ d1irud_ d1irue_ d3mi01_
d1rypf_ d1iruh_ d2z3ba_ d1iruc_ d3mka2_ d1iru1_ d1ny721 d1ny722
d1a6ca3 d1a6ca2 d1pgl22 d1pgl21 d1pgl11 d1ny711 d1mqta_ d1d4m3_
d1fmd3_ d2mev1_ g1aym.1 d1ev13_ d4q4w1_ d1r1a3_ d2x5ia_ d1rhi1_
g1r1a.1 g1d4m.1 g1rhi.1 d1b35b_ d1oopa_ d1tme1_ d4iv1a_ g1pvc.1
g1fpn.1 d1fpn1_ d4gb31_ d1d4m1_ d1fmd1_ d1ev11_ d2wzr3_ g1cov.1
d1qqp3_ g1fmd.1 d2x5ic_ d1rhi3_ g1oop.1 d1hxs1_ d1fpn3_ d1bev1_
g2mev.1 d1eah3_ d1aym1_ d1pvc3_ d1qqp1_ d1zba3_ d1h8ta_ d3vbhc_
g1b35.1 g1bev.1 d1hxs3_ g1ev1.1 d1bev3_ g1tmf.1 d1tmf1_ d1b35a_
d2mev3_ g1eah.1 g1h8t.1 d1eah1_ d4q4w3_ d1r1a1_ d1aym3_ d1pvc1_
d1zba1_ d1h8tc_ d1oopc_ d1tme3_ g1qqp.1 d4iv1c_ g1tme.1 d1y14b1
d2nn6i1 d1i94l_ d2zjra2 d1smxa_ d2qamc2 d1hh2p1 d2r7da1 d1rl2a2
d1wfqa1 d3bzka4 d1x6oa2 d3a0ja_ d2j01d2 d2ja9a1 d4ql5a_ d1hr0w_
d1luza_ d2n3sa_ d2vnud1 d1h95a_ d1go3e1 d2nn6h1 d1ne3a_ d2mo1a_
d1iz6a2 d1jjga_ d1e3pa2 d2ix0a3 d2ix0a2 d2lxja_ d1ueba3 d1ueba2
d2z0sa1 d1u0la1 d2ix0a1 d2eifa2 d2je6i1 d3i2za1 d2c35b1 d2ahob2
d1t9ha1 d3i4oa_ d2qall1 d1jt8a_ d2nn6g1 d1g6pa_ d3cama_ d1i94q_
d1c9oa_ d1d7qa_ d2ba0a1 d2vnud2 d2vnud3 d1khia2 d2qalq1 d1wi5a1
d1xtda2 d1ny4a_ d1eixa_ d3exra_ d2ffca1 d2fdsa1 d3n3ma1 d2czda_
d1q6oa_ d2yyua_ d1vqta1 d1dbta_ d3ldva1 d4lt9a1 d3tc7a1 d3hoja_
d1i4na_ d3vnda_ d3nl8a1 d1geqa_ d3nava1 d1v5xa_ d4a2ra1 d3tc6a1
d1ujpa_ d4ijba_ d1xcfa_ d1vc4a_ d1rd5a_ d1qopa_ d3b5va_ d2r80b_
d1cqxa1 d1cg5a_ d3boma_ d2h8fb_ d2raoa_ d3bj1a_ d4hrrb_ d2r50a_
d3d1kb_ d2aa1b1 d3zhwa_ d3hf4b_ d3gdja_ d1jebb_ d3bj1b_ d1x9fb_

d1wmub_ d1gcva_ d3bcqb_ d3d4xb_ d1gvha1 d2dc3a_ d3hf4a_ d4yu3a_
d3k8ba_ d1qpwb_ d2r80a_ d3bcqa_ d1v4wa_ d3wtgb_ d1jl7a_ d4esaa_
d1a9we_ d3d1ab_ d1fsla_ d4h2la_ d1v4wb_ d1mwca_ d1vhba_ d1jeba_
d1spgb_ d1h97a_ d1q1fa_ d1tu9a1 d3d1aa_ d1hdsb_ d3hyub_ d2zfbb_
d3fs4a_ d1oj6a_ d1shrb_ d4h2lb_ d4yu3b_ d1spga_ d3hrwa_ d3qqra_
d3at5a_ d4wchd_ d3hyua_ d1cg5b_ d1uc3a_ d1hbra_ d2raob_ d1outb_
d4esab_ d3gdjb_ d3qqqa_ d1x9fc_ d3vrfa_ d1itha_ d3mkba_ d3d4xa_
d3mjpa_ d1jl6a_ d3fh9a_ d1outa_ d2dc3b_ d1it2a_ d3eoka_ d3g46a_
d1qpwa_ d3vrfb_ d1ch4a_ d1x9fd_ d1hdsa_ d2zfba_ d3mkbb_ d1or4a_
d3wtga_ d1a4fa_ d3fh9b_ d3bomb_ d3cy5a_ d3at5b_ d1x9fa_ d2oifa_
d4lmxb_ d1cpcb_ d3v57a_ d1kn1a_ d1cpca_ d4f0ta_ d2vjha_ d4po5b_
d1eyxa_ d1alla_ d4f0tb_ d1b33b_ d1b8db_ d4h0mb_ d2vjhb_ d4lm6b_
d2vmlb_ d1allb_ d2vjta_ d3dbja_ d2vmla_ d2vjtb_ d4f0ub_ d1f99b_
d4rmpa1 d1liaa_ d1eyxb_ d2uulb_ d4lmsb_ d4f0ua_ d2bv8a_ d1f99a_
d2bv8b_ d2c7lb_ d3dbjb_ d4rmpb1 d1b8da_ d4h0ma_ d3v57b_ d1kn1b_
d1liab_ d2uula_ d2j96a_ d3ia2a_ d4dgqa1 d1hkha_ d1zoia_ d3nqyb_
d4gera_ d1azwa_ d1mtza_ d2xqfa_ d1ea5a_ d1f6wa_ d1dx4a_ d1qe3a_
d1k4ya_ d4qwwa_ d2ha2a_ d3c8da2 d2pbla1 d1jkma_ d1jjia_ d1lzla_
d2qvba_ d2v9za_ d4kafa1 d4iuaa3 d4bvva_ d5hpga_ d3k65a_ d1ki0a3
d1ki0a2 d1ki0a1 d1a0ha1 d1pmla_ d3sp8a3 d2feba1 d1gvfa_ d3qm3a1
d2fjka_ d1f74a_ d3l21a_ d2e1da_ d1to3a1 d1w3ia_ d1sfla_ d1n7ka_
d1vcva1 d4imea_ d1wa3a_ d2ocza1 d3m16a_ d2yxga_ d1ub3a_ d3a5fa_
d1fbaa_ d1l6wa_ d1xfba1 d1wx0a1 d1vpxa1 d1ojxa_ d1mxsa_ d2a4aa1
d1xkya1 d3cq0a1 d3s1xa_ d1f05a_ d1fdja_ d3cpra1 d4d2ja_ d1cg2a1
d1yloa2 d1y7ea2 d1y0ya3 d1lfwa1 d1vixa1 d1fnoa4 d1vhoa2 d1ysja1
d1vhea2 d1z2la1 d2fvga2 d1vgya1 d1r3na1 d2grea2 d1xmba1 g1q7l.1
d3rp2a_ g1ucy.1 d1fuja_ d2zchp_ d3w94a_ d1si5h_ d1lo6a_ g2pka.1
d1cgha_ d1kigh_ d1a5ia_ d3s9ba_ d1hj8a_ d1ao5a_ d2anya_ d1q3xa1
d1gvza_ d1azza_ d4gsoa_ d2f91a1 d1iaua_ d1gdna_ d1eufa_ g1fiw.1
d1eq9a_ d4nfea_ d2rdla_ d1spja_ d1elva1 d1a7sa_ d1ekbb_ d1pfxc_
d1yc0a_ d2aiqa_ g1fiz.1 d1fi8a_ d4k60a_ d4dgja_ d1mzaa_ d1op0a_
d3tjva_ d1pytd_ d1sgfg_ g1gg6.1 d2eeka_ d1a0ja_ d3g01a_ d1pytc_
d4e7na_ d1ddja_ d1z8ga1 d2hlca_ d1npma_ d1brup_ g1jou.1 d1sgfa_
d1autc_ g2hnt.1 g1kdq.1 d1orfa_ d3s69a_ d4crga_ d1fxya_ d1bqya_
d1svpa_ d1mbma_ d3su6a1 d4agka_ d1vcpa_ d1q31a_ g1n1l.1 d3e90b_
d1ep5a_ d3u1jb_ d1dzka_ d4tlja_ d2a2ua_ d4wfva_ d3t1da_ d4iaxa_
d3zq3a_ d2nnda_ d1i4ua_ d1kxoa_ d1bbpa_ d1kt7a_ d1exsa_ d3s26a1
d1lf7a_ d1qfta_ d4gh7a_ d1z24a1 d1qabe_ d1xkia_ d1ew3a_ d3bx7a_
d4iawa1 d1n0sa_ d3kzaa_ d2k23a_ d2l9ca1 d2joza1 d1gm6a_ d3kffa_
d3saoa1 d1e5pa_ d1yupa1 d1iiua_ d1e69a_ d1mv5a_ d3g60a4 g1ii8.1
d1ewqa2 d1ji0a_ d1qhla_ d1v43a3 d2wmfa1 d1vpla_ d1g2912 d4a82a2
g1f2t.1 d2d62a1 d3tuic1 d2bbsa1 d3g60a3 d2ixea_ d1g6ha_ d1sgwa1
d2qi9c_ d3rlfa1 d3b60a1 d3d31a2 d2pzea1 g1xew.1 d1jj7a_ d1oxxk2
d2onka1 d1w1wa_ d1iwea_ d1qf5a_ d1vmaa2 d1nija1 d1np6a_ d2p67a1

d3ihla_ d1okkd2 d1ls1a2 d1s1ma2 d1iona_ d2v40a_ d3hida_ d2vo1a1
d2g0ta1 d1vcoa2 d1j8mf2 d1g3qa_ d1xjca_ d1p9ba_ d1qzxa3 d1ihua2
d1ihua1 d2qm8a_ d1dj2a_ d4ioja_ d1cp2a_ d1yrba2 d1hyqa_ d3gqba2
d1skye3 d1xp8a1 d1tf7a2 d1tf7a1 d1ye8a1 d1skyb3 d2qe7d2 d4blta_
d4huta_ d3ldaa2 d4hyya_ d2i3ba1 d3j9tb2 d3ntua2 d1vdza1 d2z43a_
d1e9ra_ d1nlfa_ d3ziaa2 d1n0wa_ d1g6oa_ d3ziad2 d3j9ta2 d1pzna2
d3gqbb2 d2i49a_ d1nnfa_ d1xvxa_ d1ursa_ d1h3da1 d1iuda_ d4m1va1
d1twya_ d3e13x_ d2nxoa1 d4pfua_ d3ttma_ d1elja_ d2qmwa1 d1us5a_
d1y3na1 d1jeta_ d1uqwa_ d4f19a1 d2esna2 d3thia_ d3fasa1 d2uyeb_
d2xwva1 d1r9la_ d2fyia1 d1pb7a_ d2ozza1 d1sw5a_ d1nh8a1 d1vr5a1
d2g29a_ d1xs5a_ d3lswa_ d2z23a_ d3w9va_ d3tcha_ d1q35a_ d1z7me1
d3q27a_ d1p99a_ d1ve4a1 d2i6ea1 d4mcoa1 d4n8ya_ d1eu8a_ d1zbma1
d1hsla_ d1ixca2 d1j1na_ d1xvya_ d1y9ua_ d2z22a1 d1xoca1 d4oera_
d1wdna_ d5f1qa1 d1xt8a1 d1i6aa_ d4gwoa_ d4igta1 d1pc3a_ d4mmpa1
d1o63a_ d2czla1 d2onsa2 d4mija1 d4ok5a1 d2bmfa1 d2bmfa2 d1yksa1
d1yksa2 d1jr6a_ d2qeqa1 d2jlqa1 d1a1va2 d1eh3a_ d3skpa_ d1dtza1
d1dtza2 d1ieja_ d1jnfa2 d1ce2a1 d1jnfa1 d1b1xa2 d1b1xa1 d1jw1a1
d1jw1a2 d1ryoa_ d1h76a1 d1h76a2 d1c4oa1 d2p6ra3 d2p6ra4 d1oywa2
d1oywa3 d1wrba1 d1w36b2 d1w36b1 d1c4oa2 d3bora_ d1t6na_ d2eyqa5
d2eyqa3 d2eyqa2 d2zu6a2 d1veca_ d1uaaa1 d1w36d1 d2g9na1 d1z3ix2
d2rb4a_ d1hv8a1 d1hv8a2 d1t5ia_ d1w36c2 d2j0sa2 d2j0sa1 d1fuka_
d3hjha1 d1z5za1 d1qdea_ d1z63a1 d1s2ma2 d1s2ma1 d1wp9a1 d1wp9a2
d1gm5a4 d3dina4 d3dina3 d1q0ua_ d1w36c1 d1z3ix1 d1nkta3 d1nkta4
d1uaaa2 d1w36d2 g1qhh.1 d2fwra2 d2fwra1 d3q3ma_ d1za0a1 d1mxra_
d2inca_ d1jk0a_ d2incb_ d1mhyb_ d3hf1b_ d1mtyb_ d1r2fa_ d2uw1a_
d3n37a_ d1afra_ d1oquc_ d1syya_ d1mtyd_ d2o1za_ d1uzra_ d1smsa_
d2fkza_ d1zuja1 d1s3qa1 d2fjca1 d1z6om1 d1veia_ d4dyya_ d1jiga_
d3gvya_ d2pyba_ d4cy9a1 d3bvfa1 d1r03a_ d2cf7a_ d1nf4a_ d1zs3a1
d4iwka_ d3ka8a_ d3np0x_ d1n1qa_ d1lb3a_ d4e6ka_ d1z6oa1 d2fzfa1
d4dyua_ d3ge4a_ d1jgca_ d4am4a_ d2z6ma_ d1ji5a_ d1vlga_ d1lkoa1
d1krqa_ d3qz3a_ d1o9ra_ d2ffxj_ d1euma_ d1yv1a1 d2v2pa_ d1j30a_
d4di0a_ d1dpsa_ d1oewa_ d2qzxa_ d2asia_ d1psoe_ d1miqa_ d1smra_
d2h6ta_ d1pfza_ d1izda_ d3hd8a_ d1j71a_ d1dpja_ d4aa9a_ d1t6ex_
g1b5f.1 g1lya.1 g1htr.1 d1qdma2 d1bxoa_ d1wkra_ d1tzsa_ d1ibqa_
d2qzwa_ d1h4ra3 d4yl8a1 d2al6a3 d1l7ya_ d1wgda1 d1wgga1 d4q5eb_
d1wjna1 d1se9a_ d3n3kb_ d1wz0a1 d1we6a1 d2m0xa_ d2gbka_ d3rt3b1
d1wh3a1 d1sifa_ d2ekec2 d4ii2b1 d4hk2a1 d1c3ta_ d1m94a_ d1wgha1
d1ttna1 d1j8ca_ d1wx8a1 d3v6cb1 d2ma9b_ d4i6lb1 d1bt0a_ d2jvca1
d1iyfa_ d2k1fa_ d1wxva1 d1wf9a1 d1t0ya_ d2io0b2 d1x1ma1 d2gbma_
d1zw7a1 d2m17a_ d1we7a1 d2lwpa_ d2gbja_ d1wjua1 d1v6ea1 d1v86a1
d4eewa_ d1wiaa1 d4icva_ d4gu2a_ d1wx7a1 d1wy8a1 d4hjka1 d2faza1
d1v5oa1 d3ruib_ d1eo6a_ d3waob_ d2r2qa1 d3m95a_ d3wana_ d2al3a1
d2cr5a1 d1wj4a1 d1s3sg_ d3ubra_ d1qo8a1 d1d4ca1 d2k3va_ d1qdba_
d1m1qa_ d2j7aa_ d1h21a_ d1ogyb_ d4fasa_ d1jnia_ d1oaha_ d1sp3a_

d1um8a_ d1ixza_ d1qvra2 d1qvra3 d1qzma_ d1sxjc2 d1jr3d2 d2p65a_
d1l8qa2 d1g8pa_ d3hu3a3 d1sxjd2 d1njfa_ d1in4a2 d1sxja2 d1tuea1
d2ce7a2 d1w5sa2 d1sxjb2 d1d2na_ d1ny5a2 d2gnoa2 d2a5yb3 d1fnna2
d1u0ja1 d1lv7a_ d1iqpa2 d2fnaa2 d1ixsb2 d4gita_ d1sxje2 d1g41a_
d2e3fa_ d1mm0a_ d2e3ea_ d2llda_ d2ny8x_ d2e3ga_ d2ny9x_ d2nz3a_
d2lj7a_ d1n4na_ d2m8bb1 d2kyqa_ d3psma_ d1jkza_ d1jxca_ d2gl1a_
d4uj0a_ d2k4ua_ d1hp2a_ d2lu9a_ d1baha_ d2meoa1 d1wm7a_ d1hlya_
d1n8ma_ d1jlza_ d1wpda_ d1c55a_ d1pvza_ d1wt7a_ d1rjia_ d1wz5a_
d1m2sa_ d1pjva_ d1c49a_ d1qkya_ d2ktca1 d1wmta_ d1du9a_ d1j5ja_
d1r1ga_ d1t0za_ d2i61a_ d1t1ta_ d1sn4a_ d1kv0a_ d2lkba_ d1npia_
d1wwna_ d1pe4a_ d2kbka_ d2li7a_ d1djta_ d2kbha_ d1dq7a_ d1sega_
d1omya_ d1zywa1 d1jzaa_ d1n9wa1 d1bbua1 d1e1oa1 d1c0aa1 d1b8aa1
d1l0wa1 d3a74a1 d1iyjb4 d1iyjb5 d1iyjb3 d1o7ia_ d4ipca1 d1x3ea_
d4apva_ d1jb7a1 d1jb7a2 d1jb7a3 d3kdfa_ d1ue1b_ d1wjja1 d3eiva_
d1xjva2 d1xjva1 d2ccza2 d1se8a_ d1l1oc_ d1jb7b_ d1qvca_ d3ulla_
d1qzga_ d1miua5 d1miua3 d3g48a1 d1gd7a_ d1mkha_ d1jjcb3 d1pyba_
d3ubub_ d1egia_ d1qo3c_ d1v7pa_ d1tdqb_ d3ubua_ d1c3ab_ d1h8ua_
d3bx4b_ d1t8ca1 d1sb2a1 d1qdda_ d2ziba1 d3cada_ d1byfa_ d1j34b_
d1jwia_ d1jzna_ d1c3aa_ d1wmza_ d3bdwb_ d1hq8a_ d1fifa1 d1dv8a_
d3hupa_ d1wk1a1 d1umra_ d2afpa_ d3bx4a_ d1gz2a_ d1sl6a_ d1v7pb_
d3g8ka_ d1ixxa_ d1j34a_ d1jwib_ d1g1sa1 d1y17a1 d1ja3a_ d1fvub_
d3bdwa_ d1sb2b1 d1fvua_ d1umrc_ d1oz7a_ d4m17a_ d3cfwa1 d1ypqa1
d1g1ta1 d3fd4a_ d3l9jc_ d4dn8a1 d1v4lb_ d1rdl1_ d2msba_ d1dgsa2
d1fvia1 d1ckma1 d1x9na2 d1rpma_ d2shpa1 d1yfoa_ d1l8ka_ d2ooqa1
d1g4us2 d1p15a_ d1lara2 d1lara1 d1wcha_ d2fh7a2 d2fh7a1 d1ohea2
d2f4da_ d1rxda_ d2hxpa_ d1xria_ d1d5ra2 d1fpza_ d3ezza_ d4y2ea_
d1i9sa_ d1c1la_ d4lbqa_ d1slta_ d4no4a_ d3zsja_ d3duia_ d1g86a_
d1is3a_ d1qmja_ d1hlca_ d1tdha3 d1q3ba3 d1ee8a3 d1k82a3 g1qmo.1
g1len.1 d3zyra_ d1n47a_ g2ltn.1 d1avba_ d3usua_ d1dhkb_ d1f9ka_
d1ukga_ d1g8wa_ d1g9fa_ d1wbfa_ d3wcsa_ d1g7ya_ g1loe.1 d1mvqa_
d1hqla_ d1v6ia_ d1dbna_ d1qnwa_ d1fx5a_ d4l8qa_ d1ioaa_ d3n35a_
d1epwa1 d3h33a_ d1rwja_ d19hca_ d1os6a_ d3bxua_ d3h4na_ d1i77a_
d3caoa_ d1j0pa_ d2ctha_ d1duwa_ d1ofwa_ d2e84a_ d1hh5a_ d3h34a_
d1dszb_ d1a6ya_ d3cbba_ d1ynwa1 d1r4ia_ d1hcqa_ d2hana_ d2hanb_
d2enva1 d1kb4b_ d2nllb_ d1lo1a_ d1cita_ d2dara2 d2dara1 d1wyha2
d2dloa2 d1rutx4 d1rutx3 d2cuqa2 d1x4la2 d1x4la1 d1x3ha2 d1x3ha1
d2d8za1 d1x61a2 d1x6aa1 d1x6aa2 d2cora2 d2cora1 d1v6ga1 d1v6ga2
d2d8za2 d1x64a1 d1x64a2 d1b8ta1 d2co8a1 d1x63a2 d2dj7a2 d2dj7a1
d2cu8a2 d2cu8a1 d1g47a2 d1g47a1 d1j2oa1 d1wyha1 d2dloa1 d1wiga1
d1wiga2 d2cupa2 d2cupa3 d1x68a1 d2d8ya2 d2d8ya1 d1m3va2 d1x62a1
d1x68a2 d1x61a1 d1b8ta3 d1b8ta2 d2d8xa2 d2d8xa1 d1x4ka1 d1x4ka2
d2cuqa1 d1m3va1 d2co8a2 d1j2oa2 d1x63a1 d2cura1 d2cura2 d1x62a2
d1op9a_ d1y0la1 d1etzb1 d1egjl1 d2gsib1 d1nj9a1 d4jfhd1 d1aqkl1
d1t2ja_ d1lo4h1 d1zvya1 d3v4vl1 d4enec1 d1kxtb_ d5e8ea1 d2pxya_

d4weud_ d1xiwc_ d2esvd1 d1qnzh_ d1ogae1 d1f4xh1 d4n1hb_ d3ezjb1
d3mlwl1 d1kfah1 d1etza1 d1b4jh1 d3cfic_ d4w68a_ d2arja1 d1ek3a_
d2ok0l1 d1vera_ d1uwgh1 d1blnb1 d1fpth1 d1rzga1 d2giya1 d4m3kb1
d1nc2a1 d2nyyd1 d1ncna_ d1fbih1 d5dxwa_ d4fqqa1 d1dqdh1 d1jfql1
d1kelh1 d1faih1 d1frgl1 d1figl1 d4m6nl1 d1fo0a_ d4hxbl1 d3b5ga_
d3quyd1 d2h9gb1 d1i8kb_ d2dd8h1 d2ck0h1 d1eaja_ d4w6wb1 d2a6ib3
d1t66d1 d2e27h_ d1ob1b1 d1f11b1 d2v7na1 d1ad0a1 d2xt1b1 d1pskh1
d4i13b1 d2op4l1 d1a0ql1 d1uwel1 d1za3a1 d1ncbl1 d4uu9a_ d1bm3h1
d1cloh1 d1nqba2 d1cf8h1 d4y5va_ d1mhph1 d5anma1 d4cdgc1 d4ldeb_
d1y0lb1 d2pcpb1 d1z3gl1 d1dfbl1 d1fh5h1 d3g5xa1 d1tzib1 d4dn3l1
d3zhka1 d2b1hh1 d2uyla1 d1f3dh1 d1nj9b1 d2agjh1 d2h1pl1 d1q72h1
d8fabb1 d1ndmb1 d1rjca1 d4u6hb1 d3upca_ d2fbjl1 d1osph1 d1tvda_
d2cjvh_ d4fhbd1 d1bd2d1 d1rz7h1 d3nn8a_ d1blna1 d1knoa1 d1nfda1
d2bmkb1 d1dqll_ d1igyb1 d1igfl1 d12e8h1 d3utza1 d1nc2b1 d1rihh1
d4mwfb1 d4jvpa1 d1mieh1 d1rhfa1 d1fgnl1 d1sbsh1 d1lp9e1 d1dn0a1
d4lqfl1 d1iaih1 d1kgcd1 d1mh5b1 d1gpol1 d2g60h3 d1f8tl1 d2jixd1
d4grwh_ d1fo0b_ d1bfob1 d1axsa1 d1u9ka_ d1nmbh_ d1h8na1 d1h8na2
d1jguh1 d4eura1 d3sdyl1 d35c8h1 d1w72l1 d1mvub_ d1i9rh1 d1ob1a1
d4wenb_ d1yeeh1 d3ietc1 d1f58h1 d1ad0b1 d1mqkl_ d1nbvh1 d4b50a_
d3r8bb_ d1bafl1 d1mamh1 d4rgne1 d2gsgb_ d35c8l1 d1bafh1 d1ai1h1
d2pcpa1 d2fath1 d2x1pa_ d1orsb1 d1ibgl1 d1shma_ d1a2yb_ d1u0qa_
d2coqa_ d1hilb1 d1qfwi_ d1jv5b_ d1fn4b1 d5e0qa_ d1nsnh1 d1plgl1
d4ocxl1 d1kb5h1 d1rzfh1 d1pg7x1 d2fx7h1 d2oi9c_ d1l7il1 d3eyva1
d1d5il1 d4ffyl_ d1tetl1 d1a6wh_ d1ac6a_ d3dvfa_ d1nfde1 d1mimh1
d1ri8a1 d1jhlh_ d3dura_ d1fskb1 d1f97a1 d3so3b1 d2ak4d1 d1lp9f1
d4z7wf1 d1l7th1 d4y5ya_ d4x7ec1 d1emth1 d1ikfl1 d1nlbh1 d4utal1
d1bjma1 d1eapb1 d1u6ah1 d2dtgd1 d1kj2b_ d1xeda1 d1c1eh1 d1fgvh_
d2bnub1 d2fb4h1 d2dtgb1 d1ktrh_ d1jpth1 d1qkzl1 d4hepg_ d2atpb1
d1flrh1 d2hrpl1 d1mvfa_ d1iaim1 d3k80a1 d2jb5h1 d3zkxc_ d4grwe_
d1it9h1 d1rhhb1 d3w9ec_ d1e4wh1 d1sy6h1 d2j6eh1 d3qxta_ d1jglh1
d1p7ka1 d1tzhb1 d4n8pa1 d1gafl1 d2jelh1 d1wioa2 d4i0cc1 d1cicb1
d1lmka2 d1lmka1 d1iqwh1 d1orqb1 d1ejoh1 d1jnlh1 d4zg1a_ d43c9b_
d1ggbl1 d1wejh1 d3q6fa1 d1f3dj1 d3c6la1 d1bqlh1 d3p9wb_ d1a3rl1
d1h0db1 d2uzil_ d1eo8l1 d1qfwl_ d1n7ml1 d1igja1 d1c5ch1 d2a9mh_
d1rmfh1 d1fj1b1 d4heme_ d1ccza1 d1a5fh1 d1ogad1 d1dlfh_ d1qa9b_
d1vgel1 d3qskb_ d1sjxa_ d4ozfg1 d1sq2n_ d1qd0a_ d1kcvh1 d1c5db1
d3qcul1 d3bn9d1 d1mjuh1 d4y8dc_ d7fabh1 d3dvgb1 d1lo0l1 d1a6tb1
d1gigh1 d1kcrh1 d2hwzl1 d1yjdc1 d3clfl1 d4krna_ d1dl7l_ d1e6ol1
d1mexh1 d3r0ma_ d4kkna_ d1fnsl1 d1oaqh_ d1rzia1 d1ucbh1 d1ncwl1
d4b41a_ d1nfdf1 d2mpah1 d2d7tl1 d4grwf_ d4b5ea_ d1lk3h1 d3u79a_
d4u3xa_ d1ghfl1 d1bvkb_ d1mcww1 d1b88a_ d1jrhh1 d32c2b1 d4wemb_
d1dsfh_ d1op3h1 d1ce1l1 d1iqda1 d1qoka1 d1qoka2 d1qfuh1 d2crya1
d1ay1h1 d1oaql_ d3ab0b_ d1rurl1 d1t4kb1 d1bz7b1 d1n0xh1 d1forl1
d1ucbl1 d4w6yb_ d43c9a_ d2aabl1 d1tzia1 d4n1ca_ d1k6qh1 d3g9ab_

d4tvsa1 d1igmh_ d1pz5b1 d3dx9a1 d3wxwh_ d1nakh1 d1kcul1 d3eaka_
d4qlra1 d1cr9l1 d1d9ka_ d1f4xl1 d1deea1 d2i27n2 d1c12a1 d2orbl1
d1j8hd1 d1mcph1 d1c5da1 d1kenh1 d1bd2e1 d1h5ba_ d4x7fc1 d3upaa_
d1t3fa1 d1indh1 d2dtgc1 d4hc1l1 d1zv5a1 d4wgvb_ d1uwgl1 d4f37k1
d1aqkh1 d1vesa_ d3zkqd_ d1fptl1 d1lo4l1 d1knob1 d3rrqa_ d1ct8b1
d4orzc_ d1mlbb1 d4hgkc1 d1dqdl1 d1bogb1 d4ozhe1 d1dr9a1 d1i7za1
d1dn0b1 d1kell1 d1rzib1 d1ypzf1 d1wtla_ d1hkfa_ d1fail1 d1figh1
d2vyre_ d1sjva_ d1ohqa_ d1bfoa1 d1pkqb1 d1mjul1 d1b4jl1 d1nsnl1
d2arjb1 d1p4bh_ d4aj0a_ d3nn8b_ d2rcja1 d1f2xk_ d4v1dd_ d1adqh1
d2eksa_ d4k3ga_ d1jfqh1 d1uweh1 d1frgh1 d4gftb_ d1uz8b1 d4n1ea_
d1bm3l1 d1clol1 d3mcka1 d2vq1a1 d1ae6h1 d4aq1b1 d1uj3b1 d1nbqa1
d1fh5l1 d2ck0l1 d2ywza_ d1q72l1 d4nbxb_ d1hila1 d4hzlb1 d2j88l1
d1zmya1 d1cd0a_ d1a4ka1 d1pskl1 d1a0qh1 d4krmb_ d1ncbh1 d2fbjh1
d1j8he1 d1i3ua_ d1cf8l1 d1fe8h1 d2p1ya1 d1eeqa_ d2x89a_ d1ol0a_
d2r0lh1 d1ub5a1 d1dfbh1 d3eyvb1 d4jdvb1 d4p2cg_ d2gsia1 d2agjl1
d1u3ha1 d2h1ph1 d1rihl1 d1fskc1 d1boga1 d2a77l1 d1ospl1 d1qfoa_
d3dwta_ d2fx7l1 d1rz7l1 d3stba1 d1pkqa1 d1b6da1 d1dzba2 d1dzba1
d1kj2a_ d4l1ha_ d1rzgb1 d1dqlh_ d12e8l1 d1miel1 d1jmaa_ d1sbsl1
d1fgnh1 d1f58l1 d1mqkh_ d1ndgb1 d3qegl1 d1iail1 d2aw2a1 d1tqbc1
d3fo2a1 d4dkaa_ d1f8th1 d2g60l1 d2ywya_ d1uj3a1 d1yc7a_ d3bd3a_
d4w6xb1 d1nmbl_ d1i8ka_ d1rjlb1 d1ypze1 d1p7kb1 d1kxqe_ d1gpoh1
d2z4qa1 d1w72h1 d1i9rl1 d4qgya1 d4xt1c_ d2qqnh1 d1cica1 d3c08h1
d2cd0a_ d2bdnh1 d1hnga1 d1maml1 d1nldh1 d1cr9h1 d4lcih1 d3bkyh1
d3qg7l1 d1ai1l1 d1kxvc_ d4iosd_ d1l7ih1 d2x1qa_ d1ub6b1 d3k74b_
d1ibgh1 d1teth1 d1igya1 d1hxma1 d2x6ma_ d2e7la_ d5e03a_ d1qfwm_
d2r0kh1 d1igjb1 d1rurh1 d1plgh1 d1mj8h1 d1yuhb1 d3fmgl1 d1rzfl1
d1emtl1 d1ad9b1 d1igtb1 d1nlbl1 d1d5ih1 d3k7ua_ d4ffyh_ d1bwwa_
d3dvga1 d1a6ta1 d1fgvl_ d4qxgl1 d1miml1 d1jhll_ d2hffb1 d2hh0l1
d1qkzh1 d1l7tl1 d1afvh1 d1iaii1 d2cjuh_ d1um5h1 d2ghwb2 d2ghwb1
d1kgce1 d2fgwh1 d1ikfh1 d1dvfd_ d2i24n1 d2xtjb_ d1za3b1 d2yc1a_
d2hh0h1 d2x1oa_ d1bvka_ d1rjla1 d5e7ba1 d1igta1 d1il1b1 d2fb4l1
d1mvua_ d1jptl1 d32c2a1 d1fvca_ d1iqdb1 d2hrph1 d2p4ab_ d4qo1a_
d1p2cb1 d1wt5c_ d1t4ka1 d5c1mb_ d1it9l1 d1bz7a1 d1b2wh1 d1e4wl1
d1jnll1 d1yedb1 d1za6b1 d3u7aa_ d1igfh1 d3gnml1 d1orsa1 d1wejl1
d1a2ya_ d1hxmb1 d1gafh1 d2j4wh1 d1pz5a1 d1j05a_ d1a3rh1 d4fqll1
d1qfwh_ d1eo8h1 d1n7mh1 d4s11a_ d1kegh1 d5da4a_ d1iqwl1 d3whxb1
d1vgeh1 d1deeb1 d1c12b1 d1t2ql1 d1ad9a1 d3gsnb1 d3b2uc1 d1ggbh1
d1t3fb1 d1nakl1 d4ocmc_ d1lila1 d2v7hb3 d2esve1 d1mcoh1 d2qr0b1
d2q3aa_ d1ct8a1 d1kcrl1 d2pxyb_ d2uzih_ d5c3ld_ d3durb_ d1c5cl1
d2ak4e1 d1dl7h_ d1xiwd_ d1mlba1 d1rmfl1 d1i7zb1 d1dlfl_ d2xa3a_
d5bq7b1 d1dqqb1 d3h42l1 d1egjh1 d1kcvl1 d1fe8l1 d2j6el1 d1f3rb2
d1lk3l1 d1sm3h1 d1q0xh1 d1ghfh1 d7fabl1 d1igch1 d1lo0h1 d4idla_
d5c2bl_ d1jrhl1 d1il1a1 d3zhda1 d4kfzc_ d1b0wa_ d1e6oh1 d1mexl1
d4krpb_ d1fnsh1 d1ncwh1 d4hgma_ d4eigb1 d4rzca_ d2mpal1 d4y5xa_

d5en2b1 d3lrga1 d4ej1c1 d1rhha1 d1j05b_ d1forh1 d1q9wb1 d2wzpd_ d2aq2a1 d1h0da1 d3revb1 d2ij0c1 d1tzha1 d4m7jl1 d1k6ql1 d3b9kc1 d1igml_ d3qeua1 d1dsfl_ d1ce1h1 d1kcuh1 d1zvha1 d1zvoa1 d1tqbb1 d1ay1l1 d2qscl1 d2cgrh1 d1nl0h1 d1kenl1 d3eo0a1 d4n9ob1 d1n0xl1 d3k1kc_ d2aq2b1 d1ck1a1 d1enfa1 d1i4pa1 d1v1oa1 d1et9a1 d1esfa1 d2g9hd1 d4ohja1 d1klud1 d1bxta1 d1eu3a1 d1ty0a1 d1fnua1 d2cqva1 d1fhga_ d2k1ma_ d2dl2a1 d1biha3 d1biha1 d1biha4 d3qp3a1 d1g1ca_ d1gxea_ d1cvsc2 d1cvsc1 d1xmwa2 d1m4ka2 d2otpa2 d1biha2 d1rhfa2 d2fcba2 d2fcba1 d1wwbx_ d1gsma2 d1epfa1 d1epfa2 d2dava1 d1waaa_ d1f97a2 d1x44a1 d1vcaa2 d1fnla2 d1fnla1 d3b5ha2 d3h8na2 d3h8na1 d1jbja1 d1jbja2 d1pd6a_ d1f2qa2 d1f2qa1 d3cx2a_ d1nbqa2 d3b5ha1 d1n26a1 d1xiwb_ d1xaua_ d1fltx_ d2ifga1 d1olla1 d1olla2 d1cs6a4 d1cs6a3 d1cs6a2 d1cs6a1 d2gw5a2 d1sy6a1 d1xiwa_ d1ucta2 d1ucta1 d1ry7b2 d1gl4b_ d1qz1a3 d1olza1 d2c9aa1 d1wwca_

**Family-59 (c.3.1.5)**

**Positive dataset contains 10 protein sequences.**

d2jk6a2 d2jk6a1 d1xhca1 d1xhca2 d1w4xa1 d1w4xa2 d1xdia1 d2gv8a2 d2gv8a1 d3dk9a1

**Negative dataset contains 4041 protein sequences.**

d1m32a_ d1ibja_ d1c7na_ d1iuga_ d4q75a_ d2bkwa1 d1t3ia_ d1n8pa_ d1qz9a_ d1jf9a_ d1eg5a_ d3kgwa_ d1cl1a_ d2ctza1 d1vjoa_ d2rfva_ d1gc0a_ d1cs1a_ d1d2fa_ d2ch1a1 d1qgna_ d1pffa_ d1elua_ d1sefa_ d1sfna_ d1sq4a_ d1js3a_ d3rcha_ d3k40a_ d2cfba1 d1dfoa_ d1o69a_ d1bj4a_ d3k28a_ d2bwpa_ d3lg0a_ d2fnua_ d3ou5a_ d1z7da1 d1iaya_ d4msoa_ d1bs0a_ d1s0aa_ d1sffa_ d3tqxa_ d1mdoa_ d3qboa_ d3pgya_ d1bjna_ d2dkja_ d1ejia_ d1vefa1 d4wxga_ d3bs8a_ d3gbxa1 d1w23a_ d1u08a_ d1lc5a_ d1b5pa_ d2ay1a_ d3pd6a_ d7aata_ d2csta_ d1j32a_ d1fg7a_ d4wlha_ d1o4sa_ d1v2da_ d1gdea_ d1m6sa_ d1v72a1 d1svva_ d1ajsa_ d1wsta1 d2f8ja2 d1lk9a_ d1vp4a1 d1xi9a1 d2gb3a1 d1yaaa_ d1r0ka3 d1j5pa3 d1vkoa2 d1vm6a2 d3qvsa2 d1gr0a2 d2tbvc_ d1x36a_ d1smvc_ d1ng0c_ d4sbvc_ d1opoa_ d1f2nc_ d1fwxa1 d1ibya_ d1f56a_ d1bypa_ d1azca_ d1ws8a_ d3u25a_ d1cuoa_ d1id2a_ d1rkra_ d2iaac_ d4dp1x_ d1nwpa_ d2gima_ d2i7sa_ d1qhqa_ d1gyca3 d1hfua2 d1kbva1 d1kbva2 d1sdda1 d1sdda2 d1gyca2 d1mzya1 d2bw4a1 d1aoza1 d1aoza3 d1aoza2 d1kyaa2 d1kyaa3 d1kyaa1 d1mzya2 d4e9sa1 d1sddb1 d1sddb2 d1hfua1 d1hfua3 d1oe1a2 d1oe1a1 d1v10a2 d1v10a3 d1v10a1 d1a3qa1 d1iknc_ d3brda1 d1p7hl1 d1oy3c_ d1imhc1 d1zk9a1 d2cxka1 d2bema_ d1pama1 d1ea9c1 d1qhoa1 d2fhfa2 d2fhfa1 d1k3ia1 d1j0ha1 d1cxla1 d2bhua1 d1edqa1 d1f1sa2 d1ut9a2 d1gvia1 d1txka1 d1zx5a1 d1qwra_ d2phla2 d2phla1 d1fxza2 d1fxza1 d1lrha_ d1uija1 g1dgw.1 d2v09a_ d1uika2 d1uika1 d1dgwa_ d1ep0a_ d2pa7a1 d1wlta1 d1nxma_ d2c0za1 d1ds6b_ d1fstb_ d3us3a3 d3us3a1 d1v70a_ d2f4pa1 d1yhfa1 d2b5ea2 d2b5ea3 d2b5ea1 d2b5ea4 d2kp1a1 d2kp2a1 d1hyua3 d1hyua4 d1j08a1 d1j08a2 d1yloa2 d1y7ea2 d1y0ya3 d1lfwa1 d1vixa1 d1fnoa4 d1vhoa2

d1ysja1 d1vhea2 d1z2la1 d2fvga2 d1vgya1 d1r3na1 d2grea2 d1xmba1
g1q7l.1 d1rtqa_ d1h32a1 d2bjca1 d2hsga1 d1qpza1 d1efaa1 d1mqta_
d1d4m3_ d1fmd3_ d2mev1_ g1aym.1 d1ev13_ d4q4w1_ d1r1a3_ d2x5ia_
d1rhi1_ g1r1a.1 g1d4m.1 g1rhi.1 d1b35b_ d1oopa_ d1tme1_ d4iv1a_
g1pvc.1 g1fpn.1 d1fpn1_ d4gb31_ d1d4m1_ d1fmd1_ d1ev11_ d2wzr3_
d1qqp3_ g1fmd.1 d2x5ic_ d1rhi3_ g1oop.1 d1hxs1_ g1hxs.1 d1fpn3_
d1bev1_ g2mev.1 d1eah3_ d1aym1_ d1pvc3_ d1qqp1_ d1zba3_ d1h8ta_
d3vbhc_ g1b35.1 g1bev.1 d1hxs3_ g1ev1.1 d1bev3_ g1tmf.1 d1tmf1_
d1b35a_ d2mev3_ g1eah.1 g1h8t.1 d1eah1_ d4q4w3_ d1r1a1_ d1aym3_
d1pvc1_ d1zba1_ d1h8tc_ d1oopc_ d1tme3_ g1qqp.1 d4iv1c_ g1tme.1
d1e3oc2 d1ic8a2 d2ovga_ d1sq8a_ d1rzsa_ d3kz3a_ d3orca_ d3gpdg2
d2q49a2 d1hdgo2 d2g17a2 d2cvoa2 d2czca1 d3pwka2 d1obfo2 d1i32a2
d1zyao2 d2pkqo2 d1b7go2 d2hjsa2 d1cf2o2 d1vkna2 d1nvmb2 d2m9wa1
d3dfxa_ d2msya_ d2djoa1 d2r5za_ d1au7a1 d1f43a_ d2lmda1 d1akhb_
d1s7ea1 d3nara_ d1zq3p1 d2craa1 d1mija_ d4uuta_ d3a01a_ d1x58a1
d3rkqa1 d3cmya1 d2cqxa1 d2da6a1 d1e3oc1 d1fjla_ d1mh3a1 d1ig7a_
d1x41a1 d1wh7a1 d1gt0c1 d2mw8a_ d1lfup_ d1x2ma1 d1b72a_ d1wi3a1
d1jgga_ d1wjha1 d1x2na1 d1mnmc_ d2cuea1 d3k2aa_ d2hosa_ d2jwta2
d1pufa_ d1octc1 d2hi3a_ d1b8ia_ d4j19a_ d3lnqa_ d1wh5a1 d1b8ib_
d1fexa_ d2cqra1 d1guua_ d1ug2a1 d2cqqa1 d2ajea1 d4a69c_ d1h8ac1
d2crga1 d2iw5b1 d1xc5a1 d2cu7a1 d1wgxa1 d2cjja1 d2ckxa1 d1gvda_
d1hcra_ d1tc3c_ d1gdta1 d1u78a2 d6paxa2 d1k78a1 d1k78a2 d1igna2
d2iu5a1 d1zk8a1 d2g7sa1 d2fd5a1 d2np5a1 d1z0xa1 d2id6a1 d2d6ya1
d2i10a1 d1vi0a1 d2oi8a1 d3c07a1 d2ns7a1 d2fq4a1 d2yvea1 d1t33a1
d2o7ta1 d1rkta1 d2np3b1 d2g7ga1 d2gena1 d2fbqa1 d2g7la1 d2gfna1
d2g3ba1 d2jj7a1 d2hyja1 d1sgma1 d1ui5a1 d2hkua1 d2id3a1 d4jyka1
d1bl0a2 d1d5ya1 d1d5ya2 d1prtc2 d3lv8a_ d1s3ga1 d1zaka1 d1s4qa_
d3tvta2 d1uf9a_ d1khta_ d1ckea_ d1osna_ d1p5zb_ d4tmka_ d3cm0a_
d1tmka_ d1jaga_ d3h86a_ d1t0hb_ d1nn5a_ d2h92a_ d3kb2a1 d2c95a1
d1yj5a2 d1kgda_ d1ly1a_ d2ak3a1 d1vyva2 d1deka_ d2grja1 d1p3ja1
d1y63a_ d1e4ya1 d1s96a_ d3iija1 d1lvga_ d1nksa_ d1vyua2 d4e22a_
d1qf9a_ d1jjva_ d1q3ta_ d1ki9a_ d1lw7a2 d1teva_ d1kjwa2 d1gsia_
d1g3ma_ d1j99a_ d1t8ta1 d1fmja_ d1zrha_ d1ls6a_ d2a3ra_ d1aqua_
d2zptx_ d3ckla_ d1texa_ d1q44a_ d2gwha_ d1odfa_ d4gi7a_ d2jeoa1
d1rz3a_ d1z0ja1 d3umra2 d3gj0a1 d2gaoa1 d1yzga1 d1x3sa1 d1g7sa4
d1puja_ d1udxa2 d1kk1a3 d2o52a_ d1jwyb_ d1zcaa2 d1zcba2 d2fv8a1
d2cjwa_ d3dz8a1 d1xzpa2 d1vg8a_ d4qxaa_ d2ew1a1 d1x1ra1 d2bcgy_
d3doea1 d3cona_ d3rega_ d3wyga_ d1upta_ d2b6ha1 d1zd9a1 d1lnza2
d1r5ba3 d1n0ua2 d1r2qa_ d2f9la1 d2rhda_ d3q3jb_ d3q85a_ d1efma1
d1r8qa_ d2gf9a1 d2akab1 d1ek0a_ d1z08a1 d2gcoa1 d2dy1a2 d4g01b_
d2cxxa1 d1svia_ d3q72a_ d1ni3a1 d4ac9a4 d2erxa1 d2bmea_ d1wxqa1
d2gf0a1 d4ldja1 d1zunb3 d1d5ca_ d4lv5b_ d1efca3 d1kmqa_ d2g6ba1
d3lrpa_ d1tada2 d2y8ea_ d4rd4a1 d1z06a1 d2heia_ d3a58b_ d1u0la2
d1d2ea3 d1zj6a1 d2ocba_ d3rwoa_ d1mr3f_ d2e9sa1 d4u5xa1 d2iwra_

| | | | | | | | |
|---|---|---|---|---|---|---|---|
| d2fn4a1 | d3bwdd_ | d2nzja1 | d2atva1 | d2ksqa_ | d3l43a_ | d1mkya2 | d1mkya1 |
| d1wmsa_ | d1jnya3 | d2rmka_ | d1f6ba_ | d1f60a3 | d1gota2 | d1jala1 | d2fh5b1 |
| d1g16a_ | d2wblc_ | d1m7ba_ | d1wf3a1 | d1f5na2 | d1t9ha2 | d1z6xa1 | d1z0aa1 |
| d2fg5a1 | d2a5ja1 | d2bcjq2 | d1s0ua3 | d1ky3a_ | d4y0va_ | d4lhwa_ | d2qmea1 |
| d2atxa1 | d1egaa1 | d1ryha_ | d1moza_ | d1nrjb_ | d2erya1 | d3bfka_ | d1puia_ |
| d1fzqa_ | d3j3az1 | d3t5ga_ | d1shza2 | d3defa_ | d2f7sa1 | d3raba_ | d3tsoa_ |
| d3aq4a_ | d2al7a1 | d3sfva1 | d3bc1a_ | d2ncda_ | d1sdma_ | d3h4sa1 | d2heha_ |
| d2grya_ | g3kin.1 | d1kk8a2 | d1br2a2 | d1goja_ | d1f9va_ | d2mysa2 | d1lkxa_ |
| d3t0qa_ | d4db1a1 | d3gbja_ | d1ry6a_ | d1v8ka_ | d1k8ka2 | d1k8ka1 | d3uleb1 |
| d3i33a2 | d3i33a1 | d1dkgd2 | d1dkgd1 | d4ci6a2 | d1jcfa1 | d4a2ba2 | d2fxua1 |
| d1yaga2 | d1yaga1 | d4cbua2 | d1bupa2 | d1g99a2 | d2e1za2 | d2e1za1 | d1saza1 |
| d1saza2 | d1iqca2 | d1iqca1 | d1eb7a2 | d1eb7a1 | d1nmla2 | d3h3no1 | d3h3no2 |
| d3ezwa2 | d1zc6a1 | d1zbsa1 | d1zbsa2 | d1zc6a2 | d2ch5a2 | d2ch5a1 | d1zxoa2 |
| d1zxoa1 | d2d0oa3 | d2d0oa2 | d1nbwa2 | d1hroa_ | d4gedb_ | d3zooa_ | d1mz4a_ |
| d1gu2a_ | d1f1fa_ | d1dvva_ | d1c6ra_ | d1c75a_ | d1e29a_ | d1gdva_ | d1cnoa_ |
| d1h32b_ | d1co6a_ | d2bh4x_ | d2aiua_ | d5cytr_ | d1jdla_ | d1i8oa_ | d2zboa_ |
| d3dmia_ | d1ls9a_ | d1wvec_ | d2yk3a_ | d3ph2b_ | d1dw0a_ | d5aura_ | d1ql3a_ |
| d1qn2a_ | d1f1ca_ | d4zzva_ | d2xspa_ | d1eg1a_ | d4v20a_ | d4ipma1 | d1gpia_ |
| d4csia_ | d3pfza_ | d3ovwa_ | d2yoka_ | d2nqya_ | d1h4ga_ | d1hixa_ | d1oa2a_ |
| d1olra_ | d3b7ma_ | d4h7ma1 | d3wp3a_ | d2vgda_ | d1igoa_ | d3zsea_ | d3wx5a_ |
| d1oa3a_ | d1pvxa_ | d1m4wa_ | d1f5ja_ | d1oa4a_ | d1ks5a_ | d4ixla1 | d1evsa_ |
| d1cnt1_ | d1pvhb_ | d1z7ca_ | d1huwa_ | d1bgea_ | d1rhga_ | d1f6fa_ | d1f45b_ |
| d1i1rb_ | d1axia_ | d1etea_ | d3tgxb_ | d1scfa_ | d2z3qa1 | d3qb1a_ | d1m48a_ |
| d4nkqb_ | d1eera_ | d1v7mv_ | d5e4ea_ | d2o27a_ | d3b5ka_ | d2psma1 | d4zf7a_ |
| d3uf5a_ | d4x51a_ | d4ypgc1 | d3oq3a_ | d1lqsl_ | d1n1fa_ | d1au1a_ | d1wu3i_ |
| d3besl_ | d3ux9a_ | d3rbaa_ | d2qtra_ | d1nupa_ | d3l92a_ | d1f9aa_ | d1qjca_ |
| d2h29a_ | d1vlha_ | d1od6a_ | d1lw7a1 | d1mrza2 | d4ruka_ | d3wdpp1 | d3wq5a_ |
| d1gnxa_ | d4gxpa_ | d4hz8a_ | d1e4mm_ | d3aiua_ | d1wcga1 | d3ahxa_ | d1v02a_ |
| d2dgaa_ | d1pbga_ | d1vffa1 | d1qoxa_ | d4ha4a_ | d2el7a_ | d1iq0a2 | d1irxa2 |
| d1r6ta2 | d1ivsa4 | d2cyba_ | d1rqga2 | d1j09a2 | d2yxna_ | d1f7ua2 | d1h3fa1 |
| d1ffya3 | d2g36a1 | d1nzja_ | d1n3la_ | d1i6la_ | d1pfva2 | d1h3na3 | d3n9ia_ |
| d3sz3a1 | d3sw2a_ | d1moxc_ | d1dx5i3 | d1edmb_ | d1autl2 | d1autl1 | d1dval1 |
| d1szba2 | d1kigl_ | d2y5fl_ | d1ijqa2 | d1gk5a_ | d1g1sa2 | d1nt0a3 | d1xdtr_ |
| d1lmja2 | d1lmja1 | d1nzia2 | d2wphe_ | d1klil_ | d1pfxl2 | d1pfxl1 | d1p9ja_ |
| d1g1ta2 | d1f7ea_ | d4yzub_ | d2y5ia_ | d1psra_ | d3n22a_ | d2egda_ | d4ggfc_ |
| d2wcea_ | d2luca_ | d4aqia_ | d3nsia_ | d2lp3a_ | d1j55a_ | d1a4pa_ | d1k8ua_ |
| d1nsha_ | d1exra_ | d1tiza1 | d1nyaa_ | d1g8ia_ | d1ggwa_ | d1wdcc_ | d3fwba_ |
| d1ggza_ | d2o5ga_ | d1ctaa_ | d1m45a_ | d3i5gc_ | d1sl8a_ | d1fpwa_ | d2scpa_ |
| d2roaa_ | d4aqra_ | d3pn7b_ | d1br1b_ | d2mysb_ | d1s6ca_ | d1bjfa_ | d2obha_ |
| d1jfka_ | d1jf0a_ | d1qv0a_ | d1lkja_ | d2mlfc_ | d2m97a_ | d2d8na_ | d4by5a_ |
| d1jbaa_ | d4nqga_ | d1wdcb_ | d2mysc_ | d1j7qa_ | d1s6ia_ | d1uhka2 | d1r2ua_ |
| d1dtla_ | d3kf9a_ | d3pn7c_ | d1gjya_ | d1y1xa_ | d1hqva_ | d1dvia_ | d1df0a1 |
| d1qxpa2 | d4usla_ | d1k94a_ | d1jfxa_ | d1wkya2 | d1uuqa_ | d3wh9a_ | d4m1ra_ |
| d1xyza_ | d4xuya_ | d2je8a5 | d4bf7a1 | d3cuia_ | d1ur4a_ | d1qw9a2 | d1odza_ |

d2vzsa5 d1bqca_ d2x2ya1 d1hjqa_ d1qnra_ d4y7ea1 d1vjza_ d4k68a_
d2v3ga_ d4hpga_ d1nofa2 d1nq6a_ d1h4pa_ d1aq0a_ d3ziza_ d4bs0a_
d1ecea_ d1h1na_ d1ta3b_ d1w91a2 d1i1wa_ d1uhva2 d1us3a2 d1egza_
d7a3ha_ d1tvna1 d3nyda1 d1ghsa_ d2cyga1 d4xv0a_ d2c8na2 d1w32a_
d3emca_ d1v6ya1 d1foba_ d2uwfa1 d2fgla_ d3niya1 d2c0ha1 d1rh9a1
d1yq2a5 d1szna2 d1fa2a_ d1pama4 d5do8a1 d1ea9c3 d2fhfa5 d1qhoa4
d1avaa2 d1m53a2 d4m8ua1 d2gjpa2 d1lwha2 d4howa1 d1ktba2 d1j0ha3
d3vm5a1 d2x1ia_ d1wzaa2 d1uasa2 d2diea1 d1mxga2 d1ht6a2 d1eswa_
d1iv8a2 d5cpsa_ d1ud2a2 d1zjaa1 d3gbea1 d1kwga2 d1wdpa_ d1gcya2
d1g5aa2 d1hvxa2 d3dhpa2 d2bhua3 d4do4a1 d3qgva1 d1e43a2 d1g94a2
d2d3na2 d1gjwa2 d1gvia3 d1x1na1 d1r7aa2 d1tz7a1 d3ljqa_ g1k2x.1
d4c7ga2 d2gjxa1 d1nowa1 d1jaka1 d1yhta1 d4toqa_ d1wb0a1 d1w9pa1
d1eoka_ d1itxa1 d1kfwa1 d1goia2 d1edqa2 d2gsja_ d1hjxa1 d1vf8a1
d1etob_ d1umqa_ d1g2ha_ d1t77a2 d1mixa2 d1e5wa2 d1j19a2 d1isna2
d2al6b2 d3syxa1 d1mkea1 d2p8va_ d1xoda1 d1i7aa_ d1evha_ d1egxa_
d2hmta_ d1lssa_ d1iuka_ d2csua1 d3q9ub_ d2yv1a1 d3q9na_ d2d59a_
d2yv2a1 d1euca1 d1y81a1 d1wgqa1 d1v89a1 d2coda1 d1zc3b_ d1wg7a1
d1u5ea1 d1v61a1 d2coca1 d2ys1a1 d1v5pa1 d1ntya2 d1wjma1 d1wi1a1
d1u29a_ d1xx0a1 d1unqa_ d2zkmx3 d1v88a1 d2adza1 d1x1fa1 d1p6sa_
d2coaa1 d1btka_ d1v5ma1 d1faoa_ d2x18a_ d1dyna_ d1x1ga1 d4kaxb_
d2j59m1 d1ki1b2 d1xcga2 d1txda2 d1u5da1 d1dbha2 d1w1ha_ d1upqa_
d1v5ua1 d1foea2 d2fjla1 d1eaza_ d2dfka2 d2cofa1 d1kz7a2 d1h6ta1
d9ldta1 d2ewda3 d1up7a1 d2dfda1 d1s6ya1 d1vjta1 d7mdha1 d1hyea1
d1t2da1 d1ojsa3 d1ldna1 d1hyha1 d1civa1 d2a92a1 d1ez4a1 d1i0za1
d1y6ja1 d1gv0a1 d1ur5a1 d1llda1 d1obba1 d1v8ba1 d1gdha1 d1l7da1
d2dlda1 d1mx3a1 d1ygya1 d1qp8a1 d1li4a1 d1j4aa1 d1npya1 d3t4ea2
d1a4ia1 d1o0sa1 d2hk9a2 d2egga2 d1b26a1 d1bvua1 d1gq2a1 d1c1da1
d1leha1 d1gtma1 d1euza1 d1nvta1 d3dona2 d1edza1 d1vi2a1 d1p77a1
d1nyta1 d1b0aa1 d1vl6a1 d1v9la1 d1txga2 d3f47a1 d1mv8a2 d2pv7a2
d1xc2a3 d2ahra2 d4xiya1 d2f1ka2 d1pgja2 d1i36a2 d1np3a2 d1lj8a4
d1teaa3 d1qmga2 d2i76a2 d2g5ca2 d1wdka3 d1dlja2 d3cuma3 d1kola2
d1f8fa2 d1qora2 d1jqba2 d1vj0a2 d1cdoa2 d1gu7a2 d1d1ta2 d1xa0a2
d2dm6a2 d1jvba2 d1rjwa2 d1h2ba2 d1tt7a2 d1piwa2 d1o89a2 d1iz0a2
d1e3ja2 d1yb5a2 d1uufa2 d1vj1a2 d1e3ia2 d3gpdg1 d1vc2a1 d2nvwa1
d1u8fo1 d1ydwa1 d1hdgo1 d2g17a1 d1cf2o1 d2czca2 d1r0ka2 d1obfo1
d2cvoa1 d1ebfa1 d1h6da1 d1i32a1 d1zh8a1 d2q49a1 d1qkia1 d3pzra1
d1xeaa1 d1vkoa1 d1vm6a3 d3qvsa1 d2pkqo1 d1b7go1 d1gr0a1 d1p1ja1
d1lc0a1 d1j0xo1 d3pwka1 d2hjsa1 d1zyao1 d1vkna1 d1tlta1 d1nvmb1
d3sjua_ d3ojfa1 d1xu9a1 d2jjya1 d1n7ha_ d1y1pa1 d1h5qa_ d1jtva_
d4fs3a_ d2bkaa1 d1gy8a_ d2c5aa1 d1ulsa_ d1ooea_ d2b69a1 d1y5ma1
d1spxa_ d1ae1a_ d1ydea1 d1gega_ d1hdoa_ d1o5ia_ d1e6ua_ d1snya1
d2rhca_ d3zv4a_ d5ceja_ d2d1ya1 d1fjha_ d3w8ea_ d2fmua1 d1x1ta_
d1rpna_ d3ay7a1 d1k2wa_ d1eq2a_ d2hrba1 d3ruaa_ d2ew8a1 d4hwka_
d1xgka_ d2bd0a1 d1yo6a1 d2a35a1 d2ntva_ d1vl8a_ d1hdca_ d4bkua_

d2ag5a1 d2q45a_ d1nffa_ d4iy1a_ d1zbqa1 d1kewa_ d1ek6a_ d1ja9a_
d2q46a2 d1iy8a_ d1cyda_ d2ptga_ d1hxha_ d1uzma1 d3lyla_ d4m89a_
d3wyea_ d2blla1 d1w6ua_ d1sb8a_ d2q2va_ d4nr0a_ d1e6wa_ d2gasa_
d3oiga1 d2bgka1 d1mxha_ d1vl0a1 d1rkxa_ d1r6da_ d1wmaa1 d1e7wa_
d2c07a1 d1xkqa_ d3tzha_ d1g0oa_ d3lz6a_ d1qyca_ d1edoa_ d1gz6a_
d1oc2a_ d1t2aa_ d1zema1 d2xoxa_ d1z45a2 d1geea_ d1p33a_ d3lt0a_
d4e6pa_ d2wyua_ d1db3a_ d4q9na_ d1xhla_ d4o1ma_ d1fmca_ d2uvda_
d2gdza1 d1uaya_ d2z1ma_ d2o23a2 d2fr1a2 d2fr1a1 d1qsga_ d1qyda_
d4zjua_ d2a4ka1 d1yb1a_ d1wvga1 d1yxma1 d3f4ba_ d1mg5a_ d1xg5a1
g1y43.1 d3nqyb_ d4gera_ d1bqba_ d1cmxa_ d1xd3a_ d3ifwa_ d1g0da4
d2f4ma1 d1x3za_ d3i2ka1 d1lnsa3 d1mpxa2 d2w0pa_ d2diaa1 d2e9ia2
d2e9ja1 d2dj4a1 d2eeba1 d2diba1 d2dmba1 d2dmca1 d2d7oa1 d2ee6a1
d2d7pa1 d2eeaa1 d2di8a1 d1wlha1 d2d7na1 d2nqca_ d2d7ma1 d2dica1
d3cnka_ d2k3ta1 d2eeca1 d1qfha1 d1qfha2 d2bp3a1 d1v05a_ d2ee9a1
d2eeda1 d2di9a1 d3ioqa_ d1fh0a_ d1o0ea_ d1khqa_ d1m6da_ d2cb5a_
d1cs8a_ g8pch.1 d1s4va_ d2pnsa_ d1vsna1 d1mira_ d1iwda_ d2oula_
d7pcka_ d3bwka_ d3bcna_ d2p86a_ d1pvja_ d1pcia_ d1gece_ d1deua_
d4bs6a_ d1xkga1 d1cqda_ d1jqpa2 d2c0ya_ d2bdza_ d3p5ua_ d1pv8a_
d1w5qa_ d2c1ha_ d1ohla_ d1zbfa1 d2z1ha_ d2etja1 d3qioa1 d1jl1a_
d2e4la_ d1jl2a_ d2lsna1 d1io2a_ d1uaxa_ d2yv0x_ d1mu2a1 d1ekea_
d1i39a_ d1s5ja1 d2hbja2 d2igia_ d1wlja_ d1qhta1 d4dlea1 d2f96a1
d3tr8a1 d3mxma_ d1q8ia1 d1y97a1 d1w0ha_ d1kfsa1 d2py5a1 d1noya_
d1d5aa1 d1yt3a3 d2guia_ d3mwpa1 d4kb1a_ d1b9ra_ d1offa_ d1frra_
d3wcqa_ d1e9ma_ d1l5pa_ d1wria_ d1i7ha_ d3av8a_ d1czpa_ d1rfka_
d3p1ma1 d3b2ga_ d2bt6a2 d3nvva1 d1krha3 d2h88b1 d1n62a2 d1t3qa2
d1kf6b2 d1zoyb1 d1jq4a_ d1dgja2 d1rm6c2 d1jmxa2 d1pbya1 d1pbya2
d1r7ha_ d1faaa_ d1wika1 d1xfla_ d1ttza1 d2f51a1 d1ti3a_ d1nhoa_
d1sena_ d2i4aa_ d1ep7a_ d1zmaa1 d1fo5a_ d3lefa1 d3diea1 d2yana1
d1z6na1 d1oaza_ d3zywa1 d4aj8a_ d1nm3a1 d4fiwa_ d1wmja_ d4hs1a1
d1iloa_ d1xwaa1 d1wjka1 d2vm1a_ d1fb6a_ d1gh2a_ d1h75a_ d1nw2a_
d1kx5b_ d3a6na_ d4m6ba2 d2nqbd_ d3wkjd_ d2yfwe_ d4h9na_ d5bnxa_
d1kx5c_ d3r45a_ d1eqzb_ d4jjnb_ d1f66c_ d1eqza_ d4wnnb_ d1kx5a_
d1u35c1 d1id3a_ d2nqbc_ d1kx5d_ d1f1ea_ d1b67a_ d1bh9b_ d1tafb_
d1jfib_ d1q9ca_ d1h3ob_ d2byka1 d1n1ja_ d4g92b_ d1bh9a_ d1jfia_
d1h3oa_ d2bykb1 d1n1jb_ d1pd212 d1nhya2 d2a2ra2 d1k0dd2 d1f2ea2
d1gsua2 d1f3ba2 d2fhea2 d2pvqa1 d1tw9a2 d1oyja2 d1e6ba2 d1k0ma2
d3ik7a1 d4is0a1 d1r5aa2 d1jlva2 d1m0ua2 d3gtub2 d1z9ha2 d1okta2
d1axda2 d2gsra2 d1tu7a2 d1k3ya2 d1zgma1 d1b48a2 d1gnwa2 d1gwca2
d2gsta2 d1glqa2 d1oe8a2 d1pn9a2 d1v2aa2 d1fw1a2 d1duga2 d1ovna2
d1ig8a1 d1czan2 d1czan3 d1czan1 d1ig8a2 d1czan4 d1bg3a4 d1bg3a3
d1bg3a2 d1nira1 d1qksa1 d3h1jd1 d1qqya_ d1f6ra_ d1ivma_ d2gv0a_
d1iiza_ d2z2fa_ d1j8xa_ d1ghla_ d1b9oa_ d174la_ d169la_ d2o4wa_
d1p37a_ d1k28a3 d1xjta1 d1jtma_ d2f2qa_ d4e97a1 d1iyka2 d1rxta1
d1iica2 d1iica1 d2k4ua_ d1hp2a_ d2lu9a_ d1baha_ d2meoa1 d1wm7a_

d1hlya_ d1n8ma_ d1jlza_ d1wpda_ d1c55a_ d1pvza_ d1wt7a_ d1wz5a_
d1m2sa_ d1pjva_ d1c49a_ d1qkya_ d2ktca1 d1wmta_ d1du9a_ d1j5ja_
d1r1ga_ d1mpya2 d1kw3b2 d1kw3b1 d4ghga1 d1tfza1 d3isqa1 d3isqa2
d1sqia1 d1sqia2 d1lkda2 d1lkda1 d1mpya1 d1cjxa2 d1sp9b2 d1t47a1
d1t47a2 d1f1ua2 d1sp8a1 d1sp8a2 d1f1ua1 d4iuaa3 d4bvva_ d3k65a_
d1ki0a3 d1ki0a2 d1ki0a1 d1a0ha1 d1pmla_ d3sp8a3 d2feba1 d1qsta_
d2beia1 d4m3sa_ d1u6ma1 d1ygha_ d1yk3a1 d3dr6a_ d1xmta_ d1z4ea1
d2ge3a1 d1bo4a_ d2fiaa1 d2jdca1 d1y9wa1 d3to7a_ d1wwza1 d2fl4a1
d2ozua1 d2b5ga_ d2cy2a1 d2p0wa_ d1cm0a_ d4pswa_ d1yrea1 d1yr0a1
d1m4ia_ d2gana1 d1yvka1 d2g3aa1 d1y7ra1 d2euia1 d1q2ya_ d2fsra1
d1xeba_ d2ae6a1 d1cjwa_ d1qsma_ d2aj6a1 d1y9ka1 d1i12a_ d1vkca_
d2atra1 d1n71a_ d2d4pa1 d2fiwa1 d2fe7a1 d1tiqa1 d2ou2a_ d1yx0a1
d2fcka1 d1p0ha_ d2j8ma_ d2pq8a_ d1z4ra1 d1s3za1 d1ufha_ d1xvqa1
d1pn0a2 d3hy2a_ d2cvba1 d1o8xa_ d1lu4a_ d1we0a1 d2bmxa1 d2a4va1
d1psqa_ d1xiya1 d1q98a_ d1n8ja_ d1zofa1 d2ggta_ d3tjja_ d4je1a1
d1o73a_ d3s9fa_ d2zcta_ d1qmva_ d1jfua_ d2b1ka_ d5dvba1 d2b5xa1
d2v2ga_ d1st9a_ d3tb2a_ d4llra_ d4fh8a1 d1e2ya_ d1knga_ d2h30a_
d1i5ga_ d2z9sa_ d2f8aa1 d1tp9a1 d2b7ka_ d1nm3a2 d3hvva_ d2wfca1
d4kcea_ d1xvwa1 d4gqca1 d3qpma_ d4k1fa_ d1zzoa1 d1gp1a_ d1prxa_
d4od7a1 d1z6ma1 d4mcua1 d4dvca1 d3l9sa_ d1un2a_ d3rppa_ d1zsqa1
d2m5ta_ d1cqqa_ d1wqsa_ d5bpea_ d2ipha_ d1lvoa_ d2bhga1 d3tnta_
d3sjia_ d3d23a1 d2fyqa_ d3tloa_ d3zvea1 d2hrva_ d3q3ya1 d4asha1
d1e88a1 d1l6ja5 d1l6ja4 d1eaka4 d1eaka3 d1l6ja3 d1h8pa1 d1w52x1
d2ppla1 d1hpla2 d1lpbb2 d2oxea1 d1bu8a2 d1rp1a2 d1ys1x_ d4hs9a_
d1ispa_ d1ex9a_ d1ku0a_ d3qmma_ d1n9wa1 d1bbua1 d1e1oa1 d1c0aa1
d1l0wa1 d3a74a1 d1eova1 d1lgya_ d4l3wa_ d1llfa_ d1gz7a_ d1ukca_
d1cr6a2 d1bvsa3 d1ixra2 d3groa_ d1pjaa_ d1thta_ d1svpa_ d1mbma_
d3su6a1 d1cu1a1 d4agka_ d1vcpa_ d1q31a_ g1n1l.1 d3e90b_ d1ep5a_
d3u1jb_ d3zxha_ d1cgla_ d1rm8a_ d1eaka2 d1hy7a_ d2jsda_ d1cxva_
d1hv5a_ d1i76a_ d3p0ya1 d1igra1 d1igra2 d1n8yc2 d1n8yc1 d1m6ba2
d1m6ba1 d1dzka_ d4tlja_ d2a2ua_ d4wfva_ d3t1da_ d4iaxa_ d3zq3a_
d2nnda_ d1i4ua_ d1kxoa_ d1bbpa_ d1kt7a_ d1exsa_ d3s26a1 d1lf7a_
d1qfta_ d4gh7a_ d1z24a1 d1qabe_ d1xkia_ d1ew3a_ d3bx7a_ d4iawa1
d1n0sa_ d1gkab_ d3kzaa_ d2k23a_ d2l9ca1 d2joza1 d1gm6a_ d3kffa_
d3saoa1 d1yupa1 d1iiua_ d1lmra_ d1cixa_ d1y29a_ d1v7fa_ d2jtba_
d1d1ha_ d1g9pa_ d1lupa_ d1mb6a_ d2a2va_ d1niya_ d1i25a_ d1hvwa_
d2mpqa_ d1ie6a_ d1kqha_ d1la4a_ d2mxoa1 d1dl0a_ d1koza_ d1qk6a_
d1emxa_ d1lqra_ d1c6wa_ d1rmka_ d1ixta_ d1eyoa_ d1cnna_ d1fu3a_
d1g1pa_ d1f3ka_ d1rypd_ d1irua_ d1rypc_ d3unfk_ d1rypa_ d1j2pa_
d1irui_ d1j2qh_ d3unfh_ d1m4ya_ d1rypb_ d1irug_ d1iru2_ d1q5rh_
d1rypg_ d1g3ka_ d1iruf_ d1q5qa_ d1e94a_ d1irub_ d1ryph_ d3ty6a_
d1iruj_ d1iruk_ d1rype_ d1irud_ d1irue_ d3mi01_ d1rypf_ d1iruh_
d2z3ba_ d1iruc_ d3mka2_ d1iru1_ d1jgta2 d1ofda3 d1ea0a3 d1ecfa2
d1q15a2 d1ct9a2 d1te5a_ d3ubub_ d1egia_ d1qo3c_ d1v7pa_ d1tdqb_

| | | | | | | | |
|---|---|---|---|---|---|---|---|
| d3ubua_ | d1c3ab_ | d1h8ua_ | d3bx4b_ | d1t8ca1 | d1sb2a1 | d1qdda_ | d2ziba1 |
| d3cada_ | d1byfa_ | d1j34b_ | d1jwia_ | d1jzna_ | d1c3aa_ | d1oz7b_ | d1wmza_ |
| d3bdwb_ | d1hq8a_ | d1fifa1 | d1dv8a_ | d3hupa_ | d1wk1a1 | d1umra_ | d2afpa_ |
| d3bx4a_ | d1gz2a_ | d1sl6a_ | d1v7pb_ | d3g8ka_ | d1ixxa_ | d1j34a_ | d1jwib_ |
| d1g1sa1 | d1y17a1 | d1ja3a_ | d1fvub_ | d3bdwa_ | d1sb2b1 | d1fvua_ | d1umrc_ |
| d1oz7a_ | d4m17a_ | d3cfwa1 | d1ypqa1 | d1g1ta1 | d3fd4a_ | d3l9jc_ | d4dn8a1 |
| d1v4lb_ | d1rdl1_ | d2msba_ | d4w9ta_ | d1jvna1 | d4x2ra1 | d1ka9f_ | d1qo2a_ |
| d4axka1 | d1thfd_ | d4u28a_ | d2y88a_ | d4wd0a_ | d1vzwa1 | d3exra_ | d2ffca1 |
| d2fdsa1 | d3n3ma1 | d2czda_ | d1q6oa_ | d2yyua_ | d1vqta1 | d1dbta_ | d3ldva1 |
| d1rw1a_ | d1z3ea1 | d3l78a_ | d4lt9a1 | d3tc7a1 | d3hoja_ | d1i4na_ | d3vnda_ |
| d3nl8a1 | d1geqa_ | d3nava1 | d1v5xa_ | d4a2ra1 | d3tc6a1 | d1ujpa_ | d4ijba_ |
| d1xcfa_ | d1vc4a_ | d1rd5a_ | d1qopa_ | d3b5va_ | d2r80b_ | d1cqxa1 | d1cg5a_ |
| d1gcvb_ | d3boma_ | d2h8fb_ | d2raoa_ | d3bj1a_ | d4hrrb_ | d2r50a_ | d3d1kb_ |
| d2aa1b1 | d3zhwa_ | d3hf4b_ | d3gdja_ | d1jebb_ | d3bj1b_ | d1x9fb_ | d1wmub_ |
| d1gcva_ | d3bcqb_ | d3d4xb_ | d1gvha1 | d2dc3a_ | d3hf4a_ | d4yu3a_ | d3k8ba_ |
| d1qpwb_ | d2r80a_ | d3bcqa_ | d1v4wa_ | d3wtgb_ | d1jl7a_ | d4esaa_ | d1a9we_ |
| d3d1ab_ | d1fsla_ | d4h2la_ | d1v4wb_ | d1mwca_ | d1vhba_ | d1jeba_ | d1spgb_ |
| d1h97a_ | d1q1fa_ | d1tu9a1 | d3d1aa_ | d1hdsb_ | d3hyub_ | d2zfbb_ | d3fs4a_ |
| d1oj6a_ | d1shrb_ | d4h2lb_ | d4yu3b_ | d1spga_ | d3hrwa_ | d3qqra_ | d3at5a_ |
| d4wchd_ | d3hyua_ | d1cg5b_ | d1uc3a_ | d1hbra_ | d2raob_ | d1outb_ | d4esab_ |
| d3gdjb_ | d3qqqa_ | d1x9fc_ | d3vrfa_ | d1itha_ | d3mkba_ | d3d4xa_ | d3mjpa_ |
| d1jl6a_ | d3fh9a_ | d1outa_ | d2dc3b_ | d1it2a_ | d3eoka_ | d3g46a_ | d1qpwa_ |
| d3vrfb_ | d1ch4a_ | d1x9fd_ | d1hdsa_ | d2zfba_ | d3mkbb_ | d1or4a_ | d3wtga_ |
| d1a4fa_ | d3fh9b_ | d3bomb_ | d3cy5a_ | d3at5b_ | d1x9fa_ | d2oifa_ | d4lmxb_ |
| d1cpcb_ | d3v57a_ | d1kn1a_ | d1cpca_ | d4f0ta_ | d2vjha_ | d4po5b_ | d1eyxa_ |
| d1alla_ | d4f0tb_ | d1b33b_ | d1b8db_ | d4h0mb_ | d1b33a_ | d2vjhb_ | d4lm6b_ |
| d2vmlb_ | d1allb_ | d2vjta_ | d3dbja_ | d2vmla_ | d2vjtb_ | d4f0ub_ | d1f99b_ |
| d4rmpa1 | d1liaa_ | d1eyxb_ | d2uulb_ | d4lmsb_ | d4f0ua_ | d2bv8a_ | d1f99a_ |
| d2bv8b_ | d2c7lb_ | d3dbjb_ | d4rmpb1 | d1b8da_ | d4h0ma_ | d3v57b_ | d1kn1b_ |
| d2uula_ | d2j96a_ | d1kyqa1 | g1gxs.1 | d1mtza_ | d1k8qa_ | d2xqfa_ | d1f6wa_ |
| d1dx4a_ | d1qe3a_ | d1k4ya_ | d4qwwa_ | d2ha2a_ | d1sfra_ | d1f0na_ | d4qeka_ |
| d3c8da2 | d2pbla1 | d1jkma_ | d1jjia_ | d1lzla_ | d2qvba_ | d2v9za_ | d4kafa1 |
| d1s5ja2 | d1qhta2 | d4dlea2 | d1tgoa2 | d3py8a2 | d1q8ia2 | d1kfsa2 | d2py5a2 |
| d1d5aa2 | d3u1ra1 | d1g9ka2 | d1k7ia2 | d1f74a_ | d3l21a_ | d2e1da_ | d1to3a1 |
| d1w3ia_ | d1sfla_ | d1n7ka_ | d1vcva1 | d4imea_ | d1wa3a_ | d2ocza1 | d3m16a_ |
| d2yxga_ | d1ub3a_ | d3a5fa_ | d1fbaa_ | d1l6wa_ | d1xfba1 | d1wx0a1 | d1vpxa1 |
| d1ojxa_ | d1mxsa_ | d2a4aa1 | d1xkya1 | d3cq0a1 | d3s1xa_ | d1f05a_ | d1fdja_ |
| d3cpra1 | d4d2ja_ | d4mh8a_ | d4i7ga1 | d3rp2a_ | g1ucy.1 | d1fuja_ | d2zchp_ |
| d3w94a_ | d1si5h_ | d1lo6a_ | g2pka.1 | d1cgha_ | d1kigh_ | d1a5ia_ | d3s9ba_ |
| d1hj8a_ | d1ao5a_ | d2anya_ | d1q3xa1 | d1gvza_ | d1azza_ | d4gsoa_ | d2f91a1 |
| d1iaua_ | d1gdna_ | d1eufa_ | g1fiw.1 | d1eq9a_ | d4nfea_ | d2rdla_ | d1spja_ |
| d1elva1 | d1a7sa_ | d1ekbb_ | d1pfxc_ | d1yc0a_ | d2aiqa_ | g1fiz.1 | d1fi8a_ |
| d4k60a_ | d4dgja_ | d1mzaa_ | d1op0a_ | d3tjva_ | d1pytd_ | d1sgfg_ | g1gg6.1 |
| d2eeka_ | g1rtf.1 | d1a0ja_ | d3g01a_ | d1pytc_ | d4e7na_ | d1ddja_ | d1z8ga1 |

d2hlca_ d1npma_ d1brup_ g1jou.1 d1sgfa_ d1autc_ g2hnt.1 g1kdq.1
d1orfa_ d3s69a_ d4crga_ d1fxya_ d2ftba_ d1pmpa_ d2rcqa1 d1fdqa_
d4lkpa_ d1sa8a_ d1vyfa_ d1lpja_ d3vg7a1 d4azqa1 d5bvqa_ d1p6pa_
d2qo4a_ d1opba_ d1yiva1 d1o1va_ d4bvma1 d4tkba_ d4i3ba_ d1bwya_
d1ftpa_ d1ggla_ d4gkca_ d1cbia_ d3p6da1 d1kqwa_ d2a0aa1 d1m4la_
d2bk7a_ d3fjua_ d1z5ra1 d1kwma1 d1uwya2 d2c1ca1 d1dtda_ d1h8la2
d3lgia_ d3i77a_ d2z9ia2 d4nsva_ d1lcya2 d4jcna_ d1agja_ d1os8a_
d1p3ca_ d1l1ja_ d1qtfa_ d1e69a_ d1mv5a_ d3g60a4 g1ii8.1 d1ewqa2
d1ji0a_ d1qhla_ d1v43a3 d2wmfa1 d1vpla_ d1g2912 d4a82a2 g1f2t.1
d2d62a1 d3tuic1 d2bbsa1 d3g60a3 d2ixea_ d1g6ha_ d1sgwa1 d2qi9c_
d3rlfa1 d3b60a1 d3d31a2 d2pzea1 g1xew.1 d1jj7a_ d1oxxk2 d2onka1
d1w1wa_ d1iwea_ d1qf5a_ d1vmaa2 d1nija1 d1np6a_ d2p67a1 d3ihla_
d1okkd2 d1ls1a2 d1s1ma2 d1iona_ d2v40a_ d1dj3a_ d3hida_ d2vo1a1
d2g0ta1 d1vcoa2 d1j8mf2 d1g3qa_ d1xjca_ d1p9ba_ d1qzxa3 d1ihua2
d1ihua1 d2qm8a_ d1dj2a_ d4ioja_ d1cp2a_ d1yrba2 d3gqba2 d1skye3
d1xp8a1 d1tf7a2 d1tf7a1 d1ye8a1 d1skyb3 d2qe7d2 d4blta_ d4huta_
d3ldaa2 d4hyya_ d2i3ba1 d1cr1a_ d3j9tb2 d3ntua2 d1vdza1 d2z43a_
d1e9ra_ d1nlfa_ d3ziaa2 d1n0wa_ d1g6oa_ d3ziad2 d3j9ta2 d1pzna2
d3gqbb2 d2i49a_ d1nnfa_ d1xvxa_ d1ursa_ d1h3da1 d1iuda_ d4m1va1
d1twya_ d3e13x_ d2nxoa1 d4pfua_ d3ttma_ d1elja_ d2qmwa1 d1us5a_
d1y3na1 d1jeta_ d1uqwa_ d4f19a1 d2esna2 d3thia_ d3fasa1 d2uyeb_
d2xwva1 d1r9la_ d2fyia1 d1pb7a_ d2ozza1 d1sw5a_ d1nh8a1 d1vr5a1
d2g29a_ d1xs5a_ d3lswa_ d2z23a_ d3w9va_ d3tcha_ d1q35a_ d1z7me1
d3q27a_ d1p99a_ d1ve4a1 d2i6ea1 d4mcoa1 d4n8ya_ d1eu8a_ d1zbma1
d1hsla_ d1ixca2 d1j1na_ d1xvya_ d1y9ua_ d2z22a1 d1xoca1 d4oera_
d1wdna_ d5f1qa1 d1xt8a1 d1i6aa_ d4gwoa_ d4igta1 d1pc3a_ d4mmpa1
d1o63a_ d2czla1 d2onsa2 d4mija1 d4ok5a1 d2bmfa1 d2bmfa2 d1yksa1
d1yksa2 d2qeqa1 d2jlqa1 d1a1va2 d1eh3a_ d3skpa_ d1dtza1 d1dtza2
d1ieja_ d1jnfa2 d1ce2a1 d1jnfa1 d1b1xa2 d1b1xa1 d1jw1a1 d1jw1a2
d1ryoa_ d1h76a1 d1h76a2 d1c4oa1 d2p6ra3 d2p6ra4 d1oywa2 d1oywa3
d1wrba1 d1w36b2 d1w36b1 d1c4oa2 d3bora_ d1t6na_ d2eyqa5 d2eyqa3
d2eyqa2 d2zu6a2 d1veca_ d1uaaa1 d1w36d1 d2g9na1 d1z3ix2 d2rb4a_
d1hv8a1 d1hv8a2 d1t5ia_ d1w36c2 d2j0sa2 d2j0sa1 d1fuka_ d3hjha1
d1z5za1 d1qdea_ d1z63a1 d1s2ma2 d1s2ma1 d1wp9a1 d1wp9a2 d1gm5a3
d3dina4 d3dina3 d1q0ua_ d1w36c1 d1z3ix1 d1nkta3 d1nkta4 d1uaaa2
d1w36d2 g1qhh.1 d2fwra2 d2fwra1 d3q3ma_ d1za0a1 d1mxra_ d2inca_
d1jk0a_ d2incb_ d1mhyb_ d3hf1b_ d1r2fa_ d2uw1a_ d1mhyd_ d3n37a_
d1afra_ d1oquc_ d1syya_ d1mtyd_ d2o1za_ d1uzra_ d1smsa_ d2fkza_
d1zuja1 d1s3qa1 d2fjca1 d1z6om1 d1veia_ d4dyya_ d1jiga_ d3gvya_
d2pyba_ d4cy9a1 d3bvfa1 d1r03a_ d2cf7a_ d1nf4a_ d1zs3a1 d4iwka_
d3ka8a_ d3np0x_ d1n1qa_ d1lb3a_ d4e6ka_ d1z6oa1 d2fzfa1 d4dyua_
d3ge4a_ d1jgca_ d4am4a_ d2z6ma_ d1ji5a_ d1vlga_ d1lkoa1 d1krqa_
d3qz3a_ d1o9ra_ d2ffxj_ d1euma_ d1yv1a1 d2v2pa_ d1j30a_ d4di0a_
d1dpsa_ d2cbia2 d1gqia1 d2choa2 d1oewa_ d2qzxa_ d2asia_ d1psoe_

d1miqa_ d1smra_ d2h6ta_ d1pfza_ d1izda_ d3hd8a_ d1j71a_ d1dpja_
d4aa9a_ d1t6ex_ g1b5f.1 g1lya.1 g1htr.1 d1qdma2 d1bxoa_ d1wkra_
d1tzsa_ d1ibqa_ d2qzwa_ d1um8a_ d1ixza_ d1qvra2 d1qvra3 d1qzma_
d1sxjc2 d1jr3d2 d2p65a_ d1l8qa2 d3hu3a3 d1sxjd2 d1njfa_ d1in4a2
d1sxja2 d1tuea1 d2ce7a2 d1w5sa2 d1sxjb2 d1d2na_ d1ny5a2 d1ofha_
d2gnoa2 d2a5yb3 d1fnna2 d1u0ja1 d1lv7a_ d1iqpa2 d2fnaa2 d1ixsb2
d4gita_ d1sxje2 d1g41a_ d1ux8a_ d4i0va_ d2bkma_ d1dlwa_ d2bmma_
d2za0a_ d2c21a1 d1k8rb_ d1wgra1 d1wfya1 d1e8ya3 d1wgya1 d1lfda_
d2cs4a1 d1wxma1 d2byea1 d3kh0a1 d2byfa1 d1wxaa1 d1e7ua3 d1h4ra3
d4yl8a1 d2al6a3 d1l7ya_ d1wgda1 d1wgga1 d4q5eb_ d1wjna1 d1se9a_
d3n3kb_ d1wz0a1 d1we6a1 d2m0xa_ d2gbka_ d3rt3b1 d1wh3a1 d1sifa_
d2ekec2 d4ii2b1 d4hk2a1 d1m94a_ d1wgha1 d1ttna1 d1j8ca_ d1wx8a1
d3v6cb1 d2ma9b_ d4i6lb1 d1bt0a_ d2jvca1 d1iyfa_ d2k1fa_ d1wxva1
d1wf9a1 d1t0ya_ d2io0b2 d1x1ma1 d2gbma_ d1zw7a1 d2m17a_ d1ud7a_
d1we7a1 d2lwpa_ d2gbja_ d1wjua1 d1v6ea1 d1v86a1 d4eewa_ d1wiaa1
d4icva_ d4gu2a_ d1wx7a1 d1wy8a1 d4hjka1 d2faza1 d1v5oa1 d2al3a1
d2cr5a1 d1wj4a1 d1s3sg_ d4lu0a_ d3unda1 d3e9aa1 d1vr6a2 d1n8fa_
d2qkfa_ d1d9ea_ d1o60a_ d1dszb_ d1a6ya_ d3cbba_ d1ynwa1 d1r4ia_
d1hcqa_ d2hana_ d2hanb_ d2enva1 d1kb4b_ d2nllb_ d1lo1a_ d1cita_
d2dara2 d2dara1 d1wyha2 d2dloa2 d1rutx4 d1rutx3 d2cuqa2 d1x4la2
d1x4la1 d1x3ha2 d1x3ha1 d2d8za1 d1x61a2 d1x6aa1 d1x6aa2 d2cora2
d2cora1 d1v6ga1 d1v6ga2 d2d8za2 d1x64a1 d1x64a2 d1b8ta1 d2co8a1
d1x63a2 d2dj7a2 d2dj7a1 d2cu8a2 d2cu8a1 d1g47a2 d1g47a1 d1j2oa1
d1wyha1 d2dloa1 d1wiga1 d1wiga2 d2cupa2 d2cupa3 d1x68a1 d2d8ya2
d2d8ya1 d1x62a1 d1x68a2 d1x61a1 d1b8ta3 d1b8ta2 d2d8xa2 d2d8xa1
d1x4ka1 d1x4ka2 d2cuqa1 d1m3va1 d2co8a2 d1j2oa2 d1x63a1 d2cura1
d2cura2 d1x62a2 d2e3fa_ d1mm0a_ d2e3ea_ d2llda_ d2ny8x_ d2e3ga_
d2ny9x_ d2nz3a_ d2lj7a_ d2m8bb1 d2kyqa_ d3psma_ d1jkza_ d1jxca_
d2gl1a_ d1ugla_ d4uj0a_ d1js9c_ d1f15b_ d1za7a_ d1t0za_ d2i61a_
d1t1ta_ d1sn4a_ d1kv0a_ d2lkba_ d1npia_ d1wwna_ d1pe4a_ d2kbka_
d2li7a_ d1djta_ d2kbha_ d1dq7a_ d1sega_ d1omya_ d1zywa1 d1jzaa_
d1bnla_ d1t61c1 d1iyjb4 d1iyjb5 d1iyjb3 d1o7ia_ d4ipca1 d1x3ea_
d4apva_ d1jb7a1 d1jb7a2 d1jb7a3 d3kdfa_ d1ue1b_ d1wjja1 d3eiva_
d1xjva2 d1xjva1 d2ccza2 d1se8a_ d1l1oc_ d1jb7b_ d1qvca_ d1qzga_
d1miua4 d1miua5 d1miua3 d1y14b1 d2nn6i1 d1i94l_ d2zjra2 d1smxa_
d2qamc2 d1hh2p1 d2r7da1 d1rl2a2 d1wfqa1 d3bzka4 d1x6oa2 d3a0ja_
d2j01d2 d2ja9a1 d4ql5a_ d1hr0w_ d1luza_ d2n3sa_ d2vnud1 d1h95a_
d1go3e1 d2nn6h1 d1ne3a_ d2mo1a_ d1iz6a2 d1jjga_ d1e3pa2 d2ix0a3
d2ix0a2 d2lxja_ d1ueba3 d1ueba2 d2z0sa1 d1u0la1 d2ix0a1 d2eifa2
d2je6i1 d3i2za1 d2c35b1 d2ahob2 d1t9ha1 d3i4oa_ d2qall1 d1jt8a_
d2nn6g1 d1g6pa_ d3cama_ d1i94q_ d1c9oa_ d1d7qa_ d2ba0a1 d2vnud2
d2vnud3 d1khia2 d2qalq1 d1wi5a1 d1xtda2 d1ny4a_ d1fvia1 d1ckma1
d1p16a1 d1x9na2 d2aq2b1 d1ck1a1 d1enfa1 d1i4pa1 d1v1oa1 d1et9a1
d1esfa1 d2g9hd1 d4ohja1 d1klud1 d1bxta1 d1eu3a1 d1ty0a1 d1fnua1

d1ohea1 d1ohea2 d2f4da_ d1rxda_ d2hxpa_ d1xria_ d1d5ra2 d1fpza_
d3ezza_ d4y2ea_ d1i9sa_ d1c1la_ d4lbqa_ d1a78a_ d1slta_ d4no4a_
d3zsja_ d3duia_ d1is3a_ d1qmja_ d1hlca_ d1tdha3 d1q3ba3 d1ee8a3
g1qmo.1 g1len.1 d3zyra_ d1fnya_ d1n47a_ g2ltn.1 d1avba_ d3usua_
d1dhkb_ d1f9ka_ d1ukga_ d1g8wa_ d1g9fa_ d3wcsa_ d1g7ya_ g1loe.1
d1mvqa_ d1hqla_ d1v6ia_ d1dbna_ d1qnwa_ d1fx5a_ d4l8qa_ d1ioaa_
d3n35a_ d3ubra_ d1qo8a1 d1d4ca1 d2k3va_ d1ft5a_ d1qdba_ d1m1qa_
d2j7aa_ d1h21a_ d1ogyb_ d4fasa_ d1jnia_ d1oaha_ d1sp3a_ d3h33a_
d1rwja_ d19hca_ d1os6a_ d3bxua_ d3h4na_ d1i77a_ d3caoa_ d1j0pa_
d2ctha_ d1duwa_ d2e84a_ d1hh5a_ d1gyoa_ d3h34a_ d1ccza2 d1vcaa1
d1dr9a2 d1ic1a1 d2d9qb1 d2nxyb2 d1adqa1 d1fnga1 d1uvqb1 d1zs8a1
d2vola1 d2vola2 d1mfbl2 d2jelh2 d1uwel2 d3qq4b1 d1kjvb_ d1k5na1
d1osph2 d4odhl2 d1ow0a1 d1ow0a2 d1bfob2 d3l9ra2 d3jtth1 d2fath2
d4elkb2 d1fn4b2 d2xfxb_ d1pg7x2 d1cqka_ d4k71b2 d1lk2a1 d1eapb2
d4lfhg2 d1i4fa1 d2nw2a2 d1kfah2 d1mhca1 d1h3ua1 d1wejh2 d1h0db2
d1a5fh2 d1kcvh2 d1mjuh2 d1qqda1 d1kcrh2 d1k8ia1 d3huja2 d2nnab1
d1nfdf2 d3mffb2 d1s9va1 d1lk3h2 d1q0xl2 d1fv1b1 d1neza1 d4rfeb2
d1r3ha1 d1a6tb2 d1kjva1 d1pz5b2 d1onqa1 d2bc4a2 d1de4a1 d1k8da1
d1tcra2 d1dn0b2 d1nfde2 d2xwtb2 d1bfoa2 d1mexh2 d1iakb1 d1ypzf2
d4f7ea2 d1q72l2 d2fbjh2 d1fe8h2 d1clzh2 d1fngb1 d1uvqa1 d1vgka1
d1c16a1 d4j12a2 d4x99b2 d1igtb3 d1je6a1 d1q72h2 d1nldh2 d1teth2
d2hmic2 d1i1ca2 d1i1ca1 d1k8ib1 d1q0yh2 d1kfal2 d3frua1 d1pg7w2
d1u58a1 d1igyb4 d1igyb3 d1hyrc1 d1klub1 d1deeb2 d1c12b2 d2bc4b2
d2ig2h2 d1kcvl2 d3wn5b1 d1lk2b_ d1lk3l2 d1mjul2 d1sm3h2 d1q0xh2
d1zvsa1 d1ydpa1 d1u8ib2 d1ypze2 d1kcuh2 d1op9a_ d1y0la1 d1etzb1
d1egjl1 d2gsib1 d1nj9a1 d4jfhd1 d1aqkl1 d1t2ja_ d1lo4h1 d1zvya1
d3v4vl1 d4enec1 d1kxtb_ d5e8ea1 d2pxya_ d4weud_ d1xiwc_ d2esvd1
d1qnzh_ d1ogae1 d1f4xh1 d4n1hb_ d3ezjb1 d3mlwl1 d1kfah1 d1etza1
d1b4jh1 d3cfic_ d4w68a_ d2arja1 d1ek3a_ d2ok0l1 d1vera_ d1uwgh1
d1blnb1 d1fpth1 d1rzga1 d2giya1 d4m3kb1 d1nc2a1 d2nyyd1 d1ncna_
d1fbih1 d5dxwa_ d4fqqa1 d1dqdh1 d1jfql1 d1kelh1 d1faih1 d1frgl1
d1figl1 d4m6nl1 d1fo0a_ d4hxbl1 d3b5ga_ d3quyd1 d2h9gb1 d1i8kb_
d2dd8h1 d2ck0h1 d1eaja_ d4w6wb1 d2a6ib3 d1t66d1 d2e27h_ d1ob1b1
d1f11b1 d2v7na1 d1ad0a1 d2xt1b1 d1pskh1 d4i13b1 d2op4l1 d1a0ql1
d1uwel1 d1za3a1 d1ncbl1 d4uu9a_ d1bm3h1 d1cloh1 d1nqba2 d1cf8h1
d4y5va_ d1mhph1 d5anma1 d4cdgc1 d4ldeb_ d1y0lb1 d2pcpb1 d1z3gl1
d1dfbl1 d1fh5h1 d3g5xa1 d1tzib1 d4dn3l1 d3zhka1 d2b1hh1 d2uyla1
d1f3dh1 d1nj9b1 d2agjh1 d2h1pl1 d1q72h1 d8fabb1 d1ndmb1 d1rjca1
d4u6hb1 d3upca_ d2fbjl1 d1osph1 d1tvda_ d2cjvh_ d4fhbd1 d1bd2d1
d1rz7h1 d3nn8a_ d1blna1 d1knoa1 d1nfda1 d2bmkb1 d1dqll_ d1igyb1
d1igfl1 d12e8h1 d3utza1 d1nc2b1 d1rihh1 d4mwfb1 d4jvpa1 d1mieh1
d1rhfa1 d1fgnl1 d1sbsh1 d1lp9e1 d1dn0a1 d4lqfl1 d1iaih1 d1kgcd1
d1mh5b1 d1gpol1 d2g60h3 d1f8tl1 d2jixd1 d4grwh_ d1fo0b_ d1bfob1
d1axsa1 d1u9ka_ d1nmbh_ d1h8na1 d1h8na2 d1jguh1 d4eura1 d3sdyl1

d35c8h1 d1w72l1 d1mvub_ d1i9rh1 d1ob1a1 d4wenb_ d1yeeh1 d3ietc1
d1f58h1 d1ad0b1 d1mqkl_ d1nbvh1 d4b50a_ d3r8bb_ d1bafl1 d1mamh1
d4rgne1 d2gsgb_ d35c8l1 d1bafh1 d1ai1h1 d2pcpa1 d2fath1 d2x1pa_
d1orsb1 d1ibgl1 d1shma_ d1a2yb_ d1u0qa_ d2coqa_ d1hilb1 d1qfwi_
d1jv5b_ d1fn4b1 d5e0qa_ d1nsnh1 d1plgl1 d4ocxl1 d1kb5h1 d1rzfh1
d1pg7x1 d2fx7h1 d2oi9c_ d1l7il1 d3eyva1 d1d5il1 d4ffyl_ d1tetl1
d1a6wh_ d1ac6a_ d3dvfa_ d1nfde1 d1mimh1 d1ri8a1 d1jhlh_ d3dura_
d1fskb1 d1f97a1 d3so3b1 d2ak4d1 d1lp9f1 d4z7wf1 d1l7th1 d4y5ya_
d4x7ec1 d1emth1 d1ikfl1 d1nlbh1 d4utal1 d1bjma1 d1eapb1 d1u6ah1
d2dtgd1 d1kj2b_ d1xeda1 d1c1eh1 d1fgvh_ d2bnub1 d2fb4h1 d2dtgb1
d1ktrh_ d1jpth1 d1qkzl1 d4hepg_ d2atpb1 d1flrh1 d2hrpl1 d1mvfa_
d1iaim1 d3k80a1 d2jb5h1 d3zkxc_ d4grwe_ d1it9h1 d1rhhb1 d3w9ec_
d1e4wh1 d1sy6h1 d2j6eh1 d3qxta_ d1jglh1 d1p7ka1 d1tzhb1 d4n8pa1
d1gafl1 d2jelh1 d1wioa2 d4i0cc1 d1cicb1 d1lmka2 d1lmka1 d1iqwh1
d1orqb1 d1ejoh1 d1jnlh1 d4zg1a_ d43c9b_ d1ggbl1 d1wejh1 d3q6fa1
d1f3dj1 d3c6la1 d1bqlh1 d3p9wb_ d1a3rl1 d1h0db1 d2uzil_ d1eo8l1
d1qfwl_ d1n7ml1 d1igja1 d1c5ch1 d2a9mh_ d1rmfh1 d1fj1b1 d4heme_
d1ccza1 d1a5fh1 d1ogad1 d1dlfh_ d1qa9b_ d1vgel1 d3qskb_ d1sjxa_
d4ozfg1 d1sq2n_ d1qd0a_ d1kcvh1 d1c5db1 d3qcul1 d3bn9d1 d1mjuh1
d4y8dc_ d7fabh1 d3dvgb1 d1lo0l1 d1a6tb1 d1gigh1 d1kcrh1 d2hwzl1
d1yjdc1 d3clfl1 d4krna_ d1dl7l_ d1e6ol1 d1mexh1 d3r0ma_ d4kkna_
d1fnsl1 d1oaqh_ d1rzia1 d1ucbh1 d1ncwl1 d4b41a_ d1nfdf1 d2mpah1
d2d7tl1 d4grwf_ d4b5ea_ d1lk3h1 d3u79a_ d4u3xa_ d1ghfl1 d1bvkb_
d1mcww1 d1b88a_ d1jrhh1 d32c2b1 d4wemb_ d1dsfh_ d1op3h1 d1ce1l1
d1iqda1 d1qoka1 d1qoka2 d1qfuh1 d2crya1 d1ay1h1 d1oaql_ d3ab0b_
d1rurl1 d1t4kb1 d1bz7b1 d1n0xh1 d1forl1 d1ucbl1 d4w6yb_ d43c9a_
d2aabl1 d1tzia1 d4n1ca_ d1k6qh1 d3g9ab_ d4tvsa1 d1igmh_ d1pz5b1
d3dx9a1 d3wxwh_ d1nakh1 d1kcul1 d3eaka_ d4qlra1 d1cr9l1 d1d9ka_
d1f4xl1 d1deea1 d2i27n2 d1c12a1 d2orbl1 d1j8hd1 d1mcph1 d1c5da1
d1kenh1 d1bd2e1 d1h5ba_ d4x7fc1 d3upaa_ d1t3fa1 d1indh1 d2dtgc1
d4hc1l1 d1zv5a1 d4wgvb_ d1uwgl1 d4f37k1 d1aqkh1 d1vesa_ d3zkqd_
d1fptl1 d1lo4l1 d1knob1 d3rrqa_ d1ct8b1 d4orzc_ d1mlbb1 d4hgkc1
d1dqdl1 d1bogb1 d4ozhe1 d1dr9a1 d1i7za1 d1dn0b1 d1kell1 d1rzib1
d1ypzf1 d1wtla_ d1hkfa_ d1fail1 d1figh1 d2vyre_ d1sjva_ d1ohqa_
d1bfoa1 d1pkqb1 d1mjul1 d1b4jl1 d1nsnl1 d2arjb1 d1p4bh_ d4aj0a_
d3nn8b_ d2rcja1 d1f2xk_ d4v1dd_ d1adqh1 d2eksa_ d4k3ga_ d1jfqh1
d1uweh1 d1frgh1 d4gftb_ d1uz8b1 d4n1ea_ d1bm3l1 d1clol1 d3mcka1
d2vq1a1 d1ae6h1 d4aq1b1 d1uj3b1 d1nbqa1 d1fh5l1 d2ck0l1 d2ywza_
d1q72l1 d4nbxb_ d1hila1 d4hzlb1 d2j88l1 d1zmya1 d1cd0a_ d1a4ka1
d1pskl1 d1a0qh1 d4krmb_ d1ncbh1 d2fbjh1 d1j8he1 d1i3ua_ d1cf8l1
d1fe8h1 d2p1ya1 d1eeqa_ d2x89a_ d1ol0a_ d2r0lh1 d1ub5a1 d1dfbh1
d3eyvb1 d4jdvb1 d4p2cg_ d2gsia1 d2agjl1 d1u3ha1 d2h1ph1 d1rihl1
d1fskc1 d1boga1 d2a77l1 d1ospl1 d1qfoa_ d3dwta_ d2fx7l1 d1rz7l1
d3stba1 d1pkqa1 d1b6da1 d1dzba2 d1dzba1 d1kj2a_ d4l1ha_ d1rzgb1

d1dqlh_ d12e8l1 d1miel1 d1jmaa_ d1sbsl1 d1fgnh1 d1f58l1 d1mqkh_
d1ndgb1 d3qegl1 d1iail1 d2aw2a1 d1tqbc1 d3fo2a1 d4dkaa_ d1f8th1
d2g60l1 d2ywya_ d1uj3a1 d1yc7a_ d3bd3a_ d4w6xb1 d1nmbl_ d1i8ka_
d1rjlb1 d1ypze1 d1p7kb1 d1kxqe_ d1gpoh1 d2z4qa1 d1w72h1 d1i9rl1
d4qgya1 d4xt1c_ d2qqnh1 d1cica1 d3c08h1 d2cd0a_ d2bdnh1 d1hnga1
d1maml1 d1nldh1 d1cr9h1 d4lcih1 d3bkyh1 d3qg7l1 d1ai1l1 d1kxvc_
d4iosd_ d1l7ih1 d2x1qa_ d1ub6b1 d3k74b_ d1ibgh1 d1teth1 d1igya1
d1hxma1 d2x6ma_ d2e7la_ d5e03a_ d1qfwm_ d2r0kh1 d1igjb1 d1rurh1
d1plgh1 d1mj8h1 d1yuhb1 d3fmgl1 d1rzfl1 d1emtl1 d1ad9b1 d1igtb1
d1nlbl1 d1d5ih1 d3k7ua_ d4ffyh_ d1bwwa_ d3dvga1 d1a6ta1 d1fgvl_
d4qxgl1 d1miml1 d1jhll_ d2hffb1 d2hh0l1 d1qkzh1 d1l7tl1 d1afvh1
d1iaii1 d2cjuh_ d1um5h1 d2ghwb2 d2ghwb1 d1kgce1 d2fgwh1 d1ikfh1
d1dvfd_ d2i24n1 d2xtjb_ d1za3b1 d2yc1a_ d2hh0h1 d2x1oa_ d1bvka_
d1rjla1 d5e7ba1 d1igta1 d1il1b1 d2fb4l1 d1mvua_ d1jptl1 d32c2a1
d1fvca_ d1iqdb1 d2hrph1 d2p4ab_ d4qo1a_ d1p2cb1 d1wt5c_ d1t4ka1
d5c1mb_ d1it9l1 d1bz7a1 d1b2wh1 d1e4wl1 d1jnll1 d1yedb1 d1za6b1
d3u7aa_ d1igfh1 d3gnml1 d1orsa1 d1wejl1 d1a2ya_ d1hxmb1 d1gafh1
d2j4wh1 d1pz5a1 d1j05a_ d1a3rh1 d4fqll1 d1qfwh_ d1eo8h1 d1n7mh1
d4s11a_ d1kegh1 d5da4a_ d1iqwl1 d3whxb1 d1vgeh1 d1deeb1 d1c12b1
d1t2ql1 d1ad9a1 d3gsnb1 d3b2uc1 d1ggbh1 d1t3fb1 d1nakl1 d4ocmc_
d1lila1 d2v7hb3 d2esve1 d1mcoh1 d2qr0b1 d2q3aa_ d1ct8a1 d1kcrl1
d2pxyb_ d2uzih_ d5c3ld_ d3durb_ d1c5cl1 d2ak4e1 d1dl7h_ d1xiwd_
d1mlba1 d1rmfl1 d1i7zb1 d1dlfl_ d2xa3a_ d5bq7b1 d1dqqb1 d3h42l1
d1egjh1 d1kcvl1 d1fe8l1 d2j6el1 d1f3rb2 d1lk3l1 d1sm3h1 d1q0xh1
d1ghfh1 d7fabl1 d1igch1 d1lo0h1 d4idla_ d5c2bl_ d1jrhl1 d1il1a1
d3zhda1 d4kfzc_ d1b0wa_ d1e6oh1 d1mexl1 d4krpb_ d1fnsh1 d1ncwh1
d4hgma_ d4eigb1 d4rzca_ d2mpal1 d4y5xa_ d5en2b1 d3lrga1 d4ej1c1
d1rhha1 d1j05b_ d1forh1 d1q9wb1 d2wzpd_ d2aq2a1 d1h0da1 d3revb1
d2ij0c1 d1tzha1 d4m7jl1 d1k6ql1 d3b9kc1 d1igml_ d3qeua1 d1dsfl_
d1ce1h1 d1kcuh1 d1zvha1 d1zvoa1 d1tqbb1 d1ay1l1 d2qscl1 d2cgrh1
d1nl0h1 d1kenl1 d3eo0a1 d4n9ob1 d1n0xl1 d3k1kc_ d2cqva1 d1fhga_
d2k1ma_ d2dl2a1 d1biha3 d1biha1 d1biha4 d3qp3a1 d1g1ca_ d1gxea_
d1cvsc2 d1cvsc1 d1xmwa2 d1m4ka2 d2otpa2 d1biha2 d1rhfa2 d2fcba2
d2fcba1 d1wwbx_ d1gsma2 d1epfa1 d1epfa2 d2dava1 d1waaa_ d1f97a2
d1x44a1 d1vcaa2 d1fnla2 d1fnla1 d3b5ha2 d3h8na2 d3h8na1 d1jbja1
d1jbja2 d1pd6a_ d1f2qa2 d1f2qa1 d3cx2a_ d1nbqa2 d3b5ha1 d1n26a1
d1xiwb_ d1xaua_ d1fltx_ d2ifga1 d1olla1 d1olla2 d1cs6a4 d1cs6a3
d1cs6a2 d1cs6a1 d2gw5a2 d1sy6a1 d1xiwa_ d1ucta2 d1ucta1 d1ry7b2
d1gl4b_ d1qz1a3 d1olza1 d2c9aa1 d1wwca_ d1c4qa_ d1prtc1 d2wv6d_
d1prtf_ d1ltrd_ d4l63b1 d1r4pb_ d1prtd_ d1tl0f_ d2bosa_ d1prtb1
d3chbd_

**Family-60 (c.37.1.1)**

**Positive dataset contains 17 protein sequences.**

d3lv8a_ d3tvta2 d3cm0a_ d3h86a_ d1t0hb_ d2h92a_ d3kb2a1 d2c95a1

d1yj5a2 d1vyva2 d2grja1 d1y63a_ d3iija1 d1vyua2 d4e22a_ d1q3ta_ d1teva_

**Negative dataset contains 3651 protein sequences.**

d1m32a_ d1ibja_ d1c7na_ d1iuga_ d4q75a_ d2bkwa1 d1t3ia_ d1qz9a_ d1jf9a_ d1eg5a_ d1p3wa_ d3kgwa_ d1cl1a_ d2ctza1 d1vjoa_ d2rfva_ d1gc0a_ d1cs1a_ d1d2fa_ d2ch1a1 d1qgna_ d1pffa_ d1elua_ d1u08a_ d1lc5a_ d1b5pa_ d2ay1a_ d1bw0a_ d3pd6a_ d7aata_ d2csta_ d1j32a_ d1fg7a_ d4wlha_ d1o4sa_ d1v2da_ d1gdea_ d1m6sa_ d1v72a1 d1svva_ d1wsta1 d2f8ja2 d1lk9a_ d1vp4a1 d1xi9a1 d2gb3a1 d1yaaa_ d3rcha_ d3k40a_ d1pmma_ d1xkta1 d2hfka_ d1jmkc_ d5d3ka_ d2cfba1 d1dfoa_ d1o69a_ d1bj4a_ d3k28a_ d2bwpa_ d3lg0a_ d2fnua_ d3ou5a_ d1z7da1 d1b9ha_ d4msoa_ d1bs0a_ d1s0aa_ d1sffa_ d3tqxa_ d1mdoa_ d3qboa_ d3pgya_ d1bjna_ d2dkja_ d1ejia_ d1vefa1 d4wxga_ d3bs8a_ d3gbxa1 d1w23a_ d1h6ua2 d1sefa_ d1sfna_ d1rc6a_ d2bnma2 d1f56a_ d1bypa_ d1azca_ d1ws8a_ d3u25a_ d1cuoa_ d1id2a_ d1rkra_ d2iaac_ d4dp1x_ d1nwpa_ d2gima_ d2i7sa_ d1qhqa_ d3ehbb1 d1fftb1 d1gyca3 d1hfua2 d1kbva1 d1kbva2 d1sdda1 d1sdda2 d1gyca1 d1gyca2 d1mzya1 d2bw4a1 d1aoza1 d1aoza3 d1aoza2 d1kyaa2 d1kyaa3 d1kyaa1 d1mzya2 d4e9sa1 d1sddb1 d1sddb2 d1hfua1 d1hfua3 d1oe1a2 d1oe1a1 d1v10a2 d1v10a3 d1v10a1 d1rypd_ d1irua_ d1rypc_ d3unfk_ d1rypa_ d1j2pa_ d1irui_ d1j2qh_ d3unfh_ d1m4ya_ d1rypb_ d1irul_ d1irug_ d1iru2_ d1q5rh_ d1rypg_ d1g3ka_ d1iruf_ d1q5qa_ d1e94a_ d1irub_ d1ryph_ d3ty6a_ d1iruj_ d1iruk_ d1rype_ d1irud_ d1irue_ d3mi01_ d1rypf_ d1iruh_ d2z3ba_ d1iruc_ d3mka2_ d1iru1_ d3sxwa1 d1j3qa_ d4luka_ d1a3qa1 d1iknc_ d3brda1 d1p7hl1 d1oy3c_ d1imhc1 d1gjia1 d1zk9a1 d2cxka1 d2bema_ d1pama1 d1ea9c1 d1qhoa1 d2fhfa2 d2fhfa1 d1k3ia1 d1j0ha1 d1cxla1 d2bhua1 d1edqa1 d1f1sa2 d1ut9a2 d1gvia1 d1txka1 d1zx5a1 d1mqta_ d1d4m3_ d1fmd3_ d2mev1_ g1aym.1 d1ev13_ d4q4w1_ d1r1a3_ d2x5ia_ d1rhi1_ g1r1a.1 g1d4m.1 g1rhi.1 d1b35b_ d1oopa_ d1tme1_ d4iv1a_ g1pvc.1 g1fpn.1 d1fpn1_ d4gb31_ d1d4m1_ d1fmd1_ d1ev11_ d2wzr3_ g1cov.1 d1qqp3_ g1fmd.1 d2x5ic_ d1rhi3_ g1oop.1 d1hxs1_ g1hxs.1 d1fpn3_ d1bev1_ g2mev.1 d1eah3_ d1aym1_ d1pvc3_ d1qqp1_ d1zba3_ d1h8ta_ d3vbhc_ g1b35.1 g1bev.1 d1hxs3_ g1ev1.1 d1bev3_ g1tmf.1 d1tmf1_ d2mev3_ g1eah.1 g1h8t.1 d1eah1_ d4q4w3_ d1r1a1_ d1aym3_ d1pvc1_ d1zba1_ d1h8tc_ d1oopc_ d1tme3_ g1qqp.1 d4iv1c_ g1tme.1 d1ds6b_ d1fstb_ d1ju8a_ d4alac_ d1pjwa_ d1rera1 d1s6na_ d3uzqb_ d1tafa_ d1bh9b_ d1tafb_ d1jfib_ d1h3ob_ d2byka1 d1n1ja_ d4g92b_ d1bh9a_ d1jfia_ d1h3oa_ d2bykb1 d1n1jb_ d1eixa_ d3exra_ d2ffca1 d2fdsa1 d3n3ma1 d2czda_ d2yyua_ d1vqta1 d1dbta_ d3ldva1 d2bjca1 d2hsga1 d1qpza1 d1efaa1 d1ydwa2 d2nvwa2 d1h6da2 d1zh8a2 d1xeaa2 d1tlta2 d1r0ka3 d1vkoa2 d1vm6a2 d3qvsa2 d1gr0a2 d1p1ja2 d2ovga_ d1sq8a_ d1rzsa_ d3kz3a_ d3orca_ d3gpdg2 d2q49a2 d1hdgo2 d2g17a2 d2cvoa2 d2czca1 d3pwka2 d1obfo2 d1i32a2 d1zyao2 d2pkqo2 d1b7go2 d2hjsa2 d1cf2o2 d1vkna2 d1nvmb2 d1ux8a_ d1dlya_ d4i0va_

d2bkma_ d1dlwa_ d2bmma_ d2msya_ d2djoa1 d2r5za_ d1au7a1 d1f43a_
d2lmda1 d1akhb_ d1s7ea1 d3nara_ d1zq3p1 d2craa1 d1mija_ d4uuta_
d3a01a_ d1x58a1 d3rkqa1 d3cmya1 d2cqxa1 d2da6a1 d1e3oc1 d1fjla_
d1mh3a1 d1ig7a_ d1x41a1 d1wh7a1 d1gt0c1 d2mw8a_ d1lfup_ d1x2ma1
d1b72a_ d1wi3a1 d1jgga_ d1wjha1 d1x2na1 d1mnmc_ d2cuea1 d3k2aa_
d2hosa_ d2jwta2 d1pufa_ d1octc1 d2hi3a_ d4j19a_ d3lnqa_ d1wh5a1
d1b8ib_ d1hcra_ d1tc3c_ d1gdta1 d1u78a2 d2iu5a1 d1zk8a1 d2g7sa1
d2fd5a1 d2np5a1 d1z0xa1 d2id6a1 d2d6ya1 d2i10a1 d1vi0a1 d2oi8a1
d3c07a1 d2ns7a1 d2fq4a1 d2yvea1 d1t33a1 d2o7ta1 d1rkta1 d2np3b1
d2g7ga1 d2gena1 d2fbqa1 d2g7la1 d2gfna1 d2g3ba1 d2jj7a1 d2hyja1
d1sgma1 d1ui5a1 d2hkua1 d2id3a1 d4jyka1 d1kb0a1 d1k8ka2 d1k8ka1
d3uleb1 d3i33a2 d3i33a1 d1bupa1 d1dkgd2 d1dkgd1 d4ci6a2 d1jcfa1
d4a2ba2 d2fxua1 d1yaga2 d1yaga1 d4cbua2 d1bupa2 d1iqca2 d1iqca1
d1eb7a2 d1nmla2 d1nmla1 d1qksa1 d3h1jd1 d1hroa_ d4gedb_ d3zooa_
d1mz4a_ d1gu2a_ d1f1fa_ d1dvva_ d1c6ra_ d1c75a_ d1e29a_ d1gdva_
d1cnoa_ d1h32b_ d1co6a_ d2bh4x_ d2aiua_ d5cytr_ d1jdla_ d1i8oa_
d2zboa_ d3dmia_ d1ls9a_ d1wvec_ d2yk3a_ d3ph2b_ d1dw0a_ d5aura_
d1ql3a_ d1qn2a_ d1f1ca_ d2nqya_ d1h4ga_ d1hixa_ d1oa2a_ d1olra_
d3b7ma_ d4h7ma1 d3wp3a_ d2vgda_ d1igoa_ d3zsea_ d2nlra_ d3wx5a_
d1oa3a_ d1pvxa_ d1f5ja_ d1oa4a_ d1ks5a_ d4ixla1 d1evsa_ d1cnt1_
d1pvhb_ d1z7ca_ d1huwa_ d1bgea_ d1rhga_ d1f6fa_ d1f45b_ d1i1rb_
d1axia_ d1etea_ d3tgxb_ d1scfa_ d2z3qa1 d3qb1a_ d1m48a_ d4nkqb_
d1eera_ d1v7mv_ d5e4ea_ d2o27a_ d3b5ka_ d2psma1 d4zf7a_ d3uf5a_
d4dnxa2 d1m8pa2 d1g8fa2 d1jhda2 d1x6va2 d2b7la_ d3rbaa_ d2qtra_
d1nupa_ d3l92a_ d1f9aa_ d1qjca_ d2h29a_ d1vlha_ d1od6a_ d1lw7a1
d1mrza2 d4ruka_ d3sw2a_ d1moxc_ d1dx5i3 d1edmb_ d1autl2 d1autl1
d1dval1 d1szba2 d1kigl_ d2y5fl_ d1ijqa2 d1gk5a_ d1g1sa2 d1nt0a3
d1xdtr_ d1lmja2 d1lmja1 d1nzia2 d2wphe_ d1klil_ d1pfxl2 d1pfxl1
d1p9ja_ d1g1ta2 d1f7ea_ d4yzub_ d2mgpa1 d1b9wa2 d2mgpa2 d1ob1c1
d1ob1c2 d1n1ia2 d1n1ia1 d2npra2 d2npra1 d1m1xb4 d1qx2a_ d1ig5a_
d2y5ia_ d1qlsa_ d1psra_ d3n22a_ d2egda_ d4ggfc_ d2wcea_ d2luca_
d4aqia_ d3nsia_ d2lp3a_ d1j55a_ d1a4pa_ d1k8ua_ d1nsha_ d1pvaa_
d3fs7a_ d2pvba_ d1ttxa_ d1rwya_ d2kyfa_ d1rk9a_ d1a75a_ d1exra_
d1tiza1 d1nyaa_ d1g8ia_ d1ggwa_ d1wdcc_ d3fwba_ d1ggza_ d2o5ga_
d1ctaa_ d1m45a_ d3i5gc_ d1sl8a_ d1fpwa_ d2scpa_ d2roaa_ d4aqra_
d3pn7b_ d1br1b_ d2mysb_ d1s6ca_ d1bjfa_ d2obha_ d1jfka_ d1jf0a_
d1qv0a_ d1lkja_ d2mlfc_ d2m97a_ d2d8na_ d4by5a_ d1jbaa_ d4nqga_
d1wdcb_ d2mysc_ d1j7qa_ d1s6ia_ d1uhka2 d1r2ua_ d1dtla_ d3kf9a_
d3pn7c_ d1fi6a_ d1c07a_ d5d67d_ d1tuza_ d1h8ba_ d1eg3a2 d2zkmx1
d1snla_ d1gjya_ d1y1xa_ d1hqva_ d1dvia_ d1df0a1 d1qxpa2 d4usla_
d1k94a_ d1wkya2 d1uuqa_ d3wh9a_ d4m1ra_ d1xyza_ d4xuya_ d2je8a5
d4bf7a1 d3cuia_ d1ur4a_ d1qw9a2 d1odza_ d2vzsa5 d1bqca_ d2x2ya1
d1hjqa_ d1qnra_ d4y7ea1 d1vjza_ d4k68a_ d2v3ga_ d4hpga_ d1nofa2
d1nq6a_ d1h4pa_ d1aq0a_ d3ziza_ d4bs0a_ d1ecea_ d1h1na_ d1ta3b_

d1w91a2 d1i1wa_ d1uhva2 d1us3a2 d1egza_ d7a3ha_ d1tvna1 d3nyda1
d1ghsa_ d2cyga1 d4xv0a_ d2c8na2 d1w32a_ d3emca_ d1v6ya1 d1foba_
d2uwfa1 d2fgla_ d3niya1 d2c0ha1 d1rh9a1 d1yq2a5 d1szna2 d1fa2a_
d1pama4 d5do8a1 d1hx0a2 d2fhfa5 d1qhoa4 d1avaa2 d1m53a2 d4m8ua1
d2gjpa2 d1lwha2 d4howa1 d1ktba2 d1j0ha3 d3vm5a1 d2x1ia_ d1wzaa2
d1uasa2 d2diea1 d1mxga2 d1ht6a2 d1eswa_ d1iv8a2 d5cpsa_ d1ud2a2
d1zjaa1 d3gbea1 d1kwga2 d1wdpa_ d1gcya2 d1g5aa2 d1hvxa2 d3dhpa2
d2bhua3 d4do4a1 d3qgva1 d1e43a2 d1g94a2 d2d3na2 d1gjwa2 d1gvia3
d1x1na1 d1r7aa2 d1tz7a1 d3wdpp1 d3wq5a_ d1gnxa_ d4gxpa_ d4hz8a_
d1e4mm_ d3aiua_ d1wcga1 d1qvba_ d3ahxa_ d1v02a_ d2dgaa_ d1vffa1
d1qoxa_ d4ha4a_ d1mixa2 d1e5wa2 d1isna2 d2al6b2 d1xl4a1 d1n9pa_
d3vsqa_ d2gixa1 d2v76a_ d1wj1a1 d1qqga2 d1qqga1 d1ntva_ d1n3ha_
d4dbba_ d3f0wa1 d1p3ra_ d1wgua1 d2cy4a1 d3hqca_ d1wvha1 d1xr0b1
d1wgqa1 d1v89a1 d2coda1 d1zc3b_ d1wg7a1 d1u5ea1 d1v61a1 d2coca1
d2ys1a1 d1v5pa1 d1ntya2 d1wjma1 d1wi1a1 d1u29a_ d1xx0a1 d1unqa_
d2zkmx3 d1fhoa_ d1v88a1 d2adza1 d1x1fa1 d1p6sa_ d2coaa1 d1btka_
d1v5ma1 d1faoa_ d2x18a_ d1dyna_ d1x1ga1 d4kaxb_ d2j59m1 d1ki1b2
d1xcga2 d1txda2 d1u5da1 d1w1ha_ d1upqa_ d1v5ua1 d1foea2 d2fjla1
d1eaza_ d2dfka2 d2cofa1 d1kz7a2 d1h6ua1 d9ldta1 d2ewda3 d1up7a1
d2dfda1 d1s6ya1 d1vjta1 d7mdha1 d1hyea1 d1t2da1 d1ojsa3 d1b8pa1
d1ldna1 d1hyha1 d1civa1 d2a92a1 d1ez4a1 d1i0za1 d1y6ja1 d1gv0a1
d1ur5a1 d1llda1 d1obba1 d1npya1 d3t4ea2 d1a4ia1 d1o0sa1 d2hk9a2
d2egga2 d1b26a1 d1bvua1 d1gq2a1 d1c1da1 d1leha1 d1gtma1 d1euza1
d3dona2 d1edza1 d1gpja2 d1vi2a1 d1p77a1 d1nyta1 d1b0aa1 d1vl6a1
d1v9la1 d1etob_ d1umqa_ d1g2ha_ d1kola2 d1f8fa2 d1qora2 d1jqba2
d1vj0a2 d1cdoa2 d1gu7a2 d1d1ta2 d1xa0a2 d2dm6a2 d1jvba2 d1rjwa2
d1llua2 d1h2ba2 d1tt7a2 d1piwa2 d1o89a2 d1iz0a2 d1e3ja2 d1yb5a2
d1vj1a2 d1e3ia2 d3gpdg1 d1vc2a1 d2nvwa1 d1u8fo1 d1ydwa1 d1hdgo1
d2g17a1 d1cf2o1 d2czca2 d1r0ka2 d1obfo1 d2cvoa1 d1ebfa1 d1h6da1
d1i32a1 d1zh8a1 d2q49a1 d1qkia1 d1j5pa4 d3pzra1 d1xeaa1 d1vkoa1
d1vm6a3 d3qvsa1 d2pkqo1 d1b7go1 d1gr0a1 d1lc0a1 d1j0xo1 d3pwka1
d2hjsa1 d1zyao1 d1vkna1 d1tlta1 d1nvmb1 d3sjua_ d3ojfa1 d1xu9a1
d2jjya1 d1n7ha_ d1y1pa1 d1h5qa_ d1jtva_ d4fs3a_ d2bkaa1 d1gy8a_
d2c5aa1 d1ulsa_ d1ooea_ d2b69a1 d1y5ma1 d1spxa_ d1ae1a_ d1bxka_
d1ydea1 d1gega_ d1hdoa_ d1o5ia_ d1e6ua_ d1snya1 d2rhca_ d3zv4a_
d5ceja_ d2d1ya1 d1fjha_ d3w8ea_ d2fmua1 d1x1ta_ d1rpna_ d3ay7a1
d1k2wa_ d1eq2a_ d2hrba1 d3ruaa_ d2ew8a1 d4hwka_ d1xgka_ d2bd0a1
d1yo6a1 d2a35a1 d2ntva_ d1vl8a_ d1hdca_ d4bkua_ d2ag5a1 d2q45a_
d1nffa_ d4iy1a_ d1zbqa1 d1kewa_ d1ek6a_ d1ja9a_ d2q46a2 d1iy8a_
d1cyda_ d2ptga_ d1hxha_ d1uzma1 d3lyla_ d4m89a_ d3wyea_ d2blla1
d1w6ua_ d1sb8a_ d2q2va_ d4nr0a_ d1e6wa_ d2gasa_ d3oiga1 d2bgka1
d1mxha_ d1vl0a1 d1rkxa_ d1r6da_ d1wmaa1 d1e7wa_ d2c07a1 d1xkqa_
d3tzha_ d1g0oa_ d3lz6a_ d1qyca_ d1edoa_ d1oc2a_ d1t2aa_ d1zema1
d2xoxa_ d1z45a2 d1geea_ d1p33a_ d3lt0a_ d4e6pa_ d2wyua_ d1db3a_

d4q9na_ d1xhla_ d4o1ma_ d1fmca_ d2uvda_ d2gdza1 d1uaya_ d2z1ma_
d2o23a2 d2fr1a2 d2fr1a1 d1qsga_ d1qyda_ d4zjua_ d2a4ka1 d1yb1a_
d1wvga1 d1yxma1 d3f4ba_ d1mg5a_ d1xg5a1 d1r55a1 d3gboa_ d1atla_
d1kufa_ d4q1la_ d1quaa_ d1yp1a_ d1wnia_ d2gfoa1 d1vjva_ d2ayna1
d3nhea_ d4ekfa_ d2ckga_ d1th0a_ d2bkra_ d1xd3a_ d3ifwa_ d3i2ka1
d1mpxa2 d3ioqa_ d1fh0a_ d1o0ea_ d1khqa_ d1m6da_ d2cb5a_ d1cs8a_
g8pch.1 d1s4va_ d2pnsa_ d1vsna1 d1mira_ d1iwda_ d2oula_ d7pcka_
d3bwka_ d3bcna_ d2p86a_ d1pvja_ d1pcia_ d1gece_ d1deua_ d4bs6a_
d1xkga1 d1cqda_ d1jqpa2 d2c0ya_ d2bdza_ d3p5ua_ d2o36a_ d1k9xa_
d1zoya1 d1d4ca2 d1chua2 d1jnra2 d1kf6a2 d2jk6a2 d2jk6a1 d1feca1
d1feca2 d1lpfa1 d1lpfa2 d1xhca1 d1xhca2 d1dxla2 d1dxla1 d1gera2
d1m6ia2 d1onfa1 d1onfa2 d3lada2 d3lada1 d1w4xa1 d1w4xa2 d1gesa2
d1mo9a1 d1xdia1 d1fl2a1 d1fcda1 d1fcda2 d1h6va2 d1h6va1 d1mo9a2
d1gv4a2 d1gv4a1 d2gv8a2 d2gv8a1 d1aoga1 d1aoga2 d1hyua1 d1ebda1
d1ebda2 d3dk9a1 d1q1ra2 d1q1ra1 d1gesa1 d1zbfa1 d2z1ha_ d2etja1
d3qioa1 d1jl1a_ d2e4la_ d1jl2a_ d2lsna1 d1uaxa_ d2yv0x_ d1mu2a1
d1ekea_ d1i39a_ d1o94a2 d1lqta1 d1cjca1 d1pn0a1 d1reoa1 d1ju2a1
d2f5va1 d3c96a1 d2gmha1 d1pj5a2 d1gpea1 d1o5wa1 d1n4wa1 d1k0ia1
d2dw4a2 d2ivda1 d1seza1 d2voua1 d1s5ja1 d2hbja2 d2igia_ d1wlja_
d1qhta1 d4dlea1 d1tgoa1 d2f96a1 d3tr8a1 d3mxma_ d1q8ia1 d1y97a1
d1w0ha_ d1kfsa1 d2py5a1 d1noya_ d1d5aa1 d1yt3a3 d2guia_ d3mwpa1
d4kb1a_ d1iv0a_ d2d5ra1 d1js9c_ d1f15b_ d1za7a_ d1laja_ d2csua1
d3q9ub_ d2yv1a1 d3q9na_ d2d59a_ d1oi7a1 d2yv2a1 d1euca1 d1y81a1
d1v58a1 d1t3ba1 d4ilfa2 d4npba2 d3nvva1 d1krha3 d2h88b1 d1n62a2
d1t3qa2 d1kf6b2 d1zoyb1 d1jq4a_ d1dgja2 d1rm6c2 d1jmxa2 d1jmxa1
d1pbya1 d1r7ha_ d1faaa_ d1wika1 d1xfla_ d1ttza1 d2f51a1 d1ti3a_
d1nhoa_ d1sena_ d2i4aa_ d1ep7a_ d1zmaa1 d1fo5a_ d3lefa1 d3diea1
d2yana1 d1z6na1 d1oaza_ d3zywa1 d4aj8a_ d1nm3a1 d4fiwa_ d1wmja_
d4hs1a1 d1iloa_ d1xwaa1 d1wjka1 d2vm1a_ d1fb6a_ d1gh2a_ d1h75a_
d1nw2a_ d1kx5b_ d3a6na_ d4m6ba2 d2nqbd_ d3wkjd_ d2yfwe_ d4h9na_
d5bnxa_ d1kx5c_ d3r45a_ d1eqzb_ d4jjnb_ d1f66c_ d1eqza_ d4wnnb_
d1kx5a_ d1u35c1 d1id3a_ d2nqbc_ d1kx5d_ d1f1ea_ d1b67a_ d2b5ea2
d2b5ea3 d2b5ea1 d2b5ea4 d2kp1a1 d2kp2a1 d1hyua4 d1j08a1 d1j08a2
d1pd212 d1nhya2 d2a2ra2 d1k0dd2 d1f2ea2 d1gsua2 d1f3ba2 d2fhea2
d2pvqa1 d1tw9a2 d1oyja2 d1e6ba2 d1k0ma2 d3ik7a1 d4is0a1 d1r5aa2
d1jlva2 d1m0ua2 d3gtub2 d1z9ha2 d1okta2 d1axda2 d2gsra2 d1tu7a2
d1k3ya2 d1zgma1 d1b48a2 d1gnwa2 d1gwca2 d2gsta2 d1glqa2 d1oe8a2
d1pn9a2 d1v2aa2 d1fw1a2 d1duga2 g1f8v.1 d1novc_ d2bbvc_ d1txga2
d3f47a1 d2pv7a2 d1xc2a3 d2ahra2 d4xiya1 d2f1ka2 d1pgja2 d1i36a2
d1np3a2 d1lj8a4 d1teaa3 d1qmga2 d2i76a2 d2g5ca2 d1wdka3 d1dlja2
d3cuma3 d1qqya_ d1gd6a_ d1f6ra_ d1ivma_ d2gv0a_ d1iiza_ d2z2fa_
d1j8xa_ d1ghla_ d1b9oa_ d174la_ d169la_ d2o4wa_ d1p37a_ d1k28a3
d1xjta1 d1jtma_ d2f2qa_ d4e97a1 d1iyka2 d1iyka1 d1rxta1 d1iica2
d2pjsa1 d1klla_ d2i7ra1 d1ecsa_ d1xqaa1 d5cj3a1 d1jifa_ d2p7oa_

d1r9ca_ d1nkia_ d1mpya2 d1kw3b2 d1kw3b1 d4ghga1 d1tfza1 d3isqa1
d3isqa2 d1sqia1 d1sqia2 d1lkda2 d1lkda1 d1mpya1 d1cjxa2 d1cjxa1
d1sp9b2 d1t47a1 d1t47a2 d1f1ua2 d1sp8a1 d1sp8a2 d1s5ja2 d1qhta2
d4dlea2 d1tgoa2 d3py8a2 d1q8ia2 d2py5a2 d1d5aa2 d1qsta_ d2beia1
d4m3sa_ d1u6ma1 d1ygha_ d1yk3a1 d3dr6a_ d1xmta_ d1z4ea1 d2ge3a1
d1bo4a_ d2fiaa1 d2jdca1 d1y9wa1 d3to7a_ d1wwza1 d2fl4a1 d2ozua1
d2b5ga_ d2cy2a1 d2p0wa_ d1cm0a_ d4pswa_ d1yrea1 d1yr0a1 d1m4ia_
d2gana1 d1yvka1 d2g3aa1 d1y7ra1 d2euia1 d1q2ya_ d2fsra1 d1xeba_
d2ae6a1 d1cjwa_ d1qsma_ d2aj6a1 d1y9ka1 d1i12a_ d1vkca_ d2atra1
d1n71a_ d2d4pa1 d2fiwa1 d2fe7a1 d1tiqa1 d2ou2a_ d1yx0a1 d2fcka1
d1p0ha_ d2j8ma_ d2pq8a_ d1z4ra1 d1s3za1 d1ufha_ d2hqya1 d2hqya2
d1lrza2 d1ne9a1 d1lrza3 d1xvqa1 d1pn0a2 d3hy2a_ d2cvba1 d1o8xa_
d1lu4a_ d1we0a1 d2bmxa1 d2a4va1 d1psqa_ d1xiya1 d1q98a_ d1n8ja_
d1zofa1 d2ggta_ d3tjja_ d4je1a1 d1o73a_ d3s9fa_ d2zcta_ d1qmva_
d1jfua_ d2b1ka_ d5dvba1 d2b5xa1 d2v2ga_ d1st9a_ d3tb2a_ d4llra_
d4fh8a1 d1knga_ d2h30a_ d1i5ga_ d2z9sa_ d2f8aa1 d1tp9a1 d2b7ka_
d1nm3a2 d3hvva_ d2wfca1 d4kcea_ d1xvwa1 d4gqca1 d3qpma_ d4k1fa_
d1zzoa1 d1gp1a_ d1prxa_ d4od7a1 d1z6ma1 d4mcua1 d4dvca1 d1r4wa_
d3l9sa_ d1un2a_ d3rppa_ d1zsqa1 d2phla2 d2phla1 d1fxza2 d1fxza1
d1lrha_ d1uija1 g1dgw.1 d2v09a_ d1uika2 d1uika1 d1e88a1 d1l6ja5
d1l6ja4 d1eaka4 d1eaka3 d1l6ja3 d1h8pa2 d1w52x1 d2ppla1 d1hpla2
d1lpbb2 d2oxea1 d1bu8a2 d1rp1a2 d1ys1x_ d4hs9a_ d1ispa_ d1ex9a_
d1ku0a_ d3qmma_ d1lgya_ d4l3wa_ d1llfa_ d1gz7a_ d1ukca_ d1bvsa3
d1ixra2 d1svpa_ d1mbma_ d3su6a1 d4agka_ d1vcpa_ d1q31a_ g1n1l.1
d3e90b_ d1ep5a_ d3u1jb_ d1xwdc1 d3kj4a_ d1w8aa_ d2ifga3 d1p9ag_
d1xkua_ d1g12a_ d1m4la_ d2bk7a_ d3fjua_ d1z5ra1 d1kwma1 d1uwya2
d1jqga1 d2c1ca1 d1dtda_ d1h8la2 d2tbvc_ d1c8nc_ d1x36a_ d1smvc_
d1ng0c_ d4sbvc_ d1opoa_ d1f2nc_ d1cixa_ d1y29a_ d1v7fa_ d2jtba_
d1d1ha_ d1g9pa_ d1lupa_ d1qk7a_ d1mb6a_ d2a2va_ d1niya_ d1hvwa_
d2mpqa_ d1ie6a_ d1kqha_ d1la4a_ d2mxoa1 d1dl0a_ d1koza_ d1qk6a_
d1emxa_ d1lqra_ d1c6wa_ g1apy.1 d3ljqa_ d3lgia_ d3i77a_ d2z9ia2
d4nsva_ d1lcya2 d4jcna_ d1agja_ d1os8a_ d1hpga_ d1p3ca_ d1qtfa_
d1ny721 d1ny722 d1a6ca3 d1a6ca1 d1pgl22 d1pgl21 d1pgl11 d1ny711
d1jgta2 d1ofda3 d1ea0a3 d1q15a2 d1ct9a2 d1ao0a2 d1te5a_ d1y14b1
d2nn6i1 d1i94l_ d2zjra2 d1smxa_ d2qamc2 d1hh2p1 d2r7da1 d1rl2a2
d1wfqa1 d3bzka4 d1x6oa2 d3a0ja_ d2j01d2 d2ja9a1 d4ql5a_ d1luza_
d2n3sa_ d2vnud1 d1h95a_ d1go3e1 d2nn6h1 d1ne3a_ d2mo1a_ d1iz6a2
d1jjga_ d1e3pa2 d2ix0a3 d2ix0a2 d2lxja_ d1ueba3 d1ueba2 d2z0sa1
d1u0la1 d2ix0a1 d2eifa2 d2je6i1 d3i2za1 d2c35b1 d2ahob2 d1t9ha1
d3i4oa_ d2qall1 d1jt8a_ d2nn6g1 d1g6pa_ d3cama_ d1i94q_ d1c9oa_
d1d7qa_ d2ba0a1 d2vnud2 d2vnud3 d1khia2 d2qalq1 d1wi5a1 d1xtda2
d1ny4a_ d3ia2a_ d1a88a_ d4dgqa1 d1zoia_ d1rw1a_ d1z3ea1 d3l78a_
d4lt9a1 d3tc7a1 d3hoja_ d1i4na_ d3vnda_ d3nl8a1 d1geqa_ d3nava1
d1v5xa_ d4a2ra1 d3tc6a1 d1ujpa_ d4ijba_ d1xcfa_ d1vc4a_ d1rd5a_

d1qopa_ d3b5va_ d2r80b_ d1cqxa1 d1cg5a_ d1gcvb_ d3boma_ d2h8fb_
d2raoa_ d3bj1a_ d4hrrb_ d2r50a_ d3d1kb_ d2aa1b1 d3zhwa_ d3hf4b_
d3gdja_ d1jebb_ d3bj1b_ d1x9fb_ d1wmub_ d1gcva_ d3bcqb_ d3d4xb_
d1gvha1 d2dc3a_ d3hf4a_ d4yu3a_ d3k8ba_ d1qpwb_ d2r80a_ d3bcqa_
d1v4wa_ d3wtgb_ d1jl7a_ d4esaa_ d1a9we_ d3d1ab_ d1fsla_ d4h2la_
d1v4wb_ d1mwca_ d1vhba_ d1jeba_ d1spgb_ d1h97a_ d1q1fa_ d1tu9a1
d3d1aa_ d1hdsb_ d3hyub_ d2zfbb_ d3fs4a_ d1oj6a_ d1shrb_ d4h2lb_
d4yu3b_ d1spga_ d3hrwa_ d3qqra_ d3at5a_ d4wchd_ d3hyua_ d1cg5b_
d1uc3a_ d1hbra_ d2raob_ d1outb_ d4esab_ d3gdjb_ d3qqqa_ d1x9fc_
d3vrfa_ d1itha_ d3mkba_ d3d4xa_ d3mjpa_ d1jl6a_ d3fh9a_ d1outa_
d2dc3b_ d1it2a_ d3eoka_ d3g46a_ d1qpwa_ d3vrfb_ d1ch4a_ d1x9fd_
d1hdsa_ d2zfba_ d3mkbb_ d1or4a_ d3wtga_ d1a4fa_ d3fh9b_ d3bomb_
d3cy5a_ d3at5b_ d1x9fa_ d2oifa_ d4lmxb_ d1cpcb_ d3v57a_ d1kn1a_
d1cpca_ d4f0ta_ d2vjha_ d4po5b_ d1eyxa_ d1alla_ d4f0tb_ d1b33b_
d1b8db_ d4h0mb_ d1b33a_ d2vjhb_ d4lm6b_ d2vmlb_ d2vjta_ d3dbja_
d2vmla_ d2vjtb_ d4f0ub_ d1f99b_ d4rmpa1 d1liaa_ d1eyxb_ d2uulb_
d4lmsb_ d4f0ua_ d2bv8a_ d1f99a_ d2bv8b_ d2c7lb_ d3dbjb_ d4rmpb1
d1b8da_ d4h0ma_ d3v57b_ d1kn1b_ d1liab_ d2uula_ d2j96a_ d1pfsa_
d2a1ka_ d1je5a_ g1gxs.1 g1wht.1 d1azwa_ d1mtza_ d1hlga_ d2xqfa_
d1ea5a_ d1f6wa_ d1dx4a_ d1qe3a_ d1k4ya_ d4qwwa_ d2ha2a_ d1offa_
d1frra_ d3wcqa_ d1e9ma_ d1l5pa_ d1wria_ d1i7ha_ d3av8a_ d1czpa_
d1rfka_ d3p1ma1 d3b2ga_ d2bt6a2 d4iuaa3 d4bvva_ d5hpga_ d3k65a_
d1ki0a3 d1ki0a2 d1ki0a1 d1a0ha1 d1pmla_ d3sp8a3 d2feba1 d3u1ra1
d1g9ka2 d1kapp2 d1f74a_ d3l21a_ d2e1da_ d1to3a1 d1w3ia_ d1sfla_
d1n7ka_ d1vcva1 d4imea_ d1wa3a_ d2ocza1 d1f2ja_ d3m16a_ d2yxga_
d1ub3a_ d3a5fa_ d1fbaa_ d1l6wa_ d1xfba1 d1wx0a1 d1vpxa1 d1ojxa_
d1mxsa_ d2a4aa1 d1xkya1 d3cq0a1 d3s1xa_ d1f05a_ d1fdja_ d3cpra1
d4d2ja_ d1cg2a1 d1yloa2 d1y7ea2 d1y0ya3 d1lfwa1 d1vixa1 d1fnoa4
d1vhoa2 d1ysja1 d1vhea2 d1z2la1 d2fvga2 d1vgya1 d2grea2 d1xmba1
g1q7l.1 d1rtqa_ d3rp2a_ g1ucy.1 d1fuja_ d2zchp_ d3w94a_ d1si5h_
d1lo6a_ g2pka.1 d1cgha_ d1kigh_ d1a5ia_ d3s9ba_ d1hj8a_ d1ao5a_
d2anya_ d1q3xa1 d1gvza_ d1azza_ d4gsoa_ d2f91a1 d1iaua_ d1gdna_
d1eufa_ g1fiw.1 d1eq9a_ d4nfea_ d2rdla_ d1spja_ d1elva1 d1a7sa_
d1ekbb_ d1pfxc_ d1yc0a_ d2aiqa_ g1fiz.1 d1fi8a_ d4k60a_ d4dgja_
d1mzaa_ d1op0a_ d3tjva_ d1pytd_ d1sgfg_ g1gg6.1 d2eeka_ g1rtf.1
d1a0ja_ d3g01a_ d1pytc_ d4e7na_ d1ddja_ d1z8ga1 d2hlca_ d1npma_
g1jou.1 d1sgfa_ d1autc_ g2hnt.1 g1kdq.1 d1orfa_ d3s69a_ d4crga_
d1fxya_ d1bqya_ d2ftba_ d1pmpa_ d2rcqa1 d1fdqa_ d4lkpa_ d1sa8a_
d1vyfa_ d1lpja_ d3vg7a1 d4azqa1 d1o8va_ d5bvqa_ d2qo4a_ d1opba_
d1yiva1 d1o1va_ d4bvma1 d4tkba_ d4i3ba_ d1bwya_ d1ftpa_ d1ggla_
d4gkca_ d1cbia_ d3p6da1 d1kqwa_ d2a0aa1 d1dzka_ d4tlja_ d2a2ua_
d4wfva_ d3t1da_ d4iaxa_ d3zq3a_ d2nnda_ d1i4ua_ d1kxoa_ d1bbpa_
d1kt7a_ d1exsa_ d3s26a1 d1lf7a_ d1qfta_ d4gh7a_ d1z24a1 d1qabe_
d1xkia_ d1ew3a_ d3bx7a_ d4iawa1 d1n0sa_ d1gkab_ d3kzaa_ d2k23a_

| | | | | | | | |
|---|---|---|---|---|---|---|---|
| d2l9ca1 | d2joza1 | d1gm6a_ | d3kffa_ | d3saoa1 | d1e5pa_ | d1yupa1 | d1iiua_ |
| d2i49a_ | d1nnfa_ | d1xvxa_ | d1ursa_ | d1h3da1 | d1iuda_ | d4m1va1 | d1twya_ |
| d3e13x_ | d2nxoa1 | d4pfua_ | d3ttma_ | d1elja_ | d2qmwa1 | d1us5a_ | d1y3na1 |
| d1jeta_ | d1uqwa_ | d4f19a1 | d2esna2 | d3thia_ | d3fasa1 | d2uyeb_ | d2xwva1 |
| d1r9la_ | d2fyia1 | d1pb7a_ | d2ozza1 | d1sw5a_ | d1nh8a1 | d1vr5a1 | d2g29a_ |
| d1xs5a_ | d3lswa_ | d2z23a_ | d3w9va_ | d3tcha_ | d1q35a_ | d1z7me1 | d3q27a_ |
| d1p99a_ | d1ve4a1 | d2i6ea1 | d4mcoa1 | d4n8ya_ | d1eu8a_ | d1zbma1 | d1hsla_ |
| d1ixca2 | d1j1na_ | d1xvya_ | d1y9ua_ | d2z22a1 | d1xoca1 | d4oera_ | d1wdna_ |
| d5f1qa1 | d1xt8a1 | d1i6aa_ | d4gwoa_ | d4igta1 | d1pc3a_ | d4mmpa1 | d1o63a_ |
| d2czla1 | d2onsa2 | d4mija1 | d1eh3a_ | d3skpa_ | d1dtza1 | d1dtza2 | d1ieja_ |
| d1jnfa2 | d1ce2a1 | d1jnfa1 | d1b1xa2 | d1jw1a1 | d1jw1a2 | d1ryoa_ | d1h76a1 |
| d1h76a2 | d3q3ma_ | d1za0a1 | d1mxra_ | d2inca_ | d1jk0a_ | d2incb_ | d1mhyb_ |
| d3hf1b_ | d1r2fa_ | d2uw1a_ | d1mhyd_ | d3n37a_ | d1afra_ | d1oquc_ | d1syya_ |
| d1mtyd_ | d2o1za_ | d1uzra_ | d1smsa_ | d2fkza_ | d1zuja1 | d1s3qa1 | d2fjca1 |
| d1z6om1 | d1veia_ | d4dyya_ | d1jiga_ | d3gvya_ | d2pyba_ | d4cy9a1 | d3bvfa1 |
| d1r03a_ | d2cf7a_ | d1nf4a_ | d1zs3a1 | d4iwka_ | d3ka8a_ | d3np0x_ | d1n1qa_ |
| d1lb3a_ | d4e6ka_ | d1z6oa1 | d2fzfa1 | d4dyua_ | d3ge4a_ | d1jgca_ | d4am4a_ |
| d2z6ma_ | d1ji5a_ | d1vlga_ | d1lkoa1 | d1krqa_ | d3qz3a_ | d1o9ra_ | d2ffxj_ |
| d1euma_ | d1yv1a1 | d2v2pa_ | d1j30a_ | d4di0a_ | d1dpsa_ | d1oewa_ | d2qzxa_ |
| d2asia_ | d1psoe_ | d1smra_ | d2h6ta_ | d1pfza_ | d1izda_ | d3hd8a_ | d1j71a_ |
| d1dpja_ | d4aa9a_ | d1t6ex_ | g1b5f.1 | g1lya.1 | g1htr.1 | d1qdma2 | d1bxoa_ |
| d1wkra_ | d1tzsa_ | d1ibqa_ | d2qzwa_ | d1bdqa_ | d3ggta_ | d3ixoa_ | d1mrxa_ |
| d2haha_ | d1tw7a_ | d4njsa_ | d1sgua_ | d2p3da_ | d1nsoa_ | d3d3ta_ | d2rkfa_ |
| d1c6ya_ | d2rspa_ | d3ogpa_ | d3ekwa_ | d2o4na_ | d2j9ja_ | d1baia_ | d3ggua_ |
| d3lzua_ | d4npua_ | d4j55a_ | d2r8na_ | d2fxda_ | d2p3ca_ | d4dqea_ | d3ttpa_ |
| d3t3ca_ | d2p3aa_ | d2hc0a_ | d2fdda_ | d3mwsa_ | d4yl8a1 | d2al6a3 | d1wgda1 |
| d1wgga1 | d4q5eb_ | d1wjna1 | d1se9a_ | d3n3kb_ | d1wz0a1 | d1we6a1 | d2m0xa_ |
| d2gbka_ | d3rt3b1 | d1wh3a1 | d1sifa_ | d2ekec2 | d4ii2b1 | d4hk2a1 | d1c3ta_ |
| d1m94a_ | d1wgha1 | d1ttna1 | d1j8ca_ | d1wx8a1 | d3v6cb1 | d2ma9b_ | d4i6lb1 |
| d1bt0a_ | d2jvca1 | d1iyfa_ | d2k1fa_ | d1wxva1 | d1wf9a1 | d1t0ya_ | d2io0b2 |
| d1x1ma1 | d2gbma_ | d1zw7a1 | d2m17a_ | d1ud7a_ | d1we7a1 | d2lwpa_ | d2gbja_ |
| d1wjua1 | d1v6ea1 | d1v86a1 | d4eewa_ | d1wiaa1 | d4icva_ | d4gu2a_ | d1wx7a1 |
| d1wy8a1 | d4hjka1 | d2faza1 | d1v5oa1 | d3ruib_ | d1eo6a_ | d3waob_ | d2r2qa1 |
| d3m95a_ | d3wana_ | d2al3a1 | d2cr5a1 | d1wj4a1 | d1g66a_ | d5ajha1 | d1dszb_ |
| d1a6ya_ | d3cbba_ | d1ynwa1 | d1r4ia_ | d1hcqa_ | d2hana_ | d2hanb_ | d2enva1 |
| d1kb4b_ | d2nllb_ | d1lo1a_ | d1cita_ | d1adqa1 | d1fnga1 | d1uvqb1 | d1zs8a1 |
| d2vola1 | d2vola2 | d1mfbl2 | d2jelh2 | d1uwel2 | d3qq4b1 | d1kjvb_ | d1k5na1 |
| d1osph2 | d4odhl2 | d1ow0a1 | d1ow0a2 | d1bfob2 | d3l9ra2 | d3jtth1 | d2fath2 |
| d4elkb2 | d1fn4b2 | d2xfxb_ | d1pg7x2 | d1cqka_ | d4k71b2 | d1lk2a1 | d1eapb2 |
| d4lfhg2 | d1i4fa1 | d2nw2a2 | d1kfah2 | d1mhca1 | d1h3ua1 | d1wejh2 | d1h0db2 |
| d1a5fh2 | d1kcvh2 | d1mjuh2 | d1qqda1 | d1kcrh2 | d1k8ia1 | d3huja2 | d2nnab1 |
| d1nfdf2 | d3mffb2 | d1s9va1 | d1lk3h2 | d1q0xl2 | d1fv1b1 | d1neza1 | d4rfeb2 |
| d1r3ha1 | d1a6tb2 | d1kjva1 | d1pz5b2 | d1onqa1 | d2bc4a2 | d1de4a1 | d1k8da1 |
| d1tcra2 | d1dn0b2 | d1nfde2 | d2xwtb2 | d1bfoa2 | d1mexh2 | d1iakb1 | d1ypzf2 |

d4f7ea2 d1q72l2 d2fbjh2 d1fe8h2 d1clzh2 d1fngb1 d1uvqa1 d1vgka1
d1c16a1 d4j12a2 d4x99b2 d1igtb3 d1je6a1 d1q72h2 d1nldh2 d1teth2
d2hmic2 d1i1ca2 d1i1ca1 d1k8ib1 d1q0yh2 d1kfal2 d3frua1 d1pg7w2
d1u58a1 d1igyb4 d1igyb3 d1hyrc1 d1klub1 d1deeb2 d1c12b2 d2bc4b2
d1kcvl2 d3wn5b1 d1lk2b_ d1lk3l2 d1mjul2 d1sm3h2 d1q0xh2 d1zvsa1
d1ydpa1 d1u8ib2 d1ypze2 d1kcuh2 d2lj7a_ d1n4na_ d2m8bb1 d2kyqa_
d3psma_ d1jkza_ d1jxca_ d2gl1a_ d4uj0a_ d2k4ua_ d1hp2a_ d2lu9a_
d1baha_ d2meoa1 d1wm7a_ d1hlya_ d1n8ma_ d1jlza_ d1wpda_ d1c55a_
d1pvza_ d1wt7a_ d1rjia_ d1wz5a_ d1m2sa_ d1pjva_ d1qkya_ d2ktca1
d1wmta_ d1du9a_ d1j5ja_ d1r1ga_ d1e3oc2 d1ic8a2 d1t0za_ d2i61a_
d1t1ta_ d1sn4a_ d1kv0a_ d2lkba_ d1npia_ d1wwna_ d1pe4a_ d2kbka_
d2li7a_ d1djta_ d2kbha_ d1dq7a_ d1sega_ d1omya_ d1zywa1 d1jzaa_
d2jcqa_ d1n9wa1 d1bbua1 d1e1oa1 d1b8aa1 d1l0wa1 d3a74a1 d1eova1
d1t61c1 d1iyjb4 d1iyjb5 d1iyjb3 d1o7ia_ d4ipca1 d1x3ea_ d4apva_
d1jb7a1 d1jb7a2 d1jb7a3 d3kdfa_ d1ue1b_ d1wjja1 d3eiva_ d1xjva2
d1xjva1 d2ccza2 d1se8a_ d1l1oc_ d1jb7b_ d1qvca_ d3ulla_ d1qzga_
d1miua4 d1miua5 d1miua3 d3g48a1 d1gd7a_ d1mkha_ d1jjcb3 d1pyba_
d3ubub_ d1egia_ d1v7pa_ d1tdqb_ d3ubua_ d1c3ab_ d1h8ua_ d3bx4b_
d1t8ca1 d1sb2a1 d1qdda_ d2ziba1 d3cada_ d1byfa_ d1j34b_ d1jwia_
d1jzna_ d1c3aa_ d1oz7b_ d1wmza_ d3bdwb_ d1hq8a_ d1fifa1 d1dv8a_
d3hupa_ d1wk1a1 d1umra_ d2afpa_ d3bx4a_ d1gz2a_ d1sl6a_ d1v7pb_
d3g8ka_ d1ixxa_ d1j34a_ d1jwib_ d1g1sa1 d1y17a1 d1ja3a_ d1fvub_
d3bdwa_ d1sb2b1 d1fvua_ d1umrc_ d1oz7a_ d4m17a_ d3cfwa1 d1ypqa1
d1g1ta1 d3fd4a_ d3l9jc_ d4dn8a1 d1v4lb_ d1rdl1_ d2msba_ d1prtc2
d1prtb2 d3zxha_ d1cgla_ d1rm8a_ d1eaka2 d1hy7a_ d2jsda_ d1cxva_
d1hv5a_ d1i76a_ d1rpma_ d2shpa1 d1yfoa_ d1l8ka_ d2ooqa1 d1g4us2
d1p15a_ d1lara2 d1lara1 d1wcha_ d2fh7a2 d2fh7a1 d1ohea1 d1ohea2
d2f4da_ d1rxda_ d2hxpa_ d1xria_ d1fpza_ d3ezza_ d4y2ea_ d1i9sa_
d1k9ua_ d2opoa_ d3azya_ d3wvja_ d1upsa1 d1ajoa_ d2hyka_ d1ajka_
d2vy0a_ d1o4ya_ d3o5sa_ d3d6ea_ d3atga_ d1dypa_ d1umza_ d3i4ia_
d1c1la_ d4lbqa_ d1a78a_ d1slta_ d4no4a_ d3zsja_ d3duia_ d1g86a_
d1is3a_ d1qmja_ d1hlca_ g1qmo.1 g1len.1 d3zyra_ d1fnya_ d1n47a_
g2ltn.1 d1avba_ d3usua_ d1dhkb_ d1f9ka_ d1ukga_ d1g8wa_ d1g9fa_
d1wbfa_ d3wcsa_ d1g7ya_ g1loe.1 d1mvqa_ d1hqla_ d1dbna_ d1qnwa_
d1fx5a_ d4l8qa_ d1ioaa_ d3n35a_ d3ubra_ d1qo8a1 d1d4ca1 d2k3va_
d1ft5a_ d1qdba_ d2j7aa_ d1h21a_ d1ogyb_ d4fasa_ d1jnia_ d1oaha_
d1sp3a_ d3h33a_ d1rwja_ d19hca_ d1os6a_ d3bxua_ d3h4na_ d1i77a_
d3caoa_ d1j0pa_ d2ctha_ d1duwa_ d1ofwa_ d2e84a_ d1hh5a_ d1gyoa_
d3h34a_ d1wioa4 d1ccza2 d1vcaa1 d1ic1a1 d2d9qb1 d2nxyb2 d2dara2
d2dara1 d1wyha2 d2dloa2 d1rutx4 d1rutx3 d2cuqa2 d1x4la2 d1x4la1
d1x3ha2 d1x3ha1 d2d8za1 d1x61a2 d1x6aa1 d1x6aa2 d2cora2 d2cora1
d1v6ga1 d1v6ga2 d2d8za2 d1x64a1 d1x64a2 d1b8ta1 d2co8a1 d1x63a2
d2dj7a2 d2dj7a1 d2cu8a2 d2cu8a1 d1g47a2 d1g47a1 d1j2oa1 d1wyha1
d2dloa1 d1wiga1 d1wiga2 d2cupa2 d2cupa3 d1x68a1 d2d8ya2 d2d8ya1

d1m3va2 d1x62a1 d1x68a2 d1x61a1 d1b8ta3 d1b8ta2 d2d8xa2 d2d8xa1
d1x4ka1 d1x4ka2 d2cuqa1 d1m3va1 d2co8a2 d1j2oa2 d1x63a1 d2cura1
d2cura2 d1x62a2 d1op9a_ d1y0la1 d1etzb1 d1egjl1 d2gsib1 d1nj9a1
d4jfhd1 d1aqkl1 d1t2ja_ d1lo4h1 d1zvya1 d3v4vl1 d4enec1 d1kxtb_
d5e8ea1 d2pxya_ d4weud_ d1xiwc_ d2esvd1 d1qnzh_ d1ogae1 d1f4xh1
d4n1hb_ d3ezjb1 d3mlwl1 d1kfah1 d1etza1 d1b4jh1 d3cfic_ d4w68a_
d2arja1 d1ek3a_ d2ok0l1 d1vera_ d1uwgh1 d1blnb1 d1fpth1 d1rzga1
d2giya1 d4m3kb1 d1nc2a1 d2nyyd1 d1ncna_ d1fbih1 d5dxwa_ d4fqqa1
d1dqdh1 d1jfql1 d1kelh1 d1faih1 d1frgl1 d4m6nl1 d1fo0a_ d4hxbl1
d3b5ga_ d3quyd1 d2h9gb1 d1i8kb_ d2dd8h1 d2ck0h1 d1eaja_ d4w6wb1
d2a6ib3 d1t66d1 d2e27h_ d1ob1b1 d1f11b1 d2v7na1 d1ad0a1 d2xt1b1
d1pskh1 d4i13b1 d2op4l1 d1a0ql1 d1uwel1 d1za3a1 d1ncbl1 d4uu9a_
d1bm3h1 d1cloh1 d1nqba2 d1cf8h1 d4y5va_ d1mhph1 d5anma1 d4cdgc1
d4ldeb_ d1y0lb1 d2pcpb1 d1z3gl1 d1dfbl1 d1fh5h1 d3g5xa1 d1tzib1
d4dn3l1 d3zhka1 d2b1hh1 d2uyla1 d1f3dh1 d1nj9b1 d2agjh1 d2h1pl1
d1q72h1 d8fabb1 d1ndmb1 d1rjca1 d4u6hb1 d3upca_ d2fbjl1 d1osph1
d1tvda_ d2cjvh_ d4fhbd1 d1bd2d1 d1rz7h1 d3nn8a_ d1blna1 d1knoa1
d1nfda1 d2bmkb1 d1dqll_ d1igyb1 d1igfl1 d12e8h1 d3utza1 d1nc2b1
d1rihh1 d4mwfb1 d4jvpa1 d1mieh1 d1rhfa1 d1fgnl1 d1sbsh1 d1lp9e1
d1dn0a1 d4lqfl1 d1iaih1 d1kgcd1 d1mh5b1 d1gpol1 d2g60h3 d1f8tl1
d2jixd1 d4grwh_ d1fo0b_ d1bfob1 d1axsa1 d1u9ka_ d1nmbh_ d1h8na1
d1h8na2 d1jguh1 d4eura1 d3sdyl1 d35c8h1 d1w72l1 d1mvub_ d1i9rh1
d1ob1a1 d4wenb_ d1yeeh1 d3ietc1 d1f58h1 d1ad0b1 d1mqkl_ d1nbvh1
d4b50a_ d3r8bb_ d1bafl1 d1mamh1 d4rgne1 d2gsgb_ d35c8l1 d1bafh1
d1ai1h1 d2pcpa1 d2fath1 d2x1pa_ d1orsb1 d1ibgl1 d1shma_ d1a2yb_
d1u0qa_ d2coqa_ d1hilb1 d1qfwi_ d1jv5b_ d1fn4b1 d5e0qa_ d1nsnh1
d1plgl1 d4ocxl1 d1kb5h1 d1rzfh1 d1pg7x1 d2fx7h1 d2oi9c_ d1l7il1
d3eyva1 d1d5il1 d4ffyl_ d1tetl1 d1a6wh_ d1ac6a_ d3dvfa_ d1nfde1
d1mimh1 d1ri8a1 d1jhlh_ d3dura_ d1fskb1 d1f97a1 d3so3b1 d2ak4d1
d1lp9f1 d4z7wf1 d1l7th1 d4y5ya_ d4x7ec1 d1emth1 d1ikfl1 d1nlbh1
d4utal1 d1bjma1 d1eapb1 d1u6ah1 d2dtgd1 d1kj2b_ d1xeda1 d1c1eh1
d1fgvh_ d2bnub1 d2fb4h1 d2dtgb1 d1ktrh_ d1jpth1 d1qkzl1 d4hepg_
d2atpb1 d1flrh1 d2hrpl1 d1mvfa_ d1iaim1 d3k80a1 d2jb5h1 d3zkxc_
d4grwe_ d1it9h1 d1rhhb1 d3w9ec_ d1e4wh1 d1sy6h1 d2j6eh1 d3qxta_
d1jglh1 d1p7ka1 d1tzhb1 d4n8pa1 d1gafl1 d2jelh1 d1wioa2 d4i0cc1
d1cicb1 d1lmka2 d1lmka1 d1iqwh1 d1orqb1 d1ejoh1 d1jnlh1 d4zg1a_
d43c9b_ d1ggbl1 d1wejh1 d3q6fa1 d1f3dj1 d3c6la1 d1bqlh1 d3p9wb_
d1a3rl1 d1h0db1 d2uzil_ d1eo8l1 d1qfwl_ d1n7ml1 d1igja1 d1c5ch1
d2a9mh_ d1rmfh1 d1fj1b1 d4heme_ d1ccza1 d1a5fh1 d1ogad1 d1dlfh_
d1qa9b_ d1vgel1 d3qskb_ d1sjxa_ d4ozfg1 d1sq2n_ d1qd0a_ d1kcvh1
d1c5db1 d3qcul1 d8faba1 d3bn9d1 d1mjuh1 d4y8dc_ d7fabh1 d3dvgb1
d1lo0l1 d1a6tb1 d1gigh1 d1kcrh1 d2hwzl1 d1yjdc1 d3clfl1 d4krna_
d1dl7l_ d1e6ol1 d1mexh1 d3r0ma_ d4kkna_ d1fnsl1 d1oaqh_ d1rzia1
d1ucbh1 d1ncwl1 d4b41a_ d1nfdf1 d2mpah1 d2d7tl1 d4grwf_ d4b5ea_

d1lk3h1 d3u79a_ d4u3xa_ d1ghfl1 d1bvkb_ d1mcww1 d1b88a_ d1jrhh1
d32c2b1 d4wemb_ d1dsfh_ d1op3h1 d1ce1l1 d1iqda1 d1qoka1 d1qoka2
d1qfuh1 d2crya1 d1ay1h1 d1oaql_ d3ab0b_ d1rurl1 d1t4kb1 d1bz7b1
d1n0xh1 d1forl1 d1ucbl1 d4w6yb_ d43c9a_ d2aabl1 d1tzia1 d4n1ca_
d1k6qh1 d3g9ab_ d4tvsa1 d1igmh_ d1pz5b1 d3dx9a1 d3wxwh_ d1nakh1
d1kcul1 d3eaka_ d4qlra1 d1cr9l1 d1d9ka_ d1f4xl1 d1deea1 d2i27n2
d1c12a1 d2orbl1 d1j8hd1 d1mcph1 d1c5da1 d1kenh1 d1bd2e1 d1h5ba_
d4x7fc1 d3upaa_ d1t3fa1 d1indh1 d2dtgc1 d4hc1l1 d1zv5a1 d4wgvb_
d1uwgl1 d4f37k1 d1aqkh1 d1vesa_ d3zkqd_ d1fptl1 d1lo4l1 d1knob1
d3rrqa_ d1ct8b1 d4orzc_ d1mlbb1 d4hgkc1 d1dqdl1 d1bogb1 d4ozhe1
d1dr9a1 d1i7za1 d1dn0b1 d1kell1 d1rzib1 d1ypzf1 d1wtla_ d1hkfa_
d1fail1 d1figh1 d2vyre_ d1sjva_ d1ohqa_ d1bfoa1 d1pkqb1 d1mjul1
d1b4jl1 d1nsnl1 d2arjb1 d1p4bh_ d4aj0a_ d3nn8b_ d2rcja1 d1f2xk_
d4v1dd_ d1adqh1 d2eksa_ d4k3ga_ d1jfqh1 d1uweh1 d1frgh1 d4gftb_
d1uz8b1 d4n1ea_ d1bm3l1 d1clol1 d3mcka1 d2vq1a1 d1ae6h1 d4aq1b1
d1uj3b1 d1nbqa1 d1fh5l1 d2ck0l1 d2ywza_ d1q72l1 d4nbxb_ d1hila1
d4hzlb1 d2j88l1 d1zmya1 d1cd0a_ d1a4ka1 d1pskl1 d1a0qh1 d4krmb_
d1ncbh1 d2fbjh1 d1j8he1 d1i3ua_ d1cf8l1 d1fe8h1 d2p1ya1 d1eeqa_
d2x89a_ d1ol0a_ d2r0lh1 d1ub5a1 d1dfbh1 d3eyvb1 d4jdvb1 d4p2cg_
d2gsia1 d2agjl1 d1u3ha1 d2h1ph1 d1rihl1 d1fskc1 d1boga1 d2a77l1
d1ospl1 d1qfoa_ d3dwta_ d2fx7l1 d1rz7l1 d3stba1 d1pkqa1 d1b6da1
d1dzba2 d1dzba1 d1kj2a_ d4l1ha_ d1rzgb1 d1dqlh_ d12e8l1 d1miel1
d1jmaa_ d1sbsl1 d1fgnh1 d1f58l1 d1mqkh_ d1ndgb1 d3qegl1 d1iail1
d2aw2a1 d1tqbc1 d3fo2a1 d4dkaa_ d1f8th1 d2g60l1 d2ywya_ d1uj3a1
d1yc7a_ d3bd3a_ d4w6xb1 d1nmbl_ d1i8ka_ d1rjlb1 d1ypze1 d1p7kb1
d1kxqe_ d1gpoh1 d2z4qa1 d1w72h1 d1i9rl1 d4qgya1 d4xt1c_ d2qqnh1
d1cica1 d3c08h1 d2cd0a_ d2bdnh1 d1hnga1 d1maml1 d1nldh1 d1cr9h1
d4lcih1 d3bkyh1 d3qg7l1 d1ai1l1 d1kxvc_ d4iosd_ d1l7ih1 d2x1qa_
d1ub6b1 d3k74b_ d1ibgh1 d1teth1 d1igya1 d1hxma1 d2x6ma_ d2e7la_
d5e03a_ d1qfwm_ d2r0kh1 d1igjb1 d1rurh1 d1plgh1 d1mj8h1 d1yuhb1
d3fmgl1 d1rzfl1 d1emtl1 d1ad9b1 d1igtb1 d1nlbl1 d1d5ih1 d3k7ua_
d4ffyh_ d1bwwa_ d3dvga1 d1a6ta1 d1fgvl_ d4qxgl1 d1miml1 d1jhll_
d2hffb1 d2hh0l1 d1qkzh1 d1l7tl1 d1afvh1 d1iaii1 d2cjuh_ d1um5h1
d2ghwb2 d2ghwb1 d1kgce1 d2fgwh1 d1ikfh1 d1dvfd_ d2i24n1 d2xtjb_
d1za3b1 d2yc1a_ d2hh0h1 d2x1oa_ d1bvka_ d1rjla1 d5e7ba1 d1igta1
d1il1b1 d2fb4l1 d1mvua_ d1jptl1 d32c2a1 d1fvca_ d1iqdb1 d2hrph1
d2p4ab_ d4qo1a_ d1p2cb1 d1wt5c_ d1t4ka1 d5c1mb_ d1it9l1 d1bz7a1
d1b2wh1 d1e4wl1 d1jnll1 d1yedb1 d1za6b1 d3u7aa_ d1igfh1 d3gnml1
d1orsa1 d1wejl1 d1a2ya_ d1hxmb1 d1gafh1 d2j4wh1 d1pz5a1 d1j05a_
d1a3rh1 d4fqll1 d1qfwh_ d1eo8h1 d1n7mh1 d4s11a_ d1kegh1 d5da4a_
d1iqwl1 d3whxb1 d1vgeh1 d1deeb1 d1c12b1 d1t2ql1 d1ad9a1 d3gsnb1
d3b2uc1 d1ggbh1 d1t3fb1 d1nakl1 d4ocmc_ d1lila1 d2v7hb3 d2esve1
d1mcoh1 d2qr0b1 d2q3aa_ d1ct8a1 d1kcrl1 d2pxyb_ d2uzih_ d5c3ld_
d3durb_ d1c5cl1 d2ak4e1 d1dl7h_ d1xiwd_ d1mlba1 d1rmfl1 d1i7zb1

d1dlfl_ d2xa3a_ d5bq7b1 d1dqqb1 d3h42l1 d1egjh1 d1kcvl1 d1fe8l1 d2j6el1 d1f3rb2 d1lk3l1 d1sm3h1 d1q0xh1 d1ghfh1 d7fabl1 d1igch1 d1lo0h1 d4idla_ d5c2bl_ d1jrhl1 d1il1a1 d3zhda1 d4kfzc_ d1b0wa_ d1e6oh1 d1mexl1 d4krpb_ d1fnsh1 d1ncwh1 d4hgma_ d4eigb1 d4rzca_ d2mpal1 d4y5xa_ d5en2b1 d3lrga1 d4ej1c1 d1rhha1 d1j05b_ d1forh1 d1q9wb1 d2wzpd_ d2aq2a1 d1h0da1 d3revb1 d2ij0c1 d1tzha1 d4m7jl1 d1k6ql1 d3b9kc1 d1igml_ d3qeua1 d1dsfl_ d1ce1h1 d1kcuh1 d1zvha1 d1zvoa1 d1tqbb1 d1ay1l1 d2qscl1 d2cgrh1 d1nl0h1 d1kenl1 d3eo0a1 d4n9ob1 d1n0xl1 d3k1kc_ d1c4qa_ d1prtc1 d2wv6d_ d1prtf_ d1ltrd_ d4l63b1 d1r4pb_ d1prtd_ d1tl0f_ d2bosa_ d1prtb1 d3chbd_ d2aq2b1 d1ck1a1 d1enfa1 d1i4pa1 d1v1oa1 d1et9a1 d1esfa1 d2g9hd1 d4ohja1 d1bxta1 d1eu3a1 d1ty0a1 d1fnua1 d2cqva1 d1fhga_ d2k1ma_ d2dl2a1 d1biha3 d1biha1 d1biha4 d3qp3a1 d1g1ca_ d1gxea_ d1cvsc2 d1cvsc1 d1xmwa2 d1m4ka2 d2otpa2 d1biha2 d1rhfa2 d2fcba2 d2fcba1 d1wwbx_ d1gsma2 d1epfa1 d1epfa2 d2dava1 d1waaa_ d1f97a2 d1x44a1 d1vcaa2 d1fnla2 d1fnla1 d3b5ha2 d3h8na2 d3h8na1 d1jbja1 d1jbja2 d1pd6a_ d1f2qa2 d1f2qa1 d3cx2a_ d1nbqa2 d3b5ha1 d1n26a1 d1xiwb_ d1xaua_ d1fltx_ d2ifga1 d1olla1 d1olla2 d1cs6a4 d1cs6a3 d1cs6a2 d1cs6a1 d2gw5a2 d1sy6a1 d1xiwa_ d1ucta2 d1ucta1 d1ry7b2 d1gl4b_ d1qz1a3 d1olza1 d2c9aa1 d1wwca_

**Family-61 (c.37.1.10)**

**Positive dataset contains 13 protein sequences.**

d1vmaa2 d2p67a1 d3ihla_ d1s1ma2 d2v40a_ d3hida_ d2vo1a1 d2g0ta1 d1vcoa2 d1xjca_ d2qm8a_ d4ioja_ d1yrba2

**Negative dataset contains 3717 protein sequences.**

d1m32a_ d1ibja_ d1c7na_ d1iuga_ d4q75a_ d2bkwa1 d1t3ia_ d1n8pa_ d1qz9a_ d1eg5a_ d1p3wa_ d3kgwa_ d1cl1a_ d2ctza1 d1vjoa_ d2rfva_ d1gc0a_ d1cs1a_ d1d2fa_ d2ch1a1 d1qgna_ d1pffa_ d1elua_ d1c7ga_ d2v1pa1 d2ez1a_ d1u08a_ d1lc5a_ d1b5pa_ d2ay1a_ d1bw0a_ d3pd6a_ d7aata_ d2csta_ d1j32a_ d1fg7a_ d4wlha_ d1o4sa_ d1v2da_ d1gdea_ d1m6sa_ d1v72a1 d1svva_ d1ajsa_ d1wsta1 d2f8ja2 d1lk9a_ d1vp4a1 d1xi9a1 d2gb3a1 d3i2ka1 d1mpxa2 d1xkta1 d2hfka_ d1jmkc_ d5d3ka_ d2cfba1 d1dfoa_ d1o69a_ d1bj4a_ d3k28a_ d2bwpa_ d3lg0a_ d2fnua_ d3ou5a_ d1z7da1 d1b9ha_ d1iaya_ d4msoa_ d1bs0a_ d1s0aa_ d1sffa_ d3tqxa_ d1mdoa_ d3qboa_ d3pgya_ d2dkja_ d1ejia_ d1vefa1 d4wxga_ d3bs8a_ d3gbxa1 d1w23a_ d3lgia_ d3i77a_ d2z9ia2 d4nsva_ d1lcya2 d4jcna_ d1agja_ d1os8a_ d1p3ca_ d1l1ja_ d1qtfa_ d1ddla_ d1auyb_ d1qnia1 d1fwxa1 d2wo3b_ d1shxa_ d1f56a_ d1bypa_ d1azca_ d1ws8a_ d3u25a_ d1cuoa_ d1id2a_ d1rkra_ d2iaac_ d4dp1x_ d1nwpa_ d2gima_ d2i7sa_ d1qhqa_ d1gyca3 d1hfua2 d1kbva1 d1kbva2 d1sdda1 d1sdda2 d1gyca1 d1gyca2 d1mzya1 d2bw4a1 d1aoza1 d1aoza3 d1aoza2 d1kyaa2 d1kyaa3 d1kyaa1 d1mzya2 d4e9sa1 d1sddb1 d1sddb2 d1hfua1 d1hfua3 d1oe1a2 d1oe1a1 d1v10a2 d1v10a3 d1v10a1 g1f8v.1 d1novc_ d2bbvc_ d3sxwa1 d4luka_ d1iknc_ d3brda1 d1p7hl1 d1oy3c_ d1imhc1 d1gjia1

d1zk9a1 d2cxka1 d2h0va_ d4alac_ d1rera1 d1s6na_ d3uzqb_ d2phla2
d2phla1 d1fxza2 d1fxza1 d1lrha_ d1uija1 g1dgw.1 d2v09a_ d1uika2
d1uika1 d1dgwa_ d1ogpa1 d1ds6b_ d1fstb_ d1ex0a1 d1g0da1 d3us3a3
d3us3a1 d1tafa_ d1bh9b_ d1jfib_ d1q9ca_ d1h3ob_ d2byka1 d1n1ja_
d4g92b_ d1bh9a_ d1jfia_ d1h3oa_ d2bykb1 d1n1jb_ d1k9ua_ d2opoa_
d1r0ka3 d1j5pa3 d1vkoa2 d1vm6a2 d3qvsa2 d1p1ja2 d2ovga_ d1sq8a_
d1rzsa_ d3kz3a_ d3orca_ d3gpdg2 d2q49a2 d1hdgo2 d2g17a2 d2cvoa2
d2czca1 d3pwka2 d1obfo2 d1i32a2 d1zyao2 d2pkqo2 d1b7go2 d2hjsa2
d1cf2o2 d1vkna2 d1nvmb2 d2msya_ d2djoa1 d2r5za_ d1au7a1 d1f43a_
d2lmda1 d1akhb_ d1s7ea1 d3nara_ d1zq3p1 d2craa1 d1mija_ d4uuta_
d3a01a_ d1x58a1 d3rkqa1 d3cmya1 d2cqxa1 d2da6a1 d1e3oc1 d1fjla_
d1mh3a1 d1ig7a_ d1x41a1 d1wh7a1 d1gt0c1 d2mw8a_ d1lfup_ d1x2ma1
d1b72a_ d1wi3a1 d1jgga_ d1wjha1 d1x2na1 d1mnmc_ d2cuea1 d3k2aa_
d2hosa_ d2jwta2 d1pufa_ d1octc1 d2hi3a_ d1b8ia_ d4j19a_ d3lnqa_
d1wh5a1 d1b8ib_ d2iu5a1 d1zk8a1 d2g7sa1 d2fd5a1 d2np5a1 d1z0xa1
d2id6a1 d2d6ya1 d2i10a1 d1vi0a1 d2oi8a1 d3c07a1 d2ns7a1 d2fq4a1
d2yvea1 d1t33a1 d2o7ta1 d1rkta1 d2np3b1 d2g7ga1 d2gena1 d2fbqa1
d2g7la1 d2gfna1 d2g3ba1 d2jj7a1 d2hyja1 d1sgma1 d1ui5a1 d2hkua1
d2id3a1 d4jyka1 d2av4a1 d1s5ja2 d1qhta2 d4dlea2 d1tgoa2 d3py8a2
d1q8ia2 d1kfsa2 d2py5a2 d1d5aa2 d1k8ka2 d1k8ka1 d3uleb1 d3i33a2
d3i33a1 d1bupa1 d1dkgd2 d1dkgd1 d4ci6a2 d1jcfa1 d4a2ba2 d2fxua1
d1yaga2 d1yaga1 d4cbua2 d1bupa2 d1g99a1 d2e1za2 d2e1za1 d1saza1
d1saza2 d1ig8a1 d1czan2 d1czan3 d1czan1 d1ig8a2 d1czan4 d1bg3a3
d1bg3a2 d1bg3a1 d3h3no1 d3h3no2 d3ezwa2 d1zc6a1 d1zbsa1 d1zbsa2
d1zc6a2 d2ch5a2 d2ch5a1 d1zxoa2 d1zxoa1 d1hroa_ d4gedb_ d3zooa_
d1mz4a_ d1gu2a_ d1f1fa_ d1dvva_ d1c6ra_ d1c75a_ d1e29a_ d1gdva_
d1cnoa_ d1h32b_ d1co6a_ d2bh4x_ d2aiua_ d5cytr_ d1jdla_ d1i8oa_
d2zboa_ d3dmia_ d1wvec_ d2yk3a_ d3ph2b_ d1dw0a_ d5aura_ d1ql3a_
d1qn2a_ d1f1ca_ d4zzva_ d2xspa_ d1eg1a_ d4v20a_ d4ipma1 d1gpia_
d4csia_ d3pfza_ d3ovwa_ d2yoka_ d1prtc2 d1prtb2 d2ah2a1 d1h32a1
d1evsa_ d1cnt1_ d1pvhb_ d1z7ca_ d1huwa_ d1bgea_ d1f6fa_ d1f45b_
d1i1rb_ d1axia_ d1etea_ d3tgxb_ d1scfa_ d2z3qa1 d3qb1a_ d1hula_
d1m48a_ d4nkqb_ d1eera_ d1v7mv_ d5e4ea_ d2o27a_ d3b5ka_ d2psma1
d4zf7a_ d3uf5a_ d4x51a_ d1d9ca_ d4ypgc1 d3oq3a_ d1lqsl_ d1n1fa_
d1au1a_ d1wu3i_ d3besl_ d3ux9a_ d3rbaa_ d2qtra_ d1nupa_ d3l92a_
d1f9aa_ d1qjca_ d2h29a_ d1vlha_ d1od6a_ d1lw7a1 d1mrza2 d4ruka_
d2el7a_ d1iq0a2 d1irxa2 d1r6ta2 d1ivsa4 d2cyba_ d1rqga2 d1j09a2
d2yxna_ d1f7ua2 d1h3fa1 d1ffya3 d2g36a1 d1nzja_ d1jila_ d1n3la_
d1i6la_ d1pfva2 d1h3na3 d3n9ia_ d3sz3a1 d3sw2a_ d1moxc_ d1dx5i3
d1edmb_ d1autl2 d1autl1 d1nqlb_ d1dval1 d1szba2 d1kigl_ d2y5fl_
d1ijqa2 d1gk5a_ d1g1sa2 d1nt0a3 d1lmja2 d1lmja1 d1nzia2 d2wphe_
d1klil_ d1pfxl2 d1pfxl1 d1p9ja_ d1g1ta2 d1f7ea_ d4yzub_ d1m1xb5
d2y5ia_ d1psra_ d3n22a_ d2egda_ d4ggfc_ d2wcea_ d2luca_ d4aqia_
d3nsia_ d2lp3a_ d1j55a_ d1a4pa_ d1k8ua_ d1nsha_ d1pvaa_ d3fs7a_

d2pvba_ d1ttxa_ d1rwya_ d2kyfa_ d1rk9a_ d1exra_ d1tiza1 d1nyaa_
d1g8ia_ d1ggwa_ d1wdcc_ d3fwba_ d1ggza_ d2o5ga_ d1ctaa_ d1m45a_
d3i5gc_ d1sl8a_ d1fpwa_ d2scpa_ d2roaa_ d4aqra_ d3pn7b_ d1br1b_
d2mysb_ d1s6ca_ d1bjfa_ d2obha_ d1jfka_ d1jf0a_ d1qv0a_ d1lkja_
d2mlfc_ d2m97a_ d2d8na_ d4by5a_ d1jbaa_ d4nqga_ d1wdcb_ d2mysc_
d1j7qa_ d1s6ia_ d1uhka2 d1r2ua_ d1dtla_ d3kf9a_ d3pn7c_ d1gjya_
d1y1xa_ d1hqva_ d1dvia_ d1df0a1 d1qxpa2 d4usla_ d1k94a_ d1wkya2
d1uuqa_ d3wh9a_ d4m1ra_ d1xyza_ d4xuya_ d2je8a5 d4bf7a1 d3cuia_
d1ur4a_ d1qw9a2 d1odza_ d2vzsa5 d1bqca_ d2x2ya1 d1qnra_ d4y7ea1
d1vjza_ d4k68a_ d2v3ga_ d4hpga_ d1nofa2 d1nq6a_ d1h4pa_ d1aq0a_
d3ziza_ d4bs0a_ d1ecea_ d1h1na_ d1ta3b_ d1w91a2 d1i1wa_ d1uhva2
d1us3a2 d1egza_ d7a3ha_ d1tvna1 d3nyda1 d1ghsa_ d2cyga1 d4xv0a_
d2c8na2 d1w32a_ d3emca_ d1v6ya1 d1foba_ d2uwfa1 d2fgla_ d3niya1
d2c0ha1 d1rh9a1 d1yq2a5 d1szna2 d1fa2a_ d1pama4 d5do8a1 d1ea9c3
d1hx0a2 d2fhfa5 d1qhoa4 d1avaa2 d1m53a2 d4m8ua1 d2gjpa2 d1lwha2
d4howa1 d1ktba2 d1j0ha3 d3vm5a1 d2x1ia_ d1wzaa2 d1uasa2 d2diea1
d1mxga2 d1ht6a2 d1eswa_ d1iv8a2 d5cpsa_ d1ud2a2 d1zjaa1 d3gbea1
d1kwga2 d1wdpa_ d1gcya2 d1g5aa2 d1hvxa2 d3dhpa2 d2bhua3 d4do4a1
d3qgva1 d1e43a2 d1g94a2 d2d3na2 d1gjwa2 d1gvia3 d1x1na1 d1r7aa2
d1tz7a1 g1apy.1 d3ljqa_ d4toqa_ d1wb0a1 d1w9pa1 d1eoka_ d1itxa1
d1kfwa1 d1goia2 d1edqa2 d2gsja_ d1hjxa1 d1jnda1 d1vf8a1 d3wdpp1
d3wq5a_ d1gnxa_ d4gxpa_ d4hz8a_ d1e4mm_ d3aiua_ d1wcga1 d1qvba_
d3ahxa_ d1v02a_ d2dgaa_ d1pbga_ d1vffa1 d1qoxa_ d4ha4a_ d2w0pa_
d2diaa1 d2e9ia2 d2e9ja1 d2dj4a1 d2eeba1 d2diba1 d2dmba1 d2dmca1
d2d7oa1 d2ee6a1 d2d7pa1 d2eeaa1 d2di8a1 d1wlha1 d2d7na1 d2nqca_
d2d7ma1 d2dica1 d3cnka_ d2k3ta1 d2eeca1 d1qfha1 d2bp3a1 d1v05a_
d2ee9a1 d2eeda1 d2di9a1 d3syxa1 d1mkea1 d2p8va_ d1xoda1 d1i7aa_
d1evha_ d1egxa_ d1id1a_ d2hmta_ d1lssa_ d1iuka_ d2csua1 d3q9ub_
d2yv1a1 d3q9na_ d2d59a_ d1oi7a1 d2yv2a1 d1euca1 d1y81a1 d3nvva1
d2h88b1 d1n62a2 d1t3qa2 d1kf6b2 d1zoyb1 d1jq4a_ d1ffva2 d1dgja2
d1rm6c2 d1d4ua2 d9ldta1 d2ewda3 d1up7a1 d2dfda1 d1s6ya1 d1vjta1
d7mdha1 d1hyea1 d1t2da1 d1ojsa3 d1b8pa1 d1ldna1 d1hyha1 d1civa1
d2a92a1 d1ez4a1 d1i0za1 d1y6ja1 d1gv0a1 d1ur5a1 d1llda1 d1obba1
d1npya1 d3t4ea2 d1a4ia1 d1o0sa1 d2hk9a2 d2egga2 d1b26a1 d1bvua1
d1gq2a1 d1c1da1 d1leha1 d1gtma1 d1euza1 d1nvta1 d3dona2 d1edza1
d1vi2a1 d1p77a1 d1nyta1 d1b0aa1 d1vl6a1 d1v9la1 d1txga2 d3f47a1
d1mv8a2 d2pv7a2 d1xc2a3 d2ahra2 d4xiya1 d2f1ka2 d1pgja2 d1i36a2
d1np3a2 d1lj8a4 d1teaa3 d1qmga2 d2i76a2 d2g5ca2 d1wdka3 d1dlja2
d3cuma3 d1kola2 d1f8fa2 d1qora2 d1jqba2 d1vj0a2 d1cdoa2 d1gu7a2
d1d1ta2 d1xa0a2 d2dm6a2 d1jvba2 d1rjwa2 d1h2ba2 d1tt7a2 d1piwa2
d1o89a2 d1iz0a2 d1e3ja2 d1yb5a2 d1uufa2 d1vj1a2 d1e3ia2 d3gpdg1
d1vc2a1 d2nvwa1 d1u8fo1 d1ydwa1 d1hdgo1 d2g17a1 d1cf2o1 d2czca2
d1r0ka2 d1obfo1 d2cvoa1 d1ebfa1 d1h6da1 d1i32a1 d1zh8a1 d2q49a1
d1qkia1 d1j5pa4 d3pzra1 d1xeaa1 d1vkoa1 d1vm6a3 d3qvsa1 d2pkqo1

d1b7go1 d1gr0a1 d1p1ja1 d1lc0a1 d1j0xo1 d3pwka1 d2hjsa1 d1zyao1
d1vkna1 d1tlta1 d1nvmb1 d3sjua_ d3ojfa1 d1xu9a1 d2jjya1 d1y1pa1
d1h5qa_ d1jtva_ d4fs3a_ d2bkaa1 d1gy8a_ d2c5aa1 d1ulsa_ d1ooea_
d2b69a1 d1y5ma1 d1spxa_ d1ae1a_ d1bxka_ d1ydea1 d1gega_ d1hdoa_
d1o5ia_ d1e6ua_ d1snya1 d2rhca_ d3zv4a_ d5ceja_ d2d1ya1 d1fjha_
d3w8ea_ d2fmua1 d1x1ta_ d1rpna_ d3ay7a1 d1k2wa_ d1eq2a_ d2hrba1
d3ruaa_ d2ew8a1 d4hwka_ d1xgka_ d2bd0a1 d1yo6a1 d2a35a1 d2ntva_
d1vl8a_ d1hdca_ d4bkua_ d2ag5a1 d2q45a_ d1nffa_ d4iy1a_ d1zbqa1
d1kewa_ d1ek6a_ d1ja9a_ d2q46a2 d1iy8a_ d1cyda_ d2ptga_ d1hxha_
d1uzma1 d3lyla_ d4m89a_ d3wyea_ d2blla1 d1w6ua_ d1sb8a_ d2q2va_
d4nr0a_ d1e6wa_ d2gasa_ d3oiga1 d2bgka1 d1mxha_ d1vl0a1 d1rkxa_
d1r6da_ d1wmaa1 d1e7wa_ d2c07a1 d1xkqa_ d3tzha_ d1g0oa_ d3lz6a_
d1qyca_ d1edoa_ d1gz6a_ d1oc2a_ d1t2aa_ d1zema1 d2xoxa_ d1z45a2
d1geea_ d1p33a_ d3lt0a_ d4e6pa_ d2wyua_ d1db3a_ d4q9na_ d1xhla_
d4o1ma_ d1fmca_ d2uvda_ d2gdza1 d1uaya_ d2z1ma_ d2o23a2 d2fr1a2
d2fr1a1 d1qsga_ d1qyda_ d4zjua_ d2a4ka1 d1yb1a_ d1wvga1 d1yxma1
d3f4ba_ d1mg5a_ d1xg5a1 d1r55a1 d3gboa_ d1atla_ d1kufa_ d1buda_
d4q1la_ d1quaa_ d1yp1a_ d1wnia_ g1y43.1 d4ekfa_ d2ckga_ d1th0a_
d2bkra_ d1cmxa_ d1xd3a_ d3ifwa_ d4c5pa_ d2bsza1 d4bgfa_ d1w4ta1
d1mixa2 d1e5wa2 d1j19a2 d1isna2 d2al6b2 d3ioqa_ d1fh0a_ d1khqa_
d1m6da_ d2cb5a_ d1cs8a_ g8pch.1 d1s4va_ d2pnsa_ d1vsna1 d1mira_
d1iwda_ d2oula_ d7pcka_ d3bwka_ d3bcna_ d2p86a_ d1pvja_ d1pcia_
d1gece_ d1deua_ d4bs6a_ d1xkga1 d1cqda_ d1jqpa2 d2c0ya_ d2bdza_
d3p5ua_ d2o36a_ d1ex4a2 d1c6va_ d2jk6a2 d2jk6a1 d1feca1 d1feca2
d1lpfa1 d1lpfa2 d1xhca1 d1xhca2 d1dxla2 d1dxla1 d1gera2 d1m6ia2
d1onfa1 d3lada2 d3lada1 d1w4xa1 d1w4xa2 d1gesa2 d1mo9a1 d1xdia1
d1fl2a2 d1fl2a1 d1fcda1 d1fcda2 d1h6va2 d1h6va1 d1mo9a2 d1gv4a2
d1gv4a1 d2gv8a2 d2gv8a1 d1aoga1 d1aoga2 d1hyua1 d1ebda1 d1ebda2
d3dk9a1 d1q1ra2 d1q1ra1 d1gesa1 d1zbfa1 d2z1ha_ d2etja1 d3qioa1
d1jl1a_ d2e4la_ d1jl2a_ d2lsna1 d1io2a_ d1uaxa_ d2yv0x_ d1mu2a1
d1ekea_ d1i39a_ d1ep0a_ d2pa7a1 d1wlta1 d1nxma_ d2c0za1 d1pn0a1
d1reoa1 d1ju2a1 d2f5va1 d3c96a1 d2gmha1 d1pj5a2 d1gpea1 d1o5wa1
d1n4wa1 d1k0ia1 d2dw4a2 d2ivda1 d1seza1 d2voua1 d1kdga1 d1s5ja1
d2hbja2 d2igia_ d1wlja_ d1qhta1 d4dlea1 d1tgoa1 d2f96a1 d3tr8a1
d3mxma_ d1q8ia1 d1y97a1 d1w0ha_ d1kfsa1 d2py5a1 d1noya_ d1d5aa1
d1yt3a3 d2guia_ d3mwpa1 d4kb1a_ d2y8ga_ d1xkea1 d2y8fa_ d2v76a_
d1wj1a1 d1aqca_ d1qqga2 d1qqga1 d1ntva_ d1n3ha_ d4dbba_ d3f0wa1
d1p3ra_ d1wgua1 d2cy4a1 d3hqca_ d1wvha1 d1xr0b1 d1kb0a1 d1wgqa1
d1v89a1 d2coda1 d1zc3b_ d1wg7a1 d1u5ea1 d1v61a1 d2coca1 d2ys1a1
d1v5pa1 d1ntya2 d1wjma1 d1wi1a1 d1u29a_ d1xx0a1 d1unqa_ d2zkmx3
d1fhoa_ d1v88a1 d2adza1 d1x1fa1 d1p6sa_ d2coaa1 d1btka_ d1v5ma1
d1faoa_ d2x18a_ d1dyna_ d1x1ga1 d4kaxb_ d2j59m1 d1ki1b2 d1xcga2
d1txda2 d1u5da1 d1dbha2 d1w1ha_ d1upqa_ d1v5ua1 d2fjla1 d1eaza_
d2dfka2 d2cofa1 d1kz7a2 d1faaa_ d1wika1 d1xfla_ d1ttza1 d2f51a1

| | | | | | | | |
|---|---|---|---|---|---|---|---|
| d1ti3a_ | d1nhoa_ | d1sena_ | d2i4aa_ | d1ep7a_ | d1zmaa1 | d1fo5a_ | d3lefa1 |
| d3diea1 | d2yana1 | d1z6na1 | d1oaza_ | d3zywa1 | d4aj8a_ | d1nm3a1 | d4fiwa_ |
| d1wmja_ | d4hs1a1 | d1iloa_ | d1xwaa1 | d1wjka1 | d2vm1a_ | d1fb6a_ | d1gh2a_ |
| d1h75a_ | d1nw2a_ | d1ku5a_ | d1f1ea_ | d1b67a_ | d2b5ea2 | d2b5ea3 | d2b5ea1 |
| d2b5ea4 | d2kp1a1 | d2kp2a1 | d1hyua3 | d1hyua4 | d1j08a1 | d1j08a2 | d1pd212 |
| d1nhya2 | d2a2ra2 | d1k0dd2 | d1gsua2 | d1f3ba2 | d2fhea2 | d2pvqa1 | d1tw9a2 |
| d1oyja2 | d1e6ba2 | d1k0ma2 | d3ik7a1 | d4is0a1 | d1r5aa2 | d1jlva2 | d1m0ua2 |
| d3gtub2 | d1z9ha2 | d1okta2 | d1axda2 | d2gsra2 | d1tu7a2 | d1k3ya2 | d1zgma1 |
| d1b48a2 | d1gnwa2 | d1gwca2 | d2gsta2 | d1glqa2 | d1oe8a2 | d1pn9a2 | d1v2aa2 |
| d1fw1a2 | d1duga2 | d3vrda1 | d3vrda2 | d1fcdc2 | d1fcdc1 | d1h1oa2 | d1h1oa1 |
| d1m70a2 | d1nira1 | d1qksa1 | d3h1jd1 | d4ilya_ | d4olta_ | d1qgia_ | d1qqya_ |
| d1gd6a_ | d1f6ra_ | d1ivma_ | d2gv0a_ | d1iiza_ | d2z2fa_ | d1j8xa_ | d1ghla_ |
| d1b9oa_ | d174la_ | d169la_ | d2o4wa_ | d1p37a_ | d1k28a3 | d1xjta1 | d1jtma_ |
| d2f2qa_ | d4e97a1 | d1iyka2 | d1iyka1 | d1rxta1 | d1iica1 | d2za0a_ | d2c21a1 |
| d1f9za_ | d2pjsa1 | d1klla_ | d2i7ra1 | d1xqaa1 | d5cj3a1 | d1npba_ | d1jifa_ |
| d2p7oa_ | d1r9ca_ | d1nkia_ | d1mpya2 | d1kw3b2 | d1kw3b1 | d4ghga1 | d1tfza1 |
| d3isqa1 | d3isqa2 | d1sqia1 | d1sqia2 | d1lkda2 | d1lkda1 | d1mpya1 | d1cjxa2 |
| d1cjxa1 | d1sp9b2 | d1t47a1 | d1t47a2 | d1f1ua2 | d1sp8a1 | d1sp8a2 | d1f1ua1 |
| d2bema_ | d1pama1 | d1ea9c1 | d1qhoa1 | d2fhfa2 | d2fhfa1 | d1k3ia1 | d1j0ha1 |
| d1cxla1 | d2bhua1 | d1edqa1 | d1f1sa2 | d1ut9a2 | d1gvia1 | d1txka1 | d1w5qa_ |
| d2c1ha_ | d1ohla_ | d1l6sa_ | d1qsta_ | d2beia1 | d4m3sa_ | d1u6ma1 | d1ygha_ |
| d1yk3a1 | d3dr6a_ | d1xmta_ | d1z4ea1 | d2ge3a1 | d1bo4a_ | d2fiaa1 | d2jdca1 |
| d1y9wa1 | d3to7a_ | d1wwza1 | d2fl4a1 | d2ozua1 | d2b5ga_ | d2cy2a1 | d2p0wa_ |
| d1cm0a_ | d4pswa_ | d1yrea1 | d1yr0a1 | d1m4ia_ | d2gana1 | d1yvka1 | d1ghea_ |
| d2g3aa1 | d1y7ra1 | d2euia1 | d1q2ya_ | d2fsra1 | d1xeba_ | d2ae6a1 | d1cjwa_ |
| d1qsma_ | d2aj6a1 | d1y9ka1 | d1i12a_ | d1vkca_ | d2atra1 | d1n71a_ | d2d4pa1 |
| d2fiwa1 | d2fe7a1 | d1tiqa1 | d2ou2a_ | d1yx0a1 | d2fcka1 | d2j8ma_ | d2pq8a_ |
| d1z4ra1 | d1s3za1 | d1ufha_ | d1dgsa2 | d1fvia1 | d1p16a1 | d1x9na2 | d2nqya_ |
| d1h4ga_ | d1hixa_ | d1oa2a_ | d1olra_ | d3b7ma_ | d4h7ma1 | d3wp3a_ | d2vgda_ |
| d3zsea_ | d2nlra_ | d3wx5a_ | d1oa3a_ | d1pvxa_ | d1m4wa_ | d1f5ja_ | d1oa4a_ |
| d1ks5a_ | d4ixla1 | d1xl4a1 | d3vsqa_ | d2gixa1 | d1p7ba1 | d1xvqa1 | d1pn0a2 |
| d3hy2a_ | d2cvba1 | d1o8xa_ | d1lu4a_ | d1we0a1 | d2bmxa1 | d2a4va1 | d1psqa_ |
| d1xiya1 | d1q98a_ | d1n8ja_ | d1zofa1 | d2ggta_ | d3tjja_ | d4je1a1 | d1o73a_ |
| d3s9fa_ | d2zcta_ | d1jfua_ | d2b1ka_ | d5dvba1 | d2b5xa1 | d2v2ga_ | d1st9a_ |
| d3tb2a_ | d4llra_ | d4fh8a1 | d1e2ya_ | d1knga_ | d2h30a_ | d1i5ga_ | d2z9sa_ |
| d2f8aa1 | d1tp9a1 | d2b7ka_ | d1nm3a2 | d3hvva_ | d2wfca1 | d4kcea_ | d1xvwa1 |
| d4gqca1 | d3qpma_ | d4k1fa_ | d1zzoa1 | d1gp1a_ | d1prxa_ | d4od7a1 | d1z6ma1 |
| d4mcua1 | d4dvca1 | d3l9sa_ | d1un2a_ | d3rppa_ | d1rmka_ | d1ixta_ | d1eyoa_ |
| d1cnna_ | d1fu3a_ | d1g1pa_ | d1fyga_ | d1e88a1 | d1l6ja5 | d1l6ja4 | d1eaka4 |
| d1eaka3 | d1l6ja3 | d1h8pa2 | d1etha2 | d1w52x1 | d2ppla1 | d1lpbb2 | d2oxea1 |
| d1bu8a2 | d1rp1a2 | d1ys1x_ | d4hs9a_ | d1ispa_ | d1ex9a_ | d1ku0a_ | d3qmma_ |
| d2h1ia1 | d2r8ba1 | d3b5ea1 | d3cn9a_ | d1auoa_ | d4l3wa_ | d1llfa_ | d1gz7a_ |
| d1ukca_ | d2hqya1 | d2hqya2 | d1lrza2 | d1ne9a1 | d1lrza3 | d1c4xa_ | d1j1ia_ |
| d2rhwa_ | d3groa_ | d1pjaa_ | d1thta_ | d1ra6a_ | d1muka_ | d3gsza_ | d1khva_ |

d1te9a_ d2xwha_ d3uqsa_ d3hkwa_ d3n6ma_ d2cjqa_ d2xyma_ d1teba_
d1te8a_ d1s48a_ d1u09a1 d4wfxa_ d3tyqa_ d2pgga1 d3zxha_ d1cgla_
d1rm8a_ d1eaka2 d2jsda_ d1cxva_ d1hv5a_ d1i76a_ d3p0ya1 d1igra1
d1igra2 d1n8yc2 d1n8yc1 d1m6ba2 d1m6ba1 d1jx4a2 d3gv5b1 d1t94a2
d3bq0a2 d2tbvc_ d1c8nc_ d1x36a_ d1smvc_ d1ng0c_ d4sbvc_ d1f2nc_
d1cixa_ d1y29a_ d1v7fa_ d2jtba_ d1g9pa_ d1lupa_ d1qk7a_ d1mb6a_
d2a2va_ d1niya_ d1i25a_ d1hvwa_ d2mpqa_ d1ie6a_ d1kqha_ d1la4a_
d2mxoa1 d1dl0a_ d1koza_ d1qk6a_ d1emxa_ d1lqra_ d1c6wa_ d1js9c_
d1f15b_ d1za7a_ d1rypd_ d1irua_ d1rypc_ d3unfk_ d1rypa_ d1j2pa_
d1irui_ d1j2qh_ d3unfh_ d1m4ya_ d1rypb_ d1irul_ d1irug_ d1iru2_
d1q5rh_ d1rypg_ d1g3ka_ d1q5qa_ d1e94a_ d1irub_ d1ryph_ d3ty6a_
d1iruj_ d1iruk_ d1rype_ d1irud_ d1irue_ d3mi01_ d1rypf_ d1iruh_
d2z3ba_ d1iruc_ d3mka2_ d1iru1_ d1ny721 d1a6ca3 d1a6ca2 d1a6ca1
d1pgl22 d1pgl21 d1pgl11 d1ny711 d1mqta_ d1d4m3_ d1fmd3_ d2mev1_
g1aym.1 d1ev13_ d4q4w1_ d1r1a3_ d2x5ia_ d1rhi1_ g1r1a.1 g1d4m.1
g1rhi.1 d1b35b_ d1oopa_ d4iv1a_ g1pvc.1 g1fpn.1 d1fpn1_ d4gb31_
d1d4m1_ d1fmd1_ d1ev11_ d2wzr3_ g1cov.1 d1qqp3_ g1fmd.1 d2x5ic_
d1rhi3_ g1oop.1 d1hxs1_ g1hxs.1 d1fpn3_ d1bev1_ g2mev.1 d1eah3_
d1aym1_ d1pvc3_ d1qqp1_ d1zba3_ d1h8ta_ d3vbhc_ g1b35.1 g1bev.1
d1hxs3_ g1ev1.1 d1bev3_ g1tmf.1 d1tmf1_ d1b35a_ d2mev3_ g1eah.1
g1h8t.1 d1eah1_ d4q4w3_ d1r1a1_ d1aym3_ d1pvc1_ d1zba1_ d1h8tc_
d1oopc_ d1tme3_ g1qqp.1 d4iv1c_ g1tme.1 d3ubub_ d1egia_ d1qo3c_
d1v7pa_ d1tdqb_ d3ubua_ d1c3ab_ d1h8ua_ d3bx4b_ d1t8ca1 d1sb2a1
d1qdda_ d2ziba1 d3cada_ d1byfa_ d1j34b_ d1jwia_ d1jzna_ d1c3aa_
d1oz7b_ d1wmza_ d3bdwb_ d1hq8a_ d1fifa1 d1dv8a_ d3hupa_ d1wk1a1
d1umra_ d2afpa_ d3bx4a_ d1gz2a_ d1sl6a_ d1v7pb_ d3g8ka_ d1ixxa_
d1j34a_ d1jwib_ d1g1sa1 d1y17a1 d1ja3a_ d1fvub_ d3bdwa_ d1sb2b1
d1fvua_ d1umrc_ d1oz7a_ d4m17a_ d3cfwa1 d1ypqa1 d1g1ta1 d3fd4a_
d3l9jc_ d4dn8a1 d1v4lb_ d2msba_ d4lt9a1 d3tc7a1 d3hoja_ d1i4na_
d3vnda_ d3nl8a1 d1geqa_ d3nava1 d1v5xa_ d4a2ra1 d3tc6a1 d1ujpa_
d4ijba_ d1xcfa_ d1rd5a_ d1qopa_ d3b5va_ d2r80b_ d1cqxa1 d1cg5a_
d1gcvb_ d3boma_ d2h8fb_ d2raoa_ d3bj1a_ d4hrrb_ d2r50a_ d3d1kb_
d2aa1b1 d3zhwa_ d3hf4b_ d3gdja_ d1jebb_ d3bj1b_ d1x9fb_ d1wmub_
d1gcva_ d3bcqb_ d3d4xb_ d1gvha1 d2dc3a_ d3hf4a_ d4yu3a_ d3k8ba_
d1qpwb_ d2r80a_ d3bcqa_ d1v4wa_ d3wtgb_ d1jl7a_ d4esaa_ d1a9we_
d3d1ab_ d1fsla_ d4h2la_ d1v4wb_ d1mwca_ d1vhba_ d1jeba_ d1spgb_
d1h97a_ d1q1fa_ d1tu9a1 d3d1aa_ d1hdsb_ d3hyub_ d2zfbb_ d3fs4a_
d1oj6a_ d1shrb_ d4h2lb_ d4yu3b_ d1spga_ d3hrwa_ d3qqra_ d3at5a_
d4wchd_ d3hyua_ d1cg5b_ d1uc3a_ d1hbra_ d2raob_ d1outb_ d4esab_
d3gdjb_ d3qqqa_ d1x9fc_ d3vrfa_ d1itha_ d3mkba_ d3d4xa_ d3mjpa_
d1jl6a_ d3fh9a_ d1outa_ d2dc3b_ d1it2a_ d3eoka_ d3g46a_ d1qpwa_
d3vrfb_ d1ch4a_ d1x9fd_ d1hdsa_ d2zfba_ d3mkbb_ d1or4a_ d3wtga_
d1a4fa_ d3fh9b_ d3bomb_ d3cy5a_ d3at5b_ d1x9fa_ d2oifa_ d4lmxb_
d1cpcb_ d3v57a_ d1kn1a_ d1cpca_ d4f0ta_ d2vjha_ d4po5b_ d1eyxa_

d1alla_ d4f0tb_ d1b33b_ d1b8db_ d4h0mb_ d1b33a_ d2vjhb_ d4lm6b_
d2vmlb_ d1allb_ d2vjta_ d3dbja_ d2vmla_ d2vjtb_ d4f0ub_ d1f99b_
d4rmpa1 d1liaa_ d1eyxb_ d2uulb_ d4lmsb_ d4f0ua_ d2bv8a_ d1f99a_
d2bv8b_ d2c7lb_ d3dbjb_ d4rmpb1 d1b8da_ d4h0ma_ d3v57b_ d1liab_
d2uula_ d2j96a_ d1kyqa1 g1gxs.1 g1wht.1 d1mtza_ d1hlga_ d2xqfa_
d1f6wa_ d1dx4a_ d1qe3a_ d1k4ya_ d4qwwa_ d2ha2a_ d2qvba_ d2v9za_
d4kafa1 d1jmxa4 d1jmxa3 d1pbya4 d1b9ra_ d1offa_ d1frra_ d3wcqa_
d1e9ma_ d1wria_ d1i7ha_ d3av8a_ d1czpa_ d1rfka_ d3p1ma1 d3b2ga_
d2bt6a2 d4iuaa3 d4bvva_ d5hpga_ d3k65a_ d1ki0a3 d1ki0a2 d1ki0a1
d1a0ha1 d1pmla_ d3sp8a3 d2feba1 d1f74a_ d3l21a_ d2e1da_ d1to3a1
d1w3ia_ d1sfla_ d1n7ka_ d1vcva1 d4imea_ d1wa3a_ d2ocza1 d1f2ja_
d3m16a_ d2yxga_ d1ub3a_ d3a5fa_ d1fbaa_ d1l6wa_ d1xfba1 d1wx0a1
d1vpxa1 d1ojxa_ d1mxsa_ d2a4aa1 d1xkya1 d3cq0a1 d3s1xa_ d1f05a_
d1fdja_ d3cpra1 d4d2ja_ d4mh8a_ d1mu2a2 d4i7ga1 d3rp2a_ g1ucy.1
d1fuja_ d2zchp_ d3w94a_ d1si5h_ d1lo6a_ g2pka.1 d1cgha_ d1kigh_
d1a5ia_ d3s9ba_ d1hj8a_ d1ao5a_ d2anya_ d1q3xa1 d1gvza_ d1azza_
d4gsoa_ d2f91a1 d1iaua_ d1gdna_ d1eufa_ g1fiw.1 d1eq9a_ d4nfea_
d2rdla_ d1spja_ d1elva1 d1a7sa_ d1ekbb_ d1pfxc_ d1yc0a_ d2aiqa_
g1fiz.1 d1fi8a_ d4k60a_ d4dgja_ d1mzaa_ d1op0a_ d3tjva_ d1pytd_
d1sgfg_ g1gg6.1 d2eeka_ g1rtf.1 d1a0ja_ d3g01a_ d1pytc_ d4e7na_
d1ddja_ d1z8ga1 d2hlca_ d1npma_ d1brup_ g1jou.1 d1sgfa_ d1autc_
g2hnt.1 g1kdq.1 d3s69a_ d4crga_ d1fxya_ d1bqya_ d1svpa_ d1mbma_
d3su6a1 d1cu1a1 d4agka_ d1vcpa_ d1q31a_ g1n1l.1 d3e90b_ d1ep5a_
d3u1jb_ d1m4la_ d2bk7a_ d3fjua_ d1z5ra1 d1kwma1 d1jqga1 d2c1ca1
d1dtda_ d1h8la2 d4lu0a_ d1of8a_ d3unda1 d3e9aa1 d1vr6a2 d1n8fa_
d2qkfa_ d1d9ea_ d2i49a_ d1nnfa_ d1xvxa_ d1ursa_ d1h3da1 d1iuda_
d4m1va1 d1twya_ d3e13x_ d2nxoa1 d4pfua_ d3ttma_ d1elja_ d2qmwa1
d1us5a_ d1y3na1 d1jeta_ d1uqwa_ d4f19a1 d2esna2 d3thia_ d3fasa1
d2uyeb_ d2xwva1 d1r9la_ d2fyia1 d1pb7a_ d2ozza1 d1sw5a_ d1nh8a1
d1vr5a1 d2g29a_ d1xs5a_ d3lswa_ d2z23a_ d3w9va_ d3tcha_ d1q35a_
d1z7me1 d3q27a_ d1p99a_ d1ve4a1 d2i6ea1 d4mcoa1 d4n8ya_ d1eu8a_
d1zbma1 d1hsla_ d1ixca2 d1j1na_ d1xvya_ d1y9ua_ d2z22a1 d1xoca1
d4oera_ d1wdna_ d5f1qa1 d1xt8a1 d1i6aa_ d4gwoa_ d4igta1 d1pc3a_
d4mmpa1 d1o63a_ d2czla1 d2onsa2 d4mija1 d1eh3a_ d3skpa_ d1dtza1
d1dtza2 d1ieja_ d1jnfa2 d1ce2a1 d1jnfa1 d1b1xa2 d1jw1a1 d1jw1a2
d1ryoa_ d1h76a1 d1h76a2 d3q3ma_ d1za0a1 d1mxra_ d2inca_ d1jk0a_
d2incb_ d1mhyb_ d3hf1b_ d1mtyb_ d1r2fa_ d2uw1a_ d1mhyd_ d3n37a_
d1oquc_ d1syya_ d1mtyd_ d2o1za_ d1uzra_ d1smsa_ d2fkza_ d1zuja1
d1s3qa1 d2fjca1 d1z6om1 d1veia_ d4dyya_ d1jiga_ d3gvya_ d2pyba_
d4cy9a1 d3bvfa1 d1r03a_ d2cf7a_ d1nf4a_ d1zs3a1 d4iwka_ d3ka8a_
d3np0x_ d1n1qa_ d1lb3a_ d4e6ka_ d1z6oa1 d2fzfa1 d4dyua_ d3ge4a_
d4am4a_ d2z6ma_ d1ji5a_ d1vlga_ d1lkoa1 d1krqa_ d3qz3a_ d1o9ra_
d2ffxj_ d1euma_ d1yv1a1 d2v2pa_ d1j30a_ d4di0a_ d1dpsa_ d1oewa_
d2qzxa_ d2asia_ d1psoe_ d1miqa_ d1smra_ d2h6ta_ d1pfza_ d1izda_

d3hd8a_ d1j71a_ d1dpja_ d4aa9a_ d1t6ex_ g1b5f.1 g1lya.1 g1htr.1
d1qdma2 d1bxoa_ d1wkra_ d1tzsa_ d1ibqa_ d2qzwa_ d1orva2 d1xfda2
d1h6ua1 d1bdqa_ d3ggta_ d3ixoa_ d1mrxa_ d2haha_ d1tw7a_ d4njsa_
d1sgua_ d2p3da_ d1nsoa_ d3d3ta_ d2rkfa_ d1c6ya_ d2rspa_ d3ogpa_
d3ekwa_ d2o4na_ d2j9ja_ d3ggua_ d3lzua_ d4npua_ d4j55a_ d2r8na_
d2fxda_ d2p3ca_ d4dqea_ d3ttpa_ d3t3ca_ d2p3aa_ d2hc0a_ d2fdda_
d3mwsa_ d1sr9a2 d1rqba2 d3rcha_ d3k40a_ d1pmma_ d1k8rb_ d1wgra1
d1ef5a_ d1wfya1 d1e8ya3 d1wgya1 d1lfda_ d2cs4a1 d1wxma1 d2byea1
d3kh0a1 d2byfa1 d1wxaa1 d1e7ua3 d1h4ra3 d4yl8a1 d2al6a3 d1wgda1
d1wgga1 d4q5eb_ d1wjna1 d1se9a_ d3n3kb_ d1wz0a1 d1we6a1 d2m0xa_
d2gbka_ d3rt3b1 d1wh3a1 d1sifa_ d2ekec2 d4ii2b1 d4hk2a1 d1c3ta_
d1m94a_ d1wgha1 d1ttna1 d1j8ca_ d1wx8a1 d3v6cb1 d2ma9b_ d4i6lb1
d2jvca1 d1iyfa_ d2k1fa_ d1wxva1 d1wf9a1 d1t0ya_ d2io0b2 d1x1ma1
d2gbma_ d1zw7a1 d2m17a_ d1ud7a_ d1we7a1 d2lwpa_ d2gbja_ d1wjua1
d1v6ea1 d1v86a1 d4eewa_ d1wiaa1 d4icva_ d4gu2a_ d1wx7a1 d1wy8a1
d4hjka1 d2faza1 d1v5oa1 d3ruib_ d1eo6a_ d3waob_ d2r2qa1 d3m95a_
d3wana_ d2al3a1 d2cr5a1 d1wj4a1 d1s3sg_ d1ps9a2 d1o94a2 d1cjca1
d4wfia_ d4cg1a1 d3visa_ d2ftba_ d1pmpa_ d2rcqa1 d4lkpa_ d1sa8a_
d1vyfa_ d1lpja_ d3vg7a1 d4azqa1 d1o8va_ d5bvqa_ d1p6pa_ d2qo4a_
d1opba_ d1yiva1 d1o1va_ d4bvma1 d4tkba_ d4i3ba_ d1bwya_ d1ftpa_
d1ggla_ d4gkca_ d1cbia_ d3p6da1 d1kqwa_ d2a0aa1 d1dszb_ d1a6ya_
d3cbba_ d1ynwa1 d1r4ia_ d1hcqa_ d2hana_ d2hanb_ d2enva1 d1kb4b_
d2nllb_ d1lo1a_ d1cita_ d1adqa1 d1fnga1 d1uvqb1 d1zs8a1 d2vola1
d2vola2 d1mfbl2 d2jelh2 d1uwel2 d3qq4b1 d1kjvb_ d1k5na1 d1osph2
d4odhl2 d1ow0a1 d1ow0a2 d1bfob2 d3l9ra2 d3jtth1 d2fath2 d4elkb2
d1fn4b2 d2xfxb_ d1pg7x2 d1cqka_ d4k71b2 d1lk2a1 d1eapb2 d4lfhg2
d1i4fa1 d2nw2a2 d1kfah2 d1mhca1 d1h3ua1 d1wejh2 d1h0db2 d1a5fh2
d1kcvh2 d1mjuh2 d1qqda1 d1kcrh2 d1k8ia1 d3huja2 d2nnab1 d1nfdf2
d3mffb2 d1s9va1 d1lk3h2 d1q0xl2 d1fv1b1 d1neza1 d4rfeb2 d1r3ha1
d1a6tb2 d1kjva1 d1pz5b2 d1onqa1 d2bc4a2 d1de4a1 d1k8da1 d1tcra2
d1dn0b2 d1nfde2 d2xwtb2 d1bfoa2 d1mexh2 d1iakb1 d1ypzf2 d4f7ea2
d1q72l2 d2fbjh2 d1fe8h2 d1clzh2 d1fngb1 d1uvqa1 d1vgka1 d1c16a1
d4j12a2 d4x99b2 d1igtb3 d1je6a1 d1q72h2 d1nldh2 d1teth2 d2hmic2
d1i1ca2 d1i1ca1 d1q0yh2 d1kfal2 d3frua1 d1pg7w2 d1u58a1 d1igyb4
d1igyb3 d1hyrc1 d1klub1 d1deeb2 d1c12b2 d2bc4b2 d2ig2h2 d1kcvl2
d3wn5b1 d1lk2b_ d1lk3l2 d1mjul2 d1sm3h2 d1q0xh2 d1zvsa1 d1ydpa1
d1u8ib2 d1ypze2 d1kcuh2 d2e3fa_ d1mm0a_ d2e3ea_ d2llda_ d2ny8x_
d2e3ga_ d2ny9x_ d2nz3a_ d2lj7a_ d1n4na_ d2m8bb1 d2kyqa_ d3psma_
d1jkza_ d1jxca_ d2gl1a_ d1ugla_ d4uj0a_ d2k4ua_ d1hp2a_ d2lu9a_
d1baha_ d2meoa1 d1wm7a_ d1hlya_ d1n8ma_ d1jlza_ d1wpda_ d1c55a_
d1pvza_ d1wt7a_ d1rjia_ d1wz5a_ d1m2sa_ d1pjva_ d1c49a_ d1qkya_
d2ktca1 d1wmta_ d1du9a_ d1j5ja_ d1r1ga_ d1c4qa_ d1prtc1 d2wv6d_
d1prtf_ d1ltrd_ d4l63b1 d1r4pb_ d1prtd_ d1tl0f_ d2bosa_ d1prtb1
d1t0za_ d2i61a_ d1t1ta_ d1sn4a_ d1kv0a_ d2lkba_ d1npia_ d1wwna_

d1pe4a_ d2kbka_ d2li7a_ d1djta_ d2kbha_ d1dq7a_ d1sega_ d1zywa1
d1jzaa_ d1dy2a_ d1t61c1 d1y14b1 d2nn6i1 d1i94l_ d2zjra2 d1smxa_
d2qamc2 d1hh2p1 d2r7da1 d1rl2a2 d1wfqa1 d3bzka4 d1x6oa2 d3a0ja_
d2j01d2 d2ja9a1 d4ql5a_ d1hr0w_ d1luza_ d2n3sa_ d2vnud1 d1h95a_
d1go3e1 d2nn6h1 d1ne3a_ d2mo1a_ d1iz6a2 d1jjga_ d1e3pa2 d2ix0a3
d2ix0a2 d2lxja_ d1ueba3 d1ueba2 d2z0sa1 d1u0la1 d2ix0a1 d2eifa2
d2je6i1 d3i2za1 d2c35b1 d2ahob2 d1t9ha1 d3i4oa_ d2qall1 d1jt8a_
d2nn6g1 d1g6pa_ d3cama_ d1i94q_ d1c9oa_ d1d7qa_ d2ba0a1 d2vnud2
d2vnud3 d1khia2 d2qalq1 d1wi5a1 d1xtda2 d1ny4a_ d1dzka_ d4tlja_
d2a2ua_ d4wfva_ d3t1da_ d4iaxa_ d3zq3a_ d2nnda_ d1i4ua_ d1kxoa_
d1bbpa_ d1kt7a_ d1exsa_ d3s26a1 d1lf7a_ d4gh7a_ d1z24a1 d1qabe_
d1xkia_ d1ew3a_ d3bx7a_ d4iawa1 d1n0sa_ d1gkab_ d3kzaa_ d2k23a_
d2l9ca1 d2joza1 d1gm6a_ d3kffa_ d3saoa1 d1e5pa_ d1yupa1 d1iiua_
d1rpma_ d2shpa1 d1yfoa_ d1l8ka_ d2ooqa1 d1g4us2 d1p15a_ d1lara2
d1lara1 d1wcha_ d2fh7a2 d2fh7a1 d1ohea1 d2f4da_ d1rxda_ d2hxpa_
[truncated: 637,757 more chars]
