# Supplementary material for: Protein remote homology detection based on bidirectional long short-term memory
Source: BMC Bioinformatics. 2017 Oct 10;18:443. doi: 10.1186/s12859-017-1842-2 (PMC5634958; doi:10.1186/s12859-017-1842-2)
Supplement: Supplementary file 2 — The source code and its document of ProDec-BLSTM. (ZIP 316 kb) [file 12859_2017_1842_MOESM2_ESM.zip › ProDec-BLSTM/README.pdf]

ProDec-BLSTM

Background: Protein remote homology detection plays a vital role in studies of protein structures and functions. Almost all of the traditional machine leaning methods require fixed length features to represent the protein sequences. However, it is never an easy task to extract the discriminative features with limited knowledge of proteins. On the other hand, deep learning technique has demonstrated its advantage in automatic learning representations. It is worthwhile to explore the applications of deep learning techniques to the protein remote homology detection.

Results: In this study, we employ the Bidirectional Long Short-Term Memory (BLSTM) to learn effective features from pseudo protein sequences, also propose a predictor called ProDec-BLSTM: it includes input layer, bidirectional LSTM, time distributed dense layer and output layer. This neural network can automatically extract the discriminative features by using bidirectional LSTM and the time distributed dense layer.

Conclusion: Experimental results on a widely-used benchmark dataset show that ProDec-BLSTM outperforms other related methods in terms of both the mean ROC and mean ROC50 scores. This promising result shows that ProDec-BLSTM is a useful tool for protein remote homology detection. Furthermore, the hidden patterns learnt by ProDec-BLSTM can be interpreted and visualized, and therefore, additional useful information can be obtained.

Dependency

NCBI-BLAST-2.4.0 ftp://ftp.ncbi.nlm.nih.gov/blast/executables/blast+/2.4.0/
Keras 2.0.6
Theano 0.9.0
Numpy 1.11.2
Biopython 1.68

Content

data/: the training dataset and testing dataset in FASTA format. All of the samples should be converted to pseudo proteins based on PSSM [1, 2].
results/: the prediction results of ProDec-BLSTM.

Usage

python ProDec-BLSTM.py [-family_index <family index>] [-train FALSE] [-test

FALSE] [-pos_train_dir] [<dir>] [-neg_train_dir] [<dir>] [-pos_test_dir] [<dir>] [-neg_test_dir] [<dir>] [-model_dir] [<dir>] [-weights_dir] [<dir>]

-family_index: family index
-train: bool. train a ProDec-BLSTM model
-test: bool. load the trained ProDec-BLSTM model
-model_dir: the directory of the trained model json file of ProDec-BLSTM. If test is false, this argument can be empty.
-weights_dir: the directory of the trained model weight file of ProDec-BLSTM. If test is false, this argument can be empty.
-pos_train_dir: the directory of positive training dataset
-neg_train_dir: the directory of negative training dataset
-pos_test_dir: the directory of positive testing dataset
-neg_test_dir: the directory of negative testing dataset
-h: show this usage

The parameters of ProDec-BLSTM can be set in Paramters.py.

Use example

1. Generate the pseudo proteins of training and testing samples based on PSSM [1, 2] and put them in the directory of data.
2. Training the model of ProDec-BLSTM for family a.138.1.3:

python ProDec-BLSTM.py -family_index a.138.1.3 -train True -pos_train_dir YOUR_POS_TRAIN_DIR -neg_train_dir YOUR_NEG_TRAIN_DIR -pos_test_dir YOUR_POS_TEST_DIR -neg_test_dir YOUR_NEG_TEST_DIR

3. Testing the trained model of ProDec-BLSTM for family a.138.1.3:

python ProDec-BLSTM.py -family_index a.138.1.3 -test True -pos_test_dir YOUR_POS_TEST_DIR -neg_test_dir YOUR_NEG_TEST_DIR -model_dir YOUR_MODEL_JSON_FILE_DIR -weights_dir YOUR_MODEL_WEIGHTS_FILE_DIR

Reference

1. Liu B, Zhang D, Xu R, Xu J, Wang X, Chen Q, Dong Q, Chou K-C: **Combining evolutionary information extracted from frequency profiles with sequence-based kernels for protein remote homology detection**. *Bioinformatics (Oxford, England)* 2014, **30**(4):472-479.
2. Chen J, Long R, Wang X, Liu B, Chou K-C: **dRHP-PseRA: detecting remote homology proteins using profile based pseudo protein sequence and rank aggregation**. *Scientific Reports* 2016, **6**:32333.
